# Global, regional, and national age-sex-specific mortality and life expectancy, 1950–2017: a systematic analysis for the Global Burden of Disease Study 2017

**DOI:** 10.1016/S0140-6736(18)31891-9

**Published:** 2018-11-10

**Authors:** GBD 2017 Mortality Collaborators, Daniel Dicker, Grant Nguyen, Degu Abate, Kalkidan Hassen Abate, Solomon M Abay, Cristiana Abbafati, Nooshin Abbasi, Hedayat Abbastabar, Foad Abd-Allah, Jemal Abdela, Ahmed Abdelalim, Omar Abdel-Rahman, Alireza Abdi, Ibrahim Abdollahpour, Rizwan Suliankatchi Abdulkader, Ahmed Abdulahi Abdurahman, Haftom Temesgen Abebe, Molla Abebe, Zegeye Abebe, Teshome Abuka Abebo, Victor Aboyans, Haftom Niguse Abraha, Aklilu Roba Abrham, Laith Jamal Abu-Raddad, Niveen ME Abu-Rmeileh, Manfred Mario Kokou Accrombessi, Pawan Acharya, Oladimeji M Adebayo, Isaac Akinkunmi Adedeji, Rufus Adesoji Adedoyin, Victor Adekanmbi, Olatunji O Adetokunboh, Beyene Meressa Adhena, Tara Ballav Adhikari, Mina G Adib, Arsène Kouablan Adou, Jose C Adsuar, Mohsen Afarideh, Ashkan Afshin, Gina Agarwal, Rakesh Aggarwal, Sargis Aghasi Aghayan, Sutapa Agrawal, Anurag Agrawal, Mehdi Ahmadi, Alireza Ahmadi, Hamid Ahmadieh, Mohamed Lemine Cheikh brahim Ahmed, Sayem Ahmed, Muktar Beshir Ahmed, Amani Nidhal Aichour, Ibtihel Aichour, Miloud Taki Eddine Aichour, Ali S Akanda, Mohammad Esmaeil Akbari, Mohammed Akibu, Rufus Olusola Akinyemi, Tomi Akinyemiju, Nadia Akseer, Fares Alahdab, Ziyad Al-Aly, Khurshid Alam, Animut Alebel, Alicia V Aleman, Kefyalew Addis Alene, Ayman Al-Eyadhy, Raghib Ali, Mehran Alijanzadeh, Reza Alizadeh-Navaei, Syed Mohamed Aljunid, Ala'a Alkerwi, François Alla, Peter Allebeck, Christine A Allen, Jordi Alonso, Rajaa M Al-Raddadi, Ubai Alsharif, Khalid Altirkawi, Nelson Alvis-Guzman, Azmeraw T Amare, Erfan Amini, Walid Ammar, Yaw Ampem Amoako, Nahla Hamed Anber, Catalina Liliana Andrei, Sofia Androudi, Megbaru Debalkie Animut, Mina Anjomshoa, Degefaye Zelalem Anlay, Hossein Ansari, Ansariadi Ansariadi, Mustafa Geleto Ansha, Carl Abelardo T Antonio, Seth Christopher Yaw Appiah, Olatunde Aremu, Habtamu Abera Areri, Johan Ärnlöv, Megha Arora, Al Artaman, Krishna K Aryal, Mohsen Asadi-Lari, Hamid Asayesh, Ephrem Tsegay Asfaw, Solomon Weldegebreal Asgedom, Reza Assadi, Zerihun Ataro, Tesfay Mehari Mehari Atey, Seyyed Shamsadin Athari, Suleman Atique, Sachin R Atre, Madhu Sudhan Atteraya, Engi F Attia, Marcel Ausloos, Leticia Avila-Burgos, Euripide F G A Avokpaho, Ashish Awasthi, Baffour Awuah, Beatriz Paulina Ayala Quintanilla, Henok Tadesse Ayele, Yohanes Ayele, Rakesh Ayer, Tambe B Ayuk, Peter S Azzopardi, Natasha Azzopardi-Muscat, Hamid Badali, Alaa Badawi, Kalpana Balakrishnan, Ayele Geleto Bali, Maciej Banach, Amrit Banstola, Aleksandra Barac, Miguel A Barboza, Simon Barquera, Lope H Barrero, Huda Basaleem, Quique Bassat, Arindam Basu, Sanjay Basu, Bernhard T Baune, Shahrzad Bazargan-Hejazi, Neeraj Bedi, Ettore Beghi, Masoud Behzadifar, Meysam Behzadifar, Yannick Béjot, Bayu Begashaw Bekele, Abate Bekele Belachew, Aregawi Gebreyesus Belay, Ezra Belay, Saba Abraham Belay, Yihalem Abebe Belay, Michelle L Bell, Aminu K Bello, Derrick A Bennett, Isabela M Bensenor, Adugnaw Berhane, Adam E Berman, Eduardo Bernabe, Robert S Bernstein, Gregory J Bertolacci, Mircea Beuran, Tina Beyranvand, Neeraj Bhala, Eesh Bhatia, Samir Bhatt, Suraj Bhattarai, Soumyadeeep Bhaumik, Zulfiqar A Bhutta, Belete Biadgo, Ali Bijani, Boris Bikbov, Nigus Bililign, Muhammad Shahdaat Bin Sayeed, Sait Mentes Birlik, Charles Birungi, Donal Bisanzio, Tuhin Biswas, Tone Bjørge, Archie Bleyer, Berrak Bora Basara, Dipan Bose, Cristina Bosetti, Soufiane Boufous, Rupert Bourne, Oliver J Brady, Nicola Luigi Bragazzi, Luisa C Brant, Alexandra Brazinova, Nicholas J K Breitborde, Hermann Brenner, Gabrielle Britton, Traolach Brugha, Kristin E Burke, Reinhard Busse, Zahid A Butt, Lucero Cahuana-Hurtado, Charlton S K H Callender, Ismael R Campos-Nonato, Julio Cesar Campuzano Rincon, Jorge Cano, Mate Car, Rosario Cárdenas, Giulia Carreras, Juan J Carrero, Austin Carter, Félix Carvalho, Carlos A Castañeda-Orjuela, Jacqueline Castillo Rivas, Franz Castro, Ferrán Catalá-López, Alanur Çavlin, Ester Cerin, Yazan Chaiah, Ana Paula Champs, Hsing-Yi Chang, Jung-Chen Chang, Aparajita Chattopadhyay, Pankaj Chaturvedi, Wanqing Chen, Peggy Pei-Chia Chiang, Odgerel Chimed-Ochir, Ken Lee Chin, Vesper Hichilombwe Chisumpa, Abdulaal Chitheer, Jee-Young J Choi, Hanne Christensen, Devasahayam J Christopher, Sheng-Chia Chung, Flavia M Cicuttini, Liliana G Ciobanu, Massimo Cirillo, Rafael M Claro, Aaron J Cohen, Daniel Collado-Mateo, Maria-Magdalena Constantin, Sara Conti, Cyrus Cooper, Leslie Trumbull Cooper, Paolo Angelo Cortesi, Monica Cortinovis, Ewerton Cousin, Michael H Criqui, Elizabeth A Cromwell, Christopher Stephen Crowe, John A Crump, Alexandra Cucu, Matthew Cunningham, Alemneh Kabeta Daba, Berihun Assefa Dachew, Abel Fekadu Dadi, Lalit Dandona, Rakhi Dandona, Anh Kim Dang, Paul I Dargan, Ahmad Daryani, Siddharth K Das, Rajat Das Gupta, José das Neves, Tamirat Tesfaye Dasa, Aditya Prasad Dash, Nicole Davis Weaver, Dragos Virgil Davitoiu, Kairat Davletov, Anand Dayama, Barbora de Courten, Fernando Pio De la Hoz, Diego De leo, Jan-Walter De Neve, Meaza Girma Degefa, Louisa Degenhardt, Tizta T Degfie, Selina Deiparine, Robert P Dellavalle, Gebre Teklemariam Demoz, Balem Betsu Demtsu, Edgar Denova-Gutiérrez, Kebede Deribe, Nikolaos Dervenis, Don C Des Jarlais, Getenet Ayalew Dessie, Subhojit Dey, Samath Dhamminda Dharmaratne, Meghnath Dhimal, Eric L Ding, Shirin Djalalinia, David Teye Doku, Kate A Dolan, Christl A Donnelly, E Ray Dorsey, Dirk Douwes-Schultz, Kerrie E Doyle, Thomas M Drake, Tim Robert Driscoll, Manisha Dubey, Eleonora Dubljanin, Eyasu Ejeta Duken, Bruce B Duncan, Andre R Duraes, Hedyeh Ebrahimi, Soheil Ebrahimpour, Dumessa Edessa, David Edvardsson, Anne Elise Eggen, Charbel El Bcheraoui, Maysaa El Sayed Zaki, Mohammed Elfaramawi, Ziad El-Khatib, Christian Lycke Ellingsen, Iqbal R F Elyazar, Ahmadali Enayati, Aman Yesuf Yesuf Endries, Benjamin Er, Sergey Petrovich Ermakov, Babak Eshrati, Sharareh Eskandarieh, Reza Esmaeili, Alireza Esteghamati, Sadaf Esteghamati, Mahdi Fakhar, Hamed Fakhim, Tamer Farag, Mahbobeh Faramarzi, Mohammad Fareed, Farzaneh Farhadi, Talha A Farid, Carla Sofia e Sá Farinha, Andrea Farioli, Andre Faro, Maryam S Farvid, Farshad Farzadfar, Mohammad Hosein Farzaei, Mir Sohail Fazeli, Valery L Feigin, Andrea B Feigl, Fariba Feizy, Netsanet Fentahun, Seyed-Mohammad Fereshtehnejad, Eduarda Fernandes, Joao C Fernandes, Garumma Tolu Feyissa, Daniel Obadare Fijabi, Irina Filip, Samuel Finegold, Florian Fischer, Luisa Sorio Flor, Nataliya A Foigt, John A Ford, Kyle J Foreman, Carla Fornari, Tahvi D Frank, Richard Charles Franklin, Takeshi Fukumoto, John E Fuller, Nancy Fullman, Thomas Fürst, João M Furtado, Neal D Futran, Adriana Galan, Silvano Gallus, Ketevan Gambashidze, Amiran Gamkrelidze, Fortune Gbetoho Gankpe, Alberto L Garcia-Basteiro, Miguel A Garcia-Gordillo, Teshome Gebre, Abadi Kahsu Gebre, Gebremedhin Berhe Gebregergs, Tsegaye Tewelde Gebrehiwot, Amanuel Tesfay Gebremedhin, Tilayie Feto Gelano, Yalemzewod Assefa Gelaw, Johanna M Geleijnse, Ricard Genova-Maleras, Bradford D Gessner, Sefonias Getachew, Peter W Gething, Kebede Embaye Gezae, Mohammad Rasoul Ghadami, Reza Ghadimi, Khalil Ghasemi Falavarjani, Maryam Ghasemi-Kasman, Hesam Ghiasvand, Mamata Ghimire, Aloke Gopal Ghoshal, Paramjit Singh Gill, Tiffany K Gill, Richard F Gillum, Giorgia Giussani, Shifalika Goenka, Srinivas Goli, Ricardo Santiago Gomez, Mari Carmen Gomez-Cabrera, Hector Gómez-Dantés, Philimon N Gona, Amador Goodridge, Sameer Vali Gopalani, Atsushi Goto, Alessandra C Goulart, Bárbara Niegia Garcia Goulart, Ayman Grada, Giuseppe Grosso, Harish Chander Gugnani, Andre Luiz Sena Guimaraes, Yuming Guo, Prakash C Gupta, Rahul Gupta, Rajeev Gupta, Tanush Gupta, Bishal Gyawali, Juanita A Haagsma, Vladimir Hachinski, Nima Hafezi-Nejad, Tekleberhan B Hagos, Tewodros Tesfa Hailegiyorgis, Gessessew Bugssa Hailu, Arya Haj-Mirzaian, Arvin Haj-Mirzaian, Randah R Hamadeh, Samer Hamidi, Alexis J Handal, Graeme J Hankey, Hilda L Harb, Sivadasanpillai Harikrishnan, Hamidreza Haririan, Josep Maria Haro, Mehedi Hasan, Hadi Hassankhani, Hamid Yimam Hassen, Rasmus Havmoeller, Roderick J Hay, Simon I Hay, Yihua He, Akbar Hedayatizadeh-Omran, Mohamed I Hegazy, Behzad Heibati, Mohsen Heidari, Delia Hendrie, Andualem Henok, Nathaniel J Henry, Ileana Heredia-Pi, Claudiu Herteliu, Fatemeh Heydarpour, Pouria Heydarpour, Sousan Heydarpour, Desalegn Tsegaw Hibstu, Hans W Hoek, Michael K Hole, Enayatollah Homaie Rad, Praveen Hoogar, Masako Horino, H Dean Hosgood, Seyed Mostafa Hosseini, Mehdi Hosseinzadeh, Sorin Hostiuc, Mihaela Hostiuc, Peter J Hotez, Damian G Hoy, Mohamed Hsairi, Aung Soe Htet, Guoqing Hu, John J Huang, Abdullatif Husseini, Mohammedaman Mama Hussen, Susan Hutfless, Kim Moesgaard Iburg, Ehimario U Igumbor, Chad Thomas Ikeda, Olayinka Stephen Ilesanmi, Usman Iqbal, Seyed Sina Naghibi Irvani, Oluwaseyi Oluwakemi Isehunwa, Sheikh Mohammed Shariful Islam, Farhad Islami, Leila Jahangiry, Nader Jahanmehr, Rajesh Jain, Sudhir Kumar Jain, Mihajlo Jakovljevic, Spencer L James, Mehdi Javanbakht, Sudha Jayaraman, Achala Upendra Jayatilleke, Sun Ha Jee, Panniyammakal Jeemon, Ravi Prakash Jha, Vivekanand Jha, John S Ji, Sarah Charlotte Johnson, Jost B Jonas, Ankur Joshi, Jacek Jerzy Jozwiak, Suresh Banayya Jungari, Mikk Jürisson, Madhanraj K, Zubair Kabir, Rajendra Kadel, Amaha Kahsay, Molla Kahssay, Rizwan Kalani, Umesh Kapil, Manoochehr Karami, Behzad Karami Matin, André Karch, Corine Karema, Narges Karimi, Seyed M Karimi, Hamidreza Karimi-Sari, Amir Kasaeian, Getachew Mullu Kassa, Tesfaye Dessale Kassa, Zemenu Yohannes Kassa, Nicholas J Kassebaum, Marzieh Katibeh, Srinivasa Vittal Katikireddi, Anil Kaul, Norito Kawakami, Hossein Kazemeini, Zhila Kazemi, Ali Kazemi Karyani, Prakash K C, Seifu Kebede, Peter Njenga Keiyoro, Grant Rodgers Kemp, Andre Pascal Kengne, Andre Keren, Maia Kereselidze, Yousef Saleh Khader, Morteza Abdullatif Khafaie, Alireza Khajavi, Nauman Khalid, Ibrahim A Khalil, Ejaz Ahmad Khan, Gulfaraz Khan, Muhammad Shahzeb Khan, Muhammad Ali Khan, Young-Ho Khang, Tripti Khanna, Mona M Khater, Alireza Khatony, Habibolah Khazaie, Abdullah T Khoja, Ardeshir Khosravi, Mohammad Hossein Khosravi, Jagdish Khubchandani, Aliasghar A Kiadaliri, Getiye D Dejenu Kibret, Cho-il Kim, Daniel Kim, Jun Y Kim, Young-Eun Kim, Ruth W Kimokoti, Yohannes Kinfu, Sanjay Kinra, Adnan Kisa, Katarzyna Kissimova-Skarbek, Niranjan Kissoon, Mika Kivimäki, Marcus E Kleber, Luke D Knibbs, Ann Kristin Skrindo Knudsen, Sonali Kochhar, Yoshihiro Kokubo, Tufa Kolola, Jacek A Kopec, Margaret N Kosek, Soewarta Kosen, Parvaiz A Koul, Ai Koyanagi, Michael A Kravchenko, Kewal Krishan, Sanjay Krishnaswami, Barthelemy Kuate Defo, Burcu Kucuk Bicer, Andreas A Kudom, Ernst J Kuipers, Xie Rachel Kulikoff, G Anil Kumar, Manasi Kumar, Pushpendra Kumar, Fekede Asefa Kumsa, Michael J Kutz, Sheetal D Lad, Alessandra Lafranconi, Dharmesh Kumar Lal, Ratilal Lalloo, Hilton Lam, Faris Hasan Lami, Qing Lan, Sinéad M Langan, Van C Lansingh, Sonia Lansky, Heidi Jane Larson, Dennis Odai Laryea, Zohra S Lassi, Arman Latifi, Pablo M Lavados, Avula Laxmaiah, Jeffrey V Lazarus, Georgy Lebedev, Paul H Lee, James Leigh, Cheru Tesema Leshargie, Samson Leta, Miriam Levi, Shanshan Li, Yichong Li, Xiaohong Li, Juan Liang, Xiaofeng Liang, Misgan Legesse Liben, Lee-Ling Lim, Stephen S Lim, Miteku Andualem Limenih, Shai Linn, Shiwei Liu, Yang Liu, Rakesh Lodha, Giancarlo Logroscino, Chris Lonsdale, Scott A Lorch, Stefan Lorkowski, Paulo A Lotufo, Rafael Lozano, Tim C D Lucas, Raimundas Lunevicius, Ronan A Lyons, Stefan Ma, Crispin Mabika, Erlyn Rachelle King Macarayan, Mark T Mackay, Emilie R Maddison, Ralph Maddison, Fabiana Madotto, Hassan Magdy Abd El Razek, Muhammed Magdy Abd El Razek, Dhaval P Maghavani, Marek Majdan, Reza Majdzadeh, Azeem Majeed, Reza Malekzadeh, Manzoor Ahmad Malik, Deborah Carvalho Malta, Abdullah A Mamun, Wondimu Ayele Manamo, Ana-Laura Manda, Mohammad Ali Mansournia, Lorenzo Giovanni Mantovani, Chabila Christopher Mapoma, Dadi Marami, Joemer C Maravilla, Wagner Marcenes, Shakhnazarova Marina, Jose Martinez-Raga, Sheila C O Martins, Francisco Rogerlândio Martins-Melo, Winfried März, Melvin B Marzan, Tivani Phosa Mashamba-Thompson, Felix Masiye, Benjamin Ballard Massenburg, Pallab K Maulik, Mohsen Mazidi, John J McGrath, Martin McKee, Suresh Mehata, Sanjay Madhav Mehendale, Man Mohan Mehndiratta, Ravi Mehrotra, Kala M Mehta, Varshil Mehta, Tesfa Mekonen, Tefera Chane Mekonnen, Hagazi Gebre Meles, Kidanu Gebremariam Meles, Addisu Melese, Mulugeta Melku, Peter T N Memiah, Ziad A Memish, Walter Mendoza, Desalegn Tadese Mengistu, Getnet Mengistu, George A Mensah, Seid Tiku Mereta, Atte Meretoja, Tuomo J Meretoja, Tomislav Mestrovic, Haftay Berhane Mezgebe, Yode Miangotar, Bartosz Miazgowski, Tomasz Miazgowski, Ted R Miller, G K Mini, Andreea Mirica, Erkin M Mirrakhimov, Awoke Temesgen Misganaw, Babak Moazen, Nurilign Abebe Moges, Karzan Abdulmuhsin Mohammad, Moslem Mohammadi, Noushin Mohammadifard, Maryam Mohammadi-Khanaposhtani, Mousa Mohammadnia-Afrouzi, Shafiu Mohammed, Mohammed A Mohammed, Viswanathan Mohan, Ali H Mokdad, Mariam Molokhia, Lorenzo Monasta, Ghobad Moradi, Mahmoudreza Moradi, Maziar Moradi-Lakeh, Mehdi Moradinazar, Paula Moraga, Lidia Morawska, Ilais Moreno Velásquez, Joana Morgado-da-Costa, Shane Douglas Morrison, Abbas Mosapour, Marilita M Moschos, Seyyed Meysam Mousavi, Achenef Asmamaw Muche, Kindie Fentahun Muchie, Ulrich Otto Mueller, Satinath Mukhopadhyay, Erin C Mullany, Kate Muller, Manoj Murhekar, Tasha B Murphy, G V S Murthy, Srinivas Murthy, Jonah Musa, Kamarul Imran Musa, Ghulam Mustafa, Saravanan Muthupandian, Jean B Nachega, Gabriele Nagel, Mohsen Naghavi, Aliya Naheed, Azin Nahvijou, Gurudatta Naik, Sanjeev Nair, Farid Najafi, Vinay Nangia, Jobert Richie Nansseu, Bruno Ramos Nascimento, Haseeb Nawaz, Busisiwe P Ncama, Nahid Neamati, Ionut Negoi, Ruxandra Irina Negoi, Subas Neupane, Charles Richard James Newton, Frida N Ngalesoni, Josephine W Ngunjiri, Ha Thu Nguyen, Huong Thanh Nguyen, Long Hoang Nguyen, Michele Nguyen, Trang Huyen Nguyen, Dina Nur Anggraini Ningrum, Yirga Legesse Nirayo, Muhammad Imran Nisar, Molly R Nixon, Nomonde Nolutshungu, Shuhei Nomura, Ole F Norheim, Mehdi Noroozi, Bo Norrving, Jean Jacques Noubiap, Hamid Reza Nouri, Malihe Nourollahpour Shiadeh, Mohammad Reza Nowroozi, Elaine O Nsoesie, Peter S Nyasulu, Richard Ofori-Asenso, Okechukwu Samuel Ogah, Felix Akpojene Ogbo, In-Hwan Oh, Anselm Okoro, Olanrewaju Oladimeji, Andrew T Olagunju, Tinuke O Olagunju, Pedro R Olivares, Bolajoko Olubukunola Olusanya, Jacob Olusegun Olusanya, Sok King Ong, John Nelson Opio, Eyal Oren, Justin R Ortiz, Alberto Ortiz, Erika Ota, Stanislav S Otstavnov, Simon Øverland, Mayowa Ojo Owolabi, Abayomi Samuel Oyekale, Mahesh P A, Rosana Pacella, Smita Pakhale, Abhijit P Pakhare, Adrian Pana, Basant Kumar Panda, Songhomitra Panda-Jonas, Achyut Raj Pandey, Jeyaraj Durai Pandian, Andrea Parisi, Eun-Kee Park, Charles D H Parry, Hadi Parsian, Shanti Patel, Ajay Patle, Scott B Patten, George C Patton, Deepak Paudel, Neil Pearce, Emmanuel K Peprah, Alexandre Pereira, David M Pereira, Krystle M Perez, Norberto Perico, Aslam Pervaiz, Konrad Pesudovs, William A Petri, Max Petzold, Michael R Phillips, David M Pigott, Julian David Pillay, Meghdad Pirsaheb, Farhad Pishgar, Dietrich Plass, Suzanne Polinder, Constance Dimity Pond, Svetlana Popova, Maarten J Postma, Farshad Pourmalek, Akram Pourshams, Hossein Poustchi, Dorairaj Prabhakaran, V Prakash, Swayam Prakash, Narayan Prasad, Mostafa Qorbani, D Alex Quistberg, Amir Radfar, Anwar Rafay, Alireza Rafiei, Fakher Rahim, Kazem Rahimi, Afarin Rahimi-Movaghar, Vafa Rahimi-Movaghar, Mahfuzar Rahman, Mohammad Hifz Ur Rahman, Muhammad Aziz Rahman, Sajjad ur Rahman, Rajesh Kumar Rai, Fatemeh Rajati, Sasa Rajsic, Sree Bhushan Raju, Usha Ram, Chhabi Lal Ranabhat, Prabhat Ranjan, Anna Ranta, Davide Rasella, David Laith Rawaf, Salman Rawaf, Sarah E Ray, Christian Razo-García, Maria Albertina Santiago Rego, Jürgen Rehm, Robert C Reiner, Nickolas Reinig, Cesar Reis, Giuseppe Remuzzi, Andre M N Renzaho, Serge Resnikoff, Satar Rezaei, Shahab Rezaeian, Mohammad Sadegh Rezai, Seyed Mohammad Riahi, Antonio Luiz P Ribeiro, Horacio Riojas, Maria Jesus Rios-Blancas, Kedir Teji Roba, Stephen R Robinson, Leonardo Roever, Luca Ronfani, Gholamreza Roshandel, Denis O Roshchin, Ali Rostami, Dietrich Rothenbacher, Enrico Rubagotti, George Mugambage Ruhago, Soheil Saadat, Yogesh Damodar Sabde, Perminder S Sachdev, Basema Saddik, Ehsan Sadeghi, Sahar Saeedi Moghaddam, Hosein Safari, Yahya Safari, Roya Safari-Faramani, Mahdi Safdarian, Sare Safi, Saeid Safiri, Rajesh Sagar, Amirhossein Sahebkar, Mohammad Ali Sahraian, Haniye Sadat Sajadi, Mohamadreza Salahshoor, Nasir Salam, Joseph S Salama, Payman Salamati, Raphael de Freitas Saldanha, Yahya Salimi, Hamideh Salimzadeh, Inbal Salz, Evanson Zondani Sambala, Abdallah M Samy, Juan Sanabria, Maria Dolores Sanchez-Niño, Itamar S Santos, João Vasco Santos, Milena M Santric Milicevic, Bruno Piassi Sao Jose, Mayank Sardana, Abdur Razzaque Sarker, Nizal Sarrafzadegan, Benn Sartorius, Shahabeddin Sarvi, Brijesh Sathian, Maheswar Satpathy, Miloje Savic, Arundhati R Sawant, Monika Sawhney, Sonia Saxena, Mehdi Sayyah, Vinod Scaria, Elke Schaeffner, Kathryn Schelonka, Maria Inês Schmidt, Ione J C Schneider, Ben Schöttker, Aletta Elisabeth Schutte, David C Schwebel, Falk Schwendicke, James G Scott, Mario Sekerija, Sadaf G Sepanlou, Edson Serván-Mori, Hosein Shabaninejad, Katya Anne Shackelford, Azadeh Shafieesabet, Amira A Shaheen, Masood Ali Shaikh, Raad A Shakir, Mehran Shams-Beyranvand, MohammadBagher Shamsi, Morteza Shamsizadeh, Heidar Sharafi, Kiomars Sharafi, Mehdi Sharif, Mahdi Sharif-Alhoseini, Meenakshi Sharma, Jayendra Sharma, Rajesh Sharma, Jun She, Aziz Sheikh, Kevin N Sheth, Peilin Shi, Kenji Shibuya, Girma Temam Shifa, Mekonnen Sisay Shiferaw, Mika Shigematsu, Rahman Shiri, Reza Shirkoohi, Ivy Shiue, Farhad Shokraneh, Mark G Shrime, Sharvari Rahul Shukla, Si Si, Soraya Siabani, Tariq J Siddiqi, Inga Dora Sigfusdottir, Rannveig Sigurvinsdottir, Naris Silpakit, Diego Augusto Santos Silva, João Pedro Silva, Dayane Gabriele Alves Silveira, Narayana Sarma Venkata Singam, Jasvinder A Singh, Virendra Singh, Anju Pradhan Sinha, Dhirendra Narain Sinha, Freddy Sitas, Vegard Skirbekk, Karen Sliwa, Adauto Martins Soares Filho, Badr Hasan Sobaih, Soheila Sobhani, Moslem Soofi, Joan B Soriano, Ireneous N Soyiri, Luciano A Sposato, Chandrashekhar T Sreeramareddy, Vinay Srinivasan, Rakesh Kumar Srivastava, Vladimir I Starodubov, Vasiliki Stathopoulou, Nicholas Steel, Dan J Stein, Caitlyn Steiner, Leo G Stewart, Mark A Stokes, Agus Sudaryanto, Mu'awiyyah Babale Sufiyan, Gerhard Sulo, Bruno F Sunguya, Patrick John Sur, Ipsita Sutradhar, Bryan L Sykes, P N Sylaja, Dillon O Sylte, Cassandra E I Szoeke, Rafael Tabarés-Seisdedos, Takahiro Tabuchi, Santosh Kumar Tadakamadla, Ken Takahashi, Nikhil Tandon, Aberash Abay Tassew, Segen Gebremeskel Tassew, Mohammad Tavakkoli, Nuno Taveira, Nega Yimer Tawye, Arash Tehrani-Banihashemi, Tigist Gashaw Tekalign, Merhawi Gebremedhin Tekle, Habtamu Temesgen, Mohamad-Hani Temsah, Omar Temsah, Abdullah Sulieman Terkawi, Manaye Yihune Teshale, Belay Tessema, Mebrahtu Teweldemedhin, Jarnail Singh Thakur, Kavumpurathu Raman Thankappan, Sathish Thirunavukkarasu, Laura Anne Thomas, Nihal Thomas, Amanda G Thrift, Binyam Tilahun, Quyen G To, Ruoyan Tobe-Gai, Marcello Tonelli, Roman Topor-Madry, Fotis Topouzis, Anna E Torre, Miguel Tortajada-Girbés, Marcos Roberto Tovani-Palone, Jeffrey A Towbin, Bach Xuan Tran, Khanh Bao Tran, Suryakant Tripathi, Srikanth Prasad Tripathy, Thomas Clement Truelsen, Nu Thi Truong, Afewerki Gebremeskel Tsadik, Nikolaos Tsilimparis, Lorainne Tudor Car, E Murat Tuzcu, Stefanos Tyrovolas, Kingsley Nnanna Ukwaja, Irfan Ullah, Muhammad Shariq Usman, Olalekan A Uthman, Selen Begüm Uzun, Muthiah Vaduganathan, Afsane Vaezi, Gaurang Vaidya, Pascual R Valdez, Elena Varavikova, Santosh Varughese, Tommi Juhani Vasankari, Ana Maria Nogales Vasconcelos, Narayanaswamy Venketasubramanian, Ramesh Vidavalur, Santos Villafaina, Francesco S Violante, Sergey Konstantinovitch Vladimirov, Vasily Vlassov, Stein Emil Vollset, Theo Vos, Kia Vosoughi, Isidora S Vujcic, Gregory R Wagner, Fasil Wagnew Shiferaw Wagnew, Yasir Waheed, Yanping Wang, Yuan-Pang Wang, Molla Mesele Wassie, Elisabete Weiderpass, Robert G Weintraub, Daniel J Weiss, Jordan Weiss, Fitsum Weldegebreal, Kidu Gidey Weldegwergs, Andrea Werdecker, Ronny Westerman, Harvey A Whiteford, Justyna Widecka, Katarzyna Widecka, Tissa Wijeratne, Andrea Sylvia Winkler, Charles Shey Wiysonge, Charles D A Wolfe, Sintayehu Ambachew Wondemagegn, Shouling Wu, Grant M A Wyper, Gelin Xu, Rajaram Yadav, Bereket Yakob, Tomohide Yamada, Lijing L Yan, Yuichiro Yano, Mehdi Yaseri, Yasin Jemal Yasin, Pengpeng Ye, Jamal A Yearwood, Gökalp Kadri Yentür, Alex Yeshaneh, Ebrahim M Yimer, Paul Yip, Engida Yisma, Naohiro Yonemoto, Seok-Jun Yoon, Hunter W York, Marcel Yotebieng, Mustafa Z Younis, Mahmoud Yousefifard, Chuanhua Yu, Geevar Zachariah, Vesna Zadnik, Shamsa Zafar, Zoubida Zaidi, Sojib Bin Zaman, Mohammad Zamani, Zohreh Zare, Hajo Zeeb, Mulugeta Molla Zeleke, Zerihun Menlkalew Zenebe, Taddese Alemu Zerfu, Kai Zhang, Xueying Zhang, Maigeng Zhou, Jun Zhu, Sanjay Zodpey, Inbar Zucker, Liesl Joanna J Zuhlke, Alan D Lopez, Emmanuela Gakidou, Christopher J L Murray

## Abstract

**Background:**

Assessments of age-specific mortality and life expectancy have been done by the UN Population Division, Department of Economics and Social Affairs (UNPOP), the United States Census Bureau, WHO, and as part of previous iterations of the Global Burden of Diseases, Injuries, and Risk Factors Study (GBD). Previous iterations of the GBD used population estimates from UNPOP, which were not derived in a way that was internally consistent with the estimates of the numbers of deaths in the GBD. The present iteration of the GBD, GBD 2017, improves on previous assessments and provides timely estimates of the mortality experience of populations globally.

**Methods:**

The GBD uses all available data to produce estimates of mortality rates between 1950 and 2017 for 23 age groups, both sexes, and 918 locations, including 195 countries and territories and subnational locations for 16 countries. Data used include vital registration systems, sample registration systems, household surveys (complete birth histories, summary birth histories, sibling histories), censuses (summary birth histories, household deaths), and Demographic Surveillance Sites. In total, this analysis used 8259 data sources. Estimates of the probability of death between birth and the age of 5 years and between ages 15 and 60 years are generated and then input into a model life table system to produce complete life tables for all locations and years. Fatal discontinuities and mortality due to HIV/AIDS are analysed separately and then incorporated into the estimation. We analyse the relationship between age-specific mortality and development status using the Socio-demographic Index, a composite measure based on fertility under the age of 25 years, education, and income. There are four main methodological improvements in GBD 2017 compared with GBD 2016: 622 additional data sources have been incorporated; new estimates of population, generated by the GBD study, are used; statistical methods used in different components of the analysis have been further standardised and improved; and the analysis has been extended backwards in time by two decades to start in 1950.

**Findings:**

Globally, 18·7% (95% uncertainty interval 18·4–19·0) of deaths were registered in 1950 and that proportion has been steadily increasing since, with 58·8% (58·2–59·3) of all deaths being registered in 2015. At the global level, between 1950 and 2017, life expectancy increased from 48·1 years (46·5–49·6) to 70·5 years (70·1–70·8) for men and from 52·9 years (51·7–54·0) to 75·6 years (75·3–75·9) for women. Despite this overall progress, there remains substantial variation in life expectancy at birth in 2017, which ranges from 49·1 years (46·5–51·7) for men in the Central African Republic to 87·6 years (86·9–88·1) among women in Singapore. The greatest progress across age groups was for children younger than 5 years; under-5 mortality dropped from 216·0 deaths (196·3–238·1) per 1000 livebirths in 1950 to 38·9 deaths (35·6–42·83) per 1000 livebirths in 2017, with huge reductions across countries. Nevertheless, there were still 5·4 million (5·2–5·6) deaths among children younger than 5 years in the world in 2017. Progress has been less pronounced and more variable for adults, especially for adult males, who had stagnant or increasing mortality rates in several countries. The gap between male and female life expectancy between 1950 and 2017, while relatively stable at the global level, shows distinctive patterns across super-regions and has consistently been the largest in central Europe, eastern Europe, and central Asia, and smallest in south Asia. Performance was also variable across countries and time in observed mortality rates compared with those expected on the basis of development.

**Interpretation:**

This analysis of age-sex-specific mortality shows that there are remarkably complex patterns in population mortality across countries. The findings of this study highlight global successes, such as the large decline in under-5 mortality, which reflects significant local, national, and global commitment and investment over several decades. However, they also bring attention to mortality patterns that are a cause for concern, particularly among adult men and, to a lesser extent, women, whose mortality rates have stagnated in many countries over the time period of this study, and in some cases are increasing.

**Funding:**

Bill & Melinda Gates Foundation.

**Research in context**

**Evidence before this study**

Several organisations report on aspects of all-cause mortality or life expectancy: the UN Population Division, Department of Economics and Social Affairs (UNPOP), the United States Census Bureau, and WHO. Additionally, previous iterations of the Global Burden of Disease Study (GBD) have produced these estimates on an annual basis. UNPOP reports age-specific mortality by 5-year age groups for 162 countries and for time periods that cover 5 calendar years; these estimates are updated every 2 years (most recently in June, 2017). The United States Census Bureau produces mortality assessments for 15–25 countries per year, and WHO reports periodically on life expectancy and sometimes on other measures of mortality and bases its estimates on results from the UNPOP. The most recent release of estimates by WHO was in January, 2017, based on UNPOP estimates from 2015. GBD 2016 provided comprehensive assessment of age-sex-specific mortality for 195 countries and territories from 1970 to 2016 that were compliant with the Guidelines on Accurate and Transparent Reporting of Health Estimates.

**Added value of this study**

The most important changes in GBD 2017 are the independent estimation of population and a comprehensive update on fertility, which are described in a separate paper. There are several countries with significant differences in population size between the UNPOP estimates and the new GBD estimates. Since population is the denominator for mortality calculations, this leads to substantial changes in life expectancy and age-specific mortality rates in several countries. There were four major data additions and improvements that related to the estimation of mortality. First, for the estimation of population size, we systematically searched for census data and found data from 1257 censuses, which are now used in the analysis and which enabled an extended analysis of completeness using death distribution methods in more locations than previous iterations. Second, in the estimation of adult mortality, we included data from 31 Demographic Surveillance Sites (DSS) which were adjusted based on the relationship between DSS under-5 death rates and national under-5 death rates. Third, we used published sources to create a database of fatal discontinuities from conflicts and natural disasters that extends back to 1950; each fatal discontinuity has been given a unique ID that tags the reported deaths to a location, date, and type of discontinuity. Fourth, GBD 2017 included an additional 622 data sources that were not available for GBD 2016 and which do not fall into the three categories already described. The main methodological improvements fall into two categories: the first category is enhancements to the modelling framework, which improved the estimation of both child mortality, defined as the probability of death below the age of 5 years, and adult mortality, a term we use to refer to the probability of death between ages 15 and 60 years. For child mortality, we standardised hyperparameter selection for the spatiotemporal Gaussian process regression models, which enhances the comparability of results between locations and across time. For adult mortality, we also standardised hyperparameter selection and added child mortality as a covariate to the model. These changes had minimal effect on the mean estimate but changed the width of the uncertainty intervals in small populations and locations with sparse data. The second category encompasses three substantial improvements to the GBD model life table system: first, we revised the entire database to reflect the change in population counts. Second, each life table in the database was assigned a quality score using explicit criteria related to the variance in the slope of the death rate with respect to age, reductions in mortality at older ages compared with younger ages (age >60 years), and other unexpected crossovers. On the basis of these quality scores, life tables have been assigned to three categories: high quality for universal use, acceptable quality for use in the creation of location-specific standards, and unacceptable quality. Third, we estimated complete single-year life tables for each sex, location, and year instead of abridged life tables as in previous iterations of the GBD. In GBD 2017, for the first time, we are reporting a complete time series of trends in age-specific mortality and life expectancy since 1950. The extension of the analysis back in time provides the opportunity to analyse and report on longer-term trends in age-specific mortality.

**Table:** Life expectancy at birth and at age 60 years, probability of death between birth and age 5 years, probability of death between ages 15 and 60 years, and total number of deaths, for countries and territories and subnational units in the UK, by sex, 2017

			**Probability of death between birth and age 5 years**		**Probability of death between ages 15 and 60 years**		**Life expectancy at birth (years)**		**Life expectancy at age 60 years (years)**		**Total deaths (thousands)**	
			Male	Female	Male	Female	Male	Female	Male	Female	Male	Female
**Global**			**0·04 (0·04 to 0·05)**	**0·04 (0·03 to 0·04)**	**0·17 (0·17 to 0·17)**	**0·10 (0·10 to 0·11)**	**70·48 (70·12 to 70·82)**	**75·59 (75·31 to 75·86)**	**19·39 (19·28 to 19·51)**	**22·61 (22·5 to 22·73)**	**30 387 (29 986 to 30 775)**	**25 558 (25 224 to 25 885)**
Low SDI			0·07 (0·06 to 0·08)	0·06 (0·06 to 0·07)	0·24 (0·23 to 0·25)	0·18 (0·18 to 0·19)	64·48 (63·8 to 65·13)	67·34 (66·75 to 67·95)	16·77 (16·5 to 17·04)	18·15 (17·84 to 18·45)	4806 (4685 to 4939)	4131 (4023 to 4240)
Low-middle SDI			0·06 (0·05 to 0·06)	0·05 (0·05 to 0·05)	0·22 (0·21 to 0·23)	0·16 (0·15 to 0·17)	66·27 (65·67 to 66·86)	70·08 (69·5 to 70·65)	17·28 (17·02 to 17·53)	19·4 (19·11 to 19·69)	6579 (6363 to 6813)	5656 (5465 to 5866)
Middle SDI			0·02 (0·02 to 0·02)	0·02 (0·02 to 0·02)	0·17 (0·16 to 0·17)	0·09 (0·09 to 0·09)	71·71 (71·37 to 72·09)	77·42 (77·09 to 77·7)	18·92 (18·69 to 19·18)	22·22 (21·96 to 22·46)	6067 (5911 to 6217)	4536 (4418 to 4662)
High-middle SDI			0·01 (0·01 to 0·01)	0·01 (0·01 to 0·01)	0·15 (0·14 to 0·15)	0·07 (0·07 to 0·07)	73·33 (72·98 to 73·69)	79·42 (79·13 to 79·7)	19·1 (18·85 to 19·37)	22·68 (22·44 to 22·91)	7831 (7607 to 8059)	6259 (6088 to 6439)
High SDI			0·01 (<0·01 to 0·01)	<0·01 (<0·01 to <0·01)	0·10 (0·10 to 0·10)	0·05 (0·05 to 0·06)	78·47 (78·3 to 78·65)	83·7 (83·53 to 83·86)	22·46 (22·33 to 22·58)	26·19 (26·05 to 26·32)	4997 (4922 to 5071)	4875 (4797 to 4953)
**Central Europe, eastern Europe, and central Asia**			**0·02 (0·01 to 0·02)**	**0·01 (0·01 to 0·01)**	**0·24 (0·24 to 0·25)**	**0·10 (0·10 to 0·10)**	**68·5 (68·3 to 68·68)**	**77·57 (77·41 to 77·74)**	**17·02 (16·92 to 17·12)**	**21·99 (21·89 to 22·1)**	**2427 (2398 to 2457)**	**2303 (2273 to 2332)**
Central Asia			0·03 (0·03 to 0·03)	0·02 (0·02 to 0·03)	0·22 (0·21 to 0·23)	0·11 (0·10 to 0·12)	67·37 (66·76 to 67·92)	74·83 (74·26 to 75·4)	15·82 (15·46 to 16·14)	20·30 (19·94 to 20·67)	353 (339 to 369)	277 (266 to 290)
	Armenia		0·01 (0·01 to 0·01)	0·01 (0·01 to 0·01)	0·16 (0·16 to 0·17)	0·07 (0·06 to 0·07)	72·38 (71·97 to 72·81)	78·65 (78·23 to 79·06)	17·93 (17·64 to 18·22)	21·51 (21·16 to 21·84)	14 (14 to 15)	14 (13 to 14)
	Azerbaijan		0·04 (0·03 to 0·05)	0·03 (0·03 to 0·04)	0·19 (0·17 to 0·21)	0·09 (0·08 to 0·10)	67·23 (66·2 to 68·22)	74·66 (73·74 to 75·66)	15·1 (14·51 to 15·71)	20·27 (19·56 to 21)	45 (41 to 48)	31 (28 to 33)
	Georgia		0·01 (0·01 to 0·01)	0·01 (0·01 to 0·01)	0·24 (0·23 to 0·25)	0·08 (0·08 to 0·09)	68·39 (67·96 to 68·81)	77·31 (76·89 to 77·73)	16·21 (15·99 to 16·45)	20·83 (20·53 to 21·13)	25 (25 to 26)	25 (24 to 26)
	Kazakhstan		0·02 (0·01 to 0·02)	0·01 (0·01 to 0·02)	0·26 (0·24 to 0·27)	0·10 (0·10 to 0·11)	67·46 (66·76 to 68·16)	76·38 (75·75 to 77·06)	16·24 (15·84 to 16·65)	20·93 (20·50 to 21·42)	74 (71 to 78)	61 (57 to 64)
	Kyrgyzstan		0·02 (0·02 to 0·02)	0·02 (0·02 to 0·02)	0·21 (0·20 to 0·21)	0·10 (0·09 to 0·10)	69·07 (68·7 to 69·44)	76·27 (75·88 to 76·65)	16·83 (16·6 to 17·06)	20·92 (20·64 to 21·21)	19 (18 to 19)	15 (15 to 16)
	Mongolia		0·03 (0·02 to 0·04)	0·02 (0·02 to 0·03)	0·30 (0·27 to 0·33)	0·14 (0·12 to 0·15)	64·48 (63·18 to 65·94)	73·66 (72·47 to 74·84)	14·9 (14·16 to 15·68)	19·68 (18·85 to 20·52)	13 (12 to 14)	8 (8 to 9)
	Tajikistan		0·05 (0·04 to 0·06)	0·04 (0·04 to 0·05)	0·18 (0·16 to 0·20)	0·12 (0·11 to 0·14)	67·67 (66·33 to 68·92)	73·3 (72·06 to 74·54)	17·19 (16·34 to 17·94)	20·75 (19·9 to 21·67)	28 (26 to 30)	20 (18 to 22)
	Turkmenistan		0·03 (0·03 to 0·04)	0·03 (0·02 to 0·03)	0·25 (0·23 to 0·27)	0·13 (0·12 to 0·14)	66·54 (65·42 to 67·68)	73·87 (72·72 to 74·94)	16·27 (15·66 to 16·93)	20·05 (19·26 to 20·76)	19 (17 to 20)	14 (13 to 16)
	Uzbekistan		0·03 (0·02 to 0·03)	0·02 (0·02 to 0·02)	0·21 (0·19 to 0·24)	0·12 (0·11 to 0·14)	67·12 (65·55 to 68·6)	73·75 (72·18 to 75·35)	15 (14·07 to 15·95)	19·38 (18·24 to 20·57)	116 (103 to 130)	89 (78 to 102)
Central Europe			0·01 (0·01 to 0·01)	0·01 (<0·01 to 0·01)	0·15 (0·15 to 0·16)	0·07 (0·06 to 0·07)	73·62 (73·34 to 73·92)	80·44 (80·19 to 80·70)	18·69 (18·49 to 18·88)	23·13 (22·94 to 23·34)	678 (663 to 695)	649 (633 to 665)
	Albania		0·01 (0·01 to 0·02)	0·01 (0·01 to 0·01)	0·10 (0·08 to 0·13)	0·05 (0·04 to 0·06)	74·93 (72·83 to 77·11)	82·1 (79·9 to 84·32)	19·57 (18·12 to 21·14)	25 (23·18 to 26·9)	13 (11 to 16)	8 (7 to 11)
	Bosnia and Herzegovina		0·01 (0·01 to 0·01)	0·01 (0·01 to 0·01)	0·13 (0·12 to 0·14)	0·06 (0·06 to 0·07)	74·34 (73·62 to 75·04)	79·06 (78·39 to 79·74)	18·62 (18·1 to 19·12)	21·57 (21·03 to 22·11)	19 (18 to 20)	18 (17 to 19)
	Bulgaria		0·01 (0·01 to 0·01)	0·01 (0·01 to 0·01)	0·19 (0·18 to 0·20)	0·09 (0·08 to 0·09)	71·33 (70·60 to 72·11)	78·58 (77·88 to 79·24)	17·3 (16·83 to 17·82)	22·01 (21·49 to 22·52)	56 (53 to 60)	51 (48 to 55)
	Croatia		<0·01 (<0·01 to 0·01)	<0·01 (<0·01 to <0·01)	0·12 (0·11 to 0·13)	0·05 (0·04 to 0·05)	75·39 (74·71 to 76·08)	81·61 (80·95 to 82·28)	19·28 (18·81 to 19·8)	23·57 (23·03 to 24·15)	25 (24 to 27)	26 (24 to 28)
	Czech Republic		<0·01 (<0·01 to <0·01)	<0·01 (<0·01 to <0·01)	0·11 (0·10 to 0·12)	0·05 (0·05 to 0·06)	76·31 (75·6 to 77)	81·96 (81·29 to 82·6)	19·95 (19·42 to 20·46)	24·06 (23·52 to 24·57)	56 (52 to 59)	55 (51 to 59)
	Hungary		0·01 (<0·01 to 0·01)	<0·01 (<0·01 to <0·01)	0·17 (0·15 to 0·18)	0·08 (0·07 to 0·08)	73·19 (72·42 to 73·89)	80·20 (79·5 to 80·86)	18·13 (17·59 to 18·63)	23·02 (22·47 to 23·55)	60 (57 to 64)	62 (58 to 66)
	Macedonia		0·01 (0·01 to 0·01)	0·01 (0·01 to 0·01)	0·13 (0·12 to 0·14)	0·07 (0·06 to 0·08)	73·88 (73·19 to 74·58)	79·68 (79·15 to 80·26)	18·34 (17·86 to 18·84)	22·91 (22·56 to 23·34)	12 (11 to 12)	8 (7 to 8)
	Montenegro		<0·01 (<0·01 to 0·01)	<0·01 (<0·01 to <0·01)	0·13 (0·12 to 0·15)	0·07 (0·06 to 0·08)	74·06 (72·92 to 75·15)	78·93 (78·13 to 79·72)	18·2 (17·38 to 19)	21·55 (20·93 to 22·18)	3 (3 to 4)	3 (3 to 3)
	Poland		<0·01 (<0·01 to 0·01)	<0·01 (<0·01 to <0·01)	0·16 (0·15 to 0·17)	0·06 (0·06 to 0·07)	74·07 (73·35 to 74·8)	81·85 (81·2 to 82·44)	19·3 (18·81 to 19·8)	24·32 (23·81 to 24·8)	204 (192 to 216)	191 (180 to 203)
	Romania		0·01 (0·01 to 0·01)	0·01 (0·01 to 0·01)	0·19 (0·18 to 0·21)	0·08 (0·07 to 0·08)	71·55 (70·82 to 72·26)	78·95 (78·35 to 79·61)	17·8 (17·33 to 18·27)	22·27 (21·8 to 22·77)	136 (129 to 144)	125 (117 to 132)
	Serbia		0·01 (0·01 to 0·01)	<0·01 (<0·01 to <0·01)	0·14 (0·13 to 0·15)	0·08 (0·07 to 0·08)	73·59 (72·93 to 74·24)	77·86 (77·2 to 78·54)	18·02 (17·58 to 18·48)	20·55 (20·04 to 21·09)	57 (54 to 60)	67 (62 to 71)
	Slovakia		0·01 (0·01 to 0·01)	0·01 (<0·01 to 0·01)	0·14 (0·13 to 0·15)	0·06 (0·06 to 0·07)	74·09 (73·4 to 74·77)	80·57 (79·86 to 81·27)	18·73 (18·25 to 19·22)	23·18 (22·63 to 23·74)	27 (25 to 28)	26 (24 to 28)
	Slovenia		<0·01 (<0·01 to <0·01)	<0·01 (<0·01 to <0·01)	0·10 (0·09 to 0·11)	0·04 (0·04 to 0·05)	77·92 (77·17 to 78·71)	84·22 (83·45 to 84·99)	21·31 (20·75 to 21·89)	26·01 (25·36 to 26·64)	10 (9 to 11)	10 (9 to 11)
Eastern Europe			0·01 (0·01 to 0·01)	0·01 (0·01 to 0·01)	0·30 (0·29 to 0·30)	0·11 (0·11 to 0·11)	66·49 (66·28 to 66·71)	77·24 (77·06 to 77·43)	16·14 (16·02 to 16·27)	21·66 (21·52 to 21·79)	1395 (1375 to 1415)	1376 (1355 to 1398)
	Belarus		0·01 (0·01 to 0·01)	0·01 (<0·01 to 0·01)	0·23 (0·22 to 0·25)	0·08 (0·08 to 0·09)	68·96 (68·2 to 69·68)	78·78 (78·14 to 79·45)	16·01 (15·56 to 16·45)	22·06 (21·58 to 22·57)	60 (56 to 63)	61 (58 to 65)
	Estonia		<0·01 (<0·01 to <0·01)	<0·01 (<0·01 to <0·01)	0·17 (0·14 to 0·19)	0·06 (0·05 to 0·07)	73·64 (71·97 to 75·29)	82·08 (80·69 to 83·49)	19·19 (18·11 to 20·29)	24·6 (23·52 to 25·72)	7 (6 to 8)	8 (7 to 9)
	Latvia		0·01 (<0·01 to 0·01)	<0·01 (<0·01 to <0·01)	0·23 (0·20 to 0·25)	0·08 (0·07 to 0·10)	70·13 (68·55 to 71·75)	79·85 (78·38 to 81·3)	17·22 (16·26 to 18·22)	23·07 (21·99 to 24·17)	13 (12 to 15)	15 (13 to 17)
	Lithuania		0·01 (<0·01 to 0·01)	<0·01 (<0·01 to <0·01)	0·24 (0·22 to 0·26)	0·08 (0·07 to 0·09)	69·63 (68·72 to 70·51)	80·20 (79·43 to 80·97)	17·16 (16·63 to 17·7)	23·39 (22·84 to 23·97)	20 (19 to 21)	21 (19 to 22)
	Moldova		0·02 (0·01 to 0·02)	0·01 (0·01 to 0·01)	0·25 (0·24 to 0·26)	0·10 (0·09 to 0·10)	68·2 (67·78 to 68·66)	77·42 (77·01 to 77·86)	16·34 (16·1 to 16·6)	21·64 (21·33 to 21·96)	22 (21 to 23)	20 (19 to 20)
	Russia		0·01 (0·01 to 0·01)	0·01 (0·01 to 0·01)	0·29 (0·29 to 0·30)	0·11 (0·11 to 0·11)	66·75 (66·63 to 66·89)	77·24 (77·12 to 77·36)	16·43 (16·36 to 16·5)	21·7 (21·62 to 21·78)	919 (911 to 926)	916 (907 to 925)
	Ukraine		0·01 (0·01 to 0·01)	0·01 (0·01 to 0·01)	0·33 (0·31 to 0·35)	0·11 (0·10 to 0·12)	64·65 (63·86 to 65·44)	76·52 (75·78 to 77·19)	15·24 (14·84 to 15·64)	21·2 (20·69 to 21·66)	355 (337 to 373)	335 (317 to 357)
**High income**			**0·01 (0·01 to 0·01)**	**<0·01 (<0·01 to 0·01)**	**0·10 (0·10 to 0·10)**	**0·06 (0·06 to 0·06)**	**78·43 (78·25 to 78·61)**	**83·56 (83·38 to 83·74)**	**22·51 (22·38 to 22·63)**	**26·18 (26·04 to 26·32)**	**4885 (4812 to 4959)**	**4784 (4705 to 4866)**
Australasia			<0·01 (<0·01 to 0·01)	<0·01 (<0·01 to <0·01)	0·08 (0·07 to 0·09)	0·05 (0·04 to 0·05)	80·13 (79·05 to 81·23)	84·42 (83·44 to 85·37)	23·52 (22·73 to 24·34)	26·58 (25·79 to 27·37)	106 (96 to 117)	98 (88 to 108)
	Australia		<0·01 (<0·01 to <0·01)	<0·01 (<0·01 to <0·01)	0·08 (0·07 to 0·09)	0·05 (0·04 to 0·05)	80·21 (78·94 to 81·49)	84·58 (83·42 to 85·74)	23·56 (22·66 to 24·48)	26·69 (25·74 to 27·64)	89 (79 to 100)	82 (72 to 92)
	New Zealand		<0·01 (<0·01 to 0·01)	<0·01 (<0·01 to <0·01)	0·09 (0·08 to 0·09)	0·05 (0·05 to 0·06)	79·65 (79·03 to 80·29)	83·57 (82·98 to 84·16)	23·33 (22·88 to 23·81)	26·03 (25·53 to 26·5)	17 (16 to 18)	16 (15 to 17)
High-income Asia Pacific			<0·01 (<0·01 to <0·01)	<0·01 (<0·01 to <0·01)	0·07 (0·07 to 0·08)	0·04 (0·03 to 0·04)	80·76 (80·49 to 81·03)	86·93 (86·71 to 87·15)	23·62 (23·42 to 23·82)	28·67 (28·49 to 28·84)	879 (858 to 901)	814 (796 to 832)
	Brunei		0·01 (0·01 to 0·01)	0·01 (0·01 to 0·01)	0·15 (0·13 to 0·16)	0·10 (0·09 to 0·11)	73·35 (72·31 to 74·39)	77·5 (76·63 to 78·43)	18·9 (17·88 to 19·83)	21·58 (21 to 22·21)	1 (1 to 1)	1 (1 to 1)
	Japan		<0·01 (<0·01 to <0·01)	<0·01 (<0·01 to <0·01)	0·07 (0·07 to 0·07)	0·04 (0·03 to 0·04)	81·08 (80·80 to 81·34)	87·21 (86·96 to 87·44)	23·8 (23·59 to 24)	28·93 (28·73 to 29·11)	704 (688 to 722)	668 (652 to 685)
	Singapore		<0·01 (<0·01 to <0·01)	<0·01 (<0·01 to <0·01)	0·06 (0·06 to 0·07)	0·03 (0·03 to 0·04)	81·93 (81·24 to 82·61)	87·55 (86·9 to 88·08)	24·28 (23·72 to 24·83)	29·13 (28·58 to 29·55)	11 (11 to 12)	9 (8 to 9)
	South Korea		<0·01 (<0·01 to <0·01)	<0·01 (<0·01 to <0·01)	0·08 (0·07 to 0·09)	0·04 (0·03 to 0·04)	79·52 (78·74 to 80·29)	85·48 (84·89 to 86·11)	22·64 (22·06 to 23·21)	27·23 (26·75 to 27·74)	163 (151 to 176)	136 (127 to 146)
High-income North America			0·01 (0·01 to 0·01)	0·01 (0·01 to 0·01)	0·13 (0·13 to 0·14)	0·08 (0·08 to 0·08)	76·46 (76·15 to 76·76)	81·38 (81·11 to 81·66)	21·9 (21·7 to 22·1)	24·87 (24·68 to 25·08)	1603 (1566 to 1642)	1534 (1498 to 1569)
	Canada		0·01 (0·01 to 0·01)	0·01 (<0·01 to 0·01)	0·09 (0·08 to 0·09)	0·05 (0·05 to 0·06)	79·86 (79·2 to 80·53)	83·99 (83·36 to 84·57)	23·5 (23·02 to 24)	26·47 (25·96 to 26·92)	141 (133 to 150)	138 (131 to 147)
	Greenland		0·01 (0·01 to 0·02)	0·01 (0·01 to 0·01)	0·18 (0·17 to 0·19)	0·11 (0·10 to 0·12)	70·84 (70·33 to 71·38)	77·15 (76·2 to 78·04)	17·5 (17·3 to 18·32)	22·05 (21·34 to 22·7)	<1 (<1 to <1)	<1 (<1 to <1)
	USA		0·01 (0·01 to 0·01)	0·01 (0·01 to 0·01)	0·14 (0·14 to 0·14)	0·08 (0·08 to 0·08)	76·09 (75·76 to 76·42)	81·09 (80·80 to 81·38)	21·72 (21·51 to 21·94)	24·69 (24·48 to 24·91)	1461 (1424 to 1499)	1396 (1361 to 1431)
Southern Latin America			0·01 (0·01 to 0·01)	0·01 (0·01 to 0·01)	0·13 (0·12 to 0·15)	0·07 (0·07 to 0·08)	74·51 (73·32 to 75·55)	80·36 (79·33 to 81·28)	19·86 (19·06 to 20·58)	23·77 (22·98 to 24·48)	248 (228 to 272)	230 (211 to 253)
	Argentina		0·01 (0·01 to 0·01)	0·01 (0·01 to 0·01)	0·14 (0·12 to 0·16)	0·08 (0·07 to 0·09)	73·57 (71·97 to 74·97)	79·67 (78·33 to 80·99)	19·2 (18·14 to 20·16)	23·35 (22·37 to 24·34)	172 (154 to 195)	160 (142 to 180)
	Chile		0·01 (0·01 to 0·01)	0·01 (0·01 to 0·01)	0·11 (0·10 to 0·13)	0·06 (0·05 to 0·07)	77·19 (75·73 to 78·67)	82·11 (80·81 to 83·42)	21·76 (20·72 to 22·84)	24·85 (23·81 to 25·92)	59 (52 to 66)	54 (47 to 61)
	Uruguay		0·01 (0·01 to 0·01)	0·01 (0·01 to 0·01)	0·15 (0·13 to 0·17)	0·08 (0·07 to 0·09)	73·51 (72·07 to 75·02)	80·43 (79·03 to 81·87)	19·26 (18·3 to 20·27)	23·94 (22·92 to 25·03)	17 (15 to 19)	17 (15 to 19)
Western Europe			<0·01 (<0·01 to <0·01)	<0·01 (<0·01 to <0·01)	0·08 (0·08 to 0·08)	0·05 (0·04 to 0·05)	79·53 (79·19 to 79·84)	84·21 (83·9 to 84·51)	22·65 (22·4 to 22·89)	26·21 (25·96 to 26·46)	2049 (1992 to 2111)	2108 (2046 to 2174)
	Andorra		<0·01 (<0·01 to <0·01)	<0·01 (<0·01 to <0·01)	0·08 (0·06 to 0·09)	0·04 (0·04 to 0·05)	80·55 (79·43 to 81·68)	85·06 (83·58 to 86·74)	23·48 (22·79 to 24·22)	26·85 (25·59 to 28·34)	<1 (<1 to <1)	<1 (<1 to <1)
	Austria		<0·01 (<0·01 to <0·01)	<0·01 (<0·01 to <0·01)	0·08 (0·07 to 0·09)	0·04 (0·04 to 0·05)	79·4 (78·75 to 80·07)	84·03 (83·4 to 84·62)	22·41 (21·91 to 22·92)	25·94 (25·42 to 26·43)	39 (37 to 42)	42 (40 to 45)
	Belgium		<0·01 (<0·01 to <0·01)	<0·01 (<0·01 to <0·01)	0·09 (0·08 to 0·09)	0·05 (0·05 to 0·05)	78·87 (78·22 to 79·55)	83·82 (83·14 to 84·45)	22·16 (21·66 to 22·66)	25·97 (25·43 to 26·48)	54 (51 to 57)	55 (52 to 59)
	Cyprus		<0·01 (<0·01 to <0·01)	<0·01 (<0·01 to <0·01)	0·08 (0·07 to 0·09)	0·04 (0·03 to 0·04)	78·45 (77·41 to 79·47)	85·21 (84·33 to 85·98)	21·46 (20·70 to 22·23)	26·96 (26·23 to 27·56)	5 (4 to 5)	3 (3 to 4)
	Denmark		<0·01 (<0·01 to <0·01)	<0·01 (<0·01 to <0·01)	0·08 (0·08 to 0·09)	0·05 (0·05 to 0·06)	78·81 (78·12 to 79·48)	82·69 (81·91 to 83·37)	21·86 (21·34 to 22·36)	24·83 (24·2 to 25·4)	27 (26 to 29)	27 (25 to 29)
	Finland		<0·01 (<0·01 to <0·01)	<0·01 (<0·01 to <0·01)	0·10 (0·09 to 0·10)	0·04 (0·04 to 0·05)	78·55 (77·77 to 79·23)	84·28 (83·58 to 84·94)	22·1 (21·54 to 22·59)	26·15 (25·58 to 26·7)	28 (26 to 30)	27 (26 to 29)
	France		<0·01 (<0·01 to <0·01)	<0·01 (<0·01 to <0·01)	0·09 (0·09 to 0·10)	0·05 (0·04 to 0·05)	79·82 (79·18 to 80·43)	85·72 (85·15 to 86·29)	23·38 (22·91 to 23·83)	27·84 (27·38 to 28·29)	289 (274 to 306)	290 (274 to 307)
	Germany		<0·01 (<0·01 to <0·01)	<0·01 (<0·01 to <0·01)	0·09 (0·08 to 0·11)	0·05 (0·04 to 0·06)	78·24 (76·91 to 79·49)	83·01 (81·82 to 84·2)	21·61 (20·63 to 22·55)	25·11 (24·14 to 26·09)	464 (415 to 520)	484 (429 to 544)
	Greece		<0·01 (<0·01 to 0·01)	<0·01 (<0·01 to <0·01)	0·10 (0·09 to 0·10)	0·05 (0·04 to 0·05)	78·44 (77·79 to 79·15)	83·56 (82·96 to 84·21)	22·12 (21·64 to 22·64)	25·67 (25·16 to 26·18)	63 (59 to 66)	57 (54 to 61)
	Iceland		<0·01 (<0·01 to <0·01)	<0·01 (<0·01 to <0·01)	0·07 (0·07 to 0·07)	0·04 (0·03 to 0·04)	79·83 (79·4 to 80·25)	85·94 (85·45 to 86·42)	22·63 (22·31 to 22·95)	27·57 (27·16 to 27·98)	1 (1 to 1)	1 (1 to 1)
	Ireland		<0·01 (<0·01 to <0·01)	<0·01 (<0·01 to <0·01)	0·07 (0·06 to 0·08)	0·05 (0·04 to 0·05)	80 (79·31 to 80·71)	83·68 (82·92 to 84·35)	22·83 (22·32 to 23·38)	25·6 (24·97 to 26·16)	16 (15 to 17)	15 (14 to 16)
	Israel		<0·01 (<0·01 to <0·01)	<0·01 (<0·01 to <0·01)	0·07 (0·06 to 0·07)	0·04 (0·03 to 0·04)	81·27 (80·60 to 81·92)	84·58 (83·93 to 85·25)	24·02 (23·5 to 24·52)	26·33 (25·77 to 26·9)	23 (21 to 24)	23 (22 to 25)
	Italy		<0·01 (<0·01 to <0·01)	<0·01 (<0·01 to <0·01)	0·06 (0·06 to 0·07)	0·04 (0·03 to 0·04)	80·85 (80·22 to 81·43)	85·31 (84·72 to 85·91)	23·39 (22·91 to 23·84)	26·99 (26·5 to 27·5)	299 (282 to 317)	324 (303 to 344)
	Luxembourg		<0·01 (<0·01 to <0·01)	<0·01 (<0·01 to <0·01)	0·07 (0·06 to 0·08)	0·05 (0·04 to 0·05)	80·03 (78·91 to 81·2)	83·25 (82·31 to 84·22)	22·83 (21·99 to 23·73)	25·22 (24·44 to 26·02)	2 (2 to 2)	2 (2 to 3)
	Malta		0·01 (0·01 to 0·01)	0·01 (<0·01 to 0·01)	0·07 (0·07 to 0·08)	0·04 (0·04 to 0·04)	78·91 (78·42 to 79·45)	83·02 (82·42 to 83·6)	21·99 (21·64 to 22·4)	25 (24·49 to 25·5)	2 (2 to 2)	2 (2 to 2)
	Netherlands		<0·01 (<0·01 to <0·01)	<0·01 (<0·01 to <0·01)	0·07 (0·06 to 0·07)	0·05 (0·05 to 0·06)	79·89 (79·25 to 80·50)	83·06 (82·42 to 83·71)	22·44 (21·95 to 22·92)	25·21 (24·68 to 25·74)	74 (69 to 79)	78 (73 to 84)
	Norway		<0·01 (<0·01 to <0·01)	<0·01 (<0·01 to <0·01)	0·07 (0·06 to 0·07)	0·04 (0·04 to 0·04)	80·46 (80·25 to 80·69)	84·17 (83·95 to 84·39)	23·11 (22·95 to 23·28)	25·94 (25·77 to 26·12)	20 (20 to 21)	21 (21 to 22)
	Portugal		<0·01 (<0·01 to <0·01)	<0·01 (<0·01 to <0·01)	0·10 (0·09 to 0·11)	0·04 (0·04 to 0·05)	78·51 (77·86 to 79·23)	84·22 (83·6 to 84·82)	22·15 (21·68 to 22·68)	26·11 (25·62 to 26·61)	57 (53 to 60)	57 (53 to 61)
	Spain		<0·01 (<0·01 to <0·01)	<0·01 (<0·01 to <0·01)	0·07 (0·07 to 0·08)	0·04 (0·03 to 0·04)	80·21 (79·65 to 80·80)	85·82 (85·31 to 86·34)	23·03 (22·6 to 23·48)	27·53 (27·11 to 27·97)	211 (200 to 222)	206 (195 to 218)
	Sweden		<0·01 (<0·01 to <0·01)	<0·01 (<0·01 to <0·01)	0·06 (0·06 to 0·07)	0·04 (0·04 to 0·04)	80·79 (80·22 to 81·35)	84·18 (83·65 to 84·71)	23·36 (22·92 to 23·8)	25·91 (25·47 to 26·36)	45 (42 to 47)	47 (45 to 50)
	Switzerland		<0·01 (<0·01 to <0·01)	<0·01 (<0·01 to <0·01)	0·05 (0·05 to 0·06)	0·03 (0·03 to 0·04)	82·12 (81·53 to 82·78)	85·66 (85·09 to 86·27)	24·46 (23·99 to 24·98)	27·32 (26·84 to 27·84)	31 (29 to 33)	34 (31 to 36)
	UK		<0·01 (<0·01 to 0·01)	<0·01 (<0·01 to <0·01)	0·08 (0·08 to 0·08)	0·05 (0·05 to 0·06)	79·18 (79·05 to 79·32)	82·72 (82·59 to 82·85)	22·5 (22·42 to 22·6)	25·05 (24·95 to 25·14)	299 (295 to 302)	310 (306 to 313)
		England	<0·01 (<0·01 to 0·01)	<0·01 (<0·01 to <0·01)	0·08 (0·08 to 0·08)	0·05 (0·05 to 0·05)	79·49 (79·39 to 79·59)	82·91 (82·83 to 83·01)	22·67 (22·61 to 22·75)	25·18 (25·11 to 25·26)	245 (243 to 247)	256 (254 to 258)
		Northern Ireland	0·01 (<0·01 to 0·01)	<0·01 (<0·01 to 0·01)	0·09 (0·08 to 0·1)	0·06 (0·05 to 0·06)	78·74 (77·74 to 79·77)	82·48 (81·45 to 83·44)	22·39 (21·68 to 23·15)	24·92 (24·11 to 25·69)	8 (7 to 8)	8 (7 to 9)
		Scotland	<0·01 (<0·01 to 0·01)	<0·01 (<0·01 to <0·01)	0·12 (0·1 to 0·13)	0·07 (0·06 to 0·08)	76·91 (75·95 to 77·96)	81·2 (80·33 to 82·12)	21·29 (20·63 to 22·02)	23·99 (23·34 to 24·73)	29 (26 to 31)	29 (27 to 32)
		Wales	<0·01 (<0·01 to 0·01)	<0·01 (<0·01 to <0·01)	0·1 (0·09 to 0·1)	0·06 (0·05 to 0·06)	78·27 (77·52 to 79·1)	82·47 (81·67 to 83·23)	22 (21·45 to 22·59)	24·88 (24·24 to 25·49)	17 (16 to 18)	16 (15 to 18)
**Latin America and Caribbean**			**0·02 (0·02 to 0·02)**	**0·02 (0·01 to 0·02)**	**0·17 (0·17 to 0·18)**	**0·09 (0·09 to 0·09)**	**72·79 (72·44 to 73·16)**	**78·94 (78·63 to 79·23)**	**20·94 (20·80 to 21·09)**	**23·72 (23·57 to 23·87)**	**1895 (1863 to 1928)**	**1501 (1475 to 1527)**
Andean Latin America			0·02 (0·02 to 0·03)	0·02 (0·01 to 0·02)	0·12 (0·11 to 0·13)	0·08 (0·07 to 0·09)	76·18 (74·95 to 77·35)	79·49 (78·39 to 80·58)	22·62 (21·77 to 23·44)	24·19 (23·37 to 25)	159 (146 to 174)	137 (124 to 150)
	Bolivia		0·03 (0·03 to 0·04)	0·03 (0·02 to 0·03)	0·14 (0·11 to 0·18)	0·12 (0·09 to 0·15)	71·3 (68·76 to 73·93)	74·15 (72·08 to 76·58)	18·45 (16·43 to 20·49)	20·36 (19·01 to 22·22)	35 (28 to 43)	32 (26 to 37)
	Ecuador		0·02 (0·02 to 0·02)	0·02 (0·01 to 0·02)	0·15 (0·13 to 0·17)	0·09 (0·08 to 0·10)	74·77 (73·32 to 76·08)	78·72 (77·48 to 79·92)	22·26 (21·44 to 23·06)	23·5 (22·63 to 24·36)	48 (43 to 53)	40 (36 to 44)
	Peru		0·02 (0·01 to 0·02)	0·01 (0·01 to 0·02)	0·10 (0·08 to 0·12)	0·07 (0·06 to 0·08)	78·74 (76·78 to 80·79)	81·89 (80·05 to 83·73)	24·28 (22·95 to 25·69)	25·91 (24·48 to 27·31)	77 (65 to 89)	65 (55 to 77)
Caribbean			0·04 (0·03 to 0·05)	0·03 (0·03 to 0·04)	0·18 (0·17 to 0·20)	0·12 (0·11 to 0·14)	70·35 (69·35 to 71·43)	75·39 (74·36 to 76·39)	19·78 (19·25 to 20·34)	22·63 (22·02 to 23·23)	196 (185 to 208)	164 (154 to 175)
	Antigua and Barbuda		0·01 (0·01 to 0·02)	0·01 (0·01 to 0·01)	0·13 (0·12 to 0·14)	0·09 (0·08 to 0·09)	75·28 (74·4 to 76·15)	78·74 (78·13 to 79·36)	20·97 (20·38 to 21·55)	22·48 (21·97 to 23·07)	<1 (<1 to <1)	<1 (<1 to <1)
	The Bahamas		0·01 (0·01 to 0·02)	0·01 (0·01 to 0·01)	0·22 (0·20 to 0·24)	0·13 (0·12 to 0·14)	70·84 (69·58 to 72·12)	76·58 (75·41 to 77·89)	19·81 (19·09 to 20·56)	22·35 (21·54 to 23·25)	1 (1 to 1)	1 (1 to 1)
	Barbados		0·01 (0·01 to 0·02)	0·01 (0·01 to 0·01)	0·13 (0·12 to 0·15)	0·09 (0·08 to 0·10)	75·49 (74·44 to 76·64)	78·63 (77·73 to 79·62)	21·28 (20·59 to 22·02)	23·23 (22·63 to 23·97)	1 (1 to 1)	1 (1 to 2)
	Belize		0·02 (0·02 to 0·02)	0·02 (0·01 to 0·02)	0·21 (0·21 to 0·22)	0·12 (0·11 to 0·12)	71·25 (70·67 to 71·84)	77·4 (76·87 to 77·94)	20·73 (20·42 to 21·07)	23·02 (22·68 to 23·38)	1 (1 to 1)	1 (1 to 1)
	Bermuda		0·01 (<0·01 to 0·01)	<0·01 (<0·01 to 0·01)	0·11 (0·10 to 0·12)	0·04 (0·03 to 0·04)	77·05 (76·42 to 77·6)	85·67 (84·82 to 86·53)	21·11 (20·59 to 21·45)	27·61 (26·92 to 28·31)	<1 (<1 to <1)	<1 (<1 to <1)
	Cuba		0·01 (<0·01 to 0·01)	<0·01 (<0·01 to 0·01)	0·12 (0·11 to 0·14)	0·07 (0·06 to 0·08)	76·18 (74·64 to 77·65)	80·71 (79·32 to 82·1)	20·76 (19·7 to 21·79)	23·68 (22·6 to 24·79)	55 (49 to 62)	46 (41 to 53)
	Dominica		0·03 (0·03 to 0·04)	0·03 (0·02 to 0·03)	0·17 (0·16 to 0·19)	0·10 (0·09 to 0·11)	70·42 (69·42 to 71·4)	75·36 (74·33 to 76·4)	19·01 (18·46 to 19·53)	21·55 (20·89 to 22·3)	<1 (<1 to <1)	<1 (<1 to <1)
	Dominican Republic		0·03 (0·03 to 0·04)	0·03 (0·02 to 0·03)	0·21 (0·18 to 0·24)	0·11 (0·10 to 0·13)	69·78 (67·83 to 71·9)	76·77 (75·17 to 78·47)	19·48 (18·33 to 20·86)	23·12 (22·08 to 24·32)	40 (35 to 45)	27 (24 to 31)
	Grenada		0·02 (0·01 to 0·02)	0·01 (0·01 to 0·02)	0·18 (0·17 to 0·19)	0·12 (0·11 to 0·13)	72·99 (72·31 to 73·65)	75·41 (74·68 to 76·15)	20·11 (19·67 to 20·53)	20·53 (19·98 to 21·14)	1 (1 to 1)	1 (1 to 1)
	Guyana		0·03 (0·02 to 0·03)	0·02 (0·02 to 0·02)	0·27 (0·24 to 0·31)	0·17 (0·15 to 0·20)	66·36 (64·55 to 68·16)	72·16 (70·49 to 73·9)	17·01 (16 to 18·04)	19·56 (18·49 to 20·70)	3 (3 to 3)	2 (2 to 3)
	Haiti		0·06 (0·05 to 0·08)	0·05 (0·05 to 0·06)	0·25 (0·20 to 0·30)	0·22 (0·18 to 0·27)	63·83 (61·44 to 66·42)	65·96 (63·27 to 68·75)	16·05 (15 to 17·52)	16·95 (15·36 to 18·92)	45 (39 to 52)	43 (36 to 52)
	Jamaica		0·02 (0·01 to 0·02)	0·01 (0·01 to 0·02)	0·18 (0·15 to 0·21)	0·11 (0·09 to 0·13)	71·96 (69·85 to 74·14)	77·48 (75·41 to 79·4)	19·14 (17·88 to 20·50)	22·3 (20·77 to 23·72)	11 (9 to 13)	9 (8 to 11)
	Puerto Rico		0·01 (0·01 to 0·01)	0·01 (0·01 to 0·01)	0·17 (0·16 to 0·18)	0·08 (0·07 to 0·08)	74·52 (73·69 to 75·39)	81·6 (80·88 to 82·32)	22·38 (21·84 to 22·94)	25·76 (25·2 to 26·32)	18 (17 to 20)	16 (15 to 17)
	Saint Lucia		0·02 (0·01 to 0·02)	0·01 (0·01 to 0·01)	0·18 (0·16 to 0·19)	0·10 (0·09 to 0·11)	73·12 (72·24 to 74)	78·08 (77·2 to 78·93)	20·57 (20·04 to 21·13)	22·62 (21·98 to 23·2)	1 (1 to 1)	1 (1 to 1)
	Saint Vincent and the Grenadines		0·02 (0·02 to 0·02)	0·01 (0·01 to 0·02)	0·21 (0·20 to 0·22)	0·13 (0·12 to 0·14)	69·65 (68·86 to 70·38)	75·41 (74·56 to 76·29)	18·08 (17·66 to 18·49)	21·18 (20·59 to 21·74)	1 (1 to 1)	<1 (<1 to <1)
	Suriname		0·03 (0·03 to 0·04)	0·03 (0·02 to 0·03)	0·22 (0·19 to 0·24)	0·12 (0·11 to 0·14)	68·95 (67·25 to 70·72)	75·28 (73·98 to 76·61)	18·37 (17·36 to 19·42)	21·97 (21·05 to 22·9)	2 (2 to 3)	2 (2 to 2)
	Trinidad and Tobago		0·02 (0·02 to 0·02)	0·01 (0·01 to 0·02)	0·19 (0·15 to 0·24)	0·11 (0·08 to 0·14)	71·13 (68·45 to 73·95)	77·55 (74·82 to 80·33)	19·1 (17·43 to 20·85)	22·73 (20·81 to 24·81)	6 (5 to 8)	5 (4 to 6)
	Virgin Islands		0·01 (0·01 to 0·01)	0·01 (0·01 to 0·01)	0·22 (0·19 to 0·25)	0·09 (0·08 to 0·11)	69·49 (67·94 to 71·76)	78·78 (77·23 to 80·05)	16·61 (15·8 to 18·7)	22·72 (21·66 to 23·66)	1 (1 to 1)	1 (<1 to 1)
Central Latin America			0·02 (0·01 to 0·02)	0·01 (0·01 to 0·02)	0·17 (0·17 to 0·18)	0·09 (0·08 to 0·09)	73·3 (72·79 to 73·82)	79·42 (79·01 to 79·82)	21·44 (21·17 to 21·72)	23·87 (23·6 to 24·14)	766 (742 to 792)	593 (575 to 611)
	Colombia		0·02 (0·01 to 0·02)	0·01 (0·01 to 0·01)	0·12 (0·11 to 0·14)	0·06 (0·05 to 0·07)	77·44 (75·94 to 79·03)	82·68 (81·36 to 83·95)	24·03 (23·03 to 25·03)	26·31 (25·27 to 27·32)	127 (113 to 143)	107 (95 to 121)
	Costa Rica		0·01 (0·01 to 0·01)	0·01 (0·01 to 0·01)	0·13 (0·12 to 0·14)	0·06 (0·06 to 0·07)	76·31 (75·53 to 77·13)	82·67 (81·86 to 83·4)	21·83 (21·33 to 22·36)	25·68 (25·04 to 26·25)	14 (13 to 14)	10 (9 to 11)
	El Salvador		0·01 (0·01 to 0·02)	0·01 (0·01 to 0·01)	0·26 (0·21 to 0·30)	0·10 (0·08 to 0·13)	69·29 (66·66 to 72·05)	78·3 (75·98 to 80·41)	19·96 (18·64 to 21·41)	22·92 (21·25 to 24·51)	23 (20 to 27)	18 (15 to 22)
	Guatemala		0·03 (0·02 to 0·03)	0·02 (0·02 to 0·03)	0·23 (0·20 to 0·25)	0·12 (0·10 to 0·14)	69·14 (67·44 to 70·76)	75·99 (74·53 to 77·38)	19·72 (18·87 to 20·60)	22·14 (21·17 to 23·16)	52 (46 to 58)	38 (33 to 42)
	Honduras		0·02 (0·01 to 0·02)	0·01 (0·01 to 0·02)	0·17 (0·13 to 0·21)	0·14 (0·10 to 0·17)	72·88 (70·17 to 75·6)	74·96 (72·41 to 78·18)	20·52 (19·01 to 22·22)	20·80 (19·3 to 23·23)	23 (19 to 27)	22 (17 to 26)
	Mexico		0·02 (0·01 to 0·02)	0·01 (0·01 to 0·02)	0·19 (0·18 to 0·19)	0·09 (0·09 to 0·09)	72·56 (72·27 to 72·85)	78·5 (78·22 to 78·76)	20·77 (20·64 to 20·89)	22·98 (22·84 to 23·13)	401 (396 to 407)	310 (305 to 315)
	Nicaragua		0·02 (0·01 to 0·02)	0·01 (0·01 to 0·02)	0·14 (0·12 to 0·16)	0·07 (0·06 to 0·09)	76·92 (75·26 to 78·41)	80·64 (79·36 to 82·04)	23·58 (22·5 to 24·57)	24·64 (23·71 to 25·7)	12 (11 to 14)	11 (10 to 12)
	Panama		0·02 (0·02 to 0·02)	0·02 (0·01 to 0·02)	0·12 (0·11 to 0·13)	0·07 (0·06 to 0·07)	77·01 (76·17 to 77·93)	81·7 (80·93 to 82·47)	23·58 (23·07 to 24·15)	25·89 (25·35 to 26·46)	11 (10 to 11)	8 (8 to 9)
	Venezuela		0·02 (0·01 to 0·02)	0·01 (0·01 to 0·02)	0·20 (0·17 to 0·23)	0·08 (0·07 to 0·10)	71·23 (68·89 to 73·7)	79·6 (77·73 to 81·49)	20·41 (19·11 to 21·8)	24·03 (22·63 to 25·52)	104 (87 to 121)	69 (58 to 81)
Tropical Latin America			0·02 (0·02 to 0·02)	0·02 (0·01 to 0·02)	0·19 (0·18 to 0·19)	0·09 (0·09 to 0·09)	72·03 (71·75 to 72·29)	79·07 (78·81 to 79·28)	20·35 (20·28 to 20·43)	23·72 (23·65 to 23·8)	774 (767 to 781)	608 (602 to 614)
	Brazil		0·02 (0·02 to 0·02)	0·02 (0·01 to 0·02)	0·19 (0·18 to 0·19)	0·09 (0·09 to 0·09)	71·98 (71·71 to 72·23)	79·06 (78·81 to 79·27)	20·36 (20·30 to 20·43)	23·74 (23·66 to 23·81)	755 (749 to 761)	594 (588 to 599)
	Paraguay		0·02 (0·01 to 0·02)	0·01 (0·01 to 0·01)	0·16 (0·12 to 0·19)	0·09 (0·07 to 0·11)	73·44 (70·99 to 75·98)	78·93 (76·76 to 81·19)	20·04 (18·49 to 21·72)	23·24 (21·66 to 24·95)	19 (15 to 22)	14 (11 to 17)
**North Africa and Middle East**			**0·03 (0·03 to 0·03)**	**0·02 (0·02 to 0·03)**	**0·15 (0·14 to 0·15)**	**0·10 (0·09 to 0·10)**	**72 (71·53 to 72·49)**	**76·85 (76·4 to 77·32)**	**19·32 (19·02 to 19·64)**	**22·53 (22·21 to 22·86)**	**1684 (1628 to 1742)**	**1179 (1135 to 1224)**
	Afghanistan		0·06 (0·05 to 0·06)	0·05 (0·04 to 0·06)	0·27 (0·21 to 0·32)	0·28 (0·23 to 0·34)	63·56 (61·28 to 65·89)	63·18 (60·63 to 65·85)	15·43 (14·64 to 16·33)	15·07 (14·18 to 16·33)	115 (100 to 131)	112 (96 to 130)
	Algeria		0·02 (0·02 to 0·03)	0·02 (0·02 to 0·02)	0·09 (0·09 to 0·10)	0·07 (0·07 to 0·08)	77·03 (76·39 to 77·61)	78·48 (77·89 to 79·06)	22·46 (22 to 22·88)	23·05 (22·61 to 23·5)	90 (85 to 95)	78 (74 to 83)
	Bahrain		0·01 (0·01 to 0·01)	0·01 (0·01 to 0·01)	0·06 (0·06 to 0·07)	0·05 (0·05 to 0·06)	78·8 (77·81 to 79·84)	80·44 (79·49 to 81·38)	21·67 (20·87 to 22·51)	22·82 (22·02 to 23·63)	2 (2 to 2)	1 (1 to 1)
	Egypt		0·02 (0·02 to 0·03)	0·02 (0·02 to 0·02)	0·20 (0·18 to 0·23)	0·13 (0·11 to 0·14)	67·96 (66·61 to 69·31)	74·33 (72·88 to 75·79)	15·16 (14·39 to 15·97)	19·98 (18·99 to 20·97)	316 (284 to 353)	183 (161 to 206)
	Iran		0·02 (0·02 to 0·02)	0·01 (0·01 to 0·01)	0·12 (0·12 to 0·12)	0·06 (0·06 to 0·06)	75·47 (75·38 to 75·55)	79·36 (79·28 to 79·46)	21·2 (21·15 to 21·25)	22·79 (22·73 to 22·85)	219 (217 to 220)	161 (160 to 162)
	Iraq		0·03 (0·02 to 0·03)	0·02 (0·02 to 0·03)	0·15 (0·14 to 0·16)	0·08 (0·07 to 0·08)	74·79 (73·85 to 75·6)	78·83 (78·06 to 79·65)	23·58 (22·92 to 24·2)	24·42 (23·81 to 25·01)	92 (87 to 97)	60 (56 to 65)
	Jordan		0·01 (0·01 to 0·02)	0·01 (0·01 to 0·02)	0·08 (0·07 to 0·10)	0·05 (0·04 to 0·06)	77·85 (76·34 to 79·18)	81·07 (79·84 to 82·31)	22·16 (20·89 to 23·22)	24·07 (23·12 to 25·09)	16 (15 to 18)	11 (10 to 13)
	Kuwait		0·01 (0·01 to 0·01)	0·01 (0·01 to 0·01)	0·08 (0·07 to 0·08)	0·03 (0·03 to 0·03)	80·66 (79·98 to 81·35)	87·18 (86·69 to 87·67)	24·31 (23·81 to 24·83)	29·22 (28·81 to 29·62)	6 (5 to 6)	2 (2 to 2)
	Lebanon		0·01 (0·01 to 0·01)	0·01 (0·01 to 0·01)	0·12 (0·11 to 0·13)	0·07 (0·06 to 0·08)	75·8 (75·05 to 76·38)	79·95 (79·37 to 80·71)	20·51 (19·82 to 20·99)	23·14 (22·7 to 23·74)	17 (16 to 19)	16 (15 to 17)
	Libya		0·01 (0·01 to 0·02)	0·01 (0·01 to 0·02)	0·18 (0·15 to 0·21)	0·12 (0·10 to 0·14)	71·14 (69·36 to 73·18)	74·97 (73·27 to 76·87)	18·82 (17·7 to 20·13)	20·21 (18·97 to 21·62)	20 (17 to 23)	14 (12 to 17)
	Morocco		0·02 (0·02 to 0·03)	0·02 (0·01 to 0·02)	0·13 (0·10 to 0·16)	0·12 (0·10 to 0·15)	73·23 (71 to 75·48)	74·7 (72·66 to 76·8)	19·48 (17·91 to 21·12)	20·19 (18·78 to 21·61)	113 (95 to 136)	107 (90 to 128)
	Oman		0·01 (0·01 to 0·01)	0·01 (0·01 to 0·01)	0·11 (0·08 to 0·13)	0·07 (0·06 to 0·09)	75·47 (73·25 to 77·89)	79·44 (78·21 to 81·24)	20·10 (18·48 to 21·94)	22·87 (22·05 to 24·28)	7 (6 to 9)	4 (3 to 4)
	Palestine		0·01 (0·01 to 0·02)	0·01 (0·01 to 0·01)	0·11 (0·10 to 0·11)	0·07 (0·06 to 0·07)	75·62 (74·72 to 76·43)	78 (77·32 to 78·85)	20·39 (19·58 to 21·09)	21·25 (20·70 to 21·96)	7 (7 to 8)	7 (7 to 8)
	Qatar		0·01 (0·01 to 0·01)	0·01 (0·01 to 0·01)	0·07 (0·05 to 0·08)	0·05 (0·04 to 0·06)	79·55 (77·69 to 81·55)	81·66 (79·84 to 83·51)	22·73 (21·29 to 24·29)	23·98 (22·46 to 25·58)	3 (2 to 4)	1 (1 to 1)
	Saudi Arabia		0·01 (0·01 to 0·01)	0·01 (0·01 to 0·01)	0·13 (0·10 to 0·15)	0·08 (0·07 to 0·10)	75·29 (73·87 to 76·57)	79·43 (78·04 to 80·23)	20·31 (19·39 to 20·94)	23·08 (22·24 to 23·7)	64 (55 to 74)	30 (27 to 35)
	Sudan		0·05 (0·05 to 0·06)	0·04 (0·04 to 0·05)	0·16 (0·13 to 0·21)	0·14 (0·11 to 0·18)	68·85 (66·37 to 71·45)	72·02 (69·54 to 74·68)	18·26 (16·45 to 20·17)	20·15 (18·44 to 21·92)	120 (101 to 140)	89 (75 to 104)
	Syria		0·02 (0·02 to 0·02)	0·02 (0·02 to 0·02)	0·28 (0·25 to 0·30)	0·13 (0·11 to 0·14)	65·49 (63·79 to 67·19)	75·04 (73·98 to 76·31)	18·57 (17·16 to 20·12)	22·37 (21·8 to 23·31)	76 (68 to 85)	39 (35 to 42)
	Tunisia		0·01 (0·01 to 0·01)	0·01 (0·01 to 0·01)	0·10 (0·08 to 0·13)	0·06 (0·04 to 0·07)	76·09 (73·66 to 78·57)	80·72 (78·47 to 83·03)	20·57 (18·86 to 22·46)	23·62 (21·84 to 25·54)	38 (30 to 47)	28 (22 to 35)
	Turkey		0·02 (0·01 to 0·02)	0·01 (0·01 to 0·02)	0·11 (0·10 to 0·12)	0·05 (0·05 to 0·06)	75·2 (74·14 to 76·25)	83·04 (82·04 to 84·04)	20·02 (19·24 to 20·81)	26·17 (25·35 to 26·99)	246 (225 to 269)	156 (141 to 172)
	United Arab Emirates		0·01 (0·01 to 0·01)	0·01 (0·01 to 0·01)	0·15 (0·12 to 0·19)	0·09 (0·07 to 0·12)	71·65 (69·35 to 74·05)	76·94 (74·73 to 79·19)	16·74 (15·15 to 18·43)	20·33 (18·63 to 22·11)	22 (17 to 27)	5 (4 to 6)
	Yemen		0·05 (0·04 to 0·06)	0·04 (0·04 to 0·05)	0·22 (0·18 to 0·27)	0·16 (0·13 to 0·20)	65·98 (63·58 to 68·33)	70·27 (67·58 to 72·72)	16·81 (15·48 to 18·48)	19·2 (17·29 to 20·83)	93 (77 to 113)	72 (58 to 89)
**South Asia**			**0·04 (0·04 to 0·05)**	**0·05 (0·04 to 0·05)**	**0·20 (0·20 to 0·21)**	**0·15 (0·15 to 0·16)**	**67·91 (67·4 to 68·45)**	**70·21 (69·66 to 70·74)**	**17·41 (17·19 to 17·64)**	**18·77 (18·52 to 19·02)**	**6587 (6400 to 6768)**	**5813 (5652 to 5982)**
	Bangladesh		0·03 (0·03 to 0·04)	0·03 (0·03 to 0·04)	0·15 (0·13 to 0·17)	0·11 (0·10 to 0·13)	71·8 (70·29 to 73·34)	74·6 (73·05 to 76·03)	19·49 (18·45 to 20·55)	21·24 (20·19 to 22·32)	503 (447 to 562)	384 (341 to 433)
	Bhutan		0·03 (0·02 to 0·04)	0·03 (0·02 to 0·03)	0·13 (0·11 to 0·16)	0·10 (0·08 to 0·12)	72·34 (69·83 to 74·79)	76·04 (73·93 to 78·11)	19·08 (17·08 to 20·80)	21·84 (20·23 to 23·39)	2 (2 to 3)	2 (1 to 2)
	India		0·04 (0·04 to 0·05)	0·04 (0·04 to 0·05)	0·21 (0·20 to 0·22)	0·15 (0·15 to 0·16)	67·81 (67·25 to 68·33)	70·18 (69·53 to 70·76)	17·2 (17 to 17·39)	18·6 (18·37 to 18·82)	5230 (5115 to 5360)	4680 (4564 to 4798)
	Nepal		0·03 (0·03 to 0·04)	0·03 (0·03 to 0·04)	0·18 (0·15 to 0·22)	0·13 (0·11 to 0·16)	68·72 (67·24 to 70·56)	73·28 (71·54 to 75·11)	16·58 (16·02 to 18)	20·04 (18·89 to 21·23)	103 (89 to 113)	80 (70 to 92)
	Pakistan		0·06 (0·05 to 0·07)	0·06 (0·05 to 0·07)	0·21 (0·17 to 0·25)	0·18 (0·14 to 0·21)	66·31 (63·8 to 69·1)	67·41 (65·07 to 70·12)	17·33 (15·77 to 18·97)	17·99 (16·41 to 19·74)	749 (633 to 880)	667 (566 to 775)
**Southeast Asia, east Asia, and Oceania**			**0·02 (0·02 to 0·02)**	**0·02 (0·01 to 0·02)**	**0·14 (0·13 to 0·14)**	**0·07 (0·07 to 0·07)**	**72·91 (72·54 to 73·33)**	**78·56 (78·21 to 78·9)**	**19·01 (18·75 to 19·28)**	**22·48 (22·22 to 22·75)**	**8837 (8562 to 9099)**	**6574 (6370 to 6782)**
East Asia			0·01 (0·01 to 0·02)	0·01 (0·01 to 0·01)	0·12 (0·11 to 0·12)	0·06 (0·05 to 0·06)	74·46 (73·98 to 74·94)	79·88 (79·43 to 80·30)	19·4 (19·08 to 19·74)	22·82 (22·49 to 23·15)	6375 (6121 to 6624)	4670 (4483 to 4866)
	China		0·01 (0·01 to 0·02)	0·01 (0·01 to 0·01)	0·11 (0·11 to 0·12)	0·05 (0·05 to 0·06)	74·52 (74·05 to 75·01)	79·92 (79·44 to 80·36)	19·39 (19·07 to 19·74)	22·81 (22·47 to 23·16)	6052 (5802 to 6297)	4400 (4214 to 4591)
	North Korea		0·02 (0·02 to 0·03)	0·02 (0·02 to 0·02)	0·20 (0·16 to 0·24)	0·11 (0·09 to 0·14)	68·64 (67·1 to 70·21)	75·05 (72·91 to 77·17)	16·45 (15·92 to 17·04)	20·54 (19·1 to 22·07)	113 (101 to 126)	122 (101 to 146)
	Taiwan (province of China)		0·01 (<0·01 to 0·01)	<0·01 (<0·01 to <0·01)	0·13 (0·12 to 0·14)	0·05 (0·05 to 0·06)	76·82 (76·1 to 77·51)	83·26 (82·63 to 83·87)	21·77 (21·29 to 22·23)	25·67 (25·18 to 26·17)	106 (100 to 112)	73 (69 to 78)
Oceania			0·05 (0·04 to 0·06)	0·04 (0·04 to 0·05)	0·41 (0·35 to 0·47)	0·30 (0·25 to 0·36)	58·2 (55·92 to 60·60)	63·38 (61·1 to 65·54)	13·41 (12·71 to 14·17)	15·71 (15 to 16·41)	65 (56 to 74)	45 (39 to 52)
	American Samoa		0·01 (0·01 to 0·01)	0·01 (0·01 to 0·01)	0·21 (0·19 to 0·23)	0·15 (0·14 to 0·17)	69·99 (68·51 to 71·65)	73·8 (72·94 to 74·78)	17·11 (15·92 to 18·64)	19·66 (19·24 to 20·19)	<1 (<1 to <1)	<1 (<1 to <1)
	Federated States of Micronesia		0·02 (0·02 to 0·02)	0·02 (0·01 to 0·02)	0·30 (0·25 to 0·35)	0·22 (0·17 to 0·27)	64·98 (62·8 to 67·25)	69·58 (67·15 to 71·68)	15·12 (14·39 to 15·83)	17·66 (16·48 to 18·51)	<1 (<1 to <1)	<1 (<1 to <1)
	Fiji		0·03 (0·02 to 0·03)	0·02 (0·02 to 0·03)	0·26 (0·22 to 0·29)	0·18 (0·15 to 0·21)	65·9 (64·17 to 67·7)	70·40 (68·44 to 72·51)	14·94 (13·93 to 16·02)	17·56 (16·27 to 19·02)	4 (3 to 5)	3 (3 to 4)
	Guam		0·01 (0·01 to 0·02)	0·01 (0·01 to 0·01)	0·23 (0·21 to 0·25)	0·12 (0·11 to 0·13)	70·23 (69·19 to 71·34)	76·4 (75·31 to 77·46)	18·82 (18·21 to 19·49)	21·5 (20·72 to 22·26)	1 (1 to 1)	1 (<1 to 1)
	Kiribati		0·05 (0·04 to 0·05)	0·04 (0·03 to 0·05)	0·41 (0·35 to 0·47)	0·23 (0·19 to 0·28)	58·59 (56·21 to 61·05)	66·31 (63·94 to 68·86)	13·14 (12·39 to 14·07)	16·2 (15·27 to 17·67)	1 (<1 to 1)	<1 (<1 to 1)
	Marshall Islands		0·02 (0·02 to 0·03)	0·02 (0·02 to 0·02)	0·33 (0·29 to 0·39)	0·27 (0·22 to 0·31)	62·57 (60·56 to 64·61)	66·82 (64·55 to 68·96)	13·46 (12·53 to 14·46)	16·44 (15·33 to 17·38)	<1 (<1 to <1)	<1 (<1 to <1)
	Northern Mariana Islands		0·01 (0·01 to 0·01)	0·01 (0·01 to 0·01)	0·15 (0·13 to 0·18)	0·09 (0·07 to 0·10)	73·59 (72·32 to 75·01)	79·25 (78·02 to 80·15)	19·45 (18·3 to 20·42)	22·96 (22·21 to 23·71)	<1 (<1 to <1)	<1 (<1 to <1)
	Papua New Guinea		0·06 (0·05 to 0·07)	0·05 (0·04 to 0·06)	0·45 (0·38 to 0·52)	0·34 (0·28 to 0·40)	56·23 (53·56 to 59·16)	61·23 (58·55 to 63·85)	12·6 (11·72 to 13·62)	14·49 (13·56 to 15·38)	50 (42 to 59)	34 (29 to 41)
	Samoa		0·02 (0·01 to 0·02)	0·01 (0·01 to 0·01)	0·16 (0·13 to 0·19)	0·13 (0·11 to 0·16)	71·28 (70·03 to 72·69)	74·49 (72·89 to 76·7)	17·43 (17·07 to 18·21)	19·95 (18·96 to 21·52)	1 (1 to 1)	1 (<1 to 1)
	Solomon Islands		0·03 (0·02 to 0·03)	0·02 (0·02 to 0·03)	0·30 (0·25 to 0·35)	0·24 (0·20 to 0·29)	64·12 (62 to 66·31)	67·52 (65·39 to 69·43)	14·93 (14·1 to 15·81)	16·7 (15·85 to 17·37)	2 (2 to 3)	2 (2 to 2)
	Tonga		0·02 (0·02 to 0·02)	0·01 (0·01 to 0·02)	0·22 (0·18 to 0·26)	0·12 (0·10 to 0·15)	68·62 (66·74 to 70·06)	75·14 (73·33 to 77·21)	16·57 (15·63 to 17·2)	20·35 (19·17 to 21·83)	<1 (<1 to <1)	<1 (<1 to <1)
	Vanuatu		0·03 (0·03 to 0·04)	0·03 (0·02 to 0·03)	0·34 (0·28 to 0·42)	0·23 (0·18 to 0·29)	62·11 (59·17 to 64·96)	67·75 (65·02 to 70·22)	14·21 (13·05 to 15·32)	16·67 (15·66 to 17·82)	1 (1 to 2)	1 (1 to 1)
Southeast Asia			0·03 (0·02 to 0·03)	0·02 (0·02 to 0·02)	0·19 (0·18 to 0·20)	0·11 (0·11 to 0·12)	69·45 (68·87 to 70·02)	75·76 (75·18 to 76·29)	17·57 (17·23 to 17·91)	21·4 (20·99 to 21·78)	2397 (2302 to 2496)	1859 (1781 to 1947)
	Cambodia		0·03 (0·03 to 0·04)	0·03 (0·02 to 0·03)	0·23 (0·19 to 0·27)	0·14 (0·12 to 0·17)	66·77 (65·28 to 68·26)	72·7 (70·59 to 74·24)	16·06 (15·66 to 16·45)	19·6 (18·19 to 20·56)	54 (49 to 60)	48 (43 to 56)
	Indonesia		0·03 (0·02 to 0·03)	0·02 (0·02 to 0·03)	0·18 (0·17 to 0·19)	0·13 (0·12 to 0·14)	69·21 (68·39 to 70·07)	73·87 (73·03 to 74·67)	16·69 (16·18 to 17·31)	19·89 (19·29 to 20·43)	904 (850 to 957)	738 (694 to 791)
	Laos		0·06 (0·05 to 0·08)	0·05 (0·04 to 0·06)	0·22 (0·19 to 0·26)	0·15 (0·12 to 0·18)	65·05 (62·98 to 67·11)	70·32 (68·26 to 72·28)	16·31 (15·3 to 17·53)	19·32 (17·86 to 20·59)	26 (23 to 29)	20 (17 to 23)
	Malaysia		0·01 (0·01 to 0·01)	0·01 (0·01 to 0·01)	0·16 (0·15 to 0·18)	0·09 (0·08 to 0·10)	72·4 (71·26 to 73·48)	77·34 (76·36 to 78·35)	18·2 (17·44 to 18·96)	20·80 (20·05 to 21·61)	96 (88 to 105)	69 (62 to 76)
	Maldives		0·01 (0·01 to 0·01)	0·01 (0·01 to 0·01)	0·07 (0·06 to 0·07)	0·05 (0·04 to 0·05)	79·93 (79·22 to 80·62)	83·37 (82·62 to 84·15)	23·06 (22·52 to 23·6)	25·73 (25·07 to 26·4)	1 (1 to 1)	<1 (<1 to <1)
	Mauritius		0·01 (0·01 to 0·02)	0·01 (0·01 to 0·01)	0·19 (0·17 to 0·20)	0·09 (0·08 to 0·10)	71·54 (70·65 to 72·46)	78·1 (77·23 to 78·96)	18·65 (18·11 to 19·22)	22·27 (21·63 to 22·91)	6 (5 to 6)	5 (4 to 5)
	Myanmar		0·05 (0·04 to 0·06)	0·04 (0·03 to 0·04)	0·25 (0·21 to 0·29)	0·14 (0·11 to 0·17)	64·86 (63·15 to 66·71)	72·15 (70·26 to 74·22)	15·86 (15·44 to 16·65)	20·04 (18·76 to 21·46)	229 (204 to 251)	181 (155 to 209)
	Philippines		0·03 (0·02 to 0·04)	0·02 (0·02 to 0·03)	0·24 (0·20 to 0·28)	0·13 (0·11 to 0·16)	66·58 (64·65 to 68·61)	73·1 (71·16 to 74·95)	15·87 (14·71 to 17·14)	19·55 (18·26 to 20·78)	380 (327 to 437)	287 (247 to 334)
	Sri Lanka		0·01 (0·01 to 0·01)	0·01 (0·01 to 0·01)	0·15 (0·12 to 0·18)	0·06 (0·05 to 0·08)	73·83 (71·67 to 75·96)	81·05 (79·55 to 83·32)	19·49 (18·2 to 20·83)	23·97 (22·83 to 25·89)	73 (61 to 87)	53 (41 to 61)
	Seychelles		0·01 (0·01 to 0·02)	0·01 (0·01 to 0·01)	0·21 (0·20 to 0·22)	0·10 (0·09 to 0·11)	70·11 (69·49 to 70·74)	77·69 (76·95 to 78·44)	17·57 (17·21 to 17·94)	22·12 (21·61 to 22·66)	<1 (<1 to <1)	<1 (<1 to <1)
	Thailand		0·01 (0·01 to 0·01)	0·01 (0·01 to 0·01)	0·18 (0·16 to 0·20)	0·08 (0·07 to 0·08)	74·32 (72·91 to 75·92)	81·96 (80·85 to 83·14)	22·15 (21·29 to 23·15)	25·83 (24·97 to 26·75)	273 (244 to 301)	195 (174 to 215)
	Timor-Leste		0·04 (0·03 to 0·05)	0·03 (0·03 to 0·04)	0·17 (0·15 to 0·20)	0·13 (0·11 to 0·16)	68·83 (67·27 to 70·67)	73·02 (71·29 to 74·76)	17·09 (16·19 to 18·45)	19·98 (18·85 to 21·11)	4 (4 to 5)	3 (3 to 3)
	Vietnam		0·02 (0·01 to 0·02)	0·01 (0·01 to 0·01)	0·20 (0·17 to 0·23)	0·08 (0·06 to 0·10)	69·98 (68·33 to 71·23)	79·16 (77·84 to 80·89)	17·15 (16·06 to 17·85)	22·79 (21·92 to 24·12)	349 (319 to 401)	258 (221 to 286)
**Sub-Saharan Africa**			**0·08 (0·08 to 0·09)**	**0·07 (0·07 to 0·08)**	**0·28 (0·27 to 0·29)**	**0·21 (0·20 to 0·22)**	**61·65 (60·79 to 62·42)**	**66·24 (65·38 to 67·02)**	**16·43 (16·06 to 16·74)**	**18·91 (18·47 to 19·35)**	**4072 (3922 to 4265)**	**3404 (3268 to 3563)**
Central sub-Saharan Africa			0·08 (0·07 to 0·10)	0·07 (0·06 to 0·08)	0·30 (0·27 to 0·33)	0·23 (0·21 to 0·26)	60·29 (58·66 to 62)	64·41 (62·7 to 65·98)	14·94 (14·3 to 15·86)	17·13 (16·04 to 18·17)	505 (460 to 556)	443 (404 to 488)
	Angola		0·07 (0·06 to 0·08)	0·06 (0·05 to 0·07)	0·29 (0·24 to 0·33)	0·22 (0·18 to 0·25)	61·67 (59·67 to 63·96)	66·68 (64·5 to 68·9)	15·21 (14·48 to 16·44)	18·44 (16·91 to 19·96)	100 (88 to 115)	84 (73 to 96)
	Central African Republic		0·13 (0·11 to 0·16)	0·12 (0·10 to 0·14)	0·52 (0·45 to 0·58)	0·38 (0·31 to 0·45)	49·11 (46·48 to 51·72)	54·91 (51·97 to 58·02)	11·92 (11·07 to 12·96)	14·24 (12·84 to 16·34)	36 (31 to 42)	28 (24 to 33)
	Congo (Brazzaville)		0·06 (0·05 to 0·07)	0·05 (0·04 to 0·06)	0·29 (0·24 to 0·34)	0·31 (0·26 to 0·36)	62·55 (60·39 to 64·81)	62·7 (60·20 to 65·63)	15·6 (14·92 to 16·79)	15·87 (14·81 to 17·61)	18 (16 to 21)	19 (16 to 23)
	Democratic Republic of the Congo		0·09 (0·07 to 0·10)	0·08 (0·07 to 0·09)	0·29 (0·25 to 0·34)	0·23 (0·19 to 0·27)	60·36 (58·19 to 62·67)	64·32 (62·01 to 66·69)	14·98 (14·08 to 16·33)	16·97 (15·45 to 18·43)	340 (298 to 389)	303 (266 to 345)
	Equatorial Guinea		0·06 (0·05 to 0·07)	0·05 (0·04 to 0·06)	0·26 (0·20 to 0·32)	0·26 (0·20 to 0·32)	64·26 (61·26 to 67·1)	66·42 (62·61 to 70·52)	16·85 (15·17 to 18·74)	19·35 (16·38 to 22·62)	4 (3 to 5)	4 (3 to 5)
	Gabon		0·04 (0·03 to 0·05)	0·03 (0·03 to 0·04)	0·26 (0·22 to 0·30)	0·16 (0·13 to 0·20)	65·08 (63·3 to 66·7)	72·07 (69·79 to 74·39)	15·84 (15·31 to 16·23)	19·96 (18·39 to 21·71)	6 (6 to 7)	5 (4 to 5)
Eastern sub-Saharan Africa			0·07 (0·06 to 0·08)	0·06 (0·05 to 0·06)	0·28 (0·27 to 0·29)	0·20 (0·19 to 0·21)	62·51 (61·74 to 63·26)	67·43 (66·77 to 68·11)	16·04 (15·76 to 16·32)	18·74 (18·38 to 19·09)	1412 (1365 to 1460)	1126 (1085 to 1165)
	Burundi		0·09 (0·07 to 0·10)	0·07 (0·07 to 0·08)	0·31 (0·26 to 0·36)	0·24 (0·20 to 0·29)	59·69 (57·35 to 62·16)	63·58 (61·3 to 65·88)	14·74 (13·86 to 16·19)	16·31 (14·96 to 17·97)	46 (40 to 52)	36 (32 to 41)
	Comoros		0·05 (0·04 to 0·06)	0·05 (0·04 to 0·05)	0·20 (0·16 to 0·23)	0·16 (0·13 to 0·20)	67·1 (65·04 to 69·21)	70·04 (67·84 to 72·28)	16·7 (15·69 to 18·04)	18·76 (17·22 to 20·23)	2 (2 to 3)	2 (2 to 3)
	Djibouti		0·05 (0·04 to 0·06)	0·04 (0·04 to 0·05)	0·23 (0·18 to 0·29)	0·20 (0·16 to 0·26)	66·05 (63·13 to 68·79)	68·86 (65·27 to 72·01)	16·62 (15·14 to 18·43)	18·85 (16·52 to 21·08)	4 (3 to 5)	3 (2 to 4)
	Eritrea		0·05 (0·04 to 0·06)	0·04 (0·04 to 0·05)	0·38 (0·32 to 0·45)	0·24 (0·19 to 0·30)	59·17 (56·42 to 61·93)	65·92 (63·4 to 68·97)	13·58 (12·66 to 14·79)	16·39 (15·29 to 18·17)	23 (19 to 27)	19 (16 to 22)
	Ethiopia		0·06 (0·05 to 0·07)	0·05 (0·05 to 0·06)	0·20 (0·18 to 0·21)	0·15 (0·14 to 0·17)	66·66 (65·57 to 67·74)	70·38 (69·3 to 71·51)	17·37 (16·74 to 17·96)	19·66 (18·99 to 20·35)	308 (291 to 328)	229 (214 to 244)
	Kenya		0·05 (0·04 to 0·06)	0·04 (0·03 to 0·05)	0·29 (0·28 to 0·31)	0·21 (0·20 to 0·23)	63·21 (62·44 to 63·94)	68·75 (67·94 to 69·55)	15·78 (15·53 to 16·06)	19·46 (19·05 to 19·9)	162 (156 to 167)	127 (123 to 132)
	Madagascar		0·08 (0·07 to 0·09)	0·07 (0·06 to 0·08)	0·26 (0·21 to 0·32)	0·22 (0·18 to 0·27)	62·17 (59·75 to 64·82)	64·81 (62·28 to 67·54)	15·51 (14·38 to 17·14)	16·67 (15·16 to 18·48)	97 (81 to 116)	87 (74 to 103)
	Malawi		0·07 (0·06 to 0·08)	0·06 (0·05 to 0·07)	0·34 (0·30 to 0·38)	0·22 (0·19 to 0·25)	59·6 (57·93 to 61·5)	66·93 (64·87 to 68·98)	15·09 (14·51 to 15·98)	19·42 (17·86 to 20·77)	72 (65 to 80)	57 (51 to 64)
	Mozambique		0·08 (0·07 to 0·09)	0·07 (0·06 to 0·08)	0·44 (0·40 to 0·50)	0·29 (0·25 to 0·34)	54·82 (52·67 to 57·04)	61·99 (59·39 to 64·45)	13·77 (12·85 to 14·69)	17·25 (15·74 to 19·04)	142 (126 to 160)	114 (100 to 130)
	Rwanda		0·05 (0·04 to 0·06)	0·04 (0·04 to 0·05)	0·22 (0·19 to 0·26)	0·16 (0·13 to 0·18)	65·75 (64·04 to 67·64)	70·83 (69·06 to 72·73)	16·22 (15·47 to 17·42)	19·61 (18·41 to 20·88)	36 (32 to 40)	32 (29 to 37)
	Somalia		0·11 (0·09 to 0·14)	0·09 (0·08 to 0·11)	0·34 (0·28 to 0·42)	0·27 (0·22 to 0·34)	56·52 (53·67 to 59·32)	60·59 (57·74 to 63·27)	14·08 (12·83 to 15·66)	15·34 (13·65 to 17·05)	80 (63 to 103)	65 (52 to 83)
	South Sudan		0·11 (0·09 to 0·13)	0·10 (0·08 to 0·11)	0·33 (0·27 to 0·41)	0·24 (0·19 to 0·32)	56·94 (53·94 to 59·97)	61·83 (58·63 to 65·14)	14·88 (13·34 to 16·51)	16·9 (14·82 to 18·87)	56 (47 to 67)	42 (35 to 50)
	Tanzania		0·07 (0·05 to 0·08)	0·06 (0·05 to 0·07)	0·24 (0·21 to 0·27)	0·18 (0·15 to 0·20)	64·62 (62·89 to 66·27)	68·88 (67·18 to 70·58)	17·08 (16·07 to 17·92)	19·57 (18·5 to 20·56)	185 (166 to 207)	157 (141 to 177)
	Uganda		0·07 (0·06 to 0·08)	0·05 (0·05 to 0·06)	0·28 (0·24 to 0·32)	0·17 (0·15 to 0·20)	62·28 (60·50 to 64·15)	69·17 (67·2 to 71·13)	15·71 (14·9 to 17·01)	19·68 (18·34 to 20·93)	131 (119 to 146)	102 (91 to 115)
	Zambia		0·07 (0·06 to 0·09)	0·05 (0·05 to 0·06)	0·32 (0·29 to 0·36)	0·23 (0·20 to 0·26)	60·36 (58·52 to 62·34)	66·28 (64·46 to 68·35)	15·33 (14·76 to 16·29)	18·38 (17·08 to 19·86)	68 (60 to 76)	51 (45 to 57)
Southern sub-Saharan Africa			0·04 (0·04 to 0·05)	0·04 (0·03 to 0·04)	0·37 (0·35 to 0·38)	0·25 (0·23 to 0·27)	61·5 (60·75 to 62·18)	68·49 (67·57 to 69·33)	16·81 (16·59 to 17·02)	20·98 (20·65 to 21·28)	355 (343 to 368)	305 (292 to 319)
	Botswana		0·03 (0·02 to 0·03)	0·02 (0·02 to 0·02)	0·28 (0·23 to 0·35)	0·21 (0·18 to 0·26)	67·03 (64·14 to 69·19)	70·97 (68·75 to 72·48)	18·15 (16·59 to 18·94)	20·01 (18·92 to 20·85)	7 (6 to 9)	7 (6 to 8)
	Lesotho		0·08 (0·07 to 0·09)	0·06 (0·05 to 0·07)	0·57 (0·51 to 0·62)	0·37 (0·31 to 0·43)	50·27 (48·13 to 52·65)	59·32 (56·33 to 62·67)	12·21 (11·47 to 13·06)	16·64 (14·87 to 19·24)	14 (12 to 16)	11 (9 to 14)
	Namibia		0·04 (0·04 to 0·05)	0·03 (0·03 to 0·04)	0·33 (0·28 to 0·38)	0·21 (0·16 to 0·27)	62·33 (60·28 to 64·31)	70·70 (67·46 to 73·54)	15·73 (15·17 to 16·2)	21·46 (19·66 to 23·14)	10 (9 to 11)	7 (6 to 9)
	South Africa		0·04 (0·03 to 0·04)	0·03 (0·03 to 0·04)	0·36 (0·34 to 0·38)	0·25 (0·23 to 0·27)	62·8 (61·99 to 63·56)	69·69 (68·6 to 70·62)	17·51 (17·35 to 17·66)	21·88 (21·69 to 22·06)	255 (245 to 266)	221 (210 to 233)
	Swaziland (eSwatini)		0·05 (0·04 to 0·06)	0·04 (0·03 to 0·05)	0·49 (0·43 to 0·55)	0·28 (0·23 to 0·33)	54·92 (52·57 to 57·56)	65·15 (62·13 to 68·35)	13·22 (12·38 to 14·44)	18·52 (16·35 to 20·95)	6 (5 to 7)	4 (4 to 5)
	Zimbabwe		0·06 (0·05 to 0·07)	0·05 (0·04 to 0·06)	0·40 (0·35 to 0·44)	0·27 (0·23 to 0·31)	58·15 (56·31 to 60·10)	64·39 (62·13 to 66·6)	14·13 (13·2 to 15·09)	16·97 (15·55 to 18·57)	64 (57 to 71)	54 (47 to 62)
Western sub-Saharan Africa			0·10 (0·09 to 0·11)	0·09 (0·08 to 0·10)	0·25 (0·23 to 0·28)	0·20 (0·18 to 0·23)	61·7 (60·16 to 62·94)	65·33 (63·57 to 66·85)	17·07 (16·26 to 17·69)	18·87 (17·81 to 19·87)	1801 (1674 to 1972)	1531 (1414 to 1681)
	Benin		0·09 (0·08 to 0·11)	0·08 (0·07 to 0·09)	0·24 (0·19 to 0·29)	0·18 (0·14 to 0·22)	62·61 (60·09 to 65·03)	66·63 (64·19 to 69·09)	16·4 (14·89 to 17·69)	18·4 (16·74 to 20·04)	43 (37 to 51)	37 (32 to 43)
	Burkina Faso		0·12 (0·10 to 0·14)	0·10 (0·08 to 0·11)	0·28 (0·25 to 0·32)	0·19 (0·17 to 0·22)	58·94 (56·92 to 61·04)	64·38 (62·57 to 66·3)	15·41 (14·42 to 16·4)	17·74 (16·61 to 18·89)	100 (88 to 117)	82 (73 to 94)
	Cameroon		0·08 (0·07 to 0·09)	0·07 (0·06 to 0·08)	0·30 (0·25 to 0·35)	0·24 (0·20 to 0·28)	60·97 (58·62 to 63·46)	65·1 (62·69 to 67·82)	15·7 (14·45 to 17·31)	18·22 (16·36 to 20)	104 (90 to 119)	87 (75 to 101)
	Cape Verde		0·02 (0·02 to 0·03)	0·02 (0·02 to 0·02)	0·19 (0·17 to 0·21)	0·09 (0·08 to 0·10)	72·52 (71·26 to 73·75)	79·01 (78·23 to 80·06)	21·18 (20·49 to 21·95)	23·98 (23·63 to 24·72)	2 (1 to 2)	1 (1 to 1)
	Chad		0·12 (0·11 to 0·14)	0·11 (0·10 to 0·13)	0·28 (0·24 to 0·33)	0·23 (0·19 to 0·27)	58·6 (56·43 to 60·82)	61·64 (59·19 to 64·23)	15·8 (14·55 to 16·93)	17·08 (15·68 to 18·51)	80 (71 to 91)	67 (59 to 75)
	Côte d'Ivoire		0·09 (0·08 to 0·11)	0·07 (0·06 to 0·08)	0·30 (0·26 to 0·34)	0·22 (0·19 to 0·26)	60·10 (57·82 to 62·32)	65·31 (62·84 to 67·7)	15·83 (14·5 to 17·06)	18·06 (16·59 to 19·68)	108 (95 to 122)	77 (67 to 87)
	The Gambia		0·05 (0·04 to 0·06)	0·04 (0·04 to 0·05)	0·27 (0·23 to 0·32)	0·21 (0·17 to 0·25)	63·78 (62·03 to 65·79)	67·87 (65·62 to 70·16)	15·67 (15·19 to 16·42)	17·88 (16·38 to 19·43)	7 (7 to 8)	6 (5 to 7)
	Ghana		0·06 (0·05 to 0·07)	0·05 (0·04 to 0·06)	0·28 (0·25 to 0·32)	0·20 (0·17 to 0·23)	62·59 (60·95 to 64·33)	68·4 (66·65 to 70·28)	15·36 (14·83 to 16·2)	18·64 (17·37 to 19·94)	111 (100 to 122)	91 (80 to 102)
	Guinea		0·10 (0·09 to 0·13)	0·09 (0·08 to 0·10)	0·29 (0·26 to 0·33)	0·25 (0·21 to 0·28)	59·26 (57·22 to 61·36)	62·23 (60·32 to 64·18)	15·09 (14·07 to 16·27)	16·28 (15·14 to 17·53)	58 (52 to 65)	51 (46 to 57)
	Guinea-Bissau		0·08 (0·07 to 0·10)	0·07 (0·06 to 0·07)	0·38 (0·32 to 0·43)	0·28 (0·24 to 0·32)	57·36 (55·12 to 59·67)	62·63 (60·33 to 64·94)	13·99 (12·98 to 14·89)	16·1 (14·72 to 17·86)	8 (7 to 10)	7 (6 to 8)
	Liberia		0·08 (0·07 to 0·10)	0·07 (0·06 to 0·08)	0·24 (0·20 to 0·28)	0·22 (0·18 to 0·26)	63·7 (61·52 to 65·79)	65·11 (63·13 to 67·43)	16·89 (15·5 to 17·99)	17·4 (15·96 to 19)	16 (14 to 19)	15 (13 to 17)
	Mali		0·13 (0·11 to 0·15)	0·11 (0·10 to 0·12)	0·22 (0·19 to 0·26)	0·21 (0·17 to 0·24)	60·96 (58·73 to 63·17)	62·98 (61·06 to 64·87)	17·37 (16·36 to 18·6)	17·89 (16·66 to 19·17)	101 (87 to 117)	86 (76 to 97)
	Mauritania		0·05 (0·04 to 0·06)	0·04 (0·04 to 0·05)	0·15 (0·12 to 0·18)	0·15 (0·12 to 0·19)	70·04 (68·03 to 72·26)	71·01 (68·91 to 73·02)	18·64 (17·39 to 20·26)	19·11 (17·65 to 20·48)	10 (9 to 12)	10 (8 to 11)
	Niger		0·11 (0·09 to 0·14)	0·10 (0·09 to 0·12)	0·24 (0·20 to 0·28)	0·20 (0·16 to 0·24)	61·13 (58·83 to 63·48)	63·59 (61·39 to 65·95)	16·56 (15·47 to 17·7)	17·63 (16·22 to 19·06)	92 (79 to 108)	81 (71 to 92)
	Nigeria		0·11 (0·10 to 0·12)	0·10 (0·08 to 0·11)	0·23 (0·19 to 0·28)	0·19 (0·15 to 0·25)	62·76 (59·7 to 65·2)	65·82 (62·32 to 69·11)	18·55 (16·62 to 19·91)	20·09 (17·61 to 22·73)	847 (724 to 1015)	736 (623 to 879)
	São Tomé and Príncipe		0·03 (0·03 to 0·04)	0·02 (0·02 to 0·03)	0·20 (0·17 to 0·23)	0·16 (0·13 to 0·18)	68·09 (66·51 to 69·83)	71·77 (70·06 to 73·78)	16·77 (16·09 to 17·78)	18·83 (17·7 to 20·26)	1 (<1 to 1)	<1 (<1 to 1)
	Senegal		0·05 (0·05 to 0·06)	0·04 (0·04 to 0·05)	0·21 (0·18 to 0·25)	0·17 (0·14 to 0·20)	66·14 (64·5 to 67·87)	70·05 (68·32 to 71·93)	16·45 (15·54 to 17·51)	18·83 (17·58 to 20·03)	48 (43 to 54)	40 (35 to 45)
	Sierra Leone		0·12 (0·10 to 0·14)	0·10 (0·09 to 0·12)	0·27 (0·22 to 0·31)	0·24 (0·20 to 0·29)	59·47 (57·21 to 61·72)	61·38 (59·4 to 63·73)	16·04 (14·86 to 17·16)	16·45 (15·14 to 17·89)	37 (32 to 43)	34 (30 to 38)
	Togo		0·07 (0·06 to 0·09)	0·06 (0·05 to 0·07)	0·30 (0·25 to 0·35)	0·20 (0·16 to 0·24)	61·37 (59·06 to 63·8)	67·23 (64·96 to 69·62)	15·36 (14·41 to 16·76)	18·54 (16·93 to 20·16)	27 (24 to 31)	23 (20 to 27)

Data in parentheses are 95% uncertainty intervals. Super-regions, regions, and countries are listed in alphabetical order. SDI=Socio-demographic Index.

**Implications of all the available evidence**

By using internally consistent estimates of deaths, births, and population over time, this analysis of trends in age-sex-specific death rates and summary measures such as life expectancy provides important perspectives on how mortality has been evolving since 1950. The findings of this study highlight global successes, such as the remarkable decline in under-5 mortality. This great success story reflects significant local, national, and global commitment and investment over several decades, a commitment that has intensified since the turn of the century. At the same time, our findings also bring attention to mortality patterns that are cause for concern, particularly among men aged 20–45 years and, to a lesser extent, women aged 20–45 years. In these groups, our findings show mortality rates that have stagnated over the time period covered by this study, and in some cases, are increasing. Comparing levels of mortality to those expected on the basis of development status, as measured with the Socio-demographic Index, provides insights into which countries have achieved lower and which countries are experiencing higher mortality rates than would be expected based on their level of development. Our findings show enormous variation in progress achieved across locations and ages, with countries that are performing better than expected in all regions of the world. Our results also highlight that greater emphasis needs to be placed on understanding the drivers of success for countries that have performed better than expected and that urgent attention needs to be brought to those countries that are lagging behind.

## Introduction

Measurement of mortality has always been crucial for populations, and mortality is a quantity that societies have attempted to track since ancient times.^1^, ^2^ More recently, its relevance and importance have been highlighted in the global agenda in the form of the health-related Sustainable Development Goals (SDGs), which not only include two indicators expressly focused on all-cause mortality (SDG indicators 3.2.1, under-5 mortality, and 3.2.2, neonatal mortality), but also death registration (SDG indicator 17.19.2c) and ten indicators of cause-specific or risk-attributable mortality.^3^ The prominence of mortality among the health-related SDGs intensifies the need for comparable, robust measurements of mortality that can be used for monitoring progress on mortality levels and trends across countries. National governments and international agencies alike need reliable evidence to identify and then prioritise addressing the largest challenges in improving survival, particularly during the SDG era.

Amid global gains in life expectancy and significant reductions in child mortality over the past few decades, concerning trends have surfaced in several countries and demographic groups, which have been attributed to a wide range of determinants of health.^4^, ^5^, ^6^, ^7^, ^8^ For example, although many high-income countries, including the USA and the UK, experienced large gains in life expectancy for many decades, the pace of progress has stalled in recent years, particularly in the past decade, and within-country inequalities in life expectancy have widened.^9^, ^10^, ^11^, ^12^, ^13^, ^14^ For other countries, such as Syria and Yemen, civil war has effectively erased—and reversed—years of steady gains.^14^, ^15^ In Mexico, studies have highlighted a combination of surging interpersonal violence and non-communicable diseases (NCDs) as the main factors underlying rising age-specific mortality among adult men, while in the USA, drug use disorders, suicide, cirrhosis, and diabetes are considered to be among the main culprits for plateaued mortality improvements among men.^9^, ^10^, ^11^, ^16^ Increasing rates of obesity are also viewed as a probable factor underlying the slowing of progress in female life expectancy in various countries.^17^, ^18^, ^19^ Changes in age-specific mortality rates and life expectancy can be used to track the impact of population-wide health threats, such as the HIV epidemic in sub-Saharan Africa, and also to quantify uncharacteristically high mortality experiences, such as the excess adult male mortality in central and eastern European countries during 1990s.^20^, ^21^, ^22^, ^23^ Accurate monitoring of levels and trends of mortality on a timely basis can provide crucial information for deploying resources and effective interventions at the population level.

The Global Burden of Diseases, Injuries, and Risk Factors Study (GBD) provides the only source of annually updated age-sex-specific mortality for countries across the world. Three other analytical efforts exist that provide estimates of age-specific mortality for a broad set of countries; however, we believe that these are not as comprehensive or timely as the GBD. The United Nations Population Division, Department of Economics and Social Affairs (UNPOP) has reported on life expectancy and age-specific mortality for 5-year calendar intervals by age, sex, and country since 2005 and for 201 countries. Their estimates are updated biannually; however, the estimates are not reported with uncertainty intervals (UIs).^24^ The US Census Bureau analyses only 15–25 countries per year and updates demographic estimates for them.^25^ WHO estimates of mortality are largely based on UNPOP estimates that have been interpolated to single years with some modifications for countries with complete vital registration (VR).^26^ In addition to these cross-national efforts, many countries produce their own estimates of age-specific mortality, which often differ from the international assessments.^27^, ^28^, ^29^

GBD 2017 represents the third iteration of the annual updates of the GBD.^14^, ^30^ This version of the GBD reports on trends in age-specific mortality and summary measures of mortality, such as life expectancy, with four main improvements. First, new data sources that have been released or reported since GBD 2016 have been incorporated. Second, for the first time, estimates of age-sex-specific population generated in the GBD are used in the estimation of all-cause mortality, whereas previous efforts by the GBD used the UN Population Division estimates of population by age and sex.^31^ Third, statistical methods used in different components of the analysis have been further standardised and improved. Lastly, we have extended the analysis and reporting of age-specific mortality back to 1950 to further contribute to research and analyses of long-term trends in mortality and life expectancy.

## Methods

### Overview

As with GBD 2016, this analysis adheres to the Guidelines for Accurate and Transparent Health Estimates Reporting (GATHER) standards developed by WHO and others.^32^ A table detailing our adherence to GATHER is included in appendix 1; statistical code used in the entire process is publicly available online. Analyses were done with Python versions 2.5.4 and 2.7.3, Stata version 13.1, and R version 3.1.2.

The methods used to produce estimates of age-specific mortality remain similar to those used in GBD 2016. Here we provide a broad overview and highlight the major changes since GBD 2016. All other details are included in appendix 1.

### Geographical units and time periods

The GBD is hierarchically organised by geographic units or locations, with seven super-regions, 21 regions nested within those super-regions, and 195 countries or territories within the 21 regions. Each year, GBD includes subnational analyses for a few new countries and continues to provide subnational estimates for countries that were added in previous cycles. Subnational estimation in GBD 2017 includes five new countries (Ethiopia, Iran, New Zealand, Norway, Russia) and countries previously estimated at subnational levels (GBD 2013: China, Mexico, and the UK [regional level]; GBD 2015: Brazil, India, Japan, Kenya, South Africa, Sweden, and the USA; GBD 2016: Indonesia and the UK [local government authority level]). All analyses are at the first level of administrative organisation within each country except for New Zealand (by Māori ethnicity), Sweden (by Stockholm and non-Stockholm), and the UK (by local government authorities). All subnational estimates for these countries were incorporated into model development and evaluation as part of GBD 2017. To meet data use requirements, in this publication we present all subnational estimates excluding those pending publication (Brazil, India, Japan, Kenya, Mexico, Sweden, the UK, and the USA); these results are presented in appendix tables and figures (appendix 2). Subnational estimates for countries with populations larger than 200 million (as measured with our most recent year of published estimates) that have not yet been published elsewhere are presented wherever estimates are illustrated with maps, but are not included in data tables.

### Data and data processing

In the estimation of age-specific mortality for GBD 2017, we used five types of data. These were data from VR systems, sample registration systems, household surveys (complete birth histories, summary birth histories, sibling histories), censuses (summary birth histories, household deaths), and Demographic Surveillance Sites (DSS).

The most robust source for estimating age-specific mortality is a VR system that records all deaths by age, sex, and location. Our analysis of mortality starts with collating all publicly available VR data plus data shared directly by governments or GBD collaborators from VR systems. We evaluate the completeness of VR data separately for deaths under the age of 5 years and deaths over the age of 15 years. For under-5 deaths, we statistically compare VR-based death rates with those recorded in censuses or surveys. For deaths over the age of 15 years, we apply three methods for detecting under-registration: generalised growth balance, synthetic extinct generations, and a hybrid method that uses both methods.^33^, ^34^, ^35^, ^36^, ^37^, ^38^ These methods are collectively described as death distribution methods because they use the demographic balance equation to infer completeness of registration. Age misreporting and migration affect these methods.^33^, ^38^ We used the spatiotemporal regression framework with the results of these methods for all intercensal intervals to produce a coherent time series of completeness for each location. For this step, the first stage of the model uses completeness of child death registration as a covariate and then applies time and space weights on the residuals to produce a smoothed result. In some countries, sample registration systems are operated wherein events are recorded in detail for a representative sample of communities within those countries. We used the same death distribution methods to evaluate the completeness of these sources as for VR; sample registration death counts were scaled in the death distribution methods analysis to the national level. This study considers a country to have complete VR when it used a civil registration system, vital statistics, or sample registration system that captures at least 95% of all deaths within the country. When calculating death rates for under-5 mortality, adult mortality, or empirical life tables, we used the GBD population estimates by age, sex, location, and year as the denominator.^31^

In addition to VR data, for the estimation of under-5 death rates, we use data from complete birth histories collected through household survey programmes, including the World Fertility Survey, Demographic and Health Surveys, some Multiple Indicator Cluster Surveys, and various other national surveys. A wider set of surveys and many censuses also collect data on the number of livebirths for a woman and the number of these children who are still surviving. This information is called a summary birth history and can yield an unbiased assessment of the trend in the under-5 death rate.^39^

Assessments of adult mortality, in addition to VR and sample registration data, use survey data collected on sibling histories. A sibling history means that a respondent is asked to report on the survival or death of each of their siblings; in other words, the respondent provides a complete birth history for their mother. Sibling histories are subject to survivor bias and recall bias. Sibling history data are processed for GBD using methods that address these limitations.^40^ Some surveys and some censuses also use information on deaths in a household over some recent time interval—for example, the past 12 months. Studies suggest that respondents can over-report or under-report deaths of household members.^41^ We apply death distribution methods to assess completeness, which can be greater than 100% due to telescoping of event reporting, which happens when a respondent reports an event that happened before the recall period as if it happened during the recall period.

For GBD 2017, we also included DSS data on adult mortality for the first time, specifically on the probability of death between the ages of 15 and 60 years (45q15), from local communities that are under direct surveillance. Because these DSS communities are not nationally representative, we adjusted the level of 45q15 based on the ratio of the probability of death from birth to age 5 years (5q0) from the DSS to the national 5q0, taking into account that the relationship between 5q0 and 45q15 changes as the level of 5q0 declines because, on average, there are larger declines in 5q0 than in 45q15 over time.

### New data for GBD 2017 compared to GBD 2016

In GBD 2017, we have added 458 location-years of VR data at the national level and 9 location-years of VR data at the subnational level compared with GBD 2016. We also included an additional 62 complete birth history sources at the national level, 12 complete birth history sources at the subnational level, 72 national summary birth history sources, and 16 subnational summary birth history data sources. 11 national and seven subnational sibling history surveys were also added. We included 1529 datapoints from DSSs in 15 countries. The total numbers of datapoints used were 181 625 for under-5 mortality estimation and 63 234 for adult mortality estimation. We also used 35 177 empirical life tables in the all-cause mortality database for GBD 2017. Appendix 1 provides complete lists of data availability and data sources by location; these are also available using our online source tool, the Global Health Data Exchange. The addition of these data has provided increasingly accurate mortality metrics in many countries over all years estimated in GBD.

### Estimating under-5 mortality and more detailed age intervals below 5 years

Using all the VR, complete birth history, and summary birth history data available for each country, we estimate the time trends from 1950 to 2017 for each location. We use spatiotemporal Gaussian process regression (ST-GPR) to estimate time trends. This model has four components. First, it includes three covariates: lag-distributed income (LDI) per capita, average years of schooling for women aged 15–49 years, and the crude rate of death from HIV/AIDS.^42^, ^43^, ^44^ Second, it includes random effects for each source of data in each country, where a source refers to a particular survey or census. Using the random effects, data are adjusted to the reference source for each country. The reference source is VR in countries with complete VR and complete birth histories in countries without complete VR. In some locations, reference sources are selected on the basis of expert knowledge of a country and its data sources provided by GBD collaborators. The third component of the model borrows strength over space and time by smoothing the residuals; the degree of smoothing is controlled by three hyperparameters. These hyperparameters are a time weight (lambda), a space weight (zeta), and a temporal correlation weight (scale). Additional details on the selection of the hyperparameters are included in appendix 1 section 2.2. The fourth component of the model uses the output after the first three components have been run as the mean prior in a Gaussian process regression. Gaussian process regression also includes four hyperparameters, lambda, zeta, scale, and an additional hyperparameter, amplitude. Details on these hyperparameters are included in appendix 1. In GBD 2017, to standardise our analysis further, we have opted to use the same amplitude for all locations. The value for amplitude is based on the analysis of variation over time in countries with complete VR that is not explained by the covariates.

We use a multiphase approach to generate age-specific and age-sex-specific under-5 mortality. We first model the ratio of male to female 5q0. Next, we run separate models to estimate the probability of death for each sex and age group, specifically early neonatal (0–6 days), late neonatal (7–27 days), postneonatal (28–364 days), infant mortality (<1 year), and childhood mortality (between 1 and 5 years). These are run to take advantage of greater data density for both the ratio of male to female mortality and the split between infant mortality and childhood mortality as compared with the split of infant mortality into the components of early neonatal, late neonatal, and postneonatal. Each is modelled using ST-GPR. Results of the sex-ratio model are first applied to derive sex-specific under-5 death rates (U5MR). Next, the probability of death from birth to the exact age of 1 year and from age 1 year to the exact age of 5 years are transformed to conditional probabilities and scaled to the sex-specific U5MR estimates. This is done to ensure that the value of 1 minus the probabilities from birth to the exact age of 1 year and from age 1 year to the exact age of 5 years equals the probability of death between birth and the exact age of 5 years. Lastly, early neonatal, late neonatal, and postneonatal model results are transformed to conditional probabilities and scaled in the same manner to equal the sex-specific probability of death from birth to the exact age of 1 year. More information on the models, model hyperparameters, and scaling can be found in appendix 1 section 2.2.

### Estimating the probability of death between ages 15 and 60 years

Data on the probability of death between the ages of 15 and 60 years are also modelled using ST-GPR. In the first stage model, we use LDI per capita, average years of schooling for the population aged 15–59 years, the crude rate of death from HIV/AIDS, and the under-5 mortality rate as covariates.^42^, ^43^, ^44^ Under-5 mortality rate was not used as a covariate in GBD 2016, but we found that the model, which is now estimating for a longer time period going back to 1950, performs better when this covariate is included. We model the data for males and females together and include a dummy variable for sex in the model. In GBD 2016, we had run separate models for males and females, but this had yielded implausible sex ratios of adult mortality rates in specific location-years. More details, including hyperparameters for the ST-GPR model, are described in appendix 1 section 2.3.

### GBD model life table system and the database of empirical life tables

To produce a complete set of age-specific mortality rates (an abridged life table) for each location, we used the GBD model life table system, which identifies a reference life table for each location, year, and sex, on the basis of the nearest matches found in our empirical life table database.^14^ As we have revised the population denominators used to create the empirical life tables in GBD 2017, we have substantially updated and revised the database of empirical life tables as well. In previous GBD iterations, we excluded life tables based on implausible patterns of variation in death rates in the age groups older than 40 years. As with previous GBD cycles, we have two sets of life tables that meet inclusion criteria: a universal set that is used for all locations to identify matches and a location-specific set that is used for each location along with the universal set. We have formalised the inclusion criteria for life tables for both the location-specific and the universal set, and those are listed in section 2.4 of appendix 1. Life tables that meet all of the general inclusion criteria but not all of the universal life table inclusion criteria are categorised as location-specific life tables. For each life table, within each location, we sort life tables by year and generate smoothed life tables using moving averages of widths 3, 5, and 7 adjacent years within each location. This smoothing helps to address jumps or drops in age-specific mortality in locations where small numbers of deaths resulted in high variability of mortality patterns across age. After separately categorising each life table, we keep the least-smoothed of the candidate life tables within each life table set. The smoothing process and inclusion criteria help to address implausible age patterns from countries with small populations, unstable death rates, or poor data quality.

We have also set the number of matches searched for in the databases to be 100 for all locations; to ensure that locations with high-quality data primarily rely on their own age patterns of mortality, we have modified the space-time weighting scheme through a 25-fold increase in the country-specific weights compared with GBD 2016, with an additional 15-fold increase in 0-year and 1-year lag country-specific weights and a three-fold increase in the 2-year, 3-year, and 4-year lag country-specific weights. We also generated a new geographical strata of life table weights for subnational locations that are within the same country, which were assigned the same value as the original GBD 2016 country-specific weights.

For both all-cause mortality and cause-specific mortality analyses in GBD, we amassed a comprehensive database on human mortality from full VR systems and sample VR systems such as the Sample Registration System (SRS) from India and the Disease Surveillance Point system from China. These data sources provided a total of 42 138 empirical life tables, which also include subnational locations. After applying inclusion criteria, we use 35 177 life tables, of which 10 885 are universal and 24 292 location-specific. The GBD model life tables varied in quality in accordance with the coverage of a location's VR: for locations where VR coverage was high, the standard was overwhelmingly derived from observed mortality patterns, whereas in locations where VR coverage was low, the standard was based on locations with similar under-5 and adult mortality rates, with more weight given to life tables that were closer geographically and temporally. The selection of geographically and temporally similar locations helped to capture differences in mortality patterns by age due to specific causes of death.

### Single-year life tables

To support the estimation of single-year population for each location-age-sex-year, we have also generated single-year life tables for all locations from the abridged life tables after the HIV/AIDS mortality reconcilliation process and the addition of fatal discontinuities. Our method for generating single-year probabilities of death that are consistent with the abridged life table probabilities of death and known data on single-year patterns is described in the GBD 2017 population and fertility publication.^31^

### Fatal discontinuities

Fatal discontinuities are idiosyncratic increases in mortality that would affect long-term mortality trends if modelled using the all-cause mortality estimation process, and as a result, are estimated separately. Events categorised as fatal discontinuities are epidemics (such as Ebola virus disease or cholera); natural disasters, major technological or transport accidents, and war and terrorism. The specific data sources used to compile fatal discontinuities can be explored using the online source tool, the Global Health Data Exchange, and are described in detail in appendix 1 section 4. Estimates from high-quality VR systems were included instead of estimates from other sources in the event that conflicting sources were identified for a fatal discontinuity, with few exceptions when there was evidence to suggest that the VR system was compromised by the event. Regional, cause-specific UIs were used to estimate uncertainty for events where only point-estimate mortality data were available.

For GBD 2017, we have recoded the locations of all events using a new suite of software developed in-house to match differently coded locations in the fatal discontinuities database to GBD locations, taking advantage of detailed location information that was presented in non-standardised ways—eg, sources that included the name of a city or village instead of latitude and longitude. We first overlaid the portions of the database with latitude-longitude coordinates to the most detailed GBD location. When coordinates were not available, we used three web-based geocoding services—the Google Maps, OpenStreetMap, and Geonames geocoding application programming interfaces—to get a set of possible latitude and longitude coordinates from the location, overlaid those coordinates on to GBD locations, and then used the most common result from the three services to assign a GBD location.

Since discontinuities for recent years are not well tracked in the available databases, we have supplemented these databases with online searches. For GBD 2017, we systematised the identification of events missing from our database by mining Twitter accounts of major news providers for common terms associated with such events, like “earthquake” and “casualties.” This provided 62 events. Once events were identified, news reports of death totals, location, and date were used.

The age pattern of deaths is rarely identified in databases of fatal discontinuities. In order to estimate an age and sex distribution, events were first assigned to a GBD cause. Events were then split based on both the global age and sex distribution of that cause of death and the age and sex distribution of the population in the GBD location of the event, following the GBD causes of death age-sex-splitting algorithm. The main effect of this effort is that we are much less likely to miss shocks or allocate them to the wrong subnational location.

### HIV/AIDS in countries with large epidemics and incomplete VR

We produced estimates of adult HIV/AIDS incidence and prevalence using the estimation and projection package (EPP), a Bayesian model developed by UNAIDS.^45^ Our implementation of EPP made use of GBD-estimated demographic parameters, mortality rates for people on and off antiretroviral therapy, and CD4 progression rates to fit a model to HIV/AIDS prevalence data from surveillance sites and representative surveys. EPP-generated incidence and prevalence time series were used as inputs into Spectrum, a compartmental HIV/AIDS progression model originally developed by UNAIDS. Spectrum generated a full set of age-sex-specific HIV/AIDS mortality rates using detailed demographic parameters that align with those used for EPP. In countries with VR data, we adjusted age-specific and sex-specific incidence rates to produce mortality estimates that better fit observed deaths. In parallel, the GBD model life table process produced a separate set of HIV/AIDS death estimates, which were reconciled with Spectrum outputs to produce final mortality estimates. For countries with high-quality VR systems, mortality estimates were generated using ST-GPR on VR data.

### Analysing the relationship between age-specific mortality rates and development status

To characterise development status, we used the Socio-demographic Index (SDI), a composite measure based on the total fertility under the age of 25 years (TFU25), average educational attainment in those aged 15 years or older, and LDI. Compared with GBD 2016, the SDI calculation in GBD 2017 has been refined to use TFU25 instead of the total fertility rate because TFU25 does not show a U-shaped pattern with development at higher levels of development status and is a better proxy for the status of women in society.^31^ Aggregate SDI groupings were generated by applying quintile cutoffs from the distribution of national-level SDI for countries with populations greater than 1 million in 2017 to estimates of SDI for all GBD locations in 2017. The SDI analysis is described in further detail in appendix 1 (section 3); additional detail on correlation for the weighted scores is also provided.

To evaluate the average relationship between SDI and all-cause mortality, we fit a generalised additive model with a Loess smoother on SDI by age and sex group using GBD 2017 estimates from 1950 to 2017. The expected value is based solely on SDI status and does not vary over time. Examination of how the ratio of observed death rates to expected death rates changes over time allows us to explore the impact of how the relationships are changing over time. The expected age-sex-specific mortality rates were subsequently used to generate a complete life table expected on the basis of SDI alone.

### Uncertainty analysis

We estimate uncertainty systematically throughout the all-cause mortality estimation process. We generated 1000 draws for each all-cause mortality metric, and 95% UIs are calculated using the 2·5th and 97·5th percentiles of the draw-level values. Analytical steps are connected at the draw level, and the uncertainty of key mortality metrics is propagated throughout the all-cause mortality estimation process. Uncertainty in under-5 mortality and adult mortality rate estimation and completeness synthesis are estimated using non-sampling error and sampling error by data source. For the model life table step and HIV/AIDS-specific mortality calculations, uncertainty was estimated from uncertainty in the life table standard and from the regression parameters and sampling error in the EPP, respectively.

### Role of the funding source

The funders of the study had no role in study design, data collection, data analysis, data interpretation, or writing of the report. All authors had full access to the data in the study and had final responsibility for the decision to submit for publication.

## Results

### Levels and trends in death registration

**Figure 1:**
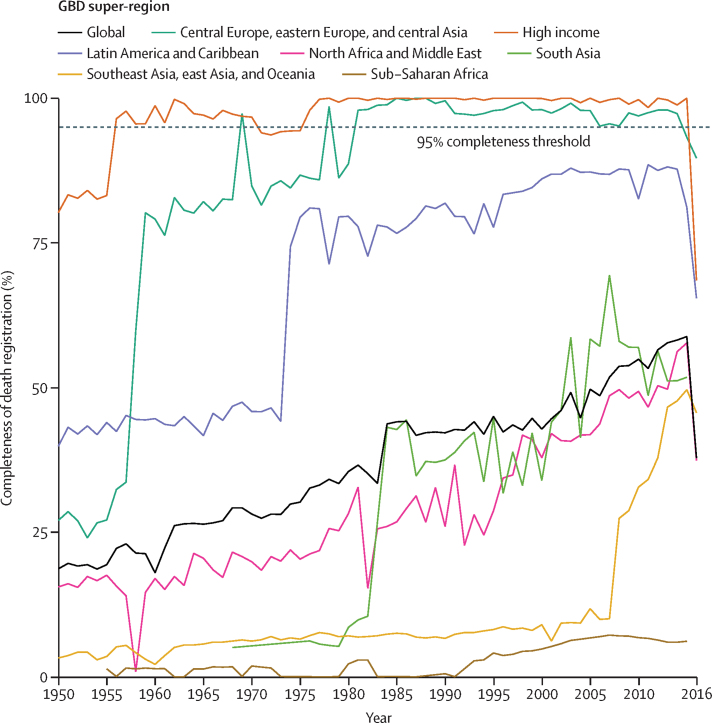
Estimated proportion of deaths that are registered and reported globally and by GBD super-region, for both sexes combined, 1950–2016 Each line represents the proportion of deaths that are registered and reported for a given GBD super-region or globally from 1950 to 2016. The reason for the dips in the most recent years is that lags in reporting mean that estimated deaths are higher than what is reported, resulting in a huge drop in completeness from 2015, where the reported deaths are more complete. GBD=Global Burden of Diseases, Injuries, and Risk Factors Study.

The proportion of deaths that are registered and reported through VR and civil registration systems globally and by super-region are shown in figure 1, with detailed information on each location shown in appendix 1. The updated results of the application of all three death distribution methods and the synthesised time series of completeness are available online. Globally, 18·7% (95% UI 18·4–19·0) of deaths were registered in 1950 and that number has been steadily increasing, with 58·8% (58·2–59·3) of all deaths registered in 2015, the most recent year with the highest reported rate. 2015 is the peak year of completeness of death registration and reporting globally, which partly reflects the substantial lag between when deaths occur and when they get reported through existing systems. Two super-regions, the high-income countries and central Europe, eastern Europe, and central Asia have had complete registration since 1985, with completeness considered to be above 95%. The Latin America and the Caribbean region has also had high registration since the late 1970s, which has been increasing and reached a high of 88·5% (88·0–89·0) in 2011 and staying around that level since. Substantial progress was seen in southeast Asia, east Asia, and Oceania in the past decade, with registration having increased from less than 10% as recently as 2006, to 49·6% (48·7–50·6) in 2015. South Asia and north Africa and the Middle East had improvements throughout this time period; south Asia reached a maximum completeness of 69·4% (68·4–70·5) in 2007 and was at 51·1% in both 2013 and 2014, and north Africa and the Middle East attained a 57·7% (56·5–58·9) completeness in 2015. Globally, 134 of 195 countries and territories had increases in completeness since the first year for which we have reported VR. Notable increases were for Iran, which increased from 13·4% (13·1–13·8) in 1974 to 89·8% (89·4–90·1) in 2016, Turkey, which increased from 28·9% (27·5–30·5) in 1978 to 100% (100–100) in 2016, and South Korea, which was 39·0% (35·0–43·2) in 1957 and increased to 97·9% (94·5–100·0) in 2016. Despite the increases over time, figure 1 also shows that 62·2% of all deaths did not get reported or registered as recently as 2016. Sub-Saharan Africa stands out as the region with the lowest rate of death registration and reporting. While substantial progress has been made in all other super-regions, sub-Saharan Africa remains at very low levels of death reporting and registration. 56 countries had registration that was complete or at its highest level in 2016. In other countries, the lag between registration and reporting is even longer; only 81 countries were regarded as having complete registration (>95%) for at least 1 year in the past 5 years, which were mainly in western Europe, central Europe, and the Caribbean.

### Trends in number of age-specific deaths and death rates at the global level since 1950

**Figure 2:**
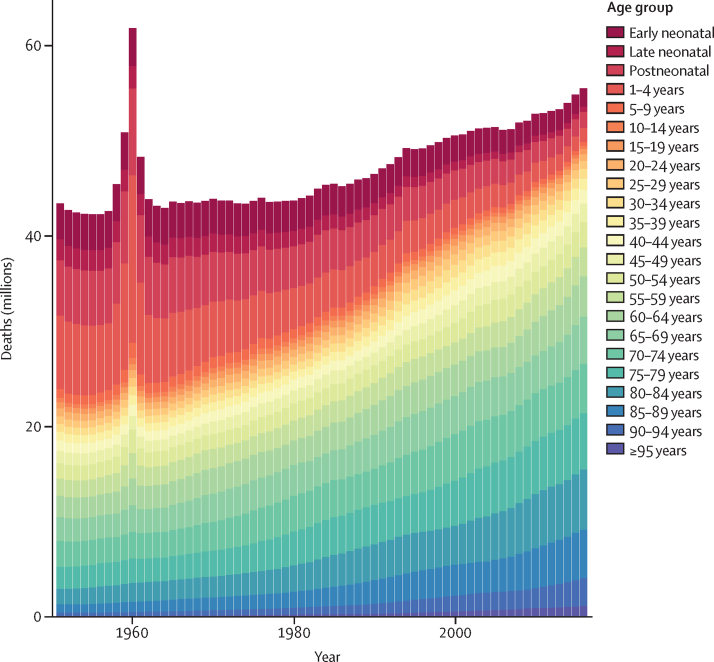
Total number of deaths by age, globally, for both sexes combined, 1950–2017 Each stacked bar represents the total number of deaths in the given year attributable to each age group, from 1950 to 2017, for both sexes combined. The early neonatal age group is 0–6 days, late neonatal is 7–27 days, and postneonatal 28–364 days.

Figure 2 shows the total number of deaths over time by age and for both sexes combined (sex-specific results are available in appendix 2). There were 43·7 million (95% UI 43·0–44·3) deaths in the world in 1950, and that number had increased to 55·9 million (55·4–56·5) by 2017. This relatively small increase of 28·1% (25·9–30·4) in the total number of global deaths is even more impressive when taken in the context of population growth. Despite the huge increase in the global population, from 2·57 billion (2·52–2·62) in 1950 to 7·64 billion (7·39–7·87) in 2017—increase of 297·2% (293·6–299·9)—the number of deaths has remained comparatively constant. The highest number of deaths, 61·8 million (61·4–62·3), occurred in 1960. The excess number of deaths in 1960 compared with adjacent years was due to the Great Leap Forward in China. Overall, for both men and women (figures by sex shown in appendix 2), there has been a huge decrease in childhood deaths across all of the four age groups that refer to under-5 mortality (figure 2). As a proportion of total deaths, deaths before the age of 5 years have decreased from 44·9% (44·2–45·7) in 1950 to 9·6% (9·3–10·0) in 2017. Conversely, deaths at ages older than 75 years have increased substantially, going from 11·9% (11·8–11·9) of total deaths in 1950 to 39·2% (39·1–39·4) of total deaths in 2017.

The trends in age-specific deaths during this time period are shown in more detail in appendix 2. Broadly, the trends over time in the number of deaths fall into three categories, aside from the large spike in deaths in 1960 due to the Great Leap Forward in China. First, the age groups younger than 5 years have had consistent declines in the numbers of deaths since 1950. The largest declines were in the four age groups pertaining to ages younger than 5 years for both boys and girls. Globally, for both sexes combined between 1950 and 2017, the number of deaths declined in the early neonatal period from 3·7 million (95% UI 3·6–4·0) to 1·9 million (1·8–1·9); in the late neonatal period from 2·2 million (2·1–2·3) to 0·5 million (0·5–0·5); in the postneonatal period from 5·8 million (5·6–6·1) to 1·6 million (1·6–1·7); and at ages 1–4 years from 7·8 million (7·6–8·1) to 1·4 million (1·3–1·5). This substantial decline in the total number of under-5 deaths, from 19·6 million (19·1–20·2) to 5·4 million (5·2–5·6), also needs to be considered in the context of the number of births, which has increased by 49·9% (43·5–56·5) from 92·6 million (88·9–96·4) to 138·8 million (130·0–149·1) during the same period. Second are the age groups starting at age 5 years and up to age 49 years, for which the numbers of deaths have remained relatively constant between 1950 and 2017. For example, for the 20–24 years age group there were 892 000 (879 000–909 000) deaths in 1950 and 710 000 (697 000–725 000) in 2017. Third, in the older age groups (ie, those older than 50 years), the number of deaths has steadily increased since 1950; these increases are most notable in the age groups older than 80 years.

**Figure 3:**
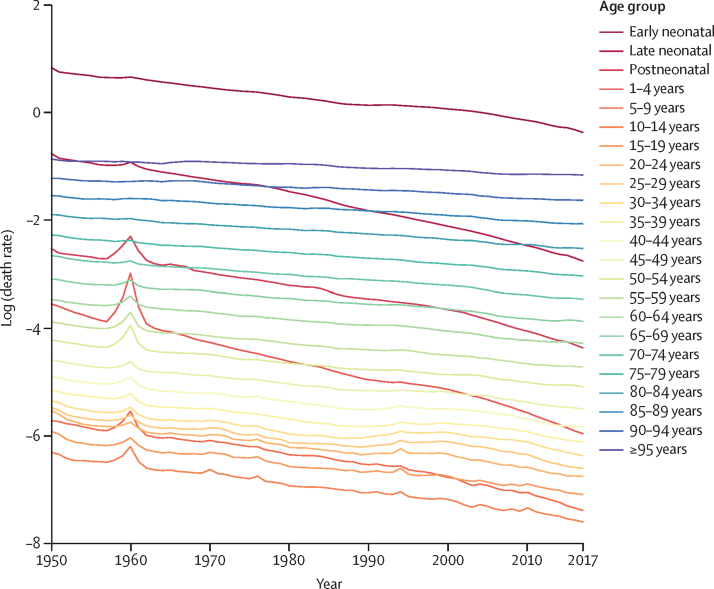
Natural logarithm of age-specific mortality rates, globally, for both sexes combined, 1950–2017 Each line represents the natural logarithm of the global death rate for a single year by age group, from 1950 to 2017, for both sexes combined. The early neonatal age group is 0–6 days, late neonatal is 7–27 days, and postneonatal 28–364 days.

Figure 3 shows depicts the trends in age-specific death rates since 1950 on a natural log scale. Death rates in the younger age groups, especially those younger than 5 years, have declined faster than those in adult age groups for both men and women. For some age groups, particularly those older than 80 years, death rates have not changed much over the past 68 years, suggesting that the large increase in the absolute number of deaths shown in figure 2 is driven by increases in the populations of those age groups over time, and not by increases in age-specific death rates.

**Figure 4:**
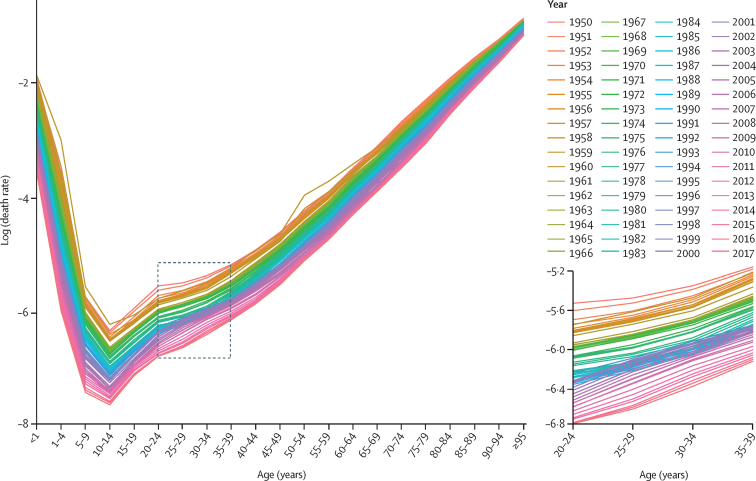
Global log (death rate) age-pattern for both sexes combined, by year, 1950–2017 Each line represents the logarithm of global age-specific mortality rates for a given year between 1950 and 2017 and for both sexes combined. The inset shows a closer view of age groups from 20 to 39 years.

Figure 4 shows the age-specific mortality rate curves for all years since 1950. On a natural log scale, the same difference on the y-axis represents the same percentage decline. This representation highlights the remarkable progress in age-specific mortality rates over time. The exception here is the period between 1958 and 1961, which reflects the impact of the Great Leap Forward in China, seen as higher mortality rates for all age groups under the age of 15 years and higher mortality rates than other years for ages 50–64 years. Outside of that period, the younger age groups that compose under-5 mortality have steady progress over time, with the mortality rate for 1–4 year olds dropping from 2554·7 deaths (95% UI 2330·7–2788·5) per 100 000 to 264·7 (239·6–293·3) between 1950 and 2017. What is less visible in the previous figures is the steady progress in the age groups 5–9 years and 10–14 years, for which mortality rates have dropped from 330·4 (325·9–335·4) per 100 000 to 62·3 (61·2–63·5) per 100 000 and 183·4 (181·3–186·0) per 100 000 to 50·3 (49·5–51·1) per 100 000, respectively. Progress in age-specific mortality rates occurred across all ages, but become less pronounced for the older age groups. Despite overall progress, in the younger adult age groups (ages 20–45 years) the curves from the early 2000s cross over those for the 1990s, indicating a reversal in decades of progress on young adult mortality (see insert in figure 4). Other than this period of reversal at the global level, progress has been remarkably consistent in global death rates, albeit with very different relative changes by age group.

### Global, regional, and national trends in life expectancy since 1950

**Figure 5:**
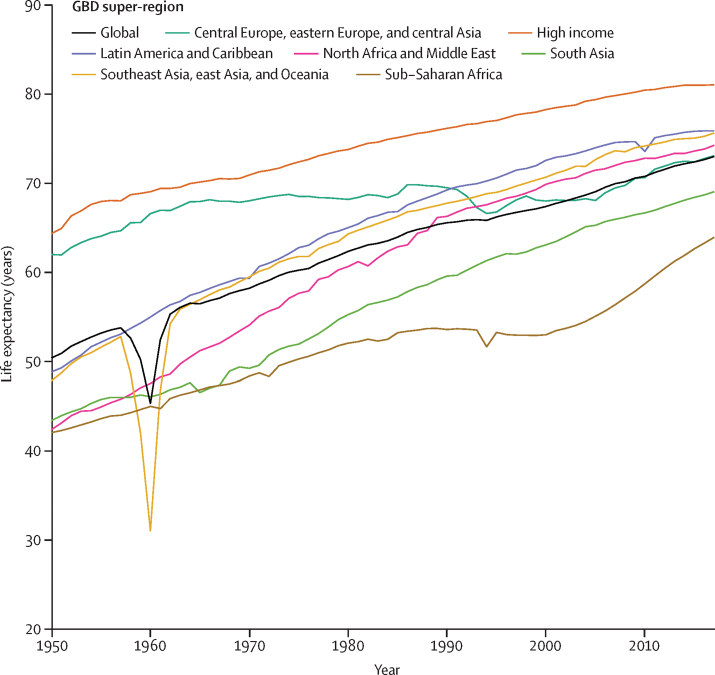
Life expectancy at birth and by GBD super-region for both sexes combined, 1950–2017 GBD=Global Burden of Diseases, Injuries, and Risk Factors Study.

Taking into account trends in age-specific mortality rates over time, figure 5 shows global and regional trends for both sexes combined in life expectancy at birth since 1950 (sex-specific figures are available in appendix 2). Globally, life expectancy at birth has increased from 48·1 years (95% UI 46·5–49·6) in 1950 to 70·5 years (70·1–70·8) in 2017 for men and from 52·9 years (51·7–54·0) in 1950 to 75·6 years (75·3–75·9) in 2017 for women. The huge impact of the Great Leap Forward in China in 1960 is shown clearly at both the global and regional level. Globally, life expectancy dropped by 5·1 years (3·9–6·2) as a result of the famine. Other than this massive fatal discontinuity, the trend in life expectancy at the global level has been one of steady increases. The smallest gain at the global level was during the 1990s and is partly explained by the fact that two super-regions, central Europe, eastern Europe, and central Asia, and sub-Saharan Africa, experienced declines during this decade. At the super-region level, the largest gains in life expectancy since 1950 were in north Africa and the Middle East, where life expectancy increased from 42·4 years (40·6–44·1) to 74·2 years (73·9–74·6). At the other end of the scale, the smallest net gains in life expectancy were in central Europe, eastern Europe, and central Asia, where life expectancy has increased by a total of only 11·1 years (10·3–11·9) since 1950, including periods where life expectancy decreased. Progress has been made in this super-region since 2000, with increases of 5·6 years (5·4–5·8) for men and 4·2 years (4·1–4·4) for women in life expectancy up to 2017.

Sub-Saharan Africa had the lowest levels of life expectancy in 2017, at 63·9 years (95% UI 63·1–64·6) for both sexes combined, which is where the global average was in the mid-1980s. Although the net increase during the 68-year period since 1950 has been the smallest for sub-Saharan Africa, with declines occurring during the 1990s for both men and women, the region has also shown the greatest gains in life expectancy since 2000. Life expectancy has increased by 10·9 years (10·1–11·7) since 2000, a much faster rate of increase than in previous decades. High-income regions had the highest life expectancy in 2017; however, the gains since 2010 have been very small at 0·6 years (0·5–0·7).

**Figure 6:**
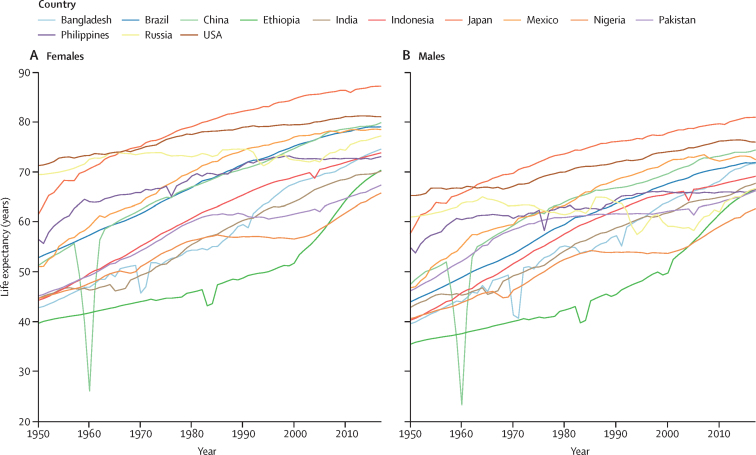
Life expectancy at birth for the countries with population greater than 100 million, 2017 Each line represents life expectancy at birth from 1950 to 2017 for females (A) and males (B).

Figure 6 shows changes in life expectancy at birth for women (figure 6A) and men (figure 6B) for the 13 countries with a population greater than 100 million in 2017, which jointly represent 62·5% (95% UI 61·5–63·4) of the global population. Among these 13 countries, Russia stands out for having the smallest net gain over the 68-year period of the study at only 5·7 years (4·8–6·7) for men and 7·7 years (7·0—8·6) for women. Men in Ethiopia and Pakistan have similar life expectancies to men in Russia in 2017 at 66·7 years (65·6–67·7) and 66·4 years (63·8–69·1), respectively; however, men in both Ethiopia and Pakistan have had significant increases in life expectancy since 1950, adding up to a total gain of 31·1 years (28·0–34·3) in Ethiopia and 20·1 years (16·1–23·9) in Pakistan, with particularly pronounced increases since 2000 for Ethiopia. While not as noticeable as for men, gains in life expectancy have also been small for women in Russia, with the most pronounced declines seen in the early 1990s. Furthermore, whereas Russia was ranked second among this group of countries in both men and women in 1950, following the USA, by 2017, it had fallen to tenth place for men and sixth place for women.

The largest gains in life expectancy among the 13 most populous countries were in Bangladesh (32·1 years [95% UI 29·3 to 35·2] for men and 31·7 years [28·4 to 35·0] for women) and Ethiopia (31·1 years [28·0 to 34·3] for men and 30·6 years [28·3 to 32·9] for women). The Philippines stands out as the country in this group in which the gap between female and male life expectancy has grown the most since 1950. The gap was only 1·6 years (–0·1 to 3·3) in 1950 and has grown to 6·5 years (3·6 to 9·1) in 2017. In 1950, life expectancy in the Philippines was only 2·7 years (0·7 to 5·0) lower for men and 5·0 years (3·5–6·6) lower for women compared with Japan. By 2017, the gap between these two countries had increased to 14·5 years (12·5–16·5) for men and 14·0 years (12·3–16·0) for women because the Philippines has experienced much smaller gains compared with most other large countries.

Despite the massive setback around the famine in 1960, China has made steady progress and, in 2017, life expectancy was 74·5 years (95% UI 74·1 to 75·0) for men and 79·9 years (79·4 to 80·4) for women. Among the world's most populous countries, Japan has had the highest life expectancy for men and women since 1963 and continues to do so in 2017. A worrying finding shown in figure 6B is that, in both Mexico and the USA, men have had declines in life expectancy since 2012. Women in these two countries have not had declines, although their gains in life expectancy have not been significantly different from zero (0·01 years [–0·28 to 0·26] in Mexico and −0·11 years [–0·4 to 0·18] in the USA) since 2010.

**Figure 7:**
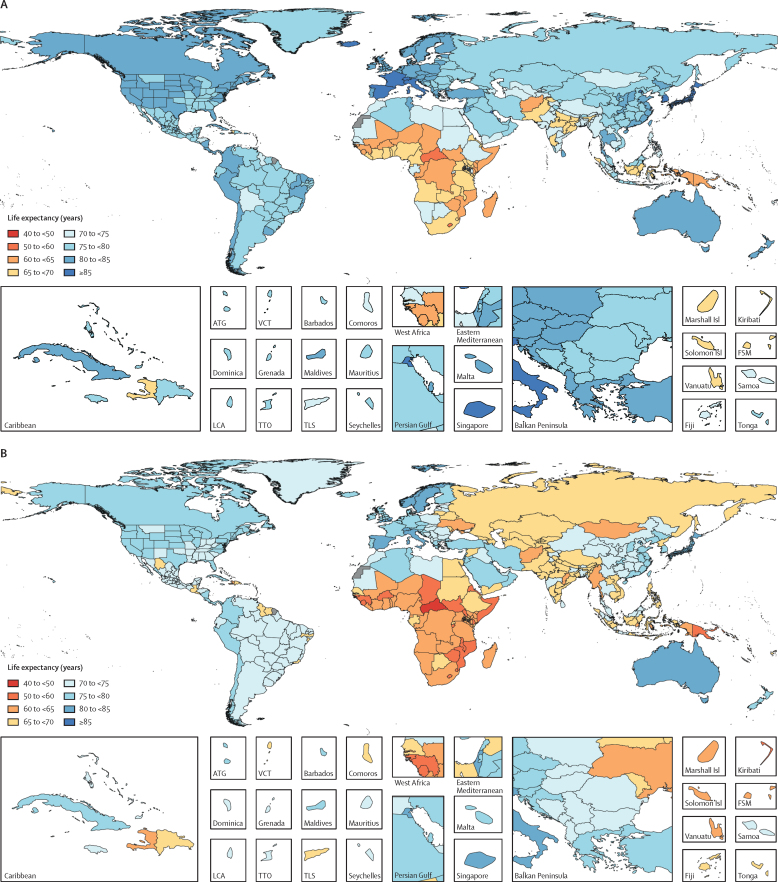

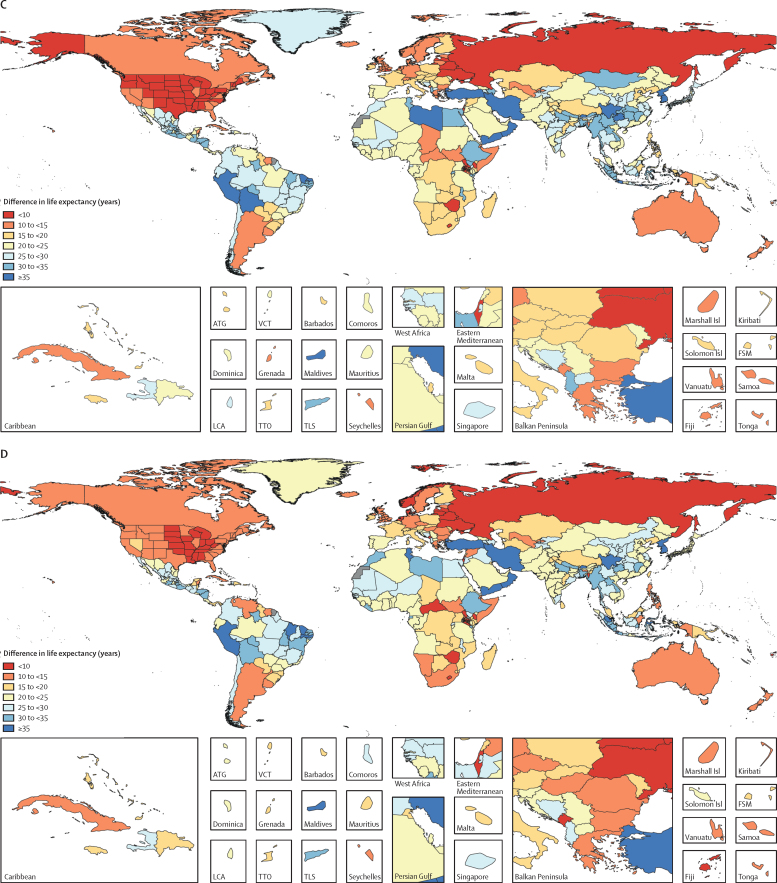
Life expectancy at birth, by location, for females (A) and males (B), 2017, and difference in life expectancy at birth, by location, for females (C) and males (D) between 2017 and 1950 ATG=Antigua and Barbuda. FSM=Federated States of Micronesia. LCA=Saint Lucia. TLS=Timor-Leste. TTO=Trinidad and Tobago. VCT=Saint Vincent and the Grenadines.

Figure 7 shows life expectancy at birth in 2017 and the net change in life expectancy at birth between 1950 and 2017, by sex. Country-specific estimates for 2017 are also shown in the table, which provides several summary measures of mortality in 2017. Appendix 2 includes results for 5-year age groups. There was enormous variation in life expectancy and death rates around the world in 2017. Across all countries, life expectancy at birth ranges from 49·1 years (95% UI 46·5–51·7) in the Central African Republic to 82·1 years (81·5–82·8) in Switzerland among men and from 54·9 years (52·0–58·0) in Central African Republic to 87·6 years (86·9–88·1) in Singapore among women. Three countries, the Central African Republic, Lesotho, and Mozambique, had life expectancies in 2017 that were lower than that of Singapore in 1950 for both men (53·5 years) and women (60·2 years).

There has also been marked heterogeneity across the world in gains in life expectancy at birth since 1950, as shown in figure 7C (females) and figure 7D (males). For men, 13 countries have achieved gains in life expectancy of 35 years or more since 1950: North Korea, Maldives, Myanmar, Timor-Leste, South Korea, Peru, Iran, Jordan, Oman, Tunisia, Turkey, Yemen, and Bhutan. The countries that have achieved gains in life expectancy of less than 10 years since 1950 for men include six countries in eastern and central Europe (Montenegro, Belarus, Latvia, Lithuania, Russia, Ukraine), as well as Fiji, Andorra, Denmark, Israel, the Netherlands, Uruguay, Central African Republic, Seychelles, Lesotho, South Africa, Zimbabwe, American Samoa, Guam, and the Northern Mariana Islands. For women, 11 countries have achieved gains in life expectancy of 35 years or more since 1950: these are the Maldives, Timor-Leste, South Korea, Bolivia, Peru, Nicaragua, Iran, Jordan, Oman, Yemen, and Bhutan. The countries that have achieved gains in life expectancy of less than 10 years for women since 1950 include Belarus, Latvia, Russia, Ukraine, Andorra, the USA, Lesotho, Zimbabwe, American Samoa, Guam, and the Northern Mariana Islands.

### Differences in mortality experience between women and men

**Figure 8:**
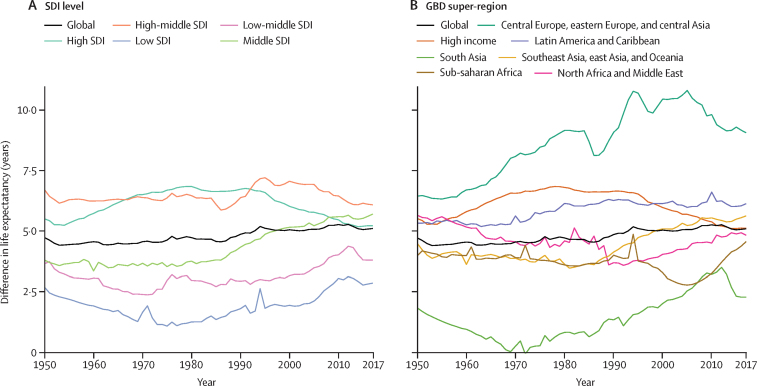
Difference in life expectancy at birth between females and males for SDI (A) and GBD super-region (B), 1950–2017 Each line represents the difference in life expectancy between females and males (female life expectancy minus male life expectancy) for a given SDI level in 2017 (A) and GBD super-region (B), for each year between 1950 and 2017. SDI=Socio-demographic Index. GBD=Global Burden of Diseases, Injuries, and Risk Factors Study.

Figure 8 shows the trend over time in the gap between women and men for life expectancy at birth by SDI quintiles (figure 8A) and by super-region (figure 8B). Globally, the gap has remained fairly stable over the past 68 years, increasing from 4·7 years (95% UI 4·2–5·4) in 1950 to 5·1 years (4·9–5·3) in 2017, but with a lot of variation across SDI quintiles and regions. Across all SDI quintiles, female life expectancy was higher than male life expectancy for all years since 1950, with the gap being larger as level of development increases in 1950, but with variable trends across SDI quintiles over time. Overall, there appears to be an increasing gap between women and men that at some point starts to shrink. The shrinking occurs at different points in time across levels of development: for high-SDI countries, the gap between women and men has been decreasing since 1990, for high-middle SDI countries since 2000, and in the lower three quintiles of SDI, the gap has been decreasing since about 2010. In 2017, the gap ranged between 6·1 years (5·6–6·5) among high-middle SDI countries to 2·9 years (2·3–3·4) among low-SDI countries.

Figure 8B shows the heterogeneous trends in the gap between female and male life expectancy over time and across super-regions. The male disadvantage is substantially larger in central Europe, eastern Europe, and central Asia than in other super-regions, and has been consistently so since 1950; the gap in this region increased between 1950 and 2005, and has been decreasing since then but remains the largest across all regions: in 2017, women in central Europe, eastern Europe, and central Asia had a life expectancy that was 9·1 years (95% UI 8·9 to 9·3) greater than that of men. The next largest gap was in Latin America and the Caribbean, where female life expectancy was 6·1 years (5·8 to 6·5) greater than that of males in 2017. The gap had been increasing between 1950 and 2010 in this region, but has remained relatively constant since 2000. A similar pattern occurred in high-income countries, but the shrinking of the gap occurred earlier in this region, peaking in 1979 when it was 6·8 years (6·8 to 6·9) and dropping to 5·1 years (4·9 to 5·4) in 2017. Sub-Saharan Africa had very little change in the gap between female and male life expectancy between 1950 and 1997, followed by a reduction until 2005 and then a steady increase since then. In North Africa and the Middle East the gap between female and male life expectancy had been decreasing continuously between 1950 and 1989 but has been increasing since. Male disadvantage in southeast Asia, east Asia, and Oceania tended to decrease between 1950 and 1981, and has been increasing or staying flat since. South Asia, the region with the smallest gap throughout this time period, has had two reversals in the trend. The smallest gap was in 1972, with a difference of only −0·04 years (–0·8 to 0·8) between female and male life expectancy, which then increased until 2012 and has decreased since; in 2017 the gap in south Asia, which is the smallest of any of the super-regions, was 2·3 years (1·9 to 2·8).

### Observed versus expected life expectancy

**Figure 9:**
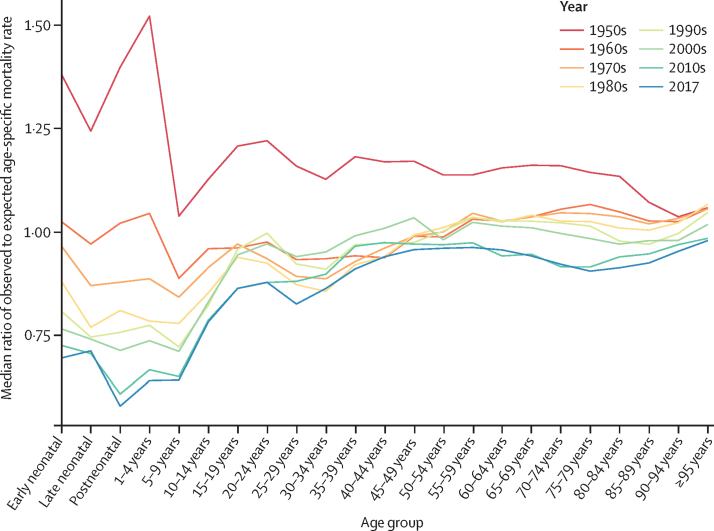
Median observed to expected ratio of age-specific mortality rate, globally, for both sexes combined, in the 1950s, 1960s, 1970s, 1980s, 1990s, 2000s, 2010s, and 2017 Each line represents the median ratio across locations for each decade of the observed age-specific mortality rate to that expected on the basis of SDI, for each of the 23 GBD age groups. The early neonatal age group is 0–6 days, late neonatal is 7–27 days, and postneonatal 28–364 days. SDI=Socio-demographic Index. GBD=Global Burden of Diseases, Injuries, and Risk Factors Study.

Figure 9 shows the median value within each decade of the ratio between the observed age-specific mortality rates and what we refer to as expected mortality rates, which represent the mortality rates that would be anticipated on the basis of development status approximated by SDI, for each age group, and how that ratio has changed over time. For the younger age groups, particularly those younger than 5 years, there is a wide range in the ratio of observed to expected mortality rates across the eight time periods shown in the figure. In the earlier time periods, starting at 1950, the ratio of observed to expected mortality was highest for children younger than 10 years, suggesting that more children were dying than would be expected based on the level of development. Over time, that ratio shrinks substantially. The biggest change over time was in the 1–4 years age group, for which the median ratio of observed to expected life expectancy went from 1·52 (95% UI 1·04–2·03) in 1950 to 0·77 (0·63–0·90) in 1990 and 0·64 (0·52–0·77) in 2017. The effect of time was much more pronounced for the younger age groups. Among young adults and older adults (about 15–64 years), the trend over time was less clear and the range in the ratio of observed to expected age-specific mortality rates was much smaller than for younger age groups. Among the older age groups, the ratio has been close to 1 and only small decreases and increases have occurred after age 80 years over the 68 years covered by this study.

**Figure 10:**
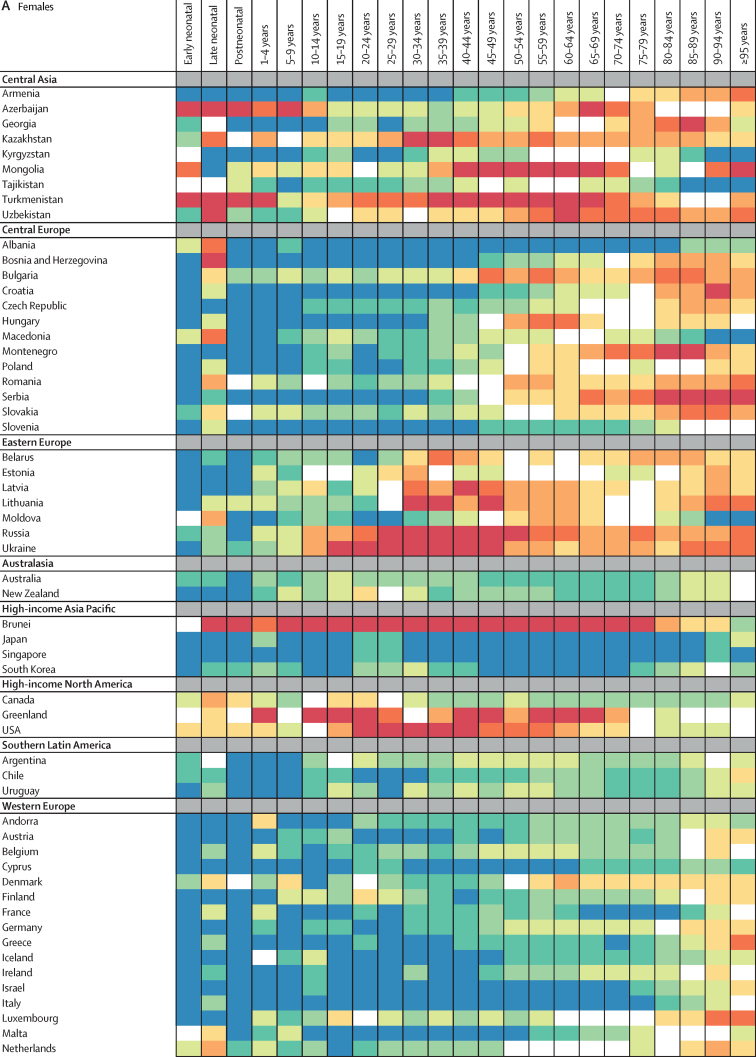

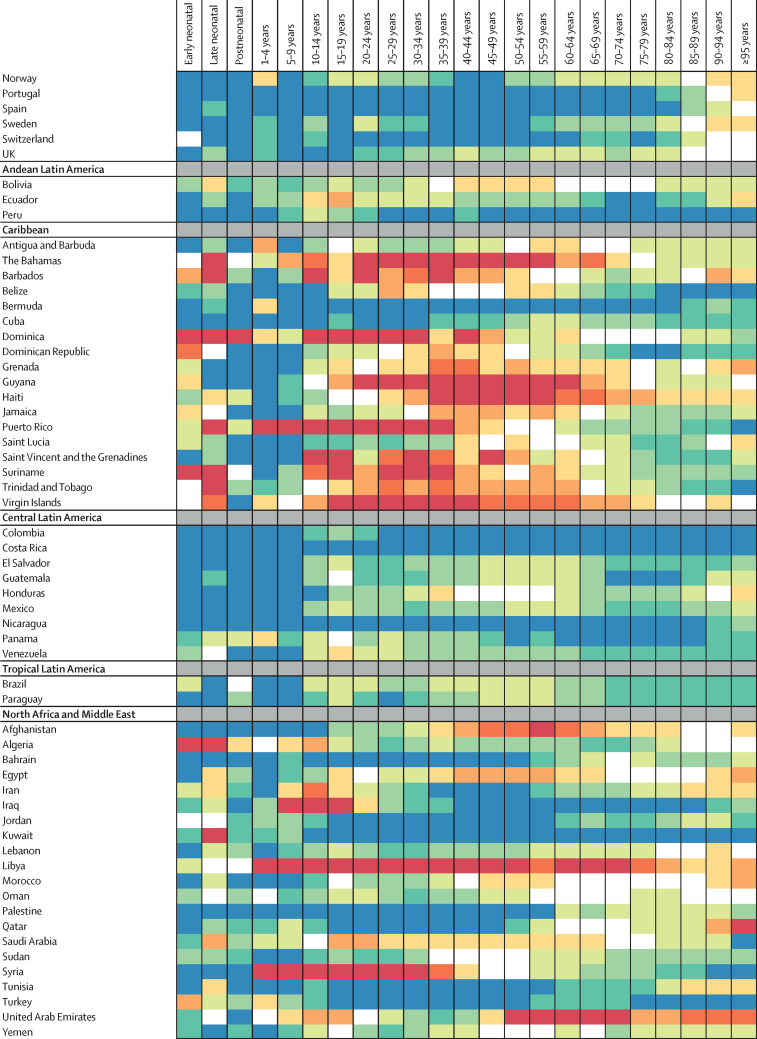

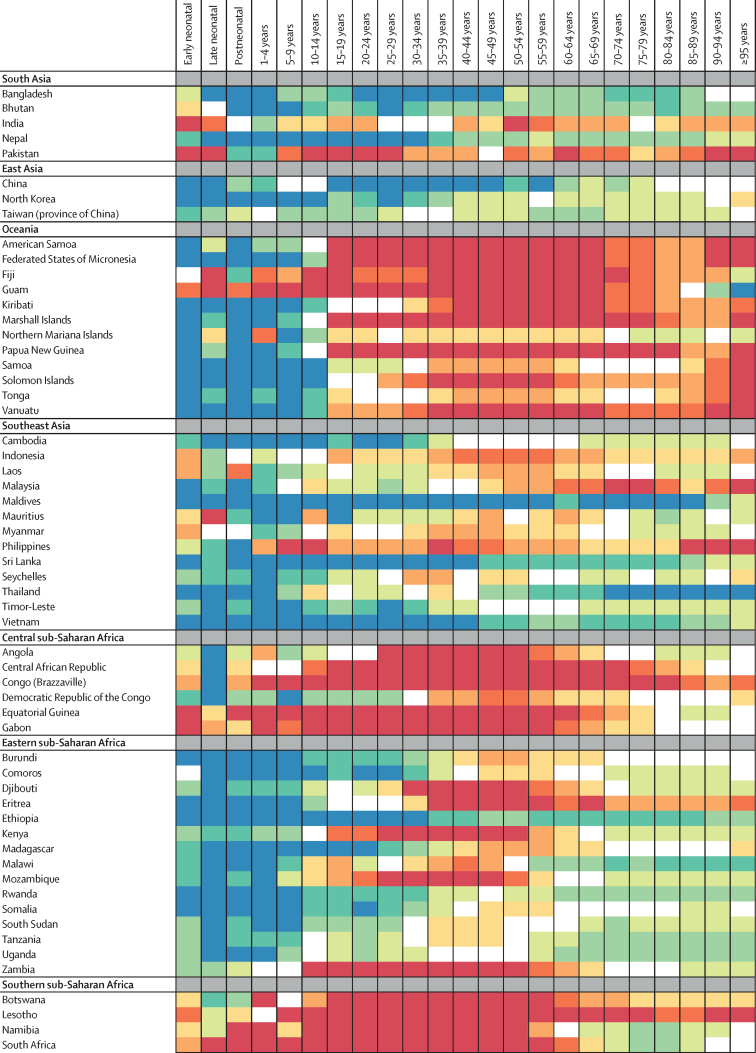

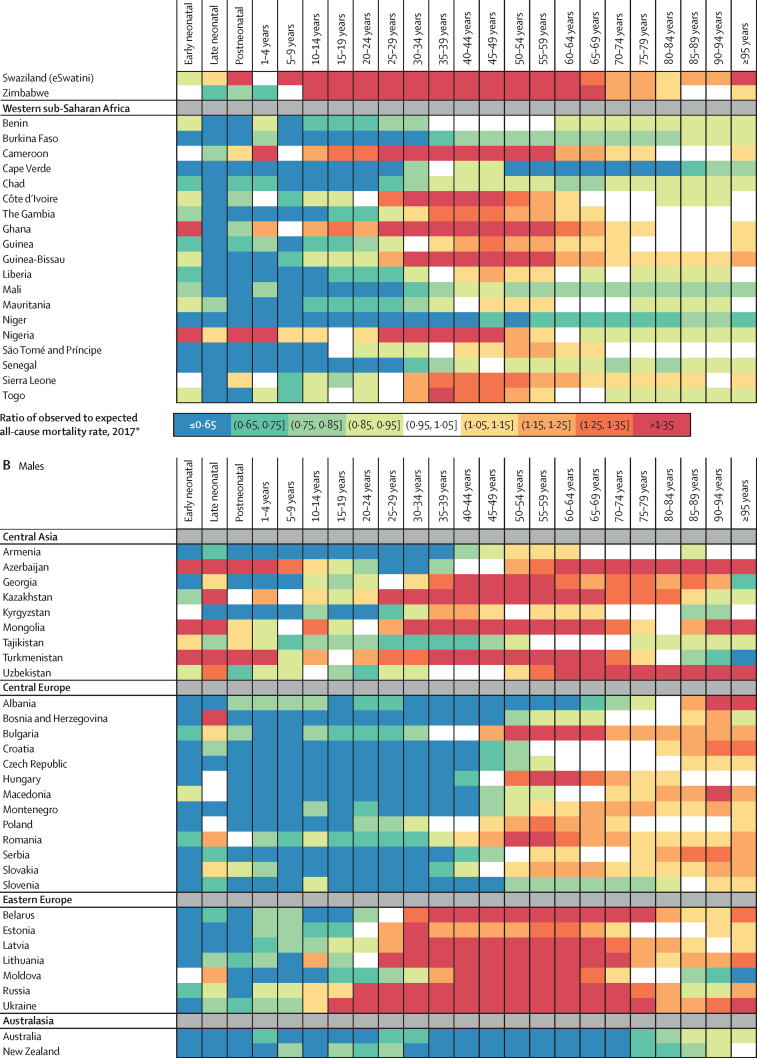

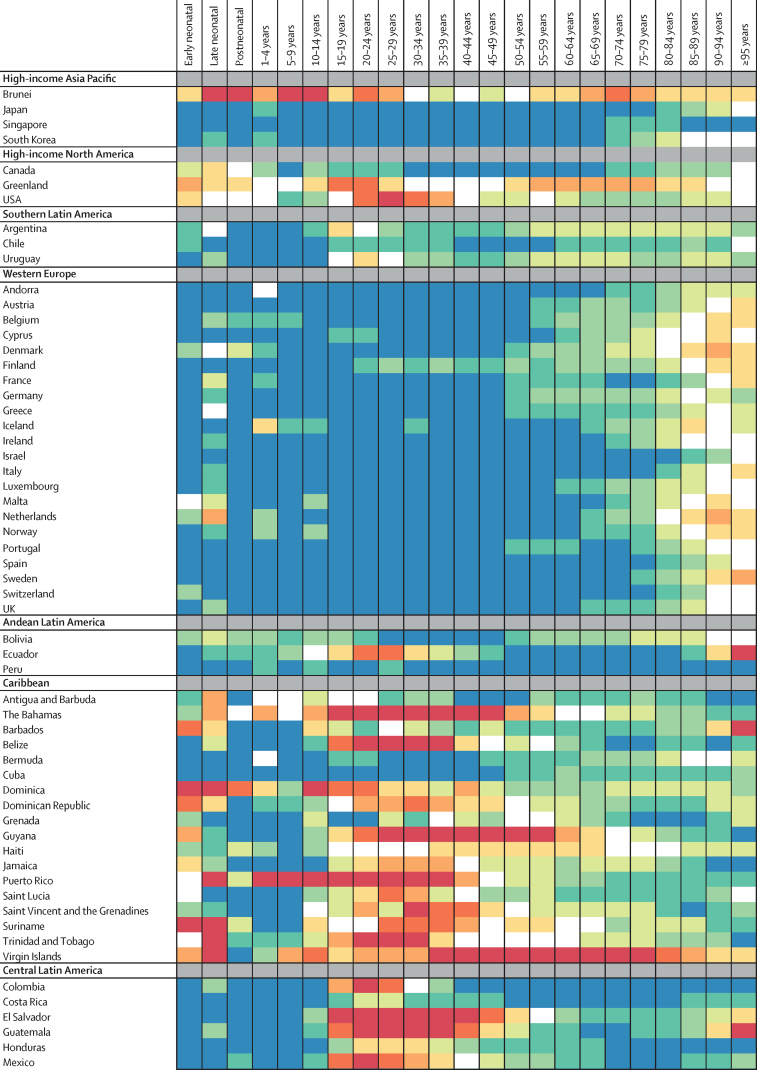

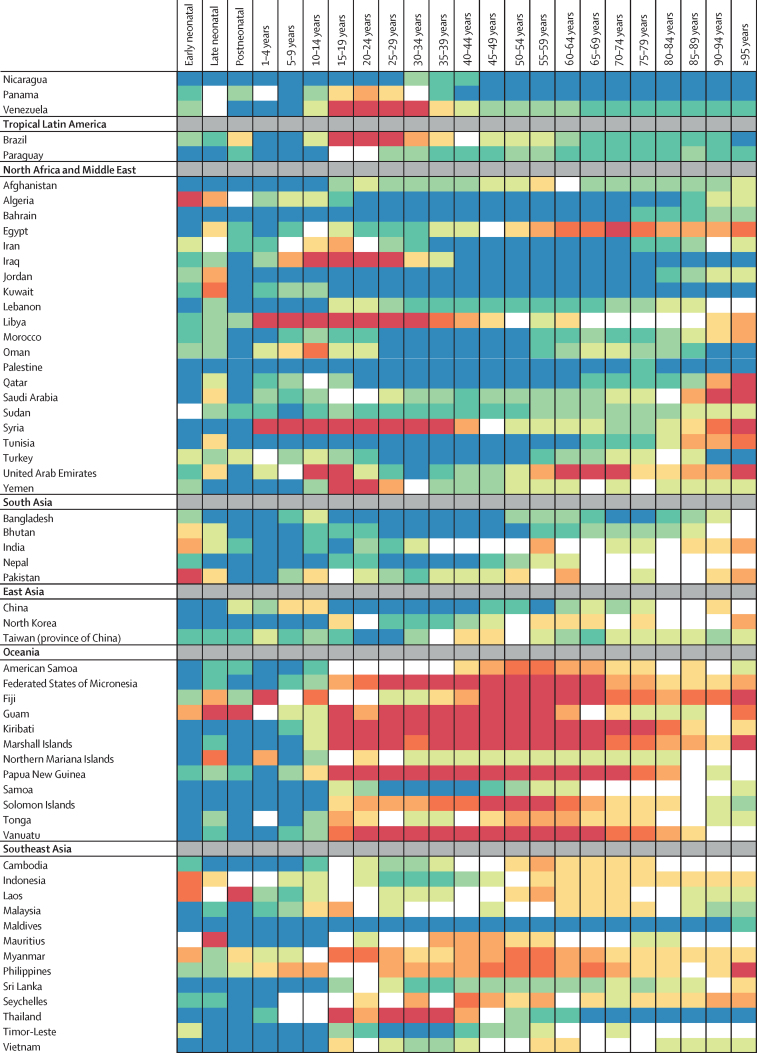

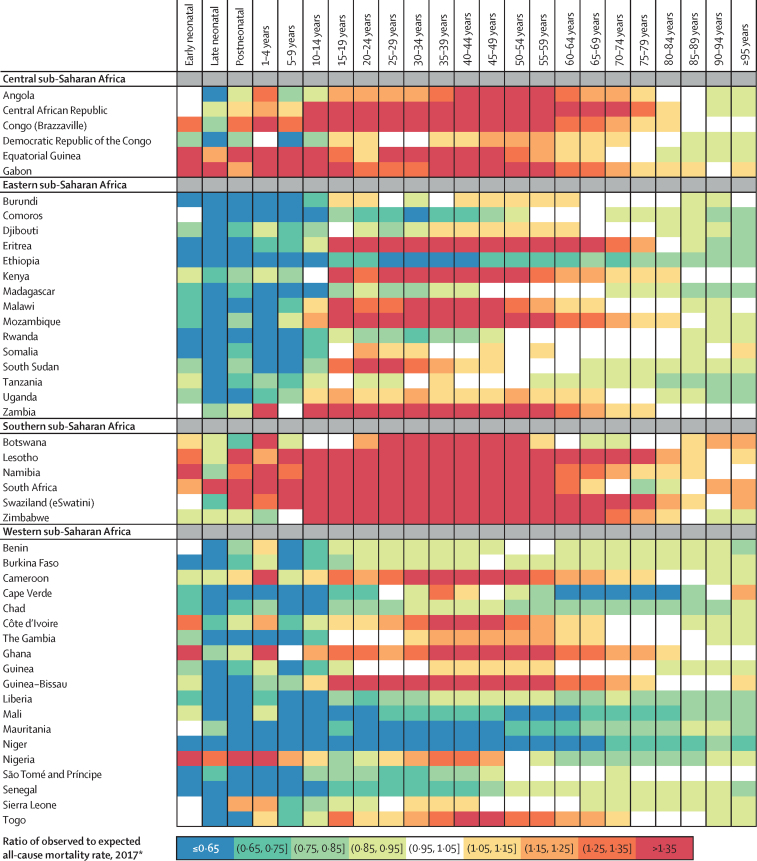
Age-specific, all-cause mortality rate with the ratio of observed to expected by SDI, 2017 Each square represents the ratio of the observed all-cause mortality rate to that expected on the basis of SDI for females (A) and males (B) for that given location, sex, and age group. The early neonatal age group is 0–6 days, late neonatal is 7–27 days, and postneonatal 28–364 days. SDI=Socio-demographic Index. *Round brackets indicate excluded endpoints whereas square brackets indicate included endpoints.

As well as varying over time, the way in which observed mortality levels correspond to those expected on the basis of development also varied substantially across countries, as represented in figure 10, which shows the 2017 values.

Figure 10A explores the relationship of observed mortality rate with SDI for women. There are 24 high-performing countries for women, where the ratio of observed to expected mortality rate was lower than 0·95 across all 23 age groups in the analysis. The list overlaps heavily with that for men and has several countries that performed better for women than men, including Colombia, Mexico, and Uruguay. China did very well for women, except between the ages of 5 and 14 years, but in Indonesia, age-specific mortality rates for women are higher than expected for most age groups, with the exception of child age groups, whereas for men, Indonesia performed much better. India had higher than expected mortality for almost all age groups, and particularly for early neonatal and reproductive ages. The USA stands out as having higher than expected rates in most age groups for women, particularly during the neonatal period and during reproductive years, and mortality rates were higher than expected until the age of 70 years. As also seen among men, much higher than expected mortality rates occurred in adult women aged 15–69 years in several countries in Oceania, including Fiji, Kiribati, the Marshall Islands, Micronesia, Papua New Guinea, the Solomon Islands, and Vanuatu. In several countries in eastern Europe, adult women had mortality rates that were more than 1·35 times what would be expected. In the Ukraine and Russia, the high mortality rates start at the age of 10 years and continue throughout adulthood. A similarly worrying pattern was present in many countries in sub-Saharan Africa, where women had much higher mortality rates than expected for the reproductive age groups. In some countries, such as Congo (Brazzaville), Equatorial Guinea, Gabon, Ghana, Nigeria, Lesotho, and South Africa, mortality rates were also high for girls under the age of 5 years.

In figure 10B, which shows the results for males, there are 16 high-performing countries for which the ratio of observed to expected mortality rates was lower than 0·95 across the 23 age groups in the analysis. These countries include some high-income countries that are known to have low mortality, such as Australia and Singapore, as well as countries in other regions that have low observed to expected ratios but do not necessarily have low mortality across all ages, such as Cuba, Nicaragua, and Peru in Latin America and the Caribbean; Ethiopia, Chad, Liberia, Niger, and Senegal in sub-Saharan Africa; the Maldives in southeast Asia; and Bahrain and Palestine in north Africa and the Middle East. Figure 10B also shows that several countries in eastern Europe and sub-Saharan Africa had very high mortality rates for young adult males, greater than 1·35 times what would be expected from their level of development. The Central African Republic and Lesotho stand out as two countries with age-specific mortality rates that are as expected or higher than expected for their level of development across all 23 age groups. In sub-Saharan Africa, while there was variation across countries, the ages most affected were 15–59 years, whereas in Europe, these were mostly ages 25–69 years. Mortality rates were also very high among young adult men in Oceania, including Kiribati, the Marshall Islands, Micronesia, Papua New Guinea, and Vanuatu, as well as some Caribbean and Latin American countries such as the Bahamas, Belize, Guyana, Trinidad and Tobago, Puerto Rico, El Salvador, Guatemala, Venezuela, and Brazil. Among the most populous countries, China had lower than expected mortality rates in all age groups except children aged 5–9 years and adults older than 80 years. Conversely, the USA has higher than expected mortality rates between the ages of 20 and 40 years, with ratios greater than 1·05 for these age groups. The results for India and Pakistan were generally as expected except for early neonatal ages in Pakistan and early neonatal to young adult ages in India, whereas Bangladesh performed better than or as expected for all age groups. Nigeria performed worse than expected in under-5 mortality and in young adults and better than expected in older age groups.

## Discussion

### Main findings

This study represents the first comprehensive analysis of age-sex-specific death rates for single calendar years and single-year age groups for 195 countries and territories from 1950 to 2017. Our results show the remarkable variation in mortality rates over time and across countries. The decline in death rates has been the greatest in the age groups younger than 5 years, followed by young adults, and has been slower among older adults. Rising death rates have occurred in conflicts, natural disasters, large HIV epidemics, and in several locations such as eastern Europe, some countries in southeast Asia and Latin America, and most recently, in the USA. Increases in adult mortality rates even as child death rates fall are stark reminders that the drivers of adult mortality can be complex. Because of the celebrated progress in many locations, many people have come to expect age-specific death rates to always decline; however, there is nothing inevitable about the trajectory of death rates, particularly in adults.

### Cross-cutting themes

A long-running theme in the demographic literature has been the balance between development and technology innovation as determinants of mortality change. Thomas McKeown,^46^ examining declines in mortality in the UK in the first half of the 20th century, argued that health technology played little role and mortality decline in that setting was driven by improvements in the standard of living. Samuel Preston,^47^ in a series of classic studies, roughly assigned one-third of life expectancy improvement to rising income per capita, one-third to improvements in educational attainment, and one-third to changes correlated with time, which he assigned to technology improvement. Given the wide array of drugs, vaccines, and procedures and understanding of risk factors that have emerged in the past 50 years, often with strong causal evidence such as randomised trials, major temporal shifts unexplained by development should be expected. These reductions in mortality with time, even after controlling for development, might be due to a broader array of social and other determinants aside from health-enhancing technologies. Declines are not inevitable: adverse trends such as the obesity epidemic, the opioid crisis, or the rise of drug-related violence in some locations could lead to shifts over time in the opposite direction.^9^, ^10^, ^11^, ^16^ Our analysis of observed and expected death rates provides some insight into these shifts. On average, we found considerable temporal shifts toward lower death rates, controlling for SDI, in age groups younger than 20 years. The analysis of observed death rates to those expected on the basis of SDI alone suggests that child death rates are nearly a third lower today than they were in 1950; this is most likely because of technology shifts or risk factor changes correlated with time. At older ages—ie, older than 60 years—the technology shift is more modest. In the range from ages 20 to 50 years, there was much less evidence of major changes over time, and in fact during the 1990s, the trend was in the opposite direction, possibly because of the HIV epidemic, the rise of mortality in the former Soviet Union, and increases in adult death rates in other countries, possibly related to drug use and suicide. By examining the role of temporal shifts, which can be a combination of technology change, the development of risk factor trends by age, and other social determinants instead of life expectancy, we have highlighted that progress for younger adults has not been as sensitive to new technologies. Understanding these patterns is important for prioritising the investments by societies in innovation as a means to improve health in different age groups.

Our study shows how remarkable the decline in under-5 mortality since 1950 has been at the global level. For the first time, in 2017, the age group younger than 5 years had fewer deaths than some of other 5-year age groups, like 75–79 years. The decline in the numbers of under-5 deaths might, however, slow down in the coming years. The global number of births per year has remained consistently around 135–140 million births since 1990, but a growing proportion of these births are occurring in low-income countries in sub-Saharan Africa that have the highest under-5 death rates.^31^ This shift creates a demographic challenge that might require increased funding efforts for effective child health programmes to sustain progress. At the very least, the shift of the global birth cohort toward more disadvantaged settings warrants careful monitoring if the ambitious goals of the SDGs are to be achieved. The use of internally consistent populations, fertility, birth, and death numbers in the current GBD iteration, GBD 2017, has also resulted in a revision of the total number of global under-5 deaths. GBD 2016 noted that, in 2016, the number of under-5 deaths had dropped below 5 million for the first time. The revised estimates in GBD 2017 indicate that this milestone has not been yet met, as the number of under-5 deaths in 2017 is now estimated to be at 5·4 million (95% UI 5·2–5·6).

By contrast with under-5 deaths, the number of deaths occurring globally at ages older than 65 years has been steadily increasing and has more than tripled since 1950. The increase in the numbers of deaths in the setting of declining or stagnant death rates is due to a shift in the mean age of the world's population and an increase in the average age group of death from 20–24 years in 1950 to 65–69 years in 2017. As of 2008, more than 50% of deaths have occurred among individuals older than 65 years. Monitoring age-sex-specific mortality rates in these older age groups will be increasingly important to evaluate progress in improving health. However, measurement of death rates in these age groups has two fundamental challenges. First, in locations with working VR systems, both the numerator and the denominator are affected by age misreporting; the GBD analysis of population detected considerable evidence of age misreporting at the population level,^31^ and it is likely that this is also true for death registration. Second, in settings without death registration, survey data on sibling histories do not provide information on death rates at these older ages. Household recall of deaths in surveys and censuses is uncertain, since telescoping or incomplete recall can lead to biases in either direction. As the world continues to age, more emphasis will be needed on improving the assessment and recording of age in both death registration and census or registry counts.

For the first time, the GBD assessment of age-specific mortality and life expectancy is based on the GBD population estimates, not annual interpolations of the UNPOP estimates. The shift to the GBD population estimates has had an important effect on locations with VR data and where the age-specific assessments of population by the GBD differ from those of UNPOP.^31^ Differences in age-specific estimates are largest in some older age groups in some countries. For example, in Costa Rica, which has complete death registration, the life expectancy at age 15 years in the year 2015 changed from 66·92 years in GBD 2016 to 65·77 years (95% UI 65·6–65·9) in GBD 2017 due to the change in population denominators. Populations have also changed considerably in some smaller countries such as Bermuda. In countries without VR data, changes in the population denominators have had a much smaller effect since the mortality rates derived from summary birth histories, complete birth histories, sibling histories, and household deaths are not affected by population numbers. In these settings, changes in the population age-structure have resulted in changes in the estimated number of deaths but not the death rates.

Our assessment of the role of fatal discontinuities and their effect on age-specific mortality highlights the enormous impact that conflicts, natural disasters, famines, and some epidemics can have on the number of deaths from year to year, on top of longer-term trends. The most notable fatal discontinuity in the past 68 years was the Great Leap Forward in China, which had a demonstrable impact on global deaths between 1959 and 1961. Reconstructing the impact of the Great Leap Forward on death rates is particularly challenging. Many studies have been published on the effects of the Great Leap Forward, with estimates ranging from 2 million to 55 million deaths.^48^, ^49^ Other fatal discontinuities, such as the Rwanda genocide and the conflict in Syria, have also had large effects on mortality in specific locations since 1990.

Sex differences in life expectancy have a characteristic pattern with level of development: differences tend to increase across the first four quintiles of SDI such that women's advantage increases steadily. For the highest quintile of SDI, particularly in the past 20 years, the women's life expectancy advantage is shrinking. Our analysis also shows that, above and beyond the level expected on the basis of SDI, in the central Europe, eastern Europe, and central Asia super-region, women's life expectancies are much higher than those of men. These spatial and temporal patterns in the sex gap in life expectancies highlight the complex set of social, cultural, and economic factors that explain life expectancy differences. Purely biological differences in survival are likely to be small, less than 1–2 years of life expectancy;^50^ differences of this magnitude are seen in the subnational locations with the highest levels of SDI in the UK, the USA, and Japan. In some countries, particularly those with low SDI, the differences are smaller than this biological component or there might even be higher life expectancies for men than women. In these situations, disadvantages in the status of women, access to health care, and other factors might contribute to explain the disadvantage for women; maternal mortality in these settings is also an important contributor. In many locations, however, the male disadvantage is much larger than what it would be based purely on the biological difference, and over the past seven decades, the gap between female and male life expectancy has remained surprisingly constant at the global level, despite massive increases in the average life expectancy of both sexes. Understanding how much of this male disadvantage is due to known risk factors, including risk-taking behaviour associated with masculine social roles and occupational risks, how much is differential use of health care or low adherence to treatment, and how much is other determinants will be an important step to quantifying how to reduce this sex difference in life expectancies.

### Improving estimation

In GBD 2017, we extended the analysis of age-specific mortality rates back to 1950, adding two decades of assessment. In some lower-SDI regions, particularly those in west, east, and central sub-Saharan Africa, the data for mortality measurement are sparser for these earlier decades than after 1970. Beginning in the 1970s, the World Fertility Survey started collecting complete birth histories, followed by the Demographic and Health Surveys and in some countries the Multiple Indicator Cluster Surveys. Before 1970, evidence from these regions on levels of child mortality came exclusively from summary birth histories collected in censuses. Information on levels of adult mortality in these regions in the 1950s and 1960s is extremely limited, coming from a few surveys or censuses with household death recall. Estimates of adult mortality in this time period are highly dependent on the covariates used in the first-stage model of the ST-GPR—namely, LDI per capita, educational attainment, total fertility rate, and under-5 death rate. Given the more limited empirical basis for estimation in this period, model specification matters; models that include the under-5 death rate, for example, generally lead to higher estimates in this period than those without the under-5 death rate. In-sample fits with and without the under-5 death rate were quite similar, suggesting that, in future iterations of the GBD, we should consider using an ensemble of the models with fits similar to the available data.

Our model for under-5 mortality includes bias corrections for certain data sources, during which we adjust non-reference sources to the level of designated references sources using a mixed effects regression model. This approach has not been used for the analysis of adult mortality largely because there are fewer overlapping data sources in these age groups. In future work, we will explore whether such an approach should be used for settings with VR and sibling history data.

Following standard demographic approaches, we use death distribution methods to assess the completeness of VR data. We use variants of the three standard death distribution methods, synthetic extinct generations, generalised growth balance, and the hybrid of the two, that were developed and validated using simulation data and data in settings where registration is known to be complete.^33^ Although we use optimised versions of these methods, the results of these methods are accompanied by large uncertainty intervals, as shown in studies using simulated data.^33^ Moreover, in GBD 2017, the entire time series of results of the death distribution methods is evaluated in a coherent fashion to generate a time series of completeness. In the era of ensemble modelling and machine learning, it might be time to revisit death distribution methods in detail, looking to see if better predictions of death registration completeness can be developed and validated in simulation environments. Analysing all the different age periods of death reporting, called in this literature “age trims”,^33^ in a time series manner could provide alternative ways to estimate completeness over time. Even the optimised death distribution methods have not been formulated as statistical models; there are likely to be opportunities to develop Bayesian versions of these models that could enhance performance. Given the importance of these methods for using incomplete VR in demographic analyses, more research on the death distribution methods is warranted.

The development of GBD population estimates^31^ also led to a complete overhaul of the empirical life table database used for the GBD model life table system. This database is used to identify the reference standard for the probability of death at each age. For this cycle, we defined objective criteria to assign life tables based on observed deaths (corrected for under-reporting as required) and estimated populations into three groups: high-quality life tables used in the universal database, lower-quality life tables used for only the locations from which that life table was measured, and life tables that were rejected as too low quality. To deal with small number issues, we also considered life tables computed on 3-year, 5-year, and 7-year moving averages of observed death rates. Of 42 138 life tables evaluated, 25·8% were in the high-quality category, 57·5% were in the lower-quality category, and 16·7% were rejected. Because of the large number of candidate life tables, we developed objective criteria that could be applied in an automated fashion based on the variance of the slope of the log death rate versus age curve, mortality declines over the age of 65 years, crossovers in the probability of death between 1 and 5 years (4q1) and between 0 and 1 year (1q0), and large swings in death rates in adjacent age groups. In future work, these criteria could be enhanced by the addition of other screening criteria, particularly at older ages where death rates approach very high levels above the age of 85 years. This more formalised approach to the life table database is necessary because, with each iteration of estimation, the population denominators can change, and thus the shape of the life tables can change. Application of this approach means that we have a much richer database of high-quality life tables than is reflected in the older demographic model life table systems; Ansley Coale and Paul Demeny (1966) used only 192 life tables, whereas the UN Model Life Table System (1955) used only 158 life tables.^51^, ^52^ Our larger database of life tables captures many more nuanced patterns that relate to the preponderance of different causes of death in different locations such as infectious diseases, injuries, and some non-communicable causes.

We estimate uncertainty in death rates, and this uncertainty currently reflects sampling error, non-sampling error, and estimation error for the under-5 death rate, the probability of death between 15 and 60 years, and the model life table system, along with uncertainty due to HIV in countries with epidemics with a peak prevalence greater than 0·5%. GBD population estimates used as the denominators for calculating death rates have 95% UIs. However, our uncertainty intervals in this analysis do not incorporate uncertainty in population estimates. Incorporating uncertainty into the population denominator in each step of the GBD mortality analysis would not be trivial. Given the multiple analytical steps that go into our mortality estimation process, there are several challenges. We do not have the computational resources to repeat the entire GBD estimation process multiple times, sampling from the distribution of population by age and sex. Further research is needed to find efficient methods for including the population uncertainty in the final results. Another existing issue is the uncertainty in spatial aggregates such as regional, super-region, or global mortality indicators. Currently, we assume that uncertainty in estimates in different locations is uncorrelated. But this is likely to be an oversimplification because model specification of the first stage in ST-GPR for the under-5 death rate and 45q15 are likely correlated over space. By underestimating this correlation, we are likely to be underestimating the UI for regional, super-region, and global values of life expectancy or other life table indicators. To date, we have not identified any principled basis for estimating the spatial correlation of UIs. To some extent, if we adopted an ensemble modelling approach in the future for the first stage of the ST-GPR, we would automatically incorporate the spatial correlation due to specification uncertainty, which might be another reason for future iterations to explore that strategy.

As part of each annual GBD update, we have re-estimated the entire time series of age-specific mortality by location covered by the study to ensure that we produce an internally consistent time series using the latest methods. Changes from cycle to cycle can be due to the use of new mortality data, changes in population, changes in covariate values, changes in which covariates are used, and changes in model specification including hyperparameter values. It would be useful in future iterations of the GBD to provide some decomposition of changes in the final results due to each of these factors for each location and year. Such a full decomposition of change would be highly computationally intensive and is beyond our current capabilities. Future work will explore ways in which this more formal analysis of change can be efficiently implemented.

### Comparison to UNPOP

The main alternative provider of mortality estimates is UNPOP. Scatterplots of estimates of life expectancy at birth from UNPOP compared to GBD in 1950, 1960, 1970, 1980, 1990, 2000, 2010, and 2017, for both sexes combined, are shown in appendix 2. Life expectancy from UNPOP is reported for 5-year intervals, so these figures are based on linear interpolation—eg, 1960 is the interpolation of the values for 1955–1960 with the values for 1960–1965. The concordance correlation coefficient between the two sets of estimates ranged from 0·86 in the year 1950 to 0·97 in the year 2003. The concordance correlation coefficient in 2017 was 0·93. While these concordance coefficients appear to be high, there are considerable differences in many locations. Differences in life expectancy were larger in the earlier periods, and concordance gets higher in more recent time periods, most likely as a result of increased data availability over time. However, there are some substantial differences between the UNPOP and GBD estimates even for the summary measure of life expectancy at birth. These differences are not restricted to sub-Saharan Africa, where data are more scarce, but are also noticeable in other regions, including Latin America and the Caribbean. It is challenging to disentangle the main drivers of the differences, because the methods used by the UNPOP to estimate mortality for each location are not sufficiently documented. For some locations, the UNPOP estimates of life expectancy are based on levels of child mortality and selection of a Coale and Demeny Model Life Table or UN Model Life Table. In west Africa, the differences stem in part from our analysis using sibling history data as one of the key inputs along with Demographic Surveillance Site data where available. In other regions such as Central America, differences seem to stem from very different assessments of the completeness of VR, although the method used by the UNPOP to come to these judgments is not documented. Given the widespread use of life expectancy as both a summary measure of mortality and as a general measure of the progress of nations, such large differences in life expectancy between UNPOP and GBD estimates warrant broader scientific debate.

### Limitations

This study has several limitations. First, we use sibling history data corrected for survivor bias and recall bias.^40^, ^53^ Although the validity of sibling histories has been challenged, empirical studies to date have not provided a clear answer on a consistent direction of the bias.^40^, ^54^ We believe it is appropriate to use the sibling history data that are available, but these data suggest much lower levels of adult mortality in some parts of west Africa than would be predicted from child mortality alone. Second, estimates of the completeness of VR are based on the application of death distribution methods, which are highly uncertain as shown in studies using simulated data.^33^ Third, in some locations where there is evidence of substantial age misreporting at older ages, the GBD estimates of the age structure of the population in the terminal age group, which might be as young as 65 years or older, use estimated death rates as an input. In locations with VR data, this creates a circularity where population distribution within the terminal age group depends on death rates in those age groups that depend on population denominators. We deal with this circularity by looping through the entire population estimation process several times to reach a stable estimate of population and death rates. Nevertheless, this interdependence means that estimates of death rates with partial or complete VR in locations with substantial age misreporting might be biased. Fourth, our estimates of death rates at ages 5–9 and 10–14 years in settings without VR data depend on the selection of a model life table reference using the under-5 death rates, the adult mortality rate, and the database of empirical mortality patterns in the life table database. We have not used direct measurement of adolescent mortality calculated from birth histories because we have not yet been able to evaluate the impact of recall bias on these estimates. For future GBD iterations, we will continue to explore the incorporation of this information and DSS data on death rates in 5–9 and 10–14 year olds. Fifth, we continue to use modelled HIV/AIDS death rates based on the analysis of HIV/AIDS prevalence data using Spectrum to revise adult mortality estimates in locations without VR data and with peak HIV/AIDS prevalence greater than 0·5%. However, the estimation of death rates from HIV/AIDS on the basis of prevalence data and the scale-up of antiretroviral therapy are dependent on many assumptions and might still tend to overestimate mortality in these locations. Sixth, we are not able to capture all fatal discontinuities, particularly the smaller ones. We will continue to explore data on fatal discontinuities and incorporate them for further iterations of the GBD. Moreover, the magnitude of fatal discontinuities in many locations is fundamentally based on media reports of events on a particular day in a particular place. These media reports, even when highly detailed, might not be accurate. Seventh, we have not explored multiple data density scores for the selection of hyperparameters in the ST-GPR, but hope to explore this further in future GBD iterations.

### Conclusion

This analysis of age-sex-specific mortality shows that, against a general background of consistent declines in death rates for most age groups over the past 68 years, there are remarkably complex patterns across countries. Levels and trends are particularly heterogeneous in younger adults, which suggests that drivers of health outcomes are more varied and complex in these age groups. Given that the SDGs have been framed to be ambitious and refer to health across the lifespan, careful monitoring of age-sex-specific levels of mortality will remain crucial. The GBD provides the only annually revised, GATHER-compliant set of estimates for the assessment of performance against local, national, and global goals. As several reversals in formerly declining mortality rates demonstrate, continued reductions in age-specific mortality should not be taken for granted. Monitoring trends in the future is critical as progress in many locations and certain age groups has stagnated.

Correspondence to: Prof Christopher J L Murray, Institute for Health Metrics and Evaluation, Seattle, WA 98121, USA cjlm@uw.edu

## Data sharing

To download the data used in these analyses, please visit the Global Health Data Exchange at http://ghdx.healthdata.org/gbd–2017.
