# Department of Error

**DOI:** 10.1016/S0140-6736(19)31046-3

**Published:** 2019-06-22

*GBD 2017 Mortality Collaborators. Global, regional, and national age-sex-specific mortality and life expectancy, 1950–2017: a systematic analysis for the Global Burden of Disease Study 2017.* Lancet *2018; **392:** 1684–735—*In this Global Health Metrics paper, Mohammad Reza Salahshoor's name has been corrected; affiliation details have been amended for Mina Anjomshoa, Yasin Jemal Yasin, and Mohammad Reza Salahshoor; and the declaration of interests statement has been amended for Boris Bikbov. Additionally, appendix 2 has been updated. These corrections have been made to the online version as of June 20, 2019.

