# Supplementary material for: Global, regional, and national age-sex-specific mortality and life expectancy, 1950–2017: a systematic analysis for the Global Burden of Disease Study 2017
Source: Lancet. 2018 Nov 10;392(10159):1684–735. doi: 10.1016/S0140-6736(18)31891-9 (PMC6227504; doi:10.1016/S0140-6736(18)31891-9)
Supplement: Supplementary appendix 2 [file mmc2.pdf]

THE LANCET

Supplementary appendix 2

This appendix formed part of the original submission and has been peer reviewed. We post it as supplied by the authors.

Supplement to: GBD 2017 Mortality Collaborators. Global, regional, and national age-sex-specific mortality and life expectancy, 1950–2017: a systematic analysis for the Global Burden of Disease Study 2017. *Lancet* 2018; **392:** 1684–735.

Supplementary results appendix for Global, regional, and national age-specific mortality and life expectancy, 1950-2017: a systematic analysis for the Global Burden of Disease Study 2017

Table of Contents

SR Figure 1: Number of deaths over time by age group, by sex, 1950-2017………………………………………….3

SR Figure 2: Comparison of life expectancy at birth by location in GBD 2016 vs GBD 2017…………………….4

SR Figure 3: Comparison of life expectancy at birth by location in GBD 2016 vs GBD 2017…………………….5

SR Figure 4: Comparison of life expectancy at birth for countries in GBD 2017 vs WPP 2017 for 1950, (a), 1960 (b), 1970 (c), 1980 (d), 1990 (e), 2000 (f), 2010 (g), 2017 (h), both sexes combined….…………….………6

SR Table 1: Age-standardised mortality rates by location, year, and sex, 1950-2017……………..…………..…14

SR Table 2: Under-5 death numbers by location, year, 1950-2017, both sexes……………………………………35

SR Table 3: Under-5 death numbers by location, year, 1950-2017, females…………..……………………………..42

SR Table 4: Under-5 death numbers by location, year, 1950-2017, males…………………………………………..49

SR Table 5: Life expectancy at birth in 2015, by sex and location, from UN, WHO, census, and GBD 2017………56

SR Table 6: Estimates of life expectancy at birth by location and year, 1950-2017, both sexes…………….59

SR Table 7: Estimates of life expectancy at birth by location and year, 1950-2017, females………….………66

SR Table 8: Estimates of life expectancy at birth by location and year, 1950-2017, males…………..……….73

SR Table 9: Estimates of life expectancy at age 65 by location and year, 1950-2017, both sexes…..………79

SR Table 10: Estimates of life expectancy at age 65 by location and year, 1950-2017, females….…………. 87

SR Table 11: Estimates of life expectancy at age 65 by location and year, 1950-2017, males……………….94

SR Table 12: Under-5 mortality rates by location and year, 1950-2017, both sexes…………………………….101

SR Table 13: Under-5 mortality rates by location and year, 1950-2017, females.………………….……………..108

SR Table 14: Under-5 mortality rates by location and year, 1950-2017, males……………………….…………..115

SR Table 15: Ratio of adjusted to unadjusted 5q0 values for scaled locations by year……………………….122

SR Figure 1: Number of deaths over time by age group, 1950−2017

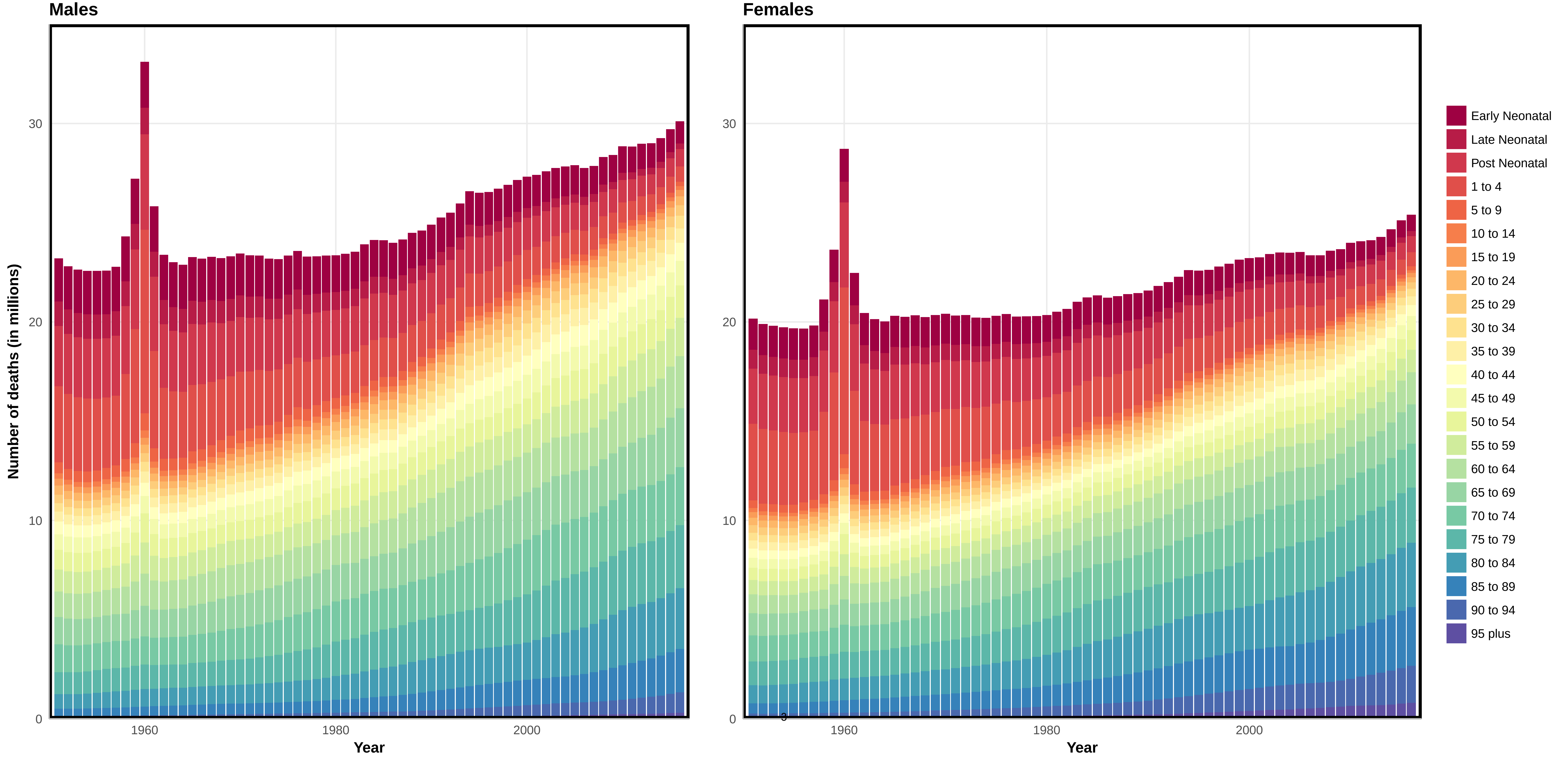

Figure 2 Comparison of estimates of under−5 mortality from GBD 2017 and IGME 2016

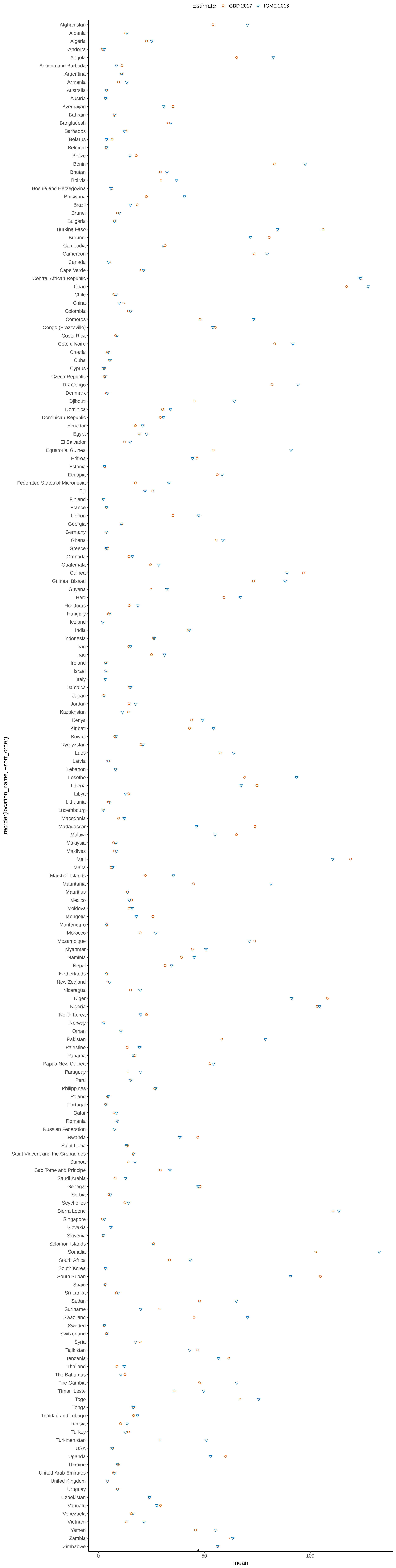

Figure 3 Comparison of life expectancy at birth by location in GBD 2016 vs GBD 2017

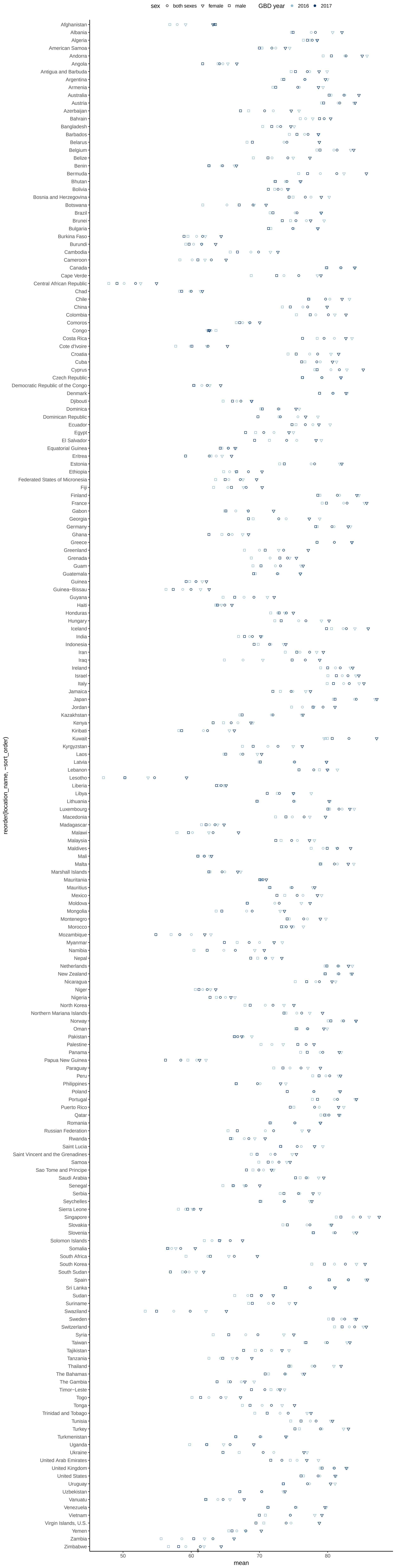

Figure 4 Comparison of life expectancy at birth for countries in GBD 2017 vs WPP 2017 for 1950, both sexes combined CCC: 0.8615292

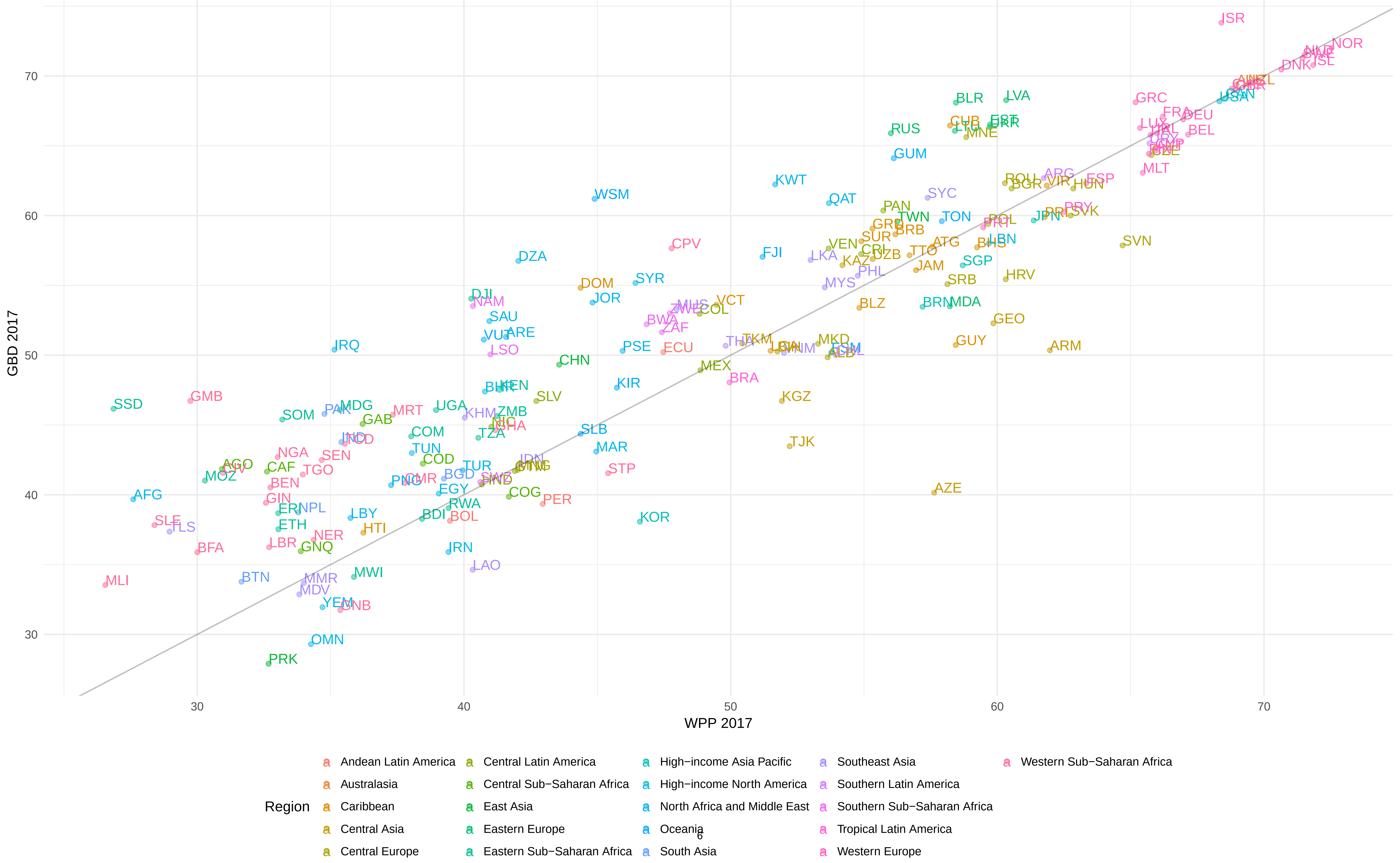

Comparison of life expectancy at birth for countries in GBD 2017 vs WPP 2017 for 1960, both sexes combined

CCC: 0.9040162

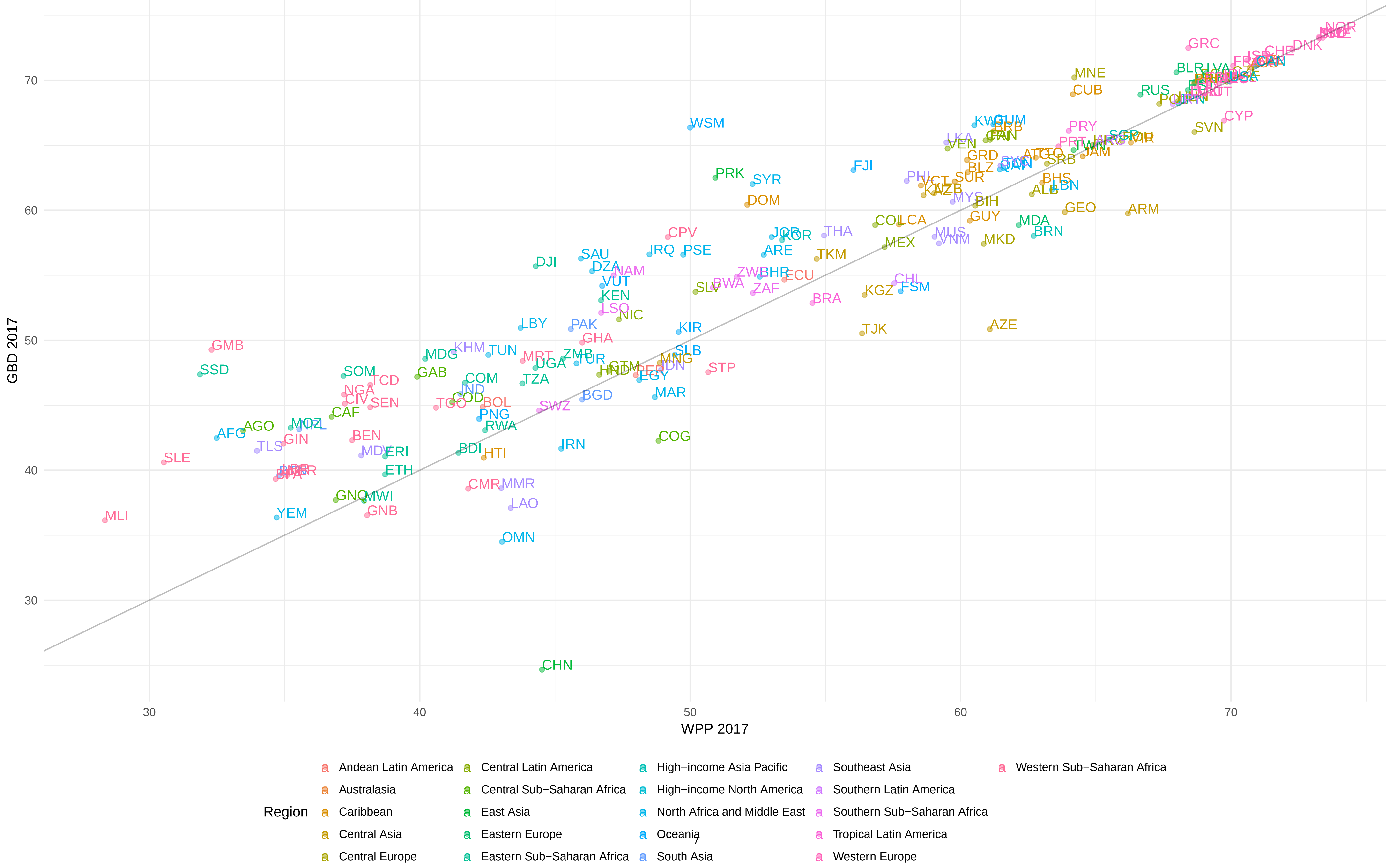

Comparison of life expectancy at birth for countries in GBD 2017 vs WPP 2017 for 1970, both sexes combined

CCC: 0.9317709

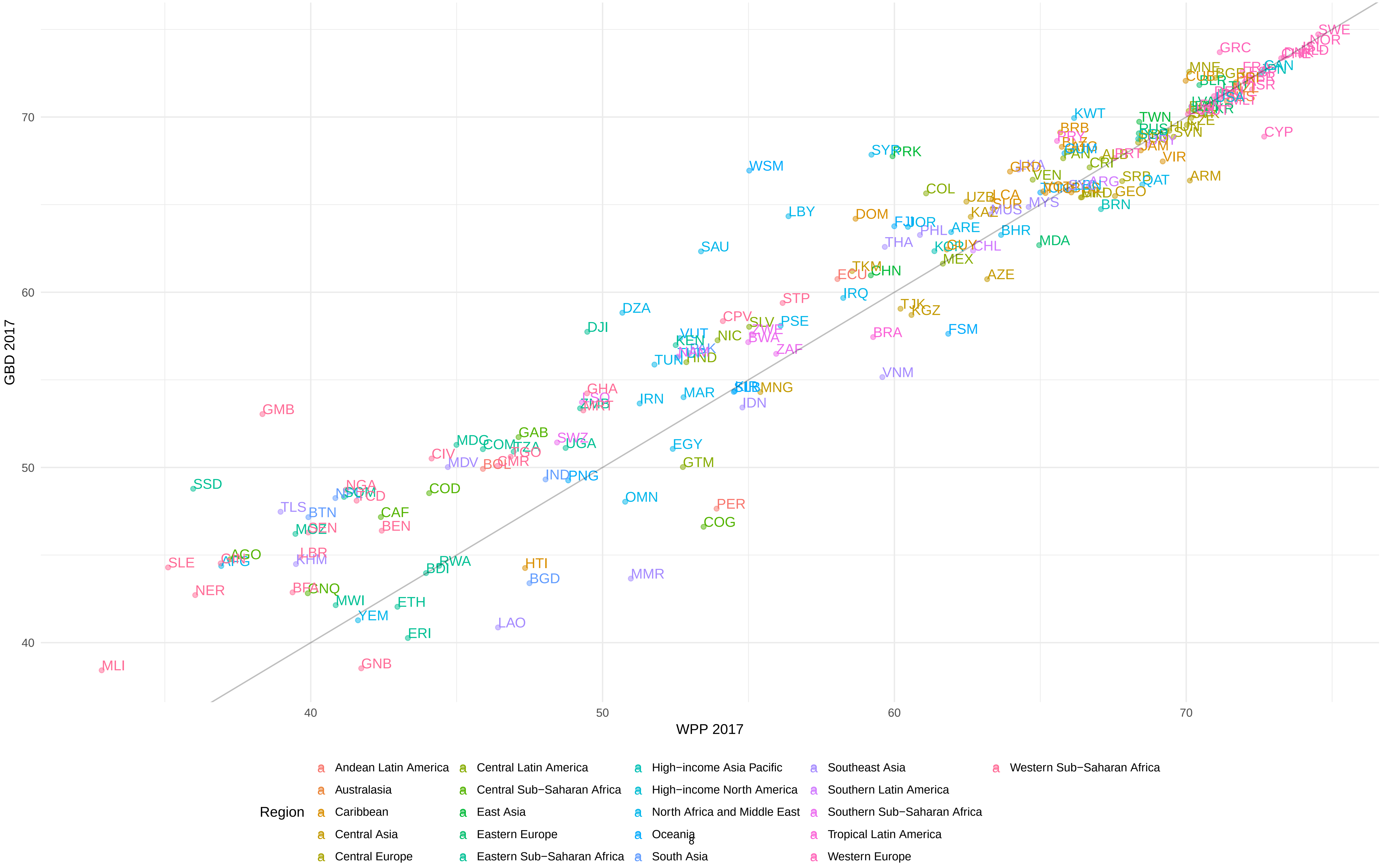

Comparison of life expectancy at birth for countries in GBD 2017 vs WPP 2017 for 1980, both sexes combined

CCC: 0.9292399

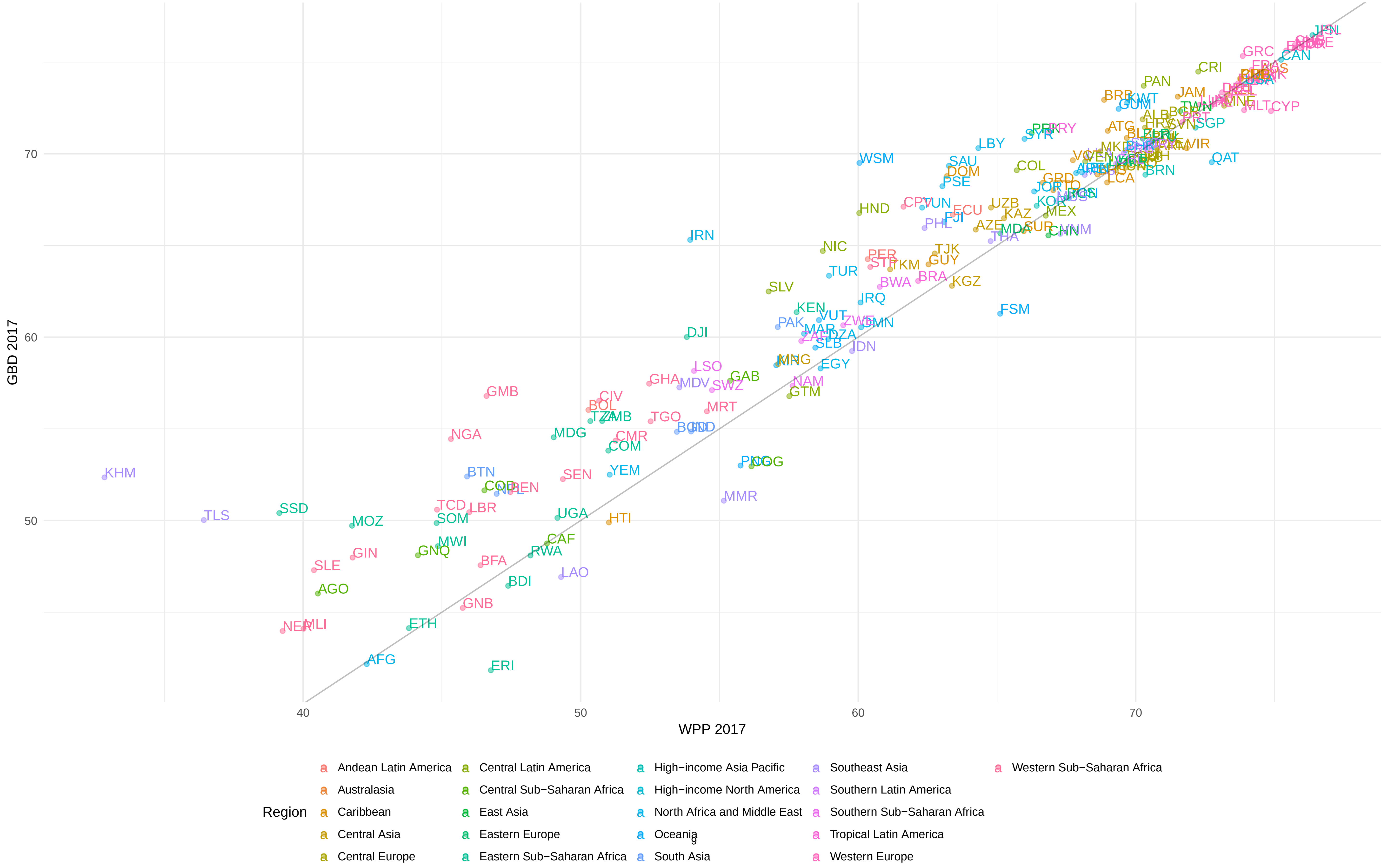

Comparison of life expectancy at birth for countries in GBD 2017 vs WPP 2017 for 1990, both sexes combined

CCC: 0.9383326

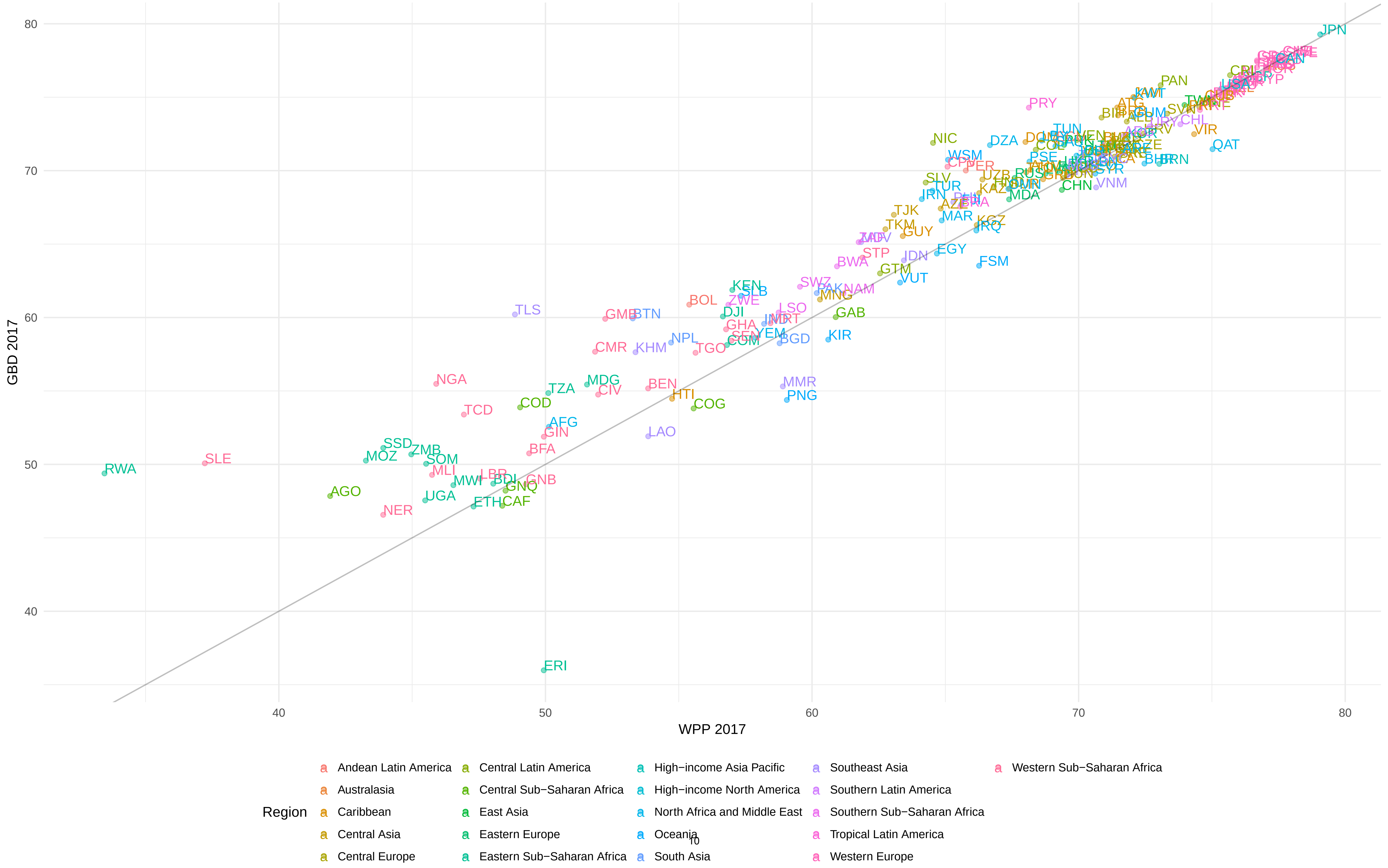

Comparison of life expectancy at birth for countries in GBD 2017 vs WPP 2017 for 2000, both sexes combined

CCC: 0.9699195

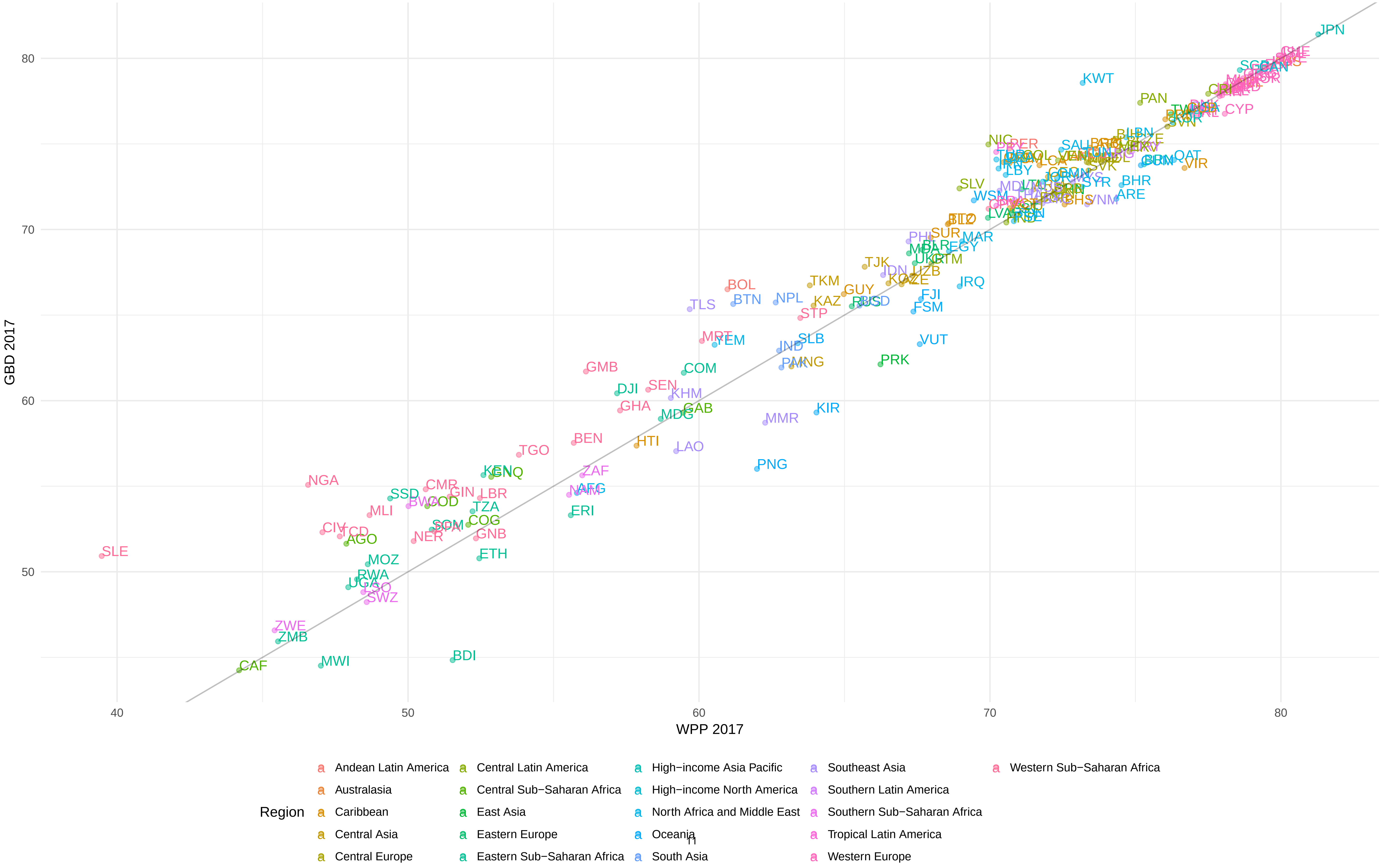

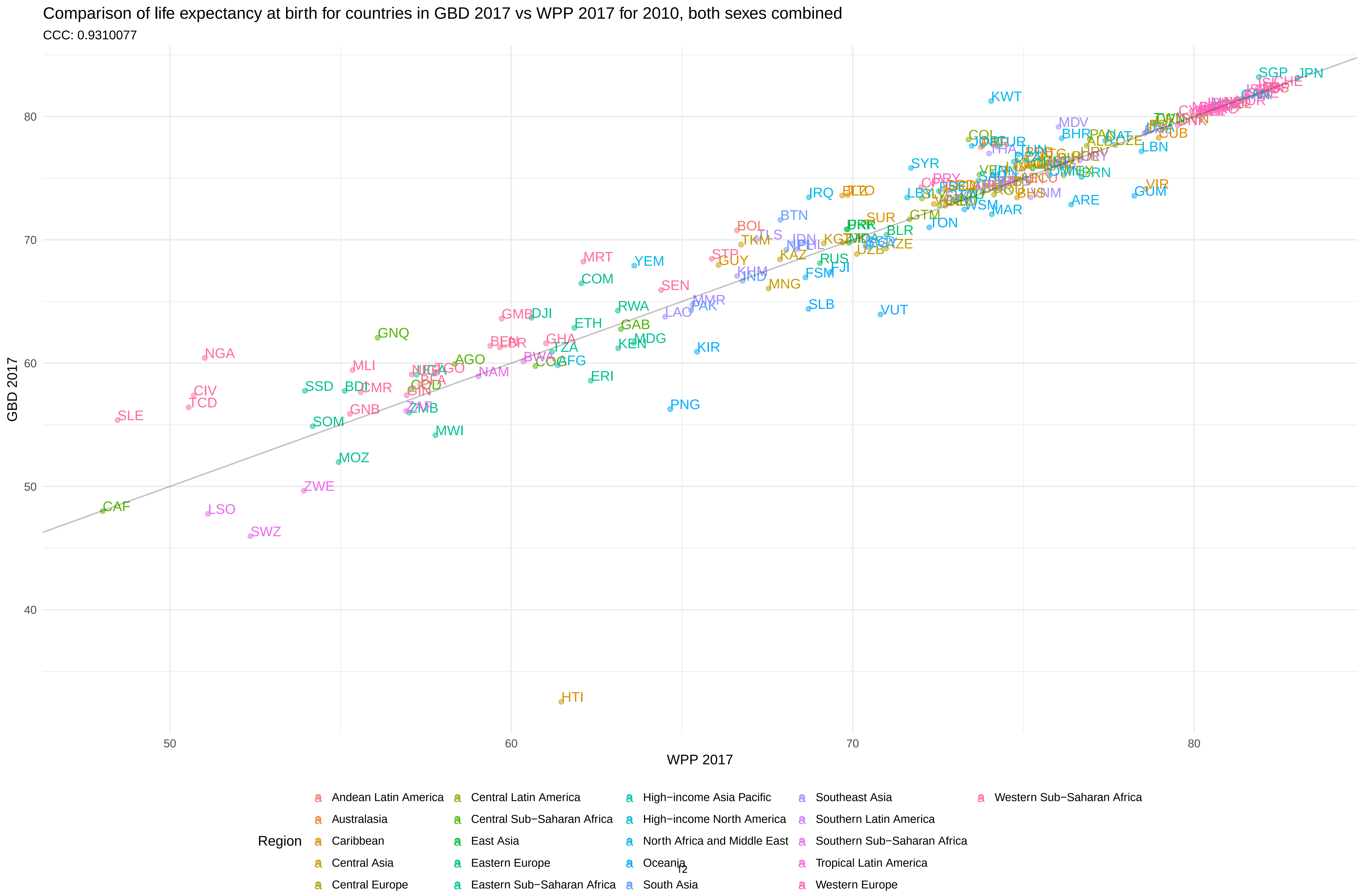
Comparison of life expectancy at birth for countries in GBD 2017 vs WPP 2017 for 2010, both sexes combined
CCC: 0.9310077
GBD 2017
WPP 2017
80
70
60
50
40
50
60
70
80
SGP
JPN
KWT
MDV
BHR
PAN
QAT
CUB
LBN
COL
SYR
IRQ
BLZ
LBY
VIR
GUM
ARE
BTN
BOL
TLS
TKM
IDN
KGZ
SUR
GTM
TON
MAR
WSM
BLR
PRK
AZE
UZB
KAZ
RUS
FJI
FSM
MNG
KHM
IND
STP
GUY
MRT
YEM
COM
SEN
SLB
VUT
MMR
PAK
LAO
RWA
GAB
GMB
DJI
ETH
MDG
KEN
KIR
GNQ
GHA
TZA
AGO
BWA
COG
AFG
NGA
MLI
NAM
ERI
SSD
BDI
CMR
CIV
TCD
SLE
GNB
ZMB
SOM
MWI
PNG
MOZ
ZWE
CAF
LSO
SWZ
HTI
Region
Andean Latin America
Australasia
Caribbean
Central Asia
Central Europe
Central Latin America
Central Sub−Saharan Africa
East Asia
Eastern Europe
Eastern Sub−Saharan Africa
High−income Asia Pacific
High−income North America
North Africa and Middle East
Oceania
South Asia
Southeast Asia
Southern Latin America
Southern Sub−Saharan Africa
Tropical Latin America
Western Europe
Western Sub−Saharan Africa

Comparison of life expectancy at birth for countries in GBD 2017 vs WPP 2017 for 2017, both sexes combined

CCC: 0.92815

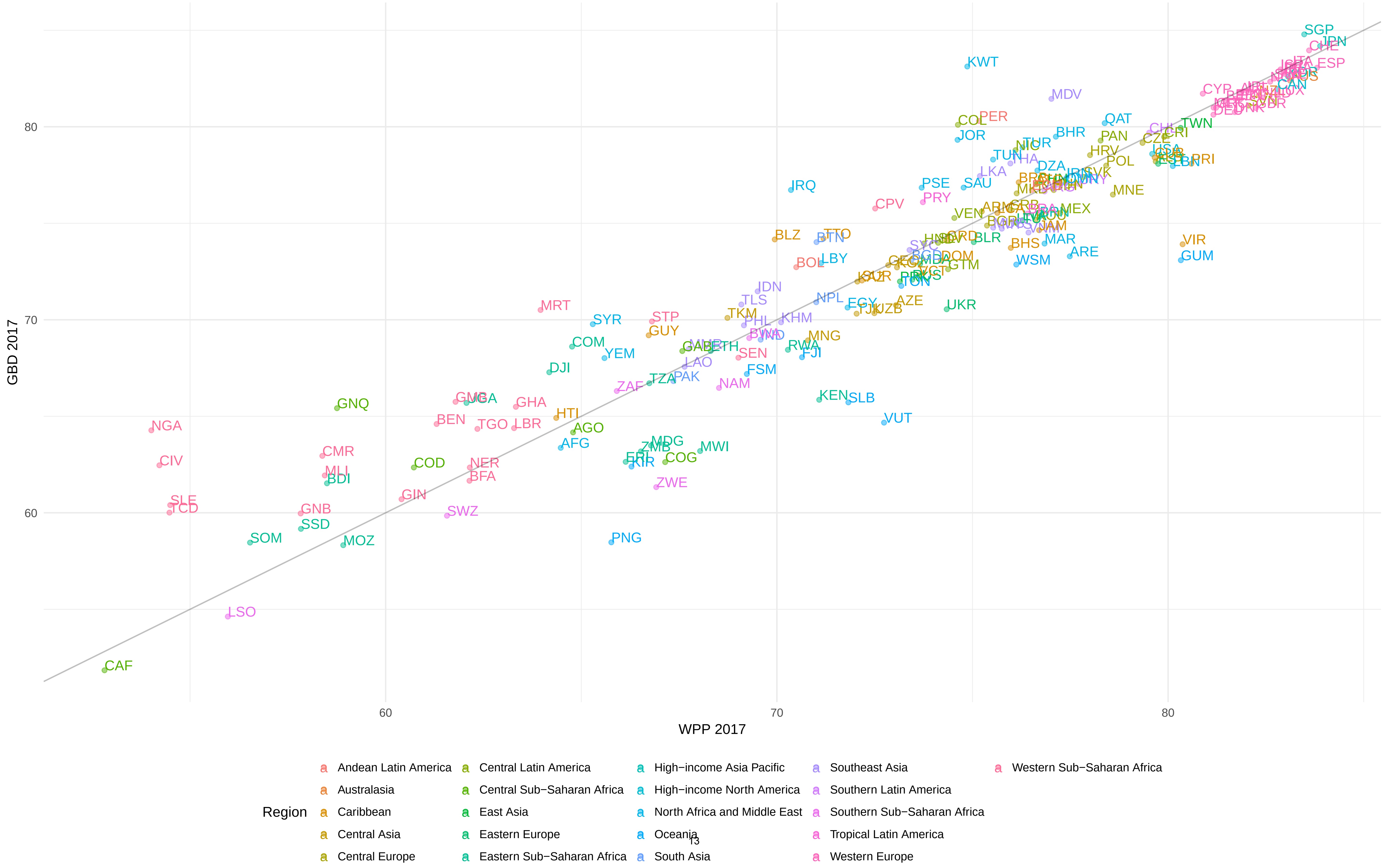

Table 1 Age-standardised mortality rates by location and year for males for 1950, 1955...2005, 2010, 2017

Location	1950	1955	1960	1965	1970	1975	1980	1985	1990	1995	2000	2005	2010	2017
Global	2293 (2259.5-2329.1)	2041.6 (2015.8-2067.4)	2473 (2451-2496.9)	1867.7 (1847.2-1888.5)	1765.9 (1747.8-1784)	1646.3 (1630.4-1662.3)	1539.9 (1525.5-1554.6)	1443.2 (1431.8-1455)	1363.3 (1353.6-1373.6)	1331.1 (1322.8-1339.6)	1249.3 (1241.2-1257)	1157 (1149.8-1164.6)	1070.8 (1063.1-1079)	959.7 (947.8-971.2)
Low SDI	3130.6 (2992.2-3281.5)	2868.3 (2742.5-3005.2)	2766.2 (2642.9-2894.9)	2662.9 (2555.1-2769.9)	2525.9 (2433.7-2612)	2303.6 (2227-2386.4)	2166 (2099.4-2241.6)	2076.2 (2015.3-2142)	1971.4 (1911.3-2025.9)	1860.7 (1813.2-1904)	1746.3 (1714.3-1778.5)	1588.6 (1563.6-1612.1)	1521.2 (1498.3-1545.1)	1308.5 (1272-1346.4)
Low-middle SDI	3034.9 (2939.7-3133.6)	2706 (2622.6-2792.7)	2858.7 (2781.3-2942.7)	2414.9 (2347.1-2491.1)	2226.4 (2165.8-2292)	2097.5 (2041.3-2151.5)	1895.2 (1843-1945.4)	1749.2 (1704-1795.4)	1667.1 (1627.5-1705.5)	1619.9 (1583.7-1655.9)	1586.4 (1552.3-1623.9)	1482.2 (1447.8-1519.5)	1410.4 (1375.2-1447.7)	1253.8 (1215.5-1292.6)
Middle SDI	1916.1 (1860.7-1972.9)	1651.7 (1604.5-1696.5)	3142.6 (3094.9-3192.1)	1466.6 (1427.3-1507.1)	1325.7 (1292.3-1358.3)	1179.9 (1152.6-1209.7)	1088.2 (1064.6-1112.9)	1021.4 (1002.6-1041.4)	962.8 (948.5-976.2)	918.4 (907.6-929.5)	874.3 (863.5-885.6)	827 (817.7-836.2)	792.3 (781.7-803.9)	729.8 (711.3-747.1)
High-middle SDI	2572.9 (2478.8-2670.4)	2305.2 (2218.3-2394)	2586.6 (2512.4-2663.9)	2074.2 (2008.1-2145.7)	1974.5 (1923.6-2030.4)	1879.4 (1832.6-1925.8)	1844.5 (1804.9-1885.4)	1770.1 (1737.3-1805)	1719.8 (1691.2-1747.1)	1818.9 (1796.3-1840.6)	1665 (1646.3-1683.9)	1557.1 (1540.7-1573.1)	1367.1 (1349-1385.8)	1205.3 (1171.3-1239.5)
High SDI	1532.7 (1497.5-1572.7)	1353.6 (1337.6-1368.9)	1308 (1300-1316.4)	1277.4 (1269.6-1285.1)	1249.1 (1242.4-1255.7)	1167.7 (1163.7-1171.2)	1095 (1093.1-1097)	1010.2 (1008.4-1012)	934.4 (932.6-936.4)	873 (871.3-874.7)	778.8 (777.5-780.3)	703 (701.9-704.2)	634.5 (633.2-635.9)	591.7 (583-600.4)
Central Europe, Eastern Europe, and Central Asia	1794.6 (1751.8-1840.8)	1588.3 (1557.1-1624.2)	1436.2 (1423.6-1448.3)	1415.8 (1401.9-1429.7)	1481.8 (1469.5-1494.5)	1481.3 (1468.7-1494.1)	1552.8 (1542.8-1564.3)	1525.5 (1519.6-1531.6)	1466.6 (1463.8-1469.5)	1733.5 (1730.3-1736.9)	1616.9 (1613.8-1620.1)	1622.4 (1618.6-1625.6)	1396.1 (1393.2-1399.2)	1188.7 (1175.1-1202.6)
Central Asia	2575 (2428-2717.7)	2179.2 (2104.4-2256.6)	1927.4 (1876.2-1983.1)	1788.1 (1721.9-1863)	1666.6 (1639.2-1695.5)	1563 (1519.1-1608.6)	1452.5 (1435.5-1469)	1411.2 (1399.6-1422.9)	1369.5 (1358.2-1381.2)	1606.6 (1593.6-1620.4)	1515.1 (1498.2-1533.3)	1589.5 (1566.4-1611.6)	1474.3 (1454.8-1493.2)	1401.8 (1351.3-1460.4)
ARM Armenia	2231.6 (1914.8-2607)	1804.5 (1651-1974.9)	1516.1 (1409.9-1626.4)	1383.5 (1253.8-1521.5)	1228.7 (1167.9-1296)	1209.9 (1106.2-1313.7)	1097.8 (1048.5-1146.7)	1131.3 (1094.7-1166.4)	1277.5 (1235.8-1317.9)	1394.2 (1380.2-1407.9)	1178.9 (1166.2-1191.3)	1151.6 (1141.3-1162.7)	1113.1 (1102.9-1122.6)	993 (960.2-1027)
AZE Azerbaijan	3245 (2823.5-3655.2)	2813.2 (2570.2-3060.2)	2426.5 (2260.8-2603.8)	2079.7 (1891.9-2272.7)	1821.4 (1741.8-1907)	1625.7 (1523.3-1733.2)	1447.9 (1410.3-1484.7)	1353.7 (1328.4-1381.1)	1327.8 (1274.6-1384)	1809.7 (1743.2-1878.8)	1606.6 (1549.4-1667.7)	1678.3 (1617.3-1745.7)	1553.9 (1490.4-1617.1)	1707.2 (1590.3-1826.1)
GEO Georgia	2395.6 (2027.2-2781.9)	2094.1 (1897.1-2302.3)	1840 (1717.4-1976.2)	1675.2 (1525.8-1835.7)	1523.2 (1457.3-1592.5)	1465.7 (1337.9-1603.3)	1365.7 (1290.5-1442.8)	1337.6 (1280.8-1400.7)	1347.6 (1287.4-1404.5)	1284.3 (1229.8-1344)	1220.4 (1170.7-1271.4)	1172.6 (1160.7-1185.1)	1251.6 (1241.8-1262.2)	1293.5 (1257-1330.3)
KAZ Kazakhstan	2396.3 (2068.7-2731.9)	2093.4 (1912.6-2271.8)	1879.1 (1764.1-1999.8)	1848.6 (1679.3-2012.5)	1799 (1744.4-1855.3)	1684.6 (1585.7-1791.6)	1567.9 (1546.3-1591.6)	1532.7 (1522.4-1543.2)	1503.8 (1494.4-1513.6)	1954.5 (1944.2-1964.9)	1830.9 (1821.1-1842.1)	1833.6 (1824.4-1844.8)	1580.8 (1572.1-1590)	1300.9 (1243.5-1361.3)
KGZ Kyrgyzstan	3276.1 (2844.7-3766.3)	2808.9 (2530.6-3093.5)	2471.1 (2247-2700)	2271.4 (2022.6-2529.6)	2106.7 (1922.5-2300.3)	1960.4 (1769.7-2150.4)	1829.9 (1713.7-1942.4)	1642.3 (1560.1-1715.5)	1561.6 (1490-1637.2)	1653.5 (1575.9-1735.9)	1495.3 (1423.2-1570.7)	1577.6 (1526.3-1626.3)	1393.7 (1384.8-1403)	1143.1 (1113.9-1173.6)
MNG Mongolia	3857.3 (3368.1-4450.8)	3057.1 (2713.1-3410.7)	2578.9 (2343.6-2823.6)	2192.6 (2056.8-2337.9)	2131.3 (2017.8-2260.3)	1919.8 (1818.2-2021.5)	1936.2 (1840-2034)	1889.6 (1810.5-1975.4)	1842.8 (1777.2-1912.2)	2035.3 (1962.7-2099.9)	2029.9 (1967.7-2090.2)	1925.4 (1866.7-1989.6)	1788.5 (1724.8-1851.4)	1541.8 (1408.1-1675.8)
TJK Tajikistan	2654.1 (2294.7-3050.1)	2418.6 (2228-2624.3)	2241.3 (2099.8-2393.2)	2004.5 (1836.3-2188.1)	1808.6 (1730.5-1893.9)	1636.8 (1539.3-1740.3)	1413.9 (1376.2-1453.7)	1238.2 (1216.4-1263)	1234.2 (1206.5-1262)	1433.6 (1371.2-1508.3)	1211.4 (1166.3-1256.8)	1170.8 (1121.7-1233.5)	1177.7 (1109-1248.2)	1148.5 (1067.5-1252.1)
TKM Turkmenistan	2773.6 (2417.2-3155.5)	2255 (2072.8-2448.6)	1964 (1826-2110.9)	1822.1 (1656-1985.8)	1682.3 (1608.5-1765.7)	1646.4 (1536.5-1764.9)	1539.3 (1495.8-1591.3)	1520.7 (1494-1550.9)	1468.2 (1447.2-1489)	1706.6 (1684.4-1730.6)	1548.2 (1525.2-1572.9)	1708.9 (1687.5-1732)	1281.9 (1264.8-1300.4)	1226.9 (1137.5-1319.2)
UZB Uzbekistan	2303.5 (1982.7-2653.8)	1802.6 (1651.5-1976.2)	1620.4 (1505-1744.5)	1536 (1398.3-1683.1)	1451.8 (1395.3-1507.7)	1377.9 (1288.2-1466.4)	1308.4 (1285.4-1333.3)	1312.5 (1298.9-1327)	1204.8 (1194-1216.1)	1369.3 (1357.8-1381.8)	1433.1 (1387.1-1483.9)	1713.3 (1639-1787.1)	1699 (1637.9-1760.3)	1774.5 (1597.8-1969.1)
Central Europe	1815.4 (1775.6-1861)	1526.6 (1513.3-1541.9)	1392.2 (1386.1-1398.8)	1346.2 (1340-1353)	1396 (1390.1-1402)	1343.8 (1339.9-1348.1)	1411.8 (1408.2-1415.5)	1415 (1412.4-1417.7)	1387 (1384.4-1389.3)	1362.7 (1357.9-1367.8)	1214.5 (1210.8-1218.4)	1138.5 (1136.2-1141)	1034.4 (1031.8-1037.2)	917.3 (896.9-937.9)
ALB Albania	2016 (1807.8-2233.7)	1568.4 (1457.3-1687.1)	1344.5 (1267-1421.7)	1237.1 (1132.2-1344.7)	1147.9 (980.4-1333.6)	1118.1 (939.3-1318.7)	1113.7 (953.1-1285.5)	1125.1 (1066-1188.5)	1098.3 (1085.1-1111.6)	1022.9 (1010.3-1035.3)	1034.7 (986.3-1089.5)	997.8 (946.4-1048.9)	911.6 (825.9-1003.1)	893.9 (738.7-1069.2)
BIH Bosnia and Herzegovina	2279.7 (2168-2406.2)	1771.8 (1719.5-1828.7)	1582.4 (1547.4-1618.3)	1550.5 (1520.5-1585.8)	1563.4 (1538.6-1589.4)	1388.5 (1369.5-1408.8)	1388.5 (1373.5-1404)	1224.7 (1212.2-1236.1)	1099.9 (1088.5-1110.4)	1567 (1405-1745.4)	999.2 (904.8-1102)	974.2 (962.5-986)	920.9 (909.3-933.4)	911 (857.9-969.5)
BGR Bulgaria	1511.8 (1486.9-1540.8)	1255.3 (1246.1-1265.3)	1128.4 (1118.9-1138.2)	1062.8 (1054.2-1071.9)	1127.1 (1117.5-1136.6)	1225.2 (1215.6-1234.6)	1243.5 (1235.2-1252.8)	1261.1 (1252.6-1269.8)	1392.3 (1383.1-1401.9)	1425.1 (1416.6-1434.3)	1328.2 (1319.5-1337)	1260.6 (1252.4-1269.2)	1150.8 (1141.8-1160)	1080.8 (1018.3-1142.5)
HRV Croatia	2365 (2267.8-2480.4)	1801.4 (1761.5-1844.9)	1575.4 (1552.4-1600)	1437.4 (1420.2-1455.6)	1462.9 (1449.4-1477)	1351.7 (1338.6-1364.1)	1383.5 (1371.8-1395.3)	1393.6 (1381.6-1405)	1325.8 (1313.9-1337.5)	1215.9 (1204.4-1227.5)	1150 (1137.8-1161.5)	1107.1 (1096.1-1118.5)	984.7 (972.8-996.6)	848.8 (798.8-898.6)
CZE Czech Republic	1625.1 (1613.1-1636.8)	1486.4 (1476.8-1496)	1430 (1420-1439.2)	1444.7 (1435.3-1454.1)	1582.4 (1573.2-1592.1)	1528.2 (1518.4-1538.3)	1566.9 (1558.3-1576.3)	1503.4 (1494.1-1512.3)	1502.4 (1493.4-1511.1)	1265.3 (1257.2-1273.7)	1108 (1100.3-1115.7)	1001.9 (993.9-1009.6)	885.4 (878.6-891.9)	771.4 (727-819.9)
HUN Hungary	1743.6 (1725.9-1761.8)	1421 (1411.4-1430.6)	1417.2 (1407.1-1426.7)	1384.6 (1376-1393.2)	1450.4 (1441.4-1460)	1453.3 (1443.7-1462.1)	1570.1 (1561.6-1578.3)	1609.4 (1601.3-1617.7)	1544.4 (1535.9-1552.4)	1517.5 (1510.1-1525.3)	1340.9 (1333.6-1348.3)	1263.9 (1256.2-1271.5)	1136.8 (1129-1144.2)	943.7 (892.5-1002.4)
MKD Macedonia	1822.3 (1641.9-2015.8)	1589.8 (1462.5-1723.8)	1501.2 (1381.4-1631.7)	1383.6 (1278.3-1507.3)	1258.4 (1202-1321.5)	1122.4 (1100.8-1144.8)	1129.2 (1110-1148.7)	1181.3 (1164.2-1198.3)	1184.1 (1168.2-1200.9)	1233.9 (1216.2-1251.4)	1179.5 (1162.2-1196.3)	1160 (1141.7-1176.9)	1073.5 (1056.1-1089.4)	974.5 (917.2-1031.9)
MNE Montenegro	1349 (1252-1450.7)	1088.4 (1031-1144.4)	1089.6 (1030.3-1151.2)	1142 (1085.7-1199.3)	1153.5 (1093.1-1214.5)	1142.8 (1086.5-1205.7)	1181.6 (1157.1-1204.5)	1173.4 (1154-1193)	1068.3 (1045-1090.9)	1047.5 (1023.9-1072.7)	1042.1 (1014.1-1068.9)	1015.3 (964.6-1069.8)	923.2 (875.9-972.4)	929.6 (846.8-1022.5)
POL Poland	1954.9 (1940.8-1974.3)	1608.7 (1602.3-1614.8)	1349.4 (1344-1355.1)	1289.3 (1283.9-1294.7)	1335.6 (1330.7-1340.6)	1299.4 (1294.7-1303.9)	1403.4 (1399-1407.8)	1405.2 (1400.7-1409.7)	1391.6 (1387.4-1395.8)	1323.7 (1319.5-1327.9)	1171.5 (1167.7-1175.3)	1082.3 (1078.6-1086.1)	978 (975-981.4)	864 (816.5-913)
ROU Romania	1661.6 (1424.7-1938.1)	1524.3 (1453.3-1597.4)	1397.5 (1390.1-1404.8)	1350.5 (1343.7-1357.1)	1360.6 (1353.9-1367.1)	1294.7 (1288.4-1300.7)	1375.9 (1370-1382.1)	1415.5 (1409.9-1421)	1352.9 (1347.7-1358.3)	1448.5 (1442.8-1453.8)	1280.2 (1274.7-1285.3)	1223.5 (1218.1-1228.5)	1147.2 (1142.1-1152.1)	1039.2 (983.5-1097.6)
SRB Serbia	1983.9 (1942.4-2036)	1596.5 (1583.9-1610.2)	1489.8 (1478.7-1501.8)	1473.1 (1462.2-1483.4)	1603.7 (1593.3-1614.5)	1420.4 (1410.5-1430.8)	1465.6 (1456.3-1474.9)	1500.5 (1491.2-1509.4)	1430.6 (1422.5-1438.6)	1415.3 (1406.8-1423.6)	1332.8 (1321.2-1346.3)	1258.7 (1249.1-1268.8)	1127.4 (1118.6-1135.7)	989.6 (936.5-1043.8)
SVK Slovakia	1675.1 (1507.2-1845)	1430 (1312.5-1552.1)	1345.1 (1235-1465.9)	1364.1 (1246.4-1485.5)	1411 (1306.2-1516.4)	1386.9 (1375.9-1398.7)	1421.4 (1409.6-1433.6)	1411.6 (1400.4-1423.1)	1435.2 (1424.3-1445.6)	1288.9 (1277.9-1299.1)	1234.6 (1224.4-1244.6)	1167.1 (1157.2-1176.5)	1058.1 (1048.2-1067.8)	902.3 (853.1-954.5)
SVN Slovenia	2202.1 (2169.2-2237.2)	1775 (1755.6-1795.1)	1610.3 (1590.8-1631)	1479.7 (1459.9-1499.8)	1502.2 (1485.1-1520.4)	1381.8 (1364.2-1399.9)	1362.3 (1345.7-1378.2)	1365.2 (1348-1381.9)	1221 (1205-1237)	1136.8 (1121.7-1151.5)	1041 (1026-1056.3)	906.2 (892.2-920.4)	786.2 (774.3-799.2)	675 (629-721.2)
Eastern Europe	1615.8 (1546.2-1696.4)	1495.3 (1434.7-1563.8)	1343 (1323.4-1361.2)	1357.7 (1337.4-1377)	1475.5 (1452.8-1497.5)	1535.7 (1513.9-1557.6)	1643.8 (1625.2-1664.6)	1598.8 (1587.9-1610.2)	1512.2 (1508.5-1516.1)	1967.9 (1964.2-1971.4)	1868.9 (1866.8-1871)	1923.9 (1922.6-1925.3)	1607.5 (1606.3-1608.9)	1321.2 (1302.2-1339.4)
BLR Belarus	1273.6 (1068-1514.9)	1186.7 (1027.2-1354.4)	1112.7 (1070.4-1159.8)	1085.3 (1065.4-1107.4)	1157.6 (1143.6-1172.3)	1230.1 (1217.8-1243)	1328.9 (1318.1-1340)	1367.3 (1357-1377.8)	1339.7 (1330.7-1349)	1627.6 (1617.8-1637.1)	1618 (1608.5-1626.9)	1689.2 (1680.5-1697.7)	1553.5 (1545.6-1561.9)	1247.4 (1185-1315.8)
EST Estonia	1648.6 (1369.6-1955.8)	1597.4 (1382.6-1847.6)	1485.7 (1463.2-1509.7)	1417.2 (1395.7-1438)	1482.4 (1462.8-1503.3)	1519.2 (1501.3-1537.7)	1582.8 (1564.6-1600.8)	1584.6 (1565.8-1604)	1529.8 (1511.2-1546.7)	1749.7 (1730.9-1767.1)	1449.3 (1433-1467.6)	1323.5 (1305.9-1341.4)	1087.4 (1071.3-1105.5)	886 (781.9-1001.9)
LVA Latvia	1451.5 (1194.8-1724.6)	1356.3 (1125.5-1572.8)	1309.1 (1287.7-1331.5)	1287.3 (1270.9-1302.6)	1334.7 (1318.7-1349.9)	1491.9 (1477.1-1507.1)	1525 (1510.4-1539.2)	1517.1 (1503.1-1530.9)	1522 (1507.5-1536.1)	1842.5 (1828.4-1857.5)	1473.7 (1459.5-1487)	1523.7 (1507.8-1539.3)	1274.3 (1259.8-1289.9)	1114 (994.3-1242.8)
LTU Lithuania	1402.1 (1160-1667.5)	1285.3 (1095.5-1497.9)	1143.9 (1130.3-1159)	1094.6 (1082.5-1107.6)	1189.1 (1175.1-1201.9)	1239.5 (1226.8-1252.1)	1330.1 (1318.2-1342.3)	1350.3 (1337.7-1362.8)	1317 (1304.9-1328.5)	1553.1 (1541.3-1565.5)	1299.8 (1287.9-1311.8)	1408.3 (1396.3-1420.1)	1282.4 (1271-1295.1)	1145.9 (1078.5-1217.8)
MDA Moldova	2476.4 (2134-2835.9)	2215.4 (2029.2-2429.7)	2042.8 (1914-2187.9)	1923.3 (1758.4-2106.9)	1783.6 (1711.4-1860.3)	1690.8 (1595.3-1803.5)	1592.3 (1569.6-1620.4)	1612.4 (1597.4-1629)	1465.9 (1452.8-1479.1)	1764.1 (1750.4-1778.7)	1503.5 (1490.1-1517)	1528.2 (1517.2-1539.9)	1458.3 (1446-1470.7)	1170.6 (1137.5-1204)
RUS Russian Federation	1670.2 (1621.5-1719.1)	1540.7 (1498.1-1586.5)	1397.1 (1367.4-1426.9)	1424.7 (1391.1-1456.5)	1556.2 (1518.6-1591.4)	1622 (1587.4-1657.5)	1750.9 (1721.6-1783.6)	1682.8 (1666.7-1699.8)	1562.2 (1560.2-1564.3)	2055.1 (2053.5-2056.8)	1986.8 (1985.4-1988.2)	2032.2 (2030.8-2033.6)	1672.6 (1671.3-1673.9)	1279.1 (1268.4-1289)
UKR Ukraine	1533.5 (1293.6-1814.7)	1440.2 (1235.4-1662.3)	1242.7 (1221.9-1266)	1256.3 (1241.6-1273.6)	1374 (1360.1-1388.7)	1429.6 (1414.3-1444.6)	1514.3 (1498.5-1530.5)	1479.1 (1463.6-1494.8)	1450.1 (1436.1-1463.3)	1874.9 (1861.2-1888.6)	1729.1 (1721.8-1736.1)	1774 (1770.4-1777.8)	1494.6 (1490.9-1498.7)	1517.3 (1446.8-1593)
High-income	1568 (1529.9-1610.1)	1401.4 (1384.3-1418)	1362.6 (1354-1371.1)	1337 (1328.9-1345)	1303.4 (1296.6-1310.4)	1215.2 (1211.1-1219.2)	1128.4 (1127.4-1129.6)	1035.6 (1034.7-1036.4)	953.1 (952.4-953.9)	890.4 (889.6-891.1)	799.1 (798.4-799.8)	720.6 (720-721.3)	653.7 (653-654.4)	612.4 (603.2-621.7)
Australasia	1373.3 (1364-1383)	1340.2 (1332-1348.5)	1304.3 (1296.9-1312.1)	1346.3 (1339-1353.9)	1363.1 (1356.2-1370.4)	1258.7 (1251.8-1264.9)	1114.7 (1108.6-1121)	1027.8 (1022.2-1033.6)	913.1 (907.6-918.5)	827.4 (821.9-832.4)	720.8 (716.2-725.4)	621.5 (617.3-625.5)	565.9 (561.9-569.7)	537.5 (485.7-592.1)
AUS Australia	1387.9 (1377.7-1397.9)	1361.5 (1352.1-1370.2)	1315.4 (1306.8-1324.2)	1357.7 (1349.6-1366.4)	1373 (1365.5-1381)	1262 (1254.2-1269.1)	1103.4 (1096.7-1110.6)	1014.8 (1008.3-1021)	905.9 (899.8-911.9)	813.2 (807-819)	711 (706-716.4)	612.4 (607.6-617.1)	559 (554.6-563.2)	533.9 (474.6-596.7)
NZL New Zealand	1316 (1292.1-1341.2)	1256.2 (1237.8-1275.7)	1255.9 (1238.2-1274.5)	1296.1 (1276.9-1315.1)	1316.7 (1298.8-1335.1)	1241.8 (1225-1259.6)	1164.6 (1150.7-1177.9)	1089 (1075.8-1103)	950.3 (938.9-963.2)	900.3 (889.4-911.7)	771.5 (761.3-782.1)	669.7 (661.5-678.2)	602.5 (594.2-610.8)	557.3 (525-589.6)
High-income Asia-Pacific	2239.9 (2161.6-2311.6)	1678.9 (1615.4-1742.5)	1619.5 (1559.3-1683.1)	1503.2 (1443.5-1560.2)	1367.6 (1320.4-1414.8)	1187.8 (1166.2-1209.5)	1068.5 (1065.7-1071.8)	947.7 (945.6-950.2)	862.8 (860.9-864.6)	803.5 (802-805.2)	710.2 (708.8-711.7)	643.2 (642-644.4)	589.5 (587.4-591.4)	518.2 (505.3-530.9)
BRN Brunei	2663.1 (2357.2-2987.7)	2365.2 (2137.4-2634.1)	2082.8 (1908.2-2262.1)	1715.1 (1609.2-1821.2)	1540.5 (1463.2-1612)	1416.1 (1351.7-1482.9)	1329.9 (1258.9-1395)	1328.8 (1260.5-1395.5)	1340.6 (1277.9-1401.8)	1208.3 (1161.5-1257.8)	1083.3 (1034.2-1129.1)	986.1 (943.8-1035.4)	935.6 (888-989)	910.3 (828.1-1005.8)
JPN Japan	1832.5 (1780.7-1886.3)	1507.4 (1462.1-1554)	1475.5 (1431.9-1518.5)	1357.7 (1316.2-1402.1)	1236.9 (1202.4-1273.7)	1069.8 (1049.2-1091)	945 (943.2-946.7)	842 (840.5-843.5)	776.4 (774.9-777.8)	737.9 (736.5-739.4)	657.7 (656.4-659.1)	610.4 (609.1-611.7)	565.9 (564.5-567.1)	504.3 (492.3-517.5)
JPN Aichi	1759.8 (1472.2-2083.8)	1436.1 (1188.8-1709.5)	1443.2 (1183.7-1741.4)	1325.8 (1076.7-1606.8)	1200.6 (988.4-1409.1)	1046.7 (936.7-1166.6)	944.8 (937.1-952.7)	827.3 (820.6-834.3)	780.6 (773.4-787.7)	743.4 (737-750.2)	656.4 (650.8-662.5)	605.8 (600.2-611.4)	561.5 (555.8-567.7)	477.1 (419.8-544.5)
JPN Akita	1952.2 (1616.3-2323.1)	1620.2 (1327.7-1958.5)	1620.3 (1338.3-1955.8)	1542.4 (1272.9-1855.2)	1440.1 (1192.7-1699.5)	1261.1 (1120.9-1412.6)	1090.5 (1075-1105.6)	1056.9 (1041.7-1073.2)	899.7 (885.2-914.2)	857.4 (843.5-872.8)	776.8 (763.7-790.2)	723 (709.1-736.7)	662.4 (649.7-674.9)	594.8 (517.5-682.6)
JPN Aomori	2045.4 (1708.5-2425)	1687.2 (1398.2-2023.1)	1626 (1343.1-1929.5)	1502.6 (1241.5-1804.6)	1392.4 (1159.9-1660.8)	1213.6 (1095.6-1352.5)	1040.3 (1025.9-1053.8)	934.8 (921.6-947.3)	841.4 (828.8-853.7)	825.4 (811.6-837.9)	745.2 (734.6-756.8)	724 (712.3-735.5)	666.2 (654.8-677.8)	599.9 (526.4-687.5)
JPN Chiba	1789 (1491.8-2108.7)	1477.3 (1239.4-1764.2)	1481.9 (1228.4-1790.2)	1356.2 (1121.5-1636.5)	1238.6 (1042.6-1462.9)	1077.1 (964.1-1199.8)	939.2 (930.4-948.5)	830.1 (822.3-837.2)	759.1 (751.7-766.6)	733 (726.4-740)	643.9 (637.7-650.6)	603.8 (597.8-609.7)	557.7 (551.8-563.5)	503.4 (442.5-570.1)
JPN Ehime	1872.2 (1533.3-2234.1)	1561.6 (1264.2-1872.5)	1494.7 (1217.3-1823.2)	1376.6 (1125.2-1673.6)	1275.7 (1055.1-1521.5)	1111 (984.8-1248.9)	940.6 (926.9-953.8)	828.1 (816.4-839.8)	771.2 (759.6-782.7)	734.2 (721.9-746.1)	666.1 (655.1-678)	630.1 (619.4-640.3)	579.2 (568.5-590.7)	523.9 (454.7-604)
JPN Fukui	1673.2 (1376.2-1996.8)	1368.8 (1119.1-1642.1)	1389.6 (1147.5-1653.9)	1289.1 (1084.1-1520)	1173.4 (1032-1341.2)	1025.9 (951.8-1102.7)	926.3 (910.1-942.6)	812 (799.6-824)	742.6 (731.5-754.3)	692.3 (680.5-704.3)	627.8 (616.4-638.3)	585.9 (575.3-597.1)	548.6 (538.2-559.2)	492.8 (433.9-562)
JPN Fukuoka	1936.4 (1600.9-2291.2)	1601 (1312.6-1916.8)	1539.6 (1274-1840.6)	1423.3 (1177.2-1704.6)	1300 (1076.9-1531.5)	1131.8 (1011.9-1257.6)	993.9 (985.1-1002.5)	883 (874.8-890.7)	817 (809.2-824.9)	763.1 (755.6-769.9)	678.4 (671.5-685.1)	630.6 (623.3-637.2)	579.4 (572.4-586.6)	494.8 (433.4-568.2)
JPN Fukushima	1870.6 (1550.7-2223.9)	1526.3 (1262.3-1827.8)	1504.1 (1245.7-1811.1)	1397.5 (1141.3-1679.5)	1293.3 (1079.4-1534.9)	1117.8 (1003.6-1243.7)	974.7 (963-987.1)	852.3 (841.9-862.7)	768.7 (757.9-780)	731.2 (720.6-741.4)	668.9 (659.4-678.5)	636 (626.6-645.6)	586.1 (575.3-597)	528.4 (461.8-606.5)
JPN Gifu	1736.2 (1447.6-2056.8)	1418.5 (1175.6-1696.3)	1423.6 (1171.6-1701)	1312.3 (1068.8-1605.6)	1192.9 (992-1423.5)	1032.4 (921.6-1157.3)	937.5 (925-950.4)	819.5 (808.3-831.5)	743.6 (732.8-754.5)	721.2 (710.7-732.5)	643.5 (634.2-653.4)	599.4 (590-609.1)	549.1 (539.7-558.3)	515.6 (448.6-587.4)
JPN Gunma	1758.8 (1456-2086.5)	1447.1 (1196.6-1746.6)	1460.2 (1188.4-1772.1)	1361.6 (1116.6-1631.6)	1256 (1049.3-1498.5)	1092.6 (978.3-1215.8)	930.6 (917.9-944.3)	843.9 (833.4-855.5)	749.1 (738.5-760.1)	719.7 (708.5-730.6)	651.6 (641.2-662.2)	609.4 (599.8-619.2)	573 (563.5-582.3)	529.1 (461.8-606.4)
JPN Hiroshima	1866.9 (1536.5-2171.8)	1552.1 (1274.8-1846.7)	1493.9 (1228.1-1788.2)	1367.1 (1135.8-1629.5)	1254.2 (1043.1-1488.8)	1088.6 (972.1-1213.5)	944.4 (934-954.7)	829 (820.1-839.3)	762.8 (753.5-772.2)	729.2 (719.8-737.9)	649 (640.7-657.5)	599.6 (591-607.8)	553.3 (544.8-561.3)	467.9 (408.1-534.4)
JPN Hokkaidō	1894.7 (1577.6-2221.8)	1560.8 (1280.4-1859)	1523 (1259.9-1830.4)	1405.7 (1146.6-1674.2)	1296.8 (1081.3-1539)	1124.6 (1014.3-1242.1)	970.9 (962.9-978.9)	863.9 (856.4-871.5)	785.7 (778.2-792.7)	728.9 (721.3-735.6)	659.2 (653-665.8)	626.4 (620-633.2)	577.4 (571.1-583.8)	509.9 (447.9-576.6)
JPN Hyōgo	1918 (1597.8-2250)	1568.2 (1326.6-1840.1)	1531.4 (1260.9-1826.8)	1396 (1130.2-1674.1)	1259.5 (1063.7-1511.7)	1083.7 (977.5-1199.3)	982.7 (974.3-991.6)	884 (875.8-891.8)	813.3 (806-820.4)	834.6 (824.8-844.9)	674.1 (667.1-680.7)	621.1 (614.2-627.8)	565 (558.4-571.5)	499.3 (438.4-570)
JPN Ibaraki	1864.3 (1554.6-2187.3)	1534.1 (1269.4-1804)	1502 (1217.9-1819.5)	1387.8 (1112.1-1673.6)	1270 (1039.9-1520.8)	1077.7 (958.1-1210)	935.2 (924.8-945.5)	872 (861.8-882.1)	797.1 (788.3-806.7)	756.1 (747.1-765)	686.2 (677.3-694.8)	632 (623.9-640.5)	594.1 (586-601.8)	550.8 (482-625.4)
JPN Ishikawa	1668.9 (1386.7-1974.9)	1400 (1157.1-1677.2)	1392.9 (1147.4-1675.2)	1277.2 (1048.6-1538.9)	1153.7 (987.6-1353.1)	967.2 (884.9-1055.4)	813.3 (800.5-826.6)	749.9 (738.3-760.8)	715.4 (704.3-726.6)	689.9 (678.3-701.8)	634.3 (624.1-645.3)	585 (575.1-595.2)	544.7 (535.1-554.9)	497.6 (437.9-565.7)
JPN Iwate	1901.4 (1559-2241.7)	1548.9 (1261.8-1830.3)	1511 (1232-1806.7)	1405 (1151.7-1695.4)	1285 (1067.5-1522.8)	1111.9 (986.3-1243.7)	997.9 (983.6-1012.5)	881.9 (868.9-894.9)	804.7 (793.2-816.7)	751.3 (739.3-763.4)	688.5 (677.2-699)	666.2 (654-678)	618.9 (606-632.2)	526.3 (456.2-602.8)
JPN Kagawa	1724.8 (1417.8-2061.5)	1426.9 (1155.1-1704.3)	1403.3 (1136.9-1677)	1295.4 (1053.8-1542.2)	1172.9 (996.1-1363.7)	1013.4 (928.4-1106.6)	903.4 (888.9-918.6)	796.3 (784.1-808.7)	762.2 (750.6-773.7)	703.8 (692-715.8)	636.6 (625.3-647.8)	605.6 (595.8-617)	558.4 (548-569)	508.8 (447.4-579.2)
JPN Kagoshima	1948.9 (1646.2-2288.6)	1595.7 (1314.1-1901.9)	1558.4 (1267.1-1879.4)	1466 (1196.4-1787.8)	1362.8 (1133.3-1635.9)	1184.8 (1061.8-1320.9)	1008.5 (995.2-1021.8)	878.7 (866.7-891.7)	798.3 (786.1-810)	756.9 (745-768.3)	678.6 (668.2-689.1)	638 (628-648.8)	582.4 (571.4-592.9)	533 (463.5-604.3)
JPN Kanagawa	1784.4 (1480.2-2095.2)	1468.7 (1222-1762.3)	1427.1 (1171.4-1726)	1294.7 (1064.9-1563.6)	1168.8 (976.5-1402.6)	1001.9 (899.4-1107.3)	907.6 (900.5-914.9)	814.5 (808.3-820.8)	747.6 (741.8-753.6)	715.1 (709.4-721)	638 (632.6-643.6)	580.9 (575.9-585.9)	540.5 (535.3-545.7)	468.1 (409.5-531)
JPN Kōchi	1992.9 (1675.3-2354.1)	1667.4 (1390.2-1976)	1592.2 (1332.9-1908.4)	1481.3 (1230.3-1764)	1371.9 (1170.9-1593.9)	1181.7 (1084.8-1291.2)	993.3 (977.7-1009.6)	860.7 (848.2-873.2)	790.2 (777.3-804.8)	749.7 (736.9-763.5)	683.4 (671.5-695.5)	646.8 (635-658.6)	609.2 (596.7-622.3)	495.1 (432.4-567.7)
JPN Kumamoto	1885.8 (1566.2-2240.1)	1513.2 (1238.3-1841.1)	1470.4 (1218.1-1777)	1367.8 (1114.6-1642.4)	1267.7 (1048.7-1492.4)	1114.5 (986.6-1247)	940 (926.7-952.9)	812.8 (800.6-823.8)	748.8 (737.9-760)	685.7 (674.5-696.4)	616.2 (606.3-626)	572.4 (562.4-582.1)	528.8 (520-537.9)	489.1 (423.8-562.9)
JPN Kyōto	1688 (1392.1-1986.9)	1401.7 (1159.5-1678.2)	1352.3 (1097.6-1617)	1222.9 (1002.9-1522.1)	1089.6 (916.4-1310.3)	911.4 (805.9-1021.4)	810.8 (800.5-820.2)	756.4 (746-765.9)	722.9 (714-732.3)	692.2 (683.2-701.4)	643.9 (635-652.8)	588.3 (580.2-596.5)	535.3 (527.3-543.5)	450.9 (392.1-513)
JPN Mie	1765.8 (1492.8-2097.8)	1443.4 (1180.5-1737.6)	1434.6 (1177.2-1733.4)	1320.7 (1068.1-1602.3)	1199.4 (990.1-1430.3)	1032.2 (909.1-1159)	946.7 (932.2-961.2)	845.1 (833.8-857.4)	786.7 (775.4-798.4)	753.1 (741.2-764.5)	656.6 (645.9-667.5)	611.5 (602-621.8)	568.7 (558.9-579.1)	518.6 (455.8-598.8)
JPN Miyagi	1817.8 (1525.8-2160.1)	1492.4 (1251.8-1808.5)	1522.1 (1248.2-1853.8)	1418.7 (1170.1-1716)	1321.4 (1103.6-1575.2)	1173.8 (1057.5-1304.9)	1016.2 (1003.1-1029.6)	853.8 (842.7-864.4)	782.5 (772.6-793.3)	731.1 (721.1-741.2)	661.8 (652.9-670.7)	622.3 (613.8-631.3)	563.1 (553.3-572.6)	510.6 (446.2-580.1)
JPN Miyazaki	1907.1 (1562.4-2255.8)	1568.4 (1307.3-1871)	1505.2 (1240.6-1826.9)	1396.7 (1146.4-1685.1)	1269.6 (1054.6-1512.9)	1088.5 (959.9-1225.1)	969.8 (955.4-984.9)	841.8 (829.2-854.9)	780.9 (767.5-794.7)	749.7 (736.5-763)	660.2 (648.6-672.6)	603.8 (591.7-615.7)	566.9 (555.1-578.1)	528.7 (458.5-608.7)
JPN Nagano	1636.3 (1367.3-1960.1)	1345.7 (1106.3-1604.4)	1355.5 (1105.6-1632.2)	1263.1 (1035-1530.7)	1160.3 (963.1-1400.5)	1023.3 (916.5-1151.7)	845.1 (832.1-857.1)	792.3 (781.5-802.3)	698.8 (689.3-709)	658.4 (648.5-668.3)	593.8 (584.6-603)	557.3 (548.9-566.2)	511.6 (503.1-520.4)	477.5 (417.1-543.9)
JPN Nagasaki	1943.9 (1631.5-2302.4)	1605.9 (1325.9-1896.1)	1571.5 (1277-1895)	1470 (1197.1-1750.9)	1355.9 (1122.4-1620.8)	1181 (1049.9-1318.9)	1036.1 (1022.3-1050)	888.5 (875.9-900.7)	820.6 (807.3-834)	768.4 (756.5-780.3)	677.3 (666.4-689.1)	631.6 (621.1-642.4)	587.7 (576.6-599)	526.8 (460.3-600.3)
JPN Nara	1904.3 (1591.1-2255.2)	1547.2 (1281.4-1864.6)	1567.1 (1291.5-1875.2)	1446.4 (1188.4-1745)	1318.7 (1093.8-1584.2)	1144.2 (1010.4-1288.1)	982.2 (966.6-997.5)	879.8 (866-895.1)	790.2 (777.6-802.2)	724.4 (712.1-736.6)	635.5 (623.2-646.5)	603.9 (593.6-615.4)	546.8 (535.7-557.6)	494.7 (428.1-569.4)
JPN Niigata	1827.9 (1514.6-2160.3)	1493.9 (1234.8-1771)	1517.1 (1250.8-1825.6)	1427.3 (1167.8-1716.1)	1331 (1113.1-1600.4)	1181.7 (1059.2-1319.4)	998.2 (986.5-1010.6)	873 (861.7-883.5)	760.9 (750.2-770.4)	721 (710.5-731.4)	657.1 (648.2-665.9)	612.3 (603.3-621.5)	565.7 (556.8-575.2)	502.2 (437.9-572)
JPN Ōita	1833.3 (1532.4-2165.5)	1504.9 (1241.1-1805.2)	1452.2 (1184.1-1730.8)	1330.3 (1071.9-1599.1)	1188.6 (989.4-1407.6)	1011 (893.7-1137.6)	959 (944.3-973.3)	842.7 (829.4-856.2)	767.7 (754.5-781.1)	725.5 (712.8-738.6)	651.9 (639.9-664)	595.1 (584-607)	553.4 (541.9-564.9)	453.2 (395.2-521.7)
JPN Okayama	1742.4 (1447.3-2061.3)	1437.2 (1185.7-1713.1)	1402.4 (1160.5-1703.6)	1287.4 (1051.4-1540.6)	1162.5 (960.1-1383.3)	1002.4 (894.7-1121.4)	909.3 (896.2-921.5)	830.4 (818.6-842)	755.9 (744.8-767.2)	710.4 (700.6-720.5)	640.8 (630.5-650.5)	589.3 (579.5-599.2)	554.1 (544.9-563.3)	499.2 (432.8-572.9)
JPN Okinawa	1932.3 (1582.5-2276.6)	1589.3 (1311.1-1915.9)	1525.6 (1237-1826.7)	1474.2 (1192.9-1801.9)	1470.8 (1220.2-1763)	1374.5 (1231.6-1537.9)	941.3 (927.4-955.3)	743 (729.7-756.3)	711.6 (699.7-722.8)	672.5 (661.5-683.7)	626.2 (616.1-636.4)	583.9 (574.3-593.7)	567.7 (558.1-576.9)	506.1 (438.5-576.9)
JPN Ōsaka	2030.8 (1711.2-2376.1)	1654.3 (1386.1-1944.7)	1581.3 (1325.6-1860.5)	1427.4 (1191.1-1702.3)	1280.6 (1081.7-1495.8)	1097.4 (985.3-1210)	1021.5 (1014.1-1028.5)	928.5 (922.2-935.1)	860 (853.9-866.1)	802.1 (795.9-808.4)	708.4 (702.6-714.1)	645.3 (639.8-650.8)	602.3 (596.8-607.4)	540.2 (475.7-613)
JPN Saga	1845.1 (1531.1-2174.3)	1510.3 (1242.9-1799.4)	1480.8 (1222.8-1789)	1378.7 (1152.1-1638.2)	1253.5 (1070.3-1435.5)	1079.1 (988-1173.6)	962.9 (947.6-979.3)	864.1 (851.4-877.8)	795.7 (782.2-808.4)	745.8 (732.2-759.1)	681.8 (669.3-693.1)	624.7 (613.2-636.1)	579.7 (568.1-591.1)	509.7 (446.5-579.9)
JPN Saitama	1787.5 (1491.9-2097.1)	1471.2 (1234-1746.8)	1470.5 (1217.1-1766.3)	1316.8 (1081.6-1593.7)	1152 (960-1387.1)	964.9 (864.6-1075.1)	936.6 (928.3-945.2)	850.8 (843.3-858.5)	775.9 (769.3-783.1)	731.8 (725-738.9)	645.5 (639.6-651.7)	600.1 (594.2-606.1)	561.5 (556.2-567.1)	509.2 (451.2-575.6)
JPN Shiga	1694.8 (1383.1-2051.8)	1405.3 (1157.8-1679.4)	1449.8 (1187.1-1726.8)	1366.6 (1121.9-1633.5)	1256 (1061.8-1460)	1056.1 (963.5-1145)	837.9 (823.9-851.9)	768.1 (756.8-780.2)	769.4 (757.5-781.5)	723.9 (713.4-735.5)	647.6 (637.5-657.7)	593.5 (584-602.9)	526.9 (518-535.9)	470.9 (415-532)
JPN Shimane	1789.8 (1454.6-2119.6)	1482.1 (1218-1749.6)	1427.8 (1166.8-1695.9)	1324.2 (1097.3-1576.8)	1213.7 (1027.8-1404.2)	1051.5 (954.2-1155.1)	905.6 (888.5-921.1)	783.6 (770.9-796)	735.8 (722.5-748.9)	692.1 (679.3-705.4)	638.2 (626-650.7)	606.9 (596-619.5)	546.7 (535.1-558.4)	474.3 (412.6-539.2)
JPN Shizuoka	1760.9 (1457.7-2076.2)	1445.2 (1209.8-1725.8)	1423.2 (1177-1720)	1303.4 (1070.6-1560.7)	1182.3 (998-1404.1)	1026.6 (922-1134.2)	924.9 (915.6-934.5)	816 (807.2-824.5)	744.8 (736.6-752.8)	707.5 (699.1-715.6)	633.6 (625.6-641)	582.4 (575.2-589.6)	549.6 (542.3-556.8)	512.9 (448.6-586.1)
JPN Tochigi	1817 (1502.2-2166.8)	1514 (1248.9-1806.6)	1504.3 (1246.4-1816.5)	1390.8 (1137.9-1686.1)	1262.6 (1048.8-1503.3)	1077.8 (960.4-1214.8)	944.4 (933.2-957)	883.6 (871.8-895.6)	816.4 (804.8-827.5)	771.2 (760.2-782.8)	674.5 (664.4-684.9)	653.1 (643.8-663.2)	596.5 (587-605.5)	528.9 (461.2-604.6)
JPN Tokushima	1895.3 (1589.6-2234.6)	1573.7 (1297.7-1881.7)	1540.7 (1259.2-1845.6)	1428.3 (1176.3-1714.3)	1305.8 (1112.8-1517.6)	1125.8 (1031.1-1233.9)	990.2 (973.6-1007.2)	873.8 (861.3-888.1)	802.3 (789.1-815.9)	752.1 (738.7-764.8)	676.7 (665.1-688.6)	620.3 (609.1-632.4)	570.7 (558.1-582.4)	524.1 (456.8-598.3)
JPN Tōkyō	1751.7 (1467-2068.8)	1433.6 (1197.6-1714.2)	1392.4 (1144.4-1647.1)	1275.1 (1064.9-1500.4)	1156.4 (963.9-1349.8)	1004.3 (911.2-1106)	899.6 (893.5-905.4)	809.9 (804.4-815)	776.9 (771.8-782)	736.6 (731.4-741.6)	653.6 (648.8-658.3)	587.2 (582.6-592)	551.5 (547.3-555.9)	494.9 (436.2-560)
JPN Tottori	1869.4 (1558.3-2214.2)	1531 (1272.9-1819.4)	1466.9 (1233.4-1744.8)	1345.2 (1137-1577)	1223.3 (1072.3-1392.2)	1072 (997.2-1153.9)	974.7 (956.9-993.2)	862.7 (848.4-876.9)	801.2 (787.9-814.9)	764.4 (752.4-777.5)	672.9 (660.4-686)	640.4 (628.9-652.4)	580.7 (567.9-592.8)	525.1 (462.1-593.5)
JPN Toyama	1837 (1527.8-2179)	1524.5 (1254.9-1815.6)	1529.1 (1264.1-1818)	1414.2 (1172.8-1681.5)	1290.3 (1101.6-1505.9)	1098 (1005.4-1199.2)	963.3 (948.1-978.1)	864.6 (851.7-876.5)	777.9 (766.9-790.1)	719.1 (707.9-731)	636.4 (626.2-647)	595 (584.9-605.6)	564.6 (553.9-575)	519.5 (456.1-586.8)
JPN Wakayama	1949.6 (1642.3-2319.1)	1596.7 (1323.4-1889.4)	1540 (1286.2-1828.6)	1409 (1175.6-1674.1)	1282.4 (1104.6-1484.4)	1119 (1024.2-1218.8)	997.3 (983-1012.4)	911 (898.1-923.2)	835.6 (822.9-848.5)	779.8 (767.3-791.7)	682.5 (670.6-693.3)	642.2 (631.1-653.7)	621.3 (609.8-633.2)	545.6 (479.9-617.3)
JPN Yamagata	1718.7 (1403.7-2073.9)	1421.1 (1136-1713.4)	1406.4 (1135.1-1724.5)	1294.4 (1046.7-1597)	1171.2 (966.1-1397.1)	991.3 (880.2-1116.1)	871.5 (857.6-886)	768.4 (756.2-780.8)	716.7 (703.6-729.8)	691.2 (678.2-704.3)	631.9 (619.4-644.3)	609.7 (597.8-621.1)	544.2 (533.3-555)	505.7 (437.4-578.7)
JPN Yamaguchi	1931.8 (1624.1-2271.6)	1590.2 (1313.7-1883.9)	1532.7 (1278.6-1832.9)	1412.7 (1148.7-1675.6)	1284.7 (1060-1540)	1116.7 (1000.2-1241.4)	1022.3 (1008.1-1035.8)	862.1 (849.8-874.6)	795.6 (783.4-808.2)	754.6 (742.9-766.8)	687.8 (675.6-697.9)	646 (633.7-657.4)	596.5 (585.5-607.5)	523.5 (455-599.9)
JPN Yamanashi	1804.1 (1489.6-2136.8)	1493.2 (1220.7-1789.4)	1455.2 (1194.5-1750.3)	1363.8 (1133.6-1636.4)	1255.8 (1079.1-1465)	1077.2 (981.3-1184.6)	953.9 (937.2-969.4)	832.9 (819-846.6)	756.9 (744.8-769.2)	718.4 (706.6-730.6)	638.6 (628.1-649.8)	598 (587-608.9)	552.7 (542.4-564.6)	495.9 (433.2-566.6)
KOR South Korea	4124.7 (3766.4-4451.1)	2503 (2182.9-2839.2)	2276.6 (1981.9-2613.1)	2145.9 (1854.3-2453.6)	1980.2 (1728.8-2227.8)	1818.8 (1741.7-1889.6)	1771.1 (1763.3-1779.4)	1595 (1589.9-1600.7)	1376.3 (1372.4-1380.4)	1170 (1166.6-1173.6)	1023.2 (1019.9-1026.3)	825.5 (822.7-828.3)	700.5 (690.2-710.7)	588.9 (548.3-632.7)
SGP Singapore	2324.4 (2145.3-2508.1)	1915.6 (1893.2-1938.4)	1787.5 (1765.1-1810.6)	1623.3 (1603.7-1642.3)	1612.3 (1592.5-1632.3)	1544.9 (1526.5-1561.3)	1312.3 (1297.3-1328.3)	1068.8 (1056-1081.9)	915.8 (903.9-927.8)	881.3 (869.4-893.4)	746.1 (692.6-809.5)	643.9 (635.3-652.4)	537.5 (530.1-545.8)	448.2 (419.6-478.7)
High-income North America	1371.8 (1330.2-1419.5)	1279.5 (1242.8-1317.8)	1302.7 (1300.7-1304.6)	1296.8 (1295.1-1298.6)	1269.9 (1268.2-1271.6)	1160.9 (1159.4-1162.5)	1089.2 (1087.8-1090.6)	1021.6 (1020.1-1023)	941.7 (940.3-943.1)	906.2 (905-907.4)	826.2 (825.1-827.5)	765.5 (764.3-766.5)	690.9 (689.9-691.9)	688.4 (672.5-704.9)
CAN Canada	1286.5 (1278.5-1294.7)	1210.6 (1204.1-1217.7)	1194.9 (1188.3-1201.3)	1173.7 (1167.3-1179.9)	1133.7 (1128-1139.4)	1109.7 (1104.7-1115.2)	1008.6 (1003.8-1012.9)	946.1 (941.1-950.6)	876.1 (871.5-880.6)	830.4 (826.1-835)	736.1 (732.1-740.3)	658.5 (654.7-662.3)	584.9 (581.3-588.2)	543.4 (512.5-575.7)
GRL Greenland	2726.2 (2600.5-2860.2)	2354.4 (2289.5-2414.4)	1890.2 (1843.4-1939.9)	1623.8 (1576-1671.8)	1480.6 (1438-1521.5)	1671.8 (1632.4-1713.7)	1824 (1780.3-1868.4)	1888.6 (1847.4-1930.6)	1720.3 (1669.1-1768.5)	1509.7 (1464.7-1552.7)	1342 (1302.5-1381.6)	1207.5 (1179.2-1241)	1106.4 (1082.5-1133)	1061.2 (996.2-1089)

Table 1 Age-standardised mortality rates by location and year for males for 1950, 1955...2005, 2010, 2017

Location	1950	1955	1960	1965	1970	1975	1980	1985	1990	1995	2000	2005	2010	2017
USA USA	1379.1 (1333.5-1431.4)	1285.8 (1245.7-1327.7)	1312.8 (1310.8-1314.8)	1308.6 (1306.7-1310.4)	1283.1 (1281.4-1284.9)	1166.2 (1164.5-1167.8)	1097.4 (1095.8-1099)	1029.6 (1028.1-1031)	949.2 (947.8-950.5)	915.3 (914-916.6)	836.8 (835.5-838)	777.9 (776.8-779.1)	703.4 (702.4-704.5)	705.7 (688-723.7)
USA Alabama	1504.3 (1234.5-1780.8)	1381.1 (1169.7-1598.1)	1398.2 (1384.4-1412.7)	1432.8 (1419.7-1446.1)	1380 (1366.5-1393.3)	1271.7 (1260.1-1283.5)	1201.4 (1190.5-1213.1)	1134.5 (1122.9-1146.5)	1081.5 (1070.4-1092.3)	1060.1 (1049.3-1070.8)	1011.2 (1000.4-1022)	967.3 (957.5-977.5)	910.9 (901.5-919.9)	930.8 (817.9-1049.3)
USA Alaska	1462.6 (1249.3-1695.6)	1251.8 (1113.6-1401.6)	1369.4 (1340.5-1396.9)	1486.8 (1458.4-1515.4)	1442.4 (1416.8-1468.5)	1179.3 (1158.6-1199.9)	1122.4 (1100.5-1143)	1109.8 (1090.2-1128.5)	1008.1 (989.4-1027.2)	943.8 (926.3-960.5)	833.7 (817.4-848.5)	739.8 (724.8-754)	697.2 (684.5-709.9)	701.1 (639.3-774.1)
USA Arizona	1427.2 (1194.8-1683.8)	1288.3 (1090.8-1509.1)	1312.4 (1292.5-1332.5)	1269.4 (1252-1288.6)	1262.3 (1246.2-1279.1)	1100.8 (1085.7-1116)	1039.8 (1027.6-1052.2)	921.5 (910.4-933.2)	865.6 (855.6-875.6)	856.9 (846.9-866.2)	780.3 (772.3-788.6)	738.4 (730.7-746.3)	646.9 (640.2-653.9)	661.9 (581.6-756.2)
USA Arkansas	1319.1 (1098.6-1568.5)	1217.1 (1034.8-1430.1)	1227.9 (1210.5-1246.2)	1277.2 (1259.6-1295.1)	1255.5 (1240.2-1271.6)	1202.4 (1186.3-1218.3)	1111.2 (1096.8-1125.8)	1074.6 (1059.8-1089.6)	1018.7 (1004.8-1033)	1014.6 (1000.9-1027.9)	966 (953.5-979)	899.7 (888.3-912.3)	854.2 (842.7-865.2)	882.7 (767.2-1010.1)
USA California	1306.6 (1088.1-1526.1)	1207.7 (1021.8-1396.2)	1260.3 (1253.9-1267)	1250.4 (1244.3-1256.8)	1203.6 (1198.2-1209.2)	1091.1 (1086.1-1096.1)	1038.1 (1033.2-1042.8)	970.7 (966.3-975.2)	898.1 (894.2-901.9)	837.5 (833.8-841.1)	743.4 (739.9-747)	694.9 (691.7-698.1)	597.6 (594.5-600.6)	590.8 (526.9-657.7)
USA Colorado	1336.2 (1098.6-1596.6)	1241.5 (1048.2-1469.4)	1197 (1179.7-1213.4)	1183 (1168.3-1198.1)	1162.4 (1147.6-1177.7)	1058.3 (1044.6-1072.3)	990.7 (978.4-1003)	921.9 (909.7-934.4)	840.8 (828.7-852.1)	825.4 (814.5-835.2)	748.8 (739.2-758.8)	702.4 (694-711.1)	634.8 (627.3-642.9)	634.1 (556.2-715.8)
USA Connecticut	1332 (1097.4-1612.4)	1218.5 (1026.5-1430.7)	1262.1 (1246.8-1277.7)	1245.2 (1230.6-1260.6)	1172.4 (1157.1-1186)	1080.2 (1066.4-1093.4)	1044.6 (1031.2-1056.5)	960.8 (948.2-972.8)	840.1 (828.4-850.7)	833.5 (822.7-844.8)	762.1 (751.4-772.5)	688.7 (679.5-698.1)	618.3 (609.7-627.1)	619.1 (539.5-706.9)
USA Delaware	1539.8 (1301.7-1827.8)	1350.3 (1187.5-1519.3)	1374.4 (1349.2-1399.8)	1401.5 (1378.6-1425)	1384.5 (1360.7-1406.8)	1255.3 (1236.7-1276)	1174.2 (1154.6-1194.4)	1087.8 (1068.7-1107.7)	1021.9 (1003.1-1041.4)	993.7 (975.9-1013)	872.1 (856.7-887)	813.6 (800.1-827.7)	730.7 (717.7-744.2)	719.2 (653.2-790.1)
USA Washington, DC	1604.9 (1344.3-1905.6)	1506.5 (1280.3-1735.9)	1561.1 (1534.6-1586)	1629.9 (1604.6-1655.1)	1634.8 (1611.5-1659.3)	1472.1 (1447.9-1495.9)	1422 (1396.9-1447.4)	1394.7 (1371.5-1419.5)	1456.4 (1432.2-1481.1)	1396 (1373.1-1419.7)	1118.1 (1095.9-1140.5)	1018.4 (996.2-1041.2)	809.1 (791.3-829.2)	784.3 (682.7-899.6)
USA Florida	1336.7 (1119.5-1594.1)	1225.6 (1037.9-1443.1)	1241.7 (1231.1-1252.5)	1204.2 (1194.4-1213.7)	1216.7 (1207.3-1226.1)	1091.6 (1083.7-1098.9)	1035.6 (1028.8-1042.9)	934.5 (927.9-941.3)	876.5 (870.9-882.1)	851.9 (846.5-857.1)	778.2 (772.9-783.1)	732 (727.6-736.3)	655.5 (651.4-659.7)	667.4 (583.3-749.4)
USA Georgia	1552.2 (1293.1-1846.5)	1428.7 (1209.3-1662.7)	1457 (1444.1-1469.9)	1413.2 (1401.4-1425.5)	1421 (1408.9-1432.5)	1358.5 (1347-1370)	1219.7 (1209.4-1230.1)	1175.8 (1165.8-1185.6)	1084.9 (1076.1-1094)	1045.6 (1037.1-1054.2)	979.6 (971.9-988)	899.2 (892.3-906.3)	825.6 (818.9-832.1)	796.8 (700.8-910)
USA Hawaii	1114.9 (904.3-1334.8)	961.3 (790.2-1141.3)	878 (856.9-898.8)	872.6 (854.9-891.8)	917.3 (897.6-936.9)	848.1 (830-867.5)	818.8 (802.6-835.5)	760.1 (743.6-776.9)	731.2 (716.5-747)	714.4 (699.5-728.1)	652.1 (637.1-666.7)	628.7 (615.6-642.4)	588.6 (576-601.4)	572.2 (495.3-656.6)
USA Idaho	1281.9 (1046.8-1537.8)	1168.2 (981.7-1386.5)	1210.8 (1186-1235.3)	1217.8 (1192.4-1242.8)	1199.7 (1176.9-1223.4)	1090.7 (1066.8-1112.5)	980.3 (959.6-1001)	909.6 (891.3-928.5)	848.6 (829.1-867.7)	821.5 (803.1-839.5)	758.9 (743.1-775.4)	727.9 (713.7-742.5)	687.1 (673.8-700.5)	690.3 (595.4-797.5)
USA Illinois	1392.5 (1155.4-1658.2)	1315.8 (1107.9-1531)	1356.2 (1348.2-1364.8)	1360.5 (1351.9-1369)	1346.7 (1338.9-1354.4)	1231.6 (1224.5-1238.8)	1138.1 (1131.1-1145.5)	1048 (1041-1055.2)	986.2 (979.4-992.7)	958.4 (951.5-964.7)	856.9 (850.6-863.2)	779.3 (773.1-785.3)	699.6 (694.2-704.9)	682 (601.9-770.6)
USA Indiana	1353.1 (1115.7-1630.9)	1263.6 (1069.4-1483.4)	1303.9 (1291.5-1315.8)	1320.1 (1308.6-1331.8)	1286.5 (1274.9-1297.5)	1176.9 (1166.5-1187.3)	1120.3 (1110.3-1130.8)	1049.5 (1038.9-1060)	977.4 (966.9-988)	960.7 (951.9-969.7)	903.4 (894.5-912.5)	837.7 (829.1-845.9)	784.5 (776.8-792.1)	808.9 (710.3-916.2)
USA Iowa	1248.8 (1025.7-1508.1)	1195 (1003.5-1402)	1175.1 (1160.9-1190)	1201.1 (1186.4-1215.6)	1170.6 (1157.3-1185.5)	1081.8 (1068.7-1096.1)	991.1 (978.2-1004.6)	933.9 (919.9-946.6)	841.1 (828.3-855)	827.2 (814.6-839.1)	761.5 (750.2-773.9)	705.5 (695.1-716.6)	675.1 (665-684.9)	667.3 (583.2-759.5)
USA Kansas	1224.6 (998.4-1474.8)	1126.1 (954.7-1332.7)	1173.8 (1157.6-1188.6)	1160.3 (1144.4-1176.8)	1145.1 (1129.6-1159.6)	1063.7 (1049.8-1078.4)	1007.4 (992.7-1021.9)	935.1 (921.6-949.4)	871.7 (858.7-884.8)	861.1 (848.8-873.9)	808.1 (796-819.8)	762.1 (751.3-773)	728.3 (717.6-738.5)	713.1 (622.5-815)
USA Kentucky	1385.3 (1141.4-1656)	1268.2 (1075.4-1494.6)	1315.6 (1301.4-1329.5)	1319.2 (1305-1333.9)	1361.8 (1348.3-1374.7)	1256 (1242.8-1268.6)	1172.7 (1160.1-1184.7)	1115.3 (1103.2-1127.2)	1045.3 (1033.7-1057.5)	1033.1 (1021.2-1044.8)	980.9 (969.9-991.7)	929.7 (919.9-939.6)	880 (870.7-889.3)	924.1 (814.1-1044.3)
USA Louisiana	1472.9 (1221.9-1726.4)	1364.9 (1160.5-1592.6)	1379.2 (1365.1-1393.2)	1395.9 (1382.6-1409.6)	1382.9 (1370-1396.2)	1298.4 (1286.8-1310.1)	1220 (1208.1-1231.6)	1147.8 (1135.7-1158.5)	1088.7 (1078.5-1099.4)	1061.2 (1051-1071.6)	1006.5 (996.2-1016.4)	1048.6 (1039.1-1058.4)	867.9 (858.7-876.8)	888.5 (778.5-1015)
USA Maine	1437.3 (1187.4-1725.7)	1331.1 (1114-1567.6)	1328.8 (1303.8-1352.9)	1278.3 (1256.6-1301.6)	1301.2 (1277.9-1324.2)	1185.8 (1164.9-1205.6)	1101.4 (1080.8-1121.7)	1039.3 (1019.9-1059.8)	916.4 (898.3-934.7)	879.8 (862-898.1)	823.1 (806.6-840.3)	798 (782.4-814.2)	713.6 (698.7-729.2)	769.6 (666.5-893.5)
USA Maryland	1500.7 (1257.1-1784)	1393.4 (1184.9-1627.9)	1428.3 (1413.4-1443.8)	1413.8 (1399.7-1427.6)	1343.3 (1331.3-1355.1)	1223.5 (1212.3-1235.3)	1148.8 (1136.9-1160)	1096.9 (1086.2-1107.4)	997.9 (987.8-1008.3)	979 (969.5-988.8)	881.5 (872.2-890.5)	804 (795.7-812.1)	691.7 (684.1-699.8)	715.4 (623.9-818.3)
USA Massachusetts	1359.4 (1125.1-1624.6)	1255.7 (1063.9-1475.5)	1339.7 (1328.2-1352.1)	1308.7 (1297.8-1319.8)	1261.8 (1250.3-1273.1)	1149.7 (1139-1160.2)	1056.3 (1046.5-1066)	1006.9 (997.5-1016.1)	881.4 (872-890.1)	841.2 (832.9-849.6)	775.5 (767.8-783.9)	700.5 (693.6-708.2)	638.9 (632.1-646.2)	655.4 (578.6-750.4)
USA Michigan	1351.8 (1126.7-1606)	1255.4 (1052.4-1455.1)	1309.2 (1299-1318.7)	1336.4 (1327.1-1346.1)	1305.2 (1296.4-1314.1)	1201.9 (1193.8-1210.4)	1116 (1108.4-1123.8)	1079.1 (1070.9-1087.1)	967.6 (959.9-974.7)	931.3 (924-938.7)	865.9 (859.5-872.6)	800.7 (794.1-807)	746.8 (741-752.7)	756 (665.8-858.7)
USA Minnesota	1258.4 (1021.3-1521.7)	1177.2 (987.9-1395.5)	1166.4 (1152.7-1180.6)	1177.3 (1164.3-1191.3)	1139.1 (1126.8-1152.6)	1032.8 (1022-1045.6)	958.9 (947.5-970.9)	903.3 (892.3-913.8)	829.2 (819.8-839.7)	814.2 (803.9-823.9)	734.2 (725.2-743.7)	664.1 (654.9-672.5)	614.5 (606.2-622.6)	611.9 (538.3-695.4)
USA Mississippi	1481.3 (1238.1-1788.7)	1378.7 (1171.2-1602.1)	1356.1 (1339.1-1373)	1395.8 (1379.7-1412.8)	1415.8 (1400.1-1433.2)	1291.1 (1276.4-1306.1)	1210.7 (1195.9-1225)	1163.1 (1148.6-1178.7)	1099 (1084.8-1113.2)	1101.8 (1089.1-1115.6)	1048.4 (1035.6-1061.2)	1022.4 (1011.2-1033.7)	932.4 (920.7-943.7)	964.7 (851.6-1088.7)
USA Missouri	1364.2 (1124.7-1622.8)	1261.4 (1069.3-1483.5)	1285 (1272.8-1297.5)	1303.3 (1290.3-1316)	1295.2 (1283.5-1306.9)	1175.7 (1165.2-1187.1)	1105.3 (1095-1115.6)	1045.9 (1035-1056.9)	971.5 (962-981.7)	968.5 (958.3-977.9)	889.9 (880.5-899.2)	841.3 (833.2-849.5)	777.3 (769.6-785.3)	786.8 (694.5-893.3)
USA Montana	1445.1 (1194.8-1720.7)	1331.4 (1126.1-1576.6)	1323.4 (1297-1350.1)	1353.4 (1327-1380.3)	1262.4 (1235.2-1287.5)	1151.6 (1128-1174.4)	1062.7 (1041.5-1084.4)	987.6 (966.9-1008.2)	872 (851.2-892.6)	885 (864.2-905)	819.1 (800.9-837.4)	764.5 (748.8-780.6)	700 (683.4-715.3)	780.3 (680.9-887.9)
USA Nebraska	1233.8 (1014.1-1492.4)	1144.3 (964.9-1356.8)	1165.3 (1146.1-1186)	1195.7 (1175.9-1213.3)	1171.4 (1153.1-1190.1)	1073.7 (1057.3-1092.3)	1001.5 (983.2-1017.9)	945.9 (928.5-963.7)	887.7 (871.1-903.3)	834.7 (818.8-850.5)	763.6 (749.6-777.9)	715.6 (702.6-728.7)	675.4 (661.9-687.9)	677 (594.5-775.2)
USA Nevada	1823.2 (1537.3-2149.8)	1521.6 (1289.9-1776.9)	1496.6 (1459.9-1533.2)	1533.6 (1500.2-1567.7)	1388.5 (1359-1417.2)	1235.1 (1210.1-1260.6)	1194.6 (1172.5-1217.7)	1078.6 (1058.8-1098.7)	1054.4 (1035.5-1072.9)	1029.6 (1014.1-1045.5)	925.2 (911.7-939.7)	904.7 (891.8-916.9)	747.8 (737.1-758.4)	728.9 (632.3-836.8)
USA New Hampshire	1487.5 (1227.5-1800.1)	1348.2 (1122.2-1568.5)	1324 (1295-1352.8)	1326.5 (1299.3-1354.3)	1288.7 (1262.6-1315)	1190.9 (1166.7-1216)	1032.2 (1010.7-1053.5)	1009.7 (990.2-1030.6)	901.1 (882.3-920)	852.3 (834.8-870)	786.8 (769.6-803.5)	741.9 (725.1-758.5)	664.7 (649.9-679.7)	683.8 (591.1-783.9)
USA New Jersey	1403.5 (1167.6-1655.2)	1324.3 (1123.8-1552.4)	1341.1 (1330.6-1352.7)	1330.9 (1321-1341.4)	1293.8 (1284.5-1303)	1151.2 (1142.3-1160.1)	1104.5 (1095.5-1113.5)	1067.9 (1059.9-1076.6)	939.5 (931.7-947.2)	900.5 (892.9-908.1)	814 (806.8-821.5)	725 (718.1-732.4)	647.1 (640.7-653.2)	627.2 (547.7-710.7)
USA New Mexico	1359.7 (1127.1-1623)	1245.2 (1045.4-1457.3)	1217.3 (1195.3-1241.2)	1265.4 (1243-1287.5)	1224.9 (1203.6-1246.7)	1153.2 (1134-1173.7)	1059.8 (1043.9-1077.1)	963 (946.6-980.4)	888 (872.8-902.8)	876.1 (862.1-891.1)	797.8 (784-810.4)	761.6 (749.7-774.3)	710.8 (699.4-721.9)	742 (640.5-847.8)
USA New York	1379.9 (1145.7-1642.1)	1317 (1134.3-1522.4)	1364.3 (1357.4-1371.1)	1352.3 (1346.4-1358.8)	1333.4 (1326.9-1339.5)	1173.8 (1167.7-1180.1)	1122.8 (1117-1128.8)	1064.1 (1058.5-1069.5)	978.2 (972.8-983.3)	912.6 (907.2-917.6)	777.1 (772.3-782)	699.4 (694.9-703.7)	623.6 (619.1-627.8)	600.9 (525.6-681.5)
USA North Carolina	1495 (1221.2-1766.5)	1373.9 (1162.3-1603.6)	1421.8 (1409.6-1433.6)	1432.7 (1421.6-1444.3)	1404.1 (1393.7-1414.5)	1274.9 (1264.6-1285.1)	1184.1 (1174.4-1193.7)	1103.2 (1094-1113.4)	1033.6 (1024.5-1042.5)	1001 (992.8-1009.6)	940.4 (932.4-947.8)	849.8 (843.1-856.3)	775.4 (768.4-781.7)	755.5 (663.7-857.3)
USA North Dakota	1220 (1027.4-1446)	1116.5 (985.6-1254.4)	1148.1 (1127.6-1170.8)	1199.3 (1179.6-1219.4)	1168.4 (1149.7-1189.3)	1063.6 (1044-1082.8)	971.8 (954.3-988.8)	914.3 (895.7-931.1)	849.9 (833.1-867.2)	832.2 (815-849.3)	766.4 (749.6-783)	701.2 (686.7-716)	670.4 (655.8-684.9)	709.3 (644.3-775.8)
USA Ohio	1377.7 (1139.8-1629.3)	1292.5 (1107.5-1482)	1310.6 (1302-1319)	1329 (1320.8-1337.5)	1309.7 (1301.6-1317.5)	1194.3 (1186.3-1202.5)	1147.2 (1139.9-1155.1)	1059 (1051.1-1066.7)	976.6 (969.4-983.7)	959 (951.3-966.1)	886.6 (879.4-894.2)	844.8 (838.4-851.5)	771.4 (765.5-777.2)	818.2 (709.2-923.8)
USA Oklahoma	1293.4 (1059.9-1552.7)	1192.4 (995.6-1405.5)	1240.2 (1224.1-1256.3)	1225.8 (1211-1240.7)	1267 (1252.3-1282.4)	1170.4 (1156.9-1183.9)	1111.5 (1099.7-1123.7)	1053.8 (1041.3-1066.1)	957.2 (945.2-969.4)	985.4 (973.6-998)	945.3 (934.2-956.2)	924.3 (913-935.1)	871.6 (861.8-882)	882.3 (771.5-998.6)
USA Oregon	1339.6 (1100.2-1599.7)	1247.2 (1026.7-1479.2)	1250.3 (1232.2-1268.8)	1248.6 (1232.6-1266.2)	1188.2 (1172.1-1204.6)	1067.5 (1052.2-1082.2)	1032.6 (1020.6-1045.4)	961 (948-974.9)	896.8 (885.2-909.2)	873 (861-885)	778.2 (767.4-789.2)	744.2 (734.6-753.9)	677.6 (668.9-686.5)	678.8 (591.9-774.4)
USA Pennsylvania	1449.9 (1204.1-1705.8)	1373.5 (1168.2-1578)	1379.8 (1371.7-1388)	1381.5 (1372.9-1390)	1337.8 (1329.6-1346.2)	1209.8 (1202.2-1217.5)	1143.4 (1136.3-1150.9)	1069.9 (1062.6-1077.2)	964.2 (957.7-971)	938.7 (931.9-945.5)	867.4 (861.2-873.6)	809.5 (803.9-815.5)	731.5 (725.7-737.4)	749.3 (659.4-850.1)
USA Rhode Island	1409.8 (1171.8-1671.1)	1304.3 (1092.2-1522.1)	1317.9 (1291-1343.6)	1352.9 (1328.6-1379.2)	1270.4 (1248-1292.9)	1116.3 (1095.2-1137.6)	1093.6 (1073-1114.3)	1007.6 (987-1028.2)	908.6 (890.6-927.3)	827 (809.6-845.4)	789.4 (772-806)	748.8 (732.2-766.1)	690.4 (675-705.8)	710.3 (615.8-824.2)
USA South Carolina	1676.6 (1386.8-1979.4)	1525 (1287.2-1786.2)	1631.7 (1616-1648.1)	1507.4 (1491.8-1522.8)	1462 (1446.4-1477.3)	1341.3 (1328-1354)	1237 (1224.4-1249.8)	1152.1 (1139.6-1164.5)	1066.1 (1054.9-1077.4)	1042 (1030.7-1053.2)	988.8 (978.4-998.4)	909.7 (900-919.1)	838 (828.7-847)	861.1 (749.1-975.4)
USA South Dakota	1288.8 (1075.6-1522.2)	1172.9 (1034.7-1325.1)	1210.3 (1189.5-1231.5)	1194.8 (1175.8-1215.8)	1187.6 (1167.4-1207.6)	1087 (1067.8-1105.5)	1025.6 (1008.4-1043.4)	956.1 (939.3-973)	873.4 (856.3-890.9)	854.8 (839-871.3)	781.3 (766.2-796.9)	737.2 (724.1-751.5)	679.7 (666.8-693.2)	721.7 (653.2-792.2)
USA Tennessee	1372.1 (1133.4-1631.6)	1274 (1069.8-1489.6)	1317.1 (1303-1331.1)	1307.1 (1294.5-1319.9)	1341.8 (1329.1-1354.8)	1216.3 (1204.2-1227.3)	1155.1 (1143.9-1165.8)	1096 (1084.9-1106.6)	1067.9 (1057.2-1079)	1056.3 (1046.7-1065.5)	991.6 (982.9-1001)	932.2 (923.6-940.2)	863.1 (855.4-871.4)	842.4 (741.9-956.2)
USA Texas	1315.8 (1099.4-1584.2)	1240.1 (1039.5-1441.1)	1245.3 (1236.2-1253.3)	1228.9 (1220.9-1236.9)	1237.1 (1229.4-1244.8)	1142.1 (1135.3-1149.3)	1090.7 (1084.5-1097)	1049.2 (1043.3-1055.3)	977.1 (971.7-982.4)	925.1 (919.4-930)	860 (855.1-864.8)	790 (786.1-794.4)	723.4 (719.6-727.2)	707.5 (621.7-797.5)
USA Utah	1254.5 (1030.9-1502.6)	1114.9 (932.5-1337.9)	1193.5 (1170.8-1218.7)	1202.8 (1181.3-1225.5)	1132.6 (1112.1-1153.8)	1027.1 (1008.5-1046)	939 (923-955.3)	896.7 (879.7-914.4)	795.6 (781.1-811.5)	779.3 (765.7-794.1)	723.3 (711.6-735.7)	676.5 (665.6-687.4)	637.2 (627.2-647.4)	648 (567.9-738)
USA Vermont	1492.4 (1292.4-1699.8)	1320.2 (1206.8-1440.8)	1341.7 (1316.2-1367.3)	1340.9 (1319.7-1361.5)	1270.8 (1249.8-1291.2)	1169.5 (1150.5-1188.6)	1101.3 (1082.8-1119.2)	1030.8 (1012.7-1050.1)	924.3 (907.3-941.7)	868.7 (853.9-884.5)	789.2 (775-803.2)	706.4 (694.1-720.1)	654.6 (641.6-667.7)	666.5 (614.4-722.5)
USA Virginia	1480.4 (1225.7-1757.3)	1386.5 (1171.8-1620.3)	1379.4 (1365.8-1393.2)	1392.3 (1379.9-1403.9)	1331.9 (1320.8-1342.8)	1227.7 (1217.5-1238.5)	1157.8 (1147.3-1168.1)	1083.3 (1074.1-1092.3)	996.7 (987.5-1006.3)	958.6 (950-967.6)	872.7 (864.3-881.3)	795.4 (787.7-803.2)	707.7 (700.4-714.4)	690.5 (605.5-779)
USA Washington	1336.4 (1101.7-1622.6)	1272.6 (1080.4-1500.3)	1250.6 (1235.4-1264.4)	1206.4 (1192.2-1220.3)	1210.5 (1197.5-1223.1)	1073.1 (1061.7-1084.4)	1001.4 (990.5-1012)	940.4 (929.8-951)	868.4 (858.7-878.1)	824.8 (815.5-833.2)	769.5 (761-778.5)	705.4 (698.2-713.2)	648 (640.7-655)	637.8 (556.7-722.9)
USA West Virginia	1447.7 (1195.4-1729.5)	1334.1 (1129.4-1556.5)	1342.2 (1324.1-1359.7)	1378.5 (1361.2-1394.3)	1405.7 (1387.5-1422.4)	1290.6 (1274.2-1309)	1196.7 (1181.7-1213)	1123.2 (1107-1138.9)	1048 (1030.9-1064.5)	1020.6 (1004.5-1037)	980.3 (965.4-996.1)	938.2 (924.4-953.5)	898.6 (884.6-912.5)	922.2 (811.3-1043.8)
USA Wisconsin	1295.5 (1078.7-1548.9)	1200.6 (1013.2-1384.4)	1231.7 (1219.7-1246)	1239.2 (1226.6-1252.2)	1183 (1171.2-1194.6)	1079.9 (1068.3-1091.5)	1013.9 (1003.2-1024.5)	943.8 (933.8-954.2)	892.5 (882.6-902.1)	853.4 (843.3-862.9)	798.6 (789.1-808.2)	740.7 (732.1-749.5)	676.6 (669.1-684.2)	688.2 (594.8-777.4)
USA Wyoming	1404.9 (1220.3-1608.5)	1227.5 (1121.2-1350.6)	1255 (1228.4-1279.4)	1260.5 (1237.7-1283.6)	1301.8 (1279.2-1325.3)	1250 (1229.4-1270.2)	1107.2 (1089.6-1124.8)	1021.4 (1003.5-1039.9)	928.4 (911.5-944.9)	900.4 (883.6-918.2)	845.5 (829.6-861.3)	777.5 (764.9-791.5)	706.7 (694-718.9)	719.4 (659.5-782.9)
Southern Latin America	1794.6 (1773.3-1817.6)	1669.3 (1658.2-1681.5)	1622.1 (1615.9-1628.4)	1552.8 (1547.6-1557.8)	1520 (1515.1-1524.8)	1363.5 (1321.1-1405)	1292.9 (1288.8-1296.9)	1184 (1180-1187.7)	1132.6 (1129.2-1136.4)	1049.4 (1046-1052.9)	996.2 (993.9-999.5)	928.8 (925.8-931.8)	872.8 (869.7-875.9)	817.1 (751.4-893.9)
ARG Argentina	1681 (1651-1714.8)	1589.2 (1571.4-1606.9)	1518.8 (1511.3-1526.2)	1491.3 (1485.7-1496.8)	1508 (1501.5-1514.2)	1365 (1303.9-1423.3)	1299 (1293.9-1304.1)	1190.3 (1185.6-1195.1)	1150.7 (1146.1-1155.6)	1075.8 (1071.4-1080.1)	1044.1 (1040.1-1048.3)	977.2 (973.1-980.8)	922.7 (918.7-926.6)	876.4 (785.8-989.8)
CHL Chile	2272.5 (2255.3-2290.6)	2056.1 (2044.3-2068.3)	2030.7 (2019.2-2041.8)	1829.8 (1818.8-1839.9)	1654.5 (1645.6-1663.9)	1417.4 (1409.5-1425.2)	1277.8 (1270.5-1285.7)	1174.8 (1167.9-1181.3)	1072.9 (1065.8-1079.3)	955 (948.5-961.4)	852.1 (846.4-857.8)	789 (783.6-794.8)	740.4 (735-746)	671.7 (595.6-755.7)
URY Uruguay	1510.8 (1491.3-1530.6)	1316.1 (1300.3-1331.9)	1301.8 (1287.3-1317.6)	1305.6 (1290.2-1320.5)	1322.3 (1306.7-1336.5)	1259.9 (1245.1-1274.5)	1277.8 (1263.1-1293.1)	1161.2 (1147.6-1174.8)	1138.5 (1125.2-1152.2)	1102.4 (1089.2-1115)	1030.7 (1018.5-1043.6)	973.7 (960.7-987.5)	907.5 (894.8-920)	868.1 (773.3-969.7)
Western Europe	1488.7 (1425.4-1561.7)	1391.2 (1374.7-1408.3)	1324.1 (1318.1-1330.5)	1305.9 (1299.9-1312.4)	1289.3 (1283.9-1295.2)	1246.1 (1242.2-1249.9)	1156.1 (1154.5-1157.6)	1057 (1055.7-1058.2)	976.9 (975.8-978.1)	900.6 (899.5-901.6)	800.5 (799.4-801.6)	705.2 (704.1-706.4)	635.5 (634.5-636.4)	577.5 (561.6-595.1)
AND Andorra	862.7 (698.5-1044.3)	874 (708.5-1057.2)	877.9 (711.1-1037.3)	882.1 (710.3-1031.4)	874.5 (705.5-1029.2)	851.1 (713.7-979.8)	819.8 (716.5-936.2)	794.7 (709.1-886.3)	763.8 (699.9-851)	707.4 (643.6-790)	636.1 (592.8-691.1)	611 (576.1-652.2)	571.9 (537.4-605.4)	528.5 (478-580.2)
AUT Austria	1570.8 (1556.5-1585.3)	1514.2 (1502.6-1525.1)	1479.7 (1468.5-1490.8)	1435.5 (1424-1446.9)	1459.3 (1448-1471.3)	1380.1 (1369.6-1390.5)	1272.8 (1263.3-1282.5)	1163.7 (1155.3-1173.7)	1018.5 (1009.9-1026.5)	949.7 (941.5-958.1)	828.5 (820.9-836.3)	731.8 (724.5-739.3)	669.7 (663.1-677)	589 (553.6-624.7)
BEL Belgium	1727.5 (1714.2-1741.8)	1453.7 (1443.9-1463.1)	1392.6 (1382.6-1402.7)	1359.1 (1349.1-1368.6)	1358.5 (1349-1368.2)	1327.7 (1318.8-1337.4)	1239.4 (1230.8-1248.2)	1145.2 (1136.5-1153.9)	1015.9 (1007.4-1023.8)	955.1 (947.8-962.7)	872.7 (865.6-880.1)	774 (767-781)	697.7 (691.5-704)	615.5 (580.2-653)
CYP Cyprus	1558.2 (1319.2-1852.7)	1538.4 (1321.1-1813.3)	1485.6 (1283.9-1720.7)	1475.5 (1285.4-1678.3)	1432 (1306.6-1558.5)	1333.1 (1304.4-1360)	1165.4 (1148-1182.5)	1075.6 (1059.4-1091.5)	946.1 (930.4-961.5)	863.6 (826.7-904)	955.3 (902.9-1016.2)	918.3 (869.9-974.9)	749.6 (708.7-792.4)	653.6 (594.7-717.6)
DNK Denmark	1182.9 (1169.4-1196.9)	1108.7 (1097.7-1120.8)	1127.6 (1115.3-1140.4)	1175 (1163.5-1187)	1100.7 (1089.2-1112.5)	1083.5 (1073-1094.2)	1114.4 (1103-1125.9)	1069.4 (1058.6-1079.8)	1036.6 (1026.1-1047.1)	1007.5 (996.9-1017.6)	877.1 (866.4-887.1)	791.1 (781.4-800.2)	726.8 (718.1-736)	630.6 (593.2-671.3)
FIN Finland	1919.9 (1902.4-1938.8)	1645.1 (1630.5-1659.3)	1531.1 (1518.6-1543.6)	1580.8 (1568.8-1592.9)	1527.7 (1514.9-1539.9)	1390.6 (1379.3-1402.5)	1245.7 (1234.8-1257.2)	1186.3 (1175.6-1197.3)	1108.1 (1098.2-1118.1)	980.8 (971.2-990)	888.8 (880.2-898.4)	780.7 (771.8-789)	708.7 (700.8-716.8)	630 (593-673.4)
FRA France	1486.8 (1479.6-1496.6)	1459.7 (1455.3-1464.3)	1358.2 (1354-1362.4)	1362.1 (1357.6-1366.4)	1309.2 (1305.3-1313.3)	1275.2 (1271.3-1278.8)	1173.6 (1170-1176.9)	1100.6 (1097.4-1104.1)	966.4 (963.1-969.5)	897.9 (895-901)	804.8 (801.8-807.7)	712.5 (710-715.1)	647.2 (644.9-649.4)	555.9 (526.3-588)
DEU Germany	1565.5 (1298.1-1875)	1458.9 (1390.3-1531.8)	1435.8 (1432.2-1439.5)	1391 (1387.5-1394.5)	1446.3 (1442.7-1450.1)	1388.7 (1385.1-1392.2)	1268.6 (1265.6-1271.8)	1085.5 (1082.5-1088.3)	1055 (1052.1-1057.8)	942.2 (939.5-945)	805.3 (802.8-808)	698.4 (696-700.8)	650 (647.4-652.4)	632.1 (564.6-709.5)
GRC Greece	1156.6 (1096.1-1226.3)	1007 (998.4-1016.4)	999.1 (990.3-1007.8)	1009.7 (1000.9-1018.2)	992.3 (983.7-1001.3)	966.2 (957.5-974.3)	959 (951.2-967.2)	945.8 (937.6-953.6)	853 (845.5-860.8)	818.3 (811.7-825.3)	781.1 (774.8-787.6)	718.2 (712.4-724.5)	636.9 (631.1-642.6)	620.7 (583.3-657.6)
ISL Iceland	1107.6 (1065.9-1148.6)	1054.5 (1027.7-1080.7)	1038 (1014.2-1062.4)	1056.4 (1033.7-1078.6)	1073.8 (1052.4-1098)	927.1 (906.2-948.5)	902.9 (877.5-930.4)	845 (816.1-878.9)	793.3 (770.8-816.7)	774.6 (755.7-794.5)	664.8 (648.2-682)	579.8 (565.5-595.1)	565.4 (550.3-580.3)	574.4 (552.3-598)
IRL Ireland	1461.4 (1441.6-1480.5)	1358.5 (1341.6-1373.9)	1313.4 (1297.8-1330.1)	1324.7 (1308.5-1340.3)	1334.5 (1319.1-1350.5)	1295 (1280.1-1309.9)	1276.6 (1261.9-1292.4)	1239.6 (1224.7-1255.8)	1108.7 (1093.9-1123.5)	1053.4 (1039.8-1067.5)	944.7 (932.1-956.7)	760.1 (750-770.5)	638.1 (629.4-647.1)	563.4 (526.7-600.5)
ISR Israel	890.1 (867.3-914.5)	1047.4 (1030.4-1064.8)	1033.7 (1016.9-1050)	1104.2 (1088.1-1120.1)	1116.9 (1103-1131.2)	1079 (1065.5-1092)	997.4 (985.3-1010.9)	919.4 (907.3-932)	782.5 (772-792.8)	790.5 (781.3-800.1)	735.6 (726.8-744.5)	642.6 (635.3-649.7)	553.9 (546.8-561.4)	495.9 (466.5-528.1)
ITA Italy	1381.1 (1373.6-1389.8)	1300.5 (1295.9-1304.8)	1237.7 (1233.8-1241.7)	1235.7 (1231.6-1239.7)	1187.1 (1183.3-1190.9)	1197 (1193.1-1201.2)	1151.1 (1147.3-1155)	1030.2 (1026.7-1033.8)	914.9 (911.5-918.1)	843.2 (840.2-846.3)	751.6 (748.5-754.8)	668.5 (665.6-671.6)	600.9 (598-603.8)	526 (497-558.4)
LUX Luxembourg	1578.8 (1538-1621.1)	1503.7 (1479.1-1529.3)	1412.4 (1388.5-1437.9)	1421.9 (1399.3-1446.4)	1453.8 (1428.8-1478.9)	1448 (1424.6-1471.4)	1292.9 (1270.6-1315.1)	1225.7 (1203.2-1248.5)	1076.5 (1056-1096.7)	974.6 (955.7-992.6)	830.5 (814.5-848.5)	749.3 (733-765.7)	613.4 (599.4-627.4)	558.4 (500-617.7)
MLT Malta	1437.7 (1392.6-1484.4)	1426.6 (1398.1-1452.6)	1425.8 (1401-1450.4)	1372.6 (1347.8-1395.3)	1306.1 (1283.8-1328.8)	1229.8 (1205.8-1253.2)	1264.1 (1240.7-1287.8)	1099.3 (1076.3-1121.1)	929.1 (910.8-947.9)	851.6 (834.5-870.1)	809.1 (791.3-825.2)	742.5 (726.5-758.5)	672.5 (655.5-688.8)	623.5 (592.4-651.2)
NLD Netherlands	1120.4 (1110.2-1131.2)	1112.2 (1104.3-1120.5)	1077.6 (1069.4-1085.7)	1104.2 (1096.5-1111.9)	1130.8 (1123.3-1139.1)	1101.1 (1093.7-1108.7)	1029.1 (1021.9-1036)	1003.3 (996.5-1010.5)	961 (953.9-967.8)	915.8 (909.1-923)	859.9 (853.2-866.6)	743.6 (737.6-749.1)	647.8 (642.2-653.7)	584.1 (550.4-620.9)
NOR Norway	1011.8 (969.1-1056.3)	1010.2 (971.7-1051.8)	1027.1 (997.3-1060.9)	1065.8 (1045.9-1087.4)	1088 (1080.3-1095.4)	1032 (1025.8-1037.8)	1004.2 (998.4-1010.2)	993.9 (988.1-1000.2)	960.1 (954.3-966)	888.7 (883.5-894.2)	815.9 (810.7-821)	698.6 (693.9-703.2)	635.2 (630.9-639.2)	546.2 (534.5-557.5)
PRT Portugal	1744.2 (1723.1-1767.6)	1595 (1584.2-1606.5)	1483.2 (1472.3-1493.9)	1482.2 (1471.7-1492.6)	1435.3 (1425.3-1445.7)	1474.4 (1462.4-1485.8)	1260.5 (1251.9-1268.9)	1198.6 (1190.7-1207.1)	1158.5 (1150.7-1166.7)	1077.1 (1069.3-1085.1)	966.4 (959.5-974)	806 (787.4-825)	713.8 (707.2-720.1)	631.5 (592.3-669.2)
ESP Spain	1639.3 (1628.6-1655)	1373 (1367.2-1379)	1211.7 (1206.3-1217)	1179.8 (1175.2-1184.8)	1168.6 (1163.7-1173.3)	1096.7 (1092-1101.1)	966.4 (962.4-970.6)	933.8 (929.5-937.9)	906 (902.3-909.9)	848.4 (845-851.9)	767.7 (764.3-771)	704.1 (700.9-707.2)	612.9 (609.6-615.6)	550.2 (520.9-579.3)
SWE Sweden	1194.2 (1140.8-1249.2)	1107.3 (1056.7-1155.1)	1096.5 (1047.1-1143.1)	1052.4 (1004.6-1098.8)	1018.8 (974.8-1063.1)	1042.7 (1009.3-1077.5)	1007.1 (999.3-1015.7)	935 (927.9-942.5)	863.8 (855.8-871.7)	790.3 (783.1-797.2)	718.1 (711.6-725.2)	656.4 (649.7-663.3)	600 (593.8-606.6)	530.6 (501.7-560.6)
SWE Stockholm	1263.9 (979.6-1569.6)	1209.4 (946.5-1507.5)	1215.4 (961-1498.1)	1167.9 (930.8-1437.1)	1120.3 (906.5-1359.9)	1149 (967.6-1323)	1124 (1102-1145.4)	1007.6 (991.8-1024.1)	957 (937.8-976.4)	838.8 (823.3-854.1)	753.3 (739.7-767.1)	667.5 (654.4-679.8)	602.4 (590.6-613.7)	543.4 (501-585.1)
SWE Sweden except Stockholm	1183.2 (1138.8-1225.6)	1092.4 (1049.9-1131.6)	1079.9 (1040.3-1119.3)	1035.9 (995.9-1074)	1003.2 (962.8-1038.2)	1025.5 (996.3-1055)	987.4 (978.9-996.2)	922.4 (914.5-930.8)	846.9 (837.8-855.6)	781.2 (773.1-789.3)	711.6 (704.4-719.3)	654.9 (647.6-662.3)	600.2 (593.3-607.6)	528.9 (496.1-563.5)
CHE Switzerland	1348 (1333.4-1363)	1304.5 (1292.5-1316.7)	1214.7 (1202.7-1225.9)	1191.8 (1180.4-1203.5)	1155.5 (1144.4-1165.5)	1058.4 (1048.1-1068.2)	994.6 (984.7-1003.9)	913.1 (904.1-922.6)	869 (860.3-877.3)	791.4 (783.7-799.5)	703.4 (695.6-711.4)	615 (608.1-621.8)	551.5 (545-558.1)	463 (433.6-491.6)
GBR United Kingdom	1486.2 (1450.3-1524.5)	1445.1 (1410.9-1480.5)	1379.8 (1345.6-1415.5)	1368.9 (1334.1-1405.7)	1342 (1311-1376.3)	1277.5 (1256.8-1298.6)	1190.9 (1185.8-1195.4)	1100 (1097.7-1102.3)	1006 (1004.1-1008)	934.4 (932.4-936.5)	818.7 (815.6-821.9)	708.4 (706.9-710)	633.7 (632.1-635.2)	595.1 (588.2-601.9)
GBR England	1448.3 (1412.5-1485.4)	1411.2 (1374.7-1447.4)	1349 (1315.8-1384.9)	1340.1 (1306.1-1375)	1319.4 (1289.4-1352.4)	1252.7 (1233.1-1273.6)	1167.5 (1162.4-1172.7)	1078.9 (1076.8-1081)	988.2 (986.4-990)	917.3 (915.5-919.3)	802.9 (800.5-805.5)	694 (692.7-695.5)	618.6 (617.2-620)	580.9 (576-585.9)
ENG East Midlands	1479 (1331.4-1637.6)	1436.9 (1302.9-1586.1)	1366.4 (1239.9-1500.2)	1354.3 (1230-1491.5)	1322.7 (1211.5-1440.1)	1246.3 (1171.2-1324.2)	1165.5 (1147.7-1183.9)	1070.7 (1063.2-1078.1)	980.8 (974.2-987.5)	910.8 (903.8-917.9)	810.8 (801.9-820.4)	705.2 (700.1-710.4)	631.4 (626.4-637)	591.7 (574.5-608.7)
UKF Derby	1563.6 (1183.3-2034.3)	1504.9 (1149-1948.4)	1415.3 (1052.3-1822.1)	1390 (1063.6-1796)	1349.6 (1062-1689.3)	1283.3 (1084-1518.8)	1269.4 (1210-1333.1)	1149.2 (1124.5-1177)	1061.8 (1035.1-1085.8)	924.7 (902-949.5)	803.3 (776.2-830.1)	722.8 (704.8-741.3)	701.1 (682.3-719.1)	652.2 (599.1-710.2)
UKF Derbyshire	1490.1 (1138.8-1949.2)	1453.7 (1119.1-1849.2)	1394.1 (1054.1-1806.8)	1393.5 (1076.6-1823.2)	1374.3 (1087.6-1727.7)	1299.9 (1087.2-1536.2)	1193.9 (1145.9-1246.1)	1087 (1070.6-1105.1)	1006.1 (989.4-1023.9)	935 (918.2-952)	813.9 (790.2-839.1)	703.7 (690.4-716.7)	618 (605.9-631.8)	606.8 (559.1-654.6)
UKF Leicester	1612.4 (1226.6-2110.2)	1562 (1200.4-2012.4)	1471.5 (1131-1860.4)	1446.9 (1129.4-1832.3)	1385.7 (1112-1719.7)	1293.7 (1088.7-1511.3)	1207.6 (1154.2-1266.8)	1134.4 (1112.5-1157.5)	1032.7 (1012.7-1053.6)	998.1 (975.3-1020.7)	921.9 (892.4-952.5)	818.9 (801.9-837.3)	731.6 (715.6-748.9)	695.4 (643.5-751.7)
UKF Leicestershire	1322.7 (1006.9-1708.5)	1292 (982.6-1653.5)	1236.4 (928.4-1605.2)	1232.2 (943-1584.8)	1215.6 (952.9-1541.8)	1153.2 (975.3-1381.8)	1082.4 (1036.8-1132.8)	996.6 (978.8-1015.6)	909.1 (891-927.6)	828.2 (811.2-844.7)	727.6 (703.7-750.4)	633.3 (620-646.9)	577 (564.7-590.1)	531.7 (493.1-572.8)
UKF Lincolnshire	1471.7 (1134.9-1896.4)	1428.1 (1088.9-1834.4)	1358.1 (1046.6-1723.6)	1345.1 (1037.8-1700.4)	1309.1 (1040.4-1655.8)	1225.4 (1030.6-1446.1)	1128.4 (1082.2-1177.3)	1062.3 (1042.9-1082.1)	964.4 (946.9-982)	894.4 (877.7-912.1)	800 (775.6-823.9)	691.8 (678.2-705.5)	614.2 (601.4-628.7)	586.4 (538.9-631.9)
UKF Northamptonshire	1405.9 (1068.9-1824)	1362.4 (1039.1-1741.5)	1292.6 (1019.5-1659.1)	1277.7 (990-1618)	1246.7 (991.7-1562.3)	1171.1 (985.1-1401.9)	1115.8 (1068.2-1167.3)	1023.1 (1005.9-1041.6)	955.2 (936.6-972.9)	888 (870.5-905.5)	801.7 (778.3-826.9)	698.6 (684.5-712.8)	641 (628.6-653.9)	576 (531.3-620.7)
UKF Nottingham	1654.2 (1242.6-2119.6)	1620.5 (1246-2087.8)	1553.1 (1203.9-1975.5)	1557 (1206.1-1983.9)	1525.7 (1221.6-1896.6)	1440.9 (1223.4-1705.5)	1335.3 (1277.6-1394.3)	1194 (1169.5-1218.7)	1092.3 (1069.2-1117.1)	1052.7 (1030.1-1075.5)	1014.3 (983.4-1047.9)	887.4 (866.7-907.6)	763.5 (745.9-781)	711 (653.6-771.8)
UKF Nottinghamshire	1465.3 (1109.3-1906.9)	1418.4 (1080.8-1821.1)	1342 (1023.1-1729.1)	1324.1 (1026.3-1672)	1294.3 (1009.1-1595.5)	1225.7 (1037.8-1439.4)	1160.9 (1113.9-1207.4)	1066.3 (1048.3-1085.5)	974 (956.8-991.6)	916 (898.8-932.7)	810 (787.3-833.7)	702.9 (689.9-717.1)	623.1 (610.2-636.8)	590.8 (547-637.3)
UKF Rutland	1303.8 (971.3-1712.8)	1240.1 (926-1649.5)	1159.2 (867.8-1535.8)	1134.6 (849.6-1481.1)	1093 (845.8-1408.5)	1016.9 (845.9-1219.7)	967.4 (888.4-1051.1)	925 (880.6-970.5)	825.7 (785.8-867.7)	740.8 (704.1-777.8)	644.7 (609.5-683.8)	592 (561.3-624.3)	547.8 (517.8-579.4)	521.1 (463-585.5)
ENG East of England	1322.6 (1193.7-1457.7)	1288 (1161-1424.3)	1229.5 (1110.3-1369.5)	1219.6 (1104.6-1350)	1210.8 (1105.2-1320.6)	1159.9 (1084.4-1238.8)	1087.2 (1069.8-1105.7)	975 (968.8-981.8)	907.2 (900.8-912.9)	841.3 (835.2-847.3)	742.4 (733.8-751.1)	645 (640.3-649.8)	577.1 (572.7-581.3)	548.7 (532.8-565.9)
UKH Bedford	1439.3 (1084.4-1847.2)	1398.3 (1059.8-1832.2)	1332.3 (1011.1-1731.3)	1326.5 (1018.3-1708.8)	1308.1 (1049.1-1629.7)	1232.9 (1031.7-1447.5)	1105.2 (1044.1-1169.6)	994.5 (967.3-1021.7)	967 (939.2-995.7)	905.7 (878.4-933.4)	797.9 (767.3-828.3)	688.8 (667.2-710.1)	657.1 (637.9-677.8)	610.4 (558.4-668.9)
UKH Cambridgeshire	1274.5 (955.2-1639.5)	1260.8 (941.4-1637.6)	1214.6 (912-1582.6)	1218 (925.6-1560.6)	1215.4 (951.6-1517)	1160 (966-1378.8)	1077.8 (1029.1-1133.1)	953.3 (935.1-972.6)	887 (870.4-906.5)	827.3 (811.1-845.3)	712.3 (690.1-734.6)	633.1 (620.3-646.9)	556.2 (543.8-568.8)	511 (467.6-551.6)
UKH Central Bedfordshire	1327.6 (998.5-1722.9)	1280.6 (947.4-1655.8)	1212.9 (913.2-1576)	1195.4 (918.3-1519.3)	1183.4 (933.3-1476)	1138.4 (947.3-1341.1)	1114.4 (1061.3-1175.1)	1013.9 (989-1038.1)	943.8 (920.1-969.4)	860.8 (838.9-882.4)	764.1 (736.6-793.4)	674.4 (655.5-694.6)	565.1 (548.9-581.7)	505.4 (463.8-548.4)
UKH Essex	1296.2 (999.9-1645.3)	1271.2 (965-1626.2)	1224 (940.7-1564.4)	1223.8 (942.1-1562.5)	1233.3 (964.2-1523.1)	1196.5 (1005.2-1396.7)	1109.7 (1062.6-1154.2)	980.2 (964.9-996.3)	892 (878.7-906)	842.3 (828.7-856.3)	736.4 (717.3-757.6)	642.8 (631.9-653.2)	580.6 (570.6-590.7)	570.4 (527.1-614.2)
UKH Hertfordshire	1286.7 (961.5-1652.5)	1252.2 (951.5-1612.8)	1201.4 (913.9-1546.4)	1202.6 (913.4-1539.1)	1213 (955-1504.9)	1176.4 (978.5-1381.1)	1091.1 (1046.7-1135.2)	989.2 (972.9-1006.1)	912.8 (897-928.5)	823.8 (809.5-838.6)	742.5 (720.7-763.8)	621.9 (610.2-633.9)	573.3 (561.5-584.5)	499.2 (458.6-541.8)

Table 1 Age-standardised mortality rates by location and year for males for 1950, 1955...2005, 2010, 2017

Location	1950	1955	1960	1965	1970	1975	1980	1985	1990	1995	2000	2005	2010	2017
UKH Luton	1447.6 (1094-1877.8)	1383.9 (1035.2-1796.2)	1296.1 (991-1684)	1265.2 (973.6-1628.6)	1235.2 (976.8-1538.3)	1179.9 (972-1393.5)	1172.7 (1110.5-1234.6)	1013.6 (989.7-1040)	1017.1 (992.1-1044.1)	980.5 (955-1007.5)	851.7 (824.4-881.5)	765.5 (745.6-786.3)	663.7 (644.6-682)	588.4 (539.9-637.7)
UKH Norfolk	1330.8 (1021.5-1706)	1292.5 (983.4-1637.6)	1226.8 (938.9-1566.3)	1206.8 (933.5-1536.7)	1180.4 (939.3-1484.7)	1115.8 (938.3-1301.7)	1061.4 (1019.7-1106.8)	956.4 (940.9-973.1)	908.2 (892.3-924.5)	824.5 (808.7-839.9)	728.2 (706.2-749.3)	638.6 (627.1-650.3)	555.6 (545.3-566.4)	581.3 (538.9-625.9)
UKH Peterborough	1486.3 (1091.2-1928.5)	1444.9 (1071.4-1919.5)	1380.8 (1047.1-1814.3)	1378.6 (1052.8-1779.5)	1362.2 (1088.1-1713.8)	1296.2 (1078-1526)	1143.6 (1079.2-1202.8)	1049.3 (1022.1-1078.7)	974.8 (950.9-1000.7)	927.3 (901.1-955)	817.5 (788.2-849.1)	736.6 (714-758.1)	689.2 (669.1-710.7)	649.6 (597.7-707.4)
UKH Southend-on-Sea	1413.1 (1078.3-1821.6)	1354.3 (1044.5-1729.7)	1281.2 (970.6-1638.5)	1254.4 (948.7-1610.1)	1216.9 (953.4-1529.5)	1143 (953.6-1349.4)	1057.5 (994-1116.9)	968.7 (940-997.9)	911.6 (887.6-937.7)	867.2 (842.1-893.7)	807.7 (776.3-839.2)	724.5 (702.9-746.1)	642.8 (624.5-661.7)	622.3 (569.4-679.6)
UKH Suffolk	1300.5 (974.4-1660.5)	1258.2 (961.9-1645.5)	1191.2 (903.4-1542)	1167.9 (891.9-1488.6)	1143.6 (894.3-1446.5)	1086.3 (905.3-1286.2)	1040.5 (995.6-1086.1)	948.3 (929.8-966.1)	877.6 (860.5-894.6)	824.6 (808-840.9)	726.4 (704.2-747.6)	612.9 (599.9-624.8)	555.5 (543.9-568)	534.1 (492.4-579.9)
UKH Thurrock	1486.4 (1109.1-1898.8)	1454.2 (1086.6-1869.1)	1393.4 (1049.2-1800)	1389.2 (1057.9-1776.1)	1373.9 (1063.5-1721.8)	1308.1 (1097.2-1537.5)	1200.9 (1137.3-1262.8)	1066.9 (1038.6-1095.9)	985.9 (957.8-1015)	900.9 (874.4-928.8)	763.2 (732.7-794.2)	723.1 (702-746.4)	620.3 (600.8-641)	592.3 (539.9-647.5)
ENG Greater London	1418 (1354.5-1483.1)	1393.8 (1329.6-1457)	1333.9 (1275.6-1394.6)	1325.2 (1269-1384.9)	1291.1 (1242.4-1343.3)	1218.9 (1182.2-1254.8)	1154.5 (1144.4-1164.3)	1074.7 (1070.1-1079.2)	995.7 (991.6-999.7)	947.9 (943.7-951.7)	801.7 (797-806.2)	680.1 (677.1-682.9)	597.9 (595.2-600.7)	535.5 (528.2-543.4)
UKI Barking and Dagenham	1564.1 (1192-2001.4)	1536.1 (1194.7-1969.1)	1485.1 (1167.7-1877.6)	1489.8 (1192.4-1868.9)	1468.5 (1173.9-1801.9)	1398.9 (1199.6-1639.3)	1320.5 (1255.8-1383.8)	1196.9 (1169-1224.9)	1092.1 (1065.4-1119.5)	1041.2 (1013.1-1068.3)	956.1 (923.9-988.5)	820.2 (796.9-843.7)	740.2 (719.8-761.1)	747.6 (687.6-808.6)
UKI Barnet	1250.6 (974.2-1617.9)	1225.7 (954.5-1568.9)	1169.2 (904.8-1474.4)	1151.4 (890.9-1448.4)	1115 (881.3-1375.1)	1049 (883.8-1228.5)	1017.4 (969.3-1062.5)	944.8 (923.2-966.2)	885 (865.8-904.5)	826.5 (807.1-845.5)	686.3 (663.6-709)	595.3 (581.4-608.5)	532.3 (518.6-545.6)	483.8 (445.5-524)
UKI Bexley	1308.4 (999.4-1681)	1281.8 (992.5-1664.7)	1235 (962.5-1559.8)	1228.9 (965-1526.8)	1205.3 (951.7-1487.2)	1132.9 (955.1-1331.2)	1067.7 (1015.3-1119.5)	967.7 (943.8-991.8)	888 (864.7-912.4)	820.2 (798.8-841.4)	749.4 (725.3-776.9)	662.1 (643.2-680.3)	600.3 (584-618)	564.7 (519.5-614.7)
UKI Brent	1399 (1069.7-1773.1)	1364.1 (1062.7-1723.3)	1282.4 (998.7-1621.6)	1254.6 (986.9-1592.5)	1207.4 (955-1488.9)	1132 (968.7-1315.6)	1097.8 (1050.1-1150.2)	1087.1 (1064.1-1110.8)	954.7 (934.6-975.7)	919.9 (899.8-939.6)	794.6 (768.6-819.5)	660.7 (645.5-677.5)	589.4 (575.4-603.7)	530.9 (490.8-573.4)
UKI Bromley	1248.2 (944.1-1619.8)	1221.2 (935.3-1550.5)	1172.2 (890.6-1475.4)	1161.3 (900.9-1473)	1134.6 (912.6-1406.7)	1069.8 (918.1-1243.9)	1008.6 (962-1057.7)	978.1 (957.4-999.6)	884.6 (864.6-903.3)	824 (805.5-842.5)	698.5 (673.9-720.4)	567.8 (553.5-582)	521.1 (507.7-535.1)	517.5 (477.3-559)
UKI Camden	1501.8 (1157.9-1910.9)	1516.9 (1175.2-1924)	1457 (1147.9-1843.7)	1469.3 (1171.5-1857.2)	1442.5 (1154.8-1777.7)	1369.1 (1158.8-1591.8)	1290.6 (1232.5-1352.2)	1092 (1069.3-1115.7)	1110.7 (1087.3-1135.9)	1060.6 (1037.4-1083.9)	946.1 (913.4-976.5)	839 (820.2-858.1)	615.2 (599.8-631)	452.7 (415.6-491)
UKI Croydon	1302.8 (997.8-1655.7)	1265.8 (966.1-1619.7)	1201.4 (935.2-1533.8)	1177.4 (927.9-1476.5)	1146.3 (927.4-1430.6)	1086.7 (928.2-1264.2)	1080.9 (1031.5-1127.7)	1047.9 (1027-1068.8)	956.3 (936.7-975.5)	877.3 (858.5-895.8)	758.9 (736-783.1)	620.3 (606.1-633.6)	602.4 (588.3-617.1)	526.9 (488-570.5)
UKI Ealing	1458 (1092.6-1849.4)	1433.6 (1107.9-1823.4)	1375.8 (1071.4-1752.1)	1371.8 (1067.9-1710.5)	1336.2 (1054.2-1625.8)	1260.4 (1072.2-1474.1)	1176.5 (1128-1230.9)	1082.1 (1060.5-1105.1)	1011.9 (991.8-1032.8)	958.4 (938.6-978.8)	794.6 (770.3-818.7)	665.6 (650.5-680.4)	596.2 (582.5-610.3)	531.3 (488.9-575)
UKI Enfield	1333.8 (1009.8-1749.2)	1308.1 (994.7-1693.5)	1261.6 (983.4-1656)	1255.6 (976.9-1578.3)	1231.3 (994.2-1522.3)	1163.7 (985.1-1346.8)	1110.9 (1057.1-1165.9)	1028.5 (1005.7-1051.1)	940.4 (918.7-962.2)	914.3 (892.5-935.2)	762.9 (738.2-788)	620 (605.2-634.6)	552.8 (538.4-568)	563.4 (518.7-608.9)
UKI Greenwich	1460.5 (1112.8-1855.4)	1427.8 (1089.1-1811.1)	1365.2 (1051.7-1745.2)	1350.3 (1049.2-1699.5)	1313.7 (1040.9-1628.1)	1238.1 (1049.5-1451.7)	1180.2 (1125.8-1241.5)	1121.2 (1096.5-1147.2)	1077.3 (1054.4-1103.6)	1059 (1035.4-1081.7)	900.4 (870.4-929.5)	794.9 (776.3-813.9)	667.6 (651.8-683.5)	583.8 (537.1-634.7)
UKI Hackney	1484.3 (1141.2-1908.5)	1460.7 (1128.4-1846.8)	1414.4 (1110.6-1786)	1435.4 (1127.7-1788.7)	1417.3 (1133.4-1779.2)	1359.8 (1150.1-1588.2)	1254 (1197.3-1311)	1204.5 (1179.1-1230.6)	1098.2 (1074.6-1123.7)	1059.3 (1034.9-1083.7)	859.6 (832.2-887.4)	757 (739-775.3)	672.2 (656-688.1)	631.8 (582.2-682.8)
UKI Hammersmith and Fulham	1557.6 (1179.6-2000.9)	1552.4 (1200.1-1980)	1479.3 (1145.7-1879.4)	1472.4 (1143-1842.9)	1431.5 (1146.8-1764.9)	1367.1 (1177.3-1599.7)	1269.8 (1213.2-1332.3)	1200.4 (1174.2-1227.8)	1191.5 (1164.6-1219.5)	1156.1 (1129.5-1181.2)	841.1 (813.5-869.2)	714.2 (695.6-733)	655.9 (637.7-675.2)	630.1 (582.5-680.3)
UKI Haringey	1486.5 (1132.5-1882.7)	1479 (1143.9-1886.9)	1416.4 (1088.1-1796.5)	1412.8 (1111.1-1776.2)	1381 (1106.8-1696.1)	1300.9 (1108.3-1524.3)	1213.6 (1159.3-1275.4)	1090.1 (1067-1113.3)	1024.8 (1001.4-1047.5)	1011.6 (988.1-1035.1)	875.6 (847.7-904.7)	775.8 (758.7-793.8)	681.5 (665.4-699.9)	509.5 (470.1-552.1)
UKI Harrow	1240.6 (935.6-1616.6)	1207.9 (916.9-1596.4)	1152.8 (873-1485.3)	1136.3 (872.3-1440)	1099.5 (871.9-1360.5)	1024.8 (868.5-1190.9)	1009.5 (960.7-1061.1)	908.2 (886.2-931.2)	849 (826.4-870.2)	826.2 (804.1-847.6)	676.2 (651.9-699.9)	550.6 (535.1-567.3)	462.3 (449.3-475.9)	449.7 (413.7-487.5)
UKI Havering	1303.1 (980.6-1697.4)	1272 (960.1-1640.4)	1217.7 (938-1563.9)	1203.1 (921.7-1518)	1178.1 (913.3-1476.3)	1116.6 (935.8-1303.4)	1067.9 (1013.6-1125.5)	1045.5 (1020.5-1070.4)	946 (924-969.8)	874 (852.7-895)	774.3 (748.9-802.1)	632.1 (615-649.3)	612.3 (596.7-629.2)	573.5 (529-619.7)
UKI Hillingdon	1320.3 (1001.6-1740.8)	1296 (1001-1655.2)	1246.8 (968.2-1594.2)	1244.9 (976.2-1585.4)	1224.6 (993.9-1509.3)	1162.4 (987.7-1346.2)	1104.6 (1055-1159.6)	1018.7 (995.2-1042.4)	922.7 (900.7-943.7)	887.7 (867.1-909.1)	773.8 (749.2-799.2)	684.6 (668.2-700.5)	585.4 (571.1-600.7)	505.6 (464.5-548)
UKI Hounslow	1372.3 (1058.2-1774.4)	1347.2 (1043.9-1759)	1284.9 (981.5-1662.9)	1271.9 (979.2-1611.7)	1232.5 (989.6-1541.8)	1159.8 (987.8-1357)	1107.5 (1053.7-1166.9)	1064.4 (1040.7-1087)	977.3 (954.1-998.9)	982.4 (959.6-1004.7)	841.7 (814.5-869.7)	709.2 (692.6-727.4)	616.7 (600.1-632.1)	554.8 (511.4-600.1)
UKI Islington	1495.3 (1150.5-1904)	1469.2 (1143.4-1869.9)	1393.4 (1093.2-1754.8)	1384.2 (1075.7-1729)	1348.5 (1076.3-1649.3)	1298.5 (1106.3-1510.2)	1238.2 (1180.5-1294.8)	1187.1 (1161.2-1213.2)	1152.4 (1128.6-1176.1)	1106.7 (1083.8-1131.3)	978.5 (944.9-1008.7)	858.5 (838.4-878.4)	718.4 (701.4-735.8)	565.4 (520.5-612.9)
UKI Kensington and Chelsea	1506 (1147.2-1937)	1600.7 (1265.2-2000.7)	1496.7 (1183.3-1873.9)	1472.1 (1162.5-1845.8)	1427.7 (1155.2-1731.1)	1345.7 (1159-1561.2)	1275.1 (1219.3-1339.8)	1140 (1112.5-1169.1)	893.6 (871.2-916.5)	811.8 (791.7-834.2)	660.5 (636.1-685)	564 (547.1-581.7)	473.6 (458.8-489.2)	398.5 (361.5-435.8)
UKI Kingston upon Thames	1294 (987.8-1670.5)	1268.2 (956.1-1636)	1217.2 (937.3-1567)	1212.2 (945.5-1529.6)	1182.7 (934.9-1462.3)	1105.9 (945.5-1283.9)	1043.6 (987.5-1099.8)	976.9 (947.6-1005.5)	865.9 (839.9-892.2)	780.7 (757.3-807.5)	656.3 (633.5-681.4)	536.8 (520.1-553.3)	486.3 (470.7-503.7)	494.5 (455.2-534.6)
UKI Lambeth	1627.9 (1250.5-2043)	1612.2 (1245.5-2054.8)	1545.6 (1201-1960.2)	1551.7 (1222-1937.3)	1510.2 (1193.2-1858.3)	1424.8 (1216.4-1648.7)	1296 (1237.2-1352)	1213.1 (1190.4-1237)	1129.9 (1109.2-1150.2)	1129.6 (1109.3-1151.2)	904.4 (877.6-930.3)	789.5 (772.6-805.3)	701.6 (687.2-716.8)	662.3 (612.9-716.7)
UKI Lewisham	1500.4 (1161.4-1904.4)	1462.6 (1154.6-1860.1)	1383 (1090.6-1782.4)	1358.3 (1077.1-1720)	1319.7 (1074.3-1622.2)	1257.4 (1087-1451.4)	1267.6 (1211.6-1322.7)	1133.6 (1109.6-1158.3)	1078 (1055.2-1100.8)	1074.7 (1052.1-1097.5)	931.1 (902.2-958.1)	808.1 (789.5-827)	681.8 (665.9-698.2)	581.6 (534.8-628)
UKI Merton	1308.8 (978.8-1674.1)	1275 (980.7-1656.5)	1216.9 (950.2-1526.5)	1205.1 (950.9-1508.6)	1171 (943.6-1436.7)	1100.7 (942.6-1282.2)	1097.1 (1041.7-1156.2)	990.5 (965.4-1015.9)	920.4 (895.1-945.4)	841.4 (819.3-863.8)	699.7 (674.2-724.4)	602.7 (585.5-619.2)	570.8 (554.3-587.4)	539.7 (495.6-591.2)
UKI Newham	1531 (1176-1946.6)	1495.6 (1164.9-1940.1)	1431.3 (1105.6-1847.7)	1420.9 (1103.2-1786.9)	1385.5 (1113-1721.7)	1318.3 (1129.7-1538.6)	1252.2 (1195.2-1310.3)	1197.1 (1171.7-1222.8)	1124.2 (1100.4-1149.5)	1112.6 (1090-1134.9)	983.3 (954-1012.6)	798.4 (781.7-815.5)	762.1 (746-779.2)	581.2 (536.3-626.2)
UKI Redbridge	1329.5 (1018-1696.6)	1306.5 (1003.6-1675.3)	1255.1 (962.3-1589.1)	1244.1 (969.1-1577.4)	1210.7 (972.7-1487)	1133.2 (962.7-1328)	1056.9 (1006.7-1109.4)	977.4 (953.4-1002.2)	891.8 (869-914.2)	841.3 (819.6-863)	756.5 (732.2-782.9)	643.7 (628.1-659.3)	564.5 (550.5-579.6)	512.4 (471-553.9)
UKI Richmond upon Thames	1321.4 (1007.3-1717.8)	1311 (1002.2-1690.7)	1269.6 (985.2-1620.4)	1280 (998.8-1610.4)	1264 (1018.2-1557.6)	1188.9 (1004.8-1397.6)	1101.7 (1046.4-1159.3)	978 (953.1-1004.6)	844.6 (821.3-868.4)	754.4 (733.5-776.1)	598.2 (575.6-621.9)	500.3 (483.6-517)	440.8 (425.8-455.7)	452.5 (415-491.8)
UKI Southwark	1476.7 (1103.7-1912.7)	1435.9 (1076.8-1839.4)	1367.3 (1055.1-1727.3)	1359.1 (1060.1-1711.8)	1325 (1063.8-1638.7)	1274.4 (1077.6-1483.9)	1216.8 (1166.4-1269.6)	1247.6 (1222.8-1272.4)	1202.3 (1179.3-1225.8)	1093.1 (1069.3-1115.2)	882.7 (856.8-911.2)	821.6 (804.1-839.3)	681.7 (665.4-697.5)	663.3 (613.4-716.4)
UKI Sutton	1278.4 (976.6-1612.1)	1228.3 (934.4-1585.4)	1156.5 (888.1-1485.1)	1129.8 (877.7-1439)	1084.9 (872-1365.9)	1010.2 (863.6-1178.5)	962.1 (914.1-1015.1)	936.3 (912.7-960)	839.4 (814.5-863.8)	850 (827-873.8)	695.8 (670.9-720.3)	601 (584.2-618.8)	539.4 (522.9-556.3)	536.9 (492.2-583.6)
UKI Tower Hamlets	1638.7 (1285.3-2079.2)	1613.2 (1248.3-2050.6)	1571 (1235.1-1964.5)	1609.2 (1265.8-2018.5)	1599.3 (1276.6-1942.8)	1534.8 (1308.1-1776.5)	1384.2 (1321.4-1450.8)	1284.1 (1257-1311.8)	1204.3 (1177.1-1231.3)	1158.4 (1132.6-1184.1)	988.2 (955.9-1020)	849.4 (829.2-869.7)	744.1 (726.9-761.8)	562.2 (520.6-606.9)
UKI Waltham Forest	1453.9 (1118.8-1870.8)	1438.6 (1114.5-1840.5)	1392.1 (1104.7-1767.2)	1392.7 (1112.3-1757.6)	1365 (1098.1-1674.7)	1277.5 (1093.5-1495.3)	1139.7 (1081.4-1196.4)	1044.4 (1020.2-1069.4)	969.7 (946.5-992.9)	987.8 (964.5-1010.8)	908.3 (878.7-937.7)	767.5 (749.8-787)	658.5 (642.4-675)	572.7 (528.6-620.5)
UKI Wandsworth	1461 (1101.8-1873.7)	1444.5 (1097.8-1855.6)	1382.8 (1084.8-1732.1)	1374.8 (1081.2-1730.4)	1348 (1080.3-1649.1)	1293.8 (1101.2-1505.5)	1256 (1201.7-1315.6)	1185 (1162.1-1208.8)	1158.6 (1134.6-1182.5)	1071.7 (1049-1092.8)	860 (833.6-887)	710.8 (695.2-726.7)	614 (598.8-629.9)	585.8 (540.5-635.4)
UKI Westminster	1406.8 (1100.6-1772.8)	1443.2 (1128.3-1812.8)	1393.4 (1098.7-1754.9)	1407.6 (1106.7-1777.1)	1388.2 (1118.7-1702.6)	1334.2 (1137.6-1536.4)	1271.3 (1211.6-1328.2)	1124.4 (1100.8-1149.1)	1043.4 (1020.1-1066.8)	959.4 (937.5-981.5)	779.1 (754.2-805.2)	637.2 (621.6-654.4)	519.2 (505.4-533.2)	471.2 (433.1-509.4)
ENG North East England	1651.3 (1519.8-1791.6)	1606.5 (1485.5-1750.8)	1538.3 (1415.5-1667.2)	1521 (1407.5-1637.5)	1480 (1381.8-1588.3)	1374.1 (1301.5-1450.2)	1290.4 (1270.2-1309.2)	1226.1 (1217.1-1233.8)	1118 (1110.3-1125.8)	1026.1 (1018.3-1033.4)	906.6 (896.5-916.3)	790.8 (784.6-797.4)	689.2 (683.4-695.3)	656.5 (638.9-674.6)
UKC County Durham	1638 (1262.4-2069.9)	1596 (1239.9-2024.6)	1529.1 (1177.1-1945.6)	1512.4 (1166.2-1917.5)	1475.6 (1185.8-1854.2)	1375.2 (1163.7-1620.1)	1281.5 (1227.4-1336.3)	1225.4 (1204.8-1245.7)	1115.5 (1095.5-1136.6)	1053 (1033.9-1073.9)	895.8 (867.3-924)	757.2 (741.4-773.6)	685.8 (670.6-700.6)	648.5 (596.5-701.1)
UKC Darlington	1642.9 (1270.4-2081.8)	1592.7 (1225.8-2077.1)	1525.4 (1172.4-1946.6)	1511.3 (1162.7-1935.1)	1469.8 (1158.4-1839.6)	1348.6 (1115.5-1604.6)	1265.7 (1189.3-1336)	1180.6 (1144.9-1215.1)	1060 (1027.1-1094.7)	972.6 (939.7-1007.6)	879.5 (843.3-916.7)	804 (777.1-831.6)	688.9 (665.1-711.8)	611.1 (557.4-674)
UKC Gateshead	1744.6 (1346.6-2200.1)	1713.7 (1319.4-2189)	1656.3 (1268.9-2104.7)	1657.7 (1283.5-2106.6)	1626.8 (1301.5-2016.3)	1508.6 (1269.8-1776.6)	1360.5 (1293.4-1427.4)	1263.5 (1235.4-1290.5)	1152.6 (1125-1181.5)	1047.3 (1021.5-1072.1)	968.6 (934.2-1004.7)	838.4 (816.8-861.7)	727.9 (708-748)	681.9 (625.1-742.1)
UKC Hartlepool	1764.5 (1361.9-2241.7)	1705.1 (1306.1-2147.9)	1621.9 (1212.4-2029.1)	1603.8 (1200.4-1996)	1555.1 (1205.2-1953.4)	1427.4 (1185.7-1701.3)	1306.6 (1229.4-1382.3)	1229.1 (1197.1-1264.1)	1121.6 (1087.4-1156.3)	1005.9 (973.8-1040.7)	883.2 (847.4-919.6)	811.8 (783.7-840.7)	716.4 (691.2-742.9)	682 (623.1-749.8)
UKC Middlesbrough	1765.9 (1347.5-2304.2)	1704.1 (1292.6-2207.2)	1616.9 (1212.7-2076.9)	1586.5 (1198-2024.3)	1531 (1206.5-1939.9)	1421.8 (1194.9-1680.1)	1377.5 (1306.7-1446.9)	1266.2 (1235.8-1296.1)	1178.5 (1150.8-1209.1)	1066.2 (1038.2-1094.4)	965.2 (930.7-999.2)	859.8 (834.3-884.9)	771.6 (748-795.5)	710.7 (652.2-773.3)
UKC Newcastle upon Tyne	1674.4 (1295.2-2134.8)	1643.2 (1266.3-2089.2)	1574.6 (1222-1993.5)	1562.9 (1233.1-1937.6)	1514.4 (1212.1-1883.4)	1399.2 (1168.6-1668.3)	1272.1 (1218-1332.3)	1194.7 (1169.1-1217.9)	1119 (1095.1-1142.9)	1057.4 (1033.1-1081.1)	917.1 (887.8-946.4)	835.7 (815-856)	718.6 (701.3-735.9)	681.9 (631.2-738.7)
UKC North Tyneside	1578.9 (1203.1-2017.9)	1528.8 (1164.9-1936.6)	1469.6 (1127.1-1854.2)	1456.6 (1142.9-1816.7)	1424.7 (1144.6-1762)	1328.2 (1121.1-1575.7)	1268 (1200.9-1330.4)	1224.6 (1195.7-1252.5)	1086.1 (1059.3-1114.6)	1017.7 (991.6-1044.6)	910.8 (878.9-944.2)	790.8 (769.6-812.8)	693.3 (673.1-713.1)	674.2 (617.5-731.3)
UKC Northumberland	1510.8 (1149.2-1917.3)	1467 (1133.9-1869.2)	1409 (1077-1796.7)	1390.7 (1091.5-1752.4)	1355.9 (1082-1687.9)	1263.9 (1055-1492.2)	1255.2 (1197.9-1309.9)	1204.2 (1178.4-1229.2)	1068.9 (1045.9-1091.8)	954.5 (932.2-976.8)	856.4 (828.3-883.8)	694.3 (677.6-711.6)	593.4 (579-609.6)	601.6 (555.7-654.5)
UKC Redcar and Cleveland	1695.1 (1273-2146.6)	1649.1 (1244.1-2096.7)	1578.4 (1212.8-1994.2)	1561.5 (1201.6-1967.2)	1515.2 (1212.6-1904.4)	1397.8 (1174.6-1654)	1289.4 (1224-1354.2)	1205.4 (1176.1-1235.1)	1117.1 (1088.6-1147.9)	970.6 (943.3-999.5)	865.4 (831.2-898.7)	767.4 (744.1-791.9)	665.5 (642-687.8)	632.7 (577.9-693.7)
UKC South Tyneside	1717.5 (1308.9-2181.3)	1650.1 (1262.2-2074.9)	1568.8 (1210.6-1984.1)	1540.1 (1206-1926.2)	1493.6 (1193.3-1865.8)	1396.5 (1170.4-1650.9)	1307.9 (1245.4-1373.4)	1287.8 (1258.7-1317)	1142.7 (1114.3-1170.4)	1040.1 (1011-1068)	937.3 (904.6-970.7)	823.5 (800.9-847.4)	724 (702.8-745.2)	749.6 (686.9-816.4)
UKC Stockton-on-Tees	1595.3 (1201-2058.7)	1551.6 (1157-1994.5)	1485.8 (1095.5-1917.2)	1468.1 (1117.6-1878.7)	1427.8 (1127.9-1804.7)	1322.5 (1110.3-1593.6)	1245.3 (1181.4-1312)	1192.7 (1163.3-1222.2)	1107.2 (1079.7-1137)	1008.9 (983.3-1035.8)	846.8 (815.1-877.9)	789.8 (768.5-810.9)	663.1 (644-681.9)	623.9 (568.4-682.4)
UKC Sunderland	1653.2 (1276-2094)	1607.9 (1246.6-2046.8)	1536.3 (1189.2-1962.6)	1511.7 (1172.9-1880.1)	1469 (1167.5-1824.1)	1367.9 (1141.3-1606)	1310.6 (1250.6-1367.8)	1250.9 (1227.5-1275)	1172.3 (1145.8-1196.8)	1068.4 (1044.7-1092.1)	970.7 (937.1-1001.9)	866.6 (845.2-888.7)	741.1 (723.5-758.8)	692.1 (637-750.6)
ENG North West England	1594.9 (1498.7-1704.8)	1545.8 (1449.8-1654.9)	1475.7 (1384.3-1573.3)	1459.6 (1362.3-1553.4)	1415.1 (1332-1507)	1325.6 (1265.5-1386.2)	1254.5 (1240.5-1267.4)	1195.5 (1190.1-1201)	1088.6 (1083.6-1093.6)	1017.2 (1012.4-1021.8)	897.4 (890.7-904.6)	780.8 (776.6-784.7)	695.4 (691.6-699.4)	639.4 (626.8-651.8)
UKD Blackburn with Darwen	1639.9 (1266.4-2104.3)	1589 (1226.2-2058.2)	1524.7 (1184.6-1928.9)	1515.5 (1173.4-1889.7)	1473.4 (1183.6-1814.2)	1380.4 (1175.3-1616.2)	1319.2 (1257.2-1389.5)	1276.1 (1243.8-1308.3)	1186.3 (1158-1215.1)	1146.8 (1118.2-1173.6)	1005.6 (970.7-1040.5)	883.4 (860.6-908.5)	789.1 (767.6-810.9)	680.5 (624.7-739.6)
UKD Blackpool	1671.1 (1287.1-2139.9)	1607.3 (1233.4-2069.4)	1530.4 (1197.6-1956.3)	1507.7 (1177.1-1875.2)	1443.9 (1140.4-1787.1)	1319.7 (1122.9-1542.3)	1255 (1192.7-1318.9)	1180.1 (1150.9-1208.5)	1051.5 (1025.9-1078.4)	1001.8 (976.8-1026.6)	1012.9 (980.5-1050.3)	1019.9 (993.4-1046.4)	897 (872.5-922.2)	804.4 (739.6-871.1)
UKD Bolton	1626.9 (1257.2-2060.5)	1579.4 (1234.1-2017.4)	1512.6 (1173.2-1891.1)	1498.6 (1174-1860.9)	1456.9 (1168.3-1806)	1371.7 (1172-1602.6)	1299.3 (1240-1356.2)	1226.1 (1201.1-1251.5)	1100.8 (1077.5-1124.4)	1050.8 (1027.5-1074.1)	931.4 (903.3-963.2)	816.3 (797.9-835.1)	726.6 (709.1-744.8)	623.1 (575.6-673.5)
UKD Bury	1553.2 (1206.4-1996.8)	1514.5 (1170.3-1946.5)	1455.3 (1127.7-1828.1)	1444.4 (1133.1-1806.6)	1408.8 (1125.9-1735.9)	1314.1 (1113.9-1529.7)	1235 (1177.8-1297.4)	1146.6 (1117.8-1175.2)	1070.7 (1045.1-1097.7)	1028.9 (1002.6-1055.4)	844 (813.6-871.7)	745.2 (724.5-766.2)	680.9 (661.9-700.9)	683.3 (632.6-739.9)
UKD Cheshire East	1411.2 (1062.7-1828.2)	1373.2 (1046.3-1776.7)	1323.9 (1017.1-1666)	1321.9 (1016.4-1649.5)	1299.8 (1035.5-1603)	1217 (1043.4-1421.3)	1146.5 (1097.5-1197.5)	1072 (1049.6-1092.3)	956.2 (935-976.6)	883.6 (865.3-902.1)	794.3 (769.2-819.7)	641.2 (626-655.9)	558 (544.4-572.3)	548.5 (504.8-593.8)
UKD Cheshire West and Chester	1436 (1112.2-1855.1)	1394.6 (1075.5-1827.9)	1338.7 (1038.1-1694.1)	1332.1 (1050.5-1663.7)	1307.6 (1045.4-1598.3)	1232.8 (1061.3-1438.6)	1185.6 (1135.5-1236.2)	1096.2 (1075-1117.5)	1001.9 (980.8-1022.3)	921.7 (902.4-942.7)	785.9 (761.6-813.9)	652.5 (637.4-667.5)	593.6 (579.1-608)	583.6 (542.1-629.4)
UKD Cumbria	1491.1 (1149.5-1883.4)	1443.3 (1103.9-1846.7)	1378.8 (1056.6-1769.9)	1365 (1061.3-1733.6)	1326 (1038.8-1645)	1242.7 (1049.3-1448.1)	1183.7 (1134.5-1230.7)	1127.5 (1108.1-1146.2)	1038.2 (1020.2-1056.8)	940.5 (920.7-959)	818.8 (794.6-844.8)	711.4 (697.1-725.3)	620.5 (607.4-634.6)	576.8 (534.7-622.8)
UKD Halton	1622.9 (1239.7-2081.4)	1585.9 (1204.8-2065.8)	1536 (1196-1935.3)	1544.3 (1211.6-1970.4)	1511.8 (1199.4-1886)	1413.8 (1198.6-1663.7)	1323 (1254.5-1393.3)	1207.5 (1176-1239)	1080.2 (1051.5-1110.2)	1027.4 (998.6-1058.4)	933.3 (897.5-967.9)	877.6 (852.4-904.1)	740 (716.2-763.4)	653.7 (596.1-712.5)
UKD Knowsley	1644.3 (1280.5-2094.8)	1591.9 (1229.6-2045.3)	1525.1 (1192.3-1920.1)	1518.4 (1190.2-1900.6)	1472.5 (1162.9-1830.1)	1369.4 (1165.1-1610.9)	1302.8 (1240.5-1363)	1194.3 (1167.1-1222.3)	1119.2 (1091.9-1145.8)	1100.7 (1071.9-1129.1)	1046.3 (1012-1083.6)	887.9 (863-912.8)	807.3 (782.5-830.4)	762.6 (700.4-831.5)
UKD Lancashire	1541.7 (1208.2-1948.7)	1495.4 (1159.5-1893.4)	1425.7 (1113.6-1783.6)	1402 (1087.7-1795.3)	1362.2 (1057.2-1718.6)	1284.1 (1057-1553.8)	1228.5 (1179.1-1280.9)	1195.8 (1177.9-1213.1)	1054.6 (1037.2-1071.2)	970.9 (953-987.6)	860.7 (836.8-885)	739.9 (727.1-752.7)	658.4 (646.1-670.5)	613.3 (568-662.7)
UKD Liverpool	1706.3 (1316.1-2131.8)	1656.3 (1297.7-2088.4)	1577.3 (1227.4-1976.8)	1560.9 (1221.2-1946)	1501.9 (1182.9-1862.6)	1408.1 (1188.9-1664)	1316.6 (1265.6-1365.3)	1257.1 (1237.6-1278.1)	1189.5 (1171-1210)	1157.6 (1137.3-1178.9)	1044.8 (1014.4-1076.5)	918.2 (902-935.2)	818.4 (801.4-835.7)	716.1 (665.5-772)
UKD Manchester	1783.8 (1365.2-2267.4)	1727.2 (1333-2209.8)	1637.2 (1262-2075.1)	1625.6 (1280.9-2020.9)	1566.6 (1236.8-1913.9)	1472.7 (1264.1-1711.9)	1405 (1351.5-1459.8)	1334.5 (1313.5-1355.5)	1272.3 (1252-1293.1)	1220.9 (1200.2-1243.2)	1084.7 (1053.9-1114.3)	969.5 (951.4-988.3)	839.4 (823.4-854.9)	802.8 (745.6-863.8)
UKD Oldham	1656.8 (1290.1-2120.1)	1609.7 (1242.7-2050.8)	1546.4 (1189.9-1964.1)	1534.6 (1186.8-1931)	1490.7 (1183-1833.2)	1395 (1187.8-1615.2)	1332.8 (1273.7-1394.8)	1297.2 (1270.8-1322.2)	1158.8 (1132.7-1186.9)	1111.4 (1085.9-1137.1)	969.6 (939.5-999.1)	852 (831.1-872.7)	749.6 (731.6-768.1)	691.3 (636.9-749.3)
UKD Rochdale	1649.6 (1274-2105.4)	1598.1 (1241.6-2066)	1525.4 (1195.4-1925.8)	1507.1 (1209.6-1867.6)	1457.4 (1167.9-1795.3)	1360.8 (1165.1-1585.3)	1258.8 (1205.3-1314.9)	1230.5 (1204.8-1257.2)	1148.3 (1123.5-1174.8)	1059.7 (1036.4-1083.3)	928.6 (897.9-960.7)	841.4 (820.7-862.4)	754.2 (735.8-772.4)	679.1 (628.5-735.9)
UKD Salford	1772.2 (1370.9-2223)	1735.6 (1347.7-2223.6)	1669.5 (1310.5-2104.6)	1668.6 (1305.7-2099.5)	1622.4 (1290-2009.7)	1523.5 (1302.2-1775.9)	1383.3 (1323.9-1442.1)	1315.3 (1288.5-1341.2)	1185.5 (1162.2-1209.4)	1138.5 (1114.4-1163.1)	981 (947.5-1011.3)	879.2 (858.8-898.7)	801.8 (784.4-820.5)	733.4 (677.9-794.9)
UKD Sefton	1540.3 (1191-1990.3)	1482.1 (1143.6-1901.4)	1410.1 (1094.1-1787.9)	1391.3 (1100.6-1736.5)	1342.6 (1071-1650.3)	1253.5 (1065.7-1469.9)	1180.6 (1132.5-1234)	1127.2 (1105.5-1149.1)	1011.2 (990.5-1030.6)	967.6 (946.3-989.6)	863.3 (836.9-889.7)	710.5 (694.1-727.1)	691.5 (674.8-708.8)	638.5 (590.2-690.2)
UKD St Helens	1683.7 (1316.2-2135.3)	1643.2 (1264.5-2097.1)	1582.2 (1226-1978.1)	1580.6 (1226.4-1979.4)	1553.7 (1219.9-1920.1)	1471.3 (1247.5-1725.3)	1378.3 (1313.1-1440.9)	1315.6 (1286.4-1344)	1161.3 (1132.9-1188.8)	1042.5 (1017.5-1068.1)	902.3 (871.7-934.2)	805.2 (784.3-826.3)	749.5 (729-770.6)	754.8 (695.6-818.6)
UKD Stockport	1461.2 (1113.6-1855.2)	1411.4 (1093.3-1784.5)	1346.9 (1041.8-1694.3)	1330.3 (1043.9-1668.6)	1295.7 (1038.7-1574.9)	1219.5 (1041.3-1427.8)	1171.1 (1117.5-1219.3)	1125 (1102.9-1148.8)	1036.9 (1015.1-1060.2)	958.2 (936.9-979.5)	794.9 (770.1-820.7)	704.7 (687.9-721.5)	656.9 (639.7-673.6)	560.6 (516.4-607.7)
UKD Tameside	1609.3 (1248.9-2029.5)	1556.1 (1204.8-1960.1)	1484.9 (1149.2-1899.6)	1468 (1136.9-1875.4)	1420.1 (1125.2-1764.5)	1326 (1132.4-1567.2)	1258.9 (1203.9-1314.3)	1182.6 (1157.1-1208.2)	1100 (1076.2-1123.7)	1065.1 (1041-1089.4)	970.6 (937.9-1002.9)	851.7 (830.3-873.6)	767.7 (747.4-787.5)	689.1 (633.8-747.3)
UKD Trafford	1408.4 (1082.3-1787.8)	1357.4 (1044.4-1746.3)	1288.8 (988.5-1636.6)	1265.8 (984.2-1605.5)	1222 (961.6-1502.2)	1139.4 (970.9-1342.7)	1106.5 (1055.4-1161.6)	1090.8 (1066.2-1116.2)	1014.8 (991.5-1038.6)	944.9 (922.4-966.5)	800.8 (773.4-829.7)	679.3 (661.4-697.2)	589.1 (573.2-604.5)	550.7 (506.4-599.8)
UKD Warrington	1524.4 (1157.9-1973.1)	1488.5 (1130.2-1915.5)	1435.2 (1110.7-1813.1)	1432.9 (1128.5-1798.5)	1409.2 (1119.3-1782.8)	1329.3 (1133-1568.4)	1254.3 (1194.4-1313.8)	1198.6 (1171-1225.6)	1112.6 (1086-1139.8)	1033.7 (1008.1-1060.5)	848.6 (819.8-878.5)	709.3 (690.9-729.6)	656.4 (639.2-675)	675.3 (620.1-733.7)
UKD Wigan	1645.2 (1260.9-2099.6)	1601.9 (1235.8-2061.3)	1532.6 (1190.3-1969.8)	1517.3 (1175.1-1935.9)	1480.5 (1170.5-1820)	1402.1 (1197.7-1636.2)	1351.6 (1294.1-1408.1)	1274.3 (1251.5-1298.7)	1146.4 (1122.8-1168.7)	1011.4 (990.9-1033.6)	941 (911.7-970.4)	864.9 (846.7-883.6)	740.5 (724-756.8)	702.4 (650.4-757.8)
UKD Wirral	1584 (1200.9-2009.4)	1523.4 (1160.1-1946)	1443.4 (1119.9-1828)	1421.3 (1115.8-1781.6)	1372 (1083.7-1680.9)	1276 (1097.9-1497.4)	1201.5 (1149.6-1254.3)	1153.4 (1132.2-1174.2)	1087.8 (1066.4-1109.4)	1005.4 (984.9-1025.2)	880.8 (851.7-908.3)	763.2 (746.1-780.3)	684.8 (670-700.9)	643.8 (597.6-693.5)
ENG South East England	1286.9 (1186.5-1394)	1254.8 (1151.4-1366.8)	1203 (1109-1303.3)	1207.5 (1109.9-1304)	1218.2 (1134.2-1315.3)	1189 (1125.7-1253.6)	1090.5 (1075.8-1105.4)	997.3 (991.7-1003.2)	898.2 (892.8-903.4)	840.6 (835.7-845.5)	735.2 (728.5-742.5)	627.1 (623.1-631.1)	566.3 (562.4-569.9)	541.4 (528.5-555.1)
UKJ Bracknell Forest	1265.9 (951.4-1657.4)	1226.6 (926.3-1592.8)	1173.9 (879.6-1515.8)	1182.2 (903.7-1518.3)	1187 (945.5-1491.2)	1143.7 (965-1357.1)	1041.7 (978.6-1100.7)	996.7 (964.1-1028)	863.6 (837.1-892.4)	777.1 (749.8-806)	673.1 (645.9-702.5)	560.7 (541.1-581.9)	510.8 (490.9-531.8)	522.8 (473.6-576.4)
UKJ Brighton and Hove	1392.9 (1052.5-1773)	1365.1 (1043.3-1718.6)	1306.5 (999.4-1680.1)	1303.8 (1023.4-1657.7)	1296.4 (1038.5-1612.7)	1242.5 (1058.5-1443.6)	1149 (1095.5-1205.4)	1065.5 (1040.8-1088.8)	1079.3 (1056.5-1103)	1059.3 (1036.5-1082.5)	864.2 (834.9-890.7)	793.8 (776.6-811)	685.3 (668.1-701.4)	578 (533.3-627.7)
UKJ Buckinghamshire	1189.4 (909.7-1510.2)	1157.4 (892.4-1473)	1116.7 (861.3-1441.7)	1122.7 (860.8-1434.9)	1138.8 (909.9-1415.7)	1111.6 (940.5-1290.9)	1037 (993.5-1083.9)	974.9 (955.9-993.2)	886.9 (869.2-903.9)	817.7 (800.8-835.7)	667 (646.4-688.1)	570.4 (558.3-582.6)	523.4 (511.3-535.3)	455 (419.2-490.9)
UKJ East Sussex	1333.5 (1002.4-1723.3)	1294.2 (973.8-1690)	1237.4 (938.6-1613.1)	1236.5 (959.9-1588.3)	1228.5 (968.4-1539.6)	1173.4 (990.7-1389.9)	1045.2 (999.6-1097.1)	949.4 (930.9-969.2)	851.8 (834.3-869.8)	802.5 (785.1-819.5)	718.8 (695.2-740.6)	620.6 (606.8-634.3)	575.4 (561.3-589.1)	560.8 (519-605.1)
UKJ Hampshire	1208 (917.4-1585.1)	1181 (903-1544)	1136.4 (882.4-1447.9)	1144.1 (882.3-1477.8)	1154.4 (908.3-1460.8)	1124.9 (945.9-1334.2)	1046.9 (1001.4-1090)	974.4 (958.3-991)	887 (872.8-901.6)	831 (816.9-845.5)	709.9 (690.3-730)	587.7 (576.9-599.1)	541.2 (531.2-551.4)	503.2 (466.5-542.5)
UKJ Isle of Wight	1403.1 (1032.5-1814.3)	1344.9 (1006.1-1735.1)	1276 (961.6-1631.6)	1263.5 (957.2-1609.3)	1250.4 (990.8-1550.4)	1196 (1006.9-1405.2)	1094.7 (1027.9-1162.5)	1061.3 (1028.2-1094)	959.1 (929.4-989.8)	846.8 (821.2-874.1)	739 (709.4-766.7)	666.3 (645.3-689.6)	603.2 (583-624.2)	550.6 (502.3-601.8)
UKJ Kent	1325.9 (1014.7-1687.8)	1293.3 (996.8-1643.5)	1242.6 (957.1-1597.5)	1253.1 (958.1-1588.4)	1287.8 (994.2-1616.3)	1298.6 (1075.5-1543.9)	1184 (1137.7-1236.4)	1050.1 (1033.5-1066.3)	934.9 (918.8-949.7)	877.1 (861.7-892.3)	768.7 (745.6-791.7)	654.5 (643-667.2)	587.1 (576.1-598.5)	592.6 (552.1-637.6)
UKJ Medway	1369 (1026-1805.8)	1324.4 (1004.1-1724)	1249.4 (948.2-1597.6)	1230.2 (960-1576.4)	1220.4 (972.6-1538.2)	1181.2 (998.2-1379.2)	1109.3 (1057.6-1163)	1074 (1049.7-1098)	954.9 (934-974.9)	889 (869.6-910.4)	866.9 (838.7-894.5)	772.2 (753.3-791.2)	661.1 (643.9-678.8)	617.3 (565.7-666.3)
UKJ Milton Keynes	1339.6 (1008.6-1757.1)	1312.5 (998.2-1699.1)	1278.1 (969.4-1653.2)	1306.6 (1010.4-1678.4)	1328.9 (1066.6-1659.3)	1290.5 (1098.9-1510.6)	1165.7 (1103.8-1224.7)	1047.4 (1018.7-1076.6)	904.2 (880.7-926.8)	865.1 (842.8-888.4)	743 (717.8-767.9)	683.2 (665.3-702.4)	653.1 (635.5-669.8)	619.1 (570.2-672.4)
UKJ Oxfordshire	1186.6 (894.1-1551.5)	1165.9 (892.4-1516.6)	1120.6 (856.3-1438.9)	1126.7 (852.3-1449.4)	1137.7 (882.4-1419.7)	1111.3 (940.3-1306.7)	1048.3 (1005.6-1096.5)	946.3 (928-963.9)	862.3 (845.9-878.2)	813.3 (797.4-831.3)	716.5 (694.9-738.2)	615.5 (602.6-629.8)	552.7 (541.8-563.6)	497.5 (459.8-537.7)
UKJ Portsmouth	1444 (1080.6-1846.9)	1414.8 (1063.5-1812.8)	1358.8 (1036.2-1746.2)	1363.8 (1050.6-1748.8)	1374.4 (1104.9-1700.4)	1346.7 (1143.2-1576.3)	1292.6 (1236.4-1356.9)	1199.2 (1172-1225.4)	1021.6 (999.2-1045.7)	1011.5 (988.3-1034.6)	893.9 (862.6-923.7)	764.2 (744.7-782.4)	714.8 (696.3-733.3)	728 (670.1-787.2)
UKJ Reading	1382.5 (1040.4-1779.4)	1364.3 (1043.2-1724.5)	1322.1 (1006.1-1701.8)	1345.6 (1031.5-1743.4)	1357.2 (1053.5-1705.3)	1307.4 (1103.4-1546.7)	1173.7 (1112.9-1238.6)	1072.2 (1044-1100)	965.9 (940.7-990.6)	912 (887-935.7)	811.3 (783.1-840.4)	728.1 (708.1-749.3)	678.4 (659.7-697.3)	622.6 (572.8-674.7)
UKJ Slough	1364 (1028.8-1792.4)	1321.7 (1017.8-1696.1)	1265.3 (967.7-1629.1)	1270.8 (976.6-1639.1)	1271.3 (996.2-1596.8)	1225.8 (1034.2-1428.4)	1140.8 (1074.6-1203)	1138.8 (1106.7-1175.3)	997.1 (967.7-1027.4)	881.2 (856.4-906.2)	835.2 (804.3-867)	737.1 (714.5-758.7)	709.6 (688.2-732.4)	613.2 (561.2-669.7)
UKJ Southampton	1451.6 (1090.4-1903.2)	1420.6 (1073.9-1831.3)	1360.3 (1042.5-1747.5)	1367.7 (1062.9-1732.5)	1362 (1087-1692.4)	1308.3 (1110.9-1525)	1137.2 (1085.6-1190.7)	1059.3 (1033.9-1083.5)	1055.7 (1032.2-1078.9)	994.2 (972.5-1018.9)	846.2 (818.3-872)	745.4 (728.4-764.5)	671.4 (654.5-688)	656.4 (603.6-715.5)
UKJ Surrey	1185.3 (877.8-1547.7)	1154.3 (877.8-1478.7)	1109.7 (846.8-1430.1)	1115.2 (840.5-1435)	1135.5 (881.8-1421.8)	1120 (930-1319.3)	1064.7 (1021-1108.9)	963.7 (947.5-980.8)	854.2 (839.4-869.3)	797.9 (783.5-812.6)	715.2 (695.2-735.2)	584.9 (573-596.7)	509.2 (499-519.7)	507.8 (470.3-548.4)
UKJ West Berkshire	1218.7 (907.7-1573.6)	1188.8 (900.6-1534.8)	1151.8 (864.7-1496.1)	1167.3 (892.9-1501.7)	1178 (926-1483.9)	1145.5 (962.5-1350)	1037.2 (975.9-1100.9)	1064.4 (1035.2-1097.5)	858.6 (834.5-883.4)	766.1 (744.1-789.5)	661.4 (635.8-685.3)	595.7 (577.5-616.2)	525.5 (508.1-543.6)	514.4 (470.5-562.7)
UKJ West Sussex	1294.2 (994.8-1643.8)	1261 (969.6-1635.1)	1217.9 (934.7-1582.2)	1238.4 (966.7-1558.6)	1257.4 (984.1-1573.5)	1212 (1026.7-1422.7)	1073.3 (1031.5-1121.9)	963.8 (947.1-980.7)	862.3 (847.3-878.1)	806.1 (791.1-821.4)	707.9 (687.8-727.8)	614.6 (602.4-627.1)	558.6 (547.8-569.7)	555.4 (514.1-601)
UKJ Windsor and Maidenhead	1112.4 (834.9-1439.2)	1070.7 (795.5-1402.1)	1011.3 (761.2-1310.8)	999.6 (761.4-1278)	989.6 (782.5-1237.7)	948.9 (800.2-1114.2)	908 (855.6-963)	886.6 (858.4-915.4)	830.6 (804.4-856)	797.7 (771.7-822.1)	757 (727.5-787.2)	559 (539.7-578.6)	505.9 (487-525.7)	496.8 (453.5-544.3)
UKJ Wokingham	1127.6 (855.6-1482)	1093.2 (829.9-1429.7)	1039.3 (786.4-1372.5)	1039.8 (788.9-1353.9)	1037.1 (813.7-1300.9)	992.1 (834.6-1171.7)	889 (839.4-944.5)	811.9 (786-838.2)	701 (678.3-723.4)	632.5 (611.1-653.9)	531.9 (509.4-554)	490.4 (473.1-507.7)	466.8 (449.4-484.1)	520.4 (473.4-572.8)
ENG South West England	1349.4 (1248-1461.9)	1311.3 (1216.6-1412.3)	1253.3 (1156.3-1348.6)	1252.4 (1169-1347.4)	1243.3 (1159.5-1330.8)	1182.7 (1126-1239.7)	1079.8 (1065.2-1094.2)	983.6 (977.5-989.9)	907 (901.3-912.6)	830.2 (824.7-835.6)	732.9 (726.3-739.8)	633.3 (629.3-637.6)	575.1 (571.3-579.2)	554.7 (541.8-568.4)
UKK Bath and North East Somerset	1232.1 (915.2-1589.6)	1188.8 (911.8-1527.6)	1124.4 (849.8-1450.6)	1110.6 (846.7-1434.1)	1084.6 (862.4-1356.3)	1008.4 (840.3-1191.6)	918.2 (868.2-968.5)	870.8 (845.1-897)	839.1 (814-862.2)	773.2 (751.1-794.5)	669.4 (644.4-696.4)	575.7 (557.8-593.4)	540.7 (523.7-557.6)	543.7 (498.6-589)

Table 1 Age-standardised mortality rates by location and year for males for 1950, 1955...2005, 2010, 2017

Location	1950	1955	1960	1965	1970	1975	1980	1985	1990	1995	2000	2005	2010	2017
UKK Bournemouth	1365.1 (1048.6-1786)	1312 (1000.7-1714.1)	1239 (959.4-1591)	1223.5 (945.2-1537.1)	1194.3 (946.3-1474.1)	1113.8 (933.4-1311.5)	980.4 (926.2-1038)	947.9 (921.2-974.1)	981.8 (954.6-1009.2)	953 (925.7-978.8)	815 (787-846.9)	705.7 (686.2-725.5)	667.4 (649.3-687.1)	587.1 (539.4-637.6)
UKK Bristol, City of	1425.3 (1081.3-1824)	1398.6 (1056.8-1795.6)	1336.4 (1007-1704)	1334.8 (1039.2-1683.8)	1323 (1057.8-1628.6)	1271.1 (1078.2-1489.2)	1175.8 (1125.8-1232.1)	1057.4 (1036.7-1078.6)	1007.1 (986.9-1027.5)	923.6 (903.5-943.4)	828.3 (800.8-855.5)	723.2 (706.4-740.5)	652.5 (638-667.7)	556.3 (512.7-604.5)
UKK Cornwall	1415.4 (1060.6-1797.8)	1371.2 (1059.6-1752.3)	1311 (1002.3-1660.4)	1314.3 (1022.4-1676.2)	1308.2 (1046.1-1612.2)	1250.9 (1054.5-1471.3)	1130.5 (1082.7-1182.7)	1003.4 (984.1-1023.9)	931.1 (912.5-950.2)	844.8 (827.3-863.3)	748.3 (724.7-774.3)	639.5 (626.1-653.5)	587.2 (573.6-600.7)	608.8 (565.3-656.1)
UKK Devon	1339.5 (1022.3-1718.5)	1307.2 (988.7-1660.7)	1255.8 (970.1-1597.1)	1262.3 (984.2-1581)	1256.4 (994.5-1546.2)	1196.1 (1002.4-1404.2)	1057.8 (1011.8-1102.9)	942.8 (925.5-960.2)	868.8 (852.7-884.6)	795.9 (781.7-811.8)	709.3 (688.3-729.3)	607.1 (595-619.1)	559.7 (548.3-570.9)	587.3 (544.3-633.8)
UKK Dorset	1298.9 (987.8-1720.9)	1252.1 (963-1615)	1189.9 (894.5-1520.3)	1182.9 (919.4-1505.1)	1165.3 (934.3-1425.3)	1101.4 (934-1286)	1019.1 (971.8-1065.6)	931.8 (910.8-952.3)	828.6 (810.3-847.1)	761.1 (744.5-778.4)	660.5 (639.3-682.5)	558.3 (545.2-572.2)	517.3 (504.3-530.8)	500.6 (463.2-542.8)
UKK Gloucestershire	1298.3 (970.2-1649.3)	1262.4 (968.2-1601.3)	1211.9 (912.9-1552.9)	1211 (916-1547)	1212.9 (954.8-1520.2)	1166.6 (983.8-1381.9)	1115.9 (1064.7-1167.9)	1020.5 (1001.1-1040.1)	923.1 (905.3-941.8)	840.6 (822.7-857.5)	724.8 (703.4-748.7)	629.7 (617-643.6)	552.8 (540.4-565.7)	547 (506.4-590)
UKK North Somerset	1285 (971.4-1670.8)	1236 (960.8-1582)	1172.7 (889.3-1520.2)	1160.8 (894.2-1466.8)	1138.8 (915.3-1397.2)	1064.3 (891.2-1247.7)	970.4 (917.7-1028.1)	907.8 (881.1-933.1)	869.9 (846.2-893.8)	806.4 (783.9-829)	712.6 (686.5-737.2)	644.8 (627.3-664.1)	609.4 (592-627.1)	580.5 (534.3-631.5)
UKK Plymouth	1494.8 (1139.9-1919)	1461.1 (1127.5-1879.3)	1405.4 (1086.6-1796.3)	1417 (1083.3-1788.8)	1416.2 (1133.8-1764.3)	1356.3 (1143.5-1587.9)	1236.1 (1182.7-1296.5)	1101.8 (1078.2-1125.7)	1016.9 (994.9-1040.9)	990.4 (968.2-1014.7)	861.3 (833.1-890.1)	773 (754-792.3)	688.5 (671.2-708.7)	599.7 (554.6-652.5)
UKK Poole	1360.6 (1026.5-1748.7)	1316.9 (993.5-1748)	1263.5 (959.2-1661.7)	1269.3 (967.9-1614.5)	1259.1 (984.4-1554.4)	1186.7 (979.6-1405)	1045.7 (983.7-1111.3)	945.3 (915.4-975.5)	823.3 (798.7-848.6)	749.4 (726.6-772.6)	662.1 (635.3-689.8)	607.5 (588.1-626.7)	552.5 (532.9-572)	530.7 (487.4-578.9)
UKK Somerset	1310.8 (984.1-1692.3)	1278.3 (961.6-1652)	1231 (924.3-1576.9)	1238.1 (946.8-1585.9)	1239.3 (959.7-1556.4)	1182 (982.8-1394)	1081.2 (1032.3-1135.7)	986.5 (965-1007.8)	886.3 (868.1-905.4)	831.8 (813-849.8)	727.1 (704.5-749.3)	607.8 (594.7-621.7)	569.1 (555.8-583.5)	563.8 (519.8-609.9)
UKK South Gloucestershire	1277.7 (966.1-1652)	1245.2 (949.1-1615.2)	1202.3 (913.4-1572)	1217.5 (924.4-1569.3)	1225.8 (967.3-1522.5)	1166.5 (985.6-1358.5)	1050.3 (997.2-1103.1)	988.3 (964.5-1014)	879.1 (855.3-902.9)	768.3 (747.2-788.7)	699.6 (675.6-724.8)	606.1 (588.8-623.8)	521.5 (506.8-536.8)	480.6 (442.3-525.5)
UKK Swindon	1346 (1013.3-1719.5)	1311.2 (996.7-1677.4)	1263.6 (955.8-1646.4)	1277.4 (995.3-1626.6)	1289.7 (1025.1-1614)	1239.7 (1052.1-1447.5)	1152 (1090.5-1213.2)	1075.4 (1046.2-1103.3)	1001.6 (974.4-1028.2)	950.8 (925.2-976.6)	793.1 (762.9-820.8)	680.2 (662.8-699.5)	607.7 (591.4-625.6)	600.4 (550.5-653.1)
UKK Torbay	1424.9 (1086.6-1848.1)	1365.2 (1032.1-1785.2)	1300.4 (986.8-1668.4)	1295.5 (995.4-1663.8)	1274 (1007.2-1603.7)	1195.6 (1002.2-1419.1)	1093.3 (1030.4-1161.6)	1030.3 (1000.5-1061.8)	901.2 (871.9-927.6)	822.5 (797.5-848.7)	834.3 (803.2-868)	787.1 (761.9-810.4)	692.9 (670.8-716.1)	609.6 (558.8-664.1)
UKK Wiltshire	1273 (982.2-1632.6)	1244.1 (966-1582.4)	1186.1 (916.3-1510)	1179.4 (916.9-1491.3)	1175.2 (943.3-1447.8)	1128.2 (954.2-1319.8)	1076.4 (1025.5-1126.8)	1001.2 (980.7-1021.4)	938 (918.5-959.1)	797.1 (779.6-814.6)	722.5 (699.7-747.1)	626.2 (612.2-639.6)	540.1 (527.3-554.1)	493.7 (457.6-533.2)
ENG West Midlands	1516.3 (1394.9-1638)	1475.3 (1360.3-1598.2)	1410.7 (1291.3-1533.2)	1404.4 (1292.2-1522.1)	1397.4 (1297.7-1500.6)	1329.7 (1263.4-1401.5)	1229.1 (1212.1-1245.5)	1126.6 (1120.4-1132.9)	1043.5 (1037.5-1049.7)	963.8 (958.1-969.5)	840.2 (831.9-848.8)	733.7 (729.1-738.5)	641.4 (636.7-645.4)	610.5 (596.7-624.6)
UKG Birmingham	1585.9 (1260.4-1986)	1534.3 (1214-1952.6)	1460.2 (1135.8-1849.7)	1447.6 (1139.9-1829.6)	1420.7 (1118.5-1751.5)	1339.3 (1116.1-1565.9)	1286.3 (1238.8-1333.7)	1192.9 (1175.7-1210.2)	1099.9 (1084-1116.8)	1021.2 (1005.7-1037.9)	912.4 (887.4-938)	798.7 (785.2-812.1)	676.9 (665.6-688.7)	646.5 (600.6-695.2)
UKG Coventry	1524.9 (1177.2-1956.5)	1478.4 (1129.8-1863.8)	1400.6 (1084-1784.7)	1382.8 (1088.7-1735.2)	1366.1 (1074-1669.1)	1301.2 (1089.1-1516.9)	1217.1 (1164.7-1268.4)	1108.2 (1086.7-1128.8)	1079 (1058-1101.1)	983.7 (962.3-1005.3)	861.4 (834-890.3)	783.6 (766.1-801.4)	694.4 (679-711.4)	629.4 (582.6-679.1)
UKG Dudley	1473.8 (1134.3-1906.9)	1425 (1103.6-1811.7)	1353.6 (1035.7-1719.3)	1334.7 (1024.6-1697.9)	1330.8 (1063.5-1659.4)	1276.3 (1087.5-1506.8)	1215.5 (1165.8-1266.2)	1151.5 (1128.5-1175.3)	1025 (1003.4-1045.4)	964.7 (945-986)	858.1 (831.8-886.2)	744.3 (726.5-763.5)	644.5 (629-662)	609.7 (562-658.5)
UKG Herefordshire, County of	1395.9 (1057-1785.8)	1352.4 (1037.4-1760.4)	1288.9 (999.9-1668.9)	1276.2 (988.2-1632.2)	1259.7 (990.5-1588.8)	1182.5 (992.7-1387.3)	1122.5 (1063.6-1183.7)	975.6 (948.6-1001.1)	896.7 (871.3-922.2)	821.2 (797.4-846.1)	728.6 (702.2-755.2)	646.9 (627.5-666.8)	585.8 (568-603.1)	551.6 (504.2-602)
UKG Sandwell	1622.2 (1235.3-2078.1)	1570.2 (1201.1-1972.5)	1492.3 (1158.1-1898.2)	1475.9 (1153.9-1878.6)	1457.2 (1157.3-1805.2)	1379.9 (1160.9-1612.8)	1267.1 (1212.7-1321.9)	1200.5 (1176.4-1225.1)	1166.4 (1143.3-1190.1)	1087.6 (1065.2-1110.4)	979.1 (948.8-1009.9)	850.2 (830.7-869)	743.4 (726.2-761.5)	666.6 (617.6-721.1)
UKG Shropshire	1455.8 (1115.2-1854)	1428.7 (1087.7-1815.7)	1380.5 (1052.2-1774)	1387.2 (1085.9-1771.4)	1388.8 (1106.7-1699.9)	1307.9 (1097.1-1538)	1140.1 (1087-1195.6)	1050 (1026.8-1073)	956 (935.7-978.6)	911.8 (891.1-933.8)	766.3 (739.6-793.2)	671.4 (653.7-687.6)	577.3 (561.6-592.6)	538.5 (495.3-585.1)
UKG Solihull	1363 (1029.9-1776.2)	1327.3 (1012.2-1714.2)	1268.1 (959.9-1626.7)	1264.4 (979.1-1595.1)	1249.9 (995.6-1570.4)	1163.5 (971.1-1379.5)	1045.7 (994.4-1098.1)	923.8 (899.1-948.1)	829.9 (808.2-853.3)	776.5 (755.7-798.1)	684.8 (660.1-709.2)	609 (591.7-627.9)	526 (510.5-542.7)	542.4 (495.2-592.6)
UKG Staffordshire	1464.9 (1131.2-1886.6)	1437.6 (1106.3-1829.6)	1387.6 (1060.3-1768.5)	1401.2 (1073.9-1792.9)	1429.6 (1119.2-1792)	1396.6 (1173.4-1662.6)	1240.2 (1190.3-1289)	1100.2 (1080.9-1119)	1049.2 (1031.7-1067.4)	955.2 (938.1-972.9)	821.9 (798-847)	701.6 (687.7-715.1)	601.8 (589.5-613.7)	619.3 (573.4-666.3)
UKG Stoke-on-Trent	1708.5 (1306.8-2172.1)	1672.4 (1275.5-2134.7)	1609 (1218.8-2035.4)	1612.4 (1231.4-2042.8)	1613.5 (1279.2-1978.5)	1542.5 (1313.7-1794.9)	1410.3 (1349.7-1469.8)	1314.4 (1287.7-1342)	1187.2 (1162.1-1211.6)	1145.2 (1119.2-1170.1)	961.6 (932-992.9)	846.5 (826.8-866.5)	761.1 (742.9-778.9)	728.7 (670.3-784)
UKG Telford and Wrekin	1545.6 (1139-1985.1)	1518.6 (1125.5-1970.5)	1469.6 (1115.5-1888.4)	1481.8 (1156.7-1882)	1490.8 (1166.7-1847.1)	1429.7 (1186.9-1685.3)	1334.2 (1266.5-1407)	1197 (1166.4-1227.4)	1074.4 (1047.1-1105.5)	943.3 (918.3-969.4)	843.4 (813.1-873.3)	718.9 (698.2-741.9)	730.1 (709.5-751.3)	705.5 (649-767.2)
UKG Walsall	1633.1 (1230.8-2078.9)	1588.9 (1226.1-2046.2)	1520.1 (1186.3-1960.2)	1518.5 (1201.5-1916.2)	1518.5 (1204.3-1860.2)	1446.5 (1208.9-1689)	1310.9 (1252.8-1369.6)	1208.3 (1184-1233.5)	1119.1 (1095.7-1141.9)	1039.5 (1015.7-1062.9)	874.3 (845.3-903.2)	778.4 (759.9-796.7)	703 (686.6-719.7)	667.8 (615.6-720.6)
UKG Warwickshire	1400.4 (1082.6-1791)	1367.3 (1045.6-1770.8)	1311.5 (1002.1-1679.3)	1311.9 (1017.5-1694.4)	1312.8 (1045.3-1625.8)	1253.5 (1058.7-1484.5)	1145.3 (1095.8-1199)	1065.5 (1045.1-1085.8)	970.3 (951.2-989.7)	914.3 (895.3-932.2)	781.4 (756-806.7)	685.6 (670.6-700.6)	592.7 (578.8-606)	560.2 (519.5-607.6)
UKG Wolverhampton	1598.4 (1224.2-2049.2)	1549.9 (1188.4-1962.5)	1475.7 (1141.7-1894.1)	1468.3 (1163.8-1865.6)	1449.8 (1151.1-1807)	1367.4 (1151.4-1597.7)	1224 (1171.5-1281.1)	1149.1 (1125.9-1174.4)	1101.2 (1078.2-1125.3)	999.9 (978.2-1021.1)	906 (877-938.2)	879.7 (859-902.2)	740.2 (721.9-758.8)	673.1 (621.1-726.9)
UKG Worcestershire	1370.5 (1020.9-1799.3)	1334.7 (1014.7-1737.8)	1278 (967.6-1642.8)	1265.4 (965.7-1621.5)	1262.2 (992.4-1566.2)	1203.8 (1006-1415.9)	1162.3 (1111.9-1214)	1063 (1042.6-1082.6)	973.1 (955.4-993)	883.4 (866-902.9)	774.5 (750.1-798.3)	646.6 (633.2-659.9)	599.3 (585.8-613.1)	564.6 (522.8-606.9)
ENG Yorkshire and the Humber	1514.7 (1405-1641.5)	1484.8 (1381.1-1604)	1423.8 (1318-1545.7)	1415 (1313.6-1520.3)	1388.8 (1296.8-1484.6)	1320.9 (1259.7-1388.3)	1228.8 (1213.3-1244.1)	1141.8 (1136-1148)	1039.2 (1033.5-1045)	948.4 (942.9-954)	839 (831.7-846.7)	735.3 (730.7-740)	660.2 (656.3-664.3)	617.6 (603.5-631.4)
UKE Barnsley	1569.3 (1218.4-2002.3)	1538.9 (1192.2-1975.5)	1479 (1140-1878.1)	1467.4 (1134.2-1843.1)	1437.5 (1146.7-1786.6)	1361.6 (1153.4-1586.3)	1223.6 (1170.8-1283.1)	1184.9 (1161-1210.2)	1109.5 (1084.7-1136.9)	1058.7 (1033.9-1083.5)	899.1 (868.7-929.3)	779.2 (759.6-800.4)	699.4 (680.7-718.5)	681.4 (627.7-738.3)
UKE Bradford	1575.5 (1172.5-2020.1)	1526.3 (1141.3-1937.3)	1444.9 (1092.8-1824.6)	1421.5 (1102.7-1797.9)	1378.9 (1084.8-1700.1)	1307.2 (1100.7-1539.9)	1282.3 (1226.8-1336.8)	1211.4 (1190.5-1232.1)	1080.5 (1059.9-1099.6)	1028.6 (1009.4-1049.3)	898.4 (872.5-926)	807.1 (789.7-824.1)	686.4 (671.7-701.4)	615.9 (569.5-662.9)
UKE Calderdale	1582.8 (1211.3-2028.1)	1548.2 (1181.5-1993.1)	1489.4 (1134.6-1900.8)	1483.5 (1145-1869.1)	1455.9 (1157.9-1806.9)	1386.3 (1169.2-1625.3)	1258.6 (1197-1317.6)	1231.4 (1203.1-1258.5)	1095.7 (1070.5-1122.5)	1002.6 (977.7-1027.3)	862.5 (833.4-891)	753 (731.7-774.3)	698.5 (679.5-718.3)	648.7 (594.8-702.6)
UKE Doncaster	1512.5 (1158.2-1934.7)	1465.2 (1122.3-1929)	1385.6 (1046.4-1767.9)	1358.8 (1038.6-1705.9)	1324.8 (1046.6-1638.2)	1266 (1062.7-1463.4)	1241 (1185-1294.9)	1119.5 (1096.7-1142.9)	1035 (1012.5-1057.2)	1043.5 (1021.6-1064)	880.1 (852.6-908.6)	794.1 (776.8-812.3)	714.3 (697.7-730.9)	667.5 (615.7-721.9)
UKE East Riding of Yorkshire	1374.9 (1051.2-1768.5)	1340.8 (1026-1735.1)	1282.8 (977.6-1641.8)	1269 (968.2-1588.5)	1241.8 (988-1542.8)	1181.6 (1000.4-1372.6)	1097.5 (1046.5-1151.9)	1024 (1001.9-1046.4)	963.9 (942.1-985.6)	867.8 (847.1-888.1)	771.5 (746.3-795.9)	666.4 (651.7-682.7)	597.2 (581.6-612.4)	559.8 (515.7-603.2)
UKE Kingston upon Hull, City of	1558.2 (1185.3-2021.7)	1514.2 (1142.5-1957.6)	1432.1 (1089.7-1818.7)	1401.8 (1091.4-1772.2)	1359.1 (1076.7-1674.9)	1299 (1100-1521.5)	1316.5 (1262.6-1369.5)	1194.1 (1171.6-1217.9)	1108.8 (1085.6-1132.6)	1057.7 (1033.8-1081.6)	996.2 (964.5-1028.4)	870.7 (850.5-890.9)	804.2 (785.9-823.7)	778.7 (718.8-835.5)
UKE Kirklees	1582.1 (1213.3-2023.7)	1551.9 (1191-2003.2)	1497 (1146.5-1937.2)	1500.6 (1160.8-1913.6)	1489.4 (1165.7-1852.2)	1428 (1196.4-1668.6)	1314.4 (1258.2-1372.9)	1197.4 (1174.3-1220.4)	1087.8 (1066.1-1109.1)	955.3 (934.9-976)	856.7 (828.9-885.8)	764.9 (748.7-783.2)	688.3 (672.3-704.6)	595.8 (548.7-644.6)
UKE Leeds	1518.5 (1147.8-1921.2)	1503.1 (1131.5-1905.1)	1450.7 (1108.9-1852.7)	1456.5 (1133.1-1848.9)	1437.1 (1150.8-1788.7)	1367.7 (1147.6-1592.6)	1251.3 (1203.5-1298.4)	1137.4 (1118.6-1155.4)	999.1 (982.5-1015.7)	919.2 (904.1-934.6)	827.6 (804.4-851.8)	725.9 (711.5-739.4)	665.9 (653.5-678.7)	652.8 (605.7-699.7)
UKE North East Lincolnshire	1460.7 (1118.9-1882.9)	1399 (1055.2-1788.8)	1308.1 (979.8-1688.2)	1272.6 (973.1-1621.8)	1218 (967.8-1522.1)	1145.9 (968.2-1349.5)	1089.9 (1029.8-1151)	1112 (1082.3-1140.7)	1033.1 (1006.8-1060.4)	959.7 (935.1-986.3)	871.6 (840-903.9)	812.9 (789.4-835.9)	699.3 (678.2-719.8)	647.2 (592.8-705.7)
UKE North Lincolnshire	1539.5 (1160-1992.4)	1507.2 (1130.4-1978.1)	1443.9 (1082.8-1873.8)	1436.4 (1090.1-1820.7)	1408.6 (1100.1-1754.3)	1336.7 (1122.9-1563.7)	1256.5 (1193.4-1320.8)	1174.4 (1145.9-1203.1)	1054.8 (1026.6-1082.1)	959.8 (934.4-986.3)	825.3 (795.1-857)	713.2 (693.1-733.6)	659.9 (640.2-678.8)	615.3 (563.5-669.5)
UKE North Yorkshire	1391.2 (1080.4-1771.9)	1366.5 (1045.4-1747)	1313.1 (1016.9-1697)	1305.4 (1021.2-1641.4)	1282.8 (1014.7-1573.5)	1217.3 (1022.5-1419.7)	1103.6 (1055.2-1151.7)	1032.2 (1012.6-1050.2)	951.6 (933.3-969.6)	833.8 (818.2-849.3)	735.9 (714.5-759.3)	644.2 (630.4-657.9)	564.6 (551.7-578.5)	542.4 (499.7-588.1)
UKE Rotherham	1573.3 (1187.7-2028.6)	1554 (1180.9-2008.4)	1505.5 (1136-1915.2)	1507.4 (1164.7-1885.9)	1488.1 (1184.2-1849.3)	1412.3 (1191.9-1633.6)	1292.6 (1234.6-1353.2)	1170.5 (1144.8-1196.2)	1092.3 (1068.7-1116.4)	976.5 (953.6-998.7)	883.3 (854.6-914.5)	773 (755.4-791)	698.2 (680.2-716.6)	682 (627.8-737.8)
UKE Sheffield	1492.4 (1155.7-1929.6)	1465.6 (1133.8-1869.1)	1405.6 (1102.6-1774.1)	1398.7 (1101.5-1757.2)	1379.7 (1095.6-1708.1)	1318.7 (1115.7-1541)	1222.5 (1174.9-1273.5)	1135.8 (1114.8-1154.8)	1061.7 (1041.5-1081.4)	957.5 (939-976)	850.4 (825.5-876.5)	703.4 (688.5-717.6)	648.2 (633.6-661.7)	608.4 (561.2-655.9)
UKE Wakefield	1643.1 (1262.6-2113.5)	1640.4 (1251.6-2106.5)	1609.3 (1221.2-2044.2)	1633.2 (1256.4-2028.1)	1633.8 (1293.5-2016.4)	1554.8 (1305.5-1794.3)	1383.3 (1325.2-1442.5)	1271.9 (1248.5-1296.3)	1093.8 (1072.1-1119.3)	975.3 (954.6-994.6)	865.8 (840.3-894.9)	770.7 (753.7-788.1)	696.3 (680.6-712.1)	672.4 (622.9-728.1)
UKE York	1359.3 (1025.5-1783.5)	1327.9 (996.4-1718.3)	1265.6 (927.6-1627.8)	1248.2 (951.3-1582.3)	1215 (962.7-1495.8)	1146.4 (962.2-1322.6)	1084.4 (1026-1142.6)	1050 (1024.8-1076.1)	961 (936.8-986.9)	818.9 (797.6-840.4)	718.3 (692-745.2)	633.6 (615.1-653.1)	567 (550.4-584.2)	545 (499.1-592.5)
GBR Northern Ireland	1522.9 (1488.5-1559.4)	1445 (1419.5-1470.8)	1374.2 (1351.4-1398.9)	1377.7 (1355.4-1400.6)	1397.8 (1375.1-1419.4)	1477.3 (1455.4-1500.5)	1397.7 (1374.3-1418.4)	1294 (1273.4-1314.9)	1176.5 (1156-1197)	1061.3 (1043-1081)	882.2 (856.9-907.5)	761 (746.6-776)	704.3 (690.3-717.9)	613.4 (558.3-670.3)
GBR Scotland	1661.8 (1642.8-1683.1)	1614.6 (1600.2-1629.3)	1559.7 (1546.3-1574.6)	1550.8 (1537.5-1564.6)	1471.6 (1457.9-1485.7)	1425.1 (1411.6-1438.2)	1355.5 (1343.5-1368)	1250.3 (1238.6-1262.7)	1140.1 (1128.5-1151.7)	1056 (1044.8-1067.8)	939.8 (916.5-964.9)	819.6 (810.4-828.5)	745.7 (736.8-754.8)	706.6 (648.4-764.6)
GBR Wales	1719.5 (1316.7-2201.9)	1664.7 (1308.1-2096.9)	1561.1 (1214.5-1956.2)	1519.4 (1161.6-1916.3)	1460.7 (1124.2-1859)	1333 (1104.5-1592.6)	1224 (1179.8-1269.9)	1121 (1108.1-1133.4)	1010.8 (998.9-1023.3)	962.4 (950-973.7)	849.5 (827.3-871.6)	734 (724.3-743.5)	659.8 (650.7-669.6)	633.6 (588.4-677)
Latin America and Caribbean	2217.8 (2169.6-2267.7)	1977.6 (1935.5-2019.5)	1832.4 (1795-1868.4)	1688.4 (1655.2-1726.4)	1620.9 (1590.8-1653.2)	1441.6 (1420.6-1460.4)	1336 (1324.8-1348.4)	1262.4 (1255.6-1269)	1142.4 (1137-1147.7)	1087.1 (1080.7-1093.3)	964.4 (958.6-970.2)	892.7 (887.1-898.7)	910.9 (905.2-917.1)	814.5 (800.7-828.5)
Andean Latin America	2443.8 (2312-2595.5)	2077.3 (1997.7-2159.1)	1983.9 (1908.2-2058.1)	1704.7 (1635.8-1770.9)	2025.1 (1963.9-2086.2)	1361.4 (1313.6-1412)	1274.8 (1228.2-1323.5)	1196.8 (1153.2-1241.7)	1050.8 (1011.1-1090.9)	981 (939.1-1022.3)	865.2 (832.1-900.5)	808.9 (773.1-843.8)	777.9 (738-818.4)	662.2 (603.6-725.8)
BOL Bolivia	3066.4 (2857-3328.9)	2517.8 (2399.3-2653.3)	2353.9 (2245.5-2473.7)	2301.5 (2193.8-2407.7)	2181.9 (2073.7-2298.7)	2020.1 (1934.5-2114)	1826.5 (1731.2-1922.5)	1684.6 (1572.7-1797.5)	1534.7 (1428.8-1643.8)	1358.4 (1269.1-1462.5)	1238.4 (1168.5-1353.4)	1146.6 (1033.2-1267.6)	1071.9 (910.8-1239.8)	986.3 (795.8-1215.8)
ECU Ecuador	1976.6 (1737.4-2252.5)	1831.7 (1799.9-1865.8)	1719.6 (1690.7-1748.5)	1469.8 (1446.3-1495.4)	1423.9 (1402.5-1446.3)	1235.6 (1218.8-1254)	1177 (1160.9-1193)	1028 (1015.8-1041.1)	954.3 (943.8-964.8)	881.2 (870.9-891.1)	847.1 (835-859.3)	904.8 (857.9-950.2)	881.5 (835.8-926.2)	761.3 (692.2-838.5)
PER Peru	2375 (2169.5-2600.6)	2026.2 (1894.3-2162.8)	1967.9 (1840.5-2096.2)	1604 (1492.5-1714.5)	2228.6 (2136-2327)	1220.4 (1141.5-1300.9)	1164.5 (1094.3-1237.8)	1141.1 (1075.1-1209.3)	965.4 (907.8-1024.5)	923.7 (862.2-988.1)	774.6 (722.1-828.6)	685 (638.4-733.4)	665 (617.6-715.9)	545.9 (459.4-634.5)
Caribbean	1966.5 (1849.3-2085.9)	1771.7 (1673.8-1876)	1634.1 (1581-1691.4)	1495.6 (1448.7-1548.6)	1340 (1296.2-1386.6)	1240 (1203.6-1279.9)	1204.9 (1174.3-1239.6)	1180.9 (1154-1207.7)	1128 (1103.7-1153)	1124.9 (1098.7-1155.6)	1025.1 (995.5-1055.4)	974.8 (943.4-1009.6)	1561.6 (1527.7-1599.2)	934.2 (883.5-988.8)
ATG Antigua and Barbuda	2083.4 (1981.5-2195.9)	1752.6 (1697.9-1807.5)	1532.8 (1483.3-1585.7)	1393.6 (1346.2-1439.2)	1400.4 (1343.4-1457.2)	1435.6 (1372.1-1502.7)	1299.5 (1231.4-1371.1)	1148.8 (1094.2-1208.3)	1060.5 (1016.6-1107.3)	1033.2 (989.5-1077.4)	979.3 (939.1-1021.5)	875 (838.5-911.8)	783.2 (752-815.1)	715.1 (666.7-765.8)
BHS The Bahamas	1684.2 (1443.4-1927.8)	1534.5 (1347.8-1725.7)	1426.6 (1299.8-1550.6)	1492.5 (1437.5-1549.4)	1415.5 (1383.5-1447.8)	1299.8 (1273.7-1326.7)	1296 (1271.3-1320.8)	1205.1 (1180.5-1228.8)	1175.5 (1151.3-1198.8)	1194.1 (1168.7-1218.9)	1092.2 (1070-1114.9)	999 (978.9-1021.7)	981.7 (960.8-1003.2)	941.2 (864.1-1020.7)
BRB Barbados	1852.5 (1741.9-1977.2)	1611.4 (1565.6-1662.7)	1427.1 (1393.7-1464.7)	1386.4 (1355.5-1418.5)	1440.2 (1409.8-1471.8)	1430.6 (1402.7-1459.9)	1146.4 (1123.1-1172.5)	1050.5 (1027.6-1075.5)	1022.5 (1001.3-1042.4)	1014 (989.7-1041.3)	931.1 (907.4-954.8)	932.2 (911.6-952.7)	797.1 (778.3-815.9)	760.8 (696.9-822.4)
BLZ Belize	2034.5 (1868.5-2246.5)	1683.6 (1596.1-1780.4)	1249.5 (1182.4-1312.7)	1052.6 (997-1111.1)	971.5 (918.6-1025.5)	910.8 (862.5-958.4)	898.5 (852.1-947.2)	938.8 (893.4-989.2)	952.3 (906.3-1002.6)	989 (948.4-1032.2)	1161.1 (1137.4-1186.4)	1008.4 (989.4-1027.3)	895 (879.4-912)	893.5 (859.7-926.9)
BMU Bermuda	1747.8 (1686.2-1813.6)	1556.5 (1512.2-1593.1)	1391.2 (1359.1-1424.5)	1320.8 (1296.3-1350.8)	1338.1 (1310.7-1367.2)	1223.2 (1187.6-1258.5)	1178.9 (1143.6-1212.6)	1203.5 (1169.5-1237.4)	1183.2 (1141.5-1224.9)	937.8 (918-969.6)	874.1 (849.1-898.4)	748 (719.4-774.2)	708.9 (685.9-736)	704.4 (674.1-745.6)
CUB Cuba	1353 (1122.9-1612)	1432.8 (1221.7-1678)	1402.4 (1387.4-1416.5)	1226.6 (1216.9-1236.5)	979.3 (970.7-987.9)	841.4 (834.3-848.4)	878.4 (871-885.7)	912.6 (905.9-919.7)	899.7 (893-905.9)	923 (916.2-929.6)	806.1 (800.1-812.1)	769.6 (763.4-775.4)	730.6 (724.4-736.7)	725.1 (644-819.9)
DMA Dominica	2434.8 (2232-2682.4)	1822.2 (1731.4-1922.6)	1435.1 (1375-1499.2)	1255.3 (1209.5-1307.3)	1202.5 (1163.2-1242.8)	1084.6 (1050.2-1119.9)	949.8 (916.5-982.6)	942.2 (911.1-970.8)	1028.2 (1000.3-1057.3)	1036.9 (1008.6-1068.5)	964.7 (935.6-995.1)	921.7 (892.9-953)	937.2 (908.1-969.3)	985.3 (924.7-1050.3)
DOM Dominican Republic	1653.3 (1559.6-1764.5)	1397.9 (1354.5-1445.9)	1253.8 (1168.7-1342.8)	1261.7 (1191-1326.8)	1142.5 (1069-1223)	1052 (977.4-1128.7)	1001.3 (929.7-1079.9)	929.3 (860.2-994.5)	900.4 (838.9-968)	838.5 (775.5-908.7)	869.2 (795.9-943.8)	890.2 (805.4-973.4)	884.7 (806.9-966.1)	976.7 (847.7-1104.6)
GRD Grenada	1635.7 (1533.3-1749.7)	1548.3 (1461.9-1636.2)	1523.1 (1426-1620)	1592.6 (1484.2-1695)	1401.5 (1307.2-1492)	1189.6 (1116.2-1265.1)	1237 (1179.5-1302.6)	1325.5 (1276.2-1381.5)	1338.1 (1300.5-1374.1)	1095.3 (1062.7-1126.3)	949.6 (923.9-974.6)	900.2 (876.8-924.8)	891.9 (867.9-916)	810.8 (772.6-855)
GUY Guyana	2479.2 (2381.6-2588.3)	2095.2 (2045.8-2145.8)	1764.7 (1729.8-1801.2)	1767.8 (1665.3-1874.9)	1609.6 (1581.2-1641.1)	1681.6 (1640-1724.4)	1582.4 (1538-1630.7)	1438.1 (1398.7-1478.7)	1492.3 (1469.9-1516.3)	1439.5 (1419.5-1459.3)	1329.4 (1310.3-1349.3)	1365.5 (1346.1-1384)	1284.2 (1264.7-1304.9)	1198.4 (1061.3-1344.5)
HTI Haiti	3517.9 (3127.2-3932.8)	3101.5 (2768.2-3447.6)	2945.3 (2620.5-3300.4)	2832.9 (2511.5-3160.6)	2694.4 (2397.9-3011.6)	2485.6 (2219.4-2769.3)	2260.2 (2036.9-2506.3)	2049.7 (1872.3-2243.1)	1895.2 (1741.4-2065.4)	1857 (1692.9-2052.4)	1714.4 (1534-1927)	1593.7 (1399.7-1814.5)	4595.6 (4382.8-4836.6)	1399.9 (1198.2-1592.6)
JAM Jamaica	2025.1 (1954.9-2098)	1631 (1598.7-1664.3)	1439.2 (1414.2-1466.4)	1322 (1297.7-1345.6)	1120.7 (1101.4-1140.7)	1118.1 (1096.5-1141.6)	941.9 (923.7-959.3)	1043 (1023.7-1061.8)	851.3 (811.9-891.9)	941.5 (863.4-1020.3)	883.6 (870.8-896.2)	663.9 (650.9-677.9)	806.5 (794.1-820.1)	895.5 (766.3-1036.4)
PRI Puerto Rico	1515.1 (1488.9-1543)	1097.8 (1083.9-1112.8)	1085.8 (1071.8-1100.4)	1050.2 (1036.2-1064)	1036.2 (1022.9-1048.2)	1020.1 (1008.1-1032.8)	996.1 (984.9-1007.5)	1023.8 (1012.3-1035.6)	1015.1 (1004.2-1026.2)	1031.7 (1021.9-1041.5)	849.2 (839.3-858.8)	793.5 (784.2-803)	707.4 (697.8-717.3)	726.2 (684.4-769.3)
LCA Saint Lucia	2048.8 (1922.2-2195)	1710.4 (1645.5-1785.4)	1521.9 (1465.5-1585.1)	1346.3 (1294.8-1406.6)	1329.9 (1281.2-1382.8)	1368.5 (1332.1-1406.1)	1282.9 (1251.9-1315.2)	1198.4 (1170.6-1226.3)	1289.2 (1261.1-1318)	1120.4 (1096.4-1145.8)	990.8 (965.8-1014.4)	998.2 (975.7-1021.9)	840.4 (819.7-861.5)	848.5 (797.2-903.1)
VCT Saint Vincent and the Grenadines	1811.9 (1703.2-1938.3)	1683.8 (1619.9-1753.1)	1284.5 (1224.1-1345.2)	1222.3 (1169.7-1280)	1209.7 (1157.3-1265.7)	1174.4 (1133.5-1219.7)	1124 (1090.8-1159.4)	1059.4 (1028.2-1088.6)	1067.1 (1035-1097.4)	1042.3 (1011-1072.4)	1059 (1032.6-1084.7)	988.6 (964.2-1012)	935.5 (911.9-959.6)	984.7 (933-1038.1)
SUR Suriname	1950.3 (1783.8-2125.9)	1735.2 (1624.7-1846.4)	1573.9 (1458.1-1691.4)	1428.3 (1333.3-1534.8)	1487.5 (1373.3-1607.8)	1564.2 (1456.7-1669.9)	1515.8 (1427.5-1598.9)	1291.7 (1210.9-1372.6)	1204 (1136.5-1275)	1093.2 (1029.5-1157.5)	1172.2 (1105.8-1241.1)	1155.5 (1086.8-1232.1)	1043 (981.3-1108.7)	1067.9 (954.3-1195.6)
TTO Trinidad and Tobago	2179.5 (2112.8-2258.4)	1946.2 (1907.8-1985.3)	1707.6 (1670.8-1742.6)	1674.2 (1643.1-1702.6)	1578.8 (1550.6-1608)	1484.5 (1458.7-1509.2)	1453.6 (1430.1-1476.5)	1399.2 (1374.9-1423.3)	1260.6 (1239.6-1282)	1237.9 (1218.6-1257.7)	1169.8 (1151-1188.9)	1080.7 (1062.6-1099.3)	976.3 (958.4-995.1)	936.2 (769.3-1129.8)
VIR Virgin Islands	1799.6 (1735.1-1870.9)	1691.7 (1640.3-1742.4)	1593.3 (1549.6-1637)	1522.4 (1466.1-1575.1)	1435.8 (1364.5-1533)	1359.9 (1305.4-1443.3)	1315.9 (1240-1367.4)	1230.5 (1172-1289.9)	1157.3 (1110.1-1216.6)	1154.6 (1098.4-1240.7)	1141.3 (1050-1241.5)	1105.1 (976-1230.4)	1155.8 (995.4-1268.9)	1176.7 (963.4-1300.8)
Central Latin America	2096.4 (2055.1-2142.3)	1787.8 (1758.6-1818.7)	1613.4 (1586.2-1642.5)	1502.4 (1476.4-1529.5)	1439.9 (1415.4-1464.6)	1338.5 (1321-1354.8)	1249.6 (1229.7-1269.1)	1182.3 (1177.9-1187.1)	1041.7 (1037.5-1046)	1008.3 (1002.4-1014.4)	887.9 (882.7-893.2)	826.8 (821.5-832.2)	810.7 (805.8-816.2)	781.5 (756.7-806.5)
COL Colombia	1786.7 (1716.5-1866.2)	1630.8 (1594.6-1671.7)	1497.6 (1469.2-1526)	1383.5 (1363.5-1405.2)	1193.3 (1174.9-1211.9)	1246.3 (1231-1264.8)	1067.1 (984.6-1149.2)	1225.7 (1217-1234.8)	999.1 (991.6-1006.5)	1008.6 (1001.8-1015.4)	875.4 (869.3-882.4)	754.3 (748.3-761)	675.8 (670.1-681.9)	574.8 (509.8-644.1)
CRI Costa Rica	1763.2 (1721.8-1805)	1423.8 (1399.8-1447.3)	1219.9 (1200-1242)	1263.5 (1243-1284.5)	1179.9 (1162.2-1198.6)	992 (977.2-1006.9)	908 (894.5-921.6)	866.7 (854.2-879.1)	800.6 (788.7-811.9)	820.8 (809.9-831.2)	732.2 (722.2-741.6)	645.8 (636.7-654.9)	650.8 (642.5-658.7)	684.6 (643.4-725.5)
SLV El Salvador	2267.7 (2111.3-2455.3)	1984 (1901.7-2077.3)	1644.4 (1579.6-1712.1)	1573.3 (1525.2-1627.5)	1660.5 (1622.3-1700.2)	1425 (1394.7-1453.1)	1507.2 (1442.2-1569.5)	1332.7 (1315-1350.7)	1153.1 (1139-1167.5)	1105.6 (1056.8-1155.1)	1008.3 (961.9-1054.5)	1076.7 (1029.6-1128)	1015.6 (967.3-1061.8)	1020.1 (862.4-1194.3)
GTM Guatemala	2733.6 (2589.6-2916.2)	2484.5 (2410.9-2566.8)	2217.3 (2157.1-2282.1)	2168.3 (2120.6-2218.1)	2239.4 (2202-2278.9)	1952.7 (1923.7-1981.9)	1893.3 (1868.7-1918)	1621.5 (1604-1642.6)	1502.4 (1486.3-1519.8)	1393.3 (1379.2-1407.2)	1261.5 (1249.1-1274.7)	1218.1 (1206.9-1229.1)	1089.4 (1079.2-1100.7)	1029.6 (928-1144.4)
HND Honduras	2786.7 (2515.5-3079.5)	2519.5 (2330.4-2710.3)	2278.4 (2116.7-2449.7)	1984.8 (1841.4-2127.9)	1726.1 (1620.4-1828.1)	1418.1 (1327.8-1515.8)	1081.7 (1006.5-1164.8)	1018.8 (928-1123.9)	1015.4 (905.3-1131.1)	1006.6 (857.4-1190.6)	953.4 (783.6-1124.5)	907.8 (739.4-1099.8)	859 (698-1036.6)	799.5 (661.2-956.1)
MEX Mexico	2182 (2109.3-2259.9)	1803.4 (1749.3-1857.7)	1640.6 (1589-1694.2)	1513.6 (1466.8-1562.2)	1474.8 (1430.5-1520.4)	1342.9 (1309.8-1373.4)	1288.7 (1274.6-1302.8)	1163.4 (1159.4-1167.5)	1040.1 (1037.1-1043.3)	986 (983.2-988.7)	861.8 (859.5-864.3)	818.5 (816.4-820.6)	843.2 (841.3-845.2)	837.7 (826.1-849.2)
MEX Aguascalientes	1716 (1463.7-1998)	1487.8 (1285.2-1740.3)	1425.9 (1221.5-1646.8)	1403.5 (1210.9-1622.2)	1450.7 (1267.6-1652.3)	1342.8 (1231.9-1453.6)	1183.3 (1151.5-1214.2)	1102 (1079.3-1126.7)	1009.5 (988.5-1029.6)	966.5 (951-983.3)	852.4 (837.7-868)	828.7 (815.8-842.6)	801.6 (789.8-813.2)	735.3 (690.5-783.3)
MEX Baja California	2880.6 (2496.2-3311)	2103.5 (1838.2-2380.4)	1783.5 (1544.9-2058.2)	1622.7 (1388-1877.6)	1645.8 (1418.1-1901.7)	1593.4 (1440.3-1754.4)	1378.8 (1354-1403.8)	1389 (1370.1-1406.6)	1314 (1298.3-1330.7)	1282.1 (1268.4-1296.4)	1164.7 (1151.7-1178)	1045 (1033.5-1057.5)	998.6 (989-1008.4)	912.5 (859.1-969.2)
MEX Baja California Sur	1718.2 (1440.3-1999.3)	1519.2 (1283.4-1766.1)	1486.6 (1261.6-1721)	1504.1 (1311.3-1717.5)	1554.2 (1385.9-1733.2)	1494.5 (1386.8-1601.2)	1294.1 (1258.5-1331.3)	1118.6 (1095.3-1141.6)	1062.5 (1042.8-1081)	1050 (1032.1-1068.6)	915.8 (899.5-932)	883.7 (869.7-897.5)	856.7 (843.1-869.8)	796.4 (748.5-847.7)
MEX Campeche	1507.2 (1287.8-1769.3)	1278.2 (1078.4-1507.1)	1193.5 (1007.2-1405)	1112 (931.2-1306)	1096.9 (938.2-1252.2)	1025.8 (931.5-1117.8)	1005 (974.3-1037.1)	992.4 (970.5-1016.9)	925.4 (907-944.1)	885.1 (868.7-901.1)	742.3 (727.6-758)	703.6 (690.2-717.1)	743.4 (731.9-756.3)	830.2 (781.7-881.9)
MEX Chiapas	1672.2 (1419.3-1938.7)	1422.7 (1215.2-1659.8)	1316.5 (1122.5-1534.8)	1230.1 (1047.3-1433.1)	1193.1 (1020.9-1373.2)	1083.1 (978.5-1188.9)	1203.1 (1171.8-1235)	1145 (1120.2-1171)	952.8 (935.2-970.4)	928.6 (913.7-943.8)	855.4 (843.4-868)	811 (800.7-823.3)	812.6 (803.4-822.4)	832.8 (783.8-882.5)
MEX Chihuahua	2311 (2002.6-2635.9)	1880.5 (1636.5-2154.4)	1683.7 (1440.6-1943.1)	1548.9 (1306.3-1799.8)	1484.4 (1274.6-1710.1)	1338.2 (1213.1-1469.7)	1317 (1289.5-1347.4)	1292.7 (1274.2-1311.3)	1269.9 (1255.2-1284.9)	1228.2 (1213.5-1243.1)	1134.1 (1120.2-1148.3)	1006.4 (995.4-1017.8)	1319.6 (1309.5-1329.7)	1002.3 (939.4-1066.3)
MEX Coahuila	2035.4 (1745.8-2335.3)	1722 (1473.1-1967.3)	1606.8 (1347.4-1865.1)	1552.9 (1326.7-1803.5)	1582.5 (1369.9-1846.6)	1456.5 (1325.9-1597)	1348.1 (1319.8-1378.1)	1231.6 (1212.5-1251.7)	1155.4 (1140.6-1171.4)	1125.5 (1111.4-1140.3)	960.9 (947.8-974.4)	872.9 (861.2-884.7)	893 (882.8-903.4)	809.3 (761.3-860.2)
MEX Colima	2631.1 (2268.5-3020.8)	2224.4 (1954.4-2513.4)	2082.4 (1835.9-2353)	2026.3 (1781.2-2271.1)	2102.2 (1909.7-2293.2)	1931.5 (1816.1-2042.3)	1692.9 (1652.3-1736.3)	1461.2 (1436.4-1486.6)	1238.4 (1217.7-1258.3)	1059.9 (1039.5-1081.4)	873 (856.8-889.5)	832.7 (819.1-847.6)	860.1 (847.8-873.5)	919.9 (866.2-974)
MEX Mexico City	2448.9 (2125.7-2824)	1900.6 (1672.2-2151.3)	1712.2 (1503.4-1948.2)	1583.8 (1395.8-1799.6)	1512.1 (1326.9-1691.6)	1357.3 (1246.3-1468.1)	1377 (1354.8-1400.2)	1227.3 (1213.6-1241)	1088.2 (1079.2-1097.2)	1031.4 (1022.3-1041.3)	889.5 (881.9-897.3)	872.1 (865.2-879.9)	856.4 (848.9-863.7)	916.1 (864.5-970.7)
MEX Durango	2329.2 (2037.8-2662.1)	1944 (1689.4-2209.4)	1774.4 (1525.8-2025.6)	1625.1 (1392.7-1864.2)	1553.8 (1357.6-1774.5)	1384.8 (1257.1-1517.6)	1392.9 (1360.9-1425.3)	1239.7 (1219-1260.6)	1040.8 (1021.6-1058.8)	911.7 (896.9-926.6)	779 (764.5-794.2)	748.3 (735.7-760.8)	986.8 (973.5-999.7)	723.8 (677.6-769.1)
MEX Guanajuato	2227.1 (1944.1-2515.1)	1873.2 (1640.3-2116.9)	1724 (1489-1970.1)	1630.1 (1429.5-1846)	1638.9 (1448.4-1874.7)	1499.5 (1374.8-1644.7)	1274.6 (1241.6-1307.1)	1305.4 (1284.3-1327.2)	1186.8 (1170.8-1203.3)	1098.3 (1083.6-1113.1)	915 (902.5-928.7)	805.1 (795.7-814.6)	774.5 (767.3-782.3)	825.7 (777.6-874.4)
MEX Guerrero	2163 (1858.4-2476.8)	1795.8 (1557.8-2042.8)	1596.3 (1364.2-1824.3)	1429.9 (1236.1-1645.2)	1339.7 (1161-1525.6)	1121.6 (1022.6-1230.8)	1162.3 (1131.6-1195.2)	1012.4 (985.6-1040.8)	842.1 (822.7-862.9)	862.1 (843.7-881.9)	707.9 (693.9-723)	631.2 (620.3-642.5)	786.4 (776.7-796.4)	814.6 (766.4-864.7)
MEX Hidalgo	2196.6 (1931.9-2496)	1868.7 (1628.4-2124.5)	1736.4 (1498.5-1968.8)	1600.4 (1375.5-1837.3)	1585.9 (1381.7-1833.4)	1471.3 (1336-1613.6)	1541.3 (1506.7-1576.3)	1196.2 (1177.9-1216.5)	989.8 (972.6-1008.4)	874 (860.3-888.6)	763.5 (750.4-777.6)	736.4 (726.6-746.7)	762.7 (753.4-772.1)	721.4 (677.8-768.8)
MEX Jalisco	2172.4 (1864-2472.7)	1784.9 (1550.3-2028.7)	1606 (1382-1838.9)	1465 (1262.5-1697.6)	1417.8 (1227.5-1627)	1264.3 (1144.1-1377.7)	1127.2 (1104.1-1151.3)	1073.8 (1056.6-1090.7)	1037.5 (1023.9-1051.7)	1017.3 (1006.1-1028.1)	892.9 (882.3-904)	841.1 (832.8-849.8)	843.3 (836-851.2)	868.4 (816.9-921)
MEX México	1817.5 (1568.1-2082.6)	1611.5 (1409.5-1834.4)	1558.5 (1342.1-1795.2)	1502.6 (1291.8-1719.3)	1523.6 (1313-1758.6)	1431.6 (1231.9-1645.9)	1442.9 (1303-1586.8)	1354.3 (1340.9-1367.9)	1165.1 (1154.3-1175.3)	1068.2 (1058.6-1078.7)	916 (908.5-923.3)	851.4 (844-858.3)	820.1 (813.5-826.8)	878.2 (828.3-931.3)
MEX Michoacán de Ocampo	2390 (2056.6-2741.3)	1974.1 (1728.8-2239.2)	1781.6 (1543.4-2027.3)	1606.6 (1392.8-1849.8)	1516.1 (1316-1720.3)	1383.4 (1263.5-1511.3)	1268.3 (1235.5-1303.2)	1129.6 (1109.6-1149.9)	943.8 (928.9-958.2)	945.8 (933.8-958.5)	800.7 (789.8-813)	767.9 (757.6-779.5)	794.3 (784.9-804.6)	756.7 (711-803.4)
MEX Morelos	2076.1 (1789.3-2345.4)	1741.2 (1497.3-1979.3)	1601 (1372.2-1827.9)	1468.8 (1272.2-1695.8)	1428.4 (1231.8-1651.6)	1281 (1150.2-1419.8)	1284.4 (1252.9-1315.2)	1055.5 (1035.6-1075.7)	1001.9 (983.9-1021.6)	965 (950.4-980.2)	796.2 (783-809.4)	747.7 (736-760)	792.4 (781.2-803.5)	901.6 (847-961.5)
MEX Nayarit	2300 (1974.3-2626.2)	1888.8 (1644.9-2162.4)	1693.7 (1454.1-1943.9)	1521 (1304.2-1754.3)	1416.1 (1227.5-1617.9)	1206.2 (1090.4-1332.5)	1149.4 (1119.2-1180.1)	1108.9 (1087-1131.2)	972.9 (954.2-991.4)	896.8 (879.6-913.2)	736.7 (721-751.6)	785.8 (771.9-800.2)	830.2 (816.5-843.2)	740.3 (690.3-790.4)
MEX Nuevo León	1735.4 (1471.9-2005.9)	1449 (1244.8-1677.7)	1323.1 (1118.7-1537)	1234.8 (1044.2-1441.3)	1226.1 (1039-1421.1)	1127 (1017.5-1260.6)	1016.8 (997-1036.5)	960.9 (946.1-975.6)	994.2 (981.7-1007.6)	966.4 (954.8-977.8)	863.5 (853-875.2)	845.3 (836.7-854.7)	859.2 (850.9-867.7)	778.2 (732.4-826.6)

Table 1 Age-standardised mortality rates by location and year for males for 1950, 1955...2005, 2010, 2017

Location	1950	1955	1960	1965	1970	1975	1980	1985	1990	1995	2000	2005	2010	2017
MEX Oaxaca	2560.4 (2229.3-2953.2)	2120.8 (1853.1-2411.6)	1910.9 (1666.1-2171)	1765.4 (1541.1-2005.9)	1744.9 (1517.4-1982.1)	1628.8 (1493.7-1772.5)	1562.7 (1533.4-1593.2)	1296.8 (1278.4-1315.8)	1085 (1069.4-1102.2)	982.4 (968.6-995.3)	822.1 (810.7-833.4)	763.4 (752.3-774.1)	776.4 (767.5-786.2)	785.4 (736.4-833.4)
MEX Puebla	2621 (2333.5-2952.9)	2211.9 (1970.7-2490.6)	2029.2 (1776.6-2277.9)	1867.5 (1627.8-2111.2)	1804.3 (1578.1-2048.7)	1613.9 (1482.5-1752.5)	1554.1 (1519.7-1588.2)	1322 (1295.8-1349.1)	1157.4 (1141.1-1174.8)	1061.4 (1047.3-1076.4)	916.6 (904.2-929.4)	848.7 (837-862.6)	821.8 (811.8-832.4)	905.8 (854.6-956.9)
MEX Querétaro	1992.8 (1685-2303.4)	1724.1 (1480.7-1975.7)	1624.3 (1396.2-1867.5)	1550.2 (1320-1782)	1562 (1337-1815.1)	1458.8 (1253.4-1681.5)	1440.5 (1332.4-1554.1)	1182.7 (1161.8-1204.5)	1114.3 (1097.5-1132.6)	1053.1 (1036.9-1067.9)	959.5 (945.8-972.9)	853.4 (841.3-865.2)	811.4 (800.9-822.2)	853.2 (799.5-907.1)
MEX Quintana Roo	915.3 (755.1-1108.7)	900 (751.4-1070.4)	891.7 (736.8-1064.4)	974.6 (822.5-1145.2)	1121.4 (971-1286.5)	1148.4 (1052.3-1252.8)	1043.5 (1011.4-1078.6)	846.1 (823.8-868.6)	884.9 (865.6-904.8)	860.5 (844.5-877.3)	794.1 (779.1-809.8)	780.8 (769.3-793.4)	832.2 (820.9-843.1)	909.6 (857.8-961.7)
MEX San Luis Potosí	2214.7 (1905.9-2544.7)	1834.2 (1588.9-2102.4)	1672.8 (1444-1906.7)	1572 (1344.2-1820.2)	1564.5 (1361.5-1799.6)	1452.2 (1315.3-1594.4)	1131.7 (1103.2-1163.2)	1076.8 (1059.1-1095)	923 (907.8-937.3)	873.7 (860.4-888.2)	752.1 (740-765.6)	723.5 (711-736.6)	760.2 (750-771.5)	733.7 (689.7-780.1)
MEX Sinaloa	1908.1 (1632.2-2194.1)	1627.2 (1379.3-1877.1)	1511.6 (1286.7-1746.2)	1402.6 (1196.1-1618.3)	1372.6 (1177.1-1578.7)	1210 (1090.2-1334.8)	1144.2 (1118.1-1171.2)	1162.2 (1145.3-1178.5)	951.9 (938.5-966.5)	936.4 (925.1-949.1)	809.8 (797.9-822.2)	794.7 (784.9-805.5)	910 (900.5-920.1)	692.8 (647.2-738)
MEX Sonora	2032.5 (1720.4-2334.9)	1697.4 (1439-1975.6)	1567 (1340.1-1816.4)	1479.6 (1249.2-1731.9)	1486.7 (1249.2-1732.3)	1377.4 (1167.4-1607.7)	1334.1 (1202.4-1470.5)	1240.7 (1222.7-1259.6)	1186.1 (1171.2-1202.1)	1182.8 (1167.7-1198.1)	1004.1 (989.3-1017.9)	978.7 (966.6-992.6)	963.9 (953.2-974)	930.4 (874.8-986.6)
MEX Tabasco	1991.7 (1706.1-2298.3)	1725.7 (1488.1-1960.4)	1613.6 (1380-1858.9)	1526 (1312.8-1763.3)	1509.2 (1308.5-1727.6)	1422.7 (1289.8-1560.2)	1479 (1442.8-1518.7)	1104.6 (1079.1-1130.3)	952.9 (935-971.5)	970.1 (954.5-987.5)	821.8 (806.3-837.5)	815.4 (804.6-827.4)	859 (848.3-870.6)	873.7 (819.6-928.5)
MEX Tamaulipas	2192 (1894.2-2523.4)	1808.5 (1575.2-2063.2)	1628.3 (1389.5-1877.5)	1472.5 (1261.6-1695.7)	1390.4 (1200.3-1618.8)	1247.9 (1138.7-1372.4)	1346.8 (1320.8-1371.9)	1126.7 (1109.3-1145.2)	977.1 (962.3-991.1)	946.6 (930.7-963.6)	859.8 (848-872.7)	839.8 (830.5-848.7)	878.9 (869.4-888.3)	779.9 (731.7-829.5)
MEX Tlaxcala	2088.1 (1781.1-2413.7)	1763.5 (1512-2045.7)	1627.4 (1389.6-1896.4)	1534.6 (1314.4-1769.1)	1477.6 (1280.8-1689)	1336.2 (1179.6-1520)	1280.6 (1174.2-1400.4)	1165 (1138.8-1192.1)	964 (947.3-981.7)	930 (914.6-945.6)	809.3 (796.9-821.8)	728.9 (716.9-740.7)	748.3 (737.2-759.8)	781 (732.8-831.7)
MEX Veracruz de Ignacio de la Llave	2104.6 (1830.3-2416.3)	1712.6 (1490.3-1943.9)	1522.4 (1316.7-1723.9)	1365.9 (1171.2-1558.1)	1318.3 (1133.7-1503.8)	1208.5 (1098.8-1321.6)	1177.5 (1153.3-1201.9)	1014.7 (999.2-1030.9)	951 (937.1-966.1)	899.2 (889.4-909.6)	827.1 (815.6-839.3)	807.5 (799.2-815.7)	815.7 (808.5-823.2)	857.9 (810.4-907.4)
MEX Yucatán	1677.1 (1421.9-1979.3)	1445.9 (1237.1-1691.8)	1377.8 (1176.8-1615.4)	1338 (1135.8-1582.2)	1352.1 (1147.9-1573.6)	1289.2 (1158.2-1430.7)	1273.9 (1245.1-1300.1)	1207 (1185.3-1229.3)	1076.9 (1059-1095.8)	1036.1 (1021.6-1051.4)	866.5 (852.3-881.4)	791.6 (779.1-803.7)	839.9 (828.4-851.3)	911 (860.2-966.1)
MEX Zacatecas	2098.4 (1812.3-2421)	1715.6 (1487.5-1974)	1545 (1340.8-1784.2)	1419.1 (1212.5-1635.8)	1393.9 (1209.5-1588.9)	1275.2 (1163.9-1395.6)	1110.3 (1076.2-1142.1)	917.6 (895.4-939.4)	842.9 (823.7-862.9)	833.9 (818.3-849.6)	739.1 (723.9-754.3)	708.8 (696.6-720.8)	760.1 (748.4-772.4)	738.5 (689.1-786.6)
NIC Nicaragua	2205.3 (2053.8-2393.9)	1850.8 (1757.6-1947)	1791.6 (1713.8-1877.9)	1699.2 (1612.6-1782.7)	1670.5 (1527.9-1805.1)	1446.5 (1365.7-1531.7)	1237.5 (1091.7-1406.2)	1441.5 (1300.8-1588.1)	876.3 (821-934.3)	877.6 (806-947.7)	799.8 (746.3-858.1)	787.7 (737.4-839.2)	655.8 (626.6-685.8)	580.1 (518-655.7)
PAN Panama	1448.5 (1395.3-1507.8)	1381.6 (1350.4-1415.4)	1185.8 (1158-1215.1)	1145.5 (1120-1171.8)	1125.5 (1103.9-1148.5)	978.3 (958.5-1000.6)	889.6 (872.3-909.1)	842.9 (827.2-859.7)	788.3 (773.5-803.6)	819.4 (806-834.2)	717.2 (704.4-729.4)	687.7 (676.9-699.6)	691.3 (681-702)	612 (572.6-650.3)
VEN Venezuela	1923.2 (1895-1957.7)	1599.4 (1588.2-1610.4)	1392.7 (1383.3-1402.2)	1336.8 (1327.4-1346.1)	1336.4 (1327.5-1345.5)	1298.4 (1290.7-1306.5)	1207.4 (1200.2-1214.4)	1083.5 (1077.4-1089.6)	1031.5 (1026.3-1036.9)	1037.8 (1015.4-1061)	940.8 (936.2-945.5)	856.3 (851.6-860.8)	870.6 (866.5-874.4)	891.3 (755.5-1034.8)
Tropical Latin America	2392.3 (2290-2491.5)	2239 (2149.6-2331.7)	2103.4 (2015.4-2193.5)	1956.9 (1870-2043.6)	1817.6 (1745.9-1896)	1658.6 (1613.3-1704.3)	1502.1 (1487.5-1518)	1400.6 (1391.7-1410.3)	1283.3 (1276-1290.5)	1186.4 (1179.3-1192.7)	1049.9 (1043.7-1055.9)	960.8 (955.3-966.4)	910.8 (905.9-916.1)	860.6 (853.1-868)
BRA Brazil	2412.7 (2308-2514.7)	2263.4 (2171.9-2359.2)	2128.2 (2038-2220.7)	1980.6 (1892.4-2070.7)	1836.4 (1763.5-1917.7)	1672.8 (1625.5-1720)	1515.1 (1500.4-1531.2)	1411.3 (1402-1421.2)	1293.7 (1286.4-1300.6)	1194.2 (1187.5-1200.6)	1055.2 (1049.1-1061.4)	963.1 (957.7-968.8)	911.5 (906.6-916.6)	861.9 (855.1-867.9)
BRA Acre	2397.7 (2063.6-2748.9)	2272.7 (1965.1-2596.5)	2116 (1851.3-2427.1)	1963.7 (1704.4-2241.2)	1789.2 (1594.4-1997.1)	1611.4 (1489.9-1737.4)	1430.7 (1384.8-1481.6)	1452 (1419.8-1487)	1231.8 (1202.5-1261.6)	1130.6 (1103.9-1159.5)	964.2 (943.6-985.4)	887.4 (870.9-906.1)	844.1 (828-860.6)	836.6 (806.1-868)
BRA Alagoas	2716.2 (2354.6-3107.4)	2590.6 (2262.3-2927.1)	2480.1 (2176.9-2803.8)	2337.7 (2057.9-2651.6)	2211 (1958.8-2474.4)	2035.3 (1873-2206)	1811.4 (1760.9-1868)	1609.6 (1571.8-1650.6)	1335.3 (1305.9-1369)	1167.2 (1131.6-1204.1)	1028.6 (989.8-1068.3)	958.3 (926.1-989.5)	948.6 (934.4-964.4)	905.5 (877-935.4)
BRA Amapá	2453.5 (2087.6-2855.5)	2150.6 (1867.6-2455.5)	1885.9 (1644.5-2150.8)	1664.5 (1458.8-1883.6)	1445 (1294-1613.1)	1206.4 (1118.6-1297.9)	1002 (969.1-1035.1)	1027 (1003.7-1050)	960.3 (939.5-981.8)	1094.7 (1075.4-1113.4)	1111.5 (1094.9-1128.4)	898.6 (884.8-913.9)	811.1 (798.7-825.7)	862.2 (835.8-886.7)
BRA Amazonas	2009.7 (1694.6-2341)	1891.5 (1605.2-2217.8)	1797.4 (1535.7-2108.8)	1711.1 (1459.8-1994.1)	1660.1 (1437.7-1921.6)	1546.9 (1371.1-1722.9)	1275.6 (1220.8-1333.4)	1110.1 (1070.6-1154.3)	1029.3 (986.4-1074.2)	899.5 (866.7-935.4)	864.9 (854.3-876.8)	793.3 (783.9-803.3)	801 (791.4-811.3)	793 (768.2-819.8)
BRA Bahia	2460.3 (2119.8-2794.2)	2294.9 (1990.9-2606.5)	2144.3 (1868.5-2432)	1967.5 (1714.5-2239.2)	1781.4 (1553.6-2027.4)	1555.1 (1381.6-1734.1)	1269.5 (1200.4-1342.3)	1216 (1151.3-1279.5)	1063.7 (1006.1-1125.1)	965.3 (912.2-1020.5)	923.7 (869.7-983)	895.7 (843.4-949.7)	896.2 (844.4-949.7)	892.3 (868.8-915.6)
BRA Ceará	2817.8 (2456.8-3221.3)	2690.7 (2366.5-3031.6)	2497 (2185.3-2810.5)	2283.9 (2009.7-2592.6)	2069.6 (1794.8-2356.2)	1866.4 (1626.8-2139.6)	1528.5 (1346.9-1717.7)	1215.7 (1128.3-1307)	995.9 (950-1045.8)	945.4 (895.8-991.2)	904.3 (856.6-955.1)	854.7 (808.7-898.8)	772.5 (758.6-787.4)	738.9 (718.1-761.4)
BRA Distrito Federal	2194.8 (1918.6-2481.6)	2094.3 (1836.1-2373.4)	1924.8 (1659.4-2210)	1709.9 (1457.2-1997)	1564.7 (1345.6-1832.2)	1456.1 (1315.6-1612.8)	1296.6 (1278.6-1315)	1161.2 (1147.2-1175)	1302.8 (1287.6-1318.5)	1465 (1451.7-1479.2)	1263.2 (1251.4-1274.8)	1147.2 (1135.6-1158.5)	1054.9 (1044.6-1065.8)	884.4 (857.6-911.8)
BRA Espírito Santo	2158.6 (1852.3-2492.2)	2055.4 (1753.6-2374.2)	1988.4 (1701.2-2295.1)	1897.2 (1623.7-2165.4)	1823.1 (1568.3-2082.1)	1711.5 (1557-1860.6)	1556.1 (1538.2-1575.6)	1480.5 (1466.2-1496)	1347.9 (1334-1361.7)	1206.5 (1195-1218)	1062.4 (1053-1072.1)	979.1 (968.3-989.8)	926 (916.3-935.9)	799.9 (776.2-825.5)
BRA Goiás	2737.7 (2359.1-3114.4)	2573.7 (2232.6-2929.1)	2402.7 (2081.8-2727.8)	2226.6 (1930.9-2549.6)	2035.8 (1766.9-2345.3)	1776.3 (1540.8-2042.9)	1453 (1344.5-1567.3)	1362.4 (1346-1378.7)	1093.9 (1074.7-1114)	1067.4 (1056.5-1079.4)	975.4 (967.2-984)	886.9 (879.7-894.3)	873.3 (865.9-880.4)	832.2 (810.9-854.5)
BRA Maranhão	3589.1 (3194.1-4027.7)	3238.5 (2914.9-3586.6)	2975.3 (2637.7-3321.6)	2754.3 (2404-3110.7)	2544.2 (2229.9-2883.3)	2348.4 (2042.8-2685.7)	2013.9 (1760.7-2265.5)	1721.6 (1587.4-1847.9)	1300.1 (1232-1367.5)	1005.6 (951.2-1060.9)	847.1 (800.2-896.3)	886.5 (839.1-939.3)	861.4 (810-912.8)	853.7 (827.1-879.5)
BRA Mato Grosso	2420.3 (2039.9-2814.5)	2291.7 (1984.7-2620.8)	2138.1 (1838.6-2434.4)	1944.6 (1684.3-2242.3)	1706.7 (1475.5-1956.3)	1376 (1209.5-1555.4)	1100.4 (1023.8-1187.8)	1244.2 (1166.8-1330.6)	1112 (1041.4-1187.3)	993.6 (977.9-1010.1)	962.2 (951.2-972.9)	944.8 (934.5-955)	881.2 (871.6-891.1)	805.4 (781.8-831.1)
BRA Mato Grosso do Sul	1799.6 (1517.9-2104.7)	1743.5 (1480.4-2048.9)	1673.8 (1418-1954.2)	1573.1 (1292.3-1841.8)	1495.8 (1250.6-1749.7)	1396.3 (1232.7-1567.9)	1325.2 (1303.3-1348.7)	1253.2 (1237.7-1268.1)	1176.9 (1162.5-1191.9)	1212.9 (1199.2-1226.8)	1070 (1058.9-1081.6)	1003.7 (993-1015)	943.4 (933.1-954.7)	881.4 (853.2-910.4)
BRA Minas Gerais	2275.8 (1968.3-2603)	2133.3 (1839.2-2449.3)	2022.9 (1729.9-2327.5)	1911.9 (1631.6-2207.2)	1806.2 (1560.7-2065.8)	1666.4 (1527.1-1821.6)	1433 (1415.8-1451)	1389.2 (1375-1404)	1245.2 (1232.3-1259.4)	1101.8 (1090.7-1113.6)	921.6 (910.8-932.7)	885.8 (874.7-898.3)	838 (827.8-849.1)	779.5 (759.5-799.5)
BRA Pará	2056.1 (1756.1-2397.1)	1942.3 (1660.9-2252.4)	1842.4 (1576.8-2120.9)	1745.7 (1484.9-2025.7)	1650.6 (1428.9-1906.9)	1487.7 (1340.2-1654)	1209.7 (1178.4-1242.3)	1194.8 (1146.3-1245.3)	1071 (1024.6-1115.3)	933.5 (896.6-973.3)	865.1 (827.5-903)	864.6 (829.8-898.7)	818.8 (809.2-829.4)	848.5 (825.7-870.7)
BRA Paraíba	2420.2 (2085-2763.4)	2216 (1917.4-2521.2)	2041.1 (1776.9-2338.1)	1842.1 (1595.6-2124.3)	1675.6 (1451-1947.1)	1518.2 (1367.7-1681.4)	1275.2 (1240.8-1309.1)	1094.9 (1071.2-1120.7)	1002.2 (962.6-1040.5)	932.3 (896.7-971.2)	865.5 (829.9-903)	869.5 (829.9-907)	884.7 (845.3-924.8)	826.4 (761.5-897.3)
BRA Paraná	2646 (2223.6-3064.7)	2420.5 (2078.1-2773.4)	2232.3 (1907.2-2569.1)	2052.8 (1740.8-2396.4)	1879.8 (1605.2-2159.7)	1733.9 (1586.6-1891.4)	1777.7 (1761.8-1793.1)	1503.6 (1492.9-1514.3)	1388.1 (1377.6-1398.8)	1268.4 (1259.5-1277.7)	1096.4 (1089.1-1103.7)	1009 (999.6-1019.6)	976.2 (968.2-985.1)	898.8 (874.7-923.5)
BRA Pernambuco	3152.9 (2749.6-3581.8)	2899.8 (2573.3-3261.8)	2655.6 (2328-3016.1)	2393.3 (2095.7-2696.8)	2136.8 (1890-2400.5)	1824.2 (1684.2-1970.8)	1586.9 (1551.9-1622.7)	1546.7 (1520.2-1577.8)	1446.7 (1425.8-1469.6)	1218.4 (1202-1236.5)	1150.8 (1134.9-1168.2)	1013.7 (1000.3-1028)	946.7 (933.1-962.7)	994.5 (967.4-1023)
BRA Piauí	2225.5 (1918.5-2587.4)	2112.7 (1794.9-2413.1)	2017 (1735.7-2334.4)	1920 (1663-2218.7)	1831.8 (1569.5-2122.8)	1659.7 (1420.2-1901.9)	1467.8 (1347.2-1603.3)	1481.1 (1415.8-1547.3)	1099.8 (1046.6-1155.7)	866.2 (823.9-909.4)	884.7 (841.3-929.3)	877 (839.5-913.3)	760.3 (744.6-778.1)	744.4 (719.7-770.4)
BRA Rio de Janeiro	2427.6 (2103.5-2779.5)	2254.4 (1942.4-2594.2)	2113.5 (1815-2403.8)	1977.5 (1696.5-2298.3)	1904.2 (1644.8-2186.6)	1815.3 (1672.6-1972.3)	1698.5 (1683.7-1713.6)	1725.3 (1715.2-1735.7)	1679.8 (1669.7-1689.4)	1607.6 (1599.2-1615.9)	1296 (1289.8-1302.7)	1153.5 (1147.7-1159.4)	1070.6 (1064.8-1077.2)	1004.1 (981.8-1028.1)
BRA Rio Grande do Norte	2294.2 (1955.8-2638.7)	2143 (1869.8-2452.6)	1988 (1723.2-2281.2)	1804.8 (1554.2-2062.2)	1655.5 (1414.1-1913.8)	1470.5 (1294.6-1657)	1227.4 (1148-1306.2)	1071.1 (1019.5-1123.1)	957.2 (908.8-1004.4)	966.1 (915.8-1017.5)	830.1 (783.9-879.8)	766.2 (726.9-805)	735.2 (721.8-749.5)	828.7 (804-856.5)
BRA Rio Grande do Sul	2134.4 (1816.6-2474)	2057.7 (1770-2387.5)	1970.8 (1678.5-2279.1)	1870.7 (1576.6-2182.8)	1740.3 (1483.4-2019.6)	1573.5 (1434.2-1718)	1520.3 (1508.3-1533)	1409 (1399.9-1419)	1399.9 (1390.4-1408.8)	1339.5 (1331.4-1347.7)	1141.7 (1135.1-1148.3)	1015.2 (1009.1-1021.3)	947.5 (941.3-954.4)	900.8 (878.9-922.8)
BRA Rondônia	2406.2 (2093.4-2751.3)	2377.7 (2026.5-2738.1)	2309.3 (1982.6-2628.9)	2303 (1987.9-2645.5)	2253.6 (1966.2-2556.9)	2036.2 (1800.8-2293.5)	1761.6 (1622.5-1897.7)	1678.3 (1569.3-1789.1)	1418 (1322.1-1515.3)	1099.9 (1017.1-1183.1)	1039.1 (965.7-1114.9)	998.6 (929.3-1071.9)	894 (830.3-959.5)	849.3 (748.4-954.7)
BRA Roraima	2494.3 (2154.1-2832.7)	2420 (2097-2746.9)	2334.4 (2033.7-2664)	2247.8 (1964.9-2553.7)	2153.4 (1899.6-2428.5)	1899.4 (1686.1-2149.2)	1575.6 (1413.3-1748.5)	1481.3 (1346-1616.7)	1305.8 (1198.2-1425.5)	1215.1 (1112.1-1327.3)	1134.7 (1042.5-1235.7)	1078.3 (986.2-1174.9)	1009 (921.9-1102.6)	890.1 (776.1-1002.5)
BRA Santa Catarina	1937.2 (1616.1-2255.7)	1861.1 (1558.4-2174.6)	1787.2 (1517.3-2105.3)	1690.4 (1437.3-1983.4)	1561.7 (1323.4-1827.4)	1391.8 (1264.1-1532.2)	1388 (1367.9-1409.6)	1325.8 (1310.6-1341.2)	1335.5 (1321.2-1350.2)	1363 (1349.6-1376.6)	1135.7 (1125.1-1147.4)	965.8 (956.8-975.5)	903.1 (894.5-912.6)	809.9 (786.1-834.5)
BRA São Paulo	2078.2 (1776.6-2393)	1989.3 (1688.6-2306.4)	1902.5 (1601.4-2210.6)	1792.5 (1510.2-2099.3)	1677.1 (1440.8-1941.9)	1567.6 (1430.8-1703.2)	1565 (1553.3-1576.9)	1476.4 (1468.4-1485.2)	1452.9 (1444.6-1461.4)	1428.7 (1421.2-1436.6)	1227.1 (1222.2-1231.9)	1036.8 (1032.7-1041.1)	959.2 (954.2-964.5)	869 (851.1-886.5)
BRA Sergipe	2036.4 (1733.6-2365)	1916.7 (1649-2218.2)	1828.7 (1568.7-2094.9)	1725 (1486-1985.7)	1610.8 (1404.4-1858.3)	1475.6 (1343.3-1622.2)	1360.6 (1323.8-1400)	1177.2 (1149.4-1209)	1113.7 (1091.8-1135.6)	1112.4 (1093.2-1133.9)	970.1 (951.6-989.9)	886 (871-901.7)	884.7 (870.9-899.7)	908.8 (879.6-939.4)
BRA Tocantins	3004.5 (2534.5-3553.7)	2899.8 (2509.5-3311.8)	2759.4 (2404-3129.9)	2627.9 (2305.6-2984.2)	2402 (2112.1-2733.6)	2130.1 (1855.9-2430.5)	1718.6 (1501.9-1968.3)	1396.2 (1242-1559.3)	1163.2 (1099.6-1233.7)	1168.1 (1128.7-1208.5)	1046.1 (1012-1081.7)	837.1 (807.5-866.5)	770 (741.6-798.9)	739.2 (688-792.6)
PRY Paraguay	1561.4 (1407.8-1738.3)	1320.3 (1243.4-1398.7)	1208.3 (1141.2-1279.1)	1116.5 (1057.4-1179.9)	1116.7 (1057.1-1176)	1120.7 (1056.1-1182.1)	1015.5 (961.4-1074.8)	1000.1 (946.4-1058.7)	902 (850.6-957.2)	906.3 (849.3-965.5)	858.5 (808.9-911.1)	876.6 (819.8-932.6)	894.7 (841.2-951.6)	830.3 (685.7-992.1)
North Africa and Middle East	2853 (2754.5-2953.9)	2551.6 (2475.8-2622.7)	2316.3 (2250.5-2386.5)	2057.7 (1996.5-2123)	1912 (1859.3-1968.9)	1721.3 (1679.4-1768.8)	1570.1 (1541-1602.3)	1465.5 (1438.9-1493.3)	1274.1 (1250.9-1298.7)	1200 (1176.4-1222.6)	1096.9 (1076.4-1119.7)	1020.7 (1000.8-1041.9)	962.5 (942.8-984.3)	909.8 (880.2-938.8)
North Africa and Middle East	2853 (2754.5-2953.9)	2551.6 (2475.8-2622.7)	2316.3 (2250.5-2386.5)	2057.7 (1996.5-2123)	1912 (1859.3-1968.9)	1721.3 (1679.4-1768.8)	1570.1 (1541-1602.3)	1465.5 (1438.9-1493.3)	1274.1 (1250.9-1298.7)	1200 (1176.4-1222.6)	1096.9 (1076.4-1119.7)	1020.7 (1000.8-1041.9)	962.5 (942.8-984.3)	909.8 (880.2-938.8)
AFG Afghanistan	3648.1 (3234.6-4069.7)	3287.4 (2921-3655.9)	3074.2 (2726.4-3432.6)	2889.9 (2572.6-3195.2)	2735.6 (2457.4-3033.5)	2617.5 (2380.1-2855.7)	3038.7 (2796.8-3290)	3320.4 (3083.4-3570.3)	2224.6 (1976.4-2463.7)	2129.4 (1879-2357.4)	2087.4 (1848.4-2316.1)	1880.1 (1666.5-2109.6)	1700.7 (1509.5-1908.4)	1449.5 (1284.2-1616)
DZA Algeria	1842.8 (1757.4-1916.8)	1973.1 (1918.1-2034.6)	1864 (1786.6-1944)	1428.3 (1360.6-1490.1)	1530.3 (1436.4-1632.1)	1628 (1535.7-1738.9)	1621.6 (1561.9-1685.7)	1136 (1096.9-1178.6)	967.9 (909-1031.7)	916.2 (852.5-981.2)	864.7 (811.8-919.5)	780.3 (725.8-836.6)	701.7 (672.2-731.2)	629.5 (599.1-666.1)
BHR Bahrain	2608.9 (2229.2-2989.9)	2512.4 (2174.2-2840.7)	2175.7 (1859.5-2513.5)	1931.6 (1661.3-2218.9)	1703.9 (1479.6-1936.5)	1458.5 (1300.5-1612.3)	1175.2 (1101.5-1250.4)	1201.8 (1148.6-1254.4)	1272.4 (1215.8-1322.5)	1247.4 (1192.3-1299.1)	1060.9 (1018.9-1105.5)	971.6 (934.1-1013.1)	694.5 (666.3-726)	586.3 (531.6-644.3)
EGY Egypt	2989.8 (2728.6-3305.5)	2499.7 (2374.8-2635.8)	2202.8 (2131.4-2282.1)	1979.4 (1921.8-2038)	2114.8 (2065.6-2167)	2035.4 (1995.5-2080.6)	1803.8 (1773.6-1835)	1832.2 (1809.2-1855.5)	1586.9 (1570.7-1603.2)	1575.5 (1561.9-1591.3)	1463.8 (1451-1477.4)	1473.1 (1462.5-1485.3)	1450.7 (1439.8-1462.5)	1380.5 (1254.7-1517.5)
IRN Iran	3294.9 (3187-3420.9)	2987.8 (2904.7-3075.3)	2678.3 (2601.7-2751.8)	2370.6 (2304.6-2437)	1959.4 (1900.8-2016.9)	1520.7 (1474.4-1570.4)	1281.9 (1242.5-1322.6)	1162.1 (1125.1-1201.8)	1126.5 (1094.3-1161.2)	912.5 (889.8-934.7)	825.3 (814.4-836)	832.7 (825.5-840.3)	792.2 (788.1-796.7)	744.5 (739.8-749)
IRQ Iraq	2482.6 (2221.4-2773)	2078.3 (1915.8-2236.4)	1816.8 (1720.4-1913.7)	1774.2 (1698.7-1861.8)	1687.3 (1601.7-1766.7)	1666.9 (1574.3-1763.4)	1616.7 (1500.7-1749.4)	1654.3 (1492.4-1828.2)	1384.7 (1213.9-1554.8)	1411.2 (1249.1-1562.4)	1311 (1169.2-1450.3)	1061.1 (940.6-1188.2)	790.9 (718-866.8)	658.5 (621.1-698.7)
JOR Jordan	2640.8 (2284.5-2988.3)	2226.1 (1944.5-2509.3)	1882.2 (1673.4-2115.9)	1567.6 (1441.3-1701.1)	1367.5 (1301.6-1444.5)	1201.5 (1149.6-1257.9)	1177.8 (1114.7-1247.5)	1037.4 (948-1135.4)	908.2 (815-1011.8)	844.7 (754.7-944.3)	754.6 (684.4-828.1)	670.3 (633.4-710)	665.8 (638-695.8)	644.6 (572.3-732)
KWT Kuwait	1591.1 (1333.2-1857.7)	1295.9 (1099.2-1507.5)	1160.7 (1053-1290.6)	1064.8 (1044.6-1087.3)	995.8 (981-1011.4)	968.7 (955.7-981.3)	832.5 (821.3-843.6)	667.3 (658.8-676.2)	689.1 (677.2-700.4)	501.3 (493.9-508.2)	527 (520.2-534.2)	512.7 (506.1-519.5)	436.6 (431.4-442.1)	423.9 (397.4-451.9)
LBN Lebanon	1974.5 (1665.7-2303.2)	1771.7 (1496-2073.5)	1625.2 (1371.7-1892.9)	1514 (1273.2-1749.1)	1413.3 (1194.5-1600)	1692.7 (1497.9-1857.5)	1295.9 (1113.6-1400.5)	1531.1 (1385.6-1606.1)	1272.5 (1135.8-1359.2)	1024.3 (909-1137.5)	896.4 (817.5-981)	898.8 (826.1-962.3)	850.3 (778.8-918.6)	776.3 (735.1-831.9)
LBY Libya	3051.4 (2664.9-3472.7)	2407.6 (2109.5-2728.6)	2020.2 (1778.1-2302.4)	1594.2 (1432-1783.8)	1273.7 (1159.6-1388.9)	1149.8 (1063.1-1237.4)	1027.9 (931-1123.8)	976.7 (859.9-1088.6)	955.2 (836.5-1084.6)	908 (810.4-1028.5)	914.2 (832-1017.4)	854.3 (777.7-926.9)	945.2 (863.2-1028.6)	975.3 (841.7-1109)
MAR Morocco	3124.7 (2751.5-3531.3)	2797.4 (2468.9-3130.8)	2736.1 (2437.6-3046.4)	2300.8 (2009.9-2585.7)	2052.3 (1782.9-2311.3)	1881.9 (1681.7-2099.6)	1639.2 (1478.7-1800.1)	1331.2 (1226.1-1435.2)	1099.5 (1034.4-1165.4)	1098.8 (1036.8-1167.4)	1121.1 (1042.6-1204.2)	1073.4 (973.6-1181.2)	1016.1 (880.2-1161.9)	899 (754.3-1071.9)
PSE Palestine	2878.6 (2489-3253.3)	2484.7 (2147.7-2822.7)	2093.8 (1779.6-2409.8)	1739.5 (1480.2-2033.4)	1913.4 (1691.7-2170.8)	1331 (1131.8-1572.1)	1207.9 (1011.9-1425.4)	1104 (920.7-1289.4)	1054.9 (888.9-1215.4)	993.1 (869.4-1119.4)	1061.6 (1000.9-1130.2)	1003.8 (968.9-1041.6)	776.3 (751.6-802.4)	654.4 (610.3-707.6)
OMN Oman	4656 (4194-5159.1)	3906.8 (3521.2-4297.1)	3403.3 (3051.5-3769.2)	2891.2 (2555-3249)	2123.8 (1849.2-2432.9)	1735 (1498.9-2007.5)	1448.9 (1222.2-1702.8)	1271.4 (1057.3-1512)	1169.6 (961.3-1397.4)	1073.4 (876.2-1284.6)	976.1 (803.8-1164)	882.1 (728.1-1059.3)	901.1 (761-1071.4)	720.5 (588.5-863.6)
QAT Qatar	2111.9 (1785.9-2462.1)	2072.7 (1770.4-2412.3)	1980 (1689.7-2314.1)	1913.3 (1634.7-2230.4)	1805.2 (1538.8-2088.4)	1642.6 (1402.9-1902.6)	1561.7 (1345.5-1780.9)	1422.7 (1239.8-1612.1)	1384.2 (1212.7-1558.5)	1364.3 (1207.8-1540.4)	1178.2 (1038.5-1327.5)	818.6 (713.4-929.4)	753.8 (654.1-862.4)	629.9 (528.6-737.3)
SAU Saudi Arabia	1861.2 (1584.7-2169.6)	1987.2 (1717.8-2297.3)	1817.7 (1561.8-2089.1)	1599.9 (1363.5-1843.8)	1390.2 (1190.6-1605.8)	1160.1 (988.2-1345.4)	1050.1 (878.8-1232.4)	1006.1 (841.1-1192.6)	1004.2 (846.2-1174.3)	918.5 (798.7-1060.2)	868.8 (782.6-974.9)	911.5 (834.7-1001.9)	1012.8 (905.6-1099.6)	862.3 (796.6-945.8)
SDN Sudan	2418.4 (2104.1-2748.9)	2129.6 (1844.3-2429.7)	1938.2 (1665.6-2227.6)	1932.4 (1700-2182.1)	1929.7 (1725.1-2153.2)	1914.3 (1745-2095.6)	1874.6 (1731.2-2026.3)	2137 (1992.2-2293.9)	1632.2 (1486-1810.4)	1480.2 (1323-1669.3)	1373.6 (1218.2-1569.4)	1268.1 (1100.2-1461.3)	1177.7 (999-1364.6)	1071.9 (886.4-1279.6)
SYR Syria	1817.7 (1568.1-2090.3)	1522 (1309.8-1752.9)	1298.4 (1128.5-1499.1)	1148.5 (999.7-1301.9)	1003.7 (895.8-1125)	822.6 (748.2-906.6)	909 (821.7-1008)	1064.1 (962.6-1173.7)	1136.5 (1037.6-1251)	1211.9 (1095-1328.5)	1165.1 (1057.8-1270.1)	998.3 (911-1096.4)	962.4 (849.8-1079.4)	1282.3 (1128.6-1455.2)
TUN Tunisia	2849.8 (2534.9-3201.8)	2675.2 (2437.7-2927.5)	2453.6 (2291.3-2634.6)	2356.4 (2220.9-2497.1)	2145.7 (2069.7-2224.2)	1731.3 (1669.7-1795.3)	1329 (1271.7-1388.7)	1096.7 (1059-1134.4)	1047.5 (1005-1088.7)	1052.1 (1007-1098.5)	993.5 (905.9-1088.9)	913.1 (775.2-1050.1)	863 (707.5-1016)	826.4 (663.6-1009.5)
TUR Turkey	2917 (2534.4-3295.9)	2597.8 (2257-2913.5)	2339.5 (2028.2-2647.3)	2021 (1742.1-2327.6)	1778.4 (1550-2043.4)	1576.8 (1418.3-1746.6)	1408.9 (1338.3-1483.6)	1262.9 (1193.4-1338.4)	1169.3 (1117-1225.3)	1085.2 (1033.2-1141.7)	879.5 (835.3-927.1)	770.8 (734.1-810.5)	776.3 (737.5-817.2)	769.7 (705-837.9)
ARE United Arab Emirates	2146.4 (1818.9-2482.1)	2028.1 (1732-2353.4)	1886.6 (1602.9-2198.9)	1674.2 (1424.3-1954)	1466.6 (1228-1714.8)	1309.6 (1081-1545.6)	1214.1 (1003.2-1442)	1165.7 (968.8-1369.4)	1168.1 (988-1346.8)	1116.3 (966.1-1265.8)	1226.5 (1100.7-1381.1)	1087.5 (958.3-1224.7)	1113.5 (952.1-1278.7)	1084.5 (899-1297.8)
YEM Yemen	4632.9 (3988.8-5285.7)	3998.5 (3508.5-4512.1)	3574.3 (3171-4007.4)	3378.9 (2982-3820.4)	3028.4 (2679.2-3411.6)	2416.8 (2111.7-2753.3)	2060.5 (1782-2377.4)	1819.4 (1562.7-2116.7)	1665.8 (1416.7-1966.9)	1547.7 (1307.9-1845.4)	1421.5 (1208.7-1700.3)	1298.3 (1103.4-1555.7)	1215.1 (1010-1466.1)	1260.7 (1045.2-1495.4)
South Asia	2664.8 (2555.8-2774.1)	2488.6 (2385.7-2589.8)	2427.5 (2332-2530.6)	2377.9 (2293.6-2468.6)	2177.6 (2109.1-2246.4)	2017.5 (1956.5-2077.8)	1888.3 (1834.6-1947.2)	1740.6 (1694.6-1789.3)	1676 (1634.4-1716.6)	1562.8 (1531.5-1593.5)	1502.1 (1477.8-1526)	1372 (1351.5-1393.2)	1340.9 (1317.9-1365.1)	1194.1 (1163.5-1224.6)
South Asia	2664.8 (2555.8-2774.1)	2488.6 (2385.7-2589.8)	2427.5 (2332-2530.6)	2377.9 (2293.6-2468.6)	2177.6 (2109.1-2246.4)	2017.5 (1956.5-2077.8)	1888.3 (1834.6-1947.2)	1740.6 (1694.6-1789.3)	1676 (1634.4-1716.6)	1562.8 (1531.5-1593.5)	1502.1 (1477.8-1526)	1372 (1351.5-1393.2)	1340.9 (1317.9-1365.1)	1194.1 (1163.5-1224.6)
BGD Bangladesh	2670.6 (2346.8-3024.3)	2482.9 (2192.3-2811.4)	2330.2 (2051.1-2621.1)	2161.7 (1901-2430.5)	2517 (2294.5-2749)	1793.3 (1619.9-1964.9)	1642.9 (1551.1-1747.5)	1865.8 (1786.5-1949.8)	1707.5 (1614.1-1804)	1485.3 (1404.5-1561.4)	1321.9 (1260.1-1387.1)	1218.6 (1171.4-1269.3)	1136.3 (1083.8-1188.7)	935.5 (834.6-1042.9)
BTN Bhutan	3759.8 (3321.8-4231.3)	3432.4 (3047.3-3830)	3096 (2725.3-3445)	2720.3 (2381.6-3063.7)	2368.9 (2058.8-2688.7)	2143.9 (1864-2455.6)	1960.1 (1705.3-2253)	1712.5 (1501.7-1963.6)	1460.1 (1289.1-1655.6)	1336.7 (1196.7-1479.7)	1232.4 (1109.5-1339.6)	1088.6 (965.8-1193.2)	1006.2 (868-1139.8)	909 (753.9-1127)
IND India	2756 (2637.3-2880.7)	2578.6 (2465.7-2690)	2545.6 (2434.2-2657.3)	2554.7 (2452.8-2665.1)	2288.4 (2206.5-2375.6)	2183.9 (2110.5-2257.4)	2044.6 (1974.2-2116.7)	1796.3 (1738.7-1857.6)	1723.2 (1672.6-1773.7)	1593.7 (1557.7-1629.9)	1537.3 (1511.3-1565)	1377.2 (1355.9-1399.5)	1360.3 (1339.2-1382.3)	1213.4 (1188-1241.4)
IND Andhra Pradesh	2463.8 (1967.9-3027.9)	2344.2 (1874.7-2849.5)	2256.6 (1838.9-2758.8)	2200.4 (1835.2-2616.6)	1995.2 (1685.8-2344.4)	1945.6 (1661.2-2265.6)	1904.7 (1612.6-2224.6)	1719.4 (1447.8-2031.7)	1715.2 (1447.6-2019.8)	1607.8 (1357.2-1897.1)	1563.5 (1300.7-1849)	1372 (1141-1641.5)	1359.8 (1142.8-1629.5)	1216.7 (1022.4-1469.3)
IND Arunachal Pradesh	2407.7 (1918.9-2970.4)	2275.9 (1822.9-2781.7)	2291.2 (1882.6-2778.8)	2455.8 (2098.6-2873.9)	2122.4 (1812.6-2468.9)	1971.3 (1700.1-2267.5)	1853.2 (1584.3-2148.2)	1663.8 (1434.5-1943.7)	1665.4 (1422.8-1954.7)	1590.1 (1353.7-1844.7)	1582.9 (1335.8-1791)	1376.2 (1194.7-1538.7)	1333.2 (1162.5-1435.2)	1233.4 (1046.6-1325.3)
IND Assam	3102.9 (2586.2-3620.2)	2896.1 (2413.4-3397.4)	2875.7 (2416.4-3400.1)	2957.2 (2538.9-3434.9)	2618.2 (2241.3-3022.7)	2434.7 (2115.4-2794.3)	2262.4 (1941.2-2581)	1978 (1726.6-2244.1)	1895.8 (1719.6-2090.3)	1785.5 (1703.9-1866.4)	1856.4 (1783.3-1933.7)	1656.6 (1593.3-1719.7)	1617.1 (1557.7-1676.1)	1358.3 (1266.6-1453.2)
IND Bihar	2600.9 (2149.1-3076.8)	2394.7 (1963.5-2823.6)	2377 (1971.1-2819.2)	2419.2 (2073.5-2828.9)	2197.7 (1907.9-2535.1)	2155.4 (1900.5-2448.2)	2082.5 (1829.9-2383.1)	1848.9 (1621-2116.1)	1774.8 (1563.9-2022.6)	1668.2 (1494.5-1852.8)	1630.9 (1503.3-1759.4)	1380.8 (1316.7-1449.1)	1298.9 (1247-1355.8)	1060.8 (986.4-1142.9)
IND Chhattisgarh	2462.1 (2013-2966.5)	2364.6 (1940-2817.7)	2333.3 (1937.3-2757)	2421.5 (2057.9-2840.7)	2131.6 (1837.6-2457.9)	2093.4 (1820.2-2415.4)	1966.7 (1706.9-2264.5)	1739.6 (1521.7-2016.1)	1632.2 (1429.5-1872.8)	1490.7 (1334.1-1659.8)	1519.5 (1406.8-1632.9)	1576.8 (1508.7-1650.7)	1922.9 (1857.3-1995.8)	1912.1 (1816.6-2026.2)
IND Delhi	2340.5 (1927-2754.5)	2151 (1790.4-2586.4)	2038.2 (1701.9-2421)	2175.7 (1874.3-2510.7)	1749.5 (1503.5-2028.2)	1687.2 (1454.6-1938.5)	1619.9 (1384-1884.2)	1436.1 (1228.1-1686.7)	1434 (1232.7-1664.7)	1363.2 (1178.8-1552.8)	1333 (1186.6-1488.6)	1156.5 (1063-1258.7)	1077.2 (1003.1-1159.9)	946.4 (842.6-1059.8)
IND Goa	1841.6 (1505.2-2246)	1696.6 (1403.7-2058.3)	1589.3 (1321.1-1902.6)	1567 (1338.4-1840.2)	1424 (1230-1648.3)	1387.9 (1224.8-1575.8)	1351.9 (1183.9-1542.1)	1271.4 (1118.8-1445.7)	1338.7 (1184-1502.5)	1300.4 (1150.9-1462.4)	1289 (1115.7-1449.5)	1106 (940.6-1291.8)	1062.9 (897.5-1264.6)	980.5 (827.6-1192.5)
IND Gujarat	2590.4 (2191.9-3034.1)	2432.6 (2033-2861.7)	2403.2 (2019.5-2799.3)	2472.1 (2097.8-2854.9)	2206.8 (1897.6-2543.5)	2114.7 (1835.2-2383.8)	1990.4 (1730.6-2281.9)	1730.1 (1511.2-1951.3)	1665.5 (1504.9-1836.2)	1479.9 (1388.9-1576)	1520.6 (1445.1-1592.9)	1388.2 (1321.1-1455.4)	1304.1 (1241.4-1367.2)	1143 (1049.4-1249.1)
IND Haryana	2288.5 (1872.1-2707.8)	2184.4 (1783.5-2624.8)	2150 (1798.4-2569.1)	2164.3 (1827.7-2560.9)	1937.8 (1659.5-2249.1)	1773.4 (1528.9-2051)	1600 (1363.3-1856.9)	1389.9 (1208.5-1582.7)	1329.9 (1199.1-1476.2)	1270.1 (1201.8-1337.5)	1314.7 (1254.2-1373.7)	1244.8 (1190.9-1298.7)	1299 (1241.8-1360)	1130.7 (1042.4-1223.3)
IND Himachal Pradesh	2346.6 (1901.7-2814)	2202.3 (1795.2-2633.4)	2161.8 (1745.6-2566.7)	2127.2 (1764.5-2532.9)	1933.3 (1624.9-2273)	1848.3 (1555.5-2180.1)	1725.2 (1451.6-2026.6)	1525.2 (1300.5-1772)	1445.3 (1271.2-1633.3)	1312.9 (1218.8-1409.8)	1230 (1163-1302.8)	1177.2 (1113.1-1243.2)	1175.7 (1109.7-1239.6)	1211.6 (1100.3-1331.9)
IND Jammu and Kashmir	2164.2 (1790.1-2586.1)	1991.1 (1656.1-2384.3)	1897.4 (1560.2-2275.1)	1934.9 (1629.9-2277.1)	1697.1 (1448.9-1980.7)	1620.8 (1396.1-1875.6)	1579.3 (1358.9-1824.9)	1364.5 (1146.7-1574.4)	1357.4 (1165.5-1555)	1260.2 (1108-1415.9)	1164.9 (1057.1-1276.5)	932.9 (878-994.4)	1026.5 (975-1083.3)	910.8 (833.4-989.7)
IND Jharkhand	2536.9 (2100.2-2944)	2380.8 (1958.2-2798.8)	2374.3 (1976.9-2789.8)	2432.5 (2062.3-2840.4)	2204.3 (1885.4-2530.6)	2125.9 (1844.9-2433.7)	2027.3 (1753.9-2319)	1903.8 (1652.9-2189.4)	1865 (1641.5-2110.7)	1746.2 (1565.4-1942.7)	1708.2 (1583.2-1831.6)	1482.4 (1420.6-1544.8)	1381.2 (1329.4-1433.4)	1064 (991-1138.4)
IND Karnataka	2723 (2292.3-3216.2)	2569.7 (2172.9-3041.6)	2529.3 (2139.1-2954.2)	2538.7 (2175.5-2944.7)	2296.1 (1987.4-2629.7)	2160.7 (1881.9-2446.2)	1981.2 (1726.7-2259.7)	1715.1 (1501.7-1943.9)	1631 (1480.1-1800)	1478.9 (1405.3-1548.1)	1565 (1505.7-1629.2)	1375.9 (1320.4-1433.6)	1444 (1388.8-1500.9)	1420.3 (1323.2-1522.7)
IND Kerala	2523.2 (2076.7-3024.3)	2287.8 (1895.3-2704.9)	2182.5 (1801.9-2584.9)	2127.6 (1808-2514.2)	1886.6 (1617.4-2233.3)	1773.4 (1526.8-2029.9)	1664.5 (1422.4-1937.8)	1484.4 (1276.7-1703.4)	1440.2 (1282.7-1613.2)	1229.5 (1130.8-1331.8)	1350.8 (1264.9-1443.3)	1081.6 (1016-1147.8)	1082.4 (1020.8-1152.7)	1174.1 (1060.7-1312.1)
IND Madhya Pradesh	2949.4 (2481-3455.4)	2668.9 (2240.4-3125.2)	2597.8 (2185.6-3045.6)	2546.3 (2222.2-2932)	2131.4 (1879-2409.2)	2096.4 (1848.8-2361.4)	2052.3 (1786.9-2328.9)	1886.6 (1643.3-2160.3)	1819.2 (1595.6-2089.2)	1681.9 (1509.5-1883.9)	1642.2 (1514.6-1772.5)	1477.7 (1405.9-1553.6)	1514.8 (1449.2-1581.6)	1336.9 (1239-1454)
IND Maharashtra	2568.1 (2155.1-3019.2)	2402 (2017.9-2816.3)	2349.7 (1995.3-2726.7)	2363.5 (2020.3-2725)	2131.7 (1845.7-2443.1)	2012.5 (1750-2287.3)	1834.9 (1592.2-2097.4)	1612.7 (1407.5-1825.3)	1561.6 (1421.8-1704.5)	1430.1 (1365.5-1494.9)	1422.2 (1366.4-1478.6)	1186.5 (1143.1-1234)	1179.4 (1136.5-1223.2)	1009.2 (940.3-1078.3)
IND Manipur	2076.6 (1649.1-2579.6)	1933.1 (1551.4-2372.1)	1900.1 (1558.2-2320.4)	1959.7 (1665.3-2323.6)	1730.2 (1482.2-2017.5)	1655.4 (1437.2-1902.5)	1582.9 (1387.1-1820.3)	1496.3 (1321.6-1716.3)	1482.3 (1298.7-1675)	1487.4 (1307.9-1658.5)	1520.5 (1357.4-1648.9)	1383.7 (1204.7-1530.7)	1364.1 (1199-1470.2)	1275.8 (1104.9-1390.2)
IND Meghalaya	2025.2 (1604-2513.5)	1909.6 (1537.3-2345.6)	1865.4 (1518.2-2292.9)	1943.6 (1639.8-2307.7)	1735.4 (1481.4-2040.8)	1774.6 (1535-2038.9)	1733.9 (1503.7-2004.4)	1611.4 (1402.5-1869.4)	1632.2 (1406.1-1867.5)	1588.7 (1362.3-1801.9)	1575.9 (1346.6-1751.7)	1390.9 (1210.3-1562.8)	1378.3 (1204-1523.5)	1308.7 (1125.6-1434.3)
IND Mizoram	1742.5 (1394-2109.7)	1697.1 (1364.4-2019.9)	1651.2 (1328-1967.6)	1814.5 (1548.3-2092.3)	1595.8 (1354.2-1813.8)	1576.8 (1351-1799)	1565.3 (1357.8-1791.7)	1456 (1263.1-1628.6)	1467.1 (1298.4-1619.4)	1434.9 (1282-1558.8)	1440.7 (1306.4-1537.4)	1317.2 (1152.6-1469.2)	1331.4 (1165.5-1486.9)	1265.7 (1081.4-1412.3)
IND Nagaland	2119.5 (1661.5-2643)	2006.4 (1593.4-2473.2)	1981.2 (1617.5-2432.2)	2216.5 (1903.7-2587.1)	1744 (1475.5-2056.5)	1638.3 (1408.9-1907.1)	1554.1 (1337.3-1815.2)	1414.4 (1227.9-1673.7)	1455.2 (1267.3-1704.2)	1424.9 (1238.2-1649.8)	1465 (1250.9-1666.7)	1319.5 (1113.1-1517.6)	1310.2 (1109.1-1480.7)	1249.5 (1024.6-1432.2)
IND Odisha	3084.6 (2564.3-3646.3)	2890.7 (2416.7-3396)	2905.7 (2460.5-3405.8)	2941.7 (2542.1-3391.1)	2653.4 (2299.9-3010.8)	2474.2 (2161.6-2799.7)	2254.1 (1972.9-2573.3)	1930.3 (1719.2-2182.1)	1852.4 (1701.4-2009.8)	1834.2 (1757.2-1914.7)	1799.9 (1733.4-1864.7)	1536.2 (1482.4-1591.8)	1404.4 (1353.9-1455.2)	1225.1 (1151.7-1306.2)
IND Punjab	2151.9 (1782.2-2546.6)	2049.9 (1690.7-2425.9)	1979.3 (1635.6-2326.2)	1943.3 (1652.6-2263.2)	1747.6 (1478.8-2026.4)	1662.7 (1429.7-1912)	1554.6 (1343.5-1810.4)	1366.6 (1168.6-1582)	1336.3 (1200.9-1481.6)	1290 (1218-1357.1)	1228.9 (1174.9-1283.5)	1173.7 (1121.5-1227.5)	1091 (1041.4-1139.8)	905.1 (835.2-979.8)
IND Rajasthan	2676.9 (2212.8-3152.5)	2559.9 (2122-3017.6)	2566.7 (2128-2997.7)	2675.7 (2283.7-3084.1)	2399.9 (2063.3-2749.1)	2326.6 (2034.8-2652.8)	2178.5 (1895.7-2489)	1889.3 (1666.1-2126.5)	1782.1 (1603.3-1978.5)	1685.3 (1584.4-1796)	1473 (1404.4-1545.8)	1307.2 (1244.5-1366.9)	1351.7 (1287.2-1421.6)	1348.9 (1238.1-1470.9)
IND Sikkim	2134.5 (1662.3-2681.8)	2062.4 (1633.1-2544.8)	2024.9 (1638-2491.8)	2092.4 (1758.5-2490.3)	1904.5 (1606.7-2228.5)	1849.5 (1593.2-2134.2)	1749.6 (1499.8-2030.5)	1571.7 (1334.7-1815.5)	1575.5 (1333.3-1758.5)	1531.5 (1343.5-1666.6)	1490.5 (1344.9-1616.3)	1341.5 (1183.2-1429.6)	1291.1 (1126.9-1374.2)	1147.5 (964.7-1270.7)

Table 1 Age-standardised mortality rates by location and year for males for 1950, 1955...2005, 2010, 2017

Location	1950	1955	1960	1965	1970	1975	1980	1985	1990	1995	2000	2005	2010	2017
IND Tamil Nadu	3147.9 (2697.1-3631.1)	2918.1 (2491.5-3384.1)	2850.8 (2420.1-3281.7)	2832.5 (2467.4-3238.9)	2563.4 (2227.2-2908.2)	2405.1 (2102.6-2710.6)	2196.7 (1914.4-2497.9)	1903.5 (1679.2-2144.5)	1796.9 (1642.1-1972.6)	1628.6 (1552.7-1707.5)	1572.5 (1510-1635.4)	1326.5 (1274.8-1379.9)	1412.8 (1360.1-1469)	1213.2 (1126.6-1298.5)
IND Telangana	2236.1 (1752.9-2766.1)	2145.6 (1716.4-2685.5)	2106.8 (1699.9-2592.2)	2159.6 (1784-2635.9)	2059.3 (1711.8-2464.7)	1990 (1663.2-2369.6)	1837.1 (1548.6-2197.3)	1624.7 (1351.9-1929.7)	1654.1 (1358-1961.4)	1609.5 (1324.3-1908.3)	1631.3 (1349.7-1916.5)	1424.6 (1184.1-1681.4)	1287.8 (1080.8-1509.8)	1001.6 (841.7-1183.9)
IND Tripura	2394.7 (1924.7-2941.3)	2252.7 (1825.1-2727.5)	2255.1 (1858.8-2707)	2285.1 (1942.1-2665.2)	2011.1 (1726.7-2336.8)	1891.8 (1643-2164.8)	1916.8 (1673.6-2186.4)	1649.7 (1433-1906.2)	1656.7 (1430.3-1931)	1626.2 (1392.2-1845.6)	1592.1 (1377.8-1745.7)	1372.1 (1200.5-1521.4)	1373.6 (1228.1-1496.3)	1316.4 (1194.5-1407.3)
IND Uttar Pradesh	3329.7 (2851.4-3864.3)	3127 (2690.5-3611.6)	3137.8 (2725.6-3548.2)	3117.2 (2735.6-3518.2)	2738.5 (2462.3-3052.4)	2585.5 (2338.5-2861.3)	2401.7 (2154.5-2676.8)	2067.8 (1856.2-2316.3)	1926.8 (1727.4-2131.9)	1727.6 (1590.2-1865)	1546.4 (1456.7-1641.9)	1529.4 (1459.1-1601.2)	1514 (1444.9-1583.5)	1408.4 (1298.4-1531.2)
IND Uttarakhand	2633.8 (2171.1-3042.1)	2535.7 (2089.1-2939.4)	2465.3 (2030.6-2868.6)	2487.6 (2080.2-2862.9)	2270.7 (1953.1-2570.2)	2202.9 (1937.3-2460.6)	2150.2 (1882.7-2407.5)	1970.4 (1753.1-2201.8)	1935.9 (1739.7-2170.8)	1763.8 (1595-1992.6)	1663 (1500.6-1869.3)	1441.6 (1346.4-1576.8)	1363.8 (1298.7-1449.7)	1314.3 (1250.5-1381.2)
IND West Bengal	2636.8 (2193.4-3081.7)	2478 (2097.2-2923.1)	2471.1 (2096.7-2855.8)	2525.8 (2176.5-2913.1)	2284.3 (1984.7-2620.9)	2188.4 (1911.5-2511.9)	2049.3 (1798.5-2329.8)	1814.5 (1598.3-2052.1)	1762.1 (1566.2-1952.6)	1691.9 (1571.4-1817.7)	1525.2 (1442.2-1615.3)	1362.2 (1285.9-1440.5)	1279.4 (1207.6-1356.3)	1154.5 (1040.4-1268.8)
IND Union Territories other than Delhi	1451.8 (1205.2-1757.5)	1387.8 (1161.2-1668.7)	1364.3 (1155.3-1622.2)	1478.1 (1284.2-1715.7)	1332.8 (1165-1517.7)	1316.6 (1157.8-1462.4)	1326.6 (1154.9-1490.6)	1285.2 (1131.4-1452.8)	1360.7 (1199.3-1502.5)	1350.9 (1181.6-1476.5)	1348.9 (1177.5-1461.8)	1195.4 (1018.1-1352.7)	1181.9 (1000.6-1352)	1133.7 (941.5-1322.9)
NPL Nepal	3159.6 (2820.7-3512.1)	2915.4 (2598.6-3257.3)	2724.8 (2412.6-3064)	2519 (2210.5-2848.8)	2319.4 (2022.5-2637.7)	2181.4 (1899.8-2468.3)	2066.1 (1820.5-2329.9)	1893.4 (1673.6-2119)	1677.5 (1505.7-1860.9)	1476.5 (1356.1-1613.2)	1290.3 (1213.8-1393.1)	1202.4 (1129.5-1317.1)	1211 (1105.3-1341.1)	1202 (1039.3-1294.3)
PAK Pakistan	1958.7 (1723.9-2236.7)	1877.3 (1656.6-2119.4)	1728.7 (1535.5-1934.5)	1548.8 (1402.4-1710.9)	1391.7 (1279.4-1510.1)	1349.9 (1267.1-1442.9)	1364.3 (1284.9-1440.7)	1370.8 (1301.7-1435.3)	1406.8 (1340.3-1474)	1448.1 (1369.4-1527.7)	1441 (1360.5-1524.9)	1498.6 (1395-1614)	1380 (1214.9-1567.7)	1295.2 (1087.8-1529.3)
Southeast Asia, East Asia, and Oceania	2559.7 (2484.6-2640.7)	2179.2 (2111-2256.4)	3824 (3755.2-3897.4)	1982.4 (1919.4-2050)	1775.1 (1723.4-1827.6)	1613.9 (1570.2-1659.9)	1468.1 (1431.2-1505.3)	1373.6 (1344.5-1403.2)	1313.5 (1290.3-1334.5)	1250.8 (1232.5-1267)	1168.3 (1152.5-1184.2)	1086.2 (1073.1-1099)	1011.9 (996.8-1027.8)	916.5 (889.1-943)
East Asia	2496.1 (2399.7-2603.1)	2098 (2010.9-2198.2)	4455.5 (4364.8-4552.6)	1965.6 (1879.7-2056)	1704.5 (1638.4-1770.8)	1522.8 (1470.3-1579.1)	1404.9 (1358-1452.4)	1321.5 (1287.5-1356.1)	1271.2 (1243.5-1297.4)	1206.5 (1185-1226.2)	1116.8 (1097.5-1136.7)	1025.9 (1010-1041.5)	951 (933.3-969.6)	852.3 (820.4-883.4)
CHN China	2436.3 (2335.8-2548.2)	2100.5 (2012.2-2202.3)	4582.3 (4489.3-4681.1)	1978.4 (1888.1-2071.5)	1713.9 (1645.7-1782.3)	1530.4 (1476.1-1588.3)	1412.4 (1364.2-1461)	1328.5 (1293.9-1364.4)	1278.6 (1249.2-1305.3)	1204 (1181.7-1224.5)	1112.1 (1092.4-1132.4)	1028.5 (1012-1045.1)	954.3 (936.1-973.1)	851.9 (818.5-884.4)
PRK North Korea	6318.6 (5932.2-6722.6)	2105.8 (1739.1-2460.7)	1883 (1543-2181.9)	1685.8 (1379.5-1919.1)	1499 (1248.8-1672.6)	1352.9 (1143.6-1473.8)	1290.9 (1108.4-1394.3)	1262.8 (1081.5-1362.7)	1240.8 (1055.2-1335.9)	1836.7 (1645.1-1938.5)	2101.5 (1943.3-2205.4)	1436.9 (1327.3-1517.2)	1336.3 (1249.5-1404.2)	1222.9 (1128.2-1320.2)
TWN Taiwan (Province of China)	2098.1 (1834.8-2374.6)	1857.4 (1847.3-1868.4)	1687.3 (1677.8-1697)	1436.2 (1427.8-1444.6)	1335.5 (1328-1342.9)	1260 (1253.7-1266.4)	1111.3 (1105.7-1117.3)	998.1 (992.9-1003.3)	943.9 (939.1-948.8)	898.4 (893.9-903.3)	817.4 (813.2-821.5)	773.2 (769.4-777.3)	688.9 (685.2-692.7)	688.8 (651.7-728.1)
Oceania	3632.6 (3393.8-3895.6)	3268.3 (3057.5-3503.6)	2979.9 (2784.3-3194.4)	2720.3 (2539-2923.5)	2531.7 (2366.7-2707.6)	2375.4 (2204.3-2544)	2213.6 (2051.2-2375.4)	2160.5 (1985.5-2326.2)	2101.3 (1917.9-2277.1)	2057.8 (1876.4-2239.7)	2049.4 (1861.3-2236.8)	2061.7 (1863.9-2259.5)	2002.9 (1800.6-2216.7)	1885.4 (1681.1-2096.4)
ASM American Samoa	1512.8 (1366.9-1668.5)	1497.8 (1395.9-1590.1)	1537.3 (1478.3-1587.5)	1515 (1461.4-1569.5)	1439.2 (1388.7-1505.2)	1375.5 (1330.2-1419.5)	1297.5 (1231.7-1371.4)	1259.1 (1172.4-1345.4)	1282.5 (1198.9-1354.1)	1250.7 (1165.5-1336.1)	1192.6 (1129.7-1280.3)	1189 (1126.7-1272.4)	1165.2 (1085.5-1254.7)	1113.3 (957.5-1261)
FSM Federated States of Micronesia	3236.7 (2905.5-3573)	2877.6 (2575.3-3177.5)	2728.6 (2418-3024.3)	2542.5 (2263.3-2850.1)	2334.4 (2068.1-2625.8)	2118.1 (1866.1-2409)	1923.1 (1691.5-2213.1)	1787.7 (1559-2075.7)	1682.3 (1458.1-1970.7)	1599.5 (1385.8-1844.1)	1566.2 (1366.9-1770.3)	1505.4 (1319.9-1685.1)	1488.9 (1299.4-1674.3)	1490.2 (1314-1669.9)
FJI Fiji	1882.6 (1753.5-2016.5)	1681.1 (1591.6-1762)	1557.5 (1485.1-1640.4)	1568.4 (1487-1647.9)	1782.9 (1694.2-1871.8)	1729.2 (1639.9-1822.1)	1525.3 (1443.7-1607.6)	1460.9 (1380.5-1545.6)	1442.5 (1312.5-1592.5)	1589.1 (1536.1-1646.1)	1762.5 (1739.7-1784.7)	1697.1 (1673.1-1721.4)	1541.5 (1513.4-1570)	1485 (1311.7-1679)
GUM Guam	1615.5 (1546.1-1693.1)	1542.5 (1486.3-1597.4)	1429.9 (1381.9-1474.7)	1370.3 (1338.9-1404.1)	1393.2 (1367.4-1419.7)	1381.3 (1354.3-1410.1)	1193.8 (1165.9-1219.9)	1023.4 (996-1054.2)	1066.1 (1028.6-1105.9)	1100.1 (1058.8-1146.4)	1082.8 (1043.9-1128.9)	1079.3 (1036-1123.3)	1034.8 (993.2-1074.7)	1013.5 (937.8-1085.7)
KIR Kiribati	3203.7 (2853.2-3543.3)	2958.3 (2636.5-3279.4)	2798.5 (2464-3106.8)	2626.9 (2303.9-2917.2)	2458.6 (2180.7-2717.8)	2187.9 (1946.7-2417.4)	2072.7 (1872.1-2283.3)	2096.9 (1917.1-2275.2)	2088.9 (1968.5-2209.9)	2132.8 (2050.2-2220.5)	2157.8 (2051.1-2270)	2121.8 (1956.4-2272.1)	2067.8 (1870.6-2263)	1966.5 (1716.9-2199)
MHL Marshall Islands	3326.1 (2969.8-3723.2)	2993.4 (2661-3381.4)	2717.6 (2382.7-3088.6)	2500.5 (2174.8-2837.8)	2315.5 (2016-2607.5)	2144.1 (1896.7-2403.4)	2041 (1846.7-2238.5)	1990.3 (1868.8-2110.1)	1962.8 (1883.3-2042.5)	1965.6 (1886.6-2053.7)	1975.6 (1868.3-2083.9)	1926.4 (1800-2047.4)	1857.6 (1704.8-2024.9)	1718 (1511.6-1941.9)
MNP Northern Mariana Islands	1306.9 (1134.3-1410)	1285 (1129.4-1376.1)	1230.8 (1098.4-1334.4)	1169.4 (1073-1293.3)	1109.6 (1028.3-1258.7)	1069.4 (997.3-1205.6)	1026.8 (917.2-1173.9)	959 (850.4-1141.9)	895.3 (795-1086.9)	858.4 (771.2-998.3)	835 (765.9-914.9)	844.7 (780.5-906.7)	843.5 (771.1-922.2)	869.7 (780.1-966.9)
PNG Papua New Guinea	4264.9 (3936.6-4638.7)	3867.3 (3569.3-4207.6)	3553.9 (3269.1-3876.9)	3225.3 (2949.7-3531.9)	2920.5 (2664.3-3180.7)	2708.2 (2438.5-2963.7)	2546.7 (2300.2-2788.9)	2498.4 (2235.1-2753.3)	2411.7 (2129.8-2673)	2310.5 (2026-2585.1)	2270.1 (1978.3-2556.9)	2299.5 (2000.1-2586.6)	2239.4 (1933.8-2543.4)	2069.9 (1773.1-2359.1)
WSM Samoa	1593.7 (1409.4-1813.8)	1434.6 (1295.1-1595.9)	1307.4 (1203.4-1416.5)	1323.7 (1226.8-1422.7)	1331.1 (1233.2-1436.6)	1337.7 (1233.5-1453.5)	1271.4 (1149.9-1401.2)	1242.4 (1101.5-1404.2)	1193.3 (1048-1375.3)	1175.5 (1066.4-1343.9)	1138.5 (1065.5-1258.2)	1102.8 (1059.3-1165.5)	1075.7 (1011.5-1131)	1052 (966.9-1109.9)
SLB Solomon Islands	3183.4 (2819.9-3502.6)	3001.2 (2667.1-3296.1)	2827 (2507.9-3109.5)	2606.8 (2328-2888.3)	2374.6 (2111.8-2661.6)	2306.5 (2055.9-2603.7)	1931.1 (1679-2236.1)	1814.1 (1551.8-2091.2)	1755.3 (1499.3-2036.7)	1664.4 (1416-1925.7)	1612.5 (1393.3-1851.7)	1600.2 (1393.9-1812.2)	1551.6 (1346.3-1746.4)	1485.8 (1299.8-1679.4)
TON Tonga	2048.5 (1867.8-2256.4)	1858.7 (1729.6-2001.9)	1647.2 (1559.5-1738.6)	1503.3 (1426.1-1589.7)	1443.4 (1375.8-1534.4)	1401 (1334.8-1485.6)	1365.1 (1315.5-1431.9)	1306.2 (1260.1-1356.8)	1258.2 (1199.9-1303.9)	1229.6 (1169.3-1288.1)	1218.8 (1165.1-1281.2)	1226.7 (1166-1281.8)	1230.8 (1158.4-1314.6)	1198.2 (1097.5-1368)
VUT Vanuatu	2565.2 (2228.8-2908.7)	2444.9 (2117.1-2800.5)	2340.6 (2026.9-2695.9)	2226.7 (1907.6-2573.1)	2139 (1824.4-2488.4)	2028.6 (1716.1-2348.2)	1926 (1625.4-2230.9)	1880.8 (1593.6-2148.2)	1856 (1572.9-2135.1)	1806.1 (1536.3-2084.3)	1767.2 (1473.5-2034)	1763.5 (1458.2-2047.9)	1722.4 (1432.7-2029.8)	1681.1 (1417.5-1994.4)
Southeast Asia	2849.5 (2751.5-2958.2)	2445.2 (2354.4-2539.4)	2206.2 (2117.5-2295.5)	2084.8 (2003.8-2169.3)	2014 (1937.2-2091.2)	1892.5 (1826.3-1955.7)	1643.6 (1583.9-1696.5)	1528.7 (1482.9-1575.4)	1439.6 (1402.1-1479.8)	1383 (1353.8-1414.6)	1321.9 (1294.6-1348.3)	1250.6 (1225.1-1274.3)	1177.9 (1149.7-1203.6)	1093.7 (1053.4-1135.2)
KHM Cambodia	2949.5 (2591.8-3357.7)	2739.7 (2425.2-3097.8)	2550.4 (2255.6-2884.1)	2391.7 (2079.8-2721.1)	3000.7 (2695.7-3301.4)	6246.4 (5971.6-6520.1)	2384.4 (2152.3-2613.3)	2241.3 (2052.3-2428.1)	1991.1 (1839.3-2131)	1896.3 (1762.9-2013.7)	1814.3 (1673.8-1929.6)	1622.3 (1511.3-1719)	1455.1 (1372.9-1523.3)	1318.8 (1244.1-1390.1)
IDN Indonesia	3118.3 (2962-3280.8)	2740.4 (2593.6-2889.3)	2465.8 (2331.9-2601.8)	2240.2 (2110.9-2368.7)	2040.2 (1925.4-2150.4)	1837 (1736.2-1925)	1640.8 (1564.2-1717.4)	1494.5 (1432.8-1559.1)	1400.7 (1350.6-1451.8)	1317.5 (1272.8-1361.6)	1295.2 (1253.9-1336.1)	1282.1 (1235.6-1322.8)	1256.5 (1202.7-1305.3)	1184.7 (1111.3-1251.2)
LAO Laos	4421.2 (3950-4914.6)	4013.4 (3578.3-4429.1)	3794.7 (3393.5-4196.8)	3546.3 (3173.1-3950.4)	3258.9 (2888.9-3646.4)	2861 (2530.3-3225)	2641.1 (2330.8-2969.6)	2397.6 (2116.8-2676.7)	2251.7 (1993.1-2512.9)	2076.1 (1860.7-2302.9)	1884.6 (1708.9-2081.8)	1694.8 (1533.6-1862.5)	1517.8 (1348.1-1689.3)	1315 (1156.1-1481.4)
MYS Malaysia	2372.7 (2076.5-2687.5)	2013.5 (1807.2-2214.9)	1803 (1791.8-1815.4)	1731.3 (1722-1740.9)	1673.1 (1643.6-1705.6)	1458.8 (1431.7-1486.2)	1358.8 (1298.1-1419.3)	1273.2 (1267.1-1279.7)	1202.1 (1195.6-1208.5)	1201.4 (1196.2-1206.4)	1128.5 (1123.5-1133.7)	1005.1 (1001.1-1009.6)	973 (962-984.8)	925.7 (850.7-1010.1)
MDV Maldives	3521.9 (3127.5-3958.7)	3209.6 (2865.8-3578)	2854.4 (2548.4-3179)	2506.5 (2286.7-2743.3)	2121 (1984.2-2255.5)	1866.8 (1779.5-1961.3)	1631.5 (1560.2-1711.3)	1366.4 (1327.3-1406)	1259.8 (1233.1-1287.5)	1148.2 (1120.7-1175.7)	981 (958.1-1002.2)	814.3 (795-833.9)	650.7 (636.8-665.2)	536.9 (505.5-570.4)
MUS Mauritius	2512.3 (2453.8-2575.5)	2225.6 (2184.9-2266.1)	1999.8 (1961.4-2036.5)	1572.8 (1538.7-1606)	1464.7 (1434.9-1495.8)	1721.5 (1694.7-1748)	1522.4 (1499.2-1545.9)	1500.6 (1476.3-1523)	1409.4 (1387.6-1431.2)	1296.4 (1277.7-1316.6)	1158.9 (1140.9-1177.2)	1149.3 (1130.5-1167.4)	1017.7 (1001.7-1034.5)	981.1 (918.1-1045.2)
MMR Myanmar	4266.2 (3826.7-4740.3)	3855.7 (3471.4-4235.3)	3562.5 (3178.9-3937.2)	3329.5 (2964.4-3708.2)	3108.4 (2760.9-3494.4)	2745.5 (2414-3104.8)	2491.5 (2168-2818.2)	2303.7 (1996.7-2614.9)	2176.4 (1919.5-2446.6)	2078.3 (1846-2318.3)	1952.5 (1750.4-2156.3)	1802.4 (1630.7-1965.4)	1594.1 (1431-1726.6)	1394.7 (1263.2-1493.1)
PHL Philippines	1477.3 (1361.4-1607.2)	1375.5 (1275.3-1480.1)	1277.2 (1204.1-1353)	1327.5 (1256.5-1402.5)	1409.7 (1334.1-1491.1)	1329.3 (1253.5-1405.9)	1345 (1272.6-1414.5)	1425.2 (1346.4-1506.5)	1313.6 (1239.7-1389.5)	1292.8 (1217.8-1376.5)	1292.9 (1225.3-1371.2)	1346.7 (1268.4-1429.2)	1370.6 (1286.8-1455.4)	1354 (1171.6-1548.6)
LKA Sri Lanka	1642.7 (1595-1697.5)	1341.2 (1321.9-1360.7)	1167.2 (1157.4-1176.8)	1226.8 (1218.8-1235.4)	1268.5 (1260.4-1276.4)	1456.6 (1447.4-1466)	1171.3 (1162.8-1180.4)	1347.7 (1340.3-1355.2)	1377.8 (1353.1-1401.6)	1343.6 (1336.6-1350.9)	1372.9 (1366.2-1379.4)	1158.4 (1115.7-1198.7)	1042.1 (1036-1048.7)	877.1 (742.6-1023)
SYC Seychelles	1761.8 (1536.6-2000)	1611.4 (1431.4-1807.2)	1497.7 (1369.5-1634.1)	1484.6 (1393.6-1577.9)	1407.7 (1355.2-1459.1)	1278.5 (1244-1314)	1186.6 (1154.6-1217.4)	1272.1 (1240.8-1304.4)	1454.8 (1422.1-1488.8)	1496.2 (1461.9-1531.5)	1381 (1349.3-1413.1)	1249.2 (1221.6-1276.3)	1185.5 (1158.8-1213.6)	1131.5 (1084.2-1180.2)
THA Thailand	2090.2 (1956.7-2233.6)	1696.1 (1620.3-1775)	1555.2 (1494.5-1619.3)	1507.1 (1448.2-1565.3)	1489.7 (1429.8-1548.2)	1430.9 (1377.6-1486.5)	1456.9 (1399.1-1514.1)	1203.8 (1155.5-1252.8)	1169.6 (1123.6-1215.2)	1197.3 (1146.1-1249.3)	1039.6 (985.4-1092.3)	862.2 (824.1-901.7)	757.3 (722.8-793.4)	742.6 (665.8-818.1)
TLS Timor-Leste	4004.9 (3522.6-4483)	3231.7 (2857.8-3642.7)	2843.6 (2479.6-3233.1)	2529.8 (2217.1-2887.1)	2271.5 (1984.9-2590.9)	2530 (2276.8-2787.9)	2120.1 (1905.7-2351.7)	1667.2 (1494.6-1859.3)	1467.9 (1325.5-1608.9)	1379.8 (1264-1488.4)	1245.5 (1155.6-1332)	1172.6 (1094.2-1265.9)	1117.8 (1010.2-1241.7)	1148 (990.5-1276.8)
VNM Vietnam	2831.5 (2451.9-3269.1)	2154.6 (1851.3-2493.9)	1986.1 (1699-2299)	2078.4 (1801-2366.6)	2218.7 (1953.4-2475.2)	1802.9 (1550.5-2008.2)	1551.2 (1340.8-1726.8)	1450.4 (1267.6-1588.3)	1387.2 (1260-1476.6)	1346.1 (1293.3-1385.2)	1300.7 (1264.5-1361)	1252.8 (1196.2-1313.5)	1183.2 (1117.4-1270.3)	1109.6 (1039.4-1266.6)
Sub-Saharan Africa	3230.9 (3096.6-3362.8)	2931.2 (2822.9-3039.7)	2764.8 (2669.3-2858.5)	2578 (2494.8-2666.8)	2437.7 (2356-2524.9)	2259.3 (2184.3-2337.4)	2093.8 (2029.1-2163.9)	1998.3 (1936-2064.1)	1988.2 (1925.3-2056.2)	2037.2 (1970.1-2109.4)	2077.5 (2001.3-2154)	1929.3 (1857.8-2007)	1707.6 (1647.1-1779.2)	1439 (1390.9-1500.6)
Central sub-Saharan Africa	3494.4 (3198.5-3817.5)	3063.4 (2807.9-3319.3)	2921 (2674.1-3172.2)	2826.8 (2577.3-3076.1)	2664.9 (2433.8-2907.6)	2533.3 (2302.7-2754.5)	2426.4 (2208.8-2634.9)	2316.8 (2114.4-2508.3)	2260.1 (2104.2-2430.1)	2256.4 (2127.4-2402.2)	2209 (2091.6-2337.5)	2010 (1898.9-2130.4)	1869 (1744.2-1998.5)	1629.1 (1485.2-1763.5)
AGO Angola	3419 (3010.3-3839.7)	3253.4 (2892.1-3628)	3142.8 (2779.5-3513.2)	3114.8 (2757.4-3461.8)	2939.4 (2607.2-3256.7)	2820.1 (2494.7-3133.7)	2770.6 (2454.6-3093.9)	2714.1 (2407.1-3008.2)	2561.2 (2279.5-2836)	2431.4 (2173.7-2680.2)	2288.1 (2065.7-2520.6)	1987.2 (1790.1-2179.7)	1775.4 (1576-1963.3)	1548.8 (1357.2-1716.1)
CAF Central African Republic	4098.6 (3737.5-4487.7)	3738.6 (3423.6-4086.2)	3410.9 (3094.9-3752)	3124.4 (2824.7-3432.7)	2953.2 (2688.4-3219.6)	2859.8 (2635-3094.4)	2752.2 (2571.9-2947.4)	2730.6 (2562-2896)	2921.6 (2730-3131.8)	3057.9 (2818-3312.3)	3105.7 (2819.4-3402.2)	3009.6 (2702.4-3321.9)	2776.1 (2452.7-3103.2)	2582.3 (2269.2-2920.2)
COG Congo (Brazzaville)	4165.7 (3799-4552.3)	3790.1 (3451.1-4121)	3513.1 (3182.8-3816.6)	3224.5 (2899.9-3509.4)	2979.4 (2689.2-3260.7)	2713.1 (2458.1-2965.5)	2468.7 (2235.3-2682.2)	2272.5 (2051.1-2479.3)	2429.3 (2199.6-2663.6)	2445.6 (2249.4-2657.1)	2272.6 (2125.9-2434.4)	1920.4 (1745-2069)	1651.9 (1472.2-1805)	1483.3 (1297.8-1632.5)
COD DR Congo	3471.7 (3042.2-3962.3)	2907.7 (2567.9-3261.3)	2783.1 (2449.9-3126.4)	2697.6 (2371.4-3039.3)	2542.5 (2228.4-2870.2)	2414.5 (2109.9-2735.9)	2305.2 (2025.1-2589.6)	2181.9 (1912.1-2440.7)	2120.1 (1895.7-2351.3)	2141.9 (1954.4-2332.3)	2129.5 (1976.4-2299.1)	1964.3 (1810.9-2135.1)	1866.9 (1691.5-2050.1)	1620.5 (1409.6-1812.1)
GNQ Equatorial Guinea	4957.4 (4204.7-5771.6)	3950.8 (3488-4451.3)	3747.4 (3334.4-4188.1)	3448.5 (3056.4-3889.1)	3219.1 (2823.8-3672)	3033.4 (2654.6-3425.4)	2784.6 (2424.3-3149.3)	2744.2 (2414.7-3077)	2790.7 (2459.2-3123.5)	2701.7 (2338.5-3058.4)	1993.2 (1627.6-2375.8)	1533.4 (1259.5-1875.9)	1365.7 (1107.7-1698)	1284.9 (1050.4-1588.1)
GAB Gabon	4403.1 (3801.1-5094)	3317.3 (2971.2-3678.8)	3172.3 (2816.9-3516.5)	2965.6 (2629.8-3286.1)	2768.9 (2476.6-3055.1)	2470.5 (2195.9-2712)	2217 (2000.1-2420.8)	2070.1 (1906.9-2235.2)	2013.3 (1880.3-2177.9)	1939.9 (1801.5-2092.9)	1871.1 (1742.4-2040.1)	1797.5 (1672.1-1952.8)	1626.4 (1515.5-1748.6)	1401.8 (1312.3-1505.8)
Eastern sub-Saharan Africa	3378.6 (3259.2-3495.9)	3072.2 (2975.3-3164.2)	2894.9 (2809.2-2988.4)	2729.7 (2645.8-2808.5)	2584.3 (2512-2661.9)	2440.8 (2376.6-2509.6)	2296.5 (2236.5-2357.3)	2260.5 (2203.7-2325.7)	2280.2 (2220.6-2345.9)	2318.9 (2259-2375.9)	2277.6 (2224.4-2331.3)	2009 (1961-2055.2)	1711.5 (1669.5-1753)	1432.6 (1390.2-1473.8)
BDI Burundi	4098.1 (3686.8-4503.7)	3831.8 (3473.2-4190)	3585.2 (3256.2-3914.8)	3394.3 (3105.1-3676)	3259.3 (3007-3514.6)	3178.1 (2917.5-3423.2)	3040.7 (2783.7-3284.2)	2900.5 (2655.5-3131.5)	2779.9 (2542.9-3012.1)	3186.5 (2954.5-3426.5)	3097.1 (2871.5-3311.9)	2602.9 (2371.8-2794.7)	1921.6 (1680.9-2109.6)	1661.8 (1431.5-1860.3)
COM Comoros	3554.1 (3168.1-4006.9)	3228.1 (2905.8-3552.9)	2922.1 (2637.2-3217)	2614.2 (2346.1-2905.8)	2352.4 (2110.3-2639.7)	2199.4 (1955.5-2491.4)	2119.1 (1884-2407.1)	1964.2 (1745.6-2221.7)	1794.5 (1604.9-2024)	1679.2 (1504.8-1890.7)	1514.9 (1357.8-1693.7)	1324.7 (1201-1482.8)	1234.8 (1120.7-1408)	1191.1 (1033.5-1345.7)
DJI Djibouti	2417.4 (1981-2926)	2219.6 (1849.4-2670.7)	2086.6 (1732.9-2505.4)	1962.7 (1638.8-2353)	1840.5 (1533.6-2217)	1683.1 (1405.2-2002.9)	1579.5 (1326.5-1887.1)	1578.1 (1342.3-1863.6)	1541.8 (1304.6-1832.3)	1594.2 (1321-1909.6)	1560.8 (1271.5-1896.3)	1490.4 (1206.5-1855.1)	1373.3 (1106.1-1698.7)	1226.7 (983.9-1505.9)
ERI Eritrea	4409.2 (3993.8-4869.1)	4086.7 (3724.8-4478.7)	3884.9 (3506.2-4267.4)	4263.3 (3919.8-4582.1)	4054.6 (3735.8-4339.6)	4093.1 (3833.7-4353.5)	3857 (3646.8-4059.5)	3682.7 (3515-3860.3)	4735.3 (4558.5-4908.1)	3142.4 (2956.7-3328.5)	2925.7 (2705.2-3149.6)	2562.1 (2316-2815.1)	2257.5 (1992.6-2529.2)	1832.9 (1561.5-2105.7)
ETH Ethiopia	4324.2 (4046.6-4602.7)	3984.2 (3732.6-4227.8)	3803.9 (3577-4045.2)	3645.6 (3419.9-3882)	3484.5 (3264.8-3701.9)	3390.5 (3201.4-3581.6)	3167.5 (2995.4-3335.4)	3041.5 (2889.6-3182.4)	2831.9 (2705.7-2949.5)	2556.7 (2434-2676.7)	2313.3 (2199.5-2423)	1797.1 (1708.3-1889.7)	1422.6 (1356.8-1496.3)	1172.3 (1106.6-1245)
KEN Kenya	2550.5 (2465-2636.4)	2317.3 (2235.7-2395.1)	2065.3 (1991.4-2138.4)	1945.7 (1873-2021.7)	1833.6 (1765.3-1904.5)	1680 (1623-1742.8)	1518.9 (1470.4-1571.1)	1412.3 (1366.6-1461.1)	1467.2 (1418.9-1524.5)	1734.3 (1668.8-1808.2)	1990.9 (1921.5-2063)	1966.4 (1912.7-2021.8)	1711.7 (1668.2-1754.2)	1477.2 (1432.5-1519.8)
KEN Baringo	2237.7 (1726.4-2761)	2184 (1700.6-2672.4)	2122.2 (1637-2649.9)	2159.3 (1680.9-2677.4)	2234.9 (1780.7-2802.8)	2222 (1808.4-2787.6)	2104.6 (1717.6-2604.5)	1956.2 (1602.9-2413.1)	1937.7 (1565.3-2357.6)	2079.6 (1674.3-2462.3)	2179.8 (1863.2-2496.1)	1934.3 (1733-2191.6)	1742.1 (1545.6-1986.6)	1544.2 (1324.6-1758.3)
KEN Bomet	1468.2 (1244.8-1791.5)	1519 (1286.9-1812.2)	1381.8 (1146.3-1658.4)	1360.7 (1100.8-1634.3)	1325.9 (1026-1558.4)	1266.1 (983.9-1505.7)	1194.1 (935.5-1457.8)	1137.5 (881.5-1376.8)	1191.5 (929.3-1392.3)	1346.7 (1073.8-1561.6)	1592.8 (1344.8-1847.1)	1594.2 (1372.7-1827.2)	1520.8 (1278.7-1737.5)	1298.1 (1074.3-1548.7)
KEN Bungoma	3009.9 (2425.8-3604)	2633.3 (2137.8-3174.3)	2289.1 (1854-2807.4)	2131.7 (1700.9-2626.9)	1986.2 (1600.8-2458.9)	1821.4 (1476.7-2237.8)	1627.1 (1309.5-2009.6)	1508 (1223.1-1848.6)	1432.9 (1158.9-1778.3)	1444.3 (1157.4-1790.7)	1538.4 (1227.3-1853.9)	1486.3 (1224.4-1752.4)	1375.9 (1158.8-1580)	1266.8 (1021.6-1447.5)
KEN Busia	3375.6 (2845.6-3987.2)	2727.3 (2288.6-3228.1)	2394.4 (1984.5-2894.1)	2250.3 (1840.5-2698.9)	2126.2 (1747.6-2566.5)	1842 (1508.4-2225)	1589.8 (1318.6-1920.3)	1443.3 (1202.5-1701.4)	1574.9 (1284.4-1925.2)	2167.5 (1702.2-2730.5)	2591.2 (2076-3191.2)	2266.5 (1862.2-2719.4)	1678.9 (1397.8-1985.5)	1384 (1172.6-1648.7)
KEN Elgeyo Marakwet	2087.6 (1589.4-2575.3)	1952.3 (1474.7-2428.6)	1863.9 (1412.4-2311.8)	1809.4 (1360.6-2253.6)	1764.7 (1337.8-2246.8)	1701.6 (1332.5-2169.5)	1611.6 (1292.5-2017)	1510.1 (1207.7-1863.2)	1476.4 (1182.1-1803.6)	1557.3 (1240.8-1827.4)	1683 (1418-1925.1)	1684.9 (1516-1909.2)	1563.4 (1416.4-1748.6)	1481.5 (1282.4-1645.9)
KEN Embu	2528.8 (2035.5-3061.8)	2161 (1773.6-2617)	1865.9 (1520.8-2296.2)	1671.9 (1344.3-2074.5)	1467.1 (1176.8-1814.9)	1245 (1002.6-1549.5)	1084.1 (871.6-1345.6)	1009.7 (842.5-1223.3)	1136.5 (931.3-1457.4)	1418.4 (1091.5-1854.1)	1584.3 (1245-1937.9)	1548.8 (1268.5-1808.7)	1465.4 (1235.8-1679.7)	1369.1 (1103.2-1578.1)
KEN Garissa	2960.9 (2502.7-3460.9)	3142.4 (2719.5-3642.5)	2560.9 (2144.4-3038.2)	2442.4 (2029.2-2896.7)	2281.4 (1880.9-2732.5)	2104.2 (1737.6-2533.6)	1964.7 (1619.3-2331.3)	1848.5 (1533-2180)	1791.8 (1477.1-2070.1)	1849.8 (1560.8-2086.3)	1878.6 (1658.8-2100.2)	1749.6 (1589.1-1973)	1581.2 (1431-1772.1)	1475.3 (1255.9-1906.1)
KEN Homa Bay	3464.6 (2913.2-4029.6)	3026.9 (2520.9-3554)	2830.8 (2313.1-3363.4)	2769.6 (2277.8-3335.3)	2784.8 (2315.5-3341.3)	2659.6 (2215-3165.7)	2398 (2002.4-2829.6)	2218.8 (1880.6-2620.1)	2316.7 (1926.3-2727.7)	2876.5 (2363-3439.4)	3845.7 (3191.8-4609)	4187.4 (3580-4910)	3243.9 (2776.6-3718.6)	2492.8 (2063.9-3007.9)
KEN Isiolo	2377.6 (1964.7-2845.5)	2398.3 (2020.1-2860.4)	2206.9 (1841.6-2671.7)	2246.2 (1859.5-2706.1)	2326.5 (1964.1-2732)	2201 (1871.1-2582.6)	1945 (1648.2-2314.2)	1760 (1498.5-2078.1)	1682.7 (1428.1-1971.7)	1730.2 (1446.8-2045.7)	1906.6 (1616.2-2241.7)	1785 (1534.9-2072.6)	1684.9 (1452.3-1946.6)	1557.5 (1310.4-1800.6)
KEN Kajiado	2024.5 (1561-2523.8)	2012.1 (1582.7-2460.2)	1796.2 (1361.8-2237.6)	1715.5 (1323.9-2129.6)	1606.8 (1259-2011.3)	1471.3 (1156.2-1845.8)	1333.9 (1054.6-1650.8)	1258.6 (1000.8-1510.1)	1355 (1063.9-1650.1)	1659.6 (1267.9-2089.5)	1932 (1615.8-2276.3)	1905.8 (1685.7-2164.8)	1698.2 (1507.9-1904.1)	1484.1 (1205.9-1670.4)
KEN Kakamega	2701.3 (2217.7-3242.3)	2341.4 (1939.1-2819.8)	2010.8 (1638.9-2472.8)	1873.6 (1499.1-2312.8)	1757.9 (1411.5-2177.3)	1613.2 (1316.3-1985.3)	1458.1 (1198.1-1776.3)	1380.6 (1143.6-1658.2)	1517.3 (1252.7-1835.7)	1949.2 (1586.4-2365.1)	2254.2 (1849.6-2718)	2094.5 (1742.5-2444.4)	1736.8 (1446.9-2012.1)	1416.6 (1165.5-1673.2)
KEN Kericho	1615.2 (1218.1-2114)	1587.8 (1210.7-2034.1)	1444.3 (1094.9-1853.5)	1337.7 (1007.5-1744.4)	1233.4 (940.9-1612.1)	1118.9 (874.6-1459)	1021.2 (818.2-1290.6)	959.2 (777.9-1184.4)	1027.3 (821.2-1279.1)	1201.4 (940-1429.2)	1424.8 (1129.9-1651.3)	1498.1 (1218.8-1720.5)	1460.5 (1189.6-1687.6)	1326.7 (1060.9-1574.7)
KEN Kiambu	2637.2 (2174.6-3154)	2373 (1945.8-2844.6)	2129.5 (1704.4-2585.6)	1986.1 (1558.9-2419.7)	1818.7 (1422.2-2190.8)	1592.4 (1284.5-1869.6)	1497.1 (1213.8-1723.2)	1422.8 (1172.2-1609.7)	1487 (1269.8-1707.1)	1861.4 (1596.2-2272.8)	2195 (1848.8-2624.3)	2236.7 (1904.4-2594.1)	1798.9 (1552.8-2078.6)	1525.6 (1380.8-1738)
KEN Kilifi	2909 (2456.8-3420.5)	2579.1 (2166.1-3028)	2302.5 (1912.4-2757.1)	2197.9 (1799.1-2644.8)	2058.1 (1685.7-2477.6)	1818 (1484.4-2221.3)	1595.9 (1334.7-1920.8)	1403.2 (1167.2-1666.3)	1318.4 (1083.3-1578.6)	1347.4 (1095.2-1620.6)	1430 (1160.1-1739.2)	1452.9 (1206.5-1752.7)	1441.6 (1204.3-1696.6)	1308 (1042.7-1545)
KEN Kirinyaga	2687.8 (2164.6-3294.3)	2214.8 (1784.8-2767)	1955.3 (1513.7-2501.1)	1788.8 (1361.4-2307.1)	1674.5 (1275.4-2147.7)	1465.4 (1128.6-1887.2)	1239.7 (946.6-1589)	1155.8 (897.5-1432.1)	1209.4 (949.4-1471)	1355.4 (1075.8-1645.1)	1534.9 (1214.4-1823.3)	1624.7 (1318-1952.6)	1496.1 (1257.7-1799.3)	1407.9 (1160-1630.4)
KEN Kisii	2789.7 (2294.6-3338.2)	2627.2 (2176.9-3120.7)	2206.9 (1761.5-2720.6)	2051.3 (1621.2-2567.4)	1936.5 (1532.1-2417.1)	1803.2 (1428.5-2251.7)	1651.5 (1314.9-2035.7)	1543.5 (1262.9-1881.8)	1560.1 (1288.3-1894.5)	1761.7 (1434.2-2109.6)	2033 (1665.3-2358.5)	1930.5 (1684.8-2156.1)	1615.6 (1445.9-1777.8)	1441.2 (1243.1-1614.8)
KEN Kisumu	3060 (2514.4-3668.1)	2780.1 (2287.2-3321.9)	2528 (2051.1-3102.5)	2411.3 (1928.2-2942.4)	2339.1 (1884.4-2874)	2192.8 (1788.5-2675.8)	2041.8 (1690.6-2495.1)	1923.7 (1605.8-2333.2)	2222.8 (1784-2705.5)	3284.5 (2607.1-3992.6)	4186.9 (3527.6-4876)	3956.8 (3456.8-4488.3)	2848 (2455.7-3272.6)	2380.5 (1980.9-2876.9)
KEN Kitui	3144.1 (2646.6-3689.4)	2563.5 (2108.8-3094.9)	2318.2 (1861-2850.5)	2194.6 (1736.2-2743.3)	2061.4 (1630.4-2570.5)	1874.5 (1489.6-2332.6)	1638.5 (1326.5-2017.1)	1479 (1206.3-1777.6)	1456.5 (1178.8-1742.8)	1591.4 (1285.8-1907.6)	1779.2 (1464.9-2084.8)	1802.7 (1583.3-2064.5)	1668.2 (1495-1919)	1506.1 (1319.9-1762.4)
KEN Kwale	2960.7 (2428.3-3520.9)	2522.6 (2058.4-3046.8)	2197.8 (1781.6-2682.5)	2078 (1675-2551.2)	1976.9 (1592.8-2436.2)	1814.3 (1476.4-2244.1)	1592.6 (1292.3-1962.5)	1397 (1113.6-1742.7)	1452.9 (1102.9-1981.4)	1738.6 (1298.8-2391.7)	1809 (1414.4-2303.8)	1745.2 (1428.8-2139.1)	1654.5 (1369.1-1918.1)	1452.3 (1179.5-1654.2)
KEN Laikipia	2056.9 (1619.5-2583.4)	1947.5 (1550.4-2403.9)	1687.7 (1334.9-2073.1)	1505.7 (1178.3-1874.3)	1342.3 (1048.3-1699.8)	1174.2 (914.7-1506.3)	1044.7 (826.8-1327.3)	979.6 (780.7-1236.7)	1055.7 (853-1295.6)	1233.9 (982.5-1501.7)	1489.5 (1187.6-1798.6)	1571 (1277.1-1841.6)	1534.2 (1245-1768.3)	1344.9 (1129-1595.7)
KEN Lamu	3709.5 (3169.9-4322.5)	3442.1 (2992.6-3995.3)	3095.5 (2638.3-3620.7)	2870.2 (2431.3-3359.4)	2459.4 (2055.8-2893.7)	1891.1 (1561.9-2256.6)	1456.5 (1193-1787.6)	1272.3 (1034.7-1569.2)	1254.6 (1012.5-1530.2)	1319.3 (1042.7-1636.2)	1436.6 (1133.3-1784.4)	1488 (1197-1856)	1507 (1225.1-1857.2)	1461.8 (1146.9-1790.6)
KEN Machakos	2516.1 (2068.6-3023.2)	2275.5 (1873-2758.1)	1972.6 (1582.9-2449.8)	1817.7 (1441.3-2330.6)	1664.9 (1304.6-2129.7)	1478.2 (1162.5-1874.5)	1287.6 (1029.1-1612.8)	1167.1 (930.3-1454.8)	1146.5 (909.9-1430.3)	1224.6 (964.9-1525.4)	1397.3 (1109.4-1692.6)	1513.3 (1242.5-1764.9)	1544.3 (1294.1-1721.7)	1494.7 (1196.2-1695)
KEN Makueni	1941.7 (1557.7-2341.6)	1773.7 (1404.4-2194.6)	1699.2 (1316.2-2124.5)	1698.9 (1309-2119.9)	1652.7 (1272.6-2059.7)	1552.4 (1225.8-1943.6)	1436.4 (1159.1-1802.6)	1352.2 (1088.8-1682)	1379.7 (1100.2-1711.1)	1565.5 (1213.3-1924.7)	1854.2 (1440.2-2231.9)	1912.4 (1517.6-2225.7)	1669.5 (1350.6-1910.7)	1449.5 (1149-1694)
KEN Mandera	2759.2 (2287.3-3265.8)	3084.6 (2657.3-3588.4)	2440.7 (2010.9-2932.9)	2375.7 (1961.9-2882.5)	2306.7 (1903.8-2795.4)	2183.5 (1811.8-2644.8)	2040.7 (1695.3-2438.9)	1913.6 (1602.5-2286)	1902.2 (1575.5-2237.7)	1987.8 (1666.3-2279.5)	2005 (1739.6-2245.2)	1996.8 (1808.9-2193.9)	1888.3 (1670.2-2071.6)	1560.6 (1289.8-1742.6)
KEN Marsabit	3891.8 (3235.6-4658.9)	4460.5 (3836.5-5167.6)	3835.3 (3332.4-4414.1)	3412.6 (2949.9-3932.2)	2761.6 (2356.1-3224)	2142.6 (1819-2535.1)	1691.2 (1412-2045.2)	1438.1 (1206.9-1714.9)	1379.2 (1150.7-1667.4)	1477.8 (1226.2-1786)	1665.3 (1375.1-1938.1)	1795.8 (1561.9-1989)	1698.3 (1500.2-1882.6)	1562 (1316.4-1734.8)
KEN Meru	2245.4 (1801.5-2728.3)	1954 (1585.4-2385)	1684.1 (1345.4-2101.5)	1541.5 (1218.6-1946.5)	1406 (1117.7-1774.7)	1251.9 (1001.7-1573.8)	1115.6 (884.8-1403.4)	1043.5 (841.5-1295.6)	1098.8 (888.4-1352.1)	1280.8 (1013.1-1602.8)	1513.8 (1189.7-1836.1)	1588.4 (1292.1-1829.7)	1560.9 (1320.9-1785.5)	1462.9 (1176.7-1722.2)
KEN Migori	3219.8 (2667.4-3852.6)	2718.9 (2257.7-3296)	2549.5 (2092.1-3119.6)	2533.4 (2049.4-3091.9)	2500.6 (2059.6-3025.6)	2483.8 (2059.6-2975.5)	2407.3 (2026.9-2859.4)	2286.2 (1947.5-2701.8)	2250.2 (1893.2-2654.3)	2488.8 (2041.9-3009.4)	2986.1 (2425.9-3563.5)	3222.1 (2716-3736.2)	2745.1 (2347.2-3177)	2169.8 (1799.7-2605.5)
KEN Mombasa	2286.3 (1823.6-2794.8)	2382.2 (1960.5-2883)	1937.1 (1546.7-2418)	1719 (1339.1-2133.2)	1525.9 (1175.2-1896.3)	1320.5 (1031.7-1647.9)	1126.6 (893.3-1414)	1019.2 (827.2-1263.3)	1120.1 (931.8-1403.1)	1497.1 (1185.6-1949)	1693.3 (1333.9-2186.5)	1648.1 (1301.5-2055.5)	1546.1 (1225.4-1863.9)	1322.4 (1031.2-1639)
KEN Murang'a	2401.9 (1987.9-2902.9)	1972.8 (1601-2403.8)	1788.3 (1430.1-2220.1)	1693.7 (1355.6-2136.5)	1594 (1251.3-2033.7)	1452.4 (1123.5-1843.5)	1323.6 (1050.7-1603.1)	1262.4 (1023.7-1454.9)	1306.2 (1080.5-1463.2)	1432.8 (1215.7-1629.5)	1571.8 (1382.2-1801.3)	1685.2 (1494.8-1985)	1479 (1304.9-1651.3)	1326.7 (1115.2-1493.6)
KEN Nairobi	2557.2 (2080.1-3056.3)	2629.5 (2207.2-3094.6)	2068.1 (1713.1-2486.5)	1775.9 (1439.2-2170.5)	1544.3 (1229-1911.7)	1306.9 (1046.4-1622.3)	1138.1 (913.6-1405.3)	1081.9 (881.8-1310.6)	1220.7 (988.7-1506.1)	1606.6 (1274.9-2027.4)	1871.8 (1511.4-2207.1)	1833 (1658.2-2028.7)	1534.9 (1414.5-1707.7)	1301.2 (1175.6-1442.6)
KEN Nakuru	2013 (1599.3-2447.1)	1961.2 (1577.3-2378.1)	1726.9 (1384.3-2106.2)	1604.6 (1312.6-1983.3)	1508.5 (1198.3-1856.7)	1405.6 (1125.9-1698.4)	1286 (1032.6-1518.6)	1207 (971-1412.5)	1299.8 (988.4-1554.9)	1749.9 (1329.8-2206.7)	2104.5 (1638.2-2600.3)	1888 (1531.5-2211.9)	1380.9 (1182.8-1646.3)	1124.3 (999.5-1333.5)
KEN Nandi	2009.2 (1636.3-2478.9)	1870.8 (1526.1-2344.5)	1741.3 (1410.8-2202.7)	1693.5 (1365.1-2162.4)	1656.1 (1347.6-2092.8)	1607.5 (1341.9-2048.7)	1558.3 (1296.1-1933.4)	1540.4 (1288-1893.7)	1714.6 (1414.7-2160.3)	2125.4 (1678-2648.7)	2338.4 (1954.7-2722.7)	2168.1 (1914.1-2443.7)	1945 (1687.9-2176.7)	1651.4 (1400.9-1911.3)
KEN Narok	2133 (1685.5-2607.5)	2137.8 (1696.7-2595)	1917.8 (1490.5-2376.2)	1889.1 (1472-2357.8)	1883.8 (1466.2-2341.8)	1849 (1449.4-2290.8)	1758 (1411.5-2154.5)	1715.7 (1387-2065.4)	1782.4 (1467.5-2134.1)	1940.7 (1610.2-2295.9)	2084.4 (1739.3-2384.4)	2013.1 (1725-2274.9)	1803.2 (1537.9-2039.2)	1603.7 (1340.2-1888.1)
KEN Nyamira	2657.9 (2184.6-3168.8)	2376.9 (1925.5-2849.2)	2228.7 (1803.5-2729.1)	2151.2 (1724.5-2673.9)	2033.1 (1637.4-2523.6)	1924.8 (1576.9-2345.8)	1825 (1495.4-2184.3)	1725.6 (1445.7-2043.3)	1734.6 (1496.9-2093.4)	1975.7 (1607.6-2396.4)	2422.1 (2030.2-2816.7)	2604.4 (2265.1-2991.1)	2271.9 (1989.2-2595.2)	1913.6 (1594.9-2242.5)
KEN Nyandarua	2626.1 (2112.2-3155.8)	2453 (1959.4-2967.7)	2187.6 (1717.2-2681)	2050 (1624.4-2532.2)	1942 (1540-2430.4)	1805 (1462.4-2271.7)	1630.4 (1370.8-2030.8)	1536.2 (1329.8-1868.5)	1556.2 (1336.9-1871.9)	1687.5 (1425.6-2021.5)	1955.3 (1656.4-2287.4)	2208.8 (1867.3-2548.6)	2066.9 (1746-2413.4)	1849.6 (1560.3-2176.2)
KEN Nyeri	2466.1 (1989.9-3002.2)	2045.8 (1624.7-2549.2)	1822.5 (1415.8-2286.6)	1672.9 (1287.9-2064.5)	1527.3 (1189.4-1862.3)	1391.3 (1080.8-1675.2)	1277.6 (998.8-1474.4)	1206.6 (934.7-1390.5)	1235.5 (982.8-1431)	1391.6 (1137.3-1644.6)	1621 (1302.1-1940.8)	1635.3 (1310.7-1926.4)	1427.1 (1156.9-1647.5)	1302.9 (1097.7-1521.5)
KEN Samburu	1365.2 (1033.1-1789.6)	1345.6 (1023.6-1719.8)	1264.8 (952.8-1629.2)	1248.1 (940.2-1618.7)	1247.6 (962.3-1613.8)	1246.4 (1003.2-1593.2)	1221.1 (998.7-1520.1)	1187.6 (991.6-1440.2)	1280.2 (1057.2-1564.6)	1581 (1297.4-1938.3)	1842.3 (1520.8-2194)	1831.1 (1604.6-2165.5)	1752 (1540-2061.6)	1613.7 (1389.1-1903)
KEN Siaya	3208.6 (2665.7-3772.7)	2871 (2340.3-3393.1)	2498.1 (2008.1-3025.2)	2291.8 (1838.2-2802.6)	2116.9 (1674.3-2581.2)	1930.5 (1552.9-2361.5)	1733.8 (1406.2-2114.4)	1590.3 (1302.9-1921.6)	1609.3 (1313.4-1916.5)	1773.7 (1421.2-2145.5)	2120.2 (1665-2607.6)	2285.2 (1871.4-2790.9)	1764.8 (1498.9-2090.3)	1348.5 (1136.8-1628.9)
KEN Taita Taveta	3208.2 (2648.1-3822.3)	2940.5 (2404.8-3519.1)	2549.7 (2053.4-3124.4)	2333.9 (1843.7-2877.3)	2144.5 (1673.7-2670.9)	1884.5 (1471.5-2349.4)	1618.7 (1263.7-2029.3)	1414 (1104.8-1766.6)	1345.2 (1059-1692.9)	1403.9 (1093.1-1778.6)	1611.7 (1274.1-2009.8)	1771.2 (1422.7-2128)	1793.8 (1465.7-2107.6)	1553.1 (1201.1-1815.3)
KEN Tana River	3905.4 (3308.9-4491.7)	3304.3 (2766.7-3821.1)	2916.3 (2412.5-3473.6)	2732.1 (2211.6-3266.1)	2503.2 (1996.3-3024)	2228.9 (1762.4-2719)	1945.2 (1586.7-2393)	1782.6 (1450.1-2177.2)	1767.1 (1432.2-2183.8)	1939.7 (1558.6-2385.2)	2235.5 (1848.1-2700.3)	2401.9 (2012.7-2843.4)	2291.9 (1903.7-2670.2)	1998.9 (1586-2433)
KEN Tharaka Nithi	3092.5 (2598.3-3639.6)	2561.6 (2120-3097.9)	2498 (2033-3069.9)	2485.7 (2015.5-3022.4)	2419 (1988.4-2929.9)	2227.8 (1848.3-2678.1)	2036 (1702.9-2447.4)	1930 (1631.4-2313.5)	1969.4 (1656.4-2356)	2153.3 (1821-2547.2)	2330 (1982.6-2707.2)	2218.8 (1893-2523.9)	2032.8 (1737.3-2305.7)	1783.9 (1486.7-2087.2)
KEN Trans Nzoia	1639 (1292.2-2085.1)	1763.5 (1446.3-2160.7)	1549.2 (1238.5-1920)	1451.8 (1146.9-1822.8)	1347.9 (1075.4-1676.4)	1201.3 (969.2-1506.9)	1065.9 (873.5-1318.5)	989 (819.2-1192.2)	1081 (895.8-1354.1)	1405.7 (1118.7-1767)	1728.8 (1357.9-2083.6)	1727.5 (1403.3-2036.2)	1626.5 (1322.2-1875.7)	1429.8 (1127.7-1684.5)

Table 1 Age-standardised mortality rates by location and year for males for 1950, 1955...2005, 2010, 2017

Location	1950	1955	1960	1965	1970	1975	1980	1985	1990	1995	2000	2005	2010	2017
KEN Turkana	1773.5 (1362.2-2272.3)	1847 (1433.7-2322.2)	1698.5 (1287.9-2174.4)	1706.2 (1303.4-2167.3)	1787.4 (1387.4-2271.1)	1902.9 (1534.9-2362)	1935.5 (1598.5-2350.5)	1826.9 (1516.2-2218)	1800.5 (1464.7-2217.6)	1995 (1584.4-2393.2)	2468.1 (2114.8-2881.4)	2304.2 (2010.9-2685.9)	2105.9 (1864.1-2412.7)	1754.4 (1466.9-2010.3)
KEN Uasin Gishu	1577.3 (1221.2-1908.9)	1578.1 (1251.7-1866.9)	1432.1 (1115.1-1692.1)	1381.1 (1072.5-1615.6)	1319.8 (1014.3-1563.8)	1247.8 (981-1514.7)	1162.6 (910.9-1444.3)	1104 (874.3-1363.6)	1135.5 (907.8-1382.6)	1250.4 (1003.6-1490)	1422.8 (1193.6-1629.1)	1474.1 (1303.2-1620.9)	1400 (1183.4-1575.4)	1204.7 (997.1-1442.5)
KEN Vihiga	1996.3 (1557.7-2457.8)	1809.6 (1395.8-2280.7)	1719 (1292-2161.1)	1683.2 (1270.7-2130.1)	1616.2 (1237.6-2032.8)	1550.6 (1197.6-1951.2)	1464.4 (1154.7-1843.1)	1415.2 (1134.1-1765.9)	1491.4 (1189.8-1875.3)	1821.5 (1418.3-2277.6)	2192.2 (1744.4-2654.2)	2091 (1728.5-2494)	1559.8 (1265.2-1810.8)	1367.5 (1118.3-1605.2)
KEN Wajir	1985.1 (1604.4-2407.2)	2174.9 (1819.6-2612.6)	1940 (1582.8-2341.3)	1938.9 (1578.4-2339)	1936.5 (1585.6-2361.9)	1888.8 (1546.8-2297.4)	1865.7 (1544.6-2239.2)	1830 (1531.2-2184.6)	1851.6 (1537.3-2176.6)	1991.6 (1661-2295.1)	2181.2 (1895.3-2438.1)	2062.9 (1875.1-2281.8)	1864.5 (1677.2-2056.7)	1596.4 (1339.8-1840.5)
KEN West Pokot	2419.5 (1917.3-2995.5)	2201.9 (1806.7-2662.6)	1993.1 (1610.4-2435.9)	1923 (1529-2385.5)	1867.6 (1503.6-2318.7)	1712.5 (1406.6-2127.8)	1507.4 (1260-1832.6)	1369.3 (1156.1-1638.4)	1410.1 (1173.6-1685.1)	1563.3 (1301.2-1860.1)	1644.4 (1384.7-1961.8)	1613.5 (1446.3-1822.2)	1612.9 (1428.5-1834.3)	1466.5 (1224.4-1645.7)
MDG Madagascar	2424.9 (2166-2689.6)	2270.7 (2096-2459.7)	2170.7 (2038.5-2299.5)	1972.3 (1880.8-2071.9)	2014.6 (1917.8-2113.3)	1886.3 (1766.2-2005.5)	1775.9 (1651.1-1906.5)	1794.7 (1681-1930.1)	1823.8 (1715.9-1939.6)	1727.9 (1611-1850.5)	1708.3 (1566.8-1848.3)	1668.6 (1482.2-1837.5)	1575.8 (1362.7-1790.7)	1451.2 (1220-1670.9)
MWI Malawi	3848.6 (3417.9-4303.6)	3506 (3158.7-3889.1)	3256.4 (2929.7-3596.8)	3050.4 (2757.1-3341.3)	2797.7 (2568.2-3029.7)	2503 (2345.9-2671.6)	2257.7 (2139.2-2385.9)	2183.1 (2077.2-2296)	2347.1 (2182.6-2544.2)	2765.7 (2521.4-3047.3)	2949.9 (2690.8-3238.9)	2745.8 (2563.2-2957.8)	2223.2 (2092.5-2362.8)	1662.8 (1518.9-1801.4)
MOZ Mozambique	3228.2 (2838.1-3673.1)	2967.9 (2640.4-3326.2)	2815.1 (2488.4-3145.6)	2691.6 (2372.6-3005.3)	2496 (2197.7-2795.8)	2269.5 (2011.8-2529.8)	2199.1 (1998.8-2404)	2726.7 (2571.8-2896.6)	2310.3 (2168.4-2452.4)	2326.9 (2179.7-2499.6)	2454.7 (2271.9-2632.8)	2546.2 (2358.3-2735)	2488.6 (2284-2705.1)	2038.1 (1829.2-2285.4)
RWA Rwanda	3690 (3286.5-4106.3)	3270.5 (2927.4-3622.3)	3143.6 (2802.8-3492)	3098.2 (2786.8-3411.7)	2998.3 (2694.4-3294.9)	2890 (2612.7-3176.3)	2689.2 (2446.8-2942.2)	2529 (2318.5-2740)	2751 (2587.9-2904.5)	3171.8 (3014.9-3334)	2737.1 (2605.2-2876.5)	1985.7 (1856.5-2104.9)	1522 (1388.6-1641.8)	1321.5 (1163.2-1456.6)
SOM Somalia	3201.5 (2702.5-3743.7)	3032.5 (2604.3-3520.8)	2864.9 (2482.1-3300.1)	2758.2 (2369.2-3182.6)	2690.7 (2296.8-3134.8)	2755.3 (2372.1-3178.4)	2469.7 (2109.1-2866.5)	2422.9 (2096.1-2777.7)	2527.4 (2202.9-2889.7)	2508.8 (2142.5-2872.5)	2376.8 (2026.8-2718.8)	2186.6 (1841.2-2550)	2156.8 (1793.1-2526.4)	1864.7 (1531-2232.2)
SSD South Sudan	2885.9 (2403.3-3454.3)	2813.8 (2375.9-3351.2)	2702.2 (2306.5-3169.9)	2553.3 (2185.7-2948.1)	2410.4 (2057.8-2807.4)	2292 (1950.5-2673.3)	2220.1 (1890.5-2591.2)	2190.8 (1885.3-2547.6)	2204 (1889.8-2577.4)	2149 (1801.8-2523.2)	2084.9 (1728.9-2447.2)	1925.9 (1578.6-2291.8)	1836.6 (1490-2209.4)	1775.5 (1489.9-2113)
TZA Tanzania	2945.4 (2576.5-3334.9)	2589.1 (2297.9-2902.7)	2485.3 (2224.3-2759.3)	2322.2 (2117.3-2542.6)	2153.3 (1967.9-2346.3)	1991.3 (1815.7-2176.5)	1836 (1688.3-2011.4)	1759.2 (1627.9-1905.5)	1876.4 (1720.1-2059.9)	2029 (1856.2-2217.1)	1982.4 (1822.8-2161.3)	1761.3 (1634.4-1899.6)	1512.8 (1408.2-1641.4)	1266.8 (1157.1-1398.9)
UGA Uganda	3021.9 (2622.1-3444.6)	2615.7 (2295.6-2961)	2489.3 (2172.4-2814.3)	2330.2 (2026.1-2635.3)	2197.4 (1942.3-2459.1)	2103.1 (1887.1-2324.7)	2230.2 (2045.2-2417.9)	2249.2 (1975.6-2680.3)	2482 (2064.7-2969.4)	2666.2 (2386.1-2945.8)	2526.2 (2329.8-2740.4)	2182.2 (2020.5-2340.4)	1815.7 (1662-1964.3)	1460.3 (1292.7-1612.4)
ZMB Zambia	2692.7 (2346.7-3050.1)	2418 (2126.8-2724.3)	2307.8 (2037.3-2582.1)	2191 (1934.7-2459)	2015.2 (1786.1-2272.2)	1884.3 (1698.4-2107.4)	1845.5 (1685.9-2033.4)	1907.5 (1763.5-2060.3)	2241.1 (2049.9-2491)	2806.3 (2555.9-3089.9)	2944.9 (2735.2-3181.2)	2645.4 (2491.9-2832.8)	2076.3 (1953.5-2206.3)	1597.6 (1448.6-1727.4)
Southern sub-Saharan Africa	2373.9 (2262.4-2487.4)	2241 (2138.9-2349.1)	2149 (2050.5-2251.9)	2059.9 (1962.8-2155.9)	1929.2 (1838.8-2022.2)	1759.2 (1677-1836.4)	1664.3 (1594.5-1731)	1523.8 (1471.7-1576.4)	1460.1 (1408-1521.1)	1726.5 (1669.3-1796.6)	2299.8 (2231.7-2376.2)	2457.1 (2389.8-2540.1)	2118.5 (2068.1-2181.9)	1490.2 (1450.5-1534.6)
BWA Botswana	2729.6 (2421.6-3070.4)	2548.6 (2273.6-2850.3)	2458.8 (2192.4-2720.5)	2352.4 (2085.4-2607.8)	2213.7 (1979.1-2437.1)	1968.9 (1784.1-2175.5)	1787.9 (1636.8-1974.4)	1666.6 (1548.3-1843)	1768.7 (1582.3-2022.4)	2010.2 (1758.3-2303)	2380.9 (2028.2-2791.5)	2376.3 (2093.2-2700)	1755.9 (1514.4-2022.7)	1233.8 (1101.1-1432.5)
LSO Lesotho	2994.9 (2679.5-3311.3)	2828.5 (2538.8-3123.1)	2725.5 (2443.1-3028.7)	2640.2 (2360.3-2915)	2520.8 (2244.5-2790.3)	2357.7 (2100.8-2610.5)	2140.2 (1922.6-2375.9)	2009.3 (1838.8-2225.6)	1971.9 (1827.3-2139.4)	2245.4 (2055.5-2466.9)	2945.3 (2688.2-3263.4)	3430.3 (3150-3756.6)	3029.8 (2772.5-3299.2)	2458.2 (2177.6-2725.1)
NAM Namibia	2550.7 (2231.9-2888.3)	2382.6 (2111.2-2668)	2277.9 (2040.5-2551.8)	2151.3 (1923-2410.7)	2162.5 (1963.4-2396.7)	2089 (1933.1-2293.2)	2115.2 (1990.8-2263.8)	2012.4 (1911.3-2127.6)	1847.9 (1735.7-2008.5)	1967.5 (1823.3-2136.9)	2355.5 (2159.9-2562.1)	2444.1 (2213.1-2696.7)	1924.9 (1773.4-2128.5)	1525.5 (1419.6-1655.9)
ZAF South Africa	2216.7 (2089.2-2353.8)	2116.3 (1998.8-2246.4)	2044.8 (1928-2165.6)	1978 (1865-2094.8)	1849.8 (1740.6-1957.6)	1677.9 (1581.4-1773.1)	1595.1 (1509.2-1678.4)	1468.2 (1405.9-1531.9)	1345.9 (1303.9-1388.8)	1510.9 (1475.4-1541.8)	2090.6 (2036.2-2150.3)	2255.6 (2184-2343)	1979.3 (1924.2-2045.7)	1405.6 (1366.2-1448.9)
SWZ Swaziland	3740.6 (3366.4-4104.4)	3405 (3106.3-3719.6)	3201.3 (2890.3-3509.2)	2959.5 (2628.7-3246.7)	2752 (2454.9-3037.3)	2519.6 (2239.8-2785.1)	2319.5 (2084.8-2547.2)	2138.1 (1944.5-2350.3)	1938.4 (1771.7-2139.6)	2106.6 (1913.2-2313)	3048.2 (2754.1-3402.4)	3520.4 (3170.2-3899.1)	3246.5 (2921.9-3588)	2115.8 (1803.3-2379.6)
ZWE Zimbabwe	3068.6 (2732.4-3413.4)	2791.3 (2500.2-3105)	2594.9 (2328.8-2881.1)	2423.7 (2180.9-2681.5)	2250.1 (2038.9-2484.3)	2078.7 (1865.1-2262.2)	1924.7 (1738.1-2078.1)	1655.1 (1506.1-1797.9)	1775.6 (1533.1-2114.3)	2503.9 (2186-2928.3)	3149.1 (2810.3-3567.5)	3297.3 (3026.7-3634.5)	2724.7 (2486.8-2993.4)	1797.7 (1606.9-2012.3)
Western sub-Saharan Africa	3280.6 (3007.6-3581.8)	2988.6 (2776.1-3232.9)	2808 (2623.5-3004.5)	2556 (2391.6-2741.3)	2424.3 (2260.9-2610.1)	2202.3 (2045.4-2363.8)	1978.3 (1847.7-2119.3)	1841.9 (1717.6-1973.6)	1822 (1697.4-1959.7)	1831.1 (1684.3-1989.2)	1820.5 (1659.2-1980.5)	1712.3 (1558.2-1871.3)	1561.7 (1439.4-1715.6)	1378.4 (1285.1-1517.5)
BEN Benin	3519.5 (3079.4-3986.8)	3147.3 (2790.1-3528.9)	2953.3 (2617.5-3308.8)	2729.1 (2442.3-3045.5)	2541.6 (2277.7-2822.4)	2332.6 (2098.1-2568)	2132.5 (1935.9-2334.2)	1965 (1805.5-2136)	1856 (1724-2003.5)	1807.7 (1668.9-1955.5)	1787.3 (1619.5-1958.1)	1698.4 (1514.5-1905.9)	1572.4 (1379-1812.1)	1388.6 (1201-1625.3)
BFA Burkina Faso	3823.3 (3368.2-4284.8)	3530.9 (3145-3937.7)	3232 (2875.2-3627.6)	2968.9 (2635.4-3310.1)	2775.6 (2480.1-3067.4)	2587.7 (2338.6-2823.5)	2339.6 (2162.4-2526.4)	2141.8 (1999.1-2291.8)	2119 (1963.1-2269.8)	2160.4 (1989.7-2326.6)	2061.4 (1919.9-2205.2)	1899.4 (1761.1-2040)	1784 (1642.9-1920.9)	1608 (1438-1794.5)
CMR Cameroon	3537.9 (3167.4-3946.4)	3064.8 (2731.2-3427.4)	3610.1 (3288.8-3932.5)	2526.6 (2239.3-2808.1)	2292.4 (2057.1-2524.4)	2089 (1899.9-2284.5)	1939.4 (1784.2-2082.4)	1782.2 (1647.3-1911.2)	1771.7 (1651-1888)	1900.6 (1771.2-2023.8)	2000.2 (1854.7-2174.4)	1979.7 (1792.1-2190)	1801.8 (1583.7-2016.2)	1517.8 (1297-1739.7)
CPV Cape Verde	2093.4 (1836.1-2393.5)	1634.7 (1403.4-1881.9)	1572.4 (1372.8-1794.7)	1390.5 (1232-1570.3)	1464.3 (1329-1604)	1263.9 (1184.7-1356.2)	1091.8 (1051.4-1135)	1132.4 (1100.7-1162)	1089.2 (1058.6-1122)	1137.4 (1075.2-1203.5)	1100.3 (1008.4-1197.6)	1059.1 (977.4-1138.2)	954.3 (922.6-987.6)	879.4 (809.1-954.6)
TCD Chad	3333.6 (2892.6-3807.5)	2945.4 (2555.8-3363.8)	2716.9 (2354-3081)	2524.4 (2203.5-2854.8)	2449.5 (2168.5-2764.9)	2222.4 (1997-2484.5)	2161.7 (1981.6-2371)	2015 (1861.1-2190.2)	1930.4 (1790.4-2080.3)	1964.2 (1819.2-2117.1)	2077.2 (1916.4-2245.7)	1968.7 (1816.4-2136.7)	1802.8 (1629.8-1987.3)	1590.6 (1412.3-1791.8)
CIV Cote d'Ivoire	2728.9 (2393.3-3079.5)	2682 (2385.7-3021.7)	2494.5 (2196.8-2815.1)	2336.5 (2062.2-2627.3)	2170.1 (1934-2430.6)	1987.1 (1786-2204.4)	1777.6 (1629.2-1943.7)	1767.4 (1617.1-1949.4)	1986.9 (1780.9-2274.7)	2272.4 (2072.6-2500.2)	2231.9 (2055-2420.8)	2085.8 (1902.9-2266)	1856 (1666.3-2057.6)	1546.3 (1368.2-1753)
GMB The Gambia	2698.1 (2323.9-3090.9)	2779.4 (2422.7-3154.2)	2700.8 (2352.3-3031.6)	2532.5 (2195.2-2868.7)	2273.8 (1969.8-2592.1)	2083.1 (1778.2-2390.7)	1926.2 (1647.4-2213.1)	1797 (1560.6-2068)	1709.2 (1490.2-1960.2)	1657.4 (1451.4-1879.3)	1655.7 (1463.6-1833.2)	1635.1 (1472.1-1775.5)	1562.8 (1416.5-1680.3)	1451.8 (1313.9-1569.3)
GHA Ghana	2922.7 (2543-3328.8)	2612.7 (2282.2-2946.7)	2318.4 (2010.7-2642)	2094.6 (1826.2-2406.4)	1925.1 (1659.8-2214.2)	1777.2 (1530-2061.2)	1699.2 (1476-1952.8)	1687.8 (1485.3-1896)	1632 (1444.3-1831)	1620.4 (1464.2-1794.1)	1683.9 (1541.1-1849.1)	1734.3 (1590.2-1863.7)	1686.5 (1572.8-1784)	1490.1 (1351.9-1600.9)
GIN Guinea	3297.9 (2846.7-3762.9)	2952.7 (2582.8-3328.5)	2691.3 (2349.2-3039.3)	2454.2 (2137.1-2755.6)	2325.3 (2061.7-2598)	2329.9 (2109-2558.1)	2052.8 (1889-2238.1)	1918.9 (1790.1-2057.2)	1846.4 (1723.8-1966.3)	1817.9 (1708-1937.2)	1864.4 (1753.9-2002.1)	1827.7 (1683.5-1971.6)	1788 (1628.3-1948.7)	1630.8 (1461-1813.8)
GNB Guinea-Bissau	4510.3 (4045.6-5016.9)	4116.7 (3734.5-4522.9)	3885.8 (3511.7-4281.4)	4044.8 (3664-4422.2)	3817.8 (3439.4-4178.1)	3261.1 (2895.8-3629.1)	3053.2 (2714.9-3417.8)	2906.5 (2590.9-3245.2)	2725.5 (2414.8-3034.5)	2588.7 (2326-2867.7)	2475.2 (2248.3-2697.8)	2388.9 (2190.1-2577)	2190.4 (2006.8-2380)	1884.6 (1684.7-2129)
LBR Liberia	4123.7 (3658.7-4602.7)	3591.9 (3223-3966.4)	3212.3 (2869.4-3549.9)	2906.7 (2615-3205.8)	2590.2 (2326.4-2847.7)	2275.5 (2037.7-2528.7)	2015.3 (1797.1-2237)	1861.1 (1680.5-2052.4)	2271.7 (2108.3-2453.1)	2074.8 (1926.4-2250.4)	1792.7 (1651.9-1954.1)	1563.9 (1433.9-1702.9)	1426.3 (1286.9-1590.3)	1298.7 (1154-1491.2)
MLI Mali	4160 (3654-4715.6)	3744.4 (3381.1-4163.3)	3474.3 (3144.5-3809.4)	3327.7 (3039.5-3619)	3154.1 (2895.8-3394.7)	2923.8 (2722.9-3115)	2549.3 (2394.9-2713.6)	2304.5 (2181-2442.3)	2108.9 (1992.7-2223.9)	1945.5 (1832.6-2063.2)	1781.4 (1675.1-1885.8)	1591.9 (1479.8-1708.7)	1476.9 (1347.6-1608.4)	1375.6 (1215.2-1531.2)
MRT Mauritania	3286.8 (2888.6-3688.8)	2916.4 (2570.5-3304.7)	2694.9 (2373.4-3067.5)	2427.7 (2113-2764.6)	2173.1 (1897.5-2465)	2105.5 (1855.7-2366.9)	1907.6 (1682.6-2131.4)	1773.3 (1605.7-1948.1)	1613.3 (1474-1744.1)	1456.7 (1340.8-1580.3)	1294.3 (1198.4-1394)	1182.4 (1072-1271.7)	1072.9 (941.5-1174.5)	1000.4 (843.3-1144.9)
NER Niger	3716.6 (3301.8-4190.8)	3501.7 (3121.5-3927.8)	3227.6 (2848-3622.2)	2941.2 (2603.6-3287.1)	2795.4 (2530.8-3092.1)	2741.2 (2508.5-2979.2)	2556.7 (2364.4-2752.3)	2396.3 (2242.5-2559.5)	2196.2 (2057.8-2350.8)	2015.7 (1878.9-2149.7)	1834.1 (1709.8-1956.3)	1675.6 (1548.1-1804.7)	1543.7 (1406.1-1701.1)	1392.4 (1236.9-1575)
NGA Nigeria	3215.5 (2641.7-3869.8)	2910.8 (2458.1-3444.8)	2690.7 (2313.9-3122)	2478.7 (2153-2865.3)	2396.4 (2071-2781.5)	2127.5 (1830.4-2454.4)	1874.6 (1636.1-2150.1)	1737.8 (1500.5-1996.8)	1752.7 (1508.7-2025)	1756.2 (1476-2044.7)	1761.7 (1451.4-2077.6)	1627.6 (1329-1943.3)	1444.8 (1202.2-1752.4)	1257.8 (1078.5-1537.4)
STP Sao Tome and Principe	2910 (2673.8-3154.5)	2450.6 (2320.8-2578.1)	2048.5 (1968.2-2128.6)	1621.4 (1564.2-1674.5)	1462.4 (1421.8-1504.3)	1449.6 (1401-1504.7)	1347.6 (1295.9-1401.6)	1388.7 (1313.4-1467.9)	1331.2 (1238.7-1425.2)	1326.2 (1221.1-1433.2)	1338.4 (1232-1454.6)	1320 (1214.9-1431.5)	1250.6 (1137.6-1359.7)	1213.1 (1082.2-1319.3)
SEN Senegal	3357.8 (2946.9-3760.1)	3103.3 (2738.9-3455.3)	2885.1 (2551.4-3213.8)	2740.1 (2445.4-3038.1)	2603.2 (2355.1-2859.1)	2396.3 (2183.2-2622.1)	2131.3 (1953.2-2309.8)	1908.5 (1760.9-2060.6)	1743.3 (1623-1867.2)	1679.5 (1569-1779.8)	1567.2 (1471.9-1669.5)	1423.2 (1334-1522.9)	1324.4 (1220.2-1457.1)	1274.6 (1133.5-1418.9)
SLE Sierra Leone	3918.6 (3413.3-4434.2)	3561.2 (3137.9-3977.4)	3277.8 (2901.7-3674.8)	3005.7 (2636.8-3395.5)	2760.2 (2423.2-3097.6)	2563.9 (2242.3-2873.8)	2449.2 (2158.5-2722.5)	2324.1 (2086-2570.3)	2169 (1973.5-2367.3)	2215.2 (2063.3-2378.2)	2099.3 (1968.5-2233.7)	2025 (1889.6-2160.7)	1838.4 (1676.5-2014.9)	1547.3 (1372.4-1751)
TGO Togo	2446.4 (2119-2773)	2381.5 (2095.5-2663.8)	2355.9 (2062.6-2651.9)	2281 (1989.3-2566.1)	2093.4 (1830.7-2348.9)	1955.2 (1731.7-2188.1)	1830.7 (1644.4-2046.4)	1770.4 (1602.7-1952.6)	1745 (1589-1907.3)	1815.2 (1649-1973.7)	1905.5 (1719.4-2096.5)	1944.7 (1727.3-2133.7)	1825.1 (1608.6-2007.6)	1531 (1332.5-1726.7)

Table 1 Age-standardised mortality rates by location and year for both sexes for 1950, 1955...2005, 2010, 2017

Location	1950	1955	1960	1965	1970	1975	1980	1985	1990	1995	2000	2005	2010	2017
Global	2056.3 (2032.1-2080.7)	1815.2 (1795.6-1833.9)	2197.8 (2180.7-2214.8)	1623.5 (1608.7-1637.9)	1528.3 (1516-1541.5)	1419.6 (1408.4-1430.2)	1318.7 (1308.8-1328.3)	1245 (1236.8-1252.7)	1164.8 (1157.8-1171.8)	1132.2 (1126.7-1137.9)	1064.1 (1058.2-1070.1)	981 (975.6-986.8)	899.9 (894.4-906.2)	808.8 (800.6-816.7)
Low SDI	2956.9 (2851.1-3064.5)	2716.2 (2624.8-2810.8)	2627.2 (2540.5-2710.1)	2538.7 (2462-2611.7)	2413.3 (2353.1-2473.6)	2211.5 (2157.3-2269.9)	2062.5 (2014.2-2110)	1991.2 (1945.7-2035.4)	1869.4 (1829.2-1907.7)	1763.1 (1731.2-1794)	1646.1 (1621.8-1670.4)	1490.8 (1471.9-1510.2)	1400.8 (1383.8-1418.4)	1217.9 (1191.4-1243.1)
Low-middle SDI	2890 (2815.3-2968.6)	2590.4 (2523.8-2661.1)	2739.1 (2675.5-2806)	2324 (2270.5-2381.7)	2152.1 (2104.2-2203.3)	1997.5 (1954.9-2041.7)	1778.4 (1738-1817.7)	1664.1 (1629-1698.7)	1567 (1535.7-1600.4)	1514.6 (1484.7-1545.1)	1477.6 (1448.4-1509.5)	1362.2 (1333.5-1393.5)	1269.2 (1240.2-1300)	1136.3 (1106.5-1168.4)
Middle SDI	1736.9 (1695.5-1777.1)	1488.8 (1454.7-1525.2)	2867.1 (2835.9-2902.5)	1292.6 (1265.1-1322.2)	1173.6 (1150.9-1197.5)	1056.6 (1036.7-1077.6)	970.9 (954.9-986.5)	916.4 (903.6-928.8)	848.8 (839.4-857.9)	795.7 (788.6-803.4)	748.1 (741.3-755)	696.5 (690-702.5)	654.2 (647.6-660.6)	601.5 (590.5-612.5)
High-middle SDI	2187.6 (2125.5-2254.4)	1942.7 (1889.8-2002.7)	2154.1 (2106.3-2204.7)	1672.6 (1627.4-1714.8)	1590.3 (1557.2-1626.3)	1510.6 (1481-1538.8)	1474.9 (1450.7-1499.1)	1432 (1413.2-1451.6)	1374.2 (1358.7-1389.4)	1440.3 (1427.2-1453.2)	1321.5 (1310.8-1332.5)	1235.3 (1226-1244.1)	1086.7 (1076.8-1097.6)	959.9 (940.7-978.4)
High SDI	1317.9 (1296-1341.1)	1139.5 (1129.9-1148.8)	1082.1 (1077-1087.3)	1032.1 (1027.2-1037.2)	991.8 (987.8-996)	912.4 (910-914.7)	845.1 (843.8-846.3)	780.4 (779.3-781.5)	719.9 (718.9-721)	674.2 (673.3-675.2)	610 (609.3-610.8)	553.8 (553.1-554.5)	503.1 (502.2-503.9)	473.4 (467.8-478.7)
Central Europe, Eastern Europe, and Central Asia	1542.6 (1511.6-1575.7)	1339.4 (1315.1-1364.1)	1164 (1155.5-1172.7)	1117.3 (1108.5-1126.8)	1140.9 (1132.8-1149.3)	1125.7 (1117.7-1134.9)	1166.7 (1159.5-1174.2)	1157.2 (1153.1-1161.3)	1101 (1098.8-1103)	1286.2 (1284.1-1288.6)	1207.7 (1205.5-1209.9)	1200.1 (1197.7-1202.6)	1036.9 (1034.8-1039.1)	884.9 (876.9-893)
Central Asia	2243.4 (2141.1-2354.8)	1858.7 (1802.8-1914.3)	1609.5 (1572.1-1647.3)	1454.7 (1411.8-1497.2)	1324.9 (1306.3-1344.5)	1249.1 (1222.5-1278.3)	1170.4 (1160.2-1181.6)	1128.4 (1121-1136.5)	1080.1 (1072.9-1087.5)	1252.7 (1245.5-1260.6)	1202.3 (1191-1213.3)	1257.7 (1244.4-1271.6)	1153.1 (1141.5-1164)	1049.9 (1018.3-1082.8)
ARM Armenia	1984.3 (1743.3-2268)	1624.9 (1496.3-1756)	1362.7 (1276.3-1449)	1228.7 (1137.9-1324.5)	1102.9 (1056.1-1152.5)	1056.4 (992.8-1121.1)	947.7 (916.9-980)	948 (925.3-971.6)	1055.1 (1031.1-1080.9)	1117.4 (1106.8-1128.5)	982.1 (973.3-991.1)	928.9 (920.8-937.1)	897.8 (890.8-905.1)	810.6 (790.7-832)
AZE Azerbaijan	2823.8 (2501.5-3163.4)	2344.4 (2163-2528.3)	1924.4 (1794.1-2057.4)	1620.7 (1499.6-1747.9)	1411.1 (1352-1480.7)	1267.2 (1203.9-1336.1)	1164.5 (1135.5-1196.7)	1088.3 (1066.8-1111.1)	1046.3 (1013.1-1079.5)	1330.6 (1291.4-1372.1)	1262.6 (1226.9-1302.2)	1328.8 (1291.6-1367.3)	1191.2 (1155.5-1228.6)	1104.9 (1047.5-1168.5)
GEO Georgia	2002 (1736.1-2288.1)	1726.6 (1583.2-1874.7)	1489.5 (1399.9-1591)	1329.7 (1229.4-1430.1)	1185.2 (1137.2-1236.6)	1130.4 (1049.1-1209.2)	1048.9 (1003.7-1094.1)	1041.7 (1010.8-1075.8)	1050.3 (1018.1-1084.2)	979.9 (948.9-1013.3)	902.8 (875.8-931.5)	915.2 (905.7-925.2)	964.4 (956.8-972.9)	983.8 (959-1006.7)
KAZ Kazakhstan	1990.1 (1776.1-2222.5)	1683.3 (1568.3-1803.3)	1466.1 (1391.4-1550.9)	1393.2 (1295.6-1498.7)	1301 (1262.9-1337.5)	1244.1 (1181.5-1308.1)	1181.4 (1166.3-1197.3)	1148.7 (1140.6-1157.8)	1108.5 (1101.6-1115.9)	1431.3 (1424.7-1439)	1355.1 (1348.4-1362.8)	1361.2 (1354.6-1368.6)	1192.3 (1185.8-1199.4)	976.7 (942.7-1012.4)
KGZ Kyrgyzstan	2795.7 (2453.9-3149.8)	2336.5 (2149-2519.8)	2025 (1880.4-2183.2)	1837.3 (1682.4-2000.5)	1675.7 (1563-1792.5)	1534 (1421-1643.3)	1425.7 (1362.5-1490.8)	1306.1 (1259.8-1351.9)	1198.3 (1160.5-1236.4)	1273.8 (1230.5-1315.9)	1163.1 (1123.1-1206.5)	1195.9 (1166.7-1224.4)	1051.9 (1045.6-1058.6)	871.1 (852.2-890.3)
MNG Mongolia	3555.5 (3174.4-3976.6)	2832.9 (2591.3-3078.8)	2387.7 (2214.7-2562.1)	2016.4 (1919.7-2117.2)	1903.5 (1824.1-1988.4)	1716.5 (1646.5-1783.5)	1665.8 (1601.5-1729.9)	1677.7 (1627.1-1727.1)	1593.2 (1549.2-1639)	1741.7 (1697.6-1782.9)	1696.1 (1661-1733.2)	1550.4 (1514.1-1588.2)	1386.9 (1349.3-1421.8)	1147.5 (1074.4-1226.5)
TJK Tajikistan	2630.8 (2339.8-2957.7)	2262.7 (2105.6-2439.6)	1984.3 (1873.1-2103.3)	1691.9 (1579.4-1820.1)	1459 (1401.8-1522.4)	1324.6 (1257.7-1396.2)	1135.4 (1106.6-1168)	1050.4 (1031.4-1070.8)	1018.9 (998.3-1040.6)	1156.6 (1118.1-1196.9)	994.7 (964.4-1026.6)	960.1 (927.8-998)	944.6 (903.4-983.9)	924.2 (864.9-982.8)
TKM Turkmenistan	2479.4 (2205.9-2793.8)	1975.9 (1846.7-2116.5)	1701.7 (1595.6-1810.1)	1540.5 (1429.3-1659.5)	1400.6 (1343.4-1462.2)	1373.8 (1301-1450)	1298.4 (1265.9-1336.9)	1254.6 (1234.8-1278.7)	1203.1 (1187.7-1219.5)	1378.8 (1361.5-1398.2)	1262.7 (1245.3-1282.2)	1385.4 (1368.8-1404.5)	1084.4 (1070.8-1100.1)	1016.9 (966.2-1074.5)
UZB Uzbekistan	2050.8 (1799-2321.6)	1601.6 (1491.8-1719.7)	1427.1 (1350.8-1509.8)	1318.1 (1224.8-1415.8)	1227.3 (1188.8-1265.4)	1179.9 (1119.1-1238.9)	1124.9 (1108.5-1142.7)	1084.9 (1073.8-1097.2)	984.8 (976.5-994.7)	1139.5 (1130.2-1148.7)	1222.7 (1190.7-1254.8)	1412.5 (1368.2-1455.8)	1334 (1298.5-1369.4)	1245 (1144.4-1352.8)
Central Europe	1653.7 (1625.1-1686.2)	1360.8 (1351-1371.3)	1214.7 (1210.2-1219.2)	1147.1 (1143-1151.5)	1145.8 (1141.8-1149.5)	1084.9 (1082.3-1087.5)	1119.4 (1117.1-1121.9)	1112.9 (1110.9-1114.9)	1071.5 (1069.7-1073.3)	1053.3 (1050-1056.7)	945.6 (943.2-948.3)	880.3 (878.5-882.1)	797.2 (795.5-799.1)	709.2 (697.5-720.9)
ALB Albania	1973.2 (1815.5-2161.7)	1527.5 (1442.2-1623.1)	1211.8 (1154.8-1277.5)	1099.4 (1026.8-1171.4)	972.4 (871.6-1080.3)	889.3 (785.5-1001.8)	824.1 (740-919.4)	803.7 (769.8-839)	785.5 (776.7-794.5)	746.4 (737-755.9)	788.6 (760.8-816.8)	752 (721.3-782.6)	666.5 (621.4-715.8)	633 (549.7-724.7)
BIH Bosnia and Herzegovina	2071.1 (1966.1-2188)	1630 (1579.7-1682.4)	1413 (1381.7-1445.4)	1347.4 (1320.5-1378.5)	1309.8 (1288.1-1331.5)	1190.5 (1174.7-1206.9)	1158.3 (1146.6-1171.1)	1037.7 (1028-1048)	890.5 (882.9-898.6)	1226.7 (1130.1-1330.1)	800.4 (743.3-858.2)	775.6 (767.7-783.5)	778.6 (770.1-786.8)	765.2 (732-801.3)
BGR Bulgaria	1427.3 (1406.4-1451.8)	1113.7 (1106.9-1120.2)	993.7 (987.6-999.9)	914.2 (908-920.6)	943.8 (937.8-950.4)	995.9 (989.5-1002.2)	1012 (1005.8-1017.9)	1019 (1012.6-1024.8)	1073.9 (1067.8-1080.5)	1114 (1107.6-1120.5)	1076.2 (1069.3-1083)	996 (989.9-1002.7)	912.9 (906.6-918.7)	834.1 (797.9-869.3)
HRV Croatia	2145.6 (2054.5-2255.6)	1622.6 (1587.6-1660.8)	1391.8 (1372-1413.8)	1224.7 (1210-1240.5)	1202.9 (1191.6-1214.1)	1084 (1074.7-1093.7)	1058 (1049.4-1067)	1052.9 (1044.4-1061.3)	1008 (999.2-1017.4)	930.2 (922.4-938)	876.6 (868.2-884.9)	834.1 (826-842.8)	765.3 (757.5-773.2)	657.2 (627.6-689.5)
CZE Czech Republic	1465.3 (1455.9-1474.5)	1277.3 (1270.4-1283.9)	1189.2 (1182.2-1196)	1167.3 (1160.1-1174.2)	1247.3 (1240.4-1254.4)	1188.1 (1181.3-1195)	1216.2 (1209.7-1223)	1165.2 (1158.4-1171.9)	1126.4 (1120.2-1133)	982.6 (976.4-989)	860.4 (854.8-865.9)	782 (776.9-787.9)	687.1 (682.4-691.9)	607.4 (579.5-636.4)
HUN Hungary	1596.8 (1584.3-1610.8)	1263.8 (1257.3-1270.7)	1222.9 (1216.4-1228.8)	1168.8 (1161.8-1175.1)	1205.6 (1199-1212.6)	1192.2 (1185.5-1198.7)	1243.4 (1237.1-1249.6)	1246.4 (1240.1-1252.4)	1178.6 (1172.7-1184.2)	1140.2 (1134.9-1145.7)	1011.9 (1006.7-1017.1)	942.3 (937-947.5)	850.3 (844.7-855.7)	712.6 (682.7-743.4)
MKD Macedonia	1872.9 (1722.8-2025.5)	1632.7 (1525.5-1747.3)	1475.9 (1381.5-1571.3)	1336.9 (1258.3-1427.3)	1190.6 (1148.6-1237)	1059.5 (1039-1079.9)	1035.4 (1019.8-1050.2)	1033.7 (1021.7-1045.7)	988.7 (977.2-1000.8)	984 (972.6-995.9)	923.7 (911.8-935.9)	845 (835.5-854.7)	749.1 (739.9-758.1)	674.3 (646.9-702.7)
MNE Montenegro	1229.7 (1165.8-1299.8)	983.6 (950.1-1019.7)	942.5 (907-976.8)	916.4 (886.1-946)	889 (857.9-919.2)	931.6 (903.3-961.5)	945 (930.3-958.2)	944.5 (932.4-956.7)	851.4 (838.1-866.5)	850.8 (836.1-866.7)	866.8 (849.9-883.4)	854.8 (827.5-882.8)	764.7 (738.1-791.9)	775.5 (729.6-826.9)
POL Poland	1681.7 (1672.7-1692.8)	1366.4 (1362.3-1370.3)	1125.8 (1121.9-1129.5)	1054.5 (1050.8-1058.1)	1061.6 (1058-1065.1)	1020.3 (1017-1023.6)	1067 (1063.6-1070.3)	1074.1 (1070.5-1077.6)	1039.6 (1036.5-1042.8)	988.1 (985-991.1)	878.1 (875.3-880.9)	797.6 (794.9-800.2)	719.1 (716.7-721.5)	639.5 (614.3-667.1)
ROU Romania	1533.2 (1365.7-1715.5)	1389.7 (1338.3-1443)	1286.3 (1281.6-1291.2)	1213.6 (1208.7-1218.3)	1158 (1153.7-1162.5)	1102.3 (1098-1106.7)	1200.9 (1196-1205.3)	1213 (1208.9-1217.3)	1150.9 (1146.7-1155)	1195.9 (1192.1-1200.2)	1060.9 (1056.8-1065.1)	1002.9 (998.9-1006.8)	909.4 (905.4-913.3)	808.8 (776.4-838.8)
SRB Serbia	1820.9 (1798.4-1845.8)	1474.2 (1466.3-1482.1)	1348.5 (1340.7-1356.2)	1321.4 (1313.8-1328.8)	1289.7 (1282.8-1297)	1062.8 (1056.8-1069.3)	1072.4 (1066-1078.7)	1098.1 (1091.9-1104.5)	1071.8 (1065.5-1078.6)	1093.1 (1086-1100.4)	1041.8 (1031.5-1055.7)	1041.1 (1031.5-1050.6)	986.2 (979.6-993.1)	931.4 (893.9-969.9)
SVK Slovakia	1555.7 (1444.8-1669.8)	1293.3 (1213.9-1373.2)	1152.4 (1077.1-1225.1)	1125.7 (1061.1-1195.3)	1156.9 (1099.4-1219.4)	1119.3 (1110.2-1127.9)	1124.3 (1115.6-1133.2)	1109.6 (1101.8-1117.9)	1091 (1083.2-1098.5)	982.6 (975.2-991)	928.9 (921.7-936)	876.5 (869.5-884.3)	798.8 (791.7-805.7)	696.2 (665-728.9)
SVN Slovenia	1744.3 (1718.9-1770.6)	1393.3 (1379.2-1406.2)	1276.9 (1262.8-1290)	1184.3 (1171-1197.2)	1215.1 (1201.3-1229.4)	1140.4 (1127.5-1152.6)	1067.8 (1056.7-1079.8)	1038.4 (1027.1-1050.2)	904.4 (893.3-915.1)	846.9 (835.9-857.9)	766.7 (756.9-776.5)	673 (664.1-682.3)	586.1 (577.8-594.5)	504.9 (478.3-533.9)
Eastern Europe	1339.2 (1290.9-1394.2)	1215.5 (1174.7-1257.4)	1033.7 (1021.1-1046.5)	1012.5 (1000.2-1025.3)	1080.2 (1066.8-1094.4)	1106.1 (1092.7-1121)	1171.1 (1159.4-1184.2)	1165.2 (1158.5-1172.1)	1099.9 (1097.5-1102.2)	1398.3 (1395.9-1400.5)	1336 (1334.6-1337.5)	1359.6 (1358.6-1360.6)	1145.2 (1144.3-1146.2)	949.2 (939.5-960.2)
BLR Belarus	1094.2 (932.8-1262.5)	1000.6 (884.3-1116)	896.5 (868.3-927.7)	837.4 (822.4-854.6)	879.1 (868.4-890.4)	912.7 (903.5-922.3)	981.2 (972.8-989.4)	1011.7 (1004.1-1020.5)	991.6 (984.6-998.9)	1166.9 (1160-1173.8)	1147.1 (1141-1153.8)	1175.9 (1169.1-1182.3)	1068.9 (1063-1074.5)	869.5 (833.7-907.7)
EST Estonia	1353 (1170.2-1538.2)	1291.8 (1147.5-1447.7)	1136.6 (1119.9-1153)	1064.5 (1049.6-1079.7)	1104.3 (1090-1118.6)	1102 (1086.8-1115.6)	1142 (1128.3-1155)	1147.9 (1133.8-1162.6)	1104.1 (1091.3-1117.3)	1215.3 (1201.4-1228.7)	1024.5 (1012.2-1037.6)	914.4 (901.8-926.7)	744.1 (732-754.9)	631.4 (569.9-692.2)
LVA Latvia	1193.9 (1029.5-1385)	1112.8 (971.4-1258.1)	1027.9 (1012.6-1042.8)	974.6 (963.3-985.4)	1034.3 (1024-1044.5)	1087.6 (1076.5-1098.1)	1116.8 (1106.9-1126.9)	1124.2 (1113.3-1135)	1105.4 (1095.3-1115.3)	1275.4 (1264.8-1285.8)	1034.6 (1024.4-1044.5)	1033.8 (1023.2-1044)	890.9 (881.3-901)	781.6 (714.6-852.8)
LTU Lithuania	1306.7 (1127.7-1508.4)	1139.5 (1007-1282.8)	1001.2 (991.3-1011.4)	877.3 (868.8-886.7)	940.6 (931-950.3)	958.1 (950-966.8)	1003.6 (995.5-1011.6)	1026.7 (1018.4-1035.3)	991.2 (982.5-999.7)	1123 (1115.3-1131.3)	930.7 (923.1-938.8)	985.4 (977.3-993.6)	884.4 (876.5-892.4)	791 (753.6-830.3)
MDA Moldova	2163 (1903-2420.4)	1915.2 (1778.6-2060.7)	1747.5 (1651.8-1853)	1643.6 (1532.4-1778.2)	1526.8 (1473.2-1583.6)	1439.4 (1371.2-1512.7)	1332.4 (1313.3-1353.9)	1344.3 (1333.2-1356.5)	1209.1 (1199.7-1220.3)	1417.9 (1407.7-1428.5)	1176.8 (1167.1-1186.7)	1168 (1159.2-1176.5)	1070.1 (1061.7-1078.4)	865.3 (844.9-884.6)
RUS Russian Federation	1325 (1290.2-1357.8)	1203.1 (1175.1-1231.9)	1041.8 (1022.8-1060.2)	1030.1 (1011.5-1049.7)	1104.8 (1084.5-1126.3)	1133.5 (1113-1155.3)	1211.2 (1193.4-1231.5)	1197.7 (1187.5-1207.9)	1116.1 (1114.8-1117.4)	1432.6 (1431.5-1433.7)	1401.9 (1400.9-1402.9)	1432.1 (1431.2-1433.1)	1190.2 (1189.3-1191.1)	933.2 (927-939.1)
UKR Ukraine	1375.7 (1206.8-1576.1)	1256.3 (1114.8-1414.3)	999.5 (982.9-1018.2)	978.5 (966.7-992.2)	1044.3 (1033.9-1055.1)	1065 (1054.7-1075.7)	1108.2 (1097.8-1119.1)	1109.4 (1099.4-1119.5)	1076.3 (1067.4-1085)	1377.7 (1368.9-1386)	1264.4 (1259.4-1269.4)	1263.4 (1260.8-1266.2)	1075.9 (1073.2-1078.6)	1052.3 (1013.2-1095.8)
High-income	1346.6 (1321.8-1371.3)	1179.7 (1169.5-1189.7)	1127.5 (1122.2-1133)	1081.2 (1076.3-1086.4)	1034.5 (1030.1-1038.8)	948.3 (945.8-950.8)	869.5 (868.6-870.3)	797.1 (796.5-797.7)	732.3 (731.8-732.8)	684.7 (684.2-685.2)	623.2 (622.7-623.7)	566.1 (565.7-566.6)	517.5 (517-517.9)	490.4 (484.2-496.2)
Australasia	1175.3 (1168.5-1182.3)	1119.2 (1113.3-1124.6)	1074.8 (1069.5-1080.3)	1083.2 (1078-1088.5)	1080.2 (1075.5-1085.3)	977 (972.4-981.7)	856.7 (852.9-860.7)	802.7 (798.7-806.7)	715.3 (711.6-718.9)	652.4 (648.7-656)	576.2 (573.3-579.3)	503.8 (501-506.7)	465.4 (462.9-467.8)	444.5 (412.8-476.9)
AUS Australia	1176.9 (1169.7-1184)	1126.6 (1120.1-1132.4)	1076.5 (1070.5-1082.5)	1084.9 (1079.4-1090.8)	1083.9 (1078.6-1089.4)	976.4 (971.5-981.8)	843 (838.4-847.4)	790.5 (786-795.1)	704.1 (700.1-708.2)	638.8 (634.7-642.8)	567.7 (564.3-571)	493.6 (490.5-496.9)	457.4 (454.5-460.2)	440 (403.4-478.5)
NZL New Zealand	1175 (1156.5-1193.4)	1087.8 (1075.4-1102.2)	1064.8 (1052.2-1078.1)	1073.6 (1061.9-1086.4)	1062.2 (1050.1-1075.7)	978.8 (968.2-989.5)	918.8 (910-927.3)	861 (852.2-869.7)	770.8 (763.7-777.7)	721.1 (714.1-728.3)	619.8 (613.8-626.6)	557.6 (551.6-563.1)	507.9 (502.3-513.2)	468.5 (449.9-490)
High-income Asia-Pacific	1902.8 (1857-1950.7)	1435 (1394.9-1478.6)	1360.6 (1324.3-1399.2)	1248.9 (1215.6-1286.3)	1127.2 (1097.3-1157.6)	969.8 (957.8-982.1)	848.9 (846.8-851.6)	734.2 (732.7-736.2)	654.4 (653.1-655.7)	597.2 (596.2-598.3)	520.5 (519.6-521.3)	466.9 (466.2-467.6)	426.4 (425.2-427.6)	381 (374.2-388.3)
BRN Brunei	2136.2 (1924.9-2355.8)	1877.2 (1722.2-2043.7)	1622.5 (1518.2-1729.7)	1347.4 (1280.2-1414.4)	1257.3 (1209.3-1305.4)	1202.9 (1157.3-1246.5)	1166.7 (1122.2-1214.8)	1135.7 (1094.3-1181.2)	1160.4 (1121.9-1202.5)	1015.6 (981.4-1050.6)	890.7 (859.3-919.9)	816.3 (792.3-843.9)	784.2 (756.8-814.6)	768.6 (720.5-821.8)
JPN Japan	1614.7 (1580.5-1648.1)	1302 (1274.5-1331.5)	1244.2 (1217.6-1271.3)	1127.9 (1102.9-1153.9)	1016.5 (995.8-1038.5)	878.3 (866.5-889.7)	758.1 (757-759.3)	658.8 (657.9-659.7)	590.5 (589.6-591.4)	547.7 (546.8-548.6)	479 (478.3-479.8)	440.5 (439.7-441.2)	408.6 (407.8-409.3)	370.5 (363.6-377.8)
JPN Aichi	1601.9 (1413.5-1822.9)	1299.8 (1135.7-1477.6)	1257.3 (1087.6-1428.8)	1133.3 (974.6-1302.9)	1018.1 (888.4-1161.6)	885.5 (813.5-959.9)	775.6 (770-781.6)	666.2 (661.5-671)	608.6 (604.1-613.6)	561.9 (557.5-566.5)	488.9 (484.9-492.6)	448.7 (445.1-452.3)	411.5 (407.9-415.2)	365 (329.1-401.7)
JPN Akita	1609.9 (1400.6-1831.1)	1284.4 (1115.6-1461.4)	1163.2 (1010.4-1325.6)	1060.1 (915.4-1211.9)	972.8 (850.3-1090.7)	829.1 (768.5-893)	701.5 (692.6-710.2)	653.5 (645.7-661.3)	588.8 (581.1-595.9)	559.2 (552-566.2)	509.7 (502.7-516.1)	477.8 (470.4-484.5)	448.6 (442.2-455.5)	420.2 (378.7-463.7)
JPN Aomori	1722.3 (1491.9-1962.2)	1379.1 (1205.8-1580.2)	1303.4 (1122.9-1488.6)	1176.9 (1028.5-1344.8)	1061.3 (939-1202.9)	908.2 (841.7-978)	782.4 (773.1-791.3)	695.9 (688.4-703.5)	611.2 (604.1-618.4)	587.9 (580.3-595.2)	529.7 (523.1-536.4)	499 (492.7-505.5)	462.8 (456.5-469.4)	425.1 (384-469.2)
JPN Chiba	1575.8 (1361.2-1786.3)	1284.3 (1118.7-1465.2)	1255.1 (1082-1446.2)	1132.1 (972.6-1312.2)	1025.5 (896-1166.3)	900.3 (825.9-973.7)	759.6 (753.1-765.7)	653.8 (648.6-659.4)	582.9 (578-588)	550.3 (546-554.9)	479.5 (475.4-483.5)	445.7 (441.7-449.5)	412 (408.4-416.2)	376.6 (342.7-413.3)
JPN Ehime	1622.2 (1418-1836.8)	1312.4 (1145.5-1500.5)	1225.7 (1072.4-1400.2)	1105.2 (965.9-1263)	999.8 (880.7-1136.3)	867 (805.4-940.2)	741.4 (733.4-749.8)	643 (634.9-650.3)	576.1 (569.2-583)	541.9 (535-548.8)	481.5 (475-487.8)	447 (440.6-453.1)	410.6 (404.7-417.1)	368.7 (331.7-408.8)
JPN Fukui	1490.1 (1302.9-1692.7)	1208.8 (1055.8-1372.1)	1195.6 (1053.6-1348.1)	1096 (976.7-1234.5)	988.7 (897.2-1086.9)	859.1 (814.3-904.9)	743.5 (732-753.9)	639.4 (631.1-647.4)	570 (562.9-577.4)	521.1 (514.1-528)	451.1 (444.4-457.3)	422.5 (416.4-428.5)	383.6 (377.7-390.1)	358.6 (327.8-393.8)
JPN Fukuoka	1669.7 (1453.1-1896.9)	1340.5 (1163.4-1528.9)	1259.7 (1085.7-1440.3)	1144.9 (999.4-1324.3)	1036.7 (910.9-1177.4)	900.6 (829.3-977.4)	768.1 (762.3-773.8)	667.8 (662.5-673.4)	601.3 (596.3-606.2)	547.7 (543.1-552.4)	483.3 (478.8-487.6)	442.9 (438.8-447)	406.1 (401.8-410.3)	357.8 (323.3-394.4)
JPN Fukushima	1601.7 (1388.5-1812)	1274.9 (1099.8-1439.4)	1217.4 (1049.3-1399.7)	1117.4 (962.1-1290.7)	1019.6 (889.7-1148.1)	869.1 (806.3-934.3)	738.2 (730.3-745.7)	655.6 (649.4-662.3)	571.4 (565-577.8)	541.3 (535.3-547.7)	484.7 (478.8-490)	450.5 (445.3-455.6)	418.6 (412.9-424.4)	395.5 (357.1-435.5)
JPN Gifu	1600.2 (1402.7-1822.7)	1300.9 (1136.1-1484.1)	1272.1 (1106.3-1450.1)	1165.4 (1012.2-1337.5)	1059.2 (932.9-1208.9)	913.5 (849.6-986.5)	791.3 (782.9-799.3)	671.3 (664.2-678)	588.2 (582-594.6)	545.3 (538.8-551.7)	478.8 (473.2-484.3)	438.1 (432.6-443.9)	402.7 (397.3-408.1)	380 (342.7-418)
JPN Gunma	1587.2 (1385.1-1795.6)	1289.9 (1119.8-1476.5)	1267.4 (1083.7-1455.4)	1162.9 (1001.9-1341)	1059.9 (934.6-1196.6)	928.3 (862.8-994.4)	787.5 (779-796.1)	678.6 (671.6-686)	591.8 (585.1-598.6)	546.7 (539.9-552.9)	480.7 (475.4-486.4)	448.2 (442.5-453.8)	420 (414.5-425.3)	390.2 (353.2-430.7)
JPN Hiroshima	1645.2 (1441.4-1853.1)	1332.4 (1153.8-1527.4)	1246.6 (1084.3-1425)	1121.4 (978.1-1273.6)	1009.1 (884.3-1145.9)	876.9 (809.6-953.5)	750.6 (743.3-757.7)	651.8 (645.2-658.4)	579.1 (573.3-585)	533.6 (527.7-539.3)	463.7 (458.6-468.9)	425.8 (420.8-430.7)	393.5 (388.2-398.7)	348.5 (315.5-384.7)
JPN Hokkaidō	1680 (1469.4-1899.9)	1351.1 (1188.4-1537)	1289.1 (1114.7-1469.2)	1168.2 (1005-1342.2)	1059.4 (922.9-1203.1)	915.8 (843.3-993.9)	790.6 (784.7-796.5)	681.8 (676.8-686.8)	600.1 (595.5-605.2)	540.8 (536.4-545.3)	476.7 (472.7-480.7)	443.3 (439.3-447.3)	412.8 (408.9-416.4)	375 (338.9-412)
JPN Hyōgo	1692.2 (1500.6-1900.9)	1355.5 (1185.8-1535.5)	1288.1 (1112.8-1470)	1156.1 (997.6-1331.3)	1029.7 (900.4-1159.2)	884.7 (817.2-955.5)	786 (780.1-792.2)	689.4 (684.3-694.8)	621.8 (617.1-626.8)	646.5 (640-652.9)	491.5 (487.4-495.8)	448.5 (444.7-452.6)	409.2 (405.7-413.1)	365.8 (331.3-403.9)
JPN Ibaraki	1652.3 (1443.9-1856)	1332.6 (1165.6-1514.4)	1291.5 (1123.8-1486.2)	1175.5 (1005-1374)	1063 (924.3-1211.6)	915 (839.8-996.1)	786.9 (778.9-794.7)	703.4 (696.1-710.4)	624.5 (618.3-630.6)	571 (565.1-576.6)	504.4 (498.9-509.7)	463.3 (457.9-468.5)	436.2 (430.7-441.6)	409.2 (370.8-448.9)
JPN Ishikawa	1509.7 (1319.4-1721.8)	1246.8 (1083.4-1437.4)	1245.3 (1086.3-1419.7)	1145.6 (1009.6-1303.1)	1028.1 (931.9-1140.6)	857.6 (808.5-907.9)	723.8 (714.9-733.1)	612.1 (604.5-619.4)	553.6 (546.6-561.1)	519 (512.5-525.8)	461.5 (455.4-467.6)	411.1 (405.6-416.9)	388.5 (382.7-393.9)	360.9 (325.7-396.1)
JPN Iwate	1642.6 (1429.9-1877.8)	1314.7 (1138.5-1488.9)	1270.7 (1111.1-1434.2)	1157.1 (1016.9-1313)	1047.1 (934.6-1169.7)	905.4 (845.7-969.4)	787.5 (778.4-796.3)	680.1 (672.1-687.9)	605 (597.7-611.9)	548.3 (541.2-555.4)	487.6 (481.3-493.8)	463.9 (457.6-470.2)	428.8 (422.1-435.5)	390.1 (352.6-430.5)
JPN Kagawa	1528.6 (1334.1-1739.3)	1238.5 (1075-1411.6)	1180.4 (1022.3-1342.4)	1067.2 (928.7-1211.1)	954 (848.4-1061)	820.4 (770.7-869.9)	710.1 (700.8-720.8)	619.7 (612.1-626.9)	573.6 (566-581.3)	523 (515.7-530.2)	470 (463.3-476.8)	431.4 (424.8-438.2)	406.1 (399.9-412.1)	370.3 (335.8-407.3)
JPN Kagoshima	1710.3 (1502.8-1939.4)	1369 (1182.9-1559.3)	1299.4 (1117.7-1492.6)	1202.9 (1044.6-1379.5)	1105.2 (972-1241.5)	954.1 (886.3-1025)	791.4 (783.6-799.9)	677.1 (670.2-684.1)	597.5 (590.6-604)	543.6 (537.1-550.3)	475.3 (469.5-481.3)	444.4 (438.5-450.8)	413.3 (407.1-419.5)	383.3 (345-422.2)
JPN Kanagawa	1580.1 (1389.2-1789.6)	1277 (1103.7-1455)	1208.7 (1053.7-1379)	1075.8 (924-1239.7)	964.5 (838.1-1096.3)	828.9 (765.1-903)	727.3 (722.5-732.6)	638.6 (634.4-643.1)	577.1 (572.9-581.1)	541.1 (537.3-545)	474 (470.7-477.4)	429.3 (426.1-432.7)	397.9 (394.6-400.9)	359.7 (327.8-395.4)
JPN Kōchi	1731.1 (1524.8-1957.3)	1406.1 (1220.8-1601.8)	1320.6 (1143.8-1510.6)	1206.5 (1052.1-1366.6)	1097.7 (979.6-1218.7)	943.5 (885.4-1004.4)	778.9 (767.6-789.8)	652.4 (643.7-660.3)	588.4 (580-597.2)	539.4 (531.7-546.6)	484.4 (477.8-491.3)	449 (442.2-455.6)	419.8 (412.7-427.2)	359.7 (322.8-396.3)
JPN Kumamoto	1633 (1418.5-1869.9)	1290.7 (1110.9-1493.1)	1232.1 (1060.2-1413.5)	1138.3 (985.2-1304.6)	1044.8 (921-1182.6)	910 (842.8-980.7)	749.5 (741.4-757.3)	620.7 (613.7-627.8)	555.5 (549-562.2)	487.9 (482.1-494.2)	438.5 (433.2-444)	405.7 (400.7-411.1)	376.9 (371.7-382.2)	348.9 (315.1-383.9)
JPN Kyōto	1540.7 (1344.5-1756.2)	1252.6 (1089.6-1430.5)	1182.6 (1019.8-1355.4)	1049.4 (905.9-1228.3)	925.4 (807-1061.1)	783.4 (716.9-851.8)	682.7 (676.1-689.7)	616.7 (610.7-623.5)	563.1 (557.1-569.2)	517.2 (511.1-523.1)	465.5 (460.2-470.9)	424 (418.7-428.9)	388.1 (383.1-393.2)	341.3 (307.5-375.3)
JPN Mie	1561.2 (1366.4-1774.9)	1262.5 (1088-1437.7)	1224.4 (1048.3-1410.9)	1107.5 (950.3-1274.9)	991.8 (875.6-1119.9)	854.1 (791.7-923.2)	753.1 (743.6-761.9)	670.1 (662.7-677.3)	595.9 (588.9-602.9)	555 (548.2-561.6)	476.5 (470.4-482.1)	439.5 (433.8-445.5)	412.1 (406.1-417.6)	380.1 (343.4-418.7)
JPN Miyagi	1566.9 (1374.8-1786.5)	1254 (1089.1-1452.5)	1244.4 (1083.3-1436.1)	1141.2 (987.9-1308.5)	1045.6 (917-1173.9)	918.6 (849.2-987)	797 (788.9-805.6)	674.1 (667.3-680.8)	587 (581.3-593.5)	543.2 (537.4-549.3)	478.9 (473.4-483.7)	446.1 (441.2-451.1)	408.9 (403.6-414.2)	374.2 (338.7-411.3)
JPN Miyazaki	1674.7 (1455.1-1899.9)	1349.8 (1183.7-1552.5)	1275.2 (1105.4-1459.1)	1166 (1013-1332.1)	1050.4 (937.3-1180.3)	899.2 (835.5-964.5)	770.1 (760.8-778.9)	649.1 (641.7-656.7)	579.4 (572.4-587.4)	538 (530.6-545.1)	460.6 (454.6-466.7)	428.8 (422.3-435.2)	399.7 (393.1-406.2)	371.2 (332.6-410.9)
JPN Nagano	1468 (1287.5-1687)	1187.8 (1029.9-1363.5)	1162 (1001.2-1332.9)	1070 (928.3-1231)	975.3 (860.1-1098.6)	857.2 (798.1-923.6)	713.4 (705.5-721.2)	626.5 (620-633.3)	542.6 (536.4-548.9)	496.5 (490.8-502.1)	439.9 (434.5-445.6)	402.7 (397.8-407.6)	371.1 (366.1-376.3)	346.2 (312.7-381.6)
JPN Nagasaki	1674.1 (1454.8-1907.4)	1345.7 (1172.5-1534.5)	1280 (1116.8-1456.4)	1174.2 (1014.4-1337)	1067.4 (937.8-1198.9)	932.4 (863.7-1003.1)	799.9 (791.2-808.6)	672.4 (665.2-680.1)	599.2 (591.4-606.6)	546.7 (540.1-553.4)	472.7 (467.1-478.7)	439.9 (434-445.7)	407.9 (401.7-413.9)	381 (345.2-419.7)
JPN Nara	1660.5 (1451.9-1892.4)	1347.9 (1178.9-1541.9)	1335.8 (1169.8-1519.8)	1216.4 (1060.8-1389.1)	1096.5 (972-1231.4)	943.8 (876.4-1011.5)	787.1 (777.5-796.9)	689.4 (681.6-697.7)	614 (606.5-621.1)	548.5 (541.8-555.5)	468.9 (462.5-474.9)	431.8 (425.8-437.7)	393.2 (386.8-399.2)	361.9 (327.3-399.7)
JPN Niigata	1539.3 (1339.4-1749.8)	1242.6 (1072.2-1422.6)	1238.3 (1062.3-1409)	1151.9 (992-1317.4)	1062.5 (939.2-1201.9)	930 (861.9-999.5)	791.6 (783.7-799.7)	672.6 (666.6-679.2)	573.1 (567.2-578.5)	526.4 (520.9-531.8)	462.3 (457.2-467.5)	428 (422.9-433.2)	395.1 (390.2-400.3)	364.3 (329.8-399.2)
JPN Ōita	1633.6 (1442.2-1856.2)	1318.8 (1157.1-1506.5)	1258.9 (1092.1-1432.4)	1143.9 (989.5-1303.1)	1023 (906.8-1143)	871.4 (813.8-935.8)	766.7 (757.3-776.2)	665.3 (658-673.1)	587.9 (580.2-595.3)	530.6 (523.7-538.1)	471.5 (465.2-478.3)	423.8 (417.4-430.2)	390.4 (384.1-397.2)	342.3 (307.6-377.9)
JPN Okayama	1527.8 (1326.5-1735.6)	1239.4 (1072.3-1420.2)	1184.3 (1030-1365)	1077.6 (936-1232.5)	970.4 (852.9-1093.1)	839.1 (778.8-899.7)	736.9 (728.6-745.2)	637.9 (630.8-645.3)	562.3 (556.1-569)	523 (517.4-529.1)	454.3 (448.7-459.4)	415.8 (410.2-421)	392.4 (386.9-398)	357.8 (323.6-393.4)
JPN Okinawa	1669.9 (1446.8-1897.1)	1320.2 (1141.1-1506.5)	1219.1 (1061.1-1392.7)	1140.4 (983.7-1306.8)	1085.3 (958.6-1225.9)	978.4 (914.4-1053.4)	688.1 (679.4-696.9)	528.8 (521.7-535.8)	496.7 (490.3-503.5)	469.8 (463.7-475.9)	435.3 (429.7-440.9)	410.8 (405.9-416.3)	396.6 (391-401.7)	379 (341.8-418)
JPN Ōsaka	1794.7 (1573.7-2013.9)	1438.5 (1260.2-1624)	1348.3 (1177.6-1548.6)	1198.8 (1040.8-1374.4)	1068.8 (938.8-1210.5)	925.1 (853.3-998.3)	822.9 (818.1-827.9)	729.8 (725.6-734.2)	652.9 (648.7-657.3)	591.3 (587.5-595.5)	514.1 (510.7-517.6)	468.4 (465.1-471.9)	435.6 (432.5-439.1)	384.7 (349.2-422.4)
JPN Saga	1605.2 (1401.4-1815.6)	1281.8 (1114.4-1465.4)	1222 (1064.7-1404.2)	1115.3 (975.3-1271.3)	1002.8 (906.8-1114.2)	870.6 (819.3-923.6)	761.7 (751.2-772.1)	660.7 (652.7-669.2)	587.3 (579.4-595.3)	541.4 (533.5-548.9)	478.6 (471.3-485.4)	432.4 (425.9-438.9)	409.2 (402.4-415.6)	371 (335.7-407)
JPN Saitama	1599.5 (1402.6-1807.5)	1301.4 (1127.5-1471.3)	1272.7 (1110.1-1455.1)	1127.3 (975.4-1299.1)	994.8 (860.8-1132)	860 (791.5-930)	779.5 (773.4-786.5)	670.7 (665.8-675.9)	601.9 (597.2-606.6)	553.6 (548.9-558)	484.3 (480.4-488.1)	448.7 (444.9-452.3)	419.9 (416.2-423.5)	381.6 (347.7-420.3)
JPN Shiga	1515 (1314.6-1729.6)	1241.7 (1081.8-1427.5)	1239.4 (1079.6-1418.6)	1158.4 (1014-1306.6)	1059.5 (954-1175.1)	890.1 (841.9-940.1)	711.1 (701.4-720.8)	641.8 (634-649.5)	602.9 (595.4-609.8)	539.6 (533-546.4)	464.8 (458.9-470.6)	422.8 (417.4-428.6)	384.6 (379.6-390.2)	350.5 (319.1-385)
JPN Shimane	1576.3 (1371.4-1796.6)	1283.8 (1125-1465.6)	1218.7 (1068.2-1370.2)	1116.8 (985.8-1258.4)	1008.7 (903.7-1118.3)	864.9 (812.6-915.5)	719.5 (709.6-729.8)	606.3 (599.2-614.6)	537.9 (530.4-545.1)	497.3 (489.7-504.8)	463.3 (456.4-469.8)	421.5 (415.4-428.2)	384.2 (377.7-390.6)	353.3 (320.4-389.3)
JPN Shizuoka	1549.4 (1345.6-1761.4)	1246.7 (1085.3-1436.8)	1193.8 (1031.8-1375.5)	1078.7 (933.6-1238.7)	968.7 (840.2-1097.3)	838.8 (770.6-909.6)	733.2 (726.8-740)	632.9 (626.8-638.6)	566.3 (561-571.6)	527.5 (522.1-532.4)	462.9 (458.1-467.3)	420.4 (415.8-425.1)	401.5 (396.7-406.3)	369 (331.4-406.8)
JPN Tochigi	1618.2 (1396-1853.3)	1317.5 (1143.8-1517)	1276.1 (1115.7-1465.4)	1154.6 (1011.2-1321)	1033.2 (908.3-1166.6)	893.4 (828.6-964.9)	792.1 (783.8-800.2)	705.1 (697.9-712.9)	634.5 (626.7-641.4)	581.2 (574.8-587.7)	498.8 (493.4-504.6)	476 (470.6-481.7)	437.2 (431.7-442.9)	401.7 (363.5-439.4)
JPN Tokushima	1657.4 (1445.1-1897.7)	1346.2 (1164.5-1534.5)	1293.5 (1130.3-1477)	1185.5 (1040.2-1344.4)	1076.2 (962.5-1200.9)	920.1 (861.6-979.2)	794.4 (784-805.8)	676.6 (668.4-685.2)	604 (595.9-611.6)	548.5 (540.8-556.8)	480.8 (474.1-487.9)	449 (442.2-456)	410 (402.9-417.5)	392.6 (354.7-433.3)
JPN Tōkyō	1569.7 (1369-1774)	1257.5 (1097.6-1433.1)	1182.3 (1027.8-1343)	1063.6 (931.5-1216.6)	952.6 (835.5-1073.2)	826.2 (764.9-890.4)	730.3 (726.4-734.4)	642.8 (639.1-646.4)	598.2 (594.7-601.6)	557.3 (554.1-560.8)	492.8 (489.5-495.9)	441.6 (438.8-444.5)	406.5 (403.8-409.3)	358.2 (325.7-391.5)
JPN Tottori	1586.8 (1387.1-1817.5)	1271 (1106.7-1463.2)	1196.7 (1048.3-1359.1)	1087.4 (963.1-1218.1)	977.6 (893.4-1067.6)	839.7 (793.9-885.3)	735.3 (724-746.7)	658.8 (649.8-667.8)	589.8 (581.6-598.6)	539.8 (532.1-547.7)	475.6 (468.1-482.9)	449 (442.1-456.3)	409.6 (403-416.8)	377.6 (343-412.1)
JPN Toyama	1574.8 (1382.2-1799.4)	1288.4 (1133.8-1463.9)	1251.2 (1100-1426.6)	1140.1 (996.1-1286.8)	1032 (923.1-1142.2)	889.5 (839-940.6)	762.1 (752.4-771.8)	665.9 (657.6-673.6)	578.6 (571.8-585.4)	522.5 (515.8-529.4)	458.1 (452.3-464)	417.3 (411.6-423)	391.2 (385.6-397.1)	365.5 (329.5-401)
JPN Wakayama	1735.4 (1527.4-1964.8)	1405.1 (1233.5-1599.5)	1341.3 (1181.2-1518.2)	1218.7 (1074.4-1376.3)	1100.5 (988.8-1217.6)	951.2 (893.6-1009.4)	804.7 (794.9-814)	702.7 (694.7-711)	633 (625.2-640.7)	571.7 (564.5-578.9)	495.5 (489.3-502.4)	458.3 (452.2-464.4)	433.6 (427.4-440.5)	391.3 (353.2-431.3)
JPN Yamagata	1513.2 (1314.5-1736.5)	1220.6 (1051.9-1398.5)	1168.1 (1001.3-1344.6)	1080.2 (937.1-1248.2)	979.3 (872.3-1097.6)	822.3 (764.2-881.9)	665.4 (656.8-673.6)	587.7 (580.6-595.7)	548.8 (541.6-555.9)	517.9 (511.2-525.2)	464.3 (457.6-470.7)	429.3 (423.2-435.8)	397.2 (391.3-403.2)	371.4 (336.1-411.1)
JPN Yamaguchi	1655.7 (1452.4-1859.6)	1331.9 (1157-1523.5)	1249.7 (1078.5-1436.5)	1126.6 (977.4-1290.5)	1010.2 (885.8-1152.4)	875.6 (813.5-941.2)	780.4 (771.3-789.5)	655.8 (648.7-662.8)	583.3 (576.4-590.5)	542.2 (535.5-549.3)	485.5 (479.1-491.8)	451.5 (445.4-458)	422.6 (416.5-428.3)	377.4 (340.2-416.3)
JPN Yamanashi	1551.4 (1346.7-1775.6)	1251.2 (1075.6-1436)	1186.5 (1017.3-1361.8)	1085.9 (945.2-1239.9)	982.7 (880.9-1101.5)	841.4 (787.7-899.3)	746.2 (735.4-756.6)	652.3 (643.9-661)	577.3 (569.7-584.9)	520.8 (513.5-527.7)	460.1 (453.2-466.8)	429.1 (422.7-435.4)	399.8 (393.3-406.5)	366.8 (331.9-402.9)
KOR South Korea	3248 (3028.3-3492.4)	2054 (1847.9-2277.7)	1861.1 (1666-2085.1)	1750.1 (1550.9-1952.2)	1607.7 (1444.9-1775.6)	1392.6 (1345.7-1435.8)	1294.2 (1288.7-1300.2)	1128.6 (1125-1132.8)	994.8 (992-997.6)	864.6 (862.1-867.3)	763.3 (761-765.7)	619.9 (617.9-621.9)	514.1 (507.9-519.9)	434.9 (413.7-456.8)
SGP Singapore	1907.1 (1789.8-2029.6)	1553.8 (1539.9-1568.8)	1440.3 (1424.9-1456.7)	1347.5 (1333.7-1362)	1295 (1279.9-1309)	1204.4 (1192.1-1216.1)	1083.6 (1073.1-1093.9)	887.9 (878.8-897.2)	766.8 (759.1-774.4)	696.1 (688.6-703.5)	590.7 (555.8-626.5)	501 (493.3-508)	404.2 (398.7-410)	341 (324.4-359.2)
High-income North America	1172.8 (1142.6-1203.3)	1075.9 (1051.8-1099.8)	1076.9 (1075.6-1078.3)	1049.1 (1047.9-1050.3)	1005.3 (1004.2-1006.4)	897.4 (896.4-898.5)	833.1 (832.2-834.1)	782.5 (781.4-783.4)	727.2 (726.3-728)	710.8 (710-711.7)	669.9 (669.1-670.8)	628.7 (628-629.4)	571.7 (571-572.4)	571.4 (561.6-581.2)
CAN Canada	1154.8 (1148.9-1161)	1051.9 (1047.2-1056.6)	1016.9 (1012.4-1021.5)	970.7 (966.6-975.3)	910.1 (906.5-913.8)	866.4 (863-869.9)	773.6 (770.3-776.7)	724.5 (721.1-727.8)	670.1 (667.1-673.2)	642.3 (639.3-645.1)	582.4 (579.7-585.1)	531 (528.4-533.5)	476.5 (474.3-478.9)	451.7 (432.4-472.4)
GRL Greenland	2711.4 (2619.3-2811.9)	2167.4 (2119.3-2215.3)	1597.8 (1560.7-1631.7)	1348.8 (1314.2-1381.6)	1285 (1256.2-1313.5)	1417.8 (1389.8-1445.9)	1503.9 (1473.5-1532.9)	1487.4 (1457.5-1518.2)	1374.8 (1343-1406.9)	1267 (1237.8-1296)	1167.7 (1142.6-1194.9)	1065.8 (1042.3-1089)	960.4 (934.9-985)	862.2 (817.2-899)

Table 1 Age-standardised mortality rates by location and year for both sexes for 1950, 1955...2005, 2010, 2017

Location	1950	1955	1960	1965	1970	1975	1980	1985	1990	1995	2000	2005	2010	2017
USA USA	1173.8 (1141.1-1206.9)	1077.9 (1051.7-1103.9)	1082.1 (1080.8-1083.5)	1056.3 (1055.1-1057.5)	1014.2 (1012.9-1015.3)	900.5 (899.4-901.6)	838.9 (837.9-839.9)	788.4 (787.3-789.3)	733.4 (732.5-734.4)	718.7 (717.8-719.6)	679.8 (679-680.7)	639.9 (639.2-640.6)	582.9 (582.1-583.6)	585.7 (574.9-596.4)
USA Alabama	1299.1 (1134.1-1490.8)	1182.6 (1049.8-1324.4)	1137.7 (1128.1-1147.2)	1141.6 (1132.2-1150.6)	1077.8 (1069.3-1086.5)	960.1 (952.6-967.5)	898.7 (891.7-906)	851.3 (843.7-859.6)	815.8 (808.5-822.9)	807.9 (800.7-815)	795.8 (789-802.7)	786.3 (779.5-792.8)	750.4 (744-756.4)	765.1 (694.7-838.7)
USA Alaska	1315.4 (1159.7-1483.1)	1127.1 (1032.5-1225.1)	1203.7 (1182.3-1224.2)	1251.7 (1232-1270.1)	1191.6 (1176-1208.2)	1022.7 (1009.1-1036.9)	929.9 (916.2-942.6)	920 (907.3-931.8)	839.5 (828.6-851.4)	827.8 (816.4-837.8)	745.5 (735.3-755.5)	669.3 (660.2-679)	618.4 (610.5-626.1)	618.4 (580.1-659)
USA Arizona	1187.9 (1028.2-1347.5)	1066.7 (932.5-1202.7)	1045.2 (1031.9-1058.8)	1020.1 (1008.7-1031.9)	989.8 (978.5-1001.1)	854.1 (844.2-863.9)	811 (802.8-819.6)	726 (718.4-733.6)	681.9 (675-688.9)	689.2 (683-695.8)	645.2 (639.4-651)	618.5 (613.4-623.7)	541.2 (536.5-546.3)	549.9 (498.1-604.1)
USA Arkansas	1136.9 (986.7-1303)	1034.1 (915.5-1167.3)	1018.8 (1006-1030.8)	1024 (1012.1-1035.4)	986.3 (976.4-996.9)	917.8 (907.4-927.5)	842.1 (832.4-851.3)	818.8 (809-828.2)	782.4 (773.4-792)	782.5 (773.7-791.6)	771.7 (763.2-780)	731.1 (723.6-739.3)	704.4 (697.3-711.6)	734.7 (669.6-812.8)
USA California	1096.9 (953.5-1247.6)	999.2 (884.3-1116.8)	1020.9 (1016.6-1025.4)	1000.4 (996.1-1004.3)	957.1 (953.5-960.7)	856.1 (852.6-859.4)	805.2 (801.9-808.3)	754.6 (751.3-757.8)	706.9 (703.9-709.8)	662.9 (660.3-665.5)	612.8 (610.2-615.4)	574.6 (572.3-577)	497.2 (495.2-499.3)	484.9 (443.3-527.1)
USA Colorado	1122.7 (971.8-1296.2)	1025.7 (899.5-1161.8)	991.4 (979.1-1002.7)	954.8 (945.4-964.6)	918.6 (908.6-928.3)	813.6 (804.4-822.3)	763.9 (755.9-772.3)	712.8 (705-721.3)	658.6 (650.8-666.6)	662.1 (654.8-669.1)	625.7 (618.4-632.5)	585.3 (579.3-591.7)	540.3 (535.2-545.9)	538.9 (486.7-592.1)
USA Connecticut	1112.1 (970.5-1277.5)	1002 (882.2-1141.8)	1007 (997.3-1017)	1011.6 (1002.2-1020.7)	930.1 (920.4-939.2)	839.8 (831.1-848.9)	795.9 (787.3-803.7)	733.6 (725.9-740.9)	655.7 (647.7-663)	650.2 (642.7-657.8)	611.3 (604-618.9)	550.4 (543.8-556.8)	502.5 (496.5-508.7)	501.6 (451.7-555)
USA Delaware	1284.1 (1136.7-1443.6)	1145 (1051.6-1240.9)	1145.7 (1128.6-1162.6)	1129.2 (1115.6-1143.2)	1092.5 (1078.9-1106.8)	961.3 (950.1-973.5)	883.1 (871.3-894.2)	826.2 (815.1-837.6)	789.9 (778.5-801.5)	768.5 (758.5-779.5)	714.1 (704.5-723.1)	669.9 (661-678.4)	619.5 (611.7-627.6)	604.6 (565.1-647.2)
USA Washington, DC	1327.4 (1159-1499.7)	1221.4 (1104.8-1347.9)	1256.7 (1240.9-1272.3)	1298.6 (1283.8-1313.6)	1264.6 (1250.5-1277.8)	1112.8 (1099.2-1126.6)	1047.5 (1033.9-1063)	1023.5 (1010.4-1037.6)	1082.9 (1068.7-1096)	1150.4 (1135.8-1165.9)	865 (852.3-877.4)	793.4 (781.7-805.7)	660.3 (648.7-671.6)	644 (592.2-701.7)
USA Florida	1122.6 (983-1276.7)	1012 (887-1146)	1013 (1005.3-1020.1)	971.6 (965.4-978.2)	959.6 (953.8-965.8)	854.9 (849.4-860.2)	807 (802.2-811.9)	735.2 (730.8-739.4)	682.5 (678.6-686.3)	672.5 (668.8-676.1)	632.7 (629.3-635.8)	600.2 (597-603.2)	537.3 (534.4-540.2)	547.2 (497.4-598.2)
USA Georgia	1309.2 (1136.3-1502.6)	1172.5 (1034.8-1331.7)	1170.6 (1162.1-1179.5)	1118.8 (1110.7-1126.7)	1097 (1089.4-1104.6)	987 (979.6-994.1)	908 (901.7-915)	875.1 (868.7-881.7)	821.1 (815.4-827.5)	811.7 (806-817.1)	786.7 (781.3-792.2)	733.9 (728.7-738.8)	681.5 (676.9-686.1)	668 (607.9-730.9)
USA Hawaii	1052.7 (919.8-1190.3)	907 (801-1011.2)	845.3 (831.5-859.6)	810.9 (798.7-823)	808.3 (796.2-820.5)	705 (694.7-715.2)	660.6 (651.2-670.4)	617.3 (607.4-626)	588.2 (579.8-596.9)	568.9 (561-576.8)	538.7 (530.7-546.9)	505.1 (497.8-512.6)	468.5 (461.7-475.5)	456.8 (414.9-500.5)
USA Idaho	1085.4 (946.4-1244.3)	982 (879.2-1102.7)	987.9 (972.2-1004.8)	989.2 (975.5-1004.4)	949.3 (936.2-963)	851.1 (838.8-863.7)	760.7 (750.1-772.1)	716.6 (706.6-727.2)	674.2 (663.3-684.8)	661.8 (651.6-671.1)	639.2 (630.4-648.7)	617.3 (609-625.7)	582.1 (574.4-589.6)	581.5 (531.6-631.8)
USA Illinois	1192.2 (1043.7-1358.1)	1099.6 (972.5-1221.9)	1122.1 (1116.2-1127.9)	1099.3 (1093.5-1105.3)	1063.5 (1058.4-1068.7)	943.7 (938.6-948.9)	861.7 (856.8-866.8)	801.1 (796.4-806.4)	754.6 (749.8-759.1)	741 (736.3-745.4)	686.6 (682.3-690.9)	634.7 (630.6-638.7)	572.3 (568.6-576.2)	569.7 (516.1-627.9)
USA Indiana	1150.6 (999.4-1324.1)	1052.2 (931.2-1184.4)	1080.1 (1072.1-1088.3)	1066.9 (1059.1-1074.7)	1022.6 (1014.8-1029.9)	908.6 (901.6-915.4)	849.4 (842.5-856)	796.8 (790.1-803.5)	749.2 (742.6-756.1)	757.7 (751.4-764)	727.2 (721.2-733.1)	683.9 (678.5-689.6)	647 (641.5-652.4)	666.6 (605.9-734.3)
USA Iowa	1032.6 (889.9-1197)	974.6 (853-1109.8)	960.2 (949.9-970.3)	960 (950.4-970.4)	914.1 (904.6-923.6)	824.1 (815.6-832.9)	731.3 (723-740.9)	702 (694.2-710.4)	628.5 (620.4-637.5)	640.2 (632.2-648.8)	607.7 (599.9-615.4)	572.1 (564.9-579.2)	553.3 (546.5-559.5)	559.7 (504.4-615)
USA Kansas	1015.7 (881.7-1168.8)	928.6 (810.5-1053.1)	958.9 (948.1-970.1)	937.8 (927.4-949)	898.7 (889.1-909.7)	821 (812.3-830)	757 (748.1-765.7)	708.9 (700.1-718)	651.7 (644-659.5)	671.5 (662.7-680.3)	649.4 (641.2-657.9)	629.6 (621.5-637.5)	596.3 (589-603.7)	611.9 (554.7-674)
USA Kentucky	1193.1 (1037.2-1371.5)	1082.5 (961.1-1227.9)	1090.4 (1080.3-1100.2)	1072.4 (1063.2-1081.9)	1079.3 (1069.7-1088.3)	971.6 (963.4-980.4)	894.3 (885.6-901.8)	846.1 (838.8-854.2)	797.9 (790.2-805.3)	793.8 (786.8-801.5)	780.7 (773.6-788.4)	752.6 (745.9-759.5)	733 (726.3-739.5)	768 (699.6-837.3)
USA Louisiana	1270.8 (1094-1447.1)	1145.1 (1009.8-1284.4)	1143.7 (1134.2-1153.7)	1128.9 (1119.7-1137.8)	1094.2 (1085.5-1102.7)	991.3 (983.5-999.5)	929.8 (922.4-937.3)	881.9 (874.7-889.3)	839.7 (832.4-846.3)	828.1 (821.5-834.9)	812.3 (804.9-819.1)	859.3 (852.8-866)	713.3 (707.4-719.1)	726.4 (658.6-798.6)
USA Maine	1200 (1037.9-1365.7)	1097.1 (971.5-1228.4)	1086.3 (1073-1101.4)	1041.6 (1028.7-1055.3)	1018.4 (1005-1032.5)	893.4 (882.2-905.4)	822.9 (810.9-835.3)	777.7 (766.2-789.2)	704.8 (694.5-714.9)	688.2 (678.2-698.1)	670.7 (661-680.7)	655.1 (645.4-664.1)	592 (583.1-600.1)	630 (574.7-692.4)
USA Maryland	1275.8 (1112-1449.9)	1160.7 (1029.9-1301.6)	1179.9 (1169.5-1190.4)	1132.4 (1123.4-1142.1)	1058.1 (1050-1066.3)	938.5 (930.7-946.2)	872.6 (864.7-879.6)	837.2 (830.5-844.3)	775 (768.6-782)	765.9 (759.5-772.1)	722.8 (716.1-729)	657.6 (651.7-663.1)	577.3 (571.7-582.5)	576.4 (524.4-634.5)
USA Massachusetts	1141.6 (994.7-1304.3)	1052 (928.7-1199.5)	1078.3 (1070.3-1086.1)	1039.2 (1031.1-1047.3)	964.6 (957-972)	859.9 (853.3-867)	797.3 (790.4-803.8)	756.8 (750.2-762.8)	675.4 (669.7-681)	657.6 (651.9-663.4)	625.3 (619.7-631.1)	571.1 (566.3-576)	515.7 (510.6-520.4)	534.9 (485.5-591.1)
USA Michigan	1165.9 (1015.3-1332.5)	1072.6 (951.2-1205.7)	1088.8 (1081.7-1095.4)	1080.7 (1074.4-1087)	1033.2 (1027.2-1038.9)	932.6 (927.1-938)	853.1 (847.9-858)	822.3 (817.2-827.9)	753 (747.5-757.9)	738.3 (733.1-743.2)	706.7 (701.8-711.1)	665.5 (660.7-669.8)	620.8 (616.4-625)	634 (575.7-697.3)
USA Minnesota	1052.6 (897.1-1217.6)	968.7 (849.2-1098)	973.8 (964.3-982.7)	944.5 (936.1-953.8)	893.8 (885.2-902.4)	794.4 (786.5-802.3)	721.6 (714.5-728.9)	683.9 (676.8-690.7)	632.6 (626.4-639.1)	629.3 (623.2-636)	590.5 (584.4-596.6)	540.4 (534.5-546.1)	504.4 (499.1-509.4)	503.3 (456.8-556.6)
USA Mississippi	1290.9 (1118.2-1475)	1175.4 (1041.3-1320.7)	1142.8 (1131.4-1154.3)	1150.8 (1139.3-1161.4)	1118.5 (1107.7-1129.2)	991.9 (981.9-1001.8)	925.3 (915.9-934.6)	872.7 (863.6-882.2)	832.3 (823.3-841)	843 (834.3-851.9)	835.4 (826.8-844.1)	823.9 (816.1-831.9)	760.9 (752.9-768.3)	777.3 (706.2-856)
USA Missouri	1144.7 (1001.4-1310.4)	1044.1 (921.9-1179.2)	1058 (1049.6-1066.4)	1045.1 (1036.3-1053.4)	1020.3 (1012.7-1027.9)	902.9 (895.7-910.4)	840.3 (833.5-847.5)	787 (779.7-793.9)	744.7 (738.6-751.1)	754.7 (747.9-761.3)	722.5 (716.1-728.7)	690.3 (684.5-696.4)	645.4 (640-651.1)	658.5 (600.7-725.6)
USA Montana	1200.1 (1041.6-1361.1)	1103.7 (987.3-1242.5)	1091.3 (1074.6-1107.7)	1086.5 (1070.9-1101.5)	1009.4 (995.2-1023.4)	891.7 (878.4-904.3)	823.2 (811.1-835.6)	755.6 (744.2-767)	688.4 (677-699.8)	697.9 (686.6-708.5)	669.7 (659.5-679.7)	639.3 (629.5-649.3)	594.3 (584.5-603.4)	676.1 (617.8-734.7)
USA Nebraska	1005.3 (876.8-1146.4)	918.9 (816.4-1023.5)	954.5 (942.3-966.7)	959.5 (948.8-970)	923.6 (912.7-934.4)	829.7 (819.9-839.5)	755.1 (745-763.6)	707.8 (698.3-717.1)	669.1 (660.1-677.8)	651.5 (643-660.5)	617.5 (608.8-626)	579.4 (571.2-587.1)	554.9 (547.4-562.8)	571 (523-624.1)
USA Nevada	1490.6 (1301.7-1680.6)	1269.6 (1134.4-1414.4)	1222.6 (1199.3-1246.1)	1240.5 (1221.3-1261.2)	1129.8 (1112.2-1147.7)	976.8 (961.9-991.5)	951.4 (938.1-964.8)	867.3 (855.6-879.5)	835.5 (825-845.7)	833.6 (823.8-843.8)	785.2 (776.9-793.3)	771.6 (763.8-779)	646.8 (640.2-653.7)	626.3 (573-678.2)
USA New Hampshire	1193 (1042.8-1363)	1071 (962.5-1185.5)	1064.8 (1047.8-1080.5)	1065.2 (1049.6-1079.8)	1011.9 (998.4-1026)	905.5 (892.2-918.2)	794.7 (782.9-806.5)	761.7 (751.9-772.5)	704.8 (695.2-714.7)	672.8 (663.4-682.4)	648.5 (639.5-658.1)	616.3 (607.8-626)	549.1 (540.9-557.3)	584.1 (537.8-634.7)
USA New Jersey	1214.4 (1062.2-1379.9)	1122 (991.4-1269.2)	1117.1 (1109.8-1125)	1084.8 (1078.3-1092)	1028.6 (1022-1035.4)	899.1 (893.2-905.1)	852.7 (847-858.7)	814.6 (809-820.2)	735.1 (729.6-740.6)	715.3 (710.4-720.4)	658.6 (653.5-663.7)	594.6 (589.8-599.4)	529.2 (524.5-533.5)	505.7 (457.5-553.4)
USA New Mexico	1153.8 (1000.2-1328.7)	1041.2 (928.4-1158.3)	1028.7 (1014.5-1042.5)	1030.7 (1017-1043.4)	988.7 (975.6-1001.8)	895.1 (883.7-907.1)	809.4 (799.5-819.3)	746.9 (736.9-756.3)	702.4 (693.1-711.4)	696.7 (687.8-705.4)	653.2 (645.4-661.1)	629.2 (621.4-636.7)	594.2 (587-601.2)	610.9 (558.2-666.3)
USA New York	1199.3 (1047.3-1361.3)	1126.9 (1008.1-1246.5)	1137.5 (1132.9-1141.9)	1098.9 (1094.3-1103.3)	1055.8 (1051.7-1059.7)	909.2 (905-913.1)	862.8 (859.1-866.7)	817.8 (814.3-821.3)	750.4 (746.9-753.7)	711.6 (708.2-715.3)	625 (621.6-628.3)	571.6 (568.7-574.4)	508.8 (505.8-511.6)	490.4 (444.3-538.6)
USA North Carolina	1248.6 (1086.8-1427.2)	1128.3 (996.3-1255.1)	1151.5 (1143.4-1159.7)	1131.6 (1124-1138.9)	1080.5 (1073.3-1088)	957.4 (950.6-964)	878.2 (871.9-884.5)	832.1 (826-838.4)	784 (778.6-790.1)	776.4 (770.6-782.7)	756.1 (751.2-761.5)	698.7 (694.2-703.2)	640.2 (635.9-644.7)	632 (574.6-690.8)
USA North Dakota	1053.1 (937.4-1187.2)	964.8 (892.3-1050.1)	968.6 (955-984.5)	967.5 (955.1-979.9)	928.2 (916.7-940.4)	834 (823.5-845.7)	747.9 (737.5-758.4)	680.6 (670.9-690.3)	639 (629.3-648.4)	630.1 (620.3-639.8)	602.3 (593-611.5)	551.9 (543.3-560.7)	537.5 (529.2-546.4)	584.2 (544.9-623.9)
USA Ohio	1177.2 (1020.7-1332.2)	1090.9 (973.9-1219.1)	1092.2 (1086.3-1098)	1077.7 (1071.7-1083.6)	1035.3 (1030.1-1040.6)	924.8 (919.8-929.6)	874.7 (869.7-880)	806.1 (800.8-810.9)	752.8 (748-758.1)	751.2 (746.6-756.2)	717.4 (712.5-722.2)	688.4 (683.9-692.9)	640.9 (637.1-644.6)	687.3 (623.8-749.6)
USA Oklahoma	1082.9 (931.8-1252.1)	983.6 (858.7-1107.1)	1000.1 (989.1-1010.9)	958.4 (948.3-968.7)	978.4 (969.3-988.3)	884.6 (875.5-893.8)	837 (829.2-844.9)	802.6 (794.4-811)	749.7 (741.3-757.8)	774.3 (766.3-782.3)	772.7 (765-780.1)	768.2 (761-775.6)	726.9 (720.3-733.7)	739.9 (667.6-812.8)
USA Oregon	1106.1 (954.8-1276.5)	1012.3 (883.8-1147.5)	1015.9 (1003.4-1028.3)	992.3 (981.1-1004.1)	939.1 (928.1-950.2)	836.1 (826.6-845.1)	785.8 (777.2-794.4)	739.1 (730.2-747.4)	699.8 (691.8-708)	703.4 (696.1-711.1)	650.4 (642.8-657.8)	618.3 (611.4-624.9)	571.2 (565.3-577.7)	570.4 (518-630)
USA Pennsylvania	1249.2 (1088.7-1428.9)	1163 (1034.9-1290)	1147.6 (1142.2-1153.2)	1121.2 (1115.7-1126.5)	1062.5 (1057.2-1067.6)	935.2 (930.4-940)	874.1 (869.4-878.7)	814.1 (809.1-818.9)	745.8 (741.2-750.3)	727.2 (722.6-731.9)	692.6 (688.2-696.8)	657.2 (653.5-661.3)	596.4 (592.4-600.3)	617.8 (558.9-676.1)
USA Rhode Island	1189.1 (1036.2-1360.6)	1088.8 (971.5-1203.3)	1086.4 (1071.6-1100.8)	1079.6 (1064.8-1094.5)	997.9 (984.8-1011.8)	862.8 (850.8-874.3)	804.7 (793.1-817)	752.3 (741-763.7)	695.3 (685.3-705.7)	640.5 (630.6-650.2)	630.1 (620.4-640.2)	606.1 (595.7-616.8)	554.2 (545.6-563)	562.5 (515.1-614.3)
USA South Carolina	1431.4 (1251.3-1619.7)	1276.8 (1128.1-1439.7)	1274.7 (1263.3-1286.1)	1184 (1173.2-1194.1)	1128.2 (1118.9-1137.9)	1008.7 (999.8-1017.8)	928.7 (920-937)	864 (856.4-872.2)	819.5 (811.8-827.9)	808.5 (800.8-815.6)	787 (780-793.7)	734.7 (728.6-740.4)	689.8 (683.5-695.8)	705.8 (642.8-775.7)
USA South Dakota	1067.9 (951-1202.3)	976.1 (893.7-1059)	1002.5 (988.4-1016.5)	971.5 (959.3-983.9)	943.5 (932.3-956)	842.9 (832.2-853.6)	768 (757.7-779.4)	713.8 (703.9-724.5)	657.6 (648.5-668.1)	655.1 (645.5-664.8)	622 (613.2-631.1)	590 (581.4-599)	554.1 (546.2-562.1)	601.1 (561.6-641.9)
USA Tennessee	1191.1 (1038.8-1355.5)	1093.7 (963.1-1240.7)	1091.4 (1081.8-1100.5)	1064.6 (1055.8-1073.2)	1056.2 (1047.8-1064.4)	937.7 (930.2-945.4)	870.9 (864.2-878.2)	827.2 (820.5-834.4)	802.9 (796.1-809.5)	809.7 (803-816.2)	788.3 (782.4-794.8)	761.2 (755.5-767.1)	713.7 (708.3-719)	714.5 (652.5-782.1)
USA Texas	1105.5 (964.7-1268.9)	1014.2 (897.5-1148.6)	1007.7 (1001.7-1013.5)	978 (972.8-983.7)	974.5 (969.5-979.4)	882 (877.5-886.4)	839.1 (834.9-843.6)	802 (798.1-805.9)	746.7 (743-750.5)	726.7 (723-730.4)	705.2 (701.9-708.5)	655.8 (652.8-658.8)	608.8 (606-611.5)	596.9 (546.3-650.1)
USA Utah	1064.5 (920.4-1217.6)	954.8 (852.8-1074.8)	978.3 (964.3-993)	983.8 (970.2-998)	900.8 (889.4-912.5)	808.1 (797.6-818.8)	744.6 (735.9-754.7)	713.5 (702.9-723.1)	638.4 (629.6-646.4)	636.3 (628.1-644.6)	616.6 (609.3-624.2)	587.2 (580.7-594.3)	551.7 (544.9-558.4)	570.8 (525.3-620.9)
USA Vermont	1190.1 (1070.4-1308.5)	1061.7 (997-1134.4)	1082.1 (1064.9-1099.6)	1069.5 (1055.2-1083.5)	980.6 (968-993.7)	880 (869.6-891.5)	808.1 (796.9-819.2)	781.9 (770.9-793.3)	711.5 (701.2-721.3)	684.8 (675.3-695.1)	644.9 (636.3-654)	591.9 (583.8-600.9)	553.9 (544.7-562.2)	540.4 (507.8-572.2)
USA Virginia	1261.9 (1096-1448.6)	1153.8 (1023.2-1293.7)	1136.2 (1126.5-1145.5)	1105.3 (1097.7-1113.2)	1042.2 (1035.2-1049.9)	932.3 (925.1-939.3)	870.6 (863.9-877.4)	817.4 (810.9-823.6)	759.3 (752.6-765.2)	745.7 (740-751.4)	707 (701.5-712.6)	655.7 (650.4-660.8)	585.9 (581.2-590.3)	570.2 (517.3-625.9)
USA Washington	1118.1 (966.5-1285.7)	1041.2 (918.5-1176.3)	1024.5 (1014.2-1034.6)	972.4 (963.3-981.3)	963.7 (955.1-972.1)	840.4 (832.2-848)	779 (771.7-786)	732.8 (725.9-740)	680.9 (674.4-687.3)	666.4 (660.4-672.2)	632.6 (626.5-638.9)	590.4 (585-596.1)	543.8 (538.8-549.1)	537.8 (487-591)
USA West Virginia	1225.7 (1060.5-1404.4)	1133.1 (1003.9-1268.8)	1104.8 (1092.3-1117.5)	1129.9 (1118.9-1140.8)	1122.6 (1111.4-1134.1)	995.8 (985.3-1006.8)	907.6 (896.9-918.6)	849.2 (838.1-859.5)	791.9 (781.5-802)	787 (776.6-797.2)	789.9 (779.1-800.2)	767.2 (757.5-777.2)	740.9 (732-749.6)	771.7 (702.7-846.8)
USA Wisconsin	1096.3 (946.7-1265.4)	1011.9 (896.3-1140.7)	1032.1 (1023.3-1041.6)	1004 (995.7-1012.7)	940.2 (932.3-948.5)	835.9 (828.8-843)	770.8 (763.7-777.5)	713.5 (706.8-720.6)	684.6 (678.1-691.5)	663 (656.9-669.1)	639.3 (632.9-645.8)	600.2 (595-605.4)	555.8 (550.3-561.1)	552.3 (498.4-606.5)
USA Wyoming	1148.5 (1025.6-1276)	1014 (947.4-1087)	1035.7 (1017.8-1053.9)	1038.6 (1025.3-1052.8)	1022.8 (1010-1037.1)	952.2 (939.5-965.2)	840 (829-851.2)	772.6 (762.3-783.2)	708.9 (698.7-718.8)	705.6 (694.5-716)	688 (678.1-698.5)	647.1 (638-656.3)	606.7 (598.5-615)	611.5 (574.3-650.1)
Southern Latin America	1564 (1545-1584.9)	1453 (1442.3-1463.7)	1383.4 (1379-1387.9)	1292.5 (1289.2-1296.1)	1239.3 (1236.1-1242.6)	1112 (1085.6-1138)	1038.7 (1035.8-1041.5)	947.2 (944.5-949.8)	899.4 (896.9-902)	825.1 (822.7-827.5)	779.4 (777.2-781.5)	729.8 (727.6-731.9)	692.6 (690.6-694.7)	654 (617-698.1)
ARG Argentina	1473.4 (1445.5-1504.2)	1372.5 (1356.6-1389.1)	1269 (1263-1275)	1218.7 (1214.7-1222.7)	1206.9 (1202.7-1210.8)	1096.9 (1059.1-1133.8)	1025.4 (1021.9-1028.8)	943.6 (940.4-946.7)	906.3 (903.1-909.6)	839.3 (836.3-842.2)	809.8 (807.1-812.5)	760.2 (757.4-762.8)	727 (724.3-729.5)	694.3 (638.4-759.6)
CHL Chile	1991.5 (1980.5-2003.1)	1830.7 (1822.4-1839.1)	1833.2 (1825.5-1840.3)	1598 (1590.6-1604.3)	1415.3 (1409.2-1421.4)	1213.7 (1207.7-1219.4)	1104 (1098.7-1109.5)	984.3 (979.4-989.4)	889 (884.7-893.8)	782.6 (778.3-787)	693.2 (689.2-697.2)	644.2 (640.5-648.1)	607.5 (603.8-611.1)	557.1 (510.5-608.5)
URY Uruguay	1213.5 (1199.6-1227.5)	1121.9 (1110.9-1132.6)	1090.9 (1080.4-1101.9)	1087.1 (1076.8-1097.6)	1090.7 (1080.7-1100.9)	1029.5 (1019.9-1039.4)	1018.6 (1008.9-1028.4)	906.7 (897.6-916.8)	877.9 (869.1-886.8)	839.9 (831-848.6)	788.7 (780.5-796.9)	749.1 (740.8-757.6)	689.4 (681.4-698)	666.3 (610.7-724.4)
Western Europe	1274.5 (1235.9-1312.6)	1154.9 (1144.9-1165.6)	1082.7 (1078.9-1086.6)	1041.7 (1037.9-1045.5)	1006.9 (1003.5-1010.3)	956.3 (954.1-958.7)	880.9 (879.9-882)	809.9 (809-810.8)	747.2 (746.4-748)	687.7 (686.9-688.4)	619.4 (618.6-620.2)	552.2 (551.4-552.9)	504.2 (503.6-504.8)	465.4 (455.5-475.7)
AND Andorra	750 (620.5-887.8)	749.5 (626.9-881.9)	738.3 (617.8-853.8)	728.4 (614.5-829.6)	705.9 (598.1-802)	672.4 (584.2-762.1)	641.8 (566.6-722.3)	622.2 (561.4-693.9)	598.5 (546.3-661.9)	561.9 (505.7-622.3)	491.6 (451.9-542.1)	461.6 (434.6-498.8)	444 (417.9-478.7)	429.3 (391.5-469.1)
AUT Austria	1368.1 (1357.8-1379)	1278.6 (1270.3-1286.5)	1207.6 (1200.2-1215)	1155 (1148.1-1163.4)	1156.2 (1148-1164.6)	1072.3 (1064.8-1080.6)	980.3 (973.4-987.2)	890.9 (884.1-898)	784.8 (779.1-791)	721.8 (716.3-727.7)	642.5 (637.1-647.8)	572.3 (567.4-577)	524.8 (519.9-529.6)	474.4 (453.6-497)
BEL Belgium	1475.7 (1465.6-1486.7)	1224.1 (1217.7-1230.7)	1150.5 (1144-1157.2)	1107.8 (1100.9-1114.2)	1095 (1088.8-1101.5)	1046.2 (1039.7-1052.7)	948.8 (942.5-954.8)	868.6 (862.8-874.9)	766.3 (761.3-771.6)	714.5 (709.5-719.7)	662.5 (657.7-667.2)	595.8 (591.5-600.8)	549 (544.9-553.2)	488.6 (466.9-511.9)
CYP Cyprus	1242.7 (1092.7-1409.5)	1220.5 (1084.7-1365.6)	1174.7 (1053.2-1302.5)	1157.2 (1047.5-1263.7)	1121.5 (1048.3-1190.2)	1064.5 (1045.6-1081.1)	963 (951.9-973.8)	880.2 (870.2-890.1)	772.2 (761.5-782.2)	724.4 (700.9-746.4)	749.9 (720-779.6)	677 (651-705.5)	545.3 (522.1-570.1)	467 (437-500)
DNK Denmark	1118.7 (1106.9-1129.7)	1010.7 (1002-1019.1)	1012.9 (1004.2-1021.4)	1009.6 (1001.4-1018)	917.3 (909.5-924.9)	876.1 (868.7-883.3)	880.7 (873.3-887.7)	845.4 (838.7-852.5)	822.1 (815.4-829.1)	807.1 (800.5-814.1)	710.9 (704.2-717.4)	643.4 (636.7-649.7)	596.5 (590.6-602.6)	522.9 (497.6-549.1)
FIN Finland	1523.9 (1510.1-1537)	1299.8 (1290.5-1308.7)	1202.2 (1193.9-1210.9)	1200.9 (1192-1209.8)	1174 (1163.8-1183.5)	1037 (1028.5-1045.6)	905.2 (897.7-913)	853.9 (845.5-861.8)	817 (809.6-824.2)	731.8 (725.4-739.2)	668.8 (662.4-675.2)	589 (583.1-594.7)	538.8 (532.7-544.5)	488.1 (465.8-513.4)
FRA France	1268.3 (1263.1-1275)	1169.3 (1166.2-1172.5)	1094.6 (1091.3-1097.8)	1058.7 (1055.4-1061.8)	998 (995.2-1000.8)	950.8 (947.9-953.5)	854.4 (852.2-856.9)	790.7 (788.3-792.8)	696.6 (694.6-698.6)	638.9 (636.9-641)	585.4 (583.6-587.3)	525.5 (523.8-527.3)	482.6 (481-484.3)	421.2 (403.2-441.1)
DEU Germany	1246.7 (1088.1-1405.5)	1137.8 (1098.7-1175.7)	1095.8 (1093.4-1098.2)	1035.7 (1033.4-1038.1)	1030.5 (1028.4-1032.7)	973.6 (971.4-976.1)	910.5 (908.3-912.6)	826 (823.9-828.2)	798 (796-800)	722.2 (720.3-724.1)	631.3 (629.6-633.2)	558.5 (556.9-560.3)	531.6 (530-533.3)	520.7 (476.6-565.2)
GRC Greece	1048.2 (1007.5-1090.8)	908.4 (902.3-915)	881.7 (875.4-887.9)	878.6 (872.4-884.8)	848 (841.8-854.2)	794.6 (788.4-800.5)	814.2 (808.6-820.1)	788.5 (782.3-794.2)	678.4 (673.1-683.3)	649.2 (644.7-654.5)	630.5 (626.1-634.9)	614.9 (610.3-619.6)	542.1 (538-546.1)	509.3 (485.9-532.1)
ISL Iceland	1034.7 (1005.4-1062.4)	966.5 (946.8-985)	910.9 (893.3-928.3)	890.3 (874.5-905.8)	882.2 (867.8-897.8)	758.6 (745-771.8)	709.1 (695-725.5)	672.2 (654.8-690.6)	661.2 (645.9-675.3)	641.2 (628.9-653.5)	556.8 (546.8-567)	491 (481.2-501.2)	448 (438.3-457.6)	431.3 (417.9-446)
IRL Ireland	1366.1 (1352-1380.4)	1239.4 (1228.5-1250.2)	1174.2 (1162.2-1184.9)	1143.2 (1132-1155.1)	1131.5 (1120.7-1142.6)	1092.4 (1082-1103.1)	1040.2 (1029.2-1050.6)	976.2 (966.6-986.2)	883.6 (874-892.9)	832.6 (823.1-841.7)	758.4 (750.3-766.4)	613.7 (607-620.8)	520.1 (514.1-526.4)	473.9 (450.6-497.4)
ISR Israel	856 (837.2-876.6)	1001.9 (989.3-1013.9)	971.3 (958.8-983.5)	1026.2 (1015.1-1037.6)	1011.4 (1000.8-1021.4)	980.8 (970.9-990.8)	900.9 (891.9-910.3)	827.5 (818.9-836)	700.1 (692.3-708.5)	690 (682.8-697.2)	633 (626.8-639.3)	558.6 (553.1-563.7)	477.1 (471.8-482.1)	427.9 (408-449)
ITA Italy	1263.3 (1258-1271.7)	1137 (1134-1140.1)	1061.7 (1059-1064.3)	1037.2 (1034.3-1039.9)	980.5 (977.7-983.3)	959.7 (957-962.6)	900 (897.3-902.8)	796.6 (794.2-799.1)	704.6 (702.3-706.9)	644.8 (642.7-646.9)	576.7 (574.6-578.8)	512.4 (510.4-514.3)	466.1 (464.1-468)	416.8 (397.9-436.1)
LUX Luxembourg	1347.2 (1321.8-1376.9)	1285.4 (1270.4-1300.7)	1203.3 (1187.7-1219.5)	1183.1 (1168.4-1199.2)	1173.3 (1158.6-1189.6)	1138.8 (1123.8-1153.9)	1005.8 (991.8-1020.1)	928.1 (913.2-941.5)	811.4 (799.4-823.8)	747 (735.2-758.6)	665.3 (654.7-676)	591.9 (581.7-602.2)	524.8 (515.9-534.1)	489.5 (456.2-523.9)
MLT Malta	1390.2 (1353.6-1427.3)	1290.7 (1272.7-1310.5)	1270.6 (1253.7-1288.2)	1222.2 (1204.1-1239)	1163.4 (1145.1-1181.2)	1067.8 (1052.1-1084.2)	1079.9 (1063.9-1095.1)	920.5 (906-935.1)	765 (751.6-778.9)	695.3 (682.8-708.1)	652 (639.9-664.3)	604.3 (593-615.6)	539.6 (528.8-549.9)	510.3 (490.8-530.1)
NLD Netherlands	1055.1 (1047.5-1062.2)	1010.7 (1005-1016.6)	958 (952-963.8)	935.7 (930.3-941.6)	935.6 (930-941)	871.5 (866.4-876.6)	783.4 (778.7-787.9)	754 (749.3-758.5)	718.5 (713.7-722.7)	693.5 (689.4-697.8)	667 (662.8-671.1)	590.8 (586.9-594.5)	523.9 (520.4-527.6)	493.6 (471.9-517.7)
NOR Norway	950.8 (922.3-980.1)	899.7 (873.8-928.2)	899.1 (879.1-921)	895.9 (883.7-908.7)	877.8 (872.7-883)	821.2 (817-825.3)	776.5 (772.8-780.6)	760 (756.1-763.7)	734.8 (731-738.7)	677.1 (673.4-680.7)	635.3 (631.9-638.7)	552.6 (549.6-555.6)	516.3 (513.4-519.2)	453.5 (446.5-460.5)
PRT Portugal	1537.4 (1523.7-1554.4)	1390.8 (1383.6-1397.7)	1237.4 (1230.8-1244.3)	1221.4 (1214.1-1228.5)	1155.4 (1148.3-1162.6)	1146.4 (1138.1-1154.8)	1002.6 (996.8-1008.8)	943.5 (938-949.4)	907.8 (902.2-913.5)	834.2 (828.3-839.5)	751.6 (746.2-756.8)	623.1 (610.2-635.5)	555.6 (551.4-559.5)	490.9 (469-512.1)
ESP Spain	1419.9 (1410.8-1433.8)	1180.3 (1176.7-1184.1)	1028.5 (1024.9-1032)	996.2 (992.8-999.9)	969.1 (965.6-972.7)	901.2 (897.8-904.4)	779.5 (776.4-782.7)	734.5 (731.6-737.2)	699.7 (697-702.3)	641.3 (638.9-643.8)	578 (575.6-580.4)	529.4 (527.1-531.5)	462.4 (460.4-464.4)	418.7 (401.8-435.7)
SWE Sweden	1108.2 (1070.7-1144)	1008.4 (973.5-1043.9)	972 (937.2-1008.6)	905.6 (874.1-936.8)	851.1 (822.2-878.2)	835.9 (813.5-856.4)	790.5 (784.7-796)	726.3 (721.4-731.4)	676.9 (671.7-682.1)	618.3 (613.7-622.9)	577.9 (573.3-582.3)	533.2 (529.1-537.7)	490.9 (486.6-495.4)	448.6 (430.4-466.7)
SWE Stockholm	1134.3 (966.4-1332.8)	1048.1 (882.7-1231.7)	1020.8 (853.4-1215.7)	951.2 (805.5-1111.5)	888.6 (763.6-1017.7)	864.9 (770.8-955.3)	823.2 (809.7-835.4)	746.5 (735.7-756.9)	712.1 (700.8-723.4)	626.6 (617.4-635.9)	587.1 (578.4-595.8)	532.7 (524.9-540.8)	481.2 (473.6-488.7)	449.7 (423.1-473.6)
SWE Sweden except Stockholm	1103.4 (1067.6-1132.3)	1001.5 (970.1-1029.2)	963.7 (931.2-991)	897.7 (870.2-923.2)	844 (820.3-867.4)	829.8 (811.8-847.7)	783.4 (777-789.4)	721.8 (716.4-727.8)	669.4 (663.5-675.1)	616.3 (610.8-621.5)	575.9 (570.6-580.9)	533.4 (528.7-538.6)	493.1 (488.1-498.1)	448.9 (428-470.3)
CHE Switzerland	1185.3 (1174.5-1197.4)	1131.1 (1122.5-1140)	1026.9 (1019.1-1034.5)	986.4 (979-994.5)	934.6 (926.8-941.7)	829 (821.5-836)	767.3 (760.9-773.8)	694 (688.2-700.2)	653.8 (648.2-659.9)	603.3 (597.9-608.7)	544.2 (539.3-549.4)	481.5 (477-486.4)	443.4 (438.8-448.1)	383.8 (365.9-401.6)
GBR United Kingdom	1255.3 (1230.1-1282.3)	1175.2 (1151.9-1196.8)	1106.6 (1086.3-1128.4)	1064.9 (1044.9-1085.8)	1030.1 (1011.1-1048.6)	980.9 (968.6-994.2)	911 (908-914.2)	852.6 (851.3-854.1)	786.2 (784.9-787.6)	735.8 (734.6-737.1)	660.4 (658.2-662.3)	587.1 (585.9-588.1)	528.9 (527.8-529.9)	507.5 (503.1-511.9)
GBR England	1215 (1190.5-1237.9)	1140.8 (1117.6-1163.9)	1075.5 (1055.1-1095.6)	1037.2 (1018.7-1056.5)	1006.4 (989.2-1024.8)	960.1 (948.8-972.7)	892.2 (889.1-895.4)	836.1 (834.8-837.4)	772.5 (771.2-773.8)	723.2 (722-724.4)	648.2 (646.7-649.9)	575.6 (574.7-576.7)	516.8 (515.9-517.8)	496.9 (493.8-500.1)
ENG East Midlands	1268.1 (1182.5-1367.5)	1187.2 (1106-1279.9)	1115.2 (1041.9-1198.6)	1073.5 (1002.5-1146.7)	1040 (972.8-1109.2)	994.6 (948.1-1046.7)	905.8 (893.7-917.6)	833.8 (829.2-838.4)	772.5 (768-776.9)	724.6 (720.1-728.7)	654.8 (648.7-661.1)	587.2 (583.6-590.5)	526.4 (522.9-529.9)	504.4 (493.6-516.2)
UKF Derby	1320 (1073.6-1612.6)	1228.4 (995.2-1486)	1153 (933.6-1385.3)	1114.2 (914.5-1355.7)	1079.8 (908.8-1292.8)	1036.7 (906.2-1172.9)	969.6 (930.9-1009.1)	892.7 (875.7-910.5)	826.2 (810.7-842.3)	747.7 (733.2-762.4)	657.5 (639.4-675.3)	600.7 (587.8-613.7)	580 (567.7-592.2)	534.5 (501-568.5)
UKF Derbyshire	1287.4 (1060.6-1568.1)	1209.3 (1001.3-1445.3)	1140.9 (942.9-1370.1)	1098.8 (903.7-1325.3)	1068.3 (890.2-1275.7)	1023.1 (884.7-1167)	924.3 (894.2-955.7)	843.6 (832.9-855.2)	794.6 (783.5-806.2)	741.7 (730.4-751.9)	660.2 (644.5-675.1)	590.6 (581.8-599.7)	521.9 (513.8-530.4)	522.1 (492.5-553)
UKF Leicester	1339.7 (1091.1-1624.7)	1261.8 (1030-1520.6)	1193.5 (982.1-1443.2)	1162.7 (950.9-1391.6)	1125.1 (936.9-1330.2)	1071.7 (938.5-1218.1)	959.9 (923.9-997.9)	868.1 (853.7-883.2)	806.8 (792.3-821.7)	788.6 (773.6-803.6)	745.1 (726.4-764.3)	671.1 (659.1-683.9)	586.7 (576-597.9)	520.4 (489.7-551.6)
UKF Leicestershire	1169.3 (952.2-1424.5)	1098.2 (900.3-1333.2)	1031.7 (856.5-1251)	985.8 (805.2-1189.2)	956.6 (797.8-1142.3)	917.6 (801.3-1048.1)	837.9 (809.5-867.1)	773.7 (761.5-785.2)	709.3 (698-720.3)	655.4 (645-666.5)	596 (581.9-609.6)	533.2 (524.2-541.9)	475.4 (467.2-483.7)	460.7 (435.5-487.6)
UKF Lincolnshire	1271.7 (1034.5-1526.8)	1190.8 (992.4-1429.9)	1119.9 (925.4-1350.9)	1078.6 (883.7-1294.4)	1045.1 (873.3-1239.6)	995.7 (871.4-1129.4)	894.1 (864-927.8)	834.8 (822.1-848)	759.1 (748.1-770.6)	712.7 (702-724)	646.6 (630.9-661.8)	577.6 (568.8-586.3)	513.8 (505.5-523)	497.5 (469.7-524.6)
UKF Northamptonshire	1206.3 (976.7-1466.5)	1124.3 (909.6-1370.9)	1052.3 (866.5-1270.9)	1010.1 (835.3-1196)	977.1 (821.6-1146)	934.1 (817.4-1057.4)	855.6 (826.5-884.2)	807 (795.2-818.9)	755.6 (745-766.7)	713.5 (702.8-725.4)	638.3 (623.3-652.8)	580.2 (570.8-589.7)	531.7 (523.2-540.1)	505.2 (476.9-535.2)
UKF Nottingham	1357.1 (1111.2-1647.2)	1276.2 (1051.2-1557.2)	1205 (990.6-1460.7)	1174.6 (980.1-1406.3)	1144.7 (957.7-1362.5)	1102.9 (963-1266.2)	1023.9 (988-1060.2)	923.8 (907.9-939.6)	840 (824.7-855.6)	826.2 (811.3-841.5)	785.5 (766.2-805.1)	729.7 (717.3-742.4)	624.2 (612.4-635.6)	554.8 (520.9-587.8)
UKF Nottinghamshire	1260.8 (1031.9-1539.7)	1174.4 (963.5-1425.6)	1095.6 (902.3-1323.7)	1049.5 (873.8-1253.7)	1014.7 (855.3-1186)	972.2 (864.3-1098.9)	894.4 (866.1-924.8)	823.7 (811.5-834.8)	775.7 (765.1-787.4)	724.9 (714.7-735.8)	655.2 (640.4-670.2)	579 (570.5-587.7)	526.6 (517.3-535.2)	515.1 (488-544.2)
UKF Rutland	1101.7 (895.4-1371.5)	1021 (827.2-1260.9)	955.7 (776.4-1183.3)	925 (751.1-1127.2)	894.1 (736.4-1070.7)	855.3 (733.3-981.9)	795.9 (743.8-854.5)	772.6 (739.7-807.1)	690.4 (662.1-719.5)	616.4 (588.9-643.8)	535.8 (510.5-562.9)	488 (466.7-510.8)	458.4 (437.8-480.7)	452.2 (413.1-495.8)
ENG East of England	1148 (1063.9-1233)	1077.7 (1001.8-1165.2)	1012.9 (934-1094.3)	966.7 (897.1-1033)	939.3 (881.7-997.5)	900.4 (858-942.6)	840.3 (829.6-851.6)	772.8 (768.5-776.8)	719.8 (715.9-723.7)	671.4 (667.5-675.4)	602.8 (597.3-608.5)	537.9 (534.8-540.9)	484.1 (481.1-487.1)	469.5 (460.1-481)
UKH Bedford	1186.5 (953.9-1456.5)	1111.7 (901.3-1345.5)	1037.4 (840.3-1264)	992.4 (807.6-1198.5)	952.3 (793.3-1132.9)	898.1 (783-1012.4)	821.4 (783.2-863.2)	765.3 (747.7-784.3)	743.3 (725.9-762.1)	708 (688.8-726.1)	631 (611.9-649.2)	564.9 (551.3-578.6)	530.1 (517.1-543.5)	490.5 (459.5-522.5)
UKH Cambridgeshire	1101.3 (883.7-1341.1)	1042.5 (835.5-1261.4)	977.5 (792.9-1176.6)	926.9 (761.7-1104.8)	896.8 (758.6-1050.1)	861.1 (758.9-970.8)	826 (795.8-856.3)	768.9 (756.2-781.4)	692.2 (680.8-704)	662.7 (651.3-673.9)	587.6 (573.2-600.9)	522.2 (513-530.8)	456.1 (448-464.7)	436.7 (412.5-462.8)
UKH Central Bedfordshire	1150.8 (916-1431)	1078.3 (865.2-1322)	1012.9 (807.2-1243)	973.7 (791.8-1193)	942.4 (786.4-1116.4)	899.8 (784.8-1021.2)	856.2 (820.4-891.9)	799.3 (783.2-816)	750.4 (734.2-766.4)	695.2 (680.4-709.3)	607.8 (591.5-625.1)	539.1 (527.1-551.7)	470.9 (460.5-482)	417.3 (390.9-443.7)
UKH Essex	1151.6 (947.9-1384.1)	1086.6 (899.7-1303.7)	1028.4 (844.5-1240.1)	989.6 (823-1190.6)	975.8 (821.9-1141.2)	942.8 (832.1-1064.6)	852.9 (825.6-880)	770.2 (760.4-779.8)	713.4 (704.1-722.6)	662.6 (654.1-671.5)	600.6 (587.3-613.7)	536.2 (528.9-543.4)	484.7 (477.6-491.7)	493.9 (466.9-522)
UKH Hertfordshire	1122 (917.9-1373.8)	1050.6 (854-1284)	990 (796-1202.6)	946.8 (778.4-1139.3)	930.2 (777.8-1097.2)	901.6 (792.1-1022.1)	844.3 (817.3-871.7)	776.3 (766.4-787.5)	722.3 (712.4-732.2)	666.7 (657.2-676.4)	605.4 (591.5-619)	535.6 (527.8-543.3)	479.9 (472.6-487.3)	433.6 (410-461.7)

Table 1 Age-standardised mortality rates by location and year for both sexes for 1950, 1955...2005, 2010, 2017

Location	1950	1955	1960	1965	1970	1975	1980	1985	1990	1995	2000	2005	2010	2017
UKH Luton	1239.4 (984.3-1516.8)	1161.4 (938.6-1412.1)	1095.3 (899.2-1324.5)	1060.5 (868.5-1285.4)	1032.1 (853.3-1229.6)	985.1 (861.6-1123)	927.1 (887.6-969.7)	803.3 (786.5-821.6)	790.4 (773.5-807.1)	769.7 (753.4-786)	693.7 (675.5-713.3)	636.4 (622.9-650)	554.5 (542.5-567)	481.9 (451.4-512.9)
UKH Norfolk	1133.6 (918.5-1382.1)	1058.9 (871.9-1284.1)	986.7 (801.7-1191)	930.6 (767.2-1115.1)	891.1 (747.1-1059.4)	846.5 (739.2-965.1)	807.9 (778.3-836.9)	754.1 (743.8-764.8)	722.5 (711.8-732.8)	653.9 (643.8-663.8)	586.4 (572.2-599.7)	525.2 (517.6-533)	479.5 (472-486.8)	493.3 (465.5-521.8)
UKH Peterborough	1221.8 (981.2-1520)	1144.5 (921.5-1415.7)	1079.6 (863.6-1324.9)	1048.1 (855.8-1273.3)	1015.9 (849-1195.7)	969.5 (847.1-1111.6)	889.4 (847.8-930.3)	832.7 (814.3-852.5)	778.2 (760.4-796.1)	748.1 (731.9-764.8)	670.2 (651.2-689.1)	611.8 (597.2-627)	566.7 (552.9-580.4)	528.3 (493.6-564.5)
UKH Southend-on-Sea	1202.7 (968-1468)	1119.2 (901-1357.4)	1054.4 (841-1269.8)	1010.6 (825.2-1239.2)	966.9 (811.2-1153.6)	911.1 (799-1035.1)	843.9 (806.5-879.9)	781.4 (762-799.8)	735.7 (718.8-751.6)	701.4 (684.7-718.4)	650.2 (630.1-669.7)	592.9 (578.5-607)	537.5 (525.4-551.1)	522.5 (489.4-554.7)
UKH Suffolk	1142.1 (931.4-1371)	1069.1 (879.8-1313.1)	1002 (820-1223.1)	948.1 (786.6-1136.5)	913.9 (767.6-1073.1)	870.8 (756.4-987.6)	820.6 (792.5-851.7)	763 (752.3-774)	693.7 (683-704.2)	660.5 (650.7-670.4)	585.2 (569.8-598.9)	515.6 (507.2-523.3)	467.6 (460.2-475.4)	460.8 (435.2-490.5)
UKH Thurrock	1260.4 (1025.5-1538.3)	1196.7 (977.9-1461.8)	1133.1 (930.7-1369.3)	1093.7 (900.6-1328.6)	1056.8 (879.3-1242.7)	1005.4 (878.1-1143.1)	930.5 (888.7-972.3)	851.5 (832.1-870.5)	781.1 (762.4-799.3)	727.6 (709.6-747)	628.3 (609.1-647.6)	581.1 (566.2-595.6)	514.6 (501.5-527.6)	484.8 (451.4-519.6)
ENG Greater London	1146 (1105.3-1187.4)	1081.2 (1041.2-1119.4)	1023.5 (987.9-1060.1)	997.8 (966.3-1032.1)	970.3 (940.9-1001.3)	928.1 (906-950.8)	867.6 (862.1-873.4)	817.2 (814.5-819.8)	763.8 (761.1-766.3)	737 (734.3-739.5)	644.7 (641.6-647.7)	559.9 (557.8-561.9)	493.1 (491.1-495)	451.4 (446.2-456.7)
UKI Barking and Dagenham	1289.4 (1048.6-1546.6)	1211 (996.2-1450.4)	1147.8 (958.8-1363.2)	1116.1 (925.8-1332.6)	1080.4 (913.3-1253.6)	1028.2 (904.1-1155.8)	965.8 (925.3-1005.1)	899.3 (880.8-918.1)	835.8 (818.3-854.5)	809.6 (791.9-826.5)	743.4 (722.7-762.3)	661.2 (647.3-676)	590.3 (577.4-604.3)	568.8 (533.8-604.8)
UKI Barnet	1042.8 (859.3-1245.5)	976.8 (805.7-1171.7)	916.7 (764.8-1097.3)	877.4 (727.9-1049.2)	845.4 (712.7-990.1)	808 (713.7-907.5)	775.7 (743.7-807.3)	741.1 (728.2-754.1)	682.1 (669.8-694.9)	642.8 (631.4-654.2)	554.3 (539.2-568.5)	484.8 (475.7-494.3)	450.3 (441.2-459.4)	421.3 (396.3-445.7)
UKI Bexley	1089.8 (895.3-1326.6)	1022 (841.7-1238.1)	965.9 (792.8-1154)	933.8 (766-1094.6)	900.5 (760.1-1055)	849.8 (746.6-957.9)	786.4 (755.2-816.7)	730.8 (716-745.8)	681.3 (666.5-696.4)	658.8 (644.8-674)	584.8 (569.4-602.1)	510.7 (499-522.5)	468.7 (458.3-480.6)	458.9 (430.9-489)
UKI Brent	1145.3 (930.5-1380.8)	1080.4 (890.8-1302.9)	1011.5 (837.7-1209)	979.4 (816.4-1165.2)	946.3 (802.7-1114.7)	898.6 (793.9-1008.5)	821.4 (791.9-851.4)	821.1 (807-835.6)	751.4 (737.5-765.1)	739.3 (726.5-753.4)	642 (626.1-659.1)	537.4 (527.7-548)	481.1 (471.6-490.5)	421.7 (396.5-444.8)
UKI Bromley	1046.6 (852.5-1268.4)	978.1 (804.4-1176.2)	924.7 (766.8-1117.2)	890.6 (735.3-1064.3)	864.3 (729-1017.6)	826.2 (726.9-934.2)	771.3 (744.6-799)	733.9 (720.4-746.8)	675.9 (663.3-688.5)	637.2 (624.9-649.2)	558.5 (543.6-573.4)	476.5 (466.4-487)	436.5 (427.3-446.1)	432.9 (407.5-460.5)
UKI Camden	1183.3 (977.7-1420.4)	1159.7 (961.7-1381.4)	1105.6 (911.4-1319.4)	1088.9 (911.6-1308.6)	1072.6 (914.8-1252.4)	1042 (919.4-1172.3)	979.7 (941.6-1017.9)	893.8 (877.4-911.1)	864.7 (849.4-881.1)	840.3 (825.1-856.2)	741.1 (721.4-758.5)	651.5 (640.1-663.8)	513 (502.4-523.2)	376 (352.6-400.3)
UKI Croydon	1134.3 (926.4-1365.6)	1060.4 (870.7-1288.1)	999.9 (824.9-1187.4)	952 (786.9-1130.6)	928.6 (784.8-1087.8)	897.2 (790-1010.6)	858.6 (828.8-890.5)	817.2 (803-832.1)	758.7 (745.6-772)	711 (697.8-723.9)	626.8 (612.5-642.1)	535.9 (525.5-545.6)	506 (496.2-515.5)	475.7 (447.3-505.6)
UKI Ealing	1183.5 (977.3-1430.7)	1113.3 (918.5-1341.4)	1053 (864.6-1257.5)	1021.8 (858-1203)	995.4 (850.4-1156.2)	956 (846.2-1081.6)	896.1 (866.2-927)	840.5 (825.2-855)	774.6 (761.5-787.5)	747 (734.5-760.8)	638.3 (622-653.9)	548.1 (538.2-558.5)	480.3 (471-489.6)	448.2 (421.4-475.4)
UKI Enfield	1098.9 (904.7-1335.9)	1027.6 (843.4-1238.6)	970.9 (797.4-1159.3)	933.3 (764.7-1111.4)	907.2 (761.6-1064.5)	869.4 (765.1-972.3)	820.6 (789.1-852.5)	783.6 (768.9-799.1)	713.9 (701.1-728.2)	695.8 (681.9-709.7)	619.8 (603.9-636.6)	533.9 (524-544.4)	467.3 (457.5-477.9)	458.4 (431.3-486.6)
UKI Greenwich	1203.6 (993.3-1452.2)	1124.8 (914.9-1358.1)	1059.1 (862.4-1276.1)	1019.1 (845.1-1205.3)	984.8 (833.3-1147.5)	941 (825.7-1053.3)	897.5 (863.2-932)	875.2 (858.1-891.1)	823.3 (808.1-839.7)	798.5 (782.5-814.4)	734.8 (716.2-752.6)	655.7 (643.7-669)	545.1 (534-555.5)	520.2 (490-555.2)
UKI Hackney	1164.1 (952.6-1408.1)	1098.3 (907.7-1313.1)	1056.3 (877.8-1248.4)	1064.4 (890.6-1241.6)	1058.8 (894.9-1228.6)	1029.7 (910.6-1159.7)	968.4 (932.1-1004.6)	913.6 (896.6-930.2)	867.3 (850.8-884.2)	807.1 (791.2-823)	674.6 (656.1-692.6)	628.8 (616.3-640.7)	564.2 (553.3-576)	495.7 (465.4-526.3)
UKI Hammersmith and Fulham	1241.8 (1017-1491.3)	1196.5 (988.3-1453.1)	1135.8 (929.7-1356.2)	1109.1 (925.2-1322.1)	1082.7 (925.6-1265.7)	1054.1 (936.5-1186.6)	993.3 (952-1033)	960.6 (942.8-979.8)	917.4 (899.4-934.6)	890.7 (874.1-906.5)	711.3 (692.1-730.6)	600 (586.6-613.7)	519.2 (507.2-531.5)	468.9 (441.2-498.9)
UKI Haringey	1174.1 (967.8-1428.6)	1118.4 (933.2-1342.3)	1054.3 (881.5-1246.1)	1033.2 (855.1-1214.9)	1004.2 (839.4-1169.8)	956.3 (841.8-1079.3)	884.2 (851.7-917.4)	818.6 (802.9-835.2)	780.7 (766.4-793.8)	759 (744.3-772.6)	683.9 (665.8-701.9)	610.9 (598.4-623.3)	543.9 (532.9-554.9)	443.2 (417.2-470)
UKI Harrow	1044.1 (846.1-1279.6)	980.4 (792.5-1202.5)	935.8 (760.8-1143.4)	917.1 (761.3-1099.5)	892.7 (757.9-1049.7)	845.5 (750.1-964.7)	790.6 (759.7-822.9)	708.2 (693.3-723.7)	644.4 (631-658.5)	623.9 (610.2-637.5)	535.2 (520-551.4)	455.8 (444.7-466.3)	395.2 (385.4-404.2)	374.5 (351.9-400.4)
UKI Havering	1104 (906.5-1328.9)	1032.3 (847.5-1244.5)	974.5 (799-1177.4)	938.9 (774.2-1124.3)	909.8 (762.9-1068.9)	866.6 (761.7-976.8)	829 (796.2-861.7)	763.1 (748.9-778.6)	679.2 (666.5-692.7)	657.8 (644.2-671.9)	595.5 (578.2-614.5)	519.8 (508-531.8)	498.6 (488.1-509.1)	506.1 (474.3-539.5)
UKI Hillingdon	1100.1 (883-1343.6)	1031.7 (833.4-1252.9)	975.7 (807.5-1171.3)	945.2 (784.3-1116.7)	921.3 (778.2-1075.3)	884.2 (776.4-999.8)	837.7 (803.8-870.5)	788.8 (773.1-804.1)	728.1 (714.5-741)	714.2 (700.5-727.3)	626.1 (608.9-641.7)	555.8 (545-566.6)	490.4 (480.4-500.7)	459.4 (433.5-487.7)
UKI Hounslow	1124.3 (911.5-1365.2)	1060.9 (867.5-1284.9)	1005.2 (815.6-1217.5)	982.2 (804.4-1185.5)	953.7 (807.4-1117.6)	910.1 (803-1020.2)	846.8 (815-880.6)	825.5 (810.4-840.8)	746.3 (732.6-760.3)	740.7 (726.6-755)	666.8 (649.7-683.9)	585.4 (573.9-597.4)	511.7 (501-522.9)	464.3 (436.1-493.7)
UKI Islington	1200.2 (989.8-1436.1)	1139.6 (939.2-1368.5)	1080.5 (900.1-1294.8)	1064.1 (882.5-1268.5)	1044.1 (881.6-1219.2)	1016.3 (900-1145.5)	949.1 (911.2-988.6)	931 (914.2-949)	874 (858.4-891.1)	859 (843.1-875.2)	773.8 (753.3-794.1)	683.2 (669.5-697.5)	573.9 (562.3-586.4)	475.6 (447.3-503.5)
UKI Kensington and Chelsea	1154.8 (944.4-1391.3)	1203.7 (995.4-1418.2)	1115.7 (928.8-1324)	1079.8 (905-1274.2)	1048.6 (887.4-1231.5)	1010.2 (890.7-1134.4)	934.4 (897.8-969.8)	870.3 (851.5-888.5)	735.3 (720.1-751.6)	660.6 (645.5-677.1)	533.4 (517.8-549.6)	459.3 (448.2-471.1)	404 (393.7-414.9)	373.2 (349.5-399.4)
UKI Kingston upon Thames	1016 (826-1251.2)	951.3 (773.4-1159.4)	898.5 (736.4-1093.5)	873 (721.6-1044.7)	843.2 (711.3-998.8)	799.3 (702.9-902.2)	755.3 (723.4-790)	724.4 (706.2-743.1)	659.3 (644-676)	632.2 (616.4-648.7)	548.7 (532.3-565.3)	454.6 (442.3-465.7)	404.1 (392.8-415.9)	428.1 (399.9-456.4)
UKI Lambeth	1254.8 (1047.5-1509.1)	1187.4 (972.4-1427.1)	1123.9 (928-1340.9)	1111 (924.4-1317.9)	1083.2 (921.6-1262.1)	1036.3 (916-1165.8)	950.2 (917.3-984.8)	885.8 (871.6-900.3)	841.7 (829-855.5)	850.7 (836.9-864.3)	741.5 (722.5-758.7)	661.7 (650.7-672.9)	568.9 (559-579.3)	490.1 (462.5-520.3)
UKI Lewisham	1201.7 (979.8-1438.7)	1122.8 (921.1-1353)	1049.1 (854.2-1258.6)	1008.7 (832.8-1203.6)	974.9 (826.6-1142.5)	937.2 (826.2-1056.2)	908.1 (875-940)	839.2 (823.1-854.4)	806.2 (792.5-820.4)	827.9 (813.5-842.4)	756.6 (738.1-774.2)	670.2 (658.3-682.8)	559.2 (548.5-570.1)	492.7 (462.1-523.7)
UKI Merton	1064.1 (862.7-1280.3)	999 (826.6-1194.7)	951.7 (782.2-1129.8)	937.3 (782-1112.1)	909.2 (773.7-1059)	861.3 (761.3-972.8)	814.8 (780.8-849.7)	730.1 (713.9-745.3)	701.3 (686.8-716.3)	672.4 (657.6-688.2)	582.6 (566.1-598.6)	503.5 (491.2-515.1)	460.7 (450-471.6)	437.9 (410.7-469.5)
UKI Newham	1248.6 (1023.6-1507.5)	1170.7 (947-1410.5)	1112.3 (912.8-1345.3)	1088.8 (907.9-1299.1)	1060.3 (902.6-1241.6)	1013.4 (893.6-1144)	934.8 (900.6-970.1)	896.9 (881.5-913.7)	847.2 (830.7-862.7)	847.2 (832-861.4)	774.6 (755.1-792.5)	674.3 (661.6-686.4)	628.1 (616.6-639.8)	477.4 (449.9-506.7)
UKI Redbridge	1085.8 (880.3-1329.8)	1021.5 (852.9-1232.8)	969.1 (803.2-1170.9)	943 (791.1-1127.1)	913.6 (775.1-1072)	866.3 (768.2-974.9)	793.7 (763.6-823.8)	712.9 (699-726.7)	689.4 (676.1-703.6)	680.6 (667.9-694.1)	618.6 (601.8-634.9)	538.8 (527.4-549.6)	469.1 (459-479.5)	444.9 (416.3-473.7)
UKI Richmond upon Thames	1058.1 (852.4-1270)	1007.6 (823.9-1204.1)	965.1 (793.3-1155.6)	952.8 (798.9-1132)	930.3 (793.4-1090.4)	885.5 (783.1-996.9)	819.1 (784.8-853.5)	748 (732.2-764.8)	655.5 (640.7-670.7)	568.7 (555.3-581.3)	511 (495.2-525.9)	430.7 (420.1-441.5)	379.2 (369.1-389.2)	378.1 (353.9-403.6)
UKI Southwark	1158.8 (943.4-1406.8)	1080.3 (878-1300.1)	1021.2 (834.6-1236.5)	1008.6 (833.7-1207.3)	982.7 (822.8-1146.4)	951.2 (833.9-1069.9)	915.8 (883.2-951.6)	895.5 (879.5-912.2)	917.9 (901.2-933.8)	838.2 (824.3-851.9)	732.5 (715.2-749.8)	661.3 (649.2-672.9)	567.5 (556.8-578.5)	531.3 (500.1-562.2)
UKI Sutton	1059.8 (859.9-1295.3)	982.5 (796.2-1192.7)	920.1 (751.4-1117)	881.7 (729.3-1062.8)	843.6 (706.3-995.1)	797.2 (697-900.5)	751.4 (717.7-785.6)	742.8 (726.9-759.4)	683.1 (667.5-697.6)	681.8 (665.9-697.9)	584 (568-600.4)	497.1 (485.7-508.4)	446.2 (435-457.9)	467.4 (438-498)
UKI Tower Hamlets	1314.6 (1083.5-1591)	1237.4 (1011.4-1482.5)	1185.5 (977.8-1415.3)	1175.7 (980.8-1385.4)	1162.3 (984.4-1346.6)	1130 (998-1270.8)	1040.3 (1001-1080.6)	992.9 (974.5-1012.8)	929.5 (910.7-948.7)	917.5 (900.9-935.7)	801.8 (781-820.3)	709.2 (696.4-722.1)	596.3 (584.7-608)	477.7 (450.1-507.1)
UKI Waltham Forest	1198 (980.5-1448.7)	1129.7 (924.9-1359.9)	1067.4 (880.3-1276.6)	1033.3 (860.1-1234.5)	998.5 (840.9-1158.6)	944.3 (828-1061.7)	845.5 (813.1-879.2)	806.8 (790.6-822.9)	752.4 (737.9-768.1)	764.4 (749.6-780.2)	710.3 (692.5-728.6)	627.2 (615.1-639.5)	542.9 (532.1-554.3)	478.2 (450.7-507.1)
UKI Wandsworth	1175 (955.6-1409.5)	1118.2 (906.2-1336.6)	1064 (873.2-1281.6)	1046.8 (871.9-1245.7)	1027.6 (864.7-1204.2)	992.2 (875.7-1115.3)	924.4 (890.8-956.9)	888.1 (873.5-903.3)	855.9 (840.8-869.7)	818 (804.6-831.2)	696.9 (680-713.8)	591 (579.8-602)	502.4 (492.4-512.8)	457.2 (427.8-485.7)
UKI Westminster	1051.4 (866.1-1254.7)	1041.4 (859.7-1238.9)	987.9 (818.8-1177.4)	987.5 (824.9-1176.5)	981.6 (840.6-1159.1)	958.7 (849.4-1082.9)	898.7 (864-931.6)	805.6 (790.3-821)	797.4 (782.9-813.2)	756.4 (741.7-771.7)	590.9 (574.8-606.5)	515.7 (505.3-526.5)	438.1 (427.3-447.9)	398.6 (374.1-425.3)
ENG North East England	1430.3 (1339-1526.7)	1339.8 (1260.5-1429.3)	1259.4 (1186.1-1340.9)	1202.8 (1134.2-1279.6)	1149.9 (1085.3-1214)	1074.5 (1030.6-1119)	993.5 (981-1004.9)	942 (936.6-947.3)	879.3 (874.4-884.4)	808.7 (803.8-813.5)	731.5 (725.1-737.5)	658.8 (654.5-663.2)	582.2 (578.1-586.1)	562 (551-573.5)
UKC County Durham	1436.6 (1193-1713)	1348.2 (1120.6-1609.9)	1264.9 (1043.1-1527.1)	1204.1 (990.3-1446.4)	1152.2 (962.4-1352.3)	1077.1 (949.1-1213.9)	984.4 (953.3-1018.1)	945.8 (931.4-960.3)	886.5 (873.3-900.1)	822.8 (809.7-835.8)	733.7 (715.8-750.9)	645.9 (634.9-657.1)	580 (569.7-590.6)	563.8 (532.1-595.6)
UKC Darlington	1425.4 (1161.5-1732.1)	1338.2 (1095.6-1625.4)	1264.2 (1049.5-1517.8)	1214.7 (1008.4-1459.7)	1162.5 (982.3-1379.5)	1079.7 (947.9-1226)	994.1 (943.6-1040.2)	917.8 (895.4-941.8)	853.6 (830.9-876.1)	790.9 (770.2-811.6)	726.7 (704-750.8)	668.8 (651.5-688.1)	578.9 (562.4-594.7)	534.5 (496.9-575.1)
UKC Gateshead	1492.7 (1225.6-1791.8)	1407.5 (1158.2-1704.4)	1328.3 (1090.6-1611.3)	1276.3 (1047.3-1533)	1223.9 (1026.5-1441.9)	1142.5 (1002-1296.7)	1038.8 (998.3-1080.2)	962.6 (944.4-981.7)	877.7 (859.6-896.3)	812.6 (796.5-830.2)	754.9 (734-777.1)	666.7 (651.5-682)	597.7 (584.3-610.7)	581.6 (543.2-620.2)
UKC Hartlepool	1534.6 (1259.2-1843.7)	1435.6 (1177-1736.4)	1349.7 (1117.1-1629.4)	1295.3 (1071.9-1545.7)	1238.6 (1026.8-1457.4)	1152.2 (1005.4-1316.3)	1054.7 (1006.1-1102.6)	986.1 (961.5-1010.2)	907.1 (885.1-930.1)	833.4 (811.1-857.2)	761.8 (737.1-785.9)	710.7 (691.2-730.1)	614.8 (597.1-632.7)	569.7 (532.9-611.8)
UKC Middlesbrough	1539.1 (1251.2-1870.7)	1435.9 (1162-1751.8)	1354.1 (1099-1649.7)	1300.2 (1060.2-1573.7)	1247.4 (1044.2-1483.4)	1175.8 (1030-1350.1)	1110.3 (1063.5-1159.3)	1037.8 (1015-1060.2)	966 (944.4-987.2)	882.7 (863-902.7)	814.6 (791.7-838.5)	738.7 (721.9-757.5)	652.8 (637-668.3)	615.1 (574.9-659.2)
UKC Newcastle upon Tyne	1400.2 (1150.1-1700.8)	1319.2 (1093.4-1610.9)	1240.1 (1029.4-1514.7)	1189.5 (983.2-1420.6)	1136.3 (943.2-1334.2)	1060.3 (929.2-1201.5)	974.4 (939.1-1010.9)	918.3 (902.4-934.4)	884.5 (869.1-900.5)	835.1 (819.4-850.7)	746 (726.7-765.3)	681 (668.3-695.1)	600.6 (588.4-613.1)	573.4 (538.8-610.8)
UKC North Tyneside	1378.9 (1117-1668.1)	1283.1 (1064.6-1552.4)	1207.8 (993.1-1458)	1155.6 (957.2-1392)	1105.3 (927.6-1302.3)	1033.9 (906.4-1171)	969 (928.6-1008.4)	929.6 (910.9-948.2)	855.1 (839-873)	789.6 (771.9-807.1)	726 (706.3-746.7)	659.7 (645.4-674)	575.3 (562-588.6)	539 (505.8-576.7)
UKC Northumberland	1361.9 (1114-1650.6)	1275.9 (1056-1543.1)	1202.7 (987.6-1443.7)	1142.9 (944.8-1375.6)	1094.2 (917.5-1291.6)	1024.9 (903.9-1155.4)	966.7 (930.6-1002.6)	915.6 (900-930.6)	849.9 (834.4-864.5)	750.5 (735.8-764.4)	673.7 (656.2-691.8)	580.9 (569.1-593.2)	515.4 (504.7-526.5)	518.8 (488.3-554.6)
UKC Redcar and Cleveland	1453 (1179.9-1753.3)	1363.4 (1123.3-1669)	1284.4 (1050.6-1554.9)	1233.4 (1014.9-1482)	1178.6 (982.2-1403.1)	1096.4 (964.3-1253.7)	999.3 (958.5-1041.1)	912.8 (893.7-932.3)	845.7 (826.8-865.9)	767.6 (749.6-785.7)	697.8 (675.8-718.3)	651.9 (635.3-668.7)	577.4 (560.9-593.7)	563.5 (530.1-602.4)
UKC South Tyneside	1469.7 (1196.9-1767.2)	1359.7 (1105.8-1659)	1268.3 (1040.4-1524.3)	1204.5 (1001-1433.5)	1146.6 (964.8-1356.4)	1077.4 (946.7-1224.2)	1009.2 (967.6-1052.5)	982.5 (962.9-1002.4)	897 (877.7-917.8)	814.5 (795.7-835)	737.1 (716.3-759.2)	667.9 (652-684)	608 (592.4-622.6)	609.2 (571.8-649.2)
UKC Stockton-on-Tees	1368 (1107.4-1659.7)	1283.7 (1063.8-1556.1)	1209.8 (992.2-1460.7)	1160.5 (958.4-1400.8)	1107 (919.9-1310.1)	1029.5 (899.2-1164.5)	957.3 (919.5-998.2)	917.3 (898.4-936.6)	853.4 (836.2-872.4)	782.3 (765.8-799.3)	689.3 (669.5-709.3)	640.7 (626.4-655.8)	556.1 (543.9-569.3)	531.5 (497.5-568.7)
UKC Sunderland	1423.7 (1170.6-1725.9)	1330.3 (1097.5-1605.5)	1245.3 (1027.3-1509.1)	1182.1 (973.4-1421.4)	1127.3 (945-1331.5)	1055.3 (921.2-1193.9)	979.1 (942.6-1013.1)	944.1 (928.5-960.1)	901.5 (885.4-917.1)	839.7 (824.8-855.2)	769 (750.5-787.8)	713.7 (699.4-727.7)	620.2 (607.2-632.7)	603.1 (566.8-643.1)
ENG North West England	1324.1 (1263.9-1387.9)	1238 (1177.6-1300.5)	1168.3 (1111.7-1229.1)	1124.1 (1074.5-1179.6)	1080.7 (1035.3-1129.7)	1018.6 (988.5-1054.7)	961.7 (953.5-969.5)	918.7 (915.4-922.1)	848.5 (845.4-851.7)	794.2 (791.2-797.3)	720.2 (716.1-724.3)	645.6 (643-648.1)	582.3 (579.7-584.8)	555.7 (547.6-563.7)
UKD Blackburn with Darwen	1382.6 (1124.7-1665)	1292.4 (1047.2-1562.8)	1229.9 (1012.7-1502.1)	1188.8 (990-1420.7)	1143.7 (967.1-1352.9)	1077.1 (950.5-1215.7)	1016.6 (972.7-1059.5)	987.7 (967.4-1008.6)	918.6 (898.9-939.2)	882.5 (862.9-901.2)	819 (796.1-841.2)	720.8 (703.5-736.8)	640.1 (626.6-654.3)	595 (557.3-633.1)
UKD Blackpool	1368 (1117.4-1652.5)	1273.3 (1043.3-1532.4)	1209.4 (996.9-1454.6)	1179 (978-1410.1)	1130.4 (948.9-1333.8)	1047.8 (923.3-1190.7)	974.7 (935.2-1014.7)	881.8 (863.8-901.8)	853.2 (835.8-871.9)	792.4 (776.8-809.5)	845.8 (822.6-868.7)	833.3 (816.7-851)	746.4 (729.8-762.6)	663.1 (623.3-705.3)
UKD Bolton	1361.3 (1126.8-1650.8)	1271.4 (1049.8-1539.1)	1198.3 (979-1441.7)	1152.4 (949.8-1365.7)	1108 (939.1-1280.7)	1047.1 (919.8-1185)	991.6 (956-1027.3)	942.9 (928.1-959.4)	854.8 (840-870.9)	806.2 (791.4-819.8)	754.8 (736.2-774.5)	683.3 (670.5-695.7)	604.5 (593-616.4)	549.2 (517.6-581.4)
UKD Bury	1298.5 (1067.9-1582.4)	1215.1 (997.1-1460)	1139.9 (943.9-1364.9)	1083.3 (903.5-1291.2)	1036.6 (877.3-1210.1)	975 (855.4-1104.2)	928.2 (890.2-965.2)	885.3 (867.8-904.8)	819.5 (802.7-836.6)	773.1 (756.8-789.5)	680.2 (662.2-699)	626 (611.5-640)	575.3 (562.5-589)	596 (558.4-631.8)
UKD Cheshire East	1201 (985.5-1453)	1121.1 (925.5-1356.1)	1052.2 (860.5-1272.3)	994.4 (824.4-1194.3)	955.6 (810.6-1125.6)	905.4 (801.8-1023.4)	877.6 (848.6-908.7)	835.3 (821.3-848.9)	761.3 (748.5-774.6)	693.6 (682.8-706)	632.1 (615.9-647.5)	522.8 (513.1-532.4)	481.2 (471.7-490.8)	478.9 (452.5-507)
UKD Cheshire West and Chester	1238.3 (1007.4-1500.2)	1161.1 (951.9-1415)	1100.6 (909.2-1311.1)	1060.5 (872.9-1250.5)	1028 (879.1-1203.8)	972.8 (861.5-1095.3)	908.5 (878.1-939.6)	864.4 (850.4-877.7)	793.3 (779.1-806.8)	729.4 (716.7-743.1)	635.4 (620.3-651.7)	548.2 (537.9-558.2)	499.3 (489.9-508.6)	501.1 (473.3-530.6)
UKD Cumbria	1285 (1056.4-1526.8)	1199.9 (984.9-1451.4)	1131.6 (944.7-1349.2)	1081.4 (904.2-1264.7)	1040.9 (889.3-1209.5)	982.2 (875.2-1107.5)	923.6 (893-953.9)	882.7 (869.4-895.3)	812.3 (800.5-823.6)	759.4 (747.7-770.5)	656.1 (640.4-672.1)	581.9 (573-591.1)	532.6 (523.3-542.1)	504.6 (475.3-534.7)
UKD Halton	1391.5 (1136.3-1692.9)	1313.4 (1073.2-1592.5)	1254.8 (1045.7-1499.8)	1227 (1018.2-1467.2)	1188.3 (1003.8-1399.4)	1118.2 (984.8-1275.1)	1039.4 (996.3-1082.6)	974.1 (953.5-995.2)	870.8 (849.5-890.3)	835.9 (816.4-856.1)	789.2 (766.2-812.8)	738.9 (719.9-757.9)	636.9 (620.2-652.9)	584.2 (545.5-624.3)
UKD Knowsley	1350 (1112.9-1612.5)	1260.3 (1030.3-1515.3)	1200.5 (980.2-1453.5)	1187.8 (973.7-1416.8)	1148.7 (972.7-1350.7)	1076.1 (955.4-1208)	1005.4 (965.7-1045.7)	922.4 (903.7-940.7)	881 (862.6-898.1)	860.1 (841.1-879.4)	819.4 (796-841.9)	723.6 (707.1-740.5)	659.3 (644.7-675)	647.1 (609-688.1)
UKD Lancashire	1280.4 (1060.3-1527.8)	1194.6 (988.1-1434.9)	1120.3 (924.5-1342.1)	1062.5 (871.5-1266.8)	1018.7 (850.2-1217.3)	966.3 (856.1-1104.5)	944.2 (915.2-972)	916 (905-926.9)	838.1 (828.1-848.5)	753.1 (743.4-762.7)	689 (674.4-703.7)	620.5 (611.8-629.1)	548.2 (540.7-555.9)	532.6 (503.4-562.7)
UKD Liverpool	1383.2 (1140.7-1662.8)	1305.8 (1081.2-1557.2)	1250.8 (1037.2-1508.3)	1231.4 (1019.8-1463)	1197 (1020.2-1407.9)	1125.4 (992.2-1273.8)	1009.2 (977-1040.3)	965.7 (953.2-978.4)	913.9 (901.8-927.4)	894.7 (881.1-909.1)	828.1 (810.7-847)	746.1 (735-757.6)	675.3 (664.9-685.5)	616.5 (584.1-650.8)
UKD Manchester	1382.4 (1145.9-1668.3)	1290.3 (1074.7-1536.9)	1210.2 (1005.6-1440.3)	1177.5 (990.4-1390.5)	1133.9 (963.4-1321.6)	1076.3 (954.7-1213.3)	1030.8 (1000.5-1063.2)	1011.7 (997.8-1025.4)	959.5 (946.5-972.5)	930.2 (917.2-944.6)	835.6 (816.9-852.8)	787.5 (775.7-798.8)	688 (677.7-698.2)	674.9 (637.3-713.2)
UKD Oldham	1396.3 (1145.8-1669.6)	1309.2 (1068-1578.9)	1239.8 (1018.9-1487.1)	1191.9 (987.1-1414.5)	1140.7 (978.8-1333.1)	1069.4 (952.7-1202)	1004.1 (968.7-1042.5)	979.7 (962.2-996.9)	892.3 (874.8-908.9)	845.6 (828.7-863)	774.1 (753.7-793.4)	704.7 (691-718.2)	631.1 (618.6-643.9)	613.2 (579.1-648.8)
UKD Rochdale	1358.4 (1119.1-1643.5)	1265.1 (1034.8-1540.6)	1192.3 (983-1447.3)	1146.4 (961.1-1366.5)	1096.8 (931.9-1286.3)	1027.5 (903.9-1155.4)	966.2 (928.5-1001.7)	935.6 (918.6-953.3)	905.6 (887.8-923.7)	835.2 (819.8-850.6)	766.6 (746.7-785.3)	704 (689.7-717.7)	639.5 (627.6-652.4)	600.6 (564.2-635.7)
UKD Salford	1413.2 (1166.6-1712.6)	1325 (1092.1-1596)	1252.6 (1030.6-1508.1)	1216.5 (1004.4-1459.1)	1169.6 (990.8-1365.2)	1101.7 (982-1247.5)	1007.7 (972.7-1044.8)	978.9 (962.8-995.1)	923 (907.5-938.5)	903.9 (886.6-920.3)	789.1 (769.3-810.2)	723.8 (710-737)	657.7 (644.7-669.7)	624 (587.9-660.3)
UKD Sefton	1278.1 (1050.9-1538.2)	1186.8 (977.4-1428.2)	1120.3 (925.6-1342.6)	1083.6 (899.5-1296.5)	1043.7 (885.8-1229)	983.2 (867.5-1115.8)	929 (895.8-963.4)	868.2 (853.3-883.6)	784.1 (770-798)	753.9 (741.1-767.6)	691 (674.1-707.3)	584.7 (573.3-596.1)	569.2 (557.6-581.1)	541.9 (511.4-574.3)
UKD St Helens	1434.7 (1178.9-1728.6)	1349.2 (1116.3-1620.6)	1274.4 (1061.9-1510.2)	1220.2 (1012-1464.7)	1174.8 (992.8-1370.6)	1115.7 (985.3-1256.8)	1076.6 (1035.9-1119.3)	997.4 (979.2-1016.6)	889.6 (872.5-906.5)	812.1 (794.6-828.6)	729.1 (709.9-748.3)	664.9 (651.2-680.2)	624.2 (611-637.8)	635 (598.2-674.4)
UKD Stockport	1226.5 (1008.3-1475.4)	1141.1 (937.4-1372.7)	1070.9 (877.8-1278.2)	1015.6 (840.1-1218.6)	976.9 (820.3-1146.7)	928.2 (818.4-1041.9)	892.9 (860.5-924.9)	854.4 (840.3-869)	792.4 (779.3-806.8)	731.1 (717.8-745.4)	630 (614.1-646)	579.5 (568.6-590.8)	542 (531-552.5)	487.2 (458.7-517.8)
UKD Tameside	1357.1 (1133.9-1636.8)	1273 (1064.4-1543.6)	1209.4 (992.3-1479.3)	1169.6 (963.8-1403.5)	1126.9 (961.5-1326.5)	1063 (939.8-1196.1)	1003.2 (966.6-1042.3)	945.5 (927.9-963.7)	849.3 (833.4-866.2)	832.8 (816.1-848.2)	765.1 (744.7-786.1)	695.7 (680.8-711.1)	638.1 (624.6-652)	604.6 (566-640.6)
UKD Trafford	1210.6 (997.8-1480.6)	1134.1 (929.3-1368.3)	1076.1 (882.1-1290.9)	1042 (866.3-1235.5)	1004.3 (854.5-1186.6)	942.8 (835.4-1066.9)	888.6 (855.4-924.6)	851.5 (834.8-866.7)	782 (767.5-797.3)	742.7 (727.9-757.4)	660.6 (643-678.5)	563.1 (551.5-575.1)	488.9 (478.3-500)	478.4 (449.9-508.7)
UKD Warrington	1295.4 (1055.9-1562.8)	1219.3 (995.4-1474.3)	1160.3 (949.1-1392.1)	1124 (932.7-1350.6)	1088.2 (920.5-1277.4)	1029.7 (909.9-1157.7)	969 (933.1-1007.5)	941 (922.8-960.2)	870 (853-887.6)	791.1 (775.5-806.7)	682.1 (662.8-701)	605.3 (591.8-618.5)	554.3 (542-566.5)	566.2 (531-601.5)
UKD Wigan	1417.7 (1175.9-1698.4)	1329.9 (1081.4-1603.5)	1249.2 (1018.8-1503)	1182.1 (967.5-1414)	1133.5 (960.3-1329)	1077.5 (957.1-1216.1)	1037 (1002.6-1072.3)	983.8 (967.2-999)	883.4 (867.9-899.1)	818.4 (804.3-833.7)	752 (733.8-770.5)	679.8 (667.7-692.2)	621.5 (610.7-632.6)	615 (581.6-652)
UKD Wirral	1291.6 (1052.1-1541.1)	1199.5 (989.8-1446.8)	1128.6 (924.2-1360.7)	1089.8 (912.4-1311.8)	1044.2 (896.3-1224)	974.8 (865.7-1100.7)	897.4 (867.2-928.4)	857.4 (844.2-871.8)	820.2 (806.9-833.4)	778.4 (764.9-791.2)	715.7 (698-732.5)	647.8 (637-659.5)	582.6 (570.8-593.8)	580.4 (549.4-613.9)
ENG South East England	1070.6 (1003.9-1141.9)	1010 (945.6-1074.3)	958.2 (901-1018.1)	933.6 (879.6-994.4)	924.8 (873.4-979.2)	903.6 (865.6-941.3)	829.4 (821.3-838.1)	776.8 (773.4-780.4)	706.2 (702.9-709.4)	668.5 (665.5-671.7)	597.2 (592.6-601.3)	525.2 (522.5-527.9)	474 (471.6-476.4)	461.3 (453.2-469.4)
UKJ Bracknell Forest	1031.3 (828.6-1280.3)	970.1 (770.6-1198.7)	922 (732.7-1134.4)	908.1 (728.3-1101.8)	895.7 (744.5-1066.3)	866.6 (754.9-980.7)	800.2 (760.4-840.1)	755.5 (734.5-777.3)	675.2 (657.8-694.6)	624.7 (606.6-644.3)	557.5 (538.3-577.1)	487.1 (473.8-501.8)	442.9 (428.8-456.1)	413.6 (384.1-443.7)
UKJ Brighton and Hove	1094 (887.3-1340.4)	1038.3 (850.5-1244.9)	990.8 (809.2-1195)	980.9 (810.1-1165.5)	967.6 (818.2-1141.5)	936.3 (829.5-1057.7)	857.3 (823.2-891.6)	812.7 (796.9-827.9)	786.6 (771.9-801.5)	788.4 (773.5-802.8)	697.9 (680.4-715)	643 (630.1-655.3)	561.9 (550.6-573)	489.6 (460-519.1)
UKJ Buckinghamshire	1029.1 (828.8-1254.6)	969.4 (786.9-1173)	922.4 (746.8-1103.6)	894.2 (730.1-1073)	890.4 (739.5-1046.4)	873.6 (775.4-978.6)	816.3 (789.2-845)	760.2 (748-773)	701.8 (690.7-713.3)	651.5 (640.8-662.2)	560.6 (547.6-574.9)	486.8 (477.5-495.3)	442.4 (434.7-450.1)	409.2 (385.3-433.5)
UKJ East Sussex	1075.8 (867.8-1305.5)	1010.2 (828.5-1217.8)	956.6 (780.4-1152.9)	932.6 (763.4-1123.5)	914.2 (772.2-1073.3)	879.5 (779-991.2)	799.5 (771.4-830.9)	741 (729.2-753.7)	668.5 (657.5-679.4)	641.7 (630.5-652.4)	583.1 (567.4-597)	513.9 (504.8-523.2)	481.4 (472.6-490.5)	481.5 (454-509.7)
UKJ Hampshire	1042.6 (855.2-1266)	992.6 (811.6-1198.2)	950.6 (778.1-1138.6)	930.3 (761-1134.7)	928 (774.9-1107.8)	905.5 (789-1024.1)	805.4 (779.6-829.2)	758.9 (748.7-768.4)	691 (682.2-700.1)	661.4 (653-669.9)	580.4 (567.3-592.3)	495.3 (488.5-502.4)	453.9 (447.8-460.4)	437.5 (414-460.6)
UKJ Isle of Wight	1116.9 (897.4-1364.6)	1039.6 (830.8-1261.9)	979.7 (783.7-1190.6)	948.1 (775.3-1126.3)	921 (766.9-1089.8)	883.2 (774.3-1009)	818.8 (778.6-858.1)	794 (774.1-813.5)	731.8 (714.3-749.8)	675.2 (659-693.4)	597.3 (577.9-614.6)	547.5 (533.3-562.2)	503.7 (489.4-518)	463.9 (433.7-496.5)
UKJ Kent	1122.6 (927.7-1346.4)	1059.7 (878.1-1268.8)	1006.4 (830.7-1200.4)	979.3 (800.4-1166.6)	980.5 (823.6-1157.3)	975.2 (857.8-1104.5)	884.5 (857.2-911.7)	821 (811.1-831.6)	735.2 (726.2-743.9)	696.6 (687.6-706.1)	614.3 (601.1-627.3)	545.8 (538.5-553)	486.3 (479.4-493)	497.1 (469.8-523.4)
UKJ Medway	1136.3 (918.5-1388.6)	1065.9 (874.9-1276.5)	996.2 (806.7-1187.5)	960.6 (784.6-1166.2)	936.7 (789.7-1102.8)	907.1 (798.4-1030.8)	858 (825.1-891.4)	822.9 (807.9-837.1)	754.7 (740.9-769.5)	718.4 (704.1-731.9)	680.8 (663-699)	622 (608.9-634.7)	568.4 (556.7-580.5)	515.7 (484.9-546.5)
UKJ Milton Keynes	1085 (881.8-1324)	1023.2 (829.8-1255.2)	975.4 (786.9-1189.3)	963.4 (779.1-1158.8)	953.2 (795-1136.4)	926.2 (809.6-1050)	861.7 (825.4-900.2)	816.7 (798.2-833.8)	735.3 (718.5-751)	698.5 (683.6-713.4)	612.2 (595.1-628.6)	561.2 (548.5-573.6)	525.9 (514.8-537.5)	489.2 (459.9-519)
UKJ Oxfordshire	1022.3 (830.3-1264.1)	972.5 (789.4-1190.7)	926.8 (751.9-1128.6)	905.4 (735.3-1091.6)	902.2 (751.2-1074.5)	884.2 (780.6-1003.1)	821.8 (794.6-849.5)	752.6 (740.7-763.9)	686.2 (675.3-696.1)	639.7 (629.4-650)	580.7 (567.2-592.9)	513.2 (504.4-521.9)	461.5 (454.1-469.2)	436.3 (411.1-463.1)
UKJ Portsmouth	1169.7 (944.6-1413.1)	1106.8 (898.7-1345.2)	1050 (856-1264.6)	1026.2 (846.8-1237)	1012.1 (852.6-1188.2)	989.3 (870.6-1111.9)	942.2 (906.2-977.6)	891 (873-908.7)	796.9 (781.4-812.9)	799 (783-815.7)	740.1 (719.9-760.7)	646.5 (632.5-660.6)	575.8 (563.4-588.7)	537.8 (503.9-572.4)
UKJ Reading	1081.1 (874.1-1322.5)	1027.3 (838.8-1239.7)	973.6 (801.4-1175)	952.8 (787.4-1167.2)	934 (778.6-1100.8)	903.8 (791.8-1026.1)	839.6 (800.7-873.3)	807.1 (788.5-826.5)	741.5 (723.1-760.2)	713.8 (697.6-729.8)	656.7 (637-676)	592.8 (579.2-607.1)	545.6 (533.1-559.1)	527.9 (494.4-564.5)
UKJ Slough	1140 (918-1385.3)	1076.6 (868.5-1316.2)	1030.4 (832.8-1262.5)	1018.8 (826.5-1222.1)	1008.4 (846.4-1190.2)	983.3 (868.1-1107.9)	928.2 (887.7-971)	912 (889.9-934.3)	822.5 (802.7-843.5)	769.2 (751-787.6)	708.5 (688.9-730)	606.9 (591.7-621.5)	548.8 (534.1-562.1)	471.9 (440.7-502)
UKJ Southampton	1162.1 (942.2-1420.6)	1102.2 (893.6-1329)	1044.1 (855.8-1249.8)	1021.5 (843.6-1215)	998.3 (841.9-1167.9)	961 (855.9-1086.4)	870.2 (836.6-905.4)	828 (811.8-844.8)	807.7 (792.2-823.4)	775.7 (760.6-791.2)	699 (679.5-716.9)	628.7 (615.9-640.5)	562 (550.5-573.3)	528.5 (495.8-563)
UKJ Surrey	1006.7 (814.4-1224.1)	945.1 (766.7-1140.9)	895.1 (729-1077.7)	865.7 (708.8-1044.3)	862.9 (723.4-1026.5)	854.4 (748-959)	811.3 (784.5-837)	751.9 (742.2-761.7)	684.2 (675-693.3)	639.5 (630.2-648.4)	573.8 (560.8-587.1)	493.7 (486.5-500.8)	433.3 (426.6-439.7)	442.2 (419.1-467.5)
UKJ West Berkshire	993.6 (799.3-1195.8)	934.7 (749.1-1135.1)	887.1 (708.1-1086.7)	869.8 (708.6-1064.1)	856.8 (715.5-1021.1)	833.9 (731-948.9)	772.9 (736.3-809.1)	799.7 (780.7-819.8)	669.1 (653.7-686.1)	635.1 (620.2-650.8)	563.8 (546.2-580.7)	535.3 (521.1-548.9)	461.1 (448.3-474)	458.3 (427.1-491.7)
UKJ West Sussex	1059.6 (854-1280.2)	994.9 (806.2-1198.1)	943.9 (765.7-1140.7)	923.2 (747.9-1109.5)	914.2 (763.2-1068.6)	884.9 (777-1000.2)	802.8 (776.5-830.8)	749.3 (738.8-760.7)	678.9 (669-688.6)	632.1 (621.9-642.1)	576.1 (562.4-589)	515.8 (507.5-524.1)	470.9 (463.4-477.9)	470.3 (442.9-497.3)
UKJ Windsor and Maidenhead	947.3 (760.8-1173.5)	886.6 (713.5-1095.1)	839.7 (679.2-1025.5)	821.9 (675.8-995)	807.8 (675.5-969.4)	784.9 (688.1-891.3)	750.2 (715.3-788.1)	729.6 (710.5-748.6)	677 (660-695)	639.9 (623.5-656.9)	566.3 (548.5-585.1)	454.9 (441.8-468.1)	425.5 (413.3-437.3)	423.6 (396.5-453.5)
UKJ Wokingham	929.4 (748.5-1141.4)	871.1 (706.9-1060.6)	818.5 (660-990.1)	797.4 (643.1-968.8)	779.4 (647.3-931.3)	753.6 (657.7-859.7)	698.1 (664.5-732)	664.4 (647.4-683.4)	590.5 (575.4-606.2)	548.8 (534.5-564.2)	469.9 (454.4-485.1)	420.1 (407.6-432)	389.2 (377.9-400.9)	401.6 (375.2-429.3)
ENG South West England	1137.9 (1069.7-1206.3)	1067.4 (1002.4-1131.5)	1003.8 (947.5-1060.9)	969.3 (916.6-1022.9)	940.1 (893.7-987.5)	894.4 (862.7-928.7)	829.8 (821.5-838.2)	767.7 (763.8-771.4)	708.6 (704.5-712.4)	653.9 (650.7-657.1)	588.2 (583.8-592.6)	522.5 (519.7-525.3)	479 (476.5-481.5)	479.5 (471.2-487.7)
UKK Bath and North East Somerset	1030.2 (831.6-1262.7)	963.1 (782-1174.6)	900.9 (731.4-1090.4)	865.5 (712.9-1042.5)	828.6 (696.7-978.4)	775.5 (680.3-875)	715.6 (684.4-746.5)	683.4 (667.5-699.9)	652.1 (635.2-667.9)	618.2 (603.2-633.6)	545.1 (527.5-561.8)	472.6 (461-484.6)	440.2 (428.2-451.3)	464.6 (435.4-496.7)

Table 1 Age-standardised mortality rates by location and year for both sexes for 1950, 1955...2005, 2010, 2017

Location	1950	1955	1960	1965	1970	1975	1980	1985	1990	1995	2000	2005	2010	2017
UKK Bournemouth	1139.5 (917-1389.9)	1067 (860.8-1299.4)	1005.7 (821.7-1216.3)	973.3 (797.1-1166.5)	939.1 (784.2-1117.6)	886.5 (780.5-1001.7)	809.3 (772.5-844.4)	768 (750.3-786.4)	758.3 (740.4-776.3)	738.8 (722.6-755.8)	654.6 (635.2-674.9)	574.9 (561.7-588.5)	543.6 (531.1-557.1)	530.1 (496.5-563.3)
UKK Bristol, City of	1161.5 (951.9-1408.6)	1104.6 (904.1-1337.9)	1048.5 (864.9-1247.2)	1027.3 (853.2-1221.4)	1005.3 (848.9-1177.4)	964.7 (851.8-1083.7)	896.4 (866.8-930.4)	817.7 (804.7-831.6)	752.3 (739.4-765.1)	708.4 (696.6-720.2)	659.3 (642-675.8)	590.9 (580.7-602.7)	544.9 (534.8-555.3)	499.2 (470-530.4)
UKK Cornwall	1191.8 (960.1-1445.4)	1113.8 (916.5-1359.4)	1046.6 (858.7-1256.9)	1011.5 (828.7-1200.9)	981.6 (816-1155.7)	934.9 (823.5-1056.2)	857.7 (828.2-889.9)	776.7 (764.3-789.7)	718.1 (706-730.2)	666.2 (655.3-677.1)	602.2 (586.7-616.4)	535.8 (526.3-544.7)	489 (480.8-498.2)	509 (481.3-538.1)
UKK Devon	1131.3 (909.5-1367.4)	1062.9 (863.2-1278.5)	1001.1 (821.5-1209.7)	967.3 (799.2-1144.2)	939.3 (791-1104)	893.7 (791.5-1017.1)	816 (789.5-843.8)	744.6 (733.7-756.2)	679.8 (669.5-690.7)	626 (617.1-635.4)	568.1 (554.5-580.6)	500.3 (492.9-508)	462.6 (455.5-470)	488.6 (460.8-516.9)
UKK Dorset	1093.6 (880.4-1330.9)	1018 (826.9-1238.3)	953.4 (770.9-1161.9)	920.5 (752.7-1097.5)	889.2 (751.2-1038.8)	840.6 (750.6-944)	785.1 (757-815.2)	729.1 (716.4-742)	658.5 (647.1-670.3)	591.2 (580.5-602.9)	533.5 (519.6-547.1)	463 (454.4-472.4)	423.7 (415.3-432.9)	440.5 (414.9-467.3)
UKK Gloucestershire	1104.4 (880.6-1348.9)	1032.3 (829.7-1247.8)	968.2 (785-1170.2)	928.2 (765.3-1105.1)	903.4 (763.3-1063.5)	869.3 (764.8-981.9)	845.9 (816.3-876.3)	791.2 (778.7-803.7)	712.4 (701.4-723.7)	658.6 (648.3-669)	581 (567-594.4)	526.9 (518.1-535.9)	478.3 (469.8-486.9)	486.2 (461-514.4)
UKK North Somerset	1083.1 (881-1328.9)	1006.6 (821.7-1236.3)	938.6 (771.6-1132.3)	897 (738.9-1071)	856.9 (718.3-1012.3)	803.5 (703.4-903.6)	748.3 (713.8-782.7)	717 (700.1-733.1)	674.4 (659.3-689.5)	624.9 (610.3-641)	562.2 (546.6-578.6)	521.8 (509.7-533.8)	504.1 (491.5-516.6)	517 (485.6-551.6)
UKK Plymouth	1232.3 (988.9-1493.4)	1163.8 (934.1-1399.3)	1099 (895.9-1335.8)	1068.8 (876.5-1266.8)	1037 (875.1-1225.4)	987.2 (871.2-1113.4)	913 (879.9-948.7)	841.1 (825.9-855.3)	783.3 (769-798.3)	741.7 (727.1-756.3)	664 (646.3-683.3)	622.4 (609.8-634.9)	567.7 (556.1-580)	516.4 (486.1-549.5)
UKK Poole	1111.7 (895.1-1357.8)	1038.2 (851.4-1249.6)	975.5 (801.1-1171.7)	947.9 (777.4-1121)	913 (760-1085.7)	859.6 (751-976.9)	771.5 (735.9-808.3)	751.6 (732.3-769.7)	687.7 (670-705.3)	628 (612.3-644.9)	543.5 (525.9-560.5)	498.4 (485.2-511.1)	465.4 (451.9-478.9)	474.2 (444.9-504.7)
UKK Somerset	1130.1 (919.7-1362.5)	1059.2 (862.8-1268.3)	1000.5 (821.7-1205)	967.8 (791.5-1159.7)	945.8 (796.6-1127.3)	904.8 (796.1-1030.2)	837.3 (809.6-866.3)	769 (756.2-782.2)	712.4 (700.6-724.8)	654.4 (642.3-665.5)	588.3 (573.4-602.2)	511.6 (503.2-520.2)	466.4 (457.6-474.6)	468.8 (441.1-496)
UKK South Gloucestershire	1093 (878.1-1328.7)	1026.7 (826.3-1234.7)	966.2 (790.3-1153.3)	934.7 (761.4-1118.9)	912 (770.8-1076.5)	865 (762.4-976.3)	777.2 (744.2-807.3)	732.1 (716.6-746.9)	701.7 (686.3-716.8)	635.5 (620.5-651.1)	569.5 (553.5-586)	480.7 (469.8-491.9)	430.5 (419.9-441.6)	437.7 (410.9-465.1)
UKK Swindon	1170 (964.9-1432.7)	1102.8 (898.7-1337.6)	1044.7 (851.9-1276.8)	1018.1 (848.2-1222.5)	1002.3 (850.8-1178.8)	964.9 (858.5-1085.5)	915.6 (877.1-954.4)	837.4 (818.4-855.9)	753.9 (737-771.4)	719.8 (702.8-736.1)	640.1 (622.7-658.9)	560.4 (547.4-573.6)	501.4 (489.9-512)	514.8 (483.9-549.5)
UKK Torbay	1186.4 (956.1-1453.3)	1105.8 (885.3-1367.9)	1040.3 (841.2-1257.6)	1003.9 (825-1197.7)	964.9 (814.8-1138.2)	908.4 (792.9-1026.2)	839.4 (798.9-881.6)	787.8 (767.1-806.6)	709.2 (690-726.7)	681.9 (665.5-698.8)	664.5 (644.5-683.7)	623.3 (607.3-639.4)	568 (552-583)	514.3 (480.1-548.4)
UKK Wiltshire	1126.9 (918.6-1376.7)	1060.8 (863.5-1292.2)	992 (803.3-1194.3)	944.6 (786.1-1121.9)	916.3 (770.4-1081.5)	877.3 (771.6-990.6)	835.9 (804.6-869.6)	778.4 (764.7-792.1)	737.4 (724.5-750)	644.3 (633.1-655.7)	574 (559.6-588.1)	512.7 (503.4-522.7)	465.3 (456.7-474.5)	441 (415.4-466.3)
ENG West Midlands	1277.8 (1200.4-1364.3)	1200.4 (1126.1-1277.3)	1130.3 (1056.9-1201.9)	1087.4 (1025.9-1155.7)	1058.2 (1000.7-1117.9)	1006.9 (968.8-1047.4)	934.6 (924.1-944.5)	872.9 (868.7-876.9)	810.6 (806.8-814.6)	754.2 (750.2-757.7)	675.3 (670.4-680.4)	602.8 (599.6-605.9)	532.5 (529.7-535.4)	515.6 (506.9-525)
UKG Birmingham	1306.4 (1088.6-1537.3)	1221.6 (1017.1-1454.9)	1149.4 (964.7-1357.4)	1105.9 (932.3-1317.4)	1072.2 (902.3-1257.7)	1016.2 (897.3-1149.5)	963 (934.7-992.5)	920.1 (909.3-931)	851.2 (840.4-861.6)	795.9 (785.2-806)	716.9 (701.8-732.3)	636.7 (627.7-645.3)	556.2 (548.1-564.1)	536.8 (509.1-565.4)
UKG Coventry	1268.5 (1024.2-1523.6)	1192.8 (979.2-1432.7)	1123.3 (924.4-1345.1)	1093.4 (900.3-1304.3)	1066.3 (897.6-1252.9)	1011.7 (887.2-1142.7)	933 (899.7-966.1)	856.4 (843.3-870.3)	839.1 (825.7-853.7)	776.2 (762.6-789.8)	694.2 (677.5-711.7)	663.2 (652.1-675.6)	567.3 (557.4-578.3)	536.6 (505.4-569.1)
UKG Dudley	1254.6 (1032.3-1521.3)	1167.7 (966.7-1419.4)	1085.2 (885.9-1295.6)	1024.7 (841.2-1226.9)	992.1 (838.2-1164.8)	948.6 (836.9-1072.4)	895.7 (864.3-928.1)	867.7 (853-881.8)	806.7 (792.5-820.8)	744.5 (730.2-758.1)	676.1 (659.4-693.1)	597.8 (585.8-609.3)	526.1 (515.4-536.6)	515.4 (484.1-545.8)
UKG Herefordshire, County of	1179.6 (958-1439)	1109.8 (895.8-1366.7)	1055.4 (859.6-1270.7)	1037.1 (855.3-1248)	1009.2 (847.4-1190.8)	946.2 (835.3-1075.2)	848.3 (812.7-886.7)	782.1 (764.5-799.1)	706.1 (690.7-722.6)	652.8 (636.1-668.4)	582.4 (564.6-599.2)	526 (513.2-538.3)	489.4 (477.2-500.7)	479.2 (450.8-509.6)
UKG Sandwell	1320 (1069.5-1598.6)	1234.7 (1002.2-1501.7)	1165.4 (950.7-1417.6)	1132.5 (932.7-1374.6)	1099.8 (923.6-1299.2)	1039 (909.2-1173.6)	952.3 (919.6-987.2)	897.9 (883-912.2)	858.1 (843.8-871.7)	832.7 (818.4-847.7)	771.6 (751.5-792.1)	705 (691.8-717.6)	622.5 (610.7-634.8)	567 (534.1-604)
UKG Shropshire	1237.5 (1019.5-1490.2)	1168.9 (957.3-1415.7)	1103.6 (901.5-1329.7)	1060.9 (876.7-1273.1)	1030.4 (866.2-1203.3)	971.4 (848-1103.8)	878.7 (844.5-911)	819.6 (803.7-834.8)	739 (725.4-752.5)	698.2 (685.9-712.2)	601.3 (583.9-617)	536 (524.7-547.2)	482.2 (471.9-492.8)	474.3 (446.7-504.4)
UKG Solihull	1104.4 (876.8-1364.8)	1039.8 (833.4-1279.2)	978.9 (791.3-1189.3)	947.6 (782.2-1145.3)	912.4 (767.5-1072.8)	852.5 (744.7-966.3)	777.7 (745.3-811)	714 (698.7-728.7)	655.4 (641-671)	639.7 (624.4-654.1)	582 (564.8-598.1)	508 (495.8-520.9)	444.6 (433.7-455.8)	451.9 (421.8-482.5)
UKG Staffordshire	1273.8 (1040.5-1523.9)	1202.7 (983.6-1451.7)	1132.7 (931.8-1383.5)	1086 (901.2-1304.2)	1068.4 (897.1-1261)	1034.5 (907.4-1174.3)	944.7 (914.2-974.9)	863 (852.2-874.3)	816.2 (804.9-827.2)	754 (743-765.1)	668.2 (653-683)	585.4 (576.2-594.9)	509.5 (501.9-517.3)	518.7 (493-548.9)
UKG Stoke-on-Trent	1438.8 (1169.6-1733.4)	1360.4 (1118.5-1636.2)	1291.2 (1054.8-1542)	1254.8 (1044.6-1491.3)	1226 (1043.8-1430.8)	1170.1 (1034.5-1321.9)	1081.3 (1044.7-1119.8)	1008.6 (991.3-1026)	916.3 (900.2-932.3)	876 (860-891.3)	771.6 (752.3-790.9)	713.4 (699.1-727.5)	649 (636.6-661.6)	602.1 (568.6-637.9)
UKG Telford and Wrekin	1297 (1039.1-1574.2)	1235.3 (988-1506.7)	1178.4 (963.9-1421.4)	1147.1 (943.8-1368.9)	1119.2 (938.3-1326.9)	1061.8 (932.2-1203.5)	983.1 (941.3-1025.6)	904.9 (884.9-925.2)	801.4 (784.3-819.3)	736.4 (720-753.3)	682.4 (663.8-702)	612.3 (597.6-626.4)	583.8 (569.7-597.5)	576.4 (541.2-613.9)
UKG Walsall	1371.5 (1113.8-1681.6)	1282.3 (1047.9-1578.8)	1200.9 (995.3-1478.3)	1153.4 (953.9-1377.8)	1121.5 (950.2-1322.5)	1068.3 (937.1-1203)	989.6 (954.5-1026.1)	931.4 (915.1-947.6)	864.5 (848.4-879.4)	796.2 (780.8-812.3)	702.3 (683.3-722.4)	624.4 (611.5-637)	563.6 (552.5-574.3)	559 (526.3-593.4)
UKG Warwickshire	1207.3 (984-1457.9)	1138.1 (934.2-1379.9)	1072.1 (884.7-1306.3)	1028.8 (853.1-1253.3)	1004.7 (851.5-1178.7)	962.6 (851.6-1091.4)	898.8 (869.4-932.3)	846 (833.5-860)	772 (759.7-784.5)	721.5 (710.4-733.2)	625.5 (609.4-640.9)	561.2 (551.9-570.2)	491.8 (483.3-500.2)	478.1 (452.1-507.2)
UKG Wolverhampton	1318.5 (1062.2-1611.9)	1232.9 (1008.7-1495.4)	1158.9 (948.4-1406.6)	1121.6 (932.8-1330.8)	1088.4 (911.8-1276.1)	1031.2 (900.4-1175.5)	944.5 (910.1-982.9)	894.7 (878.6-910.5)	867.6 (851.5-883.3)	785.2 (771-799.3)	736.6 (717.4-756.5)	707.8 (695.5-721.8)	599.7 (587.9-611.7)	563.8 (531.2-596.5)
UKG Worcestershire	1198.4 (966.9-1467.5)	1127.6 (927.8-1363.7)	1062.7 (863.1-1282.3)	1009.2 (839-1200.9)	983.2 (824.5-1151.4)	940.2 (824.2-1062.1)	893.2 (861.7-924.6)	816.3 (804.3-829.1)	756.4 (745.6-768)	693.2 (682.3-704.2)	639.8 (624.9-655)	545.5 (535.7-554.9)	500.8 (492.5-509)	479.3 (451.8-507.4)
ENG Yorkshire and the Humber	1285.2 (1213.6-1355.9)	1208.6 (1143.6-1276.3)	1137.5 (1076.5-1202.3)	1094 (1037.6-1155.2)	1053.9 (1005-1106.4)	1000.5 (966.7-1038.3)	932.5 (923.1-941.9)	881 (876.8-885.1)	809.7 (806-813.3)	747.4 (743.8-751.1)	677.6 (673.1-682.4)	607.2 (604.1-610.3)	553 (550.5-555.8)	527.7 (518.9-536.4)
UKE Barnsley	1371.3 (1126.9-1645.8)	1291.9 (1064.7-1553)	1216.3 (1006.4-1456.9)	1169.8 (964.4-1413.3)	1120.2 (943.6-1315.8)	1057.1 (930.6-1188.7)	972.3 (933.6-1009.2)	938 (920.4-955.4)	865.7 (849.4-882.9)	825.1 (809.3-842.2)	735.7 (715.6-754.3)	655.9 (642.5-669.9)	598.8 (585.1-612.8)	561.8 (527.4-597.7)
UKE Bradford	1330.4 (1081.9-1614.4)	1241.6 (1010.1-1518.2)	1164.8 (945.8-1416.6)	1115.6 (933.4-1327.9)	1072.3 (913.2-1257.3)	1016.1 (900.5-1145.5)	965.3 (934.5-999.7)	926.5 (912.8-939.5)	844.2 (831.8-856.6)	799.2 (786.6-811.8)	725.2 (708-741.8)	658.1 (647-669.6)	579.5 (569.8-589.2)	542.4 (512-574.2)
UKE Calderdale	1323.5 (1082.4-1596.6)	1236.8 (1009.6-1502.6)	1163.7 (959.6-1395.4)	1118.4 (923.4-1331.8)	1075.7 (910.4-1263.7)	1021.9 (903.2-1152)	948.7 (913.2-989.2)	916.2 (898.4-932.3)	848.2 (831.9-865)	788.1 (772.4-804.2)	685.6 (667.3-703.7)	611.8 (597.9-625.8)	583.9 (570.3-597.2)	559.2 (526.8-596)
UKE Doncaster	1297.7 (1062.6-1567)	1206.9 (992.5-1477.8)	1118.5 (920.8-1341.5)	1065.7 (882.7-1275.5)	1016.4 (862.1-1191.2)	963.3 (845.9-1084.8)	930.3 (895.6-964.9)	879 (864.3-894.3)	829.6 (815.8-843.8)	820.7 (806.8-835.4)	720.2 (702.7-739.9)	642.3 (629.5-653.8)	588.7 (578-600.8)	568.8 (537.6-603.4)
UKE East Riding of Yorkshire	1195.5 (974.2-1453)	1119 (910.9-1372)	1049.9 (857.8-1277.3)	1005.7 (828.6-1208.2)	966.6 (812.8-1135.4)	917.5 (807.6-1034.8)	854.3 (823.9-888.6)	807.4 (792.3-822.5)	740.9 (727.8-754.4)	682.7 (670-696.1)	621.8 (605.9-637.5)	553.9 (543.6-564.9)	502.3 (492.1-512.6)	481.6 (454.5-511.2)
UKE Kingston upon Hull, City of	1295.1 (1059.7-1554.8)	1208.6 (983.2-1462.2)	1126.5 (924.6-1360.5)	1082 (890.6-1291)	1032.1 (863.5-1212.5)	976.5 (855.8-1101.8)	955.1 (920.4-990.8)	898.2 (882.9-915.3)	839.3 (824.8-855.2)	805.2 (791.1-819.8)	762 (741.7-781.9)	689.9 (677.4-702.9)	673 (660-686.4)	638.4 (603-679.5)
UKE Kirklees	1357.2 (1109.9-1626.1)	1278.1 (1060.3-1554.2)	1206.8 (997.5-1460.9)	1155.2 (960-1373.2)	1118 (943.4-1306.7)	1070.7 (949.1-1198.7)	1015.2 (979.1-1049.1)	922.4 (908-937.2)	851 (837.2-865.9)	754.5 (741.7-767.7)	693.2 (676.3-710.4)	649.8 (637.9-661)	561.5 (551.1-571.7)	500.4 (471.3-532.8)
UKE Leeds	1258.4 (1031.5-1531.6)	1190.5 (974.4-1442.7)	1127.2 (925.4-1365)	1097.6 (916.4-1320.2)	1064.3 (908.5-1246.6)	1012.7 (892.8-1143.6)	924.3 (896-955.6)	866.3 (854.7-877.1)	784.9 (775-795.9)	717.3 (707.7-727.7)	663.6 (648.2-679)	601.2 (592-610.5)	555.7 (547.4-563.8)	550.5 (521.1-581.4)
UKE North East Lincolnshire	1224.3 (989.6-1476.5)	1133.3 (918.5-1382.8)	1051.9 (852.5-1273.6)	1010.2 (827.8-1206.6)	960.9 (803.5-1122)	907.2 (796.8-1023.9)	859.5 (821.6-898)	858 (839.8-876.6)	824.1 (806.3-842.5)	769.9 (754.1-787.3)	703.1 (683.5-724.3)	648.7 (633.5-663.5)	574.8 (560.7-589.3)	535.5 (502.2-570.6)
UKE North Lincolnshire	1297.5 (1048.9-1577.9)	1220.1 (982.6-1496.6)	1145.1 (933.4-1399.5)	1105.2 (913.9-1312.8)	1056.6 (878.3-1235.9)	996.8 (875.9-1132)	940.7 (901.1-979.6)	911.7 (892.8-931.2)	829.3 (812.4-847.9)	735.5 (719.2-752.2)	684.4 (665.6-703)	601.1 (587.4-615.4)	554.9 (541.4-568.9)	528.5 (495.5-561.5)
UKE North Yorkshire	1207.7 (985.4-1463.4)	1141.1 (933.9-1377.1)	1081.4 (888.1-1297)	1038.4 (852.6-1236.8)	1003.4 (849.2-1184.7)	950 (836.9-1066.5)	862.6 (833.4-892.8)	797.8 (786.3-809.5)	740 (728.8-750.9)	679.7 (670-690.7)	609.8 (594.6-624.1)	533.8 (525.4-543.1)	476.8 (468.1-485.6)	463.8 (437.1-491.2)
UKE Rotherham	1341.4 (1094.1-1623.3)	1267.1 (1033.7-1516.2)	1192.5 (976.9-1424.9)	1143.8 (950.9-1362)	1096.5 (919.9-1284.4)	1034.2 (914.1-1164.6)	963.2 (927.7-1000.7)	901 (885.8-917.2)	840.2 (824.5-855.4)	754 (739.6-768.2)	711.6 (693.7-729.4)	626.8 (614-639.8)	583.3 (571.3-595.2)	597 (563-634.2)
UKE Sheffield	1268.8 (1044.5-1524.6)	1193.5 (972.4-1442.6)	1120.3 (915.3-1351.5)	1074.6 (887.3-1286.4)	1038.9 (875-1227.1)	992.4 (876.8-1116)	921.8 (893.9-952.3)	889.8 (876.8-902.5)	818 (806.2-829.8)	755.8 (744.8-767.7)	675.4 (659.6-691.3)	596.9 (586.6-606.6)	550.4 (541.8-559)	525 (497.5-554.7)
UKE Wakefield	1406 (1164.1-1699.8)	1339 (1114.6-1606.9)	1272.5 (1047.3-1535.7)	1230.1 (1026.2-1465.6)	1190.9 (1004.9-1387.4)	1129.1 (999.6-1265.7)	1035.8 (999.1-1073.4)	966.7 (951.8-983)	860 (845.2-875.1)	784.2 (770.9-796.6)	708.4 (690.2-725.4)	637.9 (626.7-650.6)	579.6 (568.8-591)	552.7 (521.4-585.7)
UKE York	1146.2 (917.8-1393.6)	1082.4 (877.8-1316.6)	1023.5 (828.6-1238.2)	991.2 (808-1184.6)	953.5 (800.1-1125.3)	903.8 (789.9-1024.6)	851.9 (816.8-890.1)	821 (804.5-838.2)	731.6 (715.7-748.2)	646 (630.8-661.6)	569.6 (552.4-586.7)	498.9 (486.9-511.3)	471.5 (459.4-482.1)	486.5 (456.5-517.2)
GBR Northern Ireland	1392.7 (1364.4-1421.1)	1311 (1289.1-1330.8)	1227.3 (1209.3-1244.8)	1151 (1134.9-1167.2)	1115 (1099.7-1130.6)	1149 (1133.8-1163.9)	1076.4 (1062.5-1090.8)	1003.4 (989.5-1018)	913.4 (899.3-927)	824 (811.1-836.9)	707 (690.6-723.3)	609.3 (599.6-619.4)	575.2 (565.8-584.2)	518.6 (483.8-553.8)
GBR Scotland	1443.3 (1429.5-1460.1)	1335.8 (1325.4-1346.6)	1278.1 (1267.8-1288.6)	1231 (1220.6-1240.7)	1169.2 (1160.1-1179.1)	1094.4 (1085.7-1103.5)	1041.8 (1033.1-1050.5)	976.5 (968-984.5)	892.6 (884.3-900.8)	825.4 (818-832.7)	752.7 (737.6-766.6)	677.6 (671.2-684.1)	619 (611.9-625.1)	593.7 (558-630.7)
GBR Wales	1475.9 (1227.3-1774)	1378 (1143.9-1647.9)	1274.4 (1057.5-1548.3)	1209.4 (1003.2-1465.1)	1150.2 (945.3-1382.6)	1053.4 (908.1-1209)	939.9 (912.6-968)	858.1 (850-865.8)	781 (773.4-788.6)	753 (745.3-761.1)	682 (667.8-695.5)	607.9 (601.4-615)	549.5 (543.1-555.1)	530.3 (503.1-559.9)
Latin America and Caribbean	2042.5 (2007.3-2079.4)	1803.8 (1776.9-1831.7)	1668.2 (1643.7-1692.8)	1528 (1505.1-1552.2)	1448 (1427.1-1468.4)	1270.7 (1256.8-1283.1)	1163.9 (1156.3-1171)	1096 (1091.5-1100.4)	977.2 (973.4-980.8)	927.7 (923.6-931.6)	816 (812.3-819.5)	752 (748.1-755.9)	756.6 (752.8-760.8)	674.9 (666.4-682.7)
Andean Latin America	2348.1 (2238.1-2469.8)	1998.6 (1935.4-2062.5)	1889.1 (1830.8-1945.1)	1607.2 (1556.5-1654.6)	1838.5 (1794.9-1882.4)	1252.6 (1219.1-1287.1)	1174.4 (1140.5-1207.9)	1098 (1070.8-1130.4)	946.5 (920.7-972.2)	902.3 (876-929.4)	784.1 (762-806.3)	726.5 (703-751.8)	691.9 (666.1-718.1)	592.6 (553.5-631.6)
BOL Bolivia	2929.4 (2756.2-3141.7)	2399.7 (2302.2-2514.4)	2212.3 (2124.1-2312.7)	2125 (2047-2201.2)	2000.7 (1920.9-2082.2)	1849.6 (1786.5-1914.1)	1693.9 (1632.4-1756.8)	1593 (1515.3-1671.4)	1459.9 (1390.8-1535.7)	1319.7 (1246.6-1391.4)	1161 (1097.5-1237.5)	1047.3 (967.6-1131.9)	965.3 (868.2-1070.4)	883.2 (760.8-1009.9)
ECU Ecuador	1956.1 (1776.2-2132.4)	1790.2 (1762.7-1818.2)	1688.7 (1663.8-1714.3)	1420.5 (1399.9-1443.2)	1331 (1312.5-1350.4)	1148.4 (1133.8-1163.3)	1049.4 (1036.9-1062.1)	904 (893.5-914.2)	840.8 (832.6-849)	770.4 (762.5-778.5)	735.4 (726-745.1)	775.2 (746.6-804.7)	751 (722.6-777.9)	667.1 (620.9-719)
PER Peru	2284 (2114-2475.3)	1952.9 (1850.5-2057)	1868.4 (1771.8-1960.4)	1513.1 (1433.6-1595.4)	1997.8 (1929.3-2070)	1118.4 (1063.9-1175.9)	1079.8 (1028.6-1130.8)	1047.7 (1004.1-1095.3)	855.8 (816.3-894.5)	848.2 (808.8-890.6)	704.1 (668.1-738.4)	622.5 (591.2-657.2)	598.7 (567.4-630.7)	487.7 (434.6-545.1)
Caribbean	1827.9 (1746.5-1906.9)	1624.2 (1558.9-1689.8)	1486.9 (1446.9-1527.5)	1380.3 (1346.3-1415.3)	1244.2 (1212.1-1277.2)	1143.6 (1116.4-1172.5)	1101.8 (1078.1-1127.1)	1064.3 (1044.3-1083.1)	1008.8 (991.6-1025.5)	996 (975.6-1017.2)	904.9 (883.5-925)	858.6 (834.9-883.2)	1327.4 (1302.5-1353.4)	797.2 (762.3-833.2)
ATG Antigua and Barbuda	1696.3 (1620.2-1789.3)	1459.8 (1417.6-1504.1)	1266.6 (1231.8-1300.7)	1197.2 (1165.3-1231.8)	1158.2 (1120.5-1196.7)	1065 (1025.3-1102.8)	963.9 (918.7-1005.4)	852.6 (814.4-892)	800.2 (771.2-831.7)	819 (791.6-851.7)	797.8 (776.9-820)	733.1 (709.1-757.2)	684.8 (664.7-704.4)	655.2 (617.7-689.6)
BHS The Bahamas	1550.9 (1372.8-1737.2)	1428.1 (1289.1-1566.6)	1334.4 (1244-1427.9)	1336.5 (1294.3-1379.6)	1207.3 (1184.7-1230.3)	1070.3 (1052.5-1087.7)	1055.4 (1038.9-1072.2)	982.8 (966.7-999.5)	932.8 (917.9-947.9)	951.2 (936-966.7)	905.2 (890.5-919.3)	811.5 (798.2-825.5)	810.2 (796.6-823.6)	798.3 (747.4-849.4)
BRB Barbados	1562.6 (1474.1-1673.6)	1334.6 (1297.2-1376.8)	1156.2 (1130.7-1182.5)	1123.2 (1101.8-1143.3)	1132.6 (1112.1-1152.6)	1069.3 (1050.7-1089.1)	923.8 (907.4-941.6)	850.8 (834.7-866.6)	847.3 (833.6-862.5)	825.8 (808.4-845.3)	775.1 (759.2-791.6)	745.5 (732.3-759.8)	682.2 (669.7-694.9)	671.9 (631.9-712.7)
BLZ Belize	1904.4 (1764.2-2088)	1608.6 (1537.5-1686.6)	1213.9 (1161.9-1268.7)	1028.3 (990.5-1069.6)	921.7 (884.6-958.1)	862.5 (830.7-894.8)	841.5 (808-870.9)	863.3 (833.3-893.5)	874.4 (844.5-908.5)	881.6 (855.4-908.8)	978.1 (961.4-994.1)	869.5 (856.7-882.6)	779 (767.4-790.7)	748 (726.8-771.2)
BMU Bermuda	1452.6 (1402.9-1504.2)	1283.2 (1253.9-1310.2)	1143 (1118.8-1167.4)	1073.3 (1055.8-1095.7)	1037.1 (1017.4-1054.8)	919.9 (893.9-944.9)	844.5 (824.4-864.3)	856 (835.9-877.5)	852.3 (831.2-875.1)	709.3 (692.8-743.9)	664.8 (651-682.4)	572.3 (550.7-592.9)	509.2 (489.5-529.4)	476.8 (451.7-503.4)
CUB Cuba	1220 (1060.9-1378.1)	1273.4 (1137.6-1427.9)	1235.2 (1223-1246.8)	1131.1 (1123.9-1138)	917.5 (911.7-923.9)	795.5 (790.1-800.7)	817.4 (812.3-822.9)	819.9 (815.1-825)	794.9 (790.5-799.3)	800.2 (795.7-805.1)	692 (687.5-696)	657.1 (652.8-661.4)	620.4 (616.2-624.5)	608.4 (558.7-665.2)
DMA Dominica	2118.2 (1944.9-2323.9)	1545.8 (1466.2-1627.8)	1244.1 (1199.3-1292.5)	1077.1 (1039.6-1113.3)	1002.7 (976.6-1033.2)	897.5 (876.2-921.9)	805.1 (784.8-825.7)	783.6 (762-804.2)	870.4 (850.1-890.9)	869.2 (848.3-892.4)	821.2 (801-842.6)	788.4 (766.4-808.7)	800.3 (777.6-823.3)	849.1 (804.3-896.5)
DOM Dominican Republic	1586.5 (1497.6-1689.1)	1353.4 (1315.6-1394.3)	1215.6 (1155.8-1277.3)	1175.7 (1123.7-1225.8)	1096.9 (1042.1-1155.2)	998.2 (947-1054.6)	925.3 (877.6-977.5)	840.8 (796.2-886.3)	801.3 (759.6-843.6)	728.8 (688.5-771.4)	714.9 (668.6-760.1)	755 (703.9-806.7)	728.7 (683.7-774.7)	788.1 (708.2-861.6)
GRD Grenada	1438.4 (1359.4-1528.5)	1315.6 (1254-1373.7)	1277.9 (1217-1340.6)	1323.1 (1256-1386.2)	1139.7 (1081.7-1199.9)	1026.4 (977.2-1077.9)	1059.5 (1017.9-1100.5)	1140.9 (1104.4-1178.8)	1128.5 (1103.4-1157)	971 (949.1-993.8)	818.6 (799.5-839.3)	787.9 (768.6-807.7)	823.3 (802.3-845.1)	806.2 (767.2-845.2)
GUY Guyana	2243.6 (2165-2332.7)	1889.7 (1849.8-1930.6)	1557.6 (1529.8-1583.4)	1521.4 (1447.9-1592.3)	1404.6 (1382.4-1427.5)	1495.1 (1467-1527.4)	1387.9 (1355.6-1421.5)	1229.2 (1203.3-1256.1)	1263.1 (1247.4-1278.8)	1245.9 (1231.4-1260.4)	1231.3 (1217.3-1245.4)	1182.9 (1168.8-1196)	1104.3 (1089.6-1118.9)	1045.8 (955.5-1136.8)
HTI Haiti	3230.5 (2961-3524.4)	2872.6 (2654.3-3108.2)	2746.6 (2526.8-2982.5)	2661 (2437.7-2891.8)	2550.1 (2325.8-2764.9)	2378.3 (2182.1-2575.1)	2195.5 (2028.5-2359.7)	2023.1 (1888.1-2152.6)	1900.1 (1791.4-2020.7)	1876.4 (1747.2-2011.5)	1737 (1596.2-1885.1)	1598 (1450.4-1753.6)	3974.9 (3812-4142.9)	1337.6 (1176-1504)
JAM Jamaica	1846.8 (1786.5-1908.2)	1452.2 (1425.6-1479.6)	1254.9 (1234.9-1275.9)	1163.6 (1145.1-1182.1)	1004 (988.7-1019.7)	962.5 (946.9-978.5)	818.8 (805.9-832.4)	911.1 (897.7-924.6)	760.5 (733.7-789.2)	805.4 (756.9-857.2)	803.3 (793.6-812.7)	641.5 (630.7-653.3)	705.1 (695.8-716.2)	766.5 (686.3-855.2)
PRI Puerto Rico	1467 (1448.2-1487.6)	1020.8 (1010.9-1030.8)	960 (950.3-970.3)	930.7 (921.1-940.2)	888.1 (878.9-896.5)	837.3 (828.7-846.2)	813.9 (805.5-821.7)	822.7 (815.2-831)	793.4 (786-800.7)	793.4 (786.5-800.5)	664.8 (658.3-671.4)	614.4 (608.2-620.8)	549.7 (543.6-556)	571.5 (547.6-595.7)
LCA Saint Lucia	2095.2 (1986-2223.7)	1724.9 (1666.1-1791.8)	1462.4 (1414.2-1515.3)	1257 (1215.9-1300.8)	1205.6 (1173.1-1242.7)	1204.2 (1178-1229.8)	1124.9 (1104.3-1146.2)	1049.9 (1031.3-1068.7)	1064 (1045.5-1083.2)	971.4 (952.7-990.3)	834.8 (818.7-850.1)	809.9 (794.6-825.8)	734.5 (719.4-750.4)	738.6 (704.5-774.8)
VCT Saint Vincent and the Grenadines	1746.3 (1653.7-1856)	1558.1 (1506.7-1617.6)	1257.3 (1207.9-1306.1)	1186.6 (1144.7-1232.4)	1090.4 (1049.1-1128.4)	1010.8 (981.1-1041.6)	947.4 (922.9-970.1)	918.5 (897.8-940.2)	989.7 (967.7-1011.9)	925.7 (903.7-947.9)	932 (912.8-951.7)	849.2 (832.7-866.2)	807.7 (791-824.3)	844.2 (808.6-881.8)
SUR Suriname	1698 (1593.9-1810.2)	1538.9 (1461.6-1614.9)	1390 (1315.9-1475.4)	1273.4 (1199-1352.6)	1294.2 (1209.9-1375.8)	1303.5 (1234.9-1374.1)	1292.3 (1235.3-1344.3)	1113.3 (1064.5-1162.5)	1046.9 (1003.4-1093.1)	944.3 (904.3-982.8)	971.4 (933.2-1016.9)	957.1 (916.8-1003.6)	870.6 (833.8-908.6)	873.2 (803.9-940.6)
TTO Trinidad and Tobago	1971.2 (1916.7-2036.3)	1679.1 (1649.8-1709)	1432.1 (1409.9-1455)	1439 (1417.4-1459.5)	1378.4 (1359.3-1398.3)	1293.8 (1275.5-1313.8)	1250 (1232.3-1267)	1191.7 (1175.2-1207.4)	1076.6 (1060.9-1091.6)	1080.8 (1066.7-1095.9)	1005.4 (991.3-1019.5)	892.8 (880.1-906.5)	793.4 (780.5-806.8)	765 (658.4-884.4)
VIR Virgin Islands	1541.9 (1482.9-1602.6)	1425.4 (1385.1-1466.3)	1356 (1320.6-1391.5)	1309.4 (1262.1-1352.9)	1232.3 (1169.8-1301.6)	1142.8 (1094.6-1206.1)	1069 (1026.2-1113.6)	988.5 (949.2-1028.1)	929.9 (887.8-984.5)	895.6 (859.6-940.3)	867.3 (823.1-908.9)	830.9 (774.1-884.3)	839.7 (766.5-895.9)	849.8 (752-925.7)
Central Latin America	2027 (1995.3-2061.7)	1719.9 (1697.5-1743.7)	1560.1 (1539.6-1581.9)	1434.3 (1414.6-1455.1)	1343.2 (1325.5-1361.9)	1217.4 (1205.6-1229.1)	1096.3 (1082.8-1109.5)	1038.1 (1034.9-1041.3)	903.1 (900.3-906.1)	868.5 (864.6-872.3)	759.1 (755.6-762.6)	706.7 (702.9-710.5)	679.2 (675.7-683)	652.8 (637.6-667.6)
COL Colombia	1831.1 (1770-1902.9)	1622.3 (1590.9-1657.1)	1505.4 (1481.5-1530.1)	1380.9 (1364.2-1398.8)	1130.4 (1115.1-1145.6)	1184.2 (1171-1199.2)	988.3 (931-1042.9)	1090.4 (1083.6-1098)	868.7 (862.7-875.1)	859.8 (854.3-865.6)	727 (721.6-732.5)	633.1 (628.1-638.5)	560.5 (555.9-565.5)	483.6 (443.3-526.7)
CRI Costa Rica	1720.2 (1690.8-1750.2)	1330.6 (1314.1-1347.8)	1149 (1133.9-1163.8)	1177.5 (1163.7-1192)	1115.1 (1102.2-1127.9)	906.5 (895.4-917.5)	780.6 (771.2-790.5)	759.3 (750.2-768.1)	692.1 (683.8-699.8)	707.5 (699.1-714.9)	613.9 (607.2-620.4)	544.8 (538.9-550.8)	530.6 (524.9-536.4)	537.3 (512.2-563.2)
SLV El Salvador	2195.3 (2061.3-2353.7)	1916.9 (1850.5-1991.4)	1606.5 (1552-1660.9)	1488.2 (1447.6-1531.2)	1496.6 (1465.7-1530.2)	1236.9 (1213-1260.1)	1239.5 (1200-1281.1)	1078.5 (1064.1-1093.6)	934.7 (923.5-945.3)	875.8 (850.6-903.7)	802.1 (777.8-828.2)	832.8 (805.9-862.2)	803.5 (777.1-830.3)	781.9 (686.9-887.6)
GTM Guatemala	2775.6 (2639.3-2940.9)	2501.3 (2437.9-2572.2)	2243.5 (2194-2298.1)	2144.5 (2103.4-2186.2)	2162.1 (2130.6-2194.1)	1835 (1809-1860.8)	1609.5 (1589.1-1631.5)	1502.1 (1487.1-1519.2)	1360.6 (1346.4-1374.8)	1220.8 (1210-1232.8)	1095.3 (1085.4-1105.2)	1015.7 (1006.9-1024.4)	879.4 (871.4-888.6)	829.7 (768.9-898.5)
HND Honduras	2680.1 (2459.2-2912.2)	2411.8 (2277-2558.4)	2206.4 (2081.2-2336.3)	1897 (1792.4-2003.4)	1633.6 (1561.1-1705.9)	1350.9 (1287.9-1416.5)	1023.5 (971-1076.5)	978.5 (916.3-1049.1)	924.5 (856-996.8)	957 (849-1067.2)	917 (796.3-1043.2)	876.1 (749.9-1013.8)	836.2 (718.2-967.9)	793.1 (679.7-902)
MEX Mexico	2055.6 (1999.2-2111.3)	1707.4 (1670-1747)	1563.5 (1525.2-1602.3)	1428 (1393.1-1465.5)	1371.6 (1339.6-1405.7)	1204.4 (1183.7-1225.4)	1116.9 (1108-1126.1)	1013.7 (1010.4-1017.1)	899.4 (896.9-901.9)	859 (856.8-861.2)	754.1 (752.3-755.9)	719.2 (717.5-720.8)	716.7 (715.3-718)	708.2 (700.2-715.5)
MEX Aguascalientes	1625.4 (1432.9-1817.3)	1416.9 (1269.7-1583.2)	1367.9 (1222.4-1522.7)	1321.1 (1183.3-1465.1)	1335.9 (1216.5-1456)	1195 (1128.5-1264)	1068.7 (1045.7-1090.6)	1012.3 (995.5-1030.5)	917.8 (903-932.2)	880.4 (868.9-892.1)	781.1 (770.7-791.8)	756.1 (747.3-765.1)	712.9 (704.2-721.2)	643.5 (614.5-673.9)
MEX Baja California	2675.9 (2376.6-3023.1)	1957.9 (1767.9-2163.8)	1668 (1489.9-1855.7)	1491.5 (1339.4-1671.6)	1463.5 (1316.6-1636.5)	1327.8 (1234.3-1434.4)	1129.2 (1112.8-1145.2)	1179.8 (1165.6-1194)	1086.5 (1075.3-1098.5)	1079.2 (1068.6-1089.4)	994.7 (984.3-1005.2)	895.5 (887.5-904.2)	823 (816.1-829.8)	744.3 (709.2-777.5)
MEX Baja California Sur	1522.1 (1350.4-1715.3)	1340.9 (1191.5-1504.8)	1307.2 (1166-1455.6)	1288.2 (1158.6-1412.3)	1293.9 (1181.6-1403.8)	1191 (1126.9-1256.6)	1029.3 (1003.9-1053.4)	889.9 (874-904.5)	852.7 (840-865.5)	847.2 (835.4-859.8)	772.5 (761.9-783.4)	747.4 (736.9-757.1)	729.5 (720.4-739.1)	678.3 (644.8-710.2)
MEX Campeche	1514.2 (1340.2-1707.8)	1296.1 (1150-1443.4)	1226.6 (1087.4-1379.7)	1144.7 (1018.3-1281)	1119.1 (1015.8-1228)	1004.2 (943.4-1065.4)	953.6 (929.8-977.8)	965.4 (948.4-983.1)	873.6 (859.8-887.5)	824.8 (812.4-836.4)	686.4 (676-697.7)	657.7 (648-667.6)	675.9 (667.3-684.6)	740 (703.8-775)
MEX Chiapas	1621.8 (1430.1-1803.5)	1391.9 (1233.7-1554.8)	1300.3 (1149.1-1454.8)	1204.3 (1063-1352.8)	1148.2 (1014-1272)	1003.2 (930.3-1076.1)	1114 (1089.2-1139.8)	1049.9 (1029-1070.9)	906.6 (891.7-923.1)	868 (855.3-881)	799.8 (790.6-810.1)	751.3 (743.3-761.3)	740.1 (733.6-747.4)	762.9 (728.4-796.9)
MEX Chihuahua	2230.8 (2003.2-2477.3)	1826.6 (1642.1-2014.3)	1646.9 (1477.3-1840.4)	1499.3 (1325.9-1682.6)	1431.3 (1273.2-1596.3)	1253.5 (1164.3-1340.7)	1166.8 (1146.3-1188.6)	1134.9 (1120.1-1150.5)	1089.6 (1078-1101.1)	1045.6 (1034.8-1056)	963.5 (953.1-974.3)	882.9 (874.5-891.4)	1027.4 (1019.7-1034.7)	849 (807.5-889.2)
MEX Coahuila	1968.5 (1762.7-2176.7)	1679 (1491.5-1871)	1579.8 (1401.2-1772)	1492.6 (1324-1666.2)	1491.1 (1343.2-1661.2)	1328.7 (1237.4-1429.7)	1200.6 (1178.4-1223.1)	1100.1 (1084.9-1117.2)	998.5 (986.7-1011.4)	940.3 (929.7-950.9)	834 (825.2-843.8)	789.2 (780.6-798)	779.8 (772-787.2)	717.1 (682-754.2)
MEX Colima	2358.1 (2098.9-2660.2)	1973 (1789.6-2184.2)	1834.6 (1664.9-2011.9)	1720.2 (1559.3-1883.6)	1714.7 (1593.8-1841.6)	1523 (1455.9-1591.5)	1329.1 (1299.1-1360.6)	1118.9 (1101.3-1138.1)	1071.8 (1057.2-1086.4)	949.9 (934.1-965.9)	783.9 (772.7-795)	741.9 (731.6-752.7)	743 (733.7-752.1)	777 (744.1-816)
MEX Mexico City	2168 (1893.6-2469.3)	1670.6 (1505.1-1844.5)	1499.9 (1345.5-1657.6)	1346.1 (1209.3-1489.9)	1257.9 (1132.4-1386)	1100.9 (1034.8-1166.6)	1073.7 (1058.1-1089.5)	968.7 (958.9-978.7)	868.3 (860.7-875.3)	858 (850.5-865.9)	761.6 (755.9-767.1)	748.7 (743-754.1)	722.1 (716.4-727.8)	731.6 (697.4-763)
MEX Durango	2137.5 (1911.7-2371.3)	1778.6 (1605.9-1972.7)	1624 (1463-1807)	1474.2 (1321.2-1645.8)	1405.6 (1270.5-1537.3)	1227.9 (1146.4-1303.8)	1186.2 (1162.1-1211.8)	1067.2 (1052.7-1084.1)	903.6 (889.5-917.3)	789.6 (778.7-800.8)	676.2 (665.8-687.2)	659 (650.4-667.8)	781.6 (772.7-790.3)	616.3 (586.1-645)
MEX Guanajuato	2120.1 (1917.1-2351.1)	1802 (1629.8-1989)	1680.3 (1504.5-1852.2)	1576.6 (1425.3-1738.8)	1554.4 (1409-1720.5)	1361.1 (1267.7-1457.4)	1141.1 (1114.7-1169.2)	1161.3 (1144.2-1178.2)	1027.7 (1015.3-1040.5)	988.1 (977.4-999)	819.9 (810.4-830.5)	722.4 (715.5-729.4)	675.4 (669.5-681.3)	704.8 (672.4-735.9)
MEX Guerrero	2029.4 (1802.4-2279.9)	1699.8 (1523.5-1888.7)	1536.3 (1362.5-1716.2)	1385.3 (1235.5-1541.9)	1307.6 (1180.6-1450.8)	1086.7 (1012-1166)	1006.3 (981.5-1033.1)	812.1 (788.7-837.6)	676.1 (659.5-692.7)	679.3 (664.6-696.1)	570 (559.2-582.4)	537.8 (529.1-546.5)	625 (617.5-633.4)	632 (599.4-659.5)
MEX Hidalgo	2086 (1877.6-2319.8)	1793.6 (1615.5-1984.2)	1677.3 (1519.3-1839.9)	1538.5 (1381.3-1704.8)	1503.8 (1354.8-1662.5)	1339.4 (1251-1436)	1341.8 (1316.7-1369.5)	1039.2 (1024.1-1055.7)	852.2 (838.3-867.2)	754.6 (744.3-766.1)	645.5 (635.2-655.9)	627.7 (620.9-634.8)	647.5 (640.7-654.2)	608.4 (577-638.7)
MEX Jalisco	2091.4 (1871.4-2329.9)	1735.3 (1555.3-1921.5)	1576.2 (1411.8-1747.4)	1420.6 (1276.3-1579.4)	1348.1 (1216.4-1485.6)	1150.2 (1074.7-1227)	1021.2 (1004.3-1039.2)	965.3 (951.3-978.3)	930 (919.7-941.1)	914.5 (906.5-922.5)	797.7 (789.4-806.6)	749 (742.6-755.6)	724.5 (719-729.9)	742.8 (708.7-776.3)
MEX México	1675.9 (1483.6-1875.6)	1492.8 (1333.9-1659.5)	1455 (1289.8-1615.9)	1385.5 (1236.4-1548.5)	1378.6 (1222.9-1542.2)	1242.5 (1109.1-1384.5)	1218.1 (1129.3-1314.5)	1175.9 (1165.4-1187.1)	1006.8 (998.6-1014)	935.5 (928.5-943.2)	815.9 (810.5-821.4)	764.1 (758.4-769.7)	716.7 (712.4-721.8)	746.8 (715.8-778.5)
MEX Michoacán de Ocampo	2227.8 (1991.6-2483.5)	1854.3 (1679.5-2045.3)	1689.6 (1512.8-1878.4)	1522.9 (1373.9-1694.2)	1426.3 (1288.9-1579.4)	1260.3 (1173.9-1346.9)	1150 (1124.1-1178)	1010.1 (993.5-1027.3)	825.1 (814.6-835.6)	821.7 (812-831.7)	703.5 (695.3-712.9)	663.5 (655.5-672.6)	671.7 (664.9-679.1)	629.8 (599.7-658.5)
MEX Morelos	1901.7 (1697.4-2112.7)	1600.1 (1427.8-1779.6)	1482.5 (1323.3-1650.9)	1351.5 (1212.7-1510.2)	1297 (1176.2-1443)	1116 (1036.1-1201.1)	1056.3 (1034.6-1079.6)	926.9 (911.4-943.5)	865.3 (851.8-880)	825.9 (815.7-836.8)	704.2 (694.5-713.7)	681.6 (673.2-690.5)	681.5 (674.1-688.9)	761.8 (723.5-798.9)
MEX Nayarit	2140 (1908.4-2381.1)	1762.3 (1599-1942.6)	1593.3 (1427-1758.2)	1432 (1277.2-1596.2)	1336.1 (1212-1462.9)	1117.3 (1044.9-1193.3)	1018.4 (996.8-1041.9)	973.8 (957.9-989.6)	862.1 (849.3-875.7)	771.3 (760.3-783.2)	647.1 (636.5-657.1)	657.6 (648.4-667.2)	673.5 (665.3-681.6)	619.5 (587.8-650.4)
MEX Nuevo León	1721.7 (1504-1938.3)	1442.3 (1280.7-1617.9)	1326.7 (1173.2-1487.9)	1230.9 (1089-1380.1)	1221.2 (1081.8-1366.8)	1098.7 (1020-1185.8)	941.9 (926.5-957.1)	873.1 (860.4-886.1)	865.7 (855.6-876)	841.6 (833-849.8)	756 (748-764.7)	732 (725.4-739)	717.1 (711.9-722.5)	657.2 (624.3-689.2)

Table 1 Age-standardised mortality rates by location and year for both sexes for 1950, 1955...2005, 2010, 2017

Location	1950	1955	1960	1965	1970	1975	1980	1985	1990	1995	2000	2005	2010	2017
MEX Oaxaca	2472.9 (2193.1-2796.7)	2067.7 (1873.1-2288.6)	1881.4 (1697.3-2083.3)	1735.8 (1565.2-1919.3)	1704.1 (1550.3-1876.7)	1542.6 (1449.3-1645.6)	1423.8 (1398.6-1447.9)	1199.7 (1184.1-1216.3)	974.1 (961.6-988.8)	884.7 (874.6-895.2)	717.6 (709.9-726.4)	668.2 (660.1-676.6)	657.3 (650.6-664.5)	666.5 (634.4-696)
MEX Puebla	2453.3 (2202.9-2702.2)	2066.3 (1879-2257.9)	1898.7 (1712-2085.8)	1739.9 (1562.3-1918.1)	1678.5 (1522.5-1845)	1458.9 (1365.6-1553)	1319 (1291.5-1348.4)	1161.3 (1139.7-1183.7)	1000 (986.7-1013.5)	938.9 (927.9-951.3)	792.7 (783.5-803.2)	744.9 (735.5-755.6)	719.4 (711.8-727.5)	769 (734.1-802.5)
MEX Querétaro	1855.1 (1623.5-2095.9)	1610 (1439.3-1795.5)	1533.5 (1376.2-1713.3)	1452.3 (1300.6-1621.2)	1451.7 (1310.1-1616.6)	1308.5 (1188.4-1439.4)	1266.6 (1197.5-1335)	1058.8 (1043.8-1074.5)	969.1 (957.7-980.6)	934.8 (924.3-944.8)	820.7 (811.8-829.9)	737.8 (729.5-745.7)	697.1 (690.1-704.4)	722 (686.2-754.4)
MEX Quintana Roo	871 (755.2-1009)	862.9 (754.3-984.5)	874.2 (765.9-990.3)	959.7 (846.6-1083.6)	1100.4 (990.9-1214.2)	1092.2 (1023.8-1166.6)	953.2 (928.7-979.7)	740.1 (723.3-757.7)	780.3 (766-795.9)	803.1 (790.9-815.4)	736.7 (726.3-747.9)	715.6 (706.2-725)	743.4 (735-751.2)	828.9 (791.9-864.8)
MEX San Luis Potosí	2121.7 (1886.3-2391.6)	1762.1 (1580.9-1953.6)	1612.1 (1431.2-1783.5)	1491.1 (1329.7-1652.8)	1452.5 (1308.1-1607.5)	1292.3 (1201.9-1386)	1036.8 (1014.3-1060.7)	969.2 (954.7-983.8)	824.7 (813.2-835.9)	786.7 (776.4-798.2)	665.5 (656.5-675.5)	639.3 (630.1-649.3)	648.2 (641-657.7)	624.3 (596.8-654.8)
MEX Sinaloa	1755 (1567-1954.8)	1501.1 (1342.9-1675.3)	1403.6 (1246.9-1575.4)	1295 (1146.3-1450.2)	1254.8 (1126.7-1399.6)	1071.5 (995.5-1155.3)	989.5 (969.6-1010.4)	942.1 (928.9-955.1)	811.4 (801.3-823.4)	792 (783.2-800.8)	693.2 (685.1-702.1)	676.3 (669.2-684)	711.1 (704.7-718)	568.3 (540.5-598.8)
MEX Sonora	1925.4 (1720.2-2159.8)	1626.7 (1453.4-1810.1)	1521.2 (1355.6-1688.8)	1426.6 (1259-1593.8)	1419.6 (1249.4-1596.3)	1279.2 (1131.7-1438.1)	1222.3 (1134.1-1317.2)	1111.9 (1098.6-1126.6)	1025.7 (1014.7-1036.6)	1026 (1015-1038.2)	868.5 (858.3-879.7)	846.4 (837.3-857.2)	814.1 (806.8-821.8)	774.3 (736.8-812)
MEX Tabasco	1852.8 (1638.2-2095.4)	1610.9 (1439-1788.2)	1519.8 (1368-1690.9)	1434.7 (1293.6-1594.5)	1417.5 (1280.3-1570.2)	1302.2 (1219.3-1385.8)	1276.9 (1248.5-1307.4)	1008 (989.3-1027.6)	873.9 (860.3-888.6)	902.4 (890.7-915.6)	760.8 (749.7-772.5)	728.5 (721-736.4)	749.1 (741.7-757.5)	754.9 (719-790.3)
MEX Tamaulipas	1998.1 (1755.1-2258.5)	1654.9 (1493.4-1844.6)	1508.5 (1344-1695)	1358.4 (1206.3-1512.9)	1280.7 (1143.8-1425.9)	1120.3 (1039.7-1199.1)	1110.2 (1091.3-1131.2)	961 (946.8-975.4)	842.6 (831.7-854.3)	805.9 (793.5-819.4)	733.3 (724.7-742.8)	708 (701.4-714.7)	717.6 (710.9-724.6)	647.6 (617.1-680.2)
MEX Tlaxcala	1983.7 (1748.6-2235.3)	1683.6 (1496.2-1886)	1564.5 (1390.6-1744.7)	1458.4 (1305.1-1622.9)	1389.8 (1252.7-1532.4)	1220.7 (1111.7-1334.1)	1137.3 (1070.6-1210.2)	1006.5 (986.5-1027.2)	869.2 (857.3-881.9)	832.8 (821.9-844.7)	729.9 (720.7-739.6)	663.7 (655.6-672.8)	661.4 (653.3-669.8)	674.1 (641.8-705.7)
MEX Veracruz de Ignacio de la Llave	1963 (1755.1-2174.8)	1614 (1451.2-1792.6)	1451.6 (1300.7-1614.5)	1295.2 (1151.6-1438.6)	1224.1 (1095.3-1362.9)	1070.6 (1002-1145.5)	984.3 (965.5-1003)	880.6 (867.7-894)	823.6 (812.4-835.4)	786.4 (778.3-795)	716.6 (707.8-726.3)	703.9 (697.5-710.1)	704.4 (699.4-709.3)	737.2 (703.7-771.1)
MEX Yucatán	1697.5 (1506.1-1910)	1472.4 (1302.1-1655.6)	1418 (1265.8-1590.3)	1368.8 (1211.9-1527.5)	1380 (1240.2-1526)	1284.4 (1199.9-1371.1)	1225.4 (1203.7-1247.9)	1150.9 (1134.6-1168.3)	1011.8 (998.7-1025.5)	971.2 (960.8-981.6)	800.1 (790.3-810.1)	748.4 (738.8-757.7)	764.2 (756.1-772.5)	806.3 (771.4-844.4)
MEX Zacatecas	2005 (1785.8-2259.5)	1634.8 (1464.8-1817.2)	1466.7 (1321.2-1635)	1322.5 (1189.5-1465.2)	1266.3 (1148.1-1396.2)	1109.3 (1037.9-1183.9)	968.5 (943.2-994)	829.1 (812.5-847)	748.3 (733.7-763)	753.7 (742.4-765.6)	663.3 (652.4-674.8)	639.9 (631.3-648.2)	655.4 (647.5-663.6)	642.3 (611.3-673.2)
NIC Nicaragua	2112.7 (1978.7-2275.7)	1721.6 (1642.6-1801.1)	1681.7 (1617.9-1752.5)	1494.6 (1430.5-1558.3)	1511.8 (1413.5-1607.6)	1259.5 (1204.7-1313.8)	1139.1 (1048.5-1243.4)	1212.2 (1132.1-1304.3)	785 (750.4-824)	769.2 (725.6-814.3)	687.2 (654.7-721.6)	674.7 (642.6-710)	583.8 (566.7-602.9)	533 (492.2-581.3)
PAN Panama	1390.7 (1342.9-1444.5)	1285.8 (1259.4-1312.6)	1116.7 (1095.4-1138.5)	1077.5 (1057.8-1097.9)	1064.6 (1047.3-1081.8)	888.8 (872.9-904.5)	773.6 (760.6-787.7)	745.4 (733.2-758)	690.1 (678.5-701.6)	681.4 (671-692)	605.2 (596.1-614.3)	572.1 (564.1-581.2)	561.7 (553.7-569.8)	522.9 (497.8-548)
VEN Venezuela	1849.6 (1830.1-1873.2)	1517.2 (1508.9-1525.1)	1297.4 (1290.7-1304.8)	1233.3 (1227.1-1239.9)	1202.7 (1196.9-1208.6)	1156.6 (1151.1-1161.9)	1038.4 (1033.5-1043.3)	933.5 (929.1-937.8)	875.6 (871.9-879.5)	855.4 (840.5-870.6)	752.6 (749.3-756)	683.3 (680.2-686.6)	683.4 (680.7-686.2)	699.3 (624.5-789.7)
Tropical Latin America	2062 (1990.7-2134.8)	1913.6 (1852.3-1979.4)	1792.9 (1734.2-1854)	1663.2 (1607.6-1719.3)	1534 (1485.5-1581.4)	1382 (1354.1-1410.6)	1258.3 (1249.3-1267.7)	1173.6 (1167.7-1179.4)	1058.7 (1053.9-1063.1)	979.3 (974.9-983.7)	859.7 (855.8-863.7)	779.7 (776.2-783.3)	733.4 (730.2-736.4)	690.9 (686.2-695.5)
BRA Brazil	2082.1 (2007.9-2156.9)	1939.2 (1876-2005.8)	1816.1 (1755.8-1879.6)	1684.5 (1627.2-1742.5)	1549.7 (1500-1598.4)	1392.7 (1363.4-1422.3)	1267.4 (1258-1277.3)	1181.2 (1175.2-1187.2)	1066.2 (1061.2-1070.6)	984.7 (980.4-989.1)	863 (859.1-867.1)	780.5 (777-784.2)	733.2 (730.1-736.2)	691.3 (687.1-695.5)
BRA Acre	2113.5 (1858-2382.7)	1982.9 (1765.8-2209.9)	1826.7 (1636.8-2028.5)	1683.2 (1513.7-1872.2)	1522 (1395.7-1656.1)	1359.1 (1280.1-1442.8)	1215.6 (1179.7-1258.1)	1213.6 (1189.5-1240.5)	1037.2 (1014.3-1061)	953.9 (932.6-978.5)	837.5 (822.1-854.2)	764.7 (752.1-779.2)	705 (692.9-717.5)	681.9 (662.2-702.1)
BRA Alagoas	2383.2 (2112.7-2660.3)	2265.5 (2043.8-2492.8)	2163.4 (1962-2383.3)	2028.4 (1840.1-2234.2)	1898.3 (1725.2-2067.4)	1717.3 (1604.4-1832.3)	1543.3 (1499.9-1591.6)	1372.4 (1339.3-1410.1)	1125.8 (1099.8-1154)	995.8 (971.1-1020.5)	867.9 (842.8-894.6)	795.9 (774.9-817.5)	771.3 (760.3-783.6)	745.7 (725.4-765.3)
BRA Amapá	2214.2 (1933.7-2509.8)	1931.6 (1721.1-2144.8)	1706 (1535.6-1885)	1524.6 (1387.4-1678.3)	1337 (1231.9-1444.8)	1132.8 (1072.3-1192.2)	946 (922.3-969.5)	923.5 (907.4-940)	849.1 (835.1-864.1)	918.5 (905.7-931)	895.1 (884.2-906.2)	727.3 (717.5-736.6)	633.5 (624-644)	669.9 (653.9-685.9)
BRA Amazonas	1758.6 (1537.7-1999)	1641.1 (1452.4-1852)	1545.6 (1371.5-1750.3)	1459 (1299.8-1647)	1398.7 (1255.9-1556.2)	1308.5 (1191.6-1426.6)	1093.8 (1054.2-1134.5)	976.3 (947.8-1007.6)	873.4 (844.8-904.3)	752.5 (731-775.8)	730 (722.2-738.4)	666.4 (659.7-673.6)	671.5 (663.6-679.7)	659.6 (642.7-677.1)
BRA Bahia	2079.6 (1845.4-2324.8)	1920.3 (1725.6-2120.8)	1785.2 (1606.7-1966.1)	1632.9 (1467.2-1807.5)	1472.7 (1321.5-1622)	1293.3 (1179.4-1404.6)	1078.9 (1034-1126.3)	1032.7 (993.1-1072.9)	901.2 (864.9-938.7)	835.2 (802-871.2)	785.4 (750.6-821.6)	738.1 (704.1-770.5)	701.8 (671.5-733.4)	666.3 (651.4-682.3)
BRA Ceará	2355.9 (2098.2-2641.5)	2207.8 (2003.9-2426.3)	2041.4 (1848.3-2242.3)	1869.6 (1688.2-2059.3)	1693.9 (1521.8-1874.3)	1513.5 (1370-1666.5)	1262.6 (1153.3-1379.8)	1033.1 (977.3-1091.1)	838 (806.1-872)	797.5 (767.1-828.2)	751.5 (721.8-782.1)	704.5 (677.3-732)	624.1 (612.2-637.2)	601.4 (586.1-615.3)
BRA Distrito Federal	2043.8 (1805.8-2295.2)	1909.7 (1714.8-2099.1)	1747.6 (1555.2-1943.3)	1524.2 (1345-1715.9)	1361.6 (1209.2-1521.7)	1269.8 (1168.4-1366.7)	1153.6 (1139.7-1166.9)	1063.2 (1052.1-1073.6)	1092.1 (1080.1-1103.3)	1152.1 (1142.7-1161.6)	1025.8 (1017.5-1034.7)	945.3 (937-954)	840 (832.1-847.7)	710.6 (692.8-729.8)
BRA Espírito Santo	1890.1 (1672.3-2112.9)	1793.7 (1602.4-2004.9)	1734.1 (1549.8-1929.4)	1655.6 (1483.1-1842.5)	1583.7 (1422-1754.6)	1453.1 (1354.6-1555.9)	1282.8 (1268.8-1297.3)	1290.8 (1279.5-1302.3)	1147.9 (1137.4-1158.2)	1019.4 (1011-1028.3)	862.5 (855.1-869.8)	786.7 (778.8-795.6)	736.4 (729.2-743.5)	635.6 (619.3-651.7)
BRA Goiás	2410.9 (2146.3-2694.3)	2284.1 (2056.6-2539.3)	2154.4 (1953.9-2380.3)	2013.1 (1820.2-2235.3)	1842.9 (1651.5-2046.9)	1591.5 (1428.1-1772.8)	1342.8 (1267.6-1425.5)	1328 (1315.5-1341.4)	1078.2 (1063.1-1094.4)	1053.4 (1044.6-1062.7)	874.9 (868.8-881.2)	767.4 (761.7-772.8)	736.3 (731.1-741.5)	681.3 (667.1-695.9)
BRA Maranhão	2789.7 (2506.1-3090.2)	2501.1 (2277.6-2727.1)	2306.9 (2084.1-2525.2)	2134.6 (1935.3-2337)	1961.4 (1771.6-2166.6)	1756 (1571.7-1962.9)	1495.6 (1360.1-1633.2)	1271.3 (1197.9-1342.7)	975.9 (935.5-1017.8)	811 (778.2-845.5)	714.4 (684.1-747.3)	720.4 (690.7-751)	663 (632.5-691.7)	632.7 (616.9-648.2)
BRA Mato Grosso	2117.7 (1840.1-2417.1)	1991.8 (1777.3-2228.3)	1844.4 (1638.7-2047.9)	1662.9 (1482.5-1855)	1439.9 (1290.4-1597.8)	1169.2 (1059.2-1288.6)	947.2 (890.7-1007.2)	1045.3 (993.1-1098.2)	923.1 (879.4-971)	849.5 (835.7-863)	827 (819.6-834.7)	796.2 (788.8-803.2)	742.3 (735.1-749.8)	668.1 (650.4-685.5)
BRA Mato Grosso do Sul	1600.1 (1401-1807.5)	1552.7 (1361.2-1750.8)	1497.3 (1310.5-1693.6)	1412.4 (1246.5-1600.8)	1345.6 (1181.4-1519.8)	1252.5 (1146.7-1360.7)	1170.9 (1152.6-1187.3)	1095.9 (1084.1-1106.9)	1027.3 (1016.5-1038.6)	1025.8 (1015.7-1037.4)	890 (881.4-899)	839.2 (831.7-846.5)	776.8 (769.4-784.4)	719.9 (701.6-738.7)
BRA Minas Gerais	2027.1 (1801.3-2264.3)	1913.7 (1697.8-2138.5)	1819.3 (1616.4-2046.7)	1717.6 (1526.1-1927.7)	1606.2 (1435.5-1785.6)	1458.3 (1363.7-1561.1)	1296.2 (1282.1-1311.9)	1234.9 (1223.3-1247.8)	1062.7 (1052.3-1073.9)	936 (927-945.7)	779.8 (770.4-790)	726.6 (717.4-736.8)	686.1 (677.7-695.8)	635.3 (622.1-649.5)
BRA Pará	1736 (1535.5-1956.3)	1631.2 (1452.2-1819.8)	1549.6 (1372.6-1735.7)	1474.1 (1311.2-1638.2)	1396.8 (1249-1547.1)	1255.3 (1161.9-1355.2)	1052.2 (1026.6-1080.3)	1026.2 (993.8-1058.2)	922.2 (892.8-954.2)	811.1 (785.8-837.1)	738.2 (714.4-763.1)	711.8 (688.4-734.1)	678.1 (670.1-687.2)	669.6 (653.4-684.4)
BRA Paraíba	2178.3 (1925.7-2443.8)	2007.4 (1811-2221.3)	1847.7 (1655.7-2042.3)	1658.7 (1479.1-1852.9)	1489.1 (1339.1-1665)	1316 (1222.2-1418.7)	1131.2 (1102.6-1160)	982 (960.7-1005)	884.7 (855.7-912.4)	822.8 (798.2-847.8)	735.9 (713.1-760.8)	721 (696.6-744)	717.9 (695.1-741.9)	671.2 (630.4-711.1)
BRA Paraná	2379 (2065-2682)	2177.2 (1950.1-2417.6)	2005.9 (1781.4-2247.4)	1836.3 (1626.3-2053.9)	1657.5 (1485.9-1845)	1490.9 (1389-1599.7)	1507.1 (1494.9-1519.6)	1330.4 (1322.1-1339.2)	1203.9 (1195-1212.2)	1095.4 (1088.5-1103)	939.1 (933.5-944.5)	838.7 (831.6-846.6)	795.8 (789.7-803.4)	736.4 (721.1-753.4)
BRA Pernambuco	2769.8 (2486.5-3062.8)	2543.9 (2310.3-2786.2)	2338.3 (2111.4-2582.6)	2122.2 (1921-2342.1)	1904.1 (1719.3-2081.7)	1623.7 (1524.6-1728.6)	1385.9 (1357.8-1415)	1333.9 (1311.6-1359.8)	1221.2 (1203.6-1239.9)	1029.4 (1015.4-1044.2)	940.6 (926.8-955.5)	826.2 (814.3-837.8)	761.8 (750.4-774.7)	795.9 (779.1-815.4)
BRA Piauí	1904.3 (1680.5-2142.2)	1799.1 (1607.4-1991.5)	1711.8 (1533.5-1902.1)	1622.8 (1444.9-1800.6)	1531.6 (1364.1-1710.4)	1363 (1223.4-1523.1)	1168.9 (1093.6-1250)	1156.2 (1118.5-1196.9)	848.7 (818.8-881.2)	698.1 (668.9-727.4)	726 (699.3-753.3)	713.7 (688.4-738.1)	619.1 (606.4-633.8)	597.7 (581.1-614.1)
BRA Rio de Janeiro	2030.5 (1795.9-2280.3)	1865 (1652-2086.7)	1733.9 (1539.7-1930.5)	1605.8 (1420.4-1801.5)	1525.6 (1358.4-1702.4)	1415.4 (1329.1-1509.3)	1306.3 (1294.9-1317.9)	1310.5 (1302.4-1319.7)	1288.7 (1281.1-1296.1)	1220.1 (1213.8-1226.3)	989 (984.4-993.7)	885.8 (881.5-890.1)	836.2 (831.7-841.3)	793.8 (779.9-808.2)
BRA Rio Grande do Norte	2074.8 (1832.3-2333.6)	1926.5 (1730.5-2138.5)	1779.5 (1601.9-1975.4)	1605.6 (1444.2-1782.4)	1456.5 (1305.5-1618.4)	1272.6 (1162.2-1389.8)	1049.7 (999.2-1104.5)	914.5 (881-948.6)	807.7 (774-840.9)	814.3 (781.4-848)	692 (663.1-722.7)	626.6 (601.7-650.3)	590.3 (578.8-602.7)	640.6 (624.5-657.2)
BRA Rio Grande do Sul	1823.6 (1614.6-2042.9)	1734.9 (1536.5-1947.1)	1648.1 (1451.4-1837.9)	1553.7 (1369.9-1739.2)	1426.5 (1279.8-1589)	1271 (1191-1362.2)	1234.6 (1225.2-1244)	1134.8 (1127.7-1142)	1090.9 (1084.5-1098)	1050.9 (1045.4-1057)	897.6 (892.8-902.5)	801 (796.6-805.6)	749.5 (745-754.3)	717.7 (703.6-732)
BRA Rondônia	2161.3 (1928.6-2398.2)	2106 (1870.9-2360.9)	2012 (1793-2231.5)	1972.2 (1764.6-2185.2)	1907 (1714.3-2116.9)	1718.7 (1565.4-1880.9)	1499.2 (1409.9-1584)	1401.7 (1329.8-1471.8)	1175.6 (1114.7-1232.4)	933.5 (885.1-981.8)	890.7 (846.4-940.2)	871.7 (824.4-919.6)	762 (721.7-804.2)	714.8 (656.6-778.3)
BRA Roraima	2179.4 (1942.1-2405)	2092.7 (1872.9-2313.5)	2008.2 (1805-2233)	1927.6 (1736.6-2142.7)	1841.9 (1670-2034.3)	1647.9 (1509.1-1805.7)	1415.3 (1306.5-1516)	1377.8 (1288.6-1464.1)	1211.6 (1138.3-1290.1)	1136.8 (1069.3-1207.9)	1105.7 (1038.7-1170.7)	1044.5 (982.3-1108.5)	888 (830-943.7)	790.3 (725-862.8)
BRA Santa Catarina	1681.2 (1469.8-1904.8)	1606.1 (1415.6-1801.9)	1549.4 (1374.6-1747.2)	1467.8 (1303.5-1660.6)	1358.2 (1205.3-1523.5)	1205.3 (1118.8-1295.6)	1181.3 (1165.1-1198.6)	1140.5 (1128.6-1153.4)	1108.1 (1097.7-1119.4)	1080.1 (1070.5-1090.5)	907.5 (899.2-916.3)	776.9 (769.8-784.1)	725.8 (719.2-732.6)	667.8 (652.3-683.6)
BRA São Paulo	1801.5 (1597.6-2039.9)	1711.3 (1521.2-1924.4)	1628.3 (1426.2-1827.1)	1526.7 (1345.8-1711)	1415.3 (1268-1580)	1301.3 (1221.9-1394.2)	1292.7 (1283.5-1302.1)	1213.4 (1207.6-1219.3)	1170.3 (1163.6-1177.4)	1112.6 (1106.9-1118.8)	954.3 (950.5-958.2)	819.9 (816.9-823)	761.7 (757.8-766)	699.4 (687.3-711.4)
BRA Sergipe	1844.1 (1601.6-2100.4)	1741.5 (1539.3-1943.6)	1662.4 (1482.3-1850)	1561.9 (1404.4-1725.8)	1440.1 (1306.5-1583.4)	1292.6 (1203.4-1388.1)	1210 (1176.6-1244.5)	1038 (1014.8-1063.6)	956.6 (939-974.1)	959 (944.7-974.8)	826.5 (811.9-843)	733.7 (721.6-746.5)	709 (698.9-720.7)	705.9 (685.7-727.2)
BRA Tocantins	2514.3 (2143.7-2926.4)	2406.8 (2143.6-2688.3)	2283.4 (2039.3-2543.5)	2175.1 (1946.8-2430.5)	1978.2 (1789.6-2198.4)	1724.6 (1565.8-1900.7)	1415.5 (1278.5-1557.7)	1212.5 (1114-1317.6)	1078.1 (1035.6-1125.5)	1044.8 (1018.7-1071.6)	928.9 (905.3-953.1)	746.7 (726.9-768)	658.3 (640.6-677.8)	630.2 (599.2-661)
PRY Paraguay	1345.1 (1238.9-1457.9)	1091.2 (1042.2-1145.4)	1057.9 (1014.7-1106.1)	980.8 (941.8-1023.9)	994.4 (954.5-1034.3)	983.5 (945.4-1021.9)	917.9 (879.5-955.7)	892.4 (856.3-931.2)	780.5 (749.2-813.1)	783.6 (749.3-820.1)	739.9 (708.6-773.5)	750.2 (715.9-783.7)	751.5 (715.8-787.3)	690.1 (607.7-788.5)
North Africa and Middle East	2571.8 (2492.8-2652.7)	2260.1 (2209.5-2310.9)	2052.5 (2006.6-2099.5)	1829.2 (1786.4-1874.8)	1704.9 (1668.9-1742.5)	1530.7 (1500.9-1561.1)	1397.7 (1376.4-1419.4)	1314.7 (1296.5-1333.9)	1157.8 (1140.9-1175.4)	1080.8 (1063.8-1098.4)	976 (960.3-992)	897 (882.6-912.5)	837.1 (823.2-852.2)	766.8 (747.8-786.5)
North Africa and Middle East	2571.8 (2492.8-2652.7)	2260.1 (2209.5-2310.9)	2052.5 (2006.6-2099.5)	1829.2 (1786.4-1874.8)	1704.9 (1668.9-1742.5)	1530.7 (1500.9-1561.1)	1397.7 (1376.4-1419.4)	1314.7 (1296.5-1333.9)	1157.8 (1140.9-1175.4)	1080.8 (1063.8-1098.4)	976 (960.3-992)	897 (882.6-912.5)	837.1 (823.2-852.2)	766.8 (747.8-786.5)
AFG Afghanistan	3630.2 (3315.5-3971.2)	3292.4 (3016-3558.7)	3097 (2832.5-3341.3)	2936.8 (2691.3-3163.5)	2802.8 (2595.8-3013.6)	2694.9 (2509.5-2882.8)	3000.9 (2813.6-3205.2)	3105.9 (2925.9-3296.7)	2270.5 (2081.1-2464.7)	2147.7 (1962.4-2333.5)	2095.2 (1918.8-2285)	1894.1 (1727.9-2073.3)	1717.8 (1574.2-1873.6)	1495.5 (1360.1-1628.6)
DZA Algeria	1712.8 (1645.8-1771.6)	1702.9 (1657.4-1747.4)	1612.6 (1553.6-1679)	1331.9 (1285.6-1387.3)	1446.4 (1378.8-1517.6)	1530.5 (1460.2-1607.3)	1502.3 (1460-1545.7)	1053.7 (1026.2-1081.2)	894.5 (854-934.2)	834.2 (795.6-872.8)	788.4 (753.9-825.1)	736.8 (700.6-772.5)	681.4 (659.8-702.3)	606.6 (584.4-630.8)
BHR Bahrain	2360.2 (2073.7-2634.8)	2234.8 (1993.7-2474.2)	1933.4 (1728.9-2140)	1723.2 (1547.7-1911.7)	1527.9 (1387.1-1681.6)	1323.3 (1228.3-1430.7)	1127.9 (1075.2-1182.3)	1135.5 (1099.5-1171.8)	1193.6 (1158-1229.5)	1200.6 (1163.7-1235.6)	1018.6 (991.3-1050.1)	873.6 (849.7-899)	649.6 (630.2-670.8)	556.5 (521.6-594.5)
EGY Egypt	2756 (2508.2-3060.9)	2243.1 (2131.8-2367.7)	2006.8 (1937.8-2083.1)	1789.8 (1732.9-1845.5)	1900.9 (1853.4-1951.7)	1761.7 (1725.2-1802)	1590 (1561.2-1619.3)	1638.4 (1617.1-1659.5)	1381.9 (1366.4-1398)	1293.2 (1279.1-1308.4)	1174 (1166.9-1181.2)	1188.3 (1178.6-1201.3)	1171.3 (1165.3-1178.6)	1116.3 (1033.5-1209.1)
IRN Iran	2869.4 (2786.9-2972.7)	2544.9 (2488.5-2611.9)	2261.2 (2206.1-2313.9)	1999.8 (1952.4-2046.8)	1649.8 (1608-1689.2)	1269.6 (1234.6-1303.2)	1064.1 (1036.7-1091)	1013.7 (987.4-1039.1)	1010.9 (988.1-1032.1)	837.8 (822.3-853.9)	754 (745.8-761.8)	743.1 (737.5-748.2)	720.6 (717.4-724)	671.4 (668.1-674.5)
IRQ Iraq	2140 (1956.9-2347.8)	1782.2 (1671.2-1890.3)	1562 (1489-1639.9)	1523.3 (1465.4-1592)	1467.3 (1411.9-1525.4)	1443.3 (1382-1504.7)	1390.2 (1311-1471.3)	1447.6 (1345.3-1554.7)	1290.2 (1183.4-1401.9)	1332.4 (1229.5-1437.5)	1246.5 (1157.9-1336.5)	1014.7 (938.1-1095.1)	768.2 (717.2-817.4)	587.1 (560.5-615.9)
JOR Jordan	2346.1 (2100.3-2594.2)	1894.2 (1721.7-2076.6)	1557.3 (1424.7-1696.8)	1282.9 (1202.6-1371)	1120 (1076.8-1169)	993.5 (959.7-1028.2)	987.4 (945.7-1030)	928 (870.4-987.9)	897.2 (829.8-970.7)	911.9 (846.3-985.5)	862.1 (808.6-920)	770.6 (740-802.7)	642 (622.3-664.9)	579.6 (531.8-633.2)
KWT Kuwait	1416.5 (1233.2-1609.6)	1227.8 (1098.3-1358.3)	1121.3 (1045.4-1199.8)	1048.6 (1032.1-1065.4)	915.7 (905.2-926.7)	901.9 (892.2-912)	797.8 (789.5-806.3)	650.6 (644-657.3)	657 (649.1-665.4)	498.2 (492.6-503.5)	511.7 (506.6-517)	484.1 (479.2-489.2)	405.1 (401.1-409.1)	353.3 (336.8-371.3)
LBN Lebanon	1785.1 (1570.5-2013)	1583.7 (1395.5-1773.7)	1447.3 (1276.5-1622)	1338.2 (1181.3-1501.7)	1233.1 (1095.9-1382)	1392.4 (1266.7-1518.3)	1077.3 (973-1168.1)	1217.1 (1127.2-1286.2)	1017.7 (935.1-1078.2)	867.9 (794.5-937.1)	752.2 (701.7-805.7)	712.9 (678.1-747.4)	686.6 (660.8-711.5)	666.8 (633.7-701.5)
LBY Libya	2870.7 (2586.8-3208.3)	2266 (2053.5-2498.9)	1895.5 (1729.5-2096.8)	1488.2 (1373.5-1610.3)	1178.8 (1103-1254.5)	1046.1 (988.6-1103.2)	933.3 (871.8-998.7)	894 (813.2-978.2)	887.4 (808.9-968.7)	852.4 (789.6-925.6)	859.6 (806.2-920.6)	815.1 (768.4-866.4)	877.7 (821.8-936.4)	877.7 (791.3-966.6)
MAR Morocco	2828.2 (2538.5-3136.4)	2498.3 (2275.4-2733)	2426.8 (2222.6-2648.7)	2049.4 (1858.7-2252.4)	1835.9 (1660.7-2014.3)	1698.4 (1566.2-1840.1)	1513.2 (1412-1611.3)	1291.4 (1218.9-1362.4)	1162.7 (1111.5-1216.6)	1128.6 (1084.2-1179.3)	1089.2 (1038.4-1145.7)	1023.2 (958.3-1097.1)	963.6 (883.3-1055)	867 (756.1-983.3)
PSE Palestine	2576.4 (2318-2838.9)	2193 (1981.1-2421.8)	1834.3 (1642-2018.2)	1512.9 (1345.9-1688.9)	1571.3 (1426.6-1733.7)	1148.9 (1022.8-1299.8)	1064.4 (942.4-1199.6)	995.3 (878.8-1115.9)	976.5 (871-1088)	937.5 (859.4-1024.1)	989 (950.5-1029.5)	971.9 (948.6-996.4)	827.6 (808.8-846.1)	677.5 (644.1-712.9)
OMN Oman	4285.9 (3897.9-4688.9)	3573.2 (3269.7-3869)	3101.6 (2830.1-3365.6)	2640.2 (2397-2882.2)	1942.7 (1747.2-2155.5)	1589.3 (1415.1-1783)	1313.7 (1161.8-1483.1)	1132.2 (997.7-1295.5)	1032.7 (901-1183.7)	956.1 (830.6-1082.9)	869.3 (761.6-1006)	772.6 (687.9-876.8)	771 (694.2-861.1)	659.1 (580.4-739.7)
QAT Qatar	1754.6 (1568.1-1970.1)	1669.6 (1497.2-1880.1)	1558.8 (1399.2-1748.7)	1481.6 (1315.1-1663.4)	1365.6 (1219.8-1524.5)	1287.6 (1154.9-1426.7)	1244.2 (1110.6-1374.8)	1222.1 (1102.7-1336.6)	1235 (1131.1-1345.4)	1251.7 (1143.6-1368.5)	1124.9 (1031.2-1230.8)	844.3 (768.6-931.2)	735.9 (660.3-816.8)	599.5 (523.7-679.8)
SAU Saudi Arabia	1821.4 (1617.1-2063)	1868.1 (1663.5-2084.6)	1706.7 (1524.3-1902.7)	1504.5 (1342-1679.1)	1311.5 (1163.3-1479.3)	1096.3 (973.8-1232)	984.4 (873.2-1109.1)	931.5 (824.8-1055.1)	909.7 (810.6-1022.6)	826.2 (746-917.5)	777.4 (717.9-845.9)	801 (747.3-854.6)	830.5 (766.6-885.5)	711.5 (668.3-777.3)
SDN Sudan	2303.5 (2062.5-2559.3)	2053.9 (1850.6-2278)	1843.6 (1655.3-2042.1)	1819.3 (1646-1996.1)	1823.8 (1672.1-1970.7)	1837 (1713.1-1962.8)	1797.5 (1684.5-1916.8)	2012.7 (1907.1-2122.1)	1562 (1455.3-1674.9)	1419 (1299.6-1552.4)	1308.3 (1181.3-1445.9)	1197.1 (1072.7-1331.8)	1096.3 (969.6-1222.3)	981.1 (854.6-1112.3)
SYR Syria	1671.1 (1478.6-1852.5)	1399.2 (1259.9-1539.9)	1199 (1091.1-1323.8)	1059.3 (963.2-1154.1)	945 (876-1023.9)	816.2 (765.1-873.9)	867.1 (807.3-932.7)	999.3 (927.2-1073.1)	1043.5 (978.6-1110.5)	1032.6 (965.8-1100.8)	923.8 (865.9-986.9)	764.5 (716.2-816.1)	731.7 (672.4-798.3)	965.2 (886.2-1056.4)
TUN Tunisia	2535 (2310-2785.4)	2334.3 (2180.7-2500.3)	2087.2 (1982.9-2195.7)	1983.3 (1907.4-2078.7)	1792.7 (1744.6-1845.8)	1464.7 (1424.5-1504.4)	1197.3 (1157.9-1238.4)	1011.4 (986.4-1036.3)	961.4 (935.2-988.5)	929.2 (898.8-958.2)	877.5 (825.7-931.7)	782.9 (700.6-874.2)	729.8 (633.7-836)	687 (583.7-802.6)
TUR Turkey	2396.9 (2153.4-2657.6)	2126.6 (1923.8-2320.2)	1930.5 (1741.7-2115.8)	1680.5 (1511.4-1870.1)	1479 (1335.3-1634.5)	1311.4 (1217.5-1412.6)	1174.1 (1131.9-1218.2)	1052 (1008.4-1096.7)	976.4 (942.1-1012.1)	913.4 (879.2-947.9)	733.4 (706.9-761.2)	623.6 (600.9-646.8)	596.8 (575.2-619.2)	535.4 (499.1-571.8)
ARE United Arab Emirates	1952.3 (1723.2-2193.7)	1866.4 (1661.2-2091.2)	1738.1 (1539.8-1957)	1547.1 (1374.4-1730.9)	1358.7 (1197.9-1523.6)	1223 (1070.4-1383.6)	1131.4 (997.9-1284.1)	1081 (951.9-1218.4)	1084 (956.9-1209.1)	1033.9 (924.7-1143.7)	1094.7 (1001.2-1200.4)	1020.7 (927-1122.1)	993.5 (881.4-1104.9)	966.1 (833.2-1107.9)
YEM Yemen	4143.6 (3533.2-4770.3)	3493.6 (3041.5-3980.3)	3114.3 (2739.7-3520.2)	2927 (2560.8-3353.3)	2642.1 (2316.7-3008)	2150.8 (1861.6-2474.8)	1855.7 (1594-2151.7)	1679 (1434.9-1959)	1575 (1334.7-1853.4)	1476 (1239.7-1750.7)	1352.3 (1135.6-1612.8)	1215.6 (1015-1468)	1110.9 (915.5-1357)	1110.3 (915.4-1343.7)
South Asia	2578.8 (2500.8-2664)	2418.4 (2345.1-2493.6)	2371.5 (2300.4-2441.6)	2339.3 (2273.6-2402.1)	2157.5 (2105.8-2207.8)	1978.5 (1933.4-2024.6)	1796.4 (1757.2-1835.9)	1689.5 (1656.1-1724.8)	1590.4 (1560.2-1621)	1469.7 (1447-1492.6)	1399.8 (1380.8-1418.8)	1264 (1247.7-1280.9)	1211.2 (1195-1228.7)	1113.3 (1091-1134.3)
South Asia	2578.8 (2500.8-2664)	2418.4 (2345.1-2493.6)	2371.5 (2300.4-2441.6)	2339.3 (2273.6-2402.1)	2157.5 (2105.8-2207.8)	1978.5 (1933.4-2024.6)	1796.4 (1757.2-1835.9)	1689.5 (1656.1-1724.8)	1590.4 (1560.2-1621)	1469.7 (1447-1492.6)	1399.8 (1380.8-1418.8)	1264 (1247.7-1280.9)	1211.2 (1195-1228.7)	1113.3 (1091-1134.3)
BGD Bangladesh	2562.3 (2327-2836.3)	2377.6 (2166.9-2614.7)	2228.8 (2035.5-2449)	2066.1 (1888-2250.4)	2351.9 (2193.8-2512.1)	1764 (1639.9-1884.4)	1646.5 (1572.3-1722)	1777.2 (1717.1-1840.6)	1577.1 (1514.3-1640.4)	1345.2 (1292-1397)	1188.9 (1143.7-1231.1)	1119.2 (1085.3-1154.7)	1045.5 (1008.9-1081.7)	864 (796.9-936.2)
BTN Bhutan	3588 (3239.4-3964.9)	3273.7 (2981.9-3578.1)	2974.3 (2698.5-3261)	2630.4 (2378.3-2894.8)	2305.9 (2081.2-2543.3)	2102.4 (1902.6-2328.9)	1937.6 (1747-2135.4)	1704.4 (1550-1886.4)	1461.6 (1333.4-1605.4)	1336.5 (1227.6-1449.7)	1183.9 (1097.5-1269.9)	1006.8 (930.2-1083.7)	890.9 (801.7-977.9)	783.1 (682.4-896.7)
IND India	2637.3 (2545.4-2731.7)	2480.6 (2396.1-2561.6)	2461.6 (2381.6-2541.8)	2481.7 (2403.1-2554.9)	2250.2 (2187.2-2314)	2114 (2060.8-2169.9)	1904.1 (1856.1-1953.8)	1740.7 (1699.1-1787.3)	1625.5 (1591.2-1664.1)	1489 (1463.3-1515.9)	1420.9 (1398.8-1442.5)	1256.6 (1239.1-1274.6)	1211.4 (1196-1229.5)	1123.7 (1104.7-1143.9)
IND Andhra Pradesh	2446.9 (2041-2932.2)	2343.4 (1950.3-2786.7)	2262.9 (1902.7-2682.9)	2213.8 (1887.3-2593.1)	2017.6 (1735.5-2332.1)	1924.5 (1658.7-2209.3)	1789.9 (1536.8-2072.3)	1704.7 (1452.4-1989.7)	1652.4 (1411.2-1909)	1525.4 (1298.3-1776.2)	1470.4 (1238.1-1733.5)	1303.8 (1090.2-1549.6)	1241.3 (1030.8-1494.4)	1134.3 (953.5-1382)
IND Arunachal Pradesh	2460 (2025.4-2966.3)	2329.2 (1936.2-2769.3)	2346.5 (1976.9-2761.2)	2501.7 (2160.8-2870.8)	2180.8 (1870-2524.1)	1981.8 (1719.9-2274.6)	1763.1 (1503.3-2037.6)	1620.7 (1398.7-1883.8)	1556.8 (1333.9-1815.4)	1445.7 (1235.7-1678.7)	1411.5 (1199.6-1622.9)	1218.6 (1052-1397.5)	1126.5 (973.5-1257.6)	1041.9 (895.9-1180.5)
IND Assam	3056.8 (2681-3450.5)	2872.4 (2481.4-3258.5)	2860.1 (2507-3251.9)	2962.3 (2641-3313.3)	2658.3 (2377.6-2948.8)	2424.8 (2189-2684.1)	2157.3 (1952.7-2388.1)	1945.9 (1771.1-2143.1)	1819.6 (1689.4-1955.3)	1732.2 (1666.2-1798.2)	1765.7 (1712.7-1821.8)	1555.4 (1507.6-1601.4)	1520.9 (1475.5-1562.2)	1329.2 (1261.9-1398.8)
IND Bihar	2480.7 (2175.1-2820.9)	2305.6 (2012.9-2608.4)	2311.3 (2030.9-2607.6)	2365.1 (2111-2632.8)	2199.6 (1980.3-2429.3)	2129.1 (1936.4-2350.5)	1990.4 (1798.8-2212.3)	1835 (1658.5-2042.1)	1714.6 (1561.4-1878.6)	1603.4 (1480-1725.9)	1551.7 (1466.3-1638.3)	1332.9 (1287-1385.2)	1268.9 (1228-1309.9)	1086.1 (1030.4-1148.6)
IND Chhattisgarh	2239.2 (1950.9-2571.1)	2174.6 (1903.8-2470.6)	2188.7 (1926-2498.9)	2304.9 (2065.5-2590.2)	2114 (1881.8-2344.5)	2044 (1837.7-2266.3)	1845.1 (1665.5-2033.8)	1745.3 (1573.5-1926)	1616.6 (1463.2-1764.2)	1478.9 (1370.7-1586.4)	1454.3 (1376.6-1525.6)	1367.1 (1319.8-1413.1)	1454.1 (1410.9-1498.2)	1405 (1341.9-1475.7)
IND Delhi	2356.9 (2049.1-2691.7)	2170.1 (1911-2463.8)	2057.1 (1814.3-2336.1)	2198.1 (1975.7-2450)	1773.5 (1585.9-1972.3)	1655.7 (1486.6-1840.1)	1514.6 (1349.4-1692.8)	1401.3 (1240.1-1580.1)	1358.6 (1206.3-1524.5)	1278.9 (1158.6-1407.5)	1240.7 (1140.4-1338.8)	1074.6 (1009.3-1138.9)	978.2 (923.2-1031.7)	871.7 (799.1-944.1)
IND Goa	1875.6 (1566.9-2250)	1747.1 (1481.4-2060.9)	1651.8 (1405-1952.8)	1627.2 (1400.9-1897.6)	1492 (1288.8-1732.2)	1398.1 (1226.3-1599.9)	1244.5 (1093.4-1420.2)	1190 (1044.4-1354.5)	1167.4 (1036-1315.6)	1086 (959.6-1210)	1036.7 (904.4-1164.5)	889.8 (757.4-1038.5)	819.8 (697.5-974.5)	769.7 (655.2-927.9)
IND Gujarat	2412.8 (2129-2702.5)	2276.8 (2020.6-2561.8)	2259.9 (2001.7-2526.3)	2321.5 (2067.7-2580.1)	2097.8 (1886-2324)	1973 (1790.7-2170.6)	1773.7 (1607.2-1952.5)	1605.7 (1459.1-1754.9)	1496.6 (1394.4-1607.9)	1289.3 (1230.7-1345)	1308.4 (1262.1-1359)	1176.5 (1133.2-1218.3)	1119.1 (1079.1-1159.7)	1008.1 (944.5-1074)
IND Haryana	2217.7 (1895.8-2526.7)	2131.4 (1851.7-2448.7)	2114.7 (1854.3-2424.5)	2155.9 (1918-2443.1)	1956.1 (1751.2-2187.4)	1775.5 (1593.9-1962.5)	1557.4 (1396.4-1735.6)	1403.3 (1258-1538.6)	1313 (1211.9-1414.8)	1228.1 (1176.7-1277.1)	1223 (1178.3-1267.7)	1082.2 (1045.5-1118.7)	1116.4 (1076.9-1154.5)	1038.7 (981.8-1102.4)
IND Himachal Pradesh	2279.7 (1974.4-2616.8)	2162.3 (1858.9-2486.1)	2129.1 (1821.2-2427.5)	2116.3 (1846.2-2406.8)	1946.3 (1711.3-2193.3)	1832.8 (1625.6-2060.8)	1648.6 (1452.2-1847.8)	1508.3 (1341.6-1684.9)	1387.7 (1267.2-1516.3)	1240.7 (1172.1-1310.5)	1093.9 (1046.3-1142.6)	1001.4 (956-1046.5)	957.5 (918.7-997.2)	899.1 (837.5-968.8)
IND Jammu and Kashmir	2047.2 (1772.5-2345.2)	1896.7 (1635.3-2171.2)	1823.5 (1581.3-2086.1)	1881 (1667.5-2139.1)	1666.2 (1492.2-1887.5)	1591.3 (1433.2-1770.7)	1490.2 (1328.3-1663.4)	1334.4 (1182.9-1487.1)	1283.7 (1151.3-1427.3)	1175 (1069.9-1286)	1084.7 (1009-1161.8)	908.8 (866-951)	907.1 (873.2-945.1)	827.6 (772-883.8)
IND Jharkhand	2447.8 (2132.8-2751.5)	2307.3 (2026.2-2610)	2320.1 (2042-2612.9)	2387.8 (2123.1-2667.6)	2203.2 (1980.2-2450.5)	2097.6 (1891.5-2320.5)	1943.2 (1752.8-2154.8)	1900.4 (1712.7-2097.2)	1826.9 (1661.6-2007.6)	1710 (1581.7-1859)	1664.1 (1576.2-1757.7)	1450.7 (1406-1496.6)	1324.2 (1283.5-1362.9)	1120.9 (1067.1-1183.1)
IND Karnataka	2535.1 (2230.9-2872.9)	2409.2 (2129.8-2724)	2382 (2094.5-2682.2)	2396.3 (2131-2678.2)	2181.8 (1954.6-2411.2)	2008.6 (1798.1-2213.1)	1746.9 (1570.7-1927.4)	1558.1 (1413.1-1706.8)	1439.4 (1340.9-1545.6)	1323.6 (1276.3-1370.2)	1307.9 (1268.5-1348.9)	1145.2 (1108.7-1182.4)	1201.4 (1164-1236.9)	1263.5 (1201.8-1327.4)
IND Kerala	2192.7 (1914.7-2505.5)	1999.3 (1747-2276.2)	1913.7 (1675.1-2174.8)	1879.1 (1649-2125.8)	1683 (1498.3-1877.5)	1566.9 (1397.1-1748)	1402 (1245.6-1572.5)	1289.2 (1160.6-1432.3)	1203.8 (1108.7-1317.1)	1050.1 (989.5-1111.2)	1089.5 (1038.6-1142.5)	908 (864.9-947.6)	872.7 (834.4-912.3)	899.6 (835.3-967.6)
IND Madhya Pradesh	2841 (2510.8-3193)	2571.6 (2250.4-2880.2)	2507.8 (2212.1-2816.2)	2448.7 (2202.9-2700.1)	2095 (1894-2292.1)	2007.1 (1830.9-2193.6)	1936.5 (1746.4-2126.5)	1903.4 (1730.5-2095)	1836.6 (1670.4-2017.5)	1708.3 (1579.4-1845.3)	1648.9 (1556.7-1746.7)	1431.5 (1380.4-1487.2)	1375.6 (1329.1-1422.4)	1324.8 (1249.9-1408.1)
IND Maharashtra	2384 (2111.1-2697.2)	2231.6 (1983.8-2506.8)	2188.6 (1934-2461.9)	2198 (1954.6-2444.3)	1993.9 (1790-2196.1)	1864.9 (1693.6-2044.4)	1651.3 (1496.8-1808.1)	1508.2 (1377.4-1647.4)	1414.7 (1328.4-1507.7)	1289.3 (1245.2-1333)	1293.1 (1254.2-1333.1)	1058.8 (1025.7-1092.8)	1011.5 (980-1042)	970.8 (920.1-1021.7)
IND Manipur	2114.4 (1748.6-2544.7)	1992.1 (1666.7-2388.6)	1968.7 (1668.4-2352.6)	2029.8 (1748.2-2351.5)	1814.1 (1568.2-2104.5)	1682.6 (1471.9-1933.1)	1512.6 (1324.7-1746.2)	1445.3 (1267.7-1652.4)	1370.5 (1199.2-1562.8)	1320.5 (1161.1-1493.5)	1312.5 (1166.6-1465.7)	1177.2 (1026-1323.7)	1127.4 (988.2-1263.6)	1069.8 (922.3-1209.8)
IND Meghalaya	1982.2 (1641.9-2415.1)	1883.4 (1560.5-2261.1)	1849.4 (1551.6-2210.7)	1945.9 (1668.3-2263.7)	1739.8 (1492.6-2034)	1732.6 (1504.3-1991.6)	1615.9 (1401.2-1853.7)	1533.5 (1333.2-1777)	1482.3 (1277.9-1704.8)	1395.8 (1201.9-1591.2)	1353.6 (1165.9-1533.9)	1189.9 (1037.5-1357.2)	1129.1 (980.2-1273.1)	1054.7 (903.3-1192.7)
IND Mizoram	1782 (1481.2-2121.7)	1733.8 (1438.8-2044.8)	1688.8 (1411.4-1991.4)	1843.1 (1585.3-2118)	1623.6 (1379.7-1865.4)	1557.3 (1338-1780)	1447.6 (1257.1-1652.8)	1361.2 (1185.9-1531.7)	1292 (1140.4-1438.3)	1220.8 (1087.7-1344.4)	1198.8 (1072.8-1307)	1087.3 (937.3-1225.1)	1058.1 (914.8-1198.9)	1004.9 (855.7-1148.7)
IND Nagaland	2131.9 (1725.8-2584.3)	2041.3 (1688.7-2469.6)	2020.1 (1695.2-2426.2)	2199.7 (1896.9-2547.8)	1799.5 (1533.2-2124.1)	1658.3 (1426.7-1923.4)	1490.1 (1270.7-1742.2)	1374.9 (1192-1611.3)	1343.6 (1163.3-1579)	1271.6 (1101.7-1480.1)	1273.9 (1091.7-1470.9)	1133.8 (946.2-1321.6)	1073.7 (892.6-1237.8)	1015.3 (836.8-1185.8)
IND Odisha	2977.4 (2609.9-3375.7)	2803 (2477.6-3168.3)	2826.5 (2510.4-3175.5)	2860.2 (2548.9-3179.7)	2614.9 (2358.2-2896.8)	2407.3 (2174.1-2664.2)	2098.4 (1894.2-2330.1)	1857.8 (1687.8-2042.6)	1728.3 (1617.5-1841)	1677.3 (1621-1733.5)	1692.5 (1641.1-1741.4)	1451.5 (1410.8-1491.2)	1331.6 (1295.5-1367.6)	1123.3 (1067.8-1180.9)
IND Punjab	2087.5 (1833-2362.9)	1996.1 (1748.2-2274)	1936.5 (1688.2-2202.3)	1915.2 (1705.2-2144.8)	1732.9 (1541.5-1935.7)	1620.1 (1454-1796.1)	1458.2 (1312.5-1628.1)	1315 (1179.8-1458.4)	1243.1 (1147-1339.1)	1160.3 (1112.8-1207.4)	1127.7 (1088.9-1165.8)	1041.8 (1005.4-1076.9)	980.1 (947.5-1013.9)	846.7 (798.7-893.2)
IND Rajasthan	2506.1 (2207.8-2842.7)	2398.5 (2109.9-2693.1)	2410.7 (2125.3-2719.7)	2503.3 (2236.5-2784.2)	2260.9 (2034.1-2508.5)	2147 (1942.1-2372.9)	1924 (1727.6-2115.4)	1729 (1570.7-1908.6)	1590.3 (1476.3-1720.7)	1469.1 (1402.7-1535.5)	1291 (1239.5-1340)	1166 (1122.6-1207.8)	1132.8 (1092.4-1175.1)	1149 (1083.9-1224.7)
IND Sikkim	2131.7 (1730.4-2600.9)	2068.2 (1704.1-2513.8)	2040.9 (1698.3-2454.4)	2116.7 (1798.1-2471.3)	1936.9 (1651.1-2278.1)	1837.3 (1585.6-2117.5)	1653.2 (1417.8-1919.9)	1521 (1298.6-1767.3)	1450.9 (1236.2-1658.6)	1369.5 (1193.3-1543.9)	1309.3 (1154.4-1470.3)	1158.4 (1011.1-1280.7)	1062.7 (912.2-1178.3)	947 (795.9-1073.2)

Table 1 Age-standardised mortality rates by location and year for both sexes for 1950, 1955...2005, 2010, 2017

Location	1950	1955	1960	1965	1970	1975	1980	1985	1990	1995	2000	2005	2010	2017
IND Tamil Nadu	2952.2 (2619.5-3290.2)	2763.4 (2450.6-3062.6)	2712.9 (2420.9-3001.4)	2717.8 (2461.4-2994.4)	2495.6 (2252.8-2740.1)	2306.4 (2092.2-2518.5)	2023.9 (1829.1-2232.7)	1810.5 (1645.7-1974.4)	1649 (1539.6-1760.1)	1471.1 (1422.4-1524.3)	1435.7 (1392.9-1480.5)	1207.8 (1170.4-1247.2)	1223.2 (1186.7-1261)	1096.3 (1036.6-1152.2)
IND Telangana	2192.7 (1808.6-2640.8)	2131.9 (1762.7-2548.4)	2113 (1767.5-2529.5)	2194.3 (1847.4-2611.3)	2122.6 (1806.4-2498.4)	2003.1 (1724.5-2339)	1751.8 (1507.6-2034.1)	1617.2 (1387.5-1872.9)	1582.4 (1346.9-1831.4)	1494.5 (1259.5-1743.3)	1489.5 (1264.4-1758.9)	1314.7 (1110.3-1555.4)	1155.7 (971-1368.2)	916.9 (772.5-1082.9)
IND Tripura	2429.1 (2025-2917.9)	2295.8 (1925.2-2700.9)	2300.9 (1950.9-2684.3)	2337.5 (2018.5-2678)	2063.2 (1778.7-2368.1)	1893.5 (1653.4-2151.1)	1781.5 (1547.3-2032.4)	1584.3 (1382.4-1826.8)	1517.5 (1310.3-1756)	1439.1 (1242.7-1645.2)	1369.4 (1185.7-1546.4)	1178.2 (1028.2-1330.4)	1134.8 (1000-1269)	1088.6 (974.8-1220.1)
IND Uttar Pradesh	3262.8 (2881.6-3650.9)	3066.8 (2762.2-3410.3)	3098.8 (2794.2-3405.8)	3105.5 (2828.6-3397.2)	2772 (2569.8-3000.6)	2592.6 (2418.7-2789.9)	2336 (2161.2-2522.4)	2095 (1936.4-2265.6)	1912.5 (1774.3-2052.5)	1702.9 (1596.3-1805)	1501.5 (1434.3-1569.6)	1443.1 (1395-1494.6)	1400.3 (1350.4-1451.2)	1355.6 (1279.2-1436.1)
IND Uttarakhand	2299.8 (2014.8-2589.4)	2243.2 (1933.9-2540.8)	2208.3 (1937.7-2481.4)	2256.2 (1997.5-2511.7)	2081.1 (1884.7-2293.6)	1991.4 (1811.7-2182)	1848.1 (1671.5-2020.1)	1772.9 (1609-1950.7)	1707.5 (1556.3-1884.4)	1562.7 (1427-1729.7)	1489.1 (1364.4-1625.5)	1302.2 (1217.3-1396.3)	1194.5 (1135.6-1260.8)	1116.1 (1069.5-1169.8)
IND West Bengal	2597.6 (2299.9-2925.5)	2450.2 (2162.8-2752.4)	2443.5 (2158.8-2740)	2488 (2232.7-2757.3)	2270.6 (2042.8-2511.6)	2130.1 (1933.3-2329.5)	1896.2 (1725-2076.2)	1756.9 (1609.5-1918)	1651 (1511.5-1795)	1551.5 (1468.8-1638.9)	1371.7 (1310.2-1432.2)	1215.4 (1164.1-1269.7)	1149.6 (1099.6-1200.9)	1064.6 (993.5-1143.1)
IND Union Territories other than Delhi	1520.6 (1298.6-1809.2)	1453.2 (1249.4-1721.7)	1434.3 (1236.9-1695.1)	1534.2 (1343.9-1766.6)	1376.7 (1198.3-1578.7)	1301.5 (1150.1-1459.7)	1208.2 (1065.5-1360.1)	1186.3 (1052.9-1331.4)	1191.3 (1066-1317.1)	1148 (1017.4-1257)	1119.1 (982.4-1222.2)	993.3 (848.5-1123.3)	947.7 (798.6-1087)	919 (774.7-1066.2)
NPL Nepal	2906.4 (2658.5-3168.2)	2700.4 (2491.2-2927.4)	2534.7 (2318.4-2763.1)	2356.7 (2142.7-2573.9)	2186.3 (1979.5-2394.5)	2075.9 (1871.9-2278.9)	1986 (1807-2173.7)	1834.6 (1686.8-1987.8)	1630.6 (1514.2-1750.9)	1414.7 (1330.3-1508.5)	1194.1 (1134.8-1266.3)	1083.9 (1027.2-1148.4)	1057.1 (979.2-1133.1)	1010.2 (922.1-1083.9)
PAK Pakistan	2073.6 (1883.5-2288.1)	1986.9 (1812.6-2166.9)	1859.8 (1692-2017.6)	1699.5 (1572.6-1824.2)	1537.3 (1448.8-1629.2)	1431.4 (1364.1-1503)	1371 (1311-1428)	1352.7 (1301.2-1403)	1401.1 (1353.7-1454.1)	1467 (1408.2-1526.7)	1461.8 (1402.5-1524.6)	1488.4 (1416.4-1564.9)	1377.8 (1264.8-1494.9)	1284.7 (1130.5-1447)
Southeast Asia, East Asia, and Oceania	2297.5 (2242.2-2354.4)	1954.8 (1899.7-2007.8)	3456.8 (3407.6-3508)	1724.4 (1680.5-1769.3)	1564.3 (1529.1-1601.1)	1445.4 (1415.3-1476.7)	1315.5 (1289.1-1341.6)	1237.9 (1219.1-1257.6)	1163.6 (1148.2-1178.4)	1087 (1076-1098.6)	999.9 (989.9-1010.4)	911.5 (903.1-920.4)	832.8 (823.9-842.8)	754.4 (738.6-770.6)
East Asia	2217.6 (2146.1-2292.1)	1878.7 (1812.7-1944.8)	4012.7 (3950.5-4075.3)	1683.3 (1626.8-1741.8)	1494.9 (1448.8-1543.3)	1367.9 (1330-1406.7)	1259.7 (1227.2-1292.8)	1200.5 (1177.6-1224.9)	1131.2 (1112.4-1149.2)	1051.4 (1038.3-1065.8)	954.6 (942.3-966.8)	857.2 (847.8-867.3)	777.5 (766.6-788.6)	701 (682.2-720.1)
CHN China	2178 (2103-2254)	1884.2 (1815.9-1952.5)	4133.6 (4069.7-4197.7)	1695.7 (1638.2-1757.4)	1505.6 (1457.7-1555.1)	1378.6 (1338.8-1418.1)	1269.9 (1236-1303.8)	1210.5 (1186.3-1235.5)	1140.2 (1120.8-1158.3)	1048.5 (1034.8-1062.5)	948.4 (936-960.9)	858.5 (848.8-868.8)	778.8 (767.2-790.1)	700.6 (681.3-720.2)
PRK North Korea	4667.3 (4429.2-4914.8)	1770.7 (1555.4-2002.5)	1564 (1359.9-1784.2)	1385.5 (1192.8-1573)	1215.5 (1057.4-1370.4)	1076.3 (943.8-1195.3)	1012.9 (899.6-1118.8)	984.8 (872.9-1091.8)	959.3 (852.2-1059.2)	1429.2 (1320.2-1522.4)	1584.5 (1482.8-1675)	1075.4 (986.4-1148.9)	1016.4 (937-1084.7)	957.9 (856.8-1055.2)
TWN Taiwan (Province of China)	1862.3 (1679.7-2061.1)	1600.5 (1593.4-1608.7)	1437.1 (1430.3-1443.8)	1225.1 (1219.3-1230.9)	1145.8 (1140.9-1151.2)	1070.5 (1065.7-1075.3)	965.3 (960.8-969.4)	874.4 (870.7-878.4)	821.5 (817.8-825)	769.4 (766-773)	688.9 (685.8-692)	635 (632.1-638)	555.5 (552.9-558.3)	539.6 (518.2-562.3)
Oceania	3329 (3150.4-3534.8)	2986.8 (2830.1-3154.8)	2716.9 (2575.1-2864.7)	2465.8 (2335.5-2602.1)	2278.6 (2168.7-2397.8)	2125.2 (2020.8-2235.9)	1989.7 (1888.1-2089.9)	1943.1 (1835.1-2046.7)	1890.7 (1777.4-2004.2)	1850.3 (1736-1966.5)	1843.6 (1721.9-1960.4)	1852.3 (1721.4-1979.3)	1807.4 (1673.1-1947.8)	1689.5 (1562.8-1835.3)
ASM American Samoa	1413.3 (1314-1508)	1348.4 (1286.8-1405.3)	1316.9 (1276.8-1359.9)	1283.4 (1245.5-1325.3)	1216.4 (1176.5-1265.9)	1148.3 (1113.7-1193.3)	1115.4 (1058-1168.1)	1094.6 (1043.9-1144.4)	1069.4 (1015.9-1114.4)	1052.9 (1002.2-1099.5)	1031.4 (985.7-1081.9)	1047.7 (1005.1-1093.7)	1036.2 (990.6-1081.9)	1007.5 (929.5-1085.1)
FSM Federated States of Micronesia	2736.8 (2484.8-3018.4)	2416.8 (2198.6-2646.4)	2304.7 (2087.8-2530.7)	2163.8 (1959.5-2382.6)	2010.1 (1824.8-2218.4)	1852.6 (1672.4-2046)	1712.5 (1549.4-1885.9)	1618.7 (1466.9-1795)	1545.7 (1408.9-1700.1)	1479.6 (1357.4-1609.4)	1438.6 (1324.9-1552.7)	1366.4 (1261-1475.9)	1343.6 (1238.9-1453)	1324.8 (1214.9-1451.8)
FJI Fiji	1924.5 (1833.3-2025.6)	1641.5 (1580.3-1702)	1486.1 (1435.7-1545.1)	1429.5 (1376.2-1480.9)	1558 (1503-1613.5)	1479.6 (1424-1536.4)	1333.2 (1281.7-1388.4)	1276.6 (1224.3-1332.4)	1268.7 (1177.8-1367.6)	1379.6 (1339.9-1418.7)	1500.8 (1483.8-1518)	1394.8 (1379.4-1412.3)	1330.6 (1311.5-1351.6)	1259.7 (1135.3-1384.3)
GUM Guam	1505.6 (1439.4-1568)	1400.7 (1359-1445.1)	1256.7 (1223-1293.9)	1144.2 (1118.7-1169.7)	1182 (1160.2-1202.9)	1088.4 (1068.4-1110.4)	909.3 (890.2-927.5)	805.3 (786.3-823.7)	850.3 (826.2-874.7)	872.8 (848.4-899.1)	835.3 (812.9-859.4)	805.6 (781.6-829.5)	804.6 (781.3-828.6)	834.9 (787.2-883.6)
KIR Kiribati	2794.7 (2542.1-3050.5)	2576.2 (2355.7-2802.6)	2423.5 (2208.8-2649.3)	2271.5 (2064.9-2490.1)	2133.2 (1941-2333.5)	1902.9 (1727.8-2071.3)	1812.2 (1658.4-1960.9)	1844.2 (1717.9-1963)	1834 (1751.8-1922.9)	1827.5 (1763.3-1894.1)	1801.9 (1731.7-1882.8)	1755.3 (1637.5-1862.7)	1707.1 (1566.9-1840.1)	1621.2 (1459.5-1780.4)
MHL Marshall Islands	2884.4 (2617.4-3149)	2561 (2328.1-2815.8)	2300.3 (2081.7-2544.1)	2095.7 (1878.7-2314.1)	1921.6 (1730.9-2127.6)	1761.2 (1600.5-1925.6)	1669 (1530.4-1789.3)	1647.7 (1559.8-1722.4)	1693.8 (1645.1-1740.7)	1736.1 (1687.6-1790.7)	1772 (1707-1843.8)	1738.6 (1661.1-1817.9)	1661.1 (1556.4-1767)	1520.6 (1386-1665.1)
MNP Northern Mariana Islands	1090.3 (970.5-1218)	1087.3 (963.5-1205.6)	1072.4 (945.7-1172.7)	1047.2 (928.3-1143.6)	1011.9 (898.3-1108.1)	977.6 (865.9-1076.7)	949.3 (849.6-1048)	909.5 (812-1016.1)	868.4 (784.1-980.3)	812.6 (745.4-901.1)	777 (722-835.8)	750.8 (703.6-801.2)	715.7 (666.3-767.6)	716.8 (661.4-777.5)
PNG Papua New Guinea	3877.2 (3623.4-4168.9)	3518.4 (3293-3764)	3247.4 (3036.7-3471.7)	2960.9 (2761.6-3169.8)	2688.2 (2514.4-2876)	2494.7 (2333-2667)	2354.3 (2198.5-2508.5)	2300.8 (2128.9-2457.8)	2212.1 (2039.9-2380.5)	2114.8 (1938.5-2286.2)	2083.5 (1899.3-2258.1)	2122.8 (1925.4-2314.3)	2069 (1870.5-2271.9)	1902.4 (1719.5-2098)
WSM Samoa	1458.2 (1325.7-1607.3)	1273.8 (1183.4-1372.4)	1099.3 (1035-1167.7)	1110.7 (1052.8-1173.9)	1127.6 (1068.6-1192.9)	1119.8 (1053.5-1189.9)	1052.6 (977.3-1132.1)	1031.6 (940.8-1133.2)	1006.8 (907.4-1114.3)	1010.8 (917.2-1116.1)	995.8 (914.5-1084)	967.9 (894.6-1038.7)	985.7 (906-1050.5)	965.3 (870.6-1044.6)
SLB Solomon Islands	2893.9 (2634.4-3143.3)	2734.7 (2505-2959.5)	2576.1 (2352.4-2797)	2382 (2178.1-2611.2)	2181.9 (1989.4-2397.9)	2121.4 (1942.3-2334.8)	1806.8 (1624.7-2013.6)	1709.5 (1533.4-1904.6)	1660.4 (1494.8-1839.6)	1577.6 (1431.2-1742.8)	1530.4 (1398.5-1684.5)	1521.3 (1395.5-1652.8)	1471.3 (1347-1596.7)	1396.3 (1278.1-1520.4)
TON Tonga	1820.9 (1689.7-1957.5)	1679.3 (1588.5-1779)	1542.6 (1479.3-1606.1)	1458.9 (1399.4-1522.6)	1397.1 (1323.9-1469.3)	1338 (1264.4-1410.6)	1280.7 (1220.3-1341.7)	1195.9 (1149-1244.8)	1147.5 (1101-1196.5)	1124.9 (1085.5-1164)	1115 (1078-1154.1)	1090 (1042-1132)	1068.5 (992.3-1137.1)	1020.3 (917.6-1136.2)
VUT Vanuatu	2417.9 (2085.2-2764.3)	2282.3 (1969.7-2634.7)	2159.7 (1858.1-2505)	2040.1 (1738.4-2369.1)	1943.1 (1651.7-2269.2)	1828.1 (1541-2115.3)	1728.6 (1457.5-2000.4)	1680.9 (1425.4-1917)	1658.5 (1416.1-1906.3)	1621.3 (1390.8-1873.3)	1591.4 (1339.4-1827.8)	1589.7 (1327-1833.7)	1547.3 (1295.4-1809.6)	1508.5 (1276.9-1776.3)
Southeast Asia	2568 (2492.5-2643.4)	2197.1 (2127.9-2262.4)	1974.6 (1907.9-2034.9)	1852.6 (1793.4-1910)	1770.9 (1720.6-1822.8)	1661.9 (1617.7-1709.8)	1451.8 (1416.2-1490.3)	1337 (1307.7-1368.3)	1250 (1225.8-1276)	1185.6 (1164.6-1207.2)	1127.8 (1107.2-1146.8)	1051.3 (1031.9-1068.8)	973.9 (956.4-992.3)	889.7 (865.2-916.9)
KHM Cambodia	2619.8 (2342.1-2908.7)	2429.2 (2198.3-2669.8)	2265.8 (2045-2491.1)	2126.5 (1907.9-2348.7)	2535.7 (2337.6-2739.9)	4798.9 (4610.4-4980.6)	2081.9 (1920.1-2230.2)	1957.1 (1830.7-2077.5)	1773.6 (1671.8-1865)	1686.3 (1610.1-1763)	1586.7 (1511.6-1664.6)	1381.4 (1303-1458.7)	1202.3 (1138.1-1271.5)	1067.4 (1001.3-1161.2)
IDN Indonesia	2872.1 (2763.4-2985.1)	2538.1 (2433.2-2636.7)	2275.9 (2183.3-2369.4)	2074.5 (1990.6-2163.3)	1898 (1817.3-1978.2)	1723 (1658.7-1793.2)	1549.6 (1500.4-1607.7)	1412.2 (1372.3-1457)	1311.8 (1279.6-1347)	1222 (1192.8-1253.9)	1185.4 (1158.1-1211.7)	1140.5 (1109.8-1167.6)	1086.2 (1050.5-1120.1)	1009.5 (964.8-1057.3)
LAO Laos	4003.4 (3650.4-4377.4)	3620.3 (3339.2-3927.8)	3402 (3123.4-3688.7)	3172.4 (2898.4-3449.2)	2920.7 (2652.2-3174.8)	2595 (2354.7-2832.8)	2401.5 (2179.6-2622)	2182.3 (1987.5-2376)	2049.5 (1875.9-2213.9)	1892.1 (1748.7-2040.6)	1711.2 (1596.5-1842.9)	1520.5 (1421.7-1638.2)	1335.6 (1233.4-1449.2)	1131 (1030.2-1243.7)
MYS Malaysia	2229.7 (2009.5-2460.1)	1920.5 (1777.6-2058)	1686.4 (1679-1694)	1567.7 (1561.1-1573.9)	1490.4 (1468.8-1511.5)	1283.9 (1264.4-1303.3)	1189.8 (1150-1229.8)	1119 (1114.8-1123.7)	1055.6 (1051-1060.4)	1042.9 (1039.2-1046.7)	992.1 (988.2-995.7)	902.1 (898.8-905)	869.8 (861.8-877.6)	828.1 (776.1-883.9)
MDV Maldives	3529.6 (3211.2-3896.3)	3227.2 (2954.5-3505.3)	2891.6 (2651.7-3131.6)	2563.6 (2394.2-2740.1)	2203.2 (2092.4-2314.7)	1976.1 (1904.5-2051.8)	1757 (1700.2-1820.2)	1472 (1442.1-1501.4)	1303.8 (1282.1-1324.8)	1172.3 (1151.1-1194.3)	957.7 (941.1-974.5)	770.3 (755.6-784)	578.4 (567.9-588.5)	478.1 (456.4-501.5)
MUS Mauritius	2157.4 (2107.4-2210)	1917.7 (1884.5-1949.6)	1821.8 (1793-1851.8)	1428 (1403.1-1453)	1347.7 (1324.6-1371.3)	1425.9 (1406-1445.7)	1231 (1213.8-1248.6)	1195.4 (1179.5-1210.9)	1096.8 (1081.7-1113.3)	1010.2 (997.2-1023.8)	935.3 (922.2-948.6)	904.5 (890.8-918)	815.6 (804.7-827.2)	783.1 (744.6-822.6)
MMR Myanmar	3759.1 (3417-4134.8)	3351.3 (3095.2-3627.5)	3101.1 (2828.5-3362.2)	2891.7 (2630.5-3156.7)	2693.3 (2440.5-2956.2)	2378.4 (2147.4-2630.3)	2161.2 (1938.5-2398.7)	1998.7 (1808.7-2207.9)	1894.3 (1726.6-2069.6)	1809.3 (1656.1-1973.8)	1696.3 (1560.5-1836.5)	1536.4 (1425.8-1645)	1312.9 (1216.6-1410.3)	1099.9 (1007.2-1189.8)
PHL Philippines	1581.2 (1486.1-1694.7)	1324 (1250.5-1400.6)	1179.1 (1126.1-1233.7)	1234.7 (1182.8-1287.9)	1324 (1276.1-1376.6)	1262.7 (1209.8-1313.3)	1210.8 (1165.8-1258.3)	1272.2 (1224.8-1319.7)	1164.2 (1117.4-1208.3)	1119.1 (1074-1167.8)	1114 (1071.5-1159.7)	1149.7 (1099.3-1198.6)	1157.2 (1108.5-1207.8)	1142.4 (1031-1251)
LKA Sri Lanka	1679.1 (1642-1720.7)	1355.5 (1339.7-1370.5)	1165.2 (1156.7-1172.8)	1168.7 (1163.2-1174.4)	1143.8 (1138.5-1149.4)	1266.1 (1259.1-1273.2)	1021.7 (1015.2-1028.2)	1105.2 (1099.8-1110.6)	1085.3 (1068.1-1101.5)	1043.5 (1037-1049.5)	998.9 (993.6-1004.6)	844.9 (820.9-869.8)	792.9 (785.9-800.6)	663.3 (579.1-743.7)
SYC Seychelles	1546.4 (1402.5-1694.3)	1422.8 (1307.4-1539.4)	1323.5 (1238.9-1410)	1279.5 (1217.2-1340.7)	1184.6 (1148.2-1221.5)	1059.9 (1035-1084.3)	981.9 (958.5-1007.5)	1010.1 (987.3-1032.7)	1043.4 (1021.8-1067.3)	1026.4 (1003.3-1048.9)	987.2 (965.9-1008.9)	933.9 (914.1-954.9)	902.1 (884-921.1)	866.4 (833.2-898.4)
THA Thailand	1860.5 (1749.3-1984.3)	1472.3 (1420.1-1532.1)	1383.6 (1339.5-1426.8)	1322.8 (1280.3-1364)	1251.5 (1214.6-1288.6)	1241.7 (1205.5-1281.8)	1237.5 (1201.7-1275.7)	996.6 (968.2-1027)	957.6 (929.5-985.5)	955.3 (926.1-984.7)	852.8 (821.2-882.7)	712.3 (690.6-735.7)	624 (604.9-644)	575.8 (530.6-620)
TLS Timor-Leste	3839.5 (3469.3-4211.2)	3095.1 (2802.6-3373.7)	2751.9 (2485.4-3006.8)	2469.1 (2236.7-2709.4)	2234.3 (2023.9-2445.3)	2387.7 (2196.5-2580.7)	2051.6 (1885.9-2234.8)	1664.6 (1529-1812.4)	1467.9 (1353.8-1590.1)	1363.5 (1274.8-1460.4)	1218.4 (1151.5-1287.7)	1106.4 (1049.9-1172.4)	1012.3 (946.5-1085.1)	993.9 (902.1-1080)
VNM Vietnam	2299.6 (2028-2597.1)	1779.4 (1581.8-2006.6)	1638.4 (1458-1835.8)	1674.4 (1513.3-1854.3)	1742.7 (1586.5-1908.6)	1425.5 (1285.2-1565.2)	1225 (1099.1-1339.8)	1132.4 (1035.5-1225)	1067.4 (988.1-1134.3)	1006.9 (946.1-1059)	962 (896.8-1019.2)	910.6 (853.7-973.8)	848.3 (794.5-907.5)	799.7 (739-874.3)
Sub-Saharan Africa	2967.8 (2852.1-3094.6)	2689.3 (2592.2-2789.2)	2527.5 (2443.8-2613.6)	2356.8 (2279.8-2440.9)	2225.8 (2152-2306.7)	2066.5 (1999.7-2141)	1926.8 (1869.9-1992.3)	1840.6 (1786.1-1901.2)	1818.3 (1761.6-1878.6)	1865.3 (1805.5-1928.6)	1919.8 (1849.9-1988.8)	1790.8 (1724.4-1857.6)	1564.3 (1506.2-1630.7)	1270.6 (1226.6-1328)
Central sub-Saharan Africa	3192.4 (2959.6-3439.5)	2819.3 (2638.8-3009.2)	2679.6 (2506.3-2846)	2577.1 (2408.7-2744.2)	2423.1 (2263.1-2584.8)	2296.3 (2138.5-2444)	2193.9 (2053.2-2323.8)	2080.5 (1968.3-2205.2)	2030.1 (1932.8-2141.9)	2043.7 (1954.2-2136.6)	2014.2 (1932.2-2100.6)	1864.7 (1784.5-1949.7)	1719.3 (1632.5-1814.4)	1461.7 (1364.2-1560.2)
AGO Angola	3037.9 (2730.2-3371.8)	2881.9 (2607.9-3149.3)	2790.2 (2532.2-3042)	2743.1 (2497.6-2996)	2590.5 (2356.8-2814.5)	2488.7 (2253.3-2722.3)	2452.8 (2233.7-2683.7)	2411.4 (2202.2-2628.7)	2293.5 (2090.9-2489.3)	2188.5 (2005-2370.3)	2058.8 (1900.1-2210.7)	1810.7 (1676-1946.4)	1575.1 (1444-1698.5)	1327.4 (1200-1457.8)
CAF Central African Republic	3620.4 (3319.3-3942)	3302.1 (3059.2-3570.4)	3010.3 (2784.2-3256.9)	2757.4 (2539.5-2985.2)	2613.6 (2429.6-2801.1)	2539.5 (2366.1-2706.5)	2440.4 (2297.3-2580.9)	2393.2 (2273.9-2515.5)	2572.4 (2417.8-2737.2)	2738.2 (2542.4-2957.4)	2851.8 (2597.5-3111.4)	2800.2 (2534.3-3071.3)	2559.8 (2288.9-2819)	2293.2 (2031.2-2530.2)
COG Congo (Brazzaville)	3757.3 (3453.6-4063.4)	3398.7 (3138.1-3643.1)	3137.4 (2883.2-3393.5)	2872.7 (2642-3098.5)	2648.1 (2438.6-2864.9)	2403.6 (2220.2-2602.9)	2190.4 (2024.3-2367.6)	2016.7 (1857.9-2167.8)	2201.5 (2040.9-2355.1)	2325.5 (2179.1-2472.8)	2245.7 (2120.7-2384)	1959.8 (1822.3-2088.9)	1709.6 (1558.3-1851.5)	1500 (1336.6-1659.6)
COD DR Congo	3169.8 (2836.4-3531.4)	2711.5 (2442.1-2970.2)	2585.1 (2342.9-2826.5)	2496.8 (2260.2-2734)	2347.9 (2108.7-2562.3)	2221.9 (2002.8-2424.1)	2114.8 (1925.3-2300.1)	1982.5 (1820.5-2153.5)	1916.5 (1775.9-2065.5)	1946.5 (1827.6-2070.6)	1940.5 (1827.6-2056.3)	1821.8 (1713.8-1933.4)	1722.7 (1602.2-1854.3)	1466.5 (1332.5-1607.4)
GNQ Equatorial Guinea	4364.9 (3694-5085)	3539.8 (3103.5-4034.5)	3361.6 (2965-3797.5)	3087.9 (2707-3518.7)	2869.5 (2491.2-3300.5)	2688.9 (2325.6-3070.3)	2452.4 (2112.4-2812.5)	2394.1 (2084.4-2722.6)	2453.1 (2138-2783.6)	2388 (2046.2-2740.3)	1818.2 (1460.7-2187)	1470.2 (1154.5-1835)	1343.3 (1033.9-1713.1)	1173.7 (905.3-1516)
GAB Gabon	3614.3 (3152.9-4122.2)	2718 (2469.7-2976)	2589.6 (2367.8-2833.3)	2406.6 (2188.6-2628.9)	2236.2 (2035.9-2436.1)	1981.4 (1800.7-2155.1)	1773.4 (1625.3-1923.9)	1657.6 (1539.3-1777.9)	1639.2 (1533.2-1746.9)	1609.8 (1498.9-1723.5)	1666.6 (1528.3-1819.7)	1635.8 (1494.3-1785.5)	1418.3 (1299.7-1546)	1129.1 (1035.6-1232.3)
Eastern sub-Saharan Africa	3161.4 (3067.2-3258.9)	2871.8 (2796.5-2945.6)	2702.2 (2636-2769.4)	2550.5 (2491.9-2612.3)	2410 (2354.2-2466.5)	2280.2 (2227.8-2331.5)	2150.2 (2105-2194.6)	2118.8 (2075.3-2163.5)	2116.1 (2070.4-2160.2)	2167.1 (2121.5-2210.3)	2119.2 (2074.6-2160.9)	1849.2 (1811.1-1887.9)	1539.8 (1505.8-1573.2)	1246.7 (1214.4-1276.9)
BDI Burundi	3814.6 (3502.2-4134.7)	3521.2 (3241.1-3795.8)	3260.2 (3017.2-3496.2)	3056.1 (2851.6-3254.1)	2922.7 (2743-3086.6)	2827.2 (2655-3000.1)	2691 (2514.6-2863)	2576.7 (2408.9-2752)	2480.9 (2299.6-2653.3)	2824.6 (2625.8-3023.7)	2811.7 (2621.5-2996.2)	2354.4 (2188-2517.4)	1765.3 (1603.7-1916.7)	1525.3 (1364.5-1676.1)
COM Comoros	3330.8 (3021.1-3688)	3035.9 (2805.6-3279.6)	2754.7 (2550.3-2965.8)	2467.7 (2274.1-2661.9)	2216.4 (2024.6-2411.2)	2061.6 (1885.7-2269)	1970.1 (1782-2174)	1834.6 (1666.8-2026.9)	1696.6 (1554.1-1853.9)	1597.8 (1473.9-1737.1)	1464.3 (1357.4-1583.3)	1272.9 (1178.2-1377.2)	1161.4 (1069.8-1270.9)	1081.7 (972.1-1192.8)
DJI Djibouti	2298.1 (1898.7-2751.7)	2128.5 (1781.4-2535.6)	1995.6 (1666-2381.6)	1870.9 (1560.5-2231.7)	1745.7 (1453.7-2092.5)	1594 (1324.7-1892.1)	1507.8 (1260.3-1803.8)	1528.2 (1291.4-1809.3)	1488 (1253.2-1773.3)	1527.9 (1261.4-1829.4)	1494.5 (1203.6-1817.9)	1418.1 (1125.2-1783.9)	1285.3 (1010.6-1626.5)	1127.4 (891.3-1427.1)
ERI Eritrea	3966.7 (3641.1-4300.5)	3621.4 (3345.6-3902.1)	3396.6 (3126.8-3659.1)	3592.3 (3346.5-3857.5)	3390.9 (3170.4-3619.4)	3368.2 (3186.4-3561.5)	3169.2 (3016.9-3334.7)	3021.4 (2887.4-3161.1)	3711.9 (3572.3-3850.5)	2470.5 (2301.7-2634.3)	2318 (2128.5-2518.1)	2091.7 (1880.8-2313.7)	1900.5 (1689.7-2106.8)	1615.9 (1414.4-1804.7)
ETH Ethiopia	3932 (3732.7-4148.1)	3548.2 (3376.4-3718.8)	3344.2 (3168-3508.1)	3182.5 (3017.5-3339.6)	3040.8 (2884.2-3192.5)	2964.6 (2817.4-3108.5)	2803.1 (2676.6-2928.6)	2721.7 (2611.7-2823)	2562.5 (2472.7-2651.6)	2404.9 (2317.4-2491.1)	2209.4 (2124.6-2297.3)	1735.8 (1669.9-1803.4)	1333.6 (1281.5-1385.2)	1051.1 (1002.2-1101.9)
KEN Kenya	2408.2 (2342.9-2476)	2166.1 (2107.9-2222.1)	1943.5 (1888.7-1994.5)	1835.6 (1781.6-1886.7)	1735.7 (1686.7-1783.4)	1599.7 (1552.6-1645.5)	1458.6 (1419.4-1499.1)	1368.8 (1332.6-1403.7)	1398.2 (1357.8-1436.7)	1627 (1573.8-1682.7)	1825.4 (1769-1883.4)	1764.6 (1719.9-1807.2)	1487.1 (1452.5-1519.8)	1225 (1192.3-1257.3)
KEN Baringo	2024.9 (1677.1-2393.6)	1963.6 (1623.2-2303.7)	1920.1 (1585-2260.2)	1966.6 (1624.1-2315.1)	2064.5 (1724.9-2425.6)	2091.3 (1779.6-2481.3)	2030.3 (1740.5-2384)	1931.8 (1660-2254.4)	1897.9 (1621.3-2207.8)	1972.6 (1681.8-2258.7)	1971.8 (1734-2214.1)	1766.1 (1570.7-1952.7)	1538.5 (1351.7-1734.1)	1323.3 (1143.2-1524.7)
KEN Bomet	1327.9 (1148.1-1557.2)	1328.9 (1157.6-1542.5)	1235 (1064.2-1451.3)	1218.7 (1043-1431.7)	1192.5 (1005.4-1402.2)	1147 (972.1-1353.8)	1092.4 (921.8-1278.8)	1035.4 (878.2-1204.5)	1055.6 (891.8-1228.1)	1212.6 (1021.7-1424.1)	1475.2 (1238-1730)	1491.9 (1268.7-1737.6)	1361.3 (1149.3-1574.3)	1075.7 (909.6-1278.8)
KEN Bungoma	2696.7 (2312-3126.2)	2337 (2005-2688.7)	2050.3 (1739.2-2377)	1912.2 (1614.1-2225.7)	1782.9 (1504.3-2099.6)	1644.6 (1392.8-1928.1)	1491.2 (1256.4-1744.6)	1394.9 (1176.9-1630.4)	1319.2 (1115.8-1543.4)	1315.1 (1106.1-1552.1)	1357.6 (1128.8-1603.4)	1342 (1130.1-1571.1)	1202.9 (1030.6-1386.9)	1101.4 (915.2-1280.4)
KEN Busia	3001.8 (2601.3-3475.3)	2427.2 (2093-2803.4)	2158.3 (1851.1-2505)	2036.2 (1729.8-2365.3)	1920 (1639.2-2246.5)	1664.1 (1428.1-1949.7)	1448 (1244.8-1685.6)	1334.8 (1156.6-1524.6)	1426.4 (1209.6-1653)	1929.8 (1554.4-2344.2)	2255 (1854.6-2746.8)	1922.2 (1612.1-2274.3)	1393.7 (1181.8-1617.4)	1142.2 (977.2-1323.4)
KEN Elgeyo Marakwet	2008.4 (1654.5-2335.6)	1880.2 (1523.1-2205.3)	1808.7 (1459-2154.8)	1773.2 (1434.1-2138.6)	1760 (1442.4-2116.3)	1730 (1417.2-2065.1)	1655.5 (1385-1969.5)	1568.6 (1320.2-1843.3)	1513.9 (1269.4-1776.9)	1552.3 (1304.4-1799.8)	1616.3 (1406.3-1824.8)	1610.1 (1439.4-1792.8)	1485.5 (1323.2-1634.7)	1377.8 (1218.5-1538)
KEN Embu	2508.7 (2071.7-2982.7)	2138.3 (1794-2533.4)	1860.1 (1546.2-2206.2)	1676.5 (1386.1-2000.1)	1479.1 (1229.3-1762.5)	1262.4 (1038.9-1521.9)	1102.9 (905.2-1328.3)	1029.2 (865-1228.2)	1105.1 (883.2-1367.8)	1320.6 (992.6-1711.7)	1396 (1088.6-1743.2)	1290.2 (1052.3-1563.6)	1145.3 (957.4-1392.1)	1006.1 (834.9-1218)
KEN Garissa	2789.1 (2443.5-3176.2)	2887.8 (2568.8-3233.7)	2421.6 (2108.3-2745.9)	2304.3 (1999.9-2619.5)	2150.4 (1863.9-2464.7)	1998.8 (1719.2-2275.1)	1896.2 (1656.6-2166.6)	1819.4 (1608.5-2061.5)	1759.2 (1552.3-1977.4)	1793.2 (1596.5-1993.4)	1787.6 (1614.3-1982.8)	1687.7 (1527.6-1892.4)	1582.8 (1421.9-1772.9)	1515.7 (1272.6-2053.8)
KEN Homa Bay	3232.8 (2809.1-3626.8)	2744.5 (2378.5-3114.9)	2553.9 (2198.2-2914.7)	2480.9 (2156.7-2850.9)	2479.7 (2155.5-2830.1)	2362.4 (2048.7-2685.8)	2124.9 (1852.1-2427.5)	1960.7 (1732.3-2235.7)	2006.4 (1757.1-2281.4)	2453.9 (2076.7-2909.1)	3249.2 (2713.1-3912.8)	3449.8 (2964.5-4037.7)	2603.7 (2296.3-2987.6)	1731.7 (1467.2-2067.6)
KEN Isiolo	2407.9 (2022.1-2822)	2381.5 (2037.4-2772)	2196.5 (1865.3-2561.9)	2236.2 (1916.5-2590.7)	2315.3 (2009.7-2640.6)	2193.6 (1892.2-2522.1)	1947 (1680-2253.3)	1790.4 (1547.3-2054.6)	1687.2 (1443.9-1928.3)	1672.2 (1424.3-1929.3)	1750.5 (1485.3-2027.1)	1616.9 (1364.3-1892.7)	1472 (1234.3-1746.1)	1304.3 (1099.2-1559.6)
KEN Kajiado	1953.2 (1615.5-2333.6)	1906.2 (1576.9-2238.6)	1717.2 (1390-2041.7)	1650.1 (1349.6-1969.9)	1568.2 (1297.1-1870.6)	1462.3 (1202.9-1738.8)	1341.9 (1106.8-1592.5)	1271.5 (1045.8-1486.9)	1343.1 (1098.9-1585.9)	1634 (1312-1995.1)	1848.8 (1560.6-2144.6)	1778.5 (1546.5-1999.5)	1508.9 (1310.5-1700.2)	1290.3 (1093.1-1464.9)
KEN Kakamega	2549.2 (2180.2-2986.6)	2253.8 (1928-2623.9)	1939.6 (1637.8-2295.8)	1799.5 (1506.6-2130.5)	1680.3 (1418.9-1983.8)	1528.8 (1295.1-1811.4)	1370.2 (1177.7-1602.9)	1296.3 (1116.5-1486.4)	1384.4 (1179.7-1595.8)	1737.7 (1444.6-2051.8)	1946.7 (1636.1-2289.9)	1768.5 (1512.4-2061.4)	1462.2 (1234.4-1687.8)	1199.3 (1005.2-1417.1)
KEN Kericho	1564.6 (1235.6-1950.7)	1519 (1209.1-1870.2)	1387.7 (1107-1722.3)	1295.5 (1041.9-1615)	1207.6 (975.7-1506.7)	1103 (902.8-1364.9)	1016.2 (826.6-1240.6)	961.2 (799.1-1145.5)	988.3 (814.4-1181.1)	1107.8 (900.2-1308)	1268.3 (1028-1507.3)	1307.1 (1066.5-1529.8)	1203.8 (977-1409.7)	1016.2 (844.6-1229.5)
KEN Kiambu	2353.2 (2034.7-2699.5)	2096.4 (1806.5-2408.5)	1883.6 (1594.3-2192.4)	1750.1 (1456-2052.4)	1593.9 (1321.1-1884.2)	1399.8 (1179.5-1644.9)	1317.8 (1124-1538)	1256.5 (1079.4-1453.1)	1303.3 (1135.9-1516.3)	1659.7 (1378.6-1980.7)	1939.3 (1614.4-2275.6)	1824.6 (1576.4-2096.7)	1445.1 (1255.2-1627.4)	1201.1 (1055-1362.1)
KEN Kilifi	2987.8 (2635.7-3357.6)	2624.4 (2295.1-2949.6)	2357.3 (2041.4-2675.9)	2260.1 (1959.9-2590.3)	2141.3 (1871.6-2437.3)	1928.3 (1676.7-2219.3)	1735.3 (1515.3-1989.7)	1582.1 (1372.6-1798.7)	1515.5 (1301.1-1725.9)	1565.2 (1335.6-1813.2)	1639.4 (1404.4-1887.4)	1611.7 (1399.5-1831.8)	1440.1 (1250.3-1637.6)	1189.9 (1007.9-1391.4)
KEN Kirinyaga	2525 (2099.9-2986)	2140 (1769-2544.5)	1891.7 (1546.7-2273)	1732 (1413.7-2110.9)	1618.7 (1329.2-1971.6)	1410.6 (1152.3-1754)	1179.4 (958.8-1460.8)	1092.7 (890.8-1341.3)	1110.5 (904.3-1357.4)	1209.6 (954.3-1481.5)	1317.6 (1011.6-1611.5)	1342.5 (1087.1-1657.3)	1207.8 (989.9-1482.5)	1087.1 (902.3-1324.6)
KEN Kisii	2785.4 (2413.7-3201.1)	2614.8 (2278.4-2994.3)	2236.9 (1906.5-2597.1)	2094.9 (1774.7-2443.2)	1991.5 (1668.3-2342)	1866.6 (1569.8-2196.2)	1735.4 (1459.9-2026.9)	1649.2 (1403.8-1924.5)	1661.2 (1437.7-1912.2)	1875 (1613.6-2148.4)	2142.8 (1860.2-2408.1)	2088.9 (1878.9-2295.6)	1759.1 (1590.6-1936.2)	1509.3 (1331.9-1684.8)
KEN Kisumu	2777.5 (2389.8-3209.4)	2461 (2110.3-2833.4)	2201.2 (1867.5-2546)	2074.8 (1762.8-2411.1)	2000.6 (1720.6-2314)	1862.9 (1608.7-2165.9)	1714.1 (1481.7-1976.2)	1585.9 (1375.3-1823.9)	1745 (1475.7-2061.8)	2528.1 (2024-3079.1)	3114.9 (2608.8-3666.4)	2876.3 (2524.4-3275.9)	2097 (1816.2-2396.5)	1616.2 (1364.1-1912.7)
KEN Kitui	2757 (2369.5-3158.2)	2310.8 (1977-2685.4)	2108.8 (1773.2-2475.4)	2001.5 (1694.4-2355)	1889.1 (1598.6-2238.8)	1739.7 (1469.5-2043.2)	1557.7 (1321.2-1815.8)	1436.7 (1225.5-1684.2)	1406.4 (1192.3-1626.1)	1500.1 (1265.9-1733.9)	1586.2 (1342.5-1833.4)	1464.7 (1280.2-1658.7)	1238.1 (1076.7-1414.2)	1052.2 (927.4-1205.4)
KEN Kwale	2822.9 (2405.1-3285.2)	2531.2 (2180.1-2906.5)	2229.6 (1906.4-2579.6)	2117.6 (1791.2-2462.7)	2025.3 (1731.5-2343.8)	1884.5 (1610.8-2175.3)	1691.1 (1462.6-1961.9)	1524.2 (1302.5-1762.7)	1612.2 (1295.2-2020.1)	1980.5 (1540.8-2506.3)	2007.6 (1624-2430)	1787 (1500.7-2085.6)	1524.2 (1301.1-1737.4)	1246.6 (1059.8-1436)
KEN Laikipia	2056.1 (1683.2-2475.1)	1921.4 (1593.2-2265.6)	1670.3 (1382.2-1979.6)	1502.5 (1252-1798.5)	1351.2 (1123.3-1634.5)	1181.1 (978.2-1436.5)	1051.1 (863-1277.3)	987.2 (820.2-1179.9)	1027.2 (856.9-1215.2)	1158.1 (949.4-1374)	1362.8 (1096.3-1652.1)	1432.3 (1156.4-1704.2)	1348.7 (1071.3-1583.8)	1142.8 (956-1395.8)
KEN Lamu	3474.9 (2998.3-3966.5)	3155.3 (2753.3-3586)	2887 (2489.8-3298.8)	2694 (2294-3087.9)	2331.7 (1997.6-2705.7)	1839.9 (1548-2162.1)	1472.1 (1230.5-1759.6)	1331.3 (1103.9-1582.5)	1327.2 (1103.4-1584)	1398.5 (1151.5-1678.9)	1484.5 (1207.3-1791.1)	1468.1 (1198.2-1779.3)	1386.3 (1133.4-1712.4)	1265 (1036.4-1513.1)
KEN Machakos	2336.3 (1980-2722.2)	2121.4 (1798.2-2468.7)	1845.2 (1546.9-2197.8)	1701.5 (1426.2-2054)	1565.9 (1314.9-1872.5)	1399.6 (1174.5-1658.4)	1230.2 (1044.3-1457.3)	1123.5 (955.4-1318.8)	1089 (928-1269)	1140.1 (964.5-1346.4)	1252.7 (1044.7-1491)	1319.4 (1125.6-1522.9)	1216.5 (1064.1-1381.2)	1103 (946.5-1259.7)
KEN Makueni	1737.8 (1464-2025.8)	1550.7 (1304.5-1819.3)	1494.2 (1244.5-1761.1)	1491.3 (1236.6-1770.4)	1453.2 (1217.2-1720.8)	1378 (1159.4-1625.9)	1294.6 (1094.9-1528.5)	1229.4 (1048.1-1445.8)	1231.2 (1057.3-1447.4)	1389.4 (1162.6-1657.7)	1660.3 (1364.4-1988.5)	1706.3 (1402.8-2009.2)	1446.7 (1221.9-1670.6)	1087.8 (916.1-1264.5)
KEN Mandera	2501.1 (2142.9-2857)	2630.3 (2303.9-2975.8)	2201.7 (1881.9-2547.6)	2145.9 (1839.9-2452.9)	2082.1 (1788.7-2397.6)	1988 (1716.7-2277.5)	1894.5 (1649.9-2168.5)	1826.3 (1602.7-2085.8)	1821.1 (1605.9-2067.7)	1879.8 (1649.5-2107.3)	1864 (1665.8-2060)	1873.5 (1706.8-2058.1)	1825.8 (1645.8-1999.2)	1569.4 (1355-1769.4)
KEN Marsabit	3872.7 (3273.7-4511)	4378.7 (3832.1-4977.4)	3766.9 (3334.9-4222.7)	3354.4 (2990.6-3743.2)	2702.1 (2366-3066.2)	2099.3 (1798-2428)	1676.7 (1427.2-1976.6)	1466.4 (1248.9-1705.8)	1393.8 (1175.9-1627)	1441.3 (1201.4-1691.4)	1530.5 (1268.2-1780.2)	1593.4 (1345.8-1838.3)	1491.7 (1271.4-1731.1)	1339.7 (1117.5-1585.8)
KEN Meru	2285.2 (1920.1-2663)	1989.2 (1696.5-2302.9)	1737.8 (1464.9-2030)	1603.8 (1347.1-1878.6)	1479.6 (1245-1736.1)	1338.5 (1137.8-1573.8)	1200.6 (1011.8-1416.5)	1122 (961.2-1318.1)	1139.7 (975.9-1334.7)	1282.6 (1084.1-1535.4)	1444.3 (1219.9-1717.2)	1455.4 (1266.1-1655.6)	1348.1 (1177.1-1511.3)	1197.2 (1014.6-1382)
KEN Migori	3011.1 (2605.8-3501.6)	2524.6 (2183.9-2897.6)	2381.5 (2029.7-2722.2)	2364.5 (2036.1-2707.6)	2327.8 (2010.1-2660.8)	2309.9 (2030.1-2636.1)	2240.7 (1969.6-2558.6)	2137.7 (1890-2435.3)	2077.7 (1831-2345.7)	2288.9 (1959.8-2690.8)	2726.4 (2323.4-3252.6)	2923.9 (2505.2-3391.5)	2449.4 (2083.5-2848.6)	1772.3 (1470.5-2105.9)
KEN Mombasa	2306.6 (1895.1-2715.4)	2307.8 (1932.8-2721.9)	1920.3 (1589.1-2296.4)	1725.3 (1417.4-2061.4)	1548.6 (1262.1-1860.7)	1358 (1106.3-1639.6)	1186.8 (973.7-1438.4)	1101.7 (908.4-1329.3)	1204.8 (984.6-1487.5)	1600.5 (1229-2021.1)	1758.8 (1387.6-2186.4)	1606.5 (1291.7-1964.4)	1397.4 (1141.3-1702.3)	1094.5 (877.5-1337.2)
KEN Murang'a	2073.5 (1775.1-2397.1)	1730.3 (1469.4-2011.4)	1587.7 (1320.4-1872.5)	1510 (1263.8-1792.5)	1424.4 (1197.1-1683.5)	1304.2 (1092.9-1539.5)	1191 (1015.2-1393.8)	1132.5 (973.7-1314.7)	1143.9 (986.1-1313.8)	1247.1 (1069.1-1446.3)	1352.3 (1157.3-1565.5)	1430.4 (1245.2-1650.9)	1231 (1080.9-1390)	1071.7 (923.2-1237.2)
KEN Nairobi	2600.2 (2244.5-2983.8)	2607.6 (2289.2-2947.8)	2054.7 (1782.5-2370.4)	1762.4 (1507.4-2052.6)	1537.6 (1303.3-1798.3)	1312.8 (1121.7-1538.9)	1156.1 (995-1351)	1108.9 (956.8-1272.9)	1261.3 (1075.9-1476.7)	1691.7 (1400.7-2001.8)	1941.1 (1625.9-2217.7)	1871.1 (1698.3-2032.2)	1548.7 (1427.8-1671.4)	1263.1 (1142.1-1382.4)
KEN Nakuru	1801.3 (1509.9-2095.5)	1737.1 (1473-2017.9)	1520.7 (1292.5-1802.1)	1412.4 (1204.4-1666.4)	1327.5 (1123.2-1572.2)	1232.6 (1035.7-1442.6)	1118.2 (935.4-1289.4)	1037.8 (869.4-1190.8)	1084.5 (872.7-1284.7)	1477.2 (1158.8-1843.5)	1766.8 (1395.2-2158.4)	1541.2 (1295-1801.4)	1125.2 (984-1290.5)	868 (782.8-995.7)
KEN Nandi	1674.5 (1386.4-2006.6)	1550.4 (1290.9-1841.9)	1435.2 (1189.2-1732.2)	1392 (1155.9-1680.2)	1361.7 (1147.6-1640.2)	1326 (1131.1-1577.6)	1286.1 (1096.1-1501.7)	1259.9 (1085.8-1466.6)	1371.8 (1153.1-1645.8)	1688.2 (1363.5-2064.4)	1794.3 (1514.4-2080.8)	1618.3 (1422.9-1827.1)	1376.9 (1202.1-1544.8)	1144 (993.3-1335.6)
KEN Narok	1986.6 (1655.7-2335.9)	1956.1 (1637.2-2281.2)	1795.9 (1496.3-2121.7)	1786.2 (1482.4-2112.8)	1802.5 (1495.7-2127.2)	1790.5 (1494.2-2107.5)	1734.1 (1462-2015.9)	1701.1 (1442.5-1954.6)	1738.9 (1497.5-1994.9)	1846.2 (1597.5-2105.7)	1905.2 (1653.6-2184.1)	1839.4 (1616.5-2085.9)	1578.8 (1381.8-1755.9)	1399.6 (1192-1602)
KEN Nyamira	2506.8 (2166.7-2862.4)	2219.1 (1891.2-2550)	2104.2 (1784.7-2417.7)	2045.1 (1724.7-2335.6)	1948 (1645-2237.2)	1857.9 (1567.4-2138.2)	1777.7 (1515.9-2050.4)	1702.7 (1477.8-1938.4)	1710 (1490.3-1945)	1924.4 (1672.5-2187.2)	2269.3 (1975.6-2597.9)	2440.2 (2132.6-2765.2)	2230.2 (1960.6-2518.5)	1800 (1578.7-2057.8)
KEN Nyandarua	2380.6 (2018-2737.7)	2206.3 (1863.1-2565.3)	1973.2 (1634.1-2323.9)	1849 (1525.5-2220.4)	1751.3 (1454.5-2083.3)	1635 (1364.6-1956.3)	1496.7 (1271.7-1770.3)	1432.8 (1231.2-1661.2)	1442.4 (1238.3-1662.6)	1565.1 (1358.7-1784.3)	1798 (1558-2024)	1988.6 (1733.3-2229.5)	1824.4 (1587.7-2058.9)	1535 (1306.9-1782.7)
KEN Nyeri	2612.2 (2251.2-3011.8)	2151.6 (1835.5-2499)	1941.7 (1636.6-2262.9)	1802.1 (1501.1-2109.1)	1668.5 (1388.7-1938.7)	1532.6 (1269.3-1787.1)	1408.7 (1163-1620.6)	1337.3 (1128.5-1538.5)	1364.1 (1170-1560.9)	1577.5 (1354.3-1821.6)	1855.3 (1579.5-2176.7)	1822.1 (1569.1-2097.6)	1525.7 (1319.9-1712.3)	1354.2 (1136.7-1560.8)
KEN Samburu	1307 (1035.3-1614.9)	1267.8 (1017-1540.9)	1207 (974.9-1474.9)	1205.4 (982.5-1484)	1224.8 (1007.8-1506.3)	1240.1 (1028.5-1519.7)	1236.4 (1039.1-1490.9)	1229.3 (1038.5-1444.1)	1293.9 (1091.5-1527.9)	1490.1 (1215.5-1815.1)	1666.1 (1361.6-2015.3)	1623.3 (1360.9-1943.8)	1489.7 (1258.6-1773.6)	1308.9 (1087.1-1562.5)
KEN Siaya	3056.2 (2627-3496.5)	2763.2 (2359.5-3180.3)	2410.4 (2041.7-2792.3)	2208.7 (1847.7-2578.2)	2042.6 (1714.2-2376.3)	1866.5 (1570.6-2186.5)	1674 (1421.5-1952.6)	1540 (1335-1774.7)	1535.9 (1334.5-1770.4)	1673.1 (1413.9-1967.3)	1947.2 (1572-2423.9)	2108.8 (1746.4-2537.3)	1619.1 (1369.6-1896.3)	1143.7 (974-1370.8)
KEN Taita Taveta	3141.3 (2731.4-3584.9)	2873.2 (2470.3-3294.9)	2491.7 (2129.8-2886.2)	2274.5 (1950.8-2656.2)	2093.7 (1770.8-2456.9)	1859.1 (1549.4-2186.8)	1625.7 (1346.1-1917.2)	1455.6 (1211.7-1711.2)	1396 (1161.6-1658.7)	1471 (1232.3-1749)	1664.5 (1386.9-1961.7)	1802.5 (1514.7-2098.4)	1739.2 (1489.5-1995)	1441.1 (1198.1-1648.4)
KEN Tana River	3712.2 (3258.5-4195.3)	3178.4 (2783.6-3596.9)	2804.9 (2433.4-3207.1)	2614.7 (2257.5-2991.9)	2389.6 (2047.4-2762.9)	2142.4 (1795.1-2480.6)	1906.4 (1602.4-2240.5)	1781.2 (1504.9-2071.5)	1767.3 (1494-2054.2)	1937.4 (1642.6-2237.6)	2171.1 (1852.5-2495)	2240.5 (1946.6-2529.6)	2128.2 (1831.1-2386.8)	1788.2 (1510.6-2072.7)
KEN Tharaka Nithi	2959.5 (2535.2-3418.3)	2403.7 (2053.2-2792.8)	2358.7 (2011-2727.3)	2362 (2006.8-2723.5)	2317.2 (1973.4-2686.3)	2159.8 (1842.9-2491.2)	2005.8 (1720.3-2317.1)	1928.2 (1664.3-2213)	1937.7 (1682.7-2219)	2064.3 (1812.2-2346.2)	2128.6 (1878.9-2414.1)	1940.2 (1725.2-2178.3)	1657.3 (1466.7-1855.3)	1420 (1224.1-1636.5)
KEN Trans Nzoia	1578 (1290.7-1923.3)	1666 (1393.7-1968.3)	1471.3 (1225.4-1766.3)	1395.7 (1151.1-1696)	1312 (1092.1-1579.6)	1182.2 (988-1432.8)	1061.5 (889.8-1272.4)	997.8 (846.1-1175.4)	1055.6 (882.4-1270)	1335.5 (1068.1-1643.3)	1582.7 (1250.8-1933.6)	1503.6 (1226.6-1824.6)	1314.3 (1082.3-1570.7)	1073.6 (886.7-1297.1)

Table 1 Age-standardised mortality rates by location and year for both sexes for 1950, 1955...2005, 2010, 2017

Location	1950	1955	1960	1965	1970	1975	1980	1985	1990	1995	2000	2005	2010	2017
KEN Turkana	1596.4 (1309.8-1931.2)	1647.2 (1375.1-1956.9)	1519.9 (1247.5-1824.9)	1526.6 (1264.2-1826.7)	1605.6 (1354.6-1903.6)	1713.8 (1470.4-2008.1)	1754.8 (1528.6-2012.8)	1670 (1460.7-1910.5)	1623.4 (1406.7-1865.6)	1776 (1513.7-2072.1)	2087.4 (1806.8-2421.1)	1976.1 (1713.9-2268.2)	1762.3 (1536.4-2012.5)	1401.3 (1201.4-1624.2)
KEN Uasin Gishu	1581.5 (1304.3-1847.5)	1572.9 (1310.2-1815.9)	1434.3 (1190-1662.7)	1391.8 (1159.1-1602.1)	1350 (1115.3-1574.3)	1302.9 (1075.5-1535.4)	1242.9 (1030.2-1471.6)	1202 (1005-1409.1)	1223.4 (1019.3-1428)	1317.3 (1104.7-1523.8)	1448 (1227.1-1640.1)	1419.5 (1245-1578.2)	1282.9 (1114.9-1464.8)	1028.9 (855.9-1208.4)
KEN Vihiga	1882.2 (1568-2221.1)	1685.9 (1383.4-2012)	1616.4 (1320.7-1943.1)	1587.7 (1303.5-1905.1)	1535.7 (1268.1-1856.9)	1494 (1238.9-1785.5)	1430.9 (1183.8-1694.9)	1402.7 (1178.5-1657.3)	1471.3 (1227.7-1744.7)	1812.2 (1475.4-2190.7)	2132.3 (1762.8-2517)	1967.9 (1660.9-2313.9)	1492.8 (1254-1761.1)	1302.6 (1067.2-1541.5)
KEN Wajir	1985.4 (1715.8-2293.7)	2069 (1800.9-2363.3)	1868.8 (1609.2-2157.6)	1857 (1591.9-2135.9)	1850.9 (1592-2126.5)	1811.7 (1559.2-2071.3)	1808.6 (1579-2060)	1805.3 (1595.2-2047.7)	1815.4 (1605-2050.7)	1915.5 (1694.2-2135.4)	2020.7 (1823-2219)	1936.4 (1779.6-2112.8)	1813.5 (1655.5-1992.4)	1635 (1410.1-1876.7)
KEN West Pokot	2284 (1908.9-2686.9)	2129.9 (1809.4-2472.3)	1977.5 (1679.1-2311.1)	1935.3 (1657.5-2259.4)	1906.7 (1635.2-2226.4)	1797.1 (1550.1-2088.3)	1638.3 (1414-1900.6)	1524.3 (1332.4-1753.8)	1534.8 (1336-1765.4)	1624.2 (1406.9-1868.9)	1610.1 (1404.3-1812.7)	1538.9 (1362.5-1697.2)	1489.9 (1319.2-1656.2)	1285 (1106.3-1475.1)
MDG Madagascar	2483.2 (2270.7-2698.4)	2336.5 (2200.2-2482.3)	2228.7 (2122.9-2340.2)	2064 (1981.2-2147.1)	2054.1 (1983-2123.9)	1908.5 (1826.9-1987.4)	1770.9 (1684.3-1869.6)	1798.4 (1706.4-1892.9)	1785.8 (1706.9-1870.2)	1712 (1632.8-1790.5)	1660.9 (1568.5-1757.9)	1589.1 (1470.1-1712.4)	1509.7 (1361.9-1653.2)	1396.4 (1231-1565)
MWI Malawi	3450.2 (3124-3792.7)	3189.6 (2923.4-3459.2)	2971 (2740.1-3219.8)	2779 (2578.3-2975.6)	2544.8 (2384.1-2713.5)	2279.9 (2170.5-2396.4)	2068.1 (1979-2153.2)	2016.8 (1943.9-2097.2)	2160.9 (2048.1-2301.6)	2568.7 (2371.3-2812.8)	2732 (2497-2979.7)	2465.1 (2273-2662.8)	1863.9 (1758.3-1973.4)	1313.7 (1215-1425.7)
MOZ Mozambique	2933.9 (2616-3261.2)	2700.9 (2445.4-2966.3)	2567.5 (2332.6-2797.7)	2450.5 (2225.8-2686.4)	2280.7 (2082.5-2499.9)	2095.6 (1925.2-2275.3)	2042 (1905.4-2187.3)	2440.2 (2327.3-2554.7)	2070.1 (1973.8-2172.3)	2062.5 (1955.1-2165.8)	2140.5 (2003.6-2285.9)	2191.5 (2036.9-2351.8)	2119.7 (1963.4-2287.5)	1670.1 (1509-1831.6)
RWA Rwanda	3338.5 (3021.7-3653.2)	2985.4 (2745.3-3236.3)	2867.2 (2651.9-3104.2)	2816.3 (2615.1-3042.7)	2717.1 (2519.7-2928)	2632.1 (2445.2-2825)	2458 (2292.9-2627.5)	2320.6 (2176.8-2470.8)	2474.7 (2354.9-2594.3)	2787.4 (2668.1-2911.8)	2424.4 (2312.1-2541.7)	1738.3 (1645.4-1824.4)	1285 (1204.9-1371.6)	1091.5 (996.3-1192.5)
SOM Somalia	2992.5 (2524.4-3500.2)	2834.7 (2426.9-3309.4)	2679.9 (2322.9-3092.8)	2578.2 (2217-2995.5)	2501.7 (2141.5-2925)	2537.5 (2186.7-2925)	2292.4 (1959.1-2666.1)	2275.6 (1969.5-2616.8)	2354.7 (2055.1-2705.4)	2323.2 (1985.3-2672.7)	2190.9 (1863.2-2530.4)	1998.7 (1670.9-2351.6)	1951.1 (1611.8-2325.7)	1699.8 (1388.9-2071.9)
SSD South Sudan	2754.1 (2292.6-3279.1)	2697.5 (2278.9-3202.9)	2581.9 (2205.5-3016.3)	2428.3 (2077.5-2803.6)	2271.3 (1935.4-2645.2)	2142.2 (1816.5-2500)	2071.2 (1758.5-2425.1)	2058.3 (1764.5-2397.8)	2058.7 (1759-2409.4)	2001.9 (1673.6-2350.4)	1931.8 (1591.7-2281.8)	1779.7 (1447.8-2143.2)	1680.9 (1350.4-2036.4)	1608.8 (1337.2-1932.5)
TZA Tanzania	2781.3 (2494.5-3092.2)	2472.9 (2265.9-2696)	2377.8 (2198.6-2590.5)	2221.7 (2068.3-2381.2)	2043.7 (1920.8-2177.4)	1866.9 (1748.1-1990.8)	1710.4 (1608.4-1823.3)	1647.2 (1559.2-1742.2)	1739.7 (1634.7-1864.5)	1895 (1765.8-2049.1)	1861.5 (1736.3-2002.8)	1653.3 (1539.7-1767.5)	1397.1 (1308.2-1491.1)	1116.9 (1040.5-1204)
UGA Uganda	2830.6 (2527.6-3165.3)	2462.9 (2231.7-2713)	2352.7 (2141.4-2570.4)	2207.1 (2018.7-2408.1)	2091.9 (1911.6-2266.5)	2022.8 (1873.7-2181.3)	2124.8 (2001.4-2257.4)	2109.8 (1935.1-2365.4)	2276 (1997.4-2586.8)	2433.1 (2233.4-2619.9)	2307.2 (2169.8-2447.3)	1956.7 (1839.4-2061.7)	1564.4 (1453.5-1672.8)	1190.1 (1084.1-1298.7)
ZMB Zambia	2554.6 (2288.7-2839.2)	2342.8 (2118.2-2583.1)	2234.6 (2027.5-2454.7)	2113.9 (1929.9-2313.3)	1940.2 (1768.9-2118.8)	1824.8 (1675.3-1982.4)	1799.7 (1676.7-1929.2)	1862.8 (1761.1-1969.2)	2149.7 (2010.5-2337.1)	2644.2 (2432.3-2873.9)	2700.4 (2507.9-2895.3)	2329.2 (2198.5-2486)	1795.6 (1710.4-1896.5)	1363.9 (1256.6-1469.9)
Southern sub-Saharan Africa	2102.2 (2023.4-2183.5)	1974.3 (1901.8-2049)	1889.7 (1819.3-1957.9)	1810.1 (1745.4-1880.1)	1691.7 (1633.6-1752.7)	1515.7 (1466.1-1568.6)	1437.8 (1395.1-1481.8)	1277.8 (1246.2-1310.1)	1177.8 (1145.6-1216.9)	1353.4 (1303.3-1408.5)	1937 (1873.3-2002.8)	2172.7 (2101.3-2253.3)	1872.4 (1821.1-1925.5)	1199.6 (1168.7-1231.8)
BWA Botswana	2380.1 (2150.9-2631.8)	2199.4 (1994.8-2412.5)	2113.8 (1907.9-2331.1)	2014.2 (1826.6-2206.1)	1883.4 (1727.5-2060.7)	1658.3 (1527.8-1808)	1488.4 (1380.5-1617.1)	1334.5 (1242.4-1448.3)	1354 (1229.2-1521.6)	1507.3 (1312-1735.5)	1849.9 (1564.6-2197.5)	2031.3 (1783.2-2300.2)	1563.2 (1439-1717.5)	1080.3 (999.3-1183.2)
LSO Lesotho	2386 (2163.2-2611.2)	2242.3 (2027.3-2460.8)	2155.2 (1950.8-2365.3)	2085.7 (1874.9-2282.5)	1996.4 (1795.6-2187.6)	1877.8 (1702.3-2053.9)	1698.5 (1541.8-1863.3)	1583.4 (1457.9-1721)	1536.6 (1432.1-1646.6)	1742.6 (1588-1899.8)	2446.1 (2211.5-2717.6)	3006.4 (2707.6-3315.9)	2638.9 (2396.7-2876.7)	2018 (1774.9-2260.8)
NAM Namibia	2278.8 (2036.5-2554.6)	2108.1 (1902.1-2314.9)	2011.5 (1818.5-2200.8)	1896.9 (1722.7-2082.7)	1884.8 (1733.4-2039.6)	1814.7 (1693.3-1952.7)	1799.9 (1701.2-1907.7)	1677.2 (1592.4-1766.1)	1531.3 (1445.2-1638.7)	1656.4 (1545.9-1782.1)	2077 (1917.4-2256)	2170.5 (1969.8-2388.7)	1605.3 (1462.8-1757.4)	1154.7 (1045.4-1280.7)
ZAF South Africa	1980.3 (1889.2-2079.6)	1879.6 (1795.2-1972.5)	1812.9 (1729.3-1899.5)	1753.5 (1680.1-1832.4)	1638.8 (1572-1712.6)	1457.3 (1400.1-1522)	1393.7 (1344.4-1446.8)	1240.8 (1201.5-1280.7)	1090.6 (1064.2-1117.3)	1171.3 (1149.8-1194.3)	1752.3 (1697.8-1818.9)	1995 (1914.1-2084.8)	1751.6 (1693.1-1818.3)	1123.2 (1092-1157.4)
SWZ Swaziland	3152.8 (2860.2-3469.4)	2864.9 (2624.4-3122.5)	2662.2 (2432.4-2894.9)	2424 (2203-2638.3)	2224.7 (2014.9-2422.3)	2013.6 (1817.6-2210.9)	1841.1 (1671.9-2002.1)	1693.2 (1554-1839.2)	1537.9 (1422.9-1658.1)	1670.1 (1533.7-1829.5)	2582.4 (2260-2921.8)	3157.8 (2808.8-3505.8)	2852.7 (2525-3166.7)	1636.8 (1442.1-1868.1)
ZWE Zimbabwe	2657.1 (2404.6-2933)	2394.2 (2168.9-2633.5)	2211.4 (2007.4-2430)	2060.8 (1881.4-2251.5)	1909.3 (1746.1-2084.1)	1751.4 (1615.4-1889)	1620 (1509.9-1730.1)	1390 (1305.3-1476.5)	1463.6 (1307.5-1703.9)	2063.3 (1771.1-2414.8)	2760.2 (2446.2-3079.4)	3011.7 (2749-3291.9)	2484.7 (2310.6-2672.2)	1532.7 (1394.7-1681.2)
Western sub-Saharan Africa	3000.7 (2750.5-3277.1)	2728.4 (2530.1-2954.1)	2551.4 (2385.1-2741.7)	2327.7 (2174.4-2501.4)	2207.9 (2055-2383.6)	2023.9 (1878.8-2179.8)	1841.6 (1719.1-1980.2)	1732.6 (1613.5-1861.4)	1715.7 (1591.4-1848.1)	1727.8 (1593.9-1874.6)	1723.3 (1576.2-1877.6)	1617.8 (1465-1776.3)	1459.9 (1333.9-1609.2)	1260.4 (1163.7-1392.2)
BEN Benin	3204.6 (2855.8-3571.4)	2874.1 (2594.6-3154)	2691.5 (2450.9-2920.1)	2484.9 (2267.2-2692)	2316.8 (2118.4-2508.8)	2128.1 (1965.2-2288.7)	1948.4 (1812.3-2091.4)	1790.2 (1685.3-1903.9)	1670.3 (1584.2-1765.8)	1614 (1526-1712.3)	1589.1 (1483.5-1705.1)	1511.2 (1390.3-1638.4)	1406.1 (1273.6-1547.8)	1252.4 (1115.3-1408.3)
BFA Burkina Faso	3569.1 (3222.2-3942.5)	3276.1 (2999.2-3577.3)	2988.6 (2739.9-3257)	2743.8 (2508.5-2982.3)	2563.6 (2362.8-2768.4)	2391.8 (2230-2558.8)	2166.8 (2034.9-2299.9)	2000.5 (1895.4-2096.5)	1966.9 (1868.2-2068)	1997.5 (1884.9-2113.5)	1902.7 (1803.9-2011.2)	1736.8 (1645-1836.7)	1587.5 (1492.7-1689.5)	1409.7 (1294-1533.1)
CMR Cameroon	3302.3 (3014.9-3599.4)	2899.5 (2655.5-3134.2)	3222.2 (2994.5-3441.5)	2435.7 (2246.9-2628.8)	2244.2 (2083.7-2404.2)	2071.8 (1943.6-2199)	1919.9 (1806-2030.6)	1738.8 (1647-1829.6)	1675.7 (1591.6-1757.8)	1765 (1675.1-1854.4)	1869.2 (1754.1-1984.7)	1863.1 (1717.3-2012.9)	1679.6 (1516.9-1838.3)	1361.4 (1205.9-1529.4)
CPV Cape Verde	1881.2 (1688.3-2091.1)	1492.4 (1335-1667.8)	1471.1 (1328.2-1628.2)	1340.1 (1219.9-1477.5)	1427 (1330.7-1535.3)	1227.8 (1167.3-1296.4)	1013.2 (982.6-1044.5)	949.3 (926.9-972.1)	862.9 (840.4-886.3)	893.6 (849-935.8)	834.9 (778.4-893.6)	798.7 (759.7-841.9)	747.3 (728.3-767.8)	702.4 (659.5-743.1)
TCD Chad	2998.6 (2666.5-3353.6)	2635.9 (2357.5-2918.9)	2430.2 (2173.2-2694.1)	2262.9 (2029.2-2498.9)	2193.8 (1999.9-2412.4)	2020.9 (1857.1-2205.4)	1978.6 (1846-2130.3)	1879.4 (1769.6-1995.5)	1808.6 (1708.4-1914.7)	1837.1 (1729.2-1955.1)	1935.3 (1818.1-2061.4)	1855 (1738.3-1980.7)	1699.3 (1574.7-1832.6)	1502.5 (1361.7-1649.9)
CIV Cote d'Ivoire	2502.8 (2265.9-2744.5)	2460.3 (2249.4-2686.7)	2293.6 (2099.9-2496.6)	2152.7 (1971.5-2345.2)	2003.9 (1847.1-2169.1)	1839.5 (1708.5-1980.6)	1660.1 (1550.6-1774.2)	1628.7 (1522.9-1750)	1776.4 (1626.6-1993.3)	2025 (1874.6-2194.3)	2027.6 (1885.7-2168.4)	1910.1 (1769.2-2053.4)	1691.9 (1552.1-1830.7)	1381.7 (1247.5-1530.9)
GMB The Gambia	2440.6 (2166.4-2720.3)	2465.9 (2214-2726.3)	2389.6 (2134.1-2630)	2238.9 (2001-2471)	2011.9 (1789.3-2230.1)	1846.4 (1642-2064.8)	1711.6 (1524.4-1902.6)	1605 (1442.2-1776.3)	1536.9 (1380-1698.3)	1494.7 (1353.2-1638.6)	1487.5 (1355.2-1618.5)	1474.8 (1353.3-1599)	1413.6 (1303.2-1526.9)	1301.5 (1191.5-1433.5)
GHA Ghana	2673.2 (2404.2-2955.9)	2397.2 (2170.2-2651.9)	2130.3 (1923.3-2357.6)	1922 (1733.2-2137.8)	1773.7 (1591.5-1967.3)	1649.3 (1487.2-1822.8)	1589.1 (1428.9-1754.7)	1582.7 (1441.9-1723.9)	1533.1 (1413.1-1671.4)	1531.4 (1422.3-1655.6)	1567.8 (1461.4-1675.9)	1562.9 (1465.4-1654.8)	1485.2 (1399.8-1571.9)	1260.3 (1164.7-1362.2)
GIN Guinea	3007.1 (2667.9-3360.8)	2823.2 (2542.7-3111.8)	2579.2 (2304-2837.5)	2364.7 (2120.7-2580)	2262.2 (2059.7-2457.7)	2290.6 (2118.1-2477.4)	2036.5 (1911.1-2170.8)	1932.5 (1834.1-2038.2)	1854.4 (1765.5-1943.2)	1796.1 (1712.5-1883.2)	1801.5 (1709-1895.1)	1755.8 (1654.1-1858.7)	1703.3 (1593.6-1815.6)	1547 (1423.8-1675.8)
GNB Guinea-Bissau	3962 (3587.4-4353.2)	3589.2 (3287.8-3887.9)	3385.7 (3107.3-3673.3)	3461.6 (3180-3740.2)	3260.3 (2974.5-3540.8)	2838 (2581.7-3097.7)	2665.8 (2434.1-2909.6)	2546.4 (2326.4-2764.8)	2389.9 (2195.4-2585.8)	2271.8 (2101.6-2453.9)	2183.3 (2043.3-2326.7)	2107.4 (1968-2250.9)	1958.3 (1803.1-2107.1)	1683.8 (1526.3-1846.1)
LBR Liberia	3619.1 (3285.4-3965.2)	3139.6 (2880.7-3400.3)	2816.7 (2572-3042.9)	2577.6 (2369.5-2792.1)	2332.2 (2151.8-2513.7)	2110.2 (1929.7-2295.3)	1917.4 (1762.6-2085.7)	1801.4 (1668.6-1949.1)	2126.5 (2007.4-2267)	1988.9 (1872.9-2132)	1763.1 (1647.7-1893.1)	1561.8 (1455.2-1674.8)	1425.1 (1314.2-1543.9)	1276.3 (1155.3-1409.1)
MLI Mali	3841.8 (3438.9-4286)	3542 (3220.8-3845.5)	3295.3 (3036.1-3566.3)	3165.7 (2942.6-3393.6)	3022.5 (2843.3-3211)	2832.8 (2681-2980.4)	2505.7 (2385.6-2622.3)	2287.8 (2188.5-2391.1)	2125.4 (2046.1-2206.8)	1972.6 (1891.4-2052.9)	1801.4 (1721.8-1881.2)	1586.1 (1505-1676.2)	1439.8 (1348.5-1533.8)	1331.2 (1211.2-1449.2)
MRT Mauritania	3070.6 (2780.8-3356.8)	2746.3 (2497.1-2980.4)	2550.7 (2327.2-2773.4)	2308 (2095.6-2512)	2076 (1882.6-2271.1)	2006.9 (1838.4-2184.2)	1847.7 (1701-1992.2)	1742.6 (1625.7-1867.7)	1617 (1517.1-1715.5)	1477.8 (1396-1571.1)	1331 (1259.4-1409.4)	1203.1 (1130.6-1283.2)	1074 (985.5-1163.3)	979.7 (873.6-1091)
NER Niger	3312.9 (3022.5-3647.8)	3101.5 (2829.6-3384.1)	2857.7 (2596.8-3123.9)	2615.2 (2398.4-2836.5)	2518 (2324.8-2709.2)	2505.7 (2344-2663.3)	2377.7 (2241.7-2510.4)	2256.8 (2132.5-2376.1)	2089.1 (1983.9-2202.4)	1955.9 (1857.3-2049.4)	1779 (1678.5-1870.2)	1606.8 (1508.1-1704.8)	1463.1 (1359.6-1572)	1314.6 (1196.5-1435.2)
NGA Nigeria	2929.7 (2409.2-3519.1)	2626.3 (2220.2-3108.8)	2417.8 (2075-2806.5)	2218.1 (1918.2-2562.7)	2132.1 (1837.6-2481.8)	1911.9 (1637.3-2208.7)	1717.6 (1496.9-1979.4)	1620 (1394.8-1868.2)	1645 (1411-1905.9)	1659.2 (1393.2-1939.6)	1674.2 (1375.6-1980.5)	1546.7 (1248.7-1871.1)	1360.7 (1103-1674.8)	1162.7 (969.1-1437.6)
STP Sao Tome and Principe	2945.6 (2757-3137.1)	2441.7 (2345.8-2538)	2019.4 (1953-2090)	1526.7 (1485-1570.5)	1350.8 (1318.2-1381.4)	1275.9 (1241.5-1311.9)	1197.6 (1163.8-1234.2)	1214.4 (1166.4-1265.7)	1196.1 (1133-1261.8)	1237.6 (1164-1320.2)	1257.8 (1184.4-1337.3)	1207.9 (1132.1-1281.7)	1124.5 (1046.7-1210.2)	1085.2 (986.4-1180.9)
SEN Senegal	3008.5 (2729.2-3321.5)	2784 (2546.8-3054)	2600.6 (2369.4-2832.2)	2482.6 (2272.1-2697.5)	2365.8 (2186.9-2558.4)	2185.5 (2022-2339.9)	1953.9 (1822.2-2086)	1749.7 (1641.3-1851.3)	1600 (1518.2-1682.8)	1552.3 (1480-1628)	1458.7 (1388-1529.1)	1326.3 (1256.5-1398.8)	1210.4 (1134.4-1296.9)	1136.1 (1044.4-1238.9)
SLE Sierra Leone	3431.7 (3075.7-3823.5)	3158.9 (2860.1-3468.3)	2932.7 (2634-3227.9)	2702.4 (2441.7-2974.4)	2483.2 (2241.8-2732.4)	2311.2 (2095.1-2532.9)	2218.1 (2013.3-2418.5)	2108.4 (1939.4-2270.5)	1970.8 (1833.1-2104.5)	2001.7 (1894.4-2108.8)	1955.3 (1857-2053.5)	1933.6 (1839.2-2031.2)	1792.3 (1680.9-1919.5)	1514.6 (1380.7-1665)
TGO Togo	2420.4 (2171.2-2700.4)	2291.5 (2071.8-2521.7)	2214.7 (2008.2-2433.8)	2109 (1916.6-2319.4)	1937.9 (1762.3-2125.4)	1816.6 (1664.6-1980.8)	1710.2 (1572.4-1850.4)	1656.7 (1539.8-1786.5)	1616.9 (1513.4-1727.5)	1637.8 (1527.7-1741.1)	1694.1 (1565-1833.7)	1698.8 (1554.1-1848.8)	1566.6 (1437.3-1720.2)	1284.9 (1158.6-1436.6)

Table 1 Age-standardised mortality rates by location and year for females for 1950, 1955...2005, 2010, 2017

Location	1950	1955	1960	1965	1970	1975	1980	1985	1990	1995	2000	2005	2010	2017
Global	1847.7 (1820.1-1878)	1622.1 (1599.6-1646.3)	1964.7 (1943.4-1985.4)	1428.4 (1409.8-1446.2)	1339.9 (1324.9-1355.6)	1239.7 (1226.6-1252.3)	1144.6 (1133.4-1155.6)	1086.8 (1076.8-1096.2)	1003.8 (996.4-1011.6)	967.7 (961.4-973.8)	908.6 (902.7-914.6)	832.3 (826.6-838.2)	754.5 (748.2-760.7)	678.1 (669.3-686.8)
Low SDI	2779 (2658.3-2903.8)	2561.2 (2453.7-2679.8)	2484.4 (2375.3-2591.7)	2409.2 (2309.3-2508)	2293.4 (2210.6-2372.2)	2111.7 (2040.2-2184.1)	1951 (1887.3-2012.4)	1900.4 (1840.3-1960)	1760 (1710.4-1811.2)	1659 (1622.4-1697.2)	1541.3 (1512-1572.4)	1390.6 (1366.6-1414.3)	1280.1 (1258.6-1304)	1128.9 (1096.7-1164.3)
Low-middle SDI	2736.8 (2640.4-2828.3)	2468.5 (2387-2551.9)	2612.5 (2535.9-2694.4)	2227.6 (2157.7-2303.2)	2071.8 (2010.1-2135.9)	1891.9 (1834.7-1948)	1659.5 (1610.7-1707.3)	1575.3 (1531.4-1618.3)	1464.4 (1429.6-1498)	1408.1 (1377.9-1440.9)	1368.4 (1337.9-1401.3)	1245.1 (1216.7-1277.3)	1135.4 (1105.1-1164.9)	1025.9 (991.8-1061)
Middle SDI	1582.6 (1530.7-1636.3)	1350.1 (1305.4-1394.7)	2619.6 (2579.9-2662.9)	1152.1 (1115.6-1188.1)	1048.7 (1016.1-1082.7)	951.5 (925-980.7)	869.2 (847.8-892.3)	822.6 (806.2-841.2)	746 (734.4-757.5)	685.1 (676.7-693.9)	634.1 (625-642.5)	580.7 (573.4-588.8)	533.3 (525.8-541.2)	487.9 (475.4-501.2)
High-middle SDI	1877 (1806.1-1955.2)	1658.7 (1597.5-1732.1)	1832.7 (1779.7-1891)	1390.6 (1342.8-1439.9)	1319.2 (1277.4-1361.7)	1247.5 (1213.6-1280.8)	1207 (1177.8-1237)	1180.2 (1157.6-1203.3)	1113.5 (1096-1132.4)	1142 (1128.1-1156.5)	1045.6 (1034.1-1058.5)	972.3 (962.4-982)	856 (845.1-868.4)	754.5 (734.3-775.8)
High SDI	1140.3 (1115.6-1165.9)	969.1 (957.5-980.6)	905.7 (899.6-911.7)	846.5 (840.9-853)	802.1 (797-807.2)	728.2 (725.5-731.1)	667.2 (665.7-668.8)	617.1 (615.9-618.5)	564.4 (563.3-565.6)	527.4 (526.4-528.5)	482.8 (481.8-483.8)	438.5 (437.4-439.3)	399.1 (398.3-400.1)	376.6 (370.4-382.8)
Central Europe, Eastern Europe, and Central Asia	1367.9 (1327.2-1412.1)	1173.6 (1141.1-1205.3)	991 (980.7-1001)	931.1 (920.4-942)	926.6 (916.7-937.4)	902.7 (892.2-913.3)	920.4 (911.5-929.3)	921.9 (916.6-926.7)	860.6 (858.3-862.8)	971.8 (969.4-974.3)	913.2 (911.2-915.5)	891.5 (889.4-893.9)	778 (775.9-780.2)	665.6 (656.8-674.2)
Central Asia	1971.8 (1862.5-2094.4)	1609.6 (1552.4-1669.4)	1372.7 (1332-1409.3)	1214.3 (1165-1267)	1083.1 (1063.4-1104.3)	1029.8 (997.7-1063.1)	972 (961.5-983.7)	934.9 (927.3-943.3)	878.1 (870.2-885.6)	1003.8 (995.5-1012.2)	976.4 (963.8-988.3)	1012.6 (998.7-1028.9)	920.6 (908.3-933.6)	811.2 (777.1-847)
ARM Armenia	1780.1 (1507.2-2081.3)	1476 (1332.1-1622)	1245 (1156.4-1345.5)	1110.7 (999.3-1226.4)	1007.6 (959.3-1060.1)	944.2 (861.9-1025.7)	837.5 (800.5-874.4)	812.8 (786.3-839.4)	885.3 (857-914.8)	913 (900.7-924.9)	833.7 (822.8-844.7)	763.2 (752.7-773.4)	730.9 (720.7-741.3)	666.8 (639.5-696.1)
AZE Azerbaijan	2524.8 (2174-2886.5)	2037.9 (1837.3-2222.6)	1620 (1497.9-1748.2)	1339.9 (1215.3-1485)	1153.4 (1096.2-1216.2)	1039.4 (961.5-1124.8)	975.9 (947.6-1005.3)	913 (893.3-933.2)	848.7 (814.1-883.9)	1029.5 (988.3-1072.2)	1045.1 (1004.8-1087.4)	1106.2 (1065.6-1150.9)	938.6 (899.7-978.5)	780.4 (720.8-843.1)
GEO Georgia	1676.3 (1406.1-1963.7)	1438.7 (1298.2-1581.2)	1227.5 (1141.8-1326.3)	1081.3 (973.2-1192.1)	946 (900.4-995.6)	904.4 (813.5-998.8)	844.1 (794.8-895.8)	848.3 (813.5-884.8)	848.6 (812.4-884.7)	775.7 (740.9-813.5)	709.1 (679.3-738.1)	735.8 (724.1-747.5)	748.3 (739.2-757.8)	751.6 (723.6-780.8)
KAZ Kazakhstan	1674.3 (1421.1-1943.6)	1385.6 (1249.9-1512.7)	1180.5 (1096.4-1267.1)	1089.4 (977.7-1214.4)	981 (941.2-1020.6)	959.6 (887.7-1033.8)	926.1 (910.2-942.7)	905.6 (897.5-913.8)	856.8 (850.1-863.9)	1082.1 (1075.7-1089)	1023.8 (1017.5-1030.7)	1013.3 (1006.7-1019.8)	912.5 (906.5-918.7)	747.3 (705.7-787.3)
KGZ Kyrgyzstan	2401.3 (2026.4-2769.1)	1971.2 (1777.2-2184.1)	1698.6 (1538.8-1876.2)	1528.5 (1355.7-1707.2)	1375.8 (1251.7-1503.7)	1243.7 (1113.1-1379.5)	1153.7 (1083.2-1222.1)	1083.5 (1036.4-1135.6)	957 (911.6-1003.7)	1002.3 (954.4-1047.1)	907.9 (863-951.2)	906.1 (875.1-939.9)	799.7 (791.7-807.5)	679.7 (657.3-702.5)
MNG Mongolia	3288.2 (2882.2-3721.8)	2637.7 (2352.1-2921.8)	2223.2 (2020.9-2429.5)	1868.1 (1741.1-1984.7)	1711.6 (1620.3-1810.8)	1547.5 (1462.5-1632.6)	1447.5 (1369.1-1521.6)	1503.3 (1443.4-1570.4)	1386 (1334.7-1439.4)	1503.9 (1449.2-1559.5)	1435.2 (1394.5-1481.1)	1247.3 (1207.3-1288.6)	1084 (1042.4-1125.7)	871.2 (793.3-956.5)
TJK Tajikistan	2597.4 (2282.5-2967.6)	2127.9 (1953.6-2311.4)	1791.6 (1669.7-1914.3)	1474.6 (1343.6-1614.9)	1227.1 (1170.9-1285)	1122 (1042.7-1199.1)	947.6 (919.8-977.3)	918.3 (900.3-936.4)	860.8 (836.7-884.8)	954.4 (907.3-998.1)	837.4 (798.7-879.2)	796.7 (754.5-840.8)	754.2 (707.7-802.5)	738.2 (668.4-812.1)
TKM Turkmenistan	2230 (1934.9-2574.5)	1742.7 (1587.6-1906.6)	1492.8 (1381.8-1595.9)	1325.1 (1201.8-1459.5)	1188.6 (1131.2-1244.9)	1169.3 (1087.8-1252.8)	1116.2 (1083.8-1150.2)	1062.5 (1045.2-1081.6)	1010.8 (995.1-1027.3)	1142.8 (1128.3-1159.8)	1037.5 (1021.2-1055.5)	1124.9 (1109.3-1141.9)	908.7 (895.7-923.2)	830.4 (765.2-907.5)
UZB Uzbekistan	1836.4 (1563.4-2123.5)	1430.6 (1301.8-1563.5)	1268.5 (1182.6-1352.7)	1147.6 (1033.2-1266.4)	1054.8 (1015.5-1096.2)	1029.7 (958.8-1102.2)	987 (969.3-1004.3)	919.7 (909.4-931)	823.4 (815.4-832.1)	969.5 (960.9-977.6)	1060.4 (1021.5-1096.3)	1195.6 (1146.4-1250.5)	1099.2 (1056.9-1141.1)	953.7 (837-1083.5)
Central Europe	1521.5 (1482.8-1565)	1230.8 (1217.9-1244)	1078.7 (1073.4-1084)	996.7 (991.7-1001.6)	958.6 (953.9-963.4)	892.9 (889.6-896.2)	900.9 (897.9-903.8)	888.3 (885.8-891)	837.4 (835.1-839.9)	819.6 (816-823.4)	742.6 (739.9-745.6)	685 (682.8-687.4)	616.1 (614-618.5)	548.6 (534.6-562.4)
ALB Albania	1935.7 (1745.3-2136.4)	1492.4 (1390.7-1601.9)	1112.1 (1046.1-1175)	995.5 (915.5-1086.6)	846 (729.5-980.5)	730.4 (608.3-865)	631 (539.3-735)	590.1 (551-630.3)	566.6 (557.2-577.2)	556.3 (546.3-565.4)	609.5 (579.4-642.1)	575.6 (543-610.2)	491.2 (442.4-541.1)	440 (345.6-547.4)
BIH Bosnia and Herzegovina	1914.1 (1805-2031.1)	1526.8 (1475.5-1578.5)	1288.3 (1257.4-1321.1)	1198.5 (1169.6-1229.4)	1121.3 (1097.2-1145.3)	1041.9 (1024.8-1060.2)	985.3 (969.5-999.9)	896.8 (883.7-910.3)	730.5 (719.8-742.3)	945.3 (838.5-1070.5)	654.7 (591.7-724.2)	626.1 (615.7-636.3)	660.7 (649.4-672.4)	646.1 (604.3-691.3)
BGR Bulgaria	1352.2 (1321.8-1392.1)	992.6 (983.2-1001.5)	877.3 (869.2-885.4)	784.3 (776-792.4)	785.1 (776.6-793.6)	796.5 (788.5-805.1)	808.7 (800-817.1)	806.9 (798.3-815.4)	821 (813.1-830.1)	869.1 (860-877.7)	871.5 (861.8-880.8)	783.9 (775.8-792.6)	723.4 (714.9-731.8)	641.5 (603.3-683.7)
HRV Croatia	1971.3 (1875.5-2081.7)	1486.4 (1451.5-1522.8)	1254.9 (1233.4-1278.5)	1073.7 (1057.1-1091.2)	1016.4 (1002.3-1029.9)	895.7 (884.1-909.3)	839.1 (827.8-850.3)	826.8 (815.6-838.2)	792.3 (780.2-805.1)	728.9 (718.7-739.1)	688.5 (677.2-700.5)	645.8 (635.2-656.6)	606.7 (596.2-617)	513.7 (477.2-551.5)
CZE Czech Republic	1344.5 (1331.6-1356.3)	1116.9 (1107.4-1126.4)	1008.4 (999.5-1018)	965.5 (956.1-974.3)	1008.4 (999.2-1017.9)	955.1 (945.5-964.2)	972.1 (962.7-981.4)	928.1 (918.8-937.1)	870.5 (862.2-879.4)	785.1 (776.7-793.9)	683.2 (675.4-690.6)	622.4 (615.5-629.5)	538.7 (532.1-545.2)	480.6 (449.8-515.2)
HUN Hungary	1479.5 (1461.4-1499)	1136.6 (1128.5-1146.3)	1069.3 (1060.8-1077.9)	1003.8 (994.3-1012.5)	1018.9 (1009.3-1027.8)	995.4 (987-1004.4)	1000.8 (992.4-1009.5)	982 (973.6-990)	910.3 (903.1-917.3)	863.1 (855.8-870.5)	774.5 (767.9-781.1)	714.1 (707.1-721.1)	651.4 (644.8-658.5)	549.2 (515.1-586.7)
MKD Macedonia	1921.9 (1720.1-2109.8)	1674.2 (1542.3-1815.1)	1454.2 (1332-1584.6)	1294.6 (1191.9-1403.5)	1125.9 (1069-1178.2)	999.1 (973.8-1025.2)	947.5 (929-965.1)	897.6 (880.6-914)	816.9 (801.5-831.4)	778.6 (763.6-794.1)	723.2 (708-738.6)	633.2 (621.3-645.3)	553.6 (542.7-563.9)	475.3 (449.8-498.3)
MNE Montenegro	1139.1 (1065.5-1216.9)	904.9 (864.3-947.2)	830.7 (793.3-865.5)	761.9 (730.5-794.6)	714.3 (681.2-745.5)	785.1 (755.5-816.3)	772.2 (755.1-789.6)	771.9 (757.9-785.9)	684.8 (668.8-702.3)	695.4 (677.6-714.5)	722.1 (701.8-742.6)	718.5 (690.3-747.7)	634.2 (606.8-664.2)	647.6 (597.9-699.7)
POL Poland	1482.8 (1469.7-1498.3)	1196.4 (1190.5-1202.1)	969.9 (964.7-975)	891.2 (886.3-896.1)	870.7 (865.9-875.5)	821.6 (817.3-826)	825.9 (821.9-830.2)	837 (832.4-841.6)	786.6 (782.3-790.8)	746.3 (742.3-750.1)	666.7 (662.9-670.4)	592 (588.5-595.4)	528 (524.6-531.2)	472 (444.5-502.8)
ROU Romania	1431.1 (1208.2-1681.7)	1283.6 (1219.2-1350.9)	1195 (1188.3-1201.9)	1102.4 (1095.7-1109.1)	999.6 (993.6-1005.3)	950.7 (944.8-956.6)	1052.6 (1046.1-1059)	1042.5 (1036.4-1048.6)	977.1 (971-982.6)	979.5 (973.9-985.3)	876.2 (870.9-882.1)	819.9 (814.2-825.6)	715.7 (709.8-721)	623.5 (585.2-660.2)
SRB Serbia	1692.7 (1660.3-1732.4)	1380.4 (1368.3-1392)	1240.8 (1229.5-1252.3)	1204.5 (1194-1214.9)	1058.1 (1048.8-1068)	823.6 (815.8-831.2)	809.3 (801.5-817.3)	822.2 (814.6-830.3)	824.5 (816.5-833.2)	866.3 (858.1-874.5)	829.1 (817-844.4)	865.7 (852.4-877)	842 (833.5-850.8)	842.3 (790.1-894.7)
SVK Slovakia	1464.3 (1320.3-1613)	1180.4 (1073.1-1285.2)	1000.8 (912.2-1094.1)	938.6 (854.1-1032.9)	951.7 (881.4-1024.9)	903 (891.5-915.1)	889.3 (878-901.3)	873.4 (862.8-884)	827 (816-837)	755.2 (744.6-765.8)	705.7 (695.9-715.4)	668.1 (659-677.8)	612.5 (604-621.7)	545.9 (508.8-585.1)
SVN Slovenia	1488.2 (1456.3-1521.7)	1174.1 (1157.1-1193.1)	1063.5 (1046.1-1080.4)	987.3 (969.9-1004.5)	1011.3 (993.9-1030.4)	965.5 (948-982.6)	856.6 (840.9-872.5)	812.7 (797.5-829.2)	695.2 (680.2-710.6)	654.8 (641.3-669.1)	584.4 (571.4-597.4)	515.7 (504.7-527.2)	445.1 (433.6-456.2)	378.4 (347.4-412.1)
Eastern Europe	1171.6 (1110.4-1241.1)	1052.2 (1002.1-1101.9)	863.1 (848.2-878.2)	825.8 (810.6-841.4)	861.1 (844.9-877.7)	867.1 (850.9-884)	901.3 (887.2-915.9)	915 (906.6-922.6)	848.7 (845.8-851.5)	1019.7 (1017.1-1022.3)	971 (969.3-972.7)	965.6 (964.2-966.8)	828.7 (827.5-829.9)	692 (681-702.9)
BLR Belarus	971.1 (780.6-1184.7)	877.8 (746-1023.4)	760.9 (734.6-788.7)	687.6 (674.4-701.4)	708.6 (699-718.7)	716.5 (707.2-725.6)	764.2 (755.6-772.5)	792.9 (784.8-801.4)	765.9 (757.9-773.8)	858 (850.5-865)	829.1 (821.5-836.9)	827 (819.8-834.5)	745.4 (737.8-752.2)	630.2 (591.6-668.6)
EST Estonia	1172.8 (962.9-1408.3)	1112.3 (924.9-1308.7)	941.4 (922.9-961.8)	872 (854.2-890.1)	885.4 (868.1-904.5)	859.9 (842.6-877.5)	883 (865.2-900.5)	891.9 (872.1-911.8)	838.3 (820.7-855.4)	862.2 (844.7-880.2)	750.3 (734.3-766.9)	652.8 (637.8-668.3)	531.5 (516.4-545.3)	463.2 (400-530.7)
LVA Latvia	1034.9 (847.2-1254.5)	962.2 (802.6-1152)	861.3 (844.4-877.4)	790.3 (777.2-804.3)	843.5 (830.2-856.8)	833.5 (819.3-846.5)	857.9 (844.4-870)	882.6 (868.7-896.1)	842.7 (829.4-855.9)	908.3 (895-921.8)	753 (741.4-765.7)	727.8 (714.2-740.5)	649 (636.1-661.3)	564.1 (491.2-644.9)
LTU Lithuania	1196.4 (993-1436.2)	1016.7 (858-1186.6)	893.4 (880.5-905.5)	728.6 (716.8-740.2)	759.3 (747.2-771.8)	752 (740.1-763.8)	767.5 (756.7-778.9)	798.7 (787-810.9)	760.6 (749.3-772.2)	817.9 (807.7-828.7)	678.3 (668.5-688.4)	689.1 (679.4-699.6)	617 (606.8-627.1)	552.9 (513.8-593.7)
MDA Moldova	1925.8 (1643.5-2222.3)	1691.4 (1530.2-1844.8)	1533.6 (1433.5-1643.4)	1440.1 (1304.3-1594.2)	1336.6 (1283.1-1392.5)	1253.9 (1172.4-1342.6)	1142.9 (1125.1-1161.9)	1151.3 (1140.3-1162.1)	1019.9 (1008.8-1031.3)	1167.4 (1155.9-1178.9)	948.1 (938.3-958.8)	912.3 (903.1-921.9)	800.1 (791.4-809.8)	647.7 (624.8-672)
RUS Russian Federation	1133.3 (1092-1173.1)	1021.2 (986.2-1056.9)	857.3 (834.9-879.6)	828.9 (806.5-852.5)	867.9 (843.7-894.1)	876.6 (851.2-902.8)	919.9 (898.2-942.7)	930.7 (919.1-942.1)	853.7 (852.1-855.1)	1026.2 (1025-1027.5)	1008.2 (1006.9-1009.4)	1009 (1007.8-1010.3)	853.5 (852.3-854.6)	689.1 (682.3-695.7)
UKR Ukraine	1251.1 (1044.5-1494.9)	1128.7 (960-1320.8)	856.9 (842.7-873)	820.4 (809.2-832.7)	856 (846.4-866.2)	858.7 (847.4-869.7)	874.8 (863.1-886.2)	890 (877.9-901.4)	844.6 (834.2-855.1)	1046.6 (1037-1056.6)	944.3 (938.8-950.2)	920.9 (917.4-924.5)	810.5 (807.1-814)	742.7 (702.7-789.9)
High-income	1161.1 (1134.8-1188.6)	1000.4 (988.4-1012.6)	941.2 (935.4-947.7)	884.8 (879.2-891.3)	834.1 (828.9-839.1)	754.2 (751.6-756.8)	684.7 (683.7-685.6)	627.9 (627.1-628.6)	573.1 (572.4-573.7)	534 (533.4-534.7)	491.9 (491.3-492.5)	448 (447.5-448.6)	410.8 (410.2-411.3)	391.2 (384.3-398.5)
Australasia	1002.3 (993.7-1011)	929 (921.5-936.5)	882.8 (875.6-890)	872.1 (864.9-878.8)	860.2 (853.5-866.3)	763.9 (757.9-770.2)	663.8 (658.5-668.9)	634.7 (629.3-639.7)	564 (559.3-568.4)	517.7 (513.3-522.2)	462.4 (458.6-466.3)	408.3 (404.7-412.1)	380.6 (377.4-383.8)	363.2 (326.5-403)
AUS Australia	994.5 (984.6-1003.6)	927.4 (918.6-936.1)	878.9 (871-886.9)	868.3 (861-875.6)	860.2 (853.3-866.9)	760.5 (753.9-767.3)	646.8 (640.9-652.9)	622.8 (617-628.5)	550.4 (545-555.4)	504.1 (499.1-509.3)	454 (449.6-458.2)	396.9 (392.7-401.1)	371.4 (367.8-374.8)	357.5 (314.1-404.3)
NZL New Zealand	1044.3 (1021.8-1067.8)	935.5 (919.2-953.6)	898.6 (881.7-915)	887.9 (871.9-904.3)	859.7 (843.8-876)	779 (765.8-792.3)	741.3 (730.2-752.6)	691.1 (680.3-703.1)	631.3 (622-641)	586 (576.6-594.9)	505.3 (497.3-513.5)	467.9 (459.6-476.3)	429.1 (422.2-436.6)	393 (369.6-418.6)
High-income Asia-Pacific	1633.8 (1580.4-1690.8)	1247.2 (1197.6-1294.7)	1161.9 (1116-1210.1)	1054.2 (1012.6-1101.2)	944.6 (907.2-981.4)	807.3 (793.2-822.1)	685.7 (683.6-688.2)	577.8 (576.2-579.7)	504.2 (503-505.6)	450.7 (449.5-451.8)	384.4 (383.4-385.4)	339.8 (338.9-340.6)	307.5 (306.2-308.9)	276.5 (269.7-283.7)
BRN Brunei	1629.8 (1401.3-1885.9)	1491 (1308-1700)	1322.8 (1191.6-1464.5)	1124.3 (1044.9-1206.4)	1080.2 (1024.2-1140.7)	1067.1 (1011.1-1123.8)	1043.8 (985.8-1108.2)	968.9 (914.3-1020.1)	996.2 (944-1048.8)	837.1 (790.7-884.9)	721.2 (684.1-764.7)	672.7 (648.7-702.9)	655.9 (630.2-679.9)	648.8 (599.5-699.4)
JPN Japan	1453.8 (1409.8-1501.3)	1146.6 (1112.9-1184.2)	1068.1 (1033.1-1101.2)	952.5 (920.3-987.1)	848.1 (824-876.8)	734.1 (720-747.8)	619.3 (617.9-620.8)	525 (523.9-526.2)	458.2 (457.1-459.5)	414.5 (413.4-415.5)	352.1 (351.1-353)	318.2 (317.3-319)	293.8 (292.9-294.7)	268.5 (261.5-276.2)
JPN Aichi	1493.8 (1248.7-1777.3)	1196.5 (982.7-1430.7)	1115.5 (904.7-1339.7)	985.4 (798.1-1200.6)	876.6 (718.7-1071.3)	762.3 (675-857.5)	648.6 (641.1-656.1)	545.2 (538.9-552.2)	485.3 (479.7-491.9)	433.7 (427.6-439.5)	367.8 (362.5-372.7)	332.9 (328.3-337.8)	298.6 (294.5-303)	276 (238.2-319)
JPN Akita	1382 (1120.3-1667.2)	1070.7 (860.9-1283)	904.4 (737.8-1083.6)	818.8 (676.3-976.8)	748.5 (645-870.1)	622.1 (571.1-675.4)	503.5 (494.1-514.1)	468.9 (460.9-477.2)	425.1 (416.7-433.5)	396.3 (388.6-403.9)	357.6 (350.2-364.7)	329.5 (322.4-336.7)	312.5 (305.3-319.5)	299.9 (259.1-341.4)
JPN Aomori	1469.7 (1184.8-1774.4)	1146.7 (916.3-1389)	1058.1 (855.7-1267.7)	932.5 (756.8-1136.5)	816.9 (686.8-965.6)	688.8 (620.8-756.5)	597.2 (586.1-608.6)	529.6 (520.1-539.6)	449.6 (441.2-457.7)	424.4 (416.7-432.9)	377.8 (370.3-385.4)	342.6 (335.2-350.2)	321.9 (314.2-329)	298.7 (259-342.4)
JPN Chiba	1431.3 (1162.7-1719.3)	1145.2 (934.5-1392.8)	1090 (881.8-1337.3)	966.7 (768.5-1197)	864.7 (708.5-1046)	764.9 (673.3-863)	624.1 (616.2-632.5)	523.3 (516.3-530.3)	455.9 (449.5-461.7)	418.8 (413.2-424.1)	357.7 (352.7-363.1)	326 (321.2-331.3)	301.2 (296.5-305.8)	273.7 (235.5-312.6)
JPN Ehime	1418.5 (1160.1-1707.3)	1110.6 (894.7-1339.1)	1009.9 (819.7-1221.4)	890.3 (722.1-1078.6)	783.8 (664.5-913.9)	681.5 (620.6-751.7)	592.5 (582.2-602.8)	508.4 (499-518.1)	439.2 (430.9-447.7)	408.4 (400.4-416.7)	352.9 (345.6-359.8)	318.3 (311.4-325.1)	291.2 (284.9-297.9)	255 (219.7-293)
JPN Fukui	1362.8 (1127.1-1638.9)	1091.2 (902.1-1299.2)	1054 (875.1-1241)	952.6 (807-1118.8)	849 (739-971.2)	735.1 (679.5-793.9)	611.2 (596-625.3)	517.2 (507-527.3)	448.9 (439.5-458.1)	404.2 (395.4-412.6)	333.1 (325.7-340.6)	311.7 (304.3-319.1)	271.4 (264.5-278.5)	257.1 (222.6-293.7)
JPN Fukuoka	1474.3 (1237.8-1763.4)	1149.7 (940.5-1375.7)	1052.7 (850.7-1278.3)	939.7 (758.6-1154.3)	843.6 (695.9-1021.7)	734.8 (643.4-826.4)	608.7 (601.4-616.3)	517.8 (511.1-525.3)	454 (447.8-460.1)	404.2 (398.5-410.2)	352.2 (346.8-357.6)	316.4 (311.2-321.4)	289 (284.2-293.8)	262.2 (225.2-303.1)
JPN Fukushima	1408.8 (1160.7-1705.9)	1094.2 (873.2-1338.3)	1015.2 (800.5-1235.6)	918.2 (740.4-1110.7)	823.7 (693.8-968.2)	694.7 (631.6-763)	573.5 (564.1-583.3)	515.9 (507-524)	433.4 (426-440.3)	406.1 (399-413)	352.6 (345.4-359)	316.5 (310.4-322.9)	294 (287.6-300.7)	288.2 (248.5-329.9)
JPN Gifu	1497.1 (1237.9-1796.3)	1203.7 (974.4-1453.4)	1145.8 (927.1-1372.8)	1041.7 (846.6-1257.2)	946.7 (785.8-1123.2)	815 (736.9-897.5)	674.6 (664.1-685)	555.6 (546.4-564.3)	473.4 (465.6-481.1)	419.1 (411.9-426.5)	361.5 (355.2-368.4)	320.3 (314.1-326.5)	296.4 (290.4-302.9)	277.1 (239.8-316.9)
JPN Gunma	1463.5 (1205.1-1753.9)	1170.2 (946.3-1423.5)	1119 (908.9-1361.7)	1008.6 (818.9-1229.4)	905.8 (763.4-1064.2)	798.3 (727.6-873.1)	673.9 (662.7-685.3)	553.6 (545-562.1)	474.3 (466.2-483.1)	420.2 (412.7-427.9)	356 (350-362.7)	329.6 (323-336.3)	305.2 (299.1-311.4)	280.9 (243.3-319.3)
JPN Hiroshima	1475.6 (1221.7-1756.1)	1159.6 (951-1404.9)	1052.4 (849.3-1265.8)	928.1 (759.1-1120.6)	823.7 (677.6-990.1)	715.2 (634.1-808.6)	605.9 (595.9-615.9)	518.5 (510.1-527.5)	446.6 (439.3-454.1)	395.6 (388.3-402.6)	332.2 (325.7-338.6)	303.1 (296.8-309.1)	278.9 (273.2-285.1)	259.8 (224.5-298.7)
JPN Hokkaidō	1494.9 (1216.8-1786.8)	1170.1 (960.4-1412.8)	1087.7 (888.8-1339.2)	965.5 (778.7-1190.8)	860.1 (703.9-1035.1)	744.3 (656-837.8)	643.5 (635.9-651.9)	539.9 (533.6-545.8)	460.8 (455-466.9)	403.4 (398-409.3)	344.4 (340-349.4)	312.1 (307.7-317)	295.6 (290.9-300)	277.8 (238.3-317.9)
JPN Hyōgo	1529.8 (1268.7-1816)	1196.5 (980.3-1450.3)	1104.7 (885.9-1358.2)	974.3 (768.5-1202.7)	855.4 (697.8-1023.3)	735.8 (655.4-819.7)	640.3 (632.8-648.4)	548.8 (542.2-555.7)	485.6 (479.1-492.4)	517.1 (509.2-524.4)	364.2 (359-369.2)	326.4 (321.5-331.7)	296.8 (292-301.5)	266.2 (230.3-304.9)
JPN Ibaraki	1492.3 (1218.3-1786)	1179.6 (964.9-1426.5)	1129.1 (921.1-1384.2)	1011 (810.4-1247.1)	903.1 (739.2-1074.3)	787.2 (699.8-889)	668.6 (657.9-679.9)	573.3 (563.6-583)	495 (486.5-502.8)	434.4 (427.1-441.8)	370 (363.4-377)	337.1 (331-343.7)	314.7 (308.3-321)	294 (253.4-339.7)
JPN Ishikawa	1391 (1146.6-1682.3)	1128.7 (928-1382.5)	1121.8 (928.6-1353.5)	1030 (860.3-1227.2)	916.5 (793.7-1057.8)	763.1 (708.2-825.1)	647.5 (634-660.5)	507.3 (496.8-516.6)	435.1 (425.9-443.7)	398.5 (390.8-406.2)	341 (334-347.9)	290.7 (284.6-296.8)	279.4 (273.1-285.8)	260.6 (225.8-295.9)
JPN Iwate	1441.9 (1167-1760.3)	1130.1 (893.7-1378)	1070.4 (868.6-1291.3)	964 (806.7-1142.3)	865.1 (748.8-983.4)	751.1 (693.7-812.2)	635.6 (624.8-646.6)	535.2 (526.5-544.2)	463.9 (455.8-472)	407.3 (399.8-415)	348.5 (341.6-355.2)	322.2 (316.1-328.9)	296.4 (289.6-303.3)	288.4 (250.1-329.1)
JPN Kagawa	1379.7 (1134.1-1638.7)	1091.6 (889.9-1313.7)	1007.2 (822.5-1195.9)	892.7 (738.4-1056.6)	789.5 (675.6-908.8)	680.9 (624.7-738.1)	575 (562.5-587.7)	493.8 (484.3-503.1)	440.5 (432.3-448.9)	396.9 (388.9-405.6)	353.1 (345.3-361.2)	308.2 (301-315.8)	296.1 (288.7-303.7)	262.3 (225.9-299.3)
JPN Kagoshima	1541.4 (1253.8-1877.2)	1207.6 (974.2-1469.5)	1115.8 (908.9-1358.8)	1016.5 (842.6-1231.7)	923.3 (783-1076.5)	794.7 (729.4-869.1)	643.4 (633.5-653.8)	539.5 (531.4-548.1)	464.1 (455.9-472.4)	406.3 (398.6-414.1)	341 (334.1-348.1)	313.8 (307.5-320.6)	296.7 (290-303.2)	274.5 (238.5-313.4)
JPN Kanagawa	1438.4 (1173.7-1726.8)	1137.5 (930.5-1344.1)	1048.7 (851.8-1272.1)	912.4 (729.5-1137.6)	805.4 (655.2-969.4)	699 (617.5-792.4)	593.3 (586.5-599.5)	507.3 (501.5-513.2)	451.2 (446.2-456.4)	411.6 (406.6-416.5)	351.4 (346.8-355.7)	314.3 (310.2-318.5)	288.3 (284.2-292.1)	273.1 (236.8-313.2)
JPN Kōchi	1514.8 (1226.3-1821.8)	1193.3 (960.8-1441.1)	1100.8 (891.2-1338.4)	986.4 (817.5-1184.2)	882.7 (759.1-1016.6)	761.8 (700.8-831.9)	618.1 (604-631.9)	502.3 (492.7-512.1)	447.4 (438.2-457.4)	396.8 (388.2-405.7)	348.5 (340.6-356.1)	312.5 (304.5-320)	288 (280.3-296.5)	259.7 (223.9-298.1)
JPN Kumamoto	1444.2 (1186.7-1707.5)	1122.7 (909.4-1371.7)	1051.6 (851.7-1293.3)	963.1 (774.9-1170.8)	875.1 (736.4-1017.9)	757.5 (684.7-833.8)	609.6 (599.3-619.4)	485.6 (477-494)	424.2 (416.3-432)	358.6 (352.3-365.5)	319.6 (313.1-326.2)	292.1 (286.2-298)	273.7 (268.1-279.5)	248.3 (216.8-283.3)
JPN Kyōto	1432.2 (1190.4-1716.9)	1138.7 (922.8-1392.4)	1049.8 (856.7-1300.1)	912.4 (746.2-1126.4)	795.4 (652.8-967.6)	683.3 (601-774.8)	585.5 (575.6-595.3)	512.6 (503.8-521.1)	448.5 (440.5-456.5)	395.4 (387.9-403.4)	343.3 (336.7-350)	312 (305.7-318.3)	282.4 (276.6-288.9)	257.3 (219.5-298.5)
JPN Mie	1413 (1143.2-1715.2)	1123.6 (894.2-1382.1)	1060.3 (855.3-1301.9)	941.9 (769-1144.7)	832.8 (701.8-971.8)	722.7 (658.1-786.4)	614.6 (603.8-625.7)	542.5 (532.5-551.5)	464.2 (455.6-472.6)	422.6 (414.8-430.4)	353.2 (346.4-360.3)	319.8 (313.4-325.9)	298.7 (292.2-305.4)	273.5 (236.8-312.2)
JPN Miyagi	1373.7 (1131.2-1666.6)	1082.7 (874.4-1320.6)	1052.6 (850.2-1283.6)	943.3 (764.9-1140.8)	839.8 (711.3-987.3)	737.3 (671.6-805.1)	646.6 (636.5-656.9)	543.2 (534.7-551.7)	450.3 (442.9-457.9)	412.3 (405.8-419.8)	349.2 (342.7-355.7)	319.3 (314-324.8)	297.5 (291-304)	269.5 (233.9-307.8)
JPN Miyazaki	1485 (1203.1-1804.4)	1171.1 (950.9-1429.4)	1086.7 (876.8-1334.2)	980.6 (803-1194.1)	877.8 (757.4-1006.4)	753.2 (694.9-813)	620.2 (609.1-632)	511.3 (502.4-520.5)	440.9 (433.1-450)	399 (391.3-406.9)	329 (322.8-335.7)	308.7 (301.6-314.9)	285.3 (279-291.9)	259.1 (224.2-297.4)
JPN Nagano	1350.3 (1099.2-1658.5)	1069.7 (863.4-1295.9)	1016.6 (827.4-1229.7)	922.8 (745-1121.9)	832.7 (696.5-988.3)	729.5 (659-799.9)	610 (600.4-620.1)	506.6 (498.7-515.5)	432 (424.7-439.6)	381.3 (374.5-388.6)	327.8 (321.7-334)	291.4 (285.8-296.9)	268.4 (262.6-274.9)	242.8 (209.7-277.4)
JPN Nagasaki	1475.2 (1194.3-1778.2)	1152.1 (947.4-1399.4)	1061.2 (881-1280.9)	954.5 (782.7-1136.6)	855.7 (727.7-1005.7)	754.2 (684.8-826.1)	636.6 (625.8-648.5)	522.1 (513.2-531.9)	452.4 (444-461)	401 (393.6-408.6)	338.2 (331.8-344.8)	310 (303.4-316.5)	287.4 (280.1-294.1)	276.9 (238.6-318.6)
JPN Nara	1497.1 (1217.9-1814.6)	1204.4 (978.7-1449.6)	1166 (960.2-1398.7)	1048 (870.5-1260.6)	933 (803.7-1068.4)	798.4 (737-860.7)	648 (635.8-659.3)	555.4 (546.1-565.1)	491.3 (483.3-499.3)	429.1 (421.6-437.2)	355.3 (348.4-362.2)	313.9 (307.6-320)	288.5 (282.2-294.5)	265.3 (231.1-301.9)
JPN Niigata	1354.7 (1096.3-1615.5)	1074.6 (872-1296.4)	1046.6 (835.6-1256.3)	959.9 (778.9-1161.9)	874.7 (737.1-1016.3)	754 (691.4-828.6)	643.4 (633.7-653.1)	531.3 (523.6-539.6)	445.5 (438-452.7)	392.6 (386-399.2)	326.9 (321-332.4)	298.1 (292.7-304)	273.8 (268.2-279.5)	260.5 (226.5-297.1)
JPN Ōita	1480.4 (1214.7-1775.7)	1172.4 (957.8-1400.5)	1103.3 (904.9-1315.1)	996.6 (824.3-1185.9)	893.5 (772.1-1032.2)	765.6 (712.1-825.3)	626.1 (614.8-637.2)	536.2 (527.1-545.3)	462.1 (454-471.1)	398.3 (390.4-406.4)	349.4 (342-357)	307 (300.4-313.4)	277.6 (271.6-284)	262.5 (227.9-298.7)
JPN Okayama	1361.8 (1110.8-1631.4)	1080.2 (864.9-1300.1)	1008.8 (821.4-1207.7)	909.1 (744-1093.6)	815.1 (687.6-955.7)	709.4 (643.6-776.8)	603.5 (593.6-613.6)	497 (488.3-505.7)	424.5 (417.6-431.6)	392.7 (386-399.5)	324.8 (318.8-331.4)	293.1 (287.1-298.9)	276.9 (270.8-282.8)	251 (217.6-286)
JPN Okinawa	1476.6 (1214.3-1780.8)	1136.3 (928.7-1399.1)	1018.2 (830.4-1240)	928.7 (761.3-1129.1)	841.3 (717.6-981.6)	728.2 (667.4-787.7)	534.1 (521.9-546.2)	394.4 (387.5-401.7)	359.4 (352.4-366.3)	335.8 (329-342.3)	300.2 (294.2-306)	284.3 (279-289.9)	268.1 (262.6-273.6)	276.5 (240.4-315.9)
JPN Ōsaka	1641.4 (1367.8-1945.2)	1287 (1072.8-1538.9)	1178.7 (957.5-1431.4)	1028.3 (840.5-1253.4)	908.5 (744.5-1092.5)	793.8 (707-898.4)	674.9 (668.4-681.5)	586.4 (580.6-592.3)	507.5 (502-512.7)	447.3 (442.7-452.7)	377.8 (373.4-382.4)	341.3 (337.5-345.4)	313.2 (309.1-317.3)	267.3 (229.6-306.1)
JPN Saga	1432.6 (1173.5-1722.9)	1114.5 (917.5-1356.3)	1030.6 (850.2-1258.5)	920.8 (765-1115)	817.3 (705.2-946.1)	718.1 (658.2-779.1)	616.6 (603.1-630.2)	519 (509.2-529.4)	446.8 (437.8-455.7)	406.3 (397.1-414.9)	344.6 (336.7-352.4)	301.8 (294.4-308.9)	293.5 (285.7-301.2)	269.5 (232-307.6)
JPN Saitama	1467.8 (1206.3-1740.6)	1174.6 (948.9-1393.1)	1125.6 (933.1-1376.9)	986.9 (799.8-1214.1)	875.5 (711.4-1043.6)	777 (682.3-876.4)	657.6 (649.2-666.5)	539.6 (532.8-546.1)	476.9 (471.1-482.9)	426.3 (420.5-431.9)	365.3 (360.5-369.8)	334 (329.5-338.4)	309.1 (304.5-313.6)	278.1 (241-317.2)
JPN Shiga	1395.5 (1130.9-1679.3)	1124.7 (917.8-1348)	1086.8 (879.5-1305.2)	1003.4 (832.3-1191.8)	909.8 (786.2-1053)	765.3 (706.8-833.7)	614.8 (601.9-628.4)	546.5 (536.5-556.7)	485.7 (476.5-494.5)	414.1 (405.8-422)	340.1 (332.9-346.9)	305.8 (299.5-312.4)	281.5 (275.3-287.7)	259.3 (224.7-296.5)
JPN Shimane	1406.6 (1149.2-1693.1)	1122.6 (911.1-1355.4)	1047.4 (860.7-1239.4)	946.7 (799.2-1113.9)	842.8 (740-957.6)	717.4 (670.5-773.4)	575.9 (562.2-590.2)	473.9 (465.1-483.4)	399.1 (390.7-407.7)	360.9 (352.8-369.5)	337.6 (330.4-345.3)	288.5 (281.5-295.2)	264.8 (257.9-272.1)	258.1 (223.2-295.6)
JPN Shizuoka	1390.1 (1144.8-1696.8)	1093 (893-1341.1)	1017.2 (823.1-1237)	907.8 (728.2-1116.7)	806.7 (653.6-980.6)	698 (616.1-793.1)	592.8 (584.7-601.6)	499.5 (492.1-507.1)	439.1 (432.2-445.9)	398.8 (392.2-405.3)	340.2 (334-346)	303.1 (297.9-308.8)	291.3 (285.7-296.9)	258.1 (219.9-297.1)
JPN Tochigi	1476.1 (1199.3-1777.1)	1172.1 (950.8-1425.9)	1106.5 (902.7-1352.3)	979.9 (805.3-1197.2)	862.7 (723.2-1020.6)	754.9 (682.6-828.1)	673.8 (661.9-684.8)	571.3 (561.6-581.1)	500.9 (492-509.7)	443.7 (436.1-451.7)	371 (364.8-377.2)	347.6 (341.4-353.9)	316.9 (310.3-323.1)	300.2 (257.8-343.9)
JPN Tokushima	1468.6 (1187.7-1766)	1163.2 (931.6-1417.2)	1088.3 (876.5-1304.7)	988.5 (821-1183.6)	892.6 (768.9-1029.7)	759.9 (694.8-826.2)	643.3 (630.2-657.3)	533.3 (523.8-544.3)	466.7 (456.9-476.7)	410.8 (401-420.7)	347.6 (340-355.9)	327.3 (318.9-335.3)	294.5 (286.6-303.4)	293.6 (253.7-336.5)
JPN Tōkyō	1445.2 (1188.4-1716.7)	1129.5 (939.6-1342.2)	1026.9 (827.8-1226.1)	903.3 (723.2-1103.5)	794.7 (655.1-962.4)	688.1 (611.2-770.5)	600.8 (595.6-606.1)	517.7 (513.2-522.4)	466.9 (462.3-471.2)	426.4 (422-430.8)	371 (366.7-374.8)	328.5 (325-331.9)	295.2 (292-298.5)	252.5 (220.9-285.5)
JPN Tottori	1386.4 (1121.6-1681.5)	1084.7 (877.8-1308.7)	999.7 (826.5-1197.6)	898.2 (751.1-1062.6)	798.2 (694.6-906.4)	675.2 (625.8-726.7)	568.8 (553.4-584)	515.4 (504.3-526.2)	446.8 (436.6-457.5)	389.2 (379.7-398.1)	340.7 (332.3-349.6)	317.1 (308.7-325.6)	289.3 (281.5-297.1)	266.2 (232.5-303.8)
JPN Toyama	1403.8 (1165.7-1681.8)	1125.8 (934.2-1353.9)	1058 (870.5-1259.7)	948.1 (792.6-1124.5)	849.2 (734.6-968.3)	739 (677.9-796.7)	617.1 (604.8-630.1)	528.1 (518.5-538)	442.4 (434.4-450.7)	390.2 (382.7-397.9)	336.1 (328.6-343.3)	295 (288.6-301.4)	272.4 (266.1-279.4)	255.6 (221-292.3)
JPN Wakayama	1579.4 (1292.4-1878.4)	1257.9 (1025.2-1525.7)	1184.9 (969.7-1426.1)	1067.4 (882.7-1277.6)	958.6 (829.5-1101.1)	823.1 (761-890.1)	662.5 (651.2-674.7)	555 (544.9-565.4)	492.2 (482.9-501.9)	430.6 (422.4-439.6)	365.7 (358.1-373.6)	330.2 (323.5-337.6)	303.4 (296.2-311.2)	281.2 (243.8-322.1)
JPN Yamagata	1368 (1114.9-1678.4)	1079.4 (877.3-1320.3)	1008.7 (820.4-1219.6)	928 (771.9-1106.6)	838.1 (728.2-966.6)	699.1 (646.9-759.3)	521.3 (511.2-531.8)	461.2 (452.7-470.1)	427.6 (419.3-435.7)	393.2 (385-401.5)	341.8 (334.8-348.7)	300.4 (293.7-307.4)	290.4 (283.6-296.9)	267.5 (233.3-307.2)
JPN Yamaguchi	1440.9 (1183.6-1721.4)	1128.1 (914.7-1388)	1027.3 (828.1-1254.3)	907.1 (741.3-1105.4)	803.5 (680.9-952.1)	702.4 (641.9-769.6)	614 (603.4-625.3)	509.3 (500.6-518.1)	438.8 (431.3-447.5)	399.6 (391.5-407.8)	349 (341.9-356.5)	319.1 (312-326.3)	304.1 (297.1-310.7)	273.5 (235.7-312.7)
JPN Yamanashi	1362.4 (1103-1655.4)	1068.2 (861.2-1292.6)	985.2 (800.2-1176.8)	879.3 (732.8-1040.4)	779.8 (668.6-895.8)	668 (613.2-724.3)	591.5 (577.8-606.1)	518.4 (508.2-529.1)	444.1 (434.3-453.2)	379.8 (371.7-387.9)	330.7 (323.1-338.5)	303.1 (295.9-310.6)	283.4 (276.3-291.2)	264 (227.3-300.7)
KOR South Korea	2460.5 (2178-2761.6)	1694.4 (1442.9-1979.4)	1538.8 (1293.7-1810.7)	1453.8 (1228.2-1710)	1344.3 (1148.2-1550.5)	1116.6 (1061.7-1172.9)	988.4 (983.4-994.2)	842.9 (839-847.3)	753.6 (750.4-756.8)	662.9 (659.6-666.1)	589.8 (586.8-593)	477.4 (474.9-479.9)	384.7 (378-391.7)	324.5 (301.5-347.4)
SGP Singapore	1558.2 (1406.1-1723.3)	1283.7 (1264.8-1304.4)	1178.2 (1157.3-1199.4)	1120.2 (1101.6-1138.3)	1048.6 (1029.7-1067)	956.4 (940-973)	897.8 (883.1-912.4)	725.6 (714-737.9)	634.6 (624.6-644.8)	546.3 (537.5-555.6)	463.3 (421-505.2)	386.9 (376.6-397.3)	295.5 (288.3-303.8)	252 (235.5-273.3)
High-income North America	986.5 (949.7-1025.3)	889.3 (860.8-920)	876.5 (874.8-878.2)	839.6 (838-841.3)	790.9 (789.4-792.3)	694 (692.7-695.3)	643.1 (641.9-644.3)	609.3 (608-610.6)	568.5 (567.4-569.6)	563.5 (562.4-564.6)	550.2 (549.1-551.3)	519.9 (518.9-520.8)	474.7 (473.7-475.6)	472.1 (460.4-483.4)
CAN Canada	1020.2 (1012.3-1028.3)	891.8 (885.4-898.4)	842.4 (836.1-848.2)	781.6 (776.1-787.7)	714.2 (709.2-719.2)	665.3 (660.7-669.7)	588.9 (584.7-593)	557.1 (552.9-561.4)	517.1 (513.5-521)	503.6 (499.9-507.3)	468.6 (465-472.2)	433.8 (430.4-437)	391.6 (388.5-394.8)	374.7 (353-399.3)
GRL Greenland	2694.3 (2573.2-2814.3)	2025 (1961.2-2088.7)	1390.4 (1342.7-1434.8)	1137.4 (1095.6-1179.8)	1114.6 (1074.8-1154.4)	1215.3 (1178.5-1255.5)	1243.2 (1206.2-1282.4)	1188.3 (1150.1-1230.6)	1114.3 (1072.5-1159.9)	1085.7 (1048-1127.4)	1029 (993.6-1065.9)	936.2 (904.6-969.5)	815.1 (780-853.2)	662 (614.2-719.2)

Table 1 Age-standardised mortality rates by location and year for females for 1950, 1955...2005, 2010, 2017

Location	1950	1955	1960	1965	1970	1975	1980	1985	1990	1995	2000	2005	2010	2017
USA USA	983.2 (943.2-1024.8)	889 (858.1-922.2)	879.4 (877.6-881.2)	844.7 (843-846.4)	797.7 (796.2-799.2)	696.7 (695.4-698.1)	648.1 (646.8-649.3)	614.4 (613-615.7)	573.9 (572.7-575.1)	570.1 (569-571.3)	559.2 (558.1-560.3)	529.6 (528.6-530.7)	484.4 (483.4-485.3)	483.7 (470.9-496.3)
USA Alabama	1111.2 (909.2-1354)	1005.1 (831.9-1190)	915.7 (902.9-927.5)	902.2 (889.8-913.8)	836.9 (824.8-848.6)	722.8 (713.7-732.9)	677.3 (668.1-686.7)	649.5 (639.8-658.9)	624.9 (616.1-633.7)	623.4 (614-632.6)	636.3 (627.7-644.9)	645.4 (636.1-654.3)	622.5 (614.3-630.8)	628.1 (548.9-717.8)
USA Alaska	1017.4 (872.7-1176.4)	907.3 (827.6-992.8)	942.2 (911.9-971.4)	929 (908.1-953)	900.7 (883.5-919.1)	845.7 (828.8-863.3)	731.2 (715.3-746.9)	728.4 (714.7-742.2)	677.7 (664.4-692.7)	701.4 (688.2-714.4)	653.7 (641.3-667)	594.1 (583.3-605)	535 (524.8-545)	528.5 (484.6-573.4)
USA Arizona	936.2 (757.2-1141)	839.4 (697.2-1000.2)	781.6 (765.1-798.8)	788.4 (773.6-802.8)	751.1 (737.4-765.3)	646.5 (633.9-658.9)	622.5 (611.8-634)	566.2 (557.2-576)	530.1 (521-539.1)	545 (537.4-553.9)	526.9 (518.5-534.7)	510.1 (502.8-517.6)	445.3 (438.6-451.7)	448.3 (392-513.1)
USA Arkansas	953.1 (766.8-1167.7)	853.7 (715.6-1009.3)	821.4 (805.8-837.2)	795.4 (780.6-810.1)	755.3 (741.9-769.2)	685 (672.2-697.6)	629.2 (617.6-641.4)	624.7 (613-637.1)	603.3 (591.3-615.5)	604.6 (593.4-616.2)	621.5 (610.7-632.6)	594 (583.9-604.1)	579.2 (568.7-589.3)	605.5 (525.1-694.1)
USA California	909.1 (744-1104.7)	814.5 (683.5-962.9)	814.4 (808.7-820.1)	795.1 (789.6-800.5)	762 (757.4-767)	676.7 (672-681.6)	632.6 (628.6-636.8)	595.4 (591.2-599.5)	558 (554.4-561.9)	524.5 (520.9-527.9)	509.2 (505.7-513)	476.2 (473-479.4)	414.2 (411.4-417)	394.6 (344.9-448)
USA Colorado	918.5 (737.7-1133.8)	826 (687.6-988.5)	806.6 (791-822.4)	760.4 (746.9-773.4)	717.9 (705.3-731.3)	623.7 (612.6-634.1)	596.3 (585-607.4)	561.4 (551.2-571.9)	524.1 (514.7-534.5)	536.1 (526.7-545.5)	530.3 (520.8-539.2)	490.6 (482.3-498.9)	460 (452.6-467.5)	454.9 (395.1-527.2)
USA Connecticut	919.6 (735-1128.7)	814.9 (671.2-977.5)	793.3 (780.4-806.1)	821.5 (808.1-834.2)	743 (731-754.8)	663.6 (652.9-674.4)	622.2 (612-632.3)	576.9 (566.3-587.3)	525.8 (515.7-535.2)	521.1 (512.2-531.1)	500.9 (491.3-510.8)	447.9 (438.6-456.4)	413.9 (405.6-421.8)	406.6 (349.1-470.5)
USA Delaware	1048.1 (889.1-1231)	958.9 (854.6-1057.9)	945 (922.9-967)	900.7 (882.5-919.5)	862 (845.3-879.1)	743.8 (729-759.1)	687.7 (675-701.8)	651.5 (638.9-665.4)	635 (622-648.5)	610.7 (598.8-622.7)	596 (585-608.2)	555 (544.5-565.5)	524.7 (515.2-534.9)	505.8 (466.1-551.8)
USA Washington, DC	1103.9 (918.1-1317.8)	999.8 (883.5-1124.8)	1027.9 (1011.6-1044.7)	1052 (1036.4-1067.5)	993.6 (978.7-1009.5)	857.8 (842.1-872.2)	793 (778.3-808.5)	766 (750.7-781.7)	802 (787.3-816.4)	928 (910.3-945.9)	674.5 (659.4-689.4)	630 (617-643)	554.6 (542.2-567.1)	531.4 (479.6-591.9)
USA Florida	917.5 (738.5-1117)	809.4 (671.2-966)	800.9 (791.7-810.6)	763.8 (755.4-772)	739.4 (731.6-747)	658.4 (651.5-665.2)	620.1 (614.3-626.2)	573.4 (567.9-579)	523.1 (518.1-528)	522.5 (517.6-527.3)	508.9 (504.3-513.1)	485.5 (481.2-489.4)	434.6 (430.7-438.6)	441.3 (381.1-504)
USA Georgia	1096.9 (890.3-1336.8)	955.3 (790.4-1147.6)	938.2 (926.9-950.1)	886.3 (875.7-895.4)	848.6 (838.9-858.6)	729.3 (720.6-738.4)	693 (684.9-701.9)	669.4 (661.3-678)	636 (628.5-643.5)	642.5 (635.3-650)	646.3 (639-653.7)	608.3 (601.6-614.9)	571.7 (565.2-577.8)	563.6 (493.8-644.6)
USA Hawaii	972.4 (814.9-1143.1)	835.2 (757.7-918.8)	806.6 (788.1-823.7)	746.6 (732.1-760.3)	716.6 (703.2-730.3)	568.2 (557.3-578.9)	510.4 (500.7-520.7)	482.7 (473.1-492.5)	455.6 (446.9-464.1)	435.5 (427.1-443.5)	439.6 (431.4-448)	398.1 (390.4-405.8)	364.4 (357.6-371.3)	355.5 (323.5-390.1)
USA Idaho	869.4 (723.8-1024.9)	781.8 (700.2-865.2)	762.2 (744.5-779.7)	766.1 (751.1-781.5)	721 (707-736)	645.5 (634.1-657.6)	578.8 (567.2-589.9)	562.1 (551.9-573.7)	534.5 (523.5-545.9)	529 (518.6-539)	540.3 (530.7-550.6)	522.4 (513.7-531)	491.9 (483.3-500.3)	484.8 (446.1-526.6)
USA Illinois	1006.4 (818.6-1230)	904 (750.7-1065)	918.3 (910.6-926.2)	882.2 (874.7-890.2)	839.8 (832.9-846.7)	728.7 (721.8-735.4)	665.2 (659.3-671.3)	629.5 (623.4-635.6)	591.5 (585.4-597.6)	584.9 (579.2-590.7)	563 (557.2-568.6)	524.9 (519.3-530.4)	472.9 (468.2-478.3)	477 (414.7-545.2)
USA Indiana	961.1 (768.1-1171.9)	859.5 (706.4-1033.9)	882.6 (872-894.7)	855.7 (845.5-865.6)	811.9 (802.5-821.8)	706.8 (697.8-715.8)	656.2 (647.3-664.5)	623.3 (614.9-631.5)	589.8 (581.8-598.4)	613.2 (604.6-621.6)	599.1 (591.1-607.1)	566.7 (559.2-574.4)	538.9 (531.7-546.3)	548.4 (477.1-629.4)
USA Iowa	829.8 (654.8-1018.9)	773.6 (630.6-924.7)	769.4 (755.3-783.2)	755.4 (741.5-768.3)	709.3 (697-721.7)	627.3 (615.8-639.1)	544.4 (532.1-555.3)	543.5 (533.7-553.7)	480.5 (470.3-491.1)	507.2 (496.6-518)	495.7 (486.2-506.5)	472.1 (462.9-481.6)	456.2 (447.1-464.6)	469.3 (400.8-539.1)
USA Kansas	818.2 (667.4-1004.2)	746.6 (613.6-893.7)	768 (751.9-783.4)	749.9 (735.3-764.2)	699.8 (687.3-713.1)	636.2 (624.4-649)	576.1 (564.3-587.3)	550.3 (539.2-560.9)	495.5 (484.9-505.1)	533.8 (523.2-545.4)	532.7 (521.7-544.2)	526.1 (515.4-537.1)	489.2 (478.9-499.7)	525.2 (456.3-610)
USA Kentucky	1007.8 (807.1-1237.9)	906.9 (750.4-1071.2)	884.4 (869.8-899)	855.7 (842.1-868.4)	840.9 (828.6-853.1)	742.8 (732.5-753.6)	679.5 (668.9-690.1)	648.8 (639.2-659.8)	618.2 (608.9-627.7)	620.1 (610.3-629.9)	633.5 (623.4-644.5)	615.7 (606.4-625.1)	615.7 (607-624.4)	636.7 (550.3-727.2)
USA Louisiana	1084.1 (882.5-1325.9)	947.9 (794.3-1124.1)	939.1 (926.6-951.1)	906 (894.1-918)	861.4 (850.1-873.3)	755.5 (745.5-765.6)	711.4 (702-721)	686.6 (677.7-696.2)	653.8 (644.6-662.8)	653.2 (644.2-661.7)	664 (654.3-673.1)	706.6 (697.2-715)	585.4 (577.5-593.8)	587.9 (510.7-676.9)
USA Maine	981.1 (806.9-1173.4)	886.1 (777.1-1007)	874.8 (858.1-891.4)	842.6 (826.6-859.6)	792.4 (776.2-807.7)	675.4 (662.9-688.5)	626.1 (612.3-639.1)	600 (587.8-613.1)	557.3 (545.2-569.1)	549.2 (538.1-561)	557 (545.9-568.9)	544.4 (533.7-555.7)	493.6 (483.5-504.1)	510.8 (459.1-563.2)
USA Maryland	1077.5 (864.5-1311.8)	960.3 (796.8-1147)	971.1 (958.4-984.8)	905.2 (893.9-918.4)	834.9 (823.9-845.5)	730.7 (720.6-740.2)	681.2 (671.4-690.5)	659.6 (650.5-669.1)	615.4 (606.5-624.6)	609.8 (601.9-618.7)	604.1 (595.8-612.5)	543.5 (536.6-551.1)	486.3 (479.6-493.3)	463 (398.6-533.8)
USA Massachusetts	963.2 (784.9-1173.8)	887 (724.1-1058.3)	874.4 (863.4-885.2)	837.4 (826.7-848.3)	751.3 (741.8-760.7)	659.8 (651.3-667.9)	625.2 (617.6-633.8)	592.9 (584.6-600.7)	534.7 (527.2-542.3)	529.2 (521.7-536.5)	518 (510.9-525.2)	474.7 (467.7-481.8)	422.6 (415.8-428.9)	437.7 (379.5-499.6)
USA Michigan	984.3 (804.3-1205.4)	897.9 (748.2-1072.3)	885.8 (876.8-895.1)	859.8 (851.1-868.1)	810.9 (803.4-818.4)	721.4 (714.5-728.6)	659.2 (652.2-666.2)	639.5 (633-646.7)	597.9 (591.2-604.4)	596.5 (590.5-602.8)	586.8 (580.7-593.2)	560.2 (553.7-566.5)	520.4 (514.9-525.7)	531.4 (459.5-605.6)
USA Minnesota	849.8 (677.8-1038.4)	768.7 (632.6-915.6)	794.2 (781.2-807.1)	741.4 (729.9-753.3)	689.5 (678.7-700.4)	605.9 (596.8-615.7)	544.5 (535.7-553.2)	527.9 (519.3-536.8)	493 (484.6-501.4)	495.7 (488-503.8)	484.8 (477.3-493.4)	446.7 (439.3-454.5)	416.7 (409.4-423.8)	412.1 (356.1-479)
USA Mississippi	1108.1 (895.8-1342)	985.8 (816.6-1157.7)	951.5 (936.4-966.4)	939.4 (925.5-954.7)	870.2 (856.7-884.7)	754.1 (741.4-767.4)	706 (693.9-718.3)	659 (646.8-670.9)	635.3 (623.9-646.4)	651.5 (641.2-663)	675.3 (664.6-686.7)	668.9 (659.2-679.2)	624.9 (615-634.6)	624.7 (540.8-721.8)
USA Missouri	943.5 (760-1152.7)	849.7 (712.9-1006.6)	860.5 (849-871.8)	831.5 (820.3-841.9)	800.5 (790.7-810.4)	695.8 (686.3-704.7)	647.9 (638.8-657.2)	606.8 (598.1-615.6)	582.2 (574.7-590.7)	596.4 (588.4-605.1)	596.8 (588.4-605.4)	571.1 (563.2-578.4)	538 (530.6-545.6)	549.3 (478.2-629.5)
USA Montana	919 (771.4-1090.6)	850 (763.9-940.5)	846.6 (828.5-864.9)	823.2 (808.2-838.2)	777.7 (764.4-791.7)	668.4 (655.6-681.6)	625.9 (614.4-638.4)	577.5 (565.3-589)	547.8 (535.6-559.7)	547.2 (536.7-558.6)	547 (536.4-558.7)	531.9 (521.4-542.2)	499.6 (489.5-510)	577.5 (527.4-630.9)
USA Nebraska	783.2 (646.3-951.7)	707.8 (618.3-809.8)	763.6 (749.3-777)	752.5 (739.5-766.1)	717.9 (705.3-729.7)	637.3 (626.1-647.9)	570.3 (560.1-580.5)	538.3 (527.6-548.9)	515.7 (505.9-526.1)	519.6 (509-530.4)	508.8 (499-519.2)	477.6 (468.9-487)	460.8 (452.6-469.4)	482.3 (433.7-536.4)
USA Nevada	1087.7 (891.8-1296.6)	956.8 (840.8-1091.5)	904.7 (880.9-930.2)	926.2 (905.3-947)	872.8 (854.4-892.2)	745.1 (729.4-761.6)	739.2 (724.4-754.4)	685.4 (671.5-698.5)	653.8 (642-665)	661.5 (649.7-673.6)	653.3 (642.7-664.1)	644.9 (635.3-654.3)	549.9 (542.1-558.6)	528.6 (480.6-578.2)
USA New Hampshire	932.8 (783.1-1095.8)	835.4 (749.1-920.9)	848.8 (830.9-867)	852.9 (837.7-869)	793 (778.2-808.6)	696.2 (682.8-708.9)	629.2 (617.9-640.9)	595.7 (585-606.4)	565.2 (554.2-575.9)	542 (531-552.9)	547.1 (536.7-557.3)	519.7 (510.1-529.5)	459.5 (450.1-468)	500.4 (460.3-545)
USA New Jersey	1047 (836.1-1258.3)	945.6 (786.5-1138.2)	928.7 (918.5-939.3)	887.8 (878.9-897.3)	824.5 (815.9-832.7)	713.7 (705.8-721)	674 (666.1-681.9)	639.1 (631.7-645.9)	588.9 (582.1-595.7)	579.6 (573.1-586.3)	544.5 (538-551.5)	495.1 (488.9-501.2)	438.6 (432.8-444.4)	409 (354.3-469.5)
USA New Mexico	934.3 (760.6-1125.3)	829.5 (727.8-933.9)	840.7 (826.1-856.8)	810.3 (795.1-825.5)	777.6 (763-792.1)	677 (664.8-689.3)	605.4 (594.2-616.6)	576 (565.5-586.3)	550.6 (541.1-561.5)	547.9 (537.8-557.9)	529.6 (521-538.8)	514.1 (505.3-522.9)	490.4 (482.5-498.5)	491.2 (444.1-544)
USA New York	1038.3 (854.4-1241.2)	959.7 (814.1-1122)	945.6 (939.3-951.7)	898.9 (893.1-905)	845.4 (839.7-850.7)	716.6 (711.5-721.6)	680.6 (675.7-685.7)	646.9 (642.1-651.7)	585.4 (580.8-589.8)	564.2 (559.8-568.7)	514.2 (509.5-518.5)	475.7 (471.4-479.5)	420.5 (416.6-424.2)	401.7 (351.1-460.8)
USA North Carolina	1024.6 (838.7-1242.5)	910.6 (762-1081.2)	919.6 (908.6-931)	884.6 (874.3-895.1)	823.9 (814.4-833.5)	717.4 (709-725.9)	656.1 (648.6-663.9)	640.8 (632.6-648.7)	604.5 (597.6-611.8)	612.8 (605.4-620.4)	618.2 (611.4-625.9)	580.5 (574.7-586.7)	532.8 (527-538.5)	529.5 (459.9-610.2)
USA North Dakota	863.6 (737-998.1)	797.6 (723.7-869.5)	779.2 (760.6-798.8)	738 (724-752.5)	705.6 (692.4-719.4)	629.9 (618.4-642.8)	562.2 (550.8-574)	498.9 (488.6-509.1)	477.2 (466.6-488.4)	479.9 (469.3-490.2)	476.5 (466.2-486.9)	436 (426.2-446.1)	427.3 (418.8-437.1)	473.8 (431.4-516)
USA Ohio	993.3 (806.9-1215.6)	909.1 (759-1079.5)	900.5 (892.1-908.3)	869.1 (861.3-876.8)	819.8 (813.2-826.6)	723.8 (717.7-729.9)	682.6 (676.3-689.6)	635 (628.7-641.3)	598.6 (592.1-605.1)	604.9 (598.8-611)	594.9 (588.5-601.2)	570.1 (564.3-576.3)	538.6 (533.3-544.1)	577.3 (501.5-662.4)
USA Oklahoma	876.4 (698.4-1077.3)	787.2 (645.6-945.4)	784.5 (769.8-798.8)	731.1 (718-744.2)	744.5 (731.7-757.1)	663.5 (652.1-674.1)	630.7 (620.5-640.9)	618.7 (608.7-629.3)	595.2 (585.4-605.7)	614.3 (603.7-624.1)	638.9 (628.4-648.8)	641.6 (631.6-651.7)	606.8 (597.4-616)	615.9 (529-713.7)
USA Oregon	868.6 (701.6-1063.5)	780.6 (638.7-929)	794.7 (778.4-810.2)	760.9 (746.1-775.4)	726 (712.6-740)	648.2 (636.6-659.9)	595.2 (584.4-607.1)	571.9 (561-582.7)	548.9 (538.3-559.3)	571 (561.2-581)	547.9 (538-557.6)	515.2 (506.5-524.4)	482.6 (474.5-490.9)	476.9 (411.7-546.9)
USA Pennsylvania	1066.5 (880.6-1290.2)	975.4 (814.5-1145.1)	947.8 (940.1-955.8)	909 (902.2-916.3)	848 (841.2-854.5)	730.5 (724.4-736.7)	682.4 (676.4-688.4)	637.6 (630.9-644)	592.9 (586.4-598.9)	575.4 (569.5-581.5)	564.8 (559-570.3)	540.6 (535-546.2)	490.3 (485.1-495.5)	508.9 (445.6-580.3)
USA Rhode Island	999.3 (821.3-1209.5)	909.6 (796.7-1033.5)	899.3 (880.9-917)	867.2 (849.7-884.1)	794 (778.7-809.8)	682.1 (668.1-696.3)	610.1 (596.6-624)	584.5 (571.7-596.6)	549.3 (537.4-561.6)	511.2 (499.7-523)	520.5 (508.7-532.4)	501.8 (490.4-513.8)	452.7 (442.5-463.7)	448.1 (402.6-499.4)
USA South Carolina	1218.1 (976-1497.7)	1065.6 (877.4-1277.6)	993.7 (979.1-1008.4)	928.8 (915.1-942.3)	869.6 (857.4-882.9)	762.9 (751.7-774.3)	706.7 (696-716.8)	662.4 (652.3-672.8)	640.8 (631-650.2)	634.2 (625-643.7)	632.8 (623.2-641.6)	597.1 (588.8-605.8)	568.5 (560.5-576.5)	573.2 (496.1-657.1)
USA South Dakota	829.7 (698.4-978.1)	769.5 (695.7-852)	792.9 (775.3-809.7)	758.7 (743.8-774)	727.8 (714-741.5)	637.9 (625.7-650.6)	563.5 (550.9-576)	530.7 (519.1-542.6)	494.9 (483.6-507.3)	500.6 (488.3-512.7)	494.9 (484.1-505.6)	469.8 (458.9-480.8)	449.2 (439.2-459.7)	494.6 (451.1-542.4)
USA Tennessee	1022.1 (816.1-1268.8)	929.9 (763.6-1102.7)	894.6 (881.7-905.8)	860.9 (850.1-872.2)	824.6 (814.2-834.9)	723 (713.6-733)	659.3 (650.6-668.5)	633.2 (624.7-641.7)	613.3 (604.6-621.9)	629.2 (621-637.1)	636.3 (628.5-643.9)	626.8 (619.1-634.3)	593.3 (586-600)	605.4 (524.1-690.8)
USA Texas	906.6 (727.8-1105.4)	807.4 (670-963.7)	799.5 (791.6-807.3)	766.3 (759-773.8)	758.7 (751.9-765)	678.2 (672.4-684.1)	645.2 (639.8-650.7)	616.7 (611.7-621.6)	572.6 (567.8-577.9)	575.2 (570.5-580.2)	584 (579.5-588.8)	546.6 (542.6-550.6)	512.7 (508.9-516.4)	501 (440-567.9)
USA Utah	881.1 (713-1071.1)	803.1 (696.1-916.5)	781 (763.1-799.1)	791.1 (776.3-807.4)	703.9 (690.5-719)	628.4 (616-640.3)	590 (579.5-601.5)	568.7 (557.5-580.3)	516.5 (506.3-526.1)	521.1 (511.6-530.9)	528.6 (519.8-538)	511.1 (503.1-519.2)	476.5 (468.4-484.1)	500.3 (454.2-549.1)
USA Vermont	920.1 (783.9-1058.7)	840.5 (766.1-923)	866.2 (844.3-888.9)	847.3 (829.1-865.5)	755.6 (740.8-771.9)	668.6 (655.6-682.7)	612.2 (599.8-624.9)	614 (601.5-627.6)	559.5 (546.1-572.6)	548.8 (536.6-561.6)	538.6 (527.1-550.8)	503.2 (492.3-515.1)	469.2 (457.6-480.3)	432.7 (394.1-473.2)
USA Virginia	1058.7 (855.2-1275.9)	944.7 (781.5-1122.8)	925.5 (912-938.7)	869.6 (858.6-880.2)	810.9 (801.1-820.7)	709.4 (700.1-718.1)	664.7 (656.2-673.3)	635 (626.6-643.5)	594.4 (585.7-602.3)	597.4 (590.2-605)	588.2 (580.6-595.4)	550.7 (543.7-557.6)	491.5 (485.4-497.7)	470.9 (412.4-536.3)
USA Washington	891.5 (727.5-1099.1)	810.8 (660.9-992.6)	809.3 (795.8-822.9)	762.2 (750-774.5)	754.1 (743.7-765.2)	655 (644.3-665.3)	608.8 (598.9-617.8)	579.5 (570.6-588.5)	538.8 (530.7-546.9)	542.2 (534.2-550.3)	524.4 (516.2-532.2)	496.3 (489.3-503.4)	457.5 (450.6-464.2)	451.2 (392.7-518.9)
USA West Virginia	1000.1 (805-1223.1)	934.6 (768.2-1113.7)	878.5 (862.1-895.7)	903.8 (889.2-919.2)	879.9 (864.7-895.8)	759.6 (745.9-774.3)	688.4 (675-702)	650.1 (637-664.7)	607.9 (595.8-620.6)	619.3 (605.7-632.6)	649.5 (636-662.8)	636.6 (623.6-649.7)	611.4 (598.9-623.1)	640.9 (555.9-733)
USA Wisconsin	904.3 (712-1120.7)	833 (689.2-990.7)	847.6 (834.4-861.3)	798.5 (787.7-809.8)	738.8 (727.9-749.5)	643.3 (633.9-652.8)	590.6 (581.5-599.3)	551.3 (542.6-560.4)	534.8 (525.8-544)	525.3 (517.6-533.1)	520.7 (512.6-529.1)	492.8 (484.9-500.2)	459.9 (452.4-467.1)	439 (376.6-504)
USA Wyoming	850.5 (724.5-984.8)	773.2 (702.6-846.9)	801.6 (778-825.4)	817.9 (799.9-836.2)	764.7 (749.2-782)	702.7 (686.6-718.6)	624.8 (611.5-638.3)	585.2 (571.7-597.9)	553.8 (541.8-566.9)	560.7 (548.1-573.7)	569.4 (555.9-582.9)	540.5 (529.4-551.8)	514.9 (503.5-525.8)	506 (465-550.5)
Southern Latin America	1350.9 (1333-1371.2)	1249.7 (1238.8-1260.4)	1162.7 (1157.3-1168.3)	1062.8 (1058.1-1067.3)	999.8 (995.7-1004.1)	900.3 (871.4-931.6)	832.1 (828.3-835.7)	756.4 (752.7-760)	711.7 (708.3-714.9)	645.3 (642.1-648.2)	608.7 (605.8-611.4)	573.9 (570.9-576.5)	550.6 (547.9-553.3)	524.6 (480.8-576.8)
ARG Argentina	1272.3 (1245-1302.6)	1165.3 (1149.7-1181.2)	1037.9 (1031-1045.2)	976.8 (971.2-982.2)	951.3 (946.3-956.4)	872.5 (831.1-916.4)	807.4 (802.8-812)	749.7 (745.5-753.8)	713.1 (709-717.1)	651.6 (647.7-655.5)	627.3 (623.6-630.8)	592.7 (589.2-595.9)	575.2 (571.9-578.6)	552.4 (489.5-622)
CHL Chile	1749.1 (1734.4-1764)	1624.3 (1612.8-1635.8)	1648.1 (1637.9-1658.2)	1396.7 (1387.5-1405.5)	1215.4 (1207-1224.3)	1043.5 (1035.4-1051.9)	952.6 (944.8-960.1)	822 (815.5-828.9)	735 (728.9-741.3)	640.4 (634.4-646.1)	562.9 (557.6-568.4)	524.2 (519.1-529.3)	496.2 (491.5-500.9)	460.7 (403.2-521.6)
URY Uruguay	970.2 (952.9-986.3)	950.2 (936.4-964.2)	903.8 (890.5-918)	889.6 (876.5-902.3)	884.7 (871.6-897.7)	831.4 (818.6-843.4)	809.3 (796.1-822.6)	708.8 (698-721.1)	675.3 (664-687)	636 (624.7-647.3)	599.7 (589.8-610.5)	577.8 (567.8-588.4)	524 (513.9-534.8)	514.8 (449.5-584.1)
Western Europe	1108 (1068.7-1149.8)	979.8 (966.3-992.6)	906.8 (902.1-912)	853.8 (849.4-858.5)	812.3 (808.4-816.5)	759.7 (757-762.6)	695.2 (693.9-696.5)	641.2 (640.1-642.4)	586.7 (585.6-587.8)	535.9 (534.9-537)	486.6 (485.6-487.6)	436.5 (435.5-437.4)	401.5 (400.6-402.4)	374.6 (362.7-387.2)
AND Andorra	499.4 (388.6-623.1)	497.3 (387.8-609.4)	482.8 (381-583.2)	480.5 (376.4-581)	472.8 (371.1-580.6)	461 (362.2-569.4)	452.5 (354.6-557.5)	448.4 (359.8-545)	434.9 (352.5-520.6)	416 (335.3-495.4)	351.9 (297.4-435.2)	318.4 (288.6-384.3)	321.4 (280.2-381.3)	338.4 (276.1-399.6)
AUT Austria	1211.1 (1198.7-1223.6)	1104.2 (1094-1114.4)	1013 (1003-1023)	962 (952.3-971.8)	952.3 (942-962.9)	872.1 (862.5-881.6)	791.8 (783.3-800.7)	717.6 (709.1-726.4)	632.9 (625.3-640.9)	571.4 (564.2-579.1)	514 (507-520.6)	457.3 (450.8-463.3)	415.2 (409.1-421.4)	383.9 (359.7-411.1)
BEL Belgium	1272.9 (1259.9-1285.9)	1041.9 (1033.4-1051.5)	955.5 (946-964.1)	908.9 (899.1-918.2)	892.8 (884.3-902)	840.9 (832.2-849.4)	742.1 (733.8-750)	675.5 (668.7-682.8)	596.5 (589.9-603.5)	549.5 (542.7-555.6)	514.5 (508.4-521.2)	467.7 (461.9-473.6)	438.5 (433.2-444)	389.1 (363.8-417.7)
CYP Cyprus	988.7 (830.4-1152.8)	963.2 (824-1116.4)	923.1 (789.2-1058.6)	901.7 (795.1-1003.8)	877 (809.4-947.8)	859.3 (838.4-881.2)	805.9 (792.8-819.4)	725.2 (712.7-737.1)	634.3 (621.1-647.3)	607.9 (582.9-632.8)	584.6 (555.8-613)	502.5 (474.2-530.2)	396.2 (373.1-424.6)	326.4 (299.4-360.1)
DNK Denmark	1058.7 (1043.3-1073.4)	921.3 (909.3-933.6)	910 (897.7-922.1)	867 (855.4-878.7)	765.6 (755.6-776.3)	711.6 (702-720.8)	702.2 (692.6-711.1)	678.6 (670.2-687.7)	665.1 (655.6-673.7)	663.5 (654.4-672.7)	591.7 (583.3-600.3)	534.8 (525.4-543.6)	497.4 (488.7-506.2)	437 (407.5-472.6)
FIN Finland	1249.2 (1232.7-1265.8)	1066 (1055.3-1078)	974.8 (962.6-985.9)	941.8 (929.9-953.5)	931.3 (919.3-945)	799.2 (788-810.5)	682.5 (672.4-692.9)	638.6 (628-649)	621.1 (611.4-630.7)	561 (551.9-570.3)	515.4 (506.9-523.9)	448.4 (441.2-455.9)	408.4 (400.6-415.7)	374.9 (348-404.4)
FRA France	1105.5 (1099.5-1112.4)	973.1 (969.1-977.3)	910.3 (906-914.3)	848.1 (844.1-852.1)	788.7 (785.2-792.3)	731.5 (728-734.7)	639.9 (636.9-643.2)	583.8 (580.7-586.8)	510.9 (508.4-513.6)	460.8 (458.4-463.3)	429.5 (427.1-431.9)	387.6 (385.5-389.9)	359.9 (357.8-362)	317.8 (298.4-338.6)
DEU Germany	998.4 (835-1170.5)	912.5 (865.7-958.1)	874.6 (871.7-877.6)	817.7 (814.6-820.8)	797.2 (794.5-799.9)	750.8 (747.8-753.8)	715.9 (713.1-718.7)	674.5 (671.8-677.4)	638.6 (635.7-641.4)	574.4 (571.9-576.9)	504.7 (502.3-507.1)	448.5 (446.4-450.8)	431.3 (429.1-433.7)	423.4 (372.3-479)
GRC Greece	957.3 (908-1008.7)	827.5 (819.3-836.2)	786.3 (777.5-794.3)	772.6 (764.1-781.4)	730.6 (722.5-738.7)	656.9 (648.7-664.8)	694.1 (686-702.3)	659.2 (651.1-667)	534.4 (528-541.2)	504 (497.6-510.7)	498.7 (492.3-505.4)	520.8 (513.8-527.5)	458.6 (452.8-464.7)	413 (385.4-440.7)
ISL Iceland	958.6 (920.1-998.1)	884.5 (859.8-908.7)	798.5 (776.4-822.2)	744.6 (722.4-767.8)	719.9 (699.6-739.1)	613.9 (596.6-631.2)	546.5 (527.9-565)	528.2 (509.2-547.9)	550.4 (532.8-568.1)	532.4 (518-548.2)	469.1 (455.5-482.2)	416.2 (403.8-429.4)	351.1 (340-364)	311.4 (294.8-328.6)
IRL Ireland	1273.9 (1256.2-1292.8)	1124.1 (1109.7-1140)	1047.3 (1031.3-1062.6)	982.2 (967.4-998.4)	954.6 (939.2-968.5)	917.2 (902.9-932.6)	845.9 (831.5-859)	771.4 (757.9-783.9)	712.9 (700.3-725.1)	666.6 (653.5-678.2)	617 (605.9-627)	502.9 (493.7-512.4)	426 (418.5-433.9)	398.4 (370.5-432.1)
ISR Israel	823.5 (800.8-848.6)	954.5 (937-972)	909.7 (892.9-926.8)	950.7 (933.8-966.5)	909.9 (894.6-924.9)	887.7 (874.2-902)	811.7 (798.2-825.8)	745.4 (732.9-757.2)	629.5 (618.5-640.9)	605.7 (596-615.6)	547.6 (539.2-556.4)	487.6 (480.1-495)	412.5 (405.7-419.3)	369.2 (342.5-397.2)
ITA Italy	1163.8 (1156.1-1174.9)	1003 (998.9-1007.2)	917.2 (913.3-921.1)	876.8 (873-880.5)	816.7 (812.9-820.6)	777.9 (774.2-781.6)	714.1 (710.5-717.8)	626.8 (623.3-630)	549.8 (546.4-552.7)	499.4 (496.5-502.4)	450.1 (447.3-453)	400.2 (397.7-402.9)	368.6 (365.9-371.2)	334.7 (312-357.8)
LUX Luxembourg	1153.9 (1125.5-1184.6)	1096.4 (1078.3-1114)	1022 (1004.3-1042.3)	981.8 (961.2-1002.2)	950.6 (930.2-969.9)	908.6 (889.1-929.7)	801.9 (785.1-820)	726.8 (710.5-743.7)	630.6 (615.6-646.5)	586.5 (571.6-601)	546 (532.4-560.2)	478.7 (466.1-491.8)	454.3 (441.8-467.1)	426.5 (385-469.7)
MLT Malta	1346.2 (1298.6-1390.7)	1171.6 (1149.5-1197.4)	1138.9 (1116.4-1165.5)	1094.3 (1071.3-1116.7)	1040.2 (1017.6-1064.4)	932.7 (912.5-955.6)	932.3 (911.2-952.4)	780.7 (761.4-802.1)	640.1 (623.2-658.7)	577.4 (560.1-594.7)	537.1 (521.5-553.4)	503 (487.5-517.6)	441.6 (428.7-455.2)	422 (395.6-449.9)
NLD Netherlands	994.8 (985-1004.6)	918.1 (909.6-926.4)	849.6 (841.6-858)	787.6 (780.3-795.7)	770.6 (763.8-778.2)	688.7 (682.1-695.7)	599.6 (593.9-605.6)	577.6 (572-583.1)	553.6 (548.1-558.9)	545.5 (540.3-550.8)	537.5 (532.3-542.4)	487.5 (482.8-492.3)	435.9 (431.4-440.6)	424.1 (395.3-453.6)
NOR Norway	893.4 (852.6-933.4)	802.4 (767.3-836.1)	785.4 (759.2-812.1)	748.5 (732.7-765.1)	702 (695.1-709)	649.8 (644.4-655.2)	594.7 (590.2-600)	579.5 (574.5-584.4)	565.3 (560.7-570.2)	524.3 (519.9-528.7)	504.4 (499.8-508.7)	447 (443.1-451.2)	426.9 (423.1-430.7)	379.2 (370.1-387.8)
PRT Portugal	1376.5 (1357.1-1401.7)	1233.3 (1222.4-1243.1)	1058.3 (1049.1-1067.9)	1030 (1020.3-1039.9)	955.9 (946.7-964.7)	911.5 (902.1-921.1)	814.8 (807.3-822.6)	759.1 (751.4-766.7)	724 (716.3-731.5)	653.2 (646.2-660.1)	589.6 (582.6-596.2)	481.3 (466.9-494.7)	431.7 (426.1-437)	381 (355.4-407.3)
ESP Spain	1253.7 (1241.5-1271)	1035.3 (1030.2-1040.4)	890.9 (886-895.7)	854.9 (850.3-859.8)	818.2 (813.5-823.1)	750.9 (746.6-755.5)	635 (630.7-639.3)	581.1 (577.2-584.9)	539.9 (536.2-543.4)	480.6 (477.3-483.9)	431.4 (428.2-434.5)	393.5 (390.7-396.3)	345.9 (343.2-348.4)	315.7 (297.2-335)
SWE Sweden	1032.8 (979-1091)	920.7 (870-973.3)	863.3 (812.5-914.9)	778.3 (732.3-826.8)	709.1 (668.1-750)	668.8 (644.2-695.4)	621.2 (613.7-628.5)	567.6 (561.1-574.6)	536.5 (530-542.9)	491.3 (485.2-497.8)	473.3 (467.3-479.3)	439.1 (433.2-444.9)	402.6 (397.3-408.6)	381.1 (359.3-404.1)
SWE Stockholm	1040.8 (828.1-1327.1)	934.7 (725.4-1194.3)	889.8 (690.4-1126)	806.2 (631.6-1015.6)	734.1 (585.9-903.4)	682.4 (582.9-791.1)	633.6 (617.6-648.8)	580.3 (567.5-593.2)	553.8 (540.9-568.5)	489.5 (478.6-500)	477.1 (466.6-488.6)	440.2 (430.4-450.6)	393.9 (384.3-403.8)	378.3 (347.4-410)
SWE Sweden except Stockholm	1031.1 (975.4-1073.4)	917.9 (864.1-957)	858.4 (813.8-897.1)	773.2 (733.3-810.5)	704.1 (667.3-734.7)	665.7 (641-687.2)	618.3 (610.3-626.6)	564.8 (557.5-572.4)	532.8 (525.4-539.8)	491.5 (484.6-498.7)	472.4 (465.7-479.4)	438.9 (432.4-445.8)	404.4 (398.1-411.2)	381.9 (355.9-409)
CHE Switzerland	1056.3 (1043.2-1069.7)	994.2 (982.7-1006.4)	878.4 (867.9-889.4)	829.5 (819.6-840)	767.5 (757.8-777.6)	660.7 (651.2-669.6)	603.9 (595.4-611.9)	538.5 (530.5-546.1)	501.1 (494.3-508.6)	468.7 (461.8-475.7)	429 (422.8-435.5)	384.2 (378.6-390.7)	362.4 (356.7-368.4)	321.1 (298-343.3)
GBR United Kingdom	1089.4 (1058.4-1119.5)	987.8 (960.6-1017)	918.6 (893.5-945.3)	865.5 (841.8-890.3)	830.1 (809.2-852.3)	789.9 (775.4-806.3)	730.4 (727-734.4)	687.4 (685.7-689.1)	636.7 (635-638.5)	599.6 (598.1-601.4)	545.6 (543-548.1)	494.1 (492.7-495.6)	445.6 (444.4-447)	433.8 (428.7-439.4)
GBR England	1051.3 (1021.5-1080.7)	956.8 (928.1-983.3)	890.3 (864.6-914.5)	841 (818.1-862.9)	807.6 (787.6-828.7)	772.9 (758.4-787.5)	714.1 (710.5-718.2)	673.5 (671.8-675.2)	625 (623.5-626.6)	589.6 (588.2-591.1)	535.8 (533.9-537.9)	484.8 (483.6-486)	435.8 (434.6-437)	426.1 (422.1-430)
ENG East Midlands	1111 (998.9-1235.9)	1006.1 (905.8-1113.3)	932.7 (838.5-1036.8)	878.6 (795.8-967.8)	848.3 (772-931.3)	822.6 (768.4-883.8)	729.7 (714.9-744)	669.5 (664-675.1)	623.9 (618.4-629.7)	593.3 (587.7-598.6)	539.6 (532.5-546.7)	496.2 (491.3-501.3)	442.1 (437.6-446.3)	430.5 (415.8-444.8)
UKF Derby	1161.7 (861.5-1526)	1051.2 (781.9-1390.2)	979.5 (717-1294)	934.5 (694.8-1213.2)	903.7 (697.4-1151.1)	872.3 (723.8-1047.5)	774.3 (727.2-822.2)	716.2 (694-740.6)	666.5 (645.9-689.6)	620.5 (602.5-639.1)	547.1 (524.9-569)	505 (487.8-521.4)	482.8 (466.8-499.1)	440.4 (401.1-481.1)
UKF Derbyshire	1118.8 (844.6-1505)	1014.5 (763.9-1331.4)	942.8 (708-1250.1)	883.3 (667.4-1172.3)	854 (659.3-1082.8)	830.5 (690.1-996)	740.9 (701.3-776.6)	674 (659.4-690)	641.9 (627.1-655.8)	608.8 (594.3-623.6)	547.8 (528.2-567.5)	505.2 (493.2-518)	444.7 (433.5-456.4)	452.3 (413.2-491.4)
UKF Leicester	1179.8 (861-1565)	1085 (798.1-1431)	1021.7 (761.7-1359.5)	987.1 (735.5-1285)	960.4 (733.7-1230.8)	926.4 (759.3-1115.3)	798.6 (751.7-845.7)	692.6 (674.5-711.6)	648 (628.5-666.1)	638.9 (620.6-657.8)	609.1 (583.8-633.4)	556.8 (540.5-574.1)	469.4 (455-484.6)	390.8 (356.8-426.5)
UKF Leicestershire	1053.8 (776.2-1371.5)	956.1 (704.2-1267.8)	882 (656.8-1156.7)	815.4 (604.5-1067.1)	783.8 (603.2-1007.2)	759.5 (631.5-907)	673.1 (637.9-710.2)	623.2 (608.7-638)	572.1 (558.5-586.3)	533.5 (520.3-547.1)	499.9 (483-516.5)	456.8 (445.4-467.8)	396.7 (386.5-407.7)	399.3 (366.8-437.6)
UKF Lincolnshire	1109.6 (814.2-1480.5)	1004.1 (740.9-1324.7)	933.3 (691-1202.6)	881 (666.8-1149.7)	855.2 (657.7-1109.5)	829.4 (673.1-1008.1)	723.5 (683.1-764.1)	669.5 (653-685.7)	609.7 (594.6-626.3)	584 (569.6-599.2)	531.7 (514.1-551.5)	487.6 (475.3-499.2)	431.4 (420.4-443.9)	419.9 (384.6-455.2)
UKF Northamptonshire	1063.9 (782.7-1399.8)	958.9 (706.4-1251.7)	884.8 (657.3-1147.1)	830.3 (631.5-1065.5)	798.3 (613.8-988.4)	773.2 (638.3-924.2)	680.1 (643.9-716)	653.5 (639-668.4)	613.7 (598.6-628.6)	588.9 (575.5-603.6)	518.5 (500.6-536.2)	488.9 (476.4-501.2)	444.7 (433.6-456.8)	442.8 (403.3-485.2)
UKF Nottingham	1156.7 (842.2-1548.8)	1049.4 (773.7-1424.8)	975.2 (722.9-1288.3)	933.2 (684.9-1202.2)	907.2 (696.2-1163.2)	888.5 (736.2-1071.4)	820.2 (774.2-868.5)	740.8 (720.8-761.5)	664 (645.4-683.9)	664.1 (645.2-683.1)	615.7 (593.7-638.8)	602.8 (585.7-620.6)	512.8 (498-528.3)	434.8 (398.4-476.5)
UKF Nottinghamshire	1102.8 (837-1438.2)	990.7 (741.1-1310)	910.7 (692.1-1200.6)	853.3 (648.6-1105.9)	821.4 (629.9-1041.3)	798.2 (661.1-953.9)	717.3 (680.1-755.8)	658.6 (643.4-672.6)	631.4 (618-646)	591.8 (578.2-605.5)	543.4 (524.7-562)	486 (473.8-498.2)	449.3 (437.6-461.1)	449.4 (412.9-488.8)
UKF Rutland	969.9 (706.5-1309)	877.9 (650.1-1160.6)	817.2 (591.2-1093.5)	782.6 (578-1057.5)	757 (565-998.7)	738.1 (591.7-911)	670.8 (600.3-742.1)	654.5 (608.8-700.6)	583.4 (545.8-621.8)	522.7 (485.4-558.4)	450.3 (417.1-485.4)	404.9 (376.8-437.1)	381.9 (353-412.6)	389.1 (342.8-447.6)
ENG East of England	1020.8 (913.4-1142.1)	927.9 (831.4-1039.9)	858.7 (766-953.8)	794.5 (710.9-876.4)	759.2 (684.4-833.5)	726.9 (675-781)	676.1 (662.9-689.6)	631.4 (626-636.8)	588.3 (582.8-593.5)	553.4 (548.6-558.2)	500.7 (494-507.2)	456.5 (452.6-460.5)	409.9 (405.9-413.7)	402.6 (389.2-416.3)
UKH Bedford	998.3 (717.5-1367.8)	906.7 (666.1-1234.3)	830.2 (610.2-1126.2)	769.9 (576.6-1018.7)	721.6 (552.1-916.1)	677.2 (554.2-820.7)	629.4 (586.3-677)	605.2 (582.5-629.9)	595.3 (573-618.3)	578.7 (556.3-600.7)	513.8 (489.9-537.8)	469.2 (451.6-488)	429.8 (412.8-449.1)	398.7 (361.3-442)
UKH Cambridgeshire	958.9 (702.2-1304)	870.1 (640.3-1157.8)	795.9 (583.3-1032)	719.3 (530.3-936.1)	680.2 (522-855.1)	656.7 (541.6-794.5)	650 (612.2-689.1)	630.1 (614.9-646.7)	554.7 (541-569)	546.1 (531.7-560.6)	493.8 (476.8-511.6)	434.8 (423.7-446.3)	377.2 (366.5-387.6)	374.9 (341.6-407.1)
UKH Central Bedfordshire	1025.4 (726.9-1355.7)	937.2 (684.2-1256.1)	871.3 (629.3-1163.1)	820 (608.5-1089.5)	777.3 (581.8-1010.7)	734.6 (599.2-906.8)	683.3 (639-726.5)	648 (626.4-669.1)	615.1 (593.3-635.4)	581.6 (563.4-601.5)	495 (474-516.8)	442 (427.5-458.2)	395 (380.3-409.1)	342.7 (309.3-376.2)
UKH Essex	1046.5 (776.7-1375.5)	955.8 (711.9-1238.5)	890 (676.4-1147.6)	831.3 (636.1-1061.1)	805.9 (626.2-1002.3)	774.4 (640.9-924.7)	685 (653.6-718.4)	627.9 (615.9-640.4)	587.9 (576.8-599.6)	541.4 (531.2-552)	503.1 (487.1-519.1)	457.4 (448.2-466.4)	409.3 (400.4-418.5)	429.5 (396.2-465.7)
UKH Hertfordshire	1011.3 (746.1-1341.4)	917.6 (682.5-1237)	849.5 (625.5-1140.5)	783.3 (583.2-1023.8)	753.5 (577.8-952.9)	728.6 (598.4-879.9)	685.9 (652.5-718.2)	635.1 (622.7-649.2)	593.6 (580.8-606)	561.5 (548.4-574.4)	507.7 (491.3-524.2)	469.7 (459.3-481)	407.7 (397.4-417.2)	380.8 (350.5-415.7)

Table 1 Age-standardised mortality rates by location and year for females for 1950, 1955...2005, 2010, 2017

Location	1950	1955	1960	1965	1970	1975	1980	1985	1990	1995	2000	2005	2010	2017
UKH Luton	1107.5 (791.9-1478.9)	1019.7 (741.5-1345)	959.8 (707.7-1271.2)	920.8 (685-1225.8)	891.6 (678.5-1157.3)	848.6 (699.3-1035.2)	764.2 (715.4-816.9)	651.6 (629.8-673.5)	625.8 (603.3-647.9)	613.7 (592.8-636)	563.9 (539.9-588.3)	529.1 (510.3-548.4)	462.3 (446.5-478.7)	398.3 (360.5-438.6)
UKH Norfolk	976 (725.6-1285.2)	879.2 (652.1-1156.8)	806.1 (600.4-1051.6)	735.5 (548.1-953)	694.9 (526.1-888.2)	663.6 (543.9-804.5)	640 (606-675.7)	610 (596.2-624.6)	587.4 (573.7-600.7)	530.4 (518.1-543.5)	479.9 (463.8-496.2)	438.2 (427.4-448.7)	416.1 (406.5-426.5)	416.1 (381.3-453.7)
UKH Peterborough	1020.6 (724.4-1423.4)	923.3 (657.9-1260.3)	859.7 (610.1-1153.4)	816.6 (582-1084.5)	778.2 (579.5-986.7)	741.9 (602.3-903.8)	706 (655.3-758.6)	676.7 (652.2-701.8)	639.2 (616.8-663.3)	618 (597.3-639.6)	556 (531.8-580.9)	513.7 (495.6-534.7)	469 (449.7-487.9)	438.8 (396-486)
UKH Southend-on-Sea	1078.2 (773.5-1453.9)	980.5 (704.1-1306.1)	916.2 (661.3-1201.6)	864.5 (637-1148.5)	818.4 (625.4-1053.1)	771.6 (637.5-938.5)	714.8 (668.6-763.8)	663.2 (638.3-688)	620.7 (598.5-642.6)	591.8 (569.8-614.3)	540.2 (515.9-564.4)	499.5 (480.1-518.9)	457.2 (439.1-476.2)	445.8 (405.5-489.1)
UKH Suffolk	1023.7 (754.2-1365.7)	929.8 (683.6-1252.4)	861.2 (641.8-1172.5)	791.6 (584.7-1044.7)	754.6 (573.8-957.6)	720.5 (587.1-878.1)	667.5 (632.6-705.4)	627.6 (613.5-641.9)	562.9 (549.9-576.2)	543.4 (530.9-556.6)	480.9 (462.9-498.8)	440.2 (429.2-450.5)	395 (385.8-405.4)	394.5 (361.5-429.3)
UKH Thurrock	1085.8 (785.3-1444)	1001.6 (732.4-1326.6)	934.8 (672.3-1262.3)	878 (652.4-1136.1)	832.3 (638.4-1060.8)	792 (642-960.9)	742.8 (690-793.7)	696.8 (672.8-722.9)	639.3 (613.6-662.3)	610.6 (586.8-635.5)	532.8 (508.9-555.9)	479.9 (461.6-500.1)	434.5 (416.9-452.1)	399.4 (357.5-444.5)
ENG Greater London	973.7 (920.6-1024.2)	888.8 (844-937.4)	832.1 (788.9-876)	801.5 (761-844.5)	777.3 (744.1-810)	749.3 (724.1-773.8)	690.2 (683.3-697.6)	650 (646.6-653.4)	606.4 (603.3-609.5)	589.9 (586.6-593.2)	528.7 (524.8-532.8)	464.8 (462.2-467.5)	409.3 (406.9-412)	381.4 (375.1-388.1)
UKI Barking and Dagenham	1057.8 (771.7-1382.5)	950.9 (703-1240.3)	887.8 (662-1149.3)	850.1 (630.7-1095.1)	819.7 (637.4-1036.1)	788.3 (647.1-946.2)	741.2 (695.1-790.8)	712.7 (690.7-736.5)	666.1 (644-690.1)	653.9 (633.4-675.7)	595.2 (569.3-620.4)	545.9 (527.2-566.2)	481.8 (464.6-500.1)	447.5 (405.3-494)
UKI Barnet	910 (681.6-1179.7)	822.2 (619.7-1062.6)	760.5 (591.5-970.4)	714 (545.9-914.5)	685.2 (538.9-863.8)	661 (549.2-789.8)	626.1 (590-664.9)	607 (591.2-623.5)	551.9 (536.7-568.3)	526.1 (511.4-540.9)	461.8 (443.3-479.3)	404.4 (391.9-417)	387.1 (375.7-398.6)	367.8 (336.7-401.1)
UKI Bexley	920.4 (687.4-1206.1)	829.5 (621.8-1105.4)	770.9 (581.5-1013.7)	731.5 (549-944.8)	697.6 (540.6-876.7)	663.5 (549.6-794.2)	607.3 (570.3-646.7)	577.1 (557.3-596.5)	543.9 (526.3-562.1)	544.5 (526.5-564.1)	467.7 (447.2-487.3)	402.2 (388.2-417.3)	372.4 (358.1-387.4)	375.4 (341.8-409.3)
UKI Brent	983.8 (733-1292.7)	903.8 (681.9-1215.6)	841.1 (631.3-1105)	808.9 (618.2-1039.6)	782.3 (617.9-975.3)	747.9 (623.5-883.2)	646.9 (611.6-683.5)	643.1 (625.1-659.9)	599.8 (583-617.5)	595 (576.7-613.1)	520.6 (499.7-542.5)	436.2 (423.9-449.4)	390.5 (377.7-402.2)	331.6 (302.5-363.1)
UKI Bromley	918.9 (673.8-1223)	829.3 (621.1-1071.3)	773.7 (581-1022.6)	730.7 (560.5-951.1)	704.9 (547.2-889.1)	679.1 (563.1-814.3)	626 (592.6-661.3)	581.8 (565.2-598.5)	545.7 (530.6-562)	519.7 (504.2-536.1)	465.7 (446.8-484.8)	408.7 (395.4-422.4)	372.3 (360.2-385.5)	365.5 (333.3-398.4)
UKI Camden	988.7 (750.1-1287.7)	948.3 (719.5-1216.8)	897 (681.9-1147.3)	870.5 (670.4-1134)	857 (675.2-1081.9)	841.9 (698.6-997.5)	785.5 (736.8-831.2)	743.4 (721.9-765.7)	681.1 (661.8-701.2)	664.6 (643.5-685.6)	577.7 (554.5-599.8)	504 (487.8-520.8)	429.6 (415.2-444)	310.3 (282.9-342.2)
UKI Croydon	1033.8 (771.8-1354.1)	939.6 (698-1227.3)	877.5 (662.9-1144)	819.2 (624-1067.1)	799.5 (623-1017.7)	780.8 (648.5-926.7)	723.3 (685-759.7)	673.2 (655.8-692.4)	635.7 (618.5-652.5)	598.3 (581.1-615.1)	526.3 (507.6-546.7)	463.4 (450.2-476.8)	428 (415.1-440.7)	427.3 (394.2-466.1)
UKI Ealing	1001 (739.6-1320.5)	907.9 (673.4-1192.8)	846.9 (623.9-1077.8)	806.6 (611.6-1024.8)	784.5 (620.8-981)	760.8 (640.5-907.8)	706.8 (668.8-746.2)	666.6 (649-684.8)	605.7 (588.8-621.7)	596.4 (579.2-613.7)	518.9 (499.8-540)	448.1 (434.1-462.4)	381.8 (369.6-393.6)	374.6 (341.6-410.9)
UKI Enfield	943.9 (703.1-1252.9)	849.7 (636.1-1120.6)	788.6 (588-1028)	739.8 (554.1-956.8)	714.2 (549.7-892.7)	690.9 (571.5-816)	646.5 (608.1-684.7)	626.4 (607.9-645.1)	569.1 (552.4-586.8)	555.4 (538.5-573.6)	521.2 (501-541.4)	466.6 (453.4-481)	402.2 (388.6-416.4)	377.4 (344.8-413.7)
UKI Greenwich	1021.7 (769.2-1352.5)	917.8 (674.3-1215.8)	851.3 (641.9-1110.9)	804.8 (613.5-1034.3)	775.7 (609.8-968.8)	752 (622.9-893.9)	717.1 (676.7-758.1)	708.8 (686.2-730.9)	652.8 (632.8-671.6)	632.3 (612.7-653.2)	615.8 (591.9-639.8)	549.6 (533.6-566.5)	451.3 (436-466.4)	465.3 (426.3-508.8)
UKI Hackney	968.6 (718.3-1291.3)	881.5 (663.1-1157.7)	838.9 (630.6-1068.8)	842.2 (649.2-1069.9)	840.1 (658-1041.9)	825.1 (686.2-978.4)	779.1 (737.2-826.8)	717.6 (697.7-738.8)	697.9 (676.1-718.4)	627.3 (607.9-645.5)	541.4 (519.2-565.1)	520.3 (503.9-537.1)	469.5 (453.8-484.4)	386.6 (351.9-424.7)
UKI Hammersmith and Fulham	1047.6 (776-1368)	981.7 (745.7-1306.8)	925 (700.1-1206)	891.6 (674.4-1144.7)	869 (684.1-1097.5)	853.6 (715.1-1009.3)	804.4 (755.3-851.1)	781.4 (758.7-806.7)	712.7 (690.8-735.7)	693.9 (672.7-716.5)	604 (579.1-628.7)	505.7 (488.6-523.6)	413.8 (398.5-429.3)	356.2 (322.6-390.6)
UKI Haringey	974.3 (707.7-1296.1)	896.7 (662.1-1170.7)	835.6 (627.2-1096.5)	813.2 (620-1041.2)	787.2 (617.2-987)	755.5 (628.3-895.9)	692.7 (654.2-731.3)	653.6 (634.2-672.9)	622.9 (604-641)	590 (572.1-607.7)	547.8 (527.4-569.6)	482.4 (466.4-499.3)	435.5 (421.4-449.2)	386.1 (352.9-424.5)
UKI Harrow	929.6 (681-1229.3)	848.2 (623.9-1127.5)	804.6 (592.4-1063.8)	784.2 (591.1-1022.2)	764.8 (587.8-972.2)	731 (610.7-878.1)	656.7 (617.1-697.6)	577.9 (558.2-598.8)	511.5 (495.2-529.5)	493.9 (475.8-511.2)	437.2 (418.3-457.7)	382 (368-395.4)	337.4 (324.8-349.7)	309 (281.7-340.3)
UKI Havering	964.4 (718.4-1270.3)	869.3 (660.1-1144.2)	809 (608-1088.5)	766.7 (590.6-1000)	738.8 (570.9-915.4)	708.4 (585.1-840.6)	671.4 (631.5-714.6)	587.5 (569.4-607.5)	512.6 (495.2-528.8)	517.8 (499.7-534.6)	475.3 (455.6-495.9)	437.3 (422-452.8)	415.7 (400.8-429.6)	450.5 (410.2-498.3)
UKI Hillingdon	946.3 (693.2-1249)	853.1 (634.4-1122.8)	793.1 (592.5-1027.3)	750.7 (567.2-965)	725.8 (561.1-907.9)	702.2 (586.4-843.2)	665 (625.3-706)	635.5 (616.8-656.2)	593.5 (575.3-610.3)	593.3 (575.5-611.2)	519.1 (497.7-539.8)	456.2 (441.9-471.8)	412.4 (398.8-425.8)	414.7 (379.1-450.6)
UKI Hounslow	967.3 (715.6-1280)	883 (664.3-1164.9)	828.7 (621.3-1095)	802.7 (606.9-1051.2)	779 (609.2-978.6)	750.4 (625.8-883.5)	682.3 (643.2-722.3)	667.7 (647.2-687.9)	586.6 (569.9-604.7)	564 (545.7-582.2)	535.9 (514.6-557.5)	487.1 (471.6-502.1)	430.9 (416.9-445.6)	389 (353.6-427.3)
UKI Islington	1029.7 (763.1-1338.2)	949.2 (713-1212.5)	892.9 (667.4-1141.6)	873.3 (656-1112.5)	858.7 (677.8-1071.3)	842.8 (702.2-1006.6)	773.4 (726.4-820.7)	751.6 (729.5-776.5)	669.9 (650.1-690.8)	679 (659.4-699.7)	620.4 (595.6-646.4)	542.7 (524.8-560.4)	455.6 (440.3-470.5)	399.1 (364.8-439.2)
UKI Kensington and Chelsea	969.9 (728.7-1262.8)	994.5 (771.2-1259.3)	918.5 (712.1-1157.9)	881.7 (684.8-1114.7)	850.8 (672.1-1061.4)	822.7 (685.2-979)	741.6 (697-785.4)	702.8 (680.5-726)	611.4 (591.9-631.8)	535.6 (517.3-556.2)	431.4 (412.7-451.4)	374.9 (359.8-389.1)	344 (330.4-358.4)	345 (311.7-379.5)
UKI Kingston upon Thames	845 (629.4-1134.7)	762.3 (572.2-999.6)	709.8 (529.8-944.2)	678.8 (510.8-898.7)	648.7 (500.4-828.3)	618.4 (506.8-743.7)	582.4 (538.5-625.2)	566.5 (544-589.9)	527.5 (507.6-547.9)	532.7 (512.3-554.1)	470.6 (447.9-492.1)	391.8 (375.9-407.5)	341.2 (326.3-356.5)	373.6 (336.3-413.8)
UKI Lambeth	1022.2 (773.9-1353.5)	931.5 (697.5-1211.1)	871.6 (665.9-1132.6)	856 (661.1-1100)	834.7 (653.5-1035.4)	805 (671.2-948.2)	740.2 (703.4-782.9)	681.6 (663.6-699.6)	648.6 (631.2-665.4)	646.5 (628.9-664.2)	601.8 (581-623)	545 (529.7-559.7)	457.1 (443.8-470.8)	365.5 (333.3-398.9)
UKI Lewisham	1011.6 (757.2-1335)	913.6 (683-1210.1)	844.9 (628.1-1124.2)	802.4 (610.2-1041.7)	771.7 (601.5-978)	745.7 (615.7-882.8)	697.5 (658.9-737.5)	662.9 (643.3-681)	627.4 (610.8-646)	655.9 (636.9-674.3)	626.1 (602.7-649.6)	561.3 (545-577.5)	465.7 (451.4-479.6)	422.6 (385.7-464.4)
UKI Merton	924 (681.5-1210.2)	842.6 (632-1103.6)	797.2 (603.5-1040.4)	781.7 (590-1008.7)	755.5 (596.9-956.8)	718.9 (593.2-862)	654.9 (614.6-696.6)	574.1 (553.8-594.7)	559.5 (541.1-578.5)	559.5 (540.8-578.1)	497.9 (476.5-520.5)	428 (413-442.6)	376.5 (362.7-390.9)	360.8 (327.8-397.9)
UKI Newham	1076.1 (809.8-1405.7)	974.1 (728.2-1261.3)	914.9 (683.9-1169.1)	886.5 (668.2-1135.6)	860.9 (668.9-1080.1)	826.1 (684.7-978.5)	741.6 (699.6-786.8)	698.4 (677.9-719.6)	654.8 (635.4-675)	652.5 (633.2-671.5)	615.3 (592.1-638.7)	568.3 (550.6-586.1)	513.6 (498.2-529.7)	392.3 (357.5-428.3)
UKI Redbridge	935.6 (690.3-1235.5)	851.3 (640.8-1124.9)	798.2 (609-1041.1)	767.6 (574.7-1010.6)	739.1 (577.1-926.4)	704.6 (581.1-847.5)	633.3 (596.9-671.1)	547.2 (530.4-563.6)	553 (537.1-571)	564.9 (547.6-582.2)	514.1 (492.8-535)	456.7 (441.3-471.1)	394.5 (381.5-407.7)	388.2 (350.1-428)
UKI Richmond upon Thames	894.7 (664.2-1183.9)	825 (619.9-1078.6)	782.7 (595.9-1027.6)	763 (588.2-1000.7)	737.8 (590.4-928.4)	706.7 (583.8-842.4)	650.5 (610.7-693.4)	603.9 (583.7-625.2)	533.5 (515.4-553.4)	452 (433.9-468.5)	448.9 (428.8-469.7)	377.5 (362.6-393.8)	330.5 (315.9-344.7)	318.8 (286.2-351.7)
UKI Southwark	970.9 (713.7-1287.2)	875.2 (652.9-1148.1)	818.6 (616-1075.8)	805 (608.9-1037.6)	780.4 (607.7-963.4)	756.8 (627-903.4)	721.3 (680.5-767.5)	672.1 (652-692.7)	719.5 (699.9-739.7)	655.4 (637.9-674.5)	611 (587.6-636.1)	535.7 (520.6-550.3)	476.2 (462.5-490.5)	437.3 (397.1-477.1)
UKI Sutton	931.4 (687.5-1230.4)	840 (635.5-1102.7)	779.4 (589.2-1037.4)	735.8 (567.9-959)	700.4 (549.3-888.2)	668.3 (558.2-794)	625.7 (584-666.5)	622.9 (602.2-645.7)	583.7 (564.1-603.6)	574.7 (554.9-595.3)	505.7 (484.3-527.6)	419.5 (404.2-435.4)	375.4 (361.6-390.6)	410.1 (374.2-451.7)
UKI Tower Hamlets	1064.7 (798-1395.3)	963.4 (727-1269.1)	911.2 (684.1-1203.2)	887.5 (668.4-1145.5)	876.9 (688.6-1101.3)	860.2 (710-1015.4)	808.6 (760.6-859.8)	775.9 (750.9-802.2)	711.6 (689.4-735.4)	724.9 (701.3-750.6)	641.9 (616.8-669)	571.9 (555.4-589)	463.8 (448-479.3)	400.5 (365.7-438.7)
UKI Waltham Forest	1015.3 (767.8-1343.2)	920.7 (695.9-1215.4)	854.6 (649.1-1116.9)	811.7 (618.4-1046.9)	777.3 (605.4-985.6)	741.4 (617.8-893.1)	666.5 (625.7-707.9)	652.3 (632.8-672.4)	609.7 (589.8-629.1)	614.5 (594.9-634.4)	573.3 (552-595.1)	521.7 (505.1-538.2)	453.2 (438.5-467.3)	403.4 (368.8-442.1)
UKI Wandsworth	1013.8 (760.5-1324.2)	938.9 (695.6-1214.9)	886.3 (677.1-1140.1)	866.4 (674.8-1118.8)	848.4 (666.9-1084.3)	820 (685.4-972.9)	733.7 (693.9-776.7)	709.4 (689.5-729.1)	664.1 (646.3-681.5)	660.6 (642.8-679.2)	585.8 (563.8-607.9)	501.6 (486.4-516.5)	419.8 (407.2-433.2)	369.5 (336.4-403.6)
UKI Westminster	844.5 (627.2-1108.5)	807.7 (617-1043.3)	754.3 (580.5-950.4)	752.7 (576.3-951.4)	749.8 (588.3-949.5)	735.8 (618.6-874.3)	675.7 (638.4-714.8)	601.6 (582.7-619.5)	621.3 (602.6-641.6)	594.1 (575.2-612.3)	449.8 (432-468.8)	417.9 (404.3-431.8)	364.7 (349.9-378.4)	331 (299.6-362.9)
ENG North East England	1258.2 (1142.7-1377.9)	1139.4 (1040.4-1255.7)	1052.2 (965.2-1153.3)	980.8 (897.9-1073.6)	928.8 (855.9-1003.3)	878 (822.9-932.4)	799.7 (785-815.1)	751.7 (744.4-759)	717.5 (710.9-723.9)	662.9 (656.2-669.4)	606.6 (599.2-614.2)	557 (551.7-562.9)	497.7 (492-502.9)	483.5 (469.5-499.3)
UKC County Durham	1260.9 (952.2-1631.6)	1140.7 (847.8-1495.7)	1049.2 (796.8-1350.3)	971 (733-1273.7)	921.3 (713.4-1169.5)	872.3 (716.7-1043)	785.5 (745.3-828.5)	753.7 (735.1-772.8)	731 (714.1-748.5)	671.1 (655.2-686.7)	622.2 (599.8-642.9)	560.9 (546.5-575.7)	497.7 (484.5-511.2)	494.3 (454.7-536.5)
UKC Darlington	1266 (934.3-1698.2)	1156.6 (864.6-1554.7)	1077 (809.7-1419.6)	1012.9 (775.6-1317.7)	959.9 (751.5-1232.8)	903 (747.1-1089.8)	817.6 (758.7-879.3)	741.7 (713.7-772.9)	713.4 (685.6-742.1)	669.9 (641.3-698.2)	618.5 (587.8-652.4)	565.5 (541.6-590.7)	491.6 (469.8-514.8)	464.7 (414.5-521.8)
UKC Gateshead	1277.9 (950.7-1690.8)	1159.5 (862.1-1539.2)	1071.8 (795-1405.7)	1003.4 (756.7-1312.1)	953.5 (723.6-1211)	905.3 (748-1083.7)	831.8 (780.1-882.3)	760.6 (738.6-784.2)	691.4 (668.6-714)	648.7 (626.9-672.4)	602.1 (577.9-629)	537.1 (517.4-558.8)	490.7 (473.6-508.5)	493.8 (450.2-545.1)
UKC Hartlepool	1354.6 (985.2-1800)	1229.5 (896.4-1656.2)	1140.5 (847.7-1518)	1072.1 (805-1396.7)	1019.2 (777.7-1299.2)	964.6 (783.4-1169.3)	881.2 (819.8-949.3)	810.9 (778.5-842.7)	757.9 (729.5-789.6)	714.2 (685.5-745.7)	667 (634.4-697.2)	626.7 (600.1-655.6)	533.1 (508.7-557.1)	477.8 (428.8-529.5)
UKC Middlesbrough	1390.7 (1011.4-1807.2)	1262 (929.5-1653.5)	1175.6 (872.4-1545.8)	1109 (832.1-1440.1)	1056.1 (804.2-1338.8)	1005.6 (832.8-1208.3)	927.2 (866-986.8)	864.7 (836.2-894)	808.7 (781.3-836.7)	752.6 (725.4-778.7)	697.5 (667.6-728.8)	638.4 (615.5-662.7)	557.7 (537.2-579.1)	540.5 (487.6-599.3)
UKC Newcastle upon Tyne	1207.9 (895.9-1578.4)	1099.4 (819.4-1469.4)	1015.6 (753.9-1335.1)	952 (710.5-1238.9)	902 (685-1146.2)	851.4 (701.5-1024.4)	785.8 (741.4-831.2)	736.9 (716.8-757)	723.9 (705.2-744.7)	683.3 (663.5-702.9)	616.3 (592.3-641.2)	560 (542.2-578.8)	507.6 (491.4-524.3)	486.1 (443.4-534.2)
UKC North Tyneside	1233.6 (891.9-1660.8)	1111 (816.7-1495.8)	1025.6 (751.7-1332.3)	957.3 (710.9-1237)	902.5 (694.6-1149.5)	850 (694.5-1019.6)	781.9 (735-832.4)	744.3 (720.1-767.4)	705.6 (684.6-728)	643.5 (621.5-663.9)	598 (572.1-623.7)	560.6 (541.1-579.6)	486.8 (470.1-505.4)	437.5 (395.3-480.6)
UKC Northumberland	1244.4 (918.6-1643.3)	1128.7 (849.1-1494.4)	1043.8 (786-1363.9)	963.3 (724.1-1233.1)	913.3 (698.6-1134.9)	863.9 (711.1-1022.5)	777.8 (731.7-823.5)	728 (707.4-748.1)	701.9 (682.6-721.4)	614.6 (596.4-633.3)	546.2 (525.4-569)	492.7 (477-508.5)	451.2 (436.7-465.4)	447.1 (410-489.8)
UKC Redcar and Cleveland	1273.4 (932-1692.9)	1159.1 (868.2-1556.4)	1075.2 (794.8-1418.2)	1011.5 (764.1-1320)	957 (725.3-1215.6)	898.1 (734.9-1075.5)	803.8 (749.9-857.6)	717.4 (694.4-743.1)	668.6 (645.6-692.8)	628.9 (605.5-651.8)	578.5 (553.1-603.8)	561.2 (539.5-583)	505.7 (484.7-527.3)	499.1 (449.1-549.5)
UKC South Tyneside	1270.6 (915.2-1665.7)	1138.3 (840.5-1523.5)	1044.8 (774.2-1384.1)	971.6 (705.6-1258.3)	914.6 (697.6-1166.5)	867.1 (704.1-1052.1)	805.3 (755.3-859.1)	776 (751.1-803.1)	732.2 (707-758.1)	661.5 (637-687.2)	593 (566.4-620.2)	551 (530-572.2)	519.5 (498.9-539.1)	499.4 (449.2-549.6)
UKC Stockton-on-Tees	1190 (873.7-1582.5)	1079.9 (803.4-1445.4)	1000.4 (740.2-1331.6)	939.5 (710.2-1222.6)	885.5 (689.9-1131.5)	831.9 (676.5-998.6)	761.3 (711.9-812.3)	720.1 (696.7-744)	676.8 (654.3-698.9)	626.8 (606.1-648.4)	569.8 (543.9-595.3)	524.1 (506.3-543.6)	470.8 (453.8-488.8)	450.4 (406.6-494.5)
UKC Sunderland	1246.9 (929-1648.2)	1124.7 (829.1-1496.1)	1033.9 (764.4-1342.2)	958.5 (715.4-1255.8)	906.3 (697.6-1157.5)	859.1 (704-1025.4)	776 (732.3-819.9)	747.9 (728-767.8)	722.3 (702.2-742.7)	692 (672-712.6)	631.5 (608-657.1)	601.6 (583.5-620.8)	528.5 (510.5-546.4)	533.9 (487-586.6)
ENG North West England	1142.6 (1059.4-1226.5)	1037.6 (968-1108.3)	968.8 (904.5-1037.8)	915.9 (858.7-975.8)	876.2 (822.8-929.9)	830.3 (795-867.5)	777.2 (767.4-786.6)	741.6 (737.3-745.7)	689.9 (686.2-694)	644.9 (640.9-648.9)	593.8 (588.5-599.3)	543.2 (539.7-546.8)	492.3 (488.9-495.6)	484 (473.1-494.4)
UKD Blackburn with Darwen	1221.2 (910.1-1606)	1110.6 (826.6-1423)	1046.5 (782.4-1379.2)	993.8 (760.3-1284.7)	950.3 (741.2-1193.5)	898 (745.4-1068.9)	833.3 (780.5-887.3)	807.1 (780.5-834.8)	747.3 (723.8-773.9)	718.2 (694.9-743.3)	682 (653.3-711.4)	594.6 (572.5-616.5)	521.5 (502.4-541.6)	519.5 (474.2-572.9)
UKD Blackpool	1182.8 (879.8-1540.7)	1073.2 (803.5-1393.7)	1014.3 (764.9-1333.5)	985 (757-1268.2)	945.8 (741.1-1194.1)	884.5 (739.4-1063.2)	804.3 (755.6-855.4)	699.6 (677.4-722.2)	710.9 (688-735)	642.8 (622.4-665.5)	713 (682.8-742.7)	683.9 (662.4-707)	619 (597.8-640.8)	545.6 (497.6-599.8)
UKD Bolton	1169.9 (882.5-1534.2)	1058.1 (800-1379.2)	983.7 (737.7-1283.9)	928.9 (698.4-1192.9)	888 (695.8-1114.6)	843.1 (697.4-1001.1)	791.6 (747.7-834.2)	757 (737.6-776.7)	692.7 (674-711.8)	647.9 (629.1-666.1)	628.4 (605.1-652.8)	582.2 (565.3-599.2)	504.6 (490.1-519.5)	480.8 (439-522.5)
UKD Bury	1119.6 (838.4-1467.6)	1013.3 (763.5-1311.3)	931.9 (697-1222.9)	860.4 (663.6-1123.2)	813.8 (639.2-1031.9)	771.8 (636-918.7)	737.3 (692-784.4)	714.8 (693-738.4)	654.4 (632.5-675.8)	606.4 (587.9-627.3)	565.9 (543.1-590.8)	536.6 (517.2-555.3)	492 (474.7-510.7)	523.1 (477.1-571.4)
UKD Cheshire East	1035.9 (763.6-1336.6)	933.5 (704.9-1230.9)	857.7 (644.2-1124.9)	777.8 (592.1-1011.1)	736.3 (583.8-939)	705 (587.1-842.9)	693.5 (656.7-733.4)	677 (659.8-695.3)	627.4 (611-644.1)	563.4 (548.5-579.3)	516.7 (498.4-536)	435.4 (423.1-448)	419.1 (406.4-431.9)	416.9 (383.6-455.1)
UKD Cheshire West and Chester	1096.3 (814.9-1435.7)	998.2 (745.8-1299.6)	934.6 (702.2-1218.3)	879.9 (676.1-1149.7)	846.3 (667.4-1069.4)	804.8 (668-959)	731.5 (689.8-772.5)	713.2 (695.2-731.6)	653.1 (635.9-670.7)	597.6 (581.3-614.2)	527.2 (506.8-545.9)	467.3 (453.1-481.3)	423.6 (411.4-436.7)	431.2 (394.4-471.8)
UKD Cumbria	1135.1 (867.8-1476.5)	1027.4 (795.9-1321.9)	956.9 (740.1-1219.5)	890.9 (687.2-1116.5)	853.1 (672.3-1060.1)	810.3 (683-961.3)	748.3 (711.7-785.4)	717 (700.1-733.3)	655.4 (640.4-670.4)	631.7 (616.8-647)	539.9 (520.6-558.6)	484.7 (472.8-496.4)	461.8 (450.5-474.2)	439.4 (403.2-476.3)
UKD Halton	1216.1 (904.7-1618.6)	1112.4 (824.4-1460.1)	1047.3 (775.3-1375.3)	1004.5 (758.7-1316.4)	968.5 (748.5-1252.2)	921.9 (763.1-1103)	854.4 (800-911.9)	817.8 (790.6-845.5)	724.6 (700.8-749.3)	701.5 (677.3-727.5)	679 (652-710.3)	634.5 (612.1-658.6)	556.7 (535.4-577.7)	524.3 (475.6-579.9)
UKD Knowsley	1180.9 (879.2-1531.9)	1069.2 (788.1-1417.2)	1006.8 (746.4-1337.3)	989.6 (744.1-1284.7)	952.6 (743.2-1196.4)	898.2 (745.2-1069.4)	824.5 (775.3-874.8)	746.6 (723-769.8)	722.1 (701.3-744.1)	703.6 (680.4-726.5)	665.7 (638.6-693.1)	606.7 (586.7-629)	548.4 (529.9-567.9)	558.9 (510.8-613.5)
UKD Lancashire	1090.9 (817-1429.3)	986.4 (748-1265.7)	914.1 (695.1-1171.2)	848.2 (654.6-1068.2)	808.2 (641.5-1029.9)	771.5 (646.5-920.5)	763.5 (730.6-798.4)	738.1 (725-751.2)	692.8 (680.6-705.6)	607.9 (596.9-619.8)	567.6 (550-585.2)	529.6 (519.1-540.5)	462.3 (453.6-471.2)	463.7 (426.9-500.2)
UKD Liverpool	1204.2 (896.1-1595.1)	1112.2 (851-1452.6)	1063.4 (812.7-1371.2)	1043.4 (802.1-1325.7)	1018.4 (797.8-1272.1)	958.8 (800.4-1142.1)	826 (788.5-867)	784.3 (769.3-799.9)	738.7 (723.1-755.9)	718.6 (702.8-734.8)	675.2 (651.7-699.2)	616.1 (601.7-630.9)	562.6 (550.2-575.9)	534.3 (493.1-581.2)
UKD Manchester	1124.6 (852.6-1449.4)	1019.7 (776.7-1291.5)	948.6 (721.3-1219.9)	916.5 (716.3-1166.6)	884 (701.1-1105)	845.1 (702.4-1000.5)	804.2 (766-841.8)	805.7 (787.7-822.5)	752.8 (737-769.8)	726.8 (710.7-742.4)	649 (627.4-671.5)	642.3 (628.2-656.9)	563.6 (550.6-577)	581.5 (535.8-627.3)
UKD Oldham	1212.8 (890.7-1561.2)	1104.8 (830.3-1438.3)	1032.8 (776.6-1344.1)	974.7 (742.2-1232.8)	926.6 (731.3-1149.2)	872.6 (723-1024.1)	808.3 (763.5-856.3)	785.9 (765-808)	722.1 (701.6-743.4)	664 (643-685.1)	631.2 (606.5-656.4)	592.6 (574-610.4)	538.2 (521.3-555.2)	544.1 (496.2-594.1)
UKD Rochdale	1170.2 (880.7-1551.1)	1056.6 (785.2-1389.5)	983.6 (729.1-1318.7)	929.5 (708.8-1188.8)	881.3 (683.2-1100.2)	826.1 (682.6-980.4)	777.2 (727.9-824.3)	746.2 (725.8-768.1)	748.5 (726.5-771.6)	685.1 (665.7-705.2)	643.3 (618.6-667.9)	594.3 (574-612.5)	545.6 (529.4-562)	533.5 (486.9-578.9)
UKD Salford	1165.3 (879.9-1528.5)	1055.3 (787.7-1373.7)	985.2 (733-1299.3)	942.5 (731.3-1242)	898.8 (715.3-1133.2)	848.4 (704.3-1015.9)	774.6 (734-818)	760.8 (740-782.4)	748 (727.6-769.6)	744.9 (724.5-765.5)	657.1 (632.4-681.4)	606.7 (588.3-624.1)	543.1 (526.3-559.6)	535.1 (487.9-583.4)
UKD Sefton	1126.8 (843.2-1476.9)	1019.2 (779.7-1335.1)	952.2 (721-1226.3)	908.3 (692.4-1175.7)	871.4 (688.2-1112.9)	824.1 (683.4-981.5)	768.2 (727-811)	708.3 (689.1-727.3)	640.5 (622.5-658.2)	615.2 (599.2-633)	572.6 (550.5-593.7)	494.2 (478.3-510.1)	476.5 (461.8-492.4)	464.2 (422.3-505.9)
UKD St Helens	1229.8 (910.4-1577.1)	1118.5 (840.4-1444.5)	1039.1 (773.2-1383.2)	967.1 (733.7-1263.3)	923.6 (719.3-1169)	887 (730.9-1059.9)	877 (824.3-930.7)	791.6 (768.7-815.3)	716.3 (695.3-738.3)	661.9 (640.4-682.8)	609.9 (583.7-634.6)	560.7 (542.7-580.4)	526.2 (508.1-545.5)	532.3 (486.6-582.7)
UKD Stockport	1075.3 (803.8-1414.3)	972.1 (742.5-1275.2)	898.5 (682.9-1156.8)	828.3 (635-1056.8)	789.1 (622.8-1008.2)	753.5 (628.5-903.9)	715.9 (676.8-755.9)	678.4 (659.5-697)	637.2 (619.8-656)	592 (575.6-609.9)	520.6 (502-540.5)	486.4 (471.6-502.5)	450.4 (436.5-464.9)	422.8 (386.7-462.6)
UKD Tameside	1199.4 (894.9-1566.1)	1098.4 (838.1-1437.2)	1034.8 (778.1-1358.9)	985.6 (754.9-1259.2)	945.7 (748.1-1199.7)	898.5 (745.4-1076.8)	836.8 (791.3-881.9)	781.4 (758.4-804)	680 (660.8-700.6)	672.3 (650.9-692.1)	617.3 (593.8-641.1)	578.5 (558.5-598.2)	536 (518.7-553.8)	533.5 (487.5-585.5)
UKD Trafford	1097.8 (821.5-1443.7)	1005.6 (757.3-1308.2)	946.8 (712.3-1232.6)	905.8 (688.7-1158.6)	869.9 (687.1-1107.3)	820.1 (689.6-990.9)	751.1 (707.9-795.7)	697 (676.4-717.8)	628.5 (609.4-647.8)	609.5 (589.9-629.7)	561.5 (538.2-584.7)	475.4 (460.1-491.5)	409.8 (395.2-424.9)	416.5 (377.7-454.8)
UKD Warrington	1128.4 (831.2-1468.5)	1028.7 (770.5-1339.1)	966.2 (725.7-1263.2)	917.5 (696.6-1184.3)	881 (696.4-1101.9)	840 (699.3-1001.5)	788.5 (743.7-838.1)	774.5 (750.7-799.7)	709.7 (688.2-731.6)	632.1 (611-652.6)	562.8 (540.1-586.1)	523.4 (505.6-541.2)	474.8 (457.7-492.6)	484.6 (441.3-531.7)
UKD Wigan	1243.4 (917.6-1645.3)	1129.3 (839-1490)	1044.1 (782.8-1383)	957 (738.8-1210.3)	910.5 (720.1-1136.7)	873.1 (727-1048.4)	838.8 (794.5-883.3)	797.7 (776.6-818.5)	716.8 (697.6-736.9)	694.1 (675.1-714.3)	619.4 (596.7-643)	553 (537.7-568.4)	529.2 (514.6-545.5)	543.1 (496.3-590.1)
UKD Wirral	1113.2 (825.2-1456.7)	1006.9 (756.7-1305.1)	940.7 (695.1-1208.5)	897.8 (686.4-1147.9)	856.6 (672.9-1071.6)	802.7 (668.7-943.8)	723.9 (687.4-762.8)	687.8 (671.8-705.5)	656.8 (638.8-673.3)	634.5 (617-651.5)	603 (582-625.8)	561.2 (546.9-577.2)	504.6 (489.2-519.6)	524 (481.4-570.2)
ENG South East England	923.1 (842.9-1014.6)	847.6 (773-929)	796 (726-868.6)	759 (694.8-826.9)	741.2 (682.7-801.3)	722.7 (682.8-765.8)	662.5 (652.4-673.3)	630.7 (626.4-635)	575.4 (571.4-579.3)	551 (547.1-554.7)	498.1 (492.5-503.4)	448.2 (444.8-451.4)	401.1 (398.1-404.3)	394.2 (383.8-404.5)
UKJ Bracknell Forest	881.5 (632.6-1185.6)	808.3 (590-1100.4)	758.8 (546.1-1023.5)	732.9 (538.8-966)	710.3 (542.4-907.2)	687.9 (555.8-825.4)	643 (595.5-690.3)	602.8 (575.6-630.5)	547.9 (525.6-573.3)	520.7 (496.6-545.7)	472.6 (447.8-498.2)	431.1 (412.9-450.2)	389.2 (371.5-406.7)	331 (296.1-369.4)
UKJ Brighton and Hove	925.2 (678.4-1233.5)	857.6 (635.3-1109.1)	815.3 (614.6-1049.7)	804.5 (600.5-1043.8)	787.9 (616-1019.1)	764.3 (637.3-910.7)	692.9 (650.7-733.4)	660.7 (642.1-679.9)	599.6 (582.3-619.9)	604.2 (586.2-624.2)	573.1 (550.9-595.3)	526 (508.5-543)	461.8 (446.7-476.5)	414.6 (377.5-452.7)
UKJ Buckinghamshire	919.2 (660.5-1231.5)	841.9 (618.4-1116)	788.8 (579.9-1024.7)	741.9 (558.4-975.1)	727.5 (566.8-937.4)	716 (592.4-850.7)	667.2 (635.3-702.6)	617.2 (601.6-633.8)	577.1 (562.6-592.1)	539.9 (525.9-553.7)	480.7 (463.6-500)	422.4 (410.4-433.4)	378.3 (367.5-389.3)	369.2 (337.6-400.9)
UKJ East Sussex	905 (668.1-1189.3)	828.5 (609.2-1078.3)	779.8 (572.7-994.8)	750.4 (561.8-958.6)	729.6 (564.3-935.1)	703.4 (590.2-838.4)	646.7 (612.5-680.9)	605.9 (590.4-620.8)	547.3 (535-560.5)	533.1 (521-546.9)	487.1 (469.6-506)	434 (422.5-445.7)	408 (396.9-418.9)	414.4 (382.3-450.2)
UKJ Hampshire	926.6 (687.2-1219.5)	861.2 (655.4-1138.3)	818.2 (615.5-1086.4)	783.2 (586.1-1009.8)	774.9 (599.8-989.6)	756.5 (623.7-902.4)	645.5 (615.9-675.8)	611.3 (599.5-624.3)	557 (545.9-568.3)	543.4 (532.6-553.5)	486.9 (471.9-503)	426.5 (417.2-435.6)	385.2 (376-393.6)	380.7 (351.2-412.2)
UKJ Isle of Wight	913.2 (684.1-1241)	829.2 (618.2-1101.8)	777.7 (583.5-1027.2)	743.7 (549.1-969.2)	713.5 (548-910.4)	683.7 (560.6-829.1)	639.6 (592-688.3)	612.1 (586.7-637.9)	572.7 (549.7-595.6)	555.1 (533.2-577)	493.6 (469.3-518.2)	457.9 (438.2-476.7)	423.6 (405.5-442.5)	388.6 (350.4-430.4)
UKJ Kent	976.6 (737.3-1250)	897.8 (679.2-1164.3)	844.4 (634.3-1082.5)	800.6 (616.5-1036.7)	785.1 (601.1-1000.9)	768.8 (641.7-909.7)	697.5 (666.6-729)	669.6 (657.5-682.5)	599.9 (589-610.9)	575.1 (564.3-585.6)	506.3 (490.4-523.1)	463.8 (454.5-472.7)	407.1 (398.8-415.3)	416.4 (384.3-448)
UKJ Medway	968.7 (722.4-1274.1)	882.3 (653.2-1149.3)	815 (601.8-1052.3)	773 (583.5-1008.7)	743.7 (577.4-940.5)	722.3 (595.2-857)	688.4 (648.5-726.9)	655 (636.3-675.3)	615.7 (597.3-634.8)	600.2 (580.3-618.5)	552.5 (530.5-574.5)	517.8 (502-534.2)	496.9 (480.8-513.3)	434.9 (395.9-474.5)
UKJ Milton Keynes	873.1 (638-1176.9)	792 (581.4-1081.9)	740.3 (545.6-993.6)	712.9 (520.6-949.3)	690.4 (522-896.7)	672.9 (549.3-806.7)	646.3 (602.7-693.5)	650.8 (627.9-673.1)	614.7 (594.1-636.9)	582.4 (562.5-603.5)	517 (495.7-537.5)	473.6 (458.1-489)	431.4 (417.2-446.9)	396.1 (359.9-433.4)
UKJ Oxfordshire	912.4 (676.1-1224.7)	843.2 (617-1121)	794.1 (580.4-1030.4)	757.1 (561.8-978.3)	745.4 (572.2-945.2)	731 (607.1-862.6)	669 (636-700.8)	616.2 (600.9-629.9)	563.2 (549.1-576.9)	522.4 (510.7-534.8)	484.5 (467.3-501.3)	437.4 (426.2-448.3)	390 (379.8-399.7)	383.1 (352.9-416.8)
UKJ Portsmouth	975.4 (718.2-1297.1)	896.4 (653.6-1194.3)	842.3 (623-1106.1)	809.4 (608.4-1071.6)	784.6 (599.8-1004.8)	761.8 (631.4-903.7)	718.9 (675.5-765.4)	691.8 (670.2-715.9)	641.1 (620.5-663.9)	650.2 (629.2-670.9)	625 (600-651.9)	552.3 (532.4-571.8)	465.9 (447.4-482.9)	409.1 (371.8-449.2)
UKJ Reading	855.4 (620.1-1131.9)	786.3 (584-1008)	732.6 (544.2-948.5)	699 (515.7-915.6)	670.8 (510.8-863.3)	651.1 (533.9-780.5)	625.5 (583.5-669.3)	629.3 (605.2-653.3)	585.3 (562.2-608.7)	574.6 (553.3-595.5)	540 (515.2-564.8)	486.8 (469.4-506.4)	437.7 (419.6-456)	447.3 (405.9-495.6)
UKJ Slough	994.5 (728.1-1326.1)	916.4 (675.8-1206.9)	869.3 (636.3-1162.5)	846.3 (629.7-1085.7)	827.6 (644-1049.5)	812.4 (672.6-956.7)	779 (724.6-834.1)	756.3 (726.5-786.2)	689.3 (662.2-714.8)	675.2 (650.2-700.4)	603 (574.9-629.6)	498.8 (478.6-519.2)	420.3 (403.8-438.5)	363.2 (329-399.3)
UKJ Southampton	945.7 (701.1-1248.8)	871 (637.5-1140.4)	816.3 (613.6-1070.3)	784.1 (579.6-1008.2)	756.7 (578.2-968.2)	731.3 (601.5-878.5)	688.8 (645.2-731.6)	664.4 (643.5-684.2)	629.8 (609.6-651)	621.7 (601.4-643.5)	584.9 (560.8-609)	535.6 (519.3-552.4)	472.4 (457.6-488.7)	430.4 (391.8-472.1)
UKJ Surrey	896.1 (666.5-1170.8)	817.8 (617.1-1073.1)	763 (583-1008.8)	715.8 (541.3-929.8)	699.7 (544-891.8)	691 (569.9-824.6)	653.4 (621.1-684.2)	617.4 (605.4-630.8)	568.8 (556.9-581.7)	531.8 (520.6-543)	475.2 (460.5-491.3)	425 (416.5-434.1)	372.8 (363.7-381.3)	386.8 (356.6-417.4)
UKJ West Berkshire	814.3 (590.4-1094)	740.3 (550.8-988.7)	690.7 (511.9-907.5)	661.7 (486.3-856.7)	639.8 (488.2-815.6)	622.1 (507.3-746.4)	590.2 (547.9-636.6)	616.6 (593.3-640.4)	535.4 (515.2-558.4)	539.6 (519.4-560.5)	488.3 (466.5-510.9)	484.1 (465.6-504.8)	406.8 (389.1-424.9)	407.5 (365.3-452.1)
UKJ West Sussex	894.2 (667.1-1180)	815.7 (597.9-1060.2)	764.6 (550.3-1015.5)	729 (537.1-954.5)	709.7 (545.7-908.2)	688 (572.5-820.9)	634.1 (604.6-667.5)	609.4 (595.4-623.2)	556.5 (544-568.8)	516.7 (503.9-528.5)	483.2 (466.7-500.1)	443.6 (433.5-454.6)	403.5 (394.1-413.4)	399.7 (368-433)
UKJ Windsor and Maidenhead	863.8 (631.8-1141.9)	791.8 (574.6-1048.9)	744.3 (543.1-974.3)	719.2 (541.2-933.1)	698.5 (530.8-872.1)	681.5 (558.6-815.6)	649.7 (604.6-696)	627.8 (603.6-653.1)	572.1 (551.7-595)	528.3 (507.3-549.8)	429.2 (406.9-450.6)	377.2 (360.9-396.1)	361.5 (346.1-378.6)	362.6 (326.3-400)
UKJ Wokingham	796.6 (581.1-1053.8)	724.1 (525.6-950.4)	670.4 (490.3-880.4)	638.4 (463.5-835.8)	612.8 (465.3-788.1)	597.8 (492.9-725.6)	571.7 (529.2-618.1)	562.4 (540.6-586.6)	511.5 (491.4-532.4)	488.7 (469.6-508.9)	421.5 (400.6-441.8)	366.5 (351.7-381.8)	327.5 (313-342.4)	309.5 (282.3-344.1)
ENG South West England	983.3 (901.8-1071.2)	895.1 (818.3-977.9)	829.7 (762.9-896.9)	781.8 (720.6-844.6)	745.7 (692.7-802.1)	708.7 (671.5-750.5)	665.5 (654.5-676)	620.9 (616.3-625.5)	573.1 (568.2-577.8)	531.9 (527.8-536.1)	483.2 (478-488.3)	437.9 (434.4-441.5)	402.3 (398.8-405.7)	416 (405.4-426)
UKK Bath and North East Somerset	903.8 (656.6-1211.9)	822.6 (597.7-1099.7)	758.7 (561.1-998.2)	713.2 (541.7-935.4)	671.6 (513.5-857.1)	630.8 (520.9-757.3)	587.5 (548.3-630.1)	560.5 (539.1-580.9)	526.4 (506.2-547.5)	510.9 (491.8-532.4)	456 (433.8-478)	394.5 (378.5-411.3)	360.8 (345.4-376.5)	398 (360.4-437.6)

Table 1 Age-standardised mortality rates by location and year for females for 1950, 1955...2005, 2010, 2017

Location	1950	1955	1960	1965	1970	1975	1980	1985	1990	1995	2000	2005	2010	2017
UKK Bournemouth	1012 (731-1335)	929.3 (667.4-1225.3)	870.9 (650.4-1151.6)	832.1 (625.9-1085)	796.5 (607.5-1016)	756.2 (622-904.7)	703.9 (659.5-749.5)	651.2 (627.3-676.8)	612.7 (589.5-635.8)	594.4 (573.6-616.4)	537.6 (513.4-563.9)	472.5 (455.6-490.8)	442.5 (425.8-459.7)	474.8 (430.5-523.9)
UKK Bristol, City of	993.2 (739.9-1294.7)	920.8 (690.7-1205.9)	866.9 (653.4-1108.2)	838 (641-1075.7)	810 (627.7-1019.3)	773 (641.5-931.1)	716.7 (676.9-756)	653.2 (637.3-668.8)	582.6 (567.9-598.6)	559.8 (545-573.5)	536.3 (515.6-556.4)	488.8 (475.7-502)	460.3 (446.8-473.5)	450.4 (411-490.3)
UKK Cornwall	1012.1 (743.7-1342.2)	916.5 (682.5-1211.5)	850.4 (625.9-1110.8)	802.3 (604.5-1033)	766.7 (581.2-977.5)	727.9 (601.7-863.5)	678.2 (640.3-718)	623.1 (608.2-639.6)	570.7 (556.6-585.2)	543.1 (529.5-557)	496.2 (478.5-514.5)	455.8 (443.8-467.6)	411.9 (401.6-423.1)	426.1 (391-464.8)
UKK Devon	967.6 (711-1254.3)	879.1 (636.8-1143.1)	814.8 (597.9-1069.1)	765.4 (576.6-984.8)	730.9 (571.1-920.7)	694.4 (581.9-836.7)	651 (617.9-685.4)	604.3 (591.8-618.4)	549.3 (537.3-562.1)	509.2 (497.9-520.8)	466.4 (449.2-482.8)	419 (409.6-430.1)	384.3 (374.8-393.2)	407.9 (374.6-443.6)
UKK Dorset	936.9 (671-1232.6)	845.1 (603.1-1122.7)	781.5 (571.6-1049.5)	740.3 (555.9-964)	706.8 (542.3-902.7)	668.9 (549.9-816.1)	631.3 (595-670.5)	593.8 (577.7-610.6)	540.5 (525.9-555.5)	471.4 (458-485.8)	437 (420.1-455.4)	388.3 (377.1-400.6)	347.8 (337.5-359.1)	386.7 (352.2-424.4)
UKK Gloucestershire	965.4 (697.2-1272.9)	873.3 (638.2-1152.9)	802.2 (591-1036.6)	745.3 (562.8-959.8)	709.2 (542.6-893.9)	680.9 (567.3-815.6)	669.8 (635.6-704.8)	637.5 (621.9-652.7)	571.9 (558.7-585.5)	536.3 (523.6-549.8)	477.3 (458.9-494.1)	449.6 (437.9-461.5)	417.7 (406.7-429.8)	432.4 (397.6-468.7)
UKK North Somerset	947.8 (698.6-1260.1)	856.7 (622.9-1163.1)	786.1 (586.8-1032.8)	733.1 (553.2-959)	687.6 (535-879)	646.2 (536.4-775.9)	611.2 (568.7-653.7)	593.8 (572.6-614.9)	550.4 (531.9-571)	506.4 (487.5-526.9)	455.9 (435.9-477.4)	428.2 (413-444.7)	421.3 (405.3-437.9)	463.5 (422.1-509.7)
UKK Plymouth	1025.6 (746.1-1375.1)	941.1 (671-1285.9)	876.3 (648.5-1173.1)	832.2 (610.2-1077.4)	790.6 (605.2-994)	748.9 (622.6-902.9)	704.8 (662.6-747)	665.1 (646-683.6)	620.7 (602.8-639.4)	571.9 (553.8-590.8)	525.8 (504.1-547.6)	512.6 (496.4-528.8)	472.2 (457.1-487.7)	446.4 (408.3-485.4)
UKK Poole	925.6 (676.1-1222.5)	837.8 (618.2-1111.1)	773.8 (586-1006.1)	737.4 (551.3-972.5)	696.6 (534.8-895.4)	655.5 (530.7-790.7)	594.3 (553.6-637.7)	617.4 (592.5-641)	589.2 (567-611.7)	536.5 (516.2-558.7)	453.1 (432.6-475.7)	414 (396.4-432.3)	395.5 (377.8-413.2)	427 (385.4-471.4)
UKK Somerset	994 (733.9-1333.2)	900.1 (664.9-1193.9)	835.5 (630.2-1096.7)	784.9 (593.6-1024.4)	753.8 (590.4-948.8)	721.8 (606.7-879)	672.3 (638.2-708.2)	619.7 (604.2-634.9)	590.5 (575.1-606.4)	530.3 (515.7-544.4)	489.3 (470-507.5)	437 (425.7-449.4)	386.9 (375.8-396.9)	394.4 (361.4-428.1)
UKK South Gloucestershire	953.8 (691.8-1269.2)	866.8 (627.8-1153.2)	795.7 (586.3-1038.1)	741.5 (553.8-956.9)	706.9 (533-900.3)	669.8 (547.7-804)	603.8 (563.7-643.1)	572.3 (553.2-591.6)	589.1 (569.2-608.2)	543.2 (524-562.5)	473.8 (453.5-493.6)	388.2 (373.4-402.7)	359.4 (345.4-373.9)	399.5 (363.8-437)
UKK Swindon	1026.8 (747.4-1349)	937.1 (671.5-1236.1)	871.2 (656.1-1135.8)	823.3 (629.8-1055.5)	796.3 (614.3-1005.9)	772.4 (642.7-938.4)	751.3 (702.4-803.3)	672.5 (649.3-695.3)	587.2 (565.5-607.1)	569.1 (549.4-590.5)	529.2 (507.5-553.5)	469 (450.5-486.6)	416.2 (401-432)	446.2 (403.5-493.3)
UKK Torbay	1017.2 (731.2-1371.1)	928.4 (668.6-1237.6)	866 (640.6-1136.7)	821.1 (612.6-1072.5)	779.5 (592.9-999.8)	734.9 (602.8-890.8)	682.8 (633.2-731.9)	633.9 (608.3-659.8)	582.9 (559-608.1)	582 (560.1-605.3)	539.1 (513.4-564.4)	499.7 (478.9-519.4)	469 (449.2-489.4)	436.3 (394-479.5)
UKK Wiltshire	1013.4 (733.8-1366)	922 (662.8-1253.9)	845.8 (615.7-1110.5)	777.4 (576.3-1011.8)	739.4 (565.2-932.3)	707.8 (583.8-856.7)	672.8 (634.2-712.7)	624.8 (608.5-641.5)	597.6 (582-613.4)	536.2 (521.4-551.6)	468.1 (450.3-486.7)	430.1 (417.8-442.5)	405.5 (393.8-418.3)	396.2 (363.5-432.8)
ENG West Midlands	1104.5 (1005.6-1208.8)	1006.9 (916.6-1105.8)	934.5 (849.5-1026.7)	876.5 (799.4-968.1)	837.7 (770.7-909.6)	796.2 (748.6-845.5)	741.3 (729.6-753.4)	699.5 (694.8-704.7)	650.3 (645.5-655)	610.4 (605.8-615.1)	554.8 (548.8-560.9)	501.9 (497.9-506.2)	446.3 (442.7-450)	435.6 (424.5-446.3)
UKG Birmingham	1126.1 (852.4-1439.9)	1024.7 (780.8-1331.4)	952.5 (738.3-1209.3)	897.2 (697.3-1157.2)	859.8 (678.7-1081.5)	814.8 (675.1-976.6)	764.8 (732.4-798.6)	735.2 (722.7-749.1)	679.4 (666.3-692.5)	638.8 (625.1-652.4)	570.8 (552.1-589.8)	511.4 (500.2-522.1)	460.2 (449.8-471.5)	447.9 (412.5-485.1)
UKG Coventry	1081.4 (785.2-1438.3)	989.2 (728.4-1308)	923.9 (674.5-1228.6)	889.6 (662.6-1154.9)	853.9 (655.8-1083.6)	804.3 (651.9-959.2)	725.7 (685.9-765.3)	673.3 (654.4-691.3)	663.4 (646.5-681.3)	627.9 (610.6-646.3)	570.8 (549.4-594.1)	564.5 (548.7-580.4)	463.9 (449.8-477.8)	457.6 (418-500.8)
UKG Dudley	1090.9 (800.1-1436.7)	983.7 (722.6-1323)	897.3 (669.3-1163.9)	820.4 (619.4-1056.2)	776.6 (608.5-984.7)	741 (606.9-890.1)	692.1 (651.8-732.4)	682.8 (664.7-699.6)	657.6 (639.5-675.6)	600 (582.4-617.9)	551.5 (530.3-573.3)	491.8 (476.6-507.2)	433.9 (419.8-448.4)	435.7 (398.4-477.5)
UKG Herefordshire, County of	1026.4 (748.7-1368)	940.9 (693.6-1232.1)	890.5 (674-1181.8)	870.7 (664.3-1139.1)	835.7 (642.1-1058.1)	780.4 (641.1-941.6)	665 (621.7-710.3)	644.6 (621.9-668)	571.3 (550.7-592.6)	534.7 (515.9-555.7)	476.3 (454.4-497.9)	433.4 (416.3-449.5)	411.3 (394.9-427.9)	414.9 (378.8-458.1)
UKG Sandwell	1119.4 (824-1490.2)	1016.9 (742.4-1338.2)	951.5 (703.1-1266.1)	914.3 (681.3-1211.1)	875 (669-1131.5)	824.5 (675.4-976.6)	749.7 (711.7-791.4)	695.1 (678-712.9)	655.8 (637.8-673.6)	659.9 (641.5-677.3)	626.7 (602.4-650.6)	590.9 (573.9-608.4)	526.3 (511.3-543.2)	486.3 (443.3-531.6)
UKG Shropshire	1047.7 (775.8-1363.4)	954 (707.4-1255)	883.5 (657.3-1152.4)	822.7 (621.6-1063)	785 (604.5-999.8)	746.6 (609.5-894.6)	701.9 (659.9-745.4)	660.4 (641-679.3)	589.4 (571.1-608.2)	552.3 (534.3-569.9)	483.5 (463.9-503.9)	434.3 (419.7-448.8)	405.4 (390.9-420.1)	416.4 (381.5-458.5)
UKG Solihull	931.6 (674.5-1260)	852.2 (630.8-1156.1)	790.5 (577.5-1059.8)	747.9 (565.3-984.8)	703 (538.9-892.4)	656.3 (537-786.8)	605.3 (566-644.9)	574.5 (556-594)	539 (521-558.2)	545.5 (526-563.6)	504.3 (482.7-525.9)	429.5 (412.6-445.7)	380.9 (366.7-395.1)	378.6 (341.9-417.9)
UKG Staffordshire	1112.8 (817.7-1456.5)	1014.8 (753.2-1331.4)	936.2 (707.7-1247.9)	860.7 (643.5-1140.6)	822 (630.4-1057.7)	788.9 (651.7-956.4)	739.3 (704.5-776.4)	697.8 (684-712.2)	655.9 (642.5-669.1)	618.8 (603.6-632.8)	555 (536.7-574.5)	497.3 (486.4-509.2)	437.1 (426.5-447.7)	433.9 (400.6-470.2)
UKG Stoke-on-Trent	1244 (932.2-1636.9)	1143 (853.8-1496.5)	1071.2 (798-1390.4)	1020.1 (781.4-1312.2)	980.2 (760.8-1229.4)	936.2 (769.9-1120.4)	878.6 (833.3-926.1)	814.6 (792.8-836.3)	736.6 (717.1-757.2)	690.1 (670.3-710.3)	628.6 (605.3-654.1)	606.7 (588.6-624.7)	558.7 (541.5-576.3)	499.3 (455.3-546.9)
UKG Telford and Wrekin	1073.2 (792.4-1429.5)	990.2 (732.3-1323.5)	933.3 (697-1215.2)	887.4 (665.1-1148.9)	850.7 (655.3-1101.8)	808.3 (665.2-965.4)	755.1 (708.3-808.1)	706.6 (681.7-729.4)	615.6 (593.2-638.4)	597.1 (575.5-617.9)	569.9 (545.4-595.5)	530.9 (511-549.1)	473.3 (455.7-492.2)	477.2 (431.7-526.1)
UKG Walsall	1178.7 (876.7-1591.8)	1065.8 (787.5-1440)	980.3 (734.2-1312.4)	915.1 (689.5-1199.8)	870.8 (666.8-1098.9)	830 (672.6-1000.6)	784 (740.8-828.2)	748.2 (727.9-767.8)	696.8 (677.5-716.9)	641.2 (622.9-660.6)	582.8 (559.2-608.2)	508 (491.6-524.7)	454.3 (440.4-470.1)	467.2 (427-512.5)
UKG Warwickshire	1062 (793-1389.8)	970.2 (722.6-1279.3)	898 (667.8-1189)	833.4 (627.6-1086.5)	798 (627.6-1012.4)	766.4 (631.6-913)	727.1 (691.6-767.6)	687.9 (671.7-705.4)	630.8 (616.3-645.4)	587.7 (574.1-602.3)	513.6 (495.1-532.2)	468.5 (455.9-480)	413.8 (402.5-425)	409 (375.2-446.1)
UKG Wolverhampton	1114.5 (824.2-1494.7)	1008.6 (746.9-1362.1)	936.2 (706.6-1265.1)	888.1 (664.5-1158.7)	849.8 (650.9-1109.7)	809.3 (662.5-977.3)	760.1 (715.1-807)	718 (697.3-739.4)	697.2 (677.1-718.6)	626.7 (607.6-646.5)	601.4 (578.7-626.2)	571.3 (554.1-589.5)	487.6 (472.1-504)	472.2 (428.5-514.8)
UKG Worcestershire	1080.9 (800.2-1425.2)	988 (726.8-1290.8)	916 (687.8-1205.2)	842.3 (629.4-1078.6)	806.7 (615.5-1027.9)	772.4 (635.6-921.9)	724 (686.4-761)	655.5 (640.6-671.2)	614 (599.1-628.9)	568.2 (555-582.7)	541.8 (522.8-562.7)	466 (453.4-478.9)	423.5 (412.1-434.9)	406.5 (372.5-442.2)
ENG Yorkshire and the Humber	1117.2 (1025.7-1208.5)	1014.3 (930.3-1102)	939.6 (866.3-1025.2)	883.8 (815.3-954.2)	839.9 (783.3-902.1)	795.8 (751.7-839)	740.5 (729.4-752.5)	707.8 (702.4-712.6)	655.5 (650.8-660.1)	611.4 (607-616.1)	562.6 (556.8-569)	510 (506-513.9)	468.9 (465.4-472.4)	453.8 (442.8-464)
UKE Barnsley	1190.7 (878.1-1569.3)	1078 (799.9-1437.3)	995.9 (756.9-1294.3)	936.6 (697.9-1216)	882.7 (693.7-1131.1)	834 (696.2-1001.4)	783.3 (736.2-827.9)	759.5 (738.4-782.5)	689.8 (669.1-711.7)	669.7 (647.6-691.1)	621.7 (596.6-646.3)	565.2 (547.6-584.2)	519.7 (501.4-538)	468.3 (425.4-516.4)
UKE Bradford	1185.3 (865.6-1544.1)	1076.6 (782.6-1401.1)	999.3 (739.3-1313.8)	939.7 (718.5-1197.6)	895 (717.6-1137.5)	843.8 (711.8-1013.1)	773.3 (737.7-814.3)	743.1 (726.2-760.6)	688.3 (672.5-703.8)	648 (632.1-663.2)	600.6 (579.7-621.3)	546.3 (532.9-560)	497.1 (484.6-509.8)	481.3 (443.7-524)
UKE Calderdale	1150.7 (858.7-1507)	1039.1 (776.7-1358.4)	961.2 (728.5-1254.3)	903.4 (679-1155)	856 (669.2-1093.6)	809 (671.9-965.8)	755.2 (710.4-801.7)	721.1 (699.1-743.3)	689.6 (668-710.9)	639.7 (617.3-661.5)	557.4 (535.1-581.5)	508.6 (490.9-526.5)	497.3 (480.4-515.2)	494.1 (450.8-540.6)
UKE Doncaster	1111.6 (821.9-1483.8)	992.6 (732.7-1330.8)	903.6 (676.2-1200.3)	844.3 (638.3-1076.8)	792.9 (620.8-994.3)	748.6 (625.5-891.3)	716.9 (678.4-757.3)	710.5 (690.4-729.7)	687.1 (669.5-704.8)	672.2 (652.6-692.1)	604 (580.9-629)	526.9 (510.3-544.9)	488.9 (474.6-504.5)	487.8 (445.7-531.6)
UKE East Riding of Yorkshire	1064.4 (792.1-1404.4)	963.1 (716.5-1279.2)	888.7 (665.2-1174)	831.7 (634.1-1075.5)	788.2 (613.6-999.1)	743.8 (617.7-888.5)	692.8 (652.9-735.7)	654.1 (636.5-672.6)	589.9 (573.2-606.4)	560.6 (544.7-576.8)	518.1 (498.6-538)	470.3 (456.2-485)	429.3 (416.5-443.2)	413.8 (380.8-452.6)
UKE Kingston upon Hull, City of	1120.1 (824.9-1471.5)	1012.9 (748.1-1352.4)	933.1 (692.6-1245.8)	887 (662.8-1145.1)	834.4 (647.1-1061.8)	779.5 (645.8-937.5)	735 (692.7-779.9)	712.8 (693.6-732.2)	668.7 (650.8-688.3)	641 (622.6-659.7)	599.5 (576-623.2)	553.5 (536.1-570.3)	566.3 (548.9-583.6)	531 (485.6-583.1)
UKE Kirklees	1196.6 (893.5-1543.5)	1090.6 (813.7-1441.8)	1011 (760-1332.6)	937.5 (715.5-1204.6)	892.3 (704.2-1131.7)	854.3 (709.3-1021)	825 (783.4-867.5)	744.1 (727.1-762.7)	695.2 (678.5-712.9)	621 (604.2-637)	579 (558.9-600)	560.3 (545.7-575.2)	463.2 (450.8-476.4)	424.8 (386.6-462.9)
UKE Leeds	1081.7 (806-1441.2)	986.2 (738-1311.2)	919.7 (691.5-1194.1)	878.8 (670.1-1131.2)	840 (642.4-1057.5)	797.1 (661.5-962.4)	722.4 (686.8-761.3)	693.4 (678.3-707.6)	636.7 (623.5-650.3)	579.3 (566.7-592.5)	543.4 (525.3-563.4)	503.1 (491.8-515.2)	468.6 (457.4-479.9)	469.7 (434.1-508.8)
UKE North East Lincolnshire	1070.6 (783.3-1402.8)	962.9 (692.1-1276.6)	884.8 (652-1179.5)	842.6 (631.3-1077.6)	797.7 (624.2-1018.1)	755.3 (630.5-901.4)	710.3 (663.6-757.3)	691.4 (667.5-713.4)	677 (654.7-698.8)	632.3 (611-653.4)	577 (552.8-604.2)	524.1 (505.9-543.2)	475.1 (456.7-493.1)	442.3 (398.2-485.6)
UKE North Lincolnshire	1076.5 (793.5-1441.3)	972 (705.6-1294.4)	897.8 (668.8-1192.3)	851.1 (638.6-1117.3)	799.8 (619.7-1027.7)	752.1 (612.8-900.8)	715.8 (666.2-765)	722.3 (698.9-748.2)	668.8 (645.7-692.8)	583.9 (563.3-606.4)	582.5 (556.8-607)	515.6 (496.7-535.2)	471.9 (454.7-490.8)	457.2 (414.8-501.4)
UKE North Yorkshire	1073.4 (801.2-1375.8)	980.8 (727.1-1272.9)	917.7 (699.3-1184.3)	859.2 (653.4-1117.8)	820.3 (646.3-1038.8)	774.2 (644.5-931.1)	700.9 (667.2-737)	639.8 (624.6-655.5)	595.5 (582-609.6)	572.9 (559.7-586.9)	520.2 (500-539)	450.2 (439.6-462)	409 (397.6-420.4)	396.9 (365.2-430.8)
UKE Rotherham	1124.7 (827.8-1454.4)	1015 (747.5-1319.8)	932.1 (701.4-1209.1)	868.2 (662-1134.6)	818.4 (643.5-1041.3)	774.5 (644.7-928.7)	738.2 (696.6-783.6)	714.1 (694.2-735.1)	673.8 (654.5-692.9)	608.9 (590.2-627.1)	597.2 (573.6-620.9)	525.8 (509.3-543.2)	497.2 (481-512.4)	530.3 (484.8-577.1)
UKE Sheffield	1102.4 (821.6-1459.1)	998.6 (732.9-1314.8)	920.1 (686.4-1208.1)	860.3 (641.5-1114.4)	820.9 (640.6-1038.8)	786.2 (652.5-943.2)	731.8 (696.3-773)	727.3 (711.2-744.1)	658.2 (643.6-672.6)	617.4 (602.5-632.6)	553.5 (535-573.1)	515.4 (501.8-528.4)	470.8 (459.1-482.8)	454.3 (418.7-491.9)
UKE Wakefield	1179.7 (874.5-1555.8)	1071.5 (794.4-1399.3)	993.1 (726.9-1311.8)	929.8 (699.8-1203.8)	885.1 (696.3-1116.3)	844.2 (699.7-1002.2)	802.5 (759.2-847.6)	767.8 (748.8-787.2)	709 (690.9-728.1)	657.1 (638.2-675.1)	594.1 (571.3-616.8)	536.9 (521.3-553.7)	489.7 (475.5-504.7)	460.8 (422.3-500.2)
UKE York	1008.5 (729-1345.3)	925.8 (679.9-1236.5)	866.2 (630.5-1152)	827.5 (618.9-1074.5)	787.4 (613.7-1007.5)	746.7 (606.9-909.4)	699.8 (651.1-746.8)	664.5 (642.3-687.9)	580.6 (560.2-599.8)	529.1 (509.2-549)	465.3 (443.6-486.7)	400.8 (385.9-417.4)	397.6 (382.3-412.2)	436.9 (399.7-477.5)
GBR Northern Ireland	1281.8 (1245.8-1317)	1197.6 (1173.2-1222.6)	1104.7 (1080.4-1128.4)	975.5 (955.9-996.2)	910.1 (891.6-931.4)	910.2 (893.3-929.8)	857.3 (839.4-877)	805.2 (787.8-823.9)	741.3 (723-758.9)	674.4 (657.5-690.3)	586.1 (565.7-607.5)	497.7 (484.4-510.5)	474.8 (463.2-487.6)	442.3 (400.8-489)
GBR Scotland	1272.1 (1255-1290.8)	1127.5 (1114.2-1141.7)	1073 (1060-1085.8)	1010.9 (998.2-1023.8)	966.2 (954.5-978)	877.6 (867-888.7)	846.9 (836.2-857.2)	797 (786.4-807.9)	730.3 (719.8-740.7)	670.5 (661-680.2)	617.7 (599.7-637.1)	570.1 (561-578.9)	520.1 (511.6-529.2)	500.2 (456.7-543.3)
GBR Wales	1280.2 (952.2-1673.9)	1158.6 (875.2-1527.5)	1061.9 (788-1431.5)	994.1 (745-1291.3)	943.5 (708.8-1210.8)	870.1 (694-1081.3)	756.8 (724.8-792.5)	685.7 (675.7-696.2)	626.9 (617.6-636.1)	609.5 (600-619.6)	561.5 (543.9-579.5)	511.9 (502.9-520.5)	461.5 (453.6-470.2)	441.2 (407.9-479.2)
Latin America and Caribbean	1869.9 (1832.5-1910.1)	1633.5 (1601.4-1667.1)	1506.6 (1477.8-1535.5)	1370.9 (1342.3-1400.8)	1280.6 (1254.2-1308.8)	1107.8 (1091.1-1124.4)	1001.2 (991.9-1010.1)	939.9 (934.7-945.4)	825.1 (820.7-829.6)	782.9 (778-788.1)	681.6 (677.1-686.4)	625.6 (620.7-630.7)	618.9 (614-623.5)	551.6 (542.3-561.4)
Andean Latin America	2261.3 (2126.4-2395.4)	1924.6 (1852.7-1998.7)	1802.1 (1734.3-1874.3)	1518.2 (1456.8-1578.8)	1664.6 (1613.5-1718)	1154.7 (1109.7-1199.1)	1081.1 (1042-1120.2)	1005.1 (967.9-1045.1)	849 (820.2-880.3)	827.7 (793.3-862.3)	706.3 (677.5-738.6)	647.9 (618.1-681.5)	610.3 (578-642.1)	527.3 (479.9-579.5)
BOL Bolivia	2801.7 (2619.4-2991.3)	2293.6 (2200.7-2388.9)	2088.7 (1996.3-2188.7)	1971.9 (1884.4-2062.2)	1842.9 (1749.9-1939.8)	1700.1 (1627-1780.1)	1575 (1495.2-1656.6)	1508.1 (1411.4-1603.8)	1389.7 (1299.3-1490.4)	1281.6 (1182-1384.8)	1089.4 (997.6-1188.9)	959 (859.4-1084)	873.3 (753.6-998.3)	795.5 (648.5-926.9)
ECU Ecuador	1934.6 (1698.1-2191.9)	1747.4 (1719.1-1777.2)	1653.1 (1627.3-1681.8)	1370.7 (1349.1-1393.8)	1245.1 (1225.5-1265.3)	1065.9 (1049.8-1080.5)	930.4 (918.2-943.3)	786 (775.2-796.5)	732.3 (723.8-740.6)	664.7 (657-673.1)	629.2 (619.9-638)	653.2 (620.7-687.9)	629 (597.2-660.4)	582.7 (527.4-644.4)
PER Peru	2201.1 (1998.3-2410.4)	1882.5 (1766.6-2008.6)	1779 (1669.4-1890.6)	1431.5 (1332.4-1529.8)	1778.1 (1699.4-1863.3)	1028.1 (956.1-1099.8)	1000.7 (940.6-1060.7)	959.8 (904.2-1021)	753.8 (709.1-800)	776.6 (726.7-828.4)	635.6 (593.2-684.4)	562 (522.9-606.9)	534.8 (494.1-577.5)	432 (361.8-510.8)
Caribbean	1693.6 (1603-1784.8)	1483.2 (1402.7-1564)	1350.3 (1294.2-1405.5)	1267.5 (1219.3-1315.3)	1147.4 (1105.5-1192.9)	1045.7 (1008.5-1083.4)	999 (968-1031.7)	952.4 (927.3-976.5)	897.3 (875.7-920.2)	878.1 (853.8-904.6)	795.9 (770.8-823.2)	753.7 (724.8-787)	1109.3 (1078.3-1142.3)	675.1 (632.4-722)
ATG Antigua and Barbuda	1475.5 (1397.2-1569.5)	1314.7 (1269.2-1361.8)	1145.7 (1108.4-1185.5)	1097.4 (1055.7-1141)	1025.5 (978.9-1074)	879.1 (832.7-925.7)	776.8 (727.4-830.4)	661.4 (613.1-709.6)	612.1 (574.3-651.2)	653.4 (624.4-699.9)	650.4 (632.6-665)	613.5 (582.8-645.2)	599.3 (574.6-625.6)	600.8 (560.8-641.3)
BHS The Bahamas	1444.2 (1213.6-1672.9)	1341.4 (1174.4-1525.5)	1256 (1144.9-1373.8)	1197.2 (1150.5-1248.4)	1035.3 (1007.7-1063.8)	889.7 (868.1-911.2)	866.9 (846.5-888)	803.8 (785-824.8)	745.2 (726.5-764.2)	758.8 (739.7-777.3)	751.5 (734.4-768.7)	667.4 (650.7-683.7)	667 (649.7-684.8)	673.2 (607.7-738.6)
BRB Barbados	1420.6 (1336.8-1520.3)	1198.9 (1163.2-1236.2)	1026.8 (1000.3-1053.2)	997.6 (974.2-1022)	983.4 (959.9-1006.4)	895.4 (872.8-918.5)	799.5 (779.4-820)	721.8 (703.8-740.6)	721.8 (703.6-740.7)	684 (661.8-707.5)	655.5 (634.7-677.7)	610.4 (594.9-627.2)	593.1 (577.6-608.7)	600.4 (549.3-647.3)
BLZ Belize	1780 (1653.4-1942.8)	1534.9 (1469.1-1605)	1177.9 (1124.7-1230.5)	1002.6 (954.8-1049.9)	871.9 (829.1-913.9)	812.4 (774-851.8)	782.8 (743-818.6)	786.3 (749.2-822.3)	795.5 (758.3-835.4)	773.4 (742.6-806.8)	802.1 (781-823.3)	733.6 (718.6-749.2)	663.6 (649.2-677.5)	602.6 (577-628.2)
BMU Bermuda	1216.5 (1146.7-1284.8)	1076.3 (1043.9-1108.5)	956.8 (928-988.1)	879 (858.7-904.2)	803.1 (774-822.7)	695.1 (662.7-727.5)	606.4 (582.8-631.6)	611.7 (588.4-638.4)	617.5 (595.6-640.6)	548.3 (525.7-584.5)	511.8 (493.8-539.2)	439.3 (411.3-469.6)	358 (332.6-384.4)	312.1 (281.4-343.9)
CUB Cuba	1074.6 (893.8-1289.3)	1101.8 (927.3-1282)	1069.7 (1057.1-1083.6)	1030.3 (1020.2-1039.4)	847.4 (839.5-855.4)	741 (733.5-748.2)	751 (743.4-758.1)	725.8 (719.5-732.4)	693.5 (686.9-699.9)	686.5 (680.3-693)	588.5 (582.9-594.3)	556.4 (550.8-562.5)	522.1 (516.7-527.8)	504.4 (442-573)
DMA Dominica	1891.3 (1740.1-2068.7)	1367.2 (1300.9-1435.8)	1121 (1075.2-1169.5)	958.6 (918.3-997.6)	867.2 (837.5-900.4)	765.6 (740.6-793.2)	700.2 (676-725.7)	668.3 (642.2-693.8)	751.7 (727.1-781.6)	739.3 (713.5-768.3)	701.3 (674.3-730.8)	672.4 (643.5-699.7)	678.2 (649.8-708.4)	722.8 (664.5-782.6)
DOM Dominican Republic	1512.9 (1428.7-1612.5)	1304.5 (1266.9-1343.5)	1173.9 (1101.5-1254)	1088 (1025.6-1155.6)	1047.7 (981-1125.8)	940.3 (874.5-1009.3)	845.2 (790-910)	749.9 (695.8-806.3)	704.9 (655.2-753.5)	624.6 (579.4-675.7)	569.3 (523.8-616.7)	625.6 (569.8-685)	580.8 (534.1-632.3)	613.6 (534-694.4)
GRD Grenada	1315.5 (1228.4-1405.3)	1182.6 (1113.2-1248.8)	1140.1 (1067.7-1211.5)	1171.7 (1095.1-1251.9)	995.2 (924-1071.1)	916.4 (855.1-984.1)	930.2 (876.9-985.2)	1003.1 (952.9-1052.8)	978.4 (944.5-1014.5)	870.8 (842.7-899)	712 (687.3-736.5)	689 (661.5-718.8)	750.7 (719.6-782.6)	784.4 (725.6-841.6)
GUY Guyana	2067.9 (1994.1-2146.6)	1732.9 (1695.9-1770.8)	1396.8 (1369-1425.3)	1315.4 (1222.9-1411.5)	1221.6 (1198.1-1244.2)	1309.5 (1275.6-1345.7)	1200.4 (1159.6-1242.8)	1030.7 (998-1066.8)	1050.3 (1031.9-1069.1)	1063.1 (1043.9-1082)	1135.5 (1116.9-1154)	1011.5 (994.7-1028.4)	935.8 (919.7-953.7)	902.4 (795.3-1018.3)
HTI Haiti	2952.2 (2609.6-3301.5)	2650.9 (2343.3-2969.7)	2552.4 (2238.6-2863.3)	2489.8 (2197.8-2798.9)	2402.3 (2131.4-2687.4)	2264.5 (2013.6-2528.7)	2123.1 (1900.1-2346.5)	1989.6 (1807.5-2162.9)	1900.4 (1755.3-2072.3)	1893.4 (1731.6-2085.6)	1758.6 (1571-1951.9)	1602.1 (1401.2-1828.1)	3413.1 (3190.6-3669.7)	1282.1 (1057.3-1533.4)
JAM Jamaica	1705.9 (1653.1-1765.9)	1313.5 (1289.6-1339.1)	1117.2 (1096.5-1138.7)	1043.2 (1023.9-1062.5)	903.3 (886.4-920.5)	833.6 (815.4-852)	721.2 (706.9-736.3)	804.3 (789.9-818.5)	686.2 (649.5-723.3)	689 (626.9-750.5)	726.7 (716-737.5)	617.6 (604.9-631.5)	611.4 (601.7-622.2)	644.6 (549.9-766.1)
PRI Puerto Rico	1415.1 (1389.4-1444.1)	943 (929.1-957.1)	836.6 (823.6-850.6)	805.5 (793-817.8)	743.4 (731.3-755.5)	668.1 (656-679.9)	649.6 (639.2-661.4)	645.3 (634.7-656.1)	601.6 (592-611.5)	589 (579.7-598.3)	509.6 (500.9-518.2)	465.9 (457.5-474.7)	418.6 (410.9-426.5)	441.4 (413.4-471.5)
LCA Saint Lucia	2065.4 (1958.1-2187.7)	1695.8 (1638.2-1758.5)	1402.1 (1351.2-1455.2)	1184.1 (1138.3-1234)	1109.5 (1069.8-1153.6)	1076 (1044.1-1107)	996.6 (968.1-1021.9)	926.3 (902.5-950.5)	891.6 (868.7-915.8)	849.6 (825.6-874.3)	702.2 (683-721.7)	651 (631.6-671.7)	635.6 (616.7-654.3)	633.8 (591.1-682.6)
VCT Saint Vincent and the Grenadines	1706.9 (1611.8-1814.2)	1477.7 (1425.4-1538.8)	1231 (1181-1285)	1148.5 (1102.4-1199.1)	1007.7 (966.1-1050.2)	900.7 (865.3-936.4)	825.5 (796.4-852.7)	808.1 (783.8-833)	906.1 (877.1-938.1)	823.4 (797.4-852.7)	813.9 (789.2-839.2)	716.3 (694.2-738.5)	678.4 (656.2-699.6)	695.1 (649.6-744)
SUR Suriname	1470.9 (1340.8-1603.2)	1363.8 (1275.7-1457.9)	1227.2 (1134.6-1320.7)	1141.5 (1039.1-1248.9)	1123.1 (1019.9-1244.2)	1087.7 (1006.5-1174.9)	1103.6 (1039.3-1172)	960.2 (907.8-1014.9)	908.5 (858.3-959.4)	807.7 (763.5-851.9)	791.3 (749.9-838.7)	778.7 (737.5-825)	714.2 (674.3-755.9)	706.6 (639.4-780)
TTO Trinidad and Tobago	1815.1 (1761-1873.2)	1477.5 (1447.2-1509.4)	1222.3 (1198.1-1248)	1266.1 (1240.4-1291.4)	1214.4 (1191.6-1239.6)	1138.8 (1115.1-1162.6)	1085.3 (1063.7-1107.9)	1019.4 (998.3-1039.8)	918.2 (899.1-936.3)	942.3 (923.8-960.1)	859.2 (842-876.7)	721.6 (705.7-737.5)	629.4 (614.7-644.8)	611.6 (483.9-758.3)
VIR Virgin Islands	1347.3 (1263.5-1427.4)	1213.4 (1162-1268.2)	1154.9 (1107.6-1204.7)	1119.3 (1049.3-1184.1)	1047.3 (953.3-1135.5)	952.8 (882.4-1041.5)	864.2 (816.5-930.1)	789.5 (753-829.9)	737.3 (683.9-819.7)	683.7 (651.5-745.6)	652.8 (624.9-680.3)	616.9 (576.7-644.5)	592.9 (542.4-637.6)	596.4 (530.5-687.4)
Central Latin America	1953.5 (1913.9-1993.1)	1650.1 (1620.5-1681.4)	1503.3 (1476-1532.6)	1365.3 (1338.8-1391.8)	1247.9 (1222.5-1274.6)	1099.9 (1084.8-1116)	949.7 (934-965.4)	901.6 (897.8-905.3)	773.4 (770.3-776.7)	739.4 (734.8-744.4)	640.8 (636.7-645.7)	597.4 (592.8-602.2)	560.6 (556-565)	537.6 (521.2-554.5)
COL Colombia	1844.9 (1787.4-1914.8)	1607.6 (1580.6-1637.9)	1506.8 (1484.7-1530.8)	1380.2 (1364.5-1396.6)	1074.3 (1060.9-1088.3)	1127.8 (1115.3-1141.7)	917.1 (842.3-991.9)	962.8 (955.8-970.4)	744.6 (738.7-750.8)	720.4 (714.9-726.1)	589.8 (584.6-595.1)	523.1 (518.3-527.8)	457.1 (452.7-461.9)	401.7 (356.5-455.6)
CRI Costa Rica	1675.2 (1636-1714.5)	1235.3 (1211.9-1259.9)	1077.6 (1058.8-1098.8)	1091.2 (1071.7-1110.4)	1049.7 (1031.2-1068.1)	821.2 (806.3-837.2)	656.7 (643.5-670.1)	657 (644.7-669.6)	590.6 (579.1-601.7)	601.7 (591.1-611.8)	506.4 (497.5-515.8)	452.9 (445.1-461.8)	423.7 (415.9-431.5)	411.9 (384.7-444.9)
SLV El Salvador	2118.1 (2001.8-2252.2)	1844.6 (1790.6-1904.9)	1562.9 (1516.1-1611.2)	1403.9 (1366.6-1442.2)	1342.9 (1315.9-1372.2)	1065 (1041.8-1087.1)	996.7 (950.7-1044.6)	852 (838.2-867.1)	743.1 (730.8-754.6)	677.7 (649.5-708.5)	628.7 (601.3-656.1)	634.3 (607.3-663)	635.7 (607.6-667)	600.2 (499.9-724.9)
GTM Guatemala	2815.5 (2680.2-2981.5)	2520.8 (2466.4-2580.4)	2272.8 (2229.2-2320)	2120.2 (2083.2-2160.1)	2084.2 (2054.8-2115.5)	1714.1 (1688.4-1740.1)	1325.7 (1304.9-1347.8)	1382.6 (1367-1399.9)	1222 (1207.3-1236.4)	1058.8 (1046.6-1071.2)	946.3 (935.8-956)	840.9 (832.3-849.5)	727.3 (719.4-736.4)	670.4 (596.1-749.9)
HND Honduras	2578.3 (2326.5-2850.2)	2309.7 (2136.3-2497.8)	2131.1 (1976.2-2285.6)	1811.4 (1690.1-1939.9)	1544.3 (1449.9-1644.7)	1284.3 (1196.7-1378.9)	965.2 (899.1-1037.3)	938.7 (855.2-1028.1)	837.1 (756.6-925.4)	909.6 (779.9-1063.8)	883.2 (725.4-1080.1)	848.1 (677-1040.6)	818.4 (641.3-1002.6)	795 (599.8-962.9)
MEX Mexico	1932.5 (1859.9-1998.7)	1612.8 (1556.5-1670.9)	1484.8 (1432.2-1542.1)	1342.9 (1291.1-1394.3)	1270 (1223.6-1318.5)	1070.3 (1044.1-1098.9)	953.5 (942.4-965.3)	873.6 (870.1-877.3)	769.3 (766.6-771.9)	742.1 (739.7-744.4)	654.7 (652.8-656.6)	628.3 (626.5-630.2)	602 (600.4-603.5)	591.6 (581.7-601.1)
MEX Aguascalientes	1537.7 (1300.8-1801.6)	1346.1 (1143-1583.2)	1309.2 (1114.4-1534.4)	1241.7 (1062-1450)	1227.8 (1086.8-1389.3)	1058.2 (981.1-1143.6)	963.2 (935.4-990.4)	939.9 (920.5-960.6)	839.8 (823.7-855.1)	801.6 (787.8-815.2)	715.6 (703.8-727.5)	692.8 (681.6-703.8)	636 (625.7-646.1)	563 (523.3-601.7)
MEX Baja California	2446.8 (2105.7-2829.2)	1797.9 (1545.6-2067.3)	1540.5 (1304.7-1811.3)	1353.2 (1131.9-1595)	1277.1 (1077.2-1512.3)	1068.5 (955.6-1198.2)	903 (884.5-921.4)	986.6 (970.3-1003.2)	875.7 (861.8-890.3)	885.2 (872.2-897.8)	825.5 (813.4-838.3)	752.5 (741.9-763.1)	656.1 (647.4-664.8)	583.2 (542.6-621.5)
MEX Baja California Sur	1345.7 (1126.8-1571.5)	1179 (988.3-1380.9)	1136.1 (963.6-1325.6)	1085.8 (937.1-1236.1)	1048 (924.7-1168.3)	907.4 (841.4-981)	781.9 (752.5-813.7)	692.4 (675.5-711.4)	670.5 (654.1-688)	661.1 (646.8-676.7)	629.6 (616.6-643.5)	610.3 (597.1-623)	600.5 (588.5-612.7)	557.9 (519.6-598.4)
MEX Campeche	1520.9 (1285.3-1778.7)	1317.8 (1112.4-1547)	1271.9 (1085.2-1505.9)	1195.6 (1027.6-1394.4)	1167.4 (1026.2-1318.1)	1003.4 (928.8-1083.7)	918 (888.7-945.1)	968.3 (947.8-988.9)	862.8 (845.2-880.7)	769.5 (754.7-785.8)	633.5 (620.2-646.4)	615.1 (603.1-627.6)	614.3 (603.1-626.4)	655.8 (609.3-699.6)
MEX Chiapas	1569.6 (1308-1839.8)	1361 (1136.9-1590.3)	1285.5 (1066.5-1500.1)	1178.9 (967.3-1397.5)	1102 (919-1280.9)	918.1 (829-1024.9)	1018.8 (991.5-1045.1)	950.3 (930.2-971.6)	860.3 (843.9-877.9)	810.5 (797.6-823.3)	748.1 (736.8-758.8)	694.3 (684.2-704.7)	672.3 (664.1-681.4)	700.1 (650.5-749.3)
MEX Chihuahua	2146.4 (1845.7-2487.7)	1772.5 (1511.2-2041.3)	1609.7 (1356.6-1892.6)	1447.8 (1212.8-1699.9)	1378.2 (1162.3-1605.7)	1167.1 (1050.9-1287.8)	1022.3 (999.5-1044.6)	985.4 (969.7-1003)	918.9 (905.9-933.1)	877.9 (865.1-890.2)	806.8 (795-818.5)	764.1 (753.8-774.4)	748.4 (739.1-757.7)	707.9 (656.6-758)
MEX Coahuila	1900.3 (1618.8-2186.1)	1637 (1384.6-1910.5)	1549.3 (1294.3-1808.1)	1430 (1199.8-1694.5)	1399.6 (1186.7-1636.4)	1201.5 (1080.3-1337.8)	1056.5 (1032.2-1079.3)	976.2 (959.5-995.2)	853.1 (839.1-867.6)	808.2 (794.9-821.4)	727.4 (716.6-739)	709.1 (698.7-719.4)	673 (663-682.8)	630.4 (585.2-676.5)
MEX Colima	2117.5 (1800.3-2443.4)	1748.1 (1502.2-2003.7)	1602.2 (1382.2-1835.8)	1447.3 (1253.7-1655.5)	1378.8 (1234.3-1535.7)	1171.3 (1093.1-1256.1)	1052.3 (1021.1-1085.6)	945.1 (926.2-965.1)	908.7 (891-926.8)	838.4 (820.3-856.1)	695.4 (682.2-709.3)	655.9 (642.8-670.3)	632.2 (620.7-643.7)	642.9 (600.5-685.5)
MEX Mexico City	1982.4 (1669.5-2316)	1520.2 (1316.2-1737.6)	1357.1 (1167.7-1571.9)	1194.9 (1012.2-1389.4)	1091 (933.6-1273.4)	923 (841.8-1007.7)	861.3 (844.8-878.5)	792.2 (781.6-802.8)	709.9 (701.9-717.8)	724.8 (717.2-733)	659.7 (653.5-665.9)	649.8 (643.2-656.2)	613.6 (607.3-620)	582.4 (543.5-620.2)
MEX Durango	1937.4 (1635.2-2241.6)	1608.7 (1349-1867.2)	1466.5 (1248.6-1709.9)	1316.9 (1135.7-1527)	1250 (1105.8-1414)	1065 (979.1-1149.6)	978.5 (953.5-1004.3)	900 (884.1-916.9)	770.8 (757.3-785.3)	672.3 (660.1-684.7)	577.4 (565.9-589.4)	574.1 (563.9-584.7)	589.1 (579.5-598.7)	515.7 (479.6-551.3)
MEX Guanajuato	2015.1 (1728.3-2320.7)	1733.1 (1503.8-1990.8)	1641.3 (1419.7-1918.5)	1528.8 (1313.3-1772.4)	1476.8 (1277.7-1688.5)	1229.9 (1108.8-1353.1)	1015.4 (990.4-1041.2)	1026.9 (1010.3-1044.1)	886.7 (874.3-899.6)	888.6 (876.8-900.1)	734.7 (724.6-745)	648 (640.1-656.3)	588.4 (581.5-595.9)	598.5 (558.6-638.3)
MEX Guerrero	1910.8 (1634-2248.9)	1617.3 (1390.3-1890.1)	1481.8 (1264.6-1748.8)	1350.9 (1151-1575.2)	1294.6 (1108-1492.3)	1070.8 (964.8-1179.2)	877.7 (850.1-906.8)	657.5 (636.9-681.2)	535 (520.4-550.8)	521.7 (508.1-536.8)	451.6 (441.2-462.7)	455.5 (446.9-464.4)	480.1 (471.9-488.4)	469.2 (433.9-502.9)
MEX Hidalgo	1983.2 (1696.6-2296.4)	1718.2 (1451-1993.4)	1614.8 (1368.8-1869.3)	1473.7 (1242.3-1717)	1418.6 (1209.1-1638.2)	1207 (1090.4-1333.5)	1142.6 (1116.1-1169.6)	885.4 (868.8-902.7)	719.6 (704.7-734.5)	642.1 (630.6-654.1)	538 (527.2-548.1)	531 (522.7-539.9)	544.4 (535.4-553.8)	507.3 (471.5-547.5)
MEX Jalisco	2015.4 (1728.3-2312.9)	1686.4 (1445.3-1956.8)	1547.7 (1310.9-1792.4)	1379.5 (1172.3-1587.9)	1285 (1094.9-1484.7)	1046.2 (945.2-1156.6)	924.4 (905.7-943.3)	866.1 (853-879.8)	830.6 (819.6-842.4)	818.8 (810.1-827.6)	710.5 (701.6-720)	665.7 (658.9-672.9)	617.8 (611.2-624.3)	629.2 (586.1-674.2)
MEX México	1540.5 (1289.9-1826.6)	1377.3 (1160.4-1624.2)	1350.6 (1153.9-1596.9)	1270.3 (1065.2-1488.6)	1237 (1047.4-1472.1)	1062 (897.8-1265)	1009.7 (900.8-1138.3)	1010.7 (999.4-1022.5)	861.9 (853.8-870.3)	815.6 (808.7-822.9)	723.4 (716.7-729.9)	684.9 (678.8-690.9)	625.1 (620.5-630.5)	630.6 (587.8-671.4)
MEX Michoacán de Ocampo	2068.5 (1771-2387.5)	1738.4 (1504.6-2001.8)	1603 (1363.8-1867.7)	1444.6 (1224.1-1699)	1345.1 (1147.1-1546)	1146.1 (1038.1-1259.5)	1048.1 (1021.8-1074.6)	905.5 (888.9-921.8)	718.6 (706.6-731.1)	708.7 (698.4-720.1)	615.8 (606.7-625.3)	569.8 (561.3-579.3)	561.7 (553.6-570.2)	515.5 (480.1-553.4)
MEX Morelos	1726.7 (1486.3-2008.1)	1457.8 (1243.8-1706.1)	1359.9 (1151.8-1602.4)	1232 (1051.3-1441.8)	1165.2 (1019.2-1335.6)	954.7 (876.1-1039.6)	839.9 (818.6-864.2)	805.2 (788.6-823.2)	740.2 (725.3-756.5)	700.6 (688.7-712.3)	620.3 (610.2-631)	623.1 (612-632.8)	583.8 (574.3-592.7)	638.3 (592.6-681.4)
MEX Nayarit	1975.8 (1673.4-2278)	1632.2 (1386.9-1887.1)	1487.1 (1270.7-1721.1)	1338.3 (1154.5-1540.4)	1251.9 (1114.7-1402)	1025.2 (961-1099.3)	884.4 (862.7-905.8)	837.2 (819.8-853.7)	749.3 (734.4-762.6)	646.6 (634.6-659.2)	558.6 (547.8-569.7)	533 (522.7-542.7)	522.6 (513.2-531.7)	503.3 (468.8-539.7)
MEX Nuevo León	1703.1 (1438.8-2003.1)	1434 (1207.4-1679.1)	1329.5 (1114.9-1558.7)	1229 (1038.2-1438.4)	1222.4 (1032.7-1441.1)	1080.1 (964.4-1206.8)	884.3 (866-902.7)	796 (781.3-811.1)	747.2 (734.4-758.6)	730.9 (720.9-741.4)	657.7 (648.1-667.8)	628.2 (619.3-636.5)	586 (578.5-593)	545.7 (510.4-585.7)

Table 1 Age-standardised mortality rates by location and year for females for 1950, 1955...2005, 2010, 2017

Location	1950	1955	1960	1965	1970	1975	1980	1985	1990	1995	2000	2005	2010	2017
MEX Oaxaca	2386.8 (2043.1-2761.2)	2013.3 (1715.6-2306.1)	1853.1 (1589.1-2148.3)	1709.4 (1470.7-1977.8)	1670.1 (1452.6-1907.1)	1463.3 (1326.6-1607.4)	1292.3 (1264.9-1321.5)	1114.7 (1099.1-1131.7)	877.6 (863.6-891.6)	801.7 (790.5-813.5)	625.2 (615.9-634.8)	584.6 (576-593.4)	553.9 (546.1-562.4)	564.5 (525.9-602.1)
MEX Puebla	2296.6 (1972.5-2628.4)	1929.5 (1656.9-2197.9)	1774.4 (1516.4-2025.8)	1620.5 (1396.2-1870.3)	1558.3 (1331.6-1794.5)	1311.4 (1194.3-1442.9)	1104.4 (1077.7-1131.7)	1015.8 (995.6-1037)	857.8 (845-870.4)	829.5 (818.5-841)	682.8 (673.5-693.4)	654.5 (645.2-665.4)	631.7 (623.3-639.7)	652.8 (609.9-696.5)
MEX Querétaro	1718.2 (1437.1-2006.7)	1496.1 (1268.2-1738.6)	1439.6 (1227-1679)	1354.1 (1146.4-1591)	1342.6 (1148-1556.8)	1161.4 (1023.3-1312)	1097.9 (1027.2-1170.2)	943.5 (926.5-960.8)	835.1 (820.8-849.4)	824.3 (812.1-836.7)	693.9 (683-704.8)	633.6 (623.9-642.8)	596.4 (587.4-604.9)	605.9 (566.5-644.9)
MEX Quintana Roo	813.6 (648.2-989.7)	821.1 (679.4-985)	860 (711.5-1031.1)	947.3 (808.4-1108)	1082.6 (947.6-1227.8)	1029.2 (949.9-1117.5)	852.4 (819.3-885.8)	609.9 (590.7-630.3)	655.7 (639.8-673.1)	745.1 (729.4-760.6)	689.2 (676.3-702.4)	655.3 (644-666.7)	654.7 (644.8-664.8)	746.4 (698.3-795.9)
MEX San Luis Potosí	2028 (1734.9-2361.9)	1689 (1442.7-1961.5)	1549 (1306.4-1797.5)	1407.4 (1193.5-1649)	1337.1 (1136.9-1565.3)	1129.3 (1013-1256.6)	942.3 (916.4-965.1)	863.8 (848.2-880)	730.1 (717.7-742.5)	703.4 (691.7-716.3)	582.7 (572.8-593.3)	560.3 (549.1-571)	545.2 (536.4-555.9)	524.5 (488.4-563.5)
MEX Sinaloa	1611.6 (1388-1878.2)	1381 (1177.8-1613.7)	1294.4 (1089.8-1533.5)	1185.9 (996.3-1388.3)	1132.4 (968.5-1318.6)	927.6 (829.2-1029.1)	830.4 (809.4-850.6)	727.9 (712.9-742.7)	675.6 (662.4-689)	650.3 (639-660.9)	576.7 (566.8-586.9)	560.1 (550-569.5)	519 (510.2-528)	451 (416.1-486.8)
MEX Sonora	1813.2 (1523-2107.7)	1551.3 (1302.7-1832.2)	1469.3 (1227.4-1753.7)	1369.7 (1152.3-1631.1)	1349.2 (1132.1-1619)	1179.3 (984.8-1407.3)	1110.2 (992.3-1242.6)	992.4 (976.7-1008.5)	866 (852.2-879.5)	865.8 (851.5-879.7)	732.5 (719.5-745.5)	717 (705.8-729.2)	670.4 (661-681.1)	625.4 (580.5-674.5)
MEX Tabasco	1704 (1426.9-2011.5)	1490.4 (1272.9-1728.9)	1420.5 (1206.2-1662.3)	1337.4 (1141.7-1552.1)	1320.6 (1144.6-1519.8)	1172 (1076.1-1274.5)	1057.1 (1029.1-1087.3)	910.8 (892.7-929.4)	794 (780.2-808.7)	841.5 (828.2-855.4)	707.6 (695.9-719.4)	646.1 (637.2-655.8)	645 (636.4-654.2)	643.8 (599-686.9)
MEX Tamaulipas	1804.9 (1503.6-2136)	1505.4 (1256.2-1765.2)	1387.9 (1153.9-1631.4)	1245.7 (1028.6-1480.4)	1172.1 (979.2-1387.4)	992.3 (882.6-1111.1)	881.1 (860.1-903.4)	803.4 (787.5-819.3)	716.5 (704.2-729.3)	676.9 (664.6-690.8)	619.2 (609.3-629.9)	589.7 (581.7-598.3)	570.4 (562.2-578.9)	528.3 (490.3-567.7)
MEX Tlaxcala	1879.7 (1590.9-2188.6)	1603.9 (1364.4-1861.9)	1498.4 (1267.7-1745.2)	1381.4 (1176.3-1608.8)	1302.3 (1115.6-1500.2)	1104.1 (967.9-1257.4)	997 (918.5-1086.4)	858.7 (835.8-884.2)	777.4 (762.8-791.2)	739.8 (726.5-752.4)	655.8 (644.5-667.4)	603.8 (593.6-614.2)	583.6 (574.2-593.2)	580.2 (542.1-620.5)
MEX Veracruz de Ignacio de la Llave	1820.1 (1555.5-2108.4)	1511.7 (1280.4-1778)	1375 (1156.6-1617.4)	1220.3 (1032.3-1435.8)	1127.3 (960-1317.3)	932.9 (842.9-1027.1)	800.6 (781.8-819)	753.3 (740.9-766)	702.9 (691.4-714.9)	681.1 (672.5-690.2)	614.7 (606.6-623.5)	610.5 (603.4-617.1)	604.7 (598.8-610.6)	629.9 (587.4-671.7)
MEX Yucatán	1720.3 (1481.3-1974.7)	1505.4 (1275-1764.3)	1466 (1235.2-1717.6)	1406.2 (1191.9-1630.8)	1417.7 (1228.3-1621.8)	1286.9 (1195.9-1390.7)	1181.2 (1155.7-1207.7)	1090.2 (1072.4-1110)	962.4 (947.5-976.6)	906.2 (892.8-919.1)	734.7 (723.5-745.4)	709.2 (697.8-720.8)	694.5 (684.8-704.2)	708.9 (662.3-758.7)
MEX Zacatecas	1907.9 (1625-2232.9)	1553.2 (1324.8-1818.6)	1384.9 (1172.5-1628)	1222.1 (1039.6-1422.2)	1135.8 (984.4-1297.7)	941 (869.9-1019.1)	829.5 (807-853.6)	742.7 (727-760.2)	655.3 (641.9-669.6)	675 (662.5-687.5)	591.5 (580.1-602.3)	576.3 (566-586.7)	557.6 (548.2-567.3)	552.7 (516.5-590.7)
NIC Nicaragua	2025.1 (1906.2-2169.3)	1604.8 (1536.9-1674.1)	1580.4 (1523.9-1638.8)	1313.7 (1251.5-1375.2)	1370 (1253.4-1493.7)	1094.6 (1036.2-1158.7)	1048.8 (937.2-1172.1)	1003.3 (907-1109.4)	700.2 (657.7-746.4)	669.5 (616-720)	584.9 (545.4-630.6)	571.3 (531-612.1)	517 (496.4-539.9)	491 (431.8-550.4)
PAN Panama	1325.8 (1274.5-1382.5)	1183.8 (1155.5-1211.9)	1041.8 (1019.2-1064.9)	1004.1 (981.6-1026.5)	1002.8 (983.2-1023.8)	797.8 (779.2-816.3)	658.6 (643.9-674)	647.3 (632.7-660.6)	592.1 (579-605.5)	547.1 (535.9-559.4)	496.5 (486.2-507.1)	462.8 (453.2-471.8)	439.6 (430.2-449)	436.1 (406.5-468.2)
VEN Venezuela	1778.8 (1752.2-1811.6)	1439.4 (1428-1450.5)	1210 (1199.5-1220.9)	1137.7 (1129.1-1146.8)	1081.6 (1073-1089.9)	1022.3 (1015-1029.6)	883.7 (877.2-890.5)	798.8 (793-804.4)	735.6 (730.6-741.3)	696.1 (679.4-713.7)	586.5 (582.2-591.1)	528 (524.1-532.1)	516.5 (512.8-520.2)	527.5 (443-620.3)
Tropical Latin America	1740.5 (1660-1823.9)	1599.1 (1527.3-1678.7)	1493.7 (1420.7-1566)	1382.7 (1319.5-1454.8)	1268.6 (1208.7-1329.5)	1133.1 (1098.5-1167.3)	1041.4 (1031.6-1051.3)	969.9 (963.8-976.3)	860.2 (855.1-865.5)	797.6 (793.2-802.6)	692.4 (688.2-696.8)	622.3 (618.6-626.5)	581.4 (578.2-584.5)	546.6 (541.3-552)
BRA Brazil	1757.4 (1674.5-1843.8)	1622.8 (1548.1-1706.2)	1513.1 (1437.4-1588.3)	1400.4 (1334.9-1475.3)	1280.4 (1219.3-1343.7)	1140 (1104.7-1175.2)	1046.9 (1037-1057.2)	974.8 (968.7-981.1)	865.4 (860.4-870.7)	801.1 (796.6-806.3)	694.2 (689.8-698.7)	622.1 (618.3-626.3)	580.6 (577.6-583.7)	546.5 (541.5-551.5)
BRA Acre	1655.4 (1394.6-1926.5)	1535.5 (1317-1767.2)	1402.3 (1214.5-1610.6)	1290.5 (1136.8-1453.1)	1167.1 (1057.7-1280.2)	1044.3 (975.6-1118.9)	964.8 (930.7-1002.4)	943.9 (921.4-968)	830.3 (808.3-852.8)	773.7 (754.5-797.5)	702.1 (687.2-717.8)	637.5 (624.5-652.7)	563.6 (551.7-576.1)	532.2 (514.7-553)
BRA Alagoas	2079.9 (1769.3-2411.4)	1966.1 (1700.7-2242)	1868.5 (1633.1-2126.4)	1738.6 (1507.9-2002.6)	1606.7 (1398-1813.4)	1428.9 (1303.1-1567.7)	1308.1 (1269.7-1351.3)	1162.6 (1130-1200.6)	939.8 (914.2-966.3)	847.6 (821.2-875.1)	724.2 (698.7-752.4)	651.9 (628.8-673.5)	614.4 (603.9-625.1)	606.8 (583-631.6)
BRA Amapá	1945.4 (1642.4-2288.6)	1697.3 (1459.3-1946.7)	1517.9 (1317.6-1731.1)	1378 (1212-1548.3)	1223.1 (1102.8-1346.8)	1053.3 (978.9-1131.3)	883.3 (854.5-914.1)	814.8 (794.3-836.2)	735.5 (719.1-754.3)	752.8 (738.3-768.6)	706.2 (692.5-719.6)	578 (566.9-589.5)	482 (471.8-493.2)	504.9 (487.6-524.6)
BRA Amazonas	1483.8 (1222.3-1751.6)	1370.2 (1160-1609.9)	1280.8 (1092.8-1508.2)	1202.1 (1018.3-1423.5)	1144.6 (975.2-1340.4)	1083.8 (951-1219.2)	921.9 (879-969.2)	843.9 (810.8-879.6)	721.5 (689.4-754.4)	612.7 (587.4-637.6)	598.8 (590-608.4)	542.5 (534.4-551.1)	545.7 (537.1-554.1)	529.9 (508.6-549.4)
BRA Bahia	1728.5 (1475.6-2021.3)	1579.9 (1377.9-1831.7)	1460.7 (1264.9-1683.5)	1331.9 (1147.7-1552.5)	1198.1 (1019.3-1382.4)	1067.3 (938-1207.6)	916 (861.7-971.2)	872.1 (828.9-922)	756.4 (716.1-799.1)	715.9 (676.3-757.2)	659.1 (621.3-702.8)	597 (561.9-636.2)	533.9 (501.2-567.5)	478.1 (462.1-494.9)
BRA Ceará	1934.4 (1644.4-2230)	1778.9 (1544-2042.1)	1637.2 (1416-1873.9)	1497.8 (1287.3-1707.6)	1351.6 (1163.8-1565)	1195.7 (1037.5-1372.7)	1024.5 (902.4-1163.5)	869.4 (802.6-934.7)	695.6 (660.9-729.4)	665.3 (634.1-699.8)	615.7 (584.1-649.6)	571.5 (540.4-602.9)	493.2 (482.6-504.6)	480.6 (463.4-497.6)
BRA Distrito Federal	1887.3 (1603.1-2197.4)	1668.9 (1453.4-1905.6)	1490.3 (1288.1-1718.3)	1285 (1092.1-1514.2)	1153.8 (977.1-1354.7)	1102.2 (980.5-1233.6)	1025.4 (1008-1043.3)	971 (956.3-985.1)	917.3 (903.5-930.3)	926.3 (914-939.9)	841.1 (830.1-852.5)	784.4 (773.4-795.3)	684.5 (674.8-693.8)	576.2 (552.5-600.4)
BRA Espírito Santo	1600.9 (1345.4-1866.7)	1511.1 (1296.8-1779.5)	1459 (1235.4-1709.3)	1396.3 (1169-1625.2)	1332.6 (1132.2-1545.2)	1201.1 (1080.4-1340.8)	1031 (1015.9-1046.7)	1104.3 (1091.6-1117.9)	957.1 (945.2-969.7)	842.5 (832-853.5)	678.2 (669.3-686.7)	612.2 (603-621.8)	566.8 (559.2-574.4)	493.5 (473.3-513.4)
BRA Goiás	2063.7 (1753.1-2411.3)	1956.4 (1670.4-2280.5)	1855.8 (1576.9-2174.2)	1751 (1474.8-2030.7)	1608.9 (1344.7-1881.5)	1373.7 (1180.2-1598.7)	1208.6 (1116.7-1313.4)	1275.1 (1260.6-1290)	1064.7 (1044.4-1085.9)	1057.3 (1046.6-1068.6)	773.8 (765.9-781.6)	650.9 (643.8-658.2)	607.2 (600.8-613.3)	541 (522.5-559.6)
BRA Maranhão	2031.7 (1756.1-2333.1)	1790.1 (1571.1-2053)	1638.3 (1428.7-1879.5)	1505.8 (1309.5-1743.1)	1372.3 (1194.9-1582.3)	1185.9 (1022.1-1385.1)	1012.8 (888.9-1151)	864.3 (810-926.1)	708.5 (670.1-746.5)	660.1 (625.9-699.8)	599 (567.6-636.8)	576.3 (544.7-611.4)	494.1 (466-523.8)	452 (434-471.5)
BRA Mato Grosso	1763.5 (1487.5-2056.5)	1652.8 (1434.2-1912.2)	1517.9 (1300-1749.5)	1352.9 (1154.8-1559.1)	1152.9 (980.1-1352.5)	947.7 (828-1088.4)	782.1 (722.9-840.5)	824.2 (772.5-878.6)	713.4 (666.6-767.4)	687.6 (670.9-702.7)	675.6 (665.5-685.5)	633.4 (624.3-642.1)	594.4 (585.8-603.1)	526.6 (505.5-548.7)
BRA Mato Grosso do Sul	1365.6 (1092.8-1626)	1319.5 (1065.5-1586)	1275.2 (1037.4-1515.4)	1207 (990.3-1442.3)	1155.2 (950.8-1378)	1076.4 (935.3-1222)	993.7 (971.3-1014.8)	917 (902.1-932.5)	863 (850.6-876.5)	835.2 (823.1-849.1)	711.9 (701.5-722.9)	677.2 (667.6-687)	618.2 (609-627.9)	571.8 (549.6-594.3)
BRA Minas Gerais	1768.8 (1496.9-2074.1)	1677.8 (1410.1-1980.1)	1598.5 (1340.3-1886.1)	1512.3 (1275.5-1768.9)	1402.4 (1190-1641.9)	1254.7 (1137.7-1373.5)	1158.6 (1146.1-1173.9)	1081.5 (1069.2-1094.9)	895.1 (885.5-905.3)	786.8 (778.2-796)	649.9 (641.3-659.3)	583.5 (575.1-592.5)	551 (543.6-559.7)	507.7 (491.7-524)
BRA Pará	1444.8 (1222.7-1687.1)	1345.3 (1156.3-1573.7)	1276.5 (1079.7-1483.7)	1219.1 (1023.2-1422)	1159.3 (985.7-1365.9)	1045.5 (930.4-1170.4)	903.7 (878.7-929.4)	865.6 (827.6-901.1)	778.2 (744-812.1)	693.3 (663.2-723.9)	613.7 (586.3-640.8)	563.5 (538-588.2)	539.3 (531.5-548.5)	499.8 (482.4-517.1)
BRA Paraíba	1947.2 (1660.2-2252)	1802.2 (1566.3-2059.9)	1656.2 (1424.4-1901.7)	1478.9 (1258.1-1698.8)	1309.9 (1120.2-1514.5)	1126.7 (1017.2-1256.6)	998.1 (971.7-1026.4)	878.1 (858-900.2)	778.5 (747.2-807.6)	725.5 (697.4-755)	623.9 (597.8-651.5)	593.4 (567.7-618)	573.6 (550.3-599.1)	538.4 (494.2-585.1)
BRA Paraná	2061.2 (1737.8-2419.5)	1884.1 (1602.4-2171.9)	1731.8 (1480.6-2033.6)	1578.6 (1324-1853.9)	1402.7 (1167.8-1640.8)	1231.5 (1106-1360.8)	1252.7 (1240.3-1265.5)	1152.9 (1142.8-1163.5)	1025.4 (1015.7-1034.3)	930.8 (922.4-939.4)	791.7 (784.6-798.2)	684.4 (677.5-691.9)	635.6 (629.1-642.8)	594.6 (576.1-613.4)
BRA Pernambuco	2404.3 (2084.1-2759.6)	2207 (1928.9-2517.1)	2038.5 (1761.2-2333.3)	1868.1 (1605.6-2154.2)	1689.4 (1468.3-1951.2)	1439.8 (1320-1575.3)	1205.6 (1182.1-1229.8)	1145.6 (1126.1-1168.5)	1027 (1010.5-1045)	866.4 (853.7-879.2)	758.9 (746.2-771.8)	666.5 (655.1-676.6)	607.4 (597.3-619.3)	631.7 (611.7-652.5)
BRA Piauí	1612.3 (1364.7-1886.6)	1512.5 (1293.5-1728.9)	1429.9 (1227.5-1650.7)	1348.9 (1153.1-1580.5)	1258.6 (1061.8-1462.8)	1102.5 (936-1275.8)	930.6 (843.4-1025.8)	910.9 (871-954.7)	645.5 (615.8-676.9)	557.9 (529.3-588.6)	589.6 (556.8-620)	573.3 (545.5-602.5)	494.8 (482.3-508.1)	467.9 (448.3-487.7)
BRA Rio de Janeiro	1675.1 (1407.3-1971.9)	1523.8 (1289.4-1808.1)	1408.6 (1173.7-1670.1)	1295.5 (1087.5-1530.5)	1219.5 (1032.8-1433.4)	1111.8 (1005-1221.9)	1026.8 (1013.9-1039)	1019.7 (1009.3-1029.9)	991.7 (983.7-1000)	932.4 (925.6-939.4)	759 (753.7-764.1)	681.8 (677.1-686.6)	659.5 (654.5-664.6)	631.2 (614.2-649.1)
BRA Rio Grande do Norte	1876.5 (1606.9-2190.1)	1732.6 (1503.8-1958.9)	1591.4 (1383-1815)	1424.3 (1232.4-1657.8)	1274 (1099.8-1468.6)	1091.8 (955.2-1235.7)	888.9 (835.4-953.2)	774.7 (736-814.4)	677.3 (640.4-714.2)	682.1 (645.1-722.5)	569 (536.8-605.3)	503.5 (478.1-529.8)	465.2 (454.4-476.8)	480.1 (461.1-499.7)
BRA Rio Grande do Sul	1533.7 (1284.3-1806.6)	1429.6 (1209.7-1688.4)	1350.8 (1136.7-1582.9)	1271.6 (1067.2-1506)	1159.6 (985.9-1360.1)	1027.1 (931-1141.7)	1005 (994.8-1015.7)	917.1 (909.4-924.8)	862.4 (855-869.8)	841 (834.6-847.8)	706.6 (700.7-712.2)	630.4 (625.2-635.5)	591.5 (586.4-597.1)	569.1 (552-586.4)
BRA Rondônia	1648.1 (1380.9-1935.8)	1601.5 (1331-1890.8)	1538.7 (1285.2-1821.4)	1540.9 (1290.6-1809.3)	1520.7 (1310.3-1767.8)	1386.4 (1221.1-1569.4)	1216 (1120-1309)	1078.8 (1015.4-1142)	898.5 (845.7-954.6)	750 (705.1-796.2)	723.2 (678.2-769.6)	730.8 (684.3-777.1)	630.9 (589.6-674.1)	578 (518.2-644.2)
BRA Roraima	1724.6 (1461.2-2016.1)	1654.3 (1405.5-1926.9)	1589.6 (1364.5-1838)	1530.3 (1320.4-1767.4)	1467.1 (1264.8-1684.6)	1344.4 (1181.4-1515.7)	1204 (1084.7-1326.7)	1216.4 (1112.6-1323.1)	1069 (979.6-1155.9)	1027.6 (946.1-1111)	1097 (1019-1181.8)	1042 (957.6-1135.6)	775.7 (708.7-846.7)	680.1 (602.1-765.6)
BRA Santa Catarina	1407.5 (1165.2-1675.4)	1336.2 (1108.4-1571.4)	1299.5 (1083.8-1549.7)	1238.8 (1030.2-1493.3)	1154.3 (963.7-1373.3)	1024.6 (918-1140.5)	985 (968.6-1002)	959.9 (947.4-973.1)	905.7 (894.9-916.6)	859.8 (849.8-870.1)	722.5 (713.1-732)	615.8 (607.7-623.4)	575 (568-582.4)	544.5 (526.4-562.9)
BRA São Paulo	1507 (1264.3-1788)	1424.6 (1178.6-1692.1)	1352.8 (1132.5-1614.8)	1266.8 (1068.6-1496.7)	1168.3 (984.1-1362.4)	1064.5 (971.4-1164.7)	1055.5 (1046.4-1064.4)	984.2 (978.5-990.3)	929.3 (922.8-936.5)	864.6 (859.3-870.9)	734.2 (730.1-738.2)	643.6 (640.1-647.1)	603.6 (599.6-607.6)	563.9 (550-578.2)
BRA Sergipe	1678.2 (1388.6-1991.9)	1586.8 (1355-1842.7)	1512.3 (1292.4-1751.2)	1414.4 (1213.7-1635.1)	1287.2 (1109.3-1468.1)	1131 (1023.4-1240.6)	1075 (1042.2-1112)	914.4 (891.1-938.4)	820.7 (800.7-839.5)	826 (811.5-841.5)	699.7 (685.5-716.5)	601.7 (589.4-614.9)	559.4 (548.9-570.9)	533.2 (508.7-557.5)
BRA Tocantins	1992.1 (1670.6-2351.5)	1894.8 (1628.3-2177)	1797.3 (1545.7-2047.8)	1720.5 (1474.3-1972.3)	1559.8 (1346.6-1800.9)	1349.2 (1159.9-1556)	1134.7 (994.8-1294.6)	1018.2 (917-1132.9)	971.2 (918.8-1024.9)	912.6 (884.4-941.7)	814.4 (789.6-840.7)	647.1 (625.8-670.2)	540.3 (522.9-558.2)	518.6 (488.2-550.2)
PRY Paraguay	1202.8 (1070.4-1331.9)	938.1 (879.2-1000.1)	955.7 (901.1-1014.3)	869.9 (821.8-919.9)	901.6 (851.3-953.2)	873 (823.6-923)	836 (787.9-886.1)	800.1 (752.1-849.7)	675.4 (634.6-719.8)	672.9 (629.8-715.9)	629.9 (591.1-671.8)	634.9 (593.7-677.8)	620.7 (578.7-660.5)	562.9 (457.5-675.4)
North Africa and Middle East	2278 (2196.3-2364.4)	1968 (1908.8-2028.3)	1795 (1742.6-1853.8)	1607.3 (1559-1658.6)	1499.7 (1455.3-1545.9)	1341.2 (1304.4-1376.4)	1225.5 (1200.3-1250.2)	1161.4 (1140.4-1182.6)	1038.4 (1017-1060.1)	959.5 (938.6-981.3)	854.5 (835-874.7)	772.9 (756-790.9)	711.3 (694.2-728.5)	628.7 (605.1-652.2)
North Africa and Middle East	2278 (2196.3-2364.4)	1968 (1908.8-2028.3)	1795 (1742.6-1853.8)	1607.3 (1559-1658.6)	1499.7 (1455.3-1545.9)	1341.2 (1304.4-1376.4)	1225.5 (1200.3-1250.2)	1161.4 (1140.4-1182.6)	1038.4 (1017-1060.1)	959.5 (938.6-981.3)	854.5 (835-874.7)	772.9 (756-790.9)	711.3 (694.2-728.5)	628.7 (605.1-652.2)
AFG Afghanistan	3588.1 (3178.1-4009)	3285.6 (2924.4-3658.3)	3113.5 (2784.8-3450.3)	2982.1 (2671-3272.5)	2872.2 (2599.1-3137.9)	2777.4 (2539.4-3024.4)	2963.5 (2738.7-3206.4)	2903 (2672.2-3150.7)	2323 (2076-2606.5)	2168.1 (1890-2444.5)	2102.7 (1847.7-2384.7)	1908.6 (1658.4-2159.9)	1739.8 (1495.6-1976.9)	1551 (1319-1776.8)
DZA Algeria	1581.8 (1507.7-1656.4)	1440.5 (1392-1485.9)	1377 (1306.3-1455.7)	1242.1 (1186.1-1300.8)	1364.8 (1278.3-1452.7)	1433.7 (1345.2-1523.6)	1384 (1332.8-1437.5)	971.9 (940.2-1006.4)	822.1 (772.1-878.7)	753.9 (703.6-803.3)	714.3 (669.9-761.8)	693.2 (643-745)	660.3 (632.1-690.8)	583 (552.5-615.3)
BHR Bahrain	2135.2 (1829.3-2472.9)	1965 (1694.9-2255.7)	1695.1 (1464.2-1955.3)	1510.3 (1308.7-1734.7)	1340.7 (1177-1521.8)	1173 (1063-1302.3)	1061 (995.3-1130.7)	1048.9 (1004.2-1095.8)	1096 (1049.8-1143.8)	1140.2 (1086.7-1191.2)	960.8 (920.7-1001.4)	766.5 (734.9-799.8)	599.5 (572.3-628.6)	518.3 (471.8-569.4)
EGY Egypt	2542 (2299-2848.1)	2015.9 (1913.9-2123.8)	1834.8 (1764.9-1914.6)	1623.1 (1563.8-1677.2)	1701.5 (1654.6-1750.3)	1509.9 (1475-1548.1)	1384.2 (1356.3-1413.3)	1441.9 (1420.4-1462.5)	1169.8 (1154.3-1186.9)	1005.5 (990-1021.7)	886.6 (879.3-893.1)	906.7 (898-921.9)	893.1 (886.7-899.3)	848.5 (749.6-961.3)
IRN Iran	2376.9 (2292-2467.4)	2075.1 (2011.4-2136.8)	1840.3 (1780.9-1894.6)	1631.2 (1579.4-1684.4)	1344.4 (1299.7-1390.7)	1022.8 (984.6-1062.3)	846 (814.9-878.4)	863 (829.9-895.8)	888.9 (857.3-919.8)	754.7 (733.3-776)	674.4 (664.2-685.4)	643.4 (637.4-649.4)	646.4 (642.6-650)	598.5 (594-602.8)
IRQ Iraq	1784.3 (1597.9-1987.1)	1492.5 (1388.9-1604.2)	1319.6 (1250.4-1391.3)	1282.4 (1220.7-1348.5)	1257.3 (1196.7-1320.5)	1229.9 (1157.7-1300.8)	1170.5 (1081.7-1262.8)	1239.5 (1112.5-1380.9)	1193.1 (1057.2-1353.8)	1250.7 (1118.6-1402.4)	1185.4 (1068.3-1314.6)	978.4 (876.9-1083)	761.7 (693.7-834.1)	519.6 (485.2-555.1)
JOR Jordan	2001.1 (1713.3-2335.9)	1538.6 (1336.3-1754.8)	1231.9 (1085.3-1381.9)	1008 (927.8-1099.9)	881 (835.6-932.7)	790.4 (756.5-825.6)	799.4 (751.9-849.5)	819.5 (746.9-898.1)	880.4 (790.8-987)	973.5 (871.4-1083.4)	975.1 (890.3-1064.3)	884.7 (835.1-933.6)	617.3 (588.5-646.8)	508.8 (450.1-569.3)
KWT Kuwait	1236.7 (1051-1449.9)	1130.3 (975.5-1303.9)	1065.5 (973.8-1164.5)	1022.6 (997.1-1047.2)	823.2 (809.2-838.8)	817.7 (804.8-831.5)	733.2 (721.9-745)	599.6 (589.7-609.1)	603.5 (592.9-614.6)	486 (477.6-494.3)	485.7 (477.6-493.6)	438 (431.2-445.5)	356.5 (351.1-362.1)	247.8 (233.4-262.1)
LBN Lebanon	1576.3 (1324.9-1837.5)	1379.9 (1156-1593.8)	1258.7 (1040.4-1480)	1156.7 (963.3-1354.2)	1054.5 (892.4-1237.3)	1105.3 (963.5-1271.2)	889.7 (758.2-1030.9)	956.3 (837.8-1058.2)	819.7 (719.5-894.3)	750.8 (658.5-816.3)	641.5 (584-715.6)	575.3 (544.7-608)	565.3 (541.5-585.7)	578.7 (540.7-610.3)
LBY Libya	2680.6 (2347.4-3034.1)	2118.3 (1847.9-2395)	1762.8 (1547.5-2009.2)	1372.4 (1221.3-1541.4)	1073.9 (975-1168.4)	931.7 (865.2-995.6)	827.5 (750.1-902.5)	801.1 (705.4-902.8)	813.2 (710.5-923.4)	793.2 (701-893.5)	802.3 (724.6-880.9)	773.2 (707.9-843.5)	808.2 (735-879.9)	776.8 (663.4-894.3)
MAR Morocco	2474.4 (2160.2-2808.8)	2167.5 (1898-2462.1)	2101.7 (1851.2-2395.2)	1788.7 (1565.3-2043.8)	1612.3 (1411.3-1845)	1510.4 (1343.9-1701.1)	1385.6 (1254.2-1515.8)	1251.6 (1159.9-1347.2)	1228.4 (1151.4-1308)	1159.5 (1097.2-1231.1)	1056.3 (992.4-1128)	974.4 (895.6-1058.2)	914.9 (829.2-1044.2)	834.1 (703.2-994.4)
PSE Palestine	2222.8 (1903.1-2536.3)	1883.5 (1622.1-2172.2)	1577.7 (1339.4-1839.1)	1297.9 (1094.5-1510.3)	1273.6 (1102.8-1452.9)	984.4 (829.2-1150.3)	934.4 (787-1088.7)	897.1 (753-1046.7)	906.3 (778.5-1056)	888.4 (794.7-1001.3)	915.5 (865.4-967.2)	924.4 (893.1-955)	851.1 (826.3-878.1)	682.6 (634.1-724.6)
OMN Oman	3926.2 (3470.6-4379.5)	3279.9 (2900.9-3639)	2849 (2501.5-3164.3)	2431.2 (2113.8-2733.9)	1788 (1536.5-2041)	1449.9 (1238.5-1680.3)	1173.3 (977.8-1383.2)	980.6 (817.5-1164.7)	873.2 (735.1-1024.8)	804 (664.4-925.5)	725 (600.8-892.6)	631.6 (580.9-763.3)	614.1 (564.4-662.5)	571.2 (484.1-627.6)
QAT Qatar	1424.3 (1196.9-1694.5)	1348 (1134.4-1585)	1254.5 (1064.4-1486.4)	1188.8 (1013.6-1406.2)	1081.6 (926.6-1259.2)	1037.9 (884.3-1203.7)	1001.5 (853.7-1140.1)	1068.3 (929.8-1205.9)	1124.9 (991.2-1274.5)	1166.9 (1031.5-1330.9)	1088.3 (953-1246.8)	882.6 (776.2-1008.8)	695.6 (599.9-803.3)	535.3 (443-636.6)
SAU Saudi Arabia	1783.8 (1519.9-2077.2)	1703.2 (1468.8-1973.8)	1559.8 (1333.6-1807.1)	1382.3 (1169.5-1623.3)	1212.3 (1018.6-1440.2)	1015.8 (853.8-1216.3)	901.4 (757.8-1060.4)	839 (705-977.4)	794.5 (680.7-913.6)	718.4 (626.1-828.9)	673.5 (607.1-752.9)	674.1 (611.2-745.2)	624.3 (576.5-674.8)	543.8 (505.3-611.9)
SDN Sudan	2160.2 (1861.8-2471.5)	1985.2 (1718.8-2254.6)	1713.6 (1477.6-1966)	1666.8 (1455.1-1897.8)	1695.3 (1505.3-1908.6)	1750.7 (1583.6-1922.7)	1715.6 (1566.3-1869.5)	1886.2 (1745-2031.4)	1487.4 (1337.3-1658.9)	1350.8 (1179.5-1548.7)	1234.5 (1056.5-1443.6)	1114.7 (944.7-1314.1)	1000.3 (841.2-1188.2)	875.1 (723.7-1043.2)
SYR Syria	1515.8 (1300.6-1757.6)	1275.1 (1116.7-1456.1)	1099.9 (963.7-1246)	969.4 (856.2-1099.1)	886.4 (789.6-997.7)	819.7 (746.9-893.4)	835.8 (762.9-912.4)	944.8 (850.9-1022.2)	949 (886-1022.9)	840.3 (793.1-907.3)	689 (652.3-756.4)	548.9 (506.3-573.6)	519.4 (475.4-567.6)	670.7 (608-720.6)
TUN Tunisia	2281.7 (2010.1-2565.5)	2072.7 (1875.9-2280)	1815.3 (1702.8-1946.7)	1711.1 (1606.3-1821.9)	1520 (1465.3-1578.5)	1254 (1206.7-1298)	1077.2 (1034.1-1123.5)	918.4 (888.1-948.1)	867.5 (835.2-900.5)	800.7 (766-838.8)	759.3 (685.8-833.9)	654.3 (556.4-774.8)	602.7 (492.2-731.1)	559.7 (442.7-690)
TUR Turkey	1978.8 (1720.7-2244.1)	1755.2 (1535.8-1983.5)	1612.7 (1420.3-1861.6)	1408.9 (1219.6-1624.6)	1214.8 (1046.4-1393.8)	1066.4 (957.5-1180.6)	954.2 (910.8-1002.2)	853.8 (807.7-905.5)	796.6 (760.4-837.6)	751.8 (715.4-788.9)	599.5 (569-629.9)	494.2 (468.5-519.7)	445.2 (423.1-469.2)	362.1 (329.1-398.3)
ARE United Arab Emirates	1768.1 (1492.6-2058.4)	1716.3 (1463.2-1990.9)	1597.5 (1356.4-1853.2)	1421.4 (1214.1-1662.6)	1240.6 (1046.1-1467.4)	1122.4 (928.1-1327.8)	1031.4 (852.7-1221.1)	974.6 (816.8-1149.2)	969.1 (820-1122.6)	912.7 (793-1042.6)	913.7 (805.8-1024.8)	918.5 (807.1-1045.7)	815.6 (702.4-943.1)	762.5 (626.8-921.7)
YEM Yemen	3691.7 (3121.2-4305.3)	3058.3 (2639.8-3526)	2737.6 (2386.1-3127.3)	2556 (2221.6-2952.9)	2324.1 (2020-2670.9)	1931.1 (1670.5-2233.5)	1678.8 (1436.2-1952)	1549.2 (1320.5-1813.8)	1483.5 (1255.8-1741)	1399.3 (1173.7-1650.5)	1277.1 (1061.5-1519.7)	1127.8 (925.1-1373.9)	1004.3 (818.1-1242)	964.7 (790.3-1196.4)
South Asia	2485.5 (2382.9-2594.5)	2341.8 (2255.4-2439.5)	2308.5 (2217.5-2404.4)	2294.1 (2205.2-2377.4)	2132.4 (2056.5-2202.9)	1932.9 (1871.6-1996.7)	1694.7 (1644.5-1748.5)	1632.2 (1584.5-1678.8)	1497.3 (1458.8-1534.5)	1371.3 (1344.9-1399.6)	1294.6 (1273.6-1318)	1156 (1137.6-1174)	1084.8 (1065.8-1103.7)	1035.4 (1007.6-1064)
South Asia	2485.5 (2382.9-2594.5)	2341.8 (2255.4-2439.5)	2308.5 (2217.5-2404.4)	2294.1 (2205.2-2377.4)	2132.4 (2056.5-2202.9)	1932.9 (1871.6-1996.7)	1694.7 (1644.5-1748.5)	1632.2 (1584.5-1678.8)	1497.3 (1458.8-1534.5)	1371.3 (1344.9-1399.6)	1294.6 (1273.6-1318)	1156 (1137.6-1174)	1084.8 (1065.8-1103.7)	1035.4 (1007.6-1064)
BGD Bangladesh	2435 (2114.4-2758.6)	2255.6 (1961.4-2550.4)	2111.4 (1841-2398.8)	1954.4 (1710.6-2205.6)	2158.3 (1943.8-2386.8)	1724.4 (1547.7-1894.7)	1645.4 (1541.2-1751.8)	1663.7 (1582.9-1746.1)	1418.2 (1336.7-1495.4)	1177.7 (1114.5-1244.5)	1031.4 (978.9-1084.4)	1002.2 (958.8-1044.6)	941.9 (896.3-987.3)	786.3 (699.5-886.3)
BTN Bhutan	3459.2 (3070.9-3896.1)	3150.1 (2779.3-3528.9)	2879 (2530.1-3228.9)	2558.4 (2235.6-2892.6)	2253.6 (1963.2-2602.5)	2066.5 (1798.3-2380.4)	1917.2 (1672.5-2202.9)	1696.1 (1482.8-1941.7)	1461.6 (1286.6-1657.8)	1334.8 (1187.4-1484.8)	1131.2 (1021.3-1245.1)	920.1 (829.2-1024.3)	773 (680.6-880.8)	660.5 (555.5-786.8)
IND India	2513.6 (2399.8-2635.4)	2377.6 (2272.4-2493.5)	2370.9 (2260.4-2479.9)	2402.3 (2299.4-2503.8)	2206 (2118-2288.4)	2035.8 (1961.7-2116)	1759.1 (1697-1825.3)	1680.1 (1618.8-1738.6)	1523.5 (1478-1568.2)	1382 (1350-1415.5)	1305.6 (1281.7-1331.5)	1140.5 (1120.3-1160.8)	1071.3 (1054.5-1089.1)	1040.1 (1014.8-1066)
IND Andhra Pradesh	2432 (1957.1-2949.1)	2342.9 (1886.5-2858.6)	2268.3 (1842.3-2748.2)	2225.7 (1831.6-2679)	2039.3 (1703-2384.8)	1902.1 (1599.7-2205.1)	1678.1 (1423.8-1947.4)	1686.6 (1403.5-2000.7)	1587.6 (1326.8-1876.4)	1441.8 (1209.4-1705.5)	1377.5 (1134.6-1651.2)	1233.6 (1014.1-1497)	1131.6 (927.3-1388.7)	1059.8 (869.1-1301.5)
IND Arunachal Pradesh	2524.2 (2023.3-3087.3)	2392.6 (1936.3-2914.8)	2410.6 (1982.6-2909.3)	2556 (2141.7-3013.8)	2249.5 (1889.9-2593.6)	1995.2 (1714.5-2286.1)	1671.2 (1419.4-1934.4)	1568.6 (1340.3-1816.3)	1430.6 (1221.2-1663.1)	1278.5 (1092.7-1488.7)	1219.2 (1042.7-1435.3)	1045.6 (894.5-1236.3)	921.1 (783.2-1081.2)	863.2 (731-1040.8)
IND Assam	3010.2 (2543.1-3561.5)	2851.5 (2379.7-3376)	2846.2 (2382-3344.2)	2973.5 (2523.1-3478.4)	2712.2 (2312.7-3132.8)	2416.5 (2060.8-2774.4)	2024.5 (1737.2-2325.4)	1904.6 (1654.2-2168.7)	1728.2 (1559.6-1913.9)	1668.6 (1591.5-1755.5)	1664.6 (1591.9-1733)	1449.8 (1384.9-1508.4)	1421.8 (1368.8-1477.8)	1288.3 (1194.9-1384.6)
IND Bihar	2367.2 (1970-2815.1)	2219.4 (1839.8-2649.1)	2243 (1860.1-2650.2)	2308.7 (1960.7-2707.8)	2196.1 (1882.1-2511.4)	2099.7 (1809.1-2410.1)	1899.2 (1647.9-2170.7)	1820.3 (1574.8-2093.7)	1650.8 (1446.2-1875.4)	1533 (1378.3-1702.9)	1466.9 (1361.3-1576.4)	1282.2 (1224.3-1339.1)	1236.5 (1185.3-1288.3)	1115.1 (1031.6-1203.1)
IND Chhattisgarh	2053.7 (1706-2441)	2015.7 (1663.8-2441.9)	2065.4 (1695.9-2497.2)	2207.5 (1848.6-2608.7)	2093.2 (1786.6-2442.9)	1997.2 (1721.9-2293.6)	1757 (1522.2-2007.4)	1730.6 (1494.8-1964.1)	1588.8 (1388-1802.9)	1455.3 (1310.5-1613.3)	1394.3 (1295.8-1485)	1193.4 (1141.9-1245.7)	1113.4 (1067.7-1159.9)	1097.8 (1014-1189.4)
IND Delhi	2375.5 (1983.5-2831.3)	2192 (1823-2591.9)	2079.2 (1751-2440.4)	2227.2 (1911.6-2590.3)	1805.3 (1530.7-2110.3)	1615.6 (1384-1886.2)	1388.2 (1181.1-1623.5)	1356.3 (1146.8-1600)	1267.9 (1069-1492.8)	1181.2 (1006.1-1358.5)	1138.2 (1003.1-1269.6)	985.3 (899.8-1066.8)	876.4 (812.5-939.6)	797.3 (703.5-893.9)
IND Goa	1891.4 (1559.2-2289.2)	1777.4 (1471.3-2146.4)	1693.6 (1413.3-2053.5)	1669.9 (1401-1996.9)	1542.9 (1314.6-1811.1)	1402.4 (1221.7-1616.3)	1161.2 (1010.9-1336.7)	1123.1 (983.9-1281.7)	1035.4 (917.6-1170.5)	927.9 (816.1-1040.7)	861.2 (748.4-968)	738 (628.8-856.6)	652.3 (554.4-775.2)	618.5 (526-750.5)
IND Gujarat	2239.7 (1876.1-2634.9)	2124.3 (1785.7-2495.1)	2117.7 (1775.4-2499.3)	2177 (1838.9-2533.1)	1991.1 (1717.9-2305.2)	1836.8 (1593.2-2099.9)	1584.6 (1382-1814.3)	1495.3 (1295.6-1714.7)	1353.8 (1219-1509.2)	1135.9 (1066.9-1205.6)	1134.8 (1081-1191.4)	1005.3 (957.4-1060.2)	963 (915.9-1012.8)	894.4 (820.5-978.8)
IND Haryana	2087.7 (1743.7-2506.8)	2033.9 (1680.5-2446.9)	2049.1 (1700.8-2423.8)	2138.4 (1791.6-2514.9)	1984.6 (1662.4-2318.6)	1786.6 (1514.3-2074)	1495.8 (1277.4-1744.9)	1426 (1234.2-1629.8)	1298.3 (1170.1-1442.8)	1185.2 (1122.3-1251.9)	1124.4 (1073.3-1181.1)	911.3 (870.3-952.9)	932.6 (892.4-974.9)	945.1 (869.6-1020.3)
IND Himachal Pradesh	2203.2 (1806-2634.6)	2115.2 (1717-2569.3)	2091.5 (1705.2-2548.7)	2107.4 (1719.9-2589.9)	1967.6 (1641.9-2342.3)	1813.2 (1521.7-2147.3)	1547.5 (1300.1-1823.6)	1489.6 (1247.2-1748.9)	1323.8 (1160.2-1509)	1165.5 (1078.8-1260.6)	961 (903.1-1021.9)	838.2 (789.1-886.7)	765 (722-810.1)	644.7 (583-714)
IND Jammu and Kashmir	1894.7 (1544-2296.2)	1773.4 (1434.8-2152.9)	1726 (1392.6-2104.9)	1810.2 (1489.4-2156.4)	1627.2 (1363-1936.1)	1552.6 (1302.4-1816.1)	1368.6 (1143.3-1608.3)	1292.5 (1086.4-1525.8)	1191.1 (1014.1-1391.6)	1072.7 (935.2-1217)	992.2 (899.1-1098.2)	881.2 (828-939.7)	783.2 (742.5-828.7)	746.7 (680.2-814.9)
IND Jharkhand	2373 (1970.9-2813.1)	2242.8 (1864-2663.4)	2268.5 (1899.2-2665.5)	2344 (1979.3-2755.7)	2198.5 (1884.2-2546.4)	2063.5 (1778.3-2361.3)	1854 (1590.5-2124.2)	1892.9 (1622-2162.1)	1781.4 (1536.1-2039.5)	1668.3 (1486-1867.7)	1616.2 (1498.9-1741.5)	1419.9 (1360.3-1482.6)	1268.7 (1220-1317.5)	1178.7 (1094.8-1267.4)
IND Karnataka	2340.2 (1970.9-2743.5)	2240.9 (1887.7-2658.4)	2224.9 (1846.4-2637.2)	2245 (1889.2-2648.8)	2058.4 (1769.5-2384.8)	1847.3 (1572-2115)	1518.5 (1290.7-1759.6)	1404.5 (1222.2-1606.4)	1255.7 (1139.1-1376.9)	1169.8 (1114.4-1227.7)	1075.2 (1031.5-1118.1)	943.7 (901.5-983.2)	996.9 (958.1-1039.7)	1137.7 (1057.5-1221.9)
IND Kerala	1876.6 (1575.9-2242.7)	1725.8 (1423.5-2062.8)	1658.8 (1355.6-1981.7)	1645.2 (1356.6-1948.2)	1490 (1240.9-1745.4)	1369.8 (1153.8-1623.6)	1165.8 (977.4-1379.3)	1112.7 (938.9-1306.6)	1002 (881-1140.9)	893.2 (825.4-968.9)	884.3 (828.6-942)	763.6 (714.9-814.4)	707.6 (665.5-751.5)	715.3 (649.4-780.6)
IND Madhya Pradesh	2744.2 (2322.4-3196.7)	2480.2 (2077.3-2912.8)	2416.5 (2048.5-2823.8)	2354.9 (2015-2714.1)	2048.5 (1779.1-2345)	1908 (1663.1-2157.8)	1816.6 (1577-2068.5)	1913 (1666.1-2194.8)	1847.3 (1614.6-2103.2)	1730.7 (1547.4-1921.2)	1654.8 (1535.2-1799)	1386.8 (1319.1-1463)	1242 (1189.5-1299.7)	1301 (1196.8-1408.3)
IND Maharashtra	2208.4 (1860.9-2591.7)	2068 (1734.4-2445.4)	2030.7 (1715.3-2392.9)	2038.1 (1725.8-2371.1)	1856.7 (1589-2138.7)	1713.9 (1481.7-1963.2)	1473.7 (1283.3-1669.6)	1399.8 (1221.8-1588)	1268.7 (1149.4-1400.1)	1151.9 (1098.5-1205.1)	1168.5 (1119.7-1216.9)	936.2 (896.1-977.7)	855.9 (821.1-890.2)	929.4 (863.5-996.8)
IND Manipur	2150.3 (1755.3-2622.2)	2047.1 (1668.9-2532.3)	2032 (1672.9-2487.5)	2095.9 (1750.7-2513.1)	1896.4 (1607.4-2217.6)	1709.3 (1483.3-1965.2)	1443.5 (1250.1-1669.7)	1390.5 (1209.9-1594)	1253 (1094.2-1434.5)	1148.3 (1005.1-1327.3)	1108.3 (977.9-1289.5)	981.6 (853.4-1142.7)	913.7 (789.1-1067.5)	887.2 (756.5-1055.7)
IND Meghalaya	1935.6 (1581.9-2377.9)	1858.3 (1500.7-2311.1)	1838.3 (1490.6-2261.7)	1963.2 (1631.8-2368.8)	1756.3 (1481.1-2070.4)	1690.5 (1463.8-1953.6)	1478.6 (1275.8-1717.9)	1446.4 (1247.5-1670.1)	1317.9 (1137.6-1534.5)	1192.4 (1026.5-1387)	1132.8 (979.2-1313.9)	998.3 (870-1157)	912.3 (786.2-1064)	853.6 (726.4-1004.8)
IND Mizoram	1833.3 (1486.9-2232.3)	1781.6 (1436.6-2183.2)	1736.6 (1414.2-2126.7)	1880.9 (1564.9-2245.9)	1659.8 (1387.5-1955.2)	1534.6 (1319.8-1783.7)	1319.7 (1146.4-1526.4)	1264.9 (1101.1-1442.7)	1123.3 (985.9-1275.6)	1022.2 (905.7-1152.2)	985.8 (871-1105.3)	890.7 (761.4-1017.7)	838.8 (712.6-966.6)	808.3 (682-951.4)
IND Nagaland	2153.9 (1716.6-2629.7)	2084 (1677.9-2586)	2066.3 (1679.7-2538.9)	2184.1 (1810.6-2632.7)	1870.1 (1567.9-2210.7)	1685.4 (1430.7-1966.6)	1414.2 (1192.6-1655.3)	1326.1 (1128.5-1558.4)	1211.7 (1029-1422.9)	1094.9 (938.2-1283.9)	1063.7 (906.6-1256)	937.3 (775.3-1125.9)	846.9 (687.1-1011.3)	813 (666-975.5)
IND Odisha	2882.4 (2431.2-3400.7)	2722 (2288-3209.9)	2748.2 (2275.2-3212.1)	2782.5 (2349.2-3221.4)	2573.7 (2234.7-2939.2)	2335.2 (2009.3-2701.8)	1950.9 (1680.9-2249.8)	1782.6 (1542.1-2045.7)	1606.4 (1453.2-1761.7)	1530.8 (1464-1595.7)	1587 (1526.8-1649.6)	1364.1 (1314.5-1413.4)	1259.1 (1215.1-1304.6)	1024.6 (950.1-1097.1)
IND Punjab	1992.4 (1667.3-2399.6)	1919.5 (1582.3-2322.5)	1877.1 (1560.6-2272.8)	1879.5 (1562.6-2282.1)	1718.6 (1463.8-2047.2)	1571.9 (1348-1815)	1336.6 (1143.9-1536)	1254.9 (1083.7-1431.6)	1133.4 (1021.5-1259.7)	1012.6 (955.6-1071.6)	1017.8 (971-1066.9)	902.1 (862.3-942)	864.9 (828.9-900.8)	786.8 (721.9-851.5)
IND Rajasthan	2322.5 (1955-2739.7)	2226.5 (1863.3-2619.9)	2244.4 (1886.6-2676.5)	2330.8 (1970.4-2725.7)	2119.8 (1810.1-2452.5)	1962.2 (1680.1-2258.9)	1679.2 (1454-1952)	1575.4 (1372.5-1812.6)	1410.7 (1265.8-1569.8)	1280.2 (1203.4-1360.9)	1134.8 (1076.9-1193.8)	1041.1 (993.4-1088.4)	947.8 (902.7-993.6)	979.3 (896.5-1065.4)
IND Sikkim	2131.5 (1707.1-2641.1)	2076.5 (1663-2587.7)	2059.8 (1668.2-2563.3)	2146.7 (1773.2-2622.1)	1978.7 (1668.2-2340)	1826.8 (1571.8-2122.3)	1549.6 (1317.6-1804.4)	1457.5 (1236.4-1703.5)	1302.2 (1105.3-1538.6)	1179 (998.3-1397.1)	1103.4 (947.6-1301.5)	959.5 (824.6-1117.7)	835.6 (702.9-978)	758.3 (629.5-889.6)

Table 1 Age-standardised mortality rates by location and year for females for 1950, 1955...2005, 2010, 2017

Location	1950	1955	1960	1965	1970	1975	1980	1985	1990	1995	2000	2005	2010	2017
IND Tamil Nadu	2747.1 (2331.7-3192.7)	2600 (2219.6-3041.5)	2566 (2189.5-2983.7)	2595.5 (2231-3026.2)	2421.4 (2122.3-2780.9)	2198.9 (1927.2-2498.1)	1840 (1583.9-2099.9)	1709.7 (1484.1-1938.5)	1494.8 (1359.5-1641.6)	1312.3 (1255.3-1373.5)	1299.8 (1246.4-1354.2)	1093.4 (1047.4-1139.7)	1043.9 (1001-1088.7)	988.4 (913.4-1071)
IND Telangana	2150.2 (1740.4-2669.7)	2117.8 (1692.4-2633.5)	2118.2 (1716.6-2614)	2229.1 (1792.7-2711)	2189.3 (1790.7-2648.5)	2018.6 (1699.8-2405.3)	1669.6 (1398.1-1986.9)	1609.2 (1345.1-1914.1)	1515.1 (1265.2-1780.2)	1386.6 (1146.3-1648.7)	1358.9 (1121.1-1640.8)	1211.4 (997.4-1459.9)	1038 (854.8-1253.5)	841.4 (697.3-1011.5)
IND Tripura	2469.6 (2009.2-2969)	2346.3 (1923.1-2819.7)	2353.7 (1949.5-2808.3)	2399.6 (2026.3-2801.6)	2128.2 (1818.2-2440.8)	1899.9 (1654.4-2157.5)	1631.1 (1406.3-1865.2)	1514.4 (1309.6-1732.5)	1373.1 (1186.3-1581.4)	1252.5 (1079.4-1450.7)	1157.7 (999.1-1347.7)	998.7 (866.5-1158.9)	929.3 (805.9-1077.6)	900.5 (784.7-1056)
IND Uttar Pradesh	3181.3 (2731.8-3617.8)	2994.6 (2597.7-3413)	3049.2 (2654.9-3454.9)	3085.6 (2699.9-3480.1)	2802.2 (2482-3121.3)	2593.1 (2338.2-2879.1)	2252.5 (2014.1-2498)	2121.2 (1896.9-2349.9)	1889.9 (1709.6-2080.1)	1670.1 (1531.5-1812.8)	1449.5 (1365-1536.4)	1349.2 (1286.6-1413.5)	1283.4 (1225.2-1342.1)	1303.9 (1206.3-1406.1)
IND Uttarakhand	1909.5 (1555-2296.3)	1906.9 (1565.1-2295.8)	1919.1 (1573-2320.3)	2006.9 (1674.8-2385)	1884.8 (1580-2190.5)	1776.3 (1511.7-2048.8)	1561.9 (1332.4-1817.1)	1579.7 (1344.6-1841.2)	1485.7 (1269.9-1754.3)	1362.4 (1166.8-1589.8)	1313.9 (1146.8-1518)	1160.9 (1037.4-1295.7)	1028.9 (939.8-1125.3)	931.5 (860.6-1010.3)
IND West Bengal	2564.9 (2163.6-2987.6)	2426.7 (2056.2-2866.1)	2417.3 (2023.7-2859)	2453.5 (2067.7-2875.7)	2259.6 (1928.5-2609.4)	2069.5 (1801.6-2356)	1750.3 (1523.5-1991)	1697.7 (1477-1937.5)	1542.5 (1380.2-1729.6)	1417.9 (1315-1525.2)	1228.8 (1159.1-1305.5)	1076.8 (1015.3-1144.1)	1029.1 (967.2-1092.9)	977.2 (888.4-1085.4)
IND Union Territories other than Delhi	1594 (1334.8-1922.1)	1523.4 (1273.4-1835.3)	1509.5 (1260.9-1841.9)	1595.3 (1362.8-1892.5)	1425.2 (1220.7-1668.4)	1288.7 (1132.8-1470.5)	1094.8 (971.4-1244.7)	1092.2 (975.7-1238)	1031 (924.4-1158.3)	959.5 (851.6-1068.1)	915.9 (806.4-1020.9)	817.4 (699.6-922.3)	751.6 (630.4-869.2)	741.6 (633.4-862.3)
NPL Nepal	2678.4 (2369.4-3017.2)	2509.3 (2203.7-2804.5)	2367.5 (2073.2-2654.1)	2211.4 (1915.2-2486.8)	2061 (1799.9-2341)	1968.7 (1716.8-2236.2)	1898.5 (1668.1-2154.7)	1769.1 (1561.9-2007.4)	1579.1 (1413-1767.6)	1349.5 (1234.8-1480)	1095.9 (1011.9-1186.8)	966.1 (889.3-1050.5)	910.4 (817.5-1009.2)	842.4 (736.5-960.5)
PAK Pakistan	2217.2 (1936.7-2520.4)	2125.9 (1874.6-2399.1)	2028.3 (1793.6-2283.8)	1893.9 (1711.5-2085.6)	1722.7 (1588.1-1866.9)	1532.3 (1440.8-1631.7)	1374.8 (1296.5-1451.1)	1328.6 (1261.9-1393.6)	1394.5 (1332.3-1457.9)	1489 (1415.2-1571.3)	1486.6 (1401.7-1569.5)	1478.5 (1393.2-1571)	1376.7 (1222.6-1528.6)	1273.7 (1068.3-1499.3)
Southeast Asia, East Asia, and Oceania	2067.8 (1996.2-2143.1)	1762.2 (1694.4-1828.6)	3141.7 (3074.6-3207.8)	1529.5 (1471.7-1590.7)	1403.9 (1355-1455.4)	1309.3 (1270.3-1353)	1191.2 (1157.2-1227.8)	1121.2 (1093.6-1147.8)	1032.2 (1012.9-1050.8)	941.6 (927.7-956.3)	849.3 (835.9-862.8)	757.8 (746.3-769)	676.2 (664.5-687.5)	608.8 (590.4-627.9)
East Asia	1998.5 (1903.5-2091.3)	1701.3 (1614.4-1786.3)	3661 (3580.7-3741.9)	1495 (1420.9-1568.9)	1354.1 (1295.6-1412.8)	1254.6 (1205.5-1308.9)	1149.3 (1104.4-1195.1)	1098.2 (1064-1128.4)	1009.1 (987-1030.4)	914.3 (897.6-932)	809.4 (794.6-824.9)	708.9 (696.7-721.2)	625.5 (612.4-638.5)	562.8 (541-585.5)
CHN China	1974.8 (1875.9-2072.5)	1708.2 (1618.9-1795.5)	3776.3 (3693.9-3859.8)	1507.3 (1431.3-1583.4)	1365.8 (1305.3-1426.9)	1267.4 (1216.3-1323.6)	1161.3 (1114.4-1208.2)	1109.9 (1075.5-1140.7)	1019.2 (995.8-1041)	911.2 (894.6-929.4)	802.1 (786.9-818)	709.2 (696.8-721.7)	625.2 (611.9-638.6)	562.1 (539.1-585.7)
PRK North Korea	3399 (3112.3-3733.2)	1548.1 (1287.1-1847)	1361.9 (1121.8-1641.6)	1202.5 (979.3-1458.2)	1049 (853.5-1257.6)	920.7 (758-1081.2)	861 (715.6-1003.3)	836 (690.7-974.8)	811.4 (671.9-945)	1231.8 (1098.1-1355)	1363.7 (1235.7-1481.3)	902.9 (788.4-1009.9)	846.6 (737-945.8)	791.1 (659.5-942.4)
TWN Taiwan (Province of China)	1694.4 (1449.9-1954.9)	1414.5 (1405.1-1424.8)	1256.2 (1247.5-1265.4)	1060.4 (1052.5-1068.6)	986.4 (979.4-993.9)	906.7 (900.3-913.5)	820 (813.3-826)	744 (738-749.9)	687.5 (682-692.8)	629.7 (624.7-634.7)	553.5 (549.2-557.8)	495.5 (491.4-499.6)	428 (424.2-431.7)	406.9 (381.7-433.9)
Oceania	2978.5 (2704.7-3232)	2666.9 (2424-2888.4)	2419.4 (2196.9-2618.3)	2178.5 (1995.6-2344.5)	1993.7 (1839.6-2133.6)	1847.7 (1707.6-1976.1)	1745.2 (1621.4-1873.8)	1707.2 (1573.1-1842.5)	1664.3 (1516.5-1811.2)	1628.3 (1485.1-1777.4)	1623.5 (1478.2-1779)	1628.4 (1483.1-1783.8)	1595.7 (1442.6-1772)	1478.7 (1330.1-1646.8)
ASM American Samoa	1309.6 (1194.7-1425.8)	1207.1 (1132.8-1285.9)	1112.5 (1057.5-1175.9)	1073.8 (1026.4-1129.3)	1013.8 (963.8-1081.5)	935.3 (885.8-1009.3)	918.8 (828.5-990.4)	900.7 (834.4-952.2)	838.2 (767-899.5)	852.3 (791-908.4)	872 (802-931.2)	907.3 (852.5-955.7)	908.1 (871.1-948.1)	900.5 (837.3-955.5)
FSM Federated States of Micronesia	2357.3 (2040.9-2696.6)	2071.2 (1763.6-2384.2)	1984.6 (1680.4-2276.8)	1872.2 (1587.4-2147.4)	1751.3 (1488.6-2025.2)	1630.7 (1365.1-1894.3)	1526.2 (1285.6-1752.7)	1460.3 (1244.2-1663.7)	1410.9 (1230-1583.3)	1359.4 (1203.3-1502.8)	1315.4 (1193.6-1436.9)	1237.5 (1125.8-1347.8)	1211.3 (1093.6-1351.8)	1174.4 (1041-1366.7)
FJI Fiji	1994.5 (1855.6-2128.2)	1592.9 (1511.3-1680.3)	1396.1 (1316.1-1467.5)	1258.9 (1193.4-1328.4)	1301 (1239-1369.2)	1217.9 (1150.4-1290)	1138.2 (1073.3-1203.3)	1090.4 (1028.7-1153.4)	1099 (983.7-1217.6)	1187.8 (1139.8-1235.6)	1280.1 (1261.9-1300.7)	1162.8 (1143.3-1182.1)	1166.5 (1142.6-1192.8)	1079.3 (918.2-1249.8)
GUM Guam	1377.8 (1276.2-1481.2)	1243.8 (1181.7-1310.1)	1084.6 (1037-1133.8)	940.5 (905.9-975.2)	977.4 (945.8-1010.9)	824.5 (797.4-852.1)	675.5 (650.2-698.2)	624.1 (601.8-648.1)	671.4 (641.7-702.9)	683 (654.6-717.6)	632.4 (608.5-660.2)	590.6 (565.3-618.2)	620.3 (592.6-649.9)	673.7 (616.4-738.3)
KIR Kiribati	2387.9 (2078.7-2725.8)	2221.2 (1928.8-2544.9)	2098 (1825.8-2414.5)	1972.8 (1716.8-2245)	1860.4 (1614.4-2127.1)	1666.9 (1432.4-1888.8)	1595.6 (1394.9-1791.7)	1631.3 (1470-1803.2)	1616.9 (1513.8-1735.6)	1566.9 (1488.6-1653.6)	1510.5 (1421.8-1607.9)	1459.9 (1319.4-1597.7)	1419.3 (1238.6-1595.8)	1354.9 (1130.4-1551.5)
MHL Marshall Islands	2363 (2031.7-2735.9)	2091.3 (1800.7-2421.7)	1872.4 (1588.7-2156.9)	1694.4 (1440.2-1940.6)	1537.1 (1311.2-1772.3)	1391.3 (1192.1-1598.4)	1311.6 (1139.8-1463.7)	1314.3 (1203-1410.7)	1417.6 (1373-1468.3)	1493.6 (1437-1550.1)	1557.6 (1483.6-1640.2)	1542.1 (1450.3-1648.6)	1457.5 (1330.6-1601.2)	1323.1 (1170.7-1519.5)
MNP Northern Mariana Islands	915.8 (762.1-1108.1)	919.9 (743.5-1099.6)	925.5 (727.2-1073.7)	922.6 (727.7-1059.2)	904.2 (722.4-1034.4)	874.4 (691.2-1009)	855.1 (681.9-986.4)	836.1 (688.9-952.5)	813.6 (684.5-922.7)	743.5 (648.1-841.8)	700.7 (623-785.5)	644.1 (568.9-716.8)	577.6 (537-641.7)	568.5 (521.3-630.9)
PNG Papua New Guinea	3442.7 (3060.2-3789)	3131.1 (2779.2-3452.3)	2902.2 (2573.5-3196.2)	2657.3 (2379.2-2915.3)	2418.5 (2162.6-2647.8)	2250.4 (2019.6-2452.5)	2139.7 (1922.8-2338)	2084.6 (1864.9-2299.8)	1995.2 (1763.7-2211.6)	1899.7 (1667.4-2120.9)	1873.2 (1642.2-2106.5)	1918 (1678.9-2154.3)	1868.4 (1633.8-2127.9)	1708 (1483.5-1952.1)
WSM Samoa	1313.1 (1150.8-1480.6)	1119.5 (1011.5-1245.7)	928.7 (853.5-1013.1)	940.2 (873.2-1019)	945.7 (875.4-1026.2)	911.5 (829.5-999.1)	834.4 (742.3-939.4)	816.4 (707.3-941)	825.8 (700.5-956.5)	853.1 (715.3-983.7)	859.5 (723-984.3)	843.9 (718.7-952.4)	899.4 (770.1-999.4)	882 (719.8-1005.8)
SLB Solomon Islands	2499.1 (2188.5-2823.1)	2379.2 (2079.7-2683.7)	2251.8 (1955.4-2564.9)	2095.8 (1818.6-2400.5)	1936.4 (1662.6-2218.1)	1888.1 (1634-2168.1)	1648.4 (1406.9-1886.5)	1581 (1361.7-1785)	1551.5 (1364.2-1750.1)	1485.7 (1324.7-1656.3)	1451.1 (1309.3-1610.6)	1450.4 (1320.7-1584.9)	1399.1 (1280.4-1539.2)	1310.5 (1180.8-1473.7)
TON Tonga	1604.5 (1425.9-1773)	1505.5 (1381.8-1633.9)	1433.9 (1347.5-1519.4)	1408 (1318.9-1486.1)	1346.5 (1230.2-1444.6)	1272.3 (1144.5-1378.6)	1194.6 (1081.3-1289.5)	1086.1 (999.3-1163.7)	1035.5 (971.4-1102.3)	1017.5 (974.6-1058.7)	1010 (977-1038.5)	957.3 (885.1-1008.1)	916.5 (793.1-1013.1)	859.1 (709.4-1005.1)
VUT Vanuatu	2172.8 (1849.1-2529.1)	2026.3 (1729.3-2375.4)	1889.2 (1605.2-2233.3)	1774.4 (1499.5-2088.1)	1674.2 (1406.5-1974.2)	1559.6 (1302-1820)	1471.3 (1238.9-1704.4)	1431.9 (1223.8-1633.3)	1422.7 (1227.1-1636.9)	1405.9 (1217.4-1624.1)	1391.5 (1187-1592.1)	1399.3 (1190.2-1603.8)	1360 (1147-1580.3)	1319.4 (1121.2-1539)
Southeast Asia	2293.5 (2205.1-2394.5)	1960.6 (1881.4-2051.3)	1756.7 (1683.9-1836.9)	1638 (1563.6-1716.5)	1549.1 (1483.3-1622.4)	1451.9 (1395.1-1516.1)	1281.4 (1237.4-1331.4)	1170.9 (1134.6-1209.4)	1085.5 (1053.9-1116.6)	1014.2 (984.7-1043.8)	960 (934.3-985.1)	881.1 (855.8-905.7)	802.5 (779.3-826.5)	720.5 (690.8-753.4)
KHM Cambodia	2276.5 (1954.8-2610.9)	2116.9 (1811.1-2414)	1989 (1698.9-2273.4)	1876.5 (1603.9-2137.6)	2095.1 (1846.2-2345.7)	3456.3 (3235.7-3684.9)	1842.8 (1650.5-2034.1)	1737.4 (1574.9-1909.2)	1608.3 (1483-1741.7)	1526.9 (1426.6-1632)	1414.9 (1319.6-1517.5)	1203.2 (1102.4-1313.7)	1020.5 (931.5-1117.3)	894.3 (803-1050.6)
IDN Indonesia	2603.4 (2464.5-2739.2)	2321.3 (2190.2-2450.1)	2078.8 (1962.1-2200.7)	1906.7 (1799.6-2021.9)	1756.9 (1654.4-1867.6)	1612 (1522.5-1709.6)	1462.2 (1392.9-1537.9)	1334.1 (1281.7-1392.9)	1227.5 (1185.9-1275.4)	1132 (1097.6-1169.5)	1083.7 (1052.6-1113.5)	1013.2 (978.7-1048.2)	939.7 (894.5-984.5)	860 (808.3-922.6)
LAO Laos	2961.1 (2580.1-3372.6)	2907.9 (2568-3283.7)	2898.7 (2578.3-3248.2)	2788.4 (2454-3127.8)	2613.7 (2281.9-2923.7)	2353.5 (2030.5-2654.5)	2181.8 (1892.4-2463.9)	1983.4 (1735.8-2239.1)	1861.3 (1635.7-2091.6)	1719.8 (1528.8-1920.7)	1547.5 (1392-1709.2)	1355.9 (1226-1493.4)	1165.5 (1040.4-1304.7)	962.6 (837.6-1116.3)
MYS Malaysia	2070.9 (1767.2-2386.8)	1817.4 (1641-2012.1)	1557.4 (1547.5-1568.5)	1393.6 (1384.3-1402.6)	1304.4 (1278.4-1330.1)	1112.9 (1086.9-1137.1)	1028.1 (979.8-1078.4)	972.8 (966.8-979)	919 (912.6-925.7)	895.7 (890.6-901.7)	861.7 (856.3-867.1)	796.9 (792.5-801)	761.8 (750.9-772.4)	729 (662.9-797.3)
MDV Maldives	3542.2 (3150.6-3929.6)	3265 (2916.4-3607.2)	2969.6 (2666.3-3255.3)	2686.9 (2465.6-2914.6)	2380.2 (2243-2520.7)	2210.2 (2117.4-2304.4)	2004.7 (1931.9-2080.2)	1661.8 (1623.8-1699)	1376.2 (1349.4-1403.7)	1265.5 (1235-1299)	962.5 (939.4-985)	731.9 (712.2-751.1)	497.8 (483.7-513.1)	412.3 (380.8-445.1)
MUS Mauritius	1922 (1866.6-1980.8)	1691.3 (1654-1728.5)	1659.6 (1623.9-1697.4)	1291.8 (1261.7-1322.9)	1230.4 (1203.2-1258.9)	1158.1 (1132.9-1182.6)	981 (958.1-1003.2)	949.1 (928-970.8)	848.3 (827.9-869.2)	785.7 (767.6-803.5)	759.5 (741.8-776.1)	717.5 (700-735.6)	648.3 (634.4-663.5)	622.4 (577.9-673.1)
MMR Myanmar	3292.6 (2897.8-3668.8)	2885.3 (2556.6-3194.1)	2671.3 (2353.4-2986.7)	2482.5 (2162.9-2806.2)	2306.7 (2003-2626.3)	2041.5 (1762.4-2350.8)	1862 (1605.5-2132.3)	1726.7 (1484.5-1962.5)	1646.1 (1436.7-1872.4)	1575.9 (1383.5-1787)	1477.3 (1306.6-1658)	1314.7 (1176.5-1462.4)	1087.6 (968.8-1230)	878.3 (755.9-1010)
PHL Philippines	1704.5 (1581-1835.6)	1267.1 (1166.4-1371.1)	1076.6 (1015-1139.2)	1139.9 (1079.6-1204.3)	1243 (1180.2-1306)	1209.9 (1143.8-1274.6)	1089.5 (1032.3-1146.3)	1127.6 (1068.2-1182.8)	1022.5 (966.5-1077.6)	958.8 (904.6-1015)	957.5 (903.5-1011.9)	972.4 (915.8-1031.6)	969 (914.6-1028.1)	960.2 (831.3-1109.9)
LKA Sri Lanka	1720.7 (1682.7-1766.3)	1373.3 (1357.6-1388.1)	1164.5 (1153.5-1174)	1100 (1092-1107.7)	998.1 (990.6-1005.8)	1053.2 (1044.5-1061.4)	862.5 (854.5-868.9)	850.6 (845.3-856)	793.3 (768.7-814.4)	757.2 (747-766.1)	673 (666.4-680.4)	592.4 (567.4-618)	596.2 (586.5-606.9)	498.9 (393.4-572.8)
SYC Seychelles	1383.4 (1205.6-1577.3)	1274.8 (1129.4-1425.4)	1181.8 (1069.9-1297.6)	1108.5 (1033-1189.2)	997.8 (952.4-1041.9)	877 (843.7-909.4)	812.1 (779.7-845.5)	805 (777-837)	743.5 (715.4-773.5)	690.3 (661.6-719.1)	702.3 (676-729.1)	700.4 (673.9-727.6)	685.4 (660.2-709.1)	654.8 (614.6-695.7)
THA Thailand	1659.4 (1549.3-1782.4)	1286.5 (1231.6-1351)	1233.8 (1185.7-1280.3)	1166.8 (1122.2-1214.4)	1061.2 (1021.5-1102.6)	1076.5 (1033.2-1120.3)	1043.1 (1004.7-1082.8)	824 (790.2-857.5)	779.1 (747.5-808.7)	743.8 (713.9-774)	686.4 (652.7-720.3)	578 (552-604.2)	504.1 (482-525.9)	432 (388.7-477.7)
TLS Timor-Leste	3708.5 (3276.7-4125.2)	2977.3 (2621.2-3304)	2666.4 (2314.3-2986)	2407.3 (2083.8-2738.4)	2194.5 (1897.7-2508.3)	2220.1 (1965.1-2511.9)	1975.3 (1742.7-2248.8)	1659.5 (1454.9-1896.6)	1466.7 (1297-1669.7)	1346.2 (1211.9-1491.3)	1189.7 (1082.4-1301)	1036.5 (945.8-1133)	905.1 (824.9-994.8)	845 (737.8-955.4)
VNM Vietnam	1870.1 (1580.1-2170.8)	1490.9 (1258.5-1754.7)	1373.8 (1172-1615.3)	1362 (1174.2-1581.6)	1377 (1197.6-1578.3)	1152.5 (986.1-1340.4)	997.4 (867.5-1149)	912.5 (805.8-1041.1)	846.5 (744.9-931.2)	775.1 (684.2-847.7)	727.1 (631.6-801.6)	668.4 (585.3-756.8)	607.9 (534.9-683.6)	575.6 (492.6-638.4)
Sub-Saharan Africa	2716.5 (2602.9-2839.6)	2461.7 (2368.7-2564.4)	2306.4 (2221-2398)	2152 (2076.1-2237.9)	2030.5 (1959.7-2111.6)	1888.7 (1824.8-1962)	1771.1 (1713.8-1837.2)	1692 (1638.9-1750.1)	1656.3 (1598.8-1713.3)	1700.7 (1642.6-1762.2)	1768.4 (1704.3-1832.4)	1659.6 (1596.5-1727.3)	1431.6 (1374.4-1495.1)	1121 (1074-1177.1)
Central sub-Saharan Africa	2816.7 (2572.4-3063.2)	2508 (2294.1-2718.8)	2380.7 (2179.6-2580.1)	2282.2 (2081.3-2484.1)	2148.2 (1963.1-2352.8)	2039 (1857.9-2210.4)	1954.2 (1791.4-2117.6)	1850.4 (1706.3-2002.3)	1818.1 (1687.6-1960.7)	1857.3 (1735.9-1984.8)	1850 (1732.9-1970.9)	1745.4 (1626.9-1865.2)	1596.5 (1482.2-1719.9)	1321.8 (1193.7-1471.8)
AGO Angola	2658.9 (2287.8-3023.6)	2501.5 (2164.9-2818.4)	2415.8 (2085.8-2732.4)	2340.6 (2026-2651.4)	2209.9 (1909.8-2494.5)	2129.1 (1841.7-2417.6)	2113.7 (1841.3-2395.1)	2096.3 (1828.2-2381.2)	2022.3 (1779-2291.3)	1950.1 (1721.6-2191.6)	1841.2 (1638-2056.5)	1648.2 (1472.5-1828.7)	1399.2 (1238.7-1558.2)	1147.3 (988.7-1333.9)
CAF Central African Republic	3195.3 (2837.2-3590.7)	2922 (2601.5-3276.2)	2661.7 (2362.4-2991.4)	2433.4 (2144.1-2722.7)	2307.2 (2042.8-2574.2)	2244.8 (2008.3-2474.2)	2151.5 (1963.2-2350.3)	2082.6 (1935.2-2245.8)	2252.9 (2058.6-2458.7)	2451.4 (2185.2-2754.5)	2630 (2302.3-3023.8)	2622.4 (2212.9-3043.7)	2373.2 (1950.9-2747.2)	2024.1 (1667.9-2383.1)
COG Congo (Brazzaville)	3411.3 (3025.5-3764.8)	3083.4 (2750.7-3436.3)	2843.6 (2520.7-3175)	2602.6 (2321.7-2889.7)	2394.3 (2128.3-2683.4)	2163.9 (1917-2425.2)	1968 (1751.4-2207.5)	1802.8 (1604.9-2014.3)	2004.1 (1791.2-2191)	2219.4 (2021.7-2414.5)	2219.6 (2019-2414.8)	1993.6 (1796.9-2172)	1760.7 (1527.1-1958)	1514 (1251.2-1749.6)
COD DR Congo	2773.4 (2384.3-3155.8)	2415.9 (2104-2731.6)	2303.8 (2001.3-2603.7)	2230.4 (1931.1-2556.9)	2103.7 (1818.9-2420.6)	1997 (1723-2256.7)	1909.4 (1671.9-2152.6)	1784.9 (1576.6-1998.5)	1730 (1546.5-1924.2)	1778.9 (1615.2-1950)	1786.6 (1629.6-1944.8)	1709.1 (1555.6-1864.1)	1607.3 (1449.9-1774)	1337.5 (1156.6-1560)
GNQ Equatorial Guinea	3846.2 (3255.9-4471.9)	3171.2 (2763.7-3629.5)	3012.7 (2641.6-3426)	2764.6 (2405.7-3164.5)	2560.6 (2202.5-2968.9)	2389.4 (2054.2-2748.7)	2172.3 (1859.2-2514.1)	2103.7 (1815.8-2424.8)	2178.9 (1884-2503.6)	2141.6 (1820.1-2480.5)	1684.6 (1337.7-2034.2)	1420.6 (1084.4-1797.7)	1328.4 (984.6-1748.5)	1095.5 (801.3-1462)
GAB Gabon	3008.9 (2596.8-3466.5)	2278 (1997.7-2586.9)	2165.8 (1879.6-2454.6)	2004.6 (1744.8-2285.8)	1854.2 (1620.8-2106.9)	1631.4 (1431.5-1844.8)	1452.8 (1277.6-1634.8)	1347.7 (1203.8-1508.5)	1342.8 (1207.2-1502.2)	1342 (1189.4-1501.5)	1499.8 (1307.2-1722.4)	1508.9 (1294.9-1754)	1252.7 (1085.5-1446.7)	900.4 (752.8-1061.3)
Eastern sub-Saharan Africa	2957.3 (2854.7-3072.4)	2684.8 (2594.3-2780.9)	2522 (2438.4-2611.4)	2381 (2301.8-2461.5)	2244.8 (2172.5-2319.1)	2126.1 (2058.1-2191.7)	2007.8 (1949.3-2069.2)	1980.2 (1928.7-2032.7)	1954.7 (1906.2-2001)	2015.6 (1960-2065.6)	1962.3 (1908-2014.5)	1694.8 (1645.3-1743.5)	1379.2 (1338.8-1419.8)	1078.6 (1039.1-1119.4)
BDI Burundi	3583 (3172.5-3997)	3280.6 (2915.7-3614.4)	3018.5 (2709.6-3304.1)	2812.9 (2558.8-3056.3)	2685 (2461.2-2893.4)	2584.5 (2374.5-2799.9)	2446.2 (2234.4-2667.7)	2343 (2107.6-2580.5)	2255.5 (2020-2509.3)	2526.9 (2252.8-2796.3)	2573.1 (2308.1-2850.6)	2140.9 (1913.5-2374.2)	1625.4 (1417.4-1858.3)	1387.4 (1180.6-1617.7)
COM Comoros	3116.2 (2759.6-3486.6)	2854.1 (2565.8-3135.1)	2596.5 (2341.1-2832.7)	2328.5 (2082.2-2571)	2086.3 (1840-2335.2)	1927.2 (1683-2168.8)	1822.7 (1585-2059.1)	1706.6 (1479.2-1937)	1600.7 (1404.9-1818.4)	1519.3 (1339.9-1709.2)	1416.7 (1272.8-1571.2)	1226 (1096.8-1366.5)	1097.7 (969.1-1242)	991.5 (841.4-1168.9)
DJI Djibouti	2193.5 (1825.7-2601.3)	2045.6 (1716.7-2420.2)	1913.8 (1604.9-2271.2)	1789.8 (1495.2-2127.3)	1662 (1384.7-1984.1)	1513.6 (1253.2-1796.6)	1440.8 (1199.4-1725.3)	1476.9 (1242.2-1753.8)	1429.3 (1196.9-1708.1)	1453.6 (1198.4-1741.7)	1418.6 (1132.5-1734.7)	1336 (1037.7-1710.9)	1186.4 (905.5-1549.7)	1016.1 (792.4-1325.5)
ERI Eritrea	3580.7 (3182.4-3994)	3238.3 (2894.2-3631.6)	3011.9 (2683-3366.2)	3048 (2742.6-3376.8)	2867.8 (2575.2-3186.9)	2792.8 (2542.1-3068.6)	2626.3 (2404.7-2874.9)	2492.5 (2301.4-2709.8)	2852.4 (2660.7-3064.5)	1965.5 (1752.7-2184)	1872.4 (1616.1-2137.8)	1750.4 (1472.1-2023.4)	1630.9 (1355.1-1890.6)	1430 (1170-1653.4)
ETH Ethiopia	3601.8 (3361.4-3870)	3199.5 (2976.3-3440.1)	2987.4 (2779.9-3219.5)	2831.2 (2636.7-3036.1)	2707.2 (2515.9-2904.4)	2646.1 (2453-2841.1)	2521.2 (2352-2700.6)	2464.4 (2313.1-2603.2)	2332.1 (2207.1-2447.6)	2267.4 (2141.5-2385.4)	2107.1 (1979.3-2223.2)	1666.1 (1571.9-1767.8)	1233.7 (1162.1-1308.6)	922.2 (857.8-989.1)
KEN Kenya	2236.1 (2148.7-2319.4)	1992.2 (1913.2-2071.1)	1802.7 (1725.5-1883)	1712.2 (1637.3-1787.6)	1629.4 (1561.5-1699.6)	1513.7 (1452.5-1575.5)	1395 (1340.3-1456.8)	1324.9 (1273.5-1380.6)	1330.7 (1278.4-1385.1)	1524.4 (1458.4-1594.6)	1674.2 (1597.6-1752.4)	1591.2 (1530.1-1649)	1301.7 (1255-1348.5)	1020.1 (978-1064.6)
KEN Baringo	1780 (1347.2-2280.4)	1719.4 (1283.6-2205.3)	1696.6 (1282.2-2165.3)	1758.2 (1361.8-2245.2)	1883.1 (1460.1-2345.9)	1953.7 (1537.3-2408.2)	1955.9 (1545.4-2423.9)	1915.5 (1548.9-2358.6)	1872.4 (1506.3-2293.6)	1879.6 (1509.6-2288.4)	1786.1 (1441.5-2136)	1614.5 (1326.9-1880.8)	1357.5 (1096-1622.9)	1131.3 (863.5-1446.1)
KEN Bomet	1187.1 (950-1514.5)	1149.9 (908.2-1465.5)	1094.5 (857.5-1418.6)	1087.7 (861.2-1414.5)	1074.3 (856.1-1389)	1043.4 (827-1331.6)	1003.3 (788.9-1253.8)	945.9 (752.9-1173.8)	937.9 (729.4-1181.2)	1096.1 (823.6-1374.2)	1373 (1053.1-1707.1)	1400.5 (1068.5-1737.8)	1231.9 (946.4-1522.8)	903.2 (720.8-1164.8)
KEN Bungoma	2376.2 (1946.2-2863.7)	2045.9 (1637-2490.9)	1816.9 (1430.3-2288.3)	1703.3 (1352.6-2143.4)	1592.8 (1237-1992)	1478.2 (1159-1855.5)	1361.1 (1072.3-1720.9)	1285.7 (1006.5-1608)	1213.2 (962.2-1502)	1201.1 (955.1-1509.6)	1208.2 (946.1-1505.8)	1228.6 (975.2-1508.6)	1071.9 (863-1295.9)	976.3 (764.8-1229.5)
KEN Busia	2565.3 (2104.2-3070)	2071.2 (1660.2-2542.6)	1864 (1493.2-2310.8)	1774.1 (1433-2217.5)	1673.2 (1355-2081.1)	1452.8 (1186-1788)	1285.2 (1037.7-1574.6)	1219.6 (991-1475.9)	1284.2 (1019.6-1554.2)	1706.3 (1256.2-2207.9)	1959.3 (1477.1-2491.2)	1651.9 (1262.5-2068.8)	1183.4 (952.4-1443.8)	963.1 (779.7-1181.8)
KEN Elgeyo Marakwet	1925.3 (1467.7-2454.2)	1807.3 (1371.3-2307.8)	1748.4 (1331.9-2244.4)	1732.9 (1336.8-2222.1)	1755.1 (1340.5-2229.8)	1766.3 (1356.3-2237.4)	1722.6 (1319.7-2168.9)	1663.8 (1289.6-2088.2)	1585.9 (1222.8-1976.3)	1567.1 (1192.8-1957.2)	1559.6 (1215.9-1918.2)	1547.4 (1253.4-1810.6)	1424 (1158.4-1635.5)	1293.7 (1039-1548.5)
KEN Embu	2486.5 (1975.9-3076.1)	2117.4 (1674.2-2625.3)	1854.6 (1453.3-2312.5)	1681.4 (1307.4-2101.8)	1490.9 (1144.2-1876.8)	1278.7 (984.6-1635.6)	1119.6 (862.2-1418.7)	1046.2 (826-1329.1)	1075.9 (780.6-1408.2)	1232.8 (843.2-1697)	1242.3 (904.2-1678.1)	1102.5 (842.1-1450)	927.8 (731.8-1235.6)	763.3 (614.5-1025.7)
KEN Garissa	2614.2 (2162.6-3071.2)	2653.5 (2208.9-3093.8)	2286.1 (1858.9-2728.8)	2175.8 (1768.5-2597.7)	2022 (1630.3-2455.9)	1880 (1509.8-2285.8)	1808 (1478.8-2213.2)	1780.6 (1459.8-2148.3)	1722.5 (1418.5-2070.3)	1731.4 (1439.4-2058)	1682.4 (1411.1-1995.4)	1609 (1328-1904)	1570.8 (1286-1873.1)	1543.5 (1114.1-2243.6)
KEN Homa Bay	2891.8 (2369-3420.6)	2391.9 (1919.7-2888.7)	2227.9 (1775.4-2694.8)	2167.3 (1745.6-2614.1)	2165.7 (1767.5-2590.6)	2062.6 (1708.3-2484.8)	1857.2 (1531.2-2245.5)	1722.2 (1412.4-2072.4)	1738.2 (1432.8-2093.4)	2104 (1678.5-2657)	2791.3 (2130.4-3565.6)	2955 (2387.4-3699.7)	2226.1 (1848.3-2793.8)	1276.1 (967.9-1691.4)
KEN Isiolo	2438.8 (1966.6-2981.4)	2359.4 (1923.2-2855)	2178.2 (1760.3-2668.2)	2219.2 (1795.6-2674.7)	2300 (1906.1-2732)	2185.1 (1808.9-2618.2)	1950.3 (1600.5-2357.8)	1825 (1500.3-2203.1)	1694 (1392-2012.1)	1606.9 (1303.8-1941.8)	1573.9 (1256-1930.4)	1425.2 (1114.3-1761.7)	1240.5 (942.8-1585.4)	1041.9 (783-1364.5)
KEN Kajiado	1838.6 (1413.4-2302.7)	1756.5 (1362.7-2186.4)	1601.6 (1220.1-2069.3)	1556.9 (1189.8-1989.5)	1517.4 (1165.5-1940.7)	1456.9 (1123.9-1879.8)	1367.2 (1027.7-1753.8)	1308.4 (992-1651.5)	1354.5 (1005.9-1708.3)	1629.6 (1177-2087.4)	1781.1 (1343.3-2224.9)	1660.8 (1281.1-2015.4)	1330.3 (1026.8-1635.2)	1105 (816.4-1400.9)
KEN Kakamega	2290 (1851.8-2771.9)	2075 (1672.9-2534.8)	1780.3 (1430.2-2214)	1650.8 (1325.1-2078)	1538.3 (1240.8-1924.7)	1390.2 (1126.8-1727.8)	1245.5 (1006.6-1536.9)	1195.2 (966.8-1443.4)	1247.4 (1004-1497.5)	1532.2 (1173.3-1867.2)	1670.8 (1303.6-2053.8)	1506.2 (1191.1-1828.2)	1259.7 (985.7-1531.8)	1043.6 (792.9-1314.4)
KEN Kericho	1466.2 (1075-1865.8)	1409.9 (1062-1812.4)	1295.9 (983.6-1681.6)	1230.7 (940.6-1605.3)	1175.6 (913.7-1526.7)	1090.7 (848.1-1400.4)	1023.6 (800-1299)	980.3 (777.1-1205.8)	949.1 (752.4-1173)	1003.2 (776.2-1275)	1106 (863.4-1407.4)	1127.3 (872.9-1402.8)	979.6 (741.9-1207.9)	766.7 (610.9-964.7)
KEN Kiambu	2056.9 (1651.7-2490.4)	1812.6 (1427-2244.9)	1638.1 (1272.2-2073.8)	1523 (1182.5-1928.6)	1386 (1056.3-1774.7)	1227.1 (936.2-1579.4)	1162.1 (900-1509.7)	1113.7 (887.8-1436)	1140.5 (898.9-1462.8)	1474.3 (1086.2-1902.4)	1707.5 (1316.7-2176.7)	1467.5 (1150.3-1824.5)	1136.3 (907.4-1399.6)	930.9 (731.6-1169.1)
KEN Kilifi	3077.4 (2578.2-3584.8)	2683.8 (2230.3-3148.6)	2419.8 (1980.3-2874.5)	2328.5 (1900.3-2786.9)	2231.8 (1823.2-2661.6)	2052 (1654.1-2497.4)	1903.6 (1532.3-2302.9)	1810.2 (1458.5-2160.5)	1775.8 (1454.8-2141.8)	1849.9 (1538.9-2240.6)	1896.3 (1547.2-2258.2)	1794.7 (1464.9-2114.2)	1447.7 (1171.6-1771.6)	1091.5 (873.2-1365)
KEN Kirinyaga	2361.1 (1880.5-2873.7)	2050.6 (1617.3-2492.8)	1814.4 (1424.6-2268.7)	1665.4 (1308.9-2106)	1556.8 (1224.4-1978.5)	1353.7 (1055.9-1746.5)	1120 (856.3-1461.7)	1032.5 (783.8-1360.3)	1018.7 (787.7-1317.9)	1079.5 (803.1-1406.7)	1134 (802-1495)	1115.5 (827.5-1489.9)	979.7 (748.7-1272.5)	852.2 (685.1-1109.5)
KEN Kisii	2799.1 (2276.9-3362.3)	2615 (2111.7-3158.1)	2278.7 (1785.1-2810.4)	2150.5 (1666.9-2656.6)	2061.3 (1581.9-2567.7)	1947.9 (1507.9-2451.1)	1845.3 (1430.8-2332.3)	1792.5 (1427.1-2219.7)	1803.4 (1453.1-2176.8)	2018.4 (1615.2-2404.9)	2264.2 (1873.6-2627.3)	2268.2 (1946.4-2581.2)	1940.4 (1653.5-2236.1)	1626.4 (1284.8-1920.4)
KEN Kisumu	2383.5 (1917.3-2886.5)	2056.4 (1671.5-2486)	1817.4 (1442.9-2219.2)	1710.9 (1373.3-2079.8)	1654.6 (1354.5-2018.3)	1532.1 (1253.7-1885.9)	1389.9 (1138.8-1699)	1261.6 (1031-1528.4)	1309.5 (1018-1646.8)	1910.5 (1381.2-2557.5)	2359.6 (1773-3027.5)	2198.4 (1728-2737.1)	1644.9 (1256.3-2056.2)	1105.7 (854.6-1453.2)
KEN Kitui	2385.5 (1914.5-2888.6)	2055 (1613.2-2547)	1896.9 (1476.5-2388.9)	1812.6 (1406.2-2261.7)	1725.1 (1332.7-2177.9)	1612 (1246.5-2043.9)	1482 (1150.5-1876.3)	1400.2 (1109.5-1752.4)	1367.8 (1088.5-1690.8)	1426.7 (1126.7-1753.6)	1432 (1134.6-1771.7)	1217.6 (973.4-1484.5)	945.6 (747.6-1159.9)	758.8 (604.7-950.7)
KEN Kwale	2733.2 (2225.2-3276.1)	2547.8 (2079.4-3044.4)	2261.6 (1802.2-2721.7)	2156.4 (1722.2-2627.9)	2076.3 (1665.1-2526.8)	1967.8 (1584.2-2408.3)	1821 (1473.4-2268.6)	1704.2 (1364-2095.6)	1851.9 (1420.7-2335.1)	2323.2 (1705.3-2995)	2274.6 (1724.5-2858.3)	1842.2 (1440.6-2279.3)	1389 (1094.5-1717.3)	1058.9 (818.7-1349.2)
KEN Laikipia	2024.9 (1575.3-2476.5)	1866.3 (1482.3-2281.3)	1632.8 (1298.5-2023.2)	1484.8 (1187.3-1870.6)	1352 (1082.8-1692.3)	1183.3 (941.1-1497.7)	1056.9 (836.8-1332.9)	998.1 (797.9-1223)	996.2 (799.4-1211.8)	1077.6 (842.7-1355.2)	1238.9 (945.6-1576)	1308 (1009-1648.7)	1193.9 (910.4-1482.6)	982.5 (758.8-1296)
KEN Lamu	3236.6 (2638.5-3845.8)	2871.7 (2344.9-3433.3)	2660.8 (2166.9-3177.7)	2501.4 (2029.2-3026)	2196.8 (1782.6-2684.3)	1790.7 (1401.2-2226.5)	1503.3 (1167.3-1864.2)	1424.3 (1085-1781.1)	1443.3 (1109.2-1801.3)	1523.2 (1175.4-1890.7)	1557.2 (1218.8-1928.9)	1438.3 (1114.8-1831.1)	1234.9 (935.9-1579.8)	1046.2 (799.5-1326.6)
KEN Machakos	2081.2 (1678.3-2628.4)	1893.6 (1497.1-2378.1)	1651.9 (1272.3-2089.9)	1527.2 (1165.3-1927.9)	1423.4 (1109.5-1811.2)	1295.3 (1000.6-1649.3)	1164.6 (912.2-1479.3)	1083.7 (849-1357.5)	1039.9 (817.2-1298.2)	1065.4 (827.4-1339.6)	1128.6 (865.2-1422.4)	1172.4 (917.9-1435.1)	988.2 (809.7-1221.3)	830.7 (692-1046.1)
KEN Makueni	1537.6 (1223.7-1914.5)	1342.1 (1042.8-1710.6)	1305.4 (1007.3-1665.8)	1310.2 (1028.4-1673)	1288 (1022.2-1651)	1238 (979.9-1580.6)	1180.4 (924-1492.8)	1128.9 (896.9-1410.2)	1109.3 (885.2-1379.4)	1250.9 (985.8-1570.6)	1516.9 (1164.6-1947.2)	1561.7 (1187.4-1975.6)	1296.9 (996.4-1622.1)	840.9 (674.3-1057)
KEN Mandera	2286.2 (1839.3-2731.6)	2262.8 (1820.5-2709.5)	1986.6 (1559-2433.2)	1941.7 (1532.1-2372.3)	1882.4 (1493.8-2334.2)	1808.5 (1448.5-2236.9)	1752.3 (1415.8-2182.6)	1737.1 (1415.4-2123.9)	1736 (1415.9-2094.6)	1761.9 (1450.2-2102.3)	1696.3 (1399.5-2008.3)	1701.5 (1395.3-1986.8)	1709.8 (1411.4-1995.5)	1550.5 (1234.5-1881.6)
KEN Marsabit	3822.4 (3133.8-4563.6)	4273.2 (3647.4-4952)	3676.4 (3162.2-4256)	3276.3 (2809.9-3788.9)	2624.9 (2218-3078)	2045.1 (1676.7-2494.8)	1665.7 (1329.3-2056.9)	1512.4 (1217-1872.9)	1426.1 (1137.8-1739.9)	1414 (1119-1729.6)	1395.3 (1085.9-1740.7)	1389.9 (1090.5-1734)	1282.1 (989.5-1634.6)	1112.2 (799.7-1504.9)
KEN Meru	2329.5 (1852.5-2843.5)	2030.1 (1624.6-2488.4)	1791.7 (1417.3-2240.6)	1666 (1321.1-2072.4)	1554.8 (1219.6-1963.3)	1431.7 (1125.2-1828.3)	1296.7 (1022.6-1619.8)	1213.4 (950.2-1516.8)	1190.5 (951.8-1489.3)	1288.1 (987.8-1645.5)	1375.1 (1053.9-1754.5)	1338.2 (1062-1652.1)	1175.1 (915.6-1433.5)	988.8 (754.2-1269.4)
KEN Migori	2786.2 (2271.5-3336.3)	2326.2 (1873.6-2788.9)	2208.6 (1771.9-2662.1)	2201.5 (1790.7-2641.6)	2166.6 (1797.1-2617.7)	2147.6 (1786.4-2562.5)	2086.6 (1740.4-2488.7)	2005.4 (1698.7-2401.5)	1931.7 (1622.8-2316.8)	2123.2 (1725-2618.8)	2522.3 (1972.5-3145.9)	2705.8 (2159.2-3346.7)	2240.4 (1743.5-2811.5)	1482.1 (1061.1-1949)
KEN Mombasa	2354.7 (1848.9-2899.9)	2261.6 (1787.8-2790.6)	1940.8 (1503.9-2437.7)	1773.4 (1372.2-2253)	1616.6 (1238.6-2061.9)	1445.2 (1087.8-1870.4)	1302.1 (982.5-1665.3)	1244.1 (933-1600.3)	1343.6 (974.9-1771.5)	1763.5 (1200.5-2364.8)	1861.2 (1342.4-2451.2)	1558.7 (1171-2027.5)	1226.3 (945.1-1608.6)	853 (673.1-1127.3)
KEN Murang'a	1781.3 (1419.3-2177.4)	1522.4 (1187.4-1902.1)	1418.1 (1096.3-1807.1)	1360.5 (1051.1-1736.8)	1286.5 (1006.1-1624.7)	1178.9 (911.8-1480.4)	1074.3 (840.4-1362.6)	1020.2 (819.5-1304.6)	1009.8 (813.2-1304.1)	1096.2 (834.1-1419.6)	1174.6 (877.9-1490)	1226.4 (967-1506.6)	1041.2 (832.3-1287.4)	893.2 (725.1-1131.3)
KEN Nairobi	2668.8 (2184.3-3140.1)	2583.3 (2160.3-3018.2)	2045.3 (1667.2-2469.1)	1753.5 (1383-2145)	1540.2 (1213.7-1913.3)	1329 (1049.5-1654.2)	1186.5 (927-1475.8)	1152 (917.3-1432.9)	1312.8 (1035.5-1647.5)	1804.4 (1368.6-2236.7)	2030.9 (1587.3-2415.3)	1916.8 (1580.4-2186.9)	1572.6 (1338.3-1753.8)	1237.2 (1010.6-1422.4)
KEN Nakuru	1508.4 (1194-1883.8)	1434.6 (1119.5-1804.6)	1250.5 (974.9-1623.9)	1166.3 (900.1-1516.2)	1106.1 (846.7-1424.9)	1033.3 (793.9-1323.1)	934.2 (714.7-1181.3)	858.4 (655.5-1074.6)	866.9 (660.9-1130.8)	1216.9 (886.9-1661.8)	1472.7 (1085.9-1965.3)	1262.6 (1005.4-1604.7)	933.6 (781.2-1158.6)	675.8 (576.7-829.7)
KEN Nandi	1221.8 (937.8-1571.9)	1129.6 (855.2-1457.7)	1059.7 (796.3-1370.9)	1049.6 (795.9-1361)	1048.3 (794-1347)	1033.9 (793.8-1325)	1004.5 (769.3-1273)	973.2 (754-1205.9)	1026.4 (789-1288.1)	1258.2 (939.3-1656.7)	1288.7 (994.9-1615.8)	1133.3 (916.8-1378.3)	918.7 (722.8-1104.3)	764.3 (610.7-959.9)
KEN Narok	1801.8 (1368-2291.7)	1748.8 (1320.1-2209.2)	1647.2 (1231.9-2069.3)	1662.7 (1247.5-2084.3)	1710.1 (1293.9-2119.7)	1729.9 (1324.2-2164.1)	1721.9 (1342.9-2158.8)	1712.4 (1346.9-2118.6)	1725.6 (1355.6-2088.4)	1772.2 (1422.8-2132.3)	1737.3 (1432.2-2109)	1681 (1412-2014.2)	1374.3 (1136-1616.4)	1205.9 (932-1425.2)
KEN Nyamira	2377.4 (1904.6-2853)	2079.9 (1622.6-2544.8)	1991.4 (1546.3-2416.9)	1948.1 (1520.2-2358.1)	1869.2 (1488.7-2246.2)	1795.1 (1403.5-2180)	1732.1 (1364.7-2119.7)	1681.3 (1343.1-2026.2)	1693.1 (1376-1982.1)	1887.4 (1591.9-2207.5)	2135.9 (1804.8-2522.4)	2286.2 (1900.1-2722.8)	2173.7 (1790.5-2610.3)	1689.4 (1390.8-2063.2)
KEN Nyandarua	2071.8 (1625.7-2555.5)	1909.5 (1482.9-2384.6)	1719.4 (1322.2-2211.5)	1616.9 (1223.1-2058.6)	1539.4 (1143.2-1964.9)	1455 (1080.4-1843.3)	1361.1 (1013.8-1706.8)	1332.6 (998.9-1638)	1337.4 (1032-1628)	1457.4 (1143.2-1732.3)	1664.5 (1328.5-1964.1)	1804.6 (1446.6-2133.6)	1624.4 (1308.8-1974.7)	1285.8 (981.5-1645.4)
KEN Nyeri	2752.1 (2262.9-3316.4)	2247.9 (1772.5-2748)	2048.9 (1601-2575.4)	1918.5 (1485.5-2407.8)	1797.2 (1377.8-2241.1)	1661.2 (1263.9-2073.1)	1527.2 (1153.5-1872.3)	1460.5 (1138.6-1762.2)	1490.9 (1193.2-1783.6)	1762 (1420.4-2124.8)	2090.9 (1714.1-2543.7)	2017.6 (1667.2-2440.9)	1643.6 (1324.9-1931)	1417 (1070.8-1733.9)
KEN Samburu	1246.2 (931.9-1580.3)	1190.9 (905.8-1527.5)	1151.2 (876.6-1491.2)	1166 (903.9-1515.8)	1204.4 (942.3-1542.2)	1233.6 (977.3-1562.6)	1248.2 (984.9-1552.9)	1265.9 (1009.4-1563)	1304.1 (1040.6-1605)	1401.8 (1078.2-1789.8)	1502.5 (1112.1-1921.6)	1440.3 (1092.7-1821.3)	1273.1 (974.9-1607.6)	1074.7 (776.8-1398.5)
KEN Siaya	2653.2 (2141.7-3206.3)	2450.6 (1970.1-2981.8)	2141.2 (1680.9-2605.5)	1973.4 (1544.8-2442.3)	1839.5 (1413.3-2281.7)	1692.7 (1294.7-2103.9)	1534.9 (1185.7-1929)	1447.1 (1144.8-1798)	1438.3 (1150.7-1783.1)	1552.1 (1218.3-1952.1)	1768.5 (1299.9-2365.4)	1973.7 (1531.8-2530.9)	1536.5 (1192.1-1945.4)	1011.9 (799.6-1321.7)
KEN Taita Taveta	3076.3 (2497.3-3682.1)	2795.2 (2251.7-3341.2)	2418.1 (1899.7-2931.5)	2202.8 (1730.6-2684.5)	2036.4 (1575.3-2513.7)	1831.6 (1418.1-2320.2)	1633.8 (1260.9-2075.1)	1501.2 (1162.9-1923.9)	1452 (1133.8-1837.7)	1542.4 (1215.7-1915)	1719.3 (1338.5-2129.4)	1838.2 (1449.5-2243.8)	1692.4 (1340.1-2020.8)	1330.4 (1002.9-1624.5)
KEN Tana River	3381.2 (2796.6-4031.8)	2947.4 (2393.3-3552)	2609.6 (2099.5-3178.8)	2437.8 (1960.2-2997.2)	2231.7 (1769.2-2773.9)	2022.6 (1555.2-2504.6)	1854.7 (1398.7-2309)	1788 (1374.9-2197.5)	1788 (1378.7-2190.7)	1956.7 (1557.9-2375.2)	2117.8 (1697.6-2553.3)	2082.6 (1709.7-2493.6)	1959.9 (1608.2-2288.5)	1562.8 (1206.1-1878.8)
KEN Tharaka Nithi	2862.9 (2303.3-3429.7)	2285.1 (1799.6-2848.2)	2255.6 (1750.4-2792.7)	2273.2 (1787.2-2767)	2243.4 (1762.7-2787.4)	2105.4 (1668.4-2593.9)	1973.1 (1576.5-2447.4)	1917.3 (1537.8-2344.9)	1902.8 (1544.4-2277)	1981.6 (1631.1-2343.7)	1954.7 (1622.9-2272.2)	1712.1 (1420.5-1986.3)	1367.7 (1130.5-1630.4)	1160.1 (930-1444.1)
KEN Trans Nzoia	1485.9 (1145.5-1845.1)	1539.7 (1229.9-1891.3)	1373.2 (1086.3-1721.2)	1325.6 (1057.9-1671.8)	1273.4 (1026.2-1586.9)	1171.4 (939.7-1463.2)	1074.5 (857.3-1338.6)	1026.6 (831.2-1255.1)	1034.6 (813.4-1284.8)	1252.3 (905.8-1651.5)	1420.9 (1036.2-1853.5)	1291.2 (972.9-1696.1)	1055 (845.9-1343)	819.4 (674.7-1061.7)

Table 1 Age-standardised mortality rates by location and year for females for 1950, 1955...2005, 2010, 2017

Location	1950	1955	1960	1965	1970	1975	1980	1985	1990	1995	2000	2005	2010	2017
KEN Turkana	1351 (1014.5-1710.6)	1383.2 (1068.4-1730.4)	1283.6 (985.3-1630.1)	1293.4 (1015-1652.4)	1371.5 (1094.3-1709.2)	1471.6 (1193.2-1816.2)	1533.5 (1242.7-1868.1)	1500.2 (1224.1-1803.5)	1452.9 (1173.9-1758.2)	1566.7 (1208.2-1953)	1736.2 (1308.4-2220.6)	1678.3 (1286.3-2141.7)	1462.6 (1120.3-1855.3)	1107.2 (795.8-1470.8)
KEN Uasin Gishu	1581.8 (1192-2019.1)	1561.5 (1188.4-1979.5)	1438 (1065.2-1826.5)	1412.6 (1036.5-1779)	1406.4 (1053.1-1779.6)	1398.1 (1050.4-1780.3)	1376.9 (1051.3-1736.1)	1359.9 (1050.9-1696.7)	1362 (1070.9-1680.2)	1415.9 (1088.8-1719)	1487 (1155.5-1802.1)	1370.6 (1063.2-1642.4)	1173.8 (896.3-1480.6)	873.5 (645.1-1153.2)
KEN Vihiga	1785.8 (1352.8-2227.7)	1589.4 (1199.8-2021.6)	1535.7 (1154.3-1987.4)	1516.6 (1141.8-1933.2)	1478.3 (1127.3-1888.8)	1454.4 (1130.4-1850.3)	1406.7 (1090.2-1746.1)	1393.4 (1095.4-1729.9)	1455 (1163.9-1818.8)	1803.8 (1409.3-2293.8)	2087.8 (1622.6-2551.6)	1875.1 (1486.8-2297.2)	1440.8 (1134.9-1799.3)	1249.6 (951.5-1586)
KEN Wajir	1952.1 (1590.6-2330.1)	1922.6 (1569.1-2293)	1763.5 (1413.1-2132.4)	1754.4 (1409.7-2120.5)	1753.6 (1413.9-2120.5)	1724.1 (1393.6-2092.3)	1741.5 (1427-2120.3)	1775 (1469.1-2122.4)	1773.7 (1465.2-2118.2)	1819.2 (1530.7-2137.1)	1808.4 (1543.3-2127)	1743.6 (1473.7-2034.5)	1714.4 (1427.5-1997.9)	1671.2 (1250.5-2067)
KEN West Pokot	2193.5 (1718-2718.4)	2077.6 (1627.1-2587.5)	1970.9 (1523.3-2476.5)	1957.3 (1527.1-2466.5)	1966.7 (1548.6-2481.4)	1926.5 (1531.2-2426.5)	1845.7 (1453-2336.3)	1778.6 (1396.5-2236.6)	1748.7 (1377.3-2171.7)	1737 (1363.4-2173.6)	1598.9 (1241.4-1962.2)	1492.8 (1191.6-1761.3)	1399.3 (1101.8-1680.5)	1143.7 (871.1-1468.4)
MDG Madagascar	2497.8 (2222.2-2764.6)	2361.5 (2184.6-2541.5)	2253.4 (2130.6-2378.6)	2137 (2042.8-2239.6)	2087.3 (1989.5-2177.1)	1937.3 (1828.5-2044)	1781.2 (1651.4-1914.1)	1823.1 (1690.7-1952.2)	1762 (1654.7-1872.5)	1708.7 (1602.3-1814.6)	1615.6 (1494.7-1745)	1512.4 (1363.2-1674.8)	1447.6 (1265.3-1654)	1341.8 (1114.2-1587)
MWI Malawi	3061.7 (2703.3-3445.1)	2880.3 (2572.7-3191.7)	2697.6 (2411.2-2974.1)	2525.1 (2263.6-2798.5)	2312 (2109.8-2532.4)	2076 (1927-2234.9)	1895 (1783.8-2006.8)	1864.8 (1777.2-1963.7)	1988.7 (1863.3-2133.8)	2381.1 (2164.5-2664.9)	2530.5 (2236.2-2828.2)	2226.9 (1977.5-2504.6)	1579.9 (1415.1-1743.7)	1053.4 (920.6-1218.2)
MOZ Mozambique	2714.4 (2331.2-3065)	2500.5 (2182.6-2819.9)	2377.1 (2057.9-2684.5)	2259.8 (1968.3-2563.9)	2106.8 (1838-2384.4)	1950.7 (1719.9-2188.5)	1904.8 (1722.9-2097)	2196.2 (2037.9-2359.7)	1854.1 (1729.2-1990.2)	1827.5 (1693.3-1970)	1870 (1691.2-2049)	1900.1 (1692.2-2114.1)	1824.4 (1618.6-2056.4)	1378.8 (1177.6-1599.1)
RWA Rwanda	3077.6 (2697.2-3441.3)	2781.1 (2443.9-3108)	2664.4 (2343.2-2987.9)	2603.6 (2307.9-2913.8)	2500.3 (2230.2-2793.7)	2428.7 (2165.1-2697.3)	2270.5 (2055.7-2497.2)	2148.4 (1964.3-2346.3)	2240.7 (2071.3-2420.3)	2474.6 (2319.1-2645.6)	2178.5 (2034.3-2333.9)	1557.7 (1435.1-1678.3)	1121 (1015-1228.3)	936.9 (816.8-1066.7)
SOM Somalia	2775.9 (2339.2-3255)	2630.3 (2249.4-3094.8)	2487.1 (2155.7-2892.4)	2389.5 (2055.1-2788.7)	2302.8 (1975.4-2694)	2312 (1990-2673.8)	2109.7 (1802.3-2463.6)	2124.9 (1840.6-2453.9)	2177.9 (1904.1-2516.1)	2137.7 (1829.5-2477.3)	2010.2 (1707-2345.8)	1820.3 (1511.9-2166.3)	1759 (1443.3-2124.3)	1545.1 (1257.4-1914.7)
SSD South Sudan	2617.6 (2182.8-3108)	2576.6 (2177.6-3044.8)	2457.2 (2102.3-2860.4)	2298.1 (1964.2-2646.7)	2124.8 (1807.6-2477)	1981.2 (1673.8-2316.1)	1906.2 (1613.5-2238.4)	1905.1 (1626.5-2219.9)	1882.5 (1603.3-2205.4)	1816.5 (1505-2132)	1738 (1421.7-2071.2)	1593.9 (1271.2-1953.5)	1490.5 (1186.7-1830.2)	1420.2 (1165.1-1742.1)
TZA Tanzania	2623.2 (2285.4-2974.1)	2362.8 (2107-2629.3)	2276.1 (2045.5-2520.2)	2125 (1928.8-2328)	1937.6 (1756.5-2111.8)	1746.9 (1587.1-1916.4)	1589.9 (1456-1746.2)	1540.6 (1429.9-1669.9)	1610.3 (1481.6-1746.1)	1767.3 (1608.9-1934.8)	1745.7 (1581.6-1922.5)	1552.1 (1390.1-1708.1)	1291.2 (1170.1-1425.9)	982.2 (879.5-1095.8)
UGA Uganda	2606.2 (2283.5-2959.4)	2286.7 (1999.5-2577.1)	2199 (1933.7-2480.2)	2072.1 (1816.8-2338.7)	1978.7 (1741.8-2224.8)	1939.1 (1742-2143.5)	2019.2 (1845.8-2199.2)	1976.8 (1815.5-2139.8)	2088.4 (1877.7-2310.2)	2222.8 (2013.8-2450.5)	2109 (1925.9-2310.1)	1755.1 (1593.1-1906.3)	1353.1 (1202.4-1511.3)	976.8 (855.5-1119.7)
ZMB Zambia	2409.2 (2086.6-2759.7)	2251.6 (1960.7-2564)	2145.2 (1874.7-2427)	2022 (1757.4-2293.9)	1853.5 (1639.9-2098.8)	1756.5 (1560.3-1959.5)	1747.7 (1571.1-1914.4)	1816.2 (1683.5-1954.1)	2059.9 (1914.9-2220.1)	2476.2 (2245.2-2737.7)	2441.9 (2205.5-2689)	2011.5 (1824.5-2220.9)	1534 (1398-1677.9)	1153.1 (1007-1305.1)
Southern sub-Saharan Africa	1846.8 (1755.6-1947.2)	1728.4 (1642.8-1826.3)	1654.4 (1574-1746.7)	1587.9 (1511-1671.9)	1484.3 (1410.5-1561.3)	1310.7 (1248.3-1374.2)	1248.8 (1195.1-1304.2)	1078.7 (1040.1-1120.1)	956.6 (925.8-991.9)	1072.8 (1019.6-1131.6)	1672.4 (1593.4-1755.8)	1981.9 (1893.2-2085.5)	1706 (1639.9-1774.3)	990.7 (952.5-1032.4)
BWA Botswana	2018.5 (1728.1-2372.2)	1851.2 (1584.5-2145.3)	1783 (1521.3-2074.7)	1702.6 (1462.5-1974.5)	1590.9 (1369.3-1825.4)	1393.2 (1206.5-1601.6)	1239.1 (1087.9-1406.2)	1065.6 (945-1197.2)	1024.5 (878.4-1180.8)	1123.4 (895.1-1380.9)	1475.7 (1135.9-1882.1)	1805 (1476-2147.9)	1441.1 (1272.5-1648.7)	963.7 (870-1102.4)
LSO Lesotho	1988.1 (1719.6-2284.7)	1884.2 (1639.1-2165.5)	1816.4 (1565.5-2083.2)	1749.8 (1505.7-2007.7)	1658.8 (1434.4-1906.9)	1543.1 (1335.5-1770.8)	1374.5 (1187.9-1561.7)	1262.9 (1111.7-1425.5)	1204 (1076.6-1343.4)	1355.5 (1183.4-1543.1)	2059.3 (1776.3-2388.1)	2669.4 (2287.4-3055.4)	2323.8 (1963.9-2656.4)	1667.3 (1341.1-1996.2)
NAM Namibia	2006.7 (1713.8-2295)	1845 (1574.9-2102.3)	1766.8 (1504.1-2015.7)	1670.3 (1428.5-1919)	1637.9 (1424.1-1857.7)	1573.8 (1388-1756.7)	1525.7 (1380.4-1676.6)	1391.5 (1275.4-1512.7)	1265.6 (1160.7-1385.1)	1394.5 (1256.6-1548)	1842 (1612.5-2063.8)	1947.1 (1674.6-2257.9)	1355.8 (1147.5-1577.1)	891.6 (739.6-1083.9)
ZAF South Africa	1751.9 (1645.5-1866.7)	1654.8 (1556.8-1766.7)	1596 (1496.6-1702.7)	1548.9 (1450.5-1645.7)	1451.8 (1362.1-1540.7)	1271.9 (1199-1348.2)	1226.8 (1166.6-1294.8)	1059.4 (1013.2-1110.7)	895.6 (865.4-926.9)	923.6 (900.7-947.5)	1512.5 (1450.2-1587.1)	1830.4 (1732.8-1936.8)	1605.9 (1535-1682.9)	923.8 (886.5-968)
SWZ Swaziland	2659.1 (2303.2-3014)	2419.4 (2101.1-2734.7)	2225.2 (1917.6-2515.1)	2006 (1711.5-2274.9)	1826.8 (1548.9-2084.3)	1640.3 (1403.3-1876.5)	1488.3 (1282-1691)	1359.5 (1192.5-1540.2)	1232.7 (1091.2-1379.4)	1338.7 (1180.2-1522.6)	2229.7 (1829.5-2648.2)	2870.6 (2357.9-3301.6)	2546.3 (2059-2977.4)	1279.3 (1029.9-1576.8)
ZWE Zimbabwe	2310.6 (2009.6-2663.5)	2071.3 (1799.2-2390.9)	1906.2 (1642.9-2217)	1773.8 (1540.4-2050.2)	1636.7 (1433.9-1874.6)	1489 (1324.9-1691.2)	1374.9 (1248.9-1525.8)	1169.5 (1077.2-1264.2)	1191.5 (1053.5-1376.5)	1681.3 (1364.7-2033.2)	2437 (2070.7-2822.2)	2779.7 (2450.2-3139.7)	2291.1 (2022.2-2559.5)	1326 (1137.9-1541.9)
Western sub-Saharan Africa	2748.8 (2518.5-3001.6)	2497.9 (2305.4-2715)	2324.5 (2159.6-2510.1)	2127.4 (1983.9-2294.3)	2015.7 (1873.1-2172.7)	1862.5 (1726.9-2010.6)	1714.9 (1597.4-1844.6)	1627.5 (1512.9-1757.1)	1608.6 (1489.3-1734.3)	1619.7 (1494.6-1753.9)	1619.1 (1482.2-1764.3)	1516.9 (1369.3-1672.6)	1355.1 (1220.8-1504.7)	1148.5 (1039.3-1278.6)
BEN Benin	2946.1 (2585.5-3330.9)	2654.7 (2342.1-2989.2)	2477.8 (2188.2-2771)	2280 (2014.8-2547.4)	2121.7 (1868.1-2374.9)	1944.2 (1747.2-2165.3)	1778.3 (1613.6-1956.9)	1625.7 (1482.8-1770.4)	1495.7 (1378.5-1624)	1435 (1319.8-1558.5)	1411.3 (1271.3-1552.5)	1347 (1183-1513)	1260.2 (1078.4-1444.6)	1130.7 (953.8-1334.3)
BFA Burkina Faso	3203.2 (2815.1-3637.2)	2934.9 (2595-3311)	2675.8 (2369.9-2999)	2463.8 (2172.9-2763.6)	2312.4 (2065.2-2577.3)	2172.3 (1949.3-2389.7)	1987 (1822.4-2170.9)	1863.9 (1736.6-1998.6)	1827.4 (1704.4-1949.1)	1848.2 (1708.4-1997.6)	1756.5 (1623.8-1911.2)	1588 (1456.7-1722.8)	1411.6 (1278.7-1541.3)	1238.7 (1100.3-1387.2)
CMR Cameroon	3095 (2736.4-3452.6)	2751.6 (2435.2-3057.2)	2872.4 (2576.5-3150.5)	2349 (2095.3-2599.7)	2192.7 (1983-2401.9)	2048.2 (1873.2-2218.4)	1895.4 (1739.5-2051.1)	1693.5 (1570.4-1815)	1581.6 (1469.6-1694.6)	1634 (1517.8-1754.6)	1742.2 (1591.1-1898.8)	1749.8 (1550.2-1952.7)	1562.8 (1354-1783)	1213.2 (1017.4-1445)
CPV Cape Verde	1741.2 (1535-1978.9)	1399.3 (1204.4-1619.1)	1394.6 (1216.6-1604.8)	1291.4 (1137.1-1472.2)	1386.9 (1253.5-1524)	1193.9 (1115.8-1276.8)	957.7 (923.4-996)	826.3 (802.2-849.7)	708.5 (683.6-734.3)	722.5 (675.4-772)	646.4 (588.9-712.8)	611.3 (585.5-655.9)	590.8 (571.4-611.8)	562.2 (513.4-597.6)
TCD Chad	2698.5 (2316.2-3077.6)	2368.8 (2039.9-2710.7)	2188.3 (1889.7-2504.8)	2044.6 (1771.1-2330.7)	1979.8 (1736.6-2245.9)	1851.7 (1642.5-2081.8)	1822.6 (1647.7-2007.5)	1762.3 (1620.5-1902.9)	1699.4 (1572.5-1830.2)	1717.3 (1582-1859.7)	1794.3 (1646.8-1954)	1733.9 (1578.7-1905.7)	1582.9 (1422.5-1767.1)	1401 (1219.7-1601.8)
CIV Cote d'Ivoire	2270.3 (1991.7-2608.5)	2231.6 (1954.2-2532.4)	2084.6 (1816.3-2375.6)	1959.7 (1715.1-2213.8)	1828.3 (1606.4-2053.1)	1682.1 (1500.6-1868.1)	1533.8 (1382.2-1693.6)	1475.6 (1342.2-1609.8)	1540.2 (1380.4-1714.8)	1750.1 (1565.1-1955)	1805.5 (1612.1-2008.6)	1722.1 (1536.6-1916.6)	1517.6 (1328.7-1708.8)	1204.4 (1030.2-1403)
GMB The Gambia	2157.9 (1856.6-2470.8)	2144.9 (1842.2-2445.5)	2082.9 (1795.7-2384.9)	1956.7 (1685-2240.8)	1762.5 (1507-2038.3)	1618.1 (1371-1879.6)	1497.9 (1287.8-1732.9)	1406.8 (1217.9-1623.9)	1353.7 (1174.2-1552)	1321.3 (1141.1-1497.2)	1315.9 (1154.9-1490)	1318.3 (1151.3-1487.2)	1272.1 (1112.5-1453.6)	1160.6 (987.8-1373.8)
GHA Ghana	2403.4 (2095-2723.6)	2165.2 (1892.1-2465.1)	1930 (1681.8-2214.7)	1742 (1489.2-2014.3)	1619.1 (1389.9-1883.2)	1521.2 (1310.7-1764.7)	1480.7 (1280.1-1697.4)	1480.9 (1293.4-1679.5)	1437.8 (1271.4-1622.1)	1445.2 (1294-1616.3)	1456.9 (1311.6-1611.1)	1406.5 (1279.7-1540.1)	1311.3 (1193.5-1442.9)	1074.7 (943.5-1219.5)
GIN Guinea	2751 (2382-3127.3)	2695.2 (2366.2-3036.3)	2466.8 (2151.2-2768.4)	2273.2 (2002.2-2547.2)	2195.7 (1948.4-2455.7)	2246.4 (2020.9-2479.1)	2016.1 (1848.8-2202.6)	1942.9 (1810.5-2099.4)	1859.7 (1741.4-1987.2)	1772.6 (1663.9-1896.3)	1739.3 (1612.4-1875.3)	1683.6 (1545.3-1834.1)	1616 (1477-1766.7)	1458.6 (1292.4-1632.7)
GNB Guinea-Bissau	3367.3 (2959.6-3754.2)	3025.4 (2672.2-3354.7)	2850.3 (2520.4-3164.6)	2857.9 (2559.7-3180.9)	2695.8 (2375.1-2993)	2399.5 (2101.8-2682.4)	2270.2 (1982.4-2561.3)	2187.6 (1904.8-2472.4)	2066.9 (1803.5-2324.8)	1977.9 (1756.5-2203.5)	1921.7 (1720.2-2110.5)	1862.1 (1667.6-2068.3)	1757.5 (1539.3-1980.5)	1506.1 (1277.3-1746.5)
LBR Liberia	3124 (2729.3-3514.8)	2706.4 (2389.1-3027.5)	2436.9 (2145.3-2703.5)	2254.8 (1999.5-2524.2)	2070.7 (1831.8-2320.1)	1939.7 (1704.5-2178.1)	1815.6 (1617.9-2049.9)	1738.5 (1551.2-1954.6)	1980.7 (1808.6-2177.1)	1900.4 (1746.2-2070.9)	1730.1 (1581.6-1881.5)	1559.9 (1410.6-1713.4)	1425.6 (1269.3-1592.4)	1254.6 (1070.7-1454.3)
MLI Mali	3548.6 (3132.8-3987)	3351.3 (2992.2-3719.5)	3125.6 (2798-3472.1)	3010.4 (2714-3303)	2894.4 (2646.6-3141.1)	2741.5 (2536.7-2943.7)	2459.1 (2285.6-2616.9)	2267.6 (2129.6-2408.7)	2139.3 (2031.3-2261)	1998.8 (1895.9-2111.3)	1822.8 (1712.6-1942.4)	1579.7 (1467.6-1700)	1399.9 (1277.7-1534.4)	1284 (1126-1451.7)
MRT Mauritania	2832.7 (2470.3-3185.3)	2573.2 (2249.4-2889.9)	2413.6 (2127.3-2723.9)	2200 (1913-2481.6)	1990.4 (1739.7-2243.1)	1918.7 (1699.5-2144.6)	1790.8 (1602.3-1993.1)	1708.9 (1544.1-1890.4)	1613 (1476.5-1766.7)	1491 (1367.2-1627.4)	1361.8 (1243.3-1491.7)	1221.1 (1104.5-1344.5)	1073.3 (949.8-1207.9)	957.6 (827-1122.3)
NER Niger	3017.5 (2651.4-3389)	2806.8 (2492.7-3131.2)	2584.7 (2289-2901.4)	2369.9 (2104.8-2648.3)	2302.4 (2059.9-2553.9)	2312.8 (2117.4-2512.2)	2223.5 (2051-2385.2)	2130.1 (1975.4-2290.6)	1987 (1859.7-2136.7)	1897.4 (1770.6-2037.7)	1722.6 (1590.6-1861.6)	1536.1 (1407.5-1669.4)	1382.5 (1232.2-1529.9)	1239.3 (1064.6-1417.9)
NGA Nigeria	2696.2 (2226.8-3216.8)	2399.6 (2033.3-2835.8)	2202.5 (1887.4-2557.3)	2010.6 (1739-2331.4)	1914.8 (1646.3-2235)	1729.4 (1473.2-1997)	1578.6 (1368.2-1823.2)	1508.5 (1296.2-1747.3)	1533.9 (1313.2-1780.5)	1550.9 (1304.5-1819.9)	1571.6 (1288.3-1876.1)	1452.7 (1156.5-1782.3)	1268.6 (991.6-1589.9)	1071.8 (848-1342.2)
STP Sao Tome and Principe	3016.1 (2781.7-3254.9)	2443.1 (2317.2-2565.6)	1994.6 (1910.1-2088.6)	1435.9 (1378.4-1490.8)	1248.8 (1206.4-1289.7)	1135.7 (1091.1-1181.3)	1079.1 (1035.7-1127)	1082.5 (1023.5-1146.2)	1092.5 (1003.8-1182.6)	1169.3 (1065.6-1279)	1193.4 (1091.2-1314)	1112.9 (1017.1-1217.8)	1018.1 (918.1-1139)	973.8 (832.5-1110.8)
SEN Senegal	2710.3 (2371.8-3095.9)	2511 (2200.3-2851.9)	2353.8 (2061.2-2652.8)	2252.2 (1981.9-2527.7)	2144.9 (1920.1-2384.3)	1981.8 (1785.7-2178.3)	1778.1 (1617.1-1954.4)	1590.8 (1462.1-1729.4)	1456.5 (1347.8-1572)	1425.9 (1328.9-1529.3)	1351.8 (1264.3-1453)	1231.5 (1139.7-1333.4)	1100.7 (994.8-1208.6)	1007.6 (887.5-1144)
SLE Sierra Leone	3032.3 (2665-3456.9)	2799.3 (2464.7-3151.8)	2598.9 (2281.8-2938.5)	2396.7 (2087.5-2702.5)	2201.8 (1922.3-2500.4)	2053.4 (1795.8-2313.6)	1977.9 (1745.7-2209.3)	1882.1 (1675.4-2106.8)	1766.2 (1592.5-1960.6)	1791.8 (1658.3-1949.4)	1813.5 (1678.1-1960.7)	1843.1 (1700.6-1977)	1745.3 (1576.4-1928.8)	1480.3 (1288-1684.3)
TGO Togo	2400.9 (2093.6-2720.9)	2196.2 (1906.8-2471.9)	2070.7 (1795.1-2354.1)	1942.4 (1699.9-2225.3)	1791.6 (1581.6-2041)	1688.6 (1491-1907.7)	1599.6 (1426.6-1792.9)	1551.7 (1395.7-1712.7)	1498.7 (1363.1-1647.8)	1478.1 (1334.5-1641.3)	1513.5 (1348.1-1708.1)	1502.3 (1308-1713.3)	1372.5 (1177.6-1573)	1101.6 (935.3-1294.7)

Table 2 Under-5 death numbers by location and year for 1950, 1955...2005, 2010, 2017 - both sexes

Location	1950	1955	1960	1965	1970	1975	1980	1985	1990	1995	2000	2005	2010	2017
Global	19600 (19100-20200)	19000 (18600-19300)	33100 (32800-33400)	18300 (18000-18600)	16600 (16400-16900)	15000 (14800-15200)	13500 (13300-13700)	13000 (12800-13200)	11800 (11600-11900)	10800 (10600-10900)	9680 (9540-9820)	8380 (8250-8520)	7140 (6980-7300)	5390 (5200-5610)
Low SDI	4440 (4110-4810)	4540 (4320-4780)	4740 (4540-4950)	4910 (4720-5120)	4800 (4650-4940)	4890 (4750-5030)	4880 (4760-5010)	4800 (4690-4900)	4600 (4500-4700)	4510 (4420-4610)	4170 (4080-4270)	3680 (3600-3760)	3230 (3140-3320)	2480 (2370-2590)
Low-middle SDI	5210 (4930-5520)	5130 (4940-5330)	6530 (6370-6700)	5240 (5080-5410)	4990 (4860-5120)	4870 (4750-4980)	4480 (4370-4590)	4250 (4160-4350)	3940 (3850-4040)	3760 (3680-3860)	3580 (3480-3680)	3210 (3100-3310)	2730 (2610-2860)	2100 (1930-2280)
Middle SDI	5900 (5660-6160)	5740 (5570-5910)	16200 (16000-16400)	5460 (5310-5600)	4610 (4480-4740)	3580 (3480-3680)	2850 (2780-2910)	2750 (2660-2840)	2270 (2210-2340)	1760 (1720-1800)	1370 (1350-1400)	1070 (1040-1090)	839 (818-861)	571 (551-592)
High-middle SDI	2960 (2830-3110)	2690 (2600-2780)	4870 (4780-4970)	2160 (2080-2240)	1780 (1720-1830)	1350 (1320-1390)	1070 (1050-1100)	1020 (991-1040)	790 (769-813)	603 (587-620)	458 (445-472)	344 (334-354)	274 (265-283)	182 (174-189)
High SDI	1060 (1020-1100)	783 (764-802)	660 (648-672)	493 (484-503)	389 (382-397)	263 (259-267)	200 (198-203)	155 (153-157)	130 (129-131)	98 (97-99)	82 (81-82)	74 (74-75)	65 (64-66)	57 (55-58)
Central Europe, Eastern Europe, and Central Asia	1060 (1010-1110)	760 (733-789)	579 (558-601)	401 (386-418)	324 (313-335)	302 (294-312)	285 (279-292)	273 (267-278)	213 (208-218)	164 (160-169)	122 (118-126)	103 (98-107)	92 (87-97)	70 (66-75)
Central Asia	295 (262-327)	260 (239-282)	234 (216-253)	187 (173-203)	141 (131-151)	130 (123-138)	131 (125-138)	132 (126-137)	113 (109-118)	93 (89-98)	71 (67-75)	61 (57-66)	58 (54-63)	46 (42-51)
Armenia	16 (12-22)	14 (11-17)	13 (10-15)	9 (7-11)	6 (5-8)	5 (4-6)	5 (4-6)	5 (4-5)	3 (3-4)	2 (2-2)	1 (1-1)	1 (1-1)	1 (1-1)	0 (0-0)
Azerbaijan	56 (43-72)	50 (41-60)	45 (37-54)	32 (26-41)	21 (17-26)	15 (12-18)	15 (13-18)	17 (15-19)	15 (13-17)	11 (10-13)	9 (8-10)	8 (7-9)	7 (6-9)	6 (5-8)
Georgia	27 (18-38)	22 (16-28)	18 (14-22)	13 (11-17)	10 (8-12)	8 (6-10)	6 (5-7)	5 (4-5)	4 (3-4)	3 (2-3)	2 (2-2)	1 (1-1)	1 (1-1)	1 (0-1)
Kazakhstan	48 (35-65)	44 (34-55)	41 (32-51)	33 (27-41)	25 (21-29)	23 (20-26)	22 (20-24)	19 (18-21)	15 (13-16)	11 (10-12)	8 (7-9)	8 (7-9)	8 (7-10)	5 (4-7)
Kyrgyzstan	26 (20-34)	22 (18-26)	19 (15-23)	16 (13-20)	12 (10-14)	11 (10-13)	11 (10-12)	10 (9-11)	8 (7-9)	6 (6-7)	5 (5-6)	5 (4-5)	4 (4-4)	3 (3-4)
Mongolia	14 (11-18)	12 (10-14)	11 (9-12)	8 (7-10)	7 (6-8)	7 (6-7)	7 (7-8)	8 (7-9)	7 (6-7)	5 (4-5)	4 (3-4)	3 (3-3)	3 (2-3)	2 (2-2)
Tajikistan	31 (24-39)	29 (25-35)	28 (24-33)	24 (20-28)	19 (16-22)	18 (15-21)	18 (16-20)	18 (17-20)	17 (15-19)	15 (13-16)	12 (11-14)	10 (9-11)	11 (9-12)	11 (9-14)
Turkmenistan	14 (11-19)	13 (11-16)	12 (10-15)	10 (8-13)	8 (6-10)	8 (7-10)	9 (8-11)	9 (8-11)	9 (8-10)	8 (7-9)	6 (6-7)	5 (4-6)	4 (4-5)	3 (2-4)
Uzbekistan	61 (45-80)	53 (42-65)	47 (38-58)	41 (34-49)	34 (28-40)	35 (31-40)	39 (35-43)	40 (36-44)	36 (33-40)	33 (30-36)	24 (21-28)	21 (17-25)	19 (16-23)	15 (12-18)
Central Europe	398 (384-413)	238 (232-243)	149 (146-151)	99 (97-101)	91 (89-93)	77 (76-78)	60 (60-61)	50 (50-51)	38 (38-38)	26 (25-26)	16 (16-17)	12 (12-13)	9 (9-9)	6 (6-7)
Albania	13 (11-16)	12 (10-13)	11 (9-12)	9 (7-10)	7 (6-8)	5 (5-7)	4 (4-5)	4 (4-4)	3 (3-4)	3 (3-3)	2 (2-2)	1 (1-1)	1 (1-1)	0 (0-1)
Bosnia and Herzegovina	28 (24-33)	19 (16-21)	13 (11-14)	9 (8-10)	6 (5-7)	4 (4-5)	3 (3-3)	2 (2-2)	1 (1-1)	1 (1-2)	0 (0-0)	0 (0-0)	0 (0-0)	0 (0-0)
Bulgaria	32 (30-34)	15 (15-16)	8 (7-8)	5 (4-5)	4 (4-4)	4 (4-4)	3 (3-3)	2 (2-2)	2 (2-2)	1 (1-1)	1 (1-1)	1 (1-1)	1 (1-1)	0 (0-1)
Croatia	22 (18-26)	11 (10-13)	6 (6-7)	4 (4-5)	3 (3-3)	2 (2-2)	2 (1-2)	1 (1-1)	1 (1-1)	1 (1-1)	0 (0-0)	0 (0-0)	0 (0-0)	0 (0-0)
Czech Republic	10 (9-10)	5 (5-6)	3 (3-3)	4 (4-4)	3 (3-4)	4 (4-4)	3 (3-3)	2 (2-2)	2 (1-2)	1 (1-1)	0 (0-1)	0 (0-0)	0 (0-0)	0 (0-0)
Hungary	24 (23-25)	14 (13-14)	8 (8-8)	6 (6-6)	6 (6-6)	7 (7-7)	4 (4-4)	3 (3-3)	2 (2-2)	1 (1-1)	1 (1-1)	1 (1-1)	1 (1-1)	0 (0-1)
Macedonia	14 (12-17)	10 (9-11)	7 (6-8)	5 (5-6)	4 (3-4)	3 (3-3)	2 (2-3)	2 (2-2)	1 (1-1)	1 (1-1)	0 (0-0)	0 (0-0)	0 (0-0)	0 (0-0)
Montenegro	1 (1-1)	1 (1-1)	1 (1-1)	1 (1-1)	0 (0-0)	0 (0-0)	0 (0-0)	0 (0-0)	0 (0-0)	0 (0-0)	0 (0-0)	0 (0-0)	0 (0-0)	0 (0-0)
Poland	134 (131-137)	73 (72-73)	40 (40-41)	25 (25-25)	20 (20-21)	18 (18-18)	16 (16-17)	14 (14-14)	10 (10-10)	6 (6-7)	4 (3-4)	3 (3-3)	2 (2-2)	2 (1-2)
Romania	64 (54-75)	44 (40-48)	31 (30-31)	15 (15-15)	23 (23-24)	17 (17-18)	14 (14-14)	12 (12-12)	10 (10-11)	6 (6-6)	5 (5-5)	4 (4-4)	2 (2-3)	2 (1-2)
Serbia	42 (41-44)	26 (25-26)	16 (16-17)	12 (12-13)	10 (10-10)	8 (8-8)	6 (6-6)	6 (6-6)	4 (4-4)	3 (3-3)	2 (1-2)	1 (1-1)	1 (1-1)	0 (0-0)
Slovakia	9 (9-10)	5 (5-5)	3 (3-3)	3 (3-3)	2 (2-3)	3 (3-3)	2 (2-2)	2 (2-2)	1 (1-1)	1 (1-1)	1 (0-1)	0 (0-0)	0 (0-0)	0 (0-0)
Slovenia	5 (4-5)	3 (3-3)	2 (2-2)	2 (2-2)	1 (1-1)	1 (1-1)	1 (1-1)	0 (0-0)	0 (0-0)	0 (0-0)	0 (0-0)	0 (0-0)	0 (0-0)	0 (0-0)
Eastern Europe	363 (327-411)	263 (247-282)	196 (187-206)	115 (109-121)	92 (88-97)	96 (92-100)	94 (90-97)	91 (88-94)	62 (61-64)	45 (44-46)	35 (34-35)	29 (29-29)	25 (25-25)	18 (17-18)
Belarus	18 (12-25)	14 (11-18)	11 (9-14)	7 (6-9)	5 (4-6)	4 (4-5)	4 (4-5)	4 (3-5)	3 (3-3)	2 (2-2)	2 (1-2)	1 (1-1)	1 (1-1)	1 (1-1)
Estonia	1 (1-2)	1 (1-1)	1 (1-1)	1 (0-1)	0 (0-0)	0 (0-0)	0 (0-1)	0 (0-0)	0 (0-0)	0 (0-0)	0 (0-0)	0 (0-0)	0 (0-0)	0 (0-0)
Latvia	3 (2-5)	2 (1-2)	1 (1-1)	1 (1-1)	1 (1-1)	1 (1-1)	1 (1-1)	1 (1-1)	1 (1-1)	0 (0-0)	0 (0-0)	0 (0-0)	0 (0-0)	0 (0-0)
Lithuania	7 (5-10)	4 (4-5)	3 (3-3)	2 (2-2)	1 (1-1)	1 (1-1)	1 (1-1)	1 (1-1)	1 (1-1)	1 (1-1)	0 (0-0)	0 (0-0)	0 (0-0)	0 (0-0)
Moldova	20 (14-28)	16 (12-20)	13 (10-16)	9 (7-12)	6 (5-8)	6 (4-7)	5 (4-6)	4 (4-5)	3 (3-3)	2 (2-2)	1 (1-2)	1 (1-1)	1 (1-1)	1 (0-1)
Russian Federation	228 (214-242)	164 (159-170)	123 (121-124)	66 (65-66)	54 (54-54)	59 (59-60)	60 (59-61)	62 (61-62)	41 (41-41)	30 (30-30)	25 (25-25)	21 (21-21)	17 (17-18)	12 (12-12)
Ukraine	87 (55-130)	61 (46-78)	45 (37-54)	30 (25-35)	24 (21-28)	24 (20-27)	22 (19-25)	19 (17-21)	13 (12-15)	9 (8-10)	6 (6-6)	5 (5-5)	5 (5-6)	4 (3-5)
High-income	857 (821-902)	700 (682-718)	622 (612-633)	500 (491-509)	406 (399-413)	275 (270-281)	204 (201-207)	153 (152-155)	135 (134-136)	103 (102-104)	89 (89-90)	80 (80-81)	71 (70-71)	60 (58-62)
Australasia	8 (7-8)	8 (7-8)	7 (7-8)	6 (6-6)	7 (7-7)	5 (5-5)	4 (4-4)	4 (3-4)	3 (3-3)	2 (2-2)	2 (2-2)	2 (2-2)	2 (2-2)	1 (1-2)
Australia	6 (6-6)	6 (6-6)	6 (6-6)	5 (5-5)	6 (5-6)	4 (4-4)	3 (3-3)	3 (3-3)	2 (2-2)	2 (2-2)	2 (1-2)	2 (1-2)	1 (1-2)	1 (1-2)
New Zealand	2 (2-2)	2 (2-2)	2 (2-2)	1 (1-1)	1 (1-1)	1 (1-1)	1 (1-1)	1 (1-1)	1 (1-1)	0 (0-1)	0 (0-0)	0 (0-0)	0 (0-0)	0 (0-0)
High-income Asia-Pacific	303 (270-343)	198 (182-215)	150 (139-161)	114 (105-122)	92 (85-99)	54 (50-58)	33 (31-36)	21 (19-22)	15 (14-16)	12 (12-13)	10 (10-10)	7 (7-7)	6 (5-6)	4 (4-4)
Brunei	0 (0-1)	0 (0-0)	0 (0-0)	0 (0-0)	0 (0-0)	0 (0-0)	0 (0-0)	0 (0-0)	0 (0-0)	0 (0-0)	0 (0-0)	0 (0-0)	0 (0-0)	0 (0-0)
Japan	146 (141-151)	93 (92-95)	62 (61-64)	39 (38-39)	34 (33-34)	24 (24-25)	16 (16-16)	11 (11-11)	8 (8-8)	7 (7-7)	5 (5-5)	4 (4-4)	3 (3-3)	2 (2-3)
Aichi	7 (6-8)	5 (4-5)	3 (3-3)	2 (2-2)	2 (2-2)	1 (1-1)	1 (1-1)	1 (1-1)	0 (0-0)	0 (0-0)	0 (0-0)	0 (0-0)	0 (0-0)	0 (0-0)
Akita	2 (2-2)	1 (1-1)	1 (1-1)	0 (0-0)	0 (0-0)	0 (0-0)	0 (0-0)	0 (0-0)	0 (0-0)	0 (0-0)	0 (0-0)	0 (0-0)	0 (0-0)	0 (0-0)
Aomori	2 (2-3)	2 (1-2)	1 (1-1)	1 (1-1)	1 (1-1)	0 (0-0)	0 (0-0)	0 (0-0)	0 (0-0)	0 (0-0)	0 (0-0)	0 (0-0)	0 (0-0)	0 (0-0)
Chiba	4 (3-4)	3 (2-3)	2 (2-2)	1 (1-1)	1 (1-1)	1 (1-1)	1 (1-1)	0 (0-0)	0 (0-0)	0 (0-0)	0 (0-0)	0 (0-0)	0 (0-0)	0 (0-0)
Ehime	2 (2-3)	1 (1-1)	1 (1-1)	1 (1-1)	0 (0-0)	0 (0-0)	0 (0-0)	0 (0-0)	0 (0-0)	0 (0-0)	0 (0-0)	0 (0-0)	0 (0-0)	0 (0-0)
Fukui	1 (1-1)	1 (1-1)	0 (0-0)	0 (0-0)	0 (0-0)	0 (0-0)	0 (0-0)	0 (0-0)	0 (0-0)	0 (0-0)	0 (0-0)	0 (0-0)	0 (0-0)	0 (0-0)
Fukuoka	6 (5-6)	4 (3-4)	2 (2-3)	1 (1-2)	1 (1-1)	1 (1-1)	1 (1-1)	0 (0-0)	0 (0-0)	0 (0-0)	0 (0-0)	0 (0-0)	0 (0-0)	0 (0-0)
Fukushima	3 (2-3)	2 (2-2)	1 (1-1)	1 (1-1)	1 (1-1)	0 (0-1)	0 (0-0)	0 (0-0)	0 (0-0)	0 (0-0)	0 (0-0)	0 (0-0)	0 (0-0)	0 (0-0)
Gifu	2 (2-3)	2 (1-2)	1 (1-1)	1 (1-1)	1 (1-1)	0 (0-0)	0 (0-0)	0 (0-0)	0 (0-0)	0 (0-0)	0 (0-0)	0 (0-0)	0 (0-0)	0 (0-0)
Gunma	2 (2-3)	1 (1-2)	1 (1-1)	1 (1-1)	1 (1-1)	0 (0-0)	0 (0-0)	0 (0-0)	0 (0-0)	0 (0-0)	0 (0-0)	0 (0-0)	0 (0-0)	0 (0-0)
Hiroshima	4 (3-5)	2 (2-3)	2 (1-2)	1 (1-1)	1 (1-1)	1 (1-1)	0 (0-0)	0 (0-0)	0 (0-0)	0 (0-0)	0 (0-0)	0 (0-0)	0 (0-0)	0 (0-0)
Hokkaidō	9 (7-10)	5 (5-6)	4 (3-4)	2 (2-2)	2 (2-2)	1 (1-1)	1 (1-1)	1 (1-1)	0 (0-0)	0 (0-0)	0 (0-0)	0 (0-0)	0 (0-0)	0 (0-0)
Hyōgo	7 (6-8)	4 (4-5)	3 (3-3)	2 (2-2)	1 (1-2)	1 (1-1)	1 (1-1)	0 (0-0)	0 (0-0)	0 (0-0)	0 (0-0)	0 (0-0)	0 (0-0)	0 (0-0)
Ibaraki	3 (2-3)	2 (2-2)	1 (1-1)	1 (1-1)	1 (1-1)	1 (0-1)	0 (0-0)	0 (0-0)	0 (0-0)	0 (0-0)	0 (0-0)	0 (0-0)	0 (0-0)	0 (0-0)
Ishikawa	1 (1-2)	1 (1-1)	1 (1-1)	0 (0-0)	0 (0-0)	0 (0-0)	0 (0-0)	0 (0-0)	0 (0-0)	0 (0-0)	0 (0-0)	0 (0-0)	0 (0-0)	0 (0-0)
Iwate	2 (2-3)	1 (1-2)	1 (1-1)	1 (1-1)	0 (0-1)	0 (0-0)	0 (0-0)	0 (0-0)	0 (0-0)	0 (0-0)	0 (0-0)	0 (0-0)	0 (0-0)	0 (0-0)
Kagawa	1 (1-2)	1 (1-1)	1 (0-1)	0 (0-0)	0 (0-0)	0 (0-0)	0 (0-0)	0 (0-0)	0 (0-0)	0 (0-0)	0 (0-0)	0 (0-0)	0 (0-0)	0 (0-0)
Kagoshima	2 (2-3)	1 (1-1)	1 (1-1)	1 (1-1)	1 (0-1)	0 (0-0)	0 (0-0)	0 (0-0)	0 (0-0)	0 (0-0)	0 (0-0)	0 (0-0)	0 (0-0)	0 (0-0)
Kanagawa	6 (5-7)	4 (4-5)	3 (3-3)	2 (2-2)	2 (2-2)	1 (1-1)	1 (1-1)	1 (1-1)	0 (0-1)	0 (0-0)	0 (0-0)	0 (0-0)	0 (0-0)	0 (0-0)
Kōchi	1 (1-2)	1 (1-1)	1 (0-1)	0 (0-0)	0 (0-0)	0 (0-0)	0 (0-0)	0 (0-0)	0 (0-0)	0 (0-0)	0 (0-0)	0 (0-0)	0 (0-0)	0 (0-0)
Kumamoto	2 (2-3)	1 (1-2)	1 (1-1)	1 (1-1)	1 (1-1)	0 (0-0)	0 (0-0)	0 (0-0)	0 (0-0)	0 (0-0)	0 (0-0)	0 (0-0)	0 (0-0)	0 (0-0)
Kyōto	3 (3-4)	2 (2-2)	1 (1-1)	1 (1-1)	1 (1-1)	0 (0-0)	0 (0-0)	0 (0-0)	0 (0-0)	0 (0-0)	0 (0-0)	0 (0-0)	0 (0-0)	0 (0-0)
Mie	2 (2-2)	1 (1-1)	1 (1-1)	1 (1-1)	0 (0-1)	0 (0-0)	0 (0-0)	0 (0-0)	0 (0-0)	0 (0-0)	0 (0-0)	0 (0-0)	0 (0-0)	0 (0-0)
Miyagi	2 (2-3)	2 (1-2)	1 (1-1)	1 (1-1)	1 (1-1)	0 (0-0)	0 (0-0)	0 (0-0)	0 (0-0)	0 (0-0)	0 (0-0)	0 (0-0)	0 (0-0)	0 (0-0)
Miyazaki	2 (1-2)	1 (1-1)	1 (1-1)	0 (0-0)	0 (0-0)	0 (0-0)	0 (0-0)	0 (0-0)	0 (0-0)	0 (0-0)	0 (0-0)	0 (0-0)	0 (0-0)	0 (0-0)
Nagano	3 (2-3)	2 (1-2)	1 (1-1)	1 (1-1)	1 (0-1)	0 (0-0)	0 (0-0)	0 (0-0)	0 (0-0)	0 (0-0)	0 (0-0)	0 (0-0)	0 (0-0)	0 (0-0)
Nagasaki	2 (2-3)	1 (1-1)	1 (1-1)	1 (1-1)	0 (0-0)	0 (0-0)	0 (0-0)	0 (0-0)	0 (0-0)	0 (0-0)	0 (0-0)	0 (0-0)	0 (0-0)	0 (0-0)
Nara	1 (1-1)	1 (1-1)	1 (0-1)	0 (0-0)	0 (0-0)	0 (0-0)	0 (0-0)	0 (0-0)	0 (0-0)	0 (0-0)	0 (0-0)	0 (0-0)	0 (0-0)	0 (0-0)
Niigata	4 (3-4)	2 (2-2)	1 (1-2)	1 (1-1)	1 (1-1)	0 (0-1)	0 (0-0)	0 (0-0)	0 (0-0)	0 (0-0)	0 (0-0)	0 (0-0)	0 (0-0)	0 (0-0)
Ōita	2 (1-2)	1 (1-1)	1 (1-1)	0 (0-0)	0 (0-0)	0 (0-0)	0 (0-0)	0 (0-0)	0 (0-0)	0 (0-0)	0 (0-0)	0 (0-0)	0 (0-0)	0 (0-0)
Okayama	2 (2-3)	1 (1-2)	1 (1-1)	1 (1-1)	0 (0-1)	0 (0-0)	0 (0-0)	0 (0-0)	0 (0-0)	0 (0-0)	0 (0-0)	0 (0-0)	0 (0-0)	0 (0-0)
Okinawa	1 (1-1)	1 (1-1)	0 (0-1)	0 (0-0)	0 (0-0)	0 (0-0)	0 (0-0)	0 (0-0)	0 (0-0)	0 (0-0)	0 (0-0)	0 (0-0)	0 (0-0)	0 (0-0)
Ōsaka	12 (10-14)	8 (7-8)	5 (5-5)	3 (3-3)	3 (2-3)	2 (2-2)	1 (1-1)	1 (1-1)	1 (1-1)	0 (0-1)	0 (0-0)	0 (0-0)	0 (0-0)	0 (0-0)
Saga	1 (1-1)	1 (1-1)	0 (0-1)	0 (0-0)	0 (0-0)	0 (0-0)	0 (0-0)	0 (0-0)	0 (0-0)	0 (0-0)	0 (0-0)	0 (0-0)	0 (0-0)	0 (0-0)
Saitama	5 (4-6)	3 (3-4)	2 (2-3)	2 (2-2)	1 (1-2)	1 (1-1)	1 (1-1)	0 (0-0)	0 (0-0)	0 (0-0)	0 (0-0)	0 (0-0)	0 (0-0)	0 (0-0)
Shiga	1 (1-1)	1 (1-1)	0 (0-1)	0 (0-0)	0 (0-0)	0 (0-0)	0 (0-0)	0 (0-0)	0 (0-0)	0 (0-0)	0 (0-0)	0 (0-0)	0 (0-0)	0 (0-0)
Shimane	1 (1-1)	1 (1-1)	0 (0-0)	0 (0-0)	0 (0-0)	0 (0-0)	0 (0-0)	0 (0-0)	0 (0-0)	0 (0-0)	0 (0-0)	0 (0-0)	0 (0-0)	0 (0-0)
Shizuoka	4 (3-5)	3 (2-3)	2 (2-2)	1 (1-1)	1 (1-1)	1 (1-1)	0 (0-0)	0 (0-0)	0 (0-0)	0 (0-0)	0 (0-0)	0 (0-0)	0 (0-0)	0 (0-0)
Tochigi	2 (2-3)	1 (1-2)	1 (1-1)	1 (1-1)	1 (1-1)	0 (0-0)	0 (0-0)	0 (0-0)	0 (0-0)	0 (0-0)	0 (0-0)	0 (0-0)	0 (0-0)	0 (0-0)
Tokushima	1 (1-1)	1 (1-1)	0 (0-1)	0 (0-0)	0 (0-0)	0 (0-0)	0 (0-0)	0 (0-0)	0 (0-0)	0 (0-0)	0 (0-0)	0 (0-0)	0 (0-0)	0 (0-0)
Tōkyō	15 (12-18)	9 (8-10)	6 (6-7)	4 (4-4)	3 (3-4)	2 (2-3)	1 (1-2)	1 (1-1)	1 (1-1)	1 (1-1)	1 (1-1)	1 (1-1)	0 (0-0)	0 (0-0)
Tottori	1 (1-1)	0 (0-1)	0 (0-0)	0 (0-0)	0 (0-0)	0 (0-0)	0 (0-0)	0 (0-0)	0 (0-0)	0 (0-0)	0 (0-0)	0 (0-0)	0 (0-0)	0 (0-0)
Toyama	2 (1-2)	1 (1-1)	1 (1-1)	0 (0-0)	0 (0-0)	0 (0-0)	0 (0-0)	0 (0-0)	0 (0-0)	0 (0-0)	0 (0-0)	0 (0-0)	0 (0-0)	0 (0-0)
Wakayama	2 (2-2)	1 (1-1)	1 (1-1)	0 (0-0)	0 (0-0)	0 (0-0)	0 (0-0)	0 (0-0)	0 (0-0)	0 (0-0)	0 (0-0)	0 (0-0)	0 (0-0)	0 (0-0)
Yamagata	2 (1-2)	1 (1-1)	1 (1-1)	0 (0-0)	0 (0-0)	0 (0-0)	0 (0-0)	0 (0-0)	0 (0-0)	0 (0-0)	0 (0-0)	0 (0-0)	0 (0-0)	0 (0-0)
Yamaguchi	3 (2-3)	2 (1-2)	1 (1-1)	1 (1-1)	1 (0-1)	0 (0-0)	0 (0-0)	0 (0-0)	0 (0-0)	0 (0-0)	0 (0-0)	0 (0-0)	0 (0-0)	0 (0-0)
Yamanashi	1 (1-1)	1 (1-1)	0 (0-0)	0 (0-0)	0 (0-0)	0 (0-0)	0 (0-0)	0 (0-0)	0 (0-0)	0 (0-0)	0 (0-0)	0 (0-0)	0 (0-0)	0 (0-0)
South Korea	151 (118-190)	100 (84-117)	84 (74-95)	73 (64-81)	57 (50-64)	28 (25-33)	17 (14-19)	9 (8-11)	6 (5-7)	5 (4-6)	4 (4-4)	3 (3-3)	2 (2-2)	1 (1-2)
Singapore	5 (5-6)	4 (4-4)	3 (3-3)	2 (2-2)	1 (1-1)	1 (1-1)	1 (1-1)	0 (0-0)	0 (0-0)	0 (0-0)	0 (0-0)	0 (0-0)	0 (0-0)	0 (0-0)
High-income North America	140 (137-143)	140 (139-142)	143 (142-143)	113 (113-113)	92 (92-93)	63 (63-64)	58 (58-59)	50 (50-51)	48 (48-49)	38 (38-38)	35 (35-36)	36 (36-36)	31 (30-31)	28 (27-29)
Canada	17 (17-18)	16 (16-16)	15 (14-15)	10 (10-11)	8 (8-8)	6 (6-6)	5 (4-5)	4 (3-4)	3 (3-3)	3 (3-3)	2 (2-2)	2 (2-2)	2 (2-2)	2 (2-3)
Greenland	0 (0-0)	0 (0-0)	0 (0-0)	0 (0-0)	0 (0-0)	0 (0-0)	0 (0-0)	0 (0-0)	0 (0-0)	0 (0-0)	0 (0-0)	0 (0-0)	0 (0-0)	0 (0-0)

Table 2 Under-5 death numbers by location and year for 1950, 1955...2005, 2010, 2017 - both sexes

Location	1950	1955	1960	1965	1970	1975	1980	1985	1990	1995	2000	2005	2010	2017
USA	123 (119-126)	124 (123-126)	128 (127-128)	102 (102-103)	85 (84-85)	57 (57-58)	53 (53-54)	47 (47-47)	45 (45-45)	35 (35-36)	33 (33-34)	34 (34-34)	28 (28-29)	26 (25-27)
Alabama	4 (3-4)	3 (3-4)	3 (3-3)	3 (3-3)	2 (2-2)	1 (1-1)	1 (1-1)	1 (1-1)	1 (1-1)	1 (1-1)	1 (1-1)	1 (1-1)	1 (1-1)	1 (0-1)
Alaska	0 (0-0)	0 (0-0)	0 (0-0)	0 (0-0)	0 (0-0)	0 (0-0)	0 (0-0)	0 (0-0)	0 (0-0)	0 (0-0)	0 (0-0)	0 (0-0)	0 (0-0)	0 (0-0)
Arizona	1 (1-1)	1 (1-1)	1 (1-1)	1 (1-1)	1 (1-1)	1 (1-1)	1 (1-1)	1 (1-1)	1 (1-1)	1 (1-1)	1 (1-1)	1 (1-1)	1 (1-1)	1 (0-1)
Arkansas	2 (2-2)	2 (1-2)	1 (1-1)	1 (1-1)	1 (1-1)	1 (1-1)	1 (1-1)	0 (0-1)	0 (0-0)	0 (0-0)	0 (0-0)	0 (0-0)	0 (0-0)	0 (0-0)
California	8 (7-9)	9 (8-9)	10 (10-10)	9 (9-9)	7 (7-8)	5 (5-5)	5 (5-6)	5 (5-5)	6 (5-6)	4 (4-4)	4 (3-4)	4 (4-4)	3 (3-3)	2 (2-3)
Colorado	1 (1-1)	1 (1-1)	1 (1-1)	1 (1-1)	1 (1-1)	1 (1-1)	1 (1-1)	1 (1-1)	1 (1-1)	0 (0-0)	0 (0-1)	0 (0-1)	0 (0-0)	0 (0-0)
Connecticut	1 (1-2)	1 (1-2)	1 (1-1)	1 (1-1)	1 (1-1)	1 (1-1)	1 (1-1)	1 (0-1)	0 (0-0)	0 (0-0)	0 (0-0)	0 (0-0)	0 (0-0)	0 (0-0)
Delaware	0 (0-0)	0 (0-0)	0 (0-0)	0 (0-0)	0 (0-0)	0 (0-0)	0 (0-0)	0 (0-0)	0 (0-0)	0 (0-0)	0 (0-0)	0 (0-0)	0 (0-0)	0 (0-0)
Washington, DC	1 (1-1)	1 (1-1)	1 (1-1)	1 (1-1)	1 (1-1)	0 (0-0)	0 (0-0)	0 (0-0)	0 (0-0)	0 (0-0)	0 (0-0)	0 (0-0)	0 (0-0)	0 (0-0)
Florida	3 (3-3)	4 (3-4)	4 (4-4)	4 (4-4)	3 (3-3)	2 (2-2)	3 (2-3)	2 (2-2)	2 (2-3)	2 (2-2)	2 (2-2)	2 (2-2)	2 (2-2)	2 (1-2)
Georgia	4 (3-4)	4 (4-4)	4 (4-4)	3 (3-3)	2 (2-2)	2 (2-2)	2 (2-2)	1 (1-2)	2 (2-2)	1 (1-1)	1 (1-1)	1 (1-1)	1 (1-1)	1 (1-1)
Hawaii	0 (0-1)	0 (0-0)	0 (0-0)	0 (0-0)	0 (0-0)	0 (0-0)	0 (0-0)	0 (0-0)	0 (0-0)	0 (0-0)	0 (0-0)	0 (0-0)	0 (0-0)	0 (0-0)
Idaho	0 (0-1)	0 (0-0)	0 (0-0)	0 (0-0)	0 (0-0)	0 (0-0)	0 (0-0)	0 (0-0)	0 (0-0)	0 (0-0)	0 (0-0)	0 (0-0)	0 (0-0)	0 (0-0)
Illinois	6 (6-7)	6 (6-7)	7 (6-7)	6 (6-6)	5 (5-5)	3 (3-3)	3 (3-3)	2 (2-2)	2 (2-2)	2 (2-2)	2 (2-2)	1 (1-2)	1 (1-1)	1 (1-1)
Indiana	3 (2-3)	3 (3-3)	3 (3-3)	3 (2-3)	2 (2-2)	1 (1-1)	1 (1-1)	1 (1-1)	1 (1-1)	1 (1-1)	1 (1-1)	1 (1-1)	1 (1-1)	1 (1-1)
Iowa	1 (1-2)	1 (1-2)	1 (1-1)	1 (1-1)	1 (1-1)	1 (1-1)	1 (1-1)	0 (0-0)	0 (0-0)	0 (0-0)	0 (0-0)	0 (0-0)	0 (0-0)	0 (0-0)
Kansas	1 (1-2)	1 (1-1)	1 (1-1)	1 (1-1)	1 (1-1)	1 (1-1)	0 (0-0)	0 (0-0)	0 (0-0)	0 (0-0)	0 (0-0)	0 (0-0)	0 (0-0)	0 (0-0)
Kentucky	3 (2-3)	3 (2-3)	2 (2-3)	2 (2-2)	1 (1-2)	1 (1-1)	1 (1-1)	1 (1-1)	1 (1-1)	1 (1-1)	1 (0-1)	1 (0-1)	0 (0-0)	0 (0-0)
Louisiana	3 (3-3)	3 (3-3)	3 (3-3)	2 (2-3)	2 (2-2)	1 (1-1)	1 (1-1)	1 (1-1)	1 (1-1)	1 (1-1)	1 (1-1)	1 (1-1)	1 (1-1)	1 (1-1)
Maine	1 (1-1)	1 (1-1)	1 (1-1)	0 (0-1)	0 (0-0)	0 (0-0)	0 (0-0)	0 (0-0)	0 (0-0)	0 (0-0)	0 (0-0)	0 (0-0)	0 (0-0)	0 (0-0)
Maryland	2 (2-2)	2 (2-2)	2 (2-2)	2 (2-2)	2 (2-2)	1 (1-1)	1 (1-1)	1 (1-1)	1 (1-1)	1 (1-1)	1 (1-1)	1 (1-1)	1 (1-1)	1 (0-1)
Massachusetts	3 (3-4)	3 (3-3)	3 (3-3)	2 (2-2)	2 (2-2)	1 (1-1)	1 (1-1)	1 (1-1)	1 (1-1)	1 (0-1)	0 (0-1)	0 (0-0)	0 (0-0)	0 (0-0)
Michigan	4 (4-5)	5 (4-5)	5 (5-5)	4 (4-4)	4 (4-4)	2 (2-2)	2 (2-2)	2 (2-2)	2 (2-2)	1 (1-1)	1 (1-1)	1 (1-1)	1 (1-1)	1 (1-1)
Minnesota	2 (2-2)	2 (2-2)	2 (2-2)	1 (1-1)	1 (1-1)	1 (1-1)	1 (1-1)	1 (1-1)	1 (1-1)	0 (0-0)	0 (0-0)	0 (0-0)	0 (0-0)	0 (0-0)
Mississippi	3 (3-4)	3 (3-3)	3 (3-3)	2 (2-2)	1 (1-1)	1 (1-1)	1 (1-1)	1 (1-1)	1 (1-1)	1 (1-1)	1 (1-1)	1 (1-1)	0 (0-0)	0 (0-0)
Missouri	3 (2-4)	3 (3-3)	3 (3-3)	2 (2-2)	2 (2-2)	1 (1-1)	1 (1-1)	1 (1-1)	1 (1-1)	1 (1-1)	1 (1-1)	1 (1-1)	1 (1-1)	1 (0-1)
Montana	0 (0-1)	0 (0-1)	0 (0-0)	0 (0-0)	0 (0-0)	0 (0-0)	0 (0-0)	0 (0-0)	0 (0-0)	0 (0-0)	0 (0-0)	0 (0-0)	0 (0-0)	0 (0-0)
Nebraska	1 (1-1)	1 (1-1)	1 (1-1)	1 (1-1)	0 (0-1)	0 (0-0)	0 (0-0)	0 (0-0)	0 (0-0)	0 (0-0)	0 (0-0)	0 (0-0)	0 (0-0)	0 (0-0)
Nevada	0 (0-0)	0 (0-0)	0 (0-0)	0 (0-0)	0 (0-0)	0 (0-0)	0 (0-0)	0 (0-0)	0 (0-0)	0 (0-0)	0 (0-0)	0 (0-0)	0 (0-0)	0 (0-0)
New Hampshire	0 (0-0)	0 (0-0)	0 (0-0)	0 (0-0)	0 (0-0)	0 (0-0)	0 (0-0)	0 (0-0)	0 (0-0)	0 (0-0)	0 (0-0)	0 (0-0)	0 (0-0)	0 (0-0)
New Jersey	4 (3-4)	4 (3-4)	4 (4-4)	3 (3-3)	3 (3-3)	2 (2-2)	2 (1-2)	1 (1-1)	1 (1-1)	1 (1-1)	1 (1-1)	1 (1-1)	1 (1-1)	1 (0-1)
New Mexico	1 (1-1)	1 (1-1)	1 (1-1)	1 (1-1)	1 (0-1)	0 (0-0)	0 (0-0)	0 (0-0)	0 (0-0)	0 (0-0)	0 (0-0)	0 (0-0)	0 (0-0)	0 (0-0)
New York	11 (10-12)	11 (10-11)	11 (10-11)	9 (9-9)	7 (7-7)	5 (4-5)	4 (4-4)	3 (3-4)	3 (3-3)	2 (2-2)	2 (2-2)	2 (2-2)	2 (1-2)	1 (1-2)
North Carolina	5 (4-5)	4 (4-5)	4 (4-4)	4 (3-4)	3 (3-3)	2 (2-2)	2 (1-2)	1 (1-1)	1 (1-1)	1 (1-1)	1 (1-1)	1 (1-1)	1 (1-1)	1 (1-1)
North Dakota	0 (0-1)	0 (0-0)	0 (0-0)	0 (0-0)	0 (0-0)	0 (0-0)	0 (0-0)	0 (0-0)	0 (0-0)	0 (0-0)	0 (0-0)	0 (0-0)	0 (0-0)	0 (0-0)
Ohio	5 (5-6)	6 (5-6)	6 (6-6)	5 (5-5)	4 (4-4)	3 (3-3)	2 (2-3)	2 (2-2)	2 (2-2)	2 (1-2)	1 (1-1)	1 (1-1)	1 (1-1)	1 (1-1)
Oklahoma	2 (1-2)	2 (1-2)	2 (2-2)	1 (1-1)	1 (1-1)	1 (1-1)	1 (1-1)	1 (1-1)	1 (1-1)	0 (0-1)	0 (0-1)	0 (0-1)	0 (0-0)	0 (0-1)
Oregon	1 (1-2)	1 (1-1)	1 (1-1)	1 (1-1)	1 (1-1)	1 (1-1)	1 (1-1)	0 (0-0)	0 (0-0)	0 (0-0)	0 (0-0)	0 (0-0)	0 (0-0)	0 (0-0)
Pennsylvania	8 (7-9)	7 (7-8)	7 (7-7)	6 (5-6)	5 (4-5)	3 (3-3)	3 (3-3)	2 (2-2)	2 (2-2)	1 (1-2)	1 (1-1)	1 (1-1)	1 (1-1)	1 (1-1)
Rhode Island	1 (0-1)	1 (0-1)	1 (0-1)	0 (0-0)	0 (0-0)	0 (0-0)	0 (0-0)	0 (0-0)	0 (0-0)	0 (0-0)	0 (0-0)	0 (0-0)	0 (0-0)	0 (0-0)
South Carolina	2 (2-3)	3 (2-3)	3 (3-3)	2 (2-2)	1 (1-1)	1 (1-1)	1 (1-1)	1 (1-1)	1 (1-1)	1 (1-1)	1 (1-1)	1 (1-1)	1 (0-1)	0 (0-1)
South Dakota	0 (0-1)	0 (0-1)	0 (0-0)	0 (0-0)	0 (0-0)	0 (0-0)	0 (0-0)	0 (0-0)	0 (0-0)	0 (0-0)	0 (0-0)	0 (0-0)	0 (0-0)	0 (0-0)
Tennessee	3 (3-4)	3 (3-3)	3 (3-3)	2 (2-3)	2 (2-2)	1 (1-1)	1 (1-1)	1 (1-1)	1 (1-1)	1 (1-1)	1 (1-1)	1 (1-1)	1 (1-1)	1 (1-1)
Texas	7 (6-8)	8 (7-8)	9 (8-9)	6 (6-7)	6 (5-6)	4 (4-4)	4 (4-4)	4 (3-4)	3 (3-3)	2 (2-3)	2 (2-3)	3 (3-3)	3 (2-3)	2 (2-3)
Utah	0 (0-0)	0 (0-0)	0 (0-0)	0 (0-0)	0 (0-0)	0 (0-0)	0 (0-0)	0 (0-0)	0 (0-0)	0 (0-0)	0 (0-0)	0 (0-0)	0 (0-0)	0 (0-0)
Vermont	0 (0-0)	0 (0-0)	0 (0-0)	0 (0-0)	0 (0-0)	0 (0-0)	0 (0-0)	0 (0-0)	0 (0-0)	0 (0-0)	0 (0-0)	0 (0-0)	0 (0-0)	0 (0-0)
Virginia	3 (3-4)	3 (3-4)	3 (3-3)	3 (3-3)	2 (2-2)	1 (1-1)	1 (1-1)	1 (1-1)	1 (1-1)	1 (1-1)	1 (1-1)	1 (1-1)	1 (1-1)	1 (1-1)
Washington	2 (1-2)	2 (2-2)	2 (2-2)	1 (1-1)	1 (1-1)	1 (1-1)	1 (1-1)	1 (1-1)	1 (1-1)	1 (1-1)	1 (0-1)	1 (0-1)	0 (0-0)	0 (0-1)
West Virginia	2 (1-3)	2 (1-2)	1 (1-1)	1 (1-1)	1 (1-1)	1 (1-1)	0 (0-0)	0 (0-0)	0 (0-0)	0 (0-0)	0 (0-0)	0 (0-0)	0 (0-0)	0 (0-0)
Wisconsin	2 (2-2)	2 (2-2)	2 (2-2)	2 (2-2)	1 (1-1)	1 (1-1)	1 (1-1)	1 (1-1)	1 (1-1)	1 (1-1)	1 (1-1)	1 (0-1)	0 (0-0)	0 (0-0)
Wyoming	0 (0-0)	0 (0-0)	0 (0-0)	0 (0-0)	0 (0-0)	0 (0-0)	0 (0-0)	0 (0-0)	0 (0-0)	0 (0-0)	0 (0-0)	0 (0-0)	0 (0-0)	0 (0-0)
Southern Latin America	70 (63-77)	75 (71-79)	83 (82-84)	69 (69-70)	64 (63-64)	52 (49-56)	38 (38-38)	27 (27-28)	27 (26-27)	21 (21-21)	17 (17-17)	14 (14-14)	13 (13-13)	10 (9-12)
Argentina	37 (30-44)	37 (33-41)	37 (36-38)	32 (32-33)	38 (37-38)	34 (30-38)	27 (26-27)	20 (20-20)	20 (20-20)	16 (16-16)	13 (13-14)	11 (11-11)	10 (10-11)	8 (7-10)
Chile	30 (30-31)	35 (35-36)	42 (42-43)	34 (33-34)	23 (23-23)	15 (15-15)	9 (9-9)	6 (6-6)	6 (5-6)	4 (4-4)	3 (3-3)	2 (2-2)	2 (2-2)	2 (1-2)
Uruguay	3 (2-3)	3 (3-3)	3 (3-3)	3 (3-3)	3 (3-3)	3 (3-3)	2 (2-2)	2 (2-2)	1 (1-1)	1 (1-1)	1 (1-1)	1 (1-1)	1 (0-1)	0 (0-1)
Western Europe	337 (325-351)	279 (274-284)	240 (239-241)	198 (197-199)	151 (150-152)	102 (101-103)	71 (70-71)	51 (51-51)	42 (42-43)	30 (29-30)	25 (25-25)	21 (21-22)	19 (19-20)	16 (15-17)
Andorra	0 (0-0)	0 (0-0)	0 (0-0)	0 (0-0)	0 (0-0)	0 (0-0)	0 (0-0)	0 (0-0)	0 (0-0)	0 (0-0)	0 (0-0)	0 (0-0)	0 (0-0)	0 (0-0)
Austria	6 (6-7)	6 (6-6)	6 (5-6)	4 (4-4)	3 (3-3)	2 (2-2)	2 (1-2)	1 (1-1)	1 (1-1)	1 (1-1)	0 (0-0)	0 (0-0)	0 (0-0)	0 (0-0)
Belgium	9 (8-9)	7 (7-7)	5 (5-6)	4 (4-4)	3 (3-4)	2 (2-2)	2 (2-2)	1 (1-1)	1 (1-1)	1 (1-1)	1 (1-1)	1 (1-1)	1 (1-1)	0 (0-1)
Cyprus	1 (1-1)	1 (1-1)	1 (1-1)	1 (1-1)	1 (1-1)	0 (0-1)	0 (0-0)	0 (0-0)	0 (0-0)	0 (0-0)	0 (0-0)	0 (0-0)	0 (0-0)	0 (0-0)
Denmark	3 (2-3)	2 (2-2)	2 (2-2)	2 (2-2)	1 (1-1)	1 (1-1)	1 (1-1)	1 (0-1)	1 (1-1)	0 (0-0)	0 (0-0)	0 (0-0)	0 (0-0)	0 (0-0)
Finland	5 (5-5)	3 (3-3)	2 (2-2)	2 (2-2)	1 (1-1)	1 (1-1)	1 (1-1)	0 (0-0)	0 (0-0)	0 (0-0)	0 (0-0)	0 (0-0)	0 (0-0)	0 (0-0)
France	48 (46-50)	32 (32-33)	22 (22-23)	18 (18-19)	15 (15-16)	12 (12-12)	10 (10-10)	8 (8-8)	7 (7-7)	4 (4-5)	4 (4-4)	3 (3-4)	3 (3-3)	3 (2-3)
Germany	62 (52-74)	55 (51-60)	51 (50-51)	38 (37-38)	27 (26-27)	17 (17-18)	13 (13-13)	9 (9-9)	8 (7-8)	5 (5-5)	4 (4-4)	3 (3-3)	3 (3-3)	3 (2-3)
Greece	10 (9-10)	8 (8-8)	7 (7-7)	6 (6-6)	5 (4-5)	4 (4-4)	3 (3-3)	2 (2-2)	1 (1-1)	1 (1-1)	1 (1-1)	1 (0-1)	0 (0-1)	0 (0-0)
Iceland	0 (0-0)	0 (0-0)	0 (0-0)	0 (0-0)	0 (0-0)	0 (0-0)	0 (0-0)	0 (0-0)	0 (0-0)	0 (0-0)	0 (0-0)	0 (0-0)	0 (0-0)	0 (0-0)
Ireland	3 (3-4)	3 (3-3)	2 (2-2)	2 (2-2)	1 (1-2)	1 (1-1)	1 (1-1)	1 (1-1)	0 (0-1)	0 (0-0)	0 (0-0)	0 (0-0)	0 (0-0)	0 (0-0)
Israel	3 (3-3)	2 (2-2)	2 (2-2)	2 (2-2)	2 (2-2)	3 (2-3)	2 (2-2)	1 (1-1)	1 (1-1)	1 (1-1)	1 (1-1)	1 (1-1)	1 (1-1)	1 (1-1)
Italy	60 (58-64)	53 (52-53)	48 (47-48)	41 (40-41)	30 (30-31)	19 (19-19)	11 (11-11)	7 (7-7)	5 (5-6)	4 (4-4)	3 (3-3)	2 (2-3)	2 (2-2)	1 (1-2)
Luxembourg	0 (0-0)	0 (0-0)	0 (0-0)	0 (0-0)	0 (0-0)	0 (0-0)	0 (0-0)	0 (0-0)	0 (0-0)	0 (0-0)	0 (0-0)	0 (0-0)	0 (0-0)	0 (0-0)
Malta	1 (1-1)	0 (0-1)	0 (0-0)	0 (0-0)	0 (0-0)	0 (0-0)	0 (0-0)	0 (0-0)	0 (0-0)	0 (0-0)	0 (0-0)	0 (0-0)	0 (0-0)	0 (0-0)
Netherlands	7 (6-7)	6 (6-6)	5 (5-5)	4 (4-5)	4 (4-4)	2 (2-2)	2 (2-2)	2 (2-2)	2 (2-2)	1 (1-1)	1 (1-1)	1 (1-1)	1 (1-1)	1 (1-1)
Norway	2 (2-2)	2 (2-2)	1 (1-1)	1 (1-1)	1 (1-1)	1 (1-1)	0 (0-0)	0 (0-0)	1 (1-1)	0 (0-0)	0 (0-0)	0 (0-0)	0 (0-0)	0 (0-0)
Portugal	32 (31-33)	26 (26-27)	22 (22-23)	18 (18-19)	13 (13-13)	7 (7-8)	5 (4-5)	3 (3-3)	2 (2-2)	1 (1-1)	1 (1-1)	1 (0-1)	0 (0-0)	0 (0-0)
Spain	55 (53-59)	42 (42-43)	33 (33-34)	28 (27-28)	21 (21-21)	14 (14-15)	8 (8-8)	5 (5-5)	4 (4-4)	2 (2-3)	2 (2-2)	2 (2-2)	2 (2-2)	1 (1-2)
Sweden	3 (2-3)	2 (2-2)	2 (2-2)	2 (2-2)	1 (1-2)	1 (1-1)	1 (1-1)	1 (1-1)	1 (1-1)	0 (0-0)	0 (0-0)	0 (0-0)	0 (0-0)	0 (0-0)
Stockholm	1 (0-1)	0 (0-1)	0 (0-0)	0 (0-0)	0 (0-0)	0 (0-0)	0 (0-0)	0 (0-0)	0 (0-0)	0 (0-0)	0 (0-0)	0 (0-0)	0 (0-0)	0 (0-0)
Sweden except Stockholm	2 (2-2)	2 (2-2)	2 (2-2)	2 (1-2)	1 (1-1)	1 (1-1)	1 (1-1)	1 (1-1)	1 (1-1)	0 (0-0)	0 (0-0)	0 (0-0)	0 (0-0)	0 (0-0)
Switzerland	3 (3-4)	3 (3-3)	2 (2-3)	2 (2-2)	2 (2-2)	1 (1-1)	1 (1-1)	1 (1-1)	1 (1-1)	1 (0-1)	0 (0-0)	0 (0-0)	0 (0-0)	0 (0-0)
United Kingdom	24 (23-26)	24 (23-25)	24 (24-25)	22 (22-23)	19 (19-19)	12 (12-13)	10 (10-10)	8 (8-8)	7 (7-7)	5 (5-5)	4 (4-4)	4 (4-4)	4 (4-4)	4 (3-4)
England	16 (16-17)	17 (17-17)	18 (17-18)	17 (17-17)	15 (14-15)	10 (9-10)	8 (8-8)	7 (7-7)	6 (6-6)	4 (4-4)	4 (4-4)	4 (4-4)	4 (3-4)	3 (3-3)
East Midlands	1 (1-2)	2 (1-2)	2 (1-2)	1 (1-2)	1 (1-1)	1 (1-1)	1 (1-1)	1 (1-1)	1 (0-1)	0 (0-0)	0 (0-0)	0 (0-0)	0 (0-0)	0 (0-0)
Derby	0 (0-0)	0 (0-0)	0 (0-0)	0 (0-0)	0 (0-0)	0 (0-0)	0 (0-0)	0 (0-0)	0 (0-0)	0 (0-0)	0 (0-0)	0 (0-0)	0 (0-0)	0 (0-0)
Derbyshire	0 (0-0)	0 (0-0)	0 (0-0)	0 (0-0)	0 (0-0)	0 (0-0)	0 (0-0)	0 (0-0)	0 (0-0)	0 (0-0)	0 (0-0)	0 (0-0)	0 (0-0)	0 (0-0)
Leicester	0 (0-0)	0 (0-0)	0 (0-0)	0 (0-0)	0 (0-0)	0 (0-0)	0 (0-0)	0 (0-0)	0 (0-0)	0 (0-0)	0 (0-0)	0 (0-0)	0 (0-0)	0 (0-0)
Leicestershire	0 (0-0)	0 (0-0)	0 (0-0)	0 (0-0)	0 (0-0)	0 (0-0)	0 (0-0)	0 (0-0)	0 (0-0)	0 (0-0)	0 (0-0)	0 (0-0)	0 (0-0)	0 (0-0)
Lincolnshire	0 (0-0)	0 (0-0)	0 (0-0)	0 (0-0)	0 (0-0)	0 (0-0)	0 (0-0)	0 (0-0)	0 (0-0)	0 (0-0)	0 (0-0)	0 (0-0)	0 (0-0)	0 (0-0)
Northamptonshire	0 (0-0)	0 (0-0)	0 (0-0)	0 (0-0)	0 (0-0)	0 (0-0)	0 (0-0)	0 (0-0)	0 (0-0)	0 (0-0)	0 (0-0)	0 (0-0)	0 (0-0)	0 (0-0)
Nottingham	0 (0-0)	0 (0-0)	0 (0-0)	0 (0-0)	0 (0-0)	0 (0-0)	0 (0-0)	0 (0-0)	0 (0-0)	0 (0-0)	0 (0-0)	0 (0-0)	0 (0-0)	0 (0-0)
Nottinghamshire	0 (0-0)	0 (0-0)	0 (0-0)	0 (0-0)	0 (0-0)	0 (0-0)	0 (0-0)	0 (0-0)	0 (0-0)	0 (0-0)	0 (0-0)	0 (0-0)	0 (0-0)	0 (0-0)
Rutland	0 (0-0)	0 (0-0)	0 (0-0)	0 (0-0)	0 (0-0)	0 (0-0)	0 (0-0)	0 (0-0)	0 (0-0)	0 (0-0)	0 (0-0)	0 (0-0)	0 (0-0)	0 (0-0)
East of England	2 (1-2)	2 (2-2)	2 (2-2)	2 (2-2)	1 (1-1)	1 (1-1)	1 (1-1)	1 (1-1)	1 (0-1)	0 (0-0)	0 (0-0)	0 (0-0)	0 (0-0)	0 (0-0)
Bedford	0 (0-0)	0 (0-0)	0 (0-0)	0 (0-0)	0 (0-0)	0 (0-0)	0 (0-0)	0 (0-0)	0 (0-0)	0 (0-0)	0 (0-0)	0 (0-0)	0 (0-0)	0 (0-0)
Cambridgeshire	0 (0-0)	0 (0-0)	0 (0-0)	0 (0-0)	0 (0-0)	0 (0-0)	0 (0-0)	0 (0-0)	0 (0-0)	0 (0-0)	0 (0-0)	0 (0-0)	0 (0-0)	0 (0-0)
Central Bedfordshire	0 (0-0)	0 (0-0)	0 (0-0)	0 (0-0)	0 (0-0)	0 (0-0)	0 (0-0)	0 (0-0)	0 (0-0)	0 (0-0)	0 (0-0)	0 (0-0)	0 (0-0)	0 (0-0)
Essex	0 (0-1)	0 (0-0)	0 (0-0)	0 (0-0)	0 (0-0)	0 (0-0)	0 (0-0)	0 (0-0)	0 (0-0)	0 (0-0)	0 (0-0)	0 (0-0)	0 (0-0)	0 (0-0)
Hertfordshire	0 (0-0)	0 (0-0)	0 (0-0)	0 (0-0)	0 (0-0)	0 (0-0)	0 (0-0)	0 (0-0)	0 (0-0)	0 (0-0)	0 (0-0)	0 (0-0)	0 (0-0)	0 (0-0)

Table 2 Under-5 death numbers by location and year for 1950, 1955...2005, 2010, 2017 - both sexes

Location	1950	1955	1960	1965	1970	1975	1980	1985	1990	1995	2000	2005	2010	2017
Luton	0 (0-0)	0 (0-0)	0 (0-0)	0 (0-0)	0 (0-0)	0 (0-0)	0 (0-0)	0 (0-0)	0 (0-0)	0 (0-0)	0 (0-0)	0 (0-0)	0 (0-0)	0 (0-0)
Norfolk	0 (0-0)	0 (0-0)	0 (0-0)	0 (0-0)	0 (0-0)	0 (0-0)	0 (0-0)	0 (0-0)	0 (0-0)	0 (0-0)	0 (0-0)	0 (0-0)	0 (0-0)	0 (0-0)
Peterborough	0 (0-0)	0 (0-0)	0 (0-0)	0 (0-0)	0 (0-0)	0 (0-0)	0 (0-0)	0 (0-0)	0 (0-0)	0 (0-0)	0 (0-0)	0 (0-0)	0 (0-0)	0 (0-0)
Southend-on-Sea	0 (0-0)	0 (0-0)	0 (0-0)	0 (0-0)	0 (0-0)	0 (0-0)	0 (0-0)	0 (0-0)	0 (0-0)	0 (0-0)	0 (0-0)	0 (0-0)	0 (0-0)	0 (0-0)
Suffolk	0 (0-0)	0 (0-0)	0 (0-0)	0 (0-0)	0 (0-0)	0 (0-0)	0 (0-0)	0 (0-0)	0 (0-0)	0 (0-0)	0 (0-0)	0 (0-0)	0 (0-0)	0 (0-0)
Thurrock	0 (0-0)	0 (0-0)	0 (0-0)	0 (0-0)	0 (0-0)	0 (0-0)	0 (0-0)	0 (0-0)	0 (0-0)	0 (0-0)	0 (0-0)	0 (0-0)	0 (0-0)	0 (0-0)
Greater London	3 (3-3)	3 (3-3)	3 (3-3)	3 (3-3)	2 (2-2)	2 (1-2)	1 (1-1)	1 (1-1)	1 (1-1)	1 (1-1)	1 (1-1)	1 (1-1)	1 (1-1)	1 (1-1)
Barking and Dagenham	0 (0-0)	0 (0-0)	0 (0-0)	0 (0-0)	0 (0-0)	0 (0-0)	0 (0-0)	0 (0-0)	0 (0-0)	0 (0-0)	0 (0-0)	0 (0-0)	0 (0-0)	0 (0-0)
Barnet	0 (0-0)	0 (0-0)	0 (0-0)	0 (0-0)	0 (0-0)	0 (0-0)	0 (0-0)	0 (0-0)	0 (0-0)	0 (0-0)	0 (0-0)	0 (0-0)	0 (0-0)	0 (0-0)
Bexley	0 (0-0)	0 (0-0)	0 (0-0)	0 (0-0)	0 (0-0)	0 (0-0)	0 (0-0)	0 (0-0)	0 (0-0)	0 (0-0)	0 (0-0)	0 (0-0)	0 (0-0)	0 (0-0)
Brent	0 (0-0)	0 (0-0)	0 (0-0)	0 (0-0)	0 (0-0)	0 (0-0)	0 (0-0)	0 (0-0)	0 (0-0)	0 (0-0)	0 (0-0)	0 (0-0)	0 (0-0)	0 (0-0)
Bromley	0 (0-0)	0 (0-0)	0 (0-0)	0 (0-0)	0 (0-0)	0 (0-0)	0 (0-0)	0 (0-0)	0 (0-0)	0 (0-0)	0 (0-0)	0 (0-0)	0 (0-0)	0 (0-0)
Camden	0 (0-0)	0 (0-0)	0 (0-0)	0 (0-0)	0 (0-0)	0 (0-0)	0 (0-0)	0 (0-0)	0 (0-0)	0 (0-0)	0 (0-0)	0 (0-0)	0 (0-0)	0 (0-0)
Croydon	0 (0-0)	0 (0-0)	0 (0-0)	0 (0-0)	0 (0-0)	0 (0-0)	0 (0-0)	0 (0-0)	0 (0-0)	0 (0-0)	0 (0-0)	0 (0-0)	0 (0-0)	0 (0-0)
Ealing	0 (0-0)	0 (0-0)	0 (0-0)	0 (0-0)	0 (0-0)	0 (0-0)	0 (0-0)	0 (0-0)	0 (0-0)	0 (0-0)	0 (0-0)	0 (0-0)	0 (0-0)	0 (0-0)
Enfield	0 (0-0)	0 (0-0)	0 (0-0)	0 (0-0)	0 (0-0)	0 (0-0)	0 (0-0)	0 (0-0)	0 (0-0)	0 (0-0)	0 (0-0)	0 (0-0)	0 (0-0)	0 (0-0)
Greenwich	0 (0-0)	0 (0-0)	0 (0-0)	0 (0-0)	0 (0-0)	0 (0-0)	0 (0-0)	0 (0-0)	0 (0-0)	0 (0-0)	0 (0-0)	0 (0-0)	0 (0-0)	0 (0-0)
Hackney	0 (0-0)	0 (0-0)	0 (0-0)	0 (0-0)	0 (0-0)	0 (0-0)	0 (0-0)	0 (0-0)	0 (0-0)	0 (0-0)	0 (0-0)	0 (0-0)	0 (0-0)	0 (0-0)
Hammersmith and Fulham	0 (0-0)	0 (0-0)	0 (0-0)	0 (0-0)	0 (0-0)	0 (0-0)	0 (0-0)	0 (0-0)	0 (0-0)	0 (0-0)	0 (0-0)	0 (0-0)	0 (0-0)	0 (0-0)
Haringey	0 (0-0)	0 (0-0)	0 (0-0)	0 (0-0)	0 (0-0)	0 (0-0)	0 (0-0)	0 (0-0)	0 (0-0)	0 (0-0)	0 (0-0)	0 (0-0)	0 (0-0)	0 (0-0)
Harrow	0 (0-0)	0 (0-0)	0 (0-0)	0 (0-0)	0 (0-0)	0 (0-0)	0 (0-0)	0 (0-0)	0 (0-0)	0 (0-0)	0 (0-0)	0 (0-0)	0 (0-0)	0 (0-0)
Havering	0 (0-0)	0 (0-0)	0 (0-0)	0 (0-0)	0 (0-0)	0 (0-0)	0 (0-0)	0 (0-0)	0 (0-0)	0 (0-0)	0 (0-0)	0 (0-0)	0 (0-0)	0 (0-0)
Hillingdon	0 (0-0)	0 (0-0)	0 (0-0)	0 (0-0)	0 (0-0)	0 (0-0)	0 (0-0)	0 (0-0)	0 (0-0)	0 (0-0)	0 (0-0)	0 (0-0)	0 (0-0)	0 (0-0)
Hounslow	0 (0-0)	0 (0-0)	0 (0-0)	0 (0-0)	0 (0-0)	0 (0-0)	0 (0-0)	0 (0-0)	0 (0-0)	0 (0-0)	0 (0-0)	0 (0-0)	0 (0-0)	0 (0-0)
Islington	0 (0-0)	0 (0-0)	0 (0-0)	0 (0-0)	0 (0-0)	0 (0-0)	0 (0-0)	0 (0-0)	0 (0-0)	0 (0-0)	0 (0-0)	0 (0-0)	0 (0-0)	0 (0-0)
Kensington and Chelsea	0 (0-0)	0 (0-0)	0 (0-0)	0 (0-0)	0 (0-0)	0 (0-0)	0 (0-0)	0 (0-0)	0 (0-0)	0 (0-0)	0 (0-0)	0 (0-0)	0 (0-0)	0 (0-0)
Kingston upon Thames	0 (0-0)	0 (0-0)	0 (0-0)	0 (0-0)	0 (0-0)	0 (0-0)	0 (0-0)	0 (0-0)	0 (0-0)	0 (0-0)	0 (0-0)	0 (0-0)	0 (0-0)	0 (0-0)
Lambeth	0 (0-0)	0 (0-0)	0 (0-0)	0 (0-0)	0 (0-0)	0 (0-0)	0 (0-0)	0 (0-0)	0 (0-0)	0 (0-0)	0 (0-0)	0 (0-0)	0 (0-0)	0 (0-0)
Lewisham	0 (0-0)	0 (0-0)	0 (0-0)	0 (0-0)	0 (0-0)	0 (0-0)	0 (0-0)	0 (0-0)	0 (0-0)	0 (0-0)	0 (0-0)	0 (0-0)	0 (0-0)	0 (0-0)
Merton	0 (0-0)	0 (0-0)	0 (0-0)	0 (0-0)	0 (0-0)	0 (0-0)	0 (0-0)	0 (0-0)	0 (0-0)	0 (0-0)	0 (0-0)	0 (0-0)	0 (0-0)	0 (0-0)
Newham	0 (0-0)	0 (0-0)	0 (0-0)	0 (0-0)	0 (0-0)	0 (0-0)	0 (0-0)	0 (0-0)	0 (0-0)	0 (0-0)	0 (0-0)	0 (0-0)	0 (0-0)	0 (0-0)
Redbridge	0 (0-0)	0 (0-0)	0 (0-0)	0 (0-0)	0 (0-0)	0 (0-0)	0 (0-0)	0 (0-0)	0 (0-0)	0 (0-0)	0 (0-0)	0 (0-0)	0 (0-0)	0 (0-0)
Richmond upon Thames	0 (0-0)	0 (0-0)	0 (0-0)	0 (0-0)	0 (0-0)	0 (0-0)	0 (0-0)	0 (0-0)	0 (0-0)	0 (0-0)	0 (0-0)	0 (0-0)	0 (0-0)	0 (0-0)
Southwark	0 (0-0)	0 (0-0)	0 (0-0)	0 (0-0)	0 (0-0)	0 (0-0)	0 (0-0)	0 (0-0)	0 (0-0)	0 (0-0)	0 (0-0)	0 (0-0)	0 (0-0)	0 (0-0)
Sutton	0 (0-0)	0 (0-0)	0 (0-0)	0 (0-0)	0 (0-0)	0 (0-0)	0 (0-0)	0 (0-0)	0 (0-0)	0 (0-0)	0 (0-0)	0 (0-0)	0 (0-0)	0 (0-0)
Tower Hamlets	0 (0-0)	0 (0-0)	0 (0-0)	0 (0-0)	0 (0-0)	0 (0-0)	0 (0-0)	0 (0-0)	0 (0-0)	0 (0-0)	0 (0-0)	0 (0-0)	0 (0-0)	0 (0-0)
Waltham Forest	0 (0-0)	0 (0-0)	0 (0-0)	0 (0-0)	0 (0-0)	0 (0-0)	0 (0-0)	0 (0-0)	0 (0-0)	0 (0-0)	0 (0-0)	0 (0-0)	0 (0-0)	0 (0-0)
Wandsworth	0 (0-0)	0 (0-0)	0 (0-0)	0 (0-0)	0 (0-0)	0 (0-0)	0 (0-0)	0 (0-0)	0 (0-0)	0 (0-0)	0 (0-0)	0 (0-0)	0 (0-0)	0 (0-0)
Westminster	0 (0-0)	0 (0-0)	0 (0-0)	0 (0-0)	0 (0-0)	0 (0-0)	0 (0-0)	0 (0-0)	0 (0-0)	0 (0-0)	0 (0-0)	0 (0-0)	0 (0-0)	0 (0-0)
North East England	1 (1-2)	1 (1-1)	1 (1-1)	1 (1-1)	1 (1-1)	1 (1-1)	0 (0-1)	0 (0-0)	0 (0-0)	0 (0-0)	0 (0-0)	0 (0-0)	0 (0-0)	0 (0-0)
County Durham	0 (0-0)	0 (0-0)	0 (0-0)	0 (0-0)	0 (0-0)	0 (0-0)	0 (0-0)	0 (0-0)	0 (0-0)	0 (0-0)	0 (0-0)	0 (0-0)	0 (0-0)	0 (0-0)
Darlington	0 (0-0)	0 (0-0)	0 (0-0)	0 (0-0)	0 (0-0)	0 (0-0)	0 (0-0)	0 (0-0)	0 (0-0)	0 (0-0)	0 (0-0)	0 (0-0)	0 (0-0)	0 (0-0)
Gateshead	0 (0-0)	0 (0-0)	0 (0-0)	0 (0-0)	0 (0-0)	0 (0-0)	0 (0-0)	0 (0-0)	0 (0-0)	0 (0-0)	0 (0-0)	0 (0-0)	0 (0-0)	0 (0-0)
Hartlepool	0 (0-0)	0 (0-0)	0 (0-0)	0 (0-0)	0 (0-0)	0 (0-0)	0 (0-0)	0 (0-0)	0 (0-0)	0 (0-0)	0 (0-0)	0 (0-0)	0 (0-0)	0 (0-0)
Middlesbrough	0 (0-0)	0 (0-0)	0 (0-0)	0 (0-0)	0 (0-0)	0 (0-0)	0 (0-0)	0 (0-0)	0 (0-0)	0 (0-0)	0 (0-0)	0 (0-0)	0 (0-0)	0 (0-0)
Newcastle upon Tyne	0 (0-0)	0 (0-0)	0 (0-0)	0 (0-0)	0 (0-0)	0 (0-0)	0 (0-0)	0 (0-0)	0 (0-0)	0 (0-0)	0 (0-0)	0 (0-0)	0 (0-0)	0 (0-0)
North Tyneside	0 (0-0)	0 (0-0)	0 (0-0)	0 (0-0)	0 (0-0)	0 (0-0)	0 (0-0)	0 (0-0)	0 (0-0)	0 (0-0)	0 (0-0)	0 (0-0)	0 (0-0)	0 (0-0)
Northumberland	0 (0-0)	0 (0-0)	0 (0-0)	0 (0-0)	0 (0-0)	0 (0-0)	0 (0-0)	0 (0-0)	0 (0-0)	0 (0-0)	0 (0-0)	0 (0-0)	0 (0-0)	0 (0-0)
Redcar and Cleveland	0 (0-0)	0 (0-0)	0 (0-0)	0 (0-0)	0 (0-0)	0 (0-0)	0 (0-0)	0 (0-0)	0 (0-0)	0 (0-0)	0 (0-0)	0 (0-0)	0 (0-0)	0 (0-0)
South Tyneside	0 (0-0)	0 (0-0)	0 (0-0)	0 (0-0)	0 (0-0)	0 (0-0)	0 (0-0)	0 (0-0)	0 (0-0)	0 (0-0)	0 (0-0)	0 (0-0)	0 (0-0)	0 (0-0)
Stockton-on-Tees	0 (0-0)	0 (0-0)	0 (0-0)	0 (0-0)	0 (0-0)	0 (0-0)	0 (0-0)	0 (0-0)	0 (0-0)	0 (0-0)	0 (0-0)	0 (0-0)	0 (0-0)	0 (0-0)
Sunderland	0 (0-0)	0 (0-0)	0 (0-0)	0 (0-0)	0 (0-0)	0 (0-0)	0 (0-0)	0 (0-0)	0 (0-0)	0 (0-0)	0 (0-0)	0 (0-0)	0 (0-0)	0 (0-0)
North West England	2 (2-3)	3 (2-3)	3 (3-3)	3 (3-3)	2 (2-2)	1 (1-2)	1 (1-1)	1 (1-1)	1 (1-1)	1 (1-1)	1 (1-1)	1 (1-1)	0 (0-1)	0 (0-0)
Blackburn with Darwen	0 (0-0)	0 (0-0)	0 (0-0)	0 (0-0)	0 (0-0)	0 (0-0)	0 (0-0)	0 (0-0)	0 (0-0)	0 (0-0)	0 (0-0)	0 (0-0)	0 (0-0)	0 (0-0)
Blackpool	0 (0-0)	0 (0-0)	0 (0-0)	0 (0-0)	0 (0-0)	0 (0-0)	0 (0-0)	0 (0-0)	0 (0-0)	0 (0-0)	0 (0-0)	0 (0-0)	0 (0-0)	0 (0-0)
Bolton	0 (0-0)	0 (0-0)	0 (0-0)	0 (0-0)	0 (0-0)	0 (0-0)	0 (0-0)	0 (0-0)	0 (0-0)	0 (0-0)	0 (0-0)	0 (0-0)	0 (0-0)	0 (0-0)
Bury	0 (0-0)	0 (0-0)	0 (0-0)	0 (0-0)	0 (0-0)	0 (0-0)	0 (0-0)	0 (0-0)	0 (0-0)	0 (0-0)	0 (0-0)	0 (0-0)	0 (0-0)	0 (0-0)
Cheshire East	0 (0-0)	0 (0-0)	0 (0-0)	0 (0-0)	0 (0-0)	0 (0-0)	0 (0-0)	0 (0-0)	0 (0-0)	0 (0-0)	0 (0-0)	0 (0-0)	0 (0-0)	0 (0-0)
Cheshire West and Chester	0 (0-0)	0 (0-0)	0 (0-0)	0 (0-0)	0 (0-0)	0 (0-0)	0 (0-0)	0 (0-0)	0 (0-0)	0 (0-0)	0 (0-0)	0 (0-0)	0 (0-0)	0 (0-0)
Cumbria	0 (0-0)	0 (0-0)	0 (0-0)	0 (0-0)	0 (0-0)	0 (0-0)	0 (0-0)	0 (0-0)	0 (0-0)	0 (0-0)	0 (0-0)	0 (0-0)	0 (0-0)	0 (0-0)
Halton	0 (0-0)	0 (0-0)	0 (0-0)	0 (0-0)	0 (0-0)	0 (0-0)	0 (0-0)	0 (0-0)	0 (0-0)	0 (0-0)	0 (0-0)	0 (0-0)	0 (0-0)	0 (0-0)
Knowsley	0 (0-0)	0 (0-0)	0 (0-0)	0 (0-0)	0 (0-0)	0 (0-0)	0 (0-0)	0 (0-0)	0 (0-0)	0 (0-0)	0 (0-0)	0 (0-0)	0 (0-0)	0 (0-0)
Lancashire	0 (0-0)	0 (0-0)	0 (0-0)	0 (0-0)	0 (0-0)	0 (0-0)	0 (0-0)	0 (0-0)	0 (0-0)	0 (0-0)	0 (0-0)	0 (0-0)	0 (0-0)	0 (0-0)
Liverpool	0 (0-0)	0 (0-0)	0 (0-0)	0 (0-0)	0 (0-0)	0 (0-0)	0 (0-0)	0 (0-0)	0 (0-0)	0 (0-0)	0 (0-0)	0 (0-0)	0 (0-0)	0 (0-0)
Manchester	0 (0-0)	0 (0-0)	0 (0-0)	0 (0-0)	0 (0-0)	0 (0-0)	0 (0-0)	0 (0-0)	0 (0-0)	0 (0-0)	0 (0-0)	0 (0-0)	0 (0-0)	0 (0-0)
Oldham	0 (0-0)	0 (0-0)	0 (0-0)	0 (0-0)	0 (0-0)	0 (0-0)	0 (0-0)	0 (0-0)	0 (0-0)	0 (0-0)	0 (0-0)	0 (0-0)	0 (0-0)	0 (0-0)
Rochdale	0 (0-0)	0 (0-0)	0 (0-0)	0 (0-0)	0 (0-0)	0 (0-0)	0 (0-0)	0 (0-0)	0 (0-0)	0 (0-0)	0 (0-0)	0 (0-0)	0 (0-0)	0 (0-0)
Salford	0 (0-0)	0 (0-0)	0 (0-0)	0 (0-0)	0 (0-0)	0 (0-0)	0 (0-0)	0 (0-0)	0 (0-0)	0 (0-0)	0 (0-0)	0 (0-0)	0 (0-0)	0 (0-0)
Sefton	0 (0-0)	0 (0-0)	0 (0-0)	0 (0-0)	0 (0-0)	0 (0-0)	0 (0-0)	0 (0-0)	0 (0-0)	0 (0-0)	0 (0-0)	0 (0-0)	0 (0-0)	0 (0-0)
St Helens	0 (0-0)	0 (0-0)	0 (0-0)	0 (0-0)	0 (0-0)	0 (0-0)	0 (0-0)	0 (0-0)	0 (0-0)	0 (0-0)	0 (0-0)	0 (0-0)	0 (0-0)	0 (0-0)
Stockport	0 (0-0)	0 (0-0)	0 (0-0)	0 (0-0)	0 (0-0)	0 (0-0)	0 (0-0)	0 (0-0)	0 (0-0)	0 (0-0)	0 (0-0)	0 (0-0)	0 (0-0)	0 (0-0)
Tameside	0 (0-0)	0 (0-0)	0 (0-0)	0 (0-0)	0 (0-0)	0 (0-0)	0 (0-0)	0 (0-0)	0 (0-0)	0 (0-0)	0 (0-0)	0 (0-0)	0 (0-0)	0 (0-0)
Trafford	0 (0-0)	0 (0-0)	0 (0-0)	0 (0-0)	0 (0-0)	0 (0-0)	0 (0-0)	0 (0-0)	0 (0-0)	0 (0-0)	0 (0-0)	0 (0-0)	0 (0-0)	0 (0-0)
Warrington	0 (0-0)	0 (0-0)	0 (0-0)	0 (0-0)	0 (0-0)	0 (0-0)	0 (0-0)	0 (0-0)	0 (0-0)	0 (0-0)	0 (0-0)	0 (0-0)	0 (0-0)	0 (0-0)
Wigan	0 (0-0)	0 (0-0)	0 (0-0)	0 (0-0)	0 (0-0)	0 (0-0)	0 (0-0)	0 (0-0)	0 (0-0)	0 (0-0)	0 (0-0)	0 (0-0)	0 (0-0)	0 (0-0)
Wirral	0 (0-0)	0 (0-0)	0 (0-0)	0 (0-0)	0 (0-0)	0 (0-0)	0 (0-0)	0 (0-0)	0 (0-0)	0 (0-0)	0 (0-0)	0 (0-0)	0 (0-0)	0 (0-0)
South East England	2 (2-2)	2 (2-2)	2 (2-2)	2 (2-2)	2 (2-2)	1 (1-1)	1 (1-1)	1 (1-1)	1 (1-1)	1 (1-1)	0 (0-0)	0 (0-0)	0 (0-0)	0 (0-0)
Bracknell Forest	0 (0-0)	0 (0-0)	0 (0-0)	0 (0-0)	0 (0-0)	0 (0-0)	0 (0-0)	0 (0-0)	0 (0-0)	0 (0-0)	0 (0-0)	0 (0-0)	0 (0-0)	0 (0-0)
Brighton and Hove	0 (0-0)	0 (0-0)	0 (0-0)	0 (0-0)	0 (0-0)	0 (0-0)	0 (0-0)	0 (0-0)	0 (0-0)	0 (0-0)	0 (0-0)	0 (0-0)	0 (0-0)	0 (0-0)
Buckinghamshire	0 (0-0)	0 (0-0)	0 (0-0)	0 (0-0)	0 (0-0)	0 (0-0)	0 (0-0)	0 (0-0)	0 (0-0)	0 (0-0)	0 (0-0)	0 (0-0)	0 (0-0)	0 (0-0)
East Sussex	0 (0-0)	0 (0-0)	0 (0-0)	0 (0-0)	0 (0-0)	0 (0-0)	0 (0-0)	0 (0-0)	0 (0-0)	0 (0-0)	0 (0-0)	0 (0-0)	0 (0-0)	0 (0-0)
Hampshire	0 (0-0)	0 (0-0)	0 (0-0)	0 (0-0)	0 (0-0)	0 (0-0)	0 (0-0)	0 (0-0)	0 (0-0)	0 (0-0)	0 (0-0)	0 (0-0)	0 (0-0)	0 (0-0)
Isle of Wight	0 (0-0)	0 (0-0)	0 (0-0)	0 (0-0)	0 (0-0)	0 (0-0)	0 (0-0)	0 (0-0)	0 (0-0)	0 (0-0)	0 (0-0)	0 (0-0)	0 (0-0)	0 (0-0)
Kent	0 (0-0)	0 (0-0)	0 (0-0)	0 (0-0)	0 (0-0)	0 (0-0)	0 (0-0)	0 (0-0)	0 (0-0)	0 (0-0)	0 (0-0)	0 (0-0)	0 (0-0)	0 (0-0)
Medway	0 (0-0)	0 (0-0)	0 (0-0)	0 (0-0)	0 (0-0)	0 (0-0)	0 (0-0)	0 (0-0)	0 (0-0)	0 (0-0)	0 (0-0)	0 (0-0)	0 (0-0)	0 (0-0)
Milton Keynes	0 (0-0)	0 (0-0)	0 (0-0)	0 (0-0)	0 (0-0)	0 (0-0)	0 (0-0)	0 (0-0)	0 (0-0)	0 (0-0)	0 (0-0)	0 (0-0)	0 (0-0)	0 (0-0)
Oxfordshire	0 (0-0)	0 (0-0)	0 (0-0)	0 (0-0)	0 (0-0)	0 (0-0)	0 (0-0)	0 (0-0)	0 (0-0)	0 (0-0)	0 (0-0)	0 (0-0)	0 (0-0)	0 (0-0)
Portsmouth	0 (0-0)	0 (0-0)	0 (0-0)	0 (0-0)	0 (0-0)	0 (0-0)	0 (0-0)	0 (0-0)	0 (0-0)	0 (0-0)	0 (0-0)	0 (0-0)	0 (0-0)	0 (0-0)
Reading	0 (0-0)	0 (0-0)	0 (0-0)	0 (0-0)	0 (0-0)	0 (0-0)	0 (0-0)	0 (0-0)	0 (0-0)	0 (0-0)	0 (0-0)	0 (0-0)	0 (0-0)	0 (0-0)
Slough	0 (0-0)	0 (0-0)	0 (0-0)	0 (0-0)	0 (0-0)	0 (0-0)	0 (0-0)	0 (0-0)	0 (0-0)	0 (0-0)	0 (0-0)	0 (0-0)	0 (0-0)	0 (0-0)
Southampton	0 (0-0)	0 (0-0)	0 (0-0)	0 (0-0)	0 (0-0)	0 (0-0)	0 (0-0)	0 (0-0)	0 (0-0)	0 (0-0)	0 (0-0)	0 (0-0)	0 (0-0)	0 (0-0)
Surrey	0 (0-0)	0 (0-0)	0 (0-0)	0 (0-0)	0 (0-0)	0 (0-0)	0 (0-0)	0 (0-0)	0 (0-0)	0 (0-0)	0 (0-0)	0 (0-0)	0 (0-0)	0 (0-0)
West Berkshire	0 (0-0)	0 (0-0)	0 (0-0)	0 (0-0)	0 (0-0)	0 (0-0)	0 (0-0)	0 (0-0)	0 (0-0)	0 (0-0)	0 (0-0)	0 (0-0)	0 (0-0)	0 (0-0)
West Sussex	0 (0-0)	0 (0-0)	0 (0-0)	0 (0-0)	0 (0-0)	0 (0-0)	0 (0-0)	0 (0-0)	0 (0-0)	0 (0-0)	0 (0-0)	0 (0-0)	0 (0-0)	0 (0-0)
Windsor and Maidenhead	0 (0-0)	0 (0-0)	0 (0-0)	0 (0-0)	0 (0-0)	0 (0-0)	0 (0-0)	0 (0-0)	0 (0-0)	0 (0-0)	0 (0-0)	0 (0-0)	0 (0-0)	0 (0-0)
Wokingham	0 (0-0)	0 (0-0)	0 (0-0)	0 (0-0)	0 (0-0)	0 (0-0)	0 (0-0)	0 (0-0)	0 (0-0)	0 (0-0)	0 (0-0)	0 (0-0)	0 (0-0)	0 (0-0)
South West England	1 (1-1)	1 (1-1)	1 (1-1)	1 (1-1)	1 (1-1)	1 (1-1)	1 (1-1)	1 (1-1)	0 (0-0)	0 (0-0)	0 (0-0)	0 (0-0)	0 (0-0)	0 (0-0)
Bath and North East Somerset	0 (0-0)	0 (0-0)	0 (0-0)	0 (0-0)	0 (0-0)	0 (0-0)	0 (0-0)	0 (0-0)	0 (0-0)	0 (0-0)	0 (0-0)	0 (0-0)	0 (0-0)	0 (0-0)

Table 2 Under-5 death numbers by location and year for 1950, 1955...2005, 2010, 2017 - both sexes

Location	1950	1955	1960	1965	1970	1975	1980	1985	1990	1995	2000	2005	2010	2017
Bournemouth	0 (0-0)	0 (0-0)	0 (0-0)	0 (0-0)	0 (0-0)	0 (0-0)	0 (0-0)	0 (0-0)	0 (0-0)	0 (0-0)	0 (0-0)	0 (0-0)	0 (0-0)	0 (0-0)
Bristol, City of	0 (0-0)	0 (0-0)	0 (0-0)	0 (0-0)	0 (0-0)	0 (0-0)	0 (0-0)	0 (0-0)	0 (0-0)	0 (0-0)	0 (0-0)	0 (0-0)	0 (0-0)	0 (0-0)
Cornwall	0 (0-0)	0 (0-0)	0 (0-0)	0 (0-0)	0 (0-0)	0 (0-0)	0 (0-0)	0 (0-0)	0 (0-0)	0 (0-0)	0 (0-0)	0 (0-0)	0 (0-0)	0 (0-0)
Devon	0 (0-0)	0 (0-0)	0 (0-0)	0 (0-0)	0 (0-0)	0 (0-0)	0 (0-0)	0 (0-0)	0 (0-0)	0 (0-0)	0 (0-0)	0 (0-0)	0 (0-0)	0 (0-0)
Dorset	0 (0-0)	0 (0-0)	0 (0-0)	0 (0-0)	0 (0-0)	0 (0-0)	0 (0-0)	0 (0-0)	0 (0-0)	0 (0-0)	0 (0-0)	0 (0-0)	0 (0-0)	0 (0-0)
Gloucestershire	0 (0-0)	0 (0-0)	0 (0-0)	0 (0-0)	0 (0-0)	0 (0-0)	0 (0-0)	0 (0-0)	0 (0-0)	0 (0-0)	0 (0-0)	0 (0-0)	0 (0-0)	0 (0-0)
North Somerset	0 (0-0)	0 (0-0)	0 (0-0)	0 (0-0)	0 (0-0)	0 (0-0)	0 (0-0)	0 (0-0)	0 (0-0)	0 (0-0)	0 (0-0)	0 (0-0)	0 (0-0)	0 (0-0)
Plymouth	0 (0-0)	0 (0-0)	0 (0-0)	0 (0-0)	0 (0-0)	0 (0-0)	0 (0-0)	0 (0-0)	0 (0-0)	0 (0-0)	0 (0-0)	0 (0-0)	0 (0-0)	0 (0-0)
Poole	0 (0-0)	0 (0-0)	0 (0-0)	0 (0-0)	0 (0-0)	0 (0-0)	0 (0-0)	0 (0-0)	0 (0-0)	0 (0-0)	0 (0-0)	0 (0-0)	0 (0-0)	0 (0-0)
Somerset	0 (0-0)	0 (0-0)	0 (0-0)	0 (0-0)	0 (0-0)	0 (0-0)	0 (0-0)	0 (0-0)	0 (0-0)	0 (0-0)	0 (0-0)	0 (0-0)	0 (0-0)	0 (0-0)
South Gloucestershire	0 (0-0)	0 (0-0)	0 (0-0)	0 (0-0)	0 (0-0)	0 (0-0)	0 (0-0)	0 (0-0)	0 (0-0)	0 (0-0)	0 (0-0)	0 (0-0)	0 (0-0)	0 (0-0)
Swindon	0 (0-0)	0 (0-0)	0 (0-0)	0 (0-0)	0 (0-0)	0 (0-0)	0 (0-0)	0 (0-0)	0 (0-0)	0 (0-0)	0 (0-0)	0 (0-0)	0 (0-0)	0 (0-0)
Torbay	0 (0-0)	0 (0-0)	0 (0-0)	0 (0-0)	0 (0-0)	0 (0-0)	0 (0-0)	0 (0-0)	0 (0-0)	0 (0-0)	0 (0-0)	0 (0-0)	0 (0-0)	0 (0-0)
Wiltshire	0 (0-0)	0 (0-0)	0 (0-0)	0 (0-0)	0 (0-0)	0 (0-0)	0 (0-0)	0 (0-0)	0 (0-0)	0 (0-0)	0 (0-0)	0 (0-0)	0 (0-0)	0 (0-0)
West Midlands	2 (2-2)	2 (2-2)	2 (2-2)	2 (2-2)	2 (2-2)	1 (1-1)	1 (1-1)	1 (1-1)	1 (1-1)	1 (1-1)	0 (0-0)	0 (0-0)	0 (0-0)	0 (0-1)
Birmingham	0 (0-1)	0 (0-1)	1 (0-1)	1 (0-1)	0 (0-0)	0 (0-0)	0 (0-0)	0 (0-0)	0 (0-0)	0 (0-0)	0 (0-0)	0 (0-0)	0 (0-0)	0 (0-0)
Coventry	0 (0-0)	0 (0-0)	0 (0-0)	0 (0-0)	0 (0-0)	0 (0-0)	0 (0-0)	0 (0-0)	0 (0-0)	0 (0-0)	0 (0-0)	0 (0-0)	0 (0-0)	0 (0-0)
Dudley	0 (0-0)	0 (0-0)	0 (0-0)	0 (0-0)	0 (0-0)	0 (0-0)	0 (0-0)	0 (0-0)	0 (0-0)	0 (0-0)	0 (0-0)	0 (0-0)	0 (0-0)	0 (0-0)
Herefordshire, County of	0 (0-0)	0 (0-0)	0 (0-0)	0 (0-0)	0 (0-0)	0 (0-0)	0 (0-0)	0 (0-0)	0 (0-0)	0 (0-0)	0 (0-0)	0 (0-0)	0 (0-0)	0 (0-0)
Sandwell	0 (0-0)	0 (0-0)	0 (0-0)	0 (0-0)	0 (0-0)	0 (0-0)	0 (0-0)	0 (0-0)	0 (0-0)	0 (0-0)	0 (0-0)	0 (0-0)	0 (0-0)	0 (0-0)
Shropshire	0 (0-0)	0 (0-0)	0 (0-0)	0 (0-0)	0 (0-0)	0 (0-0)	0 (0-0)	0 (0-0)	0 (0-0)	0 (0-0)	0 (0-0)	0 (0-0)	0 (0-0)	0 (0-0)
Solihull	0 (0-0)	0 (0-0)	0 (0-0)	0 (0-0)	0 (0-0)	0 (0-0)	0 (0-0)	0 (0-0)	0 (0-0)	0 (0-0)	0 (0-0)	0 (0-0)	0 (0-0)	0 (0-0)
Staffordshire	0 (0-0)	0 (0-0)	0 (0-0)	0 (0-0)	0 (0-0)	0 (0-0)	0 (0-0)	0 (0-0)	0 (0-0)	0 (0-0)	0 (0-0)	0 (0-0)	0 (0-0)	0 (0-0)
Stoke-on-Trent	0 (0-0)	0 (0-0)	0 (0-0)	0 (0-0)	0 (0-0)	0 (0-0)	0 (0-0)	0 (0-0)	0 (0-0)	0 (0-0)	0 (0-0)	0 (0-0)	0 (0-0)	0 (0-0)
Telford and Wrekin	0 (0-0)	0 (0-0)	0 (0-0)	0 (0-0)	0 (0-0)	0 (0-0)	0 (0-0)	0 (0-0)	0 (0-0)	0 (0-0)	0 (0-0)	0 (0-0)	0 (0-0)	0 (0-0)
Walsall	0 (0-0)	0 (0-0)	0 (0-0)	0 (0-0)	0 (0-0)	0 (0-0)	0 (0-0)	0 (0-0)	0 (0-0)	0 (0-0)	0 (0-0)	0 (0-0)	0 (0-0)	0 (0-0)
Warwickshire	0 (0-0)	0 (0-0)	0 (0-0)	0 (0-0)	0 (0-0)	0 (0-0)	0 (0-0)	0 (0-0)	0 (0-0)	0 (0-0)	0 (0-0)	0 (0-0)	0 (0-0)	0 (0-0)
Wolverhampton	0 (0-0)	0 (0-0)	0 (0-0)	0 (0-0)	0 (0-0)	0 (0-0)	0 (0-0)	0 (0-0)	0 (0-0)	0 (0-0)	0 (0-0)	0 (0-0)	0 (0-0)	0 (0-0)
Worcestershire	0 (0-0)	0 (0-0)	0 (0-0)	0 (0-0)	0 (0-0)	0 (0-0)	0 (0-0)	0 (0-0)	0 (0-0)	0 (0-0)	0 (0-0)	0 (0-0)	0 (0-0)	0 (0-0)
Yorkshire and the Humber	2 (2-2)	2 (2-2)	2 (2-2)	2 (2-2)	2 (2-2)	1 (1-1)	1 (1-1)	1 (1-1)	1 (1-1)	1 (0-1)	0 (0-0)	0 (0-0)	0 (0-0)	0 (0-0)
Barnsley	0 (0-0)	0 (0-0)	0 (0-0)	0 (0-0)	0 (0-0)	0 (0-0)	0 (0-0)	0 (0-0)	0 (0-0)	0 (0-0)	0 (0-0)	0 (0-0)	0 (0-0)	0 (0-0)
Bradford	0 (0-0)	0 (0-0)	0 (0-0)	0 (0-0)	0 (0-0)	0 (0-0)	0 (0-0)	0 (0-0)	0 (0-0)	0 (0-0)	0 (0-0)	0 (0-0)	0 (0-0)	0 (0-0)
Calderdale	0 (0-0)	0 (0-0)	0 (0-0)	0 (0-0)	0 (0-0)	0 (0-0)	0 (0-0)	0 (0-0)	0 (0-0)	0 (0-0)	0 (0-0)	0 (0-0)	0 (0-0)	0 (0-0)
Doncaster	0 (0-0)	0 (0-0)	0 (0-0)	0 (0-0)	0 (0-0)	0 (0-0)	0 (0-0)	0 (0-0)	0 (0-0)	0 (0-0)	0 (0-0)	0 (0-0)	0 (0-0)	0 (0-0)
East Riding of Yorkshire	0 (0-0)	0 (0-0)	0 (0-0)	0 (0-0)	0 (0-0)	0 (0-0)	0 (0-0)	0 (0-0)	0 (0-0)	0 (0-0)	0 (0-0)	0 (0-0)	0 (0-0)	0 (0-0)
Kingston upon Hull, City of	0 (0-0)	0 (0-0)	0 (0-0)	0 (0-0)	0 (0-0)	0 (0-0)	0 (0-0)	0 (0-0)	0 (0-0)	0 (0-0)	0 (0-0)	0 (0-0)	0 (0-0)	0 (0-0)
Kirklees	0 (0-0)	0 (0-0)	0 (0-0)	0 (0-0)	0 (0-0)	0 (0-0)	0 (0-0)	0 (0-0)	0 (0-0)	0 (0-0)	0 (0-0)	0 (0-0)	0 (0-0)	0 (0-0)
Leeds	0 (0-0)	0 (0-0)	0 (0-0)	0 (0-0)	0 (0-0)	0 (0-0)	0 (0-0)	0 (0-0)	0 (0-0)	0 (0-0)	0 (0-0)	0 (0-0)	0 (0-0)	0 (0-0)
North East Lincolnshire	0 (0-0)	0 (0-0)	0 (0-0)	0 (0-0)	0 (0-0)	0 (0-0)	0 (0-0)	0 (0-0)	0 (0-0)	0 (0-0)	0 (0-0)	0 (0-0)	0 (0-0)	0 (0-0)
North Lincolnshire	0 (0-0)	0 (0-0)	0 (0-0)	0 (0-0)	0 (0-0)	0 (0-0)	0 (0-0)	0 (0-0)	0 (0-0)	0 (0-0)	0 (0-0)	0 (0-0)	0 (0-0)	0 (0-0)
North Yorkshire	0 (0-0)	0 (0-0)	0 (0-0)	0 (0-0)	0 (0-0)	0 (0-0)	0 (0-0)	0 (0-0)	0 (0-0)	0 (0-0)	0 (0-0)	0 (0-0)	0 (0-0)	0 (0-0)
Rotherham	0 (0-0)	0 (0-0)	0 (0-0)	0 (0-0)	0 (0-0)	0 (0-0)	0 (0-0)	0 (0-0)	0 (0-0)	0 (0-0)	0 (0-0)	0 (0-0)	0 (0-0)	0 (0-0)
Sheffield	0 (0-0)	0 (0-0)	0 (0-0)	0 (0-0)	0 (0-0)	0 (0-0)	0 (0-0)	0 (0-0)	0 (0-0)	0 (0-0)	0 (0-0)	0 (0-0)	0 (0-0)	0 (0-0)
Wakefield	0 (0-0)	0 (0-0)	0 (0-0)	0 (0-0)	0 (0-0)	0 (0-0)	0 (0-0)	0 (0-0)	0 (0-0)	0 (0-0)	0 (0-0)	0 (0-0)	0 (0-0)	0 (0-0)
York	0 (0-0)	0 (0-0)	0 (0-0)	0 (0-0)	0 (0-0)	0 (0-0)	0 (0-0)	0 (0-0)	0 (0-0)	0 (0-0)	0 (0-0)	0 (0-0)	0 (0-0)	0 (0-0)
Northern Ireland	2 (1-2)	1 (1-2)	1 (1-1)	1 (1-1)	1 (1-1)	1 (1-1)	1 (0-1)	0 (0-0)	0 (0-0)	0 (0-0)	0 (0-0)	0 (0-0)	0 (0-0)	0 (0-0)
Scotland	4 (3-5)	4 (3-4)	3 (3-4)	3 (3-3)	2 (2-2)	1 (1-1)	1 (1-1)	1 (1-1)	1 (1-1)	0 (0-0)	0 (0-0)	0 (0-0)	0 (0-0)	0 (0-0)
Wales	2 (2-3)	2 (2-3)	2 (2-2)	2 (2-2)	1 (1-2)	1 (1-1)	1 (1-1)	0 (0-0)	0 (0-0)	0 (0-0)	0 (0-0)	0 (0-0)	0 (0-0)	0 (0-0)
Latin America and Caribbean	1410 (1350-1470)	1380 (1350-1420)	1380 (1350-1410)	1330 (1300-1350)	1230 (1210-1250)	1080 (1060-1100)	921 (905-937)	755 (743-768)	590 (580-600)	469 (460-478)	368 (361-376)	287 (280-295)	265 (258-273)	180 (171-190)
Andean Latin America	203 (175-234)	207 (192-223)	216 (201-231)	199 (186-212)	195 (184-206)	165 (155-175)	145 (136-153)	121 (114-128)	96 (90-101)	73 (69-78)	54 (51-57)	41 (37-44)	33 (30-37)	25 (21-29)
Bolivia	48 (39-59)	48 (42-55)	49 (43-55)	47 (42-52)	43 (39-47)	38 (35-41)	35 (32-37)	31 (29-34)	28 (26-30)	23 (21-26)	18 (17-21)	14 (12-16)	11 (9-14)	8 (6-11)
Ecuador	29 (25-34)	31 (29-33)	34 (32-36)	33 (31-35)	31 (29-33)	25 (23-27)	21 (20-23)	16 (15-17)	12 (11-13)	11 (9-12)	11 (9-12)	8 (6-9)	7 (6-8)	5 (4-7)
Peru	126 (101-153)	128 (114-141)	133 (119-147)	119 (108-131)	122 (112-132)	102 (92-112)	89 (80-97)	73 (67-80)	55 (51-61)	39 (36-43)	25 (22-27)	18 (17-20)	15 (14-17)	12 (9-15)
Caribbean	124 (112-136)	117 (110-125)	114 (107-120)	108 (102-114)	98 (93-103)	84 (80-89)	74 (70-79)	68 (64-72)	61 (57-64)	52 (49-56)	43 (40-47)	39 (35-43)	63 (59-67)	29 (25-34)
Antigua and Barbuda	0 (0-0)	0 (0-0)	0 (0-0)	0 (0-0)	0 (0-0)	0 (0-0)	0 (0-0)	0 (0-0)	0 (0-0)	0 (0-0)	0 (0-0)	0 (0-0)	0 (0-0)	0 (0-0)
The Bahamas	0 (0-0)	0 (0-0)	0 (0-0)	0 (0-0)	0 (0-0)	0 (0-0)	0 (0-0)	0 (0-0)	0 (0-0)	0 (0-0)	0 (0-0)	0 (0-0)	0 (0-0)	0 (0-0)
Barbados	1 (1-2)	1 (1-1)	1 (1-1)	0 (0-0)	0 (0-0)	0 (0-0)	0 (0-0)	0 (0-0)	0 (0-0)	0 (0-0)	0 (0-0)	0 (0-0)	0 (0-0)	0 (0-0)
Belize	1 (1-1)	1 (1-1)	1 (0-1)	1 (0-1)	0 (0-1)	0 (0-0)	0 (0-0)	0 (0-0)	0 (0-0)	0 (0-0)	0 (0-0)	0 (0-0)	0 (0-0)	0 (0-0)
Bermuda	0 (0-0)	0 (0-0)	0 (0-0)	0 (0-0)	0 (0-0)	0 (0-0)	0 (0-0)	0 (0-0)	0 (0-0)	0 (0-0)	0 (0-0)	0 (0-0)	0 (0-0)	0 (0-0)
Cuba	10 (7-15)	10 (8-12)	10 (9-11)	11 (11-12)	10 (10-11)	6 (6-6)	3 (3-3)	3 (3-4)	2 (2-2)	2 (2-2)	1 (1-1)	1 (1-1)	1 (1-1)	1 (0-1)
Dominica	1 (1-1)	1 (0-1)	0 (0-0)	0 (0-0)	0 (0-0)	0 (0-0)	0 (0-0)	0 (0-0)	0 (0-0)	0 (0-0)	0 (0-0)	0 (0-0)	0 (0-0)	0 (0-0)
Dominican Republic	20 (16-25)	22 (20-24)	25 (22-27)	24 (22-27)	23 (21-26)	21 (19-23)	18 (16-20)	16 (15-18)	15 (14-17)	12 (11-13)	9 (8-11)	8 (7-9)	6 (5-7)	6 (4-8)
Grenada	1 (0-1)	0 (0-0)	0 (0-0)	0 (0-0)	0 (0-0)	0 (0-0)	0 (0-0)	0 (0-0)	0 (0-0)	0 (0-0)	0 (0-0)	0 (0-0)	0 (0-0)	0 (0-0)
Guyana	3 (3-4)	3 (3-3)	3 (2-3)	2 (2-3)	2 (2-2)	2 (2-2)	1 (1-2)	1 (1-1)	1 (1-1)	1 (1-1)	1 (1-1)	1 (1-1)	0 (0-1)	0 (0-0)
Haiti	61 (51-72)	57 (51-64)	54 (49-60)	51 (46-56)	48 (43-52)	45 (41-48)	42 (38-46)	39 (36-43)	35 (33-38)	32 (29-36)	27 (25-30)	25 (22-29)	52 (48-56)	19 (15-24)
Jamaica	7 (6-9)	7 (6-8)	6 (6-7)	5 (4-6)	4 (4-4)	3 (3-3)	2 (2-3)	2 (2-2)	2 (1-2)	1 (1-2)	1 (1-1)	1 (1-1)	1 (1-1)	1 (0-1)
Puerto Rico	8 (8-9)	6 (6-6)	4 (4-4)	4 (4-4)	2 (2-2)	2 (2-2)	2 (1-2)	1 (1-1)	1 (1-1)	1 (1-1)	1 (1-1)	0 (0-1)	0 (0-0)	0 (0-0)
Saint Lucia	1 (0-1)	1 (0-1)	1 (0-1)	0 (0-0)	0 (0-0)	0 (0-0)	0 (0-0)	0 (0-0)	0 (0-0)	0 (0-0)	0 (0-0)	0 (0-0)	0 (0-0)	0 (0-0)
Saint Vincent and the Grenadines	1 (0-1)	1 (1-1)	1 (0-1)	0 (0-0)	0 (0-0)	0 (0-0)	0 (0-0)	0 (0-0)	0 (0-0)	0 (0-0)	0 (0-0)	0 (0-0)	0 (0-0)	0 (0-0)
Suriname	1 (1-1)	1 (1-1)	1 (1-1)	1 (1-1)	1 (1-1)	1 (1-1)	1 (1-1)	1 (1-1)	1 (0-1)	0 (0-1)	0 (0-0)	0 (0-0)	0 (0-0)	0 (0-0)
Trinidad and Tobago	3 (2-4)	3 (2-3)	2 (2-2)	2 (2-2)	1 (1-2)	1 (1-1)	1 (1-1)	1 (1-1)	1 (1-1)	1 (0-1)	1 (0-1)	0 (0-1)	0 (0-0)	0 (0-0)
Virgin Islands	0 (0-0)	0 (0-0)	0 (0-0)	0 (0-0)	0 (0-0)	0 (0-0)	0 (0-0)	0 (0-0)	0 (0-0)	0 (0-0)	0 (0-0)	0 (0-0)	0 (0-0)	0 (0-0)
Central Latin America	495 (469-524)	468 (454-482)	454 (442-466)	436 (425-446)	408 (398-418)	358 (349-367)	297 (289-304)	245 (240-251)	200 (196-205)	169 (165-174)	134 (130-139)	104 (100-108)	89 (85-92)	74 (68-80)
Colombia	77 (62-95)	81 (72-91)	88 (80-96)	82 (75-89)	71 (65-77)	60 (54-66)	46 (41-50)	38 (35-41)	30 (28-33)	28 (25-31)	24 (21-27)	21 (18-24)	16 (14-19)	12 (8-16)
Costa Rica	6 (5-6)	5 (5-6)	5 (5-6)	6 (6-6)	5 (4-5)	3 (2-3)	2 (2-2)	2 (2-2)	1 (1-1)	1 (1-1)	1 (1-1)	1 (1-1)	1 (1-1)	1 (0-1)
El Salvador	25 (20-31)	24 (21-28)	24 (21-27)	23 (21-26)	21 (19-24)	18 (16-20)	15 (13-16)	11 (10-12)	8 (8-9)	6 (5-7)	4 (4-4)	2 (2-3)	2 (2-2)	1 (1-2)
Guatemala	38 (30-47)	38 (34-43)	39 (35-43)	40 (36-43)	38 (35-41)	36 (33-39)	37 (34-40)	32 (29-34)	26 (24-29)	22 (20-24)	18 (16-20)	15 (13-16)	12 (10-14)	12 (9-16)
Honduras	18 (14-23)	17 (15-20)	17 (15-20)	16 (14-18)	15 (13-17)	14 (12-15)	12 (11-13)	10 (9-11)	9 (8-9)	8 (7-9)	7 (6-7)	5 (4-6)	4 (4-5)	3 (3-4)
Mexico	277 (257-296)	252 (243-261)	235 (228-243)	225 (219-231)	214 (207-220)	186 (180-192)	152 (148-156)	123 (119-127)	97 (94-99)	80 (78-83)	63 (61-66)	46 (44-47)	39 (37-40)	33 (31-34)
Aguascalientes	1 (1-2)	1 (1-1)	1 (1-2)	1 (1-2)	2 (1-2)	1 (1-2)	1 (1-1)	1 (1-1)	1 (1-1)	1 (1-1)	1 (0-1)	0 (0-0)	0 (0-0)	0 (0-0)
Baja California	5 (4-6)	4 (4-5)	4 (3-4)	3 (3-4)	3 (2-3)	2 (2-2)	1 (1-2)	1 (1-2)	1 (1-2)	1 (1-2)	1 (1-2)	1 (1-1)	1 (1-1)	1 (1-1)
Baja California Sur	0 (0-0)	0 (0-0)	0 (0-0)	0 (0-1)	0 (0-1)	1 (0-1)	0 (0-0)	0 (0-0)	0 (0-0)	0 (0-0)	0 (0-0)	0 (0-0)	0 (0-0)	0 (0-0)
Campeche	1 (1-1)	1 (1-1)	1 (1-1)	1 (1-1)	1 (1-1)	1 (1-1)	1 (1-1)	1 (1-1)	1 (1-1)	1 (0-1)	0 (0-0)	0 (0-0)	0 (0-0)	0 (0-0)
Chiapas	5 (3-6)	5 (4-5)	5 (4-5)	5 (4-6)	5 (5-6)	6 (5-6)	6 (5-7)	6 (5-7)	6 (5-7)	5 (4-6)	4 (3-4)	2 (2-3)	2 (2-2)	2 (2-2)
Chihuahua	11 (9-14)	9 (8-11)	8 (7-9)	7 (6-8)	6 (5-7)	5 (4-5)	4 (3-4)	3 (2-3)	2 (2-3)	2 (2-3)	2 (2-2)	1 (1-2)	1 (1-1)	1 (1-1)
Coahuila	6 (5-7)	5 (5-6)	5 (5-6)	5 (5-6)	6 (5-6)	5 (4-6)	4 (3-4)	3 (2-3)	2 (2-2)	2 (1-2)	1 (1-2)	1 (1-1)	1 (1-1)	1 (1-1)
Colima	2 (1-2)	2 (1-2)	1 (1-2)	1 (1-2)	1 (1-2)	1 (1-1)	1 (1-1)	1 (1-1)	0 (0-1)	0 (0-0)	0 (0-0)	0 (0-0)	0 (0-0)	0 (0-0)
Mexico City	40 (28-55)	34 (28-40)	29 (25-34)	26 (22-29)	21 (18-24)	18 (15-21)	14 (13-16)	9 (8-10)	6 (5-7)	5 (4-5)	4 (3-4)	3 (3-3)	3 (2-3)	2 (2-3)
Durango	7 (6-9)	6 (6-7)	6 (5-7)	5 (5-6)	5 (4-6)	4 (4-5)	3 (3-3)	2 (2-2)	2 (1-2)	1 (1-2)	1 (1-1)	1 (1-1)	1 (1-1)	1 (0-1)
Guanajuato	16 (13-20)	15 (14-17)	15 (14-17)	15 (14-17)	15 (13-16)	13 (11-14)	10 (9-11)	8 (7-9)	6 (5-7)	5 (4-5)	4 (3-4)	2 (2-3)	2 (2-2)	1 (1-2)
Guerrero	10 (7-13)	9 (8-10)	9 (8-10)	9 (8-10)	9 (8-11)	8 (7-9)	7 (6-8)	6 (5-7)	5 (4-6)	5 (4-5)	3 (3-4)	2 (2-2)	2 (1-2)	1 (1-2)
Hidalgo	6 (5-8)	6 (5-7)	6 (5-6)	5 (5-6)	6 (5-6)	5 (5-6)	5 (4-5)	4 (3-4)	3 (2-3)	2 (2-3)	2 (1-2)	1 (1-1)	1 (1-1)	1 (0-1)
Jalisco	23 (18-29)	20 (18-23)	19 (17-21)	17 (15-18)	15 (13-17)	12 (10-13)	9 (8-11)	8 (7-9)	6 (5-6)	5 (4-5)	4 (3-5)	3 (2-3)	2 (2-3)	2 (2-2)
México	8 (6-11)	9 (8-11)	11 (10-13)	14 (12-15)	17 (15-19)	16 (14-19)	15 (13-16)	13 (12-15)	12 (11-13)	9 (8-10)	8 (7-9)	7 (6-7)	5 (5-6)	4 (3-5)
Michoacán de Ocampo	18 (13-23)	16 (14-19)	15 (12-17)	13 (12-15)	11 (10-13)	10 (9-11)	8 (7-10)	6 (5-7)	5 (4-5)	4 (3-4)	3 (2-3)	2 (2-2)	2 (1-2)	1 (1-2)
Morelos	3 (2-3)	3 (2-3)	3 (2-3)	3 (3-3)	3 (3-3)	3 (2-3)	2 (2-3)	2 (2-2)	1 (1-2)	1 (1-1)	1 (1-1)	1 (1-1)	1 (0-1)	0 (0-1)
Nayarit	4 (3-5)	4 (3-4)	4 (3-4)	3 (3-4)	3 (3-3)	2 (2-3)	1 (1-2)	1 (1-1)	1 (1-1)	1 (1-1)	1 (0-1)	0 (0-0)	0 (0-0)	0 (0-0)
Nuevo León	7 (5-9)	7 (6-7)	6 (6-7)	6 (6-7)	6 (5-6)	5 (4-5)	4 (3-4)	3 (2-3)	2 (2-2)	2 (2-2)	2 (1-2)	1 (1-1)	1 (1-1)	1 (1-1)

Table 2 Under-5 death numbers by location and year for 1950, 1955...2005, 2010, 2017 - both sexes

Location	1950	1955	1960	1965	1970	1975	1980	1985	1990	1995	2000	2005	2010	2017
Oaxaca	16 (11-22)	13 (11-16)	11 (9-13)	10 (9-12)	10 (8-11)	9 (7-10)	6 (5-7)	5 (4-5)	4 (3-4)	3 (3-4)	2 (2-3)	1 (1-2)	1 (1-2)	1 (1-2)
Puebla	21 (16-26)	18 (16-21)	17 (14-19)	16 (14-18)	16 (14-17)	13 (11-14)	11 (9-13)	9 (8-11)	7 (6-8)	6 (5-7)	5 (4-6)	4 (3-5)	3 (3-4)	3 (2-3)
Querétaro	2 (2-3)	2 (2-3)	2 (2-3)	2 (2-3)	3 (2-3)	2 (2-3)	3 (2-3)	2 (2-2)	1 (1-2)	1 (1-1)	1 (1-1)	1 (1-1)	1 (1-1)	1 (0-1)
Quintana Roo	0 (0-0)	0 (0-0)	0 (0-0)	0 (0-0)	0 (0-0)	1 (1-1)	1 (1-1)	1 (1-1)	1 (1-1)	1 (1-1)	1 (0-1)	0 (0-1)	0 (0-0)	0 (0-0)
San Luis Potosí	10 (7-13)	8 (7-10)	8 (6-9)	7 (6-8)	6 (6-7)	5 (4-6)	4 (3-4)	3 (3-3)	2 (2-3)	2 (2-2)	2 (1-2)	1 (1-1)	1 (1-1)	1 (1-1)
Sinaloa	5 (4-7)	5 (5-6)	6 (5-6)	6 (5-7)	6 (6-7)	5 (4-6)	3 (3-4)	3 (2-3)	2 (2-2)	2 (1-2)	1 (1-1)	1 (1-1)	1 (1-1)	1 (0-1)
Sonora	5 (4-6)	4 (4-5)	4 (4-5)	4 (3-4)	4 (3-4)	3 (3-3)	2 (2-3)	2 (2-2)	1 (1-2)	1 (1-2)	1 (1-2)	1 (1-1)	1 (1-1)	1 (1-1)
Tabasco	3 (2-4)	3 (3-4)	3 (3-4)	4 (3-4)	4 (4-5)	4 (4-5)	4 (3-4)	3 (3-4)	2 (2-3)	2 (2-2)	1 (1-2)	1 (1-1)	1 (1-1)	1 (1-1)
Tamaulipas	8 (6-11)	7 (6-8)	6 (5-7)	6 (5-7)	5 (4-6)	4 (3-5)	3 (3-4)	3 (2-3)	2 (2-2)	2 (1-2)	1 (1-2)	1 (1-1)	1 (1-1)	1 (1-1)
Tlaxcala	3 (2-4)	3 (2-3)	3 (2-3)	2 (2-3)	2 (2-3)	2 (2-2)	2 (2-2)	1 (1-2)	1 (1-1)	1 (1-1)	1 (1-1)	1 (0-1)	0 (0-1)	0 (0-0)
Veracruz de Ignacio de la Llave	21 (16-26)	19 (17-22)	18 (16-20)	17 (15-19)	15 (13-17)	13 (12-15)	11 (9-12)	10 (9-11)	8 (7-9)	6 (6-7)	5 (4-6)	3 (2-3)	2 (2-2)	2 (1-2)
Yucatán	3 (3-4)	3 (3-4)	3 (3-4)	3 (3-3)	3 (3-3)	3 (2-3)	2 (2-3)	2 (2-2)	1 (1-2)	1 (1-1)	1 (1-1)	1 (1-1)	1 (0-1)	1 (0-1)
Zacatecas	9 (7-11)	8 (7-9)	7 (6-7)	6 (5-7)	6 (5-6)	5 (4-5)	4 (3-4)	2 (2-3)	2 (1-2)	1 (1-1)	1 (1-1)	1 (1-1)	0 (0-1)	0 (0-0)
Nicaragua	17 (14-21)	17 (15-20)	17 (15-20)	17 (15-19)	16 (14-17)	15 (14-16)	13 (12-14)	12 (11-13)	9 (8-10)	7 (6-7)	5 (4-5)	4 (3-4)	3 (2-3)	2 (2-3)
Panama	4 (3-4)	4 (3-4)	4 (3-4)	4 (3-4)	3 (3-3)	2 (2-3)	2 (2-2)	2 (1-2)	2 (1-2)	2 (1-2)	1 (1-2)	1 (1-2)	1 (1-2)	1 (1-2)
Venezuela	34 (32-36)	28 (28-29)	24 (24-25)	24 (24-25)	26 (26-27)	26 (25-26)	19 (19-20)	17 (16-17)	18 (18-19)	15 (15-16)	12 (11-12)	10 (9-10)	11 (11-11)	9 (7-11)
Tropical Latin America	583 (543-627)	587 (559-615)	596 (570-621)	583 (561-604)	530 (512-546)	474 (460-488)	405 (396-415)	322 (314-329)	234 (228-240)	174 (170-179)	137 (132-141)	104 (100-108)	80 (77-84)	53 (49-56)
Brazil	577 (538-622)	581 (554-610)	589 (564-615)	576 (554-597)	523 (506-540)	467 (453-481)	400 (390-410)	316 (309-324)	229 (224-235)	170 (165-175)	133 (129-137)	101 (97-105)	78 (74-81)	51 (48-54)
Acre	1 (1-2)	2 (1-2)	2 (1-2)	2 (1-2)	2 (1-2)	2 (1-2)	1 (1-2)	1 (1-1)	1 (1-1)	1 (1-1)	1 (1-1)	1 (1-1)	1 (0-1)	0 (0-0)
Alagoas	20 (16-25)	21 (18-24)	21 (19-25)	22 (20-25)	21 (20-23)	20 (18-23)	20 (19-22)	17 (15-18)	11 (10-12)	8 (7-8)	6 (5-6)	4 (3-4)	2 (2-3)	1 (1-2)
Amapá	1 (0-1)	1 (1-1)	1 (1-1)	1 (1-1)	1 (1-1)	1 (1-1)	0 (0-0)	0 (0-0)	0 (0-0)	0 (0-0)	0 (0-0)	0 (0-0)	0 (0-0)	0 (0-0)
Amazonas	5 (3-6)	5 (4-6)	5 (4-6)	5 (5-6)	6 (5-6)	6 (5-7)	6 (5-6)	4 (4-5)	3 (3-4)	3 (3-3)	3 (2-3)	2 (1-2)	2 (1-2)	1 (1-2)
Bahia	77 (62-96)	77 (67-90)	77 (67-89)	75 (67-84)	68 (62-75)	60 (55-65)	50 (46-55)	43 (39-46)	30 (28-33)	22 (20-24)	18 (16-20)	15 (12-17)	11 (9-13)	6 (5-8)
Ceará	52 (42-63)	53 (46-61)	54 (48-62)	54 (48-60)	50 (44-56)	47 (43-51)	41 (38-45)	31 (29-34)	21 (19-23)	15 (13-16)	11 (10-13)	8 (7-9)	5 (4-7)	3 (2-4)
Distrito Federal	1 (0-1)	1 (1-1)	1 (1-1)	2 (1-2)	2 (1-2)	2 (1-2)	2 (2-2)	2 (1-2)	2 (1-2)	1 (1-1)	1 (1-1)	1 (1-1)	1 (1-1)	1 (0-1)
Espírito Santo	7 (5-9)	7 (6-8)	7 (6-9)	7 (6-8)	6 (6-7)	6 (5-6)	4 (4-5)	4 (4-4)	3 (3-3)	2 (2-2)	2 (1-2)	1 (1-2)	1 (1-1)	1 (1-1)
Goiás	12 (8-15)	12 (10-15)	13 (11-15)	13 (11-16)	11 (10-13)	9 (8-10)	7 (6-8)	5 (5-6)	4 (3-4)	3 (3-4)	2 (2-2)	2 (1-2)	1 (1-2)	1 (1-2)
Maranhão	36 (29-44)	38 (32-43)	39 (34-45)	39 (35-44)	37 (34-40)	34 (30-38)	32 (30-36)	27 (25-30)	19 (17-21)	13 (12-14)	10 (9-11)	9 (7-10)	6 (5-7)	3 (3-4)
Mato Grosso	3 (3-5)	4 (3-5)	5 (4-5)	5 (4-6)	5 (4-5)	4 (3-4)	3 (3-4)	3 (3-4)	3 (2-3)	2 (2-3)	2 (2-2)	1 (1-1)	1 (1-1)	1 (1-1)
Mato Grosso do Sul	1 (1-2)	2 (1-2)	2 (2-2)	2 (2-3)	2 (2-3)	2 (2-3)	3 (2-3)	2 (2-2)	2 (2-2)	2 (2-2)	1 (1-1)	1 (1-1)	1 (1-1)	1 (1-1)
Minas Gerais	62 (47-79)	60 (50-71)	59 (50-70)	55 (47-64)	46 (40-53)	37 (33-42)	31 (28-34)	24 (22-27)	16 (14-18)	12 (11-14)	11 (9-13)	9 (7-11)	6 (5-8)	4 (3-5)
Pará	11 (8-15)	12 (10-14)	13 (11-15)	14 (12-17)	15 (13-17)	15 (13-17)	15 (13-16)	13 (12-14)	10 (9-11)	7 (7-8)	6 (5-7)	5 (5-6)	4 (4-5)	3 (2-4)
Paraíba	30 (24-37)	28 (25-33)	27 (24-32)	25 (22-28)	22 (19-25)	18 (17-21)	14 (12-15)	10 (9-11)	7 (6-7)	4 (4-5)	3 (3-4)	3 (2-3)	2 (1-2)	1 (1-1)
Paraná	32 (23-43)	32 (27-39)	33 (28-39)	32 (28-36)	26 (23-29)	19 (16-23)	15 (14-16)	11 (10-11)	8 (8-9)	6 (6-7)	5 (4-5)	4 (3-5)	3 (3-4)	2 (2-3)
Pernambuco	74 (59-91)	73 (63-83)	71 (62-81)	68 (60-76)	59 (52-67)	49 (45-54)	39 (35-42)	29 (27-32)	21 (19-23)	15 (13-16)	11 (10-13)	8 (7-10)	6 (4-7)	3 (2-4)
Piauí	13 (10-16)	13 (11-15)	14 (12-16)	14 (12-17)	14 (12-16)	12 (11-13)	10 (9-11)	8 (7-9)	6 (5-7)	5 (4-5)	4 (3-4)	3 (3-4)	2 (2-3)	1 (1-2)
Rio de Janeiro	34 (24-47)	33 (26-40)	31 (26-38)	28 (23-34)	26 (23-30)	24 (21-27)	20 (18-22)	15 (14-16)	11 (10-12)	9 (8-10)	6 (6-7)	4 (4-5)	4 (4-4)	3 (3-4)
Rio Grande do Norte	16 (13-20)	17 (14-19)	17 (15-19)	17 (15-19)	16 (14-18)	14 (13-15)	11 (10-12)	8 (7-9)	6 (6-7)	5 (4-5)	3 (3-4)	2 (2-3)	2 (1-2)	1 (1-1)
Rio Grande do Sul	24 (17-33)	23 (18-29)	22 (18-27)	21 (17-25)	17 (15-19)	13 (12-15)	10 (10-11)	7 (7-8)	5 (5-6)	5 (4-5)	4 (3-4)	2 (2-3)	2 (2-2)	2 (1-2)
Rondônia	0 (0-0)	0 (0-0)	0 (0-0)	0 (0-0)	1 (1-1)	1 (1-1)	1 (1-1)	2 (2-2)	2 (1-2)	1 (1-1)	1 (1-1)	1 (1-1)	1 (0-1)	0 (0-0)
Roraima	0 (0-0)	0 (0-0)	0 (0-0)	0 (0-0)	0 (0-0)	0 (0-0)	0 (0-0)	0 (0-0)	0 (0-0)	0 (0-0)	0 (0-0)	0 (0-0)	0 (0-0)	0 (0-0)
Santa Catarina	8 (5-11)	8 (6-10)	9 (7-10)	9 (7-10)	8 (7-9)	7 (6-8)	6 (5-7)	5 (4-6)	4 (3-4)	3 (3-4)	3 (2-3)	2 (2-2)	2 (1-2)	1 (1-2)
São Paulo	48 (34-64)	50 (40-61)	53 (44-63)	53 (45-63)	52 (45-60)	55 (49-62)	47 (44-51)	36 (34-38)	29 (26-32)	22 (21-25)	16 (15-17)	10 (9-11)	9 (8-11)	8 (7-9)
Sergipe	7 (5-9)	7 (6-8)	7 (6-8)	7 (6-8)	7 (6-8)	6 (5-7)	6 (5-7)	5 (4-5)	4 (3-4)	3 (2-3)	2 (2-3)	2 (1-2)	1 (1-2)	1 (1-1)
Tocantins	4 (3-5)	4 (4-5)	5 (4-6)	5 (4-6)	5 (4-5)	4 (4-5)	4 (3-4)	3 (3-3)	2 (2-2)	2 (1-2)	1 (1-2)	1 (1-1)	1 (1-1)	0 (0-1)
Paraguay	5 (4-7)	6 (5-7)	6 (6-7)	7 (6-7)	6 (6-7)	6 (6-7)	6 (5-6)	5 (5-6)	4 (4-5)	4 (4-4)	3 (3-4)	3 (3-4)	3 (2-3)	2 (1-2)
North Africa and Middle East	2110 (1980-2270)	1980 (1910-2060)	1890 (1830-1960)	1760 (1700-1810)	1640 (1590-1690)	1500 (1460-1550)	1340 (1300-1380)	1160 (1130-1200)	900 (873-928)	806 (780-835)	694 (670-718)	591 (568-616)	494 (470-520)	365 (338-396)
North Africa and Middle East	2110 (1980-2270)	1980 (1910-2060)	1890 (1830-1960)	1760 (1700-1810)	1640 (1590-1690)	1500 (1460-1550)	1340 (1300-1380)	1160 (1130-1200)	900 (873-928)	806 (780-835)	694 (670-718)	591 (568-616)	494 (470-520)	365 (338-396)
Afghanistan	133 (103-169)	135 (112-159)	138 (116-163)	143 (118-170)	150 (124-181)	157 (131-186)	152 (129-177)	110 (97-125)	89 (80-99)	139 (125-155)	136 (124-150)	119 (107-132)	96 (84-108)	87 (71-106)
Algeria	52 (45-60)	67 (60-75)	82 (71-94)	81 (73-91)	88 (78-100)	91 (82-102)	87 (80-95)	57 (52-62)	39 (34-43)	31 (27-35)	26 (23-30)	22 (19-25)	20 (17-23)	15 (12-19)
Bahrain	2 (1-2)	1 (1-2)	1 (1-2)	1 (1-1)	1 (1-1)	0 (0-1)	0 (0-0)	0 (0-0)	0 (0-0)	0 (0-0)	0 (0-0)	0 (0-0)	0 (0-0)	0 (0-0)
Egypt	497 (401-611)	443 (395-498)	404 (367-445)	364 (332-396)	343 (315-373)	319 (294-347)	278 (255-301)	227 (209-246)	163 (150-178)	120 (109-133)	95 (84-106)	82 (73-93)	71 (60-82)	44 (35-55)
Iran	460 (433-492)	421 (404-440)	389 (373-405)	359 (346-372)	300 (290-311)	208 (200-215)	160 (155-166)	131 (126-136)	105 (101-109)	76 (73-79)	63 (60-65)	54 (52-56)	46 (44-48)	21 (21-22)
Iraq	65 (49-85)	57 (47-69)	52 (43-62)	50 (41-60)	49 (42-57)	45 (39-50)	39 (36-43)	39 (36-43)	33 (30-36)	38 (35-42)	38 (34-42)	39 (35-43)	33 (28-38)	30 (25-37)
Jordan	5 (3-6)	5 (4-6)	6 (5-7)	6 (5-7)	6 (5-7)	6 (5-6)	5 (5-6)	5 (5-6)	5 (5-6)	5 (5-6)	5 (5-6)	5 (4-5)	5 (4-6)	4 (3-6)
Kuwait	0 (0-1)	1 (0-1)	1 (1-1)	1 (1-1)	2 (1-2)	2 (2-2)	2 (2-2)	1 (1-1)	1 (1-1)	1 (1-1)	0 (0-1)	1 (0-1)	1 (1-1)	0 (0-1)
Lebanon	10 (7-13)	9 (7-11)	8 (6-10)	7 (6-9)	7 (5-8)	10 (9-12)	4 (4-5)	5 (4-6)	4 (3-5)	3 (3-4)	2 (2-3)	2 (1-2)	1 (1-2)	1 (1-2)
Libya	22 (18-27)	19 (17-22)	18 (15-20)	15 (13-17)	10 (9-11)	8 (7-9)	7 (6-8)	6 (5-6)	5 (4-6)	4 (3-4)	3 (2-4)	2 (2-3)	2 (1-2)	1 (1-2)
Morocco	169 (140-205)	165 (147-184)	164 (152-179)	141 (131-152)	126 (118-135)	122 (113-130)	100 (93-107)	73 (68-80)	55 (50-60)	45 (40-50)	37 (31-42)	29 (23-36)	22 (17-28)	14 (10-18)
Palestine	11 (8-14)	9 (7-10)	7 (6-9)	6 (5-7)	5 (4-6)	4 (3-5)	4 (3-4)	3 (3-4)	3 (3-4)	3 (3-3)	3 (3-4)	4 (3-4)	3 (3-3)	2 (2-2)
Oman	12 (10-15)	12 (10-14)	12 (10-13)	11 (9-12)	8 (7-9)	7 (6-8)	5 (4-6)	4 (3-4)	2 (2-3)	1 (1-2)	1 (1-1)	0 (0-0)	1 (1-1)	1 (1-1)
Qatar	0 (0-0)	0 (0-0)	0 (0-0)	0 (0-0)	0 (0-0)	0 (0-0)	0 (0-0)	0 (0-0)	0 (0-0)	0 (0-0)	0 (0-0)	0 (0-0)	0 (0-0)	0 (0-0)
Saudi Arabia	40 (31-51)	40 (33-47)	39 (33-47)	38 (32-46)	38 (32-45)	36 (30-42)	36 (30-42)	37 (31-43)	34 (27-41)	25 (19-31)	17 (13-21)	11 (9-15)	8 (5-10)	4 (3-7)
Sudan	61 (47-76)	45 (38-52)	34 (29-39)	44 (39-49)	69 (64-75)	106 (97-115)	135 (122-148)	191 (178-204)	132 (122-144)	122 (111-134)	111 (102-123)	102 (93-113)	93 (81-105)	68 (52-89)
Syria	39 (32-47)	34 (30-38)	30 (26-34)	27 (24-30)	25 (22-28)	24 (21-26)	20 (17-23)	18 (15-20)	17 (15-20)	13 (11-15)	9 (9-9)	7 (7-7)	7 (7-7)	6 (5-7)
Tunisia	58 (47-71)	51 (45-57)	45 (41-49)	38 (35-41)	31 (29-34)	24 (22-27)	20 (18-22)	16 (14-17)	12 (10-13)	8 (7-9)	5 (4-6)	4 (3-4)	3 (3-4)	2 (1-2)
Turkey	359 (296-430)	350 (313-387)	347 (317-380)	309 (284-336)	269 (249-292)	233 (216-251)	191 (175-207)	148 (136-161)	110 (101-120)	88 (79-97)	66 (59-73)	43 (37-49)	28 (24-33)	17 (14-21)
United Arab Emirates	1 (1-1)	1 (1-1)	1 (1-1)	1 (1-1)	1 (1-1)	1 (1-2)	2 (1-2)	1 (1-2)	1 (1-1)	1 (1-1)	1 (1-1)	1 (1-1)	2 (1-2)	1 (1-1)
Yemen	117 (95-142)	116 (101-131)	115 (103-127)	115 (105-124)	111 (103-120)	102 (95-110)	95 (88-103)	91 (84-98)	88 (81-95)	83 (76-91)	75 (69-82)	64 (56-71)	53 (46-62)	45 (36-57)
South Asia	4810 (4460-5200)	4990 (4760-5240)	5300 (5070-5540)	5690 (5480-5930)	5170 (5030-5310)	5120 (4980-5260)	4770 (4640-4900)	4390 (4290-4500)	4010 (3900-4110)	3620 (3530-3710)	3140 (3050-3240)	2660 (2570-2750)	2180 (2080-2270)	1500 (1420-1600)
South Asia	4810 (4460-5200)	4990 (4760-5240)	5300 (5070-5540)	5690 (5480-5930)	5170 (5030-5310)	5120 (4980-5260)	4770 (4640-4900)	4390 (4290-4500)	4010 (3900-4110)	3620 (3530-3710)	3140 (3050-3240)	2660 (2570-2750)	2180 (2080-2270)	1500 (1420-1600)
Bangladesh	765 (635-902)	695 (611-779)	642 (567-725)	593 (541-649)	605 (555-657)	571 (520-623)	632 (589-677)	669 (630-715)	561 (523-598)	431 (401-461)	347 (318-377)	274 (250-297)	183 (163-204)	108 (89-130)
Bhutan	4 (3-5)	4 (3-4)	4 (3-4)	3 (3-4)	3 (3-4)	3 (2-4)	3 (2-4)	3 (2-3)	2 (2-3)	2 (1-2)	1 (1-1)	1 (1-1)	1 (1-1)	0 (0-1)
India	3440 (3150-3790)	3720 (3520-3960)	4100 (3900-4310)	4570 (4380-4790)	4000 (3870-4140)	3920 (3790-4050)	3470 (3360-3590)	3050 (2960-3140)	2810 (2730-2890)	2580 (2500-2650)	2240 (2160-2320)	1860 (1800-1920)	1530 (1470-1600)	1040 (982-1100)
Andhra Pradesh	128 (92-174)	136 (109-167)	147 (117-183)	165 (139-189)	145 (129-162)	148 (132-163)	129 (116-141)	112 (100-125)	105 (96-115)	89 (80-97)	68 (62-75)	53 (47-60)	39 (33-46)	21 (16-28)
Arunachal Pradesh	2 (1-3)	2 (2-3)	3 (2-3)	3 (3-4)	3 (3-3)	3 (3-3)	3 (2-3)	3 (2-3)	3 (2-3)	3 (2-3)	2 (2-2)	2 (1-2)	1 (1-1)	1 (1-1)
Assam	74 (56-96)	84 (70-98)	96 (83-112)	112 (96-130)	99 (87-110)	95 (86-105)	88 (80-96)	80 (72-87)	72 (65-80)	65 (58-72)	56 (50-62)	49 (44-55)	48 (42-54)	39 (33-47)
Bihar	305 (233-399)	326 (274-385)	352 (305-410)	392 (342-445)	366 (330-405)	377 (337-420)	356 (320-393)	320 (291-349)	293 (266-320)	290 (262-318)	261 (238-287)	229 (204-258)	196 (175-219)	141 (121-169)
Chhattisgarh	41 (31-54)	46 (39-54)	52 (45-61)	66 (57-74)	59 (50-69)	63 (55-72)	59 (52-68)	57 (50-64)	53 (48-59)	53 (48-59)	52 (47-57)	46 (41-51)	40 (36-45)	26 (22-30)
Delhi	16 (12-22)	17 (14-20)	18 (16-21)	24 (21-27)	18 (16-21)	19 (16-21)	18 (16-21)	17 (15-19)	18 (16-20)	19 (17-21)	18 (16-20)	13 (11-16)	10 (8-13)	9 (8-11)
Goa	3 (2-5)	3 (2-4)	3 (2-4)	3 (3-4)	3 (2-3)	3 (2-3)	2 (2-2)	1 (1-2)	1 (1-1)	1 (1-1)	1 (1-1)	1 (1-1)	1 (0-1)	0 (0-1)
Gujarat	130 (101-166)	141 (120-163)	154 (136-174)	176 (157-196)	148 (134-163)	146 (133-159)	135 (124-147)	121 (110-132)	113 (105-122)	100 (91-108)	86 (78-93)	69 (62-77)	53 (48-60)	40 (35-47)
Haryana	33 (25-43)	38 (32-45)	45 (39-52)	60 (53-67)	54 (49-60)	51 (46-57)	44 (41-48)	41 (38-46)	42 (38-45)	40 (36-45)	38 (35-42)	31 (28-35)	26 (23-29)	18 (15-21)
Himachal Pradesh	14 (11-19)	15 (12-18)	16 (14-19)	19 (16-21)	16 (14-18)	15 (14-17)	13 (12-15)	11 (10-12)	10 (9-11)	9 (7-10)	7 (6-8)	6 (5-6)	4 (4-5)	3 (3-4)
Jammu and Kashmir	22 (17-29)	22 (18-26)	22 (19-26)	26 (23-29)	21 (19-24)	23 (20-25)	21 (19-23)	19 (17-21)	18 (17-20)	17 (15-19)	14 (13-15)	12 (11-13)	10 (9-11)	7 (6-9)
Jharkhand	77 (55-104)	84 (69-101)	94 (78-112)	108 (93-124)	94 (79-112)	94 (81-110)	87 (75-100)	81 (71-91)	74 (66-82)	72 (65-79)	66 (59-72)	53 (47-60)	43 (37-50)	28 (24-34)
Karnataka	156 (121-196)	167 (142-194)	181 (158-207)	204 (178-231)	170 (152-188)	158 (143-175)	135 (123-149)	112 (103-120)	97 (89-105)	82 (75-90)	71 (66-77)	57 (51-63)	44 (39-50)	29 (25-33)
Kerala	109 (80-143)	100 (81-118)	93 (80-108)	93 (83-104)	66 (58-74)	51 (45-57)	37 (33-41)	28 (25-31)	22 (20-25)	17 (16-19)	14 (12-15)	9 (8-10)	6 (6-7)	5 (4-6)
Madhya Pradesh	203 (155-265)	225 (191-264)	255 (222-293)	295 (259-334)	266 (240-293)	278 (250-308)	260 (236-284)	235 (217-253)	221 (203-242)	213 (193-232)	186 (172-200)	157 (142-172)	132 (119-145)	80 (69-94)
Maharashtra	239 (178-313)	253 (212-301)	272 (238-313)	300 (259-344)	253 (226-284)	243 (220-267)	194 (177-214)	161 (148-176)	163 (148-178)	133 (120-146)	110 (100-121)	87 (74-101)	66 (55-79)	44 (38-52)
Manipur	4 (3-5)	4 (4-5)	5 (4-6)	6 (5-7)	5 (4-5)	5 (4-5)	4 (4-5)	4 (3-4)	4 (3-4)	4 (3-4)	3 (3-4)	3 (2-3)	2 (2-2)	2 (2-2)
Meghalaya	3 (2-4)	3 (3-4)	3 (3-4)	4 (4-5)	4 (3-4)	5 (4-5)	5 (4-6)	5 (4-5)	5 (4-5)	5 (4-5)	5 (4-5)	4 (3-5)	3 (3-4)	3 (2-4)
Mizoram	1 (0-1)	1 (1-1)	1 (1-1)	1 (1-1)	1 (1-1)	1 (1-1)	1 (1-1)	1 (1-1)	1 (1-1)	1 (1-1)	1 (1-1)	1 (1-1)	1 (1-1)	1 (1-1)
Nagaland	2 (1-2)	2 (2-3)	2 (2-3)	3 (3-4)	2 (2-3)	3 (2-3)	3 (2-3)	2 (2-3)	3 (3-3)	3 (3-3)	3 (3-3)	2 (2-3)	2 (2-2)	2 (1-2)
Odisha	162 (120-215)	178 (148-212)	197 (168-228)	216 (189-244)	193 (172-214)	186 (165-206)	160 (146-177)	137 (122-155)	126 (114-140)	111 (100-123)	92 (84-101)	76 (67-85)	61 (54-68)	41 (35-49)
Punjab	43 (33-56)	46 (39-55)	50 (43-57)	58 (52-65)	46 (40-51)	46 (41-51)	43 (39-47)	40 (36-44)	40 (37-44)	36 (32-39)	30 (28-32)	25 (23-28)	20 (18-23)	14 (12-16)
Rajasthan	158 (120-209)	179 (151-211)	206 (178-240)	242 (211-275)	215 (193-240)	220 (198-245)	210 (190-231)	198 (179-220)	187 (170-206)	176 (161-191)	152 (138-166)	129 (119-140)	115 (106-124)	82 (71-97)
Sikkim	1 (1-1)	1 (1-1)	1 (1-1)	1 (1-1)	1 (1-1)	1 (1-1)	1 (1-1)	1 (1-1)	1 (1-1)	1 (0-1)	0 (0-1)	0 (0-0)	0 (0-0)	0 (0-0)

Table 2 Under-5 death numbers by location and year for 1950, 1955...2005, 2010, 2017 - both sexes

Location	1950	1955	1960	1965	1970	1975	1980	1985	1990	1995	2000	2005	2010	2017
Tamil Nadu	290 (217-377)	289 (242-341)	293 (259-334)	311 (273-348)	264 (240-289)	234 (212-256)	176 (160-194)	129 (118-140)	102 (95-111)	81 (74-89)	65 (60-70)	47 (42-52)	31 (27-37)	17 (15-20)
Telangana	71 (50-98)	77 (61-95)	86 (67-107)	100 (83-118)	86 (76-96)	86 (76-98)	73 (63-85)	64 (55-75)	63 (54-72)	61 (52-70)	54 (47-61)	42 (36-48)	27 (22-33)	14 (11-18)
Tripura	6 (5-9)	8 (7-10)	10 (9-12)	12 (11-14)	10 (9-12)	10 (9-11)	9 (8-10)	8 (7-9)	7 (6-7)	6 (5-6)	4 (4-5)	4 (3-4)	3 (3-4)	3 (2-4)
Uttar Pradesh	908 (662-1210)	1010 (843-1190)	1140 (1000-1300)	1230 (1080-1400)	1100 (994-1200)	1070 (972-1170)	966 (877-1060)	854 (785-930)	777 (712-845)	740 (682-799)	654 (593-722)	553 (507-602)	469 (423-519)	313 (272-364)
Uttarakhand	12 (9-17)	13 (11-16)	15 (12-17)	19 (17-21)	16 (14-19)	17 (15-20)	16 (14-18)	15 (13-17)	15 (13-16)	14 (13-16)	13 (12-15)	11 (10-13)	9 (8-10)	5 (4-6)
West Bengal	227 (167-302)	251 (209-295)	283 (247-325)	316 (276-358)	274 (244-304)	265 (239-292)	223 (204-245)	191 (176-208)	168 (153-182)	138 (124-152)	109 (99-119)	86 (77-96)	66 (56-78)	45 (38-53)
Union Territories other than Delhi	2 (1-2)	2 (2-2)	2 (2-2)	3 (3-4)	2 (2-3)	2 (2-3)	2 (2-3)	2 (2-3)	2 (2-3)	3 (2-3)	2 (2-3)	2 (2-2)	2 (1-2)	2 (1-2)
Nepal	143 (119-168)	138 (122-154)	134 (117-151)	127 (113-141)	118 (107-130)	118 (109-128)	126 (116-137)	122 (113-133)	103 (96-112)	80 (74-87)	59 (54-64)	42 (38-47)	30 (25-34)	19 (15-23)
Pakistan	455 (384-535)	433 (388-480)	415 (375-454)	393 (354-431)	440 (402-481)	505 (465-549)	540 (496-589)	550 (509-593)	537 (501-578)	522 (481-568)	499 (451-547)	488 (433-550)	430 (372-496)	339 (272-414)
Southeast Asia, East Asia, and Oceania	6500 (6220-6790)	6260 (6060-6460)	20400 (20200-20600)	5700 (5530-5870)	4750 (4610-4900)	3490 (3380-3600)	2660 (2580-2740)	2790 (2690-2880)	2270 (2190-2350)	1750 (1700-1800)	1320 (1280-1350)	934 (909-959)	695 (674-717)	460 (438-484)
East Asia	4060 (3820-4300)	4040 (3870-4210)	18300 (18200-18600)	3820 (3670-3980)	3070 (2940-3200)	1980 (1890-2070)	1370 (1300-1430)	1680 (1590-1760)	1350 (1290-1420)	1010 (969-1050)	698 (674-722)	425 (411-440)	290 (279-302)	175 (167-185)
China	3880 (3640-4110)	3910 (3740-4080)	18200 (18000-18400)	3720 (3560-3860)	2980 (2860-3110)	1920 (1840-2020)	1330 (1260-1390)	1640 (1550-1720)	1320 (1250-1380)	936 (895-979)	632 (608-655)	404 (391-418)	275 (264-286)	165 (157-174)
North Korea	81 (68-97)	36 (28-47)	34 (27-43)	29 (23-36)	20 (16-26)	13 (10-17)	10 (8-13)	10 (8-13)	11 (9-14)	56 (51-61)	54 (49-58)	13 (10-16)	10 (8-13)	7 (5-9)
Taiwan (Province of China)	48 (45-54)	33 (33-34)	25 (24-25)	16 (16-16)	10 (10-10)	7 (7-7)	6 (6-6)	4 (4-4)	3 (3-3)	3 (3-3)	2 (2-2)	1 (1-1)	1 (1-1)	1 (1-1)
Oceania	25 (20-31)	24 (20-28)	23 (20-26)	20 (17-23)	16 (14-19)	14 (12-16)	13 (11-15)	13 (11-15)	13 (11-15)	13 (12-15)	15 (13-18)	17 (14-21)	18 (14-22)	16 (13-20)
American Samoa	0 (0-0)	0 (0-0)	0 (0-0)	0 (0-0)	0 (0-0)	0 (0-0)	0 (0-0)	0 (0-0)	0 (0-0)	0 (0-0)	0 (0-0)	0 (0-0)	0 (0-0)	0 (0-0)
Federated States of Micronesia	0 (0-0)	0 (0-0)	0 (0-0)	0 (0-0)	0 (0-0)	0 (0-0)	0 (0-0)	0 (0-0)	0 (0-0)	0 (0-0)	0 (0-0)	0 (0-0)	0 (0-0)	0 (0-0)
Fiji	1 (1-2)	1 (1-1)	1 (1-1)	1 (1-1)	1 (1-1)	1 (1-1)	1 (1-1)	1 (1-1)	1 (0-1)	1 (0-1)	1 (0-1)	1 (0-1)	1 (0-1)	0 (0-1)
Guam	0 (0-0)	0 (0-0)	0 (0-0)	0 (0-0)	0 (0-0)	0 (0-0)	0 (0-0)	0 (0-0)	0 (0-0)	0 (0-0)	0 (0-0)	0 (0-0)	0 (0-0)	0 (0-0)
Kiribati	0 (0-0)	0 (0-0)	0 (0-0)	0 (0-0)	0 (0-0)	0 (0-0)	0 (0-0)	0 (0-0)	0 (0-0)	0 (0-0)	0 (0-0)	0 (0-0)	0 (0-0)	0 (0-0)
Marshall Islands	0 (0-0)	0 (0-0)	0 (0-0)	0 (0-0)	0 (0-0)	0 (0-0)	0 (0-0)	0 (0-0)	0 (0-0)	0 (0-0)	0 (0-0)	0 (0-0)	0 (0-0)	0 (0-0)
Northern Mariana Islands	0 (0-0)	0 (0-0)	0 (0-0)	0 (0-0)	0 (0-0)	0 (0-0)	0 (0-0)	0 (0-0)	0 (0-0)	0 (0-0)	0 (0-0)	0 (0-0)	0 (0-0)	0 (0-0)
Papua New Guinea	19 (15-25)	18 (15-22)	17 (14-21)	15 (12-18)	12 (10-15)	10 (9-12)	10 (8-11)	10 (8-12)	10 (9-12)	11 (9-13)	12 (10-15)	14 (12-18)	15 (12-19)	14 (10-18)
Samoa	0 (0-1)	0 (0-0)	0 (0-0)	0 (0-0)	0 (0-0)	0 (0-0)	0 (0-0)	0 (0-0)	0 (0-0)	0 (0-0)	0 (0-0)	0 (0-0)	0 (0-0)	0 (0-0)
Solomon Islands	1 (1-1)	1 (1-1)	1 (1-1)	1 (1-1)	1 (1-1)	1 (1-1)	1 (0-1)	1 (0-1)	1 (0-1)	1 (0-1)	1 (0-1)	1 (1-1)	1 (0-1)	1 (0-1)
Tonga	0 (0-0)	0 (0-0)	0 (0-0)	0 (0-0)	0 (0-0)	0 (0-0)	0 (0-0)	0 (0-0)	0 (0-0)	0 (0-0)	0 (0-0)	0 (0-0)	0 (0-0)	0 (0-0)
Vanuatu	0 (0-0)	0 (0-0)	0 (0-0)	0 (0-0)	0 (0-0)	0 (0-0)	0 (0-0)	0 (0-0)	0 (0-0)	0 (0-0)	0 (0-0)	0 (0-0)	0 (0-0)	0 (0-0)
Southeast Asia	2420 (2270-2580)	2200 (2100-2300)	2040 (1950-2120)	1860 (1790-1940)	1670 (1610-1740)	1500 (1440-1550)	1280 (1230-1330)	1100 (1050-1140)	902 (870-938)	729 (702-756)	602 (580-625)	491 (471-512)	388 (370-406)	269 (250-290)
Cambodia	55 (41-73)	54 (43-66)	53 (44-63)	52 (43-63)	58 (50-67)	93 (85-101)	54 (49-60)	55 (50-59)	50 (47-54)	44 (41-47)	33 (31-37)	23 (21-26)	17 (15-19)	12 (9-15)
Indonesia	1300 (1220-1380)	1170 (1130-1210)	1070 (1030-1100)	964 (934-993)	835 (812-859)	736 (717-755)	635 (621-649)	518 (506-530)	404 (394-414)	316 (308-325)	261 (253-269)	213 (206-221)	169 (160-177)	109 (102-118)
Laos	34 (26-44)	34 (28-41)	34 (29-40)	34 (29-40)	33 (28-40)	32 (26-37)	31 (26-36)	29 (25-34)	28 (24-32)	26 (22-29)	22 (19-25)	19 (16-22)	15 (12-17)	10 (8-13)
Malaysia	36 (32-41)	33 (31-35)	31 (30-31)	23 (23-23)	19 (19-19)	16 (14-17)	12 (11-14)	10 (10-11)	8 (8-9)	7 (7-7)	5 (4-5)	4 (4-4)	3 (3-4)	4 (3-4)
Maldives	1 (1-2)	1 (1-2)	1 (1-2)	1 (1-1)	1 (1-1)	1 (1-1)	1 (1-1)	1 (0-1)	0 (0-0)	0 (0-0)	0 (0-0)	0 (0-0)	0 (0-0)	0 (0-0)
Mauritius	3 (2-3)	3 (2-3)	2 (2-3)	2 (2-2)	2 (2-2)	1 (1-2)	1 (1-1)	1 (1-1)	0 (0-1)	0 (0-0)	0 (0-0)	0 (0-0)	0 (0-0)	0 (0-0)
Myanmar	339 (255-436)	321 (263-386)	306 (254-362)	283 (232-342)	255 (210-309)	217 (179-259)	195 (159-236)	176 (142-212)	157 (132-184)	140 (117-164)	119 (101-138)	90 (77-105)	60 (50-71)	39 (30-49)
Philippines	182 (149-221)	173 (155-193)	169 (149-187)	166 (149-183)	162 (148-178)	153 (139-168)	147 (132-163)	137 (124-151)	119 (107-131)	98 (89-108)	91 (82-101)	87 (76-97)	82 (71-94)	66 (53-81)
Sri Lanka	52 (47-57)	41 (38-43)	33 (32-34)	29 (28-29)	25 (25-25)	26 (25-27)	19 (18-20)	14 (13-15)	11 (10-12)	8 (7-9)	6 (6-7)	7 (6-8)	4 (4-5)	2 (2-3)
Seychelles	0 (0-0)	0 (0-0)	0 (0-0)	0 (0-0)	0 (0-0)	0 (0-0)	0 (0-0)	0 (0-0)	0 (0-0)	0 (0-0)	0 (0-0)	0 (0-0)	0 (0-0)	0 (0-0)
Thailand	175 (139-222)	160 (140-182)	149 (135-164)	131 (119-146)	111 (100-123)	84 (75-94)	65 (57-73)	46 (40-53)	32 (28-38)	24 (21-29)	19 (16-22)	13 (11-16)	8 (7-9)	6 (5-7)
Timor-Leste	7 (5-9)	7 (5-8)	7 (5-8)	6 (5-7)	5 (4-6)	5 (4-6)	5 (5-6)	5 (4-5)	4 (4-5)	4 (3-4)	3 (3-3)	2 (2-3)	1 (1-2)	1 (1-2)
Vietnam	229 (165-314)	201 (154-256)	184 (145-226)	168 (139-199)	160 (136-185)	130 (113-149)	113 (102-124)	103 (93-114)	86 (78-94)	60 (54-66)	41 (37-46)	33 (29-37)	28 (23-33)	19 (14-24)
Sub-Saharan Africa	2890 (2690-3110)	2880 (2740-3040)	2910 (2790-3050)	2960 (2850-3070)	3100 (2990-3200)	3220 (3130-3320)	3330 (3250-3420)	3470 (3400-3550)	3660 (3580-3730)	3850 (3780-3930)	3950 (3860-4040)	3730 (3630-3830)	3350 (3230-3480)	2750 (2590-2950)
Central sub-Saharan Africa	312 (258-375)	319 (277-365)	332 (289-378)	341 (298-389)	343 (301-386)	354 (313-394)	372 (340-410)	388 (362-416)	419 (394-444)	460 (432-490)	494 (465-527)	495 (460-528)	465 (422-508)	354 (303-414)
Angola	79 (60-102)	78 (63-94)	77 (64-92)	73 (60-90)	71 (58-86)	75 (62-89)	84 (72-98)	97 (85-109)	109 (99-120)	119 (108-129)	120 (110-130)	114 (103-126)	94 (82-108)	68 (54-85)
Central African Republic	17 (12-22)	16 (13-20)	16 (13-19)	15 (13-18)	16 (14-18)	17 (14-19)	17 (14-19)	18 (16-20)	19 (17-21)	22 (19-25)	26 (22-30)	29 (24-34)	28 (23-34)	22 (17-30)
Congo (Brazzaville)	13 (9-16)	12 (10-15)	12 (10-14)	11 (9-14)	11 (9-13)	10 (9-12)	9 (8-11)	9 (8-10)	9 (9-10)	11 (10-12)	13 (11-14)	12 (10-13)	10 (8-11)	7 (5-9)
DR Congo	193 (146-252)	204 (167-245)	218 (178-261)	232 (194-280)	237 (198-280)	245 (208-284)	256 (225-291)	260 (236-286)	275 (252-298)	302 (276-329)	329 (302-359)	335 (307-366)	328 (288-368)	253 (205-310)
Equatorial Guinea	4 (3-5)	4 (3-5)	4 (3-4)	3 (3-4)	3 (2-4)	3 (2-3)	3 (2-3)	3 (2-3)	4 (3-4)	5 (4-5)	4 (3-5)	3 (2-4)	3 (2-3)	2 (1-3)
Gabon	6 (5-8)	6 (5-7)	6 (5-7)	5 (4-6)	5 (4-6)	4 (3-5)	3 (3-4)	3 (3-3)	3 (2-3)	3 (2-3)	2 (2-3)	2 (2-3)	2 (2-3)	2 (1-2)
Eastern sub-Saharan Africa	998 (927-1070)	1010 (960-1070)	1030 (981-1080)	1060 (1010-1100)	1110 (1070-1140)	1200 (1170-1240)	1270 (1250-1300)	1370 (1340-1390)	1450 (1420-1480)	1500 (1460-1520)	1440 (1410-1470)	1240 (1210-1270)	1060 (1030-1100)	849 (801-899)
Burundi	44 (34-56)	42 (35-50)	41 (34-48)	40 (35-45)	40 (36-45)	42 (37-46)	43 (39-48)	42 (38-46)	42 (39-46)	46 (43-50)	46 (42-49)	39 (36-43)	31 (28-35)	31 (25-39)
Comoros	2 (2-3)	2 (2-3)	2 (2-3)	2 (2-3)	2 (2-3)	2 (2-3)	2 (2-3)	2 (2-3)	2 (2-3)	2 (2-2)	2 (2-2)	1 (1-2)	1 (1-1)	1 (1-1)
Djibouti	0 (0-1)	1 (0-1)	1 (1-1)	1 (1-1)	1 (1-1)	1 (1-1)	1 (1-2)	2 (1-2)	2 (2-2)	2 (2-3)	2 (2-3)	2 (2-3)	2 (1-2)	1 (1-2)
Eritrea	18 (14-23)	19 (15-23)	20 (16-24)	22 (19-27)	24 (20-28)	25 (22-28)	26 (23-28)	26 (23-29)	29 (26-32)	20 (17-23)	17 (14-21)	15 (12-19)	14 (11-18)	11 (7-15)
Ethiopia	285 (246-332)	280 (253-311)	277 (251-304)	280 (256-305)	296 (277-317)	334 (318-354)	330 (314-346)	367 (352-384)	420 (403-437)	452 (433-472)	431 (412-448)	355 (337-373)	278 (260-295)	198 (179-222)
Kenya	80 (76-83)	78 (76-79)	75 (74-77)	81 (79-82)	87 (85-89)	87 (85-88)	85 (83-86)	84 (83-86)	86 (84-88)	95 (93-97)	97 (94-99)	88 (86-90)	74 (72-76)	60 (58-62)
Baringo	1 (0-1)	1 (1-1)	1 (1-1)	1 (1-1)	2 (1-2)	2 (2-2)	2 (2-2)	2 (2-2)	2 (2-2)	2 (2-2)	2 (2-2)	1 (1-2)	1 (1-1)	1 (1-1)
Bomet	0 (0-0)	0 (0-0)	0 (0-0)	0 (0-1)	1 (0-1)	1 (1-1)	1 (1-1)	1 (1-1)	1 (1-1)	1 (1-1)	1 (1-1)	1 (1-1)	1 (1-2)	1 (1-1)
Bungoma	3 (3-4)	3 (3-4)	3 (3-4)	3 (3-4)	4 (3-4)	4 (3-4)	4 (3-4)	4 (3-4)	4 (3-4)	4 (3-4)	4 (3-4)	3 (3-4)	3 (2-3)	2 (2-2)
Busia	3 (2-3)	3 (2-3)	3 (2-3)	3 (3-3)	3 (3-3)	3 (2-3)	3 (2-3)	3 (2-3)	3 (3-3)	3 (3-4)	3 (3-4)	3 (2-3)	2 (2-3)	2 (1-2)
Elgeyo Marakwet	0 (0-1)	0 (0-1)	0 (0-1)	1 (0-1)	1 (1-1)	1 (1-1)	1 (1-1)	1 (1-1)	1 (1-1)	1 (1-1)	1 (1-1)	1 (1-1)	0 (0-1)	0 (0-0)
Embu	1 (1-2)	1 (1-2)	1 (1-1)	1 (1-1)	1 (1-1)	1 (1-1)	1 (1-1)	1 (1-1)	1 (1-1)	1 (1-1)	1 (1-1)	1 (1-1)	1 (1-1)	1 (0-1)
Garissa	1 (1-1)	1 (1-1)	1 (1-1)	1 (1-1)	1 (1-1)	1 (1-1)	1 (1-1)	1 (1-1)	1 (1-1)	1 (1-1)	1 (1-1)	1 (1-2)	1 (1-2)	1 (1-1)
Homa Bay	5 (4-6)	5 (4-5)	5 (4-5)	5 (5-6)	6 (5-7)	6 (6-7)	6 (5-6)	6 (5-6)	6 (5-7)	7 (6-8)	7 (6-8)	6 (5-7)	4 (4-5)	3 (3-4)
Isiolo	0 (0-0)	0 (0-0)	0 (0-0)	1 (0-1)	1 (1-1)	1 (1-1)	1 (1-1)	1 (1-1)	1 (1-1)	1 (1-1)	1 (1-1)	1 (0-1)	0 (0-1)	0 (0-0)
Kajiado	0 (0-0)	0 (0-0)	0 (0-0)	0 (0-0)	0 (0-1)	0 (0-1)	0 (0-0)	0 (0-0)	0 (0-1)	1 (1-1)	1 (1-1)	1 (1-1)	1 (1-1)	1 (0-1)
Kakamega	5 (4-6)	5 (4-5)	5 (4-5)	5 (5-6)	5 (5-6)	6 (5-6)	6 (5-6)	6 (6-7)	7 (6-7)	7 (6-8)	7 (6-7)	6 (6-7)	5 (5-6)	4 (3-5)
Kericho	1 (1-1)	1 (1-1)	1 (1-1)	1 (1-1)	1 (1-1)	1 (1-1)	1 (1-1)	1 (1-1)	1 (1-1)	1 (1-1)	1 (1-1)	1 (1-1)	1 (1-1)	1 (1-1)
Kiambu	3 (2-3)	3 (2-3)	2 (2-3)	2 (2-3)	2 (2-3)	2 (2-2)	2 (2-2)	2 (1-2)	2 (1-2)	2 (2-2)	2 (2-3)	3 (2-3)	2 (2-3)	2 (2-3)
Kilifi	4 (4-5)	4 (4-5)	4 (4-5)	5 (4-5)	5 (4-5)	5 (4-5)	5 (4-5)	4 (4-5)	4 (3-5)	4 (4-5)	4 (4-5)	4 (3-4)	3 (3-3)	2 (2-2)
Kirinyaga	2 (1-2)	2 (1-2)	2 (1-2)	2 (1-2)	2 (2-2)	1 (1-2)	1 (1-1)	1 (1-1)	1 (1-1)	1 (1-1)	1 (1-1)	1 (1-1)	1 (1-1)	1 (1-1)
Kisii	4 (3-5)	4 (3-4)	3 (3-4)	4 (3-4)	4 (4-5)	4 (4-5)	4 (4-5)	4 (4-5)	4 (4-5)	4 (4-5)	4 (4-5)	3 (3-4)	2 (2-3)	2 (1-2)
Kisumu	5 (3-6)	5 (4-5)	4 (4-5)	5 (4-5)	5 (5-6)	5 (5-6)	5 (5-6)	5 (5-6)	5 (5-6)	6 (5-6)	5 (5-6)	4 (3-4)	3 (2-3)	2 (1-3)
Kitui	3 (2-3)	3 (2-3)	3 (2-3)	3 (3-3)	3 (3-3)	3 (3-3)	2 (2-3)	2 (2-3)	2 (2-3)	3 (2-3)	2 (2-3)	2 (2-2)	1 (1-2)	1 (1-1)
Kwale	2 (2-3)	2 (2-3)	2 (2-2)	2 (2-3)	3 (2-3)	3 (2-3)	2 (2-3)	2 (2-2)	2 (2-2)	2 (2-2)	2 (2-2)	2 (1-2)	1 (1-1)	1 (1-1)
Laikipia	0 (0-0)	0 (0-0)	0 (0-0)	0 (0-0)	0 (0-0)	0 (0-0)	0 (0-0)	0 (0-0)	0 (0-0)	1 (0-1)	1 (1-1)	1 (1-1)	0 (0-1)	0 (0-0)
Lamu	0 (0-1)	0 (0-1)	0 (0-1)	1 (0-1)	1 (0-1)	0 (0-0)	0 (0-0)	0 (0-0)	0 (0-0)	0 (0-0)	0 (0-0)	0 (0-0)	0 (0-0)	0 (0-0)
Machakos	3 (3-4)	3 (3-3)	3 (3-3)	3 (3-3)	3 (3-4)	3 (3-3)	3 (2-3)	2 (2-3)	2 (2-2)	2 (2-2)	2 (2-2)	2 (2-2)	2 (1-2)	1 (1-2)
Makueni	1 (1-1)	1 (1-1)	1 (1-1)	1 (1-1)	1 (1-2)	2 (1-2)	2 (1-2)	2 (1-2)	2 (1-2)	2 (2-2)	2 (2-2)	1 (1-1)	1 (1-1)	0 (0-1)
Mandera	1 (1-1)	1 (1-1)	1 (1-1)	1 (1-1)	1 (1-1)	1 (1-1)	1 (1-1)	1 (1-1)	1 (1-1)	1 (1-1)	1 (1-1)	2 (1-2)	3 (2-3)	3 (2-3)
Marsabit	1 (1-2)	1 (1-2)	1 (1-1)	1 (1-2)	1 (1-2)	1 (1-1)	1 (1-1)	1 (1-1)	1 (1-1)	1 (1-1)	1 (1-1)	1 (1-1)	1 (1-1)	1 (1-1)
Meru	4 (3-5)	3 (3-4)	3 (3-3)	3 (3-3)	3 (3-3)	3 (2-3)	2 (2-2)	2 (2-2)	2 (2-2)	2 (2-3)	2 (2-3)	2 (2-3)	2 (2-3)	3 (2-3)
Migori	3 (3-4)	4 (3-4)	4 (3-4)	4 (4-5)	5 (4-5)	6 (5-6)	7 (6-7)	7 (7-8)	7 (7-8)	7 (7-8)	8 (7-8)	7 (6-7)	5 (4-6)	4 (3-4)
Mombasa	2 (1-2)	2 (1-2)	2 (1-2)	1 (1-2)	1 (1-2)	1 (1-1)	1 (1-1)	1 (1-1)	1 (1-1)	1 (1-2)	2 (1-2)	2 (1-2)	2 (1-2)	1 (1-2)
Murang'a	2 (2-3)	2 (2-2)	2 (2-2)	2 (2-2)	2 (2-2)	2 (2-2)	1 (1-2)	1 (1-2)	1 (1-1)	1 (1-1)	1 (1-1)	1 (1-1)	1 (1-1)	1 (1-1)
Nairobi	5 (4-6)	4 (3-4)	3 (3-3)	3 (2-3)	2 (2-3)	2 (2-2)	2 (1-2)	2 (2-2)	3 (2-3)	4 (3-4)	4 (4-5)	4 (3-4)	3 (3-4)	2 (2-3)
Nakuru	1 (1-1)	1 (1-1)	1 (1-1)	1 (1-1)	1 (1-1)	1 (1-2)	1 (1-1)	1 (1-2)	2 (1-2)	2 (2-3)	3 (2-3)	2 (2-3)	2 (2-2)	2 (1-2)
Nandi	0 (0-1)	0 (0-1)	0 (0-1)	1 (0-1)	1 (1-1)	1 (1-1)	1 (1-1)	1 (1-1)	1 (1-1)	1 (1-1)	1 (1-1)	1 (1-1)	1 (1-1)	1 (1-1)
Narok	0 (0-0)	0 (0-0)	0 (0-0)	1 (0-1)	1 (1-1)	1 (1-1)	1 (1-1)	1 (1-1)	1 (1-2)	1 (1-2)	1 (1-1)	1 (1-1)	1 (1-1)	1 (1-1)
Nyamira	1 (1-1)	1 (1-1)	1 (1-1)	1 (1-1)	1 (1-1)	1 (1-1)	1 (1-1)	1 (1-1)	1 (1-1)	1 (1-1)	1 (1-1)	1 (1-1)	1 (1-1)	1 (0-1)
Nyandarua	1 (1-1)	1 (1-1)	1 (0-1)	1 (0-1)	1 (1-1)	1 (0-1)	0 (0-1)	0 (0-1)	0 (0-1)	1 (0-1)	1 (1-1)	1 (1-1)	1 (1-1)	1 (1-1)
Nyeri	2 (2-3)	2 (2-2)	2 (1-2)	2 (1-2)	2 (1-2)	1 (1-2)	1 (1-1)	1 (1-1)	1 (1-1)	1 (1-1)	1 (1-1)	1 (1-1)	1 (1-1)	1 (1-1)
Samburu	0 (0-0)	0 (0-0)	0 (0-0)	0 (0-0)	0 (0-0)	0 (0-0)	0 (0-1)	1 (0-1)	1 (1-1)	1 (1-1)	1 (1-1)	1 (1-1)	1 (0-1)	0 (0-1)
Siaya	3 (2-3)	3 (2-3)	3 (2-3)	3 (3-3)	3 (3-3)	3 (3-4)	3 (3-4)	3 (3-4)	4 (3-4)	4 (3-4)	4 (3-4)	3 (3-4)	2 (2-3)	2 (1-2)
Taita Taveta	1 (1-1)	1 (1-1)	1 (1-1)	1 (1-1)	1 (1-1)	1 (1-1)	1 (1-1)	1 (1-1)	1 (0-1)	1 (1-1)	1 (1-1)	1 (0-1)	0 (0-0)	0 (0-0)
Tana River	1 (1-1)	1 (1-1)	1 (1-1)	1 (1-1)	1 (1-1)	1 (0-1)	0 (0-1)	0 (0-0)	0 (0-0)	1 (1-1)	1 (1-1)	1 (1-1)	1 (1-1)	1 (1-1)
Tharaka Nithi	0 (0-1)	0 (0-1)	1 (0-1)	1 (0-1)	1 (1-1)	1 (1-1)	1 (0-1)	1 (0-1)	1 (0-1)	1 (1-1)	1 (1-1)	1 (0-1)	0 (0-0)	0 (0-0)
Trans Nzoia	1 (0-1)	1 (1-1)	1 (1-1)	1 (1-1)	1 (1-1)	1 (1-1)	1 (1-1)	1 (1-1)	1 (1-1)	1 (1-2)	1 (1-2)	1 (1-1)	1 (1-1)	1 (1-1)

Table 2 Under-5 death numbers by location and year for 1950, 1955...2005, 2010, 2017 - both sexes

Location	1950	1955	1960	1965	1970	1975	1980	1985	1990	1995	2000	2005	2010	2017
Turkana	1 (1-1)	1 (1-1)	1 (1-1)	1 (1-1)	1 (1-2)	2 (1-2)	2 (2-2)	2 (2-2)	2 (2-2)	2 (2-2)	2 (2-3)	3 (2-3)	3 (2-3)	2 (2-3)
Uasin Gishu	0 (0-1)	0 (0-1)	0 (0-1)	1 (0-1)	1 (1-1)	1 (1-1)	1 (1-1)	1 (1-1)	1 (1-1)	1 (1-1)	1 (1-1)	1 (1-1)	1 (1-1)	1 (1-1)
Vihiga	1 (0-1)	1 (0-1)	1 (0-1)	1 (1-1)	1 (1-1)	1 (1-1)	1 (1-1)	1 (1-1)	1 (1-1)	1 (1-2)	2 (1-2)	1 (1-1)	0 (0-1)	0 (0-1)
Wajir	1 (0-1)	1 (1-1)	1 (1-1)	1 (1-1)	1 (1-1)	1 (1-1)	1 (1-1)	1 (1-1)	1 (1-1)	1 (1-2)	2 (2-2)	2 (2-3)	2 (2-2)	2 (2-2)
West Pokot	1 (1-1)	1 (1-1)	1 (1-1)	1 (1-1)	1 (1-1)	1 (1-2)	1 (1-2)	1 (1-2)	1 (1-2)	2 (1-2)	1 (1-1)	1 (1-1)	1 (1-2)	1 (1-1)
Madagascar	45 (34-59)	48 (40-57)	51 (42-59)	55 (46-63)	59 (53-65)	63 (57-68)	68 (63-74)	74 (69-80)	74 (69-80)	75 (68-83)	73 (65-81)	69 (62-78)	74 (63-88)	72 (55-95)
Malawi	72 (58-89)	76 (65-88)	80 (69-92)	83 (74-92)	84 (76-92)	84 (78-90)	84 (79-89)	85 (80-91)	100 (94-106)	94 (89-101)	80 (75-85)	65 (60-69)	52 (47-57)	38 (31-48)
Mozambique	105 (80-138)	102 (82-124)	100 (82-118)	99 (82-117)	100 (84-116)	107 (93-122)	125 (113-137)	160 (151-170)	143 (133-153)	134 (125-145)	121 (111-131)	106 (96-116)	92 (80-104)	73 (59-90)
Rwanda	41 (32-51)	42 (36-48)	44 (39-49)	45 (41-49)	48 (44-52)	52 (49-56)	52 (49-55)	50 (46-53)	52 (49-55)	56 (52-60)	61 (56-65)	35 (32-39)	24 (21-27)	19 (15-25)
Somalia	32 (24-42)	32 (26-38)	33 (27-39)	36 (29-43)	40 (33-48)	51 (44-60)	67 (58-77)	65 (59-73)	69 (61-77)	73 (65-83)	76 (67-86)	70 (60-81)	73 (60-86)	56 (43-74)
South Sudan	30 (21-42)	32 (25-40)	34 (28-42)	34 (28-41)	33 (27-40)	34 (28-40)	36 (31-43)	40 (34-47)	44 (37-51)	44 (39-51)	44 (39-50)	45 (39-51)	49 (40-58)	47 (38-57)
Tanzania	127 (95-164)	132 (106-158)	139 (115-166)	137 (119-156)	143 (129-157)	153 (142-165)	156 (146-166)	163 (152-174)	173 (162-184)	182 (170-194)	171 (159-184)	149 (136-164)	138 (119-157)	117 (92-151)
Uganda	80 (61-103)	86 (72-102)	93 (79-109)	96 (84-109)	106 (95-116)	125 (115-136)	151 (141-162)	153 (144-163)	152 (143-161)	155 (146-165)	159 (149-171)	144 (133-156)	118 (107-131)	87 (72-103)
Zambia	35 (27-45)	38 (31-46)	42 (35-50)	45 (39-51)	44 (39-49)	43 (40-47)	46 (43-50)	52 (48-56)	60 (56-64)	64 (59-69)	62 (56-68)	56 (50-63)	45 (37-53)	37 (29-48)
Southern sub-Saharan Africa	128 (113-145)	133 (121-146)	140 (129-152)	154 (145-166)	151 (143-159)	143 (136-150)	141 (135-148)	129 (123-135)	112 (107-117)	111 (106-116)	125 (119-132)	131 (124-140)	102 (97-109)	65 (59-71)
Botswana	3 (2-4)	3 (2-3)	3 (2-3)	3 (2-3)	3 (2-3)	3 (2-3)	3 (2-3)	2 (2-3)	2 (2-2)	2 (2-3)	3 (2-3)	2 (2-3)	1 (1-2)	1 (1-1)
Lesotho	5 (4-6)	5 (5-6)	6 (5-6)	6 (5-7)	6 (6-7)	6 (6-7)	6 (5-7)	5 (5-6)	5 (4-6)	5 (4-6)	5 (5-6)	5 (5-6)	4 (3-4)	3 (2-3)
Namibia	3 (3-5)	3 (3-4)	3 (3-4)	3 (3-4)	3 (3-4)	4 (3-4)	4 (3-4)	4 (3-4)	3 (3-4)	3 (3-3)	3 (3-4)	4 (3-4)	3 (3-3)	2 (2-3)
South Africa	91 (79-106)	96 (86-107)	102 (91-112)	115 (106-125)	109 (102-116)	99 (93-106)	95 (89-102)	89 (83-95)	75 (71-80)	70 (65-75)	81 (75-87)	83 (76-90)	57 (53-62)	33 (30-36)
Swaziland	4 (3-5)	3 (3-4)	3 (3-4)	3 (3-4)	3 (3-4)	3 (3-4)	3 (3-3)	3 (2-3)	2 (2-2)	2 (2-3)	3 (3-3)	3 (3-3)	2 (2-3)	1 (1-2)
Zimbabwe	21 (16-28)	22 (18-27)	23 (19-28)	24 (21-28)	26 (23-29)	28 (25-31)	31 (28-33)	27 (25-29)	25 (23-27)	28 (26-30)	30 (28-33)	34 (31-37)	35 (31-38)	25 (20-30)
Western sub-Saharan Africa	1450 (1280-1660)	1420 (1290-1560)	1410 (1300-1520)	1410 (1320-1500)	1500 (1410-1580)	1520 (1450-1600)	1540 (1470-1610)	1590 (1530-1660)	1680 (1620-1740)	1780 (1720-1850)	1890 (1810-1970)	1860 (1770-1940)	1720 (1620-1840)	1490 (1340-1660)
Benin	39 (29-49)	37 (31-43)	36 (32-41)	36 (32-39)	38 (34-41)	38 (35-41)	37 (35-40)	37 (35-40)	37 (35-40)	36 (34-39)	37 (33-40)	37 (33-41)	38 (33-43)	34 (27-43)
Burkina Faso	79 (61-101)	73 (60-88)	68 (56-81)	67 (57-79)	73 (65-81)	79 (72-86)	82 (75-88)	85 (79-91)	88 (82-94)	93 (86-100)	93 (86-102)	91 (82-101)	90 (77-104)	87 (69-109)
Cameroon	77 (65-92)	71 (63-78)	70 (63-78)	62 (55-69)	62 (56-68)	63 (58-68)	64 (59-70)	61 (56-66)	62 (57-67)	69 (63-75)	74 (67-83)	80 (71-89)	74 (65-85)	55 (45-67)
Cape Verde	1 (1-2)	1 (1-2)	2 (1-2)	1 (1-1)	2 (2-2)	1 (1-2)	1 (1-1)	1 (1-1)	1 (1-1)	1 (1-1)	1 (0-1)	0 (0-1)	0 (0-0)	0 (0-0)
Chad	45 (34-57)	44 (36-53)	44 (37-52)	45 (38-53)	49 (42-56)	52 (46-58)	57 (51-63)	61 (56-67)	63 (58-68)	69 (63-74)	78 (71-86)	84 (75-92)	84 (74-94)	80 (67-96)
Cote d'Ivoire	45 (37-53)	49 (43-54)	53 (48-58)	59 (54-64)	63 (59-68)	64 (60-69)	63 (58-67)	68 (63-73)	75 (69-82)	86 (78-94)	90 (81-100)	90 (80-102)	82 (70-95)	66 (55-79)
The Gambia	3 (3-5)	4 (3-5)	4 (3-5)	4 (3-5)	4 (4-5)	4 (4-5)	5 (4-5)	5 (4-5)	5 (4-6)	5 (4-6)	5 (4-5)	4 (4-5)	4 (3-5)	3 (2-4)
Ghana	85 (68-106)	82 (71-94)	80 (72-89)	74 (68-81)	71 (66-77)	71 (65-77)	73 (68-79)	76 (72-81)	68 (63-72)	65 (61-69)	65 (60-70)	61 (57-65)	53 (47-59)	44 (35-55)
Guinea	62 (49-77)	58 (49-69)	55 (45-65)	53 (44-62)	55 (47-62)	58 (52-64)	60 (55-66)	64 (59-70)	68 (63-73)	68 (62-74)	62 (56-68)	55 (48-62)	49 (42-56)	41 (33-50)
Guinea-Bissau	13 (10-16)	12 (10-14)	12 (10-13)	11 (10-13)	11 (9-13)	11 (9-13)	11 (10-13)	11 (9-13)	10 (9-11)	9 (8-10)	9 (7-10)	8 (7-9)	6 (5-8)	5 (4-6)
Liberia	17 (13-22)	18 (15-22)	19 (16-22)	21 (18-23)	21 (19-23)	22 (20-24)	23 (21-25)	24 (22-26)	23 (21-24)	19 (18-21)	21 (19-23)	15 (14-17)	13 (11-15)	10 (8-13)
Mali	114 (89-141)	113 (94-132)	113 (97-130)	119 (105-133)	123 (112-135)	121 (112-131)	113 (105-121)	109 (102-116)	108 (101-116)	105 (98-112)	102 (95-110)	98 (89-107)	99 (86-111)	104 (85-129)
Mauritania	9 (6-11)	9 (7-11)	9 (8-10)	9 (8-10)	9 (8-10)	10 (9-11)	10 (9-11)	10 (9-11)	9 (9-10)	9 (8-10)	9 (8-10)	8 (7-9)	7 (6-7)	5 (4-6)
Niger	59 (47-74)	63 (53-74)	68 (57-79)	73 (64-84)	84 (75-94)	101 (93-110)	112 (104-121)	121 (112-129)	125 (116-134)	127 (118-138)	124 (113-136)	114 (103-128)	105 (89-120)	97 (79-120)
Nigeria	702 (537-905)	678 (559-812)	661 (560-768)	657 (574-743)	707 (628-792)	703 (632-781)	708 (640-777)	738 (679-801)	823 (765-885)	912 (848-977)	1010 (936-1090)	1010 (928-1090)	928 (832-1040)	789 (646-959)
Sao Tome and Principe	1 (1-1)	1 (1-1)	1 (1-1)	0 (0-1)	0 (0-0)	0 (0-0)	0 (0-0)	1 (0-1)	0 (0-1)	0 (0-0)	0 (0-0)	0 (0-0)	0 (0-0)	0 (0-0)
Senegal	40 (31-50)	42 (36-49)	45 (39-52)	52 (46-58)	59 (53-64)	60 (54-65)	57 (52-61)	53 (48-57)	47 (43-51)	47 (43-51)	44 (39-49)	37 (33-42)	30 (26-34)	23 (17-29)
Sierra Leone	40 (30-51)	41 (34-50)	44 (36-52)	44 (36-53)	42 (36-51)	42 (35-48)	42 (37-48)	43 (39-47)	44 (41-48)	38 (36-41)	38 (35-41)	42 (39-46)	37 (33-42)	30 (24-38)
Togo	23 (17-29)	23 (18-27)	23 (19-27)	23 (20-26)	22 (20-25)	22 (20-24)	22 (20-24)	22 (20-24)	22 (20-25)	22 (20-25)	23 (20-26)	23 (20-26)	21 (17-24)	14 (11-18)

Table 3 Under-5 death numbers by location and year for 1950, 1955...2005, 2010, 2017 - females

Location	1950	1955	1960	1965	1970	1975	1980	1985	1990	1995	2000	2005	2010	2017
Global	9260 (9010-9520)	8890 (8740-9060)	15400 (15200-15500)	8560 (8420-8690)	7720 (7610-7840)	6970 (6870-7070)	6320 (6240-6400)	6110 (6030-6190)	5530 (5460-5610)	5050 (4980-5110)	4520 (4460-4590)	3890 (3830-3960)	3300 (3230-3370)	2480 (2390-2570)
Low SDI	2080 (1930-2250)	2130 (2030-2230)	2220 (2130-2310)	2300 (2210-2400)	2260 (2190-2330)	2320 (2260-2390)	2350 (2290-2410)	2320 (2260-2370)	2220 (2170-2270)	2170 (2130-2220)	2000 (1950-2050)	1750 (1710-1790)	1530 (1480-1570)	1160 (1110-1210)
Low-middle SDI	2420 (2300-2560)	2370 (2290-2460)	3010 (2930-3090)	2420 (2340-2490)	2290 (2230-2340)	2240 (2190-2290)	2080 (2030-2130)	1980 (1940-2030)	1850 (1810-1890)	1760 (1720-1800)	1670 (1620-1720)	1490 (1440-1540)	1260 (1210-1320)	962 (888-1040)
Middle SDI	2870 (2750-2990)	2760 (2680-2840)	7570 (7460-7680)	2590 (2520-2650)	2160 (2100-2220)	1660 (1610-1700)	1310 (1280-1340)	1270 (1230-1310)	1040 (1010-1070)	795 (774-815)	614 (602-626)	469 (460-478)	367 (359-376)	249 (241-258)
High-middle SDI	1370 (1310-1430)	1240 (1210-1280)	2250 (2210-2290)	999 (962-1030)	812 (788-836)	614 (600-629)	482 (472-491)	456 (446-466)	354 (344-364)	268 (260-275)	201 (196-207)	149 (145-153)	118 (114-122)	79 (75-82)
High SDI	484 (467-505)	351 (343-360)	292 (286-298)	215 (210-219)	168 (165-172)	113 (112-115)	86 (85-88)	67 (66-68)	56 (55-56)	43 (42-43)	36 (35-36)	33 (32-33)	29 (28-29)	25 (24-26)
Central Europe, Eastern Europe, and Central Asia	476 (455-499)	342 (330-354)	259 (250-269)	179 (172-186)	143 (138-147)	132 (129-136)	124 (121-127)	118 (115-120)	91 (89-94)	70 (68-72)	52 (50-54)	44 (42-45)	39 (37-41)	30 (28-32)
Central Asia	136 (122-150)	120 (111-129)	107 (99-115)	85 (79-92)	64 (60-68)	59 (55-62)	59 (56-62)	58 (56-61)	49 (47-51)	40 (39-42)	30 (29-32)	26 (25-28)	25 (23-27)	19 (18-21)
Armenia	8 (6-10)	7 (5-8)	6 (5-7)	4 (3-5)	3 (2-3)	2 (2-3)	2 (2-3)	2 (2-2)	1 (1-2)	1 (1-1)	0 (0-1)	0 (0-0)	0 (0-0)	0 (0-0)
Azerbaijan	27 (21-34)	24 (20-28)	21 (18-25)	15 (12-19)	10 (8-12)	7 (6-9)	7 (6-8)	8 (7-8)	7 (6-7)	5 (4-6)	4 (3-4)	3 (3-4)	3 (3-4)	2 (2-3)
Georgia	12 (8-17)	10 (7-12)	8 (6-10)	6 (5-7)	4 (3-5)	3 (3-4)	2 (2-3)	2 (2-2)	2 (1-2)	1 (1-1)	1 (1-1)	1 (0-1)	0 (0-0)	0 (0-0)
Kazakhstan	22 (16-29)	20 (16-24)	18 (14-22)	14 (12-18)	11 (9-12)	10 (8-11)	9 (8-10)	8 (8-9)	6 (6-7)	5 (4-5)	3 (3-4)	3 (3-4)	3 (3-4)	2 (2-3)
Kyrgyzstan	12 (9-15)	10 (8-12)	8 (7-10)	7 (6-9)	5 (4-6)	5 (4-6)	5 (4-5)	5 (4-5)	4 (3-4)	3 (2-3)	2 (2-2)	2 (2-2)	2 (2-2)	1 (1-2)
Mongolia	7 (5-8)	6 (5-6)	5 (4-6)	4 (3-4)	3 (3-4)	3 (3-3)	3 (3-4)	4 (3-4)	3 (3-3)	2 (2-2)	2 (1-2)	1 (1-1)	1 (1-1)	1 (1-1)
Tajikistan	15 (12-19)	14 (12-17)	14 (12-16)	12 (10-14)	9 (8-10)	9 (7-10)	9 (8-9)	9 (8-9)	8 (7-8)	7 (6-7)	5 (5-6)	4 (4-5)	5 (4-5)	5 (4-6)
Turkmenistan	7 (5-8)	6 (5-7)	6 (5-7)	5 (4-6)	4 (3-4)	4 (3-5)	4 (3-5)	4 (4-5)	4 (3-4)	3 (3-4)	3 (2-3)	2 (2-2)	2 (2-2)	1 (1-2)
Uzbekistan	28 (21-36)	24 (20-29)	21 (17-26)	18 (15-22)	15 (13-18)	16 (14-18)	17 (15-19)	17 (16-19)	16 (14-17)	14 (13-15)	10 (9-12)	9 (8-10)	8 (7-10)	6 (5-8)
Central Europe	182 (175-189)	108 (105-111)	67 (66-69)	45 (44-46)	41 (40-42)	34 (33-34)	26 (26-27)	22 (21-22)	16 (16-17)	11 (11-11)	7 (7-7)	5 (5-5)	4 (4-4)	3 (2-3)
Albania	7 (5-8)	6 (5-7)	5 (5-6)	4 (4-5)	3 (3-4)	3 (2-3)	2 (2-2)	2 (2-2)	2 (1-2)	1 (1-1)	1 (1-1)	0 (0-0)	0 (0-0)	0 (0-0)
Bosnia and Herzegovina	14 (11-16)	9 (8-10)	6 (5-7)	4 (4-5)	3 (2-3)	2 (2-2)	1 (1-2)	1 (1-1)	0 (0-0)	1 (1-1)	0 (0-0)	0 (0-0)	0 (0-0)	0 (0-0)
Bulgaria	15 (13-16)	7 (7-7)	3 (3-4)	2 (2-2)	2 (2-2)	2 (2-2)	1 (1-1)	1 (1-1)	1 (1-1)	1 (1-1)	1 (0-1)	0 (0-0)	0 (0-0)	0 (0-0)
Croatia	10 (8-12)	5 (5-6)	3 (3-3)	2 (2-2)	1 (1-1)	1 (1-1)	1 (1-1)	1 (0-1)	0 (0-0)	0 (0-0)	0 (0-0)	0 (0-0)	0 (0-0)	0 (0-0)
Czech Republic	4 (4-5)	2 (2-2)	1 (1-1)	2 (2-2)	1 (1-1)	2 (2-2)	1 (1-1)	1 (1-1)	1 (1-1)	0 (0-0)	0 (0-0)	0 (0-0)	0 (0-0)	0 (0-0)
Hungary	11 (10-11)	6 (6-6)	3 (3-3)	3 (3-3)	3 (3-3)	3 (3-3)	2 (1-2)	1 (1-1)	1 (1-1)	1 (1-1)	0 (0-0)	0 (0-0)	0 (0-0)	0 (0-0)
Macedonia	7 (6-8)	5 (5-6)	4 (3-4)	3 (2-3)	2 (2-2)	2 (1-2)	1 (1-1)	1 (1-1)	1 (1-1)	0 (0-0)	0 (0-0)	0 (0-0)	0 (0-0)	0 (0-0)
Montenegro	1 (0-1)	0 (0-1)	0 (0-0)	0 (0-0)	0 (0-0)	0 (0-0)	0 (0-0)	0 (0-0)	0 (0-0)	0 (0-0)	0 (0-0)	0 (0-0)	0 (0-0)	0 (0-0)
Poland	60 (57-62)	32 (31-32)	17 (17-18)	11 (10-11)	9 (8-9)	7 (7-8)	7 (7-7)	6 (6-6)	4 (4-4)	3 (3-3)	2 (1-2)	1 (1-1)	1 (1-1)	1 (1-1)
Romania	29 (25-35)	20 (18-22)	14 (14-14)	7 (6-7)	10 (10-11)	8 (8-8)	6 (6-6)	5 (5-5)	4 (4-5)	3 (2-3)	2 (2-2)	2 (2-2)	1 (1-1)	1 (1-1)
Serbia	19 (18-21)	12 (11-12)	7 (7-7)	5 (5-6)	4 (4-5)	4 (3-4)	3 (3-3)	3 (3-3)	2 (2-2)	1 (1-1)	1 (1-1)	0 (0-1)	0 (0-0)	0 (0-0)
Slovakia	4 (4-4)	2 (2-2)	1 (1-1)	1 (1-1)	1 (1-1)	1 (1-1)	1 (1-1)	1 (1-1)	0 (0-1)	0 (0-0)	0 (0-0)	0 (0-0)	0 (0-0)	0 (0-0)
Slovenia	2 (2-2)	1 (1-1)	1 (1-1)	1 (1-1)	0 (0-0)	0 (0-0)	0 (0-0)	0 (0-0)	0 (0-0)	0 (0-0)	0 (0-0)	0 (0-0)	0 (0-0)	0 (0-0)
Eastern Europe	158 (143-177)	114 (108-122)	85 (81-89)	49 (46-51)	39 (37-40)	40 (39-42)	39 (38-40)	38 (37-39)	26 (25-26)	19 (18-19)	15 (14-15)	12 (12-12)	11 (11-11)	8 (7-8)
Belarus	7 (5-10)	6 (5-7)	5 (4-6)	3 (3-4)	2 (2-3)	2 (2-2)	2 (2-2)	2 (1-2)	1 (1-1)	1 (1-1)	1 (1-1)	1 (0-1)	0 (0-0)	0 (0-0)
Estonia	1 (0-1)	0 (0-1)	0 (0-0)	0 (0-0)	0 (0-0)	0 (0-0)	0 (0-0)	0 (0-0)	0 (0-0)	0 (0-0)	0 (0-0)	0 (0-0)	0 (0-0)	0 (0-0)
Latvia	1 (1-2)	1 (1-1)	1 (0-1)	0 (0-0)	0 (0-0)	0 (0-0)	0 (0-0)	0 (0-0)	0 (0-0)	0 (0-0)	0 (0-0)	0 (0-0)	0 (0-0)	0 (0-0)
Lithuania	3 (2-4)	2 (2-2)	1 (1-1)	1 (1-1)	1 (1-1)	1 (0-1)	0 (0-0)	0 (0-0)	0 (0-0)	0 (0-0)	0 (0-0)	0 (0-0)	0 (0-0)	0 (0-0)
Moldova	9 (6-12)	7 (5-8)	5 (4-7)	4 (3-5)	3 (2-3)	2 (2-3)	2 (2-3)	2 (2-2)	1 (1-1)	1 (1-1)	1 (1-1)	0 (0-0)	0 (0-0)	0 (0-0)
Russian Federation	100 (94-106)	72 (70-74)	54 (53-54)	28 (28-28)	23 (22-23)	25 (25-25)	25 (25-25)	26 (25-26)	17 (17-17)	13 (13-13)	10 (10-10)	9 (9-9)	7 (7-8)	5 (5-5)
Ukraine	37 (24-54)	26 (20-33)	19 (16-23)	12 (11-15)	10 (9-12)	10 (9-11)	9 (8-10)	8 (7-9)	6 (5-6)	4 (3-4)	3 (2-3)	2 (2-2)	2 (2-2)	2 (2-2)
High-income	392 (375-412)	312 (304-321)	275 (270-280)	218 (215-222)	177 (174-180)	120 (117-122)	88 (87-90)	66 (65-67)	58 (58-59)	45 (45-45)	39 (39-39)	35 (35-35)	31 (31-31)	27 (26-27)
Australasia	3 (3-4)	3 (3-3)	3 (3-3)	3 (3-3)	3 (3-3)	2 (2-2)	2 (2-2)	2 (1-2)	1 (1-1)	1 (1-1)	1 (1-1)	1 (1-1)	1 (1-1)	1 (1-1)
Australia	3 (2-3)	2 (2-3)	2 (2-3)	2 (2-2)	2 (2-2)	2 (2-2)	1 (1-1)	1 (1-1)	1 (1-1)	1 (1-1)	1 (1-1)	1 (1-1)	1 (1-1)	1 (0-1)
New Zealand	1 (1-1)	1 (1-1)	1 (1-1)	1 (1-1)	1 (0-1)	0 (0-0)	0 (0-0)	0 (0-0)	0 (0-0)	0 (0-0)	0 (0-0)	0 (0-0)	0 (0-0)	0 (0-0)
High-income Asia-Pacific	148 (133-166)	92 (85-100)	68 (64-73)	50 (47-54)	41 (38-44)	24 (22-26)	15 (14-16)	9 (9-10)	6 (6-7)	5 (5-6)	4 (4-4)	3 (3-3)	3 (2-3)	2 (2-2)
Brunei	0 (0-0)	0 (0-0)	0 (0-0)	0 (0-0)	0 (0-0)	0 (0-0)	0 (0-0)	0 (0-0)	0 (0-0)	0 (0-0)	0 (0-0)	0 (0-0)	0 (0-0)	0 (0-0)
Japan	69 (67-72)	43 (42-43)	28 (27-28)	17 (16-17)	14 (14-14)	10 (10-10)	7 (7-7)	5 (5-5)	3 (3-4)	3 (3-3)	2 (2-2)	2 (2-2)	2 (2-2)	1 (1-1)
Aichi	4 (3-4)	2 (2-2)	1 (1-2)	1 (1-1)	1 (1-1)	1 (1-1)	0 (0-0)	0 (0-0)	0 (0-0)	0 (0-0)	0 (0-0)	0 (0-0)	0 (0-0)	0 (0-0)
Akita	1 (1-1)	1 (0-1)	0 (0-0)	0 (0-0)	0 (0-0)	0 (0-0)	0 (0-0)	0 (0-0)	0 (0-0)	0 (0-0)	0 (0-0)	0 (0-0)	0 (0-0)	0 (0-0)
Aomori	1 (1-1)	1 (1-1)	0 (0-0)	0 (0-0)	0 (0-0)	0 (0-0)	0 (0-0)	0 (0-0)	0 (0-0)	0 (0-0)	0 (0-0)	0 (0-0)	0 (0-0)	0 (0-0)
Chiba	2 (2-2)	1 (1-1)	1 (1-1)	1 (0-1)	0 (0-1)	0 (0-0)	0 (0-0)	0 (0-0)	0 (0-0)	0 (0-0)	0 (0-0)	0 (0-0)	0 (0-0)	0 (0-0)
Ehime	1 (1-1)	1 (1-1)	0 (0-0)	0 (0-0)	0 (0-0)	0 (0-0)	0 (0-0)	0 (0-0)	0 (0-0)	0 (0-0)	0 (0-0)	0 (0-0)	0 (0-0)	0 (0-0)
Fukui	0 (0-1)	0 (0-0)	0 (0-0)	0 (0-0)	0 (0-0)	0 (0-0)	0 (0-0)	0 (0-0)	0 (0-0)	0 (0-0)	0 (0-0)	0 (0-0)	0 (0-0)	0 (0-0)
Fukuoka	3 (2-3)	2 (2-2)	1 (1-1)	1 (1-1)	1 (1-1)	0 (0-0)	0 (0-0)	0 (0-0)	0 (0-0)	0 (0-0)	0 (0-0)	0 (0-0)	0 (0-0)	0 (0-0)
Fukushima	1 (1-2)	1 (1-1)	0 (0-1)	0 (0-0)	0 (0-0)	0 (0-0)	0 (0-0)	0 (0-0)	0 (0-0)	0 (0-0)	0 (0-0)	0 (0-0)	0 (0-0)	0 (0-0)
Gifu	1 (1-2)	1 (1-1)	0 (0-1)	0 (0-0)	0 (0-0)	0 (0-0)	0 (0-0)	0 (0-0)	0 (0-0)	0 (0-0)	0 (0-0)	0 (0-0)	0 (0-0)	0 (0-0)
Gunma	1 (1-1)	1 (1-1)	0 (0-0)	0 (0-0)	0 (0-0)	0 (0-0)	0 (0-0)	0 (0-0)	0 (0-0)	0 (0-0)	0 (0-0)	0 (0-0)	0 (0-0)	0 (0-0)
Hiroshima	2 (2-2)	1 (1-1)	1 (1-1)	0 (0-0)	0 (0-0)	0 (0-0)	0 (0-0)	0 (0-0)	0 (0-0)	0 (0-0)	0 (0-0)	0 (0-0)	0 (0-0)	0 (0-0)
Hokkaidō	4 (3-5)	3 (2-3)	2 (1-2)	1 (1-1)	1 (1-1)	1 (1-1)	0 (0-0)	0 (0-0)	0 (0-0)	0 (0-0)	0 (0-0)	0 (0-0)	0 (0-0)	0 (0-0)
Hyōgo	3 (3-4)	2 (2-2)	1 (1-1)	1 (1-1)	1 (1-1)	0 (0-0)	0 (0-0)	0 (0-0)	0 (0-0)	0 (0-0)	0 (0-0)	0 (0-0)	0 (0-0)	0 (0-0)
Ibaraki	1 (1-2)	1 (1-1)	1 (1-1)	0 (0-0)	0 (0-0)	0 (0-0)	0 (0-0)	0 (0-0)	0 (0-0)	0 (0-0)	0 (0-0)	0 (0-0)	0 (0-0)	0 (0-0)
Ishikawa	1 (1-1)	0 (0-0)	0 (0-0)	0 (0-0)	0 (0-0)	0 (0-0)	0 (0-0)	0 (0-0)	0 (0-0)	0 (0-0)	0 (0-0)	0 (0-0)	0 (0-0)	0 (0-0)
Iwate	1 (1-1)	1 (1-1)	0 (0-0)	0 (0-0)	0 (0-0)	0 (0-0)	0 (0-0)	0 (0-0)	0 (0-0)	0 (0-0)	0 (0-0)	0 (0-0)	0 (0-0)	0 (0-0)
Kagawa	1 (0-1)	0 (0-0)	0 (0-0)	0 (0-0)	0 (0-0)	0 (0-0)	0 (0-0)	0 (0-0)	0 (0-0)	0 (0-0)	0 (0-0)	0 (0-0)	0 (0-0)	0 (0-0)
Kagoshima	1 (1-1)	1 (1-1)	0 (0-0)	0 (0-0)	0 (0-0)	0 (0-0)	0 (0-0)	0 (0-0)	0 (0-0)	0 (0-0)	0 (0-0)	0 (0-0)	0 (0-0)	0 (0-0)
Kanagawa	3 (3-3)	2 (2-2)	1 (1-1)	1 (1-1)	1 (1-1)	1 (1-1)	0 (0-0)	0 (0-0)	0 (0-0)	0 (0-0)	0 (0-0)	0 (0-0)	0 (0-0)	0 (0-0)
Kōchi	1 (0-1)	0 (0-0)	0 (0-0)	0 (0-0)	0 (0-0)	0 (0-0)	0 (0-0)	0 (0-0)	0 (0-0)	0 (0-0)	0 (0-0)	0 (0-0)	0 (0-0)	0 (0-0)
Kumamoto	1 (1-1)	1 (1-1)	0 (0-0)	0 (0-0)	0 (0-0)	0 (0-0)	0 (0-0)	0 (0-0)	0 (0-0)	0 (0-0)	0 (0-0)	0 (0-0)	0 (0-0)	0 (0-0)
Kyōto	1 (1-2)	1 (1-1)	1 (1-1)	0 (0-0)	0 (0-0)	0 (0-0)	0 (0-0)	0 (0-0)	0 (0-0)	0 (0-0)	0 (0-0)	0 (0-0)	0 (0-0)	0 (0-0)
Mie	1 (1-1)	1 (1-1)	0 (0-0)	0 (0-0)	0 (0-0)	0 (0-0)	0 (0-0)	0 (0-0)	0 (0-0)	0 (0-0)	0 (0-0)	0 (0-0)	0 (0-0)	0 (0-0)
Miyagi	1 (1-1)	1 (1-1)	0 (0-0)	0 (0-0)	0 (0-0)	0 (0-0)	0 (0-0)	0 (0-0)	0 (0-0)	0 (0-0)	0 (0-0)	0 (0-0)	0 (0-0)	0 (0-0)
Miyazaki	1 (1-1)	0 (0-0)	0 (0-0)	0 (0-0)	0 (0-0)	0 (0-0)	0 (0-0)	0 (0-0)	0 (0-0)	0 (0-0)	0 (0-0)	0 (0-0)	0 (0-0)	0 (0-0)
Nagano	1 (1-1)	1 (1-1)	0 (0-0)	0 (0-0)	0 (0-0)	0 (0-0)	0 (0-0)	0 (0-0)	0 (0-0)	0 (0-0)	0 (0-0)	0 (0-0)	0 (0-0)	0 (0-0)
Nagasaki	1 (1-1)	1 (1-1)	0 (0-0)	0 (0-0)	0 (0-0)	0 (0-0)	0 (0-0)	0 (0-0)	0 (0-0)	0 (0-0)	0 (0-0)	0 (0-0)	0 (0-0)	0 (0-0)
Nara	1 (0-1)	0 (0-0)	0 (0-0)	0 (0-0)	0 (0-0)	0 (0-0)	0 (0-0)	0 (0-0)	0 (0-0)	0 (0-0)	0 (0-0)	0 (0-0)	0 (0-0)	0 (0-0)
Niigata	2 (1-2)	1 (1-1)	1 (1-1)	0 (0-0)	0 (0-0)	0 (0-0)	0 (0-0)	0 (0-0)	0 (0-0)	0 (0-0)	0 (0-0)	0 (0-0)	0 (0-0)	0 (0-0)
Ōita	1 (1-1)	1 (0-1)	0 (0-0)	0 (0-0)	0 (0-0)	0 (0-0)	0 (0-0)	0 (0-0)	0 (0-0)	0 (0-0)	0 (0-0)	0 (0-0)	0 (0-0)	0 (0-0)
Okayama	1 (1-1)	1 (1-1)	0 (0-0)	0 (0-0)	0 (0-0)	0 (0-0)	0 (0-0)	0 (0-0)	0 (0-0)	0 (0-0)	0 (0-0)	0 (0-0)	0 (0-0)	0 (0-0)
Okinawa	0 (0-1)	0 (0-0)	0 (0-0)	0 (0-0)	0 (0-0)	0 (0-0)	0 (0-0)	0 (0-0)	0 (0-0)	0 (0-0)	0 (0-0)	0 (0-0)	0 (0-0)	0 (0-0)
Ōsaka	6 (5-7)	4 (3-4)	2 (2-2)	1 (1-1)	1 (1-1)	1 (1-1)	0 (0-1)	0 (0-0)	0 (0-0)	0 (0-0)	0 (0-0)	0 (0-0)	0 (0-0)	0 (0-0)
Saga	1 (0-1)	0 (0-0)	0 (0-0)	0 (0-0)	0 (0-0)	0 (0-0)	0 (0-0)	0 (0-0)	0 (0-0)	0 (0-0)	0 (0-0)	0 (0-0)	0 (0-0)	0 (0-0)
Saitama	2 (2-3)	1 (1-2)	1 (1-1)	1 (1-1)	1 (1-1)	0 (0-0)	0 (0-0)	0 (0-0)	0 (0-0)	0 (0-0)	0 (0-0)	0 (0-0)	0 (0-0)	0 (0-0)
Shiga	0 (0-1)	0 (0-0)	0 (0-0)	0 (0-0)	0 (0-0)	0 (0-0)	0 (0-0)	0 (0-0)	0 (0-0)	0 (0-0)	0 (0-0)	0 (0-0)	0 (0-0)	0 (0-0)
Shimane	0 (0-1)	0 (0-0)	0 (0-0)	0 (0-0)	0 (0-0)	0 (0-0)	0 (0-0)	0 (0-0)	0 (0-0)	0 (0-0)	0 (0-0)	0 (0-0)	0 (0-0)	0 (0-0)
Shizuoka	2 (2-2)	1 (1-1)	1 (1-1)	0 (0-0)	0 (0-0)	0 (0-0)	0 (0-0)	0 (0-0)	0 (0-0)	0 (0-0)	0 (0-0)	0 (0-0)	0 (0-0)	0 (0-0)
Tochigi	1 (1-1)	1 (1-1)	0 (0-0)	0 (0-0)	0 (0-0)	0 (0-0)	0 (0-0)	0 (0-0)	0 (0-0)	0 (0-0)	0 (0-0)	0 (0-0)	0 (0-0)	0 (0-0)
Tokushima	1 (0-1)	0 (0-0)	0 (0-0)	0 (0-0)	0 (0-0)	0 (0-0)	0 (0-0)	0 (0-0)	0 (0-0)	0 (0-0)	0 (0-0)	0 (0-0)	0 (0-0)	0 (0-0)
Tōkyō	7 (6-9)	4 (4-5)	3 (3-3)	2 (2-2)	1 (1-2)	1 (1-1)	1 (1-1)	0 (0-0)	0 (0-0)	0 (0-0)	0 (0-0)	0 (0-0)	0 (0-0)	0 (0-0)
Tottori	0 (0-0)	0 (0-0)	0 (0-0)	0 (0-0)	0 (0-0)	0 (0-0)	0 (0-0)	0 (0-0)	0 (0-0)	0 (0-0)	0 (0-0)	0 (0-0)	0 (0-0)	0 (0-0)
Toyama	1 (1-1)	1 (0-1)	0 (0-0)	0 (0-0)	0 (0-0)	0 (0-0)	0 (0-0)	0 (0-0)	0 (0-0)	0 (0-0)	0 (0-0)	0 (0-0)	0 (0-0)	0 (0-0)
Wakayama	1 (1-1)	1 (0-1)	0 (0-0)	0 (0-0)	0 (0-0)	0 (0-0)	0 (0-0)	0 (0-0)	0 (0-0)	0 (0-0)	0 (0-0)	0 (0-0)	0 (0-0)	0 (0-0)
Yamagata	1 (1-1)	0 (0-0)	0 (0-0)	0 (0-0)	0 (0-0)	0 (0-0)	0 (0-0)	0 (0-0)	0 (0-0)	0 (0-0)	0 (0-0)	0 (0-0)	0 (0-0)	0 (0-0)
Yamaguchi	1 (1-1)	1 (1-1)	0 (0-0)	0 (0-0)	0 (0-0)	0 (0-0)	0 (0-0)	0 (0-0)	0 (0-0)	0 (0-0)	0 (0-0)	0 (0-0)	0 (0-0)	0 (0-0)
Yamanashi	0 (0-0)	0 (0-0)	0 (0-0)	0 (0-0)	0 (0-0)	0 (0-0)	0 (0-0)	0 (0-0)	0 (0-0)	0 (0-0)	0 (0-0)	0 (0-0)	0 (0-0)	0 (0-0)
South Korea	76 (60-94)	48 (40-55)	39 (35-44)	33 (29-37)	26 (23-29)	13 (12-15)	8 (7-9)	4 (4-5)	3 (2-3)	2 (2-3)	2 (2-2)	1 (1-1)	1 (1-1)	1 (1-1)
Singapore	3 (2-3)	2 (2-2)	1 (1-1)	1 (1-1)	1 (1-1)	0 (0-0)	0 (0-0)	0 (0-0)	0 (0-0)	0 (0-0)	0 (0-0)	0 (0-0)	0 (0-0)	0 (0-0)
High-income North America	60 (59-62)	60 (59-61)	61 (60-61)	48 (48-49)	39 (39-40)	27 (27-27)	25 (25-25)	22 (22-22)	21 (21-21)	17 (17-17)	16 (15-16)	16 (16-16)	13 (13-14)	12 (12-13)
Canada	7 (7-8)	7 (7-7)	6 (6-6)	5 (4-5)	3 (3-3)	2 (2-3)	2 (2-2)	2 (2-2)	1 (1-1)	1 (1-1)	1 (1-1)	1 (1-1)	1 (1-1)	1 (1-1)
Greenland	0 (0-0)	0 (0-0)	0 (0-0)	0 (0-0)	0 (0-0)	0 (0-0)	0 (0-0)	0 (0-0)	0 (0-0)	0 (0-0)	0 (0-0)	0 (0-0)	0 (0-0)	0 (0-0)

Table 3 Under-5 death numbers by location and year for 1950, 1955...2005, 2010, 2017 - females

Location	1950	1955	1960	1965	1970	1975	1980	1985	1990	1995	2000	2005	2010	2017
USA	53 (51-54)	53 (52-54)	54 (54-55)	44 (43-44)	36 (36-36)	25 (24-25)	23 (23-23)	20 (20-20)	20 (19-20)	16 (15-16)	15 (14-15)	15 (15-15)	13 (12-13)	12 (11-12)
Alabama	2 (1-2)	1 (1-2)	1 (1-1)	1 (1-1)	1 (1-1)	1 (1-1)	0 (0-1)	0 (0-0)	0 (0-0)	0 (0-0)	0 (0-0)	0 (0-0)	0 (0-0)	0 (0-0)
Alaska	0 (0-0)	0 (0-0)	0 (0-0)	0 (0-0)	0 (0-0)	0 (0-0)	0 (0-0)	0 (0-0)	0 (0-0)	0 (0-0)	0 (0-0)	0 (0-0)	0 (0-0)	0 (0-0)
Arizona	0 (0-0)	0 (0-0)	1 (1-1)	0 (0-0)	0 (0-0)	0 (0-0)	0 (0-0)	0 (0-0)	0 (0-0)	0 (0-0)	0 (0-0)	0 (0-0)	0 (0-0)	0 (0-0)
Arkansas	1 (1-1)	1 (1-1)	1 (1-1)	1 (0-1)	0 (0-0)	0 (0-0)	0 (0-0)	0 (0-0)	0 (0-0)	0 (0-0)	0 (0-0)	0 (0-0)	0 (0-0)	0 (0-0)
California	3 (3-4)	4 (4-4)	4 (4-4)	4 (4-4)	3 (3-3)	2 (2-2)	2 (2-2)	2 (2-2)	2 (2-3)	2 (2-2)	2 (2-2)	2 (2-2)	1 (1-1)	1 (1-1)
Colorado	0 (0-1)	0 (0-1)	1 (1-1)	0 (0-0)	0 (0-0)	0 (0-0)	0 (0-0)	0 (0-0)	0 (0-0)	0 (0-0)	0 (0-0)	0 (0-0)	0 (0-0)	0 (0-0)
Connecticut	1 (0-1)	1 (0-1)	1 (1-1)	0 (0-1)	0 (0-0)	0 (0-0)	0 (0-0)	0 (0-0)	0 (0-0)	0 (0-0)	0 (0-0)	0 (0-0)	0 (0-0)	0 (0-0)
Delaware	0 (0-0)	0 (0-0)	0 (0-0)	0 (0-0)	0 (0-0)	0 (0-0)	0 (0-0)	0 (0-0)	0 (0-0)	0 (0-0)	0 (0-0)	0 (0-0)	0 (0-0)	0 (0-0)
Washington, DC	0 (0-1)	0 (0-0)	0 (0-0)	0 (0-0)	0 (0-0)	0 (0-0)	0 (0-0)	0 (0-0)	0 (0-0)	0 (0-0)	0 (0-0)	0 (0-0)	0 (0-0)	0 (0-0)
Florida	1 (1-1)	2 (1-2)	2 (2-2)	2 (2-2)	1 (1-1)	1 (1-1)	1 (1-1)	1 (1-1)	1 (1-1)	1 (1-1)	1 (1-1)	1 (1-1)	1 (1-1)	1 (1-1)
Georgia	2 (1-2)	2 (2-2)	2 (2-2)	1 (1-1)	1 (1-1)	1 (1-1)	1 (1-1)	1 (1-1)	1 (1-1)	1 (1-1)	1 (1-1)	1 (1-1)	0 (0-0)	0 (0-1)
Hawaii	0 (0-0)	0 (0-0)	0 (0-0)	0 (0-0)	0 (0-0)	0 (0-0)	0 (0-0)	0 (0-0)	0 (0-0)	0 (0-0)	0 (0-0)	0 (0-0)	0 (0-0)	0 (0-0)
Idaho	0 (0-0)	0 (0-0)	0 (0-0)	0 (0-0)	0 (0-0)	0 (0-0)	0 (0-0)	0 (0-0)	0 (0-0)	0 (0-0)	0 (0-0)	0 (0-0)	0 (0-0)	0 (0-0)
Illinois	3 (2-3)	3 (3-3)	3 (3-3)	2 (2-2)	2 (2-2)	1 (1-1)	1 (1-1)	1 (1-1)	1 (1-1)	1 (1-1)	1 (1-1)	1 (1-1)	1 (1-1)	0 (0-1)
Indiana	1 (1-1)	1 (1-1)	1 (1-1)	1 (1-1)	1 (1-1)	1 (1-1)	1 (0-1)	0 (0-0)	0 (0-0)	0 (0-0)	0 (0-0)	0 (0-0)	0 (0-0)	0 (0-0)
Iowa	1 (0-1)	1 (0-1)	1 (1-1)	0 (0-0)	0 (0-0)	0 (0-0)	0 (0-0)	0 (0-0)	0 (0-0)	0 (0-0)	0 (0-0)	0 (0-0)	0 (0-0)	0 (0-0)
Kansas	0 (0-1)	0 (0-1)	0 (0-1)	0 (0-0)	0 (0-0)	0 (0-0)	0 (0-0)	0 (0-0)	0 (0-0)	0 (0-0)	0 (0-0)	0 (0-0)	0 (0-0)	0 (0-0)
Kentucky	1 (1-2)	1 (1-1)	1 (1-1)	1 (1-1)	1 (1-1)	0 (0-0)	0 (0-0)	0 (0-0)	0 (0-0)	0 (0-0)	0 (0-0)	0 (0-0)	0 (0-0)	0 (0-0)
Louisiana	1 (1-1)	1 (1-1)	1 (1-1)	1 (1-1)	1 (1-1)	1 (1-1)	1 (1-1)	0 (0-1)	0 (0-0)	0 (0-0)	0 (0-0)	0 (0-0)	0 (0-0)	0 (0-0)
Maine	0 (0-0)	0 (0-0)	0 (0-0)	0 (0-0)	0 (0-0)	0 (0-0)	0 (0-0)	0 (0-0)	0 (0-0)	0 (0-0)	0 (0-0)	0 (0-0)	0 (0-0)	0 (0-0)
Maryland	1 (1-1)	1 (1-1)	1 (1-1)	1 (1-1)	1 (1-1)	0 (0-1)	0 (0-0)	0 (0-0)	0 (0-0)	0 (0-0)	0 (0-0)	0 (0-0)	0 (0-0)	0 (0-0)
Massachusetts	1 (1-2)	1 (1-1)	1 (1-1)	1 (1-1)	1 (1-1)	0 (0-1)	0 (0-0)	0 (0-0)	0 (0-0)	0 (0-0)	0 (0-0)	0 (0-0)	0 (0-0)	0 (0-0)
Michigan	2 (2-2)	2 (2-2)	2 (2-2)	2 (2-2)	2 (2-2)	1 (1-1)	1 (1-1)	1 (1-1)	1 (1-1)	1 (1-1)	1 (1-1)	1 (1-1)	0 (0-0)	0 (0-0)
Minnesota	1 (1-1)	1 (1-1)	1 (1-1)	1 (1-1)	0 (0-1)	0 (0-0)	0 (0-0)	0 (0-0)	0 (0-0)	0 (0-0)	0 (0-0)	0 (0-0)	0 (0-0)	0 (0-0)
Mississippi	1 (1-2)	1 (1-1)	1 (1-1)	1 (1-1)	1 (1-1)	0 (0-0)	0 (0-0)	0 (0-0)	0 (0-0)	0 (0-0)	0 (0-0)	0 (0-0)	0 (0-0)	0 (0-0)
Missouri	1 (1-1)	1 (1-1)	1 (1-1)	1 (1-1)	1 (1-1)	1 (1-1)	0 (0-0)	0 (0-0)	0 (0-0)	0 (0-0)	0 (0-0)	0 (0-0)	0 (0-0)	0 (0-0)
Montana	0 (0-0)	0 (0-0)	0 (0-0)	0 (0-0)	0 (0-0)	0 (0-0)	0 (0-0)	0 (0-0)	0 (0-0)	0 (0-0)	0 (0-0)	0 (0-0)	0 (0-0)	0 (0-0)
Nebraska	0 (0-1)	0 (0-0)	0 (0-0)	0 (0-0)	0 (0-0)	0 (0-0)	0 (0-0)	0 (0-0)	0 (0-0)	0 (0-0)	0 (0-0)	0 (0-0)	0 (0-0)	0 (0-0)
Nevada	0 (0-0)	0 (0-0)	0 (0-0)	0 (0-0)	0 (0-0)	0 (0-0)	0 (0-0)	0 (0-0)	0 (0-0)	0 (0-0)	0 (0-0)	0 (0-0)	0 (0-0)	0 (0-0)
New Hampshire	0 (0-0)	0 (0-0)	0 (0-0)	0 (0-0)	0 (0-0)	0 (0-0)	0 (0-0)	0 (0-0)	0 (0-0)	0 (0-0)	0 (0-0)	0 (0-0)	0 (0-0)	0 (0-0)
New Jersey	2 (1-2)	2 (1-2)	2 (2-2)	1 (1-1)	1 (1-1)	1 (1-1)	1 (1-1)	1 (1-1)	1 (1-1)	0 (0-0)	0 (0-0)	0 (0-0)	0 (0-0)	0 (0-0)
New Mexico	0 (0-1)	0 (0-0)	0 (0-0)	0 (0-0)	0 (0-0)	0 (0-0)	0 (0-0)	0 (0-0)	0 (0-0)	0 (0-0)	0 (0-0)	0 (0-0)	0 (0-0)	0 (0-0)
New York	5 (4-5)	5 (4-5)	5 (4-5)	4 (4-4)	3 (3-3)	2 (2-2)	2 (2-2)	1 (1-2)	1 (1-2)	1 (1-1)	1 (1-1)	1 (1-1)	1 (1-1)	1 (1-1)
North Carolina	2 (2-2)	2 (2-2)	2 (2-2)	2 (1-2)	1 (1-1)	1 (1-1)	1 (1-1)	1 (1-1)	1 (1-1)	1 (0-1)	1 (1-1)	1 (1-1)	0 (0-0)	0 (0-0)
North Dakota	0 (0-0)	0 (0-0)	0 (0-0)	0 (0-0)	0 (0-0)	0 (0-0)	0 (0-0)	0 (0-0)	0 (0-0)	0 (0-0)	0 (0-0)	0 (0-0)	0 (0-0)	0 (0-0)
Ohio	2 (2-3)	2 (2-3)	3 (3-3)	2 (2-2)	2 (2-2)	1 (1-1)	1 (1-1)	1 (1-1)	1 (1-1)	1 (1-1)	1 (1-1)	1 (1-1)	1 (1-1)	0 (0-1)
Oklahoma	1 (1-1)	1 (1-1)	1 (1-1)	1 (0-1)	0 (0-0)	0 (0-0)	0 (0-0)	0 (0-0)	0 (0-0)	0 (0-0)	0 (0-0)	0 (0-0)	0 (0-0)	0 (0-0)
Oregon	0 (0-1)	0 (0-1)	0 (0-0)	0 (0-0)	0 (0-0)	0 (0-0)	0 (0-0)	0 (0-0)	0 (0-0)	0 (0-0)	0 (0-0)	0 (0-0)	0 (0-0)	0 (0-0)
Pennsylvania	3 (3-4)	3 (3-3)	3 (3-3)	2 (2-2)	2 (2-2)	1 (1-1)	1 (1-1)	1 (1-1)	1 (1-1)	1 (1-1)	1 (1-1)	1 (1-1)	1 (0-1)	0 (0-1)
Rhode Island	0 (0-0)	0 (0-0)	0 (0-0)	0 (0-0)	0 (0-0)	0 (0-0)	0 (0-0)	0 (0-0)	0 (0-0)	0 (0-0)	0 (0-0)	0 (0-0)	0 (0-0)	0 (0-0)
South Carolina	1 (1-1)	1 (1-1)	1 (1-1)	1 (1-1)	1 (1-1)	0 (0-0)	0 (0-0)	0 (0-0)	0 (0-0)	0 (0-0)	0 (0-0)	0 (0-0)	0 (0-0)	0 (0-0)
South Dakota	0 (0-0)	0 (0-0)	0 (0-0)	0 (0-0)	0 (0-0)	0 (0-0)	0 (0-0)	0 (0-0)	0 (0-0)	0 (0-0)	0 (0-0)	0 (0-0)	0 (0-0)	0 (0-0)
Tennessee	1 (1-2)	1 (1-1)	1 (1-1)	1 (1-1)	1 (1-1)	1 (1-1)	0 (0-1)	0 (0-0)	0 (0-0)	0 (0-0)	0 (0-0)	0 (0-0)	0 (0-0)	0 (0-0)
Texas	3 (3-4)	3 (3-4)	4 (4-4)	3 (3-3)	2 (2-2)	2 (2-2)	2 (2-2)	2 (1-2)	1 (1-1)	1 (1-1)	1 (1-1)	1 (1-1)	1 (1-1)	1 (1-1)
Utah	0 (0-0)	0 (0-0)	0 (0-0)	0 (0-0)	0 (0-0)	0 (0-0)	0 (0-0)	0 (0-0)	0 (0-0)	0 (0-0)	0 (0-0)	0 (0-0)	0 (0-0)	0 (0-0)
Vermont	0 (0-0)	0 (0-0)	0 (0-0)	0 (0-0)	0 (0-0)	0 (0-0)	0 (0-0)	0 (0-0)	0 (0-0)	0 (0-0)	0 (0-0)	0 (0-0)	0 (0-0)	0 (0-0)
Virginia	1 (1-2)	1 (1-2)	1 (1-1)	1 (1-1)	1 (1-1)	1 (1-1)	1 (1-1)	1 (0-1)	1 (0-1)	0 (0-0)	0 (0-0)	0 (0-0)	0 (0-0)	0 (0-0)
Washington	1 (1-1)	1 (1-1)	1 (1-1)	1 (0-1)	1 (0-1)	0 (0-0)	0 (0-0)	0 (0-0)	0 (0-0)	0 (0-0)	0 (0-0)	0 (0-0)	0 (0-0)	0 (0-0)
West Virginia	1 (1-1)	1 (1-1)	1 (1-1)	0 (0-0)	0 (0-0)	0 (0-0)	0 (0-0)	0 (0-0)	0 (0-0)	0 (0-0)	0 (0-0)	0 (0-0)	0 (0-0)	0 (0-0)
Wisconsin	1 (1-1)	1 (1-1)	1 (1-1)	1 (1-1)	1 (1-1)	0 (0-0)	0 (0-0)	0 (0-0)	0 (0-0)	0 (0-0)	0 (0-0)	0 (0-0)	0 (0-0)	0 (0-0)
Wyoming	0 (0-0)	0 (0-0)	0 (0-0)	0 (0-0)	0 (0-0)	0 (0-0)	0 (0-0)	0 (0-0)	0 (0-0)	0 (0-0)	0 (0-0)	0 (0-0)	0 (0-0)	0 (0-0)
Southern Latin America	32 (29-36)	35 (33-36)	38 (38-39)	32 (31-32)	29 (29-29)	23 (22-25)	17 (17-17)	12 (12-12)	12 (11-12)	9 (9-9)	8 (7-8)	6 (6-6)	6 (6-6)	5 (4-5)
Argentina	17 (14-20)	17 (15-19)	17 (17-18)	15 (15-15)	17 (17-17)	15 (14-17)	12 (12-12)	9 (9-9)	9 (9-9)	7 (7-7)	6 (6-6)	5 (5-5)	5 (4-5)	4 (3-4)
Chile	14 (14-15)	16 (16-17)	20 (19-20)	15 (15-16)	11 (10-11)	7 (7-7)	4 (4-4)	3 (2-3)	2 (2-3)	2 (2-2)	1 (1-1)	1 (1-1)	1 (1-1)	1 (1-1)
Uruguay	1 (1-1)	1 (1-1)	1 (1-2)	1 (1-1)	1 (1-1)	1 (1-1)	1 (1-1)	1 (1-1)	1 (1-1)	1 (0-1)	0 (0-0)	0 (0-0)	0 (0-0)	0 (0-0)
Western Europe	149 (143-155)	122 (120-124)	104 (104-105)	85 (85-86)	65 (64-65)	43 (43-44)	30 (30-30)	22 (22-22)	18 (18-18)	13 (13-13)	11 (11-11)	9 (9-9)	9 (8-9)	7 (7-7)
Andorra	0 (0-0)	0 (0-0)	0 (0-0)	0 (0-0)	0 (0-0)	0 (0-0)	0 (0-0)	0 (0-0)	0 (0-0)	0 (0-0)	0 (0-0)	0 (0-0)	0 (0-0)	0 (0-0)
Austria	3 (3-3)	3 (2-3)	2 (2-2)	2 (2-2)	1 (1-1)	1 (1-1)	1 (1-1)	0 (0-1)	0 (0-0)	0 (0-0)	0 (0-0)	0 (0-0)	0 (0-0)	0 (0-0)
Belgium	4 (4-4)	3 (3-3)	2 (2-2)	2 (2-2)	1 (1-2)	1 (1-1)	1 (1-1)	1 (1-1)	0 (0-1)	0 (0-0)	0 (0-0)	0 (0-0)	0 (0-0)	0 (0-0)
Cyprus	0 (0-1)	0 (0-1)	0 (0-0)	0 (0-0)	0 (0-0)	0 (0-0)	0 (0-0)	0 (0-0)	0 (0-0)	0 (0-0)	0 (0-0)	0 (0-0)	0 (0-0)	0 (0-0)
Denmark	1 (1-1)	1 (1-1)	1 (1-1)	1 (1-1)	0 (0-1)	0 (0-0)	0 (0-0)	0 (0-0)	0 (0-0)	0 (0-0)	0 (0-0)	0 (0-0)	0 (0-0)	0 (0-0)
Finland	2 (2-2)	1 (1-1)	1 (1-1)	1 (1-1)	0 (0-0)	0 (0-0)	0 (0-0)	0 (0-0)	0 (0-0)	0 (0-0)	0 (0-0)	0 (0-0)	0 (0-0)	0 (0-0)
France	20 (20-22)	14 (14-14)	9 (9-10)	8 (8-8)	7 (6-7)	5 (5-5)	4 (4-4)	3 (3-3)	3 (3-3)	2 (2-2)	2 (2-2)	1 (1-2)	1 (1-2)	1 (1-1)
Germany	26 (22-32)	24 (22-26)	22 (21-22)	16 (16-16)	11 (11-11)	7 (7-7)	5 (5-6)	4 (4-4)	3 (3-3)	2 (2-2)	2 (2-2)	1 (1-1)	1 (1-1)	1 (1-2)
Greece	5 (4-5)	4 (4-4)	3 (3-3)	3 (3-3)	2 (2-2)	2 (2-2)	1 (1-1)	1 (1-1)	0 (0-1)	0 (0-0)	0 (0-0)	0 (0-0)	0 (0-0)	0 (0-0)
Iceland	0 (0-0)	0 (0-0)	0 (0-0)	0 (0-0)	0 (0-0)	0 (0-0)	0 (0-0)	0 (0-0)	0 (0-0)	0 (0-0)	0 (0-0)	0 (0-0)	0 (0-0)	0 (0-0)
Ireland	1 (1-2)	1 (1-1)	1 (1-1)	1 (1-1)	1 (1-1)	1 (1-1)	0 (0-0)	0 (0-0)	0 (0-0)	0 (0-0)	0 (0-0)	0 (0-0)	0 (0-0)	0 (0-0)
Israel	1 (1-2)	1 (1-1)	1 (1-1)	1 (1-1)	1 (1-1)	1 (1-1)	1 (1-1)	1 (1-1)	1 (1-1)	0 (0-0)	0 (0-0)	0 (0-0)	0 (0-0)	0 (0-0)
Italy	27 (26-30)	24 (23-24)	21 (21-22)	18 (17-18)	13 (13-13)	8 (8-8)	5 (5-5)	3 (3-3)	2 (2-2)	2 (2-2)	1 (1-1)	1 (1-1)	1 (1-1)	1 (1-1)
Luxembourg	0 (0-0)	0 (0-0)	0 (0-0)	0 (0-0)	0 (0-0)	0 (0-0)	0 (0-0)	0 (0-0)	0 (0-0)	0 (0-0)	0 (0-0)	0 (0-0)	0 (0-0)	0 (0-0)
Malta	0 (0-0)	0 (0-0)	0 (0-0)	0 (0-0)	0 (0-0)	0 (0-0)	0 (0-0)	0 (0-0)	0 (0-0)	0 (0-0)	0 (0-0)	0 (0-0)	0 (0-0)	0 (0-0)
Netherlands	3 (3-3)	2 (2-2)	2 (2-2)	2 (2-2)	2 (1-2)	1 (1-1)	1 (1-1)	1 (1-1)	1 (1-1)	1 (1-1)	1 (1-1)	0 (0-0)	0 (0-0)	0 (0-0)
Norway	1 (1-1)	1 (1-1)	1 (1-1)	1 (1-1)	0 (0-0)	0 (0-0)	0 (0-0)	0 (0-0)	0 (0-0)	0 (0-0)	0 (0-0)	0 (0-0)	0 (0-0)	0 (0-0)
Portugal	14 (14-16)	12 (12-12)	10 (10-10)	8 (8-8)	6 (6-6)	3 (3-3)	2 (2-2)	1 (1-1)	1 (1-1)	0 (0-0)	0 (0-0)	0 (0-0)	0 (0-0)	0 (0-0)
Spain	25 (24-27)	19 (18-19)	15 (14-15)	12 (12-12)	9 (9-9)	6 (6-6)	3 (3-4)	2 (2-2)	2 (2-2)	1 (1-1)	1 (1-1)	1 (1-1)	1 (1-1)	1 (0-1)
Sweden	1 (1-1)	1 (1-1)	1 (1-1)	1 (1-1)	1 (1-1)	0 (0-0)	0 (0-0)	0 (0-0)	0 (0-0)	0 (0-0)	0 (0-0)	0 (0-0)	0 (0-0)	0 (0-0)
Stockholm	0 (0-0)	0 (0-0)	0 (0-0)	0 (0-0)	0 (0-0)	0 (0-0)	0 (0-0)	0 (0-0)	0 (0-0)	0 (0-0)	0 (0-0)	0 (0-0)	0 (0-0)	0 (0-0)
Sweden except Stockholm	1 (1-1)	1 (1-1)	1 (1-1)	1 (1-1)	0 (0-1)	0 (0-0)	0 (0-0)	0 (0-0)	0 (0-0)	0 (0-0)	0 (0-0)	0 (0-0)	0 (0-0)	0 (0-0)
Switzerland	1 (1-2)	1 (1-1)	1 (1-1)	1 (1-1)	1 (1-1)	0 (0-0)	0 (0-0)	0 (0-0)	0 (0-0)	0 (0-0)	0 (0-0)	0 (0-0)	0 (0-0)	0 (0-0)
United Kingdom	10 (10-11)	10 (10-11)	10 (10-11)	10 (9-10)	8 (8-8)	5 (5-5)	4 (4-4)	4 (4-4)	3 (3-3)	2 (2-2)	2 (2-2)	2 (2-2)	2 (2-2)	2 (1-2)
England	7 (7-7)	7 (7-7)	8 (7-8)	7 (7-7)	6 (6-6)	4 (4-4)	3 (3-4)	3 (3-3)	3 (3-3)	2 (2-2)	2 (2-2)	2 (2-2)	2 (2-2)	1 (1-1)
East Midlands	1 (1-1)	1 (1-1)	1 (1-1)	1 (1-1)	1 (1-1)	0 (0-0)	0 (0-0)	0 (0-0)	0 (0-0)	0 (0-0)	0 (0-0)	0 (0-0)	0 (0-0)	0 (0-0)
Derby	0 (0-0)	0 (0-0)	0 (0-0)	0 (0-0)	0 (0-0)	0 (0-0)	0 (0-0)	0 (0-0)	0 (0-0)	0 (0-0)	0 (0-0)	0 (0-0)	0 (0-0)	0 (0-0)
Derbyshire	0 (0-0)	0 (0-0)	0 (0-0)	0 (0-0)	0 (0-0)	0 (0-0)	0 (0-0)	0 (0-0)	0 (0-0)	0 (0-0)	0 (0-0)	0 (0-0)	0 (0-0)	0 (0-0)
Leicester	0 (0-0)	0 (0-0)	0 (0-0)	0 (0-0)	0 (0-0)	0 (0-0)	0 (0-0)	0 (0-0)	0 (0-0)	0 (0-0)	0 (0-0)	0 (0-0)	0 (0-0)	0 (0-0)
Leicestershire	0 (0-0)	0 (0-0)	0 (0-0)	0 (0-0)	0 (0-0)	0 (0-0)	0 (0-0)	0 (0-0)	0 (0-0)	0 (0-0)	0 (0-0)	0 (0-0)	0 (0-0)	0 (0-0)
Lincolnshire	0 (0-0)	0 (0-0)	0 (0-0)	0 (0-0)	0 (0-0)	0 (0-0)	0 (0-0)	0 (0-0)	0 (0-0)	0 (0-0)	0 (0-0)	0 (0-0)	0 (0-0)	0 (0-0)
Northamptonshire	0 (0-0)	0 (0-0)	0 (0-0)	0 (0-0)	0 (0-0)	0 (0-0)	0 (0-0)	0 (0-0)	0 (0-0)	0 (0-0)	0 (0-0)	0 (0-0)	0 (0-0)	0 (0-0)
Nottingham	0 (0-0)	0 (0-0)	0 (0-0)	0 (0-0)	0 (0-0)	0 (0-0)	0 (0-0)	0 (0-0)	0 (0-0)	0 (0-0)	0 (0-0)	0 (0-0)	0 (0-0)	0 (0-0)
Nottinghamshire	0 (0-0)	0 (0-0)	0 (0-0)	0 (0-0)	0 (0-0)	0 (0-0)	0 (0-0)	0 (0-0)	0 (0-0)	0 (0-0)	0 (0-0)	0 (0-0)	0 (0-0)	0 (0-0)
Rutland	0 (0-0)	0 (0-0)	0 (0-0)	0 (0-0)	0 (0-0)	0 (0-0)	0 (0-0)	0 (0-0)	0 (0-0)	0 (0-0)	0 (0-0)	0 (0-0)	0 (0-0)	0 (0-0)
East of England	1 (1-1)	1 (1-1)	1 (1-1)	1 (1-1)	1 (1-1)	0 (0-0)	0 (0-0)	0 (0-0)	0 (0-0)	0 (0-0)	0 (0-0)	0 (0-0)	0 (0-0)	0 (0-0)
Bedford	0 (0-0)	0 (0-0)	0 (0-0)	0 (0-0)	0 (0-0)	0 (0-0)	0 (0-0)	0 (0-0)	0 (0-0)	0 (0-0)	0 (0-0)	0 (0-0)	0 (0-0)	0 (0-0)
Cambridgeshire	0 (0-0)	0 (0-0)	0 (0-0)	0 (0-0)	0 (0-0)	0 (0-0)	0 (0-0)	0 (0-0)	0 (0-0)	0 (0-0)	0 (0-0)	0 (0-0)	0 (0-0)	0 (0-0)
Central Bedfordshire	0 (0-0)	0 (0-0)	0 (0-0)	0 (0-0)	0 (0-0)	0 (0-0)	0 (0-0)	0 (0-0)	0 (0-0)	0 (0-0)	0 (0-0)	0 (0-0)	0 (0-0)	0 (0-0)
Essex	0 (0-0)	0 (0-0)	0 (0-0)	0 (0-0)	0 (0-0)	0 (0-0)	0 (0-0)	0 (0-0)	0 (0-0)	0 (0-0)	0 (0-0)	0 (0-0)	0 (0-0)	0 (0-0)
Hertfordshire	0 (0-0)	0 (0-0)	0 (0-0)	0 (0-0)	0 (0-0)	0 (0-0)	0 (0-0)	0 (0-0)	0 (0-0)	0 (0-0)	0 (0-0)	0 (0-0)	0 (0-0)	0 (0-0)

Table 3 Under-5 death numbers by location and year for 1950, 1955...2005, 2010, 2017 - females

Location	1950	1955	1960	1965	1970	1975	1980	1985	1990	1995	2000	2005	2010	2017
Luton	0 (0-0)	0 (0-0)	0 (0-0)	0 (0-0)	0 (0-0)	0 (0-0)	0 (0-0)	0 (0-0)	0 (0-0)	0 (0-0)	0 (0-0)	0 (0-0)	0 (0-0)	0 (0-0)
Norfolk	0 (0-0)	0 (0-0)	0 (0-0)	0 (0-0)	0 (0-0)	0 (0-0)	0 (0-0)	0 (0-0)	0 (0-0)	0 (0-0)	0 (0-0)	0 (0-0)	0 (0-0)	0 (0-0)
Peterborough	0 (0-0)	0 (0-0)	0 (0-0)	0 (0-0)	0 (0-0)	0 (0-0)	0 (0-0)	0 (0-0)	0 (0-0)	0 (0-0)	0 (0-0)	0 (0-0)	0 (0-0)	0 (0-0)
Southend-on-Sea	0 (0-0)	0 (0-0)	0 (0-0)	0 (0-0)	0 (0-0)	0 (0-0)	0 (0-0)	0 (0-0)	0 (0-0)	0 (0-0)	0 (0-0)	0 (0-0)	0 (0-0)	0 (0-0)
Suffolk	0 (0-0)	0 (0-0)	0 (0-0)	0 (0-0)	0 (0-0)	0 (0-0)	0 (0-0)	0 (0-0)	0 (0-0)	0 (0-0)	0 (0-0)	0 (0-0)	0 (0-0)	0 (0-0)
Thurrock	0 (0-0)	0 (0-0)	0 (0-0)	0 (0-0)	0 (0-0)	0 (0-0)	0 (0-0)	0 (0-0)	0 (0-0)	0 (0-0)	0 (0-0)	0 (0-0)	0 (0-0)	0 (0-0)
Greater London	1 (1-1)	1 (1-1)	1 (1-1)	1 (1-1)	1 (1-1)	1 (1-1)	1 (1-1)	0 (0-0)	0 (0-0)	0 (0-0)	0 (0-0)	0 (0-0)	0 (0-0)	0 (0-0)
Barking and Dagenham	0 (0-0)	0 (0-0)	0 (0-0)	0 (0-0)	0 (0-0)	0 (0-0)	0 (0-0)	0 (0-0)	0 (0-0)	0 (0-0)	0 (0-0)	0 (0-0)	0 (0-0)	0 (0-0)
Barnet	0 (0-0)	0 (0-0)	0 (0-0)	0 (0-0)	0 (0-0)	0 (0-0)	0 (0-0)	0 (0-0)	0 (0-0)	0 (0-0)	0 (0-0)	0 (0-0)	0 (0-0)	0 (0-0)
Bexley	0 (0-0)	0 (0-0)	0 (0-0)	0 (0-0)	0 (0-0)	0 (0-0)	0 (0-0)	0 (0-0)	0 (0-0)	0 (0-0)	0 (0-0)	0 (0-0)	0 (0-0)	0 (0-0)
Brent	0 (0-0)	0 (0-0)	0 (0-0)	0 (0-0)	0 (0-0)	0 (0-0)	0 (0-0)	0 (0-0)	0 (0-0)	0 (0-0)	0 (0-0)	0 (0-0)	0 (0-0)	0 (0-0)
Bromley	0 (0-0)	0 (0-0)	0 (0-0)	0 (0-0)	0 (0-0)	0 (0-0)	0 (0-0)	0 (0-0)	0 (0-0)	0 (0-0)	0 (0-0)	0 (0-0)	0 (0-0)	0 (0-0)
Camden	0 (0-0)	0 (0-0)	0 (0-0)	0 (0-0)	0 (0-0)	0 (0-0)	0 (0-0)	0 (0-0)	0 (0-0)	0 (0-0)	0 (0-0)	0 (0-0)	0 (0-0)	0 (0-0)
Croydon	0 (0-0)	0 (0-0)	0 (0-0)	0 (0-0)	0 (0-0)	0 (0-0)	0 (0-0)	0 (0-0)	0 (0-0)	0 (0-0)	0 (0-0)	0 (0-0)	0 (0-0)	0 (0-0)
Ealing	0 (0-0)	0 (0-0)	0 (0-0)	0 (0-0)	0 (0-0)	0 (0-0)	0 (0-0)	0 (0-0)	0 (0-0)	0 (0-0)	0 (0-0)	0 (0-0)	0 (0-0)	0 (0-0)
Enfield	0 (0-0)	0 (0-0)	0 (0-0)	0 (0-0)	0 (0-0)	0 (0-0)	0 (0-0)	0 (0-0)	0 (0-0)	0 (0-0)	0 (0-0)	0 (0-0)	0 (0-0)	0 (0-0)
Greenwich	0 (0-0)	0 (0-0)	0 (0-0)	0 (0-0)	0 (0-0)	0 (0-0)	0 (0-0)	0 (0-0)	0 (0-0)	0 (0-0)	0 (0-0)	0 (0-0)	0 (0-0)	0 (0-0)
Hackney	0 (0-0)	0 (0-0)	0 (0-0)	0 (0-0)	0 (0-0)	0 (0-0)	0 (0-0)	0 (0-0)	0 (0-0)	0 (0-0)	0 (0-0)	0 (0-0)	0 (0-0)	0 (0-0)
Hammersmith and Fulham	0 (0-0)	0 (0-0)	0 (0-0)	0 (0-0)	0 (0-0)	0 (0-0)	0 (0-0)	0 (0-0)	0 (0-0)	0 (0-0)	0 (0-0)	0 (0-0)	0 (0-0)	0 (0-0)
Haringey	0 (0-0)	0 (0-0)	0 (0-0)	0 (0-0)	0 (0-0)	0 (0-0)	0 (0-0)	0 (0-0)	0 (0-0)	0 (0-0)	0 (0-0)	0 (0-0)	0 (0-0)	0 (0-0)
Harrow	0 (0-0)	0 (0-0)	0 (0-0)	0 (0-0)	0 (0-0)	0 (0-0)	0 (0-0)	0 (0-0)	0 (0-0)	0 (0-0)	0 (0-0)	0 (0-0)	0 (0-0)	0 (0-0)
Havering	0 (0-0)	0 (0-0)	0 (0-0)	0 (0-0)	0 (0-0)	0 (0-0)	0 (0-0)	0 (0-0)	0 (0-0)	0 (0-0)	0 (0-0)	0 (0-0)	0 (0-0)	0 (0-0)
Hillingdon	0 (0-0)	0 (0-0)	0 (0-0)	0 (0-0)	0 (0-0)	0 (0-0)	0 (0-0)	0 (0-0)	0 (0-0)	0 (0-0)	0 (0-0)	0 (0-0)	0 (0-0)	0 (0-0)
Hounslow	0 (0-0)	0 (0-0)	0 (0-0)	0 (0-0)	0 (0-0)	0 (0-0)	0 (0-0)	0 (0-0)	0 (0-0)	0 (0-0)	0 (0-0)	0 (0-0)	0 (0-0)	0 (0-0)
Islington	0 (0-0)	0 (0-0)	0 (0-0)	0 (0-0)	0 (0-0)	0 (0-0)	0 (0-0)	0 (0-0)	0 (0-0)	0 (0-0)	0 (0-0)	0 (0-0)	0 (0-0)	0 (0-0)
Kensington and Chelsea	0 (0-0)	0 (0-0)	0 (0-0)	0 (0-0)	0 (0-0)	0 (0-0)	0 (0-0)	0 (0-0)	0 (0-0)	0 (0-0)	0 (0-0)	0 (0-0)	0 (0-0)	0 (0-0)
Kingston upon Thames	0 (0-0)	0 (0-0)	0 (0-0)	0 (0-0)	0 (0-0)	0 (0-0)	0 (0-0)	0 (0-0)	0 (0-0)	0 (0-0)	0 (0-0)	0 (0-0)	0 (0-0)	0 (0-0)
Lambeth	0 (0-0)	0 (0-0)	0 (0-0)	0 (0-0)	0 (0-0)	0 (0-0)	0 (0-0)	0 (0-0)	0 (0-0)	0 (0-0)	0 (0-0)	0 (0-0)	0 (0-0)	0 (0-0)
Lewisham	0 (0-0)	0 (0-0)	0 (0-0)	0 (0-0)	0 (0-0)	0 (0-0)	0 (0-0)	0 (0-0)	0 (0-0)	0 (0-0)	0 (0-0)	0 (0-0)	0 (0-0)	0 (0-0)
Merton	0 (0-0)	0 (0-0)	0 (0-0)	0 (0-0)	0 (0-0)	0 (0-0)	0 (0-0)	0 (0-0)	0 (0-0)	0 (0-0)	0 (0-0)	0 (0-0)	0 (0-0)	0 (0-0)
Newham	0 (0-0)	0 (0-0)	0 (0-0)	0 (0-0)	0 (0-0)	0 (0-0)	0 (0-0)	0 (0-0)	0 (0-0)	0 (0-0)	0 (0-0)	0 (0-0)	0 (0-0)	0 (0-0)
Redbridge	0 (0-0)	0 (0-0)	0 (0-0)	0 (0-0)	0 (0-0)	0 (0-0)	0 (0-0)	0 (0-0)	0 (0-0)	0 (0-0)	0 (0-0)	0 (0-0)	0 (0-0)	0 (0-0)
Richmond upon Thames	0 (0-0)	0 (0-0)	0 (0-0)	0 (0-0)	0 (0-0)	0 (0-0)	0 (0-0)	0 (0-0)	0 (0-0)	0 (0-0)	0 (0-0)	0 (0-0)	0 (0-0)	0 (0-0)
Southwark	0 (0-0)	0 (0-0)	0 (0-0)	0 (0-0)	0 (0-0)	0 (0-0)	0 (0-0)	0 (0-0)	0 (0-0)	0 (0-0)	0 (0-0)	0 (0-0)	0 (0-0)	0 (0-0)
Sutton	0 (0-0)	0 (0-0)	0 (0-0)	0 (0-0)	0 (0-0)	0 (0-0)	0 (0-0)	0 (0-0)	0 (0-0)	0 (0-0)	0 (0-0)	0 (0-0)	0 (0-0)	0 (0-0)
Tower Hamlets	0 (0-0)	0 (0-0)	0 (0-0)	0 (0-0)	0 (0-0)	0 (0-0)	0 (0-0)	0 (0-0)	0 (0-0)	0 (0-0)	0 (0-0)	0 (0-0)	0 (0-0)	0 (0-0)
Waltham Forest	0 (0-0)	0 (0-0)	0 (0-0)	0 (0-0)	0 (0-0)	0 (0-0)	0 (0-0)	0 (0-0)	0 (0-0)	0 (0-0)	0 (0-0)	0 (0-0)	0 (0-0)	0 (0-0)
Wandsworth	0 (0-0)	0 (0-0)	0 (0-0)	0 (0-0)	0 (0-0)	0 (0-0)	0 (0-0)	0 (0-0)	0 (0-0)	0 (0-0)	0 (0-0)	0 (0-0)	0 (0-0)	0 (0-0)
Westminster	0 (0-0)	0 (0-0)	0 (0-0)	0 (0-0)	0 (0-0)	0 (0-0)	0 (0-0)	0 (0-0)	0 (0-0)	0 (0-0)	0 (0-0)	0 (0-0)	0 (0-0)	0 (0-0)
North East England	1 (1-1)	1 (1-1)	1 (1-1)	1 (1-1)	0 (0-0)	0 (0-0)	0 (0-0)	0 (0-0)	0 (0-0)	0 (0-0)	0 (0-0)	0 (0-0)	0 (0-0)	0 (0-0)
County Durham	0 (0-0)	0 (0-0)	0 (0-0)	0 (0-0)	0 (0-0)	0 (0-0)	0 (0-0)	0 (0-0)	0 (0-0)	0 (0-0)	0 (0-0)	0 (0-0)	0 (0-0)	0 (0-0)
Darlington	0 (0-0)	0 (0-0)	0 (0-0)	0 (0-0)	0 (0-0)	0 (0-0)	0 (0-0)	0 (0-0)	0 (0-0)	0 (0-0)	0 (0-0)	0 (0-0)	0 (0-0)	0 (0-0)
Gateshead	0 (0-0)	0 (0-0)	0 (0-0)	0 (0-0)	0 (0-0)	0 (0-0)	0 (0-0)	0 (0-0)	0 (0-0)	0 (0-0)	0 (0-0)	0 (0-0)	0 (0-0)	0 (0-0)
Hartlepool	0 (0-0)	0 (0-0)	0 (0-0)	0 (0-0)	0 (0-0)	0 (0-0)	0 (0-0)	0 (0-0)	0 (0-0)	0 (0-0)	0 (0-0)	0 (0-0)	0 (0-0)	0 (0-0)
Middlesbrough	0 (0-0)	0 (0-0)	0 (0-0)	0 (0-0)	0 (0-0)	0 (0-0)	0 (0-0)	0 (0-0)	0 (0-0)	0 (0-0)	0 (0-0)	0 (0-0)	0 (0-0)	0 (0-0)
Newcastle upon Tyne	0 (0-0)	0 (0-0)	0 (0-0)	0 (0-0)	0 (0-0)	0 (0-0)	0 (0-0)	0 (0-0)	0 (0-0)	0 (0-0)	0 (0-0)	0 (0-0)	0 (0-0)	0 (0-0)
North Tyneside	0 (0-0)	0 (0-0)	0 (0-0)	0 (0-0)	0 (0-0)	0 (0-0)	0 (0-0)	0 (0-0)	0 (0-0)	0 (0-0)	0 (0-0)	0 (0-0)	0 (0-0)	0 (0-0)
Northumberland	0 (0-0)	0 (0-0)	0 (0-0)	0 (0-0)	0 (0-0)	0 (0-0)	0 (0-0)	0 (0-0)	0 (0-0)	0 (0-0)	0 (0-0)	0 (0-0)	0 (0-0)	0 (0-0)
Redcar and Cleveland	0 (0-0)	0 (0-0)	0 (0-0)	0 (0-0)	0 (0-0)	0 (0-0)	0 (0-0)	0 (0-0)	0 (0-0)	0 (0-0)	0 (0-0)	0 (0-0)	0 (0-0)	0 (0-0)
South Tyneside	0 (0-0)	0 (0-0)	0 (0-0)	0 (0-0)	0 (0-0)	0 (0-0)	0 (0-0)	0 (0-0)	0 (0-0)	0 (0-0)	0 (0-0)	0 (0-0)	0 (0-0)	0 (0-0)
Stockton-on-Tees	0 (0-0)	0 (0-0)	0 (0-0)	0 (0-0)	0 (0-0)	0 (0-0)	0 (0-0)	0 (0-0)	0 (0-0)	0 (0-0)	0 (0-0)	0 (0-0)	0 (0-0)	0 (0-0)
Sunderland	0 (0-0)	0 (0-0)	0 (0-0)	0 (0-0)	0 (0-0)	0 (0-0)	0 (0-0)	0 (0-0)	0 (0-0)	0 (0-0)	0 (0-0)	0 (0-0)	0 (0-0)	0 (0-0)
North West England	1 (1-1)	1 (1-1)	1 (1-1)	1 (1-1)	1 (1-1)	1 (1-1)	1 (1-1)	0 (0-0)	0 (0-0)	0 (0-0)	0 (0-0)	0 (0-0)	0 (0-0)	0 (0-0)
Blackburn with Darwen	0 (0-0)	0 (0-0)	0 (0-0)	0 (0-0)	0 (0-0)	0 (0-0)	0 (0-0)	0 (0-0)	0 (0-0)	0 (0-0)	0 (0-0)	0 (0-0)	0 (0-0)	0 (0-0)
Blackpool	0 (0-0)	0 (0-0)	0 (0-0)	0 (0-0)	0 (0-0)	0 (0-0)	0 (0-0)	0 (0-0)	0 (0-0)	0 (0-0)	0 (0-0)	0 (0-0)	0 (0-0)	0 (0-0)
Bolton	0 (0-0)	0 (0-0)	0 (0-0)	0 (0-0)	0 (0-0)	0 (0-0)	0 (0-0)	0 (0-0)	0 (0-0)	0 (0-0)	0 (0-0)	0 (0-0)	0 (0-0)	0 (0-0)
Bury	0 (0-0)	0 (0-0)	0 (0-0)	0 (0-0)	0 (0-0)	0 (0-0)	0 (0-0)	0 (0-0)	0 (0-0)	0 (0-0)	0 (0-0)	0 (0-0)	0 (0-0)	0 (0-0)
Cheshire East	0 (0-0)	0 (0-0)	0 (0-0)	0 (0-0)	0 (0-0)	0 (0-0)	0 (0-0)	0 (0-0)	0 (0-0)	0 (0-0)	0 (0-0)	0 (0-0)	0 (0-0)	0 (0-0)
Cheshire West and Chester	0 (0-0)	0 (0-0)	0 (0-0)	0 (0-0)	0 (0-0)	0 (0-0)	0 (0-0)	0 (0-0)	0 (0-0)	0 (0-0)	0 (0-0)	0 (0-0)	0 (0-0)	0 (0-0)
Cumbria	0 (0-0)	0 (0-0)	0 (0-0)	0 (0-0)	0 (0-0)	0 (0-0)	0 (0-0)	0 (0-0)	0 (0-0)	0 (0-0)	0 (0-0)	0 (0-0)	0 (0-0)	0 (0-0)
Halton	0 (0-0)	0 (0-0)	0 (0-0)	0 (0-0)	0 (0-0)	0 (0-0)	0 (0-0)	0 (0-0)	0 (0-0)	0 (0-0)	0 (0-0)	0 (0-0)	0 (0-0)	0 (0-0)
Knowsley	0 (0-0)	0 (0-0)	0 (0-0)	0 (0-0)	0 (0-0)	0 (0-0)	0 (0-0)	0 (0-0)	0 (0-0)	0 (0-0)	0 (0-0)	0 (0-0)	0 (0-0)	0 (0-0)
Lancashire	0 (0-0)	0 (0-0)	0 (0-0)	0 (0-0)	0 (0-0)	0 (0-0)	0 (0-0)	0 (0-0)	0 (0-0)	0 (0-0)	0 (0-0)	0 (0-0)	0 (0-0)	0 (0-0)
Liverpool	0 (0-0)	0 (0-0)	0 (0-0)	0 (0-0)	0 (0-0)	0 (0-0)	0 (0-0)	0 (0-0)	0 (0-0)	0 (0-0)	0 (0-0)	0 (0-0)	0 (0-0)	0 (0-0)
Manchester	0 (0-0)	0 (0-0)	0 (0-0)	0 (0-0)	0 (0-0)	0 (0-0)	0 (0-0)	0 (0-0)	0 (0-0)	0 (0-0)	0 (0-0)	0 (0-0)	0 (0-0)	0 (0-0)
Oldham	0 (0-0)	0 (0-0)	0 (0-0)	0 (0-0)	0 (0-0)	0 (0-0)	0 (0-0)	0 (0-0)	0 (0-0)	0 (0-0)	0 (0-0)	0 (0-0)	0 (0-0)	0 (0-0)
Rochdale	0 (0-0)	0 (0-0)	0 (0-0)	0 (0-0)	0 (0-0)	0 (0-0)	0 (0-0)	0 (0-0)	0 (0-0)	0 (0-0)	0 (0-0)	0 (0-0)	0 (0-0)	0 (0-0)
Salford	0 (0-0)	0 (0-0)	0 (0-0)	0 (0-0)	0 (0-0)	0 (0-0)	0 (0-0)	0 (0-0)	0 (0-0)	0 (0-0)	0 (0-0)	0 (0-0)	0 (0-0)	0 (0-0)
Sefton	0 (0-0)	0 (0-0)	0 (0-0)	0 (0-0)	0 (0-0)	0 (0-0)	0 (0-0)	0 (0-0)	0 (0-0)	0 (0-0)	0 (0-0)	0 (0-0)	0 (0-0)	0 (0-0)
St Helens	0 (0-0)	0 (0-0)	0 (0-0)	0 (0-0)	0 (0-0)	0 (0-0)	0 (0-0)	0 (0-0)	0 (0-0)	0 (0-0)	0 (0-0)	0 (0-0)	0 (0-0)	0 (0-0)
Stockport	0 (0-0)	0 (0-0)	0 (0-0)	0 (0-0)	0 (0-0)	0 (0-0)	0 (0-0)	0 (0-0)	0 (0-0)	0 (0-0)	0 (0-0)	0 (0-0)	0 (0-0)	0 (0-0)
Tameside	0 (0-0)	0 (0-0)	0 (0-0)	0 (0-0)	0 (0-0)	0 (0-0)	0 (0-0)	0 (0-0)	0 (0-0)	0 (0-0)	0 (0-0)	0 (0-0)	0 (0-0)	0 (0-0)
Trafford	0 (0-0)	0 (0-0)	0 (0-0)	0 (0-0)	0 (0-0)	0 (0-0)	0 (0-0)	0 (0-0)	0 (0-0)	0 (0-0)	0 (0-0)	0 (0-0)	0 (0-0)	0 (0-0)
Warrington	0 (0-0)	0 (0-0)	0 (0-0)	0 (0-0)	0 (0-0)	0 (0-0)	0 (0-0)	0 (0-0)	0 (0-0)	0 (0-0)	0 (0-0)	0 (0-0)	0 (0-0)	0 (0-0)
Wigan	0 (0-0)	0 (0-0)	0 (0-0)	0 (0-0)	0 (0-0)	0 (0-0)	0 (0-0)	0 (0-0)	0 (0-0)	0 (0-0)	0 (0-0)	0 (0-0)	0 (0-0)	0 (0-0)
Wirral	0 (0-0)	0 (0-0)	0 (0-0)	0 (0-0)	0 (0-0)	0 (0-0)	0 (0-0)	0 (0-0)	0 (0-0)	0 (0-0)	0 (0-0)	0 (0-0)	0 (0-0)	0 (0-0)
South East England	1 (1-1)	1 (1-1)	1 (1-1)	1 (1-1)	1 (1-1)	0 (0-0)	0 (0-0)	0 (0-0)	0 (0-0)	0 (0-0)	0 (0-0)	0 (0-0)	0 (0-0)	0 (0-0)
Bracknell Forest	0 (0-0)	0 (0-0)	0 (0-0)	0 (0-0)	0 (0-0)	0 (0-0)	0 (0-0)	0 (0-0)	0 (0-0)	0 (0-0)	0 (0-0)	0 (0-0)	0 (0-0)	0 (0-0)
Brighton and Hove	0 (0-0)	0 (0-0)	0 (0-0)	0 (0-0)	0 (0-0)	0 (0-0)	0 (0-0)	0 (0-0)	0 (0-0)	0 (0-0)	0 (0-0)	0 (0-0)	0 (0-0)	0 (0-0)
Buckinghamshire	0 (0-0)	0 (0-0)	0 (0-0)	0 (0-0)	0 (0-0)	0 (0-0)	0 (0-0)	0 (0-0)	0 (0-0)	0 (0-0)	0 (0-0)	0 (0-0)	0 (0-0)	0 (0-0)
East Sussex	0 (0-0)	0 (0-0)	0 (0-0)	0 (0-0)	0 (0-0)	0 (0-0)	0 (0-0)	0 (0-0)	0 (0-0)	0 (0-0)	0 (0-0)	0 (0-0)	0 (0-0)	0 (0-0)
Hampshire	0 (0-0)	0 (0-0)	0 (0-0)	0 (0-0)	0 (0-0)	0 (0-0)	0 (0-0)	0 (0-0)	0 (0-0)	0 (0-0)	0 (0-0)	0 (0-0)	0 (0-0)	0 (0-0)
Isle of Wight	0 (0-0)	0 (0-0)	0 (0-0)	0 (0-0)	0 (0-0)	0 (0-0)	0 (0-0)	0 (0-0)	0 (0-0)	0 (0-0)	0 (0-0)	0 (0-0)	0 (0-0)	0 (0-0)
Kent	0 (0-0)	0 (0-0)	0 (0-0)	0 (0-0)	0 (0-0)	0 (0-0)	0 (0-0)	0 (0-0)	0 (0-0)	0 (0-0)	0 (0-0)	0 (0-0)	0 (0-0)	0 (0-0)
Medway	0 (0-0)	0 (0-0)	0 (0-0)	0 (0-0)	0 (0-0)	0 (0-0)	0 (0-0)	0 (0-0)	0 (0-0)	0 (0-0)	0 (0-0)	0 (0-0)	0 (0-0)	0 (0-0)
Milton Keynes	0 (0-0)	0 (0-0)	0 (0-0)	0 (0-0)	0 (0-0)	0 (0-0)	0 (0-0)	0 (0-0)	0 (0-0)	0 (0-0)	0 (0-0)	0 (0-0)	0 (0-0)	0 (0-0)
Oxfordshire	0 (0-0)	0 (0-0)	0 (0-0)	0 (0-0)	0 (0-0)	0 (0-0)	0 (0-0)	0 (0-0)	0 (0-0)	0 (0-0)	0 (0-0)	0 (0-0)	0 (0-0)	0 (0-0)
Portsmouth	0 (0-0)	0 (0-0)	0 (0-0)	0 (0-0)	0 (0-0)	0 (0-0)	0 (0-0)	0 (0-0)	0 (0-0)	0 (0-0)	0 (0-0)	0 (0-0)	0 (0-0)	0 (0-0)
Reading	0 (0-0)	0 (0-0)	0 (0-0)	0 (0-0)	0 (0-0)	0 (0-0)	0 (0-0)	0 (0-0)	0 (0-0)	0 (0-0)	0 (0-0)	0 (0-0)	0 (0-0)	0 (0-0)
Slough	0 (0-0)	0 (0-0)	0 (0-0)	0 (0-0)	0 (0-0)	0 (0-0)	0 (0-0)	0 (0-0)	0 (0-0)	0 (0-0)	0 (0-0)	0 (0-0)	0 (0-0)	0 (0-0)
Southampton	0 (0-0)	0 (0-0)	0 (0-0)	0 (0-0)	0 (0-0)	0 (0-0)	0 (0-0)	0 (0-0)	0 (0-0)	0 (0-0)	0 (0-0)	0 (0-0)	0 (0-0)	0 (0-0)
Surrey	0 (0-0)	0 (0-0)	0 (0-0)	0 (0-0)	0 (0-0)	0 (0-0)	0 (0-0)	0 (0-0)	0 (0-0)	0 (0-0)	0 (0-0)	0 (0-0)	0 (0-0)	0 (0-0)
West Berkshire	0 (0-0)	0 (0-0)	0 (0-0)	0 (0-0)	0 (0-0)	0 (0-0)	0 (0-0)	0 (0-0)	0 (0-0)	0 (0-0)	0 (0-0)	0 (0-0)	0 (0-0)	0 (0-0)
West Sussex	0 (0-0)	0 (0-0)	0 (0-0)	0 (0-0)	0 (0-0)	0 (0-0)	0 (0-0)	0 (0-0)	0 (0-0)	0 (0-0)	0 (0-0)	0 (0-0)	0 (0-0)	0 (0-0)
Windsor and Maidenhead	0 (0-0)	0 (0-0)	0 (0-0)	0 (0-0)	0 (0-0)	0 (0-0)	0 (0-0)	0 (0-0)	0 (0-0)	0 (0-0)	0 (0-0)	0 (0-0)	0 (0-0)	0 (0-0)
Wokingham	0 (0-0)	0 (0-0)	0 (0-0)	0 (0-0)	0 (0-0)	0 (0-0)	0 (0-0)	0 (0-0)	0 (0-0)	0 (0-0)	0 (0-0)	0 (0-0)	0 (0-0)	0 (0-0)
South West England	1 (0-1)	1 (1-1)	1 (1-1)	1 (1-1)	0 (0-0)	0 (0-0)	0 (0-0)	0 (0-0)	0 (0-0)	0 (0-0)	0 (0-0)	0 (0-0)	0 (0-0)	0 (0-0)
Bath and North East Somerset	0 (0-0)	0 (0-0)	0 (0-0)	0 (0-0)	0 (0-0)	0 (0-0)	0 (0-0)	0 (0-0)	0 (0-0)	0 (0-0)	0 (0-0)	0 (0-0)	0 (0-0)	0 (0-0)

Table 3 Under-5 death numbers by location and year for 1950, 1955...2005, 2010, 2017 - females														
Location	1950	1955	1960	1965	1970	1975	1980	1985	1990	1995	2000	2005	2010	2017
Bournemouth	0 (0-0)	0 (0-0)	0 (0-0)	0 (0-0)	0 (0-0)	0 (0-0)	0 (0-0)	0 (0-0)	0 (0-0)	0 (0-0)	0 (0-0)	0 (0-0)	0 (0-0)	0 (0-0)
Bristol, City of	0 (0-0)	0 (0-0)	0 (0-0)	0 (0-0)	0 (0-0)	0 (0-0)	0 (0-0)	0 (0-0)	0 (0-0)	0 (0-0)	0 (0-0)	0 (0-0)	0 (0-0)	0 (0-0)
Cornwall	0 (0-0)	0 (0-0)	0 (0-0)	0 (0-0)	0 (0-0)	0 (0-0)	0 (0-0)	0 (0-0)	0 (0-0)	0 (0-0)	0 (0-0)	0 (0-0)	0 (0-0)	0 (0-0)
Devon	0 (0-0)	0 (0-0)	0 (0-0)	0 (0-0)	0 (0-0)	0 (0-0)	0 (0-0)	0 (0-0)	0 (0-0)	0 (0-0)	0 (0-0)	0 (0-0)	0 (0-0)	0 (0-0)
Dorset	0 (0-0)	0 (0-0)	0 (0-0)	0 (0-0)	0 (0-0)	0 (0-0)	0 (0-0)	0 (0-0)	0 (0-0)	0 (0-0)	0 (0-0)	0 (0-0)	0 (0-0)	0 (0-0)
Gloucestershire	0 (0-0)	0 (0-0)	0 (0-0)	0 (0-0)	0 (0-0)	0 (0-0)	0 (0-0)	0 (0-0)	0 (0-0)	0 (0-0)	0 (0-0)	0 (0-0)	0 (0-0)	0 (0-0)
North Somerset	0 (0-0)	0 (0-0)	0 (0-0)	0 (0-0)	0 (0-0)	0 (0-0)	0 (0-0)	0 (0-0)	0 (0-0)	0 (0-0)	0 (0-0)	0 (0-0)	0 (0-0)	0 (0-0)
Plymouth	0 (0-0)	0 (0-0)	0 (0-0)	0 (0-0)	0 (0-0)	0 (0-0)	0 (0-0)	0 (0-0)	0 (0-0)	0 (0-0)	0 (0-0)	0 (0-0)	0 (0-0)	0 (0-0)
Poole	0 (0-0)	0 (0-0)	0 (0-0)	0 (0-0)	0 (0-0)	0 (0-0)	0 (0-0)	0 (0-0)	0 (0-0)	0 (0-0)	0 (0-0)	0 (0-0)	0 (0-0)	0 (0-0)
Somerset	0 (0-0)	0 (0-0)	0 (0-0)	0 (0-0)	0 (0-0)	0 (0-0)	0 (0-0)	0 (0-0)	0 (0-0)	0 (0-0)	0 (0-0)	0 (0-0)	0 (0-0)	0 (0-0)
South Gloucestershire	0 (0-0)	0 (0-0)	0 (0-0)	0 (0-0)	0 (0-0)	0 (0-0)	0 (0-0)	0 (0-0)	0 (0-0)	0 (0-0)	0 (0-0)	0 (0-0)	0 (0-0)	0 (0-0)
Swindon	0 (0-0)	0 (0-0)	0 (0-0)	0 (0-0)	0 (0-0)	0 (0-0)	0 (0-0)	0 (0-0)	0 (0-0)	0 (0-0)	0 (0-0)	0 (0-0)	0 (0-0)	0 (0-0)
Torbay	0 (0-0)	0 (0-0)	0 (0-0)	0 (0-0)	0 (0-0)	0 (0-0)	0 (0-0)	0 (0-0)	0 (0-0)	0 (0-0)	0 (0-0)	0 (0-0)	0 (0-0)	0 (0-0)
Wiltshire	0 (0-0)	0 (0-0)	0 (0-0)	0 (0-0)	0 (0-0)	0 (0-0)	0 (0-0)	0 (0-0)	0 (0-0)	0 (0-0)	0 (0-0)	0 (0-0)	0 (0-0)	0 (0-0)
West Midlands	1 (1-1)	1 (1-1)	1 (1-1)	1 (1-1)	1 (1-1)	1 (1-1)	0 (0-0)	0 (0-0)	0 (0-0)	0 (0-0)	0 (0-0)	0 (0-0)	0 (0-0)	0 (0-0)
Birmingham	0 (0-0)	0 (0-0)	0 (0-0)	0 (0-0)	0 (0-0)	0 (0-0)	0 (0-0)	0 (0-0)	0 (0-0)	0 (0-0)	0 (0-0)	0 (0-0)	0 (0-0)	0 (0-0)
Coventry	0 (0-0)	0 (0-0)	0 (0-0)	0 (0-0)	0 (0-0)	0 (0-0)	0 (0-0)	0 (0-0)	0 (0-0)	0 (0-0)	0 (0-0)	0 (0-0)	0 (0-0)	0 (0-0)
Dudley	0 (0-0)	0 (0-0)	0 (0-0)	0 (0-0)	0 (0-0)	0 (0-0)	0 (0-0)	0 (0-0)	0 (0-0)	0 (0-0)	0 (0-0)	0 (0-0)	0 (0-0)	0 (0-0)
Herefordshire, County of	0 (0-0)	0 (0-0)	0 (0-0)	0 (0-0)	0 (0-0)	0 (0-0)	0 (0-0)	0 (0-0)	0 (0-0)	0 (0-0)	0 (0-0)	0 (0-0)	0 (0-0)	0 (0-0)
Sandwell	0 (0-0)	0 (0-0)	0 (0-0)	0 (0-0)	0 (0-0)	0 (0-0)	0 (0-0)	0 (0-0)	0 (0-0)	0 (0-0)	0 (0-0)	0 (0-0)	0 (0-0)	0 (0-0)
Shropshire	0 (0-0)	0 (0-0)	0 (0-0)	0 (0-0)	0 (0-0)	0 (0-0)	0 (0-0)	0 (0-0)	0 (0-0)	0 (0-0)	0 (0-0)	0 (0-0)	0 (0-0)	0 (0-0)
Solihull	0 (0-0)	0 (0-0)	0 (0-0)	0 (0-0)	0 (0-0)	0 (0-0)	0 (0-0)	0 (0-0)	0 (0-0)	0 (0-0)	0 (0-0)	0 (0-0)	0 (0-0)	0 (0-0)
Staffordshire	0 (0-0)	0 (0-0)	0 (0-0)	0 (0-0)	0 (0-0)	0 (0-0)	0 (0-0)	0 (0-0)	0 (0-0)	0 (0-0)	0 (0-0)	0 (0-0)	0 (0-0)	0 (0-0)
Stoke-on-Trent	0 (0-0)	0 (0-0)	0 (0-0)	0 (0-0)	0 (0-0)	0 (0-0)	0 (0-0)	0 (0-0)	0 (0-0)	0 (0-0)	0 (0-0)	0 (0-0)	0 (0-0)	0 (0-0)
Telford and Wrekin	0 (0-0)	0 (0-0)	0 (0-0)	0 (0-0)	0 (0-0)	0 (0-0)	0 (0-0)	0 (0-0)	0 (0-0)	0 (0-0)	0 (0-0)	0 (0-0)	0 (0-0)	0 (0-0)
Walsall	0 (0-0)	0 (0-0)	0 (0-0)	0 (0-0)	0 (0-0)	0 (0-0)	0 (0-0)	0 (0-0)	0 (0-0)	0 (0-0)	0 (0-0)	0 (0-0)	0 (0-0)	0 (0-0)
Warwickshire	0 (0-0)	0 (0-0)	0 (0-0)	0 (0-0)	0 (0-0)	0 (0-0)	0 (0-0)	0 (0-0)	0 (0-0)	0 (0-0)	0 (0-0)	0 (0-0)	0 (0-0)	0 (0-0)
Wolverhampton	0 (0-0)	0 (0-0)	0 (0-0)	0 (0-0)	0 (0-0)	0 (0-0)	0 (0-0)	0 (0-0)	0 (0-0)	0 (0-0)	0 (0-0)	0 (0-0)	0 (0-0)	0 (0-0)
Worcestershire	0 (0-0)	0 (0-0)	0 (0-0)	0 (0-0)	0 (0-0)	0 (0-0)	0 (0-0)	0 (0-0)	0 (0-0)	0 (0-0)	0 (0-0)	0 (0-0)	0 (0-0)	0 (0-0)
Yorkshire and the Humber	1 (1-1)	1 (1-1)	1 (1-1)	1 (1-1)	1 (1-1)	0 (0-0)	0 (0-0)	0 (0-0)	0 (0-0)	0 (0-0)	0 (0-0)	0 (0-0)	0 (0-0)	0 (0-0)
Barnsley	0 (0-0)	0 (0-0)	0 (0-0)	0 (0-0)	0 (0-0)	0 (0-0)	0 (0-0)	0 (0-0)	0 (0-0)	0 (0-0)	0 (0-0)	0 (0-0)	0 (0-0)	0 (0-0)
Bradford	0 (0-0)	0 (0-0)	0 (0-0)	0 (0-0)	0 (0-0)	0 (0-0)	0 (0-0)	0 (0-0)	0 (0-0)	0 (0-0)	0 (0-0)	0 (0-0)	0 (0-0)	0 (0-0)
Calderdale	0 (0-0)	0 (0-0)	0 (0-0)	0 (0-0)	0 (0-0)	0 (0-0)	0 (0-0)	0 (0-0)	0 (0-0)	0 (0-0)	0 (0-0)	0 (0-0)	0 (0-0)	0 (0-0)
Doncaster	0 (0-0)	0 (0-0)	0 (0-0)	0 (0-0)	0 (0-0)	0 (0-0)	0 (0-0)	0 (0-0)	0 (0-0)	0 (0-0)	0 (0-0)	0 (0-0)	0 (0-0)	0 (0-0)
East Riding of Yorkshire	0 (0-0)	0 (0-0)	0 (0-0)	0 (0-0)	0 (0-0)	0 (0-0)	0 (0-0)	0 (0-0)	0 (0-0)	0 (0-0)	0 (0-0)	0 (0-0)	0 (0-0)	0 (0-0)
Kingston upon Hull, City of	0 (0-0)	0 (0-0)	0 (0-0)	0 (0-0)	0 (0-0)	0 (0-0)	0 (0-0)	0 (0-0)	0 (0-0)	0 (0-0)	0 (0-0)	0 (0-0)	0 (0-0)	0 (0-0)
Kirklees	0 (0-0)	0 (0-0)	0 (0-0)	0 (0-0)	0 (0-0)	0 (0-0)	0 (0-0)	0 (0-0)	0 (0-0)	0 (0-0)	0 (0-0)	0 (0-0)	0 (0-0)	0 (0-0)
Leeds	0 (0-0)	0 (0-0)	0 (0-0)	0 (0-0)	0 (0-0)	0 (0-0)	0 (0-0)	0 (0-0)	0 (0-0)	0 (0-0)	0 (0-0)	0 (0-0)	0 (0-0)	0 (0-0)
North East Lincolnshire	0 (0-0)	0 (0-0)	0 (0-0)	0 (0-0)	0 (0-0)	0 (0-0)	0 (0-0)	0 (0-0)	0 (0-0)	0 (0-0)	0 (0-0)	0 (0-0)	0 (0-0)	0 (0-0)
North Lincolnshire	0 (0-0)	0 (0-0)	0 (0-0)	0 (0-0)	0 (0-0)	0 (0-0)	0 (0-0)	0 (0-0)	0 (0-0)	0 (0-0)	0 (0-0)	0 (0-0)	0 (0-0)	0 (0-0)
North Yorkshire	0 (0-0)	0 (0-0)	0 (0-0)	0 (0-0)	0 (0-0)	0 (0-0)	0 (0-0)	0 (0-0)	0 (0-0)	0 (0-0)	0 (0-0)	0 (0-0)	0 (0-0)	0 (0-0)
Rotherham	0 (0-0)	0 (0-0)	0 (0-0)	0 (0-0)	0 (0-0)	0 (0-0)	0 (0-0)	0 (0-0)	0 (0-0)	0 (0-0)	0 (0-0)	0 (0-0)	0 (0-0)	0 (0-0)
Sheffield	0 (0-0)	0 (0-0)	0 (0-0)	0 (0-0)	0 (0-0)	0 (0-0)	0 (0-0)	0 (0-0)	0 (0-0)	0 (0-0)	0 (0-0)	0 (0-0)	0 (0-0)	0 (0-0)
Wakefield	0 (0-0)	0 (0-0)	0 (0-0)	0 (0-0)	0 (0-0)	0 (0-0)	0 (0-0)	0 (0-0)	0 (0-0)	0 (0-0)	0 (0-0)	0 (0-0)	0 (0-0)	0 (0-0)
York	0 (0-0)	0 (0-0)	0 (0-0)	0 (0-0)	0 (0-0)	0 (0-0)	0 (0-0)	0 (0-0)	0 (0-0)	0 (0-0)	0 (0-0)	0 (0-0)	0 (0-0)	0 (0-0)
Northern Ireland	1 (1-1)	1 (1-1)	1 (1-1)	0 (0-1)	0 (0-0)	0 (0-0)	0 (0-0)	0 (0-0)	0 (0-0)	0 (0-0)	0 (0-0)	0 (0-0)	0 (0-0)	0 (0-0)
Scotland	2 (1-2)	1 (1-2)	1 (1-1)	1 (1-1)	1 (1-1)	1 (1-1)	0 (0-0)	0 (0-0)	0 (0-0)	0 (0-0)	0 (0-0)	0 (0-0)	0 (0-0)	0 (0-0)
Wales	1 (1-1)	1 (1-1)	1 (1-1)	1 (1-1)	1 (0-1)	0 (0-0)	0 (0-0)	0 (0-0)	0 (0-0)	0 (0-0)	0 (0-0)	0 (0-0)	0 (0-0)	0 (0-0)
Latin America and Caribbean	642 (617-670)	627 (614-643)	625 (612-639)	598 (587-611)	553 (543-563)	481 (472-490)	409 (401-416)	336 (331-342)	263 (258-267)	208 (204-212)	163 (159-166)	126 (123-130)	118 (115-121)	79 (75-83)
Andean Latin America	98 (85-112)	100 (93-107)	103 (97-110)	95 (89-101)	92 (87-97)	77 (72-82)	67 (62-71)	56 (53-59)	45 (42-47)	34 (32-36)	24 (23-26)	18 (17-19)	15 (13-16)	11 (9-13)
Bolivia	24 (19-28)	24 (21-26)	24 (21-26)	22 (20-24)	20 (18-22)	18 (17-19)	16 (15-17)	15 (14-16)	13 (12-14)	11 (10-12)	8 (8-9)	6 (6-7)	5 (4-6)	3 (3-5)
Ecuador	14 (12-17)	15 (14-16)	16 (15-17)	16 (14-17)	15 (14-16)	12 (11-12)	10 (9-11)	7 (7-8)	6 (5-6)	5 (4-5)	5 (4-5)	3 (3-4)	3 (3-4)	2 (2-3)
Peru	61 (48-74)	61 (55-67)	64 (57-70)	57 (52-62)	57 (53-62)	47 (43-52)	41 (37-45)	34 (31-37)	26 (24-28)	18 (17-20)	11 (10-12)	8 (7-9)	6 (6-7)	5 (4-6)
Caribbean	55 (51-61)	52 (49-55)	51 (48-53)	48 (46-51)	43 (41-46)	37 (36-39)	33 (31-35)	30 (29-32)	28 (26-29)	24 (22-25)	19 (18-21)	17 (16-19)	29 (28-31)	13 (11-15)
Antigua and Barbuda	0 (0-0)	0 (0-0)	0 (0-0)	0 (0-0)	0 (0-0)	0 (0-0)	0 (0-0)	0 (0-0)	0 (0-0)	0 (0-0)	0 (0-0)	0 (0-0)	0 (0-0)	0 (0-0)
The Bahamas	0 (0-0)	0 (0-0)	0 (0-0)	0 (0-0)	0 (0-0)	0 (0-0)	0 (0-0)	0 (0-0)	0 (0-0)	0 (0-0)	0 (0-0)	0 (0-0)	0 (0-0)	0 (0-0)
Barbados	1 (1-1)	0 (0-0)	0 (0-0)	0 (0-0)	0 (0-0)	0 (0-0)	0 (0-0)	0 (0-0)	0 (0-0)	0 (0-0)	0 (0-0)	0 (0-0)	0 (0-0)	0 (0-0)
Belize	0 (0-0)	0 (0-0)	0 (0-0)	0 (0-0)	0 (0-0)	0 (0-0)	0 (0-0)	0 (0-0)	0 (0-0)	0 (0-0)	0 (0-0)	0 (0-0)	0 (0-0)	0 (0-0)
Bermuda	0 (0-0)	0 (0-0)	0 (0-0)	0 (0-0)	0 (0-0)	0 (0-0)	0 (0-0)	0 (0-0)	0 (0-0)	0 (0-0)	0 (0-0)	0 (0-0)	0 (0-0)	0 (0-0)
Cuba	5 (3-7)	5 (4-6)	5 (4-5)	5 (5-5)	5 (4-5)	3 (2-3)	1 (1-1)	1 (1-2)	1 (1-1)	1 (1-1)	1 (1-1)	0 (0-0)	0 (0-0)	0 (0-0)
Dominica	0 (0-0)	0 (0-0)	0 (0-0)	0 (0-0)	0 (0-0)	0 (0-0)	0 (0-0)	0 (0-0)	0 (0-0)	0 (0-0)	0 (0-0)	0 (0-0)	0 (0-0)	0 (0-0)
Dominican Republic	9 (7-12)	10 (9-11)	11 (10-12)	11 (10-12)	11 (10-12)	10 (9-10)	8 (7-9)	7 (6-8)	7 (6-8)	5 (5-6)	4 (4-5)	4 (3-4)	3 (2-3)	2 (2-3)
Grenada	0 (0-0)	0 (0-0)	0 (0-0)	0 (0-0)	0 (0-0)	0 (0-0)	0 (0-0)	0 (0-0)	0 (0-0)	0 (0-0)	0 (0-0)	0 (0-0)	0 (0-0)	0 (0-0)
Guyana	2 (1-2)	1 (1-1)	1 (1-1)	1 (1-1)	1 (1-1)	1 (1-1)	1 (1-1)	1 (0-1)	0 (0-1)	0 (0-0)	0 (0-0)	0 (0-0)	0 (0-0)	0 (0-0)
Haiti	27 (22-31)	25 (22-28)	24 (22-26)	23 (21-25)	21 (19-23)	20 (18-21)	19 (17-21)	18 (17-19)	16 (15-18)	15 (13-16)	12 (11-14)	11 (10-13)	25 (23-26)	9 (7-11)
Jamaica	3 (3-4)	3 (3-3)	3 (3-3)	2 (2-3)	2 (2-2)	1 (1-2)	1 (1-1)	1 (1-1)	1 (1-1)	1 (1-1)	0 (0-1)	0 (0-0)	0 (0-0)	0 (0-0)
Puerto Rico	4 (3-4)	3 (3-3)	2 (2-2)	2 (2-2)	1 (1-1)	1 (1-1)	1 (1-1)	0 (0-1)	0 (0-0)	0 (0-0)	0 (0-0)	0 (0-0)	0 (0-0)	0 (0-0)
Saint Lucia	0 (0-0)	0 (0-0)	0 (0-0)	0 (0-0)	0 (0-0)	0 (0-0)	0 (0-0)	0 (0-0)	0 (0-0)	0 (0-0)	0 (0-0)	0 (0-0)	0 (0-0)	0 (0-0)
Saint Vincent and the Grenadines	0 (0-0)	0 (0-0)	0 (0-0)	0 (0-0)	0 (0-0)	0 (0-0)	0 (0-0)	0 (0-0)	0 (0-0)	0 (0-0)	0 (0-0)	0 (0-0)	0 (0-0)	0 (0-0)
Suriname	0 (0-0)	0 (0-0)	0 (0-1)	0 (0-1)	0 (0-1)	0 (0-0)	0 (0-0)	0 (0-0)	0 (0-0)	0 (0-0)	0 (0-0)	0 (0-0)	0 (0-0)	0 (0-0)
Trinidad and Tobago	1 (1-2)	1 (1-1)	1 (1-1)	1 (1-1)	1 (1-1)	1 (0-1)	0 (0-1)	0 (0-0)	0 (0-0)	0 (0-0)	0 (0-0)	0 (0-0)	0 (0-0)	0 (0-0)
Virgin Islands	0 (0-0)	0 (0-0)	0 (0-0)	0 (0-0)	0 (0-0)	0 (0-0)	0 (0-0)	0 (0-0)	0 (0-0)	0 (0-0)	0 (0-0)	0 (0-0)	0 (0-0)	0 (0-0)
Central Latin America	232 (220-245)	218 (212-224)	211 (205-216)	202 (197-207)	188 (183-192)	162 (158-166)	133 (130-136)	110 (107-112)	88 (86-91)	74 (72-76)	59 (57-61)	46 (44-47)	39 (37-40)	32 (30-35)
Colombia	36 (29-45)	38 (34-43)	41 (38-45)	38 (35-42)	33 (30-36)	27 (25-30)	20 (19-22)	17 (15-18)	13 (12-15)	12 (11-13)	10 (9-12)	9 (8-10)	7 (6-8)	5 (4-7)
Costa Rica	3 (2-3)	2 (2-3)	2 (2-3)	3 (3-3)	2 (2-2)	1 (1-1)	1 (1-1)	1 (1-1)	1 (1-1)	1 (0-1)	0 (0-0)	0 (0-0)	0 (0-0)	0 (0-0)
El Salvador	11 (9-14)	11 (10-13)	11 (10-12)	11 (9-12)	10 (9-11)	8 (7-9)	7 (6-7)	5 (4-5)	4 (3-4)	3 (2-3)	2 (2-2)	1 (1-1)	1 (1-1)	1 (0-1)
Guatemala	18 (15-23)	18 (16-20)	19 (17-21)	19 (17-21)	18 (17-20)	17 (15-18)	17 (16-19)	15 (14-16)	12 (11-13)	10 (9-11)	8 (7-9)	7 (6-7)	5 (5-6)	5 (4-7)
Honduras	8 (6-10)	8 (7-9)	8 (7-9)	8 (7-9)	7 (6-8)	6 (6-7)	5 (5-6)	5 (4-5)	4 (4-4)	4 (3-4)	3 (3-3)	2 (2-3)	2 (2-2)	1 (1-2)
Mexico	130 (122-139)	117 (113-121)	108 (105-112)	104 (101-107)	98 (95-101)	84 (81-86)	68 (66-70)	55 (53-56)	42 (41-44)	35 (34-36)	27 (27-28)	20 (19-21)	17 (16-18)	14 (14-15)
Aguascalientes	1 (0-1)	1 (1-1)	1 (1-1)	1 (1-1)	1 (1-1)	1 (1-1)	0 (0-1)	0 (0-1)	0 (0-0)	0 (0-0)	0 (0-0)	0 (0-0)	0 (0-0)	0 (0-0)
Baja California	2 (2-3)	2 (2-2)	2 (1-2)	1 (1-2)	1 (1-1)	1 (1-1)	1 (1-1)	1 (0-1)	1 (0-1)	1 (0-1)	1 (0-1)	0 (0-1)	0 (0-0)	0 (0-0)
Baja California Sur	0 (0-0)	0 (0-0)	0 (0-0)	0 (0-0)	0 (0-0)	0 (0-0)	0 (0-0)	0 (0-0)	0 (0-0)	0 (0-0)	0 (0-0)	0 (0-0)	0 (0-0)	0 (0-0)
Campeche	0 (0-0)	0 (0-0)	0 (0-0)	0 (0-0)	0 (0-0)	0 (0-1)	0 (0-0)	0 (0-0)	0 (0-0)	0 (0-0)	0 (0-0)	0 (0-0)	0 (0-0)	0 (0-0)
Chiapas	2 (1-3)	2 (2-2)	2 (2-2)	2 (2-2)	2 (2-3)	2 (2-3)	3 (2-3)	3 (2-3)	3 (2-3)	2 (2-3)	2 (1-2)	1 (1-1)	1 (1-1)	1 (1-1)
Chihuahua	5 (4-6)	4 (4-5)	4 (3-4)	3 (3-4)	3 (2-3)	2 (2-2)	2 (1-2)	1 (1-1)	1 (1-1)	1 (1-1)	1 (1-1)	1 (1-1)	1 (0-1)	1 (0-1)
Coahuila	3 (2-3)	3 (2-3)	2 (2-3)	2 (2-3)	2 (2-3)	2 (2-2)	2 (1-2)	1 (1-1)	1 (1-1)	1 (1-1)	1 (0-1)	0 (0-1)	0 (0-0)	0 (0-0)
Colima	1 (1-1)	1 (1-1)	1 (1-1)	1 (1-1)	1 (1-1)	1 (0-1)	0 (0-0)	0 (0-0)	0 (0-0)	0 (0-0)	0 (0-0)	0 (0-0)	0 (0-0)	0 (0-0)
Mexico City	18 (13-25)	15 (13-18)	13 (11-15)	11 (10-13)	9 (8-11)	8 (6-9)	6 (5-7)	4 (3-4)	3 (2-3)	2 (2-2)	2 (1-2)	1 (1-1)	1 (1-1)	1 (1-1)
Durango	3 (3-4)	3 (3-3)	3 (2-3)	2 (2-3)	2 (2-3)	2 (2-2)	1 (1-1)	1 (1-1)	1 (1-1)	1 (1-1)	0 (0-1)	0 (0-0)	0 (0-0)	0 (0-0)
Guanajuato	7 (6-9)	7 (6-8)	7 (6-8)	7 (6-8)	7 (6-7)	6 (5-7)	4 (4-5)	4 (3-4)	2 (2-3)	2 (2-2)	1 (1-2)	1 (1-1)	1 (1-1)	1 (1-1)
Guerrero	5 (4-6)	5 (4-5)	4 (4-5)	5 (4-5)	5 (4-5)	4 (4-5)	3 (3-4)	3 (2-3)	2 (2-3)	2 (2-2)	2 (1-2)	1 (1-1)	1 (1-1)	1 (1-1)
Hidalgo	3 (2-4)	3 (2-3)	3 (2-3)	3 (2-3)	3 (2-3)	2 (2-3)	2 (2-2)	2 (1-2)	1 (1-1)	1 (1-1)	1 (1-1)	0 (0-1)	0 (0-0)	0 (0-0)
Jalisco	11 (8-13)	10 (8-11)	9 (8-10)	8 (7-9)	7 (6-8)	6 (5-6)	4 (4-5)	3 (3-4)	2 (2-3)	2 (2-2)	2 (1-2)	1 (1-1)	1 (1-1)	1 (1-1)
México	4 (3-5)	4 (4-5)	5 (4-6)	6 (5-7)	8 (7-8)	7 (6-8)	6 (6-7)	6 (5-7)	5 (5-6)	4 (4-4)	3 (3-4)	3 (3-3)	2 (2-3)	2 (2-2)
Michoacán de Ocampo	8 (6-11)	7 (6-9)	7 (6-8)	6 (5-7)	5 (5-6)	5 (4-5)	4 (3-4)	3 (3-3)	2 (2-2)	2 (1-2)	1 (1-1)	1 (1-1)	1 (1-1)	1 (1-1)
Morelos	1 (1-1)	1 (1-1)	1 (1-1)	1 (1-1)	1 (1-1)	1 (1-1)	1 (1-1)	1 (1-1)	1 (0-1)	0 (0-1)	0 (0-0)	0 (0-0)	0 (0-0)	0 (0-0)
Nayarit	2 (1-2)	2 (1-2)	1 (1-2)	1 (1-2)	1 (1-1)	1 (1-1)	1 (1-1)	0 (0-1)	0 (0-0)	0 (0-0)	0 (0-0)	0 (0-0)	0 (0-0)	0 (0-0)
Nuevo León	3 (3-4)	3 (3-4)	3 (3-3)	3 (3-3)	3 (2-3)	2 (2-2)	2 (1-2)	1 (1-1)	1 (1-1)	1 (1-1)	1 (1-1)	1 (0-1)	0 (0-0)	0 (0-1)

Table 3 Under-5 death numbers by location and year for 1950, 1955...2005, 2010, 2017 - females														
Location	1950	1955	1960	1965	1970	1975	1980	1985	1990	1995	2000	2005	2010	2017
Oaxaca	8 (6-10)	6 (5-7)	5 (5-6)	5 (4-6)	5 (4-5)	4 (4-5)	3 (3-4)	2 (2-3)	2 (1-2)	1 (1-2)	1 (1-1)	1 (1-1)	1 (0-1)	1 (0-1)
Puebla	10 (8-12)	9 (8-10)	8 (7-9)	8 (7-8)	7 (6-8)	6 (5-7)	5 (4-6)	4 (3-5)	3 (3-4)	3 (2-3)	2 (2-3)	2 (1-2)	1 (1-2)	1 (1-1)
Querétaro	1 (1-2)	1 (1-1)	1 (1-1)	1 (1-1)	1 (1-1)	1 (1-1)	1 (1-1)	1 (1-1)	1 (1-1)	0 (0-1)	0 (0-0)	0 (0-0)	0 (0-0)	0 (0-0)
Quintana Roo	0 (0-0)	0 (0-0)	0 (0-0)	0 (0-0)	0 (0-0)	0 (0-0)	0 (0-0)	0 (0-0)	0 (0-0)	0 (0-0)	0 (0-0)	0 (0-0)	0 (0-0)	0 (0-0)
San Luis Potosí	5 (3-6)	4 (3-5)	4 (3-4)	3 (3-4)	3 (3-3)	2 (2-3)	2 (1-2)	1 (1-2)	1 (1-1)	1 (1-1)	1 (1-1)	1 (0-1)	0 (0-1)	0 (0-0)
Sinaloa	3 (2-3)	3 (2-3)	3 (2-3)	3 (3-3)	3 (3-3)	2 (2-3)	2 (1-2)	1 (1-1)	1 (1-1)	1 (1-1)	1 (0-1)	0 (0-0)	0 (0-0)	0 (0-0)
Sonora	2 (2-3)	2 (2-2)	2 (2-2)	2 (2-2)	2 (1-2)	1 (1-2)	1 (1-1)	1 (1-1)	1 (1-1)	1 (0-1)	1 (0-1)	0 (0-1)	0 (0-0)	0 (0-0)
Tabasco	1 (1-2)	1 (1-2)	1 (1-2)	2 (1-2)	2 (2-2)	2 (2-2)	2 (1-2)	1 (1-2)	1 (1-1)	1 (1-1)	1 (0-1)	0 (0-0)	0 (0-0)	0 (0-0)
Tamaulipas	3 (2-5)	3 (3-3)	3 (2-3)	3 (2-3)	2 (2-2)	2 (1-2)	1 (1-2)	1 (1-1)	1 (1-1)	1 (1-1)	1 (0-1)	0 (0-0)	0 (0-1)	0 (0-0)
Tlaxcala	1 (1-2)	1 (1-1)	1 (1-1)	1 (1-1)	1 (1-1)	1 (1-1)	1 (1-1)	1 (1-1)	0 (0-1)	0 (0-0)	0 (0-0)	0 (0-0)	0 (0-0)	0 (0-0)
Veracruz de Ignacio de la Llave	10 (8-12)	9 (8-10)	8 (7-9)	8 (7-9)	7 (6-8)	6 (5-7)	5 (4-5)	4 (4-5)	3 (3-4)	3 (3-3)	2 (2-2)	1 (1-1)	1 (1-1)	1 (1-1)
Yucatán	2 (1-2)	2 (1-2)	1 (1-2)	1 (1-2)	1 (1-2)	1 (1-1)	1 (1-1)	1 (1-1)	1 (1-1)	1 (0-1)	0 (0-0)	0 (0-0)	0 (0-0)	0 (0-0)
Zacatecas	4 (4-5)	4 (3-4)	3 (3-3)	3 (2-3)	3 (2-3)	2 (2-2)	2 (1-2)	1 (1-1)	1 (1-1)	1 (0-1)	0 (0-0)	0 (0-0)	0 (0-0)	0 (0-0)
Nicaragua	8 (7-10)	8 (7-9)	8 (7-9)	8 (7-8)	7 (6-8)	7 (6-7)	6 (5-6)	5 (5-6)	4 (4-4)	3 (3-3)	2 (2-2)	1 (1-2)	1 (1-1)	1 (1-1)
Panama	2 (1-2)	2 (1-2)	2 (1-2)	2 (1-2)	1 (1-2)	1 (1-1)	1 (1-1)	1 (1-1)	1 (1-1)	1 (1-1)	1 (1-1)	1 (1-1)	1 (1-1)	1 (0-1)
Venezuela	16 (15-17)	13 (13-13)	11 (11-12)	11 (11-11)	12 (12-12)	11 (11-12)	9 (8-9)	7 (7-7)	8 (8-8)	7 (6-7)	5 (5-5)	4 (4-5)	5 (5-5)	4 (3-5)
Tropical Latin America	256 (238-275)	257 (246-269)	260 (250-271)	253 (244-262)	229 (222-237)	205 (199-211)	176 (171-180)	140 (137-144)	102 (99-105)	76 (74-78)	60 (58-62)	46 (44-47)	35 (34-37)	23 (22-24)
Brazil	254 (236-272)	255 (244-266)	257 (247-268)	250 (241-259)	226 (219-234)	203 (196-209)	173 (169-178)	138 (135-141)	100 (98-103)	74 (73-77)	59 (57-60)	44 (43-46)	34 (32-36)	22 (21-24)
Acre	1 (0-1)	1 (1-1)	1 (1-1)	1 (1-1)	1 (1-1)	1 (1-1)	1 (1-1)	1 (1-1)	0 (0-0)	0 (0-0)	0 (0-0)	0 (0-0)	0 (0-0)	0 (0-0)
Alagoas	9 (7-11)	9 (8-11)	10 (9-11)	10 (9-11)	10 (9-11)	9 (8-10)	9 (8-10)	7 (7-8)	5 (4-5)	3 (3-4)	2 (2-3)	2 (1-2)	1 (1-1)	1 (0-1)
Amapá	0 (0-0)	0 (0-0)	0 (0-0)	0 (0-0)	0 (0-0)	0 (0-0)	0 (0-0)	0 (0-0)	0 (0-0)	0 (0-0)	0 (0-0)	0 (0-0)	0 (0-0)	0 (0-0)
Amazonas	2 (2-3)	2 (2-3)	2 (2-3)	3 (2-3)	3 (2-3)	3 (2-3)	2 (2-3)	2 (2-2)	2 (1-2)	1 (1-2)	1 (1-1)	1 (1-1)	1 (1-1)	1 (1-1)
Bahia	32 (26-40)	32 (28-37)	32 (28-37)	31 (27-35)	28 (26-31)	26 (23-28)	22 (20-24)	19 (17-20)	13 (12-14)	9 (9-11)	8 (7-9)	6 (5-7)	5 (4-5)	3 (2-3)
Ceará	21 (17-26)	22 (19-25)	22 (20-25)	22 (20-24)	20 (18-23)	19 (17-21)	17 (15-19)	13 (12-14)	9 (8-10)	6 (6-7)	5 (4-5)	3 (3-4)	2 (2-3)	1 (1-2)
Distrito Federal	0 (0-0)	0 (0-0)	1 (0-1)	1 (1-1)	1 (1-1)	1 (1-1)	1 (1-1)	1 (1-1)	1 (1-1)	1 (1-1)	0 (0-1)	0 (0-0)	0 (0-0)	0 (0-0)
Espírito Santo	3 (2-4)	3 (3-4)	3 (3-4)	3 (3-4)	3 (3-3)	2 (2-3)	2 (2-2)	2 (2-2)	1 (1-1)	1 (1-1)	1 (1-1)	1 (1-1)	0 (0-1)	0 (0-0)
Goiás	5 (4-7)	5 (5-6)	6 (5-7)	6 (5-7)	5 (4-6)	4 (3-4)	3 (3-3)	2 (2-3)	2 (2-2)	1 (1-2)	1 (1-1)	1 (1-1)	1 (1-1)	1 (1-1)
Maranhão	15 (12-18)	15 (13-17)	16 (14-18)	16 (14-18)	15 (14-16)	14 (12-15)	14 (12-15)	12 (11-13)	8 (7-9)	6 (5-6)	5 (4-5)	4 (3-4)	3 (2-3)	1 (1-2)
Mato Grosso	2 (1-2)	2 (2-2)	2 (2-3)	2 (2-3)	2 (2-2)	2 (2-2)	2 (1-2)	1 (1-2)	1 (1-1)	1 (1-1)	1 (1-1)	1 (1-1)	0 (0-1)	0 (0-0)
Mato Grosso do Sul	1 (0-1)	1 (1-1)	1 (1-1)	1 (1-1)	1 (1-1)	1 (1-1)	1 (1-1)	1 (1-1)	1 (1-1)	1 (1-1)	1 (1-1)	0 (0-1)	0 (0-0)	0 (0-0)
Minas Gerais	29 (22-37)	28 (24-33)	27 (23-32)	25 (22-30)	21 (18-24)	17 (15-20)	14 (13-16)	11 (10-12)	7 (6-8)	6 (5-6)	5 (4-6)	4 (3-5)	3 (2-4)	2 (1-2)
Pará	4 (3-6)	5 (4-6)	5 (4-6)	6 (5-7)	6 (5-7)	6 (6-7)	7 (6-7)	6 (5-7)	4 (4-5)	3 (3-4)	3 (2-3)	2 (2-3)	2 (2-2)	1 (1-2)
Paraíba	14 (11-17)	13 (12-15)	13 (11-15)	12 (10-13)	10 (9-12)	8 (8-9)	6 (6-7)	4 (4-5)	3 (3-3)	2 (2-2)	1 (1-2)	1 (1-1)	1 (1-1)	0 (0-1)
Paraná	14 (10-20)	15 (12-17)	15 (12-17)	14 (12-16)	11 (10-13)	9 (7-10)	7 (6-7)	5 (4-5)	4 (3-4)	3 (2-3)	2 (2-2)	2 (2-2)	1 (1-2)	1 (1-1)
Pernambuco	32 (26-39)	31 (27-36)	30 (27-34)	29 (26-32)	25 (22-28)	21 (19-23)	17 (15-18)	13 (11-14)	9 (8-10)	6 (6-7)	5 (4-6)	4 (3-4)	2 (2-3)	1 (1-2)
Piauí	6 (4-7)	6 (5-7)	6 (5-7)	6 (5-7)	6 (5-7)	5 (5-6)	4 (4-5)	3 (3-4)	3 (2-3)	2 (2-2)	2 (1-2)	1 (1-2)	1 (1-1)	1 (0-1)
Rio de Janeiro	15 (10-20)	14 (11-17)	13 (11-16)	12 (10-14)	11 (10-13)	10 (9-11)	8 (7-9)	6 (6-7)	5 (4-5)	4 (3-4)	3 (2-3)	2 (2-2)	2 (2-2)	1 (1-2)
Rio Grande do Norte	8 (7-10)	8 (7-10)	8 (7-10)	8 (7-9)	8 (7-9)	7 (6-7)	5 (4-5)	4 (3-4)	3 (2-3)	2 (2-2)	1 (1-2)	1 (1-1)	1 (1-1)	0 (0-1)
Rio Grande do Sul	11 (8-15)	11 (8-13)	10 (9-12)	10 (8-11)	8 (7-9)	6 (5-7)	5 (4-5)	3 (3-4)	2 (2-3)	2 (2-2)	2 (1-2)	1 (1-1)	1 (1-1)	1 (1-1)
Rondônia	0 (0-0)	0 (0-0)	0 (0-0)	0 (0-0)	0 (0-0)	0 (0-0)	1 (1-1)	1 (1-1)	1 (1-1)	0 (0-0)	0 (0-0)	0 (0-0)	0 (0-0)	0 (0-0)
Roraima	0 (0-0)	0 (0-0)	0 (0-0)	0 (0-0)	0 (0-0)	0 (0-0)	0 (0-0)	0 (0-0)	0 (0-0)	0 (0-0)	0 (0-0)	0 (0-0)	0 (0-0)	0 (0-0)
Santa Catarina	3 (2-5)	4 (3-4)	4 (3-4)	4 (3-4)	3 (3-4)	3 (3-3)	3 (2-3)	2 (2-2)	2 (2-2)	1 (1-2)	1 (1-1)	1 (1-1)	1 (1-1)	1 (0-1)
São Paulo	21 (15-28)	22 (18-26)	23 (19-27)	23 (20-27)	23 (20-26)	24 (22-27)	20 (19-22)	16 (15-17)	13 (12-14)	10 (9-11)	7 (7-8)	4 (4-5)	4 (4-5)	3 (3-4)
Sergipe	3 (2-5)	4 (3-4)	4 (3-4)	4 (3-4)	3 (3-4)	3 (3-4)	3 (2-3)	2 (2-2)	2 (2-2)	1 (1-1)	1 (1-1)	1 (1-1)	1 (0-1)	0 (0-0)
Tocantins	2 (1-2)	2 (1-2)	2 (2-2)	2 (2-2)	2 (2-2)	2 (2-2)	2 (1-2)	1 (1-1)	1 (1-1)	1 (1-1)	1 (1-1)	0 (0-1)	0 (0-0)	0 (0-0)
Paraguay	2 (2-3)	3 (2-3)	3 (3-3)	3 (3-3)	3 (3-3)	3 (2-3)	2 (2-3)	2 (2-3)	2 (2-2)	2 (2-2)	2 (1-2)	1 (1-2)	1 (1-1)	1 (1-1)
North Africa and Middle East	998 (938-1070)	941 (908-978)	905 (874-937)	845 (818-872)	789 (764-814)	723 (701-744)	640 (620-660)	550 (535-566)	425 (412-438)	378 (366-392)	322 (311-333)	272 (262-283)	225 (215-237)	167 (154-181)
North Africa and Middle East	998 (938-1070)	941 (908-978)	905 (874-937)	845 (818-872)	789 (764-814)	723 (701-744)	640 (620-660)	550 (535-566)	425 (412-438)	378 (366-392)	322 (311-333)	272 (262-283)	225 (215-237)	167 (154-181)
Afghanistan	71 (55-89)	71 (60-84)	73 (62-86)	75 (63-90)	79 (65-96)	83 (69-98)	80 (68-93)	57 (51-65)	45 (40-49)	69 (62-76)	67 (61-73)	57 (52-63)	46 (40-51)	41 (33-49)
Algeria	24 (21-28)	32 (28-36)	39 (34-45)	39 (35-44)	42 (38-47)	44 (39-48)	41 (38-44)	26 (24-28)	17 (15-19)	14 (12-16)	12 (11-14)	10 (9-12)	9 (8-10)	7 (6-9)
Bahrain	1 (1-1)	1 (1-1)	1 (1-1)	1 (0-1)	0 (0-0)	0 (0-0)	0 (0-0)	0 (0-0)	0 (0-0)	0 (0-0)	0 (0-0)	0 (0-0)	0 (0-0)	0 (0-0)
Egypt	242 (196-296)	218 (196-242)	201 (183-221)	183 (166-199)	175 (161-190)	161 (149-174)	138 (127-149)	111 (102-121)	82 (75-89)	59 (53-66)	44 (39-49)	39 (34-43)	32 (28-37)	20 (16-24)
Iran	215 (203-229)	197 (190-205)	182 (175-188)	168 (162-173)	140 (135-145)	97 (93-100)	74 (72-77)	60 (58-62)	48 (46-50)	34 (33-36)	28 (27-29)	24 (23-25)	20 (19-21)	9 (9-10)
Iraq	29 (22-36)	25 (21-30)	23 (19-27)	22 (18-27)	22 (19-25)	20 (18-22)	18 (16-20)	18 (17-19)	14 (13-16)	17 (15-19)	17 (15-19)	18 (16-20)	15 (13-17)	14 (12-17)
Jordan	2 (2-3)	2 (2-3)	3 (2-3)	3 (3-3)	3 (3-3)	3 (3-3)	3 (2-3)	2 (2-3)	3 (2-3)	3 (2-3)	3 (2-3)	2 (2-3)	2 (2-3)	2 (2-3)
Kuwait	0 (0-0)	0 (0-0)	0 (0-0)	0 (0-1)	1 (1-1)	1 (1-1)	1 (1-1)	1 (1-1)	0 (0-0)	0 (0-0)	0 (0-0)	0 (0-0)	0 (0-0)	0 (0-0)
Lebanon	5 (4-7)	5 (4-6)	4 (3-5)	4 (3-4)	3 (3-4)	5 (5-6)	2 (2-3)	3 (2-3)	2 (2-2)	2 (1-2)	1 (1-2)	1 (1-1)	1 (1-1)	1 (0-1)
Libya	11 (9-13)	9 (8-11)	8 (7-10)	7 (6-8)	5 (4-6)	4 (3-4)	3 (3-4)	3 (2-3)	2 (2-3)	2 (1-2)	1 (1-2)	1 (1-1)	1 (1-1)	1 (1-1)
Morocco	75 (63-90)	74 (67-81)	74 (68-79)	64 (59-68)	57 (53-60)	54 (51-58)	44 (41-48)	33 (30-36)	25 (23-27)	20 (18-22)	16 (14-19)	13 (10-15)	10 (8-12)	6 (4-7)
Palestine	5 (4-7)	4 (4-5)	4 (3-4)	3 (2-3)	2 (2-3)	2 (2-2)	2 (2-2)	2 (1-2)	2 (1-2)	1 (1-2)	2 (1-2)	2 (2-2)	1 (1-2)	1 (1-1)
Oman	6 (5-7)	6 (5-7)	6 (5-7)	5 (5-6)	4 (4-5)	3 (3-4)	3 (2-3)	2 (1-2)	1 (1-1)	1 (1-1)	0 (0-0)	0 (0-0)	0 (0-0)	0 (0-0)
Qatar	0 (0-0)	0 (0-0)	0 (0-0)	0 (0-0)	0 (0-0)	0 (0-0)	0 (0-0)	0 (0-0)	0 (0-0)	0 (0-0)	0 (0-0)	0 (0-0)	0 (0-0)	0 (0-0)
Saudi Arabia	19 (15-24)	19 (16-22)	19 (16-22)	18 (15-22)	18 (15-22)	17 (14-20)	17 (14-20)	17 (15-21)	16 (13-20)	12 (9-15)	8 (6-10)	5 (4-7)	4 (3-5)	2 (1-3)
Sudan	31 (24-39)	22 (19-26)	17 (14-19)	21 (19-23)	33 (30-35)	50 (46-54)	64 (58-70)	89 (83-95)	62 (56-67)	56 (50-61)	50 (46-56)	46 (41-50)	41 (36-46)	30 (23-39)
Syria	20 (17-25)	18 (15-20)	15 (13-17)	13 (12-15)	12 (11-13)	11 (10-12)	9 (8-10)	8 (7-9)	7 (6-9)	5 (5-6)	4 (4-4)	3 (3-3)	3 (3-3)	3 (3-3)
Tunisia	27 (22-32)	23 (21-26)	21 (19-23)	18 (16-19)	15 (14-16)	12 (11-13)	9 (8-10)	7 (7-8)	5 (5-6)	3 (3-4)	2 (2-2)	2 (1-2)	1 (1-2)	1 (1-1)
Turkey	159 (131-190)	159 (143-175)	161 (147-176)	146 (134-160)	124 (115-136)	107 (100-115)	87 (80-93)	68 (63-74)	51 (47-56)	40 (36-44)	29 (26-33)	19 (16-22)	13 (11-15)	8 (6-9)
United Arab Emirates	0 (0-1)	0 (0-0)	0 (0-0)	0 (0-0)	0 (0-1)	1 (0-1)	1 (1-1)	1 (1-1)	0 (0-1)	0 (0-0)	0 (0-0)	0 (0-0)	1 (1-1)	0 (0-1)
Yemen	55 (45-66)	55 (48-62)	54 (49-60)	54 (50-59)	52 (49-56)	48 (45-51)	45 (41-48)	43 (40-46)	42 (39-46)	40 (37-44)	36 (33-39)	30 (27-34)	25 (22-29)	21 (17-27)
South Asia	2240 (2070-2420)	2310 (2210-2430)	2440 (2350-2540)	2640 (2540-2740)	2400 (2340-2480)	2410 (2350-2480)	2310 (2250-2380)	2150 (2100-2210)	1970 (1920-2020)	1780 (1740-1830)	1550 (1500-1600)	1310 (1270-1350)	1070 (1030-1120)	738 (697-785)
South Asia	2240 (2070-2420)	2310 (2210-2430)	2440 (2350-2540)	2640 (2540-2740)	2400 (2340-2480)	2410 (2350-2480)	2310 (2250-2380)	2150 (2100-2210)	1970 (1920-2020)	1780 (1740-1830)	1550 (1500-1600)	1310 (1270-1350)	1070 (1030-1120)	738 (697-785)
Bangladesh	348 (293-410)	315 (282-349)	291 (261-321)	267 (246-289)	280 (258-302)	271 (248-294)	307 (286-328)	325 (305-346)	271 (253-289)	207 (192-223)	165 (151-179)	130 (119-141)	87 (79-96)	51 (42-60)
Bhutan	2 (1-2)	2 (1-2)	2 (1-2)	2 (1-2)	2 (1-2)	1 (1-2)	1 (1-2)	1 (1-2)	1 (1-1)	1 (1-1)	1 (1-1)	0 (0-1)	0 (0-0)	0 (0-0)
India	1600 (1460-1770)	1720 (1630-1830)	1880 (1800-1980)	2120 (2020-2210)	1850 (1790-1910)	1830 (1770-1890)	1670 (1620-1730)	1490 (1440-1540)	1380 (1340-1430)	1280 (1240-1320)	1110 (1070-1150)	924 (893-958)	764 (731-798)	516 (488-549)
Andhra Pradesh	60 (43-81)	63 (50-77)	67 (53-84)	76 (65-87)	66 (59-74)	68 (60-76)	60 (54-66)	53 (47-60)	49 (45-55)	41 (37-46)	32 (29-35)	25 (22-28)	18 (16-21)	10 (8-13)
Arunachal Pradesh	1 (1-1)	1 (1-1)	1 (1-1)	2 (1-2)	1 (1-2)	1 (1-2)	1 (1-1)	1 (1-1)	1 (1-1)	1 (1-1)	1 (1-1)	1 (1-1)	1 (0-1)	0 (0-0)
Assam	32 (24-42)	35 (29-42)	40 (35-46)	47 (40-55)	42 (38-47)	40 (36-45)	38 (34-42)	35 (32-39)	33 (29-36)	30 (27-34)	26 (24-29)	23 (20-27)	23 (20-27)	19 (16-23)
Bihar	132 (100-172)	141 (119-166)	154 (133-179)	176 (154-200)	171 (153-191)	182 (162-204)	180 (161-199)	165 (150-181)	152 (138-167)	150 (135-164)	136 (124-149)	119 (106-134)	102 (91-113)	73 (63-87)
Chhattisgarh	18 (13-23)	19 (16-23)	22 (18-25)	27 (24-31)	24 (21-29)	26 (22-29)	25 (22-28)	24 (21-27)	23 (21-25)	24 (21-26)	24 (21-26)	21 (19-24)	19 (17-21)	13 (11-15)
Delhi	8 (6-11)	9 (7-10)	9 (8-11)	12 (10-13)	9 (8-10)	9 (8-10)	9 (8-10)	8 (7-9)	9 (8-10)	9 (8-10)	8 (7-9)	6 (5-7)	5 (4-6)	4 (3-5)
Goa	2 (1-2)	1 (1-2)	1 (1-2)	1 (1-2)	1 (1-1)	1 (1-1)	1 (1-1)	1 (1-1)	1 (0-1)	0 (0-1)	0 (0-0)	0 (0-0)	0 (0-0)	0 (0-0)
Gujarat	56 (43-72)	60 (51-70)	66 (58-74)	76 (68-85)	63 (57-69)	63 (58-69)	62 (57-67)	57 (52-62)	55 (51-60)	49 (44-53)	42 (39-46)	34 (30-38)	26 (23-29)	19 (17-22)
Haryana	17 (13-22)	19 (16-23)	23 (20-26)	30 (26-33)	27 (24-30)	26 (23-29)	23 (21-25)	22 (20-24)	22 (20-24)	21 (19-23)	20 (18-22)	16 (15-18)	14 (12-15)	9 (8-11)
Himachal Pradesh	6 (5-9)	7 (5-8)	7 (6-8)	8 (7-9)	7 (6-8)	7 (6-8)	6 (5-7)	5 (4-6)	4 (4-5)	4 (3-4)	3 (3-4)	3 (2-3)	2 (2-2)	1 (1-2)
Jammu and Kashmir	10 (7-13)	10 (8-11)	10 (8-11)	12 (10-13)	9 (8-10)	10 (9-12)	10 (9-11)	9 (8-10)	9 (8-10)	8 (7-9)	7 (7-8)	6 (5-6)	5 (4-6)	4 (3-4)
Jharkhand	38 (27-51)	41 (33-49)	45 (37-53)	51 (44-59)	44 (37-53)	44 (38-52)	41 (36-48)	39 (35-43)	35 (32-39)	34 (31-38)	32 (28-35)	26 (23-29)	21 (18-24)	14 (12-17)
Karnataka	74 (58-94)	78 (67-91)	84 (73-96)	94 (82-106)	77 (69-85)	71 (64-78)	61 (55-67)	51 (47-55)	44 (40-47)	37 (34-41)	32 (30-34)	26 (23-28)	20 (18-23)	13 (11-15)
Kerala	44 (33-58)	40 (33-47)	37 (32-43)	37 (33-42)	25 (23-28)	19 (17-22)	15 (13-16)	11 (10-13)	9 (8-11)	7 (7-8)	6 (5-7)	4 (4-5)	3 (3-3)	2 (2-3)
Madhya Pradesh	100 (76-131)	110 (93-128)	123 (107-142)	142 (125-160)	127 (115-140)	132 (118-148)	126 (113-139)	116 (107-126)	111 (101-122)	106 (97-116)	93 (86-100)	78 (70-86)	65 (58-73)	39 (34-46)
Maharashtra	120 (88-159)	125 (104-148)	132 (116-152)	145 (124-168)	119 (106-133)	113 (102-124)	91 (82-100)	75 (69-81)	76 (69-83)	64 (57-70)	53 (49-58)	42 (36-49)	32 (27-38)	21 (18-25)
Manipur	2 (1-2)	2 (2-2)	2 (2-2)	3 (2-3)	2 (2-2)	2 (2-2)	2 (2-2)	2 (1-2)	2 (1-2)	2 (1-2)	1 (1-1)	1 (1-1)	1 (1-1)	1 (1-1)
Meghalaya	1 (1-2)	1 (1-2)	1 (1-2)	2 (2-2)	2 (1-2)	2 (2-2)	2 (2-2)	2 (2-2)	2 (2-2)	2 (2-2)	2 (2-2)	2 (1-2)	2 (1-2)	1 (1-2)
Mizoram	0 (0-0)	0 (0-0)	0 (0-0)	0 (0-1)	0 (0-0)	0 (0-0)	0 (0-1)	0 (0-1)	0 (0-0)	0 (0-0)	0 (0-0)	0 (0-0)	0 (0-0)	0 (0-0)
Nagaland	1 (1-1)	1 (1-1)	1 (1-1)	1 (1-2)	1 (1-1)	1 (1-1)	1 (1-1)	1 (1-1)	1 (1-1)	1 (1-2)	1 (1-2)	1 (1-1)	1 (1-1)	1 (1-1)
Odisha	74 (55-98)	80 (66-95)	87 (75-101)	96 (84-108)	85 (76-94)	82 (73-92)	72 (66-79)	62 (55-69)	56 (51-62)	49 (44-54)	40 (37-44)	33 (29-37)	27 (24-30)	18 (16-22)
Punjab	21 (16-27)	22 (19-26)	24 (21-27)	28 (24-31)	22 (19-24)	22 (20-25)	21 (19-23)	20 (18-22)	21 (19-23)	18 (17-20)	16 (15-17)	13 (12-15)	10 (9-12)	7 (6-8)
Rajasthan	81 (60-109)	91 (76-109)	103 (89-121)	120 (104-138)	105 (94-117)	107 (96-119)	104 (93-115)	99 (89-110)	95 (86-105)	90 (82-98)	78 (70-85)	66 (61-72)	59 (54-64)	42 (37-50)
Sikkim	0 (0-1)	0 (0-0)	0 (0-1)	1 (0-1)	0 (0-0)	0 (0-1)	0 (0-0)	0 (0-0)	0 (0-0)	0 (0-0)	0 (0-0)	0 (0-0)	0 (0-0)	0 (0-0)

Table 3 Under-5 death numbers by location and year for 1950, 1955...2005, 2010, 2017 - females

Location	1950	1955	1960	1965	1970	1975	1980	1985	1990	1995	2000	2005	2010	2017
Tamil Nadu	125 (94-163)	124 (104-147)	126 (111-143)	135 (120-151)	114 (104-124)	105 (95-115)	83 (76-91)	62 (56-67)	49 (45-54)	40 (36-43)	32 (29-34)	23 (20-25)	15 (13-18)	8 (7-9)
Telangana	29 (21-40)	32 (25-39)	35 (28-44)	41 (35-49)	35 (31-39)	36 (31-41)	31 (27-36)	28 (24-33)	29 (25-33)	28 (24-32)	25 (22-28)	20 (17-23)	13 (11-16)	7 (5-9)
Tripura	3 (2-4)	4 (3-5)	5 (4-6)	6 (5-6)	5 (4-5)	5 (4-5)	4 (4-5)	3 (3-4)	3 (3-3)	2 (2-3)	2 (2-2)	2 (1-2)	1 (1-2)	1 (1-2)
Uttar Pradesh	434 (316-575)	481 (403-561)	542 (480-615)	591 (516-671)	530 (479-580)	525 (476-570)	489 (446-537)	440 (404-480)	403 (369-438)	384 (353-414)	340 (308-376)	287 (264-313)	244 (219-271)	162 (141-190)
Uttarakhand	5 (4-7)	6 (5-7)	6 (5-7)	8 (7-9)	7 (6-8)	7 (6-9)	7 (6-8)	7 (6-8)	7 (6-7)	6 (6-7)	6 (6-7)	5 (5-6)	4 (4-5)	2 (2-3)
West Bengal	108 (80-144)	118 (99-138)	131 (115-150)	147 (128-167)	125 (113-140)	120 (108-134)	102 (93-112)	89 (82-97)	80 (73-88)	65 (59-72)	51 (46-55)	40 (36-45)	31 (26-36)	21 (18-25)
Union Territories other than Delhi	1 (1-1)	1 (1-1)	1 (1-1)	2 (1-2)	1 (1-1)	1 (1-1)	1 (1-1)	1 (1-1)	1 (1-1)	1 (1-1)	1 (1-1)	1 (1-1)	1 (1-1)	1 (1-1)
Nepal	68 (58-79)	66 (59-72)	64 (57-71)	61 (55-67)	56 (52-61)	57 (52-62)	61 (57-66)	60 (55-64)	50 (46-54)	39 (36-42)	28 (26-30)	20 (18-22)	14 (12-16)	9 (7-11)
Pakistan	214 (180-252)	206 (185-227)	199 (181-217)	191 (174-206)	219 (202-236)	254 (235-274)	273 (251-296)	274 (254-294)	266 (249-285)	259 (239-279)	243 (221-266)	236 (212-264)	207 (180-238)	162 (132-194)
Southeast Asia, East Asia, and Oceania	3170 (3040-3310)	3020 (2930-3110)	9520 (9400-9640)	2710 (2630-2790)	2230 (2170-2300)	1630 (1580-1680)	1230 (1190-1260)	1290 (1250-1330)	1040 (1000-1070)	790 (767-812)	581 (566-595)	398 (388-409)	293 (284-302)	192 (184-202)
East Asia	2030 (1920-2150)	2000 (1920-2080)	8590 (8480-8710)	1870 (1800-1940)	1490 (1430-1550)	957 (912-1000)	652 (623-682)	799 (760-837)	637 (608-669)	467 (448-486)	313 (303-324)	183 (177-189)	122 (117-126)	73 (69-77)
China	1940 (1820-2050)	1940 (1860-2020)	8530 (8420-8650)	1820 (1750-1890)	1450 (1390-1510)	931 (887-977)	634 (605-663)	779 (740-816)	620 (591-652)	432 (414-452)	283 (273-293)	173 (167-179)	115 (111-119)	69 (65-72)
North Korea	43 (38-51)	17 (13-22)	16 (13-20)	13 (11-16)	9 (7-12)	6 (5-8)	4 (4-6)	5 (4-6)	5 (4-6)	26 (23-28)	25 (23-27)	6 (4-7)	4 (3-6)	3 (2-4)
Taiwan (Province of China)	24 (22-27)	16 (16-17)	12 (12-12)	8 (7-8)	5 (5-5)	3 (3-3)	3 (3-3)	2 (2-2)	1 (1-1)	1 (1-1)	1 (1-1)	1 (1-1)	0 (0-0)	0 (0-0)
Oceania	11 (9-14)	10 (9-12)	10 (9-11)	9 (7-10)	7 (6-8)	6 (5-7)	5 (5-6)	6 (5-6)	5 (5-6)	6 (5-7)	7 (6-8)	8 (6-9)	8 (6-9)	7 (5-9)
American Samoa	0 (0-0)	0 (0-0)	0 (0-0)	0 (0-0)	0 (0-0)	0 (0-0)	0 (0-0)	0 (0-0)	0 (0-0)	0 (0-0)	0 (0-0)	0 (0-0)	0 (0-0)	0 (0-0)
Federated States of Micronesia	0 (0-0)	0 (0-0)	0 (0-0)	0 (0-0)	0 (0-0)	0 (0-0)	0 (0-0)	0 (0-0)	0 (0-0)	0 (0-0)	0 (0-0)	0 (0-0)	0 (0-0)	0 (0-0)
Fiji	1 (1-1)	1 (1-1)	1 (1-1)	1 (0-1)	0 (0-1)	0 (0-0)	0 (0-0)	0 (0-0)	0 (0-0)	0 (0-0)	0 (0-0)	0 (0-0)	0 (0-0)	0 (0-0)
Guam	0 (0-0)	0 (0-0)	0 (0-0)	0 (0-0)	0 (0-0)	0 (0-0)	0 (0-0)	0 (0-0)	0 (0-0)	0 (0-0)	0 (0-0)	0 (0-0)	0 (0-0)	0 (0-0)
Kiribati	0 (0-0)	0 (0-0)	0 (0-0)	0 (0-0)	0 (0-0)	0 (0-0)	0 (0-0)	0 (0-0)	0 (0-0)	0 (0-0)	0 (0-0)	0 (0-0)	0 (0-0)	0 (0-0)
Marshall Islands	0 (0-0)	0 (0-0)	0 (0-0)	0 (0-0)	0 (0-0)	0 (0-0)	0 (0-0)	0 (0-0)	0 (0-0)	0 (0-0)	0 (0-0)	0 (0-0)	0 (0-0)	0 (0-0)
Northern Mariana Islands	0 (0-0)	0 (0-0)	0 (0-0)	0 (0-0)	0 (0-0)	0 (0-0)	0 (0-0)	0 (0-0)	0 (0-0)	0 (0-0)	0 (0-0)	0 (0-0)	0 (0-0)	0 (0-0)
Papua New Guinea	8 (7-11)	8 (7-9)	8 (6-9)	6 (5-8)	5 (4-6)	5 (4-5)	4 (3-5)	4 (4-5)	4 (4-5)	5 (4-5)	5 (4-6)	6 (5-8)	6 (5-8)	6 (4-8)
Samoa	0 (0-0)	0 (0-0)	0 (0-0)	0 (0-0)	0 (0-0)	0 (0-0)	0 (0-0)	0 (0-0)	0 (0-0)	0 (0-0)	0 (0-0)	0 (0-0)	0 (0-0)	0 (0-0)
Solomon Islands	0 (0-1)	0 (0-1)	0 (0-1)	0 (0-0)	0 (0-0)	0 (0-0)	0 (0-0)	0 (0-0)	0 (0-0)	0 (0-0)	0 (0-0)	0 (0-0)	0 (0-0)	0 (0-0)
Tonga	0 (0-0)	0 (0-0)	0 (0-0)	0 (0-0)	0 (0-0)	0 (0-0)	0 (0-0)	0 (0-0)	0 (0-0)	0 (0-0)	0 (0-0)	0 (0-0)	0 (0-0)	0 (0-0)
Vanuatu	0 (0-0)	0 (0-0)	0 (0-0)	0 (0-0)	0 (0-0)	0 (0-0)	0 (0-0)	0 (0-0)	0 (0-0)	0 (0-0)	0 (0-0)	0 (0-0)	0 (0-0)	0 (0-0)
Southeast Asia	1130 (1070-1200)	1010 (970-1050)	917 (883-950)	830 (799-861)	737 (711-766)	666 (644-687)	568 (548-588)	487 (469-506)	395 (382-409)	317 (306-329)	261 (251-272)	208 (200-217)	163 (156-171)	112 (105-121)
Cambodia	25 (19-32)	24 (20-30)	24 (20-29)	24 (20-28)	27 (24-31)	47 (44-51)	25 (22-27)	25 (23-26)	23 (21-24)	20 (19-21)	15 (14-16)	10 (9-11)	7 (6-8)	5 (4-6)
Indonesia	638 (597-682)	555 (535-574)	489 (474-504)	434 (420-448)	367 (356-378)	327 (319-336)	287 (281-294)	235 (229-241)	177 (173-182)	136 (132-140)	111 (108-115)	87 (84-90)	69 (66-72)	44 (41-47)
Laos	16 (12-20)	16 (13-18)	16 (13-18)	16 (13-18)	15 (13-18)	14 (12-17)	14 (12-16)	13 (11-15)	13 (11-14)	11 (10-13)	10 (9-11)	8 (7-10)	6 (5-8)	4 (4-6)
Malaysia	16 (14-18)	14 (14-15)	13 (13-14)	10 (10-10)	8 (8-8)	7 (6-7)	5 (5-6)	5 (4-5)	4 (4-4)	3 (3-3)	2 (2-2)	2 (2-2)	2 (1-2)	2 (1-2)
Maldives	1 (0-1)	1 (1-1)	1 (1-1)	1 (0-1)	0 (0-1)	0 (0-0)	0 (0-0)	0 (0-0)	0 (0-0)	0 (0-0)	0 (0-0)	0 (0-0)	0 (0-0)	0 (0-0)
Mauritius	1 (1-2)	1 (1-1)	1 (1-1)	1 (1-1)	1 (1-1)	1 (1-1)	0 (0-0)	0 (0-0)	0 (0-0)	0 (0-0)	0 (0-0)	0 (0-0)	0 (0-0)	0 (0-0)
Myanmar	147 (113-187)	140 (117-165)	134 (113-156)	124 (103-148)	112 (93-134)	95 (79-112)	86 (71-102)	78 (63-93)	71 (60-82)	63 (53-74)	54 (47-63)	40 (35-47)	27 (23-31)	17 (13-21)
Philippines	82 (68-98)	77 (69-86)	75 (67-83)	73 (66-80)	71 (64-78)	67 (61-72)	64 (58-71)	60 (54-66)	52 (46-57)	42 (38-46)	39 (35-44)	37 (33-42)	35 (31-41)	28 (23-34)
Sri Lanka	25 (23-28)	20 (19-21)	16 (15-16)	13 (13-14)	12 (11-12)	12 (11-12)	9 (8-9)	6 (6-6)	5 (5-6)	4 (3-4)	3 (3-3)	3 (3-3)	2 (2-2)	1 (1-2)
Seychelles	0 (0-0)	0 (0-0)	0 (0-0)	0 (0-0)	0 (0-0)	0 (0-0)	0 (0-0)	0 (0-0)	0 (0-0)	0 (0-0)	0 (0-0)	0 (0-0)	0 (0-0)	0 (0-0)
Thailand	79 (63-100)	72 (63-82)	67 (60-73)	59 (55-65)	50 (45-55)	37 (33-41)	28 (25-31)	20 (17-23)	14 (12-16)	11 (9-12)	8 (7-10)	6 (5-7)	4 (3-4)	3 (2-3)
Timor-Leste	3 (3-4)	3 (3-4)	3 (3-4)	3 (2-3)	2 (2-3)	2 (2-3)	2 (2-3)	2 (2-2)	2 (2-2)	2 (2-2)	1 (1-2)	1 (1-1)	1 (1-1)	1 (0-1)
Vietnam	97 (71-131)	84 (66-105)	77 (61-94)	72 (60-84)	70 (61-80)	55 (48-63)	47 (42-51)	43 (38-47)	35 (32-38)	24 (22-27)	17 (15-18)	13 (11-15)	11 (9-13)	7 (6-9)
Sub-Saharan Africa	1350 (1260-1440)	1340 (1280-1410)	1350 (1300-1410)	1370 (1320-1410)	1420 (1380-1460)	1470 (1430-1510)	1520 (1490-1560)	1600 (1570-1640)	1690 (1660-1720)	1780 (1740-1810)	1820 (1780-1860)	1710 (1660-1750)	1520 (1470-1580)	1240 (1170-1330)
Central sub-Saharan Africa	152 (128-180)	155 (137-175)	161 (141-181)	165 (145-187)	165 (145-185)	169 (150-188)	176 (162-194)	183 (171-196)	198 (186-209)	216 (203-230)	231 (216-248)	230 (214-246)	214 (195-234)	161 (138-188)
Angola	37 (29-47)	36 (30-43)	36 (30-43)	34 (28-41)	33 (28-39)	35 (29-41)	39 (34-45)	45 (40-50)	52 (47-56)	56 (52-61)	56 (51-62)	53 (48-58)	43 (38-48)	30 (25-37)
Central African Republic	9 (6-11)	8 (7-10)	8 (7-10)	8 (6-9)	8 (7-9)	8 (7-9)	8 (7-9)	8 (8-9)	9 (8-10)	10 (9-11)	12 (10-14)	13 (11-16)	13 (11-16)	10 (8-14)
Congo (Brazzaville)	7 (5-8)	6 (5-8)	6 (5-7)	6 (5-7)	6 (5-7)	5 (4-6)	5 (4-5)	4 (4-5)	4 (4-5)	5 (5-6)	6 (5-7)	5 (5-6)	4 (4-5)	3 (2-4)
DR Congo	95 (73-121)	99 (83-118)	106 (88-126)	113 (94-135)	114 (96-135)	117 (100-136)	121 (107-138)	123 (112-134)	130 (119-140)	142 (129-156)	154 (140-169)	156 (142-171)	152 (134-171)	116 (95-141)
Equatorial Guinea	2 (2-3)	2 (2-2)	2 (1-2)	2 (1-2)	1 (1-2)	1 (1-2)	1 (1-1)	1 (1-2)	2 (1-2)	2 (2-2)	2 (1-2)	1 (1-2)	1 (1-2)	1 (1-1)
Gabon	3 (2-3)	3 (2-3)	3 (2-3)	2 (2-3)	2 (2-3)	2 (1-2)	1 (1-2)	1 (1-1)	1 (1-1)	1 (1-1)	1 (1-1)	1 (1-1)	1 (1-1)	1 (1-1)
Eastern sub-Saharan Africa	456 (425-489)	462 (439-486)	470 (449-491)	478 (461-496)	500 (485-516)	544 (531-558)	578 (565-591)	626 (614-638)	664 (651-676)	685 (670-699)	660 (647-674)	563 (549-577)	479 (464-495)	379 (359-400)
Burundi	21 (17-27)	21 (17-24)	20 (17-23)	19 (17-22)	19 (17-21)	20 (18-22)	21 (19-23)	20 (18-22)	20 (18-22)	22 (20-24)	21 (20-23)	18 (17-20)	14 (13-16)	14 (12-17)
Comoros	1 (1-1)	1 (1-1)	1 (1-1)	1 (1-1)	1 (1-1)	1 (1-1)	1 (1-1)	1 (1-1)	1 (1-1)	1 (1-1)	1 (1-1)	1 (1-1)	1 (0-1)	0 (0-0)
Djibouti	0 (0-0)	0 (0-0)	0 (0-0)	0 (0-0)	0 (0-1)	0 (0-1)	1 (1-1)	1 (1-1)	1 (1-1)	1 (1-1)	1 (1-1)	1 (1-1)	1 (1-1)	0 (0-1)
Eritrea	8 (6-10)	8 (7-10)	9 (7-11)	10 (9-12)	11 (9-12)	11 (10-13)	12 (11-13)	12 (11-13)	14 (13-15)	9 (8-10)	8 (6-9)	7 (5-8)	6 (5-8)	5 (3-6)
Ethiopia	122 (106-141)	120 (109-133)	119 (108-130)	120 (111-130)	127 (120-135)	144 (137-152)	143 (136-150)	162 (156-169)	188 (181-196)	202 (193-211)	193 (185-200)	156 (149-164)	121 (114-128)	85 (77-94)
Kenya	37 (35-38)	36 (35-36)	34 (34-35)	37 (36-38)	40 (39-41)	40 (39-41)	38 (37-39)	38 (37-39)	39 (38-40)	43 (42-44)	44 (43-45)	40 (39-41)	34 (33-35)	27 (26-28)
Baringo	0 (0-0)	0 (0-0)	0 (0-0)	0 (0-0)	1 (1-1)	1 (1-1)	1 (1-1)	1 (1-1)	1 (1-1)	1 (1-1)	1 (1-1)	1 (0-1)	0 (0-0)	0 (0-0)
Bomet	0 (0-0)	0 (0-0)	0 (0-0)	0 (0-0)	0 (0-0)	0 (0-0)	0 (0-0)	0 (0-0)	0 (0-0)	0 (0-0)	0 (0-0)	0 (0-0)	1 (0-1)	0 (0-1)
Bungoma	1 (1-2)	1 (1-1)	1 (1-1)	1 (1-2)	2 (1-2)	2 (1-2)	2 (1-2)	2 (1-2)	2 (1-2)	2 (1-2)	2 (1-2)	2 (1-2)	1 (1-1)	1 (1-1)
Busia	1 (1-1)	1 (1-1)	1 (1-1)	1 (1-1)	1 (1-1)	1 (1-1)	1 (1-1)	1 (1-1)	1 (1-1)	1 (1-2)	1 (1-2)	1 (1-1)	1 (1-1)	1 (1-1)
Elgeyo Marakwet	0 (0-0)	0 (0-0)	0 (0-0)	0 (0-0)	0 (0-0)	0 (0-0)	0 (0-0)	0 (0-0)	0 (0-0)	0 (0-0)	0 (0-0)	0 (0-0)	0 (0-0)	0 (0-0)
Embu	1 (1-1)	1 (1-1)	1 (1-1)	1 (1-1)	1 (1-1)	0 (0-1)	0 (0-0)	0 (0-0)	0 (0-0)	0 (0-0)	0 (0-0)	0 (0-0)	0 (0-0)	0 (0-0)
Garissa	0 (0-0)	0 (0-0)	0 (0-0)	0 (0-0)	0 (0-0)	0 (0-0)	0 (0-0)	0 (0-0)	0 (0-0)	0 (0-1)	1 (1-1)	1 (1-1)	1 (1-1)	1 (1-1)
Homa Bay	2 (2-3)	2 (2-2)	2 (2-2)	2 (2-3)	3 (2-3)	3 (3-3)	3 (2-3)	3 (2-3)	3 (2-3)	3 (3-3)	3 (3-4)	3 (3-3)	2 (2-2)	1 (1-2)
Isiolo	0 (0-0)	0 (0-0)	0 (0-0)	0 (0-0)	0 (0-0)	0 (0-0)	0 (0-0)	0 (0-0)	0 (0-0)	0 (0-0)	0 (0-0)	0 (0-0)	0 (0-0)	0 (0-0)
Kajiado	0 (0-0)	0 (0-0)	0 (0-0)	0 (0-0)	0 (0-0)	0 (0-0)	0 (0-0)	0 (0-0)	0 (0-0)	0 (0-0)	0 (0-0)	0 (0-1)	0 (0-0)	0 (0-0)
Kakamega	3 (2-3)	3 (2-3)	2 (2-3)	3 (2-3)	3 (3-3)	3 (2-3)	3 (2-3)	3 (3-3)	3 (3-3)	3 (3-4)	3 (3-3)	3 (3-3)	2 (2-3)	2 (2-2)
Kericho	0 (0-0)	0 (0-0)	0 (0-0)	0 (0-0)	0 (0-0)	0 (0-0)	0 (0-0)	0 (0-0)	0 (0-0)	0 (0-0)	0 (0-1)	0 (0-0)	0 (0-0)	0 (0-0)
Kiambu	1 (1-1)	1 (1-1)	1 (1-1)	1 (1-1)	1 (1-1)	1 (1-1)	1 (1-1)	1 (1-1)	1 (1-1)	1 (1-1)	1 (1-1)	1 (1-1)	1 (1-1)	1 (1-1)
Kilifi	2 (2-2)	2 (2-2)	2 (2-2)	2 (2-2)	2 (2-3)	2 (2-2)	2 (2-2)	2 (2-2)	2 (2-2)	2 (2-2)	2 (2-2)	2 (1-2)	1 (1-1)	1 (1-1)
Kirinyaga	1 (1-1)	1 (1-1)	1 (1-1)	1 (1-1)	1 (1-1)	1 (1-1)	0 (0-1)	0 (0-0)	0 (0-0)	0 (0-0)	0 (0-1)	0 (0-0)	0 (0-0)	0 (0-0)
Kisii	2 (1-2)	2 (1-2)	2 (1-2)	2 (1-2)	2 (2-2)	2 (2-2)	2 (2-2)	2 (2-2)	2 (2-2)	2 (2-2)	2 (2-2)	1 (1-2)	1 (1-1)	1 (1-1)
Kisumu	2 (2-3)	2 (2-3)	2 (2-2)	2 (2-3)	3 (2-3)	3 (2-3)	2 (2-3)	2 (2-3)	2 (2-3)	3 (2-3)	2 (2-3)	2 (1-2)	1 (1-1)	1 (1-1)
Kitui	1 (1-1)	1 (1-1)	1 (1-1)	1 (1-1)	1 (1-1)	1 (1-1)	1 (1-1)	1 (1-1)	1 (1-1)	1 (1-1)	1 (1-1)	1 (1-1)	1 (1-1)	0 (0-1)
Kwale	1 (1-1)	1 (1-1)	1 (1-1)	1 (1-1)	1 (1-1)	1 (1-1)	1 (1-1)	1 (1-1)	1 (1-1)	1 (1-1)	1 (1-1)	1 (1-1)	0 (0-1)	0 (0-0)
Laikipia	0 (0-0)	0 (0-0)	0 (0-0)	0 (0-0)	0 (0-0)	0 (0-0)	0 (0-0)	0 (0-0)	0 (0-0)	0 (0-0)	0 (0-0)	0 (0-0)	0 (0-0)	0 (0-0)
Lamu	0 (0-0)	0 (0-0)	0 (0-0)	0 (0-0)	0 (0-0)	0 (0-0)	0 (0-0)	0 (0-0)	0 (0-0)	0 (0-0)	0 (0-0)	0 (0-0)	0 (0-0)	0 (0-0)
Machakos	1 (1-2)	1 (1-1)	1 (1-1)	1 (1-1)	1 (1-2)	1 (1-1)	1 (1-1)	1 (1-1)	1 (1-1)	1 (1-1)	1 (1-1)	1 (1-1)	1 (1-1)	1 (1-1)
Makueni	0 (0-1)	0 (0-1)	1 (0-1)	1 (1-1)	1 (1-1)	1 (1-1)	1 (1-1)	1 (1-1)	1 (1-1)	1 (1-1)	1 (1-1)	1 (0-1)	0 (0-0)	0 (0-0)
Mandera	0 (0-0)	0 (0-0)	0 (0-0)	0 (0-0)	0 (0-0)	0 (0-0)	0 (0-0)	0 (0-0)	0 (0-0)	0 (0-0)	0 (0-0)	1 (1-1)	1 (1-1)	1 (1-1)
Marsabit	1 (1-1)	1 (1-1)	1 (1-1)	1 (1-1)	1 (1-1)	1 (1-1)	0 (0-1)	0 (0-0)	0 (0-0)	0 (0-0)	0 (0-0)	0 (0-0)	0 (0-0)	0 (0-0)
Meru	2 (1-2)	2 (1-2)	1 (1-2)	1 (1-2)	1 (1-2)	1 (1-1)	1 (1-1)	1 (1-1)	1 (1-1)	1 (1-1)	1 (1-1)	1 (1-1)	1 (1-1)	1 (1-1)
Migori	1 (1-2)	2 (1-2)	2 (1-2)	2 (2-2)	2 (2-2)	3 (2-3)	3 (3-3)	3 (3-4)	3 (3-3)	3 (3-4)	4 (3-4)	3 (3-3)	2 (2-3)	2 (2-2)
Mombasa	1 (1-1)	1 (1-1)	1 (1-1)	1 (1-1)	1 (0-1)	0 (0-1)	0 (0-1)	0 (0-1)	1 (0-1)	1 (1-1)	1 (1-1)	1 (1-1)	1 (1-1)	1 (1-1)
Murang'a	1 (1-1)	1 (1-1)	1 (1-1)	1 (1-1)	1 (1-1)	1 (1-1)	1 (1-1)	1 (0-1)	1 (0-1)	0 (0-1)	0 (0-0)	0 (0-0)	0 (0-0)	0 (0-0)
Nairobi	2 (2-3)	2 (2-2)	1 (1-2)	1 (1-1)	1 (1-1)	1 (1-1)	1 (1-1)	1 (1-1)	1 (1-1)	2 (1-2)	2 (2-2)	2 (1-2)	1 (1-2)	1 (1-1)
Nakuru	1 (0-1)	1 (0-1)	1 (0-1)	1 (0-1)	1 (1-1)	1 (1-1)	1 (1-1)	1 (1-1)	1 (1-1)	1 (1-1)	1 (1-2)	1 (1-1)	1 (1-1)	1 (1-1)
Nandi	0 (0-0)	0 (0-0)	0 (0-0)	0 (0-0)	0 (0-0)	0 (0-0)	0 (0-1)	0 (0-1)	0 (0-1)	0 (0-1)	0 (0-1)	0 (0-0)	0 (0-0)	0 (0-0)
Narok	0 (0-0)	0 (0-0)	0 (0-0)	0 (0-0)	0 (0-0)	0 (0-0)	0 (0-0)	1 (0-1)	1 (1-1)	1 (0-1)	0 (0-1)	0 (0-1)	0 (0-1)	0 (0-0)
Nyamira	0 (0-0)	0 (0-0)	0 (0-0)	0 (0-0)	0 (0-0)	0 (0-0)	0 (0-1)	0 (0-0)	0 (0-0)	0 (0-1)	1 (0-1)	0 (0-1)	0 (0-0)	0 (0-0)
Nyandarua	0 (0-0)	0 (0-0)	0 (0-0)	0 (0-0)	0 (0-0)	0 (0-0)	0 (0-0)	0 (0-0)	0 (0-0)	0 (0-0)	0 (0-0)	0 (0-0)	0 (0-0)	0 (0-0)
Nyeri	1 (1-1)	1 (1-1)	1 (1-1)	1 (1-1)	1 (1-1)	1 (1-1)	0 (0-1)	0 (0-0)	0 (0-0)	0 (0-0)	0 (0-0)	0 (0-0)	0 (0-0)	0 (0-0)
Samburu	0 (0-0)	0 (0-0)	0 (0-0)	0 (0-0)	0 (0-0)	0 (0-0)	0 (0-0)	0 (0-0)	0 (0-0)	0 (0-0)	0 (0-0)	0 (0-0)	0 (0-0)	0 (0-0)
Siaya	1 (1-2)	1 (1-1)	1 (1-1)	1 (1-2)	1 (1-2)	2 (1-2)	1 (1-2)	2 (1-2)	2 (2-2)	2 (2-2)	2 (2-2)	2 (1-2)	1 (1-1)	1 (1-1)
Taita Taveta	1 (0-1)	0 (0-1)	0 (0-0)	0 (0-0)	0 (0-0)	0 (0-0)	0 (0-0)	0 (0-0)	0 (0-0)	0 (0-0)	0 (0-0)	0 (0-0)	0 (0-0)	0 (0-0)
Tana River	0 (0-0)	0 (0-0)	0 (0-0)	0 (0-0)	0 (0-0)	0 (0-0)	0 (0-0)	0 (0-0)	0 (0-0)	0 (0-0)	0 (0-0)	0 (0-0)	0 (0-0)	0 (0-0)
Tharaka Nithi	0 (0-0)	0 (0-0)	0 (0-0)	0 (0-0)	0 (0-0)	0 (0-0)	0 (0-0)	0 (0-0)	0 (0-0)	0 (0-0)	0 (0-0)	0 (0-0)	0 (0-0)	0 (0-0)
Trans Nzoia	0 (0-0)	0 (0-0)	0 (0-0)	0 (0-0)	0 (0-1)	1 (0-1)	1 (0-1)	1 (1-1)	1 (0-1)	1 (1-1)	1 (1-1)	1 (0-1)	1 (0-1)	0 (0-1)

Table 3 Under-5 death numbers by location and year for 1950, 1955...2005, 2010, 2017 - females

Location	1950	1955	1960	1965	1970	1975	1980	1985	1990	1995	2000	2005	2010	2017
Turkana	0 (0-0)	0 (0-0)	0 (0-0)	0 (0-0)	1 (0-1)	1 (1-1)	1 (1-1)	1 (1-1)	1 (1-1)	1 (1-1)	1 (1-1)	1 (1-2)	1 (1-2)	1 (1-1)
Uasin Gishu	0 (0-0)	0 (0-0)	0 (0-0)	0 (0-0)	0 (0-0)	0 (0-0)	0 (0-0)	0 (0-0)	0 (0-1)	1 (0-1)	1 (0-1)	0 (0-1)	0 (0-1)	0 (0-0)
Vihiga	0 (0-0)	0 (0-0)	0 (0-0)	0 (0-0)	0 (0-0)	0 (0-0)	0 (0-0)	1 (0-1)	1 (0-1)	1 (1-1)	1 (1-1)	0 (0-1)	0 (0-0)	0 (0-0)
Wajir	0 (0-0)	0 (0-0)	0 (0-0)	0 (0-0)	0 (0-1)	1 (0-1)	1 (0-1)	1 (0-1)	0 (0-1)	1 (1-1)	1 (1-1)	1 (1-1)	1 (1-1)	1 (1-1)
West Pokot	0 (0-0)	0 (0-0)	0 (0-0)	0 (0-0)	1 (0-1)	1 (1-1)	1 (0-1)	1 (1-1)	1 (1-1)	1 (1-1)	1 (0-1)	0 (0-1)	1 (0-1)	0 (0-1)
Madagascar	21 (16-28)	22 (19-27)	24 (20-28)	25 (21-29)	27 (24-30)	29 (26-31)	32 (30-34)	35 (32-37)	35 (32-38)	35 (32-39)	34 (31-38)	32 (29-36)	35 (29-41)	33 (25-44)
Malawi	34 (28-41)	36 (31-41)	38 (33-42)	39 (35-42)	39 (36-43)	39 (36-42)	39 (36-42)	40 (38-42)	47 (45-50)	45 (42-47)	37 (35-40)	30 (28-32)	24 (21-26)	17 (14-21)
Mozambique	49 (38-63)	48 (39-57)	46 (39-55)	46 (38-54)	47 (39-54)	50 (43-57)	59 (53-64)	76 (71-80)	66 (62-71)	62 (58-67)	56 (52-61)	49 (44-54)	42 (37-48)	33 (27-41)
Rwanda	21 (17-26)	21 (18-24)	21 (19-24)	21 (20-23)	22 (20-24)	24 (23-26)	24 (23-26)	23 (22-25)	25 (23-26)	26 (24-28)	29 (27-31)	17 (15-18)	11 (10-13)	9 (7-11)
Somalia	13 (10-17)	13 (11-16)	13 (11-16)	15 (12-18)	17 (14-20)	22 (19-25)	28 (25-32)	28 (25-31)	30 (27-34)	33 (29-36)	34 (30-38)	32 (28-36)	33 (28-38)	26 (20-33)
South Sudan	14 (10-18)	15 (12-18)	16 (13-19)	15 (13-18)	15 (12-18)	15 (13-18)	17 (14-19)	18 (16-21)	20 (17-23)	20 (18-23)	20 (18-23)	21 (18-23)	22 (19-26)	21 (17-26)
Tanzania	61 (47-77)	64 (52-75)	67 (56-78)	66 (58-73)	68 (62-74)	73 (68-78)	74 (69-78)	77 (73-82)	82 (76-87)	86 (81-92)	82 (76-87)	70 (65-76)	64 (56-73)	54 (44-68)
Uganda	36 (29-46)	39 (33-45)	42 (36-49)	42 (37-47)	46 (42-51)	55 (51-60)	68 (64-73)	69 (65-74)	68 (64-72)	69 (65-74)	71 (67-76)	64 (60-70)	52 (48-57)	38 (32-45)
Zambia	17 (13-21)	18 (15-22)	20 (17-23)	21 (19-24)	21 (19-23)	21 (19-22)	22 (21-24)	25 (23-27)	28 (26-30)	30 (27-32)	28 (26-31)	25 (22-28)	19 (16-23)	16 (12-20)
Southern sub-Saharan Africa	60 (54-68)	63 (58-69)	66 (61-71)	72 (68-77)	70 (66-73)	65 (62-68)	63 (60-66)	57 (54-60)	50 (48-52)	50 (47-52)	57 (54-60)	60 (57-64)	47 (44-49)	29 (27-32)
Botswana	1 (1-2)	1 (1-2)	1 (1-2)	1 (1-2)	1 (1-2)	1 (1-1)	1 (1-1)	1 (1-1)	1 (1-1)	1 (1-1)	1 (1-1)	1 (1-1)	1 (1-1)	0 (0-1)
Lesotho	2 (2-3)	2 (2-3)	3 (2-3)	3 (3-3)	3 (3-3)	3 (3-3)	3 (2-3)	2 (2-3)	2 (2-2)	2 (2-2)	2 (2-3)	2 (2-3)	2 (1-2)	1 (1-1)
Namibia	2 (1-2)	2 (1-2)	2 (1-2)	2 (1-2)	2 (2-2)	2 (2-2)	2 (2-2)	2 (2-2)	2 (1-2)	1 (1-2)	1 (1-2)	2 (1-2)	1 (1-1)	1 (1-1)
South Africa	43 (38-50)	45 (40-50)	47 (43-52)	53 (49-58)	50 (47-53)	45 (42-48)	42 (40-45)	39 (37-42)	34 (32-36)	32 (30-34)	37 (35-40)	38 (36-42)	26 (24-29)	15 (14-17)
Swaziland	2 (1-2)	2 (1-2)	2 (1-2)	1 (1-2)	2 (1-2)	2 (1-2)	1 (1-2)	1 (1-1)	1 (1-1)	1 (1-1)	1 (1-1)	1 (1-1)	1 (1-1)	1 (0-1)
Zimbabwe	10 (8-13)	10 (9-13)	11 (9-13)	12 (10-13)	12 (10-13)	12 (11-14)	13 (12-15)	11 (11-12)	11 (10-12)	12 (11-13)	14 (12-15)	15 (14-17)	16 (14-17)	11 (9-13)
Western sub-Saharan Africa	678 (603-763)	661 (606-719)	654 (606-705)	653 (614-691)	686 (653-723)	694 (664-725)	707 (679-737)	734 (707-766)	777 (750-807)	825 (797-854)	872 (836-909)	853 (814-893)	784 (739-835)	673 (607-749)
Benin	19 (15-24)	18 (15-21)	18 (16-19)	17 (15-19)	18 (16-19)	18 (16-19)	17 (16-18)	17 (16-18)	17 (16-18)	17 (16-18)	17 (15-18)	17 (15-19)	17 (15-20)	15 (12-19)
Burkina Faso	38 (30-48)	35 (29-41)	32 (27-38)	32 (28-37)	34 (31-38)	37 (34-41)	38 (36-41)	40 (37-43)	41 (39-44)	43 (40-47)	43 (40-47)	42 (38-46)	40 (35-46)	38 (31-47)
Cameroon	35 (30-42)	32 (29-36)	33 (30-36)	29 (26-31)	29 (26-31)	29 (27-31)	30 (27-32)	28 (26-30)	28 (26-31)	32 (29-35)	34 (31-38)	37 (33-41)	34 (30-39)	25 (21-30)
Cape Verde	1 (1-1)	1 (1-1)	1 (1-1)	1 (1-1)	1 (1-1)	1 (1-1)	1 (0-1)	0 (0-1)	0 (0-0)	0 (0-0)	0 (0-0)	0 (0-0)	0 (0-0)	0 (0-0)
Chad	20 (16-25)	20 (17-24)	20 (17-24)	21 (18-24)	23 (20-26)	24 (21-27)	27 (24-30)	29 (27-32)	30 (28-32)	33 (30-35)	37 (34-41)	40 (36-44)	39 (35-44)	37 (31-44)
Cote d'Ivoire	21 (17-24)	22 (20-25)	24 (22-26)	27 (24-29)	29 (27-31)	28 (27-30)	27 (26-29)	30 (27-32)	33 (31-36)	38 (35-42)	40 (36-44)	39 (35-44)	35 (30-40)	28 (24-33)
The Gambia	2 (1-2)	2 (1-2)	2 (1-2)	2 (1-2)	2 (2-2)	2 (2-2)	2 (2-2)	2 (2-2)	2 (2-2)	2 (2-2)	2 (2-2)	2 (2-2)	2 (1-2)	1 (1-2)
Ghana	39 (32-47)	37 (33-42)	36 (33-40)	33 (30-35)	31 (29-34)	32 (29-35)	33 (31-36)	35 (33-37)	31 (29-33)	29 (27-31)	29 (27-31)	27 (25-29)	23 (20-25)	19 (15-23)
Guinea	30 (25-37)	28 (24-33)	27 (22-31)	26 (22-30)	27 (23-30)	28 (25-31)	29 (26-32)	31 (28-33)	32 (30-35)	32 (29-35)	29 (26-32)	26 (23-29)	23 (19-26)	19 (16-22)
Guinea-Bissau	5 (4-7)	5 (4-6)	5 (4-6)	5 (4-6)	5 (4-6)	5 (4-5)	5 (4-6)	5 (4-6)	4 (4-5)	4 (4-4)	4 (3-4)	4 (3-4)	3 (2-3)	2 (2-3)
Liberia	7 (6-9)	8 (7-9)	9 (7-10)	9 (8-10)	10 (9-11)	10 (9-11)	11 (10-11)	11 (10-12)	11 (10-11)	9 (8-10)	10 (9-11)	7 (6-8)	6 (5-7)	5 (4-6)
Mali	54 (44-66)	54 (46-62)	54 (47-61)	57 (51-62)	59 (53-64)	57 (53-62)	54 (50-57)	52 (49-55)	51 (48-55)	50 (47-53)	48 (45-52)	45 (41-49)	45 (40-50)	47 (39-57)
Mauritania	4 (3-5)	4 (3-5)	4 (4-5)	4 (4-5)	4 (4-5)	5 (4-5)	5 (4-5)	5 (4-5)	4 (4-5)	4 (4-4)	4 (4-4)	4 (3-4)	3 (3-3)	2 (2-3)
Niger	27 (22-33)	29 (25-34)	31 (27-36)	34 (30-38)	40 (36-44)	48 (45-52)	54 (50-58)	58 (54-63)	60 (56-65)	61 (57-66)	59 (55-65)	54 (50-60)	49 (43-56)	46 (38-55)
Nigeria	326 (252-414)	314 (262-370)	305 (262-353)	301 (266-339)	317 (285-353)	311 (282-340)	318 (290-347)	337 (311-366)	378 (352-407)	420 (392-448)	466 (431-501)	463 (425-502)	424 (380-475)	359 (296-433)
Sao Tome and Principe	1 (0-1)	0 (0-0)	0 (0-0)	0 (0-0)	0 (0-0)	0 (0-0)	0 (0-0)	0 (0-0)	0 (0-0)	0 (0-0)	0 (0-0)	0 (0-0)	0 (0-0)	0 (0-0)
Senegal	19 (15-23)	20 (18-23)	22 (19-25)	25 (23-28)	28 (26-30)	28 (26-31)	27 (24-29)	24 (22-26)	21 (20-23)	21 (20-23)	20 (18-22)	17 (15-19)	13 (11-15)	10 (8-12)
Sierra Leone	19 (15-24)	20 (17-23)	21 (18-24)	21 (18-25)	20 (17-24)	20 (17-23)	20 (18-23)	21 (19-22)	21 (20-23)	18 (17-20)	18 (16-19)	20 (18-22)	17 (16-20)	14 (11-17)
Togo	10 (8-13)	10 (8-12)	10 (9-12)	10 (9-12)	10 (9-11)	10 (9-11)	10 (9-11)	11 (10-12)	10 (10-11)	10 (9-11)	11 (9-12)	11 (9-12)	9 (8-11)	6 (5-8)

Table 4 Under-5 death numbers by location and year for 1950, 1955...2005, 2010, 2017 - males

Location	1950	1955	1960	1965	1970	1975	1980	1985	1990	1995	2000	2005	2010	2017
Global	10400 (10100-10700)	10100 (9870-10300)	17700 (17500-17900)	9780 (9620-9950)	8900 (8760-9040)	8020 (7910-8140)	7190 (7090-7290)	6890 (6790-6980)	6240 (6160-6320)	5710 (5630-5780)	5160 (5080-5230)	4490 (4420-4560)	3840 (3750-3930)	2920 (2810-3040)
Low SDI	2360 (2180-2550)	2410 (2280-2540)	2520 (2410-2640)	2600 (2490-2710)	2530 (2460-2620)	2570 (2490-2650)	2530 (2460-2600)	2480 (2420-2530)	2380 (2330-2430)	2340 (2290-2390)	2170 (2120-2220)	1930 (1890-1980)	1700 (1650-1750)	1320 (1260-1380)
Low-middle SDI	2790 (2620-2970)	2760 (2650-2880)	3520 (3430-3620)	2830 (2740-2920)	2700 (2630-2770)	2630 (2560-2690)	2400 (2340-2460)	2260 (2210-2320)	2100 (2040-2150)	2000 (1950-2060)	1910 (1860-1970)	1720 (1660-1780)	1470 (1400-1540)	1140 (1040-1240)
Middle SDI	3030 (2900-3180)	2980 (2880-3070)	8640 (8530-8760)	2870 (2790-2950)	2450 (2380-2520)	1920 (1870-1970)	1540 (1500-1570)	1480 (1430-1530)	1240 (1200-1270)	965 (940-989)	760 (743-776)	598 (585-610)	472 (460-485)	321 (309-334)
High-middle SDI	1590 (1510-1670)	1440 (1400-1490)	2620 (2570-2670)	1160 (1120-1200)	965 (935-995)	739 (721-759)	590 (576-605)	559 (545-572)	437 (425-450)	336 (327-346)	257 (250-265)	195 (189-201)	156 (151-161)	103 (99-107)
High SDI	571 (550-595)	432 (422-443)	368 (361-375)	279 (273-284)	221 (217-225)	150 (147-152)	114 (113-116)	88 (87-89)	74 (73-75)	55 (55-56)	46 (46-46)	42 (42-42)	37 (36-37)	32 (31-33)
Central Europe, Eastern Europe, and Central Asia	579 (550-612)	419 (403-436)	319 (308-332)	222 (214-232)	181 (175-188)	170 (165-176)	161 (157-165)	155 (151-158)	122 (119-124)	94 (91-96)	70 (68-73)	59 (56-62)	53 (50-56)	40 (38-43)
Central Asia	158 (140-178)	140 (128-153)	127 (116-138)	102 (94-111)	77 (72-83)	72 (67-76)	73 (69-76)	73 (70-76)	63 (61-66)	53 (51-56)	41 (38-43)	35 (33-38)	33 (31-36)	27 (24-30)
Armenia	9 (6-12)	7 (6-9)	7 (5-8)	5 (4-6)	3 (3-4)	3 (2-3)	3 (2-3)	3 (2-3)	2 (2-2)	1 (1-1)	1 (1-1)	0 (0-0)	0 (0-0)	0 (0-0)
Azerbaijan	29 (22-38)	26 (21-32)	24 (19-29)	17 (13-22)	11 (9-14)	8 (6-10)	8 (7-9)	9 (8-10)	8 (7-9)	6 (6-7)	5 (4-6)	5 (4-5)	4 (4-5)	3 (3-5)
Georgia	15 (10-21)	12 (9-16)	10 (8-12)	7 (6-9)	5 (4-7)	4 (3-5)	3 (3-4)	3 (2-3)	2 (2-2)	2 (1-2)	1 (1-1)	1 (1-1)	1 (0-1)	0 (0-0)
Kazakhstan	27 (19-37)	24 (19-31)	23 (18-29)	19 (15-23)	14 (12-17)	13 (11-15)	12 (11-14)	11 (10-12)	8 (8-9)	6 (6-7)	5 (4-5)	5 (4-5)	5 (4-6)	3 (2-4)
Kyrgyzstan	15 (11-19)	12 (10-15)	11 (8-13)	9 (7-11)	7 (5-8)	6 (5-7)	6 (5-7)	6 (5-6)	5 (4-5)	4 (3-4)	3 (3-3)	3 (3-3)	2 (2-3)	2 (2-2)
Mongolia	8 (6-10)	7 (5-8)	6 (5-7)	5 (4-5)	4 (3-5)	4 (3-4)	4 (4-4)	4 (4-5)	4 (3-4)	3 (2-3)	2 (2-2)	2 (1-2)	2 (1-2)	1 (1-1)
Tajikistan	16 (12-20)	15 (13-18)	15 (12-17)	12 (10-15)	10 (8-12)	9 (8-11)	9 (8-11)	10 (9-11)	9 (8-10)	8 (7-9)	7 (6-8)	6 (5-6)	6 (5-7)	7 (5-8)
Turkmenistan	8 (6-10)	7 (6-9)	7 (5-8)	5 (4-7)	4 (3-6)	5 (4-6)	5 (4-6)	5 (5-6)	5 (4-5)	4 (4-5)	4 (3-4)	3 (2-3)	3 (2-3)	2 (1-2)
Uzbekistan	33 (24-44)	29 (23-36)	26 (20-32)	23 (18-28)	19 (16-23)	20 (17-23)	22 (19-24)	22 (20-25)	20 (19-23)	19 (17-21)	14 (12-16)	12 (10-14)	11 (9-14)	9 (7-11)
Central Europe	216 (208-225)	130 (127-133)	81 (80-82)	54 (53-55)	50 (50-51)	43 (42-44)	34 (34-35)	29 (28-29)	22 (21-22)	14 (14-15)	9 (9-10)	7 (7-7)	5 (5-5)	3 (3-4)
Albania	7 (5-8)	6 (5-7)	5 (5-6)	4 (4-5)	4 (3-4)	3 (2-3)	2 (2-3)	2 (2-2)	2 (2-2)	2 (1-2)	1 (1-1)	1 (0-1)	0 (0-0)	0 (0-0)
Bosnia and Herzegovina	15 (12-17)	10 (8-11)	7 (6-7)	5 (4-5)	3 (3-4)	2 (2-3)	2 (1-2)	1 (1-1)	1 (1-1)	1 (1-1)	0 (0-0)	0 (0-0)	0 (0-0)	0 (0-0)
Bulgaria	17 (16-18)	8 (8-9)	4 (4-4)	3 (2-3)	2 (2-2)	2 (2-2)	2 (2-2)	1 (1-1)	1 (1-1)	1 (1-1)	1 (1-1)	1 (0-1)	0 (0-1)	0 (0-0)
Croatia	11 (9-14)	6 (5-7)	3 (3-4)	2 (2-3)	2 (1-2)	1 (1-1)	1 (1-1)	1 (1-1)	0 (0-0)	0 (0-0)	0 (0-0)	0 (0-0)	0 (0-0)	0 (0-0)
Czech Republic	6 (5-6)	3 (3-3)	2 (2-2)	2 (2-2)	2 (2-2)	2 (2-2)	2 (2-2)	1 (1-1)	1 (1-1)	0 (0-1)	0 (0-0)	0 (0-0)	0 (0-0)	0 (0-0)
Hungary	14 (13-14)	8 (7-8)	4 (4-4)	3 (3-4)	3 (3-4)	4 (4-4)	2 (2-2)	2 (2-2)	1 (1-1)	1 (1-1)	1 (1-1)	0 (0-0)	0 (0-0)	0 (0-0)
Macedonia	7 (6-8)	5 (4-5)	4 (3-4)	3 (2-3)	2 (2-2)	1 (1-2)	1 (1-1)	1 (1-1)	1 (1-1)	0 (0-0)	0 (0-0)	0 (0-0)	0 (0-0)	0 (0-0)
Montenegro	1 (0-1)	0 (0-0)	0 (0-0)	0 (0-0)	0 (0-0)	0 (0-0)	0 (0-0)	0 (0-0)	0 (0-0)	0 (0-0)	0 (0-0)	0 (0-0)	0 (0-0)	0 (0-0)
Poland	74 (72-77)	41 (40-41)	23 (22-23)	14 (14-15)	12 (12-12)	10 (10-11)	10 (9-10)	8 (8-8)	6 (6-6)	4 (4-4)	2 (2-2)	2 (1-2)	1 (1-1)	1 (1-1)
Romania	34 (28-42)	24 (22-26)	17 (16-17)	8 (8-8)	13 (13-13)	10 (9-10)	8 (8-8)	7 (7-7)	6 (6-6)	3 (3-4)	3 (3-3)	2 (2-2)	1 (1-1)	1 (1-1)
Serbia	23 (22-25)	14 (14-15)	9 (9-9)	7 (7-7)	6 (5-6)	5 (5-5)	4 (3-4)	4 (3-4)	2 (2-2)	2 (2-2)	1 (1-1)	1 (1-1)	0 (0-0)	0 (0-0)
Slovakia	5 (5-5)	3 (3-3)	2 (2-2)	2 (2-2)	1 (1-1)	2 (1-2)	1 (1-1)	1 (1-1)	1 (1-1)	0 (0-0)	0 (0-0)	0 (0-0)	0 (0-0)	0 (0-0)
Slovenia	2 (2-3)	2 (2-2)	1 (1-1)	1 (1-1)	1 (1-1)	0 (0-0)	0 (0-0)	0 (0-0)	0 (0-0)	0 (0-0)	0 (0-0)	0 (0-0)	0 (0-0)	0 (0-0)
Eastern Europe	205 (184-234)	149 (139-161)	112 (106-118)	66 (63-70)	54 (51-56)	56 (53-58)	54 (53-56)	53 (52-55)	36 (36-37)	26 (25-27)	20 (20-21)	17 (17-17)	14 (14-15)	10 (10-11)
Belarus	10 (7-15)	8 (6-10)	7 (5-8)	4 (3-5)	3 (3-4)	3 (2-3)	3 (2-3)	2 (2-3)	2 (2-2)	1 (1-1)	1 (1-1)	1 (1-1)	1 (1-1)	0 (0-1)
Estonia	1 (1-1)	1 (0-1)	0 (0-0)	0 (0-0)	0 (0-0)	0 (0-0)	0 (0-0)	0 (0-0)	0 (0-0)	0 (0-0)	0 (0-0)	0 (0-0)	0 (0-0)	0 (0-0)
Latvia	2 (1-3)	1 (1-1)	1 (1-1)	0 (0-1)	0 (0-0)	0 (0-1)	0 (0-0)	0 (0-0)	0 (0-0)	0 (0-0)	0 (0-0)	0 (0-0)	0 (0-0)	0 (0-0)
Lithuania	4 (3-6)	2 (2-3)	2 (2-2)	1 (1-1)	1 (1-1)	1 (1-1)	1 (1-1)	1 (1-1)	0 (0-0)	0 (0-0)	0 (0-0)	0 (0-0)	0 (0-0)	0 (0-0)
Moldova	11 (8-16)	9 (7-12)	7 (5-9)	5 (4-7)	4 (3-5)	3 (2-4)	3 (3-4)	3 (2-3)	2 (2-2)	1 (1-1)	1 (1-1)	1 (1-1)	1 (0-1)	0 (0-0)
Russian Federation	128 (120-136)	92 (89-95)	69 (68-70)	38 (37-38)	31 (31-31)	34 (34-35)	35 (34-35)	36 (35-36)	24 (24-24)	18 (18-18)	14 (14-15)	12 (12-12)	10 (10-10)	7 (7-7)
Ukraine	50 (31-76)	35 (26-45)	26 (21-31)	17 (15-21)	14 (12-16)	14 (12-16)	13 (11-15)	11 (10-12)	8 (7-9)	5 (5-6)	4 (3-4)	3 (3-3)	3 (3-3)	2 (2-3)
High-income	465 (445-489)	387 (378-398)	347 (342-353)	282 (277-287)	229 (226-234)	156 (153-159)	115 (114-117)	87 (86-88)	77 (76-77)	58 (58-59)	50 (50-50)	45 (45-45)	39 (39-40)	34 (33-35)
Australasia	4 (4-5)	4 (4-4)	4 (4-4)	4 (4-4)	4 (4-4)	3 (3-3)	2 (2-2)	2 (2-2)	2 (2-2)	1 (1-1)	1 (1-1)	1 (1-1)	1 (1-1)	1 (1-1)
Australia	3 (3-4)	3 (3-3)	3 (3-3)	3 (3-3)	3 (3-3)	2 (2-2)	2 (2-2)	2 (2-2)	1 (1-1)	1 (1-1)	1 (1-1)	1 (1-1)	1 (1-1)	1 (1-1)
New Zealand	1 (1-1)	1 (1-1)	1 (1-1)	1 (1-1)	1 (1-1)	1 (1-1)	0 (0-0)	0 (0-0)	0 (0-0)	0 (0-0)	0 (0-0)	0 (0-0)	0 (0-0)	0 (0-0)
High-income Asia-Pacific	155 (137-178)	106 (97-115)	81 (76-87)	63 (58-68)	51 (47-56)	30 (28-32)	18 (17-20)	11 (11-12)	8 (8-9)	7 (6-7)	5 (5-6)	4 (4-4)	3 (3-3)	2 (2-2)
Brunei	0 (0-0)	0 (0-0)	0 (0-0)	0 (0-0)	0 (0-0)	0 (0-0)	0 (0-0)	0 (0-0)	0 (0-0)	0 (0-0)	0 (0-0)	0 (0-0)	0 (0-0)	0 (0-0)
Japan	77 (74-80)	51 (50-52)	35 (34-35)	22 (22-22)	19 (19-20)	14 (14-14)	9 (9-9)	6 (6-6)	4 (4-4)	4 (4-4)	3 (3-3)	2 (2-2)	2 (2-2)	1 (1-1)
Aichi	4 (3-4)	3 (2-3)	2 (2-2)	1 (1-1)	1 (1-1)	1 (1-1)	0 (0-0)	0 (0-0)	0 (0-0)	0 (0-0)	0 (0-0)	0 (0-0)	0 (0-0)	0 (0-0)
Akita	1 (1-1)	1 (1-1)	0 (0-0)	0 (0-0)	0 (0-0)	0 (0-0)	0 (0-0)	0 (0-0)	0 (0-0)	0 (0-0)	0 (0-0)	0 (0-0)	0 (0-0)	0 (0-0)
Aomori	1 (1-2)	1 (1-1)	1 (1-1)	0 (0-0)	0 (0-0)	0 (0-0)	0 (0-0)	0 (0-0)	0 (0-0)	0 (0-0)	0 (0-0)	0 (0-0)	0 (0-0)	0 (0-0)
Chiba	2 (2-2)	1 (1-2)	1 (1-1)	1 (1-1)	1 (1-1)	0 (0-1)	0 (0-0)	0 (0-0)	0 (0-0)	0 (0-0)	0 (0-0)	0 (0-0)	0 (0-0)	0 (0-0)
Ehime	1 (1-1)	1 (1-1)	0 (0-1)	0 (0-0)	0 (0-0)	0 (0-0)	0 (0-0)	0 (0-0)	0 (0-0)	0 (0-0)	0 (0-0)	0 (0-0)	0 (0-0)	0 (0-0)
Fukui	0 (0-1)	0 (0-0)	0 (0-0)	0 (0-0)	0 (0-0)	0 (0-0)	0 (0-0)	0 (0-0)	0 (0-0)	0 (0-0)	0 (0-0)	0 (0-0)	0 (0-0)	0 (0-0)
Fukuoka	3 (2-3)	2 (2-2)	1 (1-1)	1 (1-1)	1 (1-1)	1 (0-1)	0 (0-0)	0 (0-0)	0 (0-0)	0 (0-0)	0 (0-0)	0 (0-0)	0 (0-0)	0 (0-0)
Fukushima	2 (1-2)	1 (1-1)	1 (1-1)	0 (0-0)	0 (0-0)	0 (0-0)	0 (0-0)	0 (0-0)	0 (0-0)	0 (0-0)	0 (0-0)	0 (0-0)	0 (0-0)	0 (0-0)
Gifu	1 (1-1)	1 (1-1)	1 (1-1)	0 (0-0)	0 (0-0)	0 (0-0)	0 (0-0)	0 (0-0)	0 (0-0)	0 (0-0)	0 (0-0)	0 (0-0)	0 (0-0)	0 (0-0)
Gunma	1 (1-1)	1 (1-1)	1 (1-1)	0 (0-0)	0 (0-0)	0 (0-0)	0 (0-0)	0 (0-0)	0 (0-0)	0 (0-0)	0 (0-0)	0 (0-0)	0 (0-0)	0 (0-0)
Hiroshima	2 (2-2)	1 (1-2)	1 (1-1)	1 (1-1)	0 (0-1)	0 (0-0)	0 (0-0)	0 (0-0)	0 (0-0)	0 (0-0)	0 (0-0)	0 (0-0)	0 (0-0)	0 (0-0)
Hokkaidō	5 (4-5)	3 (3-3)	2 (2-2)	1 (1-1)	1 (1-1)	1 (1-1)	1 (0-1)	0 (0-0)	0 (0-0)	0 (0-0)	0 (0-0)	0 (0-0)	0 (0-0)	0 (0-0)
Hyōgo	4 (3-4)	2 (2-3)	2 (1-2)	1 (1-1)	1 (1-1)	1 (1-1)	0 (0-0)	0 (0-0)	0 (0-0)	0 (0-0)	0 (0-0)	0 (0-0)	0 (0-0)	0 (0-0)
Ibaraki	2 (1-2)	1 (1-1)	1 (1-1)	0 (0-1)	0 (0-1)	0 (0-0)	0 (0-0)	0 (0-0)	0 (0-0)	0 (0-0)	0 (0-0)	0 (0-0)	0 (0-0)	0 (0-0)
Ishikawa	1 (1-1)	0 (0-1)	0 (0-0)	0 (0-0)	0 (0-0)	0 (0-0)	0 (0-0)	0 (0-0)	0 (0-0)	0 (0-0)	0 (0-0)	0 (0-0)	0 (0-0)	0 (0-0)
Iwate	1 (1-2)	1 (1-1)	1 (0-1)	0 (0-0)	0 (0-0)	0 (0-0)	0 (0-0)	0 (0-0)	0 (0-0)	0 (0-0)	0 (0-0)	0 (0-0)	0 (0-0)	0 (0-0)
Kagawa	1 (1-1)	0 (0-0)	0 (0-0)	0 (0-0)	0 (0-0)	0 (0-0)	0 (0-0)	0 (0-0)	0 (0-0)	0 (0-0)	0 (0-0)	0 (0-0)	0 (0-0)	0 (0-0)
Kagoshima	1 (1-1)	1 (1-1)	0 (0-1)	0 (0-0)	0 (0-0)	0 (0-0)	0 (0-0)	0 (0-0)	0 (0-0)	0 (0-0)	0 (0-0)	0 (0-0)	0 (0-0)	0 (0-0)
Kanagawa	3 (3-4)	2 (2-2)	2 (2-2)	1 (1-1)	1 (1-1)	1 (1-1)	0 (0-0)	0 (0-0)	0 (0-0)	0 (0-0)	0 (0-0)	0 (0-0)	0 (0-0)	0 (0-0)
Kōchi	1 (1-1)	0 (0-0)	0 (0-0)	0 (0-0)	0 (0-0)	0 (0-0)	0 (0-0)	0 (0-0)	0 (0-0)	0 (0-0)	0 (0-0)	0 (0-0)	0 (0-0)	0 (0-0)
Kumamoto	1 (1-2)	1 (1-1)	1 (0-1)	0 (0-0)	0 (0-0)	0 (0-0)	0 (0-0)	0 (0-0)	0 (0-0)	0 (0-0)	0 (0-0)	0 (0-0)	0 (0-0)	0 (0-0)
Kyōto	2 (1-2)	1 (1-1)	1 (1-1)	0 (0-0)	0 (0-0)	0 (0-0)	0 (0-0)	0 (0-0)	0 (0-0)	0 (0-0)	0 (0-0)	0 (0-0)	0 (0-0)	0 (0-0)
Mie	1 (1-1)	1 (1-1)	1 (0-1)	0 (0-0)	0 (0-0)	0 (0-0)	0 (0-0)	0 (0-0)	0 (0-0)	0 (0-0)	0 (0-0)	0 (0-0)	0 (0-0)	0 (0-0)
Miyagi	1 (1-2)	1 (1-1)	1 (1-1)	0 (0-0)	0 (0-0)	0 (0-0)	0 (0-0)	0 (0-0)	0 (0-0)	0 (0-0)	0 (0-0)	0 (0-0)	0 (0-0)	0 (0-0)
Miyazaki	1 (1-1)	1 (0-1)	0 (0-0)	0 (0-0)	0 (0-0)	0 (0-0)	0 (0-0)	0 (0-0)	0 (0-0)	0 (0-0)	0 (0-0)	0 (0-0)	0 (0-0)	0 (0-0)
Nagano	1 (1-2)	1 (1-1)	1 (1-1)	0 (0-0)	0 (0-0)	0 (0-0)	0 (0-0)	0 (0-0)	0 (0-0)	0 (0-0)	0 (0-0)	0 (0-0)	0 (0-0)	0 (0-0)
Nagasaki	1 (1-1)	1 (1-1)	0 (0-1)	0 (0-0)	0 (0-0)	0 (0-0)	0 (0-0)	0 (0-0)	0 (0-0)	0 (0-0)	0 (0-0)	0 (0-0)	0 (0-0)	0 (0-0)
Nara	1 (0-1)	0 (0-0)	0 (0-0)	0 (0-0)	0 (0-0)	0 (0-0)	0 (0-0)	0 (0-0)	0 (0-0)	0 (0-0)	0 (0-0)	0 (0-0)	0 (0-0)	0 (0-0)
Niigata	2 (1-2)	1 (1-1)	1 (1-1)	0 (0-0)	0 (0-0)	0 (0-0)	0 (0-0)	0 (0-0)	0 (0-0)	0 (0-0)	0 (0-0)	0 (0-0)	0 (0-0)	0 (0-0)
Ōita	1 (1-1)	1 (1-1)	0 (0-0)	0 (0-0)	0 (0-0)	0 (0-0)	0 (0-0)	0 (0-0)	0 (0-0)	0 (0-0)	0 (0-0)	0 (0-0)	0 (0-0)	0 (0-0)
Okayama	1 (1-1)	1 (1-1)	1 (0-1)	0 (0-0)	0 (0-0)	0 (0-0)	0 (0-0)	0 (0-0)	0 (0-0)	0 (0-0)	0 (0-0)	0 (0-0)	0 (0-0)	0 (0-0)
Okinawa	1 (0-1)	0 (0-0)	0 (0-0)	0 (0-0)	0 (0-0)	0 (0-0)	0 (0-0)	0 (0-0)	0 (0-0)	0 (0-0)	0 (0-0)	0 (0-0)	0 (0-0)	0 (0-0)
Ōsaka	6 (5-7)	4 (4-4)	3 (3-3)	2 (2-2)	1 (1-2)	1 (1-1)	1 (1-1)	0 (0-0)	0 (0-0)	0 (0-0)	0 (0-0)	0 (0-0)	0 (0-0)	0 (0-0)
Saga	1 (0-1)	0 (0-0)	0 (0-0)	0 (0-0)	0 (0-0)	0 (0-0)	0 (0-0)	0 (0-0)	0 (0-0)	0 (0-0)	0 (0-0)	0 (0-0)	0 (0-0)	0 (0-0)
Saitama	3 (2-3)	2 (2-2)	1 (1-1)	1 (1-1)	1 (1-1)	1 (1-1)	0 (0-0)	0 (0-0)	0 (0-0)	0 (0-0)	0 (0-0)	0 (0-0)	0 (0-0)	0 (0-0)
Shiga	1 (0-1)	0 (0-0)	0 (0-0)	0 (0-0)	0 (0-0)	0 (0-0)	0 (0-0)	0 (0-0)	0 (0-0)	0 (0-0)	0 (0-0)	0 (0-0)	0 (0-0)	0 (0-0)
Shimane	1 (0-1)	0 (0-0)	0 (0-0)	0 (0-0)	0 (0-0)	0 (0-0)	0 (0-0)	0 (0-0)	0 (0-0)	0 (0-0)	0 (0-0)	0 (0-0)	0 (0-0)	0 (0-0)
Shizuoka	2 (2-3)	2 (1-2)	1 (1-1)	1 (1-1)	1 (1-1)	0 (0-0)	0 (0-0)	0 (0-0)	0 (0-0)	0 (0-0)	0 (0-0)	0 (0-0)	0 (0-0)	0 (0-0)
Tochigi	1 (1-1)	1 (1-1)	1 (1-1)	0 (0-0)	0 (0-0)	0 (0-0)	0 (0-0)	0 (0-0)	0 (0-0)	0 (0-0)	0 (0-0)	0 (0-0)	0 (0-0)	0 (0-0)
Tokushima	1 (0-1)	0 (0-0)	0 (0-0)	0 (0-0)	0 (0-0)	0 (0-0)	0 (0-0)	0 (0-0)	0 (0-0)	0 (0-0)	0 (0-0)	0 (0-0)	0 (0-0)	0 (0-0)
Tōkyō	7 (6-9)	5 (4-5)	3 (3-4)	2 (2-2)	2 (2-2)	1 (1-1)	1 (1-1)	1 (1-1)	0 (0-0)	0 (0-0)	0 (0-0)	0 (0-0)	0 (0-0)	0 (0-0)
Tottori	0 (0-1)	0 (0-0)	0 (0-0)	0 (0-0)	0 (0-0)	0 (0-0)	0 (0-0)	0 (0-0)	0 (0-0)	0 (0-0)	0 (0-0)	0 (0-0)	0 (0-0)	0 (0-0)
Toyama	1 (1-1)	1 (1-1)	0 (0-0)	0 (0-0)	0 (0-0)	0 (0-0)	0 (0-0)	0 (0-0)	0 (0-0)	0 (0-0)	0 (0-0)	0 (0-0)	0 (0-0)	0 (0-0)
Wakayama	1 (1-1)	1 (1-1)	0 (0-0)	0 (0-0)	0 (0-0)	0 (0-0)	0 (0-0)	0 (0-0)	0 (0-0)	0 (0-0)	0 (0-0)	0 (0-0)	0 (0-0)	0 (0-0)
Yamagata	1 (1-1)	1 (0-1)	0 (0-0)	0 (0-0)	0 (0-0)	0 (0-0)	0 (0-0)	0 (0-0)	0 (0-0)	0 (0-0)	0 (0-0)	0 (0-0)	0 (0-0)	0 (0-0)
Yamaguchi	1 (1-2)	1 (1-1)	1 (1-1)	0 (0-0)	0 (0-0)	0 (0-0)	0 (0-0)	0 (0-0)	0 (0-0)	0 (0-0)	0 (0-0)	0 (0-0)	0 (0-0)	0 (0-0)
Yamanashi	0 (0-1)	0 (0-0)	0 (0-0)	0 (0-0)	0 (0-0)	0 (0-0)	0 (0-0)	0 (0-0)	0 (0-0)	0 (0-0)	0 (0-0)	0 (0-0)	0 (0-0)	0 (0-0)
South Korea	75 (58-98)	52 (44-62)	45 (39-50)	40 (35-45)	31 (27-35)	15 (13-18)	9 (8-10)	5 (4-6)	3 (3-4)	3 (2-3)	2 (2-2)	1 (1-1)	1 (1-1)	1 (1-1)
Singapore	3 (3-3)	2 (2-2)	2 (2-2)	1 (1-1)	1 (1-1)	0 (0-0)	0 (0-0)	0 (0-0)	0 (0-0)	0 (0-0)	0 (0-0)	0 (0-0)	0 (0-0)	0 (0-0)
High-income North America	80 (78-82)	80 (79-81)	82 (81-82)	65 (64-65)	53 (53-53)	36 (36-36)	33 (33-33)	29 (29-29)	27 (27-28)	21 (21-22)	20 (20-20)	20 (20-20)	17 (17-17)	16 (15-16)
Canada	10 (9-10)	9 (9-9)	8 (8-9)	6 (6-6)	4 (4-5)	3 (3-3)	3 (3-3)	2 (2-2)	2 (2-2)	1 (1-2)	1 (1-1)	1 (1-1)	1 (1-1)	1 (1-2)
Greenland	0 (0-0)	0 (0-0)	0 (0-0)	0 (0-0)	0 (0-0)	0 (0-0)	0 (0-0)	0 (0-0)	0 (0-0)	0 (0-0)	0 (0-0)	0 (0-0)	0 (0-0)	0 (0-0)

Table 4 Under-5 death numbers by location and year for 1950, 1955...2005, 2010, 2017 - males

Location	1950	1955	1960	1965	1970	1975	1980	1985	1990	1995	2000	2005	2010	2017
USA	70 (68-72)	71 (70-72)	73 (73-74)	59 (58-59)	48 (48-49)	33 (32-33)	30 (30-31)	27 (27-27)	26 (25-26)	20 (20-20)	19 (19-19)	19 (19-19)	16 (16-16)	14 (14-15)
Alabama	2 (2-2)	2 (2-2)	2 (2-2)	1 (1-2)	1 (1-1)	1 (1-1)	1 (1-1)	1 (0-1)	1 (0-1)	0 (0-0)	0 (0-0)	0 (0-0)	0 (0-0)	0 (0-0)
Alaska	0 (0-0)	0 (0-0)	0 (0-0)	0 (0-0)	0 (0-0)	0 (0-0)	0 (0-0)	0 (0-0)	0 (0-0)	0 (0-0)	0 (0-0)	0 (0-0)	0 (0-0)	0 (0-0)
Arizona	0 (0-1)	1 (1-1)	1 (1-1)	1 (1-1)	0 (0-0)	0 (0-0)	0 (0-0)	0 (0-0)	0 (0-0)	0 (0-0)	0 (0-0)	0 (0-0)	0 (0-0)	0 (0-0)
Arkansas	1 (1-1)	1 (1-1)	1 (1-1)	1 (1-1)	1 (0-1)	0 (0-0)	0 (0-0)	0 (0-0)	0 (0-0)	0 (0-0)	0 (0-0)	0 (0-0)	0 (0-0)	0 (0-0)
California	5 (4-5)	5 (5-5)	6 (6-6)	5 (5-5)	4 (4-4)	3 (3-3)	3 (3-3)	3 (3-3)	3 (3-3)	2 (2-2)	2 (2-2)	2 (2-2)	2 (2-2)	1 (1-2)
Colorado	1 (1-1)	1 (1-1)	1 (1-1)	1 (1-1)	1 (1-1)	0 (0-0)	0 (0-0)	0 (0-0)	0 (0-0)	0 (0-0)	0 (0-0)	0 (0-0)	0 (0-0)	0 (0-0)
Connecticut	1 (1-1)	1 (1-1)	1 (1-1)	1 (1-1)	1 (1-1)	0 (0-0)	0 (0-0)	0 (0-0)	0 (0-0)	0 (0-0)	0 (0-0)	0 (0-0)	0 (0-0)	0 (0-0)
Delaware	0 (0-0)	0 (0-0)	0 (0-0)	0 (0-0)	0 (0-0)	0 (0-0)	0 (0-0)	0 (0-0)	0 (0-0)	0 (0-0)	0 (0-0)	0 (0-0)	0 (0-0)	0 (0-0)
Washington, DC	1 (0-1)	1 (0-1)	1 (0-1)	0 (0-0)	0 (0-0)	0 (0-0)	0 (0-0)	0 (0-0)	0 (0-0)	0 (0-0)	0 (0-0)	0 (0-0)	0 (0-0)	0 (0-0)
Florida	2 (1-2)	2 (2-2)	2 (2-3)	2 (2-2)	2 (2-2)	1 (1-1)	1 (1-2)	1 (1-1)	1 (1-1)	1 (1-1)	1 (1-1)	1 (1-1)	1 (1-1)	1 (1-1)
Georgia	2 (2-2)	2 (2-2)	2 (2-2)	2 (2-2)	1 (1-1)	1 (1-1)	1 (1-1)	1 (1-1)	1 (1-1)	1 (1-1)	1 (1-1)	1 (1-1)	1 (1-1)	1 (0-1)
Hawaii	0 (0-0)	0 (0-0)	0 (0-0)	0 (0-0)	0 (0-0)	0 (0-0)	0 (0-0)	0 (0-0)	0 (0-0)	0 (0-0)	0 (0-0)	0 (0-0)	0 (0-0)	0 (0-0)
Idaho	0 (0-0)	0 (0-0)	0 (0-0)	0 (0-0)	0 (0-0)	0 (0-0)	0 (0-0)	0 (0-0)	0 (0-0)	0 (0-0)	0 (0-0)	0 (0-0)	0 (0-0)	0 (0-0)
Illinois	4 (3-4)	4 (3-4)	4 (4-4)	3 (3-3)	3 (3-3)	2 (2-2)	2 (2-2)	1 (1-1)	1 (1-1)	1 (1-1)	1 (1-1)	1 (1-1)	1 (1-1)	1 (0-1)
Indiana	2 (1-2)	2 (2-2)	2 (2-2)	1 (1-1)	1 (1-1)	1 (1-1)	1 (1-1)	1 (1-1)	1 (1-1)	0 (0-1)	0 (0-0)	0 (0-0)	0 (0-0)	0 (0-0)
Iowa	1 (1-1)	1 (1-1)	1 (1-1)	1 (1-1)	1 (1-1)	0 (0-0)	0 (0-0)	0 (0-0)	0 (0-0)	0 (0-0)	0 (0-0)	0 (0-0)	0 (0-0)	0 (0-0)
Kansas	1 (0-1)	1 (1-1)	1 (1-1)	1 (0-1)	0 (0-0)	0 (0-0)	0 (0-0)	0 (0-0)	0 (0-0)	0 (0-0)	0 (0-0)	0 (0-0)	0 (0-0)	0 (0-0)
Kentucky	1 (1-2)	1 (1-2)	1 (1-1)	1 (1-1)	1 (1-1)	1 (1-1)	1 (0-1)	0 (0-0)	0 (0-0)	0 (0-0)	0 (0-0)	0 (0-0)	0 (0-0)	0 (0-0)
Louisiana	2 (1-2)	2 (2-2)	2 (2-2)	1 (1-1)	1 (1-1)	1 (1-1)	1 (1-1)	1 (1-1)	1 (0-1)	0 (0-0)	0 (0-0)	0 (0-1)	0 (0-0)	0 (0-0)
Maine	0 (0-1)	0 (0-0)	0 (0-0)	0 (0-0)	0 (0-0)	0 (0-0)	0 (0-0)	0 (0-0)	0 (0-0)	0 (0-0)	0 (0-0)	0 (0-0)	0 (0-0)	0 (0-0)
Maryland	1 (1-1)	1 (1-1)	1 (1-1)	1 (1-1)	1 (1-1)	1 (1-1)	1 (1-1)	1 (0-1)	0 (0-1)	0 (0-0)	0 (0-0)	0 (0-0)	0 (0-0)	0 (0-0)
Massachusetts	2 (2-2)	2 (2-2)	2 (2-2)	1 (1-1)	1 (1-1)	1 (1-1)	1 (1-1)	1 (0-1)	0 (0-0)	0 (0-0)	0 (0-0)	0 (0-0)	0 (0-0)	0 (0-0)
Michigan	2 (2-3)	3 (2-3)	3 (3-3)	2 (2-2)	2 (2-2)	1 (1-1)	1 (1-1)	1 (1-1)	1 (1-1)	1 (1-1)	1 (1-1)	1 (1-1)	1 (0-1)	0 (0-1)
Minnesota	1 (1-1)	1 (1-1)	1 (1-1)	1 (1-1)	1 (1-1)	0 (0-1)	0 (0-0)	0 (0-0)	0 (0-0)	0 (0-0)	0 (0-0)	0 (0-0)	0 (0-0)	0 (0-0)
Mississippi	2 (2-2)	2 (2-2)	1 (1-2)	1 (1-1)	1 (1-1)	1 (0-1)	0 (0-0)	0 (0-0)	0 (0-0)	0 (0-0)	0 (0-0)	0 (0-0)	0 (0-0)	0 (0-0)
Missouri	2 (1-2)	2 (1-2)	2 (1-2)	1 (1-1)	1 (1-1)	1 (1-1)	1 (1-1)	1 (0-1)	1 (0-1)	0 (0-0)	0 (0-0)	0 (0-0)	0 (0-0)	0 (0-0)
Montana	0 (0-0)	0 (0-0)	0 (0-0)	0 (0-0)	0 (0-0)	0 (0-0)	0 (0-0)	0 (0-0)	0 (0-0)	0 (0-0)	0 (0-0)	0 (0-0)	0 (0-0)	0 (0-0)
Nebraska	0 (0-1)	0 (0-1)	0 (0-0)	0 (0-0)	0 (0-0)	0 (0-0)	0 (0-0)	0 (0-0)	0 (0-0)	0 (0-0)	0 (0-0)	0 (0-0)	0 (0-0)	0 (0-0)
Nevada	0 (0-0)	0 (0-0)	0 (0-0)	0 (0-0)	0 (0-0)	0 (0-0)	0 (0-0)	0 (0-0)	0 (0-0)	0 (0-0)	0 (0-0)	0 (0-0)	0 (0-0)	0 (0-0)
New Hampshire	0 (0-0)	0 (0-0)	0 (0-0)	0 (0-0)	0 (0-0)	0 (0-0)	0 (0-0)	0 (0-0)	0 (0-0)	0 (0-0)	0 (0-0)	0 (0-0)	0 (0-0)	0 (0-0)
New Jersey	2 (2-2)	2 (2-2)	2 (2-2)	2 (2-2)	2 (1-2)	1 (1-1)	1 (1-1)	1 (1-1)	1 (1-1)	0 (0-1)	0 (0-0)	0 (0-0)	0 (0-0)	0 (0-0)
New Mexico	0 (0-1)	1 (0-1)	1 (1-1)	0 (0-0)	0 (0-0)	0 (0-0)	0 (0-0)	0 (0-0)	0 (0-0)	0 (0-0)	0 (0-0)	0 (0-0)	0 (0-0)	0 (0-0)
New York	6 (6-7)	6 (6-7)	6 (6-6)	5 (5-5)	4 (4-4)	3 (3-3)	2 (2-2)	2 (2-2)	2 (2-2)	1 (1-1)	1 (1-1)	1 (1-1)	1 (1-1)	1 (1-1)
North Carolina	3 (2-3)	3 (2-3)	2 (2-2)	2 (2-2)	2 (2-2)	1 (1-1)	1 (1-1)	1 (1-1)	1 (1-1)	1 (1-1)	1 (1-1)	1 (1-1)	1 (1-1)	1 (0-1)
North Dakota	0 (0-0)	0 (0-0)	0 (0-0)	0 (0-0)	0 (0-0)	0 (0-0)	0 (0-0)	0 (0-0)	0 (0-0)	0 (0-0)	0 (0-0)	0 (0-0)	0 (0-0)	0 (0-0)
Ohio	3 (3-3)	3 (3-4)	4 (3-4)	3 (3-3)	2 (2-2)	2 (2-2)	1 (1-1)	1 (1-1)	1 (1-1)	1 (1-1)	1 (1-1)	1 (1-1)	1 (1-1)	1 (1-1)
Oklahoma	1 (1-1)	1 (1-1)	1 (1-1)	1 (1-1)	1 (1-1)	0 (0-0)	0 (0-0)	0 (0-0)	0 (0-0)	0 (0-0)	0 (0-0)	0 (0-0)	0 (0-0)	0 (0-0)
Oregon	1 (0-1)	1 (1-1)	1 (1-1)	0 (0-1)	0 (0-0)	0 (0-0)	0 (0-0)	0 (0-0)	0 (0-0)	0 (0-0)	0 (0-0)	0 (0-0)	0 (0-0)	0 (0-0)
Pennsylvania	4 (4-5)	4 (4-5)	4 (4-4)	3 (3-3)	3 (3-3)	2 (2-2)	1 (1-2)	1 (1-1)	1 (1-1)	1 (1-1)	1 (1-1)	1 (1-1)	1 (1-1)	1 (0-1)
Rhode Island	0 (0-0)	0 (0-0)	0 (0-0)	0 (0-0)	0 (0-0)	0 (0-0)	0 (0-0)	0 (0-0)	0 (0-0)	0 (0-0)	0 (0-0)	0 (0-0)	0 (0-0)	0 (0-0)
South Carolina	1 (1-2)	1 (1-2)	1 (1-2)	1 (1-1)	1 (1-1)	1 (1-1)	1 (1-1)	1 (0-1)	0 (0-0)	0 (0-0)	0 (0-0)	0 (0-0)	0 (0-0)	0 (0-0)
South Dakota	0 (0-0)	0 (0-0)	0 (0-0)	0 (0-0)	0 (0-0)	0 (0-0)	0 (0-0)	0 (0-0)	0 (0-0)	0 (0-0)	0 (0-0)	0 (0-0)	0 (0-0)	0 (0-0)
Tennessee	2 (2-2)	2 (2-2)	2 (2-2)	1 (1-1)	1 (1-1)	1 (1-1)	1 (1-1)	1 (1-1)	1 (1-1)	0 (0-1)	0 (0-1)	0 (0-1)	0 (0-0)	0 (0-0)
Texas	4 (4-5)	4 (4-5)	5 (5-5)	4 (4-4)	3 (3-3)	2 (2-2)	2 (2-2)	2 (2-2)	2 (2-2)	1 (1-1)	1 (1-1)	2 (2-2)	1 (1-1)	1 (1-2)
Utah	0 (0-0)	0 (0-0)	0 (0-0)	0 (0-0)	0 (0-0)	0 (0-0)	0 (0-0)	0 (0-0)	0 (0-0)	0 (0-0)	0 (0-0)	0 (0-0)	0 (0-0)	0 (0-0)
Vermont	0 (0-0)	0 (0-0)	0 (0-0)	0 (0-0)	0 (0-0)	0 (0-0)	0 (0-0)	0 (0-0)	0 (0-0)	0 (0-0)	0 (0-0)	0 (0-0)	0 (0-0)	0 (0-0)
Virginia	2 (2-2)	2 (2-2)	2 (2-2)	2 (2-2)	1 (1-1)	1 (1-1)	1 (1-1)	1 (1-1)	1 (1-1)	0 (0-1)	0 (0-0)	1 (0-1)	0 (0-0)	0 (0-0)
Washington	1 (1-1)	1 (1-1)	1 (1-1)	1 (1-1)	1 (1-1)	1 (1-1)	1 (0-1)	0 (0-0)	0 (0-0)	0 (0-0)	0 (0-0)	0 (0-0)	0 (0-0)	0 (0-0)
West Virginia	1 (1-2)	1 (1-1)	1 (1-1)	1 (1-1)	0 (0-1)	0 (0-0)	0 (0-0)	0 (0-0)	0 (0-0)	0 (0-0)	0 (0-0)	0 (0-0)	0 (0-0)	0 (0-0)
Wisconsin	1 (1-1)	1 (1-1)	1 (1-1)	1 (1-1)	1 (1-1)	1 (1-1)	0 (0-1)	0 (0-0)	0 (0-0)	0 (0-0)	0 (0-0)	0 (0-0)	0 (0-0)	0 (0-0)
Wyoming	0 (0-0)	0 (0-0)	0 (0-0)	0 (0-0)	0 (0-0)	0 (0-0)	0 (0-0)	0 (0-0)	0 (0-0)	0 (0-0)	0 (0-0)	0 (0-0)	0 (0-0)	0 (0-0)
Southern Latin America	38 (34-41)	40 (38-43)	45 (44-45)	37 (37-38)	35 (34-35)	29 (27-31)	21 (21-21)	15 (15-16)	15 (15-15)	12 (12-12)	10 (9-10)	8 (8-8)	7 (7-7)	6 (5-7)
Argentina	20 (16-24)	20 (18-22)	20 (19-21)	18 (17-18)	21 (20-21)	19 (17-21)	15 (15-15)	11 (11-11)	11 (11-11)	9 (9-9)	8 (7-8)	6 (6-6)	6 (6-6)	5 (4-5)
Chile	16 (15-17)	19 (19-19)	23 (22-23)	18 (18-18)	13 (12-13)	8 (8-9)	5 (5-5)	3 (3-3)	3 (3-3)	2 (2-2)	2 (2-2)	1 (1-1)	1 (1-1)	1 (1-1)
Uruguay	1 (1-2)	2 (2-2)	2 (2-2)	2 (2-2)	2 (2-2)	2 (2-2)	1 (1-1)	1 (1-1)	1 (1-1)	1 (1-1)	0 (0-1)	0 (0-0)	0 (0-0)	0 (0-0)
Western Europe	188 (181-196)	157 (154-160)	136 (135-136)	113 (112-113)	86 (86-87)	58 (58-59)	41 (40-41)	29 (29-30)	24 (24-25)	17 (17-17)	14 (14-14)	12 (12-12)	11 (11-11)	9 (9-10)
Andorra	0 (0-0)	0 (0-0)	0 (0-0)	0 (0-0)	0 (0-0)	0 (0-0)	0 (0-0)	0 (0-0)	0 (0-0)	0 (0-0)	0 (0-0)	0 (0-0)	0 (0-0)	0 (0-0)
Austria	4 (3-4)	3 (3-4)	3 (3-3)	2 (2-3)	2 (2-2)	1 (1-1)	1 (1-1)	1 (1-1)	0 (0-1)	0 (0-0)	0 (0-0)	0 (0-0)	0 (0-0)	0 (0-0)
Belgium	5 (5-5)	4 (4-4)	3 (3-3)	2 (2-3)	2 (2-2)	1 (1-1)	1 (1-1)	1 (1-1)	1 (1-1)	1 (0-1)	0 (0-0)	0 (0-0)	0 (0-0)	0 (0-0)
Cyprus	1 (0-1)	1 (0-1)	1 (0-1)	0 (0-1)	0 (0-0)	0 (0-0)	0 (0-0)	0 (0-0)	0 (0-0)	0 (0-0)	0 (0-0)	0 (0-0)	0 (0-0)	0 (0-0)
Denmark	2 (1-2)	1 (1-1)	1 (1-1)	1 (1-1)	1 (1-1)	1 (0-1)	0 (0-0)	0 (0-0)	0 (0-0)	0 (0-0)	0 (0-0)	0 (0-0)	0 (0-0)	0 (0-0)
Finland	3 (3-3)	2 (2-2)	1 (1-1)	1 (1-1)	1 (1-1)	0 (0-1)	0 (0-0)	0 (0-0)	0 (0-0)	0 (0-0)	0 (0-0)	0 (0-0)	0 (0-0)	0 (0-0)
France	27 (26-29)	18 (18-19)	13 (13-13)	11 (10-11)	9 (9-9)	7 (7-7)	6 (6-6)	4 (4-5)	4 (4-4)	3 (3-3)	2 (2-2)	2 (2-2)	2 (2-2)	2 (1-2)
Germany	36 (30-43)	32 (29-35)	29 (29-29)	22 (21-22)	15 (15-16)	10 (10-10)	7 (7-8)	5 (5-5)	4 (4-5)	3 (3-3)	2 (2-2)	2 (2-2)	2 (2-2)	2 (1-2)
Greece	5 (5-6)	4 (4-4)	4 (4-4)	3 (3-3)	3 (2-3)	2 (2-2)	2 (2-2)	1 (1-1)	1 (1-1)	1 (0-1)	0 (0-0)	0 (0-0)	0 (0-0)	0 (0-0)
Iceland	0 (0-0)	0 (0-0)	0 (0-0)	0 (0-0)	0 (0-0)	0 (0-0)	0 (0-0)	0 (0-0)	0 (0-0)	0 (0-0)	0 (0-0)	0 (0-0)	0 (0-0)	0 (0-0)
Ireland	2 (2-2)	2 (1-2)	1 (1-1)	1 (1-1)	1 (1-1)	1 (1-1)	1 (1-1)	0 (0-0)	0 (0-0)	0 (0-0)	0 (0-0)	0 (0-0)	0 (0-0)	0 (0-0)
Israel	1 (1-2)	1 (1-1)	1 (1-1)	1 (1-1)	1 (1-1)	1 (1-1)	1 (1-1)	1 (1-1)	1 (1-1)	1 (1-1)	0 (0-1)	0 (0-0)	0 (0-0)	0 (0-0)
Italy	33 (31-35)	29 (28-29)	26 (26-27)	23 (22-23)	17 (17-17)	11 (11-11)	6 (6-6)	4 (4-4)	3 (3-3)	2 (2-2)	2 (2-2)	1 (1-1)	1 (1-1)	1 (1-1)
Luxembourg	0 (0-0)	0 (0-0)	0 (0-0)	0 (0-0)	0 (0-0)	0 (0-0)	0 (0-0)	0 (0-0)	0 (0-0)	0 (0-0)	0 (0-0)	0 (0-0)	0 (0-0)	0 (0-0)
Malta	0 (0-0)	0 (0-0)	0 (0-0)	0 (0-0)	0 (0-0)	0 (0-0)	0 (0-0)	0 (0-0)	0 (0-0)	0 (0-0)	0 (0-0)	0 (0-0)	0 (0-0)	0 (0-0)
Netherlands	4 (4-4)	3 (3-3)	3 (3-3)	3 (2-3)	2 (2-2)	1 (1-1)	1 (1-1)	1 (1-1)	1 (1-1)	1 (1-1)	1 (1-1)	1 (1-1)	0 (0-0)	0 (0-0)
Norway	1 (1-1)	1 (1-1)	1 (1-1)	1 (1-1)	1 (1-1)	0 (0-0)	0 (0-0)	0 (0-0)	0 (0-0)	0 (0-0)	0 (0-0)	0 (0-0)	0 (0-0)	0 (0-0)
Portugal	17 (16-19)	14 (14-15)	12 (12-12)	10 (10-10)	7 (7-7)	4 (4-4)	3 (3-3)	2 (2-2)	1 (1-1)	1 (1-1)	0 (0-1)	0 (0-0)	0 (0-0)	0 (0-0)
Spain	30 (29-32)	23 (23-24)	19 (19-19)	16 (16-16)	12 (12-12)	8 (8-8)	5 (5-5)	3 (3-3)	2 (2-2)	1 (1-1)	1 (1-1)	1 (1-1)	1 (1-1)	1 (1-1)
Sweden	2 (1-2)	1 (1-1)	1 (1-1)	1 (1-1)	1 (1-1)	1 (1-1)	0 (0-0)	0 (0-0)	0 (0-0)	0 (0-0)	0 (0-0)	0 (0-0)	0 (0-0)	0 (0-0)
Stockholm	0 (0-0)	0 (0-0)	0 (0-0)	0 (0-0)	0 (0-0)	0 (0-0)	0 (0-0)	0 (0-0)	0 (0-0)	0 (0-0)	0 (0-0)	0 (0-0)	0 (0-0)	0 (0-0)
Sweden except Stockholm	1 (1-1)	1 (1-1)	1 (1-1)	1 (1-1)	1 (1-1)	0 (0-1)	0 (0-0)	0 (0-0)	0 (0-0)	0 (0-0)	0 (0-0)	0 (0-0)	0 (0-0)	0 (0-0)
Switzerland	2 (2-2)	2 (2-2)	1 (1-2)	1 (1-1)	1 (1-1)	1 (1-1)	0 (0-0)	0 (0-0)	0 (0-0)	0 (0-0)	0 (0-0)	0 (0-0)	0 (0-0)	0 (0-0)
United Kingdom	14 (13-15)	14 (13-14)	14 (14-14)	13 (13-13)	11 (11-11)	7 (7-7)	6 (6-6)	5 (5-5)	4 (4-4)	3 (3-3)	2 (2-3)	2 (2-2)	2 (2-2)	2 (2-2)
England	9 (9-10)	10 (10-10)	10 (10-10)	10 (10-10)	8 (8-8)	5 (5-6)	5 (5-5)	4 (4-4)	4 (3-4)	2 (2-2)	2 (2-2)	2 (2-2)	2 (2-2)	2 (2-2)
East Midlands	1 (1-1)	1 (1-1)	1 (1-1)	1 (1-1)	1 (1-1)	0 (0-0)	0 (0-0)	0 (0-0)	0 (0-0)	0 (0-0)	0 (0-0)	0 (0-0)	0 (0-0)	0 (0-0)
Derby	0 (0-0)	0 (0-0)	0 (0-0)	0 (0-0)	0 (0-0)	0 (0-0)	0 (0-0)	0 (0-0)	0 (0-0)	0 (0-0)	0 (0-0)	0 (0-0)	0 (0-0)	0 (0-0)
Derbyshire	0 (0-0)	0 (0-0)	0 (0-0)	0 (0-0)	0 (0-0)	0 (0-0)	0 (0-0)	0 (0-0)	0 (0-0)	0 (0-0)	0 (0-0)	0 (0-0)	0 (0-0)	0 (0-0)
Leicester	0 (0-0)	0 (0-0)	0 (0-0)	0 (0-0)	0 (0-0)	0 (0-0)	0 (0-0)	0 (0-0)	0 (0-0)	0 (0-0)	0 (0-0)	0 (0-0)	0 (0-0)	0 (0-0)
Leicestershire	0 (0-0)	0 (0-0)	0 (0-0)	0 (0-0)	0 (0-0)	0 (0-0)	0 (0-0)	0 (0-0)	0 (0-0)	0 (0-0)	0 (0-0)	0 (0-0)	0 (0-0)	0 (0-0)
Lincolnshire	0 (0-0)	0 (0-0)	0 (0-0)	0 (0-0)	0 (0-0)	0 (0-0)	0 (0-0)	0 (0-0)	0 (0-0)	0 (0-0)	0 (0-0)	0 (0-0)	0 (0-0)	0 (0-0)
Northamptonshire	0 (0-0)	0 (0-0)	0 (0-0)	0 (0-0)	0 (0-0)	0 (0-0)	0 (0-0)	0 (0-0)	0 (0-0)	0 (0-0)	0 (0-0)	0 (0-0)	0 (0-0)	0 (0-0)
Nottingham	0 (0-0)	0 (0-0)	0 (0-0)	0 (0-0)	0 (0-0)	0 (0-0)	0 (0-0)	0 (0-0)	0 (0-0)	0 (0-0)	0 (0-0)	0 (0-0)	0 (0-0)	0 (0-0)
Nottinghamshire	0 (0-0)	0 (0-0)	0 (0-0)	0 (0-0)	0 (0-0)	0 (0-0)	0 (0-0)	0 (0-0)	0 (0-0)	0 (0-0)	0 (0-0)	0 (0-0)	0 (0-0)	0 (0-0)
Rutland	0 (0-0)	0 (0-0)	0 (0-0)	0 (0-0)	0 (0-0)	0 (0-0)	0 (0-0)	0 (0-0)	0 (0-0)	0 (0-0)	0 (0-0)	0 (0-0)	0 (0-0)	0 (0-0)
East of England	1 (1-1)	1 (1-1)	1 (1-1)	1 (1-1)	1 (1-1)	0 (0-1)	0 (0-0)	0 (0-0)	0 (0-0)	0 (0-0)	0 (0-0)	0 (0-0)	0 (0-0)	0 (0-0)
Bedford	0 (0-0)	0 (0-0)	0 (0-0)	0 (0-0)	0 (0-0)	0 (0-0)	0 (0-0)	0 (0-0)	0 (0-0)	0 (0-0)	0 (0-0)	0 (0-0)	0 (0-0)	0 (0-0)
Cambridgeshire	0 (0-0)	0 (0-0)	0 (0-0)	0 (0-0)	0 (0-0)	0 (0-0)	0 (0-0)	0 (0-0)	0 (0-0)	0 (0-0)	0 (0-0)	0 (0-0)	0 (0-0)	0 (0-0)
Central Bedfordshire	0 (0-0)	0 (0-0)	0 (0-0)	0 (0-0)	0 (0-0)	0 (0-0)	0 (0-0)	0 (0-0)	0 (0-0)	0 (0-0)	0 (0-0)	0 (0-0)	0 (0-0)	0 (0-0)
Essex	0 (0-0)	0 (0-0)	0 (0-0)	0 (0-0)	0 (0-0)	0 (0-0)	0 (0-0)	0 (0-0)	0 (0-0)	0 (0-0)	0 (0-0)	0 (0-0)	0 (0-0)	0 (0-0)
Hertfordshire	0 (0-0)	0 (0-0)	0 (0-0)	0 (0-0)	0 (0-0)	0 (0-0)	0 (0-0)	0 (0-0)	0 (0-0)	0 (0-0)	0 (0-0)	0 (0-0)	0 (0-0)	0 (0-0)

Table 4 Under-5 death numbers by location and year for 1950, 1955...2005, 2010, 2017 - males

Location	1950	1955	1960	1965	1970	1975	1980	1985	1990	1995	2000	2005	2010	2017
Luton	0 (0-0)	0 (0-0)	0 (0-0)	0 (0-0)	0 (0-0)	0 (0-0)	0 (0-0)	0 (0-0)	0 (0-0)	0 (0-0)	0 (0-0)	0 (0-0)	0 (0-0)	0 (0-0)
Norfolk	0 (0-0)	0 (0-0)	0 (0-0)	0 (0-0)	0 (0-0)	0 (0-0)	0 (0-0)	0 (0-0)	0 (0-0)	0 (0-0)	0 (0-0)	0 (0-0)	0 (0-0)	0 (0-0)
Peterborough	0 (0-0)	0 (0-0)	0 (0-0)	0 (0-0)	0 (0-0)	0 (0-0)	0 (0-0)	0 (0-0)	0 (0-0)	0 (0-0)	0 (0-0)	0 (0-0)	0 (0-0)	0 (0-0)
Southend-on-Sea	0 (0-0)	0 (0-0)	0 (0-0)	0 (0-0)	0 (0-0)	0 (0-0)	0 (0-0)	0 (0-0)	0 (0-0)	0 (0-0)	0 (0-0)	0 (0-0)	0 (0-0)	0 (0-0)
Suffolk	0 (0-0)	0 (0-0)	0 (0-0)	0 (0-0)	0 (0-0)	0 (0-0)	0 (0-0)	0 (0-0)	0 (0-0)	0 (0-0)	0 (0-0)	0 (0-0)	0 (0-0)	0 (0-0)
Thurrock	0 (0-0)	0 (0-0)	0 (0-0)	0 (0-0)	0 (0-0)	0 (0-0)	0 (0-0)	0 (0-0)	0 (0-0)	0 (0-0)	0 (0-0)	0 (0-0)	0 (0-0)	0 (0-0)
Greater London	2 (1-2)	2 (2-2)	2 (2-2)	2 (2-2)	1 (1-1)	1 (1-1)	1 (1-1)	1 (1-1)	1 (1-1)	0 (0-0)	0 (0-0)	0 (0-0)	0 (0-0)	0 (0-0)
Barking and Dagenham	0 (0-0)	0 (0-0)	0 (0-0)	0 (0-0)	0 (0-0)	0 (0-0)	0 (0-0)	0 (0-0)	0 (0-0)	0 (0-0)	0 (0-0)	0 (0-0)	0 (0-0)	0 (0-0)
Barnet	0 (0-0)	0 (0-0)	0 (0-0)	0 (0-0)	0 (0-0)	0 (0-0)	0 (0-0)	0 (0-0)	0 (0-0)	0 (0-0)	0 (0-0)	0 (0-0)	0 (0-0)	0 (0-0)
Bexley	0 (0-0)	0 (0-0)	0 (0-0)	0 (0-0)	0 (0-0)	0 (0-0)	0 (0-0)	0 (0-0)	0 (0-0)	0 (0-0)	0 (0-0)	0 (0-0)	0 (0-0)	0 (0-0)
Brent	0 (0-0)	0 (0-0)	0 (0-0)	0 (0-0)	0 (0-0)	0 (0-0)	0 (0-0)	0 (0-0)	0 (0-0)	0 (0-0)	0 (0-0)	0 (0-0)	0 (0-0)	0 (0-0)
Bromley	0 (0-0)	0 (0-0)	0 (0-0)	0 (0-0)	0 (0-0)	0 (0-0)	0 (0-0)	0 (0-0)	0 (0-0)	0 (0-0)	0 (0-0)	0 (0-0)	0 (0-0)	0 (0-0)
Camden	0 (0-0)	0 (0-0)	0 (0-0)	0 (0-0)	0 (0-0)	0 (0-0)	0 (0-0)	0 (0-0)	0 (0-0)	0 (0-0)	0 (0-0)	0 (0-0)	0 (0-0)	0 (0-0)
Croydon	0 (0-0)	0 (0-0)	0 (0-0)	0 (0-0)	0 (0-0)	0 (0-0)	0 (0-0)	0 (0-0)	0 (0-0)	0 (0-0)	0 (0-0)	0 (0-0)	0 (0-0)	0 (0-0)
Ealing	0 (0-0)	0 (0-0)	0 (0-0)	0 (0-0)	0 (0-0)	0 (0-0)	0 (0-0)	0 (0-0)	0 (0-0)	0 (0-0)	0 (0-0)	0 (0-0)	0 (0-0)	0 (0-0)
Enfield	0 (0-0)	0 (0-0)	0 (0-0)	0 (0-0)	0 (0-0)	0 (0-0)	0 (0-0)	0 (0-0)	0 (0-0)	0 (0-0)	0 (0-0)	0 (0-0)	0 (0-0)	0 (0-0)
Greenwich	0 (0-0)	0 (0-0)	0 (0-0)	0 (0-0)	0 (0-0)	0 (0-0)	0 (0-0)	0 (0-0)	0 (0-0)	0 (0-0)	0 (0-0)	0 (0-0)	0 (0-0)	0 (0-0)
Hackney	0 (0-0)	0 (0-0)	0 (0-0)	0 (0-0)	0 (0-0)	0 (0-0)	0 (0-0)	0 (0-0)	0 (0-0)	0 (0-0)	0 (0-0)	0 (0-0)	0 (0-0)	0 (0-0)
Hammersmith and Fulham	0 (0-0)	0 (0-0)	0 (0-0)	0 (0-0)	0 (0-0)	0 (0-0)	0 (0-0)	0 (0-0)	0 (0-0)	0 (0-0)	0 (0-0)	0 (0-0)	0 (0-0)	0 (0-0)
Haringey	0 (0-0)	0 (0-0)	0 (0-0)	0 (0-0)	0 (0-0)	0 (0-0)	0 (0-0)	0 (0-0)	0 (0-0)	0 (0-0)	0 (0-0)	0 (0-0)	0 (0-0)	0 (0-0)
Harrow	0 (0-0)	0 (0-0)	0 (0-0)	0 (0-0)	0 (0-0)	0 (0-0)	0 (0-0)	0 (0-0)	0 (0-0)	0 (0-0)	0 (0-0)	0 (0-0)	0 (0-0)	0 (0-0)
Havering	0 (0-0)	0 (0-0)	0 (0-0)	0 (0-0)	0 (0-0)	0 (0-0)	0 (0-0)	0 (0-0)	0 (0-0)	0 (0-0)	0 (0-0)	0 (0-0)	0 (0-0)	0 (0-0)
Hillingdon	0 (0-0)	0 (0-0)	0 (0-0)	0 (0-0)	0 (0-0)	0 (0-0)	0 (0-0)	0 (0-0)	0 (0-0)	0 (0-0)	0 (0-0)	0 (0-0)	0 (0-0)	0 (0-0)
Hounslow	0 (0-0)	0 (0-0)	0 (0-0)	0 (0-0)	0 (0-0)	0 (0-0)	0 (0-0)	0 (0-0)	0 (0-0)	0 (0-0)	0 (0-0)	0 (0-0)	0 (0-0)	0 (0-0)
Islington	0 (0-0)	0 (0-0)	0 (0-0)	0 (0-0)	0 (0-0)	0 (0-0)	0 (0-0)	0 (0-0)	0 (0-0)	0 (0-0)	0 (0-0)	0 (0-0)	0 (0-0)	0 (0-0)
Kensington and Chelsea	0 (0-0)	0 (0-0)	0 (0-0)	0 (0-0)	0 (0-0)	0 (0-0)	0 (0-0)	0 (0-0)	0 (0-0)	0 (0-0)	0 (0-0)	0 (0-0)	0 (0-0)	0 (0-0)
Kingston upon Thames	0 (0-0)	0 (0-0)	0 (0-0)	0 (0-0)	0 (0-0)	0 (0-0)	0 (0-0)	0 (0-0)	0 (0-0)	0 (0-0)	0 (0-0)	0 (0-0)	0 (0-0)	0 (0-0)
Lambeth	0 (0-0)	0 (0-0)	0 (0-0)	0 (0-0)	0 (0-0)	0 (0-0)	0 (0-0)	0 (0-0)	0 (0-0)	0 (0-0)	0 (0-0)	0 (0-0)	0 (0-0)	0 (0-0)
Lewisham	0 (0-0)	0 (0-0)	0 (0-0)	0 (0-0)	0 (0-0)	0 (0-0)	0 (0-0)	0 (0-0)	0 (0-0)	0 (0-0)	0 (0-0)	0 (0-0)	0 (0-0)	0 (0-0)
Merton	0 (0-0)	0 (0-0)	0 (0-0)	0 (0-0)	0 (0-0)	0 (0-0)	0 (0-0)	0 (0-0)	0 (0-0)	0 (0-0)	0 (0-0)	0 (0-0)	0 (0-0)	0 (0-0)
Newham	0 (0-0)	0 (0-0)	0 (0-0)	0 (0-0)	0 (0-0)	0 (0-0)	0 (0-0)	0 (0-0)	0 (0-0)	0 (0-0)	0 (0-0)	0 (0-0)	0 (0-0)	0 (0-0)
Redbridge	0 (0-0)	0 (0-0)	0 (0-0)	0 (0-0)	0 (0-0)	0 (0-0)	0 (0-0)	0 (0-0)	0 (0-0)	0 (0-0)	0 (0-0)	0 (0-0)	0 (0-0)	0 (0-0)
Richmond upon Thames	0 (0-0)	0 (0-0)	0 (0-0)	0 (0-0)	0 (0-0)	0 (0-0)	0 (0-0)	0 (0-0)	0 (0-0)	0 (0-0)	0 (0-0)	0 (0-0)	0 (0-0)	0 (0-0)
Southwark	0 (0-0)	0 (0-0)	0 (0-0)	0 (0-0)	0 (0-0)	0 (0-0)	0 (0-0)	0 (0-0)	0 (0-0)	0 (0-0)	0 (0-0)	0 (0-0)	0 (0-0)	0 (0-0)
Sutton	0 (0-0)	0 (0-0)	0 (0-0)	0 (0-0)	0 (0-0)	0 (0-0)	0 (0-0)	0 (0-0)	0 (0-0)	0 (0-0)	0 (0-0)	0 (0-0)	0 (0-0)	0 (0-0)
Tower Hamlets	0 (0-0)	0 (0-0)	0 (0-0)	0 (0-0)	0 (0-0)	0 (0-0)	0 (0-0)	0 (0-0)	0 (0-0)	0 (0-0)	0 (0-0)	0 (0-0)	0 (0-0)	0 (0-0)
Waltham Forest	0 (0-0)	0 (0-0)	0 (0-0)	0 (0-0)	0 (0-0)	0 (0-0)	0 (0-0)	0 (0-0)	0 (0-0)	0 (0-0)	0 (0-0)	0 (0-0)	0 (0-0)	0 (0-0)
Wandsworth	0 (0-0)	0 (0-0)	0 (0-0)	0 (0-0)	0 (0-0)	0 (0-0)	0 (0-0)	0 (0-0)	0 (0-0)	0 (0-0)	0 (0-0)	0 (0-0)	0 (0-0)	0 (0-0)
Westminster	0 (0-0)	0 (0-0)	0 (0-0)	0 (0-0)	0 (0-0)	0 (0-0)	0 (0-0)	0 (0-0)	0 (0-0)	0 (0-0)	0 (0-0)	0 (0-0)	0 (0-0)	0 (0-0)
North East England	1 (1-1)	1 (1-1)	1 (1-1)	1 (1-1)	1 (1-1)	0 (0-0)	0 (0-0)	0 (0-0)	0 (0-0)	0 (0-0)	0 (0-0)	0 (0-0)	0 (0-0)	0 (0-0)
County Durham	0 (0-0)	0 (0-0)	0 (0-0)	0 (0-0)	0 (0-0)	0 (0-0)	0 (0-0)	0 (0-0)	0 (0-0)	0 (0-0)	0 (0-0)	0 (0-0)	0 (0-0)	0 (0-0)
Darlington	0 (0-0)	0 (0-0)	0 (0-0)	0 (0-0)	0 (0-0)	0 (0-0)	0 (0-0)	0 (0-0)	0 (0-0)	0 (0-0)	0 (0-0)	0 (0-0)	0 (0-0)	0 (0-0)
Gateshead	0 (0-0)	0 (0-0)	0 (0-0)	0 (0-0)	0 (0-0)	0 (0-0)	0 (0-0)	0 (0-0)	0 (0-0)	0 (0-0)	0 (0-0)	0 (0-0)	0 (0-0)	0 (0-0)
Hartlepool	0 (0-0)	0 (0-0)	0 (0-0)	0 (0-0)	0 (0-0)	0 (0-0)	0 (0-0)	0 (0-0)	0 (0-0)	0 (0-0)	0 (0-0)	0 (0-0)	0 (0-0)	0 (0-0)
Middlesbrough	0 (0-0)	0 (0-0)	0 (0-0)	0 (0-0)	0 (0-0)	0 (0-0)	0 (0-0)	0 (0-0)	0 (0-0)	0 (0-0)	0 (0-0)	0 (0-0)	0 (0-0)	0 (0-0)
Newcastle upon Tyne	0 (0-0)	0 (0-0)	0 (0-0)	0 (0-0)	0 (0-0)	0 (0-0)	0 (0-0)	0 (0-0)	0 (0-0)	0 (0-0)	0 (0-0)	0 (0-0)	0 (0-0)	0 (0-0)
North Tyneside	0 (0-0)	0 (0-0)	0 (0-0)	0 (0-0)	0 (0-0)	0 (0-0)	0 (0-0)	0 (0-0)	0 (0-0)	0 (0-0)	0 (0-0)	0 (0-0)	0 (0-0)	0 (0-0)
Northumberland	0 (0-0)	0 (0-0)	0 (0-0)	0 (0-0)	0 (0-0)	0 (0-0)	0 (0-0)	0 (0-0)	0 (0-0)	0 (0-0)	0 (0-0)	0 (0-0)	0 (0-0)	0 (0-0)
Redcar and Cleveland	0 (0-0)	0 (0-0)	0 (0-0)	0 (0-0)	0 (0-0)	0 (0-0)	0 (0-0)	0 (0-0)	0 (0-0)	0 (0-0)	0 (0-0)	0 (0-0)	0 (0-0)	0 (0-0)
South Tyneside	0 (0-0)	0 (0-0)	0 (0-0)	0 (0-0)	0 (0-0)	0 (0-0)	0 (0-0)	0 (0-0)	0 (0-0)	0 (0-0)	0 (0-0)	0 (0-0)	0 (0-0)	0 (0-0)
Stockton-on-Tees	0 (0-0)	0 (0-0)	0 (0-0)	0 (0-0)	0 (0-0)	0 (0-0)	0 (0-0)	0 (0-0)	0 (0-0)	0 (0-0)	0 (0-0)	0 (0-0)	0 (0-0)	0 (0-0)
Sunderland	0 (0-0)	0 (0-0)	0 (0-0)	0 (0-0)	0 (0-0)	0 (0-0)	0 (0-0)	0 (0-0)	0 (0-0)	0 (0-0)	0 (0-0)	0 (0-0)	0 (0-0)	0 (0-0)
North West England	1 (1-1)	1 (1-1)	2 (1-2)	1 (1-2)	1 (1-1)	1 (1-1)	1 (1-1)	1 (1-1)	1 (0-1)	0 (0-0)	0 (0-0)	0 (0-0)	0 (0-0)	0 (0-0)
Blackburn with Darwen	0 (0-0)	0 (0-0)	0 (0-0)	0 (0-0)	0 (0-0)	0 (0-0)	0 (0-0)	0 (0-0)	0 (0-0)	0 (0-0)	0 (0-0)	0 (0-0)	0 (0-0)	0 (0-0)
Blackpool	0 (0-0)	0 (0-0)	0 (0-0)	0 (0-0)	0 (0-0)	0 (0-0)	0 (0-0)	0 (0-0)	0 (0-0)	0 (0-0)	0 (0-0)	0 (0-0)	0 (0-0)	0 (0-0)
Bolton	0 (0-0)	0 (0-0)	0 (0-0)	0 (0-0)	0 (0-0)	0 (0-0)	0 (0-0)	0 (0-0)	0 (0-0)	0 (0-0)	0 (0-0)	0 (0-0)	0 (0-0)	0 (0-0)
Bury	0 (0-0)	0 (0-0)	0 (0-0)	0 (0-0)	0 (0-0)	0 (0-0)	0 (0-0)	0 (0-0)	0 (0-0)	0 (0-0)	0 (0-0)	0 (0-0)	0 (0-0)	0 (0-0)
Cheshire East	0 (0-0)	0 (0-0)	0 (0-0)	0 (0-0)	0 (0-0)	0 (0-0)	0 (0-0)	0 (0-0)	0 (0-0)	0 (0-0)	0 (0-0)	0 (0-0)	0 (0-0)	0 (0-0)
Cheshire West and Chester	0 (0-0)	0 (0-0)	0 (0-0)	0 (0-0)	0 (0-0)	0 (0-0)	0 (0-0)	0 (0-0)	0 (0-0)	0 (0-0)	0 (0-0)	0 (0-0)	0 (0-0)	0 (0-0)
Cumbria	0 (0-0)	0 (0-0)	0 (0-0)	0 (0-0)	0 (0-0)	0 (0-0)	0 (0-0)	0 (0-0)	0 (0-0)	0 (0-0)	0 (0-0)	0 (0-0)	0 (0-0)	0 (0-0)
Halton	0 (0-0)	0 (0-0)	0 (0-0)	0 (0-0)	0 (0-0)	0 (0-0)	0 (0-0)	0 (0-0)	0 (0-0)	0 (0-0)	0 (0-0)	0 (0-0)	0 (0-0)	0 (0-0)
Knowsley	0 (0-0)	0 (0-0)	0 (0-0)	0 (0-0)	0 (0-0)	0 (0-0)	0 (0-0)	0 (0-0)	0 (0-0)	0 (0-0)	0 (0-0)	0 (0-0)	0 (0-0)	0 (0-0)
Lancashire	0 (0-0)	0 (0-0)	0 (0-0)	0 (0-0)	0 (0-0)	0 (0-0)	0 (0-0)	0 (0-0)	0 (0-0)	0 (0-0)	0 (0-0)	0 (0-0)	0 (0-0)	0 (0-0)
Liverpool	0 (0-0)	0 (0-0)	0 (0-0)	0 (0-0)	0 (0-0)	0 (0-0)	0 (0-0)	0 (0-0)	0 (0-0)	0 (0-0)	0 (0-0)	0 (0-0)	0 (0-0)	0 (0-0)
Manchester	0 (0-0)	0 (0-0)	0 (0-0)	0 (0-0)	0 (0-0)	0 (0-0)	0 (0-0)	0 (0-0)	0 (0-0)	0 (0-0)	0 (0-0)	0 (0-0)	0 (0-0)	0 (0-0)
Oldham	0 (0-0)	0 (0-0)	0 (0-0)	0 (0-0)	0 (0-0)	0 (0-0)	0 (0-0)	0 (0-0)	0 (0-0)	0 (0-0)	0 (0-0)	0 (0-0)	0 (0-0)	0 (0-0)
Rochdale	0 (0-0)	0 (0-0)	0 (0-0)	0 (0-0)	0 (0-0)	0 (0-0)	0 (0-0)	0 (0-0)	0 (0-0)	0 (0-0)	0 (0-0)	0 (0-0)	0 (0-0)	0 (0-0)
Salford	0 (0-0)	0 (0-0)	0 (0-0)	0 (0-0)	0 (0-0)	0 (0-0)	0 (0-0)	0 (0-0)	0 (0-0)	0 (0-0)	0 (0-0)	0 (0-0)	0 (0-0)	0 (0-0)
Sefton	0 (0-0)	0 (0-0)	0 (0-0)	0 (0-0)	0 (0-0)	0 (0-0)	0 (0-0)	0 (0-0)	0 (0-0)	0 (0-0)	0 (0-0)	0 (0-0)	0 (0-0)	0 (0-0)
St Helens	0 (0-0)	0 (0-0)	0 (0-0)	0 (0-0)	0 (0-0)	0 (0-0)	0 (0-0)	0 (0-0)	0 (0-0)	0 (0-0)	0 (0-0)	0 (0-0)	0 (0-0)	0 (0-0)
Stockport	0 (0-0)	0 (0-0)	0 (0-0)	0 (0-0)	0 (0-0)	0 (0-0)	0 (0-0)	0 (0-0)	0 (0-0)	0 (0-0)	0 (0-0)	0 (0-0)	0 (0-0)	0 (0-0)
Tameside	0 (0-0)	0 (0-0)	0 (0-0)	0 (0-0)	0 (0-0)	0 (0-0)	0 (0-0)	0 (0-0)	0 (0-0)	0 (0-0)	0 (0-0)	0 (0-0)	0 (0-0)	0 (0-0)
Trafford	0 (0-0)	0 (0-0)	0 (0-0)	0 (0-0)	0 (0-0)	0 (0-0)	0 (0-0)	0 (0-0)	0 (0-0)	0 (0-0)	0 (0-0)	0 (0-0)	0 (0-0)	0 (0-0)
Warrington	0 (0-0)	0 (0-0)	0 (0-0)	0 (0-0)	0 (0-0)	0 (0-0)	0 (0-0)	0 (0-0)	0 (0-0)	0 (0-0)	0 (0-0)	0 (0-0)	0 (0-0)	0 (0-0)
Wigan	0 (0-0)	0 (0-0)	0 (0-0)	0 (0-0)	0 (0-0)	0 (0-0)	0 (0-0)	0 (0-0)	0 (0-0)	0 (0-0)	0 (0-0)	0 (0-0)	0 (0-0)	0 (0-0)
Wirral	0 (0-0)	0 (0-0)	0 (0-0)	0 (0-0)	0 (0-0)	0 (0-0)	0 (0-0)	0 (0-0)	0 (0-0)	0 (0-0)	0 (0-0)	0 (0-0)	0 (0-0)	0 (0-0)
South East England	1 (1-1)	1 (1-1)	1 (1-1)	1 (1-1)	1 (1-1)	1 (1-1)	1 (1-1)	1 (1-1)	0 (0-0)	0 (0-0)	0 (0-0)	0 (0-0)	0 (0-0)	0 (0-0)
Bracknell Forest	0 (0-0)	0 (0-0)	0 (0-0)	0 (0-0)	0 (0-0)	0 (0-0)	0 (0-0)	0 (0-0)	0 (0-0)	0 (0-0)	0 (0-0)	0 (0-0)	0 (0-0)	0 (0-0)
Brighton and Hove	0 (0-0)	0 (0-0)	0 (0-0)	0 (0-0)	0 (0-0)	0 (0-0)	0 (0-0)	0 (0-0)	0 (0-0)	0 (0-0)	0 (0-0)	0 (0-0)	0 (0-0)	0 (0-0)
Buckinghamshire	0 (0-0)	0 (0-0)	0 (0-0)	0 (0-0)	0 (0-0)	0 (0-0)	0 (0-0)	0 (0-0)	0 (0-0)	0 (0-0)	0 (0-0)	0 (0-0)	0 (0-0)	0 (0-0)
East Sussex	0 (0-0)	0 (0-0)	0 (0-0)	0 (0-0)	0 (0-0)	0 (0-0)	0 (0-0)	0 (0-0)	0 (0-0)	0 (0-0)	0 (0-0)	0 (0-0)	0 (0-0)	0 (0-0)
Hampshire	0 (0-0)	0 (0-0)	0 (0-0)	0 (0-0)	0 (0-0)	0 (0-0)	0 (0-0)	0 (0-0)	0 (0-0)	0 (0-0)	0 (0-0)	0 (0-0)	0 (0-0)	0 (0-0)
Isle of Wight	0 (0-0)	0 (0-0)	0 (0-0)	0 (0-0)	0 (0-0)	0 (0-0)	0 (0-0)	0 (0-0)	0 (0-0)	0 (0-0)	0 (0-0)	0 (0-0)	0 (0-0)	0 (0-0)
Kent	0 (0-0)	0 (0-0)	0 (0-0)	0 (0-0)	0 (0-0)	0 (0-0)	0 (0-0)	0 (0-0)	0 (0-0)	0 (0-0)	0 (0-0)	0 (0-0)	0 (0-0)	0 (0-0)
Medway	0 (0-0)	0 (0-0)	0 (0-0)	0 (0-0)	0 (0-0)	0 (0-0)	0 (0-0)	0 (0-0)	0 (0-0)	0 (0-0)	0 (0-0)	0 (0-0)	0 (0-0)	0 (0-0)
Milton Keynes	0 (0-0)	0 (0-0)	0 (0-0)	0 (0-0)	0 (0-0)	0 (0-0)	0 (0-0)	0 (0-0)	0 (0-0)	0 (0-0)	0 (0-0)	0 (0-0)	0 (0-0)	0 (0-0)
Oxfordshire	0 (0-0)	0 (0-0)	0 (0-0)	0 (0-0)	0 (0-0)	0 (0-0)	0 (0-0)	0 (0-0)	0 (0-0)	0 (0-0)	0 (0-0)	0 (0-0)	0 (0-0)	0 (0-0)
Portsmouth	0 (0-0)	0 (0-0)	0 (0-0)	0 (0-0)	0 (0-0)	0 (0-0)	0 (0-0)	0 (0-0)	0 (0-0)	0 (0-0)	0 (0-0)	0 (0-0)	0 (0-0)	0 (0-0)
Reading	0 (0-0)	0 (0-0)	0 (0-0)	0 (0-0)	0 (0-0)	0 (0-0)	0 (0-0)	0 (0-0)	0 (0-0)	0 (0-0)	0 (0-0)	0 (0-0)	0 (0-0)	0 (0-0)
Slough	0 (0-0)	0 (0-0)	0 (0-0)	0 (0-0)	0 (0-0)	0 (0-0)	0 (0-0)	0 (0-0)	0 (0-0)	0 (0-0)	0 (0-0)	0 (0-0)	0 (0-0)	0 (0-0)
Southampton	0 (0-0)	0 (0-0)	0 (0-0)	0 (0-0)	0 (0-0)	0 (0-0)	0 (0-0)	0 (0-0)	0 (0-0)	0 (0-0)	0 (0-0)	0 (0-0)	0 (0-0)	0 (0-0)
Surrey	0 (0-0)	0 (0-0)	0 (0-0)	0 (0-0)	0 (0-0)	0 (0-0)	0 (0-0)	0 (0-0)	0 (0-0)	0 (0-0)	0 (0-0)	0 (0-0)	0 (0-0)	0 (0-0)
West Berkshire	0 (0-0)	0 (0-0)	0 (0-0)	0 (0-0)	0 (0-0)	0 (0-0)	0 (0-0)	0 (0-0)	0 (0-0)	0 (0-0)	0 (0-0)	0 (0-0)	0 (0-0)	0 (0-0)
West Sussex	0 (0-0)	0 (0-0)	0 (0-0)	0 (0-0)	0 (0-0)	0 (0-0)	0 (0-0)	0 (0-0)	0 (0-0)	0 (0-0)	0 (0-0)	0 (0-0)	0 (0-0)	0 (0-0)
Windsor and Maidenhead	0 (0-0)	0 (0-0)	0 (0-0)	0 (0-0)	0 (0-0)	0 (0-0)	0 (0-0)	0 (0-0)	0 (0-0)	0 (0-0)	0 (0-0)	0 (0-0)	0 (0-0)	0 (0-0)
Wokingham	0 (0-0)	0 (0-0)	0 (0-0)	0 (0-0)	0 (0-0)	0 (0-0)	0 (0-0)	0 (0-0)	0 (0-0)	0 (0-0)	0 (0-0)	0 (0-0)	0 (0-0)	0 (0-0)
South West England	1 (1-1)	1 (1-1)	1 (1-1)	1 (1-1)	1 (1-1)	0 (0-0)	0 (0-0)	0 (0-0)	0 (0-0)	0 (0-0)	0 (0-0)	0 (0-0)	0 (0-0)	0 (0-0)
Bath and North East Somerset	0 (0-0)	0 (0-0)	0 (0-0)	0 (0-0)	0 (0-0)	0 (0-0)	0 (0-0)	0 (0-0)	0 (0-0)	0 (0-0)	0 (0-0)	0 (0-0)	0 (0-0)	0 (0-0)

Table 4 Under-5 death numbers by location and year for 1950, 1955...2005, 2010, 2017 - males

Location	1950	1955	1960	1965	1970	1975	1980	1985	1990	1995	2000	2005	2010	2017
Bournemouth	0 (0-0)	0 (0-0)	0 (0-0)	0 (0-0)	0 (0-0)	0 (0-0)	0 (0-0)	0 (0-0)	0 (0-0)	0 (0-0)	0 (0-0)	0 (0-0)	0 (0-0)	0 (0-0)
Bristol, City of	0 (0-0)	0 (0-0)	0 (0-0)	0 (0-0)	0 (0-0)	0 (0-0)	0 (0-0)	0 (0-0)	0 (0-0)	0 (0-0)	0 (0-0)	0 (0-0)	0 (0-0)	0 (0-0)
Cornwall	0 (0-0)	0 (0-0)	0 (0-0)	0 (0-0)	0 (0-0)	0 (0-0)	0 (0-0)	0 (0-0)	0 (0-0)	0 (0-0)	0 (0-0)	0 (0-0)	0 (0-0)	0 (0-0)
Devon	0 (0-0)	0 (0-0)	0 (0-0)	0 (0-0)	0 (0-0)	0 (0-0)	0 (0-0)	0 (0-0)	0 (0-0)	0 (0-0)	0 (0-0)	0 (0-0)	0 (0-0)	0 (0-0)
Dorset	0 (0-0)	0 (0-0)	0 (0-0)	0 (0-0)	0 (0-0)	0 (0-0)	0 (0-0)	0 (0-0)	0 (0-0)	0 (0-0)	0 (0-0)	0 (0-0)	0 (0-0)	0 (0-0)
Gloucestershire	0 (0-0)	0 (0-0)	0 (0-0)	0 (0-0)	0 (0-0)	0 (0-0)	0 (0-0)	0 (0-0)	0 (0-0)	0 (0-0)	0 (0-0)	0 (0-0)	0 (0-0)	0 (0-0)
North Somerset	0 (0-0)	0 (0-0)	0 (0-0)	0 (0-0)	0 (0-0)	0 (0-0)	0 (0-0)	0 (0-0)	0 (0-0)	0 (0-0)	0 (0-0)	0 (0-0)	0 (0-0)	0 (0-0)
Plymouth	0 (0-0)	0 (0-0)	0 (0-0)	0 (0-0)	0 (0-0)	0 (0-0)	0 (0-0)	0 (0-0)	0 (0-0)	0 (0-0)	0 (0-0)	0 (0-0)	0 (0-0)	0 (0-0)
Poole	0 (0-0)	0 (0-0)	0 (0-0)	0 (0-0)	0 (0-0)	0 (0-0)	0 (0-0)	0 (0-0)	0 (0-0)	0 (0-0)	0 (0-0)	0 (0-0)	0 (0-0)	0 (0-0)
Somerset	0 (0-0)	0 (0-0)	0 (0-0)	0 (0-0)	0 (0-0)	0 (0-0)	0 (0-0)	0 (0-0)	0 (0-0)	0 (0-0)	0 (0-0)	0 (0-0)	0 (0-0)	0 (0-0)
South Gloucestershire	0 (0-0)	0 (0-0)	0 (0-0)	0 (0-0)	0 (0-0)	0 (0-0)	0 (0-0)	0 (0-0)	0 (0-0)	0 (0-0)	0 (0-0)	0 (0-0)	0 (0-0)	0 (0-0)
Swindon	0 (0-0)	0 (0-0)	0 (0-0)	0 (0-0)	0 (0-0)	0 (0-0)	0 (0-0)	0 (0-0)	0 (0-0)	0 (0-0)	0 (0-0)	0 (0-0)	0 (0-0)	0 (0-0)
Torbay	0 (0-0)	0 (0-0)	0 (0-0)	0 (0-0)	0 (0-0)	0 (0-0)	0 (0-0)	0 (0-0)	0 (0-0)	0 (0-0)	0 (0-0)	0 (0-0)	0 (0-0)	0 (0-0)
Wiltshire	0 (0-0)	0 (0-0)	0 (0-0)	0 (0-0)	0 (0-0)	0 (0-0)	0 (0-0)	0 (0-0)	0 (0-0)	0 (0-0)	0 (0-0)	0 (0-0)	0 (0-0)	0 (0-0)
West Midlands	1 (1-1)	1 (1-1)	1 (1-1)	1 (1-1)	1 (1-1)	1 (1-1)	1 (1-1)	1 (0-1)	0 (0-0)	0 (0-0)	0 (0-0)	0 (0-0)	0 (0-0)	0 (0-0)
Birmingham	0 (0-0)	0 (0-0)	0 (0-0)	0 (0-0)	0 (0-0)	0 (0-0)	0 (0-0)	0 (0-0)	0 (0-0)	0 (0-0)	0 (0-0)	0 (0-0)	0 (0-0)	0 (0-0)
Coventry	0 (0-0)	0 (0-0)	0 (0-0)	0 (0-0)	0 (0-0)	0 (0-0)	0 (0-0)	0 (0-0)	0 (0-0)	0 (0-0)	0 (0-0)	0 (0-0)	0 (0-0)	0 (0-0)
Dudley	0 (0-0)	0 (0-0)	0 (0-0)	0 (0-0)	0 (0-0)	0 (0-0)	0 (0-0)	0 (0-0)	0 (0-0)	0 (0-0)	0 (0-0)	0 (0-0)	0 (0-0)	0 (0-0)
Herefordshire, County of	0 (0-0)	0 (0-0)	0 (0-0)	0 (0-0)	0 (0-0)	0 (0-0)	0 (0-0)	0 (0-0)	0 (0-0)	0 (0-0)	0 (0-0)	0 (0-0)	0 (0-0)	0 (0-0)
Sandwell	0 (0-0)	0 (0-0)	0 (0-0)	0 (0-0)	0 (0-0)	0 (0-0)	0 (0-0)	0 (0-0)	0 (0-0)	0 (0-0)	0 (0-0)	0 (0-0)	0 (0-0)	0 (0-0)
Shropshire	0 (0-0)	0 (0-0)	0 (0-0)	0 (0-0)	0 (0-0)	0 (0-0)	0 (0-0)	0 (0-0)	0 (0-0)	0 (0-0)	0 (0-0)	0 (0-0)	0 (0-0)	0 (0-0)
Solihull	0 (0-0)	0 (0-0)	0 (0-0)	0 (0-0)	0 (0-0)	0 (0-0)	0 (0-0)	0 (0-0)	0 (0-0)	0 (0-0)	0 (0-0)	0 (0-0)	0 (0-0)	0 (0-0)
Staffordshire	0 (0-0)	0 (0-0)	0 (0-0)	0 (0-0)	0 (0-0)	0 (0-0)	0 (0-0)	0 (0-0)	0 (0-0)	0 (0-0)	0 (0-0)	0 (0-0)	0 (0-0)	0 (0-0)
Stoke-on-Trent	0 (0-0)	0 (0-0)	0 (0-0)	0 (0-0)	0 (0-0)	0 (0-0)	0 (0-0)	0 (0-0)	0 (0-0)	0 (0-0)	0 (0-0)	0 (0-0)	0 (0-0)	0 (0-0)
Telford and Wrekin	0 (0-0)	0 (0-0)	0 (0-0)	0 (0-0)	0 (0-0)	0 (0-0)	0 (0-0)	0 (0-0)	0 (0-0)	0 (0-0)	0 (0-0)	0 (0-0)	0 (0-0)	0 (0-0)
Walsall	0 (0-0)	0 (0-0)	0 (0-0)	0 (0-0)	0 (0-0)	0 (0-0)	0 (0-0)	0 (0-0)	0 (0-0)	0 (0-0)	0 (0-0)	0 (0-0)	0 (0-0)	0 (0-0)
Warwickshire	0 (0-0)	0 (0-0)	0 (0-0)	0 (0-0)	0 (0-0)	0 (0-0)	0 (0-0)	0 (0-0)	0 (0-0)	0 (0-0)	0 (0-0)	0 (0-0)	0 (0-0)	0 (0-0)
Wolverhampton	0 (0-0)	0 (0-0)	0 (0-0)	0 (0-0)	0 (0-0)	0 (0-0)	0 (0-0)	0 (0-0)	0 (0-0)	0 (0-0)	0 (0-0)	0 (0-0)	0 (0-0)	0 (0-0)
Worcestershire	0 (0-0)	0 (0-0)	0 (0-0)	0 (0-0)	0 (0-0)	0 (0-0)	0 (0-0)	0 (0-0)	0 (0-0)	0 (0-0)	0 (0-0)	0 (0-0)	0 (0-0)	0 (0-0)
Yorkshire and the Humber	1 (1-1)	1 (1-1)	1 (1-1)	1 (1-1)	1 (1-1)	1 (1-1)	1 (1-1)	0 (0-0)	0 (0-0)	0 (0-0)	0 (0-0)	0 (0-0)	0 (0-0)	0 (0-0)
Barnsley	0 (0-0)	0 (0-0)	0 (0-0)	0 (0-0)	0 (0-0)	0 (0-0)	0 (0-0)	0 (0-0)	0 (0-0)	0 (0-0)	0 (0-0)	0 (0-0)	0 (0-0)	0 (0-0)
Bradford	0 (0-0)	0 (0-0)	0 (0-0)	0 (0-0)	0 (0-0)	0 (0-0)	0 (0-0)	0 (0-0)	0 (0-0)	0 (0-0)	0 (0-0)	0 (0-0)	0 (0-0)	0 (0-0)
Calderdale	0 (0-0)	0 (0-0)	0 (0-0)	0 (0-0)	0 (0-0)	0 (0-0)	0 (0-0)	0 (0-0)	0 (0-0)	0 (0-0)	0 (0-0)	0 (0-0)	0 (0-0)	0 (0-0)
Doncaster	0 (0-0)	0 (0-0)	0 (0-0)	0 (0-0)	0 (0-0)	0 (0-0)	0 (0-0)	0 (0-0)	0 (0-0)	0 (0-0)	0 (0-0)	0 (0-0)	0 (0-0)	0 (0-0)
East Riding of Yorkshire	0 (0-0)	0 (0-0)	0 (0-0)	0 (0-0)	0 (0-0)	0 (0-0)	0 (0-0)	0 (0-0)	0 (0-0)	0 (0-0)	0 (0-0)	0 (0-0)	0 (0-0)	0 (0-0)
Kingston upon Hull, City of	0 (0-0)	0 (0-0)	0 (0-0)	0 (0-0)	0 (0-0)	0 (0-0)	0 (0-0)	0 (0-0)	0 (0-0)	0 (0-0)	0 (0-0)	0 (0-0)	0 (0-0)	0 (0-0)
Kirklees	0 (0-0)	0 (0-0)	0 (0-0)	0 (0-0)	0 (0-0)	0 (0-0)	0 (0-0)	0 (0-0)	0 (0-0)	0 (0-0)	0 (0-0)	0 (0-0)	0 (0-0)	0 (0-0)
Leeds	0 (0-0)	0 (0-0)	0 (0-0)	0 (0-0)	0 (0-0)	0 (0-0)	0 (0-0)	0 (0-0)	0 (0-0)	0 (0-0)	0 (0-0)	0 (0-0)	0 (0-0)	0 (0-0)
North East Lincolnshire	0 (0-0)	0 (0-0)	0 (0-0)	0 (0-0)	0 (0-0)	0 (0-0)	0 (0-0)	0 (0-0)	0 (0-0)	0 (0-0)	0 (0-0)	0 (0-0)	0 (0-0)	0 (0-0)
North Lincolnshire	0 (0-0)	0 (0-0)	0 (0-0)	0 (0-0)	0 (0-0)	0 (0-0)	0 (0-0)	0 (0-0)	0 (0-0)	0 (0-0)	0 (0-0)	0 (0-0)	0 (0-0)	0 (0-0)
North Yorkshire	0 (0-0)	0 (0-0)	0 (0-0)	0 (0-0)	0 (0-0)	0 (0-0)	0 (0-0)	0 (0-0)	0 (0-0)	0 (0-0)	0 (0-0)	0 (0-0)	0 (0-0)	0 (0-0)
Rotherham	0 (0-0)	0 (0-0)	0 (0-0)	0 (0-0)	0 (0-0)	0 (0-0)	0 (0-0)	0 (0-0)	0 (0-0)	0 (0-0)	0 (0-0)	0 (0-0)	0 (0-0)	0 (0-0)
Sheffield	0 (0-0)	0 (0-0)	0 (0-0)	0 (0-0)	0 (0-0)	0 (0-0)	0 (0-0)	0 (0-0)	0 (0-0)	0 (0-0)	0 (0-0)	0 (0-0)	0 (0-0)	0 (0-0)
Wakefield	0 (0-0)	0 (0-0)	0 (0-0)	0 (0-0)	0 (0-0)	0 (0-0)	0 (0-0)	0 (0-0)	0 (0-0)	0 (0-0)	0 (0-0)	0 (0-0)	0 (0-0)	0 (0-0)
York	0 (0-0)	0 (0-0)	0 (0-0)	0 (0-0)	0 (0-0)	0 (0-0)	0 (0-0)	0 (0-0)	0 (0-0)	0 (0-0)	0 (0-0)	0 (0-0)	0 (0-0)	0 (0-0)
Northern Ireland	1 (1-1)	1 (1-1)	1 (1-1)	1 (1-1)	0 (0-1)	0 (0-0)	0 (0-0)	0 (0-0)	0 (0-0)	0 (0-0)	0 (0-0)	0 (0-0)	0 (0-0)	0 (0-0)
Scotland	2 (2-3)	2 (2-2)	2 (2-2)	2 (2-2)	1 (1-1)	1 (1-1)	1 (1-1)	0 (0-0)	0 (0-0)	0 (0-0)	0 (0-0)	0 (0-0)	0 (0-0)	0 (0-0)
Wales	1 (1-2)	1 (1-2)	1 (1-1)	1 (1-1)	1 (1-1)	0 (0-1)	0 (0-0)	0 (0-0)	0 (0-0)	0 (0-0)	0 (0-0)	0 (0-0)	0 (0-0)	0 (0-0)
Latin America and Caribbean	764 (731-801)	752 (732-773)	754 (736-774)	727 (712-743)	678 (664-691)	599 (588-611)	512 (503-521)	419 (412-426)	328 (322-333)	261 (256-266)	205 (201-210)	161 (157-165)	147 (143-152)	101 (96-107)
Andean Latin America	105 (90-122)	107 (99-117)	112 (104-121)	104 (97-112)	103 (97-109)	88 (83-94)	78 (73-83)	65 (61-69)	51 (48-54)	39 (37-42)	30 (28-32)	23 (21-25)	19 (17-21)	14 (11-17)
Bolivia	25 (20-31)	25 (21-29)	25 (22-29)	24 (22-27)	23 (21-25)	20 (18-22)	18 (17-20)	17 (15-18)	15 (14-16)	13 (11-14)	10 (9-11)	8 (7-9)	6 (5-8)	4 (3-6)
Ecuador	15 (13-18)	16 (15-17)	18 (16-19)	17 (16-18)	16 (15-17)	13 (12-14)	12 (11-12)	9 (8-9)	7 (6-7)	6 (5-7)	6 (5-7)	4 (3-5)	4 (3-5)	3 (2-4)
Peru	65 (52-80)	66 (59-74)	70 (62-77)	62 (56-69)	64 (59-70)	55 (50-60)	48 (43-52)	40 (36-43)	29 (27-32)	21 (19-23)	14 (12-15)	10 (9-11)	9 (8-10)	7 (5-9)
Caribbean	69 (62-76)	65 (60-69)	63 (59-67)	60 (56-63)	54 (51-57)	47 (44-50)	41 (39-44)	37 (35-40)	33 (31-35)	29 (27-31)	24 (22-26)	21 (19-24)	33 (31-36)	16 (14-19)
Antigua and Barbuda	0 (0-0)	0 (0-0)	0 (0-0)	0 (0-0)	0 (0-0)	0 (0-0)	0 (0-0)	0 (0-0)	0 (0-0)	0 (0-0)	0 (0-0)	0 (0-0)	0 (0-0)	0 (0-0)
The Bahamas	0 (0-0)	0 (0-0)	0 (0-0)	0 (0-0)	0 (0-0)	0 (0-0)	0 (0-0)	0 (0-0)	0 (0-0)	0 (0-0)	0 (0-0)	0 (0-0)	0 (0-0)	0 (0-0)
Barbados	1 (1-1)	1 (0-1)	0 (0-0)	0 (0-0)	0 (0-0)	0 (0-0)	0 (0-0)	0 (0-0)	0 (0-0)	0 (0-0)	0 (0-0)	0 (0-0)	0 (0-0)	0 (0-0)
Belize	0 (0-1)	0 (0-0)	0 (0-0)	0 (0-0)	0 (0-0)	0 (0-0)	0 (0-0)	0 (0-0)	0 (0-0)	0 (0-0)	0 (0-0)	0 (0-0)	0 (0-0)	0 (0-0)
Bermuda	0 (0-0)	0 (0-0)	0 (0-0)	0 (0-0)	0 (0-0)	0 (0-0)	0 (0-0)	0 (0-0)	0 (0-0)	0 (0-0)	0 (0-0)	0 (0-0)	0 (0-0)	0 (0-0)
Cuba	6 (4-8)	6 (4-7)	6 (5-6)	6 (6-7)	6 (6-6)	3 (3-3)	2 (2-2)	2 (2-2)	1 (1-1)	1 (1-1)	1 (1-1)	1 (0-1)	0 (0-0)	0 (0-0)
Dominica	0 (0-0)	0 (0-0)	0 (0-0)	0 (0-0)	0 (0-0)	0 (0-0)	0 (0-0)	0 (0-0)	0 (0-0)	0 (0-0)	0 (0-0)	0 (0-0)	0 (0-0)	0 (0-0)
Dominican Republic	11 (9-14)	12 (11-13)	13 (12-15)	13 (12-14)	13 (11-14)	11 (10-12)	10 (9-11)	9 (8-10)	8 (8-9)	7 (6-7)	5 (5-6)	5 (4-5)	4 (3-4)	3 (2-5)
Grenada	0 (0-0)	0 (0-0)	0 (0-0)	0 (0-0)	0 (0-0)	0 (0-0)	0 (0-0)	0 (0-0)	0 (0-0)	0 (0-0)	0 (0-0)	0 (0-0)	0 (0-0)	0 (0-0)
Guyana	2 (1-2)	2 (1-2)	1 (1-2)	1 (1-1)	1 (1-1)	1 (1-1)	1 (1-1)	1 (1-1)	1 (1-1)	0 (0-1)	0 (0-0)	0 (0-0)	0 (0-0)	0 (0-0)
Haiti	34 (28-41)	32 (28-36)	31 (27-34)	29 (26-32)	27 (24-30)	25 (23-27)	23 (21-26)	21 (19-23)	19 (18-21)	17 (15-19)	15 (13-17)	14 (12-16)	27 (25-30)	11 (8-13)
Jamaica	4 (3-5)	4 (3-4)	3 (3-4)	3 (2-3)	2 (2-2)	2 (1-2)	1 (1-1)	1 (1-1)	1 (1-1)	1 (1-1)	1 (0-1)	0 (0-1)	0 (0-1)	0 (0-1)
Puerto Rico	4 (4-5)	3 (3-3)	2 (2-2)	2 (2-2)	1 (1-1)	1 (1-1)	1 (1-1)	1 (1-1)	1 (1-1)	0 (0-1)	0 (0-0)	0 (0-0)	0 (0-0)	0 (0-0)
Saint Lucia	0 (0-0)	0 (0-0)	0 (0-0)	0 (0-0)	0 (0-0)	0 (0-0)	0 (0-0)	0 (0-0)	0 (0-0)	0 (0-0)	0 (0-0)	0 (0-0)	0 (0-0)	0 (0-0)
Saint Vincent and the Grenadines	0 (0-0)	0 (0-0)	0 (0-0)	0 (0-0)	0 (0-0)	0 (0-0)	0 (0-0)	0 (0-0)	0 (0-0)	0 (0-0)	0 (0-0)	0 (0-0)	0 (0-0)	0 (0-0)
Suriname	0 (0-1)	1 (0-1)	1 (0-1)	1 (0-1)	1 (0-1)	1 (0-1)	0 (0-0)	0 (0-0)	0 (0-0)	0 (0-0)	0 (0-0)	0 (0-0)	0 (0-0)	0 (0-0)
Trinidad and Tobago	2 (1-2)	1 (1-2)	1 (1-1)	1 (1-1)	1 (1-1)	1 (1-1)	1 (1-1)	0 (0-1)	0 (0-0)	0 (0-0)	0 (0-0)	0 (0-0)	0 (0-0)	0 (0-0)
Virgin Islands	0 (0-0)	0 (0-0)	0 (0-0)	0 (0-0)	0 (0-0)	0 (0-0)	0 (0-0)	0 (0-0)	0 (0-0)	0 (0-0)	0 (0-0)	0 (0-0)	0 (0-0)	0 (0-0)
Central Latin America	263 (249-280)	250 (242-258)	244 (237-250)	234 (228-240)	220 (215-226)	196 (190-201)	163 (159-168)	135 (132-139)	112 (109-115)	95 (92-98)	76 (73-78)	59 (56-61)	50 (48-52)	41 (38-45)
Colombia	41 (32-51)	43 (38-49)	47 (42-51)	43 (40-48)	38 (34-42)	32 (29-36)	25 (23-28)	21 (19-23)	17 (16-19)	16 (14-18)	13 (12-15)	12 (10-13)	9 (8-11)	7 (5-9)
Costa Rica	3 (3-3)	3 (3-3)	3 (3-3)	3 (3-3)	2 (2-3)	1 (1-2)	1 (1-1)	1 (1-1)	1 (1-1)	1 (1-1)	1 (1-1)	0 (0-0)	0 (0-0)	0 (0-0)
El Salvador	13 (10-17)	13 (11-15)	13 (11-15)	13 (11-14)	12 (10-13)	10 (9-11)	8 (7-9)	6 (5-7)	5 (4-5)	3 (3-4)	2 (2-2)	1 (1-2)	1 (1-1)	1 (1-1)
Guatemala	20 (16-25)	20 (17-23)	20 (18-23)	21 (19-23)	20 (18-22)	19 (17-20)	19 (18-21)	17 (16-18)	14 (13-15)	12 (11-13)	10 (9-11)	8 (7-9)	7 (6-8)	7 (5-9)
Honduras	10 (7-12)	9 (8-11)	9 (8-11)	9 (7-10)	8 (7-9)	7 (7-8)	6 (6-7)	6 (5-6)	5 (4-5)	4 (4-5)	4 (3-4)	3 (2-3)	2 (2-3)	2 (1-2)
Mexico	147 (136-158)	135 (130-140)	127 (123-131)	121 (118-124)	116 (112-119)	102 (98-105)	84 (82-87)	68 (66-70)	54 (53-56)	45 (44-47)	36 (35-37)	26 (25-27)	22 (21-23)	18 (18-19)
Aguascalientes	1 (1-1)	1 (1-1)	1 (1-1)	1 (1-1)	1 (1-1)	1 (1-1)	1 (1-1)	1 (1-1)	1 (0-1)	0 (0-0)	0 (0-0)	0 (0-0)	0 (0-0)	0 (0-0)
Baja California	3 (2-3)	2 (2-3)	2 (2-2)	2 (2-2)	1 (1-2)	1 (1-1)	1 (1-1)	1 (1-1)	1 (1-1)	1 (1-1)	1 (1-1)	1 (1-1)	1 (0-1)	0 (0-0)
Baja California Sur	0 (0-0)	0 (0-0)	0 (0-0)	0 (0-0)	0 (0-0)	0 (0-0)	0 (0-0)	0 (0-0)	0 (0-0)	0 (0-0)	0 (0-0)	0 (0-0)	0 (0-0)	0 (0-0)
Campeche	0 (0-0)	0 (0-0)	0 (0-0)	0 (0-0)	0 (0-1)	1 (0-1)	0 (0-1)	0 (0-0)	0 (0-0)	0 (0-0)	0 (0-0)	0 (0-0)	0 (0-0)	0 (0-0)
Chiapas	3 (2-4)	3 (2-3)	3 (2-3)	3 (3-3)	3 (3-4)	3 (3-4)	3 (3-4)	3 (3-4)	3 (3-4)	3 (2-3)	2 (2-2)	1 (1-2)	1 (1-1)	1 (1-1)
Chihuahua	6 (4-8)	5 (4-6)	4 (4-5)	4 (3-4)	3 (3-4)	3 (2-3)	2 (2-2)	2 (1-2)	1 (1-2)	1 (1-2)	1 (1-1)	1 (1-1)	1 (1-1)	1 (1-1)
Coahuila	3 (2-4)	3 (2-3)	3 (2-3)	3 (3-3)	3 (3-4)	3 (2-3)	2 (2-2)	1 (1-2)	1 (1-1)	1 (1-1)	1 (1-1)	1 (0-1)	0 (0-1)	1 (0-1)
Colima	1 (1-1)	1 (1-1)	1 (1-1)	1 (1-1)	1 (1-1)	1 (1-1)	0 (0-1)	0 (0-0)	0 (0-0)	0 (0-0)	0 (0-0)	0 (0-0)	0 (0-0)	0 (0-0)
Mexico City	22 (15-30)	19 (15-22)	16 (14-19)	14 (12-16)	12 (10-13)	10 (8-12)	8 (7-9)	5 (4-6)	4 (3-4)	3 (2-3)	2 (2-2)	2 (1-2)	2 (1-2)	1 (1-1)
Durango	4 (3-5)	4 (3-4)	3 (3-4)	3 (3-3)	3 (2-3)	2 (2-3)	2 (1-2)	1 (1-1)	1 (1-1)	1 (1-1)	1 (1-1)	0 (0-1)	0 (0-0)	0 (0-0)
Guanajuato	9 (7-11)	8 (7-9)	8 (7-9)	8 (7-9)	8 (7-9)	7 (6-8)	6 (5-6)	5 (4-5)	3 (3-4)	3 (2-3)	2 (2-2)	1 (1-2)	1 (1-1)	1 (1-1)
Guerrero	5 (3-6)	5 (4-5)	4 (4-5)	4 (4-5)	5 (4-5)	4 (4-5)	4 (3-4)	3 (3-4)	3 (2-3)	3 (2-3)	2 (2-2)	1 (1-1)	1 (1-1)	1 (1-1)
Hidalgo	3 (2-4)	3 (3-4)	3 (3-3)	3 (3-3)	3 (3-4)	3 (3-3)	3 (2-3)	2 (2-2)	2 (1-2)	1 (1-1)	1 (1-1)	1 (0-1)	0 (0-0)	0 (0-0)
Jalisco	12 (9-16)	11 (9-12)	10 (9-11)	9 (8-10)	8 (7-9)	6 (6-7)	5 (4-6)	4 (4-5)	3 (3-4)	3 (2-3)	2 (2-3)	2 (1-2)	1 (1-2)	1 (1-1)
México	4 (3-6)	5 (4-6)	6 (5-7)	7 (6-8)	9 (8-11)	9 (8-11)	8 (7-9)	8 (7-8)	7 (6-8)	5 (5-6)	5 (4-5)	4 (3-4)	3 (3-4)	2 (2-3)
Michoacán de Ocampo	10 (7-13)	9 (7-10)	8 (7-9)	7 (6-8)	6 (5-7)	5 (5-6)	4 (4-5)	3 (3-4)	2 (2-3)	2 (2-2)	2 (1-2)	1 (1-1)	1 (1-1)	1 (1-1)
Morelos	1 (1-2)	1 (1-2)	2 (1-2)	2 (1-2)	2 (1-2)	1 (1-2)	1 (1-2)	1 (1-1)	1 (1-1)	1 (1-1)	0 (0-1)	0 (0-0)	0 (0-0)	0 (0-0)
Nayarit	2 (2-3)	2 (2-3)	2 (2-2)	2 (2-2)	2 (2-2)	1 (1-2)	1 (1-1)	1 (1-1)	1 (0-1)	0 (0-0)	0 (0-0)	0 (0-0)	0 (0-0)	0 (0-0)
Nuevo León	3 (3-4)	3 (3-4)	3 (3-4)	3 (3-4)	3 (3-3)	3 (2-3)	2 (2-2)	2 (1-2)	1 (1-1)	1 (1-1)	1 (1-1)	1 (1-1)	1 (0-1)	1 (1-1)

Table 4 Under-5 death numbers by location and year for 1950, 1955...2005, 2010, 2017 - males

Location	1950	1955	1960	1965	1970	1975	1980	1985	1990	1995	2000	2005	2010	2017
Oaxaca	8 (6-12)	7 (6-8)	6 (5-7)	5 (5-6)	5 (4-6)	4 (4-5)	3 (3-4)	3 (2-3)	2 (2-2)	2 (1-2)	1 (1-2)	1 (1-1)	1 (1-1)	1 (1-1)
Puebla	11 (8-14)	10 (9-11)	9 (8-11)	9 (8-10)	8 (7-9)	7 (6-8)	6 (5-7)	5 (4-6)	4 (3-5)	4 (3-4)	3 (2-3)	2 (2-3)	2 (2-2)	2 (1-2)
Querétaro	1 (1-2)	1 (1-1)	1 (1-2)	1 (1-2)	1 (1-2)	1 (1-2)	1 (1-2)	1 (1-1)	1 (1-1)	1 (1-1)	0 (0-1)	0 (0-0)	0 (0-0)	0 (0-0)
Quintana Roo	0 (0-0)	0 (0-0)	0 (0-0)	0 (0-0)	0 (0-0)	0 (0-0)	0 (0-0)	0 (0-0)	0 (0-0)	0 (0-0)	0 (0-0)	0 (0-0)	0 (0-0)	0 (0-0)
San Luis Potosí	5 (4-7)	4 (4-5)	4 (3-5)	4 (3-4)	3 (3-4)	3 (2-3)	2 (2-2)	2 (1-2)	1 (1-2)	1 (1-1)	1 (1-1)	1 (1-1)	1 (0-1)	0 (0-1)
Sinaloa	3 (2-4)	3 (2-3)	3 (3-3)	3 (3-3)	3 (3-4)	3 (2-3)	2 (2-2)	1 (1-2)	1 (1-1)	1 (1-1)	1 (1-1)	0 (0-1)	0 (0-0)	0 (0-0)
Sonora	3 (2-3)	2 (2-3)	2 (2-3)	2 (2-2)	2 (2-2)	2 (1-2)	1 (1-1)	1 (1-1)	1 (1-1)	1 (1-1)	1 (1-1)	1 (0-1)	0 (0-1)	0 (0-0)
Tabasco	2 (1-2)	2 (1-2)	2 (2-2)	2 (2-2)	2 (2-3)	2 (2-3)	2 (2-2)	2 (2-2)	1 (1-2)	1 (1-1)	1 (1-1)	1 (0-1)	1 (0-1)	1 (0-1)
Tamaulipas	5 (3-6)	4 (3-5)	4 (3-4)	3 (3-4)	3 (2-3)	2 (2-3)	2 (2-2)	1 (1-2)	1 (1-1)	1 (1-1)	1 (1-1)	1 (0-1)	1 (1-1)	0 (0-1)
Tlaxcala	1 (1-2)	1 (1-2)	1 (1-2)	1 (1-2)	1 (1-1)	1 (1-1)	1 (1-1)	1 (1-1)	1 (1-1)	0 (0-1)	0 (0-0)	0 (0-0)	0 (0-0)	0 (0-0)
Veracruz de Ignacio de la Llave	11 (8-14)	10 (9-12)	10 (8-11)	9 (8-10)	8 (7-9)	7 (6-8)	6 (5-7)	5 (5-6)	4 (4-5)	4 (3-4)	3 (2-3)	2 (1-2)	1 (1-1)	1 (1-1)
Yucatán	2 (1-2)	2 (1-2)	2 (1-2)	2 (1-2)	2 (1-2)	1 (1-2)	1 (1-1)	1 (1-1)	1 (1-1)	1 (1-1)	0 (0-1)	0 (0-0)	0 (0-0)	0 (0-0)
Zacatecas	5 (3-6)	4 (3-5)	4 (3-4)	3 (3-4)	3 (3-3)	3 (2-3)	2 (2-2)	1 (1-2)	1 (1-1)	1 (1-1)	1 (0-1)	0 (0-0)	0 (0-0)	0 (0-0)
Nicaragua	9 (8-11)	9 (8-11)	10 (8-11)	9 (8-10)	9 (8-10)	8 (7-9)	7 (7-8)	6 (6-7)	5 (5-6)	4 (3-4)	3 (2-3)	2 (2-2)	2 (1-2)	1 (1-1)
Panama	2 (2-2)	2 (2-2)	2 (2-2)	2 (2-2)	2 (1-2)	1 (1-1)	1 (1-1)	1 (1-1)	1 (1-1)	1 (1-1)	1 (1-1)	1 (1-1)	1 (1-1)	1 (0-1)
Venezuela	18 (17-20)	15 (15-15)	13 (13-13)	13 (13-14)	14 (14-15)	14 (14-14)	11 (11-11)	9 (9-9)	10 (10-11)	9 (8-9)	7 (6-7)	6 (5-6)	6 (6-6)	5 (4-7)
Tropical Latin America	327 (303-353)	330 (313-347)	335 (320-351)	330 (317-342)	300 (290-311)	268 (260-277)	229 (224-235)	181 (177-186)	131 (128-135)	98 (95-101)	77 (74-79)	58 (56-61)	45 (43-47)	30 (28-31)
Brazil	324 (300-350)	326 (310-344)	332 (316-347)	326 (313-338)	297 (286-307)	265 (257-273)	226 (221-232)	178 (174-183)	129 (126-132)	95 (93-98)	75 (72-77)	57 (54-59)	44 (42-46)	29 (27-30)
Acre	1 (1-1)	1 (1-1)	1 (1-1)	1 (1-1)	1 (1-1)	1 (1-1)	1 (1-1)	1 (1-1)	1 (1-1)	0 (0-1)	0 (0-0)	0 (0-0)	0 (0-0)	0 (0-0)
Alagoas	11 (8-14)	11 (9-13)	12 (10-14)	12 (11-14)	12 (11-13)	11 (10-13)	12 (11-13)	9 (8-10)	6 (6-7)	4 (4-5)	3 (3-4)	2 (2-3)	1 (1-2)	1 (1-1)
Amapá	0 (0-0)	0 (0-0)	0 (0-0)	0 (0-1)	0 (0-0)	0 (0-0)	0 (0-0)	0 (0-0)	0 (0-0)	0 (0-0)	0 (0-0)	0 (0-0)	0 (0-0)	0 (0-0)
Amazonas	2 (2-3)	3 (2-3)	3 (2-3)	3 (2-3)	3 (3-3)	3 (3-4)	3 (3-4)	3 (2-3)	2 (2-2)	2 (1-2)	1 (1-2)	1 (1-1)	1 (1-1)	1 (1-1)
Bahia	45 (36-56)	45 (39-53)	45 (39-53)	44 (39-50)	40 (36-44)	34 (31-37)	28 (26-31)	24 (22-26)	17 (16-19)	13 (11-14)	10 (9-12)	8 (7-10)	6 (5-7)	4 (3-5)
Ceará	30 (25-37)	31 (27-36)	32 (28-37)	32 (28-36)	30 (26-34)	28 (25-30)	24 (22-27)	18 (17-20)	12 (11-13)	9 (8-9)	6 (6-7)	5 (4-5)	3 (2-4)	2 (1-2)
Distrito Federal	0 (0-0)	0 (0-1)	1 (1-1)	1 (1-1)	1 (1-1)	1 (1-1)	1 (1-1)	1 (1-1)	1 (1-1)	1 (1-1)	1 (1-1)	0 (0-1)	0 (0-0)	0 (0-0)
Espírito Santo	4 (3-5)	4 (3-5)	4 (3-5)	4 (3-5)	4 (3-4)	3 (3-4)	2 (2-3)	2 (2-2)	2 (1-2)	1 (1-1)	1 (1-1)	1 (1-1)	1 (1-1)	0 (0-1)
Goiás	6 (5-9)	7 (5-8)	7 (6-8)	7 (6-9)	6 (6-7)	5 (4-6)	4 (3-4)	3 (3-3)	2 (2-2)	2 (2-2)	1 (1-1)	1 (1-1)	1 (1-1)	1 (1-1)
Maranhão	22 (17-27)	22 (19-26)	23 (20-27)	23 (21-26)	22 (20-24)	20 (18-22)	19 (17-21)	15 (14-17)	11 (10-12)	7 (6-8)	6 (5-6)	5 (4-5)	3 (3-4)	2 (1-2)
Mato Grosso	2 (1-2)	2 (2-3)	2 (2-3)	3 (2-3)	3 (2-3)	2 (2-2)	2 (2-2)	2 (2-2)	1 (1-2)	1 (1-1)	1 (1-1)	1 (1-1)	1 (1-1)	0 (0-1)
Mato Grosso do Sul	1 (0-1)	1 (1-1)	1 (1-1)	1 (1-1)	1 (1-1)	1 (1-2)	1 (1-2)	1 (1-1)	1 (1-1)	1 (1-1)	1 (1-1)	1 (1-1)	0 (0-1)	0 (0-0)
Minas Gerais	33 (25-43)	32 (27-39)	32 (27-38)	30 (25-35)	25 (21-29)	20 (18-23)	16 (15-18)	13 (12-15)	9 (8-10)	7 (6-8)	6 (5-7)	5 (4-6)	4 (3-4)	2 (2-3)
Pará	6 (5-9)	7 (6-8)	8 (6-9)	8 (7-10)	9 (8-10)	9 (8-10)	8 (7-9)	7 (6-8)	5 (5-6)	4 (4-4)	3 (3-4)	3 (3-4)	2 (2-3)	2 (1-2)
Paraíba	16 (12-20)	15 (13-18)	15 (13-17)	13 (12-15)	12 (10-14)	10 (9-11)	8 (7-9)	5 (5-6)	4 (3-4)	3 (2-3)	2 (2-2)	2 (1-2)	1 (1-1)	1 (0-1)
Paraná	18 (13-24)	18 (15-22)	18 (15-22)	18 (15-20)	14 (12-16)	11 (9-13)	8 (8-9)	6 (6-6)	5 (4-5)	3 (3-4)	3 (2-3)	2 (2-3)	2 (1-2)	1 (1-2)
Pernambuco	42 (33-53)	41 (35-48)	41 (35-47)	39 (34-44)	34 (30-39)	28 (26-31)	22 (20-24)	17 (15-19)	12 (11-13)	8 (7-9)	6 (6-7)	5 (4-5)	3 (3-4)	2 (1-2)
Piauí	7 (5-9)	7 (6-8)	8 (7-9)	8 (7-9)	8 (7-9)	7 (6-8)	6 (5-6)	4 (4-5)	3 (3-4)	3 (2-3)	2 (2-3)	2 (2-2)	1 (1-2)	1 (1-1)
Rio de Janeiro	20 (14-27)	19 (15-23)	18 (15-22)	16 (13-20)	15 (13-17)	14 (12-16)	12 (11-13)	9 (8-9)	6 (6-7)	5 (5-5)	4 (3-4)	2 (2-3)	2 (2-3)	2 (2-2)
Rio Grande do Norte	8 (6-10)	8 (7-10)	9 (7-10)	8 (7-10)	8 (7-9)	7 (7-8)	6 (5-7)	5 (4-5)	3 (3-4)	3 (2-3)	2 (2-2)	1 (1-2)	1 (1-1)	1 (0-1)
Rio Grande do Sul	13 (9-18)	12 (10-16)	12 (10-15)	11 (9-14)	9 (8-11)	7 (6-8)	6 (5-6)	4 (4-4)	3 (3-3)	3 (2-3)	2 (2-2)	1 (1-1)	1 (1-1)	1 (1-1)
Rondônia	0 (0-0)	0 (0-0)	0 (0-0)	0 (0-0)	0 (0-0)	1 (0-1)	1 (1-1)	1 (1-1)	1 (1-1)	1 (0-1)	1 (0-1)	0 (0-0)	0 (0-0)	0 (0-0)
Roraima	0 (0-0)	0 (0-0)	0 (0-0)	0 (0-0)	0 (0-0)	0 (0-0)	0 (0-0)	0 (0-0)	0 (0-0)	0 (0-0)	0 (0-0)	0 (0-0)	0 (0-0)	0 (0-0)
Santa Catarina	4 (3-6)	5 (4-6)	5 (4-6)	5 (4-6)	4 (4-5)	4 (3-5)	3 (3-4)	3 (2-3)	2 (2-2)	2 (2-2)	1 (1-2)	1 (1-1)	1 (1-1)	1 (1-1)
São Paulo	27 (19-36)	28 (22-34)	30 (24-36)	30 (25-36)	29 (25-34)	31 (28-35)	27 (25-29)	20 (19-22)	16 (15-17)	12 (11-14)	9 (8-9)	5 (5-6)	5 (5-6)	4 (4-5)
Sergipe	3 (2-4)	3 (3-4)	3 (3-4)	4 (3-4)	3 (3-4)	3 (3-4)	3 (3-3)	3 (2-3)	2 (2-2)	1 (1-2)	1 (1-1)	1 (1-1)	1 (1-1)	0 (0-1)
Tocantins	2 (2-3)	3 (2-3)	3 (2-3)	3 (2-4)	3 (2-3)	3 (2-3)	2 (2-3)	2 (2-2)	1 (1-1)	1 (1-1)	1 (1-1)	1 (1-1)	0 (0-1)	0 (0-0)
Paraguay	3 (2-4)	3 (3-4)	4 (3-4)	4 (3-4)	4 (3-4)	3 (3-4)	3 (3-4)	3 (3-3)	3 (2-3)	2 (2-3)	2 (2-2)	2 (1-2)	1 (1-2)	1 (1-1)
North Africa and Middle East	1120 (1040-1200)	1040 (998-1080)	988 (952-1020)	912 (883-944)	852 (826-880)	783 (758-808)	703 (681-726)	614 (597-631)	475 (460-489)	428 (414-444)	372 (359-386)	318 (305-332)	269 (255-283)	198 (182-215)
North Africa and Middle East	1120 (1040-1200)	1040 (998-1080)	988 (952-1020)	912 (883-944)	852 (826-880)	783 (758-808)	703 (681-726)	614 (597-631)	475 (460-489)	428 (414-444)	372 (359-386)	318 (305-332)	269 (255-283)	198 (182-215)
Afghanistan	62 (48-79)	63 (53-75)	65 (54-77)	67 (56-80)	71 (58-86)	74 (62-88)	72 (61-84)	53 (46-60)	44 (39-50)	70 (63-78)	70 (63-77)	61 (55-68)	50 (43-57)	46 (37-57)
Algeria	28 (24-33)	35 (31-40)	43 (37-49)	43 (38-48)	46 (40-53)	48 (42-54)	46 (42-51)	31 (28-34)	22 (19-24)	17 (15-19)	15 (13-17)	12 (11-14)	11 (9-12)	8 (7-10)
Bahrain	1 (1-1)	1 (1-1)	1 (1-1)	1 (0-1)	0 (0-0)	0 (0-0)	0 (0-0)	0 (0-0)	0 (0-0)	0 (0-0)	0 (0-0)	0 (0-0)	0 (0-0)	0 (0-0)
Egypt	256 (202-319)	226 (198-256)	203 (183-225)	181 (165-199)	168 (154-184)	158 (145-174)	140 (128-152)	116 (106-126)	81 (74-89)	61 (56-68)	50 (45-56)	44 (38-50)	39 (33-45)	24 (19-31)
Iran	245 (230-264)	224 (214-235)	207 (198-217)	192 (184-199)	160 (154-166)	111 (107-115)	86 (83-89)	71 (68-74)	57 (55-59)	41 (40-43)	35 (33-36)	30 (29-31)	26 (25-27)	12 (11-12)
Iraq	36 (27-49)	32 (26-39)	29 (23-35)	28 (23-34)	27 (23-32)	25 (22-28)	22 (20-24)	21 (19-23)	18 (17-20)	21 (20-24)	21 (19-23)	21 (19-23)	18 (15-20)	16 (13-20)
Jordan	2 (2-3)	3 (2-3)	3 (2-3)	3 (3-3)	3 (3-3)	3 (3-3)	3 (3-3)	3 (2-3)	3 (3-3)	3 (3-3)	3 (2-3)	3 (2-3)	3 (2-3)	2 (2-3)
Kuwait	0 (0-0)	0 (0-0)	0 (0-0)	1 (1-1)	1 (1-1)	1 (1-1)	1 (1-1)	1 (1-1)	0 (0-0)	0 (0-0)	0 (0-0)	0 (0-0)	0 (0-0)	0 (0-0)
Lebanon	5 (3-7)	4 (3-6)	4 (3-5)	4 (3-4)	3 (3-4)	5 (4-6)	2 (2-3)	2 (2-3)	2 (2-2)	2 (1-2)	1 (1-2)	1 (1-1)	1 (0-1)	1 (0-1)
Libya	11 (9-14)	10 (8-12)	9 (8-11)	8 (7-9)	5 (5-6)	4 (4-5)	4 (3-4)	3 (3-4)	3 (2-3)	2 (2-2)	2 (1-2)	1 (1-2)	1 (1-1)	1 (1-1)
Morocco	94 (76-115)	91 (80-103)	91 (83-99)	78 (72-84)	70 (64-75)	67 (62-72)	55 (51-59)	41 (37-44)	30 (28-33)	25 (22-28)	20 (17-24)	16 (13-20)	13 (10-16)	8 (6-10)
Palestine	5 (4-7)	4 (4-5)	4 (3-4)	3 (2-3)	2 (2-3)	2 (2-2)	2 (2-2)	2 (1-2)	2 (1-2)	2 (1-2)	2 (2-2)	2 (2-2)	2 (1-2)	1 (1-1)
Oman	6 (5-8)	6 (5-7)	6 (5-7)	5 (5-6)	4 (4-5)	3 (3-4)	3 (2-3)	2 (1-2)	1 (1-2)	1 (1-1)	0 (0-0)	0 (0-0)	0 (0-0)	1 (0-1)
Qatar	0 (0-0)	0 (0-0)	0 (0-0)	0 (0-0)	0 (0-0)	0 (0-0)	0 (0-0)	0 (0-0)	0 (0-0)	0 (0-0)	0 (0-0)	0 (0-0)	0 (0-0)	0 (0-0)
Saudi Arabia	21 (16-27)	20 (17-25)	20 (17-24)	20 (16-24)	20 (16-23)	19 (15-22)	19 (16-22)	19 (16-23)	18 (14-22)	13 (10-17)	9 (7-11)	6 (5-8)	4 (3-5)	2 (2-3)
Sudan	30 (23-37)	22 (19-26)	17 (15-20)	23 (20-25)	36 (33-40)	56 (50-61)	71 (64-78)	102 (95-110)	71 (65-77)	66 (60-73)	61 (55-67)	57 (51-63)	52 (45-59)	38 (29-50)
Syria	19 (15-23)	16 (14-19)	15 (13-17)	14 (12-15)	13 (12-15)	13 (12-15)	11 (10-13)	10 (9-12)	10 (8-12)	8 (7-9)	5 (5-5)	4 (4-4)	4 (4-4)	3 (3-3)
Tunisia	32 (26-39)	28 (24-31)	24 (22-27)	20 (18-22)	16 (15-18)	13 (12-14)	10 (9-11)	9 (8-10)	6 (6-7)	4 (4-5)	3 (3-3)	2 (2-2)	2 (2-2)	1 (1-1)
Turkey	200 (163-240)	191 (170-213)	186 (169-205)	162 (149-177)	145 (133-157)	125 (115-137)	104 (95-114)	80 (73-88)	59 (54-65)	48 (43-53)	36 (32-41)	24 (20-27)	16 (13-18)	9 (8-11)
United Arab Emirates	0 (0-1)	0 (0-1)	0 (0-1)	0 (0-1)	1 (0-1)	1 (1-1)	1 (1-1)	1 (1-1)	1 (1-1)	0 (0-0)	0 (0-0)	0 (0-1)	1 (1-1)	1 (0-1)
Yemen	62 (49-75)	61 (52-69)	61 (54-67)	61 (55-66)	59 (54-64)	54 (50-59)	51 (46-55)	48 (44-52)	45 (42-49)	43 (39-47)	39 (35-43)	33 (29-37)	28 (24-33)	24 (19-30)
South Asia	2580 (2390-2790)	2680 (2550-2820)	2860 (2730-3000)	3050 (2930-3180)	2760 (2680-2840)	2700 (2630-2790)	2460 (2390-2530)	2240 (2190-2300)	2040 (1990-2090)	1830 (1790-1880)	1600 (1540-1650)	1350 (1300-1400)	1100 (1050-1150)	764 (717-818)
South Asia	2580 (2390-2790)	2680 (2550-2820)	2860 (2730-3000)	3050 (2930-3180)	2760 (2680-2840)	2700 (2630-2790)	2460 (2390-2530)	2240 (2190-2300)	2040 (1990-2090)	1830 (1790-1880)	1600 (1540-1650)	1350 (1300-1400)	1100 (1050-1150)	764 (717-818)
Bangladesh	417 (340-499)	380 (329-432)	352 (305-404)	325 (292-361)	326 (296-357)	300 (272-331)	325 (302-351)	345 (322-369)	290 (270-311)	224 (208-240)	182 (166-197)	144 (131-157)	96 (85-108)	57 (46-70)
Bhutan	2 (1-2)	2 (2-2)	2 (2-2)	2 (1-2)	2 (1-2)	2 (1-2)	2 (1-2)	1 (1-2)	1 (1-1)	1 (1-1)	1 (1-1)	0 (0-1)	0 (0-0)	0 (0-0)
India	1840 (1680-2030)	2000 (1890-2130)	2220 (2110-2340)	2460 (2350-2570)	2150 (2080-2230)	2090 (2020-2160)	1800 (1740-1860)	1560 (1510-1610)	1420 (1380-1470)	1300 (1270-1340)	1130 (1090-1170)	934 (903-964)	767 (736-799)	519 (493-550)
Andhra Pradesh	69 (49-95)	73 (59-91)	80 (63-100)	89 (75-104)	79 (70-89)	80 (72-88)	68 (62-76)	59 (53-66)	56 (51-61)	47 (43-52)	37 (33-40)	28 (25-32)	21 (18-24)	11 (9-15)
Arunachal Pradesh	1 (1-1)	1 (1-1)	1 (1-2)	2 (1-2)	2 (1-2)	2 (1-2)	1 (1-2)	1 (1-2)	1 (1-2)	1 (1-2)	1 (1-1)	1 (1-1)	1 (1-1)	1 (0-1)
Assam	43 (32-55)	49 (40-57)	56 (48-66)	64 (55-75)	56 (50-63)	54 (49-60)	50 (45-55)	45 (40-49)	40 (35-44)	35 (31-39)	30 (26-33)	26 (23-29)	25 (22-28)	20 (17-24)
Bihar	174 (132-228)	184 (155-219)	199 (171-231)	216 (188-248)	196 (174-217)	195 (174-218)	176 (158-194)	155 (141-168)	141 (128-154)	140 (126-154)	126 (113-139)	110 (98-124)	95 (84-106)	68 (58-81)
Chhattisgarh	24 (18-31)	27 (22-31)	31 (26-36)	38 (33-43)	35 (30-41)	37 (32-43)	35 (30-40)	33 (29-37)	30 (27-34)	30 (27-33)	28 (26-31)	25 (22-27)	21 (18-23)	13 (12-16)
Delhi	8 (6-11)	8 (7-10)	9 (8-11)	12 (11-14)	9 (8-10)	10 (8-11)	9 (8-11)	9 (8-10)	10 (9-11)	10 (9-12)	10 (9-11)	7 (6-9)	6 (5-7)	5 (4-6)
Goa	2 (1-3)	2 (1-2)	2 (1-2)	2 (1-2)	2 (1-2)	1 (1-2)	1 (1-1)	1 (1-1)	1 (1-1)	1 (1-1)	1 (0-1)	0 (0-0)	0 (0-0)	0 (0-0)
Gujarat	74 (57-95)	80 (68-94)	89 (78-101)	100 (88-111)	85 (77-94)	82 (75-90)	73 (67-80)	64 (58-70)	58 (53-63)	51 (46-55)	43 (39-47)	35 (31-39)	27 (24-31)	21 (18-24)
Haryana	16 (12-21)	19 (16-22)	22 (19-26)	30 (27-33)	27 (24-30)	25 (23-28)	21 (20-23)	20 (18-22)	20 (18-21)	19 (17-21)	18 (17-20)	15 (14-17)	13 (11-14)	9 (7-10)
Himachal Pradesh	8 (6-11)	8 (7-10)	9 (8-11)	10 (9-12)	9 (8-10)	8 (7-9)	7 (6-8)	6 (5-7)	5 (5-6)	5 (4-5)	4 (3-4)	3 (3-3)	2 (2-3)	2 (1-2)
Jammu and Kashmir	12 (9-17)	12 (10-15)	13 (11-15)	15 (13-16)	12 (11-13)	12 (11-14)	11 (10-12)	10 (9-11)	9 (8-10)	9 (8-10)	7 (6-8)	6 (5-6)	5 (4-6)	4 (3-4)
Jharkhand	39 (28-54)	43 (35-52)	49 (41-59)	57 (49-65)	50 (42-60)	50 (43-59)	45 (39-52)	42 (36-48)	39 (34-44)	37 (34-42)	34 (31-38)	27 (24-31)	22 (19-25)	14 (12-17)
Karnataka	81 (64-104)	89 (75-104)	98 (84-112)	110 (96-125)	92 (82-103)	87 (79-97)	74 (67-81)	61 (56-66)	53 (49-58)	45 (41-49)	39 (36-43)	31 (28-35)	24 (21-27)	16 (13-18)
Kerala	65 (47-86)	60 (48-71)	56 (48-66)	56 (50-63)	40 (35-46)	31 (28-35)	22 (20-25)	16 (15-18)	13 (11-15)	10 (9-11)	8 (7-9)	5 (5-6)	3 (3-4)	3 (2-3)
Madhya Pradesh	103 (79-134)	115 (98-135)	132 (114-154)	153 (133-175)	139 (124-153)	145 (130-161)	133 (121-146)	118 (109-128)	111 (101-121)	106 (96-116)	93 (86-101)	79 (71-86)	67 (61-73)	41 (35-48)
Maharashtra	118 (89-157)	128 (107-152)	140 (122-161)	156 (135-179)	134 (119-151)	130 (118-144)	103 (95-113)	87 (79-95)	87 (79-95)	70 (63-76)	57 (52-63)	45 (38-52)	34 (28-41)	23 (20-27)
Manipur	2 (2-3)	2 (2-3)	3 (2-3)	3 (3-4)	3 (2-3)	3 (2-3)	2 (2-3)	2 (2-2)	2 (2-2)	2 (2-2)	2 (2-2)	2 (1-2)	1 (1-1)	1 (1-1)
Meghalaya	2 (1-2)	2 (1-2)	2 (2-2)	3 (2-3)	2 (2-3)	3 (3-3)	3 (3-3)	3 (3-3)	3 (3-3)	3 (3-3)	3 (2-3)	2 (2-3)	2 (2-2)	2 (1-2)
Mizoram	0 (0-0)	0 (0-0)	0 (0-0)	1 (1-1)	0 (0-0)	0 (0-1)	0 (0-1)	1 (0-1)	1 (0-1)	1 (0-1)	0 (0-1)	0 (0-1)	0 (0-1)	0 (0-1)
Nagaland	1 (1-1)	1 (1-1)	1 (1-2)	2 (1-2)	1 (1-2)	1 (1-2)	1 (1-1)	1 (1-1)	2 (1-2)	2 (1-2)	2 (2-2)	1 (1-2)	1 (1-1)	1 (1-1)
Odisha	89 (65-118)	98 (81-117)	110 (93-129)	120 (105-136)	108 (96-120)	103 (92-115)	88 (79-97)	76 (67-86)	70 (63-77)	62 (56-69)	52 (47-57)	43 (37-48)	34 (30-38)	23 (19-27)
Punjab	22 (17-29)	24 (20-29)	26 (23-30)	30 (27-34)	24 (21-27)	24 (22-27)	22 (20-24)	20 (18-22)	20 (18-21)	17 (16-19)	15 (13-16)	12 (11-14)	10 (9-11)	7 (6-8)
Rajasthan	77 (59-100)	88 (75-104)	103 (89-120)	122 (107-138)	110 (99-122)	113 (102-126)	106 (96-116)	99 (89-110)	92 (83-101)	86 (78-94)	74 (67-81)	63 (58-68)	56 (52-60)	40 (35-47)
Sikkim	0 (0-1)	0 (0-1)	1 (0-1)	1 (1-1)	1 (0-1)	1 (1-1)	1 (0-1)	0 (0-0)	0 (0-0)	0 (0-0)	0 (0-0)	0 (0-0)	0 (0-0)	0 (0-0)

Table 4 Under-5 death numbers by location and year for 1950, 1955...2005, 2010, 2017 - males

Location	1950	1955	1960	1965	1970	1975	1980	1985	1990	1995	2000	2005	2010	2017
Tamil Nadu	165 (122-216)	165 (138-195)	168 (147-192)	176 (153-199)	150 (136-165)	129 (117-141)	93 (85-103)	67 (61-73)	53 (49-57)	41 (38-45)	33 (30-36)	24 (21-27)	16 (14-19)	9 (8-10)
Telangana	41 (29-58)	45 (36-56)	51 (40-64)	58 (48-69)	51 (45-57)	51 (45-57)	42 (36-49)	36 (30-42)	34 (29-40)	33 (28-38)	29 (25-33)	22 (19-26)	14 (12-17)	7 (6-10)
Tripura	3 (2-4)	4 (3-5)	5 (5-6)	6 (6-7)	6 (5-6)	5 (5-6)	5 (4-5)	4 (4-5)	4 (3-4)	3 (3-3)	2 (2-3)	2 (2-2)	2 (1-2)	2 (1-2)
Uttar Pradesh	474 (345-632)	529 (437-628)	599 (523-686)	643 (559-735)	570 (513-628)	548 (493-605)	477 (430-528)	414 (378-452)	374 (343-407)	356 (327-386)	314 (284-347)	265 (243-289)	225 (203-249)	151 (131-175)
Uttarakhand	7 (5-9)	7 (6-9)	8 (7-10)	11 (9-12)	9 (8-11)	10 (8-11)	9 (8-10)	8 (7-10)	8 (7-9)	8 (7-9)	7 (6-8)	6 (5-7)	5 (4-5)	3 (2-3)
West Bengal	119 (87-159)	133 (110-158)	152 (131-175)	169 (148-192)	149 (131-165)	145 (130-160)	121 (110-133)	102 (94-111)	88 (80-95)	73 (65-80)	58 (53-64)	46 (41-51)	35 (29-41)	24 (20-28)
Union Territories other than Delhi	1 (1-1)	1 (1-1)	1 (1-1)	2 (1-2)	1 (1-1)	1 (1-1)	1 (1-1)	1 (1-1)	1 (1-1)	1 (1-2)	1 (1-1)	1 (1-1)	1 (1-1)	1 (1-1)
Nepal	75 (62-89)	72 (63-81)	70 (60-80)	66 (58-75)	61 (55-68)	62 (56-67)	65 (59-71)	63 (57-68)	53 (49-58)	42 (38-45)	31 (28-34)	22 (20-25)	16 (13-18)	10 (8-12)
Pakistan	241 (202-286)	227 (202-254)	216 (193-238)	203 (181-226)	221 (200-244)	251 (229-276)	267 (243-293)	276 (253-300)	271 (249-295)	263 (240-290)	255 (229-283)	252 (223-286)	223 (190-259)	177 (140-221)
Southeast Asia, East Asia, and Oceania	3330 (3170-3480)	3240 (3130-3350)	10900 (10800-11000)	2990 (2900-3080)	2520 (2440-2600)	1860 (1800-1920)	1430 (1390-1480)	1500 (1440-1550)	1230 (1190-1280)	963 (936-992)	734 (716-754)	536 (521-550)	403 (390-416)	268 (255-282)
East Asia	2030 (1900-2160)	2040 (1950-2130)	9750 (9640-9870)	1950 (1870-2030)	1580 (1510-1650)	1020 (975-1070)	714 (678-748)	879 (829-927)	716 (681-755)	544 (521-569)	385 (371-399)	243 (235-252)	169 (162-176)	102 (97-108)
China	1940 (1820-2070)	1970 (1880-2060)	9690 (9580-9810)	1890 (1810-1980)	1530 (1470-1600)	993 (948-1040)	693 (658-727)	856 (807-904)	696 (662-734)	504 (481-529)	349 (335-362)	231 (223-239)	160 (154-167)	96 (91-102)
North Korea	38 (31-47)	19 (14-25)	18 (14-23)	15 (12-19)	11 (9-14)	7 (6-9)	5 (4-7)	6 (4-7)	6 (5-8)	30 (27-34)	29 (26-32)	7 (5-9)	6 (4-7)	4 (3-5)
Taiwan (Province of China)	24 (22-28)	17 (17-17)	13 (12-13)	8 (8-9)	5 (5-6)	4 (4-4)	3 (3-3)	2 (2-2)	2 (1-2)	2 (2-2)	1 (1-1)	1 (1-1)	1 (1-1)	0 (0-1)
Oceania	14 (11-18)	13 (11-16)	13 (11-15)	11 (10-13)	9 (8-11)	8 (7-9)	7 (6-8)	7 (7-9)	7 (6-9)	8 (7-9)	9 (7-10)	10 (8-12)	10 (8-12)	9 (7-12)
American Samoa	0 (0-0)	0 (0-0)	0 (0-0)	0 (0-0)	0 (0-0)	0 (0-0)	0 (0-0)	0 (0-0)	0 (0-0)	0 (0-0)	0 (0-0)	0 (0-0)	0 (0-0)	0 (0-0)
Federated States of Micronesia	0 (0-0)	0 (0-0)	0 (0-0)	0 (0-0)	0 (0-0)	0 (0-0)	0 (0-0)	0 (0-0)	0 (0-0)	0 (0-0)	0 (0-0)	0 (0-0)	0 (0-0)	0 (0-0)
Fiji	1 (1-1)	1 (1-1)	1 (1-1)	1 (1-1)	1 (1-1)	0 (0-1)	0 (0-1)	0 (0-0)	0 (0-0)	0 (0-0)	0 (0-0)	0 (0-0)	0 (0-0)	0 (0-0)
Guam	0 (0-0)	0 (0-0)	0 (0-0)	0 (0-0)	0 (0-0)	0 (0-0)	0 (0-0)	0 (0-0)	0 (0-0)	0 (0-0)	0 (0-0)	0 (0-0)	0 (0-0)	0 (0-0)
Kiribati	0 (0-0)	0 (0-0)	0 (0-0)	0 (0-0)	0 (0-0)	0 (0-0)	0 (0-0)	0 (0-0)	0 (0-0)	0 (0-0)	0 (0-0)	0 (0-0)	0 (0-0)	0 (0-0)
Marshall Islands	0 (0-0)	0 (0-0)	0 (0-0)	0 (0-0)	0 (0-0)	0 (0-0)	0 (0-0)	0 (0-0)	0 (0-0)	0 (0-0)	0 (0-0)	0 (0-0)	0 (0-0)	0 (0-0)
Northern Mariana Islands	0 (0-0)	0 (0-0)	0 (0-0)	0 (0-0)	0 (0-0)	0 (0-0)	0 (0-0)	0 (0-0)	0 (0-0)	0 (0-0)	0 (0-0)	0 (0-0)	0 (0-0)	0 (0-0)
Papua New Guinea	11 (8-14)	10 (8-12)	10 (8-12)	8 (7-10)	7 (6-8)	6 (5-7)	5 (5-7)	6 (5-7)	6 (5-7)	6 (5-7)	7 (6-8)	8 (6-10)	8 (7-11)	8 (6-10)
Samoa	0 (0-0)	0 (0-0)	0 (0-0)	0 (0-0)	0 (0-0)	0 (0-0)	0 (0-0)	0 (0-0)	0 (0-0)	0 (0-0)	0 (0-0)	0 (0-0)	0 (0-0)	0 (0-0)
Solomon Islands	1 (0-1)	1 (0-1)	1 (0-1)	1 (0-1)	0 (0-1)	0 (0-0)	0 (0-0)	0 (0-0)	0 (0-0)	0 (0-0)	0 (0-0)	0 (0-0)	0 (0-0)	0 (0-0)
Tonga	0 (0-0)	0 (0-0)	0 (0-0)	0 (0-0)	0 (0-0)	0 (0-0)	0 (0-0)	0 (0-0)	0 (0-0)	0 (0-0)	0 (0-0)	0 (0-0)	0 (0-0)	0 (0-0)
Vanuatu	0 (0-0)	0 (0-0)	0 (0-0)	0 (0-0)	0 (0-0)	0 (0-0)	0 (0-0)	0 (0-0)	0 (0-0)	0 (0-0)	0 (0-0)	0 (0-0)	0 (0-0)	0 (0-0)
Southeast Asia	1280 (1200-1380)	1190 (1140-1250)	1120 (1070-1170)	1030 (988-1080)	932 (896-973)	830 (798-861)	712 (684-739)	608 (585-634)	507 (488-528)	411 (396-427)	341 (329-354)	283 (271-295)	224 (214-235)	156 (145-169)
Cambodia	30 (22-40)	29 (23-36)	29 (23-35)	28 (23-34)	31 (26-36)	46 (41-50)	30 (26-33)	30 (28-33)	28 (26-30)	24 (22-26)	18 (17-20)	13 (11-15)	10 (8-11)	7 (5-9)
Indonesia	661 (623-703)	613 (592-635)	576 (554-598)	530 (513-547)	468 (453-483)	409 (397-420)	348 (340-357)	283 (276-290)	226 (221-232)	180 (175-185)	150 (145-155)	126 (122-131)	100 (95-105)	65 (61-70)
Laos	19 (14-24)	19 (15-23)	19 (15-22)	18 (15-22)	18 (15-22)	17 (14-21)	17 (14-20)	16 (13-19)	15 (13-18)	14 (12-16)	12 (11-14)	10 (9-12)	8 (7-10)	6 (5-8)
Malaysia	20 (18-23)	18 (17-19)	17 (17-18)	13 (13-13)	11 (11-11)	9 (8-10)	7 (6-8)	6 (6-6)	5 (5-5)	4 (4-4)	3 (2-3)	2 (2-2)	2 (2-2)	2 (2-3)
Maldives	1 (1-1)	1 (1-1)	1 (1-1)	1 (1-1)	1 (0-1)	0 (0-1)	0 (0-0)	0 (0-0)	0 (0-0)	0 (0-0)	0 (0-0)	0 (0-0)	0 (0-0)	0 (0-0)
Mauritius	2 (1-2)	1 (1-2)	1 (1-1)	1 (1-1)	1 (1-1)	1 (1-1)	1 (0-1)	0 (0-0)	0 (0-0)	0 (0-0)	0 (0-0)	0 (0-0)	0 (0-0)	0 (0-0)
Myanmar	191 (142-250)	181 (147-220)	172 (141-206)	159 (129-195)	143 (116-176)	122 (99-146)	110 (89-133)	98 (78-119)	87 (72-103)	77 (63-91)	65 (55-75)	49 (42-58)	33 (27-40)	22 (17-28)
Philippines	100 (81-124)	96 (86-108)	94 (82-105)	93 (83-103)	92 (83-101)	86 (77-95)	83 (74-92)	77 (69-85)	67 (61-74)	56 (50-62)	52 (47-58)	49 (43-56)	46 (40-54)	38 (30-47)
Sri Lanka	27 (23-30)	21 (20-23)	17 (17-18)	15 (15-16)	13 (13-14)	14 (13-15)	11 (10-11)	8 (7-8)	6 (6-7)	4 (4-5)	3 (3-4)	4 (3-4)	2 (2-3)	1 (1-2)
Seychelles	0 (0-0)	0 (0-0)	0 (0-0)	0 (0-0)	0 (0-0)	0 (0-0)	0 (0-0)	0 (0-0)	0 (0-0)	0 (0-0)	0 (0-0)	0 (0-0)	0 (0-0)	0 (0-0)
Thailand	96 (76-123)	88 (77-100)	83 (74-91)	72 (64-81)	61 (55-68)	47 (42-53)	37 (33-41)	26 (23-30)	19 (16-22)	14 (12-16)	10 (9-12)	7 (6-9)	5 (4-5)	3 (3-4)
Timor-Leste	4 (3-5)	3 (3-4)	3 (3-4)	3 (2-4)	3 (2-3)	3 (2-3)	3 (2-3)	2 (2-3)	2 (2-3)	2 (2-2)	2 (1-2)	1 (1-1)	1 (1-1)	1 (1-1)
Vietnam	132 (94-184)	117 (89-151)	107 (84-133)	96 (79-115)	90 (75-105)	75 (64-86)	66 (59-73)	60 (54-67)	50 (46-55)	36 (32-39)	24 (22-27)	20 (17-22)	17 (14-20)	11 (9-15)
Sub-Saharan Africa	1550 (1430-1680)	1540 (1460-1630)	1560 (1490-1640)	1590 (1530-1650)	1680 (1620-1740)	1750 (1700-1810)	1800 (1760-1860)	1870 (1830-1920)	1970 (1920-2010)	2070 (2030-2120)	2130 (2080-2180)	2020 (1970-2080)	1830 (1750-1900)	1510 (1420-1620)
Central sub-Saharan Africa	160 (131-195)	164 (141-191)	171 (148-197)	176 (153-202)	178 (156-201)	185 (163-207)	196 (178-216)	205 (190-222)	221 (207-236)	244 (228-260)	263 (247-281)	265 (246-283)	250 (226-275)	192 (164-226)
Angola	42 (31-55)	41 (33-51)	41 (34-50)	39 (31-49)	38 (30-47)	40 (32-48)	45 (38-53)	51 (45-58)	58 (52-64)	62 (57-69)	63 (58-69)	61 (55-68)	51 (44-59)	38 (29-48)
Central African Republic	8 (6-11)	8 (6-10)	8 (6-9)	7 (6-9)	8 (7-9)	8 (7-10)	8 (7-10)	9 (8-10)	10 (9-11)	12 (10-13)	14 (12-16)	15 (13-18)	15 (12-19)	12 (9-16)
Congo (Brazzaville)	6 (4-8)	6 (5-7)	6 (5-7)	5 (4-7)	5 (4-6)	5 (4-6)	5 (4-5)	4 (4-5)	5 (4-6)	6 (5-7)	7 (6-8)	6 (6-7)	5 (4-6)	4 (3-5)
DR Congo	98 (72-130)	104 (84-128)	112 (90-134)	120 (99-144)	123 (102-145)	128 (108-149)	134 (117-153)	137 (123-152)	145 (132-159)	160 (146-175)	175 (160-192)	179 (163-196)	176 (154-199)	137 (110-169)
Equatorial Guinea	2 (2-3)	2 (2-2)	2 (1-2)	2 (1-2)	2 (1-2)	1 (1-2)	1 (1-2)	2 (1-2)	2 (2-2)	2 (2-3)	2 (2-3)	2 (1-2)	1 (1-2)	1 (1-2)
Gabon	3 (2-4)	3 (3-4)	3 (3-4)	3 (2-4)	3 (2-3)	2 (2-3)	2 (2-2)	2 (1-2)	2 (1-2)	1 (1-2)	1 (1-2)	1 (1-2)	1 (1-2)	1 (1-1)
Eastern sub-Saharan Africa	542 (501-585)	549 (520-582)	562 (533-591)	576 (551-600)	606 (585-628)	659 (641-679)	696 (680-714)	741 (726-757)	783 (767-800)	811 (794-828)	783 (765-800)	679 (659-698)	585 (563-608)	470 (442-499)
Burundi	23 (17-29)	22 (18-26)	21 (17-25)	21 (18-24)	21 (18-23)	22 (19-24)	23 (20-25)	22 (20-24)	22 (20-24)	24 (22-27)	24 (22-26)	21 (19-23)	17 (15-19)	17 (13-22)
Comoros	1 (1-2)	1 (1-2)	1 (1-2)	1 (1-2)	1 (1-2)	1 (1-2)	1 (1-2)	1 (1-2)	1 (1-1)	1 (1-1)	1 (1-1)	1 (1-1)	1 (0-1)	0 (0-1)
Djibouti	0 (0-0)	0 (0-0)	0 (0-0)	0 (0-1)	1 (0-1)	1 (0-1)	1 (1-1)	1 (1-1)	1 (1-1)	1 (1-2)	1 (1-2)	1 (1-1)	1 (1-1)	1 (0-1)
Eritrea	10 (7-13)	11 (8-13)	11 (9-14)	12 (10-15)	13 (11-15)	14 (12-16)	14 (13-16)	14 (13-16)	15 (14-17)	11 (9-13)	9 (8-12)	9 (6-11)	8 (6-10)	6 (4-9)
Ethiopia	163 (140-191)	160 (144-179)	158 (143-174)	160 (146-174)	169 (157-182)	190 (180-201)	187 (178-197)	205 (196-215)	232 (222-243)	250 (239-261)	238 (227-248)	199 (189-210)	157 (147-167)	114 (102-128)
Kenya	43 (41-45)	42 (41-43)	41 (40-42)	44 (43-45)	47 (46-48)	47 (46-48)	46 (45-47)	46 (45-47)	47 (46-48)	52 (51-53)	53 (52-54)	48 (47-49)	41 (39-42)	33 (32-34)
Baringo	0 (0-1)	0 (0-1)	1 (0-1)	1 (1-1)	1 (1-1)	1 (1-1)	1 (1-1)	1 (1-1)	1 (1-1)	1 (1-1)	1 (1-1)	1 (1-1)	1 (0-1)	0 (0-0)
Bomet	0 (0-0)	0 (0-0)	0 (0-0)	0 (0-0)	0 (0-0)	0 (0-0)	0 (0-0)	0 (0-0)	0 (0-0)	0 (0-1)	1 (0-1)	1 (0-1)	1 (1-1)	1 (0-1)
Bungoma	2 (2-2)	2 (2-2)	2 (2-2)	2 (2-2)	2 (2-2)	2 (2-2)	2 (2-2)	2 (2-2)	2 (2-2)	2 (2-2)	2 (2-2)	2 (1-2)	1 (1-2)	1 (1-1)
Busia	2 (1-2)	2 (1-2)	2 (1-2)	2 (2-2)	2 (2-2)	2 (1-2)	2 (1-2)	2 (1-2)	2 (1-2)	2 (2-2)	2 (2-2)	2 (1-2)	1 (1-2)	1 (1-1)
Elgeyo Marakwet	0 (0-0)	0 (0-0)	0 (0-0)	0 (0-0)	0 (0-0)	0 (0-0)	0 (0-1)	1 (0-1)	0 (0-1)	1 (0-1)	0 (0-1)	0 (0-0)	0 (0-0)	0 (0-0)
Embu	1 (1-1)	1 (1-1)	1 (1-1)	1 (1-1)	1 (1-1)	1 (0-1)	0 (0-1)	0 (0-1)	1 (0-1)	1 (0-1)	0 (0-1)	0 (0-0)	0 (0-0)	0 (0-0)
Garissa	0 (0-0)	0 (0-0)	0 (0-0)	0 (0-0)	0 (0-1)	0 (0-0)	0 (0-1)	0 (0-0)	0 (0-0)	0 (0-1)	1 (1-1)	1 (1-1)	1 (1-1)	0 (0-1)
Homa Bay	3 (2-3)	3 (2-3)	3 (2-3)	3 (3-3)	3 (3-4)	3 (3-4)	3 (3-4)	3 (3-3)	3 (3-4)	4 (3-4)	4 (3-4)	3 (3-4)	2 (2-3)	2 (1-2)
Isiolo	0 (0-0)	0 (0-0)	0 (0-0)	0 (0-0)	0 (0-0)	0 (0-0)	0 (0-0)	0 (0-0)	0 (0-0)	0 (0-0)	0 (0-0)	0 (0-0)	0 (0-0)	0 (0-0)
Kajiado	0 (0-0)	0 (0-0)	0 (0-0)	0 (0-0)	0 (0-0)	0 (0-0)	0 (0-0)	0 (0-0)	0 (0-0)	0 (0-0)	0 (0-1)	1 (1-1)	1 (0-1)	0 (0-0)
Kakamega	2 (2-3)	2 (2-3)	2 (2-3)	3 (2-3)	3 (2-3)	3 (2-3)	3 (3-3)	3 (3-3)	3 (3-4)	4 (3-4)	3 (3-4)	3 (3-4)	3 (2-3)	2 (2-3)
Kericho	0 (0-0)	0 (0-0)	0 (0-0)	0 (0-0)	0 (0-1)	0 (0-1)	1 (0-1)	0 (0-1)	0 (0-1)	1 (0-1)	1 (1-1)	1 (0-1)	0 (0-1)	0 (0-0)
Kiambu	2 (1-2)	1 (1-2)	1 (1-2)	1 (1-2)	1 (1-2)	1 (1-1)	1 (1-1)	1 (1-1)	1 (1-1)	1 (1-1)	1 (1-2)	1 (1-2)	1 (1-2)	1 (1-1)
Kilifi	2 (2-2)	2 (2-2)	2 (2-2)	2 (2-3)	2 (2-3)	2 (2-3)	2 (2-3)	2 (2-2)	2 (2-2)	2 (2-2)	2 (2-3)	2 (2-2)	2 (1-2)	1 (1-1)
Kirinyaga	1 (1-1)	1 (1-1)	1 (1-1)	1 (1-1)	1 (1-1)	1 (1-1)	0 (0-1)	0 (0-0)	0 (0-1)	0 (0-1)	0 (0-1)	0 (0-0)	0 (0-0)	0 (0-0)
Kisii	2 (2-3)	2 (2-2)	2 (2-2)	2 (2-2)	2 (2-3)	2 (2-3)	2 (2-3)	2 (2-3)	2 (2-3)	2 (2-3)	2 (2-3)	2 (2-2)	1 (1-2)	1 (1-1)
Kisumu	2 (2-3)	2 (2-3)	2 (2-2)	2 (2-3)	3 (2-3)	3 (2-3)	3 (2-3)	3 (2-3)	3 (2-3)	3 (3-3)	3 (3-3)	2 (2-2)	1 (1-2)	1 (1-1)
Kitui	2 (1-2)	2 (1-2)	2 (1-2)	2 (2-2)	2 (2-2)	2 (1-2)	1 (1-2)	1 (1-1)	1 (1-1)	1 (1-2)	1 (1-1)	1 (1-1)	1 (1-1)	1 (0-1)
Kwale	1 (1-2)	1 (1-1)	1 (1-1)	1 (1-1)	1 (1-2)	1 (1-2)	1 (1-2)	1 (1-1)	1 (1-1)	1 (1-1)	1 (1-1)	1 (1-1)	1 (1-1)	0 (0-1)
Laikipia	0 (0-0)	0 (0-0)	0 (0-0)	0 (0-0)	0 (0-0)	0 (0-0)	0 (0-0)	0 (0-0)	0 (0-0)	0 (0-0)	0 (0-0)	0 (0-0)	0 (0-0)	0 (0-0)
Lamu	0 (0-0)	0 (0-0)	0 (0-0)	0 (0-0)	0 (0-0)	0 (0-0)	0 (0-0)	0 (0-0)	0 (0-0)	0 (0-0)	0 (0-0)	0 (0-0)	0 (0-0)	0 (0-0)
Machakos	2 (1-2)	2 (2-2)	2 (2-2)	2 (2-2)	2 (2-2)	2 (2-2)	2 (1-2)	1 (1-2)	1 (1-1)	1 (1-1)	1 (1-1)	1 (1-1)	1 (1-1)	1 (1-1)
Makueni	0 (0-1)	0 (0-1)	1 (0-1)	1 (1-1)	1 (1-1)	1 (1-1)	1 (1-1)	1 (1-1)	1 (1-1)	1 (1-1)	1 (1-1)	1 (1-1)	0 (0-0)	0 (0-0)
Mandera	0 (0-1)	0 (0-1)	0 (0-1)	0 (0-1)	0 (0-1)	0 (0-1)	0 (0-1)	0 (0-1)	0 (0-1)	1 (0-1)	1 (1-1)	1 (1-1)	2 (1-2)	2 (1-2)
Marsabit	1 (1-1)	1 (1-1)	1 (1-1)	1 (1-1)	1 (1-1)	1 (1-1)	1 (0-1)	0 (0-1)	0 (0-0)	0 (0-0)	0 (0-0)	0 (0-1)	0 (0-1)	0 (0-1)
Meru	2 (1-2)	2 (1-2)	1 (1-2)	1 (1-2)	1 (1-2)	1 (1-1)	1 (1-1)	1 (1-1)	1 (1-1)	1 (1-1)	1 (1-2)	1 (1-2)	1 (1-2)	1 (1-2)
Migori	2 (1-2)	2 (2-2)	2 (2-2)	2 (2-3)	3 (2-3)	3 (3-3)	4 (3-4)	4 (4-4)	4 (4-4)	4 (4-4)	4 (4-5)	4 (3-4)	3 (2-3)	2 (2-2)
Mombasa	1 (1-1)	1 (1-1)	1 (1-1)	1 (1-1)	1 (1-1)	1 (1-1)	1 (1-1)	1 (1-1)	1 (1-1)	1 (1-1)	1 (1-1)	1 (1-1)	1 (1-1)	1 (1-1)
Murang'a	1 (1-2)	1 (1-1)	1 (1-1)	1 (1-1)	1 (1-1)	1 (1-1)	1 (1-1)	1 (1-1)	1 (1-1)	1 (0-1)	0 (0-1)	0 (0-1)	1 (0-1)	1 (0-1)
Nairobi	2 (2-3)	2 (2-2)	2 (1-2)	1 (1-2)	1 (1-1)	1 (1-1)	1 (1-1)	1 (1-1)	1 (1-2)	2 (2-2)	2 (2-3)	2 (2-2)	2 (1-2)	1 (1-2)
Nakuru	0 (0-1)	0 (0-1)	0 (0-0)	0 (0-1)	1 (0-1)	1 (1-1)	1 (1-1)	1 (1-1)	1 (1-1)	1 (1-1)	1 (1-2)	1 (1-1)	1 (1-1)	1 (1-1)
Nandi	0 (0-0)	0 (0-0)	0 (0-0)	0 (0-0)	0 (0-0)	0 (0-0)	0 (0-1)	1 (0-1)	1 (1-1)	1 (1-1)	1 (1-1)	1 (1-1)	1 (0-1)	0 (0-1)
Narok	0 (0-0)	0 (0-0)	0 (0-0)	0 (0-0)	0 (0-0)	0 (0-1)	1 (0-1)	1 (1-1)	1 (1-1)	1 (1-1)	1 (1-1)	1 (1-1)	1 (1-1)	0 (0-1)
Nyamira	0 (0-1)	0 (0-1)	0 (0-1)	1 (0-1)	1 (0-1)	1 (1-1)	1 (1-1)	1 (1-1)	0 (0-1)	1 (0-1)	1 (1-1)	1 (0-1)	0 (0-1)	0 (0-0)
Nyandarua	0 (0-1)	0 (0-0)	0 (0-0)	0 (0-0)	0 (0-0)	0 (0-0)	0 (0-0)	0 (0-0)	0 (0-0)	0 (0-0)	0 (0-0)	0 (0-1)	0 (0-1)	0 (0-1)
Nyeri	1 (1-1)	1 (1-1)	1 (1-1)	1 (1-1)	1 (1-1)	1 (1-1)	1 (0-1)	0 (0-1)	0 (0-1)	0 (0-1)	1 (0-1)	0 (0-1)	0 (0-0)	0 (0-0)
Samburu	0 (0-0)	0 (0-0)	0 (0-0)	0 (0-0)	0 (0-0)	0 (0-0)	0 (0-0)	0 (0-0)	0 (0-0)	0 (0-0)	0 (0-0)	0 (0-0)	0 (0-0)	0 (0-0)
Siaya	1 (1-2)	1 (1-2)	1 (1-2)	2 (1-2)	2 (1-2)	2 (1-2)	2 (1-2)	2 (2-2)	2 (2-2)	2 (2-2)	2 (2-2)	2 (2-2)	1 (1-1)	1 (1-1)
Taita Taveta	1 (0-1)	1 (0-1)	1 (0-1)	1 (0-1)	1 (0-1)	0 (0-1)	0 (0-0)	0 (0-0)	0 (0-0)	0 (0-0)	0 (0-0)	0 (0-0)	0 (0-0)	0 (0-0)
Tana River	0 (0-0)	0 (0-0)	0 (0-0)	0 (0-0)	0 (0-0)	0 (0-0)	0 (0-0)	0 (0-0)	0 (0-0)	0 (0-0)	0 (0-0)	0 (0-1)	0 (0-1)	0 (0-0)
Tharaka Nithi	0 (0-0)	0 (0-0)	0 (0-0)	0 (0-0)	0 (0-0)	0 (0-0)	0 (0-0)	0 (0-0)	0 (0-0)	0 (0-0)	0 (0-0)	0 (0-0)	0 (0-0)	0 (0-0)
Trans Nzoia	0 (0-0)	0 (0-0)	0 (0-0)	0 (0-0)	1 (0-1)	1 (1-1)	1 (1-1)	1 (1-1)	1 (1-1)	1 (1-1)	1 (1-1)	1 (1-1)	1 (1-1)	1 (0-1)

Table 4 Under-5 death numbers by location and year for 1950, 1955...2005, 2010, 2017 - males

Location	1950	1955	1960	1965	1970	1975	1980	1985	1990	1995	2000	2005	2010	2017
Turkana	0 (0-1)	0 (0-1)	0 (0-1)	1 (0-1)	1 (1-1)	1 (1-1)	1 (1-1)	1 (1-1)	1 (1-1)	1 (1-1)	1 (1-1)	1 (1-2)	1 (1-2)	1 (1-2)
Uasin Gishu	0 (0-0)	0 (0-0)	0 (0-0)	0 (0-0)	0 (0-0)	0 (0-0)	0 (0-0)	0 (0-1)	1 (0-1)	1 (0-1)	1 (1-1)	1 (1-1)	1 (1-1)	1 (0-1)
Vihiga	0 (0-0)	0 (0-0)	0 (0-0)	0 (0-0)	0 (0-0)	0 (0-0)	0 (0-0)	1 (0-1)	1 (1-1)	1 (1-1)	1 (1-1)	0 (0-1)	0 (0-0)	0 (0-0)
Wajir	0 (0-0)	0 (0-0)	0 (0-0)	0 (0-0)	0 (0-0)	0 (0-1)	1 (0-1)	0 (0-1)	0 (0-1)	1 (1-1)	1 (1-1)	1 (1-1)	1 (1-1)	1 (1-1)
West Pokot	0 (0-1)	0 (0-1)	0 (0-1)	1 (0-1)	1 (1-1)	1 (1-1)	1 (1-1)	1 (1-1)	1 (1-1)	1 (1-1)	1 (1-1)	1 (1-1)	1 (1-1)	1 (1-1)
Madagascar	24 (18-31)	25 (21-31)	27 (23-32)	29 (25-34)	32 (28-36)	34 (30-37)	36 (33-40)	39 (36-43)	39 (36-43)	40 (36-44)	39 (34-43)	37 (33-42)	40 (34-46)	39 (29-51)
Malawi	38 (30-48)	40 (34-47)	43 (36-49)	44 (39-49)	45 (40-49)	45 (42-49)	45 (42-48)	45 (42-48)	53 (50-57)	50 (47-54)	43 (40-46)	35 (32-37)	28 (25-31)	21 (17-27)
Mozambique	56 (41-76)	55 (43-67)	53 (43-64)	53 (43-63)	53 (44-63)	57 (49-65)	67 (60-73)	85 (79-90)	76 (70-82)	72 (67-78)	65 (59-70)	57 (52-63)	49 (43-56)	40 (32-50)
Rwanda	20 (15-25)	21 (17-24)	22 (20-25)	24 (21-26)	26 (24-28)	28 (26-30)	28 (26-30)	26 (24-28)	27 (25-29)	29 (27-32)	32 (30-35)	19 (17-21)	13 (11-15)	11 (8-14)
Somalia	19 (14-25)	19 (15-23)	19 (16-23)	21 (17-25)	23 (19-29)	30 (25-35)	39 (34-45)	37 (33-42)	38 (34-43)	41 (35-46)	42 (36-47)	39 (32-45)	40 (32-47)	31 (23-41)
South Sudan	16 (11-23)	17 (13-22)	19 (15-23)	19 (15-22)	18 (14-22)	18 (15-22)	20 (17-24)	22 (18-26)	24 (20-28)	24 (21-28)	24 (21-27)	24 (21-28)	26 (22-32)	25 (20-31)
Tanzania	66 (48-87)	69 (54-84)	72 (58-87)	72 (61-82)	74 (66-82)	80 (74-88)	82 (76-89)	86 (79-92)	91 (85-97)	95 (89-103)	90 (83-97)	79 (71-88)	74 (63-85)	63 (49-83)
Uganda	44 (33-58)	48 (39-57)	51 (43-61)	54 (47-61)	59 (53-66)	70 (64-76)	83 (77-90)	84 (78-90)	83 (79-89)	86 (80-92)	88 (82-96)	80 (73-87)	66 (59-74)	49 (41-59)
Zambia	18 (14-24)	20 (16-24)	22 (18-26)	23 (20-27)	23 (21-26)	23 (20-25)	24 (22-26)	27 (25-29)	32 (29-34)	34 (31-37)	34 (30-37)	31 (27-36)	25 (21-30)	21 (16-28)
Southern sub-Saharan Africa	67 (59-77)	70 (64-78)	74 (68-81)	82 (77-89)	81 (76-86)	78 (74-82)	78 (75-82)	72 (69-76)	62 (59-65)	61 (58-64)	68 (65-72)	71 (67-76)	56 (52-59)	35 (32-39)
Botswana	1 (1-2)	1 (1-2)	1 (1-2)	1 (1-2)	2 (1-2)	1 (1-2)	1 (1-2)	1 (1-1)	1 (1-1)	1 (1-2)	2 (1-2)	1 (1-2)	1 (1-1)	1 (0-1)
Lesotho	3 (2-4)	3 (3-3)	3 (3-3)	3 (3-4)	3 (3-4)	3 (3-4)	3 (3-4)	3 (2-3)	3 (2-3)	3 (2-3)	3 (3-3)	3 (3-3)	2 (2-2)	2 (1-2)
Namibia	2 (1-2)	2 (1-2)	2 (1-2)	2 (1-2)	2 (1-2)	2 (2-2)	2 (2-2)	2 (2-2)	2 (2-2)	2 (2-2)	2 (2-2)	2 (2-2)	2 (1-2)	1 (1-2)
South Africa	48 (41-56)	51 (45-57)	54 (48-60)	62 (57-67)	59 (55-63)	54 (51-58)	53 (50-57)	49 (46-53)	41 (39-44)	38 (36-41)	43 (40-47)	44 (41-49)	31 (28-34)	18 (16-20)
Swaziland	2 (1-2)	2 (1-2)	2 (1-2)	2 (1-2)	2 (1-2)	2 (1-2)	2 (1-2)	1 (1-2)	1 (1-1)	1 (1-1)	2 (1-2)	2 (2-2)	1 (1-2)	1 (1-1)
Zimbabwe	11 (8-15)	12 (9-14)	12 (10-15)	13 (11-15)	14 (12-16)	16 (14-17)	17 (16-19)	15 (14-16)	14 (13-15)	16 (15-17)	17 (15-18)	19 (17-21)	19 (17-21)	13 (11-17)
Western sub-Saharan Africa	777 (673-894)	759 (687-841)	752 (690-819)	758 (707-812)	811 (763-863)	829 (783-877)	834 (792-876)	856 (821-893)	900 (864-939)	956 (918-996)	1020 (971-1060)	1000 (957-1050)	936 (878-1000)	814 (734-912)
Benin	20 (14-25)	19 (16-23)	19 (16-21)	18 (16-21)	20 (18-22)	20 (19-22)	20 (19-22)	20 (19-22)	20 (19-22)	20 (18-21)	20 (18-22)	20 (18-22)	21 (18-24)	19 (14-24)
Burkina Faso	41 (31-54)	38 (31-46)	35 (29-43)	35 (29-42)	39 (34-43)	42 (38-46)	43 (40-47)	45 (42-48)	47 (43-50)	49 (45-54)	50 (46-55)	50 (44-55)	49 (42-58)	48 (38-62)
Cameroon	42 (35-51)	38 (34-43)	37 (33-41)	33 (29-38)	33 (30-37)	34 (31-37)	35 (32-38)	33 (30-36)	33 (31-36)	37 (34-41)	40 (36-45)	43 (38-49)	40 (35-46)	30 (24-37)
Cape Verde	1 (1-1)	1 (1-1)	1 (1-1)	1 (1-1)	1 (1-1)	1 (1-1)	1 (1-1)	1 (1-1)	0 (0-0)	1 (0-1)	0 (0-0)	0 (0-0)	0 (0-0)	0 (0-0)
Chad	24 (18-32)	24 (20-29)	24 (20-29)	24 (20-29)	26 (22-30)	28 (24-31)	30 (27-33)	32 (29-35)	33 (30-36)	36 (33-39)	41 (37-45)	44 (40-49)	44 (39-50)	43 (35-51)
Cote d'Ivoire	24 (20-29)	26 (23-30)	29 (26-32)	32 (29-35)	35 (32-38)	36 (33-39)	35 (33-38)	38 (35-41)	42 (38-46)	48 (43-53)	50 (45-56)	51 (45-58)	47 (40-55)	38 (31-46)
The Gambia	2 (1-3)	2 (2-3)	2 (2-3)	2 (2-3)	2 (2-3)	2 (2-3)	3 (2-3)	3 (2-3)	3 (2-3)	3 (2-3)	3 (2-3)	2 (2-3)	2 (2-3)	2 (1-2)
Ghana	46 (36-59)	45 (38-52)	44 (39-50)	41 (37-45)	40 (37-43)	39 (36-43)	40 (37-43)	41 (39-44)	37 (34-40)	36 (33-38)	36 (33-39)	34 (32-37)	30 (27-34)	25 (20-32)
Guinea	32 (25-40)	30 (24-36)	28 (23-34)	27 (22-33)	28 (24-32)	30 (27-33)	31 (28-34)	34 (31-37)	36 (33-39)	36 (33-40)	33 (30-36)	29 (26-33)	26 (22-31)	22 (18-27)
Guinea-Bissau	7 (5-9)	7 (6-8)	7 (5-8)	6 (5-7)	6 (5-7)	6 (5-7)	6 (5-7)	6 (5-7)	5 (5-6)	5 (4-6)	5 (4-5)	4 (4-5)	4 (3-4)	3 (2-4)
Liberia	10 (7-13)	10 (8-12)	11 (9-13)	11 (10-13)	12 (10-13)	12 (10-13)	12 (11-14)	13 (12-14)	12 (11-13)	10 (10-11)	11 (10-12)	8 (7-9)	7 (6-8)	6 (4-7)
Mali	60 (45-76)	59 (48-70)	59 (50-69)	62 (54-71)	65 (58-71)	64 (58-69)	59 (55-63)	57 (53-62)	56 (53-61)	55 (51-59)	54 (50-58)	52 (47-58)	54 (47-61)	57 (45-72)
Mauritania	5 (4-6)	5 (4-6)	5 (4-6)	5 (4-5)	5 (4-5)	5 (5-6)	6 (5-6)	6 (5-6)	5 (5-6)	5 (4-5)	5 (4-5)	5 (4-5)	4 (3-4)	3 (2-4)
Niger	32 (25-41)	34 (28-41)	37 (30-44)	39 (34-46)	45 (39-51)	53 (48-58)	58 (53-63)	62 (57-67)	64 (60-70)	66 (61-72)	64 (59-71)	60 (53-68)	55 (46-64)	52 (41-65)
Nigeria	376 (282-492)	364 (296-442)	355 (298-419)	356 (308-407)	390 (345-443)	393 (348-440)	390 (351-432)	401 (368-439)	445 (411-482)	492 (455-531)	546 (505-589)	545 (501-590)	504 (447-567)	430 (349-527)
Sao Tome and Principe	1 (1-1)	0 (0-1)	0 (0-0)	0 (0-0)	0 (0-0)	0 (0-0)	0 (0-0)	0 (0-0)	0 (0-0)	0 (0-0)	0 (0-0)	0 (0-0)	0 (0-0)	0 (0-0)
Senegal	21 (16-26)	22 (18-26)	24 (20-27)	27 (23-30)	30 (27-34)	31 (28-34)	30 (28-33)	29 (26-32)	25 (23-28)	25 (23-28)	24 (21-27)	21 (18-23)	17 (14-19)	13 (10-17)
Sierra Leone	21 (15-27)	22 (17-26)	23 (19-27)	23 (18-28)	22 (18-27)	22 (18-26)	22 (19-25)	23 (20-25)	23 (21-25)	20 (19-22)	20 (18-22)	22 (20-25)	20 (17-23)	16 (12-21)
Togo	13 (9-17)	13 (10-15)	13 (10-15)	13 (11-15)	12 (11-14)	12 (10-13)	12 (10-13)	12 (11-13)	12 (11-13)	12 (11-13)	13 (11-14)	13 (11-14)	11 (9-13)	8 (6-10)

Table 5 Life expectancy at birth in 2015 from UN, WHO, census, and GBD 2017

Location	GBD 2017 Female	GBD 2017 Male	WPP 2017 Female	WPP 2017 Male	WHO Female	WHO Male	US Census Bureau Female	US Census Bureau Male
Afghanistan	61.7	60.6	64.87	62.358	64.7	61.8	52.3	49.5
Albania	81.8	74.8	80.668	77.128	78.2	74.2	81.0	75.5
Algeria	77.8	76.5	77.276	74.976	77.2	75.3	78.0	75.3
Angola	65.8	60.8	65.88	60.056	64.5	59.9	56.8	54.5
Antigua and Barbuda	78.7	75.0	78.72	73.728	77.4	72.5	78.5	74.2
Argentina	79.5	73.4	80.322	72.636	80.1	73.3	80.2	73.8
Armenia	78.4	72.0	77.744	71.408	77.9	70.9	78.0	71.1
Australia	84.6	80.4	84.794	80.822	84.5	80.6	84.7	79.7
Austria	83.9	79.0	83.894	79.012	83.8	78.9	84.2	78.8
Azerbaijan	74.4	66.9	75.27	69.606	75.5	70.2	75.5	69.2
Bahrain	80.1	78.7	77.916	76.03	79.4	78.3	81.0	76.5
Bangladesh	73.8	71.4	74.684	70.756	74	70.7	75.1	70.8
Barbados	78.5	75.5	78.22	73.378	77.9	72.9	77.6	72.8
Belarus	78.8	68.5	79.174	68.328	78.8	68.6	78.4	66.9
Belgium	83.3	78.5	83.438	78.678	83.2	78.6	83.6	78.3
Belize	77.3	71.1	72.918	67.304	73.2	67.7	70.3	67.0
Benin	65.6	61.5	62.2	59.276	62	59.3	62.9	60.1
Bhutan	75.4	71.8	70.202	70.01	70.3	70	70.5	68.6
Bosnia and Herzegovina	78.6	74.2	79.27	74.214	79.6	74.5	79.8	73.5
Botswana	68.5	65.1	70.6	62.986	67.9	62.6	52.3	56.0
Brazil	79.0	71.9	79.328	72.082	78.6	71.2	77.3	70.0
Bulgaria	78.3	71.4	78.404	71.532	78.3	71.2	77.9	71.1
Burkina Faso	63.4	58.0	61.312	60.09	60.4	59.1	57.2	53.1
Burundi	62.8	58.9	59.636	55.488	61.4	58	61.8	58.5
Cambodia	72.1	66.2	70.87	67.162	70.8	67	66.7	61.7
Cameroon	63.1	59.4	59.078	56.118	58.8	55.7	59.3	56.6
Canada	83.8	79.6	84.26	80.44	84.5	80.6	84.5	79.2
Central African Republic	53.2	48.8	53.204	49.72	53.5	51.6	53.2	50.5
Chad	60.9	57.8	54.726	52.002	54.9	52.5	51.0	48.6
Chile	82.2	77.0	81.626	76.666	82.3	76.4	81.8	75.6
China	79.3	74.0	77.776	74.854	77.7	74.8	77.7	73.4
Colombia	82.4	77.4	77.858	70.778	78.6	71.3	78.8	72.3
Comoros	69.4	66.6	65.694	62.334	65.2	62	66.2	61.6
Congo	62.2	62.2	67.05	63.606	64.8	62.4	60.0	57.6
Costa Rica	83.0	76.9	82.206	77.084	82.1	76.9	81.2	75.8
Croatia	80.5	74.6	80.89	74.282	80.6	74.3	79.0	72.5
Cuba	80.5	76.0	81.588	77.37	81	76.6	81.0	76.2
Cyprus	84.9	78.3	82.812	78.194	82.9	78.2	81.5	75.7
Democratic Republic of the Congo	63.1	59.4	61.096	58.248	61.6	58.5	58.5	55.4
Denmark	82.8	78.9	82.994	79.138	82.8	78.9	81.8	76.8
Djibouti	67.8	65.0	64.86	61.504	65.3	62	65.4	60.3
Dominican Republic	76.4	69.5	77.05	70.748	76.4	70.3	80.3	75.8
Ecuador	78.7	74.5	78.868	73.474	78.8	73.9	79.7	73.6
Egypt	73.8	67.6	73.554	69.364	72.6	68.1	73.9	71.1
El Salvador	77.4	66.5	77.95	68.76	77.9	68.7	77.9	71.1
Equatorial Guinea	65.1	63.9	59.73	56.548	61.7	57.6	65.0	62.8
Eritrea	65.1	57.9	67.236	62.948	66.7	62.5	67.1	62.1
Estonia	81.8	73.1	82.55	73.968	81.8	73	81.5	71.6
Ethiopia	69.1	65.6	68.422	64.53	66.8	63.3	63.9	59.1
Fiji	70.2	65.7	73.484	67.434	73	67	75.2	69.8
Finland	84.3	78.8	84.126	78.584	84.2	78.6	83.9	77.8
France	85.5	79.4	85.4	79.606	85.4	79.8	85.0	78.7
Gabon	70.0	64.1	68.192	64.552	67.5	64.3	52.5	51.6
Georgia	77.4	68.6	77.246	68.368	77.1	68.5	80.4	71.9
Germany	83.0	78.3	83.234	78.51	83	78.2	83.0	78.3
Ghana	67.1	61.7	63.632	61.65	64	62.2	68.7	63.8
Greece	83.6	78.1	83.62	78.368	83.4	78.4	83.2	77.8
Grenada	75.2	72.8	76.304	71.482	75.8	70.9	76.9	71.5
Guatemala	75.9	69.7	76.74	70.402	75.8	70	74.1	70.1
Guinea	60.7	57.8	59.602	59.196	59	58.1	61.7	58.6
Guinea-Bissau	61.2	56.1	58.942	55.244	60.6	57.8	52.3	48.2
Guyana	71.7	65.6	68.728	64.426	68.9	63.5	71.2	65.1
Haiti	64.9	63.0	65.716	61.362	65.6	61.1	65.9	60.8
Honduras	74.2	69.9	75.958	70.888	77.3	72.7	72.7	69.3
Hungary	78.9	71.9	79.486	72.988	78.7	71.9	79.6	72.0
Iceland	85.6	80.0	84.128	81.192	84	80.8	85.2	80.8
India	69.6	67.2	70.584	67.134	70	67.1	69.4	67.0
Indonesia	73.3	68.8	71.228	67.198	71.2	67.2	75.2	69.9
Iraq	78.2	74.5	71.59	68.13	71.8	66.9	77.2	72.6
Ireland	83.3	79.8	83.542	79.552	83.3	79.5	83.1	78.4
Israel	84.4	80.7	84.194	80.656	84	80	84.2	80.4
Italy	84.8	80.2	85.02	80.524	84.6	80.2	84.9	79.5
Jamaica	77.4	71.9	78.538	73.922	78.3	73.3	75.2	71.9
Japan	87.1	81.0	86.716	80.466	87	80.8	88.3	81.4
Jordan	80.7	77.7	76.054	72.624	75.8	72.6	75.8	73.0
Kazakhstan	76.0	67.2	75.052	66.534	74.9	66.1	75.5	65.3
Kenya	67.5	62.0	71.69	65.98	68.4	63.6	65.3	62.3
Kiribati	65.9	58.1	69.404	62.67	68.4	63.4	68.4	63.4
Kuwait	86.7	80.7	75.918	73.73	75.9	73.8	79.2	76.5

Table 5 Life expectancy at birth in 2015 from UN, WHO, census, and GBD 2017

Location	GBD 2017 Female	GBD 2017 Male	WPP 2017 Female	WPP 2017 Male	WHO Female	WHO Male	US Census Bureau Female	US Census Bureau Male
Kyrgyzstan	75.7	68.0	75.832	68.06	74.8	67.4	74.8	66.2
Latvia	79.5	69.9	79.716	70.488	79.3	69.8	79.1	69.6
Lebanon	79.8	75.5	81.554	78.024	77.6	74.8	78.7	76.2
Lesotho	53.7	47.4	57.404	51.53	53.8	50.1	53.0	52.8
Liberia	62.6	61.5	63.172	61.32	63.2	61	60.3	56.9
Libya	75.1	71.3	74.41	68.352	75.1	69.2	78.1	74.5
Lithuania	79.7	69.4	80.248	69.964	79.6	69	80.5	69.2
Luxembourg	82.9	79.9	84.218	79.978	84.7	80	84.8	79.7
Madagascar	64.2	61.6	67.404	64.346	67.2	64.2	67.1	64.1
Malawi	65.4	58.2	67.822	62.278	66	60.7	62.7	58.7
Malaysia	77.2	72.3	77.73	73.234	77.5	73	77.7	72.0
Maldives	83.1	79.7	77.824	75.86	79.6	76.9	77.8	73.1
Mali	62.2	60.1	58.202	56.91	57.8	56.9	57.3	53.5
Malta	83.0	79.3	82.52	79.078	83.2	79.6	82.7	77.9
Mauritania	70.3	69.5	64.818	62.048	65	62.4	65.0	60.4
Mauritius	77.8	71.3	78.634	71.438	78	71.2	79.0	71.9
Mexico	78.6	73.3	79.416	74.478	78.9	73.6	78.6	72.9
Mongolia	73.3	64.2	74.158	65.816	74	65.5	73.8	65.0
Montenegro	78.7	74.0	80.15	75.246	79	74.1	81.6	75.5
Morocco	74.1	72.8	76.918	75.116	76.8	74.7	79.9	73.6
Mozambique	59.8	52.8	59.928	55.524	60.9	56.9	53.7	52.2
Myanmar	71.3	64.4	69.454	64.61	68.7	64.3	68.8	63.9
Namibia	69.5	61.6	68.906	62.544	65.7	60.9	64.5	61.8
Nepal	72.1	67.3	71.836	68.568	70.5	67.5	70.5	69.0
Netherlands	83.2	79.7	83.704	80.17	83.1	79.7	83.5	79.1
New Zealand	83.2	79.8	83.59	80.238	83.7	80.3	83.2	79.0
Nicaragua	80.0	76.1	78.458	72.352	78.2	72.2	75.3	70.8
Niger	62.6	60.1	61.812	59.674	60.2	58.3	56.4	53.9
Nigeria	64.8	61.8	53.894	52.438	55.3	54.3	54.1	52.0
Norway	84.1	80.4	84.11	80.268	84.1	80.5	83.8	79.7
Oman	79.2	74.4	79.414	75.222	79.3	75.1	77.2	73.3
Pakistan	66.5	65.5	67.708	65.858	67	65.2	69.4	65.5
Panama	81.7	76.4	81.138	74.838	81.1	74.9	81.4	75.7
Papua New Guinea	60.7	55.7	67.924	63.094	68.1	63.4	69.4	64.8
Paraguay	78.5	73.2	75.52	71.3	76	72.2	79.8	74.3
Peru	81.6	78.4	77.422	72.164	78.1	73.2	75.6	71.5
Philippines	72.6	66.0	72.51	65.688	72.5	66.1	72.6	65.5
Poland	81.4	73.6	81.672	73.914	81.3	73.4	81.5	73.5
Portugal	84.4	78.5	84.13	78.168	84.3	78.2	82.6	75.9
Qatar	81.2	79.2	79.792	77.396	79.7	77.1	80.7	76.6
Romania	78.7	71.6	79.348	72.454	78.7	71.3	78.6	71.5
Rwanda	69.9	65.1	70.536	65.894	69.4 67.4	65.7 61.9	61.3	58.1
Saint Lucia	78.0	73.2	78.14	72.62	78.2	72.8	80.5	74.9
Saint Vincent and the Grenadines	75.1	69.3	75.446	71.196	75.1	69.7	77.1	73.1
Samoa	74.3	71.2	78.59	72.304	78.2	71.8	76.5	70.6
Sao Tome and Principe	71.3	67.6	68.694	64.35	70.5	66.6	65.9	63.3
Saudi Arabia	78.9	74.5	76.12	73.212	76.3	73.3	77.2	73.0
Senegal	69.4	65.7	69.814	65.412	68.3	64.4	63.4	59.3
Serbia	77.5	72.8	78.334	72.574	78.5	73.4	78.3	72.4
Seychelles	77.5	70.0	78.294	69.074	77.8	69	79.2	69.9
Sierra Leone	59.4	57.6	53.152	52.41	52	50.7	60.4	55.2
Singapore	87.2	81.6	85.036	80.864	84.8	80.6	87.5	82.1
Slovakia	80.1	73.1	80.534	73.742	80.6	73.5	80.7	73.3
Slovenia	83.9	77.6	84.012	78.6	83.5	77.6	81.9	74.4
Solomon Islands	67.2	63.8	72.508	69.556	72.4	69.5	77.9	72.5
Somalia	60.2	56.4	57.536	54.258	57.1	54.1	54.1	49.9
South Africa	65.7	60.8	67.458	59.37	66.7	60	63.9	60.8
South Sudan	61.7	57.4	57.668	55.78	59.1	57.3	62.3	59.3
Spain	85.4	79.8	85.908	80.572	85.4	79.9	84.8	78.6
Sri Lanka	80.4	73.3	78.222	71.618	78.3	72.1	80.2	73.1
Suriname	75.2	68.7	74.822	68.644	74.8	68.5	74.5	69.6
Swaziland	60.5	51.3	63.066	54.624	59.6	54.8	50.5	51.6
Sweden	84.1	80.3	84.142	80.6	84	80.3	84.0	80.1
Switzerland	85.5	81.3	85.146	81.226	84.9	80.9	84.9	80.2
Tajikistan	73.3	67.5	74.428	68.738	72.8	68.5	70.7	64.3
Thailand	81.9	74.6	79.518	71.436	79.1	71.6	77.8	71.2
Timor-Leste	72.8	68.8	70.638	66.606	70.1	66.6	69.4	66.2
Togo	65.4	60.1	61.81	60.25	61.1	59.4	67.2	61.9
Tonga	74.9	68.4	76.076	69.882	76.2	70.4	77.6	74.5
Trinidad and Tobago	77.7	71.3	74.368	67.482	75.4	68.2	75.6	69.7
Tunisia	80.3	75.7	77.184	73.484	77.8	73.9	78.1	73.8
Turkey	82.4	74.6	78.846	72.496	79.2	73	77.0	72.3
Turkmenistan	73.4	66.3	71.462	64.88	71.3	64.2	72.9	66.8
Uganda	68.1	61.1	63.124	58.232	64.5	59.7	56.4	53.5
Ukraine	75.6	63.1	77.358	68.362	76.8	67.2	76.6	66.8
United Arab Emirates	76.6	71.5	78.716	76.522	78.5	76.4	80.0	74.7
Uruguay	80.4	73.4	80.878	73.694	80.6	73	80.3	73.9
Uzbekistan	73.3	66.7	74.338	69.312	74.8	69.4	76.8	70.5
Vanuatu	67.5	61.8	74.524	70.072	73.9	69.9	74.7	71.5

Table 5 Life expectancy at birth in 2015 from UN, WHO, census, and GBD 2017								
Location	GBD 2017 Female	GBD 2017 Male	WPP 2017 Female	WPP 2017 Male	WHO Female	WHO Male	US Census Bureau Female	US Census Bureau Male
Yemen	70.5	66.7	66.428	63.65	66.2	62.8	67.4	63.1
Zambia	64.9	59.4	66.254	61.342	63.6	59.8	53.8	50.5
Zimbabwe	62.7	56.5	64.798	61.44	62.4	59	57.6	56.5

Table 6 Estimates of life expectancy at birth by location and year for 1950, 1955...2005, 2010, 2017 - both sexes

Location	1950	1955	1960	1965	1970	1975	1980	1985	1990	1995	2000	2005	2010	2017
Global	50.4 (49-51.7)	53.2 (52-54.3)	45.3 (44.5-46)	56.5 (55.8-57.1)	58.2 (57.7-58.7)	60.2 (59.8-60.6)	62.3 (62-62.7)	63.9 (63.7-64.2)	65.6 (65.3-65.8)	66.2 (66-66.4)	67.4 (67.2-67.6)	69 (68.8-69.2)	70.8 (70.5-71)	73 (72.7-73.3)
Low SDI	39.8 (37.8-41.6)	41.8 (40.1-43.3)	42.5 (40.9-43.9)	43.4 (42.2-44.5)	44.9 (44.1-45.8)	47.7 (47-48.5)	50.1 (49.5-50.7)	51.6 (51.1-52.2)	53.8 (53.4-54.3)	55.6 (55.1-56)	57.3 (56.9-57.7)	60.1 (59.7-60.5)	62 (61.6-62.4)	65.9 (65.3-66.4)
Low-middle SDI	42.8 (41.2-44.4)	45.8 (44.4-47.1)	43.1 (42-44)	48.8 (47.9-49.7)	51.2 (50.5-51.9)	53.3 (52.7-53.9)	56.7 (56.2-57.2)	58.6 (58.2-59.1)	60.4 (60-60.8)	61.2 (60.8-61.6)	61.9 (61.5-62.3)	63.6 (63.2-64)	65.5 (65.1-65.9)	68.1 (67.6-68.6)
Middle SDI	47.3 (45.6-48.7)	50.6 (49.3-51.8)	26.7 (26.3-27.2)	55.7 (54.9-56.4)	58.3 (57.7-59)	61.5 (60.9-62)	63.9 (63.5-64.3)	65.9 (65.5-66.3)	67.8 (67.4-68.1)	69.1 (68.9-69.3)	70.3 (70.1-70.4)	71.7 (71.5-71.9)	72.9 (72.7-73.1)	74.5 (74.2-74.7)
High-middle SDI	56.1 (54.8-57.5)	58.5 (57.3-59.6)	54.3 (53.5-55.2)	62.8 (62-63.6)	64.6 (64-65.1)	66.3 (65.9-66.7)	67.4 (67.1-67.8)	68.8 (68.6-69.1)	69.9 (69.7-70.1)	69.5 (69.3-69.7)	71.2 (71-71.3)	72.5 (72.4-72.6)	74.5 (74.3-74.6)	76.3 (76.1-76.5)
High SDI	64.1 (63.7-64.4)	67.8 (67.6-67.9)	69.1 (69-69.2)	70.4 (70.3-70.5)	71.2 (71.1-71.3)	72.6 (72.5-72.6)	73.8 (73.7-73.8)	75 (75-75)	76 (75.9-76)	76.8 (76.8-76.8)	78.1 (78.1-78.2)	79.3 (79.3-79.3)	80.4 (80.4-80.5)	81.1 (81-81.2)
Central Europe, Eastern Europe, and Central Asia	62 (61.1-62.8)	64 (63.4-64.6)	66.6 (66.3-66.9)	67.9 (67.6-68.2)	68 (67.8-68.2)	68.5 (68.3-68.7)	68.2 (68-68.3)	68.8 (68.7-68.9)	69.5 (69.4-69.6)	66.7 (66.7-66.8)	68 (67.9-68.1)	68 (68-68.1)	70.6 (70.6-70.7)	73.1 (72.9-73.2)
Central Asia	51.3 (49-53.3)	54.5 (52.8-56)	57.8 (56.6-58.9)	60.6 (59.5-61.5)	63 (62.3-63.7)	64.6 (64-65.2)	66.1 (65.7-66.4)	67.3 (67-67.5)	68 (67.7-68.3)	66.1 (65.9-66.4)	67.2 (66.9-67.4)	67.2 (67-67.5)	69.3 (69-69.5)	71.1 (70.6-71.5)
Armenia	50.3 (47.5-53.3)	54.9 (52.6-57.3)	59.7 (57.8-61.5)	63.4 (61.4-65.1)	66.4 (65.2-67.5)	67.8 (66.7-69)	70.2 (69.5-70.8)	71 (70.6-71.5)	70.1 (69.6-70.5)	69.9 (69.6-70.1)	72.4 (72.1-72.6)	73.4 (73.2-73.5)	74.1 (74-74.2)	75.6 (75.3-75.9)
Azerbaijan	40.2 (37.1-43.2)	44.8 (42.1-47.5)	50.8 (48.6-53.2)	56.2 (54-58.3)	60.7 (59.1-62.2)	64.1 (62.8-65.4)	65.9 (65-66.7)	67 (66.3-67.7)	67.4 (66.7-68.1)	65 (64.4-65.6)	66.8 (66.2-67.4)	67.5 (67-68.1)	69.3 (68.8-69.8)	70.8 (70-71.5)
Georgia	52.3 (49.7-55)	56 (53.9-58.2)	59.8 (58-61.5)	62.8 (61.1-64.5)	65.5 (64.4-66.5)	67.1 (66-68.3)	69.2 (68.5-69.9)	70.2 (69.6-70.7)	70 (69.5-70.5)	71.3 (70.8-71.8)	73.1 (72.6-73.5)	73.4 (73.1-73.6)	72.8 (72.6-72.9)	72.8 (72.5-73.2)
Kazakhstan	56.4 (54.3-58.7)	58.8 (57.2-60.4)	61.2 (59.9-62.4)	62.7 (61.3-64)	64.3 (63.6-65)	65.4 (64.5-66.3)	66.5 (66.1-66.8)	67.7 (67.4-68)	68.5 (68.2-68.7)	64.6 (64.4-64.9)	65.6 (65.3-65.8)	65.3 (65-65.5)	68.4 (68.2-68.6)	72 (71.5-72.5)
Kyrgyzstan	46.7 (44-49.4)	50.1 (47.9-52.6)	53.5 (51.3-55.4)	56.2 (54.3-58)	58.7 (57.1-60.1)	60.7 (59.4-62.2)	62.8 (61.9-63.7)	64.9 (64.2-65.7)	66.3 (65.7-66.9)	65.4 (64.8-66)	66.9 (66.2-67.4)	67.1 (66.7-67.5)	69.7 (69.6-69.8)	72.7 (72.4-73)
Mongolia	41.8 (38.9-44.8)	44.7 (42.1-47.3)	48.3 (46.1-50.5)	52.2 (50.5-53.8)	54.3 (53-55.6)	58 (56.9-58.9)	58.5 (57.6-59.4)	59.6 (58.8-60.4)	61.2 (60.6-61.8)	60.5 (60-61.1)	62 (61.5-62.5)	63.9 (63.4-64.3)	66 (65.6-66.5)	68.9 (67.9-69.9)
Tajikistan	43.5 (40.6-46.4)	46.5 (43.9-48.9)	50.5 (48.1-52.7)	54.7 (52.3-56.9)	59.1 (57.4-60.6)	61.5 (60.1-62.8)	64.6 (63.6-65.4)	66.2 (65.5-66.9)	67 (66.4-67.5)	65.1 (64.5-65.8)	67.8 (67.2-68.4)	69.2 (68.5-69.8)	69.8 (69-70.5)	70.3 (69.3-71.3)
Turkmenistan	50.8 (48.3-53.3)	53.3 (51.4-55.3)	56.3 (54.6-57.9)	58.9 (57.2-60.6)	61.2 (59.9-62.4)	62.4 (61.1-63.6)	63.7 (62.8-64.4)	65 (64.2-65.6)	66 (65.5-66.5)	64.8 (64.3-65.2)	66.7 (66.3-67.1)	66.1 (65.8-66.5)	69.6 (69.2-69.9)	70.1 (69.3-70.9)
Uzbekistan	56.9 (54.5-59.3)	59 (57.2-60.7)	61.3 (59.9-62.8)	63.4 (62-64.8)	65.2 (64.2-66)	66 (65.1-66.9)	67.1 (66.6-67.5)	68.2 (67.8-68.6)	69.4 (69-69.7)	67.7 (67.3-68)	67.3 (66.8-67.8)	66.8 (66.2-67.3)	68.8 (68.4-69.3)	70.3 (69.2-71.5)
Central Europe	59.5 (59.2-59.9)	63.5 (63.3-63.7)	66.6 (66.6-66.7)	68.5 (68.5-68.6)	69 (68.9-69.1)	70.3 (70.3-70.4)	70.2 (70.2-70.2)	70.5 (70.5-70.5)	71 (70.9-71)	71.2 (71.2-71.2)	73 (73-73)	74.1 (74.1-74.1)	75.5 (75.5-75.5)	77 (76.8-77.2)
Albania	49.8 (47.2-52.2)	54.5 (52.3-56.6)	61.2 (59.5-62.9)	64.3 (62.6-66)	67.6 (65.7-69.4)	70.2 (68.1-72)	71.9 (70.3-73.4)	72.4 (71.7-73.1)	73.3 (73-73.7)	74.6 (74.3-74.9)	74.9 (74.4-75.4)	76 (75.5-76.5)	77.6 (76.8-78.5)	78.2 (76.7-79.7)
Bosnia and Herzegovina	50.3 (50.1-50.5)	54.6 (54.4-54.8)	60.4 (60.2-60.5)	63.3 (63.1-63.5)	65.4 (65.3-65.6)	68.2 (68.1-68.4)	69.6 (69.5-69.8)	71.7 (71.6-71.8)	73.6 (73.5-73.7)	66.1 (65-67.1)	75.3 (74.5-76.2)	75.9 (75.8-76)	76.3 (76.1-76.4)	76.7 (76.2-77.2)
Bulgaria	61.9 (61.8-62.1)	65.7 (65.6-65.9)	70.1 (70-70.2)	72.3 (72.2-72.4)	72.2 (72.1-72.3)	72 (71.9-72.1)	72 (72-72.1)	72.2 (72.1-72.3)	71.7 (71.6-71.8)	71 (70.9-71.1)	71.6 (71.5-71.7)	72.6 (72.5-72.7)	73.8 (73.7-73.9)	74.9 (74.4-75.4)
Croatia	55.4 (55.3-55.6)	60.2 (60.1-60.4)	65 (64.9-65.2)	67.8 (67.6-67.9)	68.7 (68.6-68.9)	70.7 (70.6-70.8)	71.4 (71.3-71.5)	71.7 (71.6-71.8)	72.5 (72.4-72.6)	73.1 (73-73.2)	74.5 (74.4-74.7)	75.5 (75.4-75.6)	76.7 (76.6-76.9)	78.5 (78-79)
Czech Republic	64.3 (64.2-64.4)	68.9 (68.8-68.9)	70.3 (70.2-70.4)	70.3 (70.2-70.4)	69.6 (69.5-69.6)	70.5 (70.4-70.6)	70.3 (70.3-70.4)	71.1 (71-71.2)	71.5 (71.4-71.5)	73.3 (73.2-73.3)	75 (75-75.1)	76.3 (76.2-76.4)	77.7 (77.6-77.8)	79.2 (78.7-79.7)
Hungary	61.9 (61.8-62)	67 (66.9-67.1)	68.4 (68.3-68.5)	69.5 (69.4-69.6)	69.2 (69.1-69.3)	69.4 (69.3-69.5)	69.1 (69-69.2)	68.9 (68.8-69)	69.5 (69.4-69.6)	70 (69.9-70)	71.8 (71.7-71.9)	72.9 (72.9-73)	74.6 (74.5-74.7)	76.8 (76.3-77.3)
Macedonia	50.8 (49.7-51.9)	51.6 (50.8-52.5)	57.4 (56.6-58.3)	61.6 (60.9-62.3)	65.4 (64.9-65.9)	68.7 (68.5-69)	70.1 (69.9-70.3)	70.6 (70.4-70.8)	71.9 (71.8-72.1)	72.7 (72.5-72.8)	73.9 (73.8-74.1)	74.8 (74.7-75)	75.9 (75.7-76)	76.5 (76.1-77)
Montenegro	65.6 (65-66.3)	68.1 (67.6-68.6)	70.2 (69.7-70.7)	71.5 (71-71.9)	72.6 (72.1-73)	72.6 (72.2-73)	72.6 (72.4-72.9)	72.9 (72.8-73.1)	74.3 (74.1-74.6)	74.3 (74-74.5)	73.9 (73.6-74.2)	74.5 (74.1-74.9)	76 (75.5-76.4)	76.5 (75.8-77.2)
Poland	59.4 (59.3-59.5)	63.8 (63.8-63.9)	68.2 (68.1-68.2)	69.9 (69.9-70)	70.3 (70.3-70.4)	71.3 (71.2-71.3)	70.7 (70.7-70.7)	71 (70.9-71)	71.2 (71.2-71.2)	72.1 (72.1-72.2)	73.9 (73.9-74)	75.2 (75.1-75.2)	76.4 (76.4-76.4)	78 (77.5-78.5)
Romania	62.3 (60.2-64.5)	63.7 (63-64.4)	65.3 (65.2-65.4)	68.2 (68.1-68.3)	68.6 (68.5-68.6)	70.1 (70-70.1)	69.6 (69.6-69.7)	69.6 (69.6-69.7)	69.9 (69.8-69.9)	69.4 (69.3-69.4)	71.1 (71.1-71.2)	72.2 (72.2-72.3)	73.7 (73.6-73.7)	75.2 (74.7-75.7)
Serbia	55.1 (55-55.2)	59.8 (59.7-59.9)	63.6 (63.4-63.7)	65.5 (65.4-65.7)	66.3 (66.2-66.5)	69.4 (69.3-69.5)	69.6 (69.5-69.7)	69.5 (69.4-69.6)	70.9 (70.8-71)	71.1 (71-71.2)	72.1 (71.9-72.3)	72.7 (72.5-72.9)	74.4 (74.3-74.4)	75.7 (75.3-76.2)
Slovakia	60 (59-61.1)	67.3 (66.5-68.1)	69.9 (69.1-70.8)	70.2 (69.4-71)	69.9 (69.3-70.6)	70.4 (70.3-70.5)	70.6 (70.5-70.7)	70.9 (70.8-71)	71 (70.8-71.1)	72.5 (72.4-72.6)	73.4 (73.3-73.6)	74.3 (74.2-74.4)	75.6 (75.5-75.7)	77.4 (76.9-77.9)
Slovenia	57.9 (57.6-58.1)	62.8 (62.6-63)	66 (65.8-66.2)	68.3 (68.2-68.5)	68.9 (68.7-69)	70.3 (70.1-70.4)	71.4 (71.2-71.5)	72.1 (71.9-72.2)	73.9 (73.7-74)	74.7 (74.5-74.9)	76 (75.9-76.2)	77.7 (77.5-77.8)	79.5 (79.3-79.6)	81.1 (80.5-81.7)
Eastern Europe	65.9 (64.9-66.8)	66.7 (66-67.5)	69.1 (68.8-69.3)	70.1 (69.9-70.3)	69.4 (69.2-69.6)	69 (68.8-69.2)	68.2 (68-68.4)	68.9 (68.8-69)	69.8 (69.7-69.8)	65.5 (65.4-65.5)	66.4 (66.4-66.5)	65.9 (65.9-65.9)	69 (69-69)	71.9 (71.8-72.1)
Belarus	68.1 (66-70.2)	69.1 (67.5-70.9)	70.6 (69.9-71.3)	72.1 (71.6-72.5)	71.8 (71.5-72.2)	71.6 (71.3-71.9)	70.8 (70.6-71.1)	70.8 (70.6-71.1)	71.1 (70.9-71.3)	68.5 (68.3-68.7)	68.8 (68.6-69)	68.6 (68.5-68.8)	70.4 (70.3-70.5)	74 (73.5-74.5)
Estonia	66.5 (64.7-68.6)	67.3 (65.7-69)	69.2 (69-69.5)	70.7 (70.5-70.9)	70.4 (70.2-70.6)	70.1 (70-70.3)	69.5 (69.3-69.6)	69.7 (69.6-69.9)	70 (69.8-70.1)	67.9 (67.7-68.1)	71 (70.8-71.1)	72.9 (72.7-73.1)	76 (75.8-76.2)	78.1 (76.9-79.3)
Latvia	68.3 (66.4-70.2)	69.3 (67.6-71.3)	70.6 (70.4-70.7)	71.6 (71.4-71.7)	70.6 (70.5-70.8)	69.7 (69.5-69.8)	69.3 (69.2-69.5)	70 (69.8-70.1)	69.8 (69.7-69.9)	66.9 (66.7-67)	70.7 (70.5-70.8)	70.8 (70.7-71)	73.2 (73-73.3)	75.2 (74-76.3)
Lithuania	66.1 (64.1-68.3)	67.4 (65.7-69.3)	69.8 (69.6-69.9)	72.2 (72-72.3)	71.5 (71.3-71.6)	71.3 (71.1-71.4)	70.7 (70.6-70.8)	70.9 (70.8-71)	71.4 (71.3-71.5)	69.2 (69.1-69.3)	72.3 (72.2-72.5)	71.4 (71.3-71.5)	73.3 (73.2-73.4)	75 (74.4-75.6)
Moldova	53.5 (51.2-55.8)	56.3 (54.6-58)	58.9 (57.4-60.1)	60.8 (59.5-62.2)	62.7 (61.8-63.5)	64 (63.1-64.9)	65.7 (65.2-66.1)	66.2 (65.8-66.5)	68 (67.8-68.3)	65.8 (65.6-66.1)	68.6 (68.4-68.8)	68.7 (68.5-68.8)	69.7 (69.6-69.9)	72.9 (72.6-73.2)
Russian Federation	65.9 (65.1-66.7)	66.7 (66-67.3)	68.9 (68.6-69.1)	69.9 (69.7-70.2)	69.1 (68.8-69.3)	68.6 (68.3-68.8)	67.6 (67.4-67.8)	68.4 (68.3-68.5)	69.5 (69.5-69.5)	64.8 (64.7-64.8)	65.5 (65.5-65.5)	64.8 (64.8-64.8)	68.1 (68.1-68.1)	72.1 (72-72.2)
Ukraine	66.3 (64.3-68.3)	67.1 (65.4-68.8)	69.9 (69.2-70.5)	70.8 (70.3-71.2)	70.2 (69.8-70.6)	70 (69.7-70.4)	69.3 (69-69.6)	69.9 (69.7-70.1)	70.3 (70.1-70.5)	66.7 (66.5-66.8)	68 (68-68.1)	67.9 (67.8-67.9)	70.9 (70.8-70.9)	70.5 (70-71.1)
High-income	64.3 (64-64.6)	67.9 (67.8-68.1)	69 (68.9-69.1)	70.1 (70-70.2)	70.9 (70.8-71)	72.4 (72.3-72.4)	73.8 (73.7-73.8)	75.1 (75.1-75.1)	76.1 (76.1-76.2)	77 (77-77)	78.2 (78.2-78.3)	79.4 (79.4-79.4)	80.4 (80.4-80.4)	81 (80.9-81.2)
Australasia	69.4 (69.3-69.5)	70.3 (70.2-70.4)	71 (70.9-71.1)	71 (71-71.1)	71 (70.9-71.1)	72.4 (72.3-72.5)	74.2 (74.1-74.3)	75.2 (75.1-75.2)	76.6 (76.5-76.7)	77.9 (77.8-78)	79.4 (79.3-79.4)	80.9 (80.8-81)	81.7 (81.7-81.8)	82.3 (81.5-83)
Australia	69.4 (69.3-69.5)	70.3 (70.2-70.4)	71 (70.9-71.1)	71 (70.9-71)	70.9 (70.8-71)	72.4 (72.3-72.5)	74.4 (74.3-74.5)	75.4 (75.3-75.5)	76.9 (76.8-76.9)	78.2 (78.1-78.3)	79.6 (79.5-79.6)	81.1 (81.1-81.2)	82 (81.9-82)	82.4 (81.5-83.2)
New Zealand	69.4 (69.2-69.6)	70.6 (70.4-70.7)	71.2 (71-71.4)	71.3 (71.1-71.5)	71.4 (71.2-71.6)	72.3 (72.2-72.5)	73.3 (73.1-73.4)	74.1 (73.9-74.2)	75.4 (75.2-75.5)	76.5 (76.4-76.6)	78.4 (78.2-78.5)	79.7 (79.6-79.8)	80.7 (80.6-80.8)	81.6 (81.2-82.1)
High-income Asia-Pacific	54.4 (53.9-54.9)	63.3 (62.7-63.9)	65.4 (64.9-65.9)	67.9 (67.4-68.4)	69.9 (69.5-70.4)	72.5 (72.3-72.7)	74.4 (74.3-74.4)	76.3 (76.2-76.3)	77.8 (77.8-77.8)	78.8 (78.8-78.9)	80.4 (80.4-80.4)	81.6 (81.6-81.7)	82.7 (82.6-82.7)	83.9 (83.7-84.1)
Brunei	53.5 (51.9-55)	54.4 (53.1-55.7)	58 (56.9-59.1)	62.9 (62.1-63.7)	64.7 (64.2-65.3)	67 (66.5-67.6)	68.9 (68.3-69.4)	69.8 (69.3-70.3)	70.5 (70-70.9)	72.3 (71.9-72.8)	73.8 (73.4-74.2)	74.7 (74.3-75)	75.1 (74.7-75.5)	75.3 (74.6-76.1)
Japan	59.6 (59.3-60)	66.1 (65.8-66.4)	68.2 (67.9-68.5)	70.8 (70.5-71.1)	72.5 (72.2-72.7)	74.7 (74.5-74.8)	76.5 (76.5-76.5)	78.1 (78-78.1)	79.3 (79.3-79.3)	80 (80-80)	81.4 (81.4-81.4)	82.3 (82.3-82.3)	83.1 (83.1-83.2)	84.2 (84-84.4)
Aichi	60.9 (58.8-62.8)	67.1 (65.2-68.9)	68.8 (67.1-70.7)	71.2 (69.5-73)	72.8 (71.3-74.4)	74.9 (74-75.8)	76.5 (76.4-76.6)	78.2 (78.1-78.2)	79.2 (79.1-79.3)	79.9 (79.8-80)	81.3 (81.2-81.4)	82.2 (82.1-82.3)	83.1 (83-83.2)	84.4 (83.4-85.3)
Akita	59.1 (57-61.2)	65.7 (63.8-67.7)	68.1 (66.2-70.1)	70.9 (69.1-72.7)	72.6 (71.1-74.3)	75 (74-75.9)	77 (76.9-77.2)	78.2 (78.1-78.4)	79.3 (79.2-79.5)	79.9 (79.7-80)	80.8 (80.7-81)	81.5 (81.4-81.7)	82.3 (82.1-82.4)	83 (82-84.1)
Aomori	57.4 (55.3-59.7)	64.3 (62.4-66.3)	66.6 (64.8-68.6)	69.4 (67.7-71.1)	71.1 (69.6-72.7)	73.5 (72.6-74.4)	75.4 (75.2-75.5)	76.8 (76.7-77)	78.5 (78.3-78.6)	78.9 (78.8-79.1)	80.1 (80-80.2)	80.8 (80.6-80.9)	81.7 (81.5-81.8)	82.7 (81.6-83.7)
Chiba	60.6 (58.6-62.8)	66.9 (65-68.9)	68.7 (66.8-70.6)	71.1 (69.4-72.9)	72.6 (71.1-74.1)	74.6 (73.7-75.5)	76.7 (76.6-76.8)	78.3 (78.2-78.4)	79.6 (79.5-79.6)	80.1 (80-80.2)	81.4 (81.4-81.5)	82.2 (82.1-82.3)	83.1 (83-83.2)	84 (83.1-84.9)
Ehime	58.9 (57-61)	65.5 (63.6-67.3)	67.8 (66-69.5)	70.5 (68.8-72.2)	72.2 (70.6-73.8)	74.4 (73.4-75.3)	76.4 (76.3-76.6)	78.1 (78-78.3)	79.4 (79.3-79.5)	80 (79.9-80.2)	81.3 (81.1-81.4)	82.1 (82-82.3)	83 (82.9-83.2)	84.2 (83.1-85.2)
Fukui	61.3 (59.4-63.4)	67.4 (65.6-69.3)	69 (67.4-70.6)	71.5 (70.1-72.9)	73.2 (72-74.2)	75.2 (74.6-75.8)	76.9 (76.8-77.1)	78.6 (78.5-78.7)	79.8 (79.7-80)	80.7 (80.6-80.8)	82.1 (82-82.3)	82.8 (82.7-83)	83.8 (83.7-84)	84.5 (83.6-85.4)
Fukuoka	59.2 (57.2-61.3)	65.7 (63.7-67.7)	67.9 (66-69.9)	70.4 (68.6-72.1)	72 (70.4-73.6)	74.2 (73.2-75.1)	76.1 (76-76.2)	77.7 (77.6-77.8)	78.9 (78.8-79)	79.9 (79.8-80)	81.2 (81.1-81.3)	82.2 (82.1-82.3)	83.2 (83.1-83.3)	84.5 (83.5-85.5)
Fukushima	58.1 (56.1-60.2)	65.2 (63.3-67.3)	67.5 (65.5-69.3)	70.2 (68.3-72)	71.8 (70.3-73.5)	74.2 (73.3-75.1)	76.3 (76.1-76.4)	77.7 (77.6-77.8)	79.3 (79.1-79.4)	80 (79.8-80.1)	81.1 (81-81.3)	81.9 (81.8-82.1)	82.8 (82.7-82.9)	83.4 (82.4-84.5)
Gifu	60 (57.9-62)	66.4 (64.4-68.3)	68.2 (66.5-70)	70.7 (69-72.4)	72.3 (70.7-73.8)	74.6 (73.7-75.4)	76.4 (76.3-76.5)	78.1 (78-78.3)	79.5 (79.4-79.6)	80.3 (80.1-80.4)	81.4 (81.3-81.6)	82.4 (82.3-82.6)	83.3 (83.2-83.5)	83.9 (83-84.9)
Gunma	59.9 (58-62)	66.3 (64.4-68.2)	68.1 (66.3-70.2)	70.6 (68.9-72.5)	72.1 (70.6-73.7)	74.2 (73.4-75.1)	76.3 (76.2-76.4)	77.9 (77.8-78)	79.4 (79.3-79.5)	80.1 (80-80.2)	81.4 (81.2-81.5)	82.1 (82-82.3)	82.9 (82.7-83)	83.7 (82.7-84.7)
Hiroshima	59.2 (57.3-61.3)	65.8 (63.8-67.9)	68.2 (66.3-70.1)	70.8 (69-72.5)	72.4 (70.9-74.1)	74.6 (73.6-75.5)	76.6 (76.5-76.7)	78.2 (78.1-78.3)	79.5 (79.3-79.6)	80.2 (80.1-80.4)	81.7 (81.6-81.9)	82.7 (82.5-82.8)	83.5 (83.4-83.6)	84.8 (83.8-85.7)
Hokkaidō	58.6 (56.7-60.6)	65.3 (63.5-67.2)	67.4 (65.7-69.3)	70 (68.2-71.9)	71.7 (70.1-73.3)	73.9 (72.9-74.9)	75.8 (75.8-75.9)	77.6 (77.5-77.6)	79 (78.9-79.1)	80.1 (80-80.2)	81.4 (81.3-81.4)	82.2 (82.1-82.2)	83 (82.9-83.1)	84 (83.1-85)
Hyōgo	59.6 (57.7-61.5)	66.1 (64.4-68)	68.1 (66.4-70)	70.6 (68.9-72.4)	72.4 (71-74)	74.7 (73.8-75.5)	76.1 (76-76.2)	77.6 (77.5-77.7)	78.8 (78.7-78.9)	77.6 (77.5-77.7)	81.2 (81.1-81.3)	82.1 (82.1-82.2)	83.2 (83.1-83.3)	84.3 (83.4-85.3)
Ibaraki	58 (56.1-60)	65 (63.3-66.9)	67.2 (65.3-69.1)	70 (68.1-71.9)	71.7 (70.1-73.4)	74.1 (73.1-75)	76 (75.8-76.1)	77.4 (77.2-77.5)	78.7 (78.6-78.8)	79.6 (79.5-79.7)	80.9 (80.8-81)	81.8 (81.7-81.9)	82.5 (82.4-82.6)	83.2 (82.2-84.2)
Ishikawa	60.6 (58.6-62.7)	66.8 (64.8-68.8)	68.5 (66.8-70.2)	70.9 (69.4-72.4)	72.6 (71.3-73.8)	75 (74.3-75.7)	77 (76.9-77.2)	78.7 (78.6-78.9)	79.8 (79.6-79.9)	80.5 (80.4-80.7)	81.7 (81.6-81.9)	83 (82.8-83.1)	83.6 (83.5-83.8)	84.5 (83.6-85.4)
Iwate	58.1 (56-60.2)	65 (63.2-67)	67.1 (65.4-68.9)	69.9 (68.2-71.5)	71.7 (70.3-73.1)	74 (73.1-74.8)	75.8 (75.7-76)	77.7 (77.5-77.8)	79 (78.8-79.1)	80 (79.9-80.2)	81.2 (81.1-81.3)	81.7 (81.6-81.9)	82.5 (82.4-82.7)	83.6 (82.6-84.6)
Kagawa	60.4 (58.4-62.6)	66.8 (65-68.8)	68.8 (67.1-70.7)	71.4 (69.8-73.1)	73.2 (71.8-74.6)	75.3 (74.6-76)	77 (76.9-77.2)	78.7 (78.6-78.8)	79.5 (79.4-79.7)	80.4 (80.3-80.6)	81.5 (81.4-81.7)	82.5 (82.3-82.6)	83.1 (83-83.3)	84.1 (83.1-85.1)
Kagoshima	57 (55-59)	64 (62.2-66.1)	66.4 (64.4-68.4)	69.1 (67.4-70.9)	70.8 (69.3-72.4)	73.2 (72.3-74.1)	75.6 (75.5-75.8)	77.5 (77.4-77.6)	79 (78.9-79.1)	80 (79.8-80.1)	81.3 (81.2-81.5)	82.1 (81.9-82.2)	82.9 (82.8-83.1)	83.8 (82.8-84.8)
Kanagawa	61.4 (59.4-63.4)	67.5 (65.6-69.5)	69.4 (67.6-71.2)	71.7 (70-73.5)	73.3 (71.7-74.9)	75.5 (74.5-76.4)	77.1 (77-77.1)	78.5 (78.4-78.6)	79.6 (79.5-79.7)	80.2 (80.2-80.3)	81.6 (81.5-81.7)	82.6 (82.6-82.7)	83.5 (83.4-83.6)	84.5 (83.6-85.4)
Kōchi	57.3 (55.3-59.4)	64.2 (62.3-66.2)	66.5 (64.6-68.5)	69.2 (67.5-71)	70.9 (69.5-72.4)	73.3 (72.5-74.1)	75.7 (75.5-75.8)	77.8 (77.6-77.9)	79 (78.9-79.2)	80 (79.8-80.1)	81.2 (81-81.3)	82 (81.8-82.1)	82.8 (82.6-82.9)	84.4 (83.4-85.4)
Kumamoto	58 (55.9-60.1)	65.3 (63.2-67.3)	67.5 (65.6-69.4)	70.1 (68.4-71.9)	71.8 (70.2-73.4)	74 (73.1-75)	76.5 (76.3-76.6)	78.5 (78.4-78.7)	79.8 (79.7-80)	81.1 (81-81.2)	82.2 (82.1-82.4)	83.1 (83-83.2)	83.9 (83.8-84.1)	84.7 (83.8-85.7)
Kyōto	60.9 (58.8-62.9)	67.2 (65.3-69.2)	69.2 (67.3-71.1)	71.7 (69.7-73.4)	73.5 (71.8-75)	75.8 (74.8-76.7)	77.4 (77.3-77.5)	78.6 (78.4-78.7)	79.6 (79.4-79.7)	80.5 (80.4-80.6)	81.6 (81.5-81.7)	82.7 (82.6-82.8)	83.6 (83.5-83.8)	84.9 (84-85.9)
Mie	60.1 (58.1-62.1)	66.5 (64.7-68.6)	68.5 (66.6-70.5)	71.1 (69.3-72.9)	72.9 (71.4-74.4)	75.1 (74.2-76)	76.6 (76.5-76.8)	78 (77.9-78.1)	79.3 (79.2-79.4)	80.1 (79.9-80.2)	81.5 (81.3-81.6)	82.4 (82.2-82.5)	83.1 (83-83.2)	84 (83-84.9)
Miyagi	60.1 (58-62)	66.5 (64.5-68.4)	68.2 (66.4-69.9)	70.8 (69.2-72.5)	72.3 (70.9-73.9)	74.4 (73.5-75.2)	76.2 (76.1-76.3)	78.1 (78-78.2)	79.5 (79.4-79.6)	80.3 (80.2-80.4)	81.5 (81.4-81.6)	82.2 (82.1-82.3)	83.2 (83.1-83.3)	84.1 (83.2-85.1)
Miyazaki	57.9 (55.9-60.1)	64.7 (62.7-66.5)	67 (65.2-68.9)	69.8 (68.1-71.5)	71.6 (70.2-73.1)	74.1 (73.2-75)	76 (75.8-76.1)	77.9 (77.7-78)	79.2 (79.1-79.4)	80.1 (80-80.3)	81.7 (81.5-81.8)	82.5 (82.3-82.6)	83.3 (83.2-83.5)	84.1 (83.1-85.2)
Nagano	61 (58.9-63)	67.3 (65.5-69.3)	69.2 (67.4-71.1)	71.6 (69.9-73.3)	73.2 (71.8-74.6)	75.2 (74.3-76)	77.3 (77.2-77.4)	78.8 (78.6-78.9)	80.3 (80.2-80.4)	81.2 (81.1-81.3)	82.3 (82.2-82.5)	83.3 (83.1-83.4)	84.1 (84-84.2)	84.8 (83.9-85.8)
Nagasaki	58.9 (56.7-61)	65.3 (63.3-67.2)	67.4 (65.6-69.2)	69.9 (68.3-71.8)	71.6 (70.1-73.2)	73.8 (72.9-74.7)	75.8 (75.6-75.9)	77.7 (77.6-77.8)	79 (78.9-79.1)	79.9 (79.8-80.1)	81.4 (81.3-81.6)	82.2 (82.1-82.4)	83.1 (82.9-83.2)	83.9 (82.9-84.8)
Nara	59.4 (57.4-61.5)	65.9 (63.9-67.7)	67.7 (65.9-69.4)	70.2 (68.6-71.9)	71.9 (70.5-73.3)	74.1 (73.3-75.1)	76.3 (76.1-76.4)	77.8 (77.7-78)	79.2 (79.1-79.3)	80.3 (80.1-80.4)	81.8 (81.7-81.9)	82.6 (82.5-82.8)	83.6 (83.5-83.8)	84.5 (83.5-85.4)
Niigata	60 (58-62)	66.5 (64.6-68.5)	68.3 (66.6-70.3)	70.7 (69-72.4)	72.2 (70.7-73.7)	74.2 (73.4-75.1)	76.4 (76.2-76.5)	78.1 (78-78.2)	79.8 (79.7-79.9)	80.5 (80.4-80.6)	81.8 (81.6-81.9)	82.6 (82.5-82.7)	83.5 (83.3-83.6)	84.4 (83.5-85.3)
Ōita	58.8 (56.8-60.8)	65.5 (63.6-67.3)	67.7 (65.9-69.5)	70.4 (68.7-72.1)	72.2 (70.8-73.7)	74.6 (73.8-75.4)	76.1 (76-76.3)	77.9 (77.7-78)	79.3 (79.2-79.5)	80.4 (80.2-80.5)	81.6 (81.5-81.7)	82.7 (82.6-82.9)	83.6 (83.4-83.8)	84.9 (84-85.9)
Okayama	60.8 (58.8-63)	67.1 (65.1-69.1)	69 (67.2-70.9)	71.5 (69.8-73.2)	73.2 (71.7-74.7)	75.4 (74.5-76.2)	77 (76.9-77.1)	78.5 (78.3-78.6)	79.8 (79.6-79.9)	80.6 (80.4-80.7)	81.9 (81.8-82.1)	82.9 (82.8-83.1)	83.5 (83.4-83.7)	84.5 (83.6-85.5)
Okinawa	57.7 (55.7-60)	64.5 (62.5-66.6)	66.8 (65-68.8)	69.2 (67.4-71.1)	70.3 (68.5-72)	72.2 (71.2-73.1)	77 (76.8-77.2)	79.9 (79.8-80.1)	80.6 (80.4-80.7)	81.2 (81-81.3)	82.1 (81.9-82.2)	82.8 (82.6-82.9)	83.2 (83.1-83.4)	83.8 (82.8-84.8)
Ōsaka	59.4 (57.6-61.6)	65.9 (64.1-67.8)	67.9 (66.2-69.6)	70.4 (68.8-72)	72.1 (70.6-73.6)	74.3 (73.4-75.2)	75.7 (75.6-75.8)	77.1 (77-77.1)	78.2 (78.2-78.3)	79.2 (79.2-79.3)	80.6 (80.6-80.7)	81.7 (81.6-81.7)	82.5 (82.4-82.6)	83.8 (82.9-84.8)
Saga	59 (57.1-61.1)	65.7 (63.7-67.6)	67.9 (66-69.7)	70.5 (68.9-72.2)	72.4 (71.1-73.6)	74.6 (73.9-75.3)	76.3 (76.1-76.4)	77.9 (77.7-78)	79.2 (79-79.3)	80 (79.9-80.2)	81.3 (81.1-81.4)	82.4 (82.2-82.6)	83.1 (82.9-83.3)	84.2 (83.2-85.1)
Saitama	60.6 (58.7-62.6)	66.9 (65.1-69)	68.8 (67.1-70.5)	71.3 (69.6-73)	73.1 (71.6-74.7)	75.2 (74.4-76.2)	76.5 (76.4-76.5)	78.1 (78-78.2)	79.2 (79.2-79.3)	80 (79.9-80.1)	81.4 (81.3-81.5)	82.2 (82.1-82.3)	82.9 (82.8-83)	83.9 (83-84.8)
Shiga	60.6 (58.5-62.8)	66.8 (64.9-68.7)	68.4 (66.7-70.2)	70.8 (69.3-72.4)	72.3 (71-73.6)	74.7 (74.1-75.4)	77.2 (77.1-77.4)	78.6 (78.5-78.7)	79.4 (79.2-79.5)	80.4 (80.3-80.6)	81.8 (81.7-82)	82.8 (82.7-83)	83.8 (83.7-84)	84.8 (83.9-85.7)
Shimane	59.4 (57.3-61.5)	65.8 (64-67.6)	67.8 (66.1-69.6)	70.4 (68.9-72)	72.2 (70.8-73.6)	74.5 (73.8-75.3)	76.7 (76.5-76.8)	78.7 (78.5-78.8)	80 (79.8-80.1)	80.8 (80.6-80.9)	81.5 (81.4-81.7)	82.6 (82.4-82.7)	83.6 (83.4-83.8)	84.6 (83.6-85.5)
Shizuoka	60.8 (58.8-63.1)	67.1 (65.1-69)	69.1 (67.2-71)	71.6 (69.9-73.3)	73.2 (71.8-74.9)	75.4 (74.5-76.3)	77 (76.9-77.1)	78.6 (78.5-78.7)	79.8 (79.7-79.9)	80.5 (80.4-80.6)	81.8 (81.7-81.9)	82.8 (82.7-82.9)	83.3 (83.2-83.5)	84.2 (83.3-85.3)
Tochigi	59.2 (57.1-61.5)	65.7 (63.7-67.8)	67.7 (66-69.5)	70.4 (68.8-72.1)	72.2 (70.7-73.8)	74.4 (73.5-75.3)	76 (75.8-76.1)	77.4 (77.2-77.5)	78.5 (78.4-78.6)	79.4 (79.3-79.5)	80.9 (80.8-81.1)	81.5 (81.4-81.6)	82.4 (82.3-82.6)	83.4 (82.4-84.3)
Tokushima	58.7 (56.5-60.8)	65.2 (63.3-67.2)	67.2 (65.4-69)	69.8 (68.2-71.5)	71.5 (70.1-72.9)	73.9 (73.2-74.6)	75.7 (75.6-75.9)	77.7 (77.5-77.8)	78.9 (78.8-79.1)	79.9 (79.7-80.1)	81.3 (81.2-81.5)	82.1 (81.9-82.2)	83 (82.9-83.2)	83.6 (82.6-84.6)
Tōkyō	62.2 (60.2-64.2)	67.9 (66-69.8)	69.6 (67.9-71.4)	71.8 (70.2-73.4)	73.5 (72.1-75)	75.5 (74.7-76.4)	77 (76.9-77.1)	78.5 (78.4-78.5)	79.3 (79.2-79.3)	79.9 (79.9-80)	81.2 (81.2-81.3)	82.4 (82.3-82.4)	83.2 (83.2-83.3)	84.5 (83.7-85.4)
Tottori	59.1 (57-61.1)	65.8 (63.8-67.8)	68.1 (66.5-70)	70.9 (69.5-72.4)	72.7 (71.7-73.9)	75 (74.4-75.7)	76.6 (76.4-76.8)	78 (77.8-78.2)	79.3 (79.1-79.4)	80.1 (79.9-80.2)	81.4 (81.2-81.6)	82.1 (81.9-82.2)	83 (82.8-83.2)	83.9 (83-84.9)
Toyama	59.7 (57.6-61.7)	66.1 (64.3-68)	68.1 (66.2-69.7)	70.6 (69.1-72.2)	72.3 (71-73.6)	74.5 (73.9-75.2)	76.5 (76.4-76.7)	78 (77.9-78.2)	79.6 (79.4-79.7)	80.6 (80.5-80.7)	81.9 (81.7-82)	82.8 (82.7-83)	83.5 (83.4-83.7)	84.3 (83.3-85.3)
Wakayama	57.8 (55.8-59.9)	64.7 (62.8-66.6)	66.9 (65.1-68.6)	69.6 (68-71.2)	71.4 (70.1-72.7)	73.8 (73-74.5)	75.9 (75.7-76)	77.3 (77.2-77.5)	78.6 (78.5-78.7)	79.6 (79.5-79.8)	81.1 (80.9-81.2)	81.9 (81.8-82.1)	82.5 (82.4-82.7)	83.7 (82.7-84.7)
Yamagata	59.9 (57.7-62)	66.3 (64.4-68.3)	68.3 (66.4-70.3)	70.8 (68.9-72.5)	72.4 (71-73.9)	74.9 (74.1-75.8)	77.3 (77.2-77.5)	78.8 (78.7-79)	79.7 (79.6-79.9)	80.4 (80.3-80.6)	81.6 (81.5-81.8)	82.5 (82.3-82.6)	83.4 (83.2-83.5)	84.1 (83.1-85.1)
Yamaguchi	58.6 (56.8-60.6)	65.4 (63.4-67.4)	67.7 (65.9-69.7)	70.5 (68.8-72.3)	72.3 (70.7-74)	74.6 (73.7-75.5)	76.1 (75.9-76.2)	77.9 (77.8-78.1)	79.3 (79.2-79.5)	80 (79.9-80.1)	81.2 (81-81.3)	82 (81.9-82.2)	82.8 (82.7-83)	84 (83-85)
Yamanashi	60.1 (58-62.2)	66.4 (64.4-68.5)	68.5 (66.6-70.4)	71 (69.2-72.7)	72.6 (71.2-74)	75 (74.2-75.7)	76.5 (76.3-76.7)	78.1 (78-78.3)	79.4 (79.3-79.6)	80.5 (80.3-80.6)	81.8 (81.6-82)	82.5 (82.4-82.7)	83.4 (83.2-83.5)	84.3 (83.3-85.2)
South Korea	38.1 (36.7-39.5)	54.5 (52.7-56.4)	57.7 (55.6-59.6)	60 (58.1-62)	62.3 (60.7-64)	65.2 (64.6-65.8)	67.2 (66.9-67.4)	70 (69.8-70.1)	72.2 (72.1-72.3)	74.4 (74.3-74.4)	76.3 (76.2-76.3)	78.7 (78.7-78.7)	80.6 (80.5-80.7)	82.6 (82.1-83.1)
Singapore	56.4 (55.4-57.4)	62.4 (62.2-62.6)	65.4 (65.3-65.6)	67.4 (67.2-67.5)	68.8 (68.6-68.9)	70.1 (70-70.2)	71.4 (71.3-71.6)	73.7 (73.5-73.8)	76.1 (76-76.2)	77.3 (77.2-77.5)	79.3 (78.7-80)	81.1 (81-81.3)	83.2 (83-83.3)	84.8 (84.3-85.3)
High-income North America	68.2 (67.8-68.5)	69.8 (69.5-70.1)	70 (70-70)	70.5 (70.5-70.5)	71 (71-71)	72.7 (72.7-72.8)	73.9 (73.9-73.9)	74.9 (74.9-74.9)	75.7 (75.7-75.8)	76.1 (76.1-76.1)	77.1 (77.1-77.1)	77.8 (77.8-77.8)	79 (79-79)	78.9 (78.7-79.1)
Canada	68.4 (68.3-68.5)	70.4 (70.3-70.5)	71.1 (71-71.2)	71.9 (71.8-72)	72.7 (72.6-72.7)	73.5 (73.4-73.5)	75.1 (75.1-75.2)	76.3 (76.2-76.3)	77.3 (77.3-77.4)	78 (77.9-78)	79.2 (79.2-79.3)	80.2 (80.2-80.3)	81.4 (81.4-81.5)	82 (81.5-82.4)
Greenland	48.3 (47.3-49.4)	53.3 (52.7-53.8)	59.8 (59.3-60.3)	62.9 (62.4-63.4)	64.6 (64.1-65)	63.9 (63.5-64.2)	63 (62.6-63.4)	63.4 (63-63.8)	65.2 (64.7-65.6)	66.9 (66.6-67.3)	68.6 (68.3-69)	70.2 (69.9-70.6)	71.9 (71.5-72.2)	73.5 (73-74.1)

Table 6 Estimates of life expectancy at birth by location and year for 1950, 1955...2005, 2010, 2017 - both sexes

Location	1950	1955	1960	1965	1970	1975	1980	1985	1990	1995	2000	2005	2010	2017
USA	68.2 (67.8-68.6)	69.8 (69.5-70.1)	69.9 (69.9-70)	70.4 (70.4-70.4)	70.9 (70.9-70.9)	72.7 (72.6-72.7)	73.8 (73.8-73.8)	74.8 (74.7-74.8)	75.6 (75.6-75.6)	75.9 (75.9-76)	76.9 (76.9-76.9)	77.6 (77.5-77.6)	78.7 (78.7-78.8)	78.6 (78.4-78.8)
Alabama	65.7 (63.6-67.8)	67.5 (65.8-69.3)	68.2 (68.1-68.4)	68.2 (68.1-68.4)	69.3 (69.2-69.4)	71.2 (71-71.3)	72.4 (72.3-72.6)	73.5 (73.4-73.6)	73.9 (73.8-74)	73.9 (73.8-74)	74.4 (74.3-74.6)	74.6 (74.5-74.7)	75.3 (75.2-75.4)	74.9 (73.7-76.2)
Alaska	64 (62.2-65.8)	68.3 (66.9-69.7)	67.5 (67.3-67.8)	68.3 (68-68.5)	69.3 (69.1-69.5)	70.3 (70.1-70.5)	71.8 (71.6-72)	72.7 (72.6-72.9)	74.7 (74.5-74.9)	75.3 (75.2-75.5)	76.4 (76.2-76.5)	77.3 (77.1-77.5)	78 (77.8-78.2)	77.7 (76.8-78.5)
Arizona	66.4 (64.5-68.5)	68.8 (67.1-70.7)	69.1 (68.9-69.3)	70.2 (70-70.4)	70.7 (70.6-70.9)	73 (72.8-73.1)	74.1 (73.9-74.2)	75.6 (75.5-75.8)	76.4 (76.3-76.5)	76.2 (76.1-76.3)	77.3 (77.1-77.4)	77.7 (77.6-77.8)	79.4 (79.3-79.5)	79.1 (78-80.3)
Arkansas	68.1 (66.1-70.1)	69.9 (68.2-71.6)	70.3 (70.1-70.5)	70.2 (70-70.4)	70.9 (70.7-71.1)	72.1 (71.9-72.3)	73.5 (73.4-73.7)	74.1 (73.9-74.2)	74.5 (74.3-74.7)	74.6 (74.4-74.7)	74.9 (74.8-75.1)	75.5 (75.3-75.6)	76 (75.9-76.2)	75.4 (74-76.6)
California	69.2 (67.4-71.2)	70.9 (69.4-72.6)	70.8 (70.7-70.9)	71.2 (71.2-71.3)	71.8 (71.8-71.9)	73.5 (73.5-73.6)	74.4 (74.4-74.5)	75.4 (75.3-75.4)	76 (76-76.1)	77 (76.9-77)	78.2 (78.2-78.3)	78.9 (78.9-79)	80.7 (80.7-80.8)	80.9 (80-81.9)
Colorado	68.5 (66.5-70.5)	70.3 (68.6-72)	71 (70.9-71.2)	71.7 (71.5-71.8)	72.2 (72.1-72.4)	74.1 (73.9-74.2)	75.3 (75.2-75.4)	76.3 (76.1-76.4)	77.2 (77.1-77.4)	77.3 (77.1-77.4)	78.2 (78.1-78.4)	79 (78.8-79.1)	79.9 (79.8-80.1)	79.9 (78.8-81.1)
Connecticut	69.5 (67.6-71.5)	71.3 (69.5-72.9)	71.4 (71.3-71.5)	71.6 (71.5-71.7)	72.8 (72.6-72.9)	74.2 (74.1-74.4)	74.9 (74.8-75.1)	76 (75.9-76.1)	77.3 (77.2-77.4)	77.4 (77.2-77.5)	78.3 (78.2-78.5)	79.6 (79.4-79.7)	80.7 (80.5-80.8)	80.6 (79.4-81.8)
Delaware	66.5 (64.7-68.2)	69.2 (67.9-70.4)	69.3 (69.1-69.6)	69.6 (69.4-69.8)	70.1 (69.9-70.3)	72 (71.8-72.2)	73.3 (73.1-73.5)	74.3 (74.1-74.5)	74.9 (74.8-75.1)	75.2 (75-75.4)	76.4 (76.2-76.5)	77.1 (76.9-77.3)	78 (77.8-78.2)	78.2 (77.3-79)
Washington, DC	65 (62.9-67)	66.7 (65-68.3)	66.3 (66.1-66.6)	65.7 (65.5-65.9)	65.7 (65.5-65.9)	67.8 (67.6-68.1)	69.2 (69-69.4)	69.4 (69.2-69.6)	67.7 (67.4-67.9)	67.7 (67.5-67.9)	72.5 (72.2-72.7)	73.7 (73.5-74)	76.6 (76.4-76.8)	77.1 (75.9-78.2)
Florida	67.7 (65.7-69.7)	69.5 (67.7-71.3)	69.8 (69.6-69.9)	70.5 (70.4-70.6)	70.7 (70.6-70.8)	72.7 (72.7-72.9)	73.8 (73.7-73.9)	75.2 (75.1-75.3)	76 (76-76.1)	76.3 (76.2-76.4)	77.3 (77.2-77.4)	77.8 (77.8-77.9)	79.3 (79.2-79.4)	79.1 (78-80.2)
Georgia	65.5 (63.4-67.6)	67.5 (65.6-69.3)	67.6 (67.5-67.7)	68.3 (68.2-68.4)	68.6 (68.5-68.8)	70.8 (70.7-70.9)	72.2 (72.1-72.3)	73.1 (73-73.2)	73.8 (73.7-73.9)	74.3 (74.2-74.4)	75.1 (75-75.1)	76 (75.9-76.1)	77.1 (77-77.2)	77.2 (76-78.3)
Hawaii	69.3 (67.5-71.1)	71.1 (69.5-72.8)	72 (71.7-72.2)	72.9 (72.7-73.2)	73.7 (73.5-73.9)	75.9 (75.7-76.1)	77 (76.8-77.1)	78 (77.8-78.2)	78.5 (78.3-78.7)	78.8 (78.7-79)	79.7 (79.5-79.8)	80.4 (80.2-80.5)	81.2 (81-81.4)	81.5 (80.4-82.5)
Idaho	69.1 (67.1-71)	71.2 (69.6-72.8)	71.3 (71.1-71.5)	71.4 (71.2-71.7)	72 (71.8-72.2)	73.5 (73.3-73.7)	75.2 (75-75.4)	76.1 (76-76.3)	76.9 (76.7-77.1)	77.2 (77-77.4)	77.9 (77.7-78.1)	78.4 (78.2-78.6)	79.1 (79-79.3)	79 (78-80.1)
Illinois	68.3 (66.4-70.2)	69.7 (68.2-71.4)	69.6 (69.5-69.7)	69.9 (69.8-69.9)	70.2 (70.2-70.3)	72 (71.9-72)	73.4 (73.3-73.5)	74.6 (74.5-74.7)	75.1 (75.1-75.2)	75.5 (75.4-75.5)	76.7 (76.6-76.8)	77.7 (77.6-77.8)	79 (78.9-79.1)	79 (77.9-80.2)
Indiana	68.9 (66.9-70.8)	70.5 (68.9-72.1)	70.3 (70.2-70.5)	70.5 (70.4-70.7)	71.1 (71-71.2)	72.8 (72.7-72.9)	73.9 (73.8-74)	74.8 (74.7-75)	75.6 (75.5-75.7)	75.5 (75.4-75.6)	76.1 (76-76.2)	76.8 (76.6-76.9)	77.5 (77.4-77.6)	76.9 (75.7-78.2)
Iowa	70.6 (68.7-72.6)	71.7 (70-73.5)	72.2 (72-72.3)	72.2 (72-72.3)	72.7 (72.5-72.8)	74.2 (74.1-74.4)	75.8 (75.7-76)	76.5 (76.4-76.7)	77.9 (77.7-78)	77.6 (77.5-77.8)	78.4 (78.3-78.6)	79.1 (79-79.3)	79.5 (79.4-79.6)	79.3 (78.2-80.5)
Kansas	70.6 (68.7-72.5)	72.2 (70.5-73.9)	72 (71.8-72.1)	72.5 (72.3-72.7)	72.9 (72.7-73)	74.1 (74-74.3)	75.2 (75.1-75.4)	76.2 (76-76.3)	77.2 (77.1-77.4)	76.9 (76.8-77.1)	77.5 (77.3-77.6)	77.8 (77.7-78)	78.5 (78.3-78.6)	78.2 (77-79.4)
Kentucky	67.7 (65.7-69.7)	69.6 (67.8-71.3)	69.7 (69.5-69.8)	70.1 (70-70.3)	70.1 (69.9-70.2)	71.7 (71.5-71.8)	73 (72.8-73.1)	73.9 (73.8-74)	74.6 (74.5-74.8)	74.7 (74.6-74.9)	75.2 (75-75.3)	75.5 (75.3-75.6)	75.8 (75.7-75.9)	75 (73.8-76.2)
Louisiana	66 (64.1-68.3)	67.9 (66.3-69.7)	68.1 (68-68.2)	68.4 (68.2-68.5)	68.9 (68.8-69)	70.8 (70.6-70.9)	71.8 (71.7-71.9)	72.7 (72.6-72.8)	73.4 (73.3-73.5)	73.6 (73.4-73.7)	74.1 (74-74.2)	72.9 (72.8-73)	75.8 (75.7-75.9)	75.4 (74.1-76.7)
Maine	67.8 (66-69.8)	69.8 (68.3-71.5)	70.1 (69.9-70.3)	70.8 (70.6-71)	71 (70.8-71.2)	73 (72.9-73.2)	74.5 (74.3-74.6)	75.4 (75.2-75.6)	76.6 (76.5-76.8)	77 (76.9-77.2)	77.5 (77.4-77.7)	77.8 (77.6-77.9)	78.9 (78.8-79.1)	78 (76.8-79.1)
Maryland	67 (65.1-68.9)	68.7 (67.1-70.4)	68.6 (68.5-68.8)	69.3 (69.2-69.4)	70.3 (70.2-70.5)	72.2 (72.1-72.3)	73.4 (73.3-73.5)	74.1 (74-74.2)	75 (74.9-75.1)	75.1 (74.9-75.2)	76.2 (76-76.3)	77.2 (77.1-77.4)	78.9 (78.8-79)	78.8 (77.6-79.9)
Massachusetts	69.2 (67.4-71.1)	70.7 (68.9-72.3)	70.6 (70.5-70.7)	71.1 (71-71.2)	72 (71.9-72.2)	73.7 (73.6-73.8)	74.9 (74.8-75)	75.6 (75.5-75.7)	77 (76.9-77.1)	77.5 (77.4-77.6)	78.3 (78.2-78.4)	79.3 (79.2-79.4)	80.5 (80.4-80.6)	79.9 (78.8-81)
Michigan	68.7 (66.9-70.6)	70.2 (68.6-71.8)	70.2 (70.1-70.3)	70.5 (70.4-70.6)	70.8 (70.7-70.9)	72.3 (72.2-72.4)	73.7 (73.7-73.8)	74.3 (74.2-74.4)	75.3 (75.2-75.4)	75.7 (75.6-75.8)	76.4 (76.3-76.5)	77.1 (77-77.2)	78 (77.9-78.1)	77.6 (76.4-78.8)
Minnesota	70.3 (68.3-72.4)	71.7 (70-73.4)	71.8 (71.7-72)	72.5 (72.4-72.6)	73.1 (73-73.3)	74.9 (74.7-75)	76.1 (76-76.2)	76.9 (76.8-77.1)	77.9 (77.8-78)	78 (77.9-78.2)	79 (78.8-79.1)	80 (79.9-80.1)	80.8 (80.6-80.9)	80.7 (79.6-81.7)
Mississippi	65.1 (63-67.2)	67 (65.2-68.8)	67.7 (67.5-67.8)	67.6 (67.4-67.8)	68.4 (68.2-68.5)	70.6 (70.4-70.7)	71.8 (71.7-72)	72.9 (72.7-73)	73.4 (73.2-73.5)	73.1 (73-73.3)	73.5 (73.4-73.7)	73.5 (73.4-73.7)	74.8 (74.7-75)	74.5 (73.2-75.8)
Missouri	68.7 (66.8-70.7)	70.3 (68.7-72)	70.4 (70.3-70.5)	70.6 (70.5-70.7)	70.8 (70.7-70.9)	72.6 (72.5-72.7)	73.7 (73.6-73.8)	74.8 (74.7-74.9)	75.4 (75.3-75.5)	75.3 (75.2-75.5)	76.1 (76-76.2)	76.6 (76.4-76.7)	77.4 (77.3-77.5)	77 (75.8-78.2)
Montana	67.3 (65.4-69.3)	69.3 (67.5-70.9)	69.6 (69.3-69.8)	70 (69.7-70.2)	70.9 (70.7-71.1)	72.7 (72.4-72.9)	73.9 (73.7-74.1)	75.3 (75.1-75.5)	76.6 (76.4-76.8)	76.5 (76.3-76.7)	77.2 (77-77.4)	77.6 (77.4-77.8)	78.5 (78.3-78.7)	76.8 (75.7-78)
Nebraska	70.8 (68.9-72.8)	72.4 (70.8-74)	72.1 (71.9-72.3)	72 (71.9-72.2)	72.7 (72.5-72.8)	74.1 (73.9-74.3)	75.4 (75.2-75.6)	76.4 (76.2-76.6)	77 (76.9-77.2)	77.5 (77.3-77.6)	78.2 (78-78.3)	79 (78.8-79.1)	79.6 (79.4-79.7)	79.1 (78-80.1)
Nevada	62.1 (60.3-64.2)	66.8 (65-68.5)	67.4 (67.1-67.7)	67.3 (67.1-67.6)	69.1 (68.8-69.3)	71.7 (71.5-72)	72.3 (72.1-72.5)	74.1 (73.9-74.3)	74.4 (74.2-74.5)	74.5 (74.3-74.6)	75.5 (75.4-75.7)	75.8 (75.6-75.9)	77.8 (77.6-77.9)	77.9 (76.8-79)
New Hampshire	68 (66.1-69.9)	70.4 (68.9-71.8)	70.6 (70.4-70.8)	70.7 (70.5-70.9)	71.4 (71.2-71.6)	73.3 (73.1-73.5)	75.2 (75-75.4)	75.7 (75.5-75.9)	76.8 (76.6-76.9)	77.5 (77.4-77.7)	78.2 (78-78.3)	78.7 (78.5-78.9)	80 (79.8-80.2)	79.1 (78-80)
New Jersey	68.2 (66.5-70.1)	69.7 (68-71.3)	69.9 (69.8-70)	70.4 (70.3-70.5)	71.1 (71-71.2)	73.1 (73-73.2)	73.9 (73.9-74)	74.5 (74.4-74.6)	75.7 (75.6-75.8)	76.1 (76-76.2)	77.4 (77.3-77.5)	78.6 (78.6-78.7)	80 (80-80.2)	80.5 (79.4-81.6)
New Mexico	66.9 (64.9-68.9)	69.1 (67.5-70.9)	69.5 (69.3-69.7)	70 (69.8-70.2)	70.6 (70.4-70.8)	72.2 (72-72.4)	73.9 (73.7-74)	75.2 (75-75.3)	76 (75.8-76.2)	76.2 (76-76.3)	77.1 (77-77.3)	77.5 (77.3-77.7)	78.2 (78-78.3)	77.6 (76.4-78.7)
New York	68.4 (66.5-70.2)	69.6 (68.2-71)	69.7 (69.7-69.8)	70.3 (70.3-70.4)	70.6 (70.6-70.7)	72.7 (72.7-72.8)	73.6 (73.5-73.7)	74.1 (74.1-74.2)	75 (74.9-75)	75.8 (75.7-75.9)	77.9 (77.8-77.9)	79 (79-79.1)	80.4 (80.3-80.5)	80.8 (79.7-81.9)
North Carolina	66.6 (64.7-68.6)	68.4 (66.8-70.1)	68.3 (68.2-68.4)	68.7 (68.6-68.8)	69.4 (69.3-69.5)	71.4 (71.3-71.5)	73 (72.9-73.1)	74 (73.9-74.1)	74.7 (74.6-74.8)	75 (74.9-75.1)	75.6 (75.5-75.6)	76.5 (76.4-76.6)	77.7 (77.7-77.8)	77.8 (76.7-78.9)
North Dakota	69.8 (68.2-71.4)	71.7 (70.5-72.7)	71.7 (71.5-72)	72 (71.8-72.2)	72.8 (72.6-73)	74.2 (74-74.4)	75.6 (75.4-75.8)	76.9 (76.7-77.1)	77.7 (77.5-77.9)	77.9 (77.7-78.1)	78.5 (78.3-78.7)	79.4 (79.2-79.6)	79.6 (79.4-79.8)	78.5 (77.6-79.3)
Ohio	68.6 (66.9-70.6)	70 (68.5-71.6)	70.3 (70.2-70.3)	70.6 (70.5-70.6)	70.9 (70.9-71)	72.5 (72.4-72.6)	73.4 (73.3-73.5)	74.7 (74.6-74.8)	75.5 (75.4-75.6)	75.6 (75.5-75.7)	76.4 (76.3-76.5)	76.8 (76.7-76.8)	77.6 (77.5-77.7)	76.6 (75.4-77.8)
Oklahoma	69.2 (67.2-71.3)	71 (69.4-72.9)	70.9 (70.7-71.1)	71.7 (71.5-71.8)	71.3 (71.1-71.4)	72.7 (72.6-72.9)	73.6 (73.5-73.7)	74.3 (74.1-74.4)	75.3 (75.2-75.5)	74.8 (74.6-74.9)	75.1 (75-75.3)	75.1 (75-75.2)	75.7 (75.6-75.9)	75.3 (74-76.7)
Oregon	69.3 (67.3-71.4)	70.9 (69.1-72.8)	71 (70.8-71.2)	71.6 (71.4-71.7)	72.4 (72.2-72.5)	74.1 (73.9-74.2)	74.9 (74.7-75)	75.9 (75.7-76)	76.5 (76.4-76.7)	76.5 (76.4-76.7)	77.8 (77.7-77.9)	78.3 (78.2-78.5)	79.4 (79.2-79.5)	79.2 (78.1-80.4)
Pennsylvania	67.9 (66-69.7)	69.2 (67.8-70.7)	69.6 (69.5-69.7)	70 (69.9-70.1)	70.5 (70.5-70.6)	72.4 (72.3-72.5)	73.5 (73.4-73.6)	74.6 (74.5-74.6)	75.6 (75.5-75.7)	76 (75.9-76)	76.7 (76.6-76.8)	77.3 (77.2-77.3)	78.5 (78.4-78.5)	77.9 (76.8-79.2)
Rhode Island	68.5 (66.7-70.4)	70.4 (69-72)	70.7 (70.5-70.8)	70.9 (70.7-71.1)	71.8 (71.6-72)	73.8 (73.6-74)	74.8 (74.5-74.9)	75.7 (75.5-75.9)	76.6 (76.4-76.8)	77.7 (77.5-77.9)	78 (77.8-78.2)	78.6 (78.4-78.8)	79.6 (79.4-79.8)	79.3 (78.2-80.3)
South Carolina	63.9 (62-65.9)	65.9 (64.1-67.7)	66.1 (66-66.3)	67.2 (67-67.3)	68.2 (68-68.3)	70.1 (70-70.3)	71.8 (71.7-71.9)	73.1 (73-73.3)	73.8 (73.6-73.9)	74.2 (74.1-74.3)	74.7 (74.6-74.8)	75.6 (75.4-75.7)	76.6 (76.5-76.7)	76.1 (74.9-77.4)
South Dakota	69.2 (67.5-70.9)	71.1 (70-72.4)	70.9 (70.7-71.1)	71.7 (71.5-71.9)	72.1 (71.9-72.3)	73.5 (73.3-73.7)	74.8 (74.6-75)	76 (75.8-76.1)	77 (76.8-77.2)	77.1 (76.9-77.3)	77.8 (77.6-78)	78.4 (78.2-78.6)	79.2 (79-79.4)	78.1 (77.2-78.9)
Tennessee	67.7 (65.8-69.7)	69.3 (67.7-71.1)	69.6 (69.4-69.7)	70 (69.9-70.2)	70.1 (69.9-70.2)	72 (71.9-72.1)	73.1 (73-73.3)	74 (73.9-74.1)	74.3 (74.2-74.5)	74.3 (74.2-74.4)	74.8 (74.7-74.9)	75.2 (75.1-75.3)	76.1 (76-76.2)	76 (74.8-77.2)
Texas	68.6 (66.7-70.6)	70.2 (68.4-71.9)	70.4 (70.3-70.5)	71 (70.9-71.1)	71 (70.9-71.1)	72.7 (72.6-72.8)	73.6 (73.5-73.6)	74.3 (74.3-74.4)	75.3 (75.3-75.4)	75.9 (75.8-75.9)	76.5 (76.5-76.6)	77.4 (77.3-77.4)	78.3 (78.3-78.4)	78.5 (77.4-79.5)
Utah	69.9 (68.1-71.9)	71.9 (70.3-73.4)	71.7 (71.5-71.9)	72 (71.8-72.2)	72.9 (72.7-73.1)	74.5 (74.3-74.6)	75.7 (75.5-75.9)	76.4 (76.3-76.6)	77.8 (77.6-78)	77.8 (77.7-78)	78.5 (78.3-78.6)	79 (78.9-79.2)	79.8 (79.6-79.9)	79.3 (78.3-80.3)
Vermont	67.6 (66.3-69.1)	70.4 (69.4-71.3)	70.4 (70.1-70.7)	70.5 (70.3-70.8)	71.8 (71.7-72)	73.5 (73.3-73.6)	74.7 (74.6-74.9)	75.4 (75.2-75.6)	76.6 (76.4-76.8)	77.3 (77.1-77.4)	78.1 (78-78.3)	79.2 (79-79.3)	79.9 (79.7-80.1)	79.9 (79.2-80.6)
Virginia	66.8 (64.8-68.8)	68.5 (66.9-70.2)	68.9 (68.8-69)	69.5 (69.3-69.6)	70.3 (70.2-70.4)	72.1 (72-72.2)	73.4 (73.3-73.5)	74.5 (74.4-74.6)	75.4 (75.3-75.5)	75.8 (75.7-75.9)	76.7 (76.6-76.8)	77.6 (77.5-77.7)	79 (78.9-79.1)	79.1 (78-80.3)
Washington	69.3 (67.2-71.3)	70.6 (69-72.3)	71.1 (71-71.3)	72 (71.9-72.2)	71.9 (71.7-72)	73.9 (73.8-74)	75 (74.9-75.1)	76 (75.9-76.1)	76.9 (76.8-77)	77.3 (77.1-77.4)	78.1 (78-78.2)	78.9 (78.8-79)	79.9 (79.8-80)	79.9 (78.8-81)
West Virginia	67.5 (65.5-69.6)	69.1 (67.4-70.8)	69.7 (69.6-69.9)	69.5 (69.3-69.6)	69.4 (69.2-69.5)	71.3 (71.1-71.4)	72.7 (72.5-72.9)	73.8 (73.6-74)	74.7 (74.5-74.9)	74.9 (74.7-75)	75 (74.8-75.2)	75.2 (75-75.3)	75.5 (75.4-75.7)	74.8 (73.5-76.1)
Wisconsin	69.7 (67.8-71.6)	71.2 (69.6-72.8)	71.1 (71-71.3)	71.7 (71.6-71.9)	72.6 (72.5-72.8)	74.2 (74.1-74.3)	75.4 (75.2-75.5)	76.5 (76.3-76.6)	76.9 (76.8-77.1)	77.4 (77.3-77.5)	77.9 (77.8-78)	78.6 (78.5-78.7)	79.6 (79.5-79.7)	79.5 (78.4-80.7)
Wyoming	67.5 (66-69.2)	70.3 (69.3-71.4)	70 (69.8-70.3)	70.6 (70.3-70.8)	70.7 (70.5-71)	72.1 (71.9-72.3)	73.7 (73.6-73.9)	75 (74.8-75.2)	76.3 (76.1-76.5)	76.3 (76.1-76.5)	76.8 (76.6-77)	77.4 (77.2-77.6)	78.1 (77.9-78.3)	77.9 (77.1-78.7)
Southern Latin America	59.4 (59.4-59.5)	61.8 (61.7-61.8)	62.1 (62-62.2)	64.2 (64.2-64.3)	65.3 (65.2-65.3)	67.9 (67.5-68.3)	69.9 (69.9-70)	71.9 (71.8-71.9)	72.6 (72.6-72.7)	73.8 (73.8-73.8)	74.9 (74.8-74.9)	75.9 (75.9-76)	76.6 (76.5-76.6)	77.5 (76.7-78.2)
Argentina	62.7 (62.6-62.8)	64.2 (64.1-64.2)	65.1 (65-65.1)	66.1 (66.1-66.2)	66.1 (66-66.1)	68.2 (67.6-68.7)	70 (70-70.1)	71.8 (71.8-71.9)	72.4 (72.3-72.4)	73.4 (73.3-73.4)	74.2 (74.1-74.2)	75.3 (75.2-75.3)	75.8 (75.8-75.9)	76.6 (75.5-77.7)
Chile	50 (49.9-50.1)	54.6 (54.4-54.7)	54.4 (54.3-54.5)	59 (58.9-59.1)	62.4 (62.3-62.5)	66.5 (66.4-66.6)	69.5 (69.4-69.5)	71.9 (71.8-71.9)	73.2 (73.1-73.2)	75.1 (75-75.2)	76.9 (76.8-77)	77.9 (77.8-78)	78.6 (78.5-78.7)	79.7 (78.7-80.7)
Uruguay	65.2 (65-65.4)	67.5 (67.4-67.7)	68.2 (68-68.3)	68.3 (68.2-68.5)	68.4 (68.3-68.6)	69.3 (69.2-69.5)	70.3 (70.1-70.4)	72.3 (72.1-72.4)	73 (72.9-73.2)	73.5 (73.4-73.7)	74.6 (74.4-74.7)	75.5 (75.3-75.6)	76.4 (76.3-76.6)	77 (76-78.1)
Western Europe	66.6 (66.2-67.1)	68.9 (68.8-69.1)	70.3 (70.2-70.3)	71.2 (71.2-71.2)	71.8 (71.8-71.9)	72.8 (72.8-72.8)	74 (74-74.1)	75.3 (75.3-75.3)	76.3 (76.3-76.3)	77.4 (77.3-77.4)	78.6 (78.6-78.6)	80 (79.9-80)	81 (81-81)	81.9 (81.7-82.1)
Andorra	74.3 (72.3-76.5)	75 (73-77.1)	75.7 (73.8-77.7)	76.3 (74.6-78.3)	77 (75.4-78.9)	77.7 (76.3-79.3)	78.3 (76.9-79.6)	78.6 (77.4-79.8)	79 (77.8-80)	79.7 (78.7-80.8)	81.1 (80.2-82)	81.8 (81.1-82.4)	82.3 (81.6-82.9)	82.7 (81.8-83.6)
Austria	64.7 (64.6-64.8)	67.6 (67.5-67.7)	68.8 (68.7-68.9)	70 (69.9-70.1)	70.1 (70-70.2)	71.3 (71.2-71.4)	72.7 (72.6-72.8)	74.2 (74.1-74.2)	75.8 (75.7-75.9)	76.8 (76.8-76.9)	78.3 (78.2-78.4)	79.6 (79.5-79.7)	80.6 (80.5-80.7)	81.8 (81.3-82.2)
Belgium	65.8 (65.7-65.9)	68.7 (68.6-68.8)	69.9 (69.8-70)	70.6 (70.6-70.7)	71 (70.9-71.1)	72 (72-72.1)	73.2 (73.1-73.3)	74.5 (74.4-74.6)	76 (76-76.1)	76.9 (76.8-77)	77.8 (77.8-77.9)	79.2 (79.1-79.2)	80.1 (80-80.2)	81.4 (80.9-81.8)
Cyprus	64.8 (62.8-66.9)	65.5 (63.6-67.4)	66.9 (65.2-68.6)	67.8 (66.3-69.3)	68.9 (67.9-69.9)	70.2 (70-70.4)	72.3 (72.2-72.5)	73.9 (73.8-74.1)	75.9 (75.7-76.1)	76.8 (76.4-77.1)	76.8 (76.3-77.2)	77.9 (77.4-78.3)	80.1 (79.7-80.6)	81.7 (81-82.4)
Denmark	70.5 (70.3-70.6)	72 (71.8-72.1)	72.3 (72.2-72.5)	72.5 (72.4-72.6)	73.4 (73.3-73.5)	74.2 (74-74.3)	74.1 (74-74.2)	74.6 (74.5-74.7)	75 (74.9-75.1)	75.5 (75.4-75.6)	77 (76.9-77.1)	78.3 (78.2-78.4)	79.3 (79.2-79.4)	80.8 (80.2-81.3)
Finland	64.4 (64.3-64.6)	67.8 (67.7-67.9)	69.4 (69.3-69.5)	69.7 (69.6-69.8)	70.4 (70.3-70.5)	71.9 (71.8-72)	73.8 (73.7-74)	74.7 (74.6-74.8)	75.1 (75-75.2)	76.7 (76.6-76.8)	77.8 (77.7-77.9)	79.1 (79-79.2)	80.2 (80.1-80.3)	81.4 (80.9-81.9)
France	67.1 (67-67.1)	69.3 (69.2-69.3)	71.1 (71.1-71.2)	71.7 (71.6-71.7)	72.6 (72.6-72.6)	73.2 (73.1-73.2)	74.6 (74.5-74.6)	75.6 (75.6-75.7)	77 (77-77.1)	78.1 (78-78.1)	79.1 (79.1-79.1)	80.4 (80.4-80.4)	81.4 (81.4-81.4)	82.8 (82.4-83.3)
Germany	66.9 (64.9-68.9)	69 (68.4-69.5)	69.8 (69.7-69.8)	71.1 (71.1-71.1)	71.2 (71.2-71.2)	72.1 (72-72.1)	73.3 (73.3-73.4)	74.9 (74.9-75)	75.5 (75.5-75.6)	76.7 (76.7-76.8)	78.3 (78.3-78.3)	79.7 (79.6-79.7)	80.4 (80.4-80.4)	80.6 (79.7-81.5)
Greece	68.1 (67.4-68.8)	71.5 (71.4-71.6)	72.5 (72.4-72.6)	72.9 (72.8-73)	73.7 (73.6-73.8)	74.8 (74.7-74.9)	75.3 (75.2-75.4)	75.9 (75.9-76)	77.5 (77.4-77.6)	78.2 (78.1-78.2)	78.8 (78.8-78.9)	79.3 (79.2-79.4)	80.2 (80.2-80.3)	81 (80.5-81.5)
Iceland	70.8 (70.4-71.2)	72.4 (72.1-72.7)	73.3 (73-73.5)	73.5 (73.3-73.7)	73.7 (73.5-74)	75.4 (75.2-75.6)	76.5 (76.2-76.8)	77.3 (77-77.7)	77.8 (77.6-78.1)	78.3 (78.1-78.5)	80.1 (79.9-80.3)	81.4 (81.2-81.6)	82.4 (82.2-82.6)	82.7 (82.4-83.1)
Ireland	65.9 (65.7-66.1)	68.6 (68.5-68.8)	70.1 (70-70.3)	70.7 (70.6-70.9)	71.1 (70.9-71.2)	71.7 (71.5-71.8)	72.6 (72.4-72.7)	73.7 (73.6-73.8)	74.9 (74.7-75)	75.6 (75.5-75.7)	76.6 (76.5-76.7)	79 (78.9-79.1)	80.7 (80.6-80.8)	81.8 (81.4-82.3)
Israel	73.8 (73.5-74.1)	70.4 (70.2-70.5)	71.5 (71.4-71.7)	71.4 (71.3-71.6)	71.6 (71.4-71.7)	72.2 (72-72.3)	74.1 (74-74.2)	75.6 (75.4-75.7)	77.4 (77.3-77.5)	77.7 (77.6-77.8)	78.7 (78.6-78.8)	80.1 (80-80.2)	81.8 (81.7-81.9)	83 (82.5-83.4)
Italy	65.8 (65.7-65.8)	68.5 (68.4-68.5)	69.8 (69.7-69.8)	70.8 (70.7-70.8)	71.8 (71.7-71.8)	72.8 (72.8-72.8)	73.9 (73.9-73.9)	75.7 (75.7-75.7)	77.1 (77-77.1)	78.1 (78-78.1)	79.6 (79.5-79.6)	80.9 (80.9-81)	82 (82-82)	83.2 (82.7-83.6)
Luxembourg	66.3 (65.9-66.6)	67.4 (67.1-67.6)	68.8 (68.6-69.1)	69.6 (69.4-69.9)	70.2 (70-70.4)	71.1 (70.9-71.3)	72.7 (72.5-72.9)	73.8 (73.6-74.1)	75.4 (75.2-75.6)	76.4 (76.3-76.6)	78 (77.8-78.2)	79.4 (79.2-79.6)	80.8 (80.6-81)	81.7 (81-82.4)
Malta	63.1 (62.7-63.4)	67.2 (66.9-67.4)	68.8 (68.5-69)	69.7 (69.5-69.9)	70.7 (70.5-70.9)	71.9 (71.7-72.1)	72.4 (72.2-72.6)	74.3 (74.1-74.5)	76.5 (76.3-76.7)	77.6 (77.4-77.8)	78.5 (78.3-78.7)	79.3 (79.1-79.5)	80.4 (80.2-80.6)	81 (80.6-81.4)
Netherlands	71.5 (71.4-71.6)	72.6 (72.5-72.7)	73.3 (73.3-73.4)	73.5 (73.5-73.6)	73.6 (73.5-73.6)	74.5 (74.5-74.6)	75.8 (75.7-75.8)	76.4 (76.3-76.4)	77 (76.9-77.1)	77.6 (77.5-77.6)	78.1 (78-78.1)	79.5 (79.4-79.6)	80.8 (80.7-80.9)	81.5 (81-82)
Norway	72 (71.6-72.4)	73.5 (73.1-73.8)	73.7 (73.4-74)	73.9 (73.7-74)	74.1 (74.1-74.2)	75 (75-75.1)	75.7 (75.7-75.8)	76.1 (76-76.2)	76.6 (76.6-76.7)	77.9 (77.8-77.9)	78.6 (78.5-78.6)	80.1 (80.1-80.2)	80.9 (80.9-81)	82.3 (82.2-82.5)
Portugal	59.2 (59-59.3)	61.8 (61.7-61.9)	64.9 (64.8-65)	66.1 (66-66.2)	67.7 (67.6-67.8)	69.3 (69.1-69.5)	71.7 (71.6-71.8)	73.2 (73.1-73.3)	74.1 (74.1-74.2)	75.3 (75.2-75.4)	76.7 (76.6-76.8)	78.8 (78.5-79)	80 (80-80.1)	81.4 (81-81.9)
Spain	62.3 (62.3-62.4)	67.1 (67-67.1)	69.9 (69.8-70)	71.2 (71.2-71.3)	72.3 (72.3-72.4)	73.7 (73.6-73.7)	75.6 (75.6-75.7)	76.5 (76.5-76.6)	77 (77-77.1)	78 (78-78.1)	79.4 (79.3-79.4)	80.5 (80.4-80.5)	82 (81.9-82)	83.1 (82.7-83.5)
Sweden	71.3 (70.9-71.7)	72.7 (72.3-73.1)	73.2 (72.8-73.7)	74 (73.6-74.5)	74.7 (74.3-75.2)	75.1 (74.8-75.4)	75.8 (75.7-75.9)	76.9 (76.8-77)	77.7 (77.6-77.8)	78.9 (78.8-79)	79.8 (79.7-79.8)	80.6 (80.5-80.7)	81.5 (81.4-81.6)	82.5 (82.1-82.9)
Stockholm	70.9 (69-73)	72.3 (70.3-74.4)	72.9 (70.8-75)	73.6 (71.7-75.6)	74.2 (72.6-76.1)	74.6 (73.3-75.9)	75.2 (75-75.4)	76.6 (76.4-76.7)	77.1 (76.9-77.3)	78.7 (78.5-78.9)	79.6 (79.4-79.7)	80.7 (80.6-80.9)	81.8 (81.7-82)	82.6 (82.1-83.2)
Sweden except Stockholm	71.3 (71-71.8)	72.8 (72.4-73.2)	73.3 (73-73.7)	74.1 (73.8-74.5)	74.8 (74.5-75.2)	75.2 (74.9-75.5)	76 (75.9-76.1)	77 (76.9-77.1)	77.9 (77.8-78)	79 (78.9-79.1)	79.8 (79.7-79.9)	80.6 (80.5-80.7)	81.5 (81.3-81.6)	82.5 (82-82.9)
Switzerland	69.1 (69-69.2)	70.3 (70.2-70.4)	71.9 (71.8-72)	72.7 (72.6-72.8)	73.4 (73.3-73.5)	75 (74.8-75.1)	75.9 (75.8-76)	77.2 (77.1-77.3)	77.8 (77.7-77.9)	78.8 (78.7-78.9)	80.1 (80-80.2)	81.5 (81.4-81.6)	82.5 (82.4-82.6)	84 (83.5-84.4)
United Kingdom	69 (68.8-69.2)	70.4 (70.1-70.6)	71.1 (70.9-71.4)	71.6 (71.4-71.8)	72 (71.8-72.2)	72.8 (72.6-72.9)	73.7 (73.7-73.8)	74.7 (74.7-74.8)	75.8 (75.7-75.8)	76.7 (76.7-76.7)	78 (77.9-78)	79.3 (79.3-79.3)	80.4 (80.4-80.4)	81 (80.9-81.1)
England	69.6 (69.4-69.8)	70.8 (70.6-71.1)	71.6 (71.4-71.8)	72 (71.8-72.2)	72.4 (72.1-72.6)	73.1 (73-73.3)	74 (74-74.1)	75 (75-75)	76 (76-76)	77 (76.9-77)	78.2 (78.2-78.2)	79.5 (79.5-79.5)	80.7 (80.7-80.7)	81.2 (81.2-81.3)
East Midlands	68.8 (67.9-69.7)	70.2 (69.3-71)	71 (70.2-71.8)	71.5 (70.7-72.4)	72 (71.2-72.8)	72.8 (72.2-73.3)	73.9 (73.7-74)	75.1 (75-75.1)	76 (76-76.1)	77 (77-77.1)	78.1 (78-78.2)	79.4 (79.3-79.5)	80.5 (80.5-80.6)	81.1 (80.8-81.3)
Derby	68 (65.6-70.4)	69.4 (67.1-71.9)	70.3 (68.1-72.9)	70.8 (68.4-73.2)	71.3 (69.1-73.3)	72 (70.5-73.6)	72.8 (72.3-73.3)	74 (73.8-74.3)	75.1 (74.9-75.3)	76.5 (76.3-76.7)	77.9 (77.6-78.2)	79 (78.7-79.2)	79.4 (79.2-79.6)	80.4 (79.7-81)
Derbyshire	68.7 (66.3-71.1)	70.1 (67.8-72.3)	70.9 (68.6-73.2)	71.4 (69.1-73.7)	71.8 (69.6-74)	72.5 (70.9-74.2)	73.7 (73.3-74.1)	75 (74.8-75.2)	75.9 (75.7-76)	77 (76.8-77.1)	78.2 (77.9-78.4)	79.4 (79.3-79.6)	80.8 (80.6-80.9)	80.8 (80.2-81.4)
Leicester	67.5 (65.1-69.9)	68.9 (66.7-71.3)	69.7 (67.4-72)	70.2 (68-72.6)	70.7 (68.7-72.9)	71.5 (70-73.2)	72.8 (72.3-73.2)	74.2 (74-74.4)	75.1 (74.9-75.3)	75.7 (75.4-75.9)	76.3 (76-76.6)	77.6 (77.4-77.9)	79.1 (78.9-79.3)	80.7 (80.1-81.3)
Leicestershire	70.1 (67.7-72.5)	71.5 (69.1-73.8)	72.3 (70-74.5)	72.8 (70.6-75.1)	73.2 (71.1-75.3)	73.9 (72.4-75.4)	75 (74.6-75.4)	76.2 (76-76.3)	77.1 (77-77.3)	78.3 (78.1-78.4)	79.4 (79.1-79.6)	80.6 (80.4-80.8)	81.7 (81.5-81.8)	82 (81.4-82.5)
Lincolnshire	68.9 (66.6-71.4)	70.3 (68.1-72.5)	71.1 (68.9-73.4)	71.6 (69.4-74)	72 (69.9-74.1)	72.8 (71.3-74.4)	74.1 (73.7-74.6)	75.2 (75-75.4)	76.2 (76-76.4)	77.2 (77-77.4)	78.2 (78-78.5)	79.5 (79.3-79.7)	80.8 (80.6-80.9)	81.1 (80.5-81.7)
Northamptonshire	69.3 (66.9-71.8)	70.8 (68.5-73.2)	71.7 (69.4-74)	72.2 (70.2-74.5)	72.7 (70.8-74.8)	73.5 (72-75.1)	74.6 (74.2-74.9)	75.4 (75.2-75.5)	76.2 (76-76.4)	77.2 (77-77.4)	78.4 (78.2-78.7)	79.5 (79.3-79.7)	80.4 (80.3-80.6)	81.2 (80.6-81.8)
Nottingham	67.5 (65-70)	68.9 (66.4-71.1)	69.6 (67.3-72)	70 (67.8-72.2)	70.4 (68.3-72.6)	71.1 (69.5-72.7)	72.3 (71.8-72.7)	73.6 (73.4-73.8)	74.6 (74.3-74.8)	74.9 (74.7-75.1)	75.4 (75.2-75.8)	76.6 (76.4-76.8)	78.5 (78.3-78.7)	80 (79.4-80.7)
Nottinghamshire	69.1 (66.6-71.5)	70.5 (68.1-72.9)	71.4 (69.1-73.7)	71.9 (69.8-74.1)	72.4 (70.4-74.4)	73.1 (71.7-74.5)	74.1 (73.7-74.5)	75.2 (75-75.4)	76.1 (76-76.3)	77.1 (77-77.3)	78.2 (78-78.5)	79.6 (79.5-79.8)	80.7 (80.5-80.8)	81 (80.4-81.5)
Rutland	71.3 (68.7-73.7)	72.8 (70.3-75.2)	73.5 (71.2-75.9)	73.9 (71.7-76.1)	74.3 (72.2-76.5)	75 (73.4-76.7)	75.7 (74.9-76.5)	76.3 (75.8-76.7)	77.5 (77-78)	78.9 (78.4-79.4)	80.4 (79.8-80.9)	81.3 (80.9-81.8)	82 (81.5-82.5)	82.2 (81.3-83.1)
East of England	70.2 (69.3-71.1)	71.6 (70.7-72.4)	72.4 (71.5-73.4)	73 (72.2-73.9)	73.4 (72.6-74.2)	74.1 (73.6-74.6)	75 (74.9-75.2)	76.2 (76.1-76.2)	77.1 (77-77.1)	78 (78-78.1)	79.2 (79.1-79.3)	80.4 (80.3-80.5)	81.5 (81.4-81.5)	81.9 (81.6-82.1)
Bedford	69.4 (67-72.1)	70.9 (68.6-73.4)	71.8 (69.5-74.2)	72.4 (70.1-74.7)	72.9 (70.8-75)	73.7 (72.2-75.4)	74.9 (74.3-75.5)	76 (75.7-76.3)	76.5 (76.2-76.8)	77.2 (76.9-77.5)	78.5 (78.2-78.9)	79.7 (79.5-80)	80.4 (80.1-80.7)	81.4 (80.7-82)
Cambridgeshire	70.8 (68.5-73.3)	72.1 (69.9-74.6)	73 (70.8-75.3)	73.6 (71.5-75.9)	74 (72.1-76)	74.7 (73.2-76.1)	75.4 (75-75.8)	76.3 (76.1-76.5)	77.5 (77.3-77.7)	78.2 (78-78.4)	79.5 (79.2-79.8)	80.8 (80.6-80.9)	82.1 (81.9-82.3)	82.6 (82-83.1)
Central Bedfordshire	70.2 (67.6-72.8)	71.6 (69.2-74.1)	72.5 (70-75)	73 (70.7-75.4)	73.5 (71.5-75.5)	74.2 (72.7-75.7)	74.9 (74.4-75.4)	75.8 (75.5-76)	76.6 (76.4-76.9)	77.7 (77.4-77.9)	79.1 (78.8-79.4)	80.4 (80.2-80.6)	81.8 (81.5-82)	83 (82.4-83.7)
Essex	70.2 (68.1-72.5)	71.5 (69.4-73.8)	72.3 (70.2-74.7)	72.8 (70.5-74.9)	73 (71.2-75.1)	73.7 (72.3-75.1)	74.9 (74.5-75.3)	76.3 (76.1-76.4)	77.2 (77-77.3)	78.2 (78.1-78.3)	79.3 (79-79.5)	80.5 (80.3-80.6)	81.5 (81.3-81.6)	81.4 (80.8-82)
Hertfordshire	70.7 (68.3-73.1)	72 (69.8-74.3)	72.8 (70.6-75.3)	73.3 (71.1-75.5)	73.6 (71.6-75.6)	74.2 (72.7-75.7)	75.1 (74.7-75.5)	76.2 (76.1-76.4)	77.2 (77-77.3)	78.2 (78.1-78.4)	79.3 (79.1-79.5)	80.6 (80.4-80.7)	81.7 (81.5-81.8)	82.7 (82.2-83.3)

Table 6 Estimates of life expectancy at birth by location and year for 1950, 1955...2005, 2010, 2017 - both sexes

Location	1950	1955	1960	1965	1970	1975	1980	1985	1990	1995	2000	2005	2010	2017
Luton	68.7 (66.3-71.5)	70.1 (67.8-72.5)	71 (68.8-73.3)	71.6 (69.3-73.9)	72 (70-74.3)	72.8 (71.3-74.4)	73.5 (73-74.1)	75.2 (74.9-75.5)	75.6 (75.4-75.9)	76.2 (76-76.5)	77.3 (77-77.6)	78.3 (78.1-78.5)	79.8 (79.6-80.1)	81.5 (80.9-82.2)
Norfolk	70.1 (67.6-72.5)	71.6 (69.3-73.9)	72.6 (70.5-74.9)	73.3 (71.1-75.6)	73.9 (71.9-76)	74.8 (73.2-76.3)	75.5 (75.1-75.9)	76.4 (76.2-76.5)	77.1 (76.9-77.3)	78.2 (78-78.4)	79.4 (79.1-79.6)	80.6 (80.4-80.7)	81.5 (81.4-81.7)	81.2 (80.6-81.8)
Peterborough	69 (66.2-71.7)	70.4 (68-73)	71.3 (68.9-74)	71.8 (69.5-74.2)	72.2 (70.2-74.3)	73 (71.4-74.6)	74.1 (73.5-74.7)	75.1 (74.8-75.4)	76 (75.7-76.3)	76.6 (76.4-76.9)	77.8 (77.5-78.1)	78.8 (78.5-79.1)	79.7 (79.4-79.9)	80.7 (80-81.4)
Southend-on-Sea	69.3 (66.9-71.9)	70.8 (68.6-73.4)	71.8 (69.6-74.4)	72.5 (70.1-74.8)	73 (71-75)	73.9 (72.4-75.4)	74.8 (74.3-75.4)	75.8 (75.5-76.1)	76.7 (76.4-76.9)	77.4 (77.1-77.7)	78.2 (77.9-78.6)	79.3 (79-79.5)	80.3 (80.1-80.6)	80.7 (80.1-81.4)
Suffolk	70.3 (68.1-72.6)	71.7 (69.4-73.9)	72.6 (70.3-74.9)	73.3 (71.2-75.5)	73.8 (71.8-75.9)	74.6 (73.2-76.2)	75.4 (75-75.8)	76.3 (76.2-76.5)	77.5 (77.4-77.7)	78.3 (78.1-78.5)	79.5 (79.3-79.8)	80.9 (80.7-81.1)	81.9 (81.7-82)	82 (81.3-82.5)
Thurrock	69 (66.4-71.5)	70.3 (68-72.8)	71.1 (68.7-73.5)	71.5 (69.2-73.9)	71.9 (69.9-74.1)	72.7 (71.1-74.2)	73.7 (73.1-74.2)	74.9 (74.7-75.2)	76 (75.8-76.3)	77 (76.8-77.3)	78.6 (78.3-79)	79.5 (79.3-79.8)	80.8 (80.6-81.1)	81.5 (80.8-82.3)
Greater London	70.4 (70-70.8)	71.6 (71.1-72)	72.2 (71.7-72.6)	72.4 (72-72.8)	72.7 (72.3-73.1)	73.4 (73.1-73.7)	74.2 (74.1-74.2)	75.1 (75-75.1)	75.9 (75.8-75.9)	76.5 (76.5-76.6)	78.1 (78-78.2)	79.7 (79.7-79.8)	81.1 (81.1-81.2)	82.2 (82.1-82.3)
Barking and Dagenham	68.5 (66.1-71)	69.7 (67.6-72.1)	70.5 (68.4-72.6)	70.9 (68.7-73.2)	71.2 (69.4-73.3)	72 (70.5-73.6)	72.7 (72.2-73.2)	73.9 (73.7-74.2)	75 (74.7-75.2)	75.7 (75.5-76)	76.7 (76.4-77)	78.1 (77.8-78.3)	79.2 (79-79.5)	79.9 (79.3-80.6)
Barnet	72 (69.8-74.3)	73.2 (71.1-75.5)	73.9 (71.8-76)	74.3 (72.3-76.4)	74.7 (72.9-76.6)	75.3 (74-76.7)	75.8 (75.4-76.3)	76.6 (76.4-76.8)	77.6 (77.4-77.8)	78.4 (78.2-78.6)	80 (79.7-80.3)	81.5 (81.3-81.6)	82.2 (82-82.4)	82.9 (82.4-83.5)
Bexley	71.6 (69.3-74)	72.7 (70.5-74.9)	73.3 (71.2-75.6)	73.7 (71.8-76)	74 (72.1-76)	74.8 (73.4-76.3)	75.7 (75.3-76.2)	76.8 (76.6-77)	77.6 (77.3-77.8)	78.2 (77.9-78.4)	79.5 (79.2-79.7)	80.9 (80.7-81.2)	81.8 (81.5-82)	82 (81.4-82.7)
Brent	70.3 (67.9-72.9)	71.5 (69.3-73.9)	72.3 (70.2-74.5)	72.7 (70.4-74.8)	73 (71.1-74.9)	73.8 (72.4-75.3)	74.7 (74.2-75.1)	75 (74.8-75.2)	75.9 (75.7-76.2)	76.4 (76.2-76.6)	78.1 (77.8-78.4)	80.1 (79.9-80.3)	81.4 (81.2-81.6)	82.8 (82.3-83.4)
Bromley	72.1 (69.8-74.4)	73.2 (71.1-75.4)	73.8 (71.7-76)	74.2 (72.1-76.4)	74.5 (72.6-76.4)	75.2 (73.8-76.6)	76 (75.6-76.4)	76.8 (76.6-77)	77.8 (77.6-78)	78.6 (78.4-78.8)	80 (79.7-80.2)	81.6 (81.4-81.8)	82.5 (82.3-82.7)	82.6 (82-83.2)
Camden	70 (67.7-72.4)	71 (68.9-73.4)	71.4 (69.2-73.7)	71.4 (69.2-73.5)	71.5 (69.6-73.5)	71.9 (70.4-73.4)	72.6 (72.2-73.1)	73.8 (73.6-74)	74.1 (73.8-74.3)	74.6 (74.3-74.8)	76.4 (76.1-76.7)	77.8 (77.6-78)	80.7 (80.5-80.9)	83.8 (83.1-84.4)
Croydon	71 (68.8-73.4)	72.2 (70-74.4)	72.9 (70.8-75)	73.3 (71.4-75.4)	73.6 (71.8-75.6)	74.2 (72.9-75.6)	74.8 (74.4-75.2)	75.4 (75.2-75.7)	76.4 (76.2-76.6)	77.2 (77-77.4)	78.6 (78.3-78.8)	80.2 (80.1-80.5)	81 (80.8-81.2)	81.8 (81.2-82.4)
Ealing	70.2 (67.9-72.5)	71.3 (69.2-73.6)	71.9 (69.9-74.2)	72.2 (70.3-74.3)	72.5 (70.6-74.4)	73.1 (71.6-74.6)	73.8 (73.4-74.2)	74.8 (74.6-75)	75.7 (75.5-76)	76.4 (76.2-76.6)	78.3 (78-78.5)	80 (79.8-80.2)	81.4 (81.2-81.6)	82.4 (81.8-83)
Enfield	71.1 (68.8-73.4)	72.3 (70.1-74.6)	73 (70.8-75.2)	73.4 (71.3-75.7)	73.7 (71.8-75.7)	74.4 (73-75.9)	75.1 (74.7-75.6)	75.9 (75.7-76.1)	77 (76.7-77.2)	77.5 (77.3-77.7)	78.8 (78.5-79)	80.4 (80.1-80.6)	81.8 (81.6-82)	82.2 (81.6-82.8)
Greenwich	69.4 (67.1-71.7)	70.7 (68.5-73.1)	71.4 (69.3-73.8)	71.9 (69.8-74.2)	72.3 (70.4-74.3)	73 (71.6-74.5)	73.6 (73.1-74.1)	74.3 (74-74.5)	75.1 (74.9-75.3)	75.6 (75.4-75.9)	76.7 (76.4-76.9)	78 (77.8-78.2)	80.1 (79.9-80.3)	81.1 (80.5-81.7)
Hackney	69.8 (67.4-72.2)	70.8 (68.7-73.2)	71.3 (69.1-73.5)	71.2 (69.2-73.3)	71.2 (69.3-73.2)	71.6 (70.1-73.1)	72.3 (71.9-72.8)	73.2 (73-73.4)	73.8 (73.5-74)	74.8 (74.5-75)	76.8 (76.5-77.2)	77.8 (77.6-78)	79.2 (79-79.4)	81 (80.4-81.6)
Hammersmith and Fulham	69.6 (67.4-72)	70.7 (68.4-73)	71.1 (68.9-73.6)	71.2 (69.1-73.5)	71.4 (69.4-73.3)	71.8 (70.4-73.3)	72.4 (71.9-72.9)	73 (72.7-73.2)	73.2 (73-73.5)	74 (73.8-74.2)	76.9 (76.6-77.2)	79 (78.8-79.3)	80.7 (80.4-80.9)	82 (81.4-82.6)
Haringey	69.8 (67.5-72.2)	71 (68.7-73.2)	71.6 (69.5-73.8)	71.8 (69.8-74.1)	72.1 (70.3-74.2)	72.9 (71.4-74.4)	73.8 (73.3-74.2)	74.9 (74.6-75.1)	75.5 (75.3-75.8)	75.9 (75.7-76.1)	77.3 (77-77.6)	78.7 (78.5-78.9)	80.1 (79.9-80.3)	82.5 (81.9-83.1)
Harrow	72 (69.6-74.5)	73.1 (70.7-75.5)	73.6 (71.4-76)	73.9 (71.7-76.1)	74.2 (72.2-76.1)	74.9 (73.4-76.3)	75.7 (75.2-76.1)	77.1 (76.8-77.3)	78.1 (77.8-78.3)	78.6 (78.4-78.8)	80.2 (79.9-80.6)	82 (81.7-82.2)	83.4 (83.1-83.6)	83.9 (83.2-84.5)
Havering	71.3 (69-73.7)	72.5 (70.3-74.8)	73.2 (71-75.4)	73.5 (71.4-75.8)	73.9 (71.9-75.9)	74.6 (73.2-76.1)	75.2 (74.8-75.7)	76.3 (76.1-76.5)	77.6 (77.4-77.8)	78.2 (77.9-78.4)	79.3 (79-79.6)	80.7 (80.5-81)	81.1 (80.9-81.3)	81.1 (80.5-81.8)
Hillingdon	71.3 (69-74)	72.5 (70.2-74.9)	73 (70.8-75.2)	73.3 (71.3-75.5)	73.6 (71.7-75.5)	74.2 (72.7-75.6)	74.8 (74.4-75.3)	75.8 (75.6-76.1)	76.7 (76.5-76.9)	77.1 (76.9-77.3)	78.6 (78.3-78.9)	80 (79.8-80.2)	81.3 (81-81.5)	82.1 (81.5-82.7)
Hounslow	70.7 (68.4-73.2)	71.9 (69.7-74.3)	72.5 (70.3-74.9)	72.8 (70.6-75.2)	73.1 (71.2-75.1)	73.8 (72.4-75.2)	74.6 (74.1-75)	75.1 (74.9-75.3)	76.1 (75.9-76.4)	76.4 (76.2-76.6)	77.8 (77.5-78.1)	79.4 (79.1-79.6)	80.9 (80.6-81.1)	82 (81.4-82.6)
Islington	69.6 (67.4-72)	70.8 (68.5-73)	71.3 (69.2-73.5)	71.4 (69.3-73.6)	71.6 (69.6-73.5)	72 (70.6-73.5)	72.6 (72.1-73.1)	73.3 (73-73.5)	73.8 (73.6-74.1)	74.4 (74.2-74.7)	75.8 (75.5-76.1)	77.5 (77.3-77.7)	79.5 (79.3-79.7)	81.7 (81.1-82.3)
Kensington and Chelsea	70.4 (68.1-72.9)	71.5 (69.3-73.9)	71.9 (69.7-74.2)	71.9 (69.8-74.1)	72 (70-74)	72.4 (71-74)	73.1 (72.6-73.6)	74.4 (74.1-74.6)	76.1 (75.9-76.4)	77.6 (77.3-77.8)	80.2 (79.9-80.6)	82 (81.7-82.2)	83.3 (83-83.5)	84 (83.4-84.7)
Kingston upon Thames	72.2 (69.7-74.6)	73.4 (71-75.8)	74 (71.7-76.3)	74.3 (72.1-76.5)	74.6 (72.7-76.6)	75.3 (73.9-76.8)	76 (75.5-76.5)	76.7 (76.4-77)	77.8 (77.5-78)	78.6 (78.3-78.8)	80.1 (79.8-80.4)	82.1 (81.9-82.4)	83.3 (83-83.5)	82.8 (82.1-83.4)
Lambeth	68.3 (65.9-70.7)	69.5 (67.1-72)	70.2 (67.9-72.4)	70.3 (68.2-72.6)	70.6 (68.6-72.7)	71.3 (69.8-72.8)	72.2 (71.7-72.7)	73.4 (73.2-73.6)	73.9 (73.7-74.2)	74.1 (73.9-74.3)	76 (75.7-76.3)	77.6 (77.4-77.8)	79.5 (79.3-79.7)	81.5 (80.9-82.1)
Lewisham	69.6 (67.3-72.1)	70.9 (68.7-73.3)	71.7 (69.5-74.1)	72.1 (69.9-74.4)	72.5 (70.5-74.5)	73.2 (71.8-74.6)	73.6 (73.2-74)	74.7 (74.5-74.9)	75.1 (74.9-75.3)	75.1 (74.9-75.3)	76.2 (76-76.5)	77.8 (77.6-78)	79.8 (79.6-80)	81.5 (80.9-82.1)
Merton	71.3 (69.1-73.8)	72.5 (70.4-74.7)	73.1 (71-75.4)	73.3 (71.3-75.4)	73.6 (71.9-75.6)	74.4 (72.9-75.8)	75.1 (74.5-75.6)	76.4 (76.1-76.6)	76.9 (76.7-77.2)	77.8 (77.5-78.1)	79.4 (79.2-79.7)	81 (80.8-81.3)	81.9 (81.7-82.2)	82.5 (81.8-83.2)
Newham	68.9 (66.4-71.3)	70.1 (67.8-72.7)	70.8 (68.4-73.2)	71.1 (68.9-73.3)	71.4 (69.5-73.4)	72.1 (70.6-73.6)	72.9 (72.5-73.4)	73.8 (73.5-74)	74.4 (74.2-74.7)	74.4 (74.2-74.7)	75.7 (75.4-76)	77.6 (77.3-77.8)	78.7 (78.5-78.9)	81.6 (81-82.2)
Redbridge	71.4 (68.9-73.9)	72.5 (70.3-74.7)	73.1 (71-75.4)	73.4 (71.3-75.4)	73.7 (71.9-75.7)	74.5 (73.1-75.9)	75.5 (75-75.9)	76.8 (76.5-77)	77.3 (77.1-77.5)	77.8 (77.6-78)	78.8 (78.5-79)	80.3 (80.1-80.5)	81.7 (81.5-81.9)	82.4 (81.8-83.1)
Richmond upon Thames	71.8 (69.6-74.5)	72.8 (70.7-75.1)	73.2 (71.1-75.5)	73.3 (71.3-75.4)	73.4 (71.6-75.3)	74.1 (72.8-75.6)	75 (74.6-75.5)	76.4 (76.1-76.6)	77.9 (77.6-78.1)	79.3 (79.1-79.6)	80.6 (80.3-80.9)	82.4 (82.1-82.6)	83.7 (83.4-83.9)	83.8 (83.2-84.4)
Southwark	70.1 (67.7-72.6)	71.3 (69-73.7)	71.9 (69.7-74.3)	72.1 (69.9-74.4)	72.4 (70.4-74.5)	72.9 (71.5-74.5)	73.2 (72.7-73.6)	73.5 (73.2-73.7)	73.3 (73.1-73.5)	74.4 (74.2-74.6)	76.4 (76.1-76.7)	77.8 (77.6-78)	79.6 (79.4-79.8)	80.9 (80.3-81.5)
Sutton	71.8 (69.4-74.2)	73 (70.9-75.4)	73.8 (71.6-76.1)	74.2 (72.2-76.4)	74.7 (72.9-76.7)	75.5 (74.1-77)	76.2 (75.6-76.7)	76.5 (76.3-76.8)	77.5 (77.2-77.7)	77.8 (77.5-78)	79.4 (79.1-79.7)	81.2 (80.9-81.4)	82.3 (82-82.5)	82 (81.3-82.6)
Tower Hamlets	68.4 (66-70.7)	69.5 (67.2-71.8)	69.9 (67.8-72.2)	70 (67.8-72.3)	70 (68.1-72.1)	70.5 (69-72)	71.4 (71-71.9)	72.4 (72.2-72.7)	73.2 (73-73.5)	73.8 (73.6-74)	75.5 (75.2-75.8)	77 (76.8-77.2)	79 (78.8-79.3)	81.5 (80.9-82.1)
Waltham Forest	69.6 (67.3-72.1)	70.9 (68.5-73.3)	71.5 (69.3-73.9)	71.9 (69.7-74.2)	72.3 (70.4-74.3)	73.1 (71.8-74.6)	74.5 (74-74.9)	75.3 (75.1-75.6)	76.1 (75.9-76.3)	76.2 (76-76.4)	77.1 (76.8-77.4)	78.5 (78.3-78.7)	80.1 (79.9-80.4)	81.7 (81.1-82.3)
Wandsworth	70 (67.7-72.5)	71.1 (68.9-73.5)	71.6 (69.4-73.9)	71.7 (69.6-74)	71.9 (69.9-73.9)	72.4 (71-73.9)	73.2 (72.8-73.7)	74 (73.8-74.2)	74.5 (74.3-74.7)	75.5 (75.3-75.7)	77.4 (77.2-77.7)	79.3 (79.1-79.5)	81.1 (80.9-81.3)	82.2 (81.6-82.8)
Westminster	70.7 (68.4-73.1)	71.7 (69.4-74.1)	72.1 (69.9-74.5)	72.1 (69.8-74.3)	72.1 (70-73.9)	72.2 (70.6-73.8)	72.9 (72.5-73.4)	74.3 (74-74.5)	74.8 (74.6-75.1)	76 (75.7-76.2)	78.9 (78.6-79.2)	80.4 (80.2-80.6)	82.3 (82.1-82.5)	83.4 (82.7-84)
North East England	67 (66.2-67.8)	68.4 (67.6-69.2)	69.3 (68.6-70.1)	70 (69.3-70.7)	70.7 (70-71.4)	71.7 (71.2-72.2)	72.7 (72.6-72.9)	73.6 (73.5-73.6)	74.5 (74.4-74.5)	75.6 (75.6-75.7)	76.8 (76.7-76.9)	78.1 (78-78.1)	79.4 (79.3-79.5)	79.9 (79.6-80.1)
County Durham	66.9 (64.7-69.2)	68.3 (66.1-70.7)	69.3 (67.1-71.6)	70 (67.8-72.4)	70.7 (68.8-72.8)	71.7 (70.3-73.3)	72.8 (72.4-73.2)	73.6 (73.4-73.8)	74.4 (74.3-74.6)	75.5 (75.3-75.7)	76.8 (76.6-77.1)	78.3 (78.1-78.5)	79.5 (79.3-79.7)	79.8 (79.2-80.4)
Darlington	67 (64.6-69.5)	68.5 (66.2-70.8)	69.3 (67.1-71.5)	69.9 (67.6-72.2)	70.5 (68.3-72.6)	71.6 (70.1-73.2)	72.7 (72.2-73.3)	73.9 (73.6-74.2)	74.8 (74.5-75.2)	75.9 (75.6-76.3)	76.9 (76.5-77.3)	77.9 (77.6-78.2)	79.5 (79.2-79.8)	80.3 (79.5-81.1)
Gateshead	66.3 (63.9-68.8)	67.7 (65.3-70)	68.6 (66.2-71)	69.2 (66.8-71.6)	69.7 (67.7-71.8)	70.8 (69.3-72.5)	72 (71.5-72.5)	73.2 (73-73.5)	74.4 (74.1-74.7)	75.5 (75.2-75.7)	76.4 (76.1-76.7)	77.9 (77.6-78.1)	79.1 (78.9-79.3)	79.5 (78.8-80.2)
Hartlepool	65.6 (63.2-68.2)	67.1 (64.8-69.5)	68.1 (65.9-70.6)	68.8 (66.6-71.2)	69.4 (67.4-71.8)	70.6 (68.9-72.2)	71.7 (71.1-72.3)	72.8 (72.5-73.1)	74 (73.7-74.3)	75.2 (74.9-75.6)	76.3 (75.9-76.7)	77.2 (76.9-77.5)	78.8 (78.5-79.1)	79.6 (78.8-80.4)
Middlesbrough	65.8 (63.4-68.3)	67.3 (64.8-69.8)	68.3 (65.9-70.8)	68.9 (66.5-71.4)	69.5 (67.3-71.7)	70.5 (68.9-72.1)	71.3 (70.8-71.8)	72.3 (72-72.5)	73.2 (73-73.5)	74.5 (74.3-74.8)	75.5 (75.2-75.8)	76.7 (76.4-76.9)	78.1 (77.8-78.4)	78.9 (78.1-79.6)
Newcastle upon Tyne	67.3 (64.9-69.7)	68.6 (66.1-70.9)	69.5 (67.2-71.8)	70.1 (67.9-72.5)	70.8 (68.8-73)	71.8 (70.2-73.4)	72.9 (72.4-73.3)	73.6 (73.4-73.8)	74.1 (73.9-74.3)	75.1 (74.9-75.3)	76.4 (76.1-76.7)	77.5 (77.3-77.7)	79 (78.8-79.2)	79.6 (79-80.3)
North Tyneside	67.8 (65.4-70.3)	69.2 (67.1-71.6)	70.1 (67.9-72.5)	70.8 (68.6-73.1)	71.4 (69.4-73.5)	72.4 (70.8-73.9)	73.2 (72.7-73.7)	73.9 (73.7-74.1)	74.8 (74.6-75.1)	76 (75.8-76.3)	77 (76.7-77.3)	78.2 (77.9-78.4)	79.6 (79.4-79.9)	80.2 (79.5-80.9)
Northumberland	67.8 (65.5-70.2)	69.2 (66.9-71.4)	70.2 (68-72.5)	70.9 (68.7-73.2)	71.5 (69.5-73.6)	72.6 (71.2-74.1)	73.3 (72.9-73.8)	74.1 (73.9-74.3)	75.2 (75-75.4)	76.7 (76.5-76.9)	77.9 (77.6-78.2)	79.5 (79.3-79.7)	80.7 (80.5-81)	80.7 (80-81.4)
Redcar and Cleveland	66.5 (64.1-69.2)	68 (65.6-70.4)	68.9 (66.6-71.5)	69.6 (67.3-72)	70.2 (68.1-72.4)	71.4 (69.8-73)	72.6 (72.1-73.1)	74 (73.7-74.2)	74.9 (74.6-75.2)	76.2 (75.9-76.5)	77.4 (77-77.7)	78.3 (78-78.5)	79.6 (79.3-79.9)	79.7 (79-80.4)
South Tyneside	67.1 (64.8-69.8)	68.6 (66.3-71.1)	69.6 (67.3-72)	70.3 (68.2-72.5)	70.9 (68.9-72.9)	71.9 (70.4-73.5)	72.6 (72.1-73.1)	73.2 (73-73.5)	74.3 (74.1-74.6)	75.6 (75.4-75.9)	76.8 (76.4-77.1)	77.9 (77.7-78.2)	79 (78.7-79.3)	79.1 (78.4-79.8)
Stockton-on-Tees	67.4 (65-70)	68.9 (66.5-71.2)	69.8 (67.5-72.3)	70.5 (68.2-72.9)	71.1 (69.1-73.3)	72.2 (70.7-73.8)	73.2 (72.7-73.7)	73.9 (73.6-74.1)	74.8 (74.5-75)	76 (75.8-76.3)	77.5 (77.2-77.8)	78.4 (78.1-78.6)	79.9 (79.7-80.2)	80.5 (79.8-81.2)
Sunderland	67 (64.6-69.4)	68.4 (66.2-70.7)	69.4 (67.2-71.7)	70.2 (68-72.6)	70.9 (68.9-73.1)	72 (70.5-73.5)	72.9 (72.4-73.3)	73.6 (73.4-73.8)	74.1 (73.9-74.3)	75.2 (75-75.4)	76.3 (76-76.6)	77.3 (77.1-77.5)	78.8 (78.6-79)	79.3 (78.6-80)
North West England	68.7 (68.1-69.3)	70 (69.4-70.6)	70.7 (70-71.3)	71 (70.5-71.6)	71.5 (70.9-72)	72.3 (71.9-72.7)	73 (72.9-73.1)	73.8 (73.7-73.8)	74.8 (74.8-74.9)	75.7 (75.7-75.8)	76.9 (76.8-76.9)	78.1 (78.1-78.2)	79.3 (79.3-79.4)	79.9 (79.8-80.1)
Blackburn with Darwen	68.2 (65.9-70.6)	69.5 (67.3-71.9)	70.1 (67.8-72.5)	70.4 (68.2-72.7)	70.8 (68.8-72.9)	71.7 (70.3-73.2)	72.3 (71.8-72.8)	72.9 (72.6-73.2)	73.7 (73.5-74)	74.4 (74.2-74.7)	75.2 (74.9-75.5)	76.7 (76.5-77)	78.2 (78-78.5)	79.4 (78.7-80.1)
Blackpool	68.2 (65.7-70.7)	69.5 (67.2-71.9)	70.2 (68-72.5)	70.5 (68.4-72.8)	71 (69-73.1)	72 (70.5-73.5)	72.8 (72.3-73.3)	73.9 (73.6-74.1)	74.4 (74.1-74.7)	75.2 (74.9-75.4)	74.7 (74.4-75)	75 (74.7-75.2)	76.1 (75.9-76.4)	77.5 (76.7-78.2)
Bolton	68.3 (65.8-70.6)	69.5 (67.2-71.9)	70.2 (68.1-72.7)	70.7 (68.6-73)	71.1 (69.3-73)	71.9 (70.4-73.4)	72.5 (72-72.9)	73.3 (73.1-73.5)	74.6 (74.4-74.8)	75.6 (75.4-75.8)	76.3 (76-76.6)	77.4 (77.2-77.6)	78.9 (78.6-79.1)	80.1 (79.5-80.7)
Bury	69.1 (66.8-71.5)	70.4 (68.2-72.7)	71.1 (69-73.3)	71.5 (69.4-73.7)	71.9 (70.1-73.9)	72.8 (71.4-74.3)	73.4 (73-73.9)	74.3 (74.1-74.6)	75.3 (75.1-75.6)	76.2 (75.9-76.4)	77.7 (77.4-78)	78.6 (78.4-78.9)	79.6 (79.3-79.8)	79.5 (78.8-80.2)
Cheshire East	70.4 (68.2-72.7)	71.6 (69.3-73.9)	72.3 (70-74.5)	72.7 (70.5-75)	73.1 (71.2-75)	73.9 (72.5-75.3)	74.4 (74-74.8)	75.2 (75-75.4)	76.5 (76.3-76.7)	77.6 (77.4-77.8)	78.6 (78.4-78.9)	80.6 (80.4-80.8)	81.5 (81.3-81.7)	81.4 (80.8-82.1)
Cheshire West and Chester	70.1 (67.7-72.5)	71.2 (68.8-73.4)	71.8 (69.7-74)	72.1 (70.1-74.3)	72.4 (70.6-74.2)	73.2 (71.8-74.5)	73.9 (73.5-74.4)	74.8 (74.6-75)	75.9 (75.7-76.1)	77 (76.8-77.2)	78.5 (78.2-78.7)	80 (79.8-80.2)	81 (80.8-81.2)	81 (80.4-81.6)
Cumbria	69.2 (67-71.5)	70.5 (68.2-72.7)	71.1 (69.1-73.3)	71.6 (69.6-73.7)	72 (70.2-73.9)	72.8 (71.4-74.3)	73.5 (73.1-73.9)	74.4 (74.2-74.5)	75.4 (75.3-75.6)	76.4 (76.3-76.6)	78 (77.7-78.3)	79.4 (79.2-79.5)	80.3 (80.1-80.5)	80.8 (80.1-81.4)
Halton	68.2 (65.8-70.7)	69.5 (67.1-71.9)	70 (67.9-72.3)	70.2 (67.9-72.4)	70.5 (68.5-72.5)	71.3 (69.8-72.8)	72.1 (71.6-72.6)	73.3 (73-73.5)	74.6 (74.3-74.9)	75.3 (75-75.5)	75.9 (75.5-76.2)	76.7 (76.4-77)	78.5 (78.2-78.7)	79.6 (79-80.4)
Knowsley	68.1 (65.8-70.5)	69.4 (67.1-71.7)	70 (67.7-72.4)	70.2 (68.1-72.6)	70.5 (68.5-72.5)	71.3 (69.9-72.8)	72 (71.5-72.5)	73.2 (72.9-73.4)	74.2 (74-74.5)	74.9 (74.7-75.2)	75.5 (75.2-75.8)	76.9 (76.7-77.2)	78 (77.8-78.3)	78.5 (77.8-79.1)
Lancashire	69.2 (67-71.4)	70.5 (68.3-72.7)	71.2 (69.1-73.5)	71.8 (69.6-74.2)	72.2 (70.1-74.5)	73.1 (71.5-74.6)	73.3 (72.9-73.7)	74 (73.9-74.2)	75.1 (74.9-75.2)	76.4 (76.3-76.6)	77.4 (77.2-77.7)	78.6 (78.5-78.8)	79.9 (79.8-80.1)	80.3 (79.6-80.9)
Liverpool	67.9 (65.6-70.3)	69.1 (67-71.4)	69.6 (67.4-71.8)	69.8 (67.7-72.1)	70.1 (68.2-72.1)	70.9 (69.4-72.5)	72.1 (71.7-72.5)	72.9 (72.7-73)	73.6 (73.4-73.8)	74.1 (73.9-74.3)	75 (74.7-75.3)	76.3 (76.1-76.5)	77.6 (77.4-77.8)	78.9 (78.3-79.5)
Manchester	67.5 (65.2-70)	68.9 (66.7-71.1)	69.6 (67.4-71.9)	69.9 (67.7-72.1)	70.3 (68.3-72.2)	71.1 (69.6-72.6)	71.6 (71.2-72)	72.1 (71.9-72.3)	72.7 (72.5-72.9)	73.2 (73-73.3)	74.3 (74.1-74.6)	75.4 (75.2-75.6)	77.1 (77-77.3)	78.3 (77.7-78.9)
Oldham	67.9 (65.6-70.3)	69.2 (67-71.6)	69.9 (67.7-72.2)	70.3 (68.2-72.5)	70.8 (68.9-72.8)	71.7 (70.3-73.1)	72.5 (72.1-73)	73.1 (72.9-73.4)	74.2 (74-74.5)	74.8 (74.5-75)	75.9 (75.6-76.2)	77.1 (76.9-77.3)	78.4 (78.2-78.6)	79 (78.4-79.6)
Rochdale	68.1 (65.7-70.5)	69.4 (67.1-71.9)	70.2 (67.9-72.4)	70.6 (68.4-72.7)	71.1 (69.2-73)	72 (70.6-73.5)	72.8 (72.4-73.3)	73.4 (73.2-73.7)	74.1 (73.9-74.3)	75 (74.8-75.2)	76.1 (75.8-76.4)	77.1 (76.9-77.3)	78.3 (78.1-78.5)	79.2 (78.6-79.8)
Salford	67.6 (65.3-70.1)	68.9 (66.6-71.2)	69.5 (67.2-71.8)	69.7 (67.6-72.1)	70.1 (68.1-72.2)	71 (69.5-72.4)	72 (71.6-72.5)	72.6 (72.4-72.9)	73.5 (73.3-73.7)	74.1 (73.8-74.3)	75.6 (75.3-75.9)	76.7 (76.5-76.9)	77.9 (77.7-78.1)	78.9 (78.2-79.5)
Sefton	69.6 (67.3-71.9)	70.8 (68.6-73.1)	71.4 (69.4-73.7)	71.8 (69.6-74)	72.2 (70.2-74.1)	73 (71.6-74.4)	73.6 (73.2-74)	74.5 (74.3-74.7)	75.8 (75.6-76)	76.4 (76.2-76.6)	77.4 (77.1-77.7)	79.1 (78.9-79.4)	79.5 (79.3-79.8)	79.9 (79.2-80.5)
St Helens	68.2 (65.9-70.5)	69.4 (67.2-71.6)	70 (68-72.2)	70.3 (68.1-72.6)	70.7 (68.7-72.7)	71.4 (70-72.9)	71.9 (71.5-72.4)	73.1 (72.8-73.3)	74.5 (74.2-74.7)	75.6 (75.4-75.9)	76.8 (76.5-77.1)	77.9 (77.6-78.1)	78.7 (78.4-78.9)	78.7 (78.1-79.4)
Stockport	70.3 (68-72.5)	71.5 (69.4-73.8)	72.1 (70-74.4)	72.6 (70.4-74.8)	73 (71.1-75)	73.7 (72.4-75.1)	74.2 (73.7-74.6)	74.9 (74.7-75.1)	76 (75.8-76.2)	77 (76.8-77.2)	78.5 (78.2-78.8)	79.5 (79.2-79.7)	80.2 (80-80.4)	81.1 (80.4-81.8)
Tameside	68.3 (66-70.6)	69.6 (67.3-71.7)	70.2 (67.9-72.5)	70.6 (68.3-72.9)	71 (68.9-72.9)	71.8 (70.4-73.3)	72.5 (72.1-73)	73.5 (73.3-73.8)	74.7 (74.5-74.9)	75.2 (75-75.4)	76.2 (75.9-76.5)	77.4 (77.1-77.6)	78.4 (78.1-78.6)	79.4 (78.7-80)
Trafford	70 (67.6-72.4)	71.2 (69-73.6)	71.8 (69.7-74.1)	72.1 (70.1-74.2)	72.5 (70.5-74.3)	73.3 (71.9-74.7)	74 (73.5-74.4)	74.7 (74.5-75)	75.8 (75.6-76.1)	76.7 (76.4-76.9)	78.1 (77.8-78.4)	79.8 (79.6-80)	81.3 (81.1-81.5)	81.5 (80.8-82.1)
Warrington	69.5 (67.2-72)	70.6 (68.4-73)	71.2 (68.9-73.5)	71.4 (69.2-73.6)	71.7 (69.7-73.7)	72.5 (71.1-73.9)	73.2 (72.7-73.6)	73.8 (73.5-74)	74.7 (74.5-74.9)	76 (75.8-76.2)	77.7 (77.4-78)	79 (78.8-79.3)	80 (79.8-80.2)	80.1 (79.5-80.8)
Wigan	68.4 (66.2-70.7)	69.6 (67.4-72)	70.3 (68.2-72.7)	70.8 (68.7-73.2)	71.2 (69.1-73.2)	71.9 (70.5-73.3)	72.4 (72-72.8)	73.3 (73.1-73.4)	74.6 (74.4-74.8)	75.7 (75.5-75.9)	76.7 (76.4-76.9)	77.7 (77.5-77.9)	78.8 (78.6-79)	79.2 (78.6-79.8)
Wirral	69 (66.8-71.5)	70.3 (68.1-72.6)	71 (68.8-73.3)	71.4 (69.2-73.5)	71.9 (70-73.7)	72.8 (71.3-74.2)	73.7 (73.3-74.1)	74.5 (74.3-74.7)	75.2 (75-75.4)	76 (75.8-76.2)	76.9 (76.7-77.2)	78.1 (77.9-78.3)	79.3 (79.1-79.5)	79.4 (78.8-80)
South East England	71.5 (70.8-72.3)	72.6 (71.9-73.4)	73.3 (72.6-74)	73.5 (72.8-74.2)	73.6 (72.9-74.3)	74 (73.5-74.5)	75 (74.9-75.2)	76 (76-76.1)	77.2 (77.1-77.2)	78 (78-78.1)	79.3 (79.2-79.3)	80.6 (80.6-80.7)	81.7 (81.6-81.7)	82.1 (81.9-82.2)
Bracknell Forest	72.1 (69.5-74.7)	73.2 (70.8-75.8)	73.8 (71.5-76.3)	73.9 (71.7-76.4)	74.1 (72.1-76.2)	74.5 (73.1-76.1)	75.4 (74.9-76)	76.3 (76-76.6)	77.6 (77.3-77.9)	78.7 (78.4-79)	80 (79.6-80.3)	81.4 (81.1-81.7)	82.4 (82.1-82.7)	83.2 (82.5-83.9)
Brighton and Hove	70.8 (68.3-73.2)	71.9 (69.8-74.2)	72.5 (70.2-74.8)	72.6 (70.5-74.9)	72.8 (70.7-74.7)	73.3 (71.9-74.7)	74.2 (73.8-74.7)	75.1 (74.8-75.3)	75.5 (75.3-75.7)	75.7 (75.5-75.9)	77.1 (76.9-77.5)	78.2 (78-78.4)	79.7 (79.5-80)	81.4 (80.8-82)
Buckinghamshire	72.4 (70.1-74.9)	73.4 (71.3-75.7)	73.9 (71.9-76.3)	74.2 (72.1-76.5)	74.3 (72.4-76.4)	74.7 (73.3-76.1)	75.4 (75-75.8)	76.4 (76.3-76.6)	77.4 (77.2-77.6)	78.4 (78.2-78.6)	79.9 (79.7-80.2)	81.4 (81.2-81.6)	82.4 (82.2-82.6)	83.2 (82.7-83.8)
East Sussex	71.5 (69.2-74)	72.6 (70.4-74.9)	73.2 (71-75.6)	73.5 (71.3-75.8)	73.7 (71.8-75.7)	74.3 (72.9-75.8)	75.4 (75-75.8)	76.5 (76.3-76.7)	77.7 (77.5-77.9)	78.3 (78.1-78.5)	79.3 (79.1-79.6)	80.6 (80.5-80.8)	81.4 (81.2-81.6)	81.4 (80.8-82)
Hampshire	71.8 (69.5-74.1)	72.8 (70.6-75.1)	73.3 (71.3-75.6)	73.5 (71.1-75.8)	73.6 (71.5-75.7)	74.1 (72.6-75.6)	75.4 (75-75.7)	76.4 (76.2-76.5)	77.5 (77.4-77.7)	78.3 (78.1-78.4)	79.7 (79.5-79.9)	81.3 (81.1-81.4)	82.2 (82-82.3)	82.6 (82.1-83.1)
Isle of Wight	70.5 (67.9-73.1)	71.8 (69.5-74.5)	72.6 (70.3-75.2)	73.1 (71-75.5)	73.4 (71.4-75.5)	74 (72.4-75.5)	75 (74.4-75.6)	75.5 (75.2-75.8)	76.6 (76.3-76.9)	77.8 (77.5-78)	79.2 (78.9-79.5)	80.1 (79.9-80.4)	81 (80.7-81.3)	81.8 (81.1-82.5)
Kent	71 (68.8-73.3)	72.1 (69.9-74.3)	72.7 (70.6-74.9)	73 (70.8-75.3)	72.9 (70.9-75)	73.1 (71.6-74.7)	74.3 (74-74.7)	75.4 (75.3-75.6)	76.7 (76.5-76.8)	77.7 (77.5-77.8)	78.9 (78.7-79.2)	80.2 (80.1-80.4)	81.4 (81.3-81.6)	81.3 (80.8-81.9)
Medway	70.6 (68.1-73.2)	71.8 (69.5-74.1)	72.6 (70.4-75.1)	73 (70.8-75.4)	73.3 (71.3-75.3)	73.8 (72.3-75.3)	74.5 (74-75)	75.1 (74.9-75.3)	76.3 (76-76.5)	77.1 (76.9-77.3)	77.7 (77.4-78)	78.8 (78.6-79)	79.8 (79.5-80)	81.1 (80.5-81.7)
Milton Keynes	71 (68.6-73.6)	72.1 (69.7-74.4)	72.7 (70.3-75.2)	72.9 (70.6-75.3)	73 (70.9-75.2)	73.5 (72-75.1)	74.3 (73.8-74.8)	75.3 (75-75.5)	76.6 (76.3-76.9)	77.4 (77.2-77.7)	78.9 (78.6-79.2)	79.8 (79.6-80)	80.6 (80.3-80.8)	81.9 (81.3-82.5)
Oxfordshire	72.3 (69.8-74.8)	73.3 (71.1-75.7)	73.8 (71.5-76.2)	74 (71.8-76.3)	74.1 (72.1-76.2)	74.5 (73-75.9)	75.3 (74.9-75.7)	76.5 (76.4-76.7)	77.6 (77.5-77.8)	78.6 (78.4-78.8)	79.6 (79.4-79.9)	80.9 (80.7-81.1)	82 (81.8-82.1)	82.8 (82.2-83.3)
Portsmouth	70 (67.7-72.6)	71.2 (68.9-73.7)	71.9 (69.5-74.3)	72.1 (69.9-74.4)	72.3 (70.2-74.3)	72.7 (71.3-74.2)	73.3 (72.8-73.7)	74.1 (73.9-74.4)	75.4 (75.2-75.7)	75.8 (75.5-76)	76.7 (76.4-77)	78.2 (78-78.5)	79.5 (79.3-79.8)	80.6 (80-81.3)
Reading	71.3 (68.7-73.8)	72.3 (70.1-74.7)	72.9 (70.6-75.2)	73.1 (70.6-75.4)	73.2 (71.1-75.4)	73.7 (72.1-75.3)	74.6 (74.1-75.2)	75.4 (75.1-75.6)	76.4 (76.1-76.7)	77 (76.8-77.3)	78 (77.7-78.3)	79.1 (78.9-79.4)	80.1 (79.8-80.3)	80.8 (80.1-81.4)
Slough	70.6 (68.2-73.2)	71.7 (69.3-74.1)	72.3 (69.9-74.7)	72.4 (70.2-75)	72.5 (70.4-74.6)	72.9 (71.5-74.4)	73.6 (73.1-74.2)	74 (73.7-74.3)	75.2 (74.9-75.5)	76.2 (76-76.5)	77.2 (76.9-77.5)	78.9 (78.6-79.2)	80 (79.7-80.3)	81.7 (81.1-82.4)
Southampton	70.1 (67.5-72.7)	71.2 (68.9-73.7)	71.9 (69.7-74.2)	72.1 (70-74.4)	72.3 (70.4-74.4)	72.9 (71.4-74.3)	74.1 (73.6-74.6)	74.9 (74.7-75.1)	75.3 (75-75.5)	76.1 (75.9-76.3)	77.3 (77.1-77.7)	78.6 (78.4-78.8)	79.8 (79.6-80)	80.6 (80-81.3)
Surrey	72.4 (70.1-74.7)	73.5 (71.4-75.9)	74.1 (72.1-76.4)	74.4 (72.3-76.7)	74.4 (72.4-76.5)	74.7 (73.3-76.2)	75.4 (75.1-75.8)	76.5 (76.3-76.6)	77.7 (77.6-77.9)	78.7 (78.6-78.9)	79.9 (79.6-80.1)	81.4 (81.3-81.6)	82.7 (82.5-82.8)	82.5 (81.9-83)
West Berkshire	72.7 (70.6-75.2)	73.8 (71.5-76.2)	74.4 (72-76.9)	74.6 (72.2-76.9)	74.7 (72.6-76.7)	75.1 (73.6-76.7)	76 (75.5-76.6)	75.9 (75.6-76.2)	77.8 (77.5-78.1)	78.6 (78.3-78.8)	79.9 (79.6-80.2)	80.5 (80.3-80.8)	82 (81.7-82.3)	82.3 (81.6-82.9)
West Sussex	71.7 (69.4-74.2)	72.8 (70.5-75.1)	73.4 (71.2-75.7)	73.6 (71.5-76)	73.7 (71.8-75.8)	74.2 (72.7-75.7)	75.4 (75-75.8)	76.5 (76.3-76.6)	77.6 (77.5-77.8)	78.6 (78.4-78.8)	79.6 (79.4-79.9)	80.8 (80.6-81)	81.8 (81.6-81.9)	81.8 (81.2-82.3)
Windsor and Maidenhead	73.5 (71.1-75.9)	74.6 (72.3-76.9)	75.2 (72.9-77.4)	75.4 (73.2-77.5)	75.5 (73.4-77.4)	75.9 (74.5-77.4)	76.4 (75.9-77)	76.9 (76.6-77.2)	77.7 (77.4-78)	78.5 (78.2-78.7)	79.8 (79.4-80.1)	82 (81.8-82.3)	82.7 (82.5-83)	82.9 (82.2-83.5)
Wokingham	73.7 (71.3-76.1)	74.7 (72.5-77.1)	75.4 (73.1-77.7)	75.6 (73.4-78)	75.8 (73.7-77.8)	76.3 (74.8-77.8)	77.1 (76.6-77.7)	77.9 (77.6-78.2)	79.2 (78.9-79.5)	80.2 (79.9-80.5)	81.8 (81.5-82.1)	82.9 (82.7-83.2)	83.7 (83.4-83.9)	83.6 (83-84.3)
South West England	70.2 (69.5-71)	71.6 (70.8-72.3)	72.4 (71.8-73.1)	72.9 (72.2-73.5)	73.3 (72.7-73.9)	74 (73.6-74.5)	75 (74.9-75.1)	76.1 (76-76.2)	77.1 (77-77.2)	78.2 (78.1-78.2)	79.4 (79.3-79.5)	80.6 (80.6-80.7)	81.6 (81.5-81.6)	81.7 (81.5-81.8)
Bath and North East Somerset	71.4 (69-74)	72.7 (70.5-75.1)	73.6 (71.4-76)	74.2 (72-76.3)	74.7 (72.8-76.7)	75.6 (74.2-77.1)	76.6 (76.1-77.1)	77.4 (77.1-77.7)	78 (77.7-78.3)	78.8 (78.5-79)	80.2 (79.9-80.5)	81.7 (81.4-81.9)	82.5 (82.2-82.7)	82.1 (81.4-82.7)

Table 6 Estimates of life expectancy at birth by location and year for 1950, 1955...2005, 2010, 2017 - both sexes														
Location	1950	1955	1960	1965	1970	1975	1980	1985	1990	1995	2000	2005	2010	2017
Bournemouth	70.5 (68.1-73.1)	71.9 (69.5-74.3)	72.7 (70.6-75)	73.1 (71.1-75.4)	73.5 (71.6-75.6)	74.3 (72.9-75.8)	75.4 (74.9-75.9)	76.1 (75.8-76.4)	76.3 (76-76.6)	76.7 (76.5-77)	78.1 (77.8-78.4)	79.6 (79.3-79.8)	80.2 (79.9-80.4)	80.6 (79.9-81.3)
Bristol, City of	69.5 (67.1-71.8)	70.7 (68.5-73.2)	71.5 (69.4-73.8)	71.9 (69.8-74.1)	72.2 (70.2-74.1)	72.9 (71.4-74.3)	73.8 (73.3-74.2)	75.1 (74.9-75.3)	76.1 (75.9-76.3)	77 (76.8-77.2)	78 (77.7-78.3)	79.1 (78.9-79.3)	80.1 (79.9-80.3)	81.4 (80.7-82)
Cornwall	69.7 (67.3-72.3)	71.1 (68.8-73.3)	72 (69.8-74.3)	72.4 (70.3-74.6)	72.8 (70.7-75)	73.5 (72-75)	74.6 (74.2-75)	76 (75.8-76.2)	77 (76.8-77.2)	78 (77.8-78.2)	79.1 (78.8-79.4)	80.3 (80.1-80.5)	81.3 (81.1-81.5)	81 (80.5-81.6)
Devon	70.2 (67.9-72.8)	71.5 (69.3-74)	72.4 (70.2-74.8)	72.8 (70.8-75)	73.2 (71.2-75.2)	74 (72.5-75.5)	75.2 (74.8-75.6)	76.4 (76.2-76.6)	77.6 (77.4-77.8)	78.7 (78.5-78.8)	79.8 (79.5-80.1)	81.1 (80.9-81.3)	81.9 (81.7-82.1)	81.4 (80.8-82)
Dorset	70.7 (68.3-73.2)	72.1 (69.8-74.5)	73 (70.8-75.5)	73.5 (71.5-75.8)	74 (72.2-76)	74.8 (73.4-76.1)	75.7 (75.3-76.1)	76.8 (76.5-77)	78 (77.8-78.2)	79.2 (79-79.4)	80.4 (80.1-80.7)	81.9 (81.7-82.1)	82.7 (82.5-82.9)	82.3 (81.7-82.9)
Gloucestershire	70.7 (68.4-73.4)	72 (69.8-74.6)	72.9 (70.6-75.3)	73.4 (71.3-75.6)	73.8 (71.7-75.7)	74.4 (73-75.9)	75 (74.6-75.4)	75.9 (75.7-76.1)	77 (76.9-77.2)	78.1 (77.9-78.3)	79.5 (79.3-79.8)	80.6 (80.5-80.8)	81.6 (81.4-81.8)	81.6 (81-82.1)
North Somerset	71 (68.6-73.4)	72.4 (70-74.7)	73.3 (71.1-75.6)	73.9 (71.8-76.1)	74.4 (72.5-76.4)	75.3 (73.9-76.8)	76.2 (75.6-76.7)	76.9 (76.7-77.2)	77.7 (77.4-78)	78.7 (78.4-78.9)	79.8 (79.5-80.1)	80.6 (80.4-80.8)	81 (80.7-81.3)	80.9 (80.3-81.6)
Plymouth	69 (66.7-71.7)	70.3 (68.2-72.9)	71.1 (68.9-73.5)	71.5 (69.4-73.8)	71.9 (69.7-73.9)	72.7 (71.1-74.2)	73.7 (73.2-74.1)	74.9 (74.7-75.1)	75.8 (75.6-76)	76.6 (76.4-76.8)	77.9 (77.6-78.2)	78.7 (78.5-78.9)	79.7 (79.5-80)	80.9 (80.3-81.5)
Poole	70.6 (68.2-73.2)	71.9 (69.6-74.2)	72.7 (70.5-75.1)	73.2 (71.1-75.5)	73.6 (71.5-75.8)	74.4 (72.8-76)	75.6 (75-76.1)	76.2 (76-76.5)	77.4 (77.1-77.7)	78.5 (78.3-78.8)	80.2 (79.8-80.5)	81.1 (80.8-81.4)	81.8 (81.6-82.1)	81.8 (81.1-82.4)
Somerset	70.6 (68.3-73)	71.8 (69.7-74.2)	72.6 (70.6-74.9)	73 (70.9-75.4)	73.3 (71.3-75.4)	74.1 (72.6-75.5)	75 (74.6-75.4)	76.3 (76.1-76.5)	77.2 (77-77.4)	78.3 (78.1-78.5)	79.5 (79.2-79.8)	80.9 (80.7-81)	81.8 (81.7-82)	81.8 (81.2-82.4)
South Gloucestershire	71.1 (68.7-73.6)	72.3 (70-74.7)	73 (70.8-75.3)	73.4 (71.2-75.7)	73.7 (71.7-75.8)	74.5 (73-76)	75.7 (75.2-76.2)	76.6 (76.4-76.9)	77.4 (77.2-77.7)	78.7 (78.5-79)	80 (79.7-80.3)	81.6 (81.4-81.9)	82.7 (82.5-83)	82.7 (82.1-83.3)
Swindon	69.4 (67-71.8)	70.7 (68.4-73.1)	71.5 (69.2-74)	72 (69.8-74.2)	72.3 (70.3-74.2)	72.9 (71.5-74.4)	73.7 (73.2-74.2)	75 (74.8-75.3)	76.4 (76.2-76.7)	77.1 (76.9-77.4)	78.5 (78.1-78.8)	79.9 (79.7-80.2)	81.1 (80.9-81.4)	81.2 (80.6-81.8)
Torbay	70.1 (67.6-72.6)	71.4 (69-74)	72.3 (70-74.7)	72.8 (70.6-75)	73.2 (71.3-75.2)	74 (72.6-75.6)	75 (74.4-75.6)	75.8 (75.5-76.2)	77 (76.7-77.3)	77.6 (77.3-77.8)	77.9 (77.6-78.3)	78.6 (78.3-78.9)	79.6 (79.3-79.9)	80.6 (79.9-81.4)
Wiltshire	70.6 (68.2-73)	71.9 (69.5-74.4)	72.8 (70.6-75.1)	73.3 (71.3-75.4)	73.7 (71.8-75.7)	74.4 (73-75.9)	75.2 (74.7-75.6)	76.1 (75.9-76.3)	76.9 (76.6-77)	78.4 (78.2-78.6)	79.8 (79.5-80)	81 (80.8-81.1)	81.9 (81.8-82.1)	82.6 (82-83.1)
West Midlands	69 (68.2-69.8)	70.3 (69.5-71)	71 (70.2-71.8)	71.4 (70.7-72.1)	71.7 (71-72.4)	72.5 (72-73)	73.5 (73.4-73.6)	74.5 (74.4-74.6)	75.4 (75.3-75.5)	76.5 (76.5-76.6)	77.7 (77.6-77.8)	78.9 (78.9-79)	80.3 (80.2-80.4)	80.7 (80.5-80.9)
Birmingham	68.4 (66.4-70.6)	69.7 (67.6-71.9)	70.5 (68.4-72.5)	70.9 (68.8-73)	71.3 (69.3-73.4)	72.2 (70.8-73.8)	73.1 (72.7-73.4)	73.7 (73.6-73.9)	74.5 (74.3-74.6)	75.6 (75.4-75.7)	76.6 (76.3-76.8)	78 (77.8-78.2)	79.6 (79.5-79.8)	80.3 (79.7-80.8)
Coventry	68.6 (66.3-71.2)	69.9 (67.7-72.3)	70.6 (68.4-72.9)	71 (68.9-73.2)	71.3 (69.4-73.4)	72.1 (70.7-73.6)	73.2 (72.8-73.6)	74.2 (74-74.4)	74.6 (74.4-74.9)	75.9 (75.7-76.1)	77.2 (76.9-77.5)	77.8 (77.6-78)	79.4 (79.2-79.7)	80.3 (79.7-81)
Dudley	69.7 (67.4-72.1)	71 (68.8-73.1)	71.8 (69.7-74.1)	72.4 (70.2-74.8)	72.8 (70.8-74.8)	73.5 (72-74.9)	74.2 (73.8-74.6)	74.7 (74.5-74.9)	75.6 (75.4-75.8)	76.8 (76.6-77)	77.8 (77.5-78.1)	79.1 (78.9-79.4)	80.5 (80.3-80.7)	80.7 (80.1-81.3)
Herefordshire, County of	69.8 (67.6-72.3)	71.1 (68.7-73.6)	71.8 (69.6-74.2)	72.1 (69.9-74.3)	72.4 (70.3-74.5)	73.3 (71.8-74.8)	74.6 (74-75.1)	75.9 (75.6-76.2)	77.2 (77-77.5)	78.3 (78-78.6)	79.5 (79.2-79.8)	80.5 (80.3-80.8)	81.3 (81-81.5)	81.4 (80.7-82)
Sandwell	68 (65.6-70.5)	69.3 (66.9-71.8)	70 (67.6-72.5)	70.5 (68.2-72.8)	70.8 (68.7-72.9)	71.7 (70.2-73.3)	72.8 (72.4-73.3)	73.7 (73.5-73.9)	74.4 (74.2-74.7)	75.2 (75-75.4)	76.1 (75.8-76.4)	77 (76.8-77.2)	78.5 (78.3-78.7)	79.6 (79-80.3)
Shropshire	69.7 (67.5-72.1)	70.9 (68.7-73.2)	71.6 (69.4-73.9)	71.9 (69.7-74.2)	72.2 (70.4-74.4)	73.1 (71.7-74.7)	74.4 (74-74.9)	75.5 (75.3-75.7)	76.7 (76.5-76.9)	77.5 (77.3-77.7)	79.1 (78.8-79.4)	80.3 (80.1-80.6)	81.5 (81.2-81.7)	81.7 (81-82.3)
Solihull	70.9 (68.4-73.6)	72.1 (69.6-74.7)	72.8 (70.4-75.2)	73.1 (70.9-75.3)	73.5 (71.5-75.5)	74.4 (73-75.9)	75.6 (75.1-76)	76.9 (76.6-77.1)	77.8 (77.6-78.1)	78.4 (78.1-78.6)	79.4 (79.1-79.8)	80.9 (80.6-81.1)	82.2 (82-82.5)	81.9 (81.2-82.7)
Staffordshire	69.4 (67.2-71.9)	70.6 (68.4-73)	71.3 (69-73.4)	71.6 (69.4-73.8)	71.8 (69.7-73.9)	72.4 (70.9-73.9)	73.5 (73.2-73.9)	74.8 (74.7-75)	75.6 (75.4-75.7)	76.9 (76.7-77)	78.1 (77.8-78.3)	79.5 (79.3-79.7)	80.9 (80.8-81.1)	80.7 (80.1-81.2)
Stoke-on-Trent	67.3 (65-69.9)	68.5 (66.3-71)	69.2 (67.2-71.7)	69.5 (67.3-71.8)	69.8 (67.8-71.7)	70.5 (69.1-72)	71.5 (71.1-72)	72.7 (72.4-72.9)	73.8 (73.6-74.1)	74.7 (74.4-74.9)	76 (75.7-76.3)	76.9 (76.7-77.2)	78.1 (77.9-78.3)	78.9 (78.3-79.6)
Telford and Wrekin	68.6 (66.2-71.2)	69.8 (67.3-72.4)	70.5 (68.1-72.9)	70.8 (68.6-73.1)	71.1 (69-73.2)	71.9 (70.4-73.4)	72.9 (72.4-73.4)	74.1 (73.8-74.4)	75.6 (75.3-75.8)	76.8 (76.6-77.1)	77.7 (77.4-78)	78.9 (78.6-79.1)	79.5 (79.2-79.7)	80.1 (79.3-80.7)
Walsall	68.2 (65.8-70.6)	69.4 (67-71.9)	70.2 (67.8-72.4)	70.6 (68.4-72.9)	70.9 (68.9-73)	71.7 (70.2-73.3)	72.7 (72.3-73.1)	73.6 (73.4-73.8)	74.6 (74.4-74.8)	75.8 (75.6-76)	77.1 (76.8-77.4)	78.4 (78.2-78.6)	79.6 (79.3-79.8)	79.8 (79.1-80.4)
Warwickshire	70.2 (68-72.5)	71.3 (69-73.7)	72 (69.7-74.3)	72.4 (70-74.5)	72.6 (70.7-74.5)	73.3 (71.9-74.7)	74.2 (73.8-74.6)	75.1 (74.9-75.2)	76.2 (76-76.4)	77.2 (77.1-77.4)	78.8 (78.5-79)	79.9 (79.7-80.1)	81.3 (81.1-81.5)	81.5 (80.9-82.1)
Wolverhampton	68.6 (66.1-71.2)	69.8 (67.5-72.2)	70.5 (68.2-73)	70.9 (68.8-73.1)	71.2 (69.2-73.4)	72.1 (70.6-73.7)	73.2 (72.8-73.7)	74 (73.7-74.2)	74.4 (74.2-74.6)	75.7 (75.5-76)	76.5 (76.2-76.8)	77 (76.8-77.3)	78.9 (78.6-79.1)	79.7 (79-80.3)
Worcestershire	70.4 (68-72.8)	71.6 (69.4-73.8)	72.3 (70.1-74.6)	72.7 (70.6-74.8)	73.1 (71.2-75)	73.8 (72.4-75.3)	74.5 (74.1-74.9)	75.7 (75.5-75.8)	76.6 (76.4-76.7)	77.7 (77.5-77.9)	78.6 (78.4-78.9)	80.2 (80.1-80.4)	81.1 (80.9-81.3)	81.4 (80.8-82)
Yorkshire and the Humber	68.9 (68.3-69.6)	70.1 (69.4-70.8)	70.9 (70.2-71.5)	71.3 (70.6-72)	71.7 (71.1-72.3)	72.5 (72.1-72.9)	73.4 (73.3-73.5)	74.3 (74.2-74.4)	75.4 (75.3-75.5)	76.5 (76.4-76.6)	77.6 (77.5-77.7)	78.9 (78.8-78.9)	79.9 (79.9-80)	80.6 (80.4-80.8)
Barnsley	68.2 (65.9-70.5)	69.4 (67.2-71.7)	70.2 (68-72.4)	70.7 (68.4-72.9)	71.1 (69.1-73.2)	72 (70.6-73.6)	73 (72.6-73.5)	73.8 (73.5-74)	74.7 (74.5-74.9)	75.5 (75.3-75.7)	76.8 (76.6-77.2)	78.1 (77.9-78.4)	79.2 (79-79.4)	80 (79.4-80.7)
Bradford	68.1 (65.7-70.6)	69.4 (67.1-71.9)	70.2 (68-72.8)	70.8 (68.7-72.9)	71.3 (69.4-73.2)	72.1 (70.7-73.6)	72.7 (72.3-73.2)	73.4 (73.2-73.6)	74.6 (74.5-74.8)	75.6 (75.4-75.8)	76.6 (76.3-76.9)	77.7 (77.5-77.9)	79.2 (79.1-79.4)	80.4 (79.8-81)
Calderdale	68.4 (66-70.8)	69.7 (67.4-72.1)	70.4 (68.2-72.8)	70.9 (68.7-73.1)	71.3 (69.3-73.3)	72 (70.6-73.6)	72.9 (72.4-73.4)	73.6 (73.3-73.8)	74.7 (74.4-74.9)	75.7 (75.5-76)	77.4 (77.1-77.8)	78.7 (78.5-79)	79.3 (79.1-79.6)	80.1 (79.5-80.8)
Doncaster	68.9 (66.6-71.3)	70.2 (67.8-72.6)	71.2 (69-73.5)	71.8 (69.6-74)	72.3 (70.4-74.3)	73.1 (71.6-74.7)	73.5 (73.1-74)	74.3 (74.1-74.5)	75.2 (74.9-75.4)	75.6 (75.4-75.8)	77 (76.7-77.3)	78.2 (78-78.4)	79.2 (79-79.4)	79.7 (79.1-80.3)
East Riding of Yorkshire	70.3 (68-72.6)	71.5 (69.1-74)	72.2 (69.9-74.5)	72.7 (70.5-75)	73.1 (71.3-75.2)	73.9 (72.5-75.3)	74.7 (74.3-75.2)	75.6 (75.3-75.8)	76.7 (76.5-76.9)	77.8 (77.6-78)	78.8 (78.5-79.1)	80 (79.8-80.2)	81.1 (80.9-81.3)	81.4 (80.8-82)
Kingston upon Hull, City of	68.4 (66.1-70.9)	69.8 (67.5-72.2)	70.7 (68.5-73)	71.3 (69.2-73.6)	71.9 (70-74)	72.7 (71.2-74.2)	73.1 (72.6-73.5)	73.9 (73.7-74.2)	74.7 (74.5-74.9)	75.4 (75.2-75.6)	76.1 (75.7-76.4)	77.2 (76.9-77.4)	77.7 (77.5-78)	78.8 (78.1-79.4)
Kirklees	68.2 (66-70.5)	69.4 (67.2-71.7)	70.1 (68-72.3)	70.6 (68.4-72.9)	70.9 (69-72.9)	71.6 (70.3-73.1)	72.5 (72.1-72.9)	73.8 (73.6-74)	74.9 (74.7-75.1)	76.4 (76.2-76.6)	77.4 (77.1-77.7)	78.1 (77.9-78.3)	79.7 (79.5-79.9)	81.1 (80.5-81.7)
Leeds	69 (66.6-71.4)	70.1 (67.9-72.5)	70.8 (68.5-73.1)	71.1 (68.7-73.3)	71.4 (69.4-73.3)	72.1 (70.7-73.6)	73.3 (72.9-73.7)	74.3 (74.1-74.4)	75.5 (75.4-75.7)	76.8 (76.7-77)	77.8 (77.5-78)	78.9 (78.7-79.1)	79.9 (79.7-80.1)	80.3 (79.7-80.8)
North East Lincolnshire	69.7 (67.5-72.2)	71 (68.8-73.5)	71.9 (69.7-74.4)	72.4 (70.3-74.7)	73 (71-75.1)	73.8 (72.3-75.2)	74.3 (73.8-74.8)	74.6 (74.3-74.9)	75.2 (74.9-75.4)	76.1 (75.8-76.4)	77.1 (76.8-77.4)	78.1 (77.8-78.3)	79.5 (79.2-79.7)	80.2 (79.5-80.9)
North Lincolnshire	69 (66.5-71.6)	70.2 (67.7-72.8)	71 (68.5-73.5)	71.4 (69.3-73.7)	71.8 (69.9-74.1)	72.7 (71.2-74.2)	73.4 (72.9-73.9)	74 (73.7-74.2)	75.1 (74.8-75.3)	76.5 (76.2-76.7)	77.6 (77.3-77.9)	79 (78.8-79.3)	79.9 (79.6-80.2)	80.5 (79.8-81.2)
North Yorkshire	69.9 (67.7-72.3)	71.1 (68.9-73.3)	71.8 (69.6-74)	72.2 (70.1-74.4)	72.5 (70.6-74.5)	73.3 (71.9-74.8)	74.5 (74.1-74.9)	75.7 (75.5-75.8)	76.7 (76.6-76.9)	77.8 (77.7-78)	79 (78.8-79.3)	80.4 (80.2-80.6)	81.6 (81.4-81.8)	81.8 (81.2-82.4)
Rotherham	68.4 (66-70.9)	69.6 (67.3-72)	70.3 (68.1-72.8)	70.8 (68.7-73.1)	71.3 (69.3-73.4)	72.1 (70.7-73.6)	73 (72.6-73.5)	74.1 (73.9-74.3)	75.1 (74.8-75.3)	76.5 (76.2-76.7)	77.2 (76.9-77.5)	78.6 (78.3-78.8)	79.4 (79.2-79.6)	79.3 (78.7-80)
Sheffield	69.3 (67.1-71.6)	70.4 (68.3-72.9)	71.2 (69-73.5)	71.6 (69.5-73.9)	72 (70-74)	72.7 (71.4-74.2)	73.7 (73.3-74.1)	74.3 (74.2-74.5)	75.4 (75.2-75.5)	76.4 (76.2-76.5)	77.7 (77.4-77.9)	79.1 (78.9-79.3)	80 (79.9-80.2)	80.7 (80.1-81.2)
Wakefield	67.7 (65.3-70)	68.8 (66.6-71)	69.5 (67.2-71.9)	69.8 (67.8-72.1)	70.2 (68.2-72.2)	71 (69.7-72.5)	72.2 (71.8-72.7)	73.5 (73.3-73.7)	74.8 (74.6-75)	76 (75.8-76.2)	77.2 (76.9-77.5)	78.3 (78.1-78.5)	79.4 (79.2-79.6)	80.2 (79.6-80.8)
York	70.6 (68.3-73.1)	71.7 (69.4-74.1)	72.4 (70.1-74.9)	72.7 (70.7-75)	73.1 (71.1-75.2)	73.9 (72.4-75.4)	74.6 (74.1-75.1)	75.3 (75-75.5)	76.6 (76.4-76.9)	78.2 (78-78.5)	79.6 (79.3-80)	81 (80.8-81.3)	81.7 (81.4-81.9)	81.3 (80.7-82)
Northern Ireland	66.9 (66.7-67.1)	69.1 (68.9-69.3)	70 (69.8-70.2)	70.6 (70.5-70.8)	70.8 (70.7-71)	70.1 (69.9-70.2)	71.6 (71.4-71.7)	72.9 (72.8-73.1)	74.4 (74.3-74.6)	75.8 (75.6-76)	77.4 (77.2-77.7)	78.9 (78.7-79.1)	79.4 (79.2-79.6)	80.7 (79.9-81.4)
Scotland	66.7 (66.6-66.8)	68.5 (68.4-68.6)	69.2 (69.1-69.4)	69.7 (69.6-69.8)	70.4 (70.3-70.5)	71.2 (71.1-71.3)	72.1 (72-72.2)	73 (72.9-73.1)	74.1 (74-74.2)	75.1 (75-75.2)	76.2 (75.9-76.4)	77.4 (77.3-77.5)	78.5 (78.4-78.6)	79.1 (78.4-79.8)
Wales	65.7 (63.4-68.1)	67.3 (65-69.7)	68.4 (66.2-70.8)	69.3 (67-71.6)	70.1 (67.8-72.6)	71.6 (70-73.4)	73.2 (72.8-73.5)	74.6 (74.4-74.7)	75.8 (75.7-75.9)	76.4 (76.2-76.5)	77.5 (77.3-77.8)	78.8 (78.7-79)	79.9 (79.8-80)	80.4 (79.8-80.9)
Latin America and Caribbean	48.8 (47-50.4)	52.2 (51.2-53.1)	55 (54.2-55.7)	57.7 (57.1-58.3)	59.3 (58.8-59.8)	62.8 (62.4-63.1)	65 (64.7-65.3)	66.8 (66.6-67)	69.2 (69-69.4)	70.6 (70.4-70.7)	72.6 (72.4-72.7)	74 (73.8-74.1)	73.6 (73.4-73.7)	75.9 (75.6-76.1)
Andean Latin America	41.1 (38.7-43.3)	45.9 (44.7-47)	48.4 (47.4-49.5)	53.5 (52.6-54.4)	50.9 (50.2-51.5)	61.1 (60.3-61.7)	63 (62.4-63.7)	65.3 (64.7-65.8)	68.6 (68.1-69.1)	70.3 (69.9-70.8)	73 (72.5-73.4)	74.5 (74-75)	75.4 (74.9-75.9)	77.8 (77-78.7)
Bolivia	38.1 (35.8-40.3)	42.6 (40.5-44.5)	44.9 (43-46.6)	47 (45.5-48.4)	49.9 (48.7-51)	53.2 (52.3-54.1)	56 (55.1-56.9)	58.3 (57.3-59.2)	60.9 (60-61.8)	63.6 (62.7-64.5)	66.5 (65.5-67.4)	68.9 (67.7-70.1)	70.8 (69.3-72.3)	72.7 (71-74.6)
Ecuador	50.2 (47.7-52.7)	52.7 (51.9-53.5)	54.7 (54-55.4)	58.8 (58.1-59.4)	60.8 (60.1-61.4)	64.3 (63.8-64.8)	66.7 (66.2-67.1)	70.2 (69.9-70.5)	72 (71.8-72.2)	73.4 (73.1-73.7)	74.2 (73.8-74.5)	74 (73.5-74.6)	74.6 (74.1-75.2)	76.7 (75.7-77.6)
Peru	39.3 (36.8-41.8)	44.7 (42.9-46.5)	47.3 (45.7-49.1)	54 (52.4-55.5)	47.6 (46.7-48.7)	62.8 (61.6-64)	64.3 (63.2-65.3)	65.7 (64.8-66.5)	70 (69.2-70.8)	71.4 (70.6-72.1)	74.7 (74-75.5)	76.7 (76-77.4)	77.5 (76.8-78.2)	80.3 (78.9-81.6)
Caribbean	54.4 (53.1-55.5)	56.6 (55.6-57.5)	59.1 (58.4-59.8)	61 (60.4-61.7)	63 (62.4-63.6)	65.1 (64.5-65.6)	66.2 (65.7-66.6)	67.3 (66.9-67.7)	68.3 (68-68.7)	68.7 (68.3-69.1)	70.3 (69.9-70.8)	71.3 (70.8-71.8)	58.9 (58.5-59.4)	72.8 (72.1-73.7)
Antigua and Barbuda	57.8 (56.6-58.9)	61 (60-61.9)	63.9 (63.1-64.7)	66.3 (65.6-66.9)	68.1 (67.4-68.6)	69.5 (68.9-70.1)	71.3 (70.6-72)	73.5 (72.9-74)	74.3 (73.8-74.8)	74.1 (73.6-74.6)	74.7 (74.3-75.1)	75.8 (75.4-76.3)	76.6 (76.2-77)	77 (76.5-77.6)
The Bahamas	57.7 (55.6-60)	60.1 (58.2-62.1)	62.1 (60.7-63.5)	62.4 (61.5-63.2)	65.7 (65.1-66.3)	68.1 (67.7-68.6)	68.9 (68.5-69.2)	70.2 (69.9-70.6)	71.1 (70.8-71.5)	70.8 (70.5-71.1)	71.5 (71.2-71.7)	73.4 (73.1-73.6)	73.4 (73.1-73.7)	73.7 (72.9-74.6)
Barbados	58.7 (56.9-60.1)	61.9 (60.8-63)	66.1 (65.3-66.8)	68.1 (67.5-68.7)	69.1 (68.6-69.6)	70.5 (70.1-70.9)	72.9 (72.6-73.3)	74 (73.7-74.3)	73.8 (73.5-74)	74 (73.7-74.3)	74.8 (74.5-75.1)	75.5 (75.3-75.8)	76.7 (76.5-77)	77.1 (76.4-77.9)
Belize	53.4 (51.6-55)	56.9 (55.4-58.2)	62.9 (61.7-64.1)	66.3 (65.3-67.4)	68.3 (67.4-69.2)	69.8 (69.1-70.5)	70.9 (70.2-71.5)	71.6 (71-72.2)	72 (71.4-72.5)	72 (71.6-72.5)	70.3 (70-70.6)	72.3 (72-72.5)	73.6 (73.3-73.9)	74.2 (73.7-74.6)
Bermuda	63.4 (62.7-64)	66.1 (65.7-66.6)	68.4 (67.9-68.8)	69.4 (69.1-69.8)	70.4 (70.1-70.8)	72.4 (72-72.8)	73.7 (73.3-74)	73.8 (73.4-74.1)	73.9 (73.6-74.2)	76.4 (75.9-76.6)	77.4 (77-77.6)	79.3 (78.9-79.7)	80.5 (80.1-80.9)	81.3 (80.8-81.9)
Cuba	66.4 (64.6-68.5)	66.7 (65-68.6)	68.9 (68.8-69)	70.3 (70.2-70.4)	72.1 (72-72.2)	74.3 (74.2-74.4)	74.1 (74-74.2)	74.1 (74-74.2)	74.8 (74.7-74.9)	75.1 (75-75.1)	76.8 (76.8-76.9)	77.6 (77.5-77.7)	78.3 (78.2-78.4)	78.4 (77.4-79.4)
Dominica	49 (46.7-50.9)	55.4 (53.6-57)	61 (59.5-62.3)	65.5 (64.4-66.5)	68.2 (67.4-68.9)	71.4 (70.9-71.8)	73.8 (73.4-74.2)	74.3 (73.9-74.7)	72.9 (72.6-73.3)	72.9 (72.5-73.3)	73.7 (73.3-74.1)	74.1 (73.8-74.5)	73.6 (73.2-74.1)	72.8 (72-73.6)
Dominican Republic	54.8 (53-56.4)	58.4 (57.1-59.4)	60.4 (59.2-61.6)	61.1 (60-62.1)	64.2 (63.1-65.2)	66.6 (65.6-67.7)	68.8 (67.8-69.7)	70.9 (70.1-71.8)	72 (71.1-72.7)	73.5 (72.6-74.3)	73.9 (72.9-74.9)	73.2 (72.2-74.2)	74 (73.1-74.9)	73.1 (71.7-74.6)
Grenada	59.1 (57.6-60.5)	61.8 (60.5-63)	63.9 (62.7-64.9)	64.9 (63.9-65.9)	66.9 (65.9-67.9)	68.2 (67.3-69.1)	68.4 (67.7-69.1)	68.5 (68-69)	69.4 (69-69.8)	71.7 (71.4-72.1)	73.9 (73.6-74.3)	74.5 (74.2-74.8)	74.1 (73.7-74.4)	74.1 (73.5-74.6)
Guyana	50.7 (49.5-51.8)	54.5 (53.7-55.3)	59.2 (58.5-59.9)	60.4 (59.5-61.3)	62.4 (61.8-63)	62.3 (61.7-62.9)	64 (63.4-64.5)	65.8 (65.4-66.3)	65.5 (65.2-65.9)	66.2 (65.9-66.5)	66.2 (66-66.5)	66.6 (66.4-66.9)	68 (67.7-68.2)	69.2 (67.9-70.5)
Haiti	37.3 (34.7-39.8)	39.2 (37.3-41.3)	41 (39.2-42.9)	42.4 (40.6-44.3)	44.3 (42.5-46.1)	47 (45.5-48.7)	49.9 (48.6-51.4)	52.6 (51.3-53.9)	54.5 (53.2-55.6)	55 (53.6-56.5)	57.4 (55.9-58.9)	59.7 (58.2-61.3)	32.5 (32-33)	64.9 (63.1-66.9)
Jamaica	56.1 (55-57.1)	61 (60.2-61.6)	64.1 (63.5-64.7)	66.2 (65.7-66.7)	68.1 (67.6-68.5)	69.8 (69.4-70.3)	73.1 (72.8-73.5)	72.5 (72.2-72.8)	75 (74.5-75.5)	74.2 (73.4-75.1)	74 (73.8-74.3)	77.2 (76.9-77.4)	75.8 (75.5-76)	74.6 (73.2-76.1)
Puerto Rico	59.9 (59.7-60.1)	67.7 (67.6-67.9)	69.8 (69.6-69.9)	70.2 (70.1-70.4)	72 (71.8-72.2)	73.4 (73.3-73.6)	74.1 (74-74.2)	73.9 (73.8-74.1)	74.1 (74-74.3)	73.8 (73.7-73.9)	76.4 (76.3-76.6)	77.6 (77.4-77.7)	79 (78.8-79.1)	78.1 (77.5-78.6)
Saint Lucia	50.3 (48.5-52)	55.1 (53.6-56.4)	58.9 (57.5-60.2)	63.2 (61.9-64.3)	65.3 (64.4-66.1)	66.5 (66-67.1)	68.4 (68-68.8)	70 (69.6-70.4)	70.5 (70.2-70.8)	71.7 (71.4-72)	73.8 (73.4-74.1)	74.3 (74-74.6)	75.6 (75.3-75.8)	75.5 (74.9-76.1)
Saint Vincent and the Grenadines	53.6 (51.7-55.2)	55.2 (53.6-56.6)	61.9 (60.3-63.3)	64.3 (63-65.4)	65.8 (64.7-66.7)	67.5 (66.8-68.3)	69.6 (69-70.2)	71.2 (70.7-71.6)	71 (70.6-71.4)	71.4 (71-71.8)	71.2 (70.9-71.6)	72.4 (72.1-72.8)	72.9 (72.6-73.2)	72.3 (71.7-72.9)
Suriname	58.2 (56.6-59.6)	60.4 (59.2-61.6)	62.2 (61-63.4)	64.2 (63-65.3)	64.8 (63.6-65.9)	65.1 (64.1-66.1)	65.8 (65-66.6)	68 (67.2-68.7)	68.8 (68-69.5)	69.9 (69.2-70.6)	69.5 (68.8-70.2)	69.9 (69.1-70.6)	71.4 (70.7-72.1)	72 (70.9-73.2)
Trinidad and Tobago	57.2 (56.1-58.1)	60.7 (60-61.3)	64 (63.5-64.6)	64.9 (64.5-65.4)	65.7 (65.3-66.1)	67.1 (66.7-67.4)	68 (67.7-68.4)	68.6 (68.3-68.9)	69.9 (69.6-70.2)	69.8 (69.6-70.1)	70.4 (70.1-70.7)	71.9 (71.6-72.2)	73.6 (73.4-73.9)	74.2 (72.2-76.2)
Virgin Islands	62.2 (61.4-62.9)	64 (63.4-64.5)	65.2 (64.7-65.7)	66.2 (65.7-66.8)	67.5 (66.7-68.2)	69 (68.2-69.7)	70.3 (69.6-71)	71.6 (71-72.2)	72.5 (71.8-73.1)	73 (72.4-73.5)	73.6 (73-74.2)	74.3 (73.5-75.1)	74.1 (73.3-75.2)	73.9 (72.8-75.4)
Central Latin America	50 (48.5-51.2)	54.1 (53.3-54.7)	57.4 (56.8-58)	60 (59.6-60.5)	62 (61.7-62.4)	64.6 (64.2-64.9)	66.9 (66.6-67.2)	68.3 (68.1-68.5)	71 (70.8-71.2)	72.1 (72-72.3)	74 (73.8-74.1)	75.2 (75-75.3)	75.7 (75.5-75.8)	76.4 (76-76.7)
Colombia	53 (51.6-54.2)	56.7 (55.8-57.5)	58.9 (58.1-59.6)	61.4 (60.8-61.9)	65.6 (65.1-66.1)	66 (65.6-66.4)	69.1 (68.2-70)	67.2 (67-67.5)	71.4 (71.2-71.6)	72 (71.8-72.1)	74.1 (73.9-74.3)	76.4 (76.2-76.6)	78.1 (77.9-78.3)	80.1 (79-81.1)
Costa Rica	57.2 (56.9-57.6)	61.9 (61.6-62.1)	65.4 (65.1-65.6)	65.6 (65.4-65.8)	67.1 (66.9-67.3)	71.5 (71.3-71.7)	74.5 (74.3-74.7)	75.3 (75.1-75.4)	76.5 (76.4-76.6)	76.2 (76.1-76.4)	77.9 (77.8-78.1)	79.3 (79.2-79.4)	79.4 (79.3-79.6)	79.5 (78.9-80)
El Salvador	46.7 (44.3-48.7)	49.3 (47.3-51)	53.7 (52.1-55.2)	56.6 (55.4-57.7)	58 (57-58.9)	62.4 (61.7-63.2)	62.5 (61.7-63.2)	65.3 (64.8-65.7)	69.2 (68.8-69.5)	70.8 (70.3-71.4)	72.4 (71.9-72.9)	72.4 (71.9-72.9)	73.4 (72.9-73.9)	74 (72.2-75.8)
Guatemala	41.7 (39.6-43.5)	44.1 (42.3-45.6)	47.6 (46.2-49)	49.2 (48.1-50.3)	50 (49.1-50.9)	54.6 (53.9-55.3)	56.8 (56.1-57.4)	60.1 (59.6-60.6)	63 (62.6-63.4)	65.8 (65.4-66.1)	68 (67.7-68.3)	69.5 (69.1-69.8)	71.7 (71.3-71.9)	72.6 (71.4-73.7)
Honduras	40.7 (38.6-42.8)	43.4 (41.8-45.2)	47.3 (45.8-48.9)	51.7 (50.4-53.1)	56 (54.8-57.2)	61.1 (60.1-62.2)	66.8 (65.8-67.7)	68.1 (67-69.2)	68.9 (67.7-70.1)	69.1 (67.4-71.1)	70.4 (68.5-72.6)	71.6 (69.5-73.9)	72.8 (70.6-74.8)	74 (72.1-75.9)
Mexico	48.9 (47.3-50.3)	53.7 (52.6-54.8)	57.2 (56.3-58.1)	60.1 (59.3-60.8)	61.6 (60.9-62.3)	64.5 (63.9-65.1)	66.6 (66.2-67.1)	69.1 (68.7-69.4)	71.4 (71.1-71.7)	72.8 (72.5-73)	74.6 (74.4-74.8)	75.3 (75.1-75.5)	75.2 (75-75.4)	75.5 (75.3-75.8)
Aguascalientes	56.3 (54.1-58.5)	60 (58-62.1)	62.4 (60.4-64.3)	64.4 (62.6-66.2)	65.1 (63.4-66.7)	67 (65.9-68.1)	69.1 (68.5-69.6)	70.7 (70.3-71.1)	72.8 (72.4-73.2)	74.1 (73.8-74.4)	75.3 (75-75.6)	75.7 (75.5-75.9)	76.2 (76-76.4)	77.3 (76.7-77.9)
Baja California	48.8 (46.4-51.2)	53.8 (51.6-56)	57.5 (55.5-59.6)	60.4 (58.2-62.4)	61.5 (59.5-63.5)	64.1 (62.7-65.5)	67.6 (67.2-68)	68.4 (67.9-68.8)	70 (69.6-70.4)	70.6 (70.3-70.8)	71.4 (71.1-71.7)	72.8 (72.5-73)	73.5 (73.3-73.7)	75.2 (74.5-75.8)
Baja California Sur	52.1 (49.8-54.5)	56.7 (54.5-58.9)	59.8 (57.9-61.8)	61.7 (59.9-63.7)	63.3 (61.8-64.9)	65.7 (64.7-66.7)	69.1 (68.6-69.6)	72.8 (72.5-73.1)	73.7 (73.4-73.9)	74.1 (73.9-74.4)	75.2 (74.9-75.4)	75.4 (75.2-75.7)	75.7 (75.5-75.9)	76.8 (76.2-77.5)
Campeche	54 (51.7-56.4)	58.2 (56-60.4)	61 (58.8-63.1)	63.5 (61.4-65.8)	64.5 (62.8-66.2)	67 (65.8-68.1)	68.7 (67.9-69.4)	70 (69.4-70.5)	72.3 (71.8-72.7)	73.8 (73.4-74.1)	75.9 (75.5-76.2)	76.6 (76.3-76.9)	76.3 (76-76.5)	75.1 (74.4-75.7)
Chiapas	49.9 (47.5-52.3)	54.3 (52-56.7)	57.3 (55-60.1)	60 (57.9-62.2)	61.8 (59.7-63.9)	65.1 (63.8-66.5)	64.8 (64.1-65.6)	66.8 (66-67.5)	70.2 (69.7-70.6)	71.6 (71.1-72)	73.1 (72.7-73.4)	74.1 (73.6-74.5)	74.2 (73.9-74.6)	74.3 (73.6-74.9)
Chihuahua	48.8 (46.2-51.1)	53.7 (51.4-56)	57.3 (55.2-59.3)	60.1 (58-62.3)	61.6 (59.8-63.6)	64.3 (63-65.6)	66.3 (65.6-66.9)	67.7 (67.3-68.2)	69.2 (68.7-69.6)	70.6 (70.2-70.9)	71.8 (71.4-72.1)	72.7 (72.5-73)	68.6 (68.3-68.8)	73.1 (72.4-73.8)
Coahuila	49.9 (47.6-52.1)	54.6 (52.5-56.9)	57.9 (55.8-60)	60.5 (58.4-62.5)	61.7 (59.7-63.7)	64.1 (62.8-65.4)	66.5 (65.8-67)	69.3 (68.9-69.7)	71.4 (71-71.8)	72.9 (72.6-73.2)	74.1 (73.8-74.3)	74.6 (74.4-74.9)	74.6 (74.4-74.8)	75.5 (74.8-76.2)
Colima	48.1 (45.6-50.4)	52.6 (50.1-55.1)	55.7 (53.8-57.7)	58.2 (56-60.3)	59 (57.4-60.6)	61.8 (60.8-62.9)	65.3 (64.5-66)	69.4 (68.9-69.8)	70.7 (70.3-71)	72.5 (72-73)	75 (74.7-75.2)	75.8 (75.5-76.1)	75.5 (75.3-75.8)	75.4 (74.8-76)
Mexico City	53.8 (51.7-55.8)	58.1 (56.1-60.4)	61.1 (59.3-62.9)	63.7 (62-65.6)	65.3 (63.8-67)	67.9 (66.8-69)	68.9 (68.5-69.2)	70.8 (70.6-71)	72.5 (72.3-72.7)	73 (72.8-73.2)	74.9 (74.7-75)	75.3 (75.1-75.4)	75.6 (75.4-75.8)	75.2 (74.6-75.8)
Durango	48.1 (45.7-50.4)	53.2 (50.9-55.4)	56.9 (54.7-59)	60.1 (58-62.2)	61.9 (60-63.9)	65 (63.7-66.3)	66.3 (65.7-67)	69.2 (68.7-69.6)	71.7 (71.2-72.1)	73.7 (73.4-74)	75.8 (75.5-76.2)	76.2 (75.9-76.5)	73.5 (73.3-73.7)	76.9 (76.3-77.6)
Guanajuato	47.3 (44.9-49.8)	52.2 (49.9-54.4)	55.6 (53.5-57.7)	58.6 (56.8-60.7)	60 (58-62)	63.2 (61.8-64.5)	67 (66.3-67.7)	68.6 (68.1-69)	71.7 (71.2-72.2)	73.5 (73.2-73.8)	75.1 (74.7-75.4)	76 (75.8-76.3)	76.5 (76.3-76.7)	76.2 (75.7-76.9)
Guerrero	45.9 (43.4-48.2)	50.5 (48.1-52.8)	53.9 (51.5-56.6)	57 (54.8-59.2)	59.1 (57.2-61.1)	62.9 (61.4-64.5)	64.8 (63.9-65.6)	68.8 (67.9-69.6)	72.4 (71.9-72.9)	73.2 (72.7-73.7)	76.1 (75.6-76.5)	77 (76.6-77.4)	75 (74.7-75.4)	75.4 (74.7-76.2)
Hidalgo	48.2 (46.1-50.4)	52.4 (50.4-54.7)	55.4 (53.4-57.3)	58.1 (56.2-60)	59 (57-61.1)	61.7 (60.2-63.1)	62.5 (61.8-63.2)	68.1 (67.6-68.5)	71.7 (71.2-72.2)	73.9 (73.5-74.2)	76.2 (75.9-76.5)	76.9 (76.6-77.1)	76.7 (76.5-76.9)	77.4 (76.8-78.1)
Jalisco	49.6 (47.4-51.9)	54.4 (52.2-56.8)	57.9 (55.9-60.1)	61 (59-62.9)	62.5 (60.6-64.3)	65.7 (64.6-66.9)	68.4 (67.9-68.9)	70.5 (70-70.9)	71.5 (71-71.9)	72.7 (72.4-72.9)	74.4 (74.1-74.7)	75.4 (75.1-75.6)	75.6 (75.4-75.8)	75.4 (74.8-76.1)
México	53.6 (51.4-56)	57.2 (55-59.6)	59.6 (57.4-61.8)	61.7 (59.7-63.7)	62.7 (60.8-64.9)	65.1 (63.3-67)	66.3 (65.1-67.5)	68 (67.7-68.3)	70.3 (70-70.6)	72.2 (71.9-72.5)	74 (73.9-74.2)	75 (74.8-75.2)	75.5 (75.3-75.7)	75.1 (74.5-75.8)
Michoacán de Ocampo	46.1 (43.8-48.4)	51.1 (48.5-53.7)	54.8 (52.5-57.4)	58.1 (55.9-60.4)	60.4 (58.4-62.4)	63.6 (62.4-65)	65.6 (64.9-66.2)	68.8 (68.4-69.2)	72.4 (72-72.8)	73.2 (72.9-73.5)	75.3 (75-75.6)	76 (75.7-76.4)	75.8 (75.5-76)	76.6 (76-77.3)
Morelos	50.3 (47.9-52.6)	54.8 (52.7-57)	58 (55.9-60.3)	60.8 (58.5-62.9)	62.1 (60-64.1)	65.2 (63.9-66.6)	66.8 (66.3-67.4)	70.3 (69.9-70.8)	72.2 (71.7-72.6)	73.5 (73.2-73.8)	75.8 (75.5-76)	76.4 (76.1-76.7)	76.1 (75.9-76.3)	74.8 (74.1-75.5)
Nayarit	48.7 (46.3-51.1)	53.7 (51.5-55.8)	57.3 (55.2-59.4)	60.6 (58.3-62.8)	62.8 (60.9-64.6)	66.4 (65.1-67.6)	68.9 (68.3-69.5)	70.3 (69.8-70.7)	72.2 (71.7-72.6)	74 (73.7-74.4)	76.6 (76.2-76.9)	76.5 (76.2-76.8)	76 (75.7-76.2)	77.2 (76.5-77.8)
Nuevo León	54 (51.7-56.4)	58.6 (56.5-60.9)	61.7 (59.7-63.9)	64.1 (62.2-66.1)	65.1 (63.4-67)	67.7 (66.5-68.8)	70.9 (70.4-71.3)	72.7 (72.3-73.1)	73.1 (72.7-73.5)	74.3 (74-74.5)	75.5 (75.3-75.8)	76 (75.8-76.2)	75.9 (75.7-76)	77 (76.3-77.6)

Table 6 Estimates of life expectancy at birth by location and year for 1950, 1955...2005, 2010, 2017 - both sexes

Location	1950	1955	1960	1965	1970	1975	1980	1985	1990	1995	2000	2005	2010	2017
Oaxaca	42.6 (40.2-45)	47.6 (45.1-50.2)	51.4 (49.1-53.8)	54.1 (51.9-56.3)	55.4 (53.4-57.4)	58.2 (56.7-59.6)	60.9 (60.2-61.6)	65.3 (64.8-65.8)	69.4 (69-69.8)	71.6 (71.2-71.9)	74.5 (74.2-74.7)	75.5 (75.2-75.9)	75.8 (75.5-76.1)	75.9 (75.3-76.6)
Puebla	42.4 (40.1-44.8)	47.5 (45.3-49.6)	51.3 (48.9-53.7)	54.6 (52.5-56.7)	56.2 (54.2-58.2)	59.7 (58.3-61.1)	62.4 (61.7-63.2)	65.8 (65.1-66.5)	69.4 (69-69.8)	71.2 (70.7-71.6)	73.4 (73-73.7)	74.3 (73.9-74.7)	74.9 (74.5-75.2)	74.1 (73.4-74.8)
Querétaro	50.3 (47.9-52.6)	54.6 (52.3-57)	57.7 (55.5-59.8)	60.5 (58.4-62.6)	61.3 (59.1-63.3)	64.3 (62.6-66.1)	65.6 (64.5-66.7)	69.6 (69.3-70)	71.4 (71.1-71.6)	73.3 (73.1-73.6)	74.4 (74.2-74.6)	75.9 (75.7-76.1)	76.3 (76.1-76.5)	76.2 (75.6-76.9)
Quintana Roo	61.4 (59.2-63.8)	63.1 (61-65.2)	64.8 (62.6-66.9)	65.2 (63.1-67.2)	64.6 (62.7-66.5)	66.1 (64.9-67.3)	68.6 (67.9-69.4)	73.3 (72.7-73.8)	73.4 (72.9-73.9)	74.3 (73.9-74.6)	75.4 (75.1-75.7)	75.9 (75.6-76.1)	75.4 (75.2-75.7)	74.5 (73.9-75.1)
San Luis Potosí	47.1 (44.6-49.7)	52.2 (49.7-54.9)	55.9 (53.6-58.4)	58.8 (56.7-61.1)	60.5 (58.5-62.6)	63.6 (62.2-64.9)	68.5 (67.8-69.1)	69.8 (69.4-70.2)	72.9 (72.5-73.3)	74.2 (73.8-74.5)	76.2 (75.9-76.5)	76.8 (76.4-77.1)	76.6 (76.3-76.9)	77.1 (76.5-77.7)
Sinaloa	51.3 (49-53.6)	55.8 (53.6-58)	58.9 (56.8-61)	61.6 (59.5-63.9)	63.1 (61.1-65.1)	66.3 (65-67.6)	68.5 (67.9-69.1)	70.5 (70.1-70.8)	73.3 (72.9-73.7)	74.1 (73.8-74.3)	76.1 (75.8-76.3)	76.5 (76.3-76.7)	75 (74.9-75.2)	78.3 (77.6-78.9)
Sonora	52.9 (50.9-55.1)	57.2 (55.1-59.3)	60 (58.2-62.1)	62.5 (60.6-64.6)	63.4 (61.5-65.4)	65.6 (63.6-67.6)	67.3 (66.1-68.5)	69.5 (69.1-70)	71.2 (70.9-71.5)	71.6 (71.2-71.9)	73.6 (73.2-73.9)	74 (73.7-74.2)	74.1 (73.9-74.3)	74.7 (74-75.4)
Tabasco	51 (48.9-53.4)	55 (52.7-57.4)	57.8 (55.6-60)	59.9 (57.9-62)	61.1 (58.9-63.2)	63.1 (61.9-64.5)	64.6 (64-65.3)	69.1 (68.6-69.6)	71.9 (71.5-72.2)	72.6 (72.2-73)	74.9 (74.5-75.2)	75.5 (75.3-75.7)	74.8 (74.6-75)	74.4 (73.7-75.1)
Tamaulipas	51.9 (49.7-54)	56.4 (54.2-58.8)	59.5 (57.5-61.7)	62.4 (60.4-64.4)	63.9 (62-65.8)	66.6 (65.3-67.9)	67.6 (67-68.2)	70.4 (70-70.8)	72.7 (72.4-73)	74 (73.5-74.4)	75.4 (75.1-75.6)	75.9 (75.7-76)	75.3 (75-75.5)	76.6 (75.9-77.2)
Tlaxcala	46.5 (43.9-49)	51.6 (49.1-54.2)	55.6 (53.3-57.9)	58.9 (56.8-61.1)	61.4 (59.4-63.4)	64.8 (63.2-66.7)	67 (65.9-68.2)	69.7 (69.3-70.2)	72.1 (71.8-72.4)	73.7 (73.3-74)	75.5 (75.2-75.7)	76.5 (76.3-76.7)	76.6 (76.3-76.8)	76.3 (75.7-77)
Veracruz de Ignacio de la Llave	47.8 (45.4-50.1)	52.7 (50.5-55)	56.2 (54-58.4)	59.1 (57.2-61.2)	60.8 (58.9-62.7)	63.8 (62.6-65.2)	66.7 (66-67.3)	69.7 (69.3-70.2)	71.5 (71-71.9)	73.1 (72.8-73.4)	74.7 (74.4-75.1)	75.3 (75-75.5)	75.4 (75.2-75.6)	75 (74.4-75.6)
Yucatán	53.6 (51.4-56.1)	57.8 (55.7-60)	60.4 (58.3-62.4)	62.7 (60.6-64.8)	63.9 (62-65.7)	65.8 (64.7-67)	67.9 (67.3-68.4)	70.5 (70-70.9)	72.3 (71.8-72.7)	73.5 (73.2-73.8)	75.2 (74.9-75.5)	75.9 (75.6-76.1)	75.8 (75.5-76)	75.1 (74.4-75.7)
Zacatecas	50.4 (47.9-52.9)	55.2 (52.9-57.4)	58.7 (56.6-60.9)	61.9 (59.6-64.2)	63.6 (61.6-65.3)	66.4 (65.2-67.7)	69.1 (68.4-69.8)	71.7 (71.3-72.2)	73.8 (73.3-74.3)	74.7 (74.4-75.1)	76.4 (76.1-76.7)	76.8 (76.5-77.1)	76.5 (76.3-76.7)	76.9 (76.3-77.5)
Nicaragua	44.9 (42.3-47.2)	50 (47.8-52.2)	51.6 (49.7-53.5)	55.7 (54.1-57.2)	57.3 (55.9-58.7)	61.7 (60.8-62.7)	64.7 (63.3-66.2)	62.3 (61.1-63.5)	71.9 (71.1-72.6)	72.8 (72-73.7)	75 (74.3-75.7)	75.6 (74.9-76.3)	77.6 (77.2-78.1)	78.8 (77.7-79.8)
Panama	60.4 (59.4-61.3)	62.1 (61.4-62.8)	65.4 (64.8-66)	66.9 (66.3-67.4)	67.6 (67.2-68.1)	71.3 (70.8-71.7)	73.7 (73.3-74.1)	74.8 (74.4-75.1)	75.8 (75.5-76.1)	75.9 (75.6-76.2)	77.4 (77.1-77.7)	78.1 (77.7-78.3)	78.2 (77.9-78.5)	79.3 (78.7-79.9)
Venezuela	57.6 (57.5-57.8)	60.7 (60.6-60.8)	64.7 (64.6-64.8)	66 (65.9-66.1)	66.4 (66.3-66.5)	67.6 (67.5-67.6)	69.6 (69.5-69.7)	71.4 (71.3-71.5)	72.1 (72-72.2)	72.4 (72.1-72.7)	74 (74-74.1)	75.2 (75.2-75.3)	75.3 (75.2-75.3)	75.3 (73.6-76.8)
Tropical Latin America	48.3 (46.1-50.2)	50.7 (48.9-52.3)	53.1 (51.5-54.5)	55.5 (54.1-56.7)	57.7 (56.6-58.7)	60.7 (60-61.4)	63.3 (62.7-63.8)	65.5 (65-65.9)	67.7 (67.4-68)	69.4 (69.1-69.7)	71.5 (71.2-71.8)	73.2 (72.9-73.4)	74.4 (74.1-74.6)	75.5 (75.3-75.8)
Brazil	48 (45.9-50)	50.4 (48.6-52)	52.9 (51.2-54.3)	55.2 (53.8-56.4)	57.4 (56.3-58.4)	60.5 (59.7-61.2)	63.1 (62.5-63.6)	65.3 (64.9-65.7)	67.6 (67.2-67.9)	69.3 (68.9-69.5)	71.4 (71.1-71.7)	73.1 (72.9-73.4)	74.4 (74.1-74.6)	75.5 (75.3-75.7)
Acre	45.8 (43.6-48.1)	48.2 (45.6-51)	51.5 (49.3-54.2)	54.3 (51.9-56.7)	57 (55.1-58.9)	60.6 (59.1-62.3)	63 (61.7-64.1)	63.9 (63.2-64.6)	67.1 (66.4-67.8)	68.7 (67.9-69.5)	71 (70.5-71.5)	72.6 (72.1-73)	74.2 (73.8-74.6)	75.1 (74.6-75.6)
Alagoas	40.8 (36.6-44.6)	42.5 (39.5-45.2)	44.7 (42.1-47.5)	46.8 (44.5-49.1)	48.5 (46.5-50.4)	51.3 (49.5-53.3)	53.4 (51.8-54.9)	57 (55.7-58.2)	62.7 (61.9-63.5)	66.7 (66-67.3)	69.7 (69.1-70.3)	71.5 (70.8-72.1)	72.3 (71.9-72.7)	73.7 (73.2-74.2)
Amapá	48.5 (46.3-50.8)	51.3 (48.9-54.1)	54.8 (52.3-57.1)	57.9 (55.7-59.9)	61.4 (59.7-63)	66.1 (64.9-67.4)	70.4 (69.9-71)	71.3 (70.9-71.6)	72.7 (72.4-73)	72.1 (71.7-72.4)	72.9 (72.6-73.1)	75.5 (75.2-75.7)	76.2 (75.9-76.5)	75.3 (74.9-75.7)
Amazonas	51.8 (49.7-54.1)	53.9 (51.2-56.4)	56.4 (54-58.6)	58.6 (56.4-60.8)	59.8 (57.8-62)	61.2 (59.4-63.1)	65.1 (63.9-66.2)	68.1 (67.4-68.9)	70.1 (69.3-70.8)	72.7 (72.1-73.3)	73.8 (73.5-74)	75.6 (75.4-75.8)	75.5 (75.3-75.7)	75.7 (75.4-76.1)
Bahia	44.9 (41.1-48.7)	47.2 (44.2-50)	50.1 (47.5-52.7)	53 (50.9-55.3)	56 (54.1-57.9)	60.3 (58.8-62)	64.3 (63.3-65.3)	65.3 (64.4-66.2)	68.1 (67.3-68.9)	70.2 (69.5-71)	71.7 (70.9-72.6)	72.8 (72.1-73.6)	74.1 (73.4-74.8)	75.5 (75-76)
Ceará	36.4 (32.1-40.8)	39.1 (35.9-42.2)	42.2 (39.4-45.3)	45.2 (42.6-48)	48.3 (45.5-51)	52.6 (50.7-54.6)	57.3 (55.5-59.1)	62.7 (61.4-63.9)	67.9 (67-68.7)	70 (69.3-70.7)	71.8 (71.1-72.5)	73.4 (72.7-74.1)	75.8 (75.2-76.2)	76.8 (76.4-77.3)
Distrito Federal	46.7 (44.7-49)	49.9 (47.4-52.3)	53.2 (51.1-55.5)	56.1 (53.9-58.4)	59.5 (57.7-61.4)	63 (61.8-64.3)	66.3 (65.9-66.7)	69.6 (69.4-69.8)	69.9 (69.6-70.1)	69.7 (69.5-70)	72.1 (71.9-72.3)	73.8 (73.6-74)	75.3 (75.1-75.4)	77.4 (77-77.7)
Espírito Santo	53.9 (51.8-56.2)	55.6 (53.4-57.9)	57.3 (55.1-59.7)	59.1 (57-61.2)	60.4 (58.5-62.3)	62.6 (61.3-63.8)	66 (65.6-66.4)	67.2 (66.9-67.5)	69.5 (69.2-69.8)	70.5 (70.3-70.7)	71.9 (71.7-72)	73 (72.7-73.3)	74.2 (74-74.4)	76.6 (76.1-76.9)
Goiás	46.2 (44.2-48.3)	48.5 (46.1-51.2)	51 (48.7-53)	53.5 (51.1-56)	56.6 (54.7-58.7)	60.7 (59-62.6)	63.9 (62.8-65)	65.3 (64.8-65.7)	69 (68.5-69.4)	70.3 (69.9-70.6)	73.1 (73-73.3)	74.4 (74.2-74.5)	74.6 (74.5-74.8)	75.5 (75.1-75.8)
Maranhão	37.6 (35.3-39.9)	39.6 (37.1-42.2)	42.1 (39.4-44.9)	44.8 (42.5-47.2)	47.6 (45.5-49.7)	52.2 (50.1-54.5)	56 (54.2-58)	60 (58.7-61.2)	65.6 (64.7-66.4)	69.7 (68.9-70.4)	72.3 (71.5-73)	72.7 (72-73.4)	74.8 (74.2-75.5)	76.2 (75.8-76.6)
Mato Grosso	48.4 (46.2-50.5)	50.8 (47.9-53.4)	53.5 (51.2-55.8)	56.6 (54.1-59.1)	60.4 (58.5-62.4)	65.2 (63.5-66.9)	69.2 (68.1-70.2)	67.7 (66.8-68.7)	69.8 (69-70.7)	71.6 (71.2-72)	72.4 (72.2-72.6)	73.2 (73-73.4)	74.3 (74.1-74.5)	75.7 (75.3-76.1)
Mato Grosso do Sul	54.9 (52.8-57.3)	56.3 (53.6-58.9)	58.3 (56-60.6)	60.8 (58.3-63)	62.6 (60.5-64.7)	64.9 (63.6-66.4)	67.2 (66.8-67.6)	68.4 (68.2-68.7)	69.8 (69.6-70.1)	69.6 (69.3-69.8)	71.6 (71.3-71.8)	72.8 (72.6-73)	74.1 (73.9-74.3)	75.1 (74.8-75.5)
Minas Gerais	50.9 (48-53.7)	53 (50.6-55.4)	55.1 (52.8-57.5)	57.1 (54.9-59.5)	59.2 (57.2-61.1)	61.9 (60.7-63)	64.3 (63.7-64.8)	66.2 (65.7-66.6)	69 (68.6-69.4)	70.7 (70.3-71.1)	73.1 (72.7-73.5)	74.2 (73.8-74.6)	75.4 (75-75.7)	76.6 (76.3-77)
Pará	50.2 (48-52.5)	52.3 (49.6-55.2)	54.9 (52.3-57.8)	57 (54.6-59.4)	58.6 (56.5-60.8)	61.7 (60.1-63.2)	65.1 (64-66)	66.4 (65.6-67.2)	68.9 (68.1-69.6)	71.5 (71-72.2)	73.1 (72.5-73.8)	73.9 (73.3-74.4)	74.9 (74.5-75.2)	75.5 (75.2-75.9)
Paraíba	42.8 (39-46.7)	45.5 (42.6-48.4)	48.7 (46-51.3)	52.4 (49.8-54.9)	55.5 (53-57.9)	59 (57.3-60.5)	62.9 (61.9-63.9)	66.4 (65.6-67.2)	69.3 (68.6-69.9)	71.2 (70.6-71.7)	73.3 (72.7-73.8)	73.8 (73.2-74.4)	74.2 (73.6-74.7)	75.7 (74.9-76.6)
Paraná	51.5 (48.4-54.4)	53.6 (51.3-56.2)	55.7 (53.3-58.2)	58 (56-60.1)	60.7 (58.9-62.5)	63.4 (62-64.7)	64.5 (64.1-64.9)	67.1 (66.8-67.3)	68.8 (68.6-69)	70.3 (70-70.5)	71.8 (71.6-72)	72.8 (72.5-73.1)	73.7 (73.5-73.9)	75.1 (74.7-75.5)
Pernambuco	38.1 (34.1-42.1)	40.8 (37.7-43.6)	43.9 (41-46.7)	47 (44.5-49.4)	49.8 (47.3-52.3)	54.3 (52.9-55.6)	58.4 (57.5-59.4)	61.1 (60.1-61.9)	64 (63.4-64.5)	67.7 (67.2-68.2)	69 (68.4-69.6)	71.4 (70.9-71.9)	73.3 (72.7-73.7)	73.3 (72.8-73.7)
Piauí	48.7 (46.4-51)	50.5 (48.2-53)	52.5 (50.1-55.1)	54.1 (51.5-56.7)	55.6 (53.3-58)	59.6 (57.6-61.5)	63.6 (62.4-64.9)	65.7 (64.8-66.5)	70.4 (69.6-71)	73.6 (72.6-74.5)	73.5 (72.8-74.2)	73.9 (73.3-74.6)	76.1 (75.6-76.6)	76.8 (76.3-77.2)
Rio de Janeiro	51.8 (49.8-54)	54.2 (51.9-56.5)	56.4 (54.3-58.7)	58.6 (56.5-60.7)	59.6 (57.7-61.6)	61.4 (60.2-62.5)	63.7 (63.2-64.1)	65.1 (64.8-65.4)	65.6 (65.4-65.9)	66.5 (66.2-66.7)	69.9 (69.7-70)	71.9 (71.7-72.1)	73 (72.8-73.1)	73.7 (73.4-74)
Rio Grande do Norte	41.8 (37.8-45.7)	45 (41.6-47.8)	48.5 (45.6-51.3)	52 (49.6-54.7)	54.8 (52.3-57.4)	58.9 (57.1-60.7)	63.5 (62.2-64.7)	67.2 (66.2-68.1)	70.4 (69.6-71.1)	71.5 (70.8-72.2)	74.3 (73.6-74.9)	76 (75.3-76.6)	77.1 (76.6-77.4)	76.1 (75.6-76.5)
Rio Grande do Sul	56 (54.1-58.1)	57.7 (55.6-59.9)	59.6 (57.7-61.6)	61.4 (59.4-63.5)	63.6 (61.9-65.5)	66.1 (65-67.2)	67.2 (66.9-67.5)	69 (68.8-69.2)	70.2 (70-70.3)	70.8 (70.7-71)	72.4 (72.3-72.6)	74 (73.8-74.1)	74.9 (74.7-75)	75.4 (75.1-75.7)
Rondônia	42.5 (40.5-44.7)	43.6 (41.2-46.2)	47 (44.8-49.5)	49.5 (47-52.1)	52.2 (50.1-54.3)	56.8 (54.9-58.6)	60.9 (59.8-61.9)	63.2 (62.3-64.1)	66.2 (65.4-67.1)	70.8 (70-71.6)	71.3 (70.5-72.1)	72.8 (72-73.6)	74.1 (73.3-74.8)	74.9 (73.7-76.1)
Roraima	41.6 (39.5-43.8)	43.7 (41.1-46)	47 (44.7-49.3)	50.2 (47.8-52.6)	53.1 (51.2-55.3)	58.4 (56.7-60)	62.7 (61.4-64)	64.3 (63.2-65.4)	68.1 (67-69.2)	70 (69-70.9)	71.2 (70.3-72.1)	72.4 (71.5-73.3)	74.1 (73.1-75)	74.8 (73.6-75.9)
Santa Catarina	58 (56.1-60.2)	59.6 (57.2-61.9)	61.3 (59.3-63.4)	62.8 (60.6-64.8)	64.3 (62.6-66.1)	66.4 (65.2-67.7)	67.4 (66.7-68)	68.6 (68.2-69)	69.6 (69.3-70)	70.4 (70-70.8)	72.4 (72-72.7)	74 (73.7-74.3)	75.2 (74.9-75.4)	76.6 (76.2-76.9)
São Paulo	54.5 (52.5-56.6)	56.6 (54.3-58.9)	58.6 (56.6-60.7)	60.5 (58.4-62.6)	62.1 (60.3-64)	63.7 (62.5-64.9)	65.2 (64.8-65.6)	67.3 (67-67.5)	68 (67.8-68.2)	68.9 (68.7-69.1)	71 (70.9-71.1)	73.7 (73.6-73.9)	74.9 (74.7-75)	76.1 (75.8-76.3)
Sergipe	48.5 (44.8-51.9)	50.7 (47.5-54.2)	53.1 (50.7-55.7)	55.6 (53.5-57.9)	57.8 (56-59.7)	60.8 (59-62.5)	62.1 (60.9-63.2)	65.5 (64.7-66.2)	67.9 (67.4-68.4)	68.8 (68.4-69.2)	71.3 (70.8-71.8)	73.4 (73-73.8)	74.2 (73.9-74.6)	74.4 (73.9-75)
Tocantins	43.9 (41.6-46.3)	46 (42.9-49)	48.7 (45.9-51.4)	51.3 (48.4-53.9)	54 (51.7-56.3)	58.2 (56.2-60.4)	62.2 (60.2-64.2)	65.3 (63.6-66.9)	68.2 (67.3-69.1)	70.2 (69.5-70.7)	71.9 (71.4-72.4)	73.8 (73.3-74.3)	75.2 (74.7-75.7)	76 (75.3-76.8)
Paraguay	60.3 (58.5-61.8)	64.7 (63.7-65.7)	66.1 (65.2-67)	68.2 (67.3-68.9)	68.6 (67.9-69.3)	69.6 (68.9-70.3)	71.1 (70.5-71.8)	72.2 (71.6-72.9)	74.3 (73.7-74.9)	74 (73.4-74.6)	74.5 (73.9-75.1)	74.4 (73.8-75)	74.6 (74-75.3)	76.1 (74.4-77.7)
North Africa and Middle East	42.4 (40.6-44.1)	44.9 (43.6-46.2)	47.6 (46.4-48.6)	51.2 (50.3-52)	54.1 (53.3-54.8)	57.6 (57.1-58.2)	60.6 (60.2-61)	62.8 (62.5-63.1)	66.3 (66-66.6)	67.9 (67.6-68.2)	69.9 (69.6-70.1)	71.4 (71.2-71.7)	72.8 (72.5-73.1)	74.2 (73.9-74.6)
North Africa and Middle East	42.4 (40.6-44.1)	44.9 (43.6-46.2)	47.6 (46.4-48.6)	51.2 (50.3-52)	54.1 (53.3-54.8)	57.6 (57.1-58.2)	60.6 (60.2-61)	62.8 (62.5-63.1)	66.3 (66-66.6)	67.9 (67.6-68.2)	69.9 (69.6-70.1)	71.4 (71.2-71.7)	72.8 (72.5-73.1)	74.2 (73.9-74.6)
Afghanistan	39.7 (36.8-42.4)	41.4 (38.8-44.1)	42.5 (39.8-45.1)	43.4 (40.9-45.9)	44.4 (41.8-46.7)	45.1 (43-47.3)	42.2 (40.5-43.9)	40.3 (39-41.6)	52.6 (51-54.1)	53.3 (51.6-54.9)	54.6 (52.8-56.3)	57.3 (55.5-59.1)	59.8 (58.1-61.6)	63.4 (61.6-65.1)
Algeria	56.8 (55.8-57.7)	55.4 (54.7-56.1)	55.3 (54.2-56.4)	60.3 (59.2-61.3)	58.8 (57.5-60.2)	58.5 (57.4-59.7)	59.9 (59.2-60.6)	68.1 (67.6-68.6)	71.7 (71.1-72.4)	72.9 (72.3-73.6)	74 (73.3-74.5)	75.3 (74.6-75.9)	76.3 (76-76.7)	77.7 (77.3-78.2)
Bahrain	47.4 (44.7-50.1)	50.5 (48.1-53.1)	54.9 (52.7-57.1)	59.1 (57.2-61.2)	63.3 (61.7-64.9)	67 (65.9-68)	70.2 (69.6-70.8)	70.8 (70.5-71.2)	70.5 (70.1-70.9)	70.9 (70.6-71.3)	72.6 (72.2-72.9)	75.1 (74.8-75.4)	78.2 (77.9-78.6)	79.5 (78.8-80.2)
Egypt	40.1 (37.2-42.7)	43.6 (41.5-45.6)	46.9 (45.2-48.5)	50.7 (49.3-52.2)	51.1 (49.9-52.2)	54.3 (53.3-55.3)	58.3 (57.5-59.1)	60.1 (59.5-60.7)	64.4 (63.9-64.8)	66.6 (66.2-67)	68.7 (68.4-69)	68.8 (68.5-69.1)	69.4 (69.1-69.6)	70.6 (69.6-71.7)
Iran	35.9 (32.8-39)	38 (34.9-41)	41.7 (38.7-44.5)	46.7 (43.9-49.1)	53.7 (51.7-55.5)	61.6 (60.3-62.9)	65.3 (64.4-66.2)	67.3 (66.6-68)	68.1 (67.5-68.6)	72.1 (71.6-72.6)	73.6 (73.1-74)	74.4 (74-74.7)	75.2 (75-75.4)	77.4 (77.3-77.4)
Iraq	50.4 (48.1-52.7)	53.9 (51.8-55.9)	56.6 (54.7-58.3)	57.7 (56-59.2)	59.7 (58.5-60.8)	60.8 (59.9-61.7)	61.9 (61-62.8)	61.3 (60.1-62.4)	65.9 (64.6-67.2)	65.4 (64.2-66.7)	66.7 (65.6-67.8)	69.5 (68.5-70.7)	73.4 (72.6-74.4)	76.7 (76.1-77.3)
Jordan	53.8 (51.2-56.3)	55.1 (52.7-57.3)	57.9 (56.1-59.8)	61.4 (60-62.7)	63.7 (62.9-64.6)	66.6 (66-67.2)	67.9 (67.3-68.6)	69.7 (68.9-70.7)	71 (70-72.1)	71.6 (70.6-72.6)	72.8 (72-73.6)	74.6 (74.1-75.1)	77.6 (77.2-78)	79.3 (78.3-80.3)
Kuwait	62.2 (60-64.6)	64 (62-66)	66.5 (65.2-67.7)	67.9 (67.6-68.2)	69.9 (69.7-70.2)	70.4 (70.2-70.6)	72.8 (72.6-73)	75.8 (75.6-75.9)	74.9 (74.8-75.1)	79 (78.9-79.1)	78.6 (78.4-78.7)	79.2 (79.1-79.3)	81.2 (81.1-81.4)	83.1 (82.6-83.6)
Lebanon	58 (55.6-60.4)	59.1 (57-61.3)	61.6 (59.4-63.8)	63.8 (61.7-65.9)	65.9 (64.1-67.8)	61.7 (60.3-63.2)	69 (67.7-70.5)	65.6 (64.6-66.7)	70.3 (69.4-71.4)	73.3 (72.3-74.3)	75.4 (74.5-76.3)	76.4 (75.8-77)	77.2 (76.8-77.6)	78 (77.4-78.5)
Libya	38.3 (35.2-41.4)	45 (42.4-47.7)	50.9 (48.6-53.3)	58.1 (56.3-59.9)	64.3 (63-65.6)	67.7 (66.7-68.7)	70.3 (69.2-71.4)	71.5 (70.3-72.9)	72 (70.7-73.4)	73.1 (71.9-74.1)	73.2 (72.3-74.1)	74 (73.2-74.8)	73.4 (72.6-74.3)	72.9 (71.7-74.2)
Morocco	43.1 (40.7-45.6)	44.5 (42.5-46.6)	45.6 (44-47.3)	50.5 (48.8-52.4)	54 (52.3-55.7)	56.6 (55.2-58.2)	60.2 (59.1-61.5)	64.1 (63.2-65.1)	66.6 (65.9-67.3)	67.9 (67.2-68.6)	69.3 (68.5-70.1)	70.8 (69.9-71.8)	72.1 (70.8-73.3)	73.9 (72.4-75.6)
Palestine	50.3 (47.5-52.8)	52.8 (50.6-55.4)	56.6 (54.3-59)	60.7 (58.3-62.9)	58.1 (56.4-59.8)	66.4 (64.5-68.2)	68.2 (66.5-70)	70 (68.4-71.6)	70.6 (69.3-72)	71.9 (70.8-72.9)	70.5 (70-71)	70.9 (70.6-71.3)	73.9 (73.6-74.2)	76.8 (76.2-77.4)
Oman	29.3 (26.4-32.2)	31.7 (28.9-35)	34.5 (31.7-37.6)	38.6 (36-41.3)	48 (45.5-50.5)	54.2 (52-56.5)	60.5 (58.5-62.6)	65.7 (63.6-67.7)	68.8 (66.7-70.7)	71 (69.1-72.8)	73 (71.2-74.7)	74.9 (73.2-76.3)	75.2 (73.7-76.6)	77.1 (75.6-78.6)
Qatar	60.9 (58.7-63.1)	61.9 (59.7-64.1)	63.1 (60.9-65.2)	64.2 (62.2-66.1)	66.1 (64.3-68)	67.9 (66.3-69.6)	69.5 (68.1-71.1)	70.7 (69.4-72)	71.5 (70.3-72.7)	72.2 (70.9-73.4)	74.1 (72.9-75.3)	76.6 (75.3-77.8)	78 (76.8-79.3)	80.2 (78.7-81.6)
Saudi Arabia	52.4 (49.4-55.3)	54.3 (51.4-57)	56.3 (53.6-58.8)	59.1 (56.6-61.7)	62.3 (59.8-64.8)	66.7 (64.6-68.8)	69.3 (67.5-71.1)	70.9 (69-72.6)	71.7 (70-73.2)	73.5 (72.1-74.9)	74.7 (73.6-75.7)	74.7 (73.8-75.6)	74.8 (73.9-75.8)	76.8 (75.8-77.7)
Sudan	46.7 (43.9-49.4)	47.9 (45.5-50.4)	49.6 (47.4-51.8)	50 (48.1-51.9)	51.1 (49.7-52.7)	52.6 (51.2-54.1)	54.6 (53.3-56)	52.1 (50.9-53.2)	58.6 (57.2-59.9)	61.2 (59.7-62.7)	63.3 (61.7-65)	65.6 (64-67.4)	67.7 (66-69.5)	70.3 (68.4-72.4)
Syria	55.2 (52.5-57.6)	58.2 (56.2-60.1)	62 (60.2-63.8)	65.1 (63.6-66.8)	67.9 (66.4-69.2)	70.5 (69.5-71.5)	70.8 (69.7-71.9)	69.8 (68.8-70.9)	69.8 (68.9-70.7)	70.6 (69.6-71.5)	72.5 (71.7-73.3)	75 (74.2-75.8)	75.8 (74.8-76.8)	69.8 (68.6-70.9)
Tunisia	43 (40.3-45.3)	45.3 (43.5-47)	48.9 (47.5-50.1)	52.2 (51.1-53.2)	55.9 (55-56.7)	62.1 (61.4-62.8)	67.1 (66.5-67.8)	71 (70.5-71.5)	72.5 (72.1-73)	73.1 (72.6-73.6)	74.2 (73.5-75)	75.8 (74.5-77.1)	76.9 (75.3-78.5)	78.3 (76.5-80.1)
Turkey	41.7 (38.9-44.4)	45.5 (43.3-47.6)	48.2 (46.1-50.3)	52.3 (50.3-54.3)	56.3 (54.4-58.1)	59.8 (58.4-61.3)	63.4 (62.6-64.2)	66.5 (65.8-67.3)	68.6 (68-69.3)	70.3 (69.7-70.9)	74.1 (73.5-74.6)	76.7 (76.2-77.2)	77.6 (77.1-78.1)	78.9 (78.1-79.7)
United Arab Emirates	51.3 (48.4-54)	53.6 (50.9-56.3)	56.6 (54-59.2)	60.4 (58.1-62.7)	63.4 (61.4-65.5)	66.4 (64.5-68.4)	69 (67.1-70.8)	70.7 (69-72.4)	71.2 (69.8-72.9)	72.1 (70.8-73.5)	71.8 (70.6-72.9)	72.5 (71.2-73.7)	72.9 (71.4-74.5)	73.3 (71.4-75.2)
Yemen	31.9 (29.1-35)	33.4 (30.8-36.2)	36.4 (34.1-38.8)	37.9 (35.8-40.2)	41.3 (39-43.5)	47.8 (45.5-50.3)	52.5 (50-55.1)	56.2 (53.7-58.7)	58.6 (56.1-61.3)	60.8 (58.2-63.4)	63.3 (60.7-65.9)	65.8 (63.1-68.4)	67.9 (65.3-70.5)	68 (65.5-70.4)
South Asia	43.4 (41.7-45)	45.8 (44.2-47.3)	46.1 (44.5-47.6)	46.5 (45.3-47.7)	49.2 (48.2-50.2)	51.9 (51.1-52.8)	55.3 (54.6-55.9)	57.2 (56.7-57.8)	59.6 (59.1-60.1)	61.7 (61.3-62.1)	63.1 (62.7-63.4)	65.3 (64.8-65.7)	66.7 (66.3-67)	69 (68.6-69.5)
South Asia	43.4 (41.7-45)	45.8 (44.2-47.3)	46.1 (44.5-47.6)	46.5 (45.3-47.7)	49.2 (48.2-50.2)	51.9 (51.1-52.8)	55.3 (54.6-55.9)	57.2 (56.7-57.8)	59.6 (59.1-60.1)	61.7 (61.3-62.1)	63.1 (62.7-63.4)	65.3 (64.8-65.7)	66.7 (66.3-67)	69 (68.6-69.5)
Bangladesh	41.2 (38.8-43.3)	43.4 (41.4-45.2)	45.4 (43.6-47.2)	47.5 (45.9-49.2)	43.4 (42.2-44.6)	52.1 (50.8-53.6)	54.8 (53.8-55.9)	54.6 (53.8-55.4)	58.2 (57.4-59)	62.1 (61.4-62.9)	65.6 (64.9-66.2)	67.6 (67.1-68.1)	69.5 (68.9-70.1)	73.1 (71.9-74.2)
Bhutan	33.8 (30.9-36.9)	36.4 (33.6-39.3)	39.6 (36.8-42.4)	43.3 (40.5-46)	47.2 (44.4-49.8)	49.9 (47.4-52.5)	52.4 (50-54.7)	56 (54.1-57.8)	59.9 (58.2-61.6)	63.1 (61.6-64.6)	65.6 (64.4-66.9)	69.3 (68-70.6)	71.6 (70.3-73.1)	74 (72.4-75.8)
India	43.8 (41.7-45.8)	46.1 (44.2-47.9)	45.9 (44.2-47.6)	45.9 (44.4-47.2)	49.3 (48.1-50.5)	51.3 (50.3-52.3)	54.9 (54-55.6)	57.2 (56.5-58)	59.6 (59-60.2)	61.8 (61.2-62.3)	62.9 (62.4-63.4)	65.4 (65-65.9)	66.7 (66.2-67.1)	69 (68.4-69.5)
Andhra Pradesh	46.1 (43.2-49.4)	48 (44.5-51.7)	47.9 (43.6-52.1)	47.9 (44.6-51.2)	50.5 (48-53.2)	52.1 (49.8-54.6)	55.7 (53.3-58.3)	58.1 (55.6-60.6)	59.9 (57.6-62.4)	62 (59.5-64.4)	63.2 (60.7-65.7)	65.6 (63-68.2)	67.7 (65.3-70.1)	70.9 (68.3-73.2)
Arunachal Pradesh	44.7 (41.3-48)	46.9 (44-50)	46.4 (43.5-49.6)	45.2 (42.8-47.7)	49.7 (47.2-52.2)	52.9 (50.5-55.4)	56.9 (54.5-59.3)	58.9 (56.6-61.4)	60.8 (58.5-63.1)	62.8 (60.6-65.2)	64 (61.9-66.3)	66.9 (64.8-69)	68.8 (66.9-70.7)	70.8 (68.9-72.8)
Assam	41.1 (38.4-44)	43.5 (40.8-46.3)	43.4 (40.9-46.1)	43.3 (41.1-45.7)	47.1 (45-49.4)	49.8 (47.9-51.7)	53.3 (51.4-55.1)	55.3 (53.8-57.1)	57.2 (55.8-58.6)	58.6 (57.8-59.5)	58.5 (57.8-59.2)	61.6 (60.8-62.4)	62.7 (62-63.4)	66.8 (65.8-67.7)
Bihar	46.5 (43.7-49.3)	48.5 (45.9-51.2)	48 (45.5-50.6)	47.5 (45.2-50)	50 (48-52.1)	51.1 (49.2-53.1)	53.6 (51.6-55.5)	55.6 (53.9-57.4)	58.2 (56.7-59.7)	60.3 (59-61.6)	61.7 (60.7-62.6)	64.3 (63.5-65.1)	66.5 (65.9-67.1)	69.6 (68.7-70.5)
Chhattisgarh	45.3 (42.6-48.1)	47.4 (44.9-50.2)	47 (44.3-49.7)	46.5 (44.5-48.6)	49.9 (47.5-52.2)	51.3 (49.1-53.4)	54.5 (52.6-56.4)	56.1 (54.4-57.8)	58.8 (57.2-60.3)	61.2 (59.9-62.4)	62 (61-63)	63.2 (62.5-63.9)	62.6 (61.9-63.4)	64.5 (63.6-65.3)
Delhi	45.5 (42.6-48.4)	49.5 (46.7-52.4)	51.2 (48.9-53.7)	50.5 (48.5-52.5)	56.6 (54.6-58.6)	58.8 (57.1-60.7)	61.7 (59.8-63.6)	63.6 (61.8-65.5)	64.9 (63.2-66.6)	66.3 (64.9-67.8)	67.2 (66.1-68.4)	69.8 (69-70.7)	71.5 (70.7-72.3)	73.6 (72.5-74.7)
Goa	56.4 (53.8-59.2)	58.4 (55.8-61.4)	59 (56.1-61.8)	59.1 (56.5-61.5)	62 (59.8-64.2)	63.8 (61.7-65.8)	66.6 (64.7-68.6)	67.6 (65.7-69.5)	68.5 (66.7-70.1)	69.8 (68.1-71.5)	70.7 (69.1-72.5)	72.8 (70.9-74.7)	74 (71.9-75.8)	75.3 (73.2-77.1)
Gujarat	46.8 (44.5-49.2)	49 (46.8-51.4)	49 (46.7-51.3)	48.8 (46.8-50.8)	52.3 (50.5-54.2)	54.2 (52.5-55.9)	57.1 (55.5-58.8)	59.2 (57.8-60.9)	61.5 (60.3-62.8)	64.3 (63.5-65.2)	64.9 (64.2-65.5)	66.7 (66-67.4)	68.1 (67.5-68.8)	70.4 (69.5-71.2)
Haryana	47.1 (44.4-49.9)	49.2 (46.6-52)	49.1 (46.7-51.7)	48.8 (46.5-51)	52.7 (50.7-54.8)	55.8 (54.1-57.8)	59.8 (58-61.7)	61.9 (60.4-63.6)	63.8 (62.6-65)	65.4 (64.6-66.2)	65.5 (64.8-66.1)	67.9 (67.1-68.6)	67.3 (66.6-68)	69.2 (68.3-70.1)
Himachal Pradesh	52 (49.5-54.7)	53.5 (50.9-56.1)	53.4 (51-55.8)	53.1 (50.8-55.4)	56 (54-58.1)	57.7 (55.6-59.6)	60.6 (58.8-62.6)	62.3 (60.6-64.2)	64.3 (63-65.8)	66.3 (65.5-67.2)	68.4 (67.7-69.1)	69.9 (69.1-70.6)	70.7 (70.1-71.4)	72.3 (71.2-73.3)
Jammu and Kashmir	53.6 (51.1-56.3)	55.1 (52.6-57.8)	54.9 (52.5-57.5)	54 (51.8-56.2)	57.6 (55.6-59.4)	58.4 (56.5-60.2)	60.6 (58.9-62.4)	62.7 (61.1-64.4)	64.2 (62.6-66)	66 (64.6-67.4)	67.2 (66.2-68.2)	69.7 (69-70.5)	71 (70.3-71.6)	72.8 (71.8-73.7)
Jharkhand	47.8 (45.1-50.2)	49.5 (47-52.2)	49 (46.4-51.8)	48.4 (46.2-50.5)	51.2 (48.7-53.5)	52.5 (50.4-54.7)	54.8 (53-57)	55.6 (53.8-57.5)	57.6 (55.9-59.2)	59.5 (58.2-60.9)	60.6 (59.6-61.6)	63.3 (62.7-63.9)	65.1 (64.5-65.6)	68.6 (67.8-69.3)
Karnataka	45.4 (42.8-48.1)	47.7 (45.2-50.2)	47.6 (45.2-50.1)	47.6 (45.4-49.8)	51 (49-53.1)	53.7 (51.8-55.8)	57.7 (56.1-59.5)	60.5 (59-62)	62.8 (61.6-63.9)	64.5 (63.8-65.2)	64.8 (64.3-65.4)	67.2 (66.6-67.9)	67.2 (66.6-67.8)	67.7 (66.9-68.5)
Kerala	51.8 (49.1-54.4)	55.1 (52.5-57.7)	56.4 (54-58.7)	57.3 (55.2-59.3)	61.3 (59.3-63.3)	63.6 (61.9-65.3)	66.2 (64.6-67.9)	67.6 (66.2-69.1)	69.2 (68-70.4)	71.3 (70.5-72.1)	71.4 (70.8-72)	73.8 (73.2-74.4)	74.3 (73.7-74.8)	74.6 (73.7-75.5)
Madhya Pradesh	41.9 (39.1-44.9)	44 (41.6-46.7)	43.4 (41-46)	42.9 (40.8-45.2)	46.3 (44.4-48.4)	47.7 (45.8-49.8)	50.6 (48.8-52.4)	52.7 (51-54.4)	55.4 (53.8-57)	57.9 (56.4-59.3)	59.5 (58.4-60.5)	62.2 (61.3-63.1)	63.8 (63.1-64.5)	67.1 (66.1-68)
Maharashtra	46.8 (44.1-49.6)	49.3 (46.6-52.1)	49.6 (47.2-51.9)	49.7 (47.5-52.1)	53.1 (51-55.3)	55.2 (53.5-57)	59.4 (58-60.9)	62 (60.6-63.4)	64.1 (63.1-65.2)	66 (65.4-66.7)	65.9 (65.4-66.5)	69 (68.4-69.6)	70.2 (69.6-70.9)	71.6 (70.9-72.4)
Manipur	51.9 (48.9-54.8)	53.8 (50.8-56.7)	53.9 (50.9-56.8)	53.2 (50.7-55.7)	57.2 (54.8-59.6)	59.3 (57.2-61.4)	62.1 (60.1-64.2)	63.1 (61.1-65.2)	65 (63.1-66.9)	65.9 (64-67.9)	66.3 (64.4-68.2)	68 (66.1-69.9)	69.2 (67.3-71.1)	70.8 (69-72.6)
Meghalaya	56 (52.9-59.1)	57.3 (54.4-60.3)	57.3 (54.3-60.1)	55.8 (53.3-58.4)	58.7 (56.1-61.2)	58.1 (55.9-60.3)	60 (57.9-62.2)	61 (58.8-63.3)	62.4 (60.2-64.7)	63.9 (61.8-66)	64.9 (62.8-67.1)	67.1 (65.1-69.2)	68.2 (66.2-70.2)	69.8 (67.8-71.8)
Mizoram	59 (56-61.9)	60.2 (57.5-63.1)	60.5 (57.9-63.3)	58.6 (56.3-60.9)	62.4 (60-64.8)	62.5 (60.2-64.8)	64 (61.9-66.2)	65 (63.2-67.1)	66.3 (64.6-68.1)	67.4 (65.8-69)	67.9 (66.4-69.6)	68.9 (67.1-70.9)	69 (67.2-70.9)	70.5 (68.7-72.5)
Nagaland	49.4 (46.2-52.7)	52.2 (49-55.4)	53.1 (50-56.2)	50.3 (48-52.8)	57.6 (55-60.3)	59.7 (57.2-62.1)	62.4 (60-64.8)	63.9 (61.6-66.2)	64.7 (62.5-66.9)	65.8 (63.6-68)	65.9 (63.7-68.2)	67.6 (65.4-70.1)	68.9 (66.9-71.1)	70.8 (69-73)
Odisha	40.2 (37-43)	42.3 (39.8-45.1)	41.7 (39.3-44.3)	41.7 (39.4-44)	45 (42.9-47.1)	47.6 (45.6-49.8)	52 (50.2-53.8)	54.8 (53.1-56.4)	57.2 (56-58.5)	59.1 (58.3-60)	59.3 (58.5-60)	62.6 (61.8-63.4)	64.7 (63.9-65.4)	68.5 (67.6-69.3)
Punjab	53.5 (51-56)	54.8 (52.5-57.4)	55.4 (53.1-57.7)	55 (52.9-57.1)	58.4 (56.6-60.3)	59.9 (58-61.8)	62.4 (60.6-64.1)	63.9 (62.3-65.6)	65.2 (64-66.5)	66.8 (66.1-67.6)	67.4 (66.8-68)	69.1 (68.4-69.7)	69.9 (69.3-70.5)	72.3 (71.5-73.2)
Rajasthan	45 (42.3-47.9)	47 (44.4-49.7)	46.3 (43.8-48.8)	45.7 (43.5-47.9)	49.2 (47.3-51.3)	51.1 (49.2-53)	54.6 (52.9-56.4)	56.8 (55-58.5)	59.3 (58-60.7)	61.7 (60.8-62.7)	64 (63.2-64.9)	66.6 (65.9-67.2)	67.4 (66.7-68.1)	68.1 (67.1-69.1)
Sikkim	51.7 (48.6-54.7)	53.2 (50.3-56.2)	52.9 (50-56)	51.7 (49.1-54.3)	55.1 (52.6-57.7)	56.6 (54.3-58.9)	59.8 (57.6-62.1)	62.1 (59.7-64.4)	63.8 (61.6-66.1)	65.2 (63.1-67.5)	66.4 (64.4-68.5)	68.5 (66.7-70.6)	70 (68.3-72)	72.4 (70.6-74.5)

Table 6 Estimates of life expectancy at birth by location and year for 1950, 1955...2005, 2010, 2017 - both sexes

Location	1950	1955	1960	1965	1970	1975	1980	1985	1990	1995	2000	2005	2010	2017
Tamil Nadu	43.3 (40.8-46)	45.7 (43.3-48.7)	45.8 (43.6-48.3)	46.2 (44.1-48.4)	49.5 (47.6-51.3)	51.9 (50.1-53.8)	56 (54.3-57.8)	58.7 (57.2-60.2)	61.4 (60.4-62.6)	63.8 (63.1-64.5)	64.7 (64.2-65.3)	67.5 (66.9-68.1)	68.1 (67.5-68.7)	70.5 (69.9-71.3)
Telangana	50.1 (47.1-53.3)	51.6 (48.3-55.1)	51.6 (47.7-55.6)	51.4 (48.1-54.7)	53.7 (50.9-56.5)	55.1 (52.6-57.6)	58.2 (55.7-60.8)	59.6 (57.2-61.9)	60.7 (58.4-63)	62.5 (60.1-64.9)	63.4 (61-65.8)	65.7 (63.3-68.1)	68.1 (65.9-70.5)	71.5 (69.3-73.6)
Tripura	42.9 (39.9-46.2)	46 (42.8-49.4)	46.4 (43.5-49.4)	46.9 (44.4-49.6)	51.4 (48.9-53.9)	54.5 (52.2-57)	56.2 (54.2-58.5)	59.4 (57.2-61.8)	61.5 (59.3-63.8)	63.2 (61-65.4)	64.9 (63-67)	67.6 (65.6-69.6)	68.4 (66.5-70.3)	69.9 (68.2-71.4)
Uttar Pradesh	35.2 (32.6-38.1)	37.8 (35.1-40.9)	37.1 (34.9-39.6)	37.4 (35.1-39.8)	41.4 (39.4-43.4)	43.7 (41.9-45.6)	48 (46.3-49.8)	51.2 (49.6-52.8)	54.5 (53.2-55.9)	57.6 (56.4-58.8)	59.9 (58.8-60.9)	62.2 (61.5-63)	63.4 (62.6-64.1)	65.6 (64.5-66.7)
Uttarakhand	51.6 (49.1-54.1)	53 (50.4-55.7)	52.9 (50.4-55.5)	52.4 (50.2-54.6)	55 (52.9-57.1)	56.2 (54.3-58.1)	58.6 (56.9-60.4)	59.5 (57.6-61.3)	60.6 (59-62.3)	62.2 (60.6-63.8)	63.1 (61.7-64.6)	65.4 (64.2-66.6)	67.4 (66.5-68.3)	69.8 (68.9-70.6)
West Bengal	44.1 (41.4-46.8)	46.7 (44.1-49.5)	46.9 (44.7-49.3)	47.1 (45.1-49.3)	50.4 (48.6-52.4)	52.5 (50.8-54.4)	56.2 (54.7-57.8)	58.3 (56.9-59.8)	60.6 (59.3-62)	62.4 (61.4-63.3)	64.9 (64.2-65.6)	67.4 (66.6-68.1)	68.6 (67.8-69.5)	70.9 (70-71.9)
Union Territories other than Delhi	61.5 (58.9-63.9)	62.8 (60.1-65.2)	63.1 (60.6-65.5)	61.3 (59.1-63.2)	65.2 (63.3-67.2)	66.4 (64.6-68.3)	68.1 (66.3-69.9)	68.2 (66.3-69.8)	68.3 (66.6-70)	68.9 (67.3-70.6)	69.5 (68-71.2)	71.3 (69.5-73.2)	71.9 (70-73.9)	73.2 (71.1-75.2)
Nepal	38.7 (36.5-41.1)	40.9 (39.1-43)	43.1 (41.3-45)	45.8 (44-47.8)	48.3 (46.4-50.2)	49.7 (48-51.6)	51.5 (49.9-53.3)	54.5 (53.1-56.1)	58.3 (57.1-59.6)	62 (61-63.1)	65.7 (64.8-66.6)	67.9 (67.1-68.8)	69.2 (68.2-70.2)	70.9 (69.8-72.1)
Pakistan	45.8 (43.4-48.1)	48.1 (46.3-50)	50.9 (49.2-52.5)	53.7 (52.3-55.2)	56.5 (55.3-57.8)	58.9 (57.8-59.8)	60.5 (59.6-61.4)	61.5 (60.7-62.3)	61.7 (61-62.3)	61.2 (60.4-62)	61.9 (61.2-62.7)	61.8 (60.8-62.6)	64.3 (62.8-65.7)	66.8 (64.9-68.7)
Southeast Asia, East Asia, and Oceania	47.8 (46-49.4)	51.6 (50-53)	31 (30.4-31.7)	56.9 (55.9-57.8)	59.5 (58.6-60.3)	61.8 (61.2-62.4)	64.3 (63.8-64.8)	66.3 (65.8-66.7)	67.8 (67.4-68.1)	69 (68.7-69.2)	70.7 (70.4-70.9)	72.6 (72.5-72.8)	74.2 (74-74.3)	75.6 (75.3-75.9)
East Asia	48.8 (46.6-50.8)	52.9 (51-54.6)	25.9 (25.2-26.6)	58.3 (57-59.6)	61.2 (60.1-62.2)	63.7 (62.8-64.5)	65.8 (65.1-66.4)	67.6 (66.9-68.2)	68.8 (68.3-69.3)	70 (69.6-70.3)	71.9 (71.6-72.2)	74.2 (74-74.4)	75.7 (75.5-76)	77 (76.7-77.4)
China	49.3 (47.1-51.3)	52.6 (50.7-54.5)	24.7 (23.9-25.3)	58.1 (56.7-59.3)	61 (59.8-62)	63.5 (62.6-64.3)	65.5 (64.8-66.3)	67.4 (66.7-68)	68.7 (68.1-69.2)	70 (69.7-70.4)	72.1 (71.8-72.4)	74.2 (74-74.4)	75.8 (75.6-76)	77.1 (76.7-77.4)
North Korea	27.9 (27.1-28.7)	59.8 (57.5-62.3)	62.5 (60.4-64.9)	65 (63-67.4)	67.8 (65.9-69.9)	70.1 (68.4-72)	71.1 (69.6-72.8)	71.5 (70-73.1)	71.8 (70.2-73.4)	62.9 (61.7-64.2)	62.1 (61-63.4)	70.3 (69-71.5)	70.8 (69.7-72)	72 (70.6-73.4)
Taiwan (Province of China)	59.6 (57.5-61.5)	61.5 (61.4-61.6)	64.6 (64.5-64.7)	68 (67.9-68.1)	69.7 (69.7-69.8)	71.1 (71-71.1)	72.3 (72.3-72.4)	73.8 (73.7-73.8)	74.5 (74.4-74.5)	75.3 (75.2-75.3)	76.7 (76.7-76.8)	77.8 (77.7-77.8)	79.5 (79.4-79.6)	79.9 (79.5-80.4)
Oceania	44.1 (42.3-45.9)	45.9 (44.1-47.6)	48 (46.4-49.7)	50.4 (48.8-51.8)	52.8 (51.4-54.2)	54.8 (53.5-56.1)	56.4 (55.2-57.7)	57 (55.7-58.3)	57.8 (56.5-59.2)	58.5 (57.2-60)	58.8 (57.4-60.2)	58.4 (56.9-60)	58.9 (57.2-60.5)	60.5 (58.8-62.2)
American Samoa	63.2 (62-64.4)	64.9 (64.2-65.7)	65.9 (65.3-66.5)	66.4 (65.9-66.9)	67.5 (66.8-68.1)	68.9 (68.3-69.5)	69.9 (69.2-70.5)	70.6 (70-71.2)	70.8 (70.3-71.4)	71.1 (70.5-71.6)	71.4 (70.8-71.9)	71.2 (70.7-71.7)	71.6 (71-72.1)	71.8 (70.9-72.8)
Federated States of Micronesia	50.2 (47.9-52.4)	51.9 (49.7-54.1)	53.8 (51.7-55.8)	55.7 (53.6-57.7)	57.6 (55.6-59.5)	59.5 (57.6-61.4)	61.3 (59.4-63.1)	62.5 (60.6-64.2)	63.5 (61.9-65.1)	64.6 (63-66.1)	65.2 (63.8-66.6)	66.6 (65.2-67.9)	66.9 (65.5-68.3)	67.2 (65.6-68.8)
Fiji	57 (56-58.1)	60.7 (60-61.6)	63.1 (62.4-63.8)	64.3 (63.7-65)	63.8 (63.1-64.4)	64.5 (63.8-65.2)	66.3 (65.6-66.9)	67.3 (66.6-68)	67.7 (66.6-68.8)	66.9 (66.4-67.4)	65.9 (65.6-66.2)	67 (66.6-67.3)	67.4 (67-67.7)	68.1 (66.7-69.5)
Guam	64.1 (63.4-64.9)	64.9 (64.4-65.4)	66.6 (66.2-67)	67.8 (67.4-68.1)	67.9 (67.7-68.2)	69.4 (69.1-69.7)	72.4 (72.2-72.7)	74.2 (73.9-74.6)	73.6 (73.3-74)	73.4 (73-73.7)	73.8 (73.4-74.1)	73.9 (73.4-74.3)	73.6 (73.1-74)	73.1 (72.3-73.9)
Kiribati	47.7 (45.4-50)	48.9 (46.7-51.1)	50.6 (48.5-52.7)	52.5 (50.5-54.6)	54.4 (52.4-56.4)	57.3 (55.4-59.2)	58.5 (56.8-60.2)	58.2 (56.8-59.6)	58.5 (57.4-59.5)	58.7 (57.9-59.4)	59.3 (58.4-60.2)	60.1 (58.8-61.3)	60.9 (59.3-62.4)	62.4 (60.6-64.1)
Marshall Islands	51.5 (49.4-53.6)	53.5 (51.2-55.7)	55.6 (53.5-57.8)	57.4 (55.2-59.4)	59.1 (57.3-61)	61 (59.4-62.6)	62.3 (61-63.7)	62.9 (62.1-63.8)	62.8 (62.2-63.3)	62.4 (61.9-63)	61.7 (61-62.4)	62.1 (61.3-62.9)	63 (61.9-64.1)	64.5 (63-66)
Northern Mariana Islands	69.6 (68.1-71.4)	70.1 (68.6-71.8)	70.6 (69.1-72.4)	71.2 (69.8-72.7)	71.8 (70.4-73.2)	72.4 (71-73.9)	72.9 (71.5-74.3)	73.6 (72-75)	74.2 (72.7-75.5)	75.1 (73.9-76.2)	75.8 (74.8-76.7)	76.1 (75.3-76.9)	76.4 (75.6-77.3)	76.2 (75.3-77.2)
Papua New Guinea	40.7 (38.4-43)	42.1 (40.1-44.2)	43.9 (41.9-46)	46.4 (44.4-48.2)	49.3 (47.4-51)	51.5 (49.9-53.2)	53 (51.4-54.7)	53.4 (51.8-55.1)	54.4 (52.8-56.2)	55.5 (53.8-57.4)	56 (54.3-57.9)	55.6 (53.7-57.6)	56.3 (54.2-58.3)	58.5 (56.4-60.5)
Samoa	61.2 (59.4-62.9)	63.7 (62.2-65)	66.4 (65.2-67.5)	66.5 (65.5-67.4)	66.9 (65.9-67.9)	67.8 (66.8-68.8)	69.5 (68.4-70.7)	70.1 (68.8-71.5)	70.7 (69.2-72.3)	71.1 (69.7-72.5)	71.7 (70.5-72.8)	72.2 (71.3-73.2)	72.5 (71.6-73.5)	72.9 (71.8-74)
Solomon Islands	44.4 (42-46.7)	46.2 (44-48.4)	48.9 (46.7-51)	51.7 (49.5-53.7)	54.3 (52.3-56.3)	54.8 (52.9-56.7)	59.4 (57.4-61.4)	60.8 (58.9-62.7)	61.5 (59.6-63.2)	62.7 (60.8-64.3)	63.4 (61.7-64.8)	63.6 (62.2-65)	64.4 (63-65.8)	65.7 (64.2-67.2)
Tonga	59.6 (58.2-61.1)	61.3 (60.1-62.4)	63.3 (62.4-64)	64.7 (63.9-65.5)	65.7 (64.7-66.7)	66.6 (65.6-67.7)	67.6 (66.7-68.5)	69.1 (68.4-69.9)	70.1 (69.5-70.8)	70.6 (70.2-71.1)	70.7 (70.3-71.1)	70.8 (70.3-71.3)	71 (70.2-71.9)	71.8 (70.4-73)
Vanuatu	51.1 (48.2-53.9)	52.5 (49.7-55.3)	54.2 (51.4-56.9)	55.9 (53.1-58.6)	57.4 (54.7-60.1)	59.2 (56.4-62)	60.9 (58.2-63.8)	61.8 (59.2-64.5)	62.4 (59.7-65.1)	62.9 (60.2-65.6)	63.3 (60.7-66.1)	63.3 (60.5-66.1)	63.9 (61-66.7)	64.7 (61.8-67.4)
Southeast Asia	45 (43.8-46.1)	48.2 (47.2-49.1)	51.1 (50.3-52)	53.3 (52.5-54)	55 (54.3-55.6)	57 (56.4-57.6)	61 (60.5-61.5)	63.2 (62.8-63.6)	65.1 (64.8-65.5)	66.6 (66.3-66.9)	67.9 (67.6-68.2)	69.4 (69.1-69.7)	70.8 (70.5-71.1)	72.5 (72.1-73)
Cambodia	45.5 (43-48.1)	47 (44.4-49.5)	49.1 (46.6-51.5)	51 (48.6-53.3)	44.5 (42.8-46.2)	24.8 (24.1-25.5)	52.4 (50.9-53.8)	54.5 (53.4-55.6)	57.6 (56.7-58.6)	58.7 (57.8-59.5)	60.2 (59.1-61.1)	63.7 (62.8-64.7)	67.1 (66.1-68)	69.9 (68.6-71.1)
Indonesia	42.2 (40.6-43.8)	44.6 (43.4-45.8)	47.7 (46.6-48.9)	50.5 (49.5-51.5)	53.4 (52.6-54.4)	56.4 (55.6-57.2)	59.2 (58.5-59.9)	61.7 (61.1-62.3)	63.9 (63.4-64.4)	66 (65.6-66.5)	67.3 (66.9-67.8)	68.5 (68.1-69)	69.7 (69.2-70.2)	71.5 (70.8-72.1)
Laos	34.6 (31.8-37.5)	36.2 (33.5-39)	37.1 (34.4-39.6)	38.6 (35.8-41.2)	40.9 (38.2-43.4)	44.6 (42.1-47.1)	46.9 (44.7-49.1)	50 (48.1-51.9)	51.9 (50.3-53.6)	54.3 (52.7-55.7)	57 (55.7-58.3)	60.3 (59.1-61.6)	63.8 (62.3-65.1)	67.6 (66-69.1)
Malaysia	54.9 (53.1-56.8)	58.5 (57.4-59.7)	60.7 (60.6-60.7)	63.3 (63.2-63.4)	64.9 (64.7-65.1)	67 (66.7-67.4)	68.9 (68.3-69.4)	70.1 (70-70.1)	71.3 (71.3-71.4)	71.9 (71.9-72)	72.8 (72.7-72.8)	74 (74-74.1)	74.4 (74.3-74.5)	74.7 (73.9-75.5)
Maldives	32.9 (30-35.8)	36.8 (34.1-39.6)	41.1 (38.5-43.7)	45.6 (43.4-47.8)	50 (48.3-51.8)	53.6 (52.3-55)	57.3 (56.3-58.2)	61.7 (61-62.3)	65.1 (64.6-65.6)	68.4 (68-68.8)	72.3 (72-72.6)	75.6 (75.3-75.8)	79.2 (78.9-79.4)	81.4 (80.9-82)
Mauritius	53.3 (53-53.6)	55.6 (55.3-55.9)	58 (57.7-58.2)	62.6 (62.4-62.9)	64.4 (64.2-64.7)	64.5 (64.3-64.7)	67.4 (67.2-67.6)	68.7 (68.5-68.9)	70.1 (69.8-70.3)	71 (70.8-71.2)	72.2 (72-72.4)	72.9 (72.7-73.1)	74 (73.8-74.2)	74.8 (74.1-75.4)
Myanmar	33.7 (30.7-36.4)	35.9 (33.1-38.5)	38.6 (35.9-41.2)	41.1 (38.4-43.6)	43.7 (41.1-46.1)	47.9 (45.6-50.3)	51.1 (48.9-53.3)	53.5 (51.4-55.6)	55.3 (53.5-57.2)	56.9 (55.4-58.5)	58.7 (57.3-60.1)	61.2 (59.9-62.5)	64.7 (63.5-66)	68.5 (67.2-69.9)
Philippines	55.7 (54-57.2)	59.2 (58-60.5)	62.2 (61.1-63.3)	63.3 (62.4-64.2)	63.3 (62.4-64)	64.7 (63.9-65.5)	65.9 (65.2-66.7)	65.9 (65.2-66.6)	67.9 (67.2-68.5)	69 (68.3-69.7)	69.3 (68.7-69.9)	69.1 (68.4-69.7)	69.2 (68.6-69.9)	69.7 (68.4-71.2)
Sri Lanka	56.8 (56.7-56.9)	61 (60.9-61.1)	65.2 (65.1-65.3)	66 (65.9-66.1)	67 (66.9-67.1)	65.5 (65.5-65.6)	69.8 (69.7-69.8)	69.3 (69.3-69.4)	69.7 (69.5-69.9)	70.7 (70.7-70.8)	71.6 (71.5-71.6)	73.9 (73.5-74.2)	74.7 (74.6-74.8)	77.4 (76-79)
Seychelles	61.3 (59.6-63)	62.7 (61.3-64)	63.4 (62.3-64.5)	63.9 (63.2-64.8)	65.8 (65.3-66.4)	68.3 (67.9-68.7)	70.3 (69.9-70.7)	70.7 (70.4-71)	70.5 (70.2-70.8)	70.9 (70.6-71.2)	71.5 (71.2-71.8)	72.4 (72.1-72.7)	73.2 (72.9-73.5)	73.6 (73.1-74.1)
Thailand	50.7 (49.2-51.9)	55.6 (54.5-56.6)	58.1 (57.2-58.9)	60.5 (59.7-61.2)	62.6 (61.9-63.3)	64.3 (63.7-64.9)	65.2 (64.6-65.8)	69.4 (68.9-70)	70.7 (70.3-71.2)	70.3 (69.7-70.8)	71.7 (71.2-72.3)	74.8 (74.4-75.3)	77 (76.6-77.4)	78.1 (77.1-79.2)
Timor-Leste	37.4 (34.6-40.1)	39.1 (36.3-41.9)	41.5 (38.7-44.3)	44.5 (41.9-47.2)	47.5 (45.3-49.7)	44.9 (43.3-46.5)	50 (48.4-51.6)	56.5 (55-58)	60.2 (58.8-61.6)	62.3 (61.2-63.5)	65.3 (64.4-66.3)	67.7 (66.7-68.6)	70 (69-71.1)	70.8 (69.6-72.1)
Vietnam	50.2 (48-52.3)	55 (52.7-57.4)	57.4 (55.2-59.7)	56.5 (54.5-58.5)	55.2 (53.6-56.8)	61.5 (60-63.3)	65.6 (64.3-67.2)	67.4 (66.2-68.8)	68.9 (67.9-70)	70.2 (69.4-71.1)	71.5 (70.6-72.4)	72.4 (71.6-73.3)	73.4 (72.5-74.3)	74.5 (73.3-75.6)
Sub-Saharan Africa	42.1 (39.8-44.2)	43.6 (41.7-45.4)	45 (43.6-46.4)	46.8 (45.7-48)	48.4 (47.4-49.4)	50.3 (49.5-51)	52 (51.3-52.7)	53.2 (52.6-53.8)	53.6 (53-54.1)	53.3 (52.6-53.8)	53 (52.4-53.6)	55.1 (54.5-55.7)	58.7 (58-59.3)	63.9 (63.1-64.6)
Central sub-Saharan Africa	41.9 (39.5-44.4)	43.1 (40.9-45.4)	44.5 (42.3-46.6)	45.6 (43.4-47.6)	47.6 (45.7-49.4)	49.2 (47.5-50.8)	50.5 (49.1-51.9)	51.9 (50.8-53.2)	52.3 (51.3-53.4)	52.1 (51.2-53.1)	52.8 (51.9-53.7)	55.3 (54.4-56.2)	57.9 (57-59)	62.4 (61.1-63.6)
Angola	41.9 (39-44.7)	42.1 (39.4-44.8)	43 (40.4-45.6)	43.2 (40.4-45.8)	44.8 (42.3-47.4)	45.7 (43.5-48.3)	46 (43.8-48.4)	46.5 (44.6-48.4)	47.8 (46.1-49.6)	49.5 (47.9-51.1)	51.6 (50.2-53.1)	55.7 (54.4-57.2)	59.9 (58.5-61.4)	64.2 (62.5-65.8)
Central African Republic	41.7 (39.2-44.2)	42.5 (40.2-45)	44.1 (41.9-46.5)	45.9 (44-48)	47.2 (45.4-49)	47.8 (46.2-49.4)	48.7 (47.3-50.2)	49.1 (47.8-50.4)	47.2 (45.7-48.7)	45.4 (43.6-47.2)	44.2 (42.3-46.2)	45.2 (43.2-47.3)	48 (46.1-50.1)	51.8 (49.8-54.1)
Congo (Brazzaville)	39.9 (37.3-42.4)	40.7 (38.3-43.2)	42.3 (39.9-44.9)	44.4 (42.1-46.8)	46.6 (44.5-48.8)	49.6 (47.7-51.7)	53 (51.3-54.7)	55.9 (54.6-57.3)	53.8 (52.1-55.4)	52.2 (50.7-53.6)	52.7 (51.4-54)	56.3 (54.9-57.8)	59.8 (58.2-61.3)	62.6 (60.8-64.6)
DR Congo	42.2 (39.6-45.1)	43.8 (41.3-46.5)	45.2 (42.6-47.9)	46.4 (43.9-48.9)	48.5 (46.3-50.7)	50.2 (48.2-52.2)	51.6 (49.8-53.5)	53.4 (51.7-55)	53.9 (52.5-55.4)	53.4 (52.2-54.8)	53.8 (52.6-55)	55.9 (54.7-57.1)	57.9 (56.5-59.2)	62.3 (60.7-64.1)
Equatorial Guinea	35.9 (33-38.8)	36.4 (33.7-39.2)	37.7 (35-40.5)	40.3 (37.5-43.1)	42.8 (39.9-45.7)	45 (42.3-47.8)	48.1 (45.4-50.7)	49 (46.5-51.7)	48.2 (45.7-50.8)	48.4 (45.6-51.3)	55.5 (52.6-58.8)	60.1 (56.8-63.6)	62.1 (58.4-65.8)	65.4 (62-68.8)
Gabon	45.1 (42.3-47.6)	45.6 (43-48.1)	47.2 (44.8-49.7)	49.5 (47-52)	51.7 (49.5-53.8)	54.8 (53.1-56.6)	57.6 (56.1-59.2)	59.5 (58.2-60.9)	60 (58.8-61.2)	60.3 (59-61.6)	59.3 (57.5-60.9)	59.4 (57.6-61.1)	62.8 (61.2-64.3)	68.4 (66.9-69.7)
Eastern sub-Saharan Africa	41.3 (39.1-43.4)	42.8 (41-44.4)	44.2 (42.7-45.7)	45.6 (44.5-46.8)	47.3 (46.4-48.2)	48.7 (48-49.4)	50.1 (49.5-50.7)	50.6 (50.1-51.1)	50.7 (50.2-51.3)	50.7 (50.1-51.2)	51.5 (50.9-52)	55.1 (54.6-55.6)	59.6 (59-60.1)	64.9 (64.2-65.5)
Burundi	38.3 (35.8-40.9)	39.5 (37.1-42.1)	41.3 (39.1-43.6)	42.6 (40.8-44.5)	44 (42.4-45.7)	45.1 (43.6-46.6)	46.4 (44.9-48)	47.9 (46.5-49.5)	48.7 (47.3-50.2)	44.3 (42.7-45.8)	44.8 (43.3-46.4)	49.7 (48.4-51.1)	57.7 (56.4-59.3)	61.5 (59.7-63.4)
Comoros	44.2 (41.5-46.7)	45.4 (42.7-47.8)	46.7 (44.4-49.2)	48.9 (46.7-51.1)	51 (49.1-53.1)	52.6 (50.7-54.4)	53.8 (51.8-55.6)	56 (54.2-57.7)	58.1 (56.6-59.7)	59.8 (58.4-61.2)	61.6 (60.3-62.9)	64.5 (63.2-65.7)	66.5 (65.1-67.7)	68.6 (67.1-70.2)
Djibouti	54 (51-57.1)	54.8 (51.8-58)	55.7 (52.7-58.7)	56.7 (53.6-59.7)	57.7 (54.9-60.8)	59.3 (56.5-62.4)	60 (57.3-62.9)	60 (57.4-62.6)	60.1 (57.4-62.8)	60.3 (57.5-63.2)	60.4 (57.5-63.5)	61.5 (58.2-64.7)	63.7 (60.5-66.8)	67.3 (64.1-70.2)
Eritrea	38.7 (36.1-41.1)	39.7 (37-42.2)	41.1 (38.6-43.5)	38.3 (36.3-40.2)	40.3 (38.6-42)	40.1 (38.7-41.4)	41.8 (40.7-43)	43.3 (42.3-44.3)	36 (35.2-36.7)	51.9 (50.7-53.3)	53.3 (51.8-54.9)	56 (54.2-57.8)	58.6 (56.7-60.6)	62.6 (60.6-64.8)
Ethiopia	37.5 (35-40)	38.8 (36.4-41)	39.7 (37.6-41.8)	40.8 (39-42.7)	42 (40.4-43.8)	42.8 (41.4-44.1)	44.1 (42.9-45.3)	45.8 (44.8-46.9)	47.1 (46.2-48.1)	49.1 (48.1-50.1)	50.8 (49.9-51.6)	56.8 (56-57.6)	62.9 (62.1-63.7)	68.4 (67.5-69.3)
Kenya	47.5 (45.8-49.2)	49.7 (48.6-50.8)	53.1 (52.1-54)	55.2 (54.4-56.1)	57 (56.2-57.7)	59.2 (58.5-59.9)	61.4 (60.7-62)	62.6 (62-63.2)	61.9 (61.3-62.5)	58.1 (57.3-58.8)	55.7 (54.8-56.4)	56.9 (56.3-57.5)	61.2 (60.7-61.7)	65.9 (65.2-66.5)
Baringo	55.7 (52.9-58.9)	54.6 (51.8-57.7)	55.1 (52.5-58.3)	54.2 (51.5-57.1)	52.1 (49.4-55)	51.2 (48.4-54.1)	52 (49-54.8)	53.4 (50.9-55.9)	54.5 (52-57.2)	54.7 (52.3-57.4)	55.9 (53.7-58.3)	58.9 (56.9-61)	62.2 (60.2-64.4)	65.8 (63.6-68)
Bomet	68.5 (65.6-71)	66.7 (64.2-69.1)	69 (66.3-71.5)	69.3 (66.6-71.8)	69.6 (67-72.2)	69.9 (67.4-72.4)	70.4 (68.1-72.8)	71.3 (69.2-73.6)	71.2 (68.8-73.6)	68.1 (64.8-71.1)	63.5 (59.7-67.2)	62.5 (59.1-65.9)	64.1 (61.1-67)	68.5 (65.6-71.2)
Bungoma	44 (40.9-47.1)	46.4 (43.6-49.2)	50.6 (47.8-53.8)	53.2 (50.5-56.4)	55.4 (52.6-58.4)	57.5 (54.8-60.4)	60 (57.5-62.9)	61.3 (58.9-64.1)	62 (59.6-64.6)	61.5 (58.8-64.3)	61.1 (58.4-63.9)	62.7 (59.9-65.2)	66.2 (63.8-68.6)	69.3 (66.8-72)
Busia	38.4 (35.2-41.5)	40.1 (37.3-43)	44.5 (41.7-47.5)	47.7 (45-50.3)	50.5 (47.8-53.3)	55 (52.2-57.9)	58.7 (56-61.5)	60.7 (58.4-63.3)	58.6 (55.9-61.6)	50.3 (45.7-54.4)	47.4 (43.4-51.2)	52.2 (49-55.4)	60.9 (58.4-63.5)	66.4 (64.2-68.7)
Elgeyo Marakwet	56.3 (53.7-59.4)	56.8 (54-60.1)	57.9 (54.9-61.1)	58.5 (55.3-61.7)	58.4 (55.5-61.5)	59 (56.2-61.9)	60.3 (57.5-63.2)	61.3 (58.6-64.1)	61.9 (59.4-64.5)	61.1 (58.6-63.8)	60.4 (58-62.7)	60.9 (58.7-63)	63.8 (61.8-65.7)	67 (64.9-69.1)
Embu	47.5 (44.3-50.8)	49.3 (45.9-52.6)	53.8 (50.8-57)	57.1 (54.3-60.3)	60.7 (57.7-63.7)	64.6 (61.6-67.5)	67.3 (64.4-70)	68.4 (65.9-71)	66.4 (62.2-70.1)	62 (56.7-67.4)	61.2 (57-65.6)	63.2 (60.2-66.8)	66.1 (63.2-68.7)	69.4 (66.7-72)
Garissa	46.2 (43.6-49)	46 (43.6-48.7)	51.2 (48.3-54.1)	52.4 (49.9-55.2)	53.8 (51-56.8)	55.5 (53.1-58.1)	57 (54.7-59.4)	57.9 (55.6-60)	58.7 (56.6-60.8)	58.8 (56.7-61)	59.2 (57.2-61.2)	60.9 (58.8-62.7)	62.7 (60.5-64.6)	63.8 (54.9-67)
Homa Bay	33.2 (31.1-35.5)	36.1 (33.4-38.9)	39.6 (37.3-42.2)	42.1 (39.6-44.9)	42.8 (40.4-45.4)	45 (42.5-47.6)	49 (46.5-51.4)	51.5 (49.1-53.9)	51.1 (48.5-53.7)	46.3 (42.6-49.7)	40.2 (36.2-44.2)	39.4 (35.9-43)	46.1 (42.9-48.9)	56.2 (53.2-59.3)
Isiolo	43.1 (40-46.2)	42.7 (39.6-46.1)	44.7 (41.6-48.3)	44.9 (42-48)	44.6 (41.3-48)	45.9 (43.1-48.9)	48.7 (45.6-51.9)	50.7 (47.3-54.2)	53 (50.2-55.7)	54.7 (51.9-57.7)	54.7 (51.9-57.5)	57.6 (54.8-60.4)	60.2 (57.3-63.1)	63.7 (60.7-66.5)
Kajiado	57.8 (54.8-61)	57.5 (54.7-60.6)	60.6 (57.4-64)	61.3 (58.2-64.4)	62 (59.1-65)	63 (60.2-65.9)	64.6 (61.9-67.4)	66 (63.6-68.7)	64.7 (61.4-67.9)	59.9 (55.3-63.8)	57 (53.7-60.4)	58 (55.6-60.7)	62.3 (60.2-64.6)	66.8 (64.7-69.4)
Kakamega	45 (41.7-48.5)	46.4 (43.6-49.2)	50.8 (47.9-53.8)	53.5 (50.6-56.4)	55.5 (52.7-58.5)	58 (55.1-60.8)	60.4 (57.8-63)	61.2 (58.9-63.6)	59.4 (56.8-62.1)	53.9 (50.5-57.3)	51.5 (48.4-54.7)	54.4 (51.6-57.2)	59.5 (57.1-62.2)	64.5 (62-67.1)
Kericho	60.6 (57.4-64)	61.5 (58.5-64.9)	63.5 (60.2-66.6)	64.7 (61.5-67.8)	65.5 (62.5-68.4)	67 (64-69.8)	68.1 (65.2-70.7)	69.1 (66.6-71.6)	69.4 (67-72)	67.2 (64.5-70.1)	64.6 (61.4-68)	64.1 (60.9-67.4)	66.3 (63.4-69.5)	70.4 (67.4-73.1)
Kiambu	52.8 (50.2-55.5)	54.6 (52.1-57.5)	57.7 (55-60.7)	59.7 (56.9-62.8)	62.1 (59.4-65)	65.2 (62.5-67.8)	66.1 (63.6-68.5)	66.7 (64.5-69.1)	65.4 (62.8-67.8)	59.4 (55.7-62.9)	55.9 (52.7-59.4)	57.7 (55.3-60.3)	62.9 (60.9-65)	67.3 (65.2-69.3)
Kilifi	40.2 (37.4-43.3)	42.8 (40.5-45.6)	46.1 (43.4-48.9)	48.2 (45.4-50.9)	50.5 (48-53)	53.6 (51-56.3)	56 (53.5-58.6)	58.1 (55.8-60.6)	59.3 (56.9-61.9)	58.8 (56.4-61.4)	58.1 (55.4-61.1)	59.3 (56.7-61.9)	62.4 (60.3-64.7)	67.2 (64.8-69.5)
Kirinyaga	49.2 (46-52.4)	50.9 (48-54.1)	54.4 (51.3-57.8)	57 (53.9-60.3)	58.7 (55.4-62)	62.1 (58.8-65.3)	66.8 (63.6-69.9)	68.5 (65.4-71.4)	67.7 (64.7-70.7)	65.6 (62.3-69.2)	63.8 (60.5-67.8)	63.5 (60.1-67)	65.6 (62.4-68.9)	68.3 (65.1-71.2)
Kisii	45.2 (41.4-48.9)	47.7 (44.9-50.7)	51.3 (48.7-54.2)	53.1 (50.6-56.3)	54.7 (52-57.7)	56.4 (53.6-59.4)	57.8 (55.2-60.6)	58.6 (56.1-61.3)	58.3 (56.1-60.7)	55.9 (53.5-58.3)	54 (51.4-56.8)	55.9 (53.8-58.2)	61.3 (59.6-63)	65.8 (63.8-67.9)
Kisumu	43.3 (39.6-47.4)	45.5 (42.5-48.9)	49 (46.2-52)	51.5 (48.8-54.4)	53.2 (50.5-55.9)	55.4 (52.7-58.4)	57.5 (54.8-60.1)	58.8 (56.3-61.2)	55.4 (52.1-58.5)	45.8 (41.3-50.5)	41.7 (38.1-45.9)	44.3 (41.6-47.3)	52.3 (49.9-54.9)	59 (55.8-62.2)
Kitui	46.5 (43.4-49.7)	47.5 (44.8-50.5)	50.9 (48-53.8)	53 (50.2-55.9)	55.3 (52.5-58.2)	58 (55.4-60.7)	61.1 (58.4-63.8)	62.6 (60.1-65.2)	62.8 (60.5-65.3)	60.8 (58.4-63.7)	59.6 (57.1-62.4)	61.2 (58.7-63.7)	64.8 (62.6-67.1)	68.8 (66.5-70.9)
Kwale	41.9 (39.1-45)	44.6 (41.5-48.2)	48.2 (45.4-51.1)	50 (47.2-52.9)	51.6 (48.9-54.3)	53.8 (51-56.8)	56.6 (53.9-59.4)	58.9 (56.1-61.7)	56.8 (51.5-60.6)	51.8 (46.4-56.8)	52.6 (48.7-56.7)	56.5 (53.5-59.5)	60.9 (58.5-63.5)	66.1 (63.8-68.5)
Laikipia	58.1 (55-61.7)	58.9 (56-62.2)	62.3 (59.3-65.7)	64.2 (61.1-67.3)	65.6 (62.5-68.5)	67.4 (64.4-70.3)	69.3 (66.5-72)	70.5 (68.1-73.1)	69.5 (67-72.1)	66.6 (63.7-69.6)	63.1 (59.5-66.9)	62.2 (58.7-66)	64.2 (61.1-67.8)	68.2 (64.8-71.2)
Lamu	31.1 (28.2-34.3)	34.9 (32.3-37.9)	38.7 (35.8-42)	41.6 (38.8-44.9)	46.3 (43.5-49.2)	53.2 (50.3-56.5)	59.1 (56.1-62.1)	61.1 (58.1-64.2)	61.3 (58.4-64.2)	61 (58-63.9)	59.9 (56.9-63.5)	60.6 (57.1-64.1)	62.6 (59.2-65.8)	64.5 (61.5-67.7)
Machakos	50.6 (47.5-53.8)	52 (49.2-55)	55.4 (52.6-58.6)	57.8 (54.9-60.7)	59.9 (57-62.9)	62.5 (59.8-65.5)	65.1 (62.6-67.6)	66.7 (64.4-69.1)	67.2 (64.9-69.6)	66 (63.2-68.6)	64 (61-66.9)	63.5 (60.7-66.2)	65.8 (63.5-68.1)	68.6 (66.3-71.1)
Makueni	59.7 (56.8-62.9)	59.2 (56.2-62.2)	60.7 (57.8-63.8)	61.2 (58.1-64.1)	61.9 (59-64.9)	63 (60.2-66)	64 (61.2-66.9)	64.6 (62-67.3)	64.5 (61.9-67.1)	62.2 (58.7-65.5)	58.4 (54.2-62.4)	57.5 (53.9-61.4)	61.2 (58.2-64.3)	67.6 (65-70.2)
Mandera	42.5 (39.8-45.2)	42.4 (40.1-44.9)	48.5 (45.8-51.3)	50.1 (47.5-52.9)	52 (49.4-54.9)	54.4 (51.9-57)	56.6 (54.2-59.1)	57.7 (55.4-59.8)	57.6 (55.5-59.7)	57.3 (55.2-59.6)	58.3 (56.3-60.3)	58.3 (56.5-60.1)	59.1 (57.2-61)	62.1 (59.9-64.2)
Marsabit	32.6 (30-35.7)	33.8 (30.9-37.1)	38.8 (35.9-42)	42.6 (39.9-45.8)	47.2 (43.9-50.8)	53 (50.3-56.2)	58.1 (55.3-61.3)	60.5 (57.8-63.4)	61.7 (59.2-64.4)	61.8 (59.1-64.7)	60.7 (58.1-63.7)	59.9 (57.4-62.5)	62 (59.6-64.5)	64.5 (61.8-67.3)
Meru	50.2 (47.4-53.3)	52.6 (49.6-55.9)	56.7 (54-59.5)	59.3 (56.8-62.4)	61.7 (59-64.4)	64.1 (61.5-66.7)	66.5 (64.2-69)	67.8 (65.5-70.1)	67.1 (64.8-69.3)	64.2 (61-67.1)	61.5 (58.1-64.6)	61.6 (59.1-64.1)	63.2 (61-65.4)	65.6 (63.2-68.3)
Migori	41.8 (39.2-44.4)	43.1 (40-46.5)	45.3 (42.3-48.7)	46.2 (43.1-49.6)	47.5 (44.6-50.3)	47.4 (44.9-50.1)	47.7 (45.4-50.2)	48.6 (46.2-51)	49.6 (47.2-52.2)	47.5 (44.2-50.8)	43.9 (40-47.3)	43.8 (40.2-47.2)	49.4 (46.3-52.5)	57.6 (54.4-61)
Mombasa	44.4 (41.4-47.7)	48.6 (45.6-51.7)	53.4 (50.3-56.5)	56.4 (53.5-59.6)	59.1 (56.1-62.4)	62.3 (59.4-65.6)	65.1 (62.1-68.2)	66.5 (63.6-69.4)	63.9 (60.3-67.2)	57.1 (52.8-61.8)	55.5 (51.7-59.9)	58.2 (54.8-61.9)	61.9 (58.7-65.1)	67.6 (64.6-70.8)
Murang'a	54 (51.4-57)	56.1 (53.5-58.8)	58.8 (56.1-61.5)	60.6 (57.8-63.6)	62.4 (59.6-65.2)	64.9 (62.4-67.4)	67.3 (64.9-69.7)	68.5 (66.2-70.7)	68.4 (66.1-70.6)	66.2 (63.4-68.8)	64.3 (61.4-67)	62.9 (60.5-65.5)	65.7 (63.6-67.9)	68.8 (66.6-71.1)
Nairobi	42.1 (39.3-44.9)	46.4 (43.8-49)	52.9 (50.4-55.5)	57.1 (54.5-59.8)	60.4 (57.8-63.1)	63.8 (61.5-66.2)	66.3 (64.1-68.4)	67.2 (65.2-69.2)	64.3 (61.6-67)	57.3 (53.9-60.8)	54.8 (52-58)	57.1 (55.2-59.1)	62.7 (61.2-64.2)	68 (66.4-69.6)
Nakuru	60.9 (57.8-64)	60.7 (57.8-63.7)	63.6 (60.7-66.6)	64.8 (61.8-67.7)	65.5 (62.6-68.4)	66.5 (63.7-69.2)	68 (65.6-70.7)	69.3 (67.2-71.7)	67.5 (64.6-70.8)	59.3 (54.3-64.2)	54.9 (50.3-59.7)	58 (54.7-61.7)	64.8 (62.2-67.3)	70.7 (68.4-72.6)
Nandi	62.4 (59.4-65.7)	62.3 (59.3-65.4)	64.2 (61.1-67.5)	64.6 (61.6-67.8)	64.7 (61.7-67.7)	65 (62.1-67.6)	65.3 (62.7-68)	65.5 (62.9-68)	63.6 (59.9-66.6)	59.1 (54.4-63.2)	58 (54.7-61.4)	60.2 (57.9-62.7)	63.7 (61.6-65.9)	68 (65.6-70.3)
Narok	59.5 (56.6-62.9)	57.8 (55-60.9)	60.5 (57.5-63.8)	60.4 (57.4-63.7)	59.9 (56.9-63.1)	59.5 (56.9-62.7)	59.7 (57.1-62.5)	59.9 (57.5-62.5)	59.8 (57.4-62.5)	58.6 (56.2-61.2)	57.8 (55.2-60.4)	58.3 (55.9-60.7)	61.9 (59.7-64.2)	65.4 (63.2-67.8)
Nyamira	53.8 (51-56.5)	54.6 (51.7-57.5)	56.3 (53.5-59.6)	57.3 (54.6-60.6)	58.7 (55.8-61.9)	59.8 (57-62.8)	60.7 (58-63.5)	61.6 (59.2-64.1)	61.4 (59-63.8)	58.8 (56-61.5)	55.1 (51.9-58.4)	53.6 (50.6-56.9)	56.2 (53.6-58.7)	61.5 (58.9-63.9)
Nyandarua	53 (49.9-56.1)	54.2 (51.3-57.3)	57.2 (54.1-60.4)	58.8 (55.6-62.1)	60.2 (57-63.5)	62 (58.8-65.1)	64.2 (61.3-67)	64.9 (62.4-67.5)	64.6 (62.2-66.9)	62.9 (60.6-65.2)	59.7 (57.4-62.1)	57.2 (55-59.5)	58.7 (56.6-60.9)	62.4 (59.8-65.1)
Nyeri	49.5 (46.6-52.5)	52.6 (49.9-55.5)	56.3 (53.4-59.2)	58.9 (56.1-61.8)	61 (58.3-64)	63.2 (60.5-66.1)	65.5 (63-68.3)	66.6 (64.2-69.2)	65.7 (63.3-68.1)	61.8 (58.7-64.7)	57.6 (54.3-60.8)	58 (55.2-60.9)	62.2 (60-64.6)	65.6 (63.1-68.3)
Samburu	65.7 (62.7-68.9)	65 (62.1-68.1)	66.2 (63.2-69.2)	66.2 (63.2-69.1)	65.4 (62.5-68.2)	64.9 (61.8-67.7)	64.8 (61.8-67.5)	64.9 (62.4-67.6)	64 (61.4-66.7)	60.9 (57-64)	58.4 (54.2-62.2)	59.1 (55.5-62.4)	61.3 (58.4-64.3)	64.9 (62-67.7)
Siaya	36.3 (34-38.9)	39 (36.4-42)	43.1 (40.6-45.9)	46.2 (43.5-49.1)	48.8 (46.3-51.6)	51.4 (48.6-54.4)	54 (51.1-56.9)	55.2 (52.6-57.7)	54.3 (51.9-56.7)	52 (48.5-55)	49 (44.3-53.1)	48 (44.1-52.1)	55.4 (52.4-58.7)	64.6 (61.4-67.5)
Taita Taveta	32 (29.1-34.8)	37.1 (34.2-40.3)	42.3 (39.5-45.1)	46.4 (43.7-49.3)	50 (47.3-52.8)	54.4 (51.5-57.4)	58.5 (55.7-61.6)	61.7 (59.2-64.5)	63 (60.3-65.8)	61.7 (58.9-64.5)	58.9 (56-62)	57.4 (54.5-60.7)	59 (56.3-61.9)	64.1 (61.5-67.1)
Tana River	40.6 (37.6-43.5)	44.1 (41.1-46.9)	48 (45.1-51.2)	50.4 (47.4-53.3)	53 (49.8-56.2)	55.5 (52.5-58.8)	58.1 (55.2-61.3)	59.3 (56.4-62.5)	59.4 (56.6-62.5)	57.1 (54.2-60.2)	54.3 (51.6-57.4)	53.6 (51.2-56.3)	55.3 (53-58)	59.8 (57-62.6)
Tharaka Nithi	49.6 (46.5-52.9)	50 (46.9-53.1)	51.6 (48.4-54.8)	52.7 (49.7-56)	54.2 (51-57.4)	56.3 (53.3-59.4)	58 (55.1-61.1)	58.7 (55.8-61.5)	58.5 (55.8-61.1)	57.5 (54.9-60.2)	57.1 (54.5-59.6)	59.1 (56.8-61.5)	62.5 (60.4-64.6)	65.8 (63.3-68.2)
Trans Nzoia	63.9 (60.7-67.3)	62.6 (59.7-65.6)	65.4 (62.3-68.4)	65.8 (62.6-68.8)	65.7 (62.8-68.7)	66.3 (63.3-69)	67.4 (64.7-70.1)	68.6 (66.1-71.1)	67.7 (64.7-70.5)	62.6 (58.6-66.6)	59.1 (54.9-63.4)	60.5 (56.8-64.1)	63.6 (60.3-66.9)	68.1 (64.6-71.1)

Table 6 Estimates of life expectancy at birth by location and year for 1950, 1955...2005, 2010, 2017 - both sexes														
**Location**	**1950**	**1955**	**1960**	**1965**	**1970**	**1975**	**1980**	**1985**	**1990**	**1995**	**2000**	**2005**	**2010**	**2017**
Turkana	57.7 (54.7-60.7)	56.8 (53.7-60)	58.9 (56-61.9)	59 (56.1-62.1)	57.5 (54.6-60.5)	56.1 (53.4-58.9)	56.1 (53.6-58.7)	57.5 (55.1-59.8)	58.2 (55.9-60.8)	56.8 (53.6-59.8)	52.5 (49.2-55.4)	53.8 (50.6-57.1)	56.6 (53.9-59.4)	62.3 (59.6-65)
Uasin Gishu	64 (61.1-67)	63.6 (60.9-66.5)	65.7 (62.8-68.6)	66 (63.4-68.8)	66.3 (63.7-69)	66.6 (63.8-69.4)	67 (64.6-69.7)	67.4 (65-69.9)	67 (64.7-69.6)	65.7 (63.2-68.4)	63.4 (60.7-66.2)	63.7 (61.4-66.1)	65.8 (63.6-68)	70 (67.5-72.4)
Vihiga	54.9 (51.9-58)	55.6 (52.5-58.8)	57.4 (54.3-61)	58.5 (55.4-61.9)	59.8 (56.9-63)	60.4 (57.3-63.6)	61.4 (58.3-64.3)	61.6 (58.7-64.6)	60.4 (57.3-63.4)	55.7 (51.6-59.4)	52.5 (49-56.1)	54 (51.1-57.2)	60.7 (58-63.4)	64.5 (61.8-67.3)
Wajir	55.6 (53-58.4)	54.3 (51.8-56.9)	57.8 (55-60.7)	58.1 (55.7-60.7)	57.5 (55-60.2)	57.5 (55-60.1)	57.6 (55.3-59.9)	57.4 (55.1-59.7)	56.9 (54.8-58.9)	56 (54-58.2)	55.3 (53.6-57.3)	57.5 (55.9-59.2)	59.9 (58.2-61.6)	61.5 (58.9-64)
West Pokot	55 (52.2-57.9)	53.7 (50.9-56.7)	55.8 (53-58.7)	55.9 (53-58.7)	55.2 (52.4-58)	55.3 (52.7-57.9)	56.8 (54.1-59.7)	58.3 (56.1-60.6)	58.6 (56.2-61)	58.1 (55.8-60.3)	59.9 (57.7-62.1)	62.1 (60.2-64)	63.4 (61.5-65.3)	66.9 (64.7-69)
Madagascar	46.1 (43.9-48.3)	47.3 (45.3-48.9)	48.6 (47-50.2)	50.3 (48.9-51.7)	51.3 (50.2-52.4)	53.2 (52.1-54.2)	54.5 (53.4-55.6)	54.3 (53.1-55.3)	55.4 (54.5-56.4)	57.1 (56.1-58)	58.9 (57.9-60)	60.7 (59.5-62.1)	61.6 (60.1-63.3)	63.5 (61.6-65.4)
Malawi	34.1 (31.3-37)	35.7 (32.9-38.5)	37.6 (35.2-40.3)	39.5 (37.5-41.7)	42.1 (40.5-43.9)	45.6 (44.2-47)	48.6 (47.5-49.7)	49.7 (48.7-50.8)	48.6 (46.9-50)	45.6 (43.1-47.9)	44.5 (42-47.1)	47.5 (45.2-49.8)	54.2 (52.6-55.7)	63.2 (61.9-64.6)
Mozambique	41 (38.1-43.9)	42.1 (39.3-44.7)	43.3 (40.7-46)	44.3 (41.7-47)	46.2 (44-48.6)	48.6 (46.7-50.6)	49.7 (48.2-51.3)	45.1 (44.1-46.1)	50.3 (49.1-51.5)	50.6 (49.4-51.9)	50.4 (48.8-52)	50.5 (48.6-52.5)	52 (50.1-53.8)	58.3 (56.5-60.1)
Rwanda	39 (36.4-41.5)	40.9 (38.6-43.3)	43.1 (41.4-45)	43.8 (42.2-45.5)	44.4 (42.8-46)	45.5 (43.9-47)	48.1 (46.7-49.5)	50.8 (49.6-52)	49.4 (48.4-50.5)	46.5 (45.3-47.5)	49.6 (48.4-50.8)	57 (56-58.1)	64.3 (63.2-65.4)	68.4 (67.1-69.9)
Somalia	45.4 (42.4-48.5)	46.3 (43.4-49.2)	47.3 (44.5-50.3)	47.9 (45.2-50.9)	48.3 (45.5-51.4)	47.6 (44.9-50.4)	49.9 (47.2-52.7)	49.7 (47.2-52.3)	50 (47.6-52.6)	51 (48.4-53.6)	52.5 (49.9-55.3)	54.6 (51.8-57.6)	54.9 (52-57.7)	58.5 (55.6-61.2)
South Sudan	46.2 (43-49.9)	46.8 (43.7-50.2)	47.4 (44.3-50.6)	48 (44.8-51.2)	48.8 (45.5-52.1)	49.8 (46.6-53.1)	50.4 (47.4-53.4)	50.6 (47.9-53.4)	51.1 (48.5-53.9)	52.9 (50.3-55.7)	54.3 (51.5-57.3)	56.6 (53.4-60)	57.8 (54.5-61.1)	59.2 (56.1-62.4)
Tanzania	44.1 (41.5-46.9)	45.5 (43-48.1)	46.7 (44.5-48.9)	48.4 (46.7-50.2)	50.9 (49.5-52.5)	53.4 (52-54.7)	55.4 (54.2-56.5)	56.2 (55-57.2)	54.9 (53.2-56.3)	52.8 (50.8-54.5)	53.5 (51.7-55.3)	56.7 (55-58.4)	60.9 (59.5-62.3)	66.7 (65.5-68)
Uganda	46.1 (43.5-48.6)	46.8 (44.3-49.1)	47.9 (45.5-50.1)	49.4 (47.5-51.4)	51.1 (49.4-53)	52.1 (50.7-53.7)	50.1 (48.9-51.4)	49.6 (46.6-51.7)	47.5 (44.4-50.7)	46.8 (44.9-49)	49.1 (47.6-50.6)	53.8 (52.5-55)	59.1 (57.7-60.4)	65.7 (64.2-67.2)
Zambia	45.6 (43.1-48.3)	47.2 (44.8-49.8)	48.6 (46.1-51)	50.5 (48.6-52.6)	53.4 (51.6-55.1)	55.2 (53.6-56.7)	55.4 (54.1-56.8)	54.5 (53.3-55.5)	50.7 (48.5-52.4)	46.1 (43.9-48.4)	45.9 (43.8-48)	49.3 (47.2-51.2)	56 (54.5-57.2)	63.2 (61.7-64.6)
Southern sub-Saharan Africa	51.6 (49.8-53.1)	52.5 (51-53.9)	53.6 (52.2-54.9)	55 (53.7-56.2)	56.5 (55.6-57.5)	58.4 (57.6-59.1)	59.9 (59.2-60.6)	62.5 (61.9-63.1)	64.1 (63.2-64.7)	61.4 (60.3-62.3)	53.6 (52.5-54.5)	50.5 (49.4-51.4)	54.7 (53.8-55.4)	65 (64.3-65.7)
Botswana	52.2 (50-54.4)	53.1 (50.9-55.2)	54 (52-56.1)	55.3 (53.4-57.2)	57.2 (55.5-58.9)	60.3 (58.8-61.9)	62.7 (61.4-64)	64.6 (63.2-65.9)	63.5 (60.7-65.6)	59.8 (56.5-62.9)	53.8 (49.3-58.2)	52.6 (49.4-56.1)	60.1 (57.9-62)	69.1 (67.4-70.4)
Lesotho	50.1 (48.1-52.1)	51.1 (49.4-52.9)	52.1 (50.3-54.1)	52.6 (50.8-54.5)	53.7 (52-55.6)	55.5 (53.9-57.2)	58.2 (56.6-59.8)	59.9 (58.4-61.3)	60.3 (59-61.7)	56.6 (54.4-58.5)	48.8 (46.1-51.4)	44.3 (41.8-47)	47.8 (45.5-49.8)	54.6 (52.7-56.7)
Namibia	53.5 (51.2-55.7)	54.2 (52.1-56.4)	55 (52.9-57.1)	56.4 (54.5-58.4)	56.3 (54.8-57.9)	57.4 (56-58.7)	57.3 (56.3-58.4)	59.1 (58.2-60.1)	61.6 (60.1-62.8)	59.8 (58.1-61.2)	54.5 (52.3-56.4)	52.6 (50-55)	58.9 (56.9-60.9)	66.5 (64.6-68.2)
South Africa	51.6 (49.8-53.4)	52.6 (50.8-54.3)	53.6 (52.1-55.2)	55 (53.5-56.4)	56.5 (55.3-57.7)	58.3 (57.4-59.3)	59.8 (59-60.6)	62.4 (61.7-63.1)	65.1 (64.6-65.6)	64.4 (63.9-64.9)	55.6 (54.6-56.5)	52 (50.8-53.2)	56.1 (55.1-57.1)	66.3 (65.5-67)
Swaziland	40.9 (38.3-43.4)	42.8 (40.3-45.3)	44.6 (42.2-47.1)	48.1 (45.7-50.5)	51.4 (49.3-53.7)	54.5 (52.6-56.5)	57.1 (55.4-58.9)	59.6 (58.1-61.2)	62.1 (60.8-63.4)	59.4 (57.4-60.9)	48.2 (45.1-51.2)	43.6 (40.8-46.4)	46 (43.3-48.4)	59.8 (57.9-61.8)
Zimbabwe	53 (50.7-55.2)	53.8 (51.6-55.9)	54.9 (52.8-56.9)	56.1 (54.3-57.9)	57.6 (56-59.3)	59.2 (57.8-60.7)	60.6 (59.5-61.8)	63.6 (62.3-64.6)	60.9 (56.9-63.5)	51.9 (48-55.8)	46.6 (43.6-49.9)	45.1 (42.5-47.8)	49.6 (47.7-51.4)	61.3 (59.8-62.9)
Western sub-Saharan Africa	41.2 (38.6-43.6)	42.7 (40.4-44.9)	44 (42.2-45.7)	46.5 (45-48)	47.7 (46.3-49.1)	50.3 (48.9-51.5)	52.7 (51.4-53.8)	54.2 (53-55.3)	54.7 (53.4-55.8)	54.6 (53.3-55.9)	54.9 (53.5-56.3)	56.8 (55.4-58.3)	59.7 (58.2-61.1)	63.5 (61.9-64.8)
Benin	40.5 (37.8-43.2)	41.4 (38.8-43.8)	42.3 (40.3-44.4)	44.3 (42.4-46.2)	46.4 (44.6-48.2)	49.1 (47.5-50.7)	51.5 (50.2-52.9)	53.2 (52-54.4)	55.2 (54-56.3)	56.7 (55.6-57.9)	57.5 (56.2-58.9)	59.2 (57.8-60.7)	61.4 (59.8-63.1)	64.6 (62.7-66.5)
Burkina Faso	35.9 (32.9-38.7)	37.4 (34.5-40.5)	39.3 (36.5-42.2)	41.3 (38.9-43.9)	42.9 (40.9-44.9)	44.7 (43-46.4)	47.6 (46.2-49)	49.9 (48.8-51.2)	50.7 (49.5-52.1)	50.5 (49.1-51.9)	52.4 (51.2-53.6)	55.3 (54.2-56.5)	58.3 (56.9-59.7)	61.7 (59.9-63.2)
Cameroon	40.8 (38.4-43)	43.6 (41.7-45.4)	38.6 (37.2-39.9)	48.1 (46.5-49.8)	50.1 (48.6-51.7)	52.3 (51.1-53.6)	54.4 (53.2-55.5)	56.8 (55.8-57.9)	57.7 (56.7-58.6)	56.2 (55.2-57.2)	54.8 (53.5-56.1)	54.8 (53.3-56.4)	57.6 (56.1-59.4)	62.9 (61.1-64.9)
Cape Verde	57.7 (55.6-59.8)	58.8 (57-60.6)	57.9 (56.2-59.6)	60.6 (59-62.2)	58.4 (57.2-59.5)	62.9 (62.1-63.8)	67.1 (66.6-67.6)	68 (67.7-68.4)	70.3 (69.9-70.6)	69.2 (68.4-70)	71.2 (70.1-72.2)	72.5 (71.6-73.4)	74.3 (73.8-74.7)	75.8 (74.9-76.6)
Chad	43.6 (40.6-46.5)	45.1 (42.3-47.9)	46.6 (43.7-49.3)	47.9 (45.3-50.6)	48.1 (45.8-50.4)	50.2 (48.3-52.2)	50.6 (48.8-52.2)	52.3 (50.8-53.6)	53.4 (52.1-54.6)	53.2 (51.7-54.5)	52.1 (50.6-53.5)	53.6 (52.2-55.1)	56.4 (54.9-58)	60 (58.2-61.8)
Cote d'Ivoire	41.6 (38.9-44.1)	42.9 (40.7-45)	45.1 (43.3-47)	47.7 (46-49.4)	50.5 (49-52.2)	53.6 (52.1-55)	56.5 (55.3-57.9)	57 (55.3-58.2)	54.8 (51.8-56.6)	51.7 (49.7-53.5)	52.3 (50.8-53.9)	54.2 (52.7-55.8)	57.4 (55.8-59.1)	62.5 (60.6-64.3)
The Gambia	46.7 (44.1-49.3)	48 (45.4-50.6)	49.3 (46.7-51.7)	50.9 (48.3-53.4)	53 (50.6-55.4)	54.8 (52.6-57.1)	56.8 (54.9-58.8)	58.6 (56.7-60.4)	59.9 (58.2-61.7)	61 (59.6-62.6)	61.7 (60.3-63.2)	62.3 (61-63.7)	63.6 (62.2-65)	65.7 (64.2-67.3)
Ghana	44.6 (42.2-47.1)	46.6 (44.6-48.7)	49.8 (47.9-51.8)	52.4 (50.6-54.3)	54.2 (52.4-56.3)	56.2 (54.5-58)	57.5 (55.8-59.2)	57.9 (56.4-59.4)	59.2 (57.7-60.6)	59.5 (58.2-60.8)	59.4 (58.3-60.6)	60 (59-61.1)	61.6 (60.6-62.6)	65.5 (64.3-66.8)
Guinea	39.4 (36.5-42.3)	40.6 (37.7-43.7)	42 (39.1-45)	43.7 (40.9-46.4)	44.5 (42.1-47.1)	45.7 (43.7-47.8)	48 (46.3-49.6)	50 (48.5-51.3)	51.9 (50.7-53.1)	53.4 (52.2-54.5)	54.4 (53.2-55.5)	55.9 (54.6-57.2)	57.4 (56-58.8)	60.7 (59.1-62.3)
Guinea-Bissau	31.7 (28.8-34.7)	34.1 (31.3-36.7)	36.5 (33.7-39.2)	36.1 (33.5-38.6)	38.5 (36.2-41)	43.3 (41-45.8)	45.2 (43-47.4)	46.6 (44.7-48.4)	48.6 (46.9-50.4)	50.4 (48.9-52)	52 (50.6-53.4)	53.2 (51.9-54.6)	55.9 (54.4-57.4)	60 (58.3-61.6)
Liberia	36.2 (33.4-39.1)	38 (35.3-40.8)	39.7 (37.4-42.2)	42 (39.9-44)	44.9 (43-46.8)	47.9 (46.1-49.7)	50.4 (48.6-52.2)	52 (50.4-53.5)	49 (47.7-50.4)	49.9 (48.3-51.3)	54.3 (52.6-55.8)	58.7 (57.3-60)	61.3 (60-62.5)	64.4 (62.8-66.1)
Mali	33.5 (30.7-36.4)	34.8 (32-37.8)	36.1 (33.6-38.7)	37 (35-39.2)	38.4 (36.9-40.1)	40.6 (39.2-42.1)	44.1 (42.9-45.4)	46.8 (45.7-48)	49.3 (48.3-50.4)	51.2 (50.1-52.2)	53.3 (52.3-54.4)	56.7 (55.5-57.8)	59.4 (58.2-60.7)	61.9 (60.2-63.5)
Mauritania	45.7 (43.2-48.1)	47.3 (45.2-49.6)	48.4 (46.6-50.4)	50.7 (48.9-52.5)	53.3 (51.6-55)	53.5 (52-55.1)	56 (54.6-57.5)	57.7 (56.4-59)	59.6 (58.6-60.7)	61.4 (60.3-62.4)	63.5 (62.5-64.5)	65.7 (64.7-66.7)	68.2 (67.1-69.4)	70.5 (69-72)
Niger	36.8 (33.7-39.8)	38.1 (35.3-41.2)	39.6 (36.7-42.6)	41.8 (39.3-44.3)	42.7 (40.7-44.7)	42.6 (41-44.2)	44 (42.5-45.5)	44.7 (43.2-46.1)	46.6 (45.2-47.9)	48.5 (47.1-49.9)	51.8 (50.4-53.3)	55.6 (54.3-57)	59.1 (57.6-60.7)	62.3 (60.5-64.1)
Nigeria	42.7 (39.4-46.2)	44.2 (40.9-47.6)	45.8 (42.5-49)	48 (45.2-50.7)	48.7 (46.2-51.4)	51.8 (49.2-54.4)	54.4 (52-56.9)	55.8 (53.4-58.3)	55.5 (53-57.9)	55.3 (52.6-58.1)	55.1 (52.2-58.2)	57.2 (54.1-60.6)	60.4 (57.2-63.7)	64.3 (61-67.1)
Sao Tome and Principe	41.5 (39.3-43.6)	42.8 (41.8-43.7)	47.5 (46.7-48.4)	55.7 (55.1-56.3)	59.4 (58.8-59.9)	62.6 (61.9-63.3)	63.8 (63.2-64.5)	63 (62.3-63.7)	64.1 (63.1-65)	64 (62.9-65.1)	64.8 (63.8-65.8)	66.3 (65.4-67.3)	68.5 (67.4-69.5)	69.9 (68.7-71.2)
Senegal	42.5 (39.9-45)	43.7 (41.6-46)	44.8 (42.9-46.9)	45.3 (43.6-47.1)	46.3 (44.7-48)	48.8 (47.3-50.3)	52.3 (51-53.6)	55.6 (54.5-56.7)	58.4 (57.5-59.4)	59.1 (58.1-60)	60.6 (59.8-61.5)	63.3 (62.5-64.2)	65.9 (64.9-66.9)	68 (66.7-69.3)
Sierra Leone	37.8 (34.7-40.7)	39.2 (36.2-42.1)	40.6 (37.6-43.6)	42.4 (39.5-45.4)	44.3 (41.6-47.1)	46.2 (43.8-48.6)	47.3 (45.3-49.3)	48.4 (46.7-50.1)	50.1 (48.6-51.6)	49.7 (48.6-51)	50.9 (49.7-52.1)	52.6 (51.3-53.8)	55.4 (53.9-56.7)	60.4 (58.7-62.2)
Togo	41.5 (38.5-44.2)	43 (40.4-45.7)	44.8 (42.2-47.5)	47.2 (45.2-49.6)	50.6 (48.7-52.6)	53.2 (51.6-55)	55.4 (53.9-57)	56.7 (55.4-58.2)	57.6 (56.3-58.9)	57.4 (56.2-58.7)	56.8 (55.3-58.5)	56.9 (55.3-58.6)	59.2 (57.6-60.9)	64.3 (62.6-66.2)

Table 7 Estimates of life expectancy at birth by location and year for 1950, 1955...2005, 2010, 2017 - females

Location	1950	1955	1960	1965	1970	1975	1980	1985	1990	1995	2000	2005	2010	2017
Global	52.9 (51.7-54)	55.5 (54.5-56.4)	47.7 (47-48.4)	58.8 (58.2-59.4)	60.5 (60.1-61)	62.6 (62.2-63)	64.8 (64.5-65)	66.3 (66-66.5)	68 (67.8-68.3)	68.8 (68.6-69)	70 (69.7-70.2)	71.6 (71.4-71.8)	73.5 (73.3-73.7)	75.6 (75.3-75.9)
Low SDI	41.2 (39.6-42.7)	43 (41.5-44.5)	43.5 (42.1-44.9)	44.3 (43-45.5)	45.8 (44.9-46.8)	48.3 (47.5-49.1)	50.8 (50.1-51.6)	52.3 (51.7-53)	54.8 (54.3-55.4)	56.6 (56-57.1)	58.4 (57.9-58.8)	61.3 (60.8-61.7)	63.6 (63.2-64)	67.3 (66.7-67.9)
Low-middle SDI	44.8 (43.4-46.1)	47.4 (46.2-48.6)	44.7 (43.7-45.6)	50.2 (49.3-51.1)	52.5 (51.7-53.2)	54.8 (54.2-55.5)	58.3 (57.7-58.9)	60 (59.5-60.5)	61.9 (61.5-62.3)	62.8 (62.4-63.2)	63.5 (63.1-64)	65.4 (65-65.9)	67.6 (67.1-68.1)	70.1 (69.5-70.7)
Middle SDI	49.3 (47.9-50.6)	52.5 (51.3-53.7)	28.5 (28-29)	57.6 (56.8-58.4)	60.2 (59.5-61)	63.3 (62.7-63.9)	65.8 (65.3-66.3)	67.9 (67.5-68.3)	70 (69.7-70.3)	71.7 (71.4-71.9)	73 (72.7-73.2)	74.4 (74.2-74.6)	75.8 (75.6-76.1)	77.4 (77.1-77.7)
High-middle SDI	59.6 (58.4-60.8)	61.7 (60.6-62.8)	57.5 (56.7-58.3)	66 (65.2-66.8)	67.8 (67.2-68.4)	69.5 (69-69.9)	70.7 (70.4-71.1)	72 (71.7-72.2)	73.1 (72.9-73.4)	73.3 (73.1-73.4)	74.9 (74.7-75)	76.1 (76-76.2)	77.8 (77.6-77.9)	79.4 (79.1-79.7)
High SDI	66.9 (66.5-67.2)	70.5 (70.3-70.6)	72 (71.8-72.1)	73.5 (73.4-73.6)	74.4 (74.3-74.5)	75.9 (75.8-76)	77.2 (77.1-77.2)	78.2 (78.2-78.3)	79.3 (79.3-79.3)	80.1 (80.1-80.1)	81.1 (81.1-81.1)	82.1 (82.1-82.1)	83.1 (83.1-83.1)	83.7 (83.5-83.9)
Central Europe, Eastern Europe, and Central Asia	65 (64.2-65.7)	66.9 (66.4-67.5)	69.6 (69.4-69.9)	71.2 (70.9-71.5)	71.8 (71.6-72)	72.5 (72.3-72.7)	72.6 (72.4-72.7)	73 (72.9-73.1)	73.9 (73.8-74)	72.2 (72.2-72.3)	73.3 (73.3-73.4)	73.6 (73.6-73.7)	75.6 (75.5-75.6)	77.6 (77.4-77.7)
Central Asia	54.8 (52.9-56.7)	57.9 (56.4-59.3)	61.2 (60.2-62.2)	64.1 (63.1-65.1)	66.8 (66.2-67.4)	68.2 (67.7-68.8)	69.6 (69.3-69.9)	70.7 (70.5-71)	71.8 (71.5-72)	70.2 (70-70.4)	71 (70.7-71.3)	71.2 (70.9-71.4)	73.1 (72.8-73.3)	74.8 (74.3-75.4)
Armenia	53.4 (50.6-56.2)	57.7 (55.4-60.2)	62.4 (60.4-64.3)	66.1 (64.3-67.9)	69.1 (67.9-70.1)	70.5 (69.2-71.7)	72.7 (72.1-73.5)	73.8 (73.2-74.3)	73.3 (72.8-73.8)	73.6 (73.4-73.9)	75.2 (75-75.4)	76.4 (76.3-76.6)	77.3 (77.2-77.5)	78.7 (78.2-79.1)
Azerbaijan	42.9 (40.1-46.1)	47.7 (45-50.3)	53.9 (51.7-56.1)	59.5 (57.3-61.6)	64.2 (62.7-65.5)	67.6 (66.3-68.9)	69.1 (68.3-69.8)	70.2 (69.6-70.8)	71.2 (70.4-71.9)	69.2 (68.6-69.9)	70 (69.4-70.6)	70.6 (70-71.2)	72.8 (72.2-73.4)	74.7 (73.7-75.7)
Georgia	56.7 (54.1-59.5)	60.2 (58.1-62.3)	63.9 (62-65.5)	66.9 (65.1-68.7)	69.7 (68.8-70.6)	71.1 (69.9-72.6)	73.1 (72.3-73.8)	73.8 (73.2-74.3)	73.9 (73.4-74.4)	75.3 (74.7-75.9)	76.8 (76.3-77.3)	77 (76.8-77.2)	77.1 (76.9-77.3)	77.3 (76.9-77.7)
Kazakhstan	61 (58.4-63.6)	63.2 (61.4-65)	65.6 (64.3-66.9)	67.4 (65.8-69.2)	69.4 (68.6-70.1)	70.3 (69.2-71.4)	71.2 (70.9-71.6)	72.2 (71.9-72.4)	73.3 (73-73.5)	70.2 (69.9-70.4)	71.1 (70.8-71.3)	71.2 (70.9-71.4)	73.4 (73.2-73.6)	76.4 (75.8-77.1)
Kyrgyzstan	50.9 (48.2-53.8)	54.2 (51.9-56.7)	57.5 (55.2-59.5)	60.3 (58.2-62.3)	62.9 (61.2-64.6)	65 (63.2-66.8)	67.2 (66.2-68.2)	68.7 (67.9-69.5)	70.5 (69.7-71.1)	69.9 (69.2-70.6)	71.4 (70.7-72.1)	71.9 (71.5-72.4)	74.1 (74-74.3)	76.3 (75.9-76.6)
Mongolia	43.6 (40.9-46.6)	46.5 (43.8-49.3)	49.9 (47.6-52.4)	53.6 (52-55.3)	56.4 (54.9-57.7)	59.9 (58.8-61.1)	61.1 (60-62.1)	61.8 (60.9-62.7)	64 (63.3-64.7)	63.5 (62.9-64.1)	65.2 (64.7-65.7)	68.2 (67.7-68.7)	70.5 (70-71)	73.7 (72.5-74.8)
Tajikistan	44.9 (41.9-48.1)	48 (45.4-50.4)	52.1 (49.9-54.4)	56.5 (54.2-58.8)	61.1 (59.5-62.5)	63.2 (61.8-64.8)	66.7 (66-67.5)	68.1 (67.5-68.8)	69.5 (68.9-70.1)	68.1 (67.3-68.8)	70.4 (69.6-71.1)	71.8 (71-72.6)	72.9 (72-73.7)	73.3 (72.1-74.5)
Turkmenistan	54.2 (51.4-56.9)	56.6 (54.6-58.6)	59.5 (57.9-61.1)	62.1 (60.3-63.9)	64.6 (63.4-65.7)	65.6 (64.2-66.9)	66.9 (66.2-67.6)	68.2 (67.6-68.8)	69.3 (68.8-69.9)	68.3 (67.9-68.7)	70.6 (70.2-71)	70.2 (69.8-70.5)	73 (72.6-73.3)	73.9 (72.7-74.9)
Uzbekistan	59.8 (57.2-62.7)	61.9 (60-63.7)	64.1 (62.6-65.6)	66.3 (64.7-67.9)	68.2 (67.3-69)	68.8 (67.7-69.8)	69.8 (69.3-70.2)	71.2 (70.8-71.5)	72.6 (72.2-72.9)	70.7 (70.3-71)	70.1 (69.6-70.7)	69.7 (69.1-70.3)	71.8 (71.3-72.3)	73.7 (72.2-75.3)
Central Europe	61.5 (61-62)	65.5 (65.3-65.7)	68.8 (68.7-68.9)	70.9 (70.8-71)	71.9 (71.9-72)	73.4 (73.4-73.5)	73.7 (73.7-73.8)	74.2 (74.1-74.2)	74.9 (74.9-75)	75.3 (75.2-75.4)	76.8 (76.7-76.8)	77.8 (77.8-77.9)	79.1 (79.1-79.2)	80.4 (80.2-80.7)
Albania	50.1 (47.2-52.8)	54.7 (52.3-57)	62.2 (60.4-64)	65.6 (63.7-67.6)	69.6 (67.1-72)	72.9 (70.4-75.6)	75.4 (73.4-77.4)	76.4 (75.5-77.3)	77.4 (77-77.7)	78.2 (77.9-78.5)	78.3 (77.7-78.9)	79.3 (78.7-79.9)	81.1 (80-82.1)	82.1 (79.9-84.3)
Bosnia and Herzegovina	51.6 (51.2-52)	55.6 (55.2-55.9)	61.7 (61.5-62)	65.1 (64.9-65.3)	67.9 (67.7-68.1)	70.5 (70.3-70.7)	72.3 (72.1-72.5)	74.2 (74-74.3)	76.6 (76.4-76.8)	71.4 (69.9-72.9)	78.1 (77-79.2)	78.8 (78.7-79)	78.7 (78.6-78.9)	79.1 (78.4-79.7)
Bulgaria	63.6 (63.4-63.9)	67.9 (67.7-68.1)	72.4 (72.2-72.5)	74.7 (74.5-74.8)	75 (74.9-75.1)	75.3 (75.1-75.4)	75.4 (75.3-75.5)	75.7 (75.6-75.9)	75.5 (75.4-75.7)	75 (74.8-75.1)	75.1 (75-75.2)	76.3 (76.2-76.5)	77.4 (77.2-77.5)	78.6 (77.9-79.2)
Croatia	57.4 (57.1-57.8)	61.9 (61.7-62.2)	66.9 (66.7-67.1)	70 (69.8-70.1)	71.6 (71.4-71.8)	73.8 (73.6-73.9)	75 (74.8-75.2)	75.5 (75.3-75.6)	76.3 (76.1-76.4)	76.9 (76.7-77)	78 (77.9-78.2)	79.1 (78.9-79.3)	79.9 (79.7-80.1)	81.6 (80.9-82.3)
Czech Republic	66.5 (66.4-66.7)	71.4 (71.3-71.5)	73.2 (73-73.3)	73.5 (73.3-73.6)	73.1 (73-73.3)	74.1 (73.9-74.2)	74.1 (73.9-74.2)	74.8 (74.7-74.9)	75.5 (75.4-75.6)	76.7 (76.6-76.8)	78.3 (78.2-78.5)	79.4 (79.3-79.5)	80.8 (80.7-80.9)	82 (81.3-82.6)
Hungary	64 (63.8-64.2)	69.1 (69-69.3)	70.7 (70.5-70.8)	72 (71.8-72.1)	72.1 (72-72.3)	72.5 (72.3-72.6)	72.8 (72.7-72.9)	73 (72.9-73.1)	73.9 (73.8-74)	74.7 (74.6-74.8)	76.1 (76-76.2)	77.1 (77-77.3)	78.4 (78.3-78.5)	80.2 (79.5-80.9)
Macedonia	50.2 (48.7-51.8)	50.7 (49.6-51.9)	57 (55.9-58.2)	61.7 (60.7-62.7)	66.2 (65.6-66.8)	69.7 (69.4-70)	71.4 (71.2-71.7)	72.5 (72.3-72.8)	74.5 (74.2-74.7)	75.6 (75.4-75.9)	76.8 (76.6-77.1)	77.9 (77.7-78.1)	78.8 (78.6-79)	79.7 (79.2-80.3)
Montenegro	66.6 (65.7-67.4)	69 (68.4-69.5)	71.7 (71.1-72.3)	73.4 (72.9-74)	75.1 (74.6-75.6)	74.8 (74.3-75.2)	75.2 (74.9-75.5)	75.9 (75.7-76.1)	77.5 (77.2-77.8)	77.2 (76.9-77.5)	76.7 (76.4-77)	77.2 (76.7-77.6)	78.5 (78-79)	78.9 (78.1-79.7)
Poland	62.2 (62-62.4)	66.5 (66.4-66.6)	70.9 (70.8-71)	72.8 (72.8-72.9)	73.7 (73.6-73.8)	74.9 (74.8-74.9)	75 (74.9-75)	75.1 (75-75.2)	75.8 (75.7-75.8)	76.6 (76.5-76.7)	78.1 (78-78.1)	79.4 (79.4-79.5)	80.6 (80.5-80.7)	81.8 (81.2-82.4)
Romania	63.9 (61.2-66.7)	65.4 (64.5-66.3)	66.9 (66.8-67.1)	70.1 (70-70.2)	71 (70.9-71.1)	72.5 (72.4-72.6)	72.2 (72.1-72.3)	72.7 (72.6-72.7)	73.2 (73.1-73.3)	73.4 (73.3-73.5)	74.7 (74.6-74.8)	75.7 (75.7-75.8)	77.5 (77.4-77.6)	79 (78.3-79.6)
Serbia	57.1 (56.8-57.4)	61.4 (61.1-61.6)	65.2 (65-65.4)	67.3 (67.2-67.5)	69.4 (69.2-69.5)	72.7 (72.5-72.8)	73.2 (73-73.3)	73.4 (73.3-73.5)	74.5 (74.3-74.6)	74.4 (74.3-74.5)	75.5 (75.3-75.7)	75.7 (75.5-75.8)	77.2 (77.1-77.3)	77.9 (77.2-78.5)
Slovakia	61.7 (60.4-63.1)	69.3 (68.2-70.5)	72.3 (71.3-73.4)	73.3 (72.1-74.3)	73.3 (72.5-74.2)	74.1 (74-74.3)	74.5 (74.4-74.7)	74.9 (74.7-75)	75.5 (75.3-75.6)	76.6 (76.5-76.8)	77.6 (77.5-77.8)	78.3 (78.1-78.4)	79.3 (79.2-79.5)	80.6 (79.9-81.3)
Slovenia	60.3 (59.9-60.6)	65 (64.7-65.3)	68.5 (68.2-68.8)	71 (70.7-71.2)	72 (71.8-72.3)	73.3 (73-73.5)	75.1 (74.9-75.4)	76.1 (75.9-76.4)	77.8 (77.6-78)	78.4 (78.2-78.7)	79.7 (79.5-80)	81.1 (80.9-81.3)	82.7 (82.5-83)	84.2 (83.5-85)
Eastern Europe	69.2 (68.2-70.1)	70 (69.2-70.8)	72.5 (72.2-72.7)	73.8 (73.5-74)	73.6 (73.4-73.9)	73.6 (73.4-73.8)	73.3 (73.1-73.5)	73.6 (73.5-73.7)	74.6 (74.6-74.7)	72 (72-72.1)	73 (72.9-73)	72.7 (72.7-72.8)	74.9 (74.9-74.9)	77.2 (77.1-77.4)
Belarus	70.8 (68.2-73.8)	71.8 (69.7-74.1)	73.6 (73-74.2)	75.3 (74.9-75.7)	75.4 (75.1-75.7)	75.7 (75.5-76)	75.3 (75-75.5)	75.1 (74.9-75.4)	75.7 (75.5-75.9)	74.2 (74-74.4)	74.7 (74.5-74.8)	74.9 (74.7-75)	76.4 (76.3-76.6)	78.8 (78.1-79.4)
Estonia	69.7 (67.1-72.2)	70.5 (68.4-72.7)	72.9 (72.6-73.1)	74.3 (74.1-74.6)	74.5 (74.2-74.7)	74.7 (74.5-75)	74.3 (74-74.5)	74.3 (74.1-74.6)	75 (74.7-75.2)	74.4 (74.1-74.6)	76.3 (76-76.5)	78.2 (77.9-78.5)	80.7 (80.4-80.9)	82.1 (80.7-83.5)
Latvia	71.1 (68.5-73.7)	72.2 (69.9-74.7)	73.7 (73.5-74)	75.1 (74.9-75.4)	74.4 (74.2-74.6)	74.6 (74.4-74.8)	74.3 (74.1-74.5)	74.4 (74.2-74.6)	74.7 (74.5-74.9)	73.3 (73.1-73.5)	76.2 (76-76.4)	76.6 (76.4-76.8)	78 (77.8-78.3)	79.8 (78.4-81.3)
Lithuania	68.7 (66.2-71.4)	70.2 (68-72.5)	72.2 (72-72.4)	75.2 (75-75.4)	75.3 (75.1-75.5)	75.6 (75.4-75.8)	75.6 (75.4-75.7)	75.4 (75.3-75.6)	76.2 (76-76.3)	75.1 (74.9-75.3)	77.6 (77.5-77.8)	77.5 (77.3-77.7)	78.9 (78.7-79.1)	80.2 (79.4-81)
Moldova	56.9 (54.5-59.7)	59.7 (58.1-61.5)	62.2 (60.9-63.5)	64.1 (62.5-65.7)	65.9 (65.1-66.8)	67.1 (66.1-68.2)	68.8 (68.4-69.2)	69.3 (69-69.6)	71.4 (71.2-71.7)	69.7 (69.5-70)	72.5 (72.2-72.7)	72.9 (72.8-73.1)	74.5 (74.3-74.6)	77.4 (77-77.9)
Russian Federation	69.5 (68.7-70.2)	70.2 (69.5-70.8)	72.6 (72.3-72.9)	73.9 (73.5-74.2)	73.7 (73.3-74)	73.5 (73.1-73.8)	73.1 (72.8-73.4)	73.4 (73.2-73.5)	74.6 (74.6-74.6)	71.8 (71.8-71.8)	72.4 (72.4-72.4)	72 (72-72.1)	74.4 (74.3-74.4)	77.2 (77.1-77.4)
Ukraine	68.9 (66.4-71.4)	69.7 (67.6-71.9)	72.6 (72.1-73.1)	73.7 (73.3-74.1)	73.7 (73.3-73.9)	73.9 (73.5-74.2)	73.7 (73.4-74)	74 (73.7-74.2)	74.7 (74.5-74.9)	72.3 (72.1-72.4)	73.8 (73.7-73.9)	73.8 (73.8-73.9)	75.7 (75.7-75.8)	76.5 (75.8-77.2)
High-income	67.1 (66.8-67.5)	70.7 (70.5-70.8)	72 (71.8-72.1)	73.3 (73.1-73.4)	74.2 (74.1-74.3)	75.7 (75.7-75.8)	77.1 (77.1-77.2)	78.4 (78.3-78.4)	79.4 (79.4-79.4)	80.2 (80.2-80.3)	81.2 (81.2-81.2)	82.2 (82.1-82.2)	83.1 (83.1-83.1)	83.6 (83.4-83.7)
Australasia	71.9 (71.8-72)	73.2 (73.1-73.4)	74.1 (74-74.2)	74.3 (74.2-74.4)	74.4 (74.3-74.5)	76 (75.9-76.1)	77.7 (77.6-77.8)	78.3 (78.2-78.4)	79.7 (79.6-79.8)	80.8 (80.7-80.8)	82 (81.9-82.1)	83.3 (83.2-83.4)	83.9 (83.9-84)	84.4 (83.4-85.4)
Australia	72 (71.9-72.2)	73.3 (73.2-73.4)	74.1 (74-74.2)	74.3 (74.2-74.4)	74.4 (74.3-74.5)	76 (75.9-76.1)	78.1 (78-78.2)	78.6 (78.5-78.7)	80 (79.9-80.1)	81.1 (81-81.2)	82.2 (82.1-82.3)	83.6 (83.5-83.7)	84.2 (84.1-84.3)	84.6 (83.4-85.7)
New Zealand	71.3 (71.1-71.6)	73 (72.7-73.2)	73.9 (73.6-74.1)	74.2 (74-74.5)	74.6 (74.3-74.9)	75.6 (75.4-75.8)	76.2 (76-76.4)	77.1 (76.9-77.3)	78.1 (77.9-78.3)	79.2 (79-79.3)	80.8 (80.7-81)	81.8 (81.6-82)	82.6 (82.5-82.8)	83.6 (83-84.2)
High-income Asia-Pacific	57.8 (57.2-58.4)	65.8 (65.1-66.5)	68 (67.4-68.7)	70.7 (70-71.3)	72.7 (72.1-73.3)	75.2 (74.9-75.4)	77.4 (77.3-77.4)	79.5 (79.4-79.5)	81 (81-81.1)	82.2 (82.2-82.2)	83.8 (83.8-83.8)	85 (85-85)	85.9 (85.9-86)	86.9 (86.7-87.2)
Brunei	55.8 (53.5-58.4)	56.6 (54.6-58.6)	60 (58.3-61.7)	64.6 (63.4-65.8)	66.1 (65.2-66.9)	68 (67.3-68.8)	70.1 (69.3-70.8)	71.4 (70.8-72.2)	72.1 (71.5-72.8)	74.4 (73.7-75)	76.1 (75.4-76.7)	76.9 (76.4-77.4)	77.3 (76.9-77.8)	77.5 (76.6-78.4)
Japan	61.6 (61-62.1)	68.3 (67.9-68.8)	70.7 (70.3-71.1)	73.4 (72.9-73.8)	75.1 (74.7-75.5)	77.2 (77-77.4)	79.1 (79.1-79.1)	80.8 (80.8-80.9)	82.2 (82.2-82.2)	83.1 (83.1-83.1)	84.7 (84.6-84.7)	85.6 (85.6-85.7)	86.4 (86.4-86.4)	87.2 (87-87.4)
Aichi	61.9 (59-64.7)	68.5 (65.8-71.3)	70.7 (68.3-73.3)	73.2 (71-75.6)	74.9 (72.7-77.1)	76.9 (75.6-78.1)	78.7 (78.6-78.8)	80.6 (80.4-80.7)	81.8 (81.6-81.9)	82.8 (82.6-82.9)	84.3 (84.2-84.4)	85.3 (85.1-85.4)	86.3 (86.1-86.4)	87 (85.7-88.2)
Akita	61.9 (58.9-65.1)	68.8 (66.2-71.6)	71.8 (69.4-74.4)	74.5 (72.4-76.7)	76.2 (74.5-77.9)	78.6 (77.8-79.5)	81 (80.8-81.2)	81.9 (81.7-82.1)	82.9 (82.7-83.1)	83.5 (83.3-83.7)	84.5 (84.3-84.7)	85.3 (85.1-85.5)	85.9 (85.7-86.1)	86.3 (85.2-87.6)
Aomori	60.5 (57.4-63.7)	67.6 (65-70.6)	70.1 (67.9-72.7)	73.1 (70.8-75.5)	75.1 (73.2-77.1)	77.4 (76.4-78.5)	79 (78.8-79.2)	80.3 (80.1-80.5)	82.1 (81.9-82.3)	82.7 (82.5-82.9)	83.9 (83.7-84.1)	84.9 (84.7-85.1)	85.5 (85.3-85.7)	86.2 (85-87.4)
Chiba	62.3 (59.3-65.5)	68.9 (66.2-71.7)	71 (68.5-73.6)	73.5 (71.1-76.1)	75.1 (73-77.3)	76.9 (75.6-78.2)	79.2 (79.1-79.4)	81 (80.9-81.1)	82.3 (82.2-82.5)	83.1 (83-83.2)	84.6 (84.4-84.7)	85.4 (85.3-85.6)	86.2 (86-86.3)	87.1 (85.9-88.3)
Ehime	61.5 (58.4-64.6)	68.4 (65.7-71.3)	71 (68.5-73.6)	73.8 (71.6-76.2)	75.8 (74-77.6)	77.7 (76.7-78.7)	79.4 (79.2-79.6)	81 (80.8-81.2)	82.5 (82.3-82.7)	83.2 (83-83.4)	84.6 (84.4-84.8)	85.6 (85.4-85.8)	86.4 (86.2-86.6)	87.6 (86.5-88.9)
Fukui	62.7 (59.8-65.6)	69.1 (66.6-71.6)	71 (69-73.3)	73.6 (71.8-75.4)	75.4 (73.9-76.9)	77.4 (76.6-78.2)	79.4 (79.2-79.6)	81.1 (80.9-81.3)	82.5 (82.3-82.7)	83.4 (83.2-83.7)	85.2 (85-85.4)	85.9 (85.6-86.1)	87.1 (86.9-87.3)	87.6 (86.5-88.7)
Fukuoka	61.2 (58.3-64)	68.2 (65.6-70.9)	70.7 (68.2-73.5)	73.3 (70.9-75.8)	75 (72.9-77.2)	77 (75.7-78.4)	79.1 (79-79.3)	80.8 (80.7-81)	82.2 (82.1-82.4)	83.3 (83.1-83.4)	84.6 (84.5-84.8)	85.7 (85.5-85.8)	86.5 (86.4-86.7)	87.4 (86.2-88.7)
Fukushima	60.4 (57.5-63.4)	67.7 (64.9-70.5)	70.3 (67.7-73.1)	73.1 (70.9-75.5)	74.9 (73-76.8)	77.2 (76.2-78.2)	79.4 (79.2-79.6)	80.6 (80.4-80.8)	82.4 (82.2-82.5)	83.1 (83-83.3)	84.5 (84.4-84.7)	85.6 (85.4-85.7)	86.3 (86.1-86.5)	86.6 (85.3-87.8)
Gifu	60.9 (58-63.9)	67.6 (65-70.6)	69.8 (67.4-72.5)	72.4 (70.2-74.7)	74 (72-76.1)	76.2 (75.2-77.3)	78.5 (78.3-78.7)	80.5 (80.3-80.6)	82 (81.8-82.1)	83.1 (82.9-83.3)	84.4 (84.2-84.5)	85.6 (85.4-85.8)	86.3 (86.1-86.5)	86.9 (85.8-88.1)
Gunma	61.6 (58.6-64.6)	68.2 (65.5-71.1)	70.3 (67.8-72.9)	72.9 (70.6-75.3)	74.6 (72.7-76.5)	76.5 (75.6-77.5)	78.5 (78.3-78.7)	80.5 (80.3-80.7)	82 (81.8-82.2)	83.1 (82.9-83.2)	84.6 (84.4-84.7)	85.3 (85.1-85.5)	86 (85.9-86.2)	86.8 (85.7-88)
Hiroshima	61.2 (58.2-64.1)	68.2 (65.4-71.1)	70.9 (68.5-73.7)	73.6 (71.4-75.9)	75.4 (73.3-77.6)	77.4 (76.1-78.7)	79.3 (79.2-79.5)	81.1 (80.9-81.3)	82.5 (82.3-82.6)	83.5 (83.4-83.7)	85.2 (85-85.4)	86.1 (85.9-86.3)	86.8 (86.6-87)	87.5 (86.3-88.7)
Hokkaidō	60.6 (57.7-63.8)	67.7 (65.1-70.4)	70.1 (67.5-72.7)	72.9 (70.4-75.4)	74.8 (72.6-77)	76.8 (75.5-78.1)	78.6 (78.5-78.7)	80.5 (80.3-80.6)	82.1 (81.9-82.2)	83.3 (83.2-83.4)	84.8 (84.7-85)	85.8 (85.6-85.9)	86.3 (86.2-86.5)	86.9 (85.7-88.2)
Hyōgo	61.5 (58.6-64.5)	68.3 (65.6-71.1)	70.6 (68-73.3)	73.3 (70.8-76)	75.1 (73.1-77.4)	77.2 (76.1-78.4)	78.8 (78.7-78.9)	80.4 (80.3-80.6)	81.7 (81.6-81.9)	80.3 (80.1-80.4)	84.4 (84.3-84.5)	85.4 (85.3-85.6)	86.3 (86.2-86.4)	87.3 (86.1-88.5)
Ibaraki	60.2 (57.4-63.3)	67.4 (64.8-70.1)	69.8 (67.2-72.3)	72.6 (70.2-75.1)	74.4 (72.5-76.7)	76.5 (75.1-77.7)	78.4 (78.2-78.5)	80.1 (79.9-80.3)	81.6 (81.4-81.7)	82.8 (82.6-82.9)	84.3 (84.1-84.4)	85.1 (84.9-85.3)	85.8 (85.6-86)	86.4 (85.2-87.7)
Ishikawa	62.3 (59.3-65.3)	68.7 (65.9-71.5)	70.6 (68.2-73)	73.1 (71.1-75.1)	74.8 (73.2-76.4)	77.1 (76.3-77.9)	78.9 (78.7-79.1)	81.2 (81-81.4)	82.7 (82.5-82.9)	83.4 (83.3-83.6)	84.9 (84.7-85.1)	86.4 (86.2-86.6)	86.8 (86.6-87)	87.5 (86.4-88.6)
Iwate	60.8 (57.6-64)	67.8 (65-70.7)	70 (67.6-72.5)	72.8 (70.8-74.8)	74.6 (73.1-76.2)	76.7 (75.9-77.5)	78.7 (78.5-78.8)	80.6 (80.5-80.8)	82.1 (81.9-82.2)	83.3 (83.2-83.5)	84.8 (84.6-85)	85.5 (85.3-85.7)	86.3 (86.1-86.5)	86.6 (85.4-87.8)
Kagawa	62.4 (59.6-65.3)	69 (66.4-71.7)	71.3 (69.1-73.8)	74 (72-76.2)	75.8 (74.2-77.6)	77.8 (76.9-78.7)	79.6 (79.4-79.9)	81.4 (81.2-81.6)	82.5 (82.3-82.7)	83.5 (83.3-83.7)	84.6 (84.4-84.8)	85.9 (85.7-86.1)	86.3 (86.1-86.5)	87.4 (86.3-88.6)
Kagoshima	59.3 (56.1-62.5)	66.4 (63.7-69.4)	69 (66.5-71.7)	71.8 (69.4-74.1)	73.6 (71.8-75.5)	75.9 (74.9-76.8)	78.4 (78.3-78.6)	80.4 (80.2-80.5)	82 (81.8-82.2)	83.2 (83.1-83.4)	84.9 (84.7-85.1)	85.7 (85.5-85.9)	86.3 (86.1-86.5)	87 (85.8-88.2)
Kanagawa	63 (60-66.2)	69.4 (66.9-72.1)	71.6 (69.1-74.1)	74.1 (71.6-76.6)	75.8 (73.7-78)	77.8 (76.5-79)	79.6 (79.5-79.7)	81.2 (81.1-81.3)	82.4 (82.3-82.5)	83.2 (83.1-83.3)	84.7 (84.6-84.9)	85.8 (85.7-85.9)	86.6 (86.5-86.7)	87.1 (85.9-88.3)
Kōchi	60.1 (57.1-63.4)	67.2 (64.5-70.1)	69.7 (67.1-72.4)	72.5 (70.3-74.7)	74.4 (72.6-76.1)	76.5 (75.5-77.5)	78.9 (78.7-79.2)	81.1 (80.9-81.3)	82.3 (82.1-82.5)	83.4 (83.2-83.7)	84.7 (84.5-85)	85.8 (85.5-86)	86.5 (86.3-86.7)	87.5 (86.3-88.7)
Kumamoto	60.3 (57.6-63.3)	67.6 (65-70.5)	70 (67.4-72.6)	72.7 (70.4-75.2)	74.5 (72.7-76.4)	76.7 (75.6-77.7)	79.2 (79.1-79.4)	81.5 (81.3-81.7)	82.9 (82.7-83.1)	84.4 (84.2-84.5)	85.5 (85.3-85.7)	86.4 (86.2-86.6)	87 (86.8-87.2)	87.8 (86.7-88.9)
Kyōto	62.4 (59.6-65.3)	68.9 (66.1-71.8)	71.2 (68.5-73.7)	73.8 (71.4-76.1)	75.7 (73.5-77.8)	77.7 (76.4-79)	79.4 (79.2-79.5)	80.8 (80.7-81)	82.2 (82.1-82.4)	83.4 (83.3-83.6)	84.8 (84.6-85)	85.8 (85.6-86)	86.7 (86.5-86.9)	87.5 (86.3-88.8)
Mie	62.1 (59-65.3)	68.7 (65.7-71.7)	70.9 (68.3-73.5)	73.6 (71.4-75.8)	75.4 (73.7-77.3)	77.4 (76.5-78.3)	79.2 (79-79.4)	80.6 (80.5-80.8)	82.1 (82-82.3)	83 (82.9-83.2)	84.6 (84.4-84.8)	85.6 (85.4-85.8)	86.2 (86-86.4)	87.1 (85.9-88.2)
Miyagi	62.6 (59.6-65.6)	69.2 (66.5-72)	71 (68.6-73.6)	73.8 (71.6-76.1)	75.5 (73.7-77.3)	77.4 (76.5-78.4)	78.9 (78.7-79)	80.8 (80.6-80.9)	82.5 (82.3-82.6)	83.3 (83.1-83.5)	84.8 (84.7-85)	85.7 (85.5-85.8)	86.3 (86.1-86.5)	87.2 (86.1-88.4)
Miyazaki	60.1 (56.9-63.3)	67.1 (64.4-70.1)	69.7 (67-72.3)	72.5 (70.2-74.8)	74.4 (72.8-76.1)	76.7 (75.8-77.5)	78.9 (78.7-79.1)	80.8 (80.7-81)	82.4 (82.2-82.6)	83.5 (83.3-83.6)	85.2 (85-85.4)	85.9 (85.7-86.1)	86.6 (86.4-86.8)	87.5 (86.3-88.6)
Nagano	62.6 (59.4-65.6)	69.2 (66.5-72)	71.3 (68.9-73.8)	73.8 (71.6-76.2)	75.5 (73.5-77.5)	77.4 (76.5-78.5)	79.4 (79.3-79.6)	81.3 (81.1-81.4)	82.8 (82.6-83)	84 (83.8-84.2)	85.4 (85.2-85.5)	86.4 (86.3-86.6)	87.2 (87-87.3)	88 (86.9-89.2)
Nagasaki	61.3 (58.3-64.6)	68 (65.3-70.7)	70.4 (68.1-72.8)	73.1 (71-75.4)	74.9 (73-76.7)	76.8 (75.8-77.8)	78.8 (78.6-79)	80.8 (80.6-81)	82.3 (82.1-82.4)	83.4 (83.2-83.6)	85 (84.8-85.2)	85.8 (85.6-86)	86.5 (86.3-86.8)	86.9 (85.7-88.1)
Nara	61.1 (57.9-64.3)	67.8 (65.1-70.6)	69.9 (67.5-72.4)	72.5 (70.4-74.6)	74.3 (72.8-76)	76.5 (75.8-77.4)	78.8 (78.6-79)	80.5 (80.3-80.7)	81.8 (81.6-82)	82.9 (82.8-83.1)	84.7 (84.5-84.9)	85.8 (85.7-86)	86.6 (86.4-86.8)	87.3 (86.2-88.5)
Niigata	62.2 (59.5-65.3)	68.9 (66.3-71.8)	70.9 (68.5-73.6)	73.5 (71.3-75.8)	75 (73.4-76.9)	77.1 (76.1-78)	79.1 (78.9-79.2)	81 (80.8-81.1)	82.6 (82.4-82.7)	83.7 (83.5-83.9)	85.4 (85.2-85.5)	86.2 (86.1-86.4)	87 (86.8-87.2)	87.5 (86.4-88.6)
Ōita	60.3 (57.4-63.4)	67.4 (64.8-70.2)	69.7 (67.5-72.3)	72.5 (70.4-74.7)	74.3 (72.6-76)	76.5 (75.6-77.2)	78.8 (78.6-79)	80.6 (80.4-80.8)	82.1 (81.9-82.3)	83.5 (83.3-83.7)	84.8 (84.6-84.9)	86 (85.8-86.1)	86.9 (86.7-87.1)	87.4 (86.3-88.5)
Okayama	62.6 (59.7-65.7)	69.2 (66.6-72.2)	71.5 (69.1-74)	74 (71.9-76.3)	75.7 (73.9-77.6)	77.7 (76.7-78.7)	79.7 (79.5-79.8)	81.5 (81.3-81.6)	82.9 (82.8-83.1)	83.7 (83.5-83.9)	85.4 (85.2-85.6)	86.4 (86.2-86.6)	86.9 (86.7-87.1)	87.8 (86.7-88.9)
Okinawa	60 (56.9-63)	67.1 (64.1-69.8)	69.8 (67.1-72.4)	72.4 (70-74.9)	74.2 (72.3-76.1)	76.6 (75.7-77.5)	80.3 (80.1-80.5)	83.3 (83.1-83.5)	84.2 (84-84.4)	84.9 (84.7-85.1)	86 (85.8-86.2)	86.6 (86.4-86.7)	87.1 (86.9-87.3)	86.9 (85.6-88.1)
Ōsaka	61.1 (58.1-64)	67.8 (65.1-70.4)	70.1 (67.6-72.7)	72.7 (70.5-75)	74.5 (72.4-76.7)	76.5 (75.2-77.7)	78.4 (78.2-78.5)	79.8 (79.7-79.9)	81.2 (81.1-81.3)	82.4 (82.3-82.5)	84 (83.9-84.1)	85 (84.9-85.1)	85.8 (85.7-85.9)	87.3 (86.1-88.5)
Saga	60.9 (58-63.9)	68 (65.3-70.5)	70.5 (67.9-73)	73.4 (71.2-75.5)	75.4 (73.7-77.1)	77.3 (76.4-78.2)	79.1 (78.8-79.3)	80.9 (80.7-81.1)	82.3 (82.2-82.5)	83.2 (83-83.4)	84.8 (84.6-85)	86.1 (85.9-86.3)	86.4 (86.1-86.6)	87.2 (86-88.4)
Saitama	62.2 (59.4-65.3)	68.8 (66.3-71.8)	70.8 (68.2-73.2)	73.3 (71-75.7)	75 (73.1-77.2)	76.8 (75.5-78.1)	78.7 (78.6-78.9)	80.7 (80.6-80.8)	81.9 (81.8-82)	82.9 (82.7-83)	84.4 (84.2-84.5)	85.2 (85.1-85.3)	86 (85.8-86.1)	86.9 (85.8-88.1)
Shiga	62.2 (59.2-65.4)	68.6 (66.1-71.3)	70.6 (68.3-73.2)	73 (71-75.1)	74.6 (73-76.2)	76.9 (76-77.7)	79.3 (79-79.5)	80.7 (80.5-80.9)	81.9 (81.7-82.1)	83.3 (83.2-83.5)	85.1 (84.9-85.2)	86 (85.8-86.2)	86.8 (86.6-87)	87.5 (86.4-88.7)
Shimane	61.5 (58.5-64.6)	68.1 (65.6-70.9)	70.4 (68.1-72.9)	73.1 (71.1-75)	74.9 (73.5-76.4)	77.2 (76.4-77.9)	79.6 (79.4-79.9)	81.7 (81.5-81.9)	83.3 (83.1-83.5)	84.2 (84-84.4)	84.9 (84.7-85.1)	86.4 (86.2-86.6)	87.2 (87-87.5)	87.5 (86.3-88.7)
Shizuoka	62.9 (59.6-65.8)	69.4 (66.5-72.1)	71.6 (69.1-74.2)	74.1 (71.8-76.6)	75.9 (73.7-78.1)	77.9 (76.5-79.1)	79.6 (79.5-79.8)	81.4 (81.3-81.6)	82.6 (82.5-82.8)	83.6 (83.4-83.7)	85 (84.9-85.2)	86.1 (85.9-86.2)	86.5 (86.3-86.6)	87.5 (86.4-88.8)
Tochigi	61 (57.9-64)	67.8 (65.1-70.7)	70.2 (67.7-72.7)	73.1 (70.8-75.3)	75 (73.1-76.9)	76.9 (76-78)	78.3 (78.1-78.5)	80.1 (79.9-80.3)	81.3 (81.2-81.5)	82.5 (82.4-82.7)	84.2 (84-84.3)	84.8 (84.7-85)	85.7 (85.5-85.9)	86.2 (85-87.5)
Tokushima	60.7 (57.6-64)	67.6 (64.7-70.5)	69.9 (67.6-72.7)	72.6 (70.5-74.8)	74.3 (72.7-76.1)	76.6 (75.7-77.6)	78.6 (78.4-78.9)	80.6 (80.4-80.8)	81.9 (81.7-82.1)	83.1 (82.9-83.4)	84.8 (84.6-85)	85.4 (85.2-85.6)	86.4 (86.1-86.6)	86.4 (85.2-87.6)
Tōkyō	63.5 (60.7-66.6)	69.7 (67.2-72.2)	71.8 (69.5-74.5)	74.2 (71.9-76.7)	76 (73.9-78.1)	77.9 (76.8-79.1)	79.4 (79.3-79.5)	81 (80.9-81.1)	82.1 (82-82.2)	83 (82.8-83.1)	84.3 (84.2-84.4)	85.4 (85.3-85.5)	86.4 (86.3-86.5)	87.8 (86.8-88.8)
Tottori	61.8 (58.7-65)	68.6 (66-71.4)	71.1 (68.8-73.5)	73.9 (71.9-75.9)	75.8 (74.3-77.3)	78 (77.1-78.8)	79.9 (79.6-80.2)	81.1 (80.8-81.3)	82.5 (82.2-82.7)	83.7 (83.5-83.9)	85 (84.7-85.2)	85.7 (85.4-85.9)	86.5 (86.2-86.7)	87.2 (86.1-88.4)
Toyama	61.9 (59-64.8)	68.5 (66-71)	70.7 (68.5-73.2)	73.4 (71.4-75.4)	75.1 (73.6-76.7)	77.2 (76.4-78.1)	79.3 (79.1-79.5)	80.9 (80.7-81)	82.6 (82.5-82.8)	83.7 (83.6-83.9)	85.1 (84.9-85.3)	86.3 (86.1-86.5)	87 (86.8-87.2)	87.6 (86.5-88.8)
Wakayama	59.6 (56.8-62.8)	66.7 (63.9-69.6)	69.1 (66.6-71.7)	71.8 (69.7-74)	73.6 (72-75.3)	75.9 (75.1-76.8)	78.5 (78.3-78.7)	80.3 (80.1-80.5)	81.6 (81.4-81.8)	82.8 (82.6-83)	84.4 (84.2-84.6)	85.3 (85.1-85.5)	86.1 (85.9-86.3)	86.8 (85.6-88)
Yamagata	61.7 (58.4-64.8)	68.4 (65.6-71.2)	70.6 (68-73)	73.1 (71-75.2)	74.7 (73.1-76.3)	77.1 (76.3-77.9)	80.3 (80.1-80.5)	81.6 (81.4-81.8)	82.5 (82.4-82.7)	83.5 (83.3-83.7)	84.9 (84.7-85)	86.1 (85.9-86.3)	86.4 (86.2-86.6)	87.2 (86-88.3)
Yamaguchi	61.4 (58.6-64.5)	68.4 (65.5-71.2)	70.9 (68.4-73.6)	73.7 (71.4-76.1)	75.6 (73.6-77.4)	77.6 (76.6-78.5)	79.2 (79-79.4)	81.1 (80.9-81.2)	82.6 (82.4-82.7)	83.4 (83.2-83.6)	84.7 (84.5-84.9)	85.6 (85.4-85.8)	86.1 (85.9-86.3)	87.1 (85.9-88.3)
Yamanashi	62.8 (59.7-65.9)	69.2 (66.6-72.1)	71.6 (69.3-74.1)	74.2 (72.3-76.3)	76 (74.5-77.7)	78.1 (77.2-79)	79.5 (79.3-79.8)	81 (80.8-81.2)	82.5 (82.3-82.7)	83.9 (83.7-84.1)	85.2 (85-85.5)	86.1 (85.8-86.3)	86.7 (86.5-86.9)	87.4 (86.2-88.6)
South Korea	45 (43-47)	57.8 (55.1-60.5)	61 (58.3-63.8)	63.3 (60.7-65.9)	65.5 (63.1-67.8)	68.6 (67.9-69.4)	71.5 (71.3-71.7)	74.5 (74.3-74.6)	76.4 (76.3-76.5)	78.3 (78.2-78.3)	79.8 (79.7-79.8)	81.9 (81.8-81.9)	83.8 (83.6-84)	85.5 (84.9-86.1)
Singapore	60.2 (58.6-61.9)	65.5 (65.2-65.8)	68.7 (68.4-69)	70.5 (70.3-70.8)	72.1 (71.9-72.3)	73.5 (73.3-73.7)	74.3 (74.1-74.5)	76.7 (76.5-76.9)	78.8 (78.6-79)	80.4 (80.2-80.6)	82.1 (81.2-83)	83.8 (83.6-84.1)	86.2 (86-86.4)	87.6 (86.9-88.1)
High-income North America	71.2 (70.7-71.7)	73 (72.5-73.4)	73.4 (73.4-73.4)	74.1 (74-74.1)	74.8 (74.8-74.9)	76.6 (76.6-76.6)	77.6 (77.6-77.7)	78.4 (78.3-78.4)	79.1 (79.1-79.2)	79.3 (79.3-79.3)	79.7 (79.7-79.7)	80.3 (80.3-80.3)	81.4 (81.3-81.4)	81.4 (81.1-81.7)
Canada	70.6 (70.5-70.7)	73.1 (73-73.2)	74.2 (74.1-74.3)	75.2 (75.1-75.3)	76.2 (76.1-76.3)	77.2 (77.2-77.3)	78.8 (78.7-78.9)	79.6 (79.6-79.7)	80.6 (80.5-80.6)	80.9 (80.9-81)	81.8 (81.7-81.9)	82.5 (82.5-82.6)	83.6 (83.5-83.6)	84 (83.4-84.6)
Greenland	49 (47.9-50.2)	55.2 (54.5-55.9)	62.9 (62.2-63.6)	66.6 (65.9-67.2)	67.8 (67.2-68.4)	67 (66.5-67.5)	66.9 (66.4-67.4)	67.6 (67.1-68.2)	69 (68.4-69.6)	69.7 (69.2-70.2)	70.8 (70.3-71.3)	72.4 (71.9-72.9)	74.5 (74-75)	77.2 (76.2-78)

Table 7 Estimates of life expectancy at birth by location and year for 1950, 1955...2005, 2010, 2017 - females

Location	1950	1955	1960	1965	1970	1975	1980	1985	1990	1995	2000	2005	2010	2017
USA	71.3 (70.8-71.9)	73 (72.5-73.4)	73.3 (73.3-73.4)	74 (73.9-74)	74.7 (74.7-74.7)	76.5 (76.5-76.6)	77.5 (77.5-77.6)	78.2 (78.2-78.3)	79 (79-79)	79.1 (79.1-79.2)	79.5 (79.5-79.5)	80.1 (80.1-80.1)	81.1 (81.1-81.1)	81.1 (80.8-81.4)
Alabama	68.6 (65.8-71.4)	70.4 (68.1-73)	71.8 (71.6-72)	72.2 (72-72.4)	73.6 (73.4-73.8)	75.6 (75.5-75.8)	76.7 (76.5-76.9)	77.4 (77.2-77.6)	77.8 (77.6-78)	77.7 (77.6-77.9)	77.7 (77.5-77.8)	77.6 (77.4-77.7)	78 (77.8-78.2)	77.8 (76.1-79.4)
Alaska	70.5 (68.5-72.4)	72.4 (71.2-73.7)	71.9 (71.5-72.3)	72.6 (72.3-72.9)	74 (73.7-74.2)	74.2 (73.9-74.5)	75.9 (75.7-76.2)	76.7 (76.4-76.9)	78.1 (77.8-78.3)	78.5 (78.3-78.7)	78.9 (78.6-79.1)	79.6 (79.4-79.8)	80.4 (80.2-80.6)	80.1 (79.1-81)
Arizona	70.9 (68.1-73.7)	72.8 (70.5-75.1)	73.9 (73.6-74.2)	74.3 (74.1-74.6)	75.1 (74.9-75.4)	77.1 (76.9-77.4)	78 (77.7-78.2)	79.2 (79-79.4)	79.9 (79.7-80.1)	79.6 (79.4-79.8)	80.1 (79.9-80.3)	80.4 (80.2-80.5)	81.9 (81.7-82.1)	81.8 (80.2-83.2)
Arkansas	71.3 (68.6-74.2)	73.1 (71-75.4)	73.8 (73.5-74.1)	74.4 (74.1-74.6)	75.2 (74.9-75.4)	76.6 (76.3-76.8)	77.8 (77.6-78)	77.9 (77.7-78.1)	78.2 (78-78.5)	78.3 (78-78.5)	78 (77.8-78.2)	78.5 (78.3-78.7)	78.8 (78.6-79)	78.2 (76.5-79.9)
California	72.3 (69.7-74.9)	74.2 (72-76.3)	74.4 (74.3-74.5)	74.8 (74.7-74.9)	75.4 (75.3-75.5)	77 (76.9-77.1)	77.9 (77.8-78)	78.6 (78.6-78.7)	79.4 (79.3-79.5)	80.1 (80.1-80.2)	80.6 (80.5-80.7)	81.3 (81.3-81.4)	82.9 (82.8-82.9)	83.4 (82.1-84.7)
Colorado	72.1 (69.3-74.9)	73.9 (71.6-76.1)	74.4 (74.2-74.7)	75.2 (75-75.5)	76.1 (75.8-76.3)	77.9 (77.7-78.1)	78.7 (78.5-78.9)	79.5 (79.2-79.7)	80.3 (80-80.5)	80.1 (79.9-80.3)	80.3 (80.2-80.6)	81.2 (81-81.4)	82 (81.8-82.1)	82.1 (80.4-83.5)
Connecticut	72.7 (70.1-75.6)	74.5 (72.3-76.8)	75 (74.8-75.2)	74.9 (74.7-75.1)	76.2 (76-76.3)	77.6 (77.4-77.8)	78.4 (78.2-78.6)	79.2 (79-79.4)	80.2 (80-80.4)	80.3 (80-80.5)	80.8 (80.6-81.1)	82.1 (81.9-82.3)	83 (82.8-83.2)	83.1 (81.6-84.6)
Delaware	70.8 (68.7-73)	72.3 (71-73.8)	72.7 (72.4-73)	73.4 (73.1-73.6)	74 (73.7-74.2)	75.9 (75.7-76.2)	76.8 (76.6-77.1)	77.6 (77.3-77.8)	78 (77.7-78.2)	78.4 (78.2-78.7)	78.8 (78.6-79)	79.7 (79.4-79.9)	80.3 (80.1-80.5)	80.6 (79.6-81.5)
Washington, DC	68.8 (66-71.5)	70.4 (68.6-72.2)	69.9 (69.7-70.2)	69.6 (69.4-69.9)	70.3 (70-70.5)	72.5 (72.2-72.7)	73.9 (73.6-74.1)	74.3 (74-74.6)	73.4 (73.2-73.7)	72.7 (72.4-73)	76.6 (76.3-76.9)	77.3 (77-77.5)	79 (78.7-79.3)	79.8 (78.4-81)
Florida	71.4 (68.6-74.3)	73.3 (71-75.8)	73.7 (73.5-73.8)	74.5 (74.3-74.6)	75 (74.9-75.1)	76.9 (76.7-77)	77.8 (77.7-78)	78.8 (78.7-79)	79.8 (79.7-79.9)	79.9 (79.7-80)	80.3 (80.2-80.4)	80.7 (80.6-80.8)	82 (81.9-82.1)	81.8 (80.3-83.4)
Georgia	68.7 (65.8-71.7)	71 (68.4-73.5)	71.3 (71.1-71.5)	72.3 (72.1-72.4)	73 (72.9-73.2)	75.4 (75.2-75.5)	76.3 (76.1-76.4)	77 (76.8-77.1)	77.6 (77.4-77.7)	77.7 (77.6-77.8)	77.8 (77.7-78)	78.6 (78.5-78.7)	79.5 (79.4-79.6)	79.5 (77.9-81)
Hawaii	71.6 (69.4-73.8)	73.2 (71.9-74.5)	73.8 (73.5-74.1)	75.2 (75-75.4)	76.7 (76.5-76.9)	79.1 (78.8-79.3)	80.4 (80.2-80.6)	81.1 (80.9-81.3)	81.7 (81.5-82)	82.2 (82-82.4)	82.2 (82-82.4)	83.2 (83-83.4)	84.1 (83.9-84.3)	84.3 (83.4-85.3)
Idaho	73.2 (71.1-75.5)	74.8 (73.5-76.2)	75.2 (75-75.5)	75.6 (75.3-75.8)	76.2 (76-76.5)	77.5 (77.3-77.8)	79 (78.7-79.2)	79.4 (79.2-79.6)	80 (79.8-80.2)	80.2 (80-80.4)	80.1 (79.9-80.3)	80.6 (80.4-80.8)	81.2 (81.1-81.4)	81.3 (80.4-82.2)
Illinois	71.2 (68.5-73.9)	72.9 (70.8-75.2)	72.9 (72.7-73)	73.4 (73.3-73.5)	74 (73.9-74.1)	75.9 (75.8-76)	77.2 (77.1-77.3)	77.9 (77.8-78)	78.5 (78.4-78.7)	78.7 (78.6-78.9)	79.3 (79.2-79.4)	80.2 (80.1-80.3)	81.4 (81.3-81.5)	81.3 (79.8-82.8)
Indiana	71.9 (69.3-74.7)	73.8 (71.5-76.1)	73.6 (73.5-73.8)	74 (73.9-74.2)	74.7 (74.6-74.9)	76.5 (76.4-76.7)	77.5 (77.4-77.7)	78.2 (78-78.3)	78.8 (78.6-79)	78.4 (78.2-78.5)	78.7 (78.5-78.9)	79.3 (79.1-79.4)	79.9 (79.7-80)	79.6 (78-81.2)
Iowa	74.2 (71.6-77.1)	75.3 (73.1-77.6)	75.6 (75.4-75.8)	75.9 (75.7-76.1)	76.5 (76.3-76.7)	78.1 (77.9-78.3)	79.7 (79.5-80)	79.8 (79.6-80)	81.2 (80.9-81.4)	80.5 (80.3-80.8)	80.9 (80.7-81.1)	81.5 (81.2-81.7)	81.8 (81.6-82.1)	81.5 (80-83.2)
Kansas	74.2 (71.6-76.7)	75.6 (73.4-77.8)	75.5 (75.2-75.7)	76 (75.7-76.2)	76.7 (76.5-77)	77.8 (77.6-78)	79 (78.7-79.2)	79.5 (79.2-79.7)	80.7 (80.5-80.9)	79.9 (79.7-80.2)	80 (79.8-80.3)	80.1 (79.9-80.4)	81 (80.8-81.2)	80.2 (78.5-81.8)
Kentucky	70.9 (68.1-73.7)	72.7 (70.6-75)	73.3 (73.1-73.5)	74 (73.8-74.2)	74.3 (74.1-74.5)	75.9 (75.7-76.1)	77.1 (76.9-77.3)	77.7 (77.5-77.8)	78.2 (78-78.4)	78.2 (78-78.4)	78 (77.8-78.2)	78.3 (78.1-78.5)	78.2 (78.1-78.4)	77.7 (76.1-79.5)
Louisiana	69.1 (66.3-71.9)	71.2 (68.9-73.6)	71.5 (71.3-71.7)	72.1 (71.9-72.3)	72.9 (72.8-73.1)	75 (74.8-75.2)	75.9 (75.8-76.1)	76.5 (76.3-76.7)	77.2 (77-77.3)	77.2 (77-77.4)	77.1 (76.9-77.3)	76 (75.9-76.2)	78.6 (78.5-78.8)	78.5 (76.7-80.1)
Maine	71.5 (69.2-74)	73.2 (71.6-75)	73.6 (73.4-73.9)	74.2 (73.9-74.4)	74.9 (74.7-75.2)	77.1 (76.8-77.3)	78.2 (78-78.5)	78.8 (78.6-79.1)	79.7 (79.4-79.9)	79.9 (79.7-80.2)	79.9 (79.7-80.1)	80.2 (80-80.4)	81.2 (81-81.5)	80.8 (79.7-81.9)
Maryland	70 (67.3-72.9)	71.9 (69.5-74.3)	71.9 (71.7-72.1)	73 (72.8-73.1)	74.2 (74-74.3)	75.9 (75.8-76.1)	76.9 (76.7-77.1)	77.4 (77.3-77.6)	78.3 (78.1-78.5)	78.4 (78.2-78.5)	78.7 (78.5-78.9)	79.9 (79.7-80)	81.2 (81-81.3)	81.6 (80-83.2)
Massachusetts	72.1 (69.6-74.7)	73.5 (71.3-75.9)	73.9 (73.8-74.1)	74.4 (74.2-74.6)	75.8 (75.7-76)	77.5 (77.4-77.7)	78.3 (78.1-78.4)	78.9 (78.8-79.1)	80 (79.9-80.2)	80.3 (80.1-80.5)	80.6 (80.5-80.8)	81.6 (81.4-81.8)	82.8 (82.7-83)	82.4 (81-83.8)
Michigan	71.5 (68.9-74.1)	73.1 (70.9-75.3)	73.5 (73.3-73.6)	74 (73.9-74.1)	74.5 (74.4-74.7)	76.2 (76-76.3)	77.4 (77.2-77.5)	77.8 (77.6-77.9)	78.5 (78.3-78.6)	78.6 (78.5-78.8)	78.9 (78.8-79)	79.4 (79.3-79.6)	80.3 (80.2-80.4)	80 (78.5-81.6)
Minnesota	73.7 (71.2-76.5)	75.3 (73.2-77.5)	75.1 (74.9-75.3)	76.1 (75.9-76.3)	77 (76.8-77.1)	78.6 (78.4-78.8)	79.8 (79.6-80)	80.2 (80-80.4)	81 (80.8-81.2)	81 (80.8-81.2)	81.3 (81.1-81.5)	82.2 (82-82.4)	83 (82.8-83.1)	83 (81.5-84.5)
Mississippi	68 (65.3-71)	70.2 (67.9-72.7)	70.8 (70.6-71.1)	71.1 (70.9-71.3)	72.7 (72.5-73)	75 (74.8-75.3)	76.1 (75.9-76.3)	77 (76.8-77.3)	77.4 (77.2-77.6)	77 (76.8-77.2)	76.7 (76.5-76.9)	76.7 (76.5-76.9)	77.7 (77.5-77.9)	77.7 (75.9-79.4)
Missouri	72 (69.4-74.9)	73.7 (71.6-75.9)	73.8 (73.6-74)	74.3 (74.1-74.5)	74.7 (74.6-74.9)	76.6 (76.4-76.7)	77.5 (77.3-77.7)	78.4 (78.2-78.6)	78.8 (78.7-79)	78.7 (78.5-78.8)	78.7 (78.5-78.9)	79.2 (79-79.4)	79.9 (79.7-80.1)	79.6 (78-81.2)
Montana	72.1 (69.8-74.3)	73.5 (72.2-74.9)	73.8 (73.5-74.1)	74.5 (74.2-74.7)	75.1 (74.8-75.3)	77 (76.7-77.2)	77.9 (77.6-78.1)	78.9 (78.6-79.1)	79.6 (79.4-79.9)	79.8 (79.6-80)	79.9 (79.7-80.1)	80.1 (79.9-80.4)	80.8 (80.6-81)	79.2 (78.1-80.2)
Nebraska	74.9 (72.4-77.1)	76.3 (74.6-77.9)	75.5 (75.3-75.8)	75.9 (75.7-76.1)	76.6 (76.4-76.8)	78 (77.8-78.2)	79.2 (79-79.4)	79.9 (79.7-80.1)	80.3 (80.1-80.5)	80.3 (80.1-80.6)	80.6 (80.4-80.8)	81.3 (81.1-81.5)	81.8 (81.6-82)	81.3 (80.1-82.4)
Nevada	69.1 (66.6-71.9)	71.4 (69.6-73.2)	72.3 (71.9-72.7)	72.2 (71.9-72.5)	73.6 (73.3-73.9)	75.8 (75.5-76)	76.2 (76-76.5)	77.6 (77.4-77.9)	78 (77.7-78.2)	77.9 (77.7-78.1)	78.3 (78.1-78.5)	78.5 (78.3-78.7)	80.1 (79.9-80.2)	80.2 (79.2-81.3)
New Hampshire	72.5 (70.4-74.7)	74.2 (73-75.6)	74.2 (73.9-74.4)	74.3 (74-74.5)	75.3 (75.1-75.6)	77.1 (76.9-77.4)	78.4 (78.2-78.6)	79 (78.8-79.2)	79.7 (79.5-79.9)	80.2 (80-80.5)	80.3 (80.1-80.5)	80.9 (80.7-81.1)	82.1 (82-82.3)	81 (80.1-81.9)
New Jersey	70.9 (68.4-73.7)	72.5 (70.2-74.8)	72.9 (72.8-73)	73.6 (73.4-73.7)	74.5 (74.4-74.7)	76.5 (76.3-76.6)	77.3 (77.2-77.4)	77.9 (77.7-78)	78.7 (78.6-78.9)	79 (78.8-79.1)	79.9 (79.7-80)	81 (80.8-81.1)	82.3 (82.2-82.4)	83 (81.6-84.4)
New Mexico	70.9 (68.4-73.6)	72.9 (71.3-74.6)	73 (72.7-73.2)	74 (73.7-74.2)	74.6 (74.3-74.9)	76.6 (76.3-76.8)	78.2 (78-78.4)	78.9 (78.7-79.1)	79.5 (79.3-79.7)	79.6 (79.4-79.8)	80.1 (79.9-80.2)	80.3 (80.1-80.5)	80.8 (80.6-81)	80.7 (79.5-81.8)
New York	70.9 (68.6-73.4)	72.3 (70.3-74.3)	72.8 (72.7-72.9)	73.5 (73.4-73.6)	74.2 (74.1-74.3)	76.3 (76.2-76.4)	77 (76.9-77.1)	77.5 (77.4-77.6)	78.6 (78.5-78.7)	79.1 (79-79.2)	80.4 (80.3-80.5)	81.3 (81.2-81.4)	82.7 (82.6-82.8)	83.1 (81.7-84.5)
North Carolina	70.2 (67.5-72.9)	72.1 (69.8-74.4)	72.2 (72-72.4)	72.9 (72.7-73)	73.9 (73.8-74.1)	75.9 (75.8-76.1)	77.3 (77.2-77.5)	77.8 (77.6-77.9)	78.4 (78.3-78.6)	78.3 (78.2-78.5)	78.4 (78.2-78.5)	79.1 (79-79.2)	80.2 (80.1-80.3)	80.2 (78.5-81.7)
North Dakota	73.6 (71.7-75.5)	74.8 (73.7-75.9)	75.3 (74.9-75.5)	76.1 (75.9-76.4)	76.9 (76.7-77.2)	78.3 (78-78.5)	79.4 (79.2-79.7)	80.8 (80.5-81)	81.3 (81-81.5)	81.2 (81-81.5)	81.3 (81.1-81.5)	82.2 (81.9-82.4)	82.4 (82.1-82.6)	81.2 (80.2-82.2)
Ohio	71.5 (68.9-74.1)	73 (70.9-75.2)	73.4 (73.3-73.5)	74 (73.8-74.1)	74.6 (74.4-74.7)	76.1 (76-76.2)	76.9 (76.8-77)	77.9 (77.8-78)	78.6 (78.5-78.7)	78.6 (78.4-78.7)	78.9 (78.7-79)	79.3 (79.2-79.4)	79.9 (79.8-80)	79 (77.4-80.6)
Oklahoma	72.9 (70.2-75.7)	74.6 (72.3-77)	74.8 (74.5-75)	76 (75.7-76.2)	75.6 (75.4-75.8)	77 (76.8-77.3)	77.7 (77.5-77.9)	78 (77.8-78.2)	78.5 (78.3-78.7)	78.1 (77.9-78.3)	77.8 (77.6-78)	77.7 (77.5-77.9)	78.3 (78.1-78.5)	78 (76.2-79.9)
Oregon	73.4 (70.8-76.1)	75 (72.8-77.3)	74.9 (74.6-75.1)	75.8 (75.6-76)	76.4 (76.1-76.6)	77.8 (77.6-78)	78.7 (78.5-78.9)	79.3 (79.1-79.5)	79.8 (79.6-80)	79.4 (79.2-79.6)	80 (79.8-80.2)	80.7 (80.5-80.9)	81.5 (81.3-81.6)	81.5 (80-83.1)
Pennsylvania	70.6 (68.1-73.1)	72.1 (70.1-74.3)	72.7 (72.6-72.8)	73.3 (73.2-73.4)	74.1 (74-74.2)	76.1 (76-76.2)	77 (76.9-77.1)	77.9 (77.8-78)	78.7 (78.6-78.8)	79.2 (79-79.3)	79.4 (79.3-79.6)	79.9 (79.8-80)	81 (80.9-81.1)	80.5 (79-82)
Rhode Island	71.8 (69.5-74.3)	73.3 (71.8-75)	73.7 (73.5-74)	74.4 (74.2-74.7)	75.4 (75.1-75.6)	77.2 (77-77.5)	78.6 (78.3-78.8)	79.1 (78.8-79.3)	79.7 (79.5-80)	80.6 (80.3-80.8)	80.4 (80.2-80.7)	80.9 (80.7-81.2)	82 (81.8-82.3)	82.1 (80.9-83.2)
South Carolina	66.9 (63.8-70)	69.1 (66.4-71.9)	70.1 (69.9-70.3)	71.3 (71.1-71.5)	72.6 (72.4-72.8)	74.6 (74.4-74.9)	76.1 (75.9-76.3)	77 (76.8-77.2)	77.4 (77.2-77.6)	77.7 (77.5-77.9)	77.9 (77.7-78.1)	78.6 (78.4-78.8)	79.3 (79.2-79.5)	79.2 (77.5-80.8)
South Dakota	73.7 (71.6-75.8)	75 (73.7-76.2)	74.8 (74.5-75.1)	75.7 (75.4-75.9)	76.1 (75.8-76.3)	77.6 (77.4-77.8)	79.1 (78.8-79.4)	79.8 (79.6-80.1)	80.6 (80.4-80.9)	80.6 (80.3-80.8)	80.7 (80.5-81)	81.3 (81-81.5)	81.8 (81.6-82.1)	80.6 (79.6-81.7)
Tennessee	70.6 (67.7-73.5)	72.2 (70-74.7)	72.9 (72.8-73.1)	73.6 (73.4-73.8)	74.2 (74-74.4)	76 (75.9-76.2)	77.3 (77.1-77.5)	77.8 (77.6-78)	78.2 (78-78.3)	77.9 (77.8-78.1)	77.9 (77.7-78)	78 (77.9-78.2)	78.7 (78.6-78.9)	78.5 (76.9-80.1)
Texas	72 (69.3-74.8)	73.8 (71.6-76.2)	74.1 (73.9-74.2)	74.9 (74.8-75)	75.1 (75-75.2)	76.7 (76.6-76.8)	77.5 (77.4-77.6)	78.1 (78-78.2)	79.1 (79-79.2)	79.1 (79-79.2)	79.1 (79-79.2)	79.8 (79.7-79.9)	80.6 (80.5-80.7)	80.8 (79.4-82.2)
Utah	73.4 (71-75.9)	74.8 (73.2-76.5)	75.3 (75-75.6)	75.6 (75.3-75.8)	76.7 (76.4-76.9)	78.1 (77.9-78.3)	79 (78.7-79.2)	79.5 (79.3-79.7)	80.5 (80.3-80.7)	80.5 (80.3-80.6)	80.5 (80.3-80.6)	80.9 (80.7-81)	81.6 (81.5-81.8)	81.1 (80-82.1)
Vermont	72.6 (70.8-74.6)	74.1 (72.9-75.3)	74 (73.7-74.4)	74.4 (74.1-74.6)	75.8 (75.6-76.1)	77.4 (77.2-77.7)	78.5 (78.2-78.7)	78.6 (78.4-78.9)	79.7 (79.5-80)	80.1 (79.9-80.4)	80.4 (80.2-80.6)	81.2 (81-81.4)	82 (81.7-82.2)	82.6 (81.6-83.6)
Virginia	70 (67.5-72.9)	71.9 (69.7-74.3)	72.4 (72.2-72.5)	73.4 (73.2-73.5)	74.4 (74.2-74.6)	76.2 (76-76.4)	77.2 (77.1-77.4)	77.9 (77.8-78.1)	78.7 (78.6-78.9)	78.8 (78.7-79)	79.1 (79-79.3)	79.8 (79.7-80)	81.1 (81-81.3)	81.5 (80.1-82.9)
Washington	73.1 (70.4-75.6)	74.5 (72-77)	74.9 (74.7-75.1)	75.8 (75.6-76)	75.7 (75.5-75.9)	77.6 (77.4-77.8)	78.5 (78.3-78.7)	79.1 (78.9-79.3)	80 (79.8-80.2)	80 (79.8-80.2)	80.5 (80.3-80.7)	81.1 (80.9-81.3)	82 (81.8-82.2)	82.1 (80.6-83.5)
West Virginia	71.3 (68.6-74.1)	72.5 (70.2-75)	73.7 (73.4-73.9)	73.4 (73.1-73.6)	73.5 (73.3-73.8)	75.5 (75.2-75.7)	76.8 (76.6-77)	77.6 (77.4-77.9)	78.4 (78.1-78.6)	78.2 (77.9-78.5)	77.7 (77.5-78)	77.8 (77.6-78.1)	78.3 (78-78.5)	77.5 (75.8-79.2)
Wisconsin	72.8 (70.1-75.8)	74.3 (72.2-76.5)	74.4 (74.2-74.6)	75.3 (75.1-75.4)	76.2 (76-76.4)	77.9 (77.7-78)	78.9 (78.7-79.1)	79.7 (79.5-79.9)	80.1 (79.9-80.3)	80.3 (80.2-80.5)	80.5 (80.3-80.6)	81.1 (80.9-81.2)	81.9 (81.7-82)	82.3 (80.8-83.8)
Wyoming	73.2 (71.3-75.2)	74.7 (73.5-76)	74.4 (74-74.8)	74.6 (74.3-74.9)	75.4 (75.2-75.7)	76.5 (76.2-76.8)	78 (77.8-78.3)	78.7 (78.5-79)	79.5 (79.2-79.7)	79.4 (79.2-79.7)	79.3 (79.1-79.6)	79.8 (79.6-80.1)	80.3 (80.1-80.6)	80.5 (79.6-81.5)
Southern Latin America	62 (61.9-62.1)	64.6 (64.5-64.7)	65.3 (65.2-65.4)	67.6 (67.5-67.7)	68.9 (68.9-69)	71.4 (70.9-71.9)	73.5 (73.4-73.5)	75.3 (75.3-75.4)	76.2 (76.1-76.2)	77.4 (77.4-77.5)	78.4 (78.3-78.5)	79.2 (79.2-79.3)	79.7 (79.6-79.7)	80.4 (79.3-81.3)
Argentina	65.3 (65.2-65.4)	67.1 (67-67.2)	68.4 (68.3-68.5)	69.8 (69.7-69.9)	70 (69.9-70.1)	71.9 (71.2-72.6)	73.7 (73.6-73.8)	75.3 (75.2-75.3)	75.9 (75.8-76)	77.1 (77-77.2)	77.9 (77.8-77.9)	78.7 (78.6-78.8)	79 (78.9-79.1)	79.7 (78.3-81)
Chile	52.4 (52.2-52.7)	57.2 (57-57.5)	57.3 (57.1-57.5)	61.7 (61.6-61.9)	65.3 (65.2-65.5)	69.4 (69.3-69.5)	72.4 (72.3-72.5)	75.2 (75.1-75.3)	76.4 (76.3-76.5)	78.2 (78.1-78.3)	79.8 (79.7-79.9)	80.7 (80.6-80.8)	81.3 (81.2-81.4)	82.1 (80.8-83.4)
Uruguay	68.3 (68-68.5)	70.3 (70.1-70.5)	71.3 (71.1-71.5)	71.7 (71.5-72)	72 (71.8-72.2)	72.9 (72.7-73.1)	73.9 (73.7-74.1)	75.9 (75.7-76.1)	76.8 (76.6-77)	77.5 (77.3-77.7)	78.4 (78.2-78.6)	79 (78.8-79.2)	80.1 (79.9-80.3)	80.4 (79-81.9)
Western Europe	69.1 (68.5-69.6)	71.5 (71.4-71.7)	73 (73-73.1)	74.2 (74.1-74.3)	75 (74.9-75)	76.1 (76-76.1)	77.3 (77.3-77.3)	78.5 (78.5-78.5)	79.5 (79.5-79.5)	80.5 (80.5-80.6)	81.6 (81.5-81.6)	82.7 (82.7-82.7)	83.5 (83.5-83.6)	84.2 (83.9-84.5)
Andorra	79.8 (77.4-82.3)	80.3 (78.1-82.9)	80.8 (78.7-83.2)	81.1 (78.9-83.6)	81.5 (79.2-83.9)	81.9 (79.7-84.2)	82.2 (80.1-84.5)	82.4 (80.4-84.4)	82.6 (80.8-84.6)	83.1 (81.5-85)	84.6 (82.8-86.1)	85.5 (83.9-86.4)	85.5 (84-86.7)	85.1 (83.6-86.7)
Austria	67.2 (67-67.4)	70.3 (70.1-70.4)	71.9 (71.7-72)	73.1 (73-73.3)	73.5 (73.3-73.6)	74.7 (74.6-74.9)	76.1 (76-76.2)	77.4 (77.3-77.5)	78.9 (78.8-79)	80 (79.9-80.1)	81.1 (81-81.3)	82.3 (82.2-82.5)	83.3 (83.1-83.4)	84 (83.4-84.6)
Belgium	68.5 (68.3-68.6)	71.4 (71.2-71.5)	73 (72.9-73.1)	73.8 (73.7-74)	74.2 (74.1-74.4)	75.3 (75.2-75.4)	76.7 (76.6-76.8)	78 (77.8-78.1)	79.3 (79.2-79.4)	80.2 (80.1-80.3)	81 (80.8-81.1)	82 (81.9-82.1)	82.6 (82.5-82.7)	83.8 (83.1-84.5)
Cyprus	68.6 (66.2-71.2)	69.4 (67.2-71.8)	70.7 (68.7-72.8)	71.6 (69.9-73.4)	72.5 (71.5-73.6)	73.3 (73-73.6)	74.8 (74.6-75)	76.5 (76.3-76.7)	78.3 (78.1-78.5)	79.1 (78.7-79.5)	79.8 (79.3-80.3)	81.3 (80.7-81.8)	83.5 (82.8-84)	85.2 (84.3-86)
Denmark	71.7 (71.5-71.8)	73.6 (73.4-73.8)	74.1 (74-74.3)	74.8 (74.6-75)	76 (75.8-76.2)	77 (76.8-77.2)	77.1 (77-77.3)	77.5 (77.4-77.7)	77.8 (77.6-77.9)	78 (77.8-78.1)	79.3 (79.1-79.4)	80.5 (80.3-80.7)	81.4 (81.2-81.5)	82.7 (81.9-83.4)
Finland	68.3 (68.1-68.5)	71.3 (71.2-71.5)	73.1 (72.9-73.2)	73.8 (73.7-74)	74.5 (74.3-74.6)	76.2 (76-76.3)	78 (77.9-78.2)	78.8 (78.7-79)	79.1 (78.9-79.3)	80.3 (80.1-80.5)	81.2 (81-81.3)	82.4 (82.2-82.6)	83.4 (83.2-83.6)	84.3 (83.6-84.9)
France	69.7 (69.6-69.8)	72.2 (72.1-72.3)	74.2 (74.2-74.3)	75.3 (75.2-75.3)	76.2 (76.2-76.3)	77.1 (77.1-77.2)	78.7 (78.6-78.7)	79.7 (79.7-79.8)	81.1 (81-81.1)	82.1 (82-82.1)	82.8 (82.7-82.8)	83.9 (83.8-83.9)	84.6 (84.6-84.7)	85.7 (85.1-86.3)
Germany	70.3 (67.9-72.8)	72.1 (71.4-72.8)	72.9 (72.8-72.9)	74.3 (74.2-74.3)	74.6 (74.5-74.6)	75.5 (75.4-75.5)	76.5 (76.5-76.6)	77.7 (77.7-77.8)	78.6 (78.5-78.6)	79.8 (79.8-79.9)	81.1 (81.1-81.2)	82.3 (82.2-82.3)	82.8 (82.8-82.9)	83 (81.8-84.2)
Greece	69.8 (68.9-70.6)	73 (72.8-73.1)	74.2 (74.1-74.4)	74.9 (74.7-75)	75.9 (75.7-76)	77.4 (77.2-77.5)	77.6 (77.5-77.8)	78.4 (78.3-78.5)	80.4 (80.3-80.5)	81.3 (81.2-81.5)	81.9 (81.8-82.1)	81.8 (81.7-82)	82.8 (82.6-82.9)	83.6 (83-84.2)
Iceland	72.6 (72.1-73.1)	74.2 (73.9-74.6)	75.6 (75.2-75.9)	76.4 (76.1-76.7)	76.8 (76.5-77.1)	78.4 (78-78.7)	79.9 (79.5-80.2)	80.4 (80-80.8)	80.2 (79.8-80.5)	80.5 (80.2-80.7)	82 (81.7-82.3)	83.2 (82.9-83.5)	84.8 (84.5-85.1)	85.9 (85.5-86.4)
Ireland	67 (66.8-67.3)	70.2 (70-70.4)	71.9 (71.6-72.1)	73 (72.8-73.2)	73.5 (73.3-73.7)	74.3 (74.1-74.5)	75.5 (75.3-75.6)	76.8 (76.6-76.9)	77.6 (77.4-77.8)	78.4 (78.2-78.6)	79.2 (79-79.3)	81.3 (81.1-81.5)	82.9 (82.7-83.1)	83.7 (82.9-84.4)
Israel	74.6 (74.2-74.9)	71.3 (71-71.5)	72.6 (72.4-72.9)	72.7 (72.4-72.9)	73.5 (73.3-73.7)	73.9 (73.7-74.1)	75.8 (75.6-76)	77.2 (77-77.4)	78.9 (78.8-79.1)	79.6 (79.4-79.8)	80.7 (80.5-80.8)	81.9 (81.8-82.1)	83.5 (83.4-83.7)	84.6 (83.9-85.2)
Italy	67.4 (67.3-67.5)	70.6 (70.5-70.7)	72.2 (72.2-72.3)	73.5 (73.5-73.6)	74.7 (74.6-74.7)	76 (75.9-76)	77.2 (77.2-77.3)	78.9 (78.9-79)	80.3 (80.2-80.4)	81.2 (81.2-81.3)	82.4 (82.4-82.5)	83.6 (83.5-83.7)	84.4 (84.4-84.5)	85.3 (84.7-85.9)
Luxembourg	68.8 (68.4-69.2)	70.1 (69.9-70.4)	71.8 (71.5-72.1)	73 (72.7-73.3)	73.7 (73.4-74)	74.8 (74.5-75)	76.1 (75.8-76.3)	77.3 (77-77.6)	78.9 (78.6-79.2)	79.7 (79.4-80)	80.5 (80.3-80.8)	81.9 (81.6-82.2)	82.4 (82.2-82.7)	83.3 (82.3-84.2)
Malta	64.2 (63.7-64.7)	68.9 (68.6-69.2)	70.6 (70.3-70.9)	71.6 (71.4-71.9)	72.8 (72.5-73.1)	74.2 (73.9-74.4)	74.5 (74.3-74.8)	76.6 (76.3-76.8)	78.8 (78.5-79)	79.9 (79.6-80.2)	80.7 (80.4-81)	81.4 (81.2-81.7)	82.6 (82.3-82.9)	83 (82.4-83.6)
Netherlands	72.6 (72.5-72.8)	74.2 (74-74.3)	75.3 (75.2-75.4)	76.2 (76-76.3)	76.4 (76.3-76.5)	77.7 (77.6-77.8)	79.2 (79.1-79.3)	79.6 (79.5-79.7)	80.1 (80-80.2)	80.4 (80.3-80.5)	80.6 (80.5-80.7)	81.6 (81.5-81.7)	82.7 (82.6-82.8)	83.1 (82.4-83.7)
Norway	73.5 (72.9-74)	75.5 (75-76)	76 (75.7-76.4)	76.7 (76.5-76.9)	77.4 (77.3-77.5)	78.3 (78.2-78.4)	79.3 (79.2-79.4)	79.6 (79.5-79.7)	80 (79.9-80.1)	80.9 (80.8-81)	81.3 (81.2-81.4)	82.5 (82.4-82.6)	83 (82.9-83.1)	84.2 (84-84.4)
Portugal	61.7 (61.4-62)	64.4 (64.2-64.7)	67.8 (67.5-68)	69.2 (69-69.4)	70.9 (70.8-71.1)	73.1 (72.8-73.3)	75.1 (75-75.2)	76.5 (76.4-76.7)	77.6 (77.5-77.7)	78.9 (78.8-79)	80.1 (80-80.2)	82 (81.7-82.3)	83 (82.9-83.2)	84.2 (83.6-84.8)
Spain	64.7 (64.6-64.9)	69.4 (69.3-69.5)	72.3 (72.2-72.4)	73.8 (73.7-73.8)	75 (74.9-75.1)	76.5 (76.4-76.6)	78.6 (78.5-78.7)	79.8 (79.7-79.8)	80.5 (80.4-80.6)	81.7 (81.6-81.8)	82.9 (82.8-82.9)	83.8 (83.7-83.9)	85 (84.9-85)	85.8 (85.3-86.3)
Sweden	72.5 (71.8-73.2)	74.2 (73.5-74.9)	75.1 (74.4-75.8)	76.2 (75.6-76.9)	77.2 (76.6-77.9)	78 (77.6-78.4)	78.9 (78.8-79.1)	79.9 (79.8-80.1)	80.5 (80.4-80.6)	81.5 (81.4-81.7)	82 (81.9-82.1)	82.7 (82.6-82.9)	83.6 (83.5-83.7)	84.2 (83.7-84.7)
Stockholm	72.4 (69.4-75.2)	74.1 (71.2-77)	74.9 (72.2-77.7)	76 (73.4-78.7)	76.9 (74.6-79.4)	77.8 (76.2-79.5)	78.6 (78.4-78.9)	79.8 (79.5-80)	80.3 (80-80.5)	81.6 (81.3-81.8)	81.9 (81.7-82.1)	82.8 (82.6-83)	83.9 (83.7-84.1)	84.3 (83.6-85.1)
Sweden except Stockholm	72.5 (72-73.2)	74.2 (73.7-74.9)	75.1 (74.6-75.7)	76.3 (75.8-76.9)	77.3 (76.8-77.9)	78.1 (77.7-78.5)	79 (78.9-79.1)	80 (79.8-80.1)	80.6 (80.4-80.7)	81.5 (81.4-81.7)	82 (81.9-82.1)	82.7 (82.6-82.9)	83.6 (83.4-83.7)	84.1 (83.5-84.8)
Switzerland	71.2 (71.1-71.4)	72.6 (72.4-72.8)	74.5 (74.4-74.7)	75.4 (75.2-75.5)	76.4 (76.2-76.5)	78.1 (78-78.3)	79.1 (79-79.3)	80.4 (80.2-80.5)	81.1 (81-81.3)	81.9 (81.7-82)	82.8 (82.7-83)	84 (83.8-84.1)	84.5 (84.4-84.7)	85.7 (85.1-86.3)
United Kingdom	71.2 (70.9-71.6)	73 (72.6-73.3)	74 (73.6-74.3)	74.6 (74.3-74.9)	75.1 (74.8-75.4)	75.8 (75.6-76)	76.7 (76.6-76.8)	77.6 (77.5-77.6)	78.5 (78.4-78.5)	79.3 (79.2-79.3)	80.3 (80.2-80.3)	81.3 (81.3-81.4)	82.4 (82.3-82.4)	82.7 (82.6-82.8)
England	71.8 (71.5-72.2)	73.4 (73.1-73.8)	74.4 (74.1-74.7)	75 (74.7-75.3)	75.5 (75.2-75.8)	76.1 (75.9-76.3)	77 (76.9-77.1)	77.8 (77.8-77.8)	78.7 (78.7-78.7)	79.5 (79.4-79.5)	80.5 (80.4-80.5)	81.5 (81.5-81.6)	82.6 (82.6-82.6)	82.9 (82.8-83)
East Midlands	71 (69.8-72.3)	72.7 (71.6-74)	73.8 (72.6-75)	74.5 (73.4-75.6)	74.9 (73.8-76)	75.4 (74.6-76.2)	76.8 (76.6-77)	77.9 (77.8-78)	78.8 (78.7-78.9)	79.4 (79.3-79.5)	80.5 (80.3-80.6)	81.3 (81.2-81.4)	82.5 (82.4-82.6)	82.8 (82.5-83.2)
Derby	70.2 (66.9-73.7)	72 (68.7-75.3)	73 (69.9-76.5)	73.6 (70.5-76.9)	74 (71.3-77)	74.5 (72.5-76.7)	75.9 (75.2-76.6)	77 (76.6-77.3)	77.9 (77.6-78.3)	78.9 (78.6-79.2)	80.2 (79.8-80.7)	81.1 (80.7-81.4)	81.5 (81.2-81.9)	82.5 (81.6-83.4)
Derbyshire	71.1 (67.6-74.3)	72.8 (69.7-75.9)	73.8 (70.8-77)	74.5 (71.3-77.6)	75 (72.2-77.9)	75.4 (73.4-77.5)	76.7 (76.1-77.3)	77.9 (77.6-78.1)	78.6 (78.4-78.9)	79.3 (79.1-79.6)	80.4 (80.1-80.8)	81.2 (81-81.5)	82.5 (82.3-82.8)	82.4 (81.6-83.3)
Leicester	69.5 (66.1-73.2)	71.2 (68-74.8)	72.2 (69-75.6)	72.8 (69.7-76.1)	73.1 (70.1-76.2)	73.7 (71.6-76)	75.4 (74.7-76.1)	77.2 (76.9-77.5)	78 (77.7-78.4)	78.4 (78.1-78.7)	79 (78.6-79.4)	80 (79.7-80.3)	81.7 (81.4-82)	83.6 (82.8-84.5)
Leicestershire	71.9 (68.8-75.5)	73.6 (70.5-77)	74.7 (71.7-77.8)	75.5 (72.5-78.8)	75.9 (73.1-78.8)	76.5 (74.5-78.4)	77.8 (77.2-78.4)	78.9 (78.6-79.1)	79.7 (79.5-80)	80.6 (80.4-80.9)	81.4 (81.1-81.8)	82.3 (82-82.5)	83.5 (83.3-83.8)	83.6 (82.7-84.3)
Lincolnshire	71.2 (67.8-74.8)	73 (69.8-76.2)	74 (71.1-77.2)	74.6 (71.6-77.7)	74.9 (71.9-77.9)	75.4 (73.2-77.7)	77 (76.4-77.6)	78 (77.7-78.3)	79 (78.7-79.2)	79.6 (79.3-79.8)	80.6 (80.2-80.9)	81.5 (81.2-81.7)	82.7 (82.4-83)	83 (82.2-83.8)
Northamptonshire	71.4 (68.1-74.9)	73.2 (70.3-76.6)	74.4 (71.4-77.5)	75.1 (72.2-78.1)	75.6 (73.1-78.5)	76.1 (74.1-78.2)	77.6 (76.9-78.1)	78.1 (77.8-78.3)	78.9 (78.6-79.1)	79.6 (79.3-79.8)	80.9 (80.5-81.2)	81.5 (81.3-81.8)	82.4 (82.1-82.6)	82.7 (81.8-83.5)
Nottingham	70.1 (66.6-73.7)	71.8 (68.4-75.2)	72.9 (69.6-76.2)	73.5 (70.5-76.9)	73.8 (71-76.8)	74.3 (72.2-76.4)	75.4 (74.8-76.1)	76.5 (76.2-76.8)	77.7 (77.3-78)	77.7 (77.4-78)	78.6 (78.2-79)	79.1 (78.7-79.4)	80.8 (80.5-81.1)	82.6 (81.7-83.5)
Nottinghamshire	71.2 (68.1-74.4)	73 (69.8-76.1)	74.1 (71.1-77.2)	74.9 (72-77.9)	75.3 (72.7-78.3)	75.8 (73.8-77.8)	77 (76.4-77.5)	78 (77.8-78.3)	78.8 (78.5-79)	79.5 (79.3-79.8)	80.5 (80.1-80.8)	81.6 (81.4-81.9)	82.4 (82.2-82.7)	82.5 (81.7-83.3)
Rutland	73.5 (70-77.1)	75 (71.8-78.3)	75.9 (72.8-79.2)	76.3 (73-79.5)	76.7 (73.6-79.8)	77 (74.6-79.3)	77.9 (76.8-79.2)	78.3 (77.6-79.1)	79.6 (78.9-80.3)	80.9 (80.2-81.6)	82.4 (81.7-83.1)	83.4 (82.6-84.1)	84 (83.2-84.7)	83.8 (82.5-85)
East of England	72.1 (70.8-73.3)	73.8 (72.5-75)	74.9 (73.8-76.2)	75.8 (74.7-77)	76.3 (75.3-77.5)	77 (76.2-77.8)	77.8 (77.6-78)	78.7 (78.6-78.8)	79.5 (79.4-79.6)	80.3 (80.2-80.4)	81.3 (81.2-81.5)	82.2 (82.1-82.3)	83.3 (83.2-83.4)	83.5 (83.2-83.8)
Bedford	72.1 (68.3-75.9)	73.9 (70.4-77.2)	75.1 (71.8-78.4)	76 (72.9-79.1)	76.7 (74-79.6)	77.6 (75.5-79.7)	78.4 (77.6-79.2)	78.9 (78.5-79.4)	79.2 (78.8-79.6)	79.6 (79.2-80)	80.9 (80.4-81.4)	81.8 (81.5-82.2)	82.7 (82.3-83.1)	83.5 (82.5-84.4)
Cambridgeshire	73 (69.4-76.4)	74.7 (71.6-78)	75.9 (73.1-79.1)	77 (74.1-80.2)	77.6 (75.2-80.4)	78.2 (76.1-80.2)	78.4 (77.8-79)	78.8 (78.5-79.1)	80.1 (79.9-80.4)	80.4 (80.1-80.7)	81.5 (81.1-81.8)	82.8 (82.5-83)	84.1 (83.8-84.3)	84.1 (83.4-85)
Central Bedfordshire	72 (68.8-75.9)	73.7 (70.4-77.1)	74.8 (71.6-78.3)	75.5 (72.4-78.7)	76.1 (73.2-79.2)	76.9 (74.6-79.1)	77.7 (77-78.4)	78.4 (78-78.7)	79 (78.6-79.4)	79.7 (79.3-80)	81.4 (81-81.8)	82.5 (82.2-82.8)	83.6 (83.3-84)	85 (84.2-85.9)
Essex	71.9 (68.7-75.3)	73.6 (70.6-76.9)	74.6 (71.8-77.5)	75.3 (72.6-78.2)	75.7 (73.3-78.5)	76.3 (74.4-78.4)	77.7 (77.1-78.2)	78.8 (78.6-79)	79.5 (79.3-79.7)	80.5 (80.3-80.7)	81.3 (81-81.6)	82.2 (82-82.4)	83.3 (83.1-83.5)	82.9 (82.1-83.7)
Hertfordshire	72.4 (69.1-75.8)	74.1 (70.9-77.2)	75.1 (71.9-78.4)	76 (73-79.1)	76.5 (73.9-79.3)	77 (75-79.1)	77.8 (77.3-78.3)	78.7 (78.5-78.9)	79.5 (79.3-79.8)	80.2 (80-80.4)	81.3 (81-81.6)	82 (81.8-82.3)	83.4 (83.2-83.6)	84.1 (83.3-84.8)

Table 7 Estimates of life expectancy at birth by location and year for 1950, 1955...2005, 2010, 2017 - females

Location	1950	1955	1960	1965	1970	1975	1980	1985	1990	1995	2000	2005	2010	2017
Luton	70.5 (67-74.4)	72.2 (69.1-75.8)	73.2 (69.9-76.5)	73.8 (70.4-77.1)	74.2 (71.3-77.3)	75 (72.7-77.1)	76.1 (75.3-76.8)	78 (77.6-78.3)	78.6 (78.2-79)	79 (78.7-79.4)	79.9 (79.4-80.3)	80.5 (80.2-80.9)	81.9 (81.6-82.3)	83.5 (82.6-84.4)
Norfolk	72.2 (69-75.5)	74.1 (71.1-77.4)	75.4 (72.5-78.6)	76.5 (73.6-79.6)	77.2 (74.5-80.1)	77.9 (75.9-80)	78.4 (77.8-79)	79 (78.7-79.3)	79.6 (79.4-79.9)	80.6 (80.4-80.9)	81.7 (81.3-82)	82.6 (82.4-82.8)	83.1 (82.9-83.4)	83.1 (82.2-83.9)
Peterborough	71.6 (67.7-75.5)	73.5 (70-77.1)	74.6 (71.4-78.2)	75.4 (72.2-79)	76 (73.3-79.2)	76.7 (74.5-78.9)	77.2 (76.4-78.1)	77.8 (77.4-78.2)	78.5 (78.1-78.9)	79 (78.6-79.4)	80.1 (79.7-80.6)	80.9 (80.5-81.3)	81.9 (81.5-82.3)	82.6 (81.6-83.6)
Southend-on-Sea	71.1 (67.5-75)	72.9 (69.7-76.5)	74 (71-77.6)	74.8 (71.6-78.2)	75.4 (72.5-78.3)	76.2 (74.1-78.3)	77.1 (76.4-77.8)	78 (77.6-78.4)	78.8 (78.5-79.2)	79.5 (79.1-79.9)	80.5 (80-81)	81.3 (80.9-81.7)	82.2 (81.8-82.6)	82.5 (81.5-83.3)
Suffolk	72.2 (69-75.7)	73.9 (70.7-77.1)	75 (71.6-78.1)	75.9 (72.9-79.1)	76.5 (73.8-79.4)	77.2 (75-79.3)	78.1 (77.5-78.7)	78.8 (78.6-79.1)	80 (79.8-80.3)	80.6 (80.3-80.8)	81.7 (81.4-82.1)	82.6 (82.4-82.9)	83.7 (83.4-83.9)	83.7 (82.9-84.5)
Thurrock	71.1 (67.6-74.8)	72.8 (69.6-76.4)	73.9 (70.6-77.4)	74.7 (71.7-78)	75.3 (72.5-78.2)	75.9 (73.8-78.2)	76.7 (75.9-77.5)	77.5 (77.1-78)	78.6 (78.2-79)	79.2 (78.8-79.6)	80.6 (80.2-81.1)	81.7 (81.3-82.1)	82.7 (82.3-83.1)	83.5 (82.5-84.6)
Greater London	72.8 (72.2-73.5)	74.3 (73.7-74.9)	75.2 (74.6-75.7)	75.6 (75-76.1)	75.9 (75.4-76.4)	76.3 (76-76.7)	77.2 (77.1-77.3)	78 (78-78.1)	78.9 (78.8-78.9)	79.3 (79.3-79.4)	80.5 (80.4-80.6)	81.9 (81.9-82)	83.2 (83.1-83.3)	84 (83.8-84.2)
Barking and Dagenham	71.3 (68.1-75)	73.1 (70-76.4)	74.1 (71.1-77.4)	74.7 (71.7-78.1)	75.1 (72.4-78)	75.6 (73.5-77.8)	76.2 (75.4-77)	76.9 (76.5-77.3)	77.9 (77.5-78.3)	78.4 (78-78.7)	79.4 (78.9-79.9)	80.3 (80-80.7)	81.5 (81.2-81.9)	82.4 (81.4-83.4)
Barnet	74.1 (71.1-77.3)	75.7 (72.9-78.6)	76.6 (73.9-79.3)	77.2 (74.4-80)	77.5 (75-80.1)	78 (76.1-80)	78.6 (77.9-79.2)	79.1 (78.8-79.4)	80.1 (79.8-80.4)	80.7 (80.4-81)	82.1 (81.7-82.5)	83.4 (83.1-83.7)	83.8 (83.5-84.1)	84.4 (83.6-85.2)
Bexley	74.1 (71-77.4)	75.6 (72.4-78.7)	76.5 (73.5-79.5)	77 (74.2-80)	77.5 (74.9-80.2)	78.1 (76.1-80)	79 (78.3-79.6)	79.7 (79.3-80)	80.3 (79.9-80.6)	80.4 (80.1-80.8)	82 (81.5-82.4)	83.5 (83.1-83.8)	84.2 (83.8-84.5)	84.2 (83.3-85)
Brent	72.5 (69.3-76)	74.1 (70.7-77.3)	75 (71.9-78.2)	75.4 (72.5-78.4)	75.7 (73.2-78.4)	76.3 (74.4-78.3)	77.7 (77.1-78.4)	78.1 (77.8-78.4)	78.9 (78.6-79.2)	79.3 (79-79.6)	80.7 (80.3-81.1)	82.5 (82.2-82.8)	83.7 (83.4-84)	85.1 (84.3-85.9)
Bromley	74.1 (70.9-77.6)	75.6 (72.8-78.7)	76.4 (73.3-79.4)	76.9 (74-79.8)	77.3 (74.7-80)	77.8 (75.8-79.7)	78.6 (78-79.2)	79.6 (79.3-79.9)	80.3 (80-80.6)	80.9 (80.6-81.2)	82 (81.6-82.4)	83.3 (83-83.6)	84.2 (83.9-84.5)	84.4 (83.5-85.2)
Camden	72.6 (69.5-75.8)	73.9 (71-77.1)	74.5 (71.7-77.8)	74.7 (71.7-77.6)	74.8 (72.1-77.5)	75.1 (73.1-77.2)	75.8 (75.1-76.5)	76.6 (76.3-77)	77.6 (77.3-77.9)	78.1 (77.8-78.4)	79.6 (79.2-80.1)	81 (80.7-81.3)	82.8 (82.4-83.1)	85.6 (84.7-86.4)
Croydon	72.7 (69.6-76)	74.2 (71.2-77.4)	75 (72.1-77.9)	75.6 (72.6-78.5)	75.8 (73.1-78.5)	76.2 (74.3-78.3)	77.1 (76.5-77.7)	78 (77.7-78.3)	78.6 (78.3-78.9)	79.4 (79.1-79.7)	80.7 (80.3-81)	82 (81.7-82.3)	82.8 (82.6-83.1)	83.1 (82.3-83.9)
Ealing	72.6 (69.3-76.1)	74.2 (71.1-77.5)	75.1 (72.3-78.4)	75.6 (72.9-78.6)	75.8 (73.3-78.4)	76.3 (74.3-78.1)	77.1 (76.5-77.7)	77.9 (77.6-78.2)	78.9 (78.6-79.2)	79.3 (79-79.6)	80.7 (80.3-81.1)	82.4 (82.1-82.7)	83.9 (83.6-84.2)	84.3 (83.5-85.1)
Enfield	73.4 (70.2-76.7)	75 (71.9-78.2)	76 (73.1-79.1)	76.6 (73.7-79.7)	77 (74.5-79.8)	77.5 (75.6-79.5)	78.2 (77.6-78.8)	78.7 (78.4-79)	79.7 (79.4-80)	80.1 (79.8-80.5)	80.8 (80.4-81.2)	82 (81.7-82.3)	83.4 (83.1-83.8)	84.2 (83.3-85)
Greenwich	71.9 (68.7-75.2)	73.7 (70.5-77.1)	74.7 (71.7-77.8)	75.3 (72.4-78.3)	75.7 (73.2-78.3)	76.2 (74.2-78.2)	76.7 (76-77.3)	77.1 (76.8-77.5)	78.1 (77.8-78.4)	78.5 (78.2-78.9)	79 (78.6-79.4)	80.3 (79.9-80.6)	82.3 (81.9-82.6)	82.4 (81.5-83.2)
Hackney	72.3 (68.9-75.8)	73.8 (70.7-77)	74.5 (71.7-77.6)	74.5 (71.8-77.5)	74.5 (72-77.3)	74.8 (72.8-76.9)	75.3 (74.6-76)	76.4 (76-76.7)	76.9 (76.5-77.2)	78.1 (77.8-78.5)	79.5 (79.1-80)	80.1 (79.7-80.4)	81.4 (81.1-81.7)	83.4 (82.5-84.3)
Hammersmith and Fulham	72.3 (69.1-75.7)	73.7 (70.4-76.9)	74.3 (71.3-77.4)	74.5 (71.6-77.6)	74.7 (72-77.4)	75 (73.1-77)	75.6 (74.9-76.3)	76.1 (75.8-76.4)	77.1 (76.7-77.4)	77.7 (77.3-78)	79.2 (78.8-79.7)	81.2 (80.8-81.5)	83.2 (82.8-83.5)	84.7 (83.8-85.6)
Haringey	72.4 (68.8-76.1)	74 (70.8-77.5)	74.9 (71.7-78.2)	75.2 (72.4-78.3)	75.6 (73-78.3)	76.1 (74.1-78.2)	77 (76.4-77.7)	77.7 (77.4-78.1)	78.4 (78.1-78.8)	79.1 (78.8-79.4)	80.1 (79.6-80.5)	81.5 (81.2-81.8)	82.6 (82.3-82.9)	84 (83.1-84.8)
Harrow	73.8 (70.4-77.2)	75.2 (72-78.4)	75.8 (72.8-79.1)	76.1 (73.1-79.1)	76.3 (73.6-79.2)	76.9 (74.8-78.9)	78 (77.3-78.7)	79.5 (79.2-79.9)	80.8 (80.4-81.2)	81.3 (81-81.7)	82.5 (82-82.9)	83.9 (83.6-84.2)	85 (84.7-85.4)	85.8 (84.9-86.6)
Havering	73.5 (70.3-76.7)	75 (71.9-78)	75.9 (72.6-78.9)	76.4 (73.4-79.2)	76.7 (74.3-79.5)	77.3 (75.4-79.3)	77.9 (77.2-78.5)	79.5 (79.1-79.8)	80.8 (80.5-81.2)	80.9 (80.6-81.3)	81.8 (81.4-82.2)	82.6 (82.3-83)	83.1 (82.8-83.5)	82.5 (81.5-83.4)
Hillingdon	73.7 (70.5-77.2)	75.2 (72.1-78.4)	76 (73.2-79.1)	76.6 (73.8-79.5)	76.9 (74.4-79.6)	77.3 (75.3-79.2)	77.9 (77.2-78.5)	78.6 (78.2-78.9)	79.3 (79-79.6)	79.4 (79.1-79.7)	80.8 (80.4-81.2)	82.2 (81.9-82.5)	83.2 (82.9-83.5)	83.3 (82.5-84.2)
Hounslow	72.9 (69.6-76.5)	74.5 (71.3-77.7)	75.3 (72.1-78.5)	75.7 (72.6-78.8)	76 (73.4-78.7)	76.4 (74.6-78.4)	77.4 (76.7-78)	77.8 (77.5-78.2)	79.2 (78.8-79.5)	79.8 (79.4-80.1)	80.4 (79.9-80.8)	81.5 (81.2-81.8)	82.7 (82.4-83.1)	83.9 (83-84.8)
Islington	71.9 (68.7-75.4)	73.4 (70.5-76.7)	74.2 (71.3-77.4)	74.4 (71.6-77.5)	74.5 (72-77.2)	74.7 (72.7-76.7)	75.4 (74.7-76.2)	76.4 (76-76.7)	77.6 (77.2-77.9)	77.7 (77.4-78)	78.6 (78.2-79.1)	80.4 (80-80.7)	82.2 (81.8-82.5)	83.6 (82.7-84.4)
Kensington and Chelsea	72.8 (69.6-76.2)	74.2 (71.3-77.5)	74.8 (71.9-77.9)	75 (72.1-77.9)	75.1 (72.5-77.8)	75.4 (73.3-77.5)	76.2 (75.5-76.9)	77.3 (76.9-77.6)	78.8 (78.5-79.2)	80.4 (80.1-80.8)	82.7 (82.2-83.1)	84.1 (83.8-84.5)	85 (84.6-85.3)	85 (84.2-85.9)
Kingston upon Thames	74.8 (71.4-78.1)	76.3 (73.4-79.3)	77.2 (74.1-80.2)	77.6 (74.6-80.6)	78.1 (75.4-80.7)	78.6 (76.6-80.6)	79.3 (78.5-80.1)	79.7 (79.3-80.1)	80.4 (80-80.8)	80.6 (80.2-81)	81.9 (81.5-82.4)	83.7 (83.3-84.1)	85 (84.6-85.4)	84.2 (83.2-85.1)
Lambeth	71.4 (67.9-74.7)	73.1 (70-76.4)	73.9 (70.8-77)	74.2 (71.4-77.1)	74.5 (71.9-77.2)	75 (73-77)	75.9 (75.2-76.5)	77 (76.6-77.3)	77.5 (77.2-77.9)	77.9 (77.6-78.2)	78.9 (78.5-79.3)	80.2 (79.9-80.5)	82.1 (81.8-82.4)	84.4 (83.6-85.2)
Lewisham	72.2 (68.8-75.5)	73.9 (70.7-77.1)	74.8 (71.6-78.1)	75.4 (72.4-78.4)	75.8 (73.1-78.6)	76.3 (74.4-78.5)	77 (76.4-77.7)	77.7 (77.4-78.1)	78.4 (78-78.6)	78.1 (77.8-78.5)	78.6 (78.2-79)	80 (79.7-80.3)	81.9 (81.6-82.2)	83.1 (82.3-84)
Merton	73.4 (70.2-76.8)	74.9 (71.8-78.1)	75.6 (72.6-78.7)	75.9 (73.1-78.9)	76.2 (73.5-78.8)	76.8 (74.7-78.8)	77.8 (77-78.5)	79.3 (78.9-79.7)	79.7 (79.4-80.1)	80 (79.7-80.4)	81.3 (80.8-81.7)	82.8 (82.5-83.2)	84 (83.7-84.4)	84.5 (83.5-85.4)
Newham	71.1 (67.8-74.5)	72.8 (69.7-76.2)	73.7 (70.8-76.9)	74.1 (71.3-77.4)	74.4 (71.8-77.3)	75 (72.9-77.1)	76 (75.3-76.7)	77 (76.6-77.3)	77.8 (77.4-78.1)	77.9 (77.5-78.2)	78.7 (78.2-79.1)	79.8 (79.4-80.1)	81.1 (80.8-81.4)	83.6 (82.8-84.4)
Redbridge	73.4 (70.2-76.9)	75 (71.8-78.1)	75.8 (72.8-78.8)	76.2 (73-79.4)	76.6 (74-79.2)	77.2 (75.1-79.3)	78.3 (77.6-78.9)	79.9 (79.6-80.2)	80 (79.6-80.3)	80 (79.7-80.4)	81 (80.6-81.4)	82.2 (81.9-82.5)	83.6 (83.3-83.9)	83.9 (83-84.8)
Richmond upon Thames	74.2 (70.9-77.5)	75.5 (72.5-78.7)	76.2 (73.2-79.2)	76.4 (73.3-79.2)	76.6 (74-79)	77.1 (75.2-79.2)	78 (77.3-78.7)	79 (78.7-79.4)	80.3 (79.9-80.7)	81.9 (81.5-82.3)	82 (81.6-82.5)	83.8 (83.4-84.2)	85.1 (84.7-85.5)	85.5 (84.6-86.5)
Southwark	72.8 (69.4-76.3)	74.3 (71.2-77.6)	75.2 (72.1-78.2)	75.4 (72.5-78.5)	75.7 (73.3-78.4)	76.2 (74.2-78.2)	76.6 (75.9-77.3)	77.3 (76.9-77.6)	76.7 (76.4-77)	77.7 (77.4-78)	78.9 (78.4-79.3)	80.4 (80.1-80.7)	81.7 (81.4-82)	82.9 (82.1-83.8)
Sutton	73.7 (70.4-77.1)	75.2 (72.1-78.2)	76.2 (73.1-79)	76.7 (73.7-79.5)	77.2 (74.6-79.8)	77.8 (75.8-79.7)	78.5 (77.8-79.2)	78.8 (78.4-79.1)	79.4 (79.1-79.8)	79.8 (79.4-80.2)	81.1 (80.7-81.6)	83 (82.7-83.4)	84.1 (83.7-84.4)	83.4 (82.4-84.2)
Tower Hamlets	71.6 (68.4-74.9)	73.1 (70.1-76.2)	73.8 (70.7-77)	74.1 (71.2-77.2)	74.2 (71.6-76.9)	74.5 (72.6-76.7)	75.1 (74.5-75.9)	76 (75.6-76.4)	77 (76.6-77.4)	77.1 (76.7-77.5)	78.4 (78-78.9)	79.9 (79.6-80.2)	82 (81.7-82.3)	83.5 (82.7-84.4)
Waltham Forest	72.1 (68.7-75.4)	73.8 (70.5-77)	74.8 (71.6-77.9)	75.3 (72.4-78.3)	75.7 (73-78.5)	76.4 (74.3-78.4)	77.5 (76.8-78.2)	78 (77.7-78.3)	78.7 (78.4-79.1)	78.9 (78.6-79.3)	79.7 (79.2-80.1)	80.7 (80.4-81.1)	82.2 (81.9-82.5)	83.5 (82.7-84.4)
Wandsworth	72 (68.9-75.3)	73.5 (70.5-76.9)	74.2 (71.3-77.3)	74.4 (71.5-77.2)	74.6 (71.7-77.2)	75.1 (73.1-77.1)	76.3 (75.7-76.9)	76.9 (76.6-77.3)	77.9 (77.6-78.2)	78.2 (77.9-78.5)	79.6 (79.2-80)	81.3 (81-81.6)	83 (82.7-83.3)	84.1 (83.3-85)
Westminster	73.7 (70.4-77.1)	75.2 (72.1-78.3)	75.8 (73-78.8)	75.8 (73-78.8)	75.8 (73.1-78.6)	76 (74-78)	76.8 (76.2-77.5)	78.1 (77.8-78.5)	78.3 (77.9-78.6)	79.3 (79-79.7)	82 (81.6-82.4)	82.7 (82.4-83.1)	84.3 (83.9-84.7)	85.2 (84.4-86.1)
North East England	69.1 (68-70.3)	71 (69.8-72)	72.2 (71.1-73.2)	73.1 (72-74.1)	73.7 (72.8-74.7)	74.6 (73.9-75.3)	75.6 (75.4-75.9)	76.6 (76.5-76.7)	77.1 (77-77.2)	78.1 (78-78.3)	79.2 (79-79.3)	80.2 (80.1-80.3)	81.3 (81.2-81.4)	81.6 (81.3-81.9)
County Durham	69 (65.9-72.4)	70.9 (67.7-74.3)	72.1 (69.2-75.2)	73.1 (70-76.3)	73.8 (71.1-76.7)	74.6 (72.6-76.8)	75.8 (75.2-76.4)	76.6 (76.3-76.8)	77 (76.7-77.2)	78 (77.7-78.2)	78.9 (78.5-79.2)	80.1 (79.8-80.3)	81.3 (81.1-81.6)	81.4 (80.6-82.2)
Darlington	69 (65.4-72.6)	70.8 (67.3-74.1)	72 (68.8-75.3)	72.7 (69.7-75.8)	73.4 (70.4-76.2)	74.2 (72-76.3)	75.4 (74.6-76.3)	76.8 (76.3-77.2)	77.2 (76.8-77.7)	78.1 (77.7-78.6)	79 (78.4-79.5)	80 (79.5-80.4)	81.4 (80.9-81.9)	82 (80.8-83.2)
Gateshead	69 (65.5-72.5)	70.8 (67.5-74.2)	72 (68.8-75.4)	72.8 (69.7-76.1)	73.5 (70.7-76.5)	74.2 (72.2-76.4)	75.1 (74.4-75.9)	76.5 (76.1-76.8)	77.6 (77.2-77.9)	78.4 (78.1-78.8)	79.3 (78.8-79.7)	80.5 (80.1-80.9)	81.4 (81.1-81.8)	81.4 (80.4-82.3)
Hartlepool	67.8 (64.3-71.6)	69.7 (66.2-73.4)	70.9 (67.6-74.2)	71.7 (68.6-75)	72.4 (69.5-75.5)	73.3 (71.1-75.7)	74.4 (73.5-75.2)	75.7 (75.2-76.2)	76.5 (76.1-76.9)	77.4 (76.9-77.8)	78.2 (77.7-78.7)	78.9 (78.4-79.4)	80.6 (80.1-81)	81.7 (80.7-82.8)
Middlesbrough	67.9 (64.7-71.6)	69.7 (66.5-73.3)	70.8 (67.7-74.2)	71.6 (68.4-74.8)	72.2 (69.3-75.3)	72.9 (70.8-75.1)	73.9 (73.2-74.6)	75 (74.6-75.4)	75.8 (75.4-76.2)	76.8 (76.4-77.2)	77.7 (77.2-78.1)	78.7 (78.3-79.1)	80.1 (79.7-80.5)	80.5 (79.4-81.5)
Newcastle upon Tyne	69.5 (66.2-73)	71.2 (67.9-74.6)	72.4 (69.3-75.8)	73.4 (70.3-76.7)	74 (71.3-77.1)	74.9 (72.8-77)	75.8 (75.1-76.5)	76.6 (76.3-76.9)	76.8 (76.5-77.1)	77.7 (77.4-78)	78.9 (78.5-79.3)	80 (79.7-80.3)	81 (80.7-81.4)	81.5 (80.5-82.4)
North Tyneside	69.8 (66.2-73.7)	71.6 (68.1-75)	72.8 (69.8-76.2)	73.6 (70.6-77)	74.3 (71.5-77.3)	75.1 (73-77.4)	76.1 (75.3-76.8)	76.8 (76.5-77.1)	77.3 (77-77.7)	78.5 (78.2-78.9)	79.4 (78.9-79.9)	80.2 (79.8-80.5)	81.5 (81.2-81.9)	82.6 (81.6-83.6)
Northumberland	69.5 (66.2-73)	71.3 (68.1-74.5)	72.5 (69.3-75.7)	73.4 (70.6-76.7)	74.1 (71.6-77.1)	74.9 (72.9-77.1)	76.1 (75.5-76.8)	76.9 (76.6-77.3)	77.6 (77.3-77.9)	79 (78.7-79.3)	80.3 (79.9-80.7)	81.5 (81.2-81.8)	82.3 (82-82.7)	82.5 (81.6-83.3)
Redcar and Cleveland	68.7 (65.2-72.4)	70.6 (67-73.9)	71.8 (68.5-75.1)	72.6 (69.5-75.8)	73.3 (70.4-76.5)	74.2 (72.2-76.5)	75.6 (74.8-76.4)	77.1 (76.7-77.5)	77.9 (77.5-78.3)	78.7 (78.3-79.1)	79.7 (79.2-80.1)	80.1 (79.7-80.5)	81.2 (80.7-81.6)	81.4 (80.4-82.4)
South Tyneside	69.6 (66.3-73.4)	71.4 (68.1-74.9)	72.6 (69.4-75.9)	73.5 (70.5-77.1)	74.2 (71.4-77.3)	74.9 (72.8-77.2)	75.7 (75-76.5)	76.4 (76-76.7)	77 (76.6-77.4)	78.3 (77.8-78.7)	79.5 (79-79.9)	80.3 (79.9-80.7)	80.9 (80.5-81.3)	81.3 (80.3-82.4)
Stockton-on-Tees	69.7 (66.2-73.4)	71.5 (68.1-74.9)	72.7 (69.5-76.1)	73.6 (70.6-76.8)	74.3 (71.5-77.1)	75.2 (73.1-77.5)	76.2 (75.5-77)	77.1 (76.7-77.5)	77.8 (77.4-78.2)	78.8 (78.4-79.1)	79.9 (79.4-80.4)	80.8 (80.4-81.2)	81.9 (81.5-82.2)	82.4 (81.4-83.3)
Sunderland	69.2 (65.9-72.6)	71 (67.9-74.5)	72.3 (69.3-75.6)	73.2 (70.2-76.5)	74 (71.2-76.9)	74.8 (72.8-77)	75.9 (75.3-76.5)	76.6 (76.3-76.9)	77 (76.7-77.3)	77.7 (77.3-78)	78.8 (78.3-79.2)	79.5 (79.1-79.8)	80.7 (80.4-81.1)	80.8 (79.8-81.7)
North West England	71 (70.2-71.9)	72.6 (71.8-73.4)	73.5 (72.7-74.3)	74.1 (73.4-74.8)	74.5 (73.8-75.2)	75.2 (74.7-75.7)	75.9 (75.8-76.1)	76.7 (76.6-76.7)	77.6 (77.5-77.6)	78.4 (78.4-78.5)	79.3 (79.2-79.4)	80.3 (80.2-80.3)	81.3 (81.2-81.4)	81.5 (81.3-81.8)
Blackburn with Darwen	70.3 (67-73.7)	71.9 (69-75.2)	72.7 (69.6-76)	73.2 (70.3-76.2)	73.7 (71-76.5)	74.4 (72.4-76.5)	75.2 (74.5-75.9)	75.7 (75.3-76.1)	76.6 (76.2-76.9)	77.2 (76.8-77.5)	77.8 (77.3-78.3)	79.2 (78.8-79.6)	80.7 (80.3-81.1)	81 (80.1-82)
Blackpool	70.4 (67.1-73.9)	72.1 (69-75.4)	72.9 (69.7-76.1)	73.2 (70.3-76.2)	73.6 (70.9-76.4)	74.4 (72.3-76.5)	75.5 (74.8-76.2)	76.9 (76.5-77.3)	77.1 (76.7-77.5)	78.2 (77.8-78.6)	77.3 (76.8-77.8)	77.7 (77.4-78.1)	78.8 (78.4-79.2)	80 (78.9-81)
Bolton	70.7 (67.5-74)	72.4 (69.4-75.5)	73.3 (70.3-76.6)	73.9 (71-77)	74.3 (71.7-77)	75 (73-77.1)	75.7 (75-76.3)	76.3 (76-76.6)	77.5 (77.1-77.8)	78.3 (78-78.6)	78.7 (78.3-79.2)	79.5 (79.2-79.8)	81 (80.7-81.4)	81.7 (80.9-82.7)
Bury	71.6 (68.4-75)	73.2 (70.3-76.3)	74.2 (71.1-77.3)	74.9 (71.9-77.7)	75.5 (72.8-78.1)	76.1 (74.2-78.2)	76.6 (75.9-77.3)	77.2 (76.8-77.5)	78.3 (77.9-78.6)	79.2 (78.8-79.5)	80 (79.5-80.4)	80.5 (80.1-80.9)	81.4 (81-81.8)	81 (80.1-81.9)
Cheshire East	72.8 (69.9-76.2)	74.4 (71.3-77.5)	75.3 (72.4-78.4)	76.2 (73.3-79.1)	76.7 (74-79.2)	77.3 (75.4-79.3)	77.5 (76.9-78.1)	77.9 (77.6-78.2)	78.9 (78.6-79.2)	80.1 (79.8-80.4)	81 (80.6-81.3)	82.6 (82.3-82.9)	83 (82.7-83.3)	83 (82.1-83.8)
Cheshire West and Chester	72 (68.9-75.4)	73.5 (70.5-76.7)	74.3 (71.3-77.3)	74.8 (71.8-77.7)	75.2 (72.5-77.7)	75.8 (73.8-77.8)	76.8 (76.1-77.4)	77.3 (77-77.6)	78.3 (78.1-78.6)	79.4 (79.1-79.7)	80.7 (80.3-81.1)	81.9 (81.6-82.2)	82.9 (82.6-83.2)	82.7 (81.8-83.5)
Cumbria	71.2 (68.1-74.3)	72.8 (70-75.7)	73.7 (70.9-76.6)	74.4 (71.8-77.3)	74.8 (72.4-77.4)	75.5 (73.6-77.4)	76.4 (75.8-77)	77.1 (76.9-77.4)	78.2 (78-78.5)	78.8 (78.5-79.1)	80.4 (80-80.8)	81.5 (81.3-81.7)	82 (81.7-82.3)	82.4 (81.5-83.2)
Halton	70.5 (67.1-73.9)	72.1 (69-75.3)	72.8 (69.8-76.2)	73.2 (70.1-76.4)	73.5 (70.5-76.4)	74.1 (72.1-76.3)	74.9 (74.1-75.6)	75.7 (75.3-76.1)	77.1 (76.7-77.5)	77.6 (77.2-78)	78 (77.5-78.4)	78.7 (78.3-79)	80.1 (79.7-80.5)	80.9 (79.9-81.9)
Knowsley	70.1 (66.9-73.6)	71.8 (68.4-75.3)	72.6 (69.4-76.1)	73 (70-76.2)	73.4 (70.7-76.2)	74 (72-76.2)	74.8 (74.1-75.5)	76 (75.7-76.4)	76.9 (76.6-77.2)	77.5 (77.1-77.9)	78.2 (77.7-78.6)	79.2 (78.8-79.5)	80.3 (79.9-80.6)	80.1 (79.2-81.1)
Lancashire	71.6 (68.5-74.9)	73.2 (70.5-76.3)	74.2 (71.5-77.1)	75 (72.3-77.8)	75.5 (72.8-78)	76.2 (74.2-78.1)	76.3 (75.8-76.8)	76.9 (76.7-77.1)	77.6 (77.4-77.8)	79.1 (78.9-79.3)	79.9 (79.5-80.2)	80.6 (80.4-80.8)	81.9 (81.7-82.1)	81.9 (81.1-82.7)
Liverpool	70.1 (66.7-73.6)	71.5 (68.4-74.6)	72.1 (69.2-75.3)	72.3 (69.5-75.4)	72.6 (69.9-75.4)	73.3 (71.2-75.4)	74.9 (74.3-75.4)	75.8 (75.6-76)	76.5 (76.2-76.7)	77 (76.8-77.3)	77.7 (77.3-78.1)	78.8 (78.5-79.1)	79.9 (79.7-80.2)	80.6 (79.8-81.5)
Manchester	70.5 (67.4-73.8)	72.2 (69.5-75.4)	73.2 (70.2-76.3)	73.6 (70.8-76.4)	74 (71.4-76.7)	74.6 (72.6-76.7)	75.1 (74.6-75.7)	75.3 (75-75.6)	76.1 (75.9-76.4)	76.6 (76.4-76.9)	77.8 (77.4-78.2)	78.1 (77.9-78.4)	79.7 (79.4-79.9)	80 (79.2-80.8)
Oldham	70.3 (67.2-73.9)	71.9 (68.8-75.2)	72.8 (69.8-76)	73.4 (70.7-76.4)	73.9 (71.4-76.6)	74.7 (72.9-76.7)	75.5 (74.8-76.1)	76.1 (75.8-76.4)	77.1 (76.7-77.4)	78 (77.7-78.4)	78.6 (78.2-79.1)	79.3 (79-79.7)	80.4 (80-80.7)	80.5 (79.6-81.4)
Rochdale	70.5 (67.1-73.8)	72.2 (69-75.5)	73.2 (69.9-76.4)	73.8 (70.9-76.8)	74.4 (71.8-77.2)	75.2 (73.3-77.3)	75.9 (75.2-76.6)	76.5 (76.2-76.8)	76.6 (76.2-76.9)	77.6 (77.2-77.9)	78.4 (78-78.9)	79.3 (79-79.7)	80.3 (80-80.6)	80.7 (79.8-81.6)
Salford	70.7 (67.4-74)	72.3 (69.2-75.5)	73.1 (70-76.4)	73.6 (70.5-76.5)	74.1 (71.4-76.7)	74.8 (72.8-77)	75.8 (75.2-76.4)	76.1 (75.8-76.5)	76.5 (76.1-76.8)	76.7 (76.4-77.1)	78 (77.6-78.5)	79 (78.7-79.4)	80.3 (80-80.6)	80.6 (79.7-81.6)
Sefton	71.6 (68.3-74.9)	73.1 (70-76.1)	74 (71.1-76.9)	74.4 (71.5-77.4)	74.8 (72-77.5)	75.5 (73.5-77.5)	76.3 (75.7-76.9)	77.2 (76.9-77.5)	78.4 (78.1-78.7)	79 (78.7-79.3)	79.8 (79.4-80.2)	81.2 (80.9-81.5)	81.7 (81.3-82)	81.7 (80.8-82.7)
St Helens	70.6 (67.6-74.1)	72.2 (69.2-75.4)	73.1 (69.9-76.4)	73.7 (70.7-76.8)	74.1 (71.4-76.8)	74.7 (72.7-76.8)	74.8 (74.2-75.5)	76.1 (75.8-76.5)	77.3 (77-77.6)	78.2 (77.9-78.6)	79.1 (78.7-79.6)	80 (79.6-80.3)	80.7 (80.3-81.1)	80.9 (80-81.8)
Stockport	72.4 (69.2-75.7)	73.9 (70.9-76.9)	74.8 (72.1-77.8)	75.5 (72.8-78.4)	76 (73.3-78.6)	76.6 (74.6-78.5)	77.2 (76.6-77.8)	78 (77.7-78.3)	78.7 (78.4-79)	79.5 (79.2-79.8)	80.7 (80.3-81.1)	81.5 (81.2-81.8)	82.3 (81.9-82.6)	82.7 (81.8-83.6)
Tameside	70.2 (67-73.6)	71.8 (68.8-75)	72.6 (69.6-75.9)	73.2 (70.3-76.2)	73.6 (70.8-76.2)	74.3 (72.2-76.4)	75.1 (74.5-75.7)	76.2 (75.9-76.5)	77.6 (77.3-77.9)	78 (77.7-78.3)	79 (78.6-79.4)	79.7 (79.3-80.1)	80.5 (80.2-80.9)	80.8 (79.9-81.7)
Trafford	71.5 (68.3-75)	73 (70.1-76.3)	73.8 (70.8-76.8)	74.2 (71.4-77.2)	74.6 (71.8-77.2)	75.3 (73.2-77.2)	76.3 (75.6-76.9)	77.4 (77-77.7)	78.6 (78.3-79)	79.1 (78.7-79.4)	80 (79.6-80.5)	81.7 (81.4-82.1)	83.2 (82.9-83.6)	83 (82.1-83.9)
Warrington	71.7 (68.6-75.2)	73.2 (70.2-76.4)	74 (71-77.1)	74.4 (71.5-77.4)	74.8 (72.2-77.4)	75.4 (73.4-77.4)	76 (75.3-76.7)	76.4 (76-76.7)	77.3 (77-77.7)	78.8 (78.4-79.1)	80.1 (79.6-80.5)	80.8 (80.5-81.1)	81.8 (81.4-82.1)	81.8 (80.9-82.7)
Wigan	70.7 (67.4-74.2)	72.3 (69.2-75.5)	73.2 (70-76.4)	73.9 (71.2-76.7)	74.3 (71.9-76.9)	74.9 (72.9-76.9)	75.3 (74.7-75.9)	76.1 (75.8-76.4)	77.3 (77-77.6)	77.8 (77.5-78.1)	79.1 (78.7-79.5)	80.2 (79.9-80.5)	80.7 (80.4-81)	80.6 (79.7-81.5)
Wirral	71.1 (67.8-74.6)	72.7 (69.7-76)	73.6 (70.8-77)	74.2 (71.3-77.2)	74.7 (72.1-77.3)	75.4 (73.6-77.5)	76.5 (75.9-77.1)	77.3 (77-77.6)	78.1 (77.8-78.4)	78.6 (78.4-79)	79.2 (78.8-79.5)	80 (79.7-80.3)	81 (80.7-81.4)	80.7 (79.9-81.6)
South East England	73.7 (72.7-74.8)	75.1 (74.2-76.1)	75.9 (75-76.9)	76.4 (75.4-77.4)	76.6 (75.8-77.5)	77 (76.4-77.6)	78 (77.8-78.1)	78.6 (78.6-78.7)	79.7 (79.6-79.8)	80.3 (80.3-80.4)	81.4 (81.2-81.5)	82.4 (82.3-82.5)	83.5 (83.4-83.5)	83.7 (83.5-84)
Bracknell Forest	74.4 (71.1-78)	75.8 (72.5-79.1)	76.6 (73.3-80)	76.9 (73.9-80.1)	77.3 (74.6-80.1)	77.7 (75.7-79.9)	78.3 (77.5-79.2)	79.1 (78.6-79.6)	80.2 (79.7-80.7)	80.9 (80.4-81.4)	81.9 (81.4-82.4)	82.8 (82.3-83.2)	83.8 (83.3-84.2)	85.3 (84.4-86.3)
Brighton and Hove	73.2 (69.8-76.5)	74.5 (71.7-77.7)	75.2 (72.4-78.2)	75.5 (72.5-78.7)	75.7 (72.8-78.4)	76.1 (74.1-78.2)	77 (76.4-77.7)	77.8 (77.5-78.1)	79 (78.7-79.4)	79.2 (78.9-79.5)	79.8 (79.4-80.2)	80.7 (80.4-81)	82 (81.7-82.4)	83.2 (82.3-84.1)
Buckinghamshire	74.2 (70.8-77.8)	75.5 (72.4-78.7)	76.2 (73.4-79.4)	76.8 (73.8-79.7)	77 (74.2-79.6)	77.3 (75.4-79.2)	78 (77.5-78.5)	79 (78.7-79.2)	79.8 (79.5-80)	80.5 (80.3-80.8)	81.7 (81.3-82.1)	83 (82.7-83.2)	84 (83.7-84.3)	84.3 (83.6-85.1)
East Sussex	73.9 (70.7-77.3)	75.3 (72.4-78.6)	76.1 (73.4-79.4)	76.5 (73.8-79.7)	76.9 (74.1-79.6)	77.4 (75.4-79.2)	78.3 (77.7-78.8)	79.1 (78.8-79.4)	80.2 (79.9-80.4)	80.6 (80.3-80.8)	81.4 (81.1-81.8)	82.6 (82.3-82.8)	83.2 (82.9-83.5)	83.1 (82.3-83.9)
Hampshire	73.7 (70.6-77)	75 (71.9-77.9)	75.6 (72.6-78.6)	76 (73.1-79.1)	76.1 (73.4-78.8)	76.5 (74.6-78.6)	78.2 (77.7-78.7)	79.1 (78.8-79.3)	80.1 (79.9-80.4)	80.6 (80.4-80.8)	81.7 (81.3-82)	82.9 (82.7-83.1)	83.9 (83.7-84.1)	84.1 (83.3-84.8)
Isle of Wight	73.5 (70-76.8)	75 (71.9-78.2)	75.9 (72.9-79)	76.5 (73.6-79.7)	76.9 (74.2-79.7)	77.5 (75.3-79.6)	78.2 (77.4-79)	78.8 (78.4-79.3)	79.6 (79.2-80)	80.2 (79.8-80.6)	81.4 (80.9-82)	82.2 (81.8-82.6)	83 (82.5-83.4)	83.8 (82.8-84.8)
Kent	73.2 (70.3-76.3)	74.6 (71.7-77.6)	75.3 (72.6-78.3)	75.8 (72.9-78.6)	76 (73.3-78.9)	76.4 (74.6-78.4)	77.5 (77-77.9)	78.1 (77.9-78.3)	79.3 (79.1-79.5)	80 (79.8-80.2)	81.2 (80.9-81.5)	82.1 (81.9-82.3)	83.3 (83.1-83.5)	83.2 (82.5-84)
Medway	72.9 (69.7-76.2)	74.4 (71.5-77.7)	75.4 (72.5-78.7)	76 (73.1-79)	76.4 (73.8-79.2)	76.9 (75-79)	77.4 (76.8-78.1)	77.9 (77.6-78.3)	78.8 (78.5-79.2)	79.3 (79-79.7)	80.2 (79.8-80.6)	80.9 (80.6-81.2)	81.3 (81-81.7)	82.9 (82.1-83.8)
Milton Keynes	74.2 (70.8-77.5)	75.6 (72.2-78.8)	76.5 (73.4-79.6)	77 (73.9-80.2)	77.4 (74.5-80.3)	77.8 (75.8-79.9)	78.1 (77.4-78.9)	78.2 (77.8-78.6)	78.9 (78.5-79.3)	79.7 (79.3-80)	80.9 (80.5-81.3)	81.8 (81.4-82.1)	82.8 (82.4-83.1)	83.8 (83-84.6)
Oxfordshire	74.1 (70.7-77.4)	75.4 (72.3-78.6)	76.1 (73.2-79.3)	76.5 (73.6-79.6)	76.7 (74.1-79.5)	77 (75.2-79)	77.9 (77.4-78.5)	79 (78.8-79.3)	80 (79.8-80.3)	80.9 (80.6-81.1)	81.6 (81.3-82)	82.6 (82.4-82.9)	83.8 (83.5-84)	84.1 (83.4-84.9)
Portsmouth	72.7 (69.4-76.1)	74.2 (71-77.6)	75 (72-78.3)	75.5 (72.3-78.6)	75.8 (73-78.7)	76.3 (74.3-78.3)	76.9 (76.1-77.6)	77.4 (77-77.8)	78.3 (77.9-78.7)	78.4 (78.1-78.8)	78.9 (78.4-79.3)	80.2 (79.9-80.6)	82 (81.6-82.4)	83.3 (82.4-84.2)
Reading	74.6 (71.5-78.1)	76 (73.4-79.1)	76.9 (74.1-79.9)	77.3 (74.3-80.5)	77.7 (74.9-80.5)	78.1 (76.2-80.2)	78.5 (77.8-79.3)	78.6 (78.2-79)	79.4 (79-79.8)	79.8 (79.4-80.2)	80.4 (79.9-80.9)	81.4 (81-81.8)	82.5 (82.1-82.9)	82.6 (81.6-83.5)
Slough	72.8 (69.4-76.3)	74.1 (71.1-77.4)	74.8 (71.7-78.2)	75.2 (72.3-78.4)	75.4 (72.7-78.1)	75.7 (73.8-77.7)	76.1 (75.3-76.9)	76.5 (76.1-76.9)	77.6 (77.2-78.1)	78.1 (77.7-78.5)	79.3 (78.9-79.8)	81.2 (80.8-81.6)	82.9 (82.5-83.3)	84.4 (83.5-85.3)
Southampton	73.1 (69.9-76.5)	74.6 (71.6-77.9)	75.4 (72.5-78.5)	75.9 (73.1-79.1)	76.2 (73.5-79.1)	76.7 (74.7-78.8)	77.3 (76.7-78)	77.9 (77.5-78.2)	78.5 (78.2-78.9)	78.9 (78.6-79.3)	79.6 (79.2-80.1)	80.5 (80.2-80.8)	81.8 (81.5-82.1)	82.8 (81.9-83.7)
Surrey	74.1 (71-77.3)	75.5 (72.5-78.4)	76.4 (73.3-79.1)	77 (74.1-79.9)	77.2 (74.5-79.9)	77.5 (75.6-79.5)	78.1 (77.6-78.7)	78.8 (78.6-79)	79.9 (79.7-80.1)	80.8 (80.6-81)	82 (81.6-82.3)	83 (82.8-83.2)	84.2 (84-84.5)	83.9 (83.2-84.6)
West Berkshire	75.4 (72.1-78.9)	76.8 (73.7-79.9)	77.6 (74.7-80.7)	78.1 (75.3-81.2)	78.4 (75.8-81.2)	78.8 (76.8-80.9)	79.3 (78.5-80.1)	79 (78.6-79.4)	80.4 (80-80.8)	80.5 (80.1-80.9)	81.5 (81.1-82)	81.7 (81.3-82.1)	83.4 (82.9-83.8)	83.6 (82.6-84.5)
West Sussex	74.1 (70.9-77.4)	75.5 (72.7-79)	76.4 (73.3-79.9)	76.8 (73.9-80)	77.1 (74.4-79.9)	77.6 (75.7-79.5)	78.5 (77.9-79)	79.1 (78.9-79.4)	80.1 (79.9-80.3)	81 (80.7-81.2)	81.7 (81.3-82)	82.5 (82.2-82.7)	83.4 (83.2-83.7)	83.5 (82.7-84.3)
Windsor and Maidenhead	74.9 (71.8-78.3)	76.2 (73.2-79.5)	76.9 (74-80.2)	77.2 (74.3-80.2)	77.5 (75.1-80.4)	77.8 (75.9-79.9)	78.3 (77.5-79.1)	78.7 (78.3-79.2)	79.7 (79.3-80.1)	80.7 (80.3-81.1)	82.8 (82.3-83.3)	84 (83.6-84.4)	84.4 (84-84.8)	84.5 (83.5-85.4)
Wokingham	75.8 (72.7-79.2)	77.2 (74.3-80.4)	78 (75.2-81.1)	78.5 (75.6-81.7)	78.9 (76.2-81.6)	79.2 (77.2-81.2)	79.6 (78.8-80.4)	79.9 (79.5-80.3)	80.9 (80.5-81.3)	81.5 (81.1-81.9)	83 (82.6-83.5)	84.3 (83.9-84.7)	85.4 (85-85.8)	85.9 (85-86.7)
South West England	72.5 (71.5-73.5)	74.2 (73.2-75.2)	75.2 (74.4-76.2)	75.9 (75.1-76.8)	76.4 (75.6-77.3)	77.1 (76.5-77.7)	77.9 (77.7-78.1)	78.8 (78.7-78.9)	79.7 (79.6-79.8)	80.6 (80.5-80.7)	81.6 (81.5-81.8)	82.6 (82.5-82.7)	83.4 (83.4-83.5)	83.2 (83-83.4)
Bath and North East Somerset	73.6 (70.4-77.1)	75.2 (72-78.5)	76.2 (73.2-79.4)	76.9 (73.9-79.8)	77.6 (75-80.4)	78.4 (76.4-80.4)	79.2 (78.4-79.9)	79.9 (79.5-80.3)	80.6 (80.2-81)	81 (80.6-81.4)	82.2 (81.8-82.7)	83.6 (83.2-84)	84.5 (84.1-84.9)	83.8 (82.9-84.6)

Table 7 Estimates of life expectancy at birth by location and year for 1950, 1955...2005, 2010, 2017 - females

Location	1950	1955	1960	1965	1970	1975	1980	1985	1990	1995	2000	2005	2010	2017
Bournemouth	72.4 (69.1-76.1)	73.9 (70.9-77.5)	74.9 (71.8-78.1)	75.4 (72.4-78.5)	75.9 (73.1-78.8)	76.6 (74.6-78.7)	77.3 (76.6-78)	78.3 (77.9-78.7)	79 (78.6-79.4)	79.5 (79.1-79.8)	80.6 (80.1-81)	81.8 (81.5-82.2)	82.5 (82.1-82.9)	81.9 (80.9-82.9)
Bristol, City of	72 (68.8-75.4)	73.5 (70.3-76.7)	74.4 (71.6-77.5)	74.9 (72.1-77.9)	75.3 (72.7-78.1)	76 (73.9-78)	76.9 (76.3-77.5)	78 (77.8-78.3)	79.3 (79-79.6)	79.9 (79.7-80.2)	80.5 (80.1-80.9)	81.4 (81.1-81.7)	82.1 (81.8-82.4)	82.6 (81.7-83.4)
Cornwall	72.2 (68.9-75.7)	73.9 (70.8-77.1)	75 (72-78.3)	75.6 (72.8-78.7)	76.2 (73.4-79.1)	76.9 (74.9-78.9)	77.7 (77.1-78.3)	78.8 (78.5-79)	79.8 (79.5-80.1)	80.4 (80.1-80.6)	81.3 (81-81.7)	82.2 (81.9-82.4)	83.2 (82.9-83.4)	83 (82.1-83.8)
Devon	72.6 (69.6-76.1)	74.3 (71.4-77.8)	75.4 (72.4-78.7)	76.1 (73.3-79.2)	76.6 (74.1-79.3)	77.4 (75.3-79.2)	78.2 (77.6-78.7)	79.1 (78.8-79.3)	80.2 (79.9-80.4)	81.1 (80.8-81.3)	82 (81.7-82.4)	83 (82.8-83.3)	83.9 (83.7-84.1)	83.3 (82.5-84.1)
Dorset	72.8 (69.5-76.6)	74.6 (71.3-78.3)	75.7 (72.5-79.1)	76.4 (73.4-79.5)	77 (74.2-79.8)	77.7 (75.5-79.8)	78.4 (77.8-79.1)	79.3 (78.9-79.5)	80.3 (80-80.6)	81.7 (81.4-82)	82.6 (82.2-83)	83.7 (83.4-84)	84.7 (84.5-85)	83.7 (82.8-84.6)
Gloucestershire	73 (69.8-76.6)	74.6 (71.5-78)	75.7 (72.9-79)	76.5 (73.6-79.5)	77.1 (74.5-79.9)	77.7 (75.7-79.6)	78 (77.5-78.5)	78.6 (78.4-78.9)	79.7 (79.4-79.9)	80.5 (80.2-80.7)	81.8 (81.5-82.2)	82.4 (82.2-82.7)	83.1 (82.9-83.4)	82.9 (82.2-83.7)
North Somerset	73 (69.7-76.3)	74.7 (71.3-78.2)	75.9 (72.9-79)	76.7 (73.6-79.6)	77.3 (74.6-80)	78.1 (76.1-80)	78.8 (78-79.5)	79.3 (78.9-79.6)	80.1 (79.8-80.5)	81.1 (80.7-81.5)	82.2 (81.7-82.6)	82.8 (82.4-83.1)	83 (82.6-83.4)	82.2 (81.3-83.1)
Plymouth	72 (68.5-75.6)	73.5 (70-77.1)	74.5 (71.2-77.8)	75.2 (72.3-78.4)	75.7 (73.1-78.7)	76.4 (74.3-78.5)	77.1 (76.5-77.8)	78 (77.7-78.3)	78.8 (78.5-79.1)	79.8 (79.4-80.1)	80.7 (80.2-81.1)	81 (80.7-81.3)	81.8 (81.5-82.2)	82.5 (81.7-83.3)
Poole	73.3 (70-76.9)	74.9 (71.8-78.2)	76 (73.1-79)	76.6 (73.6-79.8)	77.3 (74.5-80)	78 (75.9-80.2)	79 (78.2-79.8)	78.8 (78.4-79.3)	79.4 (79-79.8)	80.5 (80.1-80.9)	82.3 (81.8-82.7)	83.1 (82.7-83.6)	83.6 (83.2-84)	82.9 (81.9-83.9)
Somerset	72.6 (69.2-76.1)	74.3 (71.1-77.5)	75.3 (72.4-78.2)	76 (73-79)	76.4 (73.9-79)	77 (74.9-78.9)	77.9 (77.3-78.4)	78.9 (78.7-79.2)	79.6 (79.3-79.8)	80.8 (80.5-81.1)	81.6 (81.2-82)	82.7 (82.4-82.9)	83.8 (83.6-84.1)	83.6 (82.8-84.4)
South Gloucestershire	73.1 (69.7-76.8)	74.7 (71.5-78.2)	75.7 (72.7-79)	76.5 (73.6-79.6)	77 (74.4-80)	77.8 (75.7-79.9)	78.8 (78.1-79.5)	79.5 (79.2-79.9)	79.5 (79.1-79.8)	80.5 (80.2-80.9)	82 (81.6-82.4)	83.8 (83.5-84.2)	84.6 (84.2-84.9)	83.7 (82.9-84.6)
Swindon	71.5 (68.2-75.1)	73.2 (70.1-76.9)	74.3 (71.3-77.5)	75 (72.2-78)	75.5 (72.8-78.3)	76 (73.8-78)	76.2 (75.5-77)	77.8 (77.4-78.1)	79.4 (79.1-79.8)	79.9 (79.5-80.3)	80.7 (80.2-81.1)	81.9 (81.5-82.3)	83.1 (82.8-83.5)	82.7 (81.7-83.6)
Torbay	72.5 (69-76.3)	74 (70.8-77.7)	75 (72.1-78.2)	75.6 (72.6-78.7)	76.2 (73.4-79.2)	76.9 (74.7-79)	77.7 (76.9-78.5)	78.6 (78.1-79)	79.5 (79-80)	79.7 (79.3-80.1)	80.5 (80-81)	81.2 (80.8-81.6)	81.9 (81.4-82.3)	82.4 (81.4-83.4)
Wiltshire	72.6 (69.1-76.2)	74.2 (70.8-77.8)	75.3 (72.3-78.6)	76.1 (73.2-79.3)	76.6 (74-79.5)	77.3 (75.2-79.3)	77.9 (77.3-78.6)	78.9 (78.6-79.1)	79.4 (79.2-79.7)	80.6 (80.3-80.9)	82 (81.6-82.4)	82.8 (82.5-83.1)	83.4 (83.1-83.7)	83.7 (82.8-84.4)
West Midlands	71.3 (70.3-72.4)	72.9 (71.8-73.9)	73.9 (72.8-74.9)	74.5 (73.4-75.5)	75 (74.1-76)	75.8 (75.1-76.5)	76.6 (76.5-76.8)	77.4 (77.3-77.5)	78.2 (78.1-78.3)	79.1 (79-79.2)	80.1 (80-80.2)	81.1 (81-81.2)	82.3 (82.2-82.4)	82.6 (82.3-82.8)
Birmingham	70.7 (67.8-74)	72.4 (69.4-75.3)	73.3 (70.6-76.2)	74 (71.1-76.8)	74.5 (71.9-77.1)	75.3 (73.3-77.4)	76.2 (75.7-76.7)	76.8 (76.5-76.9)	77.5 (77.2-77.7)	78.4 (78.1-78.6)	79.5 (79.1-79.8)	80.7 (80.4-80.9)	81.9 (81.6-82.1)	82.3 (81.5-83.1)
Coventry	71.1 (67.7-74.9)	72.7 (69.5-76.1)	73.6 (70.4-77.1)	74.1 (71-77.3)	74.5 (71.8-77.5)	75.4 (73.4-77.7)	76.6 (76-77.2)	77.4 (77.1-77.7)	77.7 (77.4-78)	78.5 (78.2-78.8)	79.6 (79.1-80)	79.8 (79.5-80.1)	81.8 (81.5-82.1)	82.1 (81.2-83)
Dudley	72 (68.7-75.5)	73.6 (70.4-77)	74.7 (71.9-77.8)	75.5 (72.7-78.5)	76.1 (73.5-78.8)	76.8 (74.8-78.9)	77.6 (77-78.2)	77.8 (77.5-78.1)	78.3 (78-78.6)	79.3 (79-79.6)	80.2 (79.8-80.6)	81.4 (81.1-81.7)	82.6 (82.3-83)	82.6 (81.6-83.4)
Herefordshire, County of	72 (68.6-75.6)	73.5 (70.5-76.9)	74.3 (71.1-77.4)	74.6 (71.6-77.6)	75.1 (72.4-78)	75.9 (73.8-78.1)	77.6 (76.8-78.3)	78.3 (77.9-78.7)	79.7 (79.3-80.1)	80.6 (80.2-81)	81.8 (81.3-82.2)	82.7 (82.3-83.1)	83.2 (82.8-83.6)	83 (82-83.9)
Sandwell	70.5 (67.1-74.1)	72.1 (68.9-75.8)	73.1 (69.9-76.4)	73.6 (70.3-76.9)	74.1 (71.1-77.2)	75 (73-77.3)	76.1 (75.5-76.7)	77.1 (76.8-77.4)	77.9 (77.6-78.2)	78.1 (77.9-78.4)	78.7 (78.3-79.1)	79.4 (79-79.7)	80.6 (80.3-80.9)	81.4 (80.5-82.4)
Shropshire	72.1 (69.1-75.5)	73.7 (70.8-77)	74.7 (71.7-77.9)	75.4 (72.5-78.5)	75.9 (73.3-78.8)	76.7 (74.7-78.8)	77.4 (76.7-78)	78.2 (77.9-78.5)	79.4 (79.1-79.8)	80.2 (79.9-80.6)	81.6 (81.2-82)	82.7 (82.4-83)	83.4 (83-83.7)	83.1 (82.2-84)
Solihull	73.4 (69.8-76.9)	74.9 (71.5-78.2)	75.8 (72.6-79.2)	76.4 (73.4-79.4)	77.1 (74.4-79.9)	78 (76-80)	78.9 (78.1-79.6)	79.6 (79.2-79.9)	80.2 (79.8-80.6)	80.3 (79.9-80.7)	81.2 (80.7-81.6)	82.7 (82.4-83.1)	83.9 (83.5-84.2)	83.8 (82.8-84.8)
Staffordshire	71.7 (68.6-75.1)	73.3 (70.2-76.5)	74.2 (71.1-77.2)	74.9 (71.8-78.1)	75.5 (72.6-78.3)	76.1 (74-78.2)	76.9 (76.3-77.4)	77.6 (77.3-77.8)	78.3 (78.1-78.6)	79.2 (79-79.5)	80.3 (80-80.6)	81.4 (81.2-81.6)	82.6 (82.4-82.9)	82.7 (81.9-83.4)
Stoke-on-Trent	69.8 (66.5-73.2)	71.3 (68.3-74.6)	72.2 (69.3-75.6)	72.7 (69.8-75.7)	73.1 (70.4-76.1)	73.8 (71.8-76)	74.6 (73.9-75.2)	75.6 (75.2-75.9)	76.8 (76.4-77.1)	77.8 (77.4-78.1)	78.7 (78.3-79.1)	79.1 (78.7-79.4)	79.9 (79.6-80.3)	81.1 (80.1-82)
Telford and Wrekin	71.4 (67.9-74.8)	72.9 (69.7-76.2)	73.8 (70.8-76.9)	74.4 (71.5-77.6)	74.9 (72-77.8)	75.6 (73.6-77.7)	76.4 (75.6-77.1)	77.2 (76.9-77.6)	78.8 (78.4-79.2)	79.4 (79-79.7)	79.8 (79.4-80.3)	80.6 (80.2-80.9)	81.8 (81.4-82.1)	82 (81-83)
Walsall	70.5 (66.8-73.9)	72.2 (68.7-75.6)	73.2 (70-76.4)	73.9 (70.8-77.1)	74.5 (71.8-77.4)	75.2 (73.1-77.5)	75.9 (75.2-76.5)	76.5 (76.2-76.8)	77.4 (77.1-77.7)	78.4 (78.1-78.7)	79.4 (79-79.9)	80.9 (80.6-81.3)	82.1 (81.7-82.4)	81.9 (81-82.8)
Warwickshire	72.2 (69.1-75.5)	73.7 (70.6-76.9)	74.6 (71.6-77.8)	75.3 (72.4-78.4)	75.8 (73.2-78.5)	76.4 (74.5-78.5)	77.1 (76.5-77.6)	77.7 (77.5-78)	78.7 (78.5-79)	79.7 (79.4-79.9)	81.1 (80.7-81.4)	82 (81.7-82.2)	83.2 (82.9-83.4)	83.2 (82.3-84)
Wolverhampton	71.1 (67.6-74.5)	72.7 (69.4-76.1)	73.7 (70.5-76.8)	74.3 (71.3-77.5)	74.8 (71.8-77.8)	75.5 (73.5-77.8)	76.2 (75.6-76.9)	76.9 (76.6-77.3)	77.4 (77-77.7)	78.7 (78.3-79)	79.2 (78.7-79.6)	79.7 (79.4-80.1)	81.3 (81-81.6)	81.7 (80.8-82.7)
Worcestershire	72.2 (69-75.5)	73.7 (70.8-77)	74.6 (71.7-77.6)	75.3 (72.6-78.4)	75.8 (73.2-78.6)	76.5 (74.6-78.6)	77.3 (76.7-77.8)	78.4 (78.2-78.7)	79.1 (78.9-79.4)	80 (79.8-80.3)	80.6 (80.2-81)	82.1 (81.8-82.3)	82.9 (82.7-83.2)	83.2 (82.4-84.1)
Yorkshire and the Humber	71.1 (70.2-72.2)	72.7 (71.8-73.7)	73.7 (72.8-74.6)	74.4 (73.5-75.3)	74.9 (74.2-75.7)	75.7 (75.1-76.3)	76.5 (76.3-76.7)	77.2 (77.1-77.2)	78.1 (78-78.2)	79 (78.9-79.1)	79.9 (79.8-80)	81 (80.9-81)	81.8 (81.8-81.9)	82.3 (82-82.5)
Barnsley	70.6 (67.3-74.1)	72.2 (68.9-75.7)	73.3 (70.3-76.3)	73.9 (70.9-77.2)	74.5 (71.7-77.2)	75.3 (73.3-77.3)	76 (75.4-76.7)	76.5 (76.2-76.8)	77.6 (77.3-77.9)	78.1 (77.7-78.4)	78.9 (78.5-79.4)	80 (79.6-80.3)	80.9 (80.5-81.2)	82 (81.1-83)
Bradford	70 (66.9-73.6)	71.7 (68.7-75.2)	72.7 (69.6-76)	73.4 (70.5-76.4)	73.9 (71.2-76.5)	74.8 (72.6-76.7)	75.8 (75.2-76.4)	76.4 (76.2-76.7)	77.4 (77.1-77.6)	78.2 (78-78.5)	79 (78.6-79.4)	80 (79.7-80.3)	81.1 (80.8-81.3)	81.8 (80.9-82.6)
Calderdale	70.4 (67.1-73.9)	72.1 (69-75.4)	73.2 (70.2-76.3)	73.9 (71.1-77.1)	74.5 (71.7-77.2)	75.3 (73.3-77.3)	76.1 (75.4-76.8)	76.7 (76.4-77.1)	77.4 (77.1-77.8)	78.4 (78.1-78.8)	80 (79.6-80.5)	81 (80.6-81.3)	81.2 (80.9-81.6)	81.5 (80.6-82.4)
Doncaster	71.2 (67.8-74.7)	73 (69.6-76.3)	74.1 (70.9-77.3)	74.9 (72.2-78)	75.6 (73.1-78.3)	76.4 (74.4-78.3)	76.9 (76.3-77.5)	77 (76.8-77.4)	77.5 (77.2-77.8)	78 (77.7-78.3)	79.2 (78.8-79.6)	80.6 (80.3-81)	81.4 (81.1-81.7)	81.5 (80.6-82.4)
East Riding of Yorkshire	72.1 (68.9-75.4)	73.7 (70.5-76.8)	74.6 (71.6-77.7)	75.3 (72.4-78.2)	75.9 (73.1-78.5)	76.6 (74.6-78.6)	77.4 (76.7-78)	78.2 (77.9-78.5)	79.4 (79.1-79.7)	80.2 (79.9-80.5)	80.9 (80.5-81.3)	81.9 (81.6-82.2)	82.8 (82.4-83.1)	83.1 (82.2-83.9)
Kingston upon Hull, City of	70.8 (67.5-74.3)	72.5 (69.2-75.8)	73.6 (70.3-76.8)	74.2 (71.3-77.4)	74.9 (72.1-77.7)	75.8 (73.6-77.8)	76.5 (75.8-77.1)	77 (76.7-77.3)	77.6 (77.3-77.9)	78.2 (77.9-78.6)	79 (78.6-79.5)	79.9 (79.6-80.2)	79.9 (79.6-80.2)	80.7 (79.8-81.6)
Kirklees	70.5 (67.5-73.8)	72.1 (68.9-75.3)	73 (70-76.1)	73.8 (70.9-76.7)	74.3 (71.6-77)	75 (73-77)	75.6 (75-76.2)	76.7 (76.4-77)	77.6 (77.3-77.8)	78.8 (78.6-79.1)	79.6 (79.2-80)	80 (79.7-80.3)	82 (81.7-82.2)	82.9 (82.1-83.8)
Leeds	71.3 (67.9-74.7)	72.9 (69.6-76.1)	73.8 (70.8-76.9)	74.3 (71.4-77.3)	74.8 (72.2-77.8)	75.5 (73.4-77.6)	76.6 (76-77.2)	77.2 (76.9-77.4)	78.3 (78.1-78.5)	79.5 (79.2-79.7)	80.2 (79.9-80.6)	81.1 (80.9-81.3)	81.9 (81.7-82.1)	82.1 (81.3-82.8)
North East Lincolnshire	71.7 (68.5-75.4)	73.4 (70.2-77)	74.5 (71.4-77.7)	75 (72.2-78.2)	75.6 (72.9-78.2)	76.3 (74.3-78.2)	76.9 (76.2-77.7)	77.4 (77-77.8)	77.8 (77.4-78.2)	78.8 (78.4-79.1)	79.7 (79.2-80.2)	80.7 (80.3-81.1)	81.7 (81.3-82.1)	82.4 (81.5-83.4)
North Lincolnshire	71.7 (68.1-75.2)	73.3 (70.1-76.8)	74.4 (71.2-77.6)	75 (71.9-78.1)	75.6 (72.8-78.4)	76.4 (74.4-78.7)	77 (76.3-77.8)	77.1 (76.7-77.5)	77.9 (77.5-78.3)	79.3 (78.9-79.7)	79.6 (79.2-80.1)	80.9 (80.5-81.3)	81.8 (81.4-82.2)	82.1 (81.2-83.1)
North Yorkshire	72 (69.1-75.3)	73.5 (70.5-76.8)	74.3 (71.5-77.3)	74.9 (72-77.9)	75.4 (72.7-78.1)	76.2 (74.2-78.2)	77.3 (76.8-77.9)	78.5 (78.2-78.7)	79.4 (79.2-79.7)	79.9 (79.7-80.2)	80.9 (80.5-81.3)	82.3 (82.1-82.6)	83.3 (83-83.5)	83.5 (82.7-84.3)
Rotherham	71.1 (68-74.7)	72.8 (69.8-76.3)	73.9 (71-77.1)	74.7 (71.7-77.7)	75.4 (72.6-78)	76.1 (74.1-78.1)	76.6 (75.9-77.3)	77.1 (76.7-77.4)	77.8 (77.5-78.1)	79 (78.7-79.3)	79.3 (78.9-79.7)	80.6 (80.3-81)	81.2 (80.9-81.6)	80.7 (79.8-81.6)
Sheffield	71.6 (68.3-74.9)	73.1 (70.1-76.5)	74.1 (71-77.3)	74.8 (71.9-77.9)	75.3 (72.7-78)	76 (73.9-78)	76.8 (76.2-77.3)	76.9 (76.7-77.2)	78.1 (77.8-78.3)	78.9 (78.6-79.1)	80.1 (79.7-80.4)	80.9 (80.6-81.2)	81.8 (81.6-82.1)	82.3 (81.5-83.1)
Wakefield	70.5 (67.2-74)	72.2 (69.2-75.5)	73.2 (70.1-76.5)	73.9 (70.9-77)	74.4 (71.7-77.1)	75.1 (73.1-77.2)	75.7 (75.1-76.3)	76.4 (76.1-76.7)	77.2 (76.9-77.5)	78.2 (77.9-78.6)	79.4 (79-79.8)	80.5 (80.2-80.8)	81.4 (81.1-81.7)	82.2 (81.4-83.1)
York	72.6 (69.2-76.2)	74 (70.8-77.3)	74.9 (71.8-78.3)	75.3 (72.3-78.5)	75.8 (73-78.5)	76.5 (74.3-78.7)	77.2 (76.5-78)	78 (77.6-78.4)	79.5 (79.1-79.8)	80.6 (80.2-81)	81.9 (81.5-82.4)	83.4 (83-83.8)	83.5 (83.1-83.9)	82.6 (81.7-83.5)
Northern Ireland	68.3 (68-68.6)	70.8 (70.6-71.1)	72 (71.8-72.3)	73.2 (73-73.4)	73.8 (73.5-74)	73.8 (73.6-74)	74.9 (74.6-75.1)	76.1 (75.9-76.4)	77.3 (77-77.6)	78.4 (78.1-78.6)	79.8 (79.4-80.2)	81.3 (81.1-81.6)	81.7 (81.4-81.9)	82.5 (81.5-83.4)
Scotland	68.8 (68.6-68.9)	71.2 (71-71.3)	72.1 (72-72.3)	72.7 (72.6-72.9)	73.3 (73.1-73.4)	74.4 (74.3-74.6)	75 (74.9-75.2)	75.8 (75.7-76)	76.8 (76.7-77)	77.9 (77.7-78)	78.8 (78.5-79.1)	79.7 (79.5-79.8)	80.7 (80.5-80.8)	81.2 (80.3-82.1)
Wales	68 (64.6-71.7)	69.9 (66.8-73.4)	71.3 (67.9-74.7)	72.3 (69.1-75.6)	73.1 (70.1-76.5)	74.4 (71.9-76.9)	76.1 (75.6-76.6)	77.5 (77.3-77.6)	78.6 (78.4-78.7)	79 (78.8-79.2)	79.9 (79.6-80.3)	80.9 (80.7-81.1)	81.9 (81.8-82.1)	82.5 (81.7-83.2)
Latin America and Caribbean	51.6 (50-53.1)	55 (54.1-55.8)	57.7 (57.1-58.4)	60.5 (59.9-61.1)	62.3 (61.8-62.7)	65.7 (65.4-66.1)	68.2 (67.9-68.5)	70 (69.7-70.2)	72.5 (72.3-72.6)	73.7 (73.5-73.9)	75.7 (75.5-75.8)	77 (76.8-77.2)	76.9 (76.7-77.1)	78.9 (78.6-79.2)
Andean Latin America	42 (39.9-43.9)	46.7 (45.5-48)	49.5 (48.4-50.5)	54.7 (53.7-55.7)	53.4 (52.6-54.1)	62.7 (61.9-63.5)	64.8 (64.1-65.5)	67 (66.3-67.7)	70.6 (70-71.2)	71.9 (71.3-72.6)	74.8 (74.2-75.4)	76.4 (75.8-77)	77.4 (76.7-78.1)	79.5 (78.4-80.6)
Bolivia	38.8 (36.8-40.7)	43.3 (41.6-45)	45.9 (44.1-47.4)	48.4 (47.1-49.8)	51.7 (50.5-52.9)	55.1 (54.2-56)	57.7 (56.6-58.7)	59.6 (58.3-60.9)	62.1 (60.8-63.3)	64.4 (63.2-65.7)	67.8 (66.5-69.1)	70.3 (68.7-71.9)	72.2 (70.3-74.1)	74.2 (72.1-76.6)
Ecuador	50.6 (47.7-53.6)	53.3 (52.4-54.1)	55.4 (54.6-56.2)	59.6 (58.9-60.3)	62 (61.3-62.6)	65.8 (65.3-66.3)	68.9 (68.4-69.3)	72.6 (72.3-73)	74.4 (74.1-74.6)	75.9 (75.6-76.2)	76.9 (76.5-77.2)	77 (76.4-77.6)	77.6 (76.9-78.2)	78.7 (77.5-79.9)
Peru	40.4 (38.3-42.6)	45.6 (43.6-47.6)	48.4 (46.7-50.4)	55.2 (53.4-57)	50.8 (49.6-51.9)	64.5 (63.1-65.8)	65.9 (64.7-67.1)	67.4 (66.3-68.4)	72.2 (71.3-73.2)	72.9 (72-73.9)	76.5 (75.5-77.3)	78.4 (77.4-79.3)	79.2 (78.3-80.2)	81.9 (80.1-83.7)
Caribbean	56.4 (55.1-57.5)	58.7 (57.7-59.8)	61.1 (60.3-62)	63 (62.2-63.8)	64.9 (64.2-65.7)	67.1 (66.4-67.8)	68.2 (67.6-68.8)	69.3 (68.8-69.8)	70.4 (69.9-70.8)	70.9 (70.3-71.4)	72.5 (71.8-73)	73.4 (72.7-74.1)	63 (62.4-63.6)	75.4 (74.4-76.4)
Antigua and Barbuda	60 (58.6-61.2)	62.7 (61.7-63.6)	65.5 (64.7-66.2)	67.8 (67.1-68.5)	70.1 (69.4-70.8)	72.2 (71.5-73)	74.3 (73.4-75.2)	76.9 (76.1-77.8)	77.9 (77.2-78.7)	77.3 (76.5-77.9)	77.7 (77.4-78.1)	78.5 (77.9-79.1)	78.8 (78.3-79.3)	78.7 (78.1-79.4)
The Bahamas	59.9 (57.4-62.7)	62 (59.7-64.4)	63.9 (62.3-65.5)	65.3 (64.4-66.1)	68.7 (68.1-69.3)	71.4 (70.9-71.9)	72.3 (71.9-72.7)	73.7 (73.3-74.1)	74.7 (74.3-75.1)	74.6 (74.2-75)	74.6 (74.3-75)	76.4 (76-76.7)	76.5 (76.2-76.9)	76.6 (75.4-77.9)
Barbados	60.8 (59.4-62)	64.1 (63-65)	68.1 (67.4-68.9)	70.1 (69.5-70.7)	71.5 (71-72)	73.2 (72.8-73.6)	75.1 (74.8-75.5)	76.2 (75.9-76.6)	76.1 (75.7-76.5)	76.8 (76.3-77.2)	77.2 (76.8-77.6)	78.1 (77.7-78.4)	78.6 (78.3-78.9)	78.6 (77.7-79.6)
Belize	54.8 (53.1-56.4)	58.4 (57.1-59.6)	64.1 (63-65.3)	67.4 (66.4-68.4)	69.7 (68.8-70.5)	71.2 (70.3-72)	72.4 (71.7-73.2)	73.4 (72.7-74.1)	73.8 (73.1-74.4)	74.5 (74-75)	73.7 (73.4-74.1)	75.2 (74.9-75.5)	76.3 (76-76.6)	77.4 (76.9-77.9)
Bermuda	66.7 (65.8-67.5)	69.2 (68.6-69.7)	71.3 (70.8-71.8)	72.8 (72.3-73.2)	74.5 (74.1-75)	76.5 (76-77.1)	78.3 (77.8-78.7)	78.3 (77.8-78.8)	78.3 (77.9-78.6)	79.7 (79.1-80.1)	80.7 (80.2-81.1)	82.5 (81.8-83.1)	84.4 (83.8-85)	85.7 (84.8-86.5)
Cuba	68.5 (65.9-71.1)	69 (66.8-71.4)	70.8 (70.6-70.9)	71.8 (71.7-72)	73.7 (73.5-73.8)	75.8 (75.6-75.9)	75.8 (75.7-76)	76 (75.9-76.1)	76.8 (76.6-76.9)	77.1 (77-77.3)	79 (78.8-79.1)	79.7 (79.6-79.8)	80.4 (80.3-80.5)	80.7 (79.3-82.1)
Dominica	51.5 (49.7-53.1)	57.7 (56.2-59.3)	62.9 (61.6-64.2)	67.5 (66.5-68.5)	70.5 (69.7-71.2)	73.8 (73.3-74.3)	75.8 (75.3-76.3)	76.5 (76-77)	75.3 (74.7-75.7)	75.4 (74.9-75.9)	76.1 (75.6-76.6)	76.5 (76-77)	76.2 (75.6-76.7)	75.4 (74.3-76.4)
Dominican Republic	56.5 (55.2-57.7)	59.7 (58.5-60.8)	61.6 (60.2-63)	63.3 (62.1-64.6)	65.4 (64.1-66.7)	68.1 (66.8-69.3)	70.6 (69.5-71.8)	73.1 (72-74.2)	74.4 (73.4-75.4)	76.2 (75.1-77.3)	77.5 (76.4-78.6)	76.2 (74.9-77.4)	77.4 (76.3-78.5)	76.8 (75.2-78.5)
Grenada	60.9 (59.5-62.3)	63.6 (62.3-65)	65.8 (64.5-67)	67 (65.8-68.1)	69.1 (67.9-70.2)	70.3 (69.3-71.4)	70.8 (69.9-71.7)	70.7 (70-71.4)	71.6 (71-72.1)	73.6 (73.1-74)	76.1 (75.7-76.5)	76.6 (76.1-77.1)	75.7 (75.3-76.2)	75.4 (74.7-76.2)
Guyana	52.3 (51.4-53)	56.2 (55.5-57)	61.3 (60.6-61.9)	63.2 (62.1-64.4)	65.2 (64.7-65.7)	65.1 (64.5-65.7)	67 (66.4-67.6)	69.2 (68.7-69.8)	69 (68.7-69.4)	69.4 (69-69.7)	68.2 (67.8-68.5)	69.9 (69.6-70.2)	71.3 (71-71.6)	72.2 (70.5-73.9)
Haiti	40.4 (37.5-43.3)	42.1 (39.6-44.7)	43.6 (41.4-46.2)	44.8 (42.4-47.3)	46.6 (44.4-49)	49 (47.1-51.2)	51.4 (49.7-53.4)	53.5 (52-55.2)	55 (53.6-56.5)	55.4 (53.7-57.1)	57.6 (55.7-59.4)	60.1 (57.9-62.3)	37 (36.2-37.8)	66 (63.3-68.8)
Jamaica	57.6 (56.6-58.6)	62.8 (62.1-63.5)	66.1 (65.5-66.6)	68 (67.5-68.5)	70.1 (69.6-70.5)	72.1 (71.6-72.5)	74.8 (74.4-75.2)	74.2 (73.9-74.5)	76.4 (75.7-77.1)	76.3 (75.3-77.5)	75.8 (75.5-76)	77.9 (77.7-78.2)	77.9 (77.7-78.2)	77.5 (75.4-79.4)
Puerto Rico	61.1 (60.8-61.5)	69.4 (69.1-69.7)	72.2 (72-72.5)	73.1 (72.9-73.3)	75.1 (74.9-75.3)	77.1 (76.9-77.3)	77.7 (77.5-77.9)	77.7 (77.6-77.9)	78.4 (78.2-78.6)	78.6 (78.4-78.7)	80.2 (80-80.4)	81.3 (81.1-81.5)	82.5 (82.3-82.7)	81.6 (80.9-82.3)
Saint Lucia	51.8 (50.1-53.4)	56.6 (55.2-58)	60.5 (59.2-61.7)	64.9 (63.6-66)	67.3 (66.5-68.1)	69 (68.4-69.6)	70.9 (70.5-71.4)	72.4 (72-72.8)	73.2 (72.8-73.6)	74 (73.6-74.4)	76.5 (76.2-76.9)	77.6 (77.2-78)	78 (77.6-78.3)	78.1 (77.2-78.9)
Saint Vincent and the Grenadines	54.1 (52.5-55.6)	56.4 (54.9-57.8)	62.8 (61.3-64.1)	65.5 (64.3-66.7)	67.5 (66.5-68.4)	69.7 (68.9-70.5)	71.9 (71.3-72.6)	73.3 (72.8-73.8)	72.9 (72.4-73.5)	73.5 (73-73.9)	73.7 (73.3-74.2)	75.4 (75-75.8)	75.9 (75.5-76.3)	75.4 (74.6-76.3)
Suriname	60.7 (59.2-62.4)	62.4 (61.2-63.8)	64.3 (63-65.6)	66.1 (64.7-67.5)	67.1 (65.6-68.6)	68 (66.7-69.2)	68.6 (67.7-69.4)	70.6 (69.7-71.4)	71.3 (70.5-72.2)	72.6 (71.7-73.4)	72.8 (72-73.7)	73.2 (72.4-74.1)	74.7 (73.9-75.5)	75.3 (74-76.6)
Trinidad and Tobago	58.6 (57.7-59.4)	62.7 (62.2-63.2)	66.6 (66-67)	67 (66.5-67.4)	67.9 (67.5-68.3)	69.3 (68.9-69.7)	70.4 (70-70.7)	71.2 (70.9-71.5)	72.5 (72.1-72.8)	72.1 (71.7-72.4)	72.9 (72.6-73.3)	75.3 (75-75.7)	77.1 (76.8-77.5)	77.6 (74.8-80.3)
Virgin Islands	64.8 (63.8-65.8)	67 (66.3-67.8)	68.3 (67.6-69)	69.3 (68.6-70.2)	70.7 (69.7-71.9)	72.4 (71.3-73.5)	73.9 (72.9-74.7)	75.2 (74.5-75.8)	76.2 (75.1-77)	77 (76.1-77.6)	77.6 (77.1-78.1)	78.4 (77.9-79.1)	78.9 (78-79.8)	78.8 (77.2-80.1)
Central Latin America	51.7 (50.4-52.7)	55.8 (55.1-56.4)	59 (58.4-59.6)	61.7 (61.2-62.2)	64.1 (63.6-64.5)	67 (66.7-67.4)	70.1 (69.7-70.4)	71.3 (71.1-71.5)	74 (73.9-74.2)	75.1 (75-75.2)	76.9 (76.8-77.1)	77.9 (77.8-78.1)	78.8 (78.6-78.9)	79.4 (79-79.8)
Colombia	54.4 (53.3-55.3)	57.9 (57-58.7)	60 (59.3-60.7)	62.6 (62-63.1)	67.3 (66.8-67.7)	67.8 (67.4-68.2)	71.3 (70.2-72.4)	70.3 (70.1-70.6)	74.8 (74.6-75)	75.6 (75.4-75.7)	77.9 (77.7-78)	79.5 (79.3-79.7)	81.2 (81-81.3)	82.7 (81.4-83.9)
Costa Rica	58.4 (58-58.9)	63.4 (63.1-63.8)	66.7 (66.3-67)	67.4 (67.1-67.7)	68.6 (68.3-68.9)	73.5 (73.3-73.8)	77.1 (76.9-77.4)	77.4 (77.2-77.6)	78.7 (78.5-79)	78.6 (78.4-78.8)	80.5 (80.3-80.7)	81.6 (81.4-81.8)	82.2 (82-82.4)	82.7 (81.9-83.4)
El Salvador	48.4 (46.6-49.9)	51.2 (49.7-52.6)	55.5 (54.1-56.9)	58.8 (57.7-59.8)	60.8 (59.8-61.7)	65.7 (65-66.5)	67.3 (66.5-68.1)	70.3 (69.9-70.7)	73.6 (73.2-73.9)	75.6 (74.9-76.2)	76.7 (76.1-77.3)	77 (76.5-77.6)	77.4 (76.7-77.9)	78.3 (76-80.4)
Guatemala	42.5 (40.8-43.9)	45 (43.4-46.4)	48.3 (47.1-49.5)	50.4 (49.3-51.4)	51.7 (50.8-52.6)	56.6 (55.9-57.4)	61.7 (61.1-62.4)	62.4 (61.9-62.9)	65.7 (65.3-66.2)	69.1 (68.7-69.5)	71.1 (70.8-71.5)	73.1 (72.8-73.4)	74.8 (74.6-75.1)	76 (74.5-77.4)
Honduras	41.6 (39.5-43.8)	44.5 (42.6-46.4)	48.8 (46.9-50.7)	53.1 (51.5-54.7)	57.5 (56.1-58.9)	62.7 (61.5-63.9)	68.5 (67.3-69.6)	69.4 (68-70.8)	71.2 (69.6-72.8)	70.7 (68.4-73)	71.7 (68.9-74.5)	72.9 (70.1-75.8)	73.9 (71.1-76.9)	75 (72.4-78.2)
Mexico	51.1 (49.5-52.6)	55.9 (54.8-57)	59.1 (58.2-60.1)	62 (61.2-62.8)	63.8 (63.1-64.6)	67.3 (66.7-67.9)	70.1 (69.6-70.5)	72.1 (71.8-72.5)	74.3 (74-74.6)	75.4 (75.2-75.6)	77 (76.8-77.2)	77.7 (77.4-77.8)	78.2 (78-78.4)	78.5 (78.2-78.8)
Aguascalientes	58.5 (55.7-61.6)	62.2 (59.5-65)	64.5 (62-67.2)	66.6 (64.2-69.1)	67.8 (65.9-69.7)	70.3 (68.9-71.5)	72.1 (71.5-72.6)	73.2 (72.8-73.6)	75.1 (74.7-75.5)	76.1 (75.8-76.4)	77.1 (76.8-77.3)	77.6 (77.3-77.8)	78.4 (78.2-78.6)	79.7 (78.9-80.5)
Baja California	52.2 (49.2-55.4)	57.1 (54.3-60.2)	60.6 (57.9-63.5)	63.5 (60.9-66.4)	65.2 (62.7-67.8)	68.7 (67-70.4)	71.9 (71.4-72.2)	72.2 (71.8-72.7)	73.8 (73.3-74.1)	74.3 (74-74.6)	75.2 (74.9-75.5)	76.3 (76-76.5)	77.7 (77.4-77.9)	79.1 (78.3-79.9)
Baja California Sur	55.4 (52.4-58.6)	59.9 (57.3-62.9)	63 (60.5-65.6)	65.2 (62.8-67.6)	67.4 (65.6-69.4)	70.6 (69.3-71.9)	73.9 (73.3-74.5)	76.3 (76-76.7)	77.1 (76.7-77.4)	77.6 (77.3-77.9)	78.2 (77.9-78.5)	78.5 (78.2-78.7)	78.8 (78.5-79)	79.9 (79.1-80.7)
Campeche	55.4 (52.4-58.3)	59.4 (56.6-62.2)	62 (59.3-64.7)	64.4 (61.8-67)	65.6 (63.7-67.7)	68.7 (67.4-69.9)	71 (70.2-71.7)	72.1 (71.6-72.5)	74.8 (74.3-75.2)	75.8 (75.4-76.2)	77.8 (77.5-78.1)	78.1 (77.8-78.4)	78.4 (78.1-78.7)	77.5 (76.7-78.4)
Chiapas	53 (50.2-56.4)	57.1 (54.3-60.2)	59.8 (57-63.1)	62.2 (59.3-65.4)	64.2 (61.5-67.3)	67.9 (66.1-69.6)	67.4 (66.6-68.2)	69.2 (68.4-69.8)	71.8 (71.3-72.3)	73.5 (73.1-74)	74.8 (74.5-75.1)	75.7 (75.3-76.1)	76.1 (75.8-76.4)	76.2 (75.4-77.1)
Chihuahua	50.6 (47.7-53.8)	55.4 (52.7-58.6)	58.7 (56-61.7)	61.5 (58.7-64.4)	63.1 (60.5-65.6)	66.1 (64.4-67.8)	69.2 (68.6-69.8)	70.9 (70.4-71.3)	72.6 (72.2-73.1)	73.8 (73.5-74.1)	74.9 (74.6-75.2)	75.5 (75.2-75.8)	75.2 (74.9-75.4)	76.3 (75.4-77.2)
Coahuila	51.8 (49-54.8)	56.3 (53.6-59.2)	59.4 (56.8-62.4)	62 (59.3-65)	63.5 (61-66.3)	66.4 (64.7-68.1)	69.3 (68.7-69.9)	71.6 (71.2-72)	74.1 (73.7-74.5)	75 (74.7-75.3)	76.2 (75.9-76.4)	76.4 (76.2-76.7)	77.2 (76.9-77.4)	77.7 (76.9-78.6)
Colima	51.4 (48.4-54.7)	56.2 (53.3-59.3)	59.5 (57-62.2)	62.4 (59.8-65.1)	64 (62.2-65.9)	67.4 (66.1-68.6)	70 (69.3-70.7)	72.4 (72-72.8)	74.1 (73.7-74.5)	75.1 (74.7-75.6)	77.3 (76.9-77.5)	78.2 (77.8-78.5)	78.5 (78.3-78.8)	78.6 (77.8-79.4)
Mexico City	56 (53.5-58.8)	60.3 (57.5-63.2)	63.1 (60.9-65.6)	65.8 (63.6-68.4)	67.7 (65.4-70.2)	70.8 (69.4-72.1)	72.5 (72-72.9)	73.9 (73.7-74.1)	75.6 (75.4-75.8)	75.8 (75.6-76)	77.2 (77-77.3)	77.6 (77.4-77.7)	78.3 (78.1-78.5)	78.7 (78-79.6)
Durango	51 (47.9-54.3)	56 (53.2-59)	59.5 (56.7-62.2)	62.5 (60-65.1)	64.5 (62.3-66.6)	68 (66.6-69.4)	70.3 (69.7-70.9)	72.5 (72.1-73)	75 (74.5-75.4)	76.7 (76.4-77)	78.4 (78-78.7)	78.5 (78.2-78.7)	78.2 (77.9-78.4)	79.6 (78.8-80.4)
Guanajuato	49.7 (46.8-52.8)	54.5 (51.6-57.4)	57.8 (54.9-60.7)	60.7 (58.1-63.4)	62.6 (60.1-65.3)	66.5 (64.8-68.3)	70.3 (69.6-70.9)	71.6 (71.2-72)	74.4 (73.9-74.8)	75.7 (75.4-76)	77.2 (76.9-77.4)	78.1 (77.8-78.3)	78.8 (78.6-79)	79 (78.3-79.8)
Guerrero	46.8 (43.7-49.9)	51.6 (48.6-54.8)	55 (51.7-58.4)	58 (55.1-60.8)	60 (57.4-62.8)	64.3 (62.4-66.3)	68.3 (67.4-69.1)	72.9 (72.1-73.7)	76.3 (75.8-76.8)	77.3 (76.8-77.8)	79.4 (78.9-79.8)	79.3 (78.9-79.6)	79 (78.6-79.4)	79.9 (79-80.8)
Hidalgo	49.9 (47.1-53)	54.2 (51.5-57.3)	57.1 (54.6-60)	59.8 (57.2-62.7)	61 (58.4-63.9)	64.4 (62.8-66.3)	66.4 (65.8-67.1)	71.6 (71.1-72.1)	75 (74.4-75.4)	76.7 (76.3-77)	79 (78.6-79.3)	79.4 (79.1-79.6)	79.4 (79.1-79.6)	80.2 (79.3-81)
Jalisco	51.5 (48.6-54.6)	56.2 (53.5-59.2)	59.6 (57-62.5)	62.6 (60.2-65.3)	64.6 (62.2-67.1)	68.5 (66.9-70.1)	71.1 (70.6-71.5)	73 (72.6-73.4)	74.1 (73.7-74.4)	75.1 (74.9-75.4)	76.7 (76.5-77)	77.6 (77.3-77.8)	78.4 (78.2-78.6)	78.3 (77.5-79.1)
México	56.1 (53-59)	59.6 (56.6-62.6)	61.8 (58.8-64.4)	63.9 (61.5-66.7)	65.4 (62.5-68.1)	68.5 (65.9-71.1)	70.3 (68.5-71.9)	71.4 (71.1-71.7)	73.5 (73.2-73.7)	74.8 (74.6-75.1)	76.3 (76.1-76.5)	77.2 (77-77.3)	78 (77.8-78.2)	78.1 (77.4-79)
Michoacán de Ocampo	49.1 (46-52.2)	54 (51-57.3)	57.5 (54.5-60.8)	60.6 (57.9-63.6)	63.1 (60.6-65.9)	66.9 (65.3-68.5)	69.3 (68.7-69.9)	72.2 (71.8-72.7)	75.7 (75.3-76.1)	76.3 (76-76.6)	77.9 (77.6-78.1)	78.7 (78.4-79)	78.8 (78.5-79.1)	79.9 (79-80.7)
Morelos	53.9 (51-57)	58.2 (55.3-61.2)	61.1 (58.1-64)	63.7 (60.9-66.5)	65.3 (62.9-67.6)	69 (67.5-70.5)	71.6 (71.1-72.2)	73.5 (73-73.9)	75.3 (74.8-75.7)	76.4 (76.1-76.7)	78 (77.7-78.3)	78.2 (77.9-78.5)	78.8 (78.5-79)	78 (77.2-78.9)
Nayarit	51.4 (48.4-54.6)	56.3 (53.5-59.1)	59.6 (56.7-62.3)	62.5 (59.8-65.1)	64.7 (62.6-66.8)	68.6 (67.4-69.9)	72 (71.4-72.5)	73.7 (73.2-74)	75.4 (75-75.8)	77.2 (76.9-77.5)	78.9 (78.6-79.2)	79.5 (79.2-79.8)	79.8 (79.5-80)	80.1 (79.3-81)
Nuevo León	55.5 (52.5-58.7)	59.9 (57.2-62.9)	62.8 (60.2-65.5)	65.1 (62.6-67.7)	66.2 (63.6-68.6)	69 (67.4-70.6)	73 (72.6-73.4)	74.8 (74.5-75.2)	75.7 (75.3-76)	76.5 (76.2-76.7)	77.7 (77.4-77.9)	78.3 (78.1-78.5)	79 (78.8-79.1)	79.8 (78.9-80.5)

Table 7 Estimates of life expectancy at birth by location and year for 1950, 1955...2005, 2010, 2017 - females

Location	1950	1955	1960	1965	1970	1975	1980	1985	1990	1995	2000	2005	2010	2017
Oaxaca	44.1 (41.1-47.2)	49.2 (46.2-52.5)	52.9 (50.1-56.1)	55.5 (52.7-58.3)	57 (54.4-59.7)	60.4 (58.5-62.3)	64.2 (63.4-64.9)	68.2 (67.7-68.7)	72.3 (72-72.7)	74.4 (74-74.7)	77.1 (76.8-77.4)	78 (77.7-78.3)	78.7 (78.4-79)	78.7 (77.9-79.6)
Puebla	44.4 (41.6-47.5)	49.6 (46.9-52.6)	53.3 (50.5-56.6)	56.3 (53.5-59.3)	58 (55.3-60.9)	62 (60.2-63.9)	66.5 (65.7-67.2)	69.2 (68.4-69.9)	72.5 (72-72.9)	73.9 (73.5-74.3)	76.1 (75.8-76.3)	76.7 (76.3-77.1)	77.3 (77-77.6)	77.1 (76.2-77.9)
Querétaro	52.7 (49.7-56)	57 (54.2-60.1)	59.8 (57-62.6)	62.5 (59.8-65.3)	63.6 (60.9-66.3)	67.3 (65.1-69.4)	69.1 (68.1-70.3)	72.8 (72.4-73.2)	74.4 (74.1-74.7)	75.9 (75.6-76.2)	77.4 (77.1-77.6)	78.5 (78.3-78.7)	79 (78.7-79.2)	79.2 (78.4-79.9)
Quintana Roo	62.9 (60.1-65.9)	64.6 (62.1-67.3)	65.9 (63.3-68.4)	66.2 (63.6-68.6)	65.8 (63.7-67.9)	68 (66.6-69.5)	71.7 (70.9-72.5)	76.4 (75.9-76.9)	76.4 (75.9-76.9)	76.4 (76-76.7)	77.6 (77.2-77.9)	78.1 (77.9-78.4)	78.1 (77.9-78.4)	76.9 (76.1-77.6)
San Luis Potosí	48.9 (45.9-52)	54 (50.9-57.3)	57.6 (54.8-61)	60.6 (57.7-63.8)	62.8 (60.1-65.8)	66.7 (64.9-68.4)	70.8 (70.2-71.5)	72.5 (72-72.9)	75.5 (75.1-75.8)	76.4 (76.1-76.8)	78.4 (78.2-78.7)	78.9 (78.5-79.2)	79.2 (78.9-79.4)	79.8 (78.9-80.6)
Sinaloa	54 (51.1-57)	58.4 (55.5-61.4)	61.4 (58.5-64.2)	64 (61.3-66.8)	65.9 (63.2-68.6)	69.7 (68-71.4)	72.3 (71.8-72.9)	74.8 (74.4-75.2)	76.3 (75.9-76.7)	77.3 (77-77.6)	79 (78.7-79.3)	79.4 (79.2-79.7)	80.2 (80-80.4)	81.5 (80.7-82.3)
Sonora	54.8 (51.9-58.1)	59 (56.2-61.9)	61.7 (58.8-64.5)	64 (61.3-66.8)	65.3 (62.7-67.9)	68 (65.5-70.7)	70 (68.5-71.6)	72.6 (72.2-73)	74.3 (74-74.7)	74.7 (74.4-75.1)	76.6 (76.2-76.9)	76.8 (76.6-77.1)	77.5 (77.3-77.7)	78.2 (77.4-79)
Tabasco	53.8 (50.7-56.8)	57.7 (54.9-60.7)	60.2 (57.4-63.1)	62.2 (59.5-64.9)	63.4 (60.9-65.9)	66 (64.6-67.5)	68.8 (68.1-69.5)	71.9 (71.3-72.4)	74.3 (73.9-74.7)	75 (74.6-75.4)	76.9 (76.6-77.3)	77.9 (77.7-78.1)	77.6 (77.4-77.8)	77.4 (76.6-78.3)
Tamaulipas	54.2 (51.2-57.5)	58.6 (55.8-61.9)	61.5 (58.8-64.5)	64.1 (61.5-67.1)	65.7 (63.1-68.5)	68.9 (67.1-70.5)	71.6 (70.9-72.1)	73.7 (73.3-74.1)	75.6 (75.3-75.9)	76.8 (76.3-77.1)	77.9 (77.6-78.1)	78.5 (78.3-78.7)	78.8 (78.5-79)	79.5 (78.6-80.5)
Tlaxcala	48.2 (45.3-51.7)	53.4 (50.2-56.6)	57.2 (54.3-60.3)	60.4 (57.5-63.3)	63 (60.3-65.7)	67 (64.8-69.3)	69.6 (68.3-70.9)	72.4 (71.9-72.9)	74.4 (74-74.7)	75.9 (75.6-76.2)	77.5 (77.2-77.7)	78.2 (78-78.5)	78.7 (78.4-79)	78.8 (78-79.6)
Veracruz de Ignacio de la Llave	50.6 (47.6-53.9)	55.4 (52.6-58.5)	58.7 (55.7-61.8)	61.5 (58.7-64.3)	63.5 (60.9-66.2)	67.3 (65.7-68.9)	71 (70.4-71.5)	72.9 (72.5-73.4)	74.4 (74-74.9)	75.7 (75.4-76)	77.3 (77-77.6)	77.6 (77.4-77.9)	78 (77.8-78.2)	77.7 (76.9-78.6)
Yucatán	53.8 (51-56.9)	57.9 (55.2-61)	60.4 (57.7-63.2)	62.6 (60-65.4)	63.9 (61.7-66.3)	66.5 (65.2-67.8)	69.3 (68.7-69.8)	71.7 (71.2-72.1)	74 (73.5-74.4)	75.3 (75-75.6)	77 (76.7-77.2)	77.3 (77-77.6)	77.7 (77.5-78)	77.4 (76.6-78.2)
Zacatecas	53 (49.7-56.1)	57.7 (54.6-60.7)	61 (58-64)	64.2 (61.4-67)	66.4 (64.2-68.7)	69.9 (68.6-71.3)	72.4 (71.7-73)	74.2 (73.7-74.7)	76.3 (75.8-76.7)	76.9 (76.6-77.2)	78.4 (78.1-78.8)	78.7 (78.4-79)	79.1 (78.9-79.4)	79.4 (78.7-80.2)
Nicaragua	46.6 (44.1-48.8)	52.1 (50.1-54.1)	53.5 (51.8-55.3)	58.7 (57.2-60)	59.7 (58.1-61.4)	64.8 (63.7-65.8)	66.7 (64.9-68.5)	66.9 (65.3-68.4)	74.1 (73.2-74.9)	75.2 (74.2-76.3)	77.5 (76.6-78.4)	78.3 (77.4-79.1)	79.8 (79.2-80.3)	80.6 (79.4-82)
Panama	61.5 (60.5-62.5)	63.7 (62.9-64.4)	66.7 (66.1-67.3)	68.3 (67.6-68.9)	68.8 (68.3-69.3)	73.1 (72.6-73.5)	76.1 (75.6-76.4)	76.9 (76.5-77.2)	78.1 (77.7-78.4)	79 (78.7-79.3)	80.2 (79.8-80.5)	80.9 (80.5-81.1)	81.4 (81.1-81.7)	81.7 (80.9-82.5)
Venezuela	58.7 (58.5-59)	62.1 (61.9-62.3)	66.3 (66.1-66.5)	67.7 (67.6-67.9)	68.5 (68.4-68.7)	70.1 (70-70.2)	72.6 (72.5-72.7)	74.1 (74-74.2)	75.1 (75-75.2)	75.7 (75.4-76.1)	78 (77.9-78.1)	79.3 (79.2-79.4)	79.7 (79.6-79.7)	79.6 (77.7-81.5)
Tropical Latin America	53.1 (51-55)	55.4 (53.6-56.9)	57.7 (56.2-59.1)	59.9 (58.5-61.1)	61.9 (60.8-63)	64.7 (64-65.4)	67.1 (66.6-67.6)	69.3 (68.9-69.7)	71.7 (71.4-72)	73.2 (72.9-73.4)	75.3 (75-75.5)	76.9 (76.6-77.1)	78 (77.8-78.2)	79.1 (78.8-79.3)
Brazil	52.9 (50.8-54.9)	55.1 (53.3-56.7)	57.4 (55.9-58.8)	59.6 (58.3-60.9)	61.7 (60.5-62.8)	64.5 (63.8-65.2)	66.9 (66.4-67.4)	69.2 (68.8-69.6)	71.6 (71.2-71.9)	73.1 (72.8-73.4)	75.2 (74.9-75.5)	76.9 (76.6-77.1)	78 (77.8-78.3)	79.1 (78.8-79.3)
Acre	51.6 (49.1-54.2)	54 (51.2-57)	57.2 (54.5-60)	59.7 (57.3-62.2)	62.2 (60.3-64.2)	65.3 (63.8-66.9)	67.3 (66.1-68.4)	68.8 (68.1-69.5)	71.3 (70.6-72)	72.6 (71.8-73.3)	74.5 (74-74.9)	75.9 (75.4-76.3)	77.7 (77.3-78.1)	79 (78.4-79.5)
Alagoas	44.6 (40.5-49.1)	46.3 (43.2-49.4)	48.6 (45.5-52)	50.7 (48-53.8)	52.5 (50.3-55)	55.6 (53.6-57.7)	57.6 (56-58.9)	61.1 (59.9-62.2)	66.5 (65.7-67.3)	69.8 (69.1-70.5)	73 (72.4-73.6)	75.1 (74.4-75.7)	76.5 (76.1-76.8)	77.4 (76.8-77.9)
Amapá	52 (49.4-54.7)	54.5 (51.7-57.5)	57.6 (55-60.5)	60.4 (58.1-62.8)	63.6 (61.8-65.4)	68 (66.7-69.3)	72.5 (71.9-73.1)	74.4 (74-74.8)	76 (75.7-76.4)	76 (75.7-76.3)	77.1 (76.8-77.4)	79.1 (78.9-79.4)	80.1 (79.8-80.4)	79.4 (78.8-79.8)
Amazonas	53.9 (51.2-56.8)	56.2 (53.2-59.3)	58.9 (56.3-61.5)	61.3 (58.4-64.1)	62.5 (59.8-65.3)	63.8 (61.6-65.9)	67.8 (66.7-69)	71 (70.2-71.8)	73.6 (72.8-74.3)	76.2 (75.5-76.8)	77 (76.7-77.2)	78.8 (78.5-79)	78.7 (78.5-79)	79.2 (78.7-79.7)
Bahia	51.1 (47.4-54.9)	53.2 (50.2-56.3)	55.7 (52.9-58.6)	58.3 (55.6-61.1)	60.9 (58.5-63.4)	64.4 (62.4-66.4)	67.7 (66.6-68.7)	68.8 (67.9-69.8)	71.5 (70.6-72.4)	73.2 (72.4-74.1)	74.9 (74-75.8)	76.4 (75.4-77.2)	78.2 (77.3-79)	80.1 (79.5-80.6)
Ceará	43.8 (39.7-47.9)	46.1 (42.9-49.3)	49 (46-52)	51.7 (48.8-54.7)	54.4 (51.2-57.4)	58.4 (56.1-60.8)	62.3 (60.2-64.5)	66.7 (65.4-68.1)	71.6 (70.7-72.5)	73.4 (72.6-74.1)	75.3 (74.5-76.1)	76.9 (76.1-77.7)	79.4 (78.9-79.9)	80.3 (79.8-80.8)
Distrito Federal	50.5 (48.1-53.2)	53.6 (50.8-56.5)	56.9 (54.3-59.5)	59.5 (56.7-62.2)	62.4 (60-65.1)	65.5 (64-67.2)	68.7 (68.3-69.2)	72 (71.7-72.2)	73.2 (72.9-73.4)	73.4 (73.1-73.7)	75.6 (75.4-75.9)	77.2 (77-77.4)	78.5 (78.3-78.7)	80.6 (80.1-81)
Espírito Santo	57.9 (55.3-60.6)	59.6 (56.7-62.3)	61.2 (58.4-64.1)	62.8 (60.3-65.6)	64 (61.5-66.5)	66.1 (64.3-67.7)	69.8 (69.4-70.2)	70.7 (70.4-71)	73.3 (73-73.6)	74.3 (74-74.6)	76.1 (75.9-76.4)	77.3 (77-77.6)	78.6 (78.3-78.8)	80.3 (79.8-80.8)
Goiás	50.5 (48-53)	52.7 (49.7-55.7)	55.2 (52.5-58.1)	57.5 (54.6-60.4)	60.5 (58.1-63.3)	64.4 (62.1-66.9)	67.1 (65.8-68.4)	68.3 (67.8-68.8)	71.9 (71.5-72.3)	73.1 (72.8-73.4)	76.3 (76.1-76.4)	77.6 (77.4-77.8)	78 (77.8-78.1)	79.2 (78.8-79.6)
Maranhão	46.8 (44.4-49.2)	48.6 (45.6-51.4)	51 (48.1-54.1)	53.5 (50.7-56.2)	56.2 (53.7-58.7)	60.7 (58.2-63.3)	64 (62-66.3)	67.6 (66.3-68.9)	71.6 (70.7-72.5)	73.2 (72.4-74)	75.1 (74.2-75.9)	75.8 (75-76.6)	78.3 (77.5-79.1)	79.9 (79.4-80.4)
Mato Grosso	52.5 (50.1-54.9)	54.9 (51.7-57.9)	57.5 (54.7-60.4)	60.6 (57.7-63.5)	64.3 (61.6-66.9)	68.7 (66.6-70.8)	72.4 (71.2-73.5)	71.8 (70.8-72.9)	74.1 (73.1-75.1)	75.3 (74.8-75.7)	75.9 (75.7-76.2)	77.1 (76.8-77.3)	78 (77.8-78.2)	79.4 (78.9-79.9)
Mato Grosso do Sul	58.9 (56.3-62)	60.2 (57.4-63.6)	62.1 (59.4-65)	64.4 (61.6-67.3)	65.9 (63.3-68.5)	68 (66.2-69.9)	70.3 (69.9-70.8)	72 (71.7-72.3)	73.3 (73-73.6)	73.5 (73.2-73.8)	75.4 (75.1-75.6)	76.5 (76.2-76.7)	77.8 (77.5-78)	78.6 (78.1-79.1)
Minas Gerais	54.6 (51.4-58.2)	56.6 (53.7-59.5)	58.6 (55.8-61.7)	60.6 (58-63.3)	62.6 (60.1-65.2)	65.3 (63.8-66.8)	67.2 (66.7-67.7)	69.3 (68.8-69.8)	72.4 (72-72.8)	74 (73.5-74.3)	76.3 (75.9-76.6)	77.8 (77.4-78.2)	78.8 (78.4-79.1)	80 (79.5-80.4)
Pará	55.7 (53.2-58.2)	57.6 (54.8-60.8)	59.9 (57.1-62.8)	61.7 (58.9-64.7)	63 (60.5-65.6)	65.5 (63.6-67.4)	68.2 (67.2-69.1)	69.7 (68.8-70.7)	72.1 (71.3-72.9)	74.4 (73.7-75)	76.2 (75.5-76.9)	77.5 (76.9-78.1)	78.6 (78.3-78.9)	79.8 (79.3-80.2)
Paraíba	45.9 (41.7-50.2)	48.6 (45.3-51.8)	51.8 (48.9-54.8)	55.5 (52.7-58.6)	58.6 (55.8-61.4)	62.4 (60.5-64.3)	65.8 (64.8-66.7)	69.1 (68.3-69.8)	71.9 (71.2-72.6)	73.7 (73.1-74.3)	76.2 (75.6-76.8)	77.1 (76.5-77.8)	78.1 (77.5-78.6)	79.3 (78.3-80.2)
Paraná	55.4 (52.2-58.6)	57.5 (54.8-60.3)	59.4 (56.8-62.2)	61.6 (59-64.2)	64.3 (62-66.8)	66.9 (65.2-68.6)	67.7 (67.3-68.2)	70.2 (69.9-70.5)	72 (71.8-72.3)	73.6 (73.3-73.8)	75.1 (75-75.3)	76.5 (76.2-76.7)	77.5 (77.2-77.7)	78.5 (78-79)
Pernambuco	43.7 (39.5-47.7)	46 (42.8-49.2)	48.8 (46.1-51.8)	51.6 (48.7-54.6)	54.1 (51.3-56.8)	58.1 (56.4-59.6)	62.2 (61.3-63.1)	65.1 (64.2-65.9)	68 (67.5-68.5)	71.4 (71-71.9)	73.4 (72.9-74)	75.4 (74.9-75.8)	77.2 (76.6-77.6)	77.3 (76.8-77.8)
Piauí	52.6 (50.1-55.1)	54.4 (51.7-57.2)	56.4 (53.3-59.5)	57.9 (54.8-61.1)	59.5 (56.7-62.5)	63.6 (61.3-66)	67.6 (66.1-69)	69.4 (68.5-70.2)	74.5 (73.7-75.2)	76.9 (75.9-77.7)	76.6 (75.8-77.4)	77.2 (76.5-77.9)	79.4 (78.9-79.9)	80.4 (79.9-80.9)
Rio de Janeiro	56.9 (54.2-59.6)	59 (56-61.7)	61.2 (58.5-64)	63.2 (60.6-66)	64.2 (61.8-66.9)	66 (64.7-67.5)	68.1 (67.7-68.6)	69.8 (69.6-70.1)	70.8 (70.6-71.1)	71.7 (71.4-71.9)	74.5 (74.4-74.7)	76.3 (76.2-76.5)	76.8 (76.6-77)	77.4 (77.1-77.8)
Rio Grande do Norte	42.9 (38.1-47.4)	46.4 (42.7-49.7)	50.1 (47-53.4)	53.8 (50.7-57)	56.8 (53.8-60)	61.4 (59.2-63.5)	66.5 (65.1-67.9)	70.4 (69.3-71.4)	73.5 (72.7-74.4)	74.5 (73.7-75.2)	77.4 (76.6-78.2)	79.4 (78.7-80.1)	80.6 (80.2-81)	80.4 (79.9-81)
Rio Grande do Sul	60.2 (57.8-62.9)	61.8 (59.2-64.5)	63.6 (61.3-66.2)	65.3 (62.8-67.9)	67.4 (65.2-69.7)	69.7 (68.3-71.1)	70.8 (70.5-71.1)	72.7 (72.5-72.9)	74.1 (73.9-74.3)	74.6 (74.4-74.7)	76.3 (76.1-76.4)	77.7 (77.5-77.8)	78.5 (78.3-78.6)	78.9 (78.5-79.2)
Rondônia	47.3 (44.9-49.8)	48.4 (45.5-51.4)	51.6 (48.9-54.7)	53.7 (50.8-56.8)	56.1 (53.8-58.8)	60.5 (58.3-62.5)	64.5 (63.3-65.8)	68 (67.2-68.9)	71 (70.1-71.9)	74.3 (73.6-75.1)	75 (74.2-75.8)	76.5 (75.8-77.3)	77.7 (76.9-78.5)	78.4 (77.2-79.7)
Roraima	47.7 (45.3-50.2)	49.6 (46.8-52.3)	52.6 (50-55.1)	55.5 (52.7-58.1)	58.1 (55.8-60.5)	62.8 (60.9-64.8)	66.7 (65.2-68.2)	68.1 (66.9-69.4)	71.8 (70.8-73)	73.4 (72.3-74.4)	74.3 (73.3-75.3)	75.5 (74.4-76.6)	77.6 (76.5-78.8)	78 (76.6-79.4)
Santa Catarina	62.3 (59.9-64.9)	63.7 (61-66.6)	65.1 (62.7-67.8)	66.4 (63.8-69.1)	67.7 (65.4-70.1)	69.5 (68.1-71.1)	70.8 (70.2-71.3)	72.1 (71.6-72.5)	73.2 (72.9-73.5)	74.2 (73.8-74.5)	76 (75.6-76.3)	77.7 (77.4-77.9)	78.7 (78.4-78.9)	79.6 (79.2-80)
São Paulo	59.1 (56.5-61.8)	61 (58.1-63.9)	62.8 (60.3-65.3)	64.6 (62-67.3)	66.1 (63.7-68.5)	67.4 (66-68.9)	69.1 (68.7-69.5)	71.4 (71.2-71.6)	72.5 (72.2-72.7)	73.5 (73.3-73.7)	75.5 (75.4-75.7)	77.6 (77.4-77.7)	78.4 (78.2-78.5)	79.2 (78.9-79.5)
Sergipe	49.5 (45.6-53.5)	51.9 (48-55.7)	54.4 (51.4-57.5)	57.1 (54.4-59.8)	59.5 (57.2-61.9)	62.8 (60.8-65)	64.3 (63.1-65.4)	68 (67.2-68.7)	70.8 (70.2-71.3)	71.7 (71.2-72.1)	74.4 (73.9-74.8)	76.8 (76.3-77.1)	78.1 (77.7-78.4)	79.1 (78.4-79.7)
Tocantins	50.5 (48-53.3)	52.4 (49.1-55.6)	54.8 (52-57.9)	57.2 (54.2-60.2)	59.8 (57.1-62.5)	63.6 (61.2-66.2)	67.1 (65-69.1)	69.5 (67.8-71.1)	71.6 (70.7-72.5)	73.2 (72.6-73.8)	74.5 (74-75)	76.6 (76.1-77.1)	78.5 (78.1-78.9)	79.3 (78.6-80)
Paraguay	62.2 (60.3-64.1)	67 (65.8-68.2)	67.9 (66.8-68.9)	70.2 (69.2-71.2)	70.3 (69.3-71.2)	71.6 (70.8-72.5)	72.8 (71.9-73.6)	73.9 (73.1-74.7)	76.4 (75.6-77.2)	76.4 (75.6-77.2)	77 (76.2-77.8)	76.9 (76.1-77.7)	77.5 (76.7-78.3)	78.9 (76.8-81.2)
North Africa and Middle East	45.5 (44.1-47)	48 (46.8-49.2)	50.4 (49.3-51.4)	53.8 (52.8-54.7)	56.6 (55.9-57.3)	60.1 (59.5-60.6)	63.1 (62.7-63.5)	65.2 (64.9-65.6)	68.2 (67.9-68.6)	70 (69.6-70.3)	72 (71.7-72.4)	73.7 (73.4-74.1)	75.2 (74.9-75.6)	76.8 (76.4-77.3)
North Africa and Middle East	45.5 (44.1-47)	48 (46.8-49.2)	50.4 (49.3-51.4)	53.8 (52.8-54.7)	56.6 (55.9-57.3)	60.1 (59.5-60.6)	63.1 (62.7-63.5)	65.2 (64.9-65.6)	68.2 (67.9-68.6)	70 (69.6-70.3)	72 (71.7-72.4)	73.7 (73.4-74.1)	75.2 (74.9-75.6)	76.8 (76.4-77.3)
Afghanistan	38.9 (35.8-42.1)	40 (37-43.2)	41 (38-44.1)	41.9 (39-45)	42.8 (39.8-45.5)	43.6 (41-46)	42.5 (40.5-44.5)	42.6 (41-44.3)	52 (49.9-54.3)	53.1 (50.8-55.4)	54.6 (52.1-57.1)	57.3 (54.9-59.8)	60 (57.6-62.6)	63.2 (60.6-65.8)
Algeria	58.4 (57.3-59.5)	59.3 (58.5-60)	58.9 (57.6-60.1)	61.6 (60.5-62.7)	60 (58.7-61.2)	59.8 (58.5-61)	61.5 (60.8-62.2)	69.6 (69-70.2)	73.2 (72.3-74.1)	74.6 (73.8-75.5)	75.5 (74.7-76.3)	76.4 (75.5-77.2)	77 (76.5-77.5)	78.5 (77.9-79.1)
Bahrain	49.8 (47.1-52.8)	52.9 (50.4-55.7)	57.2 (54.8-59.8)	61.4 (59.3-63.8)	65.5 (63.8-67.3)	69 (67.8-70.3)	71.6 (70.9-72.4)	72.3 (71.8-72.8)	71.9 (71.4-72.4)	72.1 (71.6-72.6)	73.9 (73.4-74.3)	76.6 (76.1-77)	79.2 (78.7-79.7)	80.4 (79.5-81.4)
Egypt	42 (39.8-44.1)	45.5 (43.6-47.3)	48.3 (46.5-50)	52.1 (50.5-53.7)	52.8 (51.5-54.1)	56.8 (55.7-57.8)	60.5 (59.6-61.4)	62.2 (61.5-62.8)	66.6 (66.1-67.1)	69.8 (69.3-70.2)	72.3 (71.9-72.6)	72.4 (72.1-72.7)	73 (72.8-73.3)	74.3 (72.9-75.8)
Iran	41.6 (39.2-44.1)	43.5 (40.8-46.2)	46.9 (44.2-49.5)	51.6 (49.1-53.8)	58.1 (56.4-59.8)	65.5 (64.2-66.8)	69.2 (68.2-70.1)	70.4 (69.6-71.1)	70.8 (70.1-71.4)	74.2 (73.7-74.7)	75.7 (75.3-76.1)	76.9 (76.5-77.2)	77.5 (77.3-77.7)	79.4 (79.3-79.5)
Iraq	54.4 (52.2-56.6)	57.7 (55.8-59.5)	60 (58.3-61.6)	61.4 (59.6-62.9)	62.7 (61.5-63.9)	64 (62.9-65.1)	65.5 (64.3-66.8)	65 (63.4-66.6)	67.6 (65.8-69.3)	67 (65.4-68.7)	68.1 (66.6-69.7)	70.9 (69.5-72.3)	74.5 (73.4-75.6)	78.8 (78.1-79.6)
Jordan	56.5 (53.5-59.3)	57.8 (55-60.5)	60.5 (58.4-62.8)	63.9 (62.4-65.4)	66.7 (65.9-67.7)	69.6 (68.9-70.3)	70.9 (70-71.7)	71.5 (70.3-72.7)	71.7 (70.3-73)	71.4 (70.1-72.8)	71.9 (70.9-73)	73.7 (73.1-74.3)	78.5 (78-79)	81.1 (79.8-82.3)
Kuwait	64.3 (61.7-66.9)	65.8 (63.4-68.1)	67.9 (66.4-69.3)	69 (68.6-69.4)	72.2 (71.9-72.5)	72.6 (72.3-72.9)	74.7 (74.5-75)	77.6 (77.4-77.8)	77.1 (76.9-77.4)	80.1 (79.9-80.3)	80.2 (80.1-80.4)	81.4 (81.2-81.5)	83.5 (83.3-83.6)	87.2 (86.7-87.7)
Lebanon	59.8 (57-62.9)	61.1 (58.4-64.1)	63.5 (60.8-66.5)	65.9 (63.3-68.7)	68.1 (65.6-70.6)	66.2 (64.1-68.3)	71.5 (69.6-73.6)	69.8 (68.2-71.5)	73.4 (72.2-75)	75.2 (74.1-76.7)	77.5 (76.2-78.5)	79 (78.4-79.7)	79.6 (79.1-80.1)	80 (79.4-80.7)
Libya	39.4 (36.6-42.4)	46.1 (43.3-49.4)	52.2 (49.4-55)	59.5 (57.3-61.8)	65.9 (64.2-67.5)	69.5 (68.3-70.7)	72.2 (70.9-73.6)	73.3 (71.6-74.9)	73.5 (71.7-75.2)	74.2 (72.7-75.8)	74.3 (73.1-75.6)	75 (73.9-76)	74.8 (73.7-75.9)	75 (73.3-76.9)
Morocco	46.8 (44.2-49.5)	48 (45.7-50.7)	49.2 (46.9-51.5)	53.6 (51.2-55.9)	56.8 (54.6-59.2)	59.1 (57-61.2)	62.1 (60.5-63.7)	65 (63.8-66.2)	66.2 (65.3-67.2)	67.9 (67-68.7)	69.9 (68.9-70.8)	71.5 (70.3-72.7)	72.8 (71-74.2)	74.7 (72.7-76.8)
Palestine	52.8 (49.8-55.8)	55.5 (52.6-58.5)	59.1 (56.2-62.1)	63.2 (60.6-66.2)	62.5 (60.3-64.8)	68.7 (66.4-71.2)	70.3 (68.2-72.7)	72 (69.9-74.2)	72.6 (70.7-74.5)	73.8 (72.3-75.1)	73.1 (72.4-73.8)	73.3 (72.9-73.7)	75 (74.6-75.4)	78 (77.3-78.9)
Oman	30.1 (27.3-33.1)	32.5 (29.4-35.8)	35.4 (32.4-38.6)	39.6 (36.7-42.9)	49.2 (46.6-52.3)	55.8 (53.1-58.6)	62.3 (59.6-65.2)	68 (65.3-70.6)	71.4 (69.2-73.7)	73.8 (71.9-76.1)	75.9 (73.6-78.2)	77.9 (75.8-79.1)	78.4 (77.3-79.6)	79.4 (78.2-81.2)
Qatar	63.9 (61.1-66.7)	64.8 (62.1-67.6)	66.1 (63.4-68.8)	67.2 (64.7-69.6)	69.3 (67.1-71.6)	71 (69-73.2)	72.7 (71-74.7)	72.6 (71.1-74.2)	72.8 (71.2-74.3)	73.3 (71.7-74.7)	74.8 (73.2-76.3)	77.1 (75.6-78.5)	79.3 (77.8-80.9)	81.7 (79.8-83.5)
Saudi Arabia	55 (52-58.1)	56.7 (53.6-59.6)	58.5 (55.4-61.4)	61.1 (58.2-64.1)	64.1 (61.3-67)	68.5 (65.7-71.1)	71.1 (68.7-73.5)	72.6 (70.5-75.1)	73.6 (71.6-75.8)	75.3 (73.6-77.1)	76.4 (75.1-77.7)	76.8 (75.7-78)	77.8 (76.8-78.8)	79.4 (78-80.2)
Sudan	47.7 (44.4-51)	49.1 (46.2-51.7)	51 (48.4-53.7)	51.9 (49.6-54.4)	52.9 (50.9-55)	54 (52.2-55.9)	55.9 (54.2-57.8)	53.6 (52.1-55.1)	59.9 (58-61.7)	62.5 (60.3-64.6)	64.8 (62.5-67.3)	67.2 (64.7-69.8)	69.3 (66.8-71.9)	72 (69.5-74.7)
Syria	56.7 (53.9-59.5)	59.5 (56.9-62)	63.4 (61.1-65.8)	66.8 (64.8-68.9)	69.5 (67.8-71.2)	71.7 (70.4-73)	72.7 (71.3-74)	72.2 (71-73.6)	72.3 (71.2-73.4)	73.5 (72.4-74.3)	75.5 (74.3-76.2)	77.9 (77.3-78.8)	78.9 (77.9-79.8)	75 (74-76.3)
Tunisia	46.1 (43.6-48.6)	47.9 (45.9-49.8)	51.2 (49.7-52.7)	53.8 (52.5-55)	57.4 (56.4-58.3)	63.6 (62.9-64.5)	68.7 (68-69.4)	72.8 (72.3-73.3)	74.5 (73.9-75)	75.5 (74.9-76)	76.6 (75.5-77.7)	78.2 (76.3-80)	79.3 (77.3-81.5)	80.7 (78.5-83)
Turkey	47.1 (44-50.3)	50.2 (47.5-52.9)	52.3 (49.8-54.8)	56 (53.6-58.4)	60.3 (58-62.7)	63.8 (62.1-65.6)	67.2 (66.3-68.1)	70.1 (69.2-71)	72.1 (71.3-72.8)	73.7 (73-74.5)	77.4 (76.7-78)	80 (79.4-80.7)	81.3 (80.7-81.9)	83 (82-84)
United Arab Emirates	53.4 (50.6-56.4)	55.6 (52.8-58.7)	58.4 (55.5-61.4)	62.1 (59.3-65)	65.2 (62.4-67.8)	68 (65.5-70.7)	70.6 (68.3-73.1)	72.3 (70.3-74.5)	73 (71.2-75)	74 (72.4-75.7)	74.2 (72.8-75.7)	74.5 (73-76)	76 (74.3-77.8)	76.9 (74.7-79.2)
Yemen	34.8 (31.8-38)	36.1 (33.4-39.2)	39 (36.5-41.7)	41 (38.5-43.5)	44.3 (41.8-46.7)	50.2 (47.8-52.8)	54.7 (52.1-57.3)	57.9 (55.3-60.4)	59.8 (57.3-62.6)	61.8 (59.3-64.6)	64.4 (61.9-67.1)	67.2 (64.4-70)	69.4 (66.7-72.2)	70.3 (67.6-72.7)
South Asia	44.4 (43-45.7)	46.5 (45.1-48)	46.6 (45-48.1)	46.9 (45.6-48.2)	49.4 (48.3-50.5)	52.1 (51.2-53.1)	55.7 (55-56.5)	57.6 (57-58.3)	60.3 (59.7-61)	62.6 (62.1-63.1)	64.2 (63.7-64.6)	66.6 (66.2-67)	68.4 (67.9-68.8)	70.2 (69.7-70.7)
South Asia	44.4 (43-45.7)	46.5 (45.1-48)	46.6 (45-48.1)	46.9 (45.6-48.2)	49.4 (48.3-50.5)	52.1 (51.2-53.1)	55.7 (55-56.5)	57.6 (57-58.3)	60.3 (59.7-61)	62.6 (62.1-63.1)	64.2 (63.7-64.6)	66.6 (66.2-67)	68.4 (67.9-68.8)	70.2 (69.7-70.7)
Bangladesh	42.9 (40.1-45.7)	44.9 (42.5-47.7)	47 (44.7-49.5)	49.1 (46.9-51.4)	45.8 (44.1-47.6)	52.3 (50.5-54.2)	54.4 (53.2-55.7)	55.2 (54.2-56.2)	59.5 (58.5-60.6)	64 (63-64.9)	67.6 (66.8-68.5)	69.3 (68.6-69.9)	71.1 (70.4-71.8)	74.6 (73.1-76)
Bhutan	34.9 (32.2-37.9)	37.4 (34.8-40.4)	40.4 (37.6-43.4)	44 (41.1-46.8)	47.7 (44.6-50.7)	50.2 (47.5-53.4)	52.5 (49.8-55.4)	56 (53.5-58.5)	59.9 (57.8-62.1)	63.1 (61.3-65)	66.4 (64.8-68)	70.5 (68.9-72.1)	73.4 (71.6-75.2)	76 (73.9-78.1)
India	44.8 (42.9-46.5)	46.8 (45-48.6)	46.4 (44.7-48.2)	46.2 (44.7-47.8)	49.4 (48.1-50.7)	51.6 (50.5-52.8)	55.6 (54.6-56.5)	57.6 (56.8-58.5)	60.4 (59.6-61.1)	62.7 (62.1-63.3)	64.1 (63.5-64.6)	66.9 (66.4-67.4)	68.5 (68-69)	70.2 (69.5-70.8)
Andhra Pradesh	46.3 (43.1-49.8)	48.1 (44.3-52.1)	47.9 (43.3-52.4)	47.7 (44-51.6)	50 (46.9-53.2)	51.9 (49.3-54.7)	56.1 (53.5-59)	58.4 (55.8-61.2)	60.9 (58.4-63.6)	63.4 (60.8-66)	64.9 (62.4-67.5)	67.2 (64.7-69.9)	69.5 (66.9-72)	72.3 (69.6-74.8)
Arunachal Pradesh	43.5 (39.9-47)	45.7 (42.4-49.4)	45.2 (41.8-48.9)	44.1 (41.2-47.3)	48.6 (45.8-51.4)	52.4 (49.6-55.1)	57.4 (54.8-60)	59.7 (57.4-62.3)	62.4 (60.1-64.9)	65 (62.8-67.5)	66.6 (64.3-68.9)	69.5 (67.3-71.6)	71.7 (69.6-73.8)	73.5 (71.1-75.5)
Assam	40.6 (37.1-43.9)	42.9 (39.5-46.4)	42.8 (39.3-46.5)	42.8 (39.6-46.1)	46.4 (43.4-49.3)	49.9 (47.3-52.7)	54 (51.6-56.6)	55.8 (53.6-58.2)	57.9 (56.1-59.7)	59.1 (58.2-60.1)	59.4 (58.5-60.3)	62.5 (61.6-63.3)	64.1 (63.3-64.9)	68.1 (67-69.3)
Bihar	47.7 (44.3-51.1)	49.2 (46.2-52.7)	48.4 (45.2-52)	47.6 (44.8-50.7)	49.4 (46.8-52.2)	50.5 (48.1-53.4)	53.1 (50.6-55.7)	54.8 (52.6-57.3)	57.9 (55.8-60.2)	60.3 (58.6-62)	61.9 (60.7-63)	64.5 (63.6-65.3)	66.8 (66.1-67.5)	69 (67.8-70.1)
Chhattisgarh	48 (44.9-51.4)	49.7 (46.5-53.1)	49.2 (45.9-52.8)	48.4 (45.6-51.4)	51.3 (48.8-54.2)	53.1 (50.7-55.6)	56.3 (54-58.8)	57.3 (55.2-59.5)	59.7 (57.7-61.7)	61.7 (60.1-63.5)	62.8 (61.6-64)	65.2 (64.4-66)	65.9 (65-66.7)	67 (65.9-68.2)
Delhi	44.6 (41.3-48.2)	48.7 (45.3-52.3)	50.6 (47.5-53.7)	50 (47.3-52.9)	55.9 (53.2-58.8)	58.9 (56.4-61.6)	62.6 (59.9-65.3)	64.2 (61.8-66.6)	66 (63.7-68.5)	67.7 (65.8-69.8)	68.7 (67.2-70.3)	71.3 (70.2-72.5)	73.3 (72.3-74.2)	75 (73.6-76.5)
Goa	55.9 (53.2-58.9)	57.9 (54.8-61.2)	58.5 (55.3-61.7)	58.7 (55.7-61.5)	61.6 (59.3-63.9)	64 (61.7-66.1)	67.7 (65.7-69.8)	68.7 (66.8-70.6)	70.3 (68.6-72)	72.1 (70.4-73.8)	73.3 (71.8-75.1)	75.4 (73.7-77.2)	76.9 (75-78.7)	78.1 (76-79.8)
Gujarat	48.7 (45.9-51.8)	50.6 (47.7-53.8)	50.3 (47.2-53.5)	50 (47.3-52.7)	53.2 (50.7-55.5)	55.3 (52.9-57.4)	58.5 (56.3-60.8)	60.3 (58.2-62.5)	62.9 (61.1-64.6)	66 (64.9-67.1)	67 (66.2-67.9)	69.2 (68.2-70)	70.6 (69.8-71.3)	72.2 (71-73.4)
Haryana	47.7 (44.2-51.1)	49.5 (46.2-53)	49.1 (45.8-52.3)	48.4 (45.6-51.4)	52 (49.4-55)	55.4 (53-58.1)	59.9 (57.4-62.4)	61.6 (59.4-63.8)	63.8 (62.1-65.4)	65.9 (64.9-66.8)	67 (66.1-67.8)	70.3 (69.5-71.2)	70 (69.2-70.8)	71 (69.9-72.1)
Himachal Pradesh	52.9 (49.7-56.3)	54.3 (50.8-58)	54.1 (50.5-57.4)	53.6 (50.2-56.9)	56.1 (53.2-59)	57.9 (55-60.7)	61.3 (58.6-64)	62.7 (60.2-65.3)	65.1 (63.1-67.1)	67.4 (66.3-68.5)	70.2 (69.3-71.1)	72.5 (71.6-73.4)	74 (73.2-74.8)	76.8 (75.6-78)
Jammu and Kashmir	55.6 (52.2-59.2)	56.8 (53.6-60.3)	56.5 (53.4-59.8)	55.2 (52.3-58.2)	58.4 (55.6-61.1)	59 (56.6-61.7)	61.8 (59.3-64.6)	63.4 (61-65.9)	65.3 (63-67.8)	67.3 (65.4-69.2)	68.5 (67.2-69.9)	70.3 (69.4-71.3)	72.3 (71.5-73.1)	73.6 (72.4-74.8)
Jharkhand	49 (45.8-52.2)	50.6 (47.5-53.9)	50 (46.9-53.3)	49.2 (46.2-52.2)	51.7 (49-54.6)	53.2 (50.6-56)	55.8 (53.3-58.4)	56.2 (54-58.8)	58.3 (56.1-60.6)	60.2 (58.4-61.9)	61.3 (60-62.5)	64 (63.2-64.7)	65.9 (65.2-66.6)	68 (67-69.1)
Karnataka	46.6 (43.4-49.7)	48.8 (45.7-51.9)	48.6 (45.6-51.9)	48.6 (45.5-51.8)	51.9 (49.1-54.8)	55 (52.6-57.8)	59.7 (57.3-62.2)	62.1 (60-64.3)	64.9 (63.4-66.4)	66.5 (65.7-67.3)	67.9 (67.2-68.5)	70.2 (69.5-71)	70.1 (69.4-70.7)	69.3 (68.3-70.3)
Kerala	55.5 (52.2-58.8)	58.5 (55.3-61.8)	59.7 (56.6-62.9)	60.3 (57.6-63.3)	64 (61.6-66.8)	66.5 (64-68.9)	69.4 (67.1-71.6)	70.4 (68.3-72.4)	72.3 (70.7-73.8)	74.1 (73.2-75)	74.6 (73.8-75.4)	76.6 (75.9-77.3)	77.3 (76.7-78)	77.4 (76.4-78.5)
Madhya Pradesh	42.1 (39-45.3)	44.1 (41-47.5)	43.4 (40.2-46.5)	42.8 (40-45.8)	46 (43.4-48.7)	48.1 (45.7-50.8)	50.9 (48.6-53.6)	52.6 (50.4-54.7)	55.4 (53.4-57.5)	58 (56.2-59.8)	59.8 (58.5-61)	63.1 (62-64.1)	65.5 (64.7-66.3)	68.4 (67.3-69.6)
Maharashtra	47.5 (44.4-50.7)	49.9 (46.6-53.5)	50.2 (47-53.3)	50.4 (47.3-53.6)	53.7 (51.1-56.5)	56.1 (53.8-58.6)	60.8 (58.7-63)	63.4 (61.4-65.2)	66 (64.5-67.4)	67.9 (67.2-68.7)	67.6 (67-68.3)	71.1 (70.3-71.8)	72.8 (72-73.5)	72.7 (71.8-73.6)
Manipur	51.9 (48.8-55.2)	53.7 (50.3-57)	53.7 (50.2-57)	53.1 (50.1-56.1)	56.8 (54.1-59.4)	59.4 (57.1-61.6)	62.9 (60.8-65.1)	64.4 (62.3-66.4)	66.6 (64.6-68.6)	68.4 (66.3-70.4)	69.3 (67.2-71.1)	71.1 (69.1-73)	72.4 (70.4-74.3)	73.7 (71.6-75.6)
Meghalaya	55.9 (52.4-59.1)	57.2 (53.8-60.5)	57.2 (53.7-60.3)	55.8 (52.6-58.7)	58.6 (56-61.3)	58.9 (56.5-61.2)	61.6 (59.3-64)	62.7 (60.6-65)	64.9 (62.7-67.2)	67 (64.9-69.1)	68.2 (66.1-70.4)	70.3 (68.2-72.4)	71.7 (69.6-73.7)	73.1 (70.9-75.1)
Mizoram	58.6 (55.1-61.9)	59.8 (56.4-63.1)	60.1 (56.8-63.3)	58.3 (55.3-61)	62 (59.3-64.7)	62.8 (60.3-65.2)	65 (62.8-67.3)	66.2 (64.3-68.2)	68.4 (66.7-70.4)	70.1 (68.4-71.8)	70.9 (69.3-72.7)	71.9 (70.1-73.9)	72.3 (70.4-74.4)	73.5 (71.4-75.5)
Nagaland	47.8 (44.4-51.5)	50.7 (47.1-54.4)	51.6 (48.1-55.3)	50.3 (47.5-53.4)	56.2 (53.5-59.3)	59 (56.5-61.5)	62.7 (60.2-65.4)	64.5 (62.2-67)	66.4 (64-68.7)	68.2 (65.9-70.5)	68.7 (66.4-71)	70.6 (68.2-73.1)	72.2 (70-74.7)	73.9 (71.9-76.2)
Odisha	41.6 (38.1-45.1)	43.5 (40.4-46.8)	42.8 (39.7-46.4)	42.6 (39.8-45.8)	45.6 (42.8-48.4)	48.4 (45.7-51.1)	53.2 (50.7-55.8)	55.9 (53.5-58.3)	58.6 (57-60.2)	60.5 (59.6-61.4)	60.4 (59.6-61.2)	64.1 (63.2-64.9)	66 (65.2-66.7)	69.8 (68.7-70.9)
Punjab	54.1 (51.1-57.6)	55.4 (52.1-58.5)	55.7 (52.5-58.9)	55.2 (52.1-58.2)	58.4 (55.7-61.1)	60.4 (58.1-62.8)	63.4 (61.2-65.8)	64.7 (62.7-66.8)	66.6 (65-68.2)	68.7 (67.7-69.5)	69.1 (68.4-69.9)	71.3 (70.5-72)	72.3 (71.6-73)	73.7 (72.5-74.8)
Rajasthan	45.1 (41.7-48.9)	47.1 (43.8-50.6)	46.3 (42.9-49.4)	45.6 (42.5-48.8)	49.2 (46.5-51.9)	51.5 (48.9-54.2)	55.6 (53.2-58.2)	57.6 (55.4-59.8)	60.5 (58.9-62.3)	63.2 (62.1-64.4)	65.5 (64.5-66.5)	68.1 (67.3-68.9)	69.9 (69.1-70.7)	70.6 (69.4-71.8)
Sikkim	51.7 (48.3-55.1)	53.2 (49.5-56.7)	52.7 (49.3-56.1)	51.5 (48.2-54.5)	54.8 (52-57.6)	56.7 (54.3-59.2)	60.6 (58.3-63.2)	62.9 (60.4-65.2)	65.5 (63.1-67.9)	67.6 (65.2-69.9)	69.1 (66.8-71.2)	71.4 (69.3-73.4)	73.3 (71.4-75.3)	75.4 (73.6-77.4)

Table 7 Estimates of life expectancy at birth by location and year for 1950, 1955...2005, 2010, 2017 - females

Location	1950	1955	1960	1965	1970	1975	1980	1985	1990	1995	2000	2005	2010	2017
Tamil Nadu	45.7 (42.7-49.2)	47.8 (44.6-51.2)	47.7 (44.8-50.9)	47.8 (44.8-50.7)	50.6 (48.2-53)	53.2 (50.9-55.6)	57.4 (55.1-60)	59.9 (57.9-62)	63.1 (61.7-64.6)	65.5 (64.6-66.3)	66.5 (65.8-67.1)	69.2 (68.5-69.9)	70.7 (70-71.4)	72.3 (71.3-73.3)
Telangana	51.6 (48-54.9)	52.7 (49-56.4)	52.6 (48.3-56.9)	52.2 (48.6-56)	54.3 (51-57.5)	55.9 (53-58.8)	59.3 (56.6-62.2)	60.4 (57.6-63.1)	61.7 (59.2-64.3)	63.9 (61.4-66.7)	65 (62.4-67.6)	67.2 (64.7-69.7)	69.8 (67.4-72.3)	72.9 (70.7-75.3)
Tripura	43.2 (39.7-46.6)	46 (42.4-49.7)	46.4 (42.9-49.9)	46.9 (44-50)	51.2 (48.5-53.9)	54.7 (52.4-57.3)	58 (55.7-60.5)	60.6 (58.4-63)	63.5 (61.3-65.8)	65.7 (63.6-68)	68 (65.9-70.2)	70.6 (68.6-72.5)	71.7 (69.8-73.6)	73 (71-74.6)
Uttar Pradesh	35.8 (33.1-38.7)	38 (35-41.6)	37 (34.2-40)	36.9 (34.2-40.2)	40.4 (38.1-43)	42.7 (40.4-44.9)	47.2 (45-49.6)	50 (48.1-52)	53.8 (51.9-55.6)	57.1 (55.6-58.7)	59.6 (58.4-60.9)	62.6 (61.7-63.5)	64.5 (63.6-65.3)	66.1 (64.8-67.5)
Uttarakhand	54.9 (51.7-58.3)	56.1 (52.8-59.1)	55.8 (52.6-59.2)	55.1 (52-57.9)	57.4 (54.7-60.1)	58.9 (56.3-61.3)	61.7 (59.2-64.3)	62.2 (59.6-64.6)	63.5 (61-65.8)	65.2 (62.9-67.5)	66.1 (63.9-68)	68.3 (66.7-69.9)	70.6 (69.4-72)	73.3 (72.2-74.4)
West Bengal	43.9 (40.7-46.9)	46.5 (43.4-49.8)	46.7 (43.8-50)	46.9 (44.1-50)	50 (47.5-52.9)	52.7 (50.3-55.3)	57 (54.8-59.3)	58.8 (56.7-61)	61.4 (59.6-63.1)	63.5 (62.4-64.6)	66.4 (65.5-67.3)	69.1 (68.2-70)	70.2 (69.3-71.2)	72.2 (70.9-73.4)
Union Territories other than Delhi	60.7 (57.9-63.2)	62 (59.2-64.7)	62.3 (59.4-65)	60.7 (58.1-63)	64.6 (62.4-66.8)	66.4 (64.4-68.3)	69 (67.1-70.8)	69 (67.2-70.7)	70.1 (68.3-71.8)	71.3 (69.8-73)	72.3 (70.8-73.9)	74.1 (72.5-75.9)	75.1 (73.3-77.1)	76.1 (74.2-77.9)
Nepal	40.4 (37.7-43.2)	42.5 (40.1-45.3)	44.6 (42.5-47)	47.2 (45-49.9)	49.5 (47.2-52.1)	50.9 (48.6-53.3)	52.3 (50.1-54.7)	55.2 (53.2-57.4)	59 (57.3-60.9)	62.9 (61.5-64.4)	67.2 (65.9-68.4)	69.7 (68.5-70.9)	71.3 (69.8-72.8)	73.3 (71.5-75.1)
Pakistan	45.3 (42.9-47.8)	47.1 (44.7-49.4)	49.3 (47.2-51.6)	51.8 (50-53.6)	54.3 (52.8-55.9)	57.3 (56-58.6)	60.1 (59-61.3)	61.5 (60.6-62.5)	61.6 (60.8-62.4)	60.6 (59.7-61.5)	61.6 (60.6-62.7)	62.1 (61-63.2)	64.7 (62.9-66.6)	67.4 (65.1-70.1)
Southeast Asia, East Asia, and Oceania	50.2 (48.7-51.8)	53.7 (52.2-55.1)	33 (32.3-33.6)	59 (58-59.9)	61.5 (60.6-62.3)	63.8 (63.1-64.4)	66.2 (65.6-66.7)	68.1 (67.7-68.6)	69.9 (69.5-70.3)	71.4 (71.2-71.7)	73.3 (73.1-73.6)	75.4 (75.2-75.6)	77.1 (76.9-77.3)	78.6 (78.2-78.9)
East Asia	51 (49.1-52.9)	54.7 (52.8-56.5)	27.5 (26.8-28.2)	60.1 (58.7-61.4)	62.8 (61.6-63.9)	65.2 (64.3-66.1)	67.3 (66.5-68.1)	69.2 (68.6-69.8)	70.8 (70.3-71.3)	72.3 (72-72.7)	74.5 (74.2-74.8)	76.9 (76.6-77.1)	78.6 (78.4-78.8)	79.9 (79.4-80.3)
China	51.3 (49.3-53.3)	54.5 (52.4-56.3)	26.2 (25.5-26.9)	59.8 (58.4-61.2)	62.5 (61.3-63.6)	65 (64-65.9)	67.1 (66.3-67.9)	69 (68.4-69.6)	70.7 (70.1-71.2)	72.4 (72-72.8)	74.7 (74.4-75)	76.9 (76.6-77.1)	78.7 (78.4-78.9)	79.9 (79.4-80.4)
North Korea	35.7 (34.4-36.9)	62.2 (59.2-65.3)	64.9 (61.9-67.8)	67.4 (64.7-70.3)	70.2 (67.6-73)	72.5 (70.2-75.1)	73.6 (71.4-75.9)	73.9 (71.8-76.3)	74.3 (72.1-76.5)	65.5 (64-67.2)	64.9 (63.4-66.5)	73.1 (71.5-74.8)	73.8 (72.2-75.5)	75 (72.9-77.2)
Taiwan (Province of China)	61.3 (58.8-63.9)	63.4 (63.3-63.6)	66.7 (66.6-66.8)	70.3 (70.2-70.4)	72.1 (72-72.2)	73.7 (73.6-73.8)	75 (74.9-75.1)	76.3 (76.2-76.4)	77.3 (77.2-77.4)	78.3 (78.2-78.4)	79.8 (79.7-79.9)	81.1 (81-81.1)	82.7 (82.6-82.8)	83.3 (82.6-83.9)
Oceania	47.4 (45.3-49.7)	49.2 (47.2-51.4)	51.3 (49.2-53.5)	53.6 (51.7-55.7)	56.1 (54.2-58)	58 (56.4-59.7)	59.4 (57.6-61.1)	60 (58.2-61.8)	60.7 (58.8-62.6)	61.4 (59.4-63.5)	61.6 (59.6-63.6)	61.2 (59-63.2)	61.6 (59.3-63.6)	63.4 (61.1-65.5)
American Samoa	65.2 (63.9-66.6)	67.1 (66.2-68.1)	68.9 (68-69.7)	69.7 (68.9-70.4)	70.8 (69.9-71.6)	72.4 (71.4-73.3)	73.3 (72.4-74.5)	74.1 (73.4-74.9)	74.9 (74.2-75.8)	74.7 (74-75.4)	74.4 (73.7-75.3)	74 (73.4-74.7)	74 (73.4-74.5)	73.8 (72.9-74.8)
Federated States of Micronesia	52.9 (50.2-55.7)	54.4 (51.7-57.6)	56.1 (53.5-59.1)	58 (55.4-60.9)	59.9 (57.1-62.8)	61.7 (59.1-64.5)	63.4 (60.8-66.1)	64.5 (62.3-67)	65.5 (63.4-67.7)	66.6 (64.6-68.5)	67.4 (65.7-69)	68.8 (67.1-70.5)	69.2 (67.2-71)	69.6 (67.2-71.7)
Fiji	56.9 (55.6-58.3)	61.6 (60.7-62.5)	64.4 (63.5-65.3)	66.5 (65.6-67.4)	66.6 (65.7-67.3)	67.6 (66.8-68.5)	69 (68.1-69.8)	70.1 (69.3-70.9)	70.1 (68.7-71.7)	69.2 (68.6-69.8)	68.2 (67.9-68.5)	69.3 (69-69.6)	69.3 (68.9-69.7)	70.4 (68.4-72.5)
Guam	66.1 (65.1-67.2)	67.4 (66.6-68.1)	69.4 (68.8-70)	71 (70.5-71.5)	71.3 (70.9-71.8)	73.8 (73.3-74.2)	76.6 (76.2-77.1)	77.8 (77.3-78.2)	77 (76.4-77.5)	76.8 (76.2-77.3)	77.5 (77-78)	78 (77.4-78.5)	77.2 (76.7-77.8)	76.4 (75.3-77.5)
Kiribati	51.2 (48.5-54.1)	52.3 (49.5-55.1)	53.8 (51.2-56.5)	55.6 (53.2-58.2)	57.3 (54.9-59.9)	60.1 (57.9-62.6)	61.2 (59-63.5)	61 (59.1-62.7)	61.4 (60-62.6)	62.2 (61.3-63.2)	63.3 (62.2-64.3)	64.2 (62.7-65.7)	65 (63.1-67.1)	66.3 (63.9-68.9)
Marshall Islands	56.5 (53.7-59.2)	58.4 (55.6-61.1)	60.4 (57.8-63.2)	62.2 (59.9-64.8)	63.9 (61.6-66.4)	65.8 (63.6-68.2)	67 (65.3-69.1)	67.4 (66.2-68.6)	66.4 (65.7-67)	65.2 (64.4-65.8)	63.9 (62.9-64.8)	64.1 (62.9-65.2)	65.1 (63.5-66.6)	66.8 (64.5-69)
Northern Mariana Islands	71.9 (69.3-74.5)	72.3 (69.8-75.1)	72.8 (70.6-75.6)	73.2 (71.2-76)	73.8 (71.8-76.3)	74.5 (72.4-77.1)	75 (73-77.6)	75.5 (73.7-77.8)	76 (74.3-78)	77.2 (75.6-78.7)	77.9 (76.5-79.2)	78.7 (77.5-80)	79.4 (78.3-80.2)	79.2 (78-80.2)
Papua New Guinea	44.4 (41.8-47.2)	45.7 (43.2-48.5)	47.4 (44.9-50.2)	49.8 (47.4-52.2)	52.5 (50.2-54.9)	54.6 (52.6-56.9)	55.9 (53.7-58.1)	56.4 (54.2-58.8)	57.3 (55-59.9)	58.4 (55.9-61.1)	58.8 (56.3-61.5)	58.3 (55.7-60.8)	58.9 (56.2-61.5)	61.2 (58.6-63.9)
Samoa	63.5 (61.3-65.8)	66.1 (64.2-67.9)	69 (67.4-70.4)	69.2 (67.9-70.4)	69.8 (68.5-71)	71.1 (69.6-72.5)	73 (71.3-74.6)	73.7 (71.8-75.5)	73.8 (71.8-75.9)	73.9 (71.9-76)	74.2 (72.4-76.3)	74.5 (72.9-76.3)	74.1 (72.8-75.8)	74.5 (72.9-76.7)
Solomon Islands	47.7 (45.1-50.2)	49.4 (46.8-51.9)	51.8 (49.2-54.3)	54.4 (51.8-57)	56.9 (54.3-59.6)	57.5 (55-60)	61.6 (59-64.3)	62.9 (60.5-65.5)	63.5 (61-65.8)	64.6 (62.4-66.6)	65.2 (63.3-67.1)	65.3 (63.6-67.1)	66.1 (64.3-67.8)	67.5 (65.4-69.4)
Tonga	62.5 (60.8-64.4)	63.9 (62.6-65.3)	65.2 (64.2-66.3)	66 (65-67.1)	67 (65.7-68.4)	68.1 (66.7-69.6)	69.3 (68.1-70.7)	71.1 (70-72.2)	72.1 (71.2-73.1)	72.6 (72-73.2)	72.8 (72.3-73.3)	73.7 (73-74.5)	74.3 (73-75.8)	75.1 (73.3-77.2)
Vanuatu	54.4 (51.5-57.2)	55.7 (52.8-58.5)	57.4 (54.7-60.2)	59.1 (56.4-61.8)	60.8 (58.1-63.5)	62.8 (60.1-65.6)	64.6 (61.9-67.2)	65.4 (62.9-68)	65.8 (63.1-68.3)	66.1 (63.4-68.6)	66.4 (63.9-68.9)	66.3 (63.6-68.9)	67 (64.2-69.6)	67.8 (65-70.2)
Southeast Asia	47.9 (46.8-49)	51 (49.9-51.9)	53.9 (52.9-54.7)	56.1 (55.3-56.9)	58 (57.3-58.8)	60.2 (59.5-60.9)	63.7 (63.1-64.3)	65.8 (65.3-66.3)	67.8 (67.4-68.3)	69.5 (69.1-69.9)	70.7 (70.3-71.1)	72.4 (72-72.7)	74 (73.6-74.4)	75.8 (75.2-76.3)
Cambodia	48.9 (46.1-51.9)	50.1 (47.2-53.2)	52.1 (49.2-55.1)	53.9 (51.1-56.9)	49.8 (47.7-52.2)	32.6 (31.7-33.6)	55.4 (53.7-57.4)	57.4 (56-58.9)	59.8 (58.6-61.1)	60.9 (59.8-62)	62.6 (61.5-63.8)	66.4 (65.1-67.6)	70 (68.7-71.3)	72.7 (70.6-74.2)
Indonesia	44.4 (42.7-46)	46.7 (45.3-48)	49.8 (48.4-51.1)	52.4 (51.2-53.6)	55.2 (54.1-56.3)	58.1 (57.1-59.2)	60.8 (59.9-61.6)	63.1 (62.4-63.8)	65.4 (64.8-66)	67.6 (67.1-68.1)	69 (68.6-69.5)	70.5 (70-71)	71.9 (71.3-72.6)	73.9 (73-74.7)
Laos	37.7 (35.1-40.7)	39.2 (36.4-42.2)	40.2 (37.4-42.9)	41.7 (39.1-44.6)	43.9 (41.3-46.7)	47.2 (44.6-50.1)	49.4 (47-52.2)	52.4 (50.1-54.8)	54.3 (52.4-56.5)	56.6 (54.7-58.7)	59.5 (57.8-61.3)	62.9 (61.4-64.6)	66.4 (64.7-68.1)	70.3 (68.3-72.3)
Malaysia	56.7 (54.2-59.2)	59.8 (58.2-61.5)	62.2 (62-62.3)	65.2 (65.1-65.4)	67.1 (66.8-67.4)	69.4 (69.1-69.8)	71.4 (70.7-72)	72.5 (72.4-72.6)	73.7 (73.6-73.8)	74.5 (74.4-74.6)	75.2 (75.2-75.3)	76.3 (76.2-76.3)	76.8 (76.7-77)	77.3 (76.4-78.4)
Maldives	33.7 (31.3-36.4)	37.4 (34.9-40.2)	41.4 (39-44.2)	45.5 (43.2-47.9)	49.5 (47.8-51.2)	52.7 (51.4-54)	56 (55.1-56.9)	60.4 (59.8-61)	64.6 (64.1-65)	68.5 (68-68.9)	73 (72.7-73.4)	76.9 (76.6-77.3)	81.4 (81-81.7)	83.4 (82.6-84.1)
Mauritius	54.6 (54.1-55.1)	57.6 (57.2-58)	59.6 (59.2-60)	64.7 (64.3-65.1)	66.5 (66.1-66.8)	68.4 (68.1-68.8)	71.4 (71-71.7)	72.5 (72.2-72.8)	74.2 (73.9-74.5)	74.9 (74.6-75.2)	75.4 (75.1-75.7)	76.3 (76-76.6)	77.4 (77.1-77.7)	78.1 (77.2-79)
Myanmar	38.4 (35.7-41.2)	40.4 (37.5-43.4)	43 (40.5-46.1)	45.5 (42.7-48.5)	47.9 (45.2-50.7)	51.9 (49.2-54.5)	54.7 (52.2-57.5)	56.8 (54.4-59.6)	58.4 (56.1-60.8)	59.8 (57.8-61.8)	61.6 (59.7-63.5)	64.2 (62.6-66)	68.1 (66.4-69.8)	72.2 (70.3-74.2)
Philippines	56.6 (55.1-58.1)	60.8 (59.3-62.3)	64.1 (62.9-65.2)	65.2 (64.1-66.3)	65.9 (65-66.8)	67.2 (66.3-68)	69.2 (68.4-70.1)	69.4 (68.6-70.2)	71.4 (70.7-72.2)	72.6 (71.9-73.4)	72.9 (72.1-73.6)	72.6 (71.8-73.4)	72.8 (72-73.5)	73.1 (71.2-75)
Sri Lanka	56.6 (56.3-56.8)	61 (60.8-61.3)	65.5 (65.3-65.7)	67.3 (67.1-67.4)	69.3 (69.1-69.4)	68.5 (68.4-68.6)	72.5 (72.4-72.6)	73.5 (73.4-73.6)	74.8 (74.5-75.2)	75.9 (75.8-76.1)	77.4 (77.3-77.5)	78.8 (78.4-79.3)	78.8 (78.6-78.9)	81.1 (79.6-83.3)
Seychelles	63.5 (61.3-65.8)	64.9 (63.1-66.7)	65.7 (64.2-67.3)	66.7 (65.6-67.8)	69 (68.4-69.8)	71.8 (71.3-72.4)	73.7 (73.2-74.2)	74.3 (73.9-74.8)	75.6 (75.1-76.1)	76.8 (76.3-77.3)	76.7 (76.3-77.2)	76.7 (76.3-77.2)	77.1 (76.7-77.6)	77.7 (77-78.4)
Thailand	53.3 (51.8-54.7)	58.3 (57.2-59.4)	60.5 (59.6-61.4)	62.9 (62.1-63.6)	65.5 (64.7-66.2)	67.3 (66.6-68)	68.8 (68.2-69.5)	72.7 (72.1-73.3)	74.3 (73.8-74.8)	74.9 (74.3-75.4)	75.7 (75-76.3)	78.3 (77.7-78.8)	80.2 (79.7-80.7)	82 (80.9-83.1)
Timor-Leste	38.2 (35.6-41)	40 (37.3-43)	42.4 (39.4-45.5)	45.2 (42.4-48.3)	48.1 (45.4-50.9)	47.5 (45.4-49.9)	51.5 (49.3-53.9)	57.1 (54.9-59.2)	60.7 (58.8-62.9)	63.1 (61.5-64.8)	66.1 (64.7-67.4)	68.9 (67.6-70.2)	71.7 (70.3-73.1)	73 (71.3-74.8)
Vietnam	55.6 (52.9-58.3)	59.4 (56.8-62.3)	61.6 (59-64.3)	61.6 (59.2-64)	61.1 (59.1-63.2)	66 (63.9-68.2)	69.4 (67.4-71.4)	71.1 (69.3-72.9)	72.7 (71.4-74.3)	74.3 (73.1-75.8)	75.7 (74.5-77.3)	77.1 (75.6-78.6)	78.3 (76.9-79.8)	79.2 (77.8-80.9)
Sub-Saharan Africa	44.2 (42.4-45.9)	45.7 (44.1-47.2)	47.1 (45.8-48.3)	48.9 (47.8-50)	50.4 (49.4-51.3)	52.3 (51.5-53)	53.9 (53.3-54.6)	55.1 (54.5-55.7)	55.7 (55-56.3)	55.2 (54.6-55.8)	54.7 (54.1-55.3)	56.5 (55.9-57.1)	60.4 (59.7-61.1)	66.2 (65.4-67)
Central sub-Saharan Africa	44.3 (42-46.8)	45.5 (43.3-47.9)	46.9 (44.5-49.2)	48.1 (45.7-50.4)	50 (47.8-52.2)	51.6 (49.5-53.5)	52.8 (51.1-54.7)	54.4 (53-56.1)	54.6 (53.4-56.1)	54.1 (52.9-55.5)	54.6 (53.5-55.9)	56.7 (55.5-58)	59.5 (58.3-60.8)	64.4 (62.7-66)
Angola	44.9 (42.2-48)	45.1 (42.4-48.3)	46.1 (43.2-49.4)	46.7 (43.9-49.8)	48.3 (45.5-51.3)	49.3 (46.6-52.1)	49.4 (46.9-52.2)	49.7 (47.4-52.3)	50.6 (48.5-52.9)	51.9 (49.8-54.4)	54.1 (52.2-56.2)	57.7 (55.9-59.6)	62.2 (60.4-64.1)	66.7 (64.5-68.9)
Central African Republic	43.6 (40.6-46.7)	44.4 (41.5-47.6)	46.1 (43.5-49.3)	48.1 (45.6-50.8)	49.4 (47.3-51.8)	50.2 (48.1-52.4)	51.2 (49.4-53.1)	52 (50.5-53.5)	50.1 (48.4-51.8)	47.6 (45.3-49.9)	45.8 (43.1-48.4)	46.4 (43.7-49.2)	49.5 (46.8-52.5)	54.9 (52-58)
Congo (Brazzaville)	40.9 (38.1-43.8)	41.6 (39-44.8)	43.4 (40.7-46.7)	45.7 (43.1-48.6)	48 (45.6-50.7)	51.3 (49.1-53.6)	54.9 (52.9-56.9)	58.4 (56.5-60.2)	56.2 (54.2-58.1)	53.5 (51.7-55.4)	53.3 (51.5-55.2)	56.1 (54.3-58.1)	59.4 (57.4-61.7)	62.7 (60.2-65.6)
DR Congo	44.6 (41.7-47.7)	46.1 (43.2-49.2)	47.5 (44.4-50.6)	48.6 (45.4-51.6)	50.6 (47.8-53.5)	52.2 (49.5-54.8)	53.6 (51.3-56.1)	55.5 (53.4-57.8)	56 (54.1-58)	55.3 (53.6-57.2)	55.6 (54-57.3)	57.3 (55.7-59)	59.4 (57.7-61.2)	64.3 (62-66.7)
Equatorial Guinea	38.4 (35.7-41.2)	38.9 (36.3-41.6)	40.1 (37.3-43)	42.7 (39.8-45.6)	45.3 (42.4-48.2)	47.5 (44.9-50.2)	50.6 (47.9-53.3)	51.8 (49.3-54.4)	50.8 (48.3-53.5)	50.9 (48.2-53.7)	57.1 (54.1-60.5)	60.8 (57.3-64.8)	62.1 (58.2-66.7)	66.4 (62.6-70.5)
Gabon	49.2 (46.3-52)	49.7 (47-52.7)	51.3 (48.6-54.3)	53.6 (50.7-56.5)	55.8 (53.2-58.5)	58.8 (56.6-61.2)	61.5 (59.7-63.4)	63.5 (61.9-65.2)	64 (62.3-65.7)	64 (62.1-65.8)	61.4 (58.7-63.9)	60.8 (58-63.4)	64.7 (62.4-67.1)	72.1 (69.8-74.4)
Eastern sub-Saharan Africa	43.1 (41.4-44.9)	44.5 (43-46.1)	46 (44.6-47.3)	47.4 (46.3-48.5)	49 (48.1-49.9)	50.4 (49.7-51.2)	51.8 (51.1-52.5)	52.4 (51.9-53)	52.8 (52.3-53.4)	52.4 (51.8-53)	53.1 (52.5-53.7)	56.6 (56.1-57.3)	61.5 (60.9-62.1)	67.4 (66.8-68.1)
Burundi	39.4 (36.9-42.1)	40.7 (38.1-43.6)	42.5 (40-45)	43.8 (41.8-45.9)	45.1 (43.3-47)	46.4 (44.6-48.2)	47.9 (46.2-49.9)	49.6 (47.7-51.6)	50.5 (48.7-52.4)	47 (45-49.1)	47.1 (45-49.1)	51.9 (50.1-53.7)	59.3 (57.2-61.3)	63.6 (61.3-65.9)
Comoros	46.8 (44.4-49.5)	47.7 (45.3-50.3)	48.9 (46.6-51.5)	50.9 (48.6-53.4)	52.8 (50.6-55.2)	54.2 (52-56.5)	55.5 (53.3-57.9)	57.5 (55.4-59.8)	59.4 (57.5-61.6)	61 (59.3-63)	62.6 (60.9-64.3)	65.5 (63.9-67.2)	67.6 (65.9-69.5)	70 (67.8-72.3)
Djibouti	55.5 (52.5-58.3)	56.2 (53.3-59.3)	57 (54.1-60)	57.9 (55.1-60.9)	59 (56.3-62)	60.6 (57.9-63.7)	61.3 (58.6-64.2)	61.1 (58.5-63.8)	61.4 (58.8-64.1)	61.7 (59-64.6)	61.8 (58.9-64.9)	62.7 (59.4-66.2)	65.1 (61.6-68.7)	68.9 (65.3-72)
Eritrea	41.5 (39.1-44.1)	42.6 (39.9-45.3)	43.9 (41.4-46.6)	42.5 (40.4-44.8)	44.5 (42.4-46.6)	45.1 (43.3-46.8)	46.9 (45.3-48.3)	48.5 (47.1-49.9)	43.6 (42.4-44.7)	57.6 (55.6-59.6)	58.8 (56.5-61.2)	60.5 (58.1-63.2)	62.6 (60.1-65.5)	65.9 (63.4-69)
Ethiopia	39.8 (37.7-41.9)	41 (38.8-43)	41.8 (39.7-44)	42.9 (41-44.9)	44.1 (42.3-45.9)	44.8 (43.3-46.2)	46 (44.6-47.4)	47.5 (46.3-48.7)	48.8 (47.8-49.9)	50.1 (49-51.3)	51.8 (50.7-52.9)	57.5 (56.5-58.5)	64.3 (63.3-65.2)	70.4 (69.3-71.5)
Kenya	49.3 (47.6-51)	51.5 (50.4-52.7)	54.6 (53.5-55.7)	56.5 (55.6-57.6)	58.1 (57.2-59)	60.1 (59.3-60.9)	62.2 (61.4-63)	63.5 (62.8-64.2)	63.2 (62.4-63.9)	59.6 (58.7-60.4)	57.1 (56.2-58)	58.4 (57.7-59.2)	63.2 (62.6-63.9)	68.8 (67.9-69.5)
Baringo	58.7 (54.9-63.2)	57.9 (54-62.3)	58.1 (54.2-62.5)	57.1 (53.3-61.2)	55.1 (51.7-59.3)	54.1 (50.7-58.2)	54.6 (51-58.5)	55.6 (52.5-59.2)	56.7 (53.8-60.2)	56.8 (53.7-60.3)	58.3 (55.4-61.7)	61 (58.3-63.8)	64.8 (61.9-67.9)	68.7 (65.4-72.1)
Bomet	70.7 (66.9-74.2)	69.6 (65.9-73.3)	71.1 (67.2-74.8)	71.3 (67.2-74.9)	71.4 (67.5-74.8)	71.6 (68-74.9)	71.9 (68.6-75.2)	73 (69.8-76.1)	73.2 (69.9-76.4)	70 (65.8-74.4)	64.8 (60-69.5)	63.8 (59.2-68.5)	65.7 (61.6-70.1)	71.3 (67.2-74.9)
Bungoma	48.3 (44.6-52.4)	50.5 (46.8-54.7)	54 (50-58.3)	56.2 (52.4-60.4)	58 (54.1-62.2)	59.7 (56-63.7)	61.6 (58-65.3)	62.8 (59.2-66.4)	63.6 (60.2-67)	63.1 (59.5-66.5)	62.9 (59.2-66.4)	63.8 (60.6-67.3)	67.6 (64.7-70.8)	70.8 (67.5-74.2)
Busia	43.5 (40-47.6)	45.2 (41.5-49.1)	48.9 (45.5-52.9)	51.5 (47.8-55.2)	53.9 (50.3-57.4)	57.9 (54.5-61.6)	60.7 (57.5-64.4)	62.4 (59.4-65.8)	60.8 (57.7-64.6)	52.6 (47.4-58.4)	49.4 (44.5-55)	54.4 (50.2-59.1)	63.4 (60.1-66.8)	69.1 (66-72)
Elgeyo Marakwet	56.9 (52.9-61.2)	57.4 (53.5-61.7)	58.5 (54.4-62.6)	58.9 (54.8-63)	58.7 (55-62.9)	59.1 (55.2-63.2)	60.5 (56.7-64.7)	61.6 (58.1-65.5)	62.5 (58.9-66.2)	61.9 (58.2-65.8)	61.4 (57.9-65)	61.9 (59-65.2)	64.8 (62.1-67.9)	68.2 (65-71.4)
Embu	46.4 (42.7-50.8)	48.6 (44.6-53.1)	53 (49.3-57.2)	56.3 (52.5-60.5)	60 (56.4-64.3)	64.1 (60.3-68.1)	67.1 (63.5-70.8)	68.5 (65.1-71.9)	67.5 (63.2-72.3)	63.5 (57.4-70)	63 (57.9-68.6)	65.4 (61.2-70)	69 (64.9-72.6)	73.4 (69.5-76.2)
Garissa	46.1 (42.7-49.9)	47.1 (43.7-50.9)	51.2 (47.5-55.3)	52.4 (49.1-56.4)	53.8 (50.2-58)	55.5 (52.3-59.2)	57.1 (53.8-60.4)	57.8 (54.7-61)	58.9 (56.1-61.9)	59.4 (56.5-62.5)	60.3 (57.3-63.1)	62.1 (59.3-65.1)	63.5 (60.5-66.6)	63.8 (53.1-69)
Homa Bay	38.3 (35.4-41.7)	40.7 (37.4-44.6)	43.9 (40.5-47.5)	46 (42.7-50)	46.6 (43.6-50.1)	48.5 (45.3-51.8)	52.2 (48.8-55.6)	54.5 (51.3-58)	54.2 (50.9-57.5)	49.3 (44.9-53.6)	42.5 (37.6-47.7)	41.2 (36.6-46)	48.1 (43.7-52.3)	62 (57.6-66.6)
Isiolo	42 (38.2-45.9)	42 (38.3-46)	43.8 (40.1-48.5)	44 (40.6-48)	43.6 (39.9-47.9)	45 (41.5-48.7)	47.8 (44.1-52.1)	49.9 (46.2-53.9)	53 (49.8-56.5)	55.8 (52.5-59.6)	56.8 (53.3-60.7)	59.6 (55.9-63.6)	62.9 (59.2-67.2)	67.4 (63.6-71.4)
Kajiado	58.7 (54.9-63.1)	59 (55.4-63.3)	61.5 (57.4-65.9)	62.1 (58.1-66.4)	62.6 (58.8-66.7)	63.3 (59.5-67.4)	65.1 (61.3-69.2)	66.5 (63.1-70.4)	65.6 (61.4-70.1)	60.5 (55.4-65.9)	57.8 (53.3-62.7)	59.4 (55.8-63.5)	64.6 (61.2-68.6)	69.4 (66.1-73.6)
Kakamega	45.4 (41.6-49.5)	47 (43.6-50.9)	51.2 (47.5-55.2)	53.8 (50.3-57.6)	55.9 (52.3-59.5)	58.5 (54.8-62)	60.9 (57.6-64.4)	61.8 (58.7-65.2)	60.7 (57.8-64.1)	55.3 (51.4-59.7)	53.2 (49.3-57.5)	56.1 (52.6-60.2)	61.1 (58-65)	66 (62.6-70)
Kericho	62.6 (58.8-66.9)	63.3 (59.6-67.5)	65 (61.2-69)	66 (62.1-69.8)	66.7 (63-70.3)	68 (64.5-71.5)	68.9 (65.6-72.3)	69.8 (66.8-72.9)	70.7 (67.8-73.7)	69.1 (65.8-72.5)	66.7 (62.8-70.5)	66.3 (62.5-70.2)	69.3 (65.5-73.4)	74.4 (70.9-77.3)
Kiambu	56 (52.6-59.8)	57.8 (54.4-61.7)	60.4 (56.7-64.6)	62.2 (58.3-66.3)	64.4 (60.6-68.6)	67.2 (63.4-71.2)	68.1 (64.4-71.7)	68.8 (65.4-72.1)	68 (64.5-71.4)	61.9 (57.2-66.9)	58.4 (53.9-63.4)	61.7 (58.1-65.9)	67.5 (64.4-71.1)	71.7 (68.3-75.1)
Kilifi	38.1 (34.7-41.9)	41.1 (38.1-44.7)	44.3 (40.9-48.2)	46.4 (42.7-50.1)	48.6 (45.3-52.5)	51.6 (48.2-55.5)	54.3 (51.1-58.1)	56.2 (53.2-59.9)	57.6 (54.7-60.8)	57.5 (54.5-60.5)	57 (53.7-60.4)	58.2 (55.4-61.6)	62.7 (59.6-65.7)	68.9 (65.6-72)
Kirinyaga	49.3 (45.6-53.5)	51.1 (47.6-55.1)	54.5 (50.7-58.6)	57.1 (53.3-61.1)	58.8 (55.1-62.8)	62.4 (58.5-66.5)	67.5 (63.6-71.5)	69.5 (65.7-73.4)	69.4 (65.8-73)	67.7 (63.8-72)	66.3 (62.2-71)	66.4 (62.3-70.7)	69 (65.4-72.6)	72.1 (68.4-75.2)
Kisii	46.3 (42.1-50.8)	48.8 (45.1-53)	51.8 (48.1-56.1)	53.4 (49.7-57.7)	54.7 (50.9-59.1)	56.3 (52.8-60.4)	57.5 (53.7-61.4)	58.2 (54.9-61.9)	58.1 (55.2-61.4)	55.4 (52.4-58.8)	53.3 (50.4-56.7)	54.7 (52.1-57.6)	60.3 (57.9-62.9)	65.8 (63-69.1)
Kisumu	45.5 (40.5-50.8)	48.1 (44.1-52.9)	51.7 (48.1-55.5)	54.3 (51-58.2)	55.9 (52.5-59.5)	58.3 (54.6-62.2)	61 (57.5-64.6)	63 (59.7-66.4)	60.9 (56.8-65.3)	50.8 (45-57.6)	46.1 (40.9-52.7)	48.7 (44.4-53.8)	56.7 (52.8-61.4)	66.4 (61.6-71)
Kitui	50.1 (46.4-54.5)	51.1 (47.5-55.3)	53.9 (50.3-58)	55.7 (52-59.7)	57.5 (53.9-61.8)	59.7 (56-63.8)	62.1 (58.4-66.1)	63.3 (60-66.8)	63.7 (60.5-67.3)	61.9 (58.6-65.6)	61.1 (57.7-65)	64 (60.4-67.7)	69 (65.5-72.6)	73.8 (70.8-76.7)
Kwale	41.1 (37.5-44.9)	44 (40.3-48.3)	47.4 (43.9-51.4)	49.3 (45.9-53.3)	50.8 (47.3-54.5)	52.7 (49.1-56.7)	55.5 (52-59.3)	57.8 (54.4-61.5)	56.1 (51.7-60.7)	51 (45.7-56.7)	51.7 (47.4-56.7)	56.5 (52.9-60.6)	62.7 (59.4-66.2)	69.1 (65.8-72.6)
Laikipia	58 (54.3-62.6)	59.2 (55.7-63.4)	62.4 (58.6-66.7)	64.3 (60.6-68.1)	65.7 (62.2-69.2)	67.5 (64-71.1)	69.5 (66.2-72.9)	70.8 (68-73.9)	70.4 (67.6-73.5)	67.9 (64.4-71.7)	64.7 (60.2-69.3)	63.5 (59.1-68.2)	65.8 (62-70.2)	70.4 (66.1-74.5)
Lamu	37.1 (33.8-41)	40.1 (36.7-44.1)	43.2 (39.6-47.4)	45.6 (42.2-49.7)	49.5 (46-53.5)	55.3 (51.7-59.4)	60.2 (56.6-64.1)	61.7 (58.2-66.1)	61.6 (58.2-65.7)	60.9 (57.4-64.9)	59.9 (56.3-64.2)	61 (57.1-65.2)	64.3 (60.4-68.6)	67.8 (64.4-71.5)
Machakos	53.2 (49-57.4)	54.8 (50.6-59.1)	57.9 (53.9-62.2)	59.9 (56.2-64.2)	61.7 (58.1-65.7)	64 (60.4-68)	66.4 (63-69.9)	67.8 (64.5-71.2)	68.5 (65.4-71.9)	67.5 (63.7-71.2)	65.6 (61.8-69.7)	65 (61.5-69)	68.7 (65.2-72)	73 (69.5-75.8)
Makueni	61.7 (58.1-65.5)	61.4 (57.6-65.3)	62.6 (58.8-66.4)	62.9 (58.9-66.9)	63.5 (59.5-67.4)	64.4 (60.8-68.1)	65.2 (61.7-68.8)	66 (62.5-69.6)	66.5 (63.4-70)	64.2 (59.8-68.2)	59.9 (54.7-64.9)	58.8 (54.4-64)	62.8 (58.7-67.2)	71.9 (68.5-75)
Mandera	46.5 (43.2-50.3)	46.9 (43.7-50.7)	51.7 (48-55.7)	53.1 (49.6-57.1)	54.7 (51.5-58.7)	56.7 (53.4-60.3)	58.6 (55.3-62.1)	59.5 (56.2-62.7)	59.7 (56.7-62.8)	59.7 (56.8-62.7)	60.9 (58.1-63.9)	61 (58.4-64)	61.3 (58.8-64.1)	63.4 (60.4-66.7)
Marsabit	32.5 (29.2-36.3)	34 (30.2-37.7)	38.8 (35-42.9)	42.5 (38.8-46.4)	47 (43.2-51.5)	52.8 (49-56.9)	57.8 (53.9-61.7)	59.8 (56.3-63.5)	61.7 (58.5-65.2)	62.5 (59.2-66.2)	62.5 (59-66.5)	62.3 (58.7-66.2)	64.3 (60.8-68.2)	67.4 (63.1-71.9)
Meru	48.8 (45-52.9)	51.6 (47.8-56)	55.6 (52-59.5)	58.2 (54.6-62.2)	60.4 (56.7-64.3)	62.7 (59.2-66.6)	65.2 (61.9-68.9)	66.8 (63.7-70.1)	66.9 (63.8-70.1)	64.4 (60.2-68.5)	62.1 (57.7-66.5)	62.4 (58.8-66.3)	64.8 (61.5-68.6)	68.3 (64.6-72.4)
Migori	43.9 (40.6-47.6)	45.4 (41.9-49.6)	47.2 (43.6-51.4)	48 (44.2-52.3)	49 (45.6-52.7)	48.9 (45.8-52.4)	49.2 (46.3-52.5)	50.1 (47-53.3)	51.2 (48-54.4)	48.9 (44.9-53)	44.8 (40.4-49.4)	44.5 (40-48.9)	50.5 (46.2-54.9)	61 (56.5-66.3)
Mombasa	47 (43.6-51.2)	50.3 (46.8-54.4)	54.4 (50.7-58.5)	56.9 (53.1-61)	59.3 (55.5-63.6)	62.2 (58.3-66.4)	64.6 (60.9-68.8)	65.7 (62.1-69.8)	63.5 (59.5-68.4)	56.5 (51.7-63.2)	55 (50.4-60.4)	58.6 (54.5-63.6)	63.8 (59.6-67.9)	71.5 (67.5-75)
Murang’a	57.6 (54-61.5)	59.5 (55.9-63.3)	61.6 (57.8-65.5)	63.1 (59.2-66.9)	64.6 (61-68.5)	66.9 (63.4-70.6)	69.2 (65.9-72.7)	70.4 (66.9-73.5)	70.5 (67-73.6)	68.4 (64.5-72.3)	66.6 (62.7-71)	65.6 (62.2-69.4)	68.5 (65.2-71.9)	71.4 (67.8-74.6)
Nairobi	44.2 (40.7-48)	47.9 (44.8-51.5)	54 (50.8-57.6)	57.9 (54.6-61.9)	60.9 (57.5-64.7)	64 (60.7-67.4)	66.4 (63.3-69.9)	67.4 (64.2-70.5)	64.9 (61.5-68.5)	57.4 (53.5-61.9)	54.9 (51.4-59)	57.2 (54.6-60.5)	63.4 (61.4-65.9)	69.7 (67.5-72.3)
Nakuru	64.1 (60.3-68.2)	64.2 (60.4-68.3)	66.9 (62.8-71.1)	68 (64.1-72.1)	68.6 (64.8-72.6)	69.3 (65.8-73.2)	70.9 (67.7-74.6)	72.5 (69.6-75.8)	71.3 (67.5-75.1)	62.6 (56.4-68.6)	57.6 (51.7-64.1)	60.9 (56.4-65.6)	67.4 (63.6-70.6)	74 (71-76.3)
Nandi	67.6 (63.7-71.7)	67.6 (63.7-71.6)	69.1 (65.4-73.3)	69.4 (65.6-73.3)	69.2 (65.7-73.2)	69.4 (65.8-73.2)	69.9 (66.7-73.7)	70.6 (67.6-74.1)	69.5 (65.5-73.3)	65.1 (59.7-70.2)	64.4 (60.2-68.9)	67 (63.8-70.5)	70.9 (68-74.4)	74.6 (71.6-77.4)
Narok	61.5 (57-66)	60.4 (56.2-64.9)	62.3 (58.2-66.9)	62.1 (58.1-66.6)	61.4 (57.5-65.8)	60.8 (57-64.9)	60.5 (56.9-64.5)	60.8 (57.3-64.5)	61.2 (57.8-64.9)	60.1 (56.6-63.8)	59.7 (56.2-63.2)	60.1 (56.6-63.3)	64.7 (61.7-67.6)	68.5 (65.7-71.9)
Nyamira	56.1 (52.1-60.5)	56.9 (52.6-61.2)	58.2 (54.2-62.8)	59 (55.2-63.4)	60.2 (56.5-64.2)	61 (57.2-65.3)	61.8 (58-65.8)	62.7 (59.1-66.2)	62.6 (59.4-66.1)	59.8 (56.5-63.2)	56.4 (52.5-60.5)	54.8 (51-59.1)	57.1 (53.4-60.7)	63.7 (60.3-67.2)
Nyandarua	55.8 (51.8-60.2)	57 (53.3-61.3)	59.8 (55.7-63.8)	61.3 (57.2-65.6)	62.6 (58.6-66.9)	64.2 (60.3-68.5)	66.2 (62.4-70.4)	66.9 (63.5-70.9)	66.9 (63.7-70.4)	65.3 (62.2-68.7)	62.3 (59.1-65.7)	60.2 (57-63.7)	62.2 (59.1-65.7)	66.5 (62.9-70.5)
Nyeri	47.7 (44.3-51.6)	51.1 (47.3-55.1)	54.6 (50.6-58.8)	57.2 (53-61.2)	59.3 (55.2-63.5)	61.6 (57.7-66)	64 (60.5-68.2)	65.2 (62-69)	64.7 (61.5-68.1)	60.5 (57-64)	55.9 (52.1-59.9)	56.5 (53-60.1)	61.4 (58.4-64.8)	65.2 (61.7-69.3)
Samburu	67.3 (63.7-71.5)	66.8 (63.2-70.7)	67.6 (63.9-71.6)	67.5 (63.8-71.1)	66.8 (63.2-70.3)	66.3 (62.8-69.8)	66.1 (62.8-69.6)	66 (63-69.4)	65.4 (62.2-68.7)	63 (59-67.2)	60.4 (55.6-65.2)	61 (56.4-65.4)	63.7 (60-67.9)	67.7 (63.9-72.1)
Siaya	38.5 (35.4-42)	41.3 (37.8-45.2)	45.1 (41.5-48.9)	47.8 (44.3-52.1)	50.1 (46.5-54.4)	52.5 (48.8-56.6)	55 (51.2-59.1)	55.9 (52.5-59.4)	55 (51.8-58.6)	52.6 (48.7-56.5)	49.5 (44.2-55)	48 (43.3-53.1)	55.5 (51.1-60.3)	66.6 (62.3-70.5)
Taita Taveta	31.7 (28.1-35.6)	37.3 (33.8-41.4)	42.6 (39.2-46.9)	46.8 (43.2-51.2)	50.3 (46.7-54.7)	54.6 (50.7-58.7)	58.8 (55-62.8)	62 (58.2-65.7)	63.4 (59.9-66.9)	62.1 (58.7-65.7)	59.1 (55.6-63.2)	57.4 (53.8-61.4)	59.6 (56.1-63.5)	65.9 (62.4-70.2)
Tana River	40.6 (37-44.7)	44.6 (40.9-48.8)	48.4 (44.5-52.7)	50.8 (47-54.9)	53.4 (49.5-57.4)	55.8 (51.9-60.1)	58.3 (54.5-62.9)	59.5 (55.9-63.6)	60 (56.3-64.4)	58 (54.2-62.1)	55.5 (51.8-59.4)	55.4 (52-58.8)	57.2 (54.1-60.5)	63 (59.7-67)
Tharaka Nithi	50.1 (45.9-54.4)	50.6 (46.5-55)	52.1 (48-56.5)	53.2 (49-57.5)	54.5 (50.3-58.7)	56.5 (52.4-60.7)	58.2 (54.2-62.2)	58.9 (55.2-62.6)	59.3 (55.9-62.7)	58.7 (55.5-62.1)	58.8 (55.8-62.2)	61.5 (58.7-64.7)	66.3 (63.3-69.2)	69.6 (66.3-72.8)
Trans Nzoia	65.7 (62-70.1)	64.5 (61-68.4)	66.9 (63.3-70.7)	67.2 (63.6-70.8)	67.1 (63.7-70.6)	67.5 (64.1-70.9)	68.4 (65.3-71.7)	69.3 (66.4-72.4)	68.8 (65.6-72.4)	63.9 (59.2-69.1)	60.4 (55.6-65.7)	62.3 (57.6-67.2)	66.4 (62.1-70.2)	71.6 (67.6-74.6)

Table 7 Estimates of life expectancy at birth by location and year for 1950, 1955...2005, 2010, 2017 - females

Location	1950	1955	1960	1965	1970	1975	1980	1985	1990	1995	2000	2005	2010	2017
Turkana	61.5 (57.7-65.9)	60.7 (57.1-64.6)	62.4 (58.6-66.3)	62.4 (58.5-66.2)	60.9 (57.1-64.7)	59.5 (56-63.2)	59.3 (55.9-62.9)	60.2 (57.1-63.6)	60.5 (57.5-63.8)	58.6 (54.7-63)	55.7 (51-60.7)	56 (51.6-61.1)	59.2 (54.9-64)	65.6 (61.3-70.5)
Uasin Gishu	63.6 (59.8-68.2)	63.6 (59.6-67.9)	65.3 (61.3-70)	65.6 (61.7-70.3)	65.6 (61.9-70)	65.6 (61.8-69.7)	66 (62.4-69.8)	66.3 (62.8-70)	66.1 (62.9-69.6)	64.9 (61.5-68.7)	62.9 (59.4-66.5)	64.1 (60.7-67.8)	67 (63.6-70.9)	72.3 (68.7-76.3)
Vihiga	55 (51.4-59.4)	55.8 (52.2-60.2)	57.5 (53.6-62.2)	58.5 (54.4-62.9)	59.7 (55.8-63.8)	60.1 (56.2-64.3)	61.1 (57.4-65.1)	61.4 (57.8-65)	60.5 (56.6-64.3)	55.7 (51.2-60.1)	52.5 (48.5-57)	54.5 (50.9-58.5)	61.1 (57.7-64.6)	65.3 (61.9-69)
Wajir	56.1 (52.6-60)	55.6 (52.2-59.5)	58.3 (54.4-62.5)	58.4 (55-62.4)	57.7 (54.3-61.5)	57.6 (54.3-61.4)	57.7 (54.6-61.2)	57.5 (54.4-60.9)	57.4 (54.6-60.5)	57.1 (54.5-60)	57.7 (55.3-60.2)	59.9 (57.4-62.5)	61.9 (59.4-64.6)	62.1 (58.5-66.5)
West Pokot	56.1 (52.3-60.6)	55.5 (51.6-60.2)	57 (53.1-61.7)	57 (53.2-61.4)	56.2 (52.3-60.5)	55.9 (52.4-60)	57 (53.2-61)	58.1 (54.7-62)	58.6 (55.3-62.5)	58.7 (55.3-62.4)	60.9 (57.5-64.7)	63.3 (60.3-66.9)	65 (61.8-68.6)	69 (65.4-72.7)
Madagascar	47.6 (45.2-49.9)	48.8 (46.7-50.6)	50 (48.4-51.5)	51.5 (50.1-53)	52.7 (51.5-53.8)	54.5 (53.3-55.7)	55.9 (54.6-57.3)	55.5 (54.2-56.8)	57 (55.8-58.2)	58.4 (57.3-59.6)	60.2 (59-61.5)	61.9 (60.4-63.5)	62.9 (60.8-64.9)	64.8 (62.3-67.5)
Malawi	36.2 (33.6-38.8)	37.8 (35.1-40.6)	39.7 (37.4-42.3)	41.7 (39.4-44)	44.3 (42.3-46.3)	47.7 (46.1-49.4)	50.6 (49.4-51.9)	51.5 (50.3-52.8)	50.5 (48.9-52)	47.2 (44.3-49.6)	45.6 (42.7-48.8)	48.7 (45.8-51.7)	56.7 (54.3-58.9)	66.9 (64.9-69)
Mozambique	43.1 (40.3-45.9)	44.1 (41.4-47.2)	45.2 (42.4-48.3)	46.3 (43.3-49.4)	48.1 (45.5-50.7)	50.1 (47.8-52.5)	51.2 (49.3-53.2)	47.7 (46.4-49.1)	52.6 (51.3-54.1)	53.1 (51.4-54.6)	52.9 (50.8-55)	52.8 (50.3-55.4)	54.4 (52-56.9)	62 (59.4-64.5)
Rwanda	39.6 (36.9-42.5)	41.6 (38.9-44.3)	44.2 (41.9-46.8)	45.5 (43.3-47.9)	46.3 (44.2-48.6)	47.2 (45.4-49.5)	49.6 (47.8-51.5)	52.2 (50.7-53.8)	51.4 (50.1-52.7)	49 (47.6-50.4)	51.5 (50-53)	58.7 (57.2-60.1)	66.3 (65-68)	70.8 (69.1-72.7)
Somalia	48.6 (45.8-51.5)	49.4 (46.6-52.2)	50.2 (47.4-53)	50.8 (48.1-53.6)	51.2 (48.5-54)	50.4 (47.9-53.2)	52.6 (50-55.3)	52 (49.5-54.6)	52.4 (49.9-55)	53.2 (50.6-55.8)	54.5 (51.9-57.3)	56.7 (54-59.6)	57.1 (54.3-60)	60.6 (57.7-63.3)
South Sudan	48 (45-51.4)	48.6 (45.6-51.9)	49.1 (46.1-52.4)	49.8 (46.6-53)	50.7 (47.5-54)	51.8 (48.6-55.2)	52.5 (49.4-55.6)	52.7 (49.9-55.5)	53.5 (50.8-56.3)	55.2 (52.5-58)	56.7 (53.8-59.9)	58.8 (55.5-62.4)	60.1 (56.8-63.7)	61.8 (58.6-65.1)
Tanzania	45.1 (42.4-47.9)	46.4 (43.9-49.1)	47.6 (45.3-50)	49.3 (47.3-51.3)	51.8 (50.2-53.7)	54.5 (52.9-56.2)	56.7 (55-58.3)	57.4 (56-58.7)	56.5 (54.9-58)	54.2 (52.1-56.2)	54.7 (52.4-56.8)	57.7 (55.6-60.1)	62.2 (60.3-64)	68.9 (67.2-70.6)
Uganda	48.3 (45.5-51.1)	48.9 (46.2-51.8)	49.9 (47.3-52.5)	51.5 (49.1-54)	53 (50.8-55.3)	53.8 (51.8-55.8)	52.3 (50.8-53.8)	52.7 (51-54.4)	51.1 (48.8-53.4)	49.9 (47.4-52.2)	51.7 (49.5-53.7)	56.2 (54.3-58.2)	61.7 (59.8-63.8)	69.2 (67.2-71.1)
Zambia	46.9 (44.1-49.7)	48.4 (45.6-51.2)	49.7 (47.3-52.6)	51.6 (49.2-54.2)	54.4 (52.1-56.8)	55.9 (54-58)	55.9 (54.4-57.8)	55.2 (53.9-56.5)	52.1 (50.2-53.7)	47.6 (45-50.1)	47.7 (44.9-50.3)	51.5 (48.7-54.2)	58.5 (56.4-60.6)	66.3 (64.5-68.3)
Southern sub-Saharan Africa	54.4 (52.7-56)	55.4 (53.9-56.9)	56.5 (55.1-57.9)	57.8 (56.4-59.1)	59.4 (58.3-60.5)	61.4 (60.4-62.3)	62.8 (62-63.6)	65.8 (65.1-66.5)	67.9 (67.1-68.6)	65.7 (64.4-66.9)	56.2 (54.9-57.4)	51.7 (50.4-53)	56.1 (55-57.2)	68.5 (67.6-69.3)
Botswana	55.9 (53.2-58.7)	56.7 (54.1-59.5)	57.6 (54.9-60.2)	58.8 (56.3-61.1)	60.6 (58.4-63)	63.8 (61.7-66)	66.3 (64.4-68.2)	68.9 (67.2-70.7)	68.8 (66.3-71.3)	65.2 (60.9-69.5)	57.7 (51.8-63.8)	54.3 (49.8-59.2)	61.3 (57.9-64.1)	71 (68.8-72.5)
Lesotho	54 (51.7-56.6)	54.9 (52.6-57.4)	55.7 (53.4-58.2)	56.2 (53.8-58.7)	57.4 (55-59.9)	59.3 (57.1-61.7)	62.3 (60.2-64.6)	64.3 (62.3-66.4)	65.2 (63.5-67)	61.6 (58.9-64.2)	52.2 (48.7-55.6)	46.7 (43.5-50.2)	50.6 (47.5-53.7)	59.3 (56.3-62.7)
Namibia	55.7 (53-58.7)	56.3 (53.7-59.3)	57 (54.4-60)	58.5 (56.1-61.3)	59 (57.1-61.3)	60.3 (58.6-62.3)	60.8 (59.4-62.4)	63 (61.7-64.3)	65.4 (63.9-66.7)	63.2 (61.2-65.2)	57 (54.1-59.7)	54.6 (51-58)	62.1 (58.7-65.1)	70.7 (67.5-73.5)
South Africa	54.4 (52.5-56.3)	55.4 (53.6-57.2)	56.5 (54.8-58.2)	57.8 (56.2-59.4)	59.2 (57.8-60.6)	61.2 (60-62.4)	62.5 (61.5-63.5)	65.5 (64.7-66.4)	68.7 (68.1-69.4)	68.7 (68.2-69.3)	58.3 (57-59.4)	53.1 (51.6-54.6)	57.3 (56-58.5)	69.7 (68.6-70.6)
Swaziland	44 (41.2-47)	46.1 (43.4-49.3)	48 (45.1-51.2)	51.7 (48.8-54.8)	55.2 (52.5-58.1)	58.4 (56-61)	61.2 (59.1-63.6)	64 (62.2-65.9)	66.5 (64.9-68.2)	63.7 (61.2-65.9)	51.1 (47-55.2)	45.2 (41.7-49.4)	48.1 (44.4-52)	65.1 (62.1-68.4)
Zimbabwe	55.9 (53.2-58.6)	56.7 (53.9-59.4)	57.7 (55.1-60.4)	58.9 (56.5-61.2)	60.6 (58.3-62.8)	62.3 (60.5-64.1)	63.7 (62.2-65.1)	66.7 (65.5-67.9)	64.7 (61.2-67.3)	55.5 (50.5-60.3)	48.4 (44.6-52.7)	46.3 (43-49.7)	51.2 (48.5-53.9)	64.4 (62.1-66.6)
Western sub-Saharan Africa	43.2 (41.1-45.2)	44.8 (42.8-46.8)	46.2 (44.4-47.7)	48.4 (47-49.9)	49.8 (48.4-51.1)	52.2 (50.9-53.4)	54.3 (53.1-55.4)	55.7 (54.4-56.8)	56.2 (55-57.4)	56.2 (54.8-57.4)	56.4 (54.9-57.7)	58.3 (56.7-59.8)	61.2 (59.7-62.8)	65.3 (63.6-66.9)
Benin	41.7 (38.9-44.6)	42.6 (39.9-45.3)	43.7 (41.5-46.1)	45.9 (43.6-48.3)	48.2 (45.9-50.6)	51.1 (49.1-53.3)	53.7 (51.9-55.5)	55.5 (53.8-57.1)	57.5 (56-58.9)	59 (57.5-60.5)	59.8 (58.2-61.6)	61.4 (59.6-63.5)	63.6 (61.5-66)	66.6 (64.2-69.1)
Burkina Faso	37.8 (35.1-40.5)	39.3 (36.3-42.4)	41.2 (38.4-44.2)	43.2 (40.7-45.9)	44.8 (42.6-47.1)	46.6 (44.5-48.7)	49.3 (47.5-50.9)	51.4 (49.9-52.9)	52.4 (51-53.8)	52.2 (50.6-53.9)	54.1 (52.5-55.8)	57.3 (55.7-58.9)	60.7 (59.1-62.5)	64.4 (62.6-66.3)
Cameroon	43 (40.7-45.4)	45.4 (43.1-47.7)	42.6 (40.8-44.5)	49.4 (47.4-51.6)	51.1 (49.3-53)	53.1 (51.6-54.8)	55.2 (53.7-56.7)	57.9 (56.6-59.3)	59.2 (58-60.4)	58 (56.7-59.3)	56.3 (54.7-58)	56.1 (54.1-58.3)	58.9 (56.8-61.3)	65.1 (62.7-67.8)
Cape Verde	59.5 (56.8-62.3)	60.4 (58-62.9)	59.4 (57.1-61.6)	61.7 (59.7-63.8)	59.4 (57.8-61)	63.8 (62.7-64.9)	68.3 (67.7-68.9)	70.5 (70.1-70.9)	73.5 (73-74)	72.8 (71.9-73.8)	75.3 (73.9-76.5)	76.7 (75.6-77.4)	77.9 (77.3-78.3)	79 (78.2-80.1)
Chad	46.7 (43.8-49.8)	47.8 (45-51)	49.2 (46.1-52.2)	50.3 (47.4-53.3)	50.6 (48-53.2)	52.2 (49.8-54.5)	52.4 (50.4-54.4)	53.6 (51.8-55.2)	54.7 (53.3-56.2)	54.6 (53-56.2)	53.7 (51.9-55.4)	55.1 (53.1-57)	58 (56-60)	61.6 (59.2-64.2)
Cote d'Ivoire	44.4 (41.8-46.9)	45.6 (43.3-48.1)	47.8 (45.4-50.3)	50.2 (47.8-52.6)	53 (51-55.2)	56 (54-58.1)	58.7 (56.9-60.6)	59.5 (57.9-61)	58 (55.5-59.9)	54.7 (52.2-57)	54.6 (52.5-56.9)	56.4 (54.4-58.5)	59.7 (57.8-62)	65.3 (62.8-67.7)
The Gambia	50.9 (48-53.7)	51.9 (48.8-54.7)	52.9 (50.1-55.9)	54.4 (51.4-57.3)	56.4 (53.6-59.2)	58 (55.4-60.9)	59.8 (57.4-62.5)	61.5 (59.1-63.9)	62.5 (60.4-64.8)	63.5 (61.5-65.7)	64 (62.2-66.2)	64.5 (62.5-66.5)	65.5 (63.6-67.5)	67.9 (65.6-70.2)
Ghana	47.2 (44.5-49.9)	49 (46.5-51.6)	52.1 (49.6-54.7)	54.7 (52.1-57.3)	56.4 (53.9-58.9)	58 (55.7-60.6)	59 (56.8-61.2)	59.3 (57.4-61.6)	60.7 (58.8-62.6)	60.8 (59-62.7)	61 (59.3-62.7)	62 (60.5-63.6)	63.9 (62.5-65.4)	68.4 (66.7-70.3)
Guinea	40.3 (37.3-43.6)	41.5 (38.4-44.6)	42.9 (40.1-46)	44.4 (41.7-47.4)	45.2 (42.6-47.9)	46.2 (43.9-48.6)	48.5 (46.5-50.3)	50.3 (48.6-52)	52.3 (50.8-53.8)	54.1 (52.7-55.4)	55.5 (54.1-56.9)	57 (55.4-58.6)	58.7 (57-60.3)	62.2 (60.3-64.2)
Guinea-Bissau	36.9 (34.2-39.7)	39 (36.3-41.8)	41.3 (38.7-44.2)	41.4 (39.1-44.2)	43.7 (41.1-46.4)	47.5 (44.9-50.4)	49.2 (46.8-51.9)	50.3 (48-52.7)	52.1 (49.9-54.5)	53.6 (51.6-55.8)	54.8 (53.1-56.8)	55.9 (54.2-57.7)	58.3 (56.3-60.3)	62.6 (60.3-64.9)
Liberia	40.5 (38-43.3)	42.1 (39.5-45)	43.6 (41-46.4)	45.3 (42.7-47.7)	47.7 (45.3-50.2)	49.9 (47.7-52.3)	51.9 (49.7-54.1)	53.2 (51.1-55.2)	51.3 (49.5-53.1)	51.5 (49.7-53.1)	54.9 (53.2-56.7)	59 (57.4-60.8)	61.6 (59.9-63.5)	65.1 (63.1-67.4)
Mali	34.7 (32.1-37.7)	36 (33.4-38.8)	37.2 (34.6-39.9)	38.1 (36-40.3)	39.4 (37.6-41.4)	41.4 (39.8-43.3)	44.8 (43.3-46.4)	47.3 (46.1-48.6)	49.5 (48.3-50.6)	51.3 (50.1-52.5)	53.5 (52.3-54.8)	57.2 (55.8-58.6)	60.4 (58.7-61.9)	63 (61.1-64.9)
Mauritania	48.1 (45.5-50.8)	49.4 (47-52.1)	50.1 (47.7-52.4)	52 (49.7-54.6)	54.4 (52.3-56.7)	54.9 (52.8-57.1)	57.1 (55.3-59.1)	58.6 (57-60.3)	60.2 (58.7-61.7)	61.8 (60.4-63.1)	63.7 (62.4-65)	65.9 (64.5-67.4)	68.6 (66.9-70.3)	71 (68.9-73)
Niger	39.4 (36.6-42.3)	40.6 (37.8-43.7)	42.1 (39.4-45)	44.2 (41.6-46.9)	44.6 (42.5-46.9)	44.1 (42.3-46.1)	45.1 (43.3-47)	45.7 (43.8-47.3)	47.4 (45.8-49)	49.2 (47.6-50.7)	52.5 (50.8-54.1)	56.6 (55-58.3)	60.2 (58.5-62.2)	63.6 (61.4-66)
Nigeria	44.7 (41.8-47.9)	46.2 (43.1-49.4)	47.8 (44.5-50.8)	50 (47.2-52.9)	51.1 (48.6-54)	54.1 (51.4-56.8)	56.3 (53.9-58.8)	57.4 (54.9-59.9)	57.1 (54.5-59.5)	57 (54.1-59.7)	56.6 (53.6-59.9)	58.6 (55.2-62.3)	61.8 (58.4-65.7)	65.8 (62.3-69.1)
Sao Tome and Principe	41.9 (40-43.8)	42.9 (41.7-44)	48 (46.9-49)	57 (56.3-57.8)	61.1 (60.4-61.8)	64.8 (63.9-65.7)	65.8 (65.1-66.6)	64.9 (63.9-65.9)	65.6 (64.3-67)	64.9 (63.5-66.4)	65.7 (64.3-67)	67.7 (66.4-69)	70.1 (68.7-71.5)	71.8 (70.1-73.8)
Senegal	45 (42.3-47.6)	45.7 (43.3-48.3)	46.7 (44.3-49.3)	47 (44.7-49.4)	48.1 (46-50.4)	50.6 (48.9-52.8)	54.2 (52.5-55.9)	57.7 (56.2-59.1)	60.4 (59-61.8)	60.9 (59.6-62.1)	62.3 (61.1-63.5)	64.9 (63.7-66.1)	67.7 (66.3-69.2)	70 (68.3-71.9)
Sierra Leone	40.1 (37.2-43)	41.5 (38.4-44.6)	43 (39.9-46.6)	44.8 (41.9-48)	46.6 (43.9-49.5)	48.5 (45.9-51.4)	49.6 (47.3-52)	50.7 (48.4-52.9)	52.3 (50.4-54.3)	52.3 (50.8-54)	52.7 (51.3-54.1)	53.8 (52.3-55.4)	56.3 (54.7-58.1)	61.4 (59.4-63.7)
Togo	45 (42.1-48.1)	46.3 (43.6-49.2)	47.8 (45-50.6)	50 (47.4-52.6)	52.8 (50.4-55.2)	55 (52.8-57.3)	56.9 (54.8-59)	58.2 (56.5-60)	59.3 (57.7-60.9)	59.5 (57.7-61.3)	59 (56.7-61.1)	59.2 (56.8-61.5)	61.6 (59.3-63.9)	67.2 (65-69.6)

Table 8 Estimates of life expectancy at birth by location and year for 1950, 1955...2005, 2010, 2017 - males

Location	1950	1955	1960	1965	1970	1975	1980	1985	1990	1995	2000	2005	2010	2017
Global	48.1 (46.5-49.6)	51 (49.7-52.3)	43.1 (42.3-43.9)	54.3 (53.5-55)	56 (55.4-56.5)	58 (57.5-58.4)	60 (59.6-60.4)	61.7 (61.4-62)	63.2 (62.9-63.4)	63.7 (63.5-63.9)	64.9 (64.7-65.2)	66.6 (66.3-66.8)	68.2 (67.9-68.4)	70.5 (70.1-70.8)
Low SDI	38.5 (36.1-40.7)	40.8 (38.8-42.6)	41.6 (39.8-43.2)	42.6 (41.2-44)	44.2 (43.2-45.2)	47.2 (46.3-48)	49.6 (48.8-50.2)	51 (50.3-51.6)	53 (52.4-53.6)	54.7 (54.2-55.2)	56.4 (56-56.8)	59.1 (58.7-59.6)	60.6 (60.1-61)	64.5 (63.8-65.1)
Low-middle SDI	41.1 (39.1-42.9)	44.3 (42.7-45.9)	41.6 (40.4-42.8)	47.6 (46.5-48.6)	50.1 (49.2-50.9)	51.9 (51.1-52.6)	55.3 (54.7-55.9)	57.3 (56.8-57.8)	59 (58.5-59.4)	59.8 (59.4-60.2)	60.4 (59.9-60.8)	61.9 (61.5-62.4)	63.6 (63.1-64)	66.3 (65.7-66.9)
Middle SDI	45.5 (43.6-47.1)	48.8 (47.4-50.3)	25.2 (24.7-25.7)	53.9 (53-54.8)	56.6 (55.7-57.4)	59.7 (59.1-60.4)	62.1 (61.5-62.6)	64.1 (63.6-64.5)	65.7 (65.3-66.1)	66.8 (66.5-67.1)	67.8 (67.6-68)	69.2 (69-69.4)	70.2 (70-70.5)	71.7 (71.4-72.1)
High-middle SDI	52.9 (51.2-54.4)	55.4 (54.1-56.6)	51.3 (50.3-52.2)	59.7 (58.7-60.5)	61.4 (60.7-62)	63.1 (62.6-63.6)	64.3 (63.9-64.7)	65.8 (65.5-66.2)	66.8 (66.5-67)	66.1 (65.9-66.3)	67.8 (67.6-68)	69.2 (69-69.3)	71.3 (71.2-71.5)	73.3 (73-73.7)
High SDI	61.4 (60.9-61.8)	65.1 (64.9-65.3)	66.2 (66.1-66.4)	67.3 (67.2-67.4)	67.9 (67.8-68)	69.2 (69.1-69.3)	70.3 (70.3-70.3)	71.6 (71.6-71.6)	72.6 (72.5-72.6)	73.5 (73.5-73.5)	75.1 (75-75.1)	76.3 (76.3-76.4)	77.6 (77.6-77.7)	78.5 (78.3-78.6)
Central Europe, Eastern Europe, and Central Asia	58.5 (57.5-59.4)	60.6 (59.9-61.3)	62.9 (62.5-63.3)	64.1 (63.7-64.4)	63.7 (63.4-64)	64 (63.8-64.3)	63.4 (63.2-63.6)	64.3 (64.2-64.4)	64.8 (64.7-64.9)	61.5 (61.4-61.6)	62.9 (62.8-63)	62.8 (62.7-62.9)	65.8 (65.7-65.8)	68.5 (68.3-68.7)
Central Asia	47.8 (45.3-50.2)	51 (49.2-52.8)	54.2 (52.8-55.6)	56.8 (55.5-58)	59 (58.2-59.7)	60.7 (60-61.4)	62.2 (61.8-62.6)	63.5 (63.2-63.8)	64.1 (63.7-64.4)	62.1 (61.8-62.4)	63.4 (63.1-63.7)	63.4 (63.1-63.7)	65.6 (65.3-65.9)	67.4 (66.8-67.9)
Armenia	47.4 (44-50.9)	52 (49.4-54.6)	56.9 (54.8-58.8)	60.4 (58.3-62.6)	63.5 (62.1-64.7)	64.9 (63.4-66.4)	67.3 (66.5-68.1)	68.1 (67.4-68.6)	66.7 (66.2-67.2)	66 (65.8-66.3)	69.3 (69-69.6)	70.1 (69.9-70.3)	70.6 (70.5-70.8)	72.4 (72-72.8)
Azerbaijan	37.4 (33.9-40.8)	41.8 (39-44.9)	47.6 (45-50)	52.6 (50.1-54.9)	56.9 (55-58.5)	60.3 (58.8-61.7)	62.2 (61.2-63.1)	63.4 (62.6-64.1)	63.4 (62.6-64.3)	60.9 (60.2-61.7)	63.6 (63-64.4)	64.5 (63.9-65.2)	66 (65.4-66.7)	67.2 (66.2-68.2)
Georgia	48 (44.9-51.5)	51.7 (49.2-54.1)	55.5 (53.5-57.5)	58.5 (56.3-60.4)	60.9 (59.7-62.1)	62.7 (61.2-64.2)	64.9 (64-65.8)	66.2 (65.4-66.8)	65.9 (65.2-66.6)	67 (66.3-67.7)	69 (68.3-69.7)	69.5 (69.2-69.7)	68.3 (68.1-68.5)	68.4 (68-68.8)
Kazakhstan	51.9 (49.4-54.7)	54.2 (52.3-56.1)	56.4 (54.9-57.9)	57.7 (56-59.4)	59 (58.2-59.7)	60.2 (59.1-61.3)	61.4 (61-61.8)	62.8 (62.5-63.1)	63.4 (63.1-63.7)	59.4 (59.1-59.7)	60.3 (60-60.6)	59.8 (59.5-60)	63.5 (63.2-63.7)	67.5 (66.8-68.2)
Kyrgyzstan	42.7 (39.5-46.1)	46 (43.4-48.7)	49.3 (46.8-51.6)	51.9 (49.5-54.4)	54.3 (52.4-56.2)	56.2 (54.5-58)	58.2 (57.1-59.4)	60.9 (60.1-61.8)	62 (61.2-62.8)	61 (60.2-61.8)	62.5 (61.6-63.3)	62.6 (62-63.1)	65.4 (65.3-65.5)	69.1 (68.7-69.4)
Mongolia	39.9 (36.5-43.5)	43 (39.9-46.2)	46.7 (44-49.4)	50.7 (48.8-52.6)	52.3 (50.8-53.8)	56.1 (54.8-57.3)	56.1 (55-57.2)	57.4 (56.5-58.4)	58.6 (57.9-59.4)	57.8 (57.1-58.4)	59.1 (58.5-59.6)	60 (59.3-60.5)	62 (61.3-62.6)	64.5 (63.2-65.9)
Tajikistan	42 (38.6-45.2)	44.9 (42.2-47.6)	48.8 (46.3-51.3)	52.9 (50.1-55.2)	57 (55.2-58.6)	59.5 (58.1-61.1)	62.2 (61.2-63.1)	64.1 (63.4-64.8)	64.5 (63.8-65.1)	62.3 (61.5-63.2)	65.4 (64.7-66.1)	66.7 (65.9-67.5)	67 (66.1-68)	67.7 (66.3-68.9)
Turkmenistan	47.7 (44.7-50.9)	50.1 (47.7-52.4)	52.9 (51-54.9)	55.5 (53.6-57.6)	57.7 (56.2-59)	59 (57.4-60.5)	60.3 (59.3-61.2)	61.5 (60.7-62.3)	62.6 (62-63.1)	61.3 (60.8-61.8)	63 (62.5-63.4)	62.4 (62-62.8)	66.4 (66-66.7)	66.5 (65.4-67.7)
Uzbekistan	53.9 (51.1-56.9)	56.1 (54-58.2)	58.3 (56.6-60.1)	60.3 (58.4-62.3)	62 (60.8-62.9)	62.9 (61.7-64.1)	64 (63.5-64.6)	65 (64.5-65.4)	66 (65.6-66.4)	64.6 (64.2-65)	64.5 (63.8-65.1)	63.9 (63.2-64.6)	65.9 (65.3-66.6)	67.1 (65.5-68.6)
Central Europe	57.5 (57-57.9)	61.4 (61.2-61.6)	64.4 (64.3-64.5)	66.1 (66-66.2)	66 (66-66.1)	67.2 (67.2-67.3)	66.8 (66.7-66.8)	66.9 (66.9-67)	67.2 (67.1-67.2)	67.3 (67.2-67.3)	69.3 (69.3-69.4)	70.4 (70.4-70.5)	71.9 (71.8-71.9)	73.6 (73.3-73.9)
Albania	49.5 (46.9-52.1)	54.3 (51.8-56.5)	60.1 (58.4-61.7)	62.9 (61.1-64.8)	65.6 (63.2-68)	67.5 (65-69.9)	68.6 (66.7-70.8)	69 (68.2-69.7)	69.8 (69.5-70.2)	71.3 (71-71.6)	71.8 (71.2-72.4)	73 (72.4-73.7)	74.6 (73.5-75.8)	74.9 (72.8-77.1)
Bosnia and Herzegovina	48.8 (48.5-49.2)	53.5 (53.2-53.8)	58.8 (58.6-59.1)	61.4 (61.1-61.6)	62.9 (62.7-63.1)	65.8 (65.7-66)	66.9 (66.7-67.1)	69.1 (69-69.3)	70.5 (70.4-70.7)	61.3 (59.9-62.8)	72.4 (71.1-73.6)	72.9 (72.7-73)	73.7 (73.6-73.9)	74.3 (73.6-75)
Bulgaria	60.4 (60.2-60.6)	63.6 (63.4-63.8)	67.9 (67.8-68.1)	70 (69.8-70.1)	69.6 (69.5-69.8)	69 (68.9-69.1)	69.1 (68.9-69.2)	69 (68.9-69.1)	68.2 (68.1-68.3)	67.4 (67.3-67.5)	68.3 (68.2-68.4)	69 (68.9-69.1)	70.4 (70.3-70.5)	71.3 (70.6-72.1)
Croatia	53.3 (53-53.6)	58.4 (58.1-58.6)	63 (62.8-63.2)	65.4 (65.2-65.6)	65.7 (65.5-65.9)	67.4 (67.2-67.6)	67.7 (67.6-67.8)	67.8 (67.7-68)	68.7 (68.6-68.9)	69.2 (69.1-69.4)	70.9 (70.8-71.1)	71.9 (71.7-72)	73.5 (73.4-73.6)	75.4 (74.7-76.1)
Czech Republic	62.1 (62-62.3)	66.2 (66.1-66.3)	67.4 (67.3-67.5)	67 (66.9-67.2)	66 (65.9-66.1)	67 (66.9-67.1)	66.7 (66.6-66.8)	67.4 (67.4-67.5)	67.6 (67.5-67.6)	69.8 (69.7-69.9)	71.7 (71.6-71.7)	73.1 (73-73.2)	74.5 (74.4-74.6)	76.3 (75.6-77)
Hungary	59.8 (59.7-60)	64.9 (64.8-65)	66 (65.9-66.2)	66.9 (66.8-67)	66.3 (66.2-66.4)	66.3 (66.2-66.4)	65.5 (65.4-65.6)	65 (64.9-65.1)	65.3 (65.2-65.4)	65.5 (65.4-65.5)	67.5 (67.5-67.6)	68.7 (68.6-68.8)	70.7 (70.6-70.8)	73.2 (72.4-73.9)
Macedonia	51.4 (49.8-53.1)	52.5 (51.4-53.6)	57.8 (56.7-58.9)	61.5 (60.4-62.5)	64.7 (64-65.3)	67.9 (67.6-68.1)	68.9 (68.7-69.1)	68.8 (68.6-69)	69.6 (69.4-69.8)	70 (69.8-70.2)	71.3 (71.1-71.5)	72 (71.9-72.2)	73.2 (73.1-73.4)	73.9 (73.2-74.6)
Montenegro	64.5 (63.5-65.4)	67 (66.4-67.8)	68.5 (67.8-69.3)	69.4 (68.8-70)	70 (69.3-70.7)	70.4 (69.7-71)	70 (69.7-70.3)	70 (69.7-70.2)	71.1 (70.8-71.5)	71.3 (70.9-71.6)	71.1 (70.8-71.5)	71.8 (71.1-72.5)	73.4 (72.7-74)	74.1 (72.9-75.2)
Poland	56.4 (56.3-56.6)	60.9 (60.8-61)	65.2 (65.1-65.2)	66.8 (66.7-66.8)	66.9 (66.8-66.9)	67.6 (67.5-67.6)	66.5 (66.4-66.5)	66.9 (66.8-66.9)	66.8 (66.8-66.9)	67.8 (67.7-67.8)	69.8 (69.7-69.8)	70.9 (70.8-70.9)	72.2 (72.1-72.2)	74.1 (73.3-74.8)
Romania	60.6 (58-63.4)	62 (61.1-62.8)	63.6 (63.5-63.8)	66.3 (66.2-66.4)	66.1 (66-66.2)	67.6 (67.5-67.7)	67 (66.9-67.1)	66.7 (66.6-66.8)	66.7 (66.6-66.8)	65.6 (65.6-65.7)	67.7 (67.6-67.8)	68.8 (68.7-68.9)	70 (69.9-70)	71.5 (70.8-72.3)
Serbia	53.1 (52.8-53.4)	58.2 (57.9-58.4)	61.9 (61.7-62.1)	63.7 (63.6-63.9)	63.5 (63.4-63.7)	66.3 (66.1-66.4)	66.3 (66.2-66.4)	66 (65.9-66.1)	67.6 (67.4-67.7)	68 (67.9-68.1)	69 (68.7-69.2)	69.9 (69.7-70.1)	71.6 (71.5-71.7)	73.6 (72.9-74.2)
Slovakia	58.2 (56.8-59.8)	65.3 (64.1-66.4)	67.4 (66.3-68.6)	67.3 (66.1-68.5)	66.7 (65.7-67.8)	66.9 (66.8-67.1)	66.9 (66.7-67)	67 (66.9-67.2)	66.7 (66.6-66.8)	68.5 (68.4-68.6)	69.3 (69.2-69.4)	70.3 (70.2-70.4)	71.8 (71.7-71.9)	74.1 (73.4-74.8)
Slovenia	55.4 (55.1-55.7)	60.4 (60.2-60.7)	63.3 (63.1-63.6)	65.5 (65.2-65.7)	65.5 (65.3-65.7)	67 (66.8-67.2)	67.4 (67.3-67.6)	67.9 (67.7-68.1)	69.7 (69.6-69.9)	70.7 (70.6-70.9)	72.1 (71.9-72.3)	74.1 (73.9-74.3)	76.1 (75.9-76.2)	77.9 (77.2-78.7)
Eastern Europe	61.5 (60.3-62.6)	62.5 (61.5-63.3)	64.5 (64.2-64.8)	65.3 (65.1-65.6)	64.2 (63.9-64.4)	63.6 (63.3-63.8)	62.5 (62.2-62.7)	63.7 (63.5-63.8)	64.5 (64.5-64.6)	59.4 (59.3-59.4)	60.4 (60.4-60.4)	59.6 (59.6-59.7)	63.2 (63.2-63.2)	66.5 (66.3-66.7)
Belarus	64.6 (61.7-67.3)	65.6 (63.4-68)	66.7 (65.8-67.5)	67.9 (67.4-68.4)	67.4 (66.9-67.7)	66.7 (66.4-67.1)	65.7 (65.4-66)	65.9 (65.7-66.2)	66.1 (65.8-66.3)	62.9 (62.7-63.1)	63.2 (63-63.4)	62.8 (62.6-62.9)	64.5 (64.4-64.6)	69 (68.2-69.7)
Estonia	62.5 (59.7-65.4)	63.2 (60.8-65.5)	64.7 (64.4-64.9)	66.2 (66-66.5)	65.5 (65.3-65.7)	64.9 (64.7-65.2)	64.2 (64-64.4)	64.7 (64.5-64.9)	64.7 (64.5-64.9)	61.7 (61.5-61.9)	65.5 (65.3-65.7)	67.4 (67.2-67.6)	70.9 (70.6-71.1)	73.6 (72-75.3)
Latvia	64.6 (61.8-67.5)	65.6 (63.2-68.5)	66.5 (66.3-66.8)	67.2 (67-67.4)	66.2 (66-66.4)	64.3 (64.1-64.5)	64 (63.8-64.1)	65 (64.9-65.2)	64.6 (64.4-64.8)	60.7 (60.6-60.9)	65 (64.9-65.2)	65 (64.8-65.2)	68 (67.8-68.2)	70.1 (68.6-71.7)
Lithuania	63.1 (60.2-66.1)	64.2 (61.9-66.8)	67 (66.7-67.2)	68.6 (68.4-68.8)	67.3 (67.2-67.5)	66.7 (66.5-66.8)	65.7 (65.5-65.8)	66.1 (66-66.3)	66.4 (66.2-66.5)	63.4 (63.2-63.5)	66.9 (66.7-67)	65.5 (65.3-65.6)	67.6 (67.5-67.8)	69.6 (68.7-70.5)
Moldova	49.8 (47.1-52.8)	52.6 (50.4-54.7)	55.2 (53.6-56.8)	57.3 (55.6-59.1)	59.2 (58.1-60.1)	60.6 (59.4-61.8)	62.3 (61.6-62.8)	62.8 (62.4-63.2)	64.5 (64.2-64.7)	61.9 (61.7-62.2)	64.6 (64.4-64.9)	64.4 (64.2-64.6)	65.1 (64.9-65.3)	68.2 (67.8-68.7)
Russian Federation	61.1 (60.1-62)	61.9 (61.2-62.7)	63.8 (63.5-64.2)	64.7 (64.3-65)	63.3 (63-63.7)	62.7 (62.4-63)	61.5 (61.2-61.8)	62.9 (62.7-63)	64 (64-64)	58.4 (58.3-58.4)	59.2 (59.2-59.2)	58.4 (58.3-58.4)	62.1 (62.1-62.1)	66.8 (66.6-66.9)
Ukraine	62.9 (60-65.8)	63.7 (61.4-66.2)	66.2 (65.5-66.9)	66.9 (66.4-67.4)	65.9 (65.5-66.3)	65.4 (65-65.8)	64.2 (63.9-64.6)	65.2 (65-65.5)	65.5 (65.3-65.7)	61.3 (61.1-61.4)	62.5 (62.4-62.5)	62.1 (62.1-62.2)	65.8 (65.7-65.8)	64.7 (63.9-65.4)
High-income	61.6 (61.2-62)	65.3 (65-65.5)	66.1 (66-66.3)	67 (66.9-67.1)	67.6 (67.5-67.7)	69 (68.9-69.1)	70.3 (70.3-70.3)	71.7 (71.7-71.8)	72.8 (72.7-72.8)	73.7 (73.7-73.7)	75.2 (75.2-75.2)	76.5 (76.4-76.5)	77.7 (77.7-77.7)	78.4 (78.2-78.6)
Australasia	67.1 (67-67.2)	67.6 (67.6-67.7)	68.2 (68.1-68.3)	68 (67.9-68.1)	67.9 (67.8-67.9)	69.1 (69-69.1)	70.8 (70.8-70.9)	72.1 (72-72.2)	73.6 (73.5-73.6)	75 (75-75.1)	76.7 (76.6-76.8)	78.5 (78.4-78.6)	79.5 (79.4-79.6)	80.1 (79.1-81.2)
Australia	67 (66.9-67.1)	67.5 (67.4-67.6)	68.1 (68-68.2)	67.9 (67.8-68)	67.7 (67.6-67.8)	69 (68.9-69.1)	71 (70.9-71)	72.3 (72.2-72.4)	73.8 (73.7-73.9)	75.3 (75.2-75.4)	76.9 (76.8-77)	78.7 (78.6-78.8)	79.7 (79.6-79.8)	80.2 (78.9-81.5)
New Zealand	67.6 (67.3-67.8)	68.3 (68.1-68.5)	68.7 (68.5-69)	68.5 (68.3-68.8)	68.5 (68.2-68.7)	69.3 (69-69.5)	70.4 (70.3-70.6)	71.1 (71-71.3)	72.6 (72.4-72.8)	73.8 (73.6-73.9)	75.8 (75.6-75.9)	77.5 (77.3-77.6)	78.7 (78.5-78.8)	79.7 (79-80.3)
High-income Asia-Pacific	51.2 (50.5-52)	60.9 (60.2-61.7)	62.9 (62.1-63.6)	65.3 (64.6-65.9)	67.2 (66.6-67.8)	69.7 (69.5-70)	71.3 (71.2-71.4)	73 (73-73.1)	74.4 (74.4-74.5)	75.4 (75.3-75.4)	76.8 (76.8-76.9)	78.1 (78.1-78.1)	79.2 (79.2-79.3)	80.8 (80.5-81)
Brunei	51.8 (49.9-53.8)	52.6 (50.9-54.3)	56.4 (55-57.8)	61.5 (60.6-62.5)	63.7 (63-64.5)	66.2 (65.6-66.9)	67.9 (67.2-68.6)	68.5 (67.8-69.2)	69.1 (68.5-69.8)	70.6 (70-71.2)	71.8 (71.3-72.4)	72.6 (72-73.1)	73 (72.4-73.6)	73.3 (72.3-74.4)
Japan	57.8 (57.3-58.3)	64 (63.5-64.4)	65.8 (65.4-66.2)	68.3 (67.8-68.6)	69.8 (69.4-70.2)	72.1 (71.8-72.3)	73.7 (73.7-73.8)	75.1 (75.1-75.2)	76.2 (76.2-76.2)	76.7 (76.7-76.8)	77.9 (77.9-78)	78.8 (78.8-78.9)	79.8 (79.7-79.8)	81.1 (80.8-81.3)
Aichi	59.8 (57.2-62.5)	65.7 (63.1-68.2)	67 (64.6-69.6)	69.2 (66.9-71.7)	70.7 (68.8-73)	72.8 (71.6-74.1)	74.2 (74.1-74.3)	75.6 (75.5-75.7)	76.5 (76.4-76.6)	77 (76.9-77.1)	78.1 (78-78.2)	79.1 (79-79.2)	80 (79.8-80.1)	81.7 (80.4-83)
Akita	56.3 (53.5-59.3)	62.7 (60-65.6)	64.7 (62.2-67.2)	67.2 (64.8-69.8)	68.8 (66.6-71.3)	71.2 (69.7-72.6)	72.9 (72.7-73.1)	74.3 (74.1-74.5)	75.4 (75.2-75.6)	75.9 (75.7-76.1)	76.9 (76.7-77.1)	77.4 (77.2-77.7)	78.4 (78.2-78.6)	79.5 (77.9-81)
Aomori	54.7 (51.9-57.4)	61.3 (58.6-64)	63.2 (60.8-65.8)	65.8 (63.3-68.4)	67.3 (64.9-69.7)	69.6 (68.2-71)	71.7 (71.5-71.8)	73.2 (73-73.4)	74.6 (74.4-74.7)	74.9 (74.7-75.1)	76 (75.9-76.2)	76.5 (76.3-76.7)	77.7 (77.4-77.9)	78.9 (77.4-80.4)
Chiba	58.9 (56.3-61.5)	64.9 (62.2-67.3)	66.4 (63.9-69)	68.7 (66.4-71)	70.1 (68-72.2)	72.2 (70.9-73.5)	74 (73.9-74.1)	75.5 (75.4-75.6)	76.7 (76.6-76.8)	77 (76.9-77.1)	78.3 (78.2-78.4)	79 (78.9-79.1)	80 (79.9-80.1)	81.1 (79.7-82.4)
Ehime	56.4 (53.6-59.5)	62.7 (60.2-65.8)	64.8 (61.9-67.6)	67.3 (64.7-69.9)	68.8 (66.4-71.2)	71 (69.4-72.5)	73.3 (73.1-73.5)	75 (74.8-75.2)	76 (75.9-76.2)	76.6 (76.4-76.8)	77.6 (77.4-77.8)	78.3 (78.1-78.5)	79.4 (79.1-79.6)	80.6 (79-82.1)
Fukui	59.9 (57.2-62.8)	65.7 (63.1-68.4)	67 (64.8-69.5)	69.3 (67.2-71.4)	70.9 (69.2-72.5)	73 (72.1-73.9)	74.3 (74.1-74.5)	75.9 (75.7-76.1)	76.9 (76.7-77.1)	77.7 (77.5-77.9)	78.7 (78.5-78.9)	79.5 (79.3-79.7)	80.3 (80.1-80.6)	81.4 (80-82.7)
Fukuoka	57.2 (54.5-60.1)	63.2 (60.6-66.1)	65.2 (62.6-67.8)	67.4 (65.1-69.9)	69 (66.8-71.4)	71.2 (69.9-72.6)	72.9 (72.8-73)	74.3 (74.2-74.4)	75.4 (75.3-75.5)	76.2 (76.1-76.3)	77.5 (77.4-77.6)	78.4 (78.3-78.5)	79.5 (79.3-79.6)	81.3 (79.8-82.6)
Fukushima	55.9 (53.3-58.6)	62.7 (60-65.3)	64.7 (62.2-67.3)	67.2 (64.9-69.8)	68.7 (66.6-71)	71.1 (69.7-72.4)	73 (72.8-73.2)	74.6 (74.4-74.7)	75.9 (75.8-76.1)	76.6 (76.4-76.8)	77.6 (77.4-77.7)	78.2 (78-78.3)	79.2 (79-79.4)	80.4 (78.9-81.8)
Gifu	59.2 (56.5-61.8)	65.2 (62.7-67.7)	66.6 (64.2-69.1)	69 (66.5-71.5)	70.6 (68.4-72.8)	72.9 (71.6-74.2)	74.2 (74.1-74.4)	75.7 (75.5-75.9)	76.9 (76.8-77.1)	77.3 (77.1-77.5)	78.3 (78.1-78.5)	79.1 (78.9-79.3)	80.2 (80-80.4)	80.9 (79.5-82.3)
Gunma	58.3 (55.7-61)	64.4 (61.7-67)	66 (63.3-68.7)	68.3 (66-70.8)	69.7 (67.5-71.9)	71.9 (70.6-73.2)	74 (73.8-74.2)	75.2 (75.1-75.4)	76.6 (76.5-76.8)	77 (76.9-77.2)	78.1 (77.9-78.2)	78.9 (78.7-79)	79.6 (79.4-79.8)	80.6 (79.1-82)
Hiroshima	57.3 (54.9-60.3)	63.6 (61-66.4)	65.6 (63.1-68.3)	68 (65.6-70.4)	69.4 (67.2-71.8)	71.7 (70.4-73.1)	73.7 (73.5-73.8)	75.2 (75.1-75.3)	76.3 (76.1-76.4)	76.8 (76.6-76.9)	78.1 (77.9-78.2)	79 (78.9-79.2)	80 (79.8-80.1)	81.9 (80.5-83.2)
Hokkaidō	56.8 (54.3-59.6)	63.2 (60.6-66)	65 (62.4-67.5)	67.4 (65.1-70)	68.8 (66.6-71.2)	71.1 (69.9-72.4)	73.1 (73-73.2)	74.6 (74.5-74.7)	75.9 (75.8-76)	76.7 (76.6-76.8)	77.7 (77.6-77.8)	78.3 (78.2-78.5)	79.4 (79.3-79.6)	80.9 (79.6-82.2)
Hyōgo	57.7 (55.1-60.6)	63.9 (61.6-66.3)	65.7 (63.3-68.2)	68.1 (65.8-70.7)	69.7 (67.4-71.9)	72 (70.8-73.3)	73.3 (73.2-73.4)	74.6 (74.5-74.7)	75.7 (75.6-75.8)	74.8 (74.6-74.9)	77.7 (77.6-77.9)	78.7 (78.5-78.8)	79.8 (79.7-79.9)	81.2 (79.8-82.5)
Ibaraki	55.8 (53.3-58.5)	62.6 (60.2-65.3)	64.7 (62.1-67.6)	67.4 (64.9-70.2)	69 (66.6-71.5)	71.6 (70.2-73)	73.4 (73.3-73.6)	74.5 (74.4-74.7)	75.8 (75.7-75.9)	76.4 (76.3-76.6)	77.5 (77.3-77.6)	78.4 (78.3-78.6)	79.2 (79.1-79.4)	80.1 (78.7-81.5)
Ishikawa	58.8 (56.3-61.6)	64.8 (62.2-67.4)	66.3 (63.9-68.9)	68.6 (66.3-71.1)	70.2 (68.2-72.2)	72.8 (71.7-73.8)	75 (74.8-75.1)	76 (75.8-76.2)	76.6 (76.4-76.8)	77.3 (77.1-77.5)	78.3 (78.1-78.4)	79.3 (79.1-79.5)	80.2 (80-80.4)	81.3 (80-82.7)
Iwate	55.5 (53-58.6)	62.4 (59.9-65.2)	64.4 (61.9-67.2)	67 (64.6-69.6)	68.8 (66.6-71.1)	71.2 (69.8-72.7)	72.8 (72.7-73)	74.5 (74.3-74.7)	75.7 (75.5-75.8)	76.5 (76.3-76.7)	77.4 (77.2-77.6)	77.8 (77.5-78)	78.6 (78.4-78.9)	80.5 (79-82)
Kagawa	58.5 (55.7-61.5)	64.7 (62.1-67.6)	66.3 (63.8-69.1)	68.7 (66.4-71.3)	70.4 (68.5-72.5)	72.7 (71.6-73.8)	74.2 (74-74.4)	75.8 (75.6-75.9)	76.3 (76.1-76.4)	77.1 (76.9-77.3)	78.2 (78-78.4)	78.8 (78.6-79)	79.8 (79.6-80)	80.9 (79.5-82.2)
Kagoshima	54.8 (52.3-57.4)	61.6 (59.1-64.4)	63.6 (60.9-66.3)	66.2 (63.6-68.9)	67.8 (65.3-70.2)	70.3 (68.9-71.7)	72.5 (72.4-72.7)	74.2 (74-74.4)	75.6 (75.5-75.8)	76.3 (76.1-76.5)	77.4 (77.2-77.6)	78.1 (77.9-78.3)	79.3 (79.1-79.5)	80.4 (78.9-81.9)
Kanagawa	59.9 (57.3-62.6)	65.5 (62.9-68.1)	67.2 (64.7-69.7)	69.4 (67.1-71.8)	70.9 (68.7-73.1)	73.2 (72-74.4)	74.5 (74.4-74.6)	75.8 (75.7-75.9)	76.8 (76.7-76.9)	77.2 (77.1-77.3)	78.5 (78.4-78.6)	79.5 (79.4-79.6)	80.3 (80.2-80.5)	81.9 (80.6-83.2)
Kōchi	54.7 (52.1-57.4)	61.4 (58.8-64)	63.4 (60.8-65.9)	65.9 (63.5-68.5)	67.5 (65.4-69.6)	70 (68.8-71.1)	72.3 (72-72.5)	74.2 (74-74.4)	75.5 (75.2-75.7)	76.2 (75.9-76.4)	77.3 (77.1-77.5)	77.9 (77.7-78.1)	78.8 (78.6-79)	81.1 (79.7-82.6)
Kumamoto	55.8 (53.1-58.6)	62.9 (60.2-65.8)	64.9 (62.4-67.4)	67.5 (65-70)	69 (66.8-71.4)	71.2 (69.8-72.8)	73.5 (73.3-73.6)	75.3 (75.1-75.4)	76.4 (76.2-76.6)	77.4 (77.3-77.6)	78.6 (78.4-78.7)	79.4 (79.2-79.6)	80.5 (80.3-80.7)	81.4 (79.9-82.8)
Kyōto	59.3 (56.7-62.2)	65.3 (62.8-68.1)	67.1 (64.7-69.9)	69.5 (66.7-71.9)	71.2 (68.9-73.3)	73.7 (72.3-75.1)	75.2 (75-75.3)	76 (75.9-76.2)	76.6 (76.5-76.8)	77.2 (77.1-77.4)	78.1 (77.9-78.3)	79.3 (79.2-79.5)	80.3 (80.2-80.5)	82.1 (80.8-83.5)
Mie	58.1 (55.5-60.7)	64.4 (61.8-67.2)	66.1 (63.6-68.7)	68.6 (66.2-71.2)	70.3 (68.1-72.6)	72.7 (71.3-74.2)	73.9 (73.7-74.1)	75.1 (75-75.3)	76.2 (76-76.4)	76.8 (76.7-77)	78.1 (77.9-78.3)	79 (78.8-79.1)	79.8 (79.6-80)	80.8 (79.3-82.2)
Miyagi	57.7 (55.1-60.2)	63.9 (61.3-66.4)	65.3 (62.8-67.9)	67.9 (65.5-70.3)	69.3 (67-71.5)	71.3 (70-72.6)	73.5 (73.3-73.6)	75.2 (75.1-75.4)	76.3 (76.2-76.5)	77.1 (76.9-77.3)	78 (77.8-78.1)	78.6 (78.4-78.8)	79.9 (79.7-80.1)	80.9 (79.6-82.3)
Miyazaki	55.9 (53.2-58.9)	62.4 (59.8-65.1)	64.5 (61.8-67.1)	67 (64.5-69.6)	68.8 (66.5-71.2)	71.3 (69.8-72.9)	72.8 (72.6-73)	74.6 (74.4-74.8)	75.7 (75.5-75.9)	76.4 (76.2-76.6)	77.7 (77.5-78)	78.7 (78.5-79)	79.7 (79.5-79.9)	80.5 (79-82.1)
Nagano	59.4 (56.6-62)	65.4 (63-68.1)	67 (64.6-69.5)	69.3 (66.9-71.7)	70.8 (68.5-73)	72.8 (71.4-74.1)	75 (74.9-75.2)	76.1 (75.9-76.2)	77.6 (77.4-77.7)	78.2 (78-78.4)	79.1 (79-79.3)	79.9 (79.7-80.1)	80.9 (80.7-81.1)	81.6 (80.3-83)
Nagasaki	56.5 (53.7-59.1)	62.6 (60.2-65.3)	64.4 (61.8-67.2)	66.8 (64.4-69.4)	68.3 (65.9-70.7)	70.6 (69.2-72.1)	72.5 (72.4-72.7)	74.2 (74.1-74.4)	75.4 (75.2-75.6)	76.1 (75.9-76.3)	77.5 (77.3-77.7)	78.2 (78-78.4)	79.2 (79-79.4)	80.5 (79.1-82)
Nara	57.8 (55.1-60.5)	64 (61.3-66.5)	65.5 (63.1-68)	67.9 (65.5-70.3)	69.4 (67-71.7)	71.6 (70.2-73.1)	73.5 (73.3-73.7)	75 (74.7-75.1)	76.3 (76.2-76.5)	77.3 (77.1-77.5)	78.6 (78.4-78.8)	79.2 (79-79.4)	80.4 (80.2-80.6)	81.4 (79.9-82.9)
Niigata	57.7 (55.2-60.4)	64 (61.6-66.6)	65.6 (63-68.1)	67.9 (65.6-70.4)	69.2 (66.9-71.4)	71.2 (69.9-72.6)	73.5 (73.3-73.6)	75 (74.9-75.2)	76.7 (76.5-76.8)	77.1 (76.9-77.3)	78 (77.8-78.1)	78.8 (78.6-79)	79.8 (79.6-80)	81.2 (79.7-82.6)
Ōita	57.4 (54.8-60.1)	63.7 (61.1-66.3)	65.6 (63.1-68.4)	68.1 (65.8-70.9)	70 (67.8-72.3)	72.6 (71.1-74.1)	73.3 (73.1-73.5)	74.9 (74.7-75.1)	76.2 (76-76.4)	76.9 (76.7-77.1)	78.1 (77.9-78.3)	79.1 (78.9-79.3)	80 (79.8-80.3)	82.2 (80.7-83.5)
Okayama	59.1 (56.5-61.9)	65 (62.4-67.7)	66.7 (64-69.2)	69 (66.7-71.5)	70.6 (68.5-73)	72.9 (71.5-74.2)	74.2 (74.1-74.4)	75.3 (75.1-75.5)	76.4 (76.2-76.6)	77.2 (77-77.4)	78.2 (78-78.4)	79.2 (79-79.4)	80 (79.8-80.2)	81.2 (79.7-82.6)
Okinawa	55.3 (52.6-58.5)	61.7 (59-64.6)	63.5 (60.9-66.5)	65.6 (62.8-68.5)	66.1 (63.5-68.7)	67.7 (66.1-69.2)	73.2 (73-73.4)	76 (75.8-76.2)	76.4 (76.2-76.6)	77.1 (76.9-77.3)	77.8 (77.7-78)	78.8 (78.6-79)	79.3 (79.1-79.5)	80.7 (79.2-82.3)
Ōsaka	57.8 (55.3-60.5)	63.9 (61.4-66.5)	65.7 (63.5-68)	68 (65.8-70.2)	69.6 (67.7-71.7)	72 (70.8-73.2)	72.9 (72.9-73)	74.1 (74-74.2)	75.1 (75-75.1)	75.9 (75.8-76)	77.1 (77-77.2)	78.2 (78.1-78.3)	79.1 (79-79.2)	80.3 (78.9-81.7)
Saga	57.1 (54.6-59.9)	63.3 (60.8-66)	65.1 (62.6-67.6)	67.6 (65.3-69.9)	69.3 (67.5-71.3)	71.7 (70.7-72.8)	73.2 (73-73.4)	74.5 (74.4-74.7)	75.6 (75.4-75.8)	76.4 (76.2-76.7)	77.4 (77.2-77.6)	78.4 (78.2-78.6)	79.5 (79.2-79.7)	81 (79.5-82.3)
Saitama	59 (56.5-61.6)	65.1 (62.6-67.7)	66.8 (64.4-69.3)	69.2 (66.8-71.6)	71.1 (68.9-73.2)	73.6 (72.4-74.9)	74.1 (74-74.2)	75.4 (75.3-75.5)	76.5 (76.4-76.6)	77.1 (77-77.2)	78.3 (78.2-78.4)	79.1 (79-79.2)	79.9 (79.8-80)	81 (79.7-82.3)
Shiga	58.9 (56-61.9)	64.9 (62.3-67.5)	66.3 (63.9-68.8)	68.5 (66.3-70.8)	69.9 (68.1-72)	72.4 (71.4-73.5)	75 (74.8-75.2)	76.2 (76-76.4)	76.6 (76.4-76.7)	77.3 (77.1-77.5)	78.4 (78.2-78.6)	79.5 (79.3-79.6)	80.7 (80.6-80.9)	82 (80.7-83.2)
Shimane	57.3 (54.6-60.4)	63.5 (61.2-66.3)	65.4 (63.1-68.1)	67.8 (65.5-70.2)	69.4 (67.5-71.6)	71.8 (70.6-73)	73.6 (73.4-73.8)	75.5 (75.3-75.7)	76.4 (76.2-76.6)	77 (76.8-77.2)	77.9 (77.7-78.1)	78.6 (78.3-78.8)	79.8 (79.6-80.1)	81.5 (80.1-83)
Shizuoka	58.9 (56.2-61.8)	64.9 (62.3-67.5)	66.6 (64.1-69.2)	69 (66.8-71.5)	70.6 (68.5-72.7)	72.8 (71.6-74.1)	74.2 (74.1-74.3)	75.6 (75.5-75.8)	76.8 (76.6-76.9)	77.3 (77.2-77.4)	78.5 (78.3-78.6)	79.4 (79.3-79.6)	80.1 (80-80.3)	81 (79.5-82.4)
Tochigi	57.5 (54.8-60.3)	63.6 (61.1-66.2)	65.3 (62.7-67.7)	67.8 (65.3-70.3)	69.4 (67.1-71.8)	71.8 (70.4-73.2)	73.5 (73.3-73.6)	74.5 (74.4-74.7)	75.5 (75.4-75.7)	76.2 (76-76.4)	77.6 (77.4-77.8)	78.2 (78-78.3)	79.2 (79-79.3)	80.5 (79.1-82)
Tokushima	56.8 (54.2-59.4)	62.9 (60.3-65.6)	64.7 (62.1-67.5)	67.1 (64.7-69.7)	68.7 (66.7-70.8)	71.1 (69.9-72.2)	72.7 (72.5-72.9)	74.5 (74.3-74.7)	75.6 (75.4-75.8)	76.4 (76.2-76.6)	77.6 (77.4-77.8)	78.5 (78.3-78.7)	79.5 (79.3-79.8)	80.6 (79.1-82)
Tōkyō	60.8 (58.2-63.6)	66.1 (63.7-68.8)	67.5 (65.4-70)	69.5 (67.5-71.7)	71 (69.1-73.1)	73.1 (71.9-74.2)	74.5 (74.4-74.6)	75.7 (75.7-75.8)	76.3 (76.2-76.4)	76.8 (76.7-76.9)	78 (78-78.1)	79.2 (79.1-79.3)	80 (79.9-80.1)	81.3 (79.9-82.6)
Tottori	56.4 (53.7-59.1)	63 (60.6-65.6)	65.2 (62.8-67.5)	67.9 (65.9-70.1)	69.6 (67.9-71.3)	71.9 (71-72.8)	73.1 (72.9-73.4)	74.7 (74.5-74.9)	75.7 (75.5-75.9)	76.2 (76-76.4)	77.5 (77.3-77.8)	78.2 (78-78.4)	79.3 (79.1-79.6)	80.5 (79.1-81.9)
Toyama	57.4 (54.9-60.2)	63.7 (61.3-66.5)	65.4 (63.1-67.9)	67.8 (65.6-70.2)	69.4 (67.4-71.3)	71.8 (70.7-72.8)	73.6 (73.4-73.8)	75 (74.8-75.2)	76.3 (76.1-76.4)	77.2 (77-77.4)	78.3 (78.2-78.5)	79.1 (78.9-79.3)	79.8 (79.6-80)	80.8 (79.5-82.2)
Wakayama	56.1 (53.3-58.7)	62.7 (60.3-65.4)	64.7 (62.3-67.3)	67.4 (65.1-69.7)	69.1 (67.2-71)	71.4 (70.4-72.5)	73 (72.8-73.2)	74.1 (74-74.3)	75.3 (75.1-75.5)	76.1 (76-76.3)	77.5 (77.3-77.7)	78.3 (78.1-78.5)	78.8 (78.6-79)	80.3 (79-81.7)
Yamagata	58 (55.1-61)	64.2 (61.5-67.2)	65.8 (63-68.6)	68.3 (65.7-70.9)	70 (67.8-72.4)	72.5 (71.1-73.9)	74.2 (74-74.4)	75.8 (75.6-76)	76.7 (76.5-76.9)	77.2 (77-77.4)	78.2 (78-78.4)	78.7 (78.5-78.9)	80.1 (79.9-80.3)	80.9 (79.5-82.4)
Yamaguchi	56.1 (53.4-58.8)	62.7 (60.1-65.3)	64.7 (62.2-67.3)	67.4 (65.1-70.1)	69.1 (66.7-71.6)	71.5 (70.2-72.9)	72.8 (72.7-73)	74.6 (74.4-74.8)	75.8 (75.6-76)	76.3 (76.1-76.5)	77.3 (77.2-77.5)	78.2 (78-78.4)	79.2 (79-79.5)	80.7 (79.3-82.2)
Yamanashi	57.5 (54.8-60.4)	63.7 (61-66.5)	65.5 (62.9-68.1)	67.8 (65.4-70.2)	69.3 (67.3-71.3)	71.8 (70.6-72.9)	73.4 (73.2-73.6)	75.1 (74.9-75.3)	76.2 (76-76.4)	76.9 (76.7-77.1)	78.2 (78-78.4)	78.9 (78.7-79.1)	80 (79.7-80.2)	81.2 (79.8-82.6)
South Korea	33.1 (31.6-34.6)	51.6 (49.1-54.2)	54.7 (52-57.3)	56.9 (54.5-59.5)	59.4 (57.4-61.6)	61.9 (61.1-62.5)	63 (62.8-63.3)	65.6 (65.4-65.7)	68 (67.9-68.1)	70.4 (70.3-70.5)	72.6 (72.6-72.6)	75.3 (75.2-75.3)	77.2 (77-77.3)	79.5 (78.7-80.3)
Singapore	53.5 (52.3-54.7)	59.7 (59.5-59.9)	62.6 (62.4-62.8)	64.7 (64.5-64.9)	65.9 (65.7-66.1)	67.2 (67-67.3)	68.8 (68.7-69)	70.8 (70.7-71)	73.5 (73.3-73.7)	74.5 (74.3-74.6)	76.6 (75.6-77.5)	78.4 (78.3-78.6)	80.2 (80-80.3)	81.9 (81.2-82.6)
High-income North America	65.4 (64.9-65.9)	66.9 (66.5-67.3)	66.9 (66.9-67)	67.2 (67.2-67.2)	67.4 (67.4-67.4)	69 (69-69)	70.2 (70.2-70.2)	71.4 (71.4-71.4)	72.3 (72.3-72.3)	72.9 (72.9-72.9)	74.4 (74.4-74.4)	75.2 (75.2-75.2)	76.6 (76.6-76.6)	76.5 (76.2-76.8)
Canada	66.4 (66.3-66.5)	68 (67.9-68.1)	68.5 (68.4-68.6)	69 (68.9-69)	69.5 (69.4-69.6)	70 (70-70.1)	71.6 (71.6-71.7)	73 (72.9-73)	74.1 (74-74.2)	74.9 (74.9-75)	76.6 (76.5-76.6)	77.8 (77.7-77.8)	79.1 (79.1-79.2)	79.9 (79.2-80.5)
Greenland	47.7 (46.5-48.9)	51.4 (50.7-52.1)	56.8 (56.2-57.4)	59.5 (58.9-60.2)	61.7 (61.1-62.2)	61 (60.5-61.5)	59.8 (59.3-60.3)	59.9 (59.4-60.4)	62.1 (61.6-62.6)	64.6 (64.1-65)	66.7 (66.3-67.2)	68.4 (68-68.8)	69.7 (69.3-70.1)	70.8 (70.3-71.4)

Table 8 Estimates of life expectancy at birth by location and year for 1950, 1955...2005, 2010, 2017 - males

Location	1950	1955	1960	1965	1970	1975	1980	1985	1990	1995	2000	2005	2010	2017
USA	65.4 (64.8-65.9)	66.8 (66.4-67.3)	66.8 (66.8-66.8)	67 (67-67.1)	67.2 (67.2-67.2)	68.9 (68.9-68.9)	70.1 (70-70.1)	71.2 (71.2-71.2)	72.1 (72.1-72.1)	72.6 (72.6-72.7)	74.2 (74.1-74.2)	74.9 (74.9-75)	76.3 (76.3-76.3)	76.1 (75.8-76.4)
Alabama	63 (60.3-66.1)	64.8 (62.6-67.3)	64.9 (64.7-65)	64.5 (64.3-64.6)	65.2 (65-65.4)	66.8 (66.7-67)	68.2 (68.1-68.4)	69.5 (69.3-69.7)	69.9 (69.7-70)	70 (69.9-70.2)	71.1 (71-71.3)	71.6 (71.5-71.8)	72.6 (72.4-72.7)	72 (70.3-73.9)
Alaska	61 (58.8-63.5)	66 (64.2-67.8)	65 (64.7-65.4)	65.5 (65.2-65.9)	66.2 (65.8-66.5)	67.3 (67-67.6)	68.6 (68.3-68.9)	69.6 (69.3-69.8)	71.9 (71.6-72.2)	72.7 (72.4-72.9)	74.1 (73.9-74.4)	75.2 (75-75.5)	75.9 (75.7-76.2)	75.6 (74.3-76.8)
Arizona	62.8 (60.3-65.5)	65.5 (63.1-68)	65.3 (65-65.5)	66.6 (66.4-66.8)	66.8 (66.6-67)	69.1 (68.9-69.3)	70.4 (70.2-70.5)	72.2 (72-72.4)	73 (72.8-73.1)	72.9 (72.8-73.1)	74.5 (74.4-74.6)	75 (74.9-75.2)	76.9 (76.7-77)	76.6 (74.7-78.3)
Arkansas	65.2 (62.5-68.1)	67 (64.6-69.4)	67.1 (66.9-67.4)	66.4 (66.2-66.6)	66.9 (66.7-67.2)	67.9 (67.7-68.1)	69.4 (69.2-69.6)	70.3 (70.1-70.5)	70.8 (70.6-71)	70.9 (70.7-71.1)	71.9 (71.7-72)	72.5 (72.3-72.7)	73.3 (73.1-73.5)	72.6 (70.6-74.6)
California	66.4 (64.1-69)	67.9 (65.8-70.2)	67.6 (67.5-67.6)	67.9 (67.8-68)	68.4 (68.3-68.5)	70.1 (70-70.1)	71 (70.9-71)	72.1 (72-72.1)	72.7 (72.7-72.8)	73.8 (73.7-73.8)	75.8 (75.7-75.8)	76.5 (76.4-76.5)	78.5 (78.4-78.5)	78.5 (77.2-79.9)
Colorado	65.3 (62.5-68.1)	67.1 (64.6-69.5)	67.9 (67.7-68.1)	68.4 (68.2-68.6)	68.6 (68.4-68.8)	70.4 (70.2-70.6)	71.9 (71.7-72.1)	73 (72.8-73.1)	74.1 (73.9-74.3)	74.4 (74.2-74.5)	76 (75.8-76.2)	76.7 (76.5-76.8)	77.9 (77.7-78)	77.7 (76.2-79.3)
Connecticut	66.6 (63.9-69.2)	68.3 (66-70.7)	68.1 (67.9-68.3)	68.4 (68.2-68.6)	69.4 (69.2-69.5)	70.7 (70.6-70.9)	71.4 (71.2-71.6)	72.6 (72.4-72.7)	74.1 (74-74.3)	74.3 (74.1-74.4)	75.6 (75.4-75.8)	76.9 (76.7-77)	78.2 (78-78.4)	78 (76.3-79.7)
Delaware	62.5 (60-64.9)	66.3 (64.5-68.2)	66.3 (66-66.5)	66.2 (65.9-66.4)	66.4 (66.2-66.7)	68.1 (67.9-68.4)	69.7 (69.4-69.9)	70.9 (70.6-71.1)	71.8 (71.5-72)	72 (71.7-72.2)	73.8 (73.6-74)	74.4 (74.2-74.7)	75.6 (75.4-75.9)	75.7 (74.4-76.9)
Washington, DC	61.1 (58.2-63.9)	62.9 (60.5-65.6)	62.7 (62.4-63)	61.7 (61.5-62)	61.1 (60.9-61.4)	63 (62.8-63.3)	64.4 (64.1-64.7)	64.4 (64.1-64.8)	62.1 (61.7-62.4)	62.8 (62.5-63.2)	68.1 (67.8-68.5)	70 (69.6-70.3)	73.9 (73.5-74.2)	74.3 (72.2-76.2)
Florida	64.4 (61.7-67.2)	66.1 (63.7-68.6)	66.2 (66.1-66.4)	66.9 (66.8-67)	66.7 (66.5-66.8)	68.8 (68.7-68.9)	69.9 (69.8-70)	71.6 (71.5-71.7)	72.3 (72.2-72.4)	72.8 (72.7-72.9)	74.3 (74.3-74.4)	75 (74.9-75.1)	76.6 (76.5-76.7)	76.3 (74.8-78.1)
Georgia	62.5 (59.8-65.3)	64.1 (61.7-66.6)	64 (63.9-64.2)	64.5 (64.4-64.7)	64.4 (64.3-64.6)	66.4 (66.2-66.5)	68.1 (68-68.2)	69.1 (68.9-69.2)	70 (69.9-70.1)	70.7 (70.6-70.9)	72.1 (72-72.2)	73.2 (73.1-73.3)	74.6 (74.5-74.7)	74.7 (72.9-76.4)
Hawaii	67.5 (64.9-70.4)	69.5 (67.1-72.3)	70.5 (70.2-70.9)	71.2 (70.9-71.5)	71.4 (71-71.6)	73.3 (73-73.5)	74 (73.7-74.3)	75.2 (74.9-75.5)	75.6 (75.3-75.8)	75.7 (75.5-76)	77.2 (76.9-77.5)	77.5 (77.3-77.8)	78.3 (78-78.6)	78.7 (76.9-80.4)
Idaho	65.7 (63-68.6)	68.3 (65.8-70.9)	68.1 (67.8-68.4)	67.9 (67.6-68.3)	68.3 (68-68.5)	69.9 (69.6-70.2)	71.7 (71.4-71.9)	73 (72.7-73.3)	74 (73.7-74.2)	74.3 (74-74.6)	75.7 (75.5-76)	76.2 (76-76.5)	77.1 (76.8-77.3)	76.8 (74.9-78.6)
Illinois	65.6 (63.1-68.4)	66.8 (64.6-69.2)	66.6 (66.5-66.7)	66.5 (66.4-66.6)	66.6 (66.5-66.7)	68.1 (68-68.2)	69.6 (69.5-69.7)	71.1 (71-71.2)	71.6 (71.5-71.7)	72.1 (72-72.2)	73.9 (73.8-74)	75.1 (75-75.2)	76.4 (76.3-76.5)	76.7 (75.1-78.2)
Indiana	66.1 (63.4-68.9)	67.5 (65.3-69.8)	67.3 (67.2-67.5)	67.3 (67.1-67.4)	67.6 (67.4-67.7)	69.2 (69-69.3)	70.2 (70-70.3)	71.4 (71.3-71.6)	72.2 (72.1-72.3)	72.4 (72.3-72.5)	73.4 (73.2-73.5)	74.1 (74-74.3)	75 (74.8-75.1)	74.3 (72.6-76.1)
Iowa	67.5 (64.9-70.2)	68.5 (66.3-71)	69 (68.8-69.2)	68.7 (68.6-68.9)	69.1 (68.9-69.3)	70.4 (70.3-70.6)	71.9 (71.8-72.1)	73.1 (73-73.3)	74.4 (74.2-74.6)	74.5 (74.3-74.7)	75.8 (75.6-76)	76.7 (76.5-76.9)	77.1 (76.9-77.3)	77.2 (75.6-78.8)
Kansas	67.4 (64.8-70.2)	69.1 (66.7-71.5)	68.7 (68.6-69)	69.3 (69-69.5)	69.2 (69-69.4)	70.5 (70.3-70.7)	71.5 (71.3-71.7)	72.8 (72.6-73)	73.7 (73.4-73.9)	73.8 (73.7-74)	74.8 (74.6-75)	75.4 (75.2-75.6)	75.9 (75.8-76.1)	76.1 (74.4-77.9)
Kentucky	64.9 (62.2-67.8)	66.8 (64.3-69.1)	66.4 (66.2-66.6)	66.7 (66.5-66.8)	66.2 (66-66.3)	67.7 (67.5-67.8)	69 (68.8-69.2)	70.2 (70-70.3)	71 (70.9-71.2)	71.3 (71.1-71.4)	72.2 (72.1-72.4)	72.6 (72.4-72.7)	73.3 (73.1-73.4)	72.3 (70.5-74.1)
Louisiana	63.2 (60.7-66)	64.8 (62.5-67.3)	64.9 (64.7-65.1)	64.8 (64.7-65)	65.1 (64.9-65.2)	66.6 (66.5-66.8)	67.7 (67.5-67.8)	68.9 (68.8-69.1)	69.5 (69.4-69.7)	69.9 (69.7-70)	71 (70.8-71.1)	69.7 (69.6-69.9)	72.9 (72.8-73.1)	72.4 (70.4-74.3)
Maine	64.5 (61.8-67.1)	66.6 (64.3-69.1)	67 (66.7-67.2)	67.6 (67.3-67.8)	67.3 (67-67.5)	69.1 (68.9-69.4)	70.7 (70.5-71)	72 (71.7-72.2)	73.5 (73.2-73.7)	74 (73.7-74.3)	75 (74.8-75.3)	75.3 (75-75.5)	76.6 (76.3-76.9)	75.3 (73.3-77.2)
Maryland	64.1 (61.5-66.7)	65.8 (63.5-68.1)	65.6 (65.5-65.8)	65.9 (65.8-66.1)	66.7 (66.5-66.8)	68.5 (68.3-68.6)	69.8 (69.6-69.9)	70.7 (70.5-70.8)	71.6 (71.4-71.7)	71.6 (71.5-71.8)	73.5 (73.3-73.6)	74.4 (74.3-74.6)	76.5 (76.4-76.7)	75.9 (74-77.6)
Massachusetts	66.3 (63.7-69)	67.8 (65.6-70.1)	67.3 (67.2-67.4)	67.7 (67.6-67.8)	68.2 (68.1-68.3)	69.7 (69.6-69.8)	71.3 (71.1-71.4)	72.1 (72-72.2)	73.6 (73.5-73.8)	74.4 (74.3-74.5)	75.7 (75.6-75.8)	76.8 (76.7-76.9)	78 (77.8-78.1)	77.4 (75.6-78.9)
Michigan	66.3 (63.8-68.9)	67.7 (65.6-70)	67.3 (67.2-67.4)	67.3 (67.2-67.4)	67.4 (67.3-67.5)	68.6 (68.5-68.7)	70.2 (70.1-70.3)	70.8 (70.7-70.9)	72 (71.9-72.1)	72.7 (72.6-72.8)	73.8 (73.7-73.9)	74.7 (74.6-74.8)	75.6 (75.5-75.7)	75.2 (73.4-76.9)
Minnesota	67.3 (64.6-70.2)	68.6 (66.2-71)	68.9 (68.7-69.1)	69.2 (69.1-69.4)	69.6 (69.4-69.8)	71.3 (71.1-71.4)	72.5 (72.3-72.6)	73.6 (73.5-73.8)	74.7 (74.6-74.9)	75 (74.8-75.2)	76.4 (76.3-76.6)	77.6 (77.4-77.8)	78.5 (78.3-78.7)	78.4 (76.8-79.9)
Mississippi	62.3 (59.4-65.1)	64.1 (61.8-66.6)	64.7 (64.5-64.9)	64.2 (64-64.4)	64.3 (64.1-64.5)	66.3 (66.1-66.5)	67.6 (67.5-67.8)	68.7 (68.5-68.9)	69.3 (69.1-69.5)	69.2 (69-69.4)	70.3 (70.1-70.5)	70.3 (70.2-70.5)	71.9 (71.8-72.1)	71.3 (69.5-73.1)
Missouri	65.7 (63.2-68.5)	67.2 (64.9-69.6)	67.2 (67.1-67.4)	67.1 (67-67.3)	67 (66.9-67.2)	68.7 (68.6-68.9)	69.9 (69.8-70.1)	71.2 (71-71.3)	71.8 (71.7-72)	71.9 (71.8-72.1)	73.3 (73.2-73.5)	73.8 (73.7-74)	74.9 (74.7-75)	74.5 (72.8-76.2)
Montana	63.6 (61-66.5)	66 (63.5-68.6)	66.3 (65.9-66.6)	66.3 (66-66.6)	67.4 (67-67.7)	68.9 (68.6-69.2)	70.3 (70-70.6)	71.8 (71.6-72.2)	73.6 (73.3-73.9)	73.4 (73.1-73.7)	74.5 (74.2-74.8)	75.2 (74.9-75.5)	76.2 (76-76.6)	74.7 (72.9-76.6)
Nebraska	67.3 (64.6-70.1)	69 (66.6-71.4)	69 (68.7-69.2)	68.5 (68.3-68.8)	68.9 (68.7-69.2)	70.4 (70.1-70.6)	71.6 (71.4-71.9)	72.9 (72.6-73.1)	73.7 (73.4-73.9)	74.5 (74.2-74.7)	75.6 (75.4-75.9)	76.5 (76.3-76.7)	77.3 (77-77.5)	77 (75.2-78.6)
Nevada	57 (54.4-59.7)	63.6 (61.3-66.2)	64.1 (63.7-64.5)	63.8 (63.4-64.1)	65.5 (65.2-65.9)	68.4 (68-68.7)	68.9 (68.6-69.2)	71 (70.7-71.3)	71.2 (70.9-71.4)	71.4 (71.2-71.6)	73.1 (72.9-73.3)	73.3 (73.1-73.5)	75.6 (75.4-75.8)	75.6 (73.8-77.5)
New Hampshire	63.9 (61.2-66.6)	66.9 (64.6-69.4)	67.3 (67-67.6)	67.4 (67.1-67.7)	67.7 (67.4-68)	69.5 (69.2-69.8)	71.8 (71.5-72.1)	72.4 (72.1-72.6)	73.7 (73.4-74)	74.7 (74.5-74.9)	75.9 (75.6-76.2)	76.5 (76.2-76.8)	77.8 (77.5-78.1)	77.1 (75.3-78.9)
New Jersey	65.8 (63.4-68.3)	67 (64.8-69.3)	67.1 (67-67.3)	67.4 (67.3-67.5)	67.7 (67.6-67.8)	69.6 (69.5-69.7)	70.5 (70.4-70.6)	71 (70.9-71.1)	72.5 (72.3-72.6)	73 (72.9-73.1)	74.8 (74.6-74.9)	76.1 (76-76.2)	77.6 (77.5-77.7)	77.8 (76.2-79.4)
New Mexico	63.5 (60.8-66.5)	66 (63.5-68.7)	66.5 (66.2-66.8)	66.6 (66.3-66.9)	66.9 (66.6-67.2)	68.1 (67.8-68.4)	69.8 (69.5-70)	71.6 (71.3-71.8)	72.5 (72.3-72.8)	72.8 (72.5-73)	74.2 (74-74.4)	74.7 (74.5-74.9)	75.6 (75.3-75.8)	74.7 (72.8-76.8)
New York	65.9 (63.3-68.6)	66.9 (64.9-69)	66.8 (66.7-66.9)	67.2 (67.1-67.3)	67.1 (67-67.2)	69.1 (69-69.1)	70 (69.9-70)	70.6 (70.5-70.6)	71.2 (71.1-71.2)	72.3 (72.3-72.4)	75.1 (75-75.2)	76.5 (76.4-76.6)	77.9 (77.8-78)	78.3 (76.7-79.9)
North Carolina	63.3 (60.7-66.4)	65 (62.6-67.5)	64.7 (64.6-64.9)	64.8 (64.7-64.9)	65.1 (65-65.3)	67.1 (66.9-67.2)	68.6 (68.5-68.8)	70.2 (70-70.3)	70.9 (70.8-71)	71.5 (71.4-71.6)	72.6 (72.5-72.7)	73.8 (73.7-73.9)	75.2 (75.1-75.3)	75.3 (73.6-77)
North Dakota	66.8 (64.4-69.3)	69.1 (67.4-70.8)	68.9 (68.6-69.1)	68.6 (68.4-68.9)	69.3 (69-69.6)	70.6 (70.4-70.9)	72.1 (71.8-72.3)	73.3 (73.1-73.6)	74.3 (74-74.5)	74.7 (74.5-75)	75.7 (75.4-76)	76.6 (76.4-76.9)	76.9 (76.6-77.2)	76 (74.7-77.2)
Ohio	66 (63.6-68.8)	67.3 (65.3-69.4)	67.3 (67.2-67.4)	67.4 (67.3-67.5)	67.4 (67.3-67.5)	68.9 (68.8-69)	69.8 (69.7-69.9)	71.4 (71.2-71.5)	72.2 (72.1-72.3)	72.5 (72.4-72.6)	73.7 (73.6-73.8)	74.1 (74-74.2)	75.2 (75.1-75.3)	74.1 (72.4-76)
Oklahoma	66.1 (63.4-69)	67.9 (65.4-70.4)	67.4 (67.2-67.6)	67.8 (67.6-68)	67.2 (67-67.4)	68.6 (68.5-68.8)	69.6 (69.4-69.7)	70.6 (70.4-70.8)	72 (71.8-72.2)	71.4 (71.3-71.6)	72.4 (72.2-72.6)	72.5 (72.3-72.7)	73.2 (73-73.3)	72.8 (71-74.6)
Oregon	65.9 (63.3-68.7)	67.5 (65.1-70.3)	67.7 (67.5-67.9)	67.9 (67.7-68.1)	68.7 (68.5-68.9)	70.5 (70.3-70.7)	71.2 (71-71.4)	72.5 (72.3-72.7)	73.3 (73.1-73.5)	73.7 (73.5-73.9)	75.5 (75.4-75.7)	76 (75.8-76.1)	77.2 (77-77.4)	77 (75.2-78.7)
Pennsylvania	65.3 (63-68)	66.4 (64.4-68.7)	66.7 (66.6-66.8)	66.8 (66.7-66.9)	67.1 (67-67.2)	68.7 (68.6-68.8)	69.9 (69.8-70)	71.1 (71-71.2)	72.2 (72.1-72.3)	72.6 (72.5-72.7)	73.8 (73.7-73.9)	74.5 (74.4-74.6)	75.8 (75.7-75.9)	75.4 (73.7-77.1)
Rhode Island	65.4 (63.1-67.9)	67.5 (65.4-69.8)	67.7 (67.4-67.9)	67.6 (67.3-67.8)	68.3 (68-68.5)	70.2 (70-70.5)	70.8 (70.5-71)	72.1 (71.8-72.4)	73.2 (72.9-73.4)	74.5 (74.3-74.8)	75.4 (75.1-75.6)	76 (75.7-76.3)	77 (76.7-77.2)	76.4 (74.4-78.2)
South Carolina	61 (58.4-63.9)	62.8 (60.4-65.4)	62.5 (62.3-62.7)	63.3 (63.1-63.4)	64 (63.8-64.2)	65.8 (65.6-66)	67.5 (67.4-67.7)	69.1 (69-69.3)	70 (69.9-70.2)	70.5 (70.4-70.7)	71.5 (71.3-71.6)	72.5 (72.3-72.6)	73.8 (73.6-73.9)	73.2 (71.4-75.1)
South Dakota	65.6 (63.2-68.3)	68 (66.3-69.9)	67.6 (67.4-67.9)	68.2 (68-68.5)	68.6 (68.3-68.8)	69.8 (69.5-70)	70.9 (70.6-71.1)	72.3 (72-72.5)	73.5 (73.2-73.7)	73.7 (73.4-73.9)	74.9 (74.6-75.1)	75.6 (75.3-75.8)	76.6 (76.3-76.8)	75.6 (74.3-76.9)
Tennessee	65 (62.4-67.9)	66.6 (64.3-69.1)	66.4 (66.2-66.6)	66.6 (66.5-66.8)	66.1 (66-66.3)	68 (67.8-68.1)	69 (68.9-69.2)	70.1 (70-70.3)	70.4 (70.3-70.6)	70.6 (70.4-70.7)	71.7 (71.5-71.8)	72.3 (72.2-72.4)	73.5 (73.3-73.6)	73.6 (71.8-75.4)
Texas	65.7 (62.9-68.4)	66.9 (64.7-69.5)	67.1 (67-67.2)	67.5 (67.4-67.6)	67.2 (67.1-67.3)	68.8 (68.7-68.9)	69.7 (69.6-69.8)	70.6 (70.5-70.7)	71.6 (71.5-71.7)	72.6 (72.5-72.6)	73.9 (73.8-74)	74.8 (74.8-74.9)	76 (75.9-76.1)	76.1 (74.6-77.8)
Utah	66.7 (64.1-69.5)	69.3 (66.7-71.8)	68.5 (68.2-68.7)	68.8 (68.5-69.1)	69.4 (69.1-69.7)	71 (70.8-71.3)	72.5 (72.3-72.7)	73.4 (73.1-73.6)	75 (74.8-75.3)	75.1 (74.9-75.4)	76.4 (76.2-76.6)	77.2 (77-77.4)	77.9 (77.7-78.1)	77.5 (75.9-79.1)
Vermont	63.2 (61.4-65.3)	67 (65.7-68.3)	67 (66.7-67.4)	67 (66.7-67.3)	68.1 (67.8-68.3)	69.6 (69.4-69.9)	71 (70.8-71.2)	72.1 (71.9-72.4)	73.4 (73.1-73.6)	74.3 (74-74.5)	75.7 (75.5-75.9)	77 (76.8-77.2)	77.7 (77.5-78)	77.2 (76.2-78.2)
Virginia	64 (61.4-66.8)	65.4 (63.1-67.8)	65.7 (65.6-65.9)	65.9 (65.7-66)	66.5 (66.3-66.6)	68.1 (67.9-68.2)	69.5 (69.4-69.7)	71 (70.8-71.1)	71.9 (71.8-72.1)	72.7 (72.6-72.8)	74.1 (74-74.2)	75.1 (75-75.3)	76.7 (76.5-76.8)	76.7 (75.1-78.3)
Washington	66.2 (63.5-69)	67.4 (65.1-69.8)	68 (67.8-68.1)	68.8 (68.6-69)	68.4 (68.2-68.6)	70.5 (70.3-70.6)	71.6 (71.5-71.8)	72.9 (72.7-73)	73.8 (73.6-73.9)	74.5 (74.4-74.6)	75.7 (75.6-75.8)	76.7 (76.5-76.8)	77.8 (77.6-77.9)	77.8 (76.2-79.4)
West Virginia	64.4 (61.6-67.3)	66.1 (63.7-68.5)	66.3 (66.1-66.5)	65.9 (65.7-66.1)	65.5 (65.4-65.8)	67.3 (67.1-67.5)	68.7 (68.5-68.9)	70 (69.8-70.2)	71 (70.7-71.2)	71.4 (71.2-71.7)	72.2 (71.9-72.4)	72.5 (72.3-72.7)	72.8 (72.6-73)	72.2 (70.4-74)
Wisconsin	67 (64.5-69.5)	68.4 (66.5-70.7)	68.2 (68.1-68.4)	68.5 (68.4-68.7)	69.3 (69.2-69.5)	70.7 (70.5-70.8)	71.9 (71.7-72)	73.1 (73-73.3)	73.7 (73.6-73.9)	74.4 (74.2-74.5)	75.2 (75.1-75.4)	76.1 (76-76.2)	77.2 (77.1-77.4)	76.8 (75.3-78.6)
Wyoming	63.4 (61.3-65.5)	67.1 (65.7-68.5)	66.6 (66.3-66.9)	67.2 (66.9-67.5)	66.8 (66.5-67.1)	68.3 (68-68.5)	69.9 (69.7-70.2)	71.6 (71.3-71.8)	73.1 (72.9-73.4)	73.3 (73.1-73.6)	74.4 (74.1-74.6)	75.1 (74.8-75.3)	76 (75.8-76.3)	75.5 (74.3-76.7)
Southern Latin America	57.2 (57.1-57.3)	59.3 (59.2-59.4)	59.3 (59.2-59.4)	61.2 (61.1-61.3)	62 (61.9-62.1)	64.6 (64.1-65.1)	66.6 (66.5-66.7)	68.5 (68.5-68.6)	69.2 (69.1-69.2)	70.3 (70.2-70.3)	71.4 (71.3-71.4)	72.6 (72.5-72.6)	73.4 (73.4-73.5)	74.5 (73.3-75.5)
Argentina	60.5 (60.4-60.6)	61.7 (61.6-61.8)	62.2 (62.1-62.3)	63 (62.9-63.1)	62.6 (62.5-62.7)	64.8 (64-65.5)	66.6 (66.5-66.7)	68.5 (68.4-68.6)	68.9 (68.9-69)	69.7 (69.6-69.8)	70.5 (70.5-70.6)	71.8 (71.7-71.8)	72.6 (72.5-72.6)	73.6 (72-75)
Chile	47.8 (47.6-48.1)	52.1 (51.9-52.2)	51.7 (51.5-51.8)	56.3 (56.2-56.5)	59.6 (59.4-59.7)	63.7 (63.6-63.9)	66.6 (66.5-66.7)	68.6 (68.5-68.7)	69.9 (69.8-70)	72 (71.9-72.1)	74 (73.9-74.1)	75.1 (75-75.2)	75.9 (75.8-76)	77.2 (75.7-78.7)
Uruguay	62.6 (62.3-62.8)	65.1 (64.9-65.3)	65.3 (65.1-65.6)	65.3 (65.1-65.5)	65.2 (65-65.4)	66 (65.8-66.3)	66.9 (66.7-67.1)	68.8 (68.6-69)	69.4 (69.2-69.5)	69.7 (69.5-69.9)	70.8 (70.6-71)	71.9 (71.7-72.1)	72.7 (72.5-72.9)	73.5 (72.1-75)
Western Europe	64.1 (63.5-64.8)	66.3 (66.1-66.4)	67.4 (67.3-67.5)	68.1 (68-68.2)	68.6 (68.5-68.7)	69.4 (69.4-69.5)	70.7 (70.6-70.7)	72 (72-72)	73 (73-73)	74.1 (74.1-74.1)	75.6 (75.6-75.6)	77.1 (77.1-77.1)	78.4 (78.4-78.4)	79.5 (79.2-79.8)
Andorra	72.2 (69.6-74.8)	72.7 (70.2-75.4)	73.2 (70.8-75.8)	73.6 (71.4-76.3)	74.1 (72-76.7)	74.8 (72.9-77)	75.4 (73.7-77.1)	75.7 (74.2-77.3)	76.1 (74.7-77.3)	77.1 (75.7-78.1)	78.3 (77.3-79.1)	78.8 (78.1-79.5)	79.6 (78.9-80.4)	80.5 (79.4-81.7)
Austria	62 (61.9-62.2)	64.7 (64.5-64.8)	65.4 (65.3-65.6)	66.6 (66.5-66.7)	66.5 (66.4-66.6)	67.6 (67.5-67.7)	69 (68.9-69.1)	70.5 (70.4-70.6)	72.4 (72.3-72.5)	73.4 (73.3-73.5)	75.2 (75-75.3)	76.7 (76.6-76.8)	77.8 (77.6-77.9)	79.4 (78.8-80.1)
Belgium	63.3 (63.1-63.4)	66 (65.9-66.2)	66.9 (66.8-67)	67.6 (67.4-67.7)	67.9 (67.8-68)	68.9 (68.8-69)	69.7 (69.6-69.8)	71.1 (71-71.2)	72.7 (72.6-72.8)	73.5 (73.4-73.6)	74.6 (74.5-74.8)	76.2 (76.1-76.3)	77.4 (77.3-77.5)	78.9 (78.2-79.5)
Cyprus	61.5 (58.9-64.1)	62.1 (59.6-64.4)	63.6 (61.2-65.8)	64.5 (62.4-66.6)	65.6 (64.3-67)	67.3 (67-67.6)	69.9 (69.7-70.2)	71.4 (71.2-71.7)	73.6 (73.4-73.8)	74.5 (74-75.1)	73.9 (73.2-74.6)	74.8 (74.1-75.4)	76.9 (76.3-77.6)	78.5 (77.4-79.5)
Denmark	69.3 (69.1-69.4)	70.4 (70.2-70.5)	70.6 (70.4-70.7)	70.2 (70.1-70.4)	70.9 (70.8-71.1)	71.4 (71.3-71.5)	71.2 (71-71.3)	71.7 (71.6-71.9)	72.2 (72.1-72.4)	72.9 (72.8-73.1)	74.7 (74.5-74.8)	76 (75.9-76.2)	77.2 (77-77.3)	78.8 (78.1-79.5)
Finland	60.5 (60.3-60.6)	64.1 (64-64.3)	65.6 (65.4-65.7)	65.5 (65.4-65.6)	66.3 (66.2-66.4)	67.6 (67.4-67.7)	69.4 (69.3-69.6)	70.3 (70.2-70.4)	71 (70.9-71.2)	72.9 (72.8-73.1)	74.3 (74.1-74.4)	75.7 (75.5-75.8)	76.9 (76.8-77)	78.5 (77.8-79.2)
France	64.4 (64.3-64.5)	66.2 (66.2-66.3)	67.9 (67.8-67.9)	68.1 (68-68.1)	68.9 (68.9-69)	69.3 (69.3-69.4)	70.5 (70.5-70.6)	71.6 (71.5-71.6)	73 (72.9-73)	74 (74-74.1)	75.4 (75.3-75.4)	76.8 (76.8-76.9)	78.1 (78-78.1)	79.8 (79.2-80.4)
Germany	63.5 (60.9-66.3)	65.7 (65-66.4)	66.4 (66.4-66.5)	67.6 (67.6-67.7)	67.5 (67.5-67.6)	68.3 (68.3-68.3)	69.7 (69.7-69.8)	71.7 (71.7-71.8)	72.1 (72.1-72.2)	73.4 (73.3-73.4)	75.2 (75.2-75.3)	76.9 (76.9-77)	77.9 (77.9-78)	78.2 (76.9-79.5)
Greece	66.4 (65.4-67.3)	70 (69.8-70.1)	70.6 (70.5-70.8)	70.9 (70.8-71)	71.5 (71.3-71.6)	72.3 (72.1-72.4)	73 (72.9-73.1)	73.5 (73.4-73.6)	74.7 (74.6-74.8)	75.1 (75-75.2)	75.9 (75.8-76)	76.8 (76.7-76.9)	77.8 (77.7-77.9)	78.4 (77.8-79.1)
Iceland	69 (68.5-69.6)	70.6 (70.2-71)	71 (70.7-71.4)	70.8 (70.5-71.1)	70.9 (70.6-71.2)	72.6 (72.3-72.9)	73.4 (73-73.8)	74.5 (74-74.9)	75.6 (75.2-75.9)	76.1 (75.8-76.4)	78.1 (77.8-78.4)	79.6 (79.3-79.9)	80 (79.7-80.2)	79.8 (79.4-80.2)
Ireland	64.9 (64.7-65.1)	67.2 (67-67.4)	68.6 (68.4-68.7)	68.7 (68.5-68.9)	68.8 (68.7-69)	69.3 (69.1-69.5)	70 (69.8-70.1)	70.9 (70.7-71)	72.2 (72.1-72.4)	72.9 (72.8-73.1)	74 (73.9-74.2)	76.7 (76.5-76.8)	78.5 (78.3-78.6)	80 (79.3-80.7)
Israel	73.1 (72.8-73.4)	69.5 (69.3-69.8)	70.5 (70.3-70.7)	70.3 (70-70.5)	69.8 (69.6-70)	70.5 (70.3-70.7)	72.4 (72.3-72.6)	73.9 (73.7-74)	75.8 (75.6-76)	75.7 (75.6-75.9)	76.5 (76.4-76.7)	78.2 (78.1-78.3)	80 (79.8-80.1)	81.3 (80.6-81.9)
Italy	64.1 (64-64.2)	66.3 (66.2-66.4)	67.3 (67.2-67.4)	68 (68-68.1)	68.9 (68.8-68.9)	69.6 (69.6-69.7)	70.6 (70.5-70.6)	72.4 (72.3-72.4)	73.7 (73.7-73.8)	74.8 (74.8-74.8)	76.5 (76.5-76.6)	78 (78-78.1)	79.4 (79.3-79.4)	80.8 (80.2-81.4)
Luxembourg	63.9 (63.4-64.3)	64.8 (64.5-65.1)	66 (65.7-66.3)	66.5 (66.2-66.8)	66.8 (66.6-67.1)	67.5 (67.3-67.8)	69.3 (69-69.5)	70.3 (70.1-70.6)	71.8 (71.6-72.1)	73 (72.8-73.3)	75.3 (75-75.5)	76.8 (76.5-77)	79 (78.8-79.3)	80 (78.9-81.2)
Malta	61.9 (61.4-62.4)	65.5 (65.2-65.8)	66.8 (66.6-67.1)	67.7 (67.5-68)	68.6 (68.3-68.9)	69.7 (69.4-70)	70.2 (70-70.4)	72.1 (71.8-72.3)	74.2 (73.9-74.4)	75.2 (75-75.4)	76.1 (75.9-76.4)	77.1 (76.9-77.4)	78.1 (77.9-78.4)	78.9 (78.4-79.5)
Netherlands	70.4 (70.3-70.5)	71 (70.9-71.1)	71.4 (71.3-71.5)	71 (70.9-71.1)	70.8 (70.7-70.9)	71.4 (71.3-71.5)	72.4 (72.3-72.5)	73 (73-73.1)	73.8 (73.7-73.9)	74.6 (74.5-74.7)	75.5 (75.4-75.5)	77.2 (77.1-77.3)	78.7 (78.6-78.8)	79.9 (79.2-80.5)
Norway	70.6 (70-71.2)	71.5 (71-72)	71.5 (71.1-71.9)	71.2 (70.9-71.4)	71.1 (71-71.2)	71.9 (71.8-72)	72.3 (72.2-72.4)	72.7 (72.7-72.8)	73.4 (73.3-73.5)	74.8 (74.8-74.9)	75.8 (75.7-75.9)	77.7 (77.6-77.8)	78.8 (78.7-78.8)	80.5 (80.2-80.7)
Portugal	56.5 (56.3-56.8)	59 (58.8-59.3)	61.9 (61.7-62.1)	62.9 (62.7-63.1)	64.3 (64.2-64.5)	65.5 (65.3-65.8)	68.3 (68.2-68.4)	69.8 (69.6-69.9)	70.7 (70.5-70.8)	71.7 (71.6-71.8)	73.3 (73.2-73.4)	75.4 (75.1-75.7)	76.9 (76.8-77)	78.5 (77.9-79.2)
Spain	59.8 (59.7-60)	64.6 (64.6-64.7)	67.4 (67.3-67.5)	68.5 (68.5-68.6)	69.6 (69.5-69.6)	70.8 (70.8-70.9)	72.6 (72.5-72.6)	73.3 (73.2-73.3)	73.5 (73.4-73.5)	74.4 (74.3-74.4)	75.9 (75.9-76)	77.1 (77.1-77.2)	78.9 (78.9-79)	80.2 (79.7-80.8)
Sweden	70.1 (69.5-70.7)	71.2 (70.6-71.8)	71.5 (70.9-72.1)	71.9 (71.3-72.5)	72.3 (71.7-72.9)	72.3 (71.8-72.7)	72.9 (72.8-73)	74 (73.8-74.1)	74.9 (74.8-75.1)	76.3 (76.2-76.4)	77.5 (77.4-77.6)	78.5 (78.4-78.6)	79.4 (79.3-79.5)	80.8 (80.2-81.4)
Stockholm	69.4 (66.5-72.5)	70.4 (67.5-73.5)	70.6 (67.9-73.6)	71 (68.3-73.8)	71.3 (68.8-74)	71.1 (69.3-73.3)	71.6 (71.4-71.9)	73.1 (72.9-73.3)	73.7 (73.5-74)	75.6 (75.4-75.8)	77 (76.8-77.2)	78.5 (78.3-78.7)	79.6 (79.4-79.8)	80.8 (80-81.6)
Sweden except Stockholm	70.2 (69.7-70.7)	71.3 (70.9-71.9)	71.6 (71.1-72.2)	72.1 (71.6-72.6)	72.5 (72-73.1)	72.5 (72.1-72.9)	73.1 (73-73.3)	74.1 (74-74.2)	75.2 (75.1-75.3)	76.4 (76.3-76.6)	77.6 (77.4-77.7)	78.5 (78.3-78.6)	79.4 (79.2-79.5)	80.8 (80.1-81.4)
Switzerland	66.9 (66.7-67.1)	68 (67.8-68.1)	69.2 (69.1-69.4)	69.9 (69.8-70)	70.3 (70.2-70.5)	71.7 (71.6-71.9)	72.6 (72.5-72.8)	73.8 (73.7-74)	74.4 (74.3-74.5)	75.6 (75.5-75.8)	77.2 (77.1-77.4)	78.9 (78.8-79.1)	80.2 (80.1-80.4)	82.1 (81.5-82.8)
United Kingdom	66.7 (66.3-67)	67.6 (67.3-67.9)	68.2 (67.9-68.5)	68.5 (68.2-68.8)	68.8 (68.5-69.1)	69.7 (69.5-69.9)	70.7 (70.6-70.7)	71.8 (71.8-71.8)	72.9 (72.9-73)	74.1 (74-74.1)	75.5 (75.4-75.6)	77.1 (77.1-77.1)	78.4 (78.4-78.4)	79.2 (79-79.3)
England	67.2 (66.9-67.5)	68.1 (67.8-68.4)	68.6 (68.3-68.9)	68.9 (68.5-69.2)	69.2 (68.9-69.4)	70 (69.8-70.2)	71 (70.9-71)	72.1 (72.1-72.1)	73.2 (73.1-73.2)	74.3 (74.3-74.4)	75.8 (75.7-75.8)	77.4 (77.4-77.4)	78.7 (78.7-78.7)	79.5 (79.4-79.6)
East Midlands	66.6 (65.3-67.8)	67.6 (66.3-68.8)	68.2 (67.1-69.4)	68.6 (67.4-69.8)	69.1 (68-70.1)	70.1 (69.3-70.8)	71 (70.8-71.2)	72.2 (72.1-72.3)	73.3 (73.2-73.4)	74.5 (74.5-74.6)	75.7 (75.6-75.9)	77.3 (77.3-77.4)	78.6 (78.5-78.6)	79.3 (79-79.6)
Derby	65.5 (62.1-69.1)	66.7 (63.4-69.9)	67.5 (64.3-71.1)	68 (64.8-71.2)	68.5 (65.7-71.4)	69.4 (67.3-71.4)	69.8 (69.2-70.4)	71.1 (70.8-71.4)	72.3 (72-72.6)	74 (73.7-74.3)	75.5 (75.1-75.9)	76.8 (76.5-77.1)	77.3 (77-77.6)	78.2 (77.3-79.1)
Derbyshire	66.5 (63-69.8)	67.5 (64.5-70.6)	68.1 (64.9-71.5)	68.4 (65-71.5)	68.7 (65.9-71.6)	69.7 (67.7-71.8)	70.8 (70.3-71.3)	72.2 (71.9-72.4)	73.1 (72.9-73.3)	74.5 (74.3-74.7)	75.8 (75.5-76.2)	77.5 (77.3-77.7)	78.9 (78.7-79.1)	79.1 (78.3-80)
Leicester	65.1 (61.6-68.5)	66.2 (63.1-69.5)	67 (64-70.2)	67.4 (64.4-70.5)	68.1 (65.4-70.8)	69.2 (67.2-71.3)	70.1 (69.5-70.7)	71.1 (70.9-71.4)	72.1 (71.8-72.3)	72.9 (72.6-73.1)	73.6 (73.2-74)	75.3 (75-75.5)	76.5 (76.2-76.8)	77.8 (77-78.7)
Leicestershire	68.2 (64.9-71.4)	69.1 (66.1-72.4)	69.7 (66.5-73.2)	70 (67-73.2)	70.4 (67.5-73.3)	71.3 (69.2-73.3)	72.2 (71.7-72.7)	73.4 (73.2-73.6)	74.5 (74.3-74.7)	75.8 (75.6-76.1)	77.2 (76.9-77.6)	78.8 (78.6-79)	79.7 (79.4-79.9)	80.4 (79.6-81.2)
Lincolnshire	66.7 (63.4-69.9)	67.8 (64.6-71)	68.4 (65.5-71.6)	68.8 (65.8-71.9)	69.3 (66.4-72.1)	70.3 (68.3-72.4)	71.3 (70.8-71.9)	72.4 (72.2-72.6)	73.4 (73.2-73.7)	74.8 (74.5-75)	75.8 (75.5-76.2)	77.5 (77.2-77.7)	78.8 (78.5-79)	79.1 (78.3-80.1)
Northamptonshire	67.1 (63.8-70.5)	68.2 (65.2-71.4)	68.9 (65.9-71.8)	69.3 (66.3-72.4)	69.8 (67-72.7)	70.8 (68.6-72.8)	71.6 (71-72.1)	72.6 (72.4-72.8)	73.5 (73.3-73.7)	74.8 (74.6-75.1)	75.9 (75.5-76.2)	77.5 (77.2-77.7)	78.4 (78.2-78.6)	79.7 (78.9-80.5)
Nottingham	64.9 (61.7-68.5)	65.9 (62.7-69.1)	66.4 (63.4-69.6)	66.6 (63.6-69.8)	67 (64.2-69.7)	68 (65.9-70)	69.1 (68.6-69.7)	70.6 (70.4-70.9)	71.5 (71.2-71.7)	72.1 (71.9-72.4)	72.5 (72.1-72.9)	74.2 (73.9-74.5)	76.2 (75.9-76.4)	77.6 (76.7-78.5)
Nottinghamshire	66.9 (63.5-70.4)	67.9 (64.8-71.2)	68.6 (65.4-71.9)	69 (66.1-72.1)	69.5 (66.8-72.4)	70.4 (68.4-72.4)	71.2 (70.7-71.7)	72.4 (72.2-72.6)	73.5 (73.3-73.7)	74.6 (74.4-74.9)	75.9 (75.6-76.2)	77.5 (77.3-77.7)	78.8 (78.6-79)	79.4 (78.6-80.2)
Rutland	68.9 (65.5-72.5)	70.3 (66.9-73.7)	71 (67.6-74.5)	71.4 (68.1-74.9)	71.9 (68.8-74.9)	72.8 (70.6-75.1)	73.5 (72.6-74.5)	74.2 (73.6-74.7)	75.4 (74.8-76)	76.9 (76.3-77.5)	78.3 (77.7-79)	79.3 (78.7-79.8)	80.1 (79.5-80.7)	80.6 (79.4-81.9)
East of England	68.2 (67-69.4)	69.1 (68-70.4)	69.8 (68.5-71)	70.1 (68.9-71.3)	70.4 (69.4-71.5)	71.2 (70.4-72)	72.2 (72-72.4)	73.5 (73.5-73.6)	74.5 (74.4-74.6)	75.7 (75.6-75.7)	76.9 (76.8-77.1)	78.5 (78.4-78.5)	79.6 (79.5-79.7)	80.2 (79.8-80.5)
Bedford	66.8 (63.6-70.4)	67.9 (64.6-71.2)	68.5 (65.3-72)	68.8 (65.7-72.1)	69.1 (66.5-71.9)	70.1 (68.1-72.3)	71.5 (70.8-72.2)	73 (72.6-73.4)	73.7 (73.3-74.1)	74.7 (74.4-75.1)	76.1 (75.7-76.5)	77.5 (77.2-77.9)	78.1 (77.7-78.4)	79.2 (78.2-80.1)
Cambridgeshire	68.7 (65.7-72.3)	69.6 (66.4-73)	70.1 (67-73.5)	70.3 (67.3-73.6)	70.5 (67.8-73.4)	71.3 (69.3-73.4)	72.4 (71.8-72.9)	73.8 (73.6-74)	74.9 (74.6-75.1)	75.9 (75.6-76.1)	77.4 (77-77.7)	78.7 (78.4-78.9)	80.1 (79.8-80.3)	81 (80.2-81.9)
Central Bedfordshire	68.2 (64.9-71.6)	69.3 (66.2-72.7)	70 (66.8-73.4)	70.4 (67.5-73.5)	70.7 (68.1-73.5)	71.5 (69.6-73.6)	72.1 (71.5-72.7)	73.2 (72.9-73.5)	74.2 (73.9-74.5)	75.5 (75.2-75.8)	76.8 (76.4-77.2)	78.2 (77.9-78.5)	79.9 (79.5-80.2)	81.1 (80.2-82)
Essex	68.4 (65.4-71.5)	69.3 (66.3-72.6)	69.9 (66.9-73)	70.1 (67.1-73.3)	70.3 (67.7-73.1)	70.9 (69.1-73)	72.1 (71.6-72.6)	73.6 (73.4-73.8)	74.7 (74.5-74.8)	75.7 (75.6-75.9)	77.1 (76.8-77.4)	78.6 (78.4-78.7)	79.6 (79.4-79.8)	79.8 (79-80.6)
Hertfordshire	68.7 (65.7-72.3)	69.7 (66.7-73)	70.2 (67.2-73.5)	70.5 (67.5-73.7)	70.6 (67.9-73.4)	71.2 (69.4-73.4)	72.3 (71.8-72.8)	73.6 (73.4-73.8)	74.7 (74.5-74.9)	76.1 (75.9-76.3)	77.2 (76.9-77.5)	78.9 (78.7-79.1)	79.8 (79.6-80)	81.3 (80.5-82.2)

Table 8 Estimates of life expectancy at birth by location and year for 1950, 1955...2005, 2010, 2017 - males

Location	1950	1955	1960	1965	1970	1975	1980	1985	1990	1995	2000	2005	2010	2017
Luton	66.6 (63.2-70.1)	67.8 (64.6-71.4)	68.7 (65.5-72)	69.3 (66.2-72.4)	69.7 (67.2-72.5)	70.6 (68.6-73)	71 (70.4-71.7)	72.5 (72.1-72.8)	72.8 (72.4-73.1)	73.6 (73.3-73.9)	74.9 (74.5-75.3)	76.2 (75.9-76.5)	77.7 (77.4-78.1)	79.5 (78.6-80.5)
Norfolk	68 (64.9-71.2)	69 (66.1-72.3)	69.8 (66.7-73)	70.2 (67.3-73.3)	70.7 (67.9-73.4)	71.6 (69.8-73.6)	72.5 (72-73)	73.7 (73.5-73.9)	74.5 (74.3-74.7)	75.7 (75.5-75.9)	77 (76.7-77.4)	78.5 (78.3-78.7)	79.8 (79.6-80)	79.2 (78.4-80.1)
Peterborough	66.5 (63.2-70.4)	67.6 (64.1-71.2)	68.2 (64.9-71.7)	68.5 (65.3-71.8)	68.8 (65.9-71.6)	69.6 (67.5-71.9)	71.1 (70.4-71.8)	72.5 (72.1-72.8)	73.5 (73.2-73.8)	74.2 (73.8-74.5)	75.5 (75-75.9)	76.7 (76.3-77)	77.4 (77.1-77.8)	78.8 (77.9-79.7)
Southend-on-Sea	67.1 (63.9-70.5)	68.3 (65.4-71.5)	69.1 (66.2-72.3)	69.7 (66.6-73.1)	70.2 (67.4-73.2)	71.2 (69.1-73.3)	72.2 (71.5-72.9)	73.3 (72.9-73.6)	74.2 (73.8-74.5)	75 (74.7-75.4)	75.8 (75.3-76.2)	77.1 (76.7-77.4)	78.3 (78-78.7)	78.9 (78-79.9)
Suffolk	68.2 (65.1-71.8)	69.4 (66.1-72.6)	70.1 (67-73.5)	70.7 (67.7-73.8)	71.1 (68.3-74.1)	72 (70-74.1)	72.7 (72.2-73.2)	73.8 (73.5-74)	74.9 (74.7-75.2)	75.9 (75.7-76.1)	77.2 (76.8-77.5)	79 (78.8-79.3)	80 (79.8-80.2)	80.2 (79.3-81.1)
Thurrock	66.9 (63.9-70.6)	67.9 (64.7-71.5)	68.4 (65.3-71.9)	68.6 (65.6-71.9)	68.8 (66-72)	69.6 (67.6-71.8)	70.7 (70.1-71.4)	72.3 (71.9-72.6)	73.4 (73.1-73.8)	74.7 (74.4-75.1)	76.4 (76-76.9)	77.2 (76.9-77.5)	78.9 (78.5-79.2)	79.5 (78.6-80.5)
Greater London	67.7 (67.1-68.3)	68.5 (67.9-69)	68.9 (68.3-69.4)	69.1 (68.5-69.6)	69.4 (68.9-69.8)	70.2 (69.9-70.6)	71 (70.8-71.1)	72 (71.9-72)	72.8 (72.7-72.8)	73.6 (73.5-73.7)	75.6 (75.5-75.7)	77.4 (77.4-77.5)	79 (78.9-79)	80.4 (80.2-80.5)
Barking and Dagenham	66 (62.8-69.4)	66.8 (63.7-69.9)	67.3 (64.4-70.2)	67.4 (64.5-70.3)	67.6 (65-70.4)	68.5 (66.5-70.3)	69.3 (68.7-69.9)	70.8 (70.5-71.1)	71.9 (71.6-72.2)	72.9 (72.6-73.2)	73.9 (73.5-74.3)	75.6 (75.3-75.9)	76.7 (76.4-77)	77.3 (76.5-78.3)
Barnet	69.4 (66.2-72.5)	70.2 (67.3-73.2)	70.7 (67.9-73.8)	71 (68.2-74.1)	71.4 (68.9-74.2)	72.3 (70.4-74.3)	72.8 (72.3-73.4)	73.8 (73.5-74.1)	74.7 (74.5-75)	75.8 (75.6-76.1)	77.7 (77.3-78)	79.2 (79-79.5)	80.4 (80.1-80.7)	81.3 (80.5-82.2)
Bexley	69.1 (65.9-72.4)	69.8 (66.6-72.9)	70.2 (67.3-73.2)	70.4 (67.7-73.2)	70.6 (68.1-73.5)	71.5 (69.6-73.5)	72.4 (71.8-73)	73.7 (73.5-74)	74.7 (74.4-75)	75.7 (75.4-76)	76.8 (76.4-77.2)	78.2 (77.9-78.5)	79.2 (78.9-79.5)	79.7 (78.8-80.7)
Brent	67.8 (64.8-71.2)	68.7 (65.8-71.7)	69.3 (66.4-72.3)	69.7 (66.7-72.6)	70.1 (67.6-72.9)	71.1 (69.2-72.9)	71.5 (70.9-72.1)	71.9 (71.6-72.1)	72.9 (72.6-73.2)	73.6 (73.3-73.9)	75.5 (75.1-75.9)	77.7 (77.4-77.9)	79.1 (78.9-79.4)	80.5 (79.7-81.3)
Bromley	69.7 (66.4-73.1)	70.4 (67.5-73.5)	70.8 (68.1-74)	71.1 (68.2-74.1)	71.4 (68.8-73.9)	72.3 (70.5-74.1)	73.1 (72.5-73.6)	73.8 (73.5-74)	75 (74.7-75.2)	76 (75.7-76.2)	77.6 (77.3-78)	79.7 (79.4-80)	80.6 (80.3-80.9)	80.7 (79.8-81.6)
Camden	67.1 (64-70.5)	67.8 (64.8-71)	67.9 (65-71)	67.8 (64.8-70.7)	67.9 (65.2-70.7)	68.6 (66.7-70.6)	69.3 (68.7-69.9)	70.9 (70.6-71.2)	70.6 (70.3-70.9)	71.1 (70.8-71.4)	73.2 (72.9-73.7)	74.7 (74.5-75)	78.6 (78.3-78.9)	81.9 (81-82.8)
Croydon	68.8 (65.8-72.1)	69.7 (66.7-72.9)	70.3 (67.3-73.2)	70.7 (68-73.6)	71.1 (68.4-73.6)	71.9 (70.1-73.8)	72.2 (71.7-72.7)	72.6 (72.4-72.9)	73.8 (73.6-74.1)	74.9 (74.6-75.1)	76.3 (76-76.7)	78.4 (78.1-78.6)	78.9 (78.7-79.2)	80.3 (79.5-81.1)
Ealing	67.6 (64.6-71.1)	68.3 (65.4-71.4)	68.7 (65.6-71.7)	68.8 (66-71.9)	69 (66.6-71.9)	69.8 (67.9-71.8)	70.5 (70-71.1)	71.6 (71.4-71.9)	72.6 (72.3-72.8)	73.6 (73.3-73.8)	75.7 (75.3-76.1)	77.6 (77.4-77.9)	78.9 (78.6-79.1)	80.4 (79.6-81.3)
Enfield	68.4 (65.1-71.8)	69.2 (66-72.5)	69.7 (66.3-72.7)	69.9 (67-72.9)	70.2 (67.6-72.7)	71.1 (69.3-73)	71.9 (71.3-72.4)	72.9 (72.6-73.2)	74 (73.7-74.2)	74.7 (74.4-75)	76.5 (76.2-76.9)	78.6 (78.3-78.8)	80 (79.7-80.2)	80 (79.2-80.9)
Greenwich	66.7 (63.7-70.1)	67.6 (64.7-71)	68.2 (65.2-71.4)	68.5 (65.6-71.6)	68.8 (66.2-71.7)	69.7 (67.8-71.7)	70.4 (69.8-71)	71.3 (71-71.6)	72 (71.7-72.2)	72.6 (72.3-72.8)	74.2 (73.8-74.6)	75.7 (75.4-75.9)	77.8 (77.6-78.1)	79.8 (78.9-80.6)
Hackney	67.1 (63.8-70.3)	67.7 (64.8-70.9)	67.9 (65-70.9)	67.8 (65-70.9)	67.8 (65-70.6)	68.4 (66.4-70.5)	69.3 (68.8-69.9)	70.1 (69.8-70.4)	70.7 (70.4-71)	71.4 (71.1-71.8)	74 (73.6-74.5)	75.5 (75.2-75.8)	77.2 (76.9-77.4)	78.8 (78-79.6)
Hammersmith and Fulham	66.6 (63.4-70.2)	67.4 (64.4-70.6)	67.7 (64.6-70.9)	67.7 (64.9-70.9)	67.9 (65.3-70.7)	68.6 (66.6-70.5)	69.1 (68.5-69.7)	69.8 (69.5-70.1)	69.5 (69.1-69.8)	70.5 (70.2-70.8)	74.6 (74.2-75)	76.8 (76.5-77.1)	78 (77.7-78.3)	79.1 (78.3-79.9)
Haringey	67 (64-70.5)	67.8 (64.7-70.9)	68.1 (65.2-71.4)	68.2 (65.3-71.2)	68.5 (65.9-71.2)	69.4 (67.5-71.4)	70.4 (69.8-71)	71.8 (71.6-72.1)	72.5 (72.2-72.8)	72.7 (72.4-72.9)	74.4 (74.1-74.9)	75.8 (75.5-76.1)	77.5 (77.2-77.8)	80.9 (80-81.7)
Harrow	69.8 (66.5-73.2)	70.5 (67.2-73.8)	71 (68-74.3)	71.3 (68.4-74.4)	71.7 (69.2-74.4)	72.6 (70.9-74.6)	73 (72.4-73.7)	74.3 (74-74.6)	75.1 (74.8-75.4)	75.7 (75.4-76)	77.8 (77.3-78.2)	79.9 (79.6-80.2)	81.6 (81.3-81.9)	81.9 (81.1-82.8)
Havering	68.9 (65.5-72.4)	69.7 (66.7-73)	70.2 (67.2-73.3)	70.6 (67.7-73.7)	70.9 (68.3-73.9)	71.8 (69.9-73.8)	72.4 (71.8-73)	73.1 (72.8-73.3)	74.2 (73.9-74.5)	75.3 (75-75.5)	76.6 (76.3-77)	78.6 (78.3-78.9)	78.9 (78.6-79.2)	79.6 (78.7-80.5)
Hillingdon	68.8 (65.3-72.2)	69.5 (66.4-72.6)	69.9 (66.9-72.9)	70 (67-73)	70.2 (67.7-72.8)	71 (69.2-72.9)	71.8 (71.2-72.3)	73 (72.7-73.3)	74 (73.7-74.3)	74.7 (74.4-74.9)	76.1 (75.8-76.5)	77.6 (77.3-77.9)	79.3 (79-79.5)	80.9 (80-81.8)
Hounslow	68.2 (65-71.5)	69 (65.8-72.1)	69.5 (66.4-72.8)	69.7 (66.8-72.8)	70.1 (67.4-72.8)	71 (69-72.9)	71.6 (71-72.2)	72.3 (72-72.6)	73 (72.7-73.3)	73.2 (72.9-73.5)	75.2 (74.8-75.6)	77.2 (76.9-77.4)	78.9 (78.6-79.2)	80.1 (79.3-81)
Islington	66.9 (63.6-70.2)	67.7 (64.7-70.8)	68.1 (65.2-71)	68.2 (65.5-71.3)	68.5 (65.9-71.3)	69.1 (67.2-71)	69.5 (68.9-70.2)	70.1 (69.8-70.4)	70.2 (69.9-70.5)	71.2 (70.9-71.5)	73 (72.6-73.4)	74.7 (74.4-74.9)	76.9 (76.6-77.2)	79.8 (79-80.7)
Kensington and Chelsea	67.3 (64-70.7)	68 (64.9-71.2)	68.2 (65.2-71.3)	68.1 (65.1-71)	68.2 (65.7-70.9)	69 (67.1-70.8)	69.7 (69.1-70.3)	71.2 (70.8-71.5)	73.2 (72.9-73.6)	74.6 (74.2-74.9)	77.6 (77.2-78.1)	79.7 (79.4-80)	81.5 (81.1-81.8)	83.1 (82.1-84.1)
Kingston upon Thames	69.4 (66.1-72.7)	70.1 (67-73.4)	70.4 (67.4-73.5)	70.5 (67.7-73.5)	70.8 (68.3-73.6)	71.7 (69.9-73.6)	72.5 (71.8-73.1)	73.4 (73.1-73.8)	74.8 (74.4-75.1)	76.2 (75.9-76.6)	78.1 (77.7-78.5)	80.3 (80-80.6)	81.3 (81-81.7)	81.2 (80.4-82.1)
Lambeth	65.2 (62.2-68.6)	66 (62.8-69.2)	66.3 (63.4-69.5)	66.4 (63.6-69.4)	66.6 (64-69.5)	67.5 (65.6-69.4)	68.6 (68-69.2)	69.8 (69.6-70.1)	70.4 (70.1-70.6)	70.6 (70.3-70.8)	73.2 (72.8-73.6)	75 (74.8-75.3)	76.9 (76.7-77.2)	78.7 (77.8-79.5)
Lewisham	66.8 (63.8-70.1)	67.7 (64.8-70.6)	68.3 (65.2-71.2)	68.6 (65.7-71.5)	69 (66.5-71.6)	69.9 (68.1-71.6)	70 (69.5-70.6)	71.5 (71.2-71.8)	71.8 (71.5-72)	72.1 (71.8-72.3)	73.7 (73.4-74.1)	75.5 (75.2-75.8)	77.6 (77.3-77.9)	79.7 (78.8-80.5)
Merton	68.7 (65.5-72.2)	69.6 (66.4-72.7)	70.1 (67.2-73)	70.3 (67.5-73.2)	70.7 (68.2-73.3)	71.6 (69.7-73.5)	72.1 (71.4-72.7)	73.1 (72.8-73.4)	73.8 (73.5-74.1)	75.3 (75-75.6)	77.4 (77-77.8)	79 (78.7-79.3)	79.7 (79.3-80)	80.5 (79.5-81.3)
Newham	66.3 (63.2-69.6)	67.2 (64-70.2)	67.7 (64.6-70.9)	67.9 (65.1-71.1)	68.3 (65.6-71)	69.1 (67.2-71)	69.8 (69.2-70.4)	70.6 (70.3-70.9)	71.2 (70.9-71.4)	71.2 (71-71.5)	72.9 (72.5-73.3)	75.4 (75.1-75.6)	76.3 (76.1-76.6)	79.7 (78.9-80.5)
Redbridge	68.9 (65.8-72.2)	69.6 (66.6-72.8)	70 (67.1-73.3)	70.2 (67.3-73.3)	70.5 (68-73.1)	71.5 (69.5-73.4)	72.4 (71.8-73)	73.4 (73.2-73.7)	74.5 (74.2-74.8)	75.3 (75-75.6)	76.4 (76-76.8)	78.2 (78-78.5)	79.7 (79.4-80)	80.9 (80.1-81.8)
Richmond upon Thames	69.1 (65.7-72.4)	69.7 (66.6-73)	69.9 (66.9-72.9)	69.8 (66.9-72.8)	69.9 (67.4-72.5)	70.8 (68.8-72.8)	71.8 (71.1-72.4)	73.3 (73-73.7)	75 (74.7-75.4)	76.5 (76.2-76.8)	78.9 (78.5-79.3)	80.8 (80.4-81.1)	82 (81.7-82.4)	81.9 (81-82.8)
Southwark	67.1 (63.7-70.7)	67.9 (64.8-71.4)	68.3 (65.4-71.5)	68.5 (65.7-71.6)	68.8 (66.2-71.6)	69.5 (67.6-71.5)	69.7 (69.2-70.3)	69.7 (69.4-70)	70 (69.7-70.2)	71.1 (70.8-71.4)	73.9 (73.4-74.2)	75.1 (74.9-75.4)	77.5 (77.2-77.7)	78.8 (78-79.6)
Sutton	69.3 (66.4-72.6)	70.3 (67.2-73.5)	70.9 (67.9-73.9)	71.3 (68.3-74.3)	71.8 (69-74.4)	72.7 (70.9-74.6)	73.4 (72.8-74)	73.9 (73.6-74.2)	75.1 (74.8-75.5)	75.4 (75.1-75.7)	77.5 (77.1-77.9)	79.1 (78.8-79.4)	80.2 (79.9-80.6)	80.4 (79.6-81.3)
Tower Hamlets	65.5 (62.4-68.7)	66.2 (63.3-69.4)	66.4 (63.5-69.4)	66.2 (63.3-69.1)	66.2 (63.7-69)	66.8 (65-68.9)	68.1 (67.5-68.7)	69.3 (69-69.5)	69.9 (69.6-70.2)	70.9 (70.6-71.2)	72.8 (72.4-73.2)	74.5 (74.2-74.8)	76.4 (76.1-76.7)	79.5 (78.7-80.4)
Waltham Forest	67.1 (63.9-70.4)	67.8 (64.7-71)	68.2 (65.2-71)	68.4 (65.5-71.2)	68.7 (66.2-71.3)	69.7 (67.8-71.6)	71.3 (70.7-71.9)	72.5 (72.2-72.8)	73.3 (73-73.6)	73.4 (73.1-73.7)	74.4 (74-74.8)	76.2 (75.9-76.5)	78 (77.7-78.2)	79.8 (79-80.7)
Wandsworth	67.4 (64.2-70.9)	68.1 (65-71.5)	68.5 (65.6-71.4)	68.6 (65.8-71.5)	68.8 (66.3-71.4)	69.5 (67.7-71.4)	69.9 (69.4-70.5)	70.8 (70.5-71)	71.1 (70.8-71.3)	72.6 (72.3-72.8)	75.1 (74.7-75.5)	77.2 (76.9-77.4)	78.9 (78.6-79.2)	80.1 (79.3-80.9)
Westminster	67.4 (64.4-70.5)	68 (65-71.1)	68.2 (65.2-71.2)	68.1 (65.1-71.2)	68.1 (65.4-70.8)	68.3 (66.5-70.3)	69 (68.4-69.6)	70.5 (70.2-70.8)	71.4 (71.1-71.7)	72.7 (72.4-73)	75.8 (75.4-76.2)	78.1 (77.8-78.3)	80.3 (80-80.6)	81.5 (80.7-82.4)
North East England	64.8 (63.8-65.9)	65.9 (64.9-66.9)	66.6 (65.5-67.7)	67.1 (66.1-68)	67.6 (66.8-68.5)	68.9 (68.2-69.5)	69.8 (69.6-70)	70.5 (70.4-70.6)	71.7 (71.6-71.8)	73 (72.9-73.1)	74.4 (74.3-74.5)	75.9 (75.8-76)	77.4 (77.3-77.5)	78.1 (77.8-78.4)
County Durham	65 (62-68.3)	66.1 (63-69.2)	66.7 (63.8-69.9)	67.2 (64.2-70.4)	67.8 (65-70.4)	69 (67-71.1)	69.9 (69.4-70.5)	70.6 (70.4-70.8)	71.9 (71.6-72.1)	72.9 (72.6-73.1)	74.7 (74.3-75)	76.4 (76.1-76.7)	77.6 (77.3-77.9)	78.2 (77.3-79.2)
Darlington	64.9 (61.6-68.2)	66 (62.7-69.2)	66.7 (63.6-70)	67.1 (64-70.4)	67.6 (64.8-70.6)	69 (66.8-71.3)	69.9 (69.2-70.7)	71 (70.6-71.4)	72.3 (71.9-72.7)	73.6 (73.2-74)	74.7 (74.2-75.2)	75.6 (75.2-76)	77.4 (77-77.8)	78.5 (77.4-79.6)
Gateshead	63.8 (60.7-67.2)	64.8 (61.8-68.2)	65.4 (62.3-68.7)	65.7 (62.6-69)	66.2 (63.4-69.1)	67.5 (65.5-69.6)	68.8 (68.2-69.4)	70 (69.7-70.3)	71.2 (70.9-71.5)	72.5 (72.2-72.8)	73.5 (73.1-73.9)	75.2 (74.8-75.5)	76.8 (76.4-77.1)	77.6 (76.6-78.5)
Hartlepool	63.4 (60.3-66.8)	64.7 (61.8-68.1)	65.4 (62.5-69)	65.9 (63.2-69.4)	66.5 (63.6-69.7)	67.9 (65.7-70.2)	69 (68.2-69.8)	70 (69.7-70.4)	71.4 (71-71.8)	73 (72.6-73.4)	74.3 (73.8-74.8)	75.4 (74.9-75.8)	76.9 (76.4-77.3)	77.5 (76.4-78.5)
Middlesbrough	63.5 (60-66.9)	64.7 (61.5-68.2)	65.6 (62.5-69)	66.2 (63.1-69.8)	66.8 (63.8-69.9)	68.1 (66-70.3)	68.7 (68.1-69.4)	69.6 (69.3-69.9)	70.6 (70.3-70.9)	72.2 (71.8-72.5)	73.2 (72.8-73.7)	74.6 (74.3-75)	76 (75.7-76.4)	77.3 (76.2-78.3)
Newcastle upon Tyne	65 (61.9-68.3)	66 (63.1-69.2)	66.6 (63.7-69.7)	66.9 (64.1-69.8)	67.4 (64.7-70.2)	68.6 (66.5-70.8)	69.8 (69.2-70.3)	70.5 (70.2-70.8)	71.2 (70.9-71.5)	72.4 (72.1-72.6)	73.8 (73.4-74.2)	75 (74.7-75.3)	76.9 (76.6-77.2)	77.8 (76.9-78.7)
North Tyneside	65.6 (62.4-69)	66.7 (63.7-70)	67.3 (64.5-70.6)	67.8 (65-70.8)	68.3 (65.7-71)	69.5 (67.4-71.5)	70.1 (69.6-70.8)	70.8 (70.5-71.1)	72.1 (71.8-72.4)	73.3 (73-73.6)	74.5 (74.1-74.9)	76 (75.7-76.3)	77.5 (77.2-77.8)	77.7 (76.8-78.7)
Northumberland	66 (62.9-69.5)	67.1 (64.1-70.2)	67.8 (64.8-71.1)	68.4 (65.4-71.3)	69 (66.4-71.7)	70.2 (68.2-72.3)	70.5 (70-71.1)	71.2 (71-71.5)	72.7 (72.5-73)	74.3 (74-74.5)	75.4 (75-75.8)	77.4 (77.1-77.7)	79 (78.7-79.3)	78.9 (78-79.8)
Redcar and Cleveland	64.3 (61.1-67.9)	65.5 (62.4-69)	66.2 (63.2-69.5)	66.6 (63.7-69.8)	67.3 (64.4-70)	68.6 (66.5-70.8)	69.8 (69.1-70.4)	70.9 (70.6-71.2)	71.9 (71.5-72.2)	73.5 (73.1-73.8)	74.9 (74.5-75.4)	76.3 (75.9-76.6)	77.8 (77.5-78.2)	78 (76.9-79.1)
South Tyneside	64.8 (61.7-68.4)	66 (63.1-69.3)	66.7 (63.8-70)	67.1 (64.2-70.2)	67.7 (64.9-70.5)	68.8 (66.8-71)	69.5 (68.9-70.1)	70.1 (69.8-70.4)	71.6 (71.3-71.9)	72.9 (72.6-73.2)	74 (73.6-74.4)	75.5 (75.1-75.8)	76.9 (76.6-77.3)	76.8 (75.8-77.7)
Stockton-on-Tees	65.2 (61.9-68.7)	66.3 (63.2-69.9)	67 (63.7-70.6)	67.5 (64.4-70.9)	68 (65.1-70.9)	69.3 (67-71.4)	70.1 (69.5-70.8)	70.7 (70.4-71)	71.7 (71.4-72.1)	73.2 (72.9-73.5)	75 (74.6-75.5)	75.9 (75.6-76.2)	77.9 (77.6-78.2)	78.6 (77.6-79.6)
Sunderland	64.8 (61.8-68.1)	66 (62.9-69)	66.7 (63.7-69.7)	67.2 (64.4-70.3)	67.8 (65.2-70.6)	69.1 (67.1-71.3)	69.7 (69.2-70.3)	70.5 (70.2-70.7)	71.2 (70.9-71.5)	72.7 (72.4-72.9)	73.8 (73.4-74.2)	75.1 (74.8-75.4)	76.7 (76.4-77)	77.8 (76.8-78.7)
North West England	66.3 (65.4-67.1)	67.2 (66.4-68)	67.7 (66.9-68.4)	67.9 (67.1-68.7)	68.3 (67.5-69)	69.2 (68.7-69.8)	69.9 (69.7-70)	70.8 (70.7-70.9)	71.9 (71.9-72)	73 (72.9-73)	74.3 (74.2-74.4)	75.9 (75.8-75.9)	77.2 (77.2-77.3)	78.3 (78-78.5)
Blackburn with Darwen	65.7 (62.5-69)	66.6 (63.5-69.9)	67.1 (64.1-70.2)	67.3 (64.6-70.5)	67.8 (65.2-70.5)	68.8 (66.8-70.7)	69.3 (68.7-69.9)	70 (69.7-70.3)	70.9 (70.5-71.2)	71.7 (71.4-72)	72.6 (72.2-73.1)	74.2 (73.9-74.6)	75.8 (75.5-76.1)	77.8 (76.9-78.7)
Blackpool	65.7 (62.4-69.1)	66.7 (63.5-70)	67.2 (64.1-70.3)	67.5 (64.7-70.7)	68 (65.3-70.9)	69.2 (67.3-71.2)	69.8 (69.2-70.5)	70.6 (70.2-70.9)	71.6 (71.2-71.9)	72.1 (71.7-72.4)	72.1 (71.6-72.5)	72.3 (71.9-72.6)	73.6 (73.2-73.9)	75.1 (74.1-76.2)
Bolton	65.8 (62.7-69.1)	66.7 (63.7-69.8)	67.2 (64.4-70.3)	67.4 (64.7-70.4)	67.7 (65-70.4)	68.6 (66.7-70.6)	69.2 (68.7-69.8)	70.2 (69.9-70.5)	71.7 (71.4-71.9)	72.7 (72.4-73)	73.7 (73.3-74.1)	75.3 (75-75.6)	76.7 (76.4-77)	78.5 (77.6-79.4)
Bury	66.5 (63.3-69.7)	67.4 (64.2-70.6)	67.8 (64.9-70.9)	68 (65.2-70.9)	68.3 (65.7-71)	69.3 (67.5-71.3)	70.1 (69.5-70.7)	71.3 (71-71.6)	72.3 (72-72.6)	73.1 (72.8-73.4)	75.2 (74.8-75.6)	76.6 (76.3-76.9)	77.6 (77.3-78)	77.9 (77-78.7)
Cheshire East	68.1 (64.8-71.6)	68.9 (65.7-72.1)	69.2 (66.4-72.3)	69.3 (66.6-72.5)	69.5 (66.9-72.3)	70.5 (68.6-72.3)	71.1 (70.6-71.7)	72.4 (72.2-72.7)	73.9 (73.6-74.1)	75 (74.8-75.3)	76.2 (75.8-76.5)	78.4 (78.2-78.7)	79.8 (79.5-80.1)	79.8 (78.9-80.7)
Cheshire West and Chester	68 (64.8-71.2)	68.8 (65.5-71.9)	69.2 (66.3-72.2)	69.3 (66.6-72.1)	69.5 (67.1-72.2)	70.5 (68.6-72.2)	71 (70.5-71.6)	72.1 (71.9-72.4)	73.3 (73-73.5)	74.4 (74.2-74.7)	76.1 (75.7-76.5)	78 (77.7-78.3)	79.1 (78.8-79.4)	79.2 (78.4-80)
Cumbria	67.1 (64.1-70.3)	68 (64.9-71.2)	68.5 (65.4-71.7)	68.7 (65.8-71.8)	69.1 (66.4-72.1)	70.1 (68.2-72.1)	70.5 (70-71.1)	71.5 (71.3-71.7)	72.6 (72.4-72.8)	73.9 (73.7-74.2)	75.5 (75.1-75.9)	77.1 (76.9-77.4)	78.6 (78.3-78.8)	79.2 (78.3-80)
Halton	66 (62.8-69.3)	66.9 (63.7-70.2)	67.2 (64.3-70.3)	67.2 (64.1-70.3)	67.5 (64.7-70.3)	68.5 (66.5-70.5)	69.3 (68.6-70)	70.7 (70.4-71.1)	72 (71.6-72.3)	72.8 (72.5-73.2)	73.7 (73.3-74.2)	74.6 (74.3-75)	76.7 (76.3-77.1)	78.3 (77.3-79.3)
Knowsley	65.8 (62.6-68.9)	66.7 (63.6-69.9)	67.1 (64.3-70.2)	67.2 (64.3-70.2)	67.6 (64.9-70.4)	68.5 (66.4-70.5)	69 (68.4-69.6)	70.2 (69.9-70.5)	71.4 (71.1-71.7)	72.1 (71.8-72.5)	72.6 (72.2-73)	74.5 (74.2-74.8)	75.6 (75.3-76)	76.7 (75.7-77.6)
Lancashire	66.7 (63.7-69.8)	67.6 (64.7-70.8)	68.2 (65.3-71.2)	68.5 (65.5-71.6)	68.9 (66-71.9)	69.8 (67.5-72.1)	70.2 (69.7-70.8)	71.1 (70.9-71.2)	72.4 (72.1-72.6)	73.6 (73.4-73.9)	74.9 (74.6-75.2)	76.6 (76.3-76.8)	77.8 (77.6-78.1)	78.6 (77.7-79.5)
Liverpool	65.4 (62.4-68.7)	66.3 (63.3-69.3)	66.7 (63.9-69.9)	66.9 (64.1-69.9)	67.4 (64.7-70.3)	68.3 (66.3-70.4)	69 (68.6-69.6)	69.8 (69.6-70)	70.6 (70.3-70.8)	71.1 (70.8-71.3)	72.2 (71.8-72.6)	73.8 (73.5-74)	75.3 (75.1-75.6)	77.2 (76.3-78)
Manchester	64.7 (61.4-68.1)	65.6 (62.4-68.8)	66 (63-69.3)	66.2 (63.4-69.2)	66.6 (64-69.5)	67.5 (65.6-69.4)	68.1 (67.6-68.6)	68.9 (68.7-69.1)	69.4 (69.2-69.6)	69.8 (69.6-70.1)	71.1 (70.7-71.5)	72.8 (72.5-73)	74.6 (74.4-74.9)	76.6 (75.8-77.4)
Oldham	65.5 (62.3-68.6)	66.4 (63.3-69.6)	66.9 (63.9-70.2)	67.2 (64.3-70.3)	67.6 (65.1-70.4)	68.7 (66.9-70.7)	69.5 (68.9-70)	70.1 (69.9-70.4)	71.3 (71-71.5)	71.5 (71.2-71.8)	73.1 (72.7-73.5)	74.8 (74.5-75.1)	76.4 (76.1-76.7)	77.5 (76.5-78.3)
Rochdale	65.5 (62.4-68.7)	66.4 (63.2-69.6)	67 (64.2-69.9)	67.2 (64.6-70.1)	67.7 (65.1-70.3)	68.7 (66.8-70.6)	69.6 (69-70.1)	70.3 (70-70.6)	71.5 (71.2-71.8)	72.4 (72.1-72.6)	73.7 (73.3-74.1)	74.8 (74.5-75.1)	76.2 (75.9-76.5)	77.7 (76.7-78.5)
Salford	64.7 (61.7-68)	65.6 (62.4-68.7)	65.9 (62.9-69)	65.9 (63-69)	66.2 (63.5-69.1)	67.2 (65.3-69.1)	68.3 (67.7-68.8)	69.2 (68.9-69.4)	70.5 (70.2-70.8)	71.3 (71.1-71.6)	73.1 (72.7-73.5)	74.4 (74.1-74.7)	75.6 (75.3-75.9)	77.1 (76.2-78)
Sefton	67.1 (63.8-70.3)	68 (64.9-71.1)	68.4 (65.5-71.5)	68.7 (66-71.5)	69.1 (66.6-71.8)	70.1 (68.1-72)	70.6 (70-71.1)	71.5 (71.3-71.7)	72.8 (72.6-73.1)	73.5 (73.2-73.8)	74.7 (74.3-75.1)	76.8 (76.5-77.1)	77.2 (76.9-77.5)	77.9 (77-78.8)
St Helens	65.9 (62.8-69.1)	66.8 (63.7-70)	67.1 (64.3-70.3)	67.2 (64.4-70.3)	67.4 (64.7-70.3)	68.2 (66.3-70.3)	69 (68.5-69.6)	70 (69.7-70.3)	71.5 (71.3-71.8)	72.9 (72.6-73.1)	74.3 (73.9-74.7)	75.6 (75.3-76)	76.6 (76.2-76.9)	76.7 (75.7-77.6)
Stockport	67.8 (64.7-71.2)	68.6 (65.8-71.7)	69.1 (66.3-72.1)	69.3 (66.5-72.2)	69.6 (67.2-72.3)	70.5 (68.6-72.4)	70.9 (70.4-71.5)	71.7 (71.5-72)	73 (72.8-73.3)	74.2 (74-74.5)	76 (75.6-76.4)	77.2 (77-77.5)	78 (77.7-78.3)	79.5 (78.6-80.4)
Tameside	66.1 (63.1-69.3)	67 (64.2-70.1)	67.5 (64.4-70.7)	67.7 (64.7-71)	68.2 (65.4-71)	69.2 (67.2-71.1)	69.8 (69.3-70.3)	70.7 (70.5-71)	71.7 (71.4-71.9)	72.3 (72.1-72.6)	73.4 (73-73.8)	75 (74.7-75.3)	76.2 (75.9-76.5)	77.9 (77-78.8)
Trafford	68.1 (65.1-71.3)	68.9 (65.9-72.1)	69.4 (66.5-72.5)	69.7 (66.8-72.7)	70.1 (67.5-73)	71 (69.1-72.9)	71.4 (70.8-72)	71.9 (71.6-72.2)	72.9 (72.6-73.2)	74.1 (73.8-74.4)	75.9 (75.5-76.3)	77.7 (77.4-78)	79.2 (78.9-79.5)	79.9 (78.9-80.8)
Warrington	67.2 (63.9-70.6)	68 (65-71.4)	68.3 (65.5-71.4)	68.4 (65.6-71.3)	68.7 (65.8-71.4)	69.6 (67.6-71.5)	70.3 (69.7-70.9)	71.1 (70.8-71.4)	72 (71.7-72.3)	73.2 (72.9-73.5)	75.3 (74.9-75.7)	77.2 (76.8-77.5)	78.1 (77.8-78.4)	78.4 (77.5-79.3)
Wigan	66.2 (63-69.5)	67 (63.9-70.3)	67.5 (64.3-70.5)	67.6 (64.6-70.7)	68 (65.4-70.8)	68.8 (67-70.8)	69.4 (68.9-69.9)	70.3 (70.1-70.5)	71.8 (71.6-72)	73.4 (73.1-73.6)	74.1 (73.7-74.5)	75.2 (75-75.5)	76.8 (76.6-77.1)	77.8 (77-78.6)
Wirral	66.6 (63.5-70.1)	67.6 (64.5-70.9)	68.1 (65.1-71.2)	68.3 (65.5-71.3)	68.8 (66.2-71.7)	69.8 (67.8-71.6)	70.5 (70-71.1)	71.3 (71.1-71.6)	72.1 (71.9-72.3)	73.1 (72.8-73.3)	74.5 (74.1-74.9)	76 (75.7-76.3)	77.4 (77.1-77.6)	78 (77.1-78.9)
South East England	69.1 (68.1-70.1)	69.9 (68.9-70.9)	70.3 (69.4-71.3)	70.4 (69.5-71.4)	70.4 (69.4-71.3)	70.9 (70.2-71.5)	72 (71.8-72.1)	73.2 (73.2-73.3)	74.5 (74.4-74.5)	75.6 (75.5-75.6)	77 (76.9-77.1)	78.7 (78.6-78.8)	79.8 (79.7-79.9)	80.3 (80.1-80.6)
Bracknell Forest	69.5 (66.1-72.9)	70.4 (67.2-73.7)	70.8 (67.6-74.2)	70.8 (67.7-74)	70.8 (67.9-73.5)	71.3 (69.3-73.3)	72.5 (71.8-73.2)	73.3 (72.9-73.6)	74.8 (74.5-75.2)	76.3 (75.9-76.7)	77.8 (77.3-78.3)	79.8 (79.4-80.2)	80.9 (80.4-81.3)	81 (80-81.9)
Brighton and Hove	67.9 (64.8-71.4)	68.7 (65.9-72)	69.1 (66-72.4)	69.2 (66.2-72.1)	69.3 (66.5-71.9)	70 (68.1-72)	71 (70.4-71.6)	72 (71.7-72.3)	71.9 (71.6-72.2)	72.1 (71.8-72.4)	74.4 (74-74.8)	75.7 (75.4-75.9)	77.4 (77.2-77.7)	79.6 (78.7-80.4)
Buckinghamshire	70.4 (67.4-73.6)	71.1 (68.2-74.2)	71.4 (68.3-74.5)	71.5 (68.5-74.6)	71.4 (68.8-74.1)	72 (70.2-73.9)	72.8 (72.3-73.3)	73.8 (73.6-74)	75 (74.7-75.2)	76.1 (75.9-76.3)	78 (77.7-78.4)	79.7 (79.4-79.9)	80.6 (80.4-80.9)	82 (81.2-82.8)
East Sussex	68.8 (65.5-72.3)	69.5 (66.3-73)	70 (66.7-73.4)	70.1 (66.9-73.2)	70.2 (67.3-73.1)	70.8 (68.7-72.9)	72.2 (71.6-72.8)	73.5 (73.3-73.8)	74.8 (74.6-75.1)	75.7 (75.5-76)	77 (76.6-77.3)	78.5 (78.2-78.8)	79.4 (79.2-79.7)	79.6 (78.7-80.5)
Hampshire	69.6 (66.2-72.9)	70.4 (67.1-73.6)	70.8 (67.9-73.9)	70.9 (67.9-73.9)	70.9 (68.1-73.8)	71.5 (69.5-73.5)	72.4 (71.9-72.9)	73.5 (73.4-73.8)	74.8 (74.6-75)	75.9 (75.7-76.1)	77.5 (77.2-77.9)	79.5 (79.3-79.7)	80.4 (80.2-80.6)	81 (80.3-81.8)
Isle of Wight	67.6 (64.2-71.4)	68.7 (65.5-72.2)	69.2 (66.2-72.7)	69.5 (66.5-72.9)	69.7 (67-72.5)	70.4 (68.3-72.5)	71.6 (70.8-72.3)	72.1 (71.8-72.5)	73.4 (73-73.8)	75.2 (74.8-75.5)	76.7 (76.3-77.2)	77.9 (77.6-78.3)	79 (78.6-79.3)	79.8 (78.8-80.8)
Kent	68.6 (65.6-71.9)	69.4 (66.5-72.5)	69.8 (66.7-73)	69.9 (66.9-73.1)	69.7 (66.9-72.8)	69.8 (67.7-72.1)	71.1 (70.6-71.6)	72.7 (72.5-72.9)	73.9 (73.7-74.1)	75.2 (75-75.4)	76.5 (76.2-76.9)	78.2 (78-78.4)	79.4 (79.2-79.6)	79.4 (78.6-80.1)
Medway	68.2 (64.7-71.8)	69.2 (66-72.5)	69.8 (66.7-73.2)	70 (67-73)	70.2 (67.4-72.9)	70.7 (68.8-72.7)	71.6 (71-72.2)	72.2 (72-72.5)	73.6 (73.3-73.8)	74.8 (74.5-75)	75.2 (74.8-75.6)	76.5 (76.3-76.8)	78.1 (77.8-78.4)	79.3 (78.5-80.2)
Milton Keynes	68.3 (64.8-71.7)	69 (65.9-72.2)	69.3 (66.2-72.6)	69.2 (66.1-72.3)	69.1 (66.3-71.8)	69.6 (67.6-71.6)	70.7 (70.1-71.4)	72.3 (72-72.7)	74.1 (73.8-74.5)	75 (74.7-75.3)	76.7 (76.3-77.1)	77.7 (77.4-78)	78.3 (78-78.6)	80 (79.1-80.8)
Oxfordshire	70.3 (67-73.7)	71 (67.9-74.1)	71.4 (68.4-74.5)	71.4 (68.4-74.7)	71.4 (68.7-74.4)	71.9 (70-73.9)	72.7 (72.2-73.2)	74 (73.8-74.2)	75.2 (74.9-75.4)	76.2 (76-76.5)	77.5 (77.1-77.8)	79 (78.7-79.2)	80.1 (79.9-80.3)	81.4 (80.6-82.1)
Portsmouth	67.4 (64.1-71)	68.2 (65.2-71.7)	68.6 (65.4-71.9)	68.7 (65.6-71.9)	68.7 (66-71.4)	69.1 (67.2-71.2)	69.8 (69.2-70.3)	70.8 (70.5-71.1)	72.4 (72.1-72.7)	73 (72.8-73.3)	74.4 (74-74.8)	76.2 (76-76.5)	77.1 (76.8-77.4)	78 (77.1-78.8)
Reading	68.2 (65-71.7)	68.9 (65.9-72.1)	69.1 (66-72.5)	69 (65.7-72.2)	68.9 (66-72)	69.5 (67.4-71.6)	70.8 (70.2-71.5)	72.1 (71.8-72.5)	73.3 (73-73.6)	74.3 (74-74.6)	75.5 (75.1-75.9)	76.8 (76.4-77.1)	77.7 (77.4-78)	79 (78.1-79.9)
Slough	68.3 (64.8-71.8)	69.2 (66-72.3)	69.6 (66.4-72.8)	69.7 (66.4-72.9)	69.7 (66.9-72.7)	70.2 (68.3-72.3)	71.2 (70.5-71.9)	71.5 (71.1-71.9)	72.7 (72.3-73.1)	74.4 (74-74.7)	75.1 (74.7-75.6)	76.6 (76.3-77)	77.2 (76.9-77.5)	79.1 (78.1-80)
Southampton	67.3 (63.8-70.9)	68.1 (64.8-71.5)	68.5 (65.4-71.9)	68.6 (65.6-71.6)	68.6 (65.9-71.4)	69.3 (67.4-71.3)	70.8 (70.3-71.4)	71.9 (71.6-72.2)	72 (71.8-72.3)	73.3 (73-73.6)	75 (74.7-75.4)	76.6 (76.3-76.8)	77.8 (77.5-78.1)	78.4 (77.5-79.3)
Surrey	70.3 (67.1-73.9)	71.1 (68.1-74.3)	71.4 (68.4-74.7)	71.5 (68.4-74.8)	71.3 (68.7-74.4)	71.7 (69.7-73.9)	72.5 (72-73)	73.9 (73.7-74.1)	75.3 (75.1-75.5)	76.4 (76.2-76.6)	77.6 (77.3-77.9)	79.6 (79.4-79.8)	81 (80.8-81.2)	81 (80.2-81.8)
West Berkshire	70.3 (67.1-73.8)	71 (67.9-74.2)	71.2 (68.1-74.6)	71.1 (68.1-74.3)	71.1 (68.3-74)	71.6 (69.6-73.7)	72.8 (72-73.5)	72.9 (72.6-73.2)	75.1 (74.7-75.4)	76.5 (76.2-76.8)	78.1 (77.7-78.5)	79.3 (78.9-79.6)	80.6 (80.2-80.9)	81 (80-81.8)
West Sussex	69 (66-72.3)	69.8 (66.6-72.9)	70.1 (66.8-73.3)	70 (67.1-73.1)	69.9 (67.2-72.9)	70.5 (68.5-72.5)	72 (71.5-72.5)	73.6 (73.3-73.8)	74.9 (74.7-75.1)	75.9 (75.7-76.2)	77.4 (77.1-77.7)	78.9 (78.7-79.1)	80 (79.7-80.2)	79.9 (79-80.7)
Windsor and Maidenhead	71.4 (68.3-74.8)	72.3 (69.1-75.7)	72.8 (69.7-76.1)	73 (70-76.1)	73.1 (70.4-75.9)	73.7 (71.8-75.7)	74.3 (73.6-75)	74.8 (74.4-75.1)	75.4 (75.1-75.8)	76 (75.7-76.4)	76.7 (76.3-77.2)	79.9 (79.5-80.2)	80.9 (80.5-81.3)	81.2 (80.2-82.1)
Wokingham	71.3 (68-74.6)	72 (68.8-75.2)	72.4 (69.1-75.7)	72.5 (69.3-75.7)	72.5 (69.8-75.3)	73.1 (71.1-75.2)	74.4 (73.7-75.1)	75.6 (75.3-76)	77.3 (76.9-77.7)	78.7 (78.3-79.1)	80.5 (80-80.9)	81.4 (81-81.8)	81.8 (81.5-82.2)	81.4 (80.4-82.4)
South West England	67.8 (66.8-68.8)	68.8 (67.9-69.8)	69.4 (68.5-70.4)	69.7 (68.8-70.5)	70 (69.1-70.8)	70.8 (70.2-71.4)	72 (71.8-72.1)	73.3 (73.2-73.3)	74.3 (74.3-74.4)	75.6 (75.5-75.6)	77 (76.8-77.1)	78.5 (78.5-78.6)	79.6 (79.5-79.6)	80.1 (79.8-80.3)
Bath and North East Somerset	68.9 (65.8-72.4)	69.9 (66.8-73.1)	70.6 (67.6-74)	71.1 (67.9-74.3)	71.5 (68.9-74.3)	72.5 (70.4-74.6)	73.8 (73.1-74.4)	74.7 (74.3-75)	75.2 (74.9-75.5)	76.3 (76-76.6)	78 (77.5-78.4)	79.6 (79.3-79.9)	80.3 (79.9-80.6)	80.4 (79.5-81.3)

Table 8 Estimates of life expectancy at birth by location and year for 1950, 1955...2005, 2010, 2017 - males

Location	1950	1955	1960	1965	1970	1975	1980	1985	1990	1995	2000	2005	2010	2017
Bournemouth	68.1 (64.7-71.3)	69.2 (65.8-72.3)	69.8 (66.7-73)	70.2 (67.4-73.3)	70.6 (68-73.5)	71.6 (69.5-73.7)	72.9 (72.2-73.7)	73.5 (73.2-73.9)	73.3 (73-73.7)	73.7 (73.4-74.1)	75.5 (75-75.9)	77.2 (76.9-77.5)	77.9 (77.5-78.2)	79.3 (78.4-80.2)
Bristol, City of	66.7 (63.5-70.2)	67.7 (64.6-71)	68.3 (65.4-71.8)	68.6 (65.7-71.7)	68.8 (66.2-71.5)	69.6 (67.6-71.6)	70.6 (70-71.2)	72 (71.7-72.2)	72.8 (72.5-73)	74 (73.7-74.2)	75.4 (75-75.8)	76.8 (76.5-77.1)	78.1 (77.9-78.4)	80.1 (79.2-81)
Cornwall	67.4 (64.3-71)	68.4 (65.4-71.5)	69 (66.1-72.4)	69.2 (66.1-72.3)	69.4 (66.7-72.2)	70.2 (68.1-72.3)	71.4 (70.9-72)	73 (72.8-73.3)	74.1 (73.9-74.4)	75.4 (75.2-75.7)	76.7 (76.3-77.1)	78.3 (78.1-78.6)	79.3 (79.1-79.6)	79 (78.2-79.9)
Devon	67.8 (64.6-71.2)	68.8 (65.8-72.2)	69.4 (66.4-72.5)	69.6 (66.8-72.6)	69.8 (67.2-72.7)	70.6 (68.7-72.8)	72.1 (71.5-72.6)	73.6 (73.3-73.8)	74.8 (74.6-75)	76.1 (75.9-76.3)	77.3 (77-77.7)	79 (78.8-79.2)	79.8 (79.6-80.1)	79.4 (78.6-80.2)
Dorset	68.6 (65.1-72)	69.6 (66.4-72.7)	70.2 (67.2-73.6)	70.5 (67.6-73.6)	70.9 (68.4-73.6)	71.8 (69.9-73.8)	72.8 (72.3-73.4)	74.1 (73.8-74.4)	75.5 (75.2-75.7)	76.6 (76.3-76.8)	78 (77.7-78.4)	79.9 (79.6-80.1)	80.6 (80.4-80.9)	80.8 (79.9-81.6)
Gloucestershire	68.3 (65.3-71.9)	69.3 (66.3-72.4)	69.9 (66.9-73.3)	70.1 (67.1-73.5)	70.4 (67.6-73.2)	71.1 (69.1-73.1)	71.9 (71.3-72.4)	73.1 (72.8-73.3)	74.2 (74-74.5)	75.5 (75.3-75.8)	77.1 (76.7-77.4)	78.7 (78.5-78.9)	80 (79.8-80.2)	80.2 (79.4-81)
North Somerset	68.6 (65.4-72.1)	69.7 (66.7-72.7)	70.4 (67.2-73.8)	70.7 (67.9-73.9)	71.1 (68.6-73.7)	72.1 (70.1-74.3)	73.2 (72.5-73.9)	74.3 (73.9-74.6)	75 (74.7-75.3)	76 (75.7-76.3)	77.2 (76.8-77.6)	78.3 (78-78.6)	78.9 (78.6-79.2)	79.6 (78.7-80.5)
Plymouth	66.3 (63.1-69.8)	67.3 (64.1-70.5)	67.8 (64.7-70.9)	68 (65.1-71.3)	68.2 (65.4-70.9)	69 (67-71.1)	70.3 (69.8-70.9)	71.8 (71.5-72.1)	72.7 (72.4-73)	73.4 (73.1-73.7)	75 (74.6-75.3)	76.3 (76-76.5)	77.6 (77.2-77.8)	79.3 (78.3-80.1)
Poole	67.9 (64.7-71.5)	68.9 (65.4-72.3)	69.4 (66-72.8)	69.6 (66.6-72.8)	69.8 (67.1-72.8)	70.6 (68.5-72.9)	71.9 (71.1-72.7)	73.4 (73.1-73.8)	75.1 (74.8-75.5)	76.4 (76-76.7)	77.9 (77.4-78.3)	78.9 (78.6-79.3)	79.9 (79.6-80.3)	80.5 (79.6-81.4)
Somerset	68.4 (65.1-71.9)	69.3 (66.1-72.7)	69.8 (66.8-73.3)	70 (66.8-73.2)	70.2 (67.4-73.3)	71 (69.1-73.2)	72.1 (71.5-72.7)	73.5 (73.3-73.8)	74.7 (74.5-75)	75.7 (75.5-76)	77.2 (76.9-77.6)	78.9 (78.7-79.2)	79.8 (79.5-80)	80 (79.1-80.8)
South Gloucestershire	68.9 (65.8-72.4)	69.8 (66.5-73)	70.2 (66.8-73.5)	70.3 (67.2-73.5)	70.4 (67.7-73.2)	71.2 (69.4-73.2)	72.6 (72-73.2)	73.7 (73.4-74)	75.2 (74.9-75.5)	76.8 (76.5-77.1)	77.8 (77.5-78.2)	79.3 (79-79.6)	80.8 (80.5-81.1)	81.7 (80.8-82.5)
Swindon	67.3 (64.2-70.8)	68.3 (65.3-71.6)	68.9 (65.7-72.3)	69.1 (66.1-72.2)	69.2 (66.4-72.1)	70 (68.1-72)	71.1 (70.4-71.7)	72.3 (72-72.6)	73.4 (73.1-73.8)	74.4 (74.1-74.7)	76.1 (75.7-76.6)	77.8 (77.5-78.1)	79.1 (78.8-79.4)	79.7 (78.9-80.6)
Torbay	67.5 (64.2-70.8)	68.6 (65.3-72)	69.3 (66.1-72.6)	69.5 (66.3-72.8)	69.9 (66.9-72.8)	70.8 (68.6-72.9)	71.9 (71.1-72.6)	72.8 (72.4-73.1)	74.2 (73.8-74.6)	75.3 (74.9-75.6)	75.2 (74.8-75.7)	75.9 (75.6-76.3)	77.3 (76.9-77.7)	78.8 (77.9-79.8)
Wiltshire	68.5 (65.3-71.6)	69.5 (66.5-72.5)	70.1 (67.1-73.3)	70.5 (67.6-73.5)	70.8 (68.2-73.4)	71.6 (69.7-73.5)	72.3 (71.8-72.9)	73.2 (73-73.5)	74.2 (73.9-74.4)	76.1 (75.8-76.4)	77.4 (77-77.7)	78.9 (78.7-79.2)	80.3 (80.1-80.6)	81.4 (80.6-82.2)
West Midlands	66.7 (65.7-67.7)	67.6 (66.6-68.5)	68.1 (67.1-69.1)	68.2 (67.2-69.3)	68.4 (67.5-69.3)	69.3 (68.7-69.9)	70.4 (70.3-70.6)	71.6 (71.5-71.7)	72.5 (72.5-72.6)	73.9 (73.8-73.9)	75.2 (75.1-75.3)	76.7 (76.6-76.8)	78.2 (78.1-78.3)	78.8 (78.5-79)
Birmingham	65.9 (63.1-68.8)	66.9 (64-69.8)	67.4 (64.6-70.6)	67.7 (64.8-70.6)	68 (65.5-70.9)	69 (67.2-71.2)	70 (69.6-70.5)	70.7 (70.5-70.9)	71.5 (71.3-71.7)	72.7 (72.5-72.9)	73.7 (73.3-74)	75.3 (75-75.5)	77.3 (77.1-77.5)	78.2 (77.4-79)
Coventry	66.2 (63-69.5)	67.2 (64.3-70.5)	67.8 (64.8-70.9)	68 (65.3-70.9)	68.2 (65.7-71.2)	69.1 (67.2-71.2)	70 (69.5-70.5)	71.2 (70.9-71.4)	71.6 (71.4-71.9)	73.2 (72.9-73.5)	74.7 (74.3-75.1)	75.8 (75.5-76)	77.1 (76.8-77.4)	78.5 (77.6-79.4)
Dudley	67.4 (64.1-70.8)	68.3 (65.3-71.4)	68.9 (66-72.1)	69.2 (66.3-72.3)	69.4 (66.9-72.1)	70.2 (68.3-72)	70.9 (70.5-71.4)	71.6 (71.4-71.9)	72.9 (72.7-73.1)	74.1 (73.9-74.4)	75.3 (74.9-75.6)	76.8 (76.5-77.1)	78.3 (78-78.5)	78.8 (77.9-79.7)
Herefordshire, County of	67.6 (64.5-71)	68.6 (65.4-71.9)	69.2 (66-72.3)	69.4 (66.5-72.6)	69.7 (66.9-72.6)	70.7 (68.7-72.7)	71.6 (71-72.3)	73.4 (73.1-73.7)	74.7 (74.3-75)	75.9 (75.5-76.2)	77 (76.6-77.5)	78.3 (77.9-78.6)	79.3 (79-79.6)	79.7 (78.7-80.7)
Sandwell	65.4 (62.2-68.9)	66.4 (63.6-69.7)	67 (63.9-70.1)	67.3 (64.3-70.3)	67.6 (64.9-70.4)	68.5 (66.6-70.6)	69.6 (69-70.1)	70.3 (70.1-70.6)	71.1 (70.8-71.3)	72.2 (72-72.5)	73.4 (73.1-73.8)	74.7 (74.4-74.9)	76.4 (76.1-76.7)	77.8 (76.9-78.7)
Shropshire	67.6 (64.5-70.9)	68.4 (65.4-71.7)	68.7 (65.6-71.9)	68.7 (65.7-71.7)	68.8 (66.3-71.6)	69.8 (67.9-71.9)	71.5 (70.9-72.1)	72.8 (72.5-73)	73.9 (73.6-74.1)	74.8 (74.6-75.1)	76.6 (76.2-76.9)	77.9 (77.7-78.2)	79.5 (79.2-79.8)	80.2 (79.3-81.1)
Solihull	68.3 (64.9-71.7)	69.1 (65.9-72.4)	69.5 (66.4-72.8)	69.6 (66.7-72.7)	69.8 (67-72.5)	70.8 (68.8-72.9)	72.1 (71.6-72.8)	74 (73.7-74.3)	75.2 (74.9-75.5)	76.3 (75.9-76.6)	77.5 (77.1-78)	78.8 (78.5-79.1)	80.4 (80-80.7)	80 (79-80.9)
Staffordshire	67.2 (64-70.4)	68 (65.1-71.2)	68.4 (65.5-71.7)	68.4 (65.5-71.6)	68.3 (65.6-71.3)	68.9 (66.9-71)	70.4 (69.9-70.9)	72.1 (71.9-72.3)	72.8 (72.6-73)	74.4 (74.2-74.6)	75.8 (75.4-76.1)	77.5 (77.3-77.7)	79.1 (78.9-79.4)	78.7 (77.9-79.5)
Stoke-on-Trent	64.8 (61.7-68.3)	65.7 (62.7-69.2)	66.2 (63.3-69.7)	66.3 (63.4-69.6)	66.4 (63.8-69.4)	67.3 (65.4-69.3)	68.5 (67.9-69.1)	69.7 (69.4-70)	70.9 (70.6-71.2)	71.6 (71.3-71.9)	73.2 (72.8-73.6)	74.8 (74.5-75)	76.2 (75.9-76.5)	76.9 (76.1-77.9)
Telford and Wrekin	66.4 (63.1-70.2)	67.3 (64.1-71)	67.6 (64.6-71)	67.7 (64.7-70.7)	67.7 (65.2-70.6)	68.5 (66.5-70.8)	69.7 (69-70.4)	71.1 (70.8-71.4)	72.4 (72-72.7)	74.3 (73.9-74.6)	75.5 (75.1-75.9)	77.1 (76.7-77.4)	77.2 (76.9-77.5)	78.1 (77.2-79)
Walsall	65.9 (62.6-69.4)	66.8 (63.5-69.9)	67.2 (64.1-70.3)	67.3 (64.5-70.2)	67.4 (64.9-70.2)	68.3 (66.4-70.5)	69.6 (69-70.1)	70.7 (70.5-71)	71.8 (71.5-72)	73.1 (72.8-73.3)	74.6 (74.2-75)	75.8 (75.5-76.1)	77 (76.7-77.3)	77.7 (76.8-78.6)
Warwickshire	68.1 (64.9-71.3)	68.9 (65.7-72.1)	69.3 (66.3-72.6)	69.4 (66.2-72.4)	69.5 (67-72.3)	70.3 (68.3-72.3)	71.4 (70.8-71.9)	72.4 (72.1-72.6)	73.6 (73.3-73.8)	74.8 (74.6-75)	76.4 (76-76.7)	77.8 (77.5-78)	79.3 (79.1-79.6)	79.7 (78.8-80.6)
Wolverhampton	66.1 (62.9-69.5)	67 (64-70.2)	67.5 (64.3-70.6)	67.6 (64.7-70.6)	67.8 (65.1-70.6)	68.8 (66.9-70.8)	70.2 (69.6-70.7)	71 (70.7-71.2)	71.5 (71.2-71.7)	72.8 (72.6-73.1)	73.9 (73.4-74.3)	74.4 (74.1-74.7)	76.4 (76.1-76.8)	77.6 (76.8-78.5)
Worcestershire	68.3 (64.9-71.9)	69.2 (66-72.5)	69.7 (66.6-72.9)	70 (67-73.2)	70.2 (67.6-73.1)	71 (69.1-73.1)	71.7 (71.2-72.2)	72.8 (72.6-73)	73.9 (73.7-74.1)	75.2 (75-75.5)	76.5 (76.1-76.9)	78.3 (78.1-78.6)	79.2 (79-79.4)	79.6 (78.8-80.4)
Yorkshire and the Humber	66.6 (65.6-67.5)	67.4 (66.5-68.3)	67.9 (66.9-68.9)	68.2 (67.3-69.1)	68.5 (67.6-69.3)	69.3 (68.7-69.9)	70.2 (70.1-70.4)	71.3 (71.3-71.4)	72.6 (72.5-72.6)	73.9 (73.8-73.9)	75.2 (75.1-75.3)	76.6 (76.6-76.7)	77.9 (77.9-78)	78.8 (78.6-79.1)
Barnsley	66.1 (62.9-69.2)	66.9 (63.8-70)	67.4 (64.5-70.6)	67.7 (65-70.9)	68.1 (65.4-70.9)	69 (67.2-71)	70.2 (69.6-70.7)	71.1 (70.8-71.3)	71.8 (71.6-72.1)	72.9 (72.6-73.1)	74.6 (74.2-75)	76.2 (75.9-76.5)	77.4 (77.1-77.7)	78 (77.1-78.9)
Bradford	65.7 (62.5-69.3)	66.7 (63.7-70.2)	67.4 (64.5-70.8)	67.9 (64.9-71)	68.3 (65.8-71.3)	69.2 (67.2-71.3)	69.6 (69-70.1)	70.3 (70.1-70.5)	71.8 (71.5-72)	72.8 (72.5-73)	74 (73.7-74.4)	75.3 (75-75.5)	77.3 (77.1-77.6)	78.9 (78.1-79.8)
Calderdale	66.1 (62.9-69.5)	66.9 (63.8-70.2)	67.4 (64.2-70.7)	67.6 (64.7-70.8)	67.8 (65.2-70.7)	68.6 (66.7-70.7)	69.6 (69-70.2)	70.3 (70-70.6)	71.8 (71.5-72)	72.9 (72.6-73.2)	74.7 (74.3-75.1)	76.3 (76-76.7)	77.3 (77-77.6)	78.7 (77.8-79.6)
Doncaster	66.9 (63.7-70.2)	67.8 (64.5-71)	68.5 (65.4-71.9)	68.9 (66.1-72.2)	69.3 (66.7-72.1)	70 (68.3-72.1)	70.4 (69.9-71)	71.6 (71.4-71.9)	72.7 (72.4-73)	73.1 (72.9-73.3)	74.7 (74.3-75.1)	75.8 (75.5-76)	77 (76.7-77.3)	77.9 (77-78.8)
East Riding of Yorkshire	68.4 (65.2-71.7)	69.2 (66-72.3)	69.7 (66.7-73)	70 (67.2-73.2)	70.3 (67.7-73.1)	71.1 (69.3-73.1)	72 (71.4-72.6)	72.9 (72.6-73.1)	73.9 (73.7-74.2)	75.3 (75.1-75.6)	76.6 (76.2-76.9)	78.1 (77.8-78.3)	79.3 (79-79.5)	79.7 (78.9-80.6)
Kingston upon Hull, City of	65.9 (62.6-69.3)	66.9 (63.9-70.3)	67.7 (64.7-71)	68.2 (65.2-71.2)	68.6 (66.1-71.4)	69.4 (67.5-71.4)	69.6 (69.1-70.1)	70.8 (70.5-71)	71.7 (71.4-71.9)	72.4 (72.2-72.7)	73.1 (72.7-73.5)	74.5 (74.2-74.8)	75.6 (75.3-75.9)	76.8 (76-77.7)
Kirklees	65.8 (62.6-69.2)	66.6 (63.4-69.8)	67.1 (63.9-70.3)	67.3 (64.3-70.4)	67.5 (64.8-70.4)	68.3 (66.3-70.4)	69.3 (68.8-69.9)	70.7 (70.5-71)	72.1 (71.9-72.4)	73.8 (73.5-74)	75 (74.6-75.4)	76.1 (75.8-76.4)	77.5 (77.2-77.7)	79.3 (78.5-80.2)
Leeds	66.5 (63.5-70)	67.2 (64.2-70.7)	67.6 (64.6-70.8)	67.7 (64.7-70.8)	67.9 (65.1-70.6)	68.7 (66.8-70.8)	69.8 (69.3-70.3)	71.2 (71-71.4)	72.6 (72.4-72.8)	74 (73.8-74.2)	75.2 (74.8-75.5)	76.6 (76.4-76.8)	77.8 (77.6-78)	78.5 (77.7-79.3)
North East Lincolnshire	67.4 (64.1-70.7)	68.4 (65.4-71.8)	69.2 (66-72.6)	69.6 (66.6-72.9)	70.2 (67.4-72.9)	71 (69-73)	71.5 (70.7-72.1)	71.7 (71.4-72)	72.4 (72.1-72.7)	73.4 (73.1-73.7)	74.4 (73.9-74.9)	75.4 (75.1-75.8)	77.2 (76.8-77.5)	78 (77-79)
North Lincolnshire	66.8 (63.6-70.4)	67.6 (64.4-71.2)	68.1 (64.9-71.5)	68.3 (65.3-71.7)	68.5 (65.7-71.6)	69.3 (67.4-71.4)	70.1 (69.5-70.8)	71 (70.7-71.3)	72.3 (72-72.7)	73.6 (73.3-74)	75.4 (75-75.8)	77 (76.7-77.4)	78 (77.6-78.3)	78.8 (77.9-79.8)
North Yorkshire	67.8 (64.8-70.9)	68.6 (65.7-71.8)	69.1 (65.8-72.2)	69.3 (66.4-72.3)	69.5 (67-72.4)	70.4 (68.6-72.5)	71.6 (71-72.1)	72.7 (72.5-73)	73.9 (73.7-74.1)	75.6 (75.4-75.8)	77 (76.6-77.3)	78.4 (78.2-78.6)	79.8 (79.6-80.1)	80 (79.1-80.9)
Rotherham	66 (62.8-69.6)	66.8 (63.7-70.1)	67.2 (64.2-70.8)	67.4 (64.6-70.6)	67.7 (65.1-70.5)	68.6 (66.7-70.6)	69.6 (69-70.2)	71.1 (70.9-71.4)	72.3 (72-72.6)	73.8 (73.5-74.1)	75 (74.6-75.4)	76.3 (76.1-76.6)	77.5 (77.2-77.8)	77.9 (77.1-78.8)
Sheffield	66.9 (63.7-70.2)	67.7 (64.8-70.8)	68.2 (65.4-71.2)	68.4 (65.6-71.3)	68.7 (66.1-71.5)	69.5 (67.6-71.5)	70.5 (70-71)	71.6 (71.4-71.8)	72.5 (72.3-72.7)	73.7 (73.5-74)	75.2 (74.8-75.5)	77.2 (76.9-77.4)	78.2 (77.9-78.4)	79 (78.2-79.9)
Wakefield	65.4 (62.2-68.7)	66.1 (63-69.4)	66.4 (63.4-69.7)	66.4 (63.7-69.7)	66.5 (63.9-69.4)	67.4 (65.6-69.5)	68.9 (68.4-69.5)	70.6 (70.3-70.8)	72.3 (72-72.5)	73.6 (73.4-73.9)	74.8 (74.4-75.2)	76.1 (75.8-76.3)	77.4 (77.1-77.6)	78.2 (77.3-79.1)
York	68.3 (64.9-71.8)	69.1 (66-72.5)	69.6 (66.6-73.4)	69.9 (67-73.2)	70.2 (67.7-73)	71.1 (69.4-73.1)	71.8 (71.2-72.5)	72.4 (72.1-72.7)	73.6 (73.3-73.9)	75.6 (75.3-75.9)	77.1 (76.7-77.5)	78.4 (78.1-78.8)	79.6 (79.3-80)	80 (79.1-81)
Northern Ireland	65.5 (65.2-65.7)	67.3 (67.1-67.6)	67.9 (67.7-68.1)	68.1 (67.9-68.3)	67.9 (67.7-68.1)	66.5 (66.3-66.7)	68.3 (68.1-68.5)	69.8 (69.6-70)	71.5 (71.3-71.7)	73 (72.8-73.2)	74.9 (74.6-75.3)	76.3 (76-76.5)	77 (76.7-77.2)	78.7 (77.7-79.8)
Scotland	64.6 (64.5-64.8)	65.8 (65.6-65.9)	66.3 (66.1-66.4)	66.6 (66.5-66.7)	67.4 (67.2-67.5)	67.9 (67.8-68)	69 (68.9-69.2)	70 (69.9-70.1)	71.2 (71-71.3)	72.1 (72-72.3)	73.4 (73.1-73.7)	75 (74.9-75.2)	76.2 (76.1-76.3)	76.9 (76-78)
Wales	63.5 (60.3-67)	64.7 (61.9-67.8)	65.7 (62.8-68.8)	66.4 (63.4-69.7)	67.2 (64.2-70.4)	68.8 (66.6-71.1)	70.2 (69.8-70.7)	71.6 (71.4-71.7)	72.9 (72.8-73.1)	73.6 (73.4-73.7)	75 (74.7-75.3)	76.7 (76.5-76.8)	77.8 (77.7-78)	78.3 (77.5-79.1)
Latin America and Caribbean	46.3 (44.3-48.1)	49.6 (48.4-50.7)	52.5 (51.5-53.3)	55.2 (54.4-55.9)	56.6 (56-57.2)	60 (59.5-60.5)	62 (61.6-62.4)	63.8 (63.6-64.1)	66.2 (66-66.4)	67.6 (67.4-67.7)	69.5 (69.3-69.7)	71 (70.8-71.2)	70.3 (70.1-70.5)	72.8 (72.4-73.2)
Andean Latin America	40.2 (37.3-42.9)	45 (43.6-46.4)	47.4 (46.2-48.6)	52.3 (51.2-53.4)	48.5 (47.8-49.3)	59.5 (58.6-60.3)	61.4 (60.6-62.1)	63.6 (62.9-64.3)	66.7 (66.1-67.3)	68.7 (68.1-69.4)	71.1 (70.6-71.8)	72.6 (72-73.3)	73.5 (72.8-74.2)	76.2 (74.9-77.4)
Bolivia	37.5 (34.6-40)	41.9 (39.5-44)	43.9 (41.8-45.8)	45.5 (43.8-47.2)	48.2 (46.8-49.4)	51.3 (50.3-52.5)	54.4 (53.3-55.4)	57 (55.8-58.2)	59.7 (58.5-61)	62.7 (61.5-63.9)	65.2 (63.9-66.3)	67.5 (66-69.1)	69.3 (67.4-71.5)	71.3 (68.8-73.9)
Ecuador	49.8 (46.8-52.8)	52 (51.2-52.8)	53.9 (53.2-54.6)	57.9 (57.2-58.6)	59.6 (58.9-60.2)	62.9 (62.4-63.5)	64.6 (64.1-65.1)	68 (67.6-68.3)	69.7 (69.5-70)	71 (70.7-71.3)	71.6 (71.2-72)	71.2 (70.4-72)	71.9 (71.1-72.7)	74.8 (73.3-76.1)
Peru	38.3 (35.1-41.4)	43.7 (41.8-45.8)	46.2 (44.4-48)	52.8 (51.1-54.5)	44.8 (43.8-45.9)	61.2 (59.8-62.7)	62.7 (61.4-64)	64 (62.9-65.1)	67.9 (66.9-68.9)	69.8 (68.8-70.9)	73.1 (72.1-74.1)	75.2 (74.2-76.2)	75.8 (74.8-76.8)	78.7 (76.8-80.8)
Caribbean	52.6 (50.9-54.1)	54.6 (53.3-55.8)	57.2 (56.4-58.1)	59.2 (58.4-60)	61.3 (60.5-62)	63.2 (62.5-63.9)	64.3 (63.7-64.9)	65.3 (64.8-65.9)	66.4 (65.9-66.8)	66.7 (66.1-67.2)	68.3 (67.7-68.8)	69.3 (68.6-69.9)	55.2 (54.7-55.7)	70.3 (69.3-71.4)
Antigua and Barbuda	55 (53.7-56.2)	58.8 (57.7-59.7)	61.7 (60.8-62.5)	64.2 (63.4-64.9)	65.4 (64.7-66.1)	66.4 (65.7-67.2)	68 (67.2-68.8)	70 (69.3-70.7)	70.8 (70.1-71.4)	70.9 (70.3-71.5)	71.7 (71.1-72.3)	73.1 (72.5-73.7)	74.3 (73.8-74.9)	75.3 (74.4-76.2)
The Bahamas	55.4 (52.7-58.3)	58 (55.7-60.4)	60.3 (58.5-62)	59.6 (58.5-60.5)	62.7 (62.1-63.4)	64.9 (64.3-65.4)	65.4 (65-65.9)	66.7 (66.3-67.2)	67.6 (67.2-68)	67.1 (66.7-67.5)	68.2 (67.9-68.6)	70.3 (69.9-70.6)	70.3 (69.9-70.7)	70.8 (69.6-72.1)
Barbados	55.8 (53.7-57.4)	59.1 (57.7-60.3)	63.2 (62.3-64.1)	65.5 (64.9-66.2)	66.3 (65.7-66.8)	67.4 (66.9-67.9)	70.4 (70-70.8)	71.4 (71-71.8)	71.2 (70.8-71.5)	71 (70.6-71.4)	72.2 (71.8-72.6)	72.8 (72.5-73.1)	74.7 (74.3-75)	75.5 (74.4-76.6)
Belize	52 (50.1-54)	55.4 (53.7-56.9)	61.7 (60.3-63.1)	65.3 (64-66.5)	67 (65.8-68.1)	68.5 (67.5-69.5)	69.4 (68.5-70.2)	70 (69.2-70.8)	70.3 (69.5-71)	69.8 (69.1-70.5)	67.3 (66.9-67.7)	69.6 (69.3-70)	71.1 (70.7-71.4)	71.2 (70.7-71.8)
Bermuda	60.2 (59.4-61)	63.2 (62.7-63.7)	65.5 (65-66)	66.2 (65.8-66.7)	66.7 (66.3-67.1)	68.4 (68-68.9)	69.3 (68.9-69.7)	69.4 (69-69.9)	69.7 (69.3-70.2)	72.9 (72.5-73.2)	73.8 (73.5-74.2)	76 (75.6-76.5)	76.6 (76.2-77)	77.1 (76.4-77.6)
Cuba	64.8 (62-67.5)	65 (62.7-67.3)	67.4 (67.3-67.5)	69.1 (69-69.2)	70.7 (70.5-70.8)	72.9 (72.8-73.1)	72.5 (72.4-72.6)	72.4 (72.3-72.5)	73 (72.9-73.1)	73.1 (73-73.2)	74.8 (74.7-74.9)	75.6 (75.5-75.7)	76.2 (76.1-76.4)	76.2 (74.6-77.7)
Dominica	46.3 (43.6-48.6)	52.7 (50.8-54.6)	58.6 (57.1-60.1)	63.1 (61.9-64.2)	65.5 (64.6-66.3)	68.7 (68-69.3)	71.5 (71-72)	71.9 (71.4-72.4)	70.4 (70-70.8)	70.3 (69.9-70.8)	71.4 (70.9-71.9)	71.9 (71.4-72.4)	71.3 (70.8-71.8)	70.4 (69.4-71.4)
Dominican Republic	53.4 (51-55.3)	57.1 (55.8-58.3)	59.3 (57.9-60.8)	59 (57.7-60.2)	63.1 (61.7-64.4)	65.3 (64.1-66.6)	67.1 (66-68.3)	68.9 (67.8-70.1)	69.6 (68.4-70.7)	70.8 (69.6-72)	70.6 (69.3-72)	70.4 (69-72)	71 (69.6-72.4)	69.8 (67.8-71.9)
Grenada	56.7 (54.9-58.4)	59.3 (57.9-60.7)	61.3 (60-62.7)	62.3 (61.1-63.5)	64.2 (63-65.3)	65.7 (64.6-66.8)	65.8 (64.8-66.6)	66.1 (65.5-66.8)	67.1 (66.6-67.6)	69.8 (69.4-70.2)	71.6 (71.2-72)	72.4 (72-72.8)	72.5 (72.1-72.8)	73 (72.3-73.6)
Guyana	49.2 (47.7-50.5)	52.9 (51.9-53.8)	57.2 (56.4-57.9)	57.8 (56.6-59)	59.9 (59.1-60.5)	59.7 (59-60.4)	61.2 (60.5-61.9)	62.8 (62.2-63.3)	62.4 (62-62.8)	63.3 (62.9-63.6)	64.4 (64-64.7)	63.6 (63.3-64)	64.9 (64.5-65.2)	66.4 (64.6-68.2)
Haiti	34.4 (31.6-37.1)	36.5 (34.3-39)	38.5 (36.3-41)	40.1 (37.9-42.3)	42.1 (39.8-44.4)	45.1 (43-47.3)	48.5 (46.7-50.5)	51.7 (49.9-53.5)	53.9 (52.3-55.5)	54.7 (52.9-56.5)	57.2 (55.2-59.2)	59.4 (57.2-61.6)	28.8 (28.2-29.2)	63.8 (61.4-66.4)
Jamaica	54.5 (53.3-55.6)	59 (58.3-59.7)	62.1 (61.5-62.7)	64.4 (63.8-65)	66.1 (65.6-66.6)	67.6 (67.1-68.1)	71.4 (71-71.8)	70.9 (70.5-71.2)	73.6 (73-74.3)	72.2 (71-73.4)	72.2 (71.9-72.5)	76.5 (76.1-76.8)	73.7 (73.4-73.9)	72 (69.8-74.1)
Puerto Rico	58.7 (58.4-59)	66.2 (65.9-66.4)	67.5 (67.3-67.7)	67.6 (67.3-67.8)	69.1 (68.9-69.3)	70 (69.8-70.2)	70.7 (70.5-70.8)	70.3 (70.1-70.5)	70 (69.8-70.1)	69.2 (69.1-69.4)	72.6 (72.5-72.8)	73.8 (73.6-73.9)	75.3 (75.1-75.5)	74.5 (73.7-75.4)
Saint Lucia	49 (47-50.8)	53.7 (52.1-55.1)	57.2 (55.5-58.6)	61.3 (59.8-62.5)	63 (62-64)	63.8 (63.1-64.5)	65.8 (65.3-66.3)	67.5 (67.1-68)	67.8 (67.4-68.2)	69.4 (68.9-69.8)	71 (70.6-71.4)	71.2 (70.8-71.6)	73.2 (72.8-73.6)	73.1 (72.2-74)
Saint Vincent and the Grenadines	52.8 (50.5-54.7)	53.5 (51.8-55.1)	60.9 (59.2-62.5)	62.9 (61.5-64.2)	63.7 (62.4-64.8)	65 (64-65.9)	67 (66.3-67.7)	68.9 (68.4-69.4)	69.1 (68.5-69.6)	69.2 (68.7-69.7)	68.8 (68.4-69.3)	69.7 (69.2-70.1)	70.2 (69.8-70.7)	69.6 (68.9-70.4)
Suriname	56 (54.1-57.8)	58.6 (57.1-59.9)	60.3 (58.8-61.6)	62.4 (60.9-63.8)	62.7 (61.2-64.1)	62.5 (61.3-63.8)	63.2 (62.2-64.3)	65.5 (64.5-66.6)	66.4 (65.4-67.4)	67.5 (66.4-68.5)	66.6 (65.6-67.5)	66.9 (65.8-67.9)	68.4 (67.3-69.4)	68.9 (67.2-70.7)
Trinidad and Tobago	55.7 (54.4-56.8)	58.8 (58-59.5)	61.7 (61.1-62.3)	63 (62.5-63.4)	63.5 (63.1-64)	64.9 (64.5-65.3)	65.8 (65.4-66.2)	66.2 (65.8-66.5)	67.5 (67.2-67.8)	67.7 (67.4-68.1)	68 (67.6-68.4)	68.9 (68.5-69.2)	70.4 (70.1-70.7)	71.1 (68.4-74)
Virgin Islands	59.5 (58.6-60.4)	61 (60.5-61.6)	62.3 (61.8-62.8)	63.4 (62.7-64.1)	64.5 (63.4-65.4)	65.7 (64.6-66.7)	66.8 (66-67.8)	68.1 (67.3-69)	69 (68.2-69.7)	69.2 (68.4-69.9)	69.8 (68.9-70.7)	70.3 (69.2-71.7)	69.9 (68.7-71.5)	69.5 (67.9-71.8)
Central Latin America	48.3 (46.6-49.8)	52.4 (51.5-53.2)	55.8 (55.2-56.5)	58.4 (57.9-58.9)	60.1 (59.6-60.6)	62.2 (61.8-62.6)	63.9 (63.5-64.2)	65.4 (65.2-65.6)	68.1 (67.9-68.3)	69.2 (69-69.4)	71.1 (70.9-71.3)	72.4 (72.2-72.6)	72.6 (72.4-72.7)	73.3 (72.8-73.8)
Colombia	51.7 (50-53.2)	55.6 (54.6-56.5)	57.8 (57-58.6)	60.2 (59.6-60.8)	64 (63.4-64.6)	64.2 (63.8-64.7)	67 (65.7-68.3)	64.3 (64-64.5)	68.1 (67.9-68.4)	68.5 (68.3-68.7)	70.4 (70.2-70.6)	73.3 (73.1-73.5)	75 (74.8-75.2)	77.4 (75.9-79)
Costa Rica	56.1 (55.7-56.5)	60.4 (60.1-60.8)	64.1 (63.8-64.4)	63.9 (63.6-64.2)	65.7 (65.4-66)	69.7 (69.4-69.9)	72 (71.8-72.2)	73.2 (73-73.4)	74.4 (74.2-74.6)	74 (73.8-74.2)	75.5 (75.3-75.7)	77 (76.8-77.2)	76.7 (76.5-76.9)	76.3 (75.5-77.1)
El Salvador	45.2 (42.3-47.5)	47.5 (45.2-49.5)	52 (50.1-53.7)	54.4 (53.1-55.7)	55.4 (54.3-56.4)	59.3 (58.5-60.1)	58 (57.1-59)	60.5 (60-60.9)	64.9 (64.5-65.2)	66.1 (65.3-66.9)	68 (67.2-68.8)	67.6 (66.8-68.4)	69 (68.3-69.8)	69.3 (66.7-72)
Guatemala	41 (38.5-43.1)	43.2 (41.3-44.9)	47 (45.4-48.6)	48.2 (46.9-49.4)	48.5 (47.5-49.4)	52.7 (52-53.4)	52.5 (51.9-53.1)	57.9 (57.3-58.4)	60.4 (59.9-60.9)	62.5 (62.1-62.9)	64.9 (64.5-65.2)	65.8 (65.5-66.1)	68.4 (68-68.7)	69.1 (67.4-70.8)
Honduras	39.9 (37.5-42.6)	42.4 (40.3-44.4)	45.9 (44.2-47.9)	50.3 (48.7-52)	54.6 (53.3-55.9)	59.5 (58.2-60.9)	65 (63.7-66.4)	66.9 (65.2-68.5)	66.6 (64.8-68.6)	67.6 (64.8-70.1)	69.1 (66.3-72.2)	70.4 (67.4-73.5)	71.7 (68.7-74.6)	72.9 (70.2-75.6)
Mexico	46.9 (45-48.4)	51.7 (50.4-52.9)	55.3 (54.2-56.3)	58.3 (57.4-59.2)	59.6 (58.7-60.4)	61.9 (61.2-62.6)	63.4 (62.9-63.9)	66.1 (65.7-66.5)	68.6 (68.2-68.9)	70.1 (69.8-70.4)	72.2 (71.9-72.4)	73 (72.8-73.2)	72.3 (72-72.5)	72.6 (72.3-72.9)
Aguascalientes	54.1 (51.1-57.1)	57.8 (55.1-60.7)	60.3 (57.6-63.1)	62.2 (59.5-64.9)	62.4 (59.9-64.9)	63.8 (62.3-65.4)	66.1 (65.5-66.7)	68.3 (67.8-68.7)	70.5 (70-71)	72 (71.7-72.4)	73.4 (73.1-73.8)	73.8 (73.5-74)	73.9 (73.6-74.1)	74.9 (74-75.7)
Baja California	45.9 (43.1-49)	50.9 (48.1-53.8)	54.8 (52-57.7)	57.6 (54.8-60.4)	58.3 (55.6-60.9)	60.1 (58.3-62)	63.8 (63.2-64.3)	64.9 (64.3-65.4)	66.7 (66.2-67.1)	67.2 (66.9-67.6)	68 (67.6-68.3)	69.6 (69.3-69.9)	69.8 (69.5-70)	71.7 (70.7-72.5)
Baja California Sur	49.3 (46.4-52.1)	53.8 (51-56.8)	56.9 (54.4-59.7)	58.8 (56.3-61.4)	60 (58.1-62)	61.9 (60.6-63.2)	65.2 (64.6-65.8)	69.8 (69.4-70.2)	70.8 (70.5-71.2)	71.2 (70.9-71.5)	72.5 (72.2-72.8)	72.8 (72.5-73.1)	73 (72.7-73.3)	74.1 (73.3-75)
Campeche	52.8 (50-55.9)	57 (54.2-60)	60 (57.2-63.1)	62.7 (59.7-65.8)	63.4 (61.1-66)	65.3 (63.8-66.9)	66.5 (65.6-67.4)	68 (67.4-68.6)	70 (69.4-70.5)	71.8 (71.4-72.3)	74 (73.6-74.4)	75.1 (74.7-75.4)	74.2 (73.9-74.6)	72.7 (71.8-73.6)
Chiapas	47.1 (44-50.3)	51.6 (48.8-54.7)	55.1 (52.2-58.5)	57.8 (55.2-60.9)	59.6 (57-62.3)	62.6 (61-64.4)	62.5 (61.7-63.4)	64.7 (63.8-65.5)	68.6 (68-69)	69.7 (69.2-70.1)	71.4 (71-71.7)	72.4 (71.9-72.9)	72.3 (71.9-72.7)	72.3 (71.4-73.2)
Chihuahua	47 (44.2-50)	52.1 (49.2-55.2)	55.8 (53.1-58.7)	58.8 (56-61.9)	60.2 (57.7-62.8)	62.5 (60.9-64.3)	63.4 (62.7-64.2)	64.8 (64.3-65.2)	66 (65.5-66.5)	67.6 (67.2-68)	68.9 (68.5-69.2)	70.1 (69.8-70.4)	62.9 (62.6-63.1)	70.1 (69.2-71.1)
Coahuila	48.1 (45.2-51.1)	52.9 (50.3-56)	56.4 (53.7-59.4)	59.1 (56.4-61.8)	60 (57.3-62.6)	61.9 (60.3-63.5)	63.8 (63.1-64.5)	67.1 (66.7-67.6)	68.9 (68.5-69.3)	70.9 (70.5-71.2)	72.1 (71.7-72.4)	72.9 (72.6-73.1)	72.1 (71.9-72.3)	73.4 (72.5-74.2)
Colima	45 (42.4-47.9)	49.3 (46.5-52.5)	52.3 (49.8-55.2)	54.6 (51.8-57.4)	54.8 (52.7-57)	57.2 (55.9-58.7)	61.1 (60.3-61.9)	66.6 (66.1-67.1)	67.5 (67.1-67.9)	70 (69.5-70.5)	72.7 (72.4-73.1)	73.6 (73.2-73.9)	72.7 (72.4-73)	72.3 (71.5-73.1)
Mexico City	51.4 (48.6-53.9)	55.8 (53.2-58.5)	58.9 (56.4-61.5)	61.5 (59.2-63.9)	62.8 (60.7-65.2)	64.8 (63.4-66.3)	65.1 (64.7-65.5)	67.5 (67.3-67.7)	69.2 (69-69.4)	70.1 (69.8-70.3)	72.4 (72.2-72.5)	72.7 (72.5-72.9)	72.8 (72.5-73)	71.5 (70.7-72.4)
Durango	45.6 (42.8-48.3)	50.7 (47.8-53.6)	54.5 (51.8-57.5)	57.9 (55.4-60.9)	59.6 (57-62.3)	62.4 (60.6-64.1)	62.8 (62.1-63.6)	66 (65.5-66.5)	68.6 (68-69)	70.8 (70.4-71.2)	73.3 (72.9-73.7)	74 (73.6-74.3)	69.2 (68.9-69.5)	74.3 (73.5-75.2)
Guanajuato	45.1 (42.2-48)	50 (47.1-52.9)	53.6 (51-56.6)	56.6 (54.1-59.3)	57.6 (54.9-60.1)	60.1 (58.3-61.9)	63.8 (63.1-64.6)	65.6 (65.1-66.2)	69.1 (68.5-69.6)	71.3 (70.9-71.6)	72.8 (72.4-73.2)	73.9 (73.6-74.2)	74 (73.8-74.3)	73.3 (72.5-74.2)
Guerrero	45 (42.1-48.2)	49.4 (46.5-52.4)	52.8 (49.7-56)	56.1 (53.3-59)	58.1 (55.6-60.8)	61.6 (59.8-63.6)	61.5 (60.5-62.4)	64.9 (64-65.8)	68.7 (68.1-69.2)	69.4 (68.7-69.9)	72.8 (72.3-73.3)	74.7 (74.2-75.1)	71.1 (70.6-71.5)	71.1 (70.1-72.2)
Hidalgo	46.6 (43.9-49.4)	50.7 (48-53.4)	53.7 (51.3-56.4)	56.5 (53.8-59.3)	57.1 (54.3-59.8)	59.1 (57.2-61.1)	59 (58.2-59.8)	64.8 (64.3-65.3)	68.6 (68.1-69.1)	71.1 (70.7-71.5)	73.5 (73.1-73.9)	74.3 (74.1-74.6)	74 (73.7-74.2)	74.6 (73.7-75.4)
Jalisco	47.8 (44.9-50.8)	52.7 (49.6-55.6)	56.3 (53.6-59.1)	59.3 (56.5-62.2)	60.5 (57.9-63.1)	63.1 (61.4-64.7)	65.7 (65.1-66.3)	67.9 (67.4-68.3)	68.8 (68.4-69.3)	70.2 (69.8-70.5)	72.1 (71.7-72.4)	73.1 (72.8-73.4)	72.8 (72.6-73)	72.5 (71.7-73.4)
México	51.5 (48.6-54.3)	55 (52.2-57.8)	57.5 (54.8-60.6)	59.7 (57-62.5)	60.3 (57.7-63)	62.1 (59.6-64.6)	62.7 (61.1-64.4)	64.7 (64.4-65.1)	67.2 (66.9-67.5)	69.6 (69.3-69.9)	71.8 (71.6-72)	72.8 (72.6-73)	73 (72.8-73.2)	72.1 (71.3-73)
Michoacán de Ocampo	43.3 (40.6-46.4)	48.4 (45.4-51.8)	52.2 (49.3-55.3)	55.8 (52.7-58.7)	57.8 (55.2-60.8)	60.5 (58.8-62.4)	62 (61.3-62.6)	65.5 (65-65.9)	69.1 (68.7-69.5)	70.1 (69.7-70.4)	72.7 (72.3-73)	73.3 (72.9-73.6)	72.7 (72.3-73)	73.3 (72.4-74.2)
Morelos	47.1 (44.2-50)	51.7 (48.9-54.7)	55.1 (52.3-58.2)	58 (55.2-60.9)	59.1 (56.2-61.8)	61.7 (59.8-63.8)	62.5 (61.8-63.2)	67.3 (66.8-67.8)	69.1 (68.6-69.6)	70.6 (70.2-71)	73.4 (73.1-73.7)	74.5 (74.2-74.8)	73.4 (73.1-73.6)	71.5 (70.5-72.4)
Nayarit	46.2 (43.3-49.2)	51.3 (48.5-54.3)	55.2 (52.6-58.1)	58.8 (55.7-61.8)	61 (58.4-63.7)	64.4 (62.6-66.2)	66 (65.4-66.8)	67.2 (66.7-67.7)	69.1 (68.6-69.6)	71.1 (70.7-71.5)	74.3 (73.9-74.7)	73.8 (73.4-74.2)	72.6 (72.3-72.8)	74.4 (73.4-75.3)
Nuevo León	52.7 (49.8-55.7)	57.4 (54.6-60.2)	60.7 (58.1-63.6)	63.2 (60.8-65.9)	64.1 (61.8-66.7)	66.4 (64.7-68)	68.8 (68.3-69.3)	70.6 (70.2-71)	70.6 (70.2-71)	72.2 (71.9-72.5)	73.5 (73.1-73.7)	73.8 (73.6-74.1)	72.9 (72.7-73.1)	74.3 (73.4-75.1)

Table 8 Estimates of life expectancy at birth by location and year for 1950, 1955...2005, 2010, 2017 - males														
Location	1950	1955	1960	1965	1970	1975	1980	1985	1990	1995	2000	2005	2010	2017
Oaxaca	41.2 (38.5-44.1)	46 (43.1-49.3)	49.9 (47-53.1)	52.8 (50.1-55.7)	53.8 (51.2-56.7)	56 (54.2-57.7)	57.8 (57.1-58.5)	62.5 (61.9-63)	66.4 (66-66.9)	68.7 (68.3-69.1)	71.7 (71.4-72.1)	73 (72.5-73.3)	72.7 (72.4-73.1)	72.9 (72-73.8)
Puebla	40.5 (37.8-43.2)	45.4 (42.8-48.2)	49.4 (46.7-52.3)	52.9 (50.2-55.6)	54.4 (52-57.1)	57.5 (55.8-59.3)	58.7 (58-59.5)	62.5 (61.8-63.3)	66.4 (65.9-66.9)	68.4 (67.9-68.8)	70.6 (70.2-71)	71.8 (71.3-72.2)	72.3 (71.9-72.7)	71 (70.1-71.9)
Querétaro	48 (45.1-51)	52.4 (49.4-55.5)	55.6 (52.9-58.5)	58.6 (55.8-61.6)	59.2 (56.3-62.2)	61.6 (59-64.2)	62.3 (60.9-63.7)	66.7 (66.3-67.1)	68.4 (68.1-68.8)	70.8 (70.5-71.1)	71.5 (71.2-71.7)	73.3 (73-73.5)	73.7 (73.4-73.9)	73.3 (72.4-74.2)
Quintana Roo	60.1 (57.1-63.3)	61.8 (59-64.5)	63.8 (61-66.6)	64.4 (61.6-67.1)	63.6 (61.2-66.2)	64.5 (62.9-66.1)	66.1 (65.3-67)	70.8 (70.2-71.4)	71 (70.4-71.6)	72.4 (72-72.9)	73.5 (73.1-74)	73.8 (73.5-74.2)	73 (72.8-73.3)	72.2 (71.4-73.1)
San Luis Potosí	45.5 (42.5-48.5)	50.5 (47.5-53.6)	54.3 (51.3-57.5)	57.2 (54.3-60.1)	58.5 (55.8-61.1)	60.8 (59-62.7)	66.2 (65.5-66.9)	67.2 (66.8-67.7)	70.4 (70-70.9)	72 (71.6-72.4)	74 (73.7-74.4)	74.7 (74.3-75)	74 (73.6-74.3)	74.4 (73.5-75.3)
Sinaloa	49 (46-51.8)	53.4 (50.7-56.4)	56.6 (53.9-59.6)	59.5 (56.5-62.6)	60.7 (58.1-63.6)	63.3 (61.4-65.1)	65.2 (64.5-65.9)	66.8 (66.4-67.2)	70.6 (70.2-71.1)	71.1 (70.8-71.4)	73.4 (73-73.7)	73.8 (73.5-74)	70.4 (70.2-70.6)	75.2 (74.3-76.1)
Sonora	51.2 (48.2-54.2)	55.5 (52.6-58.5)	58.5 (55.7-61.3)	61 (58.4-63.8)	61.6 (59-64.6)	63.5 (60.8-66.2)	64.8 (63.2-66.5)	66.9 (66.4-67.3)	68.5 (68.1-68.9)	68.7 (68.3-69.1)	70.9 (70.5-71.2)	71.4 (71.1-71.6)	71 (70.7-71.2)	71.5 (70.6-72.4)
Tabasco	48.7 (45.8-51.7)	52.6 (49.8-55.7)	55.6 (52.8-58.8)	57.9 (55.5-60.8)	59 (56.2-61.8)	60.6 (58.8-62.5)	61.1 (60.3-61.8)	66.6 (66-67.1)	69.6 (69.1-70)	70.3 (69.8-70.8)	72.9 (72.4-73.2)	73.3 (73-73.5)	72.1 (71.8-72.4)	71.5 (70.5-72.5)
Tamaulipas	49.9 (47.1-52.6)	54.3 (51.8-57.4)	57.7 (55.1-60.5)	60.7 (58.3-63.4)	62.2 (59.6-64.8)	64.5 (62.8-66.1)	64.1 (63.5-64.7)	67.3 (66.8-67.7)	69.9 (69.5-70.3)	71.3 (70.8-71.8)	73 (72.7-73.3)	73.4 (73.2-73.5)	71.9 (71.7-72.2)	73.7 (72.8-74.6)
Tlaxcala	44.8 (41.9-47.8)	49.9 (46.8-53.1)	54.1 (51.1-57.2)	57.5 (54.9-60.3)	59.8 (57.2-62.5)	62.8 (60.4-65.1)	64.6 (63-66.1)	67.2 (66.7-67.7)	69.9 (69.5-70.3)	71.5 (71.1-71.8)	73.4 (73.1-73.7)	74.8 (74.5-75.1)	74.3 (74-74.6)	73.7 (72.8-74.6)
Veracruz de Ignacio de la Llave	45.3 (42.5-48.2)	50.2 (47.5-53)	53.9 (51.4-56.7)	56.9 (54.4-59.7)	58.2 (55.8-60.9)	60.7 (59-62.4)	62.8 (62.1-63.5)	66.7 (66.2-67.2)	68.6 (68.1-69.1)	70.6 (70.2-70.9)	72.1 (71.7-72.5)	72.9 (72.6-73.1)	72.7 (72.5-73)	72.2 (71.4-73.1)
Yucatán	53.4 (50.5-56.5)	57.6 (54.9-60.5)	60.4 (57.5-63.1)	62.8 (59.9-65.5)	63.7 (61.3-66.5)	65.2 (63.5-66.9)	66.5 (65.9-67.2)	69.3 (68.8-69.7)	70.6 (70.2-71.1)	71.7 (71.4-72.1)	73.5 (73.2-73.8)	74.4 (74-74.7)	73.8 (73.5-74.1)	72.7 (71.8-73.6)
Zacatecas	48.1 (45.2-51.2)	53 (50-55.7)	56.6 (53.7-59.3)	59.8 (56.8-62.9)	61.1 (58.4-63.6)	63.3 (61.5-65)	66 (65.1-66.8)	69.3 (68.7-69.8)	71.3 (70.7-71.8)	72.5 (72.1-72.9)	74.3 (73.9-74.7)	74.9 (74.5-75.2)	73.9 (73.6-74.2)	74.3 (73.5-75.2)
Nicaragua	43.3 (40.6-45.8)	48 (45.5-50.4)	49.8 (47.5-51.9)	52.9 (51.1-54.6)	54.9 (53.2-56.7)	58.8 (57.6-60.1)	62.7 (60.5-64.8)	57.9 (56.2-59.7)	69.7 (68.6-70.7)	70.4 (69.2-71.7)	72.5 (71.5-73.5)	73 (72-73.9)	75.5 (74.8-76.1)	76.9 (75.3-78.4)
Panama	59.3 (58.3-60.4)	60.8 (60-61.5)	64.3 (63.6-65)	65.6 (65-66.2)	66.6 (66-67.1)	69.6 (69.1-70.1)	71.6 (71.1-72)	72.9 (72.5-73.3)	73.8 (73.4-74.1)	73.2 (72.8-73.6)	74.9 (74.5-75.2)	75.5 (75.1-75.8)	75.2 (74.9-75.5)	77 (76.2-77.9)
Venezuela	56.6 (56.4-56.8)	59.4 (59.2-59.5)	63.2 (63.1-63.4)	64.3 (64.1-64.4)	64.4 (64.2-64.5)	65.2 (65-65.3)	66.7 (66.6-66.8)	68.9 (68.8-69)	69.2 (69.1-69.3)	69.2 (68.8-69.5)	70.2 (70.1-70.3)	71.3 (71.3-71.4)	71.1 (71.1-71.2)	71.2 (68.9-73.7)
Tropical Latin America	44.3 (42-46.3)	46.7 (44.8-48.6)	49.3 (47.5-50.9)	51.7 (50.2-53.1)	54 (52.7-55.2)	57.1 (56.2-58)	59.8 (59.2-60.4)	61.9 (61.5-62.4)	64 (63.7-64.4)	65.8 (65.4-66.1)	67.8 (67.5-68.2)	69.6 (69.3-69.9)	70.8 (70.5-71.1)	72 (71.8-72.3)
Brazil	44 (41.8-46)	46.4 (44.4-48.3)	49 (47.1-50.6)	51.4 (49.9-52.8)	53.7 (52.4-54.9)	56.9 (56-57.8)	59.6 (58.9-60.2)	61.7 (61.2-62.2)	63.8 (63.4-64.2)	65.6 (65.3-65.9)	67.7 (67.4-68)	69.5 (69.2-69.8)	70.8 (70.5-71)	72 (71.7-72.2)
Acre	42.1 (39.3-45)	44.5 (41.3-47.6)	47.8 (45-50.9)	50.5 (47.8-53.4)	53.3 (51-55.8)	57.2 (55.4-59.1)	59.7 (58.3-60.9)	60.1 (59.3-60.9)	63.6 (62.8-64.5)	65.4 (64.5-66.2)	68 (67.4-68.5)	69.7 (69.1-70.3)	71.1 (70.7-71.5)	71.7 (71-72.2)
Alagoas	37.3 (33.4-41.1)	39 (35.7-42.3)	41.2 (38.2-44.4)	43.3 (40.6-46)	44.9 (42.6-47.4)	47.5 (45.4-49.8)	49.6 (47.8-51.2)	53.2 (51.9-54.4)	59.1 (58.2-60)	63.6 (62.8-64.4)	66.4 (65.6-67.1)	68 (67.1-68.7)	68.2 (67.7-68.6)	70 (69.4-70.7)
Amapá	45.8 (43.1-48.8)	48.7 (45.6-52.1)	52.3 (49.5-55.4)	55.6 (52.9-58.3)	59.4 (57.4-61.6)	64.4 (62.9-65.9)	68.5 (67.9-69.2)	68.4 (68-68.8)	69.6 (69.2-70)	68.4 (68-68.8)	69 (68.7-69.3)	72 (71.8-72.4)	72.6 (72.3-72.9)	71.6 (71.1-72.1)
Amazonas	50.3 (47.7-53.1)	52.2 (49.2-55.4)	54.6 (51.5-57.5)	56.7 (53.9-59.5)	57.7 (55.2-60.3)	59.1 (57-61.5)	62.6 (61.3-64)	65.6 (64.7-66.5)	66.9 (66-67.9)	69.6 (68.8-70.3)	70.9 (70.6-71.2)	72.7 (72.5-73)	72.6 (72.3-72.8)	72.5 (72-73)
Bahia	39.5 (35.3-43.8)	42.1 (38.7-45.6)	45.1 (42.3-48.3)	48.3 (45.8-51)	51.6 (49.2-54)	56.6 (54.6-58.7)	61.1 (59.8-62.4)	62 (60.9-63.2)	64.8 (63.8-65.8)	67.3 (66.3-68.3)	68.6 (67.6-69.7)	69.4 (68.4-70.5)	70.2 (69.2-71.3)	71.2 (70.6-71.7)
Ceará	30.5 (26-35.2)	33.2 (29.5-36.6)	36.4 (33.2-39.6)	39.7 (36.7-43)	42.9 (39.8-46.3)	47.6 (44.9-50.1)	52.7 (50.4-55)	58.8 (57.3-60.3)	64.3 (63.3-65.4)	66.7 (65.8-67.7)	68.3 (67.3-69.3)	70 (68.9-70.9)	72.1 (71.4-72.6)	73.3 (72.7-73.8)
Distrito Federal	44.2 (41.7-46.9)	47.6 (44.7-50.5)	51 (48.3-53.8)	53.8 (50.9-56.7)	57.1 (54.7-59.6)	60.6 (59-62.2)	63.9 (63.4-64.3)	67.3 (67-67.5)	66.6 (66.3-66.9)	66.1 (65.8-66.3)	68.4 (68.2-68.7)	70.3 (70.1-70.5)	71.8 (71.6-72)	74 (73.5-74.4)
Espírito Santo	50.7 (48.1-53.7)	52.5 (49.7-55.5)	54.2 (51.5-57.3)	56 (53.4-58.8)	57.4 (55-60)	59.7 (58.2-61.4)	62.8 (62.3-63.3)	64.1 (63.8-64.5)	66.1 (65.8-66.4)	67 (66.7-67.2)	67.9 (67.7-68.1)	69 (68.6-69.3)	70.1 (69.8-70.3)	72.9 (72.3-73.4)
Goiás	42.7 (40.4-45.4)	45.1 (42.3-48.3)	47.7 (45.1-50.4)	50.3 (47.4-53.2)	53.6 (51.1-56.2)	57.8 (55.4-60.3)	61.2 (59.9-62.5)	62.6 (62.1-63.1)	66.4 (65.9-66.8)	67.7 (67.3-68)	70.2 (70-70.4)	71.3 (71.1-71.5)	71.5 (71.3-71.7)	72 (71.5-72.4)
Maranhão	31.1 (28.7-33.8)	33.1 (30.5-35.8)	35.8 (32.8-38.7)	38.5 (36-41.2)	41.3 (39-44)	46.2 (43.8-48.9)	50 (47.8-52.5)	54 (52.4-55.5)	60.3 (59.2-61.4)	66.3 (65.3-67.3)	69.6 (68.6-70.6)	69.9 (68.9-70.9)	71.6 (70.7-72.6)	72.8 (72.3-73.4)
Mato Grosso	45.4 (42.5-48.3)	47.9 (44.4-51.2)	50.6 (47.9-53.3)	53.7 (50.8-56.5)	57.6 (55.1-60.2)	62.6 (60.6-64.9)	66.8 (65.4-68.1)	64.7 (63.5-65.9)	66.6 (65.5-67.7)	68.6 (68.2-69.1)	69.4 (69.1-69.6)	70 (69.7-70.2)	71.2 (70.9-71.4)	72.4 (71.9-72.9)
Mato Grosso do Sul	51.8 (49.1-54.9)	53.3 (50.1-56.5)	55.4 (52.7-58.4)	58 (55.1-61.3)	60 (57.4-62.8)	62.6 (60.7-64.6)	64.7 (64.2-65.2)	65.5 (65.2-65.8)	66.9 (66.6-67.2)	66.2 (65.9-66.5)	68.3 (68-68.5)	69.5 (69.2-69.7)	70.7 (70.5-70.9)	71.9 (71.4-72.4)
Minas Gerais	47.8 (44.7-50.9)	49.9 (47.2-52.9)	52.1 (49.2-55.1)	54.1 (51.4-57.1)	56.2 (53.8-58.8)	58.9 (57.4-60.4)	61.6 (60.9-62.2)	63.2 (62.7-63.6)	65.8 (65.3-66.3)	67.6 (67.1-68.1)	70 (69.5-70.4)	70.6 (70.2-71.1)	72 (71.6-72.4)	73.4 (72.9-73.8)
Pará	45.5 (42.6-48.3)	47.8 (44.6-51.1)	50.6 (47.5-54)	53 (50.2-56.1)	54.7 (52.2-57.4)	58.3 (56.4-60.2)	62.3 (61.2-63.2)	63.4 (62.5-64.4)	66 (65.2-66.9)	69 (68.2-69.7)	70.3 (69.6-71.2)	70.7 (70-71.4)	71.5 (71.1-71.8)	71.8 (71.4-72.3)
Paraíba	39.8 (35.8-44)	42.6 (39.3-46)	45.8 (42.7-48.8)	49.5 (46.3-52.5)	52.5 (49.5-55.6)	55.9 (53.7-57.9)	60.1 (58.9-61.1)	63.6 (62.7-64.5)	66.6 (65.8-67.4)	68.5 (67.8-69.2)	70.3 (69.5-71)	70.5 (69.6-71.3)	70.3 (69.5-71.1)	72.1 (70.9-73.4)
Paraná	48.4 (45.2-51.8)	50.7 (47.9-53.8)	52.9 (49.9-55.9)	55.2 (52.4-57.8)	57.9 (55.6-60.2)	60.6 (58.9-62.3)	61.8 (61.3-62.2)	64.3 (64-64.6)	65.9 (65.6-66.2)	67.2 (66.9-67.5)	68.6 (68.4-68.8)	69.3 (69-69.7)	70.1 (69.8-70.3)	71.8 (71.3-72.3)
Pernambuco	33.3 (29.1-37.5)	36.1 (32.7-39.3)	39.4 (35.9-42.7)	42.7 (39.8-45.9)	45.8 (42.8-48.9)	50.7 (48.9-52.5)	54.8 (53.7-55.9)	57.1 (56.1-58.1)	60 (59.3-60.6)	64 (63.4-64.5)	64.7 (64.1-65.3)	67.3 (66.8-67.9)	69.3 (68.7-69.8)	69.2 (68.7-69.8)
Piauí	45.3 (42.5-48.1)	47.2 (44.3-50.4)	49.2 (46.3-52.3)	50.8 (47.6-53.8)	52.2 (49.4-55.3)	56.1 (53.7-58.6)	60.1 (58.5-61.7)	62.4 (61.3-63.3)	66.6 (65.7-67.5)	70.4 (69.3-71.5)	70.5 (69.6-71.4)	70.7 (69.9-71.5)	72.9 (72.3-73.4)	73.1 (72.6-73.7)
Rio de Janeiro	47.6 (45-50.3)	50 (47.2-53)	52.3 (49.5-55.1)	54.4 (51.5-57.2)	55.4 (52.8-57.9)	57.2 (55.7-58.7)	59.5 (59-60)	60.7 (60.4-61)	60.7 (60.4-61)	61.5 (61.2-61.7)	65.2 (65.1-65.4)	67.4 (67.2-67.6)	69 (68.8-69.2)	69.9 (69.5-70.3)
Rio Grande do Norte	40.9 (37.1-45)	43.8 (40.4-47.1)	47 (43.8-50.2)	50.3 (47.7-53.4)	52.9 (50.1-56.1)	56.6 (54.4-58.9)	60.7 (59.2-62.2)	64.1 (62.9-65.2)	67.4 (66.4-68.2)	68.5 (67.6-69.5)	71.1 (70.1-72.1)	72.6 (71.8-73.4)	73.5 (73.1-74)	71.8 (71.2-72.3)
Rio Grande do Sul	52.5 (50-55.4)	54.3 (51.7-57.1)	56.2 (53.7-59)	58.1 (55.4-60.8)	60.3 (58-63)	62.9 (61.4-64.3)	63.9 (63.5-64.2)	65.6 (65.3-65.8)	66.5 (66.3-66.7)	67.2 (67-67.4)	68.7 (68.5-68.8)	70.2 (70.1-70.4)	71.2 (71.1-71.4)	71.9 (71.5-72.2)
Rondônia	39.7 (37.1-42.3)	40.8 (38-43.9)	44.2 (41.5-47)	46.5 (43.6-49.5)	49.2 (46.7-51.8)	53.9 (51.7-56.2)	58.2 (56.8-59.6)	59.9 (58.7-61.1)	62.7 (61.5-64)	68 (66.8-69.2)	68.3 (67.2-69.5)	69.7 (68.6-70.8)	71 (69.8-72.1)	71.9 (70.1-73.7)
Roraima	37.2 (34.6-40.1)	39.5 (36.6-42.4)	43 (40.3-45.8)	46.4 (43.7-49.3)	49.5 (47-52.2)	55.1 (53-57.3)	59.7 (58.1-61.5)	61.6 (60.1-63.2)	65.5 (64-66.9)	67.4 (66-68.8)	68.6 (67.2-69.9)	69.7 (68.4-71.1)	71.1 (69.7-72.4)	72 (70.2-73.9)
Santa Catarina	54.7 (52-57.6)	56.3 (53.4-59.5)	58.1 (55.4-61)	59.7 (57.1-62.4)	61.4 (59.1-63.8)	63.7 (62-65.3)	64.3 (63.7-65)	65.5 (65-65.9)	66.4 (66-66.8)	67 (66.5-67.4)	69 (68.6-69.3)	70.6 (70.2-70.9)	71.8 (71.5-72)	73.6 (73.2-74)
São Paulo	50.8 (48.2-53.4)	53 (50.2-55.9)	55 (52.4-57.9)	57 (54.3-59.9)	58.7 (56.3-61.2)	60.3 (58.8-61.8)	61.8 (61.3-62.2)	63.5 (63.2-63.8)	63.9 (63.7-64.2)	64.6 (64.4-64.9)	66.7 (66.6-66.9)	70 (69.8-70.1)	71.3 (71.2-71.5)	72.8 (72.5-73.2)
Sergipe	47.5 (44-51.2)	49.5 (46.1-53.2)	51.8 (49-54.9)	54.1 (51.5-56.8)	56.2 (53.8-58.6)	58.7 (56.7-60.7)	59.9 (58.7-61)	63 (62.2-63.7)	65 (64.5-65.6)	66 (65.5-66.4)	68.3 (67.7-68.8)	70 (69.6-70.5)	70.4 (70-70.7)	69.8 (69.2-70.5)
Tocantins	38.7 (36-41.6)	40.9 (37.4-44.5)	43.8 (40.7-47.1)	46.5 (43.3-49.7)	49.5 (46.6-52.2)	54 (51.3-56.7)	58.2 (55.5-60.7)	61.7 (59.5-63.9)	65.3 (64.2-66.4)	67.5 (66.8-68.2)	69.6 (69-70.3)	71.3 (70.7-72)	72.3 (71.6-72.9)	73.1 (72-74.2)
Paraguay	58.3 (56-60.4)	62.3 (61.2-63.6)	64.3 (63.2-65.4)	66.1 (65-67.1)	66.9 (66.1-67.8)	67.6 (66.7-68.5)	69.5 (68.6-70.4)	70.6 (69.8-71.4)	72.3 (71.4-73)	71.7 (70.7-72.6)	72.1 (71.3-73)	71.9 (71.1-72.9)	71.9 (71-72.8)	73.4 (71-76)
North Africa and Middle East	39.9 (37.8-41.9)	42.4 (40.9-43.9)	45.2 (43.9-46.4)	49.1 (48-50)	52 (51-52.9)	55.6 (54.8-56.3)	58.5 (58-59)	60.8 (60.4-61.1)	64.5 (64.1-64.9)	66.2 (65.8-66.5)	68 (67.6-68.3)	69.4 (69.1-69.8)	70.7 (70.3-71)	72 (71.5-72.5)
North Africa and Middle East	39.9 (37.8-41.9)	42.4 (40.9-43.9)	45.2 (43.9-46.4)	49.1 (48-50)	52 (51-52.9)	55.6 (54.8-56.3)	58.5 (58-59)	60.8 (60.4-61.1)	64.5 (64.1-64.9)	66.2 (65.8-66.5)	68 (67.6-68.3)	69.4 (69.1-69.8)	70.7 (70.3-71)	72 (71.5-72.5)
Afghanistan	41.7 (38.6-44.5)	42.8 (40.1-45.7)	43.9 (41.1-46.7)	44.9 (42.2-47.7)	45.9 (43.2-48.4)	46.7 (44.4-49.2)	41.9 (40.1-43.6)	38.2 (36.8-39.6)	53.1 (51-55.2)	53.4 (51.4-55.6)	54.6 (52.4-56.8)	57.3 (55.1-59.7)	59.7 (57.2-61.9)	63.6 (61.3-65.9)
Algeria	55.3 (54.2-56.4)	52.1 (51.3-52.8)	52.1 (51-53.3)	59 (57.7-60.2)	57.7 (56.1-59.4)	57.3 (55.8-58.7)	58.4 (57.5-59.2)	66.7 (66-67.3)	70.3 (69.4-71.2)	71.3 (70.5-72.3)	72.4 (71.6-73.3)	74.3 (73.3-75.2)	75.6 (75.1-76.2)	77 (76.4-77.6)
Bahrain	45.7 (42.5-48.9)	48.9 (46.1-51.9)	53.2 (50.4-56)	57.5 (55-60.1)	61.6 (59.6-63.9)	65.5 (64.1-67.1)	69.2 (68.4-70.1)	69.9 (69.3-70.4)	69.4 (69-70)	70.1 (69.6-70.6)	71.7 (71.2-72.2)	74 (73.5-74.4)	77.5 (77-77.9)	78.8 (77.8-79.8)
Egypt	38.4 (35-41.4)	42 (39.6-44.1)	45.6 (44-47.1)	49.4 (48.1-50.8)	49.6 (48.3-50.7)	52.3 (51.2-53.3)	56.4 (55.6-57.2)	58.5 (57.9-59.1)	62.6 (62.1-63)	64.4 (64-64.7)	66.2 (65.9-66.5)	66.3 (66-66.5)	66.8 (66.5-67)	68 (66.6-69.3)
Iran	31.8 (28.2-35.4)	33.9 (30.4-37.1)	37.7 (34.5-40.7)	42.9 (40-45.4)	50.2 (48-52.2)	58.5 (57.1-59.9)	62.2 (61.2-63.1)	64.6 (63.8-65.4)	65.7 (65-66.3)	70.3 (69.7-70.8)	71.7 (71.2-72.1)	72.1 (71.7-72.5)	73.1 (72.8-73.3)	75.5 (75.4-75.6)
Iraq	47.1 (44.2-49.8)	50.7 (48.2-53)	53.7 (51.6-55.6)	54.6 (52.8-56.3)	57.1 (55.7-58.4)	58 (56.9-59.1)	58.8 (57.6-60)	58.1 (56.6-59.6)	64.4 (62.5-66.4)	64 (62.3-65.9)	65.3 (63.7-67.1)	68.3 (66.7-70.1)	72.5 (71.2-73.9)	74.8 (73.9-75.6)
Jordan	51.9 (49-54.9)	53.3 (50.5-56)	56.1 (53.9-58.3)	59.5 (57.9-61.2)	61.4 (60.4-62.5)	64.2 (63.5-65)	65.6 (64.7-66.5)	68.2 (66.9-69.5)	70.5 (69-71.9)	72 (70.4-73.5)	73.9 (72.7-75.1)	75.8 (75.1-76.5)	76.8 (76.2-77.4)	77.8 (76.3-79.2)
Kuwait	60.9 (58.2-64.1)	62.9 (60.3-65.6)	65.6 (63.8-67.1)	67 (66.7-67.4)	68.2 (67.9-68.5)	68.7 (68.5-68.9)	71.4 (71.2-71.6)	74.6 (74.4-74.8)	73.3 (73.1-73.6)	78.3 (78.1-78.5)	77.5 (77.3-77.7)	77.9 (77.7-78)	79.8 (79.7-80)	80.7 (80-81.3)
Lebanon	56.8 (53.8-59.7)	57.7 (55.1-60.6)	60.1 (57.5-62.9)	62.2 (59.8-64.9)	64 (61.7-66.6)	57.9 (56.3-59.7)	66.7 (65.1-68.9)	61.8 (60.6-63.2)	67.3 (66.1-68.8)	71.3 (70-72.8)	73.2 (72.1-74.3)	73.7 (72.8-74.6)	74.6 (73.8-75.4)	75.8 (75.1-76.4)
Libya	37.5 (33.8-41)	44.1 (40.9-47.4)	50 (47.2-52.8)	57 (54.8-59.3)	63.1 (61.4-64.7)	66.2 (65-67.4)	68.7 (67.3-70.2)	70.1 (68.5-71.9)	70.8 (69-72.6)	72 (70.3-73.6)	72.1 (70.7-73.4)	73.1 (71.9-74.3)	72.2 (71-73.4)	71.1 (69.4-73.2)
Morocco	40.2 (37.1-43.3)	41.6 (39.1-44.2)	42.6 (40.6-44.7)	47.9 (45.6-50.3)	51.6 (49.4-53.9)	54.5 (52.6-56.3)	58.5 (56.9-60.2)	63.3 (62.1-64.6)	67.1 (66.1-68.1)	68 (67-68.9)	68.8 (67.7-69.8)	70.2 (68.7-71.5)	71.4 (69.6-73.2)	73.2 (71-75.5)
Palestine	48.6 (45.5-51.8)	50.8 (48.2-53.8)	54.4 (51.6-57.4)	58.4 (55.6-61.3)	53.7 (51.6-55.8)	64.1 (61.5-66.6)	66.1 (63.8-68.6)	67.8 (65.7-70.3)	68.5 (66.7-70.7)	70 (68.5-71.5)	67.9 (67.2-68.6)	68.7 (68.2-69.1)	72.9 (72.5-73.3)	75.6 (74.7-76.4)
Oman	28.7 (25.5-32.1)	31 (27.9-34.5)	33.7 (30.9-37)	37.8 (34.9-40.7)	47 (44.1-50)	53 (50.3-55.7)	59.1 (56.5-61.8)	63.9 (61.5-66.6)	66.9 (64.4-69.4)	69 (66.6-71.7)	71 (68.6-73.4)	72.7 (70.4-75.2)	73 (70.8-75)	75.5 (73.3-77.9)
Qatar	58.4 (55.8-61.3)	59.4 (56.7-62.2)	60.7 (58-63.5)	61.9 (59.3-64.5)	63.9 (61.5-66.3)	65.9 (63.7-68.2)	67.7 (65.7-69.8)	69.5 (67.8-71.4)	70.7 (69.1-72.4)	71.5 (69.9-73.2)	73.6 (72-75.2)	76.4 (74.7-78.1)	77.4 (75.8-79.1)	79.6 (77.7-81.6)
Saudi Arabia	50.9 (47.4-54.2)	52.8 (49.3-56)	54.8 (51.5-58)	57.7 (54.6-60.9)	61 (58.3-63.9)	65.4 (62.9-68)	68 (65.5-70.5)	69.6 (67.1-72)	70.3 (68-72.5)	72.3 (70.4-74.1)	73.4 (71.9-74.7)	73.2 (72.1-74.4)	73 (71.8-74.4)	75.3 (73.9-76.6)
Sudan	46.2 (43.4-49.1)	47.1 (44.4-49.9)	48.7 (46.3-51.2)	48.7 (46.5-51)	49.8 (47.9-51.7)	51.5 (49.8-53.2)	53.4 (51.8-54.9)	50.7 (49.3-52.1)	57.4 (55.6-59.1)	60 (57.9-61.9)	62 (59.8-64.1)	64.3 (62.1-66.4)	66.3 (64.1-68.7)	68.8 (66.4-71.5)
Syria	53.9 (50.7-57)	57 (54.3-59.6)	60.7 (58.3-63.1)	63.6 (61.5-66)	66.3 (64.3-68.3)	69.4 (67.8-70.9)	69.1 (67.6-70.7)	67.7 (66.3-69.3)	67.7 (66.3-69)	68.2 (66.9-69.6)	70.2 (69.1-71.5)	72.8 (71.6-74)	73.6 (72.1-75.1)	65.5 (63.8-67.2)
Tunisia	40.2 (37.1-43.3)	42.9 (40.8-45.2)	46.9 (45.1-48.4)	50.9 (49.6-52.1)	54.8 (53.9-55.7)	60.9 (60.1-61.7)	65.7 (65-66.5)	69.5 (68.9-70.1)	70.8 (70.3-71.5)	71 (70.4-71.7)	72.1 (71-73.3)	73.6 (71.8-75.6)	74.7 (72.6-77.1)	76.1 (73.7-78.6)
Turkey	37.3 (34.3-40.4)	41.5 (39-44.1)	44.6 (42.1-47.1)	49.1 (46.7-51.7)	52.8 (50.5-55.1)	56.4 (54.5-58.2)	59.9 (59-61)	63.3 (62.4-64.3)	65.6 (64.8-66.3)	67.1 (66.4-67.9)	70.9 (70.1-71.6)	73.4 (72.7-74.1)	74.1 (73.4-74.7)	75.2 (74.1-76.3)
United Arab Emirates	49.3 (45.9-52.9)	51.8 (48.7-55)	55 (52-58.2)	59 (56.2-61.9)	62.2 (59.6-64.9)	65.3 (62.9-68.1)	67.9 (65.5-70.4)	69.6 (67.4-72)	70.2 (68.2-72.4)	71.1 (69.4-72.9)	70.5 (69-71.9)	71.4 (69.8-73)	71.3 (69.5-73.3)	71.7 (69.3-74)
Yemen	29.7 (26.9-32.7)	31 (28.5-33.8)	34 (31.8-36.3)	35.2 (33.1-37.4)	38.6 (36.6-40.7)	45.5 (43.4-48)	50.5 (48.1-52.9)	54.6 (52.2-57.1)	57.5 (55-60.1)	59.8 (57.3-62.4)	62.3 (59.5-64.8)	64.7 (61.9-67.1)	66.5 (63.9-69.1)	66 (63.6-68.3)
South Asia	42.5 (40.4-44.5)	45.1 (43.3-46.8)	45.6 (43.9-47.2)	46.2 (44.9-47.5)	49.1 (48-50.1)	51.8 (51-52.7)	54.9 (54.1-55.6)	56.9 (56.3-57.6)	59 (58.4-59.5)	61 (60.5-61.4)	62.1 (61.7-62.5)	64.1 (63.6-64.5)	65.1 (64.7-65.5)	67.9 (67.4-68.4)
South Asia	42.5 (40.4-44.5)	45.1 (43.3-46.8)	45.6 (43.9-47.2)	46.2 (44.9-47.5)	49.1 (48-50.1)	51.8 (51-52.7)	54.9 (54.1-55.6)	56.9 (56.3-57.6)	59 (58.4-59.5)	61 (60.5-61.4)	62.1 (61.7-62.5)	64.1 (63.6-64.5)	65.1 (64.7-65.5)	67.9 (67.4-68.4)
Bangladesh	39.6 (37.2-42.2)	42 (39.6-44.3)	44 (41.7-46.4)	46.1 (44-48.2)	41.4 (39.9-42.9)	52 (50.2-53.7)	55.3 (54.1-56.4)	54.2 (53.3-55.2)	57.3 (56.4-58.3)	60.8 (60-61.7)	64 (63.3-64.8)	66.3 (65.6-67)	68.2 (67.5-68.9)	71.8 (70.3-73.3)
Bhutan	32.7 (29.2-36.5)	35.4 (31.9-38.8)	38.7 (35.4-42.2)	42.6 (39.3-46)	46.7 (43.6-49.9)	49.7 (46.8-52.7)	52.3 (49.6-55.1)	56 (53.6-58.5)	60 (57.9-62.3)	63.1 (61.2-65.1)	65 (63.4-66.6)	68.2 (66.6-70)	70.1 (68.3-72.1)	72.3 (69.8-74.8)
India	42.9 (40.4-45.3)	45.4 (43.3-47.4)	45.4 (43.5-47.2)	45.5 (43.9-47)	49.3 (47.9-50.5)	51.1 (50-52.1)	54.3 (53.4-55.2)	56.9 (56-57.7)	58.9 (58.3-59.6)	60.9 (60.4-61.5)	61.9 (61.4-62.4)	64.2 (63.7-64.6)	65 (64.5-65.5)	67.8 (67.2-68.3)
Andhra Pradesh	45.9 (42.2-49.9)	47.9 (44-52.6)	48 (43.7-52.5)	48.1 (44.6-51.7)	51 (48.3-53.7)	52.3 (49.7-55)	55.3 (52.8-58.1)	57.8 (55.1-60.4)	59.1 (56.5-61.8)	60.7 (58-63.4)	61.6 (59-64.3)	64 (61.3-66.9)	65.9 (63.4-68.5)	69.5 (66.8-71.9)
Arunachal Pradesh	45.8 (42.1-49.4)	47.9 (44.5-51.3)	47.4 (44.2-50.9)	46 (43.5-48.7)	50.7 (48.2-53.3)	53.3 (51-55.9)	56.5 (54.2-58.9)	58.2 (55.9-60.6)	59.5 (57.3-61.9)	61.1 (59-63.5)	62.1 (60-64.4)	64.9 (62.9-66.9)	66.4 (64.7-68.3)	68.5 (66.9-70.7)
Assam	41.5 (38-45.2)	44 (40.8-47.5)	43.9 (40.6-47.2)	43.8 (41.1-46.8)	47.8 (45-50.7)	49.7 (47.2-52.2)	52.7 (50.5-55.2)	55 (53-57.2)	56.7 (55-58.4)	58.2 (57.3-59.2)	57.8 (57-58.6)	60.8 (59.9-61.7)	61.6 (60.7-62.4)	65.5 (64.3-66.7)
Bihar	45.4 (42-49.2)	47.8 (44.5-51.5)	47.6 (44.4-51.1)	47.4 (44.6-50.2)	50.6 (48.2-53.1)	51.7 (49.5-54)	54.1 (51.7-56.5)	56.3 (54.2-58.6)	58.5 (56.4-60.5)	60.4 (58.8-62.1)	61.5 (60.4-62.8)	64.2 (63.3-65.1)	66.3 (65.6-67)	70.2 (69-71.3)
Chhattisgarh	43 (39.5-46.7)	45.3 (42.1-48.8)	44.9 (41.8-48.5)	44.6 (42.1-47.6)	48.5 (45.5-51.6)	49.5 (46.7-52.3)	52.6 (50.2-55.2)	54.9 (52.7-57.1)	57.9 (56-60)	60.6 (59-62.3)	61.2 (60-62.5)	61.3 (60.5-62.1)	59.8 (58.9-60.5)	62.1 (61-63.1)
Delhi	46.2 (42.8-49.7)	50.1 (46.7-53.7)	51.8 (48.8-55)	50.9 (48.4-53.5)	57.2 (54.6-59.8)	58.8 (56.5-61.2)	61.1 (58.8-63.5)	63.2 (60.8-65.6)	64 (61.8-66.2)	65.2 (63.2-67.3)	66 (64.4-67.5)	68.5 (67.4-69.8)	70 (69-71.1)	72.4 (70.8-74)
Goa	57.1 (54-60.4)	59.1 (56-62.4)	59.6 (56.6-62.8)	59.5 (56.9-62.1)	62.6 (60.3-64.8)	63.6 (61.6-65.7)	65.4 (63.6-67.5)	66.5 (64.6-68.3)	66.6 (64.9-68.3)	67.5 (65.9-69.2)	68.1 (66.5-69.9)	70.1 (68.2-72.2)	71 (69-73)	72.5 (70.3-74.5)
Gujarat	45.2 (42.1-48.2)	47.6 (44.7-50.6)	47.7 (44.9-50.9)	47.7 (45.2-50.4)	51.5 (49.3-54.1)	53.2 (51.1-55.7)	55.8 (53.8-58.1)	58.3 (56.3-60.4)	60.3 (58.7-61.9)	62.7 (61.7-63.8)	62.9 (62.1-63.7)	64.5 (63.6-65.4)	65.9 (65-66.7)	68.6 (67.3-69.8)
Haryana	46.9 (43.7-50.5)	49.2 (45.9-52.7)	49.4 (46.4-52.5)	49.2 (46.3-52.1)	53.4 (50.9-56.1)	56.4 (53.9-58.9)	59.9 (57.6-62.4)	62.3 (60.3-64.5)	63.8 (62.3-65.5)	65 (64-65.9)	64.2 (63.4-64.9)	65.8 (65-66.6)	65 (64.2-65.8)	67.6 (66.4-68.8)
Himachal Pradesh	51.1 (47.7-54.8)	52.8 (49.6-56.3)	52.8 (49.7-56.2)	52.6 (49.6-55.6)	55.9 (53.3-58.6)	57.5 (55-60.3)	60.2 (57.6-62.7)	62 (59.7-64.3)	63.6 (61.7-65.6)	65.2 (64.1-66.3)	66.7 (65.8-67.6)	67.5 (66.6-68.4)	67.8 (67-68.7)	68.4 (67-69.9)
Jammu and Kashmir	51.9 (48.7-55.4)	53.7 (50.7-56.9)	53.7 (50.5-57)	52.9 (50.2-55.9)	56.9 (54.5-59.4)	57.9 (55.6-60.3)	59.9 (57.7-62.2)	62.3 (60.2-64.6)	63.4 (61.5-65.7)	65 (63.4-66.9)	66.1 (64.8-67.5)	69.2 (68.3-70.1)	69.9 (69.2-70.7)	72 (70.8-73.3)
Jharkhand	46.7 (43.4-50.2)	48.5 (45.5-51.9)	48.1 (44.8-51.7)	47.6 (44.9-50.6)	50.8 (47.9-53.7)	51.8 (49.2-54.7)	54 (51.7-56.6)	55.1 (52.8-57.6)	57 (55-59.1)	59 (57.3-60.7)	60.1 (58.9-61.3)	62.7 (62-63.5)	64.3 (63.6-64.9)	69.2 (68.1-70.2)
Karnataka	44.4 (41.2-47.8)	46.8 (43.7-50.2)	46.8 (43.8-49.9)	46.8 (44.3-49.4)	50.4 (48-53)	52.6 (50.3-55.1)	56.2 (54.2-58.3)	59.1 (57.2-61)	61 (59.5-62.5)	62.7 (61.9-63.7)	62.2 (61.5-62.9)	64.6 (63.9-65.3)	64.6 (63.8-65.3)	66.2 (65.1-67.3)
Kerala	48.6 (45.3-51.8)	52.1 (49-55.4)	53.5 (50.5-56.5)	54.5 (51.9-57.2)	58.9 (56.3-61.5)	61.1 (58.9-63.3)	63.4 (61.4-65.7)	65.1 (63.1-67.2)	66.4 (64.8-68)	68.6 (67.5-69.7)	68.4 (67.5-69.2)	70.9 (70.1-71.8)	71.1 (70.4-71.9)	71.7 (70.5-73)
Madhya Pradesh	41.6 (38.2-45.3)	43.9 (40.9-47.3)	43.4 (40.6-46.5)	43 (40.5-45.7)	46.7 (44.5-49)	47.5 (45.4-49.9)	50.4 (48.2-52.7)	52.9 (50.7-55.1)	55.4 (53.3-57.5)	57.8 (56-59.6)	59.2 (57.9-60.5)	61.5 (60.4-62.4)	62.2 (61.4-63.1)	65.8 (64.6-67)
Maharashtra	46.3 (43.2-49.8)	48.8 (45.6-52)	49.1 (46.3-51.8)	49.3 (46.6-51.9)	52.7 (50.3-55.1)	54.5 (52.3-56.8)	58.3 (56.3-60.5)	60.8 (58.9-62.7)	62.5 (61.1-63.9)	64.4 (63.6-65.2)	64.4 (63.8-65.1)	67.1 (66.4-67.8)	68 (67.3-68.6)	70.7 (69.7-71.7)
Manipur	51.9 (48.5-55.5)	54 (50.7-57.4)	54.1 (50.7-57.4)	53.4 (50.9-56.3)	57.6 (55.3-60.1)	59.2 (57.2-61.4)	61.4 (59.3-63.5)	62 (60-64)	63.6 (61.6-65.6)	63.8 (62-65.8)	63.7 (61.9-65.6)	65.2 (63.3-67.2)	66.4 (64.6-68.3)	68.2 (66.5-70.1)
Meghalaya	56.1 (52.8-59.8)	57.4 (54.2-60.7)	57.3 (53.9-60.7)	55.8 (53.1-58.5)	58.7 (56.1-61.3)	57.3 (55.1-59.6)	58.6 (56.5-60.9)	59.5 (57.2-61.8)	60.3 (58.1-62.6)	61.4 (59.3-63.6)	62.1 (60.1-64.4)	64.3 (62.2-66.5)	65.1 (63.2-67.1)	66.8 (64.9-68.9)
Mizoram	59.3 (56.2-62.8)	60.5 (57.6-63.9)	60.9 (57.9-64)	58.7 (56.3-61.2)	62.6 (60.4-65.1)	62.3 (60.1-64.6)	63.3 (61.2-65.4)	64 (62.1-66.2)	64.4 (62.8-66.3)	65.1 (63.5-66.8)	65.4 (63.9-67.1)	66.3 (64.6-68.2)	66.1 (64.5-68)	67.7 (66-69.8)
Nagaland	51 (47.5-54.9)	53.7 (50.2-57.4)	54.5 (51.2-58)	50.3 (48-52.7)	58.8 (56.2-61.5)	60.3 (58.1-62.7)	62.3 (60-64.5)	63.5 (61.1-65.7)	63.5 (61.2-65.7)	64.1 (62-66.3)	63.8 (61.7-66.1)	65.3 (63.2-67.7)	66.3 (64.4-68.3)	68.2 (66.4-70.6)
Odisha	38.8 (35.4-42.6)	41.3 (37.9-44.6)	40.7 (37.5-43.9)	40.8 (37.8-43.6)	44.5 (42.1-46.9)	46.9 (44.4-49.4)	50.9 (48.7-53.3)	53.9 (51.8-56)	56.1 (54.6-57.6)	57.9 (56.9-59)	58.3 (57.4-59.1)	61.3 (60.4-62.2)	63.5 (62.6-64.3)	67.2 (66.1-68.2)
Punjab	53.1 (49.8-56.4)	54.5 (51.2-58)	55.2 (52.2-58.4)	54.9 (52.2-57.7)	58.4 (55.9-61.1)	59.6 (57.1-62.2)	61.7 (59.1-64)	63.3 (61-65.6)	64.1 (62.4-65.7)	65.4 (64.5-66.3)	66 (65.2-66.7)	67.2 (66.4-68)	67.8 (67-68.6)	71.1 (69.8-72.3)
Rajasthan	45.2 (42-48.4)	47.1 (44-50.4)	46.6 (43.8-49.9)	45.9 (43.5-48.7)	49.4 (47.2-51.9)	50.8 (48.4-53.1)	53.8 (51.6-56.1)	56.1 (54.1-58.1)	58.4 (56.8-60.1)	60.5 (59.4-61.6)	62.7 (61.8-63.7)	65.1 (64.4-65.9)	65.2 (64.3-66)	65.9 (64.6-67.2)
Sikkim	51.6 (48.1-55.4)	53.3 (50-56.9)	53 (49.7-56.6)	51.8 (49-54.5)	55.4 (52.8-58.1)	56.4 (54.1-58.9)	59.2 (57-61.5)	61.4 (59-63.9)	62.5 (60.3-64.9)	63.5 (61.5-65.9)	64.5 (62.5-66.6)	66.5 (64.8-68.6)	67.6 (66-69.5)	70 (68.3-72.1)

Table 8 Estimates of life expectancy at birth by location and year for 1950, 1955...2005, 2010, 2017 - males														
Location	1950	1955	1960	1965	1970	1975	1980	1985	1990	1995	2000	2005	2010	2017
Tamil Nadu	41.1 (37.9-44.4)	43.9 (40.8-47.3)	44.1 (41.6-47.1)	44.8 (42.3-47.4)	48.4 (46.1-50.8)	50.8 (48.6-53.3)	54.8 (52.6-57.2)	57.6 (55.7-59.7)	60 (58.6-61.6)	62.4 (61.5-63.2)	63.2 (62.5-63.9)	65.9 (65.2-66.6)	65.8 (65.1-66.5)	68.8 (67.9-69.9)
Telangana	48.7 (45.1-52.6)	50.5 (46.6-54.6)	50.6 (46.5-55.1)	50.6 (47.2-54.3)	53.2 (50.3-56.2)	54.3 (51.4-57.2)	57.1 (54.2-60.1)	58.9 (56-61.6)	59.7 (57-62.5)	61.2 (58.5-64)	61.9 (59.3-64.6)	64.2 (61.7-66.9)	66.4 (64.1-68.8)	70 (67.7-72.3)
Tripura	42.7 (39-46.6)	45.9 (42.3-49.6)	46.4 (43.1-49.8)	47 (44.2-49.8)	51.5 (48.9-54.1)	54.3 (51.9-56.8)	54.7 (52.6-56.9)	58.4 (56.1-60.7)	59.8 (57.6-62)	61 (58.9-63.3)	62.2 (60.3-64.4)	65 (63.1-66.9)	65.6 (63.7-67.4)	67.2 (65.7-68.7)
Uttar Pradesh	34.8 (31.5-38.3)	37.6 (34.4-41)	37.2 (34.5-40.2)	37.8 (35.1-40.7)	42.3 (40.1-44.5)	44.7 (42.6-46.7)	48.8 (46.8-50.8)	52.3 (50.4-54.3)	55.3 (53.6-57)	58.1 (56.7-59.5)	60.1 (58.9-61.3)	61.9 (61.1-62.7)	62.4 (61.5-63.3)	65.1 (63.7-66.4)
Uttarakhand	49 (45.8-52.6)	50.6 (47.2-54.1)	50.5 (47.1-54)	50.1 (47.1-53.3)	53 (50.2-55.8)	53.9 (51.4-56.4)	56 (53.8-58.5)	57.1 (55-59.4)	58.1 (56.1-60.2)	59.6 (57.5-61.7)	60.4 (58.5-62.5)	62.8 (61.1-64.4)	64.5 (63.2-65.7)	66.6 (65.5-67.8)
West Bengal	44.2 (41.1-47.5)	46.9 (43.6-50.2)	47.1 (44.4-50.1)	47.3 (44.8-49.8)	50.7 (48.5-52.9)	52.3 (50.3-54.6)	55.5 (53.6-57.5)	58 (56.1-59.8)	60 (58.4-61.7)	61.4 (60.2-62.6)	63.7 (62.8-64.5)	65.8 (64.9-66.8)	67.2 (66.2-68.2)	69.8 (68.5-71.1)
Union Territories other than Delhi	62.2 (59.4-65.1)	63.5 (60.7-66.2)	63.9 (61.1-66.5)	61.8 (59.6-63.8)	65.8 (63.9-67.7)	66.5 (64.8-68.3)	67.3 (65.7-69.1)	67.4 (65.6-69.1)	66.8 (65.2-68.6)	66.9 (65.3-68.7)	67.2 (65.7-68.9)	68.8 (67.1-70.8)	69.1 (67.3-71.2)	70.5 (68.4-72.8)
Nepal	37.2 (34.5-39.7)	39.4 (37-41.9)	41.8 (39.4-44.2)	44.6 (42.1-47.1)	47.1 (44.8-49.5)	48.8 (46.6-51.2)	50.7 (48.6-53.1)	54 (52-56)	57.7 (56-59.5)	61.2 (59.7-62.6)	64.4 (63.2-65.5)	66.3 (65.1-67.4)	67.3 (65.8-68.7)	68.7 (67.2-70.6)
Pakistan	46.2 (43.3-49.3)	49 (46.7-51.5)	52.2 (50.1-54.4)	55.5 (53.7-57.3)	58.6 (57.1-60.1)	60.2 (59-61.4)	61 (59.9-62.1)	61.5 (60.6-62.4)	61.7 (60.9-62.6)	61.7 (60.8-62.7)	62.2 (61.3-63.2)	61.5 (60.2-62.7)	64 (61.9-66)	66.3 (63.8-69.1)
Southeast Asia, East Asia, and Oceania	45.7 (43.7-47.5)	49.6 (48-51.2)	29.3 (28.6-30)	54.9 (53.8-55.9)	57.6 (56.6-58.5)	59.9 (59.2-60.6)	62.5 (61.9-63.1)	64.5 (63.9-65)	65.8 (65.3-66.2)	66.7 (66.4-67)	68.2 (67.9-68.5)	70.1 (69.9-70.4)	71.5 (71.3-71.8)	72.9 (72.5-73.3)
East Asia	46.8 (44.5-49)	51.1 (49-53)	24.5 (23.8-25.2)	56.6 (55.1-57.9)	59.6 (58.4-60.8)	62.2 (61.2-63.1)	64.3 (63.4-65.1)	66 (65.3-66.7)	67 (66.4-67.6)	67.9 (67.4-68.3)	69.6 (69.2-69.9)	71.8 (71.5-72.1)	73.2 (72.9-73.5)	74.5 (74-74.9)
China	47.5 (45-49.8)	50.9 (48.7-52.9)	23.3 (22.6-24)	56.3 (54.9-57.7)	59.4 (58.2-60.7)	62 (61-63)	64.1 (63.2-64.9)	65.9 (65.2-66.6)	66.9 (66.3-67.5)	67.9 (67.5-68.4)	69.7 (69.3-70.1)	71.8 (71.6-72.1)	73.3 (73-73.5)	74.5 (74.1-75)
North Korea	22.7 (21.9-23.4)	57.1 (54.3-60.2)	59.7 (57-62.7)	62.2 (59.6-65.2)	64.8 (62.6-67.6)	67.1 (65.2-69.6)	68 (66.1-70.4)	68.3 (66.5-70.5)	68.5 (66.7-70.7)	59.8 (58.2-61.6)	59 (57.5-60.6)	66.9 (65.5-68.5)	67.5 (66.1-68.9)	68.6 (67.1-70.2)
Taiwan (Province of China)	57.9 (55.5-60.3)	59.7 (59.5-59.8)	62.7 (62.5-62.8)	65.9 (65.8-66)	67.7 (67.6-67.7)	68.9 (68.8-69)	70.1 (70.1-70.2)	71.7 (71.6-71.7)	72.1 (72.1-72.2)	72.8 (72.7-72.8)	74.1 (74-74.2)	74.9 (74.8-75)	76.6 (76.5-76.7)	76.8 (76.1-77.5)
Oceania	41.6 (39.3-43.8)	43.3 (41.2-45.5)	45.4 (43.2-47.4)	47.8 (45.8-49.7)	50.3 (48.4-52.1)	52.2 (50.5-54)	54 (52.3-55.8)	54.5 (52.8-56.5)	55.3 (53.5-57.3)	56.2 (54.3-58.1)	56.4 (54.5-58.5)	56.1 (54.1-58.4)	56.6 (54.4-58.9)	58.2 (55.9-60.6)
American Samoa	61.5 (59.9-63.1)	62.9 (61.9-64)	63.3 (62.5-64.1)	63.6 (62.9-64.2)	64.6 (63.8-65.3)	65.8 (65-66.6)	67 (66.2-67.8)	67.8 (66.9-68.6)	67.6 (66.9-68.4)	68.1 (67.3-68.9)	68.7 (68-69.4)	68.8 (68.1-69.4)	69.4 (68.6-70.2)	70 (68.5-71.7)
Federated States of Micronesia	47.9 (45.2-50.5)	49.7 (47.1-52.3)	51.6 (48.9-54.3)	53.7 (50.9-56.4)	55.7 (53-58.4)	57.6 (54.9-60.3)	59.4 (56.7-62.1)	60.7 (58-63.2)	61.7 (59.2-64.2)	62.7 (60.3-65)	63.2 (61.1-65.3)	64.5 (62.3-66.6)	64.8 (62.6-67)	65 (62.8-67.2)
Fiji	57.1 (55.7-58.4)	60.1 (59.1-61.1)	62.1 (61.1-63)	62.7 (61.8-63.6)	61.6 (60.7-62.4)	62 (61.1-62.9)	64 (63.1-64.9)	64.9 (64-65.7)	65.6 (64-67)	64.8 (64.2-65.4)	63.9 (63.6-64.2)	64.9 (64.5-65.2)	65.7 (65.3-66)	65.9 (64.2-67.7)
Guam	62.8 (61.8-63.6)	63.2 (62.6-63.8)	64.6 (64-65.1)	65.3 (64.9-65.7)	65.4 (65.1-65.7)	66.1 (65.8-66.4)	69 (68.7-69.4)	71.1 (70.7-71.5)	70.8 (70.3-71.3)	70.5 (70-71)	70.5 (70-71)	70.3 (69.8-70.9)	70.3 (69.8-70.9)	70.2 (69.2-71.3)
Kiribati	44.7 (42-47.3)	45.9 (43.5-48.5)	47.7 (45.2-50.4)	49.6 (47.2-52.3)	51.6 (49.1-54.3)	54.6 (52.2-57.1)	55.8 (53.7-58)	55.5 (53.8-57.3)	55.7 (54.5-57)	55.4 (54.5-56.3)	55.6 (54.5-56.7)	56.3 (54.7-57.8)	57.1 (55.1-59.1)	58.6 (56.2-61)
Marshall Islands	48 (45.4-50.8)	49.9 (47.3-52.6)	51.9 (49.2-54.6)	53.7 (51-56.2)	55.4 (53.1-57.8)	57.2 (55.2-59.3)	58.5 (56.9-60.3)	59.3 (58.3-60.4)	59.9 (59.2-60.6)	60.3 (59.5-60.9)	60 (59.1-60.9)	60.5 (59.5-61.7)	61.3 (59.9-62.7)	62.6 (60.6-64.6)
Northern Mariana Islands	67.7 (65.9-69.9)	68.2 (66.3-70.4)	68.8 (67.1-70.9)	69.3 (67.7-71.1)	70 (68.2-71.7)	70.6 (68.7-72.1)	71.3 (69.5-72.7)	72.1 (70-73.7)	72.9 (70.7-74.6)	73.5 (71.6-75)	74 (72.7-75.2)	73.9 (73-75)	73.9 (72.9-75.1)	73.6 (72.3-75)
Papua New Guinea	37.9 (35.2-40.6)	39.4 (36.8-42)	41.2 (38.6-43.6)	43.7 (41.3-46)	46.6 (44.4-48.9)	48.9 (46.9-51.3)	50.6 (48.5-53)	51 (48.8-53.5)	52 (49.7-54.5)	53.2 (50.8-55.8)	53.7 (51.3-56.4)	53.4 (50.9-56.2)	54.1 (51.5-57)	56.2 (53.6-59.2)
Samoa	59.2 (56.9-61.6)	61.6 (59.7-63.5)	64 (62.6-65.6)	64.1 (62.8-65.4)	64.5 (63.2-65.8)	65.1 (63.7-66.3)	66.6 (65-68.3)	67.3 (65.4-69.2)	68.1 (66-70.2)	68.6 (66.8-70.3)	69.4 (67.9-70.7)	70.1 (69-71.1)	70.8 (69.9-72.1)	71.3 (70-72.7)
Solomon Islands	41.9 (39.2-44.8)	43.9 (41.2-46.8)	46.6 (44.1-49.3)	49.6 (47-52.3)	52.3 (49.8-54.9)	52.7 (50.2-55)	57.7 (55-60.4)	59.1 (56.4-61.8)	59.9 (57.2-62.5)	61.1 (58.5-63.8)	61.8 (59.5-64.1)	62.1 (59.9-64.2)	62.9 (60.9-65)	64.1 (62-66.3)
Tonga	57 (55.2-59)	59 (57.5-60.4)	61.5 (60.4-62.5)	63.5 (62.4-64.5)	64.5 (63.2-65.6)	65.3 (64-66.4)	66 (65-67)	67.4 (66.5-68.2)	68.3 (67.6-69.1)	68.8 (68.2-69.4)	68.7 (68.3-69.2)	68.2 (67.6-68.8)	68 (67-69)	68.6 (66.7-70.1)
Vanuatu	49.1 (46.2-51.8)	50.4 (47.5-53.1)	52 (49.1-54.7)	53.6 (50.8-56.3)	55 (52.2-57.8)	56.7 (53.8-59.5)	58.3 (55.5-61.3)	59.2 (56.5-61.9)	59.7 (57-62.6)	60.4 (57.6-63.3)	60.8 (58.2-63.8)	60.8 (57.9-63.8)	61.4 (58.4-64.3)	62.1 (59.2-65)
Southeast Asia	42.4 (41-43.8)	45.7 (44.4-46.9)	48.7 (47.6-49.8)	50.7 (49.8-51.6)	52.1 (51.3-52.9)	54 (53.2-54.7)	58.5 (57.8-59.1)	60.7 (60.1-61.3)	62.6 (62-63)	63.8 (63.4-64.2)	65.1 (64.7-65.5)	66.5 (66.2-66.9)	67.9 (67.5-68.3)	69.4 (68.9-70)
Cambodia	42.9 (39.9-46)	44.4 (41.4-47.3)	46.5 (43.6-49.5)	48.5 (45.8-51.3)	40.1 (38.3-42)	20.5 (19.9-21.2)	49.2 (47.3-51)	51.5 (50-53)	55.3 (54-56.6)	56.3 (55.1-57.5)	57.5 (56.2-58.9)	60.8 (59.6-62.1)	63.9 (62.7-65.2)	66.8 (65.3-68.3)
Indonesia	40.4 (38.7-42.2)	42.8 (41.4-44.3)	45.9 (44.6-47.2)	48.7 (47.5-49.9)	51.7 (50.6-52.9)	54.8 (53.8-55.8)	57.7 (56.9-58.6)	60.2 (59.5-61)	62.4 (61.8-63)	64.5 (64-65.1)	65.7 (65.2-66.2)	66.6 (66.1-67.2)	67.6 (67-68.2)	69.2 (68.4-70.1)
Laos	31.9 (28.6-35.3)	33.6 (30.3-36.8)	34.3 (31.2-37.5)	35.7 (32.7-38.9)	38.1 (34.9-41.1)	42.2 (39-45.3)	44.6 (41.8-47.5)	47.7 (45.3-50.1)	49.6 (47.4-51.7)	52 (50.1-53.9)	54.8 (53.1-56.5)	58 (56.3-59.7)	61.3 (59.5-63.2)	65 (63-67.1)
Malaysia	53.3 (51.1-55.7)	57.4 (55.9-58.9)	59.4 (59.2-59.5)	61.7 (61.6-61.8)	63 (62.7-63.3)	65 (64.6-65.3)	66.6 (65.9-67.3)	67.8 (67.7-67.9)	69.2 (69.1-69.2)	69.6 (69.5-69.6)	70.6 (70.5-70.6)	72 (71.9-72)	72.3 (72.1-72.4)	72.4 (71.3-73.5)
Maldives	32.2 (28.6-35.8)	36.3 (33.1-39.5)	40.8 (37.7-44)	45.5 (43-48.1)	50.3 (48.3-52.2)	54.3 (52.8-55.7)	58.2 (57-59.3)	62.6 (61.8-63.4)	65.5 (64.9-66.1)	68.3 (67.8-68.7)	71.6 (71.2-72)	74.5 (74.1-74.8)	77.4 (77.2-77.7)	79.9 (79.2-80.6)
Mauritius	52.1 (51.7-52.5)	53.8 (53.4-54.2)	56.5 (56.1-56.8)	60.7 (60.4-61.1)	62.5 (62.2-62.9)	61.1 (60.8-61.4)	63.9 (63.7-64.1)	65.3 (65-65.5)	66.3 (66.1-66.5)	67.3 (67.1-67.5)	69.1 (68.9-69.3)	69.7 (69.4-69.9)	70.7 (70.4-70.9)	71.5 (70.6-72.5)
Myanmar	30 (26.7-33.4)	32.1 (28.9-35.3)	34.9 (31.9-38.1)	37.5 (34.5-40.5)	40 (37.1-43)	44.5 (41.7-47.5)	47.9 (45.1-50.8)	50.4 (47.9-53.2)	52.5 (50-54.9)	54.1 (52-56.2)	56 (54.1-57.9)	58.3 (56.6-60)	61.5 (59.9-63.3)	64.9 (63.2-66.7)
Philippines	55 (52.8-57)	57.8 (56.1-59.3)	60.6 (59.3-61.8)	61.5 (60.4-62.6)	60.8 (59.7-61.8)	62.4 (61.4-63.5)	63 (62-64)	62.8 (61.8-63.8)	64.6 (63.7-65.6)	65.8 (64.8-66.7)	66.1 (65.2-66.9)	65.9 (64.9-66.8)	66 (65.1-66.9)	66.6 (64.7-68.6)
Sri Lanka	57 (56.8-57.2)	60.9 (60.7-61.1)	64.9 (64.7-65.1)	65 (64.8-65.1)	65.2 (65-65.3)	63.2 (63.1-63.3)	67.5 (67.3-67.6)	66 (65.9-66.1)	65.6 (65.3-65.9)	66.5 (66.4-66.6)	66.8 (66.7-66.8)	69.4 (68.9-69.9)	70.7 (70.7-70.8)	73.8 (71.7-76)
Seychelles	58.9 (56.7-61.4)	60.4 (58.6-62.2)	61.1 (59.7-62.5)	61.2 (60.2-62.3)	62.8 (62.1-63.4)	65 (64.5-65.5)	67.1 (66.6-67.5)	67.2 (66.8-67.6)	66.1 (65.7-66.5)	65.8 (65.5-66.2)	66.9 (66.5-67.2)	68.5 (68.2-68.8)	69.6 (69.3-69.9)	70.1 (69.5-70.7)
Thailand	48.3 (46.7-49.7)	53 (51.9-54.2)	55.8 (54.8-56.8)	58.2 (57.3-59.1)	59.9 (59.1-60.8)	61.5 (60.7-62.2)	61.9 (61.1-62.6)	66.3 (65.6-67)	67.4 (66.7-68.1)	65.9 (65.2-66.7)	68 (67.1-68.9)	71.5 (70.8-72.2)	73.9 (73.2-74.5)	74.3 (72.9-75.9)
Timor-Leste	36.4 (33-39.8)	38.2 (35-41.5)	40.7 (37.5-43.9)	43.7 (41-46.8)	46.9 (44.3-49.5)	42.8 (41-44.7)	48.7 (46.7-50.6)	55.9 (54-57.7)	59.7 (58-61.4)	61.6 (60.2-63.1)	64.7 (63.4-65.9)	66.6 (65.4-67.7)	68.5 (67.1-70)	68.8 (67.3-70.7)
Vietnam	45.3 (42.8-48.2)	50.8 (48-54.1)	53.4 (50.6-56.4)	51.6 (49.3-54.1)	49.6 (47.7-51.7)	57 (55-59.3)	61.8 (59.8-64.1)	63.6 (61.8-65.6)	64.9 (63.5-66.5)	66.1 (65.1-67.2)	67.2 (66.2-68.1)	67.9 (66.9-68.9)	68.7 (67.6-69.8)	70 (68.3-71.2)
Sub-Saharan Africa	40.2 (37.6-42.7)	41.7 (39.5-43.8)	43.1 (41.4-44.6)	45 (43.6-46.3)	46.5 (45.4-47.6)	48.4 (47.6-49.3)	50.3 (49.5-51.1)	51.4 (50.8-52.1)	51.6 (51-52.3)	51.4 (50.8-52.1)	51.4 (50.8-52)	53.7 (53.1-54.4)	57.1 (56.4-57.8)	61.7 (60.8-62.4)
Central sub-Saharan Africa	40.2 (37.5-43)	41.5 (38.8-44.1)	42.8 (40.2-45.3)	43.7 (41.3-45.9)	45.7 (43.5-47.6)	47.2 (45.1-49)	48.4 (46.6-50.2)	49.6 (48.1-51.3)	50.1 (48.8-51.4)	50.1 (48.9-51.3)	51 (49.9-52)	53.8 (52.8-54.8)	56.3 (55.1-57.7)	60.3 (58.7-62)
Angola	39.2 (35.9-42.4)	39.5 (36.3-42.6)	40.5 (37.3-43.6)	40.3 (37.3-43.5)	42 (39.1-45.2)	43 (40.4-46)	43.3 (40.9-46.1)	43.8 (41.4-46.3)	45.5 (43.3-47.6)	47.4 (45.5-49.3)	49.4 (47.6-51.2)	53.8 (52.1-55.7)	57.8 (56-59.8)	61.7 (59.7-64)
Central African Republic	40.1 (37.5-42.8)	40.8 (38.2-43.4)	42.4 (39.9-45)	44.1 (41.7-46.6)	45.2 (43-47.4)	45.7 (43.8-47.6)	46.4 (44.8-48.2)	46.5 (44.9-48)	44.6 (42.9-46.3)	43.3 (41.5-45.2)	42.8 (40.9-44.9)	44 (41.8-46.4)	46.5 (44-49)	49.1 (46.5-51.7)
Congo (Brazzaville)	39.1 (36.3-41.8)	39.8 (37.1-42.6)	41.2 (38.8-43.9)	43.1 (40.6-45.8)	45.1 (42.7-47.9)	47.9 (45.6-50.5)	51 (48.9-53.2)	53.4 (51.6-55.4)	51.5 (49.4-53.5)	50.8 (49.1-52.6)	52.2 (50.6-53.6)	56.5 (54.8-58.3)	60.1 (58.3-62.1)	62.6 (60.4-64.8)
DR Congo	40.9 (37.7-44.2)	42.4 (39.4-45.8)	43.8 (40.8-46.8)	44.8 (41.8-47.6)	47 (44.2-49.5)	48.5 (45.9-50.9)	49.9 (47.6-52.3)	51.4 (49.3-53.7)	51.8 (50-53.7)	51.5 (50-53.2)	52 (50.7-53.4)	54.4 (53-55.9)	56.3 (54.6-58)	60.4 (58.2-62.7)
Equatorial Guinea	33.9 (30.6-36.8)	34.4 (31.5-37.2)	35.7 (32.9-38.6)	38.3 (35.5-41.2)	40.7 (37.7-43.7)	42.8 (39.9-45.7)	45.8 (43.1-48.4)	46.5 (43.8-49.1)	45.6 (43-48.3)	46 (43.2-48.7)	53.9 (51.1-57)	59.4 (56.5-62.5)	62.1 (58.9-65.1)	64.3 (61.3-67.1)
Gabon	41.3 (38.1-44.5)	41.9 (38.7-44.9)	43.5 (40.7-46.5)	45.8 (43.1-48.7)	48.1 (45.6-50.8)	51.2 (48.9-53.7)	54.1 (52.1-56.3)	55.9 (54.1-57.7)	56.4 (54.8-58)	57 (55.5-58.4)	57.3 (55.6-58.9)	58.1 (56.4-59.8)	60.9 (59.1-62.6)	65.1 (63.3-66.7)
Eastern sub-Saharan Africa	39.6 (37-42)	41.1 (38.9-43.1)	42.6 (40.9-44.3)	44 (42.6-45.4)	45.6 (44.6-46.8)	47.1 (46.2-48)	48.5 (47.7-49.2)	48.9 (48.1-49.6)	48.8 (48-49.5)	49 (48.4-49.6)	50 (49.4-50.6)	53.7 (53.1-54.3)	57.8 (57.1-58.5)	62.5 (61.7-63.3)
Burundi	37.1 (34.1-40.4)	38.4 (35.8-41.4)	40.2 (37.7-42.9)	41.4 (39.2-43.6)	42.8 (40.8-44.9)	43.7 (41.8-45.8)	44.9 (42.8-46.8)	46.2 (44.4-48.3)	46.9 (45-48.8)	41.6 (40-43.3)	42.7 (41.1-44.3)	47.6 (46.1-49.3)	56.3 (54.4-58.5)	59.7 (57.3-62.2)
Comoros	41.7 (38.5-44.7)	43.1 (39.9-46)	44.6 (41.6-47.5)	47.1 (44.4-49.8)	49.4 (47-52)	51.1 (48.7-53.4)	52.3 (49.9-54.6)	54.5 (52.3-56.7)	56.9 (54.9-58.8)	58.6 (56.8-60.4)	60.7 (58.9-62.3)	63.4 (61.7-65)	65.3 (63.4-66.9)	67.1 (65-69.2)
Djibouti	52.5 (49.3-55.6)	53.4 (50.3-56.8)	54.4 (51.3-57.6)	55.4 (52.4-58.6)	56.5 (53.6-59.6)	58.1 (55.3-61.2)	58.8 (56.1-61.7)	58.9 (56.4-61.6)	58.9 (56.3-61.5)	59 (56.2-61.9)	59.3 (56.2-62.3)	60.4 (57.1-63.6)	62.5 (59.5-65.5)	66 (63.1-68.8)
Eritrea	35.8 (32.7-39.1)	37 (33.8-40.2)	38.4 (35.3-41.6)	34.7 (32.3-37.1)	36.6 (34.6-38.7)	36 (34.5-37.7)	37.6 (36.3-39)	38.9 (37.8-40.1)	30.6 (29.9-31.4)	47 (45.6-48.6)	48.3 (46.6-50.2)	51.5 (49.5-53.7)	54.5 (52.1-57)	59.2 (56.4-61.9)
Ethiopia	35.5 (32.6-38.3)	36.8 (34.1-39.4)	37.6 (35.4-40.1)	38.8 (36.8-41)	40.1 (38.1-42.1)	41 (39.3-42.7)	42.4 (40.9-43.9)	44.3 (43.1-45.6)	45.6 (44.4-46.8)	48.3 (47.1-49.3)	49.8 (48.8-50.9)	56.2 (55.2-57.2)	61.7 (60.7-62.7)	66.7 (65.6-67.7)
Kenya	46 (44.2-47.7)	48.1 (46.9-49.2)	51.8 (50.7-52.9)	54 (53.1-55)	55.9 (55-56.8)	58.2 (57.4-59)	60.5 (59.8-61.3)	61.8 (61.1-62.5)	60.6 (59.9-61.3)	56.7 (55.7-57.6)	54.3 (53.4-55.1)	55.5 (54.8-56.1)	59.3 (58.7-60)	63.2 (62.4-63.9)
Baringo	53.1 (49.4-57.2)	51.7 (48.1-56)	52.4 (48.8-56.6)	51.5 (48-55.5)	49.4 (46-53.1)	48.6 (45.2-52.2)	49.6 (46-53.6)	51.3 (48-54.7)	52.5 (49.2-56)	52.6 (49.5-56)	53.7 (50.9-56.6)	56.9 (54.5-59.3)	59.9 (57.6-62.1)	63.2 (60.4-66.1)
Bomet	66.6 (62.7-69.9)	63.9 (60.5-66.9)	67.1 (63.5-70.4)	67.4 (63.8-70.9)	67.8 (64.5-71.5)	68.3 (65.1-71.8)	68.9 (65.7-72.3)	69.7 (66.8-73.2)	69.1 (66.3-72.6)	66.3 (62.8-69.9)	62.1 (58.5-65.8)	61.3 (58.1-64.4)	62.4 (59.5-65.4)	65.8 (62.8-68.7)
Bungoma	40.4 (36.7-44.4)	42.7 (39.2-46.5)	47.6 (44.1-51.5)	50.5 (47-54.3)	53 (49.3-56.8)	55.5 (51.9-59)	58.6 (55.1-62.2)	59.9 (56.6-63.3)	60.4 (57.3-63.8)	59.9 (56.7-63.4)	59.4 (56.2-62.8)	61.4 (58.6-64.5)	64.7 (62.1-67.4)	67.7 (64.9-70.9)
Busia	33.9 (30.4-37.7)	35.7 (32.5-38.8)	40.9 (37.5-44.3)	44.3 (41.3-47.6)	47.4 (44.4-50.8)	52.4 (49-55.9)	56.7 (53.4-60.1)	59.1 (56.2-62.1)	56.4 (52.8-60)	48.2 (43.7-52.6)	45.6 (41.7-49.5)	50.1 (46.8-53.7)	58.3 (55.6-61.1)	63.5 (60.8-66.1)
Elgeyo Marakwet	55.9 (52-60.1)	56 (51.9-60.2)	57.4 (53.3-61.4)	58 (53.8-62.4)	58.2 (54.2-62.2)	58.9 (55.1-62.8)	59.9 (56.3-63.8)	60.9 (57.6-64.6)	61.3 (58-64.7)	60.3 (57.5-63.6)	59.5 (56.8-62.4)	60 (57.5-62.3)	62.8 (60.5-64.9)	65.8 (63.3-68.6)
Embu	48.5 (45.2-52.2)	50 (46.3-53.7)	54.6 (51.3-58.2)	57.9 (54.4-61.6)	61.4 (58-64.9)	65.1 (61.8-68.4)	67.5 (64.4-70.7)	68.4 (65.4-71)	65.3 (60-68.9)	60.5 (54.8-65.7)	59.5 (55.5-63.7)	61.1 (58.1-64.4)	63.2 (60.8-65.8)	65.6 (63-68.7)
Garissa	46.5 (43.4-49.9)	44.9 (42-47.9)	51.2 (47.8-54.8)	52.4 (49-56.1)	53.8 (50.3-57.5)	55.6 (52.1-59)	57.2 (54-60.5)	58.1 (55.1-61)	58.5 (55.8-61.7)	58.3 (55.7-61.2)	58.4 (56-60.8)	60.1 (57.9-62.1)	62.2 (60-64.2)	63.9 (56.8-67.3)
Homa Bay	29.6 (27.1-32.5)	32.2 (29.3-35.5)	36 (33.2-39.3)	38.6 (35.5-42.1)	39.4 (36.2-42.6)	41.7 (38.7-45)	45.9 (42.8-49.1)	48.5 (45.6-51.5)	47.9 (45.1-51.3)	43.5 (39.8-47.4)	38.3 (34.7-41.9)	37.8 (34.4-41.4)	44.1 (41-47.3)	50.8 (47.4-54.6)
Isiolo	44.2 (40.9-47.6)	43.5 (40.3-47.1)	45.7 (42.2-49.6)	45.9 (42.5-49.3)	45.5 (42.2-49.3)	46.8 (43.7-50.3)	49.5 (46.4-52.9)	51.4 (47.7-55.2)	52.9 (49.9-55.8)	53.8 (50.5-57)	52.9 (50-55.7)	56.1 (53.4-58.8)	58.1 (55.5-60.9)	60.8 (57.8-63.8)
Kajiado	57.2 (53.5-61.4)	56.4 (52.8-60.3)	60 (55.8-64.5)	60.7 (56.8-64.8)	61.5 (57.7-65.5)	62.6 (58.8-66.3)	64 (60.7-67.5)	65.4 (62.3-68.6)	63.9 (59.8-67.8)	59.3 (54.4-63.9)	56.3 (52.7-60.1)	56.8 (53.9-59.8)	60.4 (57.9-62.8)	64.7 (62-67.9)
Kakamega	45.5 (41.8-49.6)	46.2 (42.9-49.7)	50.9 (47.4-54.6)	53.5 (49.9-57.3)	55.5 (52-59.2)	57.7 (54.1-61.3)	60 (56.6-63.4)	60.6 (57.5-63.6)	58.2 (54.9-61.5)	52.5 (48.8-56.2)	50 (46.8-53.6)	52.8 (49.9-55.9)	58 (55.3-60.9)	62.9 (60-65.9)
Kericho	59.2 (55.4-63.2)	60.1 (56.4-64)	62.2 (58.5-66.1)	63.6 (59.8-67.6)	64.5 (60.8-68.1)	66 (62.4-69.4)	67.2 (64-70.3)	68.4 (65.7-71.2)	68.3 (65.4-71.3)	65.7 (62.7-69)	62.9 (59.7-66.5)	62.2 (59-65.6)	63.8 (61-67)	67.1 (63.9-70.3)
Kiambu	50.2 (46.8-53.9)	52.1 (48.7-55.7)	55.5 (51.7-59.3)	57.7 (54-61.8)	60.2 (56.7-64.2)	63.3 (60.2-66.9)	64.2 (61.2-67.5)	64.7 (61.9-67.8)	63 (60-66)	57.2 (53.3-60.7)	53.8 (50.3-57.2)	54.3 (51.7-56.9)	59 (56.7-61.4)	63.4 (60.8-65.7)
Kilifi	42.3 (39-45.8)	44.5 (41.5-48)	47.9 (44.7-51.4)	50 (46.6-53.8)	52.5 (49.1-56.1)	55.7 (52.1-59.1)	57.8 (54.6-61.1)	60.2 (57.1-63.2)	61.3 (58.3-64.7)	60.6 (57.4-64)	59.7 (56.4-63.5)	60.6 (57.4-63.8)	62.3 (59.3-65.1)	65.4 (62.4-68.6)
Kirinyaga	49.4 (45.7-53.1)	50.8 (47.1-54.7)	54.4 (50.5-58.5)	57 (53.1-61.3)	58.6 (54.8-62.9)	61.9 (57.9-66.2)	66.2 (62.3-70.3)	67.4 (64.1-71)	66 (62.8-69.6)	63.5 (60-67.2)	61.5 (57.9-65.2)	60.8 (57.6-64.3)	62.5 (59.2-65.8)	64.8 (61.8-68)
Kisii	44.1 (39.7-48.6)	46.7 (43.1-50.4)	50.7 (47.1-54.4)	52.8 (49.1-56.8)	54.6 (51.1-58.7)	56.5 (53-60.2)	58.1 (54.9-61.7)	59.1 (55.8-62.5)	58.6 (55.6-61.6)	56.5 (53.5-59.8)	54.9 (51.9-58.3)	57.4 (54.8-60)	62.5 (60.5-64.8)	65.8 (63.6-68.5)
Kisumu	42.3 (38.4-46.6)	44.1 (40.5-47.9)	47.3 (43.7-51)	49.6 (45.9-53.2)	51.1 (47.5-54.7)	53.2 (49.6-56.9)	54.7 (51.4-58.2)	55.3 (52.1-58.6)	51 (47.2-55)	42 (38.1-46.5)	38.4 (35.3-42.1)	40.9 (38.3-43.7)	48.6 (46.1-51.5)	53.1 (49.5-56.9)
Kitui	43 (39.5-46.9)	44 (40.8-47.9)	48 (44.6-51.9)	50.5 (46.8-54.6)	53.1 (49.4-57.1)	56.3 (52.7-60.1)	59.9 (56.3-63.4)	61.9 (58.4-65.3)	61.8 (58.8-65.1)	59.6 (56.5-63.1)	58 (55-61.2)	58.5 (55.7-61.2)	60.7 (58.2-63.1)	63.7 (60.7-66.6)
Kwale	43 (39.6-46.8)	45.2 (41.7-49.5)	49 (45.5-52.7)	50.9 (47.2-54.7)	52.6 (48.9-56.4)	54.9 (51.2-58.6)	57.6 (54.1-61.4)	59.9 (56-63.5)	57.5 (50.5-62.6)	53 (46-58.6)	53.7 (48.9-58.5)	56.5 (53-60)	59.3 (56.5-62.3)	63.4 (60.5-66.7)
Laikipia	58.4 (54.6-62.5)	58.9 (55.3-62.8)	62.4 (58.5-66.6)	64.2 (60.4-68.1)	65.6 (61.8-69.3)	67.4 (63.8-70.8)	69.1 (65.7-72.4)	70.3 (67.3-73.2)	68.7 (65.8-71.5)	65.5 (62.3-68.8)	61.8 (58.2-65.6)	61 (57.7-64.6)	62.7 (59.6-66.2)	66.1 (62.7-69)
Lamu	26.6 (23.5-29.9)	30.5 (27.8-33.6)	34.9 (31.8-38.5)	38.3 (35-41.9)	43.5 (40.4-46.9)	51.3 (47.8-55)	58 (54.5-61.6)	60.5 (57.1-63.9)	60.9 (57.8-64.2)	60.8 (57.3-64.4)	59.8 (56.4-63.5)	60.3 (56.7-64)	61.4 (58-64.7)	62 (58.8-65.5)
Machakos	48.5 (45.1-52.4)	50 (46.6-53.5)	53.6 (50-57.5)	56.1 (52.3-60.1)	58.4 (54.4-62.6)	61.1 (57.5-65.2)	63.9 (60.4-67.3)	65.5 (62.3-68.9)	65.8 (62.5-69.2)	64.4 (60.9-68)	62.3 (59-65.7)	62 (59.1-65.2)	63.2 (60.6-66)	64.8 (62-68.2)
Makueni	58.1 (54.2-62.1)	57.1 (53.4-61)	59 (55.3-63.2)	59.5 (55.6-63.7)	60.3 (56.5-64.4)	61.5 (57.7-65.6)	62.5 (58.7-66.4)	63.1 (59.5-66.8)	62.2 (58.7-66)	60 (56.2-64.3)	56.8 (52.7-61.3)	56.1 (52.7-60.2)	59.4 (56.5-63)	63.2 (60-66.8)
Mandera	39.4 (36.4-42.8)	38.1 (35.4-41)	45.7 (42.1-49.5)	47.5 (44.1-50.9)	49.6 (46-53.2)	52.2 (49-55.8)	54.8 (51.6-58.1)	56 (53.1-59.2)	55.7 (53-58.7)	55.3 (52.6-58.2)	56.1 (53.7-58.5)	56.3 (54.3-58.4)	57.5 (55.5-59.7)	61.1 (58.6-63.9)
Marsabit	32.8 (30.1-36)	33.8 (30.6-37.2)	39.1 (36-42.7)	42.8 (39.6-46.2)	47.4 (43.8-51.3)	53.3 (50.1-56.6)	58.5 (55.2-62)	61.2 (58.2-64.4)	61.8 (58.8-64.9)	61 (58.1-64.1)	59.1 (56.3-62)	57.8 (55.6-60.2)	60.2 (58-62.5)	62.3 (59.8-65.3)
Meru	51.6 (48.2-55.3)	53.5 (50.1-57.1)	57.9 (54.5-61.5)	60.6 (57-64.3)	63 (59.5-66.7)	65.5 (62.2-68.8)	67.8 (64.5-71.2)	68.8 (65.8-71.8)	67.3 (64.1-70.3)	63.9 (60.1-67.6)	61 (57.4-65)	60.8 (58.1-64.2)	61.6 (59.2-64.3)	63.1 (60.1-66.5)
Migori	39.8 (36.7-43.4)	40.9 (37.3-45.2)	43.5 (39.8-47.6)	44.5 (40.8-48.4)	45.8 (42.4-49.8)	45.9 (42.8-49.5)	46.2 (43.2-49.6)	47.1 (44.1-50.1)	48 (45.1-51.2)	46.1 (42.6-49.8)	42.9 (39.3-46.6)	43.2 (39.9-46.7)	48.4 (45.3-51.7)	54.2 (50.6-58)
Mombasa	42.6 (39.3-46.2)	46.9 (43.5-50.5)	52.2 (48.7-56.1)	55.5 (52.1-59.6)	58.6 (55.1-62.6)	62 (58.4-66.1)	65.2 (61.6-68.6)	66.9 (63.5-70)	64.2 (60.2-67.3)	57.4 (52.5-62.1)	55.7 (51.2-60.3)	57.9 (54.1-61.7)	60.7 (57.3-64)	64.9 (61.4-68.4)
Murang'a	50.3 (46.9-54.1)	52.5 (48.9-56.3)	55.9 (52.2-59.5)	57.9 (54.2-61.6)	60 (56.1-63.8)	62.8 (59-66.6)	65.4 (62.1-68.7)	66.6 (63.9-69.6)	66.1 (63.5-68.9)	64 (60.7-67)	62.1 (58.9-64.9)	60.4 (57.6-63)	63 (60.7-65.5)	66.1 (63.6-69)
Nairobi	40.6 (37.4-44)	45.1 (41.9-48.3)	51.9 (48.8-55.3)	56.3 (53.1-59.8)	59.8 (56.5-63.4)	63.5 (60.4-66.9)	66.1 (63.2-69.2)	66.9 (64.2-69.7)	63.9 (60.5-67.4)	57.1 (53.1-61.3)	54.7 (51.5-58.3)	56.8 (54.5-59.2)	62.1 (60.1-63.8)	66.7 (64.7-68.7)
Nakuru	59.3 (55.3-63.4)	58.9 (55.1-62.9)	61.8 (58-65.8)	62.9 (59.1-66.5)	63.7 (59.9-67.5)	64.6 (61.1-68.2)	65.9 (62.8-69.2)	66.7 (63.9-69.9)	64.5 (60.9-68.5)	56.8 (51.8-62)	52.9 (48.4-57.7)	55.7 (52.3-59.6)	62.5 (59.5-65.5)	67.6 (64.9-69.6)
Nandi	59.7 (55.9-63.8)	59.1 (55.1-62.9)	61 (56.8-65)	61.2 (57-65.3)	61.3 (57.3-65.1)	61.6 (57.7-65.1)	61.9 (58.2-65.3)	61.7 (58.2-65)	59 (54.8-62.9)	54.6 (50-58.9)	53.4 (49.9-57)	55.2 (52.7-57.9)	58.2 (55.7-60.8)	62.5 (59.5-65.5)
Narok	58 (54.2-62.2)	55.4 (51.7-59.4)	59 (55-63.3)	59 (54.9-63.5)	58.5 (54.5-63)	58.3 (54.6-62.4)	58.8 (55.2-62.5)	58.9 (55.6-62.5)	58.5 (55.4-62)	57.2 (54.1-60.6)	56.2 (53.4-59.4)	56.7 (54.1-59.5)	59.5 (57.1-62.1)	62.7 (59.8-65.8)
Nyamira	51.7 (48.1-55.5)	52.3 (48.4-56.2)	54.4 (50.4-58.5)	55.6 (51.4-59.7)	57.3 (53-61.5)	58.5 (54.6-62.4)	59.6 (55.9-63.3)	60.6 (57.4-64)	60.3 (57-63.6)	57.7 (54.1-61.4)	54 (50.4-57.6)	52.5 (49.3-55.6)	55.5 (52.7-58.4)	59.4 (56.1-62.9)
Nyandarua	51.2 (47.2-55.4)	52.2 (48.4-56.4)	55.3 (51.3-59.4)	56.9 (52.7-60.9)	58.4 (54.1-62.6)	60.2 (56-64)	62.3 (58.2-65.9)	63 (59.5-66.3)	62.3 (58.9-65.4)	60.5 (57.3-63.7)	57.2 (54.3-60.2)	54.4 (51.8-57.4)	55.4 (52.7-58.3)	58.4 (55-61.7)
Nyeri	51.4 (47.8-55.3)	54.3 (50.5-58.3)	58.2 (54.5-62.2)	60.9 (57.2-65)	63.1 (59.5-67)	65.3 (62-69.1)	67.4 (64.5-70.9)	68.3 (65.5-71.8)	67 (64.2-70.3)	63.6 (60.1-67.1)	59.8 (56.2-63.5)	59.9 (56.8-63.3)	63.2 (60.7-66.5)	66 (63.1-68.9)
Samburu	64.3 (60.5-68.4)	63.1 (59.5-66.9)	64.8 (61.1-68.7)	64.9 (60.9-68.8)	64.1 (60.3-67.7)	63.5 (59.9-67)	63.6 (60.3-66.7)	63.9 (61-66.7)	62.8 (59.6-65.8)	58.8 (54.6-62)	56.5 (52.4-60.2)	57.4 (53.8-60.5)	59.2 (56.3-61.9)	62.4 (59.3-65.3)
Siaya	34.9 (32.2-38.3)	37.6 (34.5-41.2)	42 (39.1-45.6)	45.1 (41.8-48.9)	47.8 (44.7-51.6)	50.6 (47.3-54.4)	53.1 (49.7-56.6)	54.5 (51.4-57.7)	53.5 (50.6-56.6)	51.3 (47.7-55.1)	48.6 (44.4-53.1)	48 (44.1-52.2)	55.3 (52-58.5)	62.2 (58.5-65.5)
Taita Taveta	32.1 (28.9-35.3)	37 (33.7-40.5)	42.1 (38.7-45.6)	46.1 (42.6-50.1)	49.7 (46-53.7)	54.3 (50.5-58.4)	58.3 (54.7-62.3)	61.4 (57.9-65.4)	62.6 (59-66.4)	61.5 (57.8-65.3)	58.7 (54.8-62.6)	57.4 (53.9-61.4)	58.3 (55.1-62)	62.4 (59.1-66.5)
Tana River	41.3 (37.9-45.1)	44 (40.4-47.8)	48 (43.7-52.2)	50.2 (45.9-54.5)	52.7 (48.5-57.1)	55.2 (51-59.9)	57.9 (53.8-62.1)	59.1 (55.3-63.4)	58.8 (54.9-62.9)	56.3 (52.4-60.5)	53.3 (49.8-57)	52.1 (48.8-55.4)	53.6 (50.6-57)	57 (53.1-61)
Tharaka Nithi	48.9 (44.8-52.9)	49.2 (44.9-53.3)	50.8 (46.3-55.1)	52 (47.7-56.5)	53.7 (49.4-57.9)	56 (52-60.3)	57.8 (53.9-61.8)	58.5 (54.6-62)	57.8 (54-61.4)	56.4 (53.1-60.2)	55.4 (52.3-58.7)	56.8 (54-60)	58.9 (56.2-61.7)	61.7 (58.4-65.2)
Trans Nzoia	62.6 (58.9-66.4)	61.2 (57.7-64.8)	64.1 (60.4-68)	64.6 (60.9-68.4)	64.5 (61-68.1)	65.1 (61.5-68.3)	66.4 (63.3-69.6)	68 (65.3-70.7)	66.7 (63.2-69.7)	61.5 (57.2-65.7)	58 (53.9-62.4)	59 (55.6-62.6)	61.3 (58.1-64.8)	65 (61.8-68.4)

Table 8 Estimates of life expectancy at birth by location and year for 1950, 1955...2005, 2010, 2017 - males

Location	1950	1955	1960	1965	1970	1975	1980	1985	1990	1995	2000	2005	2010	2017
Turkana	55.2 (51.4-59.3)	54 (50.2-58.2)	56.4 (52.5-60.8)	56.5 (52.8-60.8)	55 (51.1-59)	53.5 (50-57.3)	53.5 (50.2-57.1)	55.1 (51.9-58.3)	56 (52.6-59.6)	55.1 (51.3-59)	49.7 (46.4-53)	52.2 (49-55.4)	54.7 (51.9-57.6)	59.8 (56.7-62.9)
Uasin Gishu	64.2 (60.5-68)	63.5 (60-67.2)	65.8 (62.3-69.7)	66.2 (63-70)	66.7 (63.4-70.4)	67.2 (63.9-70.6)	67.9 (64.7-71.2)	68.3 (65.3-71.6)	67.7 (64.8-70.8)	66.3 (63.5-69.4)	63.9 (60.9-66.5)	63.4 (61-65.9)	64.7 (62.6-67.4)	67.9 (65.2-70.5)
Vihiga	54.8 (50.6-59.1)	55.1 (51.3-59.6)	57.2 (53.1-61.5)	58.3 (54.2-62.7)	59.8 (55.6-63.9)	60.6 (56.4-64.7)	61.7 (57.6-65.5)	61.7 (57.8-65.3)	60.1 (56-63.8)	55.7 (51-60.3)	52.4 (48.6-56.6)	53.6 (50.5-57)	60.2 (57.3-63.5)	63.7 (60.7-66.7)
Wajir	55.5 (52.3-58.9)	53.3 (50.3-56.3)	57.5 (53.9-61.1)	57.7 (54.3-61.3)	57.2 (53.8-60.6)	57.3 (53.9-60.7)	57.5 (54.4-60.8)	57.5 (54.5-60.4)	56.4 (53.7-59.4)	55.1 (52.5-58)	53.6 (51.4-56)	56 (53.9-58.2)	58.6 (56.5-60.7)	61.2 (58.1-64.3)
West Pokot	53.5 (50-57.3)	51.8 (48.1-55.5)	54.7 (51.1-58.5)	55 (51.3-59)	54.3 (50.8-57.7)	54.7 (51.1-58.1)	56.5 (53.1-60)	58.4 (55.5-61.2)	58.5 (55.4-61.5)	57.6 (54.9-60.4)	58.8 (56-61.6)	60.9 (58.4-63.1)	61.7 (59.2-64)	64.7 (62.1-67.6)
Madagascar	44.6 (42.2-47.2)	45.9 (43.6-48)	47.4 (45.5-49.2)	49.3 (47.7-50.8)	50 (48.7-51.3)	51.9 (50.6-53.3)	53.2 (51.6-54.7)	53 (51.6-54.5)	53.9 (52.7-55.1)	55.8 (54.6-57)	57.7 (56.3-59.3)	59.5 (57.8-61.4)	60.4 (58.3-62.9)	62.2 (59.7-64.8)
Malawi	32.2 (28.9-35.7)	33.8 (30.6-37)	35.7 (32.7-38.9)	37.5 (35.1-40.2)	40.1 (38.1-42.2)	43.6 (42-45.3)	46.7 (45.4-48)	48 (46.7-49.2)	46.7 (44.9-48.4)	44.1 (41.6-46.5)	43.5 (41.1-45.8)	46.4 (44.3-48.5)	51.9 (50.2-53.4)	59.6 (57.9-61.5)
Mozambique	38.9 (35.4-42.3)	40 (36.7-43.3)	41.3 (38.2-44.4)	42.4 (39.5-45.4)	44.4 (41.8-47.1)	47.1 (44.8-49.6)	48.3 (46.4-50.3)	42.5 (41.3-43.7)	48 (46.7-49.4)	48.3 (46.8-49.7)	48.2 (46.5-49.7)	48.5 (46.6-50.4)	49.7 (47.8-51.6)	54.8 (52.7-57)
Rwanda	38.5 (35.5-41.5)	40.2 (37.4-42.9)	41.9 (39.6-44.3)	42.2 (40.2-44.5)	42.4 (40.5-44.5)	43.7 (41.8-45.7)	46.6 (44.9-48.4)	49.3 (47.8-50.9)	47.4 (46.1-48.7)	43.9 (42.7-45)	47.5 (46.3-48.8)	55.2 (54.1-56.4)	61.9 (60.6-63.3)	65.8 (64-67.6)
Somalia	42.7 (39.5-45.9)	43.7 (40.7-46.8)	44.7 (41.8-47.9)	45.5 (42.7-48.6)	45.9 (42.9-49.2)	45.1 (42.3-48.1)	47.5 (44.8-50.4)	47.6 (45.1-50.3)	48 (45.5-50.5)	49 (46.3-51.7)	50.6 (48.1-53.4)	52.7 (50-55.7)	52.8 (49.9-55.7)	56.5 (53.7-59.3)
South Sudan	44.5 (41-48.5)	45.2 (41.9-48.7)	45.7 (42.5-49)	46.4 (43.1-49.7)	47.1 (43.7-50.5)	48.1 (44.8-51.4)	48.7 (45.6-51.6)	48.9 (46.2-51.8)	49.3 (46.6-52)	51.1 (48.5-53.8)	52.5 (49.7-55.5)	54.9 (51.7-58.2)	55.9 (52.8-59.1)	56.9 (53.9-60)
Tanzania	43.1 (40-46.3)	44.6 (41.6-47.6)	45.8 (43.2-48.6)	47.6 (45.5-49.9)	50 (48.3-52)	52.3 (50.7-54.1)	54.2 (52.6-55.8)	55 (53.5-56.4)	53.2 (51.2-55.1)	51.4 (49.3-53.4)	52.4 (50.5-54.3)	55.7 (53.9-57.4)	59.7 (58.1-61.2)	64.6 (62.9-66.3)
Uganda	44.2 (41.1-47.5)	45 (41.9-47.9)	46.1 (43.2-49.1)	47.6 (45.2-50.4)	49.4 (47.1-51.8)	50.6 (48.7-52.7)	48.1 (46.6-49.7)	46.8 (42.5-49.8)	44.3 (40.3-48.4)	44 (41.7-46.6)	46.7 (45-48.4)	51.5 (50-52.9)	56.5 (55.1-58.1)	62.3 (60.5-64.2)
Zambia	44.6 (41.4-47.9)	46.2 (43.3-49.4)	47.7 (44.8-50.5)	49.7 (47.2-52.3)	52.5 (50.4-54.9)	54.5 (52.6-56.5)	55 (53.3-56.6)	53.8 (52.1-55.3)	49.3 (46.6-51.5)	44.6 (42.1-46.9)	44.4 (42.2-46.4)	47.5 (45.3-49.2)	53.7 (52.1-55.3)	60.4 (58.5-62.3)
Southern sub-Saharan Africa	49.1 (47.1-51)	50 (48.2-51.6)	51 (49.4-52.5)	52.4 (50.9-53.8)	53.9 (52.7-55)	55.6 (54.6-56.5)	57.1 (56.2-58)	59.2 (58.5-60)	60.3 (59.3-61.1)	57.3 (56.2-58.2)	51 (50-51.8)	49.1 (48.1-49.9)	53.1 (52.3-53.7)	61.5 (60.7-62.2)
Botswana	49.4 (46.7-52.5)	50.2 (47.4-52.9)	51.1 (48.5-53.9)	52.3 (49.8-54.9)	54 (51.9-56.4)	57 (55-59)	59.2 (57.4-61)	60.5 (58.3-62.4)	58.6 (55.2-61.3)	55 (51.6-58.2)	50.4 (46.1-54.3)	50.9 (47.2-54.2)	58.9 (55.5-61.6)	67 (64.1-69.2)
Lesotho	45.5 (43-48.1)	46.5 (44.2-48.8)	47.7 (45.3-49.9)	48.4 (45.9-50.7)	49.8 (47.5-52.1)	51.8 (49.6-54.2)	54.5 (52.4-56.6)	55.9 (54-57.9)	56 (54.3-57.6)	52.3 (50-54.2)	45.8 (43.1-48)	42.1 (39.8-44.3)	45.1 (42.9-47.1)	50.3 (48.1-52.7)
Namibia	51.9 (49.2-54.8)	52.5 (50-55.3)	53.3 (50.8-55.9)	54.6 (52.2-57.1)	53.9 (51.9-55.9)	54.8 (52.9-56.6)	54.2 (52.8-55.7)	55.5 (54.2-56.8)	58.2 (56-59.6)	56.5 (54.8-58.1)	52.1 (50-54)	50.6 (48.3-52.8)	55.9 (53.6-57.8)	62.3 (60.3-64.3)
South Africa	49.3 (47.2-51.4)	50.1 (48.1-52.1)	51.1 (49.1-52.8)	52.4 (50.6-54.1)	53.9 (52.5-55.4)	55.6 (54.4-56.9)	57.1 (56.1-58.2)	59.2 (58.3-60.1)	61.5 (60.8-62.1)	60.1 (59.6-60.8)	52.9 (52-53.7)	50.8 (49.7-51.7)	54.7 (53.8-55.5)	62.8 (62-63.6)
Swaziland	38.5 (35.5-41.7)	40.2 (37.4-43.1)	41.8 (38.9-44.6)	45 (42.5-47.6)	48.1 (45.6-50.9)	51 (48.7-53.5)	53.3 (51.2-55.6)	55.6 (53.7-57.6)	57.9 (56.1-59.7)	55.2 (53.3-57)	45.5 (42.6-48.1)	41.9 (39.5-44.5)	43.9 (41.4-46.3)	54.9 (52.6-57.6)
Zimbabwe	50.4 (47.6-53.3)	51.2 (48.5-53.8)	52.3 (49.7-54.8)	53.6 (51.3-55.9)	55 (52.9-57.1)	56.4 (54.6-58.4)	58 (56.4-59.7)	60.8 (59-62.3)	57.5 (52.7-60.7)	48.9 (44.9-52.6)	44.9 (41.8-47.7)	43.9 (41.4-46.3)	48.1 (45.9-50)	58.1 (56.3-60.1)
Western sub-Saharan Africa	39.3 (36.3-42.1)	40.8 (38.2-43.3)	42 (40-43.9)	44.6 (43-46.3)	45.8 (44.4-47.4)	48.5 (47.1-49.8)	51.2 (49.8-52.4)	52.9 (51.6-54)	53.3 (52-54.5)	53.2 (51.9-54.5)	53.6 (52.2-55)	55.5 (54.1-57)	58.2 (56.8-59.6)	61.7 (60.2-62.9)
Benin	39.3 (35.9-42.8)	40.1 (37.3-43.1)	40.9 (38.5-43.4)	42.8 (40.4-45.1)	44.7 (42.6-46.9)	47.2 (45.3-49.2)	49.5 (47.7-51.3)	51.1 (49.5-52.7)	53 (51.4-54.4)	54.5 (53.1-56)	55.3 (53.7-57)	57.1 (55.1-59)	59.3 (57-61.4)	62.6 (60.1-65)
Burkina Faso	34.5 (30.9-37.9)	36.1 (32.6-39.7)	37.9 (34.4-41.4)	39.8 (36.9-42.9)	41.3 (39-44)	43.2 (41.3-45.1)	46 (44.3-47.7)	48.5 (47.1-50)	49.1 (47.5-50.8)	48.8 (47.1-50.4)	50.6 (49.2-52.1)	53.4 (52-55)	55.8 (54.3-57.5)	58.9 (56.9-61)
Cameroon	38.8 (35.8-41.7)	41.9 (39.5-44.3)	35 (33.5-36.5)	46.9 (44.8-49)	49.2 (47.3-51.2)	51.6 (49.9-53.4)	53.6 (52-55.1)	55.8 (54.5-57.1)	56.2 (55-57.4)	54.5 (53.3-55.8)	53.4 (51.8-54.8)	53.7 (52-55.5)	56.4 (54.6-58.5)	61 (58.6-63.5)
Cape Verde	55.2 (52.5-58)	56.7 (54.2-59.3)	56.2 (54.1-58.5)	59.4 (57.4-61.5)	57.3 (55.8-58.9)	61.9 (60.8-63)	65.6 (64.9-66.2)	65 (64.5-65.4)	66.4 (66-66.9)	65.1 (64.1-66)	66.8 (65.4-68.2)	68.2 (67-69.4)	70.5 (70-71.1)	72.5 (71.3-73.7)
Chad	40.9 (37.4-44.6)	42.5 (39.1-46.1)	44.1 (40.8-47.6)	45.6 (42.5-48.8)	45.7 (43.1-48.5)	48.3 (46-50.7)	48.8 (46.7-50.6)	51 (49.2-52.5)	52 (50.4-53.6)	51.8 (50.2-53.3)	50.6 (49.1-52.1)	52.3 (50.7-53.9)	55.1 (53.2-57)	58.6 (56.4-60.8)
Cote d'Ivoire	39.3 (36-42.6)	40.6 (38-43.3)	42.9 (40.5-45.4)	45.5 (43.3-47.8)	48.4 (46.5-50.6)	51.6 (49.7-53.5)	54.6 (53.1-56.3)	54.8 (52.6-56.4)	52.1 (48.7-54.4)	49.3 (47.3-51.4)	50.4 (48.8-52.1)	52.4 (50.7-54.1)	55.4 (53.5-57.4)	60.1 (57.8-62.3)
The Gambia	43.5 (40.2-46.7)	44.7 (41.7-47.9)	46 (43-49.1)	47.7 (44.9-50.6)	50.1 (47.3-53)	52 (49.4-54.9)	54.2 (51.8-56.7)	56.3 (54-58.7)	57.8 (55.5-60.2)	59 (56.9-61.1)	59.7 (57.9-61.6)	60.4 (58.8-62.1)	61.8 (60.2-63.5)	63.8 (62-65.8)
Ghana	42.5 (39.7-45.5)	44.6 (42.1-47.2)	47.9 (45.5-50.5)	50.3 (48-52.8)	52.3 (49.9-54.8)	54.6 (52.2-57)	56.1 (53.8-58.3)	56.5 (54.5-58.7)	57.8 (55.9-59.8)	58.2 (56.5-59.9)	58 (56.5-59.4)	58.1 (56.9-59.5)	59.4 (58.2-60.6)	62.6 (61-64.3)
Guinea	38.5 (35.1-42.1)	39.8 (36.4-43.4)	41.3 (37.7-44.8)	43 (39.6-46.4)	44 (40.9-47)	45.3 (43-47.6)	47.6 (45.7-49.3)	49.8 (48.1-51.2)	51.6 (50.1-53)	52.7 (51.4-54.1)	53.3 (52-54.6)	54.9 (53.2-56.4)	56.2 (54.6-58)	59.3 (57.2-61.4)
Guinea-Bissau	27.8 (24.6-31.4)	30.2 (27.2-33.4)	32.7 (29.6-36.1)	31.9 (29-34.8)	34.3 (31.6-37.1)	39.8 (37.1-42.6)	41.8 (39.2-44.6)	43.2 (40.8-45.6)	45.5 (43.3-47.6)	47.4 (45.4-49.4)	49.2 (47.5-51.1)	50.6 (48.9-52.5)	53.6 (51.9-55.6)	57.4 (55.1-59.7)
Liberia	32.7 (29.3-36.3)	34.6 (31.3-38.2)	36.5 (33.7-39.5)	39.2 (36.9-41.9)	42.5 (40.5-44.9)	46.1 (43.9-48.5)	49.2 (47-51.4)	51 (49.1-53)	46.8 (45.3-48.4)	48.5 (46.8-50)	53.7 (51.9-55.5)	58.5 (56.8-60.2)	60.9 (59.1-62.6)	63.7 (61.5-65.8)
Mali	32.4 (29-36.1)	33.8 (30.3-37.3)	35.2 (32.2-38.4)	36.1 (33.7-38.7)	37.5 (35.7-39.5)	39.8 (38.1-41.4)	43.5 (42.1-44.9)	46.4 (45-47.8)	49.2 (48-50.5)	51.2 (49.9-52.5)	53.1 (51.9-54.5)	56.2 (54.9-57.7)	58.6 (57-60.2)	61 (58.7-63.2)
Mauritania	43.4 (40.2-46.4)	45.4 (42.7-48.2)	46.8 (44.5-49.3)	49.4 (47.2-51.7)	52.1 (49.8-54.5)	52.1 (50-54.4)	54.9 (53-57.1)	56.9 (55.2-58.5)	59 (57.6-60.5)	61 (59.7-62.5)	63.4 (62-64.7)	65.5 (64.2-67)	67.9 (66.5-69.8)	70 (68-72.3)
Niger	34 (30.3-37.6)	35.5 (31.7-39.5)	37.2 (33.8-41)	39.7 (36.6-42.8)	41 (38.5-43.5)	41.3 (39.3-43.4)	43 (41.4-44.8)	43.9 (42.3-45.6)	45.8 (44.3-47.3)	48 (46.4-49.5)	51.2 (49.5-52.9)	54.8 (53-56.5)	58 (56.1-59.9)	61.1 (58.8-63.5)
Nigeria	40.7 (37-44.5)	42.3 (38.8-45.9)	43.9 (40.6-47.2)	46 (43.2-48.7)	46.4 (43.9-49.1)	49.5 (47-52.1)	52.6 (50.1-55.1)	54.3 (52-56.7)	54 (51.5-56.4)	53.9 (51.4-56.6)	53.8 (50.9-56.8)	56 (53.1-59.3)	59.2 (56.2-62)	62.8 (59.7-65.2)
Sao Tome and Principe	41.1 (38.5-43.6)	42.5 (41.3-43.7)	47.1 (46.1-48.1)	54.5 (53.8-55.2)	57.7 (57.1-58.4)	60.4 (59.6-61.2)	61.8 (61-62.6)	61 (60-61.9)	62.5 (61.3-63.7)	63.1 (61.8-64.4)	64 (62.6-65.3)	64.9 (63.6-66.1)	66.8 (65.6-68.2)	68.1 (66.5-69.8)
Senegal	40.1 (37.2-43.4)	41.9 (39.3-44.7)	43.2 (40.9-45.6)	43.8 (41.8-46.1)	44.8 (42.8-46.8)	47.1 (45.3-49)	50.5 (48.9-52.1)	53.7 (52.3-55.1)	56.6 (55.4-57.8)	57.4 (56.2-58.4)	59.1 (58-60.1)	61.9 (60.8-63)	64.3 (62.8-65.5)	66.1 (64.5-67.9)
Sierra Leone	35.8 (32.1-39.3)	37.2 (33.8-40.8)	38.7 (35.3-42.1)	40.5 (37.1-44.2)	42.4 (39.1-45.8)	44.3 (41.5-47.4)	45.4 (43-48.1)	46.4 (44.4-48.5)	48.1 (46.3-50)	47.3 (45.8-48.8)	49.2 (47.7-50.6)	51.4 (49.9-52.8)	54.5 (52.7-56.3)	59.5 (57.2-61.7)
Togo	38.4 (35-41.8)	40 (36.7-43.4)	42 (39.1-45.3)	44.6 (42-47.4)	48.5 (46.2-50.9)	51.5 (49.4-53.6)	54 (52.1-56)	55.4 (53.7-57.2)	56 (54.3-57.7)	55.3 (53.8-57)	54.7 (53-56.5)	54.7 (52.8-56.7)	56.8 (54.9-58.9)	61.4 (59.1-63.8)

Table 9 Estimates of life expectancy at age 65 by location and year for 1950, 1955...2005, 2010, 2017 - both sexes

Location	1950	1955	1960	1965	1970	1975	1980	1985	1990	1995	2000	2005	2010	2017
Global	12 (11.9-12.1)	12.6 (12.5-12.7)	12.4 (12.3-12.4)	13.2 (13.1-13.3)	13.5 (13.4-13.5)	13.9 (13.8-13.9)	14.2 (14.2-14.3)	14.6 (14.5-14.6)	15 (14.9-15)	15.2 (15.2-15.3)	15.7 (15.7-15.8)	16.3 (16.2-16.3)	16.8 (16.7-16.8)	17.4 (17.3-17.4)
Low SDI	9.8 (9.6-10.1)	10.1 (9.8-10.3)	10.2 (10-10.5)	10.4 (10.1-10.6)	10.6 (10.4-10.8)	11 (10.8-11.3)	11.3 (11.1-11.6)	11.5 (11.3-11.7)	11.8 (11.6-12)	12.2 (12-12.3)	12.7 (12.5-12.8)	13.2 (13.1-13.4)	13.5 (13.4-13.6)	14.2 (14-14.4)
Low-middle SDI	10.5 (10.3-10.7)	10.9 (10.7-11.1)	10.8 (10.6-11)	11.4 (11.2-11.6)	11.7 (11.5-11.9)	12.2 (12-12.4)	12.6 (12.4-12.8)	13 (12.8-13.1)	13.2 (13-13.4)	13.4 (13.2-13.6)	13.5 (13.3-13.7)	14.1 (14-14.3)	14.5 (14.3-14.7)	15 (14.8-15.2)
Middle SDI	11.2 (11-11.5)	11.9 (11.6-12.1)	9.4 (9.3-9.6)	12.5 (12.3-12.7)	13 (12.8-13.2)	13.5 (13.3-13.7)	13.9 (13.7-14)	14.3 (14.1-14.4)	14.6 (14.5-14.7)	15.1 (15-15.1)	15.4 (15.3-15.5)	15.9 (15.8-16)	16.3 (16.2-16.4)	16.8 (16.6-17)
High-middle SDI	12.5 (12.2-12.7)	13 (12.8-13.3)	13.1 (12.8-13.3)	13.7 (13.5-13.9)	13.9 (13.7-14.1)	14.1 (13.9-14.2)	14.1 (14-14.3)	14.3 (14.2-14.4)	14.6 (14.5-14.7)	14.5 (14.4-14.6)	15.1 (15-15.2)	15.5 (15.4-15.5)	16.3 (16.2-16.4)	17.1 (17-17.3)
High SDI	13.3 (13.2-13.5)	13.9 (13.9-14)	14.2 (14.1-14.2)	14.4 (14.4-14.4)	14.6 (14.6-14.7)	15.2 (15.2-15.2)	15.7 (15.7-15.8)	16.3 (16.3-16.3)	17 (17-17)	17.5 (17.5-17.5)	18.2 (18.2-18.3)	19.1 (19.1-19.1)	19.9 (19.8-19.9)	20.4 (20.3-20.4)
Central Europe, Eastern Europe, and Central Asia	13.3 (13.1-13.5)	13.8 (13.6-13.9)	14.5 (14.5-14.6)	14.6 (14.5-14.6)	14.3 (14.3-14.4)	14.5 (14.4-14.5)	14.3 (14.2-14.3)	14.3 (14.2-14.3)	14.6 (14.6-14.6)	13.8 (13.8-13.8)	14.2 (14.2-14.2)	14.3 (14.3-14.3)	15.2 (15.2-15.2)	16.3 (16.3-16.4)
Central Asia	12 (11.7-12.3)	12.6 (12.4-12.8)	13.2 (13.1-13.4)	13.6 (13.3-13.8)	14 (13.9-14.1)	14.3 (14.2-14.5)	14.9 (14.8-15)	15 (15-15.1)	15.2 (15.1-15.2)	13.9 (13.8-13.9)	14.1 (14-14.1)	13.6 (13.6-13.7)	14.2 (14.1-14.2)	14.8 (14.6-15)
Armenia	12.9 (12-13.7)	13.8 (13.3-14.4)	14.6 (14.2-15.1)	14.9 (14.3-15.5)	15.4 (15.2-15.7)	15.4 (14.9-15.9)	16.2 (15.9-16.5)	15.9 (15.8-16.1)	14.7 (14.5-14.9)	14.1 (14-14.2)	14.8 (14.7-14.8)	15 (14.9-15)	15.3 (15.3-15.4)	16.1 (15.9-16.3)
Azerbaijan	11.3 (10.5-12)	11.9 (11.5-12.4)	12.8 (12.4-13.1)	13.4 (12.9-14)	14.1 (13.9-14.3)	14.5 (14.1-14.9)	15.1 (15-15.2)	15.6 (15.6-15.7)	15.6 (15.4-15.9)	13.2 (12.9-13.4)	13.4 (13.2-13.7)	13.1 (12.9-13.4)	13.7 (13.5-14)	14.2 (13.7-14.5)
Georgia	12.4 (11.6-13.2)	13 (12.5-13.5)	13.6 (13.2-13.9)	14 (13.4-14.6)	14.6 (14.4-14.8)	14.8 (14.2-15.3)	15.1 (14.8-15.4)	15.1 (14.9-15.3)	15.1 (14.8-15.3)	15.8 (15.5-16.1)	16.3 (16.1-16.6)	16.1 (16-16.2)	15.5 (15.4-15.5)	15.2 (15-15.3)
Kazakhstan	12.3 (11.5-13.2)	13 (12.5-13.6)	13.5 (13.1-13.9)	13.5 (12.9-14.1)	13.9 (13.7-14.1)	14.3 (13.9-14.7)	14.8 (14.7-14.9)	14.7 (14.7-14.8)	15 (14.9-15)	13 (13-13.1)	13.4 (13.4-13.4)	13.3 (13.3-13.3)	14.1 (14.1-14.1)	15.4 (15.2-15.7)
Kyrgyzstan	10.7 (10.1-11.5)	11.3 (10.7-11.9)	11.8 (11.3-12.4)	12.2 (11.5-12.9)	12.5 (12-13.1)	13.2 (12.6-13.8)	14 (13.7-14.4)	14.3 (14-14.6)	14.7 (14.4-14.9)	14 (13.7-14.3)	14.3 (14-14.6)	14 (13.8-14.2)	14.6 (14.6-14.7)	15.6 (15.4-15.7)
Mongolia	9.9 (9.3-10.5)	10.3 (9.7-11)	11 (10.4-11.5)	11.7 (11.4-12.1)	11.9 (11.6-12.2)	12 (11.7-12.3)	12.3 (12.1-12.7)	11.9 (11.7-12.1)	12.2 (12-12.4)	11.3 (11.1-11.5)	11.4 (11.2-11.5)	12.2 (12-12.4)	12.7 (12.5-12.9)	14.3 (13.8-14.8)
Tajikistan	11.5 (10.8-12.3)	11.9 (11.5-12.4)	12.6 (12.3-12.9)	13.3 (12.8-13.9)	14.1 (13.9-14.3)	14.6 (14.2-15)	15.8 (15.7-15.9)	16 (16-16.1)	15.8 (15.7-15.9)	14.5 (14.2-14.8)	15.5 (15.2-15.7)	15.5 (15.1-15.8)	15.5 (15.1-15.8)	15.4 (14.9-15.9)
Turkmenistan	11.6 (10.8-12.4)	12.3 (11.8-12.8)	12.9 (12.5-13.2)	13.1 (12.5-13.7)	13.7 (13.4-13.9)	13.6 (13.2-14)	14.1 (14-14.2)	14.2 (14.2-14.3)	14.4 (14.3-14.4)	13.2 (13.2-13.3)	14.3 (14.2-14.4)	13.4 (13.4-13.5)	15.2 (15.1-15.2)	15 (14.5-15.4)
Uzbekistan	12.8 (11.9-13.8)	13.3 (12.8-13.9)	13.8 (13.5-14.2)	14 (13.5-14.6)	14.4 (14.2-14.6)	14.6 (14.2-15)	15 (14.9-15.1)	15.4 (15.4-15.4)	15.9 (15.9-15.9)	14.3 (14.3-14.4)	13.6 (13.4-13.8)	12.5 (12.3-12.8)	13.3 (13.1-13.5)	14 (13.3-14.7)
Central Europe	12.7 (12.6-12.9)	13.2 (13.2-13.3)	13.7 (13.7-13.8)	14 (13.9-14)	13.8 (13.8-13.9)	14.2 (14.2-14.2)	13.9 (13.9-14)	14.1 (14.1-14.1)	14.4 (14.4-14.4)	14.6 (14.6-14.6)	15.2 (15.2-15.2)	15.8 (15.8-15.8)	16.5 (16.5-16.5)	17.4 (17.2-17.5)
Albania	13 (12.4-13.7)	14.6 (14.1-15)	15.9 (15.5-16.2)	16 (15.5-16.6)	16.6 (15.7-17.6)	16.8 (15.7-18)	16.9 (15.9-17.8)	16.7 (16.3-17)	16.5 (16.4-16.5)	16.9 (16.8-17)	16.6 (16.3-16.9)	16.9 (16.6-17.2)	17.8 (17.2-18.3)	17.9 (16.9-19)
Bosnia and Herzegovina	13.1 (13-13.1)	13 (13-13.1)	13.3 (13.2-13.3)	13.1 (13-13.2)	13 (12.9-13.1)	13.5 (13.5-13.6)	13.6 (13.5-13.6)	14.4 (14.3-14.4)	15.3 (15.2-15.3)	14.3 (13.5-15.1)	15.9 (15.4-16.5)	16 (15.9-16.1)	16 (15.9-16.1)	16.3 (16-16.7)
Bulgaria	14.7 (14.6-14.7)	15.2 (15.1-15.2)	15.3 (15.2-15.3)	15.5 (15.5-15.6)	15.1 (15.1-15.2)	14.7 (14.6-14.7)	14.6 (14.5-14.6)	14.6 (14.6-14.7)	14.4 (14.4-14.4)	14.1 (14.1-14.2)	14.3 (14.2-14.3)	14.8 (14.7-14.8)	15.5 (15.5-15.6)	16.3 (16-16.6)
Croatia	10.7 (10.6-10.7)	11.8 (11.8-11.9)	12.5 (12.5-12.6)	13.4 (13.3-13.4)	13.3 (13.3-13.4)	13.9 (13.9-14)	14.2 (14.1-14.2)	14.3 (14.2-14.3)	14.6 (14.5-14.6)	15.2 (15.1-15.2)	15.4 (15.3-15.5)	15.9 (15.8-16)	16.6 (16.5-16.7)	17.7 (17.3-18)
Czech Republic	12.3 (12.3-12.3)	12.9 (12.8-12.9)	13.3 (13.2-13.3)	13.4 (13.3-13.4)	12.8 (12.8-12.9)	13.2 (13.1-13.2)	13 (13-13.1)	13.3 (13.3-13.3)	13.6 (13.6-13.7)	14.6 (14.6-14.7)	15.6 (15.6-15.7)	16.4 (16.4-16.5)	17.4 (17.3-17.5)	18.2 (17.9-18.6)
Hungary	12.6 (12.6-12.7)	13.5 (13.4-13.5)	13.5 (13.4-13.5)	13.7 (13.6-13.7)	13.4 (13.3-13.4)	13.5 (13.4-13.5)	13.3 (13.2-13.3)	13.5 (13.4-13.5)	14 (13.9-14)	14.2 (14.2-14.3)	15 (15-15)	15.5 (15.4-15.5)	16.2 (16.2-16.3)	17.3 (17-17.7)
Macedonia	13.4 (12.8-14)	13.2 (12.7-13.8)	13.3 (12.8-13.8)	13.9 (13.4-14.4)	14.5 (14.2-14.8)	15.1 (15-15.2)	14.7 (14.6-14.8)	14.6 (14.5-14.7)	14.9 (14.8-15)	14.8 (14.7-14.9)	15.1 (15-15.2)	15.5 (15.4-15.6)	16 (15.9-16.1)	16.7 (16.4-17)
Montenegro	13.5 (13.2-13.9)	15.2 (14.9-15.5)	15.3 (15-15.6)	15.7 (15.4-16)	15.8 (15.5-16.1)	15.4 (15.2-15.7)	15.5 (15.4-15.6)	15.6 (15.5-15.7)	16.2 (16.1-16.3)	16 (15.8-16.1)	15.7 (15.6-15.9)	15.7 (15.4-15.9)	16.4 (16.1-16.7)	16.2 (15.7-16.6)
Poland	12.6 (12.6-12.6)	13.2 (13.1-13.2)	14.2 (14.2-14.3)	14.5 (14.5-14.6)	14.3 (14.3-14.4)	14.6 (14.6-14.7)	14.4 (14.3-14.4)	14.4 (14.3-14.4)	14.7 (14.7-14.7)	15.1 (15-15.1)	15.9 (15.8-15.9)	16.7 (16.7-16.8)	17.5 (17.5-17.5)	18.3 (18-18.7)
Romania	13 (12-14)	13.1 (12.8-13.4)	13.5 (13.5-13.5)	13.5 (13.5-13.6)	13.8 (13.8-13.9)	14.2 (14.2-14.2)	13.8 (13.7-13.8)	13.9 (13.9-13.9)	14.2 (14.2-14.3)	14 (14-14)	14.7 (14.6-14.7)	15 (15-15)	15.8 (15.7-15.8)	16.6 (16.3-16.9)
Serbia	13.1 (13-13.1)	13 (12.9-13)	13.1 (13.1-13.2)	13.2 (13.1-13.2)	13.4 (13.3-13.4)	14.5 (14.5-14.6)	14.1 (14.1-14.2)	14.1 (14-14.1)	14.2 (14.1-14.2)	14.1 (14-14.1)	14.4 (14.3-14.4)	14.4 (14.4-14.5)	15 (15-15.1)	15.6 (15.3-15.9)
Slovakia	12.4 (11.8-13)	13.1 (12.7-13.6)	13.8 (13.3-14.3)	14 (13.5-14.4)	13.7 (13.3-14.1)	14 (13.9-14)	13.9 (13.9-14)	14.1 (14-14.1)	14.2 (14.1-14.2)	14.8 (14.7-14.8)	15.2 (15.1-15.2)	15.6 (15.6-15.7)	16.4 (16.3-16.4)	17.4 (17.1-17.8)
Slovenia	11.3 (11.2-11.4)	12.8 (12.7-12.9)	13.2 (13.1-13.3)	13.8 (13.7-13.9)	13.5 (13.4-13.5)	13.8 (13.7-13.9)	14.4 (14.3-14.4)	14.6 (14.5-14.7)	15.6 (15.5-15.7)	16 (15.9-16.1)	16.7 (16.6-16.8)	17.7 (17.6-17.8)	18.8 (18.7-18.9)	19.7 (19.3-20.2)
Eastern Europe	14 (13.7-14.4)	14.3 (14-14.6)	15.3 (15.2-15.4)	15.2 (15.1-15.3)	14.7 (14.6-14.8)	14.6 (14.5-14.7)	14.4 (14.3-14.4)	14.3 (14.2-14.3)	14.7 (14.7-14.7)	13.4 (13.4-13.4)	13.7 (13.7-13.7)	13.7 (13.7-13.7)	14.7 (14.7-14.7)	16 (15.9-16)
Belarus	15.7 (14.5-17.1)	16 (15.1-17)	16.7 (16.5-16.8)	16.8 (16.8-16.9)	16.3 (16.2-16.4)	16.1 (16-16.1)	15.6 (15.6-15.7)	15.3 (15.2-15.3)	15.5 (15.4-15.5)	14.4 (14.3-14.4)	14.3 (14.3-14.4)	14.1 (14.1-14.2)	14.8 (14.8-14.9)	16 (15.6-16.3)
Estonia	13.3 (12.2-14.4)	13.4 (12.5-14.3)	14.2 (14.1-14.3)	14.5 (14.3-14.6)	14.2 (14.1-14.3)	14.4 (14.3-14.5)	14.3 (14.2-14.4)	14.2 (14.1-14.3)	14.5 (14.4-14.6)	14.3 (14.2-14.4)	15.3 (15.2-15.4)	16.1 (16-16.2)	17.4 (17.3-17.5)	18.6 (17.9-19.4)
Latvia	14.3 (13.3-15.5)	14.6 (13.7-15.8)	15 (15-15.2)	15.3 (15.2-15.4)	14.8 (14.7-14.9)	14.6 (14.5-14.7)	14.5 (14.4-14.6)	14.3 (14.2-14.4)	14.6 (14.5-14.6)	14.2 (14.1-14.2)	15.4 (15.3-15.5)	15.4 (15.3-15.5)	16.2 (16.1-16.3)	17.1 (16.5-17.8)
Lithuania	14.8 (13.6-16)	15.1 (14-16.3)	15.4 (15.3-15.5)	16.1 (16-16.2)	15.6 (15.5-15.7)	15.6 (15.5-15.7)	15.4 (15.3-15.5)	15.2 (15.1-15.2)	15.5 (15.4-15.6)	15.1 (15-15.1)	16.2 (16.2-16.3)	16.1 (16-16.1)	16.7 (16.6-16.7)	17.3 (16.9-17.6)
Moldova	11.5 (10.7-12.3)	12 (11.5-12.5)	12.3 (12-12.7)	12.5 (12-13)	12.8 (12.6-12.9)	13.1 (12.7-13.4)	13.6 (13.5-13.6)	13.2 (13.2-13.3)	14 (13.9-14)	12.6 (12.6-12.7)	14 (13.9-14)	13.9 (13.9-14)	14.5 (14.4-14.5)	16.1 (15.9-16.3)
Russian Federation	14 (13.8-14.2)	14.3 (14.1-14.5)	15.2 (15.1-15.4)	15.1 (14.9-15.2)	14.6 (14.4-14.7)	14.5 (14.3-14.7)	14.2 (14.1-14.4)	14.2 (14.1-14.2)	14.6 (14.6-14.7)	13.4 (13.4-13.4)	13.5 (13.5-13.5)	13.5 (13.5-13.5)	14.5 (14.5-14.5)	16.1 (16.1-16.2)
Ukraine	14.1 (13-15.1)	14.2 (13.2-15.2)	15.5 (15.5-15.6)	15.3 (15.2-15.3)	14.7 (14.7-14.8)	14.7 (14.7-14.8)	14.4 (14.4-14.5)	14.3 (14.3-14.4)	14.7 (14.6-14.7)	13.1 (13.1-13.2)	13.9 (13.9-13.9)	13.9 (13.9-14)	14.7 (14.7-14.7)	15.3 (15-15.6)
High-income	13.4 (13.2-13.5)	14 (13.9-14)	14.2 (14.2-14.2)	14.4 (14.4-14.4)	14.7 (14.6-14.7)	15.2 (15.2-15.2)	15.8 (15.8-15.8)	16.4 (16.4-16.4)	17.1 (17.1-17.1)	17.6 (17.6-17.7)	18.4 (18.3-18.4)	19.2 (19.2-19.2)	19.9 (19.9-19.9)	20.4 (20.3-20.5)
Australasia	13.7 (13.7-13.7)	13.9 (13.9-14)	14.2 (14.2-14.2)	14.1 (14.1-14.1)	14.1 (14.1-14.1)	14.9 (14.8-14.9)	15.8 (15.8-15.8)	16.2 (16.2-16.3)	17.1 (17-17.1)	17.7 (17.7-17.8)	18.8 (18.7-18.8)	19.9 (19.8-19.9)	20.5 (20.4-20.5)	20.8 (20.3-21.3)
Australia	13.6 (13.6-13.7)	13.8 (13.8-13.9)	14.2 (14.1-14.2)	14.1 (14-14.1)	14 (14-14.1)	14.9 (14.8-14.9)	15.9 (15.9-16)	16.3 (16.3-16.4)	17.2 (17.1-17.2)	17.9 (17.8-17.9)	18.9 (18.8-18.9)	20 (19.9-20)	20.6 (20.6-20.6)	20.9 (20.3-21.5)
New Zealand	13.9 (13.8-14)	14.3 (14.2-14.3)	14.4 (14.3-14.5)	14.3 (14.2-14.3)	14.3 (14.2-14.4)	14.9 (14.8-15)	15.2 (15.1-15.3)	15.7 (15.7-15.8)	16.6 (16.5-16.7)	17.1 (17-17.1)	18.3 (18.2-18.3)	19.1 (19-19.2)	19.8 (19.8-19.9)	20.5 (20.1-20.8)
High-income Asia-Pacific	11.7 (11.5-11.9)	12.9 (12.7-13.1)	12.9 (12.7-13.1)	13.3 (13.1-13.5)	14 (13.8-14.2)	15.1 (15-15.2)	16 (16-16.1)	17.1 (17.1-17.1)	18 (18-18)	18.7 (18.7-18.7)	19.8 (19.8-19.8)	20.6 (20.6-20.6)	21.3 (21.3-21.3)	22 (21.8-22.1)
Brunei	11 (10.2-11.8)	11.4 (10.8-12.1)	12.3 (11.8-12.8)	13.2 (12.9-13.7)	13.1 (12.8-13.4)	12.9 (12.6-13.1)	13.1 (12.8-13.4)	13.2 (13-13.5)	13.1 (12.8-13.3)	14.3 (14-14.5)	15.3 (15-15.6)	16 (15.7-16.2)	16.3 (16-16.6)	16.6 (16-17.1)
Japan	12.1 (11.9-12.2)	13.2 (13.1-13.4)	13.2 (13-13.4)	13.6 (13.5-13.8)	14.3 (14.2-14.5)	15.4 (15.3-15.5)	16.5 (16.5-16.5)	17.7 (17.7-17.7)	18.6 (18.6-18.6)	19.2 (19.2-19.2)	20.3 (20.3-20.3)	21 (21-21)	21.6 (21.6-21.6)	22.2 (22.1-22.3)
Aichi	12.2 (11.2-13.1)	13.4 (12.3-14.5)	13.1 (12.1-14.2)	13.5 (12.5-14.6)	14.2 (13.2-15.2)	15.2 (14.6-15.8)	16.2 (16.1-16.2)	17.5 (17.4-17.5)	18.2 (18.2-18.3)	18.8 (18.8-18.9)	20 (19.9-20.1)	20.7 (20.6-20.7)	21.3 (21.3-21.4)	22.2 (21.5-22.9)
Akita	12.1 (11.2-13.1)	13.3 (12.3-14.4)	13.8 (12.8-14.9)	14.3 (13.3-15.4)	14.9 (14-16)	16.1 (15.5-16.7)	17.4 (17.3-17.5)	17.9 (17.9-18)	18.8 (18.7-18.9)	19.2 (19.1-19.3)	19.9 (19.8-20)	20.6 (20.5-20.7)	21.1 (21-21.2)	21.5 (20.8-22.2)
Aomori	11.6 (10.6-12.6)	12.7 (11.7-13.8)	12.8 (11.9-13.9)	13.3 (12.3-14.3)	14 (13.1-14.9)	15.1 (14.6-15.7)	16.1 (16-16.2)	17.2 (17.1-17.2)	18.3 (18.3-18.4)	18.7 (18.6-18.8)	19.6 (19.5-19.7)	20.3 (20.2-20.4)	20.8 (20.7-20.9)	21.3 (20.6-22.1)
Chiba	12.2 (11.3-13.3)	13.4 (12.4-14.5)	13.2 (12.1-14.4)	13.6 (12.5-14.7)	14.2 (13.3-15.2)	15.2 (14.6-15.8)	16.5 (16.4-16.5)	17.7 (17.7-17.8)	18.6 (18.6-18.7)	19.1 (19-19.2)	20.2 (20.2-20.3)	20.9 (20.8-20.9)	21.5 (21.4-21.5)	22.1 (21.4-22.7)
Ehime	12 (11.1-13)	13.1 (12.1-14.2)	13.4 (12.4-14.4)	13.9 (12.9-14.9)	14.6 (13.7-15.6)	15.7 (15.1-16.2)	16.8 (16.7-16.9)	18 (18-18.1)	18.9 (18.9-19)	19.4 (19.3-19.5)	20.5 (20.4-20.6)	21.1 (21-21.2)	21.7 (21.6-21.8)	22.4 (21.7-23.1)
Fukui	12.5 (11.6-13.6)	13.7 (12.7-14.7)	13.4 (12.5-14.3)	13.8 (12.9-14.7)	14.5 (13.8-15.2)	15.5 (15.1-15.9)	16.6 (16.5-16.7)	17.8 (17.7-17.9)	18.8 (18.7-18.9)	19.5 (19.4-19.6)	20.7 (20.6-20.8)	21.2 (21.1-21.3)	21.9 (21.8-22)	22.3 (21.7-23)
Fukuoka	11.8 (10.9-12.8)	13 (12-14.1)	13.1 (12.1-14.3)	13.6 (12.5-14.6)	14.3 (13.3-15.3)	15.3 (14.7-16)	16.6 (16.5-16.6)	17.8 (17.7-17.8)	18.6 (18.5-18.7)	19.3 (19.2-19.4)	20.3 (20.2-20.4)	21 (21-21.1)	21.6 (21.6-21.7)	22.5 (21.8-23.2)
Fukushima	12 (11.1-13)	13.2 (12.3-14.4)	13.2 (12.2-14.4)	13.6 (12.6-14.8)	14.3 (13.4-15.3)	15.4 (14.9-16)	16.6 (16.5-16.7)	17.6 (17.5-17.6)	18.7 (18.6-18.8)	19.2 (19.1-19.3)	20.2 (20.1-20.3)	20.9 (20.8-21)	21.5 (21.4-21.6)	21.8 (21.1-22.5)
Gifu	12.1 (11.2-13.1)	13.2 (12.2-14.3)	13.1 (12.1-14.1)	13.5 (12.5-14.5)	14.1 (13.1-15)	15.2 (14.6-15.7)	16.2 (16.2-16.3)	17.5 (17.5-17.6)	18.6 (18.5-18.7)	19.2 (19.1-19.3)	20.2 (20.2-20.3)	21 (20.9-21.1)	21.6 (21.5-21.7)	21.9 (21.3-22.7)
Gunma	12.1 (11.2-13.1)	13.2 (12.2-14.3)	13.1 (12.1-14.3)	13.5 (12.4-14.6)	14.1 (13.2-15)	15 (14.5-15.6)	16.2 (16.1-16.3)	17.5 (17.5-17.6)	18.6 (18.5-18.6)	19.2 (19.1-19.3)	20.2 (20.2-20.3)	20.8 (20.7-20.9)	21.4 (21.3-21.5)	21.8 (21.1-22.5)
Hiroshima	12 (11.1-13)	13.2 (12.1-14.4)	13.4 (12.4-14.4)	13.9 (12.9-14.9)	14.6 (13.6-15.6)	15.6 (15-16.2)	16.8 (16.7-16.9)	18 (17.9-18)	18.9 (18.8-18.9)	19.5 (19.4-19.6)	20.6 (20.5-20.7)	21.3 (21.2-21.4)	21.8 (21.7-21.9)	22.6 (21.9-23.3)
Hokkaidō	11.7 (10.9-12.8)	12.9 (11.9-13.9)	12.9 (11.9-14)	13.4 (12.3-14.5)	14 (13.1-15.1)	15.1 (14.5-15.7)	16.2 (16.2-16.3)	17.4 (17.4-17.5)	18.4 (18.4-18.5)	19.3 (19.2-19.4)	20.5 (20.5-20.6)	21.2 (21.1-21.2)	21.7 (21.6-21.7)	22.2 (21.6-23)
Hyōgo	11.8 (11-12.8)	13 (12.1-14.1)	13 (12-14.1)	13.5 (12.4-14.6)	14.2 (13.3-15.2)	15.4 (14.8-15.9)	16.3 (16.2-16.3)	17.4 (17.3-17.4)	18.2 (18.2-18.3)	18.3 (18.2-18.4)	20.1 (20-20.1)	20.9 (20.8-20.9)	21.5 (21.4-21.6)	22.2 (21.5-22.9)
Ibaraki	11.9 (11-12.8)	13 (12-14.1)	13 (12-14.1)	13.5 (12.4-14.6)	14.1 (13.2-15.2)	15.2 (14.6-15.8)	16.3 (16.2-16.4)	17.2 (17.2-17.3)	18.3 (18.2-18.4)	19 (18.9-19)	19.9 (19.9-20)	20.6 (20.5-20.7)	21.1 (21-21.2)	21.5 (20.9-22.3)
Ishikawa	12.5 (11.5-13.5)	13.6 (12.4-14.7)	13.3 (12.3-14.4)	13.7 (12.7-14.6)	14.3 (13.6-15.1)	15.6 (15.2-16.1)	16.8 (16.7-16.9)	18 (17.9-18.1)	18.9 (18.8-19)	19.3 (19.2-19.4)	20.4 (20.3-20.5)	21.5 (21.4-21.6)	21.9 (21.8-22)	22.3 (21.7-23.1)
Iwate	11.9 (10.9-13)	13.1 (12.1-14.2)	13 (12.1-14)	13.5 (12.6-14.5)	14.2 (13.4-15.1)	15.3 (14.8-15.8)	16.3 (16.2-16.4)	17.5 (17.4-17.6)	18.5 (18.4-18.6)	19.2 (19.2-19.3)	20.3 (20.2-20.4)	20.8 (20.7-20.9)	21.5 (21.3-21.6)	22 (21.3-22.7)
Kagawa	12.4 (11.5-13.4)	13.6 (12.6-14.7)	13.7 (12.7-14.8)	14.2 (13.2-15.2)	14.9 (14.1-15.8)	16 (15.6-16.4)	17 (16.9-17.1)	18.3 (18.2-18.4)	18.9 (18.8-19)	19.6 (19.5-19.7)	20.5 (20.4-20.6)	21.2 (21.1-21.3)	21.6 (21.5-21.7)	22.3 (21.6-22.9)
Kagoshima	11.5 (10.7-12.5)	12.7 (11.7-13.7)	12.8 (11.9-14)	13.3 (12.3-14.3)	13.9 (13-14.9)	15 (14.5-15.6)	16.4 (16.4-16.5)	17.6 (17.6-17.7)	18.6 (18.5-18.7)	19.4 (19.3-19.5)	20.6 (20.5-20.7)	21.2 (21.1-21.3)	21.7 (21.6-21.8)	22.1 (21.5-22.9)
Kanagawa	12.4 (11.5-13.4)	13.6 (12.6-14.9)	13.5 (12.5-14.6)	13.9 (12.9-15.1)	14.6 (13.7-15.7)	15.8 (15.2-16.4)	16.8 (16.7-16.8)	17.9 (17.8-17.9)	18.7 (18.7-18.8)	19.2 (19.2-19.3)	20.3 (20.3-20.4)	21.1 (21.1-21.2)	21.7 (21.6-21.8)	22.4 (21.7-23)
Kōchi	11.6 (10.7-12.5)	12.7 (11.7-13.8)	13 (12-14)	13.4 (12.5-14.4)	14 (13.2-14.9)	15.1 (14.6-15.6)	16.6 (16.5-16.7)	18.1 (18-18.2)	18.9 (18.8-19)	19.6 (19.5-19.7)	20.5 (20.4-20.6)	21.2 (21.1-21.3)	21.6 (21.5-21.8)	22.6 (21.9-23.4)
Kumamoto	12 (11-13)	13.2 (12.1-14.3)	13.3 (12.3-14.4)	13.7 (12.7-14.7)	14.3 (13.4-15.3)	15.4 (14.8-15.9)	16.8 (16.7-16.9)	18.3 (18.2-18.4)	19.2 (19.1-19.3)	20.2 (20.1-20.3)	21.1 (21-21.2)	21.8 (21.7-21.8)	22.3 (22.2-22.4)	22.7 (22-23.4)
Kyōto	12.4 (11.4-13.4)	13.6 (12.6-14.7)	13.5 (12.5-14.7)	14 (12.8-15.1)	14.8 (13.8-15.9)	16 (15.4-16.7)	17.2 (17.1-17.2)	18 (17.9-18.1)	18.7 (18.6-18.8)	19.4 (19.3-19.5)	20.4 (20.3-20.5)	21.2 (21.1-21.3)	21.9 (21.8-22)	22.7 (22-23.4)
Mie	12.3 (11.3-13.3)	13.4 (12.4-14.7)	13.3 (12.3-14.5)	13.8 (12.8-14.9)	14.6 (13.6-15.5)	15.6 (15.1-16.2)	16.6 (16.5-16.7)	17.5 (17.5-17.6)	18.5 (18.4-18.6)	19 (18.9-19.1)	20.3 (20.2-20.4)	20.9 (20.8-21)	21.4 (21.3-21.5)	21.9 (21.3-22.7)
Miyagi	12.2 (11.2-13.1)	13.4 (12.3-14.5)	13.2 (12.1-14.2)	13.6 (12.6-14.6)	14.1 (13.3-15.1)	15.1 (14.6-15.7)	16.1 (16.1-16.2)	17.5 (17.4-17.6)	18.6 (18.6-18.7)	19.2 (19.1-19.3)	20.3 (20.2-20.4)	21 (20.9-21.1)	21.5 (21.4-21.6)	22.2 (21.5-22.8)
Miyazaki	11.7 (10.8-12.7)	12.8 (11.8-13.8)	13 (12-14.1)	13.5 (12.5-14.5)	14.2 (13.4-15.1)	15.4 (14.9-16)	16.6 (16.5-16.7)	18 (17.9-18.1)	18.9 (18.8-19)	19.5 (19.4-19.6)	20.8 (20.7-20.9)	21.4 (21.3-21.6)	21.9 (21.8-22)	22.4 (21.6-23.1)
Nagano	12.7 (11.7-13.6)	13.8 (12.8-15)	13.7 (12.7-14.8)	14 (13-15.1)	14.7 (13.8-15.6)	15.6 (15-16.1)	16.9 (16.8-17)	18 (17.9-18.1)	19.2 (19.1-19.3)	19.9 (19.8-20)	20.9 (20.8-21)	21.7 (21.6-21.8)	22.3 (22.2-22.4)	22.7 (22-23.4)
Nagasaki	11.7 (10.7-12.7)	12.8 (11.8-13.8)	12.9 (12-13.9)	13.4 (12.4-14.5)	14 (13.1-15)	15.1 (14.5-15.6)	16.2 (16.2-16.3)	17.7 (17.6-17.8)	18.6 (18.5-18.7)	19.3 (19.2-19.4)	20.5 (20.4-20.6)	21.2 (21.1-21.3)	21.8 (21.7-21.9)	22.1 (21.4-22.8)
Nara	11.9 (10.9-12.9)	13 (12-14.1)	12.7 (11.8-13.8)	13.1 (12.2-14.1)	13.8 (13-14.7)	14.9 (14.4-15.5)	16.3 (16.2-16.4)	17.3 (17.2-17.4)	18.3 (18.2-18.3)	19.1 (19-19.2)	20.3 (20.2-20.4)	21 (20.9-21.1)	21.7 (21.6-21.8)	22.3 (21.6-23)
Niigata	12.4 (11.4-13.4)	13.5 (12.5-14.7)	13.2 (12.3-14.4)	13.5 (12.6-14.6)	14.1 (13.2-15)	15 (14.5-15.6)	16.2 (16.1-16.3)	17.6 (17.5-17.6)	18.8 (18.7-18.9)	19.5 (19.4-19.5)	20.6 (20.6-20.7)	21.3 (21.2-21.4)	21.9 (21.8-22)	22.4 (21.7-23.1)
Ōita	11.9 (11-12.9)	13 (12.1-14.1)	13.1 (12.2-14.2)	13.6 (12.7-14.7)	14.4 (13.6-15.3)	15.6 (15-16.1)	16.6 (16.5-16.7)	17.7 (17.6-17.7)	18.7 (18.6-18.8)	19.5 (19.4-19.6)	20.4 (20.3-20.5)	21.4 (21.3-21.5)	21.9 (21.8-22)	22.8 (22.1-23.5)
Okayama	12.4 (11.5-13.5)	13.6 (12.5-14.8)	13.6 (12.6-14.7)	14.1 (13.1-15.1)	14.8 (13.9-15.8)	15.8 (15.3-16.4)	16.8 (16.8-16.9)	18 (17.9-18.1)	19.1 (19-19.2)	19.6 (19.5-19.7)	20.8 (20.7-20.9)	21.5 (21.4-21.6)	21.9 (21.8-22)	22.4 (21.8-23.1)
Okinawa	11.8 (11-12.9)	13 (12-14.1)	13.5 (12.5-14.5)	13.8 (12.8-14.9)	14.2 (13.3-15.1)	15 (14.5-15.5)	17.8 (17.7-17.9)	19.9 (19.8-20)	20.4 (20.3-20.5)	20.9 (20.8-21)	21.6 (21.5-21.7)	22.2 (22.1-22.2)	22.3 (22.2-22.4)	22.4 (21.7-23.1)
Ōsaka	11.7 (10.8-12.7)	12.8 (11.8-13.9)	12.6 (11.6-13.7)	13.1 (12.2-14.2)	13.9 (13-14.8)	15 (14.4-15.5)	15.9 (15.8-15.9)	16.9 (16.8-16.9)	17.8 (17.7-17.8)	18.5 (18.5-18.6)	19.7 (19.7-19.8)	20.5 (20.5-20.6)	21.1 (21-21.1)	21.9 (21.2-22.6)
Saga	12 (11.1-13)	13.2 (12.2-14.3)	13.3 (12.3-14.3)	13.8 (12.8-14.8)	14.5 (13.7-15.3)	15.6 (15.1-16)	16.6 (16.5-16.7)	17.8 (17.7-17.9)	18.8 (18.7-18.9)	19.3 (19.2-19.4)	20.4 (20.3-20.5)	21.3 (21.2-21.4)	21.6 (21.5-21.7)	22.2 (21.5-22.9)
Saitama	12.2 (11.3-13.2)	13.4 (12.4-14.6)	13.1 (12.1-14.2)	13.6 (12.6-14.7)	14.4 (13.4-15.5)	15.5 (14.9-16.1)	16.2 (16.1-16.2)	17.4 (17.4-17.5)	18.3 (18.2-18.4)	18.9 (18.9-19)	20.1 (20-20.1)	20.7 (20.6-20.8)	21.3 (21.2-21.3)	21.9 (21.2-22.5)
Shiga	12.4 (11.4-13.5)	13.5 (12.5-14.6)	13.2 (12.2-14.2)	13.5 (12.6-14.4)	14.1 (13.3-14.8)	15.4 (15-15.8)	17.1 (17-17.2)	17.8 (17.7-17.9)	18.4 (18.3-18.5)	19.3 (19.2-19.4)	20.3 (20.3-20.4)	21.1 (21-21.2)	21.8 (21.7-21.9)	22.4 (21.7-23.1)
Shimane	12.1 (11.2-13.1)	13.2 (12.2-14.2)	13.4 (12.5-14.4)	13.8 (12.9-14.7)	14.5 (13.7-15.3)	15.6 (15.2-16.1)	17 (16.9-17.1)	18.4 (18.3-18.5)	19.5 (19.4-19.6)	20.1 (20-20.2)	20.7 (20.6-20.8)	21.5 (21.4-21.6)	22.2 (22.1-22.3)	22.6 (21.9-23.3)
Shizuoka	12.4 (11.4-13.5)	13.6 (12.5-14.7)	13.5 (12.5-14.6)	14 (13-15.1)	14.7 (13.8-15.8)	15.8 (15.2-16.4)	16.8 (16.7-16.8)	18 (18-18.1)	18.9 (18.9-19)	19.5 (19.4-19.6)	20.5 (20.4-20.6)	21.3 (21.2-21.4)	21.6 (21.5-21.7)	22.2 (21.5-22.9)
Tochigi	11.9 (11-13)	13.1 (12-14.2)	13 (12-14)	13.5 (12.6-14.5)	14.3 (13.4-15.3)	15.3 (14.8-15.9)	16.2 (16.1-16.3)	17.2 (17.1-17.3)	18.1 (18-18.2)	18.7 (18.7-18.8)	20 (19.9-20.1)	20.4 (20.3-20.5)	21 (21-21.1)	21.6 (21-22.3)
Tokushima	11.8 (10.8-12.8)	12.9 (11.9-14)	12.9 (11.9-13.9)	13.3 (12.4-14.3)	14 (13.2-14.8)	15.2 (14.7-15.7)	16.3 (16.2-16.4)	17.6 (17.5-17.7)	18.5 (18.4-18.6)	19.3 (19.2-19.4)	20.4 (20.3-20.5)	21 (20.9-21.1)	21.6 (21.5-21.8)	21.8 (21.1-22.5)
Tōkyō	12.5 (11.5-13.6)	13.6 (12.6-14.8)	13.5 (12.6-14.6)	14 (13-15)	14.8 (13.9-15.8)	15.8 (15.3-16.4)	16.8 (16.7-16.8)	17.9 (17.8-17.9)	18.5 (18.5-18.5)	19.1 (19.1-19.1)	20.2 (20.2-20.3)	21.1 (21-21.1)	21.6 (21.6-21.7)	22.4 (21.8-23.1)
Tottori	12.2 (11.2-13.2)	13.3 (12.3-14.5)	13.6 (12.6-14.6)	14.1 (13.3-15)	14.8 (14.2-15.5)	15.9 (15.5-16.3)	16.9 (16.8-17)	17.8 (17.7-17.9)	18.8 (18.7-18.9)	19.5 (19.4-19.6)	20.5 (20.4-20.6)	21 (20.9-21.1)	21.7 (21.6-21.8)	22.2 (21.6-22.9)
Toyama	12.2 (11.3-13.3)	13.4 (12.4-14.4)	13.2 (12.3-14.3)	13.6 (12.7-14.6)	14.3 (13.5-15.1)	15.3 (14.9-15.7)	16.4 (16.4-16.5)	17.6 (17.5-17.7)	18.8 (18.7-18.9)	19.5 (19.4-19.6)	20.7 (20.6-20.8)	21.4 (21.3-21.5)	22 (21.8-22.1)	22.3 (21.7-23)
Wakayama	11.7 (10.8-12.7)	12.8 (11.8-13.8)	12.8 (11.9-13.7)	13.2 (12.3-14.1)	13.9 (13.1-14.7)	15 (14.6-15.5)	16.3 (16.2-16.4)	17.3 (17.2-17.4)	18.2 (18.1-18.3)	19 (18.9-19.1)	20.1 (20-20.2)	20.8 (20.7-20.9)	21.1 (21-21.2)	21.8 (21.1-22.5)
Yamagata	12.4 (11.4-13.5)	13.5 (12.5-14.7)	13.5 (12.5-14.7)	13.8 (12.8-14.9)	14.5 (13.6-15.3)	15.8 (15.3-16.3)	17.5 (17.4-17.6)	18.2 (18.1-18.3)	18.7 (18.6-18.8)	19.3 (19.2-19.4)	20.4 (20.3-20.5)	21.2 (21.1-21.3)	21.8 (21.7-21.9)	22.2 (21.4-22.9)
Yamaguchi	11.9 (11.1-12.9)	13.1 (12.1-14.3)	13.3 (12.3-14.5)	13.8 (12.8-14.9)	14.6 (13.6-15.6)	15.6 (15.1-16.2)	16.5 (16.4-16.6)	17.9 (17.9-18)	18.8 (18.7-18.9)	19.4 (19.3-19.5)	20.3 (20.2-20.4)	20.9 (20.8-21)	21.3 (21.2-21.5)	22.1 (21.4-22.8)
Yamanashi	12.2 (11.3-13.3)	13.4 (12.4-14.6)	13.6 (12.6-14.8)	14 (13.1-15.1)	14.7 (13.9-15.5)	15.9 (15.4-16.4)	16.7 (16.6-16.8)	17.9 (17.8-18)	18.9 (18.8-19)	19.8 (19.7-19.9)	20.8 (20.6-20.9)	21.3 (21.2-21.4)	21.8 (21.7-21.9)	22.3 (21.7-23)
South Korea	9.7 (9.1-10.4)	10.8 (10.1-11.6)	11.3 (10.5-12.1)	11.6 (10.8-12.5)	12.1 (11.4-12.9)	13 (12.8-13.2)	13.3 (13.3-13.3)	14 (14-14)	14.8 (14.8-14.9)	15.8 (15.8-15.9)	16.8 (16.7-16.8)	18.3 (18.2-18.3)	19.6 (19.6-19.7)	20.8 (20.4-21.1)
Singapore	11.5 (11-12.1)	12.3 (12.2-12.3)	12.3 (12.2-12.4)	12.6 (12.5-12.7)	12.8 (12.7-12.8)	13.1 (13-13.2)	14.1 (14-14.1)	15.4 (15.4-15.5)	16.5 (16.4-16.6)	17.1 (17.1-17.2)	18.4 (17.9-18.9)	19.8 (19.7-19.9)	21.4 (21.3-21.5)	22.5 (22.1-22.8)
High-income North America	14 (13.8-14.2)	14.5 (14.3-14.6)	14.5 (14.4-14.5)	14.8 (14.7-14.8)	15.2 (15.2-15.2)	16 (15.9-16)	16.5 (16.4-16.5)	16.8 (16.8-16.8)	17.4 (17.4-17.4)	17.6 (17.6-17.6)	17.9 (17.9-17.9)	18.6 (18.6-18.6)	19.4 (19.4-19.4)	19.6 (19.4-19.7)
Canada	14.1 (14.1-14.2)	14.6 (14.6-14.7)	14.8 (14.8-14.8)	15.1 (15.1-15.2)	15.6 (15.6-15.7)	16 (15.9-16)	16.8 (16.7-16.8)	17.1 (17.1-17.2)	17.7 (17.7-17.7)	18 (17.9-18)	18.7 (18.6-18.7)	19.5 (19.5-19.5)	20.4 (20.3-20.4)	20.9 (20.5-21.2)
Greenland	9.1 (9-9.3)	10.4 (10.3-10.6)	12.4 (12.3-12.6)	13.6 (13.4-13.8)	13.7 (13.6-13.9)	13 (12.8-13.1)	12.7 (12.5-12.8)	12.7 (12.5-12.8)	13.1 (12.9-13.2)	13.4 (13.2-13.6)	13.9 (13.7-14.1)	14.6 (14.5-14.8)	15.4 (15.2-15.6)	16 (15.7-16.6)

Table 9 Estimates of life expectancy at age 65 by location and year for 1950, 1955...2005, 2010, 2017 - both sexes

Location	1950	1955	1960	1965	1970	1975	1980	1985	1990	1995	2000	2005	2010	2017
USA	14 (13.8-14.2)	14.4 (14.3-14.6)	14.4 (14.4-14.4)	14.7 (14.7-14.7)	15.1 (15.1-15.1)	16 (15.9-16)	16.4 (16.4-16.4)	16.8 (16.8-16.8)	17.4 (17.4-17.4)	17.5 (17.5-17.5)	17.8 (17.8-17.8)	18.5 (18.5-18.5)	19.3 (19.2-19.3)	19.4 (19.3-19.5)
Alabama	13.5 (12.5-14.6)	14 (13.2-15)	14.5 (14.4-14.6)	14.5 (14.5-14.6)	14.9 (14.9-15)	15.8 (15.7-15.8)	16.1 (16-16.2)	16.3 (16.3-16.4)	16.7 (16.7-16.8)	16.9 (16.8-16.9)	16.8 (16.7-16.9)	17.1 (17.1-17.2)	17.6 (17.5-17.7)	17.7 (17-18.5)
Alaska	13.9 (12.9-15)	14.4 (13.7-15.1)	13.8 (13.7-13.9)	14 (13.9-14.1)	14.7 (14.6-14.8)	15.2 (15.1-15.3)	15.4 (15.3-15.5)	15.7 (15.6-15.8)	16.5 (16.4-16.6)	16.8 (16.7-16.9)	17.5 (17.4-17.6)	18.3 (18.2-18.4)	19 (18.9-19.1)	19.1 (18.7-19.6)
Arizona	14.4 (13.4-15.6)	14.9 (14-16)	15.1 (15-15.2)	15.3 (15.2-15.4)	15.6 (15.6-15.7)	16.8 (16.7-16.9)	17 (16.9-17.1)	17.7 (17.6-17.8)	18.2 (18.1-18.3)	18.2 (18.1-18.2)	18.5 (18.5-18.6)	19.1 (19-19.1)	20.1 (20-20.2)	20.1 (19.4-20.8)
Arkansas	14.5 (13.4-15.7)	15 (14.1-16)	15.2 (15.1-15.3)	15.3 (15.2-15.4)	15.6 (15.5-15.7)	16 (15.9-16)	16.5 (16.4-16.6)	16.6 (16.5-16.7)	17 (16.9-17.1)	17 (16.9-17.1)	17 (17-17.1)	17.8 (17.7-17.9)	18.1 (18-18.2)	17.9 (17.1-18.6)
California	14.6 (13.5-15.7)	15.1 (14.2-16.1)	14.9 (14.9-15)	15.2 (15.2-15.3)	15.7 (15.6-15.7)	16.4 (16.4-16.4)	16.8 (16.8-16.8)	17.1 (17.1-17.1)	17.8 (17.7-17.8)	18.2 (18.1-18.2)	18.5 (18.5-18.6)	19.2 (19.2-19.3)	20.4 (20.3-20.4)	20.7 (20.1-21.4)
Colorado	14.5 (13.4-15.7)	14.9 (14-16)	15.2 (15.1-15.3)	15.6 (15.5-15.7)	15.9 (15.8-16)	16.7 (16.6-16.8)	17.1 (17-17.1)	17.5 (17.4-17.6)	18 (17.9-18.1)	18 (17.9-18.1)	18.3 (18.3-18.4)	19 (19-19.1)	19.8 (19.7-19.9)	20 (19.4-20.8)
Connecticut	14.2 (13.1-15.4)	14.8 (13.7-15.8)	14.7 (14.6-14.8)	14.7 (14.6-14.8)	15.5 (15.4-15.6)	16.3 (16.2-16.4)	16.7 (16.6-16.7)	17.1 (17-17.2)	18.1 (18-18.2)	18.2 (18.1-18.3)	18.5 (18.4-18.6)	19.4 (19.3-19.5)	20.2 (20.1-20.3)	20.4 (19.7-21.2)
Delaware	13.5 (12.5-14.5)	13.8 (13.2-14.5)	13.8 (13.7-14)	14.1 (14-14.1)	14.4 (14.3-14.5)	15.3 (15.2-15.4)	15.9 (15.8-16)	16.3 (16.2-16.4)	16.7 (16.6-16.8)	17 (16.9-17.1)	17.6 (17.4-17.7)	18.3 (18.2-18.4)	19.1 (19-19.2)	19.4 (18.9-19.9)
Washington, DC	14.3 (13.3-15.4)	14.5 (13.7-15.3)	14.2 (14.1-14.3)	14.3 (14.2-14.4)	14.7 (14.6-14.8)	15.5 (15.4-15.6)	15.9 (15.8-16)	16.2 (16.1-16.3)	16.2 (16.1-16.3)	15.4 (15.3-15.5)	17.5 (17.4-17.6)	18 (17.8-18.1)	19.2 (19-19.3)	19.4 (18.8-20.1)
Florida	15.1 (14.1-16.2)	15.7 (14.7-16.8)	15.7 (15.7-15.8)	16.1 (16.1-16.2)	16.3 (16.2-16.3)	16.9 (16.9-17)	17.4 (17.3-17.4)	17.9 (17.8-17.9)	18.6 (18.5-18.6)	18.6 (18.6-18.7)	18.9 (18.8-18.9)	19.6 (19.5-19.6)	20.4 (20.3-20.4)	20.3 (19.6-21)
Georgia	13.5 (12.4-14.7)	14.2 (13.2-15.3)	14.4 (14.4-14.5)	14.8 (14.7-14.8)	14.9 (14.8-14.9)	15.5 (15.4-15.5)	16 (16-16.1)	16.1 (16-16.1)	16.7 (16.6-16.7)	16.6 (16.6-16.7)	16.7 (16.7-16.8)	17.5 (17.4-17.6)	18.1 (18.1-18.2)	18.5 (17.8-19.2)
Hawaii	15 (14-16.1)	15.2 (14.3-16.1)	15.4 (15.3-15.5)	16 (15.9-16.1)	16.5 (16.4-16.6)	17.7 (17.6-17.8)	18.3 (18.1-18.4)	18.6 (18.5-18.7)	19.3 (19.2-19.4)	19.7 (19.6-19.8)	20 (19.8-20.1)	20.7 (20.5-20.8)	21.3 (21.2-21.4)	21.5 (20.9-22.2)
Idaho	14.7 (13.7-15.9)	15.2 (14.3-16)	15.2 (15-15.3)	15.3 (15.2-15.4)	15.7 (15.6-15.8)	16.3 (16.2-16.4)	17.1 (17-17.2)	17.4 (17.2-17.5)	17.9 (17.7-18)	18 (17.9-18.1)	18.1 (18-18.2)	18.5 (18.4-18.6)	19.2 (19.1-19.3)	19.4 (18.8-20)
Illinois	13.7 (12.7-14.8)	14.2 (13.4-15.1)	14 (14-14.1)	14.4 (14.3-14.4)	14.7 (14.7-14.8)	15.6 (15.6-15.7)	16.2 (16.2-16.2)	16.6 (16.5-16.6)	17.1 (17.1-17.2)	17.2 (17.2-17.3)	17.7 (17.6-17.7)	18.4 (18.3-18.4)	19.3 (19.2-19.3)	19.5 (18.7-20.2)
Indiana	13.9 (12.9-15.1)	14.5 (13.6-15.5)	14.3 (14.3-14.4)	14.6 (14.5-14.6)	14.9 (14.8-14.9)	15.6 (15.6-15.7)	16.1 (16.1-16.2)	16.4 (16.4-16.5)	17 (16.9-17.1)	16.9 (16.8-17)	17.1 (17.1-17.2)	17.8 (17.8-17.9)	18.4 (18.3-18.5)	18.4 (17.7-19.2)
Iowa	14.8 (13.6-16)	15.1 (14.1-16.1)	15.2 (15.1-15.2)	15.3 (15.2-15.4)	15.7 (15.6-15.8)	16.4 (16.3-16.5)	17.2 (17.1-17.3)	17.4 (17.3-17.5)	18.3 (18.2-18.4)	18.2 (18.1-18.3)	18.4 (18.3-18.5)	19 (18.9-19.1)	19.4 (19.3-19.5)	19.5 (18.8-20.3)
Kansas	15 (13.9-16.2)	15.5 (14.5-16.6)	15.3 (15.2-15.4)	15.6 (15.5-15.7)	16 (15.9-16.1)	16.5 (16.4-16.6)	17.2 (17.1-17.3)	17.4 (17.3-17.5)	18.2 (18.1-18.3)	17.9 (17.8-18)	18.1 (18-18.2)	18.5 (18.4-18.6)	19.1 (19-19.1)	19 (18.2-19.7)
Kentucky	14 (12.9-15.1)	14.6 (13.6-15.5)	14.6 (14.5-14.6)	14.8 (14.7-14.9)	14.8 (14.8-14.9)	15.4 (15.3-15.5)	15.9 (15.9-16)	16.1 (16.1-16.2)	16.6 (16.5-16.7)	16.6 (16.5-16.7)	16.6 (16.6-16.7)	17.2 (17.1-17.3)	17.6 (17.5-17.6)	17.5 (16.8-18.2)
Louisiana	13.5 (12.5-14.9)	14.2 (13.2-15.2)	14.2 (14.1-14.3)	14.3 (14.2-14.4)	14.6 (14.5-14.7)	15.3 (15.2-15.3)	15.8 (15.7-15.9)	16.1 (16-16.1)	16.5 (16.5-16.6)	16.7 (16.6-16.8)	16.8 (16.7-16.8)	16.7 (16.7-16.8)	17.9 (17.9-18)	18.1 (17.4-18.8)
Maine	13.7 (12.7-14.9)	14.1 (13.3-15.1)	14.2 (14.1-14.3)	14.6 (14.5-14.7)	14.8 (14.7-14.9)	15.8 (15.7-15.9)	16.3 (16.2-16.5)	16.5 (16.4-16.6)	17.3 (17.1-17.4)	17.4 (17.3-17.5)	17.6 (17.5-17.7)	18.1 (18-18.2)	18.9 (18.8-19.1)	18.7 (18.1-19.4)
Maryland	13.3 (12.3-14.4)	13.8 (12.9-14.8)	13.7 (13.6-13.8)	14.1 (14-14.1)	14.6 (14.6-14.7)	15.6 (15.5-15.6)	16 (15.9-16.1)	16.1 (16.1-16.2)	16.9 (16.8-16.9)	17.1 (17-17.2)	17.4 (17.3-17.5)	18.3 (18.3-18.4)	19.3 (19.3-19.4)	19.6 (18.9-20.3)
Massachusetts	14 (12.9-15.1)	14.4 (13.4-15.4)	14.2 (14.1-14.2)	14.6 (14.5-14.6)	15.2 (15.2-15.3)	16.1 (16-16.2)	16.6 (16.5-16.6)	16.8 (16.8-16.9)	17.7 (17.7-17.8)	17.9 (17.8-18)	18.1 (18.1-18.2)	19 (18.9-19.1)	19.9 (19.8-20)	19.9 (19.2-20.6)
Michigan	13.8 (12.8-14.9)	14.3 (13.3-15.2)	14.1 (14.1-14.2)	14.3 (14.3-14.4)	14.9 (14.8-14.9)	15.6 (15.6-15.7)	16.2 (16.1-16.2)	16.3 (16.3-16.4)	17.1 (17-17.1)	17.2 (17.1-17.2)	17.5 (17.4-17.5)	18.2 (18.1-18.2)	18.8 (18.8-18.9)	18.9 (18.1-19.6)
Minnesota	14.6 (13.5-15.9)	15.1 (14.1-16.1)	15 (14.9-15.1)	15.4 (15.3-15.4)	15.9 (15.8-16)	16.7 (16.6-16.8)	17.3 (17.2-17.4)	17.5 (17.5-17.6)	18.1 (18.1-18.2)	18.2 (18.1-18.3)	18.6 (18.5-18.7)	19.5 (19.4-19.5)	20 (20-20.1)	20.3 (19.5-21)
Mississippi	13.8 (12.8-15)	14.3 (13.3-15.3)	14.5 (14.5-14.6)	14.6 (14.5-14.7)	14.8 (14.7-14.9)	15.5 (15.4-15.6)	16.1 (16-16.1)	16.3 (16.2-16.4)	16.7 (16.6-16.8)	16.7 (16.6-16.8)	16.6 (16.5-16.6)	17 (16.9-17.1)	17.5 (17.5-17.6)	17.6 (16.9-18.4)
Missouri	14.2 (13.1-15.3)	14.7 (13.8-15.7)	14.6 (14.5-14.7)	14.8 (14.8-14.9)	15.1 (15-15.1)	15.9 (15.8-15.9)	16.3 (16.3-16.4)	16.7 (16.6-16.8)	17.2 (17.2-17.3)	17.1 (17-17.2)	17.3 (17.3-17.4)	17.9 (17.9-18)	18.6 (18.5-18.6)	18.6 (17.9-19.3)
Montana	14.1 (13.1-15.2)	14.4 (13.4-15.2)	14.5 (14.4-14.6)	14.8 (14.6-14.9)	15.4 (15.3-15.6)	16.2 (16-16.3)	16.6 (16.5-16.7)	17.1 (17-17.3)	17.8 (17.6-17.9)	17.7 (17.6-17.8)	17.9 (17.8-18.1)	18.6 (18.4-18.7)	19.3 (19.2-19.4)	18.5 (17.9-19.1)
Nebraska	15.2 (14.1-16.3)	15.6 (14.7-16.5)	15.2 (15.1-15.3)	15.5 (15.4-15.5)	15.9 (15.8-16)	16.6 (16.5-16.7)	17.1 (17.1-17.3)	17.3 (17.2-17.4)	17.8 (17.7-17.9)	18 (17.9-18.1)	18.3 (18.2-18.5)	19 (18.9-19.1)	19.4 (19.3-19.5)	19.4 (18.7-20)
Nevada	13.1 (12.1-14.2)	13.6 (12.8-14.5)	13.9 (13.8-14)	13.7 (13.6-13.8)	14.5 (14.3-14.6)	15.5 (15.4-15.6)	15.8 (15.7-15.9)	16.2 (16.1-16.3)	16.5 (16.4-16.6)	16.4 (16.3-16.5)	16.9 (16.8-17)	17.3 (17.2-17.3)	18.6 (18.5-18.7)	18.9 (18.3-19.5)
New Hampshire	13.8 (12.8-14.9)	14.3 (13.5-15.1)	14.2 (14.1-14.4)	14.4 (14.3-14.5)	14.8 (14.7-14.9)	15.6 (15.5-15.7)	16.6 (16.5-16.8)	16.8 (16.6-16.9)	17.2 (17.1-17.3)	17.5 (17.4-17.6)	17.8 (17.6-17.9)	18.4 (18.3-18.5)	19.4 (19.3-19.5)	19.2 (18.6-19.8)
New Jersey	13.5 (12.5-14.6)	13.9 (13-14.9)	13.9 (13.9-14)	14.2 (14.2-14.3)	14.8 (14.7-14.8)	15.7 (15.7-15.8)	16.1 (16-16.1)	16.4 (16.3-16.4)	17.3 (17.2-17.3)	17.5 (17.4-17.5)	17.9 (17.8-18)	18.8 (18.8-18.9)	19.8 (19.7-19.8)	20.3 (19.6-21.1)
New Mexico	14.6 (13.5-15.7)	15 (14.2-15.9)	15.1 (15-15.2)	15.1 (15-15.2)	15.6 (15.5-15.7)	16.4 (16.3-16.5)	17.1 (17-17.2)	17.5 (17.4-17.6)	18 (17.9-18.1)	18 (17.9-18.1)	18.4 (18.3-18.5)	19 (18.9-19.1)	19.6 (19.5-19.7)	19.7 (19.1-20.3)
New York	13.7 (12.7-14.8)	14 (13.2-14.9)	13.9 (13.8-13.9)	14.3 (14.3-14.3)	14.8 (14.7-14.8)	15.9 (15.8-15.9)	16.2 (16.2-16.2)	16.6 (16.5-16.6)	17.5 (17.4-17.5)	17.8 (17.8-17.9)	18.4 (18.4-18.4)	19.2 (19.1-19.2)	20.1 (20.1-20.2)	20.6 (19.9-21.3)
North Carolina	13.8 (12.7-14.9)	14.4 (13.5-15.4)	14.3 (14.2-14.3)	14.5 (14.5-14.6)	15 (14.9-15)	15.8 (15.7-15.8)	16.3 (16.3-16.4)	16.5 (16.4-16.5)	17 (16.9-17)	17 (16.9-17.1)	17.1 (17-17.1)	17.9 (17.8-17.9)	18.6 (18.5-18.6)	18.9 (18.2-19.6)
North Dakota	14.9 (13.9-15.8)	15.2 (14.5-15.8)	15.1 (15-15.2)	15.3 (15.2-15.4)	15.8 (15.7-15.9)	16.4 (16.3-16.5)	17.2 (17.1-17.3)	17.8 (17.7-17.9)	18.3 (18.2-18.4)	18.4 (18.3-18.5)	18.7 (18.6-18.8)	19.5 (19.3-19.6)	19.8 (19.7-20)	19.5 (19-20)
Ohio	13.7 (12.8-14.8)	14.2 (13.3-15.1)	14.2 (14.2-14.3)	14.4 (14.3-14.4)	14.8 (14.7-14.8)	15.5 (15.5-15.6)	15.9 (15.8-15.9)	16.3 (16.3-16.4)	16.9 (16.9-17)	16.9 (16.9-17)	17.2 (17.2-17.3)	17.8 (17.7-17.8)	18.5 (18.5-18.6)	18.2 (17.5-19)
Oklahoma	14.7 (13.5-15.9)	15.3 (14.4-16.4)	15.3 (15.2-15.4)	15.6 (15.5-15.7)	15.5 (15.4-15.6)	16.1 (16.1-16.2)	16.6 (16.5-16.7)	16.8 (16.7-16.9)	17.1 (17.1-17.2)	17.1 (17-17.1)	16.9 (16.8-17)	17.2 (17.1-17.3)	17.8 (17.7-17.9)	17.9 (17.2-18.7)
Oregon	14.5 (13.4-15.7)	15 (14-16.1)	14.9 (14.8-15)	15.2 (15.2-15.3)	15.7 (15.6-15.8)	16.5 (16.5-16.6)	16.9 (16.8-17)	17.1 (17-17.2)	17.6 (17.5-17.7)	17.6 (17.5-17.7)	18 (17.9-18.1)	18.6 (18.5-18.7)	19.3 (19.2-19.4)	19.5 (18.8-20.3)
Pennsylvania	13.2 (12.2-14.3)	13.6 (12.8-14.6)	13.7 (13.7-13.8)	14 (13.9-14)	14.5 (14.5-14.5)	15.4 (15.4-15.4)	15.8 (15.8-15.9)	16.3 (16.2-16.3)	17 (17-17.1)	17.3 (17.2-17.3)	17.6 (17.6-17.7)	18.2 (18.2-18.3)	19 (19-19.1)	19 (18.3-19.7)
Rhode Island	13.7 (12.7-14.8)	14.1 (13.3-15)	14 (13.9-14.1)	14.2 (14.1-14.3)	15 (14.9-15.1)	16.1 (16-16.2)	16.6 (16.5-16.7)	17 (16.8-17.1)	17.6 (17.5-17.7)	18.1 (18-18.2)	18.2 (18.1-18.3)	18.6 (18.5-18.8)	19.5 (19.4-19.6)	19.6 (19-20.3)
South Carolina	12.9 (11.9-14)	13.6 (12.6-14.6)	13.8 (13.7-13.9)	14.4 (14.3-14.5)	14.6 (14.6-14.7)	15.4 (15.4-15.5)	15.9 (15.8-16)	16.3 (16.2-16.3)	16.8 (16.7-16.8)	16.8 (16.7-16.9)	17 (16.9-17)	17.7 (17.7-17.8)	18.3 (18.2-18.4)	18.3 (17.6-19)
South Dakota	14.9 (14-15.9)	15.3 (14.6-15.9)	15.1 (15-15.2)	15.4 (15.3-15.5)	15.9 (15.7-16)	16.6 (16.5-16.7)	17.2 (17.1-17.3)	17.5 (17.4-17.7)	18.2 (18-18.3)	18.2 (18.1-18.4)	18.6 (18.5-18.7)	19.2 (19.1-19.3)	19.7 (19.6-19.8)	19.4 (18.9-19.9)
Tennessee	14 (13-15.1)	14.5 (13.5-15.5)	14.5 (14.5-14.6)	14.8 (14.8-14.9)	15 (14.9-15)	15.7 (15.6-15.8)	16.3 (16.2-16.3)	16.5 (16.5-16.6)	16.7 (16.6-16.8)	16.7 (16.6-16.7)	16.8 (16.8-16.9)	17.3 (17.3-17.4)	17.9 (17.9-18)	18.1 (17.4-18.8)
Texas	14.6 (13.5-15.7)	15.1 (14.1-16.1)	15.2 (15.1-15.2)	15.5 (15.4-15.5)	15.6 (15.5-15.6)	16.2 (16.1-16.2)	16.6 (16.5-16.6)	16.8 (16.7-16.8)	17.3 (17.3-17.3)	17.4 (17.4-17.4)	17.5 (17.5-17.5)	18.2 (18.2-18.3)	18.9 (18.8-18.9)	19.2 (18.6-19.9)
Utah	14.7 (13.6-15.9)	15.2 (14.3-16.1)	15 (14.9-15.2)	15.1 (15-15.2)	15.8 (15.7-16)	16.5 (16.4-16.6)	17.1 (17-17.2)	17.3 (17.2-17.4)	18.2 (18.1-18.3)	18.3 (18.2-18.4)	18.4 (18.3-18.5)	19 (18.9-19)	19.7 (19.6-19.7)	19.5 (18.9-20.1)
Vermont	14 (13.2-14.8)	14.5 (13.9-14.9)	14.3 (14.2-14.4)	14.4 (14.3-14.5)	15 (14.9-15.1)	15.8 (15.7-15.9)	16.5 (16.4-16.6)	16.5 (16.4-16.6)	17.3 (17.2-17.4)	17.6 (17.4-17.7)	18 (17.9-18.1)	18.8 (18.7-18.9)	19.4 (19.3-19.5)	19.8 (19.4-20.3)
Virginia	13.4 (12.4-14.6)	14 (13.1-15)	14.2 (14.1-14.2)	14.4 (14.3-14.5)	14.9 (14.9-15)	15.7 (15.6-15.7)	16 (16-16.1)	16.3 (16.3-16.4)	16.9 (16.9-17)	17 (16.9-17)	17.3 (17.3-17.4)	18.1 (18.1-18.2)	19 (19-19.1)	19.5 (18.8-20.2)
Washington	14.3 (13.2-15.5)	14.7 (13.7-15.7)	14.8 (14.8-14.9)	15.2 (15.2-15.3)	15.4 (15.4-15.5)	16.4 (16.3-16.4)	16.9 (16.8-17)	17.2 (17.1-17.2)	17.8 (17.7-17.9)	17.9 (17.9-18)	18.1 (18-18.2)	18.8 (18.7-18.9)	19.6 (19.5-19.6)	19.9 (19.2-20.6)
West Virginia	13.8 (12.7-15)	14.2 (13.3-15.2)	14.4 (14.3-14.5)	14.4 (14.3-14.5)	14.5 (14.4-14.6)	15.2 (15.1-15.3)	15.8 (15.7-15.9)	16.1 (16-16.2)	16.6 (16.5-16.8)	16.6 (16.5-16.7)	16.6 (16.5-16.7)	17.1 (17-17.2)	17.6 (17.5-17.7)	17.5 (16.8-18.3)
Wisconsin	14.3 (13.1-15.4)	14.7 (13.8-15.6)	14.5 (14.5-14.6)	14.9 (14.8-15)	15.5 (15.4-15.5)	16.2 (16.1-16.3)	16.8 (16.7-16.9)	17.2 (17.2-17.3)	17.6 (17.5-17.7)	17.8 (17.8-17.9)	18.1 (18-18.2)	18.8 (18.7-18.8)	19.4 (19.3-19.5)	19.7 (19-20.4)
Wyoming	14.7 (13.8-15.6)	15.1 (14.5-15.6)	14.9 (14.7-15)	15.1 (15-15.2)	15.3 (15.2-15.4)	15.6 (15.5-15.7)	16.5 (16.4-16.7)	16.8 (16.7-16.9)	17.6 (17.5-17.7)	17.6 (17.5-17.7)	17.8 (17.7-17.9)	18.4 (18.3-18.5)	19.1 (19-19.2)	19.2 (18.8-19.7)
Southern Latin America	12.8 (12.8-12.8)	13.2 (13.2-13.3)	13.8 (13.7-13.8)	14 (14-14)	14.1 (14.1-14.2)	14.8 (14.6-15)	15 (15-15.1)	15.5 (15.5-15.6)	15.9 (15.8-15.9)	16.5 (16.5-16.5)	16.9 (16.9-16.9)	17.3 (17.3-17.3)	17.7 (17.7-17.7)	18.1 (17.6-18.5)
Argentina	12.9 (12.8-12.9)	13.3 (13.3-13.4)	13.9 (13.9-13.9)	14.1 (14.1-14.1)	14.2 (14.2-14.2)	14.9 (14.6-15.2)	15.1 (15.1-15.1)	15.6 (15.6-15.6)	15.8 (15.8-15.9)	16.5 (16.4-16.5)	16.7 (16.6-16.7)	17 (17-17.1)	17.3 (17.3-17.3)	17.6 (16.9-18.3)
Chile	11.8 (11.8-11.9)	12.2 (12.1-12.2)	12.6 (12.6-12.6)	13.2 (13.2-13.2)	13.6 (13.6-13.7)	14.4 (14.3-14.4)	14.8 (14.7-14.8)	15.2 (15.2-15.3)	15.9 (15.9-16)	16.7 (16.7-16.8)	17.6 (17.6-17.7)	18.2 (18.2-18.3)	18.8 (18.7-18.8)	19.4 (18.7-20)
Uruguay	14.4 (14.3-14.5)	14.7 (14.7-14.8)	14.9 (14.9-15)	14.7 (14.6-14.8)	14.7 (14.6-14.8)	15.1 (15.1-15.2)	15.1 (15-15.2)	15.8 (15.8-15.9)	16 (15.9-16.1)	16.3 (16.2-16.4)	16.7 (16.6-16.8)	17.1 (17-17.2)	17.8 (17.7-17.9)	18 (17.3-18.7)
Western Europe	13.4 (13.2-13.7)	14 (13.9-14.1)	14.3 (14.3-14.4)	14.5 (14.4-14.5)	14.6 (14.5-14.6)	14.9 (14.9-14.9)	15.5 (15.5-15.5)	16.2 (16.1-16.2)	16.8 (16.8-16.8)	17.4 (17.4-17.5)	18.2 (18.2-18.2)	19.1 (19.1-19.1)	19.9 (19.8-19.9)	20.4 (20.2-20.5)
Andorra	15.7 (14.3-17.3)	15.9 (14.5-17.4)	16.3 (15.1-17.8)	16.6 (15.6-18)	17 (16.1-18.4)	17.5 (16.6-18.6)	17.9 (17-18.9)	18.2 (17.4-19)	18.5 (17.7-19.2)	19 (18.2-19.8)	20 (19.3-20.7)	20.5 (19.9-21)	20.7 (20.2-21.2)	20.9 (20.3-21.6)
Austria	12.9 (12.9-12.9)	13.3 (13.3-13.4)	13.6 (13.6-13.7)	13.7 (13.6-13.7)	13.6 (13.5-13.6)	14.2 (14.1-14.2)	14.9 (14.8-14.9)	15.6 (15.6-15.7)	16.6 (16.5-16.6)	17.2 (17.2-17.3)	18 (18-18.1)	19 (18.9-19)	19.6 (19.5-19.7)	20.2 (19.8-20.5)
Belgium	12.5 (12.5-12.6)	13.6 (13.5-13.6)	13.8 (13.8-13.8)	14 (13.9-14)	13.9 (13.9-14)	14.2 (14.2-14.3)	15 (14.9-15)	15.6 (15.5-15.6)	16.6 (16.5-16.6)	17.2 (17.1-17.2)	17.8 (17.8-17.9)	18.7 (18.6-18.7)	19.3 (19.3-19.4)	20.1 (19.7-20.4)
Cyprus	13.7 (12.8-14.7)	13.8 (12.9-14.8)	13.9 (13.1-14.7)	13.9 (13.2-14.6)	14 (13.5-14.5)	14.3 (14.2-14.5)	14.8 (14.8-14.9)	15.4 (15.3-15.5)	16.2 (16.1-16.3)	16.7 (16.4-16.9)	16.4 (16.1-16.7)	17.2 (16.9-17.5)	18.8 (18.5-19.1)	19.9 (19.4-20.4)
Denmark	14 (13.9-14.1)	14.6 (14.5-14.7)	14.6 (14.5-14.6)	14.5 (14.5-14.6)	15.3 (15.2-15.4)	15.6 (15.6-15.7)	15.6 (15.6-15.7)	15.9 (15.9-16)	16.1 (16-16.1)	16.1 (16-16.1)	16.9 (16.9-17)	17.8 (17.7-17.8)	18.3 (18.3-18.4)	19.4 (19-19.7)
Finland	12 (12-12.1)	13 (12.9-13)	13.4 (13.4-13.5)	13.4 (13.3-13.4)	13.5 (13.4-13.5)	14.4 (14.4-14.5)	15.3 (15.2-15.4)	15.8 (15.7-15.8)	16.2 (16.2-16.3)	17 (16.9-17.1)	17.8 (17.7-17.9)	19 (18.9-19.1)	19.6 (19.5-19.7)	20.1 (19.7-20.4)
France	13.4 (13.4-13.5)	14 (13.9-14)	14.5 (14.5-14.6)	14.7 (14.7-14.7)	15.1 (15.1-15.1)	15.4 (15.4-15.5)	16.4 (16.4-16.4)	16.9 (16.9-16.9)	17.9 (17.9-17.9)	18.5 (18.5-18.5)	19.1 (19.1-19.2)	20 (20-20.1)	20.8 (20.8-20.8)	21.6 (21.3-21.9)
Germany	13.5 (12.6-14.6)	14 (13.8-14.3)	14.1 (14.1-14.1)	14.3 (14.2-14.3)	14 (14-14)	14.5 (14.5-14.5)	15 (14.9-15)	15.8 (15.8-15.9)	16.3 (16.3-16.3)	17.1 (17-17.1)	18 (18-18)	18.9 (18.9-19)	19.4 (19.3-19.4)	19.4 (18.8-20)
Greece	15.5 (15.1-15.8)	16.1 (16-16.1)	16 (16-16.1)	15.8 (15.8-15.9)	15.8 (15.8-15.9)	16.3 (16.2-16.3)	16.1 (16-16.1)	16.3 (16.2-16.3)	17.3 (17.3-17.4)	17.8 (17.7-17.8)	18.1 (18-18.2)	18.3 (18.3-18.4)	19.2 (19.2-19.3)	19.8 (19.5-20.2)
Iceland	14.8 (14.6-15)	15.2 (15.1-15.4)	15.8 (15.7-16)	15.9 (15.8-16)	15.9 (15.7-16)	17 (16.9-17.2)	17.4 (17.2-17.5)	17.6 (17.4-17.8)	17.6 (17.4-17.8)	17.9 (17.7-18)	18.8 (18.7-18.9)	19.7 (19.6-19.9)	20.4 (20.2-20.5)	20.7 (20.4-20.9)
Ireland	12.9 (12.9-13)	13.4 (13.3-13.4)	13.7 (13.6-13.7)	13.7 (13.6-13.8)	13.7 (13.6-13.7)	13.8 (13.7-13.9)	14.1 (14-14.2)	14.5 (14.4-14.5)	15.2 (15.1-15.2)	15.5 (15.4-15.6)	16.3 (16.2-16.4)	18 (18-18.1)	19.4 (19.3-19.5)	20 (19.6-20.3)
Israel	16.8 (16.6-16.9)	14.8 (14.7-14.9)	15 (14.9-15.1)	14.4 (14.3-14.5)	14.5 (14.4-14.5)	14.7 (14.6-14.7)	15.2 (15.1-15.3)	16 (15.9-16.1)	17.3 (17.2-17.3)	17.3 (17.2-17.3)	17.9 (17.9-18)	18.9 (18.9-19)	20.1 (20-20.2)	21 (20.6-21.3)
Italy	13.5 (13.5-13.6)	14.3 (14.3-14.3)	14.8 (14.8-14.8)	14.8 (14.7-14.8)	14.9 (14.9-15)	14.9 (14.9-15)	15.4 (15.4-15.5)	16.3 (16.3-16.3)	17.3 (17.2-17.3)	17.9 (17.9-17.9)	18.6 (18.6-18.7)	19.6 (19.5-19.6)	20.2 (20.2-20.3)	21 (20.7-21.3)
Luxembourg	12.8 (12.7-12.9)	13 (12.9-13.1)	13.5 (13.4-13.6)	13.5 (13.4-13.6)	13.5 (13.4-13.6)	13.7 (13.6-13.8)	14.6 (14.5-14.7)	15.2 (15.1-15.3)	16.3 (16.2-16.4)	16.8 (16.7-16.9)	17.6 (17.5-17.7)	18.5 (18.3-18.6)	19.3 (19.2-19.5)	19.9 (19.4-20.4)
Malta	12.6 (12.4-12.7)	13 (12.9-13.1)	12.8 (12.7-12.9)	12.9 (12.8-13)	13 (12.9-13.1)	13.6 (13.5-13.7)	13.5 (13.4-13.6)	14.7 (14.6-14.9)	16.1 (16-16.2)	16.8 (16.7-16.9)	17.3 (17.2-17.4)	17.9 (17.8-18.1)	18.9 (18.7-19)	19.3 (19.1-19.6)
Netherlands	14.3 (14.2-14.3)	14.5 (14.5-14.6)	14.9 (14.9-15)	15 (15-15.1)	15 (15-15)	15.5 (15.4-15.5)	16.3 (16.2-16.3)	16.5 (16.5-16.6)	16.9 (16.8-16.9)	17.1 (17-17.1)	17.4 (17.4-17.5)	18.4 (18.3-18.4)	19.3 (19.2-19.3)	19.7 (19.4-20)
Norway	15.4 (15.2-15.6)	15.6 (15.4-15.8)	15.5 (15.3-15.7)	15.5 (15.3-15.6)	15.6 (15.5-15.6)	16 (16-16.1)	16.4 (16.4-16.5)	16.6 (16.5-16.6)	16.8 (16.8-16.9)	17.3 (17.3-17.4)	17.9 (17.9-18)	19 (19-19)	19.4 (19.4-19.5)	20.3 (20.2-20.4)
Portugal	13.5 (13.4-13.5)	13.6 (13.6-13.7)	14.3 (14.3-14.4)	14.2 (14.1-14.2)	14.2 (14.2-14.3)	13.9 (13.9-14)	14.9 (14.8-14.9)	15.4 (15.4-15.5)	15.8 (15.7-15.8)	16.4 (16.4-16.5)	17.2 (17.1-17.2)	18.5 (18.3-18.6)	19.3 (19.2-19.3)	20.1 (19.8-20.4)
Spain	13.3 (13.3-13.3)	14.2 (14.2-14.2)	15 (14.9-15)	14.9 (14.9-15)	15 (15-15.1)	15.4 (15.4-15.5)	16.5 (16.5-16.5)	17 (16.9-17)	17.5 (17.5-17.5)	18.2 (18.2-18.2)	18.9 (18.8-18.9)	19.5 (19.5-19.5)	20.5 (20.5-20.5)	21.2 (20.9-21.5)
Sweden	14.1 (13.9-14.3)	14.7 (14.4-14.9)	14.8 (14.5-15.1)	15.2 (15-15.5)	15.7 (15.5-16)	15.8 (15.7-16)	16.3 (16.2-16.3)	16.9 (16.8-16.9)	17.4 (17.3-17.4)	18 (17.9-18)	18.5 (18.4-18.5)	19.1 (19-19.1)	19.7 (19.6-19.7)	20.4 (20.1-20.7)
Stockholm	14 (12.9-15.2)	14.6 (13.4-16)	14.7 (13.4-16.1)	15.2 (14-16.5)	15.6 (14.6-16.8)	15.8 (15-16.7)	16.2 (16.1-16.3)	16.9 (16.7-17)	17.3 (17.1-17.4)	18.1 (18-18.2)	18.4 (18.3-18.6)	19.2 (19.1-19.3)	19.9 (19.8-20.1)	20.4 (20-20.8)
Sweden except Stockholm	14.1 (13.9-14.3)	14.7 (14.5-14.9)	14.8 (14.6-15)	15.2 (15-15.5)	15.7 (15.5-15.9)	15.8 (15.7-16)	16.3 (16.2-16.3)	16.9 (16.8-16.9)	17.4 (17.3-17.5)	18 (17.9-18)	18.5 (18.4-18.5)	19.1 (19-19.1)	19.6 (19.6-19.7)	20.4 (20.1-20.8)
Switzerland	13.6 (13.5-13.7)	13.8 (13.8-13.9)	14.5 (14.5-14.6)	14.7 (14.6-14.8)	15.1 (15.1-15.2)	16 (15.9-16.1)	16.6 (16.5-16.7)	17.4 (17.3-17.4)	17.9 (17.8-18)	18.5 (18.5-18.6)	19.2 (19.2-19.3)	20.1 (20.1-20.2)	20.7 (20.6-20.8)	21.6 (21.3-22)
United Kingdom	13.1 (12.9-13.2)	13.5 (13.3-13.6)	13.9 (13.7-14)	14.1 (14-14.3)	14.3 (14.2-14.4)	14.6 (14.5-14.7)	15.1 (15.1-15.1)	15.6 (15.5-15.6)	16.1 (16.1-16.1)	16.6 (16.6-16.6)	17.5 (17.5-17.5)	18.5 (18.4-18.5)	19.4 (19.3-19.4)	19.7 (19.6-19.7)
England	13.2 (13.1-13.4)	13.6 (13.5-13.8)	14 (13.9-14.2)	14.3 (14.2-14.5)	14.5 (14.3-14.6)	14.8 (14.7-14.9)	15.2 (15.2-15.3)	15.7 (15.7-15.7)	16.2 (16.2-16.2)	16.7 (16.7-16.7)	17.6 (17.6-17.6)	18.6 (18.6-18.6)	19.5 (19.5-19.5)	19.8 (19.8-19.9)
East Midlands	13 (12.5-13.5)	13.4 (12.9-13.9)	13.8 (13.3-14.3)	14.1 (13.6-14.6)	14.2 (13.7-14.7)	14.5 (14.1-14.8)	15.1 (15-15.2)	15.7 (15.6-15.7)	16.2 (16.2-16.3)	16.6 (16.6-16.7)	17.5 (17.5-17.6)	18.4 (18.4-18.5)	19.3 (19.3-19.4)	19.7 (19.5-19.8)
Derby	12.9 (11.5-14.4)	13.3 (12-14.9)	13.7 (12.3-15.3)	13.9 (12.5-15.4)	14 (12.7-15.3)	14.3 (13.4-15.3)	14.6 (14.3-14.9)	15.3 (15.2-15.5)	15.8 (15.6-15.9)	16.5 (16.4-16.7)	17.7 (17.5-17.9)	18.3 (18.2-18.5)	18.6 (18.4-18.8)	19.2 (18.7-19.7)
Derbyshire	12.8 (11.5-14.2)	13.2 (11.9-14.6)	13.5 (12.2-15)	13.8 (12.5-15.3)	13.9 (12.6-15.3)	14.2 (13.2-15.3)	14.9 (14.6-15.1)	15.5 (15.4-15.6)	16 (15.8-16.1)	16.3 (16.2-16.5)	17.4 (17.2-17.6)	18.3 (18.2-18.4)	19.3 (19.2-19.4)	19.4 (19-19.8)
Leicester	12.6 (11.3-14.1)	13 (11.7-14.5)	13.4 (12-14.8)	13.6 (12.3-15.1)	13.7 (12.5-15.1)	14 (13.1-15.1)	14.8 (14.5-15.1)	15.5 (15.4-15.7)	16.1 (16-16.3)	16.1 (16-16.3)	16.7 (16.5-16.9)	17.5 (17.4-17.7)	18.6 (18.5-18.8)	19.3 (18.8-19.7)
Leicestershire	13.5 (12.2-15)	14 (12.6-15.5)	14.4 (12.9-15.8)	14.7 (13.3-16.2)	14.8 (13.5-16.2)	15.1 (14.1-16.1)	15.6 (15.4-15.9)	16.2 (16.1-16.4)	16.8 (16.7-16.9)	17.3 (17.2-17.4)	18.2 (18-18.4)	19 (18.9-19.2)	20 (19.9-20.2)	20.4 (20-20.8)
Lincolnshire	13 (11.8-14.5)	13.4 (12.2-14.8)	13.8 (12.4-15.2)	14 (12.7-15.6)	14.2 (12.9-15.5)	14.5 (13.6-15.5)	15.2 (15-15.5)	15.7 (15.6-15.8)	16.4 (16.2-16.5)	16.8 (16.7-16.9)	17.7 (17.5-17.9)	18.6 (18.5-18.8)	19.7 (19.5-19.8)	20 (19.6-20.4)
Northamptonshire	13.3 (12-14.9)	13.8 (12.4-15.4)	14.2 (12.9-15.8)	14.5 (13.3-16)	14.7 (13.5-16)	15 (14-16)	15.5 (15.2-15.8)	16 (15.9-16.1)	16.4 (16.3-16.6)	16.8 (16.6-16.9)	17.8 (17.6-18)	18.5 (18.4-18.6)	19.3 (19.2-19.4)	19.6 (19.2-20)
Nottingham	12.5 (11.2-14)	12.9 (11.5-14.3)	13.2 (11.9-14.6)	13.4 (12.1-14.8)	13.5 (12.2-14.9)	13.7 (12.7-14.8)	14.2 (13.9-14.5)	15 (14.8-15.1)	15.7 (15.5-15.8)	15.8 (15.6-15.9)	16.4 (16.2-16.6)	16.9 (16.8-17)	18.1 (18-18.2)	18.8 (18.3-19.2)
Nottinghamshire	13 (11.7-14.4)	13.5 (12.1-15)	13.9 (12.5-15.4)	14.2 (12.9-15.6)	14.3 (13.2-15.7)	14.6 (13.7-15.5)	15.1 (14.9-15.4)	15.8 (15.7-15.9)	16.1 (16-16.2)	16.6 (16.5-16.7)	17.5 (17.3-17.7)	18.5 (18.3-18.6)	19.2 (19.1-19.4)	19.5 (19.1-19.9)
Rutland	14.1 (12.6-15.7)	14.6 (13-16.1)	14.9 (13.4-16.5)	15.2 (13.7-16.7)	15.3 (14-16.8)	15.6 (14.6-16.8)	16.1 (15.6-16.6)	16.3 (15.9-16.6)	17.1 (16.8-17.4)	18 (17.6-18.3)	19.1 (18.7-19.5)	19.8 (19.5-20.1)	20.4 (20-20.7)	20.5 (19.8-21.1)
East of England	13.7 (13.2-14.2)	14.1 (13.5-14.7)	14.5 (13.9-15.1)	14.8 (14.3-15.4)	14.9 (14.5-15.4)	15.2 (14.9-15.6)	15.6 (15.5-15.7)	16.2 (16.2-16.3)	16.7 (16.7-16.7)	17.2 (17.2-17.2)	18.1 (18-18.2)	19.1 (19-19.1)	20 (19.9-20)	20.2 (20-20.4)
Bedford	13.6 (12.2-15.2)	14 (12.7-15.7)	14.5 (13-16.1)	14.8 (13.3-16.4)	15 (13.6-16.4)	15.3 (14.4-16.4)	15.9 (15.6-16.3)	16.4 (16.2-16.6)	16.5 (16.3-16.7)	16.9 (16.7-17.1)	17.9 (17.7-18.1)	18.8 (18.6-18.9)	19.3 (19.2-19.5)	19.9 (19.4-20.3)
Cambridgeshire	13.8 (12.4-15.4)	14.3 (12.9-15.9)	14.7 (13.3-16.3)	15.1 (13.8-16.6)	15.2 (14-16.5)	15.4 (14.5-16.4)	15.6 (15.3-15.8)	16.2 (16-16.3)	17 (16.8-17.1)	17.3 (17.1-17.4)	18.3 (18.2-18.5)	19.2 (19.1-19.4)	20.3 (20.2-20.4)	20.6 (20.2-21)
Central Bedfordshire	13.7 (12.1-15.3)	14.1 (12.7-15.8)	14.5 (13-16.2)	14.8 (13.3-16.4)	14.9 (13.6-16.3)	15.2 (14.2-16.2)	15.4 (15-15.7)	15.9 (15.7-16)	16.3 (16.1-16.4)	16.9 (16.7-17)	18 (17.8-18.2)	19 (18.8-19.1)	20.1 (19.9-20.3)	21 (20.5-21.4)
Essex	13.6 (12.3-15.1)	14 (12.7-15.5)	14.4 (13-15.9)	14.6 (13.3-16)	14.6 (13.4-15.9)	14.8 (13.9-15.7)	15.5 (15.3-15.7)	16.2 (16.1-16.3)	16.8 (16.6-16.9)	17.2 (17.1-17.3)	18 (17.9-18.2)	19 (18.9-19.1)	19.9 (19.8-20)	19.8 (19.5-20.2)
Hertfordshire	13.8 (12.4-15.3)	14.3 (12.8-15.8)	14.7 (13.2-16.3)	15 (13.6-16.5)	15 (13.8-16.4)	15.2 (14.2-16.2)	15.5 (15.3-15.8)	16.1 (16-16.2)	16.6 (16.5-16.7)	17.2 (17.1-17.3)	18 (17.8-18.2)	19 (18.9-19.1)	20 (19.8-20.1)	20.5 (20.1-20.9)

Table 9 Estimates of life expectancy at age 65 by location and year for 1950, 1955...2005, 2010, 2017 - both sexes

Location	1950	1955	1960	1965	1970	1975	1980	1985	1990	1995	2000	2005	2010	2017
Luton	13.3 (11.8-15)	13.7 (12.2-15.3)	14 (12.6-15.5)	14.2 (12.8-15.7)	14.3 (13-15.8)	14.6 (13.6-15.7)	14.9 (14.6-15.3)	16.1 (15.9-16.3)	16.1 (15.9-16.3)	16.3 (16.1-16.4)	17.2 (16.9-17.4)	17.9 (17.7-18.1)	19.1 (18.9-19.3)	20.2 (19.7-20.6)
Norfolk	13.8 (12.4-15.3)	14.3 (12.9-15.7)	14.7 (13.3-16.3)	15.2 (13.8-16.7)	15.4 (14.1-16.8)	15.7 (14.7-16.7)	15.9 (15.7-16.2)	16.5 (16.4-16.6)	16.7 (16.6-16.9)	17.5 (17.4-17.6)	18.4 (18.3-18.6)	19.4 (19.3-19.5)	20.2 (20.1-20.3)	20.2 (19.8-20.6)
Peterborough	13.2 (11.7-14.9)	13.6 (12.1-15.2)	14.1 (12.6-15.7)	14.3 (12.8-15.8)	14.4 (13.2-15.8)	14.7 (13.7-15.8)	15.3 (14.9-15.6)	15.8 (15.6-16)	16.2 (16-16.4)	16.4 (16.2-16.6)	17.4 (17.2-17.6)	18.2 (18-18.4)	18.8 (18.6-19)	19.4 (18.9-19.9)
Southend-on-Sea	13.5 (12.1-15.1)	13.9 (12.5-15.6)	14.3 (13-16.1)	14.6 (13.1-16.2)	14.9 (13.6-16.2)	15.3 (14.3-16.3)	15.7 (15.4-16.1)	16.3 (16.1-16.5)	16.7 (16.5-16.8)	17 (16.8-17.2)	17.6 (17.4-17.9)	18.3 (18.2-18.5)	19.2 (19-19.4)	19.4 (19-19.9)
Suffolk	13.7 (12.4-15.2)	14.2 (12.7-15.6)	14.6 (13.1-16.1)	15 (13.6-16.4)	15.1 (13.9-16.5)	15.4 (14.5-16.5)	15.7 (15.5-16)	16.3 (16.2-16.4)	17 (16.8-17.1)	17.4 (17.3-17.5)	18.4 (18.2-18.6)	19.4 (19.3-19.5)	20.2 (20.1-20.3)	20.5 (20-20.9)
Thurrock	13.1 (11.7-14.7)	13.5 (12-15)	13.8 (12.4-15.3)	14 (12.6-15.5)	14.1 (12.9-15.5)	14.4 (13.5-15.5)	14.9 (14.6-15.3)	15.5 (15.4-15.7)	16.1 (15.9-16.3)	16.6 (16.4-16.8)	17.8 (17.6-18.1)	18.4 (18.2-18.6)	19.4 (19.2-19.6)	19.8 (19.3-20.3)
Greater London	13.6 (13.4-13.9)	14.1 (13.8-14.3)	14.4 (14.2-14.7)	14.6 (14.4-14.9)	14.8 (14.5-15)	15.1 (14.9-15.3)	15.5 (15.5-15.6)	15.9 (15.9-15.9)	16.4 (16.4-16.5)	16.7 (16.7-16.7)	17.7 (17.7-17.8)	18.8 (18.8-18.9)	19.9 (19.8-19.9)	20.4 (20.3-20.5)
Barking and Dagenham	12.8 (11.5-14.3)	13.2 (11.9-14.6)	13.5 (12.3-14.9)	13.8 (12.5-15.3)	14 (12.9-15.3)	14.3 (13.4-15.3)	14.7 (14.4-15)	15.2 (15-15.3)	15.7 (15.5-15.8)	15.8 (15.7-16)	16.4 (16.2-16.6)	17.4 (17.2-17.6)	18.4 (18.2-18.6)	18.6 (18.1-19.1)
Barnet	14.4 (13.1-15.8)	14.9 (13.5-16.4)	15.3 (13.9-16.7)	15.6 (14.3-17.1)	15.8 (14.6-17.2)	16.1 (15.2-17.1)	16.3 (16-16.6)	16.6 (16.5-16.7)	17.2 (17.1-17.4)	17.7 (17.5-17.8)	18.8 (18.6-19.1)	19.9 (19.7-20)	20.5 (20.3-20.6)	21 (20.6-21.4)
Bexley	14 (12.6-15.4)	14.4 (13-15.8)	14.7 (13.4-16.2)	15 (13.8-16.5)	15.2 (14-16.5)	15.6 (14.7-16.6)	16.1 (15.8-16.4)	16.6 (16.4-16.7)	17.1 (16.9-17.2)	17.3 (17.2-17.5)	18.3 (18-18.5)	19.3 (19.1-19.5)	20.1 (19.9-20.2)	20.5 (20-21)
Brent	13.7 (12.3-15.3)	14.2 (12.8-15.7)	14.6 (13.2-16.1)	14.8 (13.5-16.3)	15 (13.7-16.3)	15.4 (14.5-16.4)	16 (15.7-16.3)	15.9 (15.8-16)	16.8 (16.7-17)	16.8 (16.6-16.9)	17.9 (17.7-18.1)	19.4 (19.3-19.5)	20.2 (20.1-20.3)	20.9 (20.5-21.4)
Bromley	14.3 (12.9-15.8)	14.7 (13.4-16.2)	15.1 (13.7-16.6)	15.4 (14.1-16.9)	15.6 (14.3-16.9)	15.9 (14.9-16.9)	16.3 (16-16.6)	16.6 (16.4-16.7)	17.2 (17-17.3)	17.6 (17.5-17.8)	18.7 (18.5-18.9)	20 (19.8-20.1)	20.7 (20.6-20.9)	20.9 (20.5-21.4)
Camden	13.5 (12.2-14.8)	13.8 (12.5-15.2)	14 (12.7-15.5)	14.1 (12.7-15.5)	14.1 (13-15.4)	14.3 (13.4-15.3)	14.7 (14.4-15)	15.4 (15.3-15.6)	15.7 (15.5-15.8)	16 (15.9-16.1)	16.8 (16.6-17)	17.8 (17.7-18)	19.7 (19.5-19.8)	22 (21.6-22.5)
Croydon	13.7 (12.4-15.2)	14.2 (12.8-15.7)	14.6 (13.3-16)	14.9 (13.6-16.4)	15.1 (13.9-16.4)	15.3 (14.4-16.2)	15.4 (15.2-15.7)	15.9 (15.7-16)	16.3 (16.2-16.4)	16.8 (16.7-16.9)	17.9 (17.7-18.1)	19.3 (19.1-19.4)	19.7 (19.5-19.8)	20 (19.5-20.4)
Ealing	13.4 (12-14.8)	13.8 (12.4-15.3)	14.2 (12.9-15.7)	14.4 (13.2-15.8)	14.5 (13.4-15.8)	14.8 (13.8-15.7)	15.2 (14.9-15.4)	15.7 (15.5-15.8)	16.3 (16.2-16.4)	16.5 (16.4-16.6)	17.8 (17.6-18)	19 (18.9-19.2)	20.2 (20-20.3)	20.5 (20.1-21)
Enfield	13.8 (12.5-15.3)	14.3 (12.9-15.8)	14.7 (13.4-16.3)	15.1 (13.7-16.6)	15.2 (14-16.6)	15.5 (14.6-16.5)	15.8 (15.5-16.1)	16 (15.9-16.2)	16.8 (16.6-16.9)	16.9 (16.8-17.1)	18 (17.8-18.2)	19.2 (19.1-19.3)	20.2 (20.1-20.4)	20.3 (19.9-20.8)
Greenwich	13.3 (12-14.7)	13.8 (12.4-15.3)	14.2 (12.8-15.8)	14.5 (13.2-16)	14.7 (13.6-16)	15 (14.1-16)	15.3 (15-15.6)	15.3 (15.2-15.5)	15.7 (15.6-15.9)	15.9 (15.7-16)	16.7 (16.5-16.9)	17.5 (17.4-17.7)	19.1 (19-19.3)	19.3 (18.8-19.7)
Hackney	13.5 (12.2-15)	13.9 (12.5-15.3)	14.1 (12.8-15.5)	14.1 (13-15.4)	14.1 (12.9-15.4)	14.2 (13.3-15.2)	14.7 (14.4-15)	15.1 (14.9-15.2)	15.5 (15.4-15.7)	16.1 (16-16.3)	17.5 (17.3-17.7)	17.7 (17.6-17.9)	18.7 (18.5-18.8)	19.4 (18.9-19.8)
Hammersmith and Fulham	13.1 (11.9-14.6)	13.5 (12.1-14.9)	13.8 (12.5-15.3)	13.9 (12.6-15.3)	14 (12.8-15.2)	14.1 (13.2-15)	14.5 (14.2-14.9)	14.8 (14.6-14.9)	15.2 (15.1-15.4)	15.4 (15.3-15.5)	17.1 (16.9-17.3)	18.4 (18.2-18.6)	19.5 (19.3-19.7)	20.2 (19.7-20.6)
Haringey	13.5 (12.1-14.9)	13.9 (12.5-15.3)	14.3 (13-15.7)	14.4 (13.2-15.9)	14.6 (13.4-16)	14.9 (14-15.9)	15.5 (15.2-15.8)	16 (15.8-16.1)	16.4 (16.2-16.5)	16.5 (16.4-16.7)	17.4 (17.2-17.6)	18.3 (18.1-18.4)	19.2 (19-19.3)	20.6 (20.2-21.1)
Harrow	14.3 (12.9-15.9)	14.7 (13.2-16.4)	15 (13.5-16.7)	15.2 (13.8-16.6)	15.3 (14.1-16.6)	15.7 (14.7-16.6)	16.1 (15.8-16.4)	17 (16.8-17.1)	17.7 (17.5-17.9)	18 (17.9-18.2)	19.2 (19-19.4)	20.4 (20.2-20.6)	21.6 (21.5-21.8)	22 (21.5-22.4)
Havering	13.9 (12.6-15.3)	14.3 (13-15.8)	14.7 (13.3-16.2)	15 (13.6-16.5)	15.2 (14-16.5)	15.5 (14.6-16.5)	15.6 (15.3-15.9)	16.3 (16.1-16.4)	17.2 (17-17.3)	17.4 (17.2-17.6)	18.2 (18-18.4)	19.3 (19.1-19.5)	19.7 (19.6-19.9)	19.7 (19.2-20.2)
Hillingdon	14 (12.6-15.6)	14.4 (13-16.1)	14.8 (13.4-16.3)	15 (13.7-16.5)	15.1 (13.9-16.4)	15.4 (14.4-16.4)	15.7 (15.4-16)	16.1 (15.9-16.2)	16.7 (16.5-16.8)	16.8 (16.7-17)	18 (17.8-18.2)	18.8 (18.7-19)	19.9 (19.8-20.1)	20.5 (20-20.9)
Hounslow	13.8 (12.5-15.4)	14.3 (12.9-15.8)	14.6 (13.2-16.3)	14.8 (13.4-16.4)	14.9 (13.7-16.2)	15.2 (14.4-16.2)	15.8 (15.5-16.1)	15.8 (15.7-16)	16.7 (16.6-16.9)	16.6 (16.5-16.8)	17.5 (17.3-17.7)	18.5 (18.3-18.6)	19.6 (19.4-19.7)	20.2 (19.7-20.6)
Islington	13.4 (12.1-14.8)	13.8 (12.4-15.2)	14.1 (12.7-15.5)	14.2 (12.9-15.6)	14.2 (13.1-15.6)	14.4 (13.5-15.4)	14.9 (14.6-15.2)	15.1 (14.9-15.2)	15.6 (15.4-15.7)	15.6 (15.5-15.8)	16.4 (16.2-16.7)	17.4 (17.2-17.5)	18.8 (18.6-18.9)	20.1 (19.7-20.6)
Kensington and Chelsea	13.8 (12.5-15.4)	14.2 (12.8-15.8)	14.4 (13.1-16)	14.5 (13.1-15.9)	14.5 (13.2-15.8)	14.7 (13.8-15.7)	15.2 (14.9-15.5)	15.6 (15.4-15.8)	17.1 (16.9-17.2)	17.8 (17.6-18)	19.2 (19-19.4)	20.3 (20.1-20.5)	21.4 (21.2-21.6)	22 (21.5-22.5)
Kingston upon Thames	14.5 (13-16)	14.9 (13.5-16.5)	15.3 (13.9-16.9)	15.6 (14.2-17.1)	15.7 (14.4-17.1)	16.1 (15.2-17.1)	16.5 (16.1-16.8)	16.8 (16.6-17)	17.5 (17.3-17.7)	17.8 (17.6-18)	18.9 (18.7-19.1)	20.3 (20.1-20.5)	21.3 (21.1-21.5)	20.9 (20.4-21.4)
Lambeth	13 (11.7-14.4)	13.4 (12.1-14.9)	13.8 (12.5-15.3)	13.9 (12.6-15.3)	14 (12.9-15.3)	14.4 (13.5-15.4)	15.1 (14.8-15.4)	15.5 (15.4-15.7)	16 (15.9-16.2)	15.8 (15.7-15.9)	16.9 (16.7-17.1)	17.6 (17.5-17.8)	18.9 (18.7-19)	19.8 (19.3-20.2)
Lewisham	13.3 (12-14.8)	13.8 (12.5-15.3)	14.3 (12.9-15.9)	14.6 (13.3-16.1)	14.8 (13.6-16.1)	15 (14.1-16)	15.1 (14.8-15.4)	15.8 (15.7-16)	16 (15.9-16.2)	15.7 (15.6-15.9)	16.4 (16.2-16.6)	17.3 (17.1-17.4)	18.8 (18.7-19)	19.7 (19.2-20.1)
Merton	14.2 (12.9-15.8)	14.6 (13.3-16.1)	15 (13.7-16.5)	15.1 (13.8-16.5)	15.3 (14.1-16.6)	15.6 (14.7-16.6)	15.9 (15.6-16.3)	16.8 (16.6-17)	17.1 (16.9-17.2)	17.3 (17.2-17.5)	18.5 (18.3-18.7)	19.6 (19.4-19.8)	20.3 (20.2-20.5)	20.8 (20.2-21.2)
Newham	12.9 (11.6-14.4)	13.3 (11.9-15)	13.7 (12.3-15.2)	13.9 (12.6-15.4)	14 (12.8-15.3)	14.3 (13.3-15.3)	14.8 (14.5-15.1)	15 (14.9-15.2)	15.5 (15.4-15.7)	15.5 (15.3-15.6)	16.3 (16.1-16.5)	17.5 (17.4-17.7)	17.8 (17.7-18)	19.7 (19.2-20.1)
Redbridge	14.1 (12.7-15.6)	14.5 (13.1-15.9)	14.9 (13.5-16.3)	15.1 (13.7-16.5)	15.2 (14-16.5)	15.6 (14.7-16.5)	16.2 (15.9-16.5)	16.9 (16.8-17.1)	17.1 (17-17.3)	17.2 (17.1-17.3)	17.9 (17.7-18.1)	19 (18.9-19.2)	20.2 (20-20.3)	20.5 (20-21)
Richmond upon Thames	14.3 (12.9-15.9)	14.6 (13.3-16.2)	14.9 (13.5-16.4)	15 (13.7-16.4)	15 (13.8-16.3)	15.4 (14.5-16.3)	15.9 (15.6-16.2)	16.5 (16.4-16.7)	17.5 (17.3-17.7)	18.6 (18.4-18.7)	19.2 (19-19.5)	20.5 (20.3-20.7)	21.7 (21.5-21.9)	21.7 (21.3-22.2)
Southwark	13.6 (12.2-15.1)	14 (12.7-15.6)	14.4 (13-16)	14.5 (13.2-16)	14.7 (13.5-16.1)	14.9 (14-15.9)	15.1 (14.8-15.4)	15.4 (15.2-15.5)	15.1 (14.9-15.2)	15.8 (15.6-15.9)	16.9 (16.7-17.1)	17.6 (17.4-17.7)	18.8 (18.6-18.9)	19 (18.5-19.4)
Sutton	14.3 (12.8-15.9)	14.8 (13.4-16.4)	15.2 (13.8-16.8)	15.6 (14.1-17.1)	15.8 (14.5-17.2)	16.2 (15.3-17.3)	16.6 (16.3-17)	16.6 (16.4-16.8)	17.2 (17.1-17.4)	17.1 (16.9-17.3)	18.4 (18.2-18.7)	19.6 (19.5-19.8)	20.6 (20.4-20.8)	20.3 (19.8-20.7)
Tower Hamlets	12.7 (11.4-14.1)	13 (11.8-14.5)	13.3 (12-14.8)	13.4 (12.2-14.8)	13.5 (12.4-14.7)	13.7 (12.8-14.6)	14.4 (14.1-14.7)	14.5 (14.4-14.6)	15 (14.8-15.1)	15.1 (15-15.3)	16.2 (16-16.4)	16.9 (16.7-17)	18.3 (18.2-18.5)	19.9 (19.4-20.3)
Waltham Forest	13.4 (12-14.8)	13.8 (12.4-15.3)	14.2 (12.8-15.7)	14.4 (13.1-15.9)	14.6 (13.4-15.9)	14.9 (14.1-16)	15.7 (15.4-16)	15.9 (15.8-16.1)	16.5 (16.3-16.6)	16.3 (16.2-16.5)	16.9 (16.7-17.1)	17.9 (17.7-18)	19.1 (18.9-19.2)	19.9 (19.5-20.4)
Wandsworth	13.5 (12.2-15)	13.9 (12.6-15.5)	14.2 (12.8-15.7)	14.3 (13-15.7)	14.3 (13.1-15.7)	14.6 (13.7-15.5)	15.1 (14.8-15.4)	15.4 (15.2-15.5)	15.6 (15.4-15.7)	15.9 (15.8-16)	17.1 (16.9-17.3)	18.4 (18.2-18.5)	19.7 (19.6-19.9)	20.1 (19.7-20.6)
Westminster	14.3 (13-15.8)	14.7 (13.3-16.2)	14.9 (13.6-16.5)	14.8 (13.5-16.3)	14.9 (13.6-16.1)	15.1 (14.1-16.1)	15.6 (15.3-15.9)	16.4 (16.2-16.5)	16.3 (16.1-16.4)	16.7 (16.5-16.9)	18.5 (18.3-18.8)	19.5 (19.4-19.7)	20.8 (20.7-21)	21.5 (21.1-22)
North East England	12.1 (11.6-12.5)	12.5 (12-12.9)	12.8 (12.4-13.3)	13.2 (12.7-13.6)	13.4 (13-13.9)	13.9 (13.6-14.2)	14.4 (14.3-14.5)	14.7 (14.7-14.8)	15.1 (15.1-15.1)	15.7 (15.6-15.7)	16.6 (16.5-16.7)	17.5 (17.4-17.5)	18.6 (18.6-18.7)	18.9 (18.8-19.1)
County Durham	11.9 (10.7-13.3)	12.3 (11.1-13.7)	12.7 (11.4-14.2)	13.1 (11.8-14.6)	13.3 (12.2-14.7)	13.8 (12.9-14.8)	14.4 (14.1-14.6)	14.6 (14.5-14.8)	15 (14.8-15.1)	15.4 (15.3-15.6)	16.5 (16.3-16.7)	17.6 (17.5-17.8)	18.7 (18.5-18.8)	19 (18.6-19.4)
Darlington	12.3 (11-13.8)	12.7 (11.3-14.1)	13 (11.7-14.4)	13.3 (11.9-14.7)	13.5 (12.2-14.8)	14 (13-15)	14.5 (14.1-14.9)	15 (14.8-15.2)	15.5 (15.2-15.7)	16 (15.8-16.2)	16.7 (16.5-17)	17.4 (17.2-17.6)	18.7 (18.5-18.9)	19.4 (18.8-19.9)
Gateshead	11.9 (10.6-13.4)	12.2 (10.9-13.7)	12.5 (11.2-14)	12.8 (11.5-14.3)	13 (11.8-14.3)	13.5 (12.5-14.5)	14.1 (13.8-14.4)	14.6 (14.4-14.7)	15.2 (15-15.3)	15.7 (15.6-15.9)	16.4 (16.2-16.6)	17.4 (17.3-17.6)	18.3 (18.2-18.5)	18.6 (18.1-19.1)
Hartlepool	11.8 (10.6-13.3)	12.2 (10.9-13.6)	12.5 (11.2-13.9)	12.8 (11.5-14.2)	13 (11.8-14.4)	13.5 (12.5-14.5)	14.1 (13.8-14.5)	14.5 (14.4-14.7)	15 (14.8-15.2)	15.6 (15.4-15.8)	16.5 (16.3-16.8)	17 (16.8-17.2)	18.3 (18.1-18.5)	18.9 (18.4-19.4)
Middlesbrough	11.8 (10.5-13.3)	12.2 (10.8-13.7)	12.5 (11.1-14)	12.8 (11.4-14.3)	12.9 (11.7-14.3)	13.3 (12.3-14.3)	13.6 (13.3-13.9)	14.1 (13.9-14.3)	14.5 (14.4-14.7)	15.2 (15.1-15.4)	15.9 (15.7-16.1)	16.7 (16.5-16.9)	17.8 (17.6-18)	18.3 (17.8-18.8)
Newcastle upon Tyne	12.1 (10.8-13.5)	12.5 (11.1-13.9)	12.9 (11.5-14.3)	13.2 (11.9-14.7)	13.5 (12.3-14.9)	14 (13-15)	14.6 (14.3-14.9)	15 (14.8-15.1)	15.1 (15-15.3)	15.5 (15.3-15.6)	16.6 (16.4-16.8)	17.2 (17.1-17.4)	18.3 (18.2-18.5)	18.6 (18.2-19.1)
North Tyneside	12.4 (11-13.9)	12.8 (11.5-14.2)	13.2 (11.8-14.6)	13.5 (12.2-15)	13.7 (12.5-15.1)	14.1 (13.2-15.1)	14.5 (14.2-14.9)	14.8 (14.7-15)	15.3 (15.1-15.4)	15.8 (15.6-16)	16.6 (16.4-16.8)	17.4 (17.2-17.6)	18.6 (18.4-18.8)	19.2 (18.7-19.7)
Northumberland	12.4 (11.1-13.8)	12.8 (11.5-14.2)	13.1 (11.8-14.7)	13.6 (12.2-15)	13.8 (12.5-15.1)	14.1 (13.2-15.1)	14.4 (14.2-14.7)	14.7 (14.6-14.9)	15.3 (15.2-15.4)	16.2 (16.1-16.4)	17.2 (17-17.4)	18.5 (18.3-18.6)	19.6 (19.4-19.7)	19.6 (19.1-20.1)
Redcar and Cleveland	12.1 (10.8-13.6)	12.5 (11.1-13.9)	12.8 (11.4-14.3)	13.1 (11.7-14.5)	13.3 (12-14.7)	13.8 (12.8-14.7)	14.3 (14-14.7)	15 (14.8-15.1)	15.5 (15.3-15.7)	16.2 (16-16.4)	17.1 (16.8-17.3)	17.6 (17.4-17.8)	18.7 (18.5-18.9)	19.1 (18.7-19.6)
South Tyneside	12 (10.8-13.5)	12.4 (11.1-13.9)	12.8 (11.5-14.3)	13.2 (11.9-14.6)	13.4 (12.2-14.8)	13.8 (12.9-14.8)	14.3 (13.9-14.6)	14.4 (14.2-14.5)	15 (14.8-15.1)	15.7 (15.5-15.9)	16.6 (16.3-16.8)	17.4 (17.2-17.6)	18.3 (18.1-18.5)	18.1 (17.6-18.6)
Stockton-on-Tees	12.4 (11.1-14)	12.8 (11.5-14.2)	13.2 (11.8-14.7)	13.5 (12.1-15)	13.7 (12.5-15.1)	14.2 (13.3-15.3)	14.7 (14.4-15)	15 (14.8-15.1)	15.4 (15.2-15.6)	16 (15.8-16.1)	17 (16.8-17.2)	17.6 (17.5-17.8)	18.9 (18.8-19.1)	19.3 (18.8-19.8)
Sunderland	12 (10.7-13.4)	12.4 (11.1-13.8)	12.9 (11.5-14.3)	13.3 (12-14.8)	13.5 (12.3-14.9)	14 (13-15)	14.5 (14.2-14.8)	14.7 (14.6-14.8)	14.8 (14.7-15)	15.3 (15.1-15.4)	16.1 (16-16.3)	16.8 (16.6-16.9)	18.2 (18-18.3)	18.4 (17.9-18.8)
North West England	12.6 (12.3-13)	13.1 (12.7-13.4)	13.4 (13-13.8)	13.7 (13.4-14.1)	13.9 (13.6-14.2)	14.3 (14-14.5)	14.7 (14.6-14.7)	15 (15-15)	15.5 (15.5-15.5)	16 (15.9-16)	16.9 (16.8-16.9)	17.8 (17.8-17.8)	18.7 (18.6-18.7)	19 (18.9-19.2)
Blackburn with Darwen	12.4 (11.2-13.9)	12.8 (11.5-14.3)	13.1 (11.7-14.6)	13.4 (12.1-14.8)	13.6 (12.3-14.8)	14 (13.1-14.9)	14.3 (14-14.6)	14.5 (14.4-14.7)	15 (14.8-15.1)	15.3 (15.1-15.5)	16 (15.8-16.2)	17 (16.8-17.2)	18 (17.8-18.2)	18.5 (18.1-19)
Blackpool	12.6 (11.3-14)	12.9 (11.7-14.4)	13.2 (12-14.7)	13.4 (12.1-14.8)	13.6 (12.4-15)	14.2 (13.2-15.1)	14.7 (14.4-15)	15.4 (15.3-15.6)	15.7 (15.5-15.9)	16.5 (16.4-16.7)	15.9 (15.7-16.1)	16 (15.8-16.1)	17.1 (16.9-17.2)	18 (17.5-18.4)
Bolton	12.5 (11.2-13.9)	12.9 (11.6-14.3)	13.3 (11.9-14.8)	13.6 (12.3-15)	13.7 (12.7-15)	14.1 (13.2-15.1)	14.4 (14.2-14.7)	14.8 (14.7-15)	15.5 (15.4-15.7)	16 (15.8-16.1)	16.5 (16.3-16.7)	17.4 (17.3-17.6)	18.4 (18.3-18.6)	19.1 (18.7-19.5)
Bury	12.9 (11.6-14.3)	13.3 (12-14.8)	13.7 (12.4-15.1)	14.1 (12.8-15.5)	14.3 (13.1-15.6)	14.6 (13.7-15.6)	14.9 (14.6-15.2)	15.2 (15.1-15.4)	15.7 (15.6-15.9)	16.1 (15.9-16.2)	17.3 (17.1-17.5)	17.9 (17.8-18.1)	18.7 (18.5-18.9)	18.6 (18.1-19)
Cheshire East	13.4 (12-14.8)	13.8 (12.4-15.3)	14.2 (12.8-15.7)	14.6 (13.3-16.1)	14.8 (13.5-16.1)	15.1 (14.2-16.1)	15.2 (14.9-15.5)	15.6 (15.5-15.7)	16.3 (16.1-16.4)	17 (16.8-17.1)	17.7 (17.6-17.9)	19.3 (19.2-19.5)	20 (19.9-20.2)	20.2 (19.8-20.7)
Cheshire West and Chester	13.1 (11.8-14.6)	13.5 (12.1-15)	13.8 (12.6-15.2)	14.1 (12.9-15.6)	14.2 (13-15.4)	14.6 (13.7-15.5)	15.1 (14.8-15.3)	15.4 (15.3-15.5)	15.9 (15.8-16)	16.5 (16.4-16.6)	17.7 (17.5-17.9)	19 (18.9-19.2)	19.8 (19.6-19.9)	19.9 (19.4-20.3)
Cumbria	12.8 (11.6-14.3)	13.3 (11.9-14.7)	13.6 (12.4-15)	14 (12.8-15.4)	14.2 (13.1-15.4)	14.6 (13.6-15.5)	15 (14.7-15.2)	15.3 (15.2-15.4)	15.8 (15.7-15.9)	16.1 (16-16.3)	17.6 (17.4-17.8)	18.6 (18.5-18.7)	19.4 (19.2-19.5)	20 (19.6-20.4)
Halton	12.3 (11-13.7)	12.7 (11.4-14.1)	12.9 (11.6-14.2)	13 (11.8-14.4)	13.2 (12-14.4)	13.6 (12.7-14.5)	14.1 (13.7-14.4)	14.5 (14.3-14.7)	15.3 (15.1-15.5)	15.6 (15.4-15.7)	16.1 (15.9-16.4)	16.6 (16.5-16.8)	17.9 (17.7-18.1)	18.5 (18.1-19)
Knowsley	12.5 (11.3-14)	12.9 (11.6-14.4)	13.2 (11.9-14.7)	13.3 (12-14.8)	13.5 (12.2-14.7)	13.9 (13.1-14.8)	14.4 (14.1-14.7)	15.1 (15-15.3)	15.3 (15.1-15.5)	15.3 (15.2-15.5)	15.8 (15.6-16)	16.9 (16.7-17)	17.6 (17.4-17.7)	17.7 (17.2-18.1)
Lancashire	12.8 (11.6-14.1)	13.3 (12-14.7)	13.7 (12.4-15.1)	14.1 (12.8-15.6)	14.3 (13-15.7)	14.7 (13.7-15.6)	14.7 (14.5-15)	15 (14.9-15.1)	15.6 (15.5-15.7)	16.3 (16.2-16.4)	17.2 (17-17.4)	18.1 (18-18.2)	19.2 (19.1-19.3)	19.5 (19.1-19.9)
Liverpool	12.2 (10.9-13.6)	12.6 (11.3-13.9)	12.8 (11.5-14.2)	13 (11.7-14.4)	13.1 (11.9-14.3)	13.5 (12.6-14.5)	14.4 (14.1-14.6)	14.6 (14.5-14.7)	14.9 (14.8-15)	15.1 (14.9-15.2)	15.8 (15.6-16)	16.7 (16.6-16.8)	17.5 (17.4-17.6)	18.2 (17.8-18.6)
Manchester	12.3 (11-13.6)	12.8 (11.5-14.1)	13.2 (11.9-14.6)	13.4 (12.2-14.8)	13.6 (12.5-14.9)	14 (13.1-15)	14.3 (14.1-14.6)	14.4 (14.3-14.5)	14.7 (14.6-14.8)	15 (14.9-15.2)	16.1 (15.9-16.3)	16.4 (16.3-16.5)	17.4 (17.3-17.5)	17.2 (16.7-17.6)
Oldham	12.4 (11.1-13.8)	12.7 (11.5-14.3)	13.1 (11.8-14.5)	13.4 (12.1-14.8)	13.6 (12.4-14.7)	14 (13.2-14.9)	14.4 (14.1-14.7)	14.5 (14.4-14.6)	15.2 (15.1-15.4)	15.5 (15.4-15.7)	16.3 (16.2-16.6)	17.1 (16.9-17.2)	18 (17.9-18.2)	18.3 (17.9-18.7)
Rochdale	12.5 (11.2-13.9)	12.9 (11.6-14.4)	13.3 (12-14.8)	13.7 (12.4-15)	13.9 (12.7-15.1)	14.3 (13.4-15.3)	14.7 (14.4-15)	14.9 (14.7-15)	14.9 (14.8-15.1)	15.6 (15.4-15.7)	16.4 (16.2-16.6)	17.1 (17-17.3)	18 (17.8-18.1)	18.4 (18-18.9)
Salford	12.2 (10.9-13.6)	12.6 (11.3-14)	12.9 (11.6-14.4)	13.2 (11.9-14.6)	13.4 (12.2-14.6)	13.8 (12.8-14.7)	14.5 (14.2-14.8)	14.6 (14.5-14.7)	14.9 (14.8-15.1)	15.1 (14.9-15.2)	16.3 (16.1-16.5)	17.1 (16.9-17.2)	17.8 (17.6-17.9)	18 (17.6-18.5)
Sefton	12.9 (11.6-14.3)	13.4 (12-14.8)	13.7 (12.4-15.1)	14 (12.7-15.4)	14.1 (12.9-15.4)	14.5 (13.6-15.5)	14.9 (14.6-15.2)	15.4 (15.3-15.6)	16.1 (16-16.3)	16.4 (16.3-16.6)	17.3 (17.1-17.5)	18.7 (18.6-18.9)	18.9 (18.7-19.1)	19.5 (19.1-19.9)
St Helens	12.2 (10.9-13.5)	12.5 (11.3-13.9)	12.8 (11.6-14.2)	13.1 (11.8-14.5)	13.2 (12.1-14.5)	13.6 (12.7-14.5)	13.8 (13.5-14.1)	14.4 (14.2-14.5)	15.1 (15-15.3)	15.8 (15.6-15.9)	16.8 (16.6-17)	17.5 (17.3-17.7)	18.1 (17.9-18.3)	18.1 (17.7-18.6)
Stockport	13.2 (12-14.7)	13.7 (12.4-15.2)	14.1 (12.8-15.6)	14.5 (13.1-15.9)	14.6 (13.5-16)	15 (14.1-15.9)	15.1 (14.9-15.4)	15.5 (15.4-15.6)	15.9 (15.8-16.1)	16.5 (16.3-16.6)	17.8 (17.6-18)	18.5 (18.4-18.7)	19.1 (18.9-19.2)	20.1 (19.7-20.6)
Tameside	12.5 (11.2-13.8)	12.9 (11.5-14.2)	13.2 (11.7-14.7)	13.4 (12.1-15)	13.6 (12.4-14.8)	14 (13.1-15)	14.3 (14-14.6)	14.7 (14.6-14.8)	15.4 (15.3-15.6)	15.5 (15.4-15.7)	16.4 (16.2-16.6)	17.2 (17-17.3)	17.9 (17.8-18.1)	18.3 (17.9-18.8)
Trafford	13.5 (12-14.9)	13.9 (12.5-15.4)	14.1 (12.8-15.7)	14.4 (13.1-15.8)	14.5 (13.3-15.8)	15 (14-15.9)	15.3 (15-15.6)	15.6 (15.4-15.7)	16.2 (16-16.3)	16.4 (16.3-16.6)	17.4 (17.2-17.6)	18.7 (18.5-18.9)	19.9 (19.7-20.1)	20 (19.5-20.5)
Warrington	12.8 (11.5-14.3)	13.2 (11.8-14.7)	13.4 (12.1-14.9)	13.7 (12.3-15)	13.8 (12.6-15.1)	14.2 (13.3-15.1)	14.5 (14.2-14.8)	14.7 (14.6-14.9)	15.2 (15-15.3)	15.9 (15.8-16.1)	17.2 (16.9-17.4)	18.2 (18-18.3)	18.9 (18.8-19.1)	18.8 (18.4-19.3)
Wigan	12.1 (11-13.5)	12.5 (11.2-14)	12.9 (11.6-14.4)	13.3 (12-14.8)	13.4 (12.3-14.7)	13.7 (12.8-14.6)	13.9 (13.6-14.1)	14.3 (14.2-14.4)	15 (14.8-15.1)	15.5 (15.3-15.6)	16.4 (16.2-16.6)	17.3 (17.2-17.4)	18 (17.9-18.2)	18.1 (17.7-18.6)
Wirral	12.8 (11.6-14.3)	13.3 (11.9-14.7)	13.7 (12.3-15.2)	14 (12.6-15.3)	14.2 (13-15.4)	14.6 (13.7-15.6)	15.3 (15-15.5)	15.6 (15.4-15.7)	15.7 (15.6-15.9)	16.2 (16-16.3)	17 (16.8-17.2)	17.8 (17.7-18)	18.7 (18.5-18.8)	18.8 (18.4-19.2)
South East England	14.1 (13.6-14.6)	14.5 (14-15)	14.8 (14.4-15.3)	15.1 (14.6-15.5)	15.1 (14.6-15.5)	15.2 (14.9-15.5)	15.8 (15.7-15.8)	16.2 (16.2-16.3)	16.9 (16.9-16.9)	17.3 (17.2-17.3)	18.2 (18.1-18.3)	19.3 (19.2-19.3)	20.1 (20.1-20.2)	20.3 (20.2-20.5)
Bracknell Forest	14.3 (12.8-15.9)	14.7 (13.2-16.5)	15.1 (13.6-16.9)	15.3 (13.8-17)	15.3 (14-16.8)	15.6 (14.6-16.6)	16.1 (15.7-16.5)	16.4 (16.2-16.6)	17.3 (17-17.5)	17.8 (17.6-18)	18.7 (18.5-19)	19.8 (19.6-20)	20.6 (20.4-20.8)	20.9 (20.4-21.4)
Brighton and Hove	13.9 (12.5-15.4)	14.3 (13-15.8)	14.6 (13.2-16.1)	14.7 (13.4-16.2)	14.8 (13.6-16.1)	15 (14.1-16)	15.6 (15.3-15.9)	16 (15.9-16.2)	16.2 (16-16.3)	16.2 (16.1-16.4)	17.3 (17.1-17.5)	17.8 (17.7-18)	18.9 (18.8-19.1)	19.8 (19.3-20.2)
Buckinghamshire	14.3 (12.9-15.9)	14.7 (13.4-16.3)	15 (13.7-16.6)	15.3 (14-16.9)	15.3 (14-16.7)	15.3 (14.5-16.2)	15.7 (15.4-16)	16.3 (16.1-16.4)	16.8 (16.7-16.9)	17.4 (17.2-17.5)	18.6 (18.5-18.8)	19.8 (19.7-20)	20.6 (20.5-20.7)	21.2 (20.8-21.6)
East Sussex	14.1 (12.8-15.7)	14.5 (13.2-16)	14.9 (13.5-16.4)	15.1 (13.7-16.6)	15.2 (14-16.5)	15.5 (14.6-16.4)	16.2 (15.9-16.5)	16.7 (16.6-16.9)	17.5 (17.3-17.6)	17.8 (17.6-17.9)	18.6 (18.4-18.8)	19.6 (19.5-19.8)	20.2 (20-20.3)	20.3 (19.9-20.7)
Hampshire	14.3 (12.9-15.7)	14.6 (13.2-16.1)	14.9 (13.5-16.4)	15.1 (13.6-16.6)	15 (13.7-16.4)	15.2 (14.3-16.2)	16 (15.8-16.3)	16.4 (16.3-16.5)	17 (16.9-17.1)	17.3 (17.2-17.4)	18.3 (18.2-18.5)	19.6 (19.5-19.7)	20.4 (20.3-20.5)	20.8 (20.4-21.2)
Isle of Wight	13.9 (12.5-15.5)	14.3 (12.9-16)	14.8 (13.3-16.5)	15 (13.8-16.6)	15.2 (13.9-16.6)	15.4 (14.5-16.5)	15.9 (15.6-16.3)	16.1 (15.9-16.3)	16.7 (16.5-16.9)	17.3 (17.1-17.5)	18.2 (18-18.5)	19 (18.7-19.2)	19.8 (19.6-20)	20.6 (20.1-21.1)
Kent	13.8 (12.5-15.2)	14.1 (12.9-15.5)	14.5 (13.2-15.9)	14.7 (13.4-16.2)	14.6 (13.3-15.9)	14.6 (13.7-15.6)	15.2 (15-15.5)	15.8 (15.7-15.9)	16.6 (16.5-16.7)	16.9 (16.8-17)	18 (17.9-18.2)	19 (18.9-19.1)	20 (19.9-20.1)	19.8 (19.4-20.2)
Medway	13.7 (12.3-15.2)	14.1 (12.8-15.6)	14.6 (13.3-16.2)	14.9 (13.4-16.4)	14.9 (13.7-16.3)	15.1 (14.2-16.1)	15.4 (15.1-15.7)	15.6 (15.5-15.8)	16.3 (16.2-16.5)	16.7 (16.6-16.9)	17.1 (16.9-17.3)	17.9 (17.7-18)	18.7 (18.5-18.8)	19.4 (19-19.9)
Milton Keynes	13.9 (12.5-15.4)	14.3 (12.8-15.8)	14.6 (13.2-16.2)	14.8 (13.4-16.4)	14.8 (13.5-16.2)	15 (14-16)	15.4 (15.1-15.8)	15.8 (15.6-16)	16.7 (16.5-16.9)	17 (16.8-17.1)	18.1 (17.9-18.3)	18.8 (18.6-18.9)	19.3 (19.2-19.5)	20 (19.5-20.4)
Oxfordshire	14.3 (12.9-15.9)	14.7 (13.3-16.3)	15 (13.6-16.6)	15.2 (13.8-16.8)	15.2 (13.9-16.6)	15.3 (14.3-16.2)	15.7 (15.5-16)	16.4 (16.3-16.5)	17 (16.9-17.2)	17.5 (17.4-17.7)	18.4 (18.2-18.5)	19.4 (19.3-19.5)	20.3 (20.2-20.4)	20.6 (20.2-21.1)
Portsmouth	13.4 (12.1-15)	13.8 (12.5-15.4)	14.2 (12.8-15.7)	14.4 (13-15.9)	14.4 (13.2-15.7)	14.6 (13.7-15.5)	14.8 (14.5-15.1)	15.2 (15-15.4)	16.1 (15.9-16.2)	16 (15.8-16.1)	16.6 (16.4-16.8)	17.7 (17.5-17.8)	18.7 (18.5-18.8)	19.1 (18.7-19.6)
Reading	14 (12.6-15.5)	14.4 (13-15.9)	14.8 (13.4-16.2)	15 (13.5-16.4)	15 (13.8-16.4)	15.3 (14.3-16.3)	15.7 (15.4-16.1)	16 (15.8-16.2)	16.7 (16.5-16.9)	16.9 (16.7-17.1)	17.6 (17.4-17.8)	18.4 (18.2-18.6)	19.1 (18.9-19.3)	19.2 (18.7-19.6)
Slough	13.7 (12.4-15.3)	14.1 (12.6-15.7)	14.4 (12.9-16)	14.5 (13.1-16.1)	14.5 (13.3-15.8)	14.6 (13.8-15.6)	15 (14.6-15.3)	15.1 (14.9-15.3)	15.9 (15.7-16.1)	16.4 (16.2-16.6)	17 (16.8-17.2)	18.2 (18.1-18.4)	19 (18.8-19.2)	20.1 (19.6-20.6)
Southampton	13.5 (12.1-15.1)	13.9 (12.5-15.5)	14.3 (12.9-15.8)	14.5 (13.2-15.9)	14.6 (13.4-15.9)	14.8 (13.9-15.7)	15.5 (15.2-15.8)	15.8 (15.7-16)	16 (15.8-16.1)	16.2 (16.1-16.4)	17.1 (16.9-17.3)	17.9 (17.8-18.1)	18.9 (18.7-19)	19.3 (18.8-19.7)
Surrey	14.6 (13.2-16.1)	15 (13.6-16.6)	15.4 (14-17)	15.7 (14.3-17.2)	15.6 (14.3-17)	15.6 (14.7-16.6)	15.9 (15.6-16.1)	16.5 (16.4-16.6)	17.1 (16.9-17.2)	17.5 (17.4-17.7)	18.4 (18.2-18.6)	19.6 (19.5-19.7)	20.7 (20.6-20.8)	20.5 (20.1-20.9)
West Berkshire	14.5 (13.2-16.1)	14.9 (13.5-16.6)	15.3 (13.8-17)	15.5 (14-17.1)	15.6 (14.3-17)	15.8 (14.8-16.8)	16.3 (15.9-16.7)	15.8 (15.6-16)	17.3 (17.1-17.5)	17.7 (17.5-17.8)	18.7 (18.4-18.9)	19 (18.8-19.2)	20.3 (20.1-20.5)	20.2 (19.7-20.7)
West Sussex	14.2 (12.8-15.8)	14.6 (13.3-16.1)	15 (13.6-16.6)	15.2 (13.8-16.8)	15.2 (14-16.6)	15.4 (14.5-16.4)	16.1 (15.8-16.3)	16.6 (16.5-16.7)	17.2 (17.1-17.4)	17.7 (17.6-17.8)	18.5 (18.3-18.7)	19.4 (19.3-19.6)	20.2 (20.1-20.3)	20.3 (19.9-20.7)
Windsor and Maidenhead	15 (13.5-16.6)	15.5 (13.9-17.2)	15.9 (14.3-17.5)	16 (14.6-17.5)	16.1 (14.7-17.5)	16.3 (15.3-17.3)	16.4 (16.1-16.8)	16.6 (16.4-16.8)	17.2 (17-17.4)	17.6 (17.4-17.8)	18.5 (18.2-18.7)	20.3 (20.1-20.5)	20.9 (20.7-21.1)	20.9 (20.5-21.4)
Wokingham	15.1 (13.7-16.7)	15.6 (14.1-17.1)	16 (14.5-17.6)	16.2 (14.7-17.9)	16.3 (15-17.8)	16.6 (15.6-17.6)	17.1 (16.7-17.4)	17.4 (17.2-17.6)	18.3 (18.1-18.5)	18.8 (18.6-19)	20 (19.8-20.2)	20.8 (20.6-21.1)	21.5 (21.3-21.7)	21.4 (20.9-21.8)
South West England	13.8 (13.4-14.2)	14.2 (13.8-14.6)	14.6 (14.2-15)	14.8 (14.4-15.3)	15 (14.6-15.4)	15.3 (15-15.6)	15.8 (15.7-15.9)	16.4 (16.4-16.5)	16.9 (16.9-17)	17.5 (17.5-17.6)	18.4 (18.3-18.4)	19.3 (19.3-19.4)	20.1 (20.1-20.2)	20.1 (20-20.2)
Bath and North East Somerset	14.5 (13.1-16.2)	15 (13.5-16.6)	15.4 (14-17)	15.7 (14.3-17.2)	16 (14.7-17.3)	16.4 (15.5-17.4)	17 (16.6-17.3)	17.3 (17.1-17.5)	17.5 (17.4-17.7)	18 (17.8-18.2)	19 (18.8-19.3)	20.1 (19.9-20.2)	20.7 (20.5-20.9)	20.3 (19.9-20.8)

Table 9 Estimates of life expectancy at age 65 by location and year for 1950, 1955...2005, 2010, 2017 - both sexes

Location	1950	1955	1960	1965	1970	1975	1980	1985	1990	1995	2000	2005	2010	2017
Bournemouth	13.8 (12.4-15.3)	14.2 (12.8-15.8)	14.6 (13.2-16.1)	14.8 (13.5-16.4)	15 (13.7-16.4)	15.5 (14.5-16.4)	16.1 (15.8-16.5)	16.5 (16.3-16.6)	16.4 (16.2-16.6)	16.7 (16.5-16.8)	17.7 (17.4-17.9)	18.7 (18.6-18.9)	19.2 (19-19.4)	19.4 (18.9-19.8)
Bristol, City of	13.6 (12.2-15)	13.9 (12.6-15.4)	14.3 (13-15.8)	14.5 (13.2-15.9)	14.6 (13.4-15.9)	14.8 (13.9-15.8)	15.3 (15-15.5)	16 (15.9-16.1)	16.5 (16.4-16.7)	16.9 (16.8-17.1)	17.5 (17.4-17.8)	18.5 (18.3-18.6)	19.2 (19-19.3)	19.6 (19.2-20.1)
Cornwall	13.5 (12.1-15)	13.9 (12.5-15.3)	14.3 (12.9-15.8)	14.5 (13.2-16.1)	14.7 (13.4-16.1)	15 (14.1-16)	15.6 (15.3-15.9)	16.4 (16.2-16.5)	16.8 (16.7-17)	17.4 (17.3-17.5)	18.2 (18-18.4)	19.2 (19.1-19.3)	20 (19.9-20.1)	19.8 (19.4-20.2)
Devon	13.8 (12.5-15.4)	14.2 (12.9-15.8)	14.6 (13.2-16.1)	14.9 (13.6-16.3)	15 (13.8-16.3)	15.3 (14.4-16.3)	16 (15.7-16.3)	16.7 (16.6-16.8)	17.3 (17.2-17.4)	17.9 (17.8-18)	18.7 (18.5-18.9)	19.7 (19.6-19.8)	20.4 (20.3-20.5)	20 (19.6-20.5)
Dorset	14.1 (12.7-15.7)	14.5 (13.1-16.1)	15 (13.5-16.6)	15.2 (13.9-16.8)	15.4 (14.3-16.7)	15.8 (14.9-16.7)	16.3 (16-16.6)	16.9 (16.8-17.1)	17.6 (17.4-17.7)	18.4 (18.3-18.5)	19.2 (19-19.4)	20.3 (20.2-20.5)	21.1 (21-21.3)	21.1 (20.7-21.5)
Gloucestershire	14 (12.6-15.7)	14.4 (13.1-16.1)	14.9 (13.4-16.5)	15.2 (13.8-16.7)	15.3 (14-16.6)	15.5 (14.6-16.5)	15.5 (15.3-15.8)	16.1 (16-16.2)	16.8 (16.7-17)	17.4 (17.3-17.6)	18.5 (18.3-18.7)	19.2 (19.1-19.3)	20.1 (19.9-20.2)	20 (19.6-20.4)
North Somerset	14.2 (12.8-15.8)	14.7 (13.2-16.3)	15.2 (13.8-16.7)	15.5 (14.1-17)	15.7 (14.5-17.1)	16.2 (15.3-17.2)	16.6 (16.3-17)	16.9 (16.7-17.1)	17.3 (17.1-17.5)	17.8 (17.7-18)	18.8 (18.5-19)	19.3 (19.2-19.5)	19.7 (19.6-19.9)	19.6 (19.1-20)
Plymouth	13.1 (11.8-14.8)	13.5 (12.2-15.1)	13.9 (12.5-15.5)	14.1 (12.8-15.6)	14.2 (13-15.6)	14.6 (13.7-15.5)	15.1 (14.8-15.4)	15.7 (15.6-15.9)	16.1 (16-16.3)	16.5 (16.3-16.6)	17.5 (17.3-17.7)	18 (17.9-18.2)	18.9 (18.7-19)	19.6 (19.1-20)
Poole	14 (12.5-15.6)	14.4 (13-15.9)	14.8 (13.4-16.3)	15 (13.8-16.6)	15.2 (13.9-16.6)	15.7 (14.7-16.7)	16.5 (16.1-16.9)	16.6 (16.4-16.8)	17.2 (17-17.4)	17.9 (17.7-18.1)	19 (18.8-19.3)	19.7 (19.5-19.9)	20.3 (20.1-20.5)	20.1 (19.6-20.6)
Somerset	13.8 (12.5-15.3)	14.2 (12.9-15.7)	14.6 (13.2-16.1)	14.8 (13.5-16.4)	14.9 (13.6-16.2)	15.2 (14.2-16.2)	15.7 (15.4-15.9)	16.3 (16.2-16.4)	16.9 (16.8-17)	17.5 (17.4-17.7)	18.4 (18.2-18.6)	19.5 (19.4-19.6)	20.3 (20.2-20.5)	20.4 (19.9-20.8)
South Gloucestershire	14 (12.6-15.7)	14.4 (13.1-16.1)	14.8 (13.5-16.3)	15.1 (13.7-16.6)	15.2 (13.9-16.5)	15.5 (14.6-16.5)	16.2 (15.9-16.5)	16.6 (16.5-16.8)	16.8 (16.6-17)	17.6 (17.4-17.7)	18.4 (18.2-18.6)	19.8 (19.6-19.9)	20.8 (20.6-20.9)	20.6 (20.2-21.1)
Swindon	13.6 (12.2-15.1)	14 (12.6-15.5)	14.3 (12.8-15.9)	14.5 (13.1-15.9)	14.5 (13.3-15.8)	14.8 (13.9-15.7)	15 (14.7-15.4)	15.7 (15.5-15.9)	16.4 (16.2-16.5)	16.6 (16.5-16.8)	17.7 (17.5-17.9)	18.7 (18.6-18.9)	19.7 (19.5-19.8)	19.2 (18.8-19.7)
Torbay	13.5 (12.1-15.1)	13.9 (12.5-15.6)	14.3 (12.9-15.9)	14.6 (13.3-16.1)	14.8 (13.6-16.1)	15.2 (14.3-16.2)	15.7 (15.3-16.1)	16.2 (16-16.4)	17 (16.8-17.2)	17.3 (17.2-17.5)	17.5 (17.3-17.7)	18 (17.8-18.2)	18.9 (18.7-19.1)	19.8 (19.3-20.2)
Wiltshire	13.9 (12.5-15.5)	14.3 (12.9-15.9)	14.8 (13.4-16.4)	15.1 (13.8-16.5)	15.2 (13.9-16.5)	15.4 (14.5-16.4)	15.7 (15.4-16)	16.3 (16.2-16.5)	16.6 (16.4-16.7)	17.6 (17.4-17.7)	18.5 (18.3-18.7)	19.4 (19.2-19.5)	20.3 (20.1-20.4)	20.7 (20.3-21.1)
West Midlands	12.9 (12.4-13.3)	13.3 (12.8-13.8)	13.7 (13.2-14.2)	14 (13.5-14.4)	14.1 (13.7-14.5)	14.4 (14.1-14.7)	14.8 (14.8-14.9)	15.3 (15.3-15.4)	15.8 (15.8-15.9)	16.3 (16.3-16.4)	17.3 (17.3-17.4)	18.3 (18.3-18.3)	19.4 (19.3-19.4)	19.7 (19.5-19.8)
Birmingham	12.7 (11.6-14.1)	13.2 (11.9-14.6)	13.6 (12.4-14.9)	13.9 (12.6-15.2)	14 (12.8-15.4)	14.4 (13.5-15.4)	14.6 (14.4-14.9)	15.1 (15-15.2)	15.6 (15.5-15.7)	16 (15.9-16.1)	17 (16.8-17.1)	18 (17.9-18.1)	19.2 (19-19.3)	19.3 (18.9-19.7)
Coventry	13 (11.7-14.5)	13.4 (12.1-14.9)	13.7 (12.4-15.3)	13.9 (12.6-15.4)	14.1 (12.8-15.4)	14.4 (13.5-15.4)	14.9 (14.6-15.1)	15.6 (15.5-15.7)	15.6 (15.4-15.7)	16.2 (16.1-16.4)	17.3 (17.1-17.5)	17.6 (17.4-17.7)	19 (18.9-19.2)	19.2 (18.8-19.7)
Dudley	13 (11.7-14.4)	13.5 (12.1-14.9)	13.9 (12.6-15.5)	14.4 (13-15.9)	14.5 (13.3-15.8)	14.8 (13.8-15.8)	15.1 (14.8-15.3)	15.2 (15.1-15.4)	15.8 (15.6-15.9)	16.3 (16.2-16.5)	17.2 (17-17.4)	18.4 (18.2-18.5)	19.4 (19.3-19.6)	19.8 (19.3-20.2)
Herefordshire, County of	13.5 (12.1-15)	13.9 (12.4-15.5)	14.2 (12.8-15.8)	14.3 (12.9-15.8)	14.4 (13.2-15.8)	14.9 (13.9-15.9)	15.6 (15.2-15.9)	16.2 (16-16.4)	16.8 (16.6-17)	17.5 (17.3-17.7)	18.5 (18.3-18.7)	19.3 (19.1-19.5)	20 (19.8-20.2)	20.4 (20-20.9)
Sandwell	12.7 (11.3-14.2)	13.1 (11.7-14.7)	13.5 (12-15.1)	13.7 (12.3-15.3)	13.9 (12.6-15.2)	14.3 (13.3-15.3)	14.9 (14.6-15.1)	15.3 (15.1-15.4)	15.5 (15.3-15.6)	15.6 (15.5-15.7)	16.3 (16-16.5)	17.1 (17-17.3)	18.2 (18-18.3)	18.8 (18.4-19.3)
Shropshire	13.1 (11.9-14.6)	13.5 (12.1-14.9)	13.8 (12.5-15.4)	14.1 (12.8-15.5)	14.2 (13-15.5)	14.6 (13.7-15.7)	15.3 (15-15.6)	15.8 (15.7-16)	16.5 (16.3-16.6)	16.8 (16.7-17)	18.1 (17.9-18.3)	19.1 (19-19.3)	20.1 (19.9-20.2)	20.3 (19.8-20.7)
Solihull	14 (12.5-15.7)	14.4 (12.9-16.1)	14.8 (13.3-16.5)	15 (13.6-16.5)	15.2 (14-16.6)	15.7 (14.8-16.8)	16.3 (16-16.7)	16.9 (16.8-17.1)	17.5 (17.4-17.7)	17.6 (17.4-17.8)	18.5 (18.3-18.7)	19.5 (19.3-19.7)	20.7 (20.5-20.9)	20.7 (20.2-21.2)
Staffordshire	12.9 (11.6-14.3)	13.3 (12-14.7)	13.6 (12.2-15.1)	13.9 (12.6-15.3)	13.9 (12.7-15.3)	14.1 (13.2-15.1)	14.7 (14.5-14.9)	15.3 (15.2-15.4)	15.7 (15.5-15.8)	16.2 (16.1-16.3)	17.3 (17.1-17.5)	18.4 (18.3-18.5)	19.6 (19.5-19.7)	19.6 (19.2-20)
Stoke-on-Trent	12.1 (10.9-13.6)	12.5 (11.2-13.9)	12.8 (11.5-14.3)	13 (11.8-14.4)	13.1 (11.9-14.3)	13.4 (12.5-14.3)	13.8 (13.5-14.1)	14.3 (14.2-14.4)	14.9 (14.8-15.1)	15.2 (15-15.3)	16.4 (16.2-16.6)	17 (16.9-17.2)	17.9 (17.7-18)	18.5 (18-18.9)
Telford and Wrekin	12.7 (11.4-14.2)	13 (11.6-14.6)	13.3 (11.9-14.8)	13.5 (12.3-15)	13.6 (12.4-15)	14 (13-15)	14.4 (14.1-14.7)	15 (14.8-15.1)	15.9 (15.7-16.1)	16.5 (16.4-16.7)	17.2 (17-17.4)	18.1 (17.9-18.3)	18.5 (18.3-18.7)	18.9 (18.4-19.4)
Walsall	12.5 (11.1-14)	12.9 (11.4-14.4)	13.3 (11.8-14.7)	13.6 (12.3-15)	13.7 (12.4-15)	14 (13.1-15)	14.5 (14.2-14.8)	14.9 (14.8-15.1)	15.4 (15.2-15.5)	15.9 (15.7-16)	16.9 (16.7-17.2)	18.1 (17.9-18.3)	19 (18.8-19.2)	19.1 (18.7-19.6)
Warwickshire	13.3 (12-14.7)	13.6 (12.3-15.1)	14 (12.6-15.4)	14.3 (12.9-15.7)	14.4 (13.2-15.6)	14.7 (13.8-15.6)	15.1 (14.9-15.4)	15.5 (15.4-15.6)	16.1 (16-16.3)	16.6 (16.5-16.7)	17.8 (17.7-18)	18.7 (18.6-18.9)	19.9 (19.8-20)	20.2 (19.8-20.7)
Wolverhampton	12.7 (11.3-14.2)	13.1 (11.7-14.6)	13.5 (12.1-15)	13.7 (12.5-15.1)	13.9 (12.7-15.2)	14.2 (13.3-15.3)	14.9 (14.5-15.1)	15.2 (15.1-15.4)	15.3 (15.2-15.5)	16.1 (16-16.2)	16.8 (16.6-17)	17.1 (16.9-17.2)	18.5 (18.3-18.6)	19 (18.6-19.5)
Worcestershire	13.3 (12-14.8)	13.7 (12.4-15.2)	14.1 (12.8-15.7)	14.5 (13.2-15.9)	14.6 (13.4-15.9)	14.9 (13.9-15.9)	15 (14.8-15.3)	15.8 (15.7-15.9)	16.3 (16.2-16.4)	17 (16.9-17.1)	17.7 (17.5-17.9)	19 (18.9-19.2)	19.8 (19.6-19.9)	20.3 (19.9-20.7)
Yorkshire and the Humber	12.8 (12.4-13.2)	13.2 (12.8-13.6)	13.6 (13.2-14)	13.9 (13.5-14.3)	14.1 (13.7-14.5)	14.4 (14.2-14.7)	14.9 (14.8-15)	15.3 (15.3-15.3)	15.8 (15.8-15.8)	16.4 (16.3-16.4)	17.2 (17.2-17.3)	18.2 (18.2-18.3)	19 (19-19)	19.4 (19.2-19.5)
Barnsley	12.4 (11.1-13.8)	12.7 (11.5-14.2)	13.1 (11.8-14.5)	13.4 (12-14.8)	13.6 (12.4-14.9)	14 (13.1-15)	14.5 (14.2-14.8)	14.8 (14.6-14.9)	15.2 (15.1-15.4)	15.6 (15.4-15.7)	16.5 (16.3-16.8)	17.6 (17.4-17.7)	18.3 (18.2-18.5)	18.9 (18.4-19.3)
Bradford	12.6 (11.3-14.1)	13 (11.7-14.6)	13.5 (12.1-15.1)	13.8 (12.5-15.2)	14 (12.8-15.3)	14.4 (13.4-15.3)	14.6 (14.3-14.9)	15 (14.9-15.1)	15.6 (15.5-15.7)	15.9 (15.8-16.1)	16.8 (16.6-17)	17.7 (17.5-17.8)	18.8 (18.6-18.9)	19 (18.5-19.4)
Calderdale	12.7 (11.4-14.2)	13.1 (11.7-14.6)	13.5 (12.2-14.9)	13.8 (12.5-15.3)	14 (12.8-15.3)	14.3 (13.4-15.3)	14.8 (14.5-15.2)	15 (14.9-15.2)	15.6 (15.4-15.7)	15.9 (15.8-16.1)	17.2 (17-17.4)	18.2 (18-18.4)	18.6 (18.4-18.8)	18.9 (18.4-19.3)
Doncaster	12.7 (11.4-14.1)	13.1 (11.7-14.6)	13.6 (12.3-15.1)	14 (12.7-15.5)	14.3 (13.1-15.6)	14.7 (13.7-15.7)	14.8 (14.6-15.1)	15.3 (15.1-15.4)	15.6 (15.4-15.7)	15.5 (15.4-15.6)	16.7 (16.5-16.9)	17.8 (17.6-17.9)	18.5 (18.4-18.7)	18.7 (18.3-19.2)
East Riding of Yorkshire	13.3 (12-14.8)	13.8 (12.3-15.3)	14.2 (12.7-15.7)	14.5 (13.2-16)	14.7 (13.5-16)	15.1 (14.1-16.1)	15.5 (15.2-15.8)	15.9 (15.8-16)	16.4 (16.3-16.5)	17.1 (16.9-17.2)	17.8 (17.6-18)	18.9 (18.7-19)	19.7 (19.5-19.8)	20.3 (19.8-20.7)
Kingston upon Hull, City of	12.7 (11.5-14.2)	13.2 (11.8-14.8)	13.7 (12.3-15.2)	14 (12.7-15.5)	14.2 (13-15.6)	14.6 (13.7-15.6)	14.6 (14.3-14.9)	15.2 (15-15.3)	15.6 (15.4-15.7)	15.9 (15.7-16)	16.4 (16.2-16.6)	17.4 (17.3-17.6)	17.5 (17.3-17.6)	17.8 (17.4-18.3)
Kirklees	12.5 (11.3-13.9)	12.9 (11.5-14.2)	13.2 (11.9-14.6)	13.5 (12.3-15)	13.7 (12.5-15)	14 (13.1-14.9)	14.3 (14-14.5)	15 (14.9-15.1)	15.4 (15.2-15.5)	16.3 (16.2-16.4)	17.1 (16.9-17.2)	17.7 (17.5-17.8)	19 (18.8-19.1)	19.7 (19.2-20.1)
Leeds	12.9 (11.5-14.4)	13.2 (11.9-14.7)	13.6 (12.2-15.1)	13.8 (12.5-15.3)	14 (12.8-15.2)	14.3 (13.4-15.3)	15 (14.7-15.2)	15.5 (15.4-15.6)	16.1 (15.9-16.2)	16.7 (16.6-16.8)	17.4 (17.2-17.6)	18.3 (18.2-18.5)	18.9 (18.8-19)	19 (18.6-19.4)
North East Lincolnshire	13.3 (12-14.9)	13.7 (12.3-15.3)	14.2 (12.8-15.8)	14.5 (13.2-16)	14.8 (13.6-16.2)	15.2 (14.3-16.2)	15.6 (15.3-16)	15.5 (15.4-15.7)	15.8 (15.7-16)	16.3 (16.1-16.5)	17.1 (16.9-17.3)	17.7 (17.6-17.9)	18.8 (18.6-19)	19.4 (18.9-19.9)
North Lincolnshire	12.8 (11.5-14.4)	13.2 (11.7-14.8)	13.6 (12.2-15.1)	13.8 (12.6-15.3)	14.1 (12.9-15.5)	14.5 (13.6-15.5)	14.8 (14.5-15.2)	15 (14.9-15.2)	15.8 (15.6-15.9)	16.7 (16.5-16.9)	17.2 (17-17.4)	18.3 (18.1-18.5)	19 (18.8-19.1)	19.4 (18.9-19.9)
North Yorkshire	13.3 (12-14.8)	13.7 (12.3-15.1)	14 (12.7-15.5)	14.3 (13-15.8)	14.5 (13.2-15.7)	14.8 (14-15.8)	15.5 (15.2-15.7)	16.1 (15.9-16.2)	16.5 (16.4-16.6)	17.1 (17-17.3)	18 (17.8-18.2)	19.1 (19-19.3)	20.1 (20-20.2)	20.5 (20-20.9)
Rotherham	12.5 (11.2-13.9)	12.8 (11.6-14.3)	13.2 (11.9-14.7)	13.5 (12.3-15)	13.8 (12.6-15.1)	14.2 (13.3-15.1)	14.6 (14.3-14.9)	15 (14.9-15.2)	15.4 (15.3-15.6)	16.3 (16.2-16.5)	16.8 (16.6-17)	18 (17.8-18.1)	18.6 (18.4-18.8)	18.4 (18-18.8)
Sheffield	12.8 (11.6-14.3)	13.2 (11.9-14.8)	13.6 (12.3-15.2)	14 (12.6-15.4)	14.1 (12.9-15.5)	14.5 (13.6-15.4)	15 (14.8-15.2)	15.2 (15.1-15.3)	15.7 (15.5-15.8)	16.2 (16-16.3)	17.2 (17-17.4)	18.3 (18.2-18.5)	19 (18.9-19.1)	19.4 (19-19.8)
Wakefield	12.1 (10.9-13.5)	12.4 (11.2-13.7)	12.7 (11.4-14.2)	13 (11.7-14.4)	13.1 (12-14.4)	13.5 (12.7-14.4)	14.1 (13.8-14.3)	14.4 (14.3-14.6)	15.2 (15.1-15.4)	16 (15.9-16.2)	16.9 (16.7-17.1)	17.8 (17.7-18)	18.6 (18.5-18.8)	19.1 (18.7-19.5)
York	13.7 (12.4-15.3)	14.1 (12.7-15.6)	14.4 (13-16)	14.6 (13.3-16.2)	14.8 (13.6-16.2)	15.2 (14.3-16.3)	15.6 (15.2-15.9)	15.8 (15.6-16)	16.6 (16.5-16.8)	17.6 (17.4-17.8)	18.6 (18.4-18.8)	19.7 (19.5-19.9)	20.2 (20.1-20.4)	20 (19.6-20.5)
Northern Ireland	12.8 (12.7-12.9)	12.9 (12.8-13)	13.2 (13.1-13.3)	13.6 (13.5-13.7)	13.7 (13.6-13.8)	13.8 (13.7-13.9)	14.1 (14-14.2)	14.5 (14.4-14.6)	15.3 (15.2-15.4)	16 (15.9-16.1)	17.1 (16.9-17.3)	18.4 (18.3-18.5)	18.9 (18.7-19)	19.6 (19.1-20.1)
Scotland	12.2 (12.1-12.2)	12.6 (12.6-12.7)	12.9 (12.8-12.9)	13.1 (13.1-13.2)	13.4 (13.3-13.4)	13.9 (13.8-13.9)	14.2 (14.2-14.3)	14.6 (14.5-14.7)	15.1 (15.1-15.2)	15.8 (15.7-15.8)	16.6 (16.4-16.7)	17.4 (17.4-17.5)	18.2 (18.1-18.3)	18.7 (18.2-19.1)
Wales	12.1 (10.8-13.5)	12.5 (11.2-13.9)	13 (11.6-14.4)	13.3 (11.9-14.7)	13.6 (12.2-15.1)	14.1 (13.1-15.3)	14.8 (14.6-15.1)	15.5 (15.4-15.6)	16 (16-16.1)	16.4 (16.3-16.4)	17.2 (17-17.4)	18.1 (18.1-18.2)	19 (19-19.1)	19.4 (19-19.8)
Latin America and Caribbean	12.4 (12.3-12.5)	13 (12.9-13.2)	13.4 (13.3-13.5)	13.9 (13.8-14)	14.2 (14.1-14.3)	14.9 (14.8-15)	15.4 (15.3-15.4)	15.6 (15.6-15.7)	16.3 (16.3-16.3)	16.6 (16.5-16.6)	17.5 (17.4-17.5)	17.9 (17.9-18)	18.2 (18.2-18.2)	18.6 (18.5-18.7)
Andean Latin America	12.5 (12.2-12.7)	13.5 (13.3-13.7)	13.5 (13.3-13.8)	14.6 (14.3-14.8)	14 (13.8-14.2)	16.2 (16-16.5)	16.2 (16-16.4)	16.2 (15.9-16.4)	17 (16.8-17.2)	16.9 (16.7-17.2)	17.9 (17.6-18.1)	18.3 (18-18.6)	18.5 (18.2-18.8)	19.5 (18.9-20)
Bolivia	9.9 (9.7-10.2)	11.2 (11-11.3)	11.7 (11.5-11.9)	11.7 (11.4-11.9)	11.8 (11.5-12.1)	12.2 (11.9-12.4)	12.5 (12.2-12.8)	12.8 (12.4-13.2)	13.3 (12.9-13.7)	13.8 (13.4-14.3)	14.5 (14-15)	15 (14.4-15.7)	15.3 (14.5-16.2)	15.8 (14.7-17.1)
Ecuador	12.5 (11.7-13.3)	12.8 (12.7-12.8)	12.8 (12.7-12.8)	14 (14-14.1)	14.9 (14.9-15)	16.1 (16.1-16.2)	16.9 (16.8-16.9)	17.2 (17.2-17.3)	17.2 (17.2-17.3)	17.9 (17.9-17.9)	18.5 (18.4-18.5)	18.2 (18-18.5)	18.3 (18-18.6)	19 (18.4-19.5)
Peru	13.7 (13.2-14.2)	14.9 (14.5-15.3)	14.6 (14.2-15)	16 (15.6-16.5)	14.4 (14-14.7)	17.9 (17.4-18.3)	17.2 (16.8-17.7)	16.9 (16.5-17.2)	18.2 (17.8-18.6)	17.5 (17.1-17.9)	18.7 (18.3-19.1)	19.4 (19-19.9)	19.6 (19.1-20)	21 (20.1-21.9)
Caribbean	12.4 (12.1-12.8)	13.1 (12.8-13.5)	13.7 (13.6-13.9)	14.2 (14.1-14.4)	15.1 (14.9-15.2)	15.6 (15.5-15.8)	15.8 (15.6-15.9)	15.9 (15.8-16)	16.2 (16.1-16.3)	16.3 (16.2-16.4)	17.1 (16.9-17.2)	17.4 (17.2-17.6)	16.8 (16.6-17)	17.7 (17.3-18)
Antigua and Barbuda	12.6 (12.4-12.8)	13.5 (13.4-13.7)	14.4 (14.2-14.5)	14.4 (14.1-14.6)	14.5 (14.2-14.7)	14.9 (14.6-15.2)	15.6 (15.2-15.9)	16.4 (16.1-16.8)	17 (16.7-17.2)	16.6 (16.3-16.9)	16.7 (16.5-16.9)	17.3 (17-17.6)	17.7 (17.4-17.9)	17.9 (17.6-18.4)
The Bahamas	13.3 (12.5-14.3)	13.8 (13.1-14.6)	14.3 (13.8-14.8)	14.6 (14.4-14.8)	15.1 (14.9-15.2)	15.9 (15.7-16)	15.7 (15.6-15.8)	16.1 (16-16.2)	16.4 (16.2-16.5)	16.4 (16.2-16.5)	16.7 (16.6-16.8)	17.5 (17.4-17.6)	17.4 (17.3-17.6)	17.5 (17-18)
Barbados	14.3 (14.2-14.5)	14.6 (14.5-14.7)	15 (14.9-15.1)	14.6 (14.5-14.7)	14.4 (14.3-14.5)	14.7 (14.6-14.8)	15.8 (15.7-16)	16.3 (16.1-16.4)	16.4 (16.2-16.5)	16.7 (16.5-16.8)	17.1 (16.9-17.3)	17.5 (17.4-17.6)	18.4 (18.3-18.5)	18.4 (18-18.9)
Belize	12.3 (12.1-12.6)	13.7 (13.5-13.8)	15.8 (15.6-16)	16.9 (16.6-17.2)	17.4 (17.1-17.7)	17.2 (16.9-17.5)	17 (16.7-17.3)	16.6 (16.4-16.9)	16.7 (16.4-17)	16.7 (16.5-16.9)	16 (15.9-16.1)	17 (16.8-17.1)	18.1 (18-18.2)	18.2 (18-18.5)
Bermuda	12.9 (12.7-13.2)	13.6 (13.5-13.8)	14.3 (14.2-14.4)	14.7 (14.6-14.8)	14.8 (14.7-15)	15.7 (15.5-15.9)	16.3 (16.2-16.5)	16.2 (15.9-16.4)	16.1 (15.9-16.3)	17.6 (17.1-17.7)	18 (17.8-18.1)	18.9 (18.6-19.2)	19.9 (19.5-20.2)	20.3 (19.8-20.7)
Cuba	13.9 (12.9-15)	13.5 (12.7-14.4)	13.9 (13.8-13.9)	14.7 (14.6-14.7)	16.3 (16.2-16.3)	17.1 (17.1-17.2)	16.8 (16.7-16.8)	16.7 (16.7-16.8)	16.8 (16.8-16.8)	16.7 (16.7-16.8)	17.8 (17.7-17.8)	18.1 (18-18.1)	18.3 (18.3-18.4)	18.5 (17.8-19.1)
Dominica	13.9 (13.7-14.1)	15.7 (15.5-15.8)	16.3 (16.1-16.5)	16.6 (16.4-16.8)	16.4 (16.2-16.6)	16.8 (16.6-17)	17.2 (17.1-17.4)	17.2 (17-17.3)	16 (15.8-16.2)	16.1 (15.9-16.2)	16.6 (16.4-16.8)	16.9 (16.7-17.1)	17 (16.8-17.2)	16.6 (16.2-17)
Dominican Republic	14.1 (14-14.2)	15.2 (15.2-15.3)	16.3 (15.9-16.8)	17.2 (16.7-17.6)	16.7 (16.2-17.1)	17.1 (16.7-17.6)	17.4 (16.9-17.8)	18.4 (17.9-18.8)	18.8 (18.4-19.3)	19.5 (19-19.9)	19.4 (18.9-19.9)	18.7 (18.1-19.3)	18.7 (18.2-19.2)	17.7 (17-18.6)
Grenada	15.2 (14.8-15.5)	15 (14.7-15.4)	14.6 (14.3-15)	13.7 (13.3-14.1)	15 (14.6-15.4)	16 (15.6-16.4)	15.4 (15.1-15.7)	14.2 (13.9-14.4)	14 (13.8-14.1)	15.1 (15-15.3)	16.7 (16.5-16.9)	17.1 (16.9-17.3)	16.6 (16.4-16.8)	16.8 (16.4-17.1)
Guyana	10.8 (10.7-10.9)	11.6 (11.6-11.7)	12.7 (12.6-12.8)	12.8 (12.4-13.2)	13.4 (13.3-13.4)	12.8 (12.7-12.9)	13.3 (13.1-13.4)	14.2 (14.1-14.4)	13.9 (13.8-14)	14.1 (14-14.2)	14.1 (14-14.2)	14.7 (14.6-14.8)	15 (14.9-15.1)	15.2 (14.5-15.9)
Haiti	9.6 (9-10.3)	9.8 (9.2-10.5)	10 (9.3-10.6)	10.1 (9.4-10.8)	10.2 (9.6-11)	10.5 (9.9-11.2)	10.9 (10.3-11.5)	11.2 (10.7-11.7)	11.5 (11-12)	11.8 (11.2-12.3)	12.3 (11.6-13)	12.7 (11.9-13.6)	9.2 (8.8-9.8)	13.4 (12.5-14.4)
Jamaica	12 (12-12.1)	13.7 (13.6-13.7)	14.6 (14.5-14.7)	14.7 (14.6-14.8)	16.1 (16-16.2)	15.7 (15.6-15.8)	16.8 (16.7-16.9)	15.8 (15.7-15.9)	17.1 (16.8-17.4)	16.8 (16.3-17.3)	16.9 (16.8-17)	18.7 (18.6-18.8)	17.9 (17.8-17.9)	17.1 (16.2-17.9)
Puerto Rico	13.6 (13.6-13.7)	16.3 (16.2-16.3)	16.4 (16.3-16.5)	16.4 (16.3-16.5)	16.5 (16.5-16.6)	17 (16.9-17)	17 (16.9-17.1)	16.9 (16.8-16.9)	17.3 (17.3-17.4)	17.6 (17.5-17.6)	18.5 (18.4-18.6)	19.2 (19.1-19.3)	20.1 (20-20.2)	20.2 (19.9-20.6)
Saint Lucia	11.9 (11.7-12.1)	13.2 (13.1-13.4)	14.2 (14.1-14.4)	14.7 (14.5-15)	14.5 (14.3-14.7)	14.3 (14.1-14.4)	14.7 (14.5-14.8)	15.1 (14.9-15.2)	14.8 (14.7-14.9)	15.7 (15.5-15.8)	17.1 (17-17.3)	17.3 (17.2-17.5)	18.1 (18-18.3)	17.9 (17.6-18.3)
Saint Vincent and the Grenadines	14.9 (14.7-15.2)	15.8 (15.6-15.9)	16.2 (16-16.4)	15.7 (15.5-16)	15.8 (15.6-16)	15.9 (15.7-16.1)	16.1 (15.9-16.3)	16.1 (16-16.3)	15.5 (15.3-15.7)	16 (15.8-16.2)	15.9 (15.7-16)	16.5 (16.4-16.7)	16.7 (16.5-16.8)	15.9 (15.6-16.2)
Suriname	12.4 (11.9-12.9)	12.6 (12.2-13)	13.3 (12.8-13.7)	14 (13.6-14.5)	13.7 (13.2-14.2)	13.7 (13.3-14.2)	13.7 (13.4-14.1)	14.9 (14.5-15.2)	15.5 (15.2-15.9)	16.5 (16.2-16.9)	16.1 (15.7-16.4)	16.1 (15.7-16.4)	16.8 (16.5-17.2)	16.7 (16.1-17.4)
Trinidad and Tobago	10.6 (10.5-10.7)	11.6 (11.5-11.7)	12.6 (12.5-12.7)	12.6 (12.5-12.7)	13 (12.9-13.1)	13.5 (13.4-13.6)	13.6 (13.5-13.7)	13.8 (13.7-13.9)	14.6 (14.5-14.7)	14.7 (14.6-14.8)	15.4 (15.3-15.5)	16.3 (16.2-16.4)	17.3 (17.2-17.4)	17.4 (16.2-18.6)
Virgin Islands	12.4 (12.1-12.6)	12.9 (12.7-13.1)	13.2 (13-13.4)	13.3 (13.1-13.6)	13.7 (13.3-14.1)	14.2 (13.8-14.4)	14.6 (14.3-14.9)	15.1 (14.8-15.4)	15.6 (15.1-15.9)	15.8 (15.5-16.1)	16.1 (15.7-16.5)	16.4 (15.8-17)	16.2 (15.6-17)	16.1 (15.4-17.2)
Central Latin America	12.6 (12.5-12.7)	13.5 (13.4-13.6)	13.8 (13.7-14)	14.3 (14.2-14.4)	14.6 (14.5-14.8)	15.2 (15.2-15.3)	15.8 (15.7-16)	16 (16-16)	16.8 (16.8-16.8)	16.9 (16.9-17)	17.9 (17.9-18)	18.3 (18.2-18.3)	18.7 (18.6-18.7)	18.9 (18.7-19.1)
Colombia	12.6 (12.5-12.6)	13.4 (13.3-13.4)	13.7 (13.7-13.7)	14 (13.9-14)	15.4 (15.4-15.4)	14.8 (14.8-14.8)	15.9 (15.4-16.4)	15.2 (15.2-15.2)	16.9 (16.9-17)	16.9 (16.9-16.9)	18.5 (18.5-18.5)	19.2 (19.2-19.2)	20.2 (20.2-20.2)	21.1 (20.4-21.8)
Costa Rica	12.5 (12.4-12.6)	14.2 (14.1-14.3)	14.9 (14.8-15)	14.7 (14.6-14.8)	15 (14.9-15.1)	16.4 (16.3-16.5)	17 (16.9-17.1)	17.2 (17.1-17.3)	17.8 (17.7-17.9)	17.7 (17.6-17.8)	18.7 (18.6-18.8)	19.7 (19.6-19.7)	20 (19.9-20.1)	19.8 (19.4-20.2)
El Salvador	12.3 (12.3-12.4)	13 (12.9-13)	14.3 (14.2-14.4)	14.7 (14.6-14.8)	14.2 (14.2-14.3)	15.8 (15.7-15.9)	15.9 (15.7-16.2)	17 (17-17.1)	17.5 (17.4-17.5)	17.8 (17.5-18)	18.2 (18-18.5)	17.7 (17.4-17.9)	17.7 (17.5-18)	17.9 (16.9-18.9)
Guatemala	10.6 (10.6-10.7)	11.1 (11.1-11.2)	11.9 (11.9-11.9)	12.1 (12.1-12.1)	11.8 (11.7-11.8)	12.9 (12.8-12.9)	14.2 (14.2-14.3)	13.7 (13.7-13.8)	14.4 (14.3-14.4)	15.2 (15.1-15.2)	15.9 (15.9-16)	16.5 (16.5-16.6)	17.5 (17.4-17.5)	17.3 (16.7-17.8)
Honduras	12.2 (11.6-12.8)	12.1 (11.7-12.6)	11.9 (11.5-12.3)	12.7 (12.4-13.1)	13.6 (13.3-14)	14.9 (14.5-15.3)	17.2 (16.8-17.7)	17.2 (16.6-17.8)	17.8 (17.1-18.4)	16.8 (15.9-17.7)	16.8 (15.8-18)	17 (15.8-18.2)	17.1 (16-18.4)	17 (16-18.2)
Mexico	12.9 (12.8-13.1)	13.8 (13.6-14.1)	14 (13.8-14.2)	14.6 (14.4-14.8)	14.7 (14.5-15)	15.6 (15.4-15.7)	15.9 (15.8-16)	16.3 (16.3-16.3)	16.8 (16.8-16.8)	17 (16.9-17)	17.8 (17.8-17.8)	18 (17.9-18)	18.1 (18.1-18.1)	18.2 (18.1-18.3)
Aguascalientes	14 (13-15)	14.8 (13.8-15.8)	14.8 (13.9-15.8)	15.2 (14.2-16.1)	15.1 (14.3-15.9)	15.7 (15.2-16.2)	16.1 (15.9-16.2)	16.2 (16.1-16.3)	16.8 (16.7-16.9)	17 (16.9-17)	17.4 (17.3-17.5)	17.5 (17.4-17.6)	17.9 (17.8-18)	18.8 (18.4-19.2)
Baja California	12.4 (11.6-13.3)	13.3 (12.4-14.2)	13.5 (12.5-14.5)	13.9 (12.9-14.8)	13.8 (12.9-14.7)	14.3 (13.7-14.9)	15.3 (15.2-15.4)	14.7 (14.6-14.8)	15.2 (15.1-15.3)	15.4 (15.3-15.4)	15.8 (15.8-15.9)	16.5 (16.4-16.5)	17.4 (17.3-17.5)	17.9 (17.6-18.3)
Baja California Sur	14.1 (13.1-15.1)	14.9 (13.9-15.9)	14.8 (13.9-15.8)	15 (14.1-15.9)	14.8 (14.1-15.6)	15.3 (14.9-15.8)	16.2 (16-16.4)	16.8 (16.6-16.9)	16.8 (16.7-16.9)	16.7 (16.6-16.8)	17.3 (17.2-17.4)	17.5 (17.4-17.6)	17.6 (17.5-17.7)	18.2 (17.8-18.6)
Campeche	14.6 (13.6-15.6)	15.5 (14.5-16.6)	15.6 (14.6-16.8)	16.1 (15.2-17.2)	16.2 (15.4-17.1)	17 (16.5-17.5)	17.1 (17-17.3)	16.7 (16.6-16.8)	17.4 (17.3-17.5)	17.3 (17.2-17.4)	18.6 (18.5-18.7)	18.7 (18.6-18.8)	18.5 (18.4-18.6)	17.7 (17.4-18.1)
Chiapas	13.8 (13-14.8)	14.8 (13.8-15.8)	14.9 (14-16)	15.6 (14.7-16.7)	16.1 (15.2-17.3)	17.3 (16.7-18)	16.3 (16.2-16.4)	16.8 (16.7-16.8)	17.5 (17.4-17.6)	17.8 (17.7-17.8)	17.9 (17.8-18)	18 (17.9-18)	18 (17.9-18)	17.9 (17.6-18.3)
Chihuahua	12.3 (11.5-13.2)	13.2 (12.4-14.2)	13.4 (12.4-14.4)	14 (13-15)	14.1 (13.2-15.1)	14.9 (14.3-15.5)	15.1 (15-15.2)	14.9 (14.8-15)	15.2 (15.1-15.2)	15.6 (15.5-15.7)	16 (15.9-16)	16.3 (16.3-16.4)	16.1 (16.1-16.2)	16.6 (16.3-17)
Coahuila	12.3 (11.5-13.2)	13.2 (12.3-14.2)	13.3 (12.3-14.4)	13.8 (12.8-14.8)	13.7 (12.7-14.6)	14.4 (13.8-15)	14.7 (14.6-14.8)	15.1 (15-15.2)	15.6 (15.5-15.7)	16 (16-16.1)	16.6 (16.5-16.7)	16.7 (16.7-16.8)	17 (16.9-17)	17.7 (17.3-18.1)
Colima	12.5 (11.7-13.4)	13.3 (12.4-14.2)	13.3 (12.4-14.1)	13.6 (12.8-14.4)	13.4 (12.8-14)	14.1 (13.7-14.4)	15 (14.9-15.2)	16 (15.9-16.1)	15.9 (15.8-16)	16.4 (16.3-16.5)	17.5 (17.4-17.6)	17.7 (17.6-17.8)	17.8 (17.7-17.9)	17.5 (17.1-17.8)
Mexico City	12.8 (12-13.7)	13.7 (12.8-14.6)	13.8 (12.9-14.8)	14.4 (13.5-15.4)	14.7 (13.8-15.6)	15.5 (15.1-16)	15.6 (15.5-15.6)	16.1 (16-16.2)	16.4 (16.3-16.4)	16.4 (16.4-16.4)	17.2 (17.1-17.2)	17.4 (17.3-17.4)	17.9 (17.9-18)	17.8 (17.5-18.2)
Durango	12.7 (11.9-13.6)	13.7 (12.8-14.7)	13.9 (13-14.9)	14.6 (13.6-15.6)	14.8 (14-15.7)	15.6 (15.1-16.2)	15.5 (15.4-15.6)	16.1 (16-16.2)	17 (16.9-17.1)	17.6 (17.5-17.7)	18.5 (18.4-18.6)	18.5 (18.4-18.6)	17.6 (17.5-17.6)	19.1 (18.8-19.5)
Guanajuato	13.2 (12.4-14.1)	14.1 (13.2-15)	14.2 (13.3-15.2)	14.7 (13.8-15.7)	14.7 (13.8-15.6)	15.5 (14.9-16.1)	16.4 (16.3-16.5)	16 (15.9-16.1)	16.8 (16.7-16.8)	17.1 (17.1-17.2)	17.8 (17.8-17.9)	18.2 (18.1-18.2)	18.5 (18.4-18.5)	18.4 (18-18.7)
Guerrero	13.1 (12.3-14)	14.1 (13.1-15)	14.4 (13.4-15.4)	15.2 (14.2-16.2)	15.7 (14.9-16.7)	17.2 (16.6-17.8)	17.5 (17.4-17.6)	18.7 (18.7-18.8)	19.4 (19.4-19.5)	19.2 (19.2-19.3)	20.7 (20.6-20.8)	20.7 (20.6-20.8)	20 (19.9-20)	19.9 (19.5-20.2)
Hidalgo	12.3 (11.5-13.1)	13.1 (12.3-14)	13.3 (12.6-14.2)	14 (13.2-15)	14.3 (13.5-15.2)	15.3 (14.7-15.9)	15.2 (15.1-15.2)	16.9 (16.8-16.9)	17.9 (17.8-18)	18.7 (18.6-18.8)	19.5 (19.4-19.6)	19.3 (19.2-19.3)	18.8 (18.7-18.8)	19.5 (19.1-19.8)
Jalisco	12.9 (12.1-13.8)	13.9 (12.9-14.8)	14.1 (13.2-15.1)	14.7 (13.7-15.7)	14.8 (13.9-15.7)	15.8 (15.2-16.4)	16.3 (16.2-16.4)	16.3 (16.2-16.3)	16.4 (16.3-16.4)	16.5 (16.4-16.5)	17.4 (17.3-17.4)	17.7 (17.6-17.7)	18 (17.9-18)	17.8 (17.4-18.1)
México	13.7 (12.8-14.7)	14.4 (13.5-15.4)	14.4 (13.5-15.5)	14.8 (13.8-15.9)	14.8 (13.8-15.9)	15.3 (14.3-16.3)	15.3 (14.6-15.9)	15.6 (15.6-15.7)	16.5 (16.5-16.6)	16.5 (16.5-16.6)	17.2 (17.2-17.2)	17.5 (17.5-17.6)	18 (18-18)	17.8 (17.5-18.2)
Michoacán de Ocampo	12.8 (12-13.6)	13.7 (12.9-14.7)	14 (13.1-14.9)	14.7 (13.8-15.6)	15 (14.1-15.9)	15.8 (15.2-16.4)	16.4 (16.3-16.5)	16.7 (16.6-16.7)	17.9 (17.9-18)	17.7 (17.6-17.8)	18.7 (18.7-18.8)	19 (19-19.1)	18.8 (18.8-18.9)	19.5 (19.1-19.8)
Morelos	13.6 (12.8-14.5)	14.5 (13.5-15.6)	14.7 (13.7-15.8)	15.3 (14.3-16.3)	15.5 (14.6-16.4)	16.6 (16-17.2)	16.8 (16.7-16.9)	17.2 (17.1-17.3)	17.4 (17.3-17.5)	17.4 (17.4-17.5)	18.4 (18.4-18.5)	18.4 (18.3-18.5)	18.6 (18.5-18.6)	17.7 (17.4-18.2)
Nayarit	13.2 (12.4-14.2)	14.2 (13.3-15.2)	14.4 (13.5-15.5)	15 (14.1-16.1)	15.3 (14.5-16.2)	16.3 (15.7-16.9)	16.5 (16.4-16.6)	16.7 (16.6-16.8)	17.4 (17.3-17.5)	17.8 (17.7-17.9)	19 (18.9-19.1)	18.7 (18.6-18.8)	18.7 (18.7-18.8)	19.1 (18.7-19.5)
Nuevo León	13.6 (12.7-14.7)	14.5 (13.5-15.7)	14.6 (13.6-15.7)	14.9 (13.9-16)	14.7 (13.7-15.8)	15.2 (14.6-15.8)	16.1 (16-16.2)	16.5 (16.4-16.5)	16.2 (16.1-16.3)	16.5 (16.4-16.5)	17.2 (17.1-17.2)	17.3 (17.2-17.4)	17.6 (17.6-17.7)	18.4 (18-18.7)

Table 9 Estimates of life expectancy at age 65 by location and year for 1950, 1955...2005, 2010, 2017 - both sexes

Location	1950	1955	1960	1965	1970	1975	1980	1985	1990	1995	2000	2005	2010	2017
Oaxaca	12 (11.3-12.8)	12.8 (12.1-13.7)	13.1 (12.2-14)	13.7 (12.8-14.5)	13.8 (13-14.6)	14.5 (13.9-15)	14.8 (14.7-14.9)	15.9 (15.9-16)	17.1 (17-17.1)	17.5 (17.4-17.5)	18.7 (18.6-18.8)	19 (18.9-19.1)	19.1 (19.1-19.2)	19.1 (18.8-19.5)
Puebla	12 (11.3-12.8)	12.9 (12.1-13.7)	13.1 (12.3-14)	13.8 (12.9-14.7)	14 (13.2-14.8)	14.9 (14.3-15.5)	15.6 (15.6-15.7)	16.1 (16.1-16.2)	16.8 (16.7-16.8)	17 (17-17.1)	17.9 (17.9-18)	18.1 (18.1-18.2)	18.3 (18.2-18.3)	17.8 (17.4-18.2)
Querétaro	13.5 (12.7-14.5)	14.4 (13.4-15.3)	14.5 (13.5-15.5)	14.9 (14-15.9)	14.9 (14-15.8)	15.5 (14.7-16.3)	15.7 (15.3-16.1)	16.4 (16.3-16.5)	16.7 (16.6-16.8)	17.1 (17-17.1)	17.5 (17.4-17.6)	18 (18-18.1)	18.4 (18.3-18.5)	18.2 (17.9-18.6)
Quintana Roo	17.4 (16.3-18.5)	17.8 (16.7-18.9)	17.5 (16.5-18.6)	17.2 (16.1-18.3)	16.6 (15.7-17.5)	16.7 (16.1-17.2)	17.7 (17.5-17.9)	19.1 (18.9-19.2)	17.8 (17.6-17.9)	17.4 (17.3-17.5)	17.9 (17.8-18)	18.1 (18-18.2)	17.6 (17.5-17.6)	16.7 (16.4-17.1)
San Luis Potosí	12.9 (12-13.8)	13.8 (12.9-14.9)	14 (13.1-15.1)	14.6 (13.7-15.5)	14.6 (13.7-15.5)	15.3 (14.6-15.8)	16.7 (16.6-16.8)	17.1 (17-17.1)	17.8 (17.7-17.9)	17.7 (17.6-17.8)	18.8 (18.7-18.9)	18.9 (18.8-19)	18.9 (18.8-19)	19.3 (18.9-19.7)
Sinaloa	13.6 (12.7-14.6)	14.6 (13.6-15.6)	14.7 (13.7-15.8)	15.2 (14.2-16.3)	15.4 (14.4-16.4)	16.4 (15.7-17.1)	16.5 (16.4-16.6)	16.4 (16.3-16.5)	17.3 (17.2-17.3)	17.2 (17.1-17.3)	18.1 (18-18.1)	18.1 (18-18.2)	18.5 (18.4-18.6)	19.6 (19.2-20.1)
Sonora	12.6 (11.7-13.6)	13.5 (12.5-14.5)	13.5 (12.6-14.6)	13.9 (13-15)	13.9 (12.9-15)	14.4 (13.4-15.4)	14.5 (13.9-15.1)	15 (14.9-15.1)	15.3 (15.2-15.4)	15.3 (15.2-15.4)	16.4 (16.3-16.4)	16.5 (16.4-16.6)	16.9 (16.8-17)	17.3 (17-17.7)
Tabasco	13.3 (12.5-14.2)	14.2 (13.3-15.1)	14.3 (13.4-15.2)	14.8 (13.9-15.7)	14.9 (14-15.8)	15.5 (15-16.1)	15.6 (15.5-15.7)	17.2 (17.1-17.2)	17.6 (17.5-17.7)	17 (16.9-17.1)	18.1 (18-18.2)	18.1 (18-18.2)	18.1 (18-18.2)	18 (17.6-18.4)
Tamaulipas	12.8 (11.9-13.7)	13.7 (12.8-14.7)	13.9 (12.9-14.9)	14.5 (13.6-15.5)	14.7 (13.7-15.7)	15.5 (14.9-16.2)	15.4 (15.3-15.5)	16.1 (16-16.2)	16.8 (16.8-16.9)	17.1 (17-17.2)	17.7 (17.6-17.8)	17.8 (17.8-17.9)	18 (17.9-18)	18.6 (18.2-19)
Tlaxcala	13.2 (12.3-14.1)	14.2 (13.2-15.2)	14.4 (13.4-15.4)	15 (14.1-16)	15.3 (14.5-16.2)	16.2 (15.4-17)	16.4 (15.9-16.9)	17 (16.9-17.1)	17.5 (17.4-17.6)	17.6 (17.6-17.7)	18.4 (18.3-18.5)	18.7 (18.6-18.8)	18.7 (18.6-18.8)	18.7 (18.3-19.1)
Veracruz de Ignacio de la Llave	13.4 (12.6-14.4)	14.5 (13.5-15.5)	14.7 (13.8-15.7)	15.4 (14.5-16.4)	15.7 (14.8-16.6)	16.8 (16.2-17.3)	17.2 (17.1-17.3)	17.5 (17.4-17.5)	17.5 (17.4-17.5)	17.5 (17.5-17.6)	18.3 (18.2-18.3)	18.2 (18.1-18.2)	18.2 (18.2-18.3)	18 (17.6-18.3)
Yucatán	13.6 (12.7-14.6)	14.4 (13.4-15.5)	14.4 (13.4-15.4)	14.7 (13.8-15.7)	14.5 (13.7-15.4)	14.8 (14.3-15.4)	15.1 (15-15.2)	15.7 (15.6-15.8)	16.3 (16.2-16.3)	16.4 (16.3-16.5)	17.3 (17.2-17.3)	17.5 (17.4-17.5)	17.4 (17.3-17.4)	17.3 (16.9-17.6)
Zacatecas	13.6 (12.7-14.6)	14.7 (13.7-15.7)	14.9 (14-16)	15.6 (14.7-16.6)	15.8 (15-16.7)	16.7 (16.1-17.2)	17.4 (17.3-17.5)	17.9 (17.9-18)	18.3 (18.2-18.4)	18 (17.9-18.1)	18.9 (18.8-18.9)	19 (18.9-19.1)	18.7 (18.6-18.8)	18.9 (18.5-19.3)
Nicaragua	12.9 (12.8-13)	14.4 (14.3-14.5)	14.1 (14-14.2)	14.7 (14.5-15)	14.2 (13.6-14.7)	15.5 (15.1-15.9)	15.8 (15.1-16.6)	16.4 (15.7-17.1)	18.6 (18.2-19)	18.4 (18-18.9)	19 (18.6-19.4)	18.8 (18.5-19.3)	19.8 (19.6-20.1)	20.2 (19.5-20.8)
Panama	14.8 (14.6-14.9)	14.9 (14.8-15.1)	15.8 (15.6-15.9)	15.6 (15.5-15.7)	15.3 (15.2-15.4)	16.7 (16.6-16.8)	17.6 (17.5-17.7)	17.5 (17.4-17.6)	18.3 (18.2-18.4)	18.4 (18.3-18.5)	19.5 (19.4-19.6)	19.9 (19.9-20)	20.3 (20.2-20.3)	20.6 (20.3-21)
Venezuela	11.9 (11.9-11.9)	13.1 (13-13.1)	14 (13.9-14)	14.3 (14.3-14.3)	14.6 (14.5-14.6)	14.9 (14.8-14.9)	15.4 (15.4-15.4)	15.9 (15.9-15.9)	16.6 (16.5-16.6)	16.8 (16.7-16.9)	17.9 (17.9-18)	18.6 (18.6-18.7)	18.8 (18.8-18.8)	18.5 (17.6-19.4)
Tropical Latin America	12.1 (11.8-12.3)	12.4 (12.1-12.7)	12.8 (12.5-13.1)	13.2 (12.9-13.5)	13.6 (13.3-13.9)	14 (13.8-14.2)	14.6 (14.5-14.6)	15.1 (15-15.1)	15.8 (15.7-15.8)	16.2 (16.2-16.3)	17.1 (17.1-17.1)	17.6 (17.6-17.7)	18.1 (18-18.1)	18.4 (18.3-18.4)
Brazil	12 (11.7-12.3)	12.3 (12-12.6)	12.7 (12.4-13)	13.1 (12.8-13.4)	13.5 (13.2-13.8)	14 (13.8-14.1)	14.5 (14.5-14.6)	15 (15-15.1)	15.7 (15.7-15.7)	16.2 (16.2-16.2)	17.1 (17-17.1)	17.6 (17.6-17.7)	18.1 (18-18.1)	18.4 (18.4-18.4)
Acre	12 (11.1-13)	12.4 (11.5-13.4)	13 (12-14)	13.7 (12.8-14.7)	14.3 (13.6-15.1)	14.8 (14.3-15.3)	15.7 (15.6-15.9)	15.6 (15.5-15.7)	16.7 (16.6-16.8)	17.1 (17-17.2)	17.9 (17.8-18)	18.2 (18.1-18.3)	18.7 (18.6-18.8)	18.5 (18.3-18.7)
Alagoas	12.1 (11.2-13)	12.3 (11.5-13.3)	12.7 (11.8-13.6)	13.1 (12.2-14.1)	13.3 (12.5-14.2)	13.8 (13.3-14.4)	14.8 (14.8-14.9)	15.5 (15.4-15.6)	16.5 (16.4-16.5)	16.8 (16.7-17)	17.9 (17.6-18.1)	18.2 (18-18.4)	18.2 (18.1-18.3)	17.9 (17.7-18.1)
Amapá	12.7 (11.7-13.7)	13.2 (12.2-14.2)	13.8 (12.9-14.8)	14.5 (13.7-15.4)	15 (14.3-15.7)	15.6 (15.2-16.1)	16.6 (16.5-16.8)	16.8 (16.7-17)	17.5 (17.4-17.6)	17.3 (17.2-17.4)	17.7 (17.6-17.8)	18.8 (18.7-18.9)	19.5 (19.4-19.6)	19 (18.8-19.2)
Amazonas	12.7 (11.8-13.7)	13 (12-14)	13.4 (12.4-14.5)	13.8 (12.8-14.8)	14.1 (13.2-15.1)	14.5 (13.8-15.3)	15.9 (15.6-16.1)	16.6 (16.3-16.8)	17.3 (17.1-17.6)	18.4 (18.2-18.6)	18.4 (18.3-18.5)	18.7 (18.6-18.8)	18.7 (18.6-18.8)	18.9 (18.7-19.1)
Bahia	12.8 (11.9-13.8)	13.1 (12.2-14.2)	13.6 (12.7-14.6)	14.2 (13.2-15.3)	14.7 (13.8-15.8)	15.3 (14.5-16.2)	16.6 (16.2-17)	17 (16.7-17.3)	17.9 (17.6-18.3)	18.2 (17.9-18.6)	18.6 (18.2-18.9)	18.9 (18.5-19.2)	19.1 (18.7-19.4)	19.3 (19.1-19.4)
Ceará	12.4 (11.5-13.4)	12.8 (11.9-13.8)	13.3 (12.4-14.3)	13.8 (12.9-15)	14.3 (13.3-15.4)	14.9 (13.9-15.9)	16.3 (15.4-17.3)	17.7 (17.2-18.2)	18.9 (18.6-19.2)	18.7 (18.3-19)	18.9 (18.6-19.2)	18.9 (18.6-19.2)	19.8 (19.8-19.9)	19.7 (19.5-19.9)
Distrito Federal	11.3 (10.5-12.2)	11.6 (10.8-12.5)	12.1 (11.2-13.1)	12.5 (11.6-13.5)	12.8 (11.9-13.7)	13.2 (12.6-13.8)	14.3 (14.2-14.3)	15 (14.9-15)	15.2 (15.1-15.2)	14.9 (14.8-15)	15.8 (15.8-15.9)	16.6 (16.5-16.7)	17.3 (17.2-17.4)	18.6 (18.4-18.8)
Espírito Santo	12.4 (11.6-13.3)	12.6 (11.7-13.6)	12.8 (11.9-13.8)	13.1 (12.3-14.1)	13.3 (12.4-14.2)	13.5 (13-14.1)	14.3 (14.3-14.4)	14.8 (14.7-14.9)	15.8 (15.7-15.8)	16.3 (16.2-16.4)	17 (17-17.1)	17.6 (17.6-17.7)	18.3 (18.3-18.4)	19.3 (19.1-19.5)
Goiás	10.6 (9.9-11.4)	10.8 (10.1-11.6)	11.2 (10.4-12)	11.6 (10.8-12.4)	11.9 (11.1-12.8)	12.6 (11.8-13.5)	13.7 (13.3-14.1)	13.7 (13.6-13.7)	15.2 (15.1-15.3)	15.7 (15.7-15.7)	17 (16.9-17)	17.7 (17.7-17.8)	17.9 (17.9-18)	18.6 (18.5-18.8)
Maranhão	11.1 (10.4-11.9)	11.4 (10.7-12.2)	11.8 (11-12.6)	12.2 (11.5-13.1)	12.6 (11.7-13.5)	12.8 (11.8-13.7)	14.2 (13.4-15.1)	15.7 (15.2-16.2)	18 (17.7-18.3)	18.4 (18.1-18.7)	19.2 (18.9-19.6)	18.6 (18.3-19)	19 (18.7-19.4)	19 (18.8-19.2)
Mato Grosso	12.6 (11.7-13.6)	13 (12-14)	13.4 (12.4-14.5)	13.9 (12.9-15.1)	14.5 (13.5-15.6)	15.5 (14.7-16.4)	16.9 (16.5-17.4)	16 (15.6-16.4)	16.8 (16.4-17.2)	17.5 (17.4-17.6)	17.5 (17.4-17.6)	17.5 (17.4-17.5)	17.9 (17.9-18)	18.8 (18.6-19)
Mato Grosso do Sul	12.6 (11.8-13.6)	12.8 (11.9-13.8)	13.1 (12.2-14.2)	13.5 (12.5-14.5)	13.7 (12.8-14.7)	14.1 (13.5-14.9)	14.9 (14.8-15)	15.3 (15.3-15.4)	15.7 (15.6-15.8)	15.8 (15.7-15.9)	16.8 (16.7-16.8)	17.2 (17.1-17.2)	17.7 (17.6-17.7)	18.1 (17.9-18.3)
Minas Gerais	11.6 (10.7-12.5)	11.9 (11-12.8)	12.2 (11.3-13.2)	12.6 (11.7-13.5)	12.9 (12-13.8)	13.2 (12.7-13.7)	14 (14-14.1)	14.6 (14.5-14.6)	15.6 (15.6-15.7)	16.4 (16.4-16.5)	17.8 (17.7-17.8)	18.4 (18.3-18.4)	18.8 (18.7-18.8)	19.2 (19.1-19.4)
Pará	13.2 (12.2-14.2)	13.5 (12.6-14.6)	13.9 (13-15.1)	14.3 (13.3-15.5)	14.6 (13.7-15.7)	15.1 (14.4-15.8)	16.5 (16.4-16.6)	16.5 (16.3-16.7)	17 (16.8-17.3)	17.8 (17.6-18.1)	18.5 (18.3-18.8)	18.6 (18.3-18.8)	19 (18.9-19)	18.7 (18.6-18.9)
Paraíba	12.7 (11.8-13.6)	13 (12.1-13.9)	13.4 (12.5-14.5)	14 (13-15.1)	14.5 (13.5-15.5)	15.1 (14.4-15.8)	15.9 (15.8-16)	16.9 (16.8-17)	17.4 (17.2-17.6)	17.8 (17.5-18)	18.5 (18.3-18.8)	18.5 (18.2-18.7)	18.6 (18.4-18.9)	18.7 (18.2-19.1)
Paraná	11.2 (10.4-12.1)	11.4 (10.6-12.4)	11.7 (10.8-12.7)	12.1 (11.2-13.2)	12.6 (11.7-13.5)	13.1 (12.6-13.7)	13.3 (13.2-13.3)	13.9 (13.8-13.9)	14.5 (14.4-14.5)	15.2 (15.2-15.2)	16 (15.9-16)	16.8 (16.7-16.8)	17.2 (17.1-17.2)	17.7 (17.6-17.9)
Pernambuco	11.4 (10.7-12.3)	11.8 (11-12.6)	12.2 (11.4-13.2)	12.7 (11.8-13.7)	13.2 (12.3-14.1)	13.9 (13.4-14.5)	14.8 (14.8-14.9)	14.9 (14.9-14.9)	15.6 (15.6-15.7)	16.7 (16.7-16.8)	17.1 (17-17.1)	17.7 (17.7-17.8)	18.1 (18.1-18.2)	17.4 (17.2-17.5)
Piauí	12.7 (11.8-13.7)	12.9 (12.1-14)	13.3 (12.3-14.3)	13.6 (12.6-14.6)	13.8 (12.8-14.8)	14.4 (13.4-15.3)	15.5 (14.9-16.1)	15.3 (15-15.5)	18 (17.7-18.3)	19.4 (19.1-19.7)	18.7 (18.4-19)	18.7 (18.4-19)	19.7 (19.7-19.8)	19.8 (19.6-20)
Rio de Janeiro	11.8 (11-12.6)	12.1 (11.3-13)	12.5 (11.6-13.4)	12.9 (12-13.9)	13.2 (12.3-14.1)	13.4 (13-14)	14 (14-14)	14.1 (14.1-14.2)	14.3 (14.3-14.4)	14.7 (14.7-14.7)	16 (15.9-16)	16.7 (16.6-16.7)	17 (17-17)	17.4 (17.2-17.5)
Rio Grande do Norte	13.2 (12.3-14.2)	13.6 (12.7-14.6)	14.1 (13.1-15.2)	14.7 (13.6-15.8)	15.1 (14.1-16.2)	15.8 (14.9-16.8)	16.9 (16.5-17.4)	17.8 (17.5-18.1)	18.4 (18.1-18.7)	18 (17.7-18.4)	19.3 (18.9-19.6)	19.7 (19.5-20)	20.2 (20.1-20.3)	19.3 (19.1-19.5)
Rio Grande do Sul	11.9 (11-12.8)	12.1 (11.3-13.1)	12.4 (11.6-13.4)	12.8 (11.9-13.9)	13.3 (12.4-14.2)	13.8 (13.2-14.3)	13.9 (13.8-13.9)	14.5 (14.4-14.5)	14.9 (14.9-15)	15.3 (15.2-15.3)	16.3 (16.2-16.3)	16.9 (16.9-17)	17.5 (17.5-17.5)	17.9 (17.8-18.1)
Rondônia	9.8 (9.2-10.6)	10.1 (9.4-10.9)	10.6 (9.9-11.4)	11 (10.3-11.8)	11.3 (10.6-12.1)	11.9 (11.2-12.6)	13 (12.6-13.5)	13.7 (13.3-14.1)	14.8 (14.5-15.3)	16.2 (15.8-16.6)	16.7 (16.2-17.1)	16.8 (16.3-17.2)	17.7 (17.3-18.1)	17.8 (17.1-18.5)
Roraima	10.5 (9.9-11.4)	10.8 (10.1-11.6)	11.2 (10.4-12.1)	11.6 (10.9-12.5)	12 (11.2-12.8)	12.5 (11.8-13.2)	13.7 (13.2-14.3)	14.3 (13.8-14.8)	15.3 (14.8-15.8)	16 (15.5-16.4)	16.1 (15.7-16.6)	16.6 (16.1-17.1)	17.6 (17.1-18.1)	17.8 (17.2-18.5)
Santa Catarina	12.2 (11.2-13.2)	12.4 (11.5-13.4)	12.7 (11.7-13.6)	13.1 (12.1-14)	13.4 (12.5-14.4)	14 (13.4-14.6)	14 (13.9-14)	14.4 (14.4-14.5)	14.6 (14.6-14.7)	15.2 (15.1-15.2)	16.1 (16.1-16.2)	17.3 (17.2-17.3)	17.8 (17.7-17.8)	18.3 (18.1-18.5)
São Paulo	12 (11.1-13)	12.3 (11.4-13.2)	12.6 (11.7-13.7)	13.1 (12.2-14.1)	13.6 (12.7-14.5)	14 (13.4-14.5)	13.8 (13.8-13.8)	14.4 (14.4-14.4)	14.8 (14.8-14.8)	15.3 (15.3-15.3)	16.1 (16.1-16.1)	16.9 (16.9-17)	17.5 (17.4-17.5)	18.1 (18-18.2)
Sergipe	12.8 (11.9-13.8)	13.1 (12.2-14.1)	13.5 (12.6-14.5)	13.9 (13-15)	14.4 (13.5-15.3)	15 (14.4-15.6)	15.3 (15.2-15.3)	16.4 (16.3-16.5)	16.7 (16.6-16.8)	16.6 (16.5-16.7)	17.8 (17.7-17.9)	18.6 (18.6-18.7)	18.8 (18.7-18.8)	18.6 (18.4-18.8)
Tocantins	11.3 (10.6-12.2)	11.7 (10.9-12.6)	12.1 (11.2-13)	12.6 (11.7-13.5)	12.9 (12-13.9)	13.6 (12.7-14.6)	15 (14.1-15.9)	15.9 (15.2-16.6)	16.4 (16-16.7)	16.6 (16.4-16.8)	17.5 (17.4-17.7)	18.7 (18.5-18.9)	19.4 (19.2-19.6)	19.3 (19-19.7)
Paraguay	14.6 (13.9-15.2)	16.2 (15.8-16.6)	16.4 (16-16.7)	16.9 (16.5-17.2)	16.5 (16.1-16.8)	16.4 (16.1-16.7)	16.7 (16.3-17)	16.4 (16-16.7)	17.4 (17.1-17.8)	17.4 (17-17.8)	17.9 (17.5-18.3)	17.7 (17.4-18.1)	17.4 (17.1-17.8)	17.9 (16.8-18.9)
North Africa and Middle East	11.6 (11.4-11.8)	12.1 (11.9-12.3)	12.5 (12.3-12.8)	13 (12.8-13.2)	13.1 (12.9-13.3)	13.6 (13.4-13.7)	13.9 (13.7-14)	14 (13.9-14.1)	14.6 (14.5-14.7)	14.9 (14.8-15.1)	15.6 (15.4-15.7)	16.1 (16-16.2)	16.4 (16.3-16.6)	17.1 (16.9-17.3)
North Africa and Middle East	11.6 (11.4-11.8)	12.1 (11.9-12.3)	12.5 (12.3-12.8)	13 (12.8-13.2)	13.1 (12.9-13.3)	13.6 (13.4-13.7)	13.9 (13.7-14)	14 (13.9-14.1)	14.6 (14.5-14.7)	14.9 (14.8-15.1)	15.6 (15.4-15.7)	16.1 (16-16.2)	16.4 (16.3-16.6)	17.1 (16.9-17.3)
Afghanistan	9.5 (8.9-10.1)	9.6 (9-10.2)	9.7 (9.1-10.3)	9.8 (9.2-10.4)	9.8 (9.4-10.4)	9.9 (9.4-10.3)	9.8 (9.3-10.2)	9.9 (9.4-10.4)	10.6 (10-11.3)	10.5 (10-11.2)	10.6 (10.1-11.3)	11 (10.4-11.6)	11.6 (11-12.1)	12.3 (11.8-12.9)
Algeria	12.5 (12.3-12.7)	13.3 (13.1-13.4)	14 (13.8-14.3)	14.8 (14.5-15)	13.9 (13.6-14.3)	13.2 (12.9-13.6)	13.2 (13-13.4)	15.4 (15.2-15.6)	16.3 (16-16.7)	16.9 (16.6-17.3)	17.3 (16.9-17.6)	17.5 (17.2-17.9)	18 (17.7-18.2)	18.7 (18.4-19)
Bahrain	10.5 (9.7-11.3)	10.7 (10-11.7)	11.2 (10.4-12.1)	11.6 (10.8-12.5)	12.1 (11.3-12.8)	12.6 (12.1-13.2)	13.7 (13.3-14.1)	13.4 (13.1-13.6)	12.9 (12.7-13.1)	12.6 (12.4-12.8)	14 (13.8-14.2)	15 (14.8-15.2)	17.3 (17-17.5)	18.2 (17.6-18.7)
Egypt	11.9 (11.8-11.9)	13.2 (13.1-13.3)	13.4 (13.4-13.4)	13.8 (13.8-13.9)	12.6 (12.6-12.6)	12.8 (12.8-12.8)	13.1 (13.1-13.1)	12.2 (12.2-12.2)	13.1 (13-13.1)	13.2 (13.2-13.2)	13.7 (13.6-13.7)	13.5 (13.5-13.6)	13.5 (13.4-13.5)	13.7 (13.2-14.3)
Iran	11.7 (11.4-11.9)	11.9 (11.7-12.1)	12.3 (12.1-12.6)	13 (12.8-13.2)	13.9 (13.7-14.2)	15 (14.8-15.3)	15.6 (15.4-15.8)	15.6 (15.4-15.8)	15.7 (15.5-15.9)	16.5 (16.4-16.7)	17 (16.9-17.1)	17.2 (17.1-17.2)	17.5 (17.4-17.5)	18 (17.9-18)
Iraq	12 (11.5-12.5)	12.8 (12.4-13.1)	13.5 (13.2-13.7)	13.6 (13.4-13.8)	13.6 (13.4-13.9)	13.7 (13.3-14)	14.1 (13.6-14.6)	13.8 (13.2-14.4)	13.6 (13-14.3)	13.2 (12.7-13.8)	13.5 (13-14.1)	15.1 (14.5-15.7)	17.2 (16.7-17.8)	20.2 (19.7-20.6)
Jordan	12.1 (11.3-13.1)	12.4 (11.7-13.2)	13 (12.3-13.8)	13.9 (13.3-14.4)	14.8 (14.4-15.1)	15.4 (15.1-15.6)	14.9 (14.5-15.2)	15.3 (14.8-15.8)	15.4 (14.8-16)	15.2 (14.6-15.8)	15.7 (15.2-16.2)	16.4 (16.1-16.8)	18 (17.7-18.2)	19 (18.2-19.7)
Kuwait	14.1 (13.2-15.1)	14.3 (13.5-15.2)	14.6 (14.1-15.1)	14.8 (14.7-14.9)	15.9 (15.8-16)	16.1 (16-16.2)	16.8 (16.7-16.9)	18.1 (18-18.2)	18.3 (18.2-18.4)	19.8 (19.7-19.9)	19.1 (19.1-19.2)	19.5 (19.4-19.5)	20.8 (20.7-20.9)	21.9 (21.5-22.2)
Lebanon	12.6 (11.7-13.6)	12.9 (12-13.9)	13.2 (12.3-14.3)	13.5 (12.6-14.5)	13.9 (13.1-14.8)	14 (13.3-14.9)	14.4 (13.9-15.2)	14.5 (14-15.1)	15 (14.6-15.7)	15.8 (15.2-16.5)	16.8 (16.2-17.3)	17.1 (16.7-17.5)	17.4 (17.2-17.8)	18 (17.6-18.4)
Libya	10.9 (10.1-11.7)	11.7 (10.9-12.6)	12.6 (11.8-13.4)	14 (13.2-14.7)	15.1 (14.6-15.6)	15.4 (15-15.9)	16.1 (15.6-16.7)	16.4 (15.6-17.1)	16.3 (15.6-17.1)	16.5 (15.9-17.2)	16.4 (15.9-17)	16.7 (16.2-17.1)	15.8 (15.3-16.3)	16 (15.2-16.8)
Morocco	10.8 (10-11.6)	11 (10.3-11.8)	11.1 (10.3-12)	11.8 (11-12.7)	12.3 (11.5-13.2)	12.8 (12.1-13.6)	13.3 (12.8-13.9)	14.2 (13.7-14.7)	14.7 (14.3-15.1)	14.7 (14.4-15)	14.8 (14.4-15.2)	15.1 (14.6-15.6)	15.4 (14.7-16.1)	16.2 (15.2-17.2)
Palestine	11.3 (10.6-12.2)	11.9 (11.1-12.8)	12.6 (11.7-13.6)	13.4 (12.4-14.4)	13.7 (12.7-14.7)	14.6 (13.6-15.6)	14.8 (13.9-15.8)	15 (14.1-16)	15 (14.1-16)	15 (14.3-15.8)	14.9 (14.5-15.3)	14.8 (14.6-15)	15.7 (15.5-15.9)	17.1 (16.6-17.6)
Oman	9.4 (8.9-10)	9.8 (9.2-10.5)	10.2 (9.5-10.9)	10.7 (10-11.6)	12.3 (11.4-13.2)	13.2 (12.2-14.2)	13.7 (12.7-14.6)	14.2 (13.2-15.2)	14.5 (13.5-15.6)	14.8 (13.9-15.9)	15.4 (14.3-16.5)	16.4 (15.4-17.2)	16.3 (15.4-17.1)	17.5 (16.7-18.5)
Qatar	13.1 (12.3-14.1)	13.3 (12.3-14.2)	13.4 (12.5-14.4)	13.5 (12.5-14.5)	13.7 (12.8-14.7)	13.8 (13-14.7)	13.7 (12.9-14.6)	13.7 (13-14.5)	13.7 (13-14.4)	14 (13.2-14.7)	15.1 (14.4-15.9)	17 (16.2-17.8)	17.7 (16.9-18.6)	19.2 (18.2-20.2)
Saudi Arabia	12.9 (12-13.8)	13.3 (12.3-14.3)	13.7 (12.7-14.7)	14.3 (13.3-15.3)	15 (14-16.1)	15.9 (14.9-17)	16.3 (15.3-17.4)	16.3 (15.3-17.3)	16.2 (15.2-17.1)	16.6 (15.7-17.4)	16.8 (16.1-17.5)	16.4 (15.9-17)	16 (15.5-16.7)	17.5 (16.8-17.9)
Sudan	11.2 (10.4-12)	11.3 (10.6-12.1)	11.6 (10.8-12.4)	11.8 (11-12.6)	11.9 (11.3-12.7)	12.1 (11.6-12.7)	12.5 (12-13)	12.2 (11.7-12.6)	13.2 (12.6-13.9)	13.6 (12.9-14.3)	14 (13.2-14.9)	14.5 (13.6-15.4)	15 (14.1-16)	15.5 (14.4-16.8)
Syria	14.5 (13.5-15.6)	15.2 (14.2-16.2)	15.9 (15-16.9)	16.6 (15.8-17.5)	17.2 (16.5-17.9)	18 (17.4-18.7)	16.9 (16.3-17.6)	15.4 (14.8-16)	15 (14.5-15.5)	14.7 (14.2-15.3)	15.2 (14.7-15.7)	16.4 (15.9-16.8)	16.7 (16.1-17.3)	16.6 (15.8-17.4)
Tunisia	11.1 (10.4-11.9)	11.5 (10.9-12.1)	12 (11.6-12.5)	11.5 (11.2-11.9)	12 (11.7-12.2)	13.5 (13.3-13.7)	14.9 (14.6-15.2)	16.1 (15.9-16.3)	16.2 (16-16.5)	16.1 (15.8-16.3)	16.1 (15.6-16.6)	16.9 (16-17.8)	17.5 (16.4-18.6)	18 (16.8-19.2)
Turkey	11.8 (11-12.9)	12.5 (11.6-13.5)	13 (12-14.1)	13.6 (12.5-14.7)	14.1 (13.1-15.1)	14.6 (13.9-15.4)	15 (14.7-15.3)	15.4 (15-15.7)	15.6 (15.3-15.9)	16.1 (15.8-16.4)	17.6 (17.3-18)	18.6 (18.3-18.9)	18.5 (18.2-18.9)	19.1 (18.5-19.6)
United Arab Emirates	12.3 (11.3-13.3)	12.6 (11.6-13.7)	13 (11.9-14.1)	13.5 (12.5-14.6)	13.8 (12.8-15)	13.9 (12.9-15)	14.2 (13.1-15.2)	14.3 (13.3-15.3)	14.1 (13.2-15.1)	14.5 (13.7-15.3)	13.8 (13-14.5)	14.5 (13.8-15.3)	14.8 (14-15.8)	15 (13.9-16.1)
Yemen	9.8 (9-10.8)	10 (9.1-11)	10.3 (9.3-11.3)	10.4 (9.4-11.5)	10.7 (9.6-11.8)	11.4 (10.3-12.7)	12 (10.7-13.3)	12.4 (11.1-13.7)	12.7 (11.3-14.1)	13 (11.6-14.4)	13.4 (12-14.8)	13.9 (12.5-15.3)	14.4 (12.9-15.8)	14.5 (13.2-16)
South Asia	10.2 (10-10.4)	10.4 (10.2-10.7)	10.5 (10.3-10.8)	10.6 (10.4-10.9)	11 (10.9-11.3)	11.6 (11.4-11.8)	12 (11.8-12.2)	12.3 (12.2-12.5)	12.6 (12.4-12.7)	13.1 (13-13.2)	13.3 (13.2-13.4)	14.1 (14-14.2)	14.2 (14.1-14.3)	14.7 (14.6-14.8)
South Asia	10.2 (10-10.4)	10.4 (10.2-10.7)	10.5 (10.3-10.8)	10.6 (10.4-10.9)	11 (10.9-11.3)	11.6 (11.4-11.8)	12 (11.8-12.2)	12.3 (12.2-12.5)	12.6 (12.4-12.7)	13.1 (13-13.2)	13.3 (13.2-13.4)	14.1 (14-14.2)	14.2 (14.1-14.3)	14.7 (14.6-14.8)
Bangladesh	11.2 (10.4-12.1)	11.6 (10.8-12.5)	11.9 (11-12.7)	12.2 (11.5-13.1)	11.8 (11.2-12.5)	13.1 (12.5-13.8)	14 (13.6-14.4)	12.9 (12.6-13.2)	13.2 (12.9-13.6)	14.3 (13.9-14.6)	15 (14.7-15.3)	15.2 (14.9-15.4)	15.4 (15.1-15.7)	16.8 (16.1-17.4)
Bhutan	9.4 (8.9-10.1)	9.8 (9.2-10.5)	10.2 (9.6-11)	10.8 (10.1-11.6)	11.4 (10.6-12.2)	11.8 (11-12.6)	12.2 (11.4-13)	12.8 (12-13.5)	13.5 (12.7-14.2)	14.1 (13.4-14.7)	14.8 (14.2-15.4)	15.5 (14.9-16.2)	16 (15.3-16.9)	16.6 (15.5-17.8)
India	9.8 (9.6-10.1)	10.1 (9.8-10.3)	10.1 (9.9-10.4)	10.2 (9.9-10.4)	10.6 (10.4-10.8)	11 (10.8-11.2)	11.4 (11.2-11.6)	11.9 (11.7-12.1)	12.3 (12.1-12.5)	12.9 (12.8-13)	13.1 (13-13.2)	14 (13.9-14.1)	14.1 (14-14.2)	14.5 (14.3-14.6)
Andhra Pradesh	10.1 (9-11.3)	10.3 (9.1-11.6)	10.5 (9.3-11.8)	10.6 (9.5-11.8)	10.9 (9.8-12.1)	11.4 (10.4-12.6)	11.8 (10.6-13)	12.3 (11.1-13.6)	12.5 (11.4-13.9)	13.1 (11.9-14.5)	13.4 (12.1-14.8)	14.7 (13.2-16.2)	15.1 (13.7-16.6)	15.7 (14.2-17.1)
Arunachal Pradesh	10.2 (9-11.5)	10.4 (9.3-11.7)	10.4 (9.2-11.7)	10.2 (9.2-11.4)	10.9 (9.8-12)	11.4 (10.4-12.7)	11.9 (10.7-13.2)	12.4 (11.2-13.6)	12.5 (11.2-13.8)	12.9 (11.7-14.3)	12.9 (11.9-14.3)	13.8 (12.8-14.9)	14.1 (13.5-15.2)	14.5 (13.7-15.6)
Assam	8.8 (8-9.7)	9 (8.2-10.1)	9.1 (8.3-10.1)	9.1 (8.3-10)	9.6 (8.9-10.5)	10.2 (9.4-11)	10.6 (9.8-11.4)	11.3 (10.6-12)	11.6 (11.1-12.2)	11.9 (11.7-12.2)	11.7 (11.5-11.9)	12.4 (12.2-12.7)	12.6 (12.4-12.8)	13.7 (13.2-14.1)
Bihar	10.4 (9.4-11.6)	10.6 (9.6-11.8)	10.6 (9.7-11.8)	10.6 (9.8-11.6)	11 (10.2-11.8)	11.2 (10.4-12)	11.3 (10.5-12.2)	11.8 (10.9-12.6)	12 (11.3-12.8)	12.5 (11.8-13.1)	12.4 (12-12.9)	13.5 (13.3-13.8)	13.6 (13.4-13.9)	14.6 (14.1-15)
Chhattisgarh	9.9 (8.9-10.9)	10.1 (9.2-11.1)	10.2 (9.2-11.1)	10.1 (9.3-11)	10.6 (9.8-11.5)	11 (10.2-11.8)	11.6 (10.8-12.4)	11.8 (11.1-12.7)	12.2 (11.4-13)	12.7 (12.2-13.3)	12.6 (12.2-13.1)	12.9 (12.7-13.2)	12.3 (12.1-12.5)	12.3 (11.9-12.6)
Delhi	10.4 (9.4-11.5)	10.9 (9.9-12)	11.2 (10.2-12.3)	11 (10.2-11.9)	12.1 (11.2-13)	12.5 (11.7-13.5)	12.7 (11.9-13.7)	13.1 (12.2-14.1)	13.2 (12.4-14.2)	13.7 (13-14.5)	13.8 (13.2-14.4)	14.7 (14.3-15.2)	15.5 (15.1-15.9)	16.2 (15.5-16.9)
Goa	12 (10.8-13.2)	12.2 (11-13.5)	12.4 (11.1-13.7)	12.5 (11.3-13.6)	12.9 (11.8-14)	13.6 (12.5-14.6)	14 (13-15.1)	14.2 (13.2-15.3)	14.2 (13.3-15.1)	14.7 (13.9-15.6)	14.9 (14-16)	15.9 (14.7-17.2)	16.4 (15-17.7)	16.7 (15.4-17.9)
Gujarat	10.1 (9.3-11.1)	10.4 (9.5-11.4)	10.4 (9.6-11.4)	10.4 (9.6-11.3)	10.9 (10.1-11.7)	11.3 (10.6-12.1)	11.8 (11-12.6)	12.5 (11.8-13.3)	12.9 (12.3-13.5)	14.1 (13.8-14.5)	13.6 (13.3-13.9)	14.3 (14-14.6)	14.7 (14.4-15)	15.5 (15-16)
Haryana	10.4 (9.5-11.6)	10.7 (9.7-11.8)	10.8 (9.8-11.9)	10.9 (10-11.9)	11.4 (10.6-12.3)	12.2 (11.4-13.1)	12.8 (11.9-13.7)	13.5 (12.8-14.4)	14 (13.3-14.6)	14.4 (14.1-14.8)	14.5 (14.3-14.8)	15.2 (14.9-15.5)	14.9 (14.6-15.2)	14.9 (14.5-15.4)
Himachal Pradesh	10.3 (9.3-11.5)	10.5 (9.4-11.7)	10.6 (9.6-11.9)	10.7 (9.7-11.9)	11.1 (10.2-12.1)	11.6 (10.6-12.6)	12 (11.1-13.1)	12.6 (11.8-13.6)	13.1 (12.4-13.9)	14.1 (13.7-14.6)	14.8 (14.4-15.2)	15.5 (15.2-15.9)	15.8 (15.5-16.1)	16.2 (15.5-16.8)
Jammu and Kashmir	11.1 (10-12.3)	11.3 (10.2-12.5)	11.4 (10.3-12.6)	11.2 (10.3-12.2)	11.8 (10.8-12.7)	12.1 (11.3-13)	12 (11.1-13)	12.9 (12-13.9)	13.1 (12.3-14)	13.8 (13.1-14.6)	14.4 (13.8-15)	15.7 (15.3-16.1)	15.5 (15.2-15.8)	16.6 (16.1-17.2)
Jharkhand	10.1 (9.3-11.1)	10.3 (9.4-11.3)	10.3 (9.4-11.4)	10.3 (9.4-11.2)	10.6 (9.8-11.5)	11 (10.2-11.8)	11.1 (10.3-12)	11.1 (10.4-11.9)	11.2 (10.5-12)	11.6 (11-12.2)	11.6 (11.2-12.1)	12.5 (12.3-12.8)	13.2 (13-13.5)	14.1 (13.7-14.5)
Karnataka	9.8 (8.9-10.8)	10 (9.1-11.1)	10.1 (9.2-11.1)	10.2 (9.4-11.2)	10.6 (9.8-11.5)	11.2 (10.4-12)	11.7 (11-12.6)	12.5 (11.8-13.3)	12.9 (12.4-13.5)	13.5 (13.2-13.8)	13.7 (13.5-14)	14.8 (14.6-15.1)	14.2 (13.9-14.4)	13.3 (13-13.7)
Kerala	10.8 (9.8-11.9)	11.2 (10.1-12.3)	11.4 (10.4-12.5)	11.5 (10.6-12.7)	11.9 (11.1-12.8)	12.3 (11.5-13.2)	12.6 (11.8-13.6)	13.2 (12.4-14)	13.5 (12.8-14.2)	14.5 (14.1-15)	14 (13.7-14.4)	15.4 (15-15.8)	15.7 (15.4-16.1)	15.6 (15-16.2)
Madhya Pradesh	9.7 (8.9-10.7)	10 (9.1-11.1)	9.9 (9.1-11)	9.9 (9.1-10.7)	10.3 (9.6-11.2)	10.7 (10-11.4)	10.6 (9.9-11.4)	10.9 (10.2-11.7)	11.2 (10.5-12)	11.8 (11.2-12.4)	11.9 (11.5-12.3)	13.1 (12.8-13.4)	12.9 (12.6-13.1)	13.4 (13-13.9)
Maharashtra	10.2 (9.3-11.2)	10.5 (9.6-11.5)	10.6 (9.7-11.7)	10.7 (9.9-11.6)	11.2 (10.4-12.1)	11.6 (10.9-12.4)	12 (11.3-12.8)	12.6 (11.9-13.3)	13 (12.5-13.5)	13.6 (13.3-13.9)	13.6 (13.4-13.8)	15.2 (14.9-15.4)	15.2 (15-15.5)	15.3 (14.9-15.7)
Manipur	10.8 (9.6-12)	11 (9.8-12.3)	11.2 (9.8-12.4)	11.2 (10-12.4)	11.7 (10.6-12.8)	12.3 (11.2-13.4)	12.7 (11.6-13.8)	13 (11.9-14)	13.1 (12.1-14.2)	13.4 (12.5-14.4)	13.5 (12.7-14.4)	14.4 (13.6-15.4)	14.5 (13.7-15.4)	14.6 (13.8-15.7)
Meghalaya	11 (9.8-12.4)	11.2 (10-12.6)	11.3 (10-12.7)	11.1 (10-12.4)	11.7 (10.6-12.8)	12 (10.9-13.2)	12.2 (11.1-13.4)	12.6 (11.4-13.7)	12.7 (11.6-13.9)	13.1 (12.1-14.3)	13.1 (12.2-14.3)	14 (13.1-15.1)	14.3 (13.5-15.4)	14.6 (13.9-15.7)
Mizoram	11.3 (10.1-12.6)	11.5 (10.4-12.8)	11.6 (10.5-13)	11.3 (10.4-12.5)	12 (11.1-13.2)	12.5 (11.5-13.6)	12.7 (11.7-13.8)	13.2 (12.3-14.3)	13.4 (12.6-14.4)	13.8 (13.1-14.7)	13.8 (13.3-14.7)	14.7 (13.8-15.9)	15 (14.1-16.1)	15.2 (14.2-16.3)
Nagaland	10.8 (9.5-12.1)	11.1 (9.8-12.5)	11.3 (9.9-12.7)	11.3 (10.1-12.7)	12 (10.7-13.3)	12.6 (11.4-13.9)	12.9 (11.6-14.2)	13.3 (12-14.5)	13.4 (12.1-14.6)	13.8 (12.5-14.9)	13.7 (12.6-14.9)	14.7 (13.4-16.2)	14.9 (13.9-16.4)	15.2 (14.2-16.7)
Odisha	9.2 (8.4-10.2)	9.4 (8.5-10.5)	9.4 (8.5-10.4)	9.5 (8.6-10.4)	9.9 (9.1-10.8)	10.5 (9.7-11.4)	11.2 (10.3-12.1)	11.9 (11.2-12.7)	12.3 (11.8-12.9)	12.4 (12.1-12.6)	12 (11.8-12.2)	13.1 (12.9-13.4)	13.7 (13.4-13.9)	14.4 (14-14.8)
Punjab	10.9 (10-12)	11.2 (10.2-12.3)	11.4 (10.4-12.5)	11.6 (10.7-12.6)	12.1 (11.2-13.1)	12.7 (11.9-13.6)	13.2 (12.3-14.2)	14.1 (13.3-15)	14.5 (13.9-15.2)	15 (14.7-15.4)	15.1 (14.8-15.3)	15.4 (15.2-15.7)	16.1 (15.8-16.4)	17.1 (16.6-17.5)
Rajasthan	10.1 (9.2-11.1)	10.3 (9.3-11.3)	10.3 (9.3-11.3)	10.2 (9.3-11.1)	10.7 (9.9-11.5)	11 (10.3-11.9)	11.5 (10.7-12.4)	12.2 (11.4-13)	12.6 (11.9-13.2)	13 (12.6-13.4)	13.9 (13.6-14.2)	14.7 (14.4-15)	14.7 (14.4-15)	14.4 (13.9-14.9)
Sikkim	10.7 (9.4-12.1)	10.9 (9.6-12.3)	10.9 (9.6-12.3)	10.9 (9.7-12.2)	11.3 (10.1-12.5)	11.7 (10.6-12.9)	12.1 (10.9-13.3)	12.5 (11.4-13.7)	12.6 (11.7-13.8)	13 (12.2-14)	13.1 (12.4-13.9)	13.9 (13.3-14.9)	14.4 (13.8-15.6)	15 (14.2-16.4)

Table 9 Estimates of life expectancy at age 65 by location and year for 1950, 1955...2005, 2010, 2017 - both sexes

Location	1950	1955	1960	1965	1970	1975	1980	1985	1990	1995	2000	2005	2010	2017
Tamil Nadu	9.1 (8.3-10)	9.3 (8.5-10.2)	9.3 (8.6-10.2)	9.4 (8.7-10.2)	9.7 (9-10.5)	10.2 (9.6-11)	10.7 (10-11.5)	11.3 (10.7-12.1)	11.8 (11.3-12.3)	12.5 (12.3-12.8)	12.6 (12.4-12.9)	14 (13.8-14.3)	13.6 (13.3-13.8)	14.1 (13.8-14.5)
Telangana	10.3 (9.1-11.6)	10.5 (9.3-11.9)	10.7 (9.4-12)	10.8 (9.5-12.1)	11.1 (9.9-12.3)	11.5 (10.4-12.8)	11.8 (10.6-13.1)	12 (10.8-13.2)	12 (10.9-13.2)	12.5 (11.4-13.8)	12.6 (11.4-13.8)	13.7 (12.5-15)	14.3 (13.1-15.6)	15.2 (14-16.4)
Tripura	10.1 (9-11.2)	10.4 (9.3-11.7)	10.6 (9.4-11.8)	10.6 (9.6-11.9)	11.3 (10.2-12.4)	11.9 (10.8-13)	12.2 (11.1-13.4)	12.6 (11.5-13.8)	12.7 (11.5-13.9)	12.9 (11.9-14.2)	13.1 (12.3-14.2)	14.1 (13.2-15.1)	14.3 (13.6-15.2)	14.5 (13.8-15.1)
Uttar Pradesh	9 (8.2-9.8)	9.2 (8.5-10.1)	9.3 (8.5-10.1)	9.3 (8.7-10.1)	9.8 (9.2-10.4)	10.2 (9.7-10.8)	10.5 (9.9-11.2)	11.2 (10.6-11.8)	11.6 (11-12.2)	12.4 (11.9-12.9)	13.3 (12.9-13.7)	13.2 (12.9-13.5)	13.3 (13-13.5)	13.5 (13-13.9)
Uttarakhand	9.8 (9-10.7)	9.9 (9.1-11)	10 (9.2-10.9)	10 (9.3-10.9)	10.4 (9.7-11.1)	10.8 (10.2-11.5)	11.1 (10.5-11.8)	11.5 (10.8-12.2)	11.6 (10.9-12.3)	12.1 (11.4-12.8)	12.2 (11.6-12.8)	13.2 (12.7-13.7)	14 (13.6-14.4)	14.4 (14.1-14.7)
West Bengal	9.7 (8.8-10.8)	9.9 (9-11)	10 (9.2-11)	10.1 (9.3-10.9)	10.4 (9.6-11.3)	10.8 (10-11.5)	11.1 (10.4-11.9)	11.5 (10.9-12.2)	11.8 (11.1-12.4)	12.1 (11.7-12.5)	12.9 (12.5-13.2)	13.7 (13.4-14)	14 (13.6-14.3)	14.4 (13.8-14.9)
Union Territories other than Delhi	12.5 (11.3-13.6)	12.6 (11.4-13.8)	12.8 (11.6-13.9)	12.7 (11.6-13.7)	13.1 (12.1-14.2)	13.6 (12.7-14.5)	13.8 (12.8-14.8)	13.9 (13-14.8)	13.7 (13-14.6)	14 (13.4-15)	14.1 (13.6-15.2)	15.1 (14.2-16.3)	15.4 (14.4-16.7)	15.6 (14.5-16.9)
Nepal	10.1 (9.4-10.8)	10.3 (9.7-11.1)	10.6 (9.9-11.4)	10.9 (10.2-11.8)	11.2 (10.5-12.1)	11.4 (10.6-12.2)	11.6 (10.9-12.4)	12.1 (11.4-12.8)	12.7 (12.1-13.3)	13.4 (12.9-14)	14.4 (13.9-14.8)	14.9 (14.4-15.3)	14.7 (14.2-15.3)	14.8 (14.2-15.5)
Pakistan	12.3 (11.4-13.2)	12.5 (11.7-13.4)	12.8 (12-13.7)	13.2 (12.6-13.9)	13.9 (13.3-14.4)	14.5 (14.1-15)	14.8 (14.5-15.2)	14.7 (14.4-15)	14.1 (13.8-14.4)	13.5 (13.1-13.8)	13.4 (13.1-13.8)	13.5 (13.1-14)	14.1 (13.4-14.8)	14.7 (13.7-15.7)
Southeast Asia, East Asia, and Oceania	11.1 (10.9-11.3)	11.8 (11.5-12)	10.1 (9.9-10.3)	12.3 (12-12.5)	12.8 (12.6-13)	13.1 (13-13.3)	13.5 (13.3-13.7)	13.9 (13.8-14)	14.2 (14.1-14.3)	14.8 (14.7-14.8)	15.3 (15.2-15.4)	15.7 (15.6-15.8)	16.3 (16.2-16.4)	16.9 (16.7-17.1)
East Asia	11.2 (10.9-11.5)	11.9 (11.5-12.2)	9.7 (9.5-9.9)	12.3 (12-12.6)	12.9 (12.7-13.2)	13.2 (13-13.4)	13.5 (13.3-13.7)	13.9 (13.7-14.1)	14.2 (14.1-14.3)	14.9 (14.8-15)	15.5 (15.4-15.6)	15.9 (15.8-16)	16.5 (16.4-16.6)	17.1 (16.9-17.4)
China	11.2 (10.9-11.5)	11.8 (11.5-12.2)	9.6 (9.4-9.8)	12.2 (11.9-12.5)	12.9 (12.6-13.2)	13.2 (13-13.4)	13.4 (13.2-13.7)	13.8 (13.7-14)	14.2 (14-14.3)	14.9 (14.8-15)	15.5 (15.4-15.6)	15.9 (15.8-16)	16.5 (16.4-16.6)	17.1 (16.9-17.3)
North Korea	10.3 (9.7-11)	12.8 (11.8-13.9)	13.2 (12.2-14.4)	13.7 (12.6-14.9)	14.2 (13.3-15.4)	14.8 (14.1-15.8)	15 (14.3-15.9)	15.2 (14.5-16.1)	15.3 (14.6-16.3)	13.9 (13.4-14.6)	13.7 (13.3-14.3)	15.1 (14.6-15.8)	15.2 (14.6-15.8)	15.4 (14.6-16.2)
Taiwan (Province of China)	11.6 (10.8-12.5)	12 (12-12.1)	12.6 (12.5-12.6)	13.5 (13.4-13.5)	13.7 (13.7-13.7)	14.2 (14.2-14.3)	14.9 (14.9-15)	15.5 (15.5-15.6)	16.1 (16-16.1)	16.7 (16.7-16.7)	17.6 (17.6-17.6)	18.3 (18.3-18.4)	19.4 (19.3-19.4)	19.7 (19.3-20)
Oceania	8.8 (8.6-9.1)	9.2 (9-9.5)	9.7 (9.5-9.9)	10.1 (9.9-10.4)	10.3 (10.1-10.5)	10.6 (10.4-10.8)	11 (10.8-11.2)	11 (10.8-11.3)	11.1 (10.8-11.4)	11.1 (10.8-11.4)	11.2 (10.9-11.5)	11.3 (10.9-11.6)	11.4 (11.1-11.8)	11.8 (11.4-12.2)
American Samoa	13.2 (12.7-13.8)	13.2 (12.9-13.6)	13.2 (13-13.5)	13.5 (13.3-13.8)	13.8 (13.5-14.1)	14.1 (13.8-14.3)	14.2 (13.9-14.6)	14.4 (14.1-14.7)	14.5 (14.3-14.9)	14.7 (14.4-15)	14.8 (14.5-15.1)	14.7 (14.3-15)	14.8 (14.4-15.2)	14.9 (14.3-15.6)
Federated States of Micronesia	9.8 (9.2-10.4)	10 (9.4-10.7)	10.2 (9.7-11)	10.6 (10-11.2)	10.9 (10.3-11.6)	11.3 (10.7-12)	11.7 (11.1-12.4)	12 (11.4-12.6)	12.2 (11.7-12.8)	12.5 (12-13)	12.7 (12.3-13.2)	13.1 (12.7-13.5)	13.2 (12.8-13.7)	13.3 (12.7-13.8)
Fiji	11.2 (10.9-11.6)	12.3 (12-12.6)	12.8 (12.6-13.1)	13 (12.7-13.3)	12.1 (11.8-12.3)	12.6 (12.3-12.9)	13.6 (13.3-13.9)	13.7 (13.4-14)	13.5 (12.9-14.1)	12.7 (12.5-12.9)	12.2 (12.1-12.2)	12.8 (12.8-12.9)	13 (12.9-13.1)	13.3 (12.6-14.1)
Guam	12.2 (11.9-12.6)	12.4 (12.2-12.7)	13.1 (12.9-13.3)	13.8 (13.7-14)	13.4 (13.3-13.5)	14.1 (14-14.3)	15.5 (15.4-15.7)	16.6 (16.4-16.8)	16.2 (15.9-16.4)	16 (15.7-16.2)	16.4 (16.1-16.6)	16.8 (16.6-17.1)	17.1 (16.9-17.3)	16.7 (16.3-17.1)
Kiribati	9.8 (9.2-10.5)	10 (9.3-10.7)	10.2 (9.5-10.9)	10.4 (9.7-11.1)	10.7 (10-11.4)	11.2 (10.6-11.9)	11.4 (10.9-12.1)	11.3 (10.8-11.8)	11.3 (10.9-11.6)	11.2 (11-11.5)	11.3 (11-11.6)	11.5 (11.1-11.9)	11.7 (11.2-12.2)	12.1 (11.5-12.8)
Marshall Islands	9.7 (9.2-10.4)	10.1 (9.5-10.7)	10.4 (9.8-11.1)	10.8 (10.1-11.5)	11.1 (10.5-11.8)	11.4 (10.9-12.1)	11.6 (11.1-12.2)	11.7 (11.4-12)	11.6 (11.4-11.8)	11.4 (11.2-11.6)	11.2 (11-11.5)	11.3 (11-11.6)	11.6 (11.2-12)	12.1 (11.5-12.7)
Northern Mariana Islands	14.1 (13.4-14.9)	14.1 (13.5-14.9)	14.3 (13.7-15.1)	14.5 (13.9-15.2)	14.8 (14.2-15.6)	15.1 (14.5-15.9)	15.4 (14.7-16.2)	15.8 (15-16.8)	16.2 (15.3-17)	16.7 (15.9-17.3)	17 (16.5-17.5)	17.3 (16.8-17.8)	17.5 (16.9-18)	17.3 (16.5-18)
Papua New Guinea	8.3 (8-8.7)	8.4 (8.1-8.8)	8.6 (8.3-8.9)	8.8 (8.5-9.1)	9.1 (8.8-9.4)	9.4 (9.1-9.7)	9.7 (9.4-10)	9.8 (9.4-10.1)	9.9 (9.6-10.3)	10.2 (9.8-10.6)	10.3 (9.9-10.7)	10.2 (9.8-10.7)	10.4 (9.9-10.9)	10.8 (10.3-11.4)
Samoa	14.2 (13.4-14.9)	14.9 (14.3-15.6)	16 (15.5-16.5)	15.6 (15.2-16)	15.2 (14.8-15.7)	15.2 (14.8-15.7)	15.5 (15-16.1)	15.5 (14.8-16.2)	15.6 (14.8-16.4)	15.2 (14.5-16)	15.2 (14.6-15.8)	15.3 (14.8-15.9)	15 (14.6-15.6)	15.2 (14.6-15.9)
Solomon Islands	9 (8.6-9.6)	9.2 (8.7-9.8)	9.4 (8.9-10.1)	9.8 (9.2-10.3)	10.1 (9.6-10.7)	10.4 (9.8-10.9)	11 (10.5-11.7)	11.3 (10.7-12)	11.5 (11-12.2)	11.9 (11.3-12.5)	12.1 (11.5-12.7)	12.1 (11.7-12.7)	12.4 (11.9-12.9)	12.7 (12.3-13.2)
Tonga	11.4 (10.9-11.8)	11.8 (11.4-12.1)	12.2 (12-12.5)	12.6 (12.3-12.9)	12.9 (12.5-13.2)	13.1 (12.8-13.5)	13.4 (13.1-13.7)	13.7 (13.5-14)	14 (13.7-14.3)	14.1 (13.9-14.4)	14.2 (14-14.5)	14.4 (14.2-14.8)	14.7 (14.2-15.2)	15.1 (14.4-15.8)
Vanuatu	9.6 (9-10.5)	9.8 (9.1-10.7)	10.1 (9.4-11)	10.4 (9.6-11.3)	10.6 (9.8-11.6)	11 (10.2-12)	11.3 (10.6-12.3)	11.6 (10.9-12.5)	11.7 (11-12.6)	11.9 (11.1-12.7)	12 (11.2-12.9)	12 (11.2-13)	12.1 (11.3-13.1)	12.3 (11.5-13.3)
Southeast Asia	10.9 (10.7-11.2)	11.5 (11.2-11.8)	12 (11.7-12.3)	12.2 (11.9-12.5)	12.4 (12.2-12.7)	12.8 (12.6-13.1)	13.4 (13.2-13.7)	13.8 (13.7-14)	14.1 (13.9-14.3)	14.4 (14.2-14.5)	14.6 (14.5-14.8)	15 (14.9-15.2)	15.5 (15.3-15.6)	16 (15.8-16.2)
Cambodia	10.2 (9.5-10.9)	10.4 (9.7-11.1)	10.7 (10-11.5)	11 (10.2-11.9)	10.8 (10.2-11.7)	9.3 (8.8-9.9)	11.2 (10.7-11.9)	11.5 (11-12)	11.8 (11.4-12.3)	12.1 (11.7-12.5)	12.5 (12.2-13)	13.4 (12.9-13.8)	14.1 (13.6-14.5)	14.7 (14-15.1)
Indonesia	10.2 (9.9-10.5)	10.6 (10.3-10.9)	11 (10.7-11.4)	11.4 (11-11.7)	11.8 (11.4-12.1)	12.3 (12-12.7)	12.9 (12.6-13.2)	13.4 (13.1-13.6)	13.7 (13.5-13.9)	14.1 (13.8-14.3)	14 (13.9-14.2)	14.1 (13.9-14.4)	14.4 (14.1-14.7)	14.8 (14.5-15.2)
Laos	8.9 (8.4-9.4)	9.2 (8.7-9.8)	9.3 (8.8-10)	9.6 (9-10.2)	9.8 (9.2-10.5)	10.2 (9.5-10.9)	10.4 (9.8-11.2)	10.9 (10.2-11.7)	11.2 (10.6-11.9)	11.6 (11-12.2)	12.1 (11.6-12.6)	12.8 (12.2-13.4)	13.6 (12.9-14.2)	14.5 (13.7-15.3)
Malaysia	11.8 (10.9-12.7)	12.4 (11.8-13.1)	13.4 (13.3-13.4)	13.1 (13-13.1)	12.7 (12.6-12.8)	13.5 (13.4-13.6)	13.8 (13.5-14.1)	14 (13.9-14)	14.2 (14.2-14.2)	14.2 (14.2-14.2)	14.5 (14.4-14.5)	15.2 (15.2-15.3)	15.6 (15.5-15.7)	15.8 (15.3-16.3)
Maldives	8.5 (8-9)	8.8 (8.3-9.4)	9.3 (8.7-9.9)	9.8 (9.4-10.3)	10.4 (10.1-10.8)	10.9 (10.7-11.1)	11.6 (11.3-11.8)	12.5 (12.4-12.7)	13 (12.9-13.1)	13.5 (13.3-13.6)	15.1 (14.9-15.2)	16.4 (16.3-16.6)	18.7 (18.6-18.9)	20.3 (19.9-20.7)
Mauritius	11 (10.9-11.1)	11.3 (11.2-11.4)	11.5 (11.4-11.6)	13.3 (13.2-13.4)	13.5 (13.4-13.6)	12.6 (12.5-12.7)	13.4 (13.3-13.5)	13.5 (13.4-13.6)	14.1 (14-14.3)	14.8 (14.7-14.9)	15.3 (15.2-15.4)	15.6 (15.4-15.7)	16.6 (16.5-16.7)	17 (16.6-17.4)
Myanmar	8.8 (8.4-9.4)	9 (8.5-9.6)	9.3 (8.7-9.9)	9.5 (9-10.2)	9.8 (9.2-10.5)	10.4 (9.7-11.1)	10.8 (10.1-11.6)	11.3 (10.6-12.1)	11.5 (10.9-12.3)	11.8 (11.1-12.5)	12.2 (11.5-12.9)	12.8 (12.3-13.4)	13.8 (13.2-14.4)	14.7 (14.1-15.4)
Philippines	14.8 (14.4-15.2)	16 (15.5-16.5)	16.5 (16.1-16.8)	15.6 (15.3-15.9)	14.8 (14.5-15.1)	15.1 (14.8-15.4)	15.4 (15.1-15.7)	15 (14.7-15.3)	15.3 (15-15.7)	15.1 (14.8-15.4)	15 (14.7-15.3)	14.7 (14.3-15)	14.4 (14.1-14.8)	14.4 (13.7-15.2)
Sri Lanka	12.9 (12.9-12.9)	14.1 (14.1-14.2)	14.9 (14.9-15)	14.7 (14.6-14.7)	14.7 (14.7-14.7)	13.9 (13.8-13.9)	15.3 (15.3-15.4)	14.6 (14.6-14.6)	14.8 (14.7-15)	15 (15-15.1)	15.2 (15.2-15.2)	16.4 (16.1-16.6)	16.9 (16.8-16.9)	17.9 (17.1-18.9)
Seychelles	13.6 (12.7-14.4)	13.8 (13.1-14.5)	14.1 (13.5-14.6)	14.1 (13.7-14.5)	14.5 (14.3-14.8)	15.1 (14.9-15.3)	15.6 (15.4-15.8)	15.3 (15.1-15.5)	15.1 (14.9-15.2)	15.1 (15-15.3)	15.4 (15.2-15.6)	15.7 (15.5-15.8)	15.9 (15.8-16.1)	16.3 (16-16.6)
Thailand	13.8 (13.5-14.1)	15.4 (15.2-15.6)	15.4 (15.2-15.6)	15.1 (14.9-15.3)	15.1 (14.9-15.4)	14.8 (14.6-15.1)	14.6 (14.4-14.8)	15.9 (15.7-16.1)	15.9 (15.7-16.1)	16.4 (16.2-16.6)	17.7 (17.4-18)	18.7 (18.5-19)	19.4 (19.2-19.7)	20.2 (19.6-20.8)
Timor-Leste	9.6 (9-10.3)	9.9 (9.2-10.6)	10.2 (9.6-11.1)	10.7 (10-11.6)	11.2 (10.5-12.1)	11.5 (10.7-12.3)	12.2 (11.4-13)	13 (12.3-13.8)	13.5 (12.9-14.3)	13.9 (13.3-14.5)	14.3 (13.9-14.8)	14.6 (14.1-15.1)	14.9 (14.4-15.5)	15 (14.3-15.7)
Vietnam	11.6 (10.8-12.6)	12.2 (11.3-13.1)	12.5 (11.6-13.5)	12.8 (11.8-13.7)	12.9 (12-13.8)	13.5 (12.7-14.4)	14 (13.3-14.9)	14.5 (13.9-15.2)	14.7 (14.3-15.3)	15 (14.6-15.5)	15.2 (14.8-15.8)	15.7 (15.2-16.2)	16.3 (15.8-16.8)	16.6 (15.9-17.2)
Sub-Saharan Africa	10.4 (10.1-10.6)	10.6 (10.4-10.9)	10.9 (10.6-11.2)	11.3 (11-11.5)	11.5 (11.2-11.8)	12 (11.7-12.3)	12.3 (12-12.6)	12.5 (12.2-12.8)	12.7 (12.3-13)	12.6 (12.3-12.9)	12.5 (12.1-12.8)	13 (12.6-13.3)	13.6 (13.2-13.9)	14.5 (14.1-14.7)
Central sub-Saharan Africa	9.4 (9-9.8)	9.6 (9.2-10.1)	9.8 (9.4-10.4)	10 (9.6-10.6)	10.3 (9.9-10.8)	10.6 (10.1-11.1)	10.8 (10.4-11.3)	11.2 (10.7-11.6)	11.4 (11-11.8)	11.4 (11-11.8)	11.5 (11.1-11.9)	11.9 (11.5-12.4)	12.3 (11.8-12.8)	13.1 (12.5-13.7)
Angola	9.6 (9-10.3)	9.7 (9.1-10.3)	9.8 (9.2-10.5)	10 (9.4-10.7)	10.3 (9.6-11.1)	10.5 (9.8-11.3)	10.5 (9.8-11.3)	10.6 (9.9-11.4)	10.8 (10.1-11.6)	10.9 (10.3-11.7)	11.3 (10.7-12)	12 (11.4-12.7)	12.7 (12.1-13.5)	13.7 (13-14.6)
Central African Republic	9.1 (8.7-9.6)	9.2 (8.8-9.7)	9.4 (8.9-9.9)	9.6 (9.1-10.1)	9.7 (9.2-10.2)	9.7 (9.3-10.1)	9.8 (9.4-10.2)	9.9 (9.6-10.2)	9.6 (9.3-10)	9.5 (9.1-10)	9.5 (8.9-10.2)	9.7 (9-10.5)	10.1 (9.4-11.1)	10.6 (9.9-11.6)
Congo (Brazzaville)	8.9 (8.5-9.4)	9.1 (8.6-9.6)	9.3 (8.8-9.8)	9.5 (9-10.1)	9.7 (9.2-10.2)	10 (9.4-10.5)	10.3 (9.8-10.9)	10.8 (10.3-11.3)	10.5 (10.1-11)	10.4 (10-10.8)	10.6 (10.3-11.1)	11.4 (11-11.9)	12.1 (11.5-12.8)	12.7 (12.1-13.6)
DR Congo	9.4 (8.9-10.1)	9.7 (9.1-10.4)	9.9 (9.3-10.7)	10.1 (9.5-10.9)	10.4 (9.8-11.2)	10.7 (10.1-11.5)	11 (10.3-11.8)	11.4 (10.8-12.1)	11.8 (11.1-12.4)	11.7 (11.2-12.2)	11.7 (11.2-12.2)	12.1 (11.6-12.6)	12.3 (11.7-13)	13 (12.3-13.9)
Equatorial Guinea	8.5 (8-9.1)	8.6 (8-9.1)	8.7 (8.1-9.3)	9 (8.3-9.7)	9.3 (8.5-10)	9.5 (8.8-10.3)	9.9 (9.1-10.9)	10 (9.2-11)	9.9 (9.1-10.9)	10.2 (9.3-11.3)	12 (10.7-13.7)	13.7 (11.9-15.6)	14.3 (12.1-16.5)	14.9 (12.9-17.2)
Gabon	9.9 (9.3-10.6)	10 (9.4-10.8)	10.2 (9.6-11)	10.6 (9.9-11.3)	10.9 (10.2-11.6)	11.4 (10.7-12.1)	11.8 (11.3-12.5)	12.1 (11.6-12.6)	12.2 (11.7-12.7)	12.4 (11.9-13)	12.5 (11.8-13.1)	12.8 (12.1-13.6)	13.7 (13-14.4)	14.6 (13.9-15.3)
Eastern sub-Saharan Africa	9.6 (9.4-9.8)	9.8 (9.6-10)	10.1 (9.9-10.2)	10.3 (10.2-10.5)	10.6 (10.4-10.8)	10.9 (10.7-11.1)	11.2 (11-11.3)	11.2 (11.1-11.4)	11.3 (11.2-11.5)	11.3 (11.2-11.5)	11.6 (11.5-11.8)	12.5 (12.3-12.6)	13.4 (13.2-13.6)	14.2 (14-14.4)
Burundi	9 (8.5-9.5)	9.1 (8.7-9.6)	9.3 (8.9-9.8)	9.4 (9-9.9)	9.5 (9.2-10)	9.6 (9.2-10.1)	9.7 (9.3-10.2)	9.7 (9.2-10.2)	9.8 (9.4-10.3)	9.6 (9.2-10.1)	9.9 (9.4-10.4)	11 (10.4-11.7)	12 (11.3-12.9)	12.5 (11.8-13.5)
Comoros	10.2 (9.7-10.8)	10.4 (9.9-11)	10.6 (10.1-11.2)	11 (10.4-11.6)	11.3 (10.6-12)	11.5 (10.7-12.3)	11.5 (10.7-12.3)	11.7 (10.9-12.5)	12.1 (11.4-12.8)	12.4 (11.7-13)	12.9 (12.3-13.6)	13.8 (13.1-14.4)	14.2 (13.5-14.9)	14.4 (13.7-15.3)
Djibouti	11.7 (10.6-13)	11.9 (10.7-13.1)	12.1 (10.8-13.3)	12.3 (11-13.6)	12.6 (11.3-13.9)	13 (11.7-14.3)	13.1 (11.7-14.4)	13 (11.6-14.3)	12.9 (11.5-14.3)	12.8 (11.4-14.2)	12.9 (11.4-14.5)	13.3 (11.6-15.1)	13.8 (12.1-15.6)	14.3 (12.7-16)
Eritrea	8.7 (8.2-9.2)	8.8 (8.3-9.3)	9 (8.5-9.4)	8.9 (8.4-9.4)	9.1 (8.6-9.6)	9.2 (8.7-9.6)	9.3 (8.9-9.7)	9.4 (9.1-9.8)	9.2 (8.8-9.5)	10.3 (9.8-10.8)	10.8 (10.2-11.5)	11.2 (10.5-12)	11.6 (10.9-12.6)	12.4 (11.7-13.5)
Ethiopia	8.8 (8.4-9.1)	8.9 (8.5-9.2)	8.9 (8.6-9.3)	9 (8.7-9.4)	9.1 (8.8-9.5)	9.1 (8.8-9.5)	9.2 (8.9-9.5)	9.4 (9.1-9.7)	9.7 (9.5-10)	10.2 (9.9-10.4)	11 (10.7-11.3)	12.4 (12.1-12.7)	13.9 (13.6-14.2)	15 (14.5-15.4)
Kenya	10.9 (10.6-11.1)	11.2 (11-11.4)	11.6 (11.4-11.8)	11.9 (11.7-12.2)	12.4 (12.1-12.6)	12.8 (12.6-13.1)	13.3 (13-13.5)	13.6 (13.4-13.8)	13.5 (13.3-13.8)	13.2 (12.9-13.4)	12.8 (12.5-13)	13 (12.8-13.2)	13.7 (13.5-13.9)	14.3 (14.1-14.6)
Baringo	11.2 (9.8-12.9)	11.2 (9.9-12.9)	11.3 (10-12.9)	11.3 (9.9-12.9)	11.3 (9.9-12.8)	11.4 (9.9-12.8)	11.5 (10.2-12.9)	11.6 (10.3-13)	11.5 (10.3-12.9)	11.3 (10.2-12.7)	11.2 (10.3-12.3)	11.8 (11-12.8)	12.5 (11.6-13.6)	13.2 (12.1-14.4)
Bomet	13.4 (12.3-14.4)	13.5 (12.5-14.5)	13.7 (12.7-14.8)	13.9 (12.8-15)	14.2 (12.9-15.5)	14.5 (13.3-16)	14.9 (13.7-16.4)	15.2 (13.9-16.5)	15.1 (13.9-16.5)	14.8 (13.7-16.2)	14.2 (13.3-15.6)	14.6 (13.5-16.1)	15 (13.8-16.6)	15.8 (14.3-17.1)
Bungoma	10.6 (9.4-12)	11.1 (9.9-12.6)	11.6 (10.3-13.1)	12 (10.7-13.5)	12.5 (11.1-14)	13 (11.6-14.5)	13.5 (12.2-15)	13.8 (12.5-15.3)	13.9 (12.6-15.3)	14 (12.6-15.4)	13.9 (12.5-15.4)	13.9 (12.7-15.3)	14.3 (13.2-15.6)	14.7 (13.4-16.2)
Busia	10.8 (9.5-12.3)	11.2 (9.8-12.7)	11.7 (10.3-13.2)	12.2 (10.8-13.8)	12.8 (11.3-14.3)	13.6 (12.1-15.2)	14.2 (12.8-15.7)	14.5 (13.3-15.9)	14.4 (13.1-15.9)	13.6 (11.9-15.8)	12.9 (11.2-15)	13.5 (11.9-15.5)	14.2 (12.9-15.6)	14.7 (13.5-15.9)
Elgeyo Marakwet	10.8 (9.7-12.3)	10.9 (9.8-12.6)	11.1 (10-12.8)	11.3 (10-13)	11.6 (10.2-13.2)	12 (10.6-13.6)	12.3 (11-13.8)	12.6 (11.3-14)	12.5 (11.3-14)	12.3 (11.3-13.8)	12.2 (11.4-13.3)	12.5 (11.8-13.4)	13 (12.3-13.8)	13.1 (12.3-14)
Embu	11.3 (10-12.8)	11.7 (10.3-13.3)	12.4 (10.9-14)	13 (11.5-14.6)	13.7 (12.2-15.4)	14.4 (12.9-16.1)	15.1 (13.5-16.7)	15.5 (14-16.9)	15.5 (13.9-17.3)	15.3 (13.3-17.6)	15 (13-17.2)	15 (13.3-16.9)	15.3 (13.6-16.8)	15.6 (14-17.1)
Garissa	10.2 (9.2-11.3)	10.2 (9.2-11.4)	10.8 (9.7-12)	11 (9.9-12.2)	11.2 (10.1-12.5)	11.5 (10.4-12.7)	11.8 (10.7-13)	12 (11-13)	12 (11.1-13)	11.9 (11.1-12.9)	11.8 (11.1-12.6)	12.1 (11.3-12.9)	12.5 (11.7-13.3)	12.9 (11.9-14.1)
Homa Bay	9.1 (8.3-10.2)	9.5 (8.5-10.6)	9.9 (8.9-11.1)	10.3 (9.2-11.5)	10.6 (9.5-11.9)	11.1 (10-12.3)	11.7 (10.5-12.8)	12 (10.9-13.1)	11.9 (10.8-13)	11 (9.8-12.4)	9.8 (8.6-11.2)	10 (8.9-11)	11.9 (10.4-13)	13.4 (11.7-15)
Isiolo	11.9 (10.5-13.6)	12.1 (10.7-13.7)	12.3 (10.9-14)	12.4 (11-14.1)	12.6 (11.2-14.2)	13 (11.5-14.7)	13.5 (12-15.1)	13.7 (12.3-15.1)	13.6 (12.3-15.1)	13.5 (12.1-15.2)	13.1 (11.7-14.6)	13.1 (11.8-14.6)	13.4 (12-14.9)	13.7 (12.3-15.1)
Kajiado	11.7 (10.3-13.5)	11.9 (10.5-13.7)	12.1 (10.8-14)	12.3 (10.8-14)	12.5 (11.1-14.2)	12.9 (11.5-14.5)	13.2 (11.9-14.7)	13.4 (12.3-14.8)	13.2 (12.2-14.7)	12.8 (11.7-14.4)	12.5 (11.4-13.8)	12.7 (11.8-13.9)	13.3 (12.4-14.6)	13.6 (12.7-14.9)
Kakamega	11 (9.7-12.5)	11.4 (10-12.9)	11.9 (10.5-13.5)	12.4 (11-14)	13 (11.4-14.6)	13.6 (12.1-15.2)	14.2 (12.7-15.6)	14.4 (13.2-15.8)	14.3 (13-15.7)	13.7 (12.2-15.5)	13.3 (11.8-15.1)	13.7 (12.2-15.4)	14.2 (12.9-15.9)	14.8 (13.4-16.4)
Kericho	12.5 (10.8-14.5)	12.8 (11.1-14.7)	13.1 (11.4-14.9)	13.4 (11.7-15.2)	13.9 (12.2-15.7)	14.5 (12.8-16.1)	15 (13.3-16.6)	15.2 (13.7-16.7)	14.8 (13.4-16.3)	14.4 (13.2-16)	14 (12.8-15.6)	14.3 (13.1-16)	14.8 (13.6-16.5)	15.4 (14.1-16.9)
Kiambu	10.8 (9.7-12.1)	11.2 (10-12.5)	11.6 (10.4-13)	12 (10.7-13.6)	12.4 (11.1-14.1)	13 (11.8-14.3)	13.3 (12.1-14.5)	13.5 (12.4-14.7)	13.5 (12.4-14.6)	12.8 (11.6-14.3)	12.3 (11.1-13.8)	12.5 (11.4-13.9)	13.7 (12.7-15)	14.5 (13.5-15.7)
Kilifi	10.5 (9.4-11.8)	10.9 (9.8-12.3)	11.3 (10.1-12.8)	11.6 (10.4-13)	12 (10.8-13.4)	12.5 (11.2-13.9)	13 (11.7-14.3)	13.3 (12.2-14.6)	13.4 (12.2-14.7)	13.3 (12-14.8)	13.1 (11.9-14.6)	13.1 (12-14.4)	13.6 (12.5-15)	14.3 (13-15.7)
Kirinyaga	11 (9.6-12.7)	11.4 (9.9-13.1)	11.9 (10.3-13.7)	12.4 (10.8-14.2)	13 (11.2-14.8)	13.8 (12-15.6)	14.4 (12.7-16.1)	14.6 (13-16.2)	14.5 (12.9-16.2)	14.2 (12.6-16.2)	14 (12.4-16.1)	14 (12.4-15.7)	14.7 (13.1-16.2)	15.2 (13.7-16.6)
Kisii	9.7 (8.7-10.9)	10.1 (9-11.4)	10.5 (9.4-11.9)	10.9 (9.7-12.3)	11.3 (10-12.8)	11.8 (10.5-13.3)	12.2 (10.9-13.7)	12.4 (11.2-13.8)	12.2 (11.1-13.5)	11.6 (10.4-12.8)	10.9 (10.1-12)	11.1 (10.5-11.9)	11.8 (11.1-12.6)	12.5 (11.8-13.5)
Kisumu	10.3 (9.3-11.6)	10.7 (9.5-12)	11.1 (9.9-12.7)	11.6 (10.3-13.1)	12.1 (10.8-13.6)	12.7 (11.3-14.1)	13.2 (11.8-14.7)	13.7 (12.4-15)	13.5 (12.1-15.2)	12.2 (10.6-14.3)	11.4 (9.9-13)	11.8 (10.4-13.1)	12.7 (11.3-14.4)	13.6 (12.2-15)
Kitui	10.2 (9.1-11.6)	10.6 (9.4-12)	11 (9.7-12.4)	11.3 (10-12.7)	11.8 (10.4-13.2)	12.3 (11-13.7)	12.8 (11.5-14.2)	13.1 (11.9-14.5)	13.2 (12-14.5)	13.1 (11.9-14.5)	13 (11.8-14.4)	13.7 (12.7-14.8)	14.6 (13.5-15.8)	15 (13.9-16)
Kwale	10.3 (9.2-11.6)	10.7 (9.6-12.1)	11.2 (9.9-12.7)	11.5 (10.2-13)	11.9 (10.6-13.3)	12.4 (11.1-13.8)	12.9 (11.6-14.3)	13.4 (12.1-14.8)	13.4 (11.9-15.2)	13 (11.2-15.1)	12.9 (11.4-15)	12.9 (11.6-14.5)	13.1 (12.1-14.4)	13.8 (12.7-15.1)
Laikipia	12.2 (10.6-14.1)	12.5 (11-14.4)	12.9 (11.4-14.6)	13.3 (11.7-15)	13.8 (12.1-15.4)	14.3 (12.7-16)	14.8 (13.2-16.6)	15.1 (13.6-16.6)	14.9 (13.6-16.5)	14.5 (13.2-16.2)	14 (12.6-15.7)	14 (12.7-15.8)	14.4 (13.1-16.2)	15.3 (13.7-16.6)
Lamu	10.5 (9.2-12)	10.9 (9.6-12.6)	11.4 (10-13)	11.8 (10.4-13.5)	12.5 (11-14.2)	13.4 (11.9-15.3)	14.3 (12.6-16.1)	14.5 (12.9-16.2)	14.4 (12.8-16.2)	14.2 (12.6-16.1)	13.9 (12.1-15.9)	14 (12.2-15.8)	14.1 (12.3-15.8)	14.6 (13.1-16.4)
Machakos	11.2 (9.8-12.7)	11.6 (10.2-13.2)	12.1 (10.5-13.8)	12.5 (10.9-14.2)	13.1 (11.4-14.7)	13.7 (12.1-15.3)	14.3 (12.8-15.6)	14.6 (13.3-15.9)	14.7 (13.5-16)	14.7 (13.5-16.2)	14.6 (13.1-16.1)	14.5 (13.3-16)	15.1 (13.9-16.3)	15.3 (14.1-16.7)
Makueni	12.4 (11.1-13.8)	12.6 (11.3-14.1)	12.8 (11.4-14.3)	13 (11.6-14.5)	13.3 (11.9-14.9)	13.7 (12.4-15.3)	14.1 (12.8-15.5)	14.5 (13.1-15.8)	14.5 (13.2-15.8)	14.2 (12.9-15.6)	13.5 (12.2-15.1)	13.5 (12.3-15.2)	14.5 (13.2-16)	15.5 (14.2-17)
Mandera	10.2 (9.2-11.3)	10.2 (9.1-11.4)	10.8 (9.6-12.1)	10.9 (9.8-12.2)	11.1 (10-12.4)	11.4 (10.3-12.6)	11.7 (10.6-12.9)	11.9 (10.8-12.9)	11.8 (10.7-12.8)	11.6 (10.7-12.7)	11.5 (10.8-12.4)	11.5 (10.9-12.2)	11.6 (11-12.4)	12.4 (11.6-13.6)
Marsabit	10 (8.9-11.4)	10.4 (9.2-11.8)	10.9 (9.7-12.5)	11.4 (10.2-13)	12.1 (10.7-13.7)	13 (11.5-14.6)	13.7 (12.2-15.2)	14 (12.6-15.3)	13.8 (12.5-15.2)	13.5 (12.1-15.1)	13.1 (11.8-14.6)	12.9 (11.8-14.4)	13.1 (11.9-14.4)	13.5 (12.2-14.9)
Meru	11.2 (10.1-12.5)	11.7 (10.5-13.1)	12.2 (10.9-13.6)	12.6 (11.4-14.1)	13.1 (11.8-14.5)	13.6 (12.3-15)	14.1 (12.8-15.5)	14.4 (13.1-15.6)	14.4 (13.1-15.6)	14.2 (12.9-15.6)	13.8 (12.5-15.3)	13.8 (12.8-15.1)	14.2 (13.2-15.5)	14.8 (13.5-16.2)
Migori	10.1 (9-11.3)	10.4 (9.3-11.7)	10.7 (9.6-12.2)	11 (9.8-12.4)	11.4 (10.2-12.7)	11.8 (10.5-13)	12.1 (10.8-13.3)	12.3 (11-13.6)	12.3 (11.1-13.5)	11.8 (10.5-13.3)	10.8 (9.5-12.3)	10.4 (9.3-11.7)	11.2 (10-12.7)	12.7 (11.2-14.4)
Mombasa	10.7 (9.5-12.3)	11.2 (9.8-12.9)	11.9 (10.4-13.6)	12.5 (11-14.3)	13.2 (11.6-15)	14 (12.4-15.8)	14.7 (13-16.4)	15 (13.4-16.7)	15 (13.1-16.7)	14.3 (12-16.9)	13.9 (11.7-16.4)	14.3 (12.2-16.8)	14.7 (12.8-16.8)	15.5 (13.9-17.5)
Murang'a	11.7 (10.5-13.1)	12.1 (10.8-13.5)	12.4 (11.1-13.9)	12.7 (11.3-14.2)	13.2 (11.8-14.6)	13.7 (12.3-15.1)	13.9 (12.8-15.2)	14.1 (13-15.3)	14.1 (13.1-15.2)	14 (12.9-15.2)	13.9 (12.9-15.1)	13.7 (12.6-14.9)	14.7 (13.6-15.7)	15.3 (14.2-16.5)
Nairobi	9.9 (9-11.1)	10.5 (9.5-11.6)	11.4 (10.3-12.7)	12.1 (10.9-13.5)	12.9 (11.6-14.3)	13.6 (12.3-14.8)	14.1 (12.9-15.3)	14.5 (13.4-15.7)	14.3 (13-15.7)	13.2 (11.8-15)	12.4 (11.4-13.9)	12.3 (11.7-13.1)	12.8 (12.3-13.5)	13.5 (12.9-14.3)
Nakuru	12.1 (10.9-13.5)	12.4 (11.2-13.7)	12.8 (11.6-14.1)	13.2 (11.9-14.4)	13.6 (12.2-14.9)	14 (12.8-15.4)	14.4 (13.3-15.8)	14.8 (13.8-16.2)	15.1 (13.8-16.9)	15 (13.1-17)	14.7 (12.9-16.7)	15.3 (13.9-16.9)	16.4 (15.1-17.5)	16.8 (15.7-17.6)
Nandi	12.3 (11.1-13.7)	12.4 (11.2-13.8)	12.7 (11.4-14.1)	12.9 (11.6-14.3)	13.2 (11.8-14.5)	13.5 (12.2-14.8)	13.7 (12.6-15)	13.9 (12.7-15)	13.6 (12.4-14.8)	12.8 (11.5-14.3)	12.6 (11.6-13.9)	13.3 (12.3-14.3)	14.1 (13.2-15.3)	14.7 (13.6-15.8)
Narok	11.1 (10-12.6)	11.1 (10.1-12.5)	11.4 (10.3-12.7)	11.4 (10.3-12.7)	11.4 (10.2-12.8)	11.5 (10.3-12.9)	11.6 (10.5-12.9)	11.7 (10.7-12.9)	11.6 (10.7-12.6)	11.4 (10.5-12.4)	11.4 (10.5-12.3)	11.6 (10.8-12.5)	12.3 (11.5-13.1)	12.7 (11.8-13.8)
Nyamira	9.9 (9.1-10.9)	10.2 (9.4-11.2)	10.5 (9.7-11.6)	10.7 (9.9-11.9)	11.1 (10.2-12.2)	11.4 (10.5-12.6)	11.7 (10.8-12.8)	12 (11.1-13)	11.9 (11.1-12.9)	11.4 (10.7-12.3)	10.7 (10-11.4)	10.2 (9.5-10.9)	10.5 (9.6-11.3)	11.5 (10.6-12.4)
Nyandarua	10.1 (9.2-11.5)	10.4 (9.4-11.7)	10.8 (9.7-12.2)	11.1 (10-12.5)	11.5 (10.4-12.9)	11.9 (10.7-13.3)	12.3 (11.3-13.5)	12.6 (11.7-13.6)	12.5 (11.7-13.6)	12.1 (11.3-13.2)	11.6 (10.8-12.6)	11.1 (10.4-12.1)	11.7 (10.9-12.7)	12.7 (11.6-14)
Nyeri	10.3 (9.2-11.6)	10.7 (9.6-12)	11.2 (10-12.6)	11.5 (10.3-13)	11.9 (10.8-13.4)	12.4 (11.3-13.8)	12.7 (11.8-14.1)	13 (12.1-14.2)	13 (12.1-14.1)	12.6 (11.6-13.7)	12 (10.9-13.2)	12.2 (11.2-13.3)	13 (12.2-14.2)	13.7 (12.6-15.1)
Samburu	13.8 (12.1-15.6)	13.8 (12.2-15.6)	13.8 (12.3-15.5)	13.9 (12.3-15.6)	14.1 (12.4-15.8)	14.3 (12.6-15.9)	14.4 (12.8-16)	14.4 (13-15.9)	14.1 (12.6-15.5)	13.4 (12-15.2)	13 (11.7-14.7)	13.5 (12-15)	13.8 (12.4-15.3)	14 (12.7-15.5)
Siaya	9.1 (8.1-10.4)	9.5 (8.4-10.9)	10.2 (8.9-11.6)	10.8 (9.4-12.4)	11.5 (10.1-13.2)	12.2 (10.8-13.8)	12.9 (11.5-14.3)	13.3 (12.1-14.6)	13.3 (12.1-14.6)	13.1 (11.8-14.6)	12.8 (11.4-14.6)	13.1 (11.5-14.6)	14.5 (12.8-16.2)	15.7 (14.2-17.1)
Taita Taveta	9.2 (8.2-10.3)	9.7 (8.7-11)	10.3 (9.2-11.6)	10.8 (9.6-12.1)	11.3 (10-12.8)	12 (10.7-13.5)	12.6 (11.3-14.2)	13.1 (11.8-14.6)	13.2 (11.9-14.7)	13.1 (11.7-14.5)	12.7 (11.4-14.3)	12.5 (11.3-14)	12.6 (11.6-13.9)	13.4 (12.5-14.9)
Tana River	8.8 (8-9.8)	9.2 (8.4-10.4)	9.7 (8.8-10.8)	10 (9.1-11.2)	10.4 (9.4-11.6)	10.8 (9.8-12.2)	11.3 (10.2-12.6)	11.5 (10.6-12.7)	11.6 (10.6-12.8)	11.3 (10.3-12.5)	10.9 (10-12.1)	10.9 (10-11.9)	11.1 (10.3-12.1)	11.8 (10.8-13.1)
Tharaka Nithi	9.4 (8.5-10.5)	9.7 (8.7-10.8)	9.9 (8.9-11.2)	10.2 (9.2-11.4)	10.5 (9.5-11.8)	11 (9.9-12.3)	11.4 (10.3-12.5)	11.6 (10.6-12.8)	11.6 (10.7-12.7)	11.4 (10.5-12.6)	11.3 (10.4-12.3)	11.6 (10.8-12.5)	12.4 (11.5-13.4)	13.3 (12.1-14.5)
Trans Nzoia	13.6 (11.8-15.5)	13.6 (12-15.5)	13.9 (12.2-15.7)	14.1 (12.3-16)	14.5 (12.9-16.3)	15 (13.3-16.6)	15.4 (13.8-17.1)	15.6 (14.2-17)	15.3 (13.7-16.8)	14.6 (12.8-16.7)	14 (12.5-16.3)	14.4 (13-16.4)	14.9 (13.6-16.5)	15.4 (14-17)

Table 9 Estimates of life expectancy at age 65 by location and year for 1950, 1955...2005, 2010, 2017 - both sexes

Location	1950	1955	1960	1965	1970	1975	1980	1985	1990	1995	2000	2005	2010	2017
Turkana	12.9 (11.4-14.7)	12.9 (11.4-14.7)	13.1 (11.6-14.8)	13 (11.5-14.7)	13 (11.5-14.6)	13 (11.6-14.5)	13.1 (11.8-14.5)	13.2 (11.9-14.4)	13 (11.8-14.3)	12.6 (11.4-14.1)	12.2 (11-13.5)	12.7 (11.6-14)	13.4 (12.2-14.7)	13.9 (12.7-15.3)
Uasin Gishu	12.4 (11.3-14.1)	12.5 (11.5-14.1)	12.8 (11.8-14.3)	12.9 (12-14.3)	13.1 (12-14.7)	13.4 (12.2-14.9)	13.7 (12.4-15.2)	13.8 (12.6-15.3)	13.8 (12.6-15.3)	13.5 (12.4-14.9)	13.2 (12.4-14.6)	13.6 (12.9-14.7)	14.2 (13.1-15.5)	15.2 (13.9-16.8)
Vihiga	11.3 (10.1-12.7)	11.6 (10.3-13.1)	11.9 (10.6-13.4)	12.1 (10.8-13.5)	12.4 (11-13.9)	12.7 (11.4-14.2)	13 (11.7-14.5)	13.2 (12-14.6)	13.1 (11.8-14.6)	12.5 (11.1-14.4)	11.9 (10.6-13.6)	11.9 (10.6-13.4)	12.7 (11.5-14.2)	13.1 (11.9-14.6)
Wajir	11.7 (10.6-13)	11.7 (10.5-13)	12 (10.7-13.4)	12 (10.9-13.4)	12.1 (10.9-13.4)	12.2 (11-13.5)	12.2 (11.1-13.4)	12.2 (11.1-13.3)	11.9 (10.9-13)	11.6 (10.7-12.6)	11.1 (10.4-12)	11.2 (10.6-11.8)	11.6 (10.9-12.3)	12.4 (11.5-13.4)
West Pokot	11.9 (10.5-13.5)	12 (10.6-13.6)	12.2 (10.8-13.8)	12.3 (10.8-13.9)	12.4 (11-13.9)	12.6 (11.2-14)	13 (11.7-14.3)	13.2 (12-14.5)	12.9 (11.7-14.1)	12.5 (11.3-13.8)	12.4 (11.5-13.6)	12.8 (12.1-13.7)	12.9 (12.1-13.9)	13.5 (12.5-14.7)
Madagascar	10.5 (9.9-11)	10.7 (10.2-11.1)	11.1 (10.7-11.4)	11.7 (11.5-11.9)	11.6 (11.3-11.8)	12.2 (11.9-12.5)	12.8 (12.3-13.3)	12.6 (12.2-13.1)	12.5 (12.1-12.9)	12.6 (12.2-13)	12.4 (11.9-12.9)	12.3 (11.8-13)	12.7 (11.9-13.5)	13 (12.2-14.1)
Malawi	9.4 (8.9-10.1)	9.7 (9.1-10.3)	10.1 (9.5-10.7)	10.4 (9.9-11.1)	10.9 (10.5-11.5)	11.6 (11.1-12)	12 (11.7-12.4)	12 (11.7-12.3)	11.7 (11.3-12.1)	11 (10.5-11.5)	10.9 (10.4-11.5)	11.8 (11.4-12.4)	13.3 (12.7-13.8)	14.2 (13.5-14.8)
Mozambique	10 (9.4-10.8)	10.2 (9.6-11)	10.4 (9.7-11.2)	10.6 (10-11.4)	11 (10.3-11.7)	11.4 (10.8-12.1)	11.7 (11.1-12.3)	11.2 (10.8-11.7)	11.6 (11.3-12.1)	11.6 (11.2-12)	11.5 (11.1-12)	11.6 (11.1-12)	11.8 (11.2-12.3)	12.7 (11.9-13.5)
Rwanda	9.2 (8.7-9.8)	9.4 (8.9-10)	9.6 (9.1-10.3)	9.7 (9.1-10.3)	9.8 (9.3-10.4)	9.9 (9.4-10.5)	10.2 (9.7-10.7)	10.4 (9.9-10.9)	10 (9.6-10.3)	9.6 (9.3-9.9)	10.5 (10.2-10.8)	12.5 (12.1-13)	14.1 (13.6-14.7)	14.8 (14-15.5)
Somalia	9.7 (8.9-10.7)	9.8 (8.9-10.8)	10 (9-10.9)	10.1 (9.2-11.1)	10.2 (9.2-11.2)	10.2 (9.3-11.2)	10.5 (9.5-11.6)	10.5 (9.4-11.6)	10.5 (9.4-11.6)	10.5 (9.5-11.6)	10.6 (9.6-11.9)	11 (9.9-12.4)	11.2 (10.1-12.6)	11.9 (10.6-13.3)
South Sudan	10.7 (9.7-11.9)	10.8 (9.7-12)	10.9 (9.8-12)	11 (9.9-12.2)	11.2 (10.1-12.4)	11.5 (10.4-12.7)	11.6 (10.4-12.9)	11.5 (10.4-12.8)	11.4 (10.3-12.7)	11.4 (10.2-12.8)	11.6 (10.4-13.1)	12 (10.7-13.7)	12.4 (10.9-14.1)	12.7 (11.3-14.2)
Tanzania	10.3 (9.7-11)	10.5 (9.9-11.2)	10.7 (10.1-11.4)	11.1 (10.5-11.6)	11.6 (11.1-12.1)	12.2 (11.6-12.8)	12.7 (12.1-13.2)	12.9 (12.4-13.4)	12.8 (12.3-13.3)	12.7 (12.2-13.3)	13.1 (12.5-13.6)	13.9 (13.3-14.4)	14.6 (14-15.1)	14.9 (14.3-15.5)
Uganda	10.3 (9.7-11.1)	10.5 (9.8-11.3)	10.7 (10-11.4)	10.9 (10.2-11.7)	11.3 (10.6-12.1)	11.6 (11-12.2)	11.7 (11.1-12.2)	12.1 (11.5-12.7)	12 (11.4-12.8)	11.5 (10.9-12.1)	11.4 (11-12)	12.2 (11.8-12.8)	13.5 (12.9-14.2)	14.5 (13.8-15.3)
Zambia	10.6 (9.9-11.4)	10.9 (10.2-11.7)	11.1 (10.4-11.9)	11.4 (10.6-12.2)	11.9 (11.2-12.7)	12.2 (11.5-12.9)	12.2 (11.6-12.8)	12 (11.6-12.5)	11.4 (11-11.8)	10.4 (10-10.9)	10.6 (10.3-11)	11.9 (11.6-12.3)	13 (12.6-13.4)	13.7 (13.1-14.4)
Southern sub-Saharan Africa	12.3 (12-12.5)	12.5 (12.2-12.8)	12.8 (12.5-13.1)	13.1 (12.8-13.4)	13.4 (13.1-13.7)	14.4 (14.1-14.7)	14.6 (14.3-14.8)	15.5 (15.3-15.7)	16.3 (16.2-16.5)	15.5 (15.3-15.6)	13.3 (13.1-13.4)	13.3 (13.1-13.4)	13.8 (13.7-13.9)	16 (15.8-16.1)
Botswana	10.2 (9.6-10.8)	10.4 (9.8-11.1)	10.6 (10-11.2)	10.8 (10.2-11.5)	11.2 (10.6-11.9)	12 (11.4-12.6)	12.7 (12-13.3)	13.5 (12.9-14)	14 (13.3-14.5)	14.5 (13.7-15.4)	14.6 (13.6-15.5)	13.8 (13.1-14.8)	14.4 (13.7-15.1)	15.7 (14.9-16.1)
Lesotho	10.3 (9.6-11)	10.5 (9.8-11.2)	10.7 (10-11.5)	11 (10.3-11.8)	11.3 (10.6-12.1)	11.7 (11-12.5)	12.2 (11.5-12.9)	12.5 (11.9-13.1)	12.8 (12.2-13.3)	12.7 (12.1-13.3)	11.2 (10.5-12)	10.4 (9.9-11.2)	11 (10.4-12)	12.1 (11.1-13.3)
Namibia	10.7 (10.1-11.4)	10.8 (10.2-11.6)	11 (10.4-11.8)	11.3 (10.7-12)	11.5 (10.9-12.2)	11.7 (11.1-12.2)	11.8 (11.4-12.3)	12.3 (11.9-12.6)	12.7 (12.3-13.1)	12.5 (12-13)	11.6 (11.3-12.2)	12.1 (11.5-13)	14.2 (13.5-15.3)	15.4 (14.5-16.1)
South Africa	13.1 (12.8-13.5)	13.4 (13-13.7)	13.6 (13.2-14)	13.8 (13.5-14.2)	14.1 (13.8-14.5)	15.3 (14.9-15.7)	15.4 (15.1-15.7)	16.4 (16.2-16.7)	17.4 (17.3-17.6)	16.4 (16.3-16.5)	13.9 (13.8-14)	14.1 (14-14.2)	14.5 (14.4-14.5)	16.7 (16.6-16.8)
Swaziland	9.4 (8.9-10)	9.6 (9.1-10.3)	9.8 (9.3-10.5)	10.3 (9.7-11.1)	10.8 (10.2-11.5)	11.2 (10.6-12)	11.7 (11-12.4)	12.1 (11.5-12.8)	12.6 (12-13.3)	12.6 (11.9-13.2)	11 (10.3-11.8)	10.3 (9.7-11.2)	11 (10.2-12.3)	13.3 (12.2-14.5)
Zimbabwe	10.6 (9.9-11.3)	10.8 (10-11.5)	11 (10.3-11.8)	11.2 (10.5-12)	11.5 (10.9-12.2)	12 (11.4-12.6)	12.3 (11.8-12.9)	13.3 (12.8-13.8)	13.8 (13.2-14.4)	13 (12.4-13.8)	11 (10.5-11.7)	10.3 (10-10.7)	10.9 (10.4-11.5)	12.8 (12-13.6)
Western sub-Saharan Africa	10.9 (10.4-11.4)	11.1 (10.6-11.7)	11.4 (10.8-12)	11.8 (11.1-12.4)	12 (11.4-12.7)	12.5 (11.8-13.2)	13 (12.3-13.7)	13.2 (12.5-13.9)	13.2 (12.5-13.9)	13.1 (12.4-13.9)	13.2 (12.4-14.1)	13.5 (12.7-14.5)	14 (13.2-14.8)	14.6 (13.8-15.3)
Benin	10.8 (10.1-11.6)	10.9 (10.1-11.7)	11 (10.3-11.8)	11.2 (10.5-12)	11.4 (10.7-12.2)	11.8 (11.2-12.5)	12.1 (11.5-12.7)	12.5 (11.9-13)	12.7 (12.2-13.2)	12.8 (12.3-13.3)	12.9 (12.4-13.5)	13.2 (12.5-14)	13.6 (12.7-14.4)	14.2 (13.2-15.1)
Burkina Faso	9.6 (9-10.3)	9.9 (9.3-10.5)	10.2 (9.5-10.8)	10.5 (9.8-11.2)	10.7 (10.1-11.4)	11 (10.4-11.6)	11.4 (10.9-12)	11.8 (11.4-12.3)	12.1 (11.7-12.5)	12.2 (11.7-12.6)	12.3 (11.9-12.8)	12.7 (12.2-13.2)	13 (12.5-13.5)	13.4 (12.8-14.1)
Cameroon	10.1 (9.5-10.8)	10.4 (9.8-11.2)	10.3 (9.7-11)	10.9 (10.3-11.7)	11.2 (10.6-11.8)	11.5 (11-12)	11.9 (11.4-12.4)	12.4 (11.9-12.9)	12.5 (12.1-13)	12.2 (11.8-12.7)	11.9 (11.4-12.4)	12 (11.4-12.7)	12.7 (11.9-13.7)	13.8 (12.8-14.9)
Cape Verde	13.8 (12.8-14.9)	14.1 (13.1-15.2)	14.2 (13.2-15.1)	14.7 (13.8-15.6)	15 (14.3-15.7)	16 (15.5-16.4)	17 (16.7-17.2)	17.1 (17-17.3)	17.6 (17.4-17.8)	17.6 (17.2-17.9)	17.8 (17.3-18.4)	18.1 (17.8-18.5)	18.5 (18.4-18.7)	18.8 (18.4-19.2)
Chad	11.3 (10.5-12.2)	11.5 (10.6-12.4)	11.8 (10.9-12.7)	12 (11.2-12.9)	12.1 (11.3-13)	12.4 (11.6-13.2)	12.4 (11.8-13)	12.5 (12-13.1)	12.6 (12.1-13.1)	12.5 (12-13)	12.2 (11.7-12.7)	12.4 (11.8-12.9)	12.7 (12-13.3)	13.2 (12.4-14)
Cote d'Ivoire	10.7 (10.1-11.5)	10.9 (10.2-11.7)	11.2 (10.5-11.9)	11.5 (10.8-12.3)	11.9 (11.2-12.7)	12.3 (11.7-13)	12.8 (12.2-13.4)	12.8 (12.3-13.4)	12.4 (11.8-13)	11.8 (11.3-12.4)	11.8 (11.2-12.4)	12 (11.4-12.7)	12.7 (12-13.4)	13.7 (12.9-14.5)
The Gambia	10.9 (10.1-11.6)	11 (10.3-11.9)	11.2 (10.4-12)	11.4 (10.6-12.3)	11.8 (10.9-12.8)	12.1 (11.2-13)	12.3 (11.5-13.3)	12.6 (11.7-13.5)	12.8 (12-13.6)	12.9 (12.1-13.7)	12.9 (12.2-13.7)	12.9 (12.3-13.7)	13.2 (12.6-13.8)	13.6 (12.8-14.3)
Ghana	10.7 (10-11.6)	11 (10.2-11.9)	11.5 (10.6-12.4)	11.9 (11-12.8)	12.3 (11.4-13.2)	12.6 (11.8-13.6)	12.8 (12-13.7)	12.8 (12.1-13.6)	13 (12.2-13.7)	13.1 (12.4-13.7)	12.9 (12.3-13.5)	12.8 (12.3-13.3)	13 (12.5-13.4)	13.9 (13.3-14.5)
Guinea	10.6 (9.9-11.4)	10.8 (10.1-11.6)	11 (10.3-11.9)	11.3 (10.6-12.2)	11.5 (10.8-12.3)	11.7 (11.1-12.4)	12.1 (11.6-12.7)	12.4 (12-12.9)	12.6 (12.2-13)	12.6 (12.2-13)	12.5 (12-12.9)	12.4 (11.9-12.9)	12.3 (11.8-12.9)	12.7 (12-13.4)
Guinea-Bissau	8.5 (8.1-9)	8.8 (8.4-9.3)	9 (8.5-9.7)	9.2 (8.7-9.8)	9.4 (8.9-10.1)	9.8 (9.2-10.6)	10 (9.4-10.8)	10.1 (9.5-10.8)	10.3 (9.8-11)	10.6 (10.1-11.2)	10.8 (10.4-11.3)	11.1 (10.6-11.6)	11.5 (10.9-12.1)	12.2 (11.5-13)
Liberia	10.1 (9.5-10.8)	10.4 (9.7-11.1)	10.6 (10-11.3)	10.9 (10.2-11.6)	11.3 (10.6-12)	11.9 (11.2-12.7)	12.4 (11.7-13.2)	12.7 (12-13.4)	12.5 (11.9-13.1)	12.3 (11.8-12.8)	12.7 (12.1-13.2)	13.2 (12.6-13.7)	13.4 (12.7-14.1)	13.9 (13.1-14.7)
Mali	9.5 (8.9-10.1)	9.6 (9-10.2)	9.7 (9.2-10.3)	9.8 (9.3-10.3)	9.9 (9.5-10.4)	10.1 (9.7-10.6)	10.7 (10.4-11.2)	11.2 (10.9-11.6)	11.6 (11.3-12)	12.1 (11.8-12.5)	12.8 (12.4-13.2)	13.5 (13.1-13.9)	13.9 (13.4-14.5)	14.3 (13.6-15.1)
Mauritania	10.3 (9.6-11)	10.5 (9.8-11.2)	10.7 (10-11.4)	11 (10.3-11.9)	11.5 (10.8-12.3)	11.6 (10.9-12.4)	11.8 (11.1-12.5)	12 (11.4-12.5)	12.3 (11.8-12.8)	12.9 (12.4-13.4)	13.7 (13.2-14.1)	14.3 (13.8-14.8)	14.9 (14.2-15.6)	15.3 (14.4-16.3)
Niger	10.6 (9.9-11.3)	10.8 (10.1-11.6)	11.1 (10.3-11.8)	11.4 (10.7-12.2)	11.5 (10.9-12.2)	11.4 (10.9-12)	11.6 (11.1-12.1)	11.8 (11.3-12.3)	12.3 (11.8-12.7)	12.5 (12.1-13)	12.8 (12.4-13.3)	13.2 (12.7-13.7)	13.5 (12.9-14.1)	13.8 (13.1-14.6)
Nigeria	11.5 (10.4-12.8)	11.8 (10.6-13)	12.1 (10.9-13.3)	12.5 (11.2-13.9)	12.8 (11.4-14.1)	13.3 (12.1-14.8)	13.9 (12.5-15.4)	14.2 (12.8-15.7)	13.9 (12.5-15.4)	13.8 (12.3-15.6)	13.9 (12.3-16.1)	14.5 (12.7-17)	15.2 (13.3-17.4)	15.8 (13.9-17.5)
Sao Tome and Principe	11.7 (11.2-12.2)	12.5 (12.2-12.9)	13.4 (13.1-13.7)	14.6 (14.3-14.8)	14.9 (14.7-15.1)	14.9 (14.7-15.1)	15.5 (15.2-15.7)	15.4 (15.1-15.8)	15.1 (14.7-15.6)	14.7 (14.1-15.2)	14.2 (13.7-14.7)	14.2 (13.8-14.7)	14.4 (13.9-15)	14.5 (13.9-15.2)
Senegal	10.9 (10.1-11.6)	11 (10.3-11.9)	11.2 (10.4-12)	11.2 (10.5-12)	11.3 (10.6-12)	11.5 (10.9-12.2)	12 (11.4-12.6)	12.5 (12-13)	12.9 (12.4-13.3)	13.1 (12.7-13.5)	13.5 (13.1-13.8)	13.9 (13.5-14.3)	14.2 (13.6-14.8)	14.3 (13.6-15)
Sierra Leone	10.3 (9.6-11)	10.4 (9.7-11.2)	10.6 (9.9-11.4)	10.9 (10.2-11.7)	11.3 (10.5-12.1)	11.6 (10.9-12.5)	11.8 (11.1-12.7)	12 (11.4-12.8)	12.3 (11.7-12.9)	12.2 (11.8-12.8)	12 (11.6-12.4)	11.9 (11.5-12.3)	12.2 (11.7-12.8)	13.1 (12.4-13.9)
Togo	10.5 (9.8-11.3)	10.7 (10-11.4)	10.9 (10.2-11.7)	11.3 (10.6-12.1)	11.9 (11.1-12.7)	12.2 (11.5-13)	12.6 (11.9-13.3)	12.7 (12.1-13.3)	12.7 (12.2-13.3)	12.7 (12.1-13.3)	12.7 (12-13.3)	12.8 (12.1-13.6)	13.2 (12.4-14)	14 (13.1-14.9)

Table 10 Estimates of life expectancy at age 65 by location and year for 1950, 1955...2005, 2010, 2017 - females

Location	1950	1955	1960	1965	1970	1975	1980	1985	1990	1995	2000	2005	2010	2017
Global	12.9 (12.7-13)	13.5 (13.4-13.7)	13.4 (13.3-13.5)	14.3 (14.2-14.4)	14.6 (14.5-14.7)	15 (14.9-15.1)	15.5 (15.4-15.5)	15.7 (15.6-15.7)	16.2 (16.1-16.2)	16.5 (16.5-16.6)	17 (17-17.1)	17.6 (17.5-17.6)	18.2 (18.1-18.2)	18.7 (18.6-18.8)
Low SDI	10.3 (10-10.7)	10.5 (10.2-10.8)	10.6 (10.2-11)	10.7 (10.4-11)	10.9 (10.6-11.2)	11.4 (11.1-11.7)	11.9 (11.6-12.2)	11.8 (11.5-12.1)	12.2 (12-12.5)	12.7 (12.5-12.9)	13.2 (13.1-13.4)	13.9 (13.7-14)	14.2 (14.1-14.4)	14.7 (14.5-15)
Low-middle SDI	10.9 (10.7-11.2)	11.3 (11-11.6)	11.2 (11-11.5)	11.8 (11.5-12)	12 (11.8-12.3)	12.6 (12.3-12.9)	13.4 (13.1-13.6)	13.4 (13.2-13.7)	13.8 (13.6-14)	14.1 (13.9-14.2)	14.2 (14-14.4)	15 (14.8-15.2)	15.4 (15.2-15.6)	15.8 (15.6-16.1)
Middle SDI	12 (11.6-12.3)	12.6 (12.3-13)	10 (9.7-10.2)	13.3 (12.9-13.6)	13.6 (13.3-14)	14 (13.7-14.3)	14.5 (14.2-14.7)	14.9 (14.7-15.1)	15.4 (15.3-15.6)	16.1 (15.9-16.2)	16.6 (16.4-16.7)	17.2 (17.1-17.3)	17.7 (17.6-17.8)	18.2 (18-18.4)
High-middle SDI	13.5 (13.2-13.9)	14.1 (13.8-14.5)	14.3 (14-14.6)	15 (14.7-15.3)	15.1 (14.8-15.3)	15.2 (15-15.5)	15.3 (15.1-15.5)	15.4 (15.2-15.5)	15.8 (15.6-15.9)	15.7 (15.6-15.8)	16.4 (16.3-16.5)	16.8 (16.7-16.9)	17.7 (17.6-17.8)	18.5 (18.3-18.8)
High SDI	14.3 (14.2-14.5)	15.1 (15-15.2)	15.4 (15.4-15.5)	15.8 (15.8-15.9)	16.2 (16.1-16.2)	16.8 (16.8-16.9)	17.4 (17.4-17.4)	18 (17.9-18)	18.7 (18.6-18.7)	19.2 (19.2-19.2)	19.9 (19.8-19.9)	20.7 (20.7-20.7)	21.4 (21.4-21.4)	21.9 (21.8-22)
Central Europe, Eastern Europe, and Central Asia	14.1 (13.8-14.3)	14.6 (14.4-14.8)	15.5 (15.5-15.6)	15.7 (15.6-15.8)	15.5 (15.5-15.6)	15.7 (15.6-15.8)	15.6 (15.5-15.6)	15.5 (15.4-15.5)	15.9 (15.9-15.9)	15.2 (15.2-15.2)	15.7 (15.7-15.7)	15.9 (15.9-15.9)	16.8 (16.8-16.9)	18 (17.9-18.1)
Central Asia	13.2 (12.6-13.7)	13.7 (13.4-14)	14.3 (14.1-14.6)	14.8 (14.4-15.2)	15.4 (15.2-15.5)	15.5 (15.3-15.8)	16 (15.9-16.1)	16.2 (16.1-16.3)	16.4 (16.3-16.5)	15.2 (15.2-15.3)	15.3 (15.2-15.4)	15 (14.9-15.1)	15.6 (15.5-15.8)	16.5 (16.2-16.8)
Armenia	14 (12.7-15.4)	14.6 (13.8-15.4)	15.3 (14.7-15.9)	15.6 (14.8-16.6)	15.9 (15.6-16.2)	16.1 (15.4-16.8)	16.9 (16.6-17.3)	16.8 (16.6-17.1)	15.7 (15.4-15.9)	15.3 (15.2-15.4)	15.8 (15.7-15.9)	16.2 (16.1-16.3)	16.6 (16.5-16.7)	17.3 (17-17.6)
Azerbaijan	12.6 (11.4-14)	13.4 (12.6-14.2)	14.3 (13.7-14.8)	15 (14.2-15.9)	15.7 (15.3-16)	16 (15.4-16.6)	16.4 (16.2-16.5)	16.9 (16.8-17)	16.9 (16.6-17.3)	14.8 (14.5-15.1)	14.5 (14.2-14.9)	14.3 (13.9-14.6)	15.4 (15.1-15.8)	16.4 (15.8-17.1)
Georgia	13.8 (12.5-15.2)	14.3 (13.6-15.2)	14.9 (14.3-15.5)	15.4 (14.5-16.3)	16.1 (15.8-16.4)	16.2 (15.3-17)	16.4 (16-16.9)	16.2 (15.9-16.6)	16.2 (15.9-16.5)	17.1 (16.7-17.5)	17.8 (17.4-18.2)	17.4 (17.2-17.5)	17.1 (17-17.2)	16.7 (16.5-17)
Kazakhstan	13.7 (12.3-15.2)	14.3 (13.5-15.1)	14.8 (14.2-15.4)	15.1 (14.2-16)	15.7 (15.3-16)	15.8 (15.1-16.4)	16.1 (16-16.2)	16.1 (16-16.1)	16.5 (16.4-16.5)	14.6 (14.6-14.7)	15 (14.9-15)	14.9 (14.9-15)	15.6 (15.6-15.7)	17 (16.6-17.4)
Kyrgyzstan	12 (10.9-13.3)	12.5 (11.7-13.5)	13.1 (12.3-13.9)	13.4 (12.5-14.5)	13.8 (13.1-14.6)	14.6 (13.6-15.6)	15.4 (15-16)	15.5 (15.1-15.8)	16 (15.6-16.4)	15.3 (14.9-15.7)	15.8 (15.4-16.2)	15.7 (15.4-15.9)	16.3 (16.2-16.4)	17 (16.8-17.3)
Mongolia	10.4 (9.6-11.4)	11 (10.1-12)	11.7 (11-12.5)	12.6 (12.1-13.1)	13 (12.5-13.4)	13 (12.5-13.4)	13.7 (13.2-14.1)	12.7 (12.3-13)	13.1 (12.8-13.4)	12.3 (12.1-12.6)	12.5 (12.3-12.8)	13.4 (13.2-13.7)	14.2 (13.9-14.5)	16.1 (15.4-16.9)
Tajikistan	12.4 (11.3-13.6)	12.9 (12.2-13.6)	13.6 (13.1-14.1)	14.4 (13.6-15.3)	15.3 (15-15.6)	15.6 (15-16.3)	17.2 (17.1-17.4)	16.9 (16.8-17)	16.9 (16.6-17.1)	15.7 (15.3-16.1)	16.6 (16.2-16.9)	16.7 (16.3-17.2)	17.1 (16.6-17.6)	17.1 (16.3-17.9)
Turkmenistan	12.5 (11.3-13.8)	13 (12.3-13.8)	13.6 (13.1-14.2)	14 (13.2-14.8)	14.5 (14.2-14.9)	14.6 (14-15.2)	15 (14.8-15.2)	15.3 (15.2-15.4)	15.4 (15.4-15.5)	14.5 (14.4-14.6)	15.6 (15.5-15.7)	14.8 (14.8-14.9)	16.3 (16.2-16.4)	16.4 (15.7-17.1)
Uzbekistan	13.6 (12.3-15.1)	14 (13.2-14.8)	14.4 (13.9-15)	14.9 (14-15.8)	15.4 (15.1-15.6)	15.4 (14.8-16)	15.8 (15.6-15.9)	16.5 (16.4-16.5)	17 (16.9-17)	15.3 (15.3-15.4)	14.5 (14.2-14.8)	13.8 (13.4-14.1)	14.6 (14.3-14.9)	15.8 (14.8-16.9)
Central Europe	13.2 (12.9-13.4)	13.9 (13.9-14)	14.6 (14.5-14.6)	14.9 (14.9-15)	15.1 (15.1-15.1)	15.5 (15.5-15.5)	15.3 (15.3-15.4)	15.5 (15.4-15.5)	15.9 (15.8-15.9)	16.1 (16-16.1)	16.7 (16.7-16.7)	17.3 (17.3-17.4)	18.1 (18.1-18.2)	19 (18.8-19.2)
Albania	13 (12.2-13.9)	14.6 (14-15.3)	16.8 (16.4-17.3)	17 (16.2-17.8)	17.9 (16.5-19.4)	18.6 (17-20.3)	19.2 (17.9-20.6)	19.5 (18.9-20)	19.3 (19.2-19.5)	19.2 (19.1-19.4)	18.5 (18-18.8)	18.9 (18.4-19.3)	20 (19.2-20.8)	20.6 (18.9-22.4)
Bosnia and Herzegovina	14.2 (14.1-14.2)	13.8 (13.7-13.9)	14.1 (14-14.2)	14.1 (14-14.2)	14.2 (14.1-14.3)	14.4 (14.3-14.5)	14.6 (14.5-14.7)	15.1 (15-15.2)	16.4 (16.3-16.6)	15.5 (14.4-16.6)	17.2 (16.4-18)	17.5 (17.4-17.6)	17.2 (17.1-17.3)	17.4 (16.9-17.9)
Bulgaria	14.8 (14.8-14.9)	16.3 (16.2-16.4)	16.4 (16.3-16.4)	16.7 (16.6-16.9)	16.6 (16.5-16.6)	16.4 (16.3-16.4)	16.2 (16.1-16.3)	16.2 (16.1-16.2)	16 (15.9-16.1)	15.5 (15.4-15.5)	15.5 (15.4-15.6)	16.2 (16.2-16.3)	17 (16.9-17.1)	18 (17.5-18.5)
Croatia	11.4 (11.3-11.5)	12.4 (12.4-12.5)	13.2 (13.1-13.3)	14.2 (14.1-14.3)	14.4 (14.3-14.5)	15.2 (15.1-15.3)	15.6 (15.5-15.7)	15.7 (15.6-15.8)	15.9 (15.8-16)	16.6 (16.5-16.7)	16.9 (16.8-17)	17.5 (17.4-17.6)	18.1 (18-18.2)	19.3 (18.8-19.8)
Czech Republic	12.5 (12.5-12.6)	13.7 (13.6-13.7)	14.3 (14.3-14.4)	14.6 (14.6-14.7)	14.3 (14.2-14.4)	14.7 (14.6-14.8)	14.5 (14.4-14.6)	14.8 (14.7-14.9)	15.3 (15.2-15.4)	16.1 (16-16.1)	17.1 (17-17.2)	17.9 (17.8-17.9)	18.9 (18.9-19)	19.8 (19.3-20.3)
Hungary	12.7 (12.7-12.8)	14.1 (14.1-14.2)	14.3 (14.3-14.4)	14.7 (14.6-14.8)	14.5 (14.5-14.6)	14.7 (14.7-14.8)	14.6 (14.6-14.7)	14.9 (14.8-14.9)	15.5 (15.4-15.5)	15.9 (15.8-15.9)	16.6 (16.5-16.7)	17.2 (17.1-17.2)	17.9 (17.8-18)	19.1 (18.6-19.5)
Macedonia	13.1 (12.3-14.1)	13.1 (12.3-13.8)	13.6 (12.8-14.4)	14.4 (13.7-15.1)	15.1 (14.8-15.5)	15.7 (15.5-15.8)	15.5 (15.3-15.6)	15.7 (15.5-15.8)	16.3 (16.2-16.5)	16.4 (16.3-16.6)	16.8 (16.6-16.9)	17.4 (17.3-17.5)	17.8 (17.7-18)	18.8 (18.5-19.2)
Montenegro	14.5 (13.9-15)	16.2 (15.8-16.6)	16.7 (16.3-17)	17.3 (17-17.7)	17.6 (17.3-18)	16.8 (16.5-17.1)	17.2 (17-17.3)	17 (16.9-17.2)	17.6 (17.4-17.7)	17.2 (17-17.4)	16.9 (16.7-17.1)	16.8 (16.5-17.1)	17.5 (17.2-17.9)	17.4 (16.8-18)
Poland	13.4 (13.4-13.5)	14 (14-14.1)	15.2 (15.1-15.2)	15.6 (15.6-15.7)	15.6 (15.6-15.7)	16 (16-16.1)	16 (15.9-16)	15.9 (15.8-15.9)	16.3 (16.3-16.4)	16.7 (16.7-16.7)	17.5 (17.5-17.6)	18.6 (18.5-18.6)	19.4 (19.3-19.4)	20.2 (19.7-20.7)
Romania	13.4 (12-15)	13.6 (13.2-14)	13.9 (13.9-13.9)	14.1 (14.1-14.2)	14.9 (14.9-15)	15.3 (15.2-15.3)	14.6 (14.5-14.6)	14.8 (14.8-14.8)	15.2 (15.2-15.2)	15.1 (15.1-15.2)	15.8 (15.7-15.8)	16.2 (16.2-16.3)	17.2 (17.2-17.3)	18.2 (17.8-18.7)
Serbia	13.7 (13.7-13.8)	13.5 (13.4-13.5)	13.7 (13.6-13.8)	13.7 (13.7-13.8)	14.7 (14.7-14.8)	16.2 (16.2-16.3)	16 (15.9-16.1)	16 (15.9-16.1)	15.9 (15.8-16)	15.6 (15.5-15.7)	16 (15.9-16.1)	15.7 (15.6-15.8)	16.3 (16.2-16.4)	16.5 (16-17)
Slovakia	12.5 (11.8-13.4)	13.5 (12.9-14.3)	14.6 (13.9-15.3)	15.1 (14.4-15.9)	14.9 (14.4-15.5)	15.3 (15.2-15.4)	15.4 (15.3-15.5)	15.5 (15.4-15.6)	15.8 (15.7-15.9)	16.4 (16.3-16.5)	16.9 (16.8-17)	17.3 (17.2-17.4)	18 (17.9-18.1)	19 (18.5-19.5)
Slovenia	12.7 (12.6-12.9)	14.1 (14-14.2)	14.6 (14.5-14.8)	15 (14.9-15.1)	14.6 (14.5-14.8)	14.8 (14.7-15)	15.8 (15.6-15.9)	16.1 (15.9-16.2)	17.2 (17-17.3)	17.6 (17.5-17.8)	18.5 (18.3-18.7)	19.4 (19.3-19.6)	20.5 (20.3-20.7)	21.6 (21-22.2)
Eastern Europe	14.8 (14.3-15.3)	15.1 (14.7-15.5)	16.3 (16.2-16.5)	16.3 (16.1-16.4)	15.8 (15.7-16)	15.8 (15.7-16)	15.6 (15.5-15.7)	15.4 (15.4-15.5)	15.9 (15.9-15.9)	14.8 (14.8-14.9)	15.3 (15.3-15.3)	15.4 (15.4-15.4)	16.4 (16.4-16.4)	17.7 (17.5-17.8)
Belarus	16.3 (14.6-18.3)	16.7 (15.4-18.1)	17.6 (17.3-17.8)	18 (17.9-18.1)	17.5 (17.4-17.5)	17.3 (17.3-17.4)	16.8 (16.8-16.9)	16.5 (16.4-16.6)	16.7 (16.6-16.8)	15.9 (15.8-15.9)	16 (15.9-16.1)	16 (16-16.1)	16.9 (16.8-16.9)	17.9 (17.5-18.4)
Estonia	14 (12.6-15.6)	14.2 (12.9-15.6)	15.3 (15.2-15.5)	15.6 (15.4-15.7)	15.5 (15.3-15.6)	15.7 (15.5-15.8)	15.6 (15.4-15.7)	15.5 (15.4-15.7)	15.8 (15.7-16)	16 (15.8-16.1)	17 (16.8-17.2)	18.1 (17.9-18.2)	19.4 (19.2-19.6)	20.4 (19.4-21.4)
Latvia	15 (13.4-16.6)	15.4 (14-16.9)	16.1 (15.9-16.2)	16.4 (16.3-16.6)	15.8 (15.7-15.9)	15.9 (15.8-16.1)	15.9 (15.7-16)	15.5 (15.4-15.6)	15.9 (15.8-16)	15.8 (15.7-16)	17.1 (17-17.2)	17.4 (17.3-17.5)	18 (17.9-18.2)	19 (18-20)
Lithuania	15.1 (13.6-16.7)	15.6 (14.3-17.1)	15.8 (15.7-16)	17 (16.9-17.2)	16.5 (16.4-16.6)	16.8 (16.7-16.9)	16.7 (16.6-16.8)	16.4 (16.3-16.5)	16.9 (16.8-17)	16.7 (16.6-16.8)	17.9 (17.8-18)	17.9 (17.8-18)	18.7 (18.6-18.8)	19.3 (18.8-19.8)
Moldova	12.1 (11.1-13.5)	12.7 (12-13.5)	13.1 (12.6-13.6)	13.2 (12.5-14)	13.6 (13.3-13.9)	13.8 (13.3-14.3)	14.5 (14.4-14.6)	14.1 (14-14.1)	14.9 (14.8-14.9)	13.6 (13.5-13.6)	15.2 (15.1-15.3)	15.3 (15.2-15.3)	16.1 (16.1-16.2)	17.9 (17.6-18.2)
Russian Federation	14.9 (14.6-15.2)	15.2 (14.9-15.5)	16.3 (16.1-16.5)	16.2 (16-16.5)	15.8 (15.5-16)	15.8 (15.5-16)	15.5 (15.3-15.7)	15.4 (15.3-15.5)	15.9 (15.9-15.9)	14.9 (14.9-14.9)	15.1 (15.1-15.1)	15.2 (15.2-15.2)	16.3 (16.3-16.3)	17.7 (17.7-17.8)
Ukraine	14.6 (13.2-16.1)	14.7 (13.5-16.1)	16.4 (16.3-16.5)	16.2 (16.2-16.3)	15.7 (15.7-15.8)	15.8 (15.8-15.9)	15.6 (15.5-15.7)	15.4 (15.3-15.5)	15.8 (15.7-15.9)	14.4 (14.3-14.4)	15.4 (15.3-15.4)	15.5 (15.5-15.5)	16.2 (16.2-16.3)	17.2 (16.7-17.6)
High-income	14.4 (14.2-14.6)	15.1 (15.1-15.2)	15.5 (15.4-15.5)	15.8 (15.8-15.9)	16.2 (16.2-16.3)	16.9 (16.9-16.9)	17.5 (17.5-17.5)	18.1 (18.1-18.1)	18.8 (18.8-18.8)	19.4 (19.3-19.4)	20 (20-20)	20.8 (20.8-20.8)	21.5 (21.5-21.5)	21.9 (21.8-22)
Australasia	14.9 (14.8-14.9)	15.3 (15.2-15.3)	15.7 (15.6-15.7)	15.7 (15.7-15.8)	15.8 (15.8-15.9)	16.7 (16.7-16.8)	17.7 (17.7-17.8)	18 (17.9-18.1)	18.9 (18.8-18.9)	19.5 (19.4-19.5)	20.4 (20.3-20.5)	21.3 (21.3-21.4)	21.8 (21.8-21.9)	22.2 (21.4-22.9)
Australia	14.9 (14.8-14.9)	15.3 (15.2-15.3)	15.7 (15.6-15.7)	15.7 (15.7-15.8)	15.8 (15.7-15.9)	16.7 (16.7-16.8)	17.9 (17.8-18)	18.1 (18-18.2)	19 (18.9-19.1)	19.6 (19.6-19.7)	20.5 (20.4-20.6)	21.5 (21.4-21.6)	22 (21.9-22.1)	22.3 (21.4-23.2)
New Zealand	14.8 (14.7-15)	15.4 (15.2-15.5)	15.7 (15.5-15.8)	15.7 (15.6-15.8)	15.9 (15.8-16.1)	16.7 (16.6-16.8)	16.9 (16.8-17)	17.5 (17.3-17.6)	18.2 (18.1-18.3)	18.7 (18.6-18.8)	19.9 (19.7-20)	20.4 (20.2-20.5)	21 (20.9-21.1)	21.7 (21.2-22.1)
High-income Asia-Pacific	12.8 (12.5-13.1)	14 (13.7-14.3)	14.1 (13.8-14.4)	14.6 (14.3-14.9)	15.3 (15.1-15.6)	16.4 (16.3-16.6)	17.6 (17.5-17.6)	18.8 (18.7-18.8)	19.7 (19.7-19.8)	20.6 (20.6-20.6)	21.8 (21.8-21.9)	22.8 (22.8-22.8)	23.5 (23.5-23.5)	24.1 (24-24.3)
Brunei	12.9 (11.6-14.2)	13.3 (12.2-14.4)	14.3 (13.5-15.2)	15.2 (14.6-15.8)	14.6 (14.1-15)	13.8 (13.4-14.2)	13.9 (13.4-14.3)	14.3 (13.9-14.7)	13.8 (13.4-14.2)	15.3 (14.9-15.8)	16.6 (16.1-17.1)	17.3 (17-17.6)	17.6 (17.4-17.9)	17.8 (17.2-18.4)
Japan	13 (12.8-13.3)	14.3 (14-14.5)	14.4 (14.1-14.6)	14.9 (14.6-15.2)	15.7 (15.5-15.9)	16.7 (16.6-16.9)	18 (17.9-18)	19.2 (19.2-19.3)	20.3 (20.3-20.3)	21.2 (21.2-21.2)	22.5 (22.5-22.5)	23.3 (23.3-23.3)	23.9 (23.8-23.9)	24.4 (24.2-24.6)
Aichi	12.9 (11.6-14.3)	14.1 (12.7-15.7)	14 (12.6-15.7)	14.6 (13.1-16.2)	15.4 (13.9-16.9)	16.4 (15.5-17.3)	17.5 (17.4-17.6)	18.9 (18.8-19)	19.8 (19.7-19.9)	20.7 (20.6-20.8)	22.1 (22-22.2)	22.8 (22.7-22.9)	23.6 (23.5-23.7)	24.1 (23.2-25.1)
Akita	13.4 (11.9-15.1)	14.7 (13.3-16.5)	15.9 (14.4-17.5)	16.3 (14.9-17.7)	16.9 (15.7-18)	18.2 (17.6-18.9)	19.8 (19.7-20)	20.3 (20.1-20.4)	21 (20.8-21.1)	21.5 (21.4-21.7)	22.3 (22.2-22.5)	23.1 (22.9-23.2)	23.5 (23.4-23.7)	23.8 (22.9-24.7)
Aomori	12.9 (11.5-14.6)	14.2 (12.7-16)	14.4 (13-16.1)	15 (13.6-16.6)	15.9 (14.7-17.3)	17.1 (16.4-17.9)	18 (17.9-18.2)	19 (18.8-19.1)	20.4 (20.2-20.5)	20.9 (20.7-21)	21.9 (21.8-22.1)	22.8 (22.6-22.9)	23.3 (23.2-23.5)	23.8 (22.9-24.7)
Chiba	13.1 (11.8-14.8)	14.3 (12.8-16)	14.3 (12.8-15.9)	14.8 (13.2-16.5)	15.5 (14.1-17)	16.4 (15.5-17.3)	17.9 (17.8-18)	19.3 (19.2-19.4)	20.3 (20.2-20.4)	21 (20.9-21.1)	22.3 (22.2-22.4)	23.1 (22.9-23.2)	23.7 (23.6-23.8)	24.3 (23.4-25.2)
Ehime	13.1 (11.7-14.7)	14.4 (13-16.1)	14.8 (13.4-16.5)	15.5 (14.1-17.1)	16.4 (15.2-17.7)	17.3 (16.6-18)	18.3 (18.2-18.4)	19.6 (19.4-19.7)	20.8 (20.6-20.9)	21.4 (21.2-21.5)	22.6 (22.4-22.7)	23.4 (23.3-23.5)	24 (23.9-24.2)	24.8 (23.9-25.8)
Fukui	13.4 (12-14.8)	14.5 (13.2-16)	14.3 (13.1-15.8)	14.8 (13.7-16.1)	15.6 (14.7-16.7)	16.6 (16.1-17.2)	18 (17.8-18.2)	19.3 (19.2-19.4)	20.4 (20.3-20.6)	21.3 (21.1-21.4)	22.9 (22.7-23)	23.3 (23.2-23.5)	24.3 (24.1-24.5)	24.6 (23.7-25.5)
Fukuoka	12.9 (11.6-14.3)	14.2 (12.8-15.8)	14.5 (13-16.1)	15 (13.5-16.7)	15.8 (14.3-17.3)	16.8 (15.9-17.8)	18.1 (18.1-18.2)	19.5 (19.4-19.6)	20.5 (20.4-20.6)	21.4 (21.3-21.5)	22.5 (22.4-22.6)	23.4 (23.3-23.5)	24 (23.9-24.1)	24.6 (23.7-25.6)
Fukushima	13.2 (11.7-14.6)	14.4 (12.9-16.2)	14.6 (13.2-16.5)	15.1 (13.7-16.7)	15.9 (14.7-17.2)	17.1 (16.4-17.8)	18.4 (18.3-18.6)	19.1 (19-19.2)	20.5 (20.4-20.6)	21.2 (21.1-21.3)	22.4 (22.3-22.6)	23.3 (23.2-23.5)	23.8 (23.7-24)	23.9 (23-24.9)
Gifu	12.8 (11.5-14.3)	13.9 (12.5-15.7)	13.9 (12.6-15.6)	14.3 (13-15.9)	15 (13.8-16.4)	16.1 (15.4-16.9)	17.5 (17.4-17.6)	18.9 (18.8-19)	20.1 (19.9-20.2)	21.1 (21-21.2)	22.3 (22.1-22.4)	23.2 (23.1-23.3)	23.7 (23.6-23.8)	24.2 (23.3-25.1)
Gunma	12.9 (11.6-14.4)	14 (12.5-15.7)	14 (12.6-15.6)	14.5 (13.1-16.1)	15.3 (14.1-16.6)	16.2 (15.6-16.9)	17.4 (17.3-17.5)	19 (18.9-19.1)	20.1 (20-20.2)	21.1 (20.9-21.2)	22.4 (22.2-22.5)	23 (22.8-23.1)	23.6 (23.4-23.7)	24.1 (23.3-25)
Hiroshima	13.1 (11.7-14.6)	14.4 (12.8-16)	14.7 (13.3-16.5)	15.3 (13.9-16.9)	16.1 (14.7-17.6)	17.1 (16.2-18)	18.3 (18.2-18.5)	19.6 (19.4-19.7)	20.7 (20.6-20.8)	21.6 (21.5-21.7)	22.9 (22.8-23)	23.6 (23.5-23.8)	24.2 (24-24.3)	24.6 (23.7-25.5)
Hokkaidō	12.8 (11.5-14.5)	14.1 (12.6-15.7)	14.2 (12.6-15.8)	14.8 (13.2-16.4)	15.6 (14.2-17.1)	16.6 (15.7-17.6)	17.7 (17.6-17.8)	19 (19-19.1)	20.3 (20.2-20.4)	21.4 (21.3-21.5)	22.8 (22.7-22.9)	23.7 (23.6-23.8)	24.1 (24-24.2)	24.4 (23.5-25.4)
Hyōgo	12.7 (11.5-14.2)	14 (12.5-15.7)	14.1 (12.6-15.9)	14.7 (13.2-16.5)	15.6 (14.3-17.2)	16.7 (15.9-17.6)	17.8 (17.7-17.9)	18.9 (18.9-19)	19.9 (19.8-20)	20 (19.9-20.2)	22.2 (22.1-22.3)	23.1 (23-23.2)	23.7 (23.6-23.8)	24.4 (23.5-25.3)
Ibaraki	12.7 (11.4-14.3)	14 (12.5-15.6)	13.9 (12.4-15.5)	14.5 (13-16.2)	15.3 (14-16.8)	16.2 (15.4-17.1)	17.4 (17.3-17.5)	18.6 (18.5-18.7)	19.8 (19.7-20)	20.8 (20.7-21)	22.1 (22-22.2)	22.8 (22.7-22.9)	23.3 (23.2-23.5)	23.8 (22.9-24.8)
Ishikawa	13.3 (11.9-14.8)	14.4 (12.9-16)	14.2 (12.8-15.7)	14.6 (13.3-16)	15.4 (14.3-16.4)	16.6 (16-17.2)	17.6 (17.4-17.7)	19.4 (19.3-19.6)	20.6 (20.5-20.7)	21.3 (21.1-21.4)	22.6 (22.4-22.7)	23.9 (23.8-24.1)	24.2 (24-24.3)	24.5 (23.7-25.4)
Iwate	13 (11.6-14.7)	14.3 (12.7-16.1)	14.3 (12.9-15.9)	14.8 (13.6-16.2)	15.6 (14.6-16.7)	16.6 (16-17.2)	17.7 (17.6-17.9)	19.1 (18.9-19.2)	20.3 (20.1-20.4)	21.3 (21.2-21.4)	22.6 (22.5-22.7)	23.3 (23.2-23.4)	23.9 (23.8-24.1)	24 (23.1-24.9)
Kagawa	13.3 (12.1-14.8)	14.6 (13.2-16.2)	14.9 (13.6-16.5)	15.5 (14.2-16.9)	16.3 (15.3-17.5)	17.3 (16.7-17.9)	18.5 (18.3-18.6)	19.8 (19.6-19.9)	20.7 (20.5-20.8)	21.5 (21.3-21.6)	22.4 (22.3-22.6)	23.5 (23.3-23.6)	23.7 (23.5-23.9)	24.5 (23.6-25.4)
Kagoshima	12.4 (11.1-14)	13.6 (12.2-15.3)	14 (12.6-15.6)	14.5 (13.1-15.9)	15.3 (14.1-16.5)	16.4 (15.7-17)	17.9 (17.8-18)	19.2 (19.1-19.3)	20.3 (20.2-20.4)	21.4 (21.3-21.6)	22.8 (22.7-23)	23.5 (23.3-23.6)	23.9 (23.7-24)	24.4 (23.5-25.3)
Kanagawa	13.4 (11.9-15.1)	14.6 (13.2-16.3)	14.6 (13.1-16.2)	15.2 (13.5-16.9)	16 (14.6-17.6)	17 (16.1-18)	18.2 (18.1-18.3)	19.5 (19.4-19.6)	20.4 (20.3-20.5)	21.1 (21.1-21.2)	22.5 (22.4-22.5)	23.3 (23.3-23.4)	24 (23.9-24.1)	24.3 (23.4-25.2)
Kōchi	12.7 (11.3-14.3)	13.9 (12.5-15.7)	14.2 (12.8-15.9)	14.8 (13.4-16.2)	15.6 (14.5-16.7)	16.6 (15.9-17.2)	18.1 (17.9-18.3)	19.8 (19.7-19.9)	20.7 (20.5-20.8)	21.7 (21.5-21.8)	22.7 (22.6-22.9)	23.7 (23.5-23.8)	24.3 (24.1-24.4)	24.8 (23.9-25.7)
Kumamoto	13 (11.8-14.5)	14.3 (12.8-16)	14.5 (12.9-16.1)	14.9 (13.5-16.6)	15.6 (14.5-17)	16.7 (16-17.5)	18.3 (18.2-18.4)	20 (19.9-20.1)	20.9 (20.8-21.1)	22.3 (22.2-22.5)	23.3 (23.1-23.4)	23.9 (23.8-24.1)	24.4 (24.3-24.6)	25 (24.2-25.8)
Kyōto	13.1 (11.8-14.6)	14.3 (12.8-16.1)	14.4 (12.8-16)	15 (13.5-16.6)	15.9 (14.5-17.4)	17 (16.1-18)	18.1 (18-18.2)	19.2 (19.1-19.3)	20.4 (20.2-20.5)	21.3 (21.2-21.5)	22.6 (22.4-22.7)	23.3 (23.2-23.5)	24 (23.9-24.2)	24.6 (23.6-25.6)
Mie	13.1 (11.7-14.8)	14.4 (12.8-16.2)	14.4 (12.9-16.1)	15 (13.6-16.5)	15.9 (14.7-17.2)	16.9 (16.3-17.6)	18 (17.9-18.1)	19 (18.9-19.1)	20.2 (20-20.3)	20.9 (20.8-21.1)	22.4 (22.3-22.5)	23.1 (23-23.2)	23.6 (23.4-23.7)	24.2 (23.3-25.1)
Miyagi	13.3 (11.9-14.8)	14.6 (13-16.3)	14.4 (12.9-16)	15 (13.6-16.5)	15.8 (14.6-17)	16.7 (16-17.4)	17.6 (17.5-17.7)	19 (18.8-19.1)	20.4 (20.3-20.6)	21.2 (21-21.3)	22.5 (22.3-22.6)	23.2 (23.1-23.3)	23.7 (23.5-23.8)	24.3 (23.5-25.2)
Miyazaki	12.8 (11.4-14.4)	14 (12.5-15.6)	14.2 (12.7-15.9)	14.8 (13.3-16.3)	15.6 (14.6-16.7)	16.7 (16.2-17.3)	18.1 (18-18.3)	19.6 (19.5-19.7)	20.7 (20.6-20.9)	21.5 (21.4-21.6)	23.1 (23-23.2)	23.6 (23.5-23.8)	24.2 (24-24.3)	24.7 (23.8-25.6)
Nagano	13.5 (12-15.1)	14.7 (13.2-16.4)	14.7 (13.3-16.3)	15.2 (13.8-16.8)	15.9 (14.6-17.2)	16.8 (16.1-17.5)	18.1 (18-18.2)	19.5 (19.4-19.7)	20.8 (20.7-20.9)	21.7 (21.6-21.9)	22.9 (22.8-23)	23.9 (23.7-24)	24.4 (24.3-24.6)	25 (24.2-25.9)
Nagasaki	12.7 (11.4-14.3)	14 (12.5-15.5)	14.3 (12.9-15.7)	14.9 (13.6-16.4)	15.7 (14.5-16.9)	16.6 (15.9-17.3)	17.8 (17.7-18)	19.4 (19.3-19.6)	20.5 (20.4-20.6)	21.4 (21.3-21.6)	22.9 (22.7-23)	23.5 (23.4-23.7)	24.1 (24-24.3)	24.2 (23.3-25.2)
Nara	12.8 (11.4-14.5)	13.9 (12.5-15.6)	13.8 (12.5-15.3)	14.3 (12.9-15.7)	15.1 (14.1-16.2)	16.2 (15.7-16.8)	17.7 (17.6-17.8)	18.9 (18.7-19)	19.8 (19.6-19.9)	20.9 (20.7-21)	22.3 (22.1-22.4)	23.3 (23.1-23.4)	23.9 (23.7-24)	24.4 (23.6-25.3)
Niigata	13.5 (12.2-15.1)	14.7 (13.2-16.4)	14.5 (13.1-16.2)	14.9 (13.5-16.4)	15.5 (14.4-16.8)	16.6 (15.9-17.2)	17.7 (17.6-17.8)	19.1 (19-19.2)	20.5 (20.4-20.6)	21.5 (21.4-21.6)	23 (22.9-23.1)	23.8 (23.6-23.9)	24.3 (24.2-24.5)	24.6 (23.8-25.5)
Ōita	12.9 (11.5-14.4)	14 (12.6-15.6)	14.1 (12.8-15.7)	14.6 (13.3-16.1)	15.4 (14.3-16.5)	16.5 (15.9-17)	17.9 (17.8-18.1)	19.1 (18.9-19.2)	20.3 (20.1-20.4)	21.5 (21.4-21.6)	22.5 (22.3-22.6)	23.5 (23.4-23.7)	24.2 (24-24.3)	24.5 (23.7-25.4)
Okayama	13.5 (12.2-15.1)	14.8 (13.3-16.6)	14.9 (13.5-16.5)	15.4 (14-16.9)	16.1 (15-17.4)	17.1 (16.4-17.8)	18.3 (18.1-18.4)	19.8 (19.7-19.9)	21 (20.9-21.1)	21.6 (21.5-21.7)	23.1 (23-23.2)	23.8 (23.6-23.9)	24.2 (24.1-24.3)	24.8 (23.9-25.7)
Okinawa	13 (11.6-14.4)	14.2 (12.6-15.8)	14.9 (13.3-16.5)	15.3 (13.8-16.8)	16 (14.9-17.3)	17.1 (16.5-17.7)	19.5 (19.3-19.7)	21.8 (21.7-21.9)	22.5 (22.4-22.6)	23.2 (23-23.3)	24 (23.9-24.2)	24.5 (24.4-24.6)	24.8 (24.6-24.9)	24.5 (23.6-25.4)
Ōsaka	12.6 (11.2-14)	13.7 (12.3-15.3)	13.7 (12.2-15.3)	14.3 (12.9-15.8)	15.1 (13.8-16.6)	16.1 (15.2-17)	17.3 (17.2-17.4)	18.4 (18.4-18.5)	19.5 (19.4-19.6)	20.5 (20.4-20.6)	21.9 (21.8-22)	22.8 (22.7-22.9)	23.4 (23.3-23.5)	24.4 (23.5-25.4)
Saga	13 (11.7-14.5)	14.3 (12.8-15.9)	14.6 (13.1-16.1)	15.2 (13.8-16.6)	16.1 (15-17.2)	17 (16.4-17.7)	18.1 (18-18.3)	19.4 (19.3-19.6)	20.6 (20.5-20.8)	21.4 (21.2-21.5)	22.7 (22.5-22.8)	23.7 (23.5-23.9)	23.8 (23.7-24)	24.4 (23.5-25.3)
Saitama	13 (11.8-14.6)	14.2 (12.9-16.1)	14 (12.5-15.5)	14.6 (13.1-16.2)	15.4 (14.1-16.9)	16.2 (15.3-17.2)	17.4 (17.3-17.5)	19 (18.9-19.1)	19.9 (19.8-20)	20.8 (20.7-20.9)	22.1 (22-22.2)	22.8 (22.7-22.9)	23.4 (23.3-23.5)	24.1 (23.2-25)
Shiga	13.2 (11.8-14.8)	14.3 (12.9-16)	14.2 (12.9-15.9)	14.6 (13.4-16)	15.3 (14.2-16.4)	16.5 (15.9-17.1)	18 (17.9-18.2)	18.9 (18.8-19)	20 (19.9-20.1)	21.2 (21.1-21.4)	22.6 (22.5-22.8)	23.4 (23.3-23.6)	24 (23.8-24.1)	24.5 (23.7-25.4)
Shimane	13.1 (11.8-14.6)	14.3 (12.8-15.9)	14.5 (13.2-16)	15 (13.8-16.3)	15.8 (14.9-16.8)	17 (16.4-17.5)	18.6 (18.4-18.7)	20 (19.9-20.2)	21.4 (21.3-21.6)	22.3 (22.1-22.4)	22.7 (22.6-22.9)	24 (23.8-24.2)	24.6 (24.4-24.7)	24.6 (23.8-25.5)
Shizuoka	13.4 (11.8-14.9)	14.6 (13-16.3)	14.7 (13.3-16.4)	15.3 (13.8-16.9)	16.1 (14.7-17.7)	17.1 (16.2-18)	18.3 (18.2-18.4)	19.6 (19.5-19.7)	20.7 (20.5-20.8)	21.5 (21.3-21.6)	22.7 (22.6-22.8)	23.6 (23.5-23.7)	23.9 (23.8-24)	24.6 (23.7-25.6)
Tochigi	12.8 (11.4-14.3)	14 (12.5-15.6)	14.1 (12.6-15.7)	14.7 (13.3-16.2)	15.6 (14.4-16.9)	16.5 (15.8-17.2)	17.3 (17.2-17.4)	18.6 (18.5-18.7)	19.7 (19.5-19.8)	20.6 (20.5-20.8)	22.1 (22-22.2)	22.6 (22.5-22.7)	23.3 (23.2-23.5)	23.7 (22.8-24.7)
Tokushima	12.9 (11.5-14.5)	14.1 (12.6-15.8)	14.2 (12.9-15.9)	14.7 (13.4-16.1)	15.4 (14.4-16.6)	16.6 (16-17.3)	17.8 (17.6-17.9)	19.2 (19-19.3)	20.2 (20.1-20.4)	21.3 (21.1-21.5)	22.7 (22.5-22.8)	23.1 (22.9-23.3)	23.9 (23.7-24)	23.8 (22.9-24.8)
Tōkyō	13.3 (12-14.9)	14.6 (13.1-16.1)	14.7 (13.3-16.4)	15.3 (13.8-17)	16.2 (14.7-17.6)	17.2 (16.4-18.1)	18.2 (18.1-18.3)	19.4 (19.3-19.5)	20.2 (20.1-20.3)	21 (20.9-21.1)	22.3 (22.2-22.3)	23.2 (23.1-23.2)	23.9 (23.8-24)	24.8 (24-25.7)
Tottori	13.3 (11.9-14.9)	14.6 (13.1-16.3)	15 (13.6-16.4)	15.5 (14.3-16.8)	16.4 (15.4-17.4)	17.5 (17-18.1)	18.7 (18.5-18.9)	19.5 (19.3-19.6)	20.6 (20.4-20.8)	21.7 (21.5-21.9)	22.8 (22.6-22.9)	23.3 (23.1-23.5)	24.1 (23.9-24.2)	24.5 (23.7-25.4)
Toyama	13.3 (12-14.8)	14.5 (13-16)	14.5 (13.2-16.1)	15 (13.8-16.4)	15.8 (14.8-16.9)	16.7 (16.1-17.3)	18 (17.8-18.1)	19.2 (19.1-19.3)	20.7 (20.6-20.8)	21.6 (21.4-21.7)	22.8 (22.7-23)	23.9 (23.7-24)	24.5 (24.3-24.6)	24.8 (23.9-25.6)
Wakayama	12.5 (11.2-14.1)	13.7 (12.2-15.4)	13.7 (12.3-15.3)	14.1 (12.9-15.6)	14.9 (13.9-16)	16.1 (15.5-16.6)	17.7 (17.5-17.8)	18.9 (18.7-19)	19.9 (19.7-20)	20.9 (20.8-21)	22.2 (22.1-22.4)	23.1 (23-23.3)	23.6 (23.5-23.8)	24 (23.1-25)
Yamagata	13.3 (11.9-14.9)	14.5 (13-16.2)	14.6 (13.2-16.2)	14.9 (13.6-16.3)	15.6 (14.5-16.6)	16.9 (16.3-17.5)	19.1 (19-19.3)	19.7 (19.6-19.9)	20.3 (20.2-20.5)	21.3 (21.1-21.4)	22.5 (22.3-22.6)	23.6 (23.5-23.8)	23.9 (23.8-24.1)	24.4 (23.5-25.2)
Yamaguchi	13.1 (11.8-14.7)	14.4 (12.8-16.1)	14.8 (13.3-16.5)	15.4 (13.9-17)	16.3 (15-17.5)	17.2 (16.5-17.8)	18.1 (18-18.3)	19.6 (19.5-19.7)	20.7 (20.6-20.9)	21.5 (21.3-21.6)	22.6 (22.4-22.7)	23.3 (23.1-23.4)	23.7 (23.5-23.8)	24.3 (23.4-25.2)
Yamanashi	13.3 (11.9-14.9)	14.7 (13.2-16.3)	15 (13.6-16.6)	15.6 (14.3-17)	16.4 (15.4-17.6)	17.6 (17-18.2)	18.3 (18.2-18.5)	19.5 (19.3-19.6)	20.7 (20.5-20.8)	21.9 (21.7-22)	23 (22.8-23.1)	23.6 (23.5-23.8)	24.1 (23.9-24.2)	24.5 (23.6-25.4)
South Korea	11.2 (10.2-12.4)	12.3 (11.1-13.6)	12.6 (11.3-14)	12.9 (11.6-14.2)	13.4 (12.3-14.6)	14.7 (14.3-15.1)	15.3 (15.2-15.3)	16 (16-16.1)	16.6 (16.6-16.7)	17.4 (17.4-17.5)	18.3 (18.2-18.3)	19.9 (19.8-19.9)	21.4 (21.3-21.6)	22.6 (22.2-23.1)
Singapore	13.5 (12.6-14.4)	13.9 (13.8-14)	14 (13.9-14.2)	14 (13.9-14.2)	14.5 (14.3-14.6)	14.9 (14.7-15)	15.4 (15.3-15.6)	17 (16.8-17.1)	18.1 (18-18.3)	19.1 (19-19.2)	20.3 (19.6-21)	21.7 (21.4-21.9)	23.6 (23.4-23.8)	24.6 (24.1-25)
High-income North America	15.1 (14.8-15.4)	15.8 (15.5-16)	15.9 (15.9-15.9)	16.4 (16.3-16.4)	16.9 (16.9-16.9)	17.9 (17.9-17.9)	18.4 (18.4-18.4)	18.6 (18.6-18.6)	19.2 (19.2-19.2)	19.2 (19.2-19.2)	19.3 (19.2-19.3)	19.8 (19.8-19.8)	20.6 (20.5-20.6)	20.8 (20.6-20.9)
Canada	14.9 (14.9-15)	15.8 (15.7-15.8)	16.1 (16-16.1)	16.7 (16.6-16.7)	17.4 (17.4-17.5)	17.9 (17.9-18)	18.8 (18.8-18.9)	19.1 (19-19.2)	19.6 (19.6-19.7)	19.8 (19.7-19.8)	20.3 (20.2-20.3)	20.9 (20.9-21)	21.7 (21.7-21.8)	22.1 (21.7-22.6)
Greenland	9.1 (8.9-9.4)	10.8 (10.6-11)	13.3 (13.1-13.6)	14.8 (14.5-15.1)	14.8 (14.5-15.1)	14.1 (13.8-14.3)	14 (13.7-14.2)	14.1 (13.9-14.4)	14.3 (14-14.6)	14.3 (14-14.5)	14.6 (14.3-14.9)	15.5 (15.2-15.7)	16.7 (16.3-17)	18.3 (17.6-18.9)

Table 10 Estimates of life expectancy at age 65 by location and year for 1950, 1955...2005, 2010, 2017 - females

Location	1950	1955	1960	1965	1970	1975	1980	1985	1990	1995	2000	2005	2010	2017
USA	15.1 (14.8-15.5)	15.8 (15.5-16.1)	15.9 (15.9-15.9)	16.3 (16.3-16.4)	16.9 (16.9-16.9)	17.9 (17.8-17.9)	18.3 (18.3-18.4)	18.6 (18.6-18.6)	19.1 (19.1-19.1)	19.1 (19.1-19.1)	19.2 (19.1-19.2)	19.7 (19.7-19.7)	20.4 (20.4-20.4)	20.6 (20.4-20.8)
Alabama	14.5 (13-16.2)	15.1 (13.8-16.7)	16 (15.9-16.2)	16.2 (16.1-16.3)	16.7 (16.6-16.8)	17.8 (17.7-17.9)	18.1 (18-18.2)	18.3 (18.2-18.4)	18.7 (18.5-18.8)	18.7 (18.5-18.8)	18.4 (18.2-18.5)	18.4 (18.3-18.5)	18.9 (18.8-19)	19 (18-20)
Alaska	15.2 (14.1-16.5)	15.9 (15.2-16.7)	15.7 (15.4-15.9)	16 (15.8-16.2)	16.9 (16.7-17)	16.5 (16.3-16.6)	17.3 (17.1-17.5)	17.4 (17.3-17.6)	18.1 (17.9-18.2)	18.1 (18-18.3)	18.6 (18.4-18.7)	19.3 (19.1-19.4)	20.1 (19.9-20.2)	20.1 (19.5-20.8)
Arizona	16 (14.4-17.7)	16.6 (15.2-18.1)	17.3 (17.1-17.4)	17.3 (17.1-17.4)	17.7 (17.6-17.9)	18.8 (18.6-18.9)	19 (18.8-19.1)	19.5 (19.4-19.6)	19.9 (19.7-20)	19.6 (19.5-19.7)	19.8 (19.7-19.9)	20.2 (20.1-20.3)	21.2 (21.1-21.4)	21.3 (20.3-22.3)
Arkansas	15.6 (14-17.4)	16.3 (15-17.8)	16.7 (16.5-16.9)	17.1 (16.9-17.2)	17.5 (17.3-17.6)	18 (17.9-18.2)	18.6 (18.4-18.7)	18.6 (18.4-18.7)	18.9 (18.7-19)	18.8 (18.7-19)	18.5 (18.4-18.7)	19.2 (19-19.3)	19.4 (19.2-19.5)	19.2 (18.1-20.3)
California	15.9 (14.3-17.5)	16.5 (15.2-18)	16.6 (16.5-16.6)	16.9 (16.9-17)	17.4 (17.4-17.5)	18.2 (18.1-18.3)	18.6 (18.5-18.6)	18.8 (18.7-18.8)	19.3 (19.2-19.3)	19.6 (19.6-19.7)	19.8 (19.7-19.8)	20.4 (20.3-20.4)	21.5 (21.4-21.5)	22 (21-22.9)
Colorado	15.8 (14.1-17.6)	16.4 (15-17.9)	16.6 (16.5-16.8)	17.3 (17.1-17.4)	17.7 (17.6-17.9)	18.7 (18.6-18.8)	19 (18.8-19.1)	19.2 (19.1-19.4)	19.6 (19.5-19.8)	19.4 (19.3-19.6)	19.5 (19.3-19.6)	20.2 (20-20.3)	20.8 (20.7-20.9)	21 (19.9-22.1)
Connecticut	15.5 (13.9-17.4)	16.3 (14.8-17.8)	16.5 (16.4-16.7)	16.2 (16.1-16.3)	17.2 (17-17.3)	18.1 (18-18.3)	18.5 (18.4-18.7)	18.9 (18.8-19)	19.7 (19.5-19.8)	19.7 (19.6-19.8)	19.9 (19.8-20)	20.8 (20.6-20.9)	21.3 (21.2-21.5)	21.6 (20.6-22.7)
Delaware	14.6 (13.3-15.9)	15.1 (14.3-16)	15.3 (15.1-15.4)	15.7 (15.6-15.9)	16.3 (16.1-16.4)	17.3 (17.2-17.5)	17.9 (17.8-18.1)	18.1 (17.9-18.3)	18.3 (18.1-18.4)	18.7 (18.5-18.8)	18.8 (18.7-19)	19.5 (19.4-19.7)	20.1 (19.9-20.2)	20.5 (19.9-21.1)
Washington, DC	15.4 (13.9-17)	15.9 (14.9-16.9)	15.7 (15.5-15.8)	15.8 (15.7-15.9)	16.4 (16.2-16.5)	17.4 (17.3-17.5)	17.8 (17.7-18)	18.1 (17.9-18.2)	17.8 (17.6-17.9)	16.1 (16-16.3)	19.2 (19-19.4)	19.5 (19.3-19.6)	20.3 (20.1-20.5)	20.5 (19.7-21.3)
Florida	16.5 (14.9-18.2)	17.2 (15.8-18.7)	17.4 (17.3-17.5)	17.9 (17.8-18)	18.2 (18.1-18.3)	18.9 (18.8-19)	19.3 (19.2-19.3)	19.6 (19.5-19.7)	20.3 (20.2-20.4)	20.2 (20.1-20.3)	20.2 (20.2-20.3)	20.8 (20.8-20.9)	21.7 (21.6-21.7)	21.6 (20.6-22.6)
Georgia	14.7 (13.1-16.5)	15.7 (14.2-17.3)	16.1 (16-16.2)	16.5 (16.4-16.6)	16.7 (16.6-16.8)	17.7 (17.6-17.8)	18 (17.9-18.1)	18 (17.9-18.1)	18.5 (18.4-18.6)	18.2 (18.1-18.3)	18.1 (18-18.2)	18.8 (18.7-18.8)	19.3 (19.2-19.4)	19.6 (18.6-20.6)
Hawaii	15.3 (14-16.7)	15.8 (15.1-16.6)	16 (15.8-16.2)	17 (16.8-17.1)	17.9 (17.7-18)	19.5 (19.3-19.6)	20.1 (20-20.3)	20.4 (20.3-20.6)	21.1 (21-21.2)	21.5 (21.4-21.7)	21.4 (21.2-21.5)	22.3 (22.1-22.4)	22.9 (22.7-23)	23.1 (22.4-23.7)
Idaho	16 (14.7-17.5)	16.7 (15.9-17.5)	16.9 (16.7-17.1)	17.1 (16.9-17.2)	17.7 (17.5-17.8)	18.4 (18.3-18.6)	19.1 (18.9-19.3)	19.1 (18.9-19.2)	19.6 (19.4-19.8)	19.7 (19.5-19.8)	19.3 (19.2-19.5)	19.6 (19.5-19.7)	20.3 (20.1-20.4)	20.5 (19.9-21.1)
Illinois	14.9 (13.3-16.6)	15.5 (14.2-17)	15.5 (15.4-15.5)	16 (15.9-16)	16.4 (16.4-16.5)	17.5 (17.4-17.6)	18.1 (18-18.2)	18.4 (18.3-18.5)	18.9 (18.8-19)	18.9 (18.8-19)	19.1 (19-19.2)	19.6 (19.5-19.7)	20.5 (20.4-20.6)	20.5 (19.5-21.6)
Indiana	15.2 (13.6-17)	15.9 (14.4-17.5)	15.8 (15.7-15.8)	16.2 (16.1-16.3)	16.6 (16.5-16.7)	17.6 (17.5-17.7)	18.1 (18-18.2)	18.3 (18.2-18.4)	18.8 (18.7-18.9)	18.4 (18.3-18.6)	18.5 (18.4-18.6)	19.1 (19-19.2)	19.6 (19.5-19.7)	19.7 (18.6-20.7)
Iowa	16.3 (14.7-18.2)	16.6 (15.3-18.2)	16.7 (16.5-16.8)	17 (16.9-17.2)	17.6 (17.5-17.7)	18.4 (18.3-18.6)	19.5 (19.3-19.6)	19.4 (19.2-19.5)	20.3 (20.2-20.5)	19.9 (19.8-20.1)	20 (19.8-20.1)	20.4 (20.3-20.6)	20.7 (20.6-20.9)	20.6 (19.6-21.8)
Kansas	16.5 (14.8-18.2)	17 (15.6-18.6)	16.8 (16.7-17)	17.3 (17.1-17.4)	17.8 (17.7-17.9)	18.5 (18.3-18.6)	19.2 (19.1-19.4)	19.4 (19.3-19.6)	20.2 (20.1-20.4)	19.6 (19.4-19.7)	19.5 (19.3-19.7)	19.7 (19.5-19.9)	20.3 (20.2-20.5)	20 (18.8-21)
Kentucky	15 (13.4-16.8)	15.7 (14.4-17.2)	15.9 (15.8-16)	16.4 (16.2-16.5)	16.5 (16.4-16.6)	17.4 (17.2-17.5)	17.9 (17.8-18.1)	18.1 (18-18.2)	18.5 (18.4-18.6)	18.4 (18.3-18.5)	18.1 (18-18.3)	18.5 (18.4-18.6)	18.8 (18.6-18.9)	18.7 (17.7-19.8)
Louisiana	14.5 (12.9-16.2)	15.4 (14-17)	15.6 (15.5-15.7)	15.8 (15.7-15.9)	16.3 (16.1-16.4)	17.2 (17.1-17.3)	17.7 (17.6-17.8)	17.9 (17.8-18)	18.3 (18.2-18.4)	18.3 (18.2-18.4)	18.1 (18-18.2)	18 (17.9-18.1)	19.2 (19.1-19.3)	19.4 (18.4-20.5)
Maine	15 (13.6-16.6)	15.6 (14.6-16.7)	15.8 (15.6-15.9)	16.1 (15.9-16.3)	16.6 (16.5-16.8)	17.9 (17.8-18.1)	18.3 (18.2-18.5)	18.5 (18.3-18.6)	19 (18.9-19.2)	19.1 (18.9-19.2)	18.9 (18.8-19.1)	19.2 (19.1-19.4)	20.1 (20-20.3)	20 (19.3-20.8)
Maryland	14.4 (12.8-16.1)	15.1 (13.7-16.6)	15.1 (15-15.2)	15.7 (15.6-15.8)	16.4 (16.3-16.5)	17.5 (17.4-17.6)	17.9 (17.8-18)	17.9 (17.8-18)	18.5 (18.3-18.6)	18.6 (18.5-18.7)	18.6 (18.4-18.7)	19.5 (19.4-19.6)	20.4 (20.3-20.5)	20.9 (19.9-22.1)
Massachusetts	15 (13.5-16.7)	15.5 (14.1-17.2)	15.6 (15.5-15.7)	16.1 (16-16.2)	17.1 (17-17.2)	18 (17.9-18.1)	18.4 (18.3-18.5)	18.6 (18.5-18.7)	19.4 (19.3-19.5)	19.4 (19.3-19.5)	19.5 (19.4-19.6)	20.2 (20.1-20.3)	21.1 (21-21.3)	21.1 (20.1-22.1)
Michigan	15 (13.4-16.6)	15.5 (14.1-17)	15.7 (15.6-15.7)	16 (16-16.1)	16.7 (16.6-16.8)	17.6 (17.5-17.6)	18.1 (18.1-18.2)	18.2 (18.1-18.3)	18.8 (18.7-18.9)	18.7 (18.6-18.8)	18.8 (18.7-18.8)	19.3 (19.2-19.4)	20 (19.9-20)	20 (19-21)
Minnesota	16 (14.5-17.9)	16.7 (15.3-18.2)	16.4 (16.3-16.6)	17.1 (16.9-17.2)	17.8 (17.6-17.9)	18.7 (18.6-18.9)	19.5 (19.3-19.6)	19.5 (19.4-19.7)	20 (19.9-20.2)	20 (19.9-20.1)	20.1 (19.9-20.2)	20.7 (20.6-20.9)	21.3 (21.1-21.4)	21.5 (20.4-22.6)
Mississippi	14.8 (13.3-16.6)	15.5 (14.2-17.1)	15.8 (15.7-16)	16.1 (16-16.2)	16.6 (16.5-16.7)	17.5 (17.3-17.6)	17.9 (17.8-18.1)	18.4 (18.2-18.5)	18.7 (18.5-18.8)	18.5 (18.4-18.7)	18.1 (18-18.2)	18.4 (18.3-18.5)	18.9 (18.7-19)	19.1 (18-20.2)
Missouri	15.5 (13.9-17.3)	16.1 (14.8-17.6)	16.1 (16-16.2)	16.5 (16.4-16.6)	16.9 (16.8-17)	17.8 (17.7-17.9)	18.3 (18.2-18.4)	18.6 (18.5-18.7)	19 (18.9-19.1)	18.7 (18.6-18.9)	18.7 (18.6-18.8)	19.2 (19.1-19.3)	19.7 (19.6-19.8)	19.7 (18.7-20.8)
Montana	15.8 (14.4-17.1)	16.2 (15.4-17.1)	16.3 (16.1-16.4)	16.8 (16.6-16.9)	17.4 (17.2-17.5)	18.4 (18.2-18.5)	18.7 (18.5-18.8)	19.2 (19-19.4)	19.5 (19.3-19.6)	19.4 (19.3-19.6)	19.3 (19.2-19.5)	19.7 (19.6-19.9)	20.3 (20.2-20.5)	19.3 (18.7-20)
Nebraska	16.9 (15.4-18.5)	17.4 (16.4-18.5)	16.8 (16.7-16.9)	17.3 (17.1-17.4)	17.8 (17.6-17.9)	18.6 (18.5-18.8)	19.3 (19.1-19.4)	19.5 (19.3-19.6)	19.8 (19.7-20)	19.7 (19.6-19.9)	19.8 (19.6-19.9)	20.3 (20.2-20.5)	20.6 (20.5-20.7)	20.5 (19.7-21.3)
Nevada	15 (13.6-16.6)	15.8 (14.8-16.9)	16.3 (16.1-16.6)	16.2 (16-16.3)	16.6 (16.4-16.8)	17.7 (17.6-17.9)	17.8 (17.6-17.9)	18 (17.9-18.2)	18.2 (18.1-18.3)	18 (17.9-18.2)	18.2 (18.1-18.3)	18.4 (18.3-18.6)	19.7 (19.6-19.8)	19.9 (19.2-20.6)
New Hampshire	15.3 (14-16.8)	16 (15.2-16.9)	15.8 (15.6-16)	16 (15.9-16.2)	16.6 (16.5-16.8)	17.7 (17.5-17.8)	18.5 (18.3-18.6)	18.6 (18.5-18.8)	18.8 (18.7-19)	19.1 (19-19.3)	18.9 (18.8-19.1)	19.5 (19.4-19.6)	20.5 (20.4-20.6)	20.1 (19.5-20.7)
New Jersey	14.5 (13-16.3)	15.1 (13.6-16.6)	15.2 (15.1-15.3)	15.7 (15.6-15.8)	16.4 (16.3-16.5)	17.5 (17.4-17.5)	17.8 (17.7-17.9)	18.1 (18.1-18.2)	18.8 (18.8-18.9)	18.9 (18.8-19)	19.3 (19.2-19.3)	20 (19.9-20.1)	21 (20.9-21.1)	21.6 (20.6-22.6)
New Mexico	15.8 (14.3-17.5)	16.5 (15.5-17.6)	16.4 (16.3-16.6)	16.7 (16.6-16.9)	17.2 (17.1-17.4)	18.3 (18.2-18.4)	19 (18.9-19.2)	19.2 (19.1-19.4)	19.5 (19.4-19.7)	19.6 (19.4-19.7)	19.7 (19.6-19.9)	20.2 (20-20.3)	20.6 (20.5-20.7)	20.9 (20.1-21.6)
New York	14.6 (13.2-16.2)	15 (13.8-16.3)	15.1 (15.1-15.2)	15.7 (15.6-15.7)	16.4 (16.3-16.4)	17.6 (17.6-17.7)	17.9 (17.9-18)	18.2 (18.2-18.3)	19.1 (19-19.1)	19.3 (19.2-19.4)	19.7 (19.7-19.8)	20.4 (20.3-20.4)	21.3 (21.3-21.4)	21.8 (20.8-22.8)
North Carolina	15.1 (13.5-16.7)	15.8 (14.4-17.3)	15.7 (15.6-15.8)	16.2 (16.1-16.3)	16.8 (16.7-16.9)	17.8 (17.7-17.9)	18.4 (18.3-18.5)	18.4 (18.3-18.5)	18.9 (18.8-19)	18.7 (18.6-18.8)	18.5 (18.4-18.6)	19.1 (19-19.2)	19.8 (19.7-19.9)	20 (18.9-21.1)
North Dakota	16 (14.9-17.3)	16.4 (15.7-17.2)	16.6 (16.4-16.7)	17.1 (17-17.3)	17.8 (17.6-17.9)	18.6 (18.4-18.7)	19.2 (19.1-19.4)	20.2 (20.1-20.4)	20.6 (20.4-20.7)	20.5 (20.4-20.7)	20.4 (20.3-20.6)	21.2 (21-21.3)	21.4 (21.2-21.5)	20.8 (20.2-21.5)
Ohio	14.9 (13.3-16.5)	15.4 (14.1-16.9)	15.6 (15.5-15.7)	15.9 (15.8-16)	16.5 (16.4-16.5)	17.4 (17.3-17.4)	17.8 (17.7-17.9)	18.1 (18.1-18.2)	18.6 (18.5-18.7)	18.5 (18.4-18.6)	18.6 (18.5-18.6)	19 (19-19.1)	19.6 (19.5-19.7)	19.3 (18.3-20.4)
Oklahoma	16.2 (14.5-18)	16.9 (15.4-18.5)	17 (16.9-17.1)	17.6 (17.4-17.7)	17.5 (17.4-17.6)	18.3 (18.1-18.4)	18.7 (18.5-18.8)	18.7 (18.6-18.9)	18.8 (18.7-19)	18.7 (18.6-18.8)	18.3 (18.1-18.4)	18.4 (18.3-18.5)	18.9 (18.8-19.1)	19.1 (18-20.3)
Oregon	16.2 (14.6-17.9)	16.8 (15.5-18.4)	16.6 (16.5-16.8)	17.2 (17-17.3)	17.7 (17.6-17.9)	18.5 (18.4-18.7)	19 (18.8-19.1)	19.1 (18.9-19.2)	19.3 (19.2-19.5)	19 (18.9-19.2)	19.2 (19-19.3)	19.8 (19.6-19.9)	20.4 (20.2-20.5)	20.7 (19.6-21.7)
Pennsylvania	14.2 (12.7-15.8)	14.8 (13.5-16.2)	15.1 (15-15.1)	15.5 (15.4-15.5)	16.1 (16-16.2)	17.2 (17.2-17.3)	17.7 (17.6-17.8)	18.1 (18-18.2)	18.7 (18.6-18.8)	18.9 (18.8-19)	19 (19-19.1)	19.5 (19.4-19.5)	20.3 (20.2-20.4)	20.2 (19.2-21.1)
Rhode Island	14.7 (13.3-16.3)	15.3 (14.3-16.4)	15.4 (15.2-15.5)	15.8 (15.6-15.9)	16.7 (16.5-16.8)	17.9 (17.7-18.1)	18.6 (18.5-18.8)	18.8 (18.7-19)	19.4 (19.2-19.5)	19.7 (19.5-19.9)	19.6 (19.5-19.8)	19.9 (19.7-20.1)	20.8 (20.6-21)	21 (20.2-21.8)
South Carolina	14 (12.3-15.8)	15 (13.5-16.7)	15.9 (15.7-16)	16.2 (16.1-16.3)	16.5 (16.4-16.6)	17.4 (17.3-17.5)	17.9 (17.7-18)	18.2 (18.1-18.4)	18.5 (18.4-18.6)	18.5 (18.4-18.6)	18.5 (18.4-18.6)	19.1 (19-19.2)	19.5 (19.4-19.6)	19.6 (18.6-20.7)
South Dakota	16.5 (15.2-17.9)	16.9 (16-17.7)	16.6 (16.4-16.8)	17.2 (17.1-17.4)	17.9 (17.8-18.1)	18.7 (18.6-18.9)	19.5 (19.4-19.7)	19.8 (19.7-20)	20.3 (20.1-20.5)	20.2 (20.1-20.4)	20.3 (20.1-20.4)	20.8 (20.6-21)	21.1 (20.9-21.2)	20.7 (20-21.4)
Tennessee	15 (13.3-16.8)	15.6 (14.2-17.2)	15.9 (15.8-16)	16.3 (16.2-16.4)	16.7 (16.6-16.8)	17.6 (17.4-17.7)	18.3 (18.2-18.4)	18.5 (18.4-18.6)	18.7 (18.6-18.8)	18.4 (18.3-18.5)	18.3 (18.2-18.4)	18.6 (18.5-18.7)	19.1 (19.1-19.2)	19.2 (18.1-20.3)
Texas	16 (14.3-17.8)	16.7 (15.3-18.3)	16.8 (16.8-16.9)	17.3 (17.3-17.4)	17.4 (17.4-17.5)	18.1 (18-18.2)	18.5 (18.4-18.5)	18.7 (18.6-18.7)	19.1 (19-19.2)	19 (18.9-19.1)	18.8 (18.7-18.8)	19.4 (19.4-19.5)	20 (19.9-20)	20.3 (19.3-21.2)
Utah	15.8 (14.3-17.4)	16.3 (15.3-17.4)	16.6 (16.4-16.8)	16.7 (16.5-16.8)	17.6 (17.4-17.7)	18.4 (18.2-18.5)	18.8 (18.7-19)	18.9 (18.8-19.1)	19.7 (19.6-19.9)	19.6 (19.5-19.7)	19.4 (19.3-19.6)	19.8 (19.7-19.9)	20.5 (20.4-20.6)	20.3 (19.6-21)
Vermont	15.5 (14.4-16.9)	16.1 (15.3-16.8)	15.9 (15.7-16.1)	16.1 (15.9-16.2)	16.9 (16.7-17.1)	17.9 (17.7-18.1)	18.6 (18.5-18.8)	18.3 (18.1-18.5)	19.1 (18.9-19.2)	19.1 (19-19.3)	19.3 (19.1-19.4)	19.9 (19.8-20.1)	20.4 (20.3-20.6)	21.3 (20.6-22)
Virginia	14.6 (13.1-16.4)	15.3 (14-16.9)	15.6 (15.5-15.7)	16.1 (16-16.2)	16.7 (16.6-16.8)	17.7 (17.6-17.8)	18 (17.9-18.2)	18.3 (18.2-18.4)	18.7 (18.6-18.9)	18.6 (18.5-18.7)	18.6 (18.5-18.7)	19.3 (19.2-19.4)	20.2 (20.1-20.3)	20.7 (19.7-21.6)
Washington	15.9 (14.2-17.6)	16.5 (14.9-18.2)	16.5 (16.4-16.6)	17 (16.9-17.2)	17.4 (17.2-17.5)	18.4 (18.3-18.5)	18.8 (18.7-18.9)	19 (18.8-19.1)	19.5 (19.4-19.6)	19.4 (19.3-19.5)	19.4 (19.3-19.6)	20 (19.8-20.1)	20.7 (20.5-20.8)	21 (19.9-22)
West Virginia	15 (13.5-16.8)	15.4 (14-16.9)	15.9 (15.7-16)	15.9 (15.8-16.1)	16.2 (16.1-16.3)	17.1 (17-17.3)	17.8 (17.7-18)	18.1 (17.9-18.2)	18.6 (18.5-18.8)	18.3 (18.1-18.5)	17.9 (17.7-18.1)	18.3 (18.2-18.5)	18.8 (18.7-19)	18.8 (17.7-19.9)
Wisconsin	15.5 (13.8-17.5)	16 (14.6-17.5)	15.9 (15.7-16)	16.5 (16.4-16.7)	17.2 (17.1-17.4)	18.2 (18.1-18.4)	18.8 (18.7-19)	19.2 (19.1-19.3)	19.5 (19.3-19.6)	19.6 (19.5-19.7)	19.6 (19.5-19.7)	20.1 (20-20.2)	20.7 (20.6-20.8)	21.2 (20.1-22.3)
Wyoming	16.5 (15.3-17.8)	17.1 (16.3-17.8)	16.8 (16.6-17)	16.8 (16.7-17)	17.5 (17.3-17.7)	18 (17.8-18.2)	18.7 (18.5-18.9)	18.9 (18.8-19.1)	19.4 (19.2-19.5)	19.2 (19-19.4)	19 (18.9-19.2)	19.6 (19.4-19.7)	20.1 (19.9-20.2)	20.4 (19.7-21)
Southern Latin America	14 (13.9-14)	14.4 (14.4-14.4)	15.1 (15.1-15.2)	15.5 (15.5-15.5)	15.8 (15.7-15.8)	16.3 (16-16.6)	16.6 (16.6-16.7)	17.1 (17.1-17.2)	17.5 (17.5-17.6)	18.2 (18.2-18.3)	18.7 (18.6-18.7)	19.1 (19-19.1)	19.4 (19.3-19.4)	19.7 (18.9-20.3)
Argentina	14 (13.9-14)	14.6 (14.5-14.6)	15.5 (15.5-15.5)	15.8 (15.7-15.8)	16.1 (16-16.1)	16.6 (16.2-17)	16.9 (16.8-16.9)	17.2 (17.2-17.3)	17.6 (17.5-17.6)	18.3 (18.2-18.3)	18.6 (18.5-18.6)	18.9 (18.9-19)	19.1 (19-19.1)	19.3 (18.4-20.2)
Chile	12.9 (12.9-13)	13.1 (13-13.1)	13.4 (13.3-13.4)	14.1 (14.1-14.2)	14.6 (14.5-14.6)	15.3 (15.2-15.3)	15.8 (15.7-15.9)	16.5 (16.5-16.6)	17.2 (17.1-17.3)	18.1 (18-18.2)	19.1 (19-19.1)	19.6 (19.5-19.7)	20.1 (20.1-20.2)	20.6 (19.7-21.6)
Uruguay	16.2 (16.1-16.4)	15.9 (15.8-16)	16.3 (16.1-16.4)	16.2 (16.1-16.4)	16.2 (16.1-16.4)	16.8 (16.6-16.9)	16.7 (16.6-16.9)	17.7 (17.5-17.8)	17.9 (17.8-18)	18.4 (18.2-18.5)	18.7 (18.6-18.9)	19 (18.9-19.2)	19.8 (19.7-20)	19.8 (18.9-20.8)
Western Europe	14.4 (14.2-14.7)	15.1 (15-15.2)	15.5 (15.5-15.6)	15.9 (15.8-15.9)	16.1 (16.1-16.2)	16.6 (16.5-16.6)	17.2 (17.2-17.2)	17.8 (17.7-17.8)	18.5 (18.5-18.5)	19.2 (19.1-19.2)	19.9 (19.9-19.9)	20.7 (20.7-20.7)	21.4 (21.4-21.4)	21.9 (21.6-22.1)
Andorra	19.7 (18-21.6)	19.9 (18.4-21.9)	20.1 (18.7-21.9)	20.1 (18.6-22)	20.2 (18.6-22)	20.3 (18.7-22.1)	20.5 (18.9-22.3)	20.6 (19.1-22.2)	20.7 (19.4-22.3)	21 (19.7-22.7)	22.3 (20.7-23.5)	23.1 (21.6-23.7)	22.9 (21.6-23.9)	22.4 (21.3-23.8)
Austria	13.7 (13.6-13.7)	14.3 (14.2-14.3)	14.8 (14.7-14.8)	14.9 (14.8-15)	14.9 (14.8-15)	15.6 (15.5-15.7)	16.3 (16.2-16.4)	17 (16.9-17.1)	17.9 (17.8-18)	18.7 (18.6-18.8)	19.5 (19.4-19.6)	20.4 (20.3-20.5)	21.1 (21-21.2)	21.6 (21.1-22)
Belgium	13.4 (13.4-13.5)	14.5 (14.5-14.6)	15 (14.9-15)	15.3 (15.2-15.4)	15.4 (15.3-15.4)	15.8 (15.8-15.9)	16.8 (16.7-16.9)	17.5 (17.4-17.6)	18.5 (18.4-18.6)	19.1 (19-19.2)	19.6 (19.5-19.7)	20.4 (20.3-20.4)	20.9 (20.8-21)	21.7 (21.2-22.2)
Cyprus	15.4 (14.3-16.9)	15.6 (14.4-16.9)	15.6 (14.6-16.9)	15.7 (14.9-16.8)	15.7 (15.1-16.4)	15.8 (15.6-16)	15.9 (15.8-16)	16.5 (16.4-16.6)	17.3 (17.2-17.5)	17.8 (17.5-18.1)	18.2 (17.8-18.6)	19.3 (18.9-19.7)	21.1 (20.6-21.5)	22.5 (21.8-23.1)
Denmark	14.2 (14.1-14.3)	15.2 (15.1-15.3)	15.3 (15.2-15.4)	15.6 (15.5-15.7)	16.7 (16.6-16.8)	17.3 (17.2-17.4)	17.4 (17.4-17.6)	17.7 (17.6-17.8)	17.8 (17.7-17.9)	17.6 (17.5-17.7)	18.3 (18.2-18.4)	19.1 (19-19.2)	19.6 (19.5-19.8)	20.7 (20.1-21.2)
Finland	13.2 (13.1-13.3)	14.2 (14.1-14.3)	14.7 (14.6-14.8)	14.9 (14.8-15)	14.8 (14.7-14.9)	16 (15.9-16.1)	17.1 (17-17.2)	17.5 (17.4-17.7)	17.9 (17.7-18)	18.7 (18.5-18.8)	19.4 (19.3-19.6)	20.7 (20.6-20.8)	21.3 (21.2-21.5)	21.8 (21.2-22.3)
France	14.4 (14.4-14.5)	15.2 (15.1-15.2)	15.7 (15.7-15.8)	16.2 (16.2-16.2)	16.8 (16.7-16.8)	17.3 (17.2-17.3)	18.3 (18.3-18.3)	18.9 (18.9-19)	19.9 (19.8-19.9)	20.6 (20.6-20.6)	21.2 (21.1-21.2)	22 (22-22)	22.6 (22.6-22.7)	23.5 (23-23.9)
Germany	15.4 (14.2-16.8)	15.7 (15.3-16.1)	15.6 (15.6-15.7)	15.9 (15.9-15.9)	15.9 (15.9-15.9)	16.3 (16.3-16.3)	16.6 (16.5-16.6)	17.1 (17.1-17.1)	17.7 (17.7-17.8)	18.6 (18.5-18.6)	19.5 (19.5-19.6)	20.4 (20.4-20.4)	20.8 (20.7-20.8)	20.8 (19.9-21.7)
Greece	16.1 (15.6-16.5)	16.7 (16.6-16.8)	16.8 (16.7-16.9)	16.7 (16.6-16.8)	16.8 (16.7-16.9)	17.6 (17.5-17.7)	17.1 (17-17.2)	17.4 (17.3-17.5)	18.9 (18.8-18.9)	19.5 (19.4-19.6)	19.7 (19.6-19.8)	19.4 (19.3-19.5)	20.4 (20.3-20.5)	21.3 (20.8-21.8)
Iceland	15 (14.7-15.3)	15.7 (15.5-15.9)	16.5 (16.3-16.7)	16.9 (16.7-17.1)	17.1 (16.9-17.3)	18.3 (18.1-18.5)	19.2 (18.9-19.5)	19.3 (19-19.6)	19 (18.8-19.3)	19.4 (19.2-19.6)	20.1 (19.9-20.3)	20.9 (20.6-21.1)	22.2 (21.9-22.4)	23.1 (22.7-23.5)
Ireland	13.5 (13.4-13.6)	14.1 (13.9-14.2)	14.5 (14.4-14.6)	14.8 (14.7-14.9)	15 (14.9-15.1)	15.1 (15-15.2)	15.6 (15.5-15.8)	16.3 (16.2-16.4)	16.8 (16.7-17)	17.2 (17.1-17.4)	17.9 (17.7-18)	19.5 (19.4-19.6)	20.8 (20.7-20.9)	21.2 (20.6-21.7)
Israel	17 (16.7-17.2)	15.1 (14.9-15.2)	15.5 (15.3-15.6)	15 (14.8-15.1)	15.1 (15-15.2)	15.3 (15.2-15.4)	15.9 (15.7-16)	16.6 (16.5-16.7)	18 (17.9-18.1)	18.1 (18-18.3)	18.9 (18.8-19)	19.8 (19.7-19.9)	21.1 (21-21.2)	21.9 (21.4-22.4)
Italy	13.9 (13.9-13.9)	15 (15-15.1)	15.7 (15.7-15.7)	15.8 (15.8-15.9)	16.2 (16.2-16.2)	16.5 (16.4-16.5)	17.1 (17-17.1)	18 (17.9-18)	19 (18.9-19)	19.7 (19.6-19.7)	20.4 (20.3-20.4)	21.3 (21.2-21.3)	21.9 (21.8-21.9)	22.5 (22.1-23)
Luxembourg	13.6 (13.5-13.8)	14 (13.9-14.1)	14.5 (14.3-14.6)	14.7 (14.5-14.9)	14.9 (14.8-15.1)	15.3 (15.1-15.4)	16.3 (16.1-16.4)	16.9 (16.7-17.1)	18.1 (17.9-18.2)	18.5 (18.3-18.7)	19 (18.8-19.2)	19.9 (19.7-20.1)	20.3 (20.1-20.5)	20.9 (20.2-21.6)
Malta	12.6 (12.3-12.8)	13.6 (13.4-13.7)	13.4 (13.3-13.6)	13.6 (13.5-13.8)	13.9 (13.7-14)	14.5 (14.4-14.7)	14.6 (14.4-14.8)	15.9 (15.7-16.1)	17.4 (17.2-17.6)	18.1 (17.9-18.4)	18.8 (18.5-19)	19.3 (19.1-19.5)	20.3 (20-20.5)	20.6 (20.1-21.1)
Netherlands	14.5 (14.5-14.6)	15.1 (15-15.2)	15.6 (15.6-15.7)	16.2 (16.1-16.3)	16.4 (16.3-16.5)	17.2 (17.2-17.3)	18.3 (18.3-18.4)	18.6 (18.6-18.7)	19 (18.9-19.1)	19.1 (19-19.1)	19.2 (19.1-19.3)	20 (19.9-20)	20.8 (20.7-20.9)	20.9 (20.5-21.4)
Norway	15.6 (15.3-16)	16.2 (15.9-16.5)	16.2 (16-16.5)	16.6 (16.4-16.7)	17 (16.9-17.1)	17.6 (17.5-17.7)	18.3 (18.2-18.3)	18.5 (18.5-18.6)	18.8 (18.7-18.8)	19.3 (19.2-19.3)	19.7 (19.6-19.7)	20.6 (20.5-20.7)	20.8 (20.8-20.9)	21.6 (21.4-21.7)
Portugal	14.3 (14.2-14.3)	14.5 (14.4-14.6)	15.6 (15.5-15.7)	15.4 (15.3-15.5)	15.5 (15.4-15.6)	15.5 (15.4-15.5)	16.2 (16.1-16.2)	16.8 (16.8-16.9)	17.2 (17.2-17.3)	18 (17.9-18)	18.7 (18.6-18.8)	20.1 (19.9-20.3)	20.8 (20.7-20.9)	21.7 (21.2-22.1)
Spain	14.1 (14.1-14.2)	15.2 (15.1-15.2)	16 (15.9-16)	16 (16-16.1)	16.2 (16.2-16.3)	16.7 (16.7-16.8)	17.9 (17.8-17.9)	18.5 (18.4-18.5)	19.1 (19.1-19.2)	20 (19.9-20)	20.7 (20.7-20.8)	21.4 (21.4-21.5)	22.4 (22.3-22.4)	23 (22.6-23.4)
Sweden	14.4 (14.1-14.8)	15.2 (14.8-15.6)	15.5 (15.1-16)	16.3 (15.8-16.7)	17 (16.6-17.5)	17.5 (17.2-17.7)	18 (17.9-18.1)	18.7 (18.6-18.8)	19.1 (19.1-19.2)	19.7 (19.6-19.8)	20 (19.9-20.1)	20.6 (20.5-20.6)	21.1 (21-21.2)	21.5 (21.1-21.9)
Stockholm	14.5 (12.8-16.3)	15.3 (13.4-17.3)	15.6 (13.8-17.6)	16.3 (14.6-18.2)	17 (15.4-18.8)	17.5 (16.4-18.7)	18.1 (18-18.3)	18.8 (18.6-18.9)	19.2 (19-19.3)	20 (19.8-20.2)	20 (19.9-20.2)	20.6 (20.4-20.8)	21.4 (21.2-21.5)	21.6 (21-22.2)
Sweden except Stockholm	14.4 (14.2-14.8)	15.2 (14.9-15.6)	15.5 (15.2-15.9)	16.3 (15.9-16.6)	17 (16.7-17.4)	17.4 (17.2-17.7)	18 (17.9-18.1)	18.7 (18.6-18.8)	19.1 (19-19.2)	19.7 (19.6-19.8)	20 (19.9-20.1)	20.5 (20.4-20.6)	21.1 (21-21.2)	21.5 (21-22)
Switzerland	14.3 (14.2-14.4)	14.7 (14.6-14.8)	15.5 (15.4-15.6)	15.9 (15.8-16)	16.5 (16.4-16.6)	17.6 (17.5-17.7)	18.3 (18.2-18.4)	19.2 (19.1-19.3)	19.8 (19.7-19.9)	20.3 (20.2-20.4)	20.9 (20.8-21)	21.7 (21.6-21.8)	22.1 (22-22.2)	22.8 (22.4-23.3)
United Kingdom	14.1 (13.9-14.3)	14.8 (14.5-15)	15.2 (15-15.4)	15.7 (15.5-15.9)	16 (15.8-16.2)	16.4 (16.2-16.5)	16.9 (16.8-16.9)	17.3 (17.3-17.3)	17.8 (17.7-17.8)	18.1 (18.1-18.2)	18.9 (18.9-19)	19.7 (19.7-19.7)	20.6 (20.5-20.6)	20.8 (20.7-20.9)
England	14.3 (14.1-14.5)	14.9 (14.7-15.2)	15.4 (15.2-15.7)	15.9 (15.7-16.1)	16.2 (16-16.4)	16.5 (16.4-16.6)	17 (17-17)	17.4 (17.4-17.5)	17.9 (17.9-17.9)	18.3 (18.2-18.3)	19.1 (19-19.1)	19.8 (19.8-19.9)	20.7 (20.7-20.8)	20.9 (20.8-21)
East Midlands	13.9 (13.2-14.7)	14.6 (13.9-15.4)	15.1 (14.3-15.9)	15.6 (14.8-16.3)	15.8 (15.1-16.6)	16 (15.5-16.5)	16.8 (16.7-17)	17.5 (17.4-17.5)	17.9 (17.8-18)	18.2 (18.2-18.3)	19 (18.9-19.1)	19.7 (19.6-19.7)	20.6 (20.6-20.7)	20.8 (20.5-21)
Derby	13.8 (11.9-16)	14.4 (12.5-16.7)	14.9 (12.9-17.3)	15.2 (13.3-17.4)	15.4 (13.6-17.4)	15.7 (14.3-17.1)	16.4 (15.9-16.9)	17 (16.8-17.3)	17.4 (17.1-17.7)	17.9 (17.7-18.1)	19 (18.7-19.3)	19.6 (19.3-19.8)	20 (19.7-20.2)	20.6 (19.9-21.3)
Derbyshire	13.8 (11.7-15.9)	14.5 (12.5-16.6)	15 (12.9-17.2)	15.5 (13.4-17.6)	15.7 (14-17.7)	15.9 (14.5-17.3)	16.6 (16.2-17)	17.3 (17.2-17.5)	17.6 (17.5-17.8)	17.9 (17.8-18.1)	18.8 (18.5-19)	19.4 (19.3-19.6)	20.5 (20.3-20.7)	20.4 (19.8-21)
Leicester	13.5 (11.6-15.8)	14.1 (12.1-16.4)	14.5 (12.4-16.8)	14.8 (12.8-17.1)	15 (13.1-17)	15.2 (13.8-16.7)	16.2 (15.8-16.7)	17.4 (17.2-17.6)	17.7 (17.5-18)	17.7 (17.5-17.9)	18.1 (17.8-18.5)	18.9 (18.6-19.1)	20.3 (20.1-20.5)	21.6 (20.9-22.2)
Leicestershire	14.3 (12.5-16.6)	15 (13-17.4)	15.5 (13.6-17.8)	16.1 (14.1-18.4)	16.4 (14.5-18.4)	16.6 (15.3-18)	17.4 (17-17.8)	18 (17.8-18.1)	18.5 (18.4-18.7)	19 (18.8-19.2)	19.5 (19.2-19.7)	20.2 (20-20.4)	21.4 (21.2-21.5)	21.3 (20.6-21.9)
Lincolnshire	14 (12.1-16.3)	14.7 (12.8-17)	15.2 (13.3-17.4)	15.6 (13.6-17.7)	15.8 (13.8-17.8)	16 (14.5-17.5)	16.9 (16.5-17.4)	17.5 (17.3-17.6)	18.1 (17.9-18.3)	18.4 (18.2-18.6)	19.2 (18.9-19.4)	19.9 (19.7-20.1)	20.9 (20.7-21.1)	21.1 (20.5-21.7)
Northamptonshire	14.2 (12.3-16.5)	15 (13.1-17.3)	15.5 (13.6-17.8)	16 (14.1-18.2)	16.3 (14.7-18.3)	16.5 (15.2-18)	17.4 (17-17.8)	17.7 (17.5-17.9)	18.1 (17.9-18.3)	18.3 (18.1-18.5)	19.3 (19-19.6)	19.7 (19.5-19.9)	20.6 (20.4-20.8)	20.5 (19.8-21.1)
Nottingham	13.7 (11.7-16)	14.3 (12.2-16.7)	14.8 (12.8-17.1)	15.1 (13.3-17.5)	15.3 (13.5-17.3)	15.5 (14.1-16.9)	16 (15.5-16.4)	16.7 (16.5-17)	17.5 (17.3-17.8)	17.5 (17.3-17.7)	18.2 (17.9-18.5)	18.3 (18.1-18.5)	19.6 (19.3-19.8)	20.8 (20.1-21.4)
Nottinghamshire	14 (12.1-16)	14.7 (12.7-16.9)	15.3 (13.2-17.4)	15.8 (13.8-17.9)	16 (14.3-18.1)	16.2 (14.9-17.7)	16.9 (16.5-17.3)	17.6 (17.5-17.8)	17.7 (17.5-17.8)	18.2 (18.1-18.4)	18.9 (18.7-19.2)	19.8 (19.6-20)	20.4 (20.3-20.6)	20.5 (19.9-21.1)
Rutland	15 (12.8-17.4)	15.6 (13.6-17.9)	16.1 (14-18.5)	16.4 (14.2-18.7)	16.7 (14.6-18.9)	16.9 (15.3-18.5)	17.5 (16.7-18.3)	17.6 (17.1-18.2)	18.4 (17.9-18.9)	19.2 (18.7-19.7)	20.3 (19.7-20.9)	21.1 (20.6-21.6)	21.6 (21.1-22.2)	21.5 (20.5-22.4)
East of England	14.5 (13.7-15.3)	15.2 (14.4-16)	15.7 (15-16.6)	16.3 (15.6-17.1)	16.6 (15.9-17.4)	16.9 (16.4-17.5)	17.4 (17.2-17.5)	17.9 (17.8-17.9)	18.3 (18.3-18.4)	18.8 (18.7-18.8)	19.5 (19.4-19.6)	20.3 (20.2-20.3)	21.1 (21.1-21.2)	21.3 (21.1-21.5)
Bedford	14.8 (12.6-17.4)	15.6 (13.3-17.9)	16.1 (13.9-18.5)	16.7 (14.6-18.9)	17.1 (15.3-19.2)	17.6 (16.1-19.1)	18 (17.4-18.5)	18.3 (18-18.6)	18.3 (18-18.6)	18.5 (18.2-18.8)	19.4 (19.1-19.8)	20.1 (19.8-20.4)	20.9 (20.5-21.2)	21.4 (20.7-22.1)
Cambridgeshire	14.8 (12.7-17.2)	15.6 (13.5-17.9)	16.2 (14.3-18.6)	17 (15-19.4)	17.4 (15.7-19.4)	17.6 (16.2-19.1)	17.5 (17-17.9)	17.8 (17.6-18)	18.8 (18.6-19)	18.9 (18.7-19.1)	19.6 (19.4-19.9)	20.6 (20.4-20.8)	21.7 (21.5-21.9)	21.7 (21.1-22.4)
Central Bedfordshire	14.4 (12.5-17)	15.1 (13-17.5)	15.6 (13.5-18.1)	16.1 (14-18.4)	16.5 (14.5-18.6)	16.9 (15.3-18.4)	17.3 (16.8-17.8)	17.7 (17.4-17.9)	18 (17.7-18.3)	18.3 (18.1-18.6)	19.6 (19.3-19.9)	20.5 (20.2-20.7)	21.4 (21.1-21.7)	22.4 (21.8-23.1)
Essex	14.3 (12.4-16.5)	15 (13.1-17.2)	15.4 (13.6-17.5)	15.9 (14.1-18)	16.2 (14.5-18.1)	16.5 (15.1-17.9)	17.4 (17-17.7)	17.9 (17.7-18)	18.3 (18.2-18.5)	18.9 (18.7-19)	19.4 (19.2-19.6)	20.1 (20-20.3)	21.1 (21-21.3)	20.9 (20.3-21.4)
Hertfordshire	14.6 (12.6-16.8)	15.3 (13.2-17.5)	15.8 (13.6-18.1)	16.4 (14.4-18.6)	16.7 (14.9-18.7)	16.9 (15.5-18.4)	17.2 (16.9-17.6)	17.8 (17.7-18)	18.2 (18-18.4)	18.6 (18.4-18.7)	19.4 (19.1-19.6)	20 (19.8-20.1)	21.1 (21-21.3)	21.5 (20.9-22.1)

Table 10 Estimates of life expectancy at age 65 by location and year for 1950, 1955...2005, 2010, 2017 - females

Location	1950	1955	1960	1965	1970	1975	1980	1985	1990	1995	2000	2005	2010	2017
Luton	14 (11.9-16.5)	14.6 (12.6-17)	14.9 (12.9-17.3)	15.2 (13.1-17.5)	15.5 (13.6-17.6)	15.9 (14.3-17.3)	16.5 (16-17.1)	17.8 (17.5-18)	18 (17.7-18.3)	18.1 (17.9-18.4)	18.7 (18.4-19.1)	19.2 (19-19.5)	20.4 (20.1-20.6)	21.5 (20.8-22.2)
Norfolk	14.8 (12.9-17)	15.6 (13.6-17.9)	16.2 (14.3-18.5)	16.9 (15-19.2)	17.3 (15.5-19.5)	17.7 (16.2-19.2)	17.8 (17.4-18.2)	18.2 (18-18.4)	18.3 (18.2-18.5)	19.1 (18.9-19.3)	20 (19.7-20.2)	20.7 (20.5-20.9)	21.1 (20.9-21.2)	21.3 (20.7-21.9)
Peterborough	14.5 (12.2-17)	15.2 (13-17.7)	15.7 (13.6-18.3)	16.1 (14-18.7)	16.5 (14.7-18.8)	16.9 (15.4-18.5)	17.1 (16.6-17.7)	17.5 (17.2-17.7)	17.8 (17.5-18.1)	18 (17.7-18.2)	18.9 (18.5-19.2)	19.6 (19.3-19.8)	20.2 (19.9-20.5)	20.6 (19.9-21.4)
Southend-on-Sea	14.2 (12.1-16.8)	14.9 (12.8-17.4)	15.4 (13.4-17.9)	15.8 (13.7-18.2)	16.2 (14.3-18.2)	16.6 (15.1-18)	17 (16.5-17.5)	17.6 (17.3-17.9)	17.9 (17.7-18.2)	18.2 (18-18.5)	19 (18.6-19.3)	19.6 (19.3-19.9)	20.3 (20-20.6)	20.6 (19.9-21.2)
Suffolk	14.5 (12.5-16.7)	15.1 (13-17.4)	15.7 (13.4-17.9)	16.3 (14.3-18.6)	16.6 (14.9-18.7)	16.9 (15.5-18.5)	17.4 (17-17.8)	17.8 (17.7-18)	18.6 (18.4-18.8)	18.9 (18.7-19.1)	19.8 (19.6-20.1)	20.5 (20.4-20.7)	21.4 (21.3-21.6)	21.5 (20.9-22.1)
Thurrock	14.2 (12.1-16.6)	14.8 (12.7-17.2)	15.3 (13.1-17.8)	15.7 (13.8-18)	16 (14.2-18.1)	16.4 (14.9-17.9)	16.7 (16.2-17.3)	17.1 (16.9-17.4)	17.7 (17.4-18)	18 (17.7-18.3)	19.1 (18.8-19.4)	19.9 (19.6-20.2)	20.7 (20.4-21)	21.3 (20.6-22.1)
Greater London	14.8 (14.4-15.2)	15.5 (15.1-15.9)	16 (15.6-16.4)	16.3 (15.9-16.7)	16.5 (16.2-16.8)	16.8 (16.5-17)	17.4 (17.3-17.4)	17.7 (17.7-17.8)	18.2 (18.2-18.3)	18.3 (18.3-18.4)	19.2 (19.2-19.3)	20.1 (20.1-20.2)	21.2 (21.1-21.2)	21.6 (21.5-21.7)
Barking and Dagenham	14.3 (12.4-16.7)	15 (13.1-17.3)	15.5 (13.6-17.9)	15.9 (14-18.3)	16.2 (14.4-18.2)	16.5 (15.1-18)	16.9 (16.4-17.4)	17 (16.8-17.3)	17.5 (17.2-17.7)	17.5 (17.2-17.7)	18.3 (18-18.6)	18.9 (18.6-19.2)	20 (19.7-20.2)	20.4 (19.7-21.2)
Barnet	15.3 (13.5-17.5)	16.1 (14.3-18.3)	16.7 (14.8-18.6)	17.2 (15.3-19.2)	17.5 (15.7-19.3)	17.7 (16.4-19.1)	18.1 (17.6-18.5)	18.2 (18-18.4)	18.9 (18.7-19.1)	19.1 (18.9-19.4)	20.2 (19.9-20.5)	21.2 (20.9-21.4)	21.5 (21.3-21.7)	21.8 (21.2-22.4)
Bexley	15.2 (13.3-17.4)	15.9 (13.8-18.1)	16.4 (14.4-18.6)	16.9 (14.9-19)	17.2 (15.5-19.2)	17.6 (16.3-19)	18.2 (17.8-18.7)	18.6 (18.3-18.8)	19 (18.7-19.2)	18.8 (18.6-19.1)	20.1 (19.8-20.4)	21.2 (20.9-21.4)	21.8 (21.6-22.1)	21.9 (21.3-22.6)
Brent	14.8 (12.8-17.1)	15.5 (13.3-17.7)	16 (13.9-18.2)	16.3 (14.3-18.4)	16.5 (14.8-18.4)	16.9 (15.6-18.3)	18 (17.5-18.4)	17.9 (17.7-18.1)	18.4 (18.2-18.7)	18.4 (18.1-18.6)	19.5 (19.2-19.8)	20.9 (20.6-21.1)	21.6 (21.4-21.9)	22.5 (21.9-23.1)
Bromley	15.2 (13.2-17.6)	16 (14.1-18.2)	16.4 (14.4-18.6)	16.9 (14.9-18.9)	17.2 (15.4-19.1)	17.5 (16.1-18.9)	18 (17.6-18.5)	18.5 (18.3-18.8)	19 (18.8-19.2)	19.3 (19.1-19.5)	20.1 (19.8-20.4)	21 (20.8-21.3)	21.8 (21.6-22)	22.1 (21.5-22.8)
Camden	14.7 (12.8-16.8)	15.3 (13.4-17.5)	15.6 (13.7-17.8)	15.8 (13.8-17.8)	15.9 (14.1-17.7)	16 (14.7-17.4)	16.4 (16-16.9)	16.7 (16.5-16.9)	17.3 (17.1-17.5)	17.4 (17.1-17.6)	18.5 (18.2-18.8)	19.7 (19.4-19.9)	20.8 (20.6-21.1)	23.2 (22.5-23.8)
Croydon	14.3 (12.5-16.5)	15 (13.1-17.3)	15.5 (13.6-17.6)	16 (14.1-18.1)	16.2 (14.4-18.2)	16.4 (15.1-17.8)	16.9 (16.6-17.3)	17.4 (17.2-17.6)	17.6 (17.4-17.8)	18.1 (17.9-18.3)	19.2 (18.9-19.5)	20.2 (20-20.4)	20.9 (20.6-21.1)	20.8 (20.1-21.3)
Ealing	14.6 (12.6-16.9)	15.3 (13.3-17.6)	15.8 (13.9-18.2)	16.2 (14.4-18.3)	16.4 (14.7-18.2)	16.6 (15.2-17.9)	17 (16.6-17.4)	17.5 (17.3-17.7)	18.2 (18-18.4)	18.2 (18-18.4)	19.4 (19-19.6)	20.4 (20.2-20.7)	21.7 (21.4-21.9)	21.7 (21-22.3)
Enfield	14.9 (12.8-17.1)	15.7 (13.6-17.9)	16.2 (14.2-18.5)	16.8 (14.8-19)	17.1 (15.4-19.1)	17.3 (16-18.8)	17.8 (17.3-18.2)	17.8 (17.6-18.1)	18.6 (18.3-18.8)	18.7 (18.5-18.9)	19.3 (19-19.6)	20.1 (19.9-20.3)	21.2 (21-21.5)	21.7 (21.1-22.4)
Greenwich	14.5 (12.5-16.7)	15.3 (13.2-17.6)	15.8 (13.8-18)	16.3 (14.4-18.4)	16.6 (14.9-18.5)	16.8 (15.5-18.3)	17.1 (16.6-17.6)	17 (16.8-17.3)	17.6 (17.4-17.9)	17.7 (17.4-17.9)	18 (17.7-18.3)	18.7 (18.5-19)	20.4 (20.2-20.7)	20.1 (19.4-20.7)
Hackney	14.9 (12.8-17.1)	15.5 (13.5-17.8)	15.8 (14-18)	15.8 (14-17.9)	15.9 (14.2-17.8)	16 (14.7-17.4)	16.5 (16-16.9)	17 (16.8-17.3)	17.1 (16.9-17.4)	18.1 (17.9-18.4)	19.1 (18.8-19.5)	18.9 (18.6-19.1)	19.8 (19.6-20.1)	21 (20.3-21.7)
Hammersmith and Fulham	14.3 (12.4-16.4)	14.9 (12.8-17)	15.2 (13.3-17.3)	15.5 (13.6-17.6)	15.6 (13.9-17.5)	15.8 (14.5-17.1)	16.2 (15.7-16.6)	16.3 (16.1-16.5)	17 (16.7-17.2)	17.1 (16.8-17.3)	18.2 (17.9-18.5)	19.5 (19.2-19.8)	21.1 (20.8-21.4)	22.1 (21.5-22.8)
Haringey	14.9 (12.7-17.3)	15.6 (13.5-18)	16 (13.9-18.3)	16.2 (14.3-18.4)	16.5 (14.7-18.4)	16.8 (15.5-18.2)	17.5 (17-17.9)	17.8 (17.6-18.1)	18.1 (17.9-18.3)	18.5 (18.2-18.7)	19.1 (18.8-19.4)	20 (19.7-20.2)	20.8 (20.5-21)	21.6 (20.9-22.2)
Harrow	15.1 (13.1-17.5)	15.8 (13.7-18.1)	16.1 (14.1-18.5)	16.4 (14.4-18.5)	16.6 (14.7-18.6)	16.9 (15.5-18.3)	17.7 (17.2-18.2)	18.6 (18.3-18.9)	19.5 (19.2-19.7)	19.7 (19.5-20)	20.7 (20.4-21)	21.7 (21.4-21.9)	22.7 (22.4-23)	23.3 (22.6-23.9)
Havering	14.9 (13-17.1)	15.6 (13.6-17.8)	16.1 (13.9-18.3)	16.5 (14.6-18.5)	16.8 (15.2-18.8)	17.1 (15.8-18.6)	17.3 (16.9-17.8)	18.4 (18.2-18.7)	19.5 (19.2-19.7)	19.3 (19-19.5)	20 (19.7-20.3)	20.6 (20.3-20.8)	21 (20.8-21.3)	20.4 (19.6-21.1)
Hillingdon	15.1 (13.1-17.4)	15.8 (13.8-18.1)	16.3 (14.3-18.5)	16.7 (14.8-18.8)	17 (15.3-18.9)	17.2 (15.8-18.6)	17.6 (17.1-18)	17.8 (17.6-18.1)	18.3 (18.1-18.6)	18.3 (18.1-18.6)	19.4 (19.1-19.7)	20.3 (20-20.5)	21.1 (20.9-21.4)	21.2 (20.6-21.8)
Hounslow	15 (13-17.3)	15.7 (13.6-17.9)	16.1 (14-18.3)	16.3 (14.3-18.5)	16.5 (14.8-18.4)	16.8 (15.6-18.2)	17.6 (17.2-18.1)	17.6 (17.3-17.8)	18.7 (18.4-18.9)	18.8 (18.5-19)	19.2 (18.9-19.5)	19.9 (19.6-20.1)	20.8 (20.6-21.1)	21.4 (20.7-22.1)
Islington	14.4 (12.5-16.7)	15.1 (13.3-17.3)	15.4 (13.6-17.7)	15.6 (13.8-17.8)	15.7 (14-17.5)	15.9 (14.5-17.3)	16.4 (16-16.9)	16.6 (16.3-16.8)	17.4 (17.2-17.7)	17.2 (17-17.5)	18.1 (17.8-18.4)	19 (18.7-19.2)	20.4 (20.2-20.7)	21.4 (20.7-22)
Kensington and Chelsea	15 (13.1-17.3)	15.6 (13.7-17.9)	15.9 (14-18)	15.9 (14.1-18)	16.1 (14.4-18)	16.3 (15-17.8)	17.1 (16.6-17.6)	17.3 (17-17.5)	18.2 (17.9-18.4)	19.1 (18.8-19.3)	20.7 (20.4-21.1)	21.7 (21.4-22)	22.4 (22.2-22.7)	22.3 (21.7-23)
Kingston upon Thames	15.8 (13.6-18)	16.5 (14.5-18.7)	17 (14.9-19.2)	17.4 (15.3-19.5)	17.8 (15.9-19.7)	18.1 (16.7-19.6)	18.5 (18-19.1)	18.7 (18.4-19)	19.2 (18.9-19.5)	19 (18.7-19.3)	20 (19.7-20.4)	21.3 (21.1-21.6)	22.5 (22.2-22.8)	21.7 (21-22.5)
Lambeth	14.5 (12.5-16.7)	15.2 (13.2-17.5)	15.6 (13.6-17.8)	15.8 (13.9-17.8)	16 (14.4-17.9)	16.3 (15.1-17.7)	17 (16.5-17.4)	17.5 (17.3-17.8)	17.9 (17.7-18.2)	17.7 (17.5-17.9)	18.2 (18-18.5)	18.9 (18.7-19.2)	20.4 (20.2-20.7)	22 (21.4-22.7)
Lewisham	14.6 (12.6-16.8)	15.3 (13.2-17.6)	15.9 (13.7-18.2)	16.3 (14.3-18.4)	16.6 (14.8-18.5)	16.9 (15.6-18.4)	17.3 (16.9-17.8)	17.7 (17.5-17.9)	18.1 (17.8-18.3)	17.5 (17.3-17.8)	17.9 (17.6-18.2)	18.6 (18.4-18.9)	20.2 (20-20.4)	20.7 (20-21.4)
Merton	15.2 (13.3-17.5)	15.9 (13.9-18.1)	16.3 (14.3-18.4)	16.5 (14.5-18.7)	16.7 (14.9-18.6)	17.1 (15.7-18.6)	17.8 (17.3-18.3)	18.8 (18.5-19.1)	18.9 (18.6-19.1)	18.7 (18.4-18.9)	19.6 (19.3-20)	20.7 (20.5-21)	21.8 (21.5-22.1)	22.2 (21.5-22.9)
Newham	13.9 (12-16.1)	14.6 (12.7-17)	15.1 (13.2-17.4)	15.4 (13.6-17.7)	15.7 (13.9-17.7)	16 (14.6-17.4)	16.7 (16.3-17.2)	17.1 (16.9-17.4)	17.6 (17.4-17.9)	17.5 (17.3-17.8)	18.1 (17.7-18.4)	18.6 (18.4-18.9)	19.3 (19.1-19.5)	21 (20.4-21.6)
Redbridge	15.2 (13.2-17.5)	15.9 (13.8-18.1)	16.4 (14.4-18.5)	16.7 (14.6-18.9)	16.9 (15.2-18.9)	17.3 (15.9-18.8)	18.1 (17.6-18.5)	19.2 (19-19.5)	19 (18.7-19.2)	18.7 (18.4-18.9)	19.4 (19.1-19.7)	20.2 (20-20.5)	21.4 (21.1-21.6)	21.4 (20.7-22.1)
Richmond upon Thames	15.5 (13.4-17.7)	16.1 (14.1-18.4)	16.4 (14.4-18.6)	16.6 (14.6-18.6)	16.8 (15.1-18.5)	17.2 (15.8-18.6)	17.7 (17.3-18.2)	18.2 (18-18.5)	19.1 (18.8-19.4)	20.3 (20.1-20.6)	20.1 (19.8-20.5)	21.4 (21.1-21.7)	22.6 (22.3-22.9)	22.9 (22.2-23.7)
Southwark	14.9 (12.8-17.2)	15.6 (13.6-17.9)	16 (14-18.2)	16.2 (14.3-18.3)	16.4 (14.8-18.4)	16.7 (15.4-18.1)	17 (16.6-17.5)	17.7 (17.5-18)	16.9 (16.7-17.1)	17.6 (17.4-17.8)	18.1 (17.8-18.4)	19.1 (18.9-19.3)	20 (19.8-20.2)	20.4 (19.8-21.2)
Sutton	15.2 (13.2-17.4)	15.9 (13.9-18)	16.4 (14.3-18.6)	16.9 (14.9-18.9)	17.3 (15.5-19.2)	17.6 (16.3-19)	18.1 (17.6-18.6)	18 (17.8-18.3)	18.5 (18.2-18.7)	18.5 (18.2-18.7)	19.5 (19.2-19.8)	20.9 (20.6-21.2)	21.8 (21.5-22.1)	21.2 (20.5-21.8)
Tower Hamlets	14.3 (12.4-16.4)	14.9 (12.9-17)	15.2 (13.2-17.4)	15.5 (13.6-17.6)	15.6 (13.9-17.4)	15.7 (14.5-17.2)	16.1 (15.6-16.6)	16.3 (16.1-16.6)	16.9 (16.6-17.1)	16.7 (16.4-16.9)	17.7 (17.4-18)	18.5 (18.3-18.7)	20.2 (20-20.5)	21.3 (20.6-21.9)
Waltham Forest	14.6 (12.6-16.8)	15.4 (13.3-17.6)	15.9 (13.9-18.1)	16.3 (14.3-18.4)	16.6 (14.8-18.5)	16.9 (15.5-18.4)	17.8 (17.3-18.3)	17.8 (17.5-18)	18.2 (17.9-18.5)	18.1 (17.8-18.3)	18.7 (18.4-19)	19.3 (19-19.5)	20.3 (20.1-20.6)	21 (20.4-21.7)
Wandsworth	14.6 (12.7-16.7)	15.2 (13.3-17.5)	15.5 (13.7-17.6)	15.7 (13.8-17.6)	15.9 (14-17.7)	16.1 (14.8-17.5)	17 (16.5-17.4)	17.1 (16.9-17.3)	17.5 (17.3-17.7)	17.4 (17.2-17.6)	18.4 (18.1-18.7)	19.6 (19.4-19.8)	21 (20.8-21.2)	21.7 (21-22.3)
Westminster	16.1 (14.1-18.4)	16.7 (14.7-19)	17 (15.2-19.2)	16.8 (15-19)	17 (15.1-18.9)	17.2 (15.9-18.6)	17.9 (17.5-18.4)	18.6 (18.4-18.8)	18 (17.7-18.2)	18.4 (18.2-18.6)	20.7 (20.4-21)	21 (20.7-21.2)	22 (21.8-22.3)	22.7 (22.1-23.4)
North East England	13 (12.4-13.7)	13.7 (13-14.4)	14.2 (13.5-14.9)	14.8 (14.1-15.4)	15.1 (14.5-15.8)	15.5 (15-16)	16.2 (16-16.3)	16.5 (16.4-16.6)	16.7 (16.7-16.8)	17.2 (17.2-17.3)	18 (17.9-18.1)	18.7 (18.6-18.7)	19.7 (19.6-19.8)	20 (19.7-20.2)
County Durham	12.9 (11.1-15)	13.6 (11.6-15.8)	14.2 (12.3-16.3)	14.8 (12.8-17)	15.2 (13.4-17.2)	15.5 (14.1-17)	16.3 (15.9-16.7)	16.5 (16.3-16.6)	16.5 (16.3-16.7)	17.1 (16.9-17.3)	17.8 (17.6-18.1)	18.7 (18.5-18.9)	19.7 (19.5-19.9)	19.8 (19.2-20.4)
Darlington	13.1 (11.1-15.4)	13.8 (11.7-16)	14.2 (12.3-16.4)	14.7 (12.7-16.7)	15 (13.1-16.9)	15.4 (14-16.9)	16.1 (15.6-16.7)	16.7 (16.4-17)	16.9 (16.5-17.2)	17.2 (16.9-17.5)	17.9 (17.5-18.3)	18.6 (18.2-18.9)	19.8 (19.5-20.2)	20.3 (19.4-21.1)
Gateshead	13 (11.1-15.3)	13.7 (11.7-16)	14.2 (12.2-16.5)	14.7 (12.7-16.9)	15 (13.2-17.2)	15.3 (14-16.8)	15.8 (15.4-16.3)	16.5 (16.2-16.7)	17.1 (16.9-17.4)	17.5 (17.2-17.7)	18.1 (17.8-18.4)	19 (18.7-19.3)	19.8 (19.6-20.1)	19.9 (19.1-20.5)
Hartlepool	12.7 (10.8-15)	13.3 (11.2-15.8)	13.8 (11.8-16)	14.2 (12.3-16.4)	14.5 (12.7-16.6)	14.9 (13.5-16.5)	15.5 (14.9-16)	16 (15.7-16.3)	16.4 (16-16.7)	16.7 (16.4-17)	17.3 (17-17.7)	17.8 (17.4-18.1)	19.2 (18.9-19.6)	20.1 (19.3-20.9)
Middlesbrough	12.4 (10.7-14.7)	13 (11.2-15.3)	13.5 (11.6-15.7)	13.9 (12-16.1)	14.2 (12.4-16.3)	14.5 (13.2-15.9)	15.1 (14.6-15.6)	15.5 (15.2-15.7)	15.9 (15.6-16.1)	16.3 (16-16.6)	17 (16.6-17.3)	17.7 (17.4-17.9)	18.9 (18.6-19.2)	19.2 (18.4-19.9)
Newcastle upon Tyne	13.1 (11.2-15.4)	13.8 (11.7-16.1)	14.4 (12.3-16.8)	14.9 (12.9-17.2)	15.3 (13.5-17.4)	15.7 (14.3-17.2)	16.3 (15.8-16.7)	16.6 (16.4-16.8)	16.8 (16.5-17)	17 (16.8-17.3)	17.9 (17.6-18.2)	18.6 (18.3-18.8)	19.5 (19.3-19.8)	19.9 (19.2-20.6)
North Tyneside	13.2 (11.2-15.6)	13.9 (11.8-16.2)	14.4 (12.5-16.8)	15 (13.1-17.3)	15.4 (13.6-17.4)	15.7 (14.3-17.3)	16.3 (15.9-16.8)	16.6 (16.4-16.9)	16.8 (16.6-17.1)	17.5 (17.2-17.7)	18.1 (17.8-18.5)	18.6 (18.4-18.9)	19.8 (19.6-20.1)	20.6 (19.9-21.4)
Northumberland	13 (11.1-15.2)	13.7 (11.7-15.9)	14.2 (12.3-16.4)	14.9 (13-17.1)	15.2 (13.6-17.3)	15.5 (14.3-17)	16.3 (15.8-16.7)	16.6 (16.4-16.9)	16.9 (16.7-17.1)	17.9 (17.6-18.1)	18.8 (18.5-19.1)	19.6 (19.4-19.8)	20.4 (20.2-20.6)	20.6 (20-21.2)
Redcar and Cleveland	13.1 (11.1-15.4)	13.7 (11.6-15.9)	14.2 (12.2-16.5)	14.6 (12.6-16.7)	14.9 (13.2-17.1)	15.4 (14-16.9)	16.2 (15.7-16.7)	16.9 (16.7-17.2)	17.4 (17.1-17.6)	17.7 (17.5-18)	18.5 (18.2-18.9)	18.6 (18.4-18.9)	19.6 (19.3-19.9)	19.8 (19.1-20.6)
South Tyneside	13 (11.2-15.4)	13.7 (11.7-16)	14.3 (12.3-16.6)	14.8 (12.9-17.3)	15.2 (13.4-17.3)	15.6 (14.2-17.2)	16.1 (15.6-16.6)	16.3 (16-16.5)	16.6 (16.3-16.9)	17.3 (17-17.6)	18.2 (17.9-18.5)	18.8 (18.5-19.1)	19.4 (19.1-19.7)	19.7 (19-20.5)
Stockton-on-Tees	13.5 (11.5-15.8)	14.1 (12.1-16.4)	14.6 (12.6-17)	15.1 (13.2-17.3)	15.5 (13.7-17.5)	16 (14.6-17.5)	16.6 (16.1-17.1)	16.9 (16.6-17.1)	17.3 (17.1-17.6)	17.8 (17.5-18)	18.5 (18.2-18.9)	19.2 (18.9-19.4)	20.2 (19.9-20.4)	20.6 (19.9-21.3)
Sunderland	12.9 (11-15.1)	13.7 (11.7-16)	14.3 (12.4-16.6)	14.9 (12.9-17.1)	15.3 (13.5-17.4)	15.7 (14.3-17.2)	16.5 (16.1-17)	16.6 (16.4-16.8)	16.6 (16.4-16.9)	16.8 (16.5-17)	17.6 (17.3-17.9)	18 (17.8-18.2)	19.2 (19-19.5)	19.1 (18.4-19.8)
North West England	13.7 (13.2-14.2)	14.3 (13.8-14.9)	14.8 (14.3-15.3)	15.2 (14.7-15.7)	15.5 (15.1-16)	15.9 (15.6-16.3)	16.3 (16.2-16.4)	16.7 (16.6-16.7)	17.1 (17.1-17.1)	17.6 (17.5-17.6)	18.3 (18.2-18.4)	19.1 (19-19.1)	19.9 (19.8-19.9)	20 (19.9-20.2)
Blackburn with Darwen	13.3 (11.5-15.4)	13.9 (12.2-16.1)	14.3 (12.4-16.5)	14.7 (12.8-16.7)	15 (13.3-16.9)	15.4 (14.1-16.8)	15.8 (15.4-16.3)	16.1 (15.8-16.3)	16.5 (16.3-16.8)	16.8 (16.6-17.1)	17.3 (16.9-17.6)	18.4 (18.1-18.7)	19.4 (19.1-19.7)	19.3 (18.6-20)
Blackpool	13.6 (11.8-15.8)	14.2 (12.4-16.4)	14.6 (12.6-16.7)	14.8 (12.9-16.7)	15.1 (13.3-17)	15.5 (14.2-16.9)	16.2 (15.8-16.7)	17.3 (17-17.5)	16.9 (16.7-17.2)	18 (17.7-18.3)	16.9 (16.6-17.3)	17.3 (17.1-17.6)	18.2 (17.9-18.5)	19.3 (18.6-20)
Bolton	13.6 (11.7-15.7)	14.3 (12.4-16.4)	14.7 (12.8-17)	15.2 (13.3-17.4)	15.5 (13.8-17.4)	15.8 (14.5-17.3)	16.2 (15.8-16.7)	16.5 (16.3-16.7)	17.2 (17-17.4)	17.6 (17.4-17.8)	17.8 (17.5-18.1)	18.6 (18.3-18.8)	19.8 (19.5-20)	20.1 (19.5-20.8)
Bury	13.9 (12.1-16.2)	14.7 (12.8-16.9)	15.2 (13.2-17.4)	15.8 (13.8-17.8)	16.2 (14.4-18)	16.5 (15.2-18)	16.7 (16.3-17.2)	17 (16.7-17.2)	17.6 (17.4-17.9)	18.1 (17.8-18.3)	18.6 (18.3-18.9)	19.1 (18.8-19.4)	19.8 (19.5-20.1)	19.5 (18.9-20.2)
Cheshire East	14.4 (12.6-16.6)	15.2 (13.2-17.3)	15.7 (13.8-18)	16.5 (14.5-18.6)	16.9 (15-18.7)	17.1 (15.8-18.5)	17.1 (16.7-17.5)	17.3 (17.1-17.5)	17.8 (17.6-18)	18.6 (18.4-18.8)	19.3 (19-19.5)	20.7 (20.5-20.9)	21 (20.8-21.2)	21.2 (20.5-21.8)
Cheshire West and Chester	14 (12.1-16.2)	14.6 (12.8-16.8)	15.1 (13.2-17.2)	15.5 (13.5-17.5)	15.8 (14-17.6)	16.1 (14.8-17.6)	16.9 (16.5-17.3)	16.9 (16.7-17.1)	17.5 (17.3-17.7)	18.1 (17.9-18.3)	19.2 (18.9-19.5)	20.2 (20-20.4)	21 (20.7-21.2)	20.9 (20.2-21.5)
Cumbria	13.7 (11.9-15.7)	14.4 (12.6-16.4)	14.9 (13.1-16.9)	15.4 (13.8-17.5)	15.7 (14.1-17.6)	16.1 (14.8-17.4)	16.6 (16.2-17)	16.9 (16.7-17.1)	17.5 (17.3-17.7)	17.5 (17.3-17.7)	19 (18.8-19.3)	20 (19.8-20.2)	20.3 (20.1-20.5)	20.9 (20.3-21.5)
Halton	13.3 (11.4-15.4)	13.9 (12-16.1)	14.3 (12.4-16.5)	14.5 (12.6-16.6)	14.8 (12.9-16.7)	15.1 (13.8-16.6)	15.6 (15.1-16.1)	15.9 (15.7-16.2)	16.8 (16.5-17)	16.9 (16.6-17.2)	17.2 (16.9-17.6)	17.8 (17.5-18.1)	18.9 (18.6-19.2)	19.4 (18.6-20.1)
Knowsley	13.5 (11.7-15.7)	14.1 (12.1-16.5)	14.5 (12.5-16.8)	14.7 (12.8-16.8)	14.9 (13.3-16.8)	15.4 (14.1-16.8)	16.1 (15.6-16.6)	16.9 (16.6-17.1)	16.8 (16.6-17.1)	16.9 (16.6-17.2)	17.4 (17.1-17.7)	18.2 (17.9-18.4)	19 (18.7-19.3)	18.8 (18.1-19.5)
Lancashire	13.9 (12.1-16.1)	14.7 (12.9-16.7)	15.2 (13.4-17.3)	15.8 (14-17.8)	16.1 (14.3-17.9)	16.5 (15.1-17.8)	16.3 (16-16.7)	16.7 (16.6-16.9)	17 (16.9-17.2)	18.1 (17.9-18.2)	18.7 (18.5-18.9)	19.2 (19-19.4)	20.4 (20.3-20.6)	20.5 (19.9-21.1)
Liverpool	13.1 (11.2-15.3)	13.7 (11.8-15.7)	13.9 (12.1-16)	14.1 (12.4-16.1)	14.3 (12.7-16.2)	14.8 (13.5-16.2)	16 (15.6-16.4)	16.2 (16-16.3)	16.5 (16.3-16.7)	16.7 (16.5-16.9)	17.3 (17-17.6)	18.1 (17.9-18.3)	18.8 (18.6-19)	19.3 (18.6-19.9)
Manchester	13.8 (12-15.9)	14.5 (12.8-16.6)	15 (13.1-17.2)	15.3 (13.5-17.2)	15.5 (13.9-17.4)	15.9 (14.6-17.3)	16.3 (15.9-16.6)	16.2 (16-16.3)	16.5 (16.3-16.7)	16.8 (16.6-17)	18 (17.7-18.3)	17.9 (17.7-18.1)	18.9 (18.7-19.1)	18.3 (17.7-18.9)
Oldham	13.4 (11.6-15.6)	14 (12.2-16.2)	14.5 (12.6-16.6)	14.9 (13.2-16.9)	15.2 (13.6-17)	15.6 (14.4-17.1)	16.1 (15.7-16.6)	16.3 (16.1-16.5)	16.8 (16.6-17)	17.4 (17.2-17.7)	17.8 (17.5-18.1)	18.4 (18.2-18.6)	19.2 (19-19.5)	19.2 (18.5-19.9)
Rochdale	13.5 (11.6-15.6)	14.2 (12.3-16.5)	14.7 (12.6-17)	15.2 (13.4-17.3)	15.6 (13.9-17.6)	16 (14.7-17.5)	16.4 (15.9-16.9)	16.7 (16.4-16.9)	16.5 (16.3-16.8)	17.2 (17-17.4)	17.7 (17.4-18)	18.4 (18.1-18.6)	19.1 (18.9-19.3)	19.3 (18.7-20)
Salford	13.6 (11.7-15.7)	14.3 (12.4-16.5)	14.7 (12.7-17)	15 (13-17)	15.4 (13.6-17.1)	15.8 (14.5-17.3)	16.5 (16.1-17)	16.6 (16.3-16.8)	16.5 (16.3-16.8)	16.5 (16.3-16.7)	17.6 (17.4-18)	18.3 (18.1-18.5)	19.1 (18.9-19.3)	19.2 (18.5-19.8)
Sefton	13.8 (11.9-15.9)	14.4 (12.5-16.5)	14.9 (13-16.9)	15.2 (13.3-17.3)	15.5 (13.7-17.3)	15.9 (14.6-17.3)	16.4 (16-16.8)	17 (16.8-17.2)	17.7 (17.5-17.9)	17.9 (17.7-18.1)	18.6 (18.3-18.9)	19.9 (19.6-20.1)	20.1 (19.9-20.4)	20.7 (20-21.3)
St Helens	13.3 (11.6-15.4)	13.9 (12.2-16.1)	14.4 (12.4-16.6)	14.9 (12.9-16.9)	15.2 (13.4-17.1)	15.5 (14.1-16.9)	15.4 (14.9-15.8)	16.3 (16-16.5)	16.8 (16.6-17)	17.4 (17.1-17.6)	18.1 (17.8-18.4)	18.8 (18.5-19)	19.3 (19.1-19.6)	19.5 (18.8-20.1)
Stockport	14.2 (12.3-16.3)	14.9 (12.9-16.9)	15.4 (13.5-17.4)	16 (14.2-18)	16.3 (14.5-18.1)	16.6 (15.3-18)	16.9 (16.5-17.4)	17.4 (17.2-17.6)	17.7 (17.5-17.9)	18.2 (18-18.4)	19.3 (19-19.6)	19.8 (19.6-20.1)	20.5 (20.3-20.7)	21.2 (20.5-21.8)
Tameside	13.3 (11.4-15.4)	13.9 (12.1-16)	14.3 (12.3-16.5)	14.7 (12.9-16.8)	15 (13.3-16.8)	15.4 (14-16.8)	15.7 (15.3-16.2)	16.2 (16-16.5)	17.2 (17-17.5)	17.2 (16.9-17.4)	18 (17.7-18.3)	18.5 (18.3-18.8)	19.2 (18.9-19.4)	19.2 (18.6-19.9)
Trafford	14.1 (12.2-16.3)	14.7 (12.8-16.9)	15.1 (13.2-17.3)	15.4 (13.6-17.5)	15.7 (13.9-17.5)	16.1 (14.7-17.4)	16.7 (16.2-17.1)	17.2 (17-17.4)	17.8 (17.6-18.1)	18 (17.7-18.2)	18.7 (18.4-19)	20 (19.7-20.2)	21.1 (20.9-21.4)	21.1 (20.4-21.8)
Warrington	13.8 (12-16)	14.4 (12.6-16.6)	14.8 (12.9-17)	15.2 (13.4-17.3)	15.5 (13.8-17.3)	15.8 (14.5-17.2)	16.2 (15.8-16.7)	16.3 (16.1-16.6)	16.9 (16.6-17.1)	17.7 (17.5-18)	18.6 (18.3-18.9)	19.2 (18.9-19.4)	20.1 (19.8-20.3)	20 (19.3-20.7)
Wigan	13 (11.1-15.2)	13.7 (11.7-15.9)	14.2 (12.2-16.3)	14.8 (13.1-16.8)	15.1 (13.5-16.9)	15.4 (14-16.8)	15.5 (15.1-15.9)	15.9 (15.7-16.1)	16.7 (16.5-16.9)	16.7 (16.5-16.9)	17.9 (17.6-18.2)	18.8 (18.6-19)	19.2 (19-19.4)	19 (18.4-19.7)
Wirral	13.8 (11.9-16.1)	14.5 (12.6-16.8)	15 (13.2-17.4)	15.4 (13.6-17.5)	15.7 (14-17.6)	16.2 (15-17.6)	17 (16.5-17.4)	17.3 (17.1-17.5)	17.5 (17.3-17.7)	17.7 (17.5-17.9)	18.3 (18-18.6)	18.8 (18.6-19)	19.7 (19.5-19.9)	19.4 (18.8-20.1)
South East England	15.1 (14.5-15.8)	15.8 (15.1-16.4)	16.2 (15.5-16.9)	16.6 (15.9-17.3)	16.8 (16.2-17.4)	17 (16.5-17.4)	17.5 (17.4-17.7)	17.9 (17.9-18)	18.5 (18.5-18.6)	18.7 (18.7-18.8)	19.6 (19.5-19.6)	20.4 (20.3-20.5)	21.3 (21.3-21.4)	21.4 (21.2-21.6)
Bracknell Forest	15.4 (13.3-17.8)	16 (13.8-18.4)	16.5 (14.3-18.9)	16.8 (14.8-19.1)	17.1 (15.3-19.1)	17.3 (16-18.9)	17.7 (17.2-18.3)	18.2 (17.9-18.6)	18.9 (18.5-19.2)	19.1 (18.8-19.5)	19.9 (19.5-20.3)	20.6 (20.3-21)	21.5 (21.2-21.8)	22.6 (21.9-23.4)
Brighton and Hove	15.1 (13.1-17.3)	15.7 (13.8-17.9)	16 (14.2-18.2)	16.2 (14.2-18.4)	16.4 (14.5-18.3)	16.6 (15.3-18)	17.3 (16.8-17.8)	17.6 (17.4-17.8)	18.3 (18-18.5)	18.1 (17.8-18.3)	18.5 (18.2-18.8)	19.2 (19-19.5)	20.3 (20.1-20.5)	21 (20.4-21.7)
Buckinghamshire	15.1 (13-17.5)	15.7 (13.7-18)	16.1 (14.2-18.4)	16.7 (14.7-18.8)	16.8 (15-18.7)	16.9 (15.6-18.3)	17.4 (17-17.8)	18 (17.8-18.2)	18.4 (18.2-18.6)	18.8 (18.7-19)	19.8 (19.5-20.1)	20.9 (20.7-21.1)	21.8 (21.6-22)	21.9 (21.3-22.5)
East Sussex	15.4 (13.5-17.6)	16 (14.1-18.3)	16.4 (14.6-18.7)	16.7 (14.9-18.9)	17 (15.2-18.9)	17.3 (15.9-18.6)	17.8 (17.4-18.2)	18.3 (18.2-18.5)	19.1 (18.9-19.2)	19.1 (18.9-19.3)	19.8 (19.6-20.1)	20.8 (20.6-21)	21.3 (21.2-21.5)	21.3 (20.7-21.8)
Hampshire	15.1 (13.2-17.3)	15.6 (13.6-17.7)	16 (13.9-18.1)	16.3 (14.5-18.5)	16.4 (14.6-18.4)	16.6 (15.3-18.1)	17.8 (17.4-18.1)	18.1 (17.9-18.2)	18.8 (18.6-18.9)	18.9 (18.7-19)	19.7 (19.4-19.9)	20.7 (20.6-20.9)	21.6 (21.4-21.8)	21.7 (21.1-22.2)
Isle of Wight	15.3 (13.1-17.5)	16 (13.9-18.2)	16.5 (14.4-18.7)	16.9 (14.9-19.2)	17.2 (15.3-19.2)	17.5 (16-19)	17.9 (17.3-18.5)	18.2 (17.9-18.5)	18.6 (18.3-18.9)	18.7 (18.4-19)	19.6 (19.2-20)	20.2 (19.9-20.5)	20.9 (20.6-21.3)	21.8 (21-22.5)
Kent	14.7 (13-16.8)	15.3 (13.5-17.4)	15.8 (14-17.9)	16.2 (14.3-18.2)	16.3 (14.6-18.4)	16.5 (15.2-17.8)	17.1 (16.8-17.5)	17.4 (17.3-17.5)	18.2 (18.1-18.3)	18.4 (18.2-18.5)	19.5 (19.2-19.7)	20.2 (20-20.3)	21.3 (21.1-21.4)	21 (20.5-21.6)
Medway	14.8 (12.8-17)	15.5 (13.5-17.8)	16 (14.1-18.4)	16.5 (14.5-18.6)	16.8 (15-18.7)	17 (15.7-18.5)	17.2 (16.8-17.7)	17.6 (17.3-17.8)	18 (17.8-18.3)	18.1 (17.9-18.4)	18.8 (18.5-19.1)	19.3 (19.1-19.5)	19.6 (19.4-19.9)	20.7 (20-21.3)
Milton Keynes	15.4 (13.3-17.7)	16.1 (13.9-18.4)	16.6 (14.5-18.9)	17 (14.9-19.4)	17.3 (15.4-19.4)	17.5 (16.2-19)	17.7 (17.2-18.3)	17.7 (17.4-18)	18.1 (17.8-18.3)	18.4 (18.1-18.6)	19.3 (19-19.6)	20 (19.8-20.3)	20.8 (20.5-21)	21.3 (20.6-21.9)
Oxfordshire	15.2 (13.1-17.4)	15.7 (13.7-18.1)	16.1 (14.3-18.5)	16.5 (14.6-18.8)	16.7 (14.9-18.7)	16.8 (15.6-18.2)	17.4 (17.1-17.8)	18.1 (17.9-18.3)	18.6 (18.4-18.8)	19.2 (19-19.4)	19.7 (19.5-20)	20.5 (20.3-20.7)	21.5 (21.3-21.7)	21.5 (21-22.1)
Portsmouth	14.7 (12.7-17)	15.4 (13.3-17.7)	15.8 (13.8-18.2)	16.2 (14.1-18.4)	16.4 (14.6-18.5)	16.6 (15.4-18.1)	16.9 (16.5-17.4)	17.2 (17-17.5)	17.7 (17.4-18)	17.5 (17.3-17.8)	17.9 (17.5-18.2)	18.8 (18.6-19.1)	20.2 (19.9-20.5)	21.1 (20.5-21.8)
Reading	15.7 (13.6-18)	16.3 (14.5-18.6)	16.8 (14.9-19.1)	17.2 (15.2-19.6)	17.6 (15.6-19.6)	17.8 (16.4-19.3)	18 (17.5-18.5)	17.9 (17.7-18.2)	18.4 (18.1-18.7)	18.5 (18.2-18.8)	19 (18.7-19.4)	19.8 (19.5-20.1)	20.7 (20.4-21)	20.3 (19.6-21)
Slough	14.6 (12.6-16.9)	15.2 (13.2-17.5)	15.6 (13.5-18)	15.8 (14-18.1)	16 (14.3-17.9)	16.2 (14.9-17.6)	16.3 (15.8-16.9)	16.6 (16.3-16.9)	17.2 (16.9-17.5)	17.2 (17-17.5)	18.2 (17.8-18.5)	19.6 (19.3-19.9)	21 (20.7-21.3)	22.1 (21.4-22.8)
Southampton	15 (13-17.2)	15.6 (13.6-18)	16.1 (14.1-18.2)	16.4 (14.5-18.7)	16.7 (14.9-18.7)	16.9 (15.6-18.4)	17.3 (16.8-17.8)	17.5 (17.3-17.8)	17.8 (17.6-18.1)	17.8 (17.6-18.1)	18.4 (18.1-18.7)	19.1 (18.8-19.3)	20.1 (19.9-20.4)	20.8 (20.1-21.5)
Surrey	15.4 (13.5-17.6)	16 (14-18.2)	16.5 (14.5-18.6)	17.1 (15.1-19.2)	17.3 (15.4-19.2)	17.3 (16-18.8)	17.6 (17.3-18)	18.1 (18-18.3)	18.6 (18.4-18.7)	19 (18.8-19.1)	19.8 (19.6-20.1)	20.7 (20.5-20.8)	21.7 (21.6-21.9)	21.5 (20.9-22)
West Berkshire	15.9 (13.8-18.4)	16.6 (14.5-18.8)	17.2 (15.1-19.4)	17.6 (15.6-19.8)	17.9 (16-19.9)	18.1 (16.7-19.6)	18.4 (17.8-19)	17.9 (17.7-18.2)	19.1 (18.8-19.4)	18.9 (18.6-19.2)	19.7 (19.4-20.1)	19.7 (19.4-20)	21.1 (20.8-21.5)	21 (20.2-21.7)
West Sussex	15.4 (13.4-17.6)	16 (14.2-18.4)	16.5 (14.4-19)	16.9 (14.9-19.2)	17.1 (15.3-19.1)	17.4 (16-18.8)	17.9 (17.5-18.3)	18.2 (18-18.4)	18.8 (18.6-19)	19.3 (19.1-19.5)	19.8 (19.6-20.1)	20.5 (20.4-20.7)	21.3 (21.1-21.5)	21.4 (20.9-22)
Windsor and Maidenhead	15.6 (13.6-17.9)	16.2 (14.2-18.6)	16.7 (14.7-19.1)	17 (15.1-19.1)	17.2 (15.6-19.3)	17.4 (16.1-18.9)	17.7 (17.1-18.2)	17.9 (17.6-18.2)	18.5 (18.2-18.8)	19.1 (18.8-19.4)	20.7 (20.3-21.1)	21.6 (21.3-22)	22.1 (21.7-22.4)	22 (21.3-22.8)
Wokingham	16.2 (14.2-18.5)	16.9 (14.9-19.3)	17.4 (15.4-19.7)	17.8 (15.8-20.2)	18.1 (16.3-20.2)	18.3 (16.9-19.8)	18.6 (18-19.2)	18.7 (18.4-19)	19.4 (19.1-19.7)	19.6 (19.3-19.9)	20.8 (20.4-21.1)	21.8 (21.6-22.1)	22.8 (22.5-23.1)	23.1 (22.4-23.8)
South West England	14.9 (14.2-15.5)	15.5 (14.9-16.2)	16 (15.5-16.7)	16.5 (15.9-17.1)	16.8 (16.3-17.4)	17.2 (16.7-17.6)	17.5 (17.4-17.7)	18.1 (18.1-18.2)	18.6 (18.5-18.7)	19.1 (19.1-19.2)	19.8 (19.8-19.9)	20.6 (20.5-20.7)	21.3 (21.3-21.4)	21.1 (20.9-21.3)
Bath and North East Somerset	15.4 (13.4-17.8)	16.1 (14-18.6)	16.7 (14.7-19)	17.1 (15.1-19.2)	17.6 (15.8-19.6)	18 (16.7-19.5)	18.5 (17.9-19)	18.8 (18.6-19.1)	19.2 (18.9-19.5)	19.3 (19-19.6)	20.2 (19.9-20.6)	21.3 (21-21.6)	22 (21.7-22.3)	21.4 (20.7-22)

Table 10 Estimates of life expectancy at age 65 by location and year for 1950, 1955...2005, 2010, 2017 - females														
Location	1950	1955	1960	1965	1970	1975	1980	1985	1990	1995	2000	2005	2010	2017
Bournemouth	14.5 (12.6-16.9)	15.2 (13.2-17.6)	15.6 (13.6-17.8)	16 (14-18.1)	16.3 (14.5-18.4)	16.7 (15.4-18.1)	17.1 (16.6-17.6)	17.7 (17.4-18)	18 (17.7-18.3)	18.2 (17.9-18.5)	19 (18.7-19.4)	20 (19.7-20.3)	20.6 (20.3-20.9)	20.1 (19.4-20.8)
Bristol, City of	14.7 (12.8-16.9)	15.3 (13.3-17.5)	15.7 (13.8-17.9)	16 (14.1-18)	16.2 (14.5-18.2)	16.6 (15.2-17.9)	17 (16.6-17.4)	17.8 (17.6-18)	18.5 (18.3-18.7)	18.7 (18.5-18.9)	19.1 (18.8-19.4)	19.8 (19.6-20)	20.3 (20-20.5)	20.3 (19.7-20.9)
Cornwall	14.7 (12.8-17)	15.4 (13.4-17.6)	15.9 (13.9-18.2)	16.3 (14.4-18.4)	16.6 (14.8-18.7)	16.9 (15.7-18.4)	17.3 (16.9-17.8)	18.1 (17.9-18.2)	18.7 (18.5-18.9)	19 (18.8-19.2)	19.7 (19.4-20)	20.3 (20.1-20.5)	21.2 (21-21.4)	21 (20.4-21.6)
Devon	15 (13.2-17.2)	15.7 (13.7-18.1)	16.2 (14.2-18.5)	16.6 (14.8-18.8)	17 (15.3-18.8)	17.3 (15.9-18.6)	17.7 (17.3-18.1)	18.3 (18.2-18.5)	19 (18.8-19.1)	19.4 (19.3-19.6)	20.1 (19.8-20.3)	20.9 (20.8-21.1)	21.7 (21.6-21.9)	21.4 (20.8-22)
Dorset	15.3 (13.3-17.8)	16 (13.9-18.6)	16.5 (14.3-18.9)	16.9 (14.9-19.1)	17.3 (15.4-19.3)	17.7 (16.2-19.1)	18 (17.5-18.5)	18.6 (18.3-18.8)	19.1 (18.9-19.3)	20.2 (19.9-20.4)	20.6 (20.3-20.9)	21.6 (21.4-21.8)	22.5 (22.3-22.7)	21.9 (21.2-22.5)
Gloucestershire	15 (13.1-17.4)	15.7 (13.7-18.1)	16.3 (14.4-18.6)	16.8 (14.9-19)	17.2 (15.5-19.2)	17.5 (16.1-18.8)	17.5 (17.1-17.8)	17.9 (17.7-18.1)	18.7 (18.5-18.9)	19.1 (18.9-19.3)	20 (19.7-20.3)	20.3 (20.1-20.5)	21 (20.8-21.2)	20.8 (20.2-21.3)
North Somerset	15.2 (13.2-17.5)	15.9 (13.7-18.4)	16.5 (14.4-18.8)	17 (15-19.2)	17.5 (15.6-19.4)	17.9 (16.5-19.3)	18.2 (17.7-18.8)	18.4 (18.2-18.7)	18.9 (18.6-19.1)	19.4 (19.1-19.7)	20.3 (19.9-20.6)	20.7 (20.5-21)	21 (20.7-21.3)	20.3 (19.6-21)
Plymouth	14.5 (12.4-16.9)	15.1 (12.9-17.7)	15.6 (13.5-17.9)	16 (14.1-18.3)	16.4 (14.7-18.4)	16.7 (15.3-18.1)	17 (16.6-17.5)	17.6 (17.3-17.8)	17.9 (17.7-18.2)	18.6 (18.3-18.8)	19.3 (19-19.6)	19.4 (19.2-19.7)	20.2 (20-20.4)	20.5 (19.9-21.2)
Poole	15.3 (13.3-17.7)	16 (14-18.3)	16.6 (14.6-18.7)	16.9 (14.9-19.2)	17.4 (15.4-19.4)	17.8 (16.4-19.4)	18.5 (18-19.1)	18.1 (17.8-18.4)	18.3 (18-18.6)	19 (18.7-19.3)	20.3 (20-20.7)	21 (20.7-21.3)	21.4 (21.1-21.8)	20.9 (20.2-21.6)
Somerset	14.8 (12.8-17)	15.5 (13.5-17.7)	16 (14-18.1)	16.4 (14.4-18.5)	16.7 (15-18.6)	17 (15.5-18.3)	17.4 (17-17.8)	18.1 (17.9-18.3)	18.3 (18.1-18.5)	19.2 (19-19.4)	19.7 (19.5-20)	20.6 (20.4-20.8)	21.7 (21.5-21.9)	21.6 (21-22.2)
South Gloucestershire	15 (12.9-17.5)	15.7 (13.6-18.2)	16.2 (14.2-18.6)	16.8 (14.9-19)	17.1 (15.3-19.3)	17.5 (16.1-19)	18.1 (17.6-18.7)	18.6 (18.3-18.9)	18.2 (17.9-18.4)	18.8 (18.5-19.1)	19.9 (19.6-20.2)	21.4 (21.1-21.7)	22 (21.7-22.3)	21.2 (20.5-21.8)
Swindon	14.6 (12.6-17)	15.3 (13.3-17.8)	15.7 (13.8-17.9)	16.1 (14.3-18.2)	16.4 (14.6-18.4)	16.6 (15.1-18)	16.7 (16.1-17.2)	17.6 (17.3-17.8)	18.4 (18.2-18.7)	18.6 (18.3-18.8)	19.2 (18.8-19.5)	20.1 (19.8-20.4)	21 (20.8-21.3)	20.3 (19.6-21)
Torbay	14.6 (12.5-17.1)	15.2 (13.2-17.7)	15.7 (13.7-18)	16.1 (14.1-18.3)	16.5 (14.7-18.6)	16.9 (15.5-18.4)	17.3 (16.8-17.9)	18 (17.6-18.3)	18.5 (18.1-18.8)	18.4 (18.1-18.7)	19 (18.7-19.4)	19.6 (19.4-20)	20.3 (20-20.6)	20.8 (20.2-21.5)
Wiltshire	14.8 (12.7-17.2)	15.4 (13.3-18)	16 (14-18.4)	16.5 (14.6-18.8)	16.9 (15.2-18.9)	17.2 (15.8-18.6)	17.5 (17.1-18)	18.1 (17.9-18.3)	18.2 (18-18.5)	19 (18.8-19.2)	20.1 (19.8-20.4)	20.7 (20.5-20.9)	21.2 (21-21.4)	21.4 (20.8-22)
West Midlands	13.9 (13.3-14.6)	14.6 (13.9-15.3)	15.1 (14.4-15.8)	15.6 (14.8-16.3)	15.9 (15.3-16.6)	16.3 (15.8-16.8)	16.7 (16.6-16.8)	17.2 (17.1-17.2)	17.6 (17.6-17.7)	18 (18-18.1)	18.8 (18.7-18.9)	19.6 (19.6-19.7)	20.6 (20.6-20.7)	20.9 (20.7-21.1)
Birmingham	13.8 (12.1-15.9)	14.5 (12.6-16.5)	15 (13.2-16.9)	15.4 (13.6-17.4)	15.8 (14.1-17.6)	16.2 (14.8-17.6)	16.5 (16.1-16.8)	16.9 (16.7-17)	17.4 (17.3-17.6)	17.7 (17.6-17.9)	18.6 (18.4-18.9)	19.6 (19.4-19.7)	20.4 (20.3-20.6)	20.6 (20-21.2)
Coventry	14.1 (12.1-16.6)	14.8 (12.7-17.1)	15.2 (13.1-17.6)	15.5 (13.5-17.8)	15.8 (14-17.9)	16.3 (15-17.9)	17 (16.6-17.5)	17.5 (17.3-17.8)	17.5 (17.3-17.7)	17.9 (17.7-18.1)	18.8 (18.5-19.1)	18.8 (18.6-19.1)	20.5 (20.3-20.8)	20.4 (19.7-21)
Dudley	14 (12-16.3)	14.7 (12.6-17.1)	15.3 (13.4-17.6)	16 (14.1-18.2)	16.4 (14.7-18.3)	16.8 (15.4-18.3)	17.2 (16.8-17.7)	17.2 (17-17.4)	17.4 (17.2-17.6)	18.1 (17.9-18.3)	18.8 (18.5-19.1)	19.8 (19.6-20)	20.8 (20.6-21)	20.9 (20.3-21.6)
Herefordshire, County of	14.5 (12.5-16.8)	15.1 (13.1-17.4)	15.4 (13.4-17.5)	15.6 (13.7-17.7)	16 (14.2-18)	16.5 (15.1-18)	17.7 (17.2-18.3)	17.8 (17.5-18.1)	18.6 (18.3-18.9)	19 (18.7-19.3)	19.9 (19.6-20.2)	20.7 (20.4-21)	21.2 (20.9-21.5)	21.3 (20.6-22)
Sandwell	13.9 (11.9-16.3)	14.5 (12.5-17)	15 (12.9-17.4)	15.3 (13.2-17.7)	15.7 (13.8-17.8)	16.1 (14.8-17.7)	16.8 (16.3-17.2)	17.3 (17.1-17.5)	17.7 (17.5-17.9)	17.5 (17.3-17.7)	17.9 (17.6-18.2)	18.3 (18.1-18.6)	19.3 (19.1-19.6)	19.9 (19.2-20.6)
Shropshire	14.4 (12.5-16.6)	15 (13.1-17.3)	15.5 (13.6-17.8)	16.1 (14.2-18.2)	16.4 (14.6-18.4)	16.8 (15.4-18.3)	17.1 (16.7-17.6)	17.6 (17.3-17.8)	18.4 (18.1-18.6)	18.8 (18.5-19)	19.8 (19.5-20.1)	20.7 (20.4-20.9)	21.3 (21-21.5)	21.2 (20.5-21.8)
Solihull	15.2 (13-17.6)	15.8 (13.6-18.2)	16.3 (14.2-18.8)	16.8 (14.7-18.9)	17.3 (15.4-19.3)	17.8 (16.4-19.3)	18.3 (17.8-18.9)	18.7 (18.4-18.9)	19.1 (18.9-19.4)	18.9 (18.6-19.1)	19.5 (19.2-19.8)	20.8 (20.5-21.1)	21.8 (21.5-22)	22 (21.3-22.7)
Staffordshire	13.8 (12-16.1)	14.5 (12.6-16.7)	15 (13-17.1)	15.6 (13.5-17.9)	16 (14.1-18)	16.3 (14.8-17.7)	16.6 (16.3-17)	17 (16.9-17.2)	17.4 (17.3-17.6)	17.8 (17.6-18)	18.8 (18.5-19)	19.6 (19.5-19.8)	20.7 (20.5-20.9)	21 (20.4-21.5)
Stoke-on-Trent	13.2 (11.3-15.3)	13.8 (11.9-15.9)	14.2 (12.3-16.4)	14.6 (12.7-16.6)	14.8 (13.2-16.8)	15.2 (13.9-16.6)	15.4 (15-15.8)	16 (15.8-16.2)	16.7 (16.5-17)	17.1 (16.9-17.4)	17.9 (17.6-18.2)	18.3 (18-18.5)	19 (18.7-19.2)	19.9 (19.2-20.6)
Telford and Wrekin	14 (12.1-16.2)	14.6 (12.6-16.9)	15.1 (13.1-17.3)	15.5 (13.6-17.7)	15.8 (13.9-17.8)	16.2 (14.8-17.7)	16.6 (16.1-17.1)	17 (16.8-17.3)	18.1 (17.9-18.4)	18.2 (18-18.5)	18.6 (18.3-19)	19.2 (18.9-19.5)	20.1 (19.8-20.4)	20.2 (19.5-20.9)
Walsall	13.6 (11.5-15.8)	14.3 (12.1-16.6)	14.8 (12.7-17)	15.3 (13.3-17.5)	15.7 (13.9-17.7)	16.1 (14.6-17.7)	16.4 (16-16.8)	16.8 (16.6-17)	17.2 (16.9-17.4)	17.5 (17.3-17.8)	18.3 (18-18.6)	19.6 (19.4-19.9)	20.5 (20.3-20.8)	20.4 (19.7-21.1)
Warwickshire	14.2 (12.4-16.3)	14.8 (12.9-17)	15.3 (13.3-17.6)	15.9 (14-18.1)	16.2 (14.5-18.1)	16.5 (15.2-18)	16.8 (16.4-17.2)	17.3 (17.1-17.5)	17.9 (17.7-18.1)	18.3 (18.1-18.5)	19.3 (19.1-19.6)	20.1 (19.9-20.3)	21.1 (20.9-21.3)	21.3 (20.7-21.9)
Wolverhampton	13.9 (11.8-16)	14.5 (12.4-16.8)	15 (12.9-17.2)	15.5 (13.5-17.7)	15.8 (13.8-17.9)	16.2 (14.8-17.7)	16.6 (16.1-17)	17 (16.8-17.3)	17.1 (16.9-17.4)	17.8 (17.6-18)	18.2 (17.9-18.5)	18.7 (18.4-18.9)	20 (19.7-20.2)	20.3 (19.7-21)
Worcestershire	14 (12.2-16.1)	14.7 (12.8-17)	15.2 (13.2-17.3)	15.8 (14-18)	16.1 (14.4-18.1)	16.5 (15.1-17.9)	16.8 (16.4-17.2)	17.6 (17.4-17.8)	18 (17.8-18.2)	18.6 (18.4-18.8)	18.9 (18.7-19.2)	20.2 (20-20.4)	20.9 (20.7-21.1)	21.4 (20.8-22)
Yorkshire and the Humber	13.8 (13.2-14.4)	14.5 (13.9-15.1)	15 (14.4-15.6)	15.5 (14.9-16.1)	15.9 (15.3-16.4)	16.3 (15.9-16.7)	16.7 (16.6-16.8)	17.1 (17-17.1)	17.5 (17.5-17.6)	17.9 (17.9-18)	18.7 (18.6-18.7)	19.5 (19.4-19.6)	20.2 (20.2-20.3)	20.4 (20.3-20.6)
Barnsley	13.5 (11.6-15.7)	14.1 (12.1-16.4)	14.7 (12.8-16.7)	15.1 (13.2-17.4)	15.6 (13.7-17.4)	16 (14.6-17.3)	16.3 (15.9-16.8)	16.6 (16.3-16.8)	17.2 (16.9-17.4)	17.3 (17-17.5)	17.9 (17.6-18.2)	18.7 (18.4-18.9)	19.4 (19.1-19.6)	20.2 (19.5-20.9)
Bradford	13.5 (11.6-15.8)	14.1 (12.2-16.5)	14.6 (12.6-16.9)	15.1 (13.3-17.2)	15.4 (13.7-17.2)	15.8 (14.5-17.2)	16.4 (15.9-16.7)	16.7 (16.5-16.9)	17.2 (17.1-17.4)	17.6 (17.4-17.8)	18.3 (18-18.6)	19.1 (18.9-19.3)	19.8 (19.6-20)	19.8 (19.2-20.4)
Calderdale	13.8 (11.9-15.9)	14.4 (12.5-16.7)	15 (13-17.1)	15.5 (13.6-17.7)	15.8 (14-17.8)	16.2 (14.9-17.7)	16.7 (16.2-17.1)	16.9 (16.7-17.2)	17.2 (16.9-17.4)	17.6 (17.3-17.8)	18.7 (18.4-19)	19.5 (19.3-19.8)	19.8 (19.5-20)	19.8 (19.2-20.5)
Doncaster	13.8 (11.8-16)	14.6 (12.4-16.9)	15.2 (13.2-17.5)	15.8 (14-18)	16.3 (14.6-18.2)	16.7 (15.4-18.1)	17 (16.6-17.4)	17 (16.8-17.2)	17.2 (17-17.4)	17.1 (16.9-17.4)	18.1 (17.8-18.4)	19.2 (19-19.5)	19.9 (19.7-20.1)	19.8 (19.2-20.5)
East Riding of Yorkshire	14.2 (12.3-16.4)	14.9 (12.9-17.1)	15.4 (13.4-17.7)	16 (14.1-18.1)	16.4 (14.6-18.3)	16.8 (15.4-18.2)	17.1 (16.7-17.6)	17.6 (17.4-17.8)	18.3 (18.1-18.5)	18.6 (18.4-18.8)	19.3 (19-19.6)	20.2 (19.9-20.4)	20.9 (20.6-21.1)	21.3 (20.7-21.9)
Kingston upon Hull, City of	13.8 (11.8-16.1)	14.5 (12.4-16.8)	15.1 (13-17.4)	15.5 (13.6-17.7)	16 (14.1-17.9)	16.4 (15-17.9)	16.8 (16.3-17.3)	17 (16.8-17.2)	17.5 (17.2-17.7)	17.7 (17.5-18)	18.3 (18-18.6)	19.1 (18.9-19.3)	18.8 (18.6-19)	19.1 (18.4-19.8)
Kirklees	13.4 (11.6-15.5)	14 (12.1-16.2)	14.5 (12.6-16.7)	15.1 (13.2-17.1)	15.4 (13.7-17.3)	15.7 (14.4-17.1)	15.8 (15.4-16.2)	16.7 (16.5-16.9)	17 (16.8-17.2)	17.8 (17.6-18)	18.5 (18.2-18.7)	18.7 (18.5-18.9)	20.4 (20.2-20.6)	20.9 (20.3-21.6)
Leeds	13.9 (11.9-16.1)	14.6 (12.5-16.9)	15.1 (13.1-17.3)	15.5 (13.6-17.6)	15.8 (14.1-17.9)	16.2 (14.8-17.6)	16.9 (16.5-17.3)	17.3 (17.1-17.4)	17.7 (17.5-17.9)	18.3 (18.2-18.5)	18.9 (18.6-19.2)	19.6 (19.4-19.8)	20.1 (20-20.3)	20.2 (19.6-20.7)
North East Lincolnshire	14.2 (12.3-16.6)	14.9 (12.9-17.4)	15.5 (13.4-17.8)	15.9 (14-18)	16.3 (14.4-18.1)	16.7 (15.3-18.1)	17 (16.6-17.6)	17.3 (17-17.6)	17.4 (17.1-17.6)	17.7 (17.5-18)	18.5 (18.1-18.8)	19.3 (19-19.6)	20.1 (19.8-20.4)	20.7 (20-21.5)
North Lincolnshire	14.2 (12.2-16.5)	14.9 (12.9-17.3)	15.4 (13.4-17.7)	15.8 (13.8-18.1)	16.3 (14.4-18.2)	16.8 (15.4-18.4)	17 (16.5-17.6)	16.9 (16.7-17.2)	17.5 (17.2-17.7)	18.6 (18.3-18.8)	18.4 (18.1-18.7)	19.4 (19.1-19.7)	20.2 (19.9-20.4)	20.4 (19.7-21.1)
North Yorkshire	14.1 (12.4-16.3)	14.7 (12.9-17)	15.2 (13.3-17.2)	15.7 (13.8-17.7)	16 (14.3-17.9)	16.5 (15.1-17.8)	17.1 (16.7-17.5)	17.8 (17.6-18)	18.3 (18.1-18.4)	18.4 (18.2-18.6)	19.2 (18.9-19.5)	20.4 (20.2-20.6)	21.2 (21-21.4)	21.6 (21-22.2)
Rotherham	13.8 (12-16.1)	14.5 (12.6-16.8)	15.1 (13.2-17.3)	15.6 (13.6-17.7)	16.1 (14.3-17.9)	16.5 (15.1-17.9)	16.7 (16.3-17.2)	17 (16.7-17.2)	17.3 (17.1-17.5)	18.1 (17.8-18.3)	18.1 (17.8-18.4)	19.3 (19-19.5)	19.8 (19.5-20)	19.2 (18.5-19.8)
Sheffield	13.8 (11.9-16)	14.5 (12.5-16.8)	15.1 (13.1-17.3)	15.6 (13.7-17.8)	16 (14.2-17.9)	16.3 (14.9-17.7)	16.8 (16.4-17.2)	16.8 (16.6-17)	17.4 (17.2-17.6)	17.7 (17.5-17.9)	18.7 (18.4-19)	19.3 (19.1-19.5)	20.2 (20-20.3)	20.5 (19.9-21)
Wakefield	13.4 (11.5-15.6)	14.1 (12.2-16.3)	14.6 (12.6-17)	15.1 (13.2-17.3)	15.5 (13.8-17.4)	15.9 (14.5-17.3)	16.1 (15.7-16.6)	16.4 (16.2-16.6)	16.9 (16.6-17.1)	17.4 (17.2-17.7)	18.3 (18-18.6)	19.1 (18.9-19.3)	19.9 (19.6-20.1)	20.4 (19.8-21)
York	14.6 (12.6-17)	15.2 (13.1-17.5)	15.6 (13.6-18.1)	16 (14.1-18.2)	16.4 (14.5-18.3)	16.8 (15.3-18.3)	17.1 (16.6-17.7)	17.5 (17.3-17.8)	18.5 (18.2-18.7)	19.1 (18.8-19.3)	20.1 (19.8-20.5)	21.3 (21-21.6)	21.4 (21.2-21.7)	20.8 (20.2-21.5)
Northern Ireland	13.5 (13.3-13.6)	13.6 (13.4-13.7)	14 (13.8-14.1)	14.8 (14.7-15)	15.3 (15.1-15.5)	15.5 (15.3-15.6)	15.8 (15.6-15.9)	16.2 (16-16.4)	16.9 (16.7-17.1)	17.5 (17.3-17.7)	18.5 (18.2-18.7)	19.8 (19.6-20)	20.2 (20-20.4)	20.7 (19.9-21.4)
Scotland	13 (12.9-13.1)	13.8 (13.8-13.9)	14.2 (14.1-14.3)	14.5 (14.4-14.6)	14.8 (14.7-14.9)	15.7 (15.6-15.7)	15.9 (15.8-16)	16.2 (16.1-16.3)	16.7 (16.6-16.8)	17.3 (17.2-17.4)	18 (17.7-18.2)	18.6 (18.5-18.8)	19.4 (19.3-19.5)	19.8 (19.2-20.5)
Wales	13.1 (11.2-15.5)	13.8 (11.8-16)	14.3 (12.2-16.7)	14.8 (12.8-17.1)	15.2 (13.3-17.4)	15.7 (14.1-17.4)	16.6 (16.2-16.9)	17.3 (17.1-17.4)	17.8 (17.7-17.9)	18 (17.9-18.1)	18.6 (18.4-18.9)	19.4 (19.3-19.5)	20.2 (20.1-20.4)	20.7 (20.1-21.2)
Latin America and Caribbean	12.8 (12.7-13)	13.6 (13.4-13.8)	14 (13.8-14.1)	14.5 (14.3-14.7)	15 (14.8-15.2)	15.8 (15.7-16)	16.3 (16.2-16.4)	16.5 (16.5-16.6)	17.3 (17.2-17.3)	17.5 (17.5-17.6)	18.5 (18.4-18.5)	19 (18.9-19)	19.3 (19.3-19.4)	19.7 (19.6-19.9)
Andean Latin America	12.7 (12.3-13.1)	13.9 (13.6-14.2)	14 (13.7-14.3)	15.1 (14.8-15.5)	14.6 (14.3-14.9)	16.8 (16.5-17.2)	16.8 (16.4-17.1)	16.8 (16.5-17.1)	17.8 (17.5-18.1)	17.5 (17.1-17.8)	18.5 (18.2-18.8)	19 (18.6-19.4)	19.2 (18.8-19.7)	20.2 (19.4-20.9)
Bolivia	10.4 (10-10.7)	11.7 (11.5-11.9)	12.3 (12-12.6)	12.3 (12-12.7)	12.5 (12.1-12.9)	12.8 (12.4-13.2)	13.1 (12.6-13.5)	13.2 (12.7-13.8)	13.6 (13-14.2)	14 (13.3-14.6)	15 (14.3-15.7)	15.8 (14.7-16.6)	16.1 (15.1-17.4)	16.6 (15.4-18.3)
Ecuador	12.5 (11.4-13.6)	12.9 (12.8-13)	12.9 (12.8-13)	14.4 (14.3-14.5)	15.4 (15.3-15.5)	16.4 (16.3-16.5)	17.5 (17.5-17.6)	18.1 (18-18.1)	18.1 (18-18.1)	18.7 (18.6-18.8)	19.4 (19.3-19.4)	19.1 (18.7-19.5)	19.2 (18.8-19.6)	19.5 (18.7-20.3)
Peru	13.8 (13.1-14.5)	15.2 (14.6-15.8)	15.1 (14.6-15.7)	16.6 (15.9-17.3)	15.1 (14.6-15.6)	18.6 (17.9-19.3)	17.8 (17.2-18.4)	17.6 (17-18.1)	19.2 (18.6-19.7)	18.1 (17.5-18.7)	19.3 (18.7-19.9)	20.1 (19.5-20.7)	20.3 (19.6-20.9)	21.7 (20.4-23)
Caribbean	12.9 (12.5-13.4)	13.8 (13.4-14.3)	14.5 (14.2-14.8)	14.8 (14.6-15.1)	15.6 (15.4-15.9)	16.3 (16-16.5)	16.6 (16.3-16.8)	16.7 (16.6-16.9)	17.1 (16.9-17.2)	17.3 (17.1-17.4)	18 (17.8-18.2)	18.4 (18.1-18.7)	17.9 (17.6-18.2)	18.9 (18.3-19.4)
Antigua and Barbuda	13.9 (13.6-14.3)	14.6 (14.4-14.8)	15.4 (15.2-15.7)	15 (14.6-15.3)	15.5 (15.1-15.9)	16.5 (16.1-17)	17.3 (16.8-17.8)	18.5 (18-19.1)	19 (18.6-19.5)	18.2 (17.6-18.5)	18.1 (18-18.3)	18.4 (18-18.9)	18.5 (18.1-18.9)	18.5 (18.1-19.1)
The Bahamas	13.6 (12.4-15)	14 (12.9-15.1)	14.4 (13.7-15.1)	14.7 (14.4-15)	15.8 (15.6-16)	16.9 (16.8-17.1)	16.9 (16.7-17.1)	17.3 (17.1-17.5)	17.9 (17.7-18.1)	17.9 (17.7-18.1)	17.7 (17.5-17.9)	18.6 (18.4-18.8)	18.6 (18.4-18.8)	18.5 (17.7-19.3)
Barbados	15.2 (14.9-15.4)	15.4 (15.2-15.5)	16 (15.8-16.1)	15.5 (15.3-15.7)	15.4 (15.2-15.6)	16 (15.8-16.2)	16.9 (16.7-17.1)	17.7 (17.5-17.9)	17.5 (17.3-17.7)	17.9 (17.7-18.2)	18.3 (18-18.5)	19.1 (18.9-19.2)	19.4 (19.3-19.6)	19.2 (18.6-19.9)
Belize	12.3 (12.1-12.6)	13.8 (13.6-14)	15.9 (15.6-16.2)	17 (16.6-17.4)	17.8 (17.4-18.3)	17.7 (17.3-18.1)	17.5 (17.1-17.9)	17.4 (17-17.8)	17 (16.6-17.5)	17.4 (17.1-17.8)	17.3 (17.1-17.5)	17.9 (17.7-18.1)	18.8 (18.7-19)	19.2 (18.9-19.5)
Bermuda	14.1 (13.7-14.7)	14.8 (14.6-15)	15.6 (15.3-15.8)	16.1 (15.9-16.3)	16.6 (16.4-16.9)	17.6 (17.2-17.9)	18.6 (18.3-18.9)	18.5 (18.1-18.8)	18.3 (18-18.6)	19.3 (18.7-19.6)	19.6 (19.2-19.8)	20.6 (20.1-21.1)	22.1 (21.6-22.7)	23.1 (22.5-23.8)
Cuba	15 (13.5-16.5)	14.7 (13.5-16.1)	14.9 (14.8-15)	15.4 (15.3-15.5)	17 (17-17.1)	17.9 (17.8-17.9)	17.7 (17.6-17.7)	17.7 (17.6-17.8)	17.8 (17.7-17.9)	17.8 (17.7-17.8)	18.9 (18.8-18.9)	19.2 (19.1-19.3)	19.5 (19.4-19.6)	19.7 (18.7-20.7)
Dominica	14.9 (14.6-15.3)	17 (16.8-17.3)	17.4 (17.1-17.7)	17.7 (17.4-18)	17.7 (17.4-18)	18.1 (17.8-18.3)	18.3 (18-18.5)	18.3 (18-18.6)	17 (16.7-17.2)	17.1 (16.8-17.4)	17.6 (17.3-17.9)	17.9 (17.6-18.3)	18 (17.6-18.3)	17.7 (17.1-18.4)
Dominican Republic	14.3 (14.2-14.5)	15.3 (15.2-15.5)	16.4 (15.7-17.1)	17.3 (16.7-17.9)	16.9 (16.3-17.6)	17.5 (16.9-18.2)	18.1 (17.4-18.7)	19.2 (18.5-19.8)	19.6 (19-20.2)	20.5 (19.8-21.1)	21.1 (20.4-21.8)	19.9 (19.2-20.7)	20.1 (19.4-20.8)	19.3 (18.4-20.3)
Grenada	16.3 (15.8-16.7)	16 (15.5-16.6)	15.6 (15.1-16.2)	14.7 (14.1-15.2)	16.1 (15.4-16.7)	16.8 (16.2-17.4)	16.3 (15.9-16.8)	15.2 (14.8-15.6)	15 (14.7-15.3)	15.7 (15.4-15.9)	17.6 (17.3-17.8)	17.8 (17.5-18.1)	17 (16.7-17.3)	16.7 (16.2-17.3)
Guyana	11.4 (11.2-11.5)	12.2 (12.1-12.4)	13.6 (13.4-13.7)	13.9 (13.3-14.6)	14.4 (14.3-14.6)	13.8 (13.6-14.1)	14.3 (14-14.6)	15.5 (15.3-15.8)	15.1 (15-15.3)	15 (14.9-15.1)	14.5 (14.3-14.6)	15.7 (15.6-15.9)	16.1 (15.9-16.2)	16.2 (15.2-17.2)
Haiti	10.1 (9.3-11.1)	10.2 (9.4-11.3)	10.4 (9.5-11.5)	10.4 (9.6-11.5)	10.5 (9.7-11.6)	10.7 (9.9-11.7)	11 (10.2-11.9)	11.2 (10.5-12)	11.4 (10.6-12)	11.6 (10.8-12.3)	12.1 (11.2-13.1)	12.7 (11.5-13.8)	9.6 (8.9-10.4)	13.7 (12.3-15.4)
Jamaica	12.6 (12.5-12.7)	14.5 (14.4-14.6)	15.6 (15.5-15.7)	15.7 (15.6-15.9)	16.8 (16.7-16.9)	16.9 (16.8-17.1)	18 (17.9-18.2)	17 (16.9-17.1)	18.1 (17.7-18.6)	18.1 (17.4-18.8)	17.6 (17.4-17.7)	18.9 (18.7-19)	19.1 (19-19.2)	18.5 (17.1-19.8)
Puerto Rico	13.7 (13.6-13.9)	16.8 (16.7-16.9)	17.5 (17.3-17.6)	17.3 (17.2-17.4)	17.6 (17.5-17.7)	18.2 (18.1-18.4)	18.3 (18.1-18.4)	18.2 (18.1-18.3)	18.7 (18.6-18.9)	19 (18.9-19.2)	19.9 (19.8-20.1)	20.7 (20.5-20.8)	21.5 (21.4-21.6)	21.6 (21.1-22.1)
Saint Lucia	11.5 (11.3-11.8)	12.8 (12.7-13)	14.1 (13.9-14.3)	14.9 (14.6-15.2)	15 (14.7-15.3)	15 (14.8-15.2)	15.5 (15.3-15.7)	15.9 (15.7-16.1)	16.1 (15.9-16.3)	16.5 (16.3-16.7)	18.2 (18-18.4)	18.8 (18.5-19)	18.9 (18.7-19.2)	18.8 (18.2-19.3)
Saint Vincent and the Grenadines	15.1 (14.8-15.4)	16.1 (15.9-16.3)	16.1 (15.8-16.4)	15.7 (15.4-16)	16.3 (16-16.6)	16.7 (16.4-17)	17.1 (16.8-17.3)	17 (16.8-17.3)	16.2 (15.9-16.5)	17 (16.7-17.3)	16.9 (16.7-17.2)	17.9 (17.6-18.1)	18.1 (17.9-18.3)	17.4 (16.9-17.9)
Suriname	13.6 (13-14.4)	13.7 (13.1-14.2)	14.4 (13.8-15.1)	14.9 (14.1-15.7)	14.9 (14-15.7)	15.1 (14.4-15.8)	14.7 (14.2-15.2)	15.8 (15.3-16.3)	16.4 (15.9-16.8)	17.5 (17-18)	17.5 (17.1-18)	17.5 (17-18)	18 (17.6-18.5)	18.2 (17.4-19)
Trinidad and Tobago	11.2 (11.1-11.4)	12.7 (12.5-12.8)	14 (13.9-14.2)	13.5 (13.4-13.7)	13.9 (13.8-14.1)	14.4 (14.2-14.6)	14.6 (14.4-14.7)	14.9 (14.7-15.1)	15.8 (15.6-15.9)	15.7 (15.5-15.8)	16.5 (16.4-16.7)	17.8 (17.6-17.9)	18.9 (18.8-19.1)	19 (17.3-20.9)
Virgin Islands	13.2 (12.7-13.7)	13.9 (13.6-14.3)	14.1 (13.8-14.4)	14.3 (13.8-14.8)	14.8 (14.1-15.5)	15.5 (14.8-16)	16.2 (15.6-16.6)	16.8 (16.4-17.1)	17.3 (16.5-17.9)	17.8 (17.2-18.2)	18.1 (17.8-18.4)	18.5 (18.2-19)	18.8 (18.3-19.5)	18.8 (17.7-19.6)
Central Latin America	12.6 (12.4-12.7)	13.4 (13.2-13.6)	13.8 (13.6-13.9)	14.4 (14.2-14.6)	15.1 (14.9-15.2)	15.8 (15.7-15.9)	16.6 (16.5-16.7)	16.7 (16.7-16.7)	17.6 (17.5-17.6)	17.7 (17.7-17.7)	18.7 (18.7-18.8)	19.1 (19.1-19.2)	19.6 (19.6-19.7)	19.9 (19.6-20.1)
Colombia	11.8 (11.8-11.9)	12.9 (12.8-12.9)	13.3 (13.2-13.3)	13.9 (13.8-13.9)	15.9 (15.9-16)	15.2 (15.2-15.2)	16.4 (15.7-17.1)	15.8 (15.8-15.8)	17.5 (17.4-17.5)	17.6 (17.6-17.7)	19.3 (19.2-19.3)	20.1 (20.1-20.2)	21.2 (21.2-21.3)	22.1 (21.1-23)
Costa Rica	12.5 (12.3-12.6)	14.7 (14.6-14.9)	15.4 (15.2-15.5)	15.4 (15.2-15.5)	15.4 (15.3-15.6)	16.8 (16.7-17)	18.1 (17.9-18.3)	18.1 (17.9-18.2)	18.8 (18.7-19)	18.6 (18.5-18.8)	19.8 (19.7-19.9)	20.8 (20.6-20.9)	21.4 (21.2-21.5)	21.5 (20.9-22)
El Salvador	12 (11.9-12.1)	12.7 (12.6-12.8)	14 (13.9-14.1)	14.7 (14.6-14.8)	14.7 (14.6-14.8)	16.3 (16.2-16.4)	16.7 (16.3-17.1)	17.9 (17.8-18)	18.5 (18.4-18.6)	18.8 (18.4-19.1)	19.2 (18.8-19.6)	18.9 (18.5-19.2)	18.7 (18.3-19.1)	19 (17.5-20.5)
Guatemala	10 (10-10.1)	10.5 (10.5-10.6)	11.2 (11.2-11.3)	11.7 (11.6-11.8)	11.7 (11.7-11.8)	13.2 (13.1-13.3)	15.3 (15.2-15.4)	14.2 (14.2-14.3)	15 (14.9-15)	15.9 (15.8-15.9)	16.6 (16.5-16.7)	17.5 (17.4-17.6)	18.2 (18.1-18.2)	18.4 (17.5-19.3)
Honduras	12.7 (12-13.6)	12.6 (11.9-13.3)	12.1 (11.5-12.7)	13 (12.4-13.6)	14 (13.5-14.6)	15.1 (14.4-15.7)	17.4 (16.8-18.1)	17.2 (16.4-18.1)	18.2 (17.3-19.1)	16.9 (15.6-18.2)	16.9 (15.2-18.5)	17 (15.3-18.9)	17.1 (15.5-19.1)	17 (15.7-19.3)
Mexico	13.2 (12.9-13.5)	13.9 (13.5-14.2)	14 (13.7-14.4)	14.7 (14.4-15.1)	15.1 (14.7-15.4)	16.2 (16-16.4)	16.7 (16.6-16.8)	17 (17-17)	17.6 (17.6-17.6)	17.6 (17.6-17.7)	18.5 (18.4-18.5)	18.6 (18.6-18.6)	18.9 (18.9-19)	19.1 (18.9-19.2)
Aguascalientes	14.2 (12.9-15.8)	14.8 (13.4-16.3)	14.8 (13.4-16.3)	15.4 (14-16.8)	15.6 (14.5-16.6)	16.4 (15.7-17.1)	16.4 (16.3-16.7)	16.6 (16.5-16.8)	17.3 (17.2-17.5)	17.5 (17.3-17.6)	17.8 (17.7-18)	17.9 (17.8-18.1)	18.5 (18.4-18.6)	19.4 (18.9-20)
Baja California	12.9 (11.7-14.3)	13.5 (12.2-15.1)	13.8 (12.4-15.3)	14.5 (13.1-16)	14.7 (13.3-16.2)	15.8 (14.9-16.7)	16.6 (16.5-16.8)	15.6 (15.5-15.7)	16.3 (16.2-16.4)	16.3 (16.2-16.4)	16.8 (16.7-16.9)	17.4 (17.3-17.5)	18.5 (18.4-18.6)	19.1 (18.6-19.7)
Baja California Sur	15 (13.6-16.6)	15.6 (14.1-17.2)	15.7 (14.3-17.2)	16.1 (14.9-17.4)	16.3 (15.4-17.5)	17.3 (16.6-18)	17.8 (17.5-18.1)	18.3 (18.1-18.5)	18.2 (18.1-18.4)	18.4 (18.2-18.6)	18.6 (18.4-18.8)	18.8 (18.6-19)	18.9 (18.8-19.1)	19.4 (18.9-20)
Campeche	14.4 (13-15.8)	14.9 (13.5-16.5)	15 (13.5-16.5)	15.5 (14.2-16.9)	15.7 (14.6-16.9)	16.8 (16.1-17.5)	17.1 (16.9-17.3)	16.5 (16.4-16.7)	17.7 (17.6-17.9)	17.6 (17.4-17.7)	18.7 (18.6-18.9)	18.8 (18.6-18.9)	18.7 (18.5-18.8)	18.2 (17.7-18.8)
Chiapas	13.9 (12.6-15.4)	14.5 (13.2-16.1)	14.7 (13.5-16.3)	15.6 (14.1-17.3)	16.3 (15-17.9)	17.9 (16.9-18.8)	16.7 (16.5-16.8)	17.2 (17.1-17.3)	17.5 (17.4-17.6)	18 (17.9-18.1)	18.1 (18-18.2)	18.2 (18.1-18.3)	18.2 (18.1-18.3)	18.2 (17.6-18.8)
Chihuahua	12.4 (11.1-13.7)	13 (11.7-14.5)	13.1 (11.8-14.7)	13.8 (12.4-15.4)	14.1 (12.7-15.6)	15.2 (14.3-16)	15.8 (15.7-16)	15.7 (15.6-15.8)	16.2 (16-16.3)	16.5 (16.4-16.6)	16.9 (16.8-17)	17.1 (17-17.2)	17.3 (17.2-17.4)	17.6 (17.1-18.3)
Coahuila	12.5 (11.3-14)	13.1 (11.7-14.6)	13.2 (11.9-14.9)	13.9 (12.4-15.5)	14.1 (12.6-15.6)	15 (14.1-16)	15.4 (15.3-15.6)	15.9 (15.8-16)	16.7 (16.6-16.8)	16.9 (16.8-17)	17.4 (17.3-17.5)	17.4 (17.3-17.5)	17.9 (17.8-18)	18.4 (17.8-19)
Colima	13.1 (11.8-14.5)	13.7 (12.4-15.2)	13.9 (12.6-15.3)	14.5 (13.3-15.8)	14.7 (13.8-15.8)	15.7 (15.1-16.4)	16.2 (15.9-16.4)	16.6 (16.5-16.8)	16.8 (16.7-17)	16.9 (16.7-17)	18 (17.9-18.2)	18.5 (18.3-18.6)	18.6 (18.5-18.8)	18.5 (18-19.1)
Mexico City	13.4 (12.2-14.7)	14.1 (12.9-15.4)	14.3 (12.9-15.6)	15.1 (13.6-16.5)	15.5 (14.1-16.9)	16.7 (15.9-17.4)	16.9 (16.8-17)	17.3 (17.2-17.4)	17.5 (17.4-17.5)	17.2 (17.1-17.3)	17.9 (17.8-18)	18.1 (18.1-18.2)	18.8 (18.7-18.9)	19.1 (18.6-19.6)
Durango	13.4 (12.1-14.9)	14.1 (12.7-15.9)	14.4 (13-15.9)	15.2 (13.8-16.6)	15.5 (14.4-16.6)	16.5 (15.8-17.2)	16.5 (16.4-16.7)	16.9 (16.7-17)	17.7 (17.6-17.9)	18.4 (18.3-18.6)	19.4 (19.3-19.6)	19.2 (19.1-19.3)	18.9 (18.8-19.1)	20 (19.5-20.6)
Guanajuato	13.3 (12-14.7)	13.9 (12.6-15.3)	14.1 (12.6-15.5)	14.7 (13.4-16.2)	15 (13.8-16.4)	16.2 (15.3-17.2)	17 (16.9-17.1)	16.6 (16.5-16.7)	17.5 (17.4-17.6)	17.7 (17.6-17.8)	18.4 (18.3-18.4)	18.7 (18.6-18.8)	19.2 (19.1-19.2)	19.2 (18.7-19.7)
Guerrero	13.6 (12.3-15.1)	14.2 (12.8-15.8)	14.4 (12.9-16)	15.1 (13.7-16.7)	15.5 (14.2-17)	16.7 (15.9-17.7)	17.9 (17.8-18)	20.1 (20-20.3)	20.8 (20.6-20.9)	20.7 (20.6-20.9)	22 (21.8-22.1)	21.1 (21-21.2)	20.7 (20.6-20.8)	21.2 (20.6-21.8)
Hidalgo	12.6 (11.4-13.8)	13.1 (11.9-14.6)	13.3 (12-14.7)	14.1 (12.8-15.7)	14.6 (13.3-16.1)	15.9 (15-16.8)	16 (15.8-16.1)	17.8 (17.7-17.9)	18.9 (18.8-19)	19.3 (19.2-19.5)	20.5 (20.3-20.6)	20.2 (20.1-20.4)	19.7 (19.6-19.8)	20.3 (19.7-20.9)
Jalisco	13 (11.8-14.4)	13.6 (12.3-15.2)	13.9 (12.6-15.5)	14.6 (13.4-16.1)	15 (13.7-16.5)	16.3 (15.5-17.2)	16.7 (16.6-16.9)	16.8 (16.7-16.9)	16.9 (16.8-16.9)	17 (16.9-17.1)	18 (17.9-18.1)	18.3 (18.2-18.4)	18.8 (18.7-18.9)	18.6 (18.1-19.2)
México	14.2 (12.9-15.7)	14.7 (13.3-16.2)	14.7 (13.3-16.2)	15.2 (13.9-16.8)	15.4 (13.9-16.9)	16.3 (14.8-17.8)	16.3 (15.3-17.3)	16.4 (16.3-16.5)	17.3 (17.2-17.4)	17.2 (17.2-17.3)	17.9 (17.8-17.9)	18.2 (18.1-18.2)	18.8 (18.7-18.8)	18.7 (18.2-19.3)
Michoacán de Ocampo	12.9 (11.7-14.2)	13.6 (12.2-15)	13.8 (12.4-15.3)	14.5 (13.1-16.1)	14.9 (13.7-16.3)	16 (15.2-17)	16.3 (16.2-16.4)	17.2 (17.1-17.3)	18.5 (18.4-18.7)	18.4 (18.3-18.5)	19.2 (19.1-19.3)	19.6 (19.5-19.8)	19.6 (19.5-19.7)	20.2 (19.6-20.8)
Morelos	14.1 (12.6-15.5)	14.7 (13.2-16.3)	14.8 (13.3-16.5)	15.5 (14.1-17.1)	15.9 (14.6-17.2)	17.4 (16.7-18.1)	18.1 (17.9-18.2)	17.6 (17.4-17.7)	17.9 (17.8-18.1)	18.3 (18.1-18.4)	19 (18.9-19.2)	18.8 (18.7-18.9)	19.3 (19.2-19.4)	18.4 (17.9-19)
Nayarit	13.6 (12.3-15.2)	14.3 (12.9-15.9)	14.5 (13.1-16)	15.2 (13.9-16.5)	15.5 (14.6-16.6)	16.6 (16.1-17.2)	17.2 (17-17.3)	17.4 (17.3-17.6)	18.1 (17.9-18.2)	18.9 (18.7-19)	19.7 (19.6-19.9)	20 (19.8-20.1)	20 (19.9-20.1)	20.3 (19.7-20.8)
Nuevo León	13.8 (12.3-15.4)	14.4 (12.9-16.1)	14.4 (13-16.1)	14.8 (13.4-16.3)	14.6 (13.2-16.2)	15.3 (14.3-16.3)	16.5 (16.4-16.7)	17.1 (17-17.2)	17.1 (17-17.2)	17.3 (17.2-17.4)	18 (17.9-18.1)	18.3 (18.2-18.4)	18.8 (18.7-18.9)	19.5 (18.9-20)

Table 10 Estimates of life expectancy at age 65 by location and year for 1950, 1955...2005, 2010, 2017 - females

Location	1950	1955	1960	1965	1970	1975	1980	1985	1990	1995	2000	2005	2010	2017
Oaxaca	11.9 (10.9-13.1)	12.4 (11.3-13.8)	12.6 (11.4-14)	13.3 (12.1-14.6)	13.5 (12.4-14.8)	14.5 (13.6-15.3)	15 (14.9-15.1)	16 (15.9-16.1)	17.4 (17.3-17.5)	17.8 (17.7-17.9)	19.2 (19.1-19.3)	19.5 (19.4-19.6)	19.8 (19.6-19.9)	19.6 (19.1-20.2)
Puebla	12.4 (11.4-13.7)	13 (11.8-14.5)	13.2 (12-14.7)	13.9 (12.7-15.4)	14.3 (13-15.7)	15.5 (14.6-16.4)	16.6 (16.5-16.7)	16.5 (16.4-16.6)	17.5 (17.4-17.6)	17.6 (17.5-17.7)	18.6 (18.5-18.7)	18.7 (18.6-18.7)	18.8 (18.7-18.9)	18.5 (18-19)
Querétaro	14 (12.6-15.4)	14.5 (13.1-16)	14.6 (13.2-16.1)	15.1 (13.7-16.6)	15.2 (14-16.6)	16.2 (15.1-17.3)	16.4 (15.9-17)	17 (16.9-17.1)	17.5 (17.4-17.6)	17.8 (17.6-17.9)	18.4 (18.3-18.5)	18.9 (18.8-19)	19.2 (19.1-19.4)	19.2 (18.7-19.7)
Quintana Roo	17.8 (16.2-19.4)	17.7 (16.2-19.2)	17.3 (15.8-18.8)	17 (15.6-18.3)	16.4 (15.3-17.6)	16.9 (16.1-17.6)	17.9 (17.6-18.2)	19.9 (19.6-20.1)	18.6 (18.5-18.8)	17.8 (17.7-18)	18.3 (18.2-18.5)	18.7 (18.6-18.8)	18.2 (18.1-18.4)	17.3 (16.8-17.8)
San Luis Potosí	13.1 (11.8-14.4)	13.7 (12.4-15.2)	14 (12.6-15.6)	14.7 (13.3-16.3)	15 (13.6-16.5)	16 (15.1-17)	17 (16.8-17.1)	17.4 (17.2-17.5)	18.4 (18.3-18.5)	18.3 (18.1-18.4)	19.5 (19.3-19.6)	19.6 (19.4-19.7)	19.7 (19.6-19.9)	20.1 (19.5-20.7)
Sinaloa	14.3 (12.9-15.8)	14.9 (13.4-16.6)	15.1 (13.5-16.8)	15.7 (14.2-17.3)	16 (14.6-17.5)	17.3 (16.3-18.4)	17.5 (17.3-17.7)	18 (17.8-18.1)	18.2 (18.1-18.4)	18.3 (18.1-18.4)	19.1 (18.9-19.2)	19.1 (19-19.3)	19.9 (19.7-20)	20.9 (20.3-21.5)
Sonora	13 (11.6-14.5)	13.5 (12.1-15.1)	13.6 (12.1-15.2)	14.1 (12.6-15.7)	14.3 (12.8-15.8)	15.1 (13.6-16.7)	15.3 (14.3-16.2)	16 (15.9-16.1)	16.4 (16.3-16.6)	16.4 (16.3-16.6)	17.5 (17.4-17.7)	17.6 (17.5-17.7)	18 (17.9-18.1)	18.5 (17.9-19.1)
Tabasco	13.7 (12.4-15.1)	14.2 (12.9-15.8)	14.3 (12.9-15.8)	14.9 (13.6-16.4)	15.1 (13.8-16.4)	16 (15.3-16.7)	16.6 (16.5-16.8)	17.2 (17.1-17.3)	17.8 (17.7-17.9)	17.2 (17-17.3)	18.3 (18.2-18.4)	18.6 (18.5-18.7)	18.6 (18.5-18.7)	18.7 (18.2-19.3)
Tamaulipas	13.3 (11.8-14.9)	13.9 (12.5-15.6)	14.1 (12.6-15.7)	14.7 (13.2-16.5)	15 (13.5-16.7)	16.2 (15.2-17.2)	16.8 (16.7-17)	17.1 (17-17.2)	17.8 (17.6-17.9)	18.2 (18.1-18.3)	18.7 (18.6-18.8)	18.9 (18.8-19)	19.2 (19.1-19.4)	19.8 (19.2-20.4)
Tlaxcala	13.5 (12.3-14.9)	14.2 (12.8-15.7)	14.4 (13.1-16)	15.2 (13.8-16.7)	15.6 (14.3-17.1)	16.7 (15.6-18)	17.1 (16.4-17.8)	17.7 (17.5-18)	18 (17.9-18.2)	18.2 (18.1-18.4)	18.8 (18.7-19)	19.1 (19-19.3)	19.2 (19.1-19.4)	19.6 (19.1-20.1)
Veracruz de Ignacio de la Llave	13.7 (12.5-15.2)	14.4 (13-16)	14.7 (13.2-16.3)	15.5 (14-17)	16 (14.6-17.5)	17.4 (16.6-18.4)	18.2 (18-18.3)	18.1 (18-18.2)	18.2 (18.1-18.3)	18.1 (18-18.2)	19 (18.9-19.1)	18.9 (18.8-19)	18.8 (18.7-18.9)	18.6 (18.1-19.2)
Yucatán	13.4 (12.2-14.8)	13.9 (12.5-15.3)	13.9 (12.6-15.4)	14.2 (13-15.6)	14.2 (12.9-15.4)	14.7 (14-15.3)	15.1 (15-15.3)	15.8 (15.7-15.9)	16.7 (16.6-16.8)	16.9 (16.8-17)	17.8 (17.7-17.9)	17.6 (17.4-17.7)	17.8 (17.7-18)	17.8 (17.3-18.4)
Zacatecas	14 (12.6-15.6)	14.7 (13.2-16.3)	15 (13.5-16.6)	15.9 (14.4-17.4)	16.4 (15.2-17.8)	17.7 (17-18.4)	18.1 (17.9-18.3)	18.3 (18.2-18.5)	18.9 (18.8-19)	18.5 (18.3-18.6)	19.3 (19.2-19.4)	19.4 (19.2-19.5)	19.5 (19.3-19.6)	19.6 (19.1-20.1)
Nicaragua	13.1 (13-13.3)	14.9 (14.7-15.1)	14.5 (14.4-14.6)	15.8 (15.4-16.2)	14.8 (14-15.6)	16.4 (15.9-16.9)	16.2 (15.2-17.2)	17.1 (16.2-18.2)	19.1 (18.6-19.6)	19.1 (18.5-19.8)	19.9 (19.3-20.4)	19.8 (19.2-20.4)	20.4 (20-20.7)	20.6 (19.7-21.5)
Panama	14.8 (14.6-15)	16 (15.8-16.1)	16.7 (16.6-16.9)	16.5 (16.3-16.6)	16 (15.9-16.2)	17.5 (17.4-17.7)	18.8 (18.7-19)	18.5 (18.3-18.7)	19.3 (19.2-19.5)	19.8 (19.7-20)	20.8 (20.7-21)	21.3 (21.2-21.5)	21.8 (21.6-21.9)	21.6 (21.1-22.2)
Venezuela	12 (12-12.1)	13.3 (13.2-13.4)	14.4 (14.3-14.5)	14.8 (14.7-14.8)	15.3 (15.2-15.3)	15.8 (15.7-15.8)	16.4 (16.4-16.5)	16.9 (16.9-17)	17.6 (17.6-17.7)	18 (17.8-18.2)	19.3 (19.2-19.4)	20.1 (20-20.2)	20.4 (20.3-20.5)	20 (18.7-21.3)
Tropical Latin America	13.2 (12.8-13.6)	13.5 (13.1-14)	13.9 (13.5-14.4)	14.4 (13.9-14.8)	14.8 (14.4-15.3)	15.4 (15.1-15.7)	15.8 (15.7-15.8)	16.2 (16.1-16.2)	17 (16.9-17)	17.4 (17.4-17.5)	18.4 (18.3-18.4)	19 (18.9-19)	19.4 (19.4-19.4)	19.7 (19.6-19.8)
Brazil	13.1 (12.7-13.5)	13.4 (12.9-13.8)	13.8 (13.4-14.3)	14.3 (13.8-14.7)	14.8 (14.3-15.2)	15.4 (15.1-15.6)	15.7 (15.6-15.8)	16.2 (16.1-16.2)	16.9 (16.9-17)	17.4 (17.4-17.4)	18.4 (18.3-18.4)	19 (18.9-19)	19.4 (19.4-19.5)	19.7 (19.7-19.8)
Acre	14 (12.7-15.6)	14.5 (13.2-16.1)	15.2 (13.9-16.6)	15.8 (14.6-17)	16.3 (15.5-17.3)	16.8 (16.2-17.3)	17.2 (17-17.4)	17.2 (17-17.3)	18 (17.8-18.1)	18.3 (18.1-18.4)	19 (18.8-19.1)	19.3 (19.1-19.4)	20.3 (20.2-20.4)	19.9 (19.7-20.2)
Alagoas	12.9 (11.6-14.4)	13.1 (11.8-14.6)	13.5 (12.1-15)	13.9 (12.5-15.5)	14.3 (13.1-15.9)	15 (14.1-15.9)	15.6 (15.5-15.7)	16.2 (16.1-16.3)	17.5 (17.4-17.6)	17.6 (17.3-17.9)	18.9 (18.6-19.2)	19.1 (18.8-19.4)	19 (18.9-19.1)	18.7 (18.3-19)
Amapá	13.9 (12.5-15.4)	14.3 (12.9-15.7)	14.8 (13.5-16.1)	15.3 (14.1-16.5)	15.8 (14.8-16.7)	16.3 (15.7-16.9)	17.2 (16.9-17.5)	17.8 (17.7-18.1)	18.7 (18.5-18.9)	18.9 (18.8-19.1)	19.6 (19.4-19.7)	20.3 (20.2-20.5)	21.4 (21.2-21.5)	20.7 (20.4-21)
Amazonas	14.4 (13-16)	14.7 (13.3-16.3)	15.1 (13.6-16.6)	15.5 (14-17.1)	15.8 (14.4-17.4)	16.1 (14.9-17.2)	17 (16.6-17.3)	17.3 (17-17.6)	18.5 (18.2-18.9)	19.7 (19.4-20)	19.7 (19.6-19.8)	20 (19.9-20.1)	19.9 (19.8-20)	20.1 (19.8-20.4)
Bahia	14 (12.7-15.5)	14.4 (13-15.9)	14.9 (13.4-16.4)	15.5 (14-17.1)	16.1 (14.6-17.8)	16.6 (15.4-18)	17.6 (17.1-18.2)	18 (17.5-18.5)	18.9 (18.4-19.4)	19 (18.5-19.6)	19.6 (19-20.1)	20.1 (19.5-20.6)	20.8 (20.3-21.3)	21.3 (21.1-21.5)
Ceará	13.7 (12.3-15.4)	14.1 (12.7-15.7)	14.6 (13.2-16.3)	15.2 (13.7-16.9)	15.7 (14.2-17.4)	16.5 (15-18.1)	17.6 (16.2-19.1)	18.6 (17.9-19.3)	20 (19.6-20.4)	19.7 (19.2-20.1)	19.9 (19.5-20.4)	20 (19.5-20.4)	20.9 (20.8-21)	20.7 (20.4-20.9)
Distrito Federal	12.5 (11.3-13.9)	12.9 (11.6-14.2)	13.4 (12.1-14.9)	13.8 (12.5-15.3)	14.1 (12.8-15.4)	14.1 (13.3-15)	15 (14.9-15.1)	15.5 (15.4-15.6)	16.2 (16.1-16.3)	16.1 (16-16.2)	17.1 (17-17.2)	17.8 (17.7-17.9)	18.6 (18.5-18.7)	20 (19.6-20.3)
Espírito Santo	13.5 (12.2-15)	13.8 (12.4-15.2)	14 (12.7-15.5)	14.3 (13-15.9)	14.5 (13.2-15.9)	14.9 (14-15.8)	15.8 (15.7-15.9)	15.9 (15.8-16)	17.1 (17-17.2)	17.4 (17.3-17.5)	18.5 (18.4-18.6)	19.2 (19.1-19.3)	19.8 (19.7-19.9)	20.7 (20.4-21)
Goiás	11.7 (10.5-12.9)	11.9 (10.7-13.4)	12.3 (11-13.8)	12.6 (11.4-14.2)	13.1 (11.8-14.6)	13.9 (12.6-15.2)	14.5 (13.8-15.1)	14 (14-14.1)	15.6 (15.5-15.7)	16.2 (16.1-16.3)	17.9 (17.8-18)	18.8 (18.7-18.8)	18.9 (18.8-19)	19.8 (19.5-20.1)
Maranhão	13.6 (12.2-15.1)	13.9 (12.5-15.4)	14.4 (13-15.9)	14.9 (13.4-16.5)	15.4 (13.9-17)	16.1 (14.6-17.7)	17.3 (16-18.8)	18.5 (17.9-19.1)	19.7 (19.3-20.2)	19.6 (19.1-20.1)	20.2 (19.7-20.7)	19.9 (19.4-20.3)	20.6 (20.1-21.1)	20.8 (20.5-21.1)
Mato Grosso	14.5 (13.1-16.1)	14.9 (13.3-16.6)	15.3 (13.7-17.2)	15.9 (14.4-17.7)	16.6 (14.9-18.3)	17.4 (16.1-18.8)	18.3 (17.7-19)	17.6 (17-18.1)	18.5 (17.9-19.1)	18.7 (18.5-18.8)	18.5 (18.4-18.6)	18.7 (18.6-18.8)	19.1 (19-19.2)	20 (19.7-20.3)
Mato Grosso do Sul	13.6 (12.2-15.4)	13.8 (12.4-15.6)	14.1 (12.7-15.9)	14.5 (13.1-16.1)	14.7 (13.4-16.4)	15.2 (14.1-16.3)	16 (15.8-16.2)	16.5 (16.4-16.6)	16.9 (16.8-17)	17 (16.9-17.1)	18.3 (18.2-18.4)	18.5 (18.4-18.6)	19.1 (19-19.2)	19.5 (19.1-19.8)
Minas Gerais	12.5 (11.1-13.9)	12.8 (11.3-14.3)	13.1 (11.8-14.8)	13.5 (12.2-15)	13.9 (12.6-15.3)	14.4 (13.7-15.3)	14.8 (14.8-14.9)	15.4 (15.4-15.5)	16.8 (16.7-16.8)	17.5 (17.5-17.6)	18.9 (18.8-18.9)	19.6 (19.6-19.7)	20 (19.9-20)	20.4 (20.2-20.6)
Pará	14.4 (13-15.9)	14.8 (13.4-16.3)	15.2 (13.8-16.8)	15.6 (14.1-17.3)	15.9 (14.3-17.4)	16.4 (15.4-17.5)	17.6 (17.4-17.7)	17.7 (17.4-18.1)	18.1 (17.8-18.5)	18.8 (18.4-19.2)	19.8 (19.4-20.2)	20.2 (19.8-20.5)	20.2 (20.1-20.3)	20.6 (20.3-20.9)
Paraíba	13.3 (12-14.9)	13.6 (12.4-15.1)	14.1 (12.8-15.7)	14.7 (13.3-16.4)	15.4 (14-16.9)	16.3 (15.2-17.3)	16.6 (16.5-16.8)	17.3 (17.2-17.4)	18 (17.7-18.3)	18.3 (18-18.6)	19.3 (19-19.7)	19.5 (19.2-19.8)	19.6 (19.2-19.9)	19.7 (19-20.3)
Paraná	12.2 (10.9-13.7)	12.4 (11.2-13.9)	12.8 (11.4-14.2)	13.2 (11.8-14.7)	13.8 (12.5-15.4)	14.5 (13.7-15.4)	14.4 (14.3-14.5)	14.8 (14.8-14.9)	15.5 (15.5-15.6)	16.3 (16.2-16.3)	17.1 (17.1-17.2)	18 (17.9-18.1)	18.5 (18.4-18.6)	19 (18.7-19.2)
Pernambuco	11.9 (10.8-13.3)	12.2 (11.1-13.6)	12.7 (11.4-14.2)	13.2 (11.8-14.7)	13.6 (12.3-14.9)	14.5 (13.7-15.3)	15.5 (15.5-15.6)	15.7 (15.6-15.7)	16.4 (16.4-16.5)	17.6 (17.5-17.6)	18.1 (18-18.2)	18.7 (18.7-18.8)	19.2 (19.1-19.3)	18.4 (18.2-18.7)
Piauí	14.1 (12.7-15.7)	14.4 (13.1-15.9)	14.8 (13.3-16.4)	15.1 (13.7-16.7)	15.5 (14-17.2)	16.1 (14.6-17.7)	17.1 (16.2-18)	16.9 (16.5-17.3)	19.9 (19.5-20.3)	21 (20.5-21.4)	20.1 (19.7-20.6)	20 (19.6-20.4)	21 (20.8-21.1)	21 (20.8-21.4)
Rio de Janeiro	12.9 (11.6-14.4)	13.3 (11.9-14.7)	13.7 (12.3-15.3)	14.2 (12.8-15.7)	14.6 (13.3-16)	15 (14.3-15.8)	15.4 (15.4-15.5)	15.6 (15.5-15.6)	15.8 (15.7-15.8)	16.2 (16.2-16.3)	17.6 (17.5-17.6)	18.2 (18.2-18.3)	18.5 (18.5-18.6)	18.8 (18.6-19)
Rio Grande do Norte	14.3 (12.9-15.9)	14.7 (13.3-16.3)	15.2 (13.8-17)	15.8 (14.2-17.6)	16.3 (14.8-18)	17.2 (15.8-18.6)	18.2 (17.5-18.9)	18.8 (18.3-19.2)	19.4 (19-19.9)	19.1 (18.6-19.6)	20.5 (19.9-21)	20.9 (20.5-21.4)	21.4 (21.3-21.5)	20.7 (20.4-21)
Rio Grande do Sul	13.3 (11.9-14.8)	13.5 (12.2-14.9)	13.9 (12.6-15.4)	14.3 (12.9-15.8)	14.8 (13.5-16.2)	15.4 (14.5-16.2)	15.3 (15.3-15.4)	15.9 (15.9-16)	16.5 (16.4-16.5)	16.8 (16.7-16.8)	17.9 (17.9-18)	18.5 (18.5-18.6)	19.1 (19-19.1)	19.4 (19.1-19.6)
Rondônia	11.8 (10.7-13.2)	12 (10.8-13.4)	12.4 (11.1-13.8)	12.7 (11.5-14.1)	12.9 (11.7-14.2)	13.2 (12.3-14.3)	14 (13.4-14.6)	15 (14.6-15.5)	16.3 (15.8-16.8)	17.2 (16.7-17.7)	17.6 (17.1-18.2)	18 (17.5-18.5)	18.9 (18.3-19.4)	19 (18.1-19.9)
Roraima	12.2 (11-13.5)	12.5 (11.3-13.8)	12.9 (11.7-14.2)	13.3 (12.1-14.6)	13.7 (12.5-15)	14.1 (13.1-15.3)	14.9 (14.1-15.8)	15.2 (14.5-16)	16.3 (15.7-17)	16.8 (16.2-17.5)	16.8 (16.2-17.4)	17.3 (16.6-18)	18.9 (18.2-19.7)	19 (18-19.9)
Santa Catarina	13.5 (12-15)	13.7 (12.3-15.3)	13.9 (12.4-15.4)	14.2 (12.7-15.8)	14.6 (13.2-16.1)	15.2 (14.3-16.1)	15.1 (15-15.2)	15.5 (15.4-15.6)	16 (15.9-16.1)	16.6 (16.5-16.7)	17.7 (17.6-17.7)	18.8 (18.7-18.9)	19.3 (19.2-19.4)	19.6 (19.4-19.9)
São Paulo	13.1 (11.8-14.7)	13.4 (12-15)	13.8 (12.3-15.3)	14.2 (12.8-15.7)	14.8 (13.5-16.2)	15.3 (14.6-16.1)	15.1 (15.1-15.1)	15.7 (15.6-15.7)	16.1 (16.1-16.2)	16.7 (16.7-16.7)	17.6 (17.6-17.6)	18.4 (18.4-18.5)	18.9 (18.9-19)	19.4 (19.3-19.6)
Sergipe	13.6 (12.3-15.2)	13.9 (12.6-15.5)	14.3 (12.9-15.8)	14.7 (13.4-16.3)	15.3 (14-16.8)	16.1 (15.2-17.1)	16.1 (15.9-16.2)	17.1 (17-17.2)	17.6 (17.4-17.7)	17.4 (17.2-17.5)	18.6 (18.5-18.8)	19.6 (19.5-19.7)	20 (19.9-20.1)	19.9 (19.6-20.3)
Tocantins	12.7 (11.5-14.2)	13 (11.7-14.5)	13.5 (12.2-15)	13.9 (12.6-15.5)	14.5 (13.1-16)	15.3 (14-16.8)	16.3 (15-17.6)	16.8 (15.9-17.8)	16.9 (16.4-17.3)	17.5 (17.2-17.7)	18.3 (18-18.6)	19.5 (19.2-19.8)	20.4 (20.2-20.7)	20.1 (19.6-20.6)
Paraguay	15.8 (14.9-16.7)	18 (17.4-18.5)	17.2 (16.7-17.8)	18.1 (17.6-18.6)	17.2 (16.8-17.7)	17.3 (16.9-17.8)	17.3 (16.8-17.8)	17.3 (16.8-17.8)	18.5 (18-19)	18.5 (18-19)	19.1 (18.6-19.6)	18.9 (18.4-19.5)	18.7 (18.2-19.3)	19.3 (17.8-20.8)
North Africa and Middle East	12.9 (12.6-13.2)	13.5 (13.1-13.8)	13.8 (13.4-14.1)	14.2 (13.9-14.5)	14.3 (13.9-14.6)	14.7 (14.5-15)	15 (14.8-15.1)	15 (14.8-15.1)	15.4 (15.3-15.6)	15.9 (15.7-16.1)	16.7 (16.5-16.9)	17.3 (17.2-17.5)	17.8 (17.6-18)	18.6 (18.3-18.9)
North Africa and Middle East	12.9 (12.6-13.2)	13.5 (13.1-13.8)	13.8 (13.4-14.1)	14.2 (13.9-14.5)	14.3 (13.9-14.6)	14.7 (14.5-15)	15 (14.8-15.1)	15 (14.8-15.1)	15.4 (15.3-15.6)	15.9 (15.7-16.1)	16.7 (16.5-16.9)	17.3 (17.2-17.5)	17.8 (17.6-18)	18.6 (18.3-18.9)
Afghanistan	9.8 (9-10.7)	9.8 (9.1-10.8)	9.8 (9.1-10.8)	9.8 (9.2-10.7)	9.8 (9.2-10.5)	9.8 (9.3-10.4)	9.7 (9.2-10.3)	9.8 (9.2-10.5)	10.4 (9.5-11.3)	10.3 (9.6-11.4)	10.4 (9.6-11.4)	10.8 (10.1-11.8)	11.4 (10.6-12.4)	12.1 (11.3-13.2)
Algeria	13.1 (12.8-13.5)	14.3 (14.1-14.6)	15 (14.6-15.4)	15.4 (15-15.7)	14.4 (13.9-15)	13.7 (13.2-14.3)	13.8 (13.4-14.1)	16 (15.7-16.2)	16.9 (16.3-17.4)	17.5 (17-18.1)	17.9 (17.4-18.4)	17.9 (17.3-18.5)	18.1 (17.7-18.4)	18.9 (18.5-19.3)
Bahrain	11.7 (10.5-13.1)	11.9 (10.7-13.4)	12.3 (11-13.7)	12.6 (11.5-14)	13 (11.9-14.1)	13.3 (12.5-14.1)	13.7 (13.3-14.2)	13.8 (13.5-14.2)	13.7 (13.4-14)	12.9 (12.6-13.3)	14.2 (13.9-14.6)	16 (15.6-16.3)	17.8 (17.5-18.2)	18.7 (17.9-19.4)
Egypt	12.9 (12.8-12.9)	14.5 (14.5-14.6)	14.3 (14.3-14.4)	14.8 (14.7-14.8)	13.8 (13.8-13.8)	14.4 (14.4-14.5)	14.4 (14.3-14.4)	13.3 (13.3-13.4)	14.7 (14.7-14.7)	15.6 (15.6-15.7)	16.3 (16.2-16.5)	16 (16-16.1)	15.9 (15.8-16)	16.2 (15.3-17.2)
Iran	14.1 (13.7-14.4)	14.1 (13.8-14.5)	14.5 (14.1-14.9)	15.1 (14.7-15.5)	15.9 (15.5-16.2)	16.9 (16.5-17.2)	17.3 (16.9-17.6)	16.5 (16.2-16.8)	16.3 (16-16.6)	17.1 (16.9-17.3)	17.6 (17.4-17.7)	18 (17.9-18.1)	18.1 (18.1-18.2)	18.6 (18.5-18.6)
Iraq	13.5 (12.7-14.4)	14.2 (13.7-14.8)	14.8 (14.5-15.1)	14.8 (14.5-15.1)	14.8 (14.4-15.2)	14.8 (14.3-15.3)	15.1 (14.4-15.8)	14.5 (13.6-15.5)	14.1 (13.1-15.1)	13.5 (12.7-14.4)	13.7 (12.9-14.6)	15.1 (14.3-16)	16.9 (16.2-17.7)	20.3 (19.8-20.9)
Jordan	13.7 (12.4-15.1)	13.9 (12.7-15.2)	14.5 (13.4-15.7)	15.3 (14.6-16.1)	16.2 (15.7-16.7)	17 (16.6-17.3)	16.1 (15.6-16.6)	15.9 (15.1-16.6)	15.2 (14.3-16.1)	14.4 (13.6-15.3)	14.6 (13.9-15.3)	15.1 (14.7-15.6)	18.2 (17.8-18.5)	19.9 (19-20.8)
Kuwait	14.8 (13.5-16.2)	14.9 (13.8-16.2)	15 (14.3-15.7)	15 (14.8-15.2)	16.6 (16.5-16.8)	16.7 (16.6-16.8)	17.2 (17.1-17.4)	18.6 (18.5-18.7)	18.7 (18.5-18.8)	19.9 (19.8-20.1)	19.7 (19.6-19.9)	20.5 (20.4-20.6)	22.1 (22-22.2)	24.7 (24.3-25.1)
Lebanon	13.9 (12.6-15.4)	14.1 (12.8-15.7)	14.5 (13.1-16)	14.8 (13.6-16.3)	15.3 (14-16.7)	15.5 (14.3-16.7)	16 (14.9-17.2)	16 (15.3-17.1)	16.6 (15.9-17.6)	17 (16.4-18)	18.1 (17.3-18.9)	18.9 (18.6-19.3)	18.9 (18.6-19.3)	19 (18.6-19.5)
Libya	11.5 (10.4-12.8)	12.4 (11.2-13.7)	13.3 (12.2-14.6)	14.7 (13.6-15.8)	15.9 (15.1-16.8)	16.5 (15.9-17.1)	17.1 (16.3-17.9)	17.1 (16-18.1)	16.8 (15.7-17.9)	16.9 (15.9-17.9)	16.7 (16-17.6)	16.8 (16.1-17.5)	16.2 (15.5-17)	16.6 (15.5-17.9)
Morocco	12 (10.9-13.4)	12.1 (10.9-13.5)	12.2 (10.9-13.5)	12.8 (11.6-14.2)	13.3 (12-14.5)	13.6 (12.5-14.7)	13.9 (13.1-14.7)	14.2 (13.6-14.8)	14.1 (13.6-14.6)	14.3 (13.8-14.8)	15 (14.4-15.5)	15.4 (14.8-16.1)	15.7 (14.7-16.5)	16.4 (15.1-17.7)
Palestine	12.8 (11.6-14.2)	13.3 (12-14.7)	13.9 (12.6-15.4)	14.7 (13.4-16.2)	15.1 (13.9-16.4)	16 (14.7-17.6)	16 (14.7-17.5)	16.1 (14.8-17.6)	16 (14.7-17.3)	15.8 (14.8-16.8)	15.6 (15.1-16.1)	15.1 (14.8-15.4)	15.5 (15.3-15.8)	17.4 (16.9-18)
Oman	10.5 (9.6-11.6)	10.7 (9.8-12)	11.1 (10.1-12.3)	11.6 (10.5-12.9)	13.1 (11.9-14.6)	14 (12.7-15.5)	14.8 (13.5-16.4)	15.6 (14.2-17)	16.1 (14.8-17.4)	16.4 (15.4-17.8)	17.1 (15.6-18.5)	18.2 (16.9-18.7)	18.3 (17.9-18.8)	18.8 (18.2-20)
Qatar	15.1 (13.7-16.7)	15.1 (13.7-16.6)	15.2 (13.8-16.8)	15.2 (13.8-16.7)	15.4 (14.2-16.8)	15.3 (14.1-16.6)	15.2 (14.1-16.5)	14.5 (13.6-15.7)	14.2 (13.2-15.3)	14.3 (13.3-15.4)	15.1 (13.9-16.2)	16.6 (15.4-17.7)	18.2 (17-19.4)	19.9 (18.5-21.4)
Saudi Arabia	14.1 (12.7-15.7)	14.4 (13-16)	14.7 (13.3-16.3)	15.3 (13.8-16.9)	15.9 (14.4-17.6)	16.8 (15.3-18.4)	17.2 (15.9-18.8)	17.3 (16.1-18.8)	17.3 (16-18.6)	17.6 (16.5-18.8)	17.9 (16.9-18.7)	17.7 (16.9-18.4)	18.1 (17.6-18.8)	19.1 (18.3-19.7)
Sudan	12 (10.9-13.4)	12.2 (11.1-13.5)	12.4 (11.3-13.6)	12.5 (11.4-13.6)	12.5 (11.6-13.6)	12.6 (11.8-13.5)	13 (12.2-13.8)	12.6 (11.9-13.3)	13.5 (12.6-14.5)	14 (12.8-15.1)	14.5 (13.1-15.8)	15.1 (13.7-16.4)	15.7 (14.3-17.1)	16.4 (14.9-18)
Syria	15.7 (14.3-17.3)	16.3 (14.8-17.8)	16.8 (15.5-18.2)	17.2 (16.1-18.5)	17.4 (16.4-18.5)	17.6 (16.8-18.5)	17.1 (16.4-17.9)	16.1 (15.6-17)	16 (15.4-16.4)	16.2 (15.6-16.5)	16.9 (16.2-17.3)	18.1 (17.9-18.7)	18.6 (18-19.2)	18.5 (18-19.2)
Tunisia	12.2 (11.1-13.3)	12.6 (11.8-13.6)	13.3 (12.7-14)	12.7 (12.1-13.2)	13.2 (12.9-13.5)	14.5 (14.2-14.8)	15.6 (15.2-15.9)	16.8 (16.6-17.1)	17 (16.7-17.3)	17.2 (16.8-17.5)	17.1 (16.3-17.9)	18.2 (16.8-19.5)	18.8 (17.3-20.3)	19.4 (17.8-21.2)
Turkey	13.6 (12.2-15.2)	14.2 (12.8-15.8)	14.6 (13.1-16.2)	15.2 (13.6-17)	15.8 (14.4-17.5)	16.4 (15.4-17.6)	16.7 (16.2-17.2)	17.1 (16.5-17.6)	17.2 (16.7-17.6)	17.5 (17-17.9)	19.2 (18.8-19.7)	20.5 (20.1-21)	21 (20.6-21.4)	21.9 (21.2-22.7)
United Arab Emirates	13 (11.7-14.6)	13.2 (11.9-14.9)	13.6 (12.1-15.2)	14 (12.6-15.6)	14.3 (12.9-16)	14.4 (12.9-16)	14.7 (13.3-16.4)	14.9 (13.5-16.4)	14.8 (13.6-16.2)	15.2 (14.1-16.4)	14.9 (14-16)	15.1 (14.1-16.2)	16.4 (15.1-17.6)	16.9 (15.4-18.5)
Yemen	11 (9.9-12.1)	11.1 (10-12.2)	11.3 (10.1-12.4)	11.3 (10.1-12.5)	11.5 (10.3-12.7)	12.2 (11-13.5)	12.7 (11.4-14.1)	13 (11.7-14.4)	13.2 (11.8-14.7)	13.5 (12.1-14.9)	13.9 (12.5-15.5)	14.7 (13-16.2)	15.4 (13.7-16.9)	15.6 (14-17)
South Asia	10.4 (10.1-10.8)	10.6 (10.3-11)	10.7 (10.4-11)	10.7 (10.4-11.1)	11.1 (10.8-11.4)	11.8 (11.5-12)	12.8 (12.5-13)	12.6 (12.4-12.9)	13.1 (12.9-13.3)	13.7 (13.5-13.9)	14 (13.8-14.1)	14.8 (14.7-15)	15.1 (14.9-15.2)	15.2 (15-15.4)
South Asia	10.4 (10.1-10.8)	10.6 (10.3-11)	10.7 (10.4-11)	10.7 (10.4-11.1)	11.1 (10.8-11.4)	11.8 (11.5-12)	12.8 (12.5-13)	12.6 (12.4-12.9)	13.1 (12.9-13.3)	13.7 (13.5-13.9)	14 (13.8-14.1)	14.8 (14.7-15)	15.1 (14.9-15.2)	15.2 (15-15.4)
Bangladesh	11.7 (10.6-13.2)	12.1 (10.9-13.5)	12.4 (11.2-13.7)	12.7 (11.6-14)	12.3 (11.4-13.4)	13.6 (12.7-14.5)	14.3 (13.7-14.9)	13.8 (13.3-14.2)	14.3 (13.9-14.9)	15.6 (15.1-16.1)	16.3 (15.9-16.8)	16.1 (15.7-16.5)	16.2 (15.8-16.6)	17.5 (16.6-18.5)
Bhutan	9.8 (9-10.7)	10.1 (9.2-11.1)	10.5 (9.5-11.6)	11 (10-12.1)	11.5 (10.4-12.7)	11.9 (10.7-13.2)	12.2 (11.1-13.4)	12.8 (11.7-14)	13.5 (12.4-14.5)	14.1 (13.1-15)	15.1 (14.3-16)	16.3 (15.5-17.1)	17.2 (16.1-18.2)	17.9 (16.5-19.3)
India	10.2 (9.9-10.5)	10.4 (10-10.7)	10.4 (10-10.8)	10.4 (10.1-10.7)	10.7 (10.4-11)	11.3 (11-11.6)	12.4 (12-12.7)	12.2 (12-12.5)	12.9 (12.6-13.1)	13.6 (13.4-13.8)	13.9 (13.7-14)	14.8 (14.7-15)	15.1 (14.9-15.2)	15 (14.8-15.2)
Andhra Pradesh	10.1 (8.9-11.6)	10.3 (8.9-11.9)	10.4 (9.1-12)	10.5 (9.2-12)	10.8 (9.6-12.2)	11.4 (10.3-12.8)	12.5 (11.3-14)	12.3 (10.9-13.8)	12.8 (11.4-14.3)	13.5 (12.1-15)	13.8 (12.3-15.4)	14.9 (13.3-16.6)	15.7 (14-17.4)	16.1 (14.4-17.6)
Arunachal Pradesh	10.2 (9-11.7)	10.4 (9.1-12)	10.4 (9-11.9)	10.1 (8.9-11.6)	10.7 (9.6-12)	11.5 (10.4-12.7)	12.7 (11.5-14)	12.6 (11.4-14)	13.2 (11.9-14.6)	14 (12.6-15.3)	14.1 (12.8-15.5)	15 (13.7-16.3)	15.7 (14.5-17)	15.9 (14.6-17.1)
Assam	8.8 (7.8-10)	9 (7.9-10.3)	9 (8-10.4)	9 (8-10.3)	9.5 (8.5-10.7)	10.2 (9.2-11.4)	11.4 (10.4-12.7)	11.4 (10.5-12.6)	12.1 (11.3-13)	12.3 (11.9-12.7)	12.1 (11.8-12.5)	13.1 (12.8-13.5)	13.1 (12.8-13.4)	14.1 (13.5-14.7)
Bihar	10.7 (9.3-12.2)	10.8 (9.4-12.4)	10.8 (9.5-12.4)	10.7 (9.5-12.2)	11 (9.9-12.3)	11.4 (10.3-12.8)	12.2 (11.1-13.5)	12 (10.8-13.3)	12.5 (11.5-13.7)	13 (12.1-14)	13 (12.5-13.7)	14.1 (13.7-14.4)	14 (13.7-14.3)	14.3 (13.6-14.9)
Chhattisgarh	10.2 (8.9-11.6)	10.3 (8.9-11.8)	10.3 (8.9-11.9)	10.2 (9-11.6)	10.6 (9.4-11.8)	11.1 (10.1-12.3)	12.2 (11.2-13.4)	12 (10.9-13.1)	12.4 (11.4-13.5)	13 (12.2-13.9)	13.1 (12.7-13.7)	14.1 (13.8-14.4)	14.1 (13.8-14.5)	13.5 (12.9-14.1)
Delhi	10.5 (9.3-11.9)	11 (9.7-12.5)	11.3 (10-12.8)	11.1 (9.8-12.4)	12 (10.8-13.5)	12.9 (11.6-14.2)	13.7 (12.4-15.1)	13.3 (12-14.7)	13.7 (12.4-15)	14.2 (13-15.5)	14.2 (13.3-15.2)	15.1 (14.5-15.9)	16.2 (15.6-16.7)	16.6 (15.7-17.7)
Goa	11.8 (10.4-13.2)	12 (10.6-13.5)	12.1 (10.6-13.6)	12.2 (10.8-13.7)	12.7 (11.4-13.9)	13.5 (12.3-14.6)	14.8 (13.6-15.9)	14.7 (13.6-15.7)	15.2 (14.2-16.1)	15.9 (15-16.9)	16.3 (15.4-17.3)	17.2 (16.1-18.4)	18.1 (16.7-19.3)	18.4 (17.1-19.5)
Gujarat	10.6 (9.4-12)	10.8 (9.5-12.2)	10.8 (9.5-12.3)	10.8 (9.5-12.2)	11.2 (10-12.3)	11.8 (10.7-12.9)	12.9 (11.8-14.1)	13 (11.9-14.2)	13.8 (12.8-14.7)	15.3 (14.8-15.9)	14.7 (14.3-15.1)	15.5 (15.1-15.9)	15.7 (15.2-16.1)	16.4 (15.7-17.1)
Haryana	10.9 (9.5-12.4)	11 (9.6-12.6)	11 (9.7-12.6)	10.9 (9.6-12.4)	11.2 (10-12.7)	12 (10.8-13.5)	13.3 (12.1-14.8)	13.3 (12.2-14.5)	14 (13.1-14.9)	14.6 (14.2-15.1)	15 (14.6-15.4)	16.5 (16.1-16.9)	16.1 (15.8-16.5)	15.5 (14.9-16.3)
Himachal Pradesh	10.4 (9-12.1)	10.6 (9.1-12.3)	10.6 (9.1-12.3)	10.6 (9.1-12.3)	10.9 (9.6-12.4)	11.5 (10.2-13)	12.7 (11.4-14.3)	12.6 (11.3-14.1)	13.4 (12.4-14.5)	14.4 (13.8-15.1)	16 (15.5-16.5)	16.8 (16.3-17.3)	17.4 (16.9-17.9)	18.7 (17.8-19.5)
Jammu and Kashmir	11.5 (9.9-13.4)	11.7 (10-13.5)	11.7 (10.1-13.5)	11.5 (10.1-13)	11.9 (10.6-13.4)	12.3 (11.1-13.8)	13.3 (12-14.9)	13.3 (11.9-14.8)	13.8 (12.6-15.3)	14.6 (13.5-15.7)	14.9 (14.1-15.8)	15.8 (15.3-16.3)	16.7 (16.2-17.2)	16.7 (16-17.5)
Jharkhand	10.4 (9.1-11.8)	10.5 (9.1-12.1)	10.5 (9.2-12)	10.4 (9.2-11.7)	10.6 (9.5-11.8)	11.1 (10.1-12.4)	11.8 (10.7-13)	11.1 (10.1-12.4)	11.4 (10.4-12.5)	11.8 (10.9-12.7)	11.8 (11.3-12.4)	12.7 (12.4-13)	13.6 (13.3-13.9)	13.7 (13.2-14.3)
Karnataka	10.4 (9.2-11.9)	10.6 (9.3-12.1)	10.6 (9.3-12.2)	10.7 (9.4-12.1)	11 (9.9-12.3)	11.7 (10.7-13)	13.1 (11.9-14.5)	13.2 (12.1-14.4)	14 (13.2-14.8)	14.4 (14-14.8)	15.2 (14.9-15.6)	16.2 (15.9-16.6)	15.4 (15-15.7)	13.9 (13.3-14.4)
Kerala	11.8 (10.4-13.3)	12.1 (10.6-13.7)	12.2 (10.7-13.8)	12.3 (10.9-13.9)	12.6 (11.4-14)	13 (11.7-14.4)	14 (12.7-15.4)	14 (12.8-15.3)	14.7 (13.7-15.7)	15.5 (14.9-16.1)	15.4 (14.9-15.9)	16.4 (15.9-16.9)	17.1 (16.6-17.5)	17.3 (16.7-18.1)
Madhya Pradesh	10.1 (9-11.5)	10.3 (9-11.8)	10.3 (9.1-11.7)	10.2 (9.1-11.5)	10.5 (9.5-11.7)	11.1 (10.1-12.2)	11.5 (10.5-12.7)	11 (10-12.1)	11.4 (10.4-12.5)	11.9 (11.1-12.9)	12 (11.4-12.6)	13.6 (13.1-14)	13.7 (13.3-14)	13.8 (13.2-14.4)
Maharashtra	10.9 (9.7-12.3)	11.2 (9.8-12.7)	11.2 (9.9-12.7)	11.3 (10.1-12.7)	11.7 (10.6-13)	12.2 (11.2-13.5)	13 (12-14.1)	12.9 (11.9-14)	13.7 (12.9-14.5)	14.3 (14-14.7)	14.2 (13.9-14.5)	15.8 (15.5-16.2)	16.3 (15.9-16.6)	15.4 (14.8-16)
Manipur	10.7 (9.3-12)	10.8 (9.4-12.3)	10.9 (9.4-12.4)	10.9 (9.5-12.3)	11.3 (10.2-12.5)	12 (11-13.2)	13.3 (12.2-14.5)	13.2 (12.1-14.3)	13.9 (12.8-15)	14.5 (13.4-15.6)	14.8 (13.6-15.8)	15.7 (14.5-16.8)	16.1 (14.9-17.2)	16.1 (14.9-17.3)
Meghalaya	11 (9.6-12.5)	11.1 (9.7-12.7)	11.2 (9.7-12.8)	10.9 (9.7-12.4)	11.5 (10.4-12.7)	12 (10.9-13.2)	13.2 (12-14.4)	13 (11.8-14.2)	13.6 (12.3-14.8)	14.3 (13.1-15.5)	14.5 (13.3-15.7)	15.4 (14.2-16.4)	16 (14.8-17.1)	16.3 (15.1-17.4)
Mizoram	11.2 (10-12.8)	11.4 (10.1-13.1)	11.5 (10.2-13.1)	11.3 (10.2-12.7)	12 (10.9-13.3)	12.7 (11.6-13.8)	13.9 (12.7-15)	13.8 (12.8-15)	14.6 (13.6-15.7)	15.2 (14.3-16.2)	15.4 (14.5-16.3)	16.3 (15.3-17.6)	16.9 (15.8-18.2)	16.9 (15.8-18.2)
Nagaland	10.6 (9.3-12.2)	10.9 (9.4-12.6)	11.1 (9.5-12.8)	11.1 (9.6-12.7)	11.7 (10.3-13.1)	12.5 (11.3-13.9)	13.8 (12.5-15.2)	13.7 (12.4-15)	14.3 (13-15.7)	15.1 (13.7-16.3)	15.2 (13.8-16.5)	16.2 (14.7-17.8)	16.9 (15.4-18.6)	17 (15.7-18.6)
Odisha	9.4 (8.3-10.9)	9.6 (8.3-11.1)	9.5 (8.4-11.1)	9.6 (8.4-11)	9.9 (8.9-11.1)	10.6 (9.5-11.9)	12 (10.8-13.4)	12.2 (11.1-13.5)	12.9 (12.2-13.8)	13.1 (12.8-13.5)	12.4 (12.1-12.7)	13.6 (13.3-13.9)	13.9 (13.6-14.2)	15 (14.4-15.6)
Punjab	11.3 (9.9-12.8)	11.5 (10-13.1)	11.6 (10.1-13.3)	11.8 (10.2-13.4)	12.1 (10.8-13.5)	12.9 (11.7-14.2)	14.2 (13-15.6)	14.4 (13.2-15.6)	15.3 (14.4-16.2)	16.3 (15.8-16.7)	15.8 (15.4-16.2)	16.5 (16.1-16.8)	16.6 (16.2-17)	17.4 (16.7-18.1)
Rajasthan	10.9 (9.6-12.5)	11 (9.7-12.7)	11 (9.6-12.7)	10.8 (9.6-12.3)	11.2 (10-12.6)	11.8 (10.6-13.2)	13 (11.7-14.4)	13 (11.8-14.3)	13.7 (12.8-14.7)	14.3 (13.8-14.8)	15.1 (14.7-15.6)	15.7 (15.3-16.1)	16.3 (15.9-16.7)	15.7 (15-16.4)
Sikkim	10.7 (9.2-12.2)	10.8 (9.2-12.5)	10.8 (9.2-12.4)	10.7 (9.2-12.3)	11.1 (9.8-12.4)	11.7 (10.6-13)	12.9 (11.7-14.2)	12.9 (11.6-14.1)	13.5 (12.3-14.9)	14.2 (12.9-15.5)	14.5 (13.2-15.7)	15.4 (14.2-16.6)	16.3 (15.2-17.6)	16.8 (15.7-18.2)

Table 10 Estimates of life expectancy at age 65 by location and year for 1950, 1955...2005, 2010, 2017 - females

Location	1950	1955	1960	1965	1970	1975	1980	1985	1990	1995	2000	2005	2010	2017
Tamil Nadu	9.4 (8.2-10.8)	9.5 (8.3-10.8)	9.5 (8.4-10.8)	9.5 (8.4-10.7)	9.8 (8.7-10.8)	10.4 (9.4-11.4)	11.6 (10.6-12.8)	11.6 (10.7-12.8)	12.4 (11.7-13.2)	13.4 (13-13.8)	13.3 (13-13.7)	14.6 (14.2-14.9)	14.6 (14.2-14.9)	14.7 (14.2-15.3)
Telangana	10.3 (8.9-11.8)	10.4 (9-12.1)	10.5 (9.1-12.2)	10.6 (9.2-12.2)	10.8 (9.5-12.4)	11.5 (10.2-12.9)	12.5 (11.1-14)	12 (10.7-13.5)	12.4 (11.1-13.8)	13 (11.8-14.5)	13.2 (11.8-14.7)	14.2 (12.8-15.7)	15.1 (13.6-16.6)	15.7 (14.4-17.1)
Tripura	10 (8.9-11.4)	10.3 (9.1-11.8)	10.4 (9.2-11.9)	10.4 (9.2-11.8)	11 (9.9-12.2)	11.8 (10.8-13)	13 (11.9-14.3)	12.9 (11.8-14.1)	13.4 (12.2-14.7)	14 (12.8-15.2)	14.4 (13.2-15.6)	15.4 (14.2-16.4)	15.9 (14.7-17)	16 (14.8-16.9)
Uttar Pradesh	9.3 (8.4-10.5)	9.5 (8.6-10.8)	9.5 (8.6-10.8)	9.5 (8.6-10.7)	9.9 (9.1-10.9)	10.4 (9.6-11.3)	11.4 (10.4-12.4)	11.3 (10.4-12.2)	11.9 (11.1-12.8)	12.8 (12-13.5)	13.9 (13.4-14.5)	13.9 (13.5-14.3)	14.2 (13.8-14.5)	13.8 (13.2-14.5)
Uttarakhand	10.6 (9.3-12.2)	10.7 (9.5-12.3)	10.7 (9.5-12.2)	10.6 (9.5-12)	10.9 (9.9-12.2)	11.5 (10.5-12.7)	12.5 (11.4-13.8)	12.2 (11.1-13.5)	12.6 (11.4-13.8)	13.1 (12-14.4)	13.3 (12.2-14.4)	14.3 (13.4-15.2)	15.4 (14.7-16.1)	16.1 (15.5-16.8)
West Bengal	9.9 (8.8-11.2)	10.1 (8.9-11.5)	10.2 (8.9-11.7)	10.2 (9-11.6)	10.5 (9.3-11.8)	11 (10-12.2)	12.1 (11-13.3)	11.8 (10.8-12.9)	12.4 (11.5-13.3)	12.9 (12.4-13.5)	13.8 (13.3-14.3)	14.7 (14.2-15.2)	14.6 (14.1-15.1)	15 (14.2-15.8)
Union Territories other than Delhi	12.3 (11-13.6)	12.5 (11.1-13.9)	12.6 (11.1-14)	12.5 (11.3-13.8)	13 (11.8-14.3)	13.8 (12.7-14.9)	15 (13.9-16)	14.7 (13.6-15.6)	15 (14.1-15.8)	15.5 (14.7-16.3)	15.7 (14.9-16.6)	16.5 (15.6-17.7)	17.2 (16.1-18.5)	17.2 (16.2-18.4)
Nepal	10.7 (9.8-11.9)	10.9 (10-12.2)	11.2 (10.1-12.4)	11.4 (10.4-12.7)	11.7 (10.6-12.9)	11.8 (10.7-13)	12 (10.9-13.2)	12.4 (11.3-13.5)	12.9 (12-13.9)	13.8 (13-14.5)	15.1 (14.5-15.8)	15.9 (15.3-16.5)	16.1 (15.2-16.9)	16.3 (15.3-17.4)
Pakistan	11.6 (10.4-12.9)	11.7 (10.6-13)	11.8 (10.8-12.9)	12 (11.2-12.9)	12.7 (12-13.5)	13.8 (13.3-14.4)	14.8 (14.3-15.4)	14.9 (14.4-15.4)	14.1 (13.7-14.5)	13.2 (12.7-13.7)	13.2 (12.8-13.7)	13.5 (13-14)	14.2 (13.3-15.2)	14.9 (13.5-16.4)
Southeast Asia, East Asia, and Oceania	12 (11.7-12.3)	12.7 (12.4-13.1)	11 (10.7-11.3)	13.3 (12.9-13.6)	13.6 (13.3-13.9)	13.8 (13.5-14)	14.1 (13.9-14.4)	14.5 (14.3-14.8)	15 (14.9-15.2)	15.8 (15.7-15.9)	16.5 (16.4-16.6)	17 (16.9-17.2)	17.8 (17.6-17.9)	18.4 (18.1-18.6)
East Asia	12 (11.6-12.5)	12.8 (12.4-13.3)	10.6 (10.3-10.9)	13.3 (12.9-13.8)	13.6 (13.3-14)	13.8 (13.4-14.1)	14.1 (13.8-14.4)	14.5 (14.3-14.7)	15 (14.8-15.2)	15.9 (15.8-16.1)	16.7 (16.6-16.9)	17.3 (17.2-17.4)	18 (17.9-18.2)	18.6 (18.3-18.9)
China	12 (11.6-12.5)	12.8 (12.3-13.3)	10.5 (10.2-10.8)	13.3 (12.8-13.8)	13.6 (13.2-14)	13.7 (13.4-14)	14 (13.7-14.3)	14.4 (14.2-14.7)	15 (14.8-15.1)	15.9 (15.8-16.1)	16.7 (16.6-16.9)	17.3 (17.1-17.4)	18 (17.9-18.2)	18.6 (18.3-18.9)
North Korea	11.5 (10.4-12.5)	13.9 (12.4-15.5)	14.3 (12.7-16)	14.7 (13.1-16.5)	15.3 (13.9-17.1)	16 (14.7-17.5)	16.2 (15.1-17.6)	16.4 (15.3-17.8)	16.5 (15.4-18)	15 (14.2-16.1)	14.8 (14.1-15.8)	16.3 (15.4-17.3)	16.4 (15.6-17.5)	16.7 (15.4-18.1)
Taiwan (Province of China)	12.4 (11.2-13.7)	13 (12.9-13)	13.6 (13.5-13.6)	14.5 (14.4-14.6)	14.7 (14.7-14.8)	15.3 (15.2-15.4)	15.9 (15.9-16)	16.5 (16.4-16.6)	17 (16.9-17.1)	17.8 (17.7-17.9)	18.9 (18.8-18.9)	19.8 (19.7-19.9)	20.9 (20.8-21)	21.3 (20.8-21.8)
Oceania	9.3 (8.9-10)	9.7 (9.3-10.3)	10.2 (9.8-10.7)	10.8 (10.5-11.3)	11.2 (10.9-11.6)	11.5 (11.2-11.9)	11.8 (11.5-12.2)	11.8 (11.5-12.2)	11.8 (11.4-12.3)	11.9 (11.4-12.4)	12 (11.5-12.5)	12.1 (11.6-12.6)	12.3 (11.7-12.8)	12.7 (12.1-13.3)
American Samoa	13.9 (13.2-14.6)	14.1 (13.6-14.7)	14.5 (14.1-14.9)	14.7 (14.3-15)	15 (14.5-15.3)	15.5 (15-15.9)	15.6 (15.1-16.4)	15.9 (15.5-16.4)	16.4 (15.9-17.1)	16.3 (15.8-16.8)	16.1 (15.7-16.7)	15.9 (15.5-16.3)	15.9 (15.7-16.2)	15.9 (15.6-16.4)
Federated States of Micronesia	10.3 (9.4-11.5)	10.5 (9.6-11.8)	10.7 (9.9-12)	11 (10.2-12.3)	11.4 (10.5-12.5)	11.8 (10.9-13)	12.2 (11.3-13.3)	12.5 (11.7-13.6)	12.7 (12.1-13.6)	13 (12.5-13.8)	13.4 (12.9-14)	13.9 (13.3-14.4)	14 (13.4-14.7)	14.3 (13.2-15)
Fiji	10.9 (10.4-11.4)	12.5 (12.1-12.9)	13.3 (12.9-13.8)	14 (13.6-14.5)	13.6 (13.2-13.9)	14.2 (13.7-14.6)	14.6 (14.2-15.1)	14.7 (14.3-15.2)	14.5 (13.6-15.4)	13.8 (13.5-14.1)	13.2 (13.1-13.3)	14 (13.9-14.1)	13.8 (13.6-14)	14.4 (13.3-15.7)
Guam	12.5 (12-13.1)	13 (12.6-13.4)	13.9 (13.5-14.2)	15.1 (14.8-15.4)	14.4 (14.1-14.7)	16 (15.7-16.2)	17.7 (17.5-18)	18.5 (18.2-18.8)	18 (17.6-18.3)	17.8 (17.4-18.1)	18.4 (18.1-18.7)	19 (18.7-19.4)	18.6 (18.2-19)	17.8 (17.1-18.5)
Kiribati	10.9 (9.9-12.2)	11 (9.9-12.2)	11.2 (10-12.4)	11.4 (10.3-12.5)	11.5 (10.5-12.7)	12 (11.1-13.2)	12.2 (11.3-13.3)	12 (11.3-12.8)	12 (11.5-12.5)	12.1 (11.7-12.5)	12.3 (11.8-12.7)	12.5 (11.9-13.3)	12.7 (11.9-13.7)	13 (12.2-14.3)
Marshall Islands	11.1 (10-12.3)	11.4 (10.3-12.7)	11.7 (10.8-13.1)	12.1 (11.2-13.4)	12.5 (11.6-13.8)	13 (12.1-14.2)	13.3 (12.6-14.3)	13.3 (12.8-13.9)	13 (12.8-13.2)	12.7 (12.5-13)	12.4 (12-12.7)	12.4 (11.9-12.7)	12.7 (12-13.3)	13.2 (12.3-14)
Northern Mariana Islands	15.2 (13.8-16.5)	15.2 (14-16.7)	15.3 (14.4-17.1)	15.5 (14.7-17.2)	15.9 (15.1-17.4)	16.2 (15.3-17.9)	16.5 (15.6-18.1)	16.7 (15.9-18.1)	17 (16.2-18.2)	17.6 (16.8-18.6)	18 (17.2-18.8)	18.6 (17.8-19.5)	19.1 (18.3-19.7)	19 (18.2-19.7)
Papua New Guinea	8.8 (8.3-9.7)	8.9 (8.4-9.8)	9.1 (8.6-9.8)	9.3 (8.9-10)	9.6 (9.2-10.3)	10 (9.6-10.5)	10.2 (9.8-10.8)	10.3 (9.8-10.9)	10.5 (10-11.2)	10.8 (10.2-11.5)	10.9 (10.3-11.6)	10.9 (10.2-11.5)	11.1 (10.4-11.8)	11.6 (10.8-12.3)
Samoa	15 (13.9-16.1)	16 (15.1-16.9)	17.4 (16.6-18.1)	16.7 (16.1-17.3)	16.5 (15.8-17.1)	16.8 (16-17.5)	17.2 (16.3-18.2)	17.2 (16.1-18.4)	17 (15.9-18.4)	16.7 (15.6-18.1)	16.6 (15.7-18)	16.7 (15.8-18)	15.9 (15.2-17)	16.2 (15.3-17.7)
Solomon Islands	9.8 (8.9-10.7)	9.9 (9.1-11)	10.1 (9.3-11.2)	10.4 (9.6-11.5)	10.8 (9.9-11.8)	11 (10.2-11.9)	11.6 (10.9-12.6)	11.9 (11.3-12.8)	12.1 (11.4-12.8)	12.4 (11.8-13.1)	12.6 (12.1-13.2)	12.7 (12.2-13.2)	13 (12.4-13.4)	13.4 (12.7-14)
Tonga	12.1 (11.5-12.9)	12.4 (11.9-13)	12.6 (12.3-13.1)	12.8 (12.5-13.2)	13.1 (12.7-13.7)	13.5 (13-14.3)	13.9 (13.4-14.6)	14.6 (14.1-15.2)	15 (14.6-15.5)	15.2 (14.9-15.5)	15.3 (15-15.5)	15.7 (15.3-16.2)	16 (15.3-17.1)	16.6 (15.5-18)
Vanuatu	10.4 (9.5-11.5)	10.6 (9.7-11.7)	11 (10-12)	11.3 (10.3-12.3)	11.5 (10.6-12.6)	12 (11.1-13.1)	12.4 (11.6-13.5)	12.7 (12-13.6)	12.8 (12-13.6)	12.9 (12.1-13.7)	13 (12.2-13.9)	12.9 (12.2-13.9)	13.1 (12.4-14.2)	13.4 (12.6-14.4)
Southeast Asia	11.7 (11.3-12.1)	12.3 (11.9-12.7)	12.8 (12.3-13.2)	13 (12.6-13.5)	13.3 (12.9-13.7)	13.7 (13.3-14.1)	14.3 (14-14.6)	14.8 (14.5-15)	15.1 (14.8-15.3)	15.4 (15.2-15.7)	15.8 (15.6-16)	16.3 (16.1-16.6)	16.9 (16.6-17.1)	17.5 (17.2-17.9)
Cambodia	11.1 (10.2-12.4)	11.3 (10.3-12.6)	11.6 (10.6-13)	11.9 (10.8-13.2)	11.7 (10.7-13)	10.2 (9.5-11)	12 (11.1-12.9)	12.2 (11.4-13)	12.4 (11.8-13.1)	12.8 (12.2-13.3)	13.3 (12.8-13.9)	14.4 (13.6-15.1)	15.2 (14.5-15.9)	15.9 (14.7-16.8)
Indonesia	10.9 (10.4-11.4)	11.2 (10.8-11.8)	11.6 (11.1-12.1)	11.9 (11.4-12.4)	12.2 (11.7-12.7)	12.7 (12.2-13.3)	13.3 (12.9-13.7)	13.8 (13.4-14.1)	14.2 (13.8-14.5)	14.6 (14.4-14.9)	14.8 (14.6-15)	15.1 (14.9-15.4)	15.6 (15.2-16)	16.1 (15.6-16.6)
Laos	9.7 (9-10.7)	9.9 (9.1-10.9)	10 (9.2-11)	10.2 (9.3-11.3)	10.4 (9.5-11.6)	10.8 (9.9-12.1)	11.1 (10.1-12.3)	11.5 (10.5-12.7)	11.8 (10.9-13)	12.2 (11.4-13.3)	12.8 (12-13.7)	13.6 (12.8-14.5)	14.6 (13.7-15.5)	15.7 (14.5-16.8)
Malaysia	12.3 (11.1-13.7)	12.9 (12-13.7)	14.1 (14-14.1)	14.2 (14.1-14.2)	14 (13.8-14.1)	14.7 (14.5-14.9)	14.9 (14.5-15.3)	14.9 (14.9-15)	15.1 (15-15.1)	15.1 (15.1-15.1)	15.2 (15.2-15.3)	15.9 (15.9-15.9)	16.4 (16.3-16.5)	16.9 (16.2-17.6)
Maldives	8.5 (7.8-9.3)	8.7 (8-9.5)	9 (8.3-9.8)	9.3 (8.8-10)	9.7 (9.3-10.2)	9.9 (9.6-10.2)	10.3 (10.1-10.6)	11.3 (11.2-11.5)	12.4 (12.2-12.5)	13.2 (13-13.4)	15.4 (15.2-15.6)	17.1 (16.9-17.3)	20.4 (20.1-20.6)	21.6 (21-22.2)
Mauritius	12.3 (12.2-12.5)	12.3 (12.2-12.5)	12.2 (12-12.3)	14.2 (14.1-14.4)	14.3 (14.1-14.4)	14.2 (14.1-14.4)	15.2 (15-15.4)	15.1 (15-15.3)	15.8 (15.6-16)	16.5 (16.3-16.7)	16.6 (16.4-16.8)	17.1 (16.9-17.3)	18 (17.8-18.2)	18.4 (17.8-19)
Myanmar	9.5 (8.8-10.4)	9.7 (9-10.7)	10 (9.2-11.1)	10.4 (9.5-11.5)	10.7 (9.7-11.9)	11.3 (10.2-12.6)	11.8 (10.7-13.1)	12.2 (11.2-13.5)	12.5 (11.4-13.7)	12.7 (11.7-13.8)	13.1 (12.2-14.2)	13.9 (13.1-14.9)	15.2 (14.1-16.2)	16.3 (15.2-17.6)
Philippines	14.3 (13.7-14.9)	16.6 (15.9-17.4)	17 (16.5-17.5)	15.6 (15.1-16)	15.1 (14.6-15.5)	15.3 (14.9-15.8)	16.2 (15.8-16.7)	15.9 (15.5-16.4)	16.3 (15.9-16.8)	16.3 (15.9-16.8)	16.2 (15.8-16.7)	15.9 (15.5-16.4)	15.8 (15.4-16.3)	15.8 (14.6-16.9)
Sri Lanka	12.6 (12.5-12.6)	13.9 (13.8-13.9)	14.8 (14.7-14.9)	15 (14.9-15)	15.6 (15.6-15.7)	15.1 (15.1-15.2)	16.3 (16.2-16.3)	16.3 (16.3-16.3)	17 (16.8-17.3)	17.3 (17.3-17.5)	18.2 (18.1-18.2)	19 (18.6-19.3)	18.8 (18.7-18.9)	19.7 (18.8-21.5)
Seychelles	14.4 (13.2-15.6)	14.6 (13.6-15.7)	14.9 (14.1-15.8)	15.2 (14.6-15.8)	15.8 (15.5-16.2)	16.6 (16.2-16.9)	16.9 (16.6-17.3)	16.8 (16.5-17.1)	17.4 (17-17.7)	17.8 (17.5-18.1)	17.6 (17.3-17.9)	17.5 (17.2-17.8)	17.7 (17.5-18)	18.1 (17.7-18.6)
Thailand	14.9 (14.5-15.3)	16.8 (16.5-17.1)	16.5 (16.2-16.8)	16.2 (15.8-16.5)	16.4 (16.1-16.8)	15.7 (15.3-16)	15.6 (15.3-16)	17.2 (16.8-17.5)	17.1 (16.8-17.4)	17.6 (17.3-17.9)	18.6 (18.2-19.1)	19.6 (19.3-20)	20.3 (20-20.7)	21.7 (20.9-22.5)
Timor-Leste	10 (9.2-10.9)	10.2 (9.3-11.2)	10.5 (9.6-11.7)	10.9 (9.9-12.1)	11.3 (10.3-12.6)	11.6 (10.5-12.7)	12.1 (11-13.3)	12.8 (11.7-14)	13.4 (12.3-14.5)	13.8 (13-14.7)	14.5 (13.7-15.2)	15.2 (14.4-15.9)	15.9 (15.1-16.6)	16.3 (15.3-17.3)
Vietnam	12.8 (11.5-14.2)	13.3 (11.9-14.8)	13.7 (12.3-15.1)	14 (12.7-15.3)	14.1 (12.9-15.4)	14.8 (13.6-16.1)	15.5 (14.3-16.6)	16 (15-17)	16.4 (15.7-17.4)	16.8 (16.2-17.8)	17.2 (16.5-18.3)	17.8 (16.9-18.9)	18.6 (17.7-19.5)	18.8 (18-19.9)
Sub-Saharan Africa	11 (10.7-11.3)	11.2 (10.9-11.5)	11.5 (11.2-11.8)	11.8 (11.4-12.1)	12.1 (11.7-12.4)	12.5 (12.1-12.8)	12.8 (12.4-13.2)	13 (12.7-13.4)	13.2 (12.9-13.6)	13.2 (12.9-13.6)	13.1 (12.8-13.5)	13.7 (13.3-14.1)	14.4 (14-14.8)	15.4 (15.1-15.8)
Central sub-Saharan Africa	10.4 (9.8-11.2)	10.6 (10-11.4)	10.8 (10.2-11.6)	11 (10.3-11.8)	11.2 (10.6-12)	11.5 (10.8-12.2)	11.7 (11-12.4)	12 (11.3-12.7)	12.2 (11.5-12.8)	12.1 (11.5-12.7)	12.2 (11.6-12.8)	12.5 (11.9-13.2)	13 (12.3-13.7)	13.9 (12.9-14.8)
Angola	10.7 (9.8-11.9)	10.7 (9.9-11.9)	10.9 (9.9-12.1)	11.1 (10.1-12.3)	11.3 (10.3-12.6)	11.5 (10.4-12.7)	11.5 (10.4-12.7)	11.5 (10.4-12.7)	11.6 (10.6-12.8)	11.8 (10.8-12.9)	12.1 (11.1-13.1)	12.7 (11.8-13.7)	13.8 (12.8-14.9)	15 (13.7-16.3)
Central African Republic	10 (9.2-10.9)	10.1 (9.3-11)	10.3 (9.4-11.3)	10.5 (9.6-11.5)	10.6 (9.7-11.5)	10.6 (9.8-11.5)	10.7 (10.1-11.5)	10.8 (10.2-11.4)	10.4 (9.8-11.1)	10.3 (9.3-11.2)	10.1 (9.1-11.3)	10.3 (9.1-11.8)	10.7 (9.6-12.6)	11.4 (10.2-13.2)
Congo (Brazzaville)	9.7 (9-10.6)	9.8 (9-10.7)	9.9 (9.1-10.9)	10.2 (9.4-11.1)	10.4 (9.5-11.3)	10.7 (9.8-11.7)	11.1 (10.2-12)	11.4 (10.6-12.3)	11 (10.4-11.7)	10.7 (10.1-11.3)	10.7 (10.2-11.4)	11.3 (10.8-12.2)	12 (11.2-13)	12.8 (11.9-14.4)
DR Congo	10.5 (9.6-11.7)	10.8 (9.8-11.9)	10.9 (10-12.2)	11.1 (10-12.4)	11.4 (10.3-12.7)	11.6 (10.6-12.9)	11.8 (10.9-13)	12.2 (11.3-13.3)	12.5 (11.6-13.5)	12.4 (11.6-13.3)	12.4 (11.6-13.2)	12.6 (11.9-13.5)	12.9 (12-13.9)	13.7 (12.4-15)
Equatorial Guinea	9.1 (8.4-9.8)	9.1 (8.4-9.8)	9.3 (8.5-10)	9.6 (8.8-10.5)	9.9 (9.1-10.8)	10.2 (9.3-11.2)	10.7 (9.6-11.8)	10.8 (9.7-12)	10.6 (9.5-11.8)	10.9 (9.8-12.2)	12.6 (11.1-14.4)	14.1 (12.1-16.5)	14.8 (12.3-17.8)	15.9 (13.3-18.8)
Gabon	11 (10-12.2)	11.1 (10.1-12.2)	11.3 (10.3-12.5)	11.6 (10.6-12.9)	12 (11-13.2)	12.6 (11.5-13.7)	13.1 (12.2-14.2)	13.5 (12.5-14.4)	13.5 (12.5-14.4)	13.7 (12.7-14.7)	13.3 (12.2-14.4)	13.7 (12.4-15.1)	15 (13.6-16.3)	16.3 (14.9-17.8)
Eastern sub-Saharan Africa	9.9 (9.7-10.2)	10.1 (9.9-10.4)	10.3 (10.1-10.6)	10.5 (10.3-10.8)	10.8 (10.6-11.1)	11.1 (10.9-11.4)	11.4 (11.1-11.6)	11.4 (11.2-11.7)	11.6 (11.4-11.8)	11.7 (11.5-11.9)	12.2 (11.9-12.4)	13.3 (13-13.6)	14.4 (14.2-14.7)	15.3 (15-15.6)
Burundi	9.4 (8.8-10.3)	9.5 (8.9-10.4)	9.7 (9.1-10.5)	9.8 (9.2-10.5)	9.9 (9.4-10.6)	10 (9.4-10.7)	10.1 (9.5-10.8)	10.1 (9.4-10.9)	10.2 (9.5-11.1)	10 (9.3-10.8)	10.2 (9.4-11)	11.4 (10.5-12.4)	12.6 (11.5-13.9)	13.2 (12-14.6)
Comoros	10.4 (9.7-11.3)	10.6 (9.9-11.4)	10.7 (10-11.6)	11.1 (10.3-12)	11.4 (10.5-12.5)	11.7 (10.8-12.9)	11.9 (10.9-13)	12.1 (11.1-13.3)	12.4 (11.4-13.5)	12.7 (11.8-13.8)	13.2 (12.3-14.1)	14.1 (13.3-15)	14.8 (13.7-15.8)	15.2 (13.9-16.5)
Djibouti	12 (10.9-13.3)	12.1 (10.9-13.4)	12.3 (11-13.5)	12.5 (11.2-13.8)	12.7 (11.5-14.1)	13.2 (11.9-14.6)	13.3 (11.9-14.7)	13.1 (11.7-14.6)	13.1 (11.7-14.6)	13.1 (11.6-14.6)	13.3 (11.8-15.1)	14 (12-16.1)	14.7 (12.5-16.9)	15.3 (13.3-17.3)
Eritrea	9.3 (8.4-10)	9.3 (8.5-10.1)	9.5 (8.7-10.3)	9.4 (8.6-10.3)	9.6 (8.8-10.4)	9.7 (9-10.5)	9.9 (9.2-10.7)	10.1 (9.5-10.8)	10 (9.4-10.6)	11.4 (10.6-12.3)	11.9 (11-13.1)	12.2 (11.2-13.6)	12.5 (11.5-14.1)	13.2 (12.2-14.7)
Ethiopia	9.2 (8.7-9.7)	9.3 (8.8-9.9)	9.4 (8.8-9.9)	9.4 (8.9-10)	9.5 (9-10)	9.5 (9-10)	9.6 (9.1-10.1)	9.7 (9.3-10.2)	10 (9.6-10.4)	10.3 (9.9-10.8)	11.1 (10.6-11.6)	12.7 (12.1-13.2)	14.5 (14-15.1)	16 (15.4-16.6)
Kenya	11.5 (11.1-11.8)	11.7 (11.4-12.1)	12.1 (11.7-12.5)	12.3 (11.9-12.7)	12.7 (12.3-13)	13.1 (12.7-13.4)	13.4 (13.1-13.8)	13.7 (13.4-14)	13.9 (13.5-14.2)	13.7 (13.3-14.1)	13.7 (13.3-14.2)	14.2 (13.8-14.6)	15.1 (14.7-15.5)	15.9 (15.5-16.3)
Baringo	11.8 (9.9-14.2)	11.8 (9.9-14.3)	11.8 (10-14.2)	11.7 (9.8-13.8)	11.5 (9.7-13.7)	11.4 (9.6-13.5)	11.4 (9.6-13.4)	11.5 (9.8-13.3)	11.6 (9.9-13.4)	11.7 (10.1-13.6)	12 (10.5-14)	12.5 (11.3-14.3)	13.4 (12.1-15.3)	14.4 (12.5-16.7)
Bomet	14.4 (12.7-16.1)	14.5 (12.8-16.3)	14.7 (12.8-16.5)	14.8 (12.9-16.6)	15 (13-16.9)	15.3 (13.4-17.2)	15.6 (13.7-17.5)	15.9 (14.2-17.8)	16.1 (14.3-18.1)	15.9 (14.2-18.2)	15.5 (13.9-17.9)	16 (14.3-18.7)	16.8 (14.8-19.3)	17.8 (15.7-19.5)
Bungoma	11.4 (9.7-13.5)	11.8 (10.1-14)	12.3 (10.3-14.5)	12.6 (10.7-14.8)	13 (11.2-15.3)	13.5 (11.5-15.7)	13.9 (11.9-16)	14.2 (12.3-16.3)	14.5 (12.6-16.4)	14.6 (12.7-16.7)	15 (13-17.1)	14.8 (13-16.8)	15.5 (13.8-17.2)	15.7 (13.8-17.8)
Busia	11.6 (9.9-13.9)	12 (10.1-14.3)	12.5 (10.5-14.7)	12.8 (10.8-14.9)	13.3 (11.3-15.5)	14.1 (12.2-16.2)	14.7 (12.8-16.8)	14.9 (13.2-16.9)	15.1 (13.2-17.4)	14.9 (12.4-19)	14.9 (12-18.4)	15.5 (13-18.9)	15.6 (13.8-17.6)	15.8 (14.1-17.5)
Elgeyo Marakwet	10.9 (9.3-13.1)	11.1 (9.4-13.3)	11.2 (9.5-13.3)	11.3 (9.6-13.3)	11.3 (9.7-13.5)	11.5 (9.8-13.7)	11.7 (10.1-13.9)	12 (10.4-14.1)	12.2 (10.7-14.4)	12.5 (10.9-14.8)	12.8 (11.3-14.8)	13 (11.9-14.7)	13.5 (12.6-15.1)	13.9 (12.6-15.5)
Embu	11.4 (9.7-13.6)	11.8 (10-14)	12.3 (10.5-14.6)	12.8 (11-15.2)	13.5 (11.6-15.9)	14.3 (12.2-16.5)	14.9 (12.9-17)	15.3 (13.3-17.2)	15.6 (13.4-18.3)	16 (13.1-19.2)	16.5 (13.4-19.4)	17 (14.2-19.4)	17.4 (14.7-19.4)	17.7 (15.3-19.4)
Garissa	10.9 (9.5-12.7)	11 (9.6-13)	11.4 (9.9-13.4)	11.5 (10-13.5)	11.6 (10-13.6)	11.8 (10.2-13.7)	12 (10.4-13.8)	12.1 (10.5-13.8)	12.2 (10.7-13.9)	12.2 (10.8-13.9)	12.4 (11-13.9)	12.6 (11.3-14.3)	12.8 (11.5-14.5)	13.3 (11.8-15.9)
Homa Bay	10.1 (8.7-12)	10.5 (8.9-12.4)	10.9 (9.3-13)	11.2 (9.6-13.2)	11.4 (9.9-13.4)	11.8 (10.2-13.6)	12.3 (10.6-14.1)	12.6 (11-14.4)	12.7 (11.1-14.4)	12.2 (10.4-14.6)	11.4 (9.3-14.5)	12.2 (9.9-14.1)	14.2 (11.6-16)	15.7 (12.7-18.7)
Isiolo	12.1 (10.3-14.5)	12.3 (10.4-14.6)	12.5 (10.6-14.9)	12.5 (10.6-14.9)	12.6 (10.7-14.9)	12.9 (11-15.2)	13.3 (11.4-15.6)	13.3 (11.5-15.4)	13.5 (11.8-15.5)	13.7 (12-16)	14.1 (12.1-16.5)	14.5 (12.4-16.9)	14.9 (12.8-17.7)	15.3 (13-17.8)
Kajiado	12.1 (10.2-14.6)	12.2 (10.3-14.5)	12.4 (10.5-14.8)	12.5 (10.6-14.8)	12.6 (10.7-14.8)	12.7 (10.8-14.9)	12.9 (11.2-15.2)	13.2 (11.6-15.3)	13.3 (11.8-15.6)	13.1 (11.5-15.7)	13.2 (11.4-15.8)	13.5 (12-16.1)	14.6 (12.9-17.1)	15 (13.3-17.5)
Kakamega	11.9 (10.1-14.1)	12.2 (10.3-14.6)	12.7 (10.8-14.9)	13.1 (11.1-15.3)	13.6 (11.5-15.8)	14.3 (12.3-16.4)	14.8 (12.9-16.9)	15 (13.3-16.9)	15.1 (13.4-17.3)	15.1 (13-18.1)	15.3 (12.9-18.6)	15.7 (13.5-18.4)	15.9 (13.8-18.7)	16.3 (14.1-18.9)
Kericho	12.8 (10.9-15.3)	13 (11-15.3)	13.3 (11.3-15.6)	13.6 (11.6-15.8)	14 (11.8-16)	14.5 (12.4-16.5)	14.9 (13-16.9)	15.2 (13.4-17)	15.4 (13.7-17.1)	15.4 (13.3-17.6)	15.5 (13.4-17.8)	15.9 (14-18.3)	16.8 (15.1-19.4)	17.8 (15.9-19.7)
Kiambu	11.8 (10.1-13.8)	12.1 (10.3-14.3)	12.5 (10.6-14.7)	12.8 (10.9-15)	13.2 (11.3-15.5)	13.7 (11.8-15.9)	14 (12.1-16.1)	14.3 (12.3-16.1)	14.4 (12.5-16.2)	13.7 (11.7-16.5)	13.4 (11.5-15.9)	14.5 (12.3-17)	15.7 (13.7-17.9)	16.7 (14.7-18.9)
Kilifi	10.4 (9-12.3)	10.7 (9.2-12.6)	11 (9.5-12.9)	11.2 (9.7-13.2)	11.4 (9.9-13.4)	11.7 (10.1-13.7)	11.8 (10.3-13.8)	12 (10.4-13.9)	12 (10.5-13.8)	11.8 (10.2-13.5)	11.9 (10.5-13.7)	12.4 (11-14.3)	13.8 (12-15.8)	15 (13.2-17)
Kirinyaga	11.5 (9.9-13.6)	11.8 (10.1-13.9)	12.3 (10.4-14.4)	12.7 (10.7-14.8)	13.3 (11.2-15.4)	14.1 (11.9-16.3)	14.8 (12.5-17.1)	15.1 (13-17.3)	15.3 (13.1-17.4)	15.2 (13-17.7)	15.4 (12.9-18.6)	15.8 (13.1-18.5)	16.5 (14.2-18.8)	17.4 (15.1-19.1)
Kisii	9.6 (8.2-11.4)	9.9 (8.5-11.7)	10.2 (8.8-12.3)	10.5 (9-12.7)	10.8 (9.2-12.9)	11.1 (9.5-13.2)	11.4 (9.7-13.5)	11.5 (9.9-13.4)	11.4 (10-13.2)	11 (9.7-12.9)	10.6 (9.6-12.2)	10.7 (9.8-11.9)	11.4 (10.4-12.6)	12.5 (11.3-14.3)
Kisumu	12.1 (10.3-14.5)	12.5 (10.7-14.8)	13 (11.1-15.5)	13.5 (11.5-15.9)	13.9 (11.9-16.2)	14.4 (12.4-16.6)	15 (12.9-17.1)	15.4 (13.6-17.6)	15.8 (13.5-18.5)	15.1 (12-18.9)	14.8 (11.6-18.3)	14.9 (12.1-17.8)	15.2 (12.7-18.3)	16.4 (13.9-18.7)
Kitui	10.9 (9.3-12.9)	11.2 (9.6-13.3)	11.6 (9.8-13.7)	11.8 (10-14)	12.1 (10.3-14.4)	12.5 (10.6-14.6)	12.9 (11-14.9)	13.1 (11.4-15.1)	13.4 (11.6-15.3)	13.6 (11.8-15.6)	14.2 (12.1-16.4)	15.7 (13.7-18)	17 (15.1-19.3)	17.2 (15.4-19.1)
Kwale	10.3 (8.9-12.1)	10.6 (9.2-12.6)	11 (9.5-13.1)	11.2 (9.6-13.2)	11.4 (9.8-13.4)	11.7 (10-13.6)	12 (10.2-13.8)	12.1 (10.4-14.1)	11.9 (10-14.2)	11.2 (9.4-14.3)	11.6 (9.9-14.4)	12.7 (10.9-15.2)	13.9 (12.1-16.2)	14.9 (13-17.1)
Laikipia	12.5 (10.7-15)	12.8 (10.8-15.1)	13.1 (11.1-15.4)	13.5 (11.4-15.6)	13.9 (11.8-15.9)	14.5 (12.5-16.5)	15 (13.1-16.9)	15.2 (13.5-17)	15.5 (13.8-17.3)	15.5 (13.4-17.6)	15.1 (13-17.7)	15.2 (13.3-17.7)	16 (14-18.7)	17.1 (14.7-19.3)
Lamu	10.3 (8.8-12.2)	10.6 (9.1-12.7)	11 (9.4-13.1)	11.3 (9.7-13.5)	11.9 (10.1-14.1)	12.8 (10.8-15.1)	13.5 (11.6-15.9)	13.6 (11.7-15.9)	13.5 (11.5-15.8)	13.4 (11.4-15.7)	13.6 (11.5-16)	14.5 (12.2-17.1)	15.4 (13.2-18.1)	16.1 (14-18.6)
Machakos	11.9 (10-14.1)	12.3 (10.3-14.5)	12.8 (10.7-15.2)	13.1 (11-15.5)	13.4 (11.4-15.7)	13.8 (11.8-16)	14.3 (12.3-16.3)	14.7 (12.8-16.6)	15 (13.2-16.9)	15.3 (13.4-17.3)	15.7 (13.6-18.1)	16 (14-18.6)	17.4 (15.3-19.2)	17.7 (15.7-19.3)
Makueni	13.6 (11.6-15.6)	13.8 (11.8-15.9)	13.9 (11.9-16.2)	14 (12-16.2)	14.3 (12.2-16.3)	14.5 (12.5-16.5)	14.8 (12.8-16.8)	15.1 (13.2-17)	15.3 (13.4-17.2)	15.1 (13.1-17.1)	14.5 (12.6-16.9)	14.8 (12.8-17.7)	16 (13.7-18.8)	17.4 (15.4-19.1)
Mandera	10.7 (9.3-12.5)	10.8 (9.4-12.8)	11.2 (9.7-13.2)	11.3 (9.8-13.3)	11.5 (9.8-13.4)	11.7 (10-13.5)	11.8 (10.2-13.7)	11.9 (10.4-13.6)	11.9 (10.5-13.6)	11.9 (10.6-13.6)	12.2 (11-13.7)	12.2 (11.1-13.8)	12.2 (11-13.8)	12.7 (11.3-14.7)
Marsabit	10.4 (8.9-12.4)	10.7 (9.1-12.8)	11.2 (9.5-13.3)	11.6 (9.8-13.7)	12.1 (10.3-14.3)	12.8 (10.9-15.1)	13.4 (11.6-15.7)	13.6 (11.7-15.6)	13.6 (11.9-15.6)	13.7 (11.9-15.8)	14 (12-16.5)	14.2 (12.1-16.7)	14.5 (12.3-17.2)	15 (12.6-18)
Meru	11.1 (9.5-13)	11.5 (9.9-13.5)	12 (10.2-14)	12.3 (10.5-14.3)	12.7 (10.8-14.7)	13 (11.1-15.1)	13.5 (11.7-15.5)	13.8 (12.1-15.7)	14 (12.3-15.9)	14.2 (12.3-16.4)	14.5 (12.5-17)	15 (13-17.2)	15.7 (13.7-18.3)	16.3 (14.1-19)
Migori	10.7 (9.2-12.7)	11 (9.4-13)	11.3 (9.7-13.3)	11.5 (9.9-13.5)	11.8 (10-13.6)	12 (10.3-13.9)	12.2 (10.5-14.1)	12.4 (10.7-14.2)	12.6 (10.9-14.3)	12.3 (10.4-14.6)	11.6 (9.8-14.4)	11.4 (9.5-13.8)	12.2 (10.1-15.1)	14 (11.4-17.6)
Mombasa	10.4 (8.8-12.3)	10.8 (9.1-12.9)	11.3 (9.6-13.5)	11.8 (10-14)	12.4 (10.5-14.7)	13.1 (11.1-15.4)	13.7 (11.7-16.1)	14 (12-16.4)	14 (11.5-16.9)	13.4 (10.7-17.8)	13.8 (11-17.6)	15.3 (12.2-18.9)	16.7 (13.7-19.5)	17.7 (15.1-19.7)
Murang'a	12.6 (10.8-14.7)	12.9 (11-15)	13.2 (11.2-15.3)	13.4 (11.4-15.5)	13.8 (11.9-15.8)	14.3 (12.4-16.3)	14.6 (12.8-16.6)	14.9 (13-16.6)	15.1 (13.2-16.8)	15.1 (13.2-17.5)	15.3 (13.3-18)	15.3 (13.3-17.6)	16.4 (14.5-18.4)	17.1 (15-18.9)
Nairobi	10 (8.8-11.8)	10.5 (9.2-12.2)	11.4 (9.8-13.2)	12 (10.4-14)	12.6 (10.9-14.7)	13.4 (11.6-15.3)	14 (12.3-15.9)	14.3 (12.5-16.1)	13.9 (12-16)	12.7 (11-15.5)	12.3 (11-14.9)	12.5 (11.6-14.3)	13.2 (12.5-14.4)	14.3 (13.3-15.7)
Nakuru	13.7 (11.6-15.9)	13.9 (11.8-16.2)	14.3 (12.1-16.6)	14.6 (12.4-16.9)	15 (12.8-17.2)	15.4 (13.2-17.6)	15.8 (13.9-18)	16.2 (14.5-18.4)	16.9 (14.8-19.2)	17.2 (14.1-19.5)	17.4 (14.2-19.6)	18.2 (15.5-19.7)	18.7 (16.6-20)	18.9 (17.2-20.1)
Nandi	14.6 (12.4-16.9)	14.8 (12.6-17)	14.9 (12.9-17.3)	15 (12.8-17.3)	15.1 (13-17.3)	15.2 (13.2-17.4)	15.4 (13.5-17.6)	15.6 (13.8-17.6)	15.5 (13.7-17.6)	14.9 (12.8-17.2)	15.2 (13.1-17.5)	16 (14.2-18.1)	17.3 (15.6-19.6)	17.7 (15.8-19.5)
Narok	11.5 (10-13.8)	11.6 (10.1-13.8)	11.7 (10.3-14)	11.7 (10.3-13.9)	11.5 (10.2-13.6)	11.5 (10-13.6)	11.5 (10-13.4)	11.5 (10.1-13.4)	11.6 (10.4-13.4)	11.8 (10.6-13.5)	12.3 (10.8-13.8)	12.5 (11.2-14)	13.4 (12.4-14.8)	13.9 (12.8-15.8)
Nyamira	10.2 (9.1-11.9)	10.4 (9.3-12.1)	10.7 (9.6-12.4)	10.9 (9.9-12.7)	11.2 (10.1-12.8)	11.5 (10.3-13.3)	11.7 (10.4-13.5)	11.9 (10.7-13.5)	11.9 (10.9-13.3)	11.6 (10.7-12.8)	11.3 (10.1-12.4)	10.8 (9.6-12)	10.7 (9.4-12.3)	12.1 (10.6-13.4)
Nyandarua	11 (9.4-13.1)	11.2 (9.6-13.3)	11.6 (9.9-13.6)	11.8 (10.3-14)	12.1 (10.5-14.4)	12.5 (10.9-14.8)	12.8 (11.5-15)	13 (11.7-15.1)	13.1 (11.8-15)	12.7 (11.7-14.6)	12.3 (11.3-14.1)	12.1 (11-13.9)	12.8 (11.4-14.6)	14.3 (12.3-16.7)
Nyeri	10.1 (8.7-11.8)	10.4 (8.9-12.3)	10.7 (9.2-12.7)	11 (9.5-13)	11.3 (9.9-13.4)	11.7 (10.3-13.8)	12.1 (10.9-14.2)	12.5 (11.3-14.2)	12.5 (11.3-14)	11.9 (10.6-13.3)	11.3 (10-12.8)	11.7 (10.3-13)	12.8 (11.8-14.6)	13.6 (12.2-16)
Samburu	14.1 (12.1-16.5)	14.1 (12.1-16.4)	14.2 (12.1-16.3)	14.1 (12-16.2)	14.1 (12.1-16.1)	14.2 (12.2-16.2)	14.3 (12.3-16.3)	14.2 (12.4-16.1)	14.1 (12.3-16.1)	14.1 (11.9-16.6)	14.3 (12.1-17.4)	15 (12.8-18)	15.6 (13.4-18.6)	15.9 (13.6-19.2)
Siaya	9.9 (8.5-11.8)	10.3 (8.8-12.2)	10.9 (9.3-13)	11.4 (9.7-13.6)	11.9 (10.1-14.2)	12.5 (10.6-15)	13 (11.1-15.3)	13.4 (11.5-15.6)	13.6 (11.7-15.7)	13.8 (11.6-16.3)	14.2 (11.5-18.1)	14.4 (11.7-16.9)	15.8 (13-18.5)	16.7 (14.3-18.8)
Taita Taveta	9.4 (8.2-11.2)	9.9 (8.5-11.9)	10.4 (8.9-12.5)	10.8 (9.4-12.9)	11.3 (9.6-13.4)	11.8 (9.9-14)	12.2 (10.3-14.4)	12.5 (10.7-14.6)	12.7 (11-14.8)	12.6 (10.9-14.6)	12.4 (10.7-14.7)	12.4 (10.9-14.6)	13 (11.6-15.2)	14 (12.6-16.5)
Tana River	9.5 (8.1-11.4)	9.8 (8.3-11.7)	10.2 (8.6-12.2)	10.4 (8.8-12.4)	10.7 (9.1-12.8)	11 (9.4-13.2)	11.2 (9.7-13.4)	11.3 (10-13.4)	11.4 (10.1-13.4)	11.1 (9.9-12.9)	11.1 (9.8-12.9)	11.5 (10.2-13.3)	11.8 (10.7-13.6)	12.8 (11.5-15)
Tharaka Nithi	9.8 (8.4-11.6)	10 (8.6-11.9)	10.2 (8.8-12.2)	10.4 (9-12.5)	10.7 (9.2-12.7)	11 (9.5-13)	11.3 (9.8-13.3)	11.5 (10.1-13.3)	11.6 (10.3-13.4)	11.7 (10.4-13.4)	12 (10.8-13.7)	12.6 (11.5-14.3)	13.8 (12.4-15.7)	14.8 (12.9-16.8)
Trans Nzoia	13.8 (11.9-16.3)	13.9 (11.8-16.1)	14.2 (12.1-16.4)	14.4 (12.2-16.4)	14.6 (12.5-16.5)	14.9 (13-16.9)	15.3 (13.4-17.3)	15.5 (13.7-17.3)	15.7 (13.8-17.8)	15.5 (12.9-19.1)	15.6 (12.9-19.6)	16.5 (13.9-19.6)	17.5 (15.2-19.6)	18 (15.8-19.5)

Table 10 Estimates of life expectancy at age 65 by location and year for 1950, 1955...2005, 2010, 2017 - females

Location	1950	1955	1960	1965	1970	1975	1980	1985	1990	1995	2000	2005	2010	2017
Turkana	14.2 (12.1-16.8)	14.1 (12.1-16.5)	14.2 (12.2-16.6)	14.2 (12.1-16.4)	14 (12-16.1)	14 (12-16.1)	14 (12.1-16.1)	13.9 (12.2-15.9)	13.9 (12.2-15.9)	14 (11.7-16.7)	14.1 (11.7-17.7)	15 (12.4-18.2)	15.7 (13.1-18.9)	16.2 (13.5-19.6)
Uasin Gishu	12.4 (10.6-14.8)	12.5 (10.8-14.8)	12.7 (11.1-15.1)	12.8 (11.2-15.2)	12.8 (11.2-15.2)	12.8 (11.2-15.1)	12.9 (11.4-15)	13.1 (11.5-15.1)	13.2 (11.8-15.1)	13.3 (11.9-15.4)	13.5 (12.1-15.8)	14.5 (13.2-16.9)	15.5 (13.5-18)	16.8 (14.5-19.5)
Vihiga	11.8 (10.1-14)	12 (10.3-14.1)	12.2 (10.3-14.4)	12.3 (10.6-14.5)	12.6 (10.8-14.7)	12.8 (11-14.8)	13 (11.4-15)	13.2 (11.6-15)	13.1 (11.5-14.9)	12.6 (10.8-14.9)	12.2 (10.5-14.8)	12.4 (10.7-14.8)	13 (11.4-15)	13.4 (11.6-15.7)
Wajir	12.7 (11-14.7)	12.6 (10.9-14.7)	12.8 (11.1-14.9)	12.7 (11-14.8)	12.6 (10.8-14.6)	12.5 (10.8-14.4)	12.4 (10.8-14.3)	12.3 (10.7-14)	12.2 (10.7-13.8)	12 (10.7-13.6)	12 (10.6-13.3)	12 (10.8-13.4)	12.1 (10.9-13.6)	12.5 (11-15.1)
West Pokot	11.5 (9.5-13.7)	11.5 (9.7-13.8)	11.7 (9.7-14.2)	11.7 (9.6-14.1)	11.6 (9.5-13.9)	11.6 (9.6-13.6)	11.7 (9.8-13.8)	11.8 (10-13.9)	12 (10.3-14.1)	12.1 (10.4-14.3)	12.7 (11-15)	13.2 (12.1-15.1)	13.6 (12.3-15.6)	14.5 (12.7-16.9)
Madagascar	10.1 (9.3-10.8)	10.2 (9.6-10.7)	10.4 (10.1-10.8)	10.8 (10.5-11.1)	10.9 (10.6-11.2)	11.4 (11-11.9)	12.2 (11.6-12.8)	11.9 (11.4-12.6)	12.1 (11.6-12.6)	12.1 (11.6-12.6)	12.3 (11.7-13)	12.7 (11.9-13.6)	13.1 (12-14.2)	13.5 (12.1-15)
Malawi	10 (9.2-11.1)	10.3 (9.4-11.3)	10.6 (9.8-11.7)	11 (10.1-12)	11.5 (10.7-12.4)	12.1 (11.4-12.9)	12.5 (12-13)	12.4 (12-12.9)	12.1 (11.5-12.7)	11.6 (10.9-12.4)	11.8 (11-12.8)	13.2 (12.3-14.4)	15.1 (14.1-16.3)	15.8 (14.5-17)
Mozambique	10.5 (9.6-11.7)	10.7 (9.8-11.9)	10.9 (9.9-12.1)	11.1 (10.1-12.3)	11.4 (10.4-12.7)	11.8 (10.9-12.9)	12.1 (11.2-12.9)	11.6 (11-12.3)	12.4 (11.7-13)	12.4 (11.8-13.1)	12.6 (11.9-13.5)	12.9 (12-13.9)	13.1 (12-14.1)	14 (12.6-15.5)
Rwanda	9.7 (9-10.7)	9.9 (9.1-10.9)	10.1 (9.3-11.1)	10.1 (9.3-11.1)	10.2 (9.3-11.1)	10.3 (9.5-11.2)	10.5 (9.9-11.4)	10.8 (10.1-11.5)	10.4 (9.8-11)	10.2 (9.7-10.8)	11.2 (10.6-11.7)	13.4 (12.8-14.1)	15.3 (14.4-16.2)	16 (14.9-17.1)
Somalia	10.1 (9.2-11.1)	10.2 (9.1-11.2)	10.3 (9.2-11.3)	10.4 (9.4-11.5)	10.5 (9.5-11.6)	10.6 (9.6-11.6)	10.9 (9.7-12)	10.7 (9.6-11.9)	10.8 (9.7-12)	10.8 (9.7-12.1)	11 (9.9-12.4)	11.5 (10.2-12.9)	11.8 (10.4-13.2)	12.3 (10.9-13.8)
South Sudan	11.1 (10.1-12.4)	11.2 (10.1-12.4)	11.3 (10.2-12.4)	11.4 (10.3-12.6)	11.6 (10.4-12.9)	11.9 (10.8-13.2)	12.1 (10.8-13.4)	12 (10.8-13.3)	12 (10.8-13.4)	12.1 (10.9-13.6)	12.4 (11-14.1)	13 (11.4-14.8)	13.4 (11.7-15.3)	13.7 (11.9-15.3)
Tanzania	10.7 (9.8-11.8)	10.9 (10-11.8)	11 (10.2-11.9)	11.3 (10.6-12.2)	11.9 (11.1-12.7)	12.5 (11.7-13.4)	13.1 (12.3-13.9)	13.2 (12.5-13.9)	13.1 (12.5-13.9)	13.1 (12.4-14)	13.6 (12.9-14.5)	14.6 (13.8-15.6)	15.5 (14.6-16.3)	16 (15-16.8)
Uganda	10.9 (9.9-12)	10.9 (10-12.1)	11 (10.1-12.1)	11.2 (10.3-12.4)	11.5 (10.6-12.6)	11.7 (10.9-12.7)	11.7 (11-12.5)	12 (11.3-12.8)	12 (11.3-12.8)	11.8 (10.9-12.6)	12.1 (11.3-12.8)	13.3 (12.5-14.1)	15.1 (14-16.2)	16.1 (14.9-17.1)
Zambia	11.1 (10.1-12.3)	11.3 (10.2-12.6)	11.5 (10.5-12.7)	11.7 (10.7-12.9)	12.2 (11.1-13.3)	12.4 (11.4-13.4)	12.3 (11.6-13.2)	12 (11.4-12.7)	11.5 (11-12)	10.8 (10.2-11.5)	11.5 (10.9-12.3)	13.6 (12.9-14.3)	14.6 (13.8-15.4)	14.9 (13.8-16.2)
Southern sub-Saharan Africa	13.2 (12.8-13.6)	13.4 (13-13.9)	13.7 (13.2-14.1)	13.9 (13.5-14.4)	14.3 (13.8-14.7)	15.4 (15-15.9)	15.5 (15.2-15.9)	16.7 (16.4-17)	17.8 (17.6-18.1)	17.4 (17.1-17.6)	14.6 (14.4-14.8)	14.5 (14.3-14.6)	14.9 (14.7-15.1)	17.5 (17.2-17.7)
Botswana	11.4 (10.2-12.6)	11.5 (10.4-12.8)	11.7 (10.5-13)	12 (10.8-13.2)	12.4 (11.3-13.6)	13.2 (12.1-14.4)	14 (12.9-15)	15.1 (14.1-16)	15.8 (14.8-17)	16.9 (15.3-18.8)	17.2 (15.3-18.6)	15.3 (14.1-17.2)	15.2 (14.3-16)	16.3 (15.3-17.1)
Lesotho	11.4 (10.3-12.6)	11.6 (10.5-12.9)	11.9 (10.7-13.2)	12.2 (11.1-13.5)	12.6 (11.5-13.9)	13.1 (11.9-14.3)	13.7 (12.6-14.9)	14 (13-15.1)	14.4 (13.5-15.3)	14.6 (13.4-15.8)	12.7 (11.5-14.1)	11.6 (10.6-13.1)	12.3 (11.1-14)	13.5 (11.9-15.7)
Namibia	11.8 (10.7-13.2)	11.9 (10.8-13.3)	12 (10.9-13.4)	12.3 (11.1-13.6)	12.4 (11.4-13.6)	12.6 (11.7-13.6)	12.8 (12-13.7)	13.4 (12.6-14.1)	13.9 (13.2-14.7)	13.6 (12.8-14.5)	12.6 (12-13.6)	13.5 (12.3-15)	16.2 (14.9-18.3)	17.7 (16.1-19.2)
South Africa	14 (13.5-14.6)	14.3 (13.7-14.8)	14.5 (13.9-15)	14.7 (14.1-15.3)	14.9 (14.4-15.5)	16.3 (15.8-16.9)	16.3 (15.8-16.7)	17.5 (17.1-17.9)	18.9 (18.6-19.1)	18.2 (18-18.3)	15.2 (15-15.3)	15.2 (15.1-15.4)	15.6 (15.5-15.6)	18.3 (18.1-18.4)
Swaziland	10.4 (9.5-11.6)	10.7 (9.8-11.9)	10.9 (10-12.2)	11.5 (10.5-12.9)	12.1 (11.1-13.5)	12.7 (11.6-14)	13.1 (12.1-14.4)	13.5 (12.4-14.6)	14 (13.1-15)	14.2 (13.1-15.3)	12.4 (11.3-14.1)	11.4 (10.4-13.3)	12.3 (11-14.8)	15.1 (13.2-17.2)
Zimbabwe	11.5 (10.4-12.7)	11.7 (10.5-12.9)	11.9 (10.7-13.2)	12.2 (11-13.3)	12.4 (11.3-13.5)	12.9 (11.9-13.9)	13.4 (12.5-14.1)	14.4 (13.7-15.1)	15.3 (14.5-16)	15.1 (13.9-16.7)	12.3 (11.4-13.6)	11.2 (10.6-11.8)	11.7 (11-12.7)	13.7 (12.5-15.1)
Western sub-Saharan Africa	11.6 (11-12.1)	11.8 (11.1-12.4)	12 (11.3-12.6)	12.3 (11.7-13.1)	12.6 (11.9-13.3)	13 (12.2-13.7)	13.4 (12.6-14.2)	13.6 (12.9-14.4)	13.6 (12.9-14.4)	13.6 (12.8-14.4)	13.6 (12.8-14.5)	14.1 (13.2-15)	14.7 (13.7-15.6)	15.3 (14.4-16.2)
Benin	11.5 (10.4-12.8)	11.6 (10.5-12.8)	11.6 (10.6-12.8)	11.8 (10.8-13.1)	12.1 (11.1-13.3)	12.5 (11.5-13.5)	12.7 (11.9-13.7)	13.1 (12.4-13.9)	13.5 (12.8-14.3)	13.7 (13-14.4)	13.8 (13-14.8)	14.1 (13.1-15.3)	14.4 (13.2-15.7)	14.9 (13.5-16.3)
Burkina Faso	10.5 (9.6-11.6)	10.7 (9.7-11.8)	11 (10-12.1)	11.2 (10.2-12.4)	11.5 (10.5-12.5)	11.7 (10.8-12.6)	12 (11.2-12.8)	12.3 (11.7-12.9)	12.5 (11.9-13.1)	12.6 (11.9-13.3)	12.9 (12.1-13.6)	13.3 (12.6-14.1)	13.8 (13.1-14.7)	14.3 (13.4-15.3)
Cameroon	10.5 (9.6-11.6)	10.7 (9.8-11.8)	10.6 (9.8-11.6)	11.1 (10.3-12.1)	11.3 (10.5-12.2)	11.5 (10.8-12.3)	11.9 (11.3-12.6)	12.5 (11.9-13.2)	12.9 (12.3-13.6)	12.8 (12.2-13.5)	12.5 (11.8-13.3)	12.7 (11.8-13.9)	13.5 (12.3-14.9)	14.8 (13.2-16.3)
Cape Verde	14.3 (12.9-15.8)	14.4 (13.1-15.8)	14.4 (13.1-15.7)	14.7 (13.6-15.9)	14.9 (14-15.9)	15.9 (15.3-16.6)	17.2 (16.9-17.5)	17.9 (17.7-18.2)	18.8 (18.5-19.1)	18.9 (18.3-19.4)	19.5 (18.6-20.2)	19.7 (19.1-19.8)	19.5 (19.5-19.8)	19.7 (19.4-20.4)
Chad	12.1 (10.9-13.5)	12.3 (11-13.8)	12.5 (11.2-13.9)	12.7 (11.5-14.1)	12.7 (11.6-14)	12.9 (11.9-14.1)	12.9 (12.1-13.9)	13 (12.3-13.7)	13 (12.4-13.7)	13 (12.3-13.8)	12.8 (12-13.6)	13 (12.1-13.9)	13.3 (12.3-14.3)	13.8 (12.6-15)
Cote d'Ivoire	11.5 (10.3-12.7)	11.6 (10.5-12.8)	11.8 (10.7-13.1)	12.1 (11-13.3)	12.5 (11.4-13.7)	12.9 (11.9-13.9)	13.2 (12.3-14.2)	13.4 (12.6-14.2)	13.4 (12.6-14.2)	13 (12.1-14)	12.8 (11.9-13.9)	13 (12-14.1)	13.6 (12.6-14.9)	14.7 (13.4-16)
The Gambia	11.9 (10.7-13.1)	12 (10.9-13.3)	12.2 (11-13.5)	12.4 (11.2-13.7)	12.7 (11.4-14.1)	13 (11.7-14.4)	13.2 (12-14.5)	13.5 (12.3-14.7)	13.6 (12.5-14.9)	13.7 (12.7-15)	13.8 (12.7-14.9)	13.8 (12.8-15)	14.1 (13-15.3)	14.5 (13.2-15.8)
Ghana	11.5 (10.4-12.7)	11.7 (10.6-13)	12.1 (11-13.4)	12.5 (11.2-13.9)	12.8 (11.5-14.1)	13.1 (11.8-14.4)	13.2 (12.1-14.5)	13.2 (12.1-14.4)	13.3 (12.3-14.4)	13.5 (12.5-14.5)	13.5 (12.7-14.4)	13.8 (13-14.6)	14.1 (13.2-14.9)	15.2 (14.1-16.3)
Guinea	11 (10-12.2)	11.2 (10.2-12.5)	11.4 (10.3-12.7)	11.6 (10.6-12.8)	11.7 (10.7-12.8)	11.8 (10.9-12.8)	12.1 (11.3-12.9)	12.2 (11.6-12.9)	12.4 (11.8-13)	12.6 (12-13.1)	12.6 (12-13.3)	12.7 (12-13.5)	12.8 (12-13.6)	13.1 (12.1-14.2)
Guinea-Bissau	9.5 (8.9-10.5)	9.8 (9.1-10.8)	10.1 (9.3-11.2)	10.2 (9.4-11.3)	10.5 (9.6-11.6)	10.9 (10-12)	11.1 (10.1-12.3)	11.1 (10.2-12.3)	11.3 (10.4-12.5)	11.5 (10.7-12.6)	11.7 (10.9-12.7)	12 (11.1-12.9)	12.3 (11.3-13.4)	13 (11.8-14.5)
Liberia	11.1 (10.1-12.3)	11.3 (10.3-12.6)	11.5 (10.5-12.8)	11.8 (10.7-13)	12.1 (11.1-13.3)	12.4 (11.4-13.6)	12.7 (11.6-13.8)	12.7 (11.7-13.8)	12.6 (11.7-13.5)	12.3 (11.5-13.1)	12.7 (12-13.4)	13.2 (12.4-14)	13.4 (12.5-14.5)	14.1 (12.8-15.5)
Mali	9.9 (9.1-10.9)	10 (9.1-11)	10.1 (9.2-11.1)	10.1 (9.3-11)	10.1 (9.5-10.9)	10.3 (9.7-11)	10.8 (10.3-11.4)	11.2 (10.7-11.8)	11.5 (11-12)	11.9 (11.4-12.4)	12.5 (12-13.1)	13.4 (12.8-14.1)	14.1 (13.3-14.9)	14.5 (13.4-15.6)
Mauritania	10.8 (9.9-11.9)	10.9 (10-12)	11 (10.1-12.2)	11.4 (10.4-12.6)	11.7 (10.8-12.9)	11.8 (10.9-12.9)	11.9 (11.1-12.9)	12 (11.2-12.9)	12.2 (11.5-13)	12.8 (12-13.4)	13.4 (12.6-14.2)	14.1 (13.3-15)	14.9 (13.9-15.9)	15.5 (14.3-16.7)
Niger	11.3 (10.3-12.4)	11.4 (10.4-12.6)	11.6 (10.6-12.8)	12 (10.9-13.2)	12.1 (11.1-13.2)	12 (11.2-12.9)	12.1 (11.4-12.9)	12.2 (11.5-12.9)	12.6 (11.9-13.3)	12.6 (12-13.3)	13 (12.3-13.7)	13.5 (12.8-14.3)	13.9 (13-14.9)	14.2 (13.1-15.5)
Nigeria	12.1 (11-13.5)	12.4 (11.1-13.7)	12.6 (11.4-14)	13 (11.7-14.5)	13.3 (11.9-14.7)	13.8 (12.5-15.3)	14.3 (12.9-15.8)	14.5 (13.1-16.1)	14.3 (12.8-15.9)	14.2 (12.7-16.1)	14.4 (12.6-16.6)	15 (13-17.7)	15.9 (13.6-18.7)	16.4 (14.3-18.8)
Sao Tome and Principe	11.8 (11.1-12.6)	12.4 (11.9-12.9)	13.4 (12.9-13.8)	15 (14.6-15.4)	15.4 (15.1-15.7)	15.8 (15.5-16.1)	16.2 (15.8-16.6)	16.4 (15.9-17)	15.9 (15.2-16.6)	15.1 (14.3-15.9)	14.5 (13.7-15.2)	14.8 (14-15.5)	15.1 (14.2-16)	15.3 (14.3-16.6)
Senegal	11.7 (10.6-13)	11.8 (10.7-13.1)	12 (10.9-13.3)	12 (10.9-13.3)	12.1 (11.1-13.2)	12.3 (11.4-13.2)	12.6 (11.8-13.5)	13.1 (12.4-13.9)	13.5 (12.9-14.2)	13.7 (13.1-14.3)	14 (13.4-14.5)	14.5 (13.8-15.1)	15 (14.1-15.8)	15.3 (14.2-16.3)
Sierra Leone	11.2 (10.1-12.4)	11.4 (10.3-12.6)	11.7 (10.5-12.8)	12 (10.8-13.3)	12.3 (11.2-13.6)	12.6 (11.5-13.9)	12.8 (11.8-14.1)	13 (11.9-14.2)	13.2 (12.2-14.2)	13 (12.2-13.7)	12.4 (11.8-13.1)	12.2 (11.5-12.8)	12.4 (11.6-13.3)	13.3 (12.2-14.5)
Togo	11.2 (10.1-12.4)	11.3 (10.3-12.6)	11.6 (10.5-12.7)	11.9 (10.8-13.1)	12.4 (11.3-13.6)	12.7 (11.7-13.9)	13 (12.1-14.1)	13.1 (12.2-14)	13.2 (12.4-14.1)	13.5 (12.6-14.4)	13.7 (12.7-14.8)	14 (12.8-15.4)	14.4 (13.1-15.8)	15.1 (13.7-16.5)

Table 11 Estimates of life expectancy at age 65 by location and year for 1950, 1955...2005, 2010, 2017 - males

Location	1950	1955	1960	1965	1970	1975	1980	1985	1990	1995	2000	2005	2010	2017
Global	11.1 (10.9-11.2)	11.5 (11.4-11.6)	11.2 (11.1-11.3)	11.9 (11.8-12)	12.2 (12.1-12.3)	12.6 (12.5-12.6)	12.8 (12.8-12.9)	13.3 (13.2-13.3)	13.6 (13.5-13.6)	13.8 (13.8-13.9)	14.3 (14.2-14.3)	14.8 (14.7-14.8)	15.3 (15.2-15.3)	15.9 (15.8-16)
Low SDI	9.4 (9-9.7)	9.6 (9.3-10)	9.8 (9.5-10.2)	10 (9.7-10.4)	10.3 (10.1-10.6)	10.8 (10.5-11.1)	10.9 (10.6-11.1)	11.2 (11-11.5)	11.4 (11.1-11.6)	11.8 (11.6-12)	12.2 (12-12.3)	12.7 (12.6-12.8)	12.8 (12.6-12.9)	13.7 (13.4-13.9)
Low-middle SDI	10 (9.8-10.3)	10.4 (10.2-10.7)	10.4 (10.2-10.7)	11 (10.8-11.3)	11.4 (11.2-11.6)	11.8 (11.6-12)	11.9 (11.7-12.2)	12.5 (12.3-12.7)	12.6 (12.4-12.8)	12.7 (12.6-12.9)	12.8 (12.6-13)	13.3 (13.1-13.5)	13.5 (13.3-13.7)	14.1 (13.9-14.3)
Middle SDI	10.5 (10.3-10.8)	11.1 (10.8-11.3)	8.8 (8.6-9)	11.6 (11.4-11.9)	12.3 (12.1-12.6)	12.9 (12.7-13.1)	13.2 (13-13.4)	13.6 (13.4-13.7)	13.8 (13.7-13.9)	14.1 (14-14.2)	14.2 (14.1-14.4)	14.6 (14.5-14.7)	14.9 (14.8-15)	15.4 (15.2-15.6)
High-middle SDI	11.2 (10.9-11.5)	11.7 (11.4-12)	11.5 (11.3-11.8)	12.1 (11.8-12.3)	12.4 (12.1-12.5)	12.6 (12.4-12.8)	12.6 (12.5-12.8)	12.9 (12.8-13.1)	13.2 (13-13.3)	13 (12.9-13.1)	13.6 (13.5-13.7)	13.9 (13.9-14)	14.8 (14.7-14.9)	15.6 (15.4-15.9)
High SDI	12.2 (12-12.4)	12.6 (12.5-12.7)	12.7 (12.7-12.7)	12.8 (12.7-12.8)	12.8 (12.8-12.8)	13.2 (13.2-13.2)	13.7 (13.7-13.7)	14.3 (14.3-14.3)	14.9 (14.9-14.9)	15.4 (15.4-15.4)	16.3 (16.3-16.3)	17.2 (17.2-17.2)	18 (18-18)	18.6 (18.5-18.7)
Central Europe, Eastern Europe, and Central Asia	12.2 (12-12.3)	12.5 (12.3-12.6)	13 (12.9-13)	12.8 (12.8-12.9)	12.4 (12.4-12.5)	12.5 (12.4-12.5)	12.2 (12.2-12.2)	12.2 (12.2-12.2)	12.5 (12.5-12.5)	11.6 (11.6-11.7)	12.1 (12.1-12.1)	12.1 (12.1-12.1)	13 (13-13)	14 (14-14.1)
Central Asia	10.7 (10.3-11.1)	11.4 (11.2-11.7)	12 (11.8-12.2)	12.1 (11.8-12.4)	12.4 (12.3-12.5)	12.7 (12.5-12.9)	13.2 (13.1-13.3)	13.2 (13.1-13.2)	13.2 (13.1-13.2)	12 (11.9-12)	12.4 (12.3-12.5)	11.9 (11.8-12)	12.4 (12.3-12.5)	12.8 (12.5-13)
Armenia	11.6 (10.4-12.8)	12.9 (12.2-13.7)	13.8 (13.3-14.4)	14 (13.2-14.8)	14.9 (14.5-15.2)	14.5 (13.8-15.3)	15.2 (14.8-15.5)	14.6 (14.3-14.9)	13.2 (12.9-13.4)	12.5 (12.4-12.5)	13.4 (13.3-13.5)	13.5 (13.4-13.5)	13.7 (13.7-13.8)	14.6 (14.4-14.9)
Azerbaijan	9.6 (8.9-10.5)	10.2 (9.7-10.8)	10.9 (10.6-11.4)	11.5 (10.9-12.2)	12 (11.8-12.2)	12.4 (11.8-12.9)	13.1 (13-13.2)	13.5 (13.5-13.6)	13.6 (13.2-13.9)	11.1 (10.8-11.3)	12.1 (11.8-12.4)	11.8 (11.5-12.1)	11.9 (11.6-12.2)	11.9 (11.4-12.4)
Georgia	10.9 (10-12.1)	11.5 (11-12.2)	12.1 (11.7-12.6)	12.4 (11.7-13.1)	12.8 (12.5-13)	12.9 (12.2-13.5)	13.2 (12.8-13.6)	13.2 (12.9-13.5)	13.2 (12.9-13.6)	13.8 (13.5-14.2)	14.2 (13.9-14.5)	14.3 (14.2-14.4)	13.4 (13.3-13.4)	13.1 (12.9-13.3)
Kazakhstan	10.6 (9.8-11.7)	11.5 (10.8-12.1)	11.8 (11.4-12.3)	11.4 (10.8-12.1)	11.5 (11.3-11.7)	12.1 (11.6-12.5)	12.6 (12.5-12.7)	12.3 (12.3-12.4)	12.3 (12.2-12.3)	10.6 (10.6-10.6)	11.1 (11.1-11.2)	11.2 (11.2-11.2)	11.9 (11.9-12)	13.2 (12.9-13.6)
Kyrgyzstan	9.5 (8.7-10.4)	10 (9.3-10.7)	10.4 (9.7-11.1)	10.6 (9.9-11.5)	10.8 (10.2-11.5)	11.3 (10.6-12.1)	11.9 (11.4-12.4)	12.3 (12-12.8)	12.5 (12.1-12.8)	12 (11.7-12.4)	12.4 (12-12.8)	11.9 (11.7-12.2)	12.6 (12.5-12.6)	13.7 (13.5-13.9)
Mongolia	9.2 (8.3-10.2)	9.7 (8.8-10.6)	10.2 (9.5-11)	10.9 (10.4-11.3)	10.8 (10.4-11.2)	11 (10.6-11.4)	10.9 (10.5-11.3)	10.9 (10.6-11.2)	11.1 (10.8-11.4)	10.1 (9.9-10.4)	10.1 (9.9-10.3)	10.8 (10.6-11)	11 (10.8-11.3)	12.1 (11.5-12.8)
Tajikistan	10.5 (9.6-11.6)	10.9 (10.3-11.5)	11.6 (11.1-12)	12.2 (11.5-12.9)	12.9 (12.7-13.1)	13.5 (13-14)	14.2 (14.1-14.4)	14.9 (14.8-15)	14.4 (14.3-14.6)	13.1 (12.6-13.4)	14.2 (13.9-14.5)	14.2 (13.7-14.6)	14 (13.5-14.5)	13.9 (13.2-14.6)
Turkmenistan	10.6 (9.7-11.8)	11.6 (10.9-12.2)	12.1 (11.6-12.6)	12 (11.3-12.8)	12.6 (12.3-12.8)	12.3 (11.9-12.9)	12.8 (12.7-12.9)	12.6 (12.5-12.7)	12.7 (12.6-12.7)	11.5 (11.4-11.6)	12.7 (12.6-12.7)	11.8 (11.7-11.8)	13.9 (13.8-13.9)	13.3 (12.8-13.9)
Uzbekistan	11.8 (10.8-13)	12.6 (11.9-13.3)	13.1 (12.6-13.6)	13 (12.3-13.8)	13.3 (13.1-13.6)	13.6 (13.1-14.1)	14 (13.9-14.1)	13.8 (13.8-13.9)	14.2 (14.1-14.2)	12.8 (12.8-12.8)	12.3 (12.1-12.6)	11.1 (10.8-11.4)	11.8 (11.5-12.1)	12 (11.2-12.8)
Central Europe	12.1 (11.9-12.3)	12.4 (12.3-12.4)	12.7 (12.7-12.7)	12.7 (12.7-12.8)	12.3 (12.3-12.4)	12.6 (12.6-12.6)	12.3 (12.3-12.3)	12.4 (12.4-12.4)	12.6 (12.6-12.6)	12.8 (12.7-12.8)	13.4 (13.3-13.4)	13.8 (13.8-13.8)	14.5 (14.5-14.5)	15.3 (15.2-15.5)
Albania	13 (12.3-13.9)	14.5 (13.8-15.2)	14.7 (14.2-15.2)	14.8 (14.1-15.6)	15 (13.7-16.4)	14.7 (13.3-16.2)	14.3 (13.2-15.6)	13.8 (13.4-14.2)	13.7 (13.6-13.8)	14.6 (14.5-14.6)	14.7 (14.3-15.1)	15 (14.7-15.4)	15.8 (15.1-16.5)	15.6 (14.4-17.1)
Bosnia and Herzegovina	11.7 (11.6-11.8)	12 (11.9-12.1)	12.1 (12.1-12.2)	11.9 (11.9-12)	11.7 (11.6-11.7)	12.5 (12.5-12.6)	12.4 (12.4-12.5)	13.3 (13.3-13.4)	13.6 (13.6-13.7)	12.7 (11.8-13.7)	14.3 (13.5-15)	14.2 (14.1-14.3)	14.6 (14.5-14.7)	15.1 (14.6-15.5)
Bulgaria	14.6 (14.5-14.6)	14 (13.9-14)	14.1 (14-14.1)	14.2 (14.1-14.3)	13.7 (13.6-13.7)	13 (13-13.1)	13 (13-13.1)	13.1 (13-13.1)	12.8 (12.7-12.8)	12.7 (12.6-12.7)	12.8 (12.8-12.9)	13.1 (13-13.1)	13.8 (13.7-13.9)	14.2 (13.8-14.7)
Croatia	9.8 (9.7-9.8)	11 (10.9-11.1)	11.7 (11.6-11.7)	12.2 (12.2-12.3)	12 (11.9-12)	12.3 (12.3-12.4)	12.2 (12.1-12.3)	12.2 (12.2-12.3)	12.6 (12.6-12.7)	13.2 (13.1-13.3)	13.4 (13.3-13.5)	13.8 (13.8-13.9)	14.7 (14.6-14.8)	15.7 (15.3-16.2)
Czech Republic	12 (12-12.1)	11.9 (11.8-11.9)	11.9 (11.9-12)	11.7 (11.7-11.8)	11 (11-11.1)	11.3 (11.2-11.3)	11.2 (11.1-11.2)	11.4 (11.4-11.4)	11.6 (11.5-11.6)	12.8 (12.7-12.8)	13.7 (13.7-13.8)	14.5 (14.5-14.6)	15.5 (15.4-15.5)	16.3 (15.9-16.8)
Hungary	12.6 (12.5-12.6)	12.8 (12.7-12.8)	12.4 (12.4-12.5)	12.4 (12.3-12.4)	12 (11.9-12)	12 (11.9-12)	11.6 (11.6-11.6)	11.7 (11.7-11.7)	12.1 (12.1-12.1)	12.2 (12.1-12.2)	12.9 (12.8-12.9)	13.2 (13.2-13.3)	14 (13.9-14)	15 (14.6-15.5)
Macedonia	13.7 (12.8-14.6)	13.4 (12.8-14.2)	13 (12.3-13.7)	13.5 (12.8-14.1)	13.9 (13.5-14.2)	14.4 (14.3-14.6)	14 (13.9-14.1)	13.6 (13.5-13.7)	13.6 (13.5-13.7)	13.2 (13.1-13.3)	13.5 (13.4-13.6)	13.7 (13.6-13.8)	14.2 (14.1-14.3)	14.8 (14.3-15.2)
Montenegro	12.5 (11.9-13)	14 (13.6-14.5)	13.8 (13.4-14.3)	13.9 (13.6-14.3)	13.9 (13.5-14.3)	14 (13.6-14.3)	13.7 (13.5-13.9)	13.9 (13.8-14)	14.5 (14.4-14.7)	14.5 (14.3-14.6)	14.3 (14.1-14.6)	14.4 (14-14.8)	15 (14.6-15.4)	14.8 (14-15.5)
Poland	11.5 (11.4-11.5)	11.9 (11.9-12)	12.9 (12.9-12.9)	13 (13-13)	12.6 (12.6-12.6)	12.9 (12.8-12.9)	12.3 (12.3-12.3)	12.4 (12.4-12.5)	12.6 (12.6-12.6)	12.9 (12.9-12.9)	13.7 (13.6-13.7)	14.4 (14.3-14.4)	15.1 (15-15.1)	16 (15.5-16.4)
Romania	12.4 (11.2-13.8)	12.4 (12-12.8)	12.9 (12.9-13)	12.8 (12.8-12.8)	12.6 (12.6-12.6)	13 (13-13)	12.8 (12.8-12.8)	12.8 (12.8-12.8)	13.1 (13-13.1)	12.6 (12.6-12.7)	13.3 (13.3-13.3)	13.5 (13.5-13.5)	14 (14-14.1)	14.7 (14.3-15.1)
Serbia	12.2 (12.2-12.3)	12.3 (12.2-12.3)	12.3 (12.3-12.4)	12.5 (12.4-12.5)	12 (12-12.1)	12.9 (12.8-12.9)	12.4 (12.3-12.4)	12.2 (12.1-12.2)	12.4 (12.4-12.5)	12.4 (12.4-12.5)	12.7 (12.6-12.7)	13 (12.9-13)	13.6 (13.5-13.6)	14.6 (14.2-15)
Slovakia	12.1 (11.4-13)	12.7 (12.1-13.3)	12.8 (12.2-13.5)	12.6 (12-13.3)	12.3 (11.7-12.8)	12.5 (12.4-12.5)	12.3 (12.2-12.3)	12.4 (12.3-12.5)	12.2 (12.2-12.3)	12.7 (12.7-12.8)	13 (12.9-13.1)	13.4 (13.4-13.5)	14.2 (14.1-14.2)	15.4 (14.9-15.8)
Slovenia	9.8 (9.8-9.9)	11.3 (11.3-11.4)	11.5 (11.4-11.6)	12.2 (12.1-12.3)	11.9 (11.8-12)	12.5 (12.4-12.5)	12.5 (12.4-12.6)	12.6 (12.5-12.7)	13.4 (13.3-13.5)	13.7 (13.6-13.8)	14.2 (14.1-14.3)	15.3 (15.2-15.4)	16.5 (16.3-16.6)	17.5 (17-18)
Eastern Europe	12.7 (12.3-13)	12.8 (12.5-13.2)	13.5 (13.4-13.6)	13.1 (13-13.2)	12.5 (12.4-12.6)	12.3 (12.2-12.4)	11.9 (11.9-12)	11.9 (11.9-12)	12.3 (12.2-12.3)	10.9 (10.9-10.9)	11.3 (11.3-11.3)	11.2 (11.2-11.2)	12.1 (12.1-12.1)	13.4 (13.2-13.5)
Belarus	14.6 (13.2-16.2)	14.9 (13.8-16.1)	15.1 (14.9-15.3)	14.9 (14.9-15)	14.4 (14.3-14.4)	13.9 (13.9-14)	13.5 (13.4-13.5)	13.1 (13-13.1)	13.2 (13.2-13.2)	11.9 (11.8-11.9)	11.7 (11.7-11.8)	11.3 (11.3-11.4)	11.8 (11.8-11.9)	13 (12.7-13.4)
Estonia	12 (10.7-13.4)	11.9 (10.8-13)	12.2 (12.1-12.3)	12.4 (12.2-12.5)	12.1 (12-12.2)	12.1 (12-12.1)	11.9 (11.9-12)	11.9 (11.8-12)	12.1 (12-12.2)	11.7 (11.6-11.7)	12.7 (12.6-12.7)	13.2 (13.1-13.3)	14.3 (14.2-14.5)	15.9 (15-16.9)
Latvia	13.2 (11.9-14.7)	13.4 (12.3-14.9)	13.3 (13.2-13.4)	13.3 (13.2-13.4)	13.1 (13-13.2)	12.5 (12.4-12.6)	12.3 (12.2-12.4)	12.2 (12.1-12.3)	12.3 (12.2-12.3)	11.4 (11.3-11.4)	12.6 (12.5-12.6)	12.3 (12.2-12.4)	13.4 (13.3-13.5)	14.2 (13.4-15.1)
Lithuania	14.2 (12.8-15.8)	14.3 (13-15.7)	14.7 (14.6-14.8)	14.7 (14.6-14.8)	14.3 (14.2-14.4)	14 (14-14.1)	13.5 (13.5-13.6)	13.2 (13.2-13.3)	13.4 (13.3-13.4)	12.6 (12.6-12.7)	13.6 (13.6-13.7)	13.4 (13.3-13.4)	13.7 (13.7-13.8)	14.3 (13.8-14.7)
Moldova	10.5 (9.6-11.5)	10.9 (10.3-11.4)	11.2 (10.8-11.6)	11.3 (10.7-12)	11.5 (11.3-11.8)	12 (11.6-12.4)	12.3 (12.2-12.3)	12 (11.9-12)	12.6 (12.6-12.7)	11.3 (11.2-11.3)	12.3 (12.2-12.3)	12.1 (12-12.1)	12.2 (12.2-12.3)	13.6 (13.4-13.8)
Russian Federation	12.4 (12.1-12.6)	12.6 (12.3-12.8)	13.1 (12.9-13.3)	12.7 (12.5-12.9)	12 (11.9-12.2)	11.9 (11.7-12)	11.5 (11.4-11.7)	11.6 (11.5-11.7)	12 (12-12)	10.8 (10.7-10.8)	11 (11-11)	10.9 (10.9-10.9)	11.9 (11.9-11.9)	13.6 (13.6-13.7)
Ukraine	13.1 (11.8-14.5)	13.2 (12-14.4)	14.1 (14-14.1)	13.6 (13.6-13.7)	12.9 (12.8-12.9)	12.8 (12.7-12.8)	12.3 (12.3-12.4)	12.3 (12.2-12.4)	12.5 (12.4-12.6)	11 (11-11.1)	11.7 (11.7-11.8)	11.6 (11.6-11.7)	12.4 (12.3-12.4)	12.5 (12.2-12.9)
High-income	12.2 (12-12.4)	12.6 (12.6-12.7)	12.7 (12.7-12.8)	12.7 (12.7-12.8)	12.8 (12.8-12.8)	13.2 (13.2-13.2)	13.8 (13.8-13.8)	14.4 (14.4-14.4)	15 (15-15)	15.5 (15.5-15.5)	16.4 (16.3-16.4)	17.3 (17.3-17.3)	18.1 (18.1-18.1)	18.6 (18.5-18.7)
Australasia	12.5 (12.5-12.6)	12.5 (12.5-12.6)	12.7 (12.6-12.7)	12.3 (12.3-12.4)	12.2 (12.2-12.2)	12.8 (12.8-12.9)	13.6 (13.6-13.7)	14.2 (14.2-14.3)	15.1 (15-15.1)	15.8 (15.8-15.9)	17 (16.9-17)	18.2 (18.2-18.3)	19 (18.9-19)	19.4 (18.7-20.2)
Australia	12.4 (12.4-12.5)	12.4 (12.3-12.4)	12.6 (12.5-12.6)	12.3 (12.2-12.3)	12.1 (12.1-12.2)	12.8 (12.8-12.9)	13.7 (13.7-13.8)	14.3 (14.3-14.4)	15.1 (15.1-15.2)	15.9 (15.9-16)	17.1 (17-17.1)	18.3 (18.3-18.4)	19.1 (19-19.1)	19.4 (18.6-20.3)
New Zealand	13 (12.8-13.1)	13.2 (13-13.3)	13 (12.9-13.1)	12.7 (12.6-12.8)	12.5 (12.4-12.6)	13 (12.9-13.1)	13.3 (13.2-13.4)	13.8 (13.7-13.9)	14.8 (14.6-14.9)	15.2 (15.1-15.3)	16.5 (16.3-16.6)	17.7 (17.6-17.8)	18.6 (18.4-18.7)	19.2 (18.8-19.7)
High-income Asia-Pacific	10.5 (10.3-10.7)	11.6 (11.3-11.8)	11.6 (11.3-11.8)	12 (11.7-12.2)	12.5 (12.3-12.8)	13.5 (13.4-13.6)	14.3 (14.3-14.3)	15.1 (15.1-15.2)	15.8 (15.8-15.9)	16.4 (16.3-16.4)	17.3 (17.3-17.3)	18 (18-18)	18.7 (18.7-18.7)	19.5 (19.4-19.7)
Brunei	10.1 (9.2-11.2)	10.3 (9.5-11.1)	10.7 (10.1-11.4)	11.6 (11.1-12.1)	11.9 (11.6-12.3)	12.1 (11.8-12.5)	12.4 (12.1-12.8)	12.5 (12.1-12.8)	12.5 (12.1-12.8)	13.4 (13.1-13.7)	14.1 (13.8-14.5)	14.6 (14.2-15)	15 (14.5-15.5)	15.3 (14.4-16.2)
Japan	10.9 (10.7-11.1)	12 (11.8-12.2)	11.9 (11.7-12.1)	12.3 (12-12.5)	12.8 (12.6-13)	13.9 (13.8-14.1)	14.8 (14.8-14.9)	15.8 (15.8-15.8)	16.4 (16.4-16.4)	16.8 (16.8-16.8)	17.7 (17.7-17.7)	18.3 (18.3-18.4)	18.9 (18.9-18.9)	19.7 (19.5-19.9)
Aichi	11.2 (10-12.6)	12.5 (11.2-14)	12 (10.7-13.5)	12.3 (11-13.8)	12.9 (11.8-14.3)	13.9 (13.2-14.7)	14.6 (14.6-14.7)	15.7 (15.7-15.8)	16.3 (16.2-16.3)	16.6 (16.6-16.7)	17.6 (17.5-17.6)	18.2 (18.2-18.3)	18.8 (18.7-18.9)	20 (19-20.9)
Akita	10.6 (9.4-12)	11.6 (10.2-13)	11.6 (10.3-12.9)	11.9 (10.7-13.3)	12.4 (11.3-13.8)	13.5 (12.7-14.4)	14.5 (14.4-14.6)	15.2 (15-15.3)	16 (15.9-16.2)	16.3 (16.1-16.4)	17 (16.8-17.1)	17.6 (17.5-17.7)	18.1 (18-18.3)	18.8 (17.8-19.8)
Aomori	10 (8.9-11.3)	11.1 (9.8-12.5)	11.1 (10-12.5)	11.4 (10.2-12.8)	11.9 (10.7-13.2)	12.9 (12.1-13.6)	13.9 (13.8-14)	14.9 (14.8-15)	15.8 (15.7-16)	16 (15.8-16.1)	16.8 (16.7-16.9)	17.2 (17.1-17.3)	17.8 (17.7-17.9)	18.5 (17.4-19.5)
Chiba	11 (9.9-12.4)	12.2 (10.9-13.6)	11.9 (10.6-13.3)	12.3 (11-13.6)	12.8 (11.6-14)	13.7 (13-14.6)	14.7 (14.7-14.8)	15.9 (15.8-15.9)	16.5 (16.5-16.6)	16.8 (16.7-16.9)	17.8 (17.8-17.9)	18.4 (18.3-18.5)	19.1 (19-19.1)	19.8 (18.9-20.7)
Ehime	10.8 (9.6-12.2)	11.7 (10.4-13.3)	12 (10.6-13.5)	12.4 (11-13.8)	12.8 (11.6-14.2)	13.9 (13.1-14.8)	15.1 (15-15.2)	16.2 (16.1-16.3)	16.7 (16.6-16.8)	17.1 (16.9-17.2)	17.9 (17.7-18)	18.3 (18.1-18.4)	18.9 (18.8-19)	19.6 (18.6-20.7)
Fukui	11.5 (10.3-12.9)	12.6 (11.3-14.2)	12.3 (11.1-13.7)	12.7 (11.5-13.9)	13.3 (12.3-14.2)	14.2 (13.7-14.8)	14.9 (14.8-15)	16 (15.9-16.1)	16.7 (16.6-16.8)	17.3 (17.2-17.5)	18 (17.9-18.2)	18.6 (18.5-18.8)	19.1 (19-19.3)	19.9 (18.9-20.8)
Fukuoka	10.5 (9.3-11.8)	11.5 (10.2-13)	11.6 (10.4-13)	12 (10.8-13.4)	12.6 (11.4-14)	13.7 (12.9-14.5)	14.7 (14.6-14.7)	15.7 (15.6-15.7)	16.2 (16.1-16.3)	16.6 (16.6-16.7)	17.5 (17.4-17.5)	18.1 (18-18.2)	18.7 (18.6-18.8)	19.9 (18.9-20.8)
Fukushima	10.7 (9.5-12)	11.8 (10.5-13.2)	11.7 (10.4-13)	12 (10.8-13.5)	12.5 (11.3-13.8)	13.5 (12.8-14.3)	14.5 (14.4-14.5)	15.6 (15.5-15.7)	16.5 (16.4-16.6)	16.8 (16.7-16.9)	17.5 (17.4-17.6)	18 (17.9-18.1)	18.7 (18.6-18.8)	19.4 (18.4-20.4)
Gifu	11.3 (10.1-12.6)	12.5 (11.2-14)	12.2 (10.9-13.6)	12.6 (11.2-14)	13.2 (11.9-14.5)	14.2 (13.4-15)	14.9 (14.8-15)	16 (15.9-16.1)	16.8 (16.7-16.9)	17 (16.8-17.1)	17.8 (17.7-17.9)	18.4 (18.3-18.5)	19.1 (19-19.3)	19.5 (18.6-20.5)
Gunma	11.1 (10-12.5)	12.3 (10.9-13.8)	12 (10.6-13.5)	12.3 (11-13.7)	12.7 (11.5-14.1)	13.7 (13-14.5)	14.8 (14.7-14.9)	15.8 (15.7-15.9)	16.6 (16.5-16.7)	17 (16.9-17.1)	17.7 (17.6-17.9)	18.3 (18.2-18.5)	18.9 (18.7-19)	19.3 (18.3-20.3)
Hiroshima	10.9 (9.8-12.3)	11.9 (10.7-13.4)	12 (10.7-13.5)	12.4 (11.2-13.7)	12.9 (11.7-14.2)	14 (13.2-14.8)	15 (15-15.1)	16 (15.9-16.1)	16.7 (16.6-16.8)	17 (16.9-17.1)	17.8 (17.7-17.9)	18.5 (18.4-18.6)	19.1 (19-19.2)	20.3 (19.3-21.3)
Hokkaidō	10.6 (9.6-12)	11.7 (10.5-13.2)	11.7 (10.4-13.1)	12 (10.8-13.5)	12.5 (11.3-13.9)	13.6 (12.8-14.3)	14.7 (14.6-14.7)	15.7 (15.6-15.7)	16.3 (16.3-16.4)	16.9 (16.8-17)	17.9 (17.8-17.9)	18.3 (18.2-18.4)	18.9 (18.8-19)	19.7 (18.8-20.6)
Hyōgo	10.7 (9.5-12)	11.8 (10.7-13.1)	11.7 (10.4-13.1)	12.1 (10.8-13.6)	12.7 (11.4-13.9)	13.8 (13.1-14.6)	14.6 (14.5-14.6)	15.4 (15.4-15.5)	16.1 (16.1-16.2)	16.1 (16-16.2)	17.5 (17.5-17.6)	18.2 (18.1-18.3)	18.9 (18.8-19)	19.8 (18.8-20.7)
Ibaraki	10.8 (9.7-12.1)	11.9 (10.7-13.3)	11.9 (10.6-13.4)	12.2 (11-13.8)	12.8 (11.5-14.3)	14 (13.2-14.9)	15 (14.9-15.1)	15.6 (15.5-15.6)	16.3 (16.3-16.4)	16.7 (16.6-16.8)	17.4 (17.3-17.5)	18 (17.9-18.1)	18.7 (18.6-18.8)	19.2 (18.2-20.1)
Ishikawa	11.4 (10.2-12.7)	12.4 (11.1-13.9)	12.2 (10.9-13.6)	12.5 (11.2-14)	13.1 (12-14.3)	14.5 (13.8-15.1)	15.9 (15.8-16)	16.3 (16.2-16.4)	16.7 (16.6-16.8)	16.9 (16.8-17)	17.8 (17.7-17.9)	18.5 (18.4-18.7)	19.2 (19-19.3)	19.8 (18.8-20.7)
Iwate	10.7 (9.6-12.1)	11.8 (10.6-13.3)	11.8 (10.5-13.3)	12.2 (10.9-13.6)	12.7 (11.5-14.1)	13.8 (13-14.7)	14.7 (14.6-14.8)	15.5 (15.4-15.7)	16.3 (16.2-16.4)	16.7 (16.6-16.9)	17.5 (17.4-17.6)	17.9 (17.7-18)	18.5 (18.3-18.7)	19.6 (18.6-20.7)
Kagawa	11.4 (10.1-12.8)	12.5 (11.2-14.1)	12.4 (11.2-14)	12.8 (11.6-14.3)	13.4 (12.3-14.7)	14.5 (13.9-15.1)	15.3 (15.2-15.4)	16.4 (16.3-16.5)	16.7 (16.6-16.8)	17.3 (17.2-17.4)	18 (17.9-18.2)	18.5 (18.3-18.6)	19.1 (19-19.3)	19.8 (18.8-20.7)
Kagoshima	10.4 (9.3-11.6)	11.4 (10.3-12.8)	11.5 (10.2-13)	11.9 (10.5-13.3)	12.3 (11.1-13.7)	13.4 (12.7-14.2)	14.6 (14.5-14.7)	15.6 (15.5-15.7)	16.3 (16.2-16.5)	16.8 (16.7-16.9)	17.6 (17.5-17.8)	18.2 (18.1-18.4)	18.9 (18.8-19)	19.5 (18.6-20.5)
Kanagawa	11.3 (10.1-12.7)	12.4 (11.1-13.9)	12.2 (10.8-13.7)	12.6 (11.3-14.1)	13.2 (11.9-14.6)	14.4 (13.7-15.2)	15.1 (15-15.2)	16 (16-16.1)	16.7 (16.7-16.8)	17 (16.9-17)	17.9 (17.9-18)	18.7 (18.6-18.7)	19.2 (19.1-19.3)	20.2 (19.3-21.2)
Kōchi	10.4 (9.3-11.6)	11.3 (10.2-12.7)	11.6 (10.3-12.9)	11.9 (10.7-13.3)	12.4 (11.3-13.6)	13.5 (12.8-14.1)	14.8 (14.6-14.9)	16 (15.9-16.1)	16.6 (16.4-16.7)	17 (16.9-17.1)	17.7 (17.6-17.9)	18.2 (18.1-18.3)	18.5 (18.4-18.7)	20 (19-21)
Kumamoto	10.7 (9.6-12.1)	11.9 (10.5-13.4)	11.9 (10.6-13.3)	12.3 (11.1-13.8)	12.8 (11.7-14.3)	13.8 (13-14.7)	15 (14.9-15.1)	16.2 (16.1-16.3)	16.9 (16.8-17)	17.5 (17.4-17.6)	18.3 (18.2-18.4)	19 (18.9-19.1)	19.6 (19.4-19.7)	20 (19-21.1)
Kyōto	11.4 (10.3-12.9)	12.6 (11.2-14.1)	12.4 (11.1-14)	12.8 (11.3-14.3)	13.5 (12.2-14.9)	14.9 (14.1-15.8)	16 (15.9-16.1)	16.4 (16.3-16.5)	16.6 (16.5-16.7)	16.9 (16.8-17)	17.7 (17.6-17.8)	18.5 (18.4-18.6)	19.3 (19.2-19.4)	20.4 (19.4-21.4)
Mie	11.2 (10-12.5)	12.4 (11-13.9)	12.2 (10.8-13.6)	12.5 (11.2-14.1)	13.2 (11.9-14.6)	14.2 (13.4-15.2)	14.9 (14.8-15)	15.8 (15.7-15.9)	16.4 (16.3-16.5)	16.7 (16.6-16.8)	17.7 (17.5-17.8)	18.3 (18.2-18.4)	18.9 (18.7-19)	19.5 (18.5-20.5)
Miyagi	10.9 (9.8-12.2)	12.1 (10.7-13.4)	11.7 (10.4-13.2)	12 (10.7-13.4)	12.5 (11.2-13.8)	13.3 (12.6-14.1)	14.4 (14.3-14.5)	15.7 (15.6-15.8)	16.5 (16.4-16.5)	16.9 (16.8-17)	17.7 (17.6-17.8)	18.3 (18.2-18.4)	19 (18.9-19.1)	19.7 (18.8-20.7)
Miyazaki	10.6 (9.4-12)	11.6 (10.4-13)	11.8 (10.5-13.2)	12.2 (10.9-13.6)	12.8 (11.6-14.2)	13.9 (13.1-14.9)	14.8 (14.7-14.9)	16 (15.8-16.1)	16.5 (16.4-16.7)	16.9 (16.7-17)	18 (17.8-18.1)	18.7 (18.5-18.8)	19.1 (18.9-19.2)	19.6 (18.6-20.6)
Nagano	11.7 (10.4-13)	12.8 (11.5-14.3)	12.5 (11.2-13.9)	12.8 (11.5-14.2)	13.3 (12-14.6)	14.2 (13.3-15)	15.5 (15.4-15.6)	16.2 (16.1-16.3)	17.2 (17.1-17.3)	17.6 (17.5-17.7)	18.6 (18.4-18.7)	19.1 (19-19.2)	19.7 (19.6-19.9)	20.2 (19.3-21.2)
Nagasaki	10.4 (9.2-11.6)	11.4 (10.2-12.8)	11.4 (10.1-12.9)	11.7 (10.6-13.2)	12.3 (11-13.6)	13.3 (12.5-14.2)	14.3 (14.2-14.4)	15.5 (15.3-15.6)	16.1 (16-16.2)	16.6 (16.5-16.7)	17.5 (17.4-17.7)	18.3 (18.2-18.4)	18.8 (18.7-19)	19.5 (18.5-20.5)
Nara	10.7 (9.5-12.1)	11.9 (10.6-13.3)	11.5 (10.3-12.9)	11.9 (10.6-13.3)	12.4 (11.1-13.8)	13.5 (12.7-14.4)	14.6 (14.5-14.7)	15.4 (15.3-15.5)	16.3 (16.2-16.4)	16.9 (16.8-17)	17.9 (17.8-18.1)	18.4 (18.3-18.5)	19.1 (19-19.3)	20 (19-21)
Niigata	10.9 (9.8-12.2)	12 (10.8-13.4)	11.7 (10.4-13.1)	12 (10.7-13.4)	12.4 (11.1-13.7)	13.3 (12.5-14.1)	14.4 (14.3-14.5)	15.6 (15.5-15.7)	16.6 (16.5-16.7)	17 (16.9-17.1)	17.8 (17.7-17.9)	18.4 (18.3-18.5)	19 (18.9-19.1)	19.8 (18.8-20.8)
Ōita	10.9 (9.7-12.1)	12 (10.7-13.4)	12.1 (10.9-13.6)	12.6 (11.3-14.2)	13.3 (12.1-14.7)	14.5 (13.7-15.5)	15 (14.9-15.1)	15.9 (15.8-16.1)	16.7 (16.5-16.8)	17 (16.9-17.1)	17.8 (17.7-17.9)	18.7 (18.5-18.8)	19.2 (19-19.3)	20.6 (19.6-21.6)
Okayama	11.2 (10.1-12.6)	12.4 (11.1-13.8)	12.3 (11-13.7)	12.8 (11.5-14.2)	13.4 (12.2-14.8)	14.5 (13.7-15.3)	15.2 (15.1-15.3)	15.9 (15.8-16)	16.8 (16.7-16.9)	17.2 (17.1-17.3)	18 (17.9-18.1)	18.7 (18.6-18.8)	19.1 (19-19.2)	19.8 (18.8-20.8)
Okinawa	10.5 (9.4-11.9)	11.4 (10.1-12.9)	11.7 (10.5-13.3)	11.8 (10.5-13.4)	11.8 (10.6-13.2)	12.4 (11.6-13.2)	15.3 (15.2-15.4)	17.1 (17-17.3)	17.5 (17.4-17.6)	17.9 (17.8-18)	18.5 (18.3-18.6)	19.2 (19-19.3)	19.4 (19.2-19.5)	20.1 (19.1-21.1)
Ōsaka	10.4 (9.3-11.7)	11.6 (10.4-13)	11.4 (10.3-12.8)	11.8 (10.6-13.1)	12.5 (11.4-13.7)	13.6 (12.9-14.4)	14.1 (14.1-14.2)	15 (14.9-15)	15.6 (15.6-15.7)	16.1 (16-16.2)	17.1 (17-17.1)	17.9 (17.8-18)	18.4 (18.3-18.5)	19.2 (18.2-20.1)
Saga	10.7 (9.6-12)	11.8 (10.6-13.3)	11.8 (10.5-13.2)	12.2 (11-13.5)	12.8 (11.8-14)	13.9 (13.3-14.6)	14.7 (14.6-14.8)	15.7 (15.6-15.8)	16.4 (16.3-16.5)	16.7 (16.6-16.9)	17.5 (17.4-17.6)	18.3 (18.1-18.4)	18.8 (18.6-18.9)	19.7 (18.7-20.6)
Saitama	11.2 (10-12.5)	12.4 (11.1-13.8)	12 (10.7-13.4)	12.4 (11.1-13.9)	13.2 (11.9-14.6)	14.6 (13.8-15.4)	14.7 (14.6-14.8)	15.6 (15.5-15.6)	16.3 (16.2-16.4)	16.7 (16.6-16.8)	17.7 (17.6-17.8)	18.3 (18.2-18.4)	18.9 (18.8-19)	19.6 (18.7-20.5)
Shiga	11.3 (10-12.8)	12.4 (11.1-13.9)	12 (10.8-13.4)	12.2 (11-13.6)	12.7 (11.7-14)	14 (13.4-14.7)	15.8 (15.7-15.9)	16.3 (16.2-16.4)	16.3 (16.2-16.4)	16.8 (16.7-16.9)	17.6 (17.5-17.7)	18.4 (18.3-18.5)	19.3 (19.2-19.4)	20.1 (19.2-21)
Shimane	10.9 (9.8-12.4)	12 (10.9-13.5)	12.2 (11-13.6)	12.5 (11.3-13.9)	13.1 (12.1-14.3)	14.2 (13.5-14.9)	15.2 (15.1-15.4)	16.4 (16.3-16.6)	17 (16.9-17.2)	17.4 (17.2-17.5)	18.1 (17.9-18.2)	18.5 (18.4-18.7)	19.3 (19.1-19.5)	20.3 (19.3-21.3)
Shizuoka	11.2 (10-12.7)	12.4 (11.1-13.8)	12.2 (10.9-13.6)	12.6 (11.4-14)	13.2 (12-14.5)	14.3 (13.5-15)	15 (15-15.1)	16.1 (16-16.2)	16.8 (16.8-16.9)	17.2 (17.1-17.2)	17.9 (17.8-18)	18.6 (18.5-18.7)	19.1 (19-19.2)	19.6 (18.6-20.6)
Tochigi	10.9 (9.7-12.2)	11.9 (10.6-13.3)	11.8 (10.5-13.1)	12.2 (10.9-13.6)	12.8 (11.5-14.1)	13.9 (13.1-14.8)	14.9 (14.8-15)	15.4 (15.3-15.5)	16.1 (16-16.2)	16.5 (16.4-16.6)	17.5 (17.4-17.6)	17.8 (17.6-17.9)	18.5 (18.4-18.6)	19.4 (18.4-20.4)
Tokushima	10.6 (9.5-11.8)	11.6 (10.3-13)	11.6 (10.4-13.1)	12 (10.8-13.4)	12.6 (11.5-13.7)	13.7 (13-14.3)	14.6 (14.5-14.7)	15.6 (15.5-15.8)	16.3 (16.2-16.4)	16.8 (16.7-16.9)	17.6 (17.4-17.7)	18.3 (18.2-18.5)	19 (18.8-19.1)	19.5 (18.5-20.5)
Tōkyō	11.3 (10.1-12.7)	12.5 (11.2-14)	12.3 (11.1-13.8)	12.6 (11.5-14)	13.3 (12.1-14.6)	14.3 (13.6-15)	15.1 (15.1-15.2)	16.1 (16-16.1)	16.4 (16.4-16.5)	16.8 (16.8-16.9)	17.9 (17.8-17.9)	18.7 (18.6-18.7)	19 (18.9-19.1)	19.7 (18.8-20.7)
Tottori	10.7 (9.6-12)	11.9 (10.6-13.2)	12 (10.8-13.3)	12.5 (11.4-13.7)	13.1 (12.2-14.1)	14.1 (13.6-14.6)	14.8 (14.7-14.9)	15.7 (15.6-15.8)	16.4 (16.3-16.5)	16.8 (16.6-16.9)	17.6 (17.5-17.8)	18.1 (18-18.2)	18.8 (18.7-19)	19.5 (18.6-20.5)
Toyama	10.9 (9.8-12.2)	12 (10.8-13.6)	11.7 (10.5-13.1)	12 (10.9-13.4)	12.6 (11.5-13.7)	13.7 (13.1-14.4)	14.6 (14.5-14.7)	15.6 (15.5-15.7)	16.4 (16.3-16.5)	17 (16.8-17.1)	18 (17.9-18.1)	18.5 (18.4-18.6)	19 (18.8-19.1)	19.5 (18.6-20.5)
Wakayama	10.6 (9.4-11.9)	11.7 (10.5-13.2)	11.8 (10.6-13.1)	12.2 (11-13.5)	12.8 (11.7-13.9)	13.8 (13.2-14.4)	14.6 (14.5-14.7)	15.4 (15.3-15.5)	16.1 (16-16.2)	16.5 (16.4-16.6)	17.5 (17.4-17.7)	18 (17.8-18.1)	18.3 (18.1-18.4)	19.2 (18.3-20.1)
Yamagata	11.1 (9.8-12.6)	12.2 (10.9-13.9)	12 (10.6-13.6)	12.4 (11-14)	13.1 (11.9-14.5)	14.4 (13.5-15.3)	15.5 (15.4-15.6)	16.3 (16.2-16.5)	16.6 (16.5-16.7)	17 (16.8-17.1)	17.8 (17.6-17.9)	18.3 (18.2-18.5)	19.2 (19-19.3)	19.7 (18.7-20.8)
Yamaguchi	10.6 (9.5-11.9)	11.7 (10.5-13.2)	11.8 (10.6-13.2)	12.2 (11.1-13.8)	12.8 (11.6-14.3)	14 (13.2-14.8)	14.7 (14.6-14.8)	15.9 (15.8-16)	16.4 (16.3-16.5)	16.8 (16.7-16.9)	17.4 (17.3-17.5)	18 (17.8-18.1)	18.5 (18.4-18.7)	19.5 (18.5-20.5)
Yamanashi	11 (9.8-12.3)	12 (10.7-13.5)	12.1 (10.8-13.5)	12.4 (11.1-13.7)	12.9 (11.8-14)	14.1 (13.4-14.7)	14.9 (14.7-15)	16 (15.9-16.2)	16.8 (16.7-16.9)	17.2 (17.1-17.3)	18.1 (18-18.2)	18.6 (18.5-18.8)	19.2 (19-19.3)	20 (19-21)
South Korea	8.4 (7.8-9.2)	9.3 (8.6-10.2)	9.7 (8.9-10.7)	10 (9.2-11)	10.5 (9.7-11.4)	10.8 (10.6-11.1)	10.8 (10.8-10.8)	11.4 (11.4-11.4)	12.4 (12.4-12.4)	13.6 (13.6-13.6)	14.6 (14.6-14.7)	16.1 (16.1-16.1)	17.2 (17.1-17.4)	18.5 (18-19)
Singapore	9.3 (8.7-9.8)	10.3 (10.2-10.4)	10.3 (10.2-10.4)	11 (10.9-11.1)	10.9 (10.8-11)	11.3 (11.3-11.4)	12.6 (12.5-12.7)	13.7 (13.6-13.8)	14.7 (14.6-14.8)	15.1 (15-15.2)	16.3 (15.7-16.9)	17.8 (17.7-17.9)	19 (18.8-19.1)	20.2 (19.7-20.7)
High-income North America	12.9 (12.6-13.1)	13.2 (13-13.4)	13 (13-13)	13.1 (13.1-13.1)	13.2 (13.2-13.3)	13.8 (13.8-13.8)	14.2 (14.2-14.2)	14.6 (14.6-14.6)	15.3 (15.3-15.3)	15.6 (15.6-15.6)	16.2 (16.2-16.2)	17.1 (17.1-17.1)	18 (17.9-18)	18.2 (18-18.4)
Canada	13.4 (13.4-13.5)	13.6 (13.6-13.7)	13.6 (13.6-13.7)	13.7 (13.6-13.7)	13.8 (13.8-13.9)	14 (13.9-14)	14.6 (14.6-14.6)	14.9 (14.9-14.9)	15.5 (15.4-15.5)	15.9 (15.8-15.9)	16.8 (16.7-16.8)	17.8 (17.8-17.8)	18.8 (18.8-18.8)	19.5 (19-19.9)
Greenland	9.1 (8.9-9.4)	9.9 (9.7-10.1)	11.2 (11.1-11.4)	12.2 (12-12.4)	12.5 (12.3-12.7)	11.6 (11.5-11.8)	11.2 (11-11.4)	11.1 (10.9-11.2)	11.6 (11.4-11.8)	12.4 (12.2-12.6)	13 (12.8-13.2)	13.7 (13.5-13.9)	14.2 (14-14.3)	14.2 (14-15)

Table 11 Estimates of life expectancy at age 65 by location and year for 1950, 1955...2005, 2010, 2017 - males

Location	1950	1955	1960	1965	1970	1975	1980	1985	1990	1995	2000	2005	2010	2017
USA	12.9 (12.6-13.1)	13.2 (12.9-13.4)	13 (12.9-13)	13 (13-13)	13.2 (13.2-13.2)	13.8 (13.7-13.8)	14.2 (14.2-14.2)	14.5 (14.5-14.6)	15.3 (15.3-15.3)	15.6 (15.6-15.6)	16.1 (16.1-16.2)	17 (17-17)	17.9 (17.8-17.9)	18.1 (17.9-18.3)
Alabama	12.6 (11.3-14.1)	12.9 (11.8-14.3)	12.9 (12.8-13)	12.8 (12.7-12.9)	13 (12.9-13.1)	13.4 (13.4-13.5)	13.7 (13.6-13.8)	13.9 (13.9-14)	14.4 (14.3-14.5)	14.7 (14.6-14.7)	14.9 (14.8-15)	15.6 (15.5-15.6)	16.1 (16-16.2)	16.2 (15.3-17.2)
Alaska	13.4 (12.2-14.8)	13.7 (12.8-14.6)	12.9 (12.7-13)	12.8 (12.7-12.9)	13.3 (13.1-13.4)	14.2 (14-14.3)	13.8 (13.7-14)	14.2 (14.1-14.3)	15.1 (14.9-15.2)	15.6 (15.5-15.8)	16.4 (16.2-16.5)	17.4 (17.2-17.5)	17.9 (17.8-18.1)	18.2 (17.5-18.9)
Arizona	13.2 (11.9-14.6)	13.5 (12.3-14.9)	13.3 (13.2-13.5)	13.5 (13.4-13.6)	13.7 (13.6-13.8)	14.7 (14.6-14.8)	15 (14.9-15.1)	15.8 (15.7-15.9)	16.3 (16.2-16.4)	16.5 (16.4-16.6)	17.1 (17.1-17.2)	17.9 (17.8-17.9)	18.9 (18.8-18.9)	18.8 (17.8-19.8)
Arkansas	13.6 (12.2-15.1)	13.9 (12.6-15.2)	13.8 (13.7-13.9)	13.6 (13.5-13.7)	13.7 (13.6-13.8)	13.8 (13.7-13.9)	14.3 (14.2-14.4)	14.4 (14.3-14.5)	14.9 (14.8-15)	14.9 (14.8-15)	15.3 (15.2-15.4)	16.2 (16.1-16.3)	16.6 (16.5-16.7)	16.5 (15.5-17.6)
California	13.2 (12-14.7)	13.6 (12.5-15)	13.2 (13.2-13.3)	13.3 (13.3-13.4)	13.7 (13.6-13.7)	14.2 (14.2-14.3)	14.6 (14.6-14.7)	15.1 (15.1-15.1)	15.9 (15.9-15.9)	16.4 (16.3-16.4)	17 (17-17.1)	17.8 (17.8-17.9)	19 (19-19.1)	19.3 (18.5-20.2)
Colorado	13.3 (11.9-14.9)	13.5 (12.3-14.9)	13.8 (13.6-13.9)	13.8 (13.7-13.9)	13.9 (13.8-14)	14.4 (14.3-14.5)	14.8 (14.7-14.9)	15.4 (15.3-15.5)	16 (15.9-16.2)	16.2 (16.1-16.3)	16.9 (16.8-17)	17.7 (17.6-17.8)	18.7 (18.6-18.8)	18.9 (18-19.9)
Connecticut	12.9 (11.4-14.4)	13.2 (12-14.6)	12.8 (12.7-12.9)	13 (12.9-13.1)	13.5 (13.4-13.6)	14.1 (14-14.2)	14.3 (14.2-14.4)	14.9 (14.8-15)	16 (15.9-16.1)	16.2 (16.1-16.3)	16.8 (16.7-16.9)	17.7 (17.6-17.8)	18.7 (18.6-18.8)	19 (18-20)
Delaware	12.3 (10.9-13.7)	12.6 (11.7-13.6)	12.4 (12.3-12.5)	12.3 (12.1-12.4)	12.3 (12.2-12.4)	12.9 (12.8-13.1)	13.4 (13.3-13.6)	14.1 (13.9-14.2)	14.7 (14.6-14.9)	15.1 (14.9-15.2)	16 (15.9-16.1)	16.8 (16.7-16.9)	17.9 (17.7-18)	18.2 (17.5-18.9)
Washington, DC	12.9 (11.5-14.4)	12.8 (11.7-14.1)	12.4 (12.2-12.5)	12.3 (12.2-12.4)	12.5 (12.4-12.6)	12.9 (12.8-13)	13.3 (13.2-13.4)	13.7 (13.5-13.8)	14 (13.8-14.1)	14.4 (14.2-14.5)	15.2 (15.1-15.4)	16 (15.8-16.1)	17.6 (17.4-17.8)	18 (16.9-19.1)
Florida	13.9 (12.5-15.4)	14.3 (13-15.7)	14.2 (14.2-14.3)	14.5 (14.4-14.5)	14.4 (14.3-14.5)	14.9 (14.9-15)	15.3 (15.2-15.3)	15.9 (15.9-16)	16.6 (16.5-16.6)	16.8 (16.7-16.8)	17.3 (17.3-17.4)	18.1 (18.1-18.2)	18.9 (18.9-19)	18.9 (18-19.9)
Georgia	12.2 (10.9-13.7)	12.6 (11.4-13.9)	12.6 (12.5-12.7)	12.7 (12.7-12.8)	12.6 (12.6-12.7)	12.8 (12.7-12.8)	13.5 (13.5-13.6)	13.6 (13.5-13.6)	14.3 (14.2-14.3)	14.5 (14.5-14.6)	15 (14.9-15)	15.9 (15.9-16)	16.7 (16.6-16.7)	17.2 (16.2-18.2)
Hawaii	14.8 (13.3-16.5)	14.7 (13.3-16.2)	14.9 (14.7-15.1)	15.2 (15-15.4)	15.4 (15.2-15.5)	16.1 (16-16.3)	16.7 (16.5-16.8)	17 (16.8-17.2)	17.5 (17.4-17.7)	17.9 (17.8-18.1)	18.4 (18.2-18.6)	18.9 (18.7-19)	19.5 (19.3-19.7)	19.8 (18.8-20.9)
Idaho	13.8 (12.3-15.4)	14 (12.7-15.4)	13.7 (13.6-13.9)	13.7 (13.6-13.9)	13.9 (13.8-14.1)	14.2 (14.1-14.4)	15 (14.9-15.2)	15.5 (15.4-15.7)	16 (15.8-16.2)	16.2 (16.1-16.4)	16.7 (16.6-16.9)	17.3 (17.2-17.5)	18 (17.9-18.2)	18.3 (17.2-19.3)
Illinois	12.6 (11.4-14.1)	12.9 (11.7-14.2)	12.6 (12.5-12.6)	12.7 (12.6-12.7)	12.8 (12.8-12.8)	13.4 (13.3-13.4)	13.9 (13.8-13.9)	14.3 (14.3-14.4)	14.9 (14.9-15)	15.1 (15.1-15.2)	15.9 (15.9-16)	16.8 (16.8-16.9)	17.8 (17.7-17.8)	18.2 (17.2-19.1)
Indiana	12.8 (11.4-14.3)	13.1 (11.9-14.4)	12.9 (12.8-12.9)	12.8 (12.8-12.9)	13 (12.9-13)	13.4 (13.3-13.5)	13.8 (13.7-13.9)	14.1 (14.1-14.2)	14.7 (14.6-14.8)	14.9 (14.9-15)	15.4 (15.3-15.5)	16.3 (16.2-16.3)	17 (16.9-17)	17 (16.1-18)
Iowa	13.4 (12-14.9)	13.5 (12.4-14.9)	13.6 (13.5-13.7)	13.5 (13.4-13.6)	13.6 (13.5-13.7)	14.1 (14-14.1)	14.6 (14.5-14.7)	15 (14.9-15.1)	15.8 (15.6-15.9)	16 (15.9-16.2)	16.5 (16.4-16.6)	17.4 (17.2-17.5)	17.9 (17.7-18)	18.2 (17.2-19.2)
Kansas	13.7 (12.3-15.3)	14.1 (12.8-15.4)	13.8 (13.7-13.9)	13.9 (13.8-14)	14 (13.9-14.1)	14.3 (14.1-14.4)	14.7 (14.6-14.8)	15.1 (14.9-15.2)	15.7 (15.6-15.8)	15.8 (15.7-16)	16.4 (16.2-16.5)	17 (16.9-17.1)	17.6 (17.5-17.7)	17.9 (16.9-18.9)
Kentucky	13 (11.7-14.5)	13.5 (12.3-14.8)	13.2 (13.2-13.3)	13.2 (13.1-13.3)	13 (12.9-13.1)	13.3 (13.2-13.4)	13.7 (13.6-13.8)	13.8 (13.8-13.9)	14.3 (14.2-14.4)	14.4 (14.3-14.5)	14.8 (14.7-14.9)	15.6 (15.5-15.7)	16.2 (16.1-16.3)	16.1 (15.2-17.1)
Louisiana	12.5 (11.3-14)	12.9 (11.7-14.2)	12.7 (12.7-12.8)	12.6 (12.5-12.7)	12.7 (12.7-12.8)	13.1 (13-13.1)	13.6 (13.5-13.6)	13.9 (13.8-13.9)	14.4 (14.3-14.4)	14.7 (14.6-14.8)	15.1 (15-15.1)	15.3 (15.2-15.3)	16.4 (16.4-16.5)	16.6 (15.6-17.6)
Maine	12.5 (11.1-14)	12.7 (11.5-14.1)	12.7 (12.6-12.8)	13 (12.8-13.1)	12.8 (12.6-12.9)	13.4 (13.3-13.5)	14 (13.9-14.1)	14.1 (14-14.3)	15.1 (14.9-15.2)	15.4 (15.3-15.6)	16 (15.8-16.2)	16.7 (16.6-16.9)	17.6 (17.4-17.8)	17.3 (16.2-18.4)
Maryland	12.1 (10.8-13.5)	12.4 (11.3-13.7)	12.2 (12.1-12.2)	12.2 (12.2-12.3)	12.6 (12.6-12.7)	13.2 (13.2-13.3)	13.6 (13.6-13.7)	13.9 (13.8-14)	14.8 (14.8-14.9)	15.2 (15.1-15.3)	15.9 (15.8-16)	16.9 (16.8-17)	18 (17.9-18.1)	18.1 (17-19.1)
Massachusetts	12.7 (11.4-14.2)	13.1 (11.9-14.4)	12.5 (12.5-12.6)	12.8 (12.7-12.8)	13 (12.9-13)	13.6 (13.5-13.7)	14.1 (14.1-14.2)	14.4 (14.4-14.5)	15.5 (15.4-15.6)	15.9 (15.8-16)	16.4 (16.3-16.4)	17.5 (17.4-17.5)	18.3 (18.2-18.4)	18.5 (17.5-19.4)
Michigan	12.7 (11.5-14.2)	13.1 (12-14.5)	12.7 (12.6-12.7)	12.7 (12.6-12.7)	13 (12.9-13)	13.5 (13.4-13.5)	13.9 (13.8-14)	14.1 (14-14.1)	15 (14.9-15)	15.3 (15.2-15.3)	15.9 (15.8-15.9)	16.8 (16.7-16.9)	17.5 (17.5-17.6)	17.6 (16.6-18.6)
Minnesota	13.4 (11.9-15)	13.7 (12.4-15)	13.7 (13.6-13.8)	13.7 (13.6-13.8)	14 (13.9-14)	14.5 (14.4-14.6)	14.9 (14.8-15)	15.2 (15.1-15.3)	15.9 (15.8-16)	16.1 (16-16.2)	16.9 (16.8-17)	17.9 (17.8-18)	18.6 (18.5-18.7)	18.9 (17.9-19.8)
Mississippi	12.9 (11.5-14.3)	13.1 (12-14.5)	13.3 (13.2-13.4)	13.1 (13-13.2)	13 (12.9-13)	13.4 (13.3-13.5)	13.9 (13.8-14)	13.9 (13.8-14)	14.3 (14.2-14.4)	14.4 (14.3-14.5)	14.6 (14.5-14.7)	15.3 (15.2-15.3)	15.9 (15.8-16)	15.9 (15-16.9)
Missouri	12.9 (11.6-14.4)	13.3 (12.1-14.6)	13.1 (13-13.2)	13.1 (13-13.2)	13.1 (13-13.2)	13.6 (13.5-13.7)	14 (13.9-14)	14.3 (14.2-14.4)	15 (14.9-15.1)	15.1 (15-15.2)	15.7 (15.6-15.7)	16.4 (16.3-16.5)	17.2 (17.2-17.3)	17.4 (16.4-18.3)
Montana	12.9 (11.6-14.5)	13.1 (11.8-14.4)	13.1 (13-13.3)	13.1 (12.9-13.2)	13.7 (13.5-13.8)	14 (13.8-14.2)	14.5 (14.3-14.6)	15 (14.8-15.1)	15.8 (15.7-16)	15.8 (15.7-16)	16.4 (16.3-16.6)	17.3 (17.1-17.4)	18.2 (18-18.3)	17.6 (16.6-18.6)
Nebraska	13.7 (12.3-15.3)	13.9 (12.7-15.3)	13.7 (13.6-13.9)	13.7 (13.6-13.8)	13.9 (13.8-14)	14.3 (14.2-14.4)	14.7 (14.6-14.8)	14.8 (14.7-15)	15.4 (15.3-15.6)	15.9 (15.8-16.1)	16.6 (16.5-16.8)	17.3 (17.2-17.5)	17.9 (17.8-18.1)	18.1 (17.1-19.1)
Nevada	11.9 (10.6-13.5)	12.2 (11.1-13.5)	12.3 (12.1-12.4)	12 (11.8-12.1)	12.7 (12.6-12.9)	13.5 (13.3-13.7)	13.9 (13.7-14)	14.4 (14.2-14.5)	14.8 (14.7-14.9)	14.8 (14.6-14.9)	15.6 (15.5-15.7)	16.1 (16-16.2)	17.5 (17.4-17.6)	17.8 (16.7-18.9)
New Hampshire	12.3 (10.8-13.8)	12.6 (11.4-14)	12.6 (12.5-12.8)	12.5 (12.4-12.7)	12.8 (12.6-12.9)	13.2 (13-13.3)	14.4 (14.2-14.5)	14.4 (14.3-14.6)	15.1 (15-15.3)	15.5 (15.4-15.7)	16.3 (16.1-16.5)	17.1 (16.9-17.3)	18.1 (17.9-18.3)	18.2 (17.2-19.3)
New Jersey	12.5 (11.2-13.9)	12.7 (11.5-14)	12.5 (12.4-12.6)	12.6 (12.6-12.7)	12.9 (12.8-13)	13.6 (13.5-13.7)	13.9 (13.8-14)	14.2 (14.1-14.2)	15.2 (15.2-15.3)	15.6 (15.5-15.7)	16.2 (16.1-16.3)	17.3 (17.2-17.4)	18.2 (18.2-18.3)	18.7 (17.8-19.7)
New Mexico	13.6 (12.2-15.2)	13.8 (12.6-15.2)	13.9 (13.8-14.1)	13.7 (13.5-13.8)	14.1 (13.9-14.2)	14.4 (14.3-14.6)	15.1 (14.9-15.2)	15.5 (15.4-15.7)	16.3 (16.1-16.4)	16.3 (16.2-16.5)	17 (16.8-17.1)	17.7 (17.6-17.8)	18.5 (18.3-18.6)	18.4 (17.4-19.5)
New York	12.8 (11.4-14.2)	12.9 (11.8-14)	12.5 (12.5-12.5)	12.7 (12.7-12.8)	12.9 (12.9-13)	13.7 (13.7-13.8)	14 (14-14.1)	14.5 (14.4-14.5)	15.4 (15.3-15.4)	15.9 (15.8-15.9)	16.7 (16.6-16.7)	17.6 (17.6-17.7)	18.6 (18.5-18.6)	19.1 (18.1-20.1)
North Carolina	12.6 (11.3-14.2)	13 (11.8-14.3)	12.8 (12.7-12.8)	12.7 (12.7-12.8)	12.9 (12.8-12.9)	13.3 (13.3-13.4)	13.8 (13.7-13.8)	14.1 (14-14.1)	14.6 (14.5-14.7)	14.8 (14.8-14.9)	15.3 (15.2-15.3)	16.3 (16.3-16.4)	17.1 (17-17.2)	17.5 (16.6-18.5)
North Dakota	14 (12.7-15.4)	14.2 (13.3-15.2)	13.9 (13.8-14)	13.7 (13.6-13.9)	14 (13.9-14.1)	14.4 (14.2-14.5)	15.1 (14.9-15.2)	15.3 (15.2-15.5)	15.9 (15.8-16.1)	16.1 (15.9-16.2)	16.7 (16.5-16.9)	17.5 (17.4-17.7)	18.1 (17.9-18.3)	18 (17.3-18.7)
Ohio	12.6 (11.4-14.1)	12.9 (11.9-14.1)	12.8 (12.8-12.9)	12.7 (12.7-12.8)	12.8 (12.8-12.9)	13.3 (13.3-13.4)	13.6 (13.5-13.6)	14.1 (14-14.1)	14.7 (14.7-14.8)	14.9 (14.9-15)	15.5 (15.4-15.6)	16.2 (16.2-16.3)	17.2 (17.1-17.2)	17 (16-18)
Oklahoma	13.4 (12-15)	13.8 (12.6-15.3)	13.6 (13.5-13.7)	13.6 (13.5-13.7)	13.3 (13.3-13.4)	13.8 (13.7-13.9)	14.2 (14.1-14.3)	14.5 (14.4-14.6)	15.1 (15-15.2)	15.1 (15-15.2)	15.3 (15.2-15.4)	15.8 (15.7-15.9)	16.5 (16.4-16.6)	16.6 (15.7-17.6)
Oregon	13.2 (11.9-14.8)	13.4 (12.2-15)	13.4 (13.3-13.5)	13.4 (13.3-13.5)	13.8 (13.7-13.9)	14.4 (14.3-14.5)	14.6 (14.5-14.7)	15 (14.8-15.1)	15.6 (15.5-15.7)	15.9 (15.8-16)	16.6 (16.5-16.7)	17.3 (17.2-17.4)	18.1 (18-18.2)	18.3 (17.4-19.3)
Pennsylvania	12.2 (11-13.7)	12.5 (11.4-13.7)	12.4 (12.3-12.4)	12.4 (12.3-12.4)	12.7 (12.6-12.7)	13.2 (13.2-13.3)	13.6 (13.5-13.6)	14 (13.9-14.1)	14.9 (14.9-15)	15.2 (15.2-15.3)	15.8 (15.8-15.9)	16.6 (16.6-16.7)	17.5 (17.5-17.6)	17.6 (16.6-18.5)
Rhode Island	12.5 (11.2-14)	12.7 (11.5-14.1)	12.5 (12.4-12.7)	12.4 (12.3-12.5)	12.9 (12.8-13.1)	13.8 (13.7-14)	14 (13.9-14.2)	14.5 (14.3-14.6)	15.3 (15.2-15.5)	16 (15.8-16.1)	16.3 (16.2-16.5)	17 (16.8-17.2)	17.9 (17.7-18)	18 (16.8-19)
South Carolina	11.7 (10.5-13.2)	12.1 (10.9-13.4)	11.7 (11.6-11.7)	12.3 (12.2-12.4)	12.4 (12.3-12.5)	13 (12.9-13)	13.4 (13.3-13.5)	13.8 (13.7-13.9)	14.5 (14.4-14.6)	14.7 (14.6-14.8)	15.1 (15-15.2)	16.1 (16-16.2)	16.9 (16.8-17)	16.9 (15.9-17.9)
South Dakota	13.7 (12.4-15.2)	14 (13.1-15)	13.8 (13.6-13.9)	13.8 (13.7-14)	13.9 (13.7-14)	14.4 (14.3-14.5)	14.8 (14.7-14.9)	15.1 (14.9-15.2)	15.8 (15.6-15.9)	16 (15.9-16.2)	16.8 (16.6-16.9)	17.4 (17.2-17.5)	18.2 (18-18.3)	18 (17.3-18.7)
Tennessee	13.1 (11.8-14.6)	13.4 (12.2-14.8)	13.2 (13.1-13.2)	13.2 (13.1-13.3)	13 (12.9-13.1)	13.6 (13.5-13.6)	13.9 (13.8-14)	14.1 (14.1-14.2)	14.3 (14.2-14.4)	14.5 (14.4-14.5)	15 (14.9-15)	15.8 (15.7-15.8)	16.5 (16.5-16.6)	16.9 (15.9-17.9)
Texas	13.3 (11.9-14.7)	13.5 (12.4-14.9)	13.5 (13.4-13.5)	13.6 (13.5-13.6)	13.5 (13.5-13.6)	14 (13.9-14)	14.3 (14.3-14.4)	14.5 (14.5-14.6)	15.1 (15.1-15.1)	15.5 (15.4-15.5)	15.9 (15.9-16)	16.8 (16.8-16.9)	17.6 (17.6-17.6)	18 (17-18.9)
Utah	13.7 (12.2-15.3)	14.1 (12.7-15.5)	13.6 (13.4-13.7)	13.6 (13.4-13.7)	14 (13.9-14.2)	14.5 (14.4-14.7)	15.2 (15.1-15.3)	15.4 (15.2-15.5)	16.4 (16.2-16.5)	16.7 (16.6-16.9)	17.3 (17.1-17.4)	18 (17.9-18.1)	18.7 (18.6-18.8)	18.7 (17.8-19.7)
Vermont	12.5 (11.5-13.7)	12.8 (12.2-13.5)	12.7 (12.5-12.8)	12.6 (12.5-12.7)	12.8 (12.7-13)	13.4 (13.3-13.5)	14 (13.9-14.1)	14.2 (14.1-14.4)	15.1 (15-15.3)	15.6 (15.5-15.8)	16.4 (16.2-16.5)	17.5 (17.3-17.6)	18.2 (18-18.3)	18.2 (17.6-18.8)
Virginia	12.3 (11-13.8)	12.6 (11.4-13.9)	12.7 (12.6-12.8)	12.5 (12.5-12.6)	12.9 (12.8-12.9)	13.3 (13.2-13.3)	13.6 (13.5-13.7)	14 (13.9-14)	14.7 (14.6-14.7)	15 (14.9-15)	15.7 (15.6-15.7)	16.6 (16.6-16.7)	17.6 (17.6-17.7)	18.1 (17.1-19.1)
Washington	13 (11.5-14.5)	13.2 (12-14.5)	13.3 (13.2-13.4)	13.5 (13.4-13.6)	13.5 (13.4-13.6)	14.2 (14.1-14.3)	14.7 (14.6-14.8)	15.1 (15-15.2)	15.8 (15.7-15.9)	16.2 (16.1-16.3)	16.6 (16.5-16.6)	17.5 (17.4-17.6)	18.3 (18.2-18.4)	18.7 (17.8-19.7)
West Virginia	12.7 (11.4-14.3)	13.2 (12-14.5)	13.1 (13-13.2)	12.9 (12.8-13)	12.7 (12.6-12.8)	13.1 (13-13.2)	13.6 (13.5-13.7)	13.8 (13.7-14)	14.2 (14.1-14.4)	14.5 (14.4-14.6)	15 (14.9-15.1)	15.6 (15.5-15.7)	16.2 (16.1-16.4)	16.2 (15.3-17.2)
Wisconsin	13.1 (11.8-14.6)	13.5 (12.4-14.8)	13.2 (13.2-13.3)	13.3 (13.2-13.4)	13.6 (13.5-13.7)	14 (14-14.1)	14.5 (14.4-14.6)	15 (14.9-15)	15.4 (15.3-15.5)	15.8 (15.7-15.8)	16.3 (16.2-16.4)	17.1 (17.1-17.2)	17.9 (17.8-18)	18.1 (17.2-19.2)
Wyoming	13.4 (12.3-14.6)	13.7 (12.9-14.3)	13.4 (13.3-13.6)	13.6 (13.4-13.7)	13.4 (13.2-13.5)	13.4 (13.3-13.5)	14.3 (14.2-14.4)	14.6 (14.4-14.7)	15.5 (15.4-15.7)	15.8 (15.6-15.9)	16.3 (16.2-16.5)	17.1 (17-17.2)	18 (17.9-18.1)	18.1 (17.4-18.7)
Southern Latin America	11.7 (11.6-11.7)	12.1 (12.1-12.2)	12.5 (12.4-12.5)	12.5 (12.5-12.5)	12.5 (12.5-12.6)	13.2 (13-13.5)	13.3 (13.3-13.3)	13.8 (13.8-13.8)	14 (14-14)	14.5 (14.5-14.6)	14.8 (14.8-14.8)	15.2 (15.2-15.3)	15.7 (15.7-15.8)	16.3 (15.6-16.9)
Argentina	11.8 (11.8-11.8)	12.2 (12.1-12.2)	12.5 (12.4-12.5)	12.5 (12.5-12.5)	12.4 (12.4-12.5)	13.1 (12.8-13.5)	13.2 (13.2-13.3)	13.8 (13.7-13.8)	13.9 (13.9-13.9)	14.4 (14.4-14.4)	14.5 (14.5-14.5)	14.8 (14.8-14.9)	15.3 (15.2-15.3)	15.7 (14.8-16.5)
Chile	10.7 (10.7-10.7)	11.2 (11.2-11.3)	11.8 (11.7-11.8)	12.2 (12.2-12.3)	12.6 (12.6-12.6)	13.4 (13.3-13.4)	13.7 (13.6-13.7)	13.8 (13.8-13.8)	14.4 (14.4-14.5)	15.2 (15.1-15.2)	16 (16-16.1)	16.6 (16.6-16.7)	17.2 (17.2-17.3)	17.9 (17-18.9)
Uruguay	12.7 (12.6-12.8)	13.5 (13.5-13.6)	13.6 (13.5-13.6)	13.2 (13.1-13.3)	13.1 (13-13.2)	13.5 (13.4-13.5)	13.3 (13.2-13.4)	13.8 (13.7-13.9)	13.9 (13.8-14)	14.1 (14-14.1)	14.4 (14.3-14.5)	14.8 (14.7-14.9)	15.4 (15.3-15.5)	15.8 (14.9-16.7)
Western Europe	12.3 (11.9-12.6)	12.6 (12.5-12.7)	12.8 (12.8-12.9)	12.7 (12.7-12.8)	12.7 (12.6-12.7)	12.9 (12.9-12.9)	13.4 (13.4-13.4)	14.1 (14.1-14.1)	14.7 (14.7-14.7)	15.3 (15.3-15.3)	16.2 (16.2-16.2)	17.2 (17.2-17.2)	18 (18-18.1)	18.7 (18.5-18.9)
Andorra	14.8 (13.3-16.5)	15 (13.4-16.7)	15 (13.6-16.7)	15.1 (13.9-16.9)	15.2 (14-17)	15.6 (14.5-16.9)	16 (14.9-17)	16.4 (15.5-17.1)	16.6 (15.8-17.2)	17.3 (16.4-17.9)	18.1 (17.5-18.6)	18.3 (17.8-18.7)	18.8 (18.4-19.2)	19.4 (18.8-20.1)
Austria	12 (11.9-12)	12.1 (12.1-12.2)	12.1 (12-12.1)	12 (11.9-12)	11.8 (11.7-11.8)	12.2 (12.2-12.3)	12.9 (12.9-13)	13.6 (13.6-13.7)	14.5 (14.4-14.6)	15.1 (15-15.1)	16 (15.9-16.1)	17.1 (17-17.2)	17.8 (17.7-17.8)	18.5 (18-18.9)
Belgium	11.6 (11.6-11.6)	12.5 (12.5-12.5)	12.5 (12.4-12.5)	12.4 (12.4-12.5)	12.2 (12.2-12.3)	12.3 (12.3-12.4)	12.9 (12.8-12.9)	13.3 (13.3-13.4)	14.3 (14.2-14.3)	14.8 (14.7-14.9)	15.6 (15.5-15.7)	16.6 (16.6-16.7)	17.4 (17.4-17.5)	18.3 (17.8-18.7)
Cyprus	12.1 (10.9-13.4)	12.2 (11-13.4)	12.2 (11.2-13.4)	12.2 (11.3-13.2)	12.3 (11.7-13)	12.8 (12.7-13)	13.7 (13.6-13.8)	14.3 (14.2-14.4)	15 (14.9-15.1)	15.4 (15.1-15.8)	14.5 (14.1-14.9)	15 (14.6-15.4)	16.4 (16-16.8)	17.5 (16.8-18.2)
Denmark	13.8 (13.7-13.8)	14 (13.9-14.1)	13.8 (13.7-13.9)	13.4 (13.3-13.5)	13.8 (13.7-13.9)	13.8 (13.8-13.9)	13.6 (13.5-13.7)	13.9 (13.8-14)	14.1 (14-14.2)	14.3 (14.2-14.3)	15.3 (15.2-15.4)	16.2 (16.1-16.3)	16.9 (16.8-17)	18 (17.5-18.4)
Finland	10.5 (10.4-10.5)	11.3 (11.3-11.4)	11.7 (11.6-11.7)	11.4 (11.4-11.5)	11.7 (11.6-11.7)	12.3 (12.2-12.3)	12.8 (12.8-12.9)	13.2 (13.1-13.3)	13.9 (13.8-13.9)	14.7 (14.6-14.7)	15.6 (15.5-15.6)	16.8 (16.7-16.9)	17.5 (17.4-17.6)	18.2 (17.7-18.7)
France	12.1 (12.1-12.2)	12.4 (12.4-12.4)	12.8 (12.8-12.8)	12.7 (12.6-12.7)	13 (13-13)	13.2 (13.2-13.2)	14 (14-14)	14.5 (14.4-14.5)	15.5 (15.4-15.5)	15.9 (15.9-16)	16.7 (16.7-16.7)	17.7 (17.7-17.7)	18.6 (18.6-18.6)	19.5 (19.1-19.9)
Germany	11.7 (10.4-13.1)	12.2 (11.8-12.5)	12.1 (12.1-12.1)	12.1 (12.1-12.1)	11.7 (11.7-11.7)	12.1 (12.1-12.1)	12.7 (12.6-12.7)	13.8 (13.8-13.9)	14.1 (14.1-14.1)	14.8 (14.8-14.9)	16 (15.9-16)	17.1 (17.1-17.2)	17.7 (17.7-17.8)	17.8 (16.9-18.7)
Greece	14.8 (14.3-15.2)	15.3 (15.2-15.4)	15.1 (15-15.2)	14.8 (14.8-14.9)	14.7 (14.6-14.8)	14.8 (14.8-14.9)	14.9 (14.8-14.9)	15 (15-15.1)	15.7 (15.6-15.7)	16 (15.9-16.1)	16.5 (16.4-16.5)	17.1 (17-17.1)	18 (18-18.1)	18.3 (17.9-18.8)
Iceland	14.7 (14.4-15)	14.7 (14.5-14.8)	15.1 (15-15.3)	14.8 (14.7-15)	14.6 (14.4-14.8)	15.6 (15.5-15.8)	15.6 (15.3-15.8)	15.8 (15.5-16.1)	16.1 (15.9-16.3)	16.2 (16.1-16.4)	17.4 (17.2-17.6)	18.4 (18.2-18.6)	18.5 (18.3-18.7)	18.4 (18.1-18.7)
Ireland	12.4 (12.3-12.5)	12.7 (12.6-12.8)	12.9 (12.8-13)	12.6 (12.6-12.7)	12.4 (12.3-12.5)	12.5 (12.4-12.5)	12.6 (12.5-12.6)	12.6 (12.5-12.7)	13.4 (13.3-13.5)	13.7 (13.6-13.8)	14.6 (14.5-14.7)	16.4 (16.3-16.5)	17.8 (17.7-17.9)	18.7 (18.2-19.2)
Israel	16.6 (16.4-16.8)	14.4 (14.3-14.6)	14.5 (14.4-14.7)	13.9 (13.7-14)	13.9 (13.8-13.9)	14.1 (14-14.2)	14.5 (14.4-14.6)	15.3 (15.2-15.4)	16.4 (16.3-16.5)	16.2 (16.1-16.3)	16.8 (16.7-16.9)	17.8 (17.8-17.9)	19 (18.9-19.1)	19.9 (19.4-20.4)
Italy	13.1 (13.1-13.1)	13.5 (13.4-13.5)	13.7 (13.7-13.7)	13.4 (13.4-13.5)	13.4 (13.4-13.5)	13.2 (13.2-13.2)	13.5 (13.5-13.5)	14.3 (14.3-14.3)	15.2 (15.2-15.2)	15.7 (15.7-15.8)	16.5 (16.5-16.6)	17.5 (17.4-17.5)	18.3 (18.3-18.3)	19.2 (18.8-19.6)
Luxembourg	11.9 (11.8-12.1)	12.1 (11.9-12.2)	12.4 (12.2-12.5)	12.3 (12.1-12.4)	11.9 (11.8-12)	11.9 (11.8-12.1)	12.6 (12.5-12.7)	13 (12.9-13.2)	13.9 (13.8-14.1)	14.6 (14.5-14.7)	15.8 (15.6-15.9)	16.6 (16.5-16.8)	18.1 (18-18.3)	18.8 (18-19.6)
Malta	12.5 (12.3-12.7)	12.4 (12.3-12.5)	12 (11.9-12.1)	12 (11.9-12.2)	12.1 (11.9-12.2)	12.5 (12.4-12.7)	12.3 (12.2-12.4)	13.5 (13.3-13.6)	14.7 (14.5-14.8)	15.3 (15.1-15.4)	15.6 (15.4-15.8)	16.3 (16.2-16.5)	17.3 (17.1-17.5)	17.9 (17.6-18.3)
Netherlands	14 (13.9-14.1)	14 (13.9-14)	14.2 (14.1-14.2)	13.9 (13.8-13.9)	13.5 (13.4-13.6)	13.5 (13.5-13.6)	13.9 (13.9-14)	14 (14-14.1)	14.4 (14.3-14.4)	14.7 (14.7-14.8)	15.3 (15.2-15.3)	16.4 (16.4-16.5)	17.5 (17.5-17.6)	18.3 (17.9-18.8)
Norway	15.1 (14.8-15.4)	14.9 (14.6-15.2)	14.6 (14.4-14.9)	14.2 (14.1-14.4)	14 (13.9-14)	14.3 (14.2-14.3)	14.4 (14.4-14.5)	14.4 (14.3-14.4)	14.7 (14.6-14.7)	15.2 (15.1-15.2)	15.9 (15.9-16)	17.2 (17.1-17.2)	17.9 (17.8-17.9)	19 (18.8-19.1)
Portugal	12.4 (12.3-12.4)	12.5 (12.5-12.6)	12.7 (12.7-12.8)	12.6 (12.6-12.7)	12.6 (12.6-12.7)	12.1 (12-12.1)	13.3 (13.2-13.3)	13.7 (13.7-13.8)	14.1 (14-14.1)	14.6 (14.6-14.7)	15.4 (15.3-15.4)	16.6 (16.4-16.8)	17.5 (17.4-17.5)	18.3 (17.9-18.8)
Spain	12.2 (12.2-12.2)	13 (12.9-13)	13.7 (13.7-13.7)	13.6 (13.5-13.6)	13.6 (13.6-13.6)	13.9 (13.9-13.9)	14.8 (14.8-14.9)	15.1 (15.1-15.1)	15.5 (15.5-15.5)	16.1 (16-16.1)	16.7 (16.7-16.7)	17.3 (17.3-17.4)	18.3 (18.3-18.4)	19.1 (18.7-19.5)
Sweden	13.7 (13.4-14)	14.1 (13.8-14.4)	14 (13.7-14.3)	14.1 (13.8-14.4)	14.3 (14-14.6)	14.1 (13.9-14.4)	14.4 (14.3-14.4)	14.8 (14.8-14.9)	15.4 (15.4-15.5)	16 (15.9-16.1)	16.7 (16.6-16.8)	17.4 (17.3-17.5)	18.1 (18-18.2)	19.2 (18.8-19.6)
Stockholm	13.4 (11.9-15.3)	13.7 (12.1-15.6)	13.5 (12-15.3)	13.6 (12.1-15.3)	13.8 (12.3-15.4)	13.5 (12.5-14.8)	13.7 (13.6-13.8)	14.4 (14.3-14.5)	14.9 (14.7-15)	15.7 (15.6-15.9)	16.4 (16.3-16.6)	17.4 (17.3-17.6)	18.3 (18.1-18.4)	19 (18.5-19.6)
Sweden except Stockholm	13.8 (13.5-14)	14.2 (13.9-14.5)	14 (13.8-14.3)	14.2 (13.9-14.5)	14.4 (14.2-14.7)	14.2 (14-14.4)	14.5 (14.4-14.5)	14.9 (14.9-15)	15.5 (15.5-15.6)	16 (16-16.1)	16.8 (16.7-16.8)	17.4 (17.3-17.5)	18.1 (18-18.1)	19.2 (18.8-19.7)
Switzerland	12.7 (12.6-12.8)	12.9 (12.8-12.9)	13.3 (13.2-13.3)	13.2 (13.2-13.3)	13.5 (13.4-13.5)	14 (14-14.1)	14.5 (14.4-14.6)	15.1 (15.1-15.2)	15.6 (15.5-15.7)	16.3 (16.2-16.4)	17.2 (17.1-17.2)	18.2 (18.1-18.3)	19 (18.9-19.1)	20.2 (19.8-20.7)
United Kingdom	11.8 (11.7-12)	11.9 (11.7-12.1)	12.1 (11.9-12.3)	12.1 (12-12.3)	12.1 (12-12.3)	12.5 (12.4-12.6)	12.9 (12.9-13)	13.5 (13.4-13.5)	14.1 (14-14.1)	14.7 (14.6-14.7)	15.8 (15.8-15.8)	17 (17-17)	18 (18-18)	18.5 (18.4-18.6)
England	12 (11.8-12.1)	12 (11.9-12.2)	12.2 (12-12.4)	12.3 (12.1-12.5)	12.3 (12.1-12.4)	12.6 (12.5-12.7)	13 (13-13.1)	13.6 (13.6-13.6)	14.2 (14.2-14.2)	14.8 (14.8-14.8)	15.9 (15.9-15.9)	17.1 (17.1-17.1)	18.1 (18.1-18.2)	18.6 (18.6-18.7)
East Midlands	11.9 (11.2-12.6)	12 (11.3-12.6)	12.2 (11.5-12.9)	12.2 (11.5-12.9)	12.3 (11.7-12.9)	12.6 (12.2-13.1)	13.1 (12.9-13.2)	13.6 (13.6-13.7)	14.3 (14.2-14.3)	14.8 (14.7-14.8)	15.9 (15.8-16)	17 (16.9-17)	17.9 (17.9-18)	18.5 (18.3-18.7)
Derby	11.7 (9.9-13.7)	11.8 (10-13.8)	12 (10.3-14.2)	12.1 (10.4-14.1)	12.2 (10.6-14)	12.5 (11.3-13.8)	12.5 (12.1-12.8)	13.3 (13.1-13.5)	13.8 (13.7-14)	14.9 (14.7-15.1)	16.1 (15.9-16.4)	16.9 (16.7-17)	17 (16.8-17.3)	17.6 (17-18.3)
Derbyshire	11.7 (9.9-13.6)	11.8 (10.2-13.7)	12 (10.2-14.1)	12 (10.2-13.9)	12 (10.4-13.7)	12.3 (11.1-13.6)	12.9 (12.6-13.2)	13.4 (13.3-13.5)	14 (13.9-14.1)	14.5 (14.4-14.6)	15.8 (15.5-16)	16.9 (16.7-17)	18 (17.8-18.2)	18.3 (17.8-18.9)
Leicester	11.4 (9.6-13.3)	11.5 (9.9-13.4)	11.7 (10.1-13.7)	11.8 (10.2-13.7)	12 (10.5-13.6)	12.5 (11.4-13.7)	12.9 (12.6-13.3)	13.3 (13.2-13.5)	14.2 (14-14.3)	14.3 (14.1-14.5)	15 (14.7-15.2)	15.9 (15.8-16.1)	16.8 (16.6-17)	17 (16.4-17.6)
Leicestershire	12.5 (10.8-14.5)	12.6 (10.9-14.7)	12.8 (11-15)	12.9 (11.1-14.9)	12.9 (11.2-14.7)	13.2 (11.9-14.5)	13.6 (13.2-13.9)	14.2 (14.1-14.3)	14.8 (14.6-14.9)	15.4 (15.2-15.5)	16.7 (16.4-16.9)	17.7 (17.5-17.8)	18.5 (18.4-18.7)	19.4 (18.8-19.9)
Lincolnshire	12 (10.3-13.8)	12.1 (10.4-14)	12.3 (10.6-14.2)	12.3 (10.7-14.2)	12.4 (10.7-14.1)	12.8 (11.6-14.1)	13.4 (13.1-13.7)	13.7 (13.6-13.8)	14.4 (14.2-14.5)	15 (14.9-15.2)	16.1 (15.9-16.3)	17.3 (17.1-17.4)	18.3 (18.1-18.5)	18.8 (18.3-19.4)
Northamptonshire	12.2 (10.4-14.2)	12.3 (10.6-14.3)	12.5 (10.8-14.3)	12.6 (11-14.6)	12.7 (11.1-14.5)	13.1 (11.8-14.4)	13.3 (13-13.6)	14 (13.9-14.1)	14.5 (14.4-14.7)	15 (14.8-15.1)	16 (15.8-16.2)	17.1 (16.9-17.2)	17.8 (17.7-18)	18.7 (18.2-19.3)
Nottingham	11.1 (9.6-13.1)	11.1 (9.5-13)	11.2 (9.6-13.1)	11.2 (9.6-13.1)	11.2 (9.7-12.8)	11.6 (10.4-12.8)	12 (11.7-12.3)	12.8 (12.7-13)	13.5 (13.3-13.6)	13.8 (13.6-14)	14.4 (14.1-14.6)	15.4 (15.2-15.5)	16.5 (16.3-16.6)	16.7 (16.1-17.3)
Nottinghamshire	11.9 (10.2-14)	12.1 (10.4-14.1)	12.3 (10.5-14.3)	12.4 (10.7-14.3)	12.4 (10.9-14.2)	12.7 (11.6-14)	13.1 (12.8-13.4)	13.6 (13.5-13.7)	14.2 (14.1-14.4)	14.7 (14.5-14.8)	15.9 (15.6-16.1)	16.9 (16.8-17.1)	17.9 (17.7-18.1)	18.5 (17.9-19)
Rutland	12.9 (11.1-15.1)	13.1 (11.1-15.2)	13.4 (11.3-15.6)	13.5 (11.6-15.7)	13.6 (11.8-15.6)	14.2 (12.8-15.6)	14.5 (13.9-15.1)	14.7 (14.4-15.1)	15.6 (15.2-16)	16.5 (16.1-16.9)	17.7 (17.3-18.1)	18.3 (17.9-18.7)	19 (18.6-19.4)	19.4 (18.6-20.3)
East of England	12.6 (11.9-13.4)	12.7 (12-13.5)	12.9 (12.1-13.7)	13 (12.2-13.7)	12.9 (12.3-13.6)	13.1 (12.7-13.6)	13.5 (13.4-13.6)	14.3 (14.2-14.3)	14.8 (14.7-14.8)	15.4 (15.3-15.4)	16.5 (16.4-16.6)	17.6 (17.6-17.7)	18.6 (18.6-18.7)	19 (18.8-19.2)
Bedford	12.3 (10.6-14.4)	12.3 (10.4-14.4)	12.4 (10.6-14.5)	12.4 (10.7-14.5)	12.4 (10.8-14.1)	12.8 (11.6-14.1)	13.6 (13.2-14)	14.2 (14-14.4)	14.5 (14.2-14.7)	15.1 (14.9-15.4)	16.1 (15.9-16.4)	17.2 (17-17.4)	17.7 (17.4-17.9)	18.2 (17.6-18.9)
Cambridgeshire	12.7 (11-14.9)	12.8 (11-15)	12.9 (11.1-15.1)	12.9 (11.2-15)	12.8 (11.2-14.6)	13.1 (11.8-14.4)	13.4 (13.1-13.8)	14.3 (14.2-14.5)	14.9 (14.8-15.1)	15.4 (15.3-15.6)	16.8 (16.6-17.1)	17.7 (17.5-17.8)	18.8 (18.6-18.9)	19.5 (18.9-20.1)
Central Bedfordshire	12.6 (10.8-14.7)	12.8 (11-15)	13 (11.1-15.1)	13.1 (11.4-15.1)	13 (11.4-14.8)	13.2 (12-14.6)	13.2 (12.8-13.6)	13.9 (13.7-14.1)	14.3 (14.1-14.5)	15.1 (14.9-15.3)	16.2 (15.9-16.4)	17.2 (17-17.5)	18.7 (18.5-18.9)	19.5 (18.9-20.1)
Essex	12.7 (11.1-14.6)	12.8 (11-14.8)	12.9 (11.1-14.9)	12.9 (11.1-14.9)	12.7 (11.2-14.5)	12.8 (11.8-14.1)	13.3 (13-13.6)	14.2 (14.1-14.3)	14.8 (14.7-14.9)	15.3 (15.1-15.4)	16.4 (16.2-16.6)	17.6 (17.5-17.7)	18.5 (18.4-18.6)	18.7 (18.2-19.3)
Hertfordshire	12.8 (11.1-15)	12.9 (11.2-15)	13.1 (11.3-15.2)	13 (11.3-15.1)	12.8 (11.3-14.7)	13 (11.9-14.4)	13.4 (13.1-13.7)	14.1 (14-14.2)	14.7 (14.5-14.8)	15.5 (15.3-15.6)	16.4 (16.1-16.6)	17.8 (17.7-18)	18.6 (18.5-18.7)	19.4 (18.8-20)

Table 11 Estimates of life expectancy at age 65 by location and year for 1950, 1955...2005, 2010, 2017 - males

Location	1950	1955	1960	1965	1970	1975	1980	1985	1990	1995	2000	2005	2010	2017
Luton	12.2 (10.4-14.3)	12.4 (10.5-14.5)	12.6 (10.8-14.7)	12.8 (11-14.7)	12.8 (11.2-14.6)	13.1 (11.9-14.6)	13 (12.7-13.5)	14.1 (13.9-14.3)	14 (13.8-14.2)	14.3 (14.1-14.5)	15.6 (15.3-15.8)	16.5 (16.3-16.7)	17.7 (17.5-17.9)	18.7 (18.1-19.4)
Norfolk	12.7 (10.9-14.6)	12.8 (11.1-14.8)	13 (11.3-15)	13.1 (11.4-15.1)	13.1 (11.5-14.9)	13.5 (12.4-14.8)	13.8 (13.5-14.1)	14.6 (14.4-14.7)	15 (14.8-15.1)	15.6 (15.5-15.8)	16.7 (16.5-17)	17.9 (17.8-18)	19.2 (19.1-19.4)	19 (18.5-19.5)
Peterborough	11.9 (10.2-14.1)	12 (10.1-14.1)	12.1 (10.3-14.2)	12.1 (10.4-14.1)	12.1 (10.5-13.7)	12.4 (11.2-13.7)	13.2 (12.9-13.7)	13.9 (13.7-14.1)	14.4 (14.2-14.6)	14.6 (14.4-14.8)	15.8 (15.5-16)	16.7 (16.5-17)	17.3 (17.1-17.5)	18 (17.4-18.6)
Southend-on-Sea	12.3 (10.6-14.3)	12.5 (10.8-14.4)	12.7 (10.9-14.8)	12.9 (11.1-15)	13 (11.3-14.8)	13.4 (12.2-14.7)	13.9 (13.5-14.4)	14.6 (14.3-14.8)	14.9 (14.7-15.1)	15.3 (15.1-15.6)	15.9 (15.6-16.2)	16.8 (16.6-17)	17.8 (17.6-18)	18.1 (17.5-18.8)
Suffolk	12.7 (11-14.9)	12.9 (11-14.9)	13.2 (11.3-15.3)	13.3 (11.5-15.3)	13.3 (11.6-15.2)	13.6 (12.4-15)	13.8 (13.5-14.2)	14.5 (14.4-14.6)	15 (14.9-15.2)	15.6 (15.5-15.8)	16.7 (16.5-16.9)	18.1 (17.9-18.2)	18.9 (18.7-19.1)	19.4 (18.8-20)
Thurrock	12 (10.4-14.1)	12 (10.3-14.2)	12.1 (10.4-14.2)	12.1 (10.4-14.1)	12 (10.4-13.9)	12.3 (11.2-13.6)	13 (12.6-13.4)	13.7 (13.5-13.9)	14.3 (14-14.5)	15 (14.7-15.2)	16.3 (16-16.6)	16.5 (16.3-16.8)	17.9 (17.7-18.1)	18.2 (17.5-18.9)
Greater London	12.1 (11.8-12.4)	12.1 (11.8-12.5)	12.3 (12-12.6)	12.4 (12.1-12.7)	12.4 (12.1-12.7)	12.8 (12.6-13.1)	13.2 (13.1-13.2)	13.6 (13.6-13.7)	14.3 (14.2-14.3)	14.7 (14.7-14.7)	16 (15.9-16)	17.3 (17.3-17.3)	18.4 (18.3-18.4)	19.1 (19-19.2)
Barking and Dagenham	11.5 (9.8-13.4)	11.5 (9.8-13.3)	11.6 (10-13.3)	11.6 (10-13.2)	11.5 (10.1-13.2)	11.9 (10.7-13)	12.2 (11.9-12.6)	12.9 (12.7-13.1)	13.5 (13.3-13.7)	13.8 (13.7-14.1)	14.2 (14-14.5)	15.5 (15.3-15.8)	16.6 (16.4-16.8)	16.5 (15.9-17.2)
Barnet	13 (11.2-14.8)	13 (11.3-14.9)	13.3 (11.6-15.3)	13.4 (11.7-15.4)	13.5 (12-15.3)	13.9 (12.8-15.2)	14 (13.7-14.4)	14.5 (14.4-14.7)	15.1 (15-15.3)	15.8 (15.6-15.9)	17.1 (16.9-17.4)	18.2 (18.1-18.4)	19.1 (19-19.3)	20 (19.4-20.6)
Bexley	12.6 (10.9-14.6)	12.6 (10.8-14.5)	12.7 (11.1-14.6)	12.8 (11.3-14.6)	12.8 (11.3-14.6)	13.2 (12.1-14.5)	13.5 (13.2-13.9)	14.1 (14-14.3)	14.8 (14.6-15)	15.5 (15.3-15.7)	16.2 (15.9-16.4)	17.1 (16.9-17.3)	18 (17.8-18.2)	18.8 (18.2-19.5)
Brent	12.3 (10.7-14.3)	12.4 (10.8-14.3)	12.7 (11-14.6)	12.8 (11.1-14.6)	13 (11.5-14.8)	13.5 (12.4-14.6)	13.6 (13.3-13.9)	13.6 (13.4-13.7)	14.9 (14.7-15.1)	15 (14.9-15.2)	16.2 (16-16.5)	17.8 (17.6-18)	18.6 (18.4-18.8)	19.2 (18.7-19.8)
Bromley	12.9 (11.1-14.9)	13 (11.3-15)	13.2 (11.5-15.2)	13.3 (11.6-15.2)	13.3 (11.7-15)	13.7 (12.6-14.9)	14.1 (13.7-14.4)	14.1 (14-14.3)	14.9 (14.7-15.1)	15.6 (15.4-15.7)	16.9 (16.7-17.2)	18.6 (18.4-18.8)	19.3 (19.2-19.5)	19.6 (19-20.2)
Camden	11.8 (10.2-13.6)	11.7 (10.1-13.6)	11.8 (10.1-13.5)	11.7 (10.1-13.4)	11.7 (10.2-13.3)	12.1 (11-13.3)	12.4 (12.1-12.8)	13.7 (13.6-13.9)	13.7 (13.5-13.9)	14.4 (14.2-14.5)	14.9 (14.7-15.2)	15.9 (15.7-16.1)	18.3 (18.1-18.5)	20.7 (20.1-21.4)
Croydon	12.6 (11-14.6)	12.8 (11-14.8)	13 (11.3-14.9)	13.2 (11.6-15)	13.3 (11.7-14.9)	13.6 (12.5-14.8)	13.5 (13.1-13.8)	13.8 (13.7-14)	14.5 (14.4-14.7)	15.2 (15-15.4)	16.3 (16.1-16.5)	18.1 (18-18.3)	18.3 (18.1-18.5)	19.1 (18.5-19.7)
Ealing	11.9 (10.3-13.9)	11.9 (10.3-13.8)	12.1 (10.4-13.9)	12.1 (10.5-13.9)	12.1 (10.7-13.9)	12.5 (11.4-13.7)	12.9 (12.6-13.3)	13.5 (13.4-13.7)	14 (13.9-14.2)	14.6 (14.4-14.7)	16 (15.8-16.3)	17.5 (17.3-17.6)	18.5 (18.3-18.7)	19.3 (18.7-19.9)
Enfield	12.4 (10.6-14.5)	12.5 (10.7-14.5)	12.6 (10.7-14.5)	12.7 (11.1-14.6)	12.7 (11.2-14.3)	13.1 (12-14.3)	13.3 (13-13.7)	13.8 (13.6-13.9)	14.5 (14.3-14.7)	14.8 (14.6-15)	16.3 (16-16.5)	18.1 (17.9-18.3)	19 (18.8-19.2)	18.7 (18.2-19.3)
Greenwich	11.9 (10.3-13.8)	12 (10.4-14)	12.2 (10.5-14.1)	12.3 (10.7-14.2)	12.3 (10.8-14.1)	12.7 (11.6-14)	13 (12.6-13.4)	13.3 (13.1-13.4)	13.5 (13.3-13.6)	13.7 (13.5-13.9)	15 (14.8-15.3)	16 (15.9-16.2)	17.6 (17.4-17.7)	18.4 (17.8-19)
Hackney	11.8 (10.1-13.7)	11.8 (10.2-13.7)	11.8 (10.2-13.6)	11.7 (10.2-13.5)	11.7 (10.1-13.3)	12 (10.9-13.2)	12.5 (12.2-12.8)	12.7 (12.6-12.9)	13.6 (13.5-13.8)	13.9 (13.8-14.1)	15.7 (15.4-15.9)	16.6 (16.4-16.8)	17.5 (17.3-17.6)	17.7 (17.2-18.3)
Hammersmith and Fulham	11.5 (9.9-13.5)	11.5 (9.9-13.4)	11.7 (10-13.5)	11.6 (10.1-13.5)	11.7 (10.2-13.3)	12 (10.9-13.1)	12.5 (12.1-12.8)	12.8 (12.6-13)	13.1 (13-13.3)	13.4 (13.3-13.6)	15.9 (15.6-16.1)	17.1 (16.9-17.3)	17.7 (17.5-18)	18 (17.5-18.6)
Haringey	11.9 (10.3-13.8)	11.8 (10.2-13.7)	12 (10.3-13.9)	12 (10.4-13.8)	12 (10.5-13.6)	12.4 (11.3-13.6)	12.9 (12.5-13.2)	13.6 (13.4-13.8)	14.2 (14-14.3)	14.2 (14.1-14.4)	15.4 (15.2-15.7)	16.4 (16.2-16.6)	17.5 (17.3-17.6)	19.6 (19-20.2)
Harrow	13 (11.2-15.1)	13.1 (11.1-15.2)	13.3 (11.5-15.5)	13.5 (11.7-15.5)	13.6 (12-15.4)	14.1 (13-15.4)	14.1 (13.7-14.4)	14.9 (14.7-15)	15.4 (15.2-15.6)	15.9 (15.7-16.1)	17.3 (17.1-17.6)	18.9 (18.7-19.2)	20.4 (20.2-20.7)	20.5 (19.9-21.2)
Havering	12.6 (10.8-14.7)	12.7 (10.9-14.7)	12.9 (11.1-14.8)	13 (11.3-15)	13 (11.4-15)	13.4 (12.3-14.7)	13.5 (13.1-13.9)	13.7 (13.6-13.9)	14.5 (14.4-14.7)	15.2 (15-15.4)	16.1 (15.9-16.4)	17.8 (17.6-18)	18.1 (17.9-18.3)	18.8 (18.3-19.4)
Hillingdon	12.6 (10.7-14.6)	12.6 (11-14.6)	12.8 (11-14.7)	12.8 (11.1-14.6)	12.8 (11.3-14.4)	13.1 (12.1-14.4)	13.5 (13.1-13.8)	13.9 (13.8-14.1)	14.7 (14.5-14.8)	15 (14.8-15.2)	16.2 (16-16.5)	17.2 (17-17.4)	18.6 (18.4-18.7)	19.7 (19.1-20.4)
Hounslow	12.3 (10.6-14.2)	12.4 (10.6-14.3)	12.6 (10.8-14.6)	12.7 (11-14.6)	12.8 (11.2-14.4)	13.2 (12.1-14.4)	13.5 (13.1-13.9)	13.7 (13.6-13.9)	14.4 (14.3-14.6)	14.3 (14.1-14.5)	15.5 (15.3-15.8)	17 (16.8-17.1)	18.1 (17.9-18.3)	18.9 (18.3-19.5)
Islington	11.8 (10.2-13.7)	11.9 (10.2-13.7)	12.1 (10.4-13.9)	12.1 (10.5-14)	12.1 (10.7-13.8)	12.5 (11.4-13.6)	12.8 (12.5-13.2)	13.2 (13-13.3)	13.4 (13.3-13.6)	13.8 (13.6-14)	14.6 (14.4-14.9)	15.6 (15.4-15.8)	17.1 (16.9-17.3)	18.8 (18.2-19.4)
Kensington and Chelsea	12 (10.3-14)	11.9 (10.3-13.8)	12 (10.4-13.8)	11.9 (10.3-13.7)	11.9 (10.5-13.5)	12.3 (11.2-13.4)	12.6 (12.2-12.9)	13.3 (13.1-13.5)	15.5 (15.3-15.7)	16.3 (16.1-16.5)	17.5 (17.2-17.8)	18.7 (18.4-18.9)	20.3 (20-20.5)	21.8 (21.1-22.4)
Kingston upon Thames	12.8 (11-14.8)	12.8 (11.1-14.9)	13 (11.2-14.9)	13 (11.3-14.9)	13 (11.5-14.8)	13.5 (12.4-14.7)	13.9 (13.5-14.3)	14.3 (14.1-14.5)	15.2 (15-15.4)	16.1 (15.9-16.4)	17.5 (17.2-17.7)	19 (18.7-19.2)	19.9 (19.7-20.1)	19.9 (19.3-20.5)
Lambeth	11.2 (9.7-13.1)	11.2 (9.6-13.1)	11.3 (9.8-13.2)	11.3 (9.8-13.1)	11.4 (10-13.2)	11.9 (10.9-13)	12.7 (12.4-13)	13.1 (12.9-13.2)	13.8 (13.6-13.9)	13.7 (13.6-13.8)	15.4 (15.2-15.7)	16.2 (16.1-16.4)	17.2 (17-17.3)	17.5 (16.9-18.1)
Lewisham	11.8 (10.2-13.6)	11.9 (10.2-13.6)	12.1 (10.4-13.9)	12.2 (10.6-14)	12.3 (10.8-13.8)	12.6 (11.6-13.7)	12.4 (12.1-12.7)	13.4 (13.3-13.6)	13.6 (13.5-13.8)	13.6 (13.5-13.8)	14.6 (14.4-14.8)	15.7 (15.5-15.8)	17.2 (17-17.3)	18.4 (17.8-19)
Merton	12.6 (10.9-14.8)	12.7 (10.9-14.7)	12.9 (11.3-14.8)	13.1 (11.4-14.9)	13.1 (11.7-14.8)	13.6 (12.4-14.8)	13.5 (13.1-13.9)	14.2 (14-14.4)	14.8 (14.6-15)	15.6 (15.4-15.8)	17 (16.7-17.2)	18.1 (17.9-18.4)	18.6 (18.4-18.8)	19.1 (18.4-19.7)
Newham	11.6 (9.9-13.4)	11.6 (9.9-13.4)	11.8 (10-13.7)	11.8 (10.3-13.7)	11.9 (10.3-13.5)	12.2 (11.1-13.3)	12.4 (12.1-12.8)	12.6 (12.5-12.8)	13.1 (13-13.3)	13.3 (13.1-13.4)	14.4 (14.2-14.6)	16.3 (16.2-16.5)	16.3 (16.1-16.5)	18.3 (17.7-18.9)
Redbridge	12.5 (10.8-14.5)	12.6 (10.8-14.5)	12.7 (11-14.7)	12.8 (11.1-14.7)	12.8 (11.4-14.5)	13.3 (12.1-14.5)	13.8 (13.4-14.1)	14.2 (14-14.4)	14.9 (14.7-15.1)	15.4 (15.2-15.6)	16.2 (15.9-16.5)	17.6 (17.4-17.8)	18.7 (18.5-18.9)	19.4 (18.8-20)
Richmond upon Thames	12.7 (10.9-14.7)	12.6 (10.9-14.6)	12.7 (10.9-14.6)	12.6 (11-14.4)	12.5 (11-14.1)	13 (11.8-14.2)	13.5 (13.1-13.8)	14.3 (14.1-14.5)	15.4 (15.1-15.6)	16.3 (16.1-16.5)	18 (17.7-18.3)	19.3 (19.1-19.6)	20.4 (20.2-20.7)	20.3 (19.7-20.9)
Southwark	11.8 (10.1-13.9)	11.9 (10.2-14)	12.1 (10.4-14)	12.1 (10.5-14)	12.2 (10.7-13.9)	12.5 (11.4-13.7)	12.7 (12.4-13.1)	12.6 (12.5-12.8)	13 (12.8-13.1)	13.7 (13.5-13.9)	15.4 (15.1-15.6)	15.9 (15.7-16)	17.3 (17.1-17.5)	17.4 (16.8-17.9)
Sutton	12.9 (11.3-14.9)	13.1 (11.3-15.1)	13.4 (11.5-15.3)	13.5 (11.8-15.5)	13.7 (12-15.4)	14.2 (13.1-15.4)	14.5 (14.1-14.9)	14.6 (14.4-14.8)	15.5 (15.2-15.7)	15.3 (15.1-15.5)	17 (16.7-17.3)	18.1 (17.8-18.3)	19.1 (18.9-19.3)	19.2 (18.6-19.8)
Tower Hamlets	11.2 (9.7-12.9)	11.1 (9.6-12.9)	11.1 (9.7-12.8)	11 (9.5-12.7)	10.9 (9.6-12.5)	11.3 (10.3-12.4)	12.2 (11.9-12.6)	12.5 (12.3-12.6)	13 (12.8-13.1)	13.5 (13.4-13.7)	14.7 (14.4-14.9)	15.4 (15.2-15.6)	16.5 (16.3-16.7)	18.4 (17.8-18.9)
Waltham Forest	11.9 (10.2-13.8)	11.9 (10.2-13.7)	12 (10.3-13.7)	12 (10.4-13.7)	12 (10.6-13.6)	12.4 (11.3-13.6)	13.2 (12.8-13.6)	13.6 (13.5-13.8)	14.3 (14.1-14.5)	14.2 (14.1-14.4)	14.9 (14.6-15.1)	16.3 (16.1-16.5)	17.6 (17.4-17.8)	18.7 (18.1-19.2)
Wandsworth	11.9 (10.3-13.9)	11.9 (10.3-13.9)	12.1 (10.5-13.9)	12.1 (10.5-13.9)	12.1 (10.7-13.8)	12.4 (11.4-13.6)	12.6 (12.3-12.9)	13.1 (13-13.2)	13.2 (13-13.3)	14 (13.8-14.1)	15.5 (15.3-15.7)	16.9 (16.8-17.1)	18.1 (18-18.3)	18.4 (17.8-19)
Westminster	12.2 (10.6-14)	12.2 (10.5-14.1)	12.2 (10.6-14.1)	12.1 (10.5-14)	12.1 (10.6-13.7)	12.3 (11.3-13.6)	12.7 (12.3-13)	13.6 (13.5-13.8)	14.2 (14-14.3)	14.8 (14.6-14.9)	16.2 (15.9-16.5)	17.9 (17.7-18.1)	19.6 (19.4-19.8)	20.3 (19.7-20.9)
North East England	11.1 (10.5-11.6)	11.1 (10.5-11.7)	11.3 (10.7-11.9)	11.4 (10.9-11.9)	11.4 (10.9-11.9)	11.9 (11.5-12.3)	12.2 (12.1-12.3)	12.5 (12.5-12.6)	13.1 (13.1-13.2)	13.8 (13.8-13.9)	14.9 (14.8-15)	16.1 (16-16.1)	17.3 (17.3-17.4)	17.8 (17.6-18)
County Durham	11 (9.5-12.8)	11.1 (9.6-12.9)	11.3 (9.7-13.2)	11.4 (9.8-13.3)	11.4 (9.8-13)	11.8 (10.7-13.1)	12.2 (11.9-12.5)	12.5 (12.4-12.7)	13.1 (13-13.3)	13.5 (13.4-13.6)	15 (14.7-15.2)	16.4 (16.2-16.6)	17.5 (17.3-17.6)	18 (17.5-18.6)
Darlington	11.3 (9.7-13.2)	11.3 (9.6-13.2)	11.5 (9.8-13.4)	11.5 (9.9-13.5)	11.5 (10-13.3)	12.1 (10.9-13.5)	12.5 (12.1-12.9)	12.9 (12.7-13.1)	13.7 (13.4-13.9)	14.4 (14.1-14.7)	15.3 (15-15.6)	16 (15.7-16.3)	17.4 (17.2-17.7)	18.4 (17.6-19)
Gateshead	10.8 (9.2-12.6)	10.7 (9.1-12.6)	10.8 (9.2-12.7)	10.8 (9.2-12.6)	10.8 (9.3-12.4)	11.2 (10.1-12.5)	11.9 (11.6-12.3)	12.3 (12.1-12.4)	12.8 (12.6-13)	13.7 (13.5-13.8)	14.5 (14.2-14.7)	15.6 (15.4-15.8)	16.7 (16.5-16.9)	17.3 (16.6-17.9)
Hartlepool	10.9 (9.3-12.7)	10.9 (9.4-12.8)	11 (9.5-13.2)	11.1 (9.7-13.2)	11.2 (9.6-13)	11.8 (10.6-13.1)	12.4 (12-12.9)	12.8 (12.6-13)	13.3 (13.1-13.5)	14.3 (14-14.5)	15.5 (15.2-15.8)	16.1 (15.9-16.4)	17.2 (16.9-17.5)	17.6 (16.9-18.3)
Middlesbrough	10.8 (9.1-12.7)	10.8 (9.2-12.8)	11 (9.4-13.1)	11.2 (9.5-13.3)	11.2 (9.6-13)	11.7 (10.5-13)	11.8 (11.4-12.2)	12.4 (12.3-12.6)	12.9 (12.7-13)	13.9 (13.7-14.1)	14.6 (14.4-14.9)	15.5 (15.3-15.8)	16.5 (16.3-16.8)	17.3 (16.7-17.9)
Newcastle upon Tyne	10.9 (9.4-12.7)	11 (9.4-12.8)	11.1 (9.5-12.9)	11.2 (9.7-12.9)	11.2 (9.7-12.8)	11.7 (10.5-13)	12.4 (12-12.7)	12.8 (12.7-13)	13.1 (12.9-13.3)	13.6 (13.4-13.8)	14.9 (14.7-15.2)	15.6 (15.4-15.8)	16.9 (16.8-17.1)	17.3 (16.7-17.9)
North Tyneside	11.3 (9.7-13.3)	11.4 (9.8-13.4)	11.5 (10-13.5)	11.6 (10.2-13.4)	11.6 (10.2-13.3)	12.1 (10.9-13.3)	12.2 (11.9-12.6)	12.5 (12.4-12.7)	13.3 (13.1-13.5)	13.7 (13.6-13.9)	14.8 (14.5-15)	15.9 (15.7-16.2)	17.1 (16.9-17.4)	17.6 (17-18.2)
Northumberland	11.6 (10-13.6)	11.7 (10.1-13.6)	11.9 (10.2-13.9)	12 (10.4-13.8)	12 (10.5-13.7)	12.5 (11.3-13.8)	12.4 (12-12.7)	12.6 (12.5-12.8)	13.5 (13.3-13.6)	14.3 (14.1-14.5)	15.3 (15.1-15.6)	17.1 (16.9-17.3)	18.6 (18.4-18.8)	18.6 (18-19.1)
Redcar and Cleveland	10.9 (9.4-12.9)	11 (9.4-13)	11.1 (9.6-13)	11.2 (9.7-13.1)	11.3 (9.7-12.9)	11.8 (10.6-13.1)	12.2 (11.8-12.5)	12.7 (12.6-12.9)	13.3 (13.1-13.5)	14.3 (14.1-14.6)	15.3 (15.1-15.6)	16.3 (16.1-16.6)	17.6 (17.4-17.9)	18.4 (17.7-19.1)
South Tyneside	10.9 (9.3-12.8)	11 (9.5-12.8)	11.1 (9.6-13)	11.2 (9.8-13)	11.3 (9.8-12.9)	11.7 (10.6-13)	12 (11.7-12.4)	12.1 (11.9-12.3)	13 (12.8-13.2)	13.8 (13.6-14)	14.6 (14.4-14.9)	15.7 (15.5-15.9)	16.9 (16.7-17.1)	16.4 (15.7-17)
Stockton-on-Tees	11.3 (9.6-13.3)	11.4 (9.7-13.5)	11.5 (9.8-13.8)	11.6 (10-13.6)	11.6 (10.1-13.4)	12.1 (10.8-13.4)	12.5 (12.1-12.9)	12.8 (12.6-12.9)	13.2 (13-13.4)	13.9 (13.7-14.1)	15.2 (14.9-15.5)	15.9 (15.7-16.1)	17.6 (17.4-17.8)	17.9 (17.3-18.6)
Sunderland	11 (9.5-12.8)	11.1 (9.5-12.9)	11.3 (9.6-13.1)	11.4 (9.9-13.2)	11.4 (10-13.1)	11.9 (10.8-13.2)	12.1 (11.8-12.4)	12.4 (12.3-12.5)	12.7 (12.5-12.8)	13.5 (13.3-13.7)	14.4 (14.1-14.6)	15.4 (15.2-15.5)	16.9 (16.7-17.1)	17.5 (16.9-18.1)
North West England	11.4 (10.9-11.8)	11.4 (11-11.9)	11.6 (11.2-12.1)	11.7 (11.2-12.2)	11.7 (11.3-12.2)	12.2 (11.9-12.5)	12.5 (12.4-12.6)	12.8 (12.8-12.9)	13.5 (13.4-13.5)	14 (14-14)	15.1 (15.1-15.2)	16.3 (16.3-16.3)	17.3 (17.3-17.3)	18 (17.8-18.1)
Blackburn with Darwen	11.2 (9.6-13)	11.2 (9.6-13.1)	11.4 (9.8-13.2)	11.4 (10-13.3)	11.5 (10.1-13.1)	12 (10.9-13.1)	12.2 (11.9-12.6)	12.5 (12.3-12.7)	13 (12.8-13.2)	13.4 (13.2-13.6)	14.4 (14.2-14.7)	15.5 (15.3-15.7)	16.4 (16.2-16.6)	17.6 (17-18.3)
Blackpool	11.2 (9.6-13)	11.2 (9.6-13.1)	11.4 (9.8-13.1)	11.5 (10-13.3)	11.7 (10.2-13.4)	12.3 (11.2-13.5)	12.6 (12.3-13)	13.1 (12.9-13.3)	14.1 (13.9-14.3)	14.7 (14.5-14.9)	14.6 (14.3-14.8)	14.5 (14.3-14.7)	15.8 (15.6-16)	16.6 (16-17.2)
Bolton	11.2 (9.7-13.1)	11.3 (9.7-13)	11.4 (9.9-13.3)	11.5 (10-13.3)	11.5 (10-13.1)	11.9 (10.8-13)	12.2 (11.9-12.5)	12.7 (12.5-12.8)	13.4 (13.3-13.6)	13.9 (13.7-14.1)	14.9 (14.7-15.2)	16 (15.9-16.2)	17 (16.8-17.2)	18 (17.4-18.6)
Bury	11.6 (10-13.4)	11.6 (10-13.5)	11.7 (10.2-13.6)	11.8 (10.2-13.6)	11.7 (10.3-13.4)	12.2 (11.1-13.4)	12.5 (12.2-12.9)	13 (12.8-13.2)	13.4 (13.2-13.6)	13.7 (13.6-13.9)	15.6 (15.4-15.9)	16.6 (16.3-16.8)	17.4 (17.2-17.6)	17.5 (17-18.1)
Cheshire East	12.2 (10.5-14.3)	12.3 (10.5-14.3)	12.4 (10.7-14.3)	12.4 (10.8-14.3)	12.3 (10.8-14)	12.7 (11.6-13.8)	13 (12.6-13.3)	13.5 (13.4-13.7)	14.4 (14.2-14.5)	15 (14.9-15.2)	16 (15.7-16.2)	17.7 (17.6-17.9)	18.9 (18.7-19.1)	19.2 (18.6-19.8)
Cheshire West and Chester	12.1 (10.4-13.9)	12.1 (10.3-14)	12.3 (10.6-14.1)	12.3 (10.8-14)	12.3 (10.8-13.9)	12.7 (11.6-13.8)	12.9 (12.6-13.2)	13.5 (13.3-13.6)	14 (13.8-14.1)	14.6 (14.5-14.8)	16 (15.7-16.2)	17.6 (17.4-17.8)	18.4 (18.3-18.6)	18.8 (18.2-19.3)
Cumbria	11.8 (10.2-13.6)	11.9 (10.2-13.8)	12.1 (10.4-14)	12.1 (10.5-14)	12.2 (10.7-14)	12.6 (11.5-13.9)	12.9 (12.6-13.2)	13.3 (13.2-13.5)	13.7 (13.6-13.9)	14.5 (14.4-14.7)	15.8 (15.6-16)	16.9 (16.8-17.1)	18.2 (18.1-18.4)	19 (18.4-19.6)
Halton	11.2 (9.6-13.1)	11.2 (9.5-13.1)	11.3 (9.8-13)	11.3 (9.7-13)	11.2 (9.7-12.9)	11.7 (10.6-12.9)	12.1 (11.7-12.5)	12.7 (12.5-12.9)	13.4 (13.2-13.7)	13.9 (13.7-14.1)	14.8 (14.6-15.1)	15.3 (15.1-15.5)	16.7 (16.5-17)	17.6 (17-18.3)
Knowsley	11.2 (9.7-13)	11.3 (9.7-13.1)	11.4 (9.8-13.1)	11.4 (9.9-13.2)	11.4 (9.9-13.2)	11.9 (10.8-13.1)	12.2 (11.9-12.6)	12.9 (12.7-13.1)	13.3 (13.2-13.5)	13.4 (13.2-13.6)	13.9 (13.6-14.1)	15.2 (15-15.5)	15.9 (15.7-16.2)	16.4 (15.8-17.1)
Lancashire	11.5 (10-13.2)	11.6 (10.1-13.4)	11.8 (10.3-13.7)	12 (10.3-13.8)	12 (10.4-13.9)	12.4 (11.1-13.8)	12.7 (12.4-13)	12.9 (12.8-13)	13.7 (13.6-13.8)	14.3 (14.2-14.4)	15.4 (15.2-15.6)	16.7 (16.6-16.8)	17.8 (17.7-18)	18.4 (17.9-19)
Liverpool	10.9 (9.4-12.6)	10.9 (9.5-12.6)	11.1 (9.6-12.9)	11.2 (9.7-12.9)	11.3 (9.8-13)	11.7 (10.6-12.9)	12.1 (11.9-12.4)	12.5 (12.4-12.6)	12.8 (12.7-12.9)	13.1 (12.9-13.2)	14 (13.8-14.2)	15.1 (14.9-15.2)	16 (15.9-16.2)	17.1 (16.5-17.6)
Manchester	10.7 (9.2-12.5)	10.8 (9.2-12.5)	10.9 (9.4-12.8)	11 (9.5-12.7)	11.1 (9.7-12.8)	11.5 (10.5-12.6)	11.8 (11.6-12.1)	12.2 (12.1-12.3)	12.6 (12.5-12.7)	13 (12.9-13.1)	13.9 (13.7-14.1)	14.7 (14.6-14.9)	15.7 (15.6-15.8)	15.9 (15.3-16.4)
Oldham	11.1 (9.5-12.8)	11.2 (9.6-13)	11.3 (9.7-13.2)	11.4 (9.8-13.3)	11.4 (10-13.1)	11.9 (10.9-13)	12.2 (11.9-12.5)	12.3 (12.2-12.5)	13.2 (13-13.4)	13.3 (13.1-13.5)	14.6 (14.3-14.8)	15.6 (15.4-15.8)	16.6 (16.4-16.8)	17.3 (16.7-17.9)
Rochdale	11.2 (9.7-13)	11.3 (9.6-13.1)	11.4 (9.9-13.2)	11.5 (10-13.1)	11.6 (10.1-13.2)	12 (10.9-13.1)	12.5 (12.2-12.8)	12.6 (12.5-12.8)	13 (12.9-13.2)	13.7 (13.5-13.8)	14.9 (14.6-15.1)	15.7 (15.5-15.9)	16.7 (16.5-16.9)	17.5 (16.9-18)
Salford	10.8 (9.3-12.5)	10.7 (9.2-12.5)	10.8 (9.3-12.5)	10.8 (9.3-12.5)	10.8 (9.3-12.4)	11.2 (10.2-12.3)	11.9 (11.6-12.2)	12.1 (12-12.3)	12.9 (12.7-13)	13.3 (13.1-13.4)	14.7 (14.5-14.9)	15.6 (15.4-15.8)	16.3 (16.1-16.4)	16.8 (16.2-17.4)
Sefton	11.6 (9.9-13.4)	11.7 (10.1-13.5)	11.9 (10.3-13.7)	12 (10.4-13.7)	12.1 (10.6-13.7)	12.5 (11.4-13.7)	12.8 (12.5-13.1)	13.2 (13.1-13.4)	14 (13.9-14.2)	14.4 (14.3-14.6)	15.7 (15.4-15.9)	17.3 (17.1-17.5)	17.4 (17.2-17.6)	18.2 (17.6-18.8)
St Helens	11 (9.5-12.7)	11 (9.5-12.9)	11.1 (9.7-12.9)	11.1 (9.6-12.9)	11 (9.6-12.8)	11.4 (10.3-12.6)	11.8 (11.5-12.2)	12.1 (12-12.3)	13 (12.8-13.2)	13.8 (13.6-14)	15.1 (14.9-15.4)	16 (15.8-16.2)	16.7 (16.5-16.9)	16.7 (16.1-17.3)
Stockport	12 (10.4-13.9)	12.1 (10.5-13.9)	12.2 (10.6-14.1)	12.3 (10.7-14.1)	12.3 (10.9-13.9)	12.7 (11.6-13.9)	12.8 (12.5-13.1)	13.1 (12.9-13.2)	13.7 (13.5-13.9)	14.3 (14.1-14.5)	15.9 (15.7-16.2)	17 (16.8-17.1)	17.4 (17.2-17.6)	19 (18.4-19.6)
Tameside	11.3 (9.8-13.1)	11.4 (9.9-13.2)	11.5 (9.9-13.4)	11.6 (10-13.5)	11.7 (10.1-13.4)	12.1 (10.9-13.3)	12.4 (12.1-12.7)	12.8 (12.6-12.9)	13.2 (13-13.4)	13.5 (13.4-13.7)	14.5 (14.3-14.8)	15.6 (15.4-15.8)	16.5 (16.3-16.7)	17.4 (16.8-18)
Trafford	12.4 (10.8-14.3)	12.5 (10.7-14.4)	12.6 (11-14.6)	12.8 (11.1-14.7)	12.9 (11.4-14.7)	13.3 (12.2-14.6)	13.5 (13.1-13.9)	13.5 (13.3-13.7)	14.1 (13.9-14.3)	14.5 (14.4-14.7)	15.9 (15.6-16.1)	17.2 (17-17.4)	18.4 (18.2-18.6)	18.8 (18.1-19.4)
Warrington	11.6 (10-13.5)	11.7 (10-13.6)	11.7 (10.2-13.6)	11.7 (10.2-13.5)	11.7 (10.1-13.4)	12.1 (10.9-13.2)	12.4 (12-12.7)	12.7 (12.5-12.9)	13.2 (13-13.3)	13.8 (13.6-14)	15.5 (15.2-15.7)	16.9 (16.7-17.1)	17.6 (17.4-17.8)	17.5 (16.9-18.1)
Wigan	11.2 (9.6-13)	11.2 (9.6-13)	11.3 (9.7-13.2)	11.4 (9.7-13.2)	11.3 (9.9-13)	11.7 (10.6-12.8)	11.8 (11.5-12.1)	12.2 (12.1-12.3)	12.8 (12.7-13)	13.8 (13.7-14)	14.6 (14.4-14.9)	15.5 (15.4-15.7)	16.7 (16.6-16.9)	17.2 (16.7-17.8)
Wirral	11.5 (9.9-13.4)	11.6 (10-13.6)	11.8 (10.2-13.7)	11.9 (10.4-13.7)	12 (10.6-13.7)	12.5 (11.4-13.6)	12.9 (12.6-13.3)	13.3 (13.1-13.4)	13.5 (13.4-13.7)	14.2 (14.1-14.4)	15.4 (15.2-15.6)	16.6 (16.4-16.8)	17.5 (17.3-17.6)	18 (17.5-18.6)
South East England	12.7 (12.2-13.3)	12.8 (12.2-13.5)	13 (12.4-13.6)	13 (12.4-13.6)	12.8 (12.3-13.3)	13 (12.6-13.4)	13.5 (13.4-13.6)	14.1 (14.1-14.2)	14.9 (14.8-14.9)	15.4 (15.4-15.5)	16.5 (16.5-16.6)	17.9 (17.8-17.9)	18.7 (18.7-18.8)	19.1 (18.9-19.3)
Bracknell Forest	12.9 (11.1-15)	13 (11.2-15)	13.2 (11.4-15.3)	13.1 (11.3-15.1)	13 (11.3-14.7)	13.3 (12-14.5)	13.9 (13.5-14.4)	14.2 (13.9-14.4)	15.2 (15-15.4)	16.1 (15.8-16.4)	17.2 (16.9-17.6)	18.7 (18.4-19)	19.5 (19.2-19.7)	19 (18.3-19.7)
Brighton and Hove	12.3 (10.6-14.3)	12.3 (10.7-14.3)	12.5 (10.7-14.5)	12.5 (10.8-14.3)	12.4 (10.9-14.1)	12.7 (11.7-14)	13.3 (12.9-13.6)	13.8 (13.6-14)	13.6 (13.5-13.8)	13.9 (13.8-14.1)	15.7 (15.5-15.9)	16.2 (16.1-16.4)	17.4 (17.2-17.6)	18.5 (17.9-19.1)
Buckinghamshire	13.3 (11.6-15.2)	13.3 (11.6-15.3)	13.5 (11.7-15.4)	13.5 (11.7-15.5)	13.3 (11.7-15)	13.4 (12.3-14.6)	13.7 (13.4-14)	14.2 (14.1-14.3)	14.9 (14.8-15.1)	15.6 (15.4-15.7)	17.2 (17-17.5)	18.6 (18.4-18.7)	19.3 (19.1-19.4)	20.3 (19.7-20.9)
East Sussex	12.6 (10.9-14.7)	12.7 (10.9-14.8)	12.9 (11-14.9)	12.9 (11.1-14.8)	12.8 (11.2-14.6)	13.2 (11.9-14.4)	14 (13.7-14.4)	14.6 (14.5-14.8)	15.4 (15.2-15.5)	16 (15.8-16.2)	16.9 (16.7-17.2)	18.2 (18-18.3)	18.8 (18.6-18.9)	19.2 (18.6-19.7)
Hampshire	13.1 (11.2-15.1)	13.2 (11.3-15.2)	13.4 (11.6-15.3)	13.4 (11.6-15.4)	13.2 (11.5-15.1)	13.4 (12.2-14.7)	13.9 (13.6-14.2)	14.4 (14.2-14.5)	15 (14.9-15.1)	15.4 (15.3-15.5)	16.7 (16.5-16.9)	18.2 (18.1-18.3)	19 (18.8-19.1)	19.7 (19.2-20.2)
Isle of Wight	12.4 (10.6-14.6)	12.5 (10.8-14.7)	12.7 (11-14.8)	12.8 (11.1-14.9)	12.7 (11.2-14.5)	13 (11.9-14.3)	13.6 (13.2-14.1)	13.8 (13.5-14)	14.5 (14.2-14.7)	15.5 (15.3-15.8)	16.6 (16.3-16.9)	17.4 (17.2-17.7)	18.5 (18.2-18.7)	19.4 (18.8-20)
Kent	12.5 (10.9-14.5)	12.6 (11-14.5)	12.8 (11-14.8)	12.8 (11.1-14.7)	12.4 (10.8-14.4)	12.3 (11.1-13.7)	13 (12.6-13.3)	13.8 (13.7-13.9)	14.7 (14.5-14.8)	15.1 (14.9-15.2)	16.3 (16.1-16.5)	17.6 (17.4-17.7)	18.5 (18.4-18.7)	18.4 (17.9-18.9)
Medway	12.4 (10.5-14.6)	12.6 (10.8-14.6)	12.8 (11-14.9)	12.9 (11.1-14.8)	12.8 (11.1-14.5)	12.9 (11.8-14.2)	13.3 (12.9-13.6)	13.3 (13.2-13.5)	14.3 (14.1-14.5)	15 (14.8-15.1)	15.1 (14.9-15.4)	16.2 (16-16.4)	17.5 (17.3-17.7)	18 (17.5-18.7)
Milton Keynes	12.5 (10.7-14.5)	12.5 (10.7-14.4)	12.5 (10.8-14.6)	12.4 (10.7-14.3)	12.2 (10.6-13.8)	12.3 (11.2-13.5)	13 (12.6-13.4)	13.6 (13.4-13.8)	14.9 (14.7-15.1)	15.2 (15-15.4)	16.5 (16.3-16.8)	17.2 (17-17.4)	17.6 (17.5-17.9)	18.6 (18-19.2)
Oxfordshire	13.2 (11.4-15.3)	13.3 (11.5-15.3)	13.5 (11.7-15.5)	13.5 (11.7-15.6)	13.3 (11.7-15.2)	13.4 (12.3-14.7)	13.8 (13.4-14.1)	14.5 (14.3-14.6)	15.2 (15-15.3)	15.6 (15.4-15.7)	16.7 (16.5-17)	18 (17.8-18.2)	18.9 (18.8-19.1)	19.7 (19.1-20.2)
Portsmouth	12 (10.3-14)	12 (10.3-14.1)	12.1 (10.4-14.1)	12.1 (10.4-14.1)	11.9 (10.5-13.6)	12.1 (11-13.3)	12.3 (12-12.6)	12.8 (12.6-12.9)	13.9 (13.8-14.1)	14.1 (13.9-14.2)	15.1 (14.9-15.4)	16.3 (16.1-16.5)	16.9 (16.8-17.1)	17 (16.5-17.6)
Reading	12.3 (10.6-14.4)	12.3 (10.7-14.3)	12.4 (10.6-14.4)	12.3 (10.5-14.2)	12.1 (10.5-13.9)	12.3 (11.1-13.6)	13.1 (12.7-13.5)	13.7 (13.5-13.9)	14.5 (14.3-14.7)	15 (14.8-15.2)	15.9 (15.6-16.2)	16.8 (16.6-17)	17.4 (17.2-17.6)	17.9 (17.3-18.6)
Slough	12.5 (10.7-14.6)	12.6 (10.8-14.5)	12.7 (10.9-14.7)	12.7 (10.9-14.7)	12.6 (11-14.4)	12.8 (11.7-14.1)	13.3 (12.9-13.8)	13.3 (13.1-13.5)	14.3 (14.1-14.5)	15.4 (15.1-15.6)	15.7 (15.4-16)	16.7 (16.5-17)	16.9 (16.7-17.2)	18 (17.3-18.6)
Southampton	12 (10.2-14.1)	12.1 (10.4-14.1)	12.2 (10.5-14.2)	12.2 (10.5-14)	12.1 (10.6-13.8)	12.4 (11.3-13.6)	13.4 (13.1-13.8)	13.8 (13.6-14)	13.8 (13.6-13.9)	14.3 (14.2-14.5)	15.6 (15.4-15.8)	16.6 (16.4-16.8)	17.5 (17.3-17.6)	17.5 (16.9-18.1)
Surrey	13.4 (11.5-15.6)	13.5 (11.7-15.5)	13.6 (11.8-15.7)	13.6 (11.7-15.7)	13.3 (11.7-15.3)	13.4 (12.2-14.8)	13.7 (13.4-14)	14.3 (14.2-14.5)	15.2 (15-15.3)	15.8 (15.6-15.9)	16.7 (16.5-16.9)	18.3 (18.1-18.4)	19.4 (19.3-19.6)	19.5 (18.9-20)
West Berkshire	13.2 (11.4-15.3)	13.2 (11.4-15.2)	13.3 (11.4-15.4)	13.2 (11.4-15.2)	13 (11.4-14.8)	13.2 (12-14.5)	13.9 (13.5-14.4)	13.4 (13.2-13.6)	15.2 (15-15.4)	16.1 (15.9-16.3)	17.4 (17.1-17.7)	18.2 (17.9-18.4)	19.3 (19-19.5)	19.3 (18.7-20)
West Sussex	12.8 (11.1-14.7)	12.8 (11.1-14.8)	12.9 (11.1-14.9)	12.8 (11.2-14.7)	12.6 (11-14.4)	12.9 (11.7-14.1)	13.7 (13.4-14)	14.5 (14.3-14.6)	15.3 (15.1-15.4)	15.8 (15.6-15.9)	16.8 (16.6-17.1)	18.1 (17.9-18.2)	18.9 (18.7-19)	19.1 (18.5-19.6)
Windsor and Maidenhead	13.9 (12-16)	14 (12.1-16.3)	14.3 (12.4-16.5)	14.4 (12.6-16.5)	14.3 (12.7-16.2)	14.6 (13.4-15.9)	14.8 (14.3-15.2)	14.9 (14.7-15.1)	15.4 (15.2-15.7)	15.8 (15.6-16.1)	16.1 (15.8-16.4)	18.7 (18.5-19)	19.6 (19.3-19.9)	19.7 (19-20.4)
Wokingham	13.8 (11.9-15.9)	13.9 (12-16)	14.1 (12.1-16.2)	14.1 (12.2-16.2)	14 (12.3-15.8)	14.3 (13.1-15.6)	15.1 (14.6-15.5)	15.7 (15.4-15.9)	16.8 (16.6-17.1)	17.7 (17.4-17.9)	19 (18.7-19.3)	19.6 (19.4-19.9)	20 (19.8-20.3)	19.6 (18.9-20.3)
South West England	12.5 (11.9-13)	12.6 (12-13.1)	12.8 (12.2-13.4)	12.8 (12.3-13.3)	12.7 (12.2-13.3)	13.1 (12.7-13.4)	13.7 (13.6-13.8)	14.4 (14.3-14.4)	14.9 (14.9-15)	15.6 (15.6-15.7)	16.6 (16.6-16.7)	17.9 (17.8-17.9)	18.7 (18.7-18.8)	19 (18.9-19.2)
Bath and North East Somerset	13.2 (11.5-15.4)	13.3 (11.5-15.3)	13.6 (11.8-15.7)	13.7 (11.8-15.8)	13.8 (12.1-15.5)	14.3 (13-15.7)	14.9 (14.5-15.3)	15.2 (15-15.4)	15.5 (15.3-15.7)	16.3 (16.1-16.5)	17.5 (17.2-17.8)	18.5 (18.3-18.8)	19.2 (18.9-19.4)	19.2 (18.6-19.8)

Table 11 Estimates of life expectancy at age 65 by location and year for 1950, 1955...2005, 2010, 2017 - males

Location	1950	1955	1960	1965	1970	1975	1980	1985	1990	1995	2000	2005	2010	2017
Bournemouth	12.5 (10.7-14.4)	12.6 (10.7-14.6)	12.9 (11.1-14.8)	13 (11.4-14.9)	13.1 (11.5-14.8)	13.6 (12.4-14.9)	14.6 (14.2-15)	14.7 (14.5-14.9)	14.3 (14.1-14.5)	14.7 (14.5-14.9)	15.9 (15.6-16.2)	17.2 (17-17.4)	17.6 (17.4-17.8)	18.6 (18-19.2)
Bristol, City of	12 (10.4-14)	12.1 (10.4-14.2)	12.3 (10.6-14.5)	12.3 (10.7-14.2)	12.3 (10.8-13.9)	12.5 (11.4-13.8)	13.1 (12.7-13.4)	13.8 (13.7-13.9)	14.2 (14-14.3)	14.8 (14.6-15)	15.7 (15.5-16)	16.8 (16.7-17)	17.9 (17.7-18.1)	18.8 (18.2-19.4)
Cornwall	12.2 (10.6-14.3)	12.3 (10.6-14.2)	12.5 (10.9-14.5)	12.5 (10.8-14.4)	12.4 (11-14.1)	12.7 (11.6-13.9)	13.5 (13.2-13.8)	14.3 (14.2-14.5)	14.7 (14.6-14.8)	15.6 (15.4-15.7)	16.5 (16.2-16.7)	17.9 (17.7-18)	18.7 (18.5-18.8)	18.5 (18-19)
Devon	12.5 (10.8-14.4)	12.6 (10.9-14.6)	12.8 (11-14.7)	12.8 (11.2-14.6)	12.7 (11.2-14.5)	13 (11.9-14.3)	14 (13.7-14.3)	14.8 (14.6-14.9)	15.3 (15.2-15.5)	16 (15.8-16.1)	17 (16.8-17.2)	18.2 (18.1-18.4)	18.9 (18.7-19)	18.6 (18-19.1)
Dorset	12.8 (10.9-14.8)	12.9 (11.1-14.9)	13.2 (11.4-15.3)	13.2 (11.5-15.2)	13.3 (11.8-14.9)	13.7 (12.5-14.9)	14.2 (13.9-14.6)	15 (14.8-15.1)	15.8 (15.6-15.9)	16.4 (16.2-16.6)	17.5 (17.2-17.7)	18.9 (18.7-19.1)	19.6 (19.4-19.8)	20.2 (19.6-20.8)
Gloucestershire	12.7 (11-14.8)	12.8 (11.1-14.8)	13 (11.2-15.2)	13 (11.3-15.1)	12.9 (11.3-14.7)	13.1 (11.9-14.4)	13.3 (12.9-13.6)	14 (13.9-14.2)	14.7 (14.5-14.8)	15.4 (15.3-15.6)	16.7 (16.5-17)	17.9 (17.7-18)	19 (18.8-19.2)	19.1 (18.6-19.7)
North Somerset	12.9 (11.1-15)	13 (11.3-14.9)	13.3 (11.4-15.4)	13.4 (11.7-15.4)	13.4 (11.9-15.1)	13.9 (12.7-15.3)	14.5 (14.1-14.9)	14.9 (14.7-15.1)	15.3 (15.1-15.5)	15.9 (15.7-16.1)	16.9 (16.7-17.2)	17.7 (17.5-17.9)	18.3 (18.1-18.5)	18.8 (18.2-19.3)
Plymouth	11.8 (10.1-13.7)	11.8 (10.1-13.6)	11.9 (10.3-13.8)	11.9 (10.3-13.9)	11.8 (10.3-13.4)	12.1 (11-13.3)	12.8 (12.4-13.1)	13.5 (13.3-13.6)	14 (13.8-14.1)	14.1 (13.9-14.3)	15.4 (15.1-15.6)	16.3 (16.2-16.5)	17.4 (17.2-17.6)	18.5 (17.9-19)
Poole	12.5 (10.8-14.6)	12.6 (10.6-14.7)	12.7 (10.8-14.8)	12.7 (11-14.7)	12.6 (11.1-14.5)	13.1 (11.8-14.5)	14 (13.5-14.4)	14.6 (14.4-14.9)	15.7 (15.5-16)	16.5 (16.3-16.7)	17.5 (17.2-17.8)	18.1 (17.9-18.4)	19 (18.7-19.2)	19.2 (18.6-19.8)
Somerset	12.6 (10.9-14.7)	12.7 (10.9-14.8)	12.9 (11.1-15)	12.9 (11.1-14.8)	12.8 (11.1-14.7)	13.1 (11.9-14.4)	13.6 (13.3-14)	14.2 (14.1-14.4)	15.2 (15-15.3)	15.6 (15.5-15.8)	16.7 (16.5-16.9)	18.2 (18.1-18.4)	18.8 (18.6-19)	19 (18.4-19.6)
South Gloucestershire	12.9 (11.1-15)	12.9 (11.1-14.9)	13 (11.1-15.1)	13 (11.2-15)	12.8 (11.2-14.6)	13.2 (12.1-14.4)	13.9 (13.5-14.3)	14.3 (14.1-14.5)	15.1 (14.9-15.3)	16.1 (15.9-16.3)	16.8 (16.5-17)	17.9 (17.7-18.2)	19.3 (19.1-19.5)	20 (19.4-20.6)
Swindon	12.5 (10.9-14.6)	12.6 (10.9-14.6)	12.7 (10.8-14.8)	12.7 (11-14.6)	12.5 (10.9-14.2)	12.8 (11.6-14)	13.2 (12.8-13.6)	13.6 (13.4-13.8)	14.1 (13.9-14.3)	14.5 (14.3-14.7)	16 (15.7-16.3)	17.2 (17-17.4)	18.2 (18-18.4)	18.1 (17.5-18.7)
Torbay	12.2 (10.5-14.1)	12.3 (10.5-14.3)	12.5 (10.8-14.6)	12.6 (10.8-14.6)	12.6 (10.9-14.3)	13 (11.8-14.3)	13.6 (13.2-14)	14 (13.7-14.2)	15.1 (14.9-15.4)	16 (15.7-16.2)	15.7 (15.4-16)	16.2 (16-16.5)	17.4 (17.2-17.7)	18.6 (18-19.2)
Wiltshire	12.8 (11.1-14.7)	13 (11.2-14.8)	13.2 (11.4-15.2)	13.2 (11.6-15.2)	13.2 (11.7-14.9)	13.4 (12.3-14.7)	13.6 (13.3-14)	14.2 (14.1-14.4)	14.6 (14.4-14.8)	15.9 (15.7-16)	16.6 (16.4-16.9)	17.8 (17.6-18)	19.1 (18.9-19.3)	19.8 (19.3-20.4)
West Midlands	11.7 (11.1-12.3)	11.7 (11.2-12.3)	11.9 (11.3-12.5)	11.9 (11.4-12.5)	11.8 (11.3-12.4)	12.2 (11.8-12.5)	12.6 (12.5-12.7)	13.2 (13.2-13.3)	13.7 (13.6-13.7)	14.4 (14.3-14.4)	15.6 (15.5-15.6)	16.7 (16.7-16.8)	17.9 (17.9-18)	18.4 (18.2-18.5)
Birmingham	11.4 (9.9-13)	11.5 (9.9-13.1)	11.7 (10.1-13.5)	11.7 (10.1-13.5)	11.7 (10.3-13.5)	12.1 (11-13.5)	12.4 (12.1-12.7)	12.9 (12.8-13)	13.4 (13.3-13.5)	14 (13.9-14.1)	15.1 (14.9-15.3)	16.2 (16.1-16.3)	17.7 (17.5-17.8)	17.8 (17.3-18.4)
Coventry	11.7 (10-13.6)	11.8 (10.2-13.8)	12 (10.3-13.9)	12 (10.5-13.9)	12 (10.6-13.8)	12.3 (11.2-13.7)	12.5 (12.2-12.9)	13.5 (13.3-13.6)	13.4 (13.3-13.6)	14.4 (14.2-14.5)	15.6 (15.3-15.8)	16.2 (16-16.3)	17.3 (17.2-17.5)	18.1 (17.5-18.6)
Dudley	11.8 (10.1-13.8)	12 (10.3-13.8)	12.2 (10.5-14.1)	12.2 (10.6-14.2)	12.1 (10.6-13.8)	12.4 (11.2-13.5)	12.5 (12.2-12.8)	12.9 (12.8-13.1)	13.7 (13.6-13.9)	14.3 (14.1-14.4)	15.3 (15.1-15.5)	16.7 (16.5-16.9)	17.9 (17.7-18.1)	18.5 (17.9-19.1)
Herefordshire, County of	12.4 (10.7-14.3)	12.4 (10.6-14.4)	12.6 (10.8-14.5)	12.7 (11-14.6)	12.6 (11-14.5)	13.1 (11.9-14.4)	13.3 (12.9-13.7)	14.3 (14.1-14.5)	14.8 (14.6-15.1)	15.7 (15.5-15.9)	16.9 (16.6-17.1)	17.7 (17.5-17.9)	18.6 (18.4-18.8)	19.4 (18.8-20.1)
Sandwell	11.3 (9.6-13.2)	11.3 (9.8-13.3)	11.5 (9.9-13.4)	11.6 (10-13.5)	11.6 (10.1-13.3)	12 (10.9-13.2)	12.5 (12.2-12.8)	12.8 (12.7-13)	12.9 (12.7-13)	13.4 (13.3-13.6)	14.3 (14.1-14.6)	15.7 (15.5-15.9)	16.9 (16.7-17)	17.6 (17-18.2)
Shropshire	11.9 (10.3-13.9)	11.9 (10.3-13.9)	12 (10.3-14)	12 (10.3-13.8)	11.9 (10.4-13.5)	12.3 (11.2-13.5)	13.2 (12.8-13.5)	13.8 (13.6-14)	14.3 (14.1-14.5)	14.7 (14.5-14.8)	16.2 (16-16.5)	17.3 (17.2-17.5)	18.7 (18.5-18.9)	19.3 (18.7-19.9)
Solihull	12.6 (10.7-14.7)	12.6 (10.8-14.6)	12.7 (11-14.8)	12.7 (11.1-14.7)	12.7 (11-14.4)	13.2 (12-14.5)	13.8 (13.5-14.2)	14.7 (14.5-14.9)	15.5 (15.3-15.7)	16 (15.8-16.2)	17.2 (16.9-17.4)	18.1 (17.8-18.3)	19.4 (19.1-19.6)	19.2 (18.6-19.9)
Staffordshire	11.8 (10.2-13.6)	11.9 (10.3-13.8)	12 (10.3-14)	11.9 (10.3-13.9)	11.6 (10.1-13.4)	11.8 (10.6-13)	12.5 (12.2-12.8)	13.3 (13.2-13.4)	13.6 (13.5-13.7)	14.4 (14.2-14.5)	15.6 (15.4-15.8)	17 (16.9-17.1)	18.3 (18.2-18.5)	18.1 (17.6-18.7)
Stoke-on-Trent	10.9 (9.4-12.8)	10.9 (9.4-12.9)	11 (9.5-13.1)	11 (9.5-13)	10.9 (9.5-12.6)	11.2 (10.1-12.3)	11.8 (11.5-12.1)	12.2 (12.1-12.3)	12.7 (12.6-12.9)	13 (12.8-13.1)	14.5 (14.3-14.7)	15.6 (15.5-15.8)	16.6 (16.4-16.8)	17 (16.5-17.6)
Telford and Wrekin	11.5 (9.9-13.7)	11.5 (9.8-13.7)	11.6 (9.9-13.6)	11.6 (10-13.4)	11.4 (9.9-13.2)	11.7 (10.5-13)	12.1 (11.7-12.4)	12.7 (12.5-12.9)	13.5 (13.2-13.6)	14.6 (14.4-14.8)	15.5 (15.2-15.8)	16.9 (16.6-17.1)	16.7 (16.5-16.9)	17.6 (16.9-18.2)
Walsall	11.3 (9.7-13.3)	11.3 (9.6-13.1)	11.4 (9.7-13.2)	11.4 (9.8-13.1)	11.3 (9.9-12.9)	11.6 (10.5-12.9)	12.3 (12-12.6)	12.7 (12.6-12.9)	13.2 (13.1-13.4)	13.8 (13.7-14)	15.3 (15-15.5)	16.4 (16.2-16.5)	17.3 (17.1-17.5)	17.7 (17.2-18.3)
Warwickshire	12.1 (10.5-14)	12.2 (10.5-14.1)	12.4 (10.6-14.3)	12.4 (10.6-14.3)	12.2 (10.7-13.9)	12.6 (11.4-13.8)	13.2 (12.8-13.5)	13.5 (13.4-13.7)	14.2 (14-14.3)	14.7 (14.5-14.8)	16.1 (15.9-16.4)	17.2 (17-17.3)	18.5 (18.4-18.7)	19.1 (18.5-19.6)
Wolverhampton	11.4 (9.8-13.3)	11.4 (9.9-13.4)	11.6 (9.9-13.5)	11.6 (10-13.3)	11.5 (10-13.2)	11.9 (10.8-13.2)	12.8 (12.4-13.1)	13.1 (12.9-13.2)	13.3 (13.1-13.4)	14.2 (14-14.3)	15.2 (14.9-15.4)	15.4 (15.2-15.6)	16.9 (16.7-17.1)	17.6 (17-18.2)
Worcestershire	12.3 (10.5-14.4)	12.4 (10.6-14.4)	12.6 (10.8-14.7)	12.7 (11-14.7)	12.6 (11-14.4)	12.9 (11.8-14.2)	13 (12.6-13.3)	13.7 (13.6-13.8)	14.3 (14.1-14.4)	15.1 (14.9-15.2)	16.2 (16-16.4)	17.7 (17.6-17.9)	18.4 (18.2-18.6)	19.1 (18.6-19.7)
Yorkshire and the Humber	11.6 (11.1-12.1)	11.6 (11.1-12.2)	11.8 (11.3-12.4)	11.9 (11.4-12.4)	11.9 (11.4-12.4)	12.2 (11.8-12.5)	12.7 (12.6-12.8)	13.1 (13.1-13.2)	13.7 (13.7-13.8)	14.5 (14.4-14.5)	15.5 (15.4-15.6)	16.7 (16.7-16.8)	17.6 (17.6-17.7)	18.1 (18-18.3)
Barnsley	11.4 (9.8-13.1)	11.4 (9.8-13.2)	11.5 (9.9-13.4)	11.6 (10.1-13.5)	11.6 (10.1-13.2)	11.9 (10.8-13.1)	12.5 (12.2-12.8)	12.8 (12.6-12.9)	13 (12.9-13.2)	13.6 (13.5-13.8)	15 (14.7-15.2)	16.3 (16.1-16.5)	17.1 (16.9-17.3)	17.4 (16.9-18)
Bradford	11.3 (9.7-13.4)	11.4 (9.9-13.5)	11.7 (10.1-13.7)	11.9 (10.3-13.8)	11.9 (10.5-13.8)	12.3 (11.1-13.5)	12.3 (12-12.6)	12.8 (12.7-12.9)	13.5 (13.4-13.7)	13.9 (13.8-14.1)	15 (14.7-15.2)	16 (15.8-16.1)	17.5 (17.3-17.6)	18 (17.4-18.6)
Calderdale	11.3 (9.7-13.2)	11.4 (9.7-13.3)	11.5 (9.9-13.5)	11.6 (10-13.5)	11.5 (10-13.2)	11.8 (10.7-13.1)	12.5 (12.1-12.8)	12.6 (12.5-12.8)	13.5 (13.3-13.7)	13.9 (13.7-14.1)	15.3 (15-15.5)	16.6 (16.4-16.8)	17.2 (17-17.4)	17.8 (17.2-18.4)
Doncaster	11.6 (10-13.5)	11.7 (10-13.6)	12 (10.3-14.1)	12.2 (10.6-14.1)	12.2 (10.7-13.9)	12.5 (11.5-13.8)	12.6 (12.3-12.9)	13.3 (13.1-13.4)	13.7 (13.6-13.9)	13.7 (13.5-13.8)	15.2 (14.9-15.4)	16.1 (16-16.3)	17 (16.8-17.2)	17.5 (16.9-18.1)
East Riding of Yorkshire	12.3 (10.6-14.2)	12.3 (10.6-14.3)	12.5 (10.8-14.5)	12.7 (11.1-14.7)	12.7 (11.1-14.4)	13 (11.9-14.2)	13.6 (13.2-13.9)	13.9 (13.8-14.1)	14.2 (14.1-14.4)	15.3 (15.1-15.4)	16 (15.8-16.3)	17.4 (17.2-17.5)	18.3 (18.1-18.5)	19.1 (18.6-19.7)
Kingston upon Hull, City of	11.4 (9.7-13.3)	11.5 (9.8-13.5)	11.8 (10.1-13.8)	12 (10.3-13.8)	12 (10.6-13.7)	12.3 (11.2-13.5)	12 (11.7-12.3)	12.8 (12.7-13)	13.3 (13.1-13.4)	13.7 (13.5-13.8)	14.2 (14-14.5)	15.5 (15.4-15.7)	16.1 (15.9-16.2)	16.5 (16-17)
Kirklees	11.3 (9.7-13.2)	11.3 (9.7-13.2)	11.5 (9.8-13.4)	11.5 (9.8-13.4)	11.4 (9.9-13.2)	11.7 (10.6-12.9)	12.2 (11.9-12.6)	12.9 (12.7-13)	13.4 (13.2-13.5)	14.4 (14.2-14.6)	15.3 (15.1-15.6)	16.4 (16.2-16.5)	17.3 (17.2-17.5)	18.3 (17.7-18.9)
Leeds	11.6 (10-13.6)	11.6 (10-13.6)	11.7 (10-13.6)	11.7 (10-13.5)	11.6 (10.1-13.2)	11.9 (10.8-13.2)	12.6 (12.3-12.8)	13.2 (13.1-13.3)	14 (13.9-14.2)	14.7 (14.6-14.8)	15.6 (15.4-15.8)	16.8 (16.7-17)	17.5 (17.4-17.7)	17.7 (17.2-18.2)
North East Lincolnshire	12 (10.4-13.9)	12.2 (10.5-14.2)	12.5 (10.7-14.6)	12.7 (11-14.7)	12.9 (11.2-14.6)	13.3 (12.1-14.6)	13.7 (13.3-14.1)	13.4 (13.2-13.6)	13.9 (13.7-14.1)	14.6 (14.4-14.8)	15.5 (15.2-15.8)	16 (15.8-16.2)	17.2 (17-17.5)	18 (17.3-18.6)
North Lincolnshire	11.6 (10-13.6)	11.6 (9.9-13.7)	11.8 (10-13.8)	11.8 (10.2-13.8)	11.8 (10.3-13.6)	12.2 (11-13.5)	12.6 (12.2-13)	13 (12.8-13.2)	13.9 (13.7-14.1)	14.6 (14.4-14.8)	15.7 (15.5-16)	17 (16.8-17.2)	17.6 (17.4-17.8)	18.3 (17.7-18.9)
North Yorkshire	12.3 (10.6-14.1)	12.3 (10.6-14.3)	12.5 (10.6-14.4)	12.5 (10.9-14.3)	12.5 (11-14.3)	12.8 (11.7-14.1)	13.4 (13.1-13.8)	13.9 (13.8-14.1)	14.4 (14.3-14.6)	15.6 (15.4-15.7)	16.5 (16.2-16.7)	17.7 (17.5-17.8)	18.8 (18.6-19)	19.2 (18.6-19.8)
Rotherham	11.4 (9.7-13.3)	11.3 (9.7-13.3)	11.4 (9.8-13.5)	11.5 (10-13.3)	11.4 (9.9-13)	11.7 (10.7-12.9)	12.3 (12-12.7)	12.9 (12.7-13)	13.3 (13.1-13.5)	14.3 (14.1-14.5)	15.2 (14.9-15.4)	16.4 (16.3-16.6)	17.2 (17-17.4)	17.5 (16.9-18.1)
Sheffield	11.7 (10-13.5)	11.7 (10.1-13.6)	11.9 (10.3-13.7)	11.9 (10.4-13.7)	11.9 (10.4-13.6)	12.2 (11.1-13.4)	12.7 (12.4-13)	13.1 (13-13.3)	13.5 (13.4-13.7)	14.3 (14.1-14.4)	15.3 (15.1-15.6)	17.1 (17-17.3)	17.7 (17.5-17.9)	18.2 (17.6-18.8)
Wakefield	11.1 (9.5-12.9)	11 (9.4-12.8)	11 (9.4-12.9)	10.9 (9.5-12.8)	10.7 (9.3-12.4)	11 (10.1-12.3)	11.8 (11.5-12.1)	12.2 (12.1-12.4)	13.3 (13.1-13.4)	14.3 (14.1-14.5)	15.2 (15-15.4)	16.3 (16.1-16.4)	17.2 (17-17.4)	17.7 (17.1-18.2)
York	12.4 (10.6-14.5)	12.5 (10.8-14.6)	12.7 (10.9-15)	12.8 (11.1-14.8)	12.8 (11.3-14.6)	13.2 (12.2-14.6)	13.6 (13.3-14)	13.7 (13.5-13.9)	14.4 (14.2-14.6)	15.7 (15.5-16)	16.7 (16.4-17)	17.8 (17.6-18.1)	18.8 (18.5-19)	19.1 (18.4-19.7)
Northern Ireland	12.1 (12-12.2)	12.1 (12-12.2)	12.3 (12.2-12.4)	12.2 (12.1-12.3)	11.9 (11.8-12)	11.8 (11.7-12)	12.1 (12-12.2)	12.6 (12.5-12.7)	13.3 (13.2-13.4)	14.1 (13.9-14.2)	15.4 (15.1-15.6)	16.7 (16.6-16.8)	17.3 (17.2-17.5)	18.3 (17.7-19)
Scotland	11.2 (11.2-11.3)	11.2 (11.2-11.3)	11.3 (11.2-11.4)	11.3 (11.2-11.4)	11.5 (11.4-11.6)	11.6 (11.6-11.7)	12.2 (12.1-12.3)	12.6 (12.5-12.7)	13.2 (13.1-13.2)	13.8 (13.8-13.9)	14.8 (14.6-15)	15.9 (15.8-16)	16.8 (16.7-16.9)	17.4 (16.8-18.1)
Wales	11 (9.4-13)	11.1 (9.6-12.9)	11.4 (9.8-13.2)	11.5 (9.9-13.5)	11.6 (10-13.6)	12.2 (10.9-13.6)	12.7 (12.4-13)	13.3 (13.2-13.4)	13.9 (13.8-14)	14.4 (14.3-14.5)	15.5 (15.3-15.7)	16.7 (16.6-16.8)	17.7 (17.6-17.8)	18.1 (17.6-18.6)
Latin America and Caribbean	11.9 (11.7-12.1)	12.5 (12.3-12.7)	12.8 (12.7-13)	13.3 (13.1-13.4)	13.4 (13.3-13.6)	14 (13.9-14.1)	14.5 (14.4-14.5)	14.7 (14.7-14.8)	15.3 (15.3-15.4)	15.6 (15.5-15.6)	16.4 (16.3-16.4)	16.8 (16.7-16.8)	17 (16.9-17)	17.4 (17.2-17.5)
Andean Latin America	12.2 (11.8-12.6)	13.1 (12.8-13.4)	13 (12.7-13.3)	13.9 (13.6-14.3)	13.3 (13.1-13.6)	15.6 (15.3-15.9)	15.6 (15.3-16)	15.5 (15.2-15.8)	16.2 (15.9-16.5)	16.4 (16.1-16.8)	17.2 (16.9-17.5)	17.6 (17.2-17.9)	17.7 (17.3-18.1)	18.8 (18-19.5)
Bolivia	9.5 (9.2-9.8)	10.7 (10.5-10.9)	11.1 (10.8-11.4)	11 (10.7-11.3)	11.1 (10.8-11.6)	11.5 (11.1-11.8)	11.9 (11.5-12.4)	12.4 (11.9-12.9)	12.9 (12.4-13.5)	13.6 (13-14.1)	14 (13.3-14.4)	14.3 (13.4-15.1)	14.5 (13.2-15.8)	14.9 (13.2-16.7)
Ecuador	12.5 (11.3-13.7)	12.6 (12.5-12.6)	12.6 (12.6-12.7)	13.6 (13.6-13.7)	14.4 (14.3-14.5)	15.9 (15.8-15.9)	16.2 (16.2-16.3)	16.4 (16.3-16.4)	16.4 (16.4-16.5)	17.1 (17-17.1)	17.6 (17.5-17.6)	17.3 (16.9-17.7)	17.4 (17-17.8)	18.4 (17.7-19.1)
Peru	13.5 (12.9-14.2)	14.5 (13.9-15.1)	14 (13.4-14.6)	15.4 (14.8-16.1)	13.6 (13.2-14.1)	17 (16.4-17.7)	16.7 (16.1-17.2)	16.2 (15.6-16.7)	17.2 (16.7-17.8)	17 (16.4-17.5)	18 (17.5-18.6)	18.8 (18.2-19.4)	18.9 (18.3-19.5)	20.2 (19-21.5)
Caribbean	11.9 (11.4-12.4)	12.5 (11.9-13)	13 (12.8-13.2)	13.6 (13.4-13.9)	14.5 (14.3-14.7)	15 (14.8-15.2)	15 (14.9-15.2)	15 (14.9-15.2)	15.4 (15.2-15.5)	15.4 (15.2-15.5)	16.1 (15.8-16.3)	16.4 (16.2-16.7)	15.6 (15.4-15.9)	16.4 (15.9-16.9)
Antigua and Barbuda	10.6 (10.3-10.8)	11.6 (11.4-11.8)	12.2 (12-12.4)	13.1 (12.9-13.3)	12.7 (12.4-13)	12.7 (12.3-13)	13.5 (13-13.9)	14.1 (13.8-14.5)	14.7 (14.4-15.1)	14.9 (14.6-15.3)	15.2 (14.9-15.5)	16 (15.7-16.3)	16.8 (16.5-17.1)	17.3 (16.8-17.8)
The Bahamas	13 (12.1-14.3)	13.6 (12.8-14.6)	14.2 (13.6-14.8)	14.3 (14.1-14.6)	14.1 (14-14.2)	14.5 (14.3-14.6)	14.2 (14.1-14.4)	14.6 (14.5-14.8)	14.5 (14.4-14.7)	14.5 (14.4-14.7)	15.4 (15.3-15.6)	16.1 (16-16.3)	16.1 (16-16.3)	16.3 (15.7-17)
Barbados	12.6 (12.4-12.8)	12.8 (12.7-12.9)	13 (12.8-13.1)	12.9 (12.7-13)	12.8 (12.7-12.9)	12.9 (12.8-13.1)	14.4 (14.3-14.6)	14.5 (14.3-14.7)	15 (14.8-15.1)	15.1 (14.9-15.3)	15.6 (15.4-15.8)	15.7 (15.5-15.8)	17.1 (16.9-17.3)	17.5 (16.9-18.2)
Belize	12.3 (12-12.7)	13.6 (13.4-13.8)	15.7 (15.4-16)	16.7 (16.3-17.2)	16.9 (16.4-17.3)	16.8 (16.3-17.3)	16.5 (16.1-17)	15.9 (15.5-16.3)	16.3 (15.9-16.7)	16 (15.7-16.3)	14.9 (14.7-15)	16.1 (16-16.2)	17.4 (17.3-17.5)	17.4 (17.2-17.7)
Bermuda	11.5 (11.3-11.7)	12.2 (12.1-12.4)	12.8 (12.6-12.9)	13 (12.9-13.1)	12.9 (12.7-13)	13.5 (13.3-13.8)	13.8 (13.6-14)	13.7 (13.5-13.9)	13.7 (13.4-14)	15.5 (15.2-15.6)	16 (15.8-16.3)	16.9 (16.6-17.2)	17.3 (17-17.5)	17.3 (16.8-17.6)
Cuba	13.1 (11.7-14.6)	12.6 (11.5-13.9)	13 (13-13.1)	14 (14-14.1)	15.6 (15.6-15.7)	16.5 (16.5-16.6)	16 (15.9-16.1)	15.8 (15.8-15.9)	15.9 (15.8-15.9)	15.7 (15.6-15.7)	16.7 (16.6-16.7)	16.9 (16.9-17)	17.2 (17.1-17.2)	17.2 (16.2-18.1)
Dominica	12.4 (12.1-12.7)	13.8 (13.6-14.1)	14.6 (14.4-14.9)	15 (14.7-15.2)	14.7 (14.5-15)	15.2 (14.9-15.4)	15.9 (15.7-16.2)	15.7 (15.5-16)	14.8 (14.6-15)	14.8 (14.6-15)	15.3 (15.1-15.5)	15.7 (15.5-16)	15.9 (15.7-16.1)	15.4 (15-15.9)
Dominican Republic	13.8 (13.7-13.9)	15.1 (15-15.2)	16.3 (15.7-17)	17 (16.4-17.6)	16.5 (15.8-17.1)	16.8 (16.1-17.5)	16.8 (16.1-17.4)	17.6 (17-18.3)	18 (17.4-18.6)	18.5 (17.8-19.2)	17.8 (17.1-18.5)	17.5 (16.7-18.4)	17.3 (16.6-18.1)	16.2 (15.2-17.4)
Grenada	13.4 (13-13.9)	13.3 (12.9-13.7)	12.9 (12.5-13.4)	12.1 (11.7-12.6)	13.3 (12.8-13.9)	14.8 (14.3-15.3)	14 (13.6-14.4)	12.9 (12.6-13.2)	12.6 (12.4-12.8)	14.4 (14.2-14.6)	15.5 (15.3-15.7)	16.1 (15.9-16.3)	16.1 (15.9-16.3)	16.9 (16.5-17.3)
Guyana	10 (9.8-10.1)	10.8 (10.7-10.9)	11.6 (11.5-11.8)	11.5 (11-11.9)	12.2 (12.1-12.3)	11.8 (11.6-11.9)	12.3 (12.1-12.5)	13 (12.8-13.2)	12.7 (12.6-12.8)	13.1 (13-13.2)	13.7 (13.6-13.8)	13.6 (13.5-13.7)	13.8 (13.7-13.9)	14.1 (13.2-15)
Haiti	9.1 (8.4-10)	9.3 (8.5-10.2)	9.5 (8.7-10.4)	9.6 (8.8-10.6)	9.9 (9-10.9)	10.3 (9.4-11.2)	10.7 (10-11.6)	11.1 (10.4-11.9)	11.6 (10.9-12.3)	12 (11.2-12.8)	12.5 (11.5-13.5)	12.8 (11.7-13.9)	8.9 (8.3-9.5)	13 (12.2-14.3)
Jamaica	11.4 (11.3-11.4)	12.7 (12.6-12.7)	13.3 (13.3-13.4)	13.5 (13.4-13.6)	15.3 (15.2-15.4)	14.4 (14.3-14.6)	15.5 (15.4-15.7)	14.5 (14.4-14.6)	16 (15.7-16.4)	15.4 (14.8-16.1)	16.2 (16.1-16.3)	18.5 (18.4-18.6)	16.6 (16.5-16.7)	15.6 (14.5-16.8)
Puerto Rico	13.5 (13.4-13.6)	15.7 (15.6-15.8)	15.4 (15.3-15.5)	15.5 (15.4-15.6)	15.5 (15.4-15.6)	15.8 (15.7-15.9)	15.8 (15.7-15.9)	15.6 (15.5-15.6)	15.9 (15.8-16)	16 (16-16.1)	17 (16.9-17.1)	17.5 (17.4-17.6)	18.5 (18.4-18.6)	18.7 (18.2-19.2)
Saint Lucia	13 (12.8-13.3)	14.2 (14.1-14.4)	14.5 (14.3-14.7)	14.5 (14.2-14.8)	13.9 (13.6-14.2)	13.3 (13.1-13.5)	13.7 (13.5-13.9)	14.1 (13.9-14.3)	13.4 (13.2-13.5)	14.8 (14.6-14.9)	15.9 (15.8-16.1)	15.9 (15.7-16)	17.3 (17.1-17.4)	17 (16.6-17.5)
Saint Vincent and the Grenadines	14.6 (14.3-15)	15.2 (15-15.4)	16.4 (16.1-16.7)	15.8 (15.5-16.1)	15 (14.7-15.3)	14.6 (14.3-14.9)	14.6 (14.4-14.8)	14.9 (14.7-15.1)	14.7 (14.5-14.9)	14.8 (14.6-15)	14.6 (14.5-14.8)	15.1 (14.9-15.2)	15.2 (15-15.4)	14.6 (14.3-15)
Suriname	11.2 (10.6-11.9)	11.6 (11.1-12.1)	12.2 (11.6-12.8)	13.1 (12.6-13.7)	12.5 (11.9-13.1)	12.4 (11.9-13)	12.7 (12.3-13.2)	13.8 (13.3-14.4)	14.7 (14.2-15.1)	15.5 (15-16)	14.7 (14.2-15.1)	14.7 (14.2-15.2)	15.5 (15-16)	15.1 (14.2-16)
Trinidad and Tobago	9.9 (9.8-10)	10.4 (10.3-10.5)	11.1 (11-11.3)	11.5 (11.3-11.6)	11.9 (11.8-12)	12.4 (12.3-12.6)	12.6 (12.5-12.7)	12.6 (12.5-12.8)	13.4 (13.3-13.5)	13.6 (13.5-13.7)	14.3 (14.1-14.4)	14.9 (14.7-15)	15.7 (15.5-15.8)	15.7 (14.3-17.3)
Virgin Islands	11.4 (11.1-11.6)	11.8 (11.6-11.9)	12.1 (11.9-12.3)	12.2 (12-12.5)	12.5 (12-12.8)	12.7 (12.3-12.9)	12.8 (12.7-13.2)	13.3 (12.9-13.7)	13.7 (13.4-14)	13.7 (13.2-14.1)	13.9 (13.2-14.6)	14.1 (13.2-15.3)	13.6 (12.9-15.1)	13.5 (12.8-15.4)
Central Latin America	12.7 (12.6-12.9)	13.6 (13.5-13.7)	13.9 (13.8-14.1)	14.2 (14-14.3)	14.2 (14.1-14.4)	14.7 (14.6-14.8)	15.1 (14.9-15.2)	15.3 (15.2-15.3)	16 (16-16.1)	16.1 (16.1-16.2)	17.1 (17-17.1)	17.4 (17.3-17.4)	17.7 (17.6-17.7)	17.8 (17.6-18.1)
Colombia	13.7 (13.7-13.8)	14 (13.9-14)	14.2 (14.2-14.2)	14.1 (14.1-14.2)	14.8 (14.8-14.9)	14.4 (14.4-14.4)	15.4 (14.8-16.2)	14.6 (14.6-14.7)	16.4 (16.4-16.5)	16.2 (16.1-16.2)	17.6 (17.6-17.7)	18.1 (18.1-18.2)	19 (19-19.1)	20.1 (19.2-21)
Costa Rica	12.5 (12.4-12.7)	13.7 (13.5-13.8)	14.5 (14.4-14.7)	14.1 (14-14.2)	14.7 (14.5-14.8)	15.9 (15.8-16)	16 (15.9-16.1)	16.3 (16.1-16.4)	16.8 (16.7-16.9)	16.7 (16.6-16.8)	17.5 (17.4-17.6)	18.5 (18.4-18.6)	18.6 (18.5-18.7)	18.1 (17.6-18.5)
El Salvador	12.7 (12.6-12.8)	13.3 (13.2-13.3)	14.7 (14.6-14.8)	14.8 (14.7-14.8)	13.8 (13.7-13.8)	15.3 (15.2-15.3)	15.1 (14.8-15.5)	16.1 (16-16.2)	16.4 (16.4-16.5)	16.7 (16.3-17)	17.2 (16.8-17.5)	16.3 (16-16.7)	16.6 (16.3-17)	16.6 (15.4-17.8)
Guatemala	11.3 (11.2-11.3)	11.8 (11.7-11.8)	12.6 (12.5-12.7)	12.5 (12.5-12.6)	11.8 (11.8-11.9)	12.6 (12.5-12.6)	13.2 (13.2-13.3)	13.3 (13.3-13.4)	13.8 (13.8-13.9)	14.5 (14.4-14.5)	15.3 (15.2-15.3)	15.5 (15.5-15.6)	16.7 (16.7-16.8)	16.1 (15.3-16.8)
Honduras	11.6 (10.9-12.5)	11.6 (11-12.2)	11.7 (11.2-12.3)	12.5 (11.9-13)	13.3 (12.8-13.7)	14.8 (14.2-15.4)	17 (16.4-17.6)	17.1 (16.2-17.9)	17.4 (16.4-18.4)	16.6 (15.3-18)	16.8 (15.5-18.4)	17 (15.5-18.6)	17.2 (15.7-18.8)	17 (15.7-18.4)
Mexico	12.6 (12.4-12.9)	13.8 (13.6-14.1)	14 (13.8-14.3)	14.4 (14.2-14.7)	14.4 (14.1-14.7)	14.9 (14.8-15.1)	15.1 (15.1-15.2)	15.6 (15.5-15.6)	16 (16-16)	16.2 (16.2-16.3)	17.1 (17-17.1)	17.3 (17.2-17.3)	17.3 (17.2-17.3)	17.3 (17.1-17.4)
Aguascalientes	13.8 (12.6-15.1)	14.8 (13.5-16.2)	14.8 (13.6-16.1)	15 (13.7-16.3)	14.6 (13.5-15.8)	15 (14.3-15.7)	15.6 (15.5-15.8)	15.8 (15.7-15.9)	16.2 (16.1-16.3)	16.4 (16.3-16.5)	17 (16.8-17.1)	17 (16.9-17.1)	17.2 (17.1-17.3)	18.1 (17.6-18.6)
Baja California	11.9 (11-13.2)	13.1 (12-14.3)	13.2 (12-14.4)	13.4 (12.3-14.7)	12.9 (11.8-14.2)	13 (12.3-13.8)	14.1 (13.9-14.2)	13.8 (13.7-13.9)	14.2 (14.1-14.3)	14.5 (14.4-14.5)	14.9 (14.8-15)	15.5 (15.5-15.6)	16.2 (16.2-16.3)	16.7 (16.2-17.2)
Baja California Sur	13.2 (12-14.6)	14.2 (12.9-15.6)	14 (12.8-15.5)	13.9 (12.8-15.2)	13.5 (12.5-14.5)	13.7 (13.1-14.3)	14.7 (14.5-14.9)	15.4 (15.2-15.5)	15.5 (15.3-15.6)	15.2 (15.1-15.3)	16.1 (16-16.3)	16.3 (16.2-16.4)	16.4 (16.3-16.5)	17 (16.5-17.5)
Campeche	14.9 (13.6-16.3)	16.1 (14.8-17.8)	16.3 (14.9-17.9)	16.8 (15.4-18.4)	16.7 (15.6-18)	17.2 (16.4-18)	17.1 (17-17.3)	16.9 (16.7-17)	17.2 (17.1-17.3)	17.1 (16.9-17.2)	18.5 (18.3-18.6)	18.7 (18.5-18.8)	18.3 (18.1-18.4)	17.3 (16.8-17.7)
Chiapas	13.8 (12.7-15.2)	15 (13.7-16.5)	15.2 (13.9-16.6)	15.7 (14.4-17.2)	16 (14.7-17.5)	16.9 (16.1-17.8)	16 (15.9-16.1)	16.4 (16.3-16.4)	17.5 (17.4-17.6)	17.6 (17.5-17.6)	17.7 (17.7-17.8)	17.8 (17.7-17.8)	17.8 (17.7-17.8)	17.7 (17.2-18.1)
Chihuahua	12.2 (11.3-13.4)	13.5 (12.3-14.7)	13.6 (12.4-15)	14.1 (12.8-15.5)	14.1 (12.9-15.4)	14.6 (13.9-15.4)	14.4 (14.3-14.5)	14.1 (14-14.2)	14.2 (14.2-14.3)	14.7 (14.7-14.8)	15.1 (15-15.1)	15.6 (15.5-15.7)	15 (14.9-15.1)	15.6 (15.1-16.1)
Coahuila	12.2 (11.1-13.4)	13.3 (12.2-14.7)	13.4 (12.1-14.9)	13.7 (12.5-15.1)	13.4 (12.1-14.7)	13.8 (13-14.6)	14 (13.9-14.1)	14.3 (14.2-14.4)	14.5 (14.4-14.6)	15.2 (15.1-15.3)	15.7 (15.7-15.8)	16 (15.9-16.1)	16.1 (16-16.2)	16.9 (16.4-17.4)
Colima	11.8 (11-12.9)	12.8 (11.8-13.9)	12.6 (11.6-13.7)	12.7 (11.7-13.7)	12.1 (11.4-13)	12.6 (12.1-13.1)	13.9 (13.8-14.1)	15.4 (15.2-15.5)	15 (14.9-15.1)	15.9 (15.8-16)	17 (16.8-17.1)	16.9 (16.8-17)	17 (16.9-17.1)	16.4 (16-16.9)
Mexico City	11.9 (10.9-13)	13 (12-14.2)	13.1 (12.1-14.3)	13.5 (12.5-14.7)	13.5 (12.6-14.6)	14.1 (13.4-14.8)	13.9 (13.8-14)	14.5 (14.5-14.6)	15 (14.9-15)	15.4 (15.3-15.4)	16.2 (16.2-16.3)	16.4 (16.3-16.4)	16.8 (16.8-16.9)	16.3 (15.9-16.8)
Durango	12.1 (11.1-13.2)	13.3 (12.2-14.6)	13.5 (12.3-14.9)	14.1 (12.9-15.5)	14.2 (13-15.5)	14.9 (14.1-15.7)	14.6 (14.4-14.7)	15.4 (15.3-15.5)	16.3 (16.2-16.4)	16.8 (16.7-17)	17.7 (17.5-17.8)	17.9 (17.8-18)	16.3 (16.2-16.4)	18.3 (17.8-18.8)
Guanajuato	13.1 (12.1-14.2)	14.2 (13.1-15.5)	14.4 (13.3-15.7)	14.7 (13.6-15.9)	14.4 (13.3-15.5)	14.9 (14.2-15.6)	15.9 (15.8-16)	15.4 (15.4-15.5)	16.1 (16-16.1)	16.6 (16.5-16.7)	17.3 (17.2-17.3)	17.5 (17.5-17.6)	17.8 (17.7-17.8)	17.5 (17-17.9)
Guerrero	12.6 (11.6-13.8)	13.9 (12.8-15.1)	14.3 (13.3-15.7)	15.2 (14.1-16.5)	16 (14.9-17.2)	17.7 (16.9-18.5)	17.1 (16.9-17.2)	17.3 (17.3-17.4)	18.1 (18-18.2)	17.8 (17.7-17.9)	19.4 (19.3-19.5)	20.3 (20.2-20.4)	19.2 (19.1-19.3)	18.5 (18-19)
Hidalgo	12 (11.1-12.9)	13.2 (12.2-14.2)	13.4 (12.4-14.5)	14 (12.9-15.2)	14 (12.9-15.2)	14.6 (13.9-15.4)	14.3 (14.2-14.4)	15.9 (15.8-16)	16.9 (16.8-17)	18 (17.9-18.1)	18.5 (18.4-18.6)	18.2 (18.1-18.3)	17.8 (17.7-17.9)	18.5 (18-19)
Jalisco	12.9 (11.8-14.1)	14.1 (13-15.5)	14.3 (13.1-15.7)	14.7 (13.5-16)	14.6 (13.4-15.9)	15.2 (14.5-16)	15.8 (15.7-15.9)	15.7 (15.6-15.8)	15.9 (15.8-15.9)	15.9 (15.9-16)	16.8 (16.7-16.8)	17 (17-17.1)	17.1 (17-17.2)	16.8 (16.4-17.3)
México	13.1 (12.1-14.3)	14.1 (13.1-15.4)	14.2 (13-15.5)	14.4 (13.3-15.8)	14.2 (13-15.4)	14.3 (13.2-15.6)	14.2 (13.4-15)	14.8 (14.7-14.8)	15.7 (15.6-15.7)	15.7 (15.7-15.8)	16.5 (16.4-16.5)	16.8 (16.8-16.9)	17.1 (17.1-17.2)	16.8 (16.3-17.3)
Michoacán de Ocampo	12.7 (11.8-13.7)	13.9 (12.9-15.1)	14.2 (13.2-15.4)	14.8 (13.7-16)	15 (13.9-16.2)	15.6 (14.8-16.4)	16.5 (16.4-16.6)	16.2 (16.1-16.2)	17.3 (17.2-17.4)	17 (16.9-17.1)	18.3 (18.2-18.3)	18.4 (18.3-18.5)	18 (17.9-18.1)	18.6 (18.2-19.1)
Morelos	13.1 (12.1-14.3)	14.3 (13.3-15.7)	14.6 (13.4-16)	15.1 (13.9-16.4)	15.1 (13.8-16.4)	15.8 (14.9-16.7)	15.5 (15.4-15.7)	16.7 (16.6-16.8)	16.8 (16.7-16.9)	16.6 (16.5-16.7)	17.8 (17.7-17.9)	18 (17.9-18.1)	17.8 (17.7-17.9)	17 (16.5-17.5)
Nayarit	12.9 (11.8-14.3)	14.2 (12.9-15.6)	14.4 (13-15.9)	14.9 (13.6-16.4)	15.1 (14-16.5)	16 (15.1-16.8)	16 (15.8-16.1)	16 (15.9-16.1)	16.8 (16.7-17)	16.8 (16.7-17)	18.2 (18.1-18.4)	17.6 (17.5-17.7)	17.6 (17.5-17.7)	18 (17.5-18.6)
Nuevo León	13.5 (12.1-15)	14.7 (13.3-16.1)	14.8 (13.5-16.4)	15.1 (13.7-16.5)	14.7 (13.4-16.2)	15.1 (14.1-16)	15.6 (15.5-15.8)	15.7 (15.6-15.8)	15.2 (15.1-15.3)	15.6 (15.5-15.7)	16.2 (16.1-16.3)	16.3 (16.2-16.3)	16.4 (16.3-16.5)	17.2 (16.7-17.7)

Table 11 Estimates of life expectancy at age 65 by location and year for 1950, 1955...2005, 2010, 2017 - males

Location	1950	1955	1960	1965	1970	1975	1980	1985	1990	1995	2000	2005	2010	2017
Oaxaca	12.1 (11.3-13.1)	13.3 (12.5-14.4)	13.6 (12.6-14.7)	14.1 (13.1-15.2)	14.1 (13.1-15.3)	14.5 (13.9-15.2)	14.6 (14.5-14.7)	15.8 (15.8-15.9)	16.7 (16.6-16.8)	17.1 (17.1-17.2)	18.2 (18.1-18.3)	18.5 (18.4-18.6)	18.5 (18.4-18.5)	18.5 (18-19)
Puebla	11.6 (10.7-12.5)	12.7 (11.8-13.7)	13 (12-14.1)	13.6 (12.6-14.7)	13.7 (12.6-14.8)	14.3 (13.6-15.1)	14.7 (14.6-14.8)	15.7 (15.6-15.7)	16 (15.9-16)	16.4 (16.4-16.5)	17.2 (17.1-17.3)	17.5 (17.4-17.6)	17.6 (17.6-17.7)	16.9 (16.5-17.4)
Querétaro	13.1 (12-14.5)	14.2 (13.1-15.6)	14.4 (13.2-15.7)	14.7 (13.6-16.1)	14.5 (13.3-15.9)	14.8 (13.6-16.1)	14.9 (14.3-15.6)	15.7 (15.6-15.8)	15.9 (15.8-16)	16.3 (16.2-16.4)	16.5 (16.4-16.6)	17.1 (17-17.2)	17.5 (17.4-17.6)	17.2 (16.7-17.7)
Quintana Roo	17.2 (15.6-18.7)	17.8 (16.4-19.4)	17.7 (16.2-19.3)	17.4 (16-18.9)	16.7 (15.5-18.1)	16.6 (15.8-17.4)	17.6 (17.4-17.8)	18.6 (18.4-18.7)	17.1 (17-17.3)	17.1 (17-17.2)	17.5 (17.4-17.6)	17.6 (17.5-17.7)	17 (16.9-17.1)	16.2 (15.8-16.7)
San Luis Potosí	12.7 (11.6-13.9)	13.9 (12.8-15.3)	14.1 (13-15.4)	14.5 (13.3-15.8)	14.2 (13.1-15.4)	14.6 (13.8-15.4)	16.5 (16.3-16.6)	16.8 (16.7-16.9)	17.2 (17.1-17.3)	17.2 (17.1-17.3)	18.1 (18-18.2)	18.3 (18.2-18.4)	18.1 (18-18.2)	18.5 (18-19)
Sinaloa	13 (11.8-14.3)	14.2 (12.9-15.6)	14.3 (13-15.8)	14.7 (13.5-16.2)	14.8 (13.5-16.2)	15.6 (14.7-16.4)	15.6 (15.5-15.8)	15.1 (15-15.2)	16.4 (16.3-16.5)	16.3 (16.2-16.4)	17.1 (17-17.2)	17.1 (17-17.2)	17.2 (17.1-17.3)	18.4 (17.9-19)
Sonora	12.3 (11.2-13.7)	13.4 (12.2-14.9)	13.5 (12.2-14.8)	13.8 (12.4-15.2)	13.5 (12.2-15)	13.8 (12.5-15.1)	13.8 (13.1-14.7)	14.1 (14-14.2)	14.3 (14.2-14.3)	14.2 (14.2-14.3)	15.3 (15.2-15.4)	15.5 (15.4-15.6)	15.8 (15.8-15.9)	16.2 (15.7-16.7)
Tabasco	13 (12-14.3)	14.1 (13.2-15.5)	14.3 (13.1-15.7)	14.7 (13.6-16)	14.8 (13.6-16)	15.2 (14.4-16)	14.8 (14.6-14.9)	17.1 (17-17.2)	17.4 (17.3-17.5)	16.9 (16.8-17)	17.9 (17.8-18)	17.7 (17.6-17.8)	17.6 (17.5-17.6)	17.4 (16.8-17.9)
Tamaulipas	12.4 (11.3-13.6)	13.5 (12.5-14.8)	13.7 (12.6-15.1)	14.3 (13.1-15.6)	14.4 (13-15.6)	15 (14.1-15.7)	14.2 (14.1-14.3)	15.2 (15.1-15.3)	15.9 (15.9-16)	16 (15.9-16.1)	16.7 (16.6-16.7)	16.7 (16.6-16.8)	16.7 (16.6-16.8)	17.4 (16.9-17.9)
Tlaxcala	12.9 (11.8-14.2)	14.1 (12.9-15.5)	14.4 (13.1-15.8)	14.9 (13.7-16.4)	15.1 (13.9-16.3)	15.7 (14.6-16.8)	15.9 (15.1-16.6)	16.3 (16.1-16.5)	16.9 (16.8-17)	17.1 (17-17.2)	17.9 (17.8-18)	18.2 (18.1-18.4)	18.1 (18-18.2)	17.8 (17.3-18.3)
Veracruz de Ignacio de la Llave	13.2 (12.2-14.3)	14.5 (13.4-15.7)	14.8 (13.8-16)	15.4 (14.3-16.7)	15.4 (14.3-16.6)	16 (15.3-16.8)	16.2 (16.1-16.3)	16.8 (16.8-16.9)	16.7 (16.6-16.7)	16.9 (16.9-17)	17.6 (17.5-17.6)	17.4 (17.4-17.5)	17.6 (17.5-17.7)	17.2 (16.8-17.7)
Yucatán	13.7 (12.4-15.1)	14.8 (13.5-16.3)	14.9 (13.5-16.3)	15.1 (13.7-16.6)	14.8 (13.5-16.3)	15 (14.1-15.8)	15.1 (14.9-15.2)	15.6 (15.5-15.7)	15.9 (15.8-16)	16 (15.9-16.1)	16.8 (16.6-16.9)	17.4 (17.3-17.5)	16.9 (16.8-17)	16.7 (16.2-17.1)
Zacatecas	13.3 (12.2-14.6)	14.6 (13.4-15.9)	14.8 (13.6-16.2)	15.3 (14.1-16.7)	15.3 (14.2-16.6)	15.9 (15.1-16.7)	16.8 (16.7-17)	17.6 (17.4-17.7)	17.7 (17.6-17.8)	17.6 (17.4-17.7)	18.4 (18.3-18.5)	18.6 (18.5-18.7)	18 (17.9-18.1)	18.2 (17.7-18.7)
Nicaragua	12.7 (12.5-12.8)	13.8 (13.7-14)	13.7 (13.6-13.8)	13.6 (13.3-14)	13.5 (12.8-14.3)	14.5 (14-15)	15.4 (14.3-16.6)	15.6 (14.7-16.8)	18 (17.4-18.6)	17.7 (17.1-18.4)	18.1 (17.5-18.6)	17.9 (17.3-18.4)	19.3 (19-19.7)	19.9 (18.9-20.7)
Panama	14.7 (14.5-14.9)	14.1 (13.9-14.2)	15 (14.8-15.1)	14.8 (14.7-15)	14.6 (14.5-14.8)	15.9 (15.8-16)	16.6 (16.4-16.7)	16.6 (16.5-16.8)	17.3 (17.2-17.5)	17.1 (17-17.2)	18.2 (18.1-18.3)	18.6 (18.5-18.7)	18.8 (18.7-18.9)	19.7 (19.2-20.2)
Venezuela	11.8 (11.7-11.8)	12.7 (12.7-12.8)	13.5 (13.4-13.6)	13.7 (13.7-13.8)	13.7 (13.7-13.8)	13.9 (13.9-14)	14.3 (14.3-14.4)	14.8 (14.8-14.9)	15.4 (15.4-15.5)	15.5 (15.4-15.7)	16.5 (16.5-16.5)	17 (17-17.1)	17.1 (17-17.1)	16.9 (15.7-18.1)
Tropical Latin America	11 (10.7-11.4)	11.4 (11-11.7)	11.7 (11.4-12.1)	12.1 (11.8-12.5)	12.4 (12-12.8)	12.7 (12.5-13)	13.4 (13.3-13.5)	13.9 (13.9-14)	14.5 (14.5-14.5)	15 (14.9-15)	15.7 (15.6-15.7)	16.1 (16.1-16.2)	16.5 (16.5-16.6)	16.9 (16.8-16.9)
Brazil	11 (10.6-11.3)	11.3 (10.9-11.6)	11.6 (11.3-12)	12.1 (11.7-12.4)	12.3 (12-12.7)	12.7 (12.4-12.9)	13.3 (13.2-13.4)	13.9 (13.8-13.9)	14.4 (14.4-14.5)	14.9 (14.9-15)	15.6 (15.6-15.7)	16.1 (16.1-16.2)	16.5 (16.5-16.6)	16.9 (16.8-16.9)
Acre	11.3 (10.2-12.5)	11.6 (10.5-12.9)	12.2 (11-13.4)	12.7 (11.6-14)	13.2 (12.2-14.3)	13.6 (13-14.3)	14.7 (14.6-14.9)	14.5 (14.4-14.6)	15.7 (15.6-15.8)	16.1 (16-16.2)	17.1 (17-17.2)	17.4 (17.3-17.5)	17.4 (17.3-17.5)	17.2 (16.9-17.4)
Alagoas	11.3 (10.2-12.5)	11.5 (10.5-12.7)	11.9 (10.8-13.2)	12.3 (11.2-13.5)	12.4 (11.4-13.5)	12.7 (12.1-13.5)	14 (13.9-14.1)	14.8 (14.7-14.9)	15.5 (15.4-15.6)	16 (15.8-16.3)	16.8 (16.4-17.1)	17.2 (17-17.5)	17.3 (17.2-17.3)	17 (16.7-17.2)
Amapá	11.8 (10.6-13.2)	12.3 (11-13.6)	13 (11.7-14.3)	13.7 (12.6-14.9)	14.3 (13.3-15.3)	15 (14.4-15.7)	16.1 (15.8-16.3)	15.8 (15.7-16)	16.3 (16.1-16.4)	15.7 (15.5-15.8)	15.8 (15.7-15.9)	17.2 (17.1-17.3)	17.7 (17.6-17.8)	17.3 (17.1-17.5)
Amazonas	11.6 (10.4-12.9)	11.8 (10.6-13.3)	12.2 (10.9-13.6)	12.6 (11.3-14)	12.8 (11.5-14)	13.3 (12.3-14.3)	14.9 (14.6-15.2)	15.9 (15.5-16.2)	16.2 (15.9-16.5)	17.1 (16.8-17.4)	17.1 (17.1-17.2)	17.4 (17.4-17.5)	17.5 (17.4-17.6)	17.6 (17.4-17.9)
Bahia	11.6 (10.5-12.9)	12 (10.8-13.4)	12.5 (11.3-13.8)	13 (11.8-14.4)	13.4 (12.2-14.8)	14 (13-15.2)	15.6 (15.1-16.1)	16 (15.5-16.4)	16.9 (16.4-17.4)	17.3 (16.8-17.8)	17.4 (16.9-17.9)	17.5 (17-18)	17.3 (16.8-17.8)	17.1 (16.9-17.3)
Ceará	11.1 (10-12.3)	11.5 (10.4-12.7)	12 (10.9-13.3)	12.6 (11.4-14)	13 (11.7-14.5)	13.4 (12-14.8)	15.2 (14-16.5)	16.9 (16.1-17.6)	17.7 (17.3-18.2)	17.6 (17.2-18.1)	17.8 (17.4-18.3)	17.7 (17.3-18.2)	18.7 (18.6-18.7)	18.6 (18.4-18.7)
Distrito Federal	10.2 (9.3-11.4)	10.6 (9.6-11.8)	11.1 (10-12.4)	11.5 (10.4-12.8)	11.7 (10.6-12.9)	12.1 (11.4-12.9)	13.4 (13.3-13.5)	14.3 (14.2-14.4)	14 (13.9-14.1)	13.6 (13.5-13.7)	14.4 (14.3-14.4)	15.2 (15.1-15.2)	15.8 (15.7-15.9)	16.8 (16.6-17.1)
Espírito Santo	11.5 (10.4-12.7)	11.7 (10.6-12.9)	11.9 (10.8-13.2)	12.2 (11.2-13.5)	12.3 (11.3-13.5)	12.4 (11.8-13.2)	13 (13-13.1)	13.8 (13.7-13.9)	14.6 (14.5-14.6)	15.1 (15.1-15.2)	15.5 (15.5-15.6)	16 (15.9-16)	16.7 (16.7-16.8)	17.8 (17.5-18)
Goiás	9.7 (8.9-10.7)	10 (9.1-11)	10.3 (9.4-11.4)	10.7 (9.8-11.8)	11.1 (10-12.1)	11.6 (10.6-12.7)	13 (12.5-13.6)	13.4 (13.4-13.5)	14.9 (14.8-15.1)	15.2 (15.2-15.3)	16.1 (16-16.1)	16.8 (16.7-16.8)	16.9 (16.8-17)	17.4 (17.2-17.6)
Maranhão	9.1 (8.4-9.9)	9.3 (8.6-10.1)	9.7 (8.9-10.6)	10.1 (9.3-11.1)	10.4 (9.5-11.5)	10.3 (9.3-11.4)	11.7 (10.8-12.8)	13.2 (12.7-13.9)	16.2 (15.8-16.6)	17.2 (16.7-17.6)	18.3 (17.8-18.7)	17.6 (17.1-18)	17.6 (17.1-18.1)	17.4 (17.1-17.6)
Mato Grosso	11.5 (10.4-12.9)	11.8 (10.6-13.1)	12.2 (10.9-13.5)	12.7 (11.5-14.1)	13.2 (12-14.5)	14.2 (13.2-15.4)	15.9 (15.3-16.5)	14.9 (14.4-15.4)	15.6 (15.1-16.2)	16.5 (16.4-16.6)	16.7 (16.6-16.8)	16.4 (16.4-16.5)	16.9 (16.9-17)	17.6 (17.4-17.9)
Mato Grosso do Sul	11.9 (10.7-13.2)	12.1 (10.9-13.3)	12.4 (11.2-13.7)	12.8 (11.6-14.4)	13 (11.9-14.4)	13.4 (12.6-14.4)	14.2 (14.1-14.3)	14.4 (14.4-14.5)	14.7 (14.6-14.8)	14.8 (14.7-14.8)	15.4 (15.4-15.5)	15.9 (15.8-16)	16.3 (16.2-16.4)	16.7 (16.4-16.9)
Minas Gerais	10.8 (9.7-11.9)	11.1 (10.1-12.3)	11.4 (10.4-12.7)	11.7 (10.7-13)	11.9 (10.9-13.1)	12.1 (11.4-12.8)	13.3 (13.2-13.3)	13.6 (13.6-13.7)	14.4 (14.4-14.4)	15.2 (15.1-15.2)	16.5 (16.4-16.5)	16.9 (16.8-16.9)	17.4 (17.3-17.4)	17.9 (17.7-18.1)
Pará	11.9 (10.7-13.2)	12.2 (11-13.7)	12.7 (11.5-14.1)	13.1 (11.8-14.6)	13.4 (12.1-14.7)	13.8 (12.9-14.7)	15.4 (15.3-15.5)	15.3 (15-15.6)	15.9 (15.6-16.3)	16.9 (16.5-17.2)	17.3 (17-17.7)	17.1 (16.7-17.4)	17.8 (17.7-17.9)	16.9 (16.8-17.1)
Paraíba	12 (10.9-13.3)	12.4 (11.3-13.6)	12.8 (11.6-14.1)	13.4 (12.1-14.7)	13.7 (12.3-15)	14 (13.1-15)	15.1 (15-15.2)	16.4 (16.4-16.5)	16.9 (16.6-17.1)	17.2 (16.8-17.5)	17.6 (17.2-17.9)	17.3 (17-17.7)	17.5 (17.1-17.8)	17.4 (16.8-18)
Paraná	10.5 (9.5-11.8)	10.8 (9.7-12.1)	11.1 (10-12.4)	11.4 (10.2-12.7)	11.7 (10.7-13)	12.1 (11.4-12.8)	12.3 (12.3-12.4)	13 (13-13.1)	13.5 (13.4-13.5)	14.2 (14.1-14.2)	14.8 (14.7-14.8)	15.5 (15.4-15.5)	15.8 (15.7-15.8)	16.4 (16.2-16.5)
Pernambuco	11 (10-12.1)	11.3 (10.3-12.5)	11.7 (10.7-13)	12.3 (11.2-13.5)	12.7 (11.6-13.9)	13.3 (12.6-14.1)	14 (14-14.1)	14.1 (14.1-14.2)	14.7 (14.7-14.8)	15.8 (15.7-15.8)	15.9 (15.8-15.9)	16.5 (16.4-16.5)	16.8 (16.7-16.8)	16 (15.8-16.2)
Piauí	11.4 (10.2-12.6)	11.6 (10.5-13)	11.9 (10.7-13.2)	12.2 (11-13.6)	12.4 (11.1-13.8)	12.9 (11.7-14.3)	14.1 (13.3-14.8)	13.9 (13.5-14.2)	16.3 (15.9-16.7)	17.9 (17.5-18.4)	17.3 (16.9-17.7)	17.3 (17-17.7)	18.4 (18.4-18.5)	18.5 (18.2-18.7)
Rio de Janeiro	10.5 (9.6-11.6)	10.8 (9.8-12)	11.2 (10.2-12.4)	11.5 (10.4-12.7)	11.6 (10.6-12.8)	11.7 (11.1-12.4)	12.4 (12.3-12.4)	12.5 (12.5-12.5)	12.6 (12.6-12.7)	12.9 (12.8-12.9)	14 (13.9-14)	14.7 (14.7-14.7)	15 (15-15.1)	15.6 (15.4-15.8)
Rio Grande do Norte	12.2 (11-13.5)	12.6 (11.4-14)	13.1 (11.9-14.5)	13.7 (12.4-15.2)	14 (12.7-15.4)	14.6 (13.5-15.9)	15.8 (15.2-16.4)	16.9 (16.4-17.3)	17.3 (16.9-17.7)	16.9 (16.5-17.4)	18 (17.6-18.5)	18.4 (18-18.8)	18.8 (18.7-18.9)	17.6 (17.4-17.8)
Rio Grande do Sul	10.6 (9.6-11.8)	10.9 (9.8-12)	11.2 (10-12.4)	11.5 (10.4-12.8)	11.8 (10.6-13)	12.2 (11.6-12.9)	12.3 (12.3-12.3)	12.9 (12.8-12.9)	13.2 (13.2-13.2)	13.5 (13.5-13.6)	14.3 (14.3-14.4)	15 (15-15.1)	15.6 (15.5-15.6)	16.2 (16-16.4)
Rondônia	9.2 (8.5-10.1)	9.4 (8.5-10.4)	9.7 (8.9-10.7)	10 (9.1-11)	10.2 (9.3-11.2)	10.8 (10-11.8)	12.2 (11.7-12.9)	12.8 (12.3-13.4)	13.9 (13.4-14.5)	15.5 (15-16.2)	15.9 (15.4-16.5)	15.9 (15.3-16.4)	16.7 (16.1-17.3)	16.8 (15.9-17.8)
Roraima	9.6 (8.8-10.6)	9.9 (9-10.9)	10.2 (9.3-11.3)	10.6 (9.7-11.7)	10.9 (10-11.9)	11.4 (10.5-12.4)	12.9 (12.1-13.7)	13.7 (13-14.4)	14.6 (14-15.3)	15.3 (14.6-16)	15.6 (15-16.2)	16 (15.4-16.7)	16.5 (15.8-17.1)	16.9 (16-17.9)
Santa Catarina	11.2 (10-12.6)	11.4 (10.2-12.7)	11.7 (10.4-13)	12.1 (10.8-13.4)	12.4 (11.2-13.8)	13 (12.2-13.8)	12.8 (12.8-12.9)	13.3 (13.2-13.3)	13.3 (13.2-13.3)	13.7 (13.6-13.7)	14.4 (14.4-14.5)	15.5 (15.5-15.6)	16 (16-16.1)	16.8 (16.6-17.1)
São Paulo	11.1 (10-12.2)	11.3 (10.3-12.6)	11.7 (10.6-13)	12.1 (10.9-13.4)	12.4 (11.3-13.6)	12.7 (12.1-13.4)	12.5 (12.5-12.5)	13.1 (13-13.1)	13.3 (13.3-13.3)	13.7 (13.7-13.8)	14.4 (14.4-14.4)	15.2 (15.2-15.2)	15.7 (15.7-15.7)	16.5 (16.3-16.6)
Sergipe	12 (10.8-13.3)	12.3 (11.2-13.6)	12.7 (11.5-14)	13.1 (11.9-14.5)	13.4 (12.2-14.7)	13.8 (13-14.7)	14.4 (14.3-14.5)	15.6 (15.5-15.7)	15.8 (15.7-15.9)	15.8 (15.7-15.9)	16.9 (16.8-17)	17.5 (17.4-17.6)	17.3 (17.2-17.4)	17 (16.8-17.3)
Tocantins	10.4 (9.5-11.5)	10.7 (9.7-11.9)	11.1 (10.1-12.2)	11.5 (10.3-12.7)	11.8 (10.6-13)	12.3 (11.1-13.6)	13.9 (12.7-15.2)	15.1 (14.2-16.1)	16 (15.5-16.4)	15.8 (15.6-16.1)	16.9 (16.6-17.1)	18 (17.8-18.3)	18.6 (18.3-18.9)	18.6 (18.1-19.2)
Paraguay	13.1 (12.3-14)	14.3 (13.9-14.8)	15.4 (14.9-15.8)	15.5 (15-16)	15.5 (15.1-16)	15.3 (14.9-15.8)	15.9 (15.4-16.3)	15.4 (14.9-15.8)	16.3 (15.8-16.7)	16.2 (15.7-16.8)	16.7 (16.2-17.2)	16.5 (16-17)	16.1 (15.6-16.6)	16.5 (15.1-18)
North Africa and Middle East	10.5 (10.3-10.8)	10.9 (10.7-11.2)	11.4 (11.2-11.7)	12 (11.7-12.2)	12.1 (11.8-12.4)	12.5 (12.3-12.7)	12.9 (12.7-13.1)	13.2 (13-13.3)	13.8 (13.6-14)	14.1 (13.9-14.2)	14.6 (14.4-14.7)	14.9 (14.8-15.1)	15.2 (15-15.4)	15.8 (15.5-16.1)
North Africa and Middle East	10.5 (10.3-10.8)	10.9 (10.7-11.2)	11.4 (11.2-11.7)	12 (11.7-12.2)	12.1 (11.8-12.4)	12.5 (12.3-12.7)	12.9 (12.7-13.1)	13.2 (13-13.3)	13.8 (13.6-14)	14.1 (13.9-14.2)	14.6 (14.4-14.7)	14.9 (14.8-15.1)	15.2 (15-15.4)	15.8 (15.5-16.1)
Afghanistan	9.2 (8.5-10.1)	9.4 (8.7-10.4)	9.6 (8.8-10.5)	9.7 (8.9-10.6)	9.8 (9.1-10.7)	9.9 (9.3-10.7)	9.9 (9.3-10.6)	9.9 (9.2-10.7)	10.7 (10-11.7)	10.7 (10-11.7)	10.7 (10-11.7)	11.1 (10.4-12.1)	11.7 (11-12.5)	12.5 (12-13.2)
Algeria	11.9 (11.6-12.3)	12.3 (12.1-12.5)	13 (12.7-13.3)	14.1 (13.8-14.5)	13.4 (12.9-13.9)	12.8 (12.2-13.2)	12.7 (12.3-12.9)	14.9 (14.6-15.2)	15.8 (15.3-16.3)	16.4 (15.8-16.9)	16.7 (16.2-17.2)	17.2 (16.6-17.7)	17.9 (17.6-18.2)	18.5 (18.1-18.9)
Bahrain	9.4 (8.5-10.5)	9.7 (8.7-10.9)	10.2 (9.1-11.5)	10.7 (9.7-12)	11.3 (10.3-12.4)	12 (11.3-12.9)	13.7 (13.2-14.2)	13 (12.7-13.3)	12.2 (12-12.6)	12.3 (12-12.6)	13.8 (13.5-14.2)	14.1 (13.8-14.4)	16.8 (16.4-17.1)	17.7 (17-18.4)
Egypt	10.9 (10.9-10.9)	11.9 (11.8-12)	12.5 (12.4-12.5)	12.9 (12.8-12.9)	11.4 (11.4-11.5)	11.4 (11.4-11.4)	12 (12-12.1)	11.3 (11.3-11.3)	11.8 (11.8-11.9)	11.5 (11.5-11.5)	11.9 (11.9-11.9)	11.8 (11.8-11.8)	11.8 (11.8-11.8)	12 (11.3-12.7)
Iran	10.2 (10-10.4)	10.4 (10.1-10.6)	10.8 (10.6-11.1)	11.6 (11.3-11.8)	12.5 (12.3-12.8)	13.7 (13.4-13.9)	14.2 (13.9-14.5)	14.8 (14.5-15.1)	15.1 (14.8-15.4)	16.1 (15.9-16.3)	16.6 (16.5-16.7)	16.5 (16.5-16.6)	16.9 (16.8-16.9)	17.4 (17.3-17.4)
Iraq	10.8 (10.2-11.5)	11.6 (11.1-12.1)	12.2 (11.9-12.6)	12.5 (12.2-12.8)	12.5 (12.2-12.9)	12.6 (12.2-13.1)	13.1 (12.4-13.7)	13 (12.1-14)	13.2 (12.3-14.2)	12.9 (12.2-13.8)	13.3 (12.6-14.2)	15 (14.1-16.1)	17.6 (16.8-18.5)	20 (19.4-20.5)
Jordan	11 (10.1-12.2)	11.4 (10.4-12.4)	12 (11.1-13)	12.9 (12.2-13.6)	13.7 (13.3-14.1)	14.1 (13.8-14.5)	13.8 (13.4-14.3)	14.8 (14-15.5)	15.6 (14.7-16.5)	16.2 (15.3-17.1)	17.1 (16.4-17.9)	18 (17.5-18.4)	17.8 (17.4-18.2)	18.2 (17.1-19.2)
Kuwait	13.5 (12.3-14.9)	13.9 (12.7-15.3)	14.2 (13.4-15)	14.5 (14.4-14.7)	15 (14.8-15.1)	15.3 (15.2-15.4)	16.2 (16.1-16.3)	17.7 (17.6-17.8)	17.9 (17.7-18)	19.7 (19.6-19.8)	18.7 (18.6-18.8)	18.8 (18.7-18.9)	20 (19.9-20.1)	20.2 (19.8-20.7)
Lebanon	11.6 (10.4-12.9)	11.8 (10.7-13.2)	12.2 (11-13.6)	12.4 (11.4-13.9)	12.7 (11.9-14.1)	12.7 (12-13.8)	13 (12.5-14.1)	12.9 (12.6-13.7)	13.3 (12.8-14.3)	14.4 (13.6-15.4)	15.2 (14.4-15.9)	15 (14.4-15.7)	15.7 (15-16.5)	16.7 (16.1-17.2)
Libya	10.4 (9.4-11.6)	11.2 (10.1-12.5)	12.1 (11-13.2)	13.4 (12.4-14.3)	14.4 (13.7-15.2)	14.6 (14.1-15.3)	15.4 (14.6-16.3)	15.7 (14.9-16.8)	15.9 (14.8-16.9)	16.2 (15.2-17.1)	16.2 (15.3-16.9)	16.5 (15.9-17.3)	15.4 (14.8-16.1)	15.4 (14.4-16.6)
Morocco	10 (9.1-11.1)	10.2 (9.3-11.3)	10.3 (9.4-11.4)	11 (10-12.2)	11.5 (10.5-12.8)	12.1 (11.1-13.1)	12.8 (12-13.7)	14.1 (13.5-14.8)	15.4 (14.9-16)	15.1 (14.6-15.6)	14.7 (14.1-15.2)	14.8 (14.1-15.6)	15.1 (14-16.2)	15.9 (14.5-17.4)
Palestine	10.3 (9.4-11.5)	10.8 (9.8-12.1)	11.5 (10.4-12.9)	12.3 (11-13.7)	12.5 (11.3-13.7)	13.4 (12.1-14.7)	13.7 (12.5-15.2)	13.9 (12.7-15.4)	14 (12.9-15.4)	14.1 (13.1-15.2)	14.1 (13.5-14.6)	14.4 (14.1-14.7)	15.8 (15.5-16.1)	16.6 (15.9-17.3)
Oman	8.5 (7.9-9.3)	8.8 (8.1-9.6)	9.2 (8.4-10.1)	9.8 (8.8-10.7)	11.2 (10.1-12.4)	12.2 (11-13.4)	12.4 (11.1-13.8)	12.9 (11.5-14.3)	13.2 (11.9-14.7)	13.6 (12.3-15.2)	14.1 (12.8-15.6)	14.9 (13.5-16.5)	14.7 (13.3-16)	16.4 (15-18.1)
Qatar	11.5 (10.3-12.9)	11.5 (10.3-12.9)	11.7 (10.5-13)	11.7 (10.5-13)	11.9 (10.8-13.2)	12.2 (11.1-13.5)	12.4 (11.3-13.6)	13 (12-14.2)	13.3 (12.3-14.3)	13.7 (12.7-14.7)	15.1 (14.1-16.2)	17.3 (16.2-18.4)	17.5 (16.3-18.7)	18.8 (17.5-20.2)
Saudi Arabia	12.1 (10.8-13.3)	12.4 (11.2-13.8)	12.9 (11.7-14.2)	13.5 (12.3-15)	14.2 (13-15.7)	15.1 (13.9-16.7)	15.5 (14.1-17.2)	15.5 (14.1-17)	15.4 (14.1-16.8)	15.8 (14.6-17)	16 (15.1-16.9)	15.5 (14.6-16.2)	14.6 (14-15.6)	16.6 (15.7-17.1)
Sudan	10.5 (9.5-11.6)	10.7 (9.7-11.8)	11.1 (10.1-12.2)	11.3 (10.4-12.3)	11.5 (10.6-12.4)	11.7 (11-12.5)	12.1 (11.4-12.8)	11.8 (11.1-12.4)	13 (12.1-13.8)	13.3 (12.3-14.3)	13.7 (12.6-14.8)	14.1 (12.9-15.2)	14.4 (13.2-15.9)	14.8 (13.3-16.5)
Syria	13.5 (12.1-15)	14.3 (12.9-15.9)	15.1 (13.7-16.5)	16 (14.8-17.4)	16.9 (15.8-18.1)	18.5 (17.6-19.5)	16.8 (15.9-17.7)	14.8 (13.9-15.7)	14.2 (13.5-15)	13.7 (12.9-14.4)	14 (13.4-14.8)	15.2 (14.4-15.9)	15.4 (14.5-16.4)	15.3 (14-16.6)
Tunisia	9.9 (9.1-10.9)	10.3 (9.6-11.1)	10.9 (10.3-11.4)	10.7 (10.2-11.2)	11.1 (10.8-11.4)	12.8 (12.5-13.1)	14.4 (14-14.8)	15.4 (15.2-15.7)	15.6 (15.3-15.9)	15.2 (14.9-15.6)	15.2 (14.5-15.9)	15.8 (14.7-17)	16.3 (15-17.9)	16.7 (15.2-18.4)
Turkey	9.9 (9-11.1)	10.5 (9.5-11.8)	11 (9.9-12.4)	11.6 (10.4-13)	12.3 (11-13.5)	12.8 (11.9-13.7)	13.2 (12.8-13.6)	13.6 (13.1-14.1)	13.9 (13.5-14.3)	14.5 (14.1-14.9)	15.8 (15.4-16.2)	16.4 (16-16.8)	16 (15.6-16.4)	16.3 (15.6-17)
United Arab Emirates	11.5 (10.3-12.9)	11.9 (10.6-13.3)	12.3 (11-13.8)	12.9 (11.5-14.3)	13.3 (12-14.8)	13.4 (12.1-15.1)	13.7 (12.3-15.3)	13.8 (12.5-15.3)	13.6 (12.5-15)	13.9 (12.9-15.1)	12.9 (12-13.8)	14.1 (13.2-15.1)	13.8 (12.7-15)	13.8 (12.4-15.3)
Yemen	8.7 (8-9.6)	8.9 (8.1-9.7)	9.2 (8.4-10)	9.3 (8.5-10.2)	9.6 (8.7-10.6)	10.4 (9.5-11.5)	11.1 (9.9-12.2)	11.7 (10.4-12.9)	12.1 (10.8-13.4)	12.5 (11.1-13.8)	12.9 (11.6-14.1)	13.3 (12-14.5)	13.6 (12.3-14.9)	13.6 (12.5-15)
South Asia	9.9 (9.6-10.3)	10.2 (9.9-10.6)	10.4 (10.1-10.7)	10.6 (10.3-10.9)	11 (10.8-11.3)	11.5 (11.2-11.7)	11.3 (11.1-11.6)	12.1 (11.8-12.3)	12.1 (11.9-12.3)	12.5 (12.4-12.7)	12.7 (12.6-12.8)	13.4 (13.3-13.5)	13.4 (13.3-13.5)	14.2 (14-14.4)
South Asia	9.9 (9.6-10.3)	10.2 (9.9-10.6)	10.4 (10.1-10.7)	10.6 (10.3-10.9)	11 (10.8-11.3)	11.5 (11.2-11.7)	11.3 (11.1-11.6)	12.1 (11.8-12.3)	12.1 (11.9-12.3)	12.5 (12.4-12.7)	12.7 (12.6-12.8)	13.4 (13.3-13.5)	13.4 (13.3-13.5)	14.2 (14-14.4)
Bangladesh	10.9 (9.8-12.1)	11.2 (10.1-12.4)	11.5 (10.4-12.7)	11.9 (10.8-13.1)	11.4 (10.6-12.4)	12.8 (12-13.7)	13.8 (13.3-14.4)	12.3 (11.9-12.6)	12.5 (12-12.9)	13.4 (12.9-13.8)	14.1 (13.7-14.5)	14.5 (14.2-14.8)	14.7 (14.4-15.1)	16.2 (15.3-17.1)
Bhutan	9 (8.3-9.8)	9.4 (8.6-10.3)	9.9 (9.1-10.9)	10.5 (9.6-11.6)	11.2 (10.2-12.3)	11.7 (10.6-12.9)	12.1 (11-13.3)	12.8 (11.7-13.9)	13.5 (12.5-14.5)	14 (13.2-15)	14.4 (13.8-15.3)	14.8 (14-15.9)	15 (14.1-16.2)	15.5 (13.7-17)
India	9.5 (9.2-9.8)	9.8 (9.4-10.1)	9.9 (9.6-10.2)	9.9 (9.6-10.3)	10.4 (10.1-10.7)	10.7 (10.5-11)	10.5 (10.2-10.8)	11.7 (11.4-11.9)	11.7 (11.5-12)	12.3 (12.1-12.5)	12.4 (12.3-12.6)	13.3 (13.1-13.4)	13.2 (13.1-13.3)	13.9 (13.7-14.1)
Andhra Pradesh	10.1 (8.8-11.7)	10.3 (8.9-12)	10.6 (9.2-12.2)	10.7 (9.4-12.2)	11.1 (9.9-12.5)	11.4 (10.2-12.7)	11.1 (9.9-12.4)	12.3 (11-13.7)	12.3 (11-13.6)	12.8 (11.5-14.2)	13 (11.7-14.5)	14.4 (12.9-15.9)	14.5 (13.2-15.8)	15.3 (13.9-16.6)
Arunachal Pradesh	10.1 (8.8-11.7)	10.4 (9-12)	10.4 (9-12)	10.2 (9-11.5)	11 (9.8-12.2)	11.4 (10.3-12.6)	11.2 (10-12.4)	12.1 (10.9-13.4)	11.9 (10.6-13.2)	12.1 (11-13.5)	12 (11.1-13.3)	12.7 (12-13.8)	12.7 (12.3-13.7)	13 (12.7-14.2)
Assam	8.7 (7.7-10.1)	9 (8-10.5)	9.1 (8-10.5)	9.2 (8.1-10.4)	9.8 (8.7-11)	10.2 (9.1-11.3)	10 (9-11.3)	11.2 (10.2-12.3)	11.2 (10.5-12)	11.7 (11.3-12)	11.3 (11-11.6)	11.8 (11.5-12.1)	12.1 (11.8-12.4)	13.2 (12.7-13.8)
Bihar	10.1 (8.8-11.8)	10.4 (9-12.2)	10.5 (9.1-12.1)	10.5 (9.2-11.8)	11 (9.8-12.2)	11 (10-12.1)	10.6 (9.5-11.7)	11.6 (10.5-12.7)	11.6 (10.6-12.7)	12 (11.2-12.9)	11.9 (11.4-12.6)	13.1 (12.7-13.5)	13.3 (13-13.6)	14.9 (14.2-15.5)
Chhattisgarh	9.6 (8.3-11.1)	9.8 (8.6-11.3)	10 (8.8-11.5)	10 (8.8-11.3)	10.7 (9.6-11.9)	10.7 (9.7-11.9)	10.6 (9.5-11.8)	11.7 (10.6-12.8)	11.8 (10.8-12.9)	12.4 (11.6-13.4)	12 (11.5-12.6)	11.7 (11.3-12)	10.5 (10.3-10.7)	10.9 (10.5-11.3)
Delhi	10.3 (9-11.9)	10.8 (9.4-12.4)	11.2 (9.8-12.7)	11 (9.8-12.2)	12.1 (10.9-13.4)	12.3 (11.2-13.5)	12 (10.8-13.2)	13 (11.8-14.3)	12.9 (11.8-14.1)	13.4 (12.3-14.6)	13.4 (12.5-14.3)	14.3 (13.7-15)	14.8 (14.3-15.4)	15.7 (14.8-16.6)
Goa	12.3 (11-13.8)	12.7 (11.2-14.1)	12.9 (11.4-14.3)	12.9 (11.6-14.2)	13.4 (12.4-14.4)	13.7 (12.7-14.6)	13.1 (12.1-14.2)	13.7 (12.6-14.7)	13 (12.2-13.9)	13.2 (12.3-14.1)	13.2 (12.3-14.3)	14.3 (13-15.5)	14.4 (13.1-15.7)	14.9 (13.4-16.1)
Gujarat	9.7 (8.5-10.9)	9.9 (8.7-11.4)	10 (8.9-11.5)	10 (8.9-11.3)	10.6 (9.5-11.8)	10.9 (10-12)	10.7 (9.7-11.8)	11.9 (11-13)	12 (11.3-12.8)	12.9 (12.4-13.4)	12.4 (12.1-12.8)	12.9 (12.6-13.3)	13.6 (13.3-14)	14.4 (13.7-15)
Haryana	10.1 (9-11.6)	10.5 (9.1-12)	10.7 (9.4-12.1)	10.9 (9.6-12.3)	11.6 (10.4-12.9)	12.3 (11.2-13.6)	12.4 (11.2-13.8)	13.7 (12.7-14.9)	13.9 (13.1-14.8)	14.2 (13.8-14.7)	14.1 (13.8-14.5)	14.1 (13.8-14.4)	13.8 (13.5-14.2)	14.4 (13.7-15)
Himachal Pradesh	10.2 (8.9-11.9)	10.4 (9.1-12.2)	10.6 (9.2-12.5)	10.8 (9.4-12.4)	11.3 (10-12.7)	11.6 (10.4-13.1)	11.5 (10.2-13)	12.7 (11.5-14)	12.9 (11.9-13.9)	13.9 (13.3-14.5)	13.7 (13.2-14.2)	14.3 (13.9-14.8)	14.2 (13.8-14.7)	13.8 (13.1-14.6)
Jammu and Kashmir	10.8 (9.4-12.4)	11 (9.6-12.7)	11.1 (9.8-12.8)	11 (9.8-12.5)	11.7 (10.5-13)	12 (10.9-13.3)	11.2 (10.1-12.4)	12.5 (11.4-13.9)	12.6 (11.5-13.8)	13.2 (12.3-14.3)	13.9 (13.1-14.7)	15.6 (15.1-16.2)	14.5 (14.1-15)	16.5 (15.8-17.2)
Jharkhand	9.8 (8.7-11.3)	10 (8.8-11.6)	10.1 (8.9-11.6)	10.1 (8.9-11.5)	10.6 (9.5-11.9)	10.9 (9.9-12.1)	10.5 (9.5-11.7)	11.1 (10.1-12.3)	11 (10.1-12)	11.4 (10.7-12.3)	11.4 (10.9-12)	12.4 (12.1-12.7)	12.8 (12.5-13.1)	14.4 (13.9-15)
Karnataka	9.2 (8.1-10.6)	9.5 (8.3-11)	9.6 (8.5-11)	9.7 (8.6-11)	10.2 (9.2-11.4)	10.6 (9.7-11.7)	10.5 (9.5-11.7)	11.8 (10.8-12.8)	11.9 (11.2-12.7)	12.7 (12.3-13.1)	12.3 (12-12.6)	13.4 (13.1-13.7)	12.9 (12.6-13.2)	12.8 (12.3-13.2)
Kerala	10 (8.6-11.5)	10.3 (9-11.9)	10.6 (9.2-12.2)	10.8 (9.5-12.2)	11.2 (9.9-12.5)	11.5 (10.5-12.7)	11.3 (10.2-12.5)	12.3 (11.2-13.4)	12.3 (11.4-13.1)	13.4 (12.8-14.1)	12.5 (12-13)	14.1 (13.7-14.6)	14.1 (13.6-14.6)	13.7 (12.9-14.5)
Madhya Pradesh	9.2 (8.1-10.5)	9.5 (8.4-10.9)	9.6 (8.5-10.9)	9.5 (8.5-10.6)	10.1 (9.2-11.2)	10.3 (9.3-11.2)	9.8 (8.9-10.9)	10.8 (9.8-11.9)	11 (10-12)	11.6 (10.8-12.5)	11.8 (11.2-12.4)	12.6 (12.2-13)	12 (11.7-12.4)	13.1 (12.4-13.6)
Maharashtra	9.6 (8.4-11)	9.9 (8.7-11.2)	10 (9-11.4)	10.1 (9.1-11.4)	10.7 (9.7-11.9)	11.1 (10.1-12.2)	11 (10-12.1)	12.2 (11.3-13.2)	12.3 (11.6-13)	12.9 (12.6-13.2)	13 (12.7-13.3)	14.4 (14.1-14.7)	14.2 (13.9-14.5)	15.2 (14.6-15.7)
Manipur	11 (9.5-12.7)	11.3 (9.9-13)	11.5 (10-13.1)	11.5 (10.2-12.9)	12.1 (11-13.4)	12.5 (11.4-13.6)	12.1 (11-13.2)	12.8 (11.7-13.8)	12.4 (11.5-13.4)	12.5 (11.7-13.5)	12.3 (11.8-13.2)	13.1 (12.4-14.2)	13 (12.6-13.9)	13.1 (12.6-14.2)
Meghalaya	11.1 (9.6-12.8)	11.3 (9.8-13)	11.4 (9.9-13.1)	11.3 (10-12.7)	11.8 (10.6-13.1)	12 (10.9-13.1)	11.5 (10.4-12.6)	12.2 (11-13.3)	11.9 (10.8-13.1)	12 (11.1-13.3)	11.9 (11.2-13.1)	12.7 (12-13.8)	12.7 (12.1-13.6)	12.9 (12.4-13.8)
Mizoram	11.3 (10.1-13)	11.5 (10.4-13.2)	11.7 (10.5-13.3)	11.4 (10.4-12.7)	12.1 (11.2-13.3)	12.3 (11.4-13.5)	11.8 (10.8-12.8)	12.5 (11.8-13.7)	12.3 (11.6-13.2)	12.5 (11.9-13.3)	12.4 (12-13.1)	13.2 (12.4-14.2)	13.1 (12.3-14.1)	13.3 (12.6-14.5)
Nagaland	10.9 (9.4-12.7)	11.3 (9.7-13.1)	11.5 (9.9-13.3)	11.6 (10.2-13.1)	12.3 (11-13.6)	12.6 (11.5-13.9)	12.2 (11-13.5)	13 (11.7-14.1)	12.6 (11.4-13.7)	12.8 (11.7-13.9)	12.5 (11.6-13.7)	13.4 (12.4-14.8)	13.3 (12.5-14.6)	13.6 (12.7-15.1)
Odisha	9 (7.8-10.4)	9.3 (8.1-10.8)	9.3 (8.1-10.7)	9.4 (8.2-10.6)	9.9 (8.8-11.1)	10.3 (9.3-11.5)	10.4 (9.3-11.5)	11.7 (10.6-12.6)	11.7 (11.1-12.5)	11.7 (11.3-12)	11.6 (11.3-11.8)	12.7 (12.4-13)	13.4 (13.1-13.7)	13.8 (13.3-14.4)
Punjab	10.7 (9.5-12.2)	11 (9.7-12.7)	11.2 (10-12.9)	11.5 (10.3-12.9)	12.1 (11-13.4)	12.6 (11.5-13.8)	12.6 (11.4-13.8)	13.9 (12.7-15.2)	14 (13.1-14.8)	14.1 (13.7-14.6)	14.4 (14.1-14.8)	14.5 (14.2-14.9)	15.7 (15.3-16)	16.7 (16.1-17.4)
Rajasthan	9.4 (8.2-10.8)	9.6 (8.4-11.2)	9.7 (8.5-11.2)	9.6 (8.6-10.9)	10.2 (9.1-11.4)	10.4 (9.4-11.5)	10.2 (9.2-11.3)	11.4 (10.5-12.4)	11.5 (10.7-12.3)	11.8 (11.4-12.3)	12.8 (12.4-13.1)	13.7 (13.4-14.1)	13.1 (12.7-13.4)	13 (12.3-13.6)
Sikkim	10.7 (9.2-12.5)	10.9 (9.4-12.7)	11 (9.5-12.7)	11 (9.6-12.5)	11.4 (10.2-12.8)	11.7 (10.6-12.9)	11.4 (10.3-12.5)	12.2 (11.2-13.4)	11.8 (11.2-13)	12 (11.6-12.9)	12.1 (11.7-12.7)	12.7 (12.4-13.6)	12.9 (12.6-13.9)	13.5 (12.9-14.8)

Location	1950	1955	1960	1965	1970	1975	1980	1985	1990	1995	2000	2005	2010	2017
**Table 11 Estimates of life expectancy at age 65 by location and year for 1950, 1955...2005, 2010, 2017 - males**														
Tamil Nadu	8.8 (7.9-10)	9.1 (8-10.3)	9.2 (8.2-10.4)	9.3 (8.3-10.5)	9.7 (8.8-10.9)	10.1 (9.2-11.2)	10 (9-11)	11.1 (10.2-12.1)	11.2 (10.5-11.9)	11.8 (11.4-12.1)	12 (11.7-12.3)	13.5 (13.2-13.8)	12.6 (12.3-12.9)	13.5 (13-14)
Telangana	10.4 (8.9-12.1)	10.6 (9-12.3)	10.8 (9.3-12.5)	11 (9.5-12.6)	11.3 (9.9-12.8)	11.6 (10.2-13)	11.2 (9.8-12.6)	11.9 (10.7-13.4)	11.6 (10.5-13.1)	12 (10.8-13.4)	12 (10.9-13.4)	13.1 (12-14.5)	13.5 (12.4-14.8)	14.5 (13.4-15.7)
Tripura	10.1 (8.8-11.7)	10.5 (9.2-12.1)	10.7 (9.3-12.2)	10.8 (9.6-12.2)	11.5 (10.3-12.7)	11.9 (10.8-13)	11.5 (10.4-12.7)	12.3 (11.2-13.5)	11.9 (10.7-13.1)	11.9 (11-13.2)	11.9 (11.3-12.9)	12.7 (12.1-13.7)	12.7 (12.2-13.4)	12.9 (12.6-13.4)
Uttar Pradesh	8.7 (7.7-9.8)	9 (7.9-10.2)	9 (8.1-10.2)	9.2 (8.3-10.3)	9.7 (8.9-10.6)	10.1 (9.3-10.9)	9.8 (9-10.8)	11.1 (10.2-12)	11.3 (10.5-12.2)	12.1 (11.5-12.8)	12.8 (12.3-13.3)	12.7 (12.3-13)	12.5 (12.1-12.8)	13.1 (12.5-13.8)
Uttarakhand	9.2 (8.4-10.5)	9.4 (8.5-10.8)	9.5 (8.5-10.9)	9.5 (8.6-10.8)	9.9 (9.1-11)	10.2 (9.5-11)	9.8 (9-10.8)	10.7 (10-11.5)	10.6 (9.9-11.3)	11.1 (10.3-11.7)	11.2 (10.4-11.8)	12.2 (11.5-12.6)	12.5 (12.1-12.8)	12.6 (12.4-12.9)
West Bengal	9.5 (8.2-10.9)	9.8 (8.6-11.1)	9.9 (8.7-11.2)	9.9 (8.8-11.1)	10.3 (9.3-11.5)	10.5 (9.5-11.6)	10.2 (9.3-11.3)	11.2 (10.3-12.2)	11.2 (10.4-12.1)	11.3 (10.8-11.9)	11.9 (11.5-12.4)	12.7 (12.2-13.1)	13.3 (12.9-13.7)	13.7 (13-14.5)
Union Territories other than Delhi	12.7 (11.3-13.9)	12.8 (11.5-14.1)	13 (11.7-14.3)	12.8 (11.7-14)	13.2 (12.2-14.2)	13.3 (12.5-14.3)	12.7 (11.8-13.8)	13.2 (12.3-14.2)	12.6 (12-13.6)	12.7 (12.1-13.8)	12.7 (12.2-13.8)	13.6 (12.7-14.9)	13.7 (12.7-15)	13.9 (12.8-15.3)
Nepal	9.4 (8.6-10.3)	9.7 (8.8-10.7)	10 (9.1-11.1)	10.4 (9.4-11.5)	10.7 (9.7-11.9)	11 (10-12.1)	11.3 (10.3-12.4)	11.8 (10.8-12.8)	12.4 (11.6-13.4)	13.1 (12.4-13.8)	13.8 (13.1-14.2)	14 (13.2-14.5)	13.6 (12.8-14.3)	13.3 (12.9-14.5)
Pakistan	12.8 (11.7-14.2)	13.2 (12-14.6)	13.6 (12.5-14.8)	14.2 (13.3-15.2)	14.8 (14.1-15.6)	15.1 (14.5-15.7)	14.8 (14.3-15.4)	14.6 (14.2-15)	14.1 (13.7-14.6)	13.7 (13.2-14.2)	13.6 (13.2-14.1)	13.6 (12.9-14.2)	14 (13-15.1)	14.5 (13.1-15.9)
Southeast Asia, East Asia, and Oceania	10.2 (9.9-10.5)	10.7 (10.4-11)	9.1 (8.9-9.3)	11.1 (10.8-11.4)	11.9 (11.6-12.1)	12.4 (12.1-12.6)	12.7 (12.5-12.9)	13.1 (13-13.3)	13.3 (13.1-13.4)	13.7 (13.6-13.8)	14.1 (13.9-14.2)	14.4 (14.3-14.5)	14.8 (14.7-14.9)	15.4 (15.2-15.7)
East Asia	10.2 (9.9-10.5)	10.7 (10.3-11.1)	8.6 (8.3-8.8)	11 (10.6-11.3)	12 (11.7-12.3)	12.5 (12.2-12.8)	12.8 (12.5-13)	13.2 (13-13.4)	13.3 (13.2-13.5)	13.8 (13.7-13.9)	14.2 (14.1-14.4)	14.6 (14.5-14.7)	15.1 (14.9-15.2)	15.7 (15.4-16)
China	10.2 (9.9-10.5)	10.7 (10.3-11.1)	8.5 (8.3-8.8)	10.9 (10.6-11.3)	12 (11.7-12.3)	12.5 (12.2-12.8)	12.8 (12.5-13)	13.2 (13-13.4)	13.3 (13.2-13.5)	13.8 (13.7-13.9)	14.2 (14.1-14.4)	14.6 (14.5-14.7)	15 (14.9-15.2)	15.7 (15.4-16)
North Korea	9 (8.2-9.9)	11.3 (10.2-12.9)	11.7 (10.6-13.4)	12.1 (11.2-13.7)	12.5 (11.8-13.9)	13 (12.4-14.1)	13.1 (12.7-14.2)	13.1 (12.7-14.3)	13.2 (12.8-14.4)	11.8 (11.5-12.7)	11.7 (11.4-12.3)	13 (12.6-13.5)	13 (12.7-13.4)	13.3 (12.9-13.8)
Taiwan (Province of China)	10.6 (9.7-11.7)	10.9 (10.8-10.9)	11.3 (11.3-11.3)	12.3 (12.2-12.3)	12.5 (12.5-12.6)	13.1 (13.1-13.1)	14 (13.9-14)	14.7 (14.6-14.7)	15.3 (15.2-15.3)	15.9 (15.8-15.9)	16.6 (16.5-16.6)	17.1 (17-17.1)	17.9 (17.9-18)	18 (17.6-18.4)
Oceania	8.5 (8.3-8.7)	8.8 (8.6-9)	9.3 (9.1-9.5)	9.6 (9.4-9.8)	9.7 (9.5-10)	9.9 (9.7-10.2)	10.3 (10.1-10.7)	10.4 (10.1-10.7)	10.4 (10.1-10.8)	10.4 (10.1-10.9)	10.5 (10.1-10.9)	10.5 (10.1-11)	10.7 (10.3-11.2)	11 (10.5-11.6)
American Samoa	12.6 (11.8-13.4)	12.3 (11.9-12.9)	12 (11.9-12.3)	12.4 (12.1-12.6)	12.6 (12.3-12.9)	12.8 (12.6-13)	13 (12.7-13.3)	13.1 (12.7-13.6)	13 (12.7-13.5)	13.1 (12.7-13.7)	13.4 (12.9-13.8)	13.5 (13-13.8)	13.7 (13.1-14.3)	13.9 (12.9-15.3)
Federated States of Micronesia	9.2 (8.6-10)	9.4 (8.8-10.2)	9.7 (9.1-10.5)	10 (9.4-10.8)	10.4 (9.7-11.1)	10.8 (10-11.6)	11.2 (10.4-12)	11.5 (10.6-12.4)	11.7 (10.8-12.7)	11.9 (11.1-12.8)	12 (11.3-12.8)	12.2 (11.6-12.9)	12.3 (11.6-13)	12.3 (11.8-12.8)
Fiji	11.5 (11-12)	12.2 (11.8-12.6)	12.5 (12.1-12.9)	12.3 (11.9-12.7)	11 (10.6-11.4)	11.3 (11-11.7)	12.7 (12.3-13.1)	12.7 (12.3-13.2)	12.6 (11.9-13.3)	11.7 (11.5-11.9)	11.1 (11.1-11.2)	11.7 (11.6-11.8)	12.1 (12-12.2)	12.1 (11.3-13.1)
Guam	11.8 (11.5-12.1)	11.8 (11.5-12)	12.1 (11.9-12.4)	12.4 (12.2-12.5)	12.3 (12.1-12.4)	12.3 (12.2-12.5)	13.4 (13.2-13.5)	14.6 (14.4-14.8)	14.3 (14.1-14.6)	14.2 (13.9-14.5)	14.5 (14.1-14.8)	14.8 (14.5-15.1)	15.5 (15.2-15.8)	15.6 (15.1-16.2)
Kiribati	8.9 (8.3-9.7)	9 (8.5-9.8)	9.2 (8.6-10.1)	9.4 (8.9-10.3)	9.7 (9.2-10.5)	10.3 (9.7-11)	10.5 (9.9-11.1)	10.4 (9.9-11)	10.4 (10-10.7)	10.2 (10-10.4)	10.1 (9.9-10.3)	10.2 (9.9-10.6)	10.4 (10-10.9)	10.7 (10.1-11.4)
Marshall Islands	8.8 (8.2-9.5)	9 (8.4-9.8)	9.3 (8.6-10.1)	9.5 (8.8-10.4)	9.7 (9-10.6)	10 (9.3-10.8)	10.2 (9.6-10.8)	10.2 (9.8-10.7)	10.3 (10-10.6)	10.2 (9.9-10.5)	10.2 (9.8-10.6)	10.4 (10-10.9)	10.7 (10.1-11.3)	11.1 (10.4-12)
Northern Mariana Islands	13 (12.6-14)	13 (12.7-14)	13.3 (12.8-14)	13.5 (13-14.1)	13.8 (13.1-14.2)	14 (13.3-14.6)	14.3 (13.5-15.5)	15 (13.7-16.1)	15.6 (14-16.4)	15.9 (14.6-16.6)	16.1 (15.4-16.8)	16.2 (15.6-16.8)	16.1 (15.3-16.8)	15.9 (14.8-16.8)
Papua New Guinea	7.9 (7.7-8.1)	8 (7.8-8.3)	8.2 (7.9-8.4)	8.4 (8.1-8.7)	8.7 (8.4-9.1)	9 (8.6-9.4)	9.2 (8.8-9.7)	9.3 (8.9-9.8)	9.4 (9-10)	9.6 (9.1-10.2)	9.7 (9.2-10.4)	9.7 (9.2-10.4)	9.9 (9.3-10.6)	10.2 (9.6-11.1)
Samoa	13.5 (12.4-14.6)	13.9 (13.1-14.9)	14.6 (13.9-15.3)	14.4 (13.8-15)	14.1 (13.5-14.7)	13.8 (13.2-14.4)	14 (13.3-14.8)	14.1 (13.2-14.9)	14.3 (13.3-15.2)	13.8 (12.9-14.4)	13.7 (13.1-14.1)	13.9 (13.5-14.1)	13.9 (13.6-14.4)	14.1 (13.8-14.7)
Solomon Islands	8.6 (8.1-9.4)	8.8 (8.3-9.6)	9 (8.5-9.8)	9.4 (8.8-10.1)	9.7 (9.2-10.4)	10 (9.4-10.7)	10.7 (10-11.6)	11 (10.3-12)	11.2 (10.4-12.2)	11.5 (10.6-12.5)	11.7 (10.9-12.7)	11.7 (11-12.6)	11.9 (11.2-12.7)	12.1 (11.4-12.8)
Tonga	10.6 (10.1-11.2)	11.1 (10.6-11.6)	11.8 (11.4-12.2)	12.4 (12-12.7)	12.6 (12.2-12.9)	12.7 (12.4-13)	12.8 (12.5-13.1)	12.9 (12.7-13.1)	13.1 (12.9-13.4)	13.2 (12.9-13.6)	13.3 (12.9-13.6)	13.3 (13-13.7)	13.3 (12.9-13.7)	13.5 (12.7-14)
Vanuatu	9.2 (8.7-9.9)	9.4 (8.8-10.1)	9.6 (9-10.4)	9.9 (9.2-10.7)	10.1 (9.4-11)	10.4 (9.7-11.3)	10.7 (10-11.6)	10.8 (10.2-11.8)	10.9 (10.2-11.8)	11.1 (10.4-12)	11.2 (10.5-12.2)	11.2 (10.4-12.3)	11.3 (10.5-12.3)	11.5 (10.6-12.4)
Southeast Asia	10.1 (9.9-10.4)	10.7 (10.3-11)	11.2 (10.8-11.5)	11.4 (11-11.8)	11.5 (11.2-11.9)	12 (11.7-12.3)	12.5 (12.3-12.8)	12.8 (12.6-13.1)	13 (12.8-13.2)	13.2 (13-13.4)	13.4 (13.2-13.5)	13.6 (13.5-13.8)	13.9 (13.7-14.1)	14.3 (14-14.6)
Cambodia	9.4 (8.6-10.3)	9.6 (8.8-10.6)	9.9 (9-10.9)	10.2 (9.2-11.3)	10 (9.1-11)	8.4 (7.9-9.1)	10.4 (9.7-11.2)	10.6 (10-11.3)	11 (10.5-11.6)	11.3 (10.8-11.9)	11.6 (11.1-12.2)	12.1 (11.7-12.6)	12.6 (12.4-12.9)	13 (12.7-13.2)
Indonesia	9.7 (9.3-10.1)	10.1 (9.7-10.6)	10.5 (10.1-11)	10.9 (10.4-11.4)	11.3 (10.9-11.8)	11.9 (11.5-12.4)	12.5 (12.1-12.9)	13 (12.6-13.4)	13.2 (12.9-13.5)	13.4 (13.1-13.7)	13.2 (13-13.5)	13.1 (12.8-13.4)	13.1 (12.8-13.5)	13.4 (13-14)
Laos	8.3 (7.8-9)	8.5 (7.9-9.3)	8.7 (8-9.5)	8.8 (8.1-9.7)	9.1 (8.3-10)	9.5 (8.7-10.4)	9.8 (9-10.7)	10.2 (9.4-11.2)	10.5 (9.7-11.5)	10.9 (10.1-11.8)	11.4 (10.7-12.2)	12 (11.3-12.8)	12.6 (11.7-13.5)	13.2 (12.4-14.3)
Malaysia	11.3 (10.3-12.5)	12 (11.2-12.9)	12.7 (12.7-12.8)	12.1 (12.1-12.2)	11.6 (11.5-11.8)	12.4 (12.3-12.5)	12.7 (12.4-13.1)	13 (13-13)	13.3 (13.3-13.3)	13.3 (13.2-13.3)	13.6 (13.6-13.7)	14.6 (14.5-14.6)	14.8 (14.7-14.9)	14.8 (14.1-15.5)
Maldives	8.5 (7.8-9.3)	8.9 (8.2-9.8)	9.4 (8.7-10.3)	10.1 (9.4-10.8)	10.8 (10.3-11.2)	11.4 (11.1-11.7)	12.3 (12-12.6)	13.3 (13.1-13.5)	13.3 (13.2-13.5)	13.6 (13.5-13.8)	14.8 (14.6-15)	15.9 (15.7-16.1)	17.5 (17.3-17.7)	19.2 (18.7-19.7)
Mauritius	9.2 (9.1-9.3)	9.8 (9.7-9.9)	10.6 (10.5-10.7)	12.2 (12-12.3)	12.5 (12.3-12.6)	10.9 (10.8-11)	11.5 (11.3-11.6)	11.7 (11.5-11.8)	12.3 (12.2-12.4)	12.9 (12.8-13)	13.7 (13.6-13.8)	13.8 (13.7-13.9)	14.9 (14.8-15)	15.3 (14.8-15.8)
Myanmar	7.9 (7.5-8.5)	8.1 (7.7-8.8)	8.4 (7.8-9.1)	8.7 (8-9.6)	9 (8.3-9.9)	9.5 (8.7-10.5)	9.9 (9-11)	10.3 (9.5-11.3)	10.5 (9.8-11.6)	10.8 (10.1-11.7)	11.1 (10.5-12)	11.6 (11-12.4)	12.2 (11.7-13)	12.8 (12.5-13.5)
Philippines	15.5 (14.9-16.1)	15.4 (14.8-16.1)	16.1 (15.6-16.5)	15.6 (15.2-16.1)	14.6 (14.1-15.1)	14.9 (14.4-15.4)	14.7 (14.2-15.1)	14 (13.6-14.5)	14.3 (13.8-14.7)	13.9 (13.4-14.3)	13.7 (13.3-14.2)	13.3 (12.8-13.8)	12.9 (12.4-13.3)	12.8 (11.8-13.9)
Sri Lanka	13.2 (13.1-13.2)	14.3 (14.3-14.4)	15 (15-15.1)	14.4 (14.4-14.5)	14 (14-14.1)	13 (13-13)	14.6 (14.5-14.6)	13.3 (13.3-13.3)	13.2 (13-13.3)	13.1 (13.1-13.2)	12.8 (12.7-12.8)	13.9 (13.6-14.1)	14.8 (14.8-14.9)	15.8 (14.7-17)
[truncated: 739,445 more chars]
