# Supplementary material for: Global, regional, and national age-sex-specific mortality and life expectancy, 1950–2017: a systematic analysis for the Global Burden of Disease Study 2017
Source: Lancet. 2018 Nov 10;392(10159):1684–735. doi: 10.1016/S0140-6736(18)31891-9 (PMC6227504; doi:10.1016/S0140-6736(18)31891-9)
Supplement: Supplementary appendix 1 [file mmc1.pdf]

THE LANCET

Supplementary appendix 1

This appendix formed part of the original submission and has been peer reviewed. We post it as supplied by the authors.

Supplement to: GBD 2017 Mortality Collaborators. Global, regional, and national age-sex-specific mortality and life expectancy, 1950–2017: a systematic analysis for the Global Burden of Disease Study 2017. *Lancet* 2018; **392:** 1684–735.

Methods appendix to “Global, regional, and national under-5 mortality, adult mortality, age-specific mortality, and life expectancy, 1950-2017: a systematic analysis for the Global Burden of Disease Study 2017”

This appendix provides further methodological detail, supplemental figures, and more detailed results for “Global, regional, and national under-5 mortality, adult mortality, age-specific mortality, and life expectancy, 1950-2017: a systematic analysis for the Global Burden of Disease Study 2017.” This appendix is organized into sections that follow the structure of the main paper.

Preamble

This appendix provides further methodological detail and more detailed results for "Global, regional, and national under-5 mortality, adult mortality, age-specific mortality, and life expectancy, 1950-2017: a systematic analysis for the Global Burden of Disease Study 2017." This study complies with the Guidelines for Accurate and Transparent Health Estimates Reporting (GATHER) recommendations. It includes detailed tables and information on data in an effort to maximize transparency in our estimation processes and provide a comprehensive description of analytical steps. We intend this appendix to be a living document, to be updated with each iteration of the Global Burden of Disease Study.

Table of Contents

List of Appendix Figures and Tables 4
Authors' Contributions i

Section 1: GBD Overview 5

Section 1.1: Geographic Locations of the Analysis 5

Section 1.2: Time Period of the Analysis 5

Section 1.3: Statement of GATHER Compliance 5

Section 1.4: List of abbreviations 6

Section 1.5: GBD results overview 8

Section 1.6: Data input sources overview 9

Section 1.7: Funding Sources 9

Section 2: GBD 2017 All-Cause Mortality and HIV Estimation Process 9

Section 2.1: Overview 9

Section 2.2: Child mortality 10

Section 2.3: Adult mortality 25

Section 2.4: Model life table system 40

Section 2.5: HIV/AIDS estimation 50

Section 2.6: Age-specific mortality estimation for all GBD age-groups: with and without HIV 54

Section 3: SDI analysis 55

Section 3.1: SDI definition 55

Section 3.2: Development of revised SDI indicator 55

Section 4: Fatal Discontinuities Estimation 57

Section 4.1: Input data 57

Section 4.2: Modeling strategy 61

Section 5: Additional Methods Information 62

Section 5.1: GBD world population age standard 62

Section 5.2: Estimating correlation 63

Section 5.3: Calculating annualised rates of change 63

Section 5.4: Gross domestic product (GDP) and lagged dependent income (LDI) 63

Section 5.5: Calculation of educational attainment covariate 64

Section 6: References 65

List of Appendix Figures and Tables

Appendix Figures:

Appendix Figure 1. Analytical flowchart for the estimation of all-cause mortality by age and sex and HIV/AIDS incidence, prevalence, and mortality for GBD 2017……………………………………………………………….69

Appendix Figure 2. Estimated completeness of death registration 1950−2017……………………………………70

Appendix Tables:

Appendix Table 1. GATHER checklist ……………………………………………………………………………………………...71

Appendix Table 2. GBD location hierarchy with levels…………………………………………………………………………..74

Appendix Table 3. Number of all-cause mortality data sources by type and location, 1950–2017…………88

Appendix Table 4. Number of all-cause mortality data sources by type and year, 1950-2017………………100

Appendix Table 5. Under-5 mortality reference sources by source date and location, 1950-2017……….102

Appendix Table 6. Distribution of empirical life tables by decade and GBD super-region, 1950-2017….175

Appendix Table 7. Locations by HIV estimation group…………………………………………………………………………176

Appendix Table 8. Socio-demographic Index groupings by geography, based on 2017 values…………….178

Appendix Table 9. Socio-demographic Index values for all estimated GBD 2017 locations, 1950-1969……….192

Appendix Table 10. Socio-demographic Index values for all estimated GBD 2017 locations, 1970–1989……….202

Appendix Table 11. Socio-demographic Index values for all estimated GBD 2017 locations, 1990-2017……….211

Appendix Table 12. GBD World population age standard………………………………………………………………….216

Appendix Table 13. New data sources added for GBD 2017 mortality estimation................................217

Appendix Table 14. Number of outliered data points for the Age/Sex model by location, source type, and age group...220

Authors' Contributions

Managing the estimation process

Ashkan Afshin, Aaron Cohen, Elizabeth Cromwell, Lalit Dandona, Rakhi Dandona, Louisa Degenhardt, Samath Dharmaratne, Daniel Dicker, Charbel El Bcheraoui, Kara Estep, Valery Feigin, Nancy Fullman, Emmanuela Gakidou, Simon Hay, Spencer James, Nicholas Kassebaum, Ibrahim Khalil, Heidi Larson, Stephen Lim, Alan Lopez, Rafael Lozano, Felix Masiye, Awoke Misganaw, Ali Mokdad, Erin Mullany, Christopher Murray, Mohsen Naghavi, Elaine Nsoesie, David Pigott, Robert Reiner, Joseph Salama, Katya Shackelford, Caitlyn Steiner, Stein Emil Vollset, Theo Vos, and Harvey Whiteford

Writing the first draft of the manuscript

Megha Arora, Emmanuela Gakidou, Yihua He, Jun Y Kim, Emilie R Maddison, Erin C Mullany, Katya A Shackelford, Leo G Stewart, and Dillon O Sylte.

Providing data or critical feedback on data sources

Kalkidan Hassen Abate, Aberash Abay, Solomon Mequanente Abay, Cristiana Abbafati, Ibrahim Abdollahpour, Zegeye Abebe, Yihalem Abebe Belay, Victor Aboyans, Aklilu Abrham Roba, Laith Abu-Raddad, Niveen Abu-Rmeileh, Manfred Accrombessi, Oladimeji Adebayo, Isaac Adedeji, Rufus A. Adedoyin, Olatunji Adetokunboh, Tara Ballav Adhikari, Mina Adib, Kouablan Arsene Adou, José C. Adsuar, Mohsen Afarideh, Rakesh Aggarwal, Sargis Aghayan, Alireza Ahmadi, Mehdi Ahmadi, Mohamed Lemine Cheikh Brahim Ahmed, Muktar Ahmed, Miloud Taki Eddine Aichour, Ali Shafqat Akanda, Mohammadesmaeil Akbari, Tomi Akinyemiju, Nadia Akseer, Fares Alahdab, Khurshid Alam, Kefyalew Addis Alene, Raghib Ali, Syed Mohamed Aljunid, François Alla, Peter Allebeck, Rajaa Al-Raddadi, Ubai Alsharif, Nelson Alvis-Guzman, Sintayehu Ambachew, Erfan Amini, Walid Ammar, Yaw Ampem Amoako, Catalina Liliana Andrei, Degefaye Anlay, Hossein Ansari, Ansariadi Ansariadi, Mustafa Geleto Ansha, Carl Abelardo Antonio, Seth Christopher Yaw Appiah, Olatunde Aremu, Al Artaman, Krishna K Aryal, Hamid Asayesh, Solomon Weldegebreal Asgedom, Reza Assadi, Tesfay Mehari Atey, Seyyed Shamsadin Athari, Suleman Atique, Marcel Ausloos, Ashish Awasthi, Beatriz Paulina Ayala Quintanilla, Henok Tadesse Ayele, Rakesh Ayer, Peter Azzopardi, Alaa Badawi, Kalpana Balakrishnan, Maciej Banach, Amrit Banstola, Aleksandra Barac, Miguel A. Barboza, Lope Barrero, Sanjay Basu, Shahrzad Bazargan-Hejazi, Neeraj Bedi, Ettore Beghi, Bayu Begashaw Bekele, Saba Abraham Belay, Aminu Bello, Isabela Bensenor, Adam Berman, Eduardo Bernabe, Robert Bernstein, Tambe Betrand Ayuk, Balem Demtsu Betsu, Mircea Beuran, Soumyadeep Bhaumik, Boris Bikbov, Nigus Bililign, Muhammad Shahdaat Bin Sayeed, Sait Mentes Birlik, Charles Birungi, Donal Bisanzio, Berrak Bora Başara, Rupert Bourne, Nicola Luigi Bragazzi, Luisa Brant, Alexandra Brazinova, Nicholas Breitborde, Hermann Brenner, Gabrielle Britton, Traolach Brugha, Kristin Burke, Charlton Callender, Ismael Campos-Nonato, Julio Cesar Campuzano, Jorge Cano, Mate Car, Giulia Carreras, Juan J Carrero, Felix Carvalho, Deborah Carvalho Malta, Carlos Castañeda-Orjuela, Franz Castro, Ferrán Catalá-López, Alanur Cavlin, Yazan Chaiah, Hsing-Yi Chang, Jung-Chen Chang, Pankaj Chaturvedi, Wanqing Chen, Peggy Pei-Chia Chiang, Abdulaal Chitheer, Devasahayam Christopher, Sheng-Chia Chung, Flavia Cicuttini, Massimo Cirillo, Rafael Claro, Daniel Collado Mateo, Maria Magdalena Constantin, Cyrus Cooper, Paolo Angelo Cortesi, Monica Cortinovis, Alexandre Costa Pereira, Michael Criqui, Christopher Crowe, John Crump, Alexandra Cucu, Lalit Dandona, Rakhi Dandona, Paul Dargan, Ahmad Daryani, Siddharth Kumar Das, José Das Neves, Tamirat Dasa, Anand Dayama, Barbora De Courten, Fernando De La Hoz, Louisa Degenhardt, Kebede Deribe, Nikolaos Dervenis, Getenet Dessie, Subhojit Dey, Samath Dharmaratne, Meghnath Dhimal, Daniel Dicker, David Teye Doku,

E. Ray Dorsey, Kerrie Doyle, Thomas M Drake, Manisha Dubey, Soheil Ebrahimpour, Anne Elise Eggen, Eyasu Ejeta, Christian Lycke Ellingsen, Iqbal Elyazar, Aman Endries, Benjamin Er, Sergey Ermakov, Babak Eshrati, Sharareh Eskandarieh, Alireza Esteghamati, Sadaf Esteghamati, Mahdi Fakhar, Mahbobeh Faramarzi, Mohammad Fareed, Carla Farinha, Andrea Farioli, Andre Faro, Farshad Farzadfar, Mohammad Hosein Farzaei, Valery Feigin, Fariba Feizy, Joao Feranandes, Seyed-Mohammad Fereshtehnejad, Eduarda Fernandes, Tilayie Feto Gelano, Daniel Fijabi, Irina Filip, Nataliya Foigt, Richard Franklin, Takeshi Fukumoto, Nancy Fullman, João M. Furtado, Neal Futran, Adriana Galan, Gbetoho Gankpe, M.A. Garcia-Gordillo, Tigist Gashaw, Teshome Gebre, Tsegaye Gebrehiwot, Amanuel Tesfay Gebremedhin, Tilayie Gelano, Johanna Geleijnse, Ayele Geleto, Ricard Genova-Maleras, Peter Gething, Kebede Embaye Gezae, Hesam Ghiasvand , Mamata Ghimire, Richard Gillum, Giorgia Giussani, Shifalika Goenka, Srinivas Goli, Hector Gómez-Dantés, Sameer Gopalani, Atsushi Goto, Bárbara Goulart, Ayman Grada, Giuseppe Grosso, Andre Guimaraes, Prakash Gupta, Rahul Gupta, Rajat Das Gupta, Rajeev Gupta, Tanush Gupta, Juanita Haagsma, Nima Hafezi Nejad, Tekleberhan Beyene Hagos, Gessessew Bugssa Hailu, Arya Haj-Mirzaian, Hilda Harb, Josep Maria Haro, Mehedi Hasan, Hadi Hassankhani, Hamid Y. Hassen, Rasmus Havmoeller, Akbar Hedayatizadeh-Omran, Mohamed Hegazy, Behzad Heibati, Delia Hendrie, Claudiu Herteliu, Fatemeh Heydarpour, Pouria Heydarpour, Sousan Heydarpour, Long Hoang Nguyen, Hans Hoek, Michael Hole, Enayatollah Homaie Rad, Praveen Hoogar, H Dean Hosgood, Mehdi Hosseinzadeh, Mihaela Hostiuc, Sorin Hostiuc, Damian Hoy, Mohamed Hsairi, Aung Soe Htet, Guoqing Hu, Abdullatif Husseini, Trang Huyen Nguyen, Kim Moesgaard Iburg, Ehimario Igumbor, Usman Iqbal, Sheikh Mohammed Shariful Islam, Farhad Islami, Nader Jahanmehr, Rajesh Jain, Sudhir Jain, Mihajlo Jakovljevic, Mehdi Javanbakht, Achala Jayatilleke, Sun Ha Jee, Panniyammakal Jeemon, John Ji, Sarah Johnson, Jost B. Jonas, Jacek Jozwiak, Mikk Jürisson, Zubair Kabir, Rajendra Kadel, Amaha Kahsay, Rizwan Kalani, Manoochehr Karami, André Karch, Corine Karema, Seyed M. Karimi, Amir Kasaeian, Getachew Mullu Kassa, Nicholas J Kassebaum, Marzieh Katibeh, Srinivasa Vittal Katikireddi, Anil Kaul, Norito Kawakami, Peter Keiyoro, Andre Keren, Morteza Abdullatif Khafaie, Nauman Khalid, Ibrahim Khalil, Muhammad Ali Khan, Young-Ho Khang, Alireza Khatony, Abdullah T. Khoja, Ardeshir Khosravi, Mohammad Hossein Khosravi, Jagdish Khubchandani, Aliasghar Kiadaliri, Cho-Il Kim, Daniel Kim, Jun Kim, Young-Eun Kim, Anh Kim Dang, Adnan Kisa, Katarzyna Kissimova-Skarbek, Niranjan Kissoon, Mika Kivimaki, Marcus Kleber, Ann Kristin Knudsen, Sonali Kochhar , Soewarta Kosen, Parvaiz A Koul, Ai Koyanagi, Michael Kravchenko, Kewal Krishan, Barthelemy Kuate Defo, Burcu Kucuk Bicer, G Anil Kumar, Manasi Kumar, Pushpendra Kumar, Michael Kutz, Sheetal Lad, Dharmesh Lal, Ratilal Lalloo, Hilton Lam, Van Lansingh, Dennis Laryea, Yirga Legesse, Misgan Legesse Liben, James Leigh, Cheru T Leshargie, Xiaohong Li, Yichong Li, Juan Liang, Xiaofeng Liang, Lee-Ling Lim, Stephen Lim, Shiwei Liu, Alan Lopez, Paulo Lotufo, Stefan Ma, Erlyn Rachelle Macarayan, Hassan Magdy Abd El Razek, Mohammed Magdy Abd El Razek, Dhaval Maghavani, Marek Majdan, Reza Majdzadeh, Azeem Majeed, Reza Malekzadeh, Mohammad Ali Mansournia, Joemer Maravilla, Wagner Marcenes, Jose Martinez-Raga, Francisco Rogerlândio Martins-Melo, Winfried März, Felix Masiye, Mohsen Mazidi, John Mcgrath, Man Mohan Mehndiratta, Ravi Mehrotra, Kala Mehta, Varshil Mehta, Tesfa Mekonen, Tefera Chane Mekonnen, Kidanu Meles, Addisu Melese, Mulugeta Melku, Peter Memiah, Ziad Memish, Walter Mendoza, Desalegn Tadese Mengistu , Getnet Mengistu , Zerihun Menlkalew Zenebe, Beyene Meressa, Atte Meretoja, Tuomo Meretoja, Tomislav Mestrovic, Haftay Berhane Mezgebe, Tomasz Miazgowski, Andreea Mirica, Erkin Mirrakhimov, Babak Moazen, Karzan Mohammad, Moslem Mohammadi, Shafiu Mohammed, Viswanathan Mohan, Ali H. Mokdad, Lorenzo Monasta, Ghobad Moradi, Mehdi Moradinazar, Lidia Morawska, Ilais Moreno Velasquez, Joana Morgado-Da-Costa, Shane Morrison,

Seyyed Meysam Mousavi, Achenef Muche, Ulrich Mueller, Manoj Murhekar, Srinivas Murthy, Kamarul Imran Musa, Jean Nachega, Gabriele Nagel, Mohden Naghavi, Seyed Sina Naghibi Irvani, Sanjeev Nair, Bruno Nascimento, Haseeb Nawaz, Ionut Negoi, Ruxandra Irina Negoi, Charles Newton, Frida Ngalesoni, Josephine Ngunjiri, Ha Nguyen, Huong Nguyen, Ana Maria Nogales Vasconcelos, Nomonde Nolutshungu, Shuhei Nomura, Mehdi Noroozi, Bo Norrving, Jean Jacques Noubiap, Hamid Reza Nouri, Malihe Nourollahpour, Okechukwu Ogah, Felix Ogbo, Anselm Okoro, Olanrewaju Oladimeji, Andrew T. Olagunju, Tinuke Olagunju, Bolajoko Olusanya, Jacob Olusanya, Sok King Ong, John Nelson Opio, Alberto Ortiz, Simon Øverland, Mayowa Owolabi, Mahesh P A, Smita Pakhale, Adrian Pana, Basant Kumar Panda, Songhomitra Panda-Jonas, Jeyaraj Pandian, Andrea Parisi, Eun-Kee Park, Shanti Patel, Scott Patten, Deepak Paudel, Neil Pearce, David Pereira, Krystle Perez, Norberto Perico, William Petri, Max Petzold, David Pigott, Dietrich Plass, Suzanne Polinder, Akram Pourshams, Hossein Poustchi, Swayam Prakash, V Prakash, Mostafa Qorbani, D. Alex Quistberg, Amir Radfar, Anwar Rafay, Alireza Rafiei, Fakher Rahim, Afarin Rahimi-Movaghar, Vafa Rahimi-Movaghar, Mahfuzar Rahman, Mohammad Hifz Ur Rahman, Sajjad Rahman, Fatemeh Rajati, Sasa Rajsic, Usha Ram, Chhabi Lal Ranabhat, Prabhat Ranjan, David Rawaf, Salman Rawaf, Christian Razo, Jürgen Rehm, Giuseppe Remuzzi, Andre Renzaho, Serge Resnikoff, Shahab Rezaeian, Horacio Riojas, María Jesús Ríos Blancas, Leonardo Roever, Luca Ronfani, Gholamreza Roshandel, Denis Roshchin, Ali Rostami, Dietrich Rothenbacher, Enrico Rubagotti, Rizwan S. Abdulkader, Soheil Saadat, Basema Saddik, Hosein Safari, Saeid Safiri, Mohammad Ali Sahraian, Mohamadreza Salahshoor, Nasir Salam, Joseph Salama, Payman Salamati, Yahya Salimi, Hamideh Salimzadeh, Evanson Z Sambala, Abdallah M. Samy, Bruno Sao Jose, Muthupandian Saravanan, Mayank Sardana, Shahabeddin Sarvi, Maheswar Satpathy, Arundhati Sawant, Monika Sawhney, Sonia Saxena, Mehdi Sayyah, Vinod Scaria, Elke Schaeffner, David C Schwebel, Falk Schwendicke, James Scott, Mario Šekerija, Sadaf Sepanlou, Edson Serván-Mori, Hosein Shabaninejad, Amira Shaheen, Masood Ali Shaikh, Mehran Shams-Beyranvand, Morteza Shamsizadeh, Mehdi Sharif, Jayendra Sharma, Meenakshi Sharma, Peilin Shi, Girma Temam Shifa, Ivy Shiue, Farhad Shokraneh, Soraya Siabani, Diego Augusto Santos Silva, João Pedro Silva, Dayane Silveira, Jasvinder Singh, Virendra Singh, Dhirendra Narain Sinha, Mekonnen Sisay, Freddy Sitas, Soheila Sobhani, Moslem Soofi, Joan B Soriano, Luisa Sorio Flor, Chandrashekhar T Sreeramareddy, Vasiliki Stathopoulou, Caitlyn Steiner, Mark Stokes, Agus Sudaryanto, Muawiyyah Babale Sufiyan, Bruno Sunguya, Bryan L Sykes, Dillon Sylte, Cassandra Szoeke, Azmeraw T. Amare, Rafael Tabarés-Seisdedos, Mohammad Tavakkoli, Nuno Taveira, Gebre Teklemariam Demoz, Mohamad-Hani Temsah, Omar Temsah, Abdullah Terkawi, Belay Tessema, Mebrahtu Teweldemedhin, Nu Thi Truong, Laura Thomas, Nihal Thomas, Marcello Tonelli, Miguel Tortajada-Girbés , Marcos Roberto Tovani-Palone, Bach Tran, Khanh Bao Tran, Thomas Truelsen, Nikolaos Tsilimparis, Stefanos Tyrovolas, Kingsley N. Ukwaja, Irfan Ullah, Olalekan Uthman, Selen Begüm Uzun, Muthiah Vaduganathan, Afsane Vaezi, Gaurang Vaidya, Pascual Valdez, Tommi Vasankari, Narayanaswamy Venketasubramanian, Ramesh Vidavalur, Santos Villafaina, Sergey Vladimirov, Vasiliy Vlassov, Fasil Wagnew, Yasir Waheed, Yanping Wang, Yuan-Pang Wang, Elisabete Weiderpass, Robert Weintraub, Inbal Weiss Salz, Andrea Werdecker, Ronny Westerman, Harvey Whiteford, Justyna Widecka, Katarzyna Widecka, Tissa Wijeratne, Charles Shey Wiysonge, Charles Wolfe, Shouling Wu, Grant Wyper, Gelin Xu, Rajaram Yadav, Bereket Yakob, Tomohide Yamada, Lijing Yan, Yasin Jemal Yasin, Pengpeng Ye, Gökalp Kadri Yentür, Alex Yeshaneh, Manaye Yihune, Ebrahim M. Yimer, Naohiro Yonemoto, Marcel Yotebieng, Mustafa Younis, Geevar Zachariah, Zoubida Zaidi, Maysaa El Zaki, Sojib Bin Zaman, Mohammad Zamani, Zohreh Zare, Kai Zhang, Xueying Zhang, Jun Zhu, Sanjay Zodpey, and Liesl Zuhlke.

Developing methods or computational machinery

Ibrahim Abdollahpour, Rufus A. Adedoyin, Sutapa Agrawal, Mohammadesmaeil Akbari, Mehran Alijanzadeh, Christine Allen, Mohsen Asadi- Lari, Suleman Atique, Neeraj Bedi, Bayu Begashaw Bekele, Muhammad Shahdaat Bin Sayeed, Sait Mentes Birlik, Donal Bisanzio, Dipan Bose, Charlton Callender, Austin Carter, Ahmad Daryani, Louisa Degenhardt, Getenet Dessie, Daniel Dicker, Mahbobeh Faramarzi, Mohammad Fareed, Farshad Farzadfar, Samuel Finegold, Kyle Foreman, Tahvi Frank, Emmanuela Gakidou, Peter Gething, Roderick Hay, Nathaniel Henry, Mehdi Hosseinzadeh, Molla Kahssay, Nicholas J Kassebaum, Muhammad Shahzeb Khan, Jun Kim, Adnan Kisa, Xie Rachel Kulikoff, Michael Kutz, Misgan Legesse Liben, James Leigh, Tim Lucas, Hassan Magdy Abd El Razek, Melvin Marzan, Sanjay Mehendale, Tefera Chane Mekonnen, Ted R Miller, Grant Nguyen, Michele Nguyen, Bolajoko Olusanya, Jacob Olusanya, Charles Parry, Chhabi Lal Ranabhat, Robert Reiner, Ehsan Sadeghi, Mohamadreza Salahshoor, Abdallah M. Samy, Maheswar Satpathy, Kathryn Schelonka, David C Schwebel, Mehdi Sharif, Tariq Jamal Siddiqi, Naris Silpakit, Virendra Singh, Vinay Srinivasan, Caitlyn Steiner, Leo Stewart, Patrick Sur, Ipsita Sutradhar, Dillon Sylte, Segen Tassew, Irfan Ullah, Muhammad Shariq Usman, Ronny Westerman, Lijing Yan, and Hunter York.

Applying analytical methods to produce estimates

Aklilu Abrham Roba, Rufus A. Adedoyin, Rakesh Aggarwal, Sutapa Agrawal, Mehdi Ahmadi, Sayem Ahmed, Amani Nidhal Aichour, Ibtihel Aichour, Miloud Taki Eddine Aichour, Syed Mohamed Aljunid, Christine Allen, Seth Christopher Yaw Appiah, Olatunde Aremu, Mohsen Asadi- Lari, Arindam Basu, Bayu Begashaw Bekele, Gregory Bertolacci, Samir Bhatt, Sait Mentes Birlik, Donal Bisanzio, Alexandra Brazinova, Charlton Callender, Austin Carter, Pankaj Chaturvedi, Devasahayam Christopher, Elizabeth Cromwell, Matthew Cunningham, Ahmad Daryani, Don Des Jarlais, Getenet Dessie, Daniel Dicker, Tim Driscoll, Dumessa Edessa, Christian Lycke Ellingsen, Aman Endries, Alireza Esteghamati, Hamed Fakhim, Garumma Feyissa, Samuel Finegold, Florian Fischer, Tahvi Frank, Peter Gething, Nima Hafezi Nejad, Hamid Y. Hassen, Roderick Hay, Nathaniel Henry, Long Hoang Nguyen, Ehimario Igumbor, Sarah Johnson, Madhanraj K , Manoochehr Karami, André Karch, Jun Kim, Anh Kim Dang, Adnan Kisa, Barthelemy Kuate Defo, Xie Rachel Kulikoff, Van Lansingh, Misgan Legesse Liben, James Leigh, Cheru T Leshargie, Samson Leta, Stephen Lim, Rafael Lozano, Tim Lucas, Melvin Marzan, Mohsen Mazidi, Tefera Chane Mekonnen, Zerihun Menlkalew Zenebe, Shafiu Mohammed, Ali H. Mokdad, Haseeb Nawaz, Grant Nguyen, Michele Nguyen, In-Hwan Oh, Bolajoko Olusanya, Jacob Olusanya, David Pigott, Alireza Rafiei, Mahfuzar Rahman, Chhabi Lal Ranabhat, Seyed Mohammad Riahi, Abdallah M. Samy, Maheswar Satpathy, Mehdi Sayyah, Masood Ali Shaikh, Mehdi Sharif, Girma Temam Shifa, Naris Silpakit, Diego Augusto Santos Silva, Virendra Singh, Karen Sliwa , Luisa Sorio Flor, Vinay Srinivasan, Leo Stewart, Patrick Sur, Dillon Sylte, Nu Thi Truong, Bach Tran, Khanh Bao Tran, Kingsley N. Ukwaja, Irfan Ullah, Theo Vos, Daniel Weiss, Ronny Westerman, Tissa Wijeratne, Jamal Yearwood, Miangotar Yode, Seok-Jun Yoon, Hunter York, Chuanhua Yu, and Zoubida Zaidi.

Providing critical feedback on methods or results

Degu Abate, Kalkidan Hassen Abate, Aberash Abay, Solomon Mequanente Abay, Cristiana Abbafati, Nooshin Abbasi, Hedayat Abbastabar, Foad Abd-Allah, Omar Abdel-Rahman, Alireza Abdi, Ibrahim Abdollahpour, Ahmed Abdurahman, Haftom Abebe, Molla Abebe, Zegeye Abebe, Yihalem Abebe Belay, Teshome Abebo, Haftom Abraha, Aklilu Abrham Roba, Niveen Abu-Rmeileh, Pawan Acharya, Oladimeji Adebayo, Isaac Adedeji, Rufus A. Adedoyin, Victor Adekanmbi, Olatunji Adetokunboh, Tara Ballav Adhikari, Mina Adib, Kouablan Arsene Adou, Mohsen Afarideh, Ashkan Afshin, Gina Agarwal, Sargis

Aghayan, Anurag Agrawal, Sutapa Agrawal, Alireza Ahmadi, Mehdi Ahmadi, Muktar Ahmed, Sayem Ahmed, Amani Nidhal Aichour, Ibtihel Aichour, Miloud Taki Eddine Aichour, Ali Shafqat Akanda, Mohammadesmaeil Akbari, Mohammed Akibu, Rufus Akinyemi, Tomi Akinyemiju, Nadia Akseer, Fares Alahdab, Ziyad Al-Aly, Khurshid Alam, Animut Alebel, Alicia Aleman, Kefyalew Addis Alene, Ayman Al-Eyadhy, Mehran Alijanzadeh, Syed Mohamed Aljunid, Ala'a Alkerwi, Peter Allebeck, Jordi Alonso, Rajaa Al-Raddadi, Ubai Alsharif, Khalid Altirkawi, Nelson Alvis-Guzman, Sintayehu Ambachew, Erfan Amini, Walid Ammar, Yaw Ampem Amoako, Catalina Liliana Andrei, Sofia Androudi, Mina Anjomshoa, Degefaye Anlay, Hossein Ansari, Mustafa Geleto Ansha, Carl Abelardo Antonio, Olatunde Aremu, Johan Ärnlöv, Al Artaman, Krishna K Aryal, Hamid Asayesh, Ephrem Tsegay Asfaw, Solomon Weldegebreal Asgedom, Reza Assadi, Zerihun Ataro, Tesfay Mehari Atey, Seyyed Shamsadin Athari, Suleman Atique, Sachin Atre, Madhu S. Atteraya, Engi F. Attia, Marcel Ausloos, Leticia Avila-Burgos, Euripide Avokpaho, Ashish Awasthi, Beatriz Paulina Ayala Quintanilla, Henok Tadesse Ayele, Wondimu Ayele, Rakesh Ayer, Peter Azzopardi, Natasha Azzopardi Muscat, Alaa Badawi, Amrit Banstola, Aleksandra Barac, Miguel A. Barboza, Simon Barquera, Lope Barrero, Huda Basaleem, Quique Bassat, Arindam Basu, Sanjay Basu, Bernhard Baune, Shahrzad Bazargan-Hejazi, Neeraj Bedi, Ettore Beghi, Masoud Behzadifar, Meysam Behzadifar, Yannick Béjot, Abate Bekele, Bayu Begashaw Bekele, Ezra Belay, Saba Abraham Belay, Michelle Bell, Aminu Bello, Derrick Bennett, Isabela Bensenor, Adugnaw Berhane, Adam Berman, Eduardo Bernabe, Robert Bernstein, Tambe Betrand Ayuk, Balem Demtsu Betsu, Mircea Beuran, Tina Beyranvand, Neeraj Bhala, Eesh Bhatia, Samir Bhatt, Suraj Bhattarai, Soumyadeep Bhaumik, Belete Biadgo, Ali Bijani, Boris Bikbov, Nigus Bililign, Muhammad Shahdaat Bin Sayeed, Sait Mentes Birlik, Charles Birungi, Tuhin Biswas, Tone Bjørge, Archie Bleyer, Berrak Bora Başara, Dipan Bose, Cristina Bosetti, Soufiane Boufous, Oliver Brady, Nicola Luigi Bragazzi, Luisa Brant, Nicholas Breitborde, Hermann Brenner, Traolach Brugha, Reinhard Busse, Zahid Butt, Alessandra C Goulart, Lucero Cahuana-Hurtado, Charlton Callender, Jorge Cano, Mate Car, Rosario Cárdenas, Juan J Carrero, Felix Carvalho, Deborah Carvalho Malta, Carlos Castañeda-Orjuela, Franz Castro, Ferrán Catalá-López, Ester Cerin, Yazan Chaiah, Ana Champs, Jung-Chen Chang, Aparajita Chattopadhyay, Peggy Pei-Chia Chiang, Odgerel Chimed-Ochir, Ken Chin, Vesper Chisumpa, Jee-Young Choi, Hanne Christensen, Devasahayam Christopher, Sheng-Chia Chung, Flavia Cicuttini, Liliana G Ciobanu, Rafael Claro, Aaron Cohen, Maria Magdalena Constantin, Sara Conti, Cyrus Cooper, Paolo Angelo Cortesi, Monica Cortinovis, Alexandre Costa Pereira, Ewerton Cousin, Michael Criqui, Christopher Crowe, John Crump, Alexandra Cucu, Alemneh Kabeta Daba, Berihun Dachew, Abel Dadi, Lalit Dandona, Rakhi Dandona, Paul Dargan, Ahmad Daryani, José Das Neves, Tamirat Dasa, Kairat Davletov, Anand Dayama, Barbora De Courten, Fernando De La Hoz, Diego De Leo, Jan-Walter De Neve, Megbaru Debalkie, Louisa Degenhardt, Tizta Degfie, Robert P. Dellavalle, Edgar Denova-Gutiérrez, Kebede Deribe, Nikolaos Dervenis, Don Des Jarlais, Getenet Dessie, Samath Dharmaratne, Meghnath Dhimal, Daniel Dicker, Eric L. Ding, Shirin Djalalinia, David Teye Doku, Kate Dolan, Christl Donnelly, E. Ray Dorsey, Kerrie Doyle, Thomas M Drake, Tim Driscoll, Manisha Dubey, Eleonora Dubljanin, Bruce Duncan, Andre Duraes, Hedyeh Ebrahimi, Soheil Ebrahimpour, Dumessa Edessa, David Edvardsson, Anne Elise Eggen, Eyasu Ejeta, Mohammed Elfaramawi, Ziad El-Khatib, Christian Lycke Ellingsen, Ahmadali Enayati, Aman Endries, Benjamin Er, Babak Eshrati, Sharareh Eskandarieh, Reza Esmaeili, Alireza Esteghamati, Sadaf Esteghamati, Hamed Fakhim, Tamer Farag, Mahbobeh Faramarzi, Farzaneh Farhadi, Talha Farid, Carla Farinha, Andrea Farioli, Andre Faro, Farshad Farzadfar, Mohammad Hosein Farzaei, Mir Sohail Fazeli, Valery Feigin, Andrea B. Feigl, Fariba Feizy, Netsanet Fentahun, Joao Feranandes, Seyed-Mohammad Fereshtehnejad, Eduarda Fernandes, Tilayie Feto Gelano, Garumma Feyissa, Daniel Fijabi, Irina Filip, Samuel Finegold, Florian Fischer, Nataliya Foigt,

John Ford, Kyle Foreman, Carla Fornari, Richard Franklin, Takeshi Fukumoto, Nancy Fullman, João M. Furtado, Neal Futran, Adriana Galan, Silvano Gallus, Alberto L. García-Basteiro, Tigist Gashaw, Abadi Kahsu Gebre, Teshome Gebre, Gebremedhin Berhe Gebregergs, Tsegaye Gebrehiwot, Amanuel Tesfay Gebremedhin, Merhawi Gebremedhin, Afewerki Gebremeskel, Aregawi Gebreyesus Belay, Tilayie Gelano, Yalemzewod Gelaw, Johanna Geleijnse, Ayele Geleto, Ricard Genova-Maleras, Bradford Gessner, Sefonis Getachew, Kebede Embaye Gezae, Reza Ghadimi, Khalil Ghasemi Falavarjani , Maryam Ghasemi-Kasman, Hesam Ghiasvand , Mamata Ghimire, Aloke Gopal Ghoshal, Kidu Gidey, Paramjit Gill, Tiffany Gill, Richard Gillum, Meaza Girma, Giorgia Giussani, Srinivas Goli, Ricardo Gomez, Mari Carmen Gomez-Cabrera, Philimon Gona, Amador Goodridge, Sameer Gopalani, Atsushi Goto, Bárbara Goulart, Ayman Grada, Giuseppe Grosso, Harish Gugnani, Andre Guimaraes, Yuming Guo, Rahul Gupta, Rajat Das Gupta, Rajeev Gupta, Tanush Gupta, Bishal Gyawali, Juanita Haagsma, Vladimir Hachinski, Nima Hafezi Nejad, Tekleberhan Beyene Hagos, Gessessew Bugssa Hailu, Arvin Haj-Mirzaian, Arya Haj-Mirzaian, Randah Hamadeh, Samer Hamidi, Graeme Hankey, Hilda Harb, Habtamu Hareri, Sivadasanpillai Harikrishnan, Hamidreza Haririan, Josep Maria Haro, Mehedi Hasan, Hadi Hassankhani, Hamid Y. Hassen, Rasmus Havmoeller, Roderick Hay, Simon Hay, Akbar Hedayatizadeh-Omran, Mohamed Hegazy, Behzad Heibati, Mohsen Heidari, Delia Hendrie, Andualem Henok, Ileana B. Heredia-Pi, Claudiu Herteliu, Fatemeh Heydarpour, Sousan Heydarpour, Long Hoang Nguyen, Michael Hole, Enayatollah Homaie Rad, Praveen Hoogar, Masako Horino, H Dean Hosgood, Mostafa Hosseini, Mihaela Hostiuc, Sorin Hostiuc, Damian Hoy, Guoqing Hu, John Huang, Abdullatif Husseini, Susan Hutfless, Trang Huyen Nguyen, Kim Moesgaard Iburg, Ehimario Igumbor, Olayinka Ilesanmi, Usman Iqbal, Oluwaseyi Isehunwa, Sheikh Mohammed Shariful Islam, Farhad Islami, Nader Jahanmehr, Mihajlo Jakovljevic, Spencer James, Mehdi Javanbakht, Sudha Jayaraman, Sun Ha Jee, Panniyammakal Jeemon, Ravi Prakash Jha, Vivekanand Jha, John Ji, Jost B. Jonas, Jacek Jozwiak, Suresh Jungari, Mikk Jürisson, Madhanraj K , Zubair Kabir, Rajendra Kadel, Amaha Kahsay, Molla Kahssay, Rizwan Kalani, Manoochehr Karami, Behzad Karami Matin, André Karch, Seyed M. Karimi, Hamidreza Karimi-Sari, Amir Kasaeian, Getachew Mullu Kassa, Tesfaye Kassa, Nicholas J Kassebaum, Marzieh Katibeh, Srinivasa Vittal Katikireddi, Vittal Katikireddi , Anil Kaul, Peter Keiyoro, Andre P Kengne, Yousef Khader, Morteza Abdullatif Khafaie, Nauman Khalid, Ibrahim Khalil, Ejaz Khan, Gulfaraz Khan, Muhammad Ali Khan, Muhammad Shahzeb Khan, Young-Ho Khang, Mona Khater, Habibolah Khazaie, Abdullah T. Khoja, Ardeshir Khosravi, Mohammad Hossein Khosravi, Aliasghar Kiadaliri, Getiye Kibret, Daniel Kim, Jun Kim, Young-Eun Kim, Anh Kim Dang, Ruth Kimokoti, Yohannes Kinfu, Sanjay Kinra, Adnan Kisa, Katarzyna Kissimova-Skarbek, Niranjan Kissoon, Mika Kivimaki, Luke Knibbs, Ann Kristin Knudsen, Sonali Kochhar , Yoshihiro Kokubo, Tufa Kolola, Jacek Kopec, Margaret Kosek, Parvaiz A Koul, Ai Koyanagi, Kewal Krishan, Sanjay Krishnaswami, Barthelemy Kuate Defo, Burcu Kucuk Bicer, Andreas Kudom, Ernst J. Kuipers, Xie Rachel Kulikoff, G Anil Kumar, Manasi Kumar, Pushpendra Kumar, Fekede Asefa Kumsa, Sheetal Lad, Alessandra Lafranconi, Dharmesh Lal, Ratilal Lalloo, Hilton Lam, Faris Lami, Qing Lan, Sinead Langan, Van Lansingh, Dennis Laryea, Zohra Lassi, Arman Latifi, Pablo Lavados, Avula Laxmaiah, Jeffrey Lazarus, Paul Lee, Yirga Legesse, Misgan Legesse Liben, James Leigh, Cheru T Leshargie, Samson Leta, Miriam Levi, Shanshan Li, Lee-Ling Lim, Stephen Lim, Miteku Limenih, Shai Linn, Yang Liu, Rakesh Lodha, Chris Lonsdale, Alan Lopez, Scott Lorch, Stefan Lorkowski, Paulo Lotufo, Raimundas Lunevicius, Ronan Lyons, Stefan Ma, Crispin Mabika Mabika, Erlyn Rachelle Macarayan, Mark Mackay, Fabiana Madotto, Hassan Magdy Abd El Razek, Mohammed Magdy Abd El Razek, Marek Majdan, Reza Majdzadeh, Azeem Majeed, Reza Malekzadeh, Manzoor Malik, Mohammedaman Mama, Abdullah Mamun, Mohammad Ali Mansournia, Lorenzo Mantovani, Chabila Mapoma, Dadi Marami, Joemer Maravilla, Wagner Marcenes, Jose Martinez-Raga, Sheila Martins,

Francisco Rogerlândio Martins-Melo, Melvin Marzan, Tivani Mashamba-Thompson, Felix Masiye, Benjamin Massenburg, Pallab K Maulik, Mohsen Mazidi, John Mcgrath, Suresh Mehata, Sanjay Mehendale, Kala Mehta, Varshil Mehta, Tesfa Mekonen, Tefera Chane Mekonnen, Hagazi Gebre Meles, Kidanu Meles, Addisu Melese, Mulugeta Melku, Peter Memiah, Walter Mendoza, Desalegn Tadese Mengistu , Getnet Mengistu , Zerihun Menlkalew Zenebe, George Mensah, Beyene Meressa, Seid Tiku Mereta, Atte Meretoja, Tuomo Meretoja, Tomislav Mestrovic, Haftay Berhane Mezgebe, Bartosz Miazgowski, Tomasz Miazgowski, Ted R Miller, Gk Mini, Andreea Mirica, Erkin Mirrakhimov, Babak Moazen, Nurilign Moges, Moslem Mohammadi, Mohammed Mohammed, Shafiu Mohammed, Ali H. Mokdad, Mulugeta Molla, Mariam Molokhia, Lorenzo Monasta, Ghobad Moradi, Mahmoudreza Moradi, Maziar Moradi-Lakeh, Mehdi Moradinazar, Paula Moraga, Lidia Morawska, Joana Morgado-Da-Costa, Shane Morrison, Marilita Moschos, Seyyed Meysam Mousavi, Achenef Muche, Kindie Fentahun Muchie, Ulrich Mueller, Satinath Mukhopadhyay, Tasha Murphy, Christopher Murray, Srinivas Murthy, Jonah Musa, Kamarul Imran Musa, Ghulam Mustafa, Jean Nachega, Gabriele Nagel, Mohden Naghavi, Seyed Sina Naghibi Irvani, Aliya Naheed, Azin Nahvijou, Gurudatta Naik, Farid Najafi, Vinay Nangia, Jobert Richie Nansseu, Bruno Nascimento, Haseeb Nawaz, Busisiwe Ncama, Ionut Negoi, Ruxandra Irina Negoi, Subas Neupane, Charles Newton, Frida Ngalesoni, Josephine Ngunjiri, Grant Nguyen, Muhammad Imran Nisar, Ana Maria Nogales Vasconcelos, Mehdi Noroozi, Bo Norrving, Jean Jacques Noubiap, Hamid Reza Nouri, Malihe Nourollahpour, Mohammad Reza Nowroozi, Dina Nur Anggraini Ningrum, Peter Nyasulu, Richard Ofori-Asenso, Okechukwu Ogah, Felix Ogbo, In-Hwan Oh, Anselm Okoro, Olanrewaju Oladimeji, Andrew T. Olagunju, Tinuke Olagunju, Pedro Olivares, Bolajoko Olusanya, Jacob Olusanya, John Nelson Opio, Eyal Oren, Alberto Ortiz, Justin R Ortiz, Erika Ota, Stanislav Otstavnov, Simon Øverland, Mayowa Owolabi, Abayomi Oyekale, Mahesh P A, Rosana Pacella, Smita Pakhale, Abhijit Pakhare, Adrian Pana, Basant Kumar Panda, Songhomitra Panda-Jonas, Achyut Raj Pandey, Jeyaraj Pandian, Eun-Kee Park, Charles Parry, Hadi Parsian, Shanti Patel, Ajay Patle, Scott Patten, George Patton, Deepak Paudel, Emmanuel Peprah, David Pereira, Norberto Perico, Aslam Pervaiz, Konrad Pesudovs, Michael R. Phillips, David Pigott, Julian Pillay, Meghdad Pirsaheb, Farhad Pishgar, Dietrich Plass, Suzanne Polinder, Constance Dimity Pond, Svetlana Popova, Maarten Postma, Farshad Pourmalek, Akram Pourshams, Hossein Poustchi, Dorairaj Prabhakaran, Swayam Prakash, V Prakash, Narayan Prasad, Mostafa Qorbani, D. Alex Quistberg, Amir Radfar, Anwar Rafay, Fakher Rahim, Kazem Rahimi, Afarin Rahimi-Movaghar, Vafa Rahimi-Movaghar, Mahfuzar Rahman, Muhammad Aziz Rahman, Sajjad Rahman, Rajesh Kumar Rai, Fatemeh Rajati, Usha Ram, Chhabi Lal Ranabhat, Prabhat Ranjan, Annemarei Ranta, Davide Rasella, David Rawaf, Salman Rawaf, Sarah Ray, Christian Razo, Maria Albertina Rego, Jürgen Rehm, Robert Reiner, Cesar Reis, Giuseppe Remuzzi, Andre Renzaho, Serge Resnikoff, Satar Rezaei, Shahab Rezaeian, Mohammad Sadegh Rezai, Seyed Mohammad Riahi, Antonio Luiz Ribeiro, Horacio Riojas, María Jesús Ríos Blancas, Kedir Teji Roba, Stephen Robinson, Leonardo Roever, Luca Ronfani, Gholamreza Roshandel, Ali Rostami, Dietrich Rothenbacher, George Ruhago, Rizwan S. Abdulkader, Yogesh Sabde, Perminder Sachdev, Basema Saddik, Hosein Safari, Roya Safari-Faramani, Mahdi Safdarian, Saeid Safiri, Rajesh Sagar, Amirhossein Sahebkar, Mohammad Ali Sahraian, Haniye Sadat Sajadi, Mohamadreza Salahshoor, Nasir Salam, Raphael Saldanha, Yahya Salimi, Hamideh Salimzadeh, Evanson Z Sambala, Abdallah M. Samy, Juan Sanabria, Maria Dolores Sanchez-Niño, Itamar Santos, João Vasco Santos, Milena Santric Milicevic, Bruno Sao Jose, Muthupandian Saravanan, Mayank Sardana, Abdur Razzaque Sarker, Benn Sartorius, Brijesh Sathian, Thirunavukkarasu Sathish, Maheswar Satpathy, Miloje Savic, Monika Sawhney, Sonia Saxena, Mehdi Sayyah, Elke Schaeffner, Maria Inês Schmidt, Ione Schneider, Ben Schöttker, Aletta Schutte, David C Schwebel, Falk Schwendicke, James Scott, Sadaf Sepanlou, Edson

Serván-Mori, Hosein Shabaninejad, Azadeh Shafieesabet, Amira Shaheen, Masood Ali Shaikh, Raad Shakir, Mehran Shams-Beyranvand, Mohammadbagher Shamsi, Heidar Sharafi, Kiomars Sharafi, Mehdi Sharif, Mahdi Sharif-Alhoseini, Rajesh Sharma, Aziz Sheikh, Kevin Sheth, Kenji Shibuya, Girma Temam Shifa, Mika Shigematsu, Rahman Shiri, Ivy Shiue, Mark Shrime, Sharvari Shukla, Si Si, Soraya Siabani, Tariq Jamal Siddiqi, Inga Dora Sigfusdottir, Rannveig Sigurvinsdottir, Diego Augusto Santos Silva, Dayane Silveira, Narayana Sarma Singam, Jasvinder Singh, Virendra Singh, Anju Sinha, Dhirendra Narain Sinha, Mekonnen Sisay, Freddy Sitas, Adauto Martins Soares Filho, Badr Sobaih, Soheila Sobhani, Moslem Soofi, Joan B Soriano, Ireneous Soyiri, Luciano Sposato, Chandrashekhar T Sreeramareddy, Rakesh Srivastava, Nicholas Steel, Dan J Stein, Caitlyn Steiner, Mark Stokes, Muawiyyah Babale Sufiyan, Gerhard Sulo, Bruno Sunguya, Ipsita Sutradhar, Bryan L Sykes, Pn Sylaja, Dillon Sylte, Cassandra Szoeke, Azmeraw T. Amare, Rafael Tabarés-Seisdedos, Takahiro Tabuchi, Santosh Tadakamadla, Ken Takahashi, Nikhil Tandon, Segen Tassew, Nuno Taveira, Arash Tehrani-Banihashemi, Gebre Teklemariam Demoz, Habtamu Temesgen, Mohamad-Hani Temsah, Omar Temsah, Tewodros Tesfa, Belay Tessema, Mebrahtu Teweldemedhin, Js Thakur, Kavumpurathu Thankappan, Nu Thi Truong, Laura Thomas, Nihal Thomas, Amanda Thrift, Binyam Tilahun, Quyen G. To, Ruoyan Tobe-Gai, Marcello Tonelli, Roman Topor-Madry, Fotis Topouzis, Miguel Tortajada-Girbés , Marcos Roberto Tovani-Palone, Jeffrey Towbin, Bach Tran, Khanh Bao Tran, Srikanth Tripathy, Thomas Truelsen, Desalegn Tsegaw, Nikolaos Tsilimparis, Lorainne Tudor Car, E Tuzcu Tuzcu, E. Murat Tuzcu, Stefanos Tyrovolas, Kingsley N. Ukwaja, Irfan Ullah, Muhammad Shariq Usman, Olalekan Uthman, Selen Begüm Uzun, Muthiah Vaduganathan, Afsane Vaezi, Pascual Valdez, Santosh Varughese, Tommi Vasankari, Narayanaswamy Venketasubramanian, Ramesh Vidavalur, Francesco S Violante, Vasiliy Vlassov, Stein Emil Vollset, Theo Vos, Kia Vosoughi, Isidora Vujcic, Gregory Wagner, Fasil Wagnew, Yasir Waheed, Yuan-Pang Wang, Molla Mesele Wassie, Elisabete Weiderpass, Robert Weintraub, Jordan Weiss, Inbal Weiss Salz, Fitsum Weldegebreal, Seifu Kebede Weldegiorgis, Andrea Werdecker, Ronny Westerman, Tissa Wijeratne, Andrea Sylvia Winkler, Charles Shey Wiysonge, Charles Wolfe, Grant Wyper, Gelin Xu, Bereket Yakob, Tomohide Yamada, Lijing Yan, Yuichiro Yano, Mehdi Yaseri, Yasin Jemal Yasin, Pengpeng Ye, Gökalp Kadri Yentür, Alex Yeshaneh, Manaye Yihune, Ebrahim M. Yimer, Nega Yimer, Zemenu Yohannes, Naohiro Yonemoto, Seok-Jun Yoon, Marcel Yotebieng, Mustafa Younis, Mahmoud Yousefifard, Chuanhua Yu, Vesna Zadnik, Shamsa Zafar, Zoubida Zaidi, Maysaa El Zaki, Sojib Bin Zaman, Mohammad Zamani, Zohreh Zare, Hajo Zeeb, Taddese Zerfu, Xueying Zhang, Inbar Zucker, and Liesl Zuhlke.

Drafting the work or revising it critically for important intellectual content

Nooshin Abbasi, Yihalem Abebe Belay, Isaac Adedeji, Olatunji Adetokunboh, Mohsen Afarideh, Hamid Ahmadieh, Mohammadesmaeil Akbari, Tomi Akinyemiju, Fares Alahdab, Reza Alizadeh-Navaei, Megha Arora, Peter Azzopardi, Hamid Badali, Bernhard Baune, Neeraj Bhala, Sait Mentes Birlik, Oliver Brady, Traolach Brugha, Juan J Carrero, Felix Carvalho, Franz Castro, Devasahayam Christopher, Cyrus Cooper, John Crump, Alemneh Kabeta Daba, Ahmad Daryani, Dragos Davitoiu, Jan-Walter De Neve, Selina Deiparine, Getenet Dessie, Daniel Dicker, Kate Dolan, Thomas M Drake, Andre Duraes, David Edvardsson, Sadaf Esteghamati, Mahbobeh Faramarzi, Mohammad Hosein Farzaei, Joao Feranandes, Seyed-Mohammad Fereshtehnejad, Eduarda Fernandes, Nataliya Foigt, Nancy Fullman, Emmanuela Gakidou, Alberto L. García-Basteiro, Merhawi Gebremedhin, Aregawi Gebreyesus Belay, Ricard Genova-Maleras, Kebede Embaye Gezae, Maryam Ghasemi-Kasman, Amador Goodridge, Rajat Das Gupta, Rajeev Gupta, Mehedi Hasan, Hadi Hassankhani, Behzad Heibati, Long Hoang Nguyen, Enayatollah Homaie Rad, Masako Horino, Farhad Islami, Ravi Prakash Jha, Jost B. Jonas, Surya Kant, Manoochehr Karami, Behzad Karami Matin, Getachew Mullu Kassa, Grant Kemp, Yousef Khader, Alireza Khajavi,

Mona Khater, Daniel Kim, Anh Kim Dang, Mika Kivimaki, Ann Kristin Knudsen, Parvaiz A Koul, Dharmesh Lal, Qing Lan, Sonia Lansky, Cheru T Leshargie, Xiaofeng Liang, Stephen Lim, Alan Lopez, Crispin Mabika Mabika, Emilie Maddison, Hassan Magdy Abd El Razek, Mohammed Magdy Abd El Razek, Azeem Majeed, Reza Malekzadeh, Ana Laura Manda, Mohammad Ali Mansournia, Joemer Maravilla, Francisco Rogerlândio Martins-Melo, Benjamin Massenburg, Sanjay Mehendale, Kidanu Meles, Tuomo Meretoja, Karzan Mohammad, Maryam Mohammadi-Khanaposhtani, Ghobad Moradi, Mehdi Moradinazar, Abbas Mosapour, Erin Mullany, Christopher Murray, Ghulam Mustafa, Nahid Neamati, Grant Nguyen, Molly Nixon, Ole F. Norheim, Andrew T. Olagunju, Justin R Ortiz, Simon Øverland, Songhomitra Panda-Jonas, Emmanuel Peprah, Konrad Pesudovs, Michael R. Phillips, Vafa Rahimi-Movaghar, Muhammad Aziz Rahman, Fatemeh Rajati, Sarah Ray, Satar Rezaei, Seyed Mohammad Riahi, Leonardo Roever, Ehsan Sadeghi, Hosein Safari, Sare Safi, Saeid Safiri, Mohamadreza Salahshoor, Milena Santric Milicevic, Bruno Sao Jose, Muthupandian Saravanan, Shahabeddin Sarvi, Maheswar Satpathy, Katya Shackelford, Mehdi Sharif, Mika Shigematsu, Inga Dora Sigfusdottir, Rannveig Sigurvinsdottir, Virendra Singh, Caitlyn Steiner, Leo Stewart, Ipsita Sutradhar, Segen Tassew, Nuno Taveira, Nu Thi Truong, Marcos Roberto Tovani-Palone, Bach Tran, Olalekan Uthman, Yuan-Pang Wang, Nicole Weaver, Robert Weintraub, Manaye Yihune, Miangotar Yode, Hunter York, Maysaa El Zaki, Inbar Zucker, and Liesl Zuhlke.

Extracting, cleaning, or cataloging data; designing or coding figures and tables

Hedayat Abbastabar, Foad Abd-Allah, Ibrahim Abdollahpour, Rakesh Aggarwal, Alireza Ahmadi, Christine Allen, Erfan Amini, Mohsen Asadi- Lari, Ephrem Tsegay Asfaw, Tesfay Mehari Atey, Henok Tadesse Ayele, Huda Basaleem, Shahrzad Bazargan-Hejazi, Bayu Begashaw Bekele, Derrick Bennett, Adugnaw Berhane, Gregory Bertolacci, Mircea Beuran, Muhammad Shahdaat Bin Sayeed, Sait Mentes Birlik, Donal Bisanzio, Julio Cesar Campuzano, Alanur Cavlin, Yazan Chaiah, Wanqing Chen, John Crump, Matthew Cunningham, Ahmad Daryani, Robert P. Dellavalle, Getenet Dessie, Daniel Dicker, Iqbal Elyazar, Aman Endries, Benjamin Er, Sergey Ermakov, Hamed Fakhim, Andre Faro, Takeshi Fukumoto, John Fuller, Nancy Fullman, Gbetoho Gankpe, Yuming Guo, Mehedi Hasan, Yihua He, Behzad Heibati, Mehdi Hosseinzadeh, Damian Hoy, Trang Huyen Nguyen, Chad Ikeda, Panniyammakal Jeemon, Vivekanand Jha, Sarah Johnson, Manoochehr Karami, André Karch, Tesfaye Kassa, Vittal Katikireddi , Norito Kawakami, Grant Kemp, Andre Keren, Morteza Abdullatif Khafaie, Mohammad Hossein Khosravi, Jagdish Khubchandani, Cho-Il Kim, Jun Kim, Young-Eun Kim, Adnan Kisa, Xie Rachel Kulikoff, Michael Kutz, Misgan Legesse Liben, James Leigh, Shanshan Li, Xiaofeng Liang, Shiwei Liu, Stefan Ma, Emilie Maddison, Dhaval Maghavani, Reza Malekzadeh, Mohammad Ali Mansournia, Wagner Marcenes, Benjamin Massenburg, Mohsen Mazidi, Kidanu Meles, Ziad Memish, Zerihun Menlkalew Zenebe, Mulugeta Molla, Lorenzo Monasta, Maziar Moradi-Lakeh, Abbas Mosapour, Ghulam Mustafa, Mohden Naghavi, Haseeb Nawaz, Ionut Negoi, Ruxandra Irina Negoi, Mohammad Reza Nowroozi, Mayowa Owolabi, Andrea Parisi, Ajay Patle, David Pereira, Max Petzold, David Pigott, Hossein Poustchi, Narayan Prasad, Anwar Rafay, Alireza Rafiei, Mahfuzar Rahman, Nickolas Reinig, Seyed Mohammad Riahi, Luca Ronfani, Enrico Rubagotti, Hosein Safari, Saeid Safiri, Payman Salamati, Abdallah M. Samy, Shahabeddin Sarvi, Maheswar Satpathy, Arundhati Sawant, Monika Sawhney, Kathryn Schelonka, Sadaf Sepanlou, Mehdi Sharif, Jun She, Peilin Shi, Farhad Shokraneh, Soraya Siabani, Virendra Singh, Dhirendra Narain Sinha, Vegard Skirbekk, Chandrashekhar T Sreeramareddy, Vinay Srinivasan, Leo Stewart, Patrick Sur, Dillon Sylte, Rafael Tabarés-Seisdedos, Mohammad Tavakkoli, Omar Temsah, Ruoyan Tobe-Gai, Anna Torre, Kingsley N. Ukwaja, Irfan Ullah, Gaurang Vaidya, Tommi Vasankari, Yasir Waheed, Inbal Weiss Salz, Shouling Wu, Nega Yimer, Naohiro Yonemoto, and Hunter York.

Managing the overall research enterprise

Ashkan Afshin, Peter Allebeck, Megha Arora, Zulfiqar Bhutta, Deborah Carvalho Malta, Aaron Cohen, Elizabeth Cromwell, Lalit Dandona, Rakhi Dandona, Louisa Degenhardt, Samath Dharmaratne, Daniel Dicker, Charbel El Bcheraoui, Tamer Farag, Valery Feigin, Kyle Foreman, Nancy Fullman, Emmanuela Gakidou, Tsegaye Gebrehiwot, Peter Gething, Simon Hay, Spencer James, Nicholas Kassebaum, Ibrahim Khalil, Heidi Larson, Xiaofeng Liang, Stephen Lim, Alan Lopez, Rafael Lozano, Felix Masiye, George Mensah, Awoke Misganaw, Ali Mokdad, Erin Mullany, Kate Muller, Christopher Murray, Mohsen Naghavi, Molly Nixon, Elaine Nsoesie, David Pigott, Robert Reiner, Joseph Salama, Benn Sartorius, Katya Shackelford, Caitlyn Steiner, Roman Topor-Madry, Stein Emil Vollset, Theo Vos, Andrea Werdecker, Harvey Whiteford, and Maigeng Zhou.

Did not provide contribution information

Dash A P, Jemal Abdela, Baffour Awuah, Sree Bhushan, Jacqueline Castillo-Rivas, Leslie Cooper, Drew Dolgert, Maryam Farvid, Amiran Gamkrelidze, Mohammad Rasoul Ghadami, Alexis Handal, Ankur Joshi, Prakash K.C., Narges Karimi, Hossein Kazemeini, Zhila Kazemi, Ali Kazemi Karyani, Ketevan Kereselidze, Maia Kereselidze, Tripti Khanna, Georgy Lebedev, Giancarlo Logroscino, Ralph Maddison, Noushin Mohammadifard, Mousa Mohammadnia-Afrouzi, Gvs Murthy, Sahar Saeedi Moghaddam, Yahya Safari, Nizal Sarrafzadegan, Marina Shakhnazarova, Reza Shirkoohi, Yohanes Ayele Wondimkun, Paul Yip, and Engida Yisma.

Section 1: GBD Overview

Section 1.1: Geographic Locations of the Analysis

The locations included in GBD 2017 were arranged into a set of hierarchical categories composed of seven super-regions and a further nested set of 21 regions containing 195 countries and territories (Appendix Table 2). Each year, the GBD Study undertakes subnational analysis for a small number of new countries, and continues to estimate subnationally any that were undertaken in previous cycles. Subnational estimation in GBD 2017 includes five new countries (Ethiopia, Iran, New Zealand, Norway, Russia), and all countries previously estimated at subnational levels (GBD 2013: China, Mexico, United Kingdom; GBD 2015: Brazil, India, Japan, Kenya, South Africa, Sweden, and the United States; GBD 2016: Indonesia, United Kingdom). All analyses are at the first level of administrative organization within each country with the exception of New Zealand separately by Maori ethnicity, Sweden which has been estimated for Stockholm and non-Stockholm, and the United Kingdom which has been estimated down to the local government authority level). All subnational estimates for these countries were incorporated into model development and evaluation as part of the GBD 2017 study. To meet data use requirements, in this publication we present only subnational estimates that have been already published elsewhere (Brazil, India, Japan, Kenya, Mexico, Sweden, United Kingdom, United States); given space constraints these results are presented in appendix tables and figures instead of the main text (see Supplementary Results Appendix). Subnational estimates for countries with populations larger than 200 million (measured using our most recent year of published estimates) that have not yet been published elsewhere are presented wherever estimates are illustrated with maps, but are not included in data tables.

Section 1.2: Time Period of the Analysis

A complete set of all-cause deaths and rates were computed for the years 1950-2017.

All GBD 2017 mortality results and online data visualizations will be made available upon paper acceptance. Results for all GBD metrics will be made available upon paper acceptance.

Section 1.3: Statement of GATHER Compliance

This study complies with the Guidelines for Accurate and Transparent Health Estimates Reporting (GATHER) recommendations. We have documented the steps involved in our analytical procedures and detailed the data sources. See Appendix Table 1 for GATHER checklist.

The GATHER recommendations can be found here: http://gather-statement.org/

Section 1.4: List of abbreviations

1q0: probability of death from birth to age 1 year

4q1: probability of death from age 1 year to age 4 years

5m0: under-5 mortality rate

5q0: probability of death from birth to age 5 years

45m15: mortality rate from age 15 years to 60 years

45q15: probability of death from age 15 years to 60 years

ACLED: Armed Conflict Location and Event Database

ART: antiretroviral therapy

BTL: International Classification of Diseases Basic Tabulation List

CBH: complete birth history

CD4: CD4+ T lymphocyte; an indicator of immune function

CIBA: cohort incidence bias adjusted

CIBA-SPECTRUM: cohort incidence bias adjusted spectrum output

CoD: causes of death

CODEm: Cause of Death Ensemble model

DALY: disability-adjusted life-year

DDM: death distribution methods

DHS: Demographic and Health Surveys

DSP: Disease Surveillance Points

EM-DAT: Centre for Research on the Epidemiology of Disasters' International Disaster Database

enn: early neonatal

EPP: Estimation and Projection Package

GATHER: Guidelines for Accurate and Transparent Health Estimates Reporting

GBD: Global Burden of Disease

GEMS: Global Enteric Multicenter Study

GGB: generalized growth balance

GGBSEG: combined generalized growth balance and synthetic extinct generation

GIDEON: Global Infectious Diseases and Epidemiology Network

GK: Gakidou-King

GPR: Gaussian process regression

HDI: human development index

HIV: human immunodeficiency virus

HIV CDR: Crude death rate due to HIV/AIDS

HMD: Human Mortality Database

ICD: International Classification of Disease

IPUMS: Integrated Public Use Microdata Series

LDI: lag-distributed income per capita

lnn: late neonatal

MAD: median absolute deviation

MCCD: Medical Certification of Causes of Death

MCMC: Markov Chain Monte Carlo

MICS: Multiple Indicator Cluster Surveys

NCD: non-communicable disease

nmx: mortality rate without fatal discontinuities

PCVA: Physician Certified Verbal Autopsy

pnn: post neonatal

PRIO: Peace Research Institute Oslo

SBH: summary birth history

SCAD: Social Conflict Analysis Database

SDI: Socio-demographic Index

SEER: Surveillance, Epidemiology, and End Results Program

SEG: synthetic extinct generation

SRS: Sample Registration System

ST-GPR: Spatiotemporal Gaussian process regression

U5MR: under-5 mortality rate:

UCDP: Uppsala Conflict Data Program

UI: uncertainty interval

UN: United Nations

UNAIDS: Joint United Nations Program on HIV/AIDS

UNICEF: United Nations International Children's Emergency Fund

USAID: United States Agency for International Development

VR: vital registration

WFS: World Fertility Surveys

WPP 15: World Population Prospects 2015

WHO: World Health Organization

YLD: years lived with disability

YLL: years of life lost

Section 1.5: GBD results overview

Results from the Global Burden of Disease Study (GBD 2017) are now measured in terabytes. Results will be made available upon manuscript acceptance in an interactive data-downloading tool on the Global Health Data exchange (GHDx).

The current version of the data download tool is available in the GHDx and will contains core summary results for the GBD 2017 upon manuscript acceptance: http://ghdx.healthdata.org/gbd-results-tool. The core summary results include deaths, YLLs, years lived with disability (YLDs), and disability-adjusted life-years (DALYs). The GHDx includes data for causes, risks, cause-risk attribution, aetiologies, and impairments.

In the GBD 2017 version, the GHDx tool also contains measures such as prevalence and incidence as well as rate of change data. Data above a certain size cannot be viewed online but can be downloaded. Depending on the size of the download, users may need to enter an email address; a download location will be sent to them when the files are prepared.

Section 1.6: Data input sources overview

GBD 2017 incorporated a large number and wide variety of input sources to estimate mortality, causes of death and illness, and risk factors for 195 countries and territories from 1990-2017. These input sources are accessible through an interactive citation tool available in IHME's GHDx.

Users can retrieve citations for a specific GBD component, cause or risk, and location by choosing from the available selection boxes. They can then view and access GHDx records for input sources and export a CSV file that includes the GHDx metadata, citations, and information about where the data were used in GBD. Additional metadata for each input source are available through the citation tool, as required by the GATHER statement.

The citation tool is accessible through the GHDx at http://ghdx.healthdata.org/gbd-2017/data-input-sources

Section 1.7: Funding Sources

Research reported in this publication was supported by the Bill & Melinda Gates Foundation, the National Institute on Aging of the National Institutes of Health (award P30AG047845), and the National Institute of Mental Health of the National Institutes of Health (award R01MH110163). The content is solely the responsibility of the authors and does not necessarily represent the official views of the Bill & Melinda Gates Foundation or the National Institutes of Health.

Section 2: GBD 2017 All-Cause Mortality and HIV Estimation Process

Section 2.1: Overview

The goal of the all-cause mortality estimation process for the GBD was to produce the most accurate time series estimates for 1950 to 2017 for all-cause deaths and death rates broken down into the GBD age groups and by sex for all 918 locations in the GBD 2017. This task was necessarily complicated by the diversity of data sources (Appendix Tables 3 and 4) available for all-cause mortality for different age groups, the known biases in some sources, and the powerful effects of the HIV epidemic on the age pattern of mortality in countries with large affected populations. In this appendix, we divide the analysis

of all-cause mortality into five distinct but interconnected areas: probability of death between birth and age 5 years (5q0), probability of death between age 15 years and 60 years (45q15), estimation of a complete set of age-specific death rates, estimation of HIV mortality, and final estimates of age-specific mortality including HIV and fatal discontinuities. While HIV was a cause of death, we present some information on the epidemiological modeling of HIV in this section because of the close interdependency between HIV mortality estimation and the all-cause mortality estimation process.

If all countries had complete vital registration (VR) systems recording the event of death and periodic censuses, the task of estimating all-cause mortality rates would have been much easier. However, many countries of lower-to-middle levels of sociodemographic development produce incomplete mortality records, and multiple sources must be used to infer levels, trends, and patterns of mortality. The analytical building blocks of the process, as shown in Appendix Figure 1, were repeated through two full iterations. This iteration resulted from the interdependence between the HIV epidemiological models on estimates of HIV-free all-cause mortality and the use of estimated HIV crude death rates as an input to the 5q0, 45q15, and age-specific mortality models. In the following sections we further discuss each analytical module, identifying primary input data and analytical processes.

Section 2.2: Child mortality

Section 2.2.1: Data sources

VR from CoD

Data were provided by the GBD causes of death (CoD) research team and were aggregated into total age-sex-specific all-cause mortality for each location-year.

Data intended for use in causes of death modeling were assessed for quality with respect to consistency of cause fractions, diagnostic accuracy, and missing data, whereas for all-cause mortality modeling it was more important that data were fully representative of the given estimation area and were consistent with other all-cause mortality data sources. Thus, there were cases in which VR data prepared for cause-specific modeling could not be used in all-cause modeling or had to be adjusted based on degree of completeness before being used.

There were instances where VR data used in cause-specific mortality analysis had been collapsed to Basic Tabulation List (BTL) format rather than in full cause classification list format (e.g., ICD9). In some of these cases, we elected to use WHO data instead.

VRs, sample registration systems, and DSPs from other sources

We endeavored to include all available data from VR systems as inputs in our all-cause mortality estimation process. To achieve this, we utilized a number of multi-country VR sources, including the WHO Mortality Database, the Human Mortality Database, United Nations Demographic Yearbooks, and OECD (Organisation for Economic Co-operation and Development) databases. These multi-country sources were regularly updated in our systems when new data were added. Beyond multi-country

sources, for all ongoing national VR systems (for example, the USA National Vital Statistics System) we cataloged all data sources from each system where possible.

Some countries that do not have well-performing VR systems implement sample registration systems that are incomplete by design. We made use of these data, paying close attention to the proper weighting of sampled data and consistency with other representative sources. We have systematically extracted data from the Sample Registration System Statistical Report series published by the Registrar General of India. For the Disease Surveillance Points (DSP) system of China, we obtained both national and provincial level DSP data through a data usage agreement with the Chinese Center for Disease Control and Prevention. Census data were systematically extracted from Demographic Yearbook series, Integrated Public Use Microdata Series (IPUMS), and statistical reports from the national statistical bureaus. Appendix Figure 2 shows more detail of percent of completeness of under 5 registered deaths from 1990 to 2017.

Under-5 populations and live births

For GBD 2017, live births were produced as part of the population and fertility estimation.[1]

CBH microdata

Complete birth histories (CBHs), the preferred method for data collection on child mortality in the absence of VR, rely on administering surveys to mothers. The questionnaires ask about all living and deceased children, including date of birth, survival status, and date of death. These modules were included in many routine survey series, including the World Fertility Surveys (WFS), Demographic and Health Surveys (DHS), Multiple Indicator Cluster Surveys (MICS), and many national survey programs. When available, we downloaded and used microdata that included individual-level survey responses as opposed to tabulated results.

CBH tabulated data

In some instances, tabulated records from reports became available before survey microdata, and we incorporated those data points into our database of 5q0 data as well. However, as microdata became available, we updated with point estimates from our processed microdata rather than the tabulated report estimates.

SBH microdata

Summary birth history (SBH) questionnaires are a shorter alternative to complete birth histories. Instead of asking in detail about each child, summary birth histories simply ask mothers how many children they have given birth to and how many of the children have died. The questionnaires are shorter and can be more easily attached to other surveys. Often, censuses and MICS surveys contain summary birth histories. For GBD, we compiled all available SBH data with microdata, which enabled us to apply the updated SBH method to produce a more accurate and timely assessment of U5MR.[2]

SBH tabulated data

In cases where we did not have access to the microdata on SBH modules from surveys and censuses, we utilized the reported estimates of U5MR from survey or census reports and outliered the first two data points based on mothers aged 15-19 and 20-24.

Under-5 age-sex patterns from VR/SRS/DSP

VR systems were the primary source of data for the under-5 age pattern of mortality in high-income countries. Often, these data were classified into several age groups: early neonatal (0-6 days), late neonatal (7-27 days), post-neonatal (28-364 days), and 1 to 4 years. Some country-years of data had other age groupings with less specificity, with the early and late neonatal age groups combined, or all of the under-1 age groups combined. Sample Registration Systems (SRS) also provided data for the age-sex patterns of under-5 mortality in several countries (notably India and Bangladesh). The DSP system in China provided data on age-sex under-5 mortality as well.

Under-5 age-sex patterns from CBH

In many countries without VR systems, CBH surveys were used to obtain age-sex patterns of mortality in under-5 age groups. These sources are described above in the "CBH microdata" section. For all CBH microdata sources, we applied direct estimation methods to obtain probabilities of death for each of the under-5 age groups. Within each survey, where each observation is a child recalled by a mother, observations were grouped into 5-year groups in time to provide a data point of probability of death for each of the under-5 age-sex groups. Recall was cut off 15 years before the survey, limiting data points estimated from the survey to the 15 years prior. All of these estimates were then put in the database of estimates for the age-sex pattern of under-5 mortality.

Section 2.2.2: VR prioritization

Our continual evaluation of VR data sources led us to develop a general hierarchy of preferred VR sources. When considering which of multiple sources to use for a given location-year, we preferred to first use WHO data from GBD cause-specific mortality estimation, then unadjusted WHO data, then Human Mortality Database (HMD) data, then UN Demographic Yearbook data. There were exceptions to this hierarchy where we had reason to believe that there were quality issues with a certain source. For instance, where available we preferred to use HMD VR over WHO data for Germany, Taiwan, and Spain due to WHO data producing mortality rates that were inconsistent with previously established trends. Single-country VR sources were evaluated based on consistency with other data sources and also VR system documentation.

Section 2.2.3: Identify VR under-enumeration for bias correction

The approach to estimating the completeness of VR systems for deaths under age 5 was the same as that of the previous three GBD studies. However, the VR-specific correction in previous GBD rounds has

been removed for GBD 2017. Most biased VR is corrected in the mixed effects non-linear model outline in Section 2.2.6.

Similar to previous GBD rounds, there were countries for which only VR data were available and the VR systems were considered biased. This was a problem particularly in English-speaking Caribbean countries, so for these countries we adjusted 5q0 estimates from VR using the regional average VR bias in a given year for those countries with both VR and survey 5q0 estimates. The countries for which VR systems were adjusted using this method include Antigua and Barbuda, Bahamas, Barbados, Bermuda, Dominica, Grenada, Saint Lucia, and Saint Vincent and the Grenadines. While there was no direct evidence on the level of VR bias in these countries, assuming they were complete when similar countries in the region exhibited under-registration seemed unwarranted.

Section 2.2.4: Biennial 5q0 estimates

Computation of CBH 5q0

Microdata (individual-level survey data) from CBH yielded direct calculation of death numbers and probabilities of death in the under-5 age group. Observations were grouped into two-year intervals such that biennial estimates of 5q0 were obtained from these survey data. In GBD 2017, we unpooled surveys for our analysis, whereas surveys were pooled by series in GBD 2013.[3] Instead of grouping observations from all DHS complete birth history questionnaires from a country into one full set of observations and all MICS observations from multiple survey years into another full set of observations, we analyzed each survey separately by location (e.g., DHS 2012, DHS 1996, MICS 2002). This allowed for a greater ability to address known data quality issues in specific surveys. To compensate for the decreased sample size and to generate greater stability in the unpooled data points, we created two-year estimates of under-5 mortality, pooling observations over two-year periods instead of single years. We restrict the years of recalled CBH data to two-year intervals with over 10,000 person-months of data as generated by months contributed by each child.

Processing of tabular CBH

In some instances, microdata from surveys were not available. If survey reports could be obtained but the microdata were not available for us to do our own calculations to obtain 5q0, we used report data point estimates. These estimates were added directly to the under-5 mortality database.

Section 2.2.5: SBH time series method

SBH method from microdata

Rajaratnam and colleagues developed an updated SBH method that was able to provide more accurate and timely estimates of U5MR from microdata on SBH from surveys and censuses.[2]

Analysis of SBH from tabular data

When only tabular data were available for the numbers of children ever born and number of children that have died by mother's age, we applied the Maternal Age Cohort model from the method developed by Rajaratnam and colleagues.[2]

Section 2.2.6: 5q0 data synthesis, model running, and bias correction

Data synthesis using ST-GPR and bias correction

We applied the child mortality estimation methodology as reported by Wang and colleagues.[3] Based on the under-5 mortality data synthesis model for the Global Burden of Disease Study 2010,[4,5] 2013,[3] 2015,[6] and 2016,[7] we incorporated data bias adjustment into the modeling process. Specifically, we included a fixed effect for source type across all locations to detect systematic differences in the level of child mortality, controlling for covariates for one source type versus another. The groups of sources used to make this adjustment are listed below. In addition, we included a random effect for each country-source. By choosing a reference source (country-by-country or using the mean of a set of sources, we adjusted on a country-by-country basis for the problem of compositional bias created by substantial source-specific non-sampling error. Reference sources were not adjusted, even if multiple sources were used as reference. Once the systematic difference in sources were removed, we were able to avoid estimating false trends due to partial overlap of sources with different levels of non-sampling variance. We then applied the combination of non-linear mixed effects model, spatiotemporal regression, and GPR to synthesize raw child mortality data after data bias adjustment to obtain consistent time series estimates of mortality with 95% uncertainty intervals for every country.

Source types used in child mortality bias correction

Data source type:

1. CBH - Demographic and Health Survey
2. CBH – AIDS Indictor Survey and Malaria Indicator Survey
3. CBH – World Fertility Survey
4. CBH – Multiple Indicator Cluster Survey
5. CBH – Census
6. CBH – Other survey series
7. SBH – Demographic and Health Survey
8. SBH – Multiple Indicator Cluster Survey
9. SBH – Other survey series

10. SBH – AIDS Indicator Survey and Malaria Indicator Survey
11. SBH – Census
12. SBH – World Fertility Survey
13. VR/Sample Registration/Surveillance - complete
14. VR/Sample Registration/Surveillance – incomplete
15. Household Death Recall – Other survey series
16. Household Death Recall – Census
17. Household Death Recall - incomplete VR/Sample Registration/Surveillance

A list of under-5 mortality reference sources is provided in Appendix Table 5.

Mixed effect non-linear model and the bias adjustment for raw U5MR sources

In this stage, we used a non-linear mixed effects regression to estimate data bias and provide first stage predictions.

The nonlinear mixed effects regression model is

$$ {}_5m_{0cys} = \exp[(\beta_1 + \gamma_{1c}) * \log\left(LDI_{cy}\right) + (\beta_2 + \gamma_{2c}) * education_{cy} + \gamma_c + \gamma_{cs} + \alpha_t] + \beta_3 * HIV_{cy} + \varepsilon_{cys} $$

Where

c is country

y is year

s is source

t is source type

${}_5m_0$ is under-5 mortality rate

LDI is lag-distributed income per capita

education is mean years of education for women of reproductive age (15-49 years)

HIV is death rate due to HIV in age groups 0-4

γ is a random effect

α is a fixed effect on source type across countries

β_i is a fixed covariate coefficient

ε is the residual

Each source was categorized into one of the 17 source types across all countries. LDI and education covariate estimation is explained in Section 5 of this appendix. The HIV death rates used in this regression are the GBD 2017 HIV estimates (Section 2.5).

For each country, we relied on expert opinion to choose a source, or combination of sources, which were believed to be the least biased. If a country had a VR system which we deemed to be complete (described in detail in 2.2.1), this was the reference source. If a country did not have a complete VR system, but had DHS estimates from CBHs, these were used as the reference source. If a country had neither of these types of data, or DHS estimates were deemed unreliable, we assigned the surveys conducted after 1950 (in combination) as the reference. Incomplete VR data were not included. Additionally, in many countries we chose alternate surveys as the reference. For accurate estimation, it was important to have local knowledge on specific data sources' accuracy. All-cause mortality experts drew from their familiarity with data quality to help us to choose the reference category.

Each data source had an associated random effect as well as a source type fixed effect. The values of these random and fixed effects for the reference sources were deemed to be the true deviation from unbiased mortality level. In countries with multiple high-quality sources, the mean of the random and fixed effects from these sources was taken as this true deviation. We adjusted all other sources by including these reference values for the random and fixed effects values instead of those estimated for each individual source.

$$adjusted_5m_{0,cys} = \exp[(\beta_1 + \gamma_{1c}) * \log(LDI_{cy}) + (\beta_2 + \gamma_{2c}) * education_{cy} + \gamma_c + \gamma_{ref,c} + \alpha_{ref,c}] + (\beta_3 + \gamma_{3c}) * HIV_{cy} + \varepsilon_{cys}$$

The exception to this correction was incomplete VR data (section 2.2.3), which was adjusted upwards using a five year rolling mean of the difference between incomplete VR and a Loess of the already-adjusted survey data.

Spatiotemporal smoothing

The spatiotemporal stage smooths the residuals between the predicted time series of 5q0 and the adjusted raw data over time and across countries in the same GBD region. The predicted time series for this smoother was obtained from the equation below; no random effects or survey type fixed effects were included.

$$predicted_5m_{0,cy} = \exp[\beta_1 * \log(LDI_{cy}) + \beta_2 * education_{cy} + \alpha_{intercept}] + \beta_3 * HIV_{cy}$$

We first found the residuals between the predicted time series, above, and the adjusted points. We then applied a combination of smoothing functions to these residuals. For each country-year, we weighted all

the data points in this region based on their proximity to this country-year in space and time. We gave 99% of the weight to in-country residuals, and 1% of the weight to out-of-country residuals. Additionally, we used a modified tricubic window, as specified below, to give more weight to points closer in time, and less weight to points further in time.

$$w_t = \left(1 - \left(\frac{|r_t - r_{est}|}{1 + argmax_t|r_t - r_{est}|}\right)^{\lambda}\right)^3$$

Where

r_t is the year of interest

r_{est} is the year of the residual being weighted

$argmax_t|r_t - r_{est}|$ is the maximum distance between the year of interest and a residual within the region

λ is the weighting function that dictates how quickly the weights fall off as the distance in time increases

A larger λ implies that the assigned weights will diminish slowly with time, while a smaller λ allows the weights to diminish more rapidly with time. λ values were chosen using the parameter selection process described below. We then created one estimate of the smoothed residuals using a linear fit to this weighted data, similar to a Loess fit. Additionally, we created a second estimate of the smoothed residuals by calculating the weighted average of this data.

We then combined these two estimates for a final estimate of the smoothed residuals. In data-dense countries, more weight was given to the local linear fit; in data sparse countries, more weight was given to the weighted average. The equation for this is as follows.

$$final\ smoothed\ residual = k * linear\ estimate + (1 - k) * weighted\ average$$

Where

$$k = \frac{number\ of\ in\ country\ data\ points}{number\ of\ in\ country\ data\ points\ +\ number\ of\ country\ years\ with\ no\ data}$$

Finally, the smoothed residuals were added back to the predictions from above; this smoothed approximation to the adjusted data was used as the prior for GPR, described below.

GPR

The output of the space-time smoothing step was used as a prior for GPR, which produced a final time series of point estimates, as well as confidence bounds. Parameters for GPR were chosen through cross-validation as described below.

The model for GPR was

$$\mu_t = f(t) + S_t$$

$$f(t) \sim GP(M, C)$$

Where

μ_t is the true $\log_{10}(5q0)$ at time t

$f(t)$ is the baseline mortality risk

S_t is excess mortality due to fatal discontinuities estimated independently of $f(t)$

M is the mean for the Gaussian process

C is the covariance for the Gaussian process

We specified a prior distribution for $f(t)$ from the spatiotemporal regression, and a likelihood function that describes the data generation process; the specified prior distributions and likelihood function are described below. We then used Markov Chain Monte Carlo (MCMC)[8] to approximate the posterior distribution of $f(t)$, which also incorporated information from the observed empirical estimates of adult mortality. An MCMC chain of length 5,000 was produced; the first 3,000 samples were discarded and the remaining 2000 were thinned by a factor of 2 for a total of 1,000 simulations retained. The reported best estimates and uncertainty intervals were generated from the mean and the 2.5th and 97.5th percentiles of the 1,000 samples, respectively.

The prior distribution of $f(t)$ can be described in terms of the mean prior—the prior for M—and the covariance prior—the prior for C. We utilized the second stage predictions as the mean prior and used a Matern covariance function to describe the covariance prior. The parameters of the Matern covariance function were selected through cross-validation and were location-specific.

Likelihood

The likelihood describes the probability of observing the data given a particular set of parameters. As shown in the equation below, we used a normal model for describing the probability of observing a particular value of log(5q0)

$$log_{10}({}_5q_{0_t}) \sim Normal(f(t), V_t)$$

Where

$f(t)$ is the mean

V_t is the data variance

Data variance was calculated for each empirical observation of 5q0 and incorporated both sampling and non-sampling variation. The method for calculating the data variance depended on the type of data:

1. For estimates derived from complete VR data, we assumed that there was no non-sampling variance and included only sampling variance as computed from a binomial model. We set N equal to the national population aged 0 to 5 years and p equal to the mortality rate, ${}_5m_0$. We calculated the variance of ${}_5m_0$ from $p(1-p)/N$ and then transformed this to the variance of $\log_{10}$(5q0) using the delta method.[9]

2. For estimates derived from incomplete VR data, we wanted to include not only sampling variance but also the non-sampling variance that arises from uncertainty in the completeness estimate. For these data, the total data variance was given by the sum of the sampling variance (calculated as for complete VR data) and the variance of the completeness estimate;

3. For estimates derived from CBHs, we generated 1,000 simulations of 5q0, converted these estimates into $\log_{10}$ space, and calculated the sampling variance from these 1,000 simulations;

4. For estimates derived from SBHs we used the standard error from the mean residuals;

5. For estimates not covered under the above four calculations, the missing data variance was determined as the maximum standard error from non-VR points in the country. If the data variance was still missing it was calculated as the maximum standard error from non-VR data in the GBD region.

6. For each source type we calculated the within-source-type variance of the source-specific random effect. This additional non-sampling variance was then converted to $\log_{10}$ space and added to the variance as calculated above for all data points not classified as complete VR.

Hyperparameter selection for under-5 mortality rate ST-GPR

For GBD 2017, hyperparameters were selected based on a newly-created data density score for a given location. The data density score was calculated for each location based on the number of deaths from VR sources as well as the number of unique CBH and SBH available. The data density score was computed using the following steps:

1. Calculate complete VR score: this component of data density was computed based on the number of deaths from an unbiased VR sources in the location. Using the death counts, we capped the number of deaths at 500 for each year and then divided that number by 500. The result was a score for each year between 0 and 1 where 1 represents a complete VR system with at least 500 registered deaths. To get the final complete VR score for a location, we added up

the score for each year across the full time series. The result was a complete VR score between 0 and 68 (the range of our full 1950-2017 estimation time series).

2. Calculate incomplete VR score: this component of data density is computed in the same manner as the complete VR score using biased VR instead of unbiased VR.
3. Total CBH sources: this is simply a count of the unique complete birth histories for a location
4. Total SBH sources this is simply a count of the unique summary birth histories for a location
5. Once the intermediate calculations were completed, the following formula was used to compute the final data density

$$data\_density = complete\_vr\_deaths' + (0.5 \times incomplete\_vr\_deaths) + (2 \times cbh\_sources) + (0.25 * sbh\_sources)$$

Once the data density for a location was calculated, we assigned hyperpameters:

Table A

Data Density	Zeta	Lambda	Scale
0 to 9	0.7	0.7	15
10 to 19	0.7	0.5	15
20 to 29	0.8	0.4	15
30 to 49	0.9	0.3	10
50 plus	0.99	0.2	5

Section 2.2.7: Identify and remove outliers

There were several important quality-control steps in reviewing child mortality data and estimates. First, data points from years in which fatal discontinuities occurred were outliered, unless they were VR data points with sufficient information that the fatal discontinuities could simply be subtracted out of the VR data. The intent was to capture the underlying mortality risk rather than large stochastic variations. These fatal discontinuities were then added on in a later step (see Section 2.6.3). Secondly, we outliered data sources with quality concerns such as the Afghanistan DHS from 2010. Our extensive collaborator network allowed for review of sources, and collaborators raised concerns over known issues with data sources about which they had expert knowledge.

Section 2.2.8: Rake subnational estimates to national level (excluding South Africa)

The estimation process for 5q0 does not enforce consistency between subnational estimates and national estimates. To ensure consistency throughout the GBD hierarchy, we rescaled the subnational estimates to the national level via population-weighting to calculate an implied national estimate from the subnational estimates. This created a scalar of the national-level estimate from GPR to the aggregated subnational estimates, which was then multiplied by all of the subnational estimates to obtain the scaled estimates. In most cases, we considered national-level estimates to be more reliable, so we chose this strategy of subnational scaling. In locations with high-quality VR data, this scaling has a

minimal effect, but the effect can be greater in locations with more subnational units and variable-quality data.

In South Africa, it was essential that the province-specific mortality patterns be consistent with HIV models, since such a large part of the trend was driven by deaths due to HIV/AIDS. In this case, instead of scaling provincial-level estimates to national-level GPR estimates, we aggregated province-level GPR estimates to generate the national-level estimates.

Section 2.2.9: Review estimates for quality

Estimates of 5q0 from the ST-GPR process were reviewed in comparison to UNICEF estimates from their 2015 revision and GBD 2015 results.[10] Any differences were traced to either changes in available data or changes induced by changes in hyperparameters or input covariates. Revisions were made through this review process and through expert consultation with the GBD mortality collaborator network.

Section 2.2.10: U5MR with HIV

The 5q0 ST-GPR process generated U5MR for all GBD 2017 locations that was inclusive of the impact of all causes of death excluding fatal discontinuities, which were added in a separate step (see section 2.6.3).

Section 2.2.11: Under-5 age and sex pattern model estimation

The process used to break down under-5 mortality into age- and sex- specific groups has been previously described.[7] The current process was largely similar but was modified to improve the accuracy of predictions for countries affected by HIV/AIDS. As pointed out by Bradshaw et al.,[11] neonatal mortality tends to be overestimated if the all-cause U5MR is used as the only predictor. We used a multi-stage modeling process to generate sex-specific estimates of early neonatal (days 0 to 6), late neonatal (days 7 to 27), post-neonatal (the remainder of the first year), under-1, and childhood (ages 1 to 4) mortality. First, the ratio of male to female 5q0 was estimated, then age- and sex-specific mortality estimates were generated using this ratio. To fit models to obtain estimates data from VR, sample VR, and CBHs were converted to mortality risks for specific age groups. Sources had differing levels of age specificity and at minimum included infant (composed of early neonatal, late neonatal, and post-neonatal) and child mortality, but could include all 4 smaller age groups. The sex-specific model was fit to the data, followed by the age-specific model.

The sex model first predicted the ratio of male 5q0 to female 5q0 in rescaled logit space for each country i in region j in year t. The data were ordered by observed 5q0, and categorized into 20 evenly sized bins. We rescaled the ratio data between 0 and 1 to between 0.8 and 1.5. Then the model was fit to the data as described in the equation below.

$$logit\ \left(\frac{Male\ {}_5q_0}{Female\ {}_5q_0}\right)_{jit} = \beta + \gamma_{{}_5q_0\ bin} + \gamma_j + \gamma_i + \varepsilon_{jit}$$

The ratio was predicted by nested location and region random effects γ_i and γ_j, a random effect on the 5q0 bin, and an intercept term, β. A Loess regression was then used to smooth the estimated $\gamma_{_5q_0 \text{ bin}}$ on 5q0, creating a continuous $\gamma'_{_5q_0 \text{ bin}}$. Then, the equation below was used to predict the ratio of male to female 5q0:

$$logit\left(\frac{Male\ {}_5q_0}{Female\ {}_5q_0}\right)_{jit} = \hat{\beta} + \gamma'_{_5q_0\ bin}\left({}_5q_{0\,jit}\right) + \hat{\gamma}_j + \hat{\gamma}_i$$

The predicted ratios were unscaled, inverse logited, and used as the first stage prediction for ST-GPR as used for the estimation of U5MR. ST-GPR hyperparameters were chosen using the same method outlined of U5MR in section 2.2.6. The male and female 5q0 values were found using the system of equations that includes the prediction from the ST-GPR described above and the equation below, where $r_{\ birth}$ is the sex-ratio at birth.

$${}_5q_0 = \left(\frac{1}{1 + r_{birth}}\right) * (female\,{}_5q_0) + \left(\frac{r_{birth}}{1 + r_{birth}}\right) * (male\,{}_5q_0)$$

Age-specific models were then fit for each age group on sex-specific data. A separate model was fit for each age group, yielding five models for each sex: early neonatal, late neonatal, postneonatal, infant, and child. The log of the probability that an under-5 death occurs in a given age group conditioned on surviving to that age group was modeled instead of the mortality risk, simplifying the scaling process and restricting risks to be between 0 and 1. Because evidence suggests HIV has differential effects on different under-5 age groups,[12,13] the crude death rates from HIV/AIDS in the under-5 age group were included in the model for age groups after neonatal, given our assumption that all HIV deaths that occur in the first year of a child's life occur in the post-neonatal stage (after 28 days). The literature on HIV in these age groups is still unclear, but seems to indicate higher mortality in the post-neonatal stage. There is no clear evidence to guide alternative methods of age-splitting under-1 deaths due to HIV.[13,14] We used the crude death rate due to HIV from the GBD 2017 model (see Section 2.2.6). The inclusion of this covariate improves both the fit and prediction of the model in countries with high HIV prevalence. We included the maternal education covariate that is also used in the 5q0 first-stage model and the completeness of the source-specific 5q0 estimate for the data-point used in the regression for that 1 to 4 age group only. This completeness measure was calculated by taking the source-specific 5q0 point estimate and dividing by the final 5q0 estimate from GPR. The functional forms of the model are below, where age y included late-neonatal, post-neonatal, and under-1 children.

$$log(Pr(death\ at\ age\ enn|u5\ death)_{jit}) = \beta_1 + \gamma_{_5q_0\ bin} + \gamma_j + \gamma_i + \varepsilon_{jit}$$

$$log(Pr(death\ at\ age\ y|u5\ death)_{jit}) = \beta_1 + \beta_2 * HIV_{it} + \gamma_{_5q_0\ bin} + \gamma_j + \gamma_i + \varepsilon_{jit}$$

$$log(Pr(death\ in\ age\ 1-4\ |u5\ death)_{jit}) = \beta_1 + \beta_2 * HIV_{it} + \beta_3 * Mat.Ed._{it} + \beta_4 * Completeness_{sit} + \gamma_{_5q_0\ bin} + \gamma_j + \gamma_i + \varepsilon_{jit}$$

Similar to the sex model, the sex-specific age prediction used 5q0 bins and smoothed the random effect on the bin using 5q0. The prediction equation for the age group 1 to 4 is shown below.

$$log(Pr(death\ in\ age\ 1-4|u5\ death)_{jit}) = \hat{\beta}_1 + \hat{\beta}_2 * HIV_{it} + \hat{\beta}_3 * Mat.Ed._{it} + \hat{\beta}_4 * 1 + \hat{\gamma}'_{{}_5q_0\ bin}({}_5q_{0_{jit}}) + \hat{\gamma}_j + \hat{\gamma}_i$$

Where

y is age

j is region

t is time

$\hat{\gamma}_i$ is nested random effects on country

$\hat{\gamma}_j$ is nested random effects on region

$\hat{\beta}_1$ is an intercept term

$\hat{\gamma}'_{{}_5q_0\ \mathrm{bin}}({}_5q_{0_{\mathrm{jit}}})$ is a smoothed random effect on 5q0 bin

$\hat{\beta}_2$ is a coefficient on the under-5 crude death rate from HIV

$\hat{\beta}_3$ is a coefficient on maternal education

$\hat{\beta}_4$ is a coefficient on completeness

Note that for prediction, the completeness coefficient was multiplied by 1 instead of a source-specific completeness, as we sought to predict based on a hypothetically complete source. Similar to the sex-ratio model discussed above, these predictions were used as the first stage prediction for ST-GPR that produced sex and age specific probabilities of death.

Once each of these predictions was made by age group, they were rescaled such that the probabilities of death in the early neonatal, late neonatal, post-neonatal, and 1 to 4-year age groups aggregated to the 5q0 estimates from the under-5 model.

Section 2.2.12: Identify and remove outliers

There were several criteria for removing outliers for the under-5 age-sex pattern model. For the sex model, non-VR (survey) data points from high-quality VR locations (as determined by the GBD VR quality rating system) were outliered to ensure the model followed the highest quality data. Additionally, un-outliered sex ratio data were adjusted to be between 0.8 and 1.5.

For the age model, the following outliering criteria were used:

VR data that were considered incomplete were marked as outliers. To be considered incomplete, the 9-year rolling average of the VR data 5q0 value was compared to the 9-year rolling average of the 5q0 estimates. Then, for a given data-year, the value of 5q0 in the raw

data was compared to our final 5q0 estimate. A value of 85% was considered incomplete and outliered, unless the ratio of the 9-year rolling average was above 85% complete.

Any data that were chosen as outliers as part of the 5q0 analysis were also marked as outliers in the age pattern analysis.

If a country has both VR and CBH data, they were typically both used. If there was a conflict between the two, VR data were used.

CBH data points more than 15 years prior to the survey were outliered.

Some data points were manually outliered. For example, the definition of live birth changed in some Eastern European countries in the 1990s, leading to inconsistencies. In this case, age group data in ages that would include childbirth deaths (early neonatal, neonatal, and ages 1-4) were outliered if the definition of live birth contains a minimum weight, as it did in some of these locations.

Section 2.2.13: Under-5 age-sex splitting model application

The prediction method from the age-specific model is described above in section 2.2.11. First, the results of the sex model were applied, yielding sex-specific 5q0 estimates. Once age-sex-specific predictions of the log conditional probability of death were made, these were exponentiated and rescaled so that they sum to 1. First, the under-1 and 1-4 conditional probabilities were scaled to add to 1. Then, the early neonatal, late neonatal, and post-neonatal conditional probabilities were scaled to the under-1 conditional probability. Then, the probabilities of death were calculated so that they properly aggregated to the final 5q0 prediction. For example, to calculate the probability of death in the early neonatal age group, the rescaled conditional probability of early neonatal death given under-5 death was multiplied by the probability of under-5 death. Then, to obtain the probability of death in the late neonatal age group, the rescaled conditional probability of death in the late neonatal age group given under-5 death was multiplied by the probability of under-5 death and then divided by the probability of survival to the beginning of the age group, and so on. Equations below represent this process, where enn represents early neonatal and lnn represents late neonatal.

$$q_{enn} = \Pr(death\ in\ enn \mid u5\ death) * 5q0$$

$$q_{lnn} = \Pr(death\ in\ lnn \mid u5\ death) * 5q0/(1 - q_{enn})$$

The older age groups were also calculated in this manner, yielding probabilities of death in each of the under-5 age-sex groups.

Section 2.2.14: Under-5 death number estimation

Assigning under-5 deaths to GBD age-sex groups

To estimate the number of under-5 deaths, we ran an estimation process that aged birth cohorts through our estimated probabilities of death. This process separated our yearly birth numbers for each location into week-sized cohorts and aged each of these cohorts through our mortality estimates in week-long steps to estimate the number of person-years and deaths in each of the early neonatal, late neonatal, post neonatal, and 1-4 years age groups.

Section 2.3: Adult mortality

Section 2.3.1: Data sources

Adult population estimates

To calculate adult mortality rate using household death recall, age specific populations in age group 15 to 59 from the corresponding survey or census sources were used. This is also true in calculating adult mortality rate using reported deaths from Sample Registration System from India and Disease Surveillance Point system from China. For data from VR systems, we used estimates from the GBD population model.

VR/SRS/DSP

See section 2.2.1 descriptions of VR sources for information on how VR, SRS, and DSP data were identified and prioritized.

Household recall of deaths

Household recall was ascertained via large survey series such as the Malawi 2010 DHS. A survey series was required to include a module asking about the number of deaths of household members within a given recall time period, along with a list of household members who have not died over this period of time. In addition, these survey series were required to be nationally representative, include survey weights (if applicable), and detail the sex and age (either current or at death) of all household members.

Sibling survival histories

Data for sibling survival histories were primarily taken from large survey series in which respondents were asked about the status of their siblings, alive and dead. Examples of sibling survival history sources included the Laos 2011-12 Multiple Indicator Cluster Survey and many DHS sources. To generate estimates of sibling survival, each questionnaire must have contained a module with a full accounting of all siblings (children born to the same mother) of all respondents, along with data on the year of death (if applicable), sex, age at death, and year of birth.

Section 2.3.2: Completeness Assessment: Death Distribution Methods and completeness estimates synthesis

VR systems may not capture all adult deaths. It was important to assess the completeness (quality) of available VR data. Demographers have long applied a suite of death distribution methods (DDMs) including generalized growth balance (GGB), synthetic extinct generation (SEG), and a combined approach (GGBSEG) to assess completeness.[15–19] These methods compare the age distribution of the population recorded in two censuses with the age distribution of deaths recorded between these two censuses and attempt to estimate completeness of VR.

Recent modifications of these DDM methods provide estimates of completeness that, based on careful simulation studies, were more accurate and robust than traditional methods. Nevertheless, these methods generate completeness estimates with substantial uncertainty intervals.[20]

For this GBD study, the process for estimating completeness of death registration for adults was based on estimates of adult completeness from three death distribution methods, as well as information about child completeness.[20] These two sources of information were combined to generate a series of estimates of source- and country-specific adult death registration completeness from 1950 to 2017 (Appendix Figure 2). The underlying assumption of this process was that completeness of systems changes gradually; consequently, assessments of completeness for a given year were informed by DDM estimates for prior and future years in that country. Completeness was likely to be similar among countries within a region, so we informed estimates of completeness with levels of completeness estimated for countries in the same region or super-region by borrowing strength over space as well as time. To do this, we used a two-stage model, whereby we first predicted adult completeness based on child completeness and then used a spatial-temporal regression model to incorporate information about adult completeness from the application of DDM methods.

Child completeness was calculated as the ratio of observed child mortality to estimated child mortality for a given source, country, and year. For a particular country-source, estimates of child completeness were only available for years where that data source was present, but a complete time series of child completeness estimates was produced based on a smoothing process. For country sources with no more than three years of data, a constant level of child completeness at the level of the mean of those years that were available was assumed. For country sources with more than three years of data, Loess regression was used to fill in the time series. In order to be conservative in our out-of-sample estimates of child completeness, instead of using the Loess predictions to forecast and backcast we simply held child completeness constant before the first observation and after the last observation. When there was a gap of more than five years we linearly interpolated between the observations on either side of this gap instead of using the Loess predictions to fill in these years.

For VR, SRS, and DSP sources, we treated completeness values at 95% or higher for a given country-year combination as 100% complete. In doing so, we scaled completeness values between 90% and 95% to account for this assumption. For a given country-source combination, if a country was complete across the full time-series, we assumed its subnational locations were fully complete as well. We made an

exception to this rule for Brazil. While we found the fully complete time-series to be accurate at the national level, there were differential completeness values across its subnational locations. Including this variation was necessary in order to accurately compute life expectancy at the subnational level.

Section 2.3.3: Sibling survival method

In countries where sources including VR and household death recall were scarce, sibling histories provided important information on the levels and trends of adult mortality rates. However, as studies have shown[21], estimates of adult mortality rate using sibling survival modules had significant biases resulting from the design and implementation of the method. Specifically, there were four different types of biases:

1. Selection bias (under representation of siblings from high mortality sibships)
2. Zero reporter bias (sibships not represented in the survey due to sex composition and/or mortality level of a sibship)
3. Sparse data
4. Recall bias (generally under reporting of death of siblings living in different places or have died in the distant past)

The sibling survival technique we employed was based on the work by Obermeyer et al.[21] with a few key differences to their methods:

1. Use of appropriate survival weights that accounted for the study design
2. Implementation of a correction to account for the mortality experience of families not represented because none of the siblings were alive and eligible to respond to the survey
3. Refinements for adjusting for recall bias.

We validated these methodological developments in a range of simulation environments and adjusted for recall bias and sparse data in survey designs where the age range of the respondents was narrower than the age range desired for estimation.[21]

Sibships with higher rates of mortality were less likely to be represented in the survey because fewer of them were likely to have survived to be selected into the sample. A method to correct for this underrepresentation, proposed by Gakidou and King,[22] incorporates a sibship-level weight:

$$W_j = \frac{B_j}{S_j}$$

Where

j is a given sibship

B_j is the original size of sibship j

S_j is the number of siblings in sibship j who survive to the time of the survey

When each observation in the dataset being analyzed was at the sibship level, the Gakidou-King (GK) weight could be used to compute a weighted average of the proportions of siblings deceased as reported by each respondent. In the absence of any sibships where all siblings have died, this correction algebraically corrected for the underrepresentation of high-mortality sibships in the survey sample.

When the dataset was expanded to the sibling level (i.e., one observation for each sibling as opposed to sibship), the number of observations listed in the dataset for each sibship corresponded to the original sibship size, B_j, and so the numerator of W_j was already accounted for. The resulting sibling-level weight was therefore

$$W_i = \frac{1}{S_j}$$

for sibling i in sibship j.[23,24]

Since the analysis reported here was carried out at the sibling level, we used W_i rather than W_j. This improved on previous applications of the method, where the sibship-level weight was inappropriately applied to data that had been expanded to the sibling level.

Further, the number of surviving siblings in the family must also be tailored to the eligibility criteria for respondents of the given survey.[23] In applying Gakidou and King's elucidation of the survivorship correction, $\frac{S_j}{B_j}$represented the probability that a sibling in sibship j survived and was eligible to be selected in the survey. For DHSs, respondents must be women between the ages of 15 and 49 and so the S_j represented the number of surviving women in a sibship j who were between the ages of 15 and 49 at the time of the survey. For our analysis, the value of S_j was chosen to be consistent with the eligibility criteria of each survey.

The sampled population excluded sibships in which there were not any eligible siblings to respond to the surveys. Thus, we cannot report on the mortality experiences of these siblings. The zero-survivor correction estimated the number of sibling deaths that were missing from the sample by age and sibship size, and then added these siblings to the observed sample before calculating age-specific mortality rates. This correction was applied to sibships with one or two females. The correction used the relationship between the true number of sibships with one (or two) females and the cumulative probability of dying before the time of the survey to estimate the number of missing sibling deaths. For one-sibling sibships

$$K_{obs}^1 = K_{true}^1 * (1 - \ {}_aq_0^1)$$

$$K_{miss}^1 = K_{true}^1 * \ {}_aq_0^1$$

Where

K_{obs}^{1} is the number of sibships with one sister that were observed in the sampled population

K_{true}^{1} is the true number of sibships with one sister in the population

K_{miss}^{1} is the number of sibships with one sister that were not represented in the sampled population due to zero-survivor bias

${}_{a}q_{0}^{1}$ is the cumulative probability of death for five-year age-group a

$(1 - {}_{a}q_{0}^{1})$ is the probability that the sister has survived to the time of the survey

From these two equations, it followed that the number of sibships with only one sister that were not represented in the population due to zero-survivor bias was equal to:

$$K_{miss}^{1} = \frac{K_{obs}^{1}}{1 - {}_{a}q_{0}^{1}} \times {}_{a}q_{0}^{1}$$

We multiplied this estimate of the number of missing sibships by the number of females in the sibship (which in this case is one) to produce an estimate of the number of females in each age group that were missing from the sample because they had died. We then expanded this number so that there was one observation per missing sibling, assigned birth and death dates to these missing siblings based on the distribution in the observed siblings, and appended them to our existing dataset. This process was also carried out for families with two sisters:

$$K_{obs}^{2} = K_{true}^{2} * (1 - {}_{a}q_{0}^{1} * {}_{a}q_{0}^{2})$$

$$K_{miss}^{2} = K_{true}^{2} * {}_{a}q_{0}^{1} * {}_{a}q_{0}^{2}$$

$$\therefore K_{miss}^{2} = \frac{K_{obs}^{2}}{1 - {}_{a}q_{0}^{1} * {}_{a}q_{0}^{2}} * {}_{a}q_{0}^{1} * {}_{a}q_{0}^{2}$$

Where

K_{obs}^{2} is the number of sibships with two sisters that were observed in the sampled population

K_{true}^{2} is the true number of sibships with two sisters in the population

K_{miss}^{2} is the number of sibships with two sisters that were not represented in the sampled population due to zero-survivor bias

${}_{a}q_{0}^{1}$ is the cumulative probability of death for the first sister in five-year age-group a

${}_{a}q_{0}^{2}$ is the cumulative probability of death for the second sister in five-year age-group a.

If there was only one sister within the 15 to 49 age range, the equations were different than above because the second sister did not contribute to the probability of the sibship being observed in the sample.

$$K_{obs}^2 = K_{true}^2 * (1 - {}_aq_0^1)$$

$$K_{miss}^2 = K_{true}^2 * {}_aq_0^1 * {}_aq_0^2 + K_{true}^2 * {}_aq_0^1 * (1 - {}_aq_0^2)$$

$$\therefore K_{miss}^2 = \frac{K_{obs}^2}{1 - {}_aq_0^1} * {}_aq_0^1 * {}_aq_0^2 * \frac{K_{obs}^2}{1 - {}_aq_0^1} * {}_aq_0^1 * (1 - {}_aq_0^2)$$

Both this analysis and Obermeyer et al. accounted for time prior to the survey in the logistic regression to model mortality.[21] The GBD analysis, however, utilized an updated method for recall bias adjustment. After 45q15 was estimated for each of the surveys, the estimates were combined and paired up for all periods where they overlapped. This overlap occurred when there were at least two surveys carried out in the same country within 15 years of each other. In GBD 2013, we estimated adult mortality from sibling histories for three five-year periods prior to the survey date. This was changed in GBD 2015 – and continued for GBD 2016 and GBD 2017 – where single-year 45q15 estimates from sibling survival were generated using the same methodology to account for the changing level and trends of 45q15 within the 15-year period covered by sibling survival module. This generated pairs of estimates in years where there were overlapping surveys. For each of these pairs, we calculated the difference in the years of recall as the interval between when the two surveys were conducted. We also calculated the magnitude of the difference between the two estimates of 45q15. We then estimated the linear regression model shown in the below equation to quantify the relationship between years of recall and level of mortality separately for each sex of sibling:

$$\Delta({}_{45}q_{15})_{i,j} = \beta \times \Delta(survey\ date)_{i,j} + \xi$$

Where

$\Delta({}_{45}q_{15})_{i,j}$ is the difference in ${}_{45}q_{15}$

$\Delta(survey\ date)_{i,j}$ is the difference in survey date

j is the survey pair

i is the country

Upper and lower uncertainty intervals were also derived. The coefficient on recall period represented the effect of recall bias and was used to adjust the 45q15 estimates to account for that bias.

Section 2.3.4: Completeness data synthesis

Once we obtained a full series of under-5 completeness estimates for each country source we fit the model described in the equation:

$$log_{10}(c_{i,s,t}^{adult}) = \alpha + \beta_1 \times log_{10}(c_{i,s,t}^{child}) + \gamma_1^{SR} + \gamma_2^{SR} \times log_{10}(c_{i,s,t}^{child}) + \gamma_1^{R} + \gamma_2^{R} * log_{10}(c_{i,s,t}^{child}) + \eta_{i,s} + \xi_{i,s,t}$$

Where

i is country

s is source

t is time

R is region

SR is super-region

$c_{i,s,t}^{adult}$ is completeness of adult deaths registration

$c_{i,s,t}^{child}$ is completeness of child deaths registration

γ terms are random effects

$\eta_{i,s}$ is a random effect at the country and source level

$\xi_{i,s,t}$ is an error term

This model related adult completeness to under-5 completeness and included super-region and region-level random effects to allow for differences in both the average level of adult completeness and the relationship between child and adult completeness at these levels. The country-source random effect captured the fundamental difference in level of completeness between different data sources.

A log_{10} transformation was employed to make over- and under-completeness symmetric (e.g., 50% complete and 200% complete were symmetric around 0 when log_{10} transformed) and to simplify calculation of the variance of completeness estimates in log_{10} space, which was needed for the adult mortality estimation process. To avoid outlying DDM-derived estimates of adult completeness from unduly influencing the predictions from the model in the equation above, for any given set of three DDM estimates (GGB, SEG, GGBSEG) calculated from a single pair of censuses, the estimate that was furthest from 1 (i.e., complete) was excluded.

For each country-source $log_{10}(c_{i,s,t}^{adult})$ was predicted from coefficients estimated in the model above and child completeness. Not every country can be used to fit this model, as DDM cannot be applied in some cases due to lack of appropriate census data. However, because the coefficients used for

prediction were at the region and super-region level, predictions from this model could be generated for all countries where estimates of child completeness were available. We did not believe that the same relationship between adult and child completeness existed for registration-based sources as for recall-based sources, so the above model was applied only to registration-based sources (primarily VR data but also sample registration systems). For sources that only included household death recall, we set an arbitrary value of 1 for the first stage values instead of making predictions from completeness from child age groups for the aforementioned reason. However, it should be noted that this set value by no means reflected the true completeness of adult age groups in the household death recall sources and it changed once we applied the spatial-temporal regression.

In the second stage, we calculated the residuals from the first stage and applied spatial-temporal smoothing to these residuals. The predicted residuals were then added back onto the first-stage predictions, generating the second-stage predictions. Spatial-temporal smoothing was carried out in the same way as in the adult mortality estimation process with three modifications:

1. The λ and ζ parameters were set to 2.0 and 0.95
2. Only the fixed effect local regression variant was used
3. The residuals were not held constant out of sample.

The registration-based sources and the non-registration-based sources were handled separately in this step.

The variance of the completeness estimates was also calculated, as this information was utilized in the adult mortality estimation process. To do this, we approximated the variance based on the median absolute deviation (MAD) compared to the second-stage estimates. We calculated variances at the regional level, and did so separately for registration-based sources and other sources. Then, for each country-source-year, we generated 10,000 simulations from a normal distribution with mean equal to the second-stage prediction for that year and variance. For non-registration-based sources, we believed that both under- and over-reporting were possible (over-reporting might have occurred due to telescoping of events outside of the recall period into the recall period), and so for these sources we exponentiated the 10,000 simulations and calculated the mean, which served as the final prediction for completeness. For registration-based sources we believed that only underreporting was possible, so for these sources we first truncated any simulations above 1 to 1. We then exponentiated the 10,000 simulations to find the truncated mean, which served as the final prediction for completeness. In both cases, before exponentiating the simulations, the variance of the simulations was calculated and used as the variance of the completeness estimates in the adult mortality estimation process.

The final completeness estimates were used to adjust, where appropriate, the corresponding country-source-years before these data were used in the adult mortality estimation process. For countries in which we believed males and females had differential completeness, Saudi Arabia and Morocco, we carried out the above process separately by sex. For a small number of data points completeness could not be estimated using the procedure described above due to a lack of appropriate census data; the original growth balance method was the only viable option.[12] In previous papers, we included a selected

number of data points derived from household recall of deaths to which the Brass growth balance method had been applied. Our simulation studies suggested this method was extremely imprecise, so we excluded these points from the analysis.

Section 2.3.5: 45q15 data synthesis using non-linear mixed effects model and ST-GPR

Overview of adult (45q15) mortality estimation

For each location, we generated a time series of 45q15 estimates. We modeled the underlying mortality risk separately from excess mortality due to fatal discontinuities. To model the underlying mortality risk we relied on a three-stage process that incorporated all data in our database for each country, after excluding data identified as outliers or that referred to years identified to contain mortality shocks from conflict or natural disaster.

1. In the first stage, we applied a nonlinear mixed effects model that used covariates to explain variation in the mortality rate for the group aged 15 to 59 (45m15).
2. In the second stage, we exploited spatial and temporal correlation in the residuals from the first stage regression by performing a smoothing process on these residuals. The smoothed residuals were then added back into the first stage regression predictions to produce an updated time-series of 45q15 for each country.
3. In the third stage, we applied GPR to synthesize information from the second stage predictions and the observed data.

After applying this procedure to generate estimates of the underlying mortality risk, we modeled abrupt changes in mortality by estimating the excess risk of mortality in years identified as containing a conflict or natural disaster. This estimated excess mortality risk was then added to the underlying mortality risk to produce our final time series of 45q15 estimates. A more complete description of the various stages of our modeling strategy is given below.

First stage nonlinear mixed effects regression

Similar to estimating 5q0, the first stage model for adult mortality is a nonlinear mixed effects regression. We use this regression to generate estimates for adult mortality rates for each location, year, and sex. In addition to the three covariates used in previous GBD rounds, lag-distributed GDP per capita, mean years of education in age group 15 to 59, and crude death rate due to HIV/AIDS in age group 15 to 59, we have added 5q0 estimates which we found improved model fit. Additionally, we added a fixed effect on sex allowing us to fit the model using males and females together as opposed to running separate models in the past. The specific equation used for the nonlinear mixed effects model is specified below.

$$ {}_{45}m_{15}^{observed} = exp(\beta_1 + \beta_2 \cdot Edu + \beta_3 \cdot ln(LDI) + \beta_4 \mu + {}_5q_0 + \alpha_{sex} + \gamma_{country}) + \beta_5 \cdot HIV + \mathcal{E} $$

Where

Edu is the mean years of education for the age group 15 to 59

LDI is lag-distributed income

${}_5q_0$ is probability of death from birth to age 5 years

α_{sex} is a fixed effect on sex

γ_{country} is a country-level random effect

HIV is the crude mortality rate from HIV for ages 15 to 59

We initialized the model with starting values for each of the β coefficients equal to 0. We tested our model with different starting values, including values from a hierarchical linear mixed effects model, and found that this model was not sensitive to starting values.

The final stage-one predictions were based on predictions from the model above, excluding the country random effect. This was done to facilitate modeling spatial trends in mortality in the second stage. The model predictions were then converted from 45m15 to 45q15 (assuming constant mortality rate within five-year age groups to convert between mx and qx) for incorporation into the second stage model.

Second stage spatial-temporal smoothing of residuals

The first stage regression model reflected the explanatory power of the set of covariates but, as might be expected, failed to explain all of the variation in 45q15. The residuals from the first stage regression were correlated in both time and space, indicating that 45q15 was correlated in time and space in ways that were not fully captured by the covariates included in this regression. We exploited this remaining pattern of variation by applying a local regression to the residuals from the first stage regression, effectively smoothing across time and space.

The local regressions were fitted separately for each of the 21 GBD regions which were constructed so that countries within each region shared similar epidemiological profiles.[25] We applied two variations of local regression to the first stage residuals, both of which utilized the same weighting scheme to incorporate temporal and spatial relatedness. In both variants, a set of weighted linear regressions, one for each country-year of interest, were undertaken. When carrying out the regression for a given country-year of interest, all residuals in the dataset were weighted with respect to this country and year. We first weighted residuals with respect to time using a weighting function similar to that utilized in Loess regression:

$$w_t = \left(1 - \left(\frac{|r_t - r_{est}|}{1 + argmax_t|r_t - r_{est}|}\right)^{\lambda}\right)^3$$

Where

r_t is the year of interest

r_{est} is the year of the residual being weighted

$argmax_t|r_t - r_{est}|$ is the maximum distance between the year of interest and a residual within the region

The λ parameter in this weighting function dictated how quickly the weights fall off as the distance in time increases: a larger λ implies that the assigned weights will diminish slowly with time, while a smaller λ allows the weights to diminish more rapidly with time.

We then weighted residuals with respect to space by modifying the time weights described below. Weights for residuals within the country of interest were multiplied by a factor of:

$$\frac{\zeta \sum_{i \notin c_{est}} w_i}{(1-\zeta) \sum_{i \in c_{est}} w_i}$$

Where

c_{est} is the country of interest

w_i is the time weight

As a result of this modification, 100·ζ % of the total weight was placed on residuals within the same country, and the remaining 100·(1-ζ) % of the weight was placed on residuals from other countries in the region. For countries with no residuals (i.e. countries with no data) the above factor was 0 and there was no re-weighting. All of the weight consequently remains, by necessity, in other countries in the region. We set λ and ζ based on the data density of each location.

The first local regression variant, which we called linear local regression, was a weighted linear regression of the residuals on year and an indicator of the residual from the country currently being estimated:

$$r_{est} = \beta_0 + \beta_1 t + \beta_2 c_{est} + \mathcal{E}$$

If this indicator cannot be estimated, i.e. because there were no residuals in a given country, it is dropped

The second variant, which we called fixed effects local regression, used a weighted linear regression with no covariates. This was equivalent to a simple weighted average of the residuals.

Linear local regression incorporated information from covariates, such as year, but extrapolation was heavily based on that covariate and in settings with sparse data this could result in implausible out-of-sample predictions. In contrast, fixed effects local regression did not incorporate an explicit time-trend

and did not suffer from this problem in extrapolation. However, it was also less adequate at fitting the data in countries with many observations. We therefore combined the estimates from both variants.

As described in the below equation, we calculated the data density (d_c) for each country we estimated (c_{est}) and then calculated a weighted average of the predictions from the linear local regression and fixed effects local regression where d_c% of the weight was assigned to the linear local regression and the rest to the fixed effects local regression. In this way, the final estimates for countries with more VR data were more heavily informed by the linear local regression and the final estimates for countries with less (or no) VR data were predominantly informed by the fixed effects local regression, as was appropriate given the strengths and weaknesses of these two variants.

$$d_c = 100 \times \left(\frac{\#\, VR\ points\ in\ c_{est}}{Maximum\ \#\, VR\ points\ in\ any\ country\ in\ the\ region}\right)$$

Residuals were logit-transformed before undergoing smoothing; once the final estimates of the smoothed residuals were obtained for every country-year, these estimates were added back into the logit transform of the first stage regression predictions. This sum was then reverse-logit transformed; by carrying out the first two stages in logit-space we restricted the predictions to between 0 and 1. These predictions were called the second-stage predictions.

Model

The third stage of our prediction method was a GPR

$$\mu_t = f(t) + S_t$$

$$f(t) \sim GP(M, C)$$

Where

μ_t is the true $\log_{10}$(45q15) at time t

$f(t)$ is the baseline mortality risk

S_t captures excess mortality due to war and disasters and is estimated independently of $f(t)$

M is the Gaussian process mean

C is the Gaussian process covariance

For the Dominican Republic, Peru, and Madagascar, a slightly different model, described in the equation below, was used. For these countries, measurements from sibling histories and from VR were at different levels and the direction of the bias in each source was unknown. We therefore used a model which included a bias term for each source (β_s).

$$\mu_t = f(t) + \beta_s + S_{t,s}$$

$$\beta_s \sim Normal(0, 0.01^2)$$

$$f(t) \sim GP(M, C)$$

GPR is a method of Bayesian inference. We specified a prior distribution for $f(t)$ and a likelihood function that described the data generation process. The specified prior distributions and likelihood function are described below. We then used MCMC to approximate the posterior distribution of $f(t)$, which also incorporated information from the observed empirical estimates of adult mortality. An MCMC chain of length 5,000 was produced. The first 3,000 samples were discarded and the remaining 2,000 were thinned by a factor of 2 for a total of 1,000 simulations retained. Best estimates and confidence intervals were generated from the mean and the 2.5th and 97.5th percentiles of the 1,000 samples, respectively.

Priors

The prior distribution of $f(t)$ can be described in terms of the mean prior—the prior for M—and the covariance prior—the prior for C. We utilized the second stage predictions as the mean prior and used a Matérn covariance function to describe the covariance prior. This covariance function incorporated three parameters: the amplitude, which controlled the amount by which realizations of the Gaussian process distribution can deviate from the mean function, the scale, which controlled the distance over which the function was correlated, and the degree of differentiability, which influenced the smoothness of the samples from the Gaussian process. These parameters were selected in the parameter selection process described below.

Likelihood

The likelihood describes the probability of observing the data given a particular set of parameters. As shown in the below equation, we used a normal model for describing the probability of observing a particular value of log(45q15):

$$log_{10}({}_{45}q_{15_t}) \sim Normal(f(t), V_t)$$

Where

$f(t)$ is the mean

V_t is the data variance

Data variance was calculated for each empirical observation of 45q15 and incorporated both sampling and non-sampling variation. The method for calculating the data variance depended on the type of data. We computed sampling variance from a binomial model. We set *N* equal to the national population aged 15 to 59 years and *p* equal to the mortality rate, 45m15. We calculated the variance of 45m15 from

$$\frac{p(1-p)}{N}$$

and then transformed this to the variance of $\log_{10}$(45q15) using the delta method. For estimates derived from both complete and incomplete VR data, we included not only sampling variance but also the non-sampling variance that arises from uncertainty in the completeness estimate. For these data, the total data variance was given by the sum of the sampling variance (calculated as for complete VR data) and the variance of the completeness estimate (calculated as described in section 2.2.6).

For estimates derived from sibling history, census, or survey data, the MAD estimator of the variance was calculated by source type with reference to the second stage predictions, as described in the equation, where s is the source type (sibling history, census, or survey):

$$\theta_s^2 = (1.4826 \cdot MAD_s)^2 = 1.4826 \times median\left(\left|\left(log_{10}\left({}_{45}q_{15}^{observed}\right) - log_{10}\left({}_{45}q_{15}^{predicted}\right)\right) - median\left(log_{10}\left({}_{45}q_{15}^{observed}\right) - log_{10}\left({}_{45}q_{15}^{predicted}\right)\right)\right|\right)^2$$

Where s is the source type (sibling history, census, or survey.

Hyperparameter selection for adult mortality rate ST-GPR

Similar to the new parameter selection in U5MR, hyperparameters for adult mortality were selected based on a newly-created data density score for a given location and sex. For adult mortality, the data density score was calculated for each location and sex based on the number of deaths and the number of sibling histories. The score was computed using the following steps:

1. Calculate death number score: this component of data density was computed based on the number of deaths calculated from the DDM process. This includes both unadjusted complete deaths and adjusted deaths from incomplete sources.
2. Sibling histories: for each sibling history for a location, year, and sex, we assigned a score of 15.
3. Next, we added the death counts and the sibling history scores together for each year and sex. We then capped the sum at 1000 for each year and sex and then divided that number 1000. The result was a score for each year and sex between 0 and 1. To get the final complete VR score for a location and sex, we added up the score for each year across the full time series.
4. Once the data density for a location was calculated, we assigned the following hyperpameters:

Table B

Data Density	Zeta	Lambda	Scale
0 to 9	0.6	0.7	25
10 to 19	0.7	0.5	20
20 to 29	0.8	0.4	15
30 to 49	0.9	0.3	10
50 plus	0.99	0.2	5

We developed the data density score through iterative testing taking into account the view that different sources such as complete VR or complete birth histories are more prone to non-sampling measurement error than others. Specific cutoffs for VR deaths were tested to take into account the sample size of the source. Weights were also iteratively tested and refined.

Section 2.3.6: Identify and remove outliers

To arrive at sensible levels and trends for the adult mortality rate, certain outliers were excluded from our ST-GPR regression process. In general, we used the following process to outlier influential raw data points that otherwise lead to erroneous 45q15 estimates:

1. Raw input 45q15 data points from years affected by war, natural disasters, and other fatal discontinuities as defined in GBD 2016 were excluded from the analysis described in section 2.4.
2. Examination of survey/registration data quality resulted in the selective exclusion of some raw data sets. Examples include the Afghanistan 2010 Mortality Survey that was not nationally representative and VR data from Serbia that excluded deaths from Kosovo.
3. Visual inspection of raw input data on 45q15 and estimated time series estimates of 45q15 from ST-GPR by GBD researchers and country experts through the GBD collaborator network. Data points were outliered when they were unexplainably different from other adjacent points from the similar source, indicating a data reporting issue and compilation error in the direct sources. Some subnational-level single year 45q15 estimates from sibling survival methods were excluded due to unreasonably high or low estimates resulting from small sample sizes.

Section 2.3.7: Rake subnational estimates to national level (excluding South Africa)

First, we randomized the order of the 1,000 subnational-level draws and the national draws separately to avoid any correlation between subnational and national draws that might have been introduced in prior processes.

GBD 2017 provided estimates of 45q15 for 918 locations at both subnational and national levels. While it was absolutely essential to use input data from the subnational level in informing the level and trend of 45q15 for the corresponding location, the national level data tended to be more robust, incorporate more data sources, and cover longer time periods. To develop consistent estimates between aggregated subnational level estimates and the separately estimated national level estimates, we raked the subnational 45q15 to match our national level 45q15 estimate using the following formulas:

$$ {}_{45}q_{15}^{s'} = 1 - e^{-45 \times {}_{45}M_{15}^{s} \times r} $$

And

$$ r = \frac{\frac{ln(1 - {}_{45}q_{15}^{N})}{-45}}{\sum_{s=1}^{n} ln(1 - {}_{45}q_{15}^{s}) \times \frac{\frac{p_S}{p_N}}{-45}} $$

Where

s is subnational locations within country N

p is population in age group 15 to 59

${}_{45}q_{15}$ is the estimate of adult mortality rate from the ST-GPR process

${}_{45}q_{15}^{s'}$ is the post-raking 45q15 for subnational locations

Section 2.3.8: Review estimates for quality

The preliminary estimates of adult mortality rates were reviewed by both the researchers who work on the demographic estimation process and the GBD all-cause mortality collaborator network. Concerns regarding quality of certain survey and data points were raised and reviewed, which lead to revision of the database where applicable.

Section 2.3.9: 45q15 estimates with HIV

The results of the adult mortality rate ST-GPR process were estimates of probability of death from all causes of death except fatal discontinuities for all locations covered in GBD 2017 for 1950 to 2017.

Section 2.4: Model life table system

Section 2.4.1: Overview

In settings without complete VR systems, data were often available for some summary measures of mortality such as 5q0 (from complete or summary birth histories) and 45q15 (from sibling surveys). Model life tables, structured relationships between levels of age-specific mortality at different ages, can be used to generate estimates of age-specific mortality for detailed age-groups from summary 5q0 and 45q15. Model life tables may also simplify mortality projection from the most recent empirically observed data. This is true even for countries with high quality VR systems, due to the fact that compilation of VR data may take several years before publication.

An ideal Model life table system has several desirable attributes. First, a model life table system should be efficient and require only a few entry parameters to generate a full life table with age specific mortality rates. Second, it should adequately capture the range of age patterns of mortality observed in real populations. Third, it should provide satisfactory estimates of age-specific mortality for countries with high levels of mortality, especially those with substantial HIV/AIDS epidemics. Finally, a model life table should generate age-specific mortality with a plausible time trend, and the partial derivative of age-specific mortality should be positive with respect to entry parameters such as 5q0 and 45q15.

A simple and somewhat flexible model life table system was proposed by Brass.[17] In general the logit of the *lx* (proportion of a hypothetical birth cohort still alive at age *x*) column in a life table could be

represented as a linear transformation of the logit of the *lx* column of a reference standard life table. Murray et al. noted that as a population moved further away from the levels of mortality in the reference standard, the assumption of linearity in logit *lx* space was violated.[26] A series of age-specific modification factors were proposed that allowed for the characteristic bending of the logit *lx* function compared to the standard. Murray et al. also assessed the ability of this modified logit life table system to predict age-specific mortality rates. This modified logit life table system, with its built-in optional mechanism of predicting 45q15 from child mortality 5q0, has been extensively used by the World Health Organization since the early 2000s.[26]

This system, however, suffers from two major limitations. First, when adult mortality was very high relative to child mortality, such as in the presence of an HIV/AIDS epidemic, the age patterns of mortality generated did not fit well to the observed data. Second, when the modified logit life table system was applied to time series of 5q0 and 45q15, paradoxical trends developed where adult and child mortality declined, but predicted age-specific death rates increased in some age groups. We have extended the modified logit life table system to deal with these two major limitations. Wang et al. provide a more complete discussion of the development of this approach.[3,5] We summarize these developments in brief here. There were four distinct steps.

Section 2.4.2: Building an empirical model life table database: data sources and quality review

Relational model life table systems critically depend on the empirical database used to generate model life table standards and test the predictive validity of model life tables. For both all-cause mortality and cause-specific mortality analyses in GBD, we amassed a comprehensive database on human mortality from full VR systems, and sample VR systems such as the SRS from India and the Disease Surveillance Point system from China. These aforementioned data sources provided a total of 42,138 abridged empirical life tables prior to outliering or smoothing (Appendix Table 6). We adjusted each source by estimated completeness in the 5q0 or DDM process, applying under-5 completeness to ages under 1 and 1 to 4, adult completeness to all ages above age 15, and a weighted combination of child and adult completeness to ages 5 to 9 and 10 to 14.

For each life table, within each location, we sort life tables by year and generate smoothed lifetables using moving averages of widths of 3, 5, and 7 within each location. This smoothing helped address jumps or drops in age-specific mortality in locations where small numbers of deaths resulted in high variability of mortality patterns across age.

As with previous cycles of the GBD, we have two sets of life tables that meet inclusion criteria: a universal set that is used for all locations to identify matches and a location-specific set that is used along with the universal set for each location. We generated inclusion criteria to help guide which of the unsmoothed or smoothed life tables had consistent enough age patterns for inclusion in the life table selection process. In particular, the most common problems with life tables were jumps in age-specific mortality due to low populations or overall low mortality (such as in age group 5-9 in British subnational locations), old-age misreporting or heaping, or implausible trends due to data extraction or reporting issues. We formalized the inclusion criteria to be as follows:

- Inclusion criteria for either life table set:
  - The variance in the change in log probability of death across age, for ages between 60-90, must be below .02.
  - For ages 20 and above, the probability of death cannot drop by more than 25% and be immediately followed by an increase of more than 50%, from age-group to age-group.
  - For ages 20 and above, the probability of death cannot double and be immediately followed by a decrease of more than 50%, from age-group to age-group.
  - There must be 1 or fewer drops in probability of death from age-to-age, for ages above 65.
  - For ages 70 and above, the probability of death cannot decrease by more than 5% from age-group to age-group.
  - For ages 80-84, 85-89, or 90-94, the probability of death must be below the 99th percentile of the age-specific probability of death values of lifetables in the HMD lifetable database.
  - The estimated DDM registration completeness must be greater than 50%.
  - The percent change of 5q75 to 5q80 must be within 20% for males, or 30% for females, to the predicted percent change from a linear regression based on high-quality VR lifetables from the Human Mortality Database, using the relationship between the value of 5q80 and the percent change from 5q75 and 5q80. This was generated to address locations where probabilities of death in older ages accelerated rapidly or stagnated from one age group to another.
  - The percent change of 5q80 to 5q85 must be within 30% of the predicted value from a linear regression of the HMD lifetable dataset using the relationship between the value of 5q85 and the percent change from 5q80 and 5q85.
  - The 5q5 must be greater than .0001, and 5q10 must be greater than .0002, which are roughly the lowest levels observed in current GBD country-level results. These occur mostly in subnational locations with low populations and resultingly low and variable young-age mortality.
- Inclusion criteria for the universal life table database (in addition to the general inclusion criteria):
  - The variance in the change in log probability of death across age, for ages between 60-90, must be below .01.

- There must be no drops in probability of death values from age-to-age, for ages above 65.
- The estimated DDM registration completeness must be greater than 85%.
- The GBD population model for the country/sex must use terminal-age data starting at age 80 or higher
- The adult HIV crude death rate must be below 0.001%.
- The relationship between 5q80 and the percent change of 5q75 to 5q80 must be within 15% of HMD predicted values for males, and 20% for females.
- The relationship between 5q85 and the percent change of 5q80 to 5q85 must be within 15% of HMD predicted values for both sexes.
- The estimated both-sex population for the country must exceed 1 million.

Life tables that met all of the general inclusion criteria but not all of the universal life table inclusion criteria are categorized as location-specific life tables. Following application of the inclusion criteria, additional life tables were excluded from the life table database based on review for implausible trends across ages or country-years of data.

Our modified life table system was originally developed using a database of 3,566 life tables covering 63 countries.[26] Using the inclusion criteria above, we have expanded this database to include 10,885 universal and 24,222 location-specific life tables, for a total of 35,107 life tables.

In addition, all life tables in our database with a crude death rate due to HIV/AIDS over 0.1% in adult age groups were excluded via the process described in Section 2.5.

Section 2.4.3: Extending age-specific mortality to age 100+

To extrapolate age-specific mortality beyond age 85, we generally used the Gompertz law of mortality and other functional model age pattern of mortality methods that assume a roughly exponential increase in mortality in old ages.[9,27] Age-group dummies and probability of dying from age 80 to 84 in logarithmic scales were used to estimate the difference in age-specific probability of dying in logit scale between two consecutive age-groups, as described in the following equation:

$$logit\left({}_5q_x^{j,t,g}\right) - logit\left({}_5q_{x+5}^{j,t,g}\right) = \alpha^g + \beta_x^g \cdot age + \gamma^g \cdot logit\left({}_5q_{80}^{j,t,g}\right) + \eta_j^g + \xi_x^{j,t,g}$$

Where

j refers to country

g refers to sex

t refers to time

Parameters were estimated using data from selected countries in the Human Mortality Database with high quality VR data in the oldest old age groups above age 80.[28] The parameters estimated from the model above were then used to generate age-specific probability of death from age 85 to 109.

Section 2.4.4: GBD relational model life table system with a flexible standard selection mechanism

Our relational model life table system was based on the logic that in order to capture the very high levels of younger adult mortality seen in populations with high HIV prevalence, we needed to develop a model life table for a counterfactual population without HIV and then add on the effects of HIV by age and sex. This system was captured in three components. We first estimated counterfactual levels of 5q0 and 45q15 in the absence of HIV. Then a full set of age-specific death rates were generated using the model life table system from the counterfactual levels of child and adult mortality. Finally, we estimated the increase in mortality specific to each age group associated with HIV.

Model for populations free of HIV/AIDS

We made several innovations in estimating a set of age-specific death rates from 5q0 and 45q15 using a relational model life table. A key change from previous relational model life table systems was the shift to modeling q_x in logit space rather than the l_x in the same space. Modeling q_x enabled us to more precisely capture the different impacts of changes from the two entry parameters (5q0 and 45q15) on different age-groups. The following equation provided the life table for populations not affected by HIV/AIDS.

$$logit({}_nq_x^c) = logit({}_nq_x^s) + \beta_x^1 \cdot \left(logit({}_5q_0^c) - logit({}_5q_0^s)\right) + \beta_x^2 \cdot \left(logit({}_{45}q_{15}^c) - logit({}_{45}q_{15}^s)\right) + \xi_x$$

Where

$logit({}_{45}q_{15}^s)$ is the logit transformation of 45q15 in the standard life table

$logit({}_{45}q_{15}^c)$ is the logit transformation of 45q15 value for a country without HIV or the counterfactual level of 45q15 in the absence of HIV in a country affected by HIV/AIDS

$logit({}_5q_0^s)$ is the logit transformation of 5q0 in the standard population

$logit({}_5q_0^c)$ is the logit transformation of 5q0 for a country without HIV or the counterfactual level of 5q0 in the absence of HIV in a country affected by HIV/AIDS

$logit({}_nq_x^s)$ is the logit of the probability of death in the standard population from age x to $x + n$

$logit({}_nq_x^c)$ is the logit transformation of the probability of death from age x to $x + n$ in a country without HIV or the counterfactual level of $_nq_x$ in the absence of HIV in a country affected by HIV/AIDS

β_x^1 and β_x^2 are coefficients that vary by age x and that measure the impact of differences in child and adult mortality rates between a target life table and the standard life table on the estimated age pattern of mortality. These coefficients determined how much the estimated age pattern of mortality deviated from the standard by age and from linearity.

This equation proposed that the logit-transformed age-specific probability of dying in a target life table (c) could be represented as a function of the corresponding logit-transformed age-specific probability of dying in a standard life table (s) and the differences in probability of dying from age 0 to 5 in logit scale and the difference in probability of dying from age 15 to 60 in logit scale between a pairs of life tables, c and s. Life table c was the estimated HIV-free life table either for a country affected by the epidemic or not. The model was based on empirical observation where the differences in age specific probabilities of dying in logit scale between two life tables were highly correlated with differences in 5q0 or 45q15 in logit scale when the HIV/AIDS epidemic was not present.

Coefficients β_x^1 and β_x^2 were estimated using the following equation:

$$logit\ {}_nq_x^c - logit\ {}_nq_x^s = \beta_x^1 \times (logit\ {}_5q_0^c - logit\ {}_5q_0^s) + \beta_x^2 \times (logit_{45}q_{15}^c - logit_{45}q_{15}^s) + \xi_x$$

In estimating the parameters, we used country-time specific and region-specific (i.e., GBD region) standards for each life table in our database not affected by HIV/AIDS (other aggregated standard life tables clustered by different geographical or epidemiological criteria were also possible). Country-time specific standard life tables were used whenever an empirical life table from the same country within a 15-year time frame was available in our database. Region specific standard life tables were generated by collapsing all zero-HIV life tables in our database from the same GBD region by sex. We then paired up all zero-HIV life tables in our database with the generated region specific life tables.

The estimated $\hat{\beta}_1$ and $\hat{\beta}_2$ are shown in the table below. We limited the effects of 5q0 and 45q15 to certain age groups to avoid implausible outputs when 5q0 and 45q15 from a population change in opposite directions. Using the values in the table below also generated full life tables for populations not affected by HIV/AIDS. The values of 5q0 and 45q15 served as points of entry (or entry parameters) for this model life table system.

Table C. Model life table coefficients

Age	Difference in 5q0 (logit scale)		Difference in 45q15 (logit scale)	
**Age**	**Male**	**Female**	**Male**	**Female**
**0**	0.993	0.982	--	--
**1-4**	1.005	1.047	--	--
**5-9**	0.823	0.766	--	--
10-14	0.468	0.368	0.386	0.507
15-19	0.134	0.147	0.823	0.776
20-24	0.042	0.136	0.828	0.865
25-29	0.029	0.121	0.775	0.925
**30-34**	0.018	0.094	0.784	0.935
**35-39**	--	--	0.871	1.064
**40-44**	--	--	0.906	0.990
**45-49**	--	--	0.924	0.921
**50-54**	--	--	0.903	0.878
**55-59**	--	--	0.855	0.860
**60-64**	--	--	0.792	0.838
**65-69**	--	--	0.786	0.866
**70-74**	--	--	0.801	0.911
**75-79**	--	--	0.799	0.914
**80-84**	--	--	0.709	0.832

Our standard life table computation procedure took into account empirical relationships between differences in geography, time, and mortality age patterns. To generate a standard, we first calculated the Mahalanobis distance between the target life table and all zero-HIV empirical life tables of the same sex in our database based on 5q0 and 45q15 (in logit scale). The Mahalanobis distance between two sets of 5q0 and 45q15 was defined as:

$$D_M^i(Q^i) = \sqrt{(Q^i - O)^\tau S^{-1}(Q^i - O)}$$

Where

O is a multivariate vector representing entry parameters 5q0 and 45q15 in logit scale

$Q^i = (logit({}_5q_0^i), logit({}_{45}q_{15}^i))$ is a multivariate vector that corresponds to an empirical life table i in our life table database

We chose Mahalanobis distance over Euclidean distance because 5q0 and 45q15 were highly correlated in logit space (the correlation coefficients were 0.58 and 0.87 for males and females, respectively), and Mahalanobis distance takes the covariance matrix of 5q0 and 45q15 in logit scale into consideration when calculating the distance between any pair of life tables. We then kept the 100 most similar life tables as measured by the Mahalanohis distance, along with all life tables within 30 years from the

location itself, and then kept 100 of those life tables that were closest in terms of geography and distance in years.

Next, instead of obtaining a simple arithmetic mean of all selected life tables, we applied empirical weights to each selected life table and computed the weighted average. We updated the weights from GBD 2016[7] to provide more relative weight to lifetables from the country or neighboring subnational locations, in part to adjust for using 100 lifetables for all locations. Specifically, we increased the location-specific weights by 25 times, the location-specific 0- and 1-year lag weights by 15 times, and the 2-, 3-, and 4-year lag weights by 3 times. The change in year-lag weights helped to preserve the age pattern of the most recent years of data when projecting mortality forwards, carrying over sudden shifts in age-specific mortality such as in young adults in the United States in recent years. In addition, we generated a new level of weights for locations within the same parent-country of life tables, to allow subnational life tables to provide more weight than life tables in the region overall.

For an example location with 30 location-specific matches and 70 subnational neighbor matches, the relative weight of the location/year-specific life table would be at least .60 for males and .61 for females, and the combined weight of the two closest location-year life tables would be at least .84 for both males and females. The relative weight for country-specific life tables depends greatly on the compositional time-lag of the country-specific life tables and the number of included subnational neighbor and regional life tables.

Figure. Empirical weights by lag in time and geographic region for males.

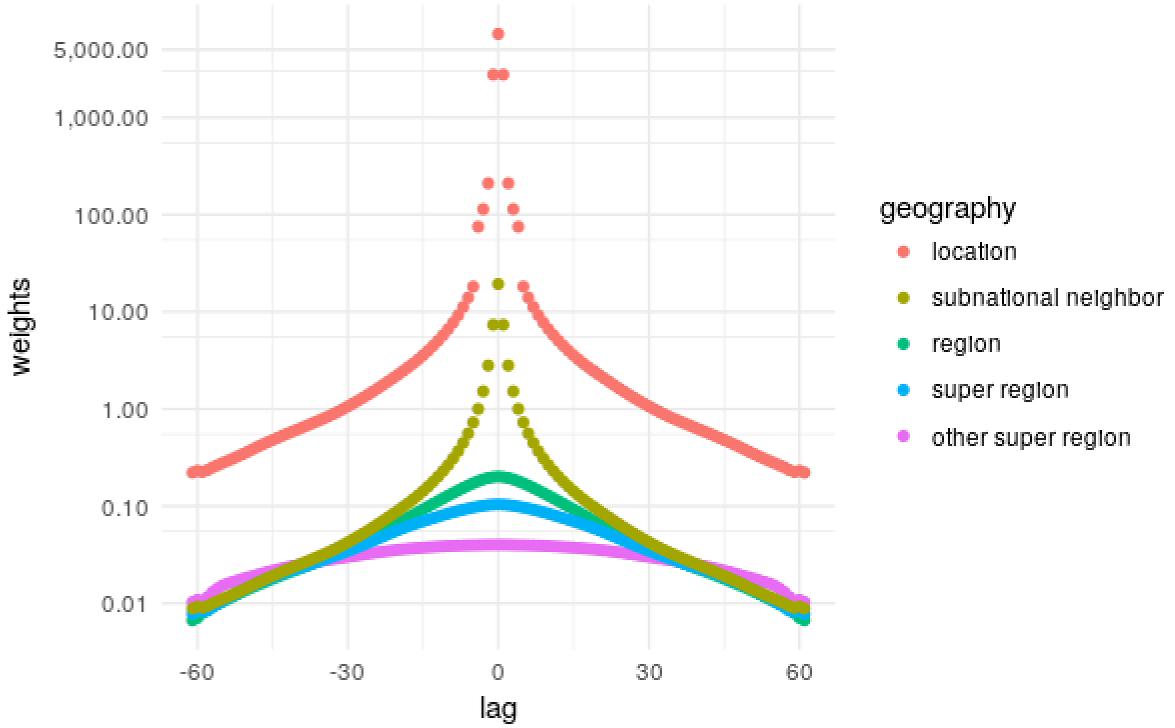

Estimating AIDS

Perhaps the most challenging issue for all existing model life table systems is providing plausible age specific mortality estimates given an HIV/AIDS epidemic. Both widely used model life table systems, the Coale-Demeny model life tables and the Modified Logit Life Table system, were largely based on empirical life tables from the pre-HIV era. In addition, they did not provide an integrated solution to the problem of incorporating deaths from HIV/AIDS.

In GBD 2016 and GBD 2017, we applied a two-step process where we first estimated the HIV/AIDS counterfactual age pattern of mortality using the method described in section 2.6, with HIV counterfactual 5q0 and 45q15 as entry parameters. Excess mortality due to HIV/AIDS in summary age groups of under-5 and age 15 to 59 were then added to the HIV-free age specific mortality.

For GBD 2016 and GBD 2017, we added excess mortality due to HIV to specific age groups by location, year, and sex. In this step, we extracted number of deaths due to HIV estimated using the Spectrum HIV model, and constructed relative risk (defined as the ratio of HIV-specific mortality between a specific age group and age group 40-44).

$$R_i = \frac{{}_nM_x^{HIV}}{{}_5M_{40}^{HIV}}$$

Where i indicates different age groups 0, 1-4, 4-9, 10-14, etc.

Because the Estimation and Projection Package (EPP)/Spectrum was run at the draw level, we had 1000 draws of relative risks of dying from HIV/AIDS by location, sex and time. This was different from the relative risks used in previous iterations of GBD that were estimated using available VR data and by types of epidemic.

Estimating all-cause mortality for South Africa

For all locations except South Africa, the estimation of all-cause mortality followed the two step process where we generated the HIV-free age pattern of mortality first, then we added the excess mortality due to HIV/AIDS. However, we leveraged the age pattern of all-cause mortality from South Africa's near-complete VR system to inform our all-cause mortality process. For this purpose, we used observed VR from South Africa and applied the two step model life table process as used for all other populations in reverse order. We first estimated with HIV mortality using observed VR, with HIV included to generate the standard life table. Then excessive mortality due to HIV was removed from the estimated all-cause mortality using the relative risk of dying from HIV estimated in the Spectrum model.

The advantage of this process was that we were able to match the completeness-adjusted VR data by age, and the age pattern of HIV-specific mortality matched the estimates in the EPP/Spectrum epidemiological model.

Uncertainty in the estimated life tables

One important attribute of GBD was the inclusion of uncertainty in every single step of the GBD mortality estimation process. We integrated uncertainty from the entry parameters (5q0 and 45q15) and the coefficients of each model based on the method illustrated by King et al.[29] Essentially, the model life table process described above was repeated 1,000 times based on randomly paired entry parameters (thus different standard life tables) and model coefficients. As a result of this process, we had wide uncertainty intervals in the estimated deaths in countries where uncertainties in estimated entry parameters were high.

Section 2.4.5: Rake subnational life tables to national level (excluding South Africa)

Since in most countries data were scarcer for subnational units than they were at the national level, estimates of subnational mortality required adjustment for consistency with national estimates. To generate consistent time series estimates of all-cause mortality, including mortality rates and death numbers, we raked subnational-level estimates to separately-estimated national level estimates. For the mortality envelope, we applied a national scalar such that the sum of the subnational location mortality envelopes matched the mortality envelope separately estimated for the national level.

The only exception to this subnational-to-national raking process was South Africa, where we aggregated subnational level estimates as our national level estimates. Based on our observation in previous iterations of GBD, the high level of heterogeneity in the HIV/AIDS epidemic at the subnational

level in South Africa introduced a challenge to accurate estimation of both all-cause and HIV cause-specific mortality in South Africa at the national level. Since our EPP-Spectrum model generated estimates at the province level for South Africa, we produced province-level all-cause mortality estimates using all available subnational sources of child and adult mortality rates and aggregated province-level results as our final national-level estimates for South Africa. The number of data sources were found to be comparable at the national and provincial levels.

Section 2.5: HIV/AIDS estimation

Section 2.5.1: Age-specific mortality (with and without HIV)

The with-HIV and without-HIV age-specific mortality rates were part of the model life table process as inputs to the HIV/AIDS estimation described in Section 2.5.

Section 2.5.2: HIV-free survival rates (for Spectrum)

The age-specific HIV-free survival probabilities from the model life table process were used to create inputs for Spectrum. We used the same method to generate single-year mortality based on 5-year abridged life tables as is later used to generate single-year mortality as an input into the population modeling process.[1] These single-year probabilities of HIV-free survival were then used as inputs to Spectrum.

Section 2.5.3: EPP and Spectrum

For the GBD analysis of HIV, we used variants of two tools developed by UNAIDS:

1. The EPP, which generated time series estimates of HIV incidence and prevalence consistent with observed prevalence data in ages 15-49 in countries with generalized epidemics. EPP uses many of the same assumptions as Spectrum but fits a simpler model to HIV prevalence data from surveillance sites and representative surveys. Antenatal care, incidence, prevalence, and treatment coverage data from UNAIDS were used in modeling for all locations. We extracted all of these data from the proprietary format used by UNAIDS.
2. The Spectrum natural history model predicted incidence, prevalence, and death by age, sex, and year using an estimated time series of HIV incidence, demographic inputs (including HIV-free mortality and population), assumptions about CD4 progression rates, and assumptions about on- and off-ART HIV death rates by age, sex, and CD4 rate. We utilized time series of treatment coverage provided in UNAIDS national HIV estimates files. For GBD 2017, we improved our sex-specific modeling strategy in Spectrum by sex-splitting incidence based on a model fit to the sex ratio of prevalence observed in countries with representative surveys. We also updated the Spectrum pediatric module to reflect changes made by UNAIDS.[30] Our child module was revised to include CD4 progression and CD4-specific mortality rates taken from a model fit to survival data from the International epidemiology Databases to Evaluate AIDS (IeDEA), an international collaboration on HIV cohorts. We also updated child initiation of ART to include data on ART distribution from IeDEA.

Both tools were modified for use in the GBD. Beginning with GBD 2013, we created an exact replica of Spectrum in Python. This enabled us to run thousands of iterations of the model at once on our computing cluster and allowed for more flexible input data structures. We also, again, ran EPP using an open-source computer program in R written by Jeffrey Eaton.[31] We ran EPP for all Group 1 countries in order produce incidence and prevalence estimates that were consistent with the demographic and epidemiological assumptions used in GBD 2017.

Section 2.5.4: HIV/Mortality Reckoning

The reckoning process was intended as a method of reconciling separate estimates of HIV mortality (and their resulting effect on estimates of HIV-free and all-cause mortality) due to two separate estimation processes within the GBD all-cause and HIV estimation framework: those from the model life table system as a way to capture the impact of HIV on age pattern of all-cause mortality, and the those from the natural history model of EPP-Spectrum as used by GBD and UNAIDS. In addition, we also utilized space-time GPR-smoothed VR data on HIV-specific mortality for countries with good quality VR instead of using mortality estimates from Spectrum.

As part of the HIV/AIDS estimation process, all GBD 2017 locations were assigned to a modeling strategy group, depending on the level of HIV within the country and the availability and quality of HIV and VR data. Group placements (Appendix Table 7) determined which sources were used for HIV-specific mortality data and how final estimates of HIV and all-cause mortality were calculated.

Group 1 included countries with greater than .25% adult prevalence of HIV and available HIV prevalence survey data and/or antenatal care (ANC) clinic data. We ran the EPP model using these available input data for all locations in Group 1. Demographic assessments for these locations depended substantially on sibling history data analysis, which had large uncertainty intervals and for which there may have been local variation in response biases. India, Sudan, and Somalia were classified as Group 1B, since they had available surveillance data but adult HIV prevalence less than .5%. In these locations, all-cause mortality estimates were taken from the model life table system while HIV-specific deaths were estimated using EPP-Spectrum. SRS data informed the age-pattern of mortality in India through adjustment of age-specific incidence in Spectrum to better match observed deaths.

Group 2A locations were classified as high quality (4 or 5 star) VR systems per the GBD VR quality rating system.[32] Because these locations had high-quality VR data, all-cause mortality estimates were driven by national data; the VR system was a robust source of data for HIV deaths.

Locations with lower quality VR comprise Group 2B. Locations without any VR data were added to Group 2C.

For HIV-specific mortality estimates, Group 2A locations used mortality output directly from the ST-GPR process due to the high quality of their VR systems. Group 1A and 1B locations used Spectrum output, while Group 2B and 2C locations used output cohort incidence bias adjusted (CIBA) deaths due to HIV/AIDS from the Spectrum model.[33]

Outputs were modeled to include an under-1 age group without the early-, late-, and post-neonatal groups. To attribute under-1 deaths from Spectrum to these neonatal groups, we assumed that all HIV deaths that occur within the first year of a child's life occur in the post-neonatal stage (after 28 days). The literature on HIV in these age groups is still unclear, but seems to indicate higher mortality in the post-neonatal stage. There is no clear evidence to guide alternative methods of age-splitting under-1 deaths due to HIV.[13,14]

Section 2.5.5: Envelope Calculation

In general, the all-cause and HIV-deleted envelopes were generated by synthesizing the results from the with-HIV and HIV-free life tables, along with selected HIV mortality from ST-GPR, Spectrum output, or cohort incidence bias adjusted Spectrum output (CIBA-Spectrum). We used with-HIV and HIV-free life tables to ascertain the implied HIV mortality from the model life table system.

For age groups under age 5, we used the under-5 results from the age-sex process described in section 2.2.14. In Group 1A and 1B locations we generated a scalar based on Spectrum results of HIV-specific and non-HIV deaths to generate HIV-deleted envelope deaths based on the all-cause results from the envelope. In other locations, we directly subtracted mortality from ST-GPR or Spectrum, after capping HIV at 90% of the all-cause envelope, to generate the HIV-deleted envelope. As mentioned previously, we made the assumption that all under-1 HIV-specific deaths occur in the post-neonatal stage.

In all groups except Group 1A, we subtracted mortality for all ages over 5 and under 15 from Spectrum or ST-GPR directly from the all-cause with-HIV envelope, after capping HIV at 90% of the all-cause envelope. In Group 1A, we directly used the HIV-free mortality and added mortality from Spectrum to generate the all-cause with-HIV envelope.

For ages above 15 and below 95, we applied separate approaches for Group 1A and 1B locations compared to Group 2A, 2B, and 2C locations. Group 1A and 1B locations used HIV mortality determined by an ensemble model where we averaged the implied HIV mortality from the model life table process and the HIV mortality output by Spectrum, which were intrinsically linked by the draw-level HIV-free mortality age pattern. Group 2A, 2B, and 2C locations used HIV mortality directly from ST-GPR and CIBA-Spectrum. For all locations except Group 1A, we subtracted the calculated HIV mortality from the all-cause envelope to generate HIV-deleted envelope deaths. In Group 1A we added the HIV mortality to HIV-free mortality from the model life table process to calculate all-cause mortality, effectively allowing all-cause mortality from the demographic estimation process to be changed based on our ensemble HIV estimates. This reflected the inherent uncertainty in all-cause mortality estimates largely based on sibling survival data in Group 1A locations and the various assumptions on mortality and program data required in both EPP and Spectrum.

For the age group 95 and above, we first approximated the over-95 mortality rate by using our life table output and divided *lx* by *Tx* from the age 95-99 values in the country-specific life tables. We then calculated a scalar from the approximated all-cause mortality rate from the life table to the all-cause mortality rate utilizing the envelope deaths/populations. We used this scalar to rescale the with-HIV and

HIV-free life tables and the implied HIV death rate to death number space. Finally, HIV-deleted and all-cause mortality were determined by the same approaches as used for other ages above 15.

Section 2.5.6: Life Table Calculation

Generally, life tables were calculated using an approach consistent with those used in calculating the envelope. However, there were a few differences in calculation particularly related to sex and location aggregation.

For Group 1A locations, we multiplied the HIV-free life table results by the ratio to obtain all-cause life table results. In all other locations, we divided the all-cause life table by the ratio to obtain the HIV-free life table results. This was consistent with their handling during the envelope process.

For aggregate locations, we generated aggregated mx and ax (the average number of years lived in the age interval, of those who died in the age interval) by weighting mx by population and ax by deaths: here, calculated by

$$mx \times population$$

Population- and death-weighted regional mx and ax values were used to generate region-level life tables.

A similar approach was applied to calculating the envelope for the under-5 age groups. However, the national-level under-5 results were substituted for the aggregated mx and ax values from the subnational units when we calculated the all-cause life table for countries with subnational locations. This approach was used for all aggregate countries except South Africa, where we aggregated the national results from subnational results (consistent with our approach in 5q0, 45q15, and the model life tables). The under-5 national aggregates were used to preserve the estimated under-5 mortality results from our 5q0 data synthesis and age/sex splitting modeling processes, which were not necessarily equivalent to those generated by the aggregated mx and ax values from the model life table process. We aggregated subnational units using the regular weighting scheme for HIV-deleted life tables.

Section 2.5.7: HIV crude death rates for under-5 and ages 15-59

Final HIV death rates were calculated based on the results of the Envelope Calculation portion of the reckoning process.

Section 2.6: Age-specific mortality estimation for all GBD age-groups: with and without HIV

Section 2.6.1: Age-specific mortality without discontinuities (with HIV/AIDS)

Age-specific mortality rates without fatal discontinuities were generated using the model life table system (Section 2.4), age-sex model (Section 2.2.11), and HIV reckoning process (Section 2.5.4) depending on the location groups.

Age-specific mortality was calculated from our age-sex model that split U5MR into mortality for age groups early neonatal (enn), late neonatal (lnn), post neonatal (pnn), and ages 1-4. Age-specific mortality rates in ages 5 and older were generated using the GBD model life table system (described in Section 2.5) for Groups 1B, 2A, 2B, and 2C locations. For Group 1A locations, age specific mortality with HIV was produced as the sum of HIV-free mortality rates generated in the first step of the model life table system (described in Section 2.5) and the results of the ensemble model for HIV-specific mortality (described in Section 2.6).

Section 2.6.2: HIV-deleted age-specific mortality

HIV-deleted age-specific mortality was used in CoD analyses in GBD 2017 to avoid the spillover effect of HIV mortality into other causes, particularly for locations affected by high HIV burden. We used the difference between the age-specific mortality with HIV without fatal discontinuities (described in Section 4.1) and the HIV-specific mortality as a result of the HIV reckoning process (Section 2.5.4).

Section 2.6.3: Add fatal discontinuities

In our mortality estimation process, we excluded data from years with fatal discontinuities to ensure that these sudden idiosyncratic increases in mortality did not affect long-term trends in mortality for a given country. This section details how we added the deaths due to fatal discontinuities to the all-cause mortality envelope and life tables. See Section 4 for more information on how fatal discontinuity death numbers were estimated.

To incorporate deaths due to fatal discontinuities into the mortality envelope, 1,000 draws of deaths due to fatal discontinuities were added pairwise to 1,000 draws of the with-HIV mortality envelope for each location, sex, and age group. Ninety-five percent uncertainty intervals were calculated as in other processes, taking the 97.5% and 2.5% quantiles of the summed draws.

To incorporate fatal discontinuity deaths into the life table, we first generated full single-year with-HIV life tables as detailed in the GBD 2017 Population and Fertility paper[1]. We calculated fatal discontinuity mortality rates by assuming the same mortality rate for each single-year age within a five-year age group, to preserve as closely as possible the five-year mortality rate after generating full life tables and re-converting to abridged life tables. We then directly added the fatal discontinuity-specific mortality rates to the mortality rates in the full life tables to regenerate full and abridged life tables with fatal discontinuities and HIV. To calculate the probability of death for the under 1 age group (1q0), we utilized

results from the under-5 age and sex pattern of mortality process (Section 2.2.14), calculated with-fatal discontinuity mx for enn, lnn, and pnn, back-calculated qx for enn, lnn, and pnn, and then aggregated to 1q0. In addition to the granular under-1 age groups, we also used the probability of death between age 1 year and age 4 years (4q1) generated by the age-sex model.

While the use of the same mortality rate for fatal discontinuities generally led to a directly corresponding increase in mx in the abridged life tables, there were cases where the differences in abridged mortality rate between the with- and without-fatal discontinuity life tables did not exactly match the input fatal discontinuity mortality rate. These cases were very limited, primarily occurring in age groups such as 90-94 with very high and age-variant mortality prior to the addition of fatal discontinuities, and where fatal discontinuity-specific mortality was relatively low compared to all-cause mortality. This created negligible differences between the mortality rate (mx) from the final abridged life tables and the mortality rates calculated using the mortality envelope and population results.

Section 2.6.4: Age-specific deaths with discontinuities and HIV/AIDS

We produced location-, sex-, year-, and age- specific death numbers including fatal discontinuities (Supplementary Results Figure 1) from the process described in Section 2.6.3.

Section 2.6.5: Life tables with HIV/AIDS and fatal discontinuities

From the process described in Section 2.6.4, we produced location-, sex-, and year-specific 5q0 (Supplementary Results Tables 2-4 and 12-14), 45q15, and life expectancy at birth (Supplementary Results Figures 2-4, Supplementary Results Tables 5-8) and age 65 (Supplementary Results Tables 9-11).

Section 3: SDI analysis

Section 3.1: SDI definition

The Socio-demographic Index (SDI) is a composite indicator of development status strongly correlated with health outcomes. In short, it is the geometric mean of 0 to 1 indices of total fertility rate under the age of 25 (TFU25), mean education for those aged 15 and older (EDU15+), and lag distributed income (LDI) per capita.

Section 3.2: Development of revised SDI indicator

SDI was originally constructed for GBD 2015 using the Human Development Index (HDI) methodology, wherein a 0 to 1 index value was determined for each of the original three covariate inputs (TFR in ages 15 to 49, EDU15+, and LDI per capita) using the observed minima and maxima over the estimation period to set the scales.[34]

In response to feedback from collaborators and the evolution of the GBD, we have refined the indicator with each GBD cycle. For GBD 2017, in conjunction with our expanded estimation of age-specific fertility, we replaced TFR with TFU25 as one of the three component indices. The TFU25 provides a better measure of women's status in society, as it focuses on ages where childbearing disrupts the pursuit of education and entrance into the workforce. In addition, we observe that in highly developed countries the TFU25 has tended to decline consistently over time, even amidst rebounds in TFR driven by increasing fertility in

older ages. The concordance correlation coefficient between SDI using the GBD 2016 method and the updated method for GBD 2017 was 0.981.

During GBD 2016 we moved from using relative index scales to absolute scales to enhance the stability of SDI's interpretation over time, as we noticed that the measure was highly sensitive to the addition of subnational units that tended to stretch the empirical minima and maxima.[7] We selected the minima and maxima of the scales by examining the relationships each of the inputs had with life expectancy at birth and under-5 mortality and identifying points of limiting returns at both high and low values, if they occurred prior to theoretical limits (e.g., a TFU25 of 0).

Thus, an index score of 0 represents the minimum level of each covariate input past which selected health outcomes can get no worse, while an index score of 1 represents the maximum level of each covariate input past which selected health outcomes cease to improve. As a composite, a location with an SDI of 0 would have a theoretical minimum level of development relevant to these health outcomes, while a location with an SDI of 1 would have a theoretical maximum level of development relevant to these health outcomes.

The final scales for GBD 2017 are summarized in table C below.

Table C. Final SDI scales

Input	Lower Bound	Upper Bound
TFU25	0	3
LDI per capita	250 USD (5.52 log USD)[a]	60,000 USD (11.00 log USD)
EDU15+	0 years	17 years

[a] The minimum for the LDI scale was originally set at the theoretical limit of 0 USD, as we did not observe an asymptotic relationship between log(LDI) and E_0 or 5q0 at lower values of log(LDI). Empirically, however, we also did not observe an LDI below 350 USD (5.86 log USD) for the estimation period 1970-2016. In log-space, this meant that approximately half of our scale was not being utilized, compressing the observed variation in LDI and diminishing its meaningful contribution to SDI. Accordingly, we set the lower limit on LDI to 250 USD (5.52 log USD) to ensure we were fully utilizing the range of the scale to capture its variation across space and time, as is the case with the other two inputs.

Using scales described above, we computed the index scores underlying SDI as follows:

$$I_{Cly} = \frac{(C_{ly} - C_{low})}{(C_{high} - C_{low})}$$

Where I_{cly} – the index for covariate *C*, location *l*, and year *y* – is equal to the difference between the value of that covariate in that location-year and the lower bound of the covariate divided by the difference between the upper and lower bounds for that covariate. If the values of input covariates fell outside the upper or lower bounds (e.g. LDI per capita greater than 60,000 USD), they were mapped to the respective upper or lower bounds. The index value for TFU25 was computed as $1 - I_{TFU25ly}$, as lower TFU25s correspond to higher levels of development, and thus higher index scores. For GBD 2017 we expanded the computation of SDI to 890 national and subnational locations spanning the time period 1950-2017.

The composite SDI was the geometric mean of these three indices for a given location-year. The cutoff values used to determine quintiles for analysis were then computed using country-level estimates of SDI

for the year 2017, excluding countries with populations less than 1 million. SDI groupings by geography are provided in Appendix Table 8; SDI values by location are provided in Appendix Tables 9-11.

Example Calculation

Below we present the calculation of SDI for a hypothetical country in the year 2010

$$TFU25 = 1.09;\ Mean\ educ\ yrs\ pc = 8.23;\ lnLDI = 9.60$$

$$I_{TFU25} = 1 - \frac{1.09 - 0}{3 - 0} = .637$$

$$I_{Educ} = \frac{8.23 - 0}{17 - 0} = .484$$

$$I_{lnLDI} = \frac{9.60 - 5.52}{11.00 - 5.52} = .744$$

$$SDI = \sqrt[3]{I_{TFU25} * I_{Educ} * I_{lnLDI}} = \sqrt[3]{.637 * .484 * .744} = .611$$

Section 4: Fatal Discontinuities Estimation

Section 4.1: Input data

Input data for fatal discontinuities were compiled from a range of sources, including country VR data; international databases that captured several cause-specific fatal discontinuities; and supplemental data in the presence of known issues with data quality, representativeness, or time lags in reporting. Below we provide more detail on the different input data sources by sub-causes of fatal discontinuities.

Subnational locations and population splitting

In locations where we produced estimates at the subnational level for GBD 2017, deaths due to all fatal discontinuity causes were assigned to the relevant subnational location(s) when that information could be obtained either through country data sources (e.g., VR) or through additional online research. In the rare case that no subnational location could be found, the deaths were split proportionally by population across all subnational locations.

In locations that have experienced boundary changes or split from other locations that we currently estimate (e.g., the former Yugoslavia, Czechoslovakia, the Soviet Union, Sudan and South Sudan), we split deaths due to events that occurred prior to boundary changes proportionally based on the populations residing within the boundaries of present-day locations unless we found documentation that clearly indicated whether the event and corresponding deaths occurred in one of the present-day GBD 2017 locations.

Choosing between multiple sources for same event

Where multiple sources reported shock deaths for the same location-year-cause, a cause-specific prioritization scheme was followed that reflected the available detail in the cause-specific datasets. For example, the Generalized Event Dataset from UCDP was prioritized above all other non-VR sources because it included detail on how deaths were distributed between multiple actors and locations in each conflict event.[35] In most cases, VR from 4- or 5-star locations (based on the GBD 2017 cause of death star-rating data quality system) was used where available.[32] In some cases, VR from 4- or 5-star locations was not chosen if there were well-known data quality issues or discrepancies in the cause of death data reporting related to a particular event (e.g., supplemental death data for Louisiana was used for Hurricane Katrina because of established data reporting issues). The process for prioritization among various sources for location-year fatal discontinuities is described more in the Modelling strategy below.

Major data sources other than country vital registration for each fatal discontinuity cause follow.

Conflict and terrorism

In GBD 2016, data for conflict and terrorism came from the Uppsala Conflict Data Program (UCDP), International Institute for Strategic Studies, and Robert S. Strauss Center for International Security and Law. For GBD 2017, data from the Global Terrorism Database (GTD), the University of Chicago Suicide Attack Database, and the RAND Database of Worldwide Terrorism Incidents were used in addition to those used in GBD 2016. The table below provides details about the various datasets we utilized from these sources, the dates they were last accessed, and the years for which we used the data provided. Where these data sources reported deaths due to gang violence, the cause was re-mapped to Physical violence by other means. Where these data sources reported deaths due to legal intervention, the cause was re-mapped to Executions and police conflict.

Table D.

Data source name	Date accessed	Years of data downloaded	Type of data included
**Uppsala Conflict Data Program**[1]			
Georeferenced Event Dataset, Version 17.1	1/16/2018	1989-2015	UCDP battles, non-state, and one-sided conflict deaths with the most disaggregated location information available
PRIO Battles Deaths Dataset, Version 3.1	1/16/2018	1970-1988	Armed conflict (civil wars, etc.)
**International Institute for Strategic Studies**			
Armed Conflict Dataset	11/17/2016	1997-2016	Insurgency, Inter-state, Intra-state conflict deaths
**Robert S. Strauss Center For International Security And Law**			
Armed Conflict Location and Event Dataset (ACLED)	6/21/2018	1997-2017	Actions of opposition groups, governments, and militias in selected locations in Africa, Asia, and the Middle East specifying the exact location and date of battle events, transfers of military control, headquarter establishment, civilian violence, and rioting
Social Conflict Analysis Database (SCAD)	1/16/2018	1990-2016	Protests, riots, strikes, inter-communal conflict, government violence against civilians, and other forms of social conflict (covers Africa, Latin America, and Asia)
**University of Maryland, Global Terrorism Database**			
Global Terrorism Database (GTD)	1/16/2018	1970-2016	Attacks aimed at attaining political, economic, religious, or social goal, includes evidence of intention to coerce, action was outside precepts of International Humanitarian Law.
**University of Chicago, Chicago Project on Security and Threats**			
Suicide Attack Database (CPOST SAD)	8/5/2017	1974-2016	Attacks in which an attacker kills him/herself in a deliberate attempt to kill others, includes only attacks perpetrated by non-state actors
**RAND National Security Research Division**			
RAND Database of Worldwide Terrorism Incidents	9/8/2017	1968-2009	Terrorism, defined by the nature of the act, not by the identity of the perpetrators or the nature of the cause; including violence, calculated to create fear/alarm, intended to coerce certain actions, motive is political, group or individual

Supplemental online research was conducted for recent conflicts where the databases above were not up-to-date. In addition, deaths due to conflict and terrorism in Iraq from 2003 to present were estimated using a combination of supplemental sources. The source found with the lowest number of deaths, Iraq Body Count (IBC)[36], was used as the lower bound of the uncertainty interval from 2003 to 2016. Estimates from the Iraq Mortality Study (IMS) by Hagopian et al[37] from 2003 to 2006, the deadliest years of the war, were used to scale deaths to generate the upper uncertainty interval limits using the following formula:

$$deaths_{GBD\ 2016,\ high} = deaths_{IBC} \cdot \overline{\left[\frac{deaths_{IMS}}{deaths_{IBC}}\right]}_{2003-2006}$$

We used the average ratio between IMS and IBC reported deaths between 2003 and 2006, multiplied by the number of deaths reported by the IBC. This high estimate is carried forward through 2017 under the assumption that the IBC similarly undercounts the number of deaths due to the ongoing civil war in Iraq. The final, best estimate for conflict and terrorism deaths in Iraq from 2003 to 2016 is the midpoint of the high and low estimates given above.

We identified four major conflicts that were not represented in these databases: 1997 civil conflict in Albania[38]; 1971 genocide in Bangladesh[39]; 1972 genocide in Burundi[40]; and 1993 genocide in Burundi[40]. In these cases, we used literature sources in order to account for these fatal discontinuities.

For country-years where multiple sources provided estimates, we prioritized sources in the following order: (1) country VR data, if death estimates were highest of all sources; (2) UCDP; (3) IISS; (4) country VR if death estimates were not the highest of all sources; (5) Robert Strauss Center; (6) Global Terrorism DB; (7) CPOST Suicide Attack Database; (8) online supplemental research.

Exposure to forces of nature, other injury causes, and protein-energy malnutrition

The Centre for Research on the Epidemiology of Disasters' International Disaster Database (EM-DAT) served as the primary non-VR source of fatal discontinuities due to exposure to forces of nature (i.e., natural disasters); other transport injuries (eg, plane, train, and boat accidents); poisonings; fire, heat, and hot substances; other exposure to mechanical forces (eg, building collapse); and protein-energy malnutrition (ie, famine or severe drought). Data from EM-DAT were last accessed February 14, 2018. Supplemental online research was conducted for events where EM-DAT was not up-to-date.

For country-years where multiple sources provided estimates, we prioritized sources in the following order: (1) country VR data, if data quality rating was 4 or 5 stars; (2) country VR data if data quality rating was less than 4 stars and death estimates were highest of all sources; (3) EM-DAT; (4) online supplemental research. Exceptions were made when it was clear that VR systems had been compromised by the event being measured.

Meningococcal meningitis and diarrheal diseases

For GBD 2017, we included fatal discontinuities due to a subset of infectious diseases: meningococcal meningitis (or meningococcal infection) and diarrheal disease caused by cholera. These two infectious diseases were first included on the fatal discontinuity cause list for GBD 2016 because (1) their current

modelling strategies with the Cause of Death Ensemble model (CODEm) does not optimally capture the potentially highly variable – or epidemic – mortality levels and trends characteristic of these two causes; and (2) they can contribute to significant total fatalities in a given location-year. Other infectious diseases for which the latter is true – high death rates in the presence of an outbreak or epidemic – are currently modelled with alternative cause of death methods (eg, natural history models for measles and yellow fever), which allow for greater variation year-over-year if or when outbreaks occur. In future iterations of the GBD, we plan to revisit the inclusion criteria for infectious diseases as fatal discontinuities and develop more of an ensemble approach to modelling causes that can be both endemic (and thus result in more uniform levels and trends over time) and epidemic (and subsequently lead to rapid increases – and decreases – in deaths for a given location-year).

The Global Infectious Diseases and Epidemiology Network (GIDEON) served as the primary data source for collating cholera, meningococcal meningitis, and meningococcal infection death reports.[41,42] For any year in which cholera or meningococcal meningitis deaths were recorded in a country or territory covered by the GBD, we directly extracted reported deaths from 1970 to 2016. When there were reporting gaps in cholera or meningococcal meningitis deaths over this period of time and the WHO annual cholera or meningitis reports included death reports for those years, the WHO reports were utilized. The primary exception were two major cholera outbreaks in Bangladesh – 1982-1983 and 1991 – which were not captured by either GIDEON or WHO. As result, we used the EM-DAT records for the 1982-1983 outbreak and literature for the 1991 outbreak.[32] For the Yemen Cholera outbreak in 2016 and 2017, we used estimates from local collaborators in the absence of other data sources.

Ebola

Since GBD 2015, outbreaks due to Ebola virus disease have been estimated using the data and methods described in the appendix of the GBD 2017 cause of death capstone and included in GBD death estimates in the same way as other fatal discontinuity causes.[32]

Section 4.2: Modeling strategy

All input data for fatal discontinuity causes were run through the cause of death data formatting and mapping process.[32]

VR de-duplication

For injury causes that also have continuous background mortality and a CODEm model, a process was established to avoid duplication of fatal discontinuity deaths in the two models. First, location-years with fatal discontinuities data from non-VR sources were identified. If these location-cause-years also had VR death estimates that were greater than 40% higher than the immediately surrounding years and could be linked to a specific fatal discontinuity event, these years were marked as outliers in the VR data and the difference between the outlier year and the average of the surrounding years was included in the relevant cause in the fatal discontinuities database. The deaths from the identified events were subtracted from the all-cause VR estimates used in the all-cause mortality estimation process.

Uncertainty analysis for input and draw-level input to age-sex splitting

Uncertainty intervals for deaths due to conflict and terrorism were generated using UCDP high and low death estimates, except in the case of Iraq 2003-2016, as explained above. In cases where low and high estimates were not included in the available data, the regional average uncertainty interval was applied to the available death estimate across all fatal discontinuity causes.

We assumed a log-normal distribution using mean death rates and standard error based on high and low estimates. In the case that standard error was less than 10e-8, the draws were set equal to the mean rate. 1,000 draws were sampled from this log-normal distribution. These 1,000 draws were then converted back to count space and used for final calculations of means and uncertainty intervals.

Age-sex splitting

All compiled data were run through the causes of death age-sex splitting process, except for where we had strong supplemental information on the age distribution of specific, large events, such as United States mortality in the Vietnam War and Iranian mortality from the Iran-Iraq conflict in the early 1980s.[32]

Changes from GBD 2016

GBD 2017 saw an effort to systematize the collection of up-to-date fatal discontinuity data through supplemental online research. New tools included use of Twitter to identify events not covered by other sources, most notably in identifying events that occurred recently (2016, 2017). This process resulted in a more comprehensive set of conflict and terrorism data for 2017, as well as large natural disasters not contained in EM-DAT or VR.

For GBD 2017, efforts were also made to improve location tagging in raw data to the GBD location hierarchy using several approaches. Identifying the correct GBD location for each event is difficult, as reports of fatal discontinuities come in many formats, often with limited metadata. The approaches used for improving the location tagging included a) utilizing the collaborator network to more accurately tag events to subnational locations when information in the data was scarce, b) automated matching with GBD location names, c) overlaying a spatial file of the most-detailed GBD geographies, d) geo-coding using precise place names, and e) for events spanning multiple GBD locations, but without detail in the raw data, deaths were split using population.

We completed a detailed review of the fatal discontinuity cause mappings for conflict and terrorism, police conflict and executions, using the text descriptions of each event when provided in the data. This exercise resulted in updating the GBD cause assigned for a number of events present in the GBD 2016 analysis, which is one contributor in the differences seen in the GBD 2016 and GBD 2017 fatal discontinuity estimates.

Section 5: Additional Methods Information

Section 5.1: GBD world population age standard

Age-standardized populations in the GBD were calculated using the GBD world population age standard. For GBD 2013, GBD 2015 and GBD 2016, the age-specific proportional distributions of all national

locations from the UNPOP World Population Prospects 2012 revision for all years from 2010 to 2035 were used to generate a standard population age structure using the non-weighted mean across all the aforementioned country-years. For GBD 2017 we have used the non-weighted mean of 2017 age-specific proportional distributions from the GBD 2017 population estimates for all national locations with a population greater than 5 million people in 2017 to generate an updated standard population age structure.[1] The values used for the age standard are found in Appendix Table 12.

Section 5.2: Estimating correlation

For the estimation of correlation, we used the Pearson correlation coefficient as estimated by Stata version 14, which uses the following function[43]:

$$\hat{\rho} = \frac{\sum_{i=1}^{n} w_i\,(x_i - \bar{x})(y_i - \bar{y})}{\sqrt{\sum_{i-1}^{n} w_i (x_i - \bar{x})^2}\,\sqrt{\sum_{i-1}^{n} w_i (y_i - \bar{y})^2}}$$

Where

w_i are the weights, if specified, or $w_i = 1$ if weights are not specified

$\bar{x} = (\sum w_i x_i)/(\sum w_i)$ is the mean of x

$\bar{y}$ is similarly defined

Section 5.3: Calculating annualised rates of change

We calculated annualized rates of change by taking the log of 5q0 in a target year, represented as $y + t$, divided by the 5q0 at the baseline year y, and divided by the difference in years between the target and baseline years, represented as t.

$$aroc_{{}_5q_0} = \frac{ln\left(\frac{{}_5q_{0\;y+t}}{{}_5q_{0\;y}}\right)}{t}$$

Section 5.4: Gross domestic product (GDP) and lagged dependent income (LDI)

Gross domestic product (GDP) per capita is based upon four commonly used GDP per capita series, World Bank World Development Indicators, International Monetary Fund World Economic Outlook report, Angus Maddison's research homepage at the University of Groningen Department of Economics, and the University of Pennsylvania (Penn) Center for International Comparisons of Production, Income, and Prices. Each of the four series are imputed separately using growth regressions and then averaged to create a unified IHME GDP per capita series. Wherever we had missing data across all series for a country, we used mixed effects regression with region-specific random effects to interpolate or extrapolate those missing observations. These methods have been previously described in James et al. [44]

Lagged dependent income (LDI) per capita is a moving average transformation of GDP per capita. We computed a 10 year lagged average of GDP to create LDI with the following formula:

$$LDI_{pc,t} = \frac{1}{5.5}\left(GDPpc_t + \sum_{i=1}^{9} GDPpc_{t-i} \cdot \left(1 - \frac{i}{10}\right)\right)$$

where LDI at time *t* is a function of current GDP (at *t*) and the previous 9 years of GDP with an inverse moving weight, and a normalization factor (sum of all the weights).

Section 5.5: Calculation of educational attainment covariate

Estimates of average years of education were based on a compilation of 2,522 censuses and household surveys. These data and the methods hereafter build on an approach used to produce a previously published dataset of international educational attainment.[45,46] Each data source included information on the distribution of educational attainment by country, year, sex, and five- or 10-year age group. Where years of schooling data were available only for multi-year bins, eg, the fraction of the population with between 6 and 9 years of completed education, we utilised a database of 1,792 sources reporting single years of completed schooling to split these binned data into single-year distributions from 0 to 18 years based on the average of the 12 closest distributions in terms of geographic proximity and year. From each of the subsequent data sources, we calculated the mean years of schooling by age and sex.

In the next step, age-cohort imputation was used to project observed cohorts through time, exploiting the relative constancy of education levels after age 25. For any data point representing a cohort aged 25 or older, we extrapolated the data forward and backward so that it was represented in all year-age combinations for that cohort. For example, a data point reflecting a cohort aged 35-39 in 2000 was projected forward for 40- to 44-year-olds in 2005, 45- to 49-year-olds in 2010, and so on. It was also projected backward for 30- to 34-year-olds in 1995 and 25- to 29-year-olds in 1990. Post-imputation, age-period models were fit on all original input data, as well as the imputed cohort data, in order to estimate a complete single-year series of educational attainment from 1950 through 2016 by age, sex, and location. Separately for each sex and GBD region, the mean level of educational attainment of the country-age-year-specific population, $Edu_{c,a,\,s,t}$, was estimated as:

$$logit\left(\frac{Edu_{c,a,s,t}}{Edu_{max_a}}\right) = \beta_{s,r} Year + \delta_{s,r}\, Age + I_{s,r} + \ \propto_{c,s} ,$$

where:

Edu_{max_a} is the maximum mean educational attainment for each age group, defined as 3 for ages 5-9, 8 for ages 10-14, 13 for ages 15-19, and 18 for all age groups 20-24 and up;

$\beta_{s,r}$is a sex- and region-specific intercept;

$\delta_{s,r}$ captures the linear secular trend for each sex and region;

$I_{s,r}$is a natural spline on age to capture the non-linear age pattern by sex and region, with knots at 15 and 25 years of age; and

$\propto_{c,s}$ is a country-sex-specific random intercept.

Finally, Gaussian process regression (GPR) was used to smooth the residuals from the age-period model, accounting for uncertainty in each data point. GPR also synthesises both data and model uncertainty to estimate uncertainty intervals.

Section 6: References

1 GBD 2017 Population and Fertility collaborators. Population and fertility by age and sex for 195 countries and territories 1950-2017: a systematic analysis for the Global Burden of Disease 2017. *The Lancet* in review.

2 Rajaratnam JK, Tran LN, Lopez AD, Murray CJL. Measuring Under-Five Mortality: Validation of New Low-Cost Methods. *PLOS Med* 2010; **7**: e1000253.

3 Wang H, Liddell CA, Coates MM, *et al.* Global, regional, and national levels of neonatal, infant, and under-5 mortality during 1990–2013: a systematic analysis for the Global Burden of Disease Study 2013. *The Lancet* 2014; **384**: 957–79.

4 Murray CJ, Ezzati M, Flaxman AD, *et al.* GBD 2010: design, definitions, and metrics. *The Lancet* 2012; **380**: 2063–6.

5 Wang H, Dwyer-Lindgren L, Lofgren KT, *et al.* Age-specific and sex-specific mortality in 187 countries, 1970–2010: a systematic analysis for the Global Burden of Disease Study 2010. *The Lancet* 2012; **380**: 2071–94.

6 Wang H, Bhutta ZA, Coates MM, *et al.* Global, regional, national, and selected subnational levels of stillbirths, neonatal, infant, and under-5 mortality, 1980–2015: a systematic analysis for the Global Burden of Disease Study 2015. *The Lancet* 2016; **388**: 1725–74.

7 Wang H, Abajobir AA, Abate KH, *et al.* Global, regional, and national under-5 mortality, adult mortality, age-specific mortality, and life expectancy, 1970–2016: a systematic analysis for the Global Burden of Disease Study 2016. *The Lancet* 2017; **390**: 1084–150.

8 Gelman A, Carlin JB, Stern HS, Rubin DB. Bayesian Data Analysis, Second Edition, 2 edition. Boca Raton, Fla: Chapman and Hall/CRC, 2003.

9 Gompertz B. On one Uniform Law of Mortality from Birth to extreme Old Age, and on the Law of Sickness. *J Inst Actuar Assur Mag* 1871; **16**: 329–44.

10 The UN Inter-agency Group for Child Mortality Estimation. Child Mortality Estimates. http://www.childmortality.org/ (accessed May 20, 2017).

11 Bradshaw D, Dorrington R. Child mortality in South Africa - we have lost touch. *S Afr Med J* 2007; **97**: 582.

12 Kerber KJ, Lawn JE, Johnson LF, *et al.* South African child deaths 1990-2011: have HIV services reversed the trend enough to meet Millennium Development Goal 4? *AIDS Lond Engl* 2013; **27**: 2637–48.

13 Kim H-Y, Kasonde P, Mwiya M, *et al.* Pregnancy loss and role of infant HIV status on perinatal mortality among HIV-infected women. *BMC Pediatr* 2012; **12**: 138.

14 Brocklehurst P, French R. The association between maternal HIV infection and perinatal outcome: a systematic review of the literature and meta-analysis. *Br J Obstet Gynaecol* 1998; **105**: 836–48.

15 Hill K. Estimating census and death registration completeness. *Asian Pac Popul Forum* 1987; **1**: 8–13, 23–4.

16 Bennett NG, Horiuchi S. Estimating the Completeness of Death Registration in a Closed Population. *Popul Index* 1981; **47**: 207–21.

17 Brass W, Coale AJ. Methods of Analysis and Estimation. In: Mathematical Demography. Springer Berlin Heidelberg, 1977: 307–13.

18 Preston SH. The changing relation between mortality and level of economic development. *Popul Stud* 1975; **29**: 231–248.

19 Preston S, Coale AJ, Trussell J, Weinstein M. Estimating the Completeness of Reporting of Adult Deaths in Populations That Are Approximately Stable. *Popul Index* 1980; **46**: 179–202.

20 Murray CJL, Rajaratnam JK, Marcus J, Laakso T, Lopez AD. What Can We Conclude from Death Registration? Improved Methods for Evaluating Completeness. *PLOS Med* 2010; **7**: e1000262.

21 Obermeyer Z, Rajaratnam JK, Park CH, *et al.* Measuring Adult Mortality Using Sibling Survival: A New Analytical Method and New Results for 44 Countries, 1974–2006. *PLOS Med* 2010; **7**: e1000260.

22 Gakidou E, King G. Death by survey: estimating adult mortality without selection bias from sibling survival data. *Demography* 2006; **43**: 569–585.

23 Masquelier B. Adult mortality from sibling survival data: a reappraisal of selection biases. *Demography* 2013; **50**: 207–28.

24 Rogers RG, Crimmins EM, editors. International Handbook of Adult Mortality. http://www.springer.com/gp/book/9789048199952 (accessed May 20, 2017).

25 Murray CJ, Ezzati M, Flaxman AD, *et al.* GBD 2010: design, definitions, and metrics. *The Lancet* 2012; **380**: 2063–6.

26 Murray CJL, Ferguson BD, Lopez AD, Guillot M, Salomon JA, Ahmad O. Modified Logit Life Table System: Principles, Empirical Validation, and Application. *Popul Stud* 2003; **57**: 165–82.

27 Thatcher AR. The long-term pattern of adult mortality and the highest attained age. *J R Stat Soc Ser A Stat Soc* 1999; **162**: 5–43.

28 Shkolnikov V, Barbieri M, Wilmoth J. Human Mortality Database. http://www.mortality.org/ (accessed May 20, 2017).

29 King G, Tomz M, Wittenberg J. Making the Most of Statistical Analyses: Improving Interpretation and Presentation. *Am J Polit Sci* 2000; **44**: 341–355.

30 Mahy M, Penazzato M, Ciaranello A, *et al.* Improving estimates of children living with HIV from the Spectrum AIDS Impact Model. *Aids* 2017; **31**: S13–S22.

31 Eaton J. epp: Implementation of EPP model for HIV epidemic estimates. 2018 https://github.com/jeffeaton/epp (accessed July 13, 2018).

32 GBD 2017 Causes of Death Collaborators. Global, regional, and national age-sex-specific mortality for 282 causes of death, 1980–2017: a systematic analysis for the Global Burden of Disease Study 2017. *The Lancet* in review.

33 Wang H, Wolock TM, Carter A, *et al.* Estimates of global, regional, and national incidence, prevalence, and mortality of HIV, 1980–2015: the Global Burden of Disease Study 2015. *Lancet HIV* 2016; **3**: e361–87.

34 Wang H, Naghavi M, Allen C, *et al.* Global, regional, and national life expectancy, all-cause mortality, and cause-specific mortality for 249 causes of death, 1980–2015: a systematic analysis for the Global Burden of Disease Study 2015. *The Lancet* 2016; **388**: 1459–544.

35 Department of Peace and Conflict Research, Uppsala University. UCDP Georeferenced Event Dataset, Version 17.1, 2016. Uppsala, Sweden: Department of Peace and Conflict Research, Uppsala University, 2017. .

36 Iraq Body Count. https://www.iraqbodycount.org/database/ (accessed May 8, 2017).

37 Hagopian A, Flaxman AD, Takaro TK, *et al.* Mortality in Iraq Associated with the 2003–2011 War and Occupation: Findings from a National Cluster Sample Survey by the University Collaborative Iraq Mortality Study. *PLOS Med* 2013; **10**: e1001533.

38 Jarvis C. The Rise and Fall of Albania's Pyramid Schemes. *Finance Dev* 2000; **37**. http://www.imf.org/external/pubs/ft/fandd/2000/03/jarvis.htm.

39 Obermeyer Z, Murray CJL, Gakidou E. Fifty years of violent war deaths from Vietnam to Bosnia: analysis of data from the world health survey programme. *BMJ* 2008; **336**: 1482–6.

40 Milton L. Rwanda, 1994: International incompetence produces genocide. 1994 https://ezproxy.uwc.edu/login?url=http://search.proquest.com/docview/234405747?accountid=42411.

41 Inc GI, Berger DS. Cholera: Global Status: 2017 edition. GIDEON Informatics Inc, 2017.

42 Inc GI, Berger DS. Bacterial Meningitis: Global Status: 2017 edition. GIDEON Informatics Inc, 2017.

43 Functions Reference Manual. 2015. http://www.stata.com/manuals13/rcorrelate.pdf (accessed May 17, 2017).

44 James SL, Gubbins P, Murray CJ, Gakidou E. Developing a comprehensive time series of GDP per capita for 210 countries from 1950 to 2015. *Popul Health Metr* 2012; **10**: 12.

45 Gakidou E, Cowling K, Lozano R, Murray CJL. Increased educational attainment and its effect on child mortality in 175 countries between 1970 and 2009: a systematic analysis. *Lancet Lond Engl* 2010; **376**: 959–74.

46 Preston SH. The changing relation between mortality and level of economic development. *Int J Epidemiol* 2007; **36**: 484–90.

Appendix Figure 1. Analytical flowchart for the estimation of all-cause mortality by age and sex and HIV/AIDS incidence, prevalence, and mortality for GBD 2017.

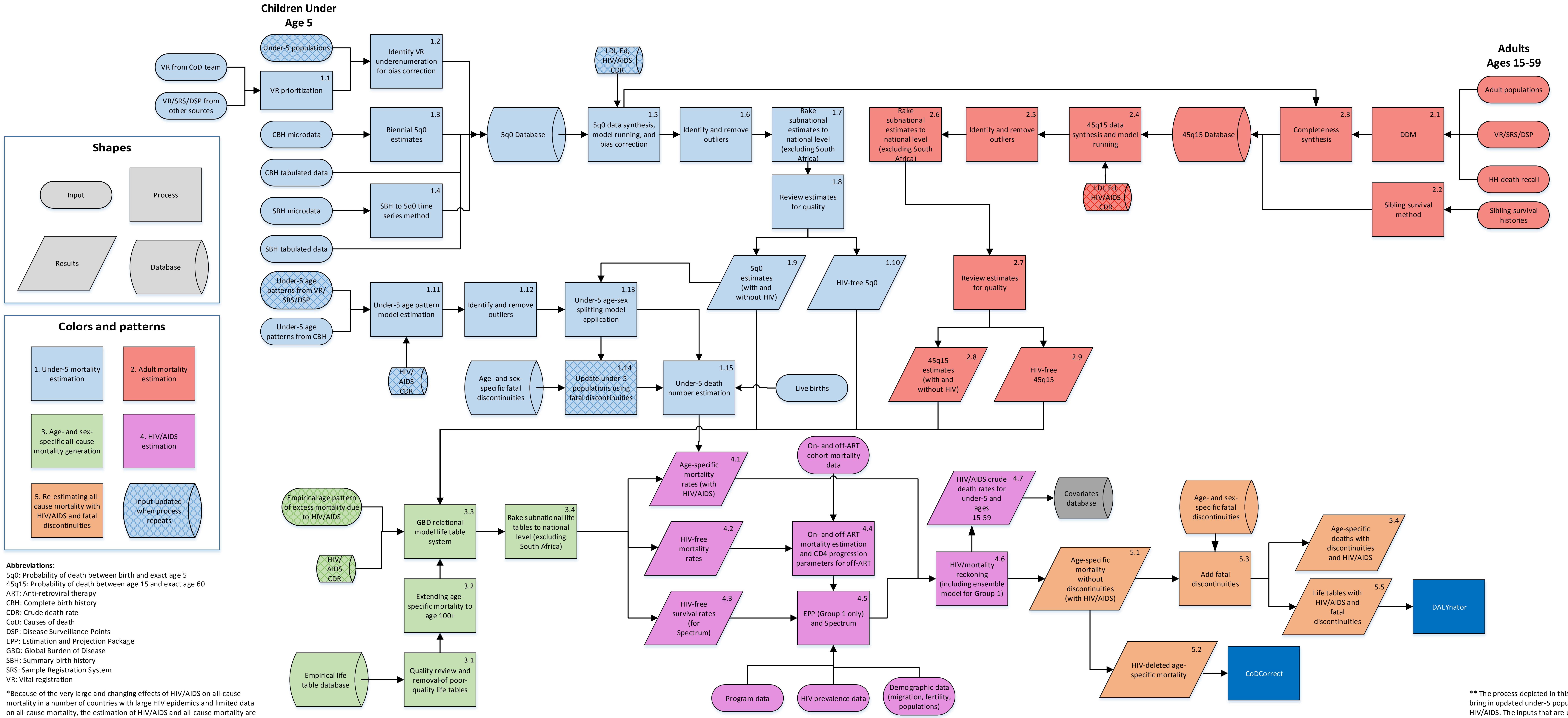

Abbreviations:
5q0: Probability of death between birth and exact age 5
45q15: Probability of death between age 15 and exact age 60
ART: Anti-retroviral therapy
CBH: Complete birth history
CDR: Crude death rate
CoD: Causes of death
DSP: Disease Surveillance Points
EPP: Estimation and Projection Package
GBD: Global Burden of Disease
SBH: Summary birth history
SRS: Sample Registration System
VR: Vital registration

*Because of the very large and changing effects of HIV/AIDS on all-cause mortality in a number of countries with large HIV epidemics and limited data on all-cause mortality, the estimation of HIV/AIDS and all-cause mortality are closely linked and presented jointly here.

** The process depicted in this flowchart is performed two times in order to bring in updated under-5 population estimates and crude death rates due to HIV/AIDS. The inputs that are updated in the second run of the process are shown using patterned boxes in this flowchart.

Appendix Figure 2. Estimated completeness of under−5 death registration, 1950−2017.

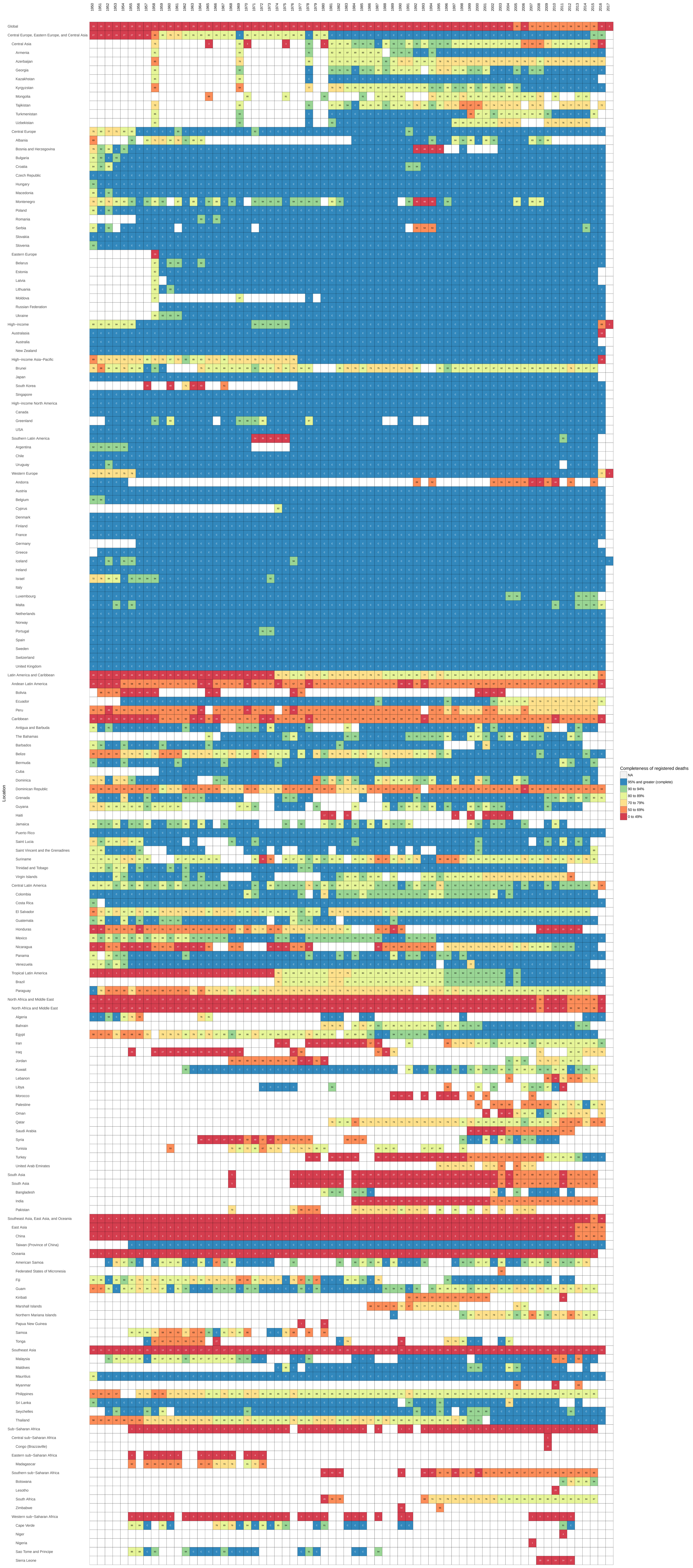

Appendix Table 1. GATHER checklist of information that should be included in reports of global health estimates, with description of compliance and location of information for GBD 2017 [Global, regional, and national under-5 mortality, adult mortality, age-specific mortality, and life expectancy, 1950-2017: a systematic analysis for the Global Burden of Disease Study 2017].

#	GATHER checklist item	Description of compliance	Reference
**Objectives and funding**			
1	Define the indicators, populations, and time periods for which estimates were made.	Narrative provided in paper and methods appendix describing indicators, definitions, and populations	Main text (Methods) and methods appendix
2	List the funding sources for the work.	Funding sources listed in paper	Main text (Summary)
**Data Inputs**			
For all data inputs from multiple sources that are synthesized as part of the study:			
3	Describe how the data were identified and how the data were accessed.	Narrative provided in paper and methods appendix describing data seeking methods	Main text (Methods) and methods appendix
4	Specify the inclusion and exclusion criteria. Identify all ad-hoc exclusions.	Narrative provided in paper and methods appendix describing inclusion and exclusion criteria by data type	Main text (Methods) and methods appendix
5	Provide information on all included data sources and their main characteristics. For each data source used, report reference information or contact name/institution, population represented, data collection method, year(s) of data collection, sex and age range, diagnostic criteria or measurement method, and sample size, as relevant.	Metadata for data sources by component, geography, cause, risk, or impairment is available through an interactive, online data source tool	Online data citation tool http://ghdx.healthdata.org/gbd-2017
6	Identify and describe any categories of input data that have potentially important biases (e.g., based on characteristics listed in item 5).	Summary of known biases by cause included in methods appendix	Methods appendix
For data inputs that contribute to the analysis but were not synthesized as part of the study:			
7	Describe and give sources for any other data inputs.	Included in online data source tool	Online data citation tool: http://ghdx.healthdata.org/gbd-2017
For all data inputs:			
8	Provide all data inputs in a file format from which data can be efficiently extracted (e.g., a spreadsheet as opposed to a PDF), including all relevant meta-data listed in item 5. For any data inputs that cannot be shared due to ethical or legal reasons, such as third-party ownership, provide a contact	Downloads of input data are available through online tools, including data visualization:	Online data visualization tools, data query tools, and the Global Health Data Exchange:

	name or the name of the institution that retains the right to the data.	http:// ghdx.healthdata.org	http:// ghdx.healthdata.org
**Data analysis**			
9	Provide a conceptual overview of the data analysis method. A diagram may be helpful.	Flow diagrams of the overall methodological processes, as well as cause-specific modelling processes, have been provided	Main text (Methods) and methods appendix
10	Provide a detailed description of all steps of the analysis, including mathematical formulae. This description should cover, as relevant, data cleaning, data pre-processing, data adjustments and weighting of data sources, and mathematical or statistical model(s).	Flow diagrams and corresponding methodological write-ups for each cause, as well as the demographics and cause of death databases and modelling processes, have been provided	Main text (Methods) and methods appendix
11	Describe how candidate models were evaluated and how the final model(s) were selected.	Details on model evaluation and finalization have been provided	Methods appendix
12	Provide the results of an evaluation of model performance, if done, as well as the results of any relevant sensitivity analysis.	Details on evaluation of model performance have been provided	Methods appendix
13	Describe methods for calculating uncertainty of the estimates. State which sources of uncertainty were, and were not, accounted for in the uncertainty analysis.	Details on uncertainty calculations have been provided	Methods appendix
14	State how analytic or statistical source code used to generate estimates can be accessed.	Access statement provided	Code is provided in an online repository [link to be added upon acceptance]
**Results and Discussion**			
15	Provide published estimates in a file format from which data can be efficiently extracted.	GBD 2017 results are available through online data visualization tools, the Global Health Data Exchange, and the online data query tool	Online data tools: http:// ghdx.healthdata.org/ gbd-2017
16	Report a quantitative measure of the uncertainty of the estimates (e.g. uncertainty intervals).	Uncertainty intervals are provided with all results	Main text, methods appendix, and online data tools: http:// ghdx.healthdata.org/ gbd-2017

17	Interpret results in light of existing evidence. If updating a previous set of estimates, describe the reasons for changes in estimates.	Discussion of methodological changes between GBD 2016 and GBD 2017 was provided	Main text (Methods and Discussion) and methods appendix
18	Discuss limitations of the estimates. Include a discussion of any modelling assumptions or data limitations that affect interpretation of the estimates.	Discussion of limitations was provided	Main text (Limitations) and methods appendix

Appendix Table 2. GBD location hierarchy with levels	
**Geography**	**Level**
Global	0
Low SDI	1
Low-middle SDI	1
Middle SDI	1
High-middle SDI	1
High SDI	1
Central Europe, Eastern Europe, and Central Asia	1
Central Asia	2
Armenia	3
Azerbaijan	3
Georgia	3
Kazakhstan	3
Kyrgyzstan	3
Mongolia	3
Tajikistan	3
Turkmenistan	3
Uzbekistan	3
Central Europe	2
Albania	3
Bosnia and Herzegovina	3
Bulgaria	3
Croatia	3
Czech Republic	3
Hungary	3
Macedonia	3
Montenegro	3
Poland	3
Romania	3
Serbia	3
Slovakia	3
Slovenia	3
Eastern Europe	2
Belarus	3
Estonia	3
Latvia	3
Lithuania	3
Moldova	3
Russian Federation	3
Ukraine	3
High-income	1
Australasia	2
Australia	3
New Zealand	3
High-income Asia Pacific	2

Appendix Table 1. GBD location hierarchy with levels

Geography	Level
Brunei	3
Japan	3
Aichi	4
Akita	4
Aomori	4
Chiba	4
Ehime	4
Fukui	4
Fukuoka	4
Fukushima	4
Gifu	4
Gunma	4
Hiroshima	4
Hokkaidō	4
Hyōgo	4
Ibaraki	4
Ishikawa	4
Iwate	4
Kagawa	4
Kagoshima	4
Kanagawa	4
Kōchi	4
Kumamoto	4
Kyōto	4
Mie	4
Miyagi	4
Miyazaki	4
Nagano	4
Nagasaki	4
Nara	4
Niigata	4
Ōita	4
Okayama	4
Okinawa	4
Ōsaka	4
Saga	4
Saitama	4
Shiga	4
Shimane	4
Shizuoka	4
Tochigi	4
Tokushima	4
Tōkyō	4
Tottori	4
Toyama	4

Appendix Table 1. GBD location hierarchy with levels	
**Geography**	**Level**
Wakayama	4
Yamagata	4
Yamaguchi	4
Yamanashi	4
South Korea	3
Singapore	3
High-income North America	2
Canada	3
Greenland	3
United States	3
Alabama	4
Alaska	4
Arizona	4
Arkansas	4
California	4
Colorado	4
Connecticut	4
Delaware	4
District of Columbia	4
Florida	4
Georgia	4
Hawaii	4
Idaho	4
Illinois	4
Indiana	4
Iowa	4
Kansas	4
Kentucky	4
Louisiana	4
Maine	4
Maryland	4
Massachusetts	4
Michigan	4
Minnesota	4
Mississippi	4
Missouri	4
Montana	4
Nebraska	4
Nevada	4
New Hampshire	4
New Jersey	4
New Mexico	4
New York	4
North Carolina	4
North Dakota	4

Appendix Table 1. GBD location hierarchy with levels	
**Geography**	**Level**
Ohio	4
Oklahoma	4
Oregon	4
Pennsylvania	4
Rhode Island	4
South Carolina	4
South Dakota	4
Tennessee	4
Texas	4
Utah	4
Vermont	4
Virginia	4
Washington	4
West Virginia	4
Wisconsin	4
Wyoming	4
Southern Latin America	2
Argentina	3
Chile	3
Uruguay	3
Western Europe	2
Andorra	3
Austria	3
Belgium	3
Cyprus	3
Denmark	3
Finland	3
France	3
Germany	3
Greece	3
Iceland	3
Ireland	3
Israel	3
Italy	3
Luxembourg	3
Malta	3
Netherlands	3
Norway	3
Portugal	3
Spain	3
Sweden	3
Stockholm	4
Sweden except Stockholm	4
Switzerland	3
United Kingdom	3

Appendix Table 1. GBD location hierarchy with levels	
**Geography**	**Level**
England	4
East Midlands	5
Derby	6
Derbyshire	6
Leicester	6
Leicestershire	6
Lincolnshire	6
Northamptonshire	6
Nottingham	6
Nottinghamshire	6
Rutland	6
East of England	5
Bedford	6
Cambridgeshire	6
Central Bedfordshire	6
Essex	6
Hertfordshire	6
Luton	6
Norfolk	6
Peterborough	6
Southend-on-Sea	6
Suffolk	6
Thurrock	6
Greater London	5
Barking and Dagenham	6
Barnet	6
Bexley	6
Brent	6
Bromley	6
Camden	6
Croydon	6
Ealing	6
Enfield	6
Greenwich	6
Hackney	6
Hammersmith and Fulham	6
Haringey	6
Harrow	6
Havering	6
Hillingdon	6
Hounslow	6
Islington	6
Kensington and Chelsea	6
Kingston upon Thames	6
Lambeth	6

Appendix Table 1. GBD location hierarchy with levels

Geography	Level
Lewisham	6
Merton	6
Newham	6
Redbridge	6
Richmond upon Thames	6
Southwark	6
Sutton	6
Tower Hamlets	6
Waltham Forest	6
Wandsworth	6
Westminster	6
North East England	5
County Durham	6
Darlington	6
Gateshead	6
Hartlepool	6
Middlesbrough	6
Newcastle upon Tyne	6
North Tyneside	6
Northumberland	6
Redcar and Cleveland	6
South Tyneside	6
Stockton-on-Tees	6
Sunderland	6
North West England	5
Blackburn with Darwen	6
Blackpool	6
Bolton	6
Bury	6
Cheshire East	6
Cheshire West and Chester	6
Cumbria	6
Halton	6
Knowsley	6
Lancashire	6
Liverpool	6
Manchester	6
Oldham	6
Rochdale	6
Salford	6
Sefton	6
St Helens	6
Stockport	6
Tameside	6
Trafford	6

Appendix Table 1. GBD location hierarchy with levels

Geography	Level
Warrington	6
Wigan	6
Wirral	6
South East England	5
Bracknell Forest	6
Brighton and Hove	6
Buckinghamshire	6
East Sussex	6
Hampshire	6
Isle of Wight	6
Kent	6
Medway	6
Milton Keynes	6
Oxfordshire	6
Portsmouth	6
Reading	6
Slough	6
Southampton	6
Surrey	6
West Berkshire	6
West Sussex	6
Windsor and Maidenhead	6
Wokingham	6
South West England	5
Bath and North East Somerset	6
Bournemouth	6
Bristol, City of	6
Cornwall	6
Devon	6
Dorset	6
Gloucestershire	6
North Somerset	6
Plymouth	6
Poole	6
Somerset	6
South Gloucestershire	6
Swindon	6
Torbay	6
Wiltshire	6
West Midlands	5
Birmingham	6
Coventry	6
Dudley	6
Herefordshire, County of	6
Sandwell	6

Appendix Table 1. GBD location hierarchy with levels	
**Geography**	**Level**
Shropshire	6
Solihull	6
Staffordshire	6
Stoke-on-Trent	6
Telford and Wrekin	6
Walsall	6
Warwickshire	6
Wolverhampton	6
Worcestershire	6
Yorkshire and the Humber	5
Barnsley	6
Bradford	6
Calderdale	6
Doncaster	6
East Riding of Yorkshire	6
Kingston upon Hull, City of	6
Kirklees	6
Leeds	6
North East Lincolnshire	6
North Lincolnshire	6
North Yorkshire	6
Rotherham	6
Sheffield	6
Wakefield	6
York	6
Northern Ireland	4
Scotland	4
Wales	4
Latin America and Caribbean	1
Andean Latin America	2
Bolivia	3
Ecuador	3
Peru	3
Caribbean	2
Antigua and Barbuda	3
The Bahamas	3
Barbados	3
Belize	3
Bermuda	3
Cuba	3
Dominica	3
Dominican Republic	3
Grenada	3
Guyana	3
Haiti	3

Appendix Table 1. GBD location hierarchy with levels	
**Geography**	**Level**
Jamaica	3
Puerto Rico	3
Saint Lucia	3
Saint Vincent and the Grenadines	3
Suriname	3
Trinidad and Tobago	3
Virgin Islands, U.S.	3
Central Latin America	2
Colombia	3
Costa Rica	3
El Salvador	3
Guatemala	3
Honduras	3
Mexico	3
Aguascalientes	4
Baja California	4
Baja California Sur	4
Campeche	4
Chiapas	4
Chihuahua	4
Coahuila	4
Colima	4
Mexico City	4
Durango	4
Guanajuato	4
Guerrero	4
Hidalgo	4
Jalisco	4
México	4
Michoacán de Ocampo	4
Morelos	4
Nayarit	4
Nuevo León	4
Oaxaca	4
Puebla	4
Querétaro	4
Quintana Roo	4
San Luis Potosí	4
Sinaloa	4
Sonora	4
Tabasco	4
Tamaulipas	4
Tlaxcala	4
Veracruz de Ignacio de la Llave	4
Yucatán	4

Appendix Table 1. GBD location hierarchy with levels	
**Geography**	**Level**
Zacatecas	4
Nicaragua	3
Panama	3
Venezuela	3
Tropical Latin America	2
Brazil	3
Acre	4
Alagoas	4
Amapá	4
Amazonas	4
Bahia	4
Ceará	4
Distrito Federal	4
Espírito Santo	4
Goiás	4
Maranhão	4
Mato Grosso	4
Mato Grosso do Sul	4
Minas Gerais	4
Pará	4
Paraíba	4
Paraná	4
Pernambuco	4
Piaui	4
Rio de Janeiro	4
Rio Grande do Norte	4
Rio Grande do Sul	4
Rondônia	4
Roraima	4
Santa Catarina	4
São Paulo	4
Sergipe	4
Tocantins	4
Paraguay	3
North Africa and Middle East	1
North Africa and Middle East	2
Afghanistan	3
Algeria	3
Bahrain	3
Egypt	3
Iran	3
Iraq	3
Jordan	3
Kuwait	3
Lebanon	3

Appendix Table 1. GBD location hierarchy with levels	
**Geography**	**Level**
Libya	3
Morocco	3
Palestine	3
Oman	3
Qatar	3
Saudi Arabia	3
Sudan	3
Syria	3
Tunisia	3
Turkey	3
United Arab Emirates	3
Yemen	3
South Asia	1
South Asia	2
Bangladesh	3
Bhutan	3
India	3
Andhra Pradesh	4
Arunachal Pradesh	4
Assam	4
Bihar	4
Chhattisgarh	4
Delhi	4
Goa	4
Gujarat	4
Haryana	4
Himachal Pradesh	4
Jammu and Kashmir	4
Jharkhand	4
Karnataka	4
Kerala	4
Madhya Pradesh	4
Maharashtra	4
Manipur	4
Meghalaya	4
Mizoram	4
Nagaland	4
Odisha	4
Punjab	4
Rajasthan	4
Sikkim	4
Tamil Nadu	4
Telangana	4
Tripura	4
Uttar Pradesh	4

Appendix Table 1. GBD location hierarchy with levels

Geography	Level
Uttarakhand	4
West Bengal	4
Union Territories other than Delhi	4
Nepal	3
Pakistan	3
Southeast Asia, East Asia, and Oceania	1
East Asia	2
China	3
North Korea	3
Taiwan	3
Oceania	2
American Samoa	3
Federated States of Micronesia	3
Fiji	3
Guam	3
Kiribati	3
Marshall Islands	3
Northern Mariana Islands	3
Papua New Guinea	3
Samoa	3
Solomon Islands	3
Tonga	3
Vanuatu	3
Southeast Asia	2
Cambodia	3
Indonesia	3
Laos	3
Malaysia	3
Maldives	3
Mauritius	3
Myanmar	3
Philippines	3
Sri Lanka	3
Seychelles	3
Thailand	3
Timor-Leste	3
Vietnam	3
Sub-Saharan Africa	1
Central Sub-Saharan Africa	2
Angola	3
Central African Republic	3
Congo	3
Democratic Republic of the Congo	3
Equatorial Guinea	3
Gabon	3

Appendix Table 1. GBD location hierarchy with levels

Geography	Level
Eastern Sub-Saharan Africa	2
Burundi	3
Comoros	3
Djibouti	3
Eritrea	3
Ethiopia	3
Kenya	3
Baringo	4
Bomet	4
Bungoma	4
Busia	4
Elgeyo-Marakwet	4
Embu	4
Garissa	4
HomaBay	4
Isiolo	4
Kajiado	4
Kakamega	4
Kericho	4
Kiambu	4
Kilifi	4
Kirinyaga	4
Kisii	4
Kisumu	4
Kitui	4
Kwale	4
Laikipia	4
Lamu	4
Machakos	4
Makueni	4
Mandera	4
Marsabit	4
Meru	4
Migori	4
Mombasa	4
Murang'a	4
Nairobi	4
Nakuru	4
Nandi	4
Narok	4
Nyamira	4
Nyandarua	4
Nyeri	4
Samburu	4
Siaya	4

Appendix Table 1. GBD location hierarchy with levels	
**Geography**	**Level**
TaitaTaveta	4
TanaRiver	4
TharakaNithi	4
TransNzoia	4
Turkana	4
UasinGishu	4
Vihiga	4
Wajir	4
WestPokot	4
Madagascar	3
Malawi	3
Mozambique	3
Rwanda	3
Somalia	3
South Sudan	3
Tanzania	3
Uganda	3
Zambia	3
Southern Sub-Saharan Africa	2
Botswana	3
Lesotho	3
Namibia	3
South Africa	3
Swaziland	3
Zimbabwe	3
Western Sub-Saharan Africa	2
Benin	3
Burkina Faso	3
Cameroon	3
Cape Verde	3
Chad	3
Cote d'Ivoire	3
The Gambia	3
Ghana	3
Guinea	3
Guinea-Bissau	3
Liberia	3
Mali	3
Mauritania	3
Niger	3
Nigeria	3
Sao Tome and Principe	3
Senegal	3
Sierra Leone	3
Togo	3

Appendix Table 3. Number of all-cause mortality data sources by type and location, 1950-2017						
**Geography**	**SBH**	**CBH**	**HH**	**SIBS**	**VR/SRS/DSP**	**DSS**
Armenia	4	4	0	0	38	0
Azerbaijan	4	1	2	0	39	0
Georgia	3	3	1	0	38	0
Kazakhstan	8	2	0	0	38	0
Kyrgyzstan	7	4	0	0	39	0
Mongolia	7	2	1	0	28	0
Tajikistan	7	1	2	0	37	0
Turkmenistan	3	1	1	0	38	0
Uzbekistan	4	2	0	0	37	0
Albania	5	2	0	0	34	0
Bosnia and Herzegovina	0	0	0	0	61	0
Bulgaria	4	0	0	0	67	0
Croatia	0	0	0	0	67	0
Czech Republic	0	0	0	0	67	0
Hungary	1	0	0	0	67	0
Macedonia	2	0	0	0	67	0
Montenegro	1	0	4	0	60	0
Poland	1	0	0	0	67	0
Romania	4	1	1	0	61	0
Serbia	2	0	4	0	60	0
Slovakia	0	0	0	0	67	0
Slovenia	0	0	0	0	67	0
Belarus	2	0	0	0	59	0
Estonia	1	0	0	0	57	0
Latvia	1	0	0	0	57	0
Lithuania	0	0	0	0	58	0
Moldova	4	2	0	0	40	0
Russian Federation	1	0	0	0	58	0
Ukraine	5	2	0	0	58	0
Australia	0	0	0	0	66	0
New Zealand	0	0	0	0	67	0
Brunei	1	0	0	0	57	0
Japan	0	0	0	0	66	0
Aichi	0	0	0	0	37	0
Akita	0	0	0	0	37	0
Aomori	0	0	0	0	37	0
Chiba	0	0	0	0	37	0
Ehime	0	0	0	0	37	0
Fukui	0	0	0	0	37	0
Fukuoka	0	0	0	0	37	0
Fukushima	0	0	0	0	37	0
Gifu	0	0	0	0	37	0
Gunma	0	0	0	0	37	0
Hiroshima	0	0	0	0	37	0
Hokkaidō	0	0	0	0	37	0
Hyōgo	0	0	0	0	36	0
Ibaraki	0	0	0	0	37	0
Ishikawa	0	0	0	0	37	0
Iwate	0	0	0	0	36	0
Kagawa	0	0	0	0	37	0
Kagoshima	0	0	0	0	37	0
Kanagawa	0	0	0	0	37	0
Kōchi	0	0	0	0	37	0

Appendix Table 3. Number of all-cause mortality data sources by type and location, 1950-2017						
**Geography**	**SBH**	**CBH**	**HH**	**SIBS**	**VR/SRS/DSP**	**DSS**
Kumamoto	0	0	0	0	37	0
Kyōto	0	0	0	0	37	0
Mie	0	0	0	0	37	0
Miyagi	0	0	0	0	36	0
Miyazaki	0	0	0	0	37	0
Nagano	0	0	0	0	37	0
Nagasaki	0	0	0	0	37	0
Nara	0	0	0	0	37	0
Niigata	0	0	0	0	37	0
Ōita	0	0	0	0	37	0
Okayama	0	0	0	0	37	0
Okinawa	0	0	0	0	37	0
Ōsaka	0	0	0	0	37	0
Saga	0	0	0	0	37	0
Saitama	0	0	0	0	37	0
Shiga	0	0	0	0	37	0
Shimane	0	0	0	0	37	0
Shizuoka	0	0	0	0	37	0
Tochigi	0	0	0	0	37	0
Tokushima	0	0	0	0	37	0
Tōkyō	0	0	0	0	37	0
Tottori	0	0	0	0	37	0
Toyama	0	0	0	0	37	0
Wakayama	0	0	0	0	37	0
Yamagata	0	0	0	0	37	0
Yamaguchi	0	0	0	0	37	0
Yamanashi	0	0	0	0	37	0
South Korea	6	1	0	0	40	0
Singapore	0	0	0	0	67	0
Canada	0	0	0	0	67	0
Greenland	0	0	0	0	60	0
United States	0	0	0	0	67	0
Alabama	0	0	0	0	57	0
Alaska	0	0	0	0	57	0
Arizona	0	0	0	0	57	0
Arkansas	0	0	0	0	57	0
California	0	0	0	0	57	0
Colorado	0	0	0	0	57	0
Connecticut	0	0	0	0	57	0
Delaware	0	0	0	0	57	0
District of Columbia	0	0	0	0	57	0
Florida	0	0	0	0	57	0
Georgia	0	0	0	0	57	0
Hawaii	0	0	0	0	57	0
Idaho	0	0	0	0	57	0
Illinois	0	0	0	0	57	0
Indiana	0	0	0	0	57	0
Iowa	0	0	0	0	57	0
Kansas	0	0	0	0	57	0
Kentucky	0	0	0	0	57	0
Louisiana	0	0	0	0	57	0
Maine	0	0	0	0	57	0
Maryland	0	0	0	0	57	0

Appendix Table 3. Number of all-cause mortality data sources by type and location, 1950-2017						
**Geography**	**SBH**	**CBH**	**HH**	**SIBS**	**VR/SRS/DSP**	**DSS**
Massachusetts	0	0	0	0	57	0
Michigan	0	0	0	0	57	0
Minnesota	0	0	0	0	57	0
Mississippi	0	0	0	0	57	0
Missouri	0	0	0	0	57	0
Montana	0	0	0	0	57	0
Nebraska	0	0	0	0	57	0
Nevada	0	0	0	0	57	0
New Hampshire	0	0	0	0	57	0
New Jersey	0	0	0	0	57	0
New Mexico	0	0	0	0	57	0
New York	0	0	0	0	57	0
North Carolina	0	0	0	0	57	0
North Dakota	0	0	0	0	57	0
Ohio	0	0	0	0	57	0
Oklahoma	0	0	0	0	57	0
Oregon	0	0	0	0	57	0
Pennsylvania	0	0	0	0	57	0
Rhode Island	0	0	0	0	57	0
South Carolina	0	0	0	0	57	0
South Dakota	0	0	0	0	57	0
Tennessee	0	0	0	0	57	0
Texas	0	0	0	0	57	0
Utah	0	0	0	0	57	0
Vermont	0	0	0	0	57	0
Virginia	0	0	0	0	57	0
Washington	0	0	0	0	57	0
West Virginia	0	0	0	0	57	0
Wisconsin	0	0	0	0	57	0
Wyoming	0	0	0	0	57	0
Argentina	3	0	0	0	61	0
Chile	4	0	0	0	66	0
Uruguay	7	0	0	0	65	0
Andorra	0	0	0	0	12	0
Austria	0	0	0	0	67	0
Belgium	2	0	0	0	67	0
Cyprus	3	0	0	0	43	0
Denmark	0	0	0	0	67	0
Finland	0	0	0	0	67	0
France	0	0	0	0	67	0
Germany	0	0	0	0	60	0
Greece	0	0	0	0	66	0
Iceland	0	0	0	0	68	0
Ireland	0	0	1	0	67	0
Israel	0	0	0	0	67	0
Italy	0	0	0	0	67	0
Luxembourg	0	0	0	0	66	0
Malta	0	0	0	0	67	0
Netherlands	0	0	0	0	67	0
Norway	0	0	0	0	67	0
Portugal	1	1	0	0	66	0
Spain	0	0	0	0	67	0
Sweden	0	0	0	0	67	0

Appendix Table 3. Number of all-cause mortality data sources by type and location, 1950-2017						
**Geography**	**SBH**	**CBH**	**HH**	**SIBS**	**VR/SRS/DSP**	**DSS**
Stockholm	0	0	0	0	34	0
Sweden except Stockholm	0	0	0	0	34	0
Switzerland	1	0	0	0	67	0
United Kingdom	0	0	0	0	66	0
England	0	0	0	0	36	0
East Midlands	0	0	0	0	36	0
Derby	0	0	0	0	36	0
Derbyshire	0	0	0	0	36	0
Leicester	0	0	0	0	36	0
Leicestershire	0	0	0	0	36	0
Lincolnshire	0	0	0	0	36	0
Northamptonshire	0	0	0	0	36	0
Nottingham	0	0	0	0	36	0
Nottinghamshire	0	0	0	0	36	0
Rutland	0	0	0	0	36	0
East of England	0	0	0	0	36	0
Bedford	0	0	0	0	36	0
Cambridgeshire	0	0	0	0	36	0
Central Bedfordshire	0	0	0	0	36	0
Essex	0	0	0	0	36	0
Hertfordshire	0	0	0	0	36	0
Luton	0	0	0	0	36	0
Norfolk	0	0	0	0	36	0
Peterborough	0	0	0	0	36	0
Southend-on-Sea	0	0	0	0	36	0
Suffolk	0	0	0	0	36	0
Thurrock	0	0	0	0	36	0
Greater London	0	0	0	0	36	0
Barking and Dagenham	0	0	0	0	36	0
Barnet	0	0	0	0	36	0
Bexley	0	0	0	0	36	0
Brent	0	0	0	0	36	0
Bromley	0	0	0	0	36	0
Camden	0	0	0	0	36	0
Croydon	0	0	0	0	36	0
Ealing	0	0	0	0	36	0
Enfield	0	0	0	0	36	0
Greenwich	0	0	0	0	36	0
Hackney	0	0	0	0	36	0
Hammersmith and Fulham	0	0	0	0	36	0
Haringey	0	0	0	0	36	0
Harrow	0	0	0	0	36	0
Havering	0	0	0	0	36	0
Hillingdon	0	0	0	0	36	0
Hounslow	0	0	0	0	36	0
Islington	0	0	0	0	36	0
Kensington and Chelsea	0	0	0	0	36	0
Kingston upon Thames	0	0	0	0	36	0
Lambeth	0	0	0	0	36	0
Lewisham	0	0	0	0	36	0
Merton	0	0	0	0	36	0
Newham	0	0	0	0	36	0
Redbridge	0	0	0	0	36	0

Appendix Table 3. Number of all-cause mortality data sources by type and location, 1950-2017						
**Geography**	**SBH**	**CBH**	**HH**	**SIBS**	**VR/SRS/DSP**	**DSS**
Richmond upon Thames	0	0	0	0	36	0
Southwark	0	0	0	0	36	0
Sutton	0	0	0	0	36	0
Tower Hamlets	0	0	0	0	36	0
Waltham Forest	0	0	0	0	36	0
Wandsworth	0	0	0	0	36	0
Westminster	0	0	0	0	36	0
North East England	0	0	0	0	36	0
County Durham	0	0	0	0	36	0
Darlington	0	0	0	0	36	0
Gateshead	0	0	0	0	36	0
Hartlepool	0	0	0	0	36	0
Middlesbrough	0	0	0	0	36	0
Newcastle upon Tyne	0	0	0	0	36	0
North Tyneside	0	0	0	0	36	0
Northumberland	0	0	0	0	36	0
Redcar and Cleveland	0	0	0	0	36	0
South Tyneside	0	0	0	0	36	0
Stockton-on-Tees	0	0	0	0	36	0
Sunderland	0	0	0	0	36	0
North West England	0	0	0	0	36	0
Blackburn with Darwen	0	0	0	0	36	0
Blackpool	0	0	0	0	36	0
Bolton	0	0	0	0	36	0
Bury	0	0	0	0	36	0
Cheshire East	0	0	0	0	36	0
Cheshire West and Chester	0	0	0	0	36	0
Cumbria	0	0	0	0	36	0
Halton	0	0	0	0	36	0
Knowsley	0	0	0	0	36	0
Lancashire	0	0	0	0	36	0
Liverpool	0	0	0	0	36	0
Manchester	0	0	0	0	36	0
Oldham	0	0	0	0	36	0
Rochdale	0	0	0	0	36	0
Salford	0	0	0	0	36	0
Sefton	0	0	0	0	36	0
St Helens	0	0	0	0	36	0
Stockport	0	0	0	0	36	0
Tameside	0	0	0	0	36	0
Trafford	0	0	0	0	36	0
Warrington	0	0	0	0	36	0
Wigan	0	0	0	0	36	0
Wirral	0	0	0	0	36	0
South East England	0	0	0	0	36	0
Bracknell Forest	0	0	0	0	36	0
Brighton and Hove	0	0	0	0	36	0
Buckinghamshire	0	0	0	0	36	0
East Sussex	0	0	0	0	36	0
Hampshire	0	0	0	0	36	0
Isle of Wight	0	0	0	0	36	0
Kent	0	0	0	0	36	0
Medway	0	0	0	0	36	0

Appendix Table 3. Number of all-cause mortality data sources by type and location, 1950-2017						
Geography	SBH	CBH	HH	SIBS	VR/SRS/DSP	DSS
Milton Keynes	0	0	0	0	36	0
Oxfordshire	0	0	0	0	36	0
Portsmouth	0	0	0	0	36	0
Reading	0	0	0	0	36	0
Slough	0	0	0	0	36	0
Southampton	0	0	0	0	36	0
Surrey	0	0	0	0	36	0
West Berkshire	0	0	0	0	36	0
West Sussex	0	0	0	0	36	0
Windsor and Maidenhead	0	0	0	0	36	0
Wokingham	0	0	0	0	36	0
South West England	0	0	0	0	36	0
Bath and North East Somerset	0	0	0	0	36	0
Bournemouth	0	0	0	0	36	0
Bristol, City of	0	0	0	0	36	0
Cornwall	0	0	0	0	36	0
Devon	0	0	0	0	36	0
Dorset	0	0	0	0	36	0
Gloucestershire	0	0	0	0	36	0
North Somerset	0	0	0	0	36	0
Plymouth	0	0	0	0	36	0
Poole	0	0	0	0	36	0
Somerset	0	0	0	0	36	0
South Gloucestershire	0	0	0	0	36	0
Swindon	0	0	0	0	36	0
Torbay	0	0	0	0	36	0
Wiltshire	0	0	0	0	36	0
West Midlands	0	0	0	0	36	0
Birmingham	0	0	0	0	36	0
Coventry	0	0	0	0	36	0
Dudley	0	0	0	0	36	0
Herefordshire, County of	0	0	0	0	36	0
Sandwell	0	0	0	0	36	0
Shropshire	0	0	0	0	36	0
Solihull	0	0	0	0	36	0
Staffordshire	0	0	0	0	36	0
Stoke-on-Trent	0	0	0	0	36	0
Telford and Wrekin	0	0	0	0	36	0
Walsall	0	0	0	0	36	0
Warwickshire	0	0	0	0	36	0
Wolverhampton	0	0	0	0	36	0
Worcestershire	0	0	0	0	36	0
Yorkshire and the Humber	0	0	0	0	36	0
Barnsley	0	0	0	0	36	0
Bradford	0	0	0	0	36	0
Calderdale	0	0	0	0	36	0
Doncaster	0	0	0	0	36	0
East Riding of Yorkshire	0	0	0	0	36	0
Kingston upon Hull, City of	0	0	0	0	36	0
Kirklees	0	0	0	0	36	0
Leeds	0	0	0	0	36	0
North East Lincolnshire	0	0	0	0	36	0
North Lincolnshire	0	0	0	0	36	0

Appendix Table 3. Number of all-cause mortality data sources by type and location, 1950-2017						
**Geography**	**SBH**	**CBH**	**HH**	**SIBS**	**VR/SRS/DSP**	**DSS**
North Yorkshire	0	0	0	0	36	0
Rotherham	0	0	0	0	36	0
Sheffield	0	0	0	0	36	0
Wakefield	0	0	0	0	36	0
York	0	0	0	0	36	0
Northern Ireland	0	0	0	0	67	0
Scotland	0	0	0	0	67	0
Wales	0	0	0	0	36	0
Bolivia	13	5	1	3	12	0
Ecuador	17	7	0	2	63	0
Peru	19	15	0	4	63	0
Antigua and Barbuda	0	0	0	0	57	0
The Bahamas	0	0	0	0	47	0
Barbados	0	0	0	0	60	0
Belize	6	3	0	0	67	0
Bermuda	1	0	0	0	66	0
Cuba	1	0	0	0	57	0
Dominica	0	0	0	0	64	0
Dominican Republic	15	12	0	2	66	0
Grenada	0	0	0	0	55	0
Guyana	7	4	0	0	45	0
Haiti	8	6	2	2	0	0
Jamaica	5	1	2	0	46	0
Puerto Rico	1	1	0	0	67	0
Saint Lucia	0	0	0	0	56	0
Saint Vincent and the Grenadines	0	0	0	0	50	0
Suriname	2	0	0	0	57	0
Trinidad and Tobago	4	2	0	0	62	0
Virgin Islands, U.S.	0	0	0	0	49	0
Colombia	13	8	0	1	64	0
Costa Rica	7	3	0	0	66	0
El Salvador	10	6	2	3	65	0
Guatemala	9	6	0	2	64	0
Honduras	9	5	2	1	44	0
Mexico	18	2	1	0	67	0
Aguascalientes	16	1	0	0	38	0
Baja California	16	1	0	0	38	0
Baja California Sur	16	1	0	0	38	0
Campeche	16	1	0	0	38	0
Chiapas	16	1	0	0	38	0
Chihuahua	16	1	0	0	38	0
Coahuila	16	1	0	0	38	0
Colima	16	1	0	0	38	0
Mexico City	16	1	0	0	38	0
Durango	16	1	0	0	38	0
Guanajuato	16	1	0	0	38	0
Guerrero	16	1	0	0	38	0
Hidalgo	15	1	0	0	38	0
Jalisco	16	1	0	0	38	0
México	16	1	0	0	38	0
Michoacán de Ocampo	16	1	0	0	38	0
Morelos	16	1	0	0	38	0
Nayarit	16	1	0	0	38	0

Appendix Table 3. Number of all-cause mortality data sources by type and location, 1950-2017						
Geography	SBH	CBH	HH	SIBS	VR/SRS/DSP	DSS
Nuevo León	16	1	0	0	38	0
Oaxaca	16	1	0	0	38	0
Puebla	16	1	0	0	38	0
Querétaro	16	1	0	0	38	0
Quintana Roo	16	1	0	0	38	0
San Luis Potosí	16	1	0	0	38	0
Sinaloa	16	1	0	0	38	0
Sonora	16	1	0	0	38	0
Tabasco	16	1	0	0	38	0
Tamaulipas	16	1	0	0	38	0
Tlaxcala	16	1	0	0	38	0
Veracruz de Ignacio de la Llave	16	1	0	0	38	0
Yucatán	16	1	0	0	38	0
Zacatecas	16	1	0	0	38	0
Nicaragua	12	5	0	1	52	0
Panama	8	1	0	0	66	0
Venezuela	4	1	1	0	64	0
Brazil	26	2	0	1	43	0
Acre	8	1	0	0	38	0
Alagoas	20	2	0	0	38	0
Amapá	8	1	0	0	38	0
Amazonas	9	1	0	0	38	0
Bahia	20	2	0	0	38	0
Ceará	20	2	0	0	38	0
Distrito Federal	20	1	0	0	38	0
Espírito Santo	20	1	0	0	38	0
Goiás	20	1	0	0	38	0
Maranhão	20	2	0	0	38	0
Mato Grosso	20	1	0	0	38	0
Mato Grosso do Sul	20	1	0	0	38	0
Minas Gerais	20	1	0	0	38	0
Pará	9	1	0	0	38	0
Paraíba	20	2	0	0	37	0
Paraná	20	1	0	0	38	0
Pernambuco	20	2	0	0	38	0
Piaui	20	2	0	0	38	0
Rio de Janeiro	20	1	0	0	38	0
Rio Grande do Norte	20	2	0	0	38	0
Rio Grande do Sul	20	1	0	0	38	0
Rondônia	8	1	0	0	38	0
Roraima	8	1	0	0	38	0
Santa Catarina	20	1	0	0	38	0
São Paulo	19	1	0	0	38	0
Sergipe	20	2	0	0	38	0
Tocantins	8	1	0	0	27	0
Paraguay	16	7	0	1	63	0
Afghanistan	6	1	1	0	0	0
Algeria	5	3	0	0	25	0
Bahrain	4	0	2	0	33	0
Egypt	13	11	2	0	64	0
Iran	5	1	9	0	32	0
Iraq	7	5	4	2	24	1
Jordan	11	6	0	1	22	0

Appendix Table 3. Number of all-cause mortality data sources by type and location, 1950-2017						
Geography	SBH	CBH	HH	SIBS	VR/SRS/DSP	DSS
Kuwait	3	0	1	0	53	0
Lebanon	4	2	1	1	8	0
Libya	4	1	0	0	15	0
Morocco	9	6	1	2	10	0
Palestine	6	4	3	0	15	0
Oman	4	0	3	0	12	0
Qatar	1	0	2	0	36	0
Saudi Arabia	2	0	4	0	14	0
Sudan	8	5	2	1	0	0
Syria	7	4	1	0	31	0
Tunisia	7	5	0	0	20	0
Turkey	14	5	1	0	36	0
United Arab Emirates	2	0	1	0	12	0
Yemen	7	5	2	0	0	0
Bangladesh	13	9	1	3	36	2
Bhutan	2	0	1	1	0	0
India	13	6	0	0	62	1
Andhra Pradesh	4	2	0	0	6	0
Arunachal Pradesh	10	4	0	0	3	0
Assam	15	5	0	0	23	0
Bihar	16	6	0	0	14	0
Chhattisgarh	12	4	0	0	15	0
Delhi	10	6	0	0	18	0
Goa	9	5	0	0	7	0
Gujarat	11	6	0	0	27	0
Haryana	12	6	0	0	22	0
Himachal Pradesh	12	6	0	0	27	0
Jammu and Kashmir	11	6	0	0	18	0
Jharkhand	12	4	0	0	13	0
Karnataka	11	6	0	0	29	0
Kerala	11	5	0	0	29	0
Madhya Pradesh	16	6	0	0	20	0
Maharashtra	11	6	0	0	25	0
Manipur	11	5	0	0	5	0
Meghalaya	11	5	0	0	6	0
Mizoram	12	5	0	0	6	0
Nagaland	9	4	0	0	5	0
Odisha	16	5	0	0	29	0
Punjab	11	6	0	0	29	0
Rajasthan	15	6	0	0	28	0
Sikkim	10	3	0	0	5	0
Tamil Nadu	11	6	0	0	29	0
Telangana	4	2	0	0	5	0
Tripura	11	5	0	0	3	0
Uttar Pradesh	16	6	0	0	17	0
Uttarakhand	11	4	0	0	3	0
West Bengal	12	6	0	0	23	0
Union Territories other than Delhi	4	1	0	0	5	0
Nepal	12	7	2	2	0	0
Pakistan	13	8	1	0	25	0
China	6	0	29	0	23	0
North Korea	0	0	1	0	0	0
Taiwan	0	0	0	0	62	0

Appendix Table 3. Number of all-cause mortality data sources by type and location, 1950-2017						
**Geography**	**SBH**	**CBH**	**HH**	**SIBS**	**VR/SRS/DSP**	**DSS**
American Samoa	1	0	0	0	52	0
Federated States of Micronesia	3	0	0	0	1	0
Fiji	7	1	0	0	54	0
Guam	0	0	0	0	65	0
Kiribati	7	0	1	0	12	0
Marshall Islands	2	0	1	0	14	0
Northern Mariana Islands	0	0	0	0	19	0
Papua New Guinea	6	1	2	0	2	0
Samoa	5	0	4	0	21	0
Solomon Islands	4	0	3	0	0	0
Tonga	3	0	2	0	18	0
Vanuatu	4	0	1	0	0	0
Cambodia	11	5	0	4	0	0
Indonesia	46	15	1	5	0	0
Laos	3	1	1	1	0	0
Malaysia	3	1	0	0	53	0
Maldives	6	1	0	0	38	0
Mauritius	0	0	0	0	67	0
Myanmar	6	1	1	1	1	0
Philippines	11	6	0	2	64	0
Sri Lanka	5	2	4	0	60	0
Seychelles	2	0	1	0	62	0
Thailand	10	2	0	0	66	0
Timor-Leste	8	6	0	4	0	0
Vietnam	16	3	2	0	0	2
Angola	4	2	0	1	0	0
Central African Republic	5	1	1	1	0	0
Congo	4	3	2	2	0	0
Democratic Republic of the Congo	5	2	0	2	0	0
Equatorial Guinea	1	0	1	0	0	0
Gabon	2	2	0	2	0	0
Burundi	9	3	1	1	0	0
Comoros	4	2	1	1	0	0
Djibouti	3	1	0	0	0	0
Eritrea	3	2	2	1	0	0
Ethiopia	8	4	1	4	0	5
Kenya	19	7	1	4	0	4
Baringo	14	5	0	1	0	0
Bomet	9	3	0	1	0	0
Bungoma	15	7	0	1	0	0
Busia	14	6	0	0	0	0
Elgeyo-Marakwet	14	6	0	1	0	0
Embu	16	7	0	0	0	0
Garissa	12	3	0	0	0	0
HomaBay	11	4	0	1	0	0
Isiolo	13	4	0	0	0	0
Kajiado	14	5	0	1	0	0
Kakamega	14	7	0	0	0	0
Kericho	15	6	0	0	0	0
Kiambu	15	6	0	1	0	0
Kilifi	15	6	0	1	0	0
Kirinyaga	14	6	0	0	0	0
Kisii	14	7	0	1	0	0

Appendix Table 3. Number of all-cause mortality data sources by type and location, 1950-2017						
Geography	SBH	CBH	HH	SIBS	VR/SRS/DSP	DSS
Kisumu	14	7	0	1	0	0
Kitui	17	7	0	1	0	0
Kwale	14	6	0	1	0	0
Laikipia	13	5	0	0	0	0
Lamu	12	4	0	0	0	0
Machakos	17	7	0	1	0	0
Makueni	11	4	0	1	0	0
Mandera	12	3	0	0	0	0
Marsabit	11	4	0	0	0	0
Meru	14	7	0	1	0	0
Migori	9	4	0	1	0	0
Mombasa	14	7	0	0	0	0
Murang’a	14	6	0	1	0	0
Nairobi	13	6	0	1	0	0
Nakuru	14	6	0	1	0	0
Nandi	15	6	0	1	0	0
Narok	14	6	0	1	0	0
Nyamira	10	4	0	1	0	0
Nyandarua	13	6	0	1	0	0
Nyeri	13	6	0	1	0	0
Samburu	10	3	0	0	0	0
Siaya	15	7	0	1	0	0
TaitaTaveta	14	6	0	1	0	0
TanaRiver	12	3	0	1	0	0
TharakaNithi	10	4	0	1	0	0
TransNzoia	14	6	0	0	0	0
Turkana	11	4	0	1	0	0
UasinGishu	14	6	0	1	0	0
Vihiga	9	3	0	1	0	0
Wajir	12	3	0	0	0	0
WestPokot	14	5	0	1	0	0
Madagascar	9	4	1	4	14	0
Malawi	19	8	6	6	0	1
Mozambique	8	4	1	3	0	1
Rwanda	13	7	1	4	0	0
Somalia	2	1	0	0	0	0
South Sudan	2	1	0	0	0	0
Tanzania	13	7	2	4	0	3
Uganda	15	7	1	5	0	1
Zambia	11	5	8	5	0	0
Botswana	6	2	3	0	6	0
Lesotho	8	4	0	3	1	0
Namibia	6	4	2	4	0	0
South Africa	6	1	4	1	30	3
Swaziland	4	3	2	1	0	0
Zimbabwe	9	8	2	6	2	0
Benin	7	6	0	2	0	0
Burkina Faso	10	4	4	3	0	2
Cameroon	8	6	2	3	0	0
Cape Verde	3	1	1	0	24	0
Chad	5	3	0	3	0	0
Cote d'Ivoire	6	9	1	3	0	1
The Gambia	6	1	1	0	0	1

Appendix Table 3. Number of all-cause mortality data sources by type and location, 1950-2017						
**Geography**	**SBH**	**CBH**	**HH**	**SIBS**	**VR/SRS/DSP**	**DSS**
Ghana	18	10	1	1	0	1
Guinea	5	3	4	3	0	0
Guinea-Bissau	4	1	0	1	0	0
Liberia	7	5	1	2	0	0
Mali	10	6	4	4	0	0
Mauritania	7	5	1	2	0	0
Niger	6	4	0	3	0	0
Nigeria	16	6	0	0	0	0
Sao Tome and Principe	6	2	1	2	20	0
Senegal	12	11	2	3	0	3
Sierra Leone	6	2	1	2	5	0
Togo	7	3	0	2	0	0

Note: CBH: Complete birth history, SBH: Summary birth history, HH: Household survey, SIBS: Sibling History, VR: Vital Registration, SRS: Sample Registration System, DSP: Disease Surveillance Points, DSS: Demographic Surveillance System

Appendix Table 4. Number of all-cause mortality data sources by type and year, 1950-2017						
Year	SBH	CBH	HH	SIBS	VR/SRS/DSP	DSS
1950	0	0	0	0	78	0
1951	0	0	0	0	77	0
1952	0	0	0	0	83	0
1953	0	0	1	0	82	0
1954	1	0	0	0	84	0
1955	0	1	0	0	91	0
1956	2	0	0	0	91	0
1957	0	1	0	0	91	0
1958	1	0	1	0	99	0
1959	0	1	0	0	147	0
1960	23	0	2	0	147	0
1961	3	1	1	0	147	0
1962	0	0	0	0	147	0
1963	1	1	0	0	148	0
1964	1	2	0	0	150	0
1965	1	1	0	0	148	0
1966	4	4	0	0	146	0
1967	1	2	1	0	144	0
1968	1	7	0	0	149	0
1969	42	3	0	0	175	0
1970	39	10	2	0	164	0
1971	39	3	3	0	165	0
1972	3	13	0	0	165	0
1973	8	4	1	0	163	1
1974	7	22	3	0	167	0
1975	13	13	0	0	167	0
1976	14	24	2	0	170	0
1977	8	11	1	0	173	0
1978	10	29	0	0	180	0
1979	48	10	1	0	270	0
1980	112	28	1	0	299	0
1981	17	6	2	0	465	0
1982	39	26	8	0	467	0
1983	5	7	1	0	466	0
1984	7	28	3	0	471	0
1985	44	11	1	0	453	0
1986	33	43	1	0	468	0
1987	38	42	7	0	473	0
1988	48	57	4	1	450	0
1989	54	16	33	1	545	0
1990	149	40	2	1	552	4
1991	113	44	7	7	552	4
1992	148	164	20	3	546	4
1993	169	34	3	5	545	6
1994	134	43	4	35	550	7

Appendix Table 4. Number of all-cause mortality data sources by type and year, 1950-2017						
Year	SBH	CBH	HH	SIBS	VR/SRS/DSP	DSS
1995	105	51	8	7	591	7
1996	120	46	39	4	627	8
1997	83	73	37	49	636	9
1998	122	100	49	3	639	9
1999	295	55	63	11	648	10
2000	288	86	104	15	653	11
2001	177	37	39	2	682	11
2002	69	108	74	36	685	11
2003	242	42	64	7	655	14
2004	78	109	89	10	706	16
2005	321	137	108	7	707	18
2006	265	64	86	5	709	21
2007	179	62	69	38	709	21
2008	203	77	69	4	710	23
2009	259	19	70	10	763	27
2010	263	20	107	6	759	31
2011	262	66	115	7	695	31
2012	160	36	97	39	703	30
2013	147	38	65	43	740	25
2014	112	88	66	13	711	22
2015	142	49	34	3	723	2
2016	95	33	38	11	497	0
2017	2	0	3	0	1	0

Note: CBH: Complete birth history, SBH: Summary birth history, HH: Household survey, SIBS: Sibling History, VR: Vital Registration, SRS: Sample Registration System, DSP: Disease Surveillance Points, DSS: Demographic Surveillance System

Appendix Table 5. Under-5 mortality reference sources by source date and location, 1950-2017			
**Location**	**Year range of data**	**Reference source**	**Data type**
Armenia	1958-1958	Vital Registration	VR/SRS/DSP
Armenia	1969-1969	Vital Registration	VR/SRS/DSP
Armenia	1975-2015	Standard demographic and health survey	CBH
Armenia	1976-2000	Census	SBH
Armenia	1976-2015	Standard demographic and health survey	SBH
Armenia	1978-1978	Vital Registration	VR/SRS/DSP
Armenia	1981-1987	Vital Registration	VR/SRS/DSP
Armenia	1989-2016	Vital Registration	VR/SRS/DSP
Armenia	2002-2002	Census	SBH
Armenia	2005-2005	Census	SBH
Armenia	2008-2008	Census	SBH
Azerbaijan	1958-1958	Vital Registration	VR/SRS/DSP
Azerbaijan	1969-1969	Vital Registration	VR/SRS/DSP
Azerbaijan	1976-2000	Multiple Indicator Cluster Survey	SBH
Azerbaijan	1978-1978	Vital Registration	VR/SRS/DSP
Azerbaijan	1981-2006	Standard demographic and health survey	CBH
Azerbaijan	1981-2016	Vital Registration	VR/SRS/DSP
Azerbaijan	1982-2006	Standard demographic and health survey	SBH
Azerbaijan	1988-1988	Reproductive Health Survey	HH
Azerbaijan	1990-1990	Census	SBH
Azerbaijan	1991-1991	Other demographic and health survey	SBH
Azerbaijan	1993-1993	Census	SBH
Azerbaijan	1993-1993	Reproductive Health Survey	HH
Azerbaijan	1994-1994	Other demographic and health survey	SBH
Azerbaijan	1997-1997	Census	SBH
Azerbaijan	1998-1998	Other demographic and health survey	SBH
Azerbaijan	1998-1998	Reproductive Health Survey	HH
Azerbaijan	1999-1999	Other demographic and health survey	HH
Azerbaijan	2000-2000	Census	SBH
Azerbaijan	2002-2002	Other demographic and health survey	SBH
Azerbaijan	2003-2003	Census	SBH
Azerbaijan	2004-2004	Other demographic and health survey	HH
Azerbaijan	2005-2005	Census	SBH
Azerbaijan	2005-2005	Other demographic and health survey	SBH
Azerbaijan	2007-2007	Other demographic and health survey	SBH
Azerbaijan	2009-2009	Other demographic and health survey	HH
Georgia	1958-1958	Vital Registration	VR/SRS/DSP
Georgia	1969-1969	Vital Registration	VR/SRS/DSP
Georgia	1975-2004	Reproductive Health Survey	SBH
Georgia	1978-1978	Vital Registration	VR/SRS/DSP
Georgia	1978-2011	Reproductive Health Survey	CBH
Georgia	1981-1992	Vital Registration	VR/SRS/DSP
Georgia	1981-2005	Multiple Indicator Cluster Survey	SBH
Georgia	1994-2016	Vital Registration	VR/SRS/DSP
Georgia	2007-2007	Reproductive Health Survey	HH
Kazakhstan	1958-1958	Vital Registration	VR/SRS/DSP
Kazakhstan	1969-1969	Vital Registration	VR/SRS/DSP
Kazakhstan	1969-1969	Census	SBH

Appendix Table 5. Under-5 mortality reference sources by source date and location, 1950-2017			
**Location**	**Year range of data**	**Reference source**	**Data type**
Kazakhstan	1971-1995	Living Standards Measurement Study	SBH
Kazakhstan	1971-1999	Standard demographic and health survey	SBH
Kazakhstan	1972-1995	Standard demographic and health survey	CBH
Kazakhstan	1973-1973	Census	SBH
Kazakhstan	1976-1976	Census	SBH
Kazakhstan	1978-1978	Vital Registration	VR/SRS/DSP
Kazakhstan	1979-1980	Census	SBH
Kazakhstan	1981-2010	Multiple Indicator Cluster Survey	SBH
Kazakhstan	1981-2015	Vital Registration	VR/SRS/DSP
Kazakhstan	1982-1983	Census	SBH
Kazakhstan	1986-1986	Census	SBH
Kazakhstan	1988-1988	Census	SBH
Kazakhstan	1990-1990	Census	SBH
Kazakhstan	1992-1993	Census	SBH
Kazakhstan	1995-1996	Census	SBH
Kazakhstan	1998-1999	Standard demographic and health survey	CBH
Kazakhstan	2000-2000	Census	SBH
Kazakhstan	2003-2003	Census	SBH
Kazakhstan	2006-2006	Census	SBH
Kyrgyzstan	1958-1958	Vital Registration	VR/SRS/DSP
Kyrgyzstan	1968-1992	Living Standards Measurement Study	SBH
Kyrgyzstan	1969-1969	Vital Registration	VR/SRS/DSP
Kyrgyzstan	1973-2012	Standard demographic and health survey	SBH
Kyrgyzstan	1974-2008	Census	SBH
Kyrgyzstan	1974-2012	Standard demographic and health survey	CBH
Kyrgyzstan	1977-1998	Living Standards Measurement Study	CBH
Kyrgyzstan	1978-1978	Vital Registration	VR/SRS/DSP
Kyrgyzstan	1981-2013	Multiple Indicator Cluster Survey	SBH
Kyrgyzstan	1981-2016	Vital Registration	VR/SRS/DSP
Kyrgyzstan	1988-2013	Multiple Indicator Cluster Survey	CBH
Mongolia	1964-1988	Census	SBH
Mongolia	1965-1965	Vital Registration	VR/SRS/DSP
Mongolia	1970-1970	Vital Registration	VR/SRS/DSP
Mongolia	1974-1999	Reproductive Health Survey	CBH
Mongolia	1975-1975	Vital Registration	VR/SRS/DSP
Mongolia	1975-2013	Multiple Indicator Cluster Survey	SBH
Mongolia	1979-2008	Reproductive Health Survey	SBH
Mongolia	1980-1980	Vital Registration	VR/SRS/DSP
Mongolia	1985-1985	Vital Registration	VR/SRS/DSP
Mongolia	1986-1986	Reproductive Health Survey	HH
Mongolia	1986-2013	Multiple Indicator Cluster Survey	CBH
Mongolia	1987-1990	Vital Registration	VR/SRS/DSP
Mongolia	1991-1991	Reproductive Health Survey	HH
Mongolia	1994-2010	Vital Registration	VR/SRS/DSP
Mongolia	1996-1996	Reproductive Health Survey	HH
Mongolia	2013-2014	Vital Registration	VR/SRS/DSP
Tajikistan	1958-1958	Vital Registration	VR/SRS/DSP
Tajikistan	1969-1969	Vital Registration	VR/SRS/DSP

Appendix Table 5. Under-5 mortality reference sources by source date and location, 1950-2017			
**Location**	**Year range of data**	**Reference source**	**Data type**
Tajikistan	1971-1971	Census	SBH
Tajikistan	1974-2006	Living Standards Measurement Study	SBH
Tajikistan	1975-1975	Census	SBH
Tajikistan	1976-2005	Multiple Indicator Cluster Survey	SBH
Tajikistan	1978-1978	Vital Registration	VR/SRS/DSP
Tajikistan	1978-1978	Census	SBH
Tajikistan	1981-1981	Census	SBH
Tajikistan	1981-1992	Vital Registration	VR/SRS/DSP
Tajikistan	1983-1983	Census	SBH
Tajikistan	1986-1986	Census	SBH
Tajikistan	1987-2012	Standard demographic and health survey	CBH
Tajikistan	1988-2012	Standard demographic and health survey	SBH
Tajikistan	1989-1989	Other survey	HH
Tajikistan	1994-2014	Vital Registration	VR/SRS/DSP
Tajikistan	1995-1995	Other survey	HH
Tajikistan	2000-2000	Other survey	HH
Tajikistan	2005-2005	Standard demographic and health survey	HH
Tajikistan	2005-2009	Other survey	HH
Tajikistan	2010-2010	Standard demographic and health survey	HH
Tajikistan	2015-2015	Standard demographic and health survey	HH
Tajikistan	2016-2016	Vital Registration	VR/SRS/DSP
Turkmenistan	1958-1958	Vital Registration	VR/SRS/DSP
Turkmenistan	1969-1969	Vital Registration	VR/SRS/DSP
Turkmenistan	1978-1978	Vital Registration	VR/SRS/DSP
Turkmenistan	1978-1978	Standard demographic and health survey	HH
Turkmenistan	1981-1981	Standard demographic and health survey	SBH
Turkmenistan	1981-1999	Vital Registration	VR/SRS/DSP
Turkmenistan	1981-2015	Multiple Indicator Cluster Survey	SBH
Turkmenistan	1983-1983	Standard demographic and health survey	HH
Turkmenistan	1985-1985	Standard demographic and health survey	SBH
Turkmenistan	1987-1988	Standard demographic and health survey	HH
Turkmenistan	1988-1988	Standard demographic and health survey	SBH
Turkmenistan	1991-1991	Standard demographic and health survey	SBH
Turkmenistan	1992-1993	Standard demographic and health survey	HH
Turkmenistan	1992-2015	Multiple Indicator Cluster Survey	CBH
Turkmenistan	1994-1994	Standard demographic and health survey	SBH
Turkmenistan	1997-1997	Standard demographic and health survey	SBH
Turkmenistan	1997-1998	Standard demographic and health survey	HH
Turkmenistan	2012-2013	Vital Registration	VR/SRS/DSP
Turkmenistan	2015-2015	Vital Registration	VR/SRS/DSP
Uzbekistan	1958-1958	Vital Registration	VR/SRS/DSP
Uzbekistan	1969-1969	Vital Registration	VR/SRS/DSP
Uzbekistan	1972-1996	Standard demographic and health survey	SBH
Uzbekistan	1973-1996	Standard demographic and health survey	CBH
Uzbekistan	1976-2005	Multiple Indicator Cluster Survey	SBH
Uzbekistan	1977-2002	Other demographic and health survey	CBH
Uzbekistan	1978-1978	Vital Registration	VR/SRS/DSP
Uzbekistan	1978-2002	Other demographic and health survey	SBH

Appendix Table 5. Under-5 mortality reference sources by source date and location, 1950-2017			
**Location**	**Year range of data**	**Reference source**	**Data type**
Uzbekistan	1981-2014	Vital Registration	VR/SRS/DSP
Albania	1950-1950	Vital Registration	VR/SRS/DSP
Albania	1955-1955	Vital Registration	VR/SRS/DSP
Albania	1957-1964	Vital Registration	VR/SRS/DSP
Albania	1978-2003	Multiple Indicator Cluster Survey	SBH
Albania	1983-2000	Reproductive Health Survey	SBH
Albania	1983-2002	Reproductive Health Survey	CBH
Albania	1983-2008	Standard demographic and health survey	CBH
Albania	1986-2006	Standard demographic and health survey	SBH
Albania	1987-2010	Vital Registration	VR/SRS/DSP
Albania	1991-1991	Census	SBH
Albania	1995-1995	Census	SBH
Albania	1999-1999	Census	SBH
Albania	2002-2002	Census	SBH
Albania	2006-2006	Census	SBH
Albania	2008-2008	Census	SBH
Bosnia and Herzegovina	1950-1991	Vital Registration	VR/SRS/DSP
Bosnia and Herzegovina	1998-2016	Vital Registration	VR/SRS/DSP
Bulgaria	1950-2016	Vital Registration	VR/SRS/DSP
Bulgaria	1951-1951	Census	SBH
Bulgaria	1954-1955	Census	SBH
Bulgaria	1957-1957	Census	SBH
Bulgaria	1959-1959	Census	SBH
Bulgaria	1961-1961	Census	SBH
Bulgaria	1965-1965	Census	SBH
Bulgaria	1969-1969	Census	SBH
Bulgaria	1971-1971	Census	SBH
Bulgaria	1974-1974	Living Standards Measurement Study	SBH
Bulgaria	1976-1976	Living Standards Measurement Study	SBH
Bulgaria	1978-1979	Living Standards Measurement Study	SBH
Bulgaria	1982-1983	Living Standards Measurement Study	SBH
Bulgaria	1985-1985	Living Standards Measurement Study	SBH
Bulgaria	1987-1987	Living Standards Measurement Study	SBH
Bulgaria	1989-1989	Living Standards Measurement Study	SBH
Bulgaria	1991-1991	Living Standards Measurement Study	SBH
Bulgaria	1993-1993	Living Standards Measurement Study	SBH
Croatia	1950-2013	Vital Registration	VR/SRS/DSP
Croatia	2015-2016	Vital Registration	VR/SRS/DSP
Czech Republic	1950-2016	Vital Registration	VR/SRS/DSP
Hungary	1950-2016	Vital Registration	VR/SRS/DSP
Hungary	1951-1951	Census	SBH
Hungary	1954-1954	Census	SBH
Hungary	1956-1956	Census	SBH
Macedonia	1950-2016	Vital Registration	VR/SRS/DSP
Macedonia	1983-2003	Multiple Indicator Cluster Survey	SBH
Macedonia	2005-2005	Multiple Indicator Cluster Survey	SBH
Macedonia	2008-2008	Multiple Indicator Cluster Survey	SBH
Montenegro	1950-1959	Vital Registration	VR/SRS/DSP

Appendix Table 5. Under-5 mortality reference sources by source date and location, 1950-2017			
Location	Year range of data	Reference source	Data type
Montenegro	1961-1969	Vital Registration	VR/SRS/DSP
Montenegro	1971-1979	Vital Registration	VR/SRS/DSP
Montenegro	1981-1989	Vital Registration	VR/SRS/DSP
Montenegro	1989-1996	Multiple Indicator Cluster Survey	SBH
Montenegro	1991-1991	Vital Registration	VR/SRS/DSP
Montenegro	1995-1996	Vital Registration	VR/SRS/DSP
Montenegro	2000-2010	Multiple Indicator Cluster Survey	SBH
Montenegro	2000-2016	Vital Registration	VR/SRS/DSP
Poland	1950-1950	Census	SBH
Poland	1950-2016	Vital Registration	VR/SRS/DSP
Poland	1953-1953	Census	SBH
Poland	1957-1957	Census	SBH
Poland	1961-1961	Census	SBH
Poland	1964-1964	Census	SBH
Poland	1966-1966	Census	SBH
Romania	1950-1995	Living Standards Measurement Study	CBH
Romania	1953-1953	Census	SBH
Romania	1956-1956	Census	SBH
Romania	1956-2016	Vital Registration	VR/SRS/DSP
Romania	1960-1960	Census	SBH
Romania	1962-1962	Census	SBH
Romania	1964-1964	Census	SBH
Romania	1968-1968	Census	SBH
Romania	1971-1971	Census	SBH
Romania	1972-1972	Living Standards Measurement Study	SBH
Romania	1973-1973	Census	SBH
Romania	1974-1974	Living Standards Measurement Study	SBH
Romania	1975-1975	Census	SBH
Romania	1978-1978	Living Standards Measurement Study	SBH
Romania	1979-1979	Census	SBH
Romania	1981-1981	Living Standards Measurement Study	SBH
Romania	1983-1983	Census	SBH
Romania	1985-1985	Living Standards Measurement Study	SBH
Romania	1986-1986	Census	SBH
Romania	1987-1987	Reproductive Health Survey	HH
Romania	1988-1988	Census	SBH
Romania	1988-1988	Living Standards Measurement Study	SBH
Romania	1992-1992	Reproductive Health Survey	HH
Romania	1997-1997	Reproductive Health Survey	HH
Serbia	1950-1952	Vital Registration	VR/SRS/DSP
Serbia	1953-1953	Census	HH
Serbia	1954-1960	Vital Registration	VR/SRS/DSP
Serbia	1961-1961	Census	HH
Serbia	1962-1970	Vital Registration	VR/SRS/DSP
Serbia	1971-1971	Census	HH
Serbia	1972-1990	Vital Registration	VR/SRS/DSP
Serbia	1984-2011	Multiple Indicator Cluster Survey	SBH
Serbia	1991-1991	Census	HH

Appendix Table 5. Under-5 mortality reference sources by source date and location, 1950-2017			
**Location**	**Year range of data**	**Reference source**	**Data type**
Serbia	1995-1997	Vital Registration	VR/SRS/DSP
Serbia	2008-2016	Vital Registration	VR/SRS/DSP
Slovakia	1950-2016	Vital Registration	VR/SRS/DSP
Slovenia	1950-2016	Vital Registration	VR/SRS/DSP
Belarus	1958-2016	Vital Registration	VR/SRS/DSP
Belarus	1969-1969	Census	SBH
Belarus	1972-1972	Census	SBH
Belarus	1976-1976	Census	SBH
Belarus	1980-1980	Census	SBH
Belarus	1981-2003	Multiple Indicator Cluster Survey	SBH
Belarus	1983-1983	Census	SBH
Belarus	1986-1986	Census	SBH
Estonia	1959-2015	Vital Registration	VR/SRS/DSP
Estonia	1968-1968	Census	SBH
Estonia	1972-1972	Census	SBH
Estonia	1976-1976	Census	SBH
Estonia	1980-1980	Census	SBH
Estonia	1983-1983	Census	SBH
Estonia	1985-1985	Census	SBH
Latvia	1960-2016	Vital Registration	VR/SRS/DSP
Latvia	1968-1968	Census	SBH
Latvia	1972-1972	Census	SBH
Latvia	1976-1976	Census	SBH
Latvia	1979-1979	Census	SBH
Latvia	1983-1983	Census	SBH
Latvia	1985-1985	Census	SBH
Lithuania	1959-2016	Vital Registration	VR/SRS/DSP
Moldova	1958-1958	Vital Registration	VR/SRS/DSP
Moldova	1969-1969	Vital Registration	VR/SRS/DSP
Moldova	1969-1969	Census	SBH
Moldova	1972-1972	Census	SBH
Moldova	1976-1976	Census	SBH
Moldova	1978-1978	Vital Registration	VR/SRS/DSP
Moldova	1979-1995	Reproductive Health Survey	SBH
Moldova	1980-1980	Census	SBH
Moldova	1980-2005	Standard demographic and health survey	CBH
Moldova	1980-2016	Vital Registration	VR/SRS/DSP
Moldova	1981-2003	Standard demographic and health survey	SBH
Moldova	1983-1983	Census	SBH
Moldova	1985-1985	Census	SBH
Moldova	1986-2011	Multiple Indicator Cluster Survey	CBH
Moldova	1988-2009	Multiple Indicator Cluster Survey	SBH
Russian Federation	1959-2016	Vital Registration	VR/SRS/DSP
Russian Federation	1969-1969	Census	SBH
Russian Federation	1972-1972	Census	SBH
Russian Federation	1976-1976	Census	SBH
Russian Federation	1979-1979	Census	SBH
Russian Federation	1983-1983	Census	SBH

Appendix Table 5. Under-5 mortality reference sources by source date and location, 1950-2017

Location	Year range of data	Reference source	Data type
Russian Federation	1985-1985	Census	SBH
Ukraine	1959-2016	Vital Registration	VR/SRS/DSP
Ukraine	1969-1969	Census	SBH
Ukraine	1972-1972	Census	SBH
Ukraine	1976-1976	Census	SBH
Ukraine	1978-1999	Reproductive Health Survey	CBH
Ukraine	1979-1980	Census	SBH
Ukraine	1982-2007	Standard demographic and health survey	CBH
Ukraine	1983-1985	Census	SBH
Ukraine	1983-2005	Standard demographic and health survey	SBH
Ukraine	1984-2010	Multiple Indicator Cluster Survey	SBH
Ukraine	1988-1988	Census	SBH
Ukraine	1991-1991	Census	SBH
Ukraine	1995-1995	Census	SBH
Ukraine	1997-1997	Census	SBH
Australia	1950-2015	Vital Registration	VR/SRS/DSP
New Zealand	1950-2016	Vital Registration	VR/SRS/DSP
Brunei	1950-1959	Vital Registration	VR/SRS/DSP
Brunei	1951-1951	Census	SBH
Brunei	1954-1955	Census	SBH
Brunei	1964-1978	Vital Registration	VR/SRS/DSP
Brunei	1982-1992	Vital Registration	VR/SRS/DSP
Brunei	1995-2015	Vital Registration	VR/SRS/DSP
Japan	1950-2015	Vital Registration	VR/SRS/DSP
Aichi	1979-2015	Vital Registration	VR/SRS/DSP
Akita	1979-2015	Vital Registration	VR/SRS/DSP
Aomori	1979-2015	Vital Registration	VR/SRS/DSP
Chiba	1979-2015	Vital Registration	VR/SRS/DSP
Ehime	1979-2015	Vital Registration	VR/SRS/DSP
Fukui	1979-2015	Vital Registration	VR/SRS/DSP
Fukuoka	1979-2015	Vital Registration	VR/SRS/DSP
Fukushima	1979-2015	Vital Registration	VR/SRS/DSP
Gifu	1979-2015	Vital Registration	VR/SRS/DSP
Gunma	1979-2015	Vital Registration	VR/SRS/DSP
Hiroshima	1979-2015	Vital Registration	VR/SRS/DSP
Hokkaidō	1979-2015	Vital Registration	VR/SRS/DSP
Hyōgo	1979-1994	Vital Registration	VR/SRS/DSP
Hyōgo	1996-2015	Vital Registration	VR/SRS/DSP
Ibaraki	1979-2015	Vital Registration	VR/SRS/DSP
Ishikawa	1979-2015	Vital Registration	VR/SRS/DSP
Iwate	1979-2010	Vital Registration	VR/SRS/DSP
Iwate	2012-2015	Vital Registration	VR/SRS/DSP
Kagawa	1979-2015	Vital Registration	VR/SRS/DSP
Kagoshima	1979-2015	Vital Registration	VR/SRS/DSP
Kanagawa	1979-2015	Vital Registration	VR/SRS/DSP
Kōchi	1979-2015	Vital Registration	VR/SRS/DSP
Kumamoto	1979-2015	Vital Registration	VR/SRS/DSP
Kyōto	1979-2015	Vital Registration	VR/SRS/DSP

Appendix Table 5. Under-5 mortality reference sources by source date and location, 1950-2017

Location	Year range of data	Reference source	Data type
Mie	1979-2015	Vital Registration	VR/SRS/DSP
Miyagi	1979-2010	Vital Registration	VR/SRS/DSP
Miyagi	2012-2015	Vital Registration	VR/SRS/DSP
Miyazaki	1979-2015	Vital Registration	VR/SRS/DSP
Nagano	1979-2015	Vital Registration	VR/SRS/DSP
Nagasaki	1979-2015	Vital Registration	VR/SRS/DSP
Nara	1979-2015	Vital Registration	VR/SRS/DSP
Niigata	1979-2015	Vital Registration	VR/SRS/DSP
Ōita	1979-2015	Vital Registration	VR/SRS/DSP
Okayama	1979-2015	Vital Registration	VR/SRS/DSP
Okinawa	1979-2015	Vital Registration	VR/SRS/DSP
Ōsaka	1979-2015	Vital Registration	VR/SRS/DSP
Saga	1979-2015	Vital Registration	VR/SRS/DSP
Saitama	1979-2015	Vital Registration	VR/SRS/DSP
Shiga	1979-2015	Vital Registration	VR/SRS/DSP
Shimane	1979-2015	Vital Registration	VR/SRS/DSP
Shizuoka	1979-2015	Vital Registration	VR/SRS/DSP
Tochigi	1979-2015	Vital Registration	VR/SRS/DSP
Tokushima	1979-2015	Vital Registration	VR/SRS/DSP
Tōkyō	1979-2015	Vital Registration	VR/SRS/DSP
Tottori	1979-2015	Vital Registration	VR/SRS/DSP
Toyama	1979-2015	Vital Registration	VR/SRS/DSP
Wakayama	1979-2015	Vital Registration	VR/SRS/DSP
Yamagata	1979-2015	Vital Registration	VR/SRS/DSP
Yamaguchi	1979-2015	Vital Registration	VR/SRS/DSP
Yamanashi	1979-2015	Vital Registration	VR/SRS/DSP
South Korea	1954-1974	World Fertility Survey	CBH
South Korea	1954-1974	World Fertility Survey	SBH
South Korea	1955-1956	Census	SBH
South Korea	1958-1959	Census	SBH
South Korea	1961-1964	Census	SBH
South Korea	1967-1974	Census	SBH
South Korea	1977-1977	Census	SBH
South Korea	1977-2016	Vital Registration	VR/SRS/DSP
South Korea	1980-1981	Census	SBH
South Korea	1983-1984	Census	SBH
South Korea	1987-1987	Census	SBH
Singapore	1950-2016	Vital Registration	VR/SRS/DSP
Canada	1950-2016	Vital Registration	VR/SRS/DSP
Greenland	1952-1965	Vital Registration	VR/SRS/DSP
Greenland	1967-1987	Vital Registration	VR/SRS/DSP
Greenland	1990-1991	Vital Registration	VR/SRS/DSP
Greenland	1994-2016	Vital Registration	VR/SRS/DSP
United States	1950-2016	Vital Registration	VR/SRS/DSP
Alabama	1959-2015	Vital Registration	VR/SRS/DSP
Alaska	1959-2015	Vital Registration	VR/SRS/DSP
Arizona	1959-2015	Vital Registration	VR/SRS/DSP
Arkansas	1959-2015	Vital Registration	VR/SRS/DSP

Appendix Table 5. Under-5 mortality reference sources by source date and location, 1950-2017			
**Location**	**Year range of data**	**Reference source**	**Data type**
California	1959-2015	Vital Registration	VR/SRS/DSP
Colorado	1959-2015	Vital Registration	VR/SRS/DSP
Connecticut	1959-2015	Vital Registration	VR/SRS/DSP
Delaware	1959-2015	Vital Registration	VR/SRS/DSP
District of Columbia	1959-2015	Vital Registration	VR/SRS/DSP
Florida	1959-2015	Vital Registration	VR/SRS/DSP
Georgia	1959-2015	Vital Registration	VR/SRS/DSP
Hawaii	1959-2015	Vital Registration	VR/SRS/DSP
Idaho	1959-2015	Vital Registration	VR/SRS/DSP
Illinois	1959-2015	Vital Registration	VR/SRS/DSP
Indiana	1959-2015	Vital Registration	VR/SRS/DSP
Iowa	1959-2015	Vital Registration	VR/SRS/DSP
Kansas	1959-2015	Vital Registration	VR/SRS/DSP
Kentucky	1959-2015	Vital Registration	VR/SRS/DSP
Louisiana	1959-2015	Vital Registration	VR/SRS/DSP
Maine	1959-2015	Vital Registration	VR/SRS/DSP
Maryland	1959-2015	Vital Registration	VR/SRS/DSP
Massachusetts	1959-2015	Vital Registration	VR/SRS/DSP
Michigan	1959-2015	Vital Registration	VR/SRS/DSP
Minnesota	1959-2015	Vital Registration	VR/SRS/DSP
Mississippi	1959-2015	Vital Registration	VR/SRS/DSP
Missouri	1959-2015	Vital Registration	VR/SRS/DSP
Montana	1959-2015	Vital Registration	VR/SRS/DSP
Nebraska	1959-2015	Vital Registration	VR/SRS/DSP
Nevada	1959-2015	Vital Registration	VR/SRS/DSP
New Hampshire	1959-2015	Vital Registration	VR/SRS/DSP
New Jersey	1959-2015	Vital Registration	VR/SRS/DSP
New Mexico	1959-2015	Vital Registration	VR/SRS/DSP
New York	1959-2015	Vital Registration	VR/SRS/DSP
North Carolina	1959-2015	Vital Registration	VR/SRS/DSP
North Dakota	1959-2015	Vital Registration	VR/SRS/DSP
Ohio	1959-2015	Vital Registration	VR/SRS/DSP
Oklahoma	1959-2015	Vital Registration	VR/SRS/DSP
Oregon	1959-2015	Vital Registration	VR/SRS/DSP
Pennsylvania	1959-2015	Vital Registration	VR/SRS/DSP
Rhode Island	1959-2015	Vital Registration	VR/SRS/DSP
South Carolina	1959-2015	Vital Registration	VR/SRS/DSP
South Dakota	1959-2015	Vital Registration	VR/SRS/DSP
Tennessee	1959-2015	Vital Registration	VR/SRS/DSP
Texas	1959-2015	Vital Registration	VR/SRS/DSP
Utah	1959-2015	Vital Registration	VR/SRS/DSP
Vermont	1959-2015	Vital Registration	VR/SRS/DSP
Virginia	1959-2015	Vital Registration	VR/SRS/DSP
Washington	1959-2015	Vital Registration	VR/SRS/DSP
West Virginia	1959-2015	Vital Registration	VR/SRS/DSP
Wisconsin	1959-2015	Vital Registration	VR/SRS/DSP
Wyoming	1959-2015	Vital Registration	VR/SRS/DSP
Argentina	1950-1956	Vital Registration	VR/SRS/DSP

Appendix Table 5. Under-5 mortality reference sources by source date and location, 1950-2017			
**Location**	**Year range of data**	**Reference source**	**Data type**
Argentina	1950-1956	Census	SBH
Argentina	1958-1970	Vital Registration	VR/SRS/DSP
Argentina	1958-1990	Census	SBH
Argentina	1976-2015	Vital Registration	VR/SRS/DSP
Chile	1950-2001	Census	SBH
Chile	1950-2015	Vital Registration	VR/SRS/DSP
Uruguay	1950-2009	Vital Registration	VR/SRS/DSP
Uruguay	1950-2011	Census	SBH
Uruguay	1988-2012	Other survey	SBH
Uruguay	2012-2015	Vital Registration	VR/SRS/DSP
Andorra	1992-1992	Vital Registration	VR/SRS/DSP
Andorra	1994-1994	Vital Registration	VR/SRS/DSP
Andorra	2002-2005	Vital Registration	VR/SRS/DSP
Andorra	2007-2010	Vital Registration	VR/SRS/DSP
Andorra	2012-2012	Vital Registration	VR/SRS/DSP
Andorra	2015-2015	Vital Registration	VR/SRS/DSP
Austria	1950-2016	Vital Registration	VR/SRS/DSP
Belgium	1950-1950	Census	SBH
Belgium	1950-2016	Vital Registration	VR/SRS/DSP
Belgium	1952-1953	Census	SBH
Belgium	1955-1955	Census	SBH
Belgium	1957-1958	Census	SBH
Belgium	1961-1961	Census	SBH
Belgium	1964-1964	Census	SBH
Belgium	1967-1967	Census	SBH
Cyprus	1951-1951	Census	SBH
Cyprus	1954-1957	Census	SBH
Cyprus	1961-1961	Census	SBH
Cyprus	1964-1964	Census	SBH
Cyprus	1967-1967	Census	SBH
Cyprus	1969-1969	Census	SBH
Cyprus	1971-1971	Census	SBH
Cyprus	1974-2016	Vital Registration	VR/SRS/DSP
Cyprus	1975-1975	Census	SBH
Cyprus	1979-1979	Census	SBH
Cyprus	1983-1983	Census	SBH
Cyprus	1986-1986	Census	SBH
Cyprus	1989-1989	Census	SBH
Denmark	1950-2016	Vital Registration	VR/SRS/DSP
Finland	1950-2016	Vital Registration	VR/SRS/DSP
France	1950-2016	Vital Registration	VR/SRS/DSP
Germany	1956-2015	Vital Registration	VR/SRS/DSP
Greece	1951-2016	Vital Registration	VR/SRS/DSP
Iceland	1950-2017	Vital Registration	VR/SRS/DSP
Ireland	1950-2016	Vital Registration	VR/SRS/DSP
Israel	1950-2016	Vital Registration	VR/SRS/DSP
Italy	1950-2016	Vital Registration	VR/SRS/DSP
Luxembourg	1950-2015	Vital Registration	VR/SRS/DSP

Appendix Table 5. Under-5 mortality reference sources by source date and location, 1950-2017			
**Location**	**Year range of data**	**Reference source**	**Data type**
Malta	1950-2016	Vital Registration	VR/SRS/DSP
Netherlands	1950-2016	Vital Registration	VR/SRS/DSP
Norway	1950-2016	Vital Registration	VR/SRS/DSP
Portugal	1950-1974	Vital Registration	VR/SRS/DSP
Portugal	1956-1974	World Fertility Survey	CBH
Portugal	1959-1974	World Fertility Survey	SBH
Portugal	1977-1977	World Fertility Survey	SBH
Portugal	1977-1979	World Fertility Survey	CBH
Portugal	1977-2004	Vital Registration	VR/SRS/DSP
Portugal	2006-2016	Vital Registration	VR/SRS/DSP
Spain	1950-2016	Vital Registration	VR/SRS/DSP
Sweden	1950-2016	Vital Registration	VR/SRS/DSP
Stockholm	1980-1986	Vital Registration	VR/SRS/DSP
Stockholm	1990-2010	Vital Registration	VR/SRS/DSP
Stockholm	2012-2016	Vital Registration	VR/SRS/DSP
Sweden except Stockholm	1980-1986	Vital Registration	VR/SRS/DSP
Sweden except Stockholm	1990-2010	Vital Registration	VR/SRS/DSP
Sweden except Stockholm	2012-2016	Vital Registration	VR/SRS/DSP
Switzerland	1950-2016	Vital Registration	VR/SRS/DSP
Switzerland	1951-1951	Census	SBH
Switzerland	1954-1954	Census	SBH
Switzerland	1957-1957	Census	SBH
United Kingdom	1950-1999	Vital Registration	VR/SRS/DSP
United Kingdom	2001-2016	Vital Registration	VR/SRS/DSP
England	1981-2016	Vital Registration	VR/SRS/DSP
East Midlands	1981-2016	Vital Registration	VR/SRS/DSP
Derby	1981-2016	Vital Registration	VR/SRS/DSP
Derbyshire	1981-2016	Vital Registration	VR/SRS/DSP
Leicester	1981-2016	Vital Registration	VR/SRS/DSP
Leicestershire	1981-2016	Vital Registration	VR/SRS/DSP
Lincolnshire	1981-2016	Vital Registration	VR/SRS/DSP
Northamptonshire	1981-2016	Vital Registration	VR/SRS/DSP
Nottingham	1981-2016	Vital Registration	VR/SRS/DSP
Nottinghamshire	1981-2016	Vital Registration	VR/SRS/DSP
Rutland	1981-2016	Vital Registration	VR/SRS/DSP
East of England	1981-2016	Vital Registration	VR/SRS/DSP
Bedford	1981-2016	Vital Registration	VR/SRS/DSP
Cambridgeshire	1981-2016	Vital Registration	VR/SRS/DSP
Central Bedfordshire	1981-2016	Vital Registration	VR/SRS/DSP
Essex	1981-2016	Vital Registration	VR/SRS/DSP
Hertfordshire	1981-2016	Vital Registration	VR/SRS/DSP
Luton	1981-2016	Vital Registration	VR/SRS/DSP
Norfolk	1981-2016	Vital Registration	VR/SRS/DSP
Peterborough	1981-2016	Vital Registration	VR/SRS/DSP
Southend-on-Sea	1981-2016	Vital Registration	VR/SRS/DSP
Suffolk	1981-2016	Vital Registration	VR/SRS/DSP
Thurrock	1981-2016	Vital Registration	VR/SRS/DSP
Greater London	1981-2016	Vital Registration	VR/SRS/DSP

Appendix Table 5. Under-5 mortality reference sources by source date and location, 1950-2017			
**Location**	**Year range of data**	**Reference source**	**Data type**
Barking and Dagenham	1981-2016	Vital Registration	VR/SRS/DSP
Barnet	1981-2016	Vital Registration	VR/SRS/DSP
Bexley	1981-2016	Vital Registration	VR/SRS/DSP
Brent	1981-2016	Vital Registration	VR/SRS/DSP
Bromley	1981-2016	Vital Registration	VR/SRS/DSP
Camden	1981-2016	Vital Registration	VR/SRS/DSP
Croydon	1981-2016	Vital Registration	VR/SRS/DSP
Ealing	1981-2016	Vital Registration	VR/SRS/DSP
Enfield	1981-2016	Vital Registration	VR/SRS/DSP
Greenwich	1981-2016	Vital Registration	VR/SRS/DSP
Hackney	1981-2016	Vital Registration	VR/SRS/DSP
Hammersmith and Fulham	1981-2016	Vital Registration	VR/SRS/DSP
Haringey	1981-2016	Vital Registration	VR/SRS/DSP
Harrow	1981-2016	Vital Registration	VR/SRS/DSP
Havering	1981-2016	Vital Registration	VR/SRS/DSP
Hillingdon	1981-2016	Vital Registration	VR/SRS/DSP
Hounslow	1981-2016	Vital Registration	VR/SRS/DSP
Islington	1981-2016	Vital Registration	VR/SRS/DSP
Kensington and Chelsea	1981-2016	Vital Registration	VR/SRS/DSP
Kingston upon Thames	1981-2016	Vital Registration	VR/SRS/DSP
Lambeth	1981-2016	Vital Registration	VR/SRS/DSP
Lewisham	1981-2016	Vital Registration	VR/SRS/DSP
Merton	1981-2016	Vital Registration	VR/SRS/DSP
Newham	1981-2016	Vital Registration	VR/SRS/DSP
Redbridge	1981-2016	Vital Registration	VR/SRS/DSP
Richmond upon Thames	1981-2016	Vital Registration	VR/SRS/DSP
Southwark	1981-2016	Vital Registration	VR/SRS/DSP
Sutton	1981-2016	Vital Registration	VR/SRS/DSP
Tower Hamlets	1981-2016	Vital Registration	VR/SRS/DSP
Waltham Forest	1981-2016	Vital Registration	VR/SRS/DSP
Wandsworth	1981-2016	Vital Registration	VR/SRS/DSP
Westminster	1981-2016	Vital Registration	VR/SRS/DSP
North East England	1981-2016	Vital Registration	VR/SRS/DSP
County Durham	1981-2016	Vital Registration	VR/SRS/DSP
Darlington	1981-2016	Vital Registration	VR/SRS/DSP
Gateshead	1981-2016	Vital Registration	VR/SRS/DSP
Hartlepool	1981-2016	Vital Registration	VR/SRS/DSP
Middlesbrough	1981-2016	Vital Registration	VR/SRS/DSP
Newcastle upon Tyne	1981-2016	Vital Registration	VR/SRS/DSP
North Tyneside	1981-2016	Vital Registration	VR/SRS/DSP
Northumberland	1981-2016	Vital Registration	VR/SRS/DSP
Redcar and Cleveland	1981-2016	Vital Registration	VR/SRS/DSP
South Tyneside	1981-2016	Vital Registration	VR/SRS/DSP
Stockton-on-Tees	1981-2016	Vital Registration	VR/SRS/DSP
Sunderland	1981-2016	Vital Registration	VR/SRS/DSP
North West England	1981-2016	Vital Registration	VR/SRS/DSP
Blackburn with Darwen	1981-2016	Vital Registration	VR/SRS/DSP
Blackpool	1981-2016	Vital Registration	VR/SRS/DSP

Appendix Table 5. Under-5 mortality reference sources by source date and location, 1950-2017			
**Location**	**Year range of data**	**Reference source**	**Data type**
Bolton	1981-2016	Vital Registration	VR/SRS/DSP
Bury	1981-2016	Vital Registration	VR/SRS/DSP
Cheshire East	1981-2016	Vital Registration	VR/SRS/DSP
Cheshire West and Chester	1981-2016	Vital Registration	VR/SRS/DSP
Cumbria	1981-2016	Vital Registration	VR/SRS/DSP
Halton	1981-2016	Vital Registration	VR/SRS/DSP
Knowsley	1981-2016	Vital Registration	VR/SRS/DSP
Lancashire	1981-2016	Vital Registration	VR/SRS/DSP
Liverpool	1981-2016	Vital Registration	VR/SRS/DSP
Manchester	1981-2016	Vital Registration	VR/SRS/DSP
Oldham	1981-2016	Vital Registration	VR/SRS/DSP
Rochdale	1981-2016	Vital Registration	VR/SRS/DSP
Salford	1981-2016	Vital Registration	VR/SRS/DSP
Sefton	1981-2016	Vital Registration	VR/SRS/DSP
St Helens	1981-2016	Vital Registration	VR/SRS/DSP
Stockport	1981-2016	Vital Registration	VR/SRS/DSP
Tameside	1981-2016	Vital Registration	VR/SRS/DSP
Trafford	1981-2016	Vital Registration	VR/SRS/DSP
Warrington	1981-2016	Vital Registration	VR/SRS/DSP
Wigan	1981-2016	Vital Registration	VR/SRS/DSP
Wirral	1981-2016	Vital Registration	VR/SRS/DSP
South East England	1981-2016	Vital Registration	VR/SRS/DSP
Bracknell Forest	1981-2016	Vital Registration	VR/SRS/DSP
Brighton and Hove	1981-2016	Vital Registration	VR/SRS/DSP
Buckinghamshire	1981-2016	Vital Registration	VR/SRS/DSP
East Sussex	1981-2016	Vital Registration	VR/SRS/DSP
Hampshire	1981-2016	Vital Registration	VR/SRS/DSP
Isle of Wight	1981-2016	Vital Registration	VR/SRS/DSP
Kent	1981-2016	Vital Registration	VR/SRS/DSP
Medway	1981-2016	Vital Registration	VR/SRS/DSP
Milton Keynes	1981-2016	Vital Registration	VR/SRS/DSP
Oxfordshire	1981-2016	Vital Registration	VR/SRS/DSP
Portsmouth	1981-2016	Vital Registration	VR/SRS/DSP
Reading	1981-2016	Vital Registration	VR/SRS/DSP
Slough	1981-2016	Vital Registration	VR/SRS/DSP
Southampton	1981-2016	Vital Registration	VR/SRS/DSP
Surrey	1981-2016	Vital Registration	VR/SRS/DSP
West Berkshire	1981-2016	Vital Registration	VR/SRS/DSP
West Sussex	1981-2016	Vital Registration	VR/SRS/DSP
Windsor and Maidenhead	1981-2016	Vital Registration	VR/SRS/DSP
Wokingham	1981-2016	Vital Registration	VR/SRS/DSP
South West England	1981-2016	Vital Registration	VR/SRS/DSP
Bath and North East Somerset	1981-2016	Vital Registration	VR/SRS/DSP
Bournemouth	1981-2016	Vital Registration	VR/SRS/DSP
Bristol, City of	1981-2016	Vital Registration	VR/SRS/DSP
Cornwall	1981-2016	Vital Registration	VR/SRS/DSP
Devon	1981-2016	Vital Registration	VR/SRS/DSP
Dorset	1981-2016	Vital Registration	VR/SRS/DSP

Appendix Table 5. Under-5 mortality reference sources by source date and location, 1950-2017

Location	Year range of data	Reference source	Data type
Gloucestershire	1981-2016	Vital Registration	VR/SRS/DSP
North Somerset	1981-2016	Vital Registration	VR/SRS/DSP
Plymouth	1981-2016	Vital Registration	VR/SRS/DSP
Poole	1981-2016	Vital Registration	VR/SRS/DSP
Somerset	1981-2016	Vital Registration	VR/SRS/DSP
South Gloucestershire	1981-2016	Vital Registration	VR/SRS/DSP
Swindon	1981-2016	Vital Registration	VR/SRS/DSP
Torbay	1981-2016	Vital Registration	VR/SRS/DSP
Wiltshire	1981-2016	Vital Registration	VR/SRS/DSP
West Midlands	1981-2016	Vital Registration	VR/SRS/DSP
Birmingham	1981-2016	Vital Registration	VR/SRS/DSP
Coventry	1981-2016	Vital Registration	VR/SRS/DSP
Dudley	1981-2016	Vital Registration	VR/SRS/DSP
Herefordshire, County of	1981-2016	Vital Registration	VR/SRS/DSP
Sandwell	1981-2016	Vital Registration	VR/SRS/DSP
Shropshire	1981-2016	Vital Registration	VR/SRS/DSP
Solihull	1981-2016	Vital Registration	VR/SRS/DSP
Staffordshire	1981-2016	Vital Registration	VR/SRS/DSP
Stoke-on-Trent	1981-2016	Vital Registration	VR/SRS/DSP
Telford and Wrekin	1981-2016	Vital Registration	VR/SRS/DSP
Walsall	1981-2016	Vital Registration	VR/SRS/DSP
Warwickshire	1981-2016	Vital Registration	VR/SRS/DSP
Wolverhampton	1981-2016	Vital Registration	VR/SRS/DSP
Worcestershire	1981-2016	Vital Registration	VR/SRS/DSP
Yorkshire and the Humber	1981-2016	Vital Registration	VR/SRS/DSP
Barnsley	1981-2016	Vital Registration	VR/SRS/DSP
Bradford	1981-2016	Vital Registration	VR/SRS/DSP
Calderdale	1981-2016	Vital Registration	VR/SRS/DSP
Doncaster	1981-2016	Vital Registration	VR/SRS/DSP
East Riding of Yorkshire	1981-2016	Vital Registration	VR/SRS/DSP
Kingston upon Hull, City of	1981-2016	Vital Registration	VR/SRS/DSP
Kirklees	1981-2016	Vital Registration	VR/SRS/DSP
Leeds	1981-2016	Vital Registration	VR/SRS/DSP
North East Lincolnshire	1981-2016	Vital Registration	VR/SRS/DSP
North Lincolnshire	1981-2016	Vital Registration	VR/SRS/DSP
North Yorkshire	1981-2016	Vital Registration	VR/SRS/DSP
Rotherham	1981-2016	Vital Registration	VR/SRS/DSP
Sheffield	1981-2016	Vital Registration	VR/SRS/DSP
Wakefield	1981-2016	Vital Registration	VR/SRS/DSP
York	1981-2016	Vital Registration	VR/SRS/DSP
Northern Ireland	1950-2016	Vital Registration	VR/SRS/DSP
Scotland	1950-2016	Vital Registration	VR/SRS/DSP
Wales	1981-2016	Vital Registration	VR/SRS/DSP
Bolivia	1951-1958	Vital Registration	VR/SRS/DSP
Bolivia	1951-2000	Census	SBH
Bolivia	1961-1961	Other survey	SBH
Bolivia	1961-2007	Standard demographic and health survey	CBH
Bolivia	1964-2016	Standard demographic and health survey	SBH

Appendix Table 5. Under-5 mortality reference sources by source date and location, 1950-2017			
**Location**	**Year range of data**	**Reference source**	**Data type**
Bolivia	1965-1965	Other survey	SBH
Bolivia	1965-1966	Vital Registration	VR/SRS/DSP
Bolivia	1968-1968	Other survey	SBH
Bolivia	1971-1971	Other survey	SBH
Bolivia	1974-1974	Other survey	SBH
Bolivia	1975-1999	Other survey	SBH
Bolivia	1976-1977	Vital Registration	VR/SRS/DSP
Bolivia	1976-2000	Multiple Indicator Cluster Survey	SBH
Ecuador	1950-2010	Census	SBH
Ecuador	1954-1979	World Fertility Survey	CBH
Ecuador	1954-2016	Vital Registration	VR/SRS/DSP
Ecuador	1955-1979	World Fertility Survey	SBH
Ecuador	1962-1986	Standard demographic and health survey	SBH
Ecuador	1963-1986	Standard demographic and health survey	CBH
Ecuador	1965-2004	Reproductive Health Survey	SBH
Ecuador	1967-2004	Reproductive Health Survey	CBH
Ecuador	1970-1997	Living Standards Measurement Study	SBH
Ecuador	1974-2012	Other survey	SBH
Ecuador	1983-2012	Other survey	CBH
Peru	1950-1964	Vital Registration	VR/SRS/DSP
Peru	1950-1977	World Fertility Survey	CBH
Peru	1953-1977	World Fertility Survey	SBH
Peru	1954-1954	Census	SBH
Peru	1957-1957	Census	SBH
Peru	1960-1960	Census	SBH
Peru	1961-1993	Living Standards Measurement Study	SBH
Peru	1961-2014	Standard demographic and health survey	CBH
Peru	1962-1962	Census	SBH
Peru	1962-2014	Standard demographic and health survey	SBH
Peru	1963-1963	Other survey	SBH
Peru	1964-1964	Census	SBH
Peru	1966-1966	Census	SBH
Peru	1966-1966	Other survey	SBH
Peru	1966-1973	Vital Registration	VR/SRS/DSP
Peru	1968-2006	Census	SBH
Peru	1969-1969	Other survey	SBH
Peru	1972-1972	Other survey	SBH
Peru	1975-1975	Other survey	SBH
Peru	1975-1992	Vital Registration	VR/SRS/DSP
Peru	1977-1977	Other survey	SBH
Peru	1984-2015	Other survey	CBH
Peru	1994-2015	Vital Registration	VR/SRS/DSP
Antigua and Barbuda	1950-1966	Vital Registration	VR/SRS/DSP
Antigua and Barbuda	1969-1978	Vital Registration	VR/SRS/DSP
Antigua and Barbuda	1983-1983	Vital Registration	VR/SRS/DSP
Antigua and Barbuda	1985-2009	Vital Registration	VR/SRS/DSP
Antigua and Barbuda	2012-2015	Vital Registration	VR/SRS/DSP
The Bahamas	1965-1965	Vital Registration	VR/SRS/DSP

Appendix Table 5. Under-5 mortality reference sources by source date and location, 1950-2017			
**Location**	**Year range of data**	**Reference source**	**Data type**
The Bahamas	1967-1969	Vital Registration	VR/SRS/DSP
The Bahamas	1971-2013	Vital Registration	VR/SRS/DSP
Barbados	1950-1995	Vital Registration	VR/SRS/DSP
Barbados	2000-2013	Vital Registration	VR/SRS/DSP
Belize	1950-2016	Vital Registration	VR/SRS/DSP
Belize	1966-1991	Census	SBH
Belize	1966-1999	Reproductive Health Survey	SBH
Belize	1973-1999	Reproductive Health Survey	CBH
Belize	1981-2011	Multiple Indicator Cluster Survey	SBH
Belize	1990-2015	Multiple Indicator Cluster Survey	CBH
Belize	1993-1993	Census	SBH
Belize	1996-1996	Census	SBH
Bermuda	1950-1950	Census	SBH
Bermuda	1950-1960	Vital Registration	VR/SRS/DSP
Bermuda	1953-1953	Census	SBH
Bermuda	1956-1956	Census	SBH
Bermuda	1964-2015	Vital Registration	VR/SRS/DSP
Cuba	1959-2015	Vital Registration	VR/SRS/DSP
Cuba	1961-1961	Census	SBH
Cuba	1964-1964	Census	SBH
Cuba	1968-1968	Census	SBH
Cuba	1971-1971	Census	SBH
Cuba	1974-1974	Census	SBH
Cuba	1977-1977	Census	SBH
Dominica	1950-1963	Vital Registration	VR/SRS/DSP
Dominica	1966-2015	Vital Registration	VR/SRS/DSP
Dominican Republic	1950-1979	World Fertility Survey	SBH
Dominican Republic	1950-1992	Vital Registration	VR/SRS/DSP
Dominican Republic	1950-2009	Census	SBH
Dominican Republic	1951-1979	World Fertility Survey	CBH
Dominican Republic	1959-2013	Standard demographic and health survey	CBH
Dominican Republic	1962-2013	Standard demographic and health survey	SBH
Dominican Republic	1976-2013	Multiple Indicator Cluster Survey	SBH
Dominican Republic	1978-2005	Other survey	CBH
Dominican Republic	1984-2013	Multiple Indicator Cluster Survey	CBH
Dominican Republic	1992-2013	Other demographic and health survey	CBH
Dominican Republic	1994-2010	Vital Registration	VR/SRS/DSP
Grenada	1950-1969	Vital Registration	VR/SRS/DSP
Grenada	1974-1978	Vital Registration	VR/SRS/DSP
Grenada	1985-1985	Vital Registration	VR/SRS/DSP
Grenada	1988-2016	Vital Registration	VR/SRS/DSP
Guyana	1950-1961	Vital Registration	VR/SRS/DSP
Guyana	1950-1974	World Fertility Survey	SBH
Guyana	1950-1975	World Fertility Survey	CBH
Guyana	1968-1992	Living Standards Measurement Study	SBH
Guyana	1969-1971	Vital Registration	VR/SRS/DSP
Guyana	1974-1974	Vital Registration	VR/SRS/DSP
Guyana	1976-2013	Multiple Indicator Cluster Survey	SBH

Appendix Table 5. Under-5 mortality reference sources by source date and location, 1950-2017

Location	Year range of data	Reference source	Data type
Guyana	1977-1977	Vital Registration	VR/SRS/DSP
Guyana	1979-1979	Vital Registration	VR/SRS/DSP
Guyana	1981-2005	AIDS Indicator Survey	SBH
Guyana	1982-2005	AIDS Indicator Survey	CBH
Guyana	1982-2009	Standard demographic and health survey	CBH
Guyana	1984-1984	Vital Registration	VR/SRS/DSP
Guyana	1984-2008	Standard demographic and health survey	SBH
Guyana	1988-2013	Vital Registration	VR/SRS/DSP
Guyana	1988-2013	Multiple Indicator Cluster Survey	CBH
Haiti	1953-1977	World Fertility Survey	SBH
Haiti	1956-1977	World Fertility Survey	CBH
Haiti	1957-2002	Census	SBH
Haiti	1970-2011	Standard demographic and health survey	SBH
Haiti	1971-2011	Standard demographic and health survey	CBH
Haiti	1975-1975	Other survey	SBH
Haiti	1978-1978	Other survey	SBH
Haiti	1981-1981	Other survey	SBH
Haiti	1983-1983	Other survey	SBH
Haiti	1985-1985	Other survey	HH
Haiti	1985-1985	Other survey	SBH
Haiti	2004-2004	Standard demographic and health survey	HH
Haiti	2005-2008	Other survey	CBH
Haiti	2009-2009	Standard demographic and health survey	HH
Haiti	2014-2014	Standard demographic and health survey	HH
Jamaica	1950-1965	Vital Registration	VR/SRS/DSP
Jamaica	1951-1975	World Fertility Survey	SBH
Jamaica	1952-1975	World Fertility Survey	CBH
Jamaica	1957-2000	Census	SBH
Jamaica	1967-1971	Vital Registration	VR/SRS/DSP
Jamaica	1975-1975	Vital Registration	VR/SRS/DSP
Jamaica	1975-2005	Multiple Indicator Cluster Survey	SBH
Jamaica	1977-1977	Vital Registration	VR/SRS/DSP
Jamaica	1980-1991	Vital Registration	VR/SRS/DSP
Jamaica	1985-1985	Reproductive Health Survey	HH
Jamaica	1990-1990	Reproductive Health Survey	HH
Jamaica	1995-1995	Reproductive Health Survey	HH
Jamaica	1999-2006	Vital Registration	VR/SRS/DSP
Jamaica	2009-2011	Vital Registration	VR/SRS/DSP
Puerto Rico	1950-2016	Vital Registration	VR/SRS/DSP
Puerto Rico	1969-1996	Reproductive Health Survey	CBH
Puerto Rico	1971-1995	Reproductive Health Survey	SBH
Saint Lucia	1950-1961	Vital Registration	VR/SRS/DSP
Saint Lucia	1963-1963	Vital Registration	VR/SRS/DSP
Saint Lucia	1968-1968	Vital Registration	VR/SRS/DSP
Saint Lucia	1972-2006	Vital Registration	VR/SRS/DSP
Saint Lucia	2008-2014	Vital Registration	VR/SRS/DSP
Saint Vincent and the Grenadines	1950-1956	Vital Registration	VR/SRS/DSP
Saint Vincent and the Grenadines	1960-1964	Vital Registration	VR/SRS/DSP

Appendix Table 5. Under-5 mortality reference sources by source date and location, 1950-2017

Location	Year range of data	Reference source	Data type
Saint Vincent and the Grenadines	1970-1972	Vital Registration	VR/SRS/DSP
Saint Vincent and the Grenadines	1974-1974	Vital Registration	VR/SRS/DSP
Saint Vincent and the Grenadines	1977-1980	Vital Registration	VR/SRS/DSP
Saint Vincent and the Grenadines	1982-1988	Vital Registration	VR/SRS/DSP
Saint Vincent and the Grenadines	1990-1990	Vital Registration	VR/SRS/DSP
Saint Vincent and the Grenadines	1992-1992	Vital Registration	VR/SRS/DSP
Saint Vincent and the Grenadines	1995-2015	Vital Registration	VR/SRS/DSP
Suriname	1950-1957	Vital Registration	VR/SRS/DSP
Suriname	1961-1966	Vital Registration	VR/SRS/DSP
Suriname	1971-1973	Vital Registration	VR/SRS/DSP
Suriname	1975-1982	Vital Registration	VR/SRS/DSP
Suriname	1975-2005	Multiple Indicator Cluster Survey	SBH
Suriname	1984-2015	Vital Registration	VR/SRS/DSP
Trinidad and Tobago	1950-2011	Vital Registration	VR/SRS/DSP
Trinidad and Tobago	1952-1976	World Fertility Survey	SBH
Trinidad and Tobago	1952-1977	World Fertility Survey	CBH
Trinidad and Tobago	1962-1987	Standard demographic and health survey	CBH
Trinidad and Tobago	1963-1987	Standard demographic and health survey	SBH
Trinidad and Tobago	1975-2005	Multiple Indicator Cluster Survey	SBH
Virgin Islands, U.S.	1950-1966	Vital Registration	VR/SRS/DSP
Virgin Islands, U.S.	1971-1973	Vital Registration	VR/SRS/DSP
Virgin Islands, U.S.	1980-1987	Vital Registration	VR/SRS/DSP
Virgin Islands, U.S.	1989-1989	Vital Registration	VR/SRS/DSP
Virgin Islands, U.S.	1993-2012	Vital Registration	VR/SRS/DSP
Colombia	1950-1977	Vital Registration	VR/SRS/DSP
Colombia	1950-2004	Census	SBH
Colombia	1951-1975	World Fertility Survey	SBH
Colombia	1951-1976	World Fertility Survey	CBH
Colombia	1961-2015	Standard demographic and health survey	CBH
Colombia	1962-2015	Standard demographic and health survey	SBH
Colombia	1979-1980	Vital Registration	VR/SRS/DSP
Colombia	1982-2015	Vital Registration	VR/SRS/DSP
Colombia	1984-2008	Other survey	SBH
Costa Rica	1950-1950	Vital Registration	VR/SRS/DSP
Costa Rica	1950-1999	Census	SBH
Costa Rica	1951-1976	World Fertility Survey	CBH
Costa Rica	1952-1976	World Fertility Survey	SBH
Costa Rica	1952-2014	Vital Registration	VR/SRS/DSP
Costa Rica	1956-1980	Other survey	SBH
Costa Rica	1961-1992	Reproductive Health Survey	SBH
Costa Rica	1969-1992	Reproductive Health Survey	CBH
El Salvador	1950-2014	Vital Registration	VR/SRS/DSP
El Salvador	1953-1953	Census	SBH
El Salvador	1957-1957	Census	SBH
El Salvador	1960-1960	Census	SBH
El Salvador	1960-1984	Standard demographic and health survey	SBH
El Salvador	1962-1962	Census	SBH
El Salvador	1963-2007	Reproductive Health Survey	SBH

Appendix Table 5. Under-5 mortality reference sources by source date and location, 1950-2017			
**Location**	**Year range of data**	**Reference source**	**Data type**
El Salvador	1965-1965	Census	SBH
El Salvador	1967-2006	Census	SBH
El Salvador	1971-2008	Reproductive Health Survey	CBH
El Salvador	1983-1984	Standard demographic and health survey	CBH
El Salvador	1984-2013	Multiple Indicator Cluster Survey	CBH
El Salvador	1989-2013	Multiple Indicator Cluster Survey	SBH
Guatemala	1950-1972	Vital Registration	VR/SRS/DSP
Guatemala	1954-2008	Reproductive Health Survey	SBH
Guatemala	1963-1963	Census	SBH
Guatemala	1963-2014	Standard demographic and health survey	SBH
Guatemala	1966-1966	Census	SBH
Guatemala	1966-2014	Standard demographic and health survey	CBH
Guatemala	1969-1969	Census	SBH
Guatemala	1972-1972	Census	SBH
Guatemala	1974-1981	Vital Registration	VR/SRS/DSP
Guatemala	1974-1998	Other demographic and health survey	SBH
Guatemala	1975-1975	Census	SBH
Guatemala	1975-1999	Living Standards Measurement Study	SBH
Guatemala	1975-2008	Reproductive Health Survey	CBH
Guatemala	1977-1977	Census	SBH
Guatemala	1983-2015	Vital Registration	VR/SRS/DSP
Honduras	1950-1983	Vital Registration	VR/SRS/DSP
Honduras	1954-1954	Other survey	SBH
Honduras	1957-1957	Other survey	SBH
Honduras	1960-1960	Other survey	SBH
Honduras	1963-1963	Other survey	SBH
Honduras	1965-1967	Other survey	SBH
Honduras	1967-2000	Reproductive Health Survey	SBH
Honduras	1969-1970	Other survey	SBH
Honduras	1970-2001	Reproductive Health Survey	CBH
Honduras	1972-1972	Reproductive Health Survey	HH
Honduras	1972-1975	Other survey	SBH
Honduras	1976-2011	Standard demographic and health survey	CBH
Honduras	1977-1977	Reproductive Health Survey	HH
Honduras	1977-1977	Other survey	SBH
Honduras	1979-1979	Other survey	SBH
Honduras	1980-1980	Reproductive Health Survey	HH
Honduras	1980-2004	Other survey	SBH
Honduras	1981-2011	Standard demographic and health survey	SBH
Honduras	1982-1982	Census	SBH
Honduras	1985-1985	Census	SBH
Honduras	1987-1990	Vital Registration	VR/SRS/DSP
Honduras	1989-1989	Census	SBH
Honduras	1991-1991	Census	SBH
Honduras	1994-1994	Census	SBH
Honduras	1997-1997	Census	SBH
Honduras	2008-2013	Vital Registration	VR/SRS/DSP
Mexico	1950-1976	World Fertility Survey	CBH

Appendix Table 5. Under-5 mortality reference sources by source date and location, 1950-2017			
**Location**	**Year range of data**	**Reference source**	**Data type**
Mexico	1950-2016	Vital Registration	VR/SRS/DSP
Mexico	1952-1976	World Fertility Survey	SBH
Mexico	1960-1987	Standard demographic and health survey	CBH
Mexico	1962-1962	Census	SBH
Mexico	1962-1986	Standard demographic and health survey	SBH
Mexico	1964-2014	Other survey	SBH
Mexico	1965-2015	Census	SBH
Mexico	1972-1972	Other survey	HH
Mexico	1977-1977	Other survey	HH
Mexico	1982-1982	Other survey	HH
Mexico	1987-1987	Other survey	HH
Mexico	1991-2015	Multiple Indicator Cluster Survey	SBH
Aguascalientes	1962-1962	Census	SBH
Aguascalientes	1964-2014	Other survey	SBH
Aguascalientes	1965-2015	Census	SBH
Aguascalientes	1971-1992	Other survey	CBH
Aguascalientes	1979-2016	Vital Registration	VR/SRS/DSP
Baja California	1961-1961	Census	SBH
Baja California	1964-2014	Other survey	SBH
Baja California	1965-2015	Census	SBH
Baja California	1973-1992	Other survey	CBH
Baja California	1979-2016	Vital Registration	VR/SRS/DSP
Baja California Sur	1961-1961	Census	SBH
Baja California Sur	1964-2014	Other survey	SBH
Baja California Sur	1965-2015	Census	SBH
Baja California Sur	1973-1992	Other survey	CBH
Baja California Sur	1979-2016	Vital Registration	VR/SRS/DSP
Campeche	1961-1961	Census	SBH
Campeche	1964-1964	Census	SBH
Campeche	1964-2014	Other survey	SBH
Campeche	1965-2015	Census	SBH
Campeche	1971-1992	Other survey	CBH
Campeche	1979-2016	Vital Registration	VR/SRS/DSP
Chiapas	1961-1961	Census	SBH
Chiapas	1964-1964	Census	SBH
Chiapas	1964-2014	Other survey	SBH
Chiapas	1965-2015	Census	SBH
Chiapas	1971-1992	Other survey	CBH
Chiapas	1979-2016	Vital Registration	VR/SRS/DSP
Chihuahua	1961-1961	Census	SBH
Chihuahua	1964-2014	Other survey	SBH
Chihuahua	1965-2015	Census	SBH
Chihuahua	1973-1992	Other survey	CBH
Chihuahua	1979-2016	Vital Registration	VR/SRS/DSP
Coahuila	1961-1961	Census	SBH
Coahuila	1964-2014	Other survey	SBH
Coahuila	1965-2015	Census	SBH
Coahuila	1971-1992	Other survey	CBH

Appendix Table 5. Under-5 mortality reference sources by source date and location, 1950-2017			
**Location**	**Year range of data**	**Reference source**	**Data type**
Coahuila	1979-2016	Vital Registration	VR/SRS/DSP
Colima	1961-1961	Census	SBH
Colima	1964-2014	Other survey	SBH
Colima	1965-2015	Census	SBH
Colima	1973-1992	Other survey	CBH
Colima	1979-2016	Vital Registration	VR/SRS/DSP
Mexico City	1960-1960	Census	SBH
Mexico City	1964-1964	Census	SBH
Mexico City	1964-2014	Other survey	SBH
Mexico City	1965-2015	Census	SBH
Mexico City	1973-1992	Other survey	CBH
Mexico City	1979-2015	Vital Registration	VR/SRS/DSP
Durango	1962-1962	Census	SBH
Durango	1964-2014	Other survey	SBH
Durango	1965-2015	Census	SBH
Durango	1971-1992	Other survey	CBH
Durango	1979-2016	Vital Registration	VR/SRS/DSP
Guanajuato	1962-1962	Census	SBH
Guanajuato	1964-2014	Other survey	SBH
Guanajuato	1965-2015	Census	SBH
Guanajuato	1971-1992	Other survey	CBH
Guanajuato	1979-2016	Vital Registration	VR/SRS/DSP
Guerrero	1961-1961	Census	SBH
Guerrero	1964-2014	Other survey	SBH
Guerrero	1965-2015	Census	SBH
Guerrero	1973-1992	Other survey	CBH
Guerrero	1979-2016	Vital Registration	VR/SRS/DSP
Hidalgo	1961-1961	Census	SBH
Hidalgo	1964-2014	Other survey	SBH
Hidalgo	1965-2014	Census	SBH
Hidalgo	1971-1992	Other survey	CBH
Hidalgo	1979-2016	Vital Registration	VR/SRS/DSP
Jalisco	1962-1962	Census	SBH
Jalisco	1964-2014	Other survey	SBH
Jalisco	1965-2015	Census	SBH
Jalisco	1971-1992	Other survey	CBH
Jalisco	1979-2016	Vital Registration	VR/SRS/DSP
México	1961-1961	Census	SBH
México	1964-2014	Other survey	SBH
México	1965-2015	Census	SBH
México	1973-1992	Other survey	CBH
México	1979-2016	Vital Registration	VR/SRS/DSP
Michoacán de Ocampo	1962-1962	Census	SBH
Michoacán de Ocampo	1964-2014	Other survey	SBH
Michoacán de Ocampo	1965-2015	Census	SBH
Michoacán de Ocampo	1971-1992	Other survey	CBH
Michoacán de Ocampo	1979-2016	Vital Registration	VR/SRS/DSP
Morelos	1961-1961	Census	SBH

Appendix Table 5. Under-5 mortality reference sources by source date and location, 1950-2017			
**Location**	**Year range of data**	**Reference source**	**Data type**
Morelos	1964-1964	Census	SBH
Morelos	1964-2014	Other survey	SBH
Morelos	1965-2015	Census	SBH
Morelos	1973-1992	Other survey	CBH
Morelos	1979-2016	Vital Registration	VR/SRS/DSP
Nayarit	1962-1962	Census	SBH
Nayarit	1964-2014	Other survey	SBH
Nayarit	1965-2015	Census	SBH
Nayarit	1971-1992	Other survey	CBH
Nayarit	1979-2016	Vital Registration	VR/SRS/DSP
Nuevo León	1961-1961	Census	SBH
Nuevo León	1964-1964	Census	SBH
Nuevo León	1964-2014	Other survey	SBH
Nuevo León	1965-2015	Census	SBH
Nuevo León	1971-1992	Other survey	CBH
Nuevo León	1979-2016	Vital Registration	VR/SRS/DSP
Oaxaca	1960-1960	Census	SBH
Oaxaca	1964-1964	Census	SBH
Oaxaca	1964-2014	Other survey	SBH
Oaxaca	1965-2015	Census	SBH
Oaxaca	1971-1992	Other survey	CBH
Oaxaca	1979-2016	Vital Registration	VR/SRS/DSP
Puebla	1961-1961	Census	SBH
Puebla	1964-2014	Other survey	SBH
Puebla	1965-2015	Census	SBH
Puebla	1971-1992	Other survey	CBH
Puebla	1979-2016	Vital Registration	VR/SRS/DSP
Querétaro	1962-1962	Census	SBH
Querétaro	1964-2014	Other survey	SBH
Querétaro	1965-2015	Census	SBH
Querétaro	1973-1992	Other survey	CBH
Querétaro	1979-2016	Vital Registration	VR/SRS/DSP
Quintana Roo	1961-1961	Census	SBH
Quintana Roo	1964-2014	Other survey	SBH
Quintana Roo	1965-2015	Census	SBH
Quintana Roo	1973-1992	Other survey	CBH
Quintana Roo	1979-2016	Vital Registration	VR/SRS/DSP
San Luis Potosí	1961-1961	Census	SBH
San Luis Potosí	1964-2014	Other survey	SBH
San Luis Potosí	1965-2015	Census	SBH
San Luis Potosí	1971-1992	Other survey	CBH
San Luis Potosí	1979-2016	Vital Registration	VR/SRS/DSP
Sinaloa	1962-1962	Census	SBH
Sinaloa	1964-2014	Other survey	SBH
Sinaloa	1965-2015	Census	SBH
Sinaloa	1971-1992	Other survey	CBH
Sinaloa	1979-2016	Vital Registration	VR/SRS/DSP
Sonora	1961-1961	Census	SBH

Appendix Table 5. Under-5 mortality reference sources by source date and location, 1950-2017			
Location	Year range of data	Reference source	Data type
Sonora	1964-2014	Other survey	SBH
Sonora	1965-2015	Census	SBH
Sonora	1973-1992	Other survey	CBH
Sonora	1979-2016	Vital Registration	VR/SRS/DSP
Tabasco	1961-1961	Census	SBH
Tabasco	1964-2014	Other survey	SBH
Tabasco	1965-2015	Census	SBH
Tabasco	1971-1992	Other survey	CBH
Tabasco	1979-2016	Vital Registration	VR/SRS/DSP
Tamaulipas	1961-1961	Census	SBH
Tamaulipas	1964-1964	Census	SBH
Tamaulipas	1964-2014	Other survey	SBH
Tamaulipas	1965-2015	Census	SBH
Tamaulipas	1973-1992	Other survey	CBH
Tamaulipas	1979-2016	Vital Registration	VR/SRS/DSP
Tlaxcala	1961-1961	Census	SBH
Tlaxcala	1964-2014	Other survey	SBH
Tlaxcala	1965-2015	Census	SBH
Tlaxcala	1971-1992	Other survey	CBH
Tlaxcala	1979-2016	Vital Registration	VR/SRS/DSP
Veracruz de Ignacio de la Llave	1961-1961	Census	SBH
Veracruz de Ignacio de la Llave	1964-1964	Census	SBH
Veracruz de Ignacio de la Llave	1964-2014	Other survey	SBH
Veracruz de Ignacio de la Llave	1965-2015	Census	SBH
Veracruz de Ignacio de la Llave	1971-1992	Other survey	CBH
Veracruz de Ignacio de la Llave	1979-2016	Vital Registration	VR/SRS/DSP
Yucatán	1960-1960	Census	SBH
Yucatán	1964-1964	Census	SBH
Yucatán	1964-2014	Other survey	SBH
Yucatán	1965-2015	Census	SBH
Yucatán	1971-1992	Other survey	CBH
Yucatán	1979-2016	Vital Registration	VR/SRS/DSP
Zacatecas	1962-1962	Census	SBH
Zacatecas	1964-2014	Other survey	SBH
Zacatecas	1965-2015	Census	SBH
Zacatecas	1971-1992	Other survey	CBH
Zacatecas	1979-2016	Vital Registration	VR/SRS/DSP
Nicaragua	1950-2004	Census	SBH
Nicaragua	1965-2006	Reproductive Health Survey	CBH
Nicaragua	1967-1967	Other survey	SBH
Nicaragua	1968-2001	Standard demographic and health survey	CBH
Nicaragua	1968-2004	Living Standards Measurement Study	SBH
Nicaragua	1968-2006	Reproductive Health Survey	SBH
Nicaragua	1970-1970	Other survey	SBH
Nicaragua	1973-1973	Other survey	SBH
Nicaragua	1973-2001	Standard demographic and health survey	SBH
Nicaragua	1976-1976	Other survey	SBH
Nicaragua	1979-1979	Other survey	SBH

Appendix Table 5. Under-5 mortality reference sources by source date and location, 1950-2017			
**Location**	**Year range of data**	**Reference source**	**Data type**
Nicaragua	1981-1981	Other survey	SBH
Nicaragua	1983-2012	Other survey	CBH
Nicaragua	1987-2011	Other demographic and health survey	SBH
Nicaragua	1988-1994	Vital Registration	VR/SRS/DSP
Nicaragua	1996-2015	Vital Registration	VR/SRS/DSP
Panama	1950-1950	Vital Registration	VR/SRS/DSP
Panama	1950-1975	World Fertility Survey	CBH
Panama	1950-2009	Census	SBH
Panama	1951-1975	World Fertility Survey	SBH
Panama	1952-2016	Vital Registration	VR/SRS/DSP
Panama	1972-2002	Living Standards Measurement Study	SBH
Venezuela	1950-1967	Vital Registration	VR/SRS/DSP
Venezuela	1952-1976	World Fertility Survey	SBH
Venezuela	1956-1977	World Fertility Survey	CBH
Venezuela	1956-2000	Census	SBH
Venezuela	1969-1994	Vital Registration	VR/SRS/DSP
Venezuela	1996-2014	Vital Registration	VR/SRS/DSP
Venezuela	1998-1998	Other survey	HH
Brazil	1950-2010	Census	SBH
Brazil	1966-1995	Standard demographic and health survey	CBH
Brazil	1967-1995	Standard demographic and health survey	SBH
Brazil	1972-1973	Other survey	SBH
Brazil	1974-2016	Vital Registration	VR/SRS/DSP
Brazil	1975-1979	Other survey	SBH
Brazil	1980-2009	Other survey	SBH
Brazil	1982-2006	Other demographic and health survey	SBH
Acre	1950-2010	Census	SBH
Acre	1971-1995	Standard demographic and health survey	SBH
Acre	1979-2016	Vital Registration	VR/SRS/DSP
Acre	1981-2009	Other survey	SBH
Acre	1984-1993	Standard demographic and health survey	CBH
Alagoas	1950-2010	Census	SBH
Alagoas	1962-1995	Standard demographic and health survey	SBH
Alagoas	1973-1973	Other survey	SBH
Alagoas	1974-1995	Standard demographic and health survey	CBH
Alagoas	1976-1979	Other survey	SBH
Alagoas	1979-2016	Vital Registration	VR/SRS/DSP
Alagoas	1980-2009	Other survey	SBH
Amapá	1950-2010	Census	SBH
Amapá	1971-1995	Standard demographic and health survey	SBH
Amapá	1979-2016	Vital Registration	VR/SRS/DSP
Amapá	1980-1981	Standard demographic and health survey	CBH
Amapá	1981-2009	Other survey	SBH
Amazonas	1950-2010	Census	SBH
Amazonas	1963-1995	Standard demographic and health survey	SBH
Amazonas	1979-2016	Vital Registration	VR/SRS/DSP
Amazonas	1980-1995	Standard demographic and health survey	CBH
Amazonas	1981-2009	Other survey	SBH

Appendix Table 5. Under-5 mortality reference sources by source date and location, 1950-2017			
**Location**	**Year range of data**	**Reference source**	**Data type**
Bahia	1950-2010	Census	SBH
Bahia	1962-1995	Standard demographic and health survey	SBH
Bahia	1973-2009	Other survey	SBH
Bahia	1976-1995	Standard demographic and health survey	CBH
Bahia	1979-2016	Vital Registration	VR/SRS/DSP
Ceará	1950-2010	Census	SBH
Ceará	1962-1995	Standard demographic and health survey	SBH
Ceará	1973-1974	Other survey	SBH
Ceará	1976-1979	Other survey	SBH
Ceará	1976-1995	Standard demographic and health survey	CBH
Ceará	1979-2016	Vital Registration	VR/SRS/DSP
Ceará	1980-2009	Other survey	SBH
Distrito Federal	1950-2010	Census	SBH
Distrito Federal	1962-1995	Standard demographic and health survey	SBH
Distrito Federal	1972-2009	Other survey	SBH
Distrito Federal	1979-2016	Vital Registration	VR/SRS/DSP
Distrito Federal	1982-1983	Standard demographic and health survey	CBH
Distrito Federal	1986-1993	Standard demographic and health survey	CBH
Espírito Santo	1950-2010	Census	SBH
Espírito Santo	1962-1995	Standard demographic and health survey	SBH
Espírito Santo	1971-1972	Other survey	SBH
Espírito Santo	1975-1979	Other survey	SBH
Espírito Santo	1978-1993	Standard demographic and health survey	CBH
Espírito Santo	1979-2016	Vital Registration	VR/SRS/DSP
Espírito Santo	1980-2009	Other survey	SBH
Goiás	1950-2010	Census	SBH
Goiás	1962-1995	Standard demographic and health survey	SBH
Goiás	1972-1973	Other survey	SBH
Goiás	1975-1979	Other survey	SBH
Goiás	1978-1979	Standard demographic and health survey	CBH
Goiás	1979-2016	Vital Registration	VR/SRS/DSP
Goiás	1980-2009	Other survey	SBH
Goiás	1982-1995	Standard demographic and health survey	CBH
Maranhão	1950-2010	Census	SBH
Maranhão	1962-1995	Standard demographic and health survey	SBH
Maranhão	1973-1973	Other survey	SBH
Maranhão	1975-1979	Other survey	SBH
Maranhão	1976-1995	Standard demographic and health survey	CBH
Maranhão	1979-2016	Vital Registration	VR/SRS/DSP
Maranhão	1980-2009	Other survey	SBH
Mato Grosso	1950-2010	Census	SBH
Mato Grosso	1962-1995	Standard demographic and health survey	SBH
Mato Grosso	1972-1972	Other survey	SBH
Mato Grosso	1974-1979	Other survey	SBH
Mato Grosso	1979-2016	Vital Registration	VR/SRS/DSP
Mato Grosso	1980-1995	Standard demographic and health survey	CBH
Mato Grosso	1980-2009	Other survey	SBH
Mato Grosso do Sul	1956-2010	Census	SBH

Appendix Table 5. Under-5 mortality reference sources by source date and location, 1950-2017			
**Location**	**Year range of data**	**Reference source**	**Data type**
Mato Grosso do Sul	1959-1995	Standard demographic and health survey	SBH
Mato Grosso do Sul	1972-1973	Other survey	SBH
Mato Grosso do Sul	1975-1979	Other survey	SBH
Mato Grosso do Sul	1978-1995	Standard demographic and health survey	CBH
Mato Grosso do Sul	1979-2016	Vital Registration	VR/SRS/DSP
Mato Grosso do Sul	1980-2009	Other survey	SBH
Minas Gerais	1950-2010	Census	SBH
Minas Gerais	1962-1995	Standard demographic and health survey	SBH
Minas Gerais	1972-1973	Other survey	SBH
Minas Gerais	1975-1976	Other survey	SBH
Minas Gerais	1978-1979	Other survey	SBH
Minas Gerais	1979-2016	Vital Registration	VR/SRS/DSP
Minas Gerais	1980-1995	Standard demographic and health survey	CBH
Minas Gerais	1980-2009	Other survey	SBH
Pará	1950-2010	Census	SBH
Pará	1962-1995	Standard demographic and health survey	SBH
Pará	1979-2016	Vital Registration	VR/SRS/DSP
Pará	1980-1995	Standard demographic and health survey	CBH
Pará	1981-2009	Other survey	SBH
Paraíba	1950-2010	Census	SBH
Paraíba	1962-1995	Standard demographic and health survey	SBH
Paraíba	1973-1974	Other survey	SBH
Paraíba	1976-1979	Other survey	SBH
Paraíba	1976-1995	Standard demographic and health survey	CBH
Paraíba	1980-2009	Other survey	SBH
Paraíba	1980-2016	Vital Registration	VR/SRS/DSP
Paraná	1950-2010	Census	SBH
Paraná	1962-1995	Standard demographic and health survey	SBH
Paraná	1972-1973	Other survey	SBH
Paraná	1975-1979	Other survey	SBH
Paraná	1979-2016	Vital Registration	VR/SRS/DSP
Paraná	1980-1987	Standard demographic and health survey	CBH
Paraná	1980-2009	Other survey	SBH
Paraná	1990-1995	Standard demographic and health survey	CBH
Pernambuco	1950-2010	Census	SBH
Pernambuco	1962-1995	Standard demographic and health survey	SBH
Pernambuco	1973-1973	Other survey	SBH
Pernambuco	1976-1977	Other survey	SBH
Pernambuco	1976-1995	Standard demographic and health survey	CBH
Pernambuco	1979-1979	Other survey	SBH
Pernambuco	1979-2016	Vital Registration	VR/SRS/DSP
Pernambuco	1980-2009	Other survey	SBH
Piaui	1950-2010	Census	SBH
Piaui	1962-1995	Standard demographic and health survey	SBH
Piaui	1973-1975	Other survey	SBH
Piaui	1976-1991	Standard demographic and health survey	CBH
Piaui	1977-1979	Other survey	SBH
Piaui	1979-2016	Vital Registration	VR/SRS/DSP

Appendix Table 5. Under-5 mortality reference sources by source date and location, 1950-2017

Location	Year range of data	Reference source	Data type
Piaui	1980-2009	Other survey	SBH
Piaui	1994-1995	Standard demographic and health survey	CBH
Rio de Janeiro	1950-2010	Census	SBH
Rio de Janeiro	1962-1995	Standard demographic and health survey	SBH
Rio de Janeiro	1971-1972	Other survey	SBH
Rio de Janeiro	1974-1979	Other survey	SBH
Rio de Janeiro	1978-1995	Standard demographic and health survey	CBH
Rio de Janeiro	1979-2016	Vital Registration	VR/SRS/DSP
Rio de Janeiro	1980-2009	Other survey	SBH
Rio Grande do Norte	1950-2010	Census	SBH
Rio Grande do Norte	1962-1995	Standard demographic and health survey	SBH
Rio Grande do Norte	1972-1972	Other survey	SBH
Rio Grande do Norte	1974-1974	Other survey	SBH
Rio Grande do Norte	1976-1979	Other survey	SBH
Rio Grande do Norte	1976-1995	Standard demographic and health survey	CBH
Rio Grande do Norte	1979-2016	Vital Registration	VR/SRS/DSP
Rio Grande do Norte	1980-2009	Other survey	SBH
Rio Grande do Sul	1950-2010	Census	SBH
Rio Grande do Sul	1962-1995	Standard demographic and health survey	SBH
Rio Grande do Sul	1971-1972	Other survey	SBH
Rio Grande do Sul	1974-1979	Other survey	SBH
Rio Grande do Sul	1978-1995	Standard demographic and health survey	CBH
Rio Grande do Sul	1979-2016	Vital Registration	VR/SRS/DSP
Rio Grande do Sul	1980-2009	Other survey	SBH
Rondônia	1950-2010	Census	SBH
Rondônia	1971-1995	Standard demographic and health survey	SBH
Rondônia	1978-1983	Standard demographic and health survey	CBH
Rondônia	1979-2016	Vital Registration	VR/SRS/DSP
Rondônia	1981-2009	Other survey	SBH
Rondônia	1986-1989	Standard demographic and health survey	CBH
Rondônia	1992-1993	Standard demographic and health survey	CBH
Roraima	1950-2010	Census	SBH
Roraima	1971-1995	Standard demographic and health survey	SBH
Roraima	1979-2016	Vital Registration	VR/SRS/DSP
Roraima	1981-2009	Other survey	SBH
Roraima	1986-1987	Standard demographic and health survey	CBH
Roraima	1992-1993	Standard demographic and health survey	CBH
Santa Catarina	1950-2010	Census	SBH
Santa Catarina	1962-1995	Standard demographic and health survey	SBH
Santa Catarina	1972-1973	Other survey	SBH
Santa Catarina	1975-1979	Other survey	SBH
Santa Catarina	1979-2016	Vital Registration	VR/SRS/DSP
Santa Catarina	1980-1983	Standard demographic and health survey	CBH
Santa Catarina	1980-2009	Other survey	SBH
Santa Catarina	1986-1993	Standard demographic and health survey	CBH
São Paulo	1950-2010	Census	SBH
São Paulo	1962-1995	Standard demographic and health survey	SBH
São Paulo	1971-1972	Other survey	SBH

Appendix Table 5. Under-5 mortality reference sources by source date and location, 1950-2017

Location	Year range of data	Reference source	Data type
São Paulo	1974-1979	Other survey	SBH
São Paulo	1979-2016	Vital Registration	VR/SRS/DSP
São Paulo	1980-1995	Standard demographic and health survey	CBH
São Paulo	1980-2009	Other survey	SBH
Sergipe	1950-2010	Census	SBH
Sergipe	1962-1995	Standard demographic and health survey	SBH
Sergipe	1973-1974	Other survey	SBH
Sergipe	1976-1979	Other survey	SBH
Sergipe	1976-1995	Standard demographic and health survey	CBH
Sergipe	1979-2016	Vital Registration	VR/SRS/DSP
Sergipe	1980-2009	Other survey	SBH
Tocantins	1967-2010	Census	SBH
Tocantins	1971-1995	Standard demographic and health survey	SBH
Tocantins	1981-2009	Other survey	SBH
Tocantins	1986-1987	Standard demographic and health survey	CBH
Tocantins	1990-1995	Standard demographic and health survey	CBH
Tocantins	1990-2016	Vital Registration	VR/SRS/DSP
Paraguay	1950-1991	Vital Registration	VR/SRS/DSP
Paraguay	1950-2001	Census	SBH
Paraguay	1953-1978	World Fertility Survey	CBH
Paraguay	1954-1978	World Fertility Survey	SBH
Paraguay	1965-1990	Standard demographic and health survey	CBH
Paraguay	1966-1990	Standard demographic and health survey	SBH
Paraguay	1970-2008	Reproductive Health Survey	CBH
Paraguay	1971-2008	Reproductive Health Survey	SBH
Paraguay	1972-1999	Other survey	SBH
Paraguay	1989-2016	Multiple Indicator Cluster Survey	CBH
Paraguay	1992-2016	Multiple Indicator Cluster Survey	SBH
Paraguay	1994-2014	Vital Registration	VR/SRS/DSP
Afghanistan	1982-2006	Other survey	SBH
Afghanistan	1983-1983	Multiple Indicator Cluster Survey	SBH
Afghanistan	1984-2015	Standard demographic and health survey	CBH
Afghanistan	1986-2010	Multiple Indicator Cluster Survey	SBH
Afghanistan	1991-2015	Standard demographic and health survey	SBH
Afghanistan	2009-2009	Other survey	SBH
Algeria	1950-1956	Vital Registration	VR/SRS/DSP
Algeria	1961-1992	Pan Arab Project for Child Development	CBH
Algeria	1964-1965	Vital Registration	VR/SRS/DSP
Algeria	1967-1991	Pan Arab Project for Child Development	SBH
Algeria	1975-2002	Pan Arab Project for Family Health	CBH
Algeria	1977-2001	Pan Arab Project for Family Health	SBH
Algeria	1980-1982	Vital Registration	VR/SRS/DSP
Algeria	1983-2012	Multiple Indicator Cluster Survey	CBH
Algeria	1985-1986	Vital Registration	VR/SRS/DSP
Algeria	1988-2012	Multiple Indicator Cluster Survey	SBH
Algeria	1990-1990	Vital Registration	VR/SRS/DSP
Algeria	1998-1998	Vital Registration	VR/SRS/DSP
Algeria	2000-2000	Vital Registration	VR/SRS/DSP

Appendix Table 5. Under-5 mortality reference sources by source date and location, 1950-2017

Location	Year range of data	Reference source	Data type
Algeria	2007-2011	Vital Registration	VR/SRS/DSP
Algeria	2014-2016	Vital Registration	VR/SRS/DSP
Bahrain	1952-1952	Census	SBH
Bahrain	1956-1956	Census	SBH
Bahrain	1959-1959	Census	SBH
Bahrain	1962-1962	Census	SBH
Bahrain	1965-1965	Census	SBH
Bahrain	1967-1967	Census	SBH
Bahrain	1972-1972	Pan Arab Project for Child Development	HH
Bahrain	1972-1972	Pan Arab Project for Child Development	SBH
Bahrain	1973-1973	Census	SBH
Bahrain	1975-1975	Census	SBH
Bahrain	1975-1975	Pan Arab Project for Child Development	SBH
Bahrain	1977-1977	Pan Arab Project for Family Health	HH
Bahrain	1977-1977	Pan Arab Project for Child Development	HH
Bahrain	1977-1977	Pan Arab Project for Child Development	SBH
Bahrain	1979-1979	Census	SBH
Bahrain	1980-1980	Pan Arab Project for Child Development	SBH
Bahrain	1980-1982	Vital Registration	VR/SRS/DSP
Bahrain	1982-1982	Census	SBH
Bahrain	1982-1982	Pan Arab Project for Family Health	HH
Bahrain	1982-1982	Pan Arab Project for Child Development	HH
Bahrain	1983-1983	Pan Arab Project for Child Development	SBH
Bahrain	1984-1993	Vital Registration	VR/SRS/DSP
Bahrain	1985-1985	Census	SBH
Bahrain	1985-1985	Pan Arab Project for Child Development	SBH
Bahrain	1987-1987	Pan Arab Project for Family Health	HH
Bahrain	1987-1987	Pan Arab Project for Child Development	HH
Bahrain	1987-1988	Census	SBH
Bahrain	1992-1992	Census	SBH
Bahrain	1992-1992	Pan Arab Project for Family Health	HH
Bahrain	1995-1995	Census	SBH
Bahrain	1995-2012	Vital Registration	VR/SRS/DSP
Bahrain	1998-1998	Census	SBH
Bahrain	2014-2014	Vital Registration	VR/SRS/DSP
Egypt	1950-1957	Vital Registration	VR/SRS/DSP
Egypt	1950-1979	World Fertility Survey	CBH
Egypt	1955-1979	World Fertility Survey	SBH
Egypt	1957-1957	Census	SBH
Egypt	1957-1990	Pan Arab Project for Child Development	CBH
Egypt	1959-1981	Vital Registration	VR/SRS/DSP
Egypt	1959-2013	Standard demographic and health survey	CBH
Egypt	1960-1960	Census	SBH
Egypt	1964-1964	Census	SBH
Egypt	1964-2013	Standard demographic and health survey	SBH
Egypt	1966-1966	Other survey	SBH
Egypt	1966-1990	Pan Arab Project for Child Development	SBH
Egypt	1967-1967	Census	SBH

Appendix Table 5. Under-5 mortality reference sources by source date and location, 1950-2017			
**Location**	**Year range of data**	**Reference source**	**Data type**
Egypt	1969-1969	Other survey	SBH
Egypt	1970-1970	Census	SBH
Egypt	1972-1972	Census	SBH
Egypt	1973-1973	Other survey	SBH
Egypt	1975-1975	Other survey	SBH
Egypt	1978-1978	Other survey	SBH
Egypt	1978-2014	Other demographic and health survey	SBH
Egypt	1980-1980	Other survey	SBH
Egypt	1983-2015	Vital Registration	VR/SRS/DSP
Egypt	1985-1986	Standard demographic and health survey	HH
Egypt	1986-2013	Multiple Indicator Cluster Survey	CBH
Egypt	1990-1991	Standard demographic and health survey	HH
Egypt	1995-1996	Standard demographic and health survey	HH
Iran	1967-2000	Other survey	CBH
Iran	1974-1974	Other survey	HH
Iran	1975-1999	Other survey	SBH
Iran	1981-2016	Census	SBH
Iran	1984-1984	Other survey	HH
Iran	1994-1994	Other survey	HH
Iran	2005-2005	Standard demographic and health survey	HH
Iran	2007-2007	Standard demographic and health survey	HH
Iran	2015-2016	Vital Registration	VR/SRS/DSP
Iraq	1969-1969	Census	SBH
Iraq	1969-2006	Other survey	CBH
Iraq	1972-1996	Census	SBH
Iraq	1973-1973	Demographic surveillance sites	DSS
Iraq	1976-1976	Other survey	HH
Iraq	1976-1977	Vital Registration	VR/SRS/DSP
Iraq	1976-2010	Multiple Indicator Cluster Survey	CBH
Iraq	1979-2005	Other survey	SBH
Iraq	1981-1981	Other survey	HH
Iraq	1981-2010	Multiple Indicator Cluster Survey	SBH
Iraq	1986-1986	Other survey	HH
Iraq	1987-1989	Vital Registration	VR/SRS/DSP
Iraq	1990-1991	Other survey	HH
Iraq	1996-1996	Other survey	HH
Iraq	2001-2001	Other survey	HH
Iraq	2008-2008	Vital Registration	VR/SRS/DSP
Iraq	2012-2016	Vital Registration	VR/SRS/DSP
Jordan	1960-1960	Census	SBH
Jordan	1963-1966	Standard demographic and health survey	CBH
Jordan	1964-1964	Census	SBH
Jordan	1964-1964	Other survey	SBH
Jordan	1966-1966	Standard demographic and health survey	SBH
Jordan	1968-1968	Census	SBH
Jordan	1968-1968	Other survey	SBH
Jordan	1968-1980	Vital Registration	VR/SRS/DSP
Jordan	1968-2012	Standard demographic and health survey	CBH

Appendix Table 5. Under-5 mortality reference sources by source date and location, 1950-2017			
**Location**	**Year range of data**	**Reference source**	**Data type**
Jordan	1968-2012	Standard demographic and health survey	SBH
Jordan	1971-1971	Census	SBH
Jordan	1971-1971	Other survey	SBH
Jordan	1973-1973	Census	SBH
Jordan	1973-1978	Other survey	SBH
Jordan	1976-1976	Census	SBH
Jordan	1980-1982	Other survey	SBH
Jordan	1984-1985	Other survey	SBH
Jordan	1985-2009	Other demographic and health survey	SBH
Jordan	1987-1987	Other survey	SBH
Jordan	1990-1990	Other survey	SBH
Jordan	1993-1993	Other survey	SBH
Jordan	1995-1995	Other survey	SBH
Jordan	2004-2006	Vital Registration	VR/SRS/DSP
Jordan	2008-2013	Vital Registration	VR/SRS/DSP
Kuwait	1956-1956	Census	SBH
Kuwait	1960-1961	Census	SBH
Kuwait	1962-1989	Vital Registration	VR/SRS/DSP
Kuwait	1963-1963	Census	SBH
Kuwait	1965-1966	Census	SBH
Kuwait	1966-1966	Pan Arab Project for Child Development	SBH
Kuwait	1968-1969	Census	SBH
Kuwait	1970-1970	Pan Arab Project for Child Development	SBH
Kuwait	1971-1971	Census	SBH
Kuwait	1973-1973	Pan Arab Project for Child Development	SBH
Kuwait	1974-1974	Census	SBH
Kuwait	1976-1976	Census	SBH
Kuwait	1976-1976	Pan Arab Project for Child Development	SBH
Kuwait	1978-1978	Pan Arab Project for Family Health	HH
Kuwait	1981-1981	Pan Arab Project for Child Development	SBH
Kuwait	1984-1984	Pan Arab Project for Family Health	HH
Kuwait	1984-1984	Pan Arab Project for Child Development	SBH
Kuwait	1988-1988	Pan Arab Project for Family Health	HH
Kuwait	1991-2015	Vital Registration	VR/SRS/DSP
Kuwait	1994-1994	Pan Arab Project for Family Health	HH
Lebanon	1966-1995	Pan Arab Project for Child Development	CBH
Lebanon	1971-1995	Pan Arab Project for Child Development	SBH
Lebanon	1979-2003	Pan Arab Project for Family Health	SBH
Lebanon	1979-2004	Pan Arab Project for Family Health	CBH
Lebanon	1998-1998	Multiple Indicator Cluster Survey	SBH
Lebanon	2004-2004	Vital Registration	VR/SRS/DSP
Lebanon	2009-2009	Multiple Indicator Cluster Survey	SBH
Lebanon	2011-2015	Vital Registration	VR/SRS/DSP
Libya	1956-1956	Census	SBH
Libya	1959-1959	Census	SBH
Libya	1962-1962	Census	SBH
Libya	1964-1995	Pan Arab Project for Child Development	CBH
Libya	1965-1965	Census	SBH

Appendix Table 5. Under-5 mortality reference sources by source date and location, 1950-2017			
**Location**	**Year range of data**	**Reference source**	**Data type**
Libya	1967-1967	Census	SBH
Libya	1969-1969	Census	SBH
Libya	1970-1994	Pan Arab Project for Child Development	SBH
Libya	1972-1976	Vital Registration	VR/SRS/DSP
Libya	1981-1981	Vital Registration	VR/SRS/DSP
Libya	1983-2007	Pan Arab Project for Family Health	SBH
Libya	1996-1996	Vital Registration	VR/SRS/DSP
Libya	2000-2000	Vital Registration	VR/SRS/DSP
Libya	2002-2002	Vital Registration	VR/SRS/DSP
Libya	2003-2003	Multiple Indicator Cluster Survey	SBH
Libya	2006-2011	Vital Registration	VR/SRS/DSP
Morocco	1953-1980	World Fertility Survey	CBH
Morocco	1955-1979	World Fertility Survey	SBH
Morocco	1957-2003	Census	SBH
Morocco	1958-2003	Standard demographic and health survey	CBH
Morocco	1963-1994	Other demographic and health survey	CBH
Morocco	1963-2003	Standard demographic and health survey	SBH
Morocco	1970-1994	Other demographic and health survey	SBH
Morocco	1970-1997	Pan Arab Project for Child Development	CBH
Morocco	1972-1996	Pan Arab Project for Child Development	SBH
Morocco	1989-1991	Vital Registration	VR/SRS/DSP
Morocco	1993-1993	Vital Registration	VR/SRS/DSP
Morocco	1995-1997	Vital Registration	VR/SRS/DSP
Morocco	1999-1999	Vital Registration	VR/SRS/DSP
Morocco	2001-2001	Vital Registration	VR/SRS/DSP
Morocco	2007-2007	Vital Registration	VR/SRS/DSP
Morocco	2011-2011	Other survey	HH
Palestine	1970-2003	Other survey	CBH
Palestine	1975-2013	Multiple Indicator Cluster Survey	SBH
Palestine	1977-2013	Multiple Indicator Cluster Survey	CBH
Palestine	1979-1979	Census	SBH
Palestine	1980-2004	Other demographic and health survey	SBH
Palestine	1983-1983	Census	SBH
Palestine	1986-1986	Census	SBH
Palestine	1988-1988	Census	SBH
Palestine	1990-1990	Census	SBH
Palestine	1992-1992	Other demographic and health survey	HH
Palestine	1992-1993	Census	SBH
Palestine	1995-1995	Census	SBH
Palestine	1998-1998	Census	SBH
Palestine	2001-2001	Census	SBH
Palestine	2001-2001	Other survey	HH
Palestine	2003-2003	Census	SBH
Palestine	2004-2004	Pan Arab Project for Family Health	HH
Palestine	2008-2016	Vital Registration	VR/SRS/DSP
Oman	1960-1960	Other survey	SBH
Oman	1963-1964	Other survey	SBH
Oman	1966-1966	Other survey	SBH

Appendix Table 5. Under-5 mortality reference sources by source date and location, 1950-2017			
**Location**	**Year range of data**	**Reference source**	**Data type**
Oman	1968-1973	Other survey	SBH
Oman	1971-1971	Pan Arab Project for Child Development	HH
Oman	1972-1972	Pan Arab Project for Child Development	SBH
Oman	1975-1975	Pan Arab Project for Child Development	SBH
Oman	1975-1976	Other survey	SBH
Oman	1976-1976	Pan Arab Project for Child Development	HH
Oman	1978-1978	Pan Arab Project for Family Health	HH
Oman	1978-1978	Pan Arab Project for Child Development	SBH
Oman	1979-1979	Census	SBH
Oman	1981-1981	Pan Arab Project for Child Development	HH
Oman	1981-1981	Pan Arab Project for Child Development	SBH
Oman	1982-1982	Census	SBH
Oman	1983-1983	Pan Arab Project for Family Health	HH
Oman	1984-1984	Pan Arab Project for Child Development	SBH
Oman	1985-1985	Census	SBH
Oman	1986-1986	Pan Arab Project for Child Development	HH
Oman	1986-1986	Pan Arab Project for Child Development	SBH
Oman	1987-1987	Pan Arab Project for Family Health	HH
Oman	1988-1988	Census	SBH
Oman	1990-1990	Census	SBH
Oman	1992-1992	Census	SBH
Oman	1993-1993	Pan Arab Project for Family Health	HH
Oman	1999-1999	Multiple Indicator Cluster Survey	HH
Oman	2004-2004	Multiple Indicator Cluster Survey	HH
Oman	2004-2014	Vital Registration	VR/SRS/DSP
Oman	2009-2009	Multiple Indicator Cluster Survey	HH
Oman	2016-2016	Vital Registration	VR/SRS/DSP
Qatar	1970-1970	Pan Arab Project for Child Development	HH
Qatar	1973-1973	Pan Arab Project for Child Development	SBH
Qatar	1975-1975	Pan Arab Project for Child Development	HH
Qatar	1975-1975	Pan Arab Project for Child Development	SBH
Qatar	1979-1979	Pan Arab Project for Family Health	HH
Qatar	1980-1980	Pan Arab Project for Child Development	HH
Qatar	1980-1980	Pan Arab Project for Child Development	SBH
Qatar	1981-2016	Vital Registration	VR/SRS/DSP
Qatar	1984-1984	Pan Arab Project for Family Health	HH
Qatar	1985-1985	Pan Arab Project for Child Development	HH
Qatar	1985-1985	Pan Arab Project for Child Development	SBH
Qatar	1989-1989	Pan Arab Project for Family Health	HH
Qatar	1994-1994	Pan Arab Project for Family Health	HH
Saudi Arabia	1971-1971	Pan Arab Project for Child Development	SBH
Saudi Arabia	1975-1975	Pan Arab Project for Child Development	SBH
Saudi Arabia	1976-1976	Other survey	SBH
Saudi Arabia	1978-1978	Pan Arab Project for Family Health	HH
Saudi Arabia	1978-1978	Pan Arab Project for Child Development	SBH
Saudi Arabia	1979-1979	Other survey	SBH
Saudi Arabia	1980-1981	Pan Arab Project for Child Development	SBH
Saudi Arabia	1982-1982	Other survey	SBH

Appendix Table 5. Under-5 mortality reference sources by source date and location, 1950-2017

Location	Year range of data	Reference source	Data type
Saudi Arabia	1984-1984	Pan Arab Project for Family Health	HH
Saudi Arabia	1984-1984	Pan Arab Project for Child Development	SBH
Saudi Arabia	1984-1984	Other survey	SBH
Saudi Arabia	1987-1987	Other survey	SBH
Saudi Arabia	1988-1988	Pan Arab Project for Family Health	HH
Saudi Arabia	1989-1989	Other survey	SBH
Saudi Arabia	1994-1994	Pan Arab Project for Family Health	HH
Saudi Arabia	2004-2004	Census	HH
Saudi Arabia	2006-2006	Other survey	HH
Saudi Arabia	2016-2016	Other survey	HH
Sudan	1955-1978	World Fertility Survey	CBH
Sudan	1962-1989	Standard demographic and health survey	CBH
Sudan	1963-1992	Pan Arab Project for Child Development	CBH
Sudan	1964-1964	Census	SBH
Sudan	1965-1989	Standard demographic and health survey	SBH
Sudan	1968-1968	Census	SBH
Sudan	1968-1992	Pan Arab Project for Child Development	SBH
Sudan	1971-1971	Census	SBH
Sudan	1974-1974	Census	SBH
Sudan	1977-1979	Census	SBH
Sudan	1981-1981	Census	SBH
Sudan	1982-2014	Multiple Indicator Cluster Survey	CBH
Sudan	1985-1985	Census	SBH
Sudan	1985-2014	Multiple Indicator Cluster Survey	SBH
Sudan	1987-1987	Census	SBH
Sudan	1990-2007	Census	SBH
Sudan	1997-1997	Multiple Indicator Cluster Survey	HH
Sudan	2002-2002	Multiple Indicator Cluster Survey	HH
Sudan	2003-2003	Pan Arab Project for Family Health	HH
Sudan	2007-2007	Multiple Indicator Cluster Survey	HH
Syria	1953-1953	Census	SBH
Syria	1953-1977	World Fertility Survey	SBH
Syria	1953-1978	World Fertility Survey	CBH
Syria	1956-1956	Census	SBH
Syria	1959-1959	Census	SBH
Syria	1961-1961	Census	SBH
Syria	1963-1992	Pan Arab Project for Child Development	CBH
Syria	1964-1964	Census	SBH
Syria	1964-1978	Vital Registration	VR/SRS/DSP
Syria	1966-1966	Census	SBH
Syria	1968-1992	Pan Arab Project for Child Development	SBH
Syria	1974-2009	Pan Arab Project for Family Health	CBH
Syria	1976-1976	Census	SBH
Syria	1976-2000	Pan Arab Project for Family Health	SBH
Syria	1980-1980	Census	SBH
Syria	1981-2005	Multiple Indicator Cluster Survey	SBH
Syria	1983-1983	Census	SBH
Syria	1983-1985	Vital Registration	VR/SRS/DSP

Appendix Table 5. Under-5 mortality reference sources by source date and location, 1950-2017

Location	Year range of data	Reference source	Data type
Syria	1985-1985	Census	SBH
Syria	1988-1988	Census	SBH
Syria	1990-1990	Census	SBH
Syria	1998-2010	Vital Registration	VR/SRS/DSP
Tunisia	1953-1978	World Fertility Survey	CBH
Tunisia	1954-1978	World Fertility Survey	SBH
Tunisia	1960-1960	Vital Registration	VR/SRS/DSP
Tunisia	1963-1988	Standard demographic and health survey	CBH
Tunisia	1964-1988	Standard demographic and health survey	SBH
Tunisia	1965-1965	Other survey	SBH
Tunisia	1965-1994	Pan Arab Project for Child Development	CBH
Tunisia	1968-1968	Other survey	SBH
Tunisia	1968-1974	Vital Registration	VR/SRS/DSP
Tunisia	1970-1970	Census	SBH
Tunisia	1970-1994	Pan Arab Project for Child Development	SBH
Tunisia	1971-1971	Other survey	SBH
Tunisia	1973-1973	Census	SBH
Tunisia	1974-1974	Other survey	SBH
Tunisia	1976-1976	Census	SBH
Tunisia	1976-1980	Vital Registration	VR/SRS/DSP
Tunisia	1976-2000	Pan Arab Project for Family Health	SBH
Tunisia	1976-2001	Pan Arab Project for Family Health	CBH
Tunisia	1977-1977	Other survey	SBH
Tunisia	1978-1978	Census	SBH
Tunisia	1979-1979	Other survey	SBH
Tunisia	1980-1980	Census	SBH
Tunisia	1986-2008	Multiple Indicator Cluster Survey	CBH
Tunisia	1987-1989	Vital Registration	VR/SRS/DSP
Tunisia	1987-2008	Multiple Indicator Cluster Survey	SBH
Tunisia	1993-1995	Vital Registration	VR/SRS/DSP
Tunisia	1998-1998	Vital Registration	VR/SRS/DSP
Tunisia	2010-2011	Multiple Indicator Cluster Survey	CBH
Tunisia	2010-2011	Multiple Indicator Cluster Survey	SBH
Turkey	1951-1951	Census	SBH
Turkey	1953-1978	World Fertility Survey	CBH
Turkey	1954-1978	World Fertility Survey	SBH
Turkey	1955-1955	Census	SBH
Turkey	1958-1958	Census	SBH
Turkey	1960-1999	Census	SBH
Turkey	1962-2010	Other survey	SBH
Turkey	1966-2008	Standard demographic and health survey	CBH
Turkey	1969-2003	Standard demographic and health survey	SBH
Turkey	1978-1979	Vital Registration	VR/SRS/DSP
Turkey	1981-1984	Vital Registration	VR/SRS/DSP
Turkey	1984-2008	Other demographic and health survey	SBH
Turkey	1987-2016	Vital Registration	VR/SRS/DSP
Turkey	2000-2000	Other demographic and health survey	HH
Turkey	2005-2005	Other demographic and health survey	HH

Appendix Table 5. Under-5 mortality reference sources by source date and location, 1950-2017			
**Location**	**Year range of data**	**Reference source**	**Data type**
Turkey	2010-2010	Other demographic and health survey	HH
Turkey	2010-2010	Other demographic and health survey	SBH
United Arab Emirates	1956-1956	Census	SBH
United Arab Emirates	1959-1959	Census	SBH
United Arab Emirates	1963-1963	Census	SBH
United Arab Emirates	1966-1966	Census	SBH
United Arab Emirates	1969-1969	Census	SBH
United Arab Emirates	1970-1970	Pan Arab Project for Child Development	SBH
United Arab Emirates	1971-1971	Census	SBH
United Arab Emirates	1974-1974	Pan Arab Project for Child Development	SBH
United Arab Emirates	1977-1977	Pan Arab Project for Family Health	HH
United Arab Emirates	1977-1977	Pan Arab Project for Child Development	SBH
United Arab Emirates	1979-1979	Pan Arab Project for Child Development	SBH
United Arab Emirates	1981-1981	Pan Arab Project for Child Development	SBH
United Arab Emirates	1982-1982	Pan Arab Project for Family Health	HH
United Arab Emirates	1987-1987	Pan Arab Project for Family Health	HH
United Arab Emirates	1992-1992	Pan Arab Project for Family Health	HH
United Arab Emirates	1995-1999	Vital Registration	VR/SRS/DSP
United Arab Emirates	2001-2007	Vital Registration	VR/SRS/DSP
Yemen	1955-1979	World Fertility Survey	SBH
Yemen	1960-1979	World Fertility Survey	CBH
Yemen	1962-2013	Standard demographic and health survey	CBH
Yemen	1967-2013	Standard demographic and health survey	SBH
Yemen	1973-2002	Pan Arab Project for Family Health	CBH
Yemen	1975-1975	Standard demographic and health survey	HH
Yemen	1976-1976	Census	SBH
Yemen	1978-2002	Pan Arab Project for Family Health	SBH
Yemen	1980-1980	Census	SBH
Yemen	1980-1980	Standard demographic and health survey	HH
Yemen	1981-2006	Multiple Indicator Cluster Survey	CBH
Yemen	1982-2006	Multiple Indicator Cluster Survey	SBH
Yemen	1983-1983	Census	SBH
Yemen	1985-1985	Standard demographic and health survey	HH
Yemen	1986-1986	Census	SBH
Yemen	1988-1988	Census	SBH
Yemen	1988-2012	Other survey	SBH
Yemen	1990-1990	Census	SBH
Yemen	1990-1990	Standard demographic and health survey	HH
Yemen	1995-1995	Standard demographic and health survey	HH
Yemen	2004-2004	Other survey	HH
Bangladesh	1950-1970	World Fertility Survey	CBH
Bangladesh	1951-1970	World Fertility Survey	SBH
Bangladesh	1955-1955	Other survey	SBH
Bangladesh	1959-1959	Other survey	SBH
Bangladesh	1962-1963	Other survey	SBH
Bangladesh	1964-1970	Standard demographic and health survey	CBH
Bangladesh	1965-1965	Other survey	SBH
Bangladesh	1967-1968	Other survey	SBH

Appendix Table 5. Under-5 mortality reference sources by source date and location, 1950-2017

Location	Year range of data	Reference source	Data type
Bangladesh	1967-1970	Other demographic and health survey	CBH
Bangladesh	1969-1970	Standard demographic and health survey	SBH
Bangladesh	1970-1970	Other survey	SBH
Bangladesh	1972-1972	Other survey	SBH
Bangladesh	1972-1973	Standard demographic and health survey	CBH
Bangladesh	1972-1973	Standard demographic and health survey	SBH
Bangladesh	1972-1973	Other demographic and health survey	CBH
Bangladesh	1972-1973	World Fertility Survey	CBH
Bangladesh	1972-1973	World Fertility Survey	SBH
Bangladesh	1975-1975	World Fertility Survey	CBH
Bangladesh	1975-1975	World Fertility Survey	SBH
Bangladesh	1975-2000	Other demographic and health survey	CBH
Bangladesh	1975-2014	Standard demographic and health survey	CBH
Bangladesh	1975-2014	Standard demographic and health survey	SBH
Bangladesh	1976-1976	Other survey	SBH
Bangladesh	1976-2000	Other demographic and health survey	SBH
Bangladesh	1980-2010	Sample Registrations System	VR/SRS/DSP
Bangladesh	1988-2012	Multiple Indicator Cluster Survey	SBH
Bangladesh	1990-1990	Census	SBH
Bangladesh	1993-1993	Census	SBH
Bangladesh	1998-1998	Census	SBH
Bangladesh	2002-2002	Census	SBH
Bangladesh	2005-2005	Census	SBH
Bangladesh	2008-2008	Census	SBH
Bangladesh	2012-2014	Sample Registrations System	VR/SRS/DSP
Bhutan	1980-2004	Census	SBH
Bhutan	1985-2009	Multiple Indicator Cluster Survey	SBH
Bhutan	1992-1993	Other survey	HH
India	1950-1964	Vital Registration	VR/SRS/DSP
India	1960-2016	Standard demographic and health survey	CBH
India	1961-1961	Census	SBH
India	1965-1965	Census	SBH
India	1968-2015	Standard demographic and health survey	SBH
India	1969-1969	Census	SBH
India	1970-1980	Sample Registrations System	VR/SRS/DSP
India	1971-1972	Census	SBH
India	1974-1975	Census	SBH
India	1974-2013	Other survey	SBH
India	1976-2013	Other survey	CBH
India	1977-1978	Census	SBH
India	1981-1982	Census	SBH
India	1983-1984	Sample Registrations System	VR/SRS/DSP
India	1984-1985	Census	SBH
India	1986-1986	Sample Registrations System	VR/SRS/DSP
India	1987-1988	Census	SBH
India	1988-1989	Sample Registrations System	VR/SRS/DSP
India	1991-1992	Census	SBH
India	1992-1992	Sample Registrations System	VR/SRS/DSP

Appendix Table 5. Under-5 mortality reference sources by source date and location, 1950-2017			
**Location**	**Year range of data**	**Reference source**	**Data type**
India	1994-1995	Census	SBH
India	1995-1995	Sample Registrations System	VR/SRS/DSP
India	1997-2016	Sample Registrations System	VR/SRS/DSP
India	1998-1998	Census	SBH
India	2002-2002	Census	SBH
India	2005-2005	Census	SBH
India	2008-2008	Census	SBH
Andhra Pradesh	1979-2007	Other survey	SBH
Andhra Pradesh	1986-2015	Standard demographic and health survey	CBH
Andhra Pradesh	1990-2005	Other survey	CBH
Andhra Pradesh	1990-2013	Standard demographic and health survey	SBH
Andhra Pradesh	2013-2015	Vital Registration	VR/SRS/DSP
Andhra Pradesh	2014-2016	Sample Registrations System	VR/SRS/DSP
Arunachal Pradesh	1968-2016	Standard demographic and health survey	SBH
Arunachal Pradesh	1974-2013	Other survey	SBH
Arunachal Pradesh	1977-2016	Standard demographic and health survey	CBH
Arunachal Pradesh	1982-1982	Census	SBH
Arunachal Pradesh	1985-1985	Census	SBH
Arunachal Pradesh	1989-1989	Census	SBH
Arunachal Pradesh	1991-1992	Census	SBH
Arunachal Pradesh	1995-1995	Census	SBH
Arunachal Pradesh	1997-1999	Census	SBH
Arunachal Pradesh	2002-2002	Census	SBH
Arunachal Pradesh	2005-2005	Census	SBH
Arunachal Pradesh	2007-2008	Census	SBH
Arunachal Pradesh	2012-2013	Vital Registration	VR/SRS/DSP
Arunachal Pradesh	2015-2015	Vital Registration	VR/SRS/DSP
Assam	1968-2015	Standard demographic and health survey	SBH
Assam	1969-2015	Standard demographic and health survey	CBH
Assam	1974-2013	Other survey	SBH
Assam	1981-1981	Census	SBH
Assam	1985-1985	Census	SBH
Assam	1988-1988	Census	SBH
Assam	1988-2005	Other survey	CBH
Assam	1991-1992	Census	SBH
Assam	1994-1995	Census	SBH
Assam	1995-2016	Sample Registrations System	VR/SRS/DSP
Assam	1998-1998	Census	SBH
Assam	2002-2002	Census	SBH
Assam	2005-2005	Census	SBH
Assam	2008-2008	Census	SBH
Assam	2015-2015	Vital Registration	VR/SRS/DSP
Bihar	1965-2015	Standard demographic and health survey	CBH
Bihar	1968-2014	Standard demographic and health survey	SBH
Bihar	1974-2013	Other survey	SBH
Bihar	1981-1981	Census	SBH
Bihar	1985-1985	Census	SBH
Bihar	1986-2013	Other survey	CBH

Appendix Table 5. Under-5 mortality reference sources by source date and location, 1950-2017			
**Location**	**Year range of data**	**Reference source**	**Data type**
Bihar	1989-1989	Census	SBH
Bihar	1991-1992	Census	SBH
Bihar	1995-1995	Census	SBH
Bihar	1998-1999	Census	SBH
Bihar	2002-2002	Census	SBH
Bihar	2004-2016	Sample Registrations System	VR/SRS/DSP
Bihar	2005-2005	Census	SBH
Bihar	2007-2008	Census	SBH
Bihar	2010-2010	Vital Registration	VR/SRS/DSP
Chhattisgarh	1979-2013	Other survey	SBH
Chhattisgarh	1980-2015	Standard demographic and health survey	CBH
Chhattisgarh	1981-1981	Census	SBH
Chhattisgarh	1981-2015	Standard demographic and health survey	SBH
Chhattisgarh	1984-1984	Census	SBH
Chhattisgarh	1987-2013	Other survey	CBH
Chhattisgarh	1988-1988	Census	SBH
Chhattisgarh	1991-1992	Census	SBH
Chhattisgarh	1994-1995	Census	SBH
Chhattisgarh	1998-1998	Census	SBH
Chhattisgarh	2002-2002	Census	SBH
Chhattisgarh	2004-2016	Sample Registrations System	VR/SRS/DSP
Chhattisgarh	2005-2005	Census	SBH
Chhattisgarh	2008-2008	Census	SBH
Chhattisgarh	2014-2015	Vital Registration	VR/SRS/DSP
Delhi	1967-2016	Standard demographic and health survey	CBH
Delhi	1968-2015	Standard demographic and health survey	SBH
Delhi	1974-2013	Other survey	SBH
Delhi	1981-1981	Census	SBH
Delhi	1984-1984	Census	SBH
Delhi	1988-1988	Census	SBH
Delhi	1990-1990	Census	SBH
Delhi	1991-1996	Other survey	CBH
Delhi	1992-1992	Census	SBH
Delhi	1994-1995	Census	SBH
Delhi	1998-1998	Census	SBH
Delhi	2000-2013	Other survey	CBH
Delhi	2002-2002	Census	SBH
Delhi	2004-2016	Sample Registrations System	VR/SRS/DSP
Delhi	2005-2005	Census	SBH
Delhi	2008-2008	Census	SBH
Delhi	2009-2013	Vital Registration	VR/SRS/DSP
Goa	1967-2012	Standard demographic and health survey	CBH
Goa	1968-2014	Standard demographic and health survey	SBH
Goa	1974-2007	Other survey	SBH
Goa	1980-1980	Census	SBH
Goa	1984-1984	Census	SBH
Goa	1988-1989	Census	SBH
Goa	1991-1991	Census	SBH

Appendix Table 5. Under-5 mortality reference sources by source date and location, 1950-2017			
**Location**	**Year range of data**	**Reference source**	**Data type**
Goa	1993-1993	Census	SBH
Goa	1993-1994	Other survey	CBH
Goa	1995-1995	Census	SBH
Goa	1997-1998	Census	SBH
Goa	1997-1998	Other survey	CBH
Goa	2001-2001	Census	SBH
Goa	2001-2002	Other survey	CBH
Goa	2005-2005	Census	SBH
Goa	2008-2008	Census	SBH
Goa	2009-2015	Vital Registration	VR/SRS/DSP
Gujarat	1967-2015	Standard demographic and health survey	CBH
Gujarat	1968-2015	Standard demographic and health survey	SBH
Gujarat	1974-2007	Other survey	SBH
Gujarat	1981-1981	Census	SBH
Gujarat	1984-1984	Census	SBH
Gujarat	1984-2013	Other survey	CBH
Gujarat	1988-1988	Census	SBH
Gujarat	1991-1992	Census	SBH
Gujarat	1994-1995	Census	SBH
Gujarat	1995-2016	Sample Registrations System	VR/SRS/DSP
Gujarat	1998-1999	Census	SBH
Gujarat	2002-2002	Census	SBH
Gujarat	2005-2005	Census	SBH
Gujarat	2008-2008	Census	SBH
Gujarat	2009-2013	Vital Registration	VR/SRS/DSP
Haryana	1968-2014	Standard demographic and health survey	SBH
Haryana	1971-2014	Standard demographic and health survey	CBH
Haryana	1974-2013	Other survey	SBH
Haryana	1981-1981	Census	SBH
Haryana	1984-1984	Census	SBH
Haryana	1985-2013	Other survey	CBH
Haryana	1988-1988	Census	SBH
Haryana	1990-1990	Census	SBH
Haryana	1992-1992	Census	SBH
Haryana	1994-1995	Census	SBH
Haryana	1995-2016	Sample Registrations System	VR/SRS/DSP
Haryana	1997-1999	Census	SBH
Haryana	2002-2002	Census	SBH
Haryana	2005-2005	Census	SBH
Haryana	2007-2008	Census	SBH
Himachal Pradesh	1968-2015	Standard demographic and health survey	SBH
Himachal Pradesh	1969-2016	Standard demographic and health survey	CBH
Himachal Pradesh	1974-2013	Other survey	SBH
Himachal Pradesh	1981-1981	Census	SBH
Himachal Pradesh	1984-1984	Census	SBH
Himachal Pradesh	1985-2012	Other survey	CBH
Himachal Pradesh	1988-1988	Census	SBH
Himachal Pradesh	1990-1992	Census	SBH

Appendix Table 5. Under-5 mortality reference sources by source date and location, 1950-2017			
**Location**	**Year range of data**	**Reference source**	**Data type**
Himachal Pradesh	1994-1995	Census	SBH
Himachal Pradesh	1995-2016	Sample Registrations System	VR/SRS/DSP
Himachal Pradesh	1998-1999	Census	SBH
Himachal Pradesh	2002-2002	Census	SBH
Himachal Pradesh	2005-2005	Census	SBH
Himachal Pradesh	2007-2008	Census	SBH
Himachal Pradesh	2009-2013	Vital Registration	VR/SRS/DSP
Jammu and Kashmir	1968-2015	Standard demographic and health survey	SBH
Jammu and Kashmir	1969-2016	Standard demographic and health survey	CBH
Jammu and Kashmir	1974-2013	Other survey	SBH
Jammu and Kashmir	1981-1981	Census	SBH
Jammu and Kashmir	1985-1985	Census	SBH
Jammu and Kashmir	1988-1988	Census	SBH
Jammu and Kashmir	1990-2013	Other survey	CBH
Jammu and Kashmir	1991-1992	Census	SBH
Jammu and Kashmir	1995-1995	Census	SBH
Jammu and Kashmir	1998-1999	Census	SBH
Jammu and Kashmir	2002-2002	Census	SBH
Jammu and Kashmir	2004-2016	Sample Registrations System	VR/SRS/DSP
Jammu and Kashmir	2005-2005	Census	SBH
Jammu and Kashmir	2007-2008	Census	SBH
Jammu and Kashmir	2009-2011	Vital Registration	VR/SRS/DSP
Jammu and Kashmir	2013-2013	Vital Registration	VR/SRS/DSP
Jammu and Kashmir	2015-2015	Vital Registration	VR/SRS/DSP
Jharkhand	1979-2013	Other survey	SBH
Jharkhand	1981-1981	Census	SBH
Jharkhand	1981-2016	Standard demographic and health survey	SBH
Jharkhand	1983-2016	Standard demographic and health survey	CBH
Jharkhand	1984-1984	Census	SBH
Jharkhand	1987-2013	Other survey	CBH
Jharkhand	1988-1988	Census	SBH
Jharkhand	1991-1992	Census	SBH
Jharkhand	1995-1995	Census	SBH
Jharkhand	1998-1998	Census	SBH
Jharkhand	2002-2002	Census	SBH
Jharkhand	2004-2016	Sample Registrations System	VR/SRS/DSP
Jharkhand	2005-2005	Census	SBH
Jharkhand	2008-2008	Census	SBH
Karnataka	1965-2014	Standard demographic and health survey	CBH
Karnataka	1968-2014	Standard demographic and health survey	SBH
Karnataka	1974-2007	Other survey	SBH
Karnataka	1980-2012	Other survey	CBH
Karnataka	1981-1981	Census	SBH
Karnataka	1984-1984	Census	SBH
Karnataka	1988-1988	Census	SBH
Karnataka	1990-1990	Census	SBH
Karnataka	1992-1992	Census	SBH
Karnataka	1994-1995	Census	SBH

Appendix Table 5. Under-5 mortality reference sources by source date and location, 1950-2017

Location	Year range of data	Reference source	Data type
Karnataka	1995-2016	Sample Registrations System	VR/SRS/DSP
Karnataka	1998-1999	Census	SBH
Karnataka	2002-2002	Census	SBH
Karnataka	2005-2005	Census	SBH
Karnataka	2007-2008	Census	SBH
Karnataka	2009-2015	Vital Registration	VR/SRS/DSP
Kerala	1967-2016	Standard demographic and health survey	CBH
Kerala	1968-2015	Standard demographic and health survey	SBH
Kerala	1974-2007	Other survey	SBH
Kerala	1980-1980	Census	SBH
Kerala	1984-1984	Census	SBH
Kerala	1986-2001	Other survey	CBH
Kerala	1988-1988	Census	SBH
Kerala	1990-1993	Census	SBH
Kerala	1995-1995	Census	SBH
Kerala	1995-2016	Sample Registrations System	VR/SRS/DSP
Kerala	1998-1998	Census	SBH
Kerala	2002-2002	Census	SBH
Kerala	2004-2005	Other survey	CBH
Kerala	2005-2005	Census	SBH
Kerala	2007-2008	Census	SBH
Kerala	2009-2015	Vital Registration	VR/SRS/DSP
Madhya Pradesh	1964-2014	Standard demographic and health survey	CBH
Madhya Pradesh	1968-2014	Standard demographic and health survey	SBH
Madhya Pradesh	1974-2013	Other survey	SBH
Madhya Pradesh	1981-1981	Census	SBH
Madhya Pradesh	1984-2013	Other survey	CBH
Madhya Pradesh	1985-1985	Census	SBH
Madhya Pradesh	1988-1988	Census	SBH
Madhya Pradesh	1991-1992	Census	SBH
Madhya Pradesh	1994-1995	Census	SBH
Madhya Pradesh	1998-1999	Census	SBH
Madhya Pradesh	2002-2002	Census	SBH
Madhya Pradesh	2004-2016	Sample Registrations System	VR/SRS/DSP
Madhya Pradesh	2005-2005	Census	SBH
Madhya Pradesh	2007-2008	Census	SBH
Madhya Pradesh	2009-2015	Vital Registration	VR/SRS/DSP
Maharashtra	1967-2015	Standard demographic and health survey	CBH
Maharashtra	1968-2014	Standard demographic and health survey	SBH
Maharashtra	1974-2007	Other survey	SBH
Maharashtra	1980-1980	Census	SBH
Maharashtra	1981-2013	Other survey	CBH
Maharashtra	1984-1984	Census	SBH
Maharashtra	1988-1988	Census	SBH
Maharashtra	1990-1992	Census	SBH
Maharashtra	1994-1995	Census	SBH
Maharashtra	1995-2016	Sample Registrations System	VR/SRS/DSP
Maharashtra	1998-1999	Census	SBH

Appendix Table 5. Under-5 mortality reference sources by source date and location, 1950-2017			
**Location**	**Year range of data**	**Reference source**	**Data type**
Maharashtra	2002-2002	Census	SBH
Maharashtra	2005-2005	Census	SBH
Maharashtra	2007-2008	Census	SBH
Maharashtra	2009-2009	Vital Registration	VR/SRS/DSP
Maharashtra	2012-2012	Vital Registration	VR/SRS/DSP
Maharashtra	2015-2015	Vital Registration	VR/SRS/DSP
Manipur	1968-2014	Standard demographic and health survey	SBH
Manipur	1974-2007	Other survey	SBH
Manipur	1975-2015	Standard demographic and health survey	CBH
Manipur	1981-1981	Census	SBH
Manipur	1985-1985	Census	SBH
Manipur	1988-1988	Census	SBH
Manipur	1991-1992	Census	SBH
Manipur	1992-1993	Other survey	CBH
Manipur	1995-1995	Census	SBH
Manipur	1996-1999	Other survey	CBH
Manipur	1998-1999	Census	SBH
Manipur	2002-2002	Census	SBH
Manipur	2002-2005	Other survey	CBH
Manipur	2005-2005	Census	SBH
Manipur	2007-2008	Census	SBH
Manipur	2009-2013	Vital Registration	VR/SRS/DSP
Meghalaya	1968-2014	Standard demographic and health survey	SBH
Meghalaya	1974-2012	Other survey	SBH
Meghalaya	1975-2015	Standard demographic and health survey	CBH
Meghalaya	1982-1982	Census	SBH
Meghalaya	1985-1985	Census	SBH
Meghalaya	1989-1989	Census	SBH
Meghalaya	1992-1992	Census	SBH
Meghalaya	1994-1995	Other survey	CBH
Meghalaya	1995-1995	Census	SBH
Meghalaya	1998-1999	Census	SBH
Meghalaya	1998-1999	Other survey	CBH
Meghalaya	2002-2002	Census	SBH
Meghalaya	2002-2005	Other survey	CBH
Meghalaya	2005-2005	Census	SBH
Meghalaya	2008-2008	Census	SBH
Meghalaya	2009-2013	Vital Registration	VR/SRS/DSP
Meghalaya	2015-2015	Vital Registration	VR/SRS/DSP
Mizoram	1968-2015	Standard demographic and health survey	SBH
Mizoram	1974-2013	Other survey	SBH
Mizoram	1975-2016	Standard demographic and health survey	CBH
Mizoram	1981-1981	Census	SBH
Mizoram	1985-1985	Census	SBH
Mizoram	1989-1989	Census	SBH
Mizoram	1991-1992	Census	SBH
Mizoram	1995-1999	Census	SBH
Mizoram	2002-2002	Census	SBH

Appendix Table 5. Under-5 mortality reference sources by source date and location, 1950-2017			
**Location**	**Year range of data**	**Reference source**	**Data type**
Mizoram	2002-2005	Other survey	CBH
Mizoram	2005-2005	Census	SBH
Mizoram	2007-2008	Census	SBH
Mizoram	2009-2013	Vital Registration	VR/SRS/DSP
Mizoram	2015-2015	Vital Registration	VR/SRS/DSP
Nagaland	1974-2013	Other survey	SBH
Nagaland	1975-2015	Standard demographic and health survey	SBH
Nagaland	1975-2016	Standard demographic and health survey	CBH
Nagaland	1982-1982	Census	SBH
Nagaland	1985-1985	Census	SBH
Nagaland	1989-1989	Census	SBH
Nagaland	1992-1993	Census	SBH
Nagaland	1995-1995	Census	SBH
Nagaland	1998-1999	Census	SBH
Nagaland	2002-2002	Census	SBH
Nagaland	2005-2005	Census	SBH
Nagaland	2007-2008	Census	SBH
Nagaland	2009-2013	Vital Registration	VR/SRS/DSP
Odisha	1967-2005	Standard demographic and health survey	CBH
Odisha	1968-2015	Standard demographic and health survey	SBH
Odisha	1974-2013	Other survey	SBH
Odisha	1981-1981	Census	SBH
Odisha	1984-1984	Census	SBH
Odisha	1984-2012	Other survey	CBH
Odisha	1988-1988	Census	SBH
Odisha	1991-1992	Census	SBH
Odisha	1994-1995	Census	SBH
Odisha	1995-2016	Sample Registrations System	VR/SRS/DSP
Odisha	1998-1999	Census	SBH
Odisha	2002-2002	Census	SBH
Odisha	2005-2005	Census	SBH
Odisha	2007-2008	Census	SBH
Odisha	2009-2015	Vital Registration	VR/SRS/DSP
Punjab	1968-2015	Standard demographic and health survey	SBH
Punjab	1970-2015	Standard demographic and health survey	CBH
Punjab	1974-2013	Other survey	SBH
Punjab	1981-1981	Census	SBH
Punjab	1984-1984	Census	SBH
Punjab	1985-2012	Other survey	CBH
Punjab	1988-1988	Census	SBH
Punjab	1991-1992	Census	SBH
Punjab	1994-1995	Census	SBH
Punjab	1995-2016	Sample Registrations System	VR/SRS/DSP
Punjab	1998-1998	Census	SBH
Punjab	2002-2002	Census	SBH
Punjab	2005-2005	Census	SBH
Punjab	2008-2008	Census	SBH
Punjab	2009-2015	Vital Registration	VR/SRS/DSP

Appendix Table 5. Under-5 mortality reference sources by source date and location, 1950-2017

Location	Year range of data	Reference source	Data type
Rajasthan	1967-2015	Standard demographic and health survey	CBH
Rajasthan	1974-2013	Other survey	SBH
Rajasthan	1975-2015	Standard demographic and health survey	SBH
Rajasthan	1981-1981	Census	SBH
Rajasthan	1983-2012	Other survey	CBH
Rajasthan	1985-1985	Census	SBH
Rajasthan	1989-1989	Census	SBH
Rajasthan	1991-1992	Census	SBH
Rajasthan	1994-1995	Census	SBH
Rajasthan	1995-2016	Sample Registrations System	VR/SRS/DSP
Rajasthan	1998-1999	Census	SBH
Rajasthan	2002-2002	Census	SBH
Rajasthan	2004-2005	Census	SBH
Rajasthan	2008-2008	Census	SBH
Rajasthan	2009-2012	Vital Registration	VR/SRS/DSP
Rajasthan	2014-2015	Vital Registration	VR/SRS/DSP
Sikkim	1974-2013	Other survey	SBH
Sikkim	1975-2014	Standard demographic and health survey	SBH
Sikkim	1981-1981	Census	SBH
Sikkim	1983-2014	Standard demographic and health survey	CBH
Sikkim	1985-1985	Census	SBH
Sikkim	1988-1988	Census	SBH
Sikkim	1991-1992	Census	SBH
Sikkim	1994-1999	Census	SBH
Sikkim	2002-2002	Census	SBH
Sikkim	2005-2005	Census	SBH
Sikkim	2007-2008	Census	SBH
Sikkim	2009-2013	Vital Registration	VR/SRS/DSP
Tamil Nadu	1966-2014	Standard demographic and health survey	CBH
Tamil Nadu	1968-2013	Standard demographic and health survey	SBH
Tamil Nadu	1974-2007	Other survey	SBH
Tamil Nadu	1980-1980	Census	SBH
Tamil Nadu	1984-1984	Census	SBH
Tamil Nadu	1984-2012	Other survey	CBH
Tamil Nadu	1988-1988	Census	SBH
Tamil Nadu	1990-1993	Census	SBH
Tamil Nadu	1995-1995	Census	SBH
Tamil Nadu	1995-2016	Sample Registrations System	VR/SRS/DSP
Tamil Nadu	1998-1998	Census	SBH
Tamil Nadu	2001-2002	Census	SBH
Tamil Nadu	2005-2005	Census	SBH
Tamil Nadu	2007-2008	Census	SBH
Tamil Nadu	2009-2015	Vital Registration	VR/SRS/DSP
Telangana	1979-2007	Other survey	SBH
Telangana	1985-2014	Standard demographic and health survey	CBH
Telangana	1989-2014	Standard demographic and health survey	SBH
Telangana	1990-2005	Other survey	CBH
Telangana	2013-2013	Vital Registration	VR/SRS/DSP

Appendix Table 5. Under-5 mortality reference sources by source date and location, 1950-2017

Location	Year range of data	Reference source	Data type
Telangana	2014-2016	Sample Registrations System	VR/SRS/DSP
Telangana	2015-2015	Vital Registration	VR/SRS/DSP
Tripura	1968-2014	Standard demographic and health survey	SBH
Tripura	1974-2013	Other survey	SBH
Tripura	1975-2014	Standard demographic and health survey	CBH
Tripura	1981-1981	Census	SBH
Tripura	1985-1985	Census	SBH
Tripura	1988-1988	Census	SBH
Tripura	1991-1992	Census	SBH
Tripura	1992-2005	Other survey	CBH
Tripura	1994-1995	Census	SBH
Tripura	1998-1998	Census	SBH
Tripura	2002-2002	Census	SBH
Tripura	2005-2005	Census	SBH
Tripura	2008-2008	Census	SBH
Tripura	2012-2013	Vital Registration	VR/SRS/DSP
Tripura	2015-2015	Vital Registration	VR/SRS/DSP
Uttar Pradesh	1963-2016	Standard demographic and health survey	CBH
Uttar Pradesh	1968-2015	Standard demographic and health survey	SBH
Uttar Pradesh	1974-2013	Other survey	SBH
Uttar Pradesh	1981-1981	Census	SBH
Uttar Pradesh	1981-2012	Other survey	CBH
Uttar Pradesh	1985-1985	Census	SBH
Uttar Pradesh	1989-1989	Census	SBH
Uttar Pradesh	1991-1992	Census	SBH
Uttar Pradesh	1995-1995	Census	SBH
Uttar Pradesh	1998-1999	Census	SBH
Uttar Pradesh	2000-2016	Sample Registrations System	VR/SRS/DSP
Uttar Pradesh	2002-2002	Census	SBH
Uttar Pradesh	2005-2005	Census	SBH
Uttar Pradesh	2008-2008	Census	SBH
Uttarakhand	1979-2013	Other survey	SBH
Uttarakhand	1981-1981	Census	SBH
Uttarakhand	1981-2014	Standard demographic and health survey	SBH
Uttarakhand	1984-1984	Census	SBH
Uttarakhand	1984-2014	Standard demographic and health survey	CBH
Uttarakhand	1988-1988	Census	SBH
Uttarakhand	1989-2012	Other survey	CBH
Uttarakhand	1991-1992	Census	SBH
Uttarakhand	1994-1995	Census	SBH
Uttarakhand	1998-1998	Census	SBH
Uttarakhand	2002-2002	Census	SBH
Uttarakhand	2005-2005	Census	SBH
Uttarakhand	2008-2008	Census	SBH
Uttarakhand	2014-2016	Sample Registrations System	VR/SRS/DSP
West Bengal	1967-2014	Standard demographic and health survey	CBH
West Bengal	1968-2014	Standard demographic and health survey	SBH
West Bengal	1974-2013	Other survey	SBH

Appendix Table 5. Under-5 mortality reference sources by source date and location, 1950-2017			
**Location**	**Year range of data**	**Reference source**	**Data type**
West Bengal	1981-1981	Census	SBH
West Bengal	1983-2013	Other survey	CBH
West Bengal	1984-1984	Census	SBH
West Bengal	1988-1988	Census	SBH
West Bengal	1990-1990	Census	SBH
West Bengal	1992-1992	Census	SBH
West Bengal	1994-1995	Census	SBH
West Bengal	1995-2016	Sample Registrations System	VR/SRS/DSP
West Bengal	1997-1999	Census	SBH
West Bengal	2001-2002	Census	SBH
West Bengal	2005-2005	Census	SBH
West Bengal	2007-2007	Census	SBH
West Bengal	2013-2013	Vital Registration	VR/SRS/DSP
Union Territories other than Delhi	1980-1980	Census	SBH
Union Territories other than Delhi	1980-2013	Other survey	SBH
Union Territories other than Delhi	1984-1984	Census	SBH
Union Territories other than Delhi	1988-1988	Census	SBH
Union Territories other than Delhi	1992-1992	Census	SBH
Union Territories other than Delhi	1992-1993	Other survey	CBH
Union Territories other than Delhi	1995-1995	Census	SBH
Union Territories other than Delhi	1996-1997	Other survey	CBH
Union Territories other than Delhi	1998-1998	Census	SBH
Union Territories other than Delhi	2002-2005	Other survey	CBH
Union Territories other than Delhi	2009-2013	Vital Registration	VR/SRS/DSP
Nepal	1950-1950	Census	SBH
Nepal	1950-1975	World Fertility Survey	CBH
Nepal	1951-1975	World Fertility Survey	SBH
Nepal	1954-1954	Census	SBH
Nepal	1958-1958	Census	SBH
Nepal	1961-1962	Census	SBH
Nepal	1964-1965	Census	SBH
Nepal	1968-1968	Census	SBH
Nepal	1968-2015	Standard demographic and health survey	CBH
Nepal	1969-1969	Other survey	HH
Nepal	1971-2015	Standard demographic and health survey	SBH
Nepal	1972-1972	Census	SBH
Nepal	1973-1973	Other survey	SBH
Nepal	1974-1974	Other survey	HH
Nepal	1975-1975	Census	SBH
Nepal	1976-1976	Other survey	SBH
Nepal	1976-2000	Census	SBH
Nepal	1979-1979	Other survey	HH
Nepal	1980-1980	Other survey	SBH
Nepal	1982-1982	Other survey	SBH
Nepal	1984-1984	Other survey	HH
Nepal	1985-1985	Other survey	SBH
Nepal	1986-2013	Multiple Indicator Cluster Survey	CBH
Nepal	1987-1987	Other survey	SBH

Appendix Table 5. Under-5 mortality reference sources by source date and location, 1950-2017			
**Location**	**Year range of data**	**Reference source**	**Data type**
Nepal	1989-1989	Other survey	HH
Nepal	1989-2013	Multiple Indicator Cluster Survey	SBH
Pakistan	1950-1972	Census	SBH
Pakistan	1950-1975	World Fertility Survey	CBH
Pakistan	1951-1975	World Fertility Survey	SBH
Pakistan	1966-2004	Standard demographic and health survey	SBH
Pakistan	1966-2004	Living Standards Measurement Study	SBH
Pakistan	1967-1967	Other survey	SBH
Pakistan	1968-1968	Sample Registrations System	VR/SRS/DSP
Pakistan	1969-2004	Standard demographic and health survey	CBH
Pakistan	1971-1971	Other survey	SBH
Pakistan	1971-2001	Other survey	CBH
Pakistan	1974-1975	Other survey	SBH
Pakistan	1975-1975	Census	SBH
Pakistan	1976-1979	Sample Registrations System	VR/SRS/DSP
Pakistan	1977-1977	Census	SBH
Pakistan	1977-1977	Other survey	SBH
Pakistan	1978-2004	Living Standards Measurement Study	CBH
Pakistan	1979-1979	Other survey	SBH
Pakistan	1981-1982	Other survey	SBH
Pakistan	1984-1993	Sample Registrations System	VR/SRS/DSP
Pakistan	1985-1985	Other survey	SBH
Pakistan	1987-1987	Other survey	SBH
Pakistan	1990-1990	Other survey	SBH
Pakistan	1994-1994	Multiple Indicator Cluster Survey	HH
Pakistan	1995-1997	Sample Registrations System	VR/SRS/DSP
Pakistan	1999-1999	Sample Registrations System	VR/SRS/DSP
Pakistan	1999-1999	Multiple Indicator Cluster Survey	HH
Pakistan	2001-2001	Sample Registrations System	VR/SRS/DSP
Pakistan	2003-2003	Sample Registrations System	VR/SRS/DSP
Pakistan	2004-2004	Multiple Indicator Cluster Survey	HH
Pakistan	2006-2007	Sample Registrations System	VR/SRS/DSP
Pakistan	2006-2007	Living Standards Measurement Study	CBH
Pakistan	2006-2007	Living Standards Measurement Study	SBH
Pakistan	2006-2012	Standard demographic and health survey	CBH
Pakistan	2006-2012	Standard demographic and health survey	SBH
Pakistan	2009-2009	Multiple Indicator Cluster Survey	HH
Pakistan	2014-2014	Multiple Indicator Cluster Survey	HH
China	1957-1999	Census	SBH
China	1974-1974	Other survey	HH
China	1981-1981	Census	HH
China	1986-1987	Other survey	HH
China	1989-1989	Census	HH
China	1991-1991	Other survey	HH
China	1991-2002	Disease surveillance points	VR/SRS/DSP
China	1994-1994	Other survey	HH
China	1995-1995	Census	HH
China	1996-2013	Other survey	HH

Appendix Table 5. Under-5 mortality reference sources by source date and location, 1950-2017			
**Location**	**Year range of data**	**Reference source**	**Data type**
China	2000-2000	Census	HH
China	2002-2002	Census	SBH
China	2004-2010	Disease surveillance points	VR/SRS/DSP
China	2005-2005	Census	HH
China	2005-2005	Census	SBH
China	2008-2008	Census	SBH
China	2010-2010	Census	HH
China	2012-2015	Disease surveillance points	VR/SRS/DSP
Taiwan	1955-2016	Vital Registration	VR/SRS/DSP
American Samoa	1952-1956	Vital Registration	VR/SRS/DSP
American Samoa	1955-1955	Other survey	SBH
American Samoa	1958-1973	Vital Registration	VR/SRS/DSP
American Samoa	1959-1959	Other survey	SBH
American Samoa	1962-1962	Other survey	SBH
American Samoa	1966-1966	Other survey	SBH
American Samoa	1969-1969	Other survey	SBH
American Samoa	1972-1972	Other survey	SBH
American Samoa	1976-1976	Vital Registration	VR/SRS/DSP
American Samoa	1982-1982	Vital Registration	VR/SRS/DSP
American Samoa	1984-1993	Vital Registration	VR/SRS/DSP
American Samoa	1997-2015	Vital Registration	VR/SRS/DSP
Federated States of Micronesia	1956-1956	Census	SBH
Federated States of Micronesia	1960-1960	Census	SBH
Federated States of Micronesia	1963-1963	Census	SBH
Federated States of Micronesia	1965-1965	Census	SBH
Federated States of Micronesia	1967-1967	Census	SBH
Federated States of Micronesia	1970-1970	Census	SBH
Federated States of Micronesia	1976-1976	Census	SBH
Federated States of Micronesia	1979-1979	Census	SBH
Federated States of Micronesia	1982-1982	Census	SBH
Federated States of Micronesia	1985-1985	Census	SBH
Federated States of Micronesia	1988-1991	Census	SBH
Federated States of Micronesia	1994-1994	Census	SBH
Federated States of Micronesia	1996-1996	Census	SBH
Federated States of Micronesia	2003-2003	Vital Registration	VR/SRS/DSP
Fiji	1950-1950	Census	SBH
Fiji	1950-1973	World Fertility Survey	CBH
Fiji	1950-1987	Vital Registration	VR/SRS/DSP
Fiji	1952-1952	Census	SBH
Fiji	1954-1971	World Fertility Survey	SBH
Fiji	1955-1955	Census	SBH
Fiji	1958-1958	Census	SBH
Fiji	1960-1962	Census	SBH
Fiji	1964-1964	Census	SBH
Fiji	1967-1967	Census	SBH
Fiji	1970-1970	Census	SBH
Fiji	1972-1972	Census	SBH
Fiji	1974-1974	Census	SBH

Appendix Table 5. Under-5 mortality reference sources by source date and location, 1950-2017

Location	Year range of data	Reference source	Data type
Fiji	1976-1977	Census	SBH
Fiji	1980-1980	Census	SBH
Fiji	1982-1983	Census	SBH
Fiji	1987-1988	Census	SBH
Fiji	1990-1992	Census	SBH
Fiji	1995-1995	Census	SBH
Fiji	1996-2009	Vital Registration	VR/SRS/DSP
Fiji	1998-1998	Census	SBH
Fiji	2001-2001	Census	SBH
Fiji	2004-2004	Census	SBH
Fiji	2011-2012	Vital Registration	VR/SRS/DSP
Guam	1950-1992	Vital Registration	VR/SRS/DSP
Guam	1994-2015	Vital Registration	VR/SRS/DSP
Kiribati	1952-1952	Census	SBH
Kiribati	1955-1955	Census	SBH
Kiribati	1957-1960	Census	SBH
Kiribati	1962-1964	Census	SBH
Kiribati	1967-1967	Census	SBH
Kiribati	1969-1969	Census	SBH
Kiribati	1972-1972	Census	SBH
Kiribati	1974-1974	Census	SBH
Kiribati	1987-1987	Census	SBH
Kiribati	1989-1990	Census	SBH
Kiribati	1991-1991	Other survey	SBH
Kiribati	1991-2001	Vital Registration	VR/SRS/DSP
Kiribati	1992-1992	Census	SBH
Kiribati	1994-1994	Other survey	SBH
Kiribati	1994-1995	Census	SBH
Kiribati	1996-1996	Other survey	HH
Kiribati	1997-1997	Other survey	SBH
Kiribati	1997-1998	Census	SBH
Kiribati	2000-2002	Census	SBH
Kiribati	2001-2001	Other survey	HH
Kiribati	2001-2001	Other survey	SBH
Kiribati	2004-2004	Census	SBH
Kiribati	2004-2004	Other survey	SBH
Kiribati	2006-2006	Other survey	HH
Kiribati	2006-2006	Other survey	SBH
Kiribati	2011-2011	Vital Registration	VR/SRS/DSP
Marshall Islands	1981-1981	Census	SBH
Marshall Islands	1984-1984	Census	SBH
Marshall Islands	1986-1997	Vital Registration	VR/SRS/DSP
Marshall Islands	1987-1987	Census	SBH
Marshall Islands	1990-1992	Census	SBH
Marshall Islands	1995-1995	Census	SBH
Marshall Islands	1995-1995	Standard demographic and health survey	HH
Marshall Islands	1999-1999	Census	SBH
Marshall Islands	2000-2000	Standard demographic and health survey	HH

Appendix Table 5. Under-5 mortality reference sources by source date and location, 1950-2017			
**Location**	**Year range of data**	**Reference source**	**Data type**
Marshall Islands	2002-2002	Census	SBH
Marshall Islands	2005-2005	Census	SBH
Marshall Islands	2005-2005	Standard demographic and health survey	HH
Marshall Islands	2005-2006	Vital Registration	VR/SRS/DSP
Marshall Islands	2008-2008	Census	SBH
Northern Mariana Islands	1998-2015	Vital Registration	VR/SRS/DSP
Papua New Guinea	1952-1952	Census	SBH
Papua New Guinea	1956-1956	Census	SBH
Papua New Guinea	1959-1959	Census	SBH
Papua New Guinea	1962-1962	Census	SBH
Papua New Guinea	1965-1965	Census	SBH
Papua New Guinea	1967-1968	Census	SBH
Papua New Guinea	1971-1971	Census	SBH
Papua New Guinea	1972-1972	Other demographic and health survey	SBH
Papua New Guinea	1974-1974	Census	SBH
Papua New Guinea	1974-1974	Other demographic and health survey	HH
Papua New Guinea	1976-1976	Census	SBH
Papua New Guinea	1976-1976	Other demographic and health survey	SBH
Papua New Guinea	1977-1977	Vital Registration	VR/SRS/DSP
Papua New Guinea	1979-1979	Other demographic and health survey	HH
Papua New Guinea	1979-1979	Other demographic and health survey	SBH
Papua New Guinea	1979-2006	Standard demographic and health survey	CBH
Papua New Guinea	1980-1980	Vital Registration	VR/SRS/DSP
Papua New Guinea	1981-1981	Census	SBH
Papua New Guinea	1982-1982	Other demographic and health survey	SBH
Papua New Guinea	1984-1984	Census	SBH
Papua New Guinea	1984-1984	Standard demographic and health survey	HH
Papua New Guinea	1984-1984	Other demographic and health survey	HH
Papua New Guinea	1985-1985	Other demographic and health survey	SBH
Papua New Guinea	1986-1986	Standard demographic and health survey	SBH
Papua New Guinea	1987-1988	Other demographic and health survey	SBH
Papua New Guinea	1988-1988	Census	SBH
Papua New Guinea	1989-1989	Standard demographic and health survey	HH
Papua New Guinea	1989-1989	Standard demographic and health survey	SBH
Papua New Guinea	1989-1989	Other demographic and health survey	HH
Papua New Guinea	1992-1992	Standard demographic and health survey	SBH
Papua New Guinea	1993-1993	Census	SBH
Papua New Guinea	1993-1993	Other demographic and health survey	SBH
Papua New Guinea	1994-1994	Standard demographic and health survey	HH
Papua New Guinea	1994-1994	Other demographic and health survey	HH
Papua New Guinea	1995-1995	Standard demographic and health survey	SBH
Papua New Guinea	1996-1996	Census	SBH
Papua New Guinea	1998-1998	Standard demographic and health survey	SBH
Papua New Guinea	1999-1999	Standard demographic and health survey	HH
Papua New Guinea	2001-2001	Standard demographic and health survey	SBH
Papua New Guinea	2003-2003	Standard demographic and health survey	SBH
Papua New Guinea	2004-2004	Standard demographic and health survey	HH
Samoa	1950-1950	Census	SBH

Appendix Table 5. Under-5 mortality reference sources by source date and location, 1950-2017

Location	Year range of data	Reference source	Data type
Samoa	1952-1953	Census	SBH
Samoa	1955-1958	Census	SBH
Samoa	1955-1969	Vital Registration	VR/SRS/DSP
Samoa	1960-1960	Census	SBH
Samoa	1962-1963	Census	SBH
Samoa	1965-1965	Census	SBH
Samoa	1967-1968	Census	SBH
Samoa	1970-1970	Census	SBH
Samoa	1972-1972	Census	SBH
Samoa	1973-1976	Vital Registration	VR/SRS/DSP
Samoa	1975-1975	Census	SBH
Samoa	1977-1977	Census	SBH
Samoa	1978-1978	Vital Registration	VR/SRS/DSP
Samoa	1980-1980	Vital Registration	VR/SRS/DSP
Samoa	1998-1998	Other survey	HH
Samoa	2002-2002	Standard demographic and health survey	HH
Samoa	2002-2002	Other survey	HH
Samoa	2007-2007	Standard demographic and health survey	HH
Samoa	2007-2007	Other survey	HH
Samoa	2012-2012	Standard demographic and health survey	HH
Solomon Islands	1952-1952	Census	SBH
Solomon Islands	1955-1955	Census	SBH
Solomon Islands	1958-1959	Census	SBH
Solomon Islands	1961-1961	Census	SBH
Solomon Islands	1964-1964	Census	SBH
Solomon Islands	1966-1967	Census	SBH
Solomon Islands	1969-1969	Census	SBH
Solomon Islands	1972-1972	Census	SBH
Solomon Islands	1981-1981	Census	SBH
Solomon Islands	1984-1984	Census	SBH
Solomon Islands	1987-1987	Census	SBH
Solomon Islands	1990-1990	Census	SBH
Solomon Islands	1993-1995	Census	SBH
Solomon Islands	1995-1995	Standard demographic and health survey	HH
Solomon Islands	1997-1997	Census	SBH
Solomon Islands	2000-2000	Census	SBH
Solomon Islands	2000-2000	Standard demographic and health survey	HH
Solomon Islands	2003-2003	Census	SBH
Solomon Islands	2003-2003	Standard demographic and health survey	HH
Solomon Islands	2005-2005	Census	SBH
Solomon Islands	2005-2005	Standard demographic and health survey	HH
Solomon Islands	2008-2008	Standard demographic and health survey	HH
Solomon Islands	2013-2013	Standard demographic and health survey	HH
Tonga	1958-1958	Census	SBH
Tonga	1958-1964	Vital Registration	VR/SRS/DSP
Tonga	1961-1961	Census	SBH
Tonga	1964-1964	Census	SBH
Tonga	1966-1966	Vital Registration	VR/SRS/DSP

Appendix Table 5. Under-5 mortality reference sources by source date and location, 1950-2017

Location	Year range of data	Reference source	Data type
Tonga	1967-1968	Census	SBH
Tonga	1970-1971	Census	SBH
Tonga	1973-1974	Census	SBH
Tonga	1977-1977	Census	SBH
Tonga	1980-1980	Census	SBH
Tonga	1982-1982	Vital Registration	VR/SRS/DSP
Tonga	1982-1982	Census	SBH
Tonga	1992-1992	Other demographic and health survey	SBH
Tonga	1996-1996	Other demographic and health survey	SBH
Tonga	2000-2000	Other demographic and health survey	HH
Tonga	2000-2000	Other demographic and health survey	SBH
Tonga	2003-2003	Other demographic and health survey	SBH
Tonga	2003-2004	Vital Registration	VR/SRS/DSP
Tonga	2005-2005	Other demographic and health survey	HH
Tonga	2006-2006	Census	HH
Tonga	2006-2006	Other demographic and health survey	SBH
Tonga	2008-2008	Other demographic and health survey	SBH
Tonga	2010-2010	Other demographic and health survey	HH
Vanuatu	1952-1952	Census	SBH
Vanuatu	1956-1956	Census	SBH
Vanuatu	1959-1959	Census	SBH
Vanuatu	1961-1961	Census	SBH
Vanuatu	1963-1963	Census	SBH
Vanuatu	1985-2005	Multiple Indicator Cluster Survey	SBH
Vanuatu	1991-1991	Census	SBH
Vanuatu	1994-1994	Census	SBH
Vanuatu	1994-1994	Other demographic and health survey	SBH
Vanuatu	1997-1997	Other demographic and health survey	SBH
Vanuatu	1998-1998	Census	SBH
Vanuatu	2001-2001	Census	SBH
Vanuatu	2001-2001	Other demographic and health survey	HH
Vanuatu	2001-2001	Other demographic and health survey	SBH
Vanuatu	2003-2003	Census	SBH
Vanuatu	2004-2004	Other demographic and health survey	SBH
Vanuatu	2006-2006	Census	SBH
Vanuatu	2006-2006	Other demographic and health survey	HH
Vanuatu	2006-2006	Other demographic and health survey	SBH
Vanuatu	2009-2009	Other demographic and health survey	SBH
Vanuatu	2011-2011	Other demographic and health survey	HH
Cambodia	1972-1974	Standard demographic and health survey	CBH
Cambodia	1972-1974	Other demographic and health survey	CBH
Cambodia	1973-1974	Census	SBH
Cambodia	1973-1974	Other demographic and health survey	SBH
Cambodia	1973-1974	Other survey	SBH
Cambodia	1979-2003	Other survey	SBH
Cambodia	1981-1997	Other demographic and health survey	CBH
Cambodia	1981-1997	Other demographic and health survey	SBH
Cambodia	1981-2007	Census	SBH

Appendix Table 5. Under-5 mortality reference sources by source date and location, 1950-2017			
**Location**	**Year range of data**	**Reference source**	**Data type**
Cambodia	1981-2014	Standard demographic and health survey	CBH
Cambodia	1981-2014	Standard demographic and health survey	SBH
Indonesia	1950-1975	World Fertility Survey	CBH
Indonesia	1950-2000	Census	CBH
Indonesia	1950-2007	Other survey	CBH
Indonesia	1950-2009	Census	SBH
Indonesia	1951-1975	World Fertility Survey	SBH
Indonesia	1958-2012	Standard demographic and health survey	CBH
Indonesia	1960-1994	Intercensal Population Survey (SUPAS)	SBH
Indonesia	1963-2011	Standard demographic and health survey	SBH
Indonesia	1968-2013	National Socio-Economic Survey (SUSENAS)	SBH
Indonesia	1969-2007	Other survey	SBH
Indonesia	2010-2010	Census	HH
Laos	1976-1976	Census	SBH
Laos	1980-1980	Census	SBH
Laos	1982-2011	Multiple Indicator Cluster Survey	CBH
Laos	1983-1983	Census	SBH
Laos	1985-1985	Reproductive Health Survey	SBH
Laos	1986-1986	Census	SBH
Laos	1987-2011	Multiple Indicator Cluster Survey	SBH
Laos	1989-1989	Census	SBH
Laos	1989-1989	Reproductive Health Survey	SBH
Laos	1991-1991	Census	SBH
Laos	1993-1993	Reproductive Health Survey	SBH
Laos	1996-1996	Reproductive Health Survey	SBH
Laos	1999-1999	Reproductive Health Survey	SBH
Laos	2002-2002	Reproductive Health Survey	HH
Laos	2002-2002	Reproductive Health Survey	SBH
Malaysia	1950-1974	World Fertility Survey	CBH
Malaysia	1950-1974	World Fertility Survey	SBH
Malaysia	1950-1979	Census	SBH
Malaysia	1952-1972	Vital Registration	VR/SRS/DSP
Malaysia	1976-1978	Vital Registration	VR/SRS/DSP
Malaysia	1984-1986	Vital Registration	VR/SRS/DSP
Malaysia	1990-2015	Vital Registration	VR/SRS/DSP
Maldives	1959-1959	Census	SBH
Maldives	1963-1963	Census	SBH
Maldives	1966-1966	Census	SBH
Maldives	1968-1969	Census	SBH
Maldives	1971-1971	Census	SBH
Maldives	1973-1974	Census	SBH
Maldives	1974-1974	Vital Registration	VR/SRS/DSP
Maldives	1976-1979	Census	SBH
Maldives	1978-2014	Vital Registration	VR/SRS/DSP
Maldives	1980-2009	Standard demographic and health survey	CBH
Maldives	1981-1981	Census	SBH
Maldives	1982-1982	Other survey	SBH

Appendix Table 5. Under-5 mortality reference sources by source date and location, 1950-2017			
**Location**	**Year range of data**	**Reference source**	**Data type**
Maldives	1983-1983	Census	SBH
Maldives	1984-2008	Standard demographic and health survey	SBH
Maldives	1985-1985	Other survey	SBH
Maldives	1986-1986	Census	SBH
Maldives	1987-1987	Other survey	SBH
Maldives	1989-1989	Census	SBH
Maldives	1990-1990	Other survey	SBH
Maldives	1991-1991	Census	SBH
Maldives	1992-1992	Other survey	SBH
Maldives	1994-1994	Census	SBH
Maldives	1994-1994	Other survey	SBH
Maldives	1996-1996	Census	SBH
Mauritius	1950-2016	Vital Registration	VR/SRS/DSP
Myanmar	1964-1964	Census	SBH
Myanmar	1968-1968	Census	SBH
Myanmar	1971-1971	Census	SBH
Myanmar	1974-1974	Census	SBH
Myanmar	1977-1977	Census	SBH
Myanmar	1979-1979	Census	SBH
Myanmar	1979-1979	Reproductive Health Survey	SBH
Myanmar	1982-1982	Reproductive Health Survey	SBH
Myanmar	1985-1986	Reproductive Health Survey	SBH
Myanmar	1988-1989	Reproductive Health Survey	SBH
Myanmar	1988-2015	Standard demographic and health survey	CBH
Myanmar	1991-1992	Reproductive Health Survey	SBH
Myanmar	1991-2015	Standard demographic and health survey	SBH
Myanmar	1994-1994	Census	SBH
Myanmar	1994-1996	Reproductive Health Survey	SBH
Myanmar	1997-1997	Census	SBH
Myanmar	1998-1998	Reproductive Health Survey	SBH
Myanmar	2001-2001	Census	SBH
Myanmar	2002-2002	Reproductive Health Survey	SBH
Myanmar	2004-2004	Reproductive Health Survey	SBH
Myanmar	2005-2005	Census	SBH
Myanmar	2008-2008	Census	SBH
Myanmar	2009-2009	Multiple Indicator Cluster Survey	HH
Myanmar	2011-2011	Census	SBH
Myanmar	2013-2013	Vital Registration	VR/SRS/DSP
Philippines	1950-1952	Census	SBH
Philippines	1950-1953	Vital Registration	VR/SRS/DSP
Philippines	1950-1977	World Fertility Survey	CBH
Philippines	1953-1977	World Fertility Survey	SBH
Philippines	1954-1955	Census	SBH
Philippines	1956-2015	Vital Registration	VR/SRS/DSP
Philippines	1958-1958	Census	SBH
Philippines	1961-1961	Census	SBH
Philippines	1964-1964	Census	SBH
Philippines	1965-1989	Census	SBH

Appendix Table 5. Under-5 mortality reference sources by source date and location, 1950-2017			
Location	Year range of data	Reference source	Data type
Philippines	1965-2013	Standard demographic and health survey	CBH
Philippines	1968-2013	Standard demographic and health survey	SBH
Philippines	1987-2011	Other demographic and health survey	SBH
Sri Lanka	1950-1975	World Fertility Survey	CBH
Sri Lanka	1950-1989	Vital Registration	VR/SRS/DSP
Sri Lanka	1951-1975	World Fertility Survey	SBH
Sri Lanka	1956-1956	Census	SBH
Sri Lanka	1959-1959	Census	SBH
Sri Lanka	1959-1986	Standard demographic and health survey	CBH
Sri Lanka	1962-1962	Census	SBH
Sri Lanka	1962-1986	Standard demographic and health survey	SBH
Sri Lanka	1965-1965	Census	SBH
Sri Lanka	1967-1967	Census	SBH
Sri Lanka	1973-1973	Other demographic and health survey	SBH
Sri Lanka	1976-1976	Other demographic and health survey	SBH
Sri Lanka	1980-1980	Other demographic and health survey	SBH
Sri Lanka	1981-1981	Census	SBH
Sri Lanka	1981-1981	Other demographic and health survey	HH
Sri Lanka	1984-1984	Other demographic and health survey	SBH
Sri Lanka	1985-1985	Census	SBH
Sri Lanka	1986-1986	Other demographic and health survey	HH
Sri Lanka	1987-1987	Standard demographic and health survey	HH
Sri Lanka	1987-1987	Other demographic and health survey	SBH
Sri Lanka	1988-1988	Census	SBH
Sri Lanka	1989-1989	Other demographic and health survey	SBH
Sri Lanka	1991-1991	Other demographic and health survey	HH
Sri Lanka	1991-1995	Vital Registration	VR/SRS/DSP
Sri Lanka	1992-1992	Census	SBH
Sri Lanka	1992-1992	Standard demographic and health survey	HH
Sri Lanka	1995-1995	Census	SBH
Sri Lanka	1995-1995	Standard demographic and health survey	HH
Sri Lanka	1997-1997	Standard demographic and health survey	HH
Sri Lanka	1997-2008	Vital Registration	VR/SRS/DSP
Sri Lanka	1998-1998	Census	SBH
Sri Lanka	1999-1999	Standard demographic and health survey	HH
Sri Lanka	2003-2004	Standard demographic and health survey	HH
Sri Lanka	2009-2009	Standard demographic and health survey	HH
Sri Lanka	2010-2010	Vital Registration	VR/SRS/DSP
Sri Lanka	2013-2013	Vital Registration	VR/SRS/DSP
Sri Lanka	2014-2014	Standard demographic and health survey	HH
Seychelles	1951-1952	Census	SBH
Seychelles	1952-1957	Vital Registration	VR/SRS/DSP
Seychelles	1954-1954	Census	SBH
Seychelles	1956-1956	Census	SBH
Seychelles	1959-1959	Vital Registration	VR/SRS/DSP
Seychelles	1959-1959	Census	SBH
Seychelles	1961-1996	Vital Registration	VR/SRS/DSP
Seychelles	1962-1962	Census	SBH

Appendix Table 5. Under-5 mortality reference sources by source date and location, 1950-2017			
**Location**	**Year range of data**	**Reference source**	**Data type**
Seychelles	1965-1965	Census	SBH
Seychelles	1967-1967	Census	SBH
Seychelles	1998-2016	Vital Registration	VR/SRS/DSP
Thailand	1950-1974	World Fertility Survey	SBH
Thailand	1950-1975	World Fertility Survey	CBH
Thailand	1950-1999	Census	SBH
Thailand	1950-2000	Vital Registration	VR/SRS/DSP
Thailand	1956-1983	Other survey	SBH
Thailand	1959-1986	Standard demographic and health survey	CBH
Thailand	1962-1986	Standard demographic and health survey	SBH
Thailand	1981-2012	Multiple Indicator Cluster Survey	SBH
Thailand	2002-2016	Vital Registration	VR/SRS/DSP
Timor-Leste	1967-2016	Standard demographic and health survey	SBH
Timor-Leste	1975-2016	Standard demographic and health survey	CBH
Timor-Leste	1976-2000	Living Standards Measurement Study	SBH
Timor-Leste	1982-2003	Other survey	CBH
Timor-Leste	1985-1985	Census	SBH
Timor-Leste	1989-1989	Census	SBH
Timor-Leste	1993-1993	Census	SBH
Timor-Leste	1996-1996	Census	SBH
Timor-Leste	1998-1998	Census	SBH
Timor-Leste	2000-2000	Census	SBH
Vietnam	1964-2008	Census	SBH
Vietnam	1969-1995	Other survey	SBH
Vietnam	1973-2002	Standard demographic and health survey	SBH
Vietnam	1974-2002	Standard demographic and health survey	CBH
Vietnam	1975-2013	Multiple Indicator Cluster Survey	SBH
Vietnam	1981-2005	AIDS Indicator Survey	SBH
Vietnam	1988-2013	Multiple Indicator Cluster Survey	CBH
Vietnam	1997-2005	Other survey	SBH
Vietnam	2007-2007	Other survey	SBH
Vietnam	2009-2009	Other survey	SBH
Angola	1976-2000	Multiple Indicator Cluster Survey	SBH
Angola	1982-2010	Malaria Indicator Survey	SBH
Angola	1985-2010	Malaria Indicator Survey	CBH
Angola	1988-2015	Standard demographic and health survey	CBH
Angola	1991-2015	Standard demographic and health survey	SBH
Central African Republic	1956-1956	Census	SBH
Central African Republic	1959-1959	Census	SBH
Central African Republic	1962-1962	Census	SBH
Central African Republic	1965-1965	Census	SBH
Central African Republic	1967-1994	Standard demographic and health survey	CBH
Central African Republic	1968-1968	Census	SBH
Central African Republic	1970-1970	Census	SBH
Central African Republic	1970-1994	Standard demographic and health survey	SBH
Central African Republic	1976-2010	Multiple Indicator Cluster Survey	SBH
Congo	1955-1955	Census	SBH
Congo	1958-1958	Census	SBH

Appendix Table 5. Under-5 mortality reference sources by source date and location, 1950-2017			
**Location**	**Year range of data**	**Reference source**	**Data type**
Congo	1962-1962	Census	SBH
Congo	1965-1965	Census	SBH
Congo	1967-1967	Census	SBH
Congo	1970-1970	Census	SBH
Congo	1978-2011	Standard demographic and health survey	CBH
Congo	1981-1996	Standard demographic and health survey	SBH
Congo	1984-1996	AIDS Indicator Survey	SBH
Congo	2000-2008	AIDS Indicator Survey	SBH
Congo	2000-2011	Standard demographic and health survey	SBH
Congo	2001-2002	Multiple Indicator Cluster Survey	HH
Congo	2006-2007	Multiple Indicator Cluster Survey	HH
Congo	2011-2012	Multiple Indicator Cluster Survey	HH
Democratic Republic of the Congo	1970-2009	Multiple Indicator Cluster Survey	SBH
Democratic Republic of the Congo	1980-2013	Standard demographic and health survey	CBH
Democratic Republic of the Congo	1982-2013	Standard demographic and health survey	SBH
Equatorial Guinea	1976-2000	Multiple Indicator Cluster Survey	SBH
Equatorial Guinea	2000-2000	Standard demographic and health survey	HH
Equatorial Guinea	2005-2005	Standard demographic and health survey	HH
Equatorial Guinea	2008-2008	Standard demographic and health survey	HH
Gabon	1973-2011	Standard demographic and health survey	CBH
Gabon	1976-2011	Standard demographic and health survey	SBH
Burundi	1953-1953	Other survey	SBH
Burundi	1957-1957	Other survey	SBH
Burundi	1960-1960	Other survey	SBH
Burundi	1962-2016	Standard demographic and health survey	SBH
Burundi	1963-1963	Other survey	SBH
Burundi	1964-2016	Standard demographic and health survey	CBH
Burundi	1966-1966	Other survey	SBH
Burundi	1968-1968	Other survey	SBH
Burundi	1972-1972	Census	SBH
Burundi	1974-1998	Other survey	SBH
Burundi	1975-2005	Multiple Indicator Cluster Survey	SBH
Burundi	1976-1976	Census	SBH
Burundi	1979-1979	Census	SBH
Burundi	1982-1982	Census	SBH
Burundi	1985-1985	Census	SBH
Burundi	1987-1987	Census	SBH
Burundi	1987-2011	Malaria Indicator Survey	SBH
Comoros	1961-1961	Census	SBH
Comoros	1964-1964	Census	SBH
Comoros	1968-1968	Census	SBH
Comoros	1971-1971	Census	SBH
Comoros	1971-2012	Standard demographic and health survey	SBH
Comoros	1973-1973	Census	SBH
Comoros	1974-2012	Standard demographic and health survey	CBH
Comoros	1976-1976	Census	SBH
Comoros	1976-2000	Multiple Indicator Cluster Survey	SBH
Djibouti	1972-1972	Other survey	SBH

Appendix Table 5. Under-5 mortality reference sources by source date and location, 1950-2017			
**Location**	**Year range of data**	**Reference source**	**Data type**
Djibouti	1975-1975	Other survey	SBH
Djibouti	1977-2001	Pan Arab Project for Family Health	SBH
Djibouti	1979-1979	Other survey	SBH
Djibouti	1982-2006	Multiple Indicator Cluster Survey	SBH
Djibouti	1983-1983	Other survey	SBH
Djibouti	1983-1996	Pan Arab Project for Family Health	CBH
Djibouti	1986-1986	Other survey	SBH
Djibouti	1989-1989	Other survey	SBH
Djibouti	1999-2002	Pan Arab Project for Family Health	CBH
Eritrea	1970-2001	Standard demographic and health survey	CBH
Eritrea	1971-2001	Standard demographic and health survey	SBH
Eritrea	1992-1992	Other survey	SBH
Eritrea	1995-1995	Other survey	SBH
Eritrea	1996-1996	Household Survey prepped via household death recall	HH
Eritrea	1999-1999	Other survey	SBH
Eritrea	2001-2001	Other survey	SBH
Eritrea	2002-2002	Household Survey prepped via household death recall	HH
Eritrea	2004-2004	Other survey	SBH
Eritrea	2007-2007	Other survey	SBH
Ethiopia	1959-2006	Census	SBH
Ethiopia	1970-2015	Standard demographic and health survey	CBH
Ethiopia	1974-1974	Other survey	SBH
Ethiopia	1975-2015	Standard demographic and health survey	SBH
Ethiopia	1977-1977	Other survey	SBH
Ethiopia	1980-1980	Other survey	SBH
Ethiopia	1983-1983	Other survey	SBH
Ethiopia	1985-1985	Other survey	SBH
Ethiopia	1987-1987	Other survey	SBH
Kenya	1950-1977	World Fertility Survey	CBH
Kenya	1950-2008	Census	SBH
Kenya	1953-1977	World Fertility Survey	SBH
Kenya	1961-2014	Standard demographic and health survey	CBH
Kenya	1964-2014	Standard demographic and health survey	SBH
Kenya	1970-2014	Other survey	SBH
Kenya	1976-2000	Multiple Indicator Cluster Survey	SBH
Kenya	1983-2014	Malaria Indicator Survey	SBH
Baringo	1950-2008	Census	SBH
Baringo	1964-2014	Standard demographic and health survey	SBH
Baringo	1970-2005	Other survey	SBH
Baringo	1976-2000	Multiple Indicator Cluster Survey	SBH
Baringo	1979-2014	Standard demographic and health survey	CBH
Baringo	1988-2003	Malaria Indicator Survey	SBH
Bomet	1970-2005	Other survey	SBH
Bomet	1975-2008	Census	SBH
Bomet	1976-2000	Multiple Indicator Cluster Survey	SBH
Bomet	1978-2014	Standard demographic and health survey	SBH
Bomet	1983-2007	Malaria Indicator Survey	SBH

Appendix Table 5. Under-5 mortality reference sources by source date and location, 1950-2017			
**Location**	**Year range of data**	**Reference source**	**Data type**
Bomet	1987-2014	Standard demographic and health survey	CBH
Bungoma	1950-2008	Census	SBH
Bungoma	1964-2014	Standard demographic and health survey	SBH
Bungoma	1970-2014	Other survey	SBH
Bungoma	1973-2014	Standard demographic and health survey	CBH
Bungoma	1976-2000	Multiple Indicator Cluster Survey	SBH
Bungoma	1983-2007	Malaria Indicator Survey	SBH
Bungoma	1996-2013	Multiple Indicator Cluster Survey	CBH
Busia	1950-2008	Census	SBH
Busia	1964-2014	Standard demographic and health survey	SBH
Busia	1970-2005	Other survey	SBH
Busia	1973-2014	Standard demographic and health survey	CBH
Busia	1976-2000	Multiple Indicator Cluster Survey	SBH
Busia	1983-2007	Malaria Indicator Survey	SBH
Elgeyo-Marakwet	1950-2008	Census	SBH
Elgeyo-Marakwet	1964-2014	Standard demographic and health survey	SBH
Elgeyo-Marakwet	1970-2005	Other survey	SBH
Elgeyo-Marakwet	1973-1974	Standard demographic and health survey	CBH
Elgeyo-Marakwet	1976-2000	Multiple Indicator Cluster Survey	SBH
Elgeyo-Marakwet	1977-2014	Standard demographic and health survey	CBH
Elgeyo-Marakwet	1983-2007	Malaria Indicator Survey	SBH
Embu	1950-2008	Census	SBH
Embu	1963-2014	Standard demographic and health survey	SBH
Embu	1970-2005	Other survey	SBH
Embu	1973-1974	Standard demographic and health survey	CBH
Embu	1976-2011	Multiple Indicator Cluster Survey	SBH
Embu	1979-2014	Standard demographic and health survey	CBH
Embu	1983-2007	Malaria Indicator Survey	SBH
Embu	1987-2008	Multiple Indicator Cluster Survey	CBH
Garissa	1950-2008	Census	SBH
Garissa	1970-2005	Other survey	SBH
Garissa	1976-2006	Multiple Indicator Cluster Survey	SBH
Garissa	1978-2014	Standard demographic and health survey	SBH
Garissa	1983-2007	Malaria Indicator Survey	SBH
Garissa	1987-2014	Standard demographic and health survey	CBH
HomaBay	1950-2008	Census	SBH
HomaBay	1964-2005	Other survey	SBH
HomaBay	1976-2000	Multiple Indicator Cluster Survey	SBH
HomaBay	1978-2014	Standard demographic and health survey	SBH
HomaBay	1983-2007	Malaria Indicator Survey	SBH
HomaBay	1985-2014	Standard demographic and health survey	CBH
HomaBay	1994-2011	Multiple Indicator Cluster Survey	CBH
Isiolo	1950-2008	Census	SBH
Isiolo	1970-2005	Other survey	SBH
Isiolo	1976-2011	Multiple Indicator Cluster Survey	SBH
Isiolo	1978-2014	Standard demographic and health survey	SBH
Isiolo	1987-1990	Standard demographic and health survey	CBH
Isiolo	1991-2008	Multiple Indicator Cluster Survey	CBH

Appendix Table 5. Under-5 mortality reference sources by source date and location, 1950-2017			
**Location**	**Year range of data**	**Reference source**	**Data type**
Isiolo	1992-2002	Malaria Indicator Survey	SBH
Isiolo	1995-2014	Standard demographic and health survey	CBH
Kajiado	1950-2008	Census	SBH
Kajiado	1958-2014	Standard demographic and health survey	SBH
Kajiado	1970-2005	Other survey	SBH
Kajiado	1975-1982	Standard demographic and health survey	CBH
Kajiado	1976-2000	Multiple Indicator Cluster Survey	SBH
Kajiado	1983-2007	Malaria Indicator Survey	SBH
Kajiado	1984-2014	Standard demographic and health survey	CBH
Kakamega	1950-2008	Census	SBH
Kakamega	1964-2014	Standard demographic and health survey	SBH
Kakamega	1970-2005	Other survey	SBH
Kakamega	1971-2014	Standard demographic and health survey	CBH
Kakamega	1976-2000	Multiple Indicator Cluster Survey	SBH
Kakamega	1983-2007	Malaria Indicator Survey	SBH
Kakamega	1998-2013	Multiple Indicator Cluster Survey	CBH
Kericho	1950-2008	Census	SBH
Kericho	1964-2014	Standard demographic and health survey	SBH
Kericho	1970-2014	Other survey	SBH
Kericho	1973-2014	Standard demographic and health survey	CBH
Kericho	1976-2000	Multiple Indicator Cluster Survey	SBH
Kericho	1983-2007	Malaria Indicator Survey	SBH
Kiambu	1950-2008	Census	SBH
Kiambu	1964-2014	Standard demographic and health survey	SBH
Kiambu	1970-2014	Other survey	SBH
Kiambu	1975-2014	Standard demographic and health survey	CBH
Kiambu	1976-2000	Multiple Indicator Cluster Survey	SBH
Kiambu	1983-2007	Malaria Indicator Survey	SBH
Kilifi	1950-2008	Census	SBH
Kilifi	1964-2014	Standard demographic and health survey	SBH
Kilifi	1970-2014	Other survey	SBH
Kilifi	1973-2014	Standard demographic and health survey	CBH
Kilifi	1976-2000	Multiple Indicator Cluster Survey	SBH
Kilifi	1983-2007	Malaria Indicator Survey	SBH
Kirinyaga	1950-2008	Census	SBH
Kirinyaga	1964-2014	Standard demographic and health survey	SBH
Kirinyaga	1970-2005	Other survey	SBH
Kirinyaga	1973-2014	Standard demographic and health survey	CBH
Kirinyaga	1976-2000	Multiple Indicator Cluster Survey	SBH
Kirinyaga	1983-2007	Malaria Indicator Survey	SBH
Kisii	1950-2008	Census	SBH
Kisii	1964-2014	Standard demographic and health survey	SBH
Kisii	1970-2005	Other survey	SBH
Kisii	1971-2014	Standard demographic and health survey	CBH
Kisii	1976-2000	Multiple Indicator Cluster Survey	SBH
Kisii	1983-2007	Malaria Indicator Survey	SBH
Kisii	1994-2011	Multiple Indicator Cluster Survey	CBH
Kisumu	1950-2008	Census	SBH

Appendix Table 5. Under-5 mortality reference sources by source date and location, 1950-2017			
**Location**	**Year range of data**	**Reference source**	**Data type**
Kisumu	1964-2014	Standard demographic and health survey	SBH
Kisumu	1970-2005	Other survey	SBH
Kisumu	1973-2014	Standard demographic and health survey	CBH
Kisumu	1976-2000	Multiple Indicator Cluster Survey	SBH
Kisumu	1983-2007	Malaria Indicator Survey	SBH
Kisumu	1994-2011	Multiple Indicator Cluster Survey	CBH
Kitui	1950-2008	Census	SBH
Kitui	1964-2014	Standard demographic and health survey	SBH
Kitui	1970-2014	Other survey	SBH
Kitui	1973-2008	Multiple Indicator Cluster Survey	CBH
Kitui	1975-2014	Standard demographic and health survey	CBH
Kitui	1976-2011	Multiple Indicator Cluster Survey	SBH
Kitui	1983-2007	Malaria Indicator Survey	SBH
Kwale	1950-2008	Census	SBH
Kwale	1960-2014	Standard demographic and health survey	SBH
Kwale	1970-2005	Other survey	SBH
Kwale	1971-2014	Standard demographic and health survey	CBH
Kwale	1976-2000	Multiple Indicator Cluster Survey	SBH
Kwale	1983-2007	Malaria Indicator Survey	SBH
Laikipia	1950-2008	Census	SBH
Laikipia	1964-2014	Standard demographic and health survey	SBH
Laikipia	1970-2005	Other survey	SBH
Laikipia	1976-2000	Multiple Indicator Cluster Survey	SBH
Laikipia	1977-1982	Standard demographic and health survey	CBH
Laikipia	1985-1986	Standard demographic and health survey	CBH
Laikipia	1993-2014	Standard demographic and health survey	CBH
Lamu	1950-2008	Census	SBH
Lamu	1970-2005	Other survey	SBH
Lamu	1972-2014	Standard demographic and health survey	SBH
Lamu	1976-2000	Multiple Indicator Cluster Survey	SBH
Lamu	1983-2007	Malaria Indicator Survey	SBH
Lamu	1987-1988	Standard demographic and health survey	CBH
Lamu	1991-1992	Standard demographic and health survey	CBH
Lamu	1997-2014	Standard demographic and health survey	CBH
Machakos	1950-2008	Census	SBH
Machakos	1964-2014	Standard demographic and health survey	SBH
Machakos	1969-2005	Other survey	SBH
Machakos	1971-2014	Standard demographic and health survey	CBH
Machakos	1976-2011	Multiple Indicator Cluster Survey	SBH
Machakos	1983-2007	Malaria Indicator Survey	SBH
Machakos	1989-2008	Multiple Indicator Cluster Survey	CBH
Makueni	1970-2005	Other survey	SBH
Makueni	1975-2008	Census	SBH
Makueni	1976-2011	Multiple Indicator Cluster Survey	SBH
Makueni	1978-2014	Standard demographic and health survey	SBH
Makueni	1983-2007	Malaria Indicator Survey	SBH
Makueni	1985-1986	Standard demographic and health survey	CBH
Makueni	1989-2008	Multiple Indicator Cluster Survey	CBH

Appendix Table 5. Under-5 mortality reference sources by source date and location, 1950-2017			
**Location**	**Year range of data**	**Reference source**	**Data type**
Makueni	1989-2014	Standard demographic and health survey	CBH
Mandera	1950-2008	Census	SBH
Mandera	1970-2005	Other survey	SBH
Mandera	1976-2006	Multiple Indicator Cluster Survey	SBH
Mandera	1978-2014	Standard demographic and health survey	SBH
Mandera	1983-2007	Malaria Indicator Survey	SBH
Mandera	1989-2014	Standard demographic and health survey	CBH
Marsabit	1950-2008	Census	SBH
Marsabit	1970-2005	Other survey	SBH
Marsabit	1973-1974	Multiple Indicator Cluster Survey	CBH
Marsabit	1978-2014	Standard demographic and health survey	SBH
Marsabit	1979-2008	Multiple Indicator Cluster Survey	CBH
Marsabit	1983-2007	Malaria Indicator Survey	SBH
Marsabit	1984-2008	Multiple Indicator Cluster Survey	SBH
Marsabit	1993-2014	Standard demographic and health survey	CBH
Meru	1950-2008	Census	SBH
Meru	1964-2014	Standard demographic and health survey	SBH
Meru	1970-2005	Other survey	SBH
Meru	1973-2014	Standard demographic and health survey	CBH
Meru	1983-2007	Malaria Indicator Survey	SBH
Meru	1984-2008	Multiple Indicator Cluster Survey	SBH
Meru	1985-2008	Multiple Indicator Cluster Survey	CBH
Migori	1970-2005	Other survey	SBH
Migori	1975-2008	Census	SBH
Migori	1976-2000	Multiple Indicator Cluster Survey	SBH
Migori	1978-2014	Standard demographic and health survey	SBH
Migori	1983-2007	Malaria Indicator Survey	SBH
Migori	1987-2014	Standard demographic and health survey	CBH
Migori	1994-2011	Multiple Indicator Cluster Survey	CBH
Mombasa	1950-2008	Census	SBH
Mombasa	1964-2014	Standard demographic and health survey	SBH
Mombasa	1970-2005	Other survey	SBH
Mombasa	1973-2014	Standard demographic and health survey	CBH
Mombasa	1976-2000	Multiple Indicator Cluster Survey	SBH
Mombasa	1983-2007	Malaria Indicator Survey	SBH
Mombasa	1991-2008	Multiple Indicator Cluster Survey	CBH
Murang'a	1950-2008	Census	SBH
Murang'a	1964-2014	Standard demographic and health survey	SBH
Murang'a	1970-2005	Other survey	SBH
Murang'a	1971-2014	Standard demographic and health survey	CBH
Murang'a	1976-2000	Multiple Indicator Cluster Survey	SBH
Murang'a	1983-2007	Malaria Indicator Survey	SBH
Nairobi	1950-2008	Census	SBH
Nairobi	1964-2008	Standard demographic and health survey	SBH
Nairobi	1967-2014	Other survey	SBH
Nairobi	1973-2014	Standard demographic and health survey	CBH
Nairobi	1976-2000	Multiple Indicator Cluster Survey	SBH
Nakuru	1950-2008	Census	SBH

Appendix Table 5. Under-5 mortality reference sources by source date and location, 1950-2017			
**Location**	**Year range of data**	**Reference source**	**Data type**
Nakuru	1964-2014	Standard demographic and health survey	SBH
Nakuru	1970-2005	Other survey	SBH
Nakuru	1975-2014	Standard demographic and health survey	CBH
Nakuru	1976-2000	Multiple Indicator Cluster Survey	SBH
Nakuru	1983-2007	Malaria Indicator Survey	SBH
Nandi	1950-2008	Census	SBH
Nandi	1964-2014	Standard demographic and health survey	SBH
Nandi	1970-2014	Other survey	SBH
Nandi	1973-2014	Standard demographic and health survey	CBH
Nandi	1976-2000	Multiple Indicator Cluster Survey	SBH
Nandi	1983-2007	Malaria Indicator Survey	SBH
Narok	1950-2008	Census	SBH
Narok	1964-2014	Standard demographic and health survey	SBH
Narok	1970-2005	Other survey	SBH
Narok	1975-1976	Standard demographic and health survey	CBH
Narok	1976-2000	Multiple Indicator Cluster Survey	SBH
Narok	1979-1980	Standard demographic and health survey	CBH
Narok	1983-2007	Malaria Indicator Survey	SBH
Narok	1983-2014	Standard demographic and health survey	CBH
Nyamira	1970-2014	Other survey	SBH
Nyamira	1973-2014	Standard demographic and health survey	SBH
Nyamira	1975-2008	Census	SBH
Nyamira	1976-2000	Multiple Indicator Cluster Survey	SBH
Nyamira	1982-2014	Standard demographic and health survey	CBH
Nyamira	1983-2007	Malaria Indicator Survey	SBH
Nyamira	1994-2011	Multiple Indicator Cluster Survey	CBH
Nyandarua	1950-2008	Census	SBH
Nyandarua	1964-2014	Standard demographic and health survey	SBH
Nyandarua	1970-2005	Other survey	SBH
Nyandarua	1975-1994	Standard demographic and health survey	CBH
Nyandarua	1976-2000	Multiple Indicator Cluster Survey	SBH
Nyandarua	1996-2014	Standard demographic and health survey	CBH
Nyeri	1950-2008	Census	SBH
Nyeri	1964-2014	Standard demographic and health survey	SBH
Nyeri	1970-2005	Other survey	SBH
Nyeri	1973-2014	Standard demographic and health survey	CBH
Nyeri	1976-2000	Multiple Indicator Cluster Survey	SBH
Samburu	1950-2008	Census	SBH
Samburu	1970-2005	Other survey	SBH
Samburu	1978-2014	Standard demographic and health survey	SBH
Samburu	1983-2007	Malaria Indicator Survey	SBH
Samburu	1989-1992	Standard demographic and health survey	CBH
Samburu	1995-2014	Standard demographic and health survey	CBH
Siaya	1950-2008	Census	SBH
Siaya	1964-2014	Standard demographic and health survey	SBH
Siaya	1970-2014	Other survey	SBH
Siaya	1973-2014	Standard demographic and health survey	CBH
Siaya	1976-2000	Multiple Indicator Cluster Survey	SBH

Appendix Table 5. Under-5 mortality reference sources by source date and location, 1950-2017			
**Location**	**Year range of data**	**Reference source**	**Data type**
Siaya	1983-2007	Malaria Indicator Survey	SBH
Siaya	1994-2011	Multiple Indicator Cluster Survey	CBH
TaitaTaveta	1950-2008	Census	SBH
TaitaTaveta	1964-2014	Standard demographic and health survey	SBH
TaitaTaveta	1970-2005	Other survey	SBH
TaitaTaveta	1975-2014	Standard demographic and health survey	CBH
TaitaTaveta	1976-2000	Multiple Indicator Cluster Survey	SBH
TaitaTaveta	1983-2007	Malaria Indicator Survey	SBH
TanaRiver	1950-2008	Census	SBH
TanaRiver	1968-2014	Standard demographic and health survey	SBH
TanaRiver	1970-2005	Other survey	SBH
TanaRiver	1976-2000	Multiple Indicator Cluster Survey	SBH
TanaRiver	1987-1990	Standard demographic and health survey	CBH
TanaRiver	1990-2003	Malaria Indicator Survey	SBH
TanaRiver	1993-2014	Standard demographic and health survey	CBH
TharakaNithi	1970-2005	Other survey	SBH
TharakaNithi	1975-2008	Census	SBH
TharakaNithi	1976-2008	Multiple Indicator Cluster Survey	SBH
TharakaNithi	1978-2014	Standard demographic and health survey	SBH
TharakaNithi	1983-2007	Malaria Indicator Survey	SBH
TharakaNithi	1991-1992	Standard demographic and health survey	CBH
TharakaNithi	1991-2008	Multiple Indicator Cluster Survey	CBH
TharakaNithi	1995-2014	Standard demographic and health survey	CBH
TransNzoia	1950-2008	Census	SBH
TransNzoia	1964-2014	Standard demographic and health survey	SBH
TransNzoia	1970-2005	Other survey	SBH
TransNzoia	1975-2014	Standard demographic and health survey	CBH
TransNzoia	1976-2000	Multiple Indicator Cluster Survey	SBH
TransNzoia	1983-2007	Malaria Indicator Survey	SBH
Turkana	1950-2008	Census	SBH
Turkana	1970-2005	Other survey	SBH
Turkana	1978-2014	Standard demographic and health survey	SBH
Turkana	1982-2006	Multiple Indicator Cluster Survey	SBH
Turkana	1983-2007	Malaria Indicator Survey	SBH
Turkana	1985-2014	Standard demographic and health survey	CBH
Turkana	1996-2013	Multiple Indicator Cluster Survey	CBH
UasinGishu	1950-2008	Census	SBH
UasinGishu	1964-2014	Standard demographic and health survey	SBH
UasinGishu	1970-2005	Other survey	SBH
UasinGishu	1973-2014	Standard demographic and health survey	CBH
UasinGishu	1976-2000	Multiple Indicator Cluster Survey	SBH
UasinGishu	1983-2007	Malaria Indicator Survey	SBH
Vihiga	1970-2005	Other survey	SBH
Vihiga	1975-2008	Census	SBH
Vihiga	1976-2000	Multiple Indicator Cluster Survey	SBH
Vihiga	1978-2014	Standard demographic and health survey	SBH
Vihiga	1983-2007	Malaria Indicator Survey	SBH
Vihiga	1987-2014	Standard demographic and health survey	CBH

Appendix Table 5. Under-5 mortality reference sources by source date and location, 1950-2017			
**Location**	**Year range of data**	**Reference source**	**Data type**
Wajir	1950-2008	Census	SBH
Wajir	1970-2005	Other survey	SBH
Wajir	1976-2006	Multiple Indicator Cluster Survey	SBH
Wajir	1978-2014	Standard demographic and health survey	SBH
Wajir	1983-2007	Malaria Indicator Survey	SBH
Wajir	1985-2014	Standard demographic and health survey	CBH
WestPokot	1950-2008	Census	SBH
WestPokot	1964-2014	Standard demographic and health survey	SBH
WestPokot	1970-2005	Other survey	SBH
WestPokot	1976-2000	Multiple Indicator Cluster Survey	SBH
WestPokot	1979-1982	Standard demographic and health survey	CBH
WestPokot	1983-2007	Malaria Indicator Survey	SBH
WestPokot	1985-1995	Standard demographic and health survey	CBH
WestPokot	1997-2014	Standard demographic and health survey	CBH
Madagascar	1955-1955	Vital Registration	VR/SRS/DSP
Madagascar	1957-1961	Vital Registration	VR/SRS/DSP
Madagascar	1964-1968	Vital Registration	VR/SRS/DSP
Madagascar	1967-2008	Standard demographic and health survey	CBH
Madagascar	1968-2008	Standard demographic and health survey	SBH
Madagascar	1970-1972	Vital Registration	VR/SRS/DSP
Madagascar	1976-2012	Multiple Indicator Cluster Survey	SBH
Madagascar	1986-2012	Malaria Indicator Survey	SBH
Malawi	1953-1953	Other survey	SBH
Malawi	1957-1957	Other survey	SBH
Malawi	1959-1959	Census	SBH
Malawi	1960-1960	Other survey	SBH
Malawi	1962-2007	Census	SBH
Malawi	1963-1969	Other survey	SBH
Malawi	1967-2015	Standard demographic and health survey	CBH
Malawi	1968-2010	Standard demographic and health survey	SBH
Malawi	1971-1972	Other survey	SBH
Malawi	1974-2001	Other survey	SBH
Malawi	1977-2013	Multiple Indicator Cluster Survey	CBH
Malawi	1982-2013	Multiple Indicator Cluster Survey	SBH
Malawi	1987-2016	Malaria Indicator Survey	SBH
Malawi	2003-2008	Other survey	CBH
Mozambique	1972-2006	Census	SBH
Mozambique	1972-2011	Standard demographic and health survey	SBH
Mozambique	1976-2011	Standard demographic and health survey	CBH
Mozambique	1979-2008	Multiple Indicator Cluster Survey	CBH
Mozambique	1984-2008	Multiple Indicator Cluster Survey	SBH
Mozambique	1985-2009	AIDS Indicator Survey	SBH
Rwanda	1952-1952	Census	SBH
Rwanda	1956-1956	Census	SBH
Rwanda	1958-1983	World Fertility Survey	CBH
Rwanda	1959-1959	Census	SBH
Rwanda	1959-1983	World Fertility Survey	SBH
Rwanda	1961-1962	Census	SBH

Appendix Table 5. Under-5 mortality reference sources by source date and location, 1950-2017

Location	Year range of data	Reference source	Data type
Rwanda	1964-1964	Census	SBH
Rwanda	1966-1993	Census	SBH
Rwanda	1967-1993	Standard demographic and health survey	CBH
Rwanda	1968-1993	Standard demographic and health survey	SBH
Rwanda	1981-1993	Other survey	SBH
Rwanda	1983-1993	Other demographic and health survey	SBH
Rwanda	1988-1993	Malaria Indicator Survey	SBH
Rwanda	1995-2005	Other survey	SBH
Rwanda	1995-2007	Other demographic and health survey	SBH
Rwanda	1995-2010	Standard demographic and health survey	SBH
Rwanda	1995-2012	Census	SBH
Rwanda	1995-2012	Malaria Indicator Survey	SBH
Rwanda	1995-2014	Standard demographic and health survey	CBH
Somalia	1982-2006	Multiple Indicator Cluster Survey	SBH
Somalia	1983-2006	Multiple Indicator Cluster Survey	CBH
South Sudan	1983-2007	Census	SBH
South Sudan	1985-2009	Multiple Indicator Cluster Survey	SBH
South Sudan	1986-2009	Multiple Indicator Cluster Survey	CBH
Tanzania	1962-2015	Standard demographic and health survey	CBH
Tanzania	1963-2002	Census	SBH
Tanzania	1967-2015	Standard demographic and health survey	SBH
Tanzania	1968-1992	Living Standards Measurement Study	SBH
Tanzania	1970-1994	Other survey	SBH
Tanzania	1978-2007	AIDS Indicator Survey	CBH
Tanzania	1979-2011	AIDS Indicator Survey	SBH
Uganda	1963-2016	Standard demographic and health survey	CBH
Uganda	1964-2016	Standard demographic and health survey	SBH
Uganda	1966-2001	Census	SBH
Uganda	1968-2015	Other survey	SBH
Uganda	1984-2014	Malaria Indicator Survey	SBH
Uganda	1985-2009	Living Standards Measurement Study	SBH
Uganda	1986-2009	Malaria Indicator Survey	CBH
Uganda	1986-2010	AIDS Indicator Survey	SBH
Zambia	1962-1962	Census	SBH
Zambia	1964-2013	Standard demographic and health survey	CBH
Zambia	1965-2009	Census	SBH
Zambia	1967-2013	Standard demographic and health survey	SBH
Zambia	1982-2011	Malaria Indicator Survey	SBH
Botswana	1964-1988	Standard demographic and health survey	SBH
Botswana	1967-1988	Standard demographic and health survey	CBH
Botswana	1971-2005	Other survey	SBH
Botswana	1973-1973	Census	SBH
Botswana	1976-1976	Census	SBH
Botswana	1976-2000	Multiple Indicator Cluster Survey	SBH
Botswana	1976-2007	Other survey	CBH
Botswana	1979-1979	Census	SBH
Botswana	1982-1982	Census	SBH
Botswana	1984-1984	Census	SBH

Appendix Table 5. Under-5 mortality reference sources by source date and location, 1950-2017

Location	Year range of data	Reference source	Data type
Botswana	1987-1987	Census	SBH
Botswana	2010-2010	Census	HH
Lesotho	1953-1977	World Fertility Survey	SBH
Lesotho	1954-1977	World Fertility Survey	CBH
Lesotho	1967-1967	Census	SBH
Lesotho	1971-1971	Census	SBH
Lesotho	1974-1974	Census	SBH
Lesotho	1975-1999	Multiple Indicator Cluster Survey	SBH
Lesotho	1977-1977	Census	SBH
Lesotho	1979-2014	Standard demographic and health survey	CBH
Lesotho	1980-1980	Census	SBH
Lesotho	1980-2014	Standard demographic and health survey	SBH
Lesotho	1982-1982	Census	SBH
Lesotho	1982-1982	Other survey	SBH
Lesotho	1985-1985	Other survey	SBH
Lesotho	1988-1988	Other survey	SBH
Lesotho	1990-1990	Other survey	SBH
Lesotho	1992-1992	Other survey	SBH
Lesotho	1994-1995	Other survey	SBH
Lesotho	1997-1998	Other survey	SBH
Lesotho	2002-2002	Other survey	SBH
Lesotho	2005-2005	Other survey	SBH
Lesotho	2008-2008	Other survey	SBH
Lesotho	2010-2010	Vital Registration	VR/SRS/DSP
Namibia	1968-2013	Standard demographic and health survey	SBH
Namibia	1969-2013	Standard demographic and health survey	CBH
Namibia	1973-1973	Census	SBH
Namibia	1976-1976	Census	SBH
Namibia	1979-1979	Census	SBH
Namibia	1982-1982	Census	SBH
Namibia	1985-1985	Census	SBH
Namibia	1987-1987	Census	SBH
Namibia	1992-1992	Census	SBH
Namibia	1995-1995	Census	SBH
Namibia	1998-1998	Census	SBH
Namibia	2001-2001	Census	SBH
Namibia	2004-2004	Census	SBH
Namibia	2007-2007	Census	SBH
South Africa	1963-1987	Other demographic and health survey	SBH
South Africa	1968-1992	Living Standards Measurement Study	SBH
South Africa	1971-1996	Census	SBH
South Africa	1971-1998	Standard demographic and health survey	CBH
South Africa	1973-1997	Standard demographic and health survey	SBH
South Africa	1980-1982	Vital Registration	VR/SRS/DSP
South Africa	1991-2015	Other survey	SBH
South Africa	1993-2015	Vital Registration	VR/SRS/DSP
South Africa	2000-2001	Census	HH
South Africa	2004-2004	Standard demographic and health survey	HH

Appendix Table 5. Under-5 mortality reference sources by source date and location, 1950-2017			
**Location**	**Year range of data**	**Reference source**	**Data type**
South Africa	2009-2009	Standard demographic and health survey	HH
South Africa	2011-2011	Census	HH
South Africa	2014-2014	Standard demographic and health survey	HH
Swaziland	1976-2014	Multiple Indicator Cluster Survey	SBH
Swaziland	1979-2006	Standard demographic and health survey	CBH
Swaziland	1982-2006	Standard demographic and health survey	SBH
Swaziland	1985-2014	Multiple Indicator Cluster Survey	CBH
Zimbabwe	1963-2015	Standard demographic and health survey	CBH
Zimbabwe	1964-2015	Standard demographic and health survey	SBH
Zimbabwe	1981-2013	Multiple Indicator Cluster Survey	CBH
Zimbabwe	1984-2013	Multiple Indicator Cluster Survey	SBH
Zimbabwe	1990-1990	Vital Registration	VR/SRS/DSP
Zimbabwe	1992-1992	Census	SBH
Zimbabwe	1995-1995	Vital Registration	VR/SRS/DSP
Zimbabwe	1995-1995	Census	SBH
Zimbabwe	1998-1998	Census	SBH
Zimbabwe	2001-2001	Census	SBH
Zimbabwe	2004-2004	Census	SBH
Zimbabwe	2007-2007	Census	SBH
Benin	1957-1981	World Fertility Survey	SBH
Benin	1959-1982	World Fertility Survey	CBH
Benin	1969-2011	Standard demographic and health survey	CBH
Benin	1972-2011	Standard demographic and health survey	SBH
Benin	1973-1973	Census	SBH
Benin	1977-1977	Census	SBH
Benin	1980-1980	Census	SBH
Benin	1983-1983	Census	SBH
Benin	1985-1985	Census	SBH
Benin	1985-2014	Multiple Indicator Cluster Survey	CBH
Benin	1988-1988	Census	SBH
Benin	1990-2014	Multiple Indicator Cluster Survey	SBH
Burkina Faso	1966-1966	Census	SBH
Burkina Faso	1967-2010	Standard demographic and health survey	CBH
Burkina Faso	1968-2010	Standard demographic and health survey	SBH
Burkina Faso	1970-1970	Census	SBH
Burkina Faso	1971-2005	Census	SBH
Burkina Faso	1981-2005	Multiple Indicator Cluster Survey	SBH
Burkina Faso	1989-2013	Malaria Indicator Survey	SBH
Burkina Faso	1991-1991	Other survey	HH
Burkina Faso	1996-1996	Census	HH
Burkina Faso	2006-2006	Census	HH
Cameroon	1951-1978	World Fertility Survey	CBH
Cameroon	1953-1977	World Fertility Survey	SBH
Cameroon	1967-2010	Standard demographic and health survey	SBH
Cameroon	1968-2011	Standard demographic and health survey	CBH
Cameroon	1976-2000	Multiple Indicator Cluster Survey	SBH
Cameroon	1980-2004	Census	SBH
Cameroon	1986-2013	Multiple Indicator Cluster Survey	CBH

Appendix Table 5. Under-5 mortality reference sources by source date and location, 1950-2017			
**Location**	**Year range of data**	**Reference source**	**Data type**
Cape Verde	1955-1957	Vital Registration	VR/SRS/DSP
Cape Verde	1959-1960	Vital Registration	VR/SRS/DSP
Cape Verde	1966-1975	Vital Registration	VR/SRS/DSP
Cape Verde	1973-1997	Reproductive Health Survey	SBH
Cape Verde	1977-1998	Reproductive Health Survey	CBH
Cape Verde	1979-1980	Vital Registration	VR/SRS/DSP
Cape Verde	1982-1982	Census	SBH
Cape Verde	1983-1985	Vital Registration	VR/SRS/DSP
Cape Verde	1985-1985	Census	SBH
Cape Verde	1985-1985	Standard demographic and health survey	SBH
Cape Verde	1988-1988	Census	SBH
Cape Verde	1988-1988	Standard demographic and health survey	SBH
Cape Verde	1990-1991	Vital Registration	VR/SRS/DSP
Cape Verde	1991-1991	Census	SBH
Cape Verde	1992-1992	Standard demographic and health survey	HH
Cape Verde	1992-1992	Standard demographic and health survey	SBH
Cape Verde	1994-1994	Census	SBH
Cape Verde	1996-1996	Census	SBH
Cape Verde	1996-1996	Standard demographic and health survey	SBH
Cape Verde	1997-1997	Standard demographic and health survey	HH
Cape Verde	1999-1999	Standard demographic and health survey	SBH
Cape Verde	2002-2002	Standard demographic and health survey	HH
Cape Verde	2002-2002	Standard demographic and health survey	SBH
Cape Verde	2011-2012	Vital Registration	VR/SRS/DSP
Chad	1969-2014	Standard demographic and health survey	CBH
Chad	1972-2014	Standard demographic and health survey	SBH
Chad	1976-2009	Multiple Indicator Cluster Survey	SBH
Cote d'Ivoire	1955-1980	World Fertility Survey	CBH
Cote d'Ivoire	1956-1980	World Fertility Survey	SBH
Cote d'Ivoire	1961-1961	Other survey	SBH
Cote d'Ivoire	1961-1986	Living Standards Measurement Study	CBH
Cote d'Ivoire	1964-1964	Other survey	SBH
Cote d'Ivoire	1967-1967	Other survey	SBH
Cote d'Ivoire	1967-2011	Standard demographic and health survey	CBH
Cote d'Ivoire	1970-1970	Other survey	SBH
Cote d'Ivoire	1970-2011	Standard demographic and health survey	SBH
Cote d'Ivoire	1972-1972	Other survey	SBH
Cote d'Ivoire	1974-1974	Other survey	SBH
Cote d'Ivoire	1980-2005	AIDS Indicator Survey	CBH
Cote d'Ivoire	1981-2005	AIDS Indicator Survey	SBH
Cote d'Ivoire	1994-1994	Multiple Indicator Cluster Survey	HH
Cote d'Ivoire	1999-1999	Multiple Indicator Cluster Survey	HH
Cote d'Ivoire	2004-2004	Multiple Indicator Cluster Survey	HH
Cote d'Ivoire	2009-2009	Multiple Indicator Cluster Survey	HH
Cote d'Ivoire	2014-2014	Multiple Indicator Cluster Survey	HH
The Gambia	1954-1954	Census	SBH
The Gambia	1958-1958	Census	SBH
The Gambia	1961-1961	Census	SBH

Appendix Table 5. Under-5 mortality reference sources by source date and location, 1950-2017			
**Location**	**Year range of data**	**Reference source**	**Data type**
The Gambia	1964-1964	Census	SBH
The Gambia	1967-1967	Census	SBH
The Gambia	1969-1969	Census	SBH
The Gambia	1972-1972	Other survey	SBH
The Gambia	1975-1975	Other survey	SBH
The Gambia	1975-2005	Multiple Indicator Cluster Survey	SBH
The Gambia	1978-1978	Other survey	HH
The Gambia	1979-1979	Other survey	SBH
The Gambia	1981-1981	Other survey	SBH
The Gambia	1983-1983	Other survey	HH
The Gambia	1983-1983	Other survey	SBH
The Gambia	1985-2012	Standard demographic and health survey	CBH
The Gambia	1986-1986	Other survey	SBH
The Gambia	1988-1988	Other survey	HH
The Gambia	1988-2012	Standard demographic and health survey	SBH
The Gambia	2007-2007	Multiple Indicator Cluster Survey	SBH
Ghana	1954-1978	World Fertility Survey	SBH
Ghana	1954-1979	World Fertility Survey	CBH
Ghana	1962-2014	Standard demographic and health survey	CBH
Ghana	1963-2014	Standard demographic and health survey	SBH
Ghana	1967-2005	Living Standards Measurement Study	SBH
Ghana	1975-2009	Census	SBH
Ghana	1980-2007	Other demographic and health survey	CBH
Ghana	1982-2011	Multiple Indicator Cluster Survey	SBH
Ghana	1983-2007	Other demographic and health survey	SBH
Ghana	1984-2011	Multiple Indicator Cluster Survey	CBH
Ghana	1990-2014	Other survey	SBH
Ghana	1992-2016	Malaria Indicator Survey	SBH
Guinea	1950-1950	Other survey	SBH
Guinea	1971-1995	Census	SBH
Guinea	1972-2012	Standard demographic and health survey	CBH
Guinea	1975-2012	Standard demographic and health survey	SBH
Guinea	1980-1980	Standard demographic and health survey	HH
Guinea	1985-1985	Standard demographic and health survey	HH
Guinea	1990-1990	Standard demographic and health survey	HH
Guinea	2004-2004	Multiple Indicator Cluster Survey	HH
Guinea	2009-2009	Multiple Indicator Cluster Survey	HH
Guinea	2014-2014	Multiple Indicator Cluster Survey	HH
Guinea-Bissau	1975-2013	Multiple Indicator Cluster Survey	SBH
Guinea-Bissau	1986-2013	Multiple Indicator Cluster Survey	CBH
Liberia	1961-1989	Standard demographic and health survey	CBH
Liberia	1961-1989	Standard demographic and health survey	SBH
Liberia	1983-1989	Census	SBH
Liberia	1983-1989	Malaria Indicator Survey	CBH
Liberia	1984-1989	Malaria Indicator Survey	SBH
Liberia	1991-2007	Census	SBH
Liberia	1991-2008	Malaria Indicator Survey	CBH
Liberia	1991-2012	Standard demographic and health survey	CBH

Appendix Table 5. Under-5 mortality reference sources by source date and location, 1950-2017			
**Location**	**Year range of data**	**Reference source**	**Data type**
Liberia	1991-2012	Standard demographic and health survey	SBH
Liberia	1991-2016	Malaria Indicator Survey	SBH
Mali	1962-2008	Census	SBH
Mali	1962-2012	Standard demographic and health survey	SBH
Mali	1964-2012	Standard demographic and health survey	CBH
Mali	1988-2015	Multiple Indicator Cluster Survey	CBH
Mali	1990-2014	Malaria Indicator Survey	SBH
Mali	1991-2015	Multiple Indicator Cluster Survey	SBH
Mauritania	1956-1981	World Fertility Survey	CBH
Mauritania	1957-1981	World Fertility Survey	SBH
Mauritania	1961-1990	Pan Arab Project for Child Development	CBH
Mauritania	1965-1989	Pan Arab Project for Child Development	SBH
Mauritania	1969-1969	Census	SBH
Mauritania	1972-1972	Census	SBH
Mauritania	1973-2000	Standard demographic and health survey	CBH
Mauritania	1976-1976	Census	SBH
Mauritania	1976-2000	Standard demographic and health survey	SBH
Mauritania	1979-1979	Census	SBH
Mauritania	1981-1981	Census	SBH
Mauritania	1982-2015	Multiple Indicator Cluster Survey	CBH
Mauritania	1983-2011	Multiple Indicator Cluster Survey	SBH
Mauritania	1984-1984	Census	SBH
Niger	1966-2011	Standard demographic and health survey	CBH
Niger	1967-2011	Standard demographic and health survey	SBH
Niger	1975-1999	Multiple Indicator Cluster Survey	SBH
Nigeria	1962-2012	Standard demographic and health survey	SBH
Nigeria	1963-2012	Standard demographic and health survey	CBH
Nigeria	1974-2016	Multiple Indicator Cluster Survey	SBH
Nigeria	1979-2009	Other survey	SBH
Nigeria	1981-2006	Census	SBH
Nigeria	1985-2010	Malaria Indicator Survey	CBH
Nigeria	1985-2014	Malaria Indicator Survey	SBH
Nigeria	1987-2016	Multiple Indicator Cluster Survey	CBH
Sao Tome and Principe	1955-1958	Vital Registration	VR/SRS/DSP
Sao Tome and Principe	1962-1962	Census	SBH
Sao Tome and Principe	1962-1971	Vital Registration	VR/SRS/DSP
Sao Tome and Principe	1965-1965	Census	SBH
Sao Tome and Principe	1969-1969	Census	SBH
Sao Tome and Principe	1971-1971	Census	SBH
Sao Tome and Principe	1973-1973	Census	SBH
Sao Tome and Principe	1976-1976	Census	SBH
Sao Tome and Principe	1976-2013	Multiple Indicator Cluster Survey	SBH
Sao Tome and Principe	1977-1979	Vital Registration	VR/SRS/DSP
Sao Tome and Principe	1980-1980	Census	SBH
Sao Tome and Principe	1982-1982	Census	SBH
Sao Tome and Principe	1984-1984	Census	SBH
Sao Tome and Principe	1984-1985	Vital Registration	VR/SRS/DSP
Sao Tome and Principe	1984-2008	Standard demographic and health survey	SBH

Appendix Table 5. Under-5 mortality reference sources by source date and location, 1950-2017

Location	Year range of data	Reference source	Data type
Sao Tome and Principe	1985-2008	Standard demographic and health survey	CBH
Sao Tome and Principe	1987-1987	Vital Registration	VR/SRS/DSP
Sao Tome and Principe	1987-1987	Census	SBH
Sao Tome and Principe	1990-2013	Multiple Indicator Cluster Survey	CBH
Senegal	1953-1978	World Fertility Survey	CBH
Senegal	1954-1978	World Fertility Survey	SBH
Senegal	1961-2014	Standard demographic and health survey	SBH
Senegal	1963-2016	Standard demographic and health survey	CBH
Senegal	1977-2001	Census	SBH
Senegal	1979-2008	Malaria Indicator Survey	CBH
Senegal	1981-2008	Malaria Indicator Survey	SBH
Senegal	2005-2005	Standard demographic and health survey	HH
Senegal	2010-2010	Standard demographic and health survey	HH
Senegal	2012-2012	Standard demographic and health survey	HH
Senegal	2014-2017	Standard demographic and health survey	HH
Sierra Leone	1975-2010	Multiple Indicator Cluster Survey	SBH
Sierra Leone	1979-2003	Census	SBH
Sierra Leone	1981-2013	Standard demographic and health survey	CBH
Sierra Leone	1983-2013	Standard demographic and health survey	SBH
Sierra Leone	2008-2012	Vital Registration	VR/SRS/DSP
Togo	1953-1953	Census	SBH
Togo	1955-1955	Census	SBH
Togo	1957-1957	Census	SBH
Togo	1964-2013	Standard demographic and health survey	SBH
Togo	1965-2013	Standard demographic and health survey	CBH
Togo	1981-2010	Multiple Indicator Cluster Survey	SBH
Togo	1992-1992	Census	SBH
Togo	1995-1995	Census	SBH
Togo	1999-1999	Census	SBH
Togo	2002-2002	Census	SBH
Togo	2005-2005	Census	SBH
Togo	2007-2007	Census	SBH

Note: CBH: Complete birth history, SBH: Summary birth history, HH: Household survey, SIBS: Sibling History, VR: Vital Registration, SRS: Sample [illegible]

Appendix Table 6. Distribution of empirical life tables by GBD super-region and decade, 1950-2017

Location	Life table type	1950-1959	1960-1969	1970-1979	1980-1989	1990-1999	2000-2009	2010-2017	All years
Southeast Asia, East Asia, and Oceania	Total	38	97	91	126	162	358	253	1125
	Location specific	29	65	72	108	157	324	238	993
	Universal	9	32	19	18	5	34	15	132
Central Europe, Eastern Europe, and Central Asia	Total	171	302	315	605	1791	1812	1355	6351
	Location specific	120	218	244	357	1043	1220	1012	4214
	Universal	51	84	71	248	748	592	343	2137
High-income	Total	604	1680	2160	4752	4598	4087	2752	20633
	Location specific	389	767	1294	3239	3009	2002	1602	12302
	Universal	215	913	866	1513	1589	2085	1150	8331
Latin America and Caribbean	Total	162	186	250	913	1190	1151	923	4775
	Location specific	149	149	198	815	1152	1147	909	4519
	Universal	13	37	52	98	38	4	14	256
North Africa and Middle East	Total	0	14	30	28	78	56	382	588
	Location specific	0	14	29	24	69	51	372	559
	Universal	0	0	1	4	9	5	10	29
South Asia	Total	0	0	7	16	221	509	451	1204
	Location specific	0	0	7	16	221	509	451	1204
	Universal	0	0	0	0	0	0	0	0
Sub-Saharan Africa	Total	2	9	6	20	74	197	123	431
	Location specific	2	9	6	20	74	197	123	431
	Universal	0	0	0	0	0	0	0	0
All locations	Total	977	2288	2859	6460	8114	8170	6239	35107
	Location specific	689	1222	1850	4579	5725	5450	4707	24222
	Universal	288	1066	1009	1881	2389	2720	1532	10885

Appendix Table 7. Locations by HIV estimation group			
**Group 1**	**Group 2A**	**Group 2B**	**Group 2C**
Angola	Antigua and Barbuda	Albania	Afghanistan
Benin	Argentina	American Samoa	Algeria
Botswana	Armenia	Azerbaijan	Andorra
Burkina Faso	Australia	Bahrain	Bangladesh
Burundi	Austria	Bosnia and Herzegovina	Bolivia
Cambodia	The Bahamas	Brunei	Bhutan
Cameroon	Barbados	China	Comoros
Cape Verde	Belarus	Cyprus	Federated States of Micronesia
Central African Republic	Belgium	Egypt	Laos
Chad	Belize	El Salvador	Lebanon
Cote D'Ivoire	Bermuda	Fiji	Libya
DR Congo	Brazil	FYR Macedonia	Marshall Islands
Djibouti	Bulgaria	Guam	Mauritania
Dominican Republic	Canada	Greenland	Morocco
Equatorial Guinea	Chile	Honduras	Nepal
Eritrea	Colombia	Indonesia	North Korea
Ethiopia	Costa Rica	Iran	Pakistan
Gabon	Croatia	Iraq	Samoa
The Gambia	Cuba	Jordan	Solomon Islands
Ghana	Czech Republic	Kiribati	Timor-Leste
Guinea	Denmark	Malaysia	Tunisia
Guinea-Bissau	Dominica	Maldives	United Arab Emirates
Haiti	Ecuador	Mongolia	Vanuatu
Kenya	Estonia	Montenegro	Yemen
Lesotho	Finland	Nicaragua	
Liberia	France	Northern Mariana Islands	
Madagascar	Georgia	Oman	
Malawi	Germany	Palestine	
Mali	Greece	Paraguay	
Mozambique	Grenada	Peru	
Myanmar	Guatemala	Qatar	
Namibia	Guyana	Sao Tome and Principe	
Niger	Hungary	Saudi Arabia	
Nigeria	Iceland	Serbia	
Papua New Guinea	Ireland	Seychelles	
Congo (Brazzaville)	Israel	Slovakia	
Rwanda	Italy	Sri Lanka	
Senegal	Jamaica	Syria	
Sierra Leone	Japan	Tajikistan	
South Africa	Kazakhstan	Thailand	
South Sudan	Kyrgyzstan	Tonga	

Appendix Table 7. Locations by HIV estimation group			
**Group 1**	**Group 2A**	**Group 2B**	**Group 2C**
Swaziland	Kuwait	Turkey	
Tanzania	Latvia	Vietnam	
Togo	Lithuania		
Uganda	Luxembourg		
Zambia	Macao		
Zimbabwe	Malta		
India (1B)	Mauritius		
Sudan (1B)	Mexico		
Somalia (1B)	Moldova		
	Netherlands		
	Norway		
	New Zealand		
	Panama		
	Philippines		
	Poland		
	Puerto Rico		
	Portugal		
	Romania		
	Russia		
	Saint Lucia		
	Saint Vincent and the Grenadines		
	Singapore		
	Slovenia		
	South Korea		
	Spain		
	Suriname		
	Sweden		
	Switzerland		
	Taiwan (province of China)		
	Trinidad and Tobago		
	Turkmenistan		
	Ukraine		
	United Kingdom		
	USA		
	Uruguay		
	Uzbekistan		
	Venezuela		
	Virgin Islands, U.S.		

Appendix Table 8. Socio-demographic Index groupings by geography, based on 2017 values		
**Geography**	**2017 SDI**	**SDI Quintile**
Global	0.652	
Central Europe, Eastern Europe, and Central Asia	0.766	
Central Asia	0.673	
Armenia	0.702	High-middle SDI
Azerbaijan	0.701	High-middle SDI
Georgia	0.7	High-middle SDI
Kazakhstan	0.735	High-middle SDI
Kyrgyzstan	0.607	Low-middle SDI
Mongolia	0.662	Middle SDI
Tajikistan	0.523	Low-middle SDI
Turkmenistan	0.696	Middle SDI
Uzbekistan	0.63	Middle SDI
Central Europe	0.814	
Albania	0.685	Middle SDI
Bosnia and Herzegovina	0.713	High-middle SDI
Bulgaria	0.792	High-middle SDI
Croatia	0.825	High SDI
Czech Republic	0.851	High SDI
Hungary	0.817	High-middle SDI
Macedonia	0.754	High-middle SDI
Montenegro	0.788	High-middle SDI
Poland	0.844	High SDI
Romania	0.784	High-middle SDI
Serbia	0.752	High-middle SDI
Slovakia	0.842	High SDI
Slovenia	0.86	High SDI
Eastern Europe	0.785	
Belarus	0.773	High-middle SDI
Estonia	0.858	High SDI
Latvia	0.825	High SDI
Lithuania	0.841	High SDI
Moldova	0.676	Middle SDI
Russian Federation	0.792	High-middle SDI
Ukraine	0.74	High-middle SDI
High-income	0.854	
Australasia	0.869	
Australia	0.873	High SDI
New Zealand	0.842	High SDI
High-income Asia-Pacific	0.869	
Brunei	0.856	High SDI
Japan	0.865	High SDI
Aichi	0.875	High SDI
Akita	0.829	High SDI
Aomori	0.825	High SDI

Appendix Table 8. Socio-demographic Index groupings by geography, based on 2017 values		
**Geography**	**2017 SDI**	**SDI Quintile**
Chiba	0.859	High SDI
Ehime	0.838	High SDI
Fukui	0.852	High SDI
Fukuoka	0.855	High SDI
Fukushima	0.831	High SDI
Gifu	0.849	High SDI
Gunma	0.851	High SDI
Hiroshima	0.863	High SDI
Hokkaidō	0.842	High SDI
Hyōgo	0.86	High SDI
Ibaraki	0.851	High SDI
Ishikawa	0.856	High SDI
Iwate	0.825	High SDI
Kagawa	0.85	High SDI
Kagoshima	0.83	High SDI
Kanagawa	0.875	High SDI
Kōchi	0.825	High SDI
Kumamoto	0.832	High SDI
Kyōto	0.873	High SDI
Mie	0.854	High SDI
Miyagi	0.85	High SDI
Miyazaki	0.823	High SDI
Nagano	0.851	High SDI
Nagasaki	0.826	High SDI
Nara	0.848	High SDI
Niigata	0.843	High SDI
Ōita	0.846	High SDI
Okayama	0.856	High SDI
Okinawa	0.818	High SDI
Ōsaka	0.872	High SDI
Saga	0.834	High SDI
Saitama	0.852	High SDI
Shiga	0.871	High SDI
Shimane	0.831	High SDI
Shizuoka	0.859	High SDI
Tochigi	0.853	High SDI
Tokushima	0.845	High SDI
Tōkyō	0.924	High SDI
Tottori	0.834	High SDI
Toyama	0.86	High SDI
Wakayama	0.84	High SDI
Yamagata	0.832	High SDI
Yamaguchi	0.849	High SDI
Yamanashi	0.854	High SDI
South Korea	0.872	High SDI

Appendix Table 8. Socio-demographic Index groupings by geography, based on 2017 values		
**Geography**	**2017 SDI**	**SDI Quintile**
Singapore	0.872	High SDI
High-income North America	0.868	
Canada	0.882	High SDI
Greenland	0.76	High-middle SDI
USA	0.867	High SDI
Alabama	0.837	High SDI
Alaska	0.861	High SDI
Arizona	0.845	High SDI
Arkansas	0.826	High SDI
California	0.872	High SDI
Colorado	0.882	High SDI
Connecticut	0.906	High SDI
Delaware	0.874	High SDI
Washington, DC	0.89	High SDI
Florida	0.864	High SDI
Georgia	0.848	High SDI
Hawaii	0.872	High SDI
Idaho	0.841	High SDI
Illinois	0.879	High SDI
Indiana	0.848	High SDI
Iowa	0.87	High SDI
Kansas	0.864	High SDI
Kentucky	0.831	High SDI
Louisiana	0.835	High SDI
Maine	0.872	High SDI
Maryland	0.896	High SDI
Massachusetts	0.913	High SDI
Michigan	0.868	High SDI
Minnesota	0.893	High SDI
Mississippi	0.819	High SDI
Missouri	0.853	High SDI
Montana	0.863	High SDI
Nebraska	0.873	High SDI
Nevada	0.847	High SDI
New Hampshire	0.904	High SDI
New Jersey	0.899	High SDI
New Mexico	0.835	High SDI
New York	0.893	High SDI
North Carolina	0.85	High SDI
North Dakota	0.88	High SDI
Ohio	0.858	High SDI
Oklahoma	0.838	High SDI
Oregon	0.871	High SDI
Pennsylvania	0.879	High SDI
Rhode Island	0.89	High SDI

Appendix Table 8. Socio-demographic Index groupings by geography, based on 2017 values

Geography	2017 SDI	SDI Quintile
South Carolina	0.846	High SDI
South Dakota	0.86	High SDI
Tennessee	0.837	High SDI
Texas	0.838	High SDI
Utah	0.856	High SDI
Vermont	0.896	High SDI
Virginia	0.885	High SDI
Washington	0.884	High SDI
West Virginia	0.825	High SDI
Wisconsin	0.878	High SDI
Wyoming	0.869	High SDI
Southern Latin America	0.72	
Argentina	0.71	High-middle SDI
Chile	0.748	High-middle SDI
Uruguay	0.707	High-middle SDI
Western Europe	0.857	
Andorra	0.902	High SDI
Austria	0.866	High SDI
Belgium	0.886	High SDI
Cyprus	0.865	High SDI
Denmark	0.918	High SDI
Finland	0.893	High SDI
France	0.865	High SDI
Germany	0.87	High SDI
Greece	0.817	High SDI
Iceland	0.907	High SDI
Ireland	0.882	High SDI
Israel	0.816	High-middle SDI
Italy	0.843	High SDI
Luxembourg	0.916	High SDI
Malta	0.836	High SDI
Netherlands	0.912	High SDI
Norway	0.911	High SDI
Portugal	0.778	High-middle SDI
Spain	0.825	High SDI
Sweden	0.883	High SDI
Stockholm	0.914	High SDI
Sweden except Stockholm	0.873	High SDI
Switzerland	0.889	High SDI
United Kingdom	0.843	High SDI
England	0.849	High SDI
East Midlands	0.83	High SDI
Derby	0.846	High SDI
Derbyshire	0.817	High SDI
Leicester	0.839	High SDI

Appendix Table 8. Socio-demographic Index groupings by geography, based on 2017 values		
**Geography**	**2017 SDI**	**SDI Quintile**
Leicestershire	0.846	High SDI
Lincolnshire	0.812	High SDI
Northamptonshire	0.829	High SDI
Nottingham	0.863	High SDI
Nottinghamshire	0.814	High SDI
Rutland	0.833	High SDI
East of England	0.84	High SDI
Bedford	0.838	High SDI
Cambridgeshire	0.871	High SDI
Central Bedfordshire	0.834	High SDI
Essex	0.832	High SDI
Hertfordshire	0.87	High SDI
Luton	0.833	High SDI
Norfolk	0.826	High SDI
Peterborough	0.818	High SDI
Southend-on-Sea	0.811	High SDI
Suffolk	0.821	High SDI
Thurrock	0.807	High SDI
Greater London	0.894	High SDI
Barking and Dagenham	0.802	High SDI
Barnet	0.865	High SDI
Bexley	0.826	High SDI
Brent	0.849	High SDI
Bromley	0.848	High SDI
Camden	0.93	High SDI
Croydon	0.833	High SDI
Ealing	0.865	High SDI
Enfield	0.839	High SDI
Greenwich	0.833	High SDI
Hackney	0.887	High SDI
Hammersmith and Fulham	0.927	High SDI
Haringey	0.854	High SDI
Harrow	0.848	High SDI
Havering	0.824	High SDI
Hillingdon	0.882	High SDI
Hounslow	0.879	High SDI
Islington	0.922	High SDI
Kensington and Chelsea	0.932	High SDI
Kingston upon Thames	0.89	High SDI
Lambeth	0.9	High SDI
Lewisham	0.843	High SDI
Merton	0.873	High SDI
Newham	0.838	High SDI
Redbridge	0.831	High SDI
Richmond upon Thames	0.902	High SDI

Appendix Table 8. Socio-demographic Index groupings by geography, based on 2017 values		
**Geography**	**2017 SDI**	**SDI Quintile**
Southwark	0.912	High SDI
Sutton	0.843	High SDI
Tower Hamlets	0.905	High SDI
Waltham Forest	0.819	High SDI
Wandsworth	0.911	High SDI
Westminster	0.927	High SDI
North East England	0.821	High SDI
County Durham	0.81	High SDI
Darlington	0.825	High SDI
Gateshead	0.826	High SDI
Hartlepool	0.793	High SDI
Middlesbrough	0.808	High SDI
Newcastle upon Tyne	0.872	High SDI
North Tyneside	0.825	High SDI
Northumberland	0.808	High SDI
Redcar and Cleveland	0.79	High SDI
South Tyneside	0.794	High SDI
Stockton-on-Tees	0.823	High SDI
Sunderland	0.815	High SDI
North West England	0.834	High SDI
Blackburn with Darwen	0.802	High SDI
Blackpool	0.781	High SDI
Bolton	0.805	High SDI
Bury	0.815	High SDI
Cheshire East	0.864	High SDI
Cheshire West and Chester	0.855	High SDI
Cumbria	0.828	High SDI
Halton	0.824	High SDI
Knowsley	0.816	High SDI
Lancashire	0.831	High SDI
Liverpool	0.852	High SDI
Manchester	0.885	High SDI
Oldham	0.79	High SDI
Rochdale	0.795	High SDI
Salford	0.838	High SDI
Sefton	0.812	High SDI
St Helens	0.803	High SDI
Stockport	0.843	High SDI
Tameside	0.797	High SDI
Trafford	0.873	High SDI
Warrington	0.86	High SDI
Wigan	0.798	High SDI
Wirral	0.803	High SDI
South East England	0.856	High SDI
Bracknell Forest	0.869	High SDI

Appendix Table 8. Socio-demographic Index groupings by geography, based on 2017 values

Geography	2017 SDI	SDI Quintile
Brighton and Hove	0.885	High SDI
Buckinghamshire	0.865	High SDI
East Sussex	0.814	High SDI
Hampshire	0.85	High SDI
Isle of Wight	0.814	High SDI
Kent	0.828	High SDI
Medway	0.809	High SDI
Milton Keynes	0.86	High SDI
Oxfordshire	0.879	High SDI
Portsmouth	0.86	High SDI
Reading	0.895	High SDI
Slough	0.859	High SDI
Southampton	0.858	High SDI
Surrey	0.883	High SDI
West Berkshire	0.872	High SDI
West Sussex	0.843	High SDI
Windsor and Maidenhead	0.889	High SDI
Wokingham	0.885	High SDI
South West England	0.841	High SDI
Bath and North East Somerset	0.875	High SDI
Bournemouth	0.858	High SDI
Bristol, City of	0.884	High SDI
Cornwall	0.817	High SDI
Devon	0.837	High SDI
Dorset	0.825	High SDI
Gloucestershire	0.85	High SDI
North Somerset	0.832	High SDI
Plymouth	0.836	High SDI
Poole	0.842	High SDI
Somerset	0.816	High SDI
South Gloucestershire	0.867	High SDI
Swindon	0.847	High SDI
Torbay	0.79	High SDI
Wiltshire	0.829	High SDI
West Midlands	0.829	High SDI
Birmingham	0.84	High SDI
Coventry	0.848	High SDI
Dudley	0.799	High SDI
Herefordshire, County of	0.828	High SDI
Sandwell	0.797	High SDI
Shropshire	0.832	High SDI
Solihull	0.855	High SDI
Staffordshire	0.826	High SDI
Stoke-on-Trent	0.804	High SDI
Telford and Wrekin	0.822	High SDI

Appendix Table 8. Socio-demographic Index groupings by geography, based on 2017 values		
**Geography**	**2017 SDI**	**SDI Quintile**
Walsall	0.791	High SDI
Warwickshire	0.857	High SDI
Wolverhampton	0.811	High SDI
Worcestershire	0.833	High SDI
Yorkshire and the Humber	0.83	High SDI
Barnsley	0.787	High SDI
Bradford	0.807	High SDI
Calderdale	0.827	High SDI
Doncaster	0.791	High SDI
East Riding of Yorkshire	0.822	High SDI
Kingston upon Hull, City of	0.813	High SDI
Kirklees	0.816	High SDI
Leeds	0.868	High SDI
North East Lincolnshire	0.804	High SDI
North Lincolnshire	0.811	High SDI
North Yorkshire	0.839	High SDI
Rotherham	0.796	High SDI
Sheffield	0.853	High SDI
Wakefield	0.806	High SDI
York	0.879	High SDI
Northern Ireland	0.835	High SDI
Scotland	0.805	High SDI
Wales	0.806	High SDI
Latin America and Caribbean	0.64	
Andean Latin America	0.628	
Bolivia	0.587	Low-middle SDI
Ecuador	0.636	Middle SDI
Peru	0.636	Middle SDI
Caribbean	0.638	
Antigua and Barbuda	0.715	High-middle SDI
The Bahamas	0.756	High-middle SDI
Barbados	0.739	High-middle SDI
Belize	0.602	Low-middle SDI
Bermuda	0.805	High-middle SDI
Cuba	0.688	Middle SDI
Dominica	0.687	Middle SDI
Dominican Republic	0.593	Low-middle SDI
Grenada	0.64	Middle SDI
Guyana	0.584	Low-middle SDI
Haiti	0.442	Low SDI
Jamaica	0.679	Middle SDI
Puerto Rico	0.813	High-middle SDI
Saint Lucia	0.653	Middle SDI
Saint Vincent and the Grenadines	0.608	Middle SDI
Suriname	0.641	Middle SDI

Appendix Table 8. Socio-demographic Index groupings by geography, based on 2017 values		
**Geography**	**2017 SDI**	**SDI Quintile**
Trinidad and Tobago	0.698	Middle SDI
Virgin Islands	0.807	High-middle SDI
Central Latin America	0.623	
Colombia	0.634	Middle SDI
Costa Rica	0.662	Middle SDI
El Salvador	0.593	Low-middle SDI
Guatemala	0.524	Low-middle SDI
Honduras	0.512	Low-middle SDI
Mexico	0.628	Middle SDI
Aguascalientes	0.659	Middle SDI
Baja California	0.657	Middle SDI
Baja California Sur	0.659	Middle SDI
Campeche	0.616	Middle SDI
Chiapas	0.533	Middle SDI
Chihuahua	0.639	Middle SDI
Coahuila	0.645	Middle SDI
Colima	0.654	Middle SDI
Mexico City	0.716	Middle SDI
Durango	0.624	Middle SDI
Guanajuato	0.621	Middle SDI
Guerrero	0.562	Middle SDI
Hidalgo	0.587	Middle SDI
Jalisco	0.649	Middle SDI
México	0.635	Middle SDI
Michoacán de Ocampo	0.586	Middle SDI
Morelos	0.635	Middle SDI
Nayarit	0.62	Middle SDI
Nuevo León	0.677	Middle SDI
Oaxaca	0.561	Middle SDI
Puebla	0.584	Middle SDI
Querétaro	0.639	Middle SDI
Quintana Roo	0.626	Middle SDI
San Luis Potosí	0.621	Middle SDI
Sinaloa	0.649	Middle SDI
Sonora	0.65	Middle SDI
Tabasco	0.611	Middle SDI
Tamaulipas	0.647	Middle SDI
Tlaxcala	0.604	Middle SDI
Veracruz de Ignacio de la Llave	0.592	Middle SDI
Yucatán	0.63	Middle SDI
Zacatecas	0.608	Middle SDI
Nicaragua	0.53	Low-middle SDI
Panama	0.677	Middle SDI
Venezuela	0.655	Middle SDI
Tropical Latin America	0.662	

Appendix Table 8. Socio-demographic Index groupings by geography, based on 2017 values		
**Geography**	**2017 SDI**	**SDI Quintile**
Brazil	0.663	Middle SDI
Acre	0.602	Low-middle SDI
Alagoas	0.556	Low-middle SDI
Amapá	0.659	Middle SDI
Amazonas	0.629	Middle SDI
Bahia	0.591	Low-middle SDI
Ceará	0.6	Low-middle SDI
Distrito Federal	0.792	High-middle SDI
Espírito Santo	0.677	Middle SDI
Goiás	0.65	Middle SDI
Maranhão	0.507	Low-middle SDI
Mato Grosso	0.662	Middle SDI
Mato Grosso do Sul	0.65	Middle SDI
Minas Gerais	0.661	Middle SDI
Pará	0.579	Low-middle SDI
Paraíba	0.574	Low-middle SDI
Paraná	0.682	Middle SDI
Pernambuco	0.594	Low-middle SDI
Piauí	0.552	Low-middle SDI
Rio de Janeiro	0.709	High-middle SDI
Rio Grande do Norte	0.605	Low-middle SDI
Rio Grande do Sul	0.693	Middle SDI
Rondônia	0.622	Middle SDI
Roraima	0.646	Middle SDI
Santa Catarina	0.702	High-middle SDI
São Paulo	0.72	High-middle SDI
Sergipe	0.616	Middle SDI
Tocantins	0.611	Middle SDI
Paraguay	0.619	Middle SDI
North Africa and Middle East	0.639	
North Africa and Middle East	0.639	
Afghanistan	0.29	Low SDI
Algeria	0.696	Middle SDI
Bahrain	0.712	High-middle SDI
Egypt	0.604	Low-middle SDI
Iran	0.7	High-middle SDI
Iraq	0.585	Low-middle SDI
Jordan	0.697	Middle SDI
Kuwait	0.786	High-middle SDI
Lebanon	0.73	High-middle SDI
Libya	0.761	High-middle SDI
Morocco	0.579	Low-middle SDI
Palestine	0.541	Low-middle SDI
Oman	0.744	High-middle SDI
Qatar	0.766	High-middle SDI

Appendix Table 8. Socio-demographic Index groupings by geography, based on 2017 values		
**Geography**	**2017 SDI**	**SDI Quintile**
Saudi Arabia	0.779	High-middle SDI
Sudan	0.478	Low-middle SDI
Syria	0.611	Middle SDI
Tunisia	0.675	Middle SDI
Turkey	0.729	High-middle SDI
United Arab Emirates	0.795	High-middle SDI
Yemen	0.43	Low SDI
South Asia	0.534	
South Asia	0.534	
Bangladesh	0.458	Low SDI
Bhutan	0.57	Low-middle SDI
India	0.55	Low-middle SDI
Andhra Pradesh	0.536	Low-middle SDI
Arunachal Pradesh	0.556	Low-middle SDI
Assam	0.53	Low-middle SDI
Bihar	0.433	Low SDI
Chhattisgarh	0.512	Low-middle SDI
Delhi	0.715	High-middle SDI
Goa	0.74	High-middle SDI
Gujarat	0.584	Low-middle SDI
Haryana	0.6	Low-middle SDI
Himachal Pradesh	0.633	Middle SDI
Jammu and Kashmir	0.59	Low-middle SDI
Jharkhand	0.487	Low-middle SDI
Karnataka	0.574	Low-middle SDI
Kerala	0.659	Middle SDI
Madhya Pradesh	0.487	Low-middle SDI
Maharashtra	0.618	Middle SDI
Manipur	0.59	Low-middle SDI
Meghalaya	0.565	Low-middle SDI
Mizoram	0.616	Middle SDI
Nagaland	0.633	Middle SDI
Odisha	0.524	Low-middle SDI
Punjab	0.622	Middle SDI
Rajasthan	0.492	Low-middle SDI
Sikkim	0.628	Middle SDI
Tamil Nadu	0.615	Middle SDI
Telangana	0.575	Low-middle SDI
Tripura	0.543	Low-middle SDI
Uttar Pradesh	0.488	Low-middle SDI
Uttarakhand	0.607	Middle SDI
West Bengal	0.538	Low-middle SDI
Union Territories other than Delhi	0.653	Middle SDI
Nepal	0.429	Low SDI
Pakistan	0.492	Low-middle SDI

Appendix Table 8. Socio-demographic Index groupings by geography, based on 2017 values		
**Geography**	**2017 SDI**	**SDI Quintile**
Southeast Asia, East Asia, and Oceania	0.685	
East Asia	0.709	
China	0.707	High-middle SDI
North Korea	0.538	Low-middle SDI
Taiwan (Province of China)	0.864	High SDI
Oceania	0.471	
American Samoa	0.702	High-middle SDI
Federated States of Micronesia	0.575	Low-middle SDI
Fiji	0.641	Middle SDI
Guam	0.794	High-middle SDI
Kiribati	0.427	Low SDI
Marshall Islands	0.55	Low-middle SDI
Northern Mariana Islands	0.758	High-middle SDI
Papua New Guinea	0.419	Low SDI
Samoa	0.576	Low-middle SDI
Solomon Islands	0.425	Low SDI
Tonga	0.625	Middle SDI
Vanuatu	0.475	Low-middle SDI
Southeast Asia	0.641	
Cambodia	0.482	Low-middle SDI
Indonesia	0.648	Middle SDI
Laos	0.519	Low-middle SDI
Malaysia	0.759	High-middle SDI
Maldives	0.655	Middle SDI
Mauritius	0.72	High-middle SDI
Myanmar	0.556	Low-middle SDI
Philippines	0.617	Middle SDI
Sri Lanka	0.68	Middle SDI
Seychelles	0.692	Middle SDI
Thailand	0.684	Middle SDI
Timor-Leste	0.505	Low-middle SDI
Vietnam	0.607	Middle SDI
Sub-Saharan Africa	0.446	
Central sub-Saharan Africa	0.457	
Angola	0.461	Low-middle SDI
Central African Republic	0.334	Low SDI
Congo (Brazzaville)	0.574	Low-middle SDI
DR Congo	0.364	Low SDI
Equatorial Guinea	0.625	Middle SDI
Gabon	0.651	Middle SDI
Eastern sub-Saharan Africa	0.387	
Burundi	0.31	Low SDI
Comoros	0.434	Low SDI
Djibouti	0.485	Low-middle SDI
Eritrea	0.409	Low SDI

Appendix Table 8. Socio-demographic Index groupings by geography, based on 2017 values		
**Geography**	**2017 SDI**	**SDI Quintile**
Ethiopia	0.334	Low SDI
Kenya	0.499	Low-middle SDI
Baringo	0.444	Low-middle SDI
Bomet	0.496	Low-middle SDI
Bungoma	0.463	Low-middle SDI
Busia	0.438	Low-middle SDI
Elgeyo Marakwet	0.496	Low-middle SDI
Embu	0.533	Low-middle SDI
Garissa	0.334	Low-middle SDI
Homa Bay	0.425	Low-middle SDI
Isiolo	0.385	Low-middle SDI
Kajiado	0.534	Low-middle SDI
Kakamega	0.45	Low-middle SDI
Kericho	0.5	Low-middle SDI
Kiambu	0.58	Low-middle SDI
Kilifi	0.456	Low-middle SDI
Kirinyaga	0.533	Low-middle SDI
Kisii	0.522	Low-middle SDI
Kisumu	0.503	Low-middle SDI
Kitui	0.461	Low-middle SDI
Kwale	0.457	Low-middle SDI
Laikipia	0.556	Low-middle SDI
Lamu	0.453	Low-middle SDI
Machakos	0.518	Low-middle SDI
Makueni	0.469	Low-middle SDI
Mandera	0.295	Low-middle SDI
Marsabit	0.34	Low-middle SDI
Meru	0.508	Low-middle SDI
Migori	0.419	Low-middle SDI
Mombasa	0.568	Low-middle SDI
Murang’a	0.528	Low-middle SDI
Nairobi	0.674	Low-middle SDI
Nakuru	0.545	Low-middle SDI
Nandi	0.501	Low-middle SDI
Narok	0.402	Low-middle SDI
Nyamira	0.544	Low-middle SDI
Nyandarua	0.534	Low-middle SDI
Nyeri	0.554	Low-middle SDI
Samburu	0.308	Low-middle SDI
Siaya	0.46	Low-middle SDI
Taita Taveta	0.529	Low-middle SDI
Tana River	0.379	Low-middle SDI
Tharaka Nithi	0.528	Low-middle SDI
Trans Nzoia	0.496	Low-middle SDI
Turkana	0.295	Low-middle SDI

Appendix Table 8. Socio-demographic Index groupings by geography, based on 2017 values		
**Geography**	**2017 SDI**	**SDI Quintile**
Uasin Gishu	0.545	Low-middle SDI
Vihiga	0.477	Low-middle SDI
Wajir	0.243	Low-middle SDI
West Pokot	0.382	Low-middle SDI
Madagascar	0.331	Low SDI
Malawi	0.349	Low SDI
Mozambique	0.34	Low SDI
Rwanda	0.407	Low SDI
Somalia	0.235	Low SDI
South Sudan	0.275	Low SDI
Tanzania	0.412	Low SDI
Uganda	0.388	Low SDI
Zambia	0.472	Low-middle SDI
Southern sub-Saharan Africa	0.64	
Botswana	0.663	Middle SDI
Lesotho	0.493	Low-middle SDI
Namibia	0.616	Middle SDI
South Africa	0.677	Middle SDI
Swaziland	0.578	Low-middle SDI
Zimbabwe	0.463	Low-middle SDI
Western sub-Saharan Africa	0.441	
Benin	0.373	Low SDI
Burkina Faso	0.284	Low SDI
Cameroon	0.482	Low-middle SDI
Cape Verde	0.549	Low-middle SDI
Chad	0.253	Low SDI
Cote d'Ivoire	0.412	Low SDI
The Gambia	0.405	Low SDI
Ghana	0.537	Low-middle SDI
Guinea	0.325	Low SDI
Guinea-Bissau	0.349	Low SDI
Liberia	0.328	Low SDI
Mali	0.267	Low SDI
Mauritania	0.471	Low-middle SDI
Niger	0.191	Low SDI
Nigeria	0.493	Low-middle SDI
Sao Tome and Principe	0.488	Low-middle SDI
Senegal	0.373	Low SDI
Sierra Leone	0.357	Low SDI
Togo	0.413	Low SDI

Appendix Table 9. Socio-demographic Index values for all estimated GBD 2017 locations, 1950-1969

Location	1950	1951	1952	1953	1954	1955	1956	1957	1958	1959	1960	1961	1962	1963	1964	1965	1966	1967	1968	1969
Global	0.359	0.361	0.364	0.366	0.368	0.371	0.372	0.373	0.374	0.376	0.378	0.381	0.385	0.389	0.393	0.398	0.402	0.406	0.411	0.417
Central Europe, Eastern Europe, and Central Asia	0.53	0.533	0.539	0.543	0.545	0.549	0.551	0.552	0.553	0.554	0.557	0.563	0.57	0.577	0.584	0.59	0.591	0.591	0.593	0.594
Central Asia	0.404	0.408	0.412	0.415	0.418	0.421	0.424	0.427	0.429	0.431	0.435	0.44	0.446	0.45	0.455	0.46	0.464	0.469	0.474	0.48
Armenia	0.378	0.381	0.386	0.391	0.396	0.402	0.409	0.416	0.422	0.428	0.436	0.445	0.452	0.457	0.464	0.471	0.477	0.484	0.489	0.495
Azerbaijan	0.381	0.384	0.39	0.395	0.4	0.406	0.413	0.419	0.425	0.43	0.436	0.444	0.451	0.456	0.465	0.473	0.481	0.488	0.496	0.503
Georgia	0.477	0.481	0.487	0.493	0.499	0.506	0.513	0.519	0.524	0.528	0.534	0.539	0.545	0.548	0.553	0.558	0.562	0.566	0.569	0.573
Kazakhstan	0.437	0.442	0.447	0.45	0.451	0.453	0.454	0.455	0.456	0.457	0.46	0.465	0.473	0.481	0.49	0.497	0.504	0.511	0.517	0.522
Kyrgyzstan	0.43	0.433	0.437	0.44	0.443	0.446	0.45	0.452	0.455	0.456	0.46	0.464	0.469	0.471	0.474	0.477	0.479	0.481	0.483	0.484
Mongolia	0.323	0.33	0.336	0.343	0.349	0.354	0.356	0.358	0.359	0.361	0.364	0.367	0.37	0.373	0.378	0.384	0.39	0.397	0.403	0.41
Tajikistan	0.271	0.273	0.276	0.278	0.281	0.284	0.288	0.292	0.295	0.297	0.301	0.306	0.309	0.309	0.312	0.316	0.32	0.322	0.324	0.326
Turkmenistan	0.421	0.424	0.428	0.431	0.434	0.437	0.44	0.442	0.444	0.445	0.447	0.45	0.453	0.457	0.463	0.469	0.475	0.481	0.486	0.491
Uzbekistan	0.331	0.332	0.334	0.336	0.337	0.338	0.34	0.341	0.343	0.344	0.347	0.352	0.355	0.358	0.362	0.367	0.371	0.376	0.38	0.384
Central Europe	0.519	0.523	0.526	0.527	0.528	0.531	0.534	0.536	0.54	0.544	0.549	0.555	0.56	0.564	0.57	0.576	0.575	0.573	0.579	0.585
Albania	0.369	0.372	0.371	0.371	0.371	0.373	0.376	0.378	0.38	0.382	0.385	0.389	0.394	0.4	0.407	0.413	0.417	0.42	0.423	0.427
Bosnia and Herzegovina	0.259	0.264	0.27	0.274	0.276	0.284	0.287	0.292	0.297	0.306	0.315	0.323	0.329	0.337	0.345	0.353	0.36	0.367	0.373	0.38
Bulgaria	0.48	0.496	0.498	0.503	0.504	0.504	0.505	0.509	0.511	0.512	0.516	0.523	0.53	0.534	0.542	0.551	0.556	0.553	0.552	0.558
Croatia	0.505	0.509	0.513	0.518	0.523	0.53	0.535	0.542	0.549	0.555	0.563	0.57	0.576	0.583	0.591	0.597	0.604	0.61	0.616	0.622
Czech Republic	0.598	0.6	0.605	0.609	0.611	0.612	0.613	0.618	0.625	0.629	0.63	0.63	0.627	0.626	0.633	0.644	0.652	0.658	0.66	0.662
Hungary	0.516	0.521	0.521	0.515	0.513	0.518	0.525	0.532	0.538	0.544	0.55	0.558	0.566	0.57	0.574	0.575	0.573	0.572	0.576	0.582
Macedonia	0.444	0.447	0.451	0.455	0.458	0.464	0.469	0.475	0.481	0.486	0.492	0.498	0.502	0.508	0.517	0.523	0.53	0.535	0.541	0.546
Montenegro	0.56	0.564	0.569	0.574	0.578	0.583	0.588	0.592	0.596	0.6	0.604	0.608	0.612	0.616	0.621	0.625	0.629	0.634	0.638	0.643
Poland	0.537	0.538	0.538	0.538	0.536	0.536	0.535	0.535	0.539	0.546	0.554	0.561	0.567	0.574	0.581	0.588	0.597	0.603	0.607	0.61
Romania	0.462	0.468	0.473	0.478	0.483	0.488	0.492	0.496	0.5	0.505	0.511	0.518	0.524	0.528	0.537	0.542	0.513	0.482	0.499	0.519
Serbia	0.52	0.524	0.527	0.527	0.53	0.538	0.545	0.547	0.545	0.541	0.539	0.542	0.545	0.547	0.547	0.549	0.553	0.556	0.56	0.566
Slovakia	0.544	0.546	0.55	0.554	0.555	0.555	0.556	0.561	0.565	0.567	0.571	0.576	0.581	0.585	0.592	0.6	0.609	0.617	0.621	0.623
Slovenia	0.571	0.578	0.584	0.589	0.594	0.597	0.601	0.606	0.611	0.615	0.619	0.621	0.622	0.623	0.623	0.625	0.631	0.638	0.645	0.648
Eastern Europe	0.545	0.549	0.558	0.563	0.566	0.572	0.574	0.573	0.573	0.575	0.576	0.583	0.593	0.601	0.611	0.617	0.62	0.622	0.621	0.618
Belarus	0.487	0.49	0.495	0.499	0.502	0.506	0.51	0.514	0.518	0.522	0.527	0.532	0.537	0.54	0.544	0.548	0.55	0.552	0.554	0.556
Estonia	0.606	0.61	0.615	0.621	0.626	0.631	0.637	0.64	0.644	0.646	0.649	0.654	0.66	0.665	0.669	0.671	0.672	0.672	0.67	0.669
Latvia	0.578	0.584	0.59	0.595	0.598	0.601	0.605	0.609	0.613	0.616	0.62	0.624	0.631	0.637	0.643	0.647	0.649	0.65	0.651	0.651
Lithuania	0.565	0.572	0.581	0.589	0.597	0.606	0.614	0.619	0.624	0.627	0.631	0.636	0.643	0.649	0.653	0.656	0.658	0.659	0.659	0.658
Moldova	0.419	0.424	0.43	0.435	0.441	0.446	0.451	0.457	0.463	0.469	0.476	0.483	0.49	0.495	0.499	0.504	0.508	0.513	0.517	0.521
Russian Federation	0.555	0.559	0.57	0.575	0.578	0.585	0.586	0.584	0.582	0.582	0.582	0.589	0.602	0.612	0.624	0.632	0.635	0.635	0.632	0.628
Ukraine	0.515	0.52	0.525	0.529	0.533	0.537	0.54	0.542	0.545	0.548	0.552	0.558	0.564	0.569	0.575	0.58	0.584	0.586	0.589	0.59
High-income	0.598	0.601	0.603	0.606	0.607	0.609	0.61	0.611	0.612	0.614	0.616	0.619	0.623	0.627	0.633	0.639	0.645	0.651	0.657	0.661
Australasia	0.621	0.616	0.612	0.612	0.611	0.609	0.607	0.606	0.606	0.607	0.608	0.612	0.621	0.633	0.642	0.649	0.654	0.656	0.658	0.661
Australia	0.628	0.622	0.618	0.617	0.616	0.614	0.612	0.611	0.611	0.613	0.614	0.618	0.626	0.636	0.647	0.654	0.659	0.66	0.662	0.665
New Zealand	0.589	0.588	0.587	0.586	0.585	0.585	0.585	0.584	0.583	0.581	0.579	0.583	0.599	0.616	0.622	0.623	0.628	0.634	0.639	0.643
High-income Asia-Pacific	0.532	0.543	0.551	0.558	0.563	0.569	0.575	0.58	0.582	0.587	0.594	0.602	0.608	0.615	0.619	0.628	0.635	0.639	0.65	0.658
Brunei	0.436	0.436	0.434	0.428	0.421	0.417	0.416	0.415	0.417	0.426	0.444	0.466	0.486	0.5	0.512	0.523	0.532	0.541	0.551	0.561
Japan	0.555	0.568	0.577	0.585	0.591	0.597	0.605	0.609	0.613	0.618	0.627	0.635	0.642	0.649	0.652	0.662	0.67	0.674	0.685	0.693
Aichi	0.563	0.576	0.586	0.593	0.599	0.605	0.613	0.617	0.62	0.625	0.633	0.641	0.648	0.654	0.656	0.667	0.674	0.676	0.689	0.696
Akita	0.527	0.539	0.548	0.555	0.56	0.565	0.573	0.577	0.579	0.584	0.592	0.599	0.606	0.613	0.615	0.626	0.633	0.636	0.648	0.656
Aomori	0.517	0.53	0.54	0.547	0.553	0.558	0.566	0.57	0.572	0.577	0.585	0.593	0.6	0.606	0.608	0.619	0.626	0.629	0.641	0.648
Chiba	0.55	0.562	0.572	0.579	0.585	0.592	0.599	0.604	0.607	0.613	0.621	0.63	0.637	0.644	0.647	0.657	0.665	0.668	0.68	0.688
Ehime	0.528	0.541	0.55	0.558	0.564	0.569	0.577	0.581	0.584	0.59	0.598	0.606	0.613	0.62	0.622	0.633	0.641	0.644	0.657	0.664
Fukui	0.532	0.545	0.555	0.562	0.568	0.574	0.581	0.586	0.588	0.593	0.601	0.609	0.616	0.622	0.624	0.635	0.643	0.645	0.658	0.666
Fukuoka	0.55	0.562	0.572	0.579	0.585	0.591	0.599	0.603	0.607	0.612	0.62	0.629	0.636	0.643	0.647	0.657	0.665	0.669	0.681	0.688
Fukushima	0.527	0.54	0.549	0.557	0.562	0.568	0.575	0.58	0.582	0.587	0.595	0.602	0.61	0.616	0.619	0.63	0.637	0.64	0.653	0.66
Gifu	0.535	0.547	0.556	0.564	0.569	0.575	0.583	0.587	0.59	0.596	0.604	0.612	0.619	0.625	0.628	0.639	0.646	0.649	0.661	0.669
Gunma	0.541	0.554	0.563	0.571	0.576	0.582	0.59	0.595	0.598	0.603	0.612	0.62	0.627	0.634	0.637	0.648	0.656	0.66	0.672	0.679
Hiroshima	0.55	0.563	0.573	0.581	0.587	0.593	0.6	0.605	0.607	0.613	0.621	0.629	0.636	0.642	0.644	0.655	0.662	0.665	0.678	0.685
Hokkaidō	0.541	0.553	0.562	0.569	0.575	0.581	0.588	0.593	0.596	0.601	0.609	0.617	0.624	0.63	0.633	0.643	0.651	0.654	0.666	0.673
Hyōgo	0.55	0.562	0.571	0.579	0.585	0.591	0.598	0.603	0.606	0.612	0.62	0.628	0.635	0.642	0.645	0.655	0.663	0.667	0.678	0.686
Ibaraki	0.538	0.551	0.56	0.568	0.574	0.58	0.588	0.593	0.595	0.601	0.609	0.617	0.624	0.631	0.634	0.645	0.652	0.656	0.668	0.676
Ishikawa	0.533	0.546	0.556	0.564	0.57	0.576	0.584	0.588	0.59	0.595	0.603	0.611	0.618	0.625	0.626	0.637	0.645	0.647	0.66	0.667
Iwate	0.517	0.53	0.539	0.546	0.552	0.558	0.565	0.569	0.571	0.577	0.585	0.592	0.599	0.606	0.608	0.619	0.626	0.629	0.641	0.648
Kagawa	0.535	0.548	0.558	0.566	0.572	0.578	0.586	0.59	0.593	0.598	0.607	0.615	0.622	0.628	0.631	0.642	0.649	0.652	0.665	0.673
Kagoshima	0.522	0.534	0.542	0.549	0.555	0.56	0.567	0.572	0.575	0.58	0.588	0.596	0.603	0.611	0.614	0.625	0.633	0.636	0.648	0.656
Kanagawa	0.572	0.584	0.593	0.601	0.607	0.613	0.62	0.625	0.628	0.634	0.642	0.65	0.657	0.664	0.668	0.677	0.685	0.689	0.7	0.708
Kōchi	0.505	0.519	0.528	0.536	0.542	0.548	0.556	0.561	0.563	0.569	0.577	0.585	0.593	0.6	0.603	0.614	0.622	0.625	0.638	0.645
Kumamoto	0.522	0.534	0.543	0.55	0.556	0.562	0.569	0.574	0.577	0.582	0.59	0.598	0.606	0.613	0.616	0.627	0.634	0.638	0.65	0.658
Kyōto	0.568	0.58	0.588	0.596	0.602	0.608	0.615	0.62	0.624	0.629	0.638	0.646	0.653	0.66	0.665	0.674	0.682	0.687	0.698	0.706
Mie	0.535	0.548	0.557	0.565	0.571	0.577	0.585	0.589	0.592	0.597	0.605	0.613	0.62	0.627	0.629	0.64	0.648	0.65	0.663	0.67
Miyagi	0.541	0.553	0.562	0.57	0.575	0.581	0.589	0.593	0.596	0.601	0.609	0.617	0.625	0.632	0.635	0.645	0.653	0.657	0.669	0.677

Appendix Table 9. Socio-demographic Index values for all estimated GBD 2017 locations, 1950-1969

Location	1950	1951	1952	1953	1954	1955	1956	1957	1958	1959	1960	1961	1962	1963	1964	1965	1966	1967	1968	1969
Miyazaki	0.515	0.528	0.538	0.545	0.551	0.556	0.564	0.568	0.571	0.576	0.584	0.592	0.599	0.606	0.608	0.619	0.627	0.63	0.642	0.65
Nagano	0.55	0.56	0.569	0.576	0.581	0.587	0.594	0.599	0.603	0.609	0.617	0.625	0.632	0.64	0.644	0.654	0.662	0.667	0.678	0.686
Nagasaki	0.525	0.537	0.546	0.553	0.558	0.564	0.571	0.575	0.578	0.584	0.592	0.599	0.607	0.614	0.617	0.627	0.635	0.639	0.651	0.658
Nara	0.535	0.546	0.555	0.563	0.569	0.575	0.582	0.587	0.591	0.597	0.606	0.614	0.622	0.629	0.634	0.644	0.652	0.656	0.668	0.676
Niigata	0.534	0.546	0.555	0.562	0.568	0.573	0.581	0.585	0.588	0.593	0.601	0.608	0.616	0.622	0.625	0.636	0.643	0.646	0.658	0.666
Ōita	0.541	0.553	0.562	0.569	0.575	0.581	0.588	0.593	0.596	0.602	0.61	0.618	0.625	0.632	0.635	0.645	0.653	0.657	0.669	0.676
Okayama	0.537	0.551	0.561	0.568	0.574	0.58	0.588	0.593	0.595	0.601	0.609	0.617	0.624	0.631	0.633	0.644	0.651	0.654	0.667	0.674
Okinawa	0.505	0.519	0.53	0.538	0.543	0.549	0.557	0.562	0.564	0.569	0.577	0.584	0.592	0.598	0.6	0.612	0.619	0.621	0.634	0.641
Ōsaka	0.572	0.585	0.594	0.602	0.608	0.614	0.621	0.626	0.629	0.634	0.642	0.65	0.657	0.664	0.667	0.677	0.684	0.687	0.699	0.706
Saga	0.527	0.539	0.548	0.555	0.561	0.566	0.574	0.579	0.582	0.587	0.595	0.603	0.611	0.618	0.621	0.632	0.64	0.644	0.656	0.664
Saitama	0.543	0.555	0.564	0.572	0.578	0.584	0.591	0.596	0.6	0.605	0.614	0.622	0.629	0.636	0.639	0.649	0.657	0.66	0.672	0.68
Shiga	0.552	0.565	0.574	0.582	0.588	0.594	0.602	0.606	0.609	0.615	0.623	0.631	0.638	0.645	0.648	0.659	0.667	0.67	0.682	0.69
Shimane	0.518	0.53	0.54	0.547	0.553	0.558	0.566	0.57	0.573	0.579	0.587	0.594	0.602	0.609	0.612	0.623	0.63	0.634	0.646	0.654
Shizuoka	0.553	0.565	0.575	0.582	0.588	0.593	0.601	0.605	0.608	0.613	0.621	0.629	0.636	0.642	0.644	0.655	0.662	0.665	0.677	0.684
Tochigi	0.543	0.556	0.565	0.573	0.579	0.584	0.592	0.596	0.599	0.604	0.612	0.62	0.627	0.634	0.637	0.648	0.655	0.659	0.671	0.678
Tokushima	0.529	0.542	0.551	0.559	0.564	0.57	0.578	0.582	0.585	0.59	0.598	0.606	0.613	0.62	0.623	0.634	0.641	0.645	0.657	0.665
Tōkyō	0.642	0.652	0.661	0.667	0.673	0.678	0.685	0.69	0.694	0.699	0.707	0.714	0.721	0.728	0.732	0.74	0.747	0.752	0.762	0.77
Tottori	0.521	0.534	0.543	0.551	0.557	0.563	0.57	0.575	0.578	0.584	0.592	0.6	0.607	0.614	0.617	0.628	0.636	0.64	0.652	0.66
Toyama	0.541	0.554	0.563	0.571	0.576	0.582	0.59	0.594	0.596	0.601	0.609	0.617	0.624	0.63	0.632	0.643	0.65	0.652	0.665	0.672
Wakayama	0.524	0.538	0.547	0.555	0.561	0.567	0.575	0.58	0.582	0.588	0.596	0.604	0.611	0.618	0.62	0.631	0.639	0.642	0.655	0.662
Yamagata	0.524	0.536	0.545	0.552	0.557	0.563	0.57	0.575	0.577	0.582	0.59	0.598	0.605	0.612	0.615	0.626	0.634	0.637	0.649	0.657
Yamaguchi	0.545	0.558	0.567	0.575	0.581	0.586	0.594	0.599	0.601	0.607	0.615	0.623	0.63	0.637	0.639	0.65	0.658	0.661	0.673	0.681
Yamanashi	0.55	0.56	0.569	0.576	0.581	0.587	0.594	0.599	0.603	0.609	0.617	0.625	0.632	0.64	0.644	0.654	0.662	0.667	0.678	0.686
South Korea	0.39	0.389	0.392	0.401	0.408	0.414	0.418	0.423	0.427	0.431	0.435	0.438	0.442	0.445	0.448	0.451	0.457	0.464	0.472	0.482
Singapore	0.419	0.414	0.406	0.403	0.407	0.412	0.416	0.422	0.428	0.443	0.458	0.467	0.476	0.486	0.499	0.509	0.518	0.533	0.548	0.559
High-income North America	0.619	0.614	0.612	0.607	0.603	0.598	0.59	0.59	0.592	0.593	0.596	0.603	0.617	0.63	0.647	0.662	0.673	0.685	0.692	0.695
Canada	0.632	0.627	0.622	0.617	0.615	0.614	0.611	0.608	0.608	0.608	0.612	0.617	0.624	0.633	0.647	0.663	0.673	0.681	0.689	0.694
Greenland	0.48	0.487	0.49	0.488	0.483	0.479	0.476	0.473	0.469	0.465	0.461	0.459	0.457	0.451	0.444	0.445	0.458	0.481	0.512	0.543
USA	0.617	0.612	0.611	0.606	0.601	0.596	0.587	0.588	0.59	0.591	0.593	0.601	0.616	0.629	0.646	0.662	0.673	0.685	0.692	0.695
Alabama	0.543	0.537	0.536	0.529	0.523	0.517	0.506	0.507	0.511	0.513	0.517	0.528	0.547	0.563	0.584	0.603	0.615	0.629	0.637	0.638
Alaska	0.609	0.601	0.599	0.588	0.578	0.565	0.544	0.54	0.539	0.535	0.535	0.545	0.568	0.587	0.614	0.637	0.652	0.669	0.679	0.68
Arizona	0.592	0.586	0.585	0.577	0.57	0.562	0.55	0.55	0.552	0.553	0.557	0.568	0.588	0.604	0.626	0.645	0.657	0.67	0.677	0.677
Arkansas	0.529	0.522	0.52	0.511	0.504	0.497	0.484	0.484	0.487	0.489	0.493	0.505	0.526	0.544	0.567	0.587	0.601	0.617	0.626	0.627
California	0.649	0.644	0.644	0.639	0.635	0.63	0.621	0.621	0.623	0.623	0.625	0.632	0.645	0.657	0.674	0.689	0.699	0.711	0.719	0.723
Colorado	0.64	0.636	0.636	0.63	0.625	0.62	0.611	0.611	0.612	0.612	0.614	0.621	0.635	0.647	0.664	0.679	0.69	0.702	0.71	0.713
Connecticut	0.672	0.669	0.669	0.666	0.663	0.66	0.654	0.653	0.653	0.653	0.654	0.658	0.668	0.677	0.691	0.704	0.714	0.726	0.734	0.74
Delaware	0.647	0.64	0.639	0.632	0.627	0.622	0.613	0.612	0.613	0.612	0.613	0.619	0.633	0.645	0.662	0.678	0.688	0.701	0.71	0.713
Washington, DC	0.663	0.657	0.654	0.647	0.641	0.633	0.621	0.62	0.621	0.62	0.62	0.627	0.643	0.657	0.674	0.69	0.702	0.716	0.727	0.73
Florida	0.604	0.596	0.596	0.59	0.585	0.58	0.57	0.572	0.575	0.578	0.581	0.591	0.607	0.621	0.639	0.654	0.664	0.675	0.681	0.68
Georgia	0.553	0.547	0.546	0.539	0.534	0.528	0.517	0.518	0.522	0.524	0.528	0.539	0.558	0.574	0.594	0.612	0.624	0.638	0.645	0.645
Hawaii	0.621	0.616	0.616	0.61	0.605	0.599	0.589	0.588	0.589	0.59	0.594	0.603	0.619	0.634	0.654	0.672	0.684	0.699	0.708	0.711
Idaho	0.588	0.579	0.577	0.567	0.558	0.547	0.532	0.53	0.53	0.528	0.53	0.54	0.56	0.578	0.602	0.624	0.638	0.654	0.663	0.665
Illinois	0.629	0.624	0.623	0.618	0.615	0.61	0.602	0.603	0.605	0.607	0.61	0.618	0.633	0.645	0.662	0.677	0.687	0.698	0.704	0.704
Indiana	0.608	0.603	0.602	0.596	0.591	0.585	0.575	0.575	0.577	0.577	0.58	0.588	0.604	0.617	0.636	0.652	0.663	0.674	0.681	0.681
Iowa	0.633	0.627	0.626	0.619	0.614	0.607	0.596	0.595	0.595	0.593	0.594	0.599	0.612	0.623	0.641	0.657	0.669	0.682	0.691	0.696
Kansas	0.625	0.619	0.619	0.613	0.608	0.601	0.59	0.588	0.589	0.588	0.589	0.596	0.61	0.623	0.641	0.658	0.67	0.684	0.693	0.698
Kentucky	0.551	0.546	0.546	0.539	0.534	0.528	0.518	0.519	0.522	0.524	0.527	0.537	0.555	0.571	0.59	0.608	0.62	0.633	0.641	0.642
Louisiana	0.545	0.537	0.535	0.527	0.519	0.51	0.496	0.495	0.496	0.495	0.497	0.505	0.524	0.541	0.564	0.584	0.599	0.616	0.628	0.633
Maine	0.6	0.593	0.592	0.585	0.579	0.573	0.562	0.561	0.562	0.561	0.563	0.571	0.587	0.601	0.62	0.638	0.65	0.663	0.671	0.673
Maryland	0.641	0.636	0.636	0.632	0.627	0.622	0.614	0.614	0.615	0.615	0.617	0.623	0.636	0.647	0.663	0.678	0.689	0.702	0.711	0.717
Massachusetts	0.676	0.673	0.673	0.67	0.668	0.665	0.66	0.66	0.661	0.662	0.664	0.67	0.679	0.688	0.701	0.713	0.721	0.731	0.738	0.742
Michigan	0.627	0.622	0.621	0.616	0.612	0.607	0.597	0.597	0.597	0.597	0.598	0.604	0.617	0.629	0.646	0.662	0.672	0.684	0.691	0.693
Minnesota	0.645	0.641	0.64	0.635	0.632	0.627	0.62	0.619	0.619	0.619	0.62	0.626	0.637	0.648	0.663	0.678	0.689	0.702	0.711	0.717
Mississippi	0.51	0.5	0.498	0.488	0.48	0.471	0.456	0.455	0.457	0.458	0.462	0.474	0.497	0.516	0.541	0.562	0.576	0.592	0.602	0.602
Missouri	0.601	0.595	0.594	0.588	0.584	0.578	0.569	0.569	0.571	0.572	0.574	0.582	0.598	0.612	0.63	0.647	0.658	0.671	0.68	0.683
Montana	0.621	0.614	0.611	0.604	0.597	0.59	0.578	0.577	0.578	0.577	0.579	0.587	0.603	0.618	0.637	0.655	0.667	0.68	0.687	0.687
Nebraska	0.634	0.626	0.625	0.618	0.613	0.607	0.596	0.596	0.597	0.596	0.598	0.605	0.619	0.632	0.649	0.666	0.677	0.69	0.698	0.701
Nevada	0.627	0.619	0.618	0.611	0.604	0.597	0.583	0.581	0.582	0.58	0.58	0.588	0.606	0.622	0.643	0.662	0.674	0.688	0.697	0.7
New Hampshire	0.633	0.628	0.627	0.622	0.618	0.613	0.604	0.604	0.604	0.604	0.605	0.612	0.625	0.636	0.653	0.669	0.679	0.692	0.7	0.704
New Jersey	0.655	0.651	0.651	0.648	0.645	0.641	0.635	0.635	0.637	0.637	0.64	0.645	0.657	0.667	0.682	0.695	0.704	0.714	0.721	0.724
New Mexico	0.574	0.565	0.563	0.554	0.546	0.537	0.522	0.521	0.523	0.523	0.525	0.536	0.556	0.573	0.597	0.618	0.631	0.647	0.656	0.657
New York	0.654	0.65	0.649	0.646	0.643	0.64	0.634	0.636	0.638	0.639	0.643	0.65	0.662	0.672	0.687	0.7	0.708	0.718	0.724	0.726
North Carolina	0.57	0.564	0.563	0.556	0.552	0.546	0.537	0.538	0.541	0.542	0.546	0.556	0.573	0.587	0.606	0.622	0.634	0.647	0.655	0.656
North Dakota	0.613	0.605	0.6	0.593	0.586	0.58	0.57	0.569	0.569	0.568	0.571	0.577	0.593	0.607	0.625	0.643	0.656	0.669	0.677	0.681
Ohio	0.624	0.62	0.62	0.616	0.612	0.608	0.6	0.601	0.603	0.605	0.608	0.616	0.629	0.641	0.657	0.671	0.68	0.69	0.695	0.695

Appendix Table 9. Socio-demographic Index values for all estimated GBD 2017 locations, 1950-1969

Location	1950	1951	1952	1953	1954	1955	1956	1957	1958	1959	1960	1961	1962	1963	1964	1965	1966	1967	1968	1969
Oklahoma	0.575	0.569	0.568	0.561	0.556	0.549	0.538	0.538	0.541	0.542	0.545	0.556	0.574	0.59	0.611	0.629	0.641	0.655	0.664	0.666
Oregon	0.645	0.639	0.638	0.632	0.627	0.621	0.611	0.609	0.609	0.608	0.609	0.615	0.628	0.639	0.657	0.673	0.684	0.698	0.707	0.712
Pennsylvania	0.636	0.632	0.632	0.629	0.626	0.622	0.617	0.618	0.621	0.622	0.626	0.633	0.645	0.656	0.67	0.683	0.691	0.701	0.706	0.706
Rhode Island	0.642	0.638	0.637	0.633	0.629	0.625	0.617	0.617	0.617	0.617	0.618	0.624	0.635	0.646	0.661	0.675	0.686	0.698	0.706	0.71
South Carolina	0.545	0.54	0.54	0.533	0.526	0.518	0.506	0.505	0.508	0.509	0.512	0.523	0.542	0.558	0.58	0.599	0.613	0.628	0.637	0.639
South Dakota	0.601	0.596	0.592	0.584	0.579	0.57	0.559	0.559	0.56	0.559	0.562	0.571	0.588	0.603	0.622	0.641	0.653	0.667	0.676	0.678
Tennessee	0.563	0.557	0.556	0.55	0.545	0.54	0.53	0.531	0.534	0.536	0.539	0.549	0.566	0.58	0.599	0.616	0.628	0.642	0.651	0.653
Texas	0.572	0.565	0.564	0.555	0.549	0.541	0.528	0.528	0.53	0.53	0.532	0.542	0.56	0.576	0.597	0.616	0.629	0.643	0.652	0.655
Utah	0.608	0.6	0.598	0.589	0.581	0.573	0.56	0.559	0.56	0.559	0.563	0.574	0.593	0.61	0.634	0.654	0.667	0.682	0.69	0.689
Vermont	0.627	0.623	0.622	0.617	0.612	0.607	0.598	0.598	0.598	0.598	0.6	0.607	0.62	0.631	0.648	0.664	0.675	0.688	0.697	0.7
Virginia	0.607	0.603	0.603	0.597	0.593	0.588	0.579	0.58	0.582	0.583	0.586	0.594	0.609	0.622	0.64	0.656	0.667	0.68	0.689	0.692
Washington	0.655	0.648	0.647	0.641	0.636	0.629	0.618	0.616	0.615	0.613	0.612	0.617	0.629	0.64	0.657	0.673	0.684	0.699	0.71	0.718
West Virginia	0.569	0.563	0.562	0.555	0.55	0.544	0.534	0.535	0.538	0.54	0.544	0.553	0.569	0.584	0.603	0.62	0.631	0.644	0.651	0.65
Wisconsin	0.638	0.634	0.634	0.629	0.624	0.619	0.612	0.611	0.612	0.611	0.613	0.619	0.63	0.64	0.656	0.671	0.681	0.693	0.701	0.705
Wyoming	0.609	0.6	0.597	0.587	0.578	0.569	0.554	0.552	0.552	0.55	0.551	0.56	0.579	0.596	0.619	0.639	0.651	0.666	0.676	0.678
Southern Latin America	0.464	0.468	0.47	0.472	0.474	0.475	0.477	0.481	0.485	0.486	0.489	0.492	0.494	0.496	0.501	0.507	0.513	0.517	0.516	0.517
Argentina	0.472	0.475	0.477	0.479	0.482	0.485	0.488	0.492	0.497	0.501	0.504	0.506	0.508	0.51	0.515	0.521	0.527	0.53	0.525	0.524
Chile	0.436	0.441	0.443	0.445	0.446	0.445	0.443	0.446	0.449	0.446	0.448	0.455	0.456	0.458	0.464	0.471	0.477	0.485	0.494	0.498
Uruguay	0.479	0.484	0.484	0.484	0.484	0.483	0.484	0.487	0.489	0.49	0.49	0.491	0.495	0.5	0.504	0.507	0.511	0.514	0.514	0.515
Western Europe	0.597	0.6	0.602	0.605	0.607	0.609	0.61	0.612	0.613	0.614	0.615	0.616	0.617	0.619	0.622	0.624	0.629	0.634	0.637	0.643
Andorra	0.721	0.724	0.728	0.733	0.737	0.741	0.745	0.75	0.754	0.758	0.762	0.767	0.771	0.776	0.781	0.786	0.79	0.794	0.798	0.801
Austria	0.651	0.654	0.654	0.656	0.656	0.655	0.654	0.653	0.652	0.647	0.645	0.649	0.652	0.655	0.658	0.659	0.658	0.657	0.659	0.665
Belgium	0.657	0.659	0.66	0.66	0.66	0.66	0.66	0.66	0.66	0.66	0.66	0.66	0.66	0.661	0.665	0.67	0.676	0.682	0.686	0.689
Cyprus	0.544	0.55	0.556	0.561	0.563	0.562	0.561	0.561	0.562	0.564	0.567	0.571	0.575	0.579	0.581	0.584	0.587	0.591	0.595	0.6
Denmark	0.693	0.692	0.689	0.688	0.688	0.688	0.689	0.691	0.694	0.697	0.7	0.701	0.702	0.704	0.705	0.706	0.714	0.73	0.744	0.753
Finland	0.633	0.637	0.637	0.637	0.638	0.64	0.643	0.648	0.652	0.653	0.656	0.658	0.659	0.663	0.671	0.678	0.682	0.688	0.697	0.707
France	0.561	0.564	0.567	0.569	0.571	0.574	0.577	0.58	0.58	0.581	0.582	0.584	0.583	0.582	0.585	0.589	0.595	0.603	0.61	0.615
Germany	0.615	0.62	0.623	0.631	0.638	0.642	0.644	0.647	0.648	0.649	0.65	0.651	0.652	0.655	0.659	0.662	0.667	0.674	0.672	0.68
Greece	0.55	0.553	0.554	0.556	0.559	0.563	0.568	0.571	0.572	0.574	0.578	0.581	0.582	0.583	0.583	0.582	0.579	0.576	0.58	0.588
Iceland	0.629	0.627	0.623	0.62	0.618	0.617	0.617	0.62	0.627	0.635	0.636	0.632	0.629	0.628	0.631	0.639	0.651	0.664	0.674	0.68
Ireland	0.609	0.611	0.615	0.62	0.623	0.626	0.628	0.629	0.629	0.629	0.629	0.628	0.628	0.629	0.631	0.634	0.637	0.639	0.641	0.641
Israel	0.525	0.526	0.527	0.531	0.533	0.536	0.542	0.551	0.561	0.566	0.571	0.578	0.583	0.587	0.592	0.6	0.61	0.619	0.623	0.624
Italy	0.578	0.583	0.587	0.588	0.59	0.592	0.595	0.598	0.601	0.604	0.607	0.609	0.611	0.617	0.619	0.617	0.621	0.625	0.629	0.633
Luxembourg	0.706	0.71	0.712	0.711	0.71	0.709	0.71	0.71	0.711	0.712	0.713	0.714	0.715	0.715	0.717	0.719	0.721	0.725	0.729	0.735
Malta	0.494	0.501	0.508	0.515	0.522	0.526	0.53	0.532	0.534	0.536	0.542	0.55	0.56	0.571	0.583	0.594	0.602	0.608	0.613	0.618
Netherlands	0.683	0.683	0.685	0.688	0.689	0.691	0.692	0.692	0.691	0.691	0.692	0.692	0.692	0.692	0.694	0.696	0.698	0.702	0.705	0.71
Norway	0.684	0.684	0.68	0.677	0.674	0.672	0.67	0.67	0.67	0.67	0.668	0.667	0.668	0.669	0.67	0.673	0.675	0.677	0.68	0.684
Portugal	0.454	0.456	0.458	0.461	0.462	0.463	0.464	0.466	0.467	0.468	0.469	0.47	0.474	0.478	0.481	0.485	0.488	0.493	0.5	0.507
Spain	0.533	0.536	0.537	0.537	0.537	0.537	0.537	0.538	0.54	0.543	0.547	0.55	0.553	0.555	0.558	0.563	0.568	0.57	0.572	0.577
Sweden	0.645	0.647	0.647	0.648	0.649	0.65	0.652	0.656	0.66	0.664	0.668	0.669	0.67	0.669	0.67	0.673	0.678	0.687	0.697	0.702
Stockholm	0.697	0.699	0.699	0.701	0.702	0.703	0.706	0.71	0.714	0.719	0.722	0.724	0.726	0.726	0.728	0.73	0.734	0.741	0.748	0.75
Sweden except Stockholm	0.634	0.636	0.636	0.637	0.638	0.638	0.64	0.644	0.648	0.652	0.655	0.657	0.657	0.656	0.656	0.659	0.664	0.674	0.684	0.69
Switzerland	0.761	0.765	0.766	0.767	0.768	0.768	0.768	0.768	0.768	0.769	0.768	0.766	0.763	0.761	0.763	0.767	0.771	0.774	0.778	0.781
United Kingdom	0.572	0.574	0.575	0.576	0.576	0.576	0.575	0.574	0.573	0.571	0.57	0.569	0.568	0.569	0.572	0.576	0.581	0.587	0.593	0.599
England	0.587	0.589	0.59	0.591	0.592	0.591	0.59	0.588	0.587	0.585	0.584	0.582	0.582	0.582	0.585	0.589	0.594	0.6	0.606	0.611
East Midlands	0.552	0.555	0.556	0.557	0.558	0.558	0.557	0.556	0.555	0.554	0.553	0.552	0.551	0.553	0.555	0.56	0.565	0.571	0.577	0.583
Derby	0.559	0.561	0.562	0.563	0.564	0.563	0.562	0.561	0.559	0.557	0.556	0.554	0.553	0.554	0.557	0.561	0.566	0.572	0.578	0.584
Derbyshire	0.542	0.545	0.546	0.547	0.548	0.548	0.547	0.546	0.545	0.544	0.543	0.542	0.542	0.543	0.546	0.55	0.556	0.562	0.568	0.574
Leicester	0.54	0.542	0.543	0.545	0.545	0.545	0.544	0.543	0.542	0.541	0.539	0.538	0.538	0.539	0.542	0.546	0.552	0.558	0.564	0.57
Leicestershire	0.57	0.572	0.573	0.574	0.575	0.575	0.575	0.574	0.573	0.572	0.571	0.571	0.571	0.572	0.575	0.579	0.585	0.591	0.597	0.603
Lincolnshire	0.546	0.548	0.549	0.55	0.551	0.551	0.55	0.549	0.548	0.547	0.546	0.545	0.545	0.546	0.549	0.553	0.558	0.564	0.571	0.577
Northamptonshire	0.555	0.557	0.558	0.559	0.56	0.56	0.559	0.558	0.557	0.556	0.554	0.553	0.553	0.554	0.557	0.561	0.567	0.573	0.579	0.585
Nottingham	0.568	0.57	0.571	0.572	0.573	0.573	0.572	0.572	0.571	0.57	0.569	0.568	0.568	0.569	0.572	0.577	0.582	0.588	0.594	0.6
Nottinghamshire	0.541	0.543	0.544	0.546	0.546	0.546	0.546	0.545	0.544	0.543	0.541	0.541	0.541	0.542	0.545	0.549	0.554	0.56	0.566	0.572
Rutland	0.57	0.573	0.574	0.575	0.576	0.576	0.575	0.574	0.573	0.572	0.571	0.57	0.57	0.571	0.574	0.579	0.584	0.59	0.596	0.602
East of England	0.567	0.57	0.571	0.572	0.573	0.573	0.572	0.572	0.571	0.57	0.568	0.568	0.568	0.569	0.572	0.576	0.581	0.587	0.594	0.6
Bedford	0.57	0.573	0.574	0.575	0.576	0.576	0.575	0.574	0.573	0.572	0.571	0.57	0.569	0.571	0.573	0.578	0.583	0.589	0.595	0.601
Cambridgeshire	0.589	0.592	0.593	0.594	0.595	0.595	0.595	0.594	0.594	0.593	0.593	0.592	0.593	0.594	0.597	0.601	0.607	0.613	0.619	0.625
Central Bedfordshire	0.563	0.565	0.567	0.568	0.569	0.569	0.568	0.567	0.566	0.565	0.564	0.563	0.562	0.563	0.566	0.571	0.576	0.582	0.588	0.595
Essex	0.557	0.559	0.561	0.562	0.563	0.563	0.562	0.562	0.561	0.56	0.559	0.558	0.558	0.56	0.563	0.567	0.572	0.578	0.584	0.591
Hertfordshire	0.592	0.594	0.595	0.597	0.598	0.598	0.597	0.596	0.595	0.594	0.593	0.593	0.592	0.594	0.597	0.601	0.606	0.612	0.618	0.624
Luton	0.551	0.553	0.554	0.556	0.556	0.556	0.555	0.554	0.552	0.55	0.549	0.547	0.546	0.547	0.55	0.554	0.56	0.566	0.573	0.579
Norfolk	0.555	0.558	0.559	0.56	0.561	0.561	0.561	0.56	0.559	0.558	0.557	0.557	0.557	0.558	0.561	0.565	0.571	0.577	0.583	0.589
Peterborough	0.557	0.559	0.56	0.562	0.562	0.562	0.561	0.559	0.558	0.556	0.554	0.553	0.552	0.552	0.555	0.56	0.565	0.572	0.578	0.584
Southend-on-Sea	0.534	0.536	0.538	0.539	0.54	0.54	0.539	0.539	0.538	0.537	0.536	0.535	0.535	0.537	0.54	0.544	0.55	0.556	0.562	0.568

Appendix Table 9. Socio-demographic Index values for all estimated GBD 2017 locations, 1950-1969																				
**Location**	**1950**	**1951**	**1952**	**1953**	**1954**	**1955**	**1956**	**1957**	**1958**	**1959**	**1960**	**1961**	**1962**	**1963**	**1964**	**1965**	**1966**	**1967**	**1968**	**1969**
Suffolk	0.556	0.559	0.56	0.561	0.562	0.562	0.561	0.56	0.559	0.557	0.556	0.555	0.554	0.555	0.558	0.562	0.568	0.574	0.58	0.586
Thurrock	0.56	0.562	0.563	0.564	0.564	0.564	0.563	0.562	0.561	0.559	0.558	0.557	0.556	0.557	0.56	0.564	0.57	0.576	0.582	0.588
Greater London	0.635	0.638	0.639	0.639	0.64	0.64	0.638	0.637	0.636	0.634	0.633	0.631	0.631	0.632	0.634	0.638	0.643	0.648	0.653	0.659
Barking and Dagenham	0.564	0.566	0.567	0.567	0.568	0.567	0.566	0.565	0.563	0.561	0.559	0.558	0.557	0.557	0.56	0.564	0.568	0.574	0.58	0.586
Barnet	0.609	0.611	0.612	0.613	0.614	0.614	0.613	0.613	0.612	0.611	0.61	0.609	0.609	0.61	0.612	0.616	0.621	0.626	0.632	0.637
Bexley	0.574	0.577	0.578	0.579	0.58	0.58	0.579	0.578	0.577	0.576	0.575	0.575	0.574	0.575	0.578	0.582	0.587	0.593	0.598	0.604
Brent	0.597	0.599	0.6	0.601	0.602	0.602	0.601	0.6	0.599	0.597	0.596	0.595	0.594	0.595	0.598	0.602	0.607	0.613	0.618	0.624
Bromley	0.606	0.608	0.609	0.611	0.611	0.611	0.611	0.61	0.609	0.608	0.607	0.606	0.606	0.607	0.609	0.613	0.618	0.624	0.629	0.635
Camden	0.709	0.712	0.713	0.714	0.715	0.715	0.715	0.715	0.714	0.714	0.714	0.713	0.714	0.715	0.718	0.722	0.726	0.731	0.735	0.739
Croydon	0.591	0.593	0.594	0.596	0.596	0.596	0.595	0.594	0.592	0.591	0.589	0.588	0.588	0.588	0.591	0.595	0.6	0.606	0.612	0.618
Ealing	0.609	0.611	0.612	0.613	0.614	0.614	0.613	0.611	0.61	0.609	0.607	0.606	0.605	0.606	0.608	0.612	0.617	0.623	0.628	0.634
Enfield	0.585	0.588	0.589	0.59	0.591	0.591	0.59	0.589	0.588	0.587	0.585	0.584	0.584	0.585	0.587	0.591	0.596	0.602	0.608	0.614
Greenwich	0.577	0.579	0.58	0.581	0.581	0.581	0.58	0.579	0.577	0.576	0.574	0.573	0.572	0.573	0.575	0.579	0.584	0.59	0.595	0.601
Hackney	0.65	0.652	0.652	0.652	0.651	0.649	0.645	0.642	0.638	0.634	0.629	0.625	0.622	0.62	0.621	0.624	0.629	0.635	0.641	0.647
Hammersmith and Fulham	0.665	0.668	0.669	0.67	0.671	0.671	0.671	0.67	0.669	0.669	0.668	0.668	0.668	0.67	0.672	0.676	0.68	0.686	0.691	0.696
Haringey	0.597	0.599	0.6	0.602	0.603	0.602	0.602	0.601	0.6	0.598	0.597	0.596	0.596	0.597	0.599	0.603	0.608	0.614	0.62	0.626
Harrow	0.597	0.599	0.6	0.602	0.602	0.602	0.602	0.601	0.6	0.598	0.597	0.596	0.596	0.597	0.599	0.603	0.608	0.614	0.62	0.625
Havering	0.579	0.581	0.582	0.584	0.584	0.584	0.583	0.582	0.582	0.58	0.579	0.579	0.578	0.579	0.582	0.586	0.591	0.596	0.602	0.607
Hillingdon	0.631	0.634	0.635	0.636	0.637	0.636	0.635	0.634	0.633	0.632	0.63	0.629	0.629	0.63	0.632	0.636	0.641	0.647	0.653	0.658
Hounslow	0.615	0.617	0.619	0.62	0.62	0.62	0.619	0.618	0.616	0.615	0.613	0.612	0.612	0.612	0.615	0.619	0.624	0.63	0.636	0.641
Islington	0.669	0.672	0.673	0.674	0.674	0.674	0.673	0.671	0.67	0.669	0.668	0.667	0.666	0.667	0.67	0.673	0.678	0.684	0.69	0.695
Kensington and Chelsea	0.688	0.69	0.691	0.693	0.694	0.694	0.695	0.695	0.695	0.695	0.695	0.695	0.696	0.698	0.7	0.704	0.708	0.713	0.719	0.724
Kingston upon Thames	0.637	0.639	0.641	0.642	0.643	0.643	0.643	0.643	0.642	0.642	0.642	0.641	0.642	0.643	0.646	0.649	0.654	0.659	0.664	0.669
Lambeth	0.616	0.618	0.619	0.62	0.621	0.62	0.619	0.617	0.616	0.614	0.613	0.611	0.611	0.612	0.614	0.618	0.623	0.628	0.634	0.64
Lewisham	0.579	0.581	0.582	0.583	0.584	0.584	0.583	0.582	0.581	0.579	0.578	0.577	0.577	0.578	0.58	0.584	0.589	0.594	0.6	0.605
Merton	0.607	0.609	0.61	0.611	0.612	0.612	0.61	0.609	0.608	0.607	0.605	0.604	0.604	0.605	0.607	0.611	0.616	0.621	0.627	0.633
Newham	0.564	0.566	0.567	0.567	0.568	0.567	0.565	0.564	0.562	0.56	0.557	0.555	0.554	0.555	0.557	0.561	0.567	0.573	0.579	0.585
Redbridge	0.581	0.583	0.584	0.585	0.586	0.586	0.585	0.584	0.583	0.582	0.581	0.58	0.58	0.581	0.583	0.587	0.592	0.597	0.603	0.609
Richmond upon Thames	0.648	0.65	0.651	0.653	0.653	0.654	0.653	0.653	0.652	0.651	0.651	0.651	0.651	0.652	0.654	0.658	0.662	0.667	0.673	0.678
Southwark	0.637	0.639	0.64	0.64	0.641	0.64	0.638	0.637	0.635	0.633	0.631	0.63	0.629	0.629	0.631	0.635	0.64	0.646	0.652	0.657
Sutton	0.584	0.587	0.588	0.589	0.59	0.59	0.589	0.589	0.588	0.587	0.585	0.585	0.584	0.586	0.588	0.592	0.597	0.603	0.609	0.615
Tower Hamlets	0.636	0.638	0.639	0.639	0.638	0.637	0.634	0.631	0.628	0.625	0.621	0.618	0.615	0.614	0.615	0.618	0.622	0.628	0.633	0.639
Waltham Forest	0.558	0.56	0.561	0.563	0.563	0.563	0.562	0.561	0.56	0.559	0.557	0.556	0.555	0.556	0.559	0.562	0.568	0.573	0.579	0.585
Wandsworth	0.634	0.636	0.637	0.639	0.639	0.639	0.638	0.638	0.637	0.636	0.635	0.634	0.634	0.635	0.637	0.641	0.645	0.651	0.656	0.661
Westminster	0.72	0.722	0.723	0.723	0.723	0.722	0.721	0.72	0.718	0.717	0.715	0.714	0.713	0.714	0.715	0.718	0.721	0.725	0.73	0.734
North East England	0.536	0.538	0.539	0.541	0.542	0.542	0.541	0.539	0.538	0.537	0.535	0.534	0.534	0.535	0.538	0.542	0.548	0.554	0.56	0.566
County Durham	0.529	0.531	0.533	0.534	0.535	0.535	0.534	0.533	0.532	0.531	0.53	0.529	0.529	0.53	0.533	0.537	0.543	0.549	0.555	0.561
Darlington	0.547	0.55	0.55	0.552	0.552	0.552	0.551	0.55	0.548	0.546	0.545	0.543	0.542	0.543	0.546	0.55	0.556	0.563	0.569	0.576
Gateshead	0.542	0.544	0.545	0.546	0.547	0.547	0.546	0.545	0.543	0.541	0.54	0.539	0.538	0.539	0.542	0.546	0.551	0.558	0.564	0.57
Hartlepool	0.522	0.524	0.525	0.527	0.527	0.527	0.525	0.524	0.522	0.52	0.518	0.516	0.515	0.516	0.519	0.523	0.529	0.535	0.542	0.548
Middlesbrough	0.526	0.528	0.529	0.53	0.531	0.531	0.529	0.528	0.526	0.524	0.522	0.521	0.52	0.521	0.523	0.528	0.534	0.54	0.547	0.553
Newcastle upon Tyne	0.566	0.569	0.57	0.571	0.572	0.573	0.572	0.572	0.571	0.571	0.57	0.57	0.57	0.572	0.575	0.579	0.585	0.591	0.597	0.603
North Tyneside	0.539	0.541	0.542	0.544	0.545	0.544	0.543	0.542	0.541	0.54	0.538	0.537	0.537	0.538	0.541	0.545	0.55	0.557	0.563	0.569
Northumberland	0.535	0.538	0.539	0.54	0.541	0.541	0.541	0.539	0.538	0.537	0.536	0.534	0.534	0.535	0.538	0.542	0.548	0.554	0.561	0.567
Redcar and Cleveland	0.513	0.516	0.517	0.518	0.519	0.518	0.517	0.516	0.514	0.513	0.511	0.51	0.509	0.509	0.512	0.517	0.522	0.529	0.535	0.541
South Tyneside	0.507	0.509	0.51	0.512	0.513	0.512	0.511	0.51	0.509	0.508	0.506	0.505	0.505	0.506	0.509	0.513	0.519	0.525	0.531	0.538
Stockton-on-Tees	0.546	0.549	0.55	0.551	0.551	0.551	0.55	0.548	0.547	0.545	0.543	0.541	0.54	0.541	0.544	0.548	0.554	0.56	0.567	0.573
Sunderland	0.528	0.53	0.531	0.532	0.533	0.533	0.532	0.53	0.529	0.527	0.525	0.524	0.523	0.523	0.526	0.531	0.536	0.543	0.549	0.556
North West England	0.567	0.57	0.571	0.572	0.572	0.572	0.571	0.57	0.568	0.567	0.565	0.564	0.563	0.564	0.567	0.571	0.576	0.582	0.588	0.594
Blackburn with Darwen	0.539	0.541	0.542	0.544	0.544	0.543	0.542	0.54	0.538	0.536	0.533	0.531	0.53	0.53	0.533	0.537	0.542	0.549	0.555	0.562
Blackpool	0.53	0.532	0.534	0.535	0.536	0.536	0.535	0.534	0.533	0.532	0.531	0.53	0.53	0.531	0.534	0.538	0.544	0.55	0.556	0.562
Bolton	0.551	0.553	0.554	0.555	0.556	0.556	0.554	0.553	0.551	0.549	0.548	0.546	0.545	0.546	0.548	0.552	0.558	0.564	0.57	0.576
Bury	0.555	0.558	0.559	0.56	0.561	0.56	0.559	0.558	0.557	0.555	0.554	0.552	0.552	0.553	0.555	0.559	0.565	0.571	0.577	0.583
Cheshire East	0.602	0.604	0.605	0.606	0.607	0.607	0.606	0.604	0.603	0.602	0.6	0.599	0.598	0.599	0.602	0.606	0.611	0.617	0.623	0.628
Cheshire West and Chester	0.594	0.596	0.597	0.598	0.599	0.598	0.597	0.596	0.594	0.593	0.591	0.59	0.589	0.59	0.592	0.596	0.602	0.607	0.613	0.619
Cumbria	0.572	0.575	0.576	0.577	0.578	0.577	0.576	0.575	0.574	0.572	0.571	0.569	0.569	0.57	0.573	0.577	0.582	0.588	0.594	0.6
Halton	0.557	0.559	0.56	0.561	0.562	0.561	0.559	0.557	0.556	0.553	0.551	0.549	0.548	0.549	0.551	0.555	0.561	0.567	0.573	0.579
Knowsley	0.547	0.549	0.55	0.551	0.551	0.55	0.549	0.547	0.545	0.543	0.541	0.54	0.539	0.539	0.542	0.546	0.551	0.557	0.562	0.568
Lancashire	0.569	0.571	0.572	0.573	0.574	0.574	0.573	0.572	0.57	0.568	0.567	0.566	0.565	0.566	0.569	0.573	0.578	0.584	0.59	0.596
Liverpool	0.567	0.569	0.57	0.571	0.572	0.572	0.571	0.57	0.569	0.568	0.567	0.566	0.566	0.567	0.57	0.574	0.579	0.585	0.591	0.596
Manchester	0.59	0.592	0.593	0.594	0.595	0.594	0.593	0.592	0.591	0.589	0.588	0.587	0.586	0.587	0.59	0.594	0.599	0.605	0.611	0.617
Oldham	0.538	0.541	0.542	0.543	0.543	0.543	0.541	0.54	0.538	0.536	0.534	0.532	0.531	0.531	0.534	0.538	0.543	0.549	0.556	0.562
Rochdale	0.54	0.542	0.543	0.544	0.545	0.544	0.543	0.541	0.539	0.538	0.536	0.534	0.533	0.533	0.536	0.54	0.545	0.552	0.558	0.564
Salford	0.562	0.564	0.565	0.566	0.566	0.565	0.564	0.562	0.561	0.559	0.557	0.555	0.554	0.555	0.557	0.561	0.567	0.573	0.579	0.585
Sefton	0.559	0.561	0.562	0.563	0.564	0.564	0.563	0.562	0.561	0.559	0.558	0.557	0.557	0.558	0.561	0.565	0.57	0.575	0.581	0.587

Appendix Table 9. Socio-demographic Index values for all estimated GBD 2017 locations, 1950-1969

Location	1950	1951	1952	1953	1954	1955	1956	1957	1958	1959	1960	1961	1962	1963	1964	1965	1966	1967	1968	1969
St Helens	0.539	0.541	0.542	0.543	0.544	0.543	0.542	0.541	0.54	0.538	0.537	0.535	0.535	0.535	0.538	0.542	0.547	0.553	0.559	0.565
Stockport	0.581	0.583	0.584	0.585	0.586	0.585	0.584	0.583	0.581	0.58	0.578	0.577	0.576	0.577	0.58	0.584	0.589	0.594	0.6	0.606
Tameside	0.544	0.547	0.547	0.549	0.549	0.549	0.548	0.546	0.545	0.543	0.542	0.54	0.539	0.54	0.543	0.547	0.552	0.558	0.564	0.57
Trafford	0.606	0.608	0.609	0.61	0.61	0.61	0.608	0.607	0.605	0.603	0.601	0.6	0.599	0.6	0.602	0.606	0.611	0.617	0.623	0.628
Warrington	0.593	0.596	0.596	0.597	0.598	0.597	0.596	0.594	0.592	0.59	0.588	0.587	0.586	0.586	0.589	0.593	0.598	0.604	0.61	0.616
Wigan	0.543	0.545	0.546	0.547	0.548	0.547	0.546	0.545	0.543	0.542	0.54	0.539	0.538	0.539	0.541	0.546	0.551	0.557	0.563	0.569
Wirral	0.549	0.551	0.552	0.553	0.554	0.553	0.552	0.551	0.549	0.547	0.546	0.544	0.544	0.544	0.547	0.551	0.556	0.562	0.568	0.574
South East England	0.608	0.611	0.611	0.612	0.613	0.613	0.611	0.61	0.609	0.607	0.605	0.604	0.603	0.604	0.607	0.611	0.616	0.621	0.627	0.633
Bracknell Forest	0.62	0.622	0.622	0.623	0.623	0.623	0.621	0.619	0.617	0.615	0.613	0.612	0.611	0.611	0.613	0.617	0.622	0.628	0.633	0.639
Brighton and Hove	0.616	0.618	0.619	0.62	0.621	0.622	0.622	0.621	0.621	0.621	0.62	0.62	0.621	0.622	0.625	0.629	0.633	0.638	0.643	0.648
Buckinghamshire	0.628	0.63	0.631	0.631	0.632	0.631	0.63	0.628	0.626	0.625	0.623	0.621	0.62	0.621	0.623	0.627	0.632	0.637	0.643	0.649
East Sussex	0.568	0.571	0.572	0.573	0.574	0.574	0.573	0.572	0.571	0.569	0.568	0.566	0.566	0.567	0.57	0.574	0.579	0.585	0.591	0.597
Hampshire	0.605	0.607	0.608	0.609	0.609	0.609	0.607	0.606	0.604	0.602	0.6	0.599	0.598	0.598	0.601	0.605	0.61	0.616	0.621	0.627
Isle of Wight	0.565	0.568	0.569	0.57	0.571	0.57	0.569	0.567	0.566	0.564	0.562	0.56	0.559	0.56	0.562	0.566	0.572	0.578	0.584	0.59
Kent	0.582	0.585	0.586	0.587	0.587	0.587	0.585	0.584	0.582	0.581	0.579	0.577	0.577	0.577	0.58	0.584	0.589	0.595	0.601	0.607
Medway	0.56	0.563	0.564	0.565	0.565	0.564	0.563	0.561	0.559	0.557	0.555	0.554	0.553	0.553	0.556	0.56	0.565	0.571	0.577	0.583
Milton Keynes	0.602	0.605	0.606	0.607	0.608	0.607	0.605	0.603	0.601	0.599	0.596	0.594	0.592	0.593	0.595	0.599	0.605	0.611	0.618	0.625
Oxfordshire	0.63	0.632	0.633	0.634	0.634	0.634	0.633	0.631	0.63	0.629	0.627	0.626	0.626	0.627	0.629	0.633	0.638	0.643	0.649	0.654
Portsmouth	0.593	0.596	0.597	0.598	0.599	0.599	0.598	0.597	0.596	0.595	0.594	0.593	0.592	0.593	0.596	0.601	0.606	0.612	0.619	0.625
Reading	0.635	0.637	0.638	0.639	0.639	0.639	0.638	0.636	0.635	0.633	0.632	0.631	0.63	0.631	0.634	0.637	0.643	0.648	0.654	0.66
Slough	0.625	0.627	0.628	0.628	0.628	0.627	0.624	0.621	0.619	0.616	0.612	0.609	0.607	0.607	0.609	0.613	0.618	0.624	0.631	0.637
Southampton	0.609	0.611	0.612	0.613	0.613	0.613	0.612	0.61	0.609	0.608	0.606	0.605	0.605	0.605	0.608	0.612	0.617	0.623	0.628	0.634
Surrey	0.636	0.638	0.639	0.64	0.641	0.64	0.639	0.638	0.637	0.635	0.634	0.633	0.633	0.634	0.636	0.64	0.645	0.65	0.655	0.661
West Berkshire	0.631	0.633	0.634	0.635	0.635	0.634	0.633	0.631	0.629	0.627	0.624	0.623	0.621	0.622	0.624	0.628	0.634	0.64	0.646	0.652
West Sussex	0.596	0.598	0.599	0.601	0.601	0.601	0.6	0.599	0.597	0.596	0.594	0.593	0.593	0.593	0.596	0.6	0.605	0.611	0.617	0.623
Windsor and Maidenhead	0.642	0.644	0.645	0.646	0.646	0.646	0.644	0.643	0.641	0.639	0.637	0.636	0.635	0.636	0.638	0.642	0.647	0.652	0.658	0.664
Wokingham	0.642	0.643	0.644	0.645	0.645	0.644	0.643	0.642	0.64	0.639	0.637	0.636	0.635	0.636	0.638	0.642	0.647	0.652	0.658	0.664
South West England	0.577	0.58	0.581	0.582	0.583	0.583	0.582	0.581	0.58	0.579	0.578	0.577	0.577	0.578	0.58	0.585	0.59	0.596	0.602	0.608
Bath and North East Somerset	0.597	0.599	0.601	0.602	0.603	0.604	0.604	0.604	0.604	0.603	0.604	0.604	0.605	0.606	0.609	0.613	0.618	0.624	0.629	0.634
Bournemouth	0.573	0.576	0.577	0.579	0.58	0.581	0.581	0.581	0.581	0.581	0.581	0.582	0.583	0.585	0.588	0.592	0.597	0.603	0.608	0.614
Bristol, City of	0.607	0.609	0.61	0.611	0.612	0.612	0.611	0.61	0.61	0.609	0.608	0.607	0.607	0.608	0.611	0.615	0.62	0.626	0.632	0.637
Cornwall	0.548	0.551	0.552	0.553	0.554	0.554	0.553	0.552	0.551	0.549	0.548	0.547	0.546	0.547	0.55	0.554	0.56	0.566	0.572	0.578
Devon	0.566	0.568	0.57	0.571	0.572	0.572	0.572	0.571	0.57	0.57	0.569	0.568	0.568	0.57	0.573	0.577	0.582	0.588	0.594	0.6
Dorset	0.568	0.57	0.571	0.573	0.573	0.573	0.573	0.572	0.571	0.569	0.568	0.567	0.567	0.568	0.571	0.575	0.58	0.586	0.593	0.599
Gloucestershire	0.584	0.587	0.588	0.589	0.59	0.59	0.589	0.587	0.586	0.585	0.584	0.583	0.582	0.583	0.586	0.59	0.595	0.601	0.607	0.613
North Somerset	0.561	0.563	0.564	0.566	0.566	0.566	0.566	0.565	0.564	0.562	0.561	0.56	0.56	0.561	0.564	0.568	0.573	0.579	0.585	0.591
Plymouth	0.56	0.563	0.564	0.566	0.567	0.567	0.566	0.566	0.565	0.564	0.563	0.562	0.562	0.563	0.566	0.571	0.576	0.582	0.588	0.594
Poole	0.575	0.578	0.579	0.58	0.581	0.581	0.58	0.579	0.578	0.576	0.575	0.574	0.574	0.575	0.578	0.582	0.587	0.593	0.6	0.606
Somerset	0.564	0.567	0.568	0.569	0.57	0.57	0.569	0.568	0.566	0.565	0.563	0.562	0.562	0.563	0.565	0.57	0.575	0.581	0.587	0.593
South Gloucestershire	0.599	0.601	0.602	0.603	0.604	0.604	0.603	0.601	0.6	0.599	0.597	0.596	0.596	0.597	0.6	0.604	0.609	0.615	0.621	0.627
Swindon	0.598	0.6	0.601	0.602	0.603	0.602	0.601	0.599	0.597	0.595	0.593	0.591	0.59	0.591	0.593	0.597	0.602	0.609	0.615	0.621
Torbay	0.542	0.545	0.546	0.548	0.549	0.549	0.549	0.548	0.548	0.547	0.546	0.545	0.545	0.546	0.549	0.554	0.559	0.565	0.571	0.578
Wiltshire	0.58	0.582	0.584	0.585	0.585	0.585	0.583	0.582	0.58	0.579	0.577	0.575	0.574	0.575	0.578	0.582	0.587	0.593	0.599	0.605
West Midlands	0.555	0.557	0.558	0.559	0.56	0.559	0.558	0.557	0.555	0.554	0.552	0.551	0.55	0.551	0.553	0.557	0.563	0.569	0.575	0.581
Birmingham	0.552	0.554	0.555	0.555	0.556	0.555	0.554	0.552	0.551	0.549	0.547	0.545	0.544	0.545	0.547	0.551	0.557	0.563	0.569	0.575
Coventry	0.57	0.572	0.572	0.573	0.574	0.573	0.572	0.57	0.569	0.567	0.565	0.564	0.563	0.564	0.567	0.571	0.576	0.582	0.588	0.594
Dudley	0.543	0.545	0.546	0.547	0.548	0.547	0.546	0.545	0.543	0.542	0.54	0.539	0.538	0.539	0.541	0.546	0.551	0.557	0.563	0.569
Herefordshire, County of	0.557	0.559	0.56	0.561	0.562	0.562	0.56	0.559	0.558	0.556	0.555	0.553	0.553	0.554	0.556	0.56	0.566	0.572	0.578	0.584
Sandwell	0.535	0.537	0.538	0.539	0.539	0.539	0.537	0.535	0.534	0.531	0.529	0.527	0.526	0.527	0.529	0.533	0.538	0.544	0.55	0.556
Shropshire	0.558	0.56	0.561	0.562	0.563	0.563	0.562	0.561	0.56	0.558	0.557	0.556	0.555	0.556	0.559	0.563	0.569	0.575	0.581	0.587
Solihull	0.59	0.592	0.593	0.594	0.595	0.594	0.593	0.591	0.59	0.588	0.586	0.585	0.584	0.585	0.587	0.591	0.596	0.602	0.608	0.614
Staffordshire	0.557	0.559	0.56	0.561	0.562	0.562	0.561	0.559	0.558	0.557	0.555	0.554	0.554	0.555	0.557	0.562	0.567	0.573	0.579	0.585
Stoke-on-Trent	0.538	0.54	0.54	0.541	0.542	0.541	0.54	0.539	0.537	0.536	0.534	0.532	0.532	0.532	0.535	0.539	0.544	0.55	0.556	0.562
Telford and Wrekin	0.561	0.564	0.564	0.565	0.566	0.565	0.564	0.562	0.56	0.558	0.556	0.554	0.553	0.554	0.556	0.56	0.566	0.572	0.579	0.585
Walsall	0.534	0.536	0.537	0.538	0.539	0.538	0.537	0.535	0.533	0.532	0.53	0.528	0.527	0.528	0.53	0.534	0.54	0.546	0.552	0.558
Warwickshire	0.582	0.584	0.585	0.586	0.586	0.586	0.585	0.584	0.582	0.581	0.579	0.578	0.577	0.578	0.581	0.585	0.59	0.596	0.602	0.608
Wolverhampton	0.544	0.546	0.547	0.547	0.548	0.547	0.546	0.544	0.542	0.541	0.539	0.537	0.536	0.536	0.539	0.543	0.548	0.554	0.56	0.566
Worcestershire	0.556	0.558	0.559	0.56	0.561	0.561	0.56	0.559	0.557	0.556	0.555	0.554	0.553	0.554	0.557	0.561	0.567	0.573	0.579	0.585
Yorkshire and the Humber	0.554	0.556	0.557	0.559	0.559	0.559	0.558	0.557	0.556	0.555	0.553	0.552	0.552	0.553	0.556	0.56	0.565	0.571	0.577	0.584
Barnsley	0.524	0.526	0.527	0.528	0.529	0.528	0.527	0.526	0.525	0.523	0.522	0.52	0.52	0.521	0.524	0.528	0.533	0.539	0.546	0.552
Bradford	0.534	0.537	0.538	0.539	0.539	0.539	0.537	0.536	0.534	0.532	0.53	0.528	0.526	0.527	0.53	0.534	0.54	0.546	0.553	0.559
Calderdale	0.55	0.552	0.553	0.555	0.555	0.555	0.554	0.552	0.551	0.549	0.547	0.546	0.545	0.545	0.548	0.553	0.558	0.565	0.571	0.578
Doncaster	0.524	0.526	0.527	0.528	0.529	0.529	0.527	0.526	0.524	0.522	0.521	0.519	0.518	0.519	0.522	0.526	0.531	0.538	0.544	0.55
East Riding of Yorkshire	0.559	0.562	0.563	0.564	0.565	0.565	0.564	0.563	0.562	0.561	0.56	0.559	0.559	0.56	0.563	0.567	0.572	0.579	0.585	0.591
Kingston upon Hull, City of	0.532	0.534	0.535	0.536	0.537	0.537	0.535	0.534	0.532	0.531	0.529	0.528	0.527	0.528	0.531	0.535	0.541	0.547	0.554	0.56

Appendix Table 9. Socio-demographic Index values for all estimated GBD 2017 locations, 1950-1969

Location	1950	1951	1952	1953	1954	1955	1956	1957	1958	1959	1960	1961	1962	1963	1964	1965	1966	1967	1968	1969
Kirklees	0.543	0.545	0.546	0.548	0.548	0.548	0.547	0.546	0.545	0.543	0.542	0.54	0.54	0.541	0.544	0.548	0.553	0.56	0.566	0.572
Leeds	0.579	0.581	0.582	0.583	0.584	0.584	0.583	0.583	0.582	0.581	0.58	0.579	0.579	0.58	0.583	0.587	0.592	0.598	0.604	0.61
North East Lincolnshire	0.547	0.549	0.55	0.551	0.552	0.551	0.549	0.547	0.545	0.543	0.541	0.538	0.537	0.538	0.54	0.545	0.55	0.557	0.564	0.57
North Lincolnshire	0.554	0.556	0.557	0.558	0.558	0.558	0.556	0.555	0.553	0.551	0.549	0.547	0.546	0.547	0.549	0.553	0.559	0.565	0.572	0.578
North Yorkshire	0.569	0.571	0.573	0.574	0.575	0.575	0.574	0.573	0.572	0.571	0.569	0.568	0.568	0.569	0.572	0.576	0.582	0.588	0.594	0.6
Rotherham	0.531	0.533	0.534	0.535	0.536	0.536	0.534	0.533	0.532	0.53	0.528	0.527	0.526	0.527	0.53	0.534	0.539	0.546	0.552	0.558
Sheffield	0.569	0.571	0.572	0.573	0.574	0.574	0.574	0.573	0.573	0.572	0.571	0.571	0.571	0.572	0.575	0.58	0.585	0.591	0.596	0.602
Wakefield	0.539	0.542	0.543	0.544	0.545	0.544	0.543	0.542	0.54	0.539	0.537	0.536	0.535	0.536	0.539	0.543	0.548	0.555	0.561	0.567
York	0.601	0.603	0.605	0.606	0.607	0.607	0.606	0.606	0.605	0.605	0.604	0.604	0.604	0.606	0.609	0.613	0.618	0.623	0.629	0.635
Northern Ireland	0.502	0.505	0.506	0.508	0.508	0.509	0.509	0.511	0.512	0.513	0.514	0.515	0.517	0.52	0.525	0.533	0.541	0.549	0.557	0.565
Scotland	0.508	0.51	0.51	0.51	0.511	0.511	0.509	0.508	0.508	0.509	0.509	0.509	0.509	0.511	0.514	0.519	0.523	0.527	0.533	0.538
Wales	0.465	0.467	0.468	0.469	0.47	0.47	0.469	0.468	0.467	0.466	0.464	0.463	0.463	0.464	0.467	0.471	0.477	0.483	0.489	0.496
Latin America and Caribbean	0.28	0.284	0.287	0.29	0.293	0.296	0.3	0.303	0.306	0.308	0.312	0.316	0.321	0.326	0.331	0.338	0.345	0.352	0.359	0.366
Andean Latin America	0.256	0.264	0.272	0.278	0.28	0.282	0.285	0.288	0.289	0.291	0.293	0.296	0.301	0.306	0.312	0.32	0.329	0.337	0.344	0.349
Bolivia	0.235	0.245	0.251	0.255	0.258	0.264	0.268	0.265	0.258	0.253	0.252	0.253	0.256	0.26	0.265	0.272	0.279	0.285	0.29	0.295
Ecuador	0.288	0.3	0.313	0.323	0.325	0.321	0.319	0.322	0.326	0.329	0.327	0.325	0.328	0.333	0.339	0.35	0.362	0.37	0.38	0.385
Peru	0.244	0.248	0.252	0.255	0.259	0.263	0.268	0.272	0.277	0.281	0.286	0.293	0.299	0.305	0.31	0.316	0.324	0.332	0.338	0.341
Caribbean	0.344	0.348	0.35	0.35	0.35	0.351	0.353	0.355	0.355	0.354	0.354	0.355	0.357	0.359	0.364	0.374	0.387	0.398	0.403	0.407
Antigua and Barbuda	0.331	0.334	0.335	0.336	0.336	0.337	0.341	0.346	0.352	0.359	0.367	0.375	0.384	0.392	0.402	0.411	0.421	0.433	0.444	0.456
The Bahamas	0.446	0.45	0.454	0.46	0.468	0.475	0.481	0.489	0.498	0.504	0.504	0.502	0.501	0.505	0.512	0.519	0.526	0.534	0.543	0.553
Barbados	0.433	0.428	0.422	0.416	0.415	0.42	0.427	0.431	0.432	0.434	0.442	0.455	0.464	0.47	0.473	0.479	0.489	0.499	0.507	0.515
Belize	0.274	0.276	0.276	0.275	0.274	0.272	0.27	0.267	0.264	0.261	0.258	0.255	0.253	0.252	0.252	0.254	0.259	0.264	0.269	0.273
Bermuda	0.451	0.458	0.463	0.468	0.473	0.478	0.484	0.489	0.496	0.502	0.508	0.514	0.522	0.533	0.545	0.556	0.568	0.58	0.591	0.6
Cuba	0.412	0.417	0.418	0.415	0.413	0.414	0.414	0.414	0.41	0.402	0.392	0.382	0.376	0.375	0.38	0.393	0.412	0.428	0.429	0.428
Dominica	0.322	0.322	0.319	0.314	0.307	0.303	0.299	0.295	0.294	0.294	0.298	0.304	0.31	0.312	0.312	0.314	0.318	0.322	0.329	0.339
Dominican Republic	0.208	0.213	0.215	0.215	0.214	0.217	0.221	0.225	0.227	0.228	0.234	0.244	0.25	0.251	0.253	0.258	0.267	0.275	0.281	0.285
Grenada	0.227	0.225	0.222	0.217	0.211	0.207	0.206	0.21	0.22	0.232	0.246	0.26	0.273	0.284	0.295	0.303	0.311	0.317	0.323	0.329
Guyana	0.28	0.282	0.284	0.285	0.285	0.285	0.285	0.285	0.286	0.287	0.291	0.297	0.305	0.313	0.323	0.335	0.348	0.362	0.374	0.384
Haiti	0.206	0.208	0.211	0.214	0.217	0.22	0.223	0.226	0.23	0.233	0.237	0.24	0.244	0.248	0.251	0.254	0.258	0.261	0.264	0.267
Jamaica	0.402	0.404	0.404	0.404	0.402	0.399	0.396	0.394	0.39	0.386	0.382	0.377	0.374	0.372	0.37	0.372	0.377	0.385	0.393	0.399
Puerto Rico	0.401	0.409	0.419	0.425	0.423	0.421	0.426	0.433	0.44	0.448	0.457	0.465	0.471	0.476	0.485	0.501	0.522	0.54	0.552	0.559
Saint Lucia	0.317	0.321	0.322	0.32	0.316	0.312	0.307	0.303	0.3	0.3	0.302	0.304	0.306	0.308	0.309	0.311	0.314	0.316	0.319	0.324
Saint Vincent and the Grenadines	0.26	0.258	0.252	0.238	0.22	0.199	0.18	0.171	0.169	0.173	0.185	0.202	0.213	0.216	0.218	0.226	0.24	0.255	0.266	0.271
Suriname	0.314	0.311	0.309	0.308	0.308	0.309	0.309	0.307	0.306	0.306	0.309	0.318	0.335	0.349	0.356	0.361	0.366	0.371	0.377	0.385
Trinidad and Tobago	0.363	0.366	0.366	0.365	0.365	0.366	0.367	0.368	0.371	0.374	0.38	0.388	0.397	0.409	0.423	0.436	0.45	0.463	0.476	0.487
Virgin Islands	0.453	0.456	0.457	0.453	0.446	0.439	0.431	0.424	0.419	0.419	0.424	0.429	0.433	0.437	0.442	0.45	0.46	0.471	0.485	0.499
Central Latin America	0.275	0.277	0.28	0.282	0.285	0.288	0.292	0.294	0.296	0.298	0.301	0.306	0.311	0.316	0.322	0.329	0.336	0.344	0.353	0.36
Colombia	0.28	0.281	0.283	0.285	0.287	0.287	0.287	0.287	0.287	0.287	0.288	0.291	0.296	0.301	0.308	0.315	0.324	0.333	0.342	0.351
Costa Rica	0.308	0.313	0.317	0.316	0.312	0.313	0.315	0.314	0.307	0.303	0.306	0.314	0.325	0.339	0.352	0.364	0.376	0.39	0.405	0.417
El Salvador	0.22	0.222	0.224	0.226	0.228	0.23	0.233	0.236	0.239	0.242	0.245	0.249	0.253	0.257	0.262	0.267	0.272	0.278	0.284	0.29
Guatemala	0.203	0.208	0.211	0.213	0.219	0.226	0.228	0.229	0.23	0.229	0.23	0.235	0.239	0.238	0.244	0.253	0.259	0.264	0.269	0.273
Honduras	0.17	0.173	0.176	0.18	0.182	0.185	0.189	0.193	0.198	0.202	0.204	0.206	0.207	0.208	0.211	0.215	0.219	0.224	0.23	0.236
Mexico	0.271	0.274	0.278	0.281	0.284	0.289	0.293	0.297	0.301	0.306	0.31	0.315	0.32	0.325	0.331	0.337	0.344	0.351	0.358	0.365
Aguascalientes	0.29	0.294	0.297	0.3	0.303	0.308	0.312	0.317	0.321	0.326	0.332	0.339	0.345	0.351	0.356	0.363	0.371	0.379	0.387	0.394
Baja California	0.297	0.302	0.306	0.309	0.313	0.318	0.322	0.327	0.331	0.336	0.341	0.347	0.352	0.359	0.366	0.373	0.381	0.39	0.399	0.408
Baja California Sur	0.303	0.307	0.31	0.313	0.317	0.321	0.325	0.329	0.332	0.336	0.34	0.345	0.35	0.356	0.361	0.368	0.375	0.383	0.392	0.4
Campeche	0.267	0.271	0.275	0.278	0.282	0.286	0.29	0.294	0.297	0.301	0.304	0.308	0.311	0.315	0.32	0.324	0.33	0.335	0.341	0.346
Chiapas	0.22	0.223	0.226	0.229	0.232	0.236	0.239	0.243	0.246	0.25	0.253	0.257	0.261	0.265	0.27	0.275	0.28	0.286	0.291	0.297
Chihuahua	0.271	0.275	0.279	0.282	0.286	0.29	0.294	0.299	0.303	0.307	0.312	0.318	0.323	0.329	0.335	0.342	0.349	0.357	0.365	0.373
Coahuila	0.295	0.299	0.303	0.307	0.311	0.315	0.319	0.324	0.328	0.333	0.338	0.343	0.348	0.354	0.36	0.367	0.373	0.38	0.387	0.394
Colima	0.272	0.275	0.279	0.282	0.286	0.291	0.295	0.299	0.304	0.308	0.314	0.319	0.325	0.33	0.337	0.344	0.351	0.358	0.366	0.373
Mexico City	0.313	0.318	0.322	0.325	0.33	0.335	0.34	0.345	0.351	0.357	0.362	0.368	0.374	0.381	0.389	0.397	0.404	0.415	0.426	0.436
Durango	0.253	0.256	0.259	0.262	0.265	0.269	0.273	0.277	0.281	0.285	0.29	0.296	0.301	0.306	0.312	0.319	0.326	0.333	0.339	0.346
Guanajuato	0.244	0.247	0.25	0.252	0.256	0.26	0.264	0.268	0.272	0.276	0.281	0.286	0.291	0.297	0.302	0.308	0.315	0.321	0.328	0.334
Guerrero	0.203	0.207	0.21	0.213	0.216	0.22	0.224	0.228	0.232	0.236	0.24	0.244	0.249	0.253	0.259	0.264	0.27	0.276	0.282	0.287
Hidalgo	0.192	0.195	0.198	0.2	0.204	0.207	0.21	0.214	0.218	0.223	0.227	0.23	0.235	0.24	0.246	0.252	0.257	0.263	0.269	0.275
Jalisco	0.278	0.282	0.286	0.289	0.293	0.298	0.303	0.307	0.312	0.317	0.323	0.329	0.335	0.341	0.347	0.354	0.361	0.368	0.375	0.382
México	0.272	0.276	0.279	0.282	0.286	0.291	0.295	0.3	0.305	0.31	0.315	0.321	0.326	0.332	0.339	0.345	0.35	0.356	0.363	0.369
Michoacán de Ocampo	0.226	0.229	0.232	0.235	0.239	0.243	0.247	0.251	0.255	0.26	0.265	0.27	0.275	0.281	0.287	0.293	0.3	0.307	0.313	0.319
Morelos	0.279	0.283	0.287	0.291	0.295	0.3	0.304	0.309	0.313	0.317	0.322	0.327	0.331	0.336	0.341	0.347	0.353	0.359	0.366	0.372
Nayarit	0.236	0.239	0.242	0.244	0.247	0.251	0.255	0.259	0.263	0.267	0.272	0.278	0.283	0.288	0.294	0.301	0.308	0.315	0.322	0.329
Nuevo León	0.317	0.321	0.325	0.328	0.332	0.337	0.341	0.345	0.349	0.354	0.359	0.364	0.37	0.376	0.383	0.39	0.398	0.407	0.416	0.425
Oaxaca	0.206	0.21	0.213	0.216	0.219	0.223	0.227	0.231	0.235	0.239	0.243	0.247	0.251	0.255	0.26	0.265	0.271	0.276	0.282	0.287
Puebla	0.217	0.22	0.223	0.226	0.23	0.234	0.237	0.242	0.246	0.251	0.255	0.259	0.264	0.27	0.276	0.282	0.287	0.293	0.3	0.307
Querétaro	0.253	0.256	0.259	0.262	0.266	0.27	0.274	0.279	0.283	0.288	0.293	0.298	0.304	0.31	0.316	0.323	0.33	0.337	0.345	0.353

Appendix Table 9. Socio-demographic Index values for all estimated GBD 2017 locations, 1950-1969

Location	1950	1951	1952	1953	1954	1955	1956	1957	1958	1959	1960	1961	1962	1963	1964	1965	1966	1967	1968	1969
Quintana Roo	0.301	0.305	0.309	0.313	0.318	0.322	0.326	0.33	0.334	0.339	0.344	0.349	0.354	0.359	0.365	0.371	0.378	0.385	0.391	0.395
San Luis Potosí	0.235	0.239	0.242	0.245	0.249	0.253	0.257	0.261	0.266	0.27	0.275	0.28	0.285	0.29	0.296	0.302	0.309	0.316	0.323	0.329
Sinaloa	0.265	0.268	0.271	0.274	0.277	0.281	0.285	0.288	0.292	0.296	0.301	0.306	0.311	0.316	0.322	0.328	0.335	0.342	0.348	0.355
Sonora	0.279	0.283	0.286	0.289	0.293	0.298	0.302	0.306	0.31	0.315	0.32	0.326	0.331	0.338	0.345	0.352	0.36	0.368	0.377	0.386
Tabasco	0.234	0.237	0.241	0.244	0.247	0.251	0.255	0.259	0.262	0.266	0.27	0.274	0.278	0.282	0.287	0.292	0.299	0.305	0.311	0.316
Tamaulipas	0.286	0.29	0.293	0.297	0.301	0.306	0.31	0.315	0.319	0.324	0.329	0.335	0.34	0.346	0.353	0.36	0.368	0.376	0.384	0.393
Tlaxcala	0.225	0.228	0.232	0.235	0.239	0.243	0.247	0.252	0.257	0.262	0.267	0.272	0.278	0.283	0.289	0.295	0.302	0.308	0.315	0.321
Veracruz de Ignacio de la Llave	0.236	0.24	0.244	0.247	0.25	0.255	0.258	0.263	0.267	0.271	0.275	0.279	0.283	0.288	0.294	0.299	0.305	0.311	0.317	0.323
Yucatán	0.274	0.278	0.281	0.284	0.288	0.293	0.297	0.301	0.306	0.311	0.315	0.318	0.322	0.327	0.332	0.337	0.341	0.346	0.352	0.357
Zacatecas	0.221	0.225	0.227	0.23	0.233	0.237	0.24	0.244	0.248	0.252	0.258	0.263	0.269	0.274	0.28	0.287	0.294	0.301	0.307	0.313
Nicaragua	0.217	0.219	0.221	0.221	0.22	0.219	0.218	0.216	0.215	0.214	0.215	0.217	0.221	0.224	0.227	0.232	0.237	0.243	0.249	0.257
Panama	0.323	0.322	0.322	0.324	0.327	0.33	0.332	0.337	0.342	0.346	0.347	0.348	0.349	0.351	0.354	0.36	0.367	0.374	0.382	0.391
Venezuela	0.342	0.34	0.337	0.333	0.336	0.344	0.351	0.353	0.345	0.334	0.334	0.345	0.356	0.362	0.37	0.379	0.384	0.396	0.412	0.422
Tropical Latin America	0.261	0.265	0.269	0.273	0.277	0.281	0.285	0.289	0.294	0.298	0.303	0.308	0.314	0.32	0.325	0.331	0.336	0.342	0.348	0.355
Brazil	0.26	0.264	0.268	0.272	0.276	0.28	0.284	0.289	0.293	0.297	0.302	0.308	0.314	0.319	0.325	0.33	0.336	0.342	0.348	0.354
Acre	0.172	0.175	0.177	0.18	0.182	0.185	0.187	0.189	0.192	0.195	0.199	0.203	0.208	0.213	0.218	0.223	0.228	0.232	0.237	0.242
Alagoas	0.149	0.151	0.154	0.156	0.159	0.161	0.163	0.166	0.168	0.171	0.174	0.177	0.181	0.184	0.188	0.191	0.195	0.2	0.205	0.209
Amapá	0.234	0.238	0.242	0.245	0.249	0.253	0.257	0.26	0.264	0.269	0.274	0.279	0.285	0.29	0.295	0.3	0.305	0.309	0.313	0.318
Amazonas	0.189	0.192	0.195	0.198	0.201	0.203	0.206	0.208	0.211	0.214	0.218	0.223	0.229	0.234	0.24	0.245	0.251	0.256	0.262	0.268
Bahia	0.184	0.187	0.19	0.193	0.196	0.2	0.203	0.206	0.209	0.213	0.217	0.222	0.226	0.231	0.235	0.24	0.244	0.248	0.252	0.256
Ceará	0.194	0.197	0.2	0.202	0.206	0.209	0.212	0.216	0.219	0.223	0.228	0.233	0.238	0.243	0.248	0.253	0.258	0.263	0.268	0.273
Distrito Federal	0.372	0.376	0.382	0.387	0.392	0.397	0.403	0.408	0.413	0.419	0.426	0.432	0.439	0.446	0.454	0.461	0.468	0.475	0.483	0.491
Espírito Santo	0.257	0.26	0.263	0.267	0.27	0.274	0.278	0.281	0.286	0.29	0.296	0.303	0.31	0.316	0.323	0.33	0.337	0.344	0.351	0.358
Goiás	0.215	0.218	0.222	0.225	0.228	0.232	0.235	0.239	0.243	0.246	0.251	0.256	0.261	0.266	0.271	0.276	0.282	0.288	0.294	0.3
Maranhão	0.127	0.129	0.132	0.134	0.136	0.138	0.14	0.142	0.144	0.147	0.15	0.154	0.158	0.162	0.167	0.17	0.173	0.176	0.179	0.183
Mato Grosso	0.232	0.235	0.238	0.242	0.245	0.248	0.252	0.255	0.258	0.262	0.266	0.27	0.275	0.28	0.285	0.29	0.296	0.302	0.31	0.318
Mato Grosso do Sul	0.229	0.232	0.235	0.237	0.24	0.243	0.246	0.249	0.253	0.256	0.262	0.268	0.274	0.28	0.286	0.292	0.299	0.306	0.314	0.321
Minas Gerais	0.26	0.264	0.268	0.272	0.276	0.28	0.284	0.288	0.293	0.297	0.302	0.307	0.313	0.318	0.323	0.329	0.334	0.339	0.345	0.351
Pará	0.204	0.207	0.21	0.214	0.217	0.22	0.224	0.227	0.23	0.234	0.239	0.244	0.249	0.254	0.259	0.263	0.268	0.273	0.277	0.282
Paraíba	0.172	0.175	0.178	0.181	0.184	0.187	0.19	0.192	0.195	0.198	0.202	0.206	0.21	0.214	0.218	0.221	0.225	0.229	0.234	0.238
Paraná	0.267	0.27	0.274	0.278	0.282	0.286	0.29	0.294	0.298	0.302	0.306	0.311	0.315	0.32	0.324	0.328	0.333	0.339	0.345	0.351
Pernambuco	0.19	0.193	0.196	0.199	0.202	0.205	0.209	0.212	0.215	0.219	0.223	0.228	0.232	0.237	0.242	0.246	0.251	0.256	0.262	0.267
Piauí	0.162	0.164	0.167	0.169	0.171	0.174	0.176	0.179	0.182	0.185	0.189	0.193	0.198	0.202	0.206	0.21	0.214	0.218	0.222	0.227
Rio de Janeiro	0.342	0.347	0.352	0.357	0.362	0.367	0.373	0.378	0.383	0.389	0.395	0.402	0.409	0.416	0.422	0.429	0.436	0.442	0.449	0.456
Rio Grande do Norte	0.184	0.187	0.19	0.193	0.196	0.199	0.202	0.205	0.208	0.211	0.215	0.219	0.223	0.227	0.231	0.235	0.24	0.244	0.25	0.254
Rio Grande do Sul	0.314	0.318	0.323	0.327	0.331	0.336	0.34	0.345	0.349	0.354	0.36	0.366	0.372	0.378	0.384	0.391	0.398	0.405	0.412	0.419
Rondônia	0.199	0.202	0.205	0.207	0.21	0.214	0.216	0.219	0.222	0.226	0.23	0.235	0.241	0.246	0.251	0.256	0.262	0.267	0.272	0.278
Roraima	0.205	0.208	0.212	0.215	0.219	0.222	0.225	0.228	0.232	0.236	0.241	0.246	0.252	0.258	0.263	0.269	0.275	0.28	0.285	0.29
Santa Catarina	0.296	0.3	0.304	0.308	0.312	0.316	0.32	0.325	0.329	0.334	0.34	0.347	0.353	0.359	0.365	0.371	0.378	0.384	0.39	0.397
São Paulo	0.323	0.327	0.332	0.337	0.341	0.346	0.351	0.356	0.361	0.366	0.372	0.378	0.384	0.39	0.397	0.403	0.409	0.416	0.423	0.429
Sergipe	0.196	0.199	0.202	0.205	0.208	0.212	0.215	0.218	0.221	0.225	0.229	0.233	0.238	0.242	0.246	0.251	0.255	0.259	0.264	0.269
Tocantins	0.175	0.178	0.181	0.185	0.188	0.191	0.193	0.196	0.199	0.202	0.205	0.209	0.214	0.218	0.222	0.226	0.229	0.232	0.236	0.239
Paraguay	0.285	0.293	0.3	0.307	0.312	0.317	0.319	0.32	0.32	0.318	0.318	0.319	0.321	0.323	0.326	0.33	0.334	0.338	0.341	0.346
North Africa and Middle East	0.182	0.186	0.19	0.195	0.199	0.204	0.209	0.214	0.219	0.224	0.23	0.236	0.242	0.248	0.255	0.261	0.269	0.276	0.283	0.29
North Africa and Middle East	0.182	0.186	0.19	0.195	0.199	0.204	0.209	0.214	0.219	0.224	0.23	0.236	0.242	0.248	0.255	0.261	0.269	0.276	0.283	0.29
Afghanistan	0.078	0.08	0.081	0.083	0.085	0.087	0.089	0.09	0.092	0.094	0.096	0.098	0.1	0.101	0.103	0.105	0.106	0.108	0.11	0.111
Algeria	0.194	0.198	0.202	0.207	0.212	0.217	0.222	0.228	0.234	0.241	0.249	0.255	0.26	0.265	0.271	0.278	0.283	0.288	0.293	0.298
Bahrain	0.226	0.232	0.238	0.244	0.25	0.257	0.264	0.272	0.28	0.289	0.298	0.309	0.321	0.334	0.347	0.36	0.372	0.384	0.393	0.404
Egypt	0.178	0.182	0.186	0.189	0.191	0.193	0.196	0.198	0.201	0.204	0.209	0.215	0.221	0.227	0.234	0.242	0.25	0.259	0.267	0.275
Iran	0.166	0.17	0.174	0.178	0.182	0.186	0.192	0.199	0.206	0.213	0.222	0.235	0.246	0.254	0.261	0.27	0.281	0.293	0.302	0.311
Iraq	0.151	0.155	0.159	0.165	0.172	0.177	0.183	0.188	0.194	0.2	0.207	0.214	0.221	0.227	0.234	0.241	0.247	0.254	0.26	0.267
Jordan	0.054	0.056	0.057	0.059	0.06	0.077	0.106	0.129	0.148	0.16	0.167	0.177	0.195	0.214	0.228	0.24	0.252	0.263	0.276	0.289
Kuwait	0.278	0.286	0.294	0.303	0.311	0.319	0.328	0.334	0.337	0.336	0.336	0.332	0.324	0.321	0.327	0.331	0.33	0.328	0.345	0.38
Lebanon	0.154	0.157	0.161	0.166	0.172	0.177	0.183	0.188	0.194	0.199	0.204	0.21	0.217	0.224	0.232	0.242	0.251	0.261	0.272	0.283
Libya	0.183	0.19	0.198	0.204	0.211	0.219	0.227	0.236	0.243	0.25	0.259	0.267	0.275	0.281	0.288	0.296	0.304	0.312	0.32	0.323
Morocco	0.136	0.138	0.141	0.144	0.146	0.149	0.152	0.154	0.157	0.16	0.163	0.167	0.172	0.177	0.183	0.189	0.195	0.202	0.209	0.216
Palestine	0.097	0.101	0.105	0.109	0.114	0.118	0.123	0.129	0.134	0.14	0.147	0.153	0.16	0.167	0.175	0.183	0.191	0.198	0.206	0.215
Oman	0.141	0.144	0.148	0.152	0.157	0.162	0.166	0.171	0.176	0.182	0.187	0.193	0.2	0.206	0.213	0.219	0.225	0.234	0.247	0.259
Qatar	0.231	0.238	0.246	0.254	0.262	0.271	0.28	0.29	0.3	0.311	0.321	0.331	0.342	0.352	0.363	0.374	0.385	0.396	0.407	0.418
Saudi Arabia	0.14	0.143	0.147	0.15	0.154	0.157	0.161	0.165	0.168	0.172	0.177	0.181	0.186	0.19	0.194	0.2	0.205	0.21	0.215	0.22
Sudan	0.072	0.074	0.077	0.079	0.082	0.085	0.09	0.096	0.101	0.105	0.109	0.113	0.116	0.119	0.121	0.124	0.125	0.127	0.129	0.131
Syria	0.154	0.157	0.163	0.168	0.174	0.179	0.185	0.191	0.196	0.201	0.206	0.211	0.217	0.222	0.227	0.233	0.237	0.241	0.243	0.245
Tunisia	0.154	0.158	0.162	0.166	0.171	0.174	0.179	0.183	0.187	0.191	0.196	0.201	0.207	0.212	0.219	0.225	0.232	0.239	0.246	0.253
Turkey	0.237	0.244	0.25	0.257	0.262	0.268	0.274	0.281	0.287	0.293	0.298	0.303	0.308	0.313	0.319	0.324	0.329	0.334	0.339	0.343
United Arab Emirates	0.205	0.212	0.219	0.228	0.237	0.244	0.251	0.26	0.273	0.282	0.284	0.283	0.287	0.298	0.307	0.306	0.301	0.302	0.316	0.33

Appendix Table 9. Socio-demographic Index values for all estimated GBD 2017 locations, 1950-1969

Location	1950	1951	1952	1953	1954	1955	1956	1957	1958	1959	1960	1961	1962	1963	1964	1965	1966	1967	1968	1969
Yemen	0.075	0.077	0.079	0.081	0.083	0.085	0.088	0.09	0.093	0.095	0.097	0.099	0.102	0.104	0.106	0.108	0.111	0.114	0.116	0.118
South Asia	0.171	0.173	0.175	0.177	0.18	0.182	0.183	0.184	0.185	0.186	0.188	0.19	0.192	0.195	0.197	0.199	0.202	0.204	0.208	0.214
South Asia	0.171	0.173	0.175	0.177	0.18	0.182	0.183	0.184	0.185	0.186	0.188	0.19	0.192	0.195	0.197	0.199	0.202	0.204	0.208	0.214
Bangladesh	0.045	0.046	0.046	0.047	0.047	0.047	0.048	0.048	0.048	0.049	0.049	0.05	0.05	0.051	0.051	0.052	0.053	0.053	0.053	0.054
Bhutan	0.112	0.115	0.118	0.122	0.126	0.13	0.134	0.138	0.142	0.146	0.151	0.156	0.161	0.165	0.17	0.175	0.181	0.186	0.191	0.197
India	0.175	0.177	0.18	0.182	0.185	0.187	0.189	0.189	0.19	0.191	0.194	0.196	0.199	0.201	0.204	0.206	0.209	0.212	0.215	0.22
Andhra Pradesh	0.14	0.142	0.143	0.145	0.147	0.148	0.148	0.146	0.144	0.143	0.143	0.145	0.146	0.146	0.146	0.146	0.148	0.149	0.152	0.158
Arunachal Pradesh	0.145	0.147	0.149	0.151	0.153	0.155	0.155	0.153	0.152	0.15	0.151	0.153	0.155	0.157	0.159	0.16	0.163	0.167	0.173	0.179
Assam	0.17	0.172	0.175	0.178	0.181	0.183	0.185	0.186	0.186	0.188	0.191	0.194	0.197	0.201	0.205	0.209	0.212	0.213	0.215	0.218
Bihar	0.153	0.155	0.157	0.159	0.162	0.164	0.166	0.167	0.168	0.17	0.172	0.174	0.177	0.179	0.181	0.183	0.185	0.187	0.189	0.193
Chhattisgarh	0.168	0.17	0.172	0.173	0.175	0.177	0.178	0.178	0.177	0.177	0.178	0.18	0.181	0.183	0.183	0.184	0.186	0.188	0.191	0.197
Delhi	0.295	0.3	0.304	0.31	0.315	0.319	0.323	0.324	0.326	0.329	0.334	0.34	0.346	0.351	0.356	0.361	0.367	0.374	0.381	0.387
Goa	0.278	0.282	0.285	0.291	0.295	0.299	0.302	0.305	0.308	0.311	0.316	0.32	0.324	0.328	0.332	0.336	0.34	0.346	0.352	0.359
Gujarat	0.189	0.192	0.194	0.197	0.2	0.202	0.204	0.206	0.207	0.208	0.211	0.214	0.217	0.22	0.223	0.226	0.229	0.231	0.235	0.239
Haryana	0.192	0.194	0.197	0.199	0.202	0.204	0.206	0.207	0.208	0.209	0.212	0.214	0.216	0.217	0.217	0.219	0.222	0.226	0.231	0.237
Himachal Pradesh	0.168	0.17	0.172	0.175	0.177	0.179	0.181	0.182	0.183	0.184	0.186	0.188	0.189	0.191	0.192	0.193	0.195	0.198	0.202	0.207
Jammu and Kashmir	0.176	0.178	0.181	0.183	0.186	0.189	0.191	0.193	0.195	0.197	0.2	0.203	0.205	0.208	0.21	0.212	0.215	0.218	0.222	0.228
Jharkhand	0.171	0.173	0.175	0.177	0.179	0.181	0.181	0.181	0.181	0.181	0.182	0.184	0.185	0.187	0.188	0.188	0.19	0.192	0.195	0.2
Karnataka	0.167	0.169	0.171	0.174	0.176	0.178	0.18	0.18	0.18	0.18	0.183	0.185	0.187	0.19	0.192	0.193	0.196	0.201	0.207	0.214
Kerala	0.231	0.235	0.238	0.244	0.248	0.252	0.256	0.259	0.263	0.267	0.272	0.277	0.281	0.286	0.291	0.296	0.3	0.305	0.308	0.312
Madhya Pradesh	0.148	0.15	0.152	0.154	0.156	0.158	0.159	0.158	0.158	0.157	0.159	0.161	0.162	0.163	0.163	0.165	0.166	0.167	0.168	0.172
Maharashtra	0.198	0.2	0.203	0.206	0.209	0.211	0.213	0.213	0.214	0.214	0.217	0.219	0.222	0.225	0.227	0.229	0.233	0.238	0.244	0.252
Manipur	0.184	0.187	0.19	0.193	0.196	0.199	0.201	0.203	0.205	0.207	0.21	0.214	0.218	0.222	0.225	0.229	0.232	0.236	0.24	0.246
Meghalaya	0.168	0.171	0.174	0.177	0.18	0.183	0.185	0.186	0.187	0.188	0.191	0.195	0.198	0.201	0.204	0.207	0.21	0.214	0.218	0.224
Mizoram	0.194	0.197	0.201	0.205	0.209	0.212	0.215	0.217	0.219	0.222	0.226	0.231	0.235	0.239	0.244	0.249	0.252	0.255	0.258	0.264
Nagaland	0.203	0.206	0.21	0.213	0.217	0.221	0.225	0.227	0.23	0.233	0.238	0.243	0.248	0.253	0.258	0.263	0.267	0.27	0.273	0.278
Odisha	0.147	0.149	0.151	0.153	0.155	0.157	0.158	0.159	0.16	0.16	0.162	0.165	0.167	0.169	0.171	0.173	0.175	0.178	0.181	0.185
Punjab	0.218	0.221	0.224	0.228	0.232	0.235	0.238	0.241	0.244	0.247	0.251	0.255	0.258	0.261	0.265	0.269	0.273	0.278	0.283	0.289
Rajasthan	0.131	0.132	0.134	0.136	0.138	0.14	0.14	0.14	0.14	0.139	0.141	0.143	0.144	0.146	0.147	0.147	0.149	0.152	0.156	0.161
Sikkim	0.16	0.163	0.165	0.167	0.17	0.171	0.173	0.173	0.173	0.173	0.176	0.178	0.181	0.183	0.186	0.188	0.191	0.194	0.198	0.204
Tamil Nadu	0.189	0.192	0.194	0.198	0.201	0.203	0.205	0.207	0.208	0.21	0.213	0.216	0.219	0.222	0.225	0.228	0.23	0.233	0.238	0.244
Telangana	0.151	0.153	0.155	0.157	0.16	0.161	0.162	0.163	0.163	0.163	0.165	0.167	0.168	0.17	0.171	0.172	0.174	0.176	0.179	0.184
Tripura	0.164	0.167	0.17	0.173	0.176	0.178	0.179	0.178	0.177	0.176	0.179	0.182	0.185	0.189	0.192	0.194	0.198	0.201	0.206	0.212
Uttar Pradesh	0.152	0.154	0.156	0.158	0.161	0.163	0.164	0.165	0.166	0.168	0.17	0.172	0.175	0.177	0.179	0.181	0.183	0.185	0.188	0.192
Uttarakhand	0.183	0.185	0.187	0.189	0.191	0.193	0.194	0.194	0.194	0.195	0.196	0.198	0.2	0.201	0.202	0.203	0.204	0.206	0.21	0.215
West Bengal	0.19	0.193	0.196	0.199	0.202	0.204	0.205	0.205	0.204	0.204	0.207	0.21	0.213	0.217	0.22	0.223	0.226	0.226	0.227	0.231
Union Territories other than Delhi	0.236	0.24	0.243	0.245	0.249	0.252	0.253	0.254	0.253	0.253	0.256	0.259	0.262	0.265	0.267	0.269	0.273	0.277	0.283	0.291
Nepal	0.118	0.12	0.122	0.124	0.126	0.127	0.129	0.131	0.133	0.135	0.137	0.139	0.141	0.142	0.145	0.147	0.149	0.151	0.153	0.154
Pakistan	0.185	0.186	0.188	0.19	0.192	0.194	0.196	0.198	0.2	0.202	0.205	0.207	0.21	0.213	0.217	0.221	0.225	0.229	0.233	0.237
Southeast Asia, East Asia, and Oceania	0.19	0.194	0.199	0.204	0.21	0.215	0.219	0.223	0.227	0.232	0.236	0.239	0.242	0.247	0.253	0.259	0.264	0.269	0.275	0.284
East Asia	0.17	0.175	0.182	0.188	0.194	0.199	0.203	0.208	0.212	0.217	0.222	0.222	0.224	0.227	0.233	0.239	0.245	0.249	0.255	0.265
China	0.165	0.171	0.177	0.183	0.189	0.194	0.198	0.202	0.207	0.212	0.216	0.216	0.218	0.221	0.226	0.233	0.238	0.242	0.248	0.258
North Korea	0.259	0.257	0.26	0.268	0.276	0.283	0.29	0.297	0.304	0.311	0.318	0.325	0.331	0.339	0.345	0.352	0.361	0.37	0.379	0.389
Taiwan (Province of China)	0.345	0.345	0.357	0.366	0.368	0.371	0.381	0.39	0.391	0.392	0.397	0.4	0.406	0.418	0.43	0.443	0.456	0.467	0.474	0.479
Oceania	0.253	0.257	0.26	0.263	0.266	0.27	0.273	0.276	0.279	0.283	0.286	0.29	0.294	0.298	0.303	0.307	0.312	0.316	0.32	0.325
American Samoa	0.452	0.458	0.465	0.471	0.477	0.483	0.488	0.491	0.493	0.495	0.497	0.497	0.497	0.497	0.498	0.501	0.505	0.512	0.52	0.529
Federated States of Micronesia	0.249	0.251	0.254	0.257	0.26	0.262	0.265	0.269	0.272	0.275	0.278	0.282	0.285	0.289	0.294	0.298	0.303	0.308	0.313	0.319
Fiji	0.313	0.315	0.317	0.32	0.321	0.323	0.324	0.326	0.328	0.331	0.335	0.341	0.348	0.358	0.369	0.377	0.386	0.394	0.402	0.409
Guam	0.551	0.549	0.542	0.531	0.517	0.501	0.487	0.478	0.467	0.458	0.463	0.482	0.505	0.526	0.544	0.559	0.573	0.583	0.59	0.592
Kiribati	0.249	0.251	0.253	0.255	0.257	0.259	0.261	0.264	0.266	0.268	0.27	0.274	0.277	0.281	0.285	0.289	0.293	0.297	0.301	0.306
Marshall Islands	0.221	0.224	0.227	0.229	0.232	0.235	0.237	0.24	0.242	0.244	0.247	0.249	0.252	0.254	0.256	0.258	0.26	0.262	0.264	0.267
Northern Mariana Islands	0.457	0.463	0.47	0.476	0.483	0.489	0.496	0.503	0.51	0.517	0.524	0.531	0.538	0.544	0.549	0.554	0.56	0.566	0.572	0.578
Papua New Guinea	0.186	0.189	0.192	0.195	0.199	0.202	0.205	0.209	0.212	0.215	0.218	0.221	0.224	0.227	0.231	0.234	0.238	0.242	0.246	0.25
Samoa	0.311	0.315	0.318	0.32	0.322	0.324	0.327	0.331	0.335	0.34	0.346	0.353	0.361	0.368	0.374	0.38	0.386	0.393	0.398	0.403
Solomon Islands	0.181	0.183	0.185	0.187	0.19	0.192	0.195	0.197	0.2	0.203	0.206	0.209	0.212	0.216	0.22	0.223	0.227	0.231	0.235	0.238
Tonga	0.278	0.282	0.287	0.292	0.297	0.302	0.307	0.313	0.318	0.322	0.328	0.333	0.339	0.344	0.349	0.354	0.359	0.363	0.368	0.372
Vanuatu	0.216	0.218	0.221	0.224	0.227	0.23	0.234	0.237	0.241	0.244	0.248	0.251	0.256	0.26	0.265	0.269	0.274	0.278	0.283	0.287
Southeast Asia	0.216	0.22	0.224	0.229	0.233	0.237	0.241	0.245	0.25	0.255	0.26	0.265	0.27	0.276	0.282	0.287	0.293	0.298	0.304	0.31
Cambodia	0.15	0.152	0.154	0.157	0.16	0.162	0.165	0.169	0.172	0.176	0.18	0.183	0.187	0.191	0.194	0.197	0.2	0.203	0.206	0.209
Indonesia	0.194	0.198	0.202	0.205	0.209	0.213	0.217	0.221	0.225	0.229	0.233	0.237	0.241	0.245	0.25	0.253	0.257	0.262	0.267	0.273
Laos	0.146	0.148	0.151	0.153	0.156	0.158	0.16	0.163	0.166	0.168	0.171	0.174	0.177	0.18	0.183	0.186	0.189	0.192	0.195	0.198
Malaysia	0.275	0.278	0.281	0.284	0.287	0.288	0.289	0.292	0.299	0.309	0.31	0.306	0.318	0.332	0.345	0.355	0.368	0.386	0.397	0.405
Maldives	0.184	0.186	0.188	0.191	0.193	0.197	0.201	0.206	0.211	0.216	0.221	0.228	0.234	0.238	0.237	0.235	0.234	0.234	0.233	0.233
Mauritius	0.247	0.252	0.259	0.265	0.269	0.272	0.275	0.28	0.284	0.288	0.29	0.294	0.298	0.306	0.316	0.329	0.343	0.357	0.369	0.38
Myanmar	0.093	0.104	0.108	0.112	0.113	0.117	0.121	0.126	0.13	0.136	0.142	0.147	0.153	0.159	0.164	0.169	0.173	0.175	0.179	0.182

Appendix Table 9. Socio-demographic Index values for all estimated GBD 2017 locations, 1950-1969																				
**Location**	**1950**	**1951**	**1952**	**1953**	**1954**	**1955**	**1956**	**1957**	**1958**	**1959**	**1960**	**1961**	**1962**	**1963**	**1964**	**1965**	**1966**	**1967**	**1968**	**1969**
Philippines	0.298	0.304	0.31	0.317	0.324	0.33	0.336	0.343	0.352	0.36	0.367	0.374	0.382	0.388	0.393	0.398	0.402	0.406	0.41	0.414
Sri Lanka	0.28	0.286	0.29	0.295	0.3	0.305	0.309	0.313	0.316	0.32	0.324	0.328	0.333	0.339	0.345	0.352	0.36	0.368	0.375	0.38
Seychelles	0.297	0.302	0.305	0.307	0.309	0.31	0.311	0.313	0.314	0.315	0.316	0.316	0.318	0.321	0.325	0.329	0.333	0.339	0.345	0.351
Thailand	0.245	0.249	0.252	0.256	0.259	0.263	0.267	0.271	0.276	0.281	0.286	0.291	0.296	0.302	0.309	0.315	0.322	0.328	0.333	0.338
Timor-Leste	0.082	0.085	0.089	0.092	0.095	0.098	0.102	0.105	0.108	0.113	0.117	0.12	0.125	0.129	0.134	0.139	0.144	0.149	0.155	0.161
Vietnam	0.194	0.197	0.201	0.205	0.209	0.213	0.217	0.221	0.226	0.23	0.235	0.24	0.246	0.252	0.258	0.264	0.269	0.273	0.276	0.28
Sub-Saharan Africa	0.14	0.143	0.146	0.149	0.152	0.155	0.158	0.161	0.165	0.168	0.172	0.176	0.18	0.184	0.188	0.191	0.195	0.199	0.203	0.207
Central sub-Saharan Africa	0.118	0.121	0.123	0.126	0.128	0.13	0.132	0.135	0.137	0.139	0.142	0.144	0.148	0.151	0.154	0.158	0.163	0.167	0.172	0.176
Angola	0.11	0.111	0.112	0.113	0.112	0.112	0.111	0.11	0.11	0.11	0.11	0.11	0.111	0.111	0.111	0.111	0.113	0.115	0.118	0.122
Central African Republic	0.107	0.11	0.112	0.114	0.116	0.118	0.121	0.122	0.124	0.126	0.128	0.131	0.134	0.135	0.137	0.139	0.143	0.145	0.147	0.149
Congo (Brazzaville)	0.137	0.14	0.144	0.147	0.151	0.155	0.158	0.162	0.166	0.17	0.174	0.179	0.183	0.188	0.193	0.197	0.202	0.207	0.211	0.216
DR Congo	0.111	0.114	0.117	0.12	0.123	0.126	0.129	0.132	0.135	0.138	0.141	0.143	0.147	0.151	0.154	0.158	0.163	0.167	0.172	0.177
Equatorial Guinea	0.055	0.057	0.057	0.057	0.056	0.053	0.049	0.04	0.032	0.033	0.034	0.036	0.037	0.039	0.04	0.042	0.043	0.045	0.046	0.048
Gabon	0.146	0.149	0.153	0.156	0.159	0.162	0.165	0.169	0.173	0.177	0.182	0.188	0.194	0.201	0.208	0.216	0.226	0.234	0.24	0.245
Eastern sub-Saharan Africa	0.099	0.102	0.103	0.105	0.107	0.11	0.112	0.115	0.118	0.12	0.123	0.126	0.129	0.133	0.135	0.138	0.141	0.144	0.148	0.152
Burundi	0.081	0.084	0.086	0.088	0.09	0.093	0.095	0.098	0.101	0.104	0.107	0.11	0.113	0.116	0.119	0.122	0.125	0.129	0.133	0.136
Comoros	0.131	0.133	0.134	0.135	0.136	0.137	0.137	0.137	0.138	0.139	0.14	0.141	0.143	0.145	0.147	0.148	0.149	0.15	0.151	0.153
Djibouti	0.14	0.142	0.145	0.147	0.15	0.154	0.158	0.163	0.167	0.17	0.172	0.174	0.176	0.178	0.18	0.181	0.184	0.187	0.19	0.194
Eritrea	0.053	0.056	0.058	0.06	0.063	0.066	0.069	0.071	0.074	0.077	0.08	0.084	0.087	0.09	0.094	0.097	0.101	0.104	0.108	0.112
Ethiopia	0.053	0.055	0.057	0.059	0.06	0.062	0.064	0.066	0.067	0.069	0.071	0.073	0.076	0.078	0.079	0.08	0.082	0.084	0.086	0.087
Kenya	0.08	0.085	0.089	0.094	0.099	0.104	0.11	0.116	0.122	0.126	0.13	0.134	0.14	0.146	0.151	0.155	0.161	0.169	0.179	0.19
Baringo	0.052	0.058	0.063	0.069	0.075	0.081	0.088	0.094	0.099	0.102	0.104	0.107	0.111	0.116	0.117	0.116	0.118	0.125	0.135	0.145
Bomet	0.047	0.048	0.049	0.05	0.051	0.052	0.053	0.054	0.055	0.056	0.057	0.058	0.059	0.06	0.061	0.062	0.063	0.087	0.112	0.133
Bungoma	0.047	0.049	0.05	0.05	0.052	0.053	0.055	0.071	0.082	0.088	0.094	0.101	0.109	0.117	0.123	0.127	0.133	0.143	0.156	0.169
Busia	0.049	0.05	0.051	0.052	0.053	0.054	0.055	0.056	0.066	0.071	0.074	0.08	0.088	0.101	0.111	0.12	0.129	0.143	0.159	0.175
Elgeyo Marakwet	0.057	0.061	0.065	0.069	0.074	0.078	0.084	0.089	0.094	0.097	0.1	0.103	0.108	0.112	0.116	0.118	0.123	0.131	0.141	0.152
Embu	0.095	0.1	0.103	0.107	0.111	0.116	0.12	0.126	0.131	0.136	0.141	0.146	0.151	0.157	0.163	0.168	0.174	0.182	0.191	0.2
Garissa	0.042	0.044	0.045	0.046	0.048	0.05	0.052	0.054	0.055	0.057	0.058	0.06	0.062	0.064	0.066	0.068	0.07	0.075	0.079	0.085
Homa Bay	0.041	0.042	0.043	0.044	0.045	0.046	0.047	0.048	0.049	0.049	0.05	0.051	0.052	0.053	0.054	0.055	0.061	0.09	0.113	0.132
Isiolo	0.101	0.104	0.106	0.109	0.112	0.115	0.118	0.122	0.126	0.129	0.132	0.135	0.139	0.143	0.148	0.152	0.157	0.164	0.172	0.18
Kajiado	0.149	0.155	0.159	0.163	0.168	0.173	0.179	0.184	0.19	0.194	0.198	0.202	0.207	0.212	0.215	0.218	0.222	0.228	0.235	0.243
Kakamega	0.047	0.049	0.049	0.05	0.051	0.052	0.066	0.077	0.086	0.092	0.097	0.104	0.111	0.119	0.126	0.131	0.138	0.149	0.162	0.175
Kericho	0.041	0.043	0.043	0.044	0.045	0.046	0.047	0.052	0.062	0.066	0.069	0.074	0.081	0.088	0.092	0.095	0.1	0.112	0.125	0.137
Kiambu	0.099	0.106	0.112	0.118	0.125	0.133	0.141	0.149	0.156	0.163	0.169	0.176	0.183	0.191	0.197	0.203	0.21	0.22	0.231	0.244
Kilifi	0.098	0.102	0.104	0.107	0.11	0.113	0.117	0.121	0.124	0.126	0.129	0.132	0.135	0.139	0.141	0.143	0.146	0.151	0.158	0.165
Kirinyaga	0.072	0.077	0.081	0.084	0.089	0.094	0.1	0.106	0.111	0.115	0.12	0.125	0.13	0.137	0.144	0.151	0.159	0.17	0.183	0.196
Kisii	0.058	0.062	0.066	0.07	0.075	0.08	0.086	0.092	0.098	0.102	0.107	0.112	0.118	0.125	0.131	0.135	0.142	0.152	0.163	0.175
Kisumu	0.043	0.045	0.046	0.046	0.047	0.048	0.049	0.058	0.066	0.067	0.069	0.073	0.08	0.088	0.094	0.097	0.105	0.119	0.135	0.15
Kitui	0.063	0.067	0.069	0.072	0.075	0.079	0.083	0.087	0.091	0.094	0.098	0.102	0.107	0.113	0.119	0.124	0.13	0.139	0.149	0.158
Kwale	0.08	0.083	0.085	0.087	0.089	0.092	0.095	0.098	0.101	0.104	0.106	0.109	0.112	0.116	0.12	0.124	0.129	0.136	0.144	0.154
Laikipia	0.038	0.04	0.041	0.045	0.053	0.06	0.067	0.074	0.08	0.083	0.087	0.091	0.096	0.103	0.108	0.111	0.118	0.129	0.142	0.155
Lamu	0.04	0.041	0.044	0.049	0.054	0.059	0.064	0.07	0.075	0.076	0.077	0.079	0.083	0.088	0.092	0.092	0.097	0.108	0.121	0.134
Machakos	0.085	0.09	0.093	0.097	0.101	0.106	0.111	0.117	0.122	0.126	0.13	0.135	0.141	0.147	0.152	0.157	0.163	0.172	0.184	0.196
Makueni	0.075	0.078	0.081	0.085	0.089	0.094	0.099	0.104	0.109	0.113	0.117	0.122	0.128	0.135	0.142	0.148	0.155	0.166	0.178	0.19
Mandera	0.03	0.031	0.032	0.033	0.034	0.036	0.037	0.039	0.04	0.04	0.041	0.041	0.042	0.044	0.045	0.046	0.047	0.052	0.057	0.063
Marsabit	0.085	0.089	0.09	0.092	0.094	0.097	0.1	0.103	0.105	0.108	0.11	0.112	0.115	0.118	0.121	0.124	0.128	0.133	0.139	0.145
Meru	0.088	0.093	0.096	0.099	0.104	0.108	0.113	0.118	0.123	0.127	0.131	0.135	0.14	0.145	0.15	0.154	0.16	0.168	0.177	0.187
Migori	0.041	0.042	0.043	0.044	0.045	0.046	0.047	0.048	0.049	0.05	0.051	0.052	0.053	0.062	0.072	0.075	0.086	0.103	0.12	0.136
Mombasa	0.103	0.111	0.113	0.116	0.12	0.125	0.13	0.136	0.141	0.146	0.151	0.156	0.162	0.168	0.174	0.18	0.187	0.195	0.204	0.214
Murang’a	0.098	0.104	0.109	0.115	0.121	0.128	0.135	0.142	0.149	0.154	0.159	0.165	0.171	0.178	0.183	0.188	0.193	0.202	0.211	0.221
Nairobi	0.13	0.14	0.146	0.151	0.159	0.167	0.176	0.185	0.194	0.202	0.21	0.219	0.228	0.237	0.246	0.254	0.265	0.277	0.289	0.302
Nakuru	0.076	0.083	0.085	0.088	0.092	0.097	0.102	0.108	0.113	0.117	0.122	0.126	0.131	0.137	0.142	0.146	0.151	0.159	0.167	0.175
Nandi	0.066	0.072	0.077	0.083	0.089	0.095	0.102	0.109	0.115	0.118	0.122	0.126	0.131	0.136	0.139	0.14	0.143	0.151	0.16	0.17
Narok	0.041	0.042	0.043	0.044	0.045	0.046	0.047	0.047	0.048	0.049	0.05	0.051	0.052	0.053	0.053	0.054	0.055	0.056	0.057	0.076
Nyamira	0.042	0.043	0.044	0.05	0.058	0.065	0.073	0.081	0.087	0.091	0.096	0.102	0.11	0.118	0.125	0.131	0.141	0.153	0.167	0.181
Nyandarua	0.045	0.047	0.048	0.048	0.05	0.051	0.052	0.053	0.054	0.055	0.056	0.057	0.063	0.079	0.091	0.101	0.113	0.131	0.149	0.167
Nyeri	0.077	0.084	0.088	0.094	0.1	0.106	0.113	0.12	0.126	0.131	0.136	0.142	0.148	0.156	0.163	0.168	0.176	0.188	0.201	0.215
Samburu	0.043	0.045	0.047	0.05	0.053	0.057	0.061	0.066	0.07	0.071	0.072	0.074	0.078	0.082	0.085	0.086	0.09	0.098	0.108	0.117
Siaya	0.01	0.01	0.01	0.011	0.011	0.012	0.014	0.021	0.027	0.031	0.036	0.041	0.046	0.053	0.059	0.064	0.072	0.082	0.091	0.102
Taita Taveta	0.105	0.111	0.114	0.116	0.121	0.125	0.129	0.134	0.139	0.142	0.146	0.15	0.154	0.159	0.162	0.165	0.17	0.175	0.182	0.19
Tana River	0.041	0.045	0.046	0.047	0.048	0.051	0.053	0.056	0.058	0.06	0.062	0.065	0.068	0.072	0.076	0.079	0.084	0.091	0.099	0.107
Tharaka Nithi	0.078	0.084	0.086	0.088	0.092	0.096	0.1	0.105	0.109	0.113	0.118	0.123	0.128	0.134	0.14	0.146	0.153	0.162	0.172	0.183
Trans Nzoia	0.089	0.094	0.098	0.102	0.107	0.112	0.118	0.124	0.129	0.132	0.135	0.138	0.142	0.147	0.15	0.152	0.156	0.164	0.174	0.185
Turkana	0.101	0.104	0.106	0.108	0.11	0.113	0.115	0.118	0.121	0.123	0.125	0.128	0.13	0.133	0.136	0.138	0.14	0.145	0.15	0.155
Uasin Gishu	0.085	0.089	0.093	0.098	0.103	0.109	0.114	0.121	0.126	0.13	0.134	0.139	0.144	0.149	0.154	0.157	0.162	0.171	0.181	0.193

Appendix Table 9. Socio-demographic Index values for all estimated GBD 2017 locations, 1950-1969

Location	1950	1951	1952	1953	1954	1955	1956	1957	1958	1959	1960	1961	1962	1963	1964	1965	1966	1967	1968	1969
Vihiga	0.064	0.071	0.078	0.085	0.092	0.099	0.107	0.114	0.121	0.126	0.13	0.136	0.142	0.148	0.154	0.158	0.164	0.173	0.184	0.195
Wajir	0.041	0.042	0.043	0.044	0.046	0.048	0.049	0.051	0.053	0.053	0.054	0.055	0.057	0.059	0.06	0.06	0.061	0.065	0.07	0.075
West Pokot	0.064	0.067	0.069	0.072	0.075	0.078	0.082	0.086	0.089	0.092	0.094	0.097	0.1	0.104	0.107	0.109	0.113	0.119	0.126	0.133
Madagascar	0.125	0.127	0.128	0.129	0.13	0.132	0.134	0.136	0.138	0.141	0.142	0.144	0.146	0.147	0.147	0.147	0.147	0.148	0.15	0.153
Malawi	0.067	0.068	0.069	0.071	0.072	0.074	0.076	0.078	0.081	0.083	0.085	0.088	0.091	0.093	0.096	0.099	0.103	0.107	0.111	0.115
Mozambique	0.054	0.056	0.057	0.059	0.061	0.063	0.064	0.066	0.069	0.071	0.074	0.076	0.079	0.082	0.085	0.088	0.09	0.093	0.096	0.1
Rwanda	0.113	0.115	0.118	0.12	0.123	0.126	0.128	0.131	0.134	0.138	0.142	0.146	0.152	0.157	0.159	0.16	0.16	0.162	0.165	0.17
Somalia	0.079	0.08	0.082	0.084	0.085	0.087	0.089	0.091	0.093	0.095	0.096	0.099	0.101	0.103	0.104	0.105	0.107	0.109	0.111	0.112
South Sudan	0.104	0.106	0.107	0.109	0.11	0.112	0.113	0.115	0.116	0.118	0.12	0.121	0.123	0.125	0.127	0.129	0.13	0.132	0.134	0.136
Tanzania	0.101	0.105	0.107	0.11	0.113	0.116	0.119	0.122	0.125	0.128	0.132	0.135	0.138	0.141	0.144	0.147	0.15	0.153	0.155	0.158
Uganda	0.085	0.085	0.084	0.083	0.083	0.082	0.082	0.082	0.083	0.084	0.084	0.085	0.087	0.089	0.091	0.093	0.094	0.097	0.099	0.101
Zambia	0.135	0.138	0.142	0.145	0.149	0.153	0.157	0.161	0.164	0.169	0.174	0.179	0.184	0.189	0.195	0.201	0.208	0.215	0.222	0.23
Southern sub-Saharan Africa	0.277	0.282	0.287	0.293	0.298	0.304	0.31	0.316	0.321	0.327	0.332	0.338	0.344	0.349	0.355	0.36	0.365	0.372	0.378	0.384
Botswana	0.166	0.17	0.174	0.178	0.182	0.187	0.191	0.196	0.2	0.205	0.209	0.213	0.217	0.221	0.225	0.228	0.232	0.237	0.242	0.247
Lesotho	0.126	0.131	0.135	0.139	0.143	0.147	0.152	0.156	0.16	0.165	0.17	0.174	0.179	0.185	0.192	0.197	0.202	0.208	0.212	0.217
Namibia	0.215	0.219	0.224	0.228	0.233	0.238	0.244	0.249	0.254	0.26	0.266	0.271	0.277	0.283	0.29	0.297	0.303	0.308	0.313	0.318
South Africa	0.31	0.316	0.321	0.326	0.332	0.339	0.345	0.351	0.356	0.362	0.368	0.374	0.38	0.385	0.391	0.396	0.402	0.409	0.416	0.422
Swaziland	0.166	0.171	0.175	0.179	0.184	0.188	0.193	0.198	0.203	0.208	0.215	0.222	0.231	0.239	0.248	0.257	0.264	0.269	0.274	0.278
Zimbabwe	0.152	0.157	0.161	0.166	0.17	0.175	0.18	0.185	0.19	0.195	0.201	0.206	0.211	0.216	0.22	0.225	0.229	0.233	0.237	0.241
Western sub-Saharan Africa	0.131	0.134	0.137	0.14	0.144	0.147	0.15	0.153	0.157	0.161	0.165	0.169	0.173	0.177	0.182	0.186	0.189	0.193	0.196	0.201
Benin	0.098	0.1	0.102	0.104	0.106	0.109	0.111	0.113	0.116	0.119	0.122	0.124	0.127	0.129	0.131	0.134	0.137	0.14	0.142	0.145
Burkina Faso	0.056	0.058	0.06	0.062	0.064	0.066	0.068	0.071	0.073	0.076	0.078	0.08	0.083	0.085	0.087	0.088	0.089	0.09	0.092	0.094
Cameroon	0.128	0.132	0.136	0.14	0.145	0.149	0.154	0.159	0.164	0.17	0.175	0.18	0.185	0.19	0.194	0.199	0.203	0.206	0.209	0.212
Cape Verde	0.123	0.124	0.125	0.125	0.126	0.127	0.128	0.128	0.13	0.133	0.136	0.14	0.144	0.147	0.152	0.157	0.163	0.17	0.177	0.185
Chad	0.064	0.065	0.067	0.068	0.07	0.071	0.073	0.075	0.076	0.078	0.08	0.081	0.083	0.085	0.086	0.088	0.089	0.091	0.092	0.093
Cote d'Ivoire	0.113	0.116	0.118	0.121	0.124	0.127	0.13	0.134	0.137	0.141	0.145	0.149	0.153	0.158	0.162	0.166	0.171	0.175	0.18	0.184
The Gambia	0.099	0.101	0.104	0.106	0.109	0.111	0.114	0.117	0.119	0.123	0.126	0.129	0.132	0.134	0.137	0.139	0.142	0.145	0.148	0.151
Ghana	0.225	0.229	0.231	0.234	0.237	0.239	0.239	0.241	0.243	0.246	0.249	0.252	0.255	0.26	0.264	0.268	0.271	0.272	0.274	0.277
Guinea	0.066	0.068	0.069	0.07	0.073	0.075	0.078	0.081	0.084	0.087	0.091	0.094	0.098	0.101	0.104	0.107	0.11	0.113	0.116	0.118
Guinea-Bissau	0.059	0.061	0.062	0.064	0.067	0.071	0.074	0.079	0.083	0.087	0.092	0.098	0.104	0.109	0.115	0.12	0.124	0.127	0.131	0.134
Liberia	0.103	0.106	0.108	0.111	0.114	0.116	0.119	0.122	0.126	0.129	0.132	0.135	0.139	0.141	0.144	0.145	0.148	0.15	0.152	0.155
Mali	0.059	0.06	0.061	0.063	0.064	0.065	0.067	0.068	0.07	0.072	0.073	0.075	0.077	0.078	0.08	0.081	0.082	0.084	0.085	0.087
Mauritania	0.124	0.128	0.131	0.135	0.138	0.142	0.146	0.15	0.154	0.159	0.164	0.168	0.172	0.175	0.18	0.185	0.189	0.193	0.196	0.2
Niger	0.056	0.057	0.058	0.059	0.06	0.061	0.062	0.063	0.065	0.066	0.067	0.068	0.07	0.071	0.071	0.072	0.073	0.074	0.075	0.076
Nigeria	0.144	0.147	0.151	0.154	0.158	0.162	0.166	0.17	0.174	0.179	0.184	0.189	0.194	0.199	0.205	0.21	0.215	0.22	0.225	0.231
Sao Tome and Principe	0.146	0.15	0.152	0.153	0.153	0.154	0.154	0.154	0.155	0.157	0.158	0.16	0.162	0.164	0.165	0.167	0.169	0.171	0.174	0.179
Senegal	0.108	0.111	0.114	0.117	0.12	0.124	0.128	0.132	0.135	0.137	0.14	0.144	0.146	0.146	0.146	0.147	0.149	0.152	0.154	0.155
Sierra Leone	0.093	0.096	0.098	0.1	0.102	0.105	0.107	0.11	0.112	0.115	0.118	0.121	0.124	0.127	0.13	0.133	0.136	0.139	0.142	0.145
Togo	0.103	0.105	0.108	0.11	0.112	0.115	0.117	0.12	0.122	0.125	0.128	0.131	0.134	0.137	0.14	0.143	0.147	0.151	0.155	0.159

Appendix Table 10. Socio-demographic Index values for all estimated GBD 2017 locations, 1970–1989																				
**Location**	**1970**	**1971**	**1972**	**1973**	**1974**	**1975**	**1976**	**1977**	**1978**	**1979**	**1980**	**1981**	**1982**	**1983**	**1984**	**1985**	**1986**	**1987**	**1988**	**1989**
Global	0.423	0.43	0.436	0.443	0.45	0.457	0.462	0.467	0.472	0.477	0.482	0.486	0.488	0.491	0.494	0.498	0.501	0.507	0.513	0.518
Central Europe, Eastern Europe, and Central Asia	0.597	0.601	0.605	0.608	0.61	0.613	0.617	0.621	0.623	0.626	0.629	0.632	0.632	0.633	0.635	0.637	0.64	0.643	0.647	0.651
Central Asia	0.486	0.49	0.494	0.499	0.503	0.508	0.514	0.519	0.525	0.529	0.532	0.536	0.538	0.541	0.543	0.545	0.548	0.552	0.556	0.56
Armenia	0.501	0.507	0.512	0.518	0.524	0.529	0.534	0.538	0.541	0.544	0.546	0.546	0.546	0.546	0.546	0.545	0.546	0.547	0.549	0.552
Azerbaijan	0.511	0.519	0.526	0.534	0.542	0.55	0.559	0.567	0.574	0.579	0.584	0.589	0.593	0.596	0.599	0.601	0.604	0.606	0.608	0.61
Georgia	0.578	0.584	0.589	0.595	0.6	0.603	0.607	0.612	0.616	0.617	0.618	0.62	0.623	0.627	0.629	0.63	0.633	0.639	0.645	0.65
Kazakhstan	0.527	0.533	0.539	0.546	0.553	0.559	0.565	0.57	0.576	0.58	0.584	0.588	0.591	0.593	0.594	0.595	0.598	0.602	0.606	0.61
Kyrgyzstan	0.485	0.487	0.492	0.499	0.504	0.509	0.515	0.52	0.525	0.529	0.531	0.532	0.533	0.536	0.54	0.543	0.547	0.552	0.557	0.56
Mongolia	0.417	0.423	0.429	0.435	0.44	0.446	0.451	0.456	0.461	0.466	0.472	0.479	0.486	0.492	0.498	0.503	0.509	0.516	0.523	0.529
Tajikistan	0.327	0.328	0.326	0.322	0.324	0.34	0.36	0.375	0.384	0.393	0.403	0.412	0.419	0.425	0.43	0.436	0.444	0.455	0.464	0.469
Turkmenistan	0.497	0.502	0.507	0.513	0.518	0.523	0.528	0.533	0.537	0.542	0.546	0.55	0.554	0.558	0.562	0.565	0.569	0.574	0.579	0.584
Uzbekistan	0.388	0.393	0.396	0.401	0.405	0.41	0.416	0.422	0.428	0.433	0.437	0.442	0.446	0.45	0.454	0.458	0.463	0.467	0.472	0.477
Central Europe	0.59	0.595	0.598	0.598	0.598	0.601	0.605	0.609	0.613	0.617	0.622	0.626	0.629	0.631	0.633	0.637	0.641	0.646	0.651	0.658
Albania	0.433	0.441	0.451	0.46	0.469	0.478	0.486	0.495	0.503	0.511	0.519	0.526	0.532	0.535	0.537	0.541	0.546	0.549	0.549	0.549
Bosnia and Herzegovina	0.386	0.393	0.4	0.406	0.413	0.419	0.425	0.432	0.438	0.445	0.452	0.458	0.465	0.47	0.475	0.48	0.484	0.489	0.492	0.495
Bulgaria	0.565	0.571	0.572	0.567	0.568	0.572	0.575	0.579	0.583	0.589	0.596	0.601	0.605	0.61	0.617	0.621	0.624	0.628	0.635	0.646
Croatia	0.628	0.633	0.637	0.64	0.644	0.647	0.651	0.656	0.661	0.667	0.672	0.678	0.683	0.688	0.692	0.697	0.702	0.707	0.713	0.719
Czech Republic	0.664	0.664	0.657	0.648	0.644	0.646	0.65	0.652	0.656	0.666	0.677	0.681	0.683	0.686	0.688	0.692	0.697	0.701	0.704	0.708
Hungary	0.586	0.591	0.593	0.587	0.58	0.583	0.59	0.598	0.605	0.615	0.624	0.633	0.642	0.648	0.65	0.653	0.658	0.664	0.67	0.675
Macedonia	0.552	0.557	0.561	0.564	0.567	0.57	0.574	0.576	0.579	0.583	0.588	0.594	0.598	0.601	0.605	0.61	0.614	0.617	0.62	0.623
Montenegro	0.647	0.652	0.656	0.661	0.665	0.669	0.674	0.677	0.681	0.685	0.687	0.689	0.691	0.692	0.694	0.696	0.698	0.699	0.701	0.704
Poland	0.612	0.615	0.619	0.621	0.623	0.623	0.626	0.631	0.633	0.633	0.636	0.635	0.63	0.63	0.633	0.639	0.645	0.649	0.654	0.659
Romania	0.532	0.542	0.55	0.549	0.548	0.555	0.559	0.564	0.569	0.573	0.58	0.589	0.601	0.604	0.604	0.604	0.606	0.611	0.62	0.636
Serbia	0.571	0.574	0.577	0.58	0.582	0.584	0.587	0.591	0.593	0.595	0.601	0.604	0.604	0.605	0.608	0.612	0.615	0.617	0.622	0.627
Slovakia	0.628	0.631	0.63	0.628	0.63	0.633	0.636	0.638	0.64	0.645	0.652	0.655	0.657	0.658	0.659	0.663	0.669	0.674	0.679	0.681
Slovenia	0.651	0.654	0.657	0.661	0.664	0.665	0.668	0.671	0.675	0.68	0.685	0.691	0.697	0.704	0.71	0.714	0.718	0.724	0.73	0.736
Eastern Europe	0.619	0.622	0.628	0.633	0.637	0.641	0.644	0.648	0.65	0.653	0.655	0.658	0.657	0.658	0.66	0.662	0.664	0.666	0.671	0.674
Belarus	0.559	0.561	0.565	0.569	0.573	0.576	0.58	0.583	0.585	0.588	0.59	0.592	0.593	0.595	0.597	0.601	0.605	0.609	0.615	0.62
Estonia	0.668	0.671	0.676	0.682	0.684	0.684	0.684	0.686	0.69	0.692	0.69	0.689	0.69	0.694	0.696	0.695	0.692	0.692	0.699	0.705
Latvia	0.651	0.655	0.659	0.664	0.668	0.669	0.672	0.675	0.678	0.68	0.681	0.681	0.68	0.681	0.683	0.683	0.683	0.685	0.689	0.693
Lithuania	0.66	0.664	0.67	0.677	0.681	0.684	0.686	0.689	0.692	0.695	0.701	0.705	0.707	0.708	0.709	0.711	0.712	0.712	0.71	0.709
Moldova	0.524	0.528	0.531	0.536	0.54	0.545	0.549	0.552	0.554	0.556	0.558	0.558	0.558	0.559	0.56	0.562	0.564	0.566	0.569	0.571
Russian Federation	0.628	0.632	0.639	0.644	0.648	0.651	0.655	0.658	0.66	0.663	0.666	0.668	0.666	0.666	0.668	0.669	0.671	0.673	0.677	0.679
Ukraine	0.593	0.596	0.599	0.604	0.609	0.613	0.617	0.62	0.624	0.627	0.63	0.632	0.633	0.636	0.639	0.643	0.646	0.65	0.655	0.659
High-income	0.666	0.673	0.681	0.688	0.694	0.7	0.705	0.71	0.715	0.718	0.723	0.728	0.734	0.74	0.744	0.749	0.754	0.758	0.762	0.765
Australasia	0.66	0.664	0.677	0.687	0.697	0.707	0.716	0.724	0.731	0.736	0.739	0.743	0.747	0.752	0.758	0.762	0.767	0.772	0.776	0.779
Australia	0.664	0.667	0.68	0.69	0.699	0.709	0.718	0.725	0.732	0.737	0.741	0.744	0.748	0.754	0.759	0.764	0.77	0.775	0.779	0.783
New Zealand	0.645	0.651	0.662	0.675	0.687	0.697	0.706	0.716	0.723	0.728	0.733	0.738	0.743	0.747	0.751	0.754	0.756	0.757	0.759	0.762
High-income Asia-Pacific	0.663	0.667	0.672	0.68	0.688	0.695	0.702	0.708	0.713	0.718	0.722	0.726	0.732	0.738	0.745	0.752	0.758	0.764	0.771	0.777
Brunei	0.57	0.584	0.601	0.619	0.639	0.654	0.657	0.654	0.653	0.656	0.66	0.668	0.677	0.685	0.691	0.697	0.704	0.712	0.718	0.724
Japan	0.696	0.697	0.701	0.707	0.714	0.722	0.73	0.737	0.743	0.749	0.754	0.759	0.762	0.766	0.771	0.777	0.781	0.787	0.792	0.798
Aichi	0.698	0.698	0.701	0.706	0.715	0.724	0.733	0.74	0.747	0.754	0.76	0.764	0.768	0.771	0.777	0.782	0.787	0.793	0.8	0.806
Akita	0.658	0.658	0.66	0.665	0.671	0.679	0.686	0.693	0.7	0.707	0.714	0.719	0.723	0.728	0.734	0.74	0.745	0.75	0.756	0.762
Aomori	0.65	0.65	0.652	0.657	0.664	0.673	0.68	0.688	0.695	0.702	0.708	0.713	0.716	0.721	0.727	0.733	0.738	0.744	0.75	0.756
Chiba	0.691	0.692	0.696	0.701	0.709	0.718	0.726	0.734	0.741	0.747	0.753	0.757	0.761	0.765	0.77	0.775	0.78	0.786	0.792	0.798
Ehime	0.667	0.668	0.671	0.677	0.685	0.693	0.701	0.708	0.714	0.721	0.727	0.731	0.735	0.739	0.744	0.749	0.754	0.759	0.765	0.771
Fukui	0.667	0.667	0.669	0.674	0.682	0.691	0.698	0.706	0.713	0.72	0.727	0.732	0.737	0.741	0.748	0.754	0.759	0.765	0.772	0.779
Fukuoka	0.692	0.695	0.699	0.705	0.713	0.72	0.727	0.734	0.739	0.745	0.75	0.754	0.757	0.761	0.766	0.771	0.775	0.781	0.787	0.792
Fukushima	0.663	0.663	0.665	0.671	0.678	0.686	0.694	0.701	0.707	0.713	0.719	0.723	0.727	0.73	0.736	0.741	0.746	0.752	0.759	0.765
Gifu	0.672	0.673	0.676	0.681	0.689	0.697	0.705	0.712	0.719	0.725	0.732	0.737	0.741	0.745	0.751	0.757	0.762	0.768	0.774	0.78
Gunma	0.683	0.685	0.689	0.695	0.702	0.71	0.717	0.724	0.729	0.735	0.74	0.745	0.748	0.751	0.756	0.761	0.766	0.771	0.776	0.782
Hiroshima	0.688	0.688	0.691	0.697	0.705	0.715	0.723	0.731	0.738	0.744	0.75	0.755	0.758	0.762	0.768	0.773	0.778	0.783	0.789	0.795
Hokkaidō	0.676	0.677	0.68	0.686	0.693	0.702	0.71	0.718	0.725	0.731	0.737	0.742	0.745	0.749	0.754	0.758	0.763	0.768	0.773	0.779
Hyōgo	0.689	0.691	0.694	0.7	0.708	0.717	0.725	0.732	0.739	0.745	0.75	0.755	0.758	0.762	0.767	0.772	0.776	0.782	0.787	0.793
Ibaraki	0.679	0.68	0.683	0.689	0.697	0.706	0.713	0.72	0.726	0.732	0.738	0.742	0.745	0.749	0.755	0.76	0.765	0.771	0.777	0.783
Ishikawa	0.669	0.668	0.67	0.675	0.682	0.691	0.7	0.707	0.715	0.722	0.729	0.734	0.739	0.744	0.751	0.757	0.763	0.77	0.777	0.783
Iwate	0.65	0.65	0.652	0.657	0.665	0.674	0.681	0.688	0.695	0.701	0.707	0.712	0.716	0.721	0.727	0.733	0.738	0.744	0.749	0.755
Kagawa	0.675	0.676	0.678	0.684	0.692	0.701	0.709	0.716	0.722	0.729	0.735	0.74	0.743	0.748	0.754	0.759	0.764	0.769	0.775	0.782
Kagoshima	0.66	0.662	0.665	0.671	0.679	0.686	0.693	0.699	0.705	0.711	0.717	0.721	0.725	0.73	0.735	0.741	0.746	0.751	0.757	0.763
Kanagawa	0.711	0.713	0.717	0.723	0.731	0.739	0.747	0.754	0.761	0.767	0.772	0.777	0.78	0.784	0.789	0.793	0.797	0.803	0.808	0.814
Kōchi	0.648	0.649	0.652	0.658	0.665	0.674	0.681	0.688	0.695	0.701	0.708	0.712	0.716	0.72	0.726	0.732	0.737	0.742	0.748	0.754
Kumamoto	0.662	0.663	0.667	0.673	0.68	0.688	0.695	0.702	0.708	0.714	0.719	0.724	0.727	0.731	0.737	0.742	0.747	0.753	0.759	0.765
Kyōto	0.71	0.714	0.718	0.724	0.731	0.738	0.745	0.751	0.757	0.763	0.768	0.772	0.775	0.779	0.783	0.788	0.792	0.797	0.802	0.808
Mie	0.673	0.673	0.676	0.681	0.689	0.698	0.706	0.713	0.72	0.726	0.732	0.737	0.741	0.744	0.75	0.756	0.761	0.767	0.774	0.781
Miyagi	0.68	0.681	0.684	0.69	0.697	0.704	0.711	0.718	0.724	0.73	0.736	0.741	0.744	0.749	0.754	0.76	0.765	0.771	0.777	0.783
Miyazaki	0.652	0.653	0.655	0.661	0.668	0.677	0.684	0.691	0.698	0.704	0.71	0.715	0.719	0.724	0.73	0.736	0.741	0.747	0.753	0.759
Nagano	0.69	0.693	0.698	0.704	0.711	0.718	0.724	0.731	0.736	0.742	0.748	0.752	0.756	0.76	0.764	0.768	0.772	0.777	0.782	0.788
Nagasaki	0.662	0.663	0.667	0.673	0.68	0.688	0.695	0.701	0.706	0.712	0.718	0.722	0.726	0.73	0.736	0.741	0.745	0.75	0.756	0.761
Nara	0.681	0.684	0.688	0.694	0.701	0.709	0.716	0.723	0.729	0.735	0.741	0.745	0.749	0.753	0.758	0.763	0.767	0.773	0.778	0.784
Niigata	0.669	0.67	0.672	0.678	0.685	0.693	0.7	0.707	0.714	0.72	0.726	0.731	0.735	0.739	0.744	0.75	0.754	0.76	0.765	0.771

Appendix Table 10. Socio-demographic Index values for all estimated GBD 2017 locations, 1970–1989

Location	1970	1971	1972	1973	1974	1975	1976	1977	1978	1979	1980	1981	1982	1983	1984	1985	1986	1987	1988	1989
Ōita	0.679	0.681	0.684	0.689	0.696	0.704	0.711	0.717	0.723	0.729	0.735	0.74	0.744	0.748	0.754	0.759	0.764	0.769	0.774	0.78
Okayama	0.677	0.677	0.68	0.685	0.694	0.703	0.711	0.719	0.726	0.732	0.737	0.742	0.745	0.749	0.755	0.761	0.766	0.772	0.778	0.784
Okinawa	0.643	0.643	0.644	0.649	0.657	0.665	0.673	0.68	0.686	0.692	0.698	0.703	0.707	0.711	0.717	0.723	0.729	0.735	0.742	0.748
Ōsaka	0.709	0.711	0.714	0.72	0.728	0.737	0.745	0.752	0.759	0.765	0.77	0.774	0.777	0.781	0.785	0.789	0.794	0.799	0.804	0.81
Saga	0.668	0.67	0.674	0.68	0.687	0.695	0.701	0.707	0.713	0.719	0.724	0.729	0.732	0.736	0.742	0.747	0.751	0.757	0.762	0.768
Saitama	0.683	0.685	0.688	0.694	0.702	0.711	0.719	0.726	0.733	0.74	0.746	0.75	0.754	0.757	0.763	0.767	0.772	0.777	0.783	0.788
Shiga	0.694	0.695	0.698	0.704	0.712	0.72	0.727	0.734	0.74	0.747	0.753	0.758	0.761	0.765	0.77	0.775	0.78	0.786	0.792	0.798
Shimane	0.658	0.659	0.662	0.668	0.676	0.684	0.69	0.697	0.702	0.708	0.713	0.718	0.722	0.725	0.731	0.736	0.74	0.746	0.751	0.757
Shizuoka	0.687	0.687	0.69	0.696	0.704	0.713	0.721	0.729	0.735	0.742	0.747	0.752	0.755	0.759	0.764	0.77	0.774	0.78	0.786	0.792
Tochigi	0.682	0.683	0.686	0.692	0.699	0.707	0.714	0.721	0.727	0.733	0.738	0.743	0.746	0.749	0.754	0.759	0.764	0.77	0.776	0.782
Tokushima	0.668	0.669	0.672	0.678	0.685	0.694	0.701	0.707	0.713	0.719	0.725	0.729	0.732	0.736	0.742	0.748	0.753	0.758	0.765	0.771
Tōkyō	0.774	0.777	0.782	0.788	0.795	0.803	0.809	0.815	0.821	0.826	0.83	0.834	0.837	0.84	0.844	0.848	0.852	0.856	0.861	0.866
Tottori	0.664	0.665	0.668	0.674	0.682	0.69	0.697	0.704	0.71	0.715	0.721	0.726	0.729	0.733	0.739	0.744	0.749	0.754	0.76	0.766
Toyama	0.674	0.673	0.675	0.68	0.687	0.696	0.704	0.712	0.719	0.726	0.733	0.738	0.743	0.747	0.753	0.759	0.765	0.771	0.778	0.785
Wakayama	0.665	0.667	0.67	0.676	0.685	0.694	0.702	0.709	0.714	0.718	0.721	0.723	0.726	0.73	0.738	0.744	0.75	0.757	0.763	0.77
Yamagata	0.66	0.661	0.663	0.669	0.675	0.683	0.689	0.696	0.702	0.709	0.715	0.72	0.725	0.729	0.734	0.74	0.744	0.75	0.756	0.761
Yamaguchi	0.684	0.685	0.688	0.694	0.702	0.71	0.717	0.724	0.73	0.736	0.742	0.746	0.75	0.753	0.759	0.764	0.768	0.774	0.78	0.786
Yamanashi	0.691	0.694	0.699	0.705	0.712	0.72	0.726	0.732	0.737	0.743	0.748	0.753	0.756	0.76	0.764	0.769	0.772	0.777	0.782	0.787
South Korea	0.494	0.508	0.523	0.542	0.557	0.568	0.578	0.587	0.595	0.601	0.605	0.611	0.623	0.636	0.647	0.659	0.671	0.682	0.692	0.702
Singapore	0.568	0.576	0.586	0.598	0.612	0.621	0.63	0.641	0.647	0.654	0.662	0.669	0.678	0.687	0.694	0.702	0.71	0.715	0.722	0.729
High-income North America	0.702	0.719	0.733	0.74	0.745	0.751	0.755	0.758	0.762	0.762	0.765	0.769	0.773	0.777	0.779	0.782	0.786	0.787	0.786	0.784
Canada	0.702	0.712	0.721	0.728	0.732	0.736	0.742	0.749	0.755	0.759	0.764	0.769	0.772	0.777	0.782	0.787	0.792	0.796	0.798	0.8
Greenland	0.57	0.592	0.609	0.622	0.631	0.636	0.642	0.648	0.653	0.657	0.662	0.666	0.671	0.674	0.675	0.676	0.676	0.675	0.673	0.673
USA	0.702	0.719	0.734	0.741	0.746	0.752	0.756	0.759	0.762	0.762	0.765	0.769	0.772	0.777	0.779	0.781	0.785	0.786	0.784	0.782
Alabama	0.643	0.659	0.675	0.684	0.69	0.699	0.705	0.71	0.716	0.718	0.723	0.729	0.733	0.739	0.742	0.745	0.749	0.75	0.748	0.745
Alaska	0.687	0.706	0.722	0.728	0.734	0.745	0.752	0.757	0.761	0.758	0.76	0.762	0.764	0.768	0.767	0.768	0.772	0.771	0.767	0.759
Arizona	0.679	0.694	0.708	0.715	0.723	0.734	0.741	0.746	0.75	0.748	0.75	0.751	0.752	0.755	0.754	0.755	0.759	0.759	0.757	0.753
Arkansas	0.631	0.646	0.659	0.667	0.673	0.683	0.689	0.695	0.701	0.702	0.706	0.711	0.716	0.721	0.723	0.726	0.729	0.729	0.727	0.723
California	0.733	0.752	0.765	0.769	0.77	0.773	0.775	0.777	0.779	0.777	0.779	0.781	0.782	0.786	0.786	0.786	0.788	0.786	0.782	0.775
Colorado	0.721	0.738	0.752	0.761	0.768	0.775	0.78	0.783	0.785	0.784	0.786	0.789	0.791	0.794	0.795	0.797	0.8	0.801	0.801	0.799
Connecticut	0.751	0.768	0.782	0.789	0.794	0.8	0.803	0.805	0.808	0.809	0.812	0.816	0.819	0.824	0.827	0.83	0.835	0.837	0.839	0.84
Delaware	0.722	0.738	0.752	0.76	0.765	0.772	0.775	0.778	0.78	0.779	0.782	0.785	0.787	0.791	0.793	0.795	0.799	0.8	0.8	0.8
Washington, DC	0.736	0.749	0.761	0.768	0.774	0.783	0.788	0.792	0.796	0.795	0.797	0.799	0.8	0.802	0.801	0.802	0.804	0.803	0.801	0.797
Florida	0.684	0.701	0.717	0.727	0.736	0.748	0.755	0.76	0.764	0.763	0.765	0.768	0.77	0.773	0.773	0.775	0.778	0.778	0.777	0.774
Georgia	0.651	0.668	0.685	0.696	0.704	0.714	0.72	0.726	0.73	0.731	0.734	0.738	0.742	0.747	0.75	0.753	0.757	0.758	0.757	0.755
Hawaii	0.717	0.731	0.744	0.75	0.756	0.764	0.768	0.772	0.775	0.774	0.776	0.778	0.779	0.782	0.782	0.783	0.787	0.787	0.786	0.782
Idaho	0.671	0.687	0.7	0.705	0.709	0.715	0.717	0.719	0.724	0.725	0.731	0.738	0.743	0.751	0.753	0.756	0.76	0.761	0.76	0.757
Illinois	0.711	0.728	0.742	0.75	0.755	0.761	0.764	0.767	0.77	0.769	0.772	0.776	0.779	0.784	0.787	0.789	0.793	0.793	0.791	0.788
Indiana	0.687	0.704	0.719	0.726	0.731	0.738	0.742	0.745	0.749	0.749	0.753	0.758	0.762	0.767	0.769	0.771	0.775	0.775	0.774	0.772
Iowa	0.706	0.724	0.738	0.746	0.75	0.755	0.756	0.758	0.762	0.763	0.767	0.772	0.777	0.783	0.785	0.789	0.793	0.794	0.794	0.793
Kansas	0.707	0.724	0.738	0.744	0.748	0.753	0.754	0.755	0.757	0.756	0.759	0.763	0.767	0.772	0.774	0.777	0.782	0.784	0.784	0.782
Kentucky	0.647	0.664	0.679	0.687	0.692	0.699	0.703	0.707	0.712	0.714	0.72	0.726	0.731	0.737	0.74	0.744	0.747	0.748	0.747	0.744
Louisiana	0.642	0.662	0.678	0.687	0.692	0.698	0.702	0.705	0.708	0.708	0.713	0.719	0.724	0.73	0.733	0.736	0.741	0.741	0.74	0.736
Maine	0.68	0.697	0.712	0.72	0.726	0.734	0.739	0.743	0.748	0.749	0.754	0.758	0.763	0.768	0.77	0.774	0.779	0.782	0.784	0.786
Maryland	0.727	0.746	0.762	0.771	0.778	0.785	0.788	0.791	0.794	0.794	0.796	0.798	0.8	0.804	0.806	0.808	0.812	0.813	0.813	0.812
Massachusetts	0.75	0.766	0.779	0.787	0.792	0.798	0.801	0.804	0.808	0.809	0.812	0.815	0.818	0.823	0.826	0.83	0.834	0.838	0.84	0.841
Michigan	0.701	0.719	0.735	0.743	0.749	0.756	0.76	0.763	0.766	0.766	0.77	0.773	0.777	0.782	0.784	0.787	0.791	0.792	0.79	0.788
Minnesota	0.728	0.744	0.756	0.761	0.765	0.769	0.77	0.772	0.776	0.777	0.781	0.786	0.791	0.797	0.801	0.806	0.811	0.814	0.816	0.816
Mississippi	0.607	0.625	0.642	0.653	0.661	0.672	0.679	0.685	0.691	0.692	0.698	0.704	0.709	0.716	0.718	0.721	0.725	0.725	0.723	0.719
Missouri	0.69	0.706	0.72	0.727	0.732	0.739	0.742	0.744	0.747	0.746	0.749	0.753	0.757	0.763	0.765	0.768	0.773	0.774	0.773	0.77
Montana	0.693	0.708	0.721	0.728	0.733	0.742	0.745	0.749	0.754	0.755	0.759	0.763	0.767	0.772	0.773	0.775	0.778	0.779	0.778	0.775
Nebraska	0.708	0.724	0.737	0.744	0.749	0.756	0.759	0.762	0.766	0.766	0.769	0.774	0.778	0.783	0.785	0.788	0.793	0.794	0.795	0.794
Nevada	0.708	0.726	0.742	0.75	0.756	0.765	0.769	0.772	0.774	0.772	0.773	0.774	0.775	0.778	0.777	0.776	0.778	0.775	0.771	0.764
New Hampshire	0.711	0.728	0.743	0.751	0.759	0.767	0.772	0.777	0.782	0.784	0.788	0.792	0.796	0.801	0.804	0.808	0.814	0.818	0.822	0.824
New Jersey	0.732	0.749	0.763	0.772	0.778	0.784	0.788	0.791	0.795	0.796	0.799	0.803	0.807	0.812	0.816	0.819	0.823	0.825	0.826	0.827
New Mexico	0.663	0.68	0.694	0.7	0.706	0.715	0.719	0.722	0.725	0.723	0.725	0.729	0.732	0.737	0.738	0.74	0.744	0.744	0.741	0.735
New York	0.735	0.753	0.767	0.773	0.776	0.781	0.784	0.787	0.792	0.793	0.796	0.8	0.802	0.806	0.808	0.811	0.814	0.815	0.816	0.815
North Carolina	0.662	0.678	0.693	0.703	0.711	0.72	0.727	0.733	0.739	0.741	0.745	0.749	0.752	0.757	0.759	0.761	0.764	0.765	0.764	0.762
North Dakota	0.687	0.703	0.717	0.728	0.735	0.743	0.745	0.748	0.753	0.753	0.756	0.761	0.765	0.771	0.774	0.778	0.783	0.787	0.788	0.788
Ohio	0.7	0.719	0.734	0.742	0.746	0.752	0.755	0.758	0.761	0.76	0.763	0.767	0.77	0.775	0.777	0.779	0.783	0.784	0.783	0.781
Oklahoma	0.672	0.688	0.703	0.71	0.716	0.723	0.726	0.729	0.732	0.73	0.734	0.738	0.742	0.748	0.75	0.752	0.757	0.758	0.755	0.751
Oregon	0.722	0.739	0.752	0.759	0.763	0.767	0.769	0.77	0.773	0.772	0.774	0.777	0.78	0.784	0.785	0.786	0.789	0.789	0.788	0.786
Pennsylvania	0.712	0.728	0.743	0.751	0.756	0.763	0.766	0.77	0.773	0.774	0.777	0.781	0.784	0.789	0.791	0.793	0.797	0.799	0.799	0.799
Rhode Island	0.719	0.735	0.749	0.758	0.764	0.771	0.775	0.779	0.782	0.783	0.786	0.79	0.793	0.797	0.799	0.802	0.807	0.809	0.811	0.812
South Carolina	0.644	0.661	0.676	0.686	0.695	0.706	0.714	0.72	0.726	0.728	0.732	0.737	0.741	0.746	0.748	0.75	0.753	0.754	0.753	0.751
South Dakota	0.684	0.698	0.711	0.719	0.724	0.731	0.733	0.735	0.739	0.74	0.743	0.748	0.752	0.757	0.76	0.763	0.767	0.769	0.769	0.768
Tennessee	0.659	0.676	0.69	0.699	0.706	0.714	0.719	0.723	0.728	0.73	0.734	0.738	0.742	0.747	0.749	0.751	0.754	0.755	0.753	0.75
Texas	0.663	0.683	0.7	0.709	0.716	0.724	0.729	0.731	0.733	0.73	0.732	0.735	0.738	0.743	0.744	0.747	0.751	0.752	0.75	0.745
Utah	0.693	0.707	0.717	0.719	0.717	0.719	0.716	0.714	0.716	0.714	0.72	0.727	0.734	0.743	0.746	0.751	0.758	0.761	0.761	0.76
Vermont	0.709	0.725	0.74	0.748	0.755	0.763	0.768	0.772	0.777	0.778	0.781	0.785	0.788	0.793	0.795	0.798	0.804	0.807	0.81	0.812
Virginia	0.7	0.717	0.731	0.739	0.745	0.754	0.759	0.765	0.77	0.772	0.777	0.781	0.785	0.789	0.791	0.794	0.798	0.8	0.8	0.799

Appendix Table 10. Socio-demographic Index values for all estimated GBD 2017 locations, 1970–1989

Location	1970	1971	1972	1973	1974	1975	1976	1977	1978	1979	1980	1981	1982	1983	1984	1985	1986	1987	1988	1989
Washington	0.732	0.752	0.767	0.773	0.776	0.78	0.781	0.782	0.784	0.782	0.785	0.788	0.79	0.794	0.795	0.797	0.8	0.801	0.8	0.797
West Virginia	0.654	0.669	0.684	0.692	0.697	0.704	0.708	0.712	0.717	0.719	0.724	0.73	0.736	0.742	0.744	0.747	0.751	0.752	0.751	0.748
Wisconsin	0.715	0.732	0.745	0.752	0.755	0.761	0.764	0.767	0.77	0.77	0.773	0.777	0.781	0.785	0.788	0.791	0.796	0.799	0.8	0.8
Wyoming	0.685	0.703	0.717	0.723	0.728	0.736	0.738	0.739	0.741	0.739	0.742	0.746	0.75	0.755	0.755	0.758	0.765	0.767	0.767	0.765
Southern Latin America	0.522	0.527	0.529	0.531	0.534	0.537	0.54	0.542	0.545	0.549	0.554	0.56	0.566	0.572	0.576	0.577	0.579	0.583	0.586	0.591
Argentina	0.531	0.536	0.538	0.539	0.538	0.537	0.537	0.537	0.538	0.543	0.55	0.557	0.563	0.569	0.572	0.572	0.575	0.579	0.584	0.589
Chile	0.501	0.506	0.509	0.517	0.527	0.536	0.544	0.55	0.554	0.557	0.558	0.562	0.569	0.575	0.579	0.583	0.586	0.587	0.589	0.593
Uruguay	0.516	0.517	0.519	0.522	0.526	0.53	0.533	0.537	0.543	0.55	0.557	0.562	0.567	0.572	0.577	0.579	0.581	0.582	0.583	0.587
Western Europe	0.649	0.653	0.66	0.667	0.674	0.68	0.685	0.691	0.697	0.701	0.707	0.714	0.72	0.727	0.732	0.737	0.743	0.748	0.753	0.758
Andorra	0.803	0.805	0.807	0.81	0.812	0.815	0.818	0.82	0.822	0.824	0.826	0.829	0.832	0.835	0.838	0.841	0.843	0.844	0.846	0.847
Austria	0.674	0.68	0.688	0.697	0.704	0.712	0.72	0.726	0.73	0.732	0.733	0.736	0.741	0.748	0.754	0.759	0.763	0.767	0.771	0.774
Belgium	0.693	0.7	0.708	0.717	0.725	0.731	0.737	0.741	0.746	0.75	0.755	0.761	0.767	0.773	0.779	0.784	0.789	0.792	0.796	0.8
Cyprus	0.604	0.609	0.619	0.632	0.64	0.643	0.645	0.646	0.646	0.648	0.652	0.658	0.664	0.67	0.677	0.685	0.694	0.702	0.71	0.718
Denmark	0.756	0.758	0.764	0.769	0.771	0.777	0.785	0.791	0.796	0.802	0.809	0.815	0.821	0.825	0.828	0.832	0.836	0.839	0.841	0.843
Finland	0.715	0.723	0.731	0.736	0.739	0.743	0.748	0.754	0.759	0.765	0.769	0.772	0.776	0.782	0.789	0.795	0.799	0.803	0.807	0.811
France	0.62	0.626	0.63	0.64	0.657	0.667	0.673	0.679	0.686	0.692	0.698	0.705	0.713	0.721	0.728	0.735	0.742	0.75	0.756	0.763
Germany	0.693	0.699	0.71	0.717	0.722	0.727	0.73	0.735	0.741	0.744	0.747	0.752	0.757	0.763	0.768	0.768	0.771	0.777	0.78	0.781
Greece	0.596	0.602	0.607	0.61	0.611	0.614	0.617	0.62	0.623	0.627	0.634	0.643	0.651	0.66	0.67	0.68	0.689	0.696	0.703	0.711
Iceland	0.682	0.684	0.69	0.7	0.711	0.72	0.729	0.736	0.741	0.747	0.754	0.762	0.771	0.78	0.788	0.794	0.798	0.802	0.805	0.809
Ireland	0.641	0.642	0.645	0.65	0.656	0.663	0.669	0.675	0.68	0.684	0.69	0.698	0.706	0.713	0.721	0.727	0.733	0.74	0.746	0.751
Israel	0.626	0.631	0.637	0.64	0.641	0.642	0.648	0.657	0.664	0.67	0.676	0.679	0.682	0.689	0.696	0.705	0.713	0.719	0.724	0.729
Italy	0.636	0.639	0.643	0.647	0.653	0.662	0.666	0.675	0.688	0.694	0.701	0.71	0.717	0.724	0.731	0.738	0.744	0.75	0.756	0.762
Luxembourg	0.744	0.753	0.76	0.768	0.774	0.779	0.783	0.787	0.791	0.794	0.798	0.802	0.808	0.813	0.818	0.823	0.828	0.831	0.835	0.84
Malta	0.623	0.627	0.632	0.635	0.639	0.646	0.656	0.665	0.673	0.68	0.687	0.694	0.7	0.706	0.71	0.714	0.717	0.72	0.723	0.726
Netherlands	0.718	0.727	0.736	0.746	0.754	0.761	0.767	0.773	0.777	0.781	0.786	0.791	0.796	0.799	0.803	0.807	0.811	0.815	0.819	0.823
Norway	0.689	0.694	0.703	0.712	0.722	0.732	0.743	0.751	0.757	0.762	0.768	0.774	0.781	0.786	0.791	0.796	0.799	0.802	0.804	0.807
Portugal	0.512	0.517	0.526	0.535	0.535	0.527	0.526	0.536	0.547	0.556	0.564	0.571	0.577	0.584	0.592	0.601	0.609	0.616	0.624	0.633
Spain	0.581	0.582	0.586	0.591	0.596	0.599	0.601	0.608	0.617	0.625	0.634	0.644	0.653	0.661	0.668	0.676	0.683	0.691	0.698	0.707
Sweden	0.703	0.705	0.71	0.715	0.721	0.73	0.738	0.744	0.749	0.753	0.757	0.763	0.768	0.773	0.776	0.779	0.78	0.781	0.781	0.781
Stockholm	0.75	0.752	0.757	0.763	0.769	0.777	0.784	0.789	0.794	0.797	0.802	0.807	0.811	0.815	0.817	0.819	0.82	0.821	0.822	0.823
Sweden except Stockholm	0.691	0.694	0.698	0.703	0.709	0.718	0.726	0.733	0.738	0.742	0.746	0.751	0.757	0.762	0.765	0.768	0.77	0.771	0.77	0.771
Switzerland	0.785	0.79	0.796	0.801	0.806	0.812	0.817	0.821	0.821	0.821	0.822	0.825	0.827	0.829	0.832	0.835	0.837	0.839	0.84	0.841
United Kingdom	0.605	0.612	0.62	0.629	0.639	0.647	0.654	0.659	0.661	0.664	0.669	0.678	0.684	0.689	0.693	0.698	0.702	0.706	0.711	0.717
England	0.618	0.625	0.633	0.642	0.652	0.66	0.667	0.672	0.673	0.676	0.682	0.689	0.696	0.701	0.705	0.709	0.713	0.716	0.722	0.727
East Midlands	0.59	0.598	0.606	0.615	0.625	0.634	0.641	0.646	0.648	0.651	0.657	0.665	0.672	0.677	0.681	0.685	0.689	0.693	0.699	0.705
Derby	0.591	0.599	0.608	0.617	0.628	0.636	0.644	0.649	0.65	0.652	0.658	0.667	0.674	0.68	0.684	0.689	0.694	0.698	0.704	0.71
Derbyshire	0.581	0.588	0.597	0.606	0.616	0.625	0.632	0.637	0.639	0.642	0.648	0.656	0.663	0.668	0.672	0.676	0.679	0.683	0.688	0.693
Leicester	0.577	0.585	0.593	0.603	0.613	0.621	0.629	0.634	0.635	0.638	0.643	0.652	0.659	0.665	0.67	0.676	0.682	0.687	0.694	0.702
Leicestershire	0.609	0.616	0.624	0.634	0.643	0.651	0.658	0.663	0.666	0.669	0.675	0.683	0.689	0.694	0.698	0.702	0.706	0.709	0.715	0.721
Lincolnshire	0.584	0.591	0.6	0.609	0.619	0.628	0.635	0.64	0.643	0.646	0.652	0.66	0.666	0.67	0.674	0.678	0.681	0.684	0.689	0.695
Northamptonshire	0.593	0.6	0.609	0.619	0.629	0.638	0.646	0.651	0.653	0.656	0.662	0.671	0.677	0.682	0.686	0.69	0.693	0.697	0.702	0.708
Nottingham	0.607	0.614	0.622	0.632	0.641	0.649	0.657	0.662	0.664	0.667	0.673	0.681	0.688	0.694	0.7	0.705	0.71	0.715	0.721	0.728
Nottinghamshire	0.579	0.587	0.595	0.604	0.614	0.622	0.629	0.634	0.637	0.64	0.645	0.653	0.66	0.664	0.668	0.672	0.676	0.68	0.685	0.691
Rutland	0.609	0.617	0.625	0.634	0.644	0.652	0.659	0.663	0.665	0.667	0.673	0.681	0.687	0.692	0.697	0.702	0.708	0.712	0.719	0.725
East of England	0.606	0.614	0.622	0.632	0.642	0.65	0.658	0.663	0.665	0.667	0.673	0.681	0.688	0.693	0.697	0.701	0.705	0.708	0.714	0.719
Bedford	0.608	0.615	0.624	0.633	0.643	0.652	0.659	0.664	0.666	0.668	0.674	0.682	0.689	0.695	0.699	0.704	0.708	0.712	0.718	0.724
Cambridgeshire	0.631	0.638	0.646	0.655	0.665	0.673	0.68	0.685	0.688	0.691	0.697	0.704	0.711	0.715	0.72	0.724	0.728	0.732	0.738	0.744
Central Bedfordshire	0.602	0.609	0.618	0.628	0.638	0.647	0.655	0.66	0.661	0.663	0.669	0.678	0.684	0.689	0.694	0.698	0.702	0.707	0.712	0.718
Essex	0.597	0.605	0.613	0.623	0.632	0.641	0.648	0.653	0.656	0.658	0.664	0.672	0.678	0.683	0.687	0.691	0.694	0.698	0.703	0.708
Hertfordshire	0.631	0.638	0.646	0.655	0.665	0.673	0.681	0.686	0.688	0.691	0.697	0.705	0.711	0.716	0.72	0.725	0.729	0.732	0.738	0.743
Luton	0.586	0.594	0.603	0.613	0.624	0.633	0.641	0.646	0.648	0.65	0.655	0.664	0.671	0.677	0.681	0.685	0.689	0.693	0.699	0.705
Norfolk	0.596	0.603	0.612	0.621	0.631	0.64	0.647	0.652	0.654	0.657	0.663	0.671	0.677	0.682	0.686	0.69	0.694	0.697	0.702	0.707
Peterborough	0.592	0.6	0.609	0.619	0.63	0.64	0.648	0.653	0.654	0.656	0.662	0.671	0.678	0.684	0.688	0.693	0.697	0.7	0.705	0.71
Southend-on-Sea	0.575	0.583	0.591	0.601	0.611	0.619	0.627	0.632	0.634	0.637	0.643	0.652	0.658	0.663	0.667	0.671	0.676	0.68	0.686	0.691
Suffolk	0.593	0.601	0.61	0.62	0.63	0.639	0.647	0.651	0.653	0.655	0.661	0.669	0.676	0.681	0.685	0.69	0.694	0.697	0.703	0.708
Thurrock	0.595	0.603	0.611	0.621	0.631	0.64	0.647	0.652	0.654	0.656	0.662	0.67	0.676	0.68	0.684	0.688	0.691	0.695	0.7	0.705
Greater London	0.665	0.672	0.68	0.688	0.697	0.705	0.712	0.717	0.719	0.721	0.727	0.735	0.741	0.746	0.75	0.755	0.759	0.763	0.768	0.774
Barking and Dagenham	0.592	0.6	0.609	0.618	0.628	0.637	0.644	0.649	0.651	0.654	0.66	0.668	0.675	0.679	0.683	0.686	0.689	0.692	0.696	0.7
Barnet	0.643	0.651	0.658	0.667	0.676	0.684	0.691	0.696	0.698	0.701	0.707	0.714	0.721	0.726	0.73	0.735	0.739	0.743	0.748	0.754
Bexley	0.611	0.618	0.626	0.635	0.645	0.653	0.66	0.665	0.667	0.67	0.676	0.683	0.689	0.694	0.697	0.7	0.704	0.706	0.711	0.716
Brent	0.631	0.639	0.647	0.656	0.666	0.675	0.682	0.687	0.689	0.691	0.697	0.705	0.712	0.717	0.722	0.727	0.732	0.736	0.742	0.748
Bromley	0.641	0.648	0.656	0.665	0.674	0.682	0.688	0.693	0.696	0.699	0.705	0.712	0.718	0.723	0.726	0.73	0.733	0.736	0.741	0.747
Camden	0.744	0.75	0.755	0.762	0.769	0.776	0.781	0.785	0.787	0.79	0.794	0.8	0.806	0.81	0.814	0.818	0.822	0.825	0.829	0.834
Croydon	0.625	0.632	0.641	0.65	0.661	0.669	0.677	0.682	0.684	0.686	0.692	0.701	0.707	0.712	0.716	0.72	0.724	0.727	0.731	0.736
Ealing	0.641	0.648	0.657	0.666	0.676	0.684	0.691	0.696	0.698	0.7	0.706	0.714	0.721	0.727	0.732	0.738	0.743	0.748	0.754	0.759
Enfield	0.621	0.629	0.637	0.647	0.657	0.665	0.672	0.677	0.68	0.683	0.689	0.697	0.703	0.707	0.711	0.715	0.719	0.722	0.727	0.732
Greenwich	0.608	0.616	0.624	0.634	0.644	0.653	0.66	0.665	0.667	0.669	0.675	0.684	0.69	0.695	0.699	0.702	0.706	0.709	0.714	0.72
Hackney	0.654	0.662	0.671	0.682	0.693	0.703	0.711	0.716	0.716	0.716	0.722	0.731	0.737	0.741	0.745	0.747	0.75	0.752	0.756	0.762
Hammersmith and Fulham	0.702	0.709	0.716	0.724	0.732	0.739	0.745	0.75	0.753	0.756	0.762	0.769	0.776	0.781	0.787	0.793	0.799	0.805	0.811	0.818
Haringey	0.633	0.641	0.649	0.659	0.669	0.677	0.684	0.689	0.692	0.695	0.701	0.709	0.716	0.721	0.725	0.73	0.734	0.738	0.744	0.749
Harrow	0.632	0.639	0.647	0.656	0.666	0.674	0.681	0.686	0.688	0.691	0.697	0.704	0.71	0.715	0.719	0.723	0.727	0.731	0.736	0.742

Appendix Table 10. Socio-demographic Index values for all estimated GBD 2017 locations, 1970–1989

Location	1970	1971	1972	1973	1974	1975	1976	1977	1978	1979	1980	1981	1982	1983	1984	1985	1986	1987	1988	1989
Havering	0.613	0.621	0.628	0.637	0.646	0.654	0.661	0.666	0.668	0.671	0.677	0.684	0.69	0.694	0.697	0.7	0.703	0.705	0.71	0.715
Hillingdon	0.665	0.672	0.681	0.69	0.699	0.708	0.715	0.72	0.722	0.725	0.731	0.739	0.746	0.751	0.755	0.759	0.763	0.766	0.771	0.776
Hounslow	0.648	0.656	0.664	0.674	0.684	0.693	0.7	0.705	0.707	0.71	0.716	0.724	0.731	0.736	0.74	0.744	0.748	0.752	0.757	0.764
Islington	0.702	0.709	0.717	0.726	0.736	0.744	0.751	0.755	0.756	0.757	0.762	0.769	0.775	0.779	0.784	0.789	0.793	0.796	0.801	0.806
Kensington and Chelsea	0.73	0.736	0.742	0.75	0.757	0.764	0.77	0.775	0.778	0.782	0.787	0.793	0.799	0.804	0.808	0.813	0.817	0.821	0.826	0.833
Kingston upon Thames	0.674	0.681	0.688	0.695	0.703	0.711	0.717	0.721	0.724	0.727	0.733	0.739	0.745	0.75	0.754	0.759	0.764	0.768	0.775	0.781
Lambeth	0.647	0.655	0.663	0.672	0.682	0.69	0.698	0.702	0.705	0.707	0.713	0.721	0.728	0.734	0.739	0.745	0.751	0.756	0.762	0.769
Lewisham	0.612	0.619	0.627	0.636	0.645	0.653	0.66	0.665	0.667	0.67	0.675	0.683	0.69	0.696	0.701	0.707	0.712	0.716	0.722	0.728
Merton	0.639	0.646	0.654	0.663	0.673	0.681	0.688	0.693	0.695	0.698	0.704	0.711	0.718	0.722	0.726	0.731	0.736	0.74	0.746	0.752
Newham	0.592	0.601	0.61	0.62	0.631	0.64	0.648	0.653	0.655	0.657	0.663	0.673	0.68	0.685	0.69	0.694	0.698	0.702	0.707	0.712
Redbridge	0.615	0.623	0.63	0.639	0.649	0.657	0.664	0.669	0.671	0.674	0.68	0.688	0.694	0.698	0.702	0.706	0.71	0.713	0.718	0.723
Richmond upon Thames	0.683	0.69	0.697	0.705	0.713	0.72	0.727	0.731	0.734	0.737	0.743	0.749	0.755	0.76	0.764	0.768	0.773	0.777	0.782	0.789
Southwark	0.664	0.672	0.68	0.69	0.7	0.708	0.715	0.72	0.722	0.725	0.731	0.74	0.746	0.753	0.758	0.763	0.769	0.774	0.78	0.787
Sutton	0.621	0.629	0.637	0.646	0.656	0.664	0.671	0.676	0.679	0.682	0.688	0.695	0.702	0.706	0.71	0.713	0.716	0.72	0.725	0.73
Tower Hamlets	0.645	0.653	0.662	0.672	0.683	0.692	0.7	0.704	0.703	0.703	0.708	0.717	0.724	0.729	0.734	0.739	0.745	0.749	0.755	0.761
Waltham Forest	0.592	0.6	0.608	0.618	0.628	0.636	0.643	0.648	0.65	0.653	0.659	0.667	0.673	0.679	0.683	0.688	0.692	0.696	0.702	0.707
Wandsworth	0.667	0.674	0.681	0.689	0.698	0.705	0.712	0.716	0.718	0.721	0.727	0.734	0.741	0.747	0.753	0.76	0.766	0.772	0.78	0.787
Westminster	0.739	0.745	0.751	0.758	0.766	0.772	0.778	0.782	0.784	0.787	0.791	0.797	0.802	0.806	0.808	0.811	0.814	0.817	0.821	0.826
North East England	0.573	0.581	0.59	0.6	0.61	0.619	0.627	0.632	0.634	0.637	0.643	0.651	0.658	0.663	0.667	0.671	0.676	0.679	0.685	0.691
County Durham	0.568	0.576	0.585	0.594	0.604	0.613	0.62	0.626	0.628	0.631	0.637	0.645	0.652	0.658	0.662	0.667	0.671	0.674	0.679	0.685
Darlington	0.583	0.591	0.6	0.611	0.622	0.631	0.639	0.644	0.646	0.649	0.655	0.664	0.671	0.676	0.68	0.684	0.687	0.689	0.693	0.698
Gateshead	0.577	0.585	0.594	0.604	0.614	0.623	0.631	0.636	0.638	0.64	0.646	0.655	0.661	0.666	0.67	0.674	0.679	0.682	0.688	0.694
Hartlepool	0.556	0.564	0.573	0.584	0.595	0.604	0.612	0.617	0.619	0.621	0.627	0.636	0.643	0.648	0.651	0.655	0.658	0.661	0.666	0.672
Middlesbrough	0.56	0.569	0.578	0.588	0.599	0.609	0.617	0.622	0.623	0.625	0.631	0.64	0.647	0.652	0.656	0.66	0.665	0.669	0.675	0.682
Newcastle upon Tyne	0.609	0.617	0.625	0.634	0.643	0.651	0.658	0.663	0.666	0.669	0.675	0.683	0.689	0.695	0.699	0.704	0.709	0.715	0.721	0.729
North Tyneside	0.576	0.584	0.592	0.602	0.612	0.621	0.629	0.634	0.636	0.639	0.645	0.653	0.659	0.664	0.668	0.671	0.675	0.679	0.685	0.691
Northumberland	0.574	0.582	0.591	0.601	0.611	0.62	0.628	0.633	0.635	0.638	0.644	0.653	0.66	0.665	0.67	0.674	0.678	0.681	0.687	0.692
Redcar and Cleveland	0.549	0.557	0.566	0.576	0.587	0.596	0.604	0.608	0.61	0.612	0.618	0.627	0.634	0.639	0.643	0.646	0.65	0.653	0.659	0.665
South Tyneside	0.545	0.553	0.562	0.572	0.582	0.591	0.599	0.604	0.606	0.609	0.615	0.623	0.629	0.633	0.636	0.639	0.643	0.646	0.652	0.659
Stockton-on-Tees	0.581	0.589	0.598	0.609	0.62	0.629	0.637	0.642	0.644	0.646	0.652	0.661	0.667	0.672	0.676	0.68	0.684	0.687	0.693	0.699
Sunderland	0.563	0.571	0.58	0.59	0.601	0.61	0.618	0.623	0.624	0.626	0.632	0.641	0.648	0.654	0.658	0.663	0.667	0.671	0.677	0.684
North West England	0.6	0.608	0.616	0.625	0.635	0.643	0.65	0.655	0.657	0.659	0.665	0.673	0.679	0.684	0.688	0.692	0.696	0.7	0.705	0.711
Blackburn with Darwen	0.569	0.577	0.586	0.597	0.608	0.617	0.625	0.63	0.631	0.633	0.639	0.648	0.655	0.661	0.665	0.669	0.673	0.676	0.681	0.687
Blackpool	0.569	0.576	0.585	0.594	0.604	0.612	0.62	0.625	0.628	0.631	0.637	0.645	0.651	0.655	0.658	0.661	0.663	0.666	0.671	0.676
Bolton	0.583	0.591	0.599	0.609	0.619	0.628	0.635	0.64	0.641	0.644	0.649	0.658	0.664	0.669	0.673	0.677	0.681	0.685	0.69	0.696
Bury	0.589	0.597	0.605	0.615	0.624	0.632	0.64	0.644	0.646	0.649	0.655	0.663	0.669	0.673	0.677	0.68	0.684	0.687	0.692	0.698
Cheshire East	0.635	0.642	0.65	0.659	0.669	0.677	0.684	0.688	0.691	0.693	0.699	0.707	0.712	0.717	0.721	0.725	0.728	0.732	0.737	0.743
Cheshire West and Chester	0.626	0.633	0.641	0.65	0.66	0.668	0.675	0.679	0.681	0.684	0.69	0.697	0.703	0.708	0.712	0.716	0.72	0.723	0.729	0.735
Cumbria	0.606	0.614	0.622	0.631	0.641	0.649	0.656	0.661	0.663	0.665	0.671	0.679	0.685	0.69	0.694	0.698	0.702	0.705	0.71	0.715
Halton	0.586	0.594	0.603	0.613	0.624	0.632	0.64	0.645	0.647	0.649	0.655	0.663	0.669	0.674	0.677	0.68	0.683	0.686	0.692	0.698
Knowsley	0.575	0.582	0.591	0.6	0.61	0.618	0.626	0.63	0.632	0.634	0.639	0.648	0.654	0.659	0.664	0.668	0.672	0.675	0.68	0.686
Lancashire	0.603	0.611	0.619	0.629	0.638	0.647	0.654	0.659	0.661	0.663	0.669	0.677	0.683	0.688	0.692	0.696	0.7	0.703	0.708	0.715
Liverpool	0.602	0.609	0.617	0.626	0.635	0.642	0.649	0.654	0.656	0.659	0.664	0.672	0.678	0.683	0.687	0.692	0.697	0.702	0.708	0.715
Manchester	0.623	0.63	0.638	0.647	0.657	0.665	0.672	0.677	0.679	0.682	0.688	0.696	0.703	0.709	0.713	0.718	0.723	0.728	0.734	0.74
Oldham	0.569	0.577	0.586	0.596	0.606	0.615	0.622	0.627	0.628	0.631	0.636	0.645	0.652	0.657	0.66	0.664	0.668	0.671	0.676	0.681
Rochdale	0.571	0.579	0.588	0.598	0.608	0.617	0.624	0.629	0.631	0.633	0.639	0.648	0.654	0.659	0.663	0.666	0.67	0.672	0.677	0.682
Salford	0.592	0.599	0.608	0.617	0.627	0.636	0.643	0.648	0.65	0.652	0.658	0.666	0.672	0.677	0.681	0.685	0.689	0.692	0.697	0.703
Sefton	0.593	0.601	0.608	0.617	0.627	0.634	0.641	0.646	0.648	0.651	0.656	0.664	0.67	0.674	0.678	0.682	0.686	0.689	0.694	0.701
St Helens	0.571	0.579	0.587	0.596	0.605	0.613	0.62	0.625	0.627	0.629	0.635	0.643	0.65	0.655	0.659	0.663	0.667	0.67	0.675	0.68
Stockport	0.613	0.62	0.628	0.637	0.646	0.654	0.661	0.666	0.668	0.67	0.676	0.683	0.69	0.694	0.698	0.702	0.706	0.71	0.715	0.722
Tameside	0.577	0.584	0.593	0.602	0.612	0.62	0.628	0.633	0.634	0.637	0.643	0.651	0.657	0.662	0.666	0.669	0.673	0.676	0.681	0.687
Trafford	0.635	0.642	0.65	0.659	0.669	0.677	0.684	0.689	0.691	0.693	0.699	0.706	0.712	0.717	0.721	0.725	0.73	0.734	0.739	0.745
Warrington	0.623	0.63	0.638	0.648	0.658	0.666	0.673	0.678	0.68	0.682	0.688	0.696	0.702	0.707	0.711	0.715	0.719	0.722	0.728	0.734
Wigan	0.575	0.583	0.591	0.601	0.61	0.619	0.626	0.631	0.632	0.635	0.64	0.648	0.655	0.66	0.664	0.668	0.672	0.676	0.681	0.687
Wirral	0.581	0.589	0.597	0.607	0.616	0.625	0.632	0.637	0.638	0.641	0.647	0.655	0.661	0.666	0.669	0.673	0.676	0.679	0.685	0.69
South East England	0.639	0.646	0.654	0.663	0.672	0.68	0.687	0.692	0.694	0.696	0.701	0.709	0.715	0.72	0.724	0.727	0.731	0.735	0.739	0.745
Bracknell Forest	0.645	0.653	0.66	0.669	0.679	0.687	0.694	0.698	0.699	0.701	0.707	0.715	0.721	0.727	0.732	0.736	0.74	0.744	0.749	0.755
Brighton and Hove	0.654	0.66	0.667	0.674	0.682	0.689	0.695	0.699	0.702	0.705	0.71	0.716	0.722	0.727	0.732	0.736	0.742	0.747	0.754	0.76
Buckinghamshire	0.655	0.662	0.67	0.679	0.688	0.696	0.702	0.707	0.708	0.711	0.716	0.724	0.73	0.735	0.739	0.743	0.747	0.75	0.754	0.759
East Sussex	0.604	0.611	0.62	0.629	0.639	0.647	0.654	0.659	0.661	0.663	0.669	0.676	0.682	0.686	0.69	0.693	0.696	0.699	0.704	0.708
Hampshire	0.634	0.641	0.649	0.659	0.668	0.676	0.683	0.688	0.689	0.691	0.697	0.704	0.71	0.715	0.719	0.723	0.726	0.73	0.735	0.74
Isle of Wight	0.596	0.604	0.612	0.622	0.632	0.64	0.647	0.652	0.653	0.655	0.66	0.668	0.674	0.678	0.681	0.684	0.688	0.691	0.695	0.7
Kent	0.614	0.622	0.63	0.639	0.649	0.658	0.665	0.67	0.671	0.674	0.68	0.687	0.693	0.698	0.701	0.704	0.707	0.71	0.714	0.719
Medway	0.59	0.598	0.606	0.616	0.626	0.635	0.642	0.646	0.648	0.65	0.655	0.663	0.669	0.673	0.677	0.68	0.684	0.687	0.692	0.698
Milton Keynes	0.632	0.64	0.65	0.66	0.672	0.681	0.689	0.694	0.695	0.697	0.702	0.711	0.717	0.722	0.726	0.73	0.734	0.738	0.744	0.749
Oxfordshire	0.66	0.667	0.674	0.683	0.691	0.699	0.705	0.71	0.712	0.714	0.72	0.727	0.733	0.739	0.743	0.747	0.751	0.755	0.76	0.765
Portsmouth	0.632	0.639	0.648	0.657	0.667	0.675	0.682	0.687	0.69	0.692	0.698	0.706	0.711	0.716	0.719	0.723	0.728	0.732	0.738	0.744
Reading	0.666	0.674	0.681	0.69	0.699	0.707	0.714	0.719	0.721	0.723	0.729	0.736	0.742	0.747	0.751	0.756	0.761	0.766	0.773	0.779
Slough	0.644	0.652	0.66	0.67	0.681	0.69	0.698	0.702	0.703	0.704	0.71	0.719	0.726	0.731	0.735	0.739	0.744	0.748	0.754	0.76
Southampton	0.64	0.647	0.655	0.664	0.673	0.68	0.687	0.692	0.694	0.696	0.702	0.709	0.715	0.72	0.724	0.728	0.732	0.736	0.741	0.747
Surrey	0.667	0.673	0.68	0.689	0.697	0.705	0.711	0.716	0.718	0.721	0.726	0.733	0.739	0.743	0.747	0.751	0.755	0.758	0.763	0.769

Appendix Table 10. Socio-demographic Index values for all estimated GBD 2017 locations, 1970–1989

Location	1970	1971	1972	1973	1974	1975	1976	1977	1978	1979	1980	1981	1982	1983	1984	1985	1986	1987	1988	1989
West Berkshire	0.659	0.667	0.675	0.685	0.695	0.703	0.711	0.715	0.717	0.719	0.724	0.733	0.739	0.743	0.747	0.751	0.755	0.758	0.764	0.769
West Sussex	0.629	0.636	0.644	0.654	0.663	0.671	0.678	0.683	0.684	0.687	0.692	0.7	0.706	0.711	0.715	0.719	0.722	0.725	0.73	0.735
Windsor and Maidenhead	0.67	0.677	0.685	0.693	0.702	0.71	0.717	0.721	0.723	0.725	0.731	0.738	0.744	0.748	0.752	0.756	0.761	0.764	0.769	0.774
Wokingham	0.67	0.677	0.684	0.693	0.701	0.709	0.715	0.72	0.722	0.724	0.729	0.736	0.741	0.745	0.749	0.753	0.757	0.761	0.767	0.773
South West England	0.614	0.622	0.63	0.639	0.648	0.656	0.664	0.668	0.67	0.673	0.679	0.687	0.693	0.698	0.702	0.705	0.709	0.713	0.718	0.724
Bath and North East Somerset	0.64	0.647	0.654	0.662	0.67	0.676	0.683	0.688	0.691	0.694	0.699	0.706	0.712	0.717	0.721	0.725	0.73	0.734	0.74	0.746
Bournemouth	0.62	0.627	0.634	0.642	0.65	0.657	0.664	0.669	0.672	0.675	0.681	0.688	0.694	0.698	0.703	0.708	0.713	0.718	0.724	0.73
Bristol, City of	0.644	0.651	0.659	0.667	0.676	0.684	0.691	0.696	0.698	0.701	0.706	0.714	0.72	0.726	0.73	0.735	0.74	0.744	0.751	0.757
Cornwall	0.585	0.593	0.602	0.611	0.621	0.63	0.637	0.642	0.644	0.646	0.652	0.66	0.666	0.67	0.674	0.677	0.681	0.684	0.689	0.695
Devon	0.606	0.614	0.622	0.631	0.64	0.648	0.655	0.66	0.662	0.665	0.671	0.679	0.685	0.69	0.693	0.697	0.701	0.704	0.709	0.715
Dorset	0.605	0.613	0.621	0.63	0.64	0.648	0.655	0.66	0.662	0.665	0.671	0.679	0.685	0.689	0.692	0.696	0.699	0.702	0.707	0.712
Gloucestershire	0.62	0.627	0.635	0.644	0.654	0.662	0.669	0.674	0.676	0.679	0.684	0.692	0.699	0.704	0.708	0.712	0.716	0.719	0.724	0.73
North Somerset	0.598	0.605	0.614	0.623	0.633	0.641	0.648	0.653	0.655	0.657	0.663	0.671	0.677	0.682	0.687	0.691	0.694	0.698	0.704	0.71
Plymouth	0.601	0.608	0.616	0.626	0.636	0.644	0.651	0.656	0.658	0.66	0.666	0.674	0.681	0.687	0.692	0.697	0.702	0.706	0.713	0.719
Poole	0.613	0.62	0.629	0.639	0.648	0.657	0.664	0.669	0.671	0.674	0.68	0.687	0.693	0.697	0.7	0.704	0.708	0.712	0.717	0.723
Somerset	0.6	0.608	0.616	0.626	0.636	0.645	0.652	0.657	0.659	0.661	0.667	0.675	0.682	0.687	0.69	0.694	0.697	0.699	0.704	0.708
South Gloucestershire	0.633	0.641	0.649	0.658	0.667	0.676	0.683	0.688	0.69	0.693	0.698	0.706	0.712	0.717	0.721	0.725	0.729	0.732	0.738	0.743
Swindon	0.628	0.636	0.644	0.654	0.664	0.673	0.681	0.686	0.687	0.689	0.695	0.703	0.71	0.715	0.719	0.723	0.727	0.73	0.736	0.742
Torbay	0.584	0.592	0.601	0.61	0.62	0.628	0.636	0.641	0.643	0.646	0.652	0.66	0.665	0.669	0.672	0.676	0.68	0.683	0.689	0.694
Wiltshire	0.612	0.62	0.628	0.638	0.648	0.656	0.664	0.668	0.67	0.672	0.677	0.686	0.693	0.698	0.702	0.706	0.71	0.713	0.718	0.722
West Midlands	0.588	0.595	0.604	0.613	0.623	0.632	0.639	0.644	0.646	0.649	0.655	0.663	0.669	0.674	0.679	0.683	0.687	0.69	0.696	0.702
Birmingham	0.582	0.59	0.598	0.608	0.618	0.627	0.635	0.639	0.641	0.643	0.649	0.658	0.665	0.67	0.675	0.68	0.685	0.689	0.695	0.702
Coventry	0.601	0.608	0.617	0.626	0.636	0.644	0.652	0.656	0.658	0.661	0.666	0.674	0.681	0.686	0.69	0.694	0.698	0.702	0.709	0.716
Dudley	0.576	0.583	0.592	0.601	0.611	0.619	0.627	0.632	0.633	0.636	0.642	0.65	0.656	0.661	0.665	0.669	0.673	0.677	0.682	0.688
Herefordshire, County of	0.591	0.599	0.607	0.617	0.627	0.635	0.643	0.648	0.65	0.653	0.659	0.667	0.674	0.678	0.682	0.685	0.688	0.691	0.695	0.701
Sandwell	0.563	0.571	0.579	0.589	0.599	0.608	0.615	0.62	0.621	0.623	0.629	0.638	0.645	0.65	0.654	0.658	0.663	0.666	0.672	0.678
Shropshire	0.594	0.601	0.61	0.619	0.629	0.637	0.645	0.65	0.652	0.655	0.661	0.669	0.675	0.679	0.683	0.687	0.69	0.693	0.698	0.704
Solihull	0.621	0.629	0.637	0.647	0.656	0.665	0.672	0.677	0.679	0.682	0.688	0.695	0.701	0.706	0.709	0.712	0.715	0.718	0.723	0.728
Staffordshire	0.592	0.599	0.607	0.617	0.627	0.635	0.642	0.647	0.649	0.652	0.658	0.666	0.672	0.677	0.68	0.684	0.688	0.691	0.696	0.702
Stoke-on-Trent	0.569	0.576	0.584	0.594	0.604	0.612	0.62	0.624	0.626	0.628	0.634	0.643	0.649	0.655	0.659	0.664	0.668	0.672	0.677	0.684
Telford and Wrekin	0.592	0.6	0.609	0.619	0.63	0.639	0.647	0.652	0.653	0.655	0.661	0.67	0.677	0.683	0.687	0.691	0.695	0.698	0.703	0.709
Walsall	0.565	0.572	0.581	0.59	0.6	0.609	0.616	0.621	0.623	0.625	0.631	0.639	0.646	0.651	0.655	0.659	0.663	0.666	0.671	0.676
Warwickshire	0.615	0.622	0.63	0.64	0.65	0.658	0.665	0.67	0.672	0.675	0.681	0.689	0.695	0.7	0.704	0.707	0.711	0.715	0.72	0.726
Wolverhampton	0.573	0.581	0.589	0.599	0.609	0.618	0.625	0.63	0.631	0.634	0.64	0.648	0.655	0.66	0.664	0.668	0.672	0.675	0.68	0.686
Worcestershire	0.591	0.599	0.607	0.617	0.626	0.635	0.642	0.647	0.65	0.653	0.659	0.666	0.673	0.678	0.681	0.685	0.688	0.692	0.697	0.702
Yorkshire and the Humber	0.59	0.598	0.606	0.616	0.626	0.634	0.642	0.647	0.649	0.652	0.658	0.666	0.672	0.677	0.681	0.686	0.69	0.694	0.699	0.705
Barnsley	0.558	0.566	0.575	0.584	0.594	0.603	0.61	0.615	0.617	0.619	0.625	0.634	0.64	0.645	0.649	0.653	0.657	0.661	0.665	0.671
Bradford	0.567	0.575	0.584	0.595	0.605	0.615	0.623	0.627	0.629	0.631	0.637	0.646	0.653	0.658	0.663	0.668	0.672	0.676	0.682	0.688
Calderdale	0.585	0.593	0.602	0.612	0.623	0.632	0.64	0.645	0.647	0.649	0.655	0.664	0.67	0.675	0.679	0.683	0.687	0.691	0.697	0.703
Doncaster	0.557	0.566	0.574	0.584	0.595	0.603	0.611	0.616	0.618	0.62	0.626	0.635	0.641	0.646	0.649	0.653	0.657	0.66	0.665	0.67
East Riding of Yorkshire	0.598	0.605	0.614	0.623	0.633	0.641	0.648	0.653	0.656	0.659	0.665	0.673	0.679	0.684	0.687	0.691	0.694	0.698	0.703	0.709
Kingston upon Hull, City of	0.567	0.576	0.584	0.594	0.605	0.613	0.621	0.626	0.628	0.631	0.637	0.645	0.652	0.657	0.66	0.664	0.668	0.672	0.677	0.683
Kirklees	0.579	0.587	0.596	0.606	0.616	0.624	0.632	0.637	0.639	0.641	0.647	0.656	0.662	0.668	0.672	0.677	0.681	0.685	0.691	0.697
Leeds	0.617	0.624	0.632	0.641	0.65	0.657	0.664	0.669	0.672	0.675	0.681	0.688	0.695	0.7	0.705	0.709	0.714	0.719	0.725	0.732
North East Lincolnshire	0.578	0.586	0.595	0.606	0.617	0.626	0.634	0.639	0.641	0.643	0.649	0.658	0.664	0.669	0.672	0.675	0.679	0.682	0.687	0.693
North Lincolnshire	0.585	0.593	0.602	0.612	0.622	0.631	0.639	0.644	0.645	0.648	0.654	0.662	0.669	0.674	0.677	0.681	0.685	0.687	0.692	0.698
North Yorkshire	0.607	0.615	0.623	0.633	0.643	0.651	0.658	0.663	0.666	0.669	0.675	0.683	0.689	0.694	0.698	0.702	0.705	0.709	0.714	0.719
Rotherham	0.565	0.573	0.582	0.592	0.602	0.61	0.618	0.623	0.624	0.627	0.633	0.641	0.648	0.653	0.656	0.66	0.664	0.667	0.672	0.677
Sheffield	0.609	0.616	0.624	0.633	0.641	0.649	0.656	0.661	0.664	0.667	0.673	0.68	0.687	0.692	0.696	0.7	0.705	0.709	0.715	0.721
Wakefield	0.574	0.582	0.591	0.601	0.611	0.62	0.627	0.632	0.634	0.637	0.643	0.651	0.658	0.662	0.666	0.67	0.674	0.677	0.682	0.688
York	0.641	0.648	0.656	0.664	0.673	0.68	0.687	0.692	0.695	0.698	0.704	0.711	0.717	0.723	0.727	0.732	0.737	0.742	0.748	0.756
Northern Ireland	0.574	0.582	0.59	0.598	0.607	0.614	0.622	0.627	0.628	0.63	0.638	0.649	0.657	0.663	0.668	0.673	0.679	0.685	0.695	0.704
Scotland	0.541	0.547	0.556	0.563	0.571	0.58	0.59	0.596	0.598	0.601	0.607	0.616	0.624	0.631	0.636	0.642	0.648	0.653	0.66	0.667
Wales	0.503	0.51	0.519	0.529	0.54	0.549	0.557	0.563	0.566	0.569	0.576	0.586	0.593	0.6	0.605	0.61	0.615	0.62	0.627	0.635
Latin America and Caribbean	0.372	0.378	0.385	0.392	0.399	0.406	0.413	0.421	0.428	0.434	0.441	0.447	0.453	0.46	0.466	0.472	0.477	0.483	0.488	0.493
Andean Latin America	0.352	0.358	0.364	0.371	0.377	0.385	0.395	0.404	0.412	0.419	0.425	0.432	0.44	0.448	0.455	0.461	0.467	0.472	0.477	0.48
Bolivia	0.302	0.31	0.318	0.324	0.329	0.332	0.332	0.332	0.335	0.34	0.346	0.352	0.36	0.366	0.371	0.375	0.38	0.388	0.396	0.4
Ecuador	0.384	0.387	0.395	0.404	0.417	0.434	0.449	0.457	0.46	0.459	0.455	0.454	0.459	0.467	0.475	0.484	0.491	0.496	0.499	0.502
Peru	0.345	0.352	0.357	0.362	0.365	0.369	0.379	0.393	0.407	0.418	0.431	0.443	0.453	0.461	0.468	0.473	0.478	0.482	0.487	0.491
Caribbean	0.408	0.41	0.417	0.427	0.437	0.445	0.454	0.464	0.472	0.479	0.484	0.487	0.491	0.497	0.503	0.51	0.516	0.518	0.521	0.525
Antigua and Barbuda	0.467	0.479	0.489	0.497	0.502	0.505	0.508	0.512	0.516	0.524	0.531	0.539	0.548	0.556	0.564	0.572	0.58	0.588	0.595	0.603
The Bahamas	0.561	0.569	0.578	0.586	0.589	0.588	0.589	0.593	0.597	0.602	0.606	0.61	0.616	0.626	0.639	0.652	0.663	0.67	0.674	0.675
Barbados	0.522	0.53	0.539	0.55	0.562	0.573	0.582	0.588	0.592	0.596	0.599	0.603	0.607	0.613	0.621	0.629	0.637	0.643	0.646	0.648
Belize	0.277	0.281	0.286	0.29	0.294	0.298	0.302	0.308	0.313	0.319	0.326	0.333	0.342	0.349	0.356	0.363	0.371	0.378	0.385	0.392
Bermuda	0.609	0.619	0.629	0.639	0.648	0.657	0.665	0.673	0.68	0.686	0.691	0.695	0.699	0.703	0.708	0.712	0.716	0.719	0.723	0.727
Cuba	0.421	0.412	0.418	0.432	0.448	0.462	0.481	0.5	0.515	0.528	0.536	0.539	0.541	0.547	0.553	0.563	0.572	0.574	0.576	0.582
Dominica	0.349	0.361	0.379	0.404	0.428	0.446	0.462	0.474	0.483	0.487	0.489	0.493	0.499	0.505	0.512	0.517	0.522	0.525	0.529	0.532
Dominican Republic	0.288	0.295	0.307	0.319	0.33	0.339	0.349	0.359	0.366	0.368	0.368	0.37	0.38	0.395	0.408	0.417	0.421	0.424	0.43	0.437
Grenada	0.335	0.342	0.349	0.357	0.366	0.375	0.384	0.391	0.398	0.405	0.409	0.412	0.413	0.415	0.418	0.422	0.427	0.433	0.44	0.449
Guyana	0.393	0.401	0.409	0.416	0.422	0.429	0.435	0.438	0.44	0.441	0.441	0.443	0.444	0.443	0.441	0.439	0.439	0.44	0.44	0.44
Haiti	0.27	0.274	0.277	0.28	0.284	0.287	0.291	0.294	0.297	0.3	0.304	0.306	0.308	0.31	0.312	0.314	0.316	0.319	0.322	0.325

Appendix Table 10. Socio-demographic Index values for all estimated GBD 2017 locations, 1970–1989

Location	1970	1971	1972	1973	1974	1975	1976	1977	1978	1979	1980	1981	1982	1983	1984	1985	1986	1987	1988	1989
Jamaica	0.406	0.416	0.429	0.442	0.453	0.465	0.476	0.486	0.493	0.498	0.502	0.508	0.513	0.519	0.524	0.531	0.537	0.54	0.542	0.543
Puerto Rico	0.564	0.572	0.579	0.586	0.593	0.596	0.597	0.6	0.61	0.62	0.628	0.637	0.648	0.657	0.664	0.668	0.672	0.675	0.677	0.68
Saint Lucia	0.333	0.342	0.352	0.362	0.373	0.383	0.395	0.406	0.417	0.426	0.432	0.436	0.44	0.445	0.451	0.461	0.471	0.482	0.491	0.5
Saint Vincent and the Grenadines	0.274	0.283	0.298	0.314	0.329	0.342	0.354	0.361	0.367	0.373	0.379	0.385	0.395	0.407	0.419	0.43	0.44	0.448	0.455	0.46
Suriname	0.395	0.406	0.415	0.422	0.43	0.436	0.438	0.443	0.45	0.455	0.457	0.458	0.461	0.468	0.478	0.491	0.504	0.512	0.517	0.523
Trinidad and Tobago	0.497	0.505	0.513	0.52	0.528	0.534	0.54	0.545	0.548	0.552	0.556	0.559	0.563	0.568	0.573	0.579	0.585	0.59	0.594	0.597
Virgin Islands	0.514	0.531	0.551	0.57	0.586	0.6	0.611	0.619	0.626	0.631	0.635	0.638	0.64	0.643	0.647	0.65	0.652	0.655	0.657	0.659
Central Latin America	0.368	0.376	0.384	0.391	0.398	0.405	0.413	0.422	0.429	0.436	0.442	0.45	0.457	0.463	0.47	0.475	0.479	0.483	0.487	0.49
Colombia	0.36	0.37	0.379	0.387	0.395	0.402	0.409	0.416	0.423	0.43	0.438	0.445	0.451	0.457	0.463	0.468	0.472	0.475	0.478	0.481
Costa Rica	0.427	0.436	0.445	0.451	0.454	0.455	0.457	0.459	0.464	0.471	0.477	0.483	0.489	0.494	0.497	0.5	0.503	0.506	0.51	0.516
El Salvador	0.296	0.3	0.304	0.309	0.315	0.322	0.329	0.339	0.349	0.357	0.363	0.37	0.377	0.383	0.388	0.392	0.395	0.397	0.4	0.402
Guatemala	0.273	0.273	0.277	0.284	0.289	0.284	0.271	0.265	0.265	0.264	0.263	0.266	0.274	0.282	0.287	0.293	0.303	0.303	0.302	0.308
Honduras	0.24	0.244	0.248	0.252	0.255	0.258	0.262	0.266	0.272	0.278	0.286	0.296	0.305	0.311	0.315	0.319	0.322	0.326	0.33	0.336
Mexico	0.372	0.379	0.387	0.396	0.404	0.413	0.424	0.435	0.443	0.449	0.456	0.463	0.47	0.477	0.483	0.489	0.494	0.499	0.504	0.509
Aguascalientes	0.402	0.412	0.422	0.432	0.442	0.453	0.464	0.475	0.484	0.491	0.498	0.506	0.513	0.519	0.526	0.532	0.538	0.543	0.549	0.554
Baja California	0.417	0.427	0.436	0.447	0.457	0.467	0.48	0.492	0.5	0.507	0.514	0.523	0.531	0.538	0.545	0.551	0.557	0.561	0.564	0.567
Baja California Sur	0.409	0.417	0.427	0.437	0.446	0.457	0.469	0.482	0.49	0.497	0.504	0.512	0.521	0.528	0.535	0.542	0.549	0.555	0.56	0.565
Campeche	0.352	0.358	0.365	0.372	0.379	0.387	0.398	0.408	0.415	0.42	0.426	0.434	0.441	0.446	0.451	0.458	0.464	0.47	0.475	0.48
Chiapas	0.302	0.309	0.315	0.321	0.327	0.334	0.344	0.352	0.358	0.362	0.367	0.372	0.377	0.38	0.384	0.388	0.391	0.394	0.397	0.4
Chihuahua	0.381	0.389	0.399	0.408	0.418	0.428	0.44	0.453	0.462	0.469	0.477	0.487	0.497	0.505	0.513	0.52	0.528	0.534	0.538	0.543
Coahuila	0.401	0.409	0.417	0.425	0.432	0.44	0.451	0.462	0.468	0.473	0.479	0.487	0.494	0.501	0.509	0.517	0.524	0.531	0.538	0.544
Colima	0.381	0.389	0.398	0.406	0.414	0.423	0.434	0.445	0.452	0.457	0.464	0.471	0.478	0.485	0.492	0.5	0.508	0.516	0.524	0.531
Mexico City	0.446	0.456	0.468	0.48	0.492	0.504	0.518	0.532	0.542	0.551	0.56	0.569	0.576	0.579	0.581	0.581	0.58	0.581	0.585	0.593
Durango	0.354	0.362	0.371	0.38	0.388	0.398	0.411	0.423	0.432	0.439	0.447	0.457	0.465	0.472	0.478	0.485	0.491	0.496	0.499	0.503
Guanajuato	0.34	0.348	0.356	0.364	0.372	0.381	0.391	0.401	0.408	0.413	0.419	0.424	0.43	0.436	0.442	0.451	0.459	0.468	0.477	0.486
Guerrero	0.294	0.3	0.308	0.315	0.322	0.33	0.34	0.351	0.358	0.364	0.371	0.379	0.386	0.392	0.398	0.404	0.409	0.414	0.418	0.423
Hidalgo	0.282	0.289	0.296	0.304	0.313	0.321	0.333	0.344	0.35	0.355	0.36	0.364	0.368	0.371	0.374	0.38	0.386	0.394	0.402	0.41
Jalisco	0.389	0.396	0.404	0.412	0.419	0.427	0.437	0.447	0.454	0.461	0.468	0.476	0.483	0.49	0.496	0.503	0.508	0.514	0.52	0.526
México	0.375	0.381	0.389	0.396	0.404	0.412	0.424	0.436	0.445	0.453	0.461	0.471	0.482	0.493	0.505	0.516	0.524	0.53	0.533	0.532
Michoacán de Ocampo	0.326	0.333	0.34	0.347	0.354	0.362	0.371	0.38	0.386	0.391	0.397	0.403	0.41	0.417	0.424	0.432	0.439	0.446	0.452	0.459
Morelos	0.378	0.385	0.393	0.4	0.408	0.415	0.425	0.435	0.441	0.446	0.451	0.458	0.465	0.472	0.479	0.486	0.495	0.503	0.51	0.518
Nayarit	0.337	0.346	0.354	0.363	0.372	0.382	0.394	0.406	0.414	0.421	0.429	0.438	0.446	0.453	0.459	0.466	0.472	0.478	0.484	0.49
Nuevo León	0.433	0.442	0.452	0.463	0.472	0.482	0.494	0.506	0.514	0.521	0.528	0.536	0.545	0.554	0.564	0.572	0.579	0.585	0.59	0.594
Oaxaca	0.292	0.299	0.305	0.312	0.318	0.326	0.335	0.345	0.352	0.357	0.364	0.372	0.38	0.387	0.393	0.4	0.407	0.413	0.419	0.425
Puebla	0.314	0.322	0.33	0.338	0.346	0.355	0.368	0.38	0.387	0.392	0.398	0.403	0.408	0.416	0.422	0.425	0.426	0.432	0.441	0.449
Querétaro	0.361	0.369	0.377	0.386	0.395	0.405	0.417	0.428	0.436	0.443	0.449	0.455	0.458	0.46	0.462	0.462	0.464	0.47	0.478	0.489
Quintana Roo	0.4	0.405	0.411	0.417	0.422	0.428	0.439	0.449	0.454	0.458	0.463	0.47	0.477	0.483	0.488	0.495	0.501	0.506	0.51	0.515
San Luis Potosí	0.336	0.344	0.351	0.359	0.366	0.375	0.386	0.396	0.404	0.41	0.418	0.426	0.434	0.44	0.447	0.454	0.46	0.465	0.471	0.477
Sinaloa	0.362	0.371	0.379	0.388	0.397	0.407	0.42	0.432	0.44	0.448	0.456	0.465	0.474	0.481	0.488	0.495	0.501	0.507	0.512	0.518
Sonora	0.395	0.403	0.413	0.423	0.432	0.442	0.454	0.465	0.473	0.48	0.488	0.495	0.503	0.512	0.52	0.527	0.533	0.538	0.543	0.549
Tabasco	0.323	0.33	0.338	0.345	0.352	0.361	0.373	0.384	0.392	0.398	0.405	0.413	0.422	0.428	0.435	0.442	0.449	0.456	0.462	0.468
Tamaulipas	0.401	0.41	0.419	0.428	0.436	0.445	0.456	0.467	0.473	0.478	0.483	0.49	0.497	0.504	0.511	0.519	0.525	0.532	0.538	0.544
Tlaxcala	0.328	0.335	0.343	0.351	0.358	0.367	0.378	0.389	0.395	0.399	0.403	0.407	0.412	0.415	0.419	0.427	0.437	0.449	0.46	0.47
Veracruz de Ignacio de la Llave	0.329	0.336	0.343	0.35	0.357	0.365	0.375	0.385	0.393	0.399	0.406	0.414	0.422	0.429	0.435	0.441	0.447	0.451	0.455	0.459
Yucatán	0.363	0.368	0.375	0.383	0.39	0.398	0.408	0.417	0.425	0.431	0.438	0.445	0.452	0.458	0.463	0.469	0.474	0.48	0.485	0.491
Zacatecas	0.32	0.328	0.337	0.344	0.352	0.362	0.373	0.384	0.392	0.399	0.407	0.416	0.425	0.432	0.439	0.447	0.455	0.462	0.469	0.476
Nicaragua	0.264	0.27	0.274	0.277	0.279	0.282	0.285	0.287	0.29	0.292	0.297	0.303	0.309	0.316	0.323	0.33	0.337	0.343	0.348	0.352
Panama	0.401	0.411	0.421	0.431	0.442	0.452	0.459	0.465	0.472	0.479	0.486	0.493	0.499	0.506	0.513	0.519	0.524	0.529	0.533	0.537
Venezuela	0.432	0.441	0.45	0.453	0.456	0.461	0.468	0.477	0.483	0.489	0.496	0.503	0.51	0.519	0.528	0.529	0.528	0.53	0.535	0.533
Tropical Latin America	0.361	0.367	0.373	0.38	0.387	0.393	0.4	0.407	0.413	0.42	0.426	0.432	0.438	0.444	0.45	0.457	0.465	0.472	0.48	0.487
Brazil	0.361	0.367	0.373	0.38	0.387	0.393	0.4	0.407	0.413	0.42	0.427	0.433	0.439	0.444	0.451	0.457	0.465	0.472	0.48	0.487
Acre	0.247	0.252	0.256	0.261	0.265	0.27	0.274	0.279	0.283	0.289	0.294	0.3	0.305	0.311	0.319	0.327	0.336	0.347	0.357	0.367
Alagoas	0.213	0.216	0.221	0.227	0.232	0.238	0.243	0.249	0.255	0.262	0.269	0.276	0.283	0.291	0.3	0.309	0.319	0.329	0.338	0.347
Amapá	0.322	0.327	0.332	0.338	0.343	0.349	0.356	0.362	0.369	0.377	0.384	0.392	0.399	0.406	0.414	0.422	0.431	0.44	0.45	0.459
Amazonas	0.272	0.277	0.282	0.288	0.293	0.298	0.303	0.308	0.315	0.323	0.33	0.339	0.348	0.358	0.369	0.381	0.393	0.405	0.416	0.427
Bahia	0.26	0.265	0.269	0.274	0.279	0.283	0.287	0.292	0.297	0.303	0.31	0.317	0.325	0.333	0.342	0.352	0.362	0.373	0.383	0.393
Ceará	0.278	0.284	0.289	0.294	0.299	0.304	0.308	0.313	0.319	0.325	0.332	0.339	0.347	0.354	0.362	0.37	0.379	0.388	0.396	0.404
Distrito Federal	0.498	0.506	0.513	0.52	0.526	0.533	0.54	0.547	0.554	0.561	0.568	0.574	0.58	0.585	0.591	0.596	0.603	0.61	0.617	0.623
Espírito Santo	0.364	0.371	0.379	0.387	0.394	0.401	0.408	0.414	0.42	0.426	0.431	0.436	0.442	0.447	0.454	0.46	0.468	0.475	0.483	0.491
Goiás	0.306	0.312	0.319	0.326	0.333	0.341	0.349	0.357	0.365	0.374	0.382	0.39	0.397	0.403	0.41	0.417	0.426	0.434	0.443	0.452
Maranhão	0.185	0.188	0.191	0.193	0.196	0.198	0.201	0.204	0.208	0.213	0.219	0.225	0.232	0.24	0.249	0.259	0.27	0.282	0.293	0.303
Mato Grosso	0.326	0.334	0.342	0.351	0.359	0.366	0.374	0.38	0.386	0.391	0.397	0.402	0.408	0.414	0.421	0.429	0.438	0.447	0.456	0.466
Mato Grosso do Sul	0.329	0.336	0.344	0.351	0.358	0.364	0.37	0.375	0.38	0.385	0.39	0.396	0.402	0.408	0.415	0.423	0.431	0.44	0.448	0.457
Minas Gerais	0.357	0.364	0.371	0.378	0.386	0.393	0.4	0.407	0.414	0.421	0.428	0.434	0.44	0.446	0.451	0.457	0.464	0.471	0.477	0.484
Pará	0.286	0.29	0.295	0.3	0.304	0.309	0.314	0.319	0.324	0.329	0.335	0.34	0.346	0.352	0.358	0.366	0.375	0.384	0.393	0.402
Paraíba	0.243	0.248	0.254	0.261	0.268	0.276	0.283	0.292	0.3	0.308	0.316	0.324	0.332	0.34	0.348	0.356	0.365	0.375	0.383	0.391
Paraná	0.357	0.364	0.372	0.38	0.389	0.399	0.408	0.418	0.428	0.438	0.447	0.455	0.463	0.47	0.476	0.482	0.489	0.495	0.501	0.507
Pernambuco	0.272	0.278	0.284	0.29	0.297	0.303	0.309	0.315	0.322	0.329	0.336	0.343	0.351	0.358	0.366	0.375	0.384	0.393	0.401	0.409
Piauí	0.231	0.236	0.241	0.247	0.252	0.258	0.264	0.271	0.277	0.284	0.29	0.297	0.304	0.311	0.318	0.326	0.334	0.342	0.35	0.358
Rio de Janeiro	0.462	0.469	0.476	0.484	0.491	0.497	0.505	0.511	0.518	0.524	0.529	0.534	0.539	0.543	0.547	0.551	0.556	0.561	0.567	0.572
Rio Grande do Norte	0.259	0.265	0.271	0.279	0.286	0.294	0.302	0.31	0.318	0.326	0.335	0.343	0.351	0.359	0.367	0.375	0.383	0.392	0.4	0.408

Appendix Table 10. Socio-demographic Index values for all estimated GBD 2017 locations, 1970–1989

Location	1970	1971	1972	1973	1974	1975	1976	1977	1978	1979	1980	1981	1982	1983	1984	1985	1986	1987	1988	1989
Rio Grande do Sul	0.426	0.433	0.44	0.447	0.454	0.46	0.466	0.472	0.477	0.483	0.488	0.493	0.498	0.502	0.507	0.513	0.519	0.526	0.532	0.538
Rondônia	0.283	0.288	0.294	0.3	0.305	0.31	0.315	0.32	0.326	0.331	0.337	0.343	0.35	0.357	0.365	0.374	0.384	0.394	0.404	0.414
Roraima	0.294	0.299	0.303	0.307	0.312	0.316	0.32	0.324	0.328	0.333	0.338	0.343	0.349	0.356	0.364	0.374	0.384	0.395	0.407	0.417
Santa Catarina	0.403	0.409	0.415	0.422	0.429	0.435	0.443	0.45	0.458	0.466	0.473	0.481	0.488	0.494	0.5	0.507	0.514	0.521	0.528	0.535
São Paulo	0.435	0.441	0.447	0.453	0.459	0.465	0.471	0.477	0.482	0.488	0.494	0.5	0.505	0.51	0.516	0.522	0.529	0.537	0.544	0.551
Sergipe	0.274	0.28	0.286	0.293	0.3	0.307	0.314	0.321	0.328	0.336	0.343	0.351	0.358	0.365	0.373	0.381	0.39	0.399	0.408	0.417
Tocantins	0.242	0.246	0.25	0.254	0.259	0.265	0.272	0.279	0.287	0.295	0.304	0.313	0.322	0.33	0.34	0.349	0.36	0.37	0.379	0.388
Paraguay	0.352	0.357	0.362	0.367	0.373	0.378	0.383	0.388	0.393	0.398	0.404	0.41	0.416	0.421	0.427	0.433	0.44	0.449	0.457	0.463
North Africa and Middle East	0.297	0.304	0.312	0.32	0.328	0.335	0.342	0.35	0.357	0.364	0.373	0.382	0.39	0.399	0.408	0.415	0.422	0.431	0.439	0.448
North Africa and Middle East	0.297	0.304	0.312	0.32	0.328	0.335	0.342	0.35	0.357	0.364	0.373	0.382	0.39	0.399	0.408	0.415	0.422	0.431	0.439	0.448
Afghanistan	0.113	0.115	0.116	0.118	0.119	0.121	0.123	0.125	0.127	0.129	0.13	0.13	0.132	0.135	0.138	0.14	0.143	0.145	0.147	0.148
Algeria	0.304	0.31	0.317	0.324	0.332	0.34	0.348	0.356	0.365	0.375	0.387	0.399	0.41	0.422	0.435	0.448	0.459	0.468	0.477	0.487
Bahrain	0.414	0.427	0.44	0.454	0.467	0.477	0.489	0.501	0.511	0.521	0.531	0.541	0.552	0.564	0.575	0.583	0.591	0.596	0.602	0.607
Egypt	0.282	0.29	0.297	0.303	0.308	0.313	0.32	0.327	0.334	0.342	0.351	0.359	0.367	0.374	0.381	0.389	0.399	0.41	0.422	0.432
Iran	0.319	0.329	0.339	0.348	0.357	0.363	0.37	0.376	0.379	0.382	0.384	0.388	0.401	0.423	0.443	0.453	0.459	0.468	0.48	0.493
Iraq	0.274	0.281	0.287	0.294	0.302	0.311	0.32	0.33	0.34	0.35	0.361	0.37	0.379	0.386	0.393	0.399	0.406	0.413	0.42	0.427
Jordan	0.302	0.316	0.329	0.343	0.357	0.371	0.385	0.399	0.414	0.429	0.446	0.463	0.477	0.49	0.503	0.515	0.526	0.535	0.542	0.547
Kuwait	0.41	0.423	0.424	0.423	0.427	0.436	0.452	0.472	0.49	0.504	0.517	0.531	0.546	0.562	0.579	0.593	0.603	0.618	0.634	0.649
Lebanon	0.295	0.307	0.32	0.332	0.351	0.363	0.372	0.383	0.383	0.395	0.407	0.419	0.429	0.439	0.452	0.466	0.48	0.494	0.505	0.512
Libya	0.318	0.31	0.308	0.326	0.357	0.39	0.423	0.456	0.489	0.519	0.545	0.563	0.58	0.594	0.604	0.611	0.616	0.622	0.628	0.636
Morocco	0.223	0.231	0.238	0.246	0.254	0.262	0.27	0.279	0.287	0.296	0.304	0.312	0.321	0.329	0.338	0.346	0.355	0.364	0.373	0.381
Palestine	0.223	0.231	0.24	0.248	0.257	0.266	0.276	0.285	0.295	0.304	0.312	0.32	0.326	0.332	0.338	0.343	0.348	0.352	0.355	0.358
Oman	0.27	0.28	0.289	0.297	0.305	0.314	0.323	0.332	0.34	0.349	0.358	0.367	0.378	0.39	0.403	0.416	0.428	0.439	0.45	0.462
Qatar	0.43	0.441	0.451	0.462	0.473	0.484	0.495	0.506	0.517	0.527	0.534	0.54	0.545	0.548	0.551	0.556	0.563	0.571	0.58	0.591
Saudi Arabia	0.226	0.232	0.238	0.245	0.251	0.257	0.261	0.266	0.271	0.276	0.28	0.285	0.292	0.303	0.318	0.334	0.352	0.371	0.393	0.415
Sudan	0.133	0.135	0.138	0.141	0.145	0.149	0.154	0.16	0.165	0.17	0.174	0.179	0.184	0.188	0.192	0.197	0.201	0.205	0.21	0.215
Syria	0.247	0.248	0.251	0.254	0.257	0.262	0.269	0.278	0.288	0.298	0.307	0.317	0.327	0.337	0.344	0.352	0.359	0.368	0.376	0.383
Tunisia	0.26	0.268	0.277	0.285	0.293	0.302	0.311	0.321	0.332	0.342	0.352	0.361	0.369	0.379	0.391	0.403	0.413	0.423	0.433	0.443
Turkey	0.349	0.355	0.362	0.369	0.377	0.385	0.393	0.402	0.411	0.419	0.426	0.434	0.441	0.448	0.455	0.462	0.47	0.479	0.489	0.498
United Arab Emirates	0.333	0.332	0.338	0.354	0.375	0.396	0.412	0.427	0.445	0.462	0.475	0.484	0.497	0.516	0.535	0.549	0.561	0.574	0.589	0.605
Yemen	0.12	0.124	0.128	0.132	0.136	0.139	0.142	0.146	0.149	0.154	0.158	0.162	0.167	0.174	0.181	0.187	0.191	0.194	0.197	0.199
South Asia	0.219	0.223	0.227	0.232	0.238	0.244	0.249	0.254	0.259	0.264	0.267	0.269	0.272	0.277	0.281	0.286	0.292	0.298	0.303	0.308
South Asia	0.219	0.223	0.227	0.232	0.238	0.244	0.249	0.254	0.259	0.264	0.267	0.269	0.272	0.277	0.281	0.286	0.292	0.298	0.303	0.308
Bangladesh	0.084	0.116	0.138	0.153	0.166	0.177	0.187	0.195	0.202	0.207	0.211	0.215	0.219	0.222	0.225	0.228	0.231	0.236	0.241	0.248
Bhutan	0.202	0.208	0.212	0.217	0.221	0.225	0.229	0.234	0.238	0.243	0.248	0.254	0.26	0.267	0.274	0.281	0.289	0.297	0.306	0.315
India	0.225	0.23	0.233	0.238	0.245	0.251	0.255	0.26	0.265	0.27	0.273	0.275	0.278	0.283	0.287	0.293	0.299	0.306	0.311	0.316
Andhra Pradesh	0.162	0.166	0.169	0.174	0.183	0.191	0.195	0.198	0.203	0.208	0.213	0.219	0.225	0.233	0.24	0.248	0.255	0.262	0.268	0.274
Arunachal Pradesh	0.185	0.19	0.196	0.202	0.209	0.214	0.216	0.221	0.228	0.234	0.236	0.24	0.247	0.254	0.261	0.269	0.279	0.288	0.298	0.305
Assam	0.223	0.228	0.231	0.235	0.241	0.245	0.248	0.25	0.255	0.259	0.261	0.264	0.269	0.276	0.283	0.289	0.296	0.306	0.314	0.321
Bihar	0.197	0.201	0.205	0.21	0.215	0.221	0.226	0.231	0.237	0.242	0.244	0.245	0.248	0.252	0.258	0.264	0.271	0.277	0.282	0.287
Chhattisgarh	0.202	0.207	0.211	0.216	0.224	0.232	0.238	0.243	0.249	0.254	0.258	0.261	0.264	0.268	0.272	0.276	0.282	0.286	0.288	0.29
Delhi	0.391	0.397	0.407	0.416	0.424	0.431	0.437	0.442	0.447	0.45	0.45	0.449	0.453	0.456	0.459	0.462	0.466	0.472	0.478	0.483
Goa	0.366	0.373	0.38	0.387	0.394	0.402	0.41	0.417	0.425	0.431	0.436	0.44	0.447	0.452	0.459	0.464	0.471	0.478	0.486	0.493
Gujarat	0.244	0.249	0.255	0.262	0.268	0.272	0.276	0.283	0.29	0.296	0.297	0.297	0.3	0.304	0.305	0.307	0.315	0.326	0.338	0.345
Haryana	0.243	0.248	0.253	0.26	0.269	0.277	0.283	0.288	0.293	0.297	0.299	0.299	0.302	0.305	0.307	0.311	0.316	0.323	0.332	0.337
Himachal Pradesh	0.213	0.218	0.224	0.23	0.238	0.245	0.251	0.259	0.267	0.275	0.279	0.283	0.287	0.291	0.296	0.302	0.31	0.317	0.326	0.333
Jammu and Kashmir	0.234	0.239	0.245	0.251	0.258	0.265	0.272	0.279	0.284	0.289	0.294	0.297	0.301	0.304	0.307	0.313	0.319	0.324	0.329	0.332
Jharkhand	0.205	0.21	0.213	0.219	0.226	0.233	0.238	0.243	0.249	0.253	0.256	0.258	0.26	0.262	0.265	0.269	0.272	0.275	0.278	0.279
Karnataka	0.22	0.223	0.226	0.231	0.237	0.242	0.245	0.249	0.253	0.257	0.259	0.261	0.266	0.271	0.277	0.283	0.291	0.3	0.309	0.316
Kerala	0.318	0.323	0.328	0.332	0.337	0.344	0.349	0.354	0.357	0.36	0.363	0.364	0.366	0.369	0.373	0.378	0.385	0.391	0.397	0.402
Madhya Pradesh	0.175	0.178	0.18	0.185	0.192	0.198	0.202	0.207	0.214	0.22	0.224	0.226	0.232	0.239	0.246	0.254	0.261	0.269	0.275	0.279
Maharashtra	0.258	0.262	0.266	0.272	0.279	0.284	0.288	0.292	0.298	0.303	0.305	0.306	0.309	0.314	0.318	0.323	0.33	0.34	0.349	0.357
Manipur	0.251	0.257	0.261	0.267	0.274	0.282	0.288	0.294	0.3	0.306	0.311	0.316	0.321	0.328	0.334	0.341	0.348	0.358	0.367	0.374
Meghalaya	0.229	0.234	0.239	0.244	0.25	0.256	0.259	0.263	0.268	0.272	0.276	0.279	0.283	0.288	0.293	0.299	0.306	0.314	0.32	0.327
Mizoram	0.27	0.275	0.28	0.284	0.29	0.297	0.303	0.307	0.313	0.318	0.322	0.325	0.329	0.333	0.34	0.351	0.363	0.376	0.384	0.389
Nagaland	0.283	0.287	0.291	0.295	0.301	0.307	0.312	0.316	0.321	0.325	0.329	0.333	0.339	0.345	0.351	0.358	0.366	0.375	0.383	0.39
Odisha	0.19	0.194	0.197	0.201	0.207	0.214	0.218	0.223	0.227	0.232	0.236	0.239	0.242	0.247	0.251	0.258	0.266	0.27	0.276	0.28
Punjab	0.294	0.299	0.303	0.308	0.313	0.318	0.324	0.33	0.334	0.339	0.341	0.342	0.345	0.348	0.351	0.355	0.359	0.367	0.374	0.38
Rajasthan	0.165	0.17	0.174	0.18	0.187	0.195	0.199	0.202	0.207	0.211	0.215	0.217	0.221	0.225	0.23	0.237	0.244	0.249	0.254	0.258
Sikkim	0.209	0.214	0.218	0.225	0.233	0.24	0.246	0.251	0.258	0.263	0.267	0.271	0.276	0.281	0.288	0.298	0.308	0.319	0.328	0.336
Tamil Nadu	0.249	0.253	0.257	0.263	0.268	0.274	0.279	0.283	0.288	0.293	0.297	0.3	0.304	0.308	0.315	0.321	0.328	0.335	0.341	0.347
Telangana	0.188	0.192	0.196	0.201	0.207	0.214	0.219	0.224	0.229	0.233	0.236	0.239	0.243	0.247	0.252	0.258	0.263	0.269	0.275	0.281
Tripura	0.217	0.223	0.228	0.236	0.245	0.253	0.257	0.26	0.264	0.269	0.274	0.28	0.286	0.292	0.297	0.303	0.31	0.316	0.323	0.329
Uttar Pradesh	0.196	0.2	0.204	0.208	0.213	0.218	0.221	0.226	0.231	0.235	0.238	0.238	0.241	0.244	0.247	0.251	0.256	0.261	0.265	0.268
Uttarakhand	0.22	0.226	0.23	0.236	0.243	0.251	0.257	0.263	0.269	0.275	0.279	0.28	0.282	0.285	0.288	0.291	0.295	0.298	0.3	0.302
West Bengal	0.235	0.238	0.24	0.244	0.252	0.259	0.263	0.266	0.272	0.278	0.283	0.287	0.292	0.296	0.301	0.307	0.314	0.319	0.321	0.322
Union Territories other than Delhi	0.299	0.306	0.313	0.322	0.332	0.343	0.35	0.357	0.365	0.371	0.376	0.378	0.381	0.385	0.389	0.392	0.396	0.399	0.402	0.404
Nepal	0.156	0.158	0.16	0.162	0.164	0.166	0.169	0.171	0.174	0.177	0.179	0.181	0.184	0.187	0.189	0.192	0.196	0.201	0.206	0.211
Pakistan	0.241	0.245	0.249	0.252	0.255	0.26	0.264	0.268	0.271	0.273	0.275	0.276	0.279	0.282	0.285	0.289	0.292	0.295	0.299	0.302
Southeast Asia, East Asia, and Oceania	0.294	0.304	0.315	0.326	0.338	0.349	0.358	0.366	0.374	0.382	0.39	0.397	0.401	0.405	0.411	0.417	0.424	0.434	0.446	0.456
East Asia	0.277	0.288	0.3	0.314	0.327	0.339	0.348	0.356	0.364	0.372	0.379	0.386	0.389	0.392	0.397	0.404	0.412	0.424	0.438	0.45

Appendix Table 10. Socio-demographic Index values for all estimated GBD 2017 locations, 1970–1989																				
**Location**	**1970**	**1971**	**1972**	**1973**	**1974**	**1975**	**1976**	**1977**	**1978**	**1979**	**1980**	**1981**	**1982**	**1983**	**1984**	**1985**	**1986**	**1987**	**1988**	**1989**
China	0.269	0.28	0.292	0.305	0.318	0.33	0.339	0.347	0.355	0.362	0.37	0.377	0.38	0.383	0.389	0.396	0.404	0.417	0.431	0.443
North Korea	0.398	0.411	0.421	0.431	0.44	0.448	0.453	0.458	0.462	0.466	0.47	0.474	0.478	0.482	0.486	0.49	0.494	0.498	0.503	0.507
Taiwan (Province of China)	0.485	0.497	0.51	0.52	0.53	0.53	0.535	0.548	0.555	0.566	0.577	0.588	0.601	0.613	0.627	0.643	0.655	0.663	0.672	0.682
Oceania	0.329	0.333	0.338	0.343	0.349	0.353	0.358	0.362	0.366	0.37	0.374	0.377	0.38	0.384	0.387	0.39	0.393	0.397	0.4	0.403
American Samoa	0.537	0.546	0.557	0.567	0.575	0.58	0.586	0.59	0.595	0.599	0.6	0.6	0.599	0.597	0.597	0.597	0.599	0.601	0.604	0.607
Federated States of Micronesia	0.325	0.331	0.337	0.344	0.351	0.358	0.365	0.373	0.38	0.387	0.395	0.402	0.408	0.415	0.422	0.429	0.436	0.443	0.449	0.456
Fiji	0.416	0.424	0.433	0.444	0.454	0.463	0.471	0.477	0.483	0.488	0.491	0.495	0.499	0.503	0.507	0.511	0.516	0.52	0.525	0.529
Guam	0.589	0.586	0.592	0.609	0.631	0.651	0.669	0.684	0.695	0.703	0.707	0.708	0.709	0.71	0.71	0.71	0.71	0.708	0.706	0.702
Kiribati	0.31	0.314	0.318	0.323	0.332	0.34	0.345	0.348	0.352	0.354	0.354	0.354	0.353	0.352	0.352	0.351	0.351	0.351	0.352	0.354
Marshall Islands	0.269	0.271	0.273	0.279	0.287	0.296	0.303	0.31	0.318	0.328	0.337	0.345	0.35	0.355	0.361	0.366	0.375	0.384	0.394	0.403
Northern Mariana Islands	0.585	0.591	0.598	0.605	0.613	0.622	0.63	0.639	0.648	0.656	0.664	0.671	0.678	0.686	0.695	0.704	0.713	0.721	0.728	0.734
Papua New Guinea	0.255	0.259	0.264	0.268	0.272	0.276	0.279	0.282	0.285	0.288	0.29	0.293	0.295	0.298	0.3	0.303	0.306	0.309	0.312	0.315
Samoa	0.411	0.422	0.431	0.442	0.451	0.458	0.467	0.475	0.483	0.492	0.499	0.503	0.507	0.51	0.514	0.518	0.522	0.527	0.531	0.535
Solomon Islands	0.241	0.244	0.247	0.25	0.254	0.258	0.262	0.266	0.271	0.277	0.282	0.286	0.29	0.294	0.297	0.3	0.303	0.306	0.309	0.312
Tonga	0.376	0.38	0.384	0.388	0.392	0.397	0.402	0.408	0.414	0.42	0.428	0.436	0.446	0.455	0.469	0.481	0.492	0.502	0.51	0.517
Vanuatu	0.292	0.296	0.3	0.305	0.313	0.32	0.326	0.331	0.337	0.342	0.346	0.35	0.353	0.357	0.361	0.365	0.369	0.372	0.374	0.377
Southeast Asia	0.316	0.323	0.329	0.336	0.343	0.351	0.359	0.367	0.374	0.382	0.39	0.398	0.406	0.414	0.422	0.43	0.437	0.444	0.451	0.459
Cambodia	0.211	0.213	0.214	0.214	0.214	0.214	0.214	0.213	0.212	0.212	0.213	0.216	0.218	0.222	0.226	0.23	0.235	0.24	0.246	0.253
Indonesia	0.28	0.287	0.294	0.303	0.312	0.321	0.33	0.339	0.347	0.357	0.366	0.376	0.385	0.395	0.405	0.414	0.424	0.433	0.443	0.454
Laos	0.201	0.204	0.207	0.211	0.215	0.219	0.223	0.227	0.231	0.235	0.24	0.246	0.253	0.259	0.266	0.273	0.28	0.287	0.293	0.3
Malaysia	0.412	0.421	0.427	0.43	0.439	0.453	0.468	0.48	0.489	0.496	0.503	0.51	0.52	0.527	0.535	0.543	0.548	0.551	0.559	0.565
Maldives	0.237	0.241	0.245	0.249	0.254	0.259	0.264	0.269	0.273	0.278	0.285	0.292	0.3	0.306	0.314	0.325	0.337	0.349	0.361	0.373
Mauritius	0.391	0.401	0.41	0.416	0.42	0.426	0.436	0.446	0.456	0.467	0.481	0.495	0.507	0.517	0.525	0.532	0.538	0.542	0.544	0.547
Myanmar	0.195	0.206	0.215	0.223	0.23	0.238	0.245	0.251	0.258	0.264	0.271	0.277	0.284	0.292	0.3	0.307	0.314	0.319	0.323	0.326
Philippines	0.417	0.421	0.425	0.43	0.433	0.437	0.442	0.449	0.455	0.463	0.47	0.477	0.484	0.49	0.494	0.497	0.498	0.5	0.502	0.505
Sri Lanka	0.385	0.393	0.399	0.401	0.405	0.41	0.415	0.418	0.422	0.425	0.429	0.433	0.439	0.444	0.45	0.456	0.462	0.469	0.477	0.484
Seychelles	0.358	0.365	0.372	0.38	0.389	0.399	0.409	0.42	0.432	0.445	0.457	0.469	0.479	0.488	0.497	0.507	0.516	0.524	0.533	0.541
Thailand	0.346	0.355	0.363	0.371	0.379	0.387	0.395	0.403	0.409	0.413	0.42	0.429	0.437	0.444	0.454	0.464	0.473	0.48	0.485	0.492
Timor-Leste	0.167	0.174	0.18	0.186	0.193	0.199	0.206	0.212	0.218	0.225	0.23	0.234	0.239	0.243	0.247	0.252	0.258	0.263	0.267	0.272
Vietnam	0.284	0.288	0.292	0.297	0.301	0.306	0.312	0.319	0.326	0.333	0.339	0.347	0.354	0.361	0.369	0.375	0.382	0.388	0.394	0.4
Sub-Saharan Africa	0.212	0.216	0.22	0.225	0.23	0.235	0.24	0.245	0.25	0.255	0.26	0.265	0.27	0.275	0.28	0.284	0.288	0.292	0.296	0.3
Central sub-Saharan Africa	0.182	0.187	0.192	0.198	0.204	0.209	0.215	0.221	0.226	0.232	0.238	0.244	0.25	0.256	0.262	0.269	0.275	0.281	0.287	0.293
Angola	0.127	0.132	0.137	0.142	0.148	0.154	0.16	0.166	0.172	0.178	0.184	0.19	0.196	0.2	0.205	0.21	0.214	0.22	0.225	0.23
Central African Republic	0.153	0.156	0.159	0.162	0.163	0.167	0.171	0.175	0.178	0.18	0.183	0.187	0.189	0.192	0.194	0.198	0.201	0.205	0.21	0.215
Congo (Brazzaville)	0.221	0.227	0.232	0.239	0.245	0.251	0.258	0.264	0.271	0.278	0.286	0.296	0.306	0.316	0.326	0.336	0.346	0.354	0.364	0.373
DR Congo	0.182	0.187	0.192	0.198	0.204	0.209	0.213	0.218	0.223	0.229	0.234	0.24	0.246	0.251	0.258	0.265	0.271	0.277	0.283	0.289
Equatorial Guinea	0.049	0.05	0.05	0.054	0.071	0.083	0.094	0.106	0.117	0.128	0.137	0.146	0.154	0.162	0.168	0.174	0.179	0.184	0.189	0.195
Gabon	0.252	0.26	0.269	0.275	0.283	0.292	0.304	0.314	0.322	0.329	0.338	0.348	0.357	0.365	0.374	0.383	0.392	0.402	0.412	0.422
Eastern sub-Saharan Africa	0.155	0.158	0.161	0.164	0.166	0.169	0.173	0.176	0.18	0.183	0.187	0.192	0.196	0.2	0.205	0.209	0.213	0.217	0.222	0.226
Burundi	0.141	0.146	0.149	0.153	0.157	0.161	0.165	0.171	0.176	0.182	0.188	0.194	0.2	0.206	0.212	0.218	0.225	0.231	0.236	0.242
Comoros	0.157	0.161	0.165	0.171	0.176	0.18	0.184	0.187	0.191	0.196	0.201	0.207	0.212	0.217	0.221	0.227	0.237	0.247	0.256	0.265
Djibouti	0.198	0.202	0.206	0.208	0.21	0.215	0.222	0.229	0.238	0.245	0.25	0.256	0.262	0.267	0.272	0.28	0.288	0.296	0.303	0.308
Eritrea	0.116	0.12	0.124	0.128	0.133	0.136	0.14	0.145	0.149	0.153	0.158	0.162	0.166	0.17	0.174	0.178	0.183	0.187	0.192	0.197
Ethiopia	0.088	0.089	0.091	0.092	0.092	0.094	0.096	0.097	0.098	0.1	0.103	0.106	0.11	0.113	0.116	0.119	0.123	0.127	0.131	0.134
Kenya	0.201	0.206	0.205	0.207	0.213	0.221	0.228	0.231	0.235	0.239	0.246	0.255	0.264	0.272	0.279	0.287	0.297	0.309	0.321	0.331
Baringo	0.155	0.154	0.14	0.124	0.12	0.131	0.135	0.127	0.111	0.093	0.09	0.109	0.125	0.139	0.151	0.164	0.183	0.206	0.227	0.242
Bomet	0.15	0.154	0.144	0.138	0.145	0.16	0.167	0.168	0.167	0.168	0.174	0.189	0.204	0.216	0.226	0.235	0.248	0.265	0.281	0.295
Bungoma	0.181	0.184	0.179	0.176	0.181	0.192	0.2	0.204	0.207	0.213	0.22	0.23	0.239	0.247	0.253	0.259	0.268	0.281	0.294	0.306
Busia	0.19	0.197	0.195	0.195	0.201	0.21	0.216	0.218	0.22	0.222	0.227	0.234	0.239	0.244	0.247	0.251	0.258	0.269	0.28	0.289
Elgeyo Marakwet	0.162	0.166	0.164	0.164	0.17	0.179	0.185	0.187	0.187	0.189	0.193	0.201	0.21	0.218	0.226	0.233	0.244	0.257	0.27	0.282
Embu	0.209	0.214	0.215	0.219	0.227	0.236	0.243	0.248	0.255	0.263	0.272	0.282	0.293	0.303	0.312	0.321	0.332	0.344	0.355	0.366
Garissa	0.09	0.093	0.091	0.091	0.093	0.096	0.098	0.098	0.097	0.098	0.1	0.103	0.106	0.109	0.111	0.114	0.119	0.127	0.137	0.145
Homa Bay	0.148	0.153	0.148	0.145	0.151	0.162	0.169	0.168	0.164	0.161	0.16	0.163	0.165	0.164	0.162	0.16	0.165	0.178	0.193	0.205
Isiolo	0.188	0.192	0.192	0.193	0.197	0.203	0.207	0.208	0.21	0.213	0.216	0.22	0.224	0.227	0.229	0.232	0.237	0.244	0.251	0.258
Kajiado	0.251	0.256	0.258	0.262	0.269	0.278	0.285	0.29	0.295	0.3	0.307	0.315	0.322	0.329	0.335	0.341	0.35	0.359	0.368	0.377
Kakamega	0.187	0.192	0.188	0.187	0.192	0.2	0.206	0.206	0.206	0.208	0.213	0.221	0.228	0.233	0.237	0.242	0.25	0.262	0.275	0.286
Kericho	0.148	0.149	0.137	0.128	0.129	0.136	0.136	0.125	0.108	0.094	0.096	0.119	0.138	0.152	0.165	0.178	0.197	0.218	0.238	0.254
Kiambu	0.256	0.263	0.264	0.268	0.276	0.286	0.294	0.3	0.306	0.313	0.323	0.335	0.347	0.358	0.369	0.379	0.39	0.402	0.415	0.425
Kilifi	0.174	0.178	0.177	0.178	0.182	0.19	0.195	0.197	0.2	0.204	0.21	0.217	0.225	0.231	0.238	0.245	0.254	0.264	0.275	0.284
Kirinyaga	0.209	0.218	0.222	0.228	0.237	0.248	0.257	0.264	0.271	0.279	0.288	0.299	0.311	0.321	0.331	0.341	0.351	0.362	0.372	0.381
Kisii	0.186	0.191	0.189	0.19	0.196	0.205	0.211	0.215	0.218	0.224	0.231	0.24	0.25	0.26	0.269	0.278	0.29	0.303	0.317	0.329
Kisumu	0.165	0.17	0.166	0.165	0.173	0.184	0.193	0.196	0.198	0.204	0.212	0.222	0.231	0.239	0.245	0.252	0.263	0.277	0.292	0.305
Kitui	0.167	0.172	0.173	0.176	0.182	0.189	0.195	0.198	0.201	0.205	0.21	0.217	0.222	0.226	0.23	0.234	0.241	0.251	0.262	0.272
Kwale	0.164	0.169	0.171	0.173	0.179	0.188	0.194	0.198	0.202	0.208	0.214	0.223	0.23	0.237	0.244	0.25	0.258	0.268	0.278	0.286
Laikipia	0.168	0.175	0.173	0.173	0.18	0.193	0.203	0.209	0.214	0.22	0.229	0.242	0.255	0.267	0.278	0.288	0.301	0.314	0.326	0.337
Lamu	0.148	0.154	0.152	0.152	0.159	0.168	0.174	0.176	0.18	0.187	0.196	0.209	0.22	0.23	0.238	0.246	0.255	0.267	0.278	0.287
Machakos	0.207	0.214	0.216	0.219	0.226	0.235	0.242	0.246	0.251	0.257	0.264	0.272	0.28	0.286	0.292	0.299	0.308	0.319	0.331	0.342
Makueni	0.201	0.207	0.207	0.209	0.215	0.223	0.229	0.232	0.234	0.237	0.241	0.247	0.252	0.255	0.259	0.262	0.268	0.276	0.285	0.293
Mandera	0.068	0.069	0.066	0.063	0.064	0.066	0.066	0.063	0.057	0.053	0.052	0.055	0.057	0.058	0.057	0.055	0.061	0.073	0.086	0.096
Marsabit	0.151	0.155	0.156	0.157	0.161	0.166	0.17	0.173	0.175	0.178	0.182	0.186	0.19	0.193	0.196	0.198	0.202	0.206	0.211	0.216
Meru	0.197	0.204	0.208	0.212	0.219	0.228	0.235	0.24	0.246	0.252	0.258	0.265	0.272	0.279	0.285	0.292	0.301	0.31	0.32	0.329

Appendix Table 10. Socio-demographic Index values for all estimated GBD 2017 locations, 1970–1989

Location	1970	1971	1972	1973	1974	1975	1976	1977	1978	1979	1980	1981	1982	1983	1984	1985	1986	1987	1988	1989
Migori	0.151	0.154	0.148	0.145	0.151	0.161	0.167	0.166	0.163	0.161	0.162	0.168	0.173	0.175	0.175	0.177	0.184	0.197	0.21	0.221
Mombasa	0.225	0.233	0.238	0.245	0.254	0.264	0.272	0.28	0.288	0.297	0.306	0.316	0.324	0.332	0.34	0.348	0.357	0.367	0.377	0.387
Murang'a	0.231	0.236	0.235	0.237	0.243	0.252	0.258	0.262	0.267	0.273	0.282	0.294	0.304	0.313	0.321	0.33	0.34	0.351	0.363	0.373
Nairobi	0.315	0.325	0.332	0.34	0.351	0.363	0.374	0.384	0.393	0.403	0.413	0.423	0.432	0.44	0.448	0.455	0.463	0.473	0.482	0.491
Nakuru	0.184	0.189	0.191	0.194	0.2	0.209	0.215	0.219	0.223	0.227	0.234	0.242	0.251	0.259	0.267	0.276	0.286	0.3	0.313	0.326
Nandi	0.179	0.181	0.172	0.166	0.168	0.177	0.182	0.181	0.179	0.178	0.184	0.196	0.21	0.224	0.236	0.249	0.264	0.281	0.297	0.311
Narok	0.093	0.086	0.061	0.062	0.063	0.064	0.065	0.066	0.067	0.068	0.069	0.07	0.071	0.072	0.101	0.121	0.144	0.169	0.191	0.206
Nyamira	0.195	0.202	0.204	0.207	0.216	0.228	0.238	0.245	0.251	0.257	0.266	0.275	0.285	0.293	0.301	0.308	0.318	0.329	0.341	0.351
Nyandarua	0.184	0.193	0.193	0.195	0.201	0.211	0.218	0.22	0.222	0.227	0.235	0.247	0.26	0.272	0.283	0.293	0.305	0.319	0.332	0.344
Nyeri	0.229	0.238	0.242	0.247	0.256	0.267	0.275	0.282	0.288	0.295	0.304	0.315	0.326	0.337	0.346	0.355	0.366	0.377	0.388	0.398
Samburu	0.126	0.128	0.122	0.117	0.12	0.127	0.13	0.128	0.124	0.12	0.122	0.13	0.139	0.146	0.152	0.158	0.167	0.179	0.19	0.199
Siaya	0.112	0.117	0.117	0.118	0.123	0.131	0.137	0.14	0.142	0.145	0.15	0.156	0.162	0.167	0.172	0.177	0.185	0.196	0.208	0.218
Taita Taveta	0.199	0.205	0.207	0.211	0.218	0.226	0.233	0.239	0.245	0.251	0.259	0.268	0.276	0.284	0.292	0.3	0.309	0.32	0.331	0.342
Tana River	0.116	0.122	0.124	0.128	0.134	0.143	0.149	0.154	0.158	0.163	0.169	0.177	0.183	0.188	0.192	0.196	0.202	0.209	0.217	0.224
Tharaka Nithi	0.193	0.201	0.207	0.213	0.221	0.23	0.238	0.244	0.251	0.259	0.266	0.274	0.281	0.288	0.294	0.301	0.309	0.317	0.326	0.334
Trans Nzoia	0.195	0.198	0.194	0.192	0.196	0.204	0.208	0.207	0.204	0.203	0.206	0.215	0.224	0.233	0.242	0.252	0.265	0.28	0.295	0.308
Turkana	0.16	0.162	0.161	0.161	0.164	0.168	0.17	0.171	0.17	0.171	0.172	0.176	0.179	0.182	0.185	0.187	0.191	0.196	0.202	0.206
Uasin Gishu	0.203	0.207	0.204	0.203	0.209	0.219	0.226	0.228	0.23	0.232	0.239	0.25	0.263	0.275	0.286	0.296	0.309	0.324	0.339	0.352
Vihiga	0.206	0.211	0.21	0.212	0.218	0.226	0.232	0.235	0.238	0.242	0.247	0.254	0.261	0.267	0.272	0.278	0.286	0.297	0.308	0.319
Wajir	0.08	0.08	0.074	0.069	0.068	0.069	0.067	0.059	0.049	0.037	0.038	0.038	0.041	0.044	0.043	0.042	0.053	0.071	0.086	0.097
West Pokot	0.14	0.142	0.138	0.134	0.137	0.143	0.147	0.145	0.141	0.137	0.136	0.14	0.146	0.152	0.156	0.161	0.17	0.183	0.195	0.205
Madagascar	0.158	0.163	0.169	0.175	0.18	0.185	0.19	0.196	0.202	0.207	0.212	0.219	0.226	0.233	0.238	0.244	0.249	0.253	0.257	0.26
Malawi	0.119	0.123	0.127	0.131	0.134	0.137	0.14	0.144	0.147	0.151	0.156	0.16	0.164	0.169	0.174	0.18	0.184	0.189	0.192	0.196
Mozambique	0.103	0.107	0.109	0.112	0.114	0.116	0.118	0.119	0.121	0.123	0.125	0.127	0.128	0.128	0.129	0.129	0.129	0.129	0.129	0.131
Rwanda	0.176	0.181	0.186	0.19	0.194	0.197	0.2	0.203	0.206	0.211	0.216	0.222	0.228	0.235	0.24	0.246	0.251	0.256	0.26	0.264
Somalia	0.113	0.115	0.117	0.119	0.12	0.123	0.125	0.128	0.13	0.132	0.134	0.134	0.136	0.137	0.138	0.14	0.141	0.144	0.147	0.15
South Sudan	0.139	0.141	0.143	0.145	0.148	0.15	0.153	0.155	0.158	0.16	0.162	0.165	0.167	0.169	0.17	0.172	0.173	0.175	0.176	0.178
Tanzania	0.16	0.163	0.167	0.171	0.176	0.18	0.186	0.193	0.2	0.208	0.216	0.222	0.229	0.235	0.241	0.248	0.254	0.259	0.265	0.27
Uganda	0.104	0.106	0.108	0.112	0.116	0.12	0.124	0.129	0.133	0.136	0.138	0.141	0.145	0.148	0.151	0.154	0.156	0.158	0.16	0.161
Zambia	0.236	0.238	0.24	0.242	0.244	0.246	0.249	0.252	0.257	0.263	0.268	0.274	0.28	0.285	0.29	0.294	0.299	0.303	0.306	0.309
Southern sub-Saharan Africa	0.388	0.393	0.4	0.409	0.417	0.422	0.428	0.436	0.445	0.452	0.457	0.463	0.471	0.48	0.487	0.492	0.496	0.503	0.511	0.517
Botswana	0.253	0.26	0.269	0.279	0.289	0.299	0.309	0.319	0.329	0.339	0.349	0.36	0.37	0.381	0.393	0.404	0.417	0.429	0.44	0.452
Lesotho	0.221	0.223	0.227	0.232	0.238	0.242	0.247	0.254	0.263	0.269	0.276	0.282	0.288	0.293	0.299	0.304	0.31	0.315	0.32	0.327
Namibia	0.324	0.329	0.335	0.342	0.349	0.356	0.364	0.371	0.379	0.386	0.393	0.401	0.408	0.414	0.421	0.427	0.432	0.438	0.444	0.449
South Africa	0.426	0.431	0.439	0.45	0.458	0.463	0.469	0.477	0.487	0.494	0.499	0.504	0.512	0.521	0.529	0.532	0.535	0.542	0.549	0.554
Swaziland	0.283	0.289	0.294	0.299	0.305	0.311	0.317	0.323	0.329	0.334	0.34	0.348	0.356	0.363	0.371	0.379	0.388	0.398	0.408	0.417
Zimbabwe	0.246	0.252	0.258	0.264	0.271	0.277	0.283	0.288	0.294	0.3	0.308	0.316	0.326	0.336	0.346	0.358	0.369	0.38	0.39	0.4
Western sub-Saharan Africa	0.206	0.211	0.216	0.221	0.227	0.233	0.239	0.244	0.25	0.255	0.259	0.264	0.268	0.271	0.275	0.279	0.282	0.284	0.287	0.29
Benin	0.147	0.15	0.153	0.156	0.159	0.162	0.165	0.168	0.171	0.174	0.178	0.182	0.185	0.189	0.193	0.197	0.201	0.206	0.21	0.214
Burkina Faso	0.095	0.095	0.096	0.098	0.1	0.101	0.102	0.103	0.105	0.108	0.111	0.113	0.115	0.117	0.119	0.123	0.126	0.129	0.132	0.136
Cameroon	0.215	0.218	0.222	0.225	0.229	0.234	0.238	0.244	0.25	0.256	0.261	0.267	0.273	0.28	0.288	0.295	0.303	0.311	0.318	0.326
Cape Verde	0.193	0.2	0.207	0.213	0.218	0.223	0.227	0.231	0.235	0.239	0.244	0.247	0.25	0.254	0.259	0.265	0.272	0.281	0.29	0.299
Chad	0.095	0.096	0.097	0.097	0.098	0.099	0.1	0.101	0.101	0.101	0.1	0.1	0.099	0.1	0.101	0.103	0.105	0.108	0.111	0.115
Cote d'Ivoire	0.19	0.195	0.199	0.204	0.208	0.213	0.217	0.219	0.222	0.224	0.226	0.228	0.23	0.233	0.236	0.24	0.245	0.252	0.259	0.266
The Gambia	0.153	0.155	0.158	0.161	0.165	0.169	0.173	0.177	0.181	0.185	0.189	0.192	0.196	0.202	0.208	0.214	0.221	0.227	0.233	0.238
Ghana	0.285	0.292	0.296	0.298	0.301	0.306	0.315	0.323	0.329	0.332	0.337	0.343	0.346	0.349	0.352	0.356	0.361	0.365	0.37	0.374
Guinea	0.12	0.122	0.123	0.125	0.128	0.131	0.133	0.136	0.138	0.14	0.143	0.145	0.147	0.148	0.15	0.152	0.155	0.157	0.159	0.161
Guinea-Bissau	0.137	0.138	0.14	0.142	0.143	0.145	0.148	0.149	0.15	0.152	0.153	0.155	0.156	0.158	0.16	0.163	0.167	0.171	0.174	0.178
Liberia	0.16	0.165	0.169	0.173	0.177	0.18	0.182	0.184	0.186	0.189	0.192	0.194	0.196	0.198	0.2	0.203	0.205	0.208	0.212	0.216
Mali	0.088	0.09	0.092	0.094	0.095	0.097	0.099	0.102	0.104	0.107	0.109	0.112	0.114	0.116	0.117	0.119	0.121	0.123	0.125	0.128
Mauritania	0.203	0.207	0.21	0.212	0.214	0.217	0.22	0.223	0.226	0.229	0.232	0.238	0.244	0.251	0.257	0.264	0.271	0.278	0.285	0.292
Niger	0.077	0.077	0.077	0.075	0.074	0.073	0.072	0.07	0.067	0.064	0.062	0.059	0.056	0.056	0.059	0.064	0.07	0.076	0.082	0.088
Nigeria	0.237	0.245	0.252	0.26	0.268	0.276	0.284	0.292	0.3	0.307	0.313	0.319	0.324	0.329	0.333	0.337	0.339	0.339	0.341	0.342
Sao Tome and Principe	0.184	0.19	0.197	0.204	0.209	0.215	0.221	0.228	0.234	0.24	0.244	0.249	0.253	0.257	0.26	0.264	0.268	0.273	0.278	0.283
Senegal	0.157	0.159	0.161	0.163	0.165	0.168	0.171	0.174	0.176	0.18	0.184	0.188	0.192	0.198	0.204	0.21	0.216	0.222	0.23	0.237
Sierra Leone	0.149	0.153	0.156	0.16	0.163	0.167	0.17	0.173	0.175	0.176	0.178	0.18	0.182	0.183	0.185	0.187	0.189	0.191	0.194	0.198
Togo	0.163	0.167	0.171	0.174	0.179	0.183	0.187	0.192	0.197	0.203	0.209	0.214	0.219	0.224	0.229	0.234	0.239	0.244	0.25	0.256

Appendix Table 11. Socio-demographic Index values for all estimated GBD 2017 locations, 1990-2017

Location	1990	1991	1992	1993	1994	1995	1996	1997	1998	1999	2000	2001	2002	2003	2004	2005	2006	2007	2008	2009	2010	2011	2012	2013	2014	2015	2016	2017
Global	0.523	0.529	0.534	0.539	0.543	0.548	0.553	0.557	0.561	0.566	0.571	0.576	0.581	0.585	0.59	0.595	0.601	0.606	0.611	0.616	0.62	0.624	0.628	0.633	0.639	0.644	0.647	0.652
Central Europe, Eastern Europe, and Central Asia	0.656	0.662	0.67	0.674	0.677	0.682	0.686	0.689	0.691	0.694	0.698	0.701	0.705	0.709	0.715	0.72	0.725	0.73	0.735	0.739	0.743	0.747	0.75	0.753	0.757	0.76	0.763	0.766
Central Asia	0.563	0.567	0.57	0.573	0.575	0.577	0.578	0.579	0.58	0.582	0.585	0.588	0.593	0.598	0.603	0.609	0.615	0.621	0.627	0.633	0.639	0.644	0.649	0.654	0.659	0.664	0.669	0.673
Armenia	0.555	0.559	0.56	0.562	0.565	0.567	0.57	0.573	0.577	0.581	0.586	0.592	0.6	0.61	0.619	0.629	0.639	0.65	0.66	0.667	0.673	0.678	0.683	0.687	0.691	0.695	0.699	0.702
Azerbaijan	0.611	0.614	0.616	0.617	0.616	0.613	0.61	0.607	0.604	0.601	0.6	0.6	0.602	0.605	0.608	0.615	0.625	0.635	0.645	0.654	0.664	0.672	0.678	0.684	0.689	0.694	0.698	0.701
Georgia	0.654	0.66	0.661	0.658	0.653	0.645	0.639	0.634	0.63	0.625	0.621	0.62	0.622	0.624	0.628	0.633	0.638	0.644	0.649	0.654	0.659	0.665	0.671	0.676	0.682	0.688	0.695	0.7
Kazakhstan	0.613	0.615	0.619	0.625	0.632	0.638	0.643	0.645	0.646	0.647	0.651	0.656	0.661	0.666	0.671	0.677	0.683	0.689	0.696	0.702	0.705	0.707	0.708	0.711	0.716	0.723	0.73	0.735
Kyrgyzstan	0.565	0.571	0.576	0.578	0.577	0.572	0.569	0.567	0.564	0.562	0.56	0.559	0.56	0.562	0.565	0.566	0.567	0.569	0.572	0.575	0.576	0.581	0.584	0.589	0.594	0.598	0.603	0.607
Mongolia	0.537	0.545	0.55	0.555	0.559	0.564	0.569	0.573	0.577	0.581	0.585	0.589	0.594	0.598	0.603	0.608	0.614	0.619	0.624	0.628	0.632	0.636	0.641	0.646	0.65	0.654	0.658	0.662
Tajikistan	0.474	0.481	0.485	0.487	0.486	0.481	0.474	0.468	0.463	0.459	0.455	0.454	0.456	0.462	0.465	0.466	0.472	0.479	0.483	0.488	0.494	0.501	0.506	0.51	0.514	0.517	0.52	0.523
Turkmenistan	0.588	0.592	0.594	0.599	0.602	0.604	0.606	0.606	0.606	0.607	0.61	0.613	0.617	0.622	0.628	0.635	0.638	0.641	0.644	0.647	0.651	0.657	0.663	0.669	0.678	0.685	0.691	0.696
Uzbekistan	0.481	0.484	0.487	0.493	0.497	0.502	0.508	0.513	0.52	0.526	0.532	0.537	0.543	0.549	0.555	0.56	0.565	0.57	0.575	0.581	0.587	0.592	0.598	0.604	0.611	0.618	0.624	0.63
Central Europe	0.665	0.671	0.677	0.683	0.69	0.698	0.705	0.711	0.717	0.723	0.731	0.738	0.745	0.751	0.757	0.762	0.767	0.772	0.776	0.782	0.788	0.793	0.797	0.802	0.805	0.808	0.811	0.814
Albania	0.548	0.545	0.542	0.541	0.542	0.546	0.552	0.558	0.566	0.577	0.584	0.593	0.602	0.611	0.619	0.627	0.635	0.642	0.648	0.653	0.658	0.661	0.665	0.668	0.672	0.676	0.681	0.685
Bosnia and Herzegovina	0.497	0.499	0.5	0.5	0.501	0.507	0.525	0.549	0.571	0.592	0.607	0.619	0.63	0.639	0.647	0.654	0.66	0.667	0.673	0.679	0.685	0.69	0.694	0.699	0.703	0.706	0.71	0.713
Bulgaria	0.658	0.668	0.676	0.684	0.693	0.699	0.705	0.706	0.704	0.703	0.708	0.715	0.721	0.726	0.731	0.736	0.741	0.746	0.751	0.757	0.765	0.771	0.775	0.778	0.781	0.784	0.788	0.792
Croatia	0.725	0.73	0.732	0.732	0.731	0.731	0.732	0.737	0.743	0.749	0.755	0.762	0.768	0.773	0.778	0.782	0.787	0.792	0.797	0.801	0.805	0.809	0.813	0.816	0.818	0.821	0.823	0.825
Czech Republic	0.711	0.717	0.726	0.74	0.757	0.769	0.777	0.783	0.788	0.794	0.799	0.804	0.809	0.814	0.819	0.823	0.827	0.83	0.833	0.836	0.84	0.843	0.846	0.847	0.848	0.848	0.849	0.851
Hungary	0.678	0.683	0.691	0.699	0.707	0.716	0.724	0.732	0.739	0.745	0.751	0.758	0.764	0.77	0.776	0.781	0.786	0.791	0.795	0.799	0.803	0.806	0.807	0.808	0.809	0.811	0.814	0.817
Macedonia	0.626	0.629	0.63	0.631	0.632	0.635	0.64	0.647	0.654	0.661	0.665	0.67	0.677	0.685	0.693	0.699	0.704	0.709	0.715	0.719	0.724	0.729	0.734	0.739	0.744	0.748	0.751	0.754
Montenegro	0.705	0.706	0.705	0.701	0.698	0.696	0.696	0.698	0.7	0.703	0.706	0.711	0.716	0.721	0.726	0.731	0.737	0.743	0.75	0.756	0.761	0.767	0.771	0.775	0.779	0.782	0.785	0.788
Poland	0.662	0.668	0.678	0.686	0.697	0.707	0.714	0.724	0.733	0.741	0.75	0.759	0.767	0.773	0.779	0.784	0.789	0.792	0.797	0.804	0.811	0.818	0.823	0.829	0.833	0.837	0.841	0.844
Romania	0.652	0.66	0.663	0.666	0.671	0.678	0.682	0.685	0.689	0.694	0.7	0.707	0.713	0.718	0.724	0.73	0.734	0.739	0.745	0.751	0.758	0.763	0.768	0.772	0.774	0.777	0.78	0.784
Serbia	0.632	0.638	0.643	0.642	0.641	0.641	0.643	0.648	0.653	0.655	0.661	0.665	0.669	0.675	0.684	0.692	0.699	0.705	0.709	0.713	0.718	0.723	0.729	0.736	0.742	0.747	0.75	0.752
Slovakia	0.684	0.69	0.699	0.71	0.722	0.732	0.74	0.748	0.756	0.764	0.772	0.779	0.784	0.788	0.793	0.798	0.804	0.809	0.814	0.818	0.823	0.828	0.832	0.834	0.836	0.838	0.839	0.842
Slovenia	0.741	0.747	0.753	0.759	0.764	0.769	0.775	0.781	0.788	0.794	0.801	0.808	0.814	0.819	0.824	0.828	0.833	0.837	0.841	0.843	0.846	0.848	0.85	0.852	0.854	0.856	0.858	0.86
Eastern Europe	0.678	0.685	0.694	0.698	0.7	0.704	0.708	0.708	0.71	0.711	0.712	0.713	0.715	0.72	0.727	0.734	0.739	0.745	0.751	0.756	0.761	0.764	0.767	0.772	0.776	0.779	0.783	0.785
Belarus	0.625	0.631	0.636	0.641	0.645	0.647	0.65	0.654	0.657	0.661	0.665	0.67	0.676	0.682	0.689	0.696	0.704	0.712	0.72	0.727	0.733	0.74	0.747	0.753	0.759	0.764	0.769	0.773
Estonia	0.711	0.719	0.728	0.736	0.742	0.746	0.75	0.755	0.761	0.766	0.772	0.778	0.783	0.788	0.794	0.799	0.806	0.813	0.82	0.826	0.832	0.838	0.843	0.847	0.851	0.854	0.856	0.858
Latvia	0.696	0.703	0.712	0.721	0.727	0.731	0.733	0.734	0.735	0.738	0.741	0.745	0.75	0.757	0.763	0.769	0.776	0.783	0.792	0.8	0.806	0.81	0.814	0.816	0.817	0.819	0.822	0.825
Lithuania	0.707	0.71	0.717	0.725	0.728	0.731	0.733	0.736	0.74	0.746	0.753	0.76	0.765	0.772	0.779	0.785	0.79	0.796	0.802	0.808	0.815	0.822	0.828	0.833	0.836	0.838	0.839	0.841
Moldova	0.575	0.578	0.58	0.582	0.583	0.584	0.584	0.582	0.58	0.577	0.574	0.574	0.577	0.582	0.588	0.595	0.602	0.61	0.618	0.624	0.632	0.64	0.647	0.654	0.66	0.666	0.671	0.676
Russian Federation	0.683	0.692	0.704	0.708	0.708	0.714	0.718	0.719	0.72	0.722	0.722	0.722	0.724	0.728	0.734	0.742	0.747	0.752	0.757	0.763	0.768	0.77	0.772	0.777	0.781	0.785	0.789	0.792
Ukraine	0.664	0.667	0.67	0.673	0.675	0.676	0.676	0.675	0.673	0.672	0.672	0.673	0.675	0.68	0.687	0.694	0.7	0.707	0.714	0.717	0.721	0.725	0.729	0.732	0.735	0.736	0.738	0.74
High-income	0.769	0.774	0.779	0.783	0.787	0.792	0.796	0.798	0.801	0.804	0.807	0.811	0.814	0.817	0.82	0.822	0.823	0.826	0.829	0.832	0.836	0.839	0.842	0.845	0.848	0.851	0.853	0.854
Australasia	0.783	0.786	0.79	0.794	0.797	0.801	0.805	0.809	0.813	0.817	0.821	0.825	0.828	0.832	0.835	0.837	0.838	0.84	0.842	0.845	0.848	0.851	0.855	0.859	0.862	0.864	0.867	0.869
Australia	0.786	0.79	0.793	0.797	0.801	0.805	0.81	0.814	0.818	0.822	0.825	0.829	0.833	0.837	0.84	0.843	0.844	0.845	0.848	0.851	0.854	0.856	0.86	0.864	0.867	0.869	0.871	0.873
New Zealand	0.765	0.768	0.771	0.774	0.777	0.78	0.783	0.786	0.79	0.794	0.798	0.802	0.805	0.807	0.809	0.811	0.811	0.811	0.813	0.816	0.819	0.823	0.828	0.832	0.835	0.838	0.84	0.842
High-income Asia-Pacific	0.783	0.789	0.794	0.799	0.804	0.809	0.813	0.817	0.82	0.823	0.826	0.83	0.833	0.836	0.839	0.842	0.844	0.846	0.849	0.851	0.853	0.856	0.858	0.861	0.863	0.865	0.867	0.869
Brunei	0.728	0.733	0.739	0.745	0.751	0.757	0.763	0.769	0.774	0.779	0.784	0.789	0.795	0.802	0.808	0.814	0.819	0.824	0.828	0.831	0.835	0.838	0.842	0.845	0.848	0.851	0.854	0.856
Japan	0.803	0.807	0.812	0.816	0.82	0.823	0.826	0.829	0.831	0.833	0.834	0.836	0.838	0.84	0.842	0.844	0.846	0.847	0.849	0.851	0.853	0.855	0.857	0.859	0.861	0.862	0.863	0.865
Aichi	0.812	0.816	0.821	0.825	0.829	0.833	0.836	0.839	0.841	0.843	0.844	0.846	0.847	0.85	0.852	0.854	0.855	0.856	0.858	0.86	0.862	0.864	0.866	0.869	0.871	0.872	0.873	0.875
Akita	0.766	0.77	0.775	0.778	0.781	0.785	0.788	0.791	0.793	0.795	0.796	0.798	0.8	0.802	0.805	0.806	0.808	0.81	0.812	0.814	0.816	0.818	0.821	0.823	0.825	0.826	0.827	0.829
Aomori	0.761	0.765	0.77	0.773	0.777	0.78	0.783	0.786	0.788	0.79	0.791	0.793	0.795	0.798	0.801	0.803	0.805	0.807	0.809	0.81	0.812	0.815	0.817	0.819	0.821	0.822	0.823	0.825
Chiba	0.803	0.807	0.812	0.816	0.82	0.823	0.827	0.829	0.831	0.832	0.833	0.834	0.836	0.837	0.839	0.841	0.842	0.843	0.845	0.846	0.848	0.85	0.852	0.854	0.856	0.857	0.858	0.859
Ehime	0.776	0.78	0.785	0.788	0.792	0.796	0.799	0.802	0.804	0.806	0.808	0.809	0.811	0.814	0.816	0.818	0.819	0.82	0.822	0.824	0.825	0.828	0.83	0.832	0.834	0.835	0.836	0.838
Fukui	0.784	0.789	0.794	0.798	0.802	0.806	0.81	0.813	0.815	0.818	0.819	0.821	0.823	0.826	0.828	0.83	0.832	0.834	0.836	0.837	0.839	0.842	0.844	0.846	0.848	0.849	0.85	0.852
Fukuoka	0.797	0.801	0.806	0.809	0.813	0.816	0.819	0.822	0.824	0.826	0.828	0.83	0.832	0.834	0.836	0.837	0.838	0.839	0.841	0.842	0.843	0.845	0.847	0.849	0.851	0.853	0.854	0.855
Fukushima	0.769	0.773	0.778	0.781	0.784	0.788	0.791	0.793	0.794	0.795	0.797	0.798	0.8	0.803	0.807	0.809	0.811	0.813	0.814	0.816	0.819	0.821	0.823	0.825	0.827	0.828	0.829	0.831
Gifu	0.786	0.79	0.795	0.799	0.803	0.807	0.81	0.813	0.815	0.817	0.818	0.82	0.821	0.824	0.826	0.828	0.829	0.831	0.833	0.835	0.837	0.839	0.841	0.843	0.845	0.846	0.847	0.849
Gunma	0.787	0.791	0.796	0.8	0.803	0.807	0.81	0.813	0.815	0.816	0.818	0.819	0.821	0.824	0.827	0.829	0.831	0.833	0.835	0.837	0.839	0.841	0.843	0.845	0.847	0.848	0.849	0.851
Hiroshima	0.8	0.805	0.809	0.813	0.817	0.821	0.825	0.828	0.83	0.832	0.833	0.835	0.836	0.839	0.841	0.842	0.843	0.844	0.846	0.847	0.849	0.852	0.854	0.856	0.858	0.86	0.861	0.863
Hokkaidō	0.783	0.788	0.792	0.796	0.799	0.803	0.806	0.808	0.809	0.811	0.812	0.813	0.815	0.818	0.82	0.822	0.824	0.825	0.827	0.828	0.83	0.832	0.834	0.836	0.838	0.839	0.84	0.842
Hyōgo	0.798	0.803	0.807	0.811	0.815	0.819	0.822	0.825	0.827	0.829	0.831	0.833	0.835	0.837	0.839	0.841	0.842	0.843	0.845	0.846	0.848	0.85	0.852	0.854	0.856	0.857	0.858	0.86
Ibaraki	0.789	0.793	0.798	0.802	0.805	0.809	0.813	0.815	0.817	0.819	0.82	0.821	0.823	0.826	0.828	0.83	0.831	0.833	0.835	0.837	0.839	0.841	0.843	0.845	0.847	0.848	0.849	0.851
Ishikawa	0.789	0.795	0.8	0.804	0.808	0.812	0.815	0.818	0.82	0.822	0.824	0.826	0.828	0.831	0.833	0.835	0.837	0.839	0.84	0.842	0.844	0.846	0.848	0.85	0.852	0.853	0.854	0.856
Iwate	0.76	0.764	0.768	0.772	0.775	0.779	0.782	0.785	0.787	0.788	0.79	0.791	0.793	0.796	0.799	0.801	0.803	0.806	0.808	0.81	0.812	0.815	0.817	0.819	0.821	0.823	0.823	0.825
Kagawa	0.787	0.792	0.797	0.801	0.804	0.808	0.811	0.814	0.816	0.817	0.819	0.82	0.822	0.825	0.827	0.829	0.831	0.832	0.834	0.835	0.837	0.839	0.842	0.844	0.846	0.847	0.848	0.85
Kagoshima	0.768	0.772	0.777	0.781	0.784	0.788	0.791	0.793	0.795	0.797	0.798	0.799	0.801	0.804	0.806	0.808	0.809	0.81	0.812	0.814	0.816	0.818	0.82	0.823	0.825	0.827	0.828	0.83
Kanagawa	0.818	0.823	0.827	0.831	0.835	0.838	0.842	0.844	0.846	0.848	0.849	0.85	0.852	0.854	0.856	0.857	0.859	0.86	0.862	0.863	0.865	0.866	0.868	0.87	0.871	0.873	0.873	0.875
Kōchi	0.759	0.763	0.767	0.771	0.775	0.779	0.783	0.786	0.788	0.791	0.793	0.795	0.797	0.8	0.803	0.805	0.806	0.808	0.81	0.812	0.813	0.816	0.818	0.82	0.822	0.823	0.824	0.825
Kumamoto	0.77	0.774	0.778	0.782	0.785	0.789	0.792	0.794	0.796	0.798	0.799	0.801	0.803	0.805	0.808	0.81	0.811	0.812	0.814	0.815	0.817	0.82	0.822	0.825	0.827	0.829	0.83	0.832
Kyōto	0.813	0.817	0.822	0.826	0.83	0.833	0.837	0.839	0.841	0.843	0.845	0.847	0.849	0.851	0.853	0.854	0.856	0.857	0.859	0.86	0.862	0.863	0.865	0.867	0.869	0.87	0.871	0.873
Mie	0.787	0.792	0.797	0.801	0.805	0.81	0.813	0.815	0.818	0.819	0.821	0.823	0.825	0.827	0.83	0.832	0.833	0.835	0.836	0.838	0.84	0.842	0.845	0.847	0.849	0.851	0.852	0.854
Miyagi	0.788	0.793	0.798	0.801	0.805	0.809	0.812	0.814	0.816	0.817	0.818	0.819	0.821	0.824	0.827	0.829	0.831	0.833	0.835	0.836	0.838	0.841	0.843	0.845	0.847	0.848	0.849	0.85
Miyazaki	0.764	0.768	0.772	0.775	0.778	0.781	0.784	0.786	0.788	0.79	0.791	0.793	0.795	0.797	0.8	0.801	0.802	0.804	0.805	0.807	0.809	0.812	0.814	0.816	0.819	0.82	0.821	0.823
Nagano	0.792	0.797	0.801	0.805	0.808	0.811	0.814	0.817	0.818	0.82	0.821	0.823	0.824	0.827	0.829	0.831	0.832	0.834	0.836	0.838	0.839	0.842	0.844	0.845	0.847	0.849	0.849	0.851
Nagasaki	0.766	0.77	0.774	0.777	0.781	0.784	0.787	0.79	0.792	0.794	0.795	0.797	0.799	0.801	0.803	0.805	0.806	0.808	0.809	0.811	0.813	0.815	0.817	0.82	0.822	0.823	0.824	0.826
Nara	0.789	0.794	0.799	0.803	0.807	0.811	0.814	0.817	0.819	0.821	0.822	0.824	0.825	0.827	0.829	0.831	0.832	0.834	0.835	0.836	0.838	0.839	0.841	0.843	0.844	0.845	0.846	0.848
Niigata	0.776	0.78	0.784	0.788	0.792	0.795	0.799	0.801	0.804	0.806	0.808	0.81	0.813	0.815	0.818	0.82	0.822	0.824	0.826	0.828	0.83	0.832	0.835	0.837	0.839	0.84	0.842	0.843
Ōita	0.785	0.789	0.795	0.802	0.808	0.809	0.809	0.81	0.812	0.813	0.815	0.816	0.819	0.821	0.824	0.825	0.827	0.828	0.83	0.831	0.833	0.835	0.838	0.84	0.842	0.843	0.844	0.846
Okayama	0.79	0.794	0.799	0.803	0.807	0.811	0.814	0.817	0.819	0.821	0.823	0.824	0.827	0.829	0.832	0.834	0.836	0.837	0.839	0.841	0.843	0.846	0.848	0.85	0.852	0.853	0.854	0.856
Okinawa	0.753	0.757	0.764	0.772	0.778	0.779	0.777	0.777	0.778	0.78	0.781	0.783	0.786	0.789	0.793	0.795	0.797	0.799	0.8	0.802	0.804	0.807	0.809	0.812	0.814	0.815	0.816	0.818
Ōsaka	0.815	0.819	0.823	0.827	0.831	0.834	0.837	0.84	0.842	0.844	0.845	0.847	0.849	0.851	0.853	0.854	0.855	0.856	0.858	0.859	0.861	0.863	0.865	0.867	0.868	0.87	0.871	0.872
Saga	0.773	0.777	0.782	0.785	0.788	0.792	0.795	0.797	0.799	0.8	0.801	0.803	0.805	0.808	0.81	0.812	0.814	0.815	0.817	0.819	0.821	0.823	0.825	0.827	0.83	0.831	0.832	0.834
Saitama	0.793	0.798	0.802	0.806	0.81	0.814	0.818	0.82	0.822	0.824	0.825	0.826	0.827	0.829	0.831	0.833	0.835	0.836	0.838	0.839	0.841	0.843	0.845	0.847	0.848	0.849	0.85	0.852
Shiga	0.804	0.809	0.814	0.819	0.823	0.827	0.83	0.833	0.836	0.838	0.84	0.841	0.843	0.846	0.848	0.85	0.852	0.853	0.855	0.857	0.858	0.861	0.863	0.865	0.867	0.868	0.869	0.871
Shimane	0.762	0.766	0.771	0.775	0.779	0.783	0.787	0.79	0.793	0.795	0.797	0.798	0.801	0.803	0.806	0.808	0.81	0.812	0.814	0.816	0.818	0.82	0.823	0.825	0.827	0.828	0.829	0.831
Shizuoka	0.798	0.802	0.807	0.81	0.814	0.818	0.821	0.823	0.825	0.826	0.827	0.828	0.83	0.833	0.835	0.837	0.838	0.84	0.842	0.843	0.846	0.848	0.85	0.853	0.855	0.856	0.857	0.859
Tochigi	0.787	0.792	0.797	0.801	0.805	0.809	0.813	0.815	0.817	0.819	0.82	0.821	0.823	0.826	0.829	0.831	0.832	0.834	0.836	0.838	0.84	0.842	0.845	0.847	0.849	0.85	0.851	0.853
Tokushima	0.776	0.781	0.786	0.79	0.794	0.798	0.802	0.805	0.807	0.809	0.812	0.814	0.816	0.819	0.822	0.824	0.826	0.828	0.83	0.831	0.833	0.835	0.837	0.839	0.841	0.842	0.843	0.845
Tōkyō	0.87	0.875	0.879	0.883	0.887	0.892	0.896	0.899	0.901	0.903	0.905	0.906	0.908	0.91	0.911	0.913	0.914	0.915	0.916	0.917	0.918	0.919	0.92	0.921	0.922	0.923	0.923	0.924
Tottori	0.77	0.775	0.78	0.783	0.787	0.791	0.794	0.797	0.799	0.801	0.802	0.804	0.806	0.808	0.811	0.813	0.814	0.816	0.818	0.819	0.821	0.824	0.826	0.828	0.831	0.832	0.833	0.834
Toyama	0.79	0.795	0.8	0.805	0.808	0.812	0.816	0.818	0.821	0.823	0.825	0.827	0.829	0.832	0.835	0.837	0.839	0.841	0.843	0.845	0.847	0.849	0.852	0.854	0.856	0.857	0.858	0.86
Wakayama	0.775	0.78	0.785	0.789	0.792	0.796	0.8	0.802	0.805	0.806	0.808	0.81	0.812	0.815	0.817	0.819	0.821	0.822	0.824	0.825	0.827	0.829	0.831	0.833	0.836	0.837	0.838	0.84
Yamagata	0.766	0.77	0.775	0.778	0.782	0.785	0.788	0.791	0.792	0.794	0.795	0.796	0.799	0.802	0.805	0.807	0.809	0.812	0.814	0.816	0.819	0.821	0.824	0.826	0.828	0.829	0.83	0.832
Yamaguchi	0.79	0.794	0.799	0.802	0.806	0.809	0.812	0.815	0.817	0.819	0.82	0.822	0.824	0.826	0.828	0.83	0.831	0.832	0.834	0.835	0.837	0.839	0.841	0.843	0.846	0.847	0.848	0.849
Yamanashi	0.791	0.796	0.8	0.804	0.808	0.812	0.815	0.818	0.82	0.822	0.823	0.825	0.827	0.829	0.832	0.834	0.836	0.837	0.839	0.841	0.842	0.844	0.846	0.848	0.85	0.851	0.853	0.854
South Korea	0.713	0.724	0.733	0.742	0.751	0.76	0.768	0.777	0.783	0.79	0.799	0.806	0.814	0.82	0.825	0.83	0.834	0.839	0.843	0.846	0.85	0.853	0.857	0.86	0.864	0.867	0.869	0.872
Singapore	0.736	0.744	0.75	0.758	0.765	0.772	0.78	0.786	0.79	0.79	0.797	0.808	0.815	0.82	0.824	0.827	0.832	0.838	0.844	0.849	0.854	0.857	0.86	0.863	0.865	0.868	0.87	0.872
High-income North America	0.784	0.786	0.789	0.793	0.796	0.8	0.805	0.807	0.809	0.812	0.815	0.82	0.824	0.827	0.829	0.83	0.829	0.832	0.837	0.843	0.848	0.852	0.856	0.859	0.861	0.865	0.867	0.868
Canada	0.802	0.805	0.808	0.811	0.814	0.818	0.823	0.828	0.832	0.836	0.841	0.846	0.85	0.853	0.857	0.859	0.861	0.862	0.864	0.867	0.871	0.874	0.877	0.878	0.879	0.88	0.881	0.882
Greenland	0.671	0.67	0.67	0.671	0.672	0.675	0.679	0.683	0.687	0.691	0.695	0.699	0.703	0.708	0.713	0.717	0.722	0.726	0.731	0.737	0.743	0.747	0.751	0.754	0.756	0.757	0.759	0.76
USA	0.781	0.784	0.787	0.79	0.793	0.798	0.803	0.804	0.806	0.809	0.812	0.817	0.821	0.823	0.826	0.826	0.826	0.829	0.834	0.84	0.846	0.85	0.853	0.857	0.859	0.863	0.866	0.867
Alabama	0.745	0.749	0.753	0.757	0.76	0.765	0.769	0.769	0.771	0.773	0.777	0.783	0.787	0.791	0.794	0.794	0.793	0.796	0.801	0.809	0.816	0.82	0.824	0.827	0.83	0.834	0.837	0.837
Alaska	0.755	0.757	0.761	0.767	0.772	0.781	0.788	0.79	0.793	0.795	0.799	0.804	0.808	0.811	0.814	0.813	0.811	0.813	0.818	0.826	0.833	0.838	0.843	0.847	0.851	0.857	0.86	0.861
Arizona	0.751	0.753	0.755	0.758	0.76	0.764	0.769	0.77	0.771	0.772	0.775	0.781	0.784	0.787	0.79	0.791	0.791	0.797	0.806	0.815	0.823	0.828	0.832	0.836	0.839	0.842	0.845	0.845
Arkansas	0.723	0.727	0.733	0.738	0.741	0.746	0.751	0.751	0.753	0.754	0.757	0.763	0.767	0.77	0.773	0.772	0.77	0.774	0.78	0.789	0.797	0.802	0.807	0.811	0.816	0.821	0.825	0.826
California	0.771	0.771	0.773	0.776	0.78	0.787	0.794	0.798	0.803	0.807	0.812	0.819	0.823	0.826	0.829	0.83	0.831	0.835	0.84	0.846	0.852	0.856	0.859	0.863	0.865	0.869	0.871	0.872
Colorado	0.798	0.801	0.804	0.807	0.81	0.815	0.818	0.819	0.82	0.821	0.824	0.83	0.833	0.836	0.839	0.84	0.84	0.844	0.849	0.855	0.861	0.865	0.869	0.872	0.875	0.879	0.881	0.882
Connecticut	0.841	0.844	0.847	0.85	0.853	0.856	0.859	0.861	0.863	0.866	0.869	0.874	0.877	0.879	0.881	0.881	0.881	0.883	0.886	0.89	0.894	0.896	0.899	0.901	0.903	0.905	0.906	0.906
Delaware	0.801	0.804	0.808	0.811	0.813	0.816	0.819	0.819	0.82	0.822	0.825	0.83	0.833	0.836	0.838	0.839	0.838	0.841	0.846	0.852	0.858	0.861	0.864	0.866	0.868	0.871	0.873	0.874
Washington, DC	0.797	0.801	0.806	0.812	0.818	0.826	0.834	0.839	0.845	0.85	0.855	0.861	0.865	0.868	0.869	0.868	0.866	0.866	0.868	0.871	0.874	0.876	0.877	0.88	0.883	0.887	0.89	0.89
Florida	0.774	0.778	0.782	0.786	0.789	0.794	0.799	0.8	0.802	0.804	0.808	0.813	0.817	0.82	0.823	0.824	0.825	0.828	0.834	0.84	0.846	0.85	0.853	0.855	0.858	0.861	0.863	0.864
Georgia	0.755	0.759	0.764	0.769	0.772	0.778	0.782	0.783	0.784	0.785	0.788	0.793	0.796	0.798	0.801	0.801	0.8	0.804	0.81	0.818	0.825	0.83	0.834	0.837	0.841	0.845	0.848	0.848
Hawaii	0.781	0.784	0.788	0.792	0.797	0.805	0.811	0.813	0.815	0.817	0.82	0.824	0.828	0.83	0.833	0.833	0.832	0.834	0.839	0.845	0.851	0.854	0.858	0.861	0.864	0.868	0.871	0.872
Idaho	0.756	0.76	0.764	0.769	0.773	0.778	0.783	0.783	0.784	0.785	0.787	0.791	0.794	0.796	0.799	0.798	0.797	0.801	0.807	0.814	0.821	0.824	0.827	0.83	0.832	0.836	0.84	0.841
Illinois	0.787	0.789	0.792	0.796	0.799	0.805	0.81	0.812	0.816	0.818	0.823	0.828	0.833	0.837	0.84	0.841	0.841	0.845	0.85	0.855	0.861	0.864	0.867	0.87	0.873	0.876	0.879	0.879
Indiana	0.772	0.774	0.778	0.781	0.783	0.787	0.791	0.791	0.793	0.794	0.797	0.802	0.806	0.808	0.81	0.809	0.807	0.809	0.813	0.819	0.825	0.829	0.832	0.836	0.839	0.844	0.847	0.848
Iowa	0.794	0.797	0.8	0.803	0.806	0.809	0.813	0.814	0.816	0.818	0.821	0.825	0.828	0.829	0.832	0.831	0.83	0.832	0.837	0.843	0.848	0.852	0.856	0.86	0.863	0.867	0.87	0.87
Kansas	0.782	0.785	0.788	0.791	0.794	0.798	0.802	0.803	0.804	0.806	0.809	0.814	0.817	0.819	0.821	0.819	0.817	0.819	0.823	0.83	0.837	0.842	0.847	0.852	0.856	0.861	0.864	0.864
Kentucky	0.743	0.747	0.751	0.755	0.759	0.763	0.768	0.769	0.77	0.772	0.776	0.782	0.786	0.789	0.791	0.79	0.787	0.789	0.793	0.799	0.806	0.81	0.814	0.817	0.821	0.827	0.83	0.831
Louisiana	0.735	0.739	0.743	0.748	0.752	0.758	0.762	0.764	0.765	0.767	0.771	0.777	0.782	0.786	0.789	0.788	0.787	0.79	0.796	0.803	0.811	0.815	0.819	0.823	0.826	0.831	0.835	0.835
Maine	0.789	0.795	0.8	0.805	0.809	0.814	0.817	0.819	0.821	0.823	0.826	0.83	0.834	0.837	0.84	0.84	0.84	0.842	0.846	0.851	0.856	0.859	0.861	0.864	0.866	0.869	0.871	0.872
Maryland	0.813	0.817	0.821	0.825	0.828	0.833	0.836	0.838	0.839	0.841	0.845	0.85	0.854	0.857	0.86	0.861	0.861	0.864	0.868	0.874	0.879	0.883	0.886	0.889	0.891	0.894	0.896	0.896
Massachusetts	0.843	0.847	0.85	0.853	0.856	0.86	0.864	0.866	0.869	0.871	0.875	0.88	0.883	0.886	0.888	0.889	0.89	0.893	0.896	0.899	0.902	0.904	0.907	0.909	0.91	0.912	0.913	0.913
Michigan	0.788	0.791	0.795	0.799	0.803	0.808	0.813	0.815	0.817	0.82	0.823	0.829	0.832	0.835	0.838	0.838	0.837	0.839	0.841	0.845	0.849	0.852	0.854	0.857	0.86	0.864	0.867	0.868
Minnesota	0.818	0.821	0.825	0.828	0.83	0.834	0.837	0.839	0.841	0.843	0.845	0.849	0.852	0.855	0.857	0.858	0.858	0.86	0.865	0.87	0.874	0.877	0.881	0.884	0.886	0.89	0.892	0.893
Mississippi	0.719	0.722	0.727	0.732	0.736	0.741	0.746	0.747	0.748	0.75	0.753	0.758	0.762	0.764	0.765	0.764	0.76	0.763	0.77	0.779	0.789	0.794	0.8	0.804	0.809	0.814	0.818	0.819
Missouri	0.77	0.773	0.777	0.781	0.784	0.789	0.793	0.795	0.797	0.799	0.802	0.806	0.81	0.812	0.813	0.812	0.811	0.813	0.818	0.825	0.831	0.835	0.839	0.842	0.846	0.85	0.853	0.853
Montana	0.775	0.779	0.783	0.789	0.793	0.799	0.804	0.805	0.807	0.808	0.81	0.814	0.816	0.819	0.822	0.822	0.821	0.823	0.827	0.833	0.839	0.843	0.847	0.851	0.855	0.859	0.862	0.863
Nebraska	0.795	0.798	0.802	0.805	0.808	0.813	0.817	0.817	0.819	0.819	0.821	0.824	0.827	0.829	0.831	0.831	0.83	0.832	0.836	0.842	0.848	0.853	0.857	0.861	0.865	0.869	0.872	0.873
Nevada	0.76	0.761	0.762	0.765	0.767	0.773	0.779	0.781	0.784	0.787	0.791	0.798	0.802	0.805	0.808	0.809	0.81	0.814	0.82	0.827	0.833	0.836	0.838	0.84	0.842	0.845	0.847	0.847
New Hampshire	0.827	0.832	0.835	0.838	0.84	0.844	0.847	0.849	0.852	0.856	0.86	0.865	0.869	0.872	0.874	0.875	0.876	0.878	0.881	0.885	0.889	0.891	0.894	0.897	0.899	0.902	0.904	0.904
New Jersey	0.828	0.831	0.834	0.838	0.841	0.845	0.849	0.85	0.852	0.854	0.857	0.861	0.864	0.867	0.87	0.87	0.871	0.873	0.876	0.881	0.885	0.888	0.89	0.893	0.895	0.897	0.899	0.899
New Mexico	0.733	0.735	0.739	0.743	0.748	0.755	0.76	0.762	0.764	0.765	0.767	0.773	0.777	0.779	0.782	0.781	0.779	0.783	0.79	0.799	0.808	0.814	0.819	0.823	0.828	0.832	0.835	0.835
New York	0.816	0.819	0.822	0.825	0.828	0.832	0.836	0.838	0.841	0.843	0.847	0.852	0.855	0.858	0.86	0.861	0.861	0.864	0.868	0.872	0.877	0.88	0.883	0.886	0.889	0.892	0.893	0.893
North Carolina	0.763	0.766	0.77	0.774	0.777	0.781	0.785	0.785	0.786	0.788	0.791	0.796	0.8	0.802	0.805	0.805	0.805	0.809	0.815	0.822	0.829	0.833	0.836	0.84	0.843	0.846	0.849	0.85
North Dakota	0.79	0.792	0.796	0.799	0.802	0.807	0.813	0.816	0.82	0.823	0.827	0.83	0.833	0.834	0.835	0.835	0.833	0.835	0.839	0.844	0.85	0.854	0.859	0.864	0.87	0.875	0.879	0.88
Ohio	0.78	0.783	0.787	0.79	0.793	0.797	0.801	0.802	0.804	0.806	0.809	0.814	0.818	0.82	0.822	0.822	0.821	0.822	0.826	0.832	0.837	0.841	0.844	0.847	0.85	0.854	0.857	0.858

Appendix Table 11. Socio-demographic Index values for all estimated GBD 2017 locations, 1990-2017

Location	1990	1991	1992	1993	1994	1995	1996	1997	1998	1999	2000	2001	2002	2003	2004	2005	2006	2007	2008	2009	2010	2011	2012	2013	2014	2015	2016	2017
Oklahoma	0.749	0.751	0.755	0.758	0.76	0.764	0.768	0.768	0.768	0.769	0.772	0.777	0.781	0.784	0.786	0.785	0.784	0.786	0.792	0.8	0.808	0.813	0.818	0.824	0.829	0.835	0.838	0.838
Oregon	0.785	0.788	0.791	0.794	0.797	0.802	0.806	0.808	0.811	0.814	0.818	0.824	0.827	0.83	0.833	0.833	0.833	0.836	0.841	0.847	0.852	0.855	0.858	0.861	0.864	0.867	0.87	0.871
Pennsylvania	0.8	0.804	0.808	0.812	0.816	0.821	0.824	0.826	0.828	0.83	0.833	0.837	0.84	0.842	0.845	0.845	0.845	0.847	0.851	0.856	0.86	0.863	0.866	0.869	0.871	0.875	0.878	0.879
Rhode Island	0.815	0.818	0.822	0.825	0.827	0.831	0.834	0.835	0.838	0.84	0.843	0.848	0.851	0.854	0.857	0.858	0.859	0.862	0.866	0.87	0.875	0.877	0.88	0.882	0.885	0.887	0.889	0.89
South Carolina	0.752	0.757	0.762	0.768	0.772	0.777	0.781	0.782	0.783	0.785	0.787	0.793	0.796	0.799	0.801	0.8	0.799	0.802	0.808	0.815	0.822	0.826	0.83	0.834	0.838	0.842	0.845	0.846
South Dakota	0.769	0.772	0.777	0.783	0.788	0.794	0.799	0.801	0.804	0.805	0.808	0.811	0.813	0.814	0.816	0.814	0.812	0.814	0.819	0.826	0.833	0.838	0.842	0.847	0.851	0.856	0.859	0.86
Tennessee	0.749	0.752	0.757	0.761	0.765	0.77	0.774	0.775	0.777	0.779	0.781	0.786	0.788	0.789	0.79	0.789	0.786	0.789	0.795	0.803	0.81	0.815	0.819	0.823	0.827	0.832	0.836	0.837
Texas	0.743	0.745	0.747	0.75	0.752	0.757	0.761	0.763	0.764	0.766	0.769	0.775	0.779	0.782	0.784	0.783	0.782	0.785	0.792	0.801	0.809	0.815	0.82	0.824	0.829	0.834	0.837	0.838
Utah	0.761	0.765	0.769	0.773	0.776	0.781	0.786	0.787	0.79	0.792	0.795	0.8	0.803	0.806	0.808	0.808	0.807	0.811	0.817	0.825	0.832	0.837	0.841	0.845	0.848	0.852	0.855	0.856
Vermont	0.815	0.819	0.823	0.827	0.83	0.833	0.837	0.839	0.841	0.844	0.848	0.853	0.857	0.86	0.864	0.866	0.866	0.869	0.872	0.876	0.88	0.882	0.885	0.887	0.89	0.893	0.895	0.896
Virginia	0.8	0.803	0.807	0.81	0.814	0.818	0.822	0.823	0.824	0.826	0.829	0.834	0.838	0.841	0.845	0.846	0.847	0.851	0.856	0.862	0.867	0.871	0.874	0.877	0.88	0.883	0.885	0.885
Washington	0.797	0.8	0.804	0.807	0.811	0.816	0.821	0.823	0.827	0.83	0.834	0.84	0.844	0.846	0.849	0.848	0.848	0.85	0.855	0.86	0.865	0.868	0.871	0.874	0.877	0.881	0.883	0.884
West Virginia	0.749	0.752	0.756	0.761	0.764	0.769	0.773	0.774	0.775	0.776	0.778	0.783	0.786	0.787	0.789	0.787	0.784	0.784	0.787	0.793	0.799	0.802	0.806	0.81	0.814	0.82	0.824	0.825
Wisconsin	0.801	0.804	0.808	0.812	0.815	0.819	0.823	0.825	0.826	0.828	0.831	0.835	0.839	0.841	0.843	0.843	0.843	0.845	0.849	0.853	0.858	0.862	0.865	0.868	0.871	0.874	0.877	0.878
Wyoming	0.766	0.771	0.777	0.782	0.786	0.792	0.796	0.797	0.799	0.801	0.804	0.809	0.813	0.816	0.818	0.819	0.819	0.823	0.831	0.838	0.846	0.851	0.855	0.858	0.862	0.866	0.869	0.869
Southern Latin America	0.594	0.6	0.607	0.613	0.619	0.626	0.632	0.638	0.643	0.648	0.652	0.655	0.658	0.662	0.667	0.673	0.677	0.679	0.682	0.685	0.69	0.695	0.7	0.704	0.707	0.713	0.717	0.72
Argentina	0.59	0.595	0.604	0.61	0.617	0.624	0.63	0.635	0.64	0.644	0.647	0.649	0.65	0.653	0.658	0.665	0.669	0.672	0.675	0.677	0.681	0.686	0.691	0.693	0.696	0.702	0.707	0.71
Chile	0.6	0.608	0.615	0.62	0.626	0.633	0.64	0.647	0.654	0.661	0.667	0.674	0.681	0.687	0.692	0.696	0.698	0.701	0.704	0.708	0.714	0.721	0.727	0.732	0.738	0.742	0.746	0.748
Uruguay	0.592	0.597	0.6	0.602	0.606	0.609	0.613	0.618	0.625	0.632	0.637	0.64	0.643	0.647	0.652	0.656	0.659	0.661	0.663	0.666	0.671	0.675	0.68	0.685	0.691	0.697	0.702	0.707
Western Europe	0.764	0.77	0.776	0.782	0.787	0.791	0.795	0.798	0.801	0.805	0.809	0.813	0.817	0.82	0.822	0.825	0.828	0.83	0.833	0.836	0.838	0.842	0.845	0.848	0.851	0.853	0.855	0.857
Andorra	0.85	0.854	0.856	0.857	0.858	0.859	0.86	0.863	0.866	0.868	0.871	0.873	0.875	0.878	0.881	0.883	0.885	0.886	0.888	0.89	0.891	0.894	0.896	0.897	0.899	0.9	0.901	0.902
Austria	0.776	0.778	0.78	0.785	0.79	0.795	0.8	0.805	0.809	0.813	0.818	0.822	0.825	0.828	0.831	0.834	0.838	0.841	0.845	0.847	0.85	0.854	0.857	0.859	0.862	0.863	0.865	0.866
Belgium	0.803	0.808	0.813	0.818	0.822	0.826	0.829	0.832	0.835	0.837	0.84	0.843	0.847	0.849	0.852	0.854	0.856	0.858	0.861	0.863	0.867	0.871	0.875	0.879	0.882	0.884	0.885	0.886
Cyprus	0.724	0.73	0.74	0.75	0.758	0.765	0.771	0.778	0.784	0.789	0.795	0.803	0.81	0.817	0.824	0.83	0.837	0.842	0.847	0.851	0.854	0.857	0.859	0.861	0.862	0.863	0.864	0.865
Denmark	0.846	0.849	0.852	0.855	0.858	0.862	0.866	0.87	0.874	0.877	0.881	0.884	0.888	0.891	0.893	0.895	0.897	0.898	0.9	0.902	0.904	0.907	0.91	0.912	0.914	0.915	0.916	0.918
Finland	0.813	0.813	0.814	0.817	0.821	0.825	0.828	0.831	0.835	0.84	0.844	0.847	0.851	0.854	0.857	0.859	0.862	0.865	0.869	0.871	0.875	0.878	0.881	0.884	0.887	0.889	0.891	0.893
France	0.769	0.776	0.783	0.79	0.793	0.795	0.802	0.806	0.808	0.813	0.816	0.819	0.824	0.827	0.83	0.833	0.836	0.838	0.84	0.842	0.845	0.848	0.851	0.854	0.857	0.86	0.863	0.865
Germany	0.787	0.796	0.801	0.805	0.809	0.811	0.812	0.813	0.813	0.814	0.818	0.823	0.827	0.829	0.832	0.835	0.838	0.842	0.846	0.848	0.851	0.855	0.858	0.861	0.864	0.866	0.868	0.87
Greece	0.717	0.723	0.731	0.738	0.744	0.75	0.755	0.761	0.767	0.773	0.778	0.782	0.787	0.792	0.796	0.8	0.803	0.806	0.809	0.812	0.815	0.818	0.819	0.82	0.819	0.818	0.817	0.817
Iceland	0.814	0.818	0.821	0.825	0.828	0.83	0.833	0.835	0.839	0.843	0.848	0.854	0.859	0.862	0.865	0.869	0.872	0.876	0.88	0.883	0.886	0.889	0.892	0.895	0.899	0.902	0.905	0.907
Ireland	0.756	0.762	0.768	0.774	0.779	0.785	0.79	0.795	0.802	0.808	0.814	0.821	0.827	0.834	0.84	0.844	0.846	0.849	0.851	0.855	0.858	0.862	0.865	0.867	0.87	0.874	0.878	0.882
Israel	0.734	0.738	0.743	0.748	0.752	0.757	0.76	0.764	0.768	0.772	0.776	0.78	0.783	0.786	0.789	0.793	0.796	0.798	0.798	0.799	0.801	0.803	0.805	0.808	0.81	0.812	0.814	0.816
Italy	0.767	0.772	0.778	0.783	0.788	0.793	0.797	0.8	0.804	0.807	0.81	0.814	0.817	0.819	0.821	0.823	0.825	0.827	0.829	0.83	0.832	0.834	0.836	0.838	0.839	0.841	0.842	0.843
Luxembourg	0.845	0.849	0.851	0.854	0.858	0.862	0.866	0.869	0.873	0.876	0.878	0.88	0.881	0.883	0.885	0.888	0.891	0.894	0.896	0.899	0.901	0.904	0.906	0.909	0.912	0.913	0.915	0.916
Malta	0.729	0.733	0.737	0.743	0.748	0.752	0.756	0.761	0.766	0.773	0.779	0.784	0.788	0.792	0.796	0.799	0.802	0.805	0.808	0.811	0.814	0.817	0.82	0.823	0.826	0.829	0.833	0.836
Netherlands	0.827	0.832	0.837	0.841	0.845	0.849	0.852	0.855	0.858	0.862	0.866	0.87	0.873	0.876	0.879	0.882	0.885	0.887	0.89	0.892	0.895	0.898	0.901	0.904	0.906	0.908	0.91	0.912
Norway	0.811	0.816	0.821	0.827	0.831	0.835	0.84	0.846	0.85	0.855	0.86	0.866	0.87	0.873	0.876	0.878	0.88	0.882	0.885	0.888	0.892	0.896	0.9	0.903	0.906	0.909	0.91	0.911
Portugal	0.642	0.65	0.659	0.667	0.675	0.682	0.688	0.694	0.699	0.705	0.71	0.716	0.722	0.727	0.732	0.736	0.741	0.744	0.748	0.751	0.755	0.76	0.764	0.768	0.771	0.773	0.775	0.778
Spain	0.715	0.723	0.731	0.738	0.745	0.752	0.758	0.763	0.768	0.773	0.778	0.782	0.786	0.79	0.794	0.797	0.799	0.802	0.805	0.809	0.812	0.815	0.818	0.819	0.82	0.822	0.823	0.825
Sweden	0.784	0.789	0.795	0.802	0.808	0.815	0.82	0.825	0.831	0.835	0.838	0.841	0.844	0.847	0.85	0.853	0.855	0.857	0.86	0.862	0.865	0.868	0.871	0.874	0.876	0.879	0.881	0.883
Stockholm	0.825	0.83	0.835	0.84	0.845	0.85	0.854	0.859	0.864	0.867	0.871	0.873	0.876	0.879	0.882	0.885	0.888	0.891	0.893	0.896	0.899	0.902	0.904	0.907	0.909	0.911	0.913	0.914
Sweden except Stockholm	0.773	0.778	0.785	0.792	0.798	0.805	0.811	0.816	0.821	0.825	0.829	0.832	0.834	0.838	0.841	0.843	0.845	0.848	0.85	0.852	0.854	0.858	0.86	0.863	0.866	0.868	0.87	0.873
Switzerland	0.841	0.842	0.844	0.846	0.847	0.848	0.849	0.85	0.851	0.852	0.854	0.857	0.859	0.86	0.862	0.863	0.865	0.868	0.871	0.873	0.875	0.877	0.88	0.882	0.884	0.886	0.887	0.889
United Kingdom	0.723	0.729	0.736	0.743	0.749	0.754	0.758	0.762	0.767	0.774	0.78	0.786	0.791	0.794	0.797	0.801	0.804	0.807	0.81	0.813	0.817	0.821	0.826	0.832	0.836	0.839	0.841	0.843
England	0.732	0.738	0.745	0.752	0.758	0.763	0.766	0.77	0.776	0.782	0.788	0.794	0.798	0.801	0.804	0.807	0.81	0.813	0.816	0.819	0.823	0.827	0.832	0.838	0.842	0.845	0.847	0.849
East Midlands	0.71	0.716	0.723	0.73	0.737	0.741	0.745	0.75	0.755	0.762	0.768	0.773	0.778	0.781	0.785	0.788	0.791	0.794	0.797	0.8	0.804	0.808	0.813	0.819	0.823	0.826	0.828	0.83
Derby	0.715	0.722	0.73	0.738	0.745	0.75	0.754	0.758	0.764	0.771	0.778	0.785	0.79	0.793	0.797	0.801	0.803	0.806	0.809	0.811	0.815	0.82	0.826	0.832	0.837	0.841	0.844	0.846
Derbyshire	0.698	0.703	0.71	0.716	0.723	0.727	0.731	0.735	0.74	0.747	0.753	0.758	0.762	0.765	0.768	0.771	0.774	0.777	0.779	0.782	0.785	0.79	0.796	0.803	0.808	0.812	0.815	0.817
Leicester	0.708	0.715	0.723	0.73	0.737	0.743	0.747	0.751	0.756	0.763	0.769	0.775	0.78	0.785	0.79	0.794	0.798	0.802	0.805	0.809	0.813	0.817	0.822	0.828	0.832	0.835	0.837	0.839
Leicestershire	0.727	0.733	0.74	0.748	0.754	0.759	0.763	0.767	0.773	0.778	0.784	0.789	0.794	0.798	0.802	0.806	0.81	0.814	0.817	0.82	0.823	0.827	0.831	0.836	0.839	0.842	0.844	0.846
Lincolnshire	0.699	0.705	0.711	0.718	0.724	0.729	0.732	0.737	0.742	0.748	0.754	0.76	0.764	0.768	0.77	0.772	0.774	0.776	0.779	0.781	0.784	0.788	0.793	0.8	0.804	0.808	0.81	0.812
Northamptonshire	0.714	0.72	0.728	0.735	0.741	0.746	0.749	0.753	0.757	0.764	0.77	0.775	0.779	0.781	0.784	0.787	0.79	0.792	0.796	0.798	0.802	0.806	0.812	0.818	0.822	0.825	0.828	0.829
Nottingham	0.735	0.742	0.749	0.757	0.763	0.769	0.774	0.78	0.786	0.793	0.8	0.806	0.812	0.817	0.822	0.826	0.829	0.832	0.835	0.839	0.842	0.847	0.852	0.856	0.859	0.861	0.862	0.863
Nottinghamshire	0.696	0.702	0.708	0.715	0.722	0.726	0.73	0.734	0.74	0.747	0.754	0.76	0.765	0.768	0.771	0.775	0.778	0.781	0.783	0.785	0.788	0.792	0.797	0.803	0.807	0.81	0.812	0.814
Rutland	0.73	0.735	0.741	0.746	0.752	0.756	0.759	0.762	0.767	0.772	0.777	0.781	0.784	0.787	0.79	0.793	0.797	0.8	0.804	0.806	0.81	0.814	0.818	0.823	0.826	0.829	0.831	0.833
East of England	0.724	0.73	0.737	0.744	0.75	0.755	0.759	0.763	0.768	0.774	0.78	0.786	0.79	0.793	0.796	0.799	0.802	0.805	0.808	0.811	0.814	0.818	0.824	0.83	0.834	0.837	0.839	0.84
Bedford	0.73	0.736	0.743	0.749	0.756	0.76	0.764	0.768	0.774	0.78	0.785	0.79	0.794	0.797	0.8	0.803	0.805	0.807	0.81	0.812	0.816	0.82	0.825	0.83	0.833	0.836	0.837	0.838
Cambridgeshire	0.75	0.757	0.764	0.771	0.777	0.782	0.786	0.791	0.797	0.803	0.809	0.815	0.82	0.823	0.827	0.83	0.833	0.836	0.84	0.842	0.846	0.85	0.855	0.86	0.864	0.867	0.869	0.871
Central Bedfordshire	0.723	0.729	0.736	0.743	0.749	0.753	0.757	0.76	0.765	0.771	0.776	0.781	0.785	0.787	0.79	0.793	0.795	0.797	0.8	0.803	0.806	0.81	0.816	0.822	0.827	0.83	0.833	0.834
Essex	0.713	0.719	0.726	0.734	0.74	0.745	0.748	0.753	0.758	0.764	0.77	0.775	0.78	0.783	0.787	0.79	0.793	0.796	0.8	0.802	0.806	0.81	0.816	0.821	0.825	0.828	0.83	0.832
Hertfordshire	0.749	0.755	0.762	0.769	0.776	0.781	0.784	0.789	0.795	0.802	0.81	0.816	0.821	0.825	0.829	0.832	0.835	0.837	0.84	0.843	0.846	0.85	0.855	0.86	0.863	0.866	0.868	0.87
Luton	0.71	0.716	0.723	0.731	0.737	0.742	0.745	0.749	0.754	0.76	0.766	0.771	0.775	0.778	0.781	0.785	0.79	0.795	0.8	0.805	0.809	0.814	0.819	0.825	0.828	0.83	0.832	0.833
Norfolk	0.712	0.717	0.724	0.731	0.737	0.741	0.745	0.749	0.754	0.761	0.767	0.772	0.776	0.779	0.781	0.784	0.787	0.79	0.792	0.795	0.798	0.803	0.808	0.814	0.819	0.822	0.824	0.826
Peterborough	0.713	0.718	0.724	0.731	0.737	0.741	0.743	0.746	0.751	0.757	0.761	0.765	0.768	0.77	0.772	0.774	0.776	0.778	0.779	0.781	0.784	0.788	0.795	0.803	0.808	0.813	0.816	0.818
Southend-on-Sea	0.696	0.701	0.707	0.714	0.72	0.724	0.727	0.731	0.736	0.743	0.75	0.755	0.761	0.765	0.768	0.77	0.773	0.775	0.778	0.781	0.785	0.79	0.795	0.801	0.805	0.808	0.81	0.811
Suffolk	0.713	0.719	0.726	0.732	0.738	0.742	0.745	0.748	0.753	0.758	0.763	0.767	0.771	0.773	0.775	0.777	0.78	0.782	0.785	0.788	0.792	0.797	0.803	0.81	0.814	0.817	0.819	0.821
Thurrock	0.71	0.716	0.723	0.73	0.737	0.741	0.745	0.749	0.754	0.759	0.763	0.767	0.769	0.77	0.772	0.774	0.775	0.776	0.778	0.781	0.784	0.789	0.793	0.799	0.802	0.804	0.806	0.807
Greater London	0.78	0.786	0.793	0.8	0.806	0.81	0.814	0.818	0.823	0.83	0.836	0.842	0.846	0.849	0.852	0.855	0.858	0.861	0.863	0.866	0.87	0.875	0.88	0.885	0.889	0.891	0.893	0.894
Barking and Dagenham	0.704	0.709	0.716	0.723	0.73	0.734	0.737	0.741	0.746	0.752	0.756	0.76	0.762	0.762	0.763	0.763	0.763	0.763	0.764	0.765	0.768	0.773	0.78	0.787	0.793	0.797	0.8	0.802
Barnet	0.759	0.765	0.771	0.777	0.783	0.788	0.792	0.796	0.802	0.809	0.814	0.82	0.824	0.827	0.829	0.831	0.834	0.835	0.838	0.84	0.843	0.847	0.852	0.856	0.859	0.862	0.864	0.865
Bexley	0.721	0.727	0.734	0.74	0.746	0.75	0.753	0.757	0.761	0.767	0.772	0.777	0.781	0.784	0.787	0.789	0.791	0.793	0.796	0.798	0.801	0.804	0.809	0.815	0.819	0.822	0.824	0.826
Brent	0.753	0.758	0.764	0.771	0.777	0.781	0.785	0.789	0.795	0.801	0.806	0.811	0.814	0.816	0.818	0.819	0.821	0.821	0.822	0.823	0.825	0.828	0.833	0.839	0.842	0.845	0.847	0.849
Bromley	0.751	0.756	0.762	0.768	0.774	0.778	0.782	0.786	0.791	0.798	0.804	0.809	0.814	0.817	0.82	0.822	0.825	0.827	0.829	0.83	0.831	0.833	0.837	0.84	0.843	0.845	0.847	0.848
Camden	0.838	0.843	0.848	0.854	0.858	0.862	0.866	0.869	0.873	0.88	0.885	0.889	0.892	0.895	0.898	0.901	0.904	0.906	0.908	0.911	0.913	0.917	0.92	0.924	0.926	0.927	0.929	0.93
Croydon	0.741	0.747	0.754	0.761	0.767	0.771	0.774	0.777	0.782	0.788	0.794	0.799	0.803	0.805	0.808	0.81	0.812	0.813	0.815	0.816	0.818	0.82	0.824	0.827	0.83	0.831	0.832	0.833
Ealing	0.764	0.77	0.776	0.783	0.789	0.794	0.797	0.801	0.807	0.814	0.82	0.825	0.828	0.831	0.833	0.834	0.835	0.836	0.837	0.837	0.839	0.843	0.848	0.853	0.857	0.86	0.863	0.865
Enfield	0.737	0.742	0.749	0.755	0.761	0.765	0.768	0.772	0.777	0.783	0.788	0.793	0.797	0.799	0.801	0.802	0.804	0.806	0.809	0.811	0.814	0.818	0.824	0.829	0.833	0.836	0.838	0.839
Greenwich	0.725	0.732	0.74	0.747	0.754	0.758	0.762	0.766	0.771	0.777	0.783	0.788	0.791	0.793	0.795	0.797	0.799	0.801	0.803	0.805	0.809	0.813	0.819	0.824	0.828	0.83	0.832	0.833
Hackney	0.767	0.774	0.781	0.789	0.795	0.798	0.8	0.801	0.805	0.811	0.817	0.822	0.825	0.828	0.831	0.834	0.838	0.843	0.848	0.853	0.858	0.864	0.871	0.877	0.882	0.884	0.886	0.887
Hammersmith and Fulham	0.825	0.833	0.839	0.845	0.851	0.856	0.86	0.864	0.869	0.875	0.879	0.883	0.887	0.889	0.892	0.895	0.898	0.901	0.904	0.907	0.91	0.914	0.917	0.921	0.923	0.924	0.925	0.927
Haringey	0.755	0.761	0.766	0.773	0.778	0.782	0.785	0.788	0.792	0.798	0.804	0.809	0.813	0.815	0.818	0.821	0.823	0.825	0.828	0.829	0.832	0.835	0.84	0.845	0.848	0.851	0.852	0.854
Harrow	0.747	0.754	0.76	0.767	0.773	0.777	0.78	0.784	0.79	0.796	0.802	0.808	0.812	0.815	0.819	0.821	0.824	0.825	0.827	0.829	0.83	0.832	0.835	0.839	0.842	0.844	0.846	0.848
Havering	0.719	0.725	0.731	0.737	0.743	0.747	0.75	0.754	0.759	0.765	0.77	0.775	0.78	0.783	0.786	0.789	0.792	0.794	0.797	0.8	0.802	0.805	0.809	0.814	0.817	0.82	0.823	0.824
Hillingdon	0.781	0.787	0.794	0.801	0.807	0.811	0.815	0.818	0.822	0.828	0.832	0.836	0.84	0.842	0.845	0.846	0.848	0.85	0.852	0.854	0.857	0.862	0.867	0.872	0.876	0.879	0.881	0.882
Hounslow	0.769	0.775	0.781	0.788	0.793	0.797	0.801	0.805	0.811	0.818	0.824	0.83	0.834	0.837	0.84	0.842	0.844	0.845	0.848	0.849	0.852	0.856	0.862	0.868	0.872	0.875	0.877	0.879
Islington	0.812	0.818	0.825	0.832	0.838	0.843	0.847	0.851	0.856	0.862	0.867	0.872	0.876	0.879	0.882	0.885	0.888	0.891	0.895	0.898	0.902	0.906	0.91	0.914	0.917	0.919	0.921	0.922
Kensington and Chelsea	0.839	0.845	0.851	0.857	0.861	0.865	0.869	0.873	0.877	0.884	0.889	0.894	0.897	0.899	0.902	0.905	0.907	0.909	0.912	0.915	0.918	0.921	0.924	0.927	0.929	0.93	0.931	0.932
Kingston upon Thames	0.787	0.794	0.8	0.807	0.812	0.817	0.821	0.826	0.831	0.838	0.844	0.849	0.854	0.858	0.861	0.864	0.867	0.869	0.872	0.874	0.876	0.879	0.882	0.885	0.887	0.888	0.889	0.89
Lambeth	0.775	0.783	0.791	0.797	0.804	0.808	0.812	0.816	0.821	0.828	0.835	0.84	0.845	0.848	0.852	0.856	0.86	0.864	0.869	0.873	0.877	0.882	0.887	0.892	0.895	0.897	0.899	0.9
Lewisham	0.733	0.74	0.746	0.753	0.759	0.763	0.766	0.77	0.775	0.781	0.787	0.793	0.797	0.799	0.802	0.804	0.806	0.808	0.811	0.813	0.816	0.821	0.826	0.832	0.836	0.839	0.841	0.843
Merton	0.758	0.765	0.771	0.779	0.785	0.79	0.795	0.799	0.805	0.812	0.819	0.824	0.828	0.83	0.833	0.835	0.837	0.839	0.841	0.843	0.845	0.849	0.855	0.86	0.865	0.868	0.871	0.873
Newham	0.716	0.722	0.728	0.734	0.74	0.743	0.746	0.748	0.753	0.76	0.765	0.771	0.775	0.777	0.78	0.781	0.783	0.785	0.788	0.792	0.799	0.806	0.815	0.823	0.829	0.833	0.836	0.838
Redbridge	0.728	0.733	0.739	0.746	0.751	0.756	0.759	0.763	0.768	0.774	0.78	0.785	0.789	0.791	0.794	0.796	0.798	0.801	0.803	0.805	0.807	0.811	0.816	0.821	0.825	0.827	0.83	0.831
Richmond upon Thames	0.794	0.801	0.807	0.813	0.819	0.824	0.827	0.832	0.837	0.844	0.85	0.856	0.86	0.864	0.867	0.87	0.872	0.874	0.876	0.878	0.88	0.883	0.887	0.891	0.895	0.897	0.9	0.902
Southwark	0.793	0.8	0.807	0.815	0.821	0.827	0.83	0.835	0.84	0.847	0.852	0.857	0.86	0.863	0.866	0.869	0.872	0.875	0.879	0.883	0.887	0.893	0.898	0.904	0.907	0.909	0.911	0.912
Sutton	0.735	0.74	0.747	0.754	0.761	0.765	0.769	0.774	0.78	0.787	0.793	0.798	0.802	0.805	0.808	0.809	0.811	0.813	0.815	0.817	0.819	0.823	0.828	0.833	0.836	0.839	0.841	0.843
Tower Hamlets	0.766	0.773	0.78	0.788	0.795	0.801	0.805	0.81	0.817	0.825	0.832	0.839	0.844	0.848	0.852	0.857	0.861	0.866	0.871	0.876	0.881	0.886	0.891	0.896	0.9	0.902	0.904	0.905
Waltham Forest	0.712	0.718	0.724	0.731	0.738	0.742	0.745	0.749	0.754	0.76	0.765	0.77	0.773	0.775	0.777	0.778	0.78	0.781	0.783	0.784	0.787	0.792	0.798	0.805	0.81	0.814	0.817	0.819
Wandsworth	0.793	0.8	0.807	0.814	0.82	0.825	0.83	0.835	0.84	0.848	0.855	0.862	0.867	0.87	0.873	0.876	0.879	0.881	0.885	0.887	0.889	0.893	0.897	0.901	0.905	0.907	0.909	0.911
Westminster	0.831	0.837	0.843	0.849	0.854	0.858	0.862	0.865	0.87	0.875	0.881	0.885	0.889	0.891	0.895	0.898	0.901	0.903	0.905	0.907	0.91	0.913	0.916	0.92	0.922	0.924	0.925	0.927
North East England	0.697	0.703	0.711	0.718	0.725	0.73	0.733	0.738	0.744	0.75	0.757	0.763	0.767	0.771	0.774	0.778	0.781	0.784	0.787	0.79	0.794	0.798	0.804	0.81	0.814	0.817	0.819	0.821
County Durham	0.69	0.696	0.703	0.71	0.717	0.721	0.725	0.73	0.735	0.742	0.748	0.754	0.758	0.762	0.765	0.767	0.77	0.772	0.776	0.778	0.782	0.786	0.792	0.798	0.802	0.806	0.808	0.81
Darlington	0.703	0.709	0.716	0.723	0.73	0.734	0.737	0.74	0.745	0.752	0.758	0.764	0.769	0.773	0.777	0.78	0.783	0.785	0.788	0.791	0.795	0.8	0.806	0.812	0.817	0.821	0.823	0.825
Gateshead	0.7	0.706	0.713	0.72	0.727	0.732	0.735	0.739	0.745	0.751	0.758	0.764	0.769	0.773	0.777	0.781	0.784	0.787	0.791	0.794	0.798	0.803	0.808	0.814	0.818	0.821	0.824	0.826
Hartlepool	0.677	0.683	0.69	0.697	0.703	0.707	0.71	0.714	0.719	0.726	0.732	0.737	0.74	0.742	0.744	0.747	0.749	0.752	0.755	0.758	0.762	0.767	0.773	0.78	0.785	0.788	0.791	0.793
Middlesbrough	0.688	0.695	0.702	0.709	0.716	0.72	0.723	0.727	0.732	0.74	0.746	0.751	0.755	0.758	0.762	0.766	0.769	0.772	0.775	0.778	0.781	0.786	0.792	0.797	0.801	0.804	0.806	0.808
Newcastle upon Tyne	0.736	0.743	0.751	0.759	0.765	0.771	0.776	0.781	0.787	0.794	0.801	0.808	0.814	0.82	0.825	0.831	0.835	0.84	0.843	0.846	0.85	0.854	0.858	0.863	0.866	0.869	0.87	0.872
North Tyneside	0.696	0.703	0.71	0.718	0.724	0.729	0.733	0.737	0.743	0.749	0.756	0.762	0.768	0.772	0.776	0.78	0.784	0.787	0.79	0.793	0.797	0.802	0.807	0.813	0.817	0.821	0.823	0.825
Northumberland	0.697	0.703	0.709	0.716	0.722	0.727	0.73	0.735	0.74	0.746	0.752	0.757	0.761	0.764	0.767	0.77	0.773	0.776	0.779	0.781	0.784	0.787	0.793	0.798	0.802	0.805	0.807	0.808
Redcar and Cleveland	0.67	0.675	0.682	0.689	0.696	0.701	0.704	0.708	0.714	0.721	0.727	0.732	0.736	0.738	0.741	0.743	0.745	0.747	0.75	0.752	0.756	0.76	0.767	0.775	0.78	0.784	0.787	0.79
South Tyneside	0.664	0.671	0.678	0.685	0.692	0.697	0.7	0.705	0.711	0.718	0.725	0.731	0.737	0.74	0.744	0.748	0.751	0.754	0.757	0.759	0.762	0.767	0.773	0.78	0.785	0.789	0.792	0.794
Stockton-on-Tees	0.705	0.712	0.72	0.728	0.735	0.74	0.744	0.748	0.754	0.761	0.767	0.772	0.775	0.777	0.779	0.781	0.784	0.785	0.788	0.791	0.794	0.798	0.804	0.811	0.815	0.818	0.821	0.823
Sunderland	0.689	0.696	0.704	0.713	0.72	0.725	0.729	0.733	0.739	0.746	0.752	0.758	0.763	0.766	0.77	0.773	0.776	0.779	0.782	0.784	0.787	0.791	0.797	0.803	0.808	0.811	0.813	0.815
North West England	0.716	0.723	0.729	0.736	0.742	0.747	0.751	0.755	0.76	0.766	0.772	0.778	0.782	0.786	0.789	0.792	0.795	0.798	0.801	0.804	0.807	0.812	0.817	0.823	0.827	0.83	0.832	0.834
Blackburn with Darwen	0.691	0.695	0.701	0.707	0.713	0.717	0.719	0.723	0.728	0.734	0.74	0.745	0.748	0.75	0.753	0.756	0.758	0.761	0.764	0.766	0.769	0.774	0.78	0.788	0.793	0.797	0.8	0.802
Blackpool	0.68	0.685	0.691	0.698	0.703	0.707	0.71	0.713	0.718	0.724	0.729	0.733	0.737	0.738	0.741	0.743	0.745	0.747	0.748	0.75	0.752	0.756	0.762	0.768	0.773	0.776	0.779	0.781
Bolton	0.701	0.707	0.714	0.72	0.726	0.73	0.733	0.736	0.741	0.746	0.751	0.756	0.759	0.761	0.763	0.765	0.768	0.769	0.772	0.774	0.777	0.781	0.787	0.794	0.798	0.801	0.803	0.805
Bury	0.703	0.709	0.716	0.723	0.73	0.734	0.738	0.742	0.747	0.752	0.758	0.762	0.766	0.768	0.771	0.774	0.777	0.779	0.781	0.783	0.786	0.791	0.797	0.803	0.807	0.81	0.813	0.815
Cheshire East	0.748	0.754	0.76	0.767	0.773	0.778	0.782	0.787	0.792	0.798	0.803	0.809	0.813	0.817	0.82	0.823	0.826	0.829	0.832	0.834	0.836	0.84	0.845	0.851	0.856	0.859	0.861	0.864
Cheshire West and Chester	0.74	0.747	0.754	0.761	0.768	0.772	0.776	0.78	0.785	0.792	0.797	0.803	0.807	0.811	0.815	0.818	0.822	0.825	0.828	0.831	0.834	0.838	0.842	0.847	0.85	0.852	0.854	0.855
Cumbria	0.719	0.724	0.731	0.737	0.743	0.748	0.751	0.755	0.76	0.765	0.77	0.774	0.777	0.779	0.782	0.784	0.787	0.789	0.792	0.794	0.798	0.802	0.808	0.815	0.819	0.823	0.826	0.828
Halton	0.702	0.708	0.715	0.723	0.729	0.734	0.737	0.74	0.745	0.75	0.756	0.762	0.766	0.769	0.772	0.775	0.778	0.78	0.784	0.787	0.791	0.797	0.803	0.81	0.815	0.819	0.822	0.824
Knowsley	0.691	0.696	0.702	0.708	0.714	0.718	0.722	0.726	0.731	0.737	0.743	0.749	0.754	0.757	0.76	0.764	0.767	0.77	0.774	0.777	0.782	0.787	0.793	0.8	0.806	0.81	0.813	0.816
Lancashire	0.72	0.726	0.734	0.741	0.747	0.752	0.756	0.76	0.765	0.77	0.775	0.78	0.784	0.786	0.789	0.792	0.794	0.797	0.799	0.802	0.805	0.809	0.815	0.82	0.824	0.827	0.829	0.831
Liverpool	0.721	0.728	0.735	0.742	0.748	0.753	0.757	0.762	0.768	0.775	0.783	0.789	0.795	0.801	0.806	0.811	0.815	0.819	0.823	0.828	0.831	0.835	0.84	0.844	0.847	0.849	0.851	0.852
Manchester	0.746	0.753	0.76	0.768	0.775	0.78	0.785	0.79	0.796	0.804	0.812	0.819	0.825	0.83	0.835	0.84	0.844	0.849	0.853	0.856	0.86	0.864	0.869	0.874	0.878	0.88	0.883	0.885
Oldham	0.685	0.69	0.697	0.703	0.708	0.711	0.713	0.715	0.719	0.724	0.729	0.733	0.737	0.739	0.741	0.744	0.746	0.749	0.752	0.754	0.758	0.763	0.77	0.778	0.782	0.786	0.788	0.79
Rochdale	0.686	0.692	0.698	0.705	0.711	0.715	0.717	0.721	0.726	0.732	0.738	0.742	0.746	0.748	0.75	0.753	0.755	0.757	0.76	0.762	0.765	0.77	0.777	0.783	0.787	0.791	0.793	0.795
Salford	0.708	0.714	0.721	0.728	0.735	0.74	0.743	0.747	0.753	0.761	0.768	0.775	0.781	0.785	0.79	0.794	0.797	0.8	0.803	0.806	0.809	0.814	0.819	0.826	0.83	0.834	0.836	0.838
Sefton	0.706	0.713	0.72	0.727	0.733	0.738	0.741	0.746	0.751	0.757	0.763	0.768	0.773	0.776	0.78	0.783	0.786	0.788	0.79	0.792	0.794	0.796	0.8	0.803	0.806	0.809	0.81	0.812

Appendix Table 11. Socio-demographic Index values for all estimated GBD 2017 locations, 1990-2017

Location	1990	1991	1992	1993	1994	1995	1996	1997	1998	1999	2000	2001	2002	2003	2004	2005	2006	2007	2008	2009	2010	2011	2012	2013	2014	2015	2016	2017
St Helens	0.684	0.69	0.696	0.703	0.709	0.713	0.716	0.72	0.725	0.731	0.738	0.744	0.749	0.753	0.756	0.759	0.762	0.765	0.768	0.771	0.774	0.779	0.785	0.791	0.795	0.798	0.801	0.803
Stockport	0.727	0.734	0.741	0.748	0.755	0.759	0.763	0.767	0.772	0.779	0.785	0.791	0.796	0.799	0.803	0.806	0.809	0.812	0.814	0.817	0.82	0.823	0.828	0.833	0.837	0.839	0.841	0.843
Tameside	0.691	0.697	0.703	0.71	0.716	0.721	0.724	0.728	0.734	0.74	0.746	0.751	0.755	0.758	0.76	0.762	0.763	0.764	0.766	0.768	0.77	0.774	0.779	0.785	0.79	0.793	0.795	0.797
Trafford	0.751	0.757	0.764	0.771	0.778	0.782	0.786	0.79	0.795	0.802	0.809	0.815	0.82	0.824	0.829	0.832	0.836	0.839	0.842	0.844	0.848	0.852	0.856	0.862	0.865	0.868	0.871	0.873
Warrington	0.739	0.745	0.752	0.759	0.765	0.769	0.773	0.776	0.782	0.788	0.795	0.801	0.807	0.811	0.814	0.818	0.822	0.825	0.827	0.83	0.833	0.838	0.843	0.849	0.853	0.856	0.858	0.86
Wigan	0.691	0.697	0.703	0.71	0.716	0.72	0.723	0.727	0.731	0.737	0.742	0.747	0.75	0.753	0.755	0.757	0.76	0.762	0.764	0.766	0.769	0.774	0.78	0.786	0.79	0.793	0.796	0.798
Wirral	0.695	0.701	0.708	0.714	0.721	0.725	0.728	0.732	0.737	0.743	0.749	0.753	0.757	0.761	0.763	0.766	0.768	0.77	0.773	0.775	0.777	0.781	0.786	0.792	0.796	0.799	0.801	0.803
South East England	0.749	0.755	0.761	0.767	0.773	0.777	0.781	0.784	0.789	0.795	0.801	0.806	0.809	0.812	0.815	0.818	0.821	0.823	0.826	0.828	0.831	0.836	0.841	0.846	0.85	0.852	0.855	0.856
Bracknell Forest	0.759	0.764	0.77	0.776	0.781	0.785	0.788	0.791	0.797	0.804	0.811	0.817	0.822	0.825	0.829	0.832	0.835	0.838	0.841	0.843	0.845	0.849	0.854	0.859	0.862	0.865	0.867	0.869
Brighton and Hove	0.766	0.772	0.779	0.785	0.791	0.796	0.801	0.806	0.811	0.816	0.822	0.827	0.833	0.838	0.843	0.847	0.851	0.855	0.859	0.863	0.865	0.868	0.872	0.876	0.879	0.881	0.883	0.885
Buckinghamshire	0.764	0.769	0.775	0.782	0.788	0.792	0.795	0.799	0.804	0.81	0.815	0.82	0.824	0.826	0.829	0.832	0.834	0.836	0.838	0.84	0.842	0.846	0.851	0.855	0.859	0.861	0.863	0.865
East Sussex	0.712	0.718	0.724	0.73	0.736	0.74	0.742	0.745	0.749	0.754	0.759	0.763	0.766	0.769	0.771	0.773	0.776	0.778	0.781	0.783	0.787	0.791	0.797	0.803	0.807	0.81	0.812	0.814
Hampshire	0.744	0.75	0.756	0.762	0.768	0.772	0.775	0.778	0.782	0.788	0.793	0.798	0.802	0.804	0.807	0.81	0.812	0.815	0.818	0.82	0.824	0.828	0.834	0.839	0.843	0.846	0.848	0.85
Isle of Wight	0.704	0.709	0.715	0.722	0.727	0.732	0.735	0.739	0.744	0.749	0.756	0.761	0.765	0.767	0.768	0.771	0.773	0.776	0.779	0.781	0.784	0.788	0.794	0.801	0.806	0.809	0.812	0.814
Kent	0.723	0.728	0.734	0.74	0.746	0.75	0.752	0.756	0.76	0.765	0.77	0.774	0.777	0.779	0.782	0.785	0.787	0.79	0.793	0.796	0.8	0.805	0.811	0.817	0.822	0.824	0.826	0.828
Medway	0.703	0.709	0.715	0.722	0.728	0.731	0.734	0.737	0.742	0.747	0.752	0.756	0.76	0.762	0.765	0.768	0.771	0.773	0.776	0.778	0.781	0.785	0.791	0.797	0.802	0.805	0.807	0.809
Milton Keynes	0.754	0.76	0.767	0.774	0.78	0.784	0.786	0.789	0.793	0.798	0.802	0.806	0.81	0.812	0.815	0.817	0.819	0.821	0.823	0.825	0.829	0.834	0.84	0.847	0.852	0.856	0.859	0.86
Oxfordshire	0.769	0.775	0.781	0.788	0.794	0.798	0.801	0.805	0.81	0.816	0.822	0.827	0.831	0.835	0.838	0.841	0.844	0.847	0.849	0.852	0.855	0.859	0.864	0.869	0.872	0.875	0.878	0.879
Portsmouth	0.75	0.756	0.763	0.77	0.776	0.781	0.785	0.79	0.795	0.8	0.805	0.81	0.815	0.818	0.822	0.824	0.827	0.829	0.832	0.835	0.838	0.842	0.846	0.851	0.854	0.857	0.858	0.86
Reading	0.785	0.791	0.797	0.803	0.809	0.813	0.817	0.821	0.827	0.834	0.84	0.847	0.852	0.856	0.86	0.864	0.866	0.868	0.87	0.872	0.874	0.877	0.882	0.887	0.89	0.892	0.894	0.895
Slough	0.764	0.77	0.777	0.784	0.79	0.793	0.796	0.799	0.802	0.806	0.81	0.813	0.815	0.816	0.817	0.818	0.819	0.82	0.822	0.825	0.829	0.834	0.841	0.848	0.852	0.855	0.858	0.859
Southampton	0.752	0.758	0.765	0.772	0.779	0.784	0.789	0.794	0.8	0.805	0.81	0.815	0.819	0.823	0.826	0.829	0.831	0.834	0.836	0.837	0.839	0.842	0.845	0.849	0.852	0.855	0.856	0.858
Surrey	0.773	0.779	0.785	0.791	0.797	0.801	0.805	0.809	0.814	0.821	0.827	0.833	0.838	0.841	0.845	0.847	0.85	0.853	0.856	0.858	0.861	0.864	0.868	0.873	0.876	0.879	0.881	0.883
West Berkshire	0.774	0.78	0.786	0.793	0.799	0.803	0.805	0.808	0.813	0.819	0.824	0.829	0.832	0.835	0.836	0.836	0.837	0.838	0.84	0.842	0.846	0.851	0.857	0.863	0.867	0.869	0.871	0.872
West Sussex	0.74	0.745	0.751	0.757	0.763	0.767	0.77	0.773	0.777	0.783	0.788	0.793	0.796	0.799	0.802	0.804	0.807	0.809	0.812	0.814	0.818	0.822	0.827	0.833	0.837	0.84	0.842	0.843
Windsor and Maidenhead	0.778	0.783	0.789	0.795	0.8	0.805	0.808	0.811	0.816	0.823	0.829	0.835	0.839	0.843	0.847	0.851	0.854	0.857	0.86	0.863	0.866	0.87	0.874	0.88	0.883	0.885	0.887	0.889
Wokingham	0.778	0.784	0.79	0.797	0.802	0.806	0.81	0.814	0.82	0.826	0.832	0.837	0.842	0.845	0.849	0.853	0.856	0.858	0.861	0.863	0.865	0.868	0.871	0.876	0.879	0.882	0.883	0.885
South West England	0.729	0.735	0.741	0.748	0.754	0.758	0.762	0.766	0.771	0.777	0.783	0.788	0.792	0.796	0.799	0.802	0.805	0.807	0.81	0.813	0.816	0.82	0.825	0.831	0.835	0.838	0.84	0.841
Bath and North East Somerset	0.752	0.758	0.764	0.77	0.777	0.782	0.786	0.79	0.796	0.803	0.809	0.816	0.822	0.828	0.833	0.838	0.842	0.846	0.85	0.853	0.856	0.859	0.863	0.867	0.869	0.872	0.874	0.875
Bournemouth	0.736	0.743	0.75	0.757	0.763	0.768	0.773	0.778	0.784	0.79	0.797	0.803	0.808	0.812	0.816	0.821	0.824	0.828	0.831	0.834	0.836	0.839	0.843	0.848	0.851	0.853	0.856	0.858
Bristol, City of	0.763	0.77	0.777	0.784	0.791	0.796	0.8	0.805	0.81	0.817	0.823	0.828	0.833	0.836	0.839	0.843	0.846	0.849	0.853	0.856	0.859	0.863	0.868	0.873	0.877	0.88	0.882	0.884
Cornwall	0.7	0.706	0.713	0.721	0.727	0.731	0.734	0.738	0.743	0.749	0.755	0.76	0.764	0.768	0.771	0.774	0.777	0.78	0.783	0.786	0.789	0.793	0.799	0.806	0.81	0.813	0.815	0.817
Devon	0.72	0.726	0.733	0.74	0.746	0.75	0.753	0.757	0.762	0.769	0.775	0.78	0.785	0.789	0.793	0.796	0.8	0.803	0.806	0.808	0.811	0.816	0.821	0.826	0.83	0.833	0.835	0.837
Dorset	0.716	0.721	0.727	0.734	0.74	0.744	0.747	0.751	0.756	0.762	0.769	0.773	0.777	0.779	0.781	0.783	0.786	0.788	0.791	0.793	0.797	0.802	0.808	0.814	0.818	0.821	0.823	0.825
Gloucestershire	0.735	0.741	0.747	0.754	0.76	0.765	0.768	0.772	0.777	0.783	0.79	0.795	0.8	0.804	0.808	0.811	0.813	0.816	0.818	0.82	0.824	0.828	0.833	0.839	0.843	0.846	0.848	0.85
North Somerset	0.714	0.72	0.727	0.733	0.739	0.743	0.746	0.75	0.755	0.76	0.766	0.771	0.776	0.78	0.783	0.786	0.789	0.792	0.795	0.798	0.801	0.806	0.813	0.819	0.824	0.827	0.83	0.832
Plymouth	0.724	0.73	0.737	0.744	0.75	0.754	0.758	0.762	0.767	0.772	0.778	0.783	0.787	0.79	0.793	0.796	0.799	0.802	0.805	0.807	0.81	0.814	0.819	0.825	0.829	0.832	0.834	0.836
Poole	0.727	0.733	0.74	0.746	0.753	0.758	0.761	0.765	0.771	0.777	0.783	0.789	0.793	0.796	0.798	0.801	0.804	0.806	0.809	0.811	0.814	0.818	0.824	0.83	0.835	0.838	0.84	0.842
Somerset	0.713	0.718	0.724	0.731	0.737	0.741	0.744	0.748	0.752	0.757	0.763	0.767	0.77	0.772	0.775	0.777	0.78	0.782	0.785	0.787	0.789	0.794	0.799	0.805	0.809	0.812	0.814	0.816
South Gloucestershire	0.747	0.752	0.758	0.765	0.771	0.775	0.779	0.783	0.789	0.796	0.802	0.808	0.813	0.817	0.821	0.824	0.827	0.831	0.834	0.837	0.84	0.844	0.849	0.855	0.859	0.862	0.865	0.867
Swindon	0.747	0.753	0.76	0.767	0.773	0.776	0.778	0.781	0.786	0.792	0.797	0.801	0.805	0.806	0.807	0.809	0.811	0.813	0.815	0.818	0.82	0.825	0.831	0.837	0.841	0.844	0.846	0.847
Torbay	0.699	0.705	0.711	0.717	0.723	0.727	0.73	0.733	0.736	0.741	0.745	0.749	0.75	0.751	0.753	0.755	0.757	0.759	0.761	0.762	0.764	0.767	0.773	0.779	0.783	0.786	0.789	0.79
Wiltshire	0.726	0.731	0.737	0.743	0.749	0.753	0.756	0.759	0.763	0.769	0.775	0.78	0.784	0.786	0.789	0.791	0.793	0.795	0.797	0.799	0.803	0.808	0.813	0.819	0.823	0.826	0.828	0.829
West Midlands	0.707	0.713	0.72	0.727	0.734	0.739	0.743	0.747	0.752	0.759	0.765	0.771	0.776	0.779	0.783	0.786	0.789	0.792	0.795	0.798	0.802	0.806	0.812	0.818	0.822	0.825	0.828	0.829
Birmingham	0.707	0.714	0.721	0.728	0.735	0.739	0.743	0.747	0.753	0.76	0.766	0.773	0.778	0.782	0.787	0.791	0.794	0.798	0.803	0.807	0.811	0.817	0.823	0.829	0.834	0.837	0.839	0.84
Coventry	0.722	0.729	0.737	0.745	0.751	0.757	0.762	0.766	0.772	0.779	0.785	0.791	0.796	0.8	0.803	0.807	0.81	0.812	0.815	0.818	0.821	0.825	0.831	0.837	0.841	0.844	0.847	0.848
Dudley	0.692	0.698	0.704	0.71	0.716	0.721	0.724	0.728	0.733	0.739	0.746	0.751	0.756	0.759	0.762	0.765	0.767	0.77	0.772	0.774	0.776	0.779	0.784	0.789	0.793	0.795	0.797	0.799
Herefordshire, County of	0.706	0.712	0.719	0.726	0.733	0.738	0.742	0.747	0.752	0.759	0.766	0.771	0.776	0.779	0.783	0.786	0.789	0.792	0.795	0.798	0.801	0.806	0.812	0.818	0.822	0.825	0.827	0.828
Sandwell	0.682	0.688	0.695	0.702	0.708	0.712	0.715	0.719	0.724	0.729	0.735	0.74	0.744	0.747	0.75	0.752	0.754	0.756	0.759	0.761	0.765	0.769	0.776	0.783	0.788	0.792	0.795	0.797
Shropshire	0.708	0.714	0.721	0.728	0.734	0.739	0.743	0.747	0.752	0.759	0.766	0.771	0.776	0.781	0.785	0.789	0.793	0.795	0.799	0.802	0.806	0.811	0.816	0.821	0.825	0.828	0.83	0.832
Solihull	0.733	0.74	0.747	0.755	0.762	0.767	0.771	0.776	0.783	0.789	0.795	0.801	0.807	0.811	0.815	0.818	0.822	0.825	0.828	0.83	0.832	0.835	0.84	0.845	0.848	0.851	0.853	0.855
Staffordshire	0.708	0.714	0.722	0.729	0.736	0.74	0.744	0.749	0.755	0.761	0.767	0.772	0.777	0.78	0.783	0.787	0.79	0.793	0.796	0.799	0.802	0.806	0.811	0.816	0.819	0.822	0.824	0.826
Stoke-on-Trent	0.689	0.696	0.703	0.71	0.716	0.721	0.724	0.728	0.733	0.738	0.743	0.747	0.75	0.751	0.753	0.754	0.756	0.757	0.76	0.763	0.767	0.772	0.78	0.788	0.794	0.798	0.801	0.804
Telford and Wrekin	0.713	0.719	0.726	0.733	0.74	0.745	0.749	0.754	0.759	0.764	0.769	0.773	0.776	0.777	0.779	0.781	0.783	0.784	0.787	0.789	0.791	0.796	0.802	0.809	0.814	0.817	0.82	0.822
Walsall	0.681	0.686	0.692	0.698	0.704	0.707	0.709	0.712	0.717	0.723	0.729	0.734	0.738	0.74	0.743	0.746	0.749	0.752	0.755	0.757	0.761	0.766	0.772	0.779	0.783	0.787	0.789	0.791
Warwickshire	0.732	0.738	0.746	0.753	0.76	0.765	0.769	0.774	0.779	0.786	0.792	0.798	0.802	0.806	0.809	0.812	0.816	0.819	0.823	0.826	0.83	0.834	0.84	0.845	0.85	0.853	0.855	0.857
Wolverhampton	0.691	0.697	0.704	0.712	0.719	0.723	0.727	0.731	0.737	0.744	0.751	0.757	0.761	0.764	0.768	0.771	0.774	0.776	0.779	0.781	0.784	0.788	0.794	0.8	0.804	0.807	0.809	0.811
Worcestershire	0.707	0.713	0.72	0.727	0.733	0.738	0.742	0.747	0.754	0.762	0.771	0.778	0.782	0.786	0.791	0.794	0.798	0.8	0.803	0.805	0.808	0.811	0.816	0.822	0.826	0.829	0.831	0.833
Yorkshire and the Humber	0.711	0.717	0.724	0.731	0.737	0.742	0.745	0.75	0.755	0.762	0.768	0.773	0.778	0.781	0.785	0.788	0.791	0.794	0.797	0.8	0.803	0.808	0.813	0.819	0.823	0.826	0.828	0.83
Barnsley	0.675	0.68	0.686	0.692	0.698	0.702	0.705	0.709	0.715	0.721	0.726	0.731	0.735	0.738	0.741	0.743	0.746	0.748	0.75	0.752	0.755	0.759	0.765	0.772	0.778	0.781	0.785	0.787
Bradford	0.693	0.699	0.705	0.712	0.718	0.721	0.723	0.725	0.729	0.736	0.741	0.747	0.751	0.753	0.756	0.759	0.762	0.765	0.768	0.771	0.775	0.78	0.787	0.794	0.799	0.803	0.805	0.807
Calderdale	0.708	0.714	0.721	0.729	0.735	0.739	0.743	0.746	0.751	0.758	0.763	0.768	0.771	0.774	0.777	0.78	0.783	0.785	0.788	0.791	0.795	0.8	0.807	0.814	0.819	0.823	0.825	0.827
Doncaster	0.674	0.679	0.685	0.692	0.697	0.701	0.704	0.707	0.712	0.718	0.724	0.729	0.733	0.736	0.74	0.743	0.746	0.749	0.752	0.755	0.759	0.764	0.771	0.778	0.783	0.786	0.789	0.791
East Riding of Yorkshire	0.714	0.72	0.727	0.734	0.741	0.745	0.749	0.753	0.758	0.764	0.769	0.774	0.778	0.781	0.784	0.787	0.79	0.792	0.794	0.797	0.799	0.802	0.807	0.812	0.815	0.818	0.82	0.822
Kingston upon Hull, City of	0.689	0.695	0.702	0.71	0.717	0.722	0.726	0.73	0.736	0.742	0.749	0.755	0.76	0.763	0.767	0.77	0.773	0.776	0.779	0.782	0.785	0.789	0.795	0.801	0.806	0.809	0.811	0.813
Kirklees	0.703	0.709	0.716	0.723	0.73	0.734	0.737	0.741	0.746	0.752	0.758	0.763	0.767	0.77	0.773	0.776	0.779	0.781	0.783	0.785	0.788	0.793	0.798	0.805	0.809	0.812	0.814	0.816
Leeds	0.739	0.746	0.753	0.761	0.768	0.773	0.778	0.783	0.789	0.797	0.803	0.81	0.816	0.82	0.825	0.83	0.834	0.837	0.841	0.843	0.846	0.849	0.854	0.858	0.862	0.864	0.867	0.868
North East Lincolnshire	0.698	0.705	0.712	0.72	0.727	0.731	0.733	0.737	0.741	0.745	0.749	0.753	0.755	0.757	0.758	0.761	0.763	0.767	0.77	0.775	0.779	0.784	0.79	0.796	0.799	0.802	0.803	0.804
North Lincolnshire	0.702	0.707	0.714	0.72	0.727	0.731	0.734	0.737	0.742	0.748	0.752	0.757	0.76	0.762	0.764	0.766	0.768	0.77	0.773	0.776	0.78	0.785	0.792	0.799	0.803	0.807	0.809	0.811
North Yorkshire	0.724	0.73	0.737	0.743	0.75	0.754	0.758	0.762	0.767	0.774	0.78	0.785	0.79	0.794	0.798	0.801	0.803	0.806	0.808	0.81	0.813	0.817	0.822	0.827	0.831	0.834	0.836	0.839
Rotherham	0.681	0.687	0.693	0.7	0.707	0.711	0.714	0.717	0.722	0.728	0.734	0.739	0.744	0.747	0.75	0.753	0.756	0.759	0.761	0.763	0.766	0.77	0.776	0.782	0.787	0.791	0.794	0.796
Sheffield	0.727	0.733	0.74	0.747	0.753	0.758	0.762	0.767	0.773	0.78	0.786	0.792	0.798	0.803	0.807	0.811	0.815	0.819	0.823	0.826	0.83	0.834	0.839	0.843	0.847	0.849	0.852	0.853
Wakefield	0.692	0.698	0.705	0.712	0.718	0.722	0.726	0.729	0.734	0.741	0.747	0.752	0.755	0.758	0.76	0.763	0.765	0.766	0.768	0.769	0.772	0.777	0.784	0.791	0.797	0.801	0.804	0.806
York	0.762	0.769	0.776	0.783	0.789	0.795	0.799	0.804	0.81	0.816	0.822	0.829	0.834	0.839	0.844	0.848	0.851	0.854	0.857	0.859	0.862	0.865	0.868	0.872	0.874	0.876	0.878	0.879
Northern Ireland	0.71	0.718	0.726	0.735	0.742	0.747	0.751	0.755	0.761	0.767	0.774	0.78	0.786	0.79	0.795	0.798	0.801	0.805	0.808	0.812	0.815	0.818	0.822	0.825	0.828	0.831	0.833	0.835
Scotland	0.672	0.678	0.686	0.693	0.701	0.706	0.711	0.716	0.722	0.73	0.737	0.743	0.748	0.752	0.757	0.761	0.764	0.768	0.772	0.777	0.781	0.785	0.789	0.793	0.797	0.8	0.803	0.805
Wales	0.642	0.65	0.659	0.668	0.676	0.683	0.688	0.694	0.702	0.711	0.719	0.727	0.734	0.739	0.745	0.75	0.755	0.76	0.765	0.77	0.774	0.78	0.786	0.792	0.797	0.8	0.803	0.806
Latin America and Caribbean	0.497	0.502	0.508	0.514	0.521	0.527	0.534	0.54	0.546	0.552	0.557	0.563	0.567	0.571	0.576	0.581	0.587	0.592	0.598	0.603	0.608	0.613	0.618	0.624	0.628	0.633	0.637	0.64
Andean Latin America	0.483	0.486	0.49	0.495	0.501	0.507	0.512	0.518	0.526	0.533	0.539	0.546	0.552	0.558	0.563	0.567	0.572	0.577	0.581	0.585	0.59	0.595	0.601	0.606	0.612	0.618	0.624	0.628
Bolivia	0.403	0.408	0.416	0.424	0.431	0.438	0.447	0.456	0.466	0.475	0.485	0.494	0.504	0.513	0.52	0.526	0.532	0.536	0.539	0.543	0.547	0.551	0.555	0.559	0.565	0.573	0.581	0.587
Ecuador	0.506	0.51	0.515	0.52	0.524	0.524	0.522	0.521	0.526	0.532	0.539	0.548	0.557	0.564	0.57	0.575	0.581	0.585	0.588	0.59	0.594	0.603	0.611	0.618	0.623	0.628	0.632	0.636
Peru	0.493	0.495	0.497	0.501	0.509	0.517	0.526	0.534	0.542	0.549	0.555	0.56	0.564	0.568	0.571	0.575	0.578	0.584	0.59	0.594	0.6	0.604	0.608	0.614	0.62	0.626	0.632	0.636
Caribbean	0.531	0.536	0.539	0.543	0.546	0.549	0.552	0.555	0.559	0.563	0.568	0.573	0.578	0.583	0.589	0.596	0.602	0.607	0.61	0.613	0.617	0.621	0.624	0.626	0.628	0.631	0.635	0.638
Antigua and Barbuda	0.61	0.616	0.622	0.627	0.631	0.633	0.636	0.64	0.644	0.649	0.653	0.657	0.661	0.666	0.67	0.675	0.68	0.685	0.69	0.694	0.696	0.698	0.7	0.703	0.706	0.709	0.712	0.715
The Bahamas	0.674	0.669	0.665	0.667	0.671	0.676	0.681	0.687	0.695	0.703	0.71	0.715	0.718	0.721	0.723	0.727	0.732	0.737	0.742	0.746	0.751	0.753	0.755	0.755	0.755	0.755	0.755	0.756
Barbados	0.65	0.655	0.661	0.667	0.672	0.675	0.678	0.68	0.682	0.683	0.686	0.689	0.693	0.698	0.703	0.707	0.71	0.713	0.717	0.72	0.723	0.726	0.729	0.732	0.734	0.736	0.738	0.739
Belize	0.402	0.414	0.429	0.445	0.458	0.471	0.482	0.49	0.496	0.501	0.506	0.511	0.518	0.525	0.533	0.541	0.549	0.557	0.563	0.569	0.576	0.581	0.586	0.59	0.594	0.598	0.6	0.602
Bermuda	0.73	0.732	0.734	0.737	0.738	0.74	0.741	0.743	0.746	0.749	0.752	0.754	0.757	0.761	0.765	0.769	0.773	0.777	0.782	0.786	0.79	0.794	0.797	0.8	0.803	0.804	0.805	0.805
Cuba	0.592	0.6	0.604	0.605	0.604	0.603	0.601	0.6	0.601	0.604	0.607	0.611	0.617	0.623	0.629	0.636	0.644	0.646	0.647	0.649	0.652	0.656	0.663	0.668	0.671	0.676	0.683	0.688
Dominica	0.534	0.535	0.539	0.546	0.555	0.563	0.57	0.577	0.584	0.592	0.599	0.606	0.614	0.621	0.63	0.636	0.641	0.645	0.65	0.656	0.662	0.668	0.673	0.677	0.68	0.682	0.684	0.687
Dominican Republic	0.442	0.443	0.445	0.449	0.454	0.459	0.464	0.469	0.475	0.48	0.484	0.49	0.495	0.499	0.511	0.525	0.536	0.547	0.555	0.562	0.568	0.572	0.575	0.576	0.578	0.583	0.589	0.593
Grenada	0.46	0.472	0.485	0.497	0.508	0.519	0.529	0.54	0.55	0.558	0.566	0.573	0.578	0.584	0.589	0.594	0.599	0.604	0.608	0.612	0.615	0.618	0.62	0.624	0.628	0.632	0.637	0.64
Guyana	0.442	0.446	0.45	0.454	0.458	0.465	0.472	0.481	0.489	0.497	0.503	0.51	0.516	0.521	0.526	0.53	0.533	0.537	0.542	0.547	0.552	0.557	0.563	0.568	0.573	0.576	0.58	0.584
Haiti	0.328	0.332	0.336	0.339	0.342	0.345	0.35	0.355	0.361	0.367	0.373	0.379	0.384	0.389	0.393	0.397	0.401	0.405	0.409	0.413	0.416	0.42	0.423	0.427	0.431	0.435	0.439	0.442
Jamaica	0.547	0.553	0.56	0.565	0.571	0.578	0.585	0.591	0.596	0.6	0.607	0.615	0.621	0.626	0.63	0.635	0.639	0.644	0.649	0.653	0.658	0.663	0.667	0.671	0.673	0.676	0.677	0.679
Puerto Rico	0.684	0.689	0.693	0.698	0.703	0.708	0.714	0.719	0.724	0.729	0.736	0.745	0.752	0.756	0.759	0.762	0.766	0.77	0.774	0.778	0.782	0.786	0.791	0.797	0.805	0.811	0.813	0.813
Saint Lucia	0.509	0.516	0.524	0.532	0.54	0.549	0.558	0.566	0.573	0.58	0.585	0.589	0.593	0.598	0.603	0.606	0.609	0.613	0.618	0.623	0.628	0.633	0.636	0.64	0.643	0.646	0.65	0.653
Saint Vincent and the Grenadines	0.465	0.472	0.479	0.486	0.492	0.499	0.506	0.512	0.518	0.524	0.53	0.537	0.543	0.549	0.553	0.557	0.561	0.566	0.571	0.575	0.579	0.584	0.588	0.593	0.597	0.601	0.605	0.608
Suriname	0.529	0.534	0.538	0.541	0.543	0.543	0.544	0.547	0.551	0.555	0.56	0.568	0.576	0.585	0.592	0.597	0.601	0.604	0.608	0.612	0.616	0.62	0.625	0.628	0.632	0.636	0.639	0.641
Trinidad and Tobago	0.601	0.604	0.608	0.611	0.615	0.619	0.624	0.629	0.634	0.64	0.646	0.652	0.657	0.663	0.669	0.675	0.68	0.685	0.689	0.691	0.693	0.694	0.695	0.696	0.697	0.698	0.698	0.698
Virgin Islands	0.673	0.683	0.691	0.699	0.706	0.713	0.719	0.725	0.73	0.734	0.737	0.74	0.748	0.755	0.762	0.768	0.774	0.78	0.786	0.79	0.795	0.799	0.802	0.804	0.805	0.806	0.806	0.807
Central Latin America	0.492	0.496	0.501	0.507	0.515	0.521	0.528	0.534	0.54	0.545	0.551	0.557	0.561	0.564	0.567	0.571	0.577	0.583	0.588	0.593	0.597	0.601	0.606	0.61	0.614	0.617	0.621	0.623
Colombia	0.482	0.483	0.485	0.49	0.496	0.503	0.511	0.518	0.524	0.528	0.533	0.537	0.541	0.544	0.548	0.553	0.558	0.565	0.573	0.581	0.588	0.596	0.604	0.611	0.617	0.624	0.629	0.634
Costa Rica	0.524	0.53	0.536	0.54	0.545	0.55	0.555	0.56	0.566	0.573	0.581	0.589	0.594	0.598	0.603	0.607	0.609	0.612	0.616	0.623	0.629	0.634	0.64	0.645	0.649	0.654	0.658	0.662
El Salvador	0.406	0.409	0.413	0.418	0.424	0.431	0.44	0.449	0.459	0.47	0.481	0.491	0.5	0.508	0.515	0.523	0.531	0.539	0.545	0.55	0.556	0.561	0.567	0.573	0.578	0.584	0.589	0.593
Guatemala	0.313	0.318	0.326	0.338	0.353	0.363	0.371	0.381	0.397	0.411	0.42	0.427	0.433	0.44	0.449	0.458	0.465	0.47	0.476	0.482	0.489	0.496	0.501	0.507	0.513	0.517	0.521	0.524
Honduras	0.34	0.344	0.347	0.352	0.358	0.365	0.372	0.381	0.39	0.4	0.41	0.419	0.428	0.437	0.445	0.452	0.459	0.465	0.47	0.475	0.481	0.486	0.49	0.495	0.499	0.504	0.508	0.512
Mexico	0.513	0.517	0.521	0.529	0.537	0.543	0.549	0.555	0.561	0.566	0.572	0.577	0.581	0.586	0.59	0.595	0.598	0.602	0.606	0.608	0.611	0.613	0.616	0.619	0.622	0.624	0.626	0.628
Aguascalientes	0.558	0.561	0.565	0.571	0.577	0.583	0.591	0.597	0.603	0.606	0.609	0.612	0.615	0.621	0.626	0.632	0.636	0.64	0.643	0.644	0.645	0.647	0.649	0.651	0.653	0.655	0.657	0.659
Baja California	0.57	0.572	0.575	0.579	0.582	0.585	0.589	0.594	0.599	0.604	0.608	0.611	0.615	0.618	0.622	0.626	0.63	0.634	0.639	0.642	0.644	0.646	0.649	0.651	0.653	0.654	0.656	0.657
Baja California Sur	0.569	0.573	0.577	0.582	0.586	0.589	0.593	0.598	0.602	0.605	0.609	0.612	0.614	0.617	0.621	0.624	0.628	0.633	0.637	0.641	0.644	0.647	0.649	0.652	0.654	0.656	0.657	0.659
Campeche	0.485	0.49	0.496	0.504	0.511	0.517	0.525	0.534	0.543	0.551	0.558	0.564	0.569	0.573	0.577	0.581	0.585	0.589	0.593	0.595	0.597	0.599	0.602	0.605	0.608	0.611	0.614	0.616
Chiapas	0.403	0.407	0.413	0.422	0.43	0.436	0.444	0.452	0.459	0.465	0.47	0.475	0.48	0.484	0.489	0.494	0.498	0.503	0.508	0.512	0.514	0.517	0.52	0.522	0.525	0.528	0.531	0.533
Chihuahua	0.546	0.549	0.552	0.555	0.558	0.559	0.562	0.566	0.57	0.574	0.577	0.581	0.585	0.589	0.594	0.599	0.604	0.61	0.615	0.619	0.622	0.625	0.628	0.63	0.633	0.635	0.637	0.639
Coahuila	0.549	0.555	0.561	0.567	0.573	0.576	0.581	0.587	0.592	0.597	0.601	0.604	0.607	0.611	0.616	0.62	0.624	0.628	0.631	0.633	0.634	0.635	0.637	0.638	0.64	0.642	0.644	0.645
Colima	0.538	0.543	0.549	0.556	0.561	0.565	0.571	0.577	0.583	0.588	0.594	0.598	0.603	0.607	0.611	0.616	0.621	0.626	0.631	0.634	0.637	0.64	0.643	0.645	0.648	0.65	0.652	0.654
Mexico City	0.602	0.606	0.61	0.617	0.629	0.64	0.65	0.656	0.661	0.666	0.671	0.676	0.681	0.685	0.689	0.693	0.696	0.698	0.7	0.701	0.702	0.704	0.706	0.708	0.71	0.712	0.714	0.716
Durango	0.506	0.508	0.511	0.516	0.52	0.523	0.528	0.535	0.542	0.55	0.556	0.562	0.568	0.574	0.581	0.586	0.592	0.597	0.602	0.605	0.607	0.609	0.612	0.614	0.617	0.619	0.622	0.624
Guanajuato	0.494	0.499	0.504	0.511	0.519	0.527	0.534	0.541	0.546	0.551	0.558	0.565	0.571	0.575	0.579	0.583	0.587	0.591	0.595	0.598	0.601	0.604	0.607	0.611	0.614	0.616	0.619	0.621
Guerrero	0.426	0.429	0.433	0.44	0.445	0.45	0.456	0.465	0.473	0.48	0.487	0.492	0.498	0.505	0.511	0.517	0.523	0.529	0.535	0.539	0.542	0.545	0.548	0.552	0.555	0.558	0.56	0.562
Hidalgo	0.417	0.424	0.433	0.444	0.454	0.462	0.471	0.481	0.491	0.501	0.511	0.52	0.527	0.531	0.535	0.538	0.543	0.548	0.554	0.558	0.562	0.567	0.572	0.576	0.58	0.583	0.585	0.587
Jalisco	0.532	0.538	0.545	0.552	0.558	0.563	0.569	0.576	0.582	0.588	0.593	0.598	0.602	0.606	0.611	0.615	0.619	0.624	0.628	0.63	0.632	0.635	0.637	0.64	0.642	0.645	0.647	0.649
México	0.529	0.524	0.525	0.539	0.558	0.568	0.574	0.577	0.58	0.585	0.59	0.596	0.6	0.603	0.607	0.611	0.613	0.614	0.614	0.614	0.615	0.617	0.621	0.625	0.628	0.631	0.633	0.635
Michoacán de Ocampo	0.464	0.47	0.476	0.483	0.489	0.494	0.501	0.508	0.515	0.522	0.527	0.532	0.537	0.542	0.546	0.551	0.555	0.559	0.563	0.566	0.568	0.571	0.573	0.576	0.579	0.581	0.584	0.586
Morelos	0.525	0.532	0.539	0.547	0.553	0.558	0.564	0.571	0.577	0.582	0.587	0.591	0.595	0.598	0.601	0.605	0.608	0.612	0.615	0.617	0.619	0.622	0.624	0.627	0.629	0.631	0.634	0.635
Nayarit	0.495	0.5	0.506	0.512	0.518	0.523	0.529	0.537	0.545	0.553	0.56	0.566	0.571	0.576	0.581	0.585	0.59	0.594	0.598	0.6	0.602	0.604	0.607	0.61	0.613	0.615	0.618	0.62
Nuevo León	0.598	0.602	0.606	0.611	0.615	0.617	0.62	0.625	0.63	0.635	0.64	0.644	0.647	0.65	0.653	0.656	0.659	0.662	0.665	0.666	0.667	0.669	0.67	0.672	0.673	0.675	0.676	0.677
Oaxaca	0.431	0.435	0.441	0.447	0.452	0.455	0.459	0.466	0.473	0.479	0.485	0.49	0.496	0.501	0.507	0.512	0.517	0.523	0.529	0.533	0.537	0.541	0.545	0.549	0.552	0.555	0.558	0.561
Puebla	0.456	0.463	0.47	0.478	0.487	0.493	0.496	0.497	0.498	0.499	0.504	0.509	0.516	0.524	0.532	0.538	0.544	0.549	0.554	0.558	0.563	0.567	0.571	0.575	0.577	0.58	0.582	0.584
Querétaro	0.497	0.502	0.508	0.518	0.53	0.543	0.556	0.566	0.575	0.583	0.591	0.597	0.602	0.606	0.609	0.612	0.616	0.619	0.623	0.624	0.626	0.629	0.631	0.633	0.635	0.636	0.638	0.639

Appendix Table 11. Socio-demographic Index values for all estimated GBD 2017 locations, 1990-2017

Location	1990	1991	1992	1993	1994	1995	1996	1997	1998	1999	2000	2001	2002	2003	2004	2005	2006	2007	2008	2009	2010	2011	2012	2013	2014	2015	2016	2017
Quintana Roo	0.52	0.526	0.533	0.541	0.548	0.555	0.562	0.57	0.577	0.582	0.587	0.591	0.594	0.596	0.599	0.601	0.605	0.608	0.612	0.614	0.616	0.618	0.62	0.621	0.623	0.624	0.625	0.626
San Luis Potosí	0.482	0.486	0.492	0.5	0.508	0.514	0.522	0.53	0.538	0.545	0.551	0.556	0.561	0.566	0.571	0.575	0.58	0.586	0.591	0.595	0.599	0.602	0.606	0.61	0.613	0.616	0.619	0.621
Sinaloa	0.523	0.528	0.533	0.539	0.544	0.549	0.555	0.562	0.57	0.577	0.583	0.589	0.594	0.599	0.604	0.609	0.614	0.619	0.623	0.627	0.63	0.633	0.636	0.639	0.642	0.644	0.646	0.649
Sonora	0.553	0.557	0.562	0.566	0.57	0.573	0.578	0.583	0.588	0.593	0.597	0.601	0.605	0.608	0.612	0.616	0.621	0.625	0.629	0.632	0.635	0.637	0.64	0.643	0.645	0.647	0.649	0.65
Tabasco	0.474	0.479	0.486	0.493	0.5	0.507	0.515	0.524	0.533	0.541	0.548	0.553	0.558	0.563	0.568	0.573	0.578	0.583	0.588	0.591	0.594	0.596	0.599	0.602	0.604	0.607	0.609	0.611
Tamaulipas	0.548	0.553	0.558	0.564	0.568	0.571	0.574	0.579	0.586	0.592	0.598	0.602	0.606	0.609	0.613	0.616	0.62	0.624	0.628	0.63	0.633	0.635	0.637	0.64	0.642	0.643	0.645	0.647
Tlaxcala	0.478	0.482	0.487	0.495	0.506	0.515	0.524	0.531	0.536	0.541	0.545	0.55	0.556	0.561	0.567	0.573	0.578	0.583	0.587	0.59	0.591	0.594	0.596	0.598	0.6	0.601	0.603	0.604
Veracruz de Ignacio de la Llave	0.461	0.463	0.467	0.472	0.477	0.48	0.485	0.492	0.5	0.509	0.517	0.523	0.529	0.534	0.54	0.546	0.551	0.557	0.563	0.567	0.571	0.574	0.578	0.581	0.584	0.587	0.59	0.592
Yucatán	0.497	0.502	0.508	0.516	0.524	0.531	0.54	0.548	0.556	0.562	0.569	0.575	0.581	0.587	0.592	0.596	0.599	0.602	0.605	0.608	0.61	0.613	0.617	0.62	0.623	0.626	0.628	0.63
Zacatecas	0.483	0.489	0.495	0.502	0.509	0.514	0.52	0.527	0.534	0.54	0.546	0.551	0.555	0.56	0.564	0.568	0.572	0.577	0.581	0.584	0.586	0.589	0.592	0.595	0.598	0.602	0.605	0.608
Nicaragua	0.357	0.363	0.368	0.374	0.381	0.389	0.397	0.406	0.415	0.424	0.432	0.439	0.446	0.453	0.46	0.466	0.47	0.475	0.481	0.486	0.492	0.499	0.504	0.509	0.514	0.52	0.525	0.53
Panama	0.542	0.546	0.55	0.555	0.56	0.565	0.569	0.573	0.578	0.583	0.589	0.595	0.6	0.604	0.608	0.611	0.614	0.618	0.622	0.626	0.63	0.635	0.641	0.648	0.656	0.664	0.671	0.677
Venezuela	0.528	0.536	0.553	0.559	0.566	0.578	0.587	0.592	0.591	0.587	0.591	0.602	0.602	0.588	0.576	0.579	0.594	0.61	0.621	0.625	0.629	0.633	0.64	0.646	0.648	0.651	0.654	0.655
Tropical Latin America	0.494	0.5	0.507	0.514	0.521	0.529	0.537	0.544	0.55	0.556	0.561	0.566	0.571	0.577	0.582	0.588	0.594	0.601	0.608	0.614	0.621	0.628	0.635	0.642	0.648	0.654	0.659	0.662
Brazil	0.494	0.501	0.508	0.515	0.522	0.53	0.537	0.545	0.551	0.556	0.562	0.567	0.572	0.577	0.583	0.589	0.595	0.602	0.608	0.615	0.622	0.629	0.636	0.643	0.649	0.655	0.66	0.663
Acre	0.376	0.386	0.395	0.405	0.415	0.424	0.435	0.445	0.453	0.46	0.466	0.472	0.479	0.485	0.492	0.5	0.508	0.517	0.527	0.536	0.546	0.556	0.565	0.575	0.583	0.591	0.597	0.602
Alagoas	0.355	0.363	0.371	0.379	0.387	0.395	0.404	0.412	0.419	0.425	0.431	0.436	0.442	0.448	0.455	0.462	0.47	0.478	0.487	0.496	0.505	0.514	0.523	0.531	0.539	0.546	0.552	0.556
Amapá	0.467	0.475	0.483	0.491	0.5	0.508	0.517	0.526	0.534	0.54	0.546	0.552	0.558	0.564	0.57	0.576	0.583	0.591	0.598	0.605	0.613	0.621	0.629	0.636	0.643	0.65	0.655	0.659
Amazonas	0.438	0.447	0.457	0.466	0.475	0.483	0.492	0.499	0.505	0.51	0.514	0.519	0.523	0.528	0.533	0.539	0.546	0.553	0.561	0.568	0.577	0.585	0.594	0.602	0.611	0.618	0.625	0.629
Bahia	0.402	0.41	0.419	0.427	0.435	0.443	0.451	0.459	0.465	0.47	0.475	0.48	0.485	0.491	0.496	0.503	0.51	0.518	0.526	0.534	0.542	0.551	0.559	0.567	0.575	0.582	0.587	0.591
Ceará	0.411	0.419	0.426	0.433	0.44	0.448	0.455	0.463	0.469	0.475	0.48	0.486	0.492	0.498	0.505	0.512	0.52	0.528	0.536	0.544	0.553	0.561	0.569	0.577	0.584	0.591	0.596	0.6
Distrito Federal	0.63	0.636	0.642	0.649	0.656	0.663	0.671	0.679	0.685	0.691	0.696	0.702	0.707	0.713	0.719	0.725	0.731	0.738	0.744	0.75	0.756	0.763	0.769	0.775	0.78	0.785	0.789	0.792
Espírito Santo	0.499	0.507	0.515	0.524	0.532	0.54	0.549	0.557	0.564	0.57	0.576	0.582	0.588	0.593	0.599	0.606	0.612	0.618	0.625	0.631	0.638	0.644	0.651	0.657	0.663	0.669	0.673	0.677
Goiás	0.46	0.468	0.476	0.484	0.493	0.501	0.51	0.518	0.526	0.532	0.538	0.545	0.551	0.558	0.564	0.571	0.579	0.586	0.594	0.601	0.608	0.616	0.623	0.63	0.636	0.642	0.647	0.65
Maranhão	0.313	0.322	0.33	0.339	0.347	0.355	0.364	0.371	0.377	0.38	0.383	0.386	0.389	0.392	0.396	0.402	0.409	0.418	0.427	0.436	0.446	0.456	0.467	0.477	0.486	0.495	0.502	0.507
Mato Grosso	0.475	0.484	0.492	0.501	0.509	0.518	0.527	0.535	0.543	0.548	0.554	0.559	0.564	0.57	0.576	0.582	0.589	0.596	0.604	0.611	0.618	0.626	0.633	0.641	0.648	0.654	0.659	0.662
Mato Grosso do Sul	0.465	0.473	0.481	0.489	0.497	0.506	0.515	0.523	0.531	0.537	0.543	0.549	0.555	0.56	0.566	0.573	0.58	0.587	0.594	0.6	0.607	0.614	0.622	0.629	0.636	0.642	0.647	0.65
Minas Gerais	0.491	0.498	0.506	0.513	0.521	0.53	0.538	0.545	0.551	0.557	0.562	0.567	0.573	0.579	0.585	0.591	0.598	0.604	0.611	0.618	0.624	0.631	0.637	0.643	0.649	0.654	0.658	0.661
Pará	0.41	0.418	0.425	0.432	0.44	0.447	0.454	0.461	0.466	0.47	0.473	0.476	0.48	0.483	0.488	0.493	0.499	0.506	0.514	0.521	0.529	0.538	0.546	0.554	0.562	0.569	0.575	0.579
Paraíba	0.399	0.406	0.413	0.42	0.427	0.434	0.441	0.447	0.453	0.457	0.461	0.465	0.469	0.474	0.48	0.486	0.493	0.501	0.509	0.517	0.526	0.535	0.543	0.551	0.559	0.565	0.571	0.574
Paraná	0.513	0.519	0.525	0.532	0.539	0.548	0.556	0.564	0.572	0.578	0.585	0.591	0.597	0.603	0.609	0.615	0.622	0.628	0.634	0.64	0.646	0.652	0.658	0.664	0.67	0.675	0.679	0.682
Pernambuco	0.416	0.423	0.43	0.437	0.444	0.451	0.458	0.466	0.472	0.477	0.481	0.486	0.492	0.497	0.503	0.51	0.517	0.525	0.533	0.54	0.548	0.556	0.564	0.572	0.579	0.585	0.59	0.594
Piauí	0.365	0.372	0.379	0.386	0.393	0.4	0.408	0.415	0.42	0.425	0.429	0.434	0.439	0.444	0.45	0.457	0.465	0.473	0.482	0.491	0.5	0.51	0.518	0.527	0.535	0.542	0.548	0.552
Rio de Janeiro	0.576	0.581	0.585	0.59	0.595	0.601	0.608	0.614	0.619	0.624	0.628	0.632	0.637	0.641	0.645	0.65	0.655	0.66	0.665	0.67	0.675	0.681	0.686	0.692	0.697	0.702	0.706	0.709
Rio Grande do Norte	0.415	0.422	0.429	0.436	0.444	0.451	0.46	0.467	0.474	0.48	0.485	0.491	0.497	0.503	0.509	0.516	0.524	0.532	0.541	0.549	0.558	0.567	0.575	0.583	0.59	0.597	0.602	0.605
Rio Grande do Sul	0.543	0.549	0.555	0.561	0.567	0.574	0.581	0.587	0.593	0.598	0.603	0.608	0.614	0.619	0.624	0.63	0.635	0.641	0.647	0.653	0.659	0.665	0.67	0.676	0.681	0.686	0.69	0.693
Rondônia	0.423	0.433	0.441	0.45	0.458	0.467	0.475	0.484	0.491	0.497	0.502	0.508	0.515	0.521	0.528	0.535	0.543	0.551	0.559	0.567	0.575	0.584	0.592	0.599	0.606	0.613	0.618	0.622
Roraima	0.428	0.438	0.447	0.456	0.465	0.474	0.483	0.492	0.499	0.504	0.509	0.514	0.521	0.527	0.534	0.543	0.552	0.562	0.572	0.581	0.591	0.601	0.611	0.62	0.628	0.636	0.642	0.646
Santa Catarina	0.541	0.548	0.554	0.56	0.567	0.574	0.582	0.589	0.595	0.601	0.606	0.612	0.618	0.623	0.629	0.635	0.641	0.647	0.653	0.659	0.665	0.672	0.678	0.684	0.69	0.695	0.699	0.702
São Paulo	0.558	0.565	0.572	0.579	0.587	0.595	0.603	0.611	0.618	0.624	0.63	0.636	0.641	0.646	0.652	0.657	0.663	0.669	0.674	0.68	0.685	0.691	0.697	0.703	0.708	0.713	0.717	0.72
Sergipe	0.425	0.433	0.441	0.448	0.456	0.464	0.473	0.481	0.488	0.494	0.5	0.506	0.512	0.518	0.524	0.531	0.538	0.546	0.554	0.562	0.57	0.578	0.586	0.594	0.601	0.607	0.612	0.616
Tocantins	0.396	0.404	0.412	0.42	0.428	0.436	0.445	0.453	0.46	0.466	0.471	0.477	0.484	0.491	0.498	0.507	0.517	0.527	0.537	0.547	0.558	0.568	0.577	0.586	0.594	0.601	0.607	0.611
Paraguay	0.467	0.471	0.475	0.48	0.485	0.491	0.497	0.504	0.512	0.519	0.525	0.532	0.538	0.544	0.548	0.553	0.558	0.562	0.566	0.569	0.573	0.577	0.582	0.589	0.598	0.606	0.613	0.619
North Africa and Middle East	0.456	0.464	0.472	0.478	0.485	0.492	0.499	0.506	0.513	0.521	0.531	0.539	0.547	0.553	0.561	0.568	0.574	0.582	0.589	0.596	0.602	0.607	0.612	0.617	0.622	0.628	0.635	0.639
North Africa and Middle East	0.456	0.464	0.472	0.478	0.485	0.492	0.499	0.506	0.513	0.521	0.531	0.539	0.547	0.553	0.561	0.568	0.574	0.582	0.589	0.596	0.602	0.607	0.612	0.617	0.622	0.628	0.635	0.639
Afghanistan	0.149	0.15	0.151	0.152	0.151	0.151	0.152	0.152	0.154	0.155	0.157	0.159	0.164	0.171	0.177	0.184	0.191	0.199	0.208	0.218	0.228	0.238	0.248	0.258	0.267	0.276	0.284	0.29
Algeria	0.495	0.504	0.513	0.522	0.531	0.54	0.549	0.558	0.567	0.574	0.583	0.591	0.599	0.608	0.617	0.625	0.633	0.641	0.648	0.653	0.659	0.665	0.67	0.675	0.68	0.685	0.691	0.696
Bahrain	0.612	0.618	0.624	0.63	0.635	0.639	0.644	0.648	0.653	0.657	0.662	0.668	0.673	0.677	0.68	0.684	0.687	0.69	0.693	0.695	0.696	0.697	0.698	0.7	0.702	0.706	0.709	0.712
Egypt	0.441	0.451	0.461	0.47	0.477	0.485	0.493	0.5	0.507	0.514	0.52	0.526	0.532	0.537	0.542	0.547	0.55	0.553	0.556	0.559	0.563	0.565	0.564	0.566	0.572	0.584	0.596	0.604
Iran	0.503	0.513	0.522	0.532	0.541	0.548	0.553	0.556	0.56	0.572	0.589	0.604	0.616	0.626	0.634	0.641	0.649	0.656	0.662	0.667	0.672	0.677	0.68	0.683	0.687	0.691	0.696	0.7
Iraq	0.433	0.433	0.437	0.441	0.444	0.446	0.45	0.455	0.463	0.473	0.481	0.488	0.494	0.496	0.502	0.507	0.512	0.517	0.522	0.528	0.535	0.542	0.551	0.559	0.566	0.572	0.58	0.585
Jordan	0.552	0.556	0.561	0.566	0.57	0.574	0.578	0.584	0.589	0.594	0.597	0.6	0.603	0.607	0.612	0.616	0.621	0.626	0.633	0.643	0.653	0.662	0.671	0.679	0.685	0.69	0.694	0.697
Kuwait	0.656	0.651	0.642	0.637	0.633	0.632	0.638	0.651	0.665	0.674	0.681	0.683	0.685	0.687	0.693	0.701	0.711	0.721	0.73	0.737	0.743	0.748	0.754	0.762	0.769	0.776	0.781	0.786
Lebanon	0.519	0.528	0.536	0.544	0.552	0.56	0.569	0.578	0.588	0.597	0.604	0.611	0.618	0.626	0.635	0.643	0.65	0.658	0.667	0.676	0.686	0.694	0.702	0.711	0.717	0.722	0.726	0.73
Libya	0.645	0.657	0.669	0.679	0.688	0.696	0.705	0.713	0.72	0.725	0.729	0.732	0.736	0.742	0.747	0.753	0.759	0.765	0.771	0.777	0.783	0.781	0.784	0.783	0.779	0.773	0.766	0.761
Morocco	0.389	0.397	0.404	0.411	0.419	0.426	0.434	0.442	0.449	0.456	0.463	0.47	0.477	0.484	0.49	0.496	0.503	0.51	0.517	0.525	0.532	0.54	0.547	0.554	0.561	0.567	0.574	0.579
Palestine	0.362	0.366	0.371	0.377	0.384	0.39	0.397	0.405	0.413	0.421	0.427	0.431	0.433	0.435	0.439	0.444	0.448	0.453	0.46	0.468	0.479	0.491	0.503	0.513	0.521	0.528	0.536	0.541
Oman	0.473	0.485	0.497	0.509	0.521	0.533	0.546	0.558	0.57	0.582	0.594	0.606	0.618	0.629	0.639	0.649	0.659	0.668	0.678	0.686	0.695	0.703	0.711	0.72	0.727	0.734	0.739	0.744
Qatar	0.603	0.615	0.626	0.637	0.647	0.656	0.665	0.674	0.681	0.688	0.695	0.701	0.706	0.712	0.717	0.721	0.725	0.728	0.731	0.735	0.738	0.743	0.747	0.751	0.755	0.759	0.762	0.766
Saudi Arabia	0.436	0.457	0.477	0.496	0.514	0.53	0.545	0.56	0.573	0.586	0.598	0.61	0.621	0.633	0.645	0.658	0.67	0.682	0.695	0.706	0.716	0.728	0.738	0.748	0.757	0.765	0.773	0.779
Sudan	0.221	0.228	0.236	0.244	0.252	0.26	0.267	0.275	0.283	0.291	0.3	0.308	0.317	0.326	0.336	0.346	0.357	0.367	0.378	0.389	0.401	0.414	0.425	0.437	0.448	0.46	0.47	0.478
Syria	0.389	0.394	0.401	0.409	0.419	0.429	0.439	0.448	0.458	0.466	0.474	0.481	0.489	0.496	0.506	0.518	0.527	0.536	0.545	0.556	0.566	0.576	0.584	0.589	0.595	0.601	0.606	0.611
Tunisia	0.453	0.463	0.473	0.482	0.491	0.501	0.511	0.521	0.532	0.543	0.553	0.563	0.572	0.582	0.592	0.6	0.609	0.617	0.625	0.632	0.639	0.645	0.65	0.656	0.661	0.666	0.671	0.675
Turkey	0.507	0.516	0.524	0.533	0.54	0.548	0.556	0.564	0.572	0.579	0.587	0.594	0.601	0.608	0.616	0.625	0.633	0.643	0.652	0.66	0.669	0.678	0.688	0.697	0.706	0.715	0.723	0.729
United Arab Emirates	0.621	0.637	0.651	0.663	0.675	0.686	0.696	0.707	0.716	0.726	0.734	0.742	0.749	0.755	0.76	0.765	0.77	0.776	0.781	0.784	0.788	0.79	0.791	0.793	0.791	0.794	0.794	0.795
Yemen	0.203	0.208	0.214	0.22	0.228	0.238	0.247	0.256	0.266	0.275	0.284	0.294	0.304	0.313	0.324	0.336	0.349	0.362	0.373	0.382	0.393	0.401	0.408	0.415	0.422	0.426	0.429	0.43
South Asia	0.312	0.317	0.324	0.331	0.338	0.345	0.352	0.358	0.363	0.371	0.38	0.388	0.395	0.403	0.412	0.421	0.43	0.438	0.446	0.455	0.462	0.468	0.476	0.489	0.504	0.517	0.526	0.534
South Asia	0.312	0.317	0.324	0.331	0.338	0.345	0.352	0.358	0.363	0.371	0.38	0.388	0.395	0.403	0.412	0.421	0.43	0.438	0.446	0.455	0.462	0.468	0.476	0.489	0.504	0.517	0.526	0.534
Bangladesh	0.256	0.264	0.272	0.281	0.289	0.298	0.306	0.314	0.322	0.329	0.335	0.34	0.345	0.35	0.356	0.362	0.369	0.377	0.385	0.393	0.401	0.41	0.418	0.427	0.435	0.443	0.451	0.458
Bhutan	0.324	0.333	0.343	0.352	0.362	0.372	0.382	0.392	0.402	0.412	0.422	0.432	0.442	0.452	0.461	0.47	0.479	0.489	0.498	0.507	0.516	0.525	0.534	0.542	0.549	0.557	0.564	0.57
India	0.32	0.324	0.331	0.339	0.346	0.353	0.36	0.365	0.37	0.378	0.387	0.395	0.403	0.412	0.422	0.431	0.44	0.448	0.457	0.465	0.472	0.477	0.485	0.5	0.517	0.532	0.542	0.55
Andhra Pradesh	0.28	0.286	0.294	0.303	0.312	0.322	0.331	0.337	0.344	0.352	0.363	0.374	0.384	0.395	0.406	0.415	0.424	0.434	0.442	0.451	0.459	0.466	0.473	0.487	0.503	0.517	0.528	0.536
Arunachal Pradesh	0.311	0.317	0.323	0.33	0.336	0.345	0.354	0.36	0.366	0.374	0.381	0.388	0.395	0.405	0.416	0.424	0.432	0.44	0.447	0.458	0.467	0.473	0.479	0.498	0.52	0.536	0.548	0.556
Assam	0.327	0.332	0.339	0.345	0.351	0.358	0.363	0.367	0.371	0.379	0.389	0.397	0.406	0.415	0.424	0.431	0.439	0.446	0.453	0.459	0.464	0.467	0.472	0.485	0.5	0.513	0.523	0.53
Bihar	0.29	0.293	0.297	0.298	0.298	0.298	0.298	0.297	0.292	0.291	0.292	0.294	0.296	0.299	0.303	0.308	0.316	0.324	0.334	0.342	0.349	0.355	0.365	0.382	0.4	0.414	0.425	0.433
Chhattisgarh	0.292	0.294	0.298	0.303	0.306	0.311	0.316	0.319	0.322	0.328	0.336	0.344	0.352	0.362	0.372	0.382	0.392	0.403	0.413	0.421	0.427	0.431	0.438	0.455	0.474	0.49	0.503	0.512
Delhi	0.486	0.493	0.504	0.516	0.522	0.526	0.531	0.535	0.537	0.545	0.555	0.565	0.575	0.584	0.593	0.602	0.612	0.62	0.628	0.637	0.644	0.649	0.656	0.67	0.686	0.7	0.709	0.715
Goa	0.499	0.505	0.512	0.519	0.526	0.534	0.542	0.55	0.56	0.571	0.58	0.588	0.597	0.606	0.616	0.626	0.634	0.643	0.655	0.665	0.674	0.683	0.691	0.702	0.714	0.724	0.733	0.74
Gujarat	0.35	0.353	0.361	0.37	0.379	0.388	0.396	0.402	0.409	0.418	0.426	0.433	0.438	0.447	0.457	0.469	0.478	0.487	0.496	0.506	0.515	0.521	0.528	0.541	0.556	0.569	0.577	0.584
Haryana	0.342	0.348	0.356	0.364	0.372	0.38	0.388	0.394	0.398	0.405	0.414	0.423	0.431	0.44	0.451	0.462	0.472	0.481	0.491	0.5	0.508	0.512	0.521	0.54	0.564	0.582	0.593	0.6
Himachal Pradesh	0.34	0.347	0.356	0.366	0.376	0.386	0.397	0.408	0.418	0.431	0.445	0.457	0.468	0.479	0.491	0.502	0.513	0.524	0.534	0.544	0.553	0.562	0.571	0.585	0.601	0.614	0.625	0.633
Jammu and Kashmir	0.334	0.337	0.341	0.349	0.356	0.363	0.37	0.38	0.389	0.402	0.417	0.43	0.442	0.454	0.466	0.476	0.485	0.493	0.503	0.511	0.519	0.527	0.535	0.547	0.562	0.574	0.582	0.59
Jharkhand	0.281	0.283	0.286	0.29	0.293	0.297	0.3	0.304	0.307	0.312	0.319	0.325	0.333	0.342	0.353	0.362	0.371	0.38	0.388	0.397	0.405	0.411	0.419	0.434	0.451	0.466	0.478	0.487
Karnataka	0.323	0.331	0.339	0.349	0.357	0.366	0.375	0.38	0.386	0.396	0.406	0.415	0.424	0.434	0.444	0.454	0.463	0.472	0.481	0.49	0.498	0.504	0.512	0.526	0.542	0.556	0.566	0.574
Kerala	0.404	0.408	0.418	0.429	0.438	0.447	0.456	0.463	0.472	0.483	0.496	0.506	0.515	0.525	0.536	0.548	0.557	0.565	0.574	0.581	0.587	0.592	0.599	0.612	0.629	0.642	0.652	0.659
Madhya Pradesh	0.284	0.288	0.293	0.299	0.303	0.309	0.315	0.317	0.319	0.326	0.334	0.342	0.348	0.355	0.363	0.369	0.373	0.378	0.384	0.39	0.394	0.398	0.406	0.425	0.447	0.465	0.477	0.487
Maharashtra	0.363	0.369	0.379	0.39	0.399	0.41	0.419	0.426	0.431	0.44	0.451	0.459	0.468	0.478	0.489	0.5	0.51	0.52	0.529	0.538	0.546	0.552	0.559	0.572	0.588	0.602	0.611	0.618
Manipur	0.381	0.387	0.393	0.4	0.405	0.411	0.417	0.421	0.427	0.436	0.445	0.454	0.461	0.469	0.479	0.49	0.498	0.505	0.512	0.52	0.526	0.532	0.539	0.549	0.561	0.572	0.582	0.59
Meghalaya	0.334	0.339	0.345	0.352	0.357	0.364	0.37	0.376	0.382	0.39	0.401	0.411	0.42	0.43	0.441	0.45	0.46	0.469	0.478	0.487	0.495	0.501	0.507	0.52	0.534	0.547	0.557	0.565
Mizoram	0.392	0.399	0.408	0.417	0.424	0.433	0.443	0.449	0.456	0.465	0.475	0.483	0.492	0.502	0.511	0.52	0.526	0.531	0.537	0.545	0.55	0.552	0.557	0.57	0.587	0.6	0.609	0.616
Nagaland	0.395	0.399	0.406	0.414	0.421	0.429	0.435	0.441	0.445	0.452	0.462	0.472	0.482	0.492	0.503	0.513	0.522	0.532	0.543	0.552	0.56	0.567	0.574	0.587	0.602	0.614	0.625	0.633
Odisha	0.283	0.287	0.292	0.299	0.305	0.314	0.32	0.325	0.331	0.34	0.349	0.357	0.364	0.373	0.384	0.393	0.404	0.415	0.425	0.434	0.441	0.447	0.456	0.472	0.49	0.504	0.515	0.524
Punjab	0.383	0.388	0.396	0.404	0.413	0.422	0.429	0.435	0.441	0.449	0.459	0.468	0.475	0.483	0.492	0.501	0.511	0.52	0.53	0.539	0.547	0.554	0.563	0.576	0.592	0.605	0.614	0.622
Rajasthan	0.263	0.268	0.274	0.28	0.287	0.295	0.303	0.309	0.314	0.322	0.332	0.342	0.349	0.359	0.369	0.377	0.385	0.391	0.398	0.404	0.41	0.415	0.421	0.438	0.458	0.473	0.484	0.492
Sikkim	0.343	0.349	0.355	0.362	0.368	0.375	0.382	0.388	0.395	0.403	0.413	0.421	0.431	0.442	0.453	0.463	0.473	0.482	0.492	0.512	0.528	0.541	0.555	0.574	0.593	0.608	0.619	0.628
Tamil Nadu	0.35	0.354	0.363	0.373	0.383	0.391	0.399	0.406	0.414	0.424	0.436	0.446	0.455	0.465	0.475	0.486	0.497	0.506	0.514	0.522	0.529	0.535	0.543	0.559	0.579	0.595	0.607	0.615
Telangana	0.288	0.294	0.3	0.307	0.315	0.323	0.331	0.337	0.344	0.352	0.363	0.373	0.383	0.395	0.407	0.418	0.429	0.441	0.454	0.465	0.478	0.488	0.5	0.517	0.536	0.552	0.565	0.575
Tripura	0.333	0.336	0.34	0.346	0.35	0.356	0.363	0.368	0.373	0.385	0.398	0.409	0.419	0.43	0.44	0.449	0.457	0.462	0.469	0.476	0.48	0.483	0.488	0.5	0.515	0.527	0.536	0.543
Uttar Pradesh	0.272	0.276	0.281	0.286	0.291	0.297	0.303	0.307	0.31	0.317	0.325	0.333	0.34	0.348	0.358	0.366	0.374	0.382	0.39	0.398	0.404	0.41	0.419	0.436	0.454	0.469	0.48	0.488
Uttarakhand	0.304	0.306	0.31	0.315	0.321	0.329	0.336	0.341	0.347	0.355	0.367	0.378	0.391	0.405	0.42	0.434	0.449	0.463	0.478	0.494	0.509	0.522	0.535	0.552	0.57	0.586	0.598	0.607
West Bengal	0.321	0.322	0.329	0.338	0.345	0.353	0.36	0.366	0.371	0.38	0.389	0.398	0.406	0.416	0.425	0.434	0.441	0.448	0.455	0.462	0.467	0.471	0.476	0.49	0.506	0.519	0.53	0.538
Union Territories other than Delhi	0.406	0.408	0.412	0.417	0.423	0.43	0.439	0.446	0.453	0.463	0.474	0.486	0.498	0.511	0.524	0.537	0.548	0.559	0.569	0.579	0.586	0.591	0.597	0.61	0.624	0.636	0.646	0.653
Nepal	0.216	0.222	0.228	0.235	0.242	0.249	0.257	0.265	0.273	0.281	0.29	0.298	0.307	0.315	0.323	0.331	0.339	0.347	0.355	0.364	0.373	0.382	0.39	0.399	0.407	0.415	0.422	0.429
Pakistan	0.305	0.31	0.314	0.319	0.324	0.33	0.337	0.343	0.35	0.359	0.367	0.375	0.382	0.389	0.396	0.404	0.412	0.42	0.428	0.436	0.443	0.449	0.458	0.466	0.474	0.48	0.486	0.492
Southeast Asia, East Asia, and Oceania	0.468	0.479	0.489	0.498	0.506	0.517	0.528	0.538	0.546	0.553	0.561	0.568	0.574	0.58	0.588	0.596	0.606	0.615	0.624	0.632	0.641	0.649	0.656	0.662	0.67	0.673	0.675	0.685
East Asia	0.463	0.476	0.486	0.495	0.504	0.516	0.528	0.54	0.55	0.559	0.568	0.577	0.584	0.591	0.599	0.61	0.621	0.632	0.642	0.652	0.663	0.672	0.679	0.685	0.694	0.696	0.696	0.709
China	0.456	0.469	0.479	0.489	0.498	0.51	0.523	0.535	0.545	0.554	0.563	0.573	0.58	0.587	0.595	0.606	0.618	0.63	0.64	0.65	0.661	0.671	0.678	0.683	0.693	0.695	0.694	0.707
North Korea	0.512	0.517	0.52	0.524	0.524	0.522	0.519	0.515	0.51	0.506	0.503	0.5	0.498	0.498	0.499	0.501	0.503	0.506	0.508	0.511	0.514	0.517	0.52	0.524	0.527	0.531	0.535	0.538
Taiwan (Province of China)	0.691	0.7	0.708	0.715	0.723	0.731	0.739	0.75	0.759	0.764	0.77	0.778	0.786	0.793	0.8	0.807	0.813	0.821	0.827	0.833	0.838	0.842	0.846	0.85	0.854	0.858	0.861	0.864
Oceania	0.406	0.408	0.41	0.412	0.415	0.418	0.421	0.423	0.426	0.428	0.43	0.431	0.432	0.433	0.435	0.436	0.438	0.44	0.442	0.444	0.446	0.449	0.452	0.455	0.459	0.463	0.467	0.471
American Samoa	0.609	0.612	0.615	0.62	0.624	0.629	0.633	0.636	0.639	0.643	0.647	0.651	0.655	0.659	0.662	0.666	0.668	0.671	0.673	0.675	0.678	0.682	0.687	0.691	0.694	0.697	0.7	0.702
Federated States of Micronesia	0.462	0.469	0.476	0.483	0.489	0.496	0.501	0.505	0.509	0.514	0.518	0.523	0.527	0.532	0.536	0.54	0.543	0.547	0.549	0.552	0.555	0.559	0.562	0.565	0.567	0.57	0.573	0.575
Fiji	0.533	0.537	0.542	0.547	0.552	0.558	0.563	0.568	0.574	0.58	0.585	0.589	0.594	0.598	0.602	0.604	0.607	0.61	0.611	0.613	0.615	0.617	0.619	0.623	0.627	0.632	0.637	0.641
Guam	0.698	0.695	0.693	0.694	0.698	0.703	0.709	0.717	0.727	0.739	0.75	0.759	0.765	0.768	0.77	0.77	0.769	0.77	0.771	0.774	0.776	0.78	0.784	0.788	0.792	0.794	0.794	0.794
Kiribati	0.355	0.357	0.359	0.361	0.363	0.365	0.368	0.37	0.373	0.376	0.379	0.382	0.385	0.388	0.39	0.393	0.395	0.397	0.398	0.399	0.401	0.403	0.406	0.41	0.414	0.418	0.423	0.427
Marshall Islands	0.413	0.421	0.429	0.436	0.444	0.451	0.457	0.462	0.465	0.469	0.473	0.477	0.482	0.485	0.489	0.493	0.498	0.503	0.507	0.512	0.518	0.523	0.528	0.533	0.538	0.542	0.547	0.55
Northern Mariana Islands	0.738	0.744	0.748	0.75	0.752	0.754	0.756	0.757	0.758	0.759	0.763	0.766	0.767	0.767	0.767	0.766	0.765	0.764	0.763	0.761	0.759	0.758	0.757	0.756	0.756	0.756	0.757	0.758
Papua New Guinea	0.318	0.321	0.324	0.33	0.335	0.34	0.344	0.348	0.351	0.354	0.356	0.358	0.36	0.361	0.363	0.365	0.368	0.371	0.375	0.379	0.383	0.388	0.393	0.398	0.404	0.409	0.415	0.419
Samoa	0.538	0.539	0.538	0.536	0.535	0.536	0.536	0.537	0.538	0.54	0.542	0.544	0.546	0.548	0.55	0.552	0.554	0.556	0.558	0.559	0.561	0.563	0.56	0.558	0.562	0.567	0.572	0.576
Solomon Islands	0.316	0.32	0.326	0.331	0.337	0.344	0.35	0.355	0.36	0.364	0.365	0.366	0.366	0.366	0.367	0.369	0.371	0.375	0.38	0.384	0.388	0.394	0.4	0.406	0.411	0.416	0.421	0.425
Tonga	0.522	0.528	0.533	0.538	0.544	0.549	0.553	0.557	0.56	0.564	0.566	0.569	0.573	0.577	0.581	0.584	0.587	0.589	0.592	0.595	0.599	0.603	0.607	0.61	0.614	0.617	0.621	0.625
Vanuatu	0.38	0.384	0.388	0.391	0.395	0.398	0.402	0.406	0.409	0.413	0.416	0.419	0.421	0.423	0.425	0.428	0.431	0.435	0.44	0.445	0.449	0.453	0.458	0.462	0.465	0.469	0.472	0.475
Southeast Asia	0.467	0.477	0.486	0.495	0.504	0.513	0.521	0.529	0.534	0.538	0.543	0.548	0.552	0.557	0.562	0.567	0.572	0.578	0.584	0.589	0.595	0.602	0.609	0.616	0.622	0.629	0.635	0.641
Cambodia	0.259	0.266	0.273	0.279	0.286	0.294	0.302	0.31	0.317	0.326	0.335	0.344	0.353	0.362	0.372	0.382	0.392	0.401	0.411	0.42	0.427	0.435	0.443	0.451	0.459	0.467	0.475	0.482
Indonesia	0.465	0.476	0.487	0.499	0.51	0.52	0.53	0.537	0.542	0.546	0.55	0.554	0.558	0.562	0.566	0.571	0.576	0.581	0.587	0.594	0.6	0.608	0.615	0.622	0.629	0.636	0.642	0.648
Laos	0.307	0.313	0.32	0.326	0.332	0.339	0.345	0.352	0.358	0.364	0.371	0.378	0.385	0.392	0.4	0.407	0.416	0.425	0.434	0.444	0.453	0.463	0.473	0.483	0.493	0.502	0.511	0.519
Malaysia	0.57	0.575	0.581	0.589	0.598	0.606	0.616	0.626	0.638	0.654	0.661	0.664	0.671	0.679	0.684	0.689	0.697	0.704	0.709	0.714	0.721	0.727	0.733	0.739	0.744	0.749	0.754	0.759
Maldives	0.386	0.399	0.412	0.424	0.435	0.446	0.46	0.477	0.493	0.507	0.518	0.528	0.537	0.545	0.554	0.56	0.567	0.575	0.584	0.593	0.602	0.611	0.62	0.629	0.636	0.643	0.65	0.655
Mauritius	0.554	0.561	0.568	0.576	0.585	0.595	0.603	0.609	0.613	0.616	0.621	0.627	0.633	0.639	0.646	0.651	0.657	0.663	0.669	0.675	0.68	0.687	0.694	0.701	0.708	0.713	0.717	0.72
Myanmar	0.33	0.333	0.337	0.341	0.347	0.353	0.36	0.367	0.375	0.383	0.392	0.402	0.412	0.423	0.434	0.446	0.458	0.47	0.481	0.492	0.501	0.51	0.518	0.527	0.535	0.542	0.549	0.556

Appendix Table 11. Socio-demographic Index values for all estimated GBD 2017 locations, 1990-2017

Location	1990	1991	1992	1993	1994	1995	1996	1997	1998	1999	2000	2001	2002	2003	2004	2005	2006	2007	2008	2009	2010	2011	2012	2013	2014	2015	2016	2017
Philippines	0.511	0.516	0.521	0.525	0.53	0.534	0.539	0.542	0.545	0.547	0.55	0.553	0.555	0.557	0.559	0.561	0.563	0.566	0.568	0.569	0.572	0.579	0.586	0.593	0.599	0.605	0.612	0.617
Sri Lanka	0.49	0.495	0.501	0.508	0.516	0.524	0.532	0.54	0.547	0.553	0.559	0.565	0.571	0.578	0.584	0.59	0.597	0.604	0.611	0.618	0.626	0.634	0.642	0.65	0.658	0.666	0.673	0.68
Seychelles	0.549	0.557	0.565	0.573	0.582	0.589	0.597	0.605	0.613	0.62	0.626	0.631	0.636	0.64	0.643	0.646	0.65	0.653	0.656	0.658	0.66	0.663	0.667	0.671	0.675	0.68	0.686	0.692
Thailand	0.502	0.514	0.525	0.534	0.542	0.552	0.561	0.567	0.569	0.572	0.579	0.587	0.594	0.6	0.605	0.61	0.616	0.623	0.629	0.635	0.641	0.647	0.654	0.66	0.667	0.673	0.679	0.684
Timor-Leste	0.276	0.283	0.29	0.296	0.302	0.307	0.314	0.321	0.325	0.321	0.32	0.325	0.332	0.345	0.362	0.379	0.4	0.419	0.437	0.449	0.46	0.471	0.481	0.49	0.495	0.5	0.504	0.505
Vietnam	0.406	0.413	0.42	0.427	0.435	0.444	0.452	0.461	0.469	0.477	0.483	0.49	0.497	0.504	0.511	0.518	0.525	0.532	0.54	0.547	0.554	0.562	0.57	0.578	0.585	0.593	0.6	0.607
Sub-Saharan Africa	0.304	0.307	0.311	0.314	0.317	0.32	0.324	0.328	0.332	0.335	0.339	0.343	0.348	0.353	0.359	0.365	0.371	0.379	0.386	0.393	0.4	0.407	0.414	0.421	0.428	0.435	0.441	0.446
Central sub-Saharan Africa	0.298	0.303	0.307	0.309	0.311	0.313	0.316	0.318	0.32	0.323	0.325	0.328	0.332	0.336	0.341	0.348	0.355	0.364	0.373	0.382	0.391	0.402	0.413	0.423	0.433	0.443	0.452	0.457
Angola	0.235	0.24	0.245	0.249	0.253	0.258	0.263	0.269	0.276	0.282	0.288	0.293	0.299	0.305	0.312	0.32	0.329	0.34	0.351	0.363	0.375	0.389	0.401	0.414	0.428	0.441	0.453	0.461
Central African Republic	0.22	0.225	0.228	0.232	0.236	0.24	0.242	0.245	0.249	0.254	0.257	0.261	0.265	0.268	0.271	0.275	0.28	0.285	0.29	0.296	0.304	0.313	0.323	0.325	0.328	0.33	0.333	0.334
Congo (Brazzaville)	0.382	0.39	0.398	0.405	0.41	0.416	0.421	0.426	0.43	0.434	0.439	0.444	0.449	0.455	0.46	0.467	0.475	0.482	0.49	0.499	0.509	0.52	0.531	0.542	0.552	0.561	0.569	0.574
DR Congo	0.293	0.296	0.298	0.298	0.296	0.294	0.291	0.288	0.283	0.279	0.274	0.269	0.265	0.263	0.262	0.264	0.264	0.265	0.267	0.27	0.278	0.288	0.3	0.315	0.33	0.344	0.356	0.364
Equatorial Guinea	0.2	0.204	0.212	0.22	0.229	0.241	0.26	0.292	0.316	0.339	0.363	0.388	0.41	0.429	0.449	0.467	0.483	0.499	0.516	0.53	0.544	0.559	0.573	0.587	0.599	0.61	0.62	0.625
Gabon	0.433	0.443	0.453	0.462	0.472	0.481	0.49	0.498	0.506	0.514	0.522	0.529	0.535	0.542	0.549	0.556	0.562	0.569	0.576	0.582	0.589	0.598	0.607	0.616	0.625	0.634	0.644	0.651
Eastern sub-Saharan Africa	0.23	0.233	0.236	0.239	0.241	0.245	0.249	0.254	0.259	0.262	0.266	0.271	0.276	0.282	0.288	0.294	0.301	0.308	0.316	0.324	0.332	0.34	0.348	0.356	0.365	0.373	0.381	0.387
Burundi	0.247	0.252	0.257	0.258	0.263	0.265	0.265	0.266	0.268	0.268	0.268	0.267	0.268	0.269	0.271	0.272	0.274	0.276	0.278	0.282	0.286	0.29	0.295	0.299	0.303	0.306	0.308	0.31
Comoros	0.272	0.279	0.286	0.293	0.298	0.303	0.306	0.31	0.314	0.319	0.325	0.331	0.338	0.344	0.351	0.358	0.365	0.372	0.378	0.384	0.39	0.396	0.403	0.41	0.417	0.423	0.429	0.434
Djibouti	0.313	0.317	0.32	0.322	0.325	0.329	0.333	0.337	0.339	0.341	0.342	0.347	0.359	0.374	0.388	0.4	0.407	0.412	0.419	0.425	0.432	0.439	0.446	0.454	0.462	0.47	0.478	0.485
Eritrea	0.202	0.214	0.223	0.234	0.247	0.26	0.272	0.285	0.296	0.306	0.315	0.323	0.331	0.337	0.343	0.348	0.353	0.357	0.36	0.364	0.368	0.372	0.378	0.383	0.39	0.396	0.403	0.409
Ethiopia	0.138	0.141	0.143	0.146	0.148	0.15	0.155	0.161	0.166	0.169	0.172	0.177	0.183	0.189	0.195	0.202	0.21	0.221	0.233	0.245	0.257	0.268	0.28	0.292	0.303	0.314	0.325	0.334
Kenya	0.341	0.349	0.357	0.364	0.372	0.377	0.382	0.387	0.392	0.398	0.401	0.403	0.406	0.411	0.416	0.42	0.425	0.432	0.438	0.445	0.452	0.459	0.465	0.473	0.481	0.488	0.494	0.499
Baringo	0.254	0.266	0.278	0.289	0.299	0.307	0.313	0.319	0.326	0.333	0.338	0.341	0.345	0.352	0.358	0.362	0.368	0.376	0.384	0.393	0.401	0.408	0.414	0.421	0.428	0.434	0.439	0.444
Bomet	0.306	0.315	0.325	0.333	0.341	0.347	0.351	0.355	0.361	0.367	0.371	0.373	0.378	0.385	0.392	0.398	0.406	0.414	0.423	0.433	0.442	0.449	0.456	0.465	0.475	0.483	0.49	0.496
Bungoma	0.316	0.325	0.333	0.341	0.348	0.353	0.357	0.36	0.365	0.37	0.373	0.373	0.376	0.38	0.384	0.387	0.391	0.397	0.403	0.41	0.417	0.423	0.429	0.436	0.445	0.451	0.458	0.463
Busia	0.297	0.304	0.312	0.32	0.327	0.332	0.336	0.339	0.344	0.349	0.352	0.353	0.356	0.361	0.367	0.37	0.375	0.381	0.386	0.393	0.4	0.404	0.409	0.415	0.423	0.428	0.434	0.438
Elgeyo Marakwet	0.292	0.302	0.312	0.321	0.329	0.336	0.342	0.348	0.355	0.362	0.368	0.372	0.378	0.386	0.394	0.4	0.408	0.417	0.425	0.435	0.443	0.451	0.458	0.467	0.475	0.483	0.49	0.496
Embu	0.375	0.384	0.393	0.4	0.407	0.413	0.417	0.422	0.427	0.431	0.434	0.437	0.44	0.444	0.449	0.452	0.458	0.464	0.47	0.478	0.486	0.493	0.499	0.507	0.514	0.521	0.527	0.533
Garissa	0.153	0.16	0.168	0.177	0.184	0.19	0.195	0.201	0.207	0.213	0.217	0.219	0.223	0.228	0.233	0.237	0.242	0.249	0.255	0.263	0.272	0.28	0.288	0.298	0.309	0.318	0.326	0.334
Homa Bay	0.214	0.222	0.232	0.243	0.253	0.26	0.265	0.271	0.279	0.288	0.292	0.293	0.297	0.305	0.313	0.319	0.328	0.338	0.346	0.356	0.366	0.374	0.382	0.392	0.403	0.411	0.419	0.425
Isiolo	0.264	0.27	0.276	0.282	0.288	0.292	0.295	0.298	0.301	0.305	0.307	0.308	0.31	0.314	0.318	0.321	0.326	0.331	0.337	0.343	0.35	0.355	0.36	0.365	0.372	0.377	0.381	0.385
Kajiado	0.384	0.392	0.4	0.407	0.414	0.42	0.425	0.429	0.434	0.438	0.442	0.445	0.448	0.452	0.456	0.46	0.464	0.47	0.475	0.481	0.486	0.492	0.498	0.506	0.514	0.521	0.528	0.534
Kakamega	0.295	0.303	0.311	0.319	0.326	0.332	0.337	0.342	0.348	0.356	0.36	0.361	0.365	0.37	0.375	0.378	0.383	0.389	0.394	0.4	0.407	0.412	0.417	0.425	0.433	0.439	0.445	0.45
Kericho	0.266	0.277	0.288	0.299	0.309	0.317	0.324	0.331	0.339	0.348	0.353	0.356	0.362	0.37	0.378	0.385	0.394	0.404	0.414	0.425	0.436	0.445	0.454	0.464	0.475	0.485	0.493	0.5
Kiambu	0.435	0.443	0.45	0.457	0.464	0.469	0.473	0.476	0.48	0.484	0.487	0.489	0.492	0.496	0.5	0.504	0.509	0.516	0.521	0.528	0.535	0.541	0.548	0.555	0.562	0.569	0.575	0.58
Kilifi	0.292	0.3	0.307	0.314	0.321	0.327	0.331	0.336	0.34	0.346	0.348	0.349	0.352	0.357	0.361	0.365	0.371	0.378	0.385	0.392	0.4	0.408	0.415	0.424	0.434	0.442	0.45	0.456
Kirinyaga	0.389	0.396	0.402	0.407	0.411	0.415	0.418	0.422	0.425	0.429	0.432	0.434	0.437	0.442	0.447	0.451	0.457	0.464	0.471	0.479	0.486	0.493	0.5	0.507	0.514	0.521	0.527	0.533
Kisii	0.34	0.35	0.36	0.368	0.377	0.383	0.389	0.395	0.401	0.407	0.411	0.414	0.418	0.424	0.429	0.434	0.44	0.448	0.454	0.463	0.471	0.479	0.486	0.495	0.503	0.51	0.517	0.522
Kisumu	0.315	0.325	0.334	0.342	0.349	0.355	0.36	0.364	0.37	0.376	0.381	0.384	0.388	0.395	0.402	0.407	0.415	0.424	0.432	0.442	0.451	0.459	0.466	0.475	0.484	0.491	0.497	0.503
Kitui	0.28	0.288	0.297	0.304	0.311	0.317	0.322	0.327	0.332	0.338	0.343	0.346	0.351	0.357	0.363	0.369	0.376	0.383	0.391	0.399	0.408	0.416	0.423	0.432	0.44	0.448	0.455	0.461
Kwale	0.294	0.301	0.308	0.314	0.321	0.326	0.33	0.334	0.338	0.342	0.344	0.346	0.348	0.352	0.357	0.36	0.366	0.374	0.381	0.39	0.399	0.407	0.414	0.424	0.433	0.442	0.45	0.457
Laikipia	0.346	0.354	0.361	0.368	0.375	0.38	0.385	0.389	0.395	0.401	0.405	0.409	0.415	0.423	0.433	0.442	0.451	0.462	0.472	0.483	0.494	0.502	0.511	0.521	0.531	0.54	0.549	0.556
Lamu	0.295	0.303	0.312	0.321	0.328	0.334	0.338	0.343	0.347	0.352	0.355	0.356	0.359	0.364	0.369	0.373	0.378	0.384	0.39	0.397	0.405	0.411	0.417	0.425	0.434	0.441	0.448	0.453
Machakos	0.352	0.362	0.372	0.38	0.389	0.396	0.403	0.411	0.418	0.426	0.431	0.435	0.44	0.444	0.449	0.452	0.456	0.461	0.465	0.471	0.476	0.482	0.487	0.493	0.5	0.507	0.512	0.518
Makueni	0.3	0.307	0.314	0.322	0.328	0.333	0.336	0.339	0.343	0.348	0.35	0.351	0.354	0.36	0.367	0.373	0.381	0.391	0.401	0.413	0.423	0.432	0.439	0.447	0.454	0.46	0.465	0.469
Mandera	0.102	0.109	0.118	0.126	0.135	0.142	0.149	0.156	0.164	0.172	0.178	0.181	0.187	0.194	0.201	0.206	0.212	0.218	0.224	0.231	0.238	0.244	0.251	0.261	0.271	0.28	0.288	0.295
Marsabit	0.22	0.223	0.227	0.231	0.236	0.239	0.241	0.244	0.247	0.252	0.255	0.256	0.259	0.264	0.268	0.271	0.276	0.281	0.286	0.293	0.299	0.304	0.309	0.315	0.323	0.329	0.335	0.34
Meru	0.338	0.346	0.354	0.362	0.369	0.376	0.381	0.387	0.393	0.399	0.403	0.407	0.411	0.417	0.423	0.428	0.434	0.441	0.447	0.454	0.462	0.469	0.475	0.482	0.49	0.496	0.503	0.508
Migori	0.229	0.237	0.246	0.255	0.262	0.267	0.27	0.274	0.279	0.285	0.287	0.286	0.289	0.296	0.303	0.307	0.315	0.325	0.333	0.344	0.355	0.364	0.372	0.383	0.395	0.404	0.412	0.419
Mombasa	0.396	0.405	0.414	0.422	0.43	0.437	0.443	0.449	0.455	0.461	0.465	0.468	0.471	0.475	0.478	0.481	0.486	0.491	0.496	0.502	0.51	0.517	0.525	0.534	0.543	0.552	0.561	0.568
Murang'a	0.381	0.389	0.396	0.402	0.408	0.413	0.418	0.424	0.429	0.436	0.441	0.444	0.449	0.454	0.46	0.465	0.47	0.476	0.482	0.488	0.494	0.499	0.504	0.51	0.515	0.52	0.524	0.528
Nairobi	0.499	0.506	0.513	0.519	0.525	0.53	0.534	0.539	0.544	0.549	0.553	0.556	0.56	0.566	0.572	0.577	0.585	0.593	0.602	0.611	0.619	0.627	0.634	0.643	0.652	0.66	0.667	0.674
Nakuru	0.337	0.347	0.357	0.367	0.375	0.383	0.389	0.394	0.399	0.405	0.409	0.413	0.417	0.423	0.43	0.436	0.445	0.454	0.464	0.474	0.484	0.493	0.502	0.511	0.521	0.53	0.538	0.545
Nandi	0.323	0.333	0.344	0.354	0.362	0.369	0.375	0.381	0.388	0.394	0.399	0.402	0.406	0.412	0.418	0.422	0.428	0.435	0.441	0.449	0.456	0.462	0.468	0.475	0.483	0.49	0.496	0.501
Narok	0.217	0.225	0.234	0.242	0.25	0.255	0.259	0.264	0.272	0.28	0.284	0.285	0.289	0.297	0.305	0.31	0.318	0.326	0.333	0.342	0.35	0.356	0.362	0.371	0.381	0.389	0.396	0.402
Nyamira	0.361	0.37	0.379	0.387	0.395	0.401	0.406	0.41	0.414	0.419	0.421	0.423	0.426	0.431	0.437	0.443	0.451	0.46	0.468	0.478	0.488	0.496	0.504	0.513	0.523	0.531	0.538	0.544
Nyandarua	0.353	0.361	0.369	0.376	0.382	0.388	0.392	0.397	0.402	0.409	0.413	0.416	0.421	0.428	0.436	0.442	0.449	0.458	0.466	0.475	0.484	0.491	0.498	0.506	0.514	0.521	0.528	0.534
Nyeri	0.407	0.414	0.421	0.427	0.434	0.439	0.444	0.449	0.454	0.459	0.463	0.466	0.47	0.475	0.479	0.484	0.489	0.496	0.502	0.509	0.516	0.522	0.527	0.533	0.539	0.544	0.549	0.554
Samburu	0.205	0.21	0.215	0.221	0.225	0.228	0.23	0.232	0.235	0.239	0.24	0.239	0.24	0.243	0.247	0.249	0.253	0.258	0.262	0.267	0.273	0.277	0.28	0.287	0.294	0.299	0.304	0.308
Siaya	0.227	0.236	0.246	0.255	0.265	0.272	0.279	0.287	0.294	0.303	0.309	0.313	0.318	0.326	0.334	0.34	0.349	0.359	0.368	0.379	0.39	0.4	0.409	0.421	0.432	0.443	0.452	0.46
Taita Taveta	0.352	0.362	0.371	0.38	0.389	0.396	0.403	0.409	0.416	0.422	0.427	0.43	0.434	0.438	0.443	0.447	0.452	0.458	0.464	0.472	0.48	0.487	0.494	0.502	0.509	0.516	0.523	0.529
Tana River	0.231	0.237	0.244	0.25	0.255	0.26	0.263	0.267	0.27	0.274	0.276	0.276	0.278	0.281	0.285	0.288	0.292	0.298	0.304	0.311	0.319	0.326	0.333	0.342	0.353	0.362	0.371	0.379
Tharaka Nithi	0.342	0.35	0.357	0.364	0.371	0.377	0.382	0.387	0.392	0.397	0.402	0.405	0.41	0.417	0.423	0.429	0.437	0.445	0.453	0.463	0.472	0.48	0.488	0.497	0.506	0.514	0.522	0.528
Trans Nzoia	0.318	0.328	0.337	0.346	0.353	0.359	0.363	0.368	0.373	0.38	0.384	0.388	0.392	0.399	0.406	0.411	0.418	0.426	0.433	0.441	0.448	0.454	0.46	0.468	0.477	0.484	0.491	0.496
Turkana	0.211	0.216	0.221	0.226	0.231	0.234	0.237	0.239	0.242	0.245	0.247	0.247	0.248	0.25	0.252	0.252	0.253	0.255	0.257	0.26	0.264	0.266	0.269	0.274	0.281	0.286	0.291	0.295
Uasin Gishu	0.363	0.373	0.382	0.391	0.399	0.405	0.41	0.416	0.421	0.428	0.433	0.436	0.441	0.447	0.453	0.458	0.465	0.472	0.48	0.488	0.496	0.503	0.51	0.517	0.526	0.533	0.539	0.545
Vihiga	0.328	0.337	0.346	0.355	0.362	0.367	0.371	0.374	0.376	0.378	0.378	0.378	0.379	0.382	0.387	0.392	0.399	0.408	0.417	0.427	0.437	0.444	0.45	0.457	0.464	0.469	0.474	0.477
Wajir	0.104	0.111	0.12	0.128	0.136	0.142	0.146	0.151	0.156	0.162	0.165	0.166	0.168	0.172	0.176	0.178	0.181	0.185	0.189	0.195	0.201	0.206	0.21	0.218	0.226	0.233	0.238	0.243
West Pokot	0.213	0.219	0.226	0.233	0.24	0.245	0.249	0.253	0.258	0.265	0.268	0.27	0.274	0.28	0.286	0.291	0.298	0.305	0.312	0.319	0.327	0.333	0.34	0.349	0.359	0.368	0.376	0.382
Madagascar	0.262	0.264	0.265	0.265	0.265	0.265	0.266	0.268	0.27	0.272	0.274	0.278	0.282	0.286	0.291	0.294	0.295	0.295	0.298	0.3	0.302	0.303	0.306	0.31	0.315	0.321	0.326	0.331
Malawi	0.199	0.202	0.204	0.206	0.208	0.212	0.216	0.221	0.225	0.23	0.234	0.238	0.244	0.249	0.256	0.262	0.27	0.279	0.288	0.296	0.305	0.313	0.32	0.327	0.333	0.339	0.345	0.349
Mozambique	0.133	0.135	0.136	0.138	0.14	0.142	0.147	0.154	0.162	0.169	0.175	0.182	0.189	0.197	0.206	0.215	0.224	0.235	0.246	0.257	0.268	0.279	0.29	0.301	0.311	0.322	0.332	0.34
Rwanda	0.267	0.269	0.272	0.274	0.268	0.267	0.269	0.272	0.276	0.28	0.284	0.29	0.297	0.304	0.311	0.319	0.327	0.334	0.342	0.349	0.356	0.363	0.371	0.378	0.386	0.393	0.401	0.407
Somalia	0.153	0.156	0.157	0.159	0.16	0.161	0.162	0.163	0.163	0.164	0.165	0.167	0.169	0.172	0.176	0.179	0.183	0.188	0.193	0.198	0.203	0.208	0.212	0.217	0.222	0.227	0.231	0.235
South Sudan	0.179	0.18	0.182	0.183	0.184	0.186	0.187	0.189	0.191	0.194	0.197	0.2	0.204	0.207	0.211	0.215	0.22	0.225	0.229	0.234	0.239	0.244	0.249	0.254	0.259	0.265	0.27	0.275
Tanzania	0.275	0.28	0.285	0.289	0.291	0.294	0.297	0.3	0.304	0.307	0.311	0.315	0.32	0.325	0.33	0.334	0.339	0.344	0.35	0.355	0.36	0.366	0.373	0.381	0.388	0.397	0.405	0.412
Uganda	0.163	0.164	0.166	0.169	0.173	0.178	0.185	0.193	0.202	0.212	0.222	0.232	0.243	0.254	0.265	0.276	0.287	0.298	0.31	0.32	0.33	0.339	0.348	0.357	0.366	0.374	0.382	0.388
Zambia	0.312	0.314	0.316	0.319	0.321	0.324	0.326	0.329	0.332	0.335	0.338	0.343	0.348	0.353	0.358	0.365	0.373	0.382	0.392	0.402	0.412	0.421	0.431	0.44	0.449	0.458	0.466	0.472
Southern sub-Saharan Africa	0.521	0.525	0.53	0.535	0.539	0.543	0.546	0.55	0.555	0.56	0.565	0.569	0.574	0.578	0.582	0.585	0.59	0.596	0.603	0.607	0.612	0.616	0.619	0.622	0.627	0.633	0.637	0.64
Botswana	0.463	0.473	0.483	0.492	0.501	0.509	0.517	0.524	0.532	0.539	0.546	0.553	0.56	0.567	0.574	0.581	0.588	0.597	0.605	0.612	0.619	0.626	0.633	0.639	0.646	0.652	0.658	0.663
Lesotho	0.333	0.34	0.347	0.354	0.362	0.369	0.376	0.383	0.389	0.396	0.402	0.407	0.413	0.417	0.422	0.426	0.431	0.436	0.441	0.446	0.452	0.458	0.464	0.47	0.476	0.482	0.488	0.493
Namibia	0.454	0.459	0.465	0.471	0.477	0.483	0.489	0.495	0.501	0.507	0.512	0.516	0.521	0.525	0.53	0.534	0.54	0.546	0.552	0.558	0.565	0.572	0.579	0.587	0.595	0.603	0.61	0.616
South Africa	0.557	0.56	0.565	0.57	0.574	0.576	0.579	0.583	0.587	0.593	0.598	0.602	0.607	0.611	0.615	0.619	0.624	0.631	0.639	0.644	0.649	0.653	0.655	0.658	0.664	0.669	0.674	0.677
Swaziland	0.426	0.435	0.444	0.452	0.459	0.466	0.473	0.478	0.483	0.489	0.494	0.499	0.503	0.508	0.513	0.518	0.523	0.528	0.533	0.538	0.543	0.547	0.552	0.557	0.563	0.569	0.574	0.578
Zimbabwe	0.408	0.417	0.424	0.43	0.436	0.44	0.446	0.45	0.453	0.456	0.457	0.459	0.458	0.456	0.453	0.449	0.443	0.438	0.43	0.426	0.424	0.425	0.43	0.436	0.444	0.451	0.458	0.463
Western sub-Saharan Africa	0.293	0.296	0.299	0.303	0.306	0.309	0.313	0.316	0.32	0.324	0.328	0.333	0.337	0.343	0.35	0.357	0.365	0.373	0.381	0.389	0.396	0.403	0.411	0.417	0.424	0.43	0.437	0.441
Benin	0.218	0.223	0.228	0.233	0.238	0.243	0.248	0.253	0.258	0.263	0.268	0.273	0.278	0.284	0.29	0.296	0.301	0.307	0.313	0.32	0.326	0.332	0.338	0.345	0.353	0.36	0.367	0.373
Burkina Faso	0.139	0.143	0.147	0.151	0.154	0.158	0.163	0.168	0.173	0.179	0.184	0.19	0.195	0.201	0.208	0.214	0.22	0.226	0.232	0.237	0.242	0.248	0.253	0.259	0.266	0.272	0.279	0.284
Cameroon	0.332	0.338	0.343	0.347	0.35	0.352	0.355	0.357	0.359	0.36	0.361	0.361	0.361	0.362	0.366	0.373	0.38	0.388	0.396	0.405	0.414	0.423	0.433	0.443	0.453	0.464	0.474	0.482
Cape Verde	0.307	0.315	0.322	0.329	0.337	0.347	0.357	0.367	0.377	0.387	0.399	0.41	0.42	0.431	0.441	0.451	0.461	0.472	0.482	0.491	0.5	0.509	0.517	0.524	0.531	0.538	0.544	0.549
Chad	0.119	0.123	0.128	0.131	0.135	0.138	0.141	0.144	0.146	0.148	0.15	0.152	0.154	0.156	0.161	0.166	0.171	0.178	0.185	0.192	0.2	0.207	0.216	0.224	0.232	0.24	0.248	0.253
Cote d'Ivoire	0.273	0.28	0.287	0.292	0.297	0.301	0.306	0.312	0.317	0.322	0.326	0.33	0.334	0.338	0.342	0.346	0.349	0.353	0.357	0.361	0.367	0.371	0.377	0.384	0.391	0.398	0.406	0.412
The Gambia	0.245	0.251	0.257	0.263	0.269	0.275	0.282	0.288	0.294	0.3	0.307	0.314	0.319	0.325	0.332	0.338	0.343	0.349	0.355	0.361	0.367	0.372	0.378	0.384	0.389	0.395	0.401	0.405
Ghana	0.38	0.386	0.392	0.396	0.4	0.403	0.408	0.413	0.416	0.418	0.422	0.425	0.43	0.435	0.439	0.443	0.45	0.459	0.467	0.474	0.482	0.49	0.498	0.505	0.513	0.521	0.53	0.537
Guinea	0.164	0.168	0.171	0.175	0.178	0.183	0.188	0.194	0.2	0.206	0.211	0.217	0.222	0.228	0.234	0.24	0.246	0.252	0.26	0.266	0.273	0.281	0.288	0.295	0.303	0.31	0.318	0.325
Guinea-Bissau	0.183	0.189	0.194	0.199	0.205	0.211	0.217	0.224	0.229	0.235	0.241	0.246	0.252	0.257	0.263	0.268	0.274	0.28	0.286	0.293	0.3	0.308	0.315	0.322	0.329	0.336	0.343	0.349
Liberia	0.207	0.209	0.21	0.206	0.199	0.19	0.178	0.174	0.175	0.183	0.198	0.214	0.228	0.234	0.239	0.245	0.251	0.257	0.264	0.27	0.276	0.284	0.292	0.301	0.31	0.318	0.324	0.328
Mali	0.13	0.134	0.137	0.141	0.145	0.148	0.152	0.157	0.161	0.166	0.171	0.176	0.181	0.187	0.192	0.198	0.203	0.209	0.215	0.22	0.226	0.232	0.238	0.243	0.249	0.255	0.262	0.267
Mauritania	0.299	0.307	0.314	0.321	0.328	0.335	0.341	0.346	0.351	0.356	0.359	0.363	0.367	0.371	0.377	0.382	0.39	0.398	0.406	0.412	0.42	0.427	0.435	0.442	0.45	0.458	0.465	0.471
Niger	0.092	0.097	0.101	0.105	0.108	0.111	0.113	0.115	0.118	0.121	0.123	0.125	0.127	0.13	0.134	0.137	0.14	0.144	0.147	0.151	0.156	0.16	0.165	0.17	0.176	0.181	0.186	0.191
Nigeria	0.344	0.346	0.347	0.349	0.352	0.355	0.358	0.362	0.365	0.369	0.374	0.379	0.385	0.391	0.4	0.41	0.419	0.429	0.437	0.445	0.452	0.459	0.466	0.473	0.478	0.484	0.49	0.493
Sao Tome and Principe	0.287	0.292	0.296	0.3	0.305	0.309	0.314	0.32	0.326	0.332	0.338	0.345	0.353	0.362	0.371	0.381	0.39	0.399	0.408	0.416	0.426	0.435	0.444	0.453	0.463	0.472	0.481	0.488
Senegal	0.244	0.25	0.257	0.262	0.267	0.272	0.277	0.281	0.286	0.291	0.295	0.299	0.303	0.307	0.311	0.316	0.319	0.324	0.328	0.333	0.338	0.342	0.347	0.352	0.357	0.362	0.368	0.373
Sierra Leone	0.202	0.205	0.207	0.211	0.216	0.219	0.222	0.225	0.226	0.228	0.23	0.233	0.238	0.244	0.249	0.256	0.262	0.27	0.278	0.286	0.294	0.302	0.312	0.325	0.336	0.344	0.351	0.357
Togo	0.263	0.27	0.276	0.281	0.286	0.292	0.297	0.303	0.307	0.311	0.315	0.318	0.321	0.325	0.329	0.333	0.337	0.343	0.348	0.353	0.36	0.366	0.373	0.381	0.39	0.398	0.406	0.413

Appendix Table 12. GBD world population age standard

Age group	Percent of Population	Rounded
ENN	0.040802107	0.04
LNN	0.121276112	0.12
PNN	1.916163425	1.92
0-1	2.078241644	2.08
1-4	8.102445249	8.1
5-9	9.677325318	9.68
10-14	8.952609678	8.95
15-19	8.382858071	8.38
20-24	8.01707612	8.02
25-29	7.778732811	7.78
30-34	7.331586983	7.33
35-39	6.775951151	6.78
40-44	6.089034035	6.09
45-49	5.465811837	5.47
50-54	4.873621621	4.87
55-59	4.251640217	4.25
60-64	3.5961672	3.6
65-69	2.914902912	2.91
70-74	2.130279933	2.13
75-79	1.608333391	1.61
80-84	1.078423497	1.08
85-89	0.603162091	0.6
90-94	0.234261019	0.23
95+	0.057535221	0.06

Appendix Table 13. New data sources added for GBD 2017 mortality estimation		
**Location**	**Year**	**Source**
Armenia	2016-2016	Armenia Vital Registration - Deaths 2016 ICD10
Azerbaijan	2014-2016	Azerbaijan Demographic Indicators 2017
Georgia	2015-2015	Georgia Vital Registration - Deaths 2015 ICD10
Georgia	2016-2016	Georgia Deaths by Age, Year and Sex 2002-2016
Kyrgyzstan	2016-2016	United Nations Demographic Yearbook
Tajikistan	2014-2014	United Nations Demographic Yearbook
Tajikistan	2016-2016	Tajikistan Vital Registration - Deaths 2016 ICD10
Tajikistan	2017-2017	Tajikistan Demographic and Health Survey 2017
Turkmenistan	2015-2015	Turkmenistan Vital Registration - Deaths 2015 ICD10
Turkmenistan	2015-2016	Turkmenistan Multiple Indicator Cluster Survey 2015-2016
Bosnia and Herzegovina	2013-2013	Bosnia and Herzegovina Demography and Social Statistics 2016
Bosnia and Herzegovina	2015-2016	Bosnia and Herzegovina Demography and Social Statistics 2016
Bulgaria	2014-2014	Bulgaria Vital Registration - Deaths 2014 ICD10
Bulgaria	2015-2016	Bulgaria Deaths by Age (5-year Age Group), Residence, Sex and Districts
Croatia	2015-2015	Croatia Vital Registration - Deaths 2015 ICD10
Croatia	2016-2016	Croatia Vital Registration - Deaths 2016 ICD10
Czech Republic	2016-2016	Czech Republic Vital Registration - Deaths 2016 ICD10
Hungary	2016-2016	Hungary Vital Registration - Deaths 2016 ICD10
Macedonia	2014-2014	United Nations Demographic Yearbook
Macedonia	2015-2016	Macedonia Deaths by Cause of Death, by Sex and Age, Short List - 65
Montenegro	1971-1971	Montenegro Deaths by Age and Sex 1961-2016
Montenegro	1981-1981	Montenegro Deaths by Age and Sex 1961-2016
Montenegro	1991-1991	Montenegro Deaths by Age and Sex 1961-2016
Montenegro	2014-2016	Montenegro Deaths by Age and Sex 1961-2016
Poland	2015-2015	Poland Vital Registration - Deaths 2015 ICD10
Poland	2016-2016	Poland Deaths by Sex and Age
Romania	2016-2016	Romania Vital Registration - Deaths 2016 ICD10
Serbia	2015-2016	Serbia Deaths by Causes of Death, Sex, and Age
Slovakia	2015-2016	Slovakia Deaths by Age and Sex - SR, Areas, Regions, Districts, Urban, Rural
Slovenia	1980-1981	Slovenia Deaths by Age and Sex, Annually
Slovenia	2016-2016	Slovenia Deaths by Age and Sex, Cohesion Regions, Annually
Belarus	2015-2016	Belarus Deaths by Region and Age
Estonia	2015-2015	Estonia Vital Registration - Deaths 2015 ICD10
Latvia	2015-2015	Latvia Vital Registration - Deaths 2015 ICD10
Latvia	2016-2016	United Nations Demographic Yearbook
Lithuania	2016-2016	Lithuania Vital Registration - Deaths 2016 ICD10
Moldova	1980-1980	Moldova Deceased by Age Groups, Years, and Genders 1980-2016
Moldova	1983-1984	Moldova Deceased by Age Groups, Years, and Genders 1980-2016
Moldova	2016-2016	Moldova Deceased by Age Groups, Years, and Genders 1980-2016
Ukraine	2015-2015	Ukraine Vital Registration - Deaths 2015
Ukraine	2016-2016	Ukraine Vital Registration - Deaths 2016
Australia	2015-2015	Australia Vital Registration - Deaths 2015 ICD10
New Zealand	2015-2016	New Zealand Deaths by age and sex (Total population)
Brunei	2015-2015	Brunei Vital Registration - Deaths 2015 ICD10
South Korea	2014-2014	South Korea Vital Registration - Deaths 2014 ICD10
South Korea	2015-2015	South Korea Vital Registration - Deaths 2015 ICD10
South Korea	2016-2016	South Korea KOSIS Database - Deaths by Age and Sex: Province
Greenland	2016-2016	United Nations Demographic Yearbook
USA	2016-2016	United States NVSS Mortality Data 2016
Argentina	2015-2015	Argentina Vital Registration - Deaths 2015 ICD10
Chile	2015-2015	Chile Vital Registration - Deaths 2015 ICD10
Uruguay	2011-2011	Uruguay Census 2011 - IPUMS
Uruguay	2015-2015	Uruguay Vital Registration - Deaths 2015 ICD10
Andorra	2015-2015	United Nations Demographic Yearbook
Austria	2015-2015	Austria Vital Registration - Deaths 2015 ICD10
Austria	2016-2016	Austria Vital Registration - Deaths 2016 ICD10
Belgium	2015-2015	Belgium Vital Registration - Deaths 2015 ICD10
Belgium	2016-2016	Belgium Mortality Tables: In Exact Age 1994-2016
Cyprus	2015-2015	Cyprus Vital Registration - Deaths 2015 ICD10
Cyprus	2016-2016	Cyprus Vital Registration - Deaths 2016 ICD10
Denmark	2015-2015	Denmark Vital Registration - Deaths 2015 ICD10
Denmark	2016-2016	Denmark Deaths by Sex and Age
Finland	2016-2016	Finland Deaths by Age (1 Year) and Sex
France	2015-2015	France Births, Deaths and Marriages in 2015 - Deaths
France	2016-2016	France Births, Deaths and Marriages in 2016 - Deaths
Germany	2015-2015	Germany Vital Registration - Deaths 2015 ICD10
Greece	2015-2016	Greece Deaths by Sex and Age of the Deceased 2000-2016
Iceland	2016-2016	Iceland Vital Registration - Deaths 2016 ICD10
Ireland	2015-2016	Ireland Deaths Occuring by Area of Residence, Sex, Age at Death and Year
Israel	2015-2015	Israel Vital Registration - Deaths 2015 ICD10
Israel	2016-2016	United Nations Demographic Yearbook
Italy	2014-2014	Italy Vital Registration - Deaths 2014 ICD10
Italy	2015-2015	Italy Deaths and Specific Mortality Rates by Age and Year, all Geographical Areas 2015
Italy	2016-2016	Italy Deaths and Specific Mortality Rates by Age and Year, all Geographical Areas 2016
Luxembourg	2015-2015	Luxembourg Vital Registration - Deaths 2015 ICD10
Malta	2015-2015	Malta Vital Registration - Deaths 2015 ICD10
Malta	2016-2016	United Nations Demographic Yearbook
Netherlands	2016-2016	Netherlands Vital Registration - Deaths 2016 ICD10
Portugal	2004-2004	Portugal Vital Registration - Deaths 2004
Portugal	2015-2016	Portugal Deaths by Place of Residence, Sex and Age Annual
Spain	2015-2015	Spain Vital Registration - Deaths 2015 ICD10
Spain	2016-2016	Spain Deaths by Age, Month, and Sex 2016
Sweden	2015-2015	Sweden Cause of Death Register 2015
Sweden	2016-2016	Sweden Cause of Death Register 2016

Appendix Table 13. New data sources added for GBD 2017 mortality estimation		
**Location**	**Year**	**Source**
Switzerland	2015-2015	Switzerland Deaths by Municipality, Sex, and Age Group, 2015-2016
Switzerland	2016-2016	Switzerland Permanent and Non-permanent Resident Population by Sex and Age
United Kingdom	2016-2016	United Kingdom - Scotland Vital Events Reference Tables 2016
Bolivia	1991-1991	Bolivia Population Estimates 1991
Bolivia	2016-2016	Bolivia Demographic and Health Survey 2016
Ecuador	2015-2015	Ecuador Vital Registration - Deaths 2015 ICD10
Ecuador	2016-2016	Ecuador General Deaths 2016
Peru	2015-2015	Peru Vital Registration - Deaths 2015 ICD10
Peru	2015-2015	Peru Demographic and Family Health Survey 2015
Peru	2016-2016	Peru Demographic and Family Health Survey 2016
Antigua and Barbuda	2015-2015	Antigua and Barbuda Vital Registration - Deaths 2015 ICD10
Belize	1981-1981	United Nations Demographic Yearbook - Historical Supplement 1997
Belize	2015-2015	Belize Vital Registration - Deaths 2015 ICD10
Belize	2015-2016	Belize Multiple Indicator Cluster Survey 2015-2016
Belize	2016-2016	United Nations Demographic Yearbook
Cuba	2015-2015	Cuba Vital Registration - Deaths 2015 ICD10
Dominica	2015-2015	Dominica Vital Registration - Deaths 2015 ICD10
Dominican Republic	1999-1999	Dominican Republic Demographic and Health Survey 1999
Dominican Republic	2007-2007	Dominican Republic Special Demographic and Health Survey 2007
Dominican Republic	2014-2014	Dominican Republic Births Occurred per Year, by Sex and Province of Birth, 2001-2016
Dominican Republic	2015-2015	Dominican Republic Births Occurring by Age Groups of the Mother at the Time of the Birth of the Child, According to the Year of Occurrence of the Birth, 2001-2016
Dominican Republic	2016-2016	Dominican Republic Deaths Occurred per Year, by Sex and Age Groups of the Deceased, 2001-2016
Grenada	2016-2016	Grenada Vital Registration - Deaths 2016 ICD10
Guyana	2013-2013	Guyana Vital Registration - Deaths 2013 ICD10
Haiti	2016-2017	Haiti Demographic and Health Survey 2016-2017
Jamaica	2000-2000	Jamaica Multiple Indicator Cluster Survey 2000
Jamaica	2011-2011	Jamaica Population and Housing Census 2011
Puerto Rico	2016-2016	United Nations Demographic Yearbook
Suriname	2015-2015	United Nations Demographic Yearbook
Trinidad and Tobago	2011-2011	Trinidad and Tobago Vital Registration - Deaths 2011 ICD10
Colombia	2008-2008	Colombia National Quality of Life Survey 2008
Colombia	2014-2014	Colombia Vital Registration - Deaths 2014 ICD10
Colombia	2015-2015	Colombia Vital Registration - Deaths 2015 ICD10
Colombia	2015-2016	Colombia Demographic and Health Survey 2015-2016
Costa Rica	2015-2015	Costa Rica Total Deaths by Age Groups, by Province of Residence and Sex 2015
Costa Rica	2016-2016	Costa Rica Infant, Neonatal and Fetal Death Statistics. Preliminary Data 2016
El Salvador	2014-2014	El Salvador Vital Registration - Deaths 2014 ICD10
Guatemala	2015-2015	Guatemala Vital Registration - Deaths 2015 ICD10
Mexico	2010-2010	Mexico Population and Housing Census 2010 - IPUMS
Mexico	2015-2015	Mexico Intercensal Survey 2015 - IPUMS
Mexico	2015-2015	Mexico Multiple Indicator Cluster Survey 2015
Mexico	2016-2016	Mexico Vital Registration - Deaths 2016
Nicaragua	2014-2014	Nicaragua Vital Registration - Deaths 2014 ICD10
Nicaragua	2015-2015	Nicaragua Vital Registration - Deaths 2015 ICD10
Panama	2015-2015	Panama Vital Registration - Deaths 2015 ICD10
Panama	2016-2016	Panama Vital Registration - Deaths 2016
Venezuela	2014-2014	United Nations Demographic Yearbook
Brazil	2016-2016	Brazil Mortality Information System - Deaths 2016
Paraguay	1972-1972	Paraguay Population and Housing Census 1972 - IPUMS
Paraguay	1982-1982	Paraguay Population and Housing Census 1982 - IPUMS
Paraguay	1992-1992	Paraguay Population and Housing Census 1992 - IPUMS
Paraguay	2002-2002	Paraguay Population and Housing Census 2002 - IPUMS
Paraguay	2016-2016	Paraguay Multiple Indicator Cluster Survey 2016
Afghanistan	2015-2016	Afghanistan Demographic and Health Survey 2015-2016
Algeria	2015-2016	United Nations Demographic Yearbook
Bahrain	2014-2014	Bahrain Vital Registration - Deaths 2014 ICD10
Egypt	2015-2015	Egypt Vital Registration - Deaths 2015 ICD10
Iran	2006-2006	Iran General Census of Population and Housing 2006
Iran	2011-2011	Iran National Population and Housing Census 2011
Iran	2016-2016	Iran Population and Housing Census 2016
Iraq	1991-1991	Iraq Infant and Child Mortality and Nutrition Survey 1991
Kuwait	2015-2015	Kuwait Annual Bulletin for Vital Statistics - Births and Deaths 2015
Lebanon	2004-2004	Lebanon Registered and Reported Deaths 2004
Libya	2009-2009	Libya Vital Statistics 2009
Libya	2010-2010	Libya Vital Statistics 2010
Libya	2011-2011	Libya Vital Statistics 2011
Palestine	2010-2010	Palestine - West Bank and Gaza Strip Vital Registration - Deaths 2010 ICD10
Oman	2014-2014	Oman Vital Registration - Deaths 2014 ICD10
Oman	2016-2016	United Nations Demographic Yearbook
Qatar	1991-1991	Qatar Vital Statistics Annual Bulletin 1991
Qatar	1998-1998	Qatar Vital Statistics Annual Bulletin 1998
Qatar	2015-2015	Qatar Vital Registration - Deaths 2015 ICD10
Qatar	2016-2016	Qatar Vital Registration - Deaths 2016 ICD10
Saudi Arabia	2016-2016	United Nations Demographic Yearbook
Syria	2006-2006	Syria Multiple Indicator Cluster Survey 2006
Syria	2009-2009	Syria Family Health Survey 2009
Turkey	2014-2016	Turkey Vital Registration - Deaths by Age and Sex 2009-2016
India	2011-2013	India Human Development Survey 2011-2013
India	2016-2016	India - Delhi Medical Certification of Cause of Death Report 2016
India	2016-2016	India SRS Statistical Report 2016
Nepal	2016-2017	Nepal Demographic and Health Survey 2016-2017
Pakistan	2016-2017	Pakistan - Gilgit-Baltistan Multiple Indicator Cluster Survey 2016-2017

Appendix Table 13. New data sources added for GBD 2017 mortality estimation		
**Location**	**Year**	**Source**
Taiwan (Province of China)	2015-2015	Taiwan Vital Registration - Deaths 2015
Taiwan (Province of China)	2016-2016	Taiwan Vital Registration - Deaths 2016
American Samoa	1955-1973	United Nations Demographic Yearbook - Historical Supplement 1997
Guam	1983-1983	United States Vital Statistics Report 1983
Guam	1984-1984	United States Vital Statistics Report 1984
Guam	1987-1987	United States Vital Statistics Report 1987
Papua New Guinea	2006-2007	Papua New Guinea Demographic and Health Survey 2006-2007
Samoa	2014-2014	Samoa Demographic and Health Survey 2014
Solomon Islands	2015-2015	Solomon Islands Demographic and Health Survey 2015
Cambodia	2004-2004	Cambodia Intercensal Population Survey 2004
Indonesia	1971-1971	Indonesia Population and Housing Census 1971
Indonesia	2010-2010	United Nations Demographic Yearbook
Malaysia	2010-2010	Malaysia Vital Registration - Deaths 2010 ICD10
Malaysia	2011-2011	Malaysia Vital Registration - Deaths 2011 ICD10
Malaysia	2013-2013	Malaysia Vital Registration - Deaths 2013 ICD10
Malaysia	2014-2015	United Nations Demographic Yearbook
Maldives	2014-2014	United Nations Demographic Yearbook
Mauritius	2015-2015	Mauritius Digest of Demographic Statistics 2015
Mauritius	2016-2016	Mauritius Digest of Demographic Statistics 2016
Myanmar	2015-2016	Myanmar Demographic and Health Survey 2015-2016
Sri Lanka	2013-2013	Sri Lanka Vital Statistics - Deaths 2013
Sri Lanka	2016-2016	Sri Lanka Demographic and Health Survey 2016
Seychelles	2015-2016	Seychelles Statistical Bulletin: Population and Vital Statistics December 2016
Thailand	2015-2015	Thailand Vital Registration - Deaths 2015 ICD10
Thailand	2016-2016	Thailand Vital Registration - Deaths 2016 ICD10
Timor-Leste	2016-2016	Timor-Leste Demographic and Health Survey 2016
Vietnam	1994-1994	Vietnam Intercensal Demographic Survey 1994
Burundi	2016-2017	Burundi Demographic and Health Survey 2016-2017
Ethiopia	1994-1997	Ethiopia Population and Housing Census 1994 - IPUMS
Kenya	2007-2007	Kenya Malaria Indicator Survey 2007
Kenya	2013-2014	Kenya - Bungoma County Multiple Indicator Survey 2013-2014
Kenya	2013-2014	Kenya - Kakamega County Multiple Indicator Survey 2013-2014
Kenya	2013-2014	Kenya - Turkana County Multiple Indicator Survey 2013-2014
Malawi	2015-2016	Malawi Demographic and Health Survey 2015-2016
Malawi	2017-2017	Malawi Malaria Indicator Survey 2017
Uganda	1992-1993	Uganda Integrated Household Survey 1992-1993
Uganda	2009-2010	Uganda Living Standards Measurement Survey - Integrated Survey on Agriculture 2009-2010
Uganda	2016-2016	Uganda Demographic and Health Survey 2016
Botswana	2007-2007	Botswana Vital Registration Death Data 2007
Botswana	2011-2011	Botswana Vital Statistics Report 2011
Botswana	2013-2013	Botswana Vital Statistics Report 2013
Botswana	2014-2014	Botswana Vital Statistics Report 2014
Botswana	2015-2015	Botswana Vital Statistics Report 2015
South Africa	1996-1996	South Africa Census 1996
South Africa	2011-2011	United Nations Demographic Yearbook
South Africa	2015-2015	South Africa Vital Registration - Causes of Death 2015
South Africa	2016-2016	South Africa Demographic and Health Survey 2016
South Africa	2016-2016	South Africa Community Survey 2016
Burkina Faso	2006-2006	Burkina Faso Population and Housing Census 2006 - IPUMS
Cameroon	2005-2005	Cameroon Population and Housing Census 2005 - IPUMS
Cote d'Ivoire	2016-2016	Cote d'Ivoire Multiple Indicator Cluster Survey 2016
Ghana	1991-1992	Ghana Living Standards Measurement Survey 1991-1992
Ghana	2016-2016	Ghana Malaria Indicator Survey 2016
Guinea	2014-2014	United Nations Demographic Yearbook
Guinea	2016-2016	Guinea Multiple Indicator Cluster Survey 2016
Liberia	2016-2016	Liberia Malaria Indicator Survey 2016
Mali	2015-2015	Mali Multiple Indicator Cluster Survey 2015
Mauritania	2015-2015	Mauritania Multiple Indicator Cluster Survey 2015
Nigeria	1986-1987	Nigeria - Ondo Special Demographic and Health Survey 1986-1987
Nigeria	2008-2010	Nigeria Living Standards Survey 2008-2010
Nigeria	2016-2017	Nigeria Multiple Indicator Cluster Survey with National Immunization Coverage Survey Supplement 2016-2017
Senegal	2016-2016	Senegal Continuous Demographic and Health Survey 2016
Sierra Leone	2008-2012	Sierra Leone Statistical Digest 2007-2013
USSR	1958-1958	USSR - Russia Mortality of the Urban and Rural Population 1958-1959
USSR	1969-1969	USSR - Russia Mortality of the Urban and Rural Population 1969-1970
USSR	1978-1978	USSR - Russia Mortality of the Urban and Rural Population 1978-1979
USSR	1983-1984	WHO Mortality Database Version March 2017
Yugoslavia	1950-1959	United Nations Demographic Yearbook - Historical Supplement 1997
Yugoslavia	1960-1990	WHO Mortality Database Version March 2017

Appendix Table 14: Number of outliers for the age-sex model by location, source type, and age group						
**Location**	**Source Type**	**Outliers Early Neonatal**	**Outliers Late Neonatal**	**Outliers Post Neonatal**	**Outliers Infant**	**Outliers Child**
Afghanistan	DHS	33	33	33	33	33
Albania	DHS	18	9	9	9	9
Albania	LSMS	15	15	15	15	15
Albania	RHS	12	4	3	3	3
Albania	VR	99	69	69	69	69
Algeria	MICS	9	9	9	9	9
Algeria	PAPFAM	9	9	9	9	9
Algeria	VR	6	6	6	6	6
Algeria	Other	12	12	12	12	12
American Samoa	VR	12	24	13	9	18
Andorra	VR	15	15	15	20	29
Angola	DHS	9	9	9	9	9
Angola	Other	9	9	9	9	9
Antigua and Barbuda	VR	39	51	38	36	40
Argentina	VR	3	3	3	3	3
Armenia	DHS	36	39	36	36	37
Armenia	VR	81	81	81	81	81
Azerbaijan	DHS	9	9	9	9	9
Azerbaijan	VR	108	108	108	108	108
Bahrain	VR	3	3	3	3	3
Bangladesh	DHS	90	90	90	90	90
Bangladesh	WFS	6	6	6	6	6
Barbados	VR	3	3	3	3	8
Belarus	VR	138	138	138	138	138
Belize	MICS	9	12	9	9	9
Belize	RHS	15	18	15	15	15
Benin	DHS	36	36	36	36	36
Benin	MICS	9	9	9	9	9
Benin	WFS	9	9	9	9	9
Bermuda	VR	48	72	64	46	73
Bolivia	DHS	54	54	45	45	45
Bolivia	VR	48	48	48	48	48
Botswana	DHS	15	6	6	6	6
Botswana	VR	18	18	18	18	18
Botswana	Other	23	21	14	14	14
Brazil	DHS	24	24	24	24	24
Brazil	VR	129	129	129	129	129
Brazil - Acre	DHS	9	9	9	9	9
Brazil - Acre	VR	114	114	114	114	114
Brazil - Alagoas	DHS	17	15	12	12	14
Brazil - Alagoas	VR	114	114	114	114	114
Brazil - Amapá	DHS	9	9	9	9	9
Brazil - Amazonas	DHS	9	14	12	9	11
Brazil - Amazonas	VR	66	66	66	66	66
Brazil - Bahia	DHS	21	22	21	21	21
Brazil - Bahia	VR	114	114	114	114	114
Brazil - Ceará	DHS	15	16	15	15	17
Brazil - Ceará	VR	114	114	114	114	114
Brazil - Distrito Federal	DHS	18	18	18	18	18
Brazil - Distrito Federal	VR	105	105	105	105	105
Brazil - Espírito Santo	DHS	21	21	21	21	21
Brazil - Espírito Santo	VR	114	114	114	114	114
Brazil - Goiás	DHS	21	21	21	21	21
Brazil - Goiás	VR	90	90	90	90	90
Brazil - Maranhão	DHS	13	17	12	12	12
Brazil - Maranhão	VR	114	114	114	114	114
Brazil - Mato Grosso	DHS	10	11	10	9	10
Brazil - Mato Grosso	VR	99	99	99	99	99
Brazil - Mato Grosso do Sul	DHS	9	13	10	9	14
Brazil - Mato Grosso do Sul	VR	75	75	75	75	75
Brazil - Minas Gerais	DHS	12	14	13	12	14
Brazil - Minas Gerais	VR	114	114	114	114	114
Brazil - Paraná	DHS	21	21	21	21	21
Brazil - Paraná	VR	105	105	105	105	105
Brazil - Paraíba	DHS	11	14	9	9	14
Brazil - Paraíba	VR	108	108	108	108	108
Brazil - Pará	DHS	13	13	9	9	10
Brazil - Pará	VR	114	114	114	114	114

Appendix Table 14: Number of outliers for the age-sex model by location, source type, and age group						
**Location**	**Source Type**	**Outliers Early Neonatal**	**Outliers Late Neonatal**	**Outliers Post Neonatal**	**Outliers Infant**	**Outliers Child**
Brazil - Pernambuco	DHS	19	18	18	18	19
Brazil - Pernambuco	VR	114	114	114	114	114
Brazil - Piauí	DHS	21	25	21	21	21
Brazil - Piauí	VR	114	114	114	114	114
Brazil - Rio Grande do Norte	DHS	13	17	12	12	14
Brazil - Rio Grande do Norte	VR	114	114	114	114	114
Brazil - Rio Grande do Sul	DHS	13	16	13	12	19
Brazil - Rio Grande do Sul	VR	42	42	42	42	42
Brazil - Rio de Janeiro	DHS	13	15	13	12	17
Brazil - Rio de Janeiro	VR	108	108	108	108	108
Brazil - Rondônia	DHS	12	12	12	12	12
Brazil - Rondônia	VR	9	9	9	9	9
Brazil - Roraima	DHS	9	9	9	9	9
Brazil - Roraima	VR	96	96	96	96	96
Brazil - Santa Catarina	DHS	18	18	18	18	18
Brazil - Santa Catarina	VR	114	114	114	114	114
Brazil - Sergipe	DHS	12	15	10	9	15
Brazil - Sergipe	VR	114	114	114	114	114
Brazil - São Paulo	DHS	15	16	15	15	19
Brazil - São Paulo	VR	63	63	63	63	63
Brazil - Tocantins	DHS	9	9	9	9	9
Brazil - Tocantins	VR	81	81	81	81	81
Burkina Faso	DHS	36	36	36	36	36
Burkina Faso	Other	9	9	9	9	9
Burundi	DHS	24	24	24	24	24
Cambodia	DHS	45	45	45	45	45
Cameroon	DHS	39	39	39	39	39
Cameroon	MICS	9	9	9	9	9
Cameroon	WFS	9	9	9	9	9
Cape Verde	RHS	6	6	6	6	6
Central African Republic	DHS	9	9	9	9	9
Chad	DHS	30	30	30	30	30
Colombia	DHS	69	69	69	69	69
Colombia	VR	105	105	105	105	105
Colombia	WFS	6	6	6	6	6
Comoros	DHS	18	18	18	18	18
Congo (Brazzaville)	DHS	48	48	48	48	48
Costa Rica	RHS	2	4	2	2	2
Costa Rica	WFS	6	6	6	6	6
Cote d'Ivoire	DHS	24	24	24	24	24
Cote d'Ivoire	LSMS	3	3	3	3	3
Cote d'Ivoire	WFS	9	9	9	9	9
Cote d'Ivoire	Other	9	9	9	9	9
Croatia	VR	3	3	3	3	3
Cyprus	VR	3	3	3	3	4
Czech Republic	RHS	15	15	15	15	15
DR Congo	DHS	21	21	21	21	21
Djibouti	PAPFAM	15	15	15	15	15
Dominica	VR	192	192	192	192	192
Dominican Republic	DHS	78	82	78	78	78
Dominican Republic	MICS	12	12	12	12	12
Dominican Republic	VR	198	198	198	198	198
Dominican Republic	WFS	33	33	33	33	33
Dominican Republic	Other	9	9	9	9	9
Ecuador	DHS	15	6	6	6	6
Ecuador	RHS	45	27	27	27	27
Ecuador	VR	174	66	66	66	66
Ecuador	WFS	18	18	18	18	18
Ecuador	Other	9	12	9	9	9
Egypt	DHS	81	81	81	81	81
Egypt	VR	168	168	168	168	168
Egypt	WFS	9	9	9	9	9
Egypt	Other	12	12	12	12	12
El Salvador	MICS	9	9	9	9	9
El Salvador	RHS	30	30	30	30	30
El Salvador	VR	195	195	195	195	195
Eritrea	DHS	18	18	18	18	18
Ethiopia	DHS	54	54	54	54	54

Appendix Table 14: Number of outliers for the age-sex model by location, source type, and age group						
**Location**	**Source Type**	**Outliers Early Neonatal**	**Outliers Late Neonatal**	**Outliers Post Neonatal**	**Outliers Infant**	**Outliers Child**
Ethiopia	Other	6	6	6	6	6
Fiji	VR	162	162	162	114	114
Fiji	WFS	15	15	15	6	6
Gabon	DHS	18	20	18	18	18
Georgia	RHS	29	29	29	29	29
Georgia	VR	69	69	69	69	69
Ghana	DHS	63	63	63	63	63
Ghana	MICS	18	18	18	18	18
Ghana	WFS	9	9	9	9	9
Greenland	VR	3	3	3	3	16
Grenada	VR	9	26	10	9	14
Guam	VR	0	8	0	0	3
Guatemala	DHS	36	36	36	36	36
Guatemala	RHS	18	18	18	18	18
Guatemala	VR	0	2	0	0	0
Guinea	DHS	27	27	27	27	27
Guinea-Bissau	MICS	9	9	9	9	9
Guyana	DHS	9	9	9	9	10
Guyana	MICS	9	9	9	9	9
Guyana	VR	135	135	135	135	135
Guyana	WFS	6	6	6	6	6
Guyana	Other	9	10	9	9	10
Haiti	DHS	36	36	36	36	36
Haiti	VR	21	21	21	21	21
Haiti	WFS	6	6	6	6	6
Haiti	Other	3	3	3	3	1
Honduras	DHS	21	21	21	21	21
Honduras	RHS	18	18	18	18	18
Honduras	VR	132	132	132	132	132
Iceland	VR	6	30	11	0	13
India	DHS	36	36	36	36	36
India	VR	45	45	45	45	45
India	Other	12	12	12	12	12
India - Andhra Pradesh	DHS	54	54	54	54	54
India - Andhra Pradesh	SRS	51	51	51	51	51
India - Andhra Pradesh	VR	9	9	9	9	9
India - Andhra Pradesh	Other	21	21	21	21	22
India - Arunachal Pradesh	DHS	12	13	12	12	12
India - Arunachal Pradesh	VR	6	6	6	6	6
India - Arunachal Pradesh	Other	9	9	9	9	9
India - Assam	DHS	24	24	24	24	24
India - Assam	SRS	3	3	3	3	3
India - Assam	VR	3	3	3	3	3
India - Assam	Other	3	3	3	3	5
India - Bihar	DHS	27	27	27	27	27
India - Bihar	VR	3	3	3	3	3
India - Bihar	Other	20	20	20	20	20
India - Chhattisgarh	DHS	9	9	9	9	9
India - Chhattisgarh	VR	6	6	6	6	6
India - Chhattisgarh	Other	20	20	20	20	20
India - Delhi	DHS	21	21	21	21	21
India - Delhi	SRS	9	9	9	9	9
India - Delhi	Other	26	26	26	26	26
India - Goa	DHS	21	24	21	21	21
India - Goa	VR	21	21	21	21	21
India - Goa	Other	9	9	9	9	9
India - Gujarat	DHS	24	24	24	24	24
India - Gujarat	VR	15	15	15	15	15
India - Gujarat	Other	20	20	20	20	20
India - Haryana	DHS	21	21	21	21	21
India - Haryana	Other	20	20	20	20	21
India - Himachal Pradesh	DHS	24	24	24	24	24
India - Himachal Pradesh	SRS	0	0	0	0	1
India - Himachal Pradesh	VR	15	15	15	15	15
India - Himachal Pradesh	Other	20	20	20	20	20
India - Jammu and Kashmir	DHS	24	24	24	24	24
India - Jammu and Kashmir	SRS	0	0	0	0	1
India - Jammu and Kashmir	VR	15	15	15	15	15

Appendix Table 14: Number of outliers for the age-sex model by location, source type, and age group

Location	Source Type	Outliers Early Neonatal	Outliers Late Neonatal	Outliers Post Neonatal	Outliers Infant	Outliers Child
India - Jammu and Kashmir	Other	8	8	8	8	10
India - Jharkhand	DHS	6	6	6	6	6
India - Jharkhand	SRS	6	6	6	6	6
India - Jharkhand	Other	14	14	14	14	14
India - Karnataka	DHS	27	27	27	27	27
India - Karnataka	VR	21	21	21	21	21
India - Karnataka	Other	22	22	22	22	22
India - Kerala	DHS	27	31	27	27	29
India - Kerala	SRS	15	15	15	15	15
India - Kerala	VR	21	21	21	21	21
India - Kerala	Other	15	15	15	15	15
India - Madhya Pradesh	DHS	27	27	27	27	27
India - Madhya Pradesh	VR	21	21	21	21	21
India - Madhya Pradesh	Other	18	18	18	18	18
India - Maharashtra	DHS	27	27	27	27	27
India - Maharashtra	SRS	21	21	21	21	21
India - Maharashtra	VR	9	9	9	9	9
India - Maharashtra	Other	18	18	18	18	18
India - Manipur	DHS	21	21	21	21	21
India - Manipur	VR	15	15	15	15	15
India - Manipur	Other	9	9	9	9	9
India - Meghalaya	DHS	15	15	15	15	15
India - Meghalaya	Other	9	9	9	9	9
India - Mizoram	DHS	15	23	15	15	15
India - Mizoram	Other	9	9	9	9	9
India - Nagaland	DHS	18	17	17	15	19
India - Nagaland	VR	15	15	15	15	15
India - Nagaland	Other	6	6	6	6	6
India - Odisha	DHS	27	27	27	27	27
India - Odisha	VR	21	21	21	21	21
India - Odisha	Other	21	21	21	21	21
India - Punjab	DHS	18	18	18	18	18
India - Punjab	SRS	6	6	6	6	6
India - Punjab	VR	21	21	21	21	21
India - Punjab	Other	20	20	20	20	21
India - Rajasthan	DHS	27	27	27	27	27
India - Rajasthan	VR	18	18	18	18	18
India - Rajasthan	Other	21	21	21	21	21
India - Sikkim	DHS	9	9	9	9	10
India - Sikkim	VR	15	15	15	15	15
India - Sikkim	Other	9	9	9	9	9
India - Tamil Nadu	DHS	27	27	27	27	27
India - Tamil Nadu	VR	21	21	21	21	21
India - Tamil Nadu	Other	20	20	20	20	20
India - Telangana	SRS	51	51	51	51	52
India - Telangana	VR	6	6	6	6	6
India - Telangana	Other	3	3	3	3	4
India - Tripura	DHS	18	20	18	18	18
India - Tripura	VR	9	9	9	9	9
India - Tripura	Other	0	0	0	0	5
India - Union Territories other than Delhi	VR	15	15	15	15	15
India - Union Territories other than Delhi	Other	9	9	9	9	9
India - Uttar Pradesh	DHS	27	27	27	27	27
India - Uttar Pradesh	Other	20	20	20	20	20
India - Uttarakhand	DHS	6	6	6	6	6
India - Uttarakhand	Other	8	8	8	8	9
India - West Bengal	DHS	27	27	27	27	27
India - West Bengal	VR	3	3	3	3	3
India - West Bengal	Other	20	20	20	20	20
Indonesia	Census	21	21	21	21	21
Indonesia	DHS	84	84	84	84	84
Indonesia	WFS	6	6	6	6	6
Indonesia	Other	33	33	33	33	33
Iran	VR	96	96	96	96	96
Iran	Other	12	12	12	12	12
Iraq	MICS	30	30	30	30	30
Iraq	VR	72	72	72	72	72
Iraq	Other	12	21	12	12	12

Appendix Table 14: Number of outliers for the age-sex model by location, source type, and age group						
**Location**	**Source Type**	**Outliers Early Neonatal**	**Outliers Late Neonatal**	**Outliers Post Neonatal**	**Outliers Infant**	**Outliers Child**
Jamaica	VR	96	96	96	96	96
Jamaica	WFS	6	6	6	6	6
Japan	VR	3	3	3	3	3
Japan - Akita	VR	1	15	2	1	0
Japan - Aomori	VR	0	5	0	0	1
Japan - Ehime	VR	0	10	0	0	0
Japan - Fukui	VR	1	13	1	0	2
Japan - Fukuoka	VR	0	2	0	0	0
Japan - Fukushima	VR	0	1	0	0	0
Japan - Gunma	VR	0	1	0	0	0
Japan - Hiroshima	VR	0	1	0	0	0
Japan - Hyōgo	VR	3	4	3	3	3
Japan - Ibaraki	VR	0	4	0	0	0
Japan - Ishikawa	VR	1	6	0	0	1
Japan - Iwate	VR	4	8	3	3	4
Japan - Kagawa	VR	0	10	0	0	0
Japan - Kagoshima	VR	0	2	0	0	0
Japan - Kumamoto	VR	0	1	0	0	0
Japan - Kyōto	VR	0	7	0	0	0
Japan - Kōchi	VR	0	13	1	0	3
Japan - Mie	VR	0	1	0	0	0
Japan - Miyagi	VR	3	5	3	3	3
Japan - Miyazaki	VR	0	1	0	0	0
Japan - Nagano	VR	0	9	0	0	0
Japan - Nara	VR	1	5	1	0	0
Japan - Niigata	VR	0	1	0	0	0
Japan - Okayama	VR	0	2	0	0	0
Japan - Saga	VR	2	14	1	0	0
Japan - Shiga	VR	0	1	0	0	1
Japan - Shimane	VR	2	13	1	0	1
Japan - Shizuoka	VR	0	2	0	0	0
Japan - Tochigi	VR	0	1	0	0	0
Japan - Tokushima	VR	1	5	1	0	1
Japan - Tottori	VR	8	20	6	3	4
Japan - Toyama	VR	0	5	0	0	0
Japan - Wakayama	VR	0	9	0	0	3
Japan - Yamagata	VR	0	4	0	0	2
Japan - Yamaguchi	VR	0	11	0	0	0
Japan - Yamanashi	VR	5	8	1	0	4
Japan - Ōita	VR	1	7	0	0	0
Jordan	DHS	54	54	54	54	54
Jordan	VR	66	66	66	66	66
Kazakhstan	DHS	21	23	21	21	21
Kazakhstan	VR	42	42	42	42	42
Kenya	DHS	57	57	57	57	57
Kenya	WFS	9	9	9	9	9
Kenya	Other	3	3	3	3	3
Kenya - Baringo	DHS	3	4	4	3	4
Kenya - Bomet	DHS	13	19	14	12	13
Kenya - Bungoma	DHS	14	17	7	6	6
Kenya - Bungoma	MICS	6	6	6	6	6
Kenya - Busia	DHS	35	44	34	33	37
Kenya - Elgeyo Marakwet	DHS	37	46	38	36	44
Kenya - Embu	DHS	49	55	48	48	49
Kenya - Embu	MICS	6	6	6	6	6
Kenya - Garissa	DHS	1	10	4	0	2
Kenya - Homa Bay	DHS	8	13	6	6	6
Kenya - Homa Bay	MICS	6	6	6	6	6
Kenya - Isiolo	DHS	21	22	21	21	23
Kenya - Isiolo	MICS	3	4	3	3	3
Kenya - Kajiado	DHS	44	52	44	40	45
Kenya - Kakamega	DHS	14	23	9	9	9
Kenya - Kakamega	MICS	6	8	6	6	6
Kenya - Kericho	DHS	20	34	19	18	23
Kenya - Kiambu	DHS	33	44	32	30	38
Kenya - Kilifi	DHS	19	32	19	18	20
Kenya - Kirinyaga	DHS	32	46	39	32	34
Kenya - Kisii	DHS	17	29	19	16	16

Appendix Table 14: Number of outliers for the age-sex model by location, source type, and age group						
**Location**	**Source Type**	**Outliers Early Neonatal**	**Outliers Late Neonatal**	**Outliers Post Neonatal**	**Outliers Infant**	**Outliers Child**
Kenya - Kisii	MICS	6	7	6	6	6
Kenya - Kisumu	DHS	11	23	3	3	4
Kenya - Kisumu	MICS	6	9	6	6	6
Kenya - Kitui	DHS	22	46	20	16	25
Kenya - Kitui	MICS	12	12	12	12	12
Kenya - Kwale	DHS	12	34	11	5	13
Kenya - Laikipia	DHS	48	48	49	48	51
Kenya - Lamu	DHS	24	27	23	23	24
Kenya - Machakos	DHS	30	37	32	30	34
Kenya - Machakos	MICS	6	9	7	6	6
Kenya - Makueni	DHS	25	27	25	24	25
Kenya - Makueni	MICS	6	6	6	6	6
Kenya - Mandera	DHS	11	17	5	3	7
Kenya - Marsabit	DHS	21	24	22	21	22
Kenya - Marsabit	MICS	21	21	21	21	21
Kenya - Meru	DHS	7	37	15	6	14
Kenya - Meru	MICS	9	9	9	9	9
Kenya - Migori	DHS	5	13	3	3	3
Kenya - Migori	MICS	3	4	3	3	3
Kenya - Mombasa	DHS	3	30	9	0	8
Kenya - Mombasa	MICS	3	5	3	3	3
Kenya - Murang'a	DHS	32	52	34	30	41
Kenya - Nairobi	DHS	11	25	10	6	9
Kenya - Nakuru	DHS	23	49	22	18	32
Kenya - Nandi	DHS	37	51	38	36	37
Kenya - Narok	DHS	37	47	35	33	37
Kenya - Nyamira	DHS	25	26	26	24	29
Kenya - Nyamira	MICS	6	8	6	6	6
Kenya - Nyandarua	DHS	49	45	44	43	46
Kenya - Nyeri	DHS	35	46	38	33	44
Kenya - Samburu	DHS	18	21	18	18	19
Kenya - Siaya	DHS	16	36	12	12	12
Kenya - Siaya	MICS	6	6	6	6	6
Kenya - Taita Taveta	DHS	36	43	39	34	38
Kenya - Tana River	DHS	27	29	27	27	27
Kenya - Tharaka Nithi	DHS	14	17	17	14	15
Kenya - Tharaka Nithi	MICS	6	10	6	6	6
Kenya - Trans Nzoia	DHS	40	47	33	31	36
Kenya - Turkana	DHS	14	24	13	12	12
Kenya - Turkana	MICS	3	5	3	3	3
Kenya - Uasin Gishu	DHS	27	35	27	24	26
Kenya - Vihiga	DHS	14	20	9	7	9
Kenya - Wajir	DHS	5	11	5	5	4
Kenya - West Pokot	DHS	37	42	37	36	41
Kiribati	VR	24	24	24	24	24
Kyrgyzstan	DHS	15	15	15	15	15
Kyrgyzstan	LSMS	6	6	6	6	6
Kyrgyzstan	MICS	9	9	9	9	9
Kyrgyzstan	VR	69	69	69	69	69
Laos	MICS	9	9	9	9	9
Lebanon	PAPFAM	9	9	9	9	12
Lebanon	VR	3	3	3	3	3
Lebanon	Other	12	12	12	12	13
Lesotho	DHS	27	27	27	27	27
Lesotho	WFS	6	6	6	6	6
Liberia	DHS	39	39	39	39	39
Liberia	Other	9	9	9	9	9
Libya	VR	6	6	6	6	6
Libya	Other	12	12	12	12	12
Luxembourg	VR	2	15	4	0	7
Madagascar	DHS	39	39	39	39	39
Madagascar	VR	39	39	39	39	39
Malawi	DHS	51	51	51	51	51
Malawi	MICS	21	21	21	21	21
Malaysia	WFS	6	6	6	6	6
Maldives	DHS	9	9	9	9	9
Maldives	VR	27	27	27	27	27
Mali	DHS	45	45	45	45	45

Appendix Table 14: Number of outliers for the age-sex model by location, source type, and age group						
**Location**	**Source Type**	**Outliers Early Neonatal**	**Outliers Late Neonatal**	**Outliers Post Neonatal**	**Outliers Infant**	**Outliers Child**
Mali	MICS	9	9	9	9	9
Mali	Other	3	3	3	3	3
Malta	VR	0	6	0	0	4
Mauritania	DHS	9	9	9	9	9
Mauritania	MICS	21	21	21	21	21
Mauritania	WFS	9	9	9	9	9
Mauritania	Other	9	9	9	9	9
Mexico	DHS	9	9	9	9	9
Mexico	VR	156	156	156	156	156
Mexico	WFS	6	6	6	6	6
Mexico - Aguascalientes	DHS	9	9	9	9	9
Mexico - Aguascalientes	VR	66	66	66	66	66
Mexico - Aguascalientes	Other	21	30	21	21	21
Mexico - Baja California	DHS	9	9	9	9	9
Mexico - Baja California	VR	87	87	87	87	87
Mexico - Baja California	Other	21	30	21	21	22
Mexico - Baja California Sur	VR	12	12	12	12	12
Mexico - Baja California Sur	Other	22	30	21	21	22
Mexico - Campeche	DHS	9	9	9	9	9
Mexico - Campeche	VR	102	102	102	102	102
Mexico - Campeche	Other	25	33	24	24	24
Mexico - Chiapas	DHS	12	12	12	12	12
Mexico - Chiapas	VR	114	114	114	114	114
Mexico - Chiapas	Other	26	33	24	24	24
Mexico - Chihuahua	DHS	9	9	9	9	9
Mexico - Chihuahua	VR	96	96	96	96	96
Mexico - Chihuahua	Other	23	30	21	21	21
Mexico - Coahuila	DHS	9	9	9	9	9
Mexico - Coahuila	VR	105	105	105	105	105
Mexico - Coahuila	Other	21	30	21	21	21
Mexico - Colima	DHS	9	9	9	9	9
Mexico - Colima	VR	99	99	99	99	99
Mexico - Colima	Other	22	30	21	21	21
Mexico - Durango	DHS	9	9	9	9	9
Mexico - Durango	VR	105	105	105	105	105
Mexico - Durango	Other	21	30	21	21	21
Mexico - Guanajuato	DHS	9	9	9	9	9
Mexico - Guanajuato	VR	54	54	54	54	54
Mexico - Guanajuato	Other	21	30	21	21	21
Mexico - Guerrero	DHS	12	12	12	12	12
Mexico - Guerrero	VR	114	114	114	114	114
Mexico - Guerrero	Other	21	30	21	21	21
Mexico - Hidalgo	DHS	9	9	9	9	9
Mexico - Hidalgo	VR	102	102	102	102	102
Mexico - Hidalgo	Other	24	33	24	24	24
Mexico - Jalisco	DHS	12	12	12	12	12
Mexico - Jalisco	VR	108	108	108	108	108
Mexico - Jalisco	Other	21	30	21	21	21
Mexico - Mexico City	DHS	12	12	12	12	12
Mexico - Mexico City	VR	15	15	15	15	15
Mexico - Mexico City	Other	21	30	21	21	21
Mexico - Michoacán de Ocampo	DHS	9	9	9	9	9
Mexico - Michoacán de Ocampo	VR	114	114	114	114	114
Mexico - Michoacán de Ocampo	Other	24	33	24	24	24
Mexico - Morelos	DHS	9	9	9	9	9
Mexico - Morelos	VR	105	105	105	105	105
Mexico - Morelos	Other	21	30	21	21	21
Mexico - México	DHS	9	9	9	9	9
Mexico - México	VR	12	12	12	12	12
Mexico - México	Other	21	30	21	21	21
Mexico - Nayarit	DHS	9	9	9	9	9
Mexico - Nayarit	VR	102	102	102	102	102
Mexico - Nayarit	Other	21	30	21	21	21
Mexico - Nuevo León	DHS	12	12	12	12	12
Mexico - Nuevo León	VR	102	102	102	102	102
Mexico - Nuevo León	Other	21	30	21	21	22
Mexico - Oaxaca	DHS	9	9	9	9	9
Mexico - Oaxaca	VR	96	96	96	96	96

Appendix Table 14: Number of outliers for the age-sex model by location, source type, and age group

Location	Source Type	Outliers Early Neonatal	Outliers Late Neonatal	Outliers Post Neonatal	Outliers Infant	Outliers Child
Mexico - Oaxaca	Other	25	33	24	24	24
Mexico - Puebla	DHS	9	9	9	9	9
Mexico - Puebla	VR	96	96	96	96	96
Mexico - Puebla	Other	24	33	24	24	24
Mexico - Querétaro	DHS	12	12	12	12	12
Mexico - Querétaro	VR	15	15	15	15	15
Mexico - Querétaro	Other	21	30	21	21	21
Mexico - Quintana Roo	VR	108	108	108	108	108
Mexico - Quintana Roo	Other	22	30	21	21	21
Mexico - San Luis Potosí	DHS	9	9	9	9	9
Mexico - San Luis Potosí	VR	114	114	114	114	114
Mexico - San Luis Potosí	Other	25	33	24	24	24
Mexico - Sinaloa	DHS	9	9	9	9	9
Mexico - Sinaloa	VR	102	102	102	102	102
Mexico - Sinaloa	Other	29	33	24	24	25
Mexico - Sonora	DHS	9	9	9	9	9
Mexico - Sonora	VR	108	108	108	108	108
Mexico - Sonora	Other	22	30	21	21	22
Mexico - Tabasco	DHS	9	9	9	9	9
Mexico - Tabasco	VR	102	102	102	102	102
Mexico - Tabasco	Other	34	36	27	27	27
Mexico - Tamaulipas	DHS	9	9	9	9	9
Mexico - Tamaulipas	VR	93	93	93	93	93
Mexico - Tamaulipas	Other	21	30	21	21	21
Mexico - Tlaxcala	DHS	9	9	9	9	9
Mexico - Tlaxcala	VR	12	12	12	12	12
Mexico - Tlaxcala	Other	24	33	24	24	24
Mexico - Veracruz de Ignacio de la Llave	DHS	12	12	12	12	12
Mexico - Veracruz de Ignacio de la Llave	VR	108	108	108	108	108
Mexico - Veracruz de Ignacio de la Llave	Other	21	30	21	21	21
Mexico - Yucatán	DHS	9	9	9	9	9
Mexico - Yucatán	VR	99	99	99	99	99
Mexico - Yucatán	Other	21	30	21	21	21
Mexico - Zacatecas	DHS	9	9	9	9	9
Mexico - Zacatecas	VR	105	105	105	105	105
Mexico - Zacatecas	Other	21	30	21	21	22
Moldova	DHS	9	13	9	9	9
Moldova	MICS	9	13	9	9	12
Moldova	VR	102	102	102	102	102
Mongolia	MICS	9	9	9	9	9
Mongolia	RHS	21	21	21	21	21
Mongolia	VR	60	60	60	60	60
Montenegro	VR	9	9	9	9	10
Morocco	DHS	42	42	42	42	42
Morocco	PAPFAM	24	24	24	24	24
Morocco	VR	24	24	24	24	24
Morocco	WFS	9	9	9	9	9
Morocco	Other	9	9	9	9	9
Mozambique	DHS	45	45	45	45	45
Mozambique	MICS	12	12	12	12	12
Mozambique	RHS	6	6	6	6	6
Myanmar	DHS	9	9	9	9	9
Myanmar	VR	3	3	3	3	3
Namibia	DHS	36	36	36	36	36
Nepal	DHS	45	45	45	45	45
Nepal	MICS	9	9	9	9	9
Nepal	WFS	6	6	6	6	6
Nicaragua	DHS	30	39	30	30	30
Nicaragua	RHS	18	18	18	18	18
Nicaragua	VR	144	144	144	144	144
Niger	DHS	36	36	36	36	36
Nigeria	DHS	43	45	42	42	42
Nigeria	MICS	12	12	12	12	12
Nigeria	VR	3	3	3	3	3
Nigeria	Other	12	12	12	12	12
Northern Mariana Islands	VR	16	29	13	11	27
Pakistan	DHS	39	39	39	39	39
Pakistan	LSMS	18	18	12	12	12

Appendix Table 14: Number of outliers for the age-sex model by location, source type, and age group						
**Location**	**Source Type**	**Outliers Early Neonatal**	**Outliers Late Neonatal**	**Outliers Post Neonatal**	**Outliers Infant**	**Outliers Child**
Pakistan	SRS	15	15	15	15	15
Pakistan	WFS	6	6	6	6	6
Pakistan	Other	36	36	18	18	18
Palestine	DHS	9	18	9	9	9
Palestine	MICS	21	21	21	21	21
Palestine	VR	45	45	45	45	45
Palestine	Other	12	12	12	12	12
Panama	VR	12	12	12	12	12
Panama	WFS	6	6	6	6	6
Papua New Guinea	DHS	18	18	18	18	18
Papua New Guinea	VR	6	6	6	6	6
Paraguay	DHS	9	9	9	9	9
Paraguay	MICS	9	10	9	9	9
Paraguay	RHS	27	28	27	27	27
Paraguay	VR	144	144	144	144	144
Paraguay	WFS	18	18	18	18	18
Peru	DHS	186	186	186	186	186
Peru	VR	189	189	189	189	189
Peru	WFS	9	9	9	9	9
Peru	Other	42	42	42	42	42
Philippines	DHS	45	45	45	45	45
Philippines	VR	189	189	189	189	189
Philippines	WFS	9	9	9	9	9
Portugal	VR	6	6	6	6	6
Portugal	WFS	9	9	9	9	9
Puerto Rico	RHS	9	9	9	9	12
Qatar	VR	54	54	54	54	54
Romania	LSMS	18	18	20	20	20
Rwanda	DHS	105	105	105	105	105
Rwanda	WFS	9	9	9	9	9
Saint Lucia	VR	0	2	0	0	6
Saint Vincent and the Grenadines	VR	6	9	7	6	10
Samoa	VR	42	42	42	42	42
Sao Tome and Principe	DHS	9	9	9	9	9
Sao Tome and Principe	MICS	9	9	9	9	9
Saudi Arabia	VR	39	39	39	39	39
Senegal	DHS	81	81	81	81	81
Senegal	WFS	9	9	9	9	9
Senegal	Other	9	9	9	9	9
Serbia	VR	30	30	30	30	30
Seychelles	VR	6	8	7	6	20
Sierra Leone	DHS	21	21	21	21	21
Sierra Leone	VR	15	15	15	15	15
Somalia	MICS	6	6	6	6	6
South Africa	DHS	9	9	9	9	9
South Africa	VR	78	78	78	78	78
South Africa	Other	18	18	18	18	18
South Korea	VR	78	78	78	78	78
South Korea	WFS	6	6	6	6	6
South Sudan	MICS	9	9	9	9	9
Sri Lanka	DHS	9	9	9	9	9
Sri Lanka	VR	6	6	6	6	6
Sri Lanka	WFS	6	6	6	6	6
Sudan	DHS	9	9	9	9	9
Sudan	MICS	18	18	18	18	18
Sudan	WFS	9	9	9	9	9
Sudan	Other	12	12	12	12	12
Suriname	VR	165	165	165	165	165
Swaziland	DHS	9	9	9	9	9
Swaziland	MICS	18	18	18	18	18
Sweden	VR	3	3	3	3	3
Sweden - Stockholm	VR	3	4	3	3	3
Sweden - Sweden except Stockholm	VR	3	3	3	3	3
Syria	PAPFAM	30	30	30	30	30
Syria	VR	54	54	54	54	54
Syria	WFS	9	9	9	9	9
Syria	Other	9	9	9	9	9
Tajikistan	DHS	9	9	9	9	9

Appendix Table 14: Number of outliers for the age-sex model by location, source type, and age group						
**Location**	**Source Type**	**Outliers Early Neonatal**	**Outliers Late Neonatal**	**Outliers Post Neonatal**	**Outliers Infant**	**Outliers Child**
Tajikistan	LSMS	20	20	20	20	20
Tajikistan	VR	99	99	99	99	99
Tanzania	DHS	54	54	54	54	54
Tanzania	Other	9	9	9	9	9
Thailand	DHS	9	9	9	9	9
Thailand	VR	171	171	171	171	171
Thailand	WFS	6	6	6	6	6
The Bahamas	VR	3	138	5	3	3
The Gambia	DHS	9	9	9	9	9
Timor-Leste	DHS	21	23	21	21	21
Timor-Leste	Other	6	6	6	6	6
Togo	DHS	24	24	24	24	24
Tonga	VR	30	30	30	30	30
Trinidad and Tobago	DHS	9	10	9	9	9
Trinidad and Tobago	VR	96	96	102	96	96
Trinidad and Tobago	WFS	9	9	9	9	9
Tunisia	DHS	9	9	9	9	9
Tunisia	MICS	9	9	9	9	9
Tunisia	PAPFAM	9	9	9	9	9
Tunisia	VR	60	60	60	60	60
Tunisia	WFS	9	9	9	9	9
Tunisia	Other	12	12	12	12	12
Turkey	DHS	36	36	36	36	36
Turkey	VR	105	105	105	105	105
Turkey	WFS	9	9	9	9	9
Turkmenistan	MICS	6	7	6	6	6
Turkmenistan	VR	48	48	48	48	48
USA - Montana	VR	0	1	0	0	0
USA - Nevada	VR	0	2	0	0	0
USA - New Hampshire	VR	0	0	0	0	3
USA - North Dakota	VR	0	4	0	0	1
USA - South Dakota	VR	0	1	0	0	0
USA - Vermont	VR	0	3	1	0	4
USA - Wyoming	VR	0	2	0	0	0
Uganda	DHS	57	57	57	57	57
Uganda	Other	9	9	9	9	9
Ukraine	DHS	20	36	18	18	18
Ukraine	RHS	9	18	9	9	11
Ukraine	VR	81	174	81	81	81
United Kingdom	VR	30	30	30	12	12
United Kingdom - England	VR	15	15	0	0	0
Uruguay	VR	3	3	3	3	3
Uzbekistan	DHS	15	15	15	15	15
Uzbekistan	VR	57	57	57	57	57
Venezuela	WFS	15	15	15	15	15
Vietnam	DHS	15	15	15	15	15
Vietnam	MICS	9	10	9	9	9
Virgin Islands	VR	3	10	3	3	16
Yemen	DHS	24	24	24	24	24
Yemen	MICS	9	9	9	9	9
Yemen	PAPFAM	9	9	9	9	9
Yemen	WFS	6	6	6	6	6
Yemen	Other	21	21	21	21	21
Zambia	DHS	48	48	48	48	48
Zambia	Other	6	6	6	6	6
Zimbabwe	DHS	54	54	54	54	54
Zimbabwe	MICS	18	18	18	18	18
Zimbabwe	VR	6	6	6	6	6
